# Supplementary material for: No phenotypic or genotypic evidence for a link between sleep duration and brain atrophy
Source: Nat Hum Behav. 2023 Oct 5;7(11):2008–22. doi: 10.1038/s41562-023-01707-5 (PMC10663160; doi:10.1038/s41562-023-01707-5)
Supplement: Supplementary file 1 — Extended background information (reviewed studies, MRI methods and sample characteristics), statistical analyses and results (cortical longitudinal, subcortical longitudinal, cortical cross-sectional, subcortical cross-sectional, meta-analysis, cortical vertex analyses and mega- versus meta-analytic approach), and genetic analyses and results (genetic analyses, genetic notes and STROBE-MR-checklist). [file 41562_2023_1707_MOESM1_ESM.pdf]

nature human behaviour

Article https://doi.org/10.1038/s41562-023-01707-5

No phenotypic or genotypic evidence for a link between sleep duration and brain atrophy

In the format provided by the authors and unedited

SI filename	Description
Extended background information	
Reviewed studies	Reference, sample description and main results of 19 studies testing sleep duration – brain morphometric relationships in adults
MRI methods	Overview of scanners and sequence parameters for each sample, description of MRI processing
Sample characteristics	Description of recruitment, inclusion/exclusion criteria and cognitive testing in each sample
Statistical analyses and results	
Cortical Longitudinal	R-code and results for each of 32 cortical regions<br>-thickness, area, volume (longitudinal)<br>-stratified by sex<br>-full range of sleep duration vs. 5-9 hours
Subcortical Longitudinal	R-code and results for each of 19 regions<br>-volume (longitudinal)<br>-full range of sleep duration vs. 5-9 hours<br>-effects of covariates: SES, BMI, depression, global sleep quality
Cortical cross-sectional	R-code and results for each of 32 cortical regions<br>-thickness, area, volume (cross-sectional)<br>-cortical clustering procedures & results for each metric
Subcortical cross-sectional	R-code and results for each of 19 regions<br>-volume (cross-sectional)<br>-raw volumes vs. controlling for ICV<br>-effects of covariates: SES, BMI, depression, global sleep quality
Meta-analysis	R-code and results for the meta-analysis<br>-subcortical volume and cortical thickness (cross-sectional)<br>-raw volumes vs. controlling for ICV
Cortical vertex analyses	Vertex wise results<br>-cortical thickness<br>-stratified by age (20-60 vs > 60 years)<br>-combined cross-sectional & longitudinal (spatiotemporal linear mixed-effect models)
Mega vs. meta-analytic approach	-compare results from mega- vs meta-analysis

Genetic analyses and results	
Genetic analyses	-GWAS procedures & results<br>-Calculation of PGSs<br>-Mendelian randomization procedures & results
Genetics notes	-Detailed Mendelian randomization results<br>-Comparisons of different Mendelian randomization methods
Genetics tables	-Detailed SNP-level results
STROBE-MR-checklist	- Checklist of recommended items to address in reports of Mendelian randomization studies
Instrumental variables	Tables of the instrumental variables for the MR analysis (ICV → sleep duration)

Supplementary Table 3 Overview of Supplemental Information adding background and details to the main manuscript, including R-code.

Study	N	Sample	Main result and method
**Cross-sectional**			
Alperin et al., 2019	69	Older	Duration correlated negatively with hippocampal and cortical volume and thickness reductions. ROI, FreeSurfer.
Branger et al., 2016	51	Older	No relationship between sleep duration and GM volume. VBM, SPM
Grau-Rivera et al., 2020	366	Middle-aged/ older	Insomnia symptoms associated with lower GM volume, not surviving corrections. VBM, SPM.
Kim et al., 2021	1353	Middle-aged/ older	No relationship in absence of sleep apnea. ROI, custom.
Lim et al., 2016	141	Older	No relationship to volume in any of 68 cortical regions. ROI, FreeSurfer.
Stoffers et al. 2012	65	Young/ middle-aged	Early morning awakenings negatively related to orbitofrontal volumes measured, not for initiating or maintain sleep. VBM, SPM.
Zitser et al., 2020	613	Middle-aged/ older	No relationship between global GM volume and sleep duration over several years. VBM, FSL.
Fjell et al., 2019	3105	Adult lifespan	No relationship between duration and hippocampal volume. ROI, FreeSurfer.
Fjell et al., 2019	21000	Middle-aged/ older (UKB)	$\leq$ 5 hours and $\geq$ 9 hours related to smaller hippocampal volume. ROI, FreeSurfer.
Aribisala et al., 2020	457	Older (LBC)	Nighttime sleep duration not associated with total brain volume. ROI, custom.
Sabeti et al. 2018	79	Older (90+)	No relationship between sleep duration and hippocampal volume. ROI, custom.
Westwood et al., 2017	2457	Older (incl dementia)	Long sleep (> 9 hours) negatively associated with total cerebral brain volume. ROI, custom.
De Looze et al., 2022	417	Older	$\geq$ sleep associated with smaller hippocampal subfields
Li et al., 2022	39692	Middle-aged/ older (UKB)	Inverse U-shaped relationship (global thickness, volume, area and subcortical structures)
**Longitudinal**			
Sexton et al. 2014[x]	147	Adult lifespan	No relationship between duration and volume reduction. Vertex-wise, ROI, FreeSurfer.
Lo et al., 2014	119	Older	No relationship between duration and change in total cerebral, inferior or superior frontal volume, pos relationship with ventricular expansion. ROI, FreeSurfer.
Spira et al., 2016	122	Older	Sleep < 7 hours and > 7 hours associated with frontotemporal loss. Vertex-wise, FreeSurfer.
Fjell et al., 2019	1299	Adult lifespan	No relationship with hippocampal volume loss. ROI, FreeSurfer.
Fjell et al., 2020[y]	2205	Adult lifespan	No relationship with thickness change. Vertex-wise, FreeSurfer.

Supplemental Table 1 Reviewed studies of sleep duration – brain structure

X: This sample is a subsample of Fjell et al. 2019. Y: This is a longitudinal subsample of the Fjell et al. 2019 study above UKB: UK Biobank imaging study. LBC: Lothian Birth Cohort study. VBM: Voxel-based morphometry. ROI: Regions of interest. SPM: Statistical parametric mapping. TBV: Total Brain Volume. TGMV: Total Gray Matter Volume. Fjell et al. 2019 is cited twice for the cross-sectional

results, since the study reports analyses from two independent samples. The sample in Fjell et al. 2020 is largely overlapping the longitudinal part of Fjell et al. 2019.

MAGNETIC RESONNANCE IMAGING METHODS

Specific information on image acquisition in the different samples

T1 weighted structural scans were acquired at Siemens, Philips and GE scanners at the various sites.

Sample	Scanner	Field strength (Tesla)	Sequence parameters
BASE-II	Tim Trio Siemens	3.0	TR: 2500 ms, TE: 4.77 ms, TI: 1100 ms, flip angle: 7°, slice thickness: 1.0 mm, FoV 256×256 mm, 176 slices
Betula	Discovery GE	3.0	TR: 8.19 ms, TE: 3.2 ms, TI: 450 ms, flip angle: 12°, slice thickness: 1 mm, FOV 250×250 mm, 180 slices
Cam-CAN	Tim Trio Siemens	3.0	TR: 2250 ms, TE: 2.98 ms, TI: 900 ms, flip angle: 9°, slice thickness 1 mm, FOV 256×240 mm, 192 slices
LCBC	Avanto Siemens	1.5	TR: 2400 ms, TE: 3.61 ms, TI: 1000 ms, flip angle: 8°, slice thickness: 1.2 mm, FoV: 240×240 m, 160 slices, iPat = 2
	Avanto Siemens	1.5	TR: 2400 ms, TE = 3.79 ms, TI = 1000 ms, flip angle = 8, slice thickness: 1.2 mm, FoV: 240 x 240 mm, 160 slices
	Skyra Siemens	3.0	TR: 2300 ms, TE: 2.98 ms, TI: 850 ms, flip angle: 8°, slice thickness: 1 mm, FoV: 256×256 mm, 176 slices
	Prisma Siemens	3.0	TR: 2400 ms, TE: 2.22 ms, TI: 1000 ms, flip angle: 8°, slice thickness: 0.8 mm, FoV: 240×256 mm, 208 slices, iPat = 2
UB	Tim Trio Siemens	3.0	TR: 2300 ms, TE: 2.98, TI: 900 ms, slice thickness 1 mm, flip angle: 9°, FoV 256×256 mm, 240 slices
WH-II	Verio Siemens	3.0	TR: 2530 ms, TE: 1.79/3.65/5.51/7.37 ms, TI: 1380 ms, flip angle: 7°, slice thickness: 1.0 mm, FOV: 256×256 mm
HCP	Connectome Skyra Siemens*	3.0	TR: 2400 ms, TE: 2.14 ms, TI: 1000 ms, flip angle: 8°, slice thickness: 0.7 mm, FOV: 224 mm, 256 slices, GRAPPA = 2
UKB	Skyra Siemens	3.0	TR: 2000 ms, TI: 880 ms, slice thickness: 1 mm, FoV: 208×256 mm, 256 slices, iPAT=2

Supplementary Table MR acquisition parameters

TR: Repetition time, TE: Echo time, TI: Inversion time, FoV: Field of View, iPat: in-plane acceleration, GRAPPA: GRAPPA acceleration factor. *Customized

Lifebrain MRI scanning and processing

MRI data originated from 11 different scanners (table above), and were processed with FreeSurfer (https://surfer.nmr.mgh.harvard.edu/) (1-4). To avoid introducing possible site-specific biases, quality control measures were imposed and no manual editing was done. We previously have reported that across-scanner consistencies in estimated hippocampal (5) and cortical (6) volumes for the scanners used in Lifebrain are high.

HCP MRI scanning and processing

Imaging data were collected and processed by the human connectome project (https://www.humanconnectome.org/study/hcp-young-adult) as described in (7). Imaging data were collected a customized Siemens 3T "Connectome Skyra" housed at Washington University in St. Louis, using a standard 32-channel Siemens receive head coil and a "body" transmission coil designed by Siemens specifically for the smaller space available using the special gradients of the WU-Minn and MGH-UCLA Connectome scanners. Anatomical T1-weighted magnetization-prepared rapid gradient echo (MPRAGE) images were obtained in the sagittal plane at 0.7mm isotropic resolution, and T2 weighted FLAIR images were acquired at identical resolution in the sagittal plane. Images were processed using a custom combination of tools from FSL and FreeSurfer (8-10).

UKB MRI scanning and processing

Imaging data were collected and processed by the UK Biobank (https://www.ukbiobank.ac.uk) as described in (11), using the FreeSurfer 6.0 software package. Imaging data were collected using 3.0 T Siemens Skyra (32-channel head coil). Anatomical T1-weighted magnetization-prepared rapid gradient echo (MPRAGE) images were obtained in the sagittal plane at 1mm isotropic resolution, and T2 weighted FLAIR images were acquired at 1.05x1x1mm resolution in the sagittal plane.

References

1. A. M. Dale, B. Fischl, M. I. Sereno, Cortical surface-based analysis. I. Segmentation and surface reconstruction. *Neuroimage* **9**, 179-194 (1999).
2. B. Fischl *et al.*, Whole brain segmentation: automated labeling of neuroanatomical structures in the human brain. *Neuron* **33**, 341-355 (2002).
3. M. Reuter, N. J. Schmansky, H. D. Rosas, B. Fischl, Within-subject template estimation for unbiased longitudinal image analysis. *Neuroimage* **61**, 1402-1418 (2012).
4. J. Jovicich *et al.*, Brain morphometry reproducibility in multi-center 3T MRI studies: a comparison of cross-sectional and longitudinal segmentations. *Neuroimage* **83**, 472-484 (2013).
5. A. M. Fjell *et al.*, Continuity and Discontinuity in Human Cortical Development and Change From Embryonic Stages to Old Age. *Cereb Cortex* **29**, 3879-3890 (2019).

6. L. Nyberg *et al.*, Individual differences in brain aging: heterogeneity in cortico-hippocampal but not caudate atrophy rates. *Cereb Cortex* 10.1093/cercor/bhac400 (2022).
7. M. F. Glasser *et al.*, The minimal preprocessing pipelines for the Human Connectome Project. *Neuroimage* **80**, 105-124 (2013).
8. B. Fischl, FreeSurfer. *Neuroimage* **62**, 774-781 (2012).
9. M. Jenkinson, P. Bannister, M. Brady, S. Smith, Improved optimization for the robust and accurate linear registration and motion correction of brain images. *Neuroimage* **17**, 825-841 (2002).
10. M. Jenkinson, C. F. Beckmann, T. E. Behrens, M. W. Woolrich, S. M. Smith, Fsl. *Neuroimage* **62**, 782-790 (2012).
11. F. Alfaro-Almagro *et al.*, Image processing and Quality Control for the first 10,000 brain imaging datasets from UK Biobank. *Neuroimage* **166**, 400-424 (2018).

SAMPLE DESCRIPTIVES

BASE II

Population, recruitment, inclusion/exclusion criteria and general description of study

Participants of the Berlin Aging Study II (BASE II) were community-dwelling older adults recruited from the greater Berlin metropolitan area through advertisements in newspapers and public areas (for cohort characteristics and additional details, see [1, 2]). The baseline sample comprised 2200 participants. Of these, 1600 were older adults aged 61–88 years (mean age 71.5, SD 3.89; 793 female), and 600 were younger adults aged 24–40 years (mean age 31.1, SD 3.38; 247 female). Participants were invited to a medical exam consisting of a 2-day protocol, and two cognitive testing sessions scheduled 1 week apart, and were tested in small groups (e.g. about 6 participants per group) on a comprehensive cognitive battery that covers key cognitive abilities measured by 21 tasks. Each session lasted about 3.5 h. 1828 had valid data to be included in the present analyses.

MR Sample: After completion of the cognitive examination of BASE-II, eligible participants were invited to take part in one MRI session within a time window of 2–4 weeks after cognitive testing, consisting of 341 older adults aged 61–82 years (mean age 70.1, SD = 3.89; 131 female) and 103 younger adults (mean age 31.4, SD = 3.7; 39 female). MR scans and cognitive scores were obtained 2012-2013. A subsample of the MR sample was later re-invited for follow-up. The different elements of the study were approved by the ethics committees of the Max Planck Institute for Human Development, the Charité University ethics committee and by the ethics committees of The German Association for Psychology (DGPs). Participants signed written informed consent and received monetary compensation for their participation in BASE-II and the MRI study. All experiments were performed in accordance with relevant guidelines and regulations.

Inclusion/exclusion criteria/ screening

Inclusion criteria for taking part in this study were age between 20 and 35 or 60 and 80 years, apparently healthy. Exclusion criteria were untreated diabetes and hypertension; prior stroke, head injuries or brain surgery; psychiatric illness; major depression; dementia with a score < 24 on the Mini-Mental State Examination. To that end, none of the participants took medication that might affect memory function or had a history of head injuries, medical (e.g., heart attack), neurological (e.g., epilepsy), or psychiatric disorders (e.g., depression). All participants reported normal or corrected to normal vision, were right-handed, and scored over 27 on the Mini-Mental Status Examination.

Betula

Population, recruitment and general description of study/ procedures

In the Betula longitudinal study on aging, memory and dementia, population-based sampling of healthy middle-aged and older adults was used for recruitment. Detailed recruitment procedures are found in [3, 4]. For the current analyses, the MRI subsample of the study is used. Participation in the neuroimaging study was offered to all participants who had remained in the study and completed cognitive testing at the 5th Betula test wave in 2008-2009, and 376 participants from underwent structural and functional MRI in 2009-2010.

Inclusion/exclusion criteria/ screening

Exclusion criteria were severe visual or auditory handicaps, intellectual or developmental disabilities, suspected dementia, having a mother tongue other than Swedish, MRI contraindications, neurological disorders, or visual/motor deficits that could interfere with fMRI data collection, MMSE <24, brain or head surgery, and substantial brain anatomical deviations. Eight participants were excluded completely post scanning due to discovered neurological conditions (Schizophrenia, Multiple Sclerosis, Parkinson’s Disease, Hydrocephalus, Alcoholism, and dementia), and an additional two participants were excluded due to MMSE scores below 24. In addition, for 29 participants MRI data only was excluded due to anatomical deviations (subdural hematoma, localized loss of brain tissue, subcortical atrophy, and previous brain or head surgery (n=2)), movement artifacts (n=21), or FreeSurfer processing failures (n=3). Three individuals had missing T1 images due to incomplete acquisition. Age at MRI-scanning, reported with a one decimal precision, was used in the analyses. Testing was performed 1-18 months prior to scanning (mean interval: 9 months).

Cam-Can

Population, recruitment and general description of study/ procedures

Recruitment was done by invitation letters based on the patient lists of general practitioners within the Cambridge City area. A population-based cohort of 3000 adults aged 18 or above was recruited to Stage 1 of the project, where they completed an interview including health and lifestyle questions, a core cognitive assessment, and a self-completed questionnaire of lifetime experiences and physical activity. Of those interviewed, ~700 participants aged 18-87 (100 per age decile) continued to Stage 2 where they undergo cognitive testing and provide measures of brain structure and function. A subset of ~250 adults returned for longitudinal follow-up data. The study is conducted in compliance with the Helsinki Declaration, and has been approved by the local ethics committee, Cambridgeshire 2

Research Ethics Committee (reference: 10/H0308/50).

Inclusion/exclusion criteria/ screening

General exclusion criteria: Term-time residents of colleges and universities, and participants whose Primary Care Physician feel are inappropriate to include. Exclusion criteria for the MRI part of the study: Not cognitively normal (MMSE < 24, memory defect, consent difficulties), communication difficulties (hearing problems [35db at 1000 Hz], insufficient English language, vision difficulties), medical problems by self-report of diagnosis (dementia diagnosis /Alzheimer's Disease, Parkinson's Disease, Motor Neurone disease, Multiple sclerosis, cancer, stroke, encephalitis, meningitis, epilepsy, head injury with serious results [coma, unconscious for >2 hours, skull fracture], recently diagnosed or uncontrolled high blood pressure, possible pregnancy, current psychiatric conditions [bipolar disorder, schizophrenia, psychosis]), mobility problems (restricted mobility which could prevent further participation, inability to walk 10 metres), substance abuse (past or current treatment for drug abuse, current drug usage), MRI/ MEG safety and comfort exclusions.

LCBC

Population, recruitment and general description of study/ procedures

Cognitively healthy, community dwelling participants across the lifespan were drawn from studies coordinated by the Research Group for Lifespan Changes in Brain and Cognition (LCBC www.oslobrains.no), approved by a Norwegian Regional Committee for Medical and Health Research Ethics. Written informed consent was obtained from all adult participants and from parents or other legal guardians for participants below age of majority. The samples were recruited by newspaper and web page adds, and part of the developmental sample was recruited through the population registry study MoBa https://www.fhi.no/en/studies/moba/ Most participants, including all children, were recruited for observational studies, while some adults were recruited to enter into cognitive training studies after baseline assessment.

Inclusion/exclusion criteria/ screening

Adult participants were screened using a standardized health interview prior to inclusion in the study. Participants with a history of self- or parent-reported neurological or psychiatric conditions, including clinically significant stroke, serious head injury, untreated hypertension, diabetes, and use of psychoactive drugs within the last two years, were excluded. Further, participants reporting worries concerning their cognitive status, including memory function, were excluded. All participants above 40 years scored ≥24 on the Mini Mental State Examination [5].

UB

Population, recruitment and general description of study/ procedures, inclusion/ exclusion criteria/ screening

Cohorts from the University of Barcelona (UB) site were collapsed across a number of substudies described below.

WAHA cohort [6]: Recruitment and selection of participants took place between May 2012 and May 2014; the trial ended May 31, 2016. Participants were healthy elderly men and women with normal cognitive and visual function. Inclusion criteria were age between 63 and 79 years, apparently healthy, and equally willing to be in either of the two groups. Exclusion criteria included inability to undergo neuropsychological testing; morbid obesity (BMI ≥ 40 kg/m^2); uncontrolled diabetes (HbA1c > 8%); uncontrolled hypertension (on-treatment blood pressure ≥ 150/100 mmHg); prior stroke, significant head trauma or brain surgery; relevant psychiatric illness; major depression; cognitive deterioration or dementia with a score < 24 on the Mini-Mental State Examination; other neurodegenerative disorders like Parkinson's disease; advanced AMD or eye-related conditions precluding ophthalmological evaluation; prior chemotherapy; chronic illness with projected shortened lifespan; allergy to walnuts; customary use of fish oil and/or tree nuts (> 2 servings/week) and/or other relevant sources of ALA, such as flaxseed oil or soy lecithin. Eligible participants were recruited via mailing study brochures (LLU) or through the non-profit organization Institute of Aging (BCN), advertisements in the study centers, and word of mouth. Interested individuals attended an informational group meeting, completed a short medical questionnaire and signed the informed consent. Next candidates had a face-to-face interview with the study clinician, who assessed potential compliance, reviewed the medical history, inclusion and exclusion criteria, and recent blood work and use of medications or supplements, and administered the MMSE. Eligible participants were scheduled to have baseline tests (neuropsychological and ophthalmologic evaluations and collection of fasting blood and urine) and were then randomized to either the control or walnut group using a computerized random number table with stratification by center, sex, and age range. Couples entering the study were treated as one number and were randomized into the same group.

CR and iTBS cohorts [7]: Healthy volunteers older than 60 were recruited via the Institute of Aging, Barcelona. Individuals willing to participate were gathered in an informal meeting to tell them about the investigation, which included repetitive transcranial magnetic stimulation (TMS). Eligible participants had a normal cognitive profile with MMSE scores ≥ 24 and performances not below 1.5

SD according to normative scores (adjusted for age and education (Peña-Casanova et al., 2009)) on a neuropsychological evaluation.

GABA cohort [8]: Participants were recruited from the Institute of Aging (Barcelona) and the University of Experience, an initiative by the University of Barcelona for students aged 55 and older, offering special one and two-year degrees. Older adults, aged ≥ 60 years (age (mean ± *SD*), 68.15 ± 4.6 years; age range: 60–79 years), naive to stimulation, participated in this study after giving informed consent, in accordance with the Declaration of Helsinki (1964, last revision 2013). None of the participants reported a diagnosis of a neurological or psychiatric disorder or any TMS contraindication. Inclusion criteria for the older subjects included a normal cognitive profile with mini-mental state examination (MMSE) scores of ≥ 24 and performance scores not more than 1.5 standard deviation (*SD*) below normative data (adjusted for age and years of education) on any of the administered neuropsychological tests (i.e., they did not fulfil the criteria for mild cognitive impairment (MCI). The neuropsychological battery included (1) a screening test for dementia, using the MMSE, and an evaluation of: (2) premorbid cognition and intelligence quotient (IQ), using the vocabulary subtest of the Wechsler Adult Intelligence Scale-III (WAIS-III) and National Adult Reading Test (NART); (3) verbal memory, using the Free and Cued Selective Reminding Test (SRT); (4) executive functions, using the phonemic fluency task and Trail Making Test B (TMTB); (5) language, using the semantic fluency task and Boston Naming Test (BNT); and (6) speed of processing, using the Symbol Digit Modalities Test (SDMT).

PD and MSA cohorts [9]: Healthy volunteers were recruited from the Aging Institute in Barcelona. Inclusion criteria were: scores within normality in a battery of neuropsychological tests described below (adjusted for age and education), IQ within normality as measured by the Vocabulary subtest (WAIS, scalar score >7). Exclusion criteria included: diagnosis of any neurological or psychiatric disease, mild cognitive impairment (MCI), any other condition that might affect cognition (chemotherapy or radiotherapy), diagnosis of fibromyalgia and any incompatibility to undergo MRI. Visuospatial and visuoperceptual functions were assessed with Benton Visual Form Discrimination (VFD) and Judgment of Line Orientation (JLO) tests; executive functions were evaluated with phonemic (words beginning with the letter “p” in 1 minute) and semantic (animals in 1 minute) fluencies; memory through total learning recall (sum of correct responses from trial I to trial V) and delayed recall (total recall after 20 min) through scores on Rey’s Auditory Verbal Learning Test (RAVLT). Attention and WM were assessed with Digit Span Forward and Backward, the Stroop Color-Word Test, Symbol Digits Modalities Tests (SDMT) and the Trail Making Test (in seconds), part A (TMTA) and part B (TMTB); and language was

assessed by the total number of correct responses in the short version of the Boston Naming Test (BNT).

Whitehall

Population, recruitment and general description of study/ procedures

The Whitehall II study, starting in 1985, includes 10.308 British civil servants followed over time, which allows exploring factors hypothesized to affect brain health and cognitive aging. At study start, phase 1 (1985-1988), the population representative cohort was in the age range 35-55 years. MRI was done in Phase 11 (2012-2013) of this study, at which time the total number participants was 6035, and the age range was 60-85 years [10]. A random sample willing and able to give informed consent to participant in the imaging sub-study of Whitehall II was included, and it is this sub-cohort of the Whitehall study which is included in the present. analyses. Ethical approval was granted generically for the "Protocol for non-invasive magnetic resonance investigations in healthy volunteers" (MSD/IDREC/2010/P17.2) by the University of Oxford Central University/ Medical Science Division Interdisciplinary Research Ethics Committee (CUREC/MSD-IDREC), who also approved the specific protocol: "Predicting MRI abnormalities with longitudinal data of the Whitehall II sub-study" (MSD-IDREC-C1-2011-71).

Inclusion/exclusion criteria/ screening

A random selection of 800 participants from the Whitehall II phase 11 study was done. Exclusion criteria were MRI contraindications, or being unable to travel to Oxford without assistance. After the exclusion of those with excess motion (N=4) and self-reported history of stroke (N=16). No participant was diagnosed with dementia.

HCP

Population, recruitment and general description of study/ procedures

The Human Connectome project (HCP) aimed to study and freely share data from 1200 young adults (ages 22-35) from families with twins and non-twin siblings, using a protocol that includes structural and functional magnetic resonance imaging (MRI, fMRI), diffusion tensor imaging (dMRI) at 3 Tesla (3T) and behavioral and genetic testing. The dataset used was the 1200 Subjects Release (https://www.humanconnectome.org/storage/app/media/documentation/s1200/HCP_S1200_Release_Reference_Manual.pdf). 243 MZ and DZ twin pairs were identified by genotyping. Age was entered as years without decimals.

Inclusion/exclusion criteria/ screening

The participant pool comes from healthy individuals born in Missouri to families that include twins, based on data from the Missouri Department of Health and Senior Services Bureau of Vital Records. Additional recruiting efforts in HCP were used to ensure that participants broadly reflect the ethnic and racial composition of the U.S. population as represented in the 2000 decennial census. 'Healthy' was broadly defined, aiming for a pool that is generally representative of the population at large, to capture a wide range of variability in healthy individuals with respect to behavioral, ethnic, and socioeconomic diversity. Sibships with individuals having severe neurodevelopmental disorders (e.g., autism), documented neuropsychiatric disorders (e.g., schizophrenia or depression) or neurologic disorders (e.g., Parkinson's disease) were excluded. Individuals with illnesses such as diabetes or high blood pressure were additionally excluded, as were non-twins (current sample) born prior to 37 weeks gestation. Individuals who were smokers, overweight, or had a history of heavy drinking or recreational drug use without having experienced severe symptoms were included. For a full list of inclusion and exclusion criteria, see [11].

UKB

Population, recruitment and general description of study/ procedures

UK Biobank (UKB) (https://www.ukbiobank.ac.uk/about-biobank-uk/) is a major national and international health resource with the aim of improving the prevention, diagnosis and treatment of a wide range of illnesses. UK Biobank recruited ≈500,000 people aged between 40-69 years in 2006-2010 from across the country to take part in this project [12]. Potential participants were identified through National Health Service (NHS) registers according to being aged 40-69 and living within a reasonable travelling distance of an assessment centre. Assessment centres (22 in total) are located in accessible and convenient locations with a large surrounding population. Participants have undergone measures and provided samples and detailed information about themselves and agreed to have their health followed. Age was calculated from year and month of birth (day of month is missing, and was set to 1 for all subjects) to date of assessment. Age was calculated at the 0_0 timepoint for participants without MRI, and 2_0 timepoint for participants with MRI.

References

1. Bertram, L., et al., *Cohort profile: The Berlin Aging Study II (BASE-II).* Int J Epidemiol, 2014. **43**(3): p. 703-12.
2. Gerstorf, D., et al., *Editorial.* Gerontology, 2016. **62**(3): p. 311-5.
3. Nilsson, L.G., et al., *The Betula prospective cohort study: Memory, health, and aging.* Aging, Neuropsychology and Cognition, 1997. **4**: p. 1-32.
4. Nilsson, L.-G., et al., *Betula: A prospective cohort study on memory, health and aging.* Aging, Neuropsychology, and Cognition, 2004. **.11**(2-3): p. pp.
5. Folstein, M.F., S.E. Folstein, and P.R. McHugh, *"Mini-mental state". A practical method for grading the cognitive state of patients for the clinician.* J Psychiatr Res, 1975. **12**(3): p. 189-98.
6. Rajaram, S., et al., *The Walnuts and Healthy Aging Study (WAHA): Protocol for a Nutritional Intervention Trial with Walnuts on Brain Aging.* Front Aging Neurosci, 2016. **8**: p. 333.
7. Vidal-Pineiro, D., et al., *Task-dependent activity and connectivity predict episodic memory network-based responses to brain stimulation in healthy aging.* Brain Stimul, 2014. **7**(2): p. 287-96.
8. Abellaneda-Perez, K., et al., *Age-related differences in default-mode network connectivity in response to intermittent theta-burst stimulation and its relationships with maintained cognition and brain integrity in healthy aging.* Neuroimage, 2019. **188**: p. 794-806.
9. Uribe, C., et al., *Patterns of cortical thinning in nondemented Parkinson's disease patients.* Mov Disord, 2016. **31**(5): p. 699-708.
10. Filippini, N., et al., *Study protocol: The Whitehall II imaging sub-study.* BMC Psychiatry, 2014. **14**: p. 159.
11. Van Essen, D.C., et al., *The WU-Minn Human Connectome Project: an overview.* Neuroimage, 2013. **80**: p. 62-79.
12. Guggenheim, J.A., C. Williams, and U.B.E.V. Consortium, *Role of Educational Exposure in the Association Between Myopia and Birth Order.* Jama Ophthalmology, 2015. **133**(12): p. 1408-1414.

Cortical measures longitudinal

Contents

Introduction **13**

Data 13

Models 13

Results per region and measure **13**

bankssts_area: Stratified by sex 13

Female: Full data 13

Male: Full data 14

bankssts_area 15

Full data 15

Restricted to sleep >= 5 and <= 9 hours 17

bankssts_thickness: Stratified by sex 18

Female: Full data 18

Male: Full data 19

bankssts_thickness 20

Full data 20

Restricted to sleep >= 5 and <= 9 hours 22

bankssts_volume: Stratified by sex 23

Female: Full data 23

Male: Full data 24

bankssts_volume 25

Full data 25

Restricted to sleep >= 5 and <= 9 hours 27

caudalanteriorcingulate_area: Stratified by sex 28

Female: Full data 28

Male: Full data 29

caudalanteriorcingulate_area 30

Full data 30

Restricted to sleep >= 5 and <= 9 hours 32

caudalanteriorcingulate_thickness: Stratified by sex 33

Female: Full data 33

Male: Full data 34

caudalanteriorcingulate_thickness 35

Full data 35

Restricted to sleep >= 5 and <= 9 hours 37

caudalanteriorcingulate_volume: Stratified by sex 38

Female: Full data 38

Male: Full data 39

caudalanteriorcingulate_volume 40

Full data 40

Restricted to sleep >= 5 and <= 9 hours 42

caudalmiddlefrontal_area: Stratified by sex 43

Female: Full data 43

Male: Full data 44

caudalmiddlefrontal_area . . . 45
Full data . . . 45
Restricted to sleep >= 5 and <= 9 hours . . . 47
caudalmiddlefrontal_thickness: Stratified by sex . . . 48
Female: Full data . . . 48
Male: Full data . . . 49
caudalmiddlefrontal_thickness . . . 50
Full data . . . 50
Restricted to sleep >= 5 and <= 9 hours . . . 52
caudalmiddlefrontal_volume: Stratified by sex . . . 53
Female: Full data . . . 53
Male: Full data . . . 54
caudalmiddlefrontal_volume . . . 55
Full data . . . 55
Restricted to sleep >= 5 and <= 9 hours . . . 57
cuneus_area: Stratified by sex . . . 58
Female: Full data . . . 58
Male: Full data . . . 59
cuneus_area . . . 60
Full data . . . 60
Restricted to sleep >= 5 and <= 9 hours . . . 62
cuneus_thickness: Stratified by sex . . . 63
Female: Full data . . . 63
Male: Full data . . . 64
cuneus_thickness . . . 65
Full data . . . 65
Restricted to sleep >= 5 and <= 9 hours . . . 67
cuneus_volume: Stratified by sex . . . 68
Female: Full data . . . 68
Male: Full data . . . 69
cuneus_volume . . . 70
Full data . . . 70
Restricted to sleep >= 5 and <= 9 hours . . . 72
entorhinal_area: Stratified by sex . . . 73
Female: Full data . . . 73
Male: Full data . . . 74
entorhinal_area . . . 75
Full data . . . 75
Restricted to sleep >= 5 and <= 9 hours . . . 77
entorhinal_thickness: Stratified by sex . . . 78
Female: Full data . . . 78
Male: Full data . . . 79
entorhinal_thickness . . . 80
Full data . . . 80
Restricted to sleep >= 5 and <= 9 hours . . . 82
entorhinal_volume: Stratified by sex . . . 83
Female: Full data . . . 83
Male: Full data . . . 84
entorhinal_volume . . . 85
Full data . . . 85
Restricted to sleep >= 5 and <= 9 hours . . . 87
frontalpole_area: Stratified by sex . . . 88
Female: Full data . . . 88
Male: Full data . . . 89

frontalpole_area . . . 90
Full data . . . 90
Restricted to sleep >= 5 and <= 9 hours . . . 92
frontalpole_thickness: Stratified by sex . . . 93
Female: Full data . . . 93
Male: Full data . . . 94
frontalpole_thickness . . . 95
Full data . . . 95
Restricted to sleep >= 5 and <= 9 hours . . . 97
frontalpole_volume: Stratified by sex . . . 98
Female: Full data . . . 98
Male: Full data . . . 99
frontalpole_volume . . . 100
Full data . . . 100
Restricted to sleep >= 5 and <= 9 hours . . . 102
fusiform_area: Stratified by sex . . . 103
Female: Full data . . . 103
Male: Full data . . . 104
fusiform_area . . . 105
Full data . . . 105
Restricted to sleep >= 5 and <= 9 hours . . . 107
fusiform_thickness: Stratified by sex . . . 108
Female: Full data . . . 108
Male: Full data . . . 109
fusiform_thickness . . . 110
Full data . . . 110
Restricted to sleep >= 5 and <= 9 hours . . . 112
fusiform_volume: Stratified by sex . . . 113
Female: Full data . . . 113
Male: Full data . . . 114
fusiform_volume . . . 115
Full data . . . 115
Restricted to sleep >= 5 and <= 9 hours . . . 117
inferiorparietal_area: Stratified by sex . . . 118
Female: Full data . . . 118
Male: Full data . . . 119
inferiorparietal_area . . . 120
Full data . . . 120
Restricted to sleep >= 5 and <= 9 hours . . . 122
inferiorparietal_thickness: Stratified by sex . . . 123
Female: Full data . . . 123
Male: Full data . . . 124
inferiorparietal_thickness . . . 125
Full data . . . 125
Restricted to sleep >= 5 and <= 9 hours . . . 127
inferiorparietal_volume: Stratified by sex . . . 128
Female: Full data . . . 128
Male: Full data . . . 129
inferiorparietal_volume . . . 130
Full data . . . 130
Restricted to sleep >= 5 and <= 9 hours . . . 132
inferiortemporal_area: Stratified by sex . . . 133
Female: Full data . . . 133
Male: Full data . . . 134

inferiortemporal_area . . . 135
    Full data . . . 135
    Restricted to sleep >= 5 and <= 9 hours . . . 137
inferiortemporal_thickness: Stratified by sex . . . 138
    Female: Full data . . . 138
    Male: Full data . . . 139
inferiortemporal_thickness . . . 140
    Full data . . . 140
    Restricted to sleep >= 5 and <= 9 hours . . . 142
inferiortemporal_volume: Stratified by sex . . . 143
    Female: Full data . . . 143
    Male: Full data . . . 144
inferiortemporal_volume . . . 145
    Full data . . . 145
    Restricted to sleep >= 5 and <= 9 hours . . . 147
insula_area: Stratified by sex . . . 148
    Female: Full data . . . 148
    Male: Full data . . . 149
insula_area . . . 150
    Full data . . . 150
    Restricted to sleep >= 5 and <= 9 hours . . . 152
insula_thickness: Stratified by sex . . . 153
    Female: Full data . . . 153
    Male: Full data . . . 154
insula_thickness . . . 155
    Full data . . . 155
    Restricted to sleep >= 5 and <= 9 hours . . . 157
insula_volume: Stratified by sex . . . 158
    Female: Full data . . . 158
    Male: Full data . . . 159
insula_volume . . . 160
    Full data . . . 160
    Restricted to sleep >= 5 and <= 9 hours . . . 162
isthmuscingulate_area: Stratified by sex . . . 163
    Female: Full data . . . 163
    Male: Full data . . . 164
isthmuscingulate_area . . . 165
    Full data . . . 165
    Restricted to sleep >= 5 and <= 9 hours . . . 167
isthmuscingulate_thickness: Stratified by sex . . . 168
    Female: Full data . . . 168
    Male: Full data . . . 169
isthmuscingulate_thickness . . . 170
    Full data . . . 170
    Restricted to sleep >= 5 and <= 9 hours . . . 172
isthmuscingulate_volume: Stratified by sex . . . 173
    Female: Full data . . . 173
    Male: Full data . . . 174
isthmuscingulate_volume . . . 175
    Full data . . . 175
    Restricted to sleep >= 5 and <= 9 hours . . . 177
lateraloccipital_area: Stratified by sex . . . 178
    Female: Full data . . . 178
    Male: Full data . . . 179

lateraloccipital_area 180
Full data 180
Restricted to sleep >= 5 and <= 9 hours 182
lateraloccipital_thickness: Stratified by sex 183
Female: Full data 183
Male: Full data 184
lateraloccipital_thickness 185
Full data 185
Restricted to sleep >= 5 and <= 9 hours 187
lateraloccipital_volume: Stratified by sex 188
Female: Full data 188
Male: Full data 189
lateraloccipital_volume 190
Full data 190
Restricted to sleep >= 5 and <= 9 hours 192
lateralorbitofrontal_area: Stratified by sex 193
Female: Full data 193
Male: Full data 194
lateralorbitofrontal_area 195
Full data 195
Restricted to sleep >= 5 and <= 9 hours 197
lateralorbitofrontal_thickness: Stratified by sex 198
Female: Full data 198
Male: Full data 199
lateralorbitofrontal_thickness 200
Full data 200
Restricted to sleep >= 5 and <= 9 hours 202
lateralorbitofrontal_volume: Stratified by sex 203
Female: Full data 203
Male: Full data 204
lateralorbitofrontal_volume 205
Full data 205
Restricted to sleep >= 5 and <= 9 hours 207
lingual_area: Stratified by sex 208
Female: Full data 208
Male: Full data 209
lingual_area 210
Full data 210
Restricted to sleep >= 5 and <= 9 hours 212
lingual_thickness: Stratified by sex 213
Female: Full data 213
Male: Full data 214
lingual_thickness 215
Full data 215
Restricted to sleep >= 5 and <= 9 hours 217
lingual_volume: Stratified by sex 218
Female: Full data 218
Male: Full data 219
lingual_volume 220
Full data 220
Restricted to sleep >= 5 and <= 9 hours 222
medialorbitofrontal_area: Stratified by sex 223
Female: Full data 223
Male: Full data 224

medialorbitofrontal_area . . . 225
Full data . . . 225
Restricted to sleep >= 5 and <= 9 hours . . . 227
medialorbitofrontal_thickness: Stratified by sex . . . 228
Female: Full data . . . 228
Male: Full data . . . 229
medialorbitofrontal_thickness . . . 230
Full data . . . 230
Restricted to sleep >= 5 and <= 9 hours . . . 232
medialorbitofrontal_volume: Stratified by sex . . . 233
Female: Full data . . . 233
Male: Full data . . . 234
medialorbitofrontal_volume . . . 235
Full data . . . 235
Restricted to sleep >= 5 and <= 9 hours . . . 237
middletemporal_area: Stratified by sex . . . 238
Female: Full data . . . 238
Male: Full data . . . 239
middletemporal_area . . . 240
Full data . . . 240
Restricted to sleep >= 5 and <= 9 hours . . . 242
middletemporal_thickness: Stratified by sex . . . 243
Female: Full data . . . 243
Male: Full data . . . 244
middletemporal_thickness . . . 245
Full data . . . 245
Restricted to sleep >= 5 and <= 9 hours . . . 247
middletemporal_volume: Stratified by sex . . . 248
Female: Full data . . . 248
Male: Full data . . . 249
middletemporal_volume . . . 250
Full data . . . 250
Restricted to sleep >= 5 and <= 9 hours . . . 252
paracentral_area: Stratified by sex . . . 253
Female: Full data . . . 253
Male: Full data . . . 254
paracentral_area . . . 255
Full data . . . 255
Restricted to sleep >= 5 and <= 9 hours . . . 257
paracentral_thickness: Stratified by sex . . . 258
Female: Full data . . . 258
Male: Full data . . . 259
paracentral_thickness . . . 260
Full data . . . 260
Restricted to sleep >= 5 and <= 9 hours . . . 262
paracentral_volume: Stratified by sex . . . 263
Female: Full data . . . 263
Male: Full data . . . 264
paracentral_volume . . . 265
Full data . . . 265
Restricted to sleep >= 5 and <= 9 hours . . . 267
parahippocampal_area: Stratified by sex . . . 268
Female: Full data . . . 268
Male: Full data . . . 269

parahippocampal_area 270
Full data 270
Restricted to sleep >= 5 and <= 9 hours 272
parahippocampal_thickness: Stratified by sex 273
Female: Full data 273
Male: Full data 274
parahippocampal_thickness 275
Full data 275
Restricted to sleep >= 5 and <= 9 hours 277
parahippocampal_volume: Stratified by sex 278
Female: Full data 278
Male: Full data 279
parahippocampal_volume 280
Full data 280
Restricted to sleep >= 5 and <= 9 hours 282
parsopercularis_area: Stratified by sex 283
Female: Full data 283
Male: Full data 284
parsopercularis_area 285
Full data 285
Restricted to sleep >= 5 and <= 9 hours 287
parsopercularis_thickness: Stratified by sex 288
Female: Full data 288
Male: Full data 289
parsopercularis_thickness 290
Full data 290
Restricted to sleep >= 5 and <= 9 hours 292
parsopercularis_volume: Stratified by sex 293
Female: Full data 293
Male: Full data 294
parsopercularis_volume 295
Full data 295
Restricted to sleep >= 5 and <= 9 hours 297
parsorbitalis_area: Stratified by sex 298
Female: Full data 298
Male: Full data 299
parsorbitalis_area 300
Full data 300
Restricted to sleep >= 5 and <= 9 hours 302
parsorbitalis_thickness: Stratified by sex 303
Female: Full data 303
Male: Full data 304
parsorbitalis_thickness 305
Full data 305
Restricted to sleep >= 5 and <= 9 hours 307
parsorbitalis_volume: Stratified by sex 308
Female: Full data 308
Male: Full data 309
parsorbitalis_volume 310
Full data 310
Restricted to sleep >= 5 and <= 9 hours 312
parstriangularis_area: Stratified by sex 313
Female: Full data 313
Male: Full data 314

parstriangularis_area . . . 315
Full data . . . 315
Restricted to sleep >= 5 and <= 9 hours . . . 317
parstriangularis_thickness: Stratified by sex . . . 318
Female: Full data . . . 318
Male: Full data . . . 319
parstriangularis_thickness . . . 320
Full data . . . 320
Restricted to sleep >= 5 and <= 9 hours . . . 322
parstriangularis_volume: Stratified by sex . . . 323
Female: Full data . . . 323
Male: Full data . . . 324
parstriangularis_volume . . . 325
Full data . . . 325
Restricted to sleep >= 5 and <= 9 hours . . . 327
pericalcarine_area: Stratified by sex . . . 328
Female: Full data . . . 328
Male: Full data . . . 329
pericalcarine_area . . . 330
Full data . . . 330
Restricted to sleep >= 5 and <= 9 hours . . . 332
pericalcarine_thickness: Stratified by sex . . . 333
Female: Full data . . . 333
Male: Full data . . . 334
pericalcarine_thickness . . . 335
Full data . . . 335
Restricted to sleep >= 5 and <= 9 hours . . . 337
pericalcarine_volume: Stratified by sex . . . 338
Female: Full data . . . 338
Male: Full data . . . 339
pericalcarine_volume . . . 340
Full data . . . 340
Restricted to sleep >= 5 and <= 9 hours . . . 342
postcentral_area: Stratified by sex . . . 343
Female: Full data . . . 343
Male: Full data . . . 344
postcentral_area . . . 345
Full data . . . 345
Restricted to sleep >= 5 and <= 9 hours . . . 347
postcentral_thickness: Stratified by sex . . . 348
Female: Full data . . . 348
Male: Full data . . . 349
postcentral_thickness . . . 350
Full data . . . 350
Restricted to sleep >= 5 and <= 9 hours . . . 352
postcentral_volume: Stratified by sex . . . 353
Female: Full data . . . 353
Male: Full data . . . 354
postcentral_volume . . . 355
Full data . . . 355
Restricted to sleep >= 5 and <= 9 hours . . . 357
posteriorcingulate_area: Stratified by sex . . . 358
Female: Full data . . . 358
Male: Full data . . . 359

posteriorcingulate_area . . . 360
Full data . . . 360
Restricted to sleep >= 5 and <= 9 hours . . . 362
posteriorcingulate_thickness: Stratified by sex . . . 363
Female: Full data . . . 363
Male: Full data . . . 364
posteriorcingulate_thickness . . . 365
Full data . . . 365
Restricted to sleep >= 5 and <= 9 hours . . . 367
posteriorcingulate_volume: Stratified by sex . . . 368
Female: Full data . . . 368
Male: Full data . . . 369
posteriorcingulate_volume . . . 370
Full data . . . 370
Restricted to sleep >= 5 and <= 9 hours . . . 372
precentral_area: Stratified by sex . . . 373
Female: Full data . . . 373
Male: Full data . . . 374
precentral_area . . . 375
Full data . . . 375
Restricted to sleep >= 5 and <= 9 hours . . . 377
precentral_thickness: Stratified by sex . . . 378
Female: Full data . . . 378
Male: Full data . . . 379
precentral_thickness . . . 380
Full data . . . 380
Restricted to sleep >= 5 and <= 9 hours . . . 382
precentral_volume: Stratified by sex . . . 383
Female: Full data . . . 383
Male: Full data . . . 384
precentral_volume . . . 385
Full data . . . 385
Restricted to sleep >= 5 and <= 9 hours . . . 387
precuneus_area: Stratified by sex . . . 388
Female: Full data . . . 388
Male: Full data . . . 389
precuneus_area . . . 390
Full data . . . 390
Restricted to sleep >= 5 and <= 9 hours . . . 392
precuneus_thickness: Stratified by sex . . . 393
Female: Full data . . . 393
Male: Full data . . . 394
precuneus_thickness . . . 395
Full data . . . 395
Restricted to sleep >= 5 and <= 9 hours . . . 397
precuneus_volume: Stratified by sex . . . 398
Female: Full data . . . 398
Male: Full data . . . 399
precuneus_volume . . . 400
Full data . . . 400
Restricted to sleep >= 5 and <= 9 hours . . . 402
rostralanteriorcingulate_area: Stratified by sex . . . 403
Female: Full data . . . 403
Male: Full data . . . 404

rostralanteriorcingulate_area 405
Full data 405
Restricted to sleep >= 5 and <= 9 hours 407
rostralanteriorcingulate_thickness: Stratified by sex 408
Female: Full data 408
Male: Full data 409
rostralanteriorcingulate_thickness 410
Full data 410
Restricted to sleep >= 5 and <= 9 hours 412
rostralanteriorcingulate_volume: Stratified by sex 413
Female: Full data 413
Male: Full data 414
rostralanteriorcingulate_volume 415
Full data 415
Restricted to sleep >= 5 and <= 9 hours 417
rostralmiddlefrontal_area: Stratified by sex 418
Female: Full data 418
Male: Full data 419
rostralmiddlefrontal_area 420
Full data 420
Restricted to sleep >= 5 and <= 9 hours 422
rostralmiddlefrontal_thickness: Stratified by sex 423
Female: Full data 423
Male: Full data 424
rostralmiddlefrontal_thickness 425
Full data 425
Restricted to sleep >= 5 and <= 9 hours 427
rostralmiddlefrontal_volume: Stratified by sex 428
Female: Full data 428
Male: Full data 429
rostralmiddlefrontal_volume 430
Full data 430
Restricted to sleep >= 5 and <= 9 hours 432
superiorfrontal_area: Stratified by sex 433
Female: Full data 433
Male: Full data 434
superiorfrontal_area 435
Full data 435
Restricted to sleep >= 5 and <= 9 hours 437
superiorfrontal_thickness: Stratified by sex 438
Female: Full data 438
Male: Full data 439
superiorfrontal_thickness 440
Full data 440
Restricted to sleep >= 5 and <= 9 hours 442
superiorfrontal_volume: Stratified by sex 443
Female: Full data 443
Male: Full data 444
superiorfrontal_volume 445
Full data 445
Restricted to sleep >= 5 and <= 9 hours 447
superiorparietal_area: Stratified by sex 448
Female: Full data 448
Male: Full data 449

superiorparietal_area 450
Full data 450
Restricted to sleep >= 5 and <= 9 hours 452
superiorparietal_thickness: Stratified by sex 453
Female: Full data 453
Male: Full data 454
superiorparietal_thickness 455
Full data 455
Restricted to sleep >= 5 and <= 9 hours 457
superiorparietal_volume: Stratified by sex 458
Female: Full data 458
Male: Full data 459
superiorparietal_volume 460
Full data 460
Restricted to sleep >= 5 and <= 9 hours 462
superiortemporal_area: Stratified by sex 463
Female: Full data 463
Male: Full data 464
superiortemporal_area 465
Full data 465
Restricted to sleep >= 5 and <= 9 hours 467
superiortemporal_thickness: Stratified by sex 468
Female: Full data 468
Male: Full data 469
superiortemporal_thickness 470
Full data 470
Restricted to sleep >= 5 and <= 9 hours 472
superiortemporal_volume: Stratified by sex 473
Female: Full data 473
Male: Full data 474
superiortemporal_volume 475
Full data 475
Restricted to sleep >= 5 and <= 9 hours 477
supramarginal_area: Stratified by sex 478
Female: Full data 478
Male: Full data 479
supramarginal_area 480
Full data 480
Restricted to sleep >= 5 and <= 9 hours 482
supramarginal_thickness: Stratified by sex 483
Female: Full data 483
Male: Full data 484
supramarginal_thickness 485
Full data 485
Restricted to sleep >= 5 and <= 9 hours 487
supramarginal_volume: Stratified by sex 488
Female: Full data 488
Male: Full data 489
supramarginal_volume 490
Full data 490
Restricted to sleep >= 5 and <= 9 hours 492
transversetemporal_area: Stratified by sex 493
Female: Full data 493
Male: Full data 494

transversetemporal_area . . . 495
Full data . . . 495
Restricted to sleep >= 5 and <= 9 hours . . . 497
transversetemporal_thickness: Stratified by sex . . . 498
Female: Full data . . . 498
Male: Full data . . . 499
transversetemporal_thickness . . . 500
Full data . . . 500
Restricted to sleep >= 5 and <= 9 hours . . . 502
transversetemporal_volume: Stratified by sex . . . 503
Female: Full data . . . 503
Male: Full data . . . 504
transversetemporal_volume . . . 505
Full data . . . 505
Restricted to sleep >= 5 and <= 9 hours . . . 507

P-values corrected for multiple testing **508**

Models per cluster **518**
Area cluster 1 . . . 518
All longitudinal data . . . 518
Participants sleeping 5-9 hours . . . 519
Thickness cluster 1 . . . 521
All longitudinal data . . . 521
Participants sleeping 5-9 hours . . . 522
Volume cluster 1 . . . 523
All longitudinal data . . . 523
Participants sleeping 5-9 hours . . . 524
Area cluster 2 . . . 525
All longitudinal data . . . 525
Participants sleeping 5-9 hours . . . 526
Thickness cluster 2 . . . 528
All longitudinal data . . . 528
Participants sleeping 5-9 hours . . . 529
Volume cluster 2 . . . 530
All longitudinal data . . . 530
Participants sleeping 5-9 hours . . . 531
Area cluster 3 . . . 532
All longitudinal data . . . 532
Participants sleeping 5-9 hours . . . 533
Thickness cluster 3 . . . 535
All longitudinal data . . . 535
Participants sleeping 5-9 hours . . . 536
Volume cluster 3 . . . 537
All longitudinal data . . . 537
Participants sleeping 5-9 hours . . . 538

```
library(tidyverse)
```

Introduction

Data

All participants with two or more timepoints were included. A count is shown below.

study	Participants	Observations
MPIB	286	572
UB	38	112
UCAM	252	504
UiO	340	1013
UKB	2854	5708
UmU	133	266

Models

The following GAMM was fitted (`icv` was not included when the outcome was thickness)

```
mod <- gamm4(
  value ~ sex + site + icv + s(bl_age, k = 10, bs = 'cr') +
    s(bl_age, by = sleep_z, bs = 'cr') +
    s(bl_age, by = time, k = 5, bs = 'cr') +
    s(sleep_z, by = time, k = 5, bs = 'cr', pc = 0),
  random = ~(1|id), data = long_dat, REML = FALSE
)
```

See the document on subcortical longitudinal analyses for an explanation of the model terms.

Results per region and measure

bankssts_area: Stratified by sex

Female: Full data Below is the row for `s(sleep_z):time` from the model output.

```
## s(sleep_z):time   1.000  1.000  2.367   0.124
```

Below are plots of a plot of `s(bl_age)`, `s(bl_age):sleep_z`, `s(bl_age):time`, and `s(sleep_z):time`. For `s(sleep_z):time`, roughly, if we cannot fit a straight line through the confidence bands, there is evidence that the effect of time depends on sleep duration. It is correct the that confidence interval is zero at one point along the axis; this happens because we do not include the main effect.

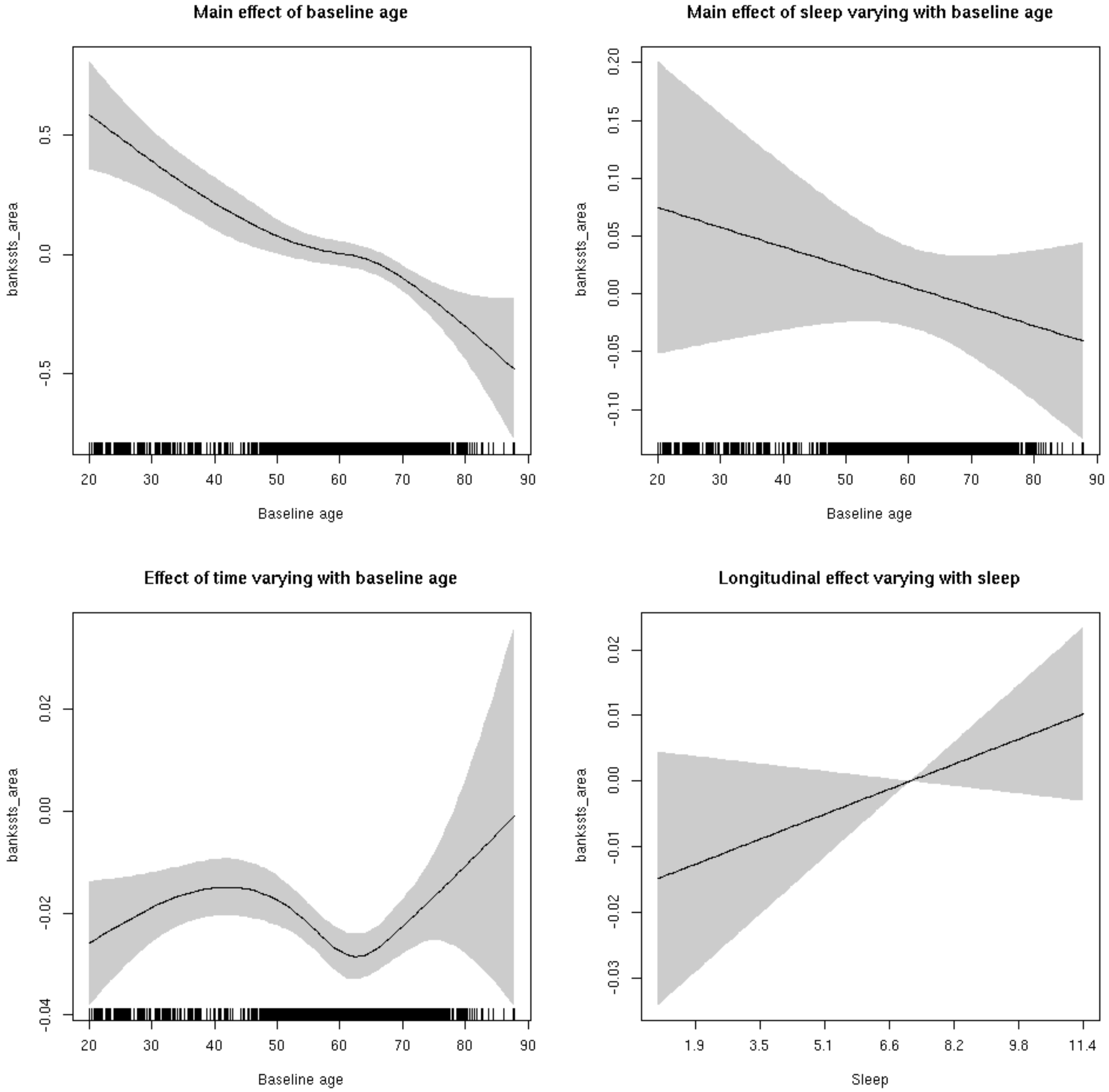

Male: Full data Below is the row for `s(sleep_z):time` from the model output.

```
## s(sleep_z):time   1.000  1.000   3.069  0.0799 .
```

Below are plots of a plot of `s(bl_age)`, `s(bl_age):sleep_z`, `s(bl_age):time`, and `s(sleep_z):time`. For `s(sleep_z):time`, roughly, if we cannot fit a straight line through the confidence bands, there is evidence that the effect of time depends on sleep duration. It is correct the that confidence interval is zero at one point along the axis; this happens because we do not include the main effect.

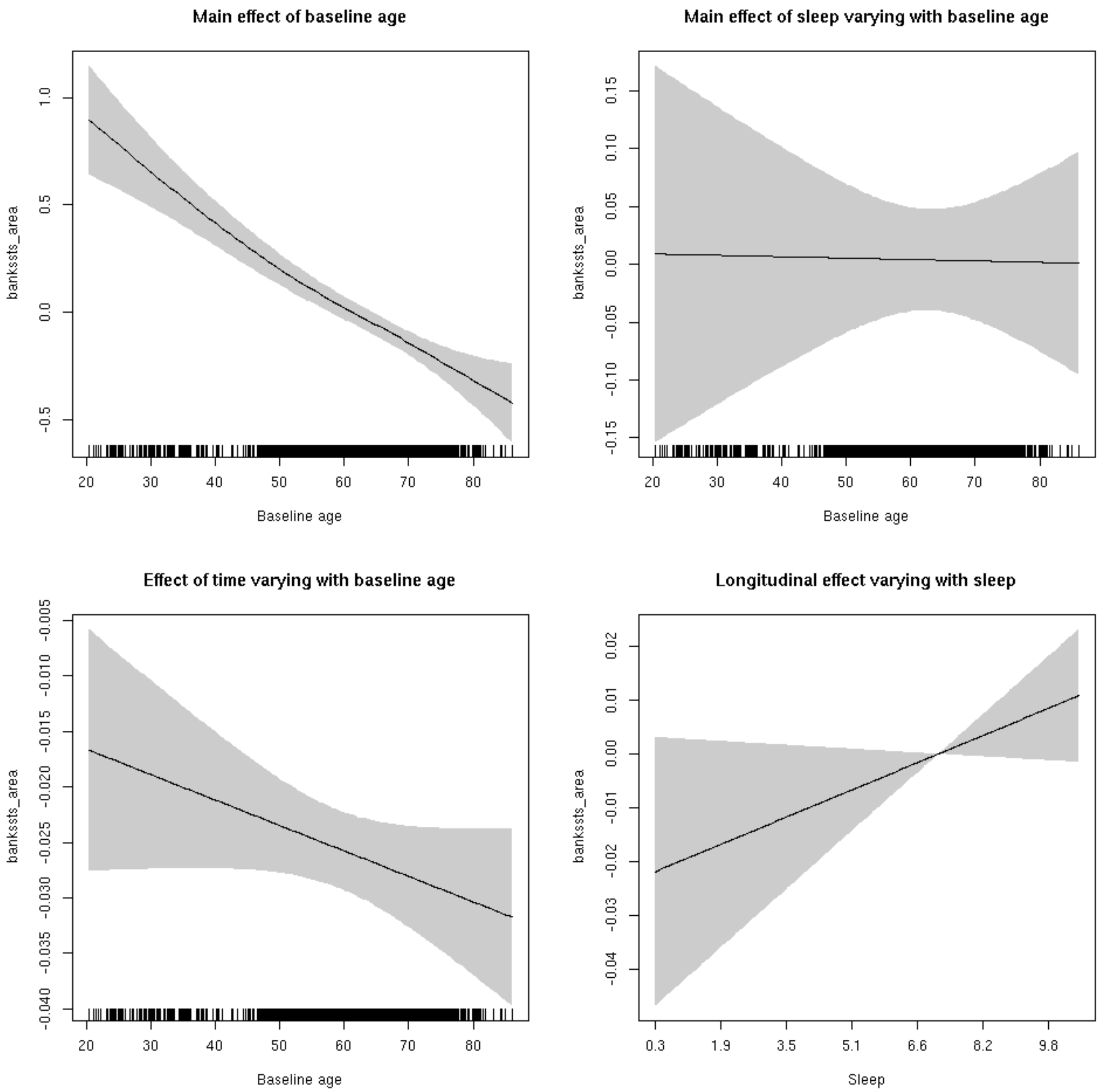

bankssts_area

Full data Below is the model output. The term `s(sleep_z):time` is probably what we care most about.

```
##
## Family: gaussian
## Link function: identity
##
## Formula:
## value ~ sex + site + icv + s(bl_age, k = 10, bs = "cr") + s(bl_age,
##     by = sleep_z, bs = "cr") + +s(bl_age, by = time, k = 5, bs = "cr") +
##     s(sleep_z, by = time, k = 5, bs = "cr", pc = 0)
##
```

```
## Parametric coefficients:
##                Estimate Std. Error t value Pr(>|t|)
## (Intercept)     0.61235    0.06302   9.717  < 2e-16 ***
## sexmale         0.08074    0.03235   2.496 0.012580 *
## siteousAvanto -0.57513    0.07673  -7.496 7.27e-14 ***
## siteousPrisma -0.73194    0.13559  -5.398 6.93e-08 ***
## siteousSkyra  -0.21024    0.07436  -2.827 0.004707 **
## siteUB        -0.55573    0.14608  -3.804 0.000143 ***
## siteUCAM      -0.57225    0.07856  -7.284 3.54e-13 ***
## siteUKB       -0.62254    0.06004 -10.369  < 2e-16 ***
## siteUmU       -0.03961    0.09031  -0.439 0.660989
## icv            0.50708    0.01571  32.279  < 2e-16 ***
## ---
## Signif. codes:  0 '***' 0.001 '**' 0.01 '*' 0.05 '.' 0.1 ' ' 1
##
## Approximate significance of smooth terms:
##                      edf Ref.df       F p-value
## s(bl_age)          3.566  3.566  43.511  <2e-16 ***
## s(bl_age):sleep_z 2.612  2.612   0.338  0.6222
## s(bl_age):time    4.044  4.044 113.147  <2e-16 ***
## s(sleep_z):time   1.000  1.000   6.019  0.0142 *
## ---
## Signif. codes:  0 '***' 0.001 '**' 0.01 '*' 0.05 '.' 0.1 ' ' 1
##
## R-sq.(adj) =  0.338
## lmer.REML = 7983.5  Scale est. = 0.020328  n = 8175
```

Below are plots of a plot of `s(bl_age)`, `s(bl_age):sleep_z`, `s(bl_age):time`, and `s(sleep_z):time`. For `s(sleep_z):time`, roughly, if we cannot fit a straight line through the confidence bands, there is evidence that the effect of time depends on sleep duration. It is correct the that confidence interval is zero at one point along the axis; this happens because we do not include the main effect.

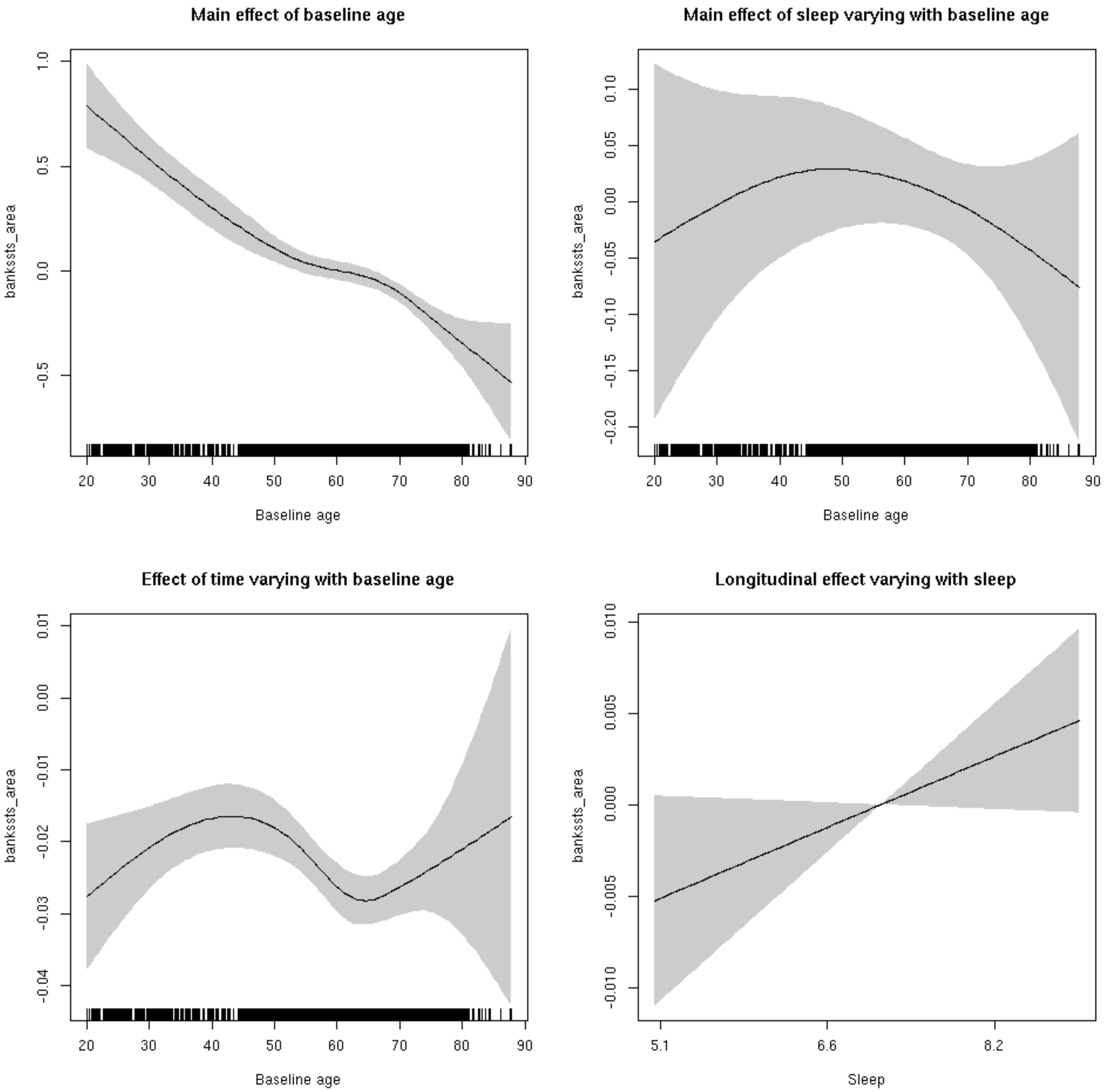

Restricted to sleep >= 5 and <= 9 hours Below is the row for `s(sleep_z):time` from the model output.

```
## s(sleep_z):time   1.000  1.000   3.339  0.0677 .
```

Below are plots of a plot of `s(bl_age)`, `s(bl_age):sleep_z`, `s(bl_age):time`, and `s(sleep_z):time`. For `s(sleep_z):time`, roughly, if we cannot fit a straight line through the confidence bands, there is evidence that the effect of time depends on sleep duration. It is correct the that confidence interval is zero at one point along the axis; this happens because we do not include the main effect.

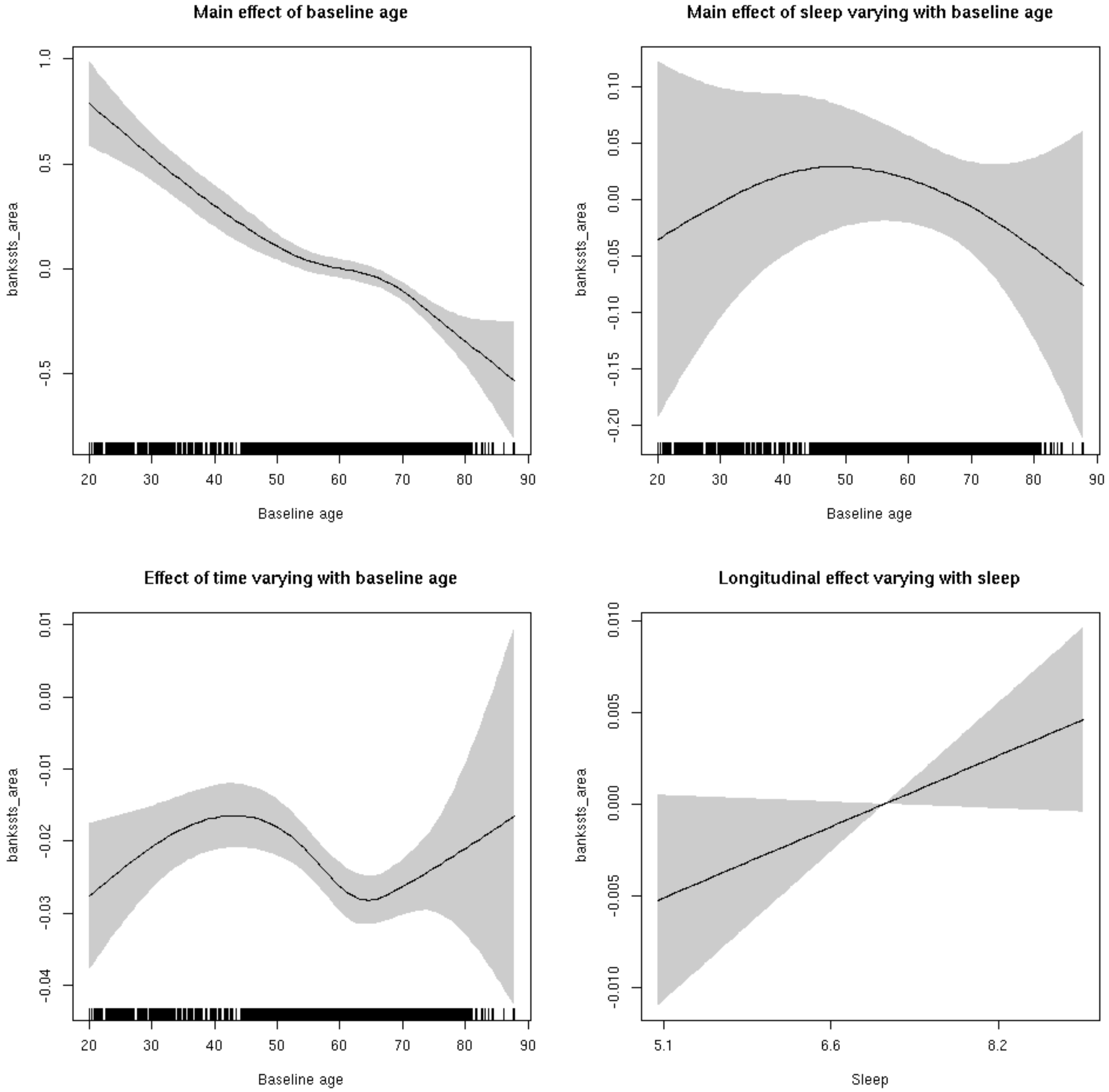

bankssts_thickness: Stratified by sex

Female: Full data Below is the row for `s(sleep_z):time` from the model output.

```
## s(sleep_z):time   1.000  1.000   0.213   0.645
```

Below are plots of a plot of `s(bl_age)`, `s(bl_age):sleep_z`, `s(bl_age):time`, and `s(sleep_z):time`. For `s(sleep_z):time`, roughly, if we cannot fit a straight line through the confidence bands, there is evidence that the effect of time depends on sleep duration. It is correct the that confidence interval is zero at one point along the axis; this happens because we do not include the main effect.

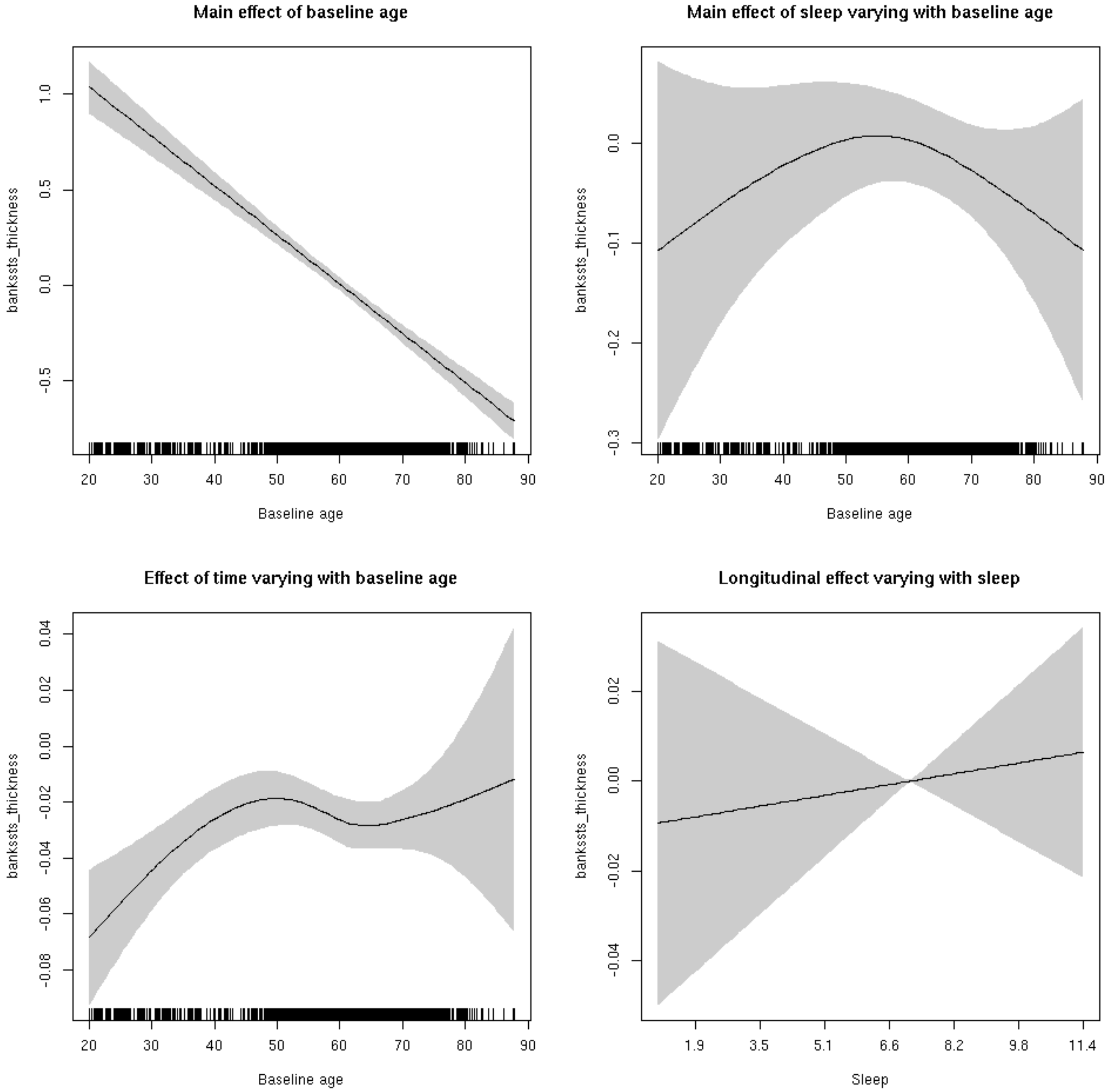

Male: Full data Below is the row for `s(sleep_z):time` from the model output.

```
## s(sleep_z):time   1.000  1.000   0.137   0.711
```

Below are plots of a plot of `s(bl_age)`, `s(bl_age):sleep_z`, `s(bl_age):time`, and `s(sleep_z):time`. For `s(sleep_z):time`, roughly, if we cannot fit a straight line through the confidence bands, there is evidence that the effect of time depends on sleep duration. It is correct the that confidence interval is zero at one point along the axis; this happens because we do not include the main effect.

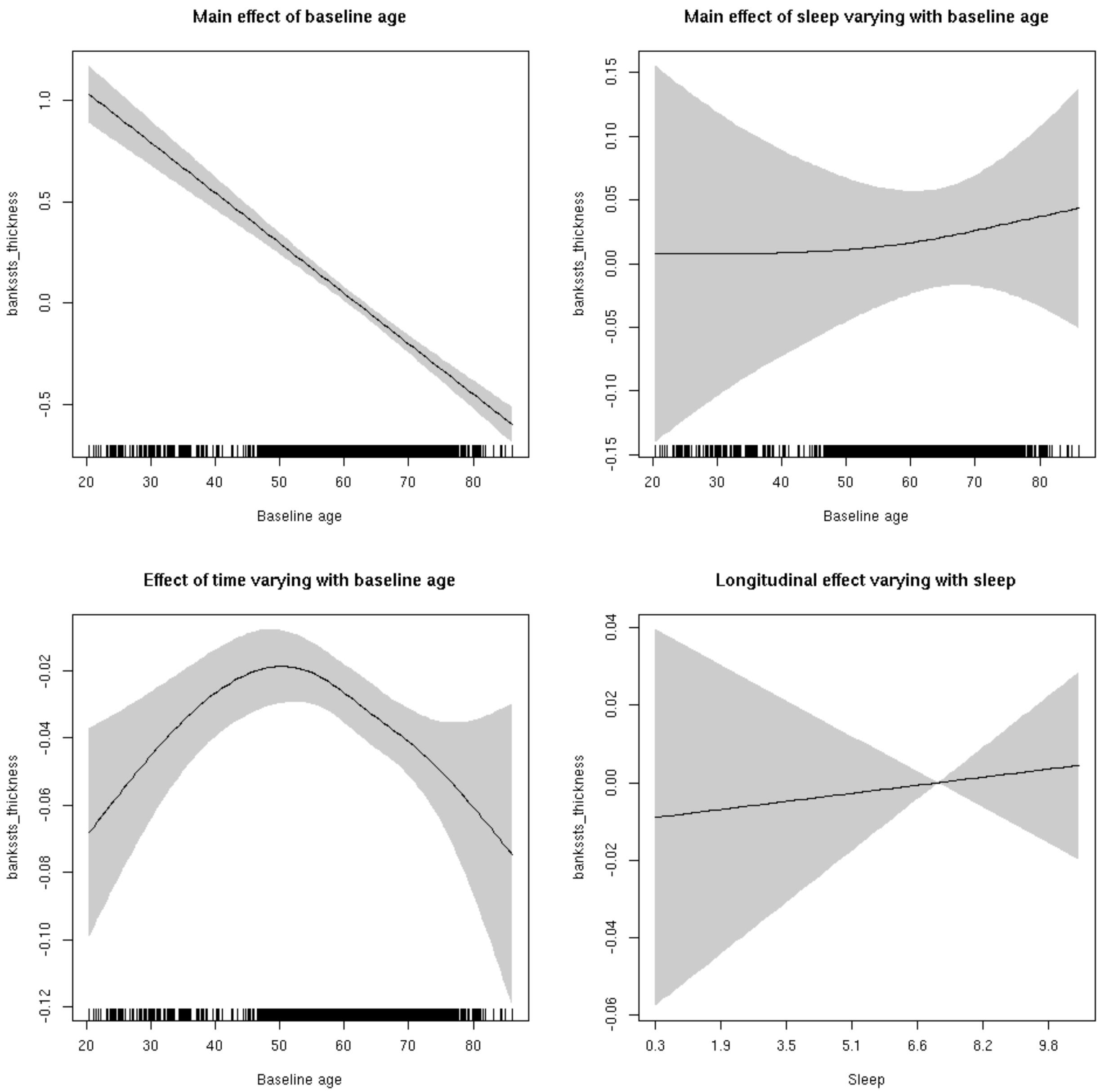

bankssts_thickness

Full data Below is the model output. The term `s(sleep_z):time` is probably what we care most about.

```
##
## Family: gaussian
## Link function: identity
##
## Formula:
## value ~ sex + site + s(bl_age, k = 10, bs = "cr") + s(bl_age,
##     by = sleep_z, bs = "cr") + +s(bl_age, by = time, k = 5, bs = "cr") +
##     s(sleep_z, by = time, k = 5, bs = "cr", pc = 0)
## <environment: 0x5630035768d8>
```

```
##
## Parametric coefficients:
##                Estimate Std. Error t value Pr(>|t|)
## (Intercept)   -1.51899    0.04786 -31.741  < 2e-16 ***
## sexmale       -0.06979    0.02463  -2.834  0.00461 **
## siteousAvanto  0.11476    0.06441   1.782  0.07482 .
## siteousPrisma  0.52722    0.22701   2.322  0.02024 *
## siteousSkyra   0.28435    0.06430   4.422 9.90e-06 ***
## siteUB         0.41214    0.13074   3.152  0.00163 **
## siteUCAM       0.42386    0.06660   6.364 2.07e-10 ***
## siteUKB        1.77664    0.04746  37.437  < 2e-16 ***
## siteUmU       -0.15292    0.08030  -1.904  0.05689 .
## ---
## Signif. codes:  0 '***' 0.001 '**' 0.01 '*' 0.05 '.' 0.1 ' ' 1
##
## Approximate significance of smooth terms:
##                      edf Ref.df       F p-value
## s(bl_age)          1.000  1.000 468.586  <2e-16 ***
## s(bl_age):sleep_z 2.000  2.000   0.008   0.992
## s(bl_age):time    3.933  3.933  50.506  <2e-16 ***
## s(sleep_z):time   1.000  1.000   0.394   0.530
## ---
## Signif. codes:  0 '***' 0.001 '**' 0.01 '*' 0.05 '.' 0.1 ' ' 1
##
## R-sq.(adj) =  0.476
## lmer.REML =  13280  Scale est. = 0.083521  n = 8166
```

Below are plots of a plot of `s(bl_age)`, `s(bl_age):sleep_z`, `s(bl_age):time`, and `s(sleep_z):time`. For `s(sleep_z):time`, roughly, if we cannot fit a straight line through the confidence bands, there is evidence that the effect of time depends on sleep duration. It is correct the that confidence interval is zero at one point along the axis; this happens because we do not include the main effect.

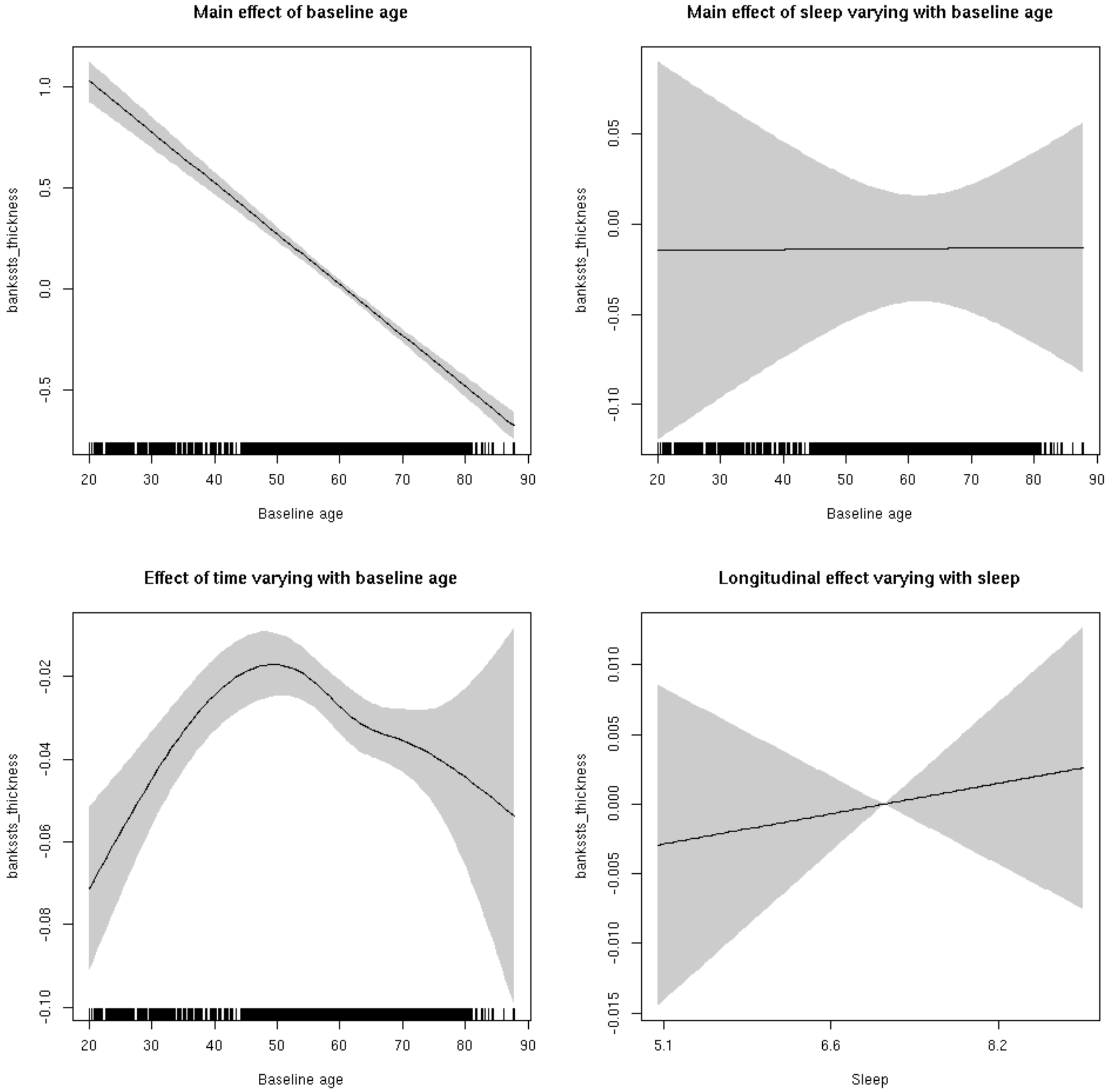

Restricted to sleep >= 5 and <= 9 hours Below is the row for `s(sleep_z):time` from the model output.

```
## s(sleep_z):time   1.00   1.00   0.262   0.608
```

Below are plots of a plot of `s(bl_age)`, `s(bl_age):sleep_z`, `s(bl_age):time`, and `s(sleep_z):time`. For `s(sleep_z):time`, roughly, if we cannot fit a straight line through the confidence bands, there is evidence that the effect of time depends on sleep duration. It is correct the that confidence interval is zero at one point along the axis; this happens because we do not include the main effect.

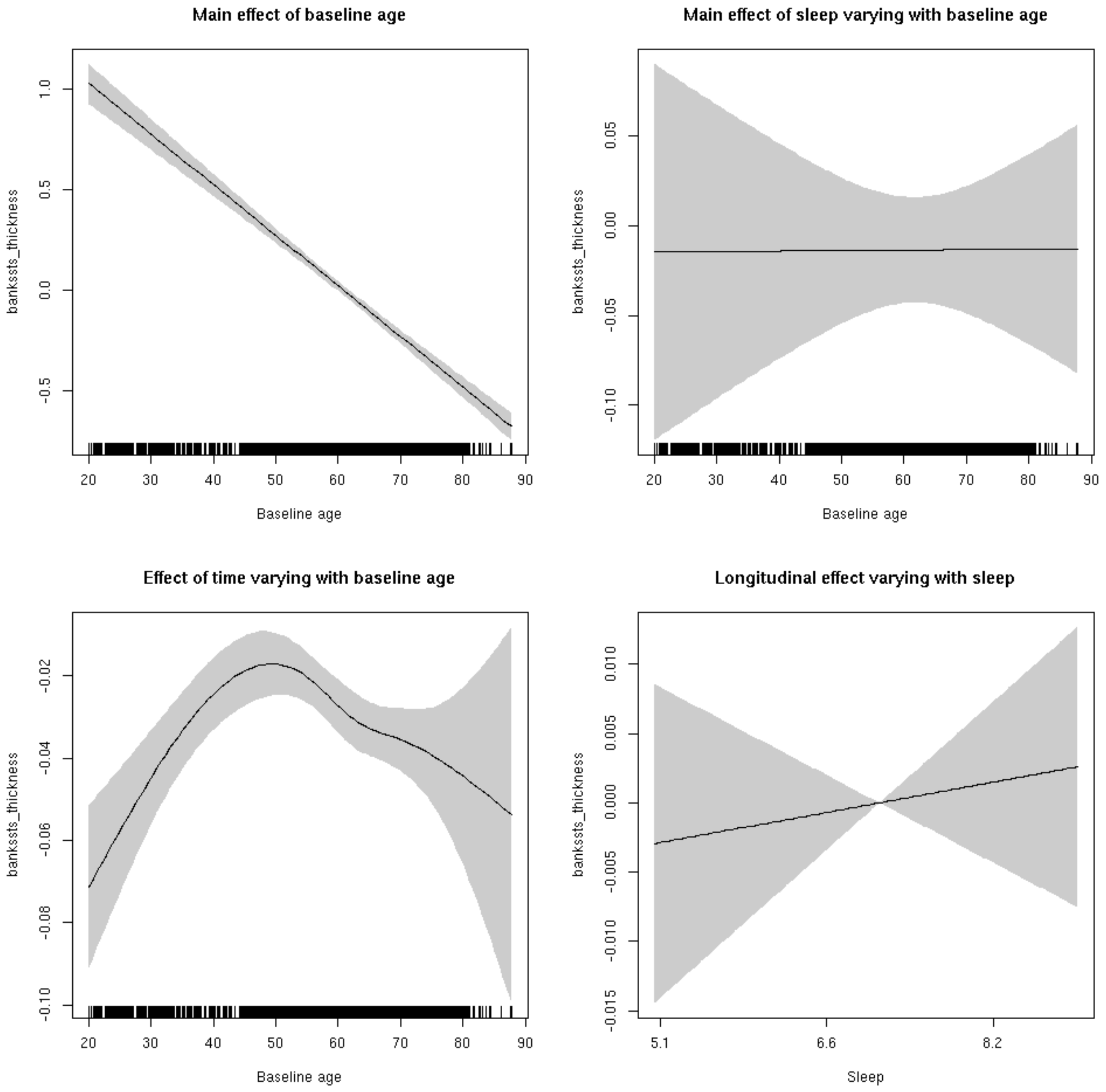

bankssts_volume: Stratified by sex

Female: Full data Below is the row for `s(sleep_z):time` from the model output.

```
## s(sleep_z):time   1.000  1.000  0.189   0.664
```

Below are plots of a plot of `s(bl_age)`, `s(bl_age):sleep_z`, `s(bl_age):time`, and `s(sleep_z):time`. For `s(sleep_z):time`, roughly, if we cannot fit a straight line through the confidence bands, there is evidence that the effect of time depends on sleep duration. It is correct the that confidence interval is zero at one point along the axis; this happens because we do not include the main effect.

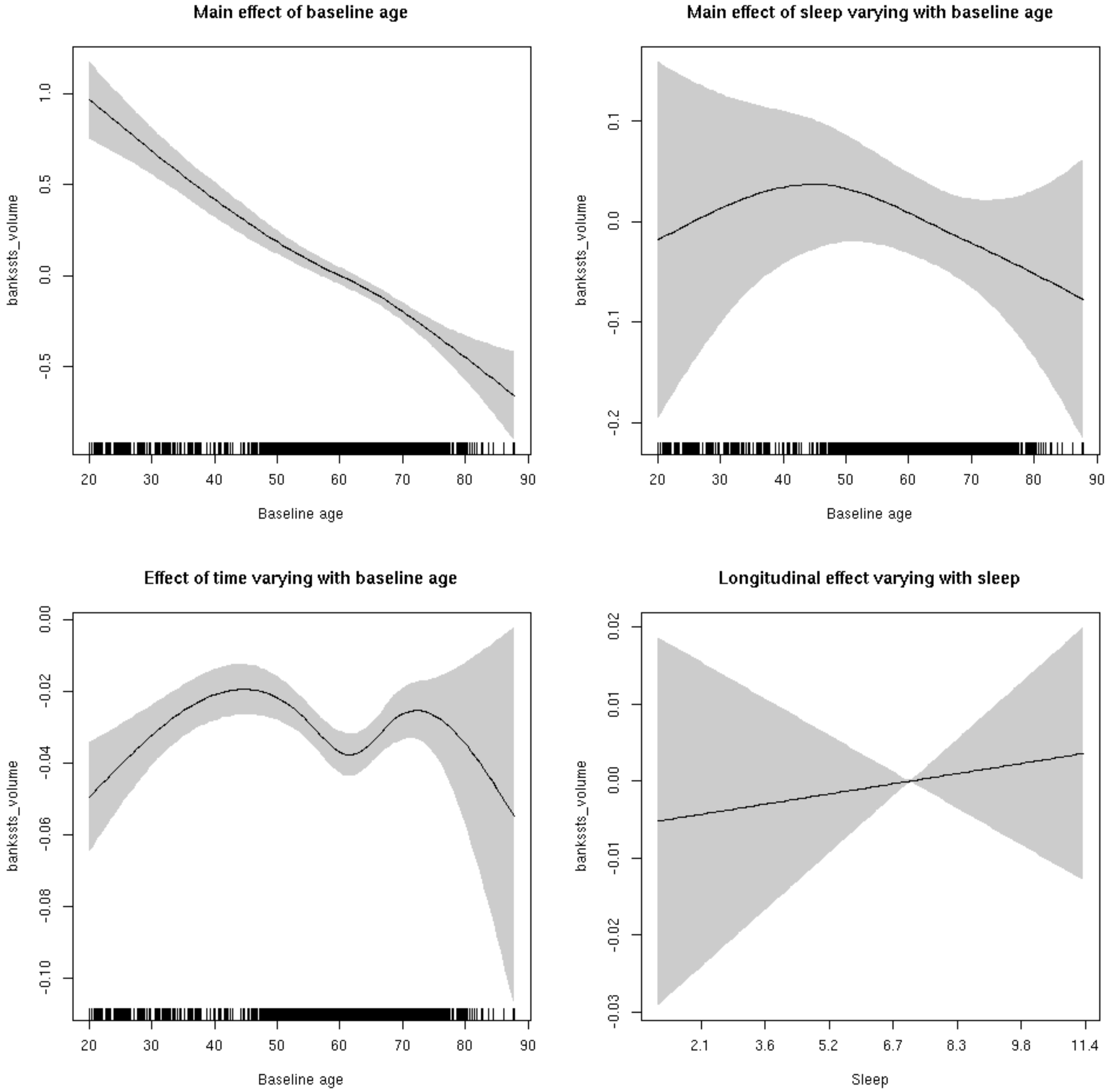

Male: Full data Below is the row for `s(sleep_z):time` from the model output.

```
## s(sleep_z):time   1.000  1.000   0.657   0.418
```

Below are plots of a plot of `s(bl_age)`, `s(bl_age):sleep_z`, `s(bl_age):time`, and `s(sleep_z):time`. For `s(sleep_z):time`, roughly, if we cannot fit a straight line through the confidence bands, there is evidence that the effect of time depends on sleep duration. It is correct the that confidence interval is zero at one point along the axis; this happens because we do not include the main effect.

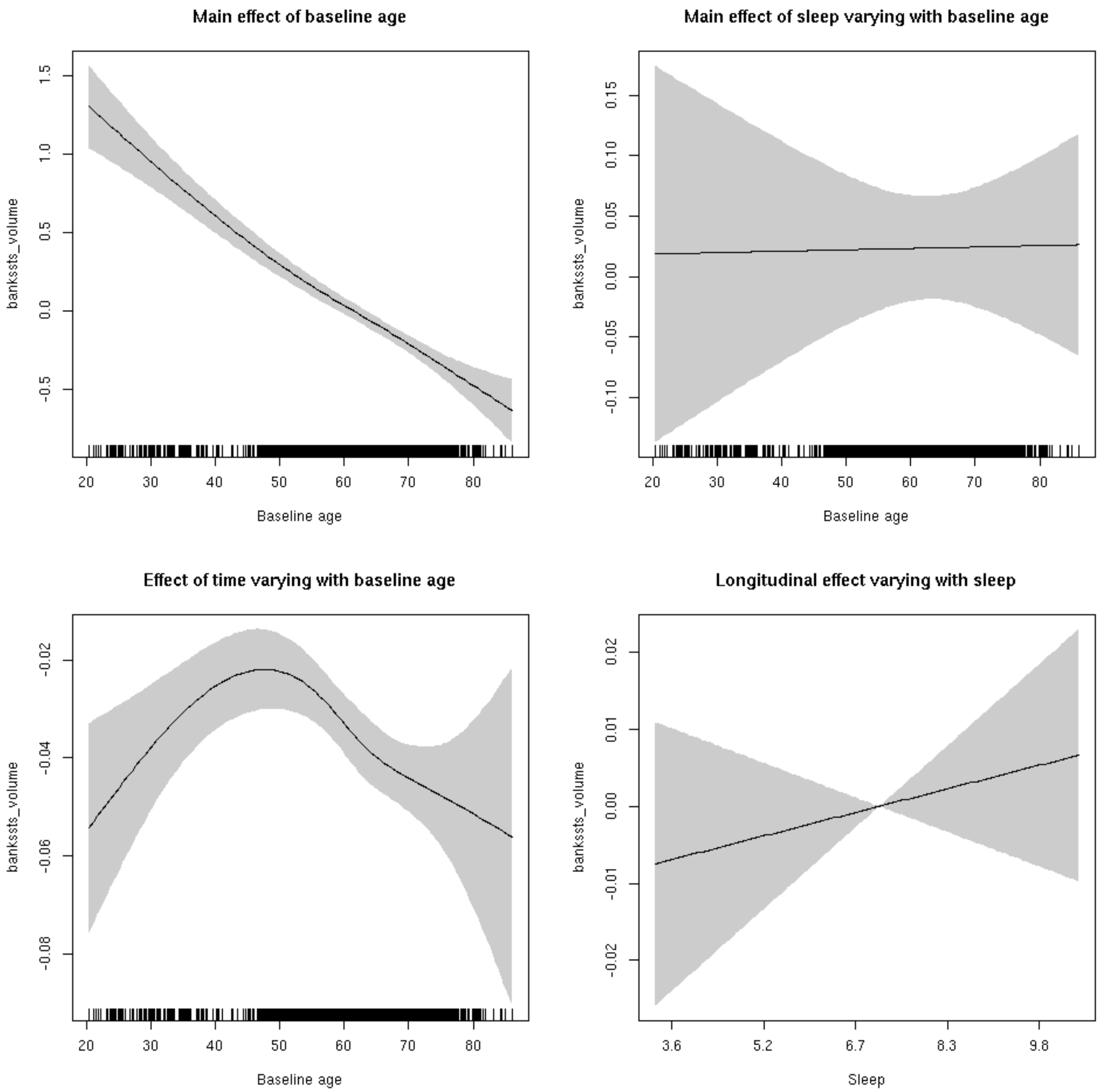

bankssts_volume

Full data Below is the model output. The term `s(sleep_z):time` is probably what we care most about.

```
##
## Family: gaussian
## Link function: identity
##
## Formula:
## value ~ sex + site + icv + s(bl_age, k = 10, bs = "cr") + s(bl_age,
##     by = sleep_z, bs = "cr") + +s(bl_age, by = time, k = 5, bs = "cr") +
##     s(sleep_z, by = time, k = 5, bs = "cr", pc = 0)
## <environment: 0x56300353d3c8>
```

```
##
## Parametric coefficients:
##               Estimate Std. Error t value Pr(>|t|)
## (Intercept)   -0.08526    0.06109  -1.396 0.162859
## sexmale        0.04572    0.03137   1.457 0.145075
## siteousAvanto -0.56471    0.07447  -7.583 3.75e-14 ***
## siteousPrisma -0.52362    0.15890  -3.295 0.000988 ***
## siteousSkyra  -0.09834    0.07211  -1.364 0.172699
## siteUB        -0.34889    0.14088  -2.476 0.013289 *
## siteUCAM      -0.39310    0.07591  -5.178 2.29e-07 ***
## siteUKB        0.17545    0.05808   3.021 0.002528 **
## siteUmU       -0.12024    0.08725  -1.378 0.168188
## icv            0.47645    0.01539  30.960  < 2e-16 ***
## ---
## Signif. codes:  0 '***' 0.001 '**' 0.01 '*' 0.05 '.' 0.1 ' ' 1
##
## Approximate significance of smooth terms:
##                     edf Ref.df       F p-value
## s(bl_age)         3.385  3.385 120.533  <2e-16 ***
## s(bl_age):sleep_z 2.788  2.788   0.581   0.487
## s(bl_age):time    4.512  4.512 120.944  <2e-16 ***
## s(sleep_z):time   1.000  1.000   0.660   0.417
## ---
## Signif. codes:  0 '***' 0.001 '**' 0.01 '*' 0.05 '.' 0.1 ' ' 1
##
## R-sq.(adj) =  0.369
## lmer.REML = 9748.9  Scale est. = 0.032785  n = 8172
```

Below are plots of a plot of `s(bl_age)`, `s(bl_age):sleep_z`, `s(bl_age):time`, and `s(sleep_z):time`. For `s(sleep_z):time`, roughly, if we cannot fit a straight line through the confidence bands, there is evidence that the effect of time depends on sleep duration. It is correct the that confidence interval is zero at one point along the axis; this happens because we do not include the main effect.

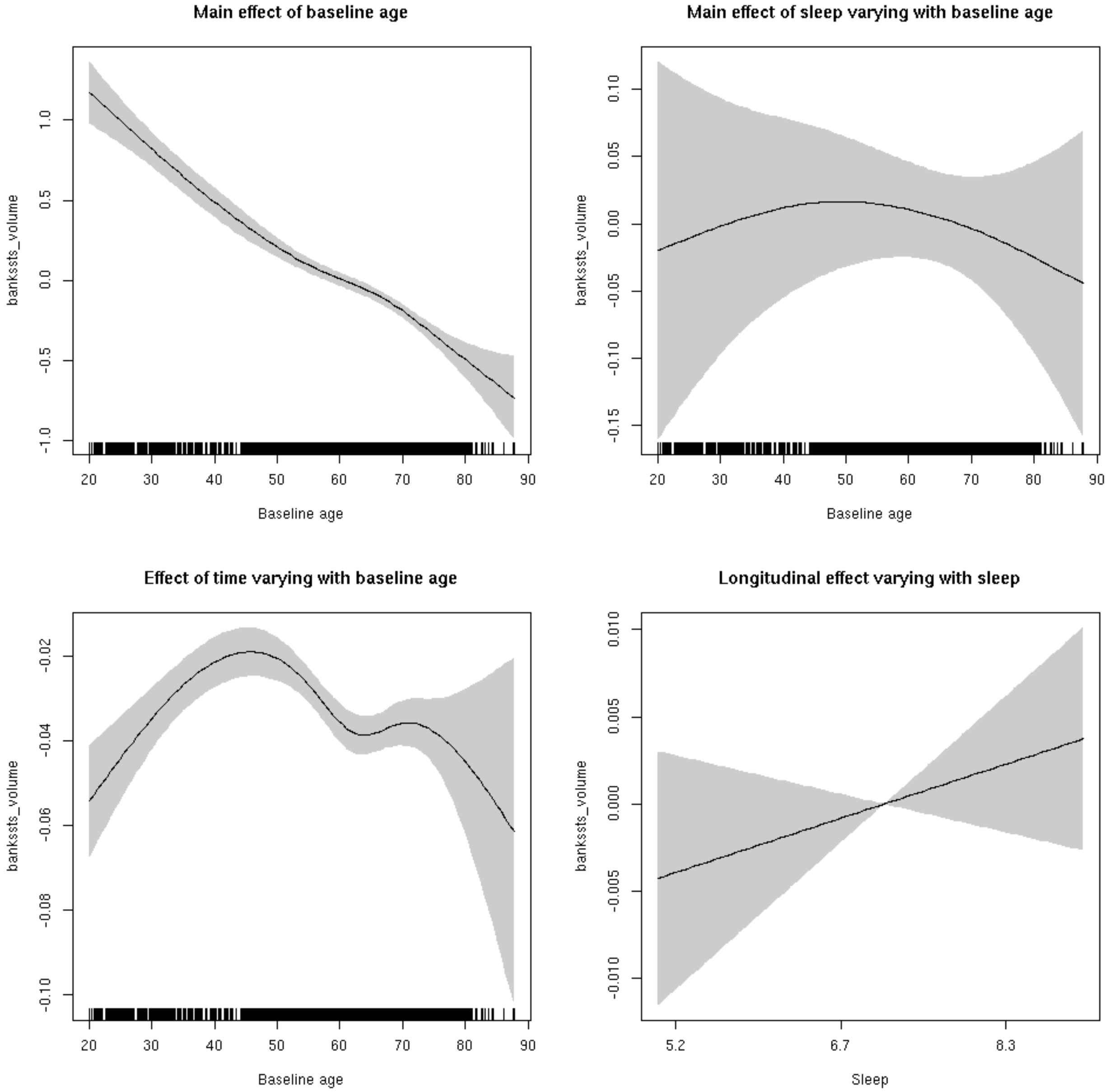

Restricted to sleep >= 5 and <= 9 hours Below is the row for `s(sleep_z):time` from the model output.

```
## s(sleep_z):time   1.000  1.000   1.364   0.243
```

Below are plots of a plot of `s(bl_age)`, `s(bl_age):sleep_z`, `s(bl_age):time`, and `s(sleep_z):time`. For `s(sleep_z):time`, roughly, if we cannot fit a straight line through the confidence bands, there is evidence that the effect of time depends on sleep duration. It is correct the that confidence interval is zero at one point along the axis; this happens because we do not include the main effect.

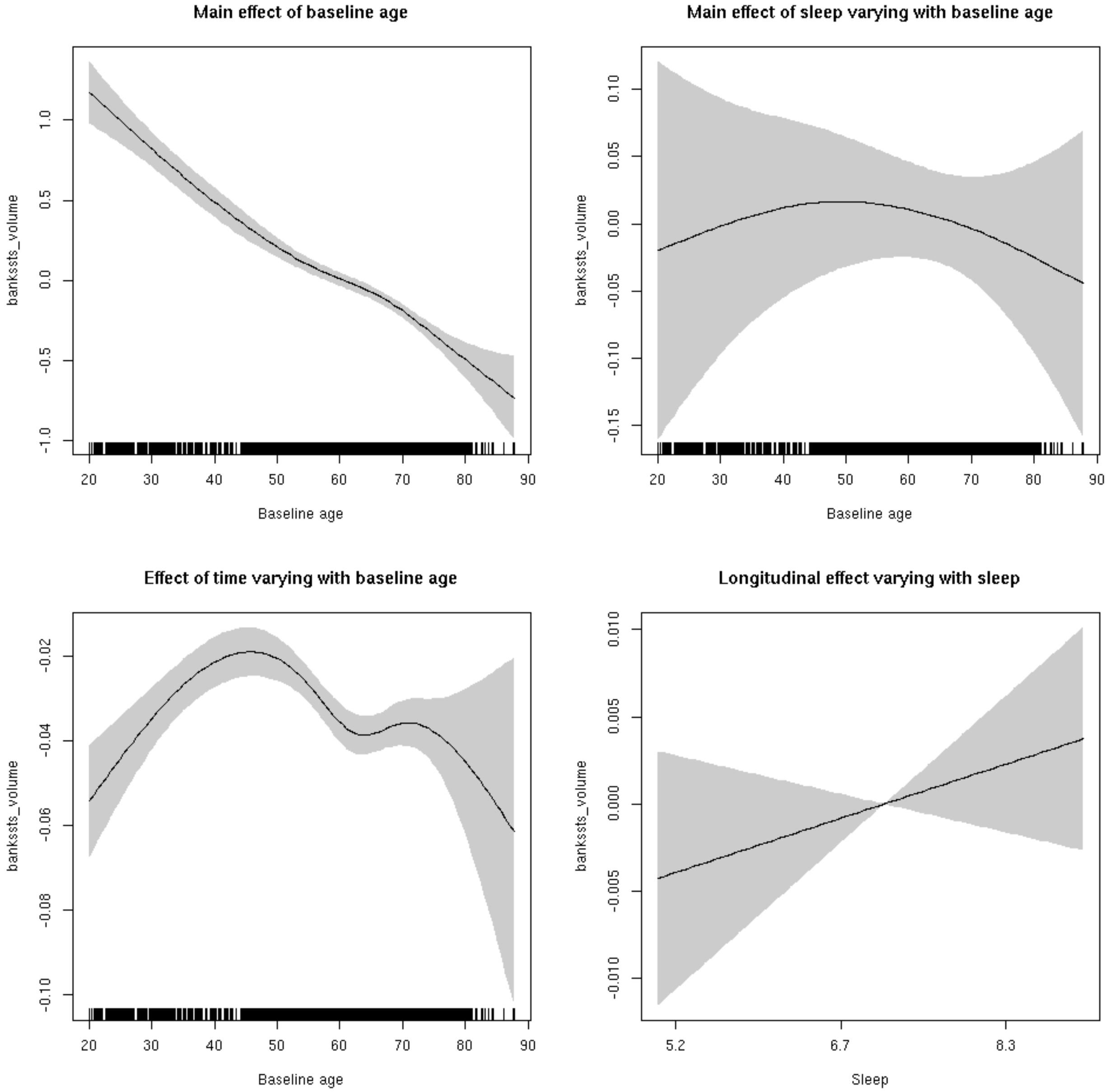

caudalanteriorcingulate_area: Stratified by sex

Female: Full data Below is the row for `s(sleep_z):time` from the model output.

```
## s(sleep_z):time   1.000  1.000  0.224    0.636
```

Below are plots of a plot of `s(bl_age)`, `s(bl_age):sleep_z`, `s(bl_age):time`, and `s(sleep_z):time`. For `s(sleep_z):time`, roughly, if we cannot fit a straight line through the confidence bands, there is evidence that the effect of time depends on sleep duration. It is correct the that confidence interval is zero at one point along the axis; this happens because we do not include the main effect.

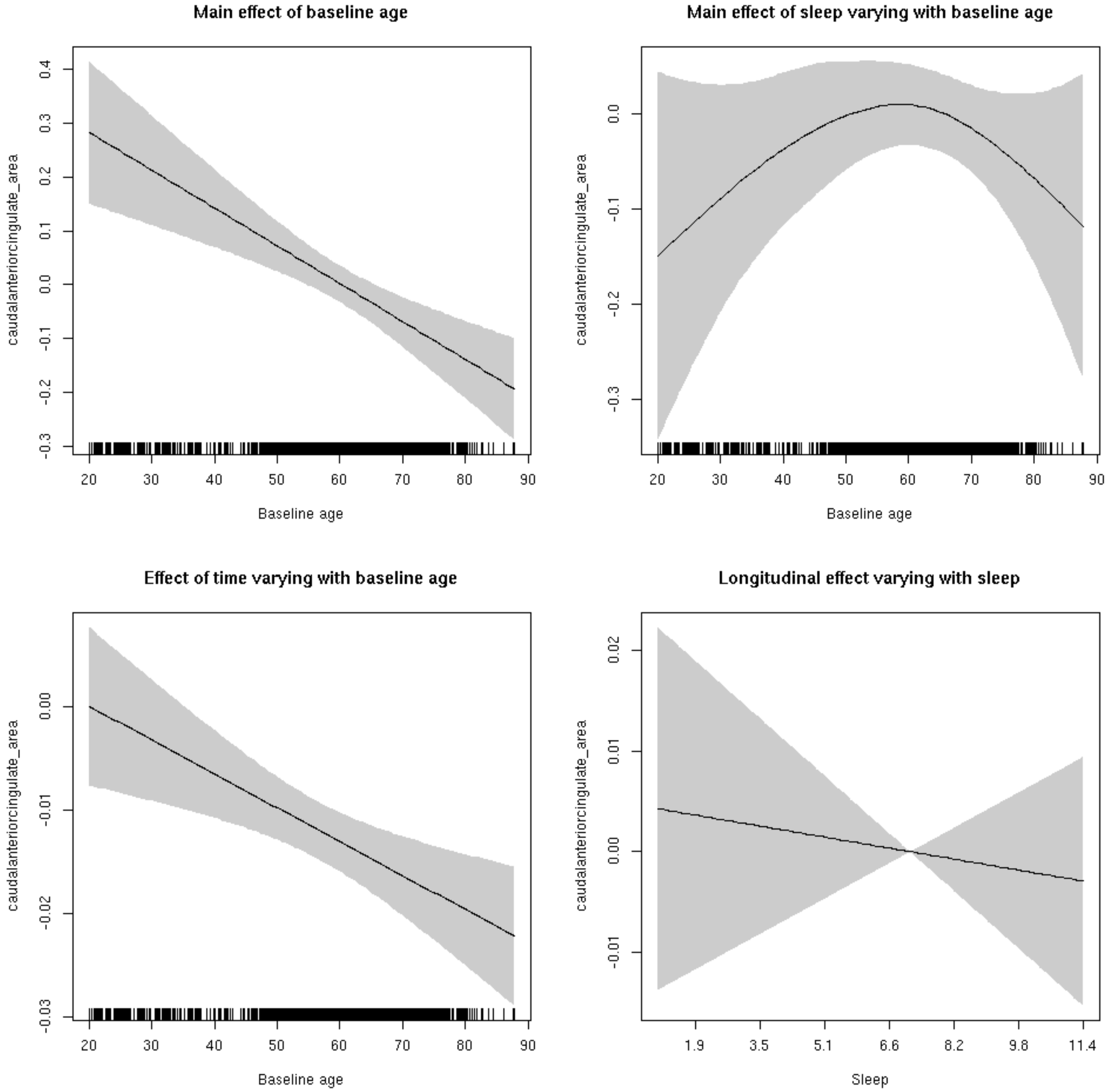

Male: Full data Below is the row for `s(sleep_z):time` from the model output.

```
## s(sleep_z):time   1.000  1.000  0.157   0.692
```

Below are plots of a plot of `s(bl_age)`, `s(bl_age):sleep_z`, `s(bl_age):time`, and `s(sleep_z):time`. For `s(sleep_z):time`, roughly, if we cannot fit a straight line through the confidence bands, there is evidence that the effect of time depends on sleep duration. It is correct the that confidence interval is zero at one point along the axis; this happens because we do not include the main effect.

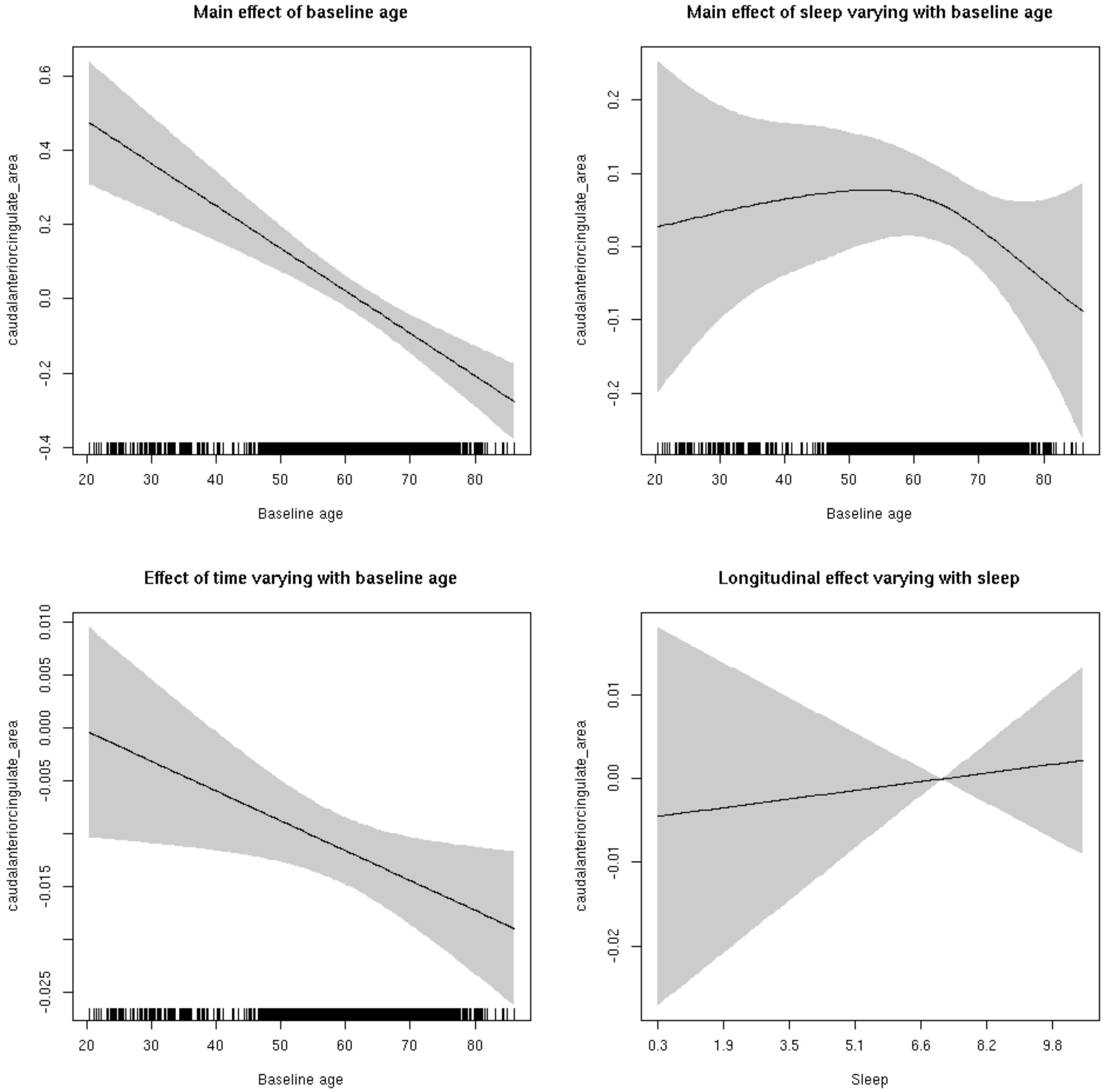

caudalanteriorcingulate_area

Full data Below is the model output. The term `s(sleep_z):time` is probably what we care most about.

```
##
## Family: gaussian
## Link function: identity
##
## Formula:
## value ~ sex + site + icv + s(bl_age, k = 10, bs = "cr") + s(bl_age,
##     by = sleep_z, bs = "cr") + +s(bl_age, by = time, k = 5, bs = "cr") +
##     s(sleep_z, by = time, k = 5, bs = "cr", pc = 0)
## <environment: 0x563003593db0>
```

```
##
## Parametric coefficients:
##                 Estimate Std. Error t value Pr(>|t|)
## (Intercept)    1.1065989  0.0615851  17.969  < 2e-16 ***
## sexmale       -0.0001814  0.0320020  -0.006 0.995478
## siteousAvanto -0.6096287  0.0757829  -8.044 9.89e-16 ***
## siteousPrisma -0.4371680  0.1281334  -3.412 0.000648 ***
## siteousSkyra  -0.3861897  0.0734294  -5.259 1.48e-07 ***
## siteUB        -0.5649774  0.1445815  -3.908 9.39e-05 ***
## siteUCAM      -0.6653884  0.0770380  -8.637  < 2e-16 ***
## siteUKB       -1.1727248  0.0575112 -20.391  < 2e-16 ***
## siteUmU       -0.2438735  0.0889675  -2.741 0.006136 **
## icv            0.4745690  0.0154794  30.658  < 2e-16 ***
## ---
## Signif. codes:  0 '***' 0.001 '**' 0.01 '*' 0.05 '.' 0.1 ' ' 1
##
## Approximate significance of smooth terms:
##                     edf Ref.df      F p-value
## s(bl_age)         1.000  1.000 54.739  <2e-16 ***
## s(bl_age):sleep_z 3.531  3.531  1.931  0.0648 .
## s(bl_age):time    2.000  2.000 69.191  <2e-16 ***
## s(sleep_z):time   1.000  1.000  0.002  0.9621
## ---
## Signif. codes:  0 '***' 0.001 '**' 0.01 '*' 0.05 '.' 0.1 ' ' 1
##
## R-sq.(adj) =  0.341
## lmer.REML = 7209.9  Scale est. = 0.017258  n = 8174
```

Below are plots of a plot of `s(bl_age)`, `s(bl_age):sleep_z`, `s(bl_age):time`, and `s(sleep_z):time`. For `s(sleep_z):time`, roughly, if we cannot fit a straight line through the confidence bands, there is evidence that the effect of time depends on sleep duration. It is correct the that confidence interval is zero at one point along the axis; this happens because we do not include the main effect.

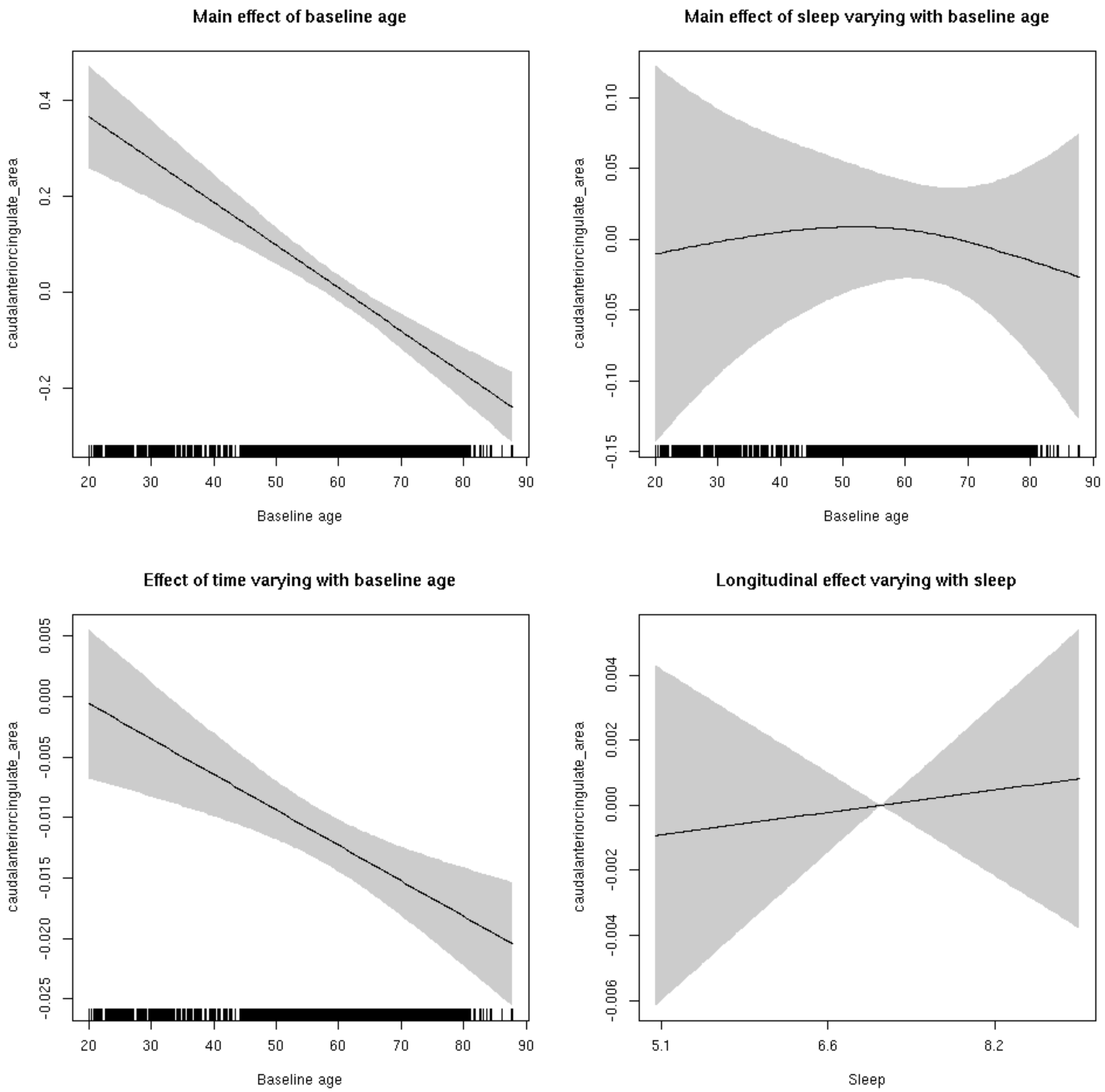

Restricted to sleep >= 5 and <= 9 hours Below is the row for `s(sleep_z):time` from the model output.

```
## s(sleep_z):time   1.000  1.000  0.126   0.723
```

Below are plots of a plot of `s(bl_age)`, `s(bl_age):sleep_z`, `s(bl_age):time`, and `s(sleep_z):time`. For `s(sleep_z):time`, roughly, if we cannot fit a straight line through the confidence bands, there is evidence that the effect of time depends on sleep duration. It is correct the that confidence interval is zero at one point along the axis; this happens because we do not include the main effect.

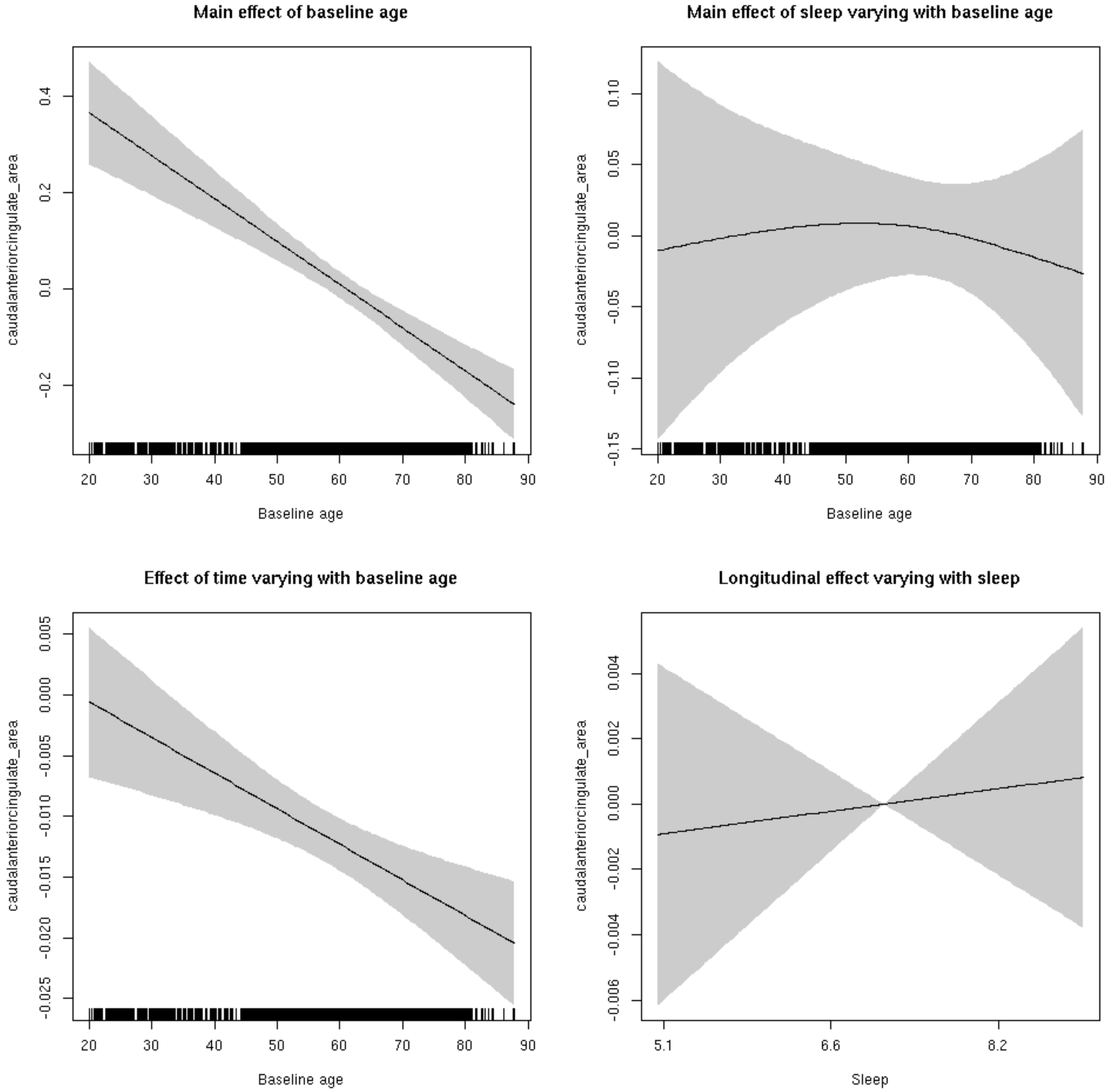

caudalanteriorcingulate_thickness: Stratified by sex

Female: Full data Below is the row for `s(sleep_z):time` from the model output.

```
## s(sleep_z):time     1       1  0.170   0.6802
```

Below are plots of a plot of `s(bl_age)`, `s(bl_age):sleep_z`, `s(bl_age):time`, and `s(sleep_z):time`. For `s(sleep_z):time`, roughly, if we cannot fit a straight line through the confidence bands, there is evidence that the effect of time depends on sleep duration. It is correct the that confidence interval is zero at one point along the axis; this happens because we do not include the main effect.

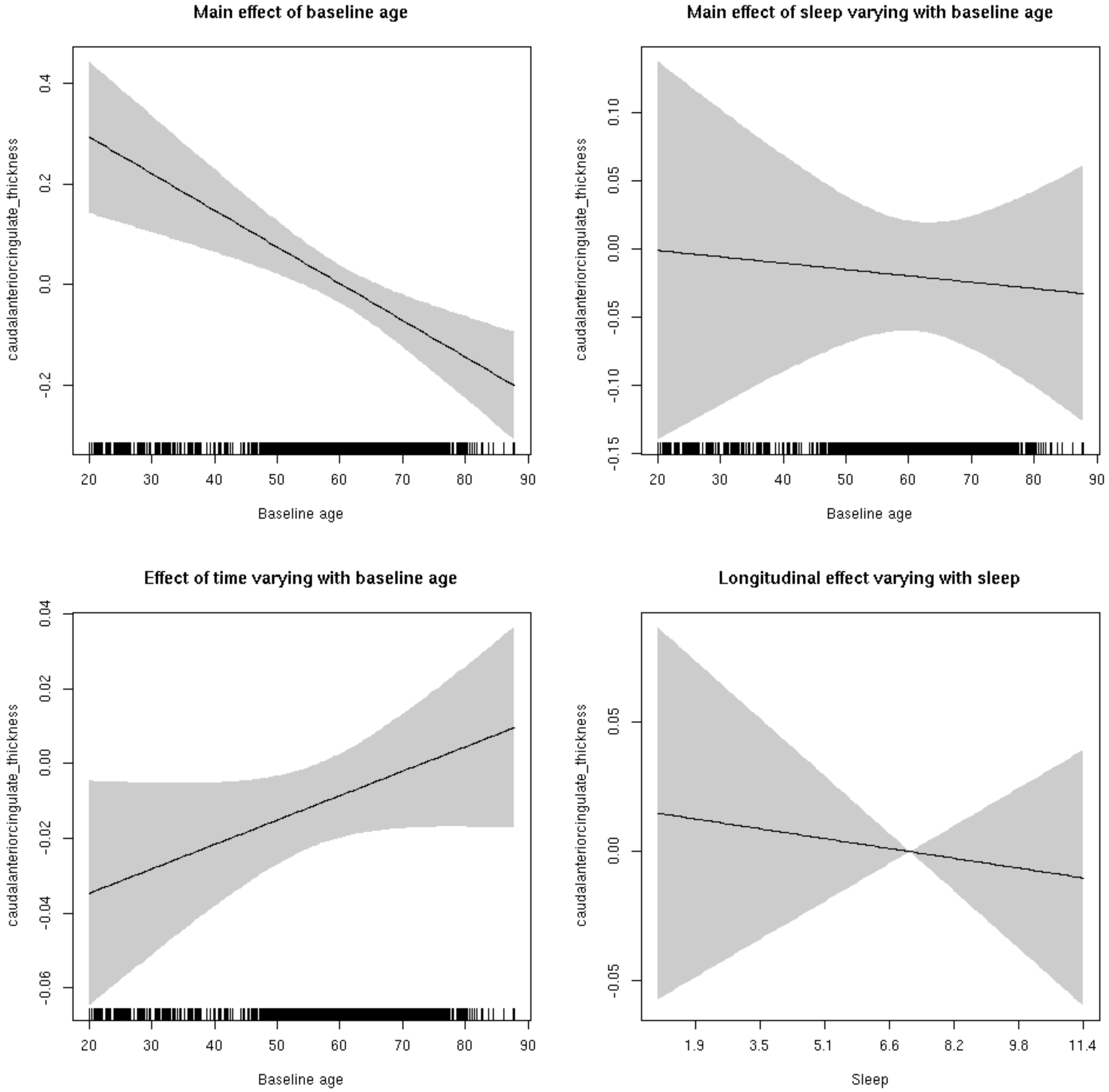

Male: Full data Below is the row for `s(sleep_z):time` from the model output.

```
## s(sleep_z):time   1.000  1.000 0.718 0.39690
```

Below are plots of a plot of `s(bl_age)`, `s(bl_age):sleep_z`, `s(bl_age):time`, and `s(sleep_z):time`. For `s(sleep_z):time`, roughly, if we cannot fit a straight line through the confidence bands, there is evidence that the effect of time depends on sleep duration. It is correct the that confidence interval is zero at one point along the axis; this happens because we do not include the main effect.

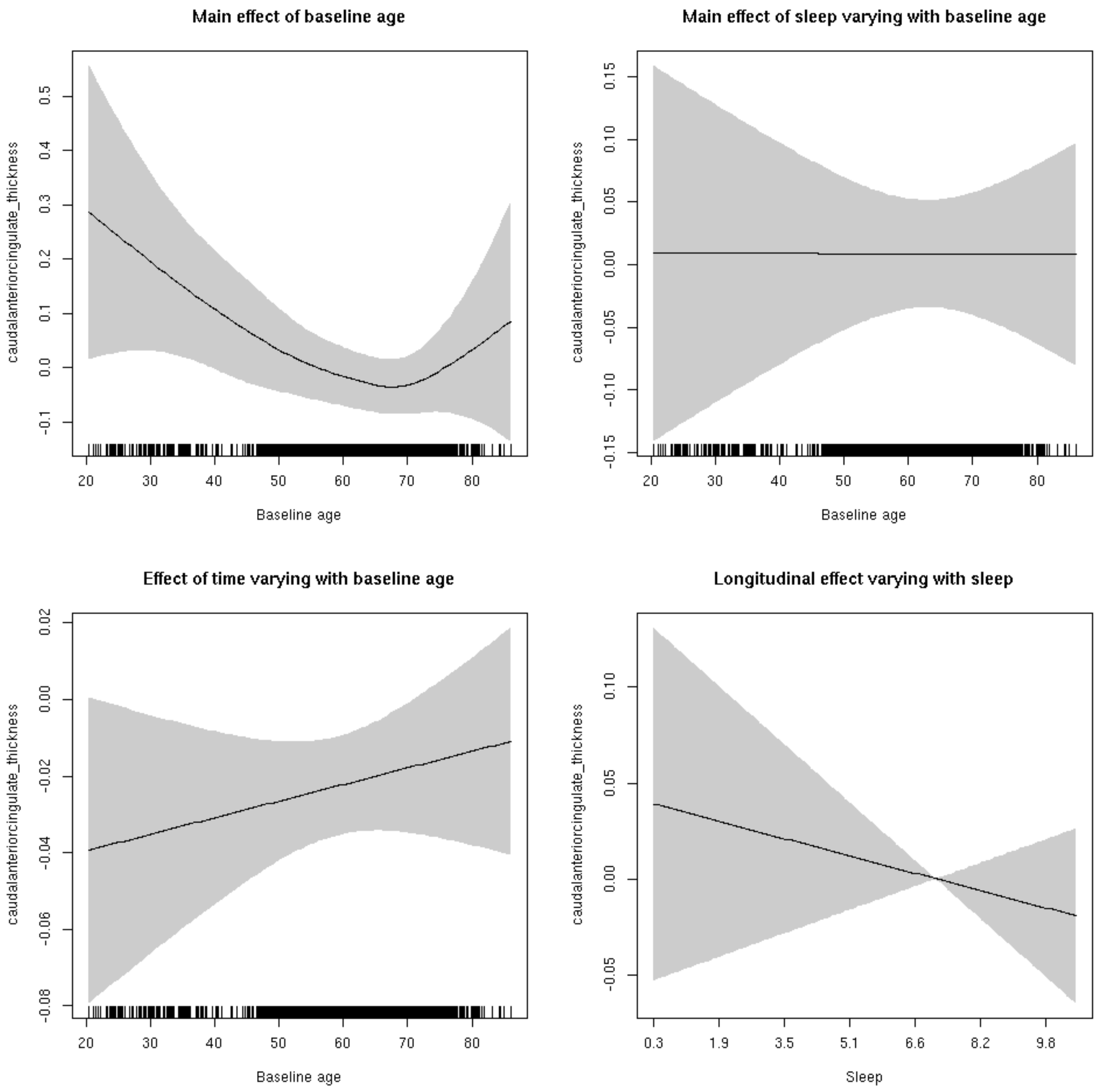

caudalanteriorcingulate_thickness

Full data Below is the model output. The term `s(sleep_z):time` is probably what we care most about.

```
##
## Family: gaussian
## Link function: identity
##
## Formula:
## value ~ sex + site + s(bl_age, k = 10, bs = "cr") + s(bl_age,
##     by = sleep_z, bs = "cr") + +s(bl_age, by = time, k = 5, bs = "cr") +
##     s(sleep_z, by = time, k = 5, bs = "cr", pc = 0)
## <environment: 0x5630035cd3e8>
```

```
##
## Parametric coefficients:
##                  Estimate Std. Error t value Pr(>|t|)
## (Intercept)    -0.4472812  0.0536713  -8.334  < 2e-16 ***
## sexmale        -0.1854118  0.0268923  -6.895  5.8e-12 ***
## siteousAvanto -0.0005201  0.0728054  -0.007  0.99430
## siteousPrisma  0.1055644  0.4003444   0.264  0.79203
## siteousSkyra  -0.1795933  0.0727280  -2.469  0.01355 *
## siteUB         0.1412205  0.1404379   1.006  0.31465
## siteUCAM      -0.0451152  0.0735808  -0.613  0.53980
## siteUKB        0.5802802  0.0541641  10.713  < 2e-16 ***
## siteUmU        0.2499192  0.0885369   2.823  0.00477 **
## ---
## Signif. codes:  0 '***' 0.001 '**' 0.01 '*' 0.05 '.' 0.1 ' ' 1
##
## Approximate significance of smooth terms:
##                      edf Ref.df     F  p-value
## s(bl_age)          2.908  2.908 7.558 8.12e-05 ***
## s(bl_age):sleep_z 2.000  2.000 0.070 0.932135
## s(bl_age):time    2.000  2.000 9.009 0.000124 ***
## s(sleep_z):time   1.000  1.000 1.058 0.303599
## ---
## Signif. codes:  0 '***' 0.001 '**' 0.01 '*' 0.05 '.' 0.1 ' ' 1
##
## R-sq.(adj) =  0.0871
## lmer.REML =  19472  Scale est. = 0.3017    n = 8178
```

Below are plots of a plot of `s(bl_age)`, `s(bl_age):sleep_z`, `s(bl_age):time`, and `s(sleep_z):time`. For `s(sleep_z):time`, roughly, if we cannot fit a straight line through the confidence bands, there is evidence that the effect of time depends on sleep duration. It is correct the that confidence interval is zero at one point along the axis; this happens because we do not include the main effect.

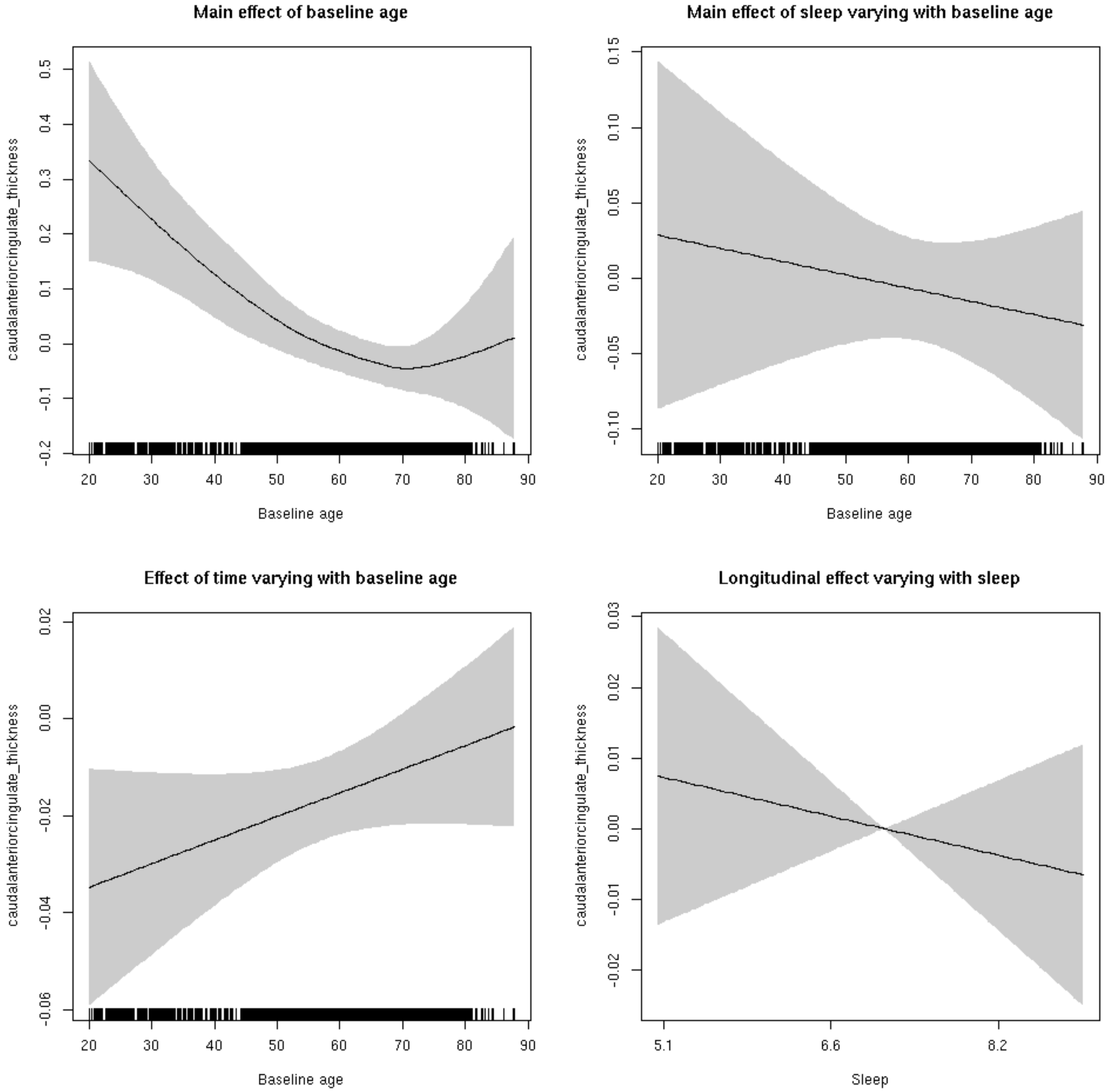

Restricted to sleep >= 5 and <= 9 hours Below is the row for `s(sleep_z):time` from the model output.

```
## s(sleep_z):time   1.000  1.000 0.502 0.478617
```

Below are plots of a plot of `s(bl_age)`, `s(bl_age):sleep_z`, `s(bl_age):time`, and `s(sleep_z):time`. For `s(sleep_z):time`, roughly, if we cannot fit a straight line through the confidence bands, there is evidence that the effect of time depends on sleep duration. It is correct the that confidence interval is zero at one point along the axis; this happens because we do not include the main effect.

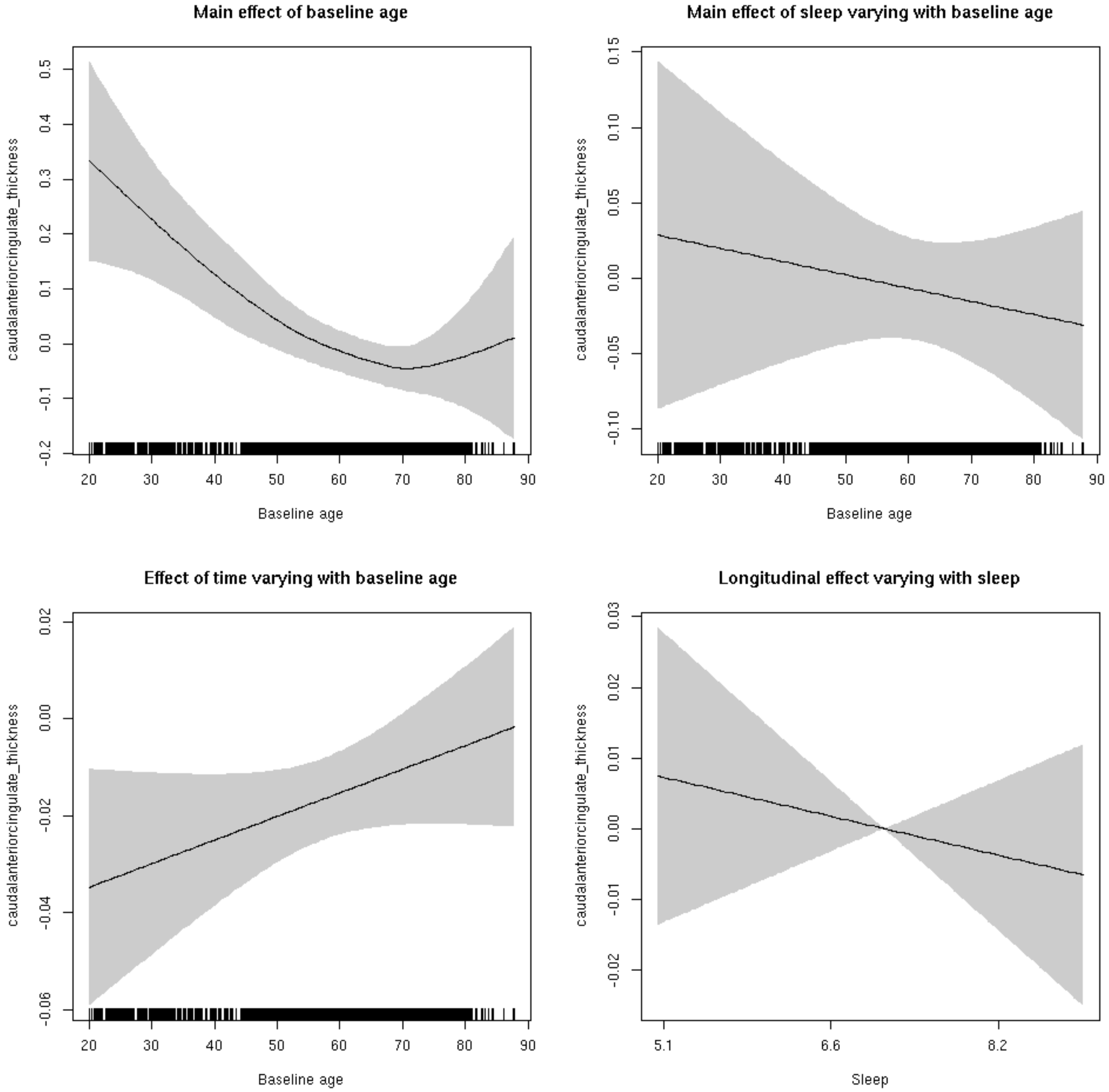

caudalanteriorcingulate_volume: Stratified by sex

Female: Full data Below is the row for `s(sleep_z):time` from the model output.

```
## s(sleep_z):time   1.000  1.000  0.923   0.337
```

Below are plots of a plot of `s(bl_age)`, `s(bl_age):sleep_z`, `s(bl_age):time`, and `s(sleep_z):time`. For `s(sleep_z):time`, roughly, if we cannot fit a straight line through the confidence bands, there is evidence that the effect of time depends on sleep duration. It is correct the that confidence interval is zero at one point along the axis; this happens because we do not include the main effect.

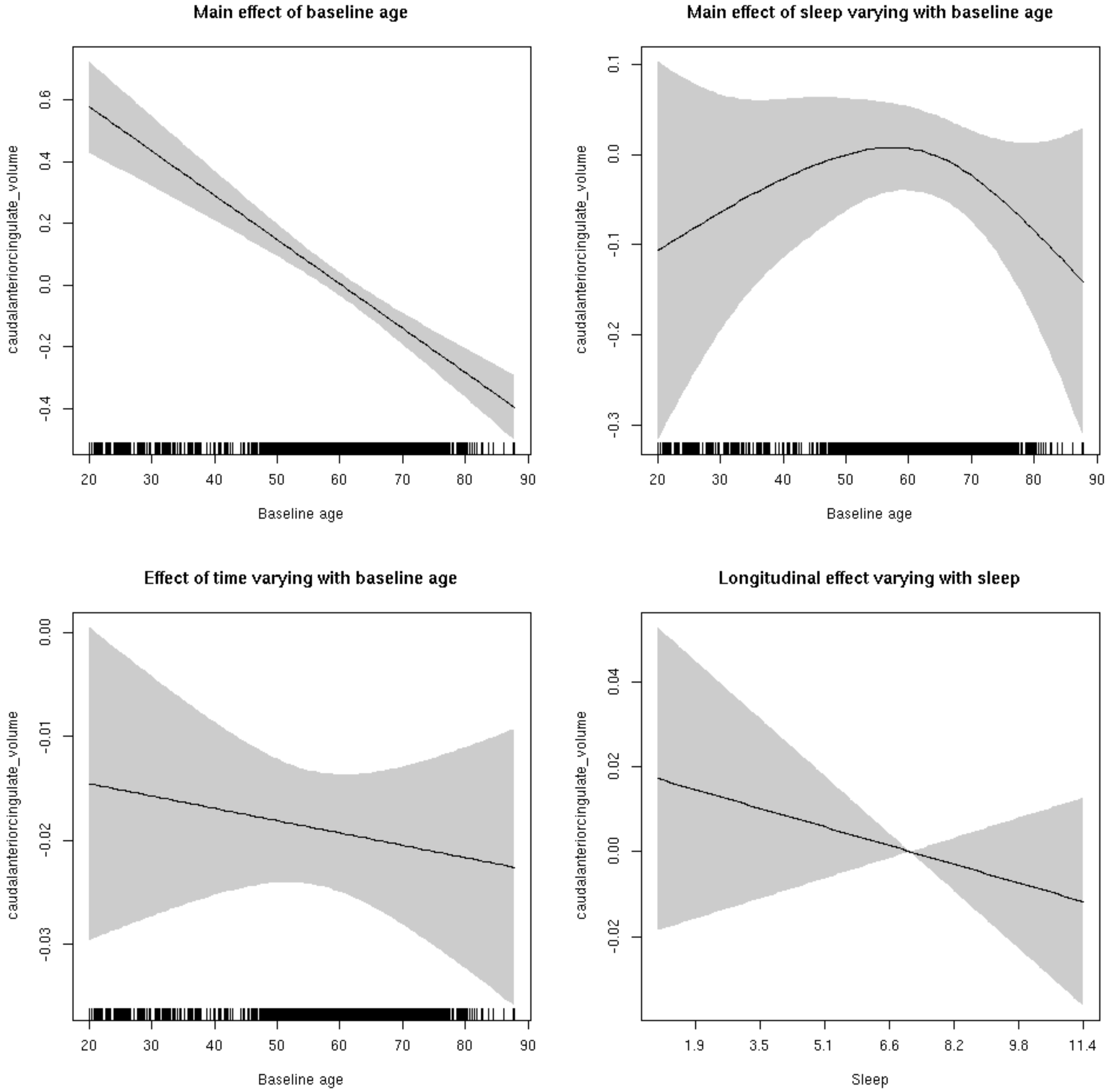

Male: Full data Below is the row for `s(sleep_z):time` from the model output.

```
## s(sleep_z):time     1       1  0.479   0.489
```

Below are plots of a plot of `s(bl_age)`, `s(bl_age):sleep_z`, `s(bl_age):time`, and `s(sleep_z):time`. For `s(sleep_z):time`, roughly, if we cannot fit a straight line through the confidence bands, there is evidence that the effect of time depends on sleep duration. It is correct the that confidence interval is zero at one point along the axis; this happens because we do not include the main effect.

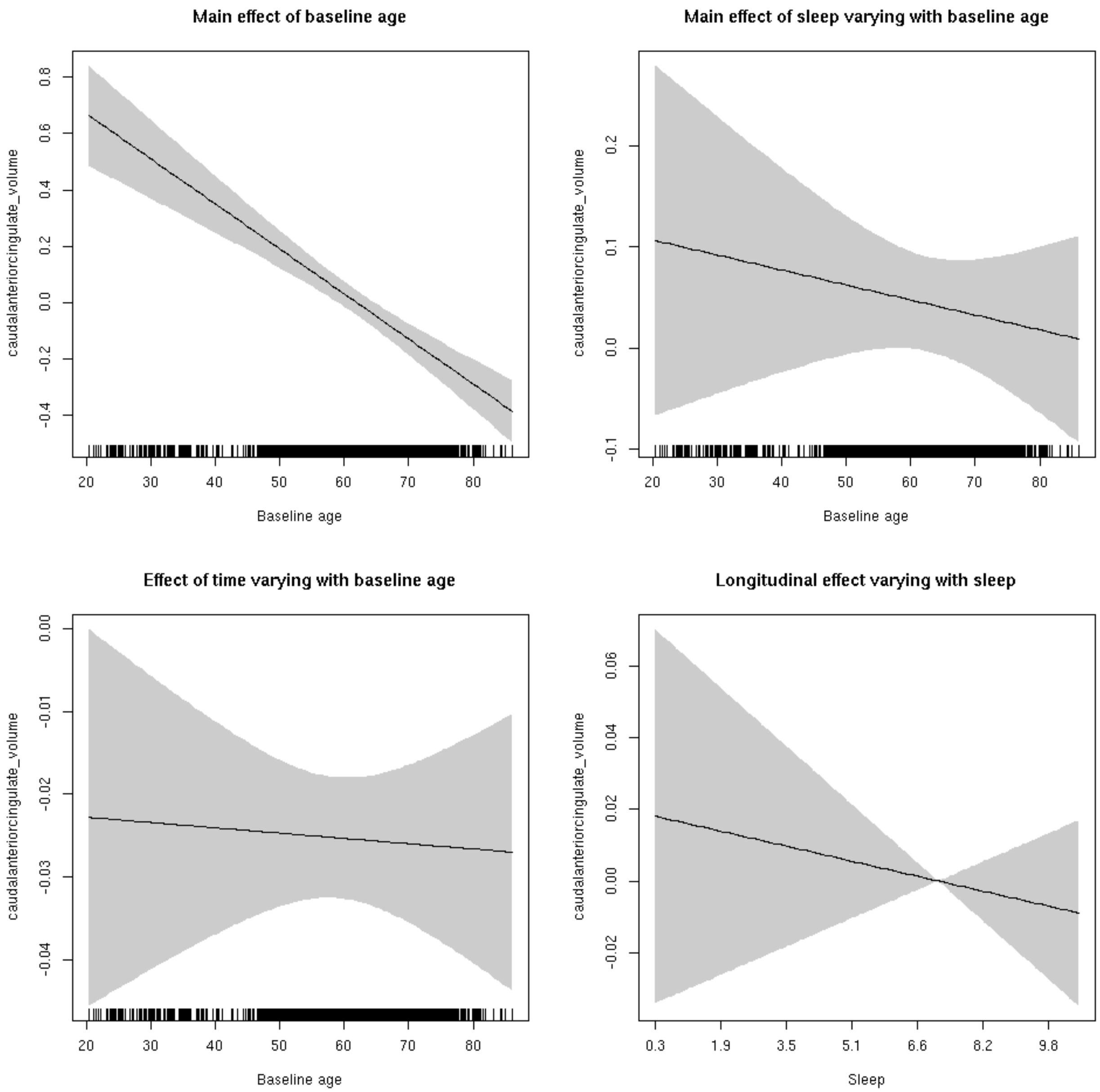

caudalanteriorcingulate__volume

Full data Below is the model output. The term `s(sleep_z):time` is probably what we care most about.

```
##
## Family: gaussian
## Link function: identity
##
## Formula:
## value ~ sex + site + icv + s(bl_age, k = 10, bs = "cr") + s(bl_age,
##     by = sleep_z, bs = "cr") + +s(bl_age, by = time, k = 5, bs = "cr") +
##     s(sleep_z, by = time, k = 5, bs = "cr", pc = 0)
## <environment: 0x5630035ac220>
```

```
##
## Parametric coefficients:
##                Estimate Std. Error t value Pr(>|t|)
## (Intercept)     0.45945    0.06733   6.824 9.50e-12 ***
## sexmale        -0.08921    0.03497  -2.551   0.0108 *
## siteousAvanto -0.44675    0.08334  -5.360 8.54e-08 ***
## siteousPrisma -0.01241    0.23287  -0.053   0.9575
## siteousSkyra  -0.37951    0.08058  -4.710 2.52e-06 ***
## siteUB        -0.29711    0.15593  -1.905   0.0568 .
## siteUCAM      -0.42192    0.08375  -5.038 4.81e-07 ***
## siteUKB       -0.39853    0.06268  -6.358 2.15e-10 ***
## siteUmU       -0.04823    0.09653  -0.500   0.6174
## icv            0.33394    0.01726  19.346  < 2e-16 ***
## ---
## Signif. codes:  0 '***' 0.001 '**' 0.01 '*' 0.05 '.' 0.1 ' ' 1
##
## Approximate significance of smooth terms:
##                    edf Ref.df       F p-value
## s(bl_age)            1      1 123.677  <2e-16 ***
## s(bl_age):sleep_z   3      3   1.328   0.260
## s(bl_age):time      2      2  47.425  <2e-16 ***
## s(sleep_z):time     1      1   1.396   0.237
## ---
## Signif. codes:  0 '***' 0.001 '**' 0.01 '*' 0.05 '.' 0.1 ' ' 1
##
## R-sq.(adj) =   0.14
## lmer.REML =  14443  Scale est. = 0.081116  n = 8176
```

Below are plots of a plot of `s(bl_age)`, `s(bl_age):sleep_z`, `s(bl_age):time`, and `s(sleep_z):time`. For `s(sleep_z):time`, roughly, if we cannot fit a straight line through the confidence bands, there is evidence that the effect of time depends on sleep duration. It is correct the that confidence interval is zero at one point along the axis; this happens because we do not include the main effect.

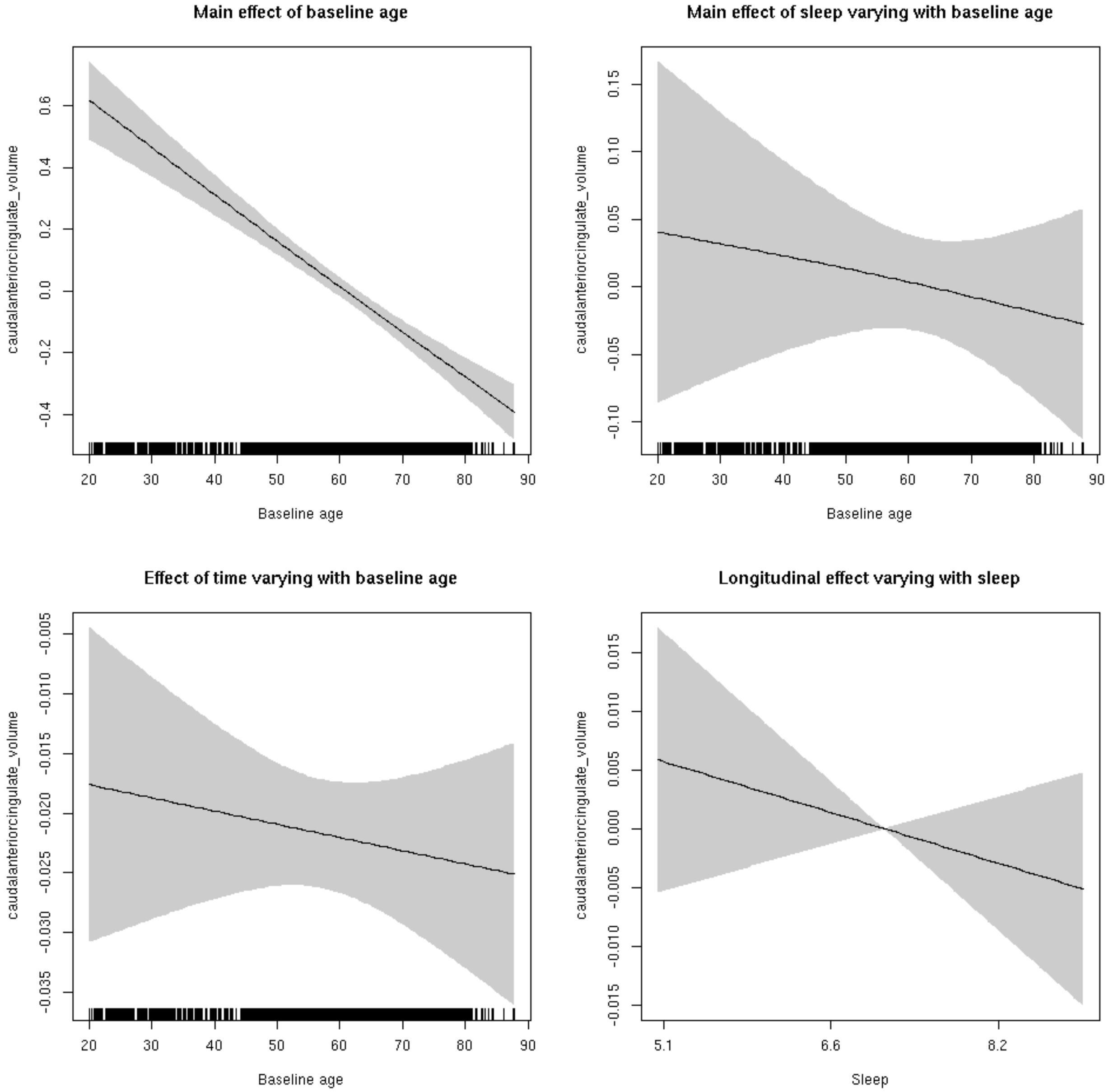

Restricted to sleep >= 5 and <= 9 hours Below is the row for `s(sleep_z):time` from the model output.

```
## s(sleep_z):time   1.000  1.000   1.075   0.300
```

Below are plots of a plot of `s(bl_age)`, `s(bl_age):sleep_z`, `s(bl_age):time`, and `s(sleep_z):time`. For `s(sleep_z):time`, roughly, if we cannot fit a straight line through the confidence bands, there is evidence that the effect of time depends on sleep duration. It is correct the that confidence interval is zero at one point along the axis; this happens because we do not include the main effect.

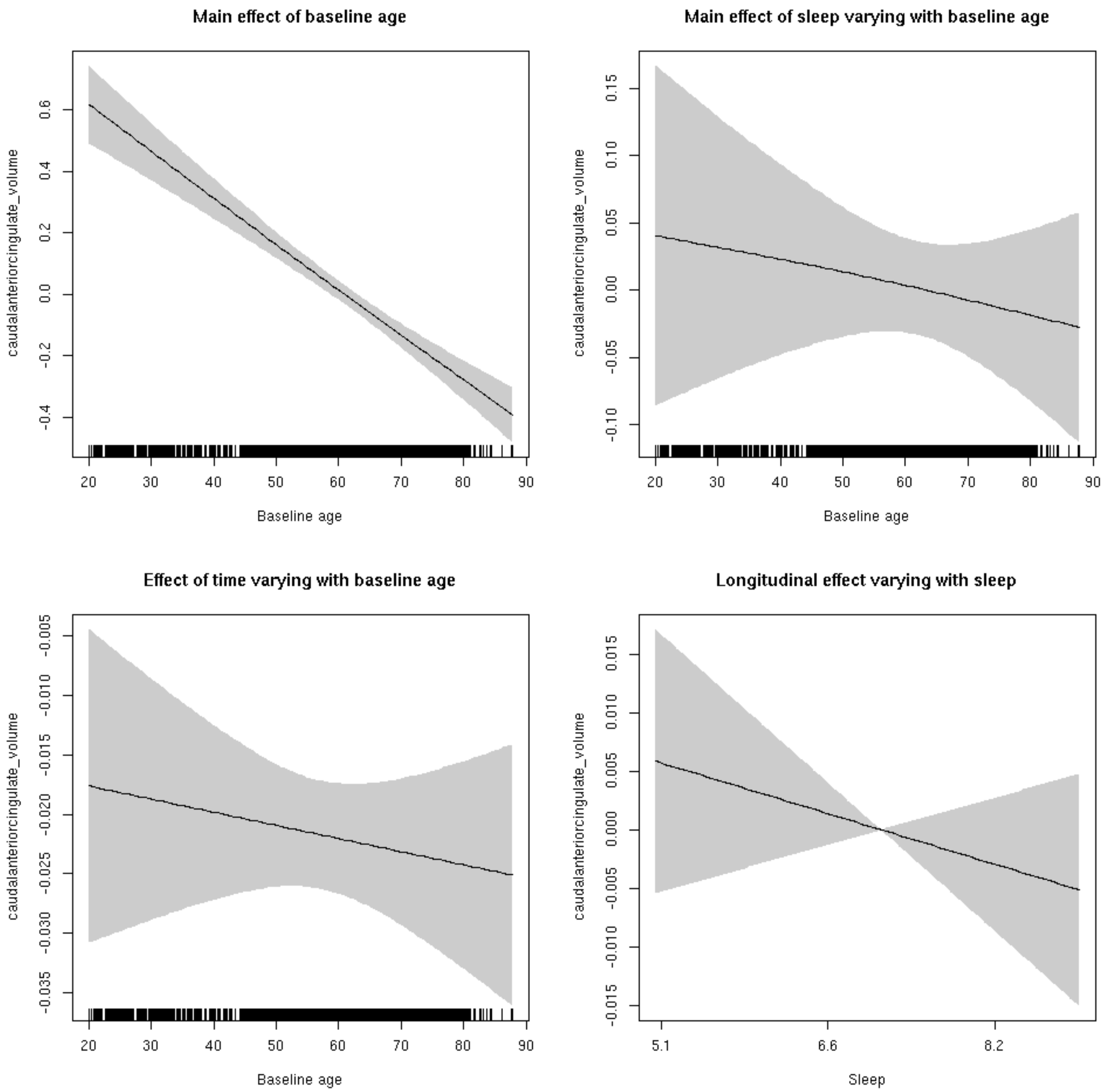

caudalmiddlefrontal_area: Stratified by sex

Female: Full data Below is the row for `s(sleep_z):time` from the model output.

```
## s(sleep_z):time   1.000  1.000  0.803    0.370
```

Below are plots of a plot of `s(bl_age)`, `s(bl_age):sleep_z`, `s(bl_age):time`, and `s(sleep_z):time`. For `s(sleep_z):time`, roughly, if we cannot fit a straight line through the confidence bands, there is evidence that the effect of time depends on sleep duration. It is correct the that confidence interval is zero at one point along the axis; this happens because we do not include the main effect.

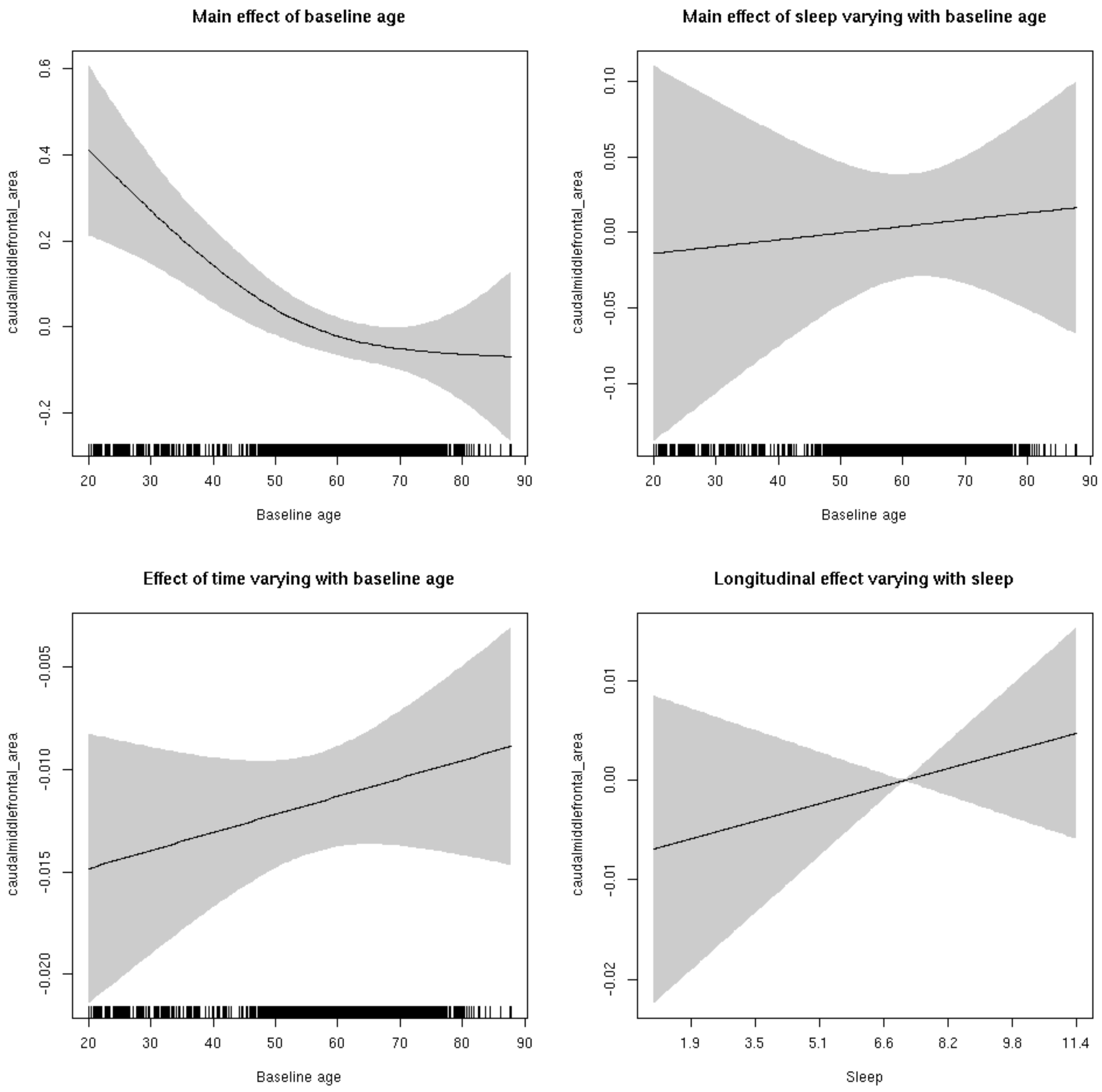

Male: Full data Below is the row for `s(sleep_z):time` from the model output.

```
## s(sleep_z):time   1.000  1.000  0.177   0.674
```

Below are plots of a plot of `s(bl_age)`, `s(bl_age):sleep_z`, `s(bl_age):time`, and `s(sleep_z):time`. For `s(sleep_z):time`, roughly, if we cannot fit a straight line through the confidence bands, there is evidence that the effect of time depends on sleep duration. It is correct the that confidence interval is zero at one point along the axis; this happens because we do not include the main effect.

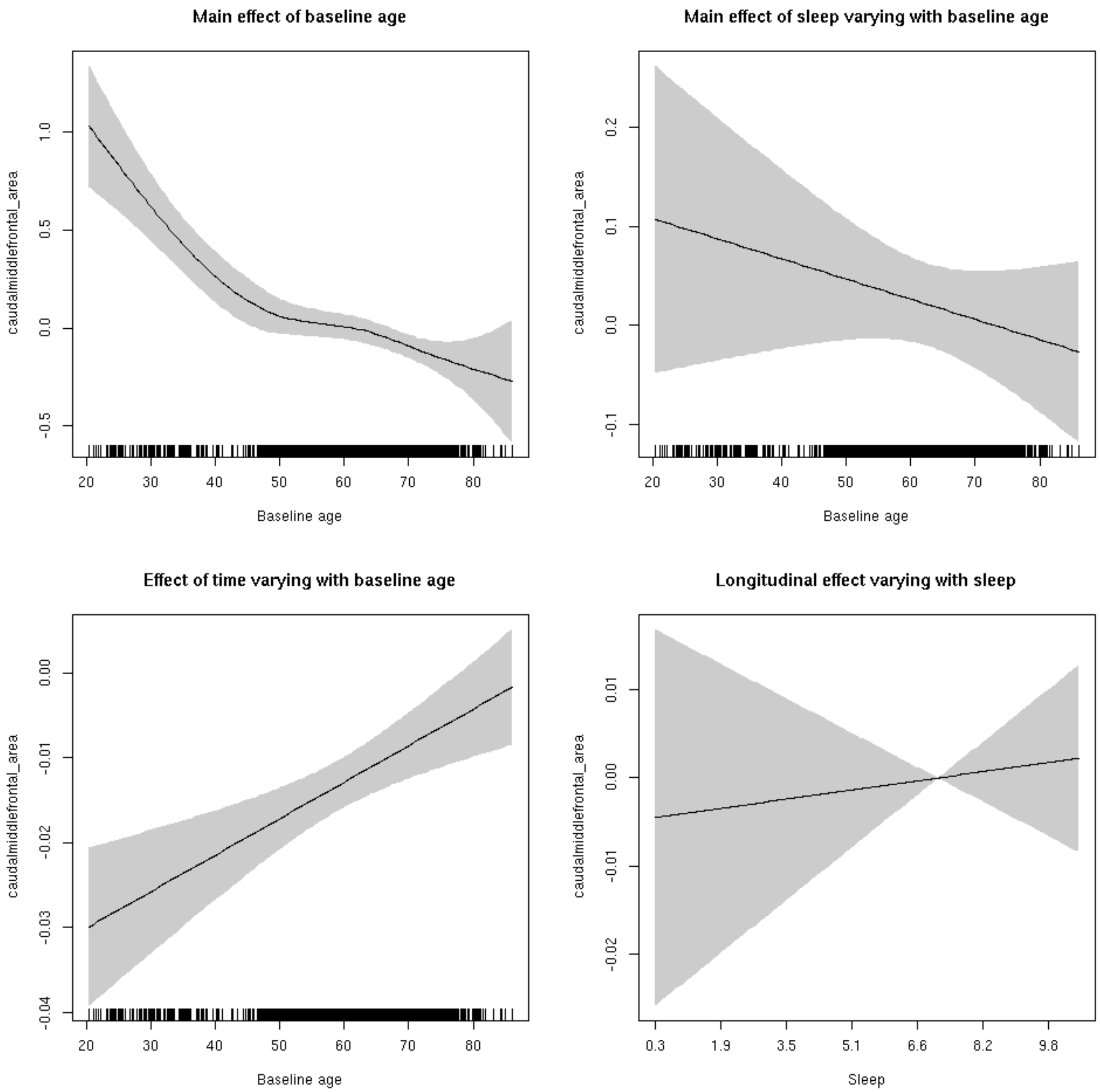

caudalmiddlefrontal_area

Full data Below is the model output. The term `s(sleep_z):time` is probably what we care most about.

```
##
## Family: gaussian
## Link function: identity
##
## Formula:
## value ~ sex + site + icv + s(bl_age, k = 10, bs = "cr") + s(bl_age,
##     by = sleep_z, bs = "cr") + +s(bl_age, by = time, k = 5, bs = "cr") +
##     s(sleep_z, by = time, k = 5, bs = "cr", pc = 0)
## <environment: 0x5630035a74b8>
```

```
##
## Parametric coefficients:
##                Estimate Std. Error t value Pr(>|t|)
## (Intercept)     0.75296    0.06074  12.397  < 2e-16 ***
## sexmale         0.03083    0.03125   0.987   0.3239
## siteousAvanto -0.68258    0.07393  -9.232  < 2e-16 ***
## siteousPrisma -0.72860    0.11929  -6.108 1.05e-09 ***
## siteousSkyra  -0.67918    0.07171  -9.472  < 2e-16 ***
## siteUB        -0.76536    0.14116  -5.422 6.06e-08 ***
## siteUCAM      -0.68013    0.07560  -8.996  < 2e-16 ***
## siteUKB       -0.74763    0.05777 -12.941  < 2e-16 ***
## siteUmU       -0.17579    0.08719  -2.016   0.0438 *
## icv            0.57455    0.01509  38.063  < 2e-16 ***
## ---
## Signif. codes:  0 '***' 0.001 '**' 0.01 '*' 0.05 '.' 0.1 ' ' 1
##
## Approximate significance of smooth terms:
##                      edf Ref.df      F p-value
## s(bl_age)          2.854  2.854 26.561  <2e-16 ***
## s(bl_age):sleep_z 2.000  2.000  0.525   0.591
## s(bl_age):time    2.000  2.000 90.682  <2e-16 ***
## s(sleep_z):time   1.000  1.000  1.124   0.289
## ---
## Signif. codes:  0 '***' 0.001 '**' 0.01 '*' 0.05 '.' 0.1 ' ' 1
##
## R-sq.(adj) =  0.368
## lmer.REML = 6106.7  Scale est. = 0.01403   n = 8140
```

Below are plots of a plot of `s(bl_age)`, `s(bl_age):sleep_z`, `s(bl_age):time`, and `s(sleep_z):time`. For `s(sleep_z):time`, roughly, if we cannot fit a straight line through the confidence bands, there is evidence that the effect of time depends on sleep duration. It is correct the that confidence interval is zero at one point along the axis; this happens because we do not include the main effect.

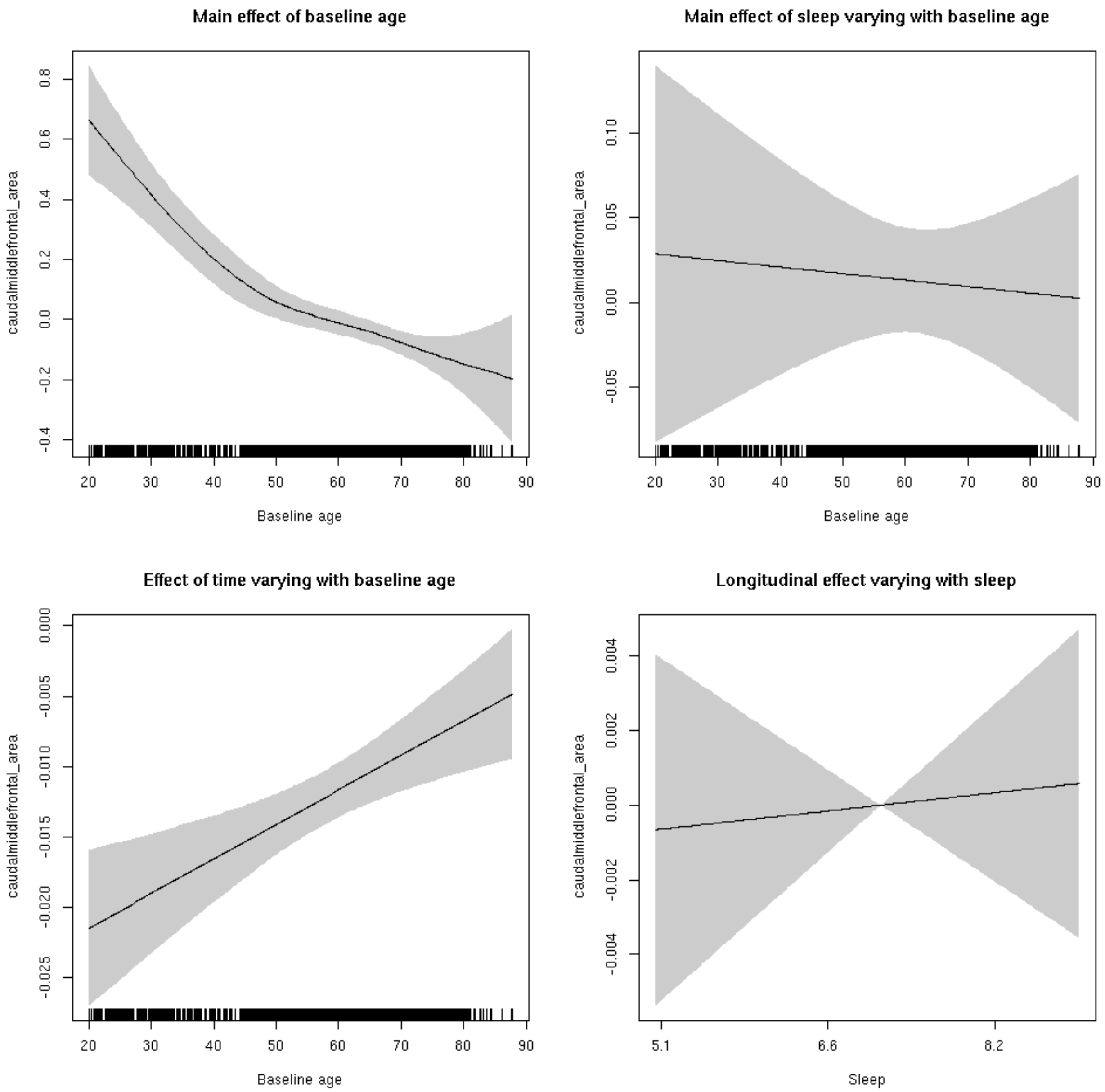

Restricted to sleep >= 5 and <= 9 hours Below is the row for `s(sleep_z):time` from the model output.

```
## s(sleep_z):time   1.000  1.000  0.079   0.779
```

Below are plots of a plot of `s(bl_age)`, `s(bl_age):sleep_z`, `s(bl_age):time`, and `s(sleep_z):time`. For `s(sleep_z):time`, roughly, if we cannot fit a straight line through the confidence bands, there is evidence that the effect of time depends on sleep duration. It is correct the that confidence interval is zero at one point along the axis; this happens because we do not include the main effect.

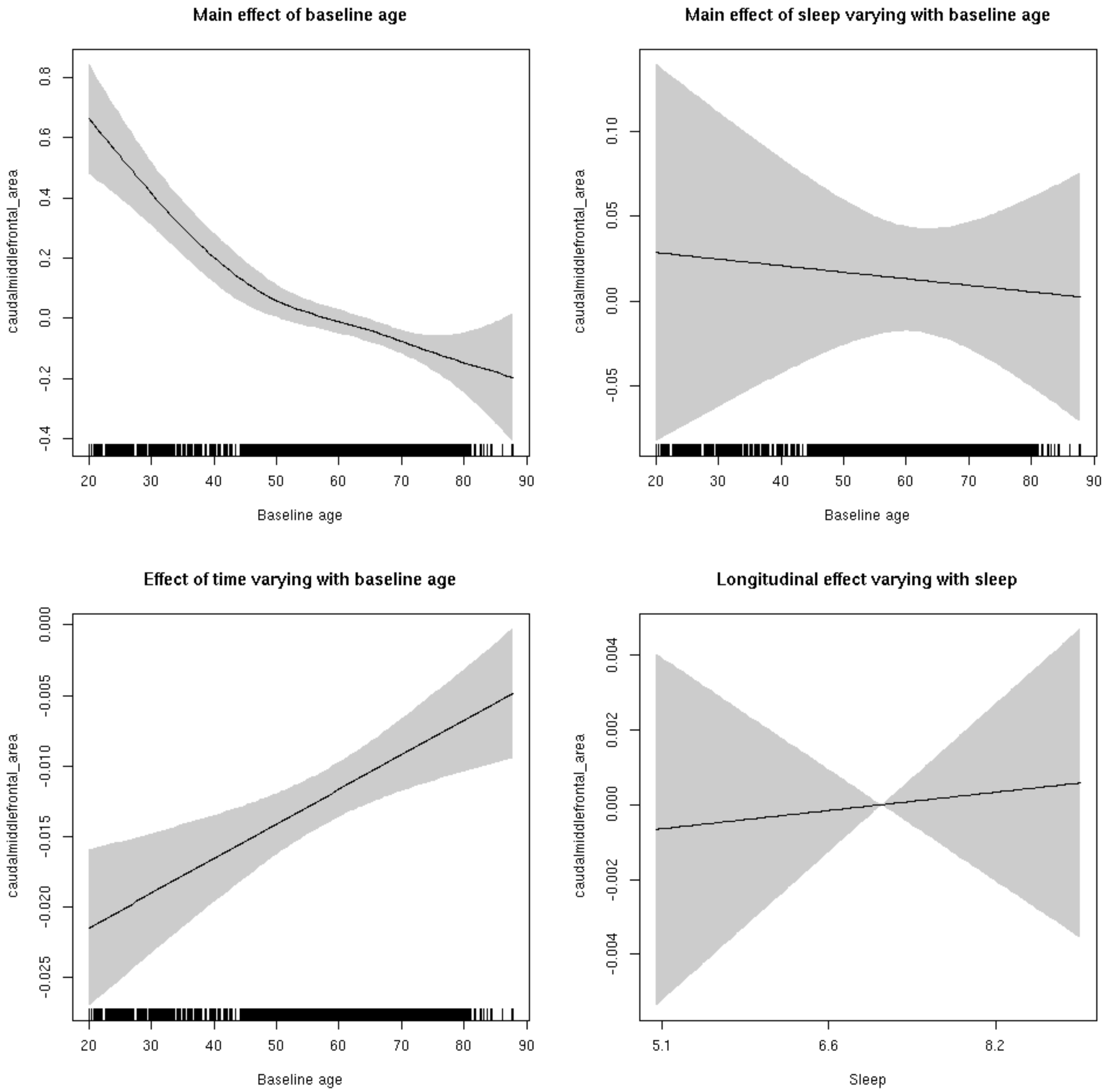

caudalmiddlefrontal_thickness: Stratified by sex

Female: Full data Below is the row for `s(sleep_z):time` from the model output.

```
## s(sleep_z):time   1.000  1.000   0.000   0.996
```

Below are plots of a plot of `s(bl_age)`, `s(bl_age):sleep_z`, `s(bl_age):time`, and `s(sleep_z):time`. For `s(sleep_z):time`, roughly, if we cannot fit a straight line through the confidence bands, there is evidence that the effect of time depends on sleep duration. It is correct the that confidence interval is zero at one point along the axis; this happens because we do not include the main effect.

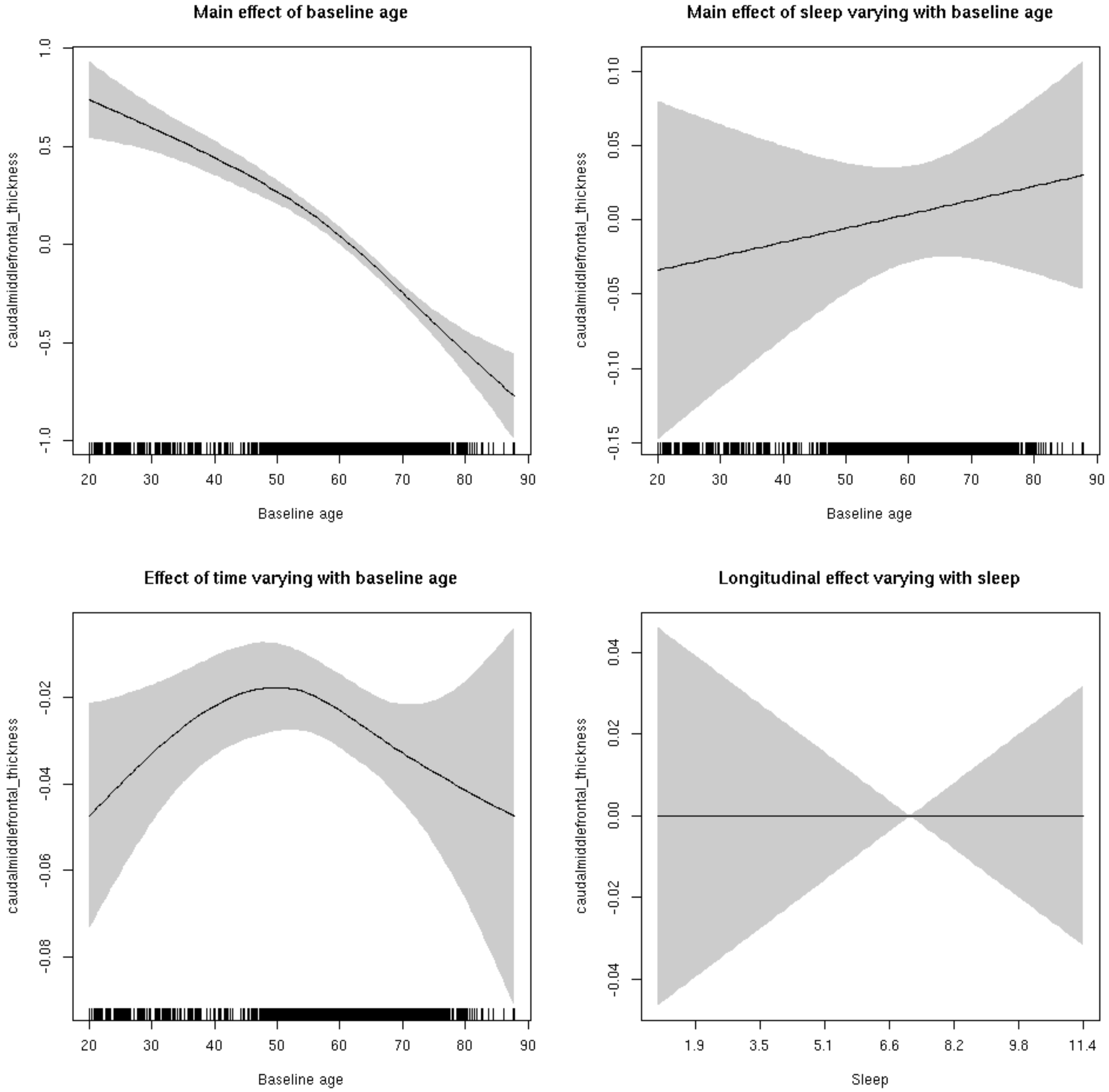

Male: Full data Below is the row for `s(sleep_z):time` from the model output.

```
## s(sleep_z):time   1.000  1.000  0.026   0.871
```

Below are plots of a plot of `s(bl_age)`, `s(bl_age):sleep_z`, `s(bl_age):time`, and `s(sleep_z):time`. For `s(sleep_z):time`, roughly, if we cannot fit a straight line through the confidence bands, there is evidence that the effect of time depends on sleep duration. It is correct the that confidence interval is zero at one point along the axis; this happens because we do not include the main effect.

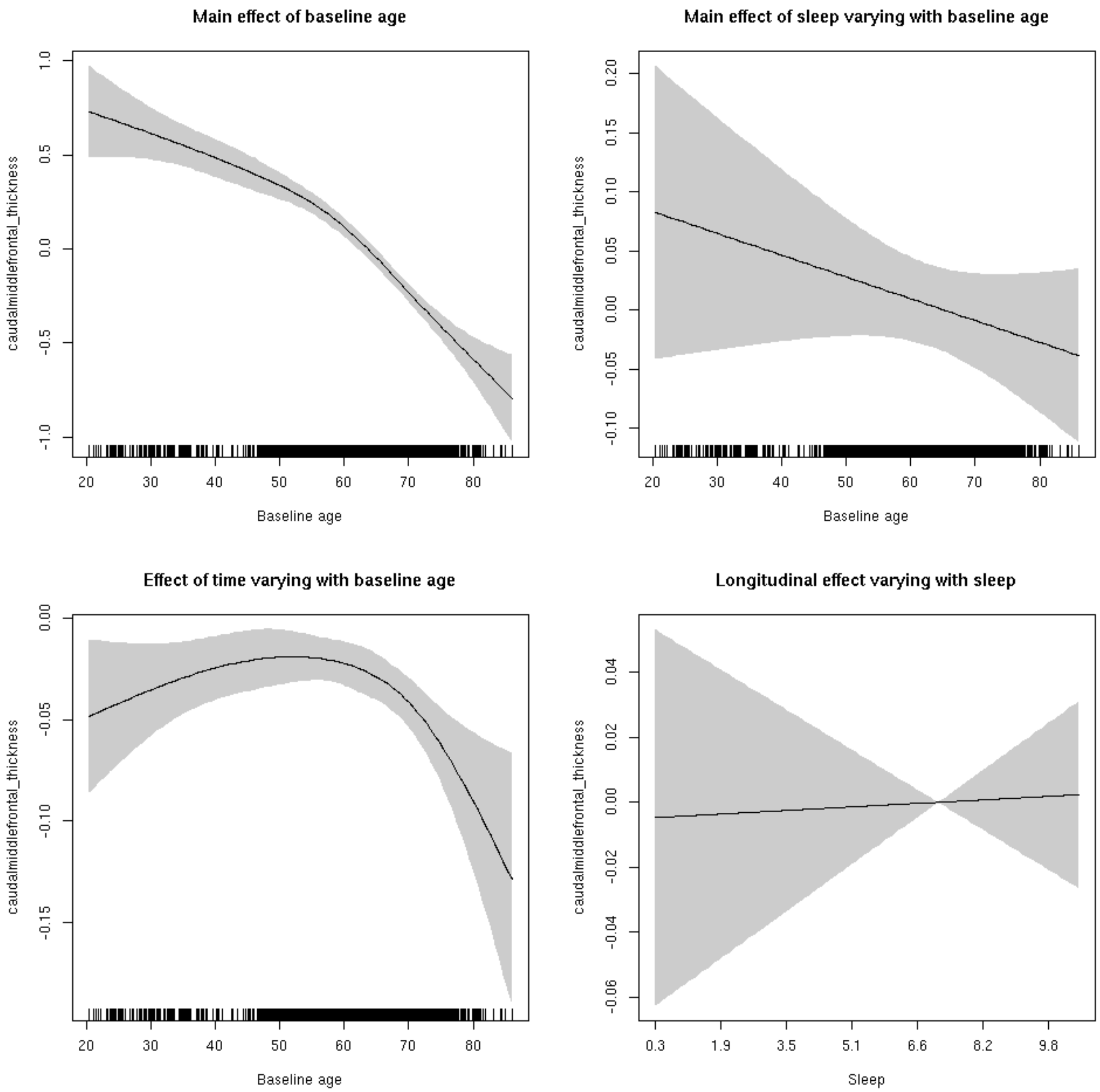

caudalmiddlefrontal__thickness

Full data Below is the model output. The term `s(sleep_z):time` is probably what we care most about.

```
##
## Family: gaussian
## Link function: identity
##
## Formula:
## value ~ sex + site + s(bl_age, k = 10, bs = "cr") + s(bl_age,
##     by = sleep_z, bs = "cr") + +s(bl_age, by = time, k = 5, bs = "cr") +
##     s(sleep_z, by = time, k = 5, bs = "cr", pc = 0)
## <environment: 0x5630035d1260>
```

```
##
## Parametric coefficients:
##                Estimate Std. Error t value Pr(>|t|)
## (Intercept)   -1.45063    0.04417 -32.841  < 2e-16 ***
## sexmale       -0.05862    0.02215  -2.647 0.008133 **
## siteousAvanto -0.08041    0.05881  -1.367 0.171616
## siteousPrisma  0.37413    0.25805   1.450 0.147132
## siteousSkyra  -0.77597    0.05879 -13.198  < 2e-16 ***
## siteUB         0.35092    0.11709   2.997 0.002735 **
## siteUCAM       0.20981    0.06074   3.454 0.000555 ***
## siteUKB        1.80423    0.04475  40.321  < 2e-16 ***
## siteUmU       -0.19011    0.07282  -2.611 0.009051 **
## ---
## Signif. codes:  0 ‘***’ 0.001 ‘**’ 0.01 ‘*’ 0.05 ‘.’ 0.1 ‘ ’ 1
##
## Approximate significance of smooth terms:
##                      edf Ref.df       F p-value
## s(bl_age)          3.508  3.508 162.148  <2e-16 ***
## s(bl_age):sleep_z 2.000  2.000   0.166   0.847
## s(bl_age):time    3.474  3.474  27.980  <2e-16 ***
## s(sleep_z):time   2.741  2.741   1.037   0.352
## ---
## Signif. codes:  0 ‘***’ 0.001 ‘**’ 0.01 ‘*’ 0.05 ‘.’ 0.1 ‘ ’ 1
##
## R-sq.(adj) =  0.623
## lmer.REML =  13842  Scale est. = 0.11531   n = 8171
```

Below are plots of a plot of `s(bl_age)`, `s(bl_age):sleep_z`, `s(bl_age):time`, and `s(sleep_z):time`. For `s(sleep_z):time`, roughly, if we cannot fit a straight line through the confidence bands, there is evidence that the effect of time depends on sleep duration. It is correct the that confidence interval is zero at one point along the axis; this happens because we do not include the main effect.

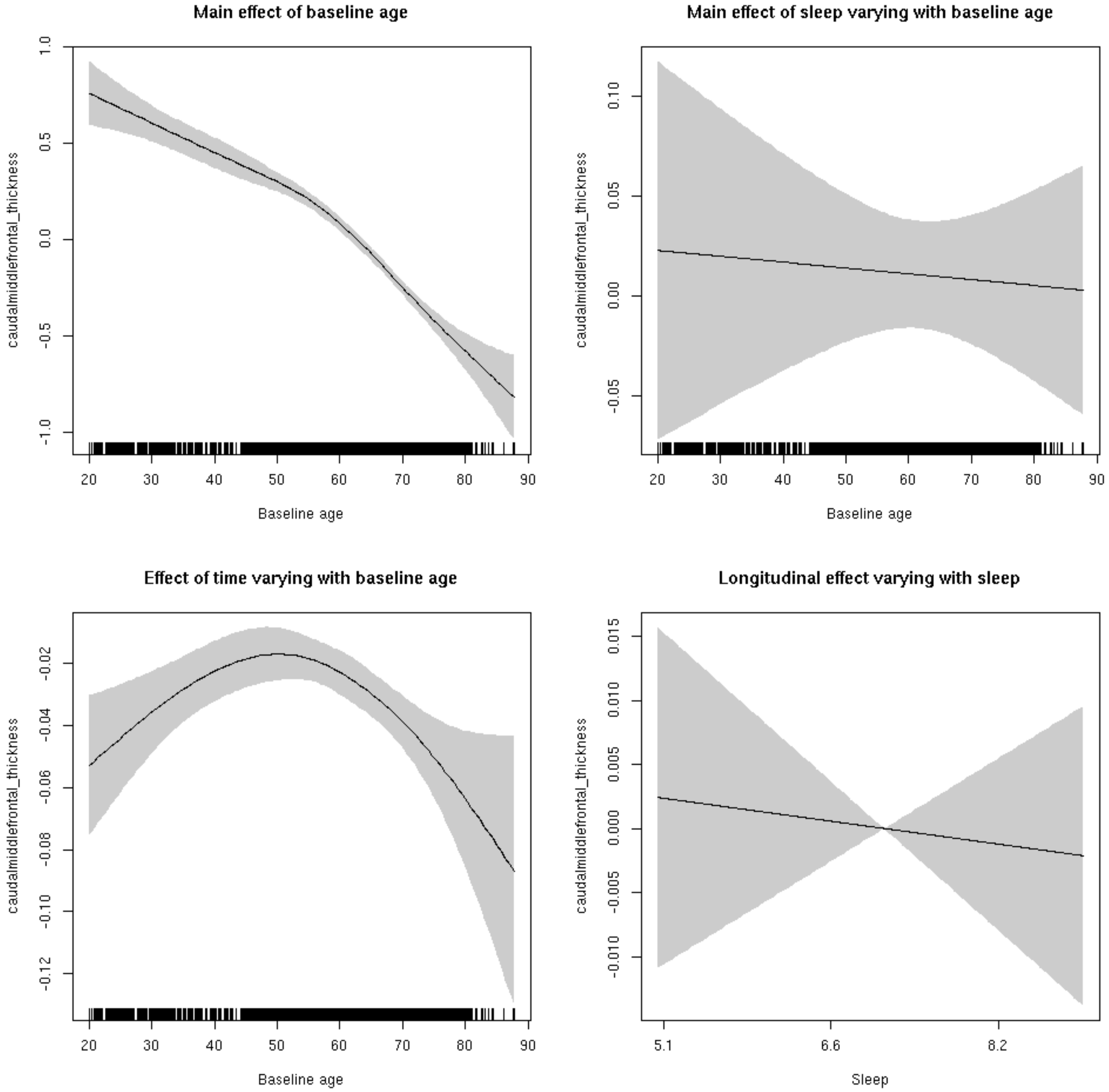

Restricted to sleep >= 5 and <= 9 hours Below is the row for `s(sleep_z):time` from the model output.

```
## s(sleep_z):time   1.000  1.000   0.132   0.716
```

Below are plots of a plot of `s(bl_age)`, `s(bl_age):sleep_z`, `s(bl_age):time`, and `s(sleep_z):time`. For `s(sleep_z):time`, roughly, if we cannot fit a straight line through the confidence bands, there is evidence that the effect of time depends on sleep duration. It is correct the that confidence interval is zero at one point along the axis; this happens because we do not include the main effect.

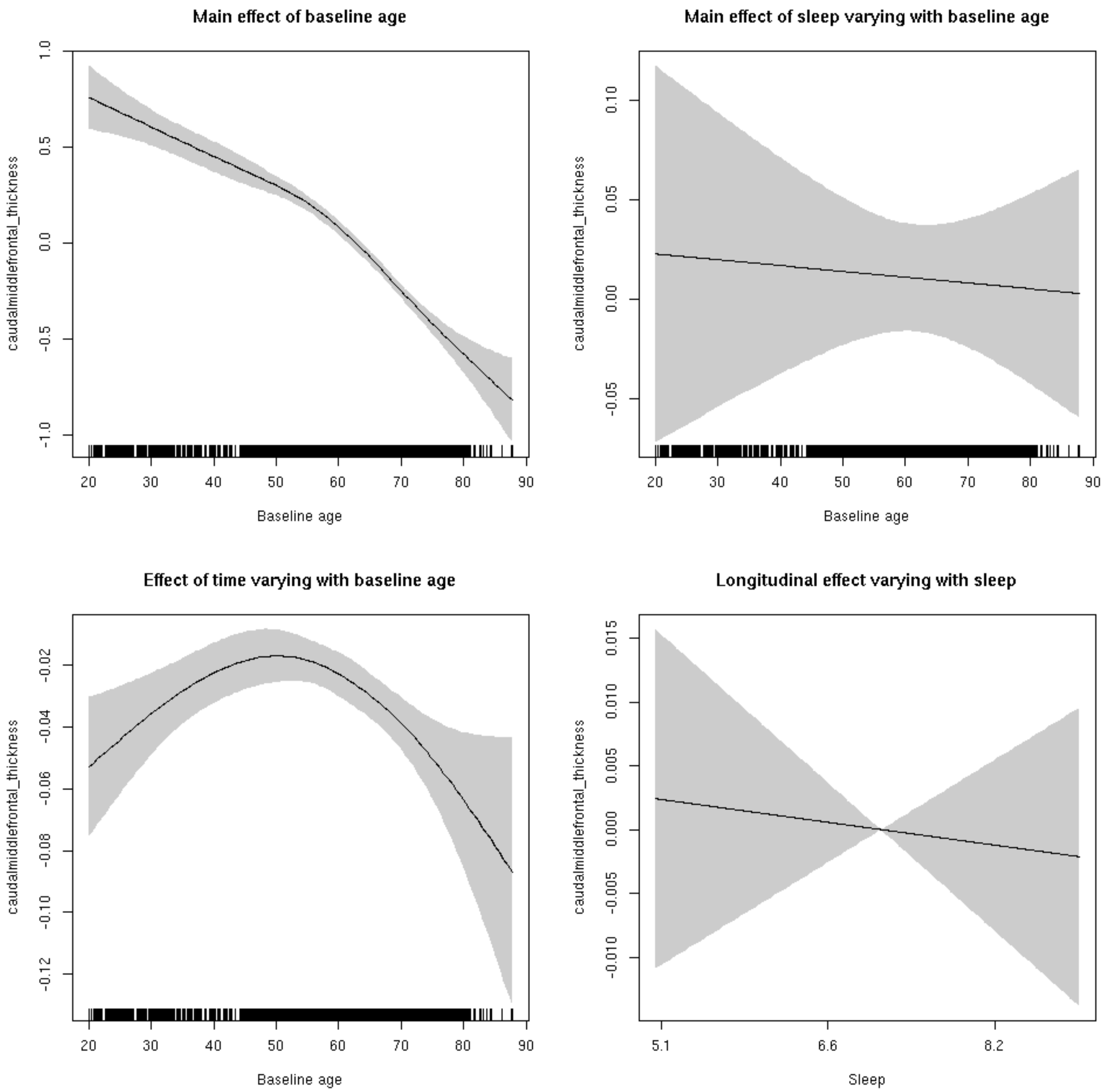

caudalmiddlefrontal_volume: Stratified by sex

Female: Full data Below is the row for `s(sleep_z):time` from the model output.

```
## s(sleep_z):time   1.000  1.000   0.015   0.903
```

Below are plots of a plot of `s(bl_age)`, `s(bl_age):sleep_z`, `s(bl_age):time`, and `s(sleep_z):time`. For `s(sleep_z):time`, roughly, if we cannot fit a straight line through the confidence bands, there is evidence that the effect of time depends on sleep duration. It is correct the that confidence interval is zero at one point along the axis; this happens because we do not include the main effect.

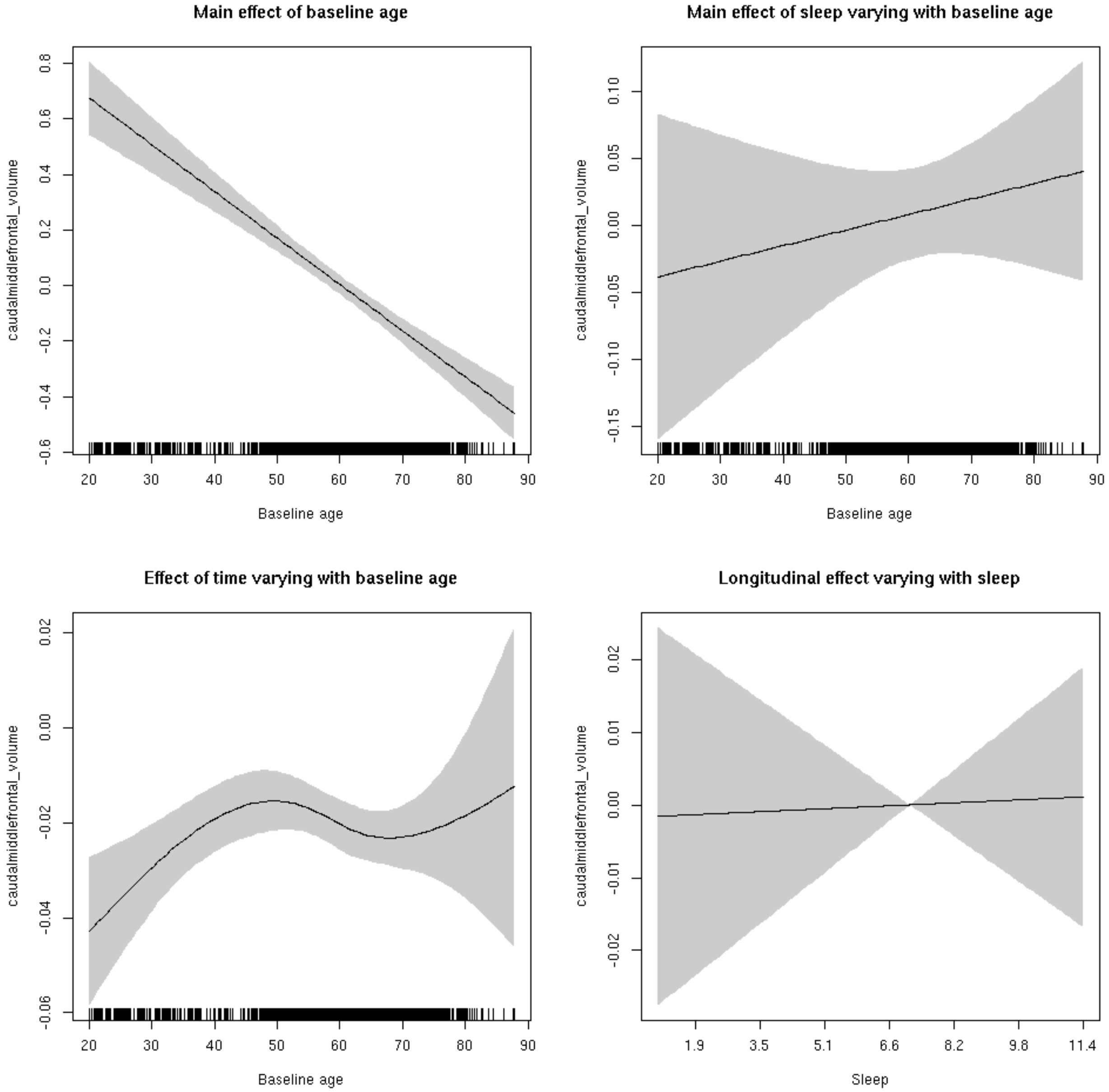

Male: Full data Below is the row for `s(sleep_z):time` from the model output.

```
## s(sleep_z):time   1.000  1.000  0.396  0.5290
```

Below are plots of a plot of `s(bl_age)`, `s(bl_age):sleep_z`, `s(bl_age):time`, and `s(sleep_z):time`. For `s(sleep_z):time`, roughly, if we cannot fit a straight line through the confidence bands, there is evidence that the effect of time depends on sleep duration. It is correct the that confidence interval is zero at one point along the axis; this happens because we do not include the main effect.

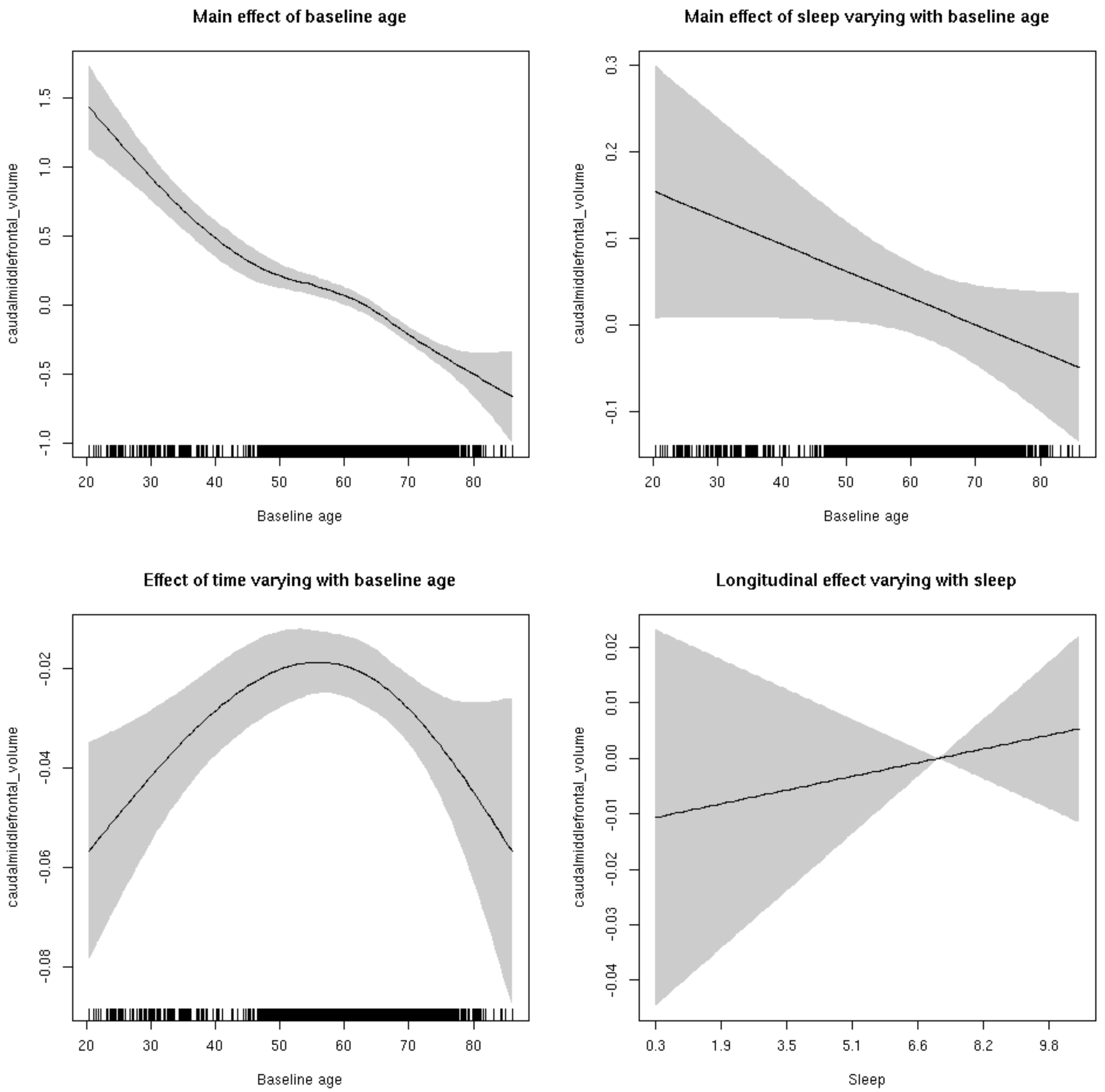

caudalmiddlefrontal_volume

Full data Below is the model output. The term `s(sleep_z):time` is probably what we care most about.

```
##
## Family: gaussian
## Link function: identity
##
## Formula:
## value ~ sex + site + icv + s(bl_age, k = 10, bs = "cr") + s(bl_age,
##     by = sleep_z, bs = "cr") + +s(bl_age, by = time, k = 5, bs = "cr") +
##     s(sleep_z, by = time, k = 5, bs = "cr", pc = 0)
## <environment: 0x5630035a36f8>
```

```
##
## Parametric coefficients:
##                 Estimate Std. Error t value Pr(>|t|)
## (Intercept)      0.30110    0.05904   5.100 3.48e-07 ***
## sexmale         -0.08521    0.03037  -2.806 0.005031 **
## siteousAvanto -0.89351    0.07206 -12.400  < 2e-16 ***
## siteousPrisma -0.57269    0.16475  -3.476 0.000511 ***
## siteousSkyra  -1.07789    0.06978 -15.447  < 2e-16 ***
## siteUB        -0.56646    0.13599  -4.166 3.14e-05 ***
## siteUCAM      -0.64851    0.07311  -8.871  < 2e-16 ***
## siteUKB       -0.09357    0.05597  -1.672 0.094601 .
## siteUmU       -0.26234    0.08425  -3.114 0.001852 **
## icv            0.58578    0.01493  39.223  < 2e-16 ***
## ---
## Signif. codes:  0 '***' 0.001 '**' 0.01 '*' 0.05 '.' 0.1 ' ' 1
##
## Approximate significance of smooth terms:
##                       edf Ref.df       F p-value
## s(bl_age)           2.724  2.724 116.214  <2e-16 ***
## s(bl_age):sleep_z 2.000  2.000   0.810   0.445
## s(bl_age):time    3.502  3.502  48.775  <2e-16 ***
## s(sleep_z):time   2.943  2.943   1.931   0.105
## ---
## Signif. codes:  0 '***' 0.001 '**' 0.01 '*' 0.05 '.' 0.1 ' ' 1
##
## R-sq.(adj) =  0.423
## lmer.REML = 9966.1  Scale est. = 0.036813  n = 8163
```

Below are plots of a plot of `s(bl_age)`, `s(bl_age):sleep_z`, `s(bl_age):time`, and `s(sleep_z):time`. For `s(sleep_z):time`, roughly, if we cannot fit a straight line through the confidence bands, there is evidence that the effect of time depends on sleep duration. It is correct the that confidence interval is zero at one point along the axis; this happens because we do not include the main effect.

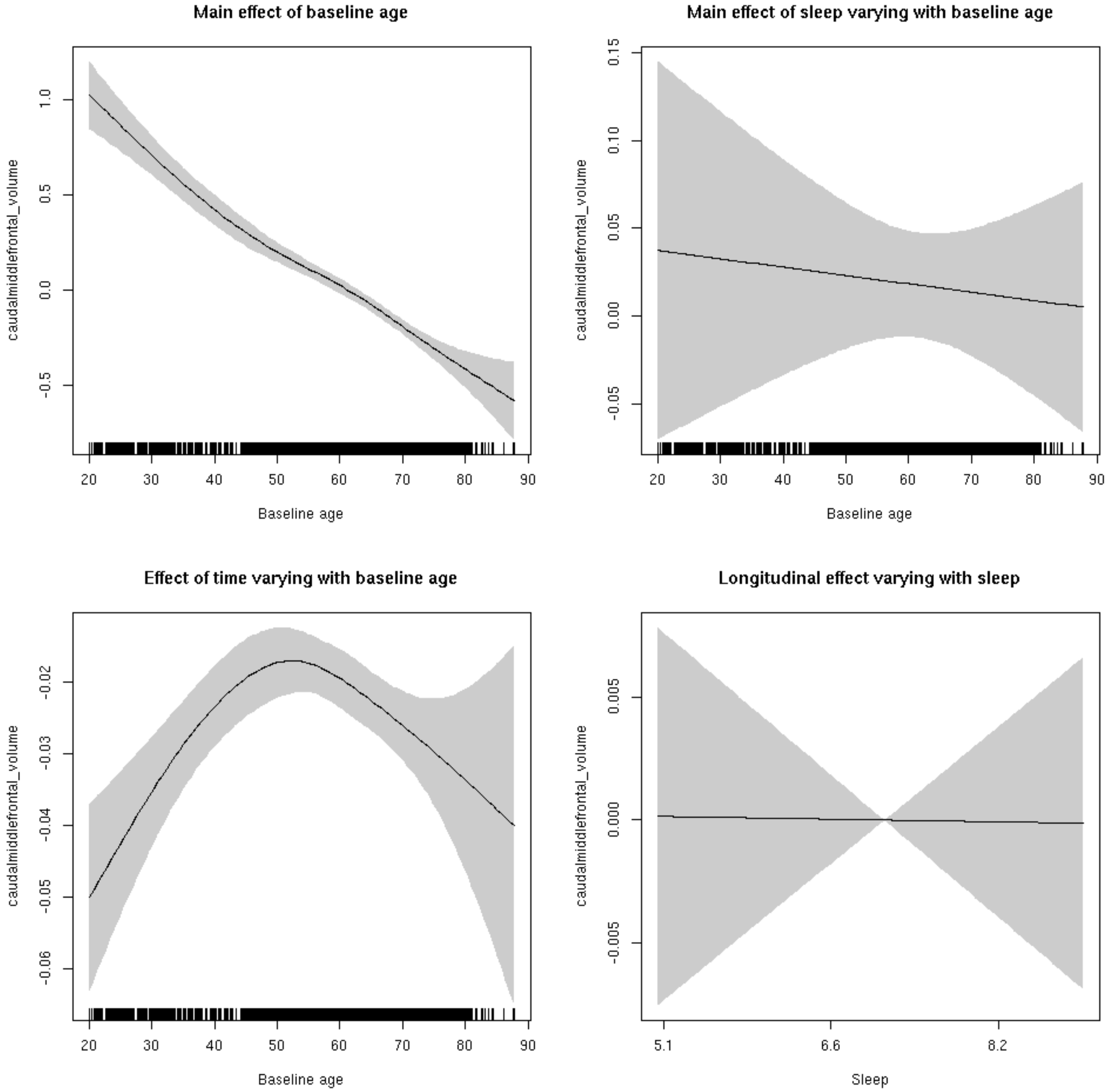

Restricted to sleep >= 5 and <= 9 hours Below is the row for `s(sleep_z):time` from the model output.

```
## s(sleep_z):time   1.000  1.000   0.001   0.969
```

Below are plots of a plot of `s(bl_age)`, `s(bl_age):sleep_z`, `s(bl_age):time`, and `s(sleep_z):time`. For `s(sleep_z):time`, roughly, if we cannot fit a straight line through the confidence bands, there is evidence that the effect of time depends on sleep duration. It is correct the that confidence interval is zero at one point along the axis; this happens because we do not include the main effect.

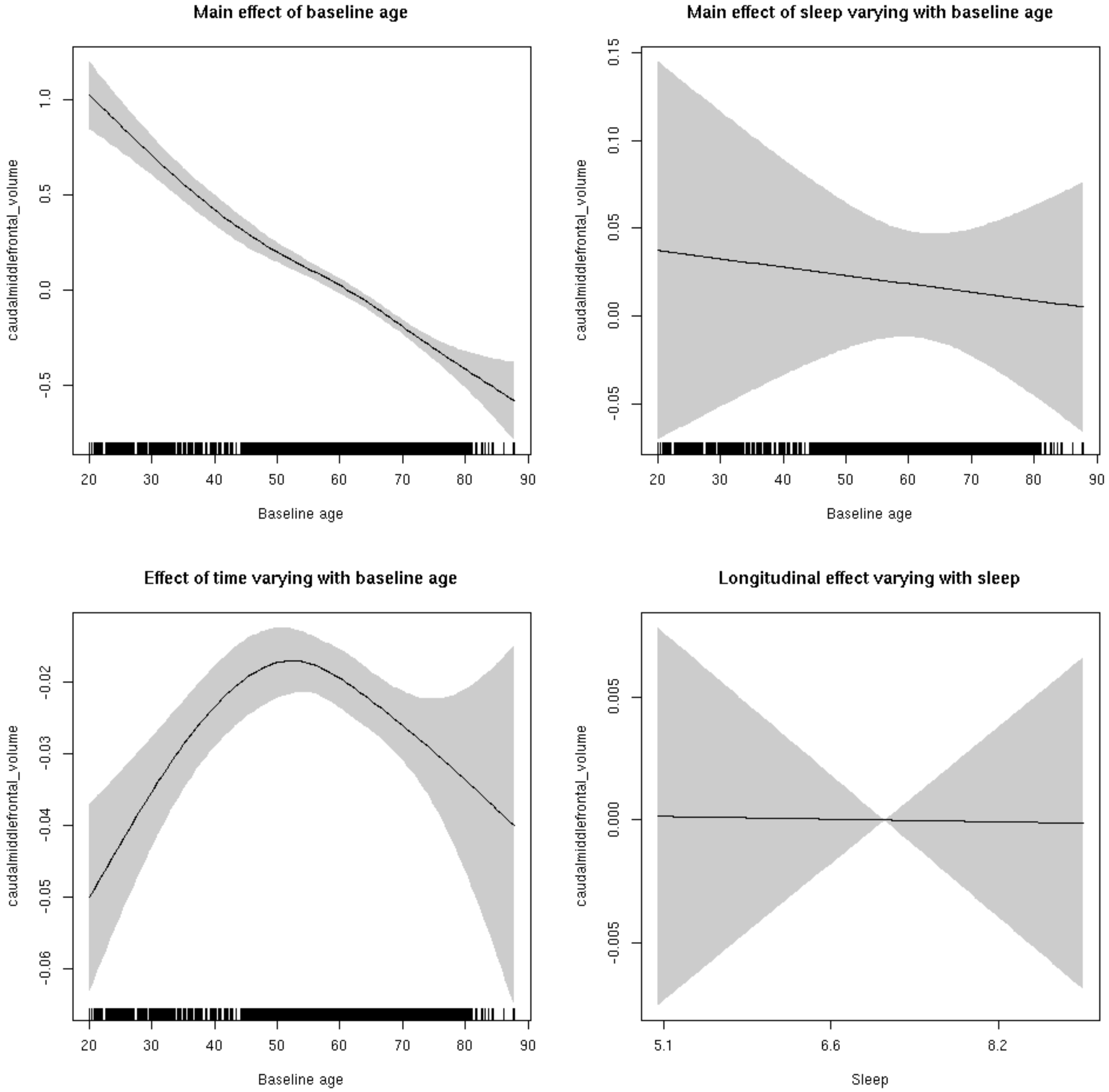

cuneus_area: Stratified by sex

Female: Full data Below is the row for `s(sleep_z):time` from the model output.

```
## s(sleep_z):time   1.000  1.000  0.965   0.326
```

Below are plots of a plot of `s(bl_age)`, `s(bl_age):sleep_z`, `s(bl_age):time`, and `s(sleep_z):time`. For `s(sleep_z):time`, roughly, if we cannot fit a straight line through the confidence bands, there is evidence that the effect of time depends on sleep duration. It is correct the that confidence interval is zero at one point along the axis; this happens because we do not include the main effect.

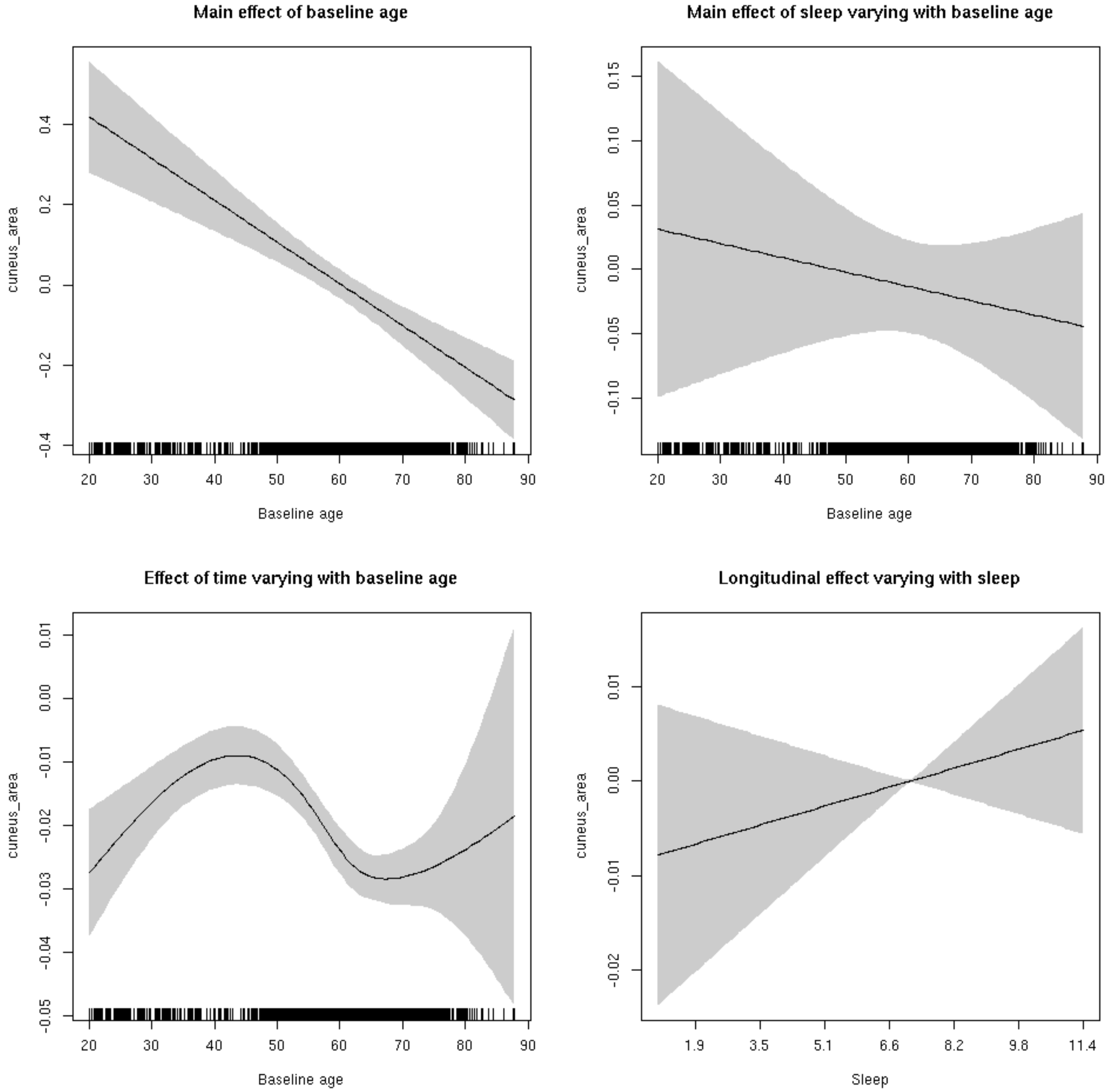

Male: Full data Below is the row for `s(sleep_z):time` from the model output.

```
## s(sleep_z):time   1.000  1.000   1.359   0.244
```

Below are plots of a plot of `s(bl_age)`, `s(bl_age):sleep_z`, `s(bl_age):time`, and `s(sleep_z):time`. For `s(sleep_z):time`, roughly, if we cannot fit a straight line through the confidence bands, there is evidence that the effect of time depends on sleep duration. It is correct the that confidence interval is zero at one point along the axis; this happens because we do not include the main effect.

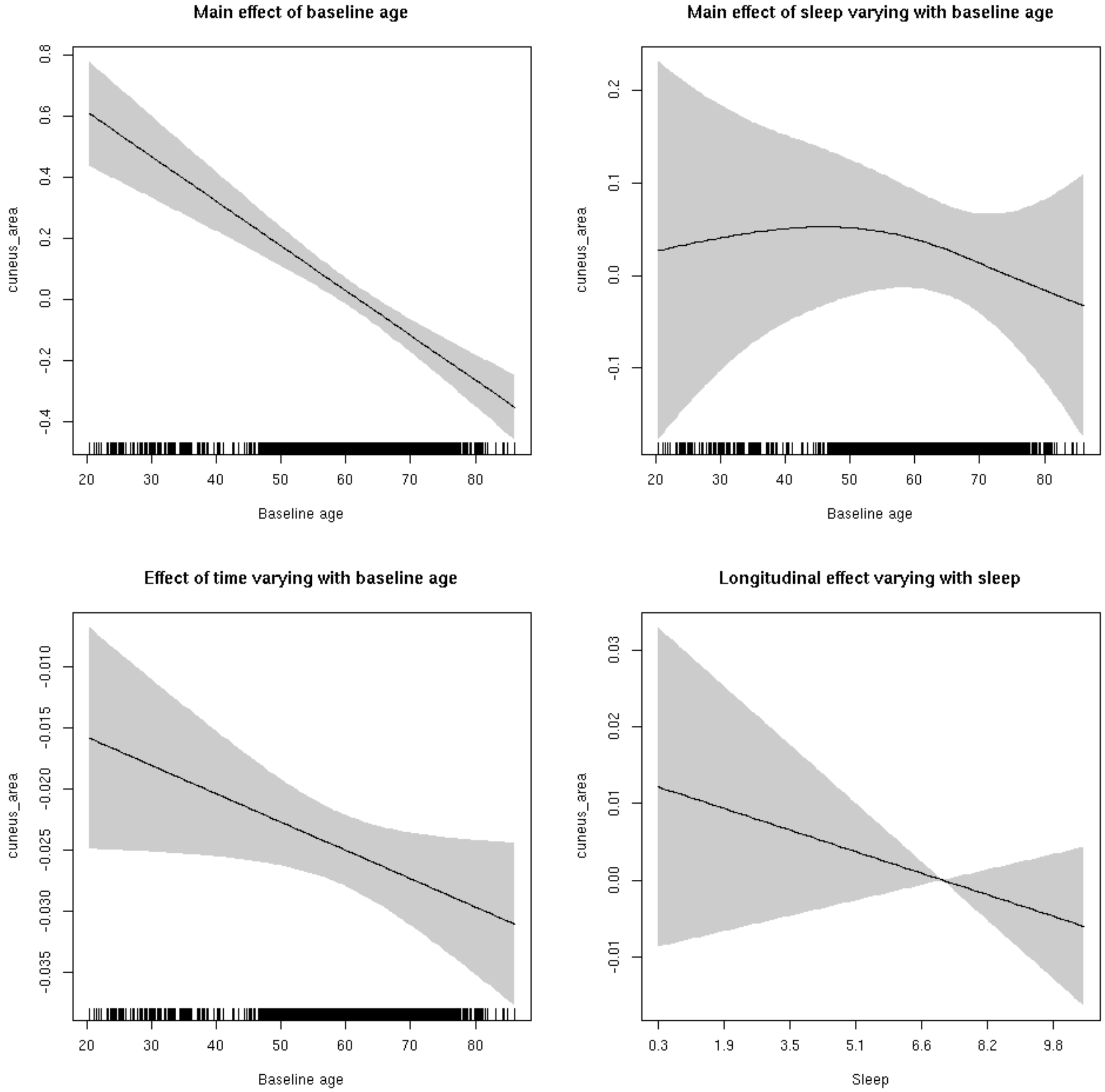

cuneus_area

Full data Below is the model output. The term `s(sleep_z):time` is probably what we care most about.

```
##
## Family: gaussian
## Link function: identity
##
## Formula:
## value ~ sex + site + icv + s(bl_age, k = 10, bs = "cr") + s(bl_age,
##     by = sleep_z, bs = "cr") + +s(bl_age, by = time, k = 5, bs = "cr") +
##     s(sleep_z, by = time, k = 5, bs = "cr", pc = 0)
## <environment: 0x5630035abbc8>
```

```
##
## Parametric coefficients:
##                Estimate Std. Error t value Pr(>|t|)
## (Intercept)   -0.02066    0.06401  -0.323  0.74686
## sexmale        0.38481    0.03325  11.573  < 2e-16 ***
## siteousAvanto -0.09491    0.07879  -1.205  0.22841
## siteousPrisma -0.31533    0.12237  -2.577  0.00999 **
## siteousSkyra   0.06836    0.07641   0.895  0.37102
## siteUB        -0.41388    0.15084  -2.744  0.00609 **
## siteUCAM      -0.15320    0.08021  -1.910  0.05617 .
## siteUKB       -0.10281    0.05986  -1.717  0.08594 .
## siteUmU       -0.24344    0.09277  -2.624  0.00870 **
## icv            0.33132    0.01594  20.789  < 2e-16 ***
## ---
## Signif. codes:  0 '***' 0.001 '**' 0.01 '*' 0.05 '.' 0.1 ' ' 1
##
## Approximate significance of smooth terms:
##                      edf Ref.df       F p-value
## s(bl_age)          1.000  1.000  92.307  <2e-16 ***
## s(bl_age):sleep_z 2.000  2.000   0.382   0.682
## s(bl_age):time    3.782  3.782 164.532  <2e-16 ***
## s(sleep_z):time   1.000  1.000   0.003   0.956
## ---
## Signif. codes:  0 '***' 0.001 '**' 0.01 '*' 0.05 '.' 0.1 ' ' 1
##
## R-sq.(adj) =  0.266
## lmer.REML = 6625.3  Scale est. = 0.013944  n = 8168
```

Below are plots of a plot of `s(bl_age)`, `s(bl_age):sleep_z`, `s(bl_age):time`, and `s(sleep_z):time`. For `s(sleep_z):time`, roughly, if we cannot fit a straight line through the confidence bands, there is evidence that the effect of time depends on sleep duration. It is correct the that confidence interval is zero at one point along the axis; this happens because we do not include the main effect.

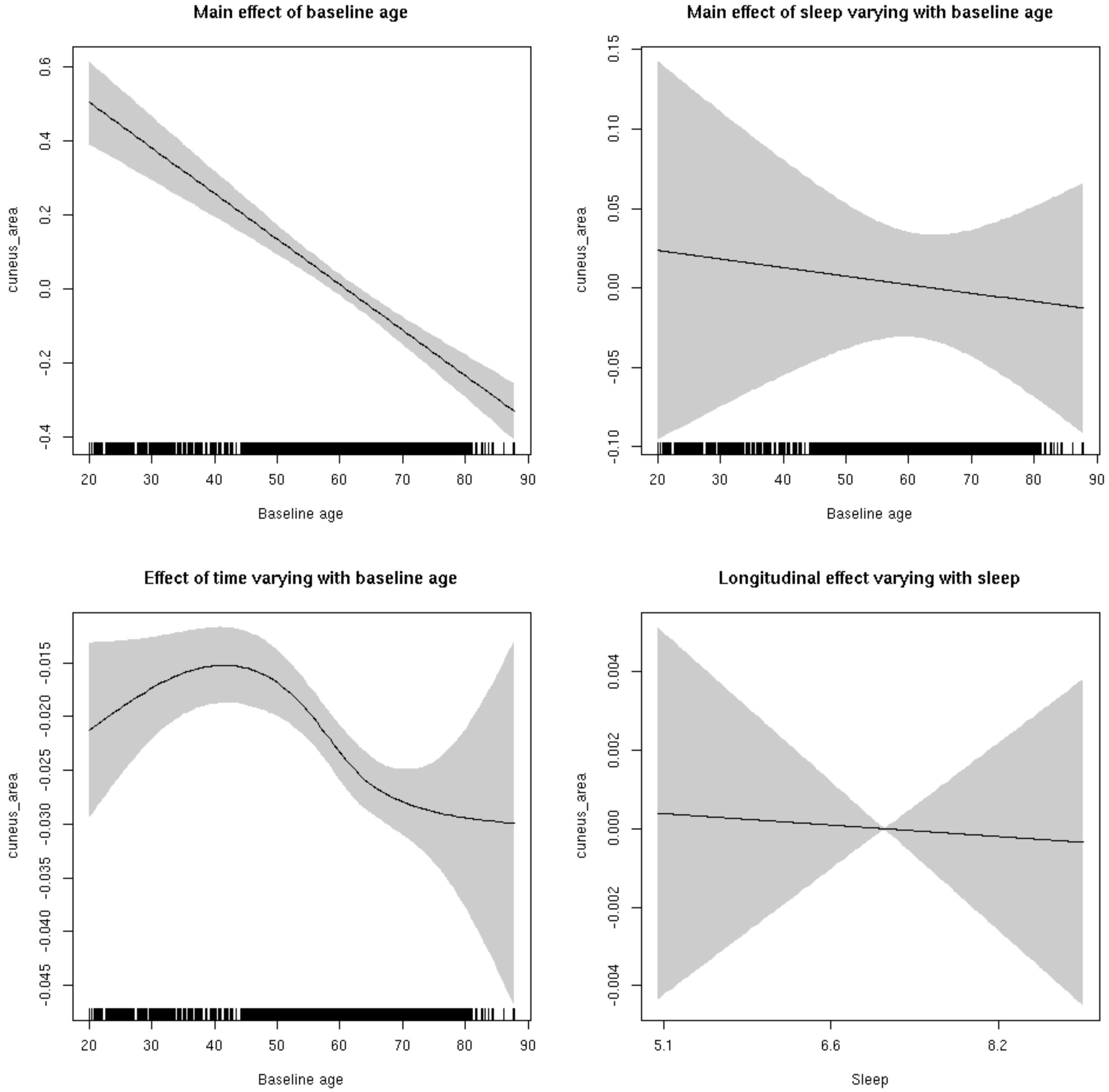

Restricted to sleep >= 5 and <= 9 hours Below is the row for `s(sleep_z):time` from the model output.

```
## s(sleep_z):time   1.000  1.000   0.028   0.867
```

Below are plots of a plot of `s(bl_age)`, `s(bl_age):sleep_z`, `s(bl_age):time`, and `s(sleep_z):time`. For `s(sleep_z):time`, roughly, if we cannot fit a straight line through the confidence bands, there is evidence that the effect of time depends on sleep duration. It is correct the that confidence interval is zero at one point along the axis; this happens because we do not include the main effect.

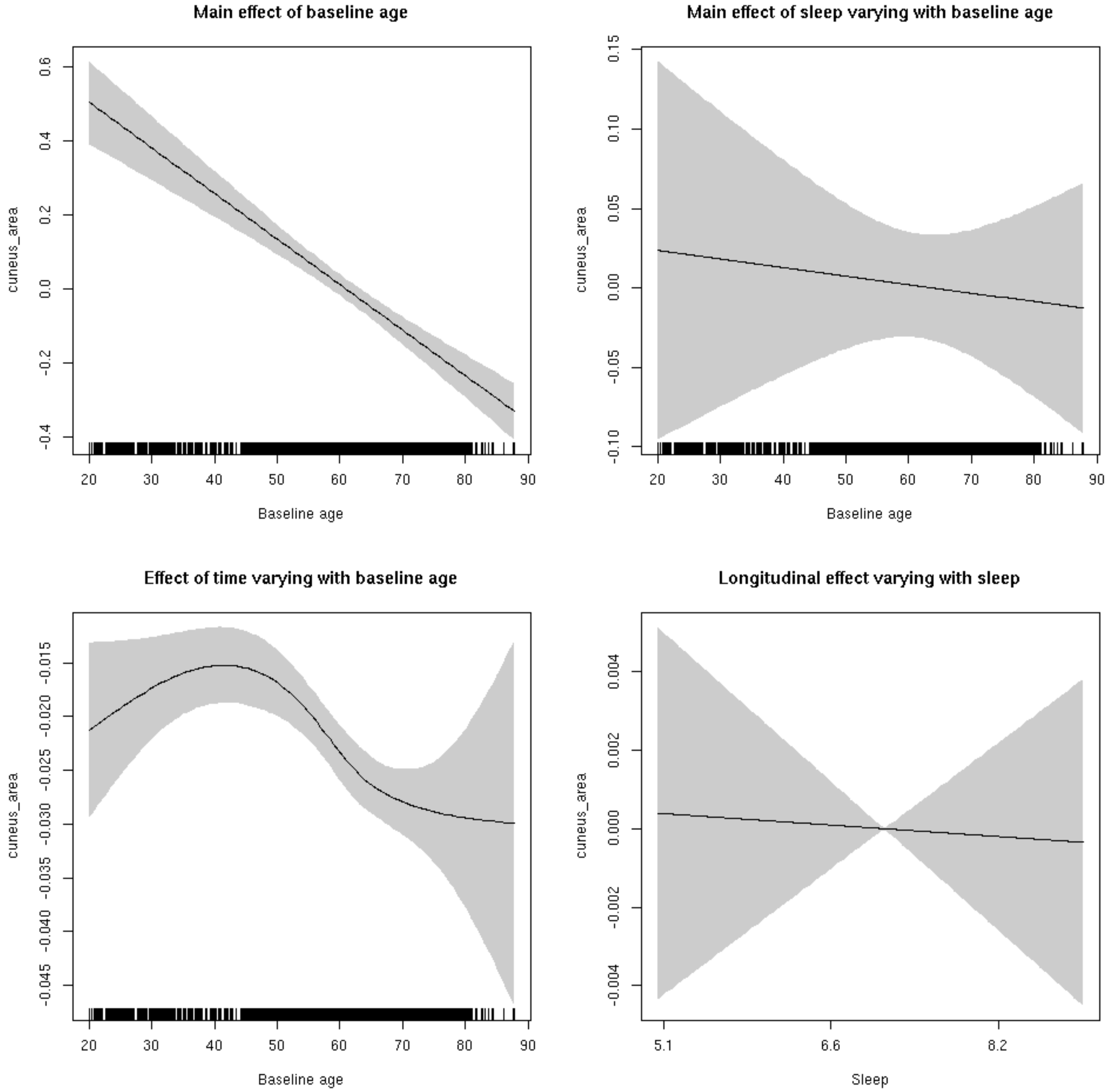

cuneus_thickness: Stratified by sex

Female: Full data Below is the row for `s(sleep_z):time` from the model output.

```
## s(sleep_z):time   1.000  1.000  0.010  0.9213
```

Below are plots of a plot of `s(bl_age)`, `s(bl_age):sleep_z`, `s(bl_age):time`, and `s(sleep_z):time`. For `s(sleep_z):time`, roughly, if we cannot fit a straight line through the confidence bands, there is evidence that the effect of time depends on sleep duration. It is correct the that confidence interval is zero at one point along the axis; this happens because we do not include the main effect.

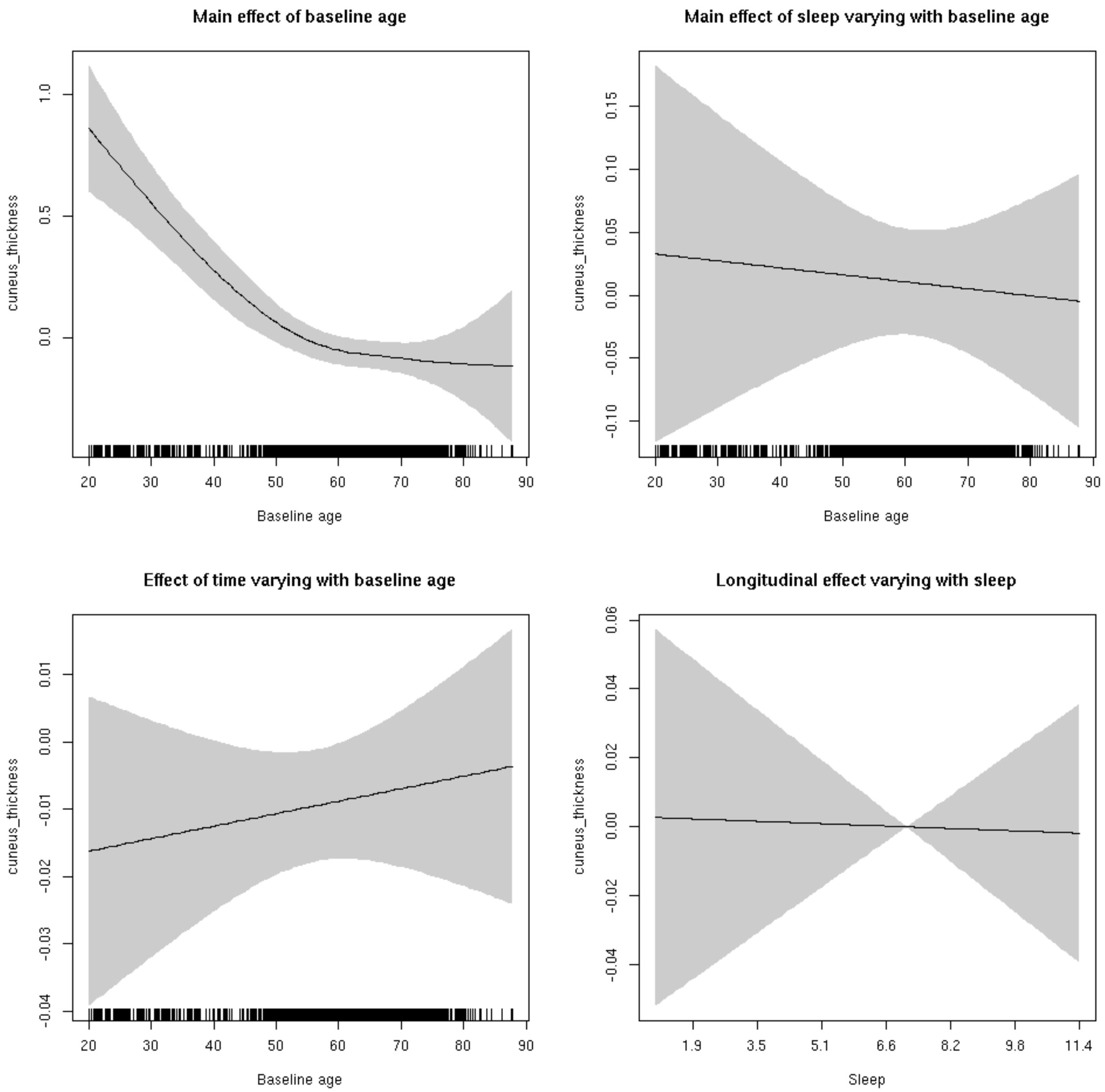

Male: Full data Below is the row for `s(sleep_z):time` from the model output.

```
## s(sleep_z):time   1.000  1.000 0.365 0.545820
```

Below are plots of a plot of `s(bl_age)`, `s(bl_age):sleep_z`, `s(bl_age):time`, and `s(sleep_z):time`. For `s(sleep_z):time`, roughly, if we cannot fit a straight line through the confidence bands, there is evidence that the effect of time depends on sleep duration. It is correct the that confidence interval is zero at one point along the axis; this happens because we do not include the main effect.

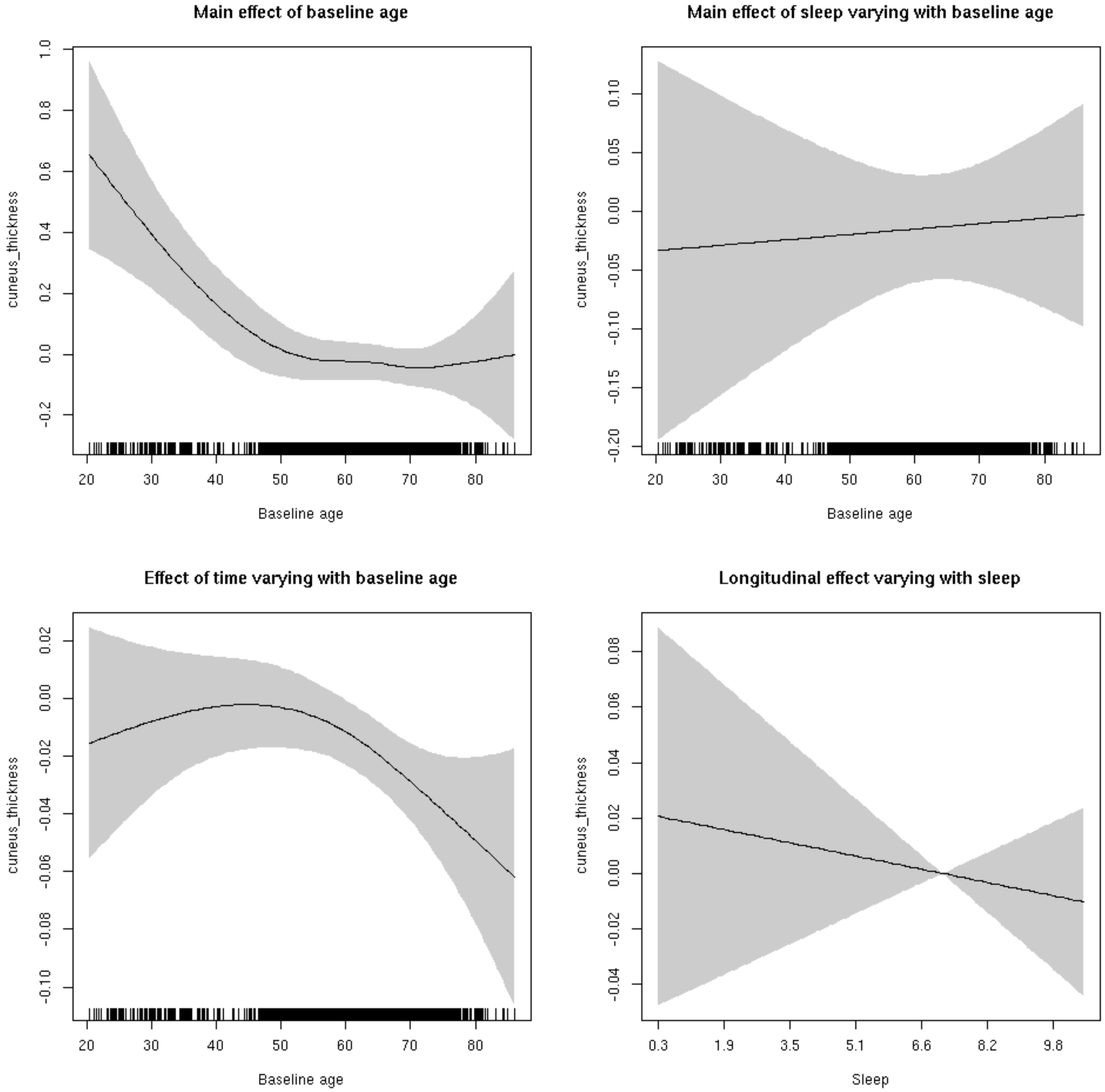

cuneus_thickness

Full data Below is the model output. The term `s(sleep_z):time` is probably what we care most about.

```
##
## Family: gaussian
## Link function: identity
##
## Formula:
## value ~ sex + site + s(bl_age, k = 10, bs = "cr") + s(bl_age,
##     by = sleep_z, bs = "cr") + +s(bl_age, by = time, k = 5, bs = "cr") +
##     s(sleep_z, by = time, k = 5, bs = "cr", pc = 0)
## <environment: 0x5630035cc6c8>
```

```
##
## Parametric coefficients:
##                Estimate Std. Error t value Pr(>|t|)
## (Intercept)   -0.74588    0.05778 -12.910  < 2e-16 ***
## sexmale        0.19011    0.02900   6.556 5.88e-11 ***
## siteousAvanto -0.33566    0.07659  -4.383 1.19e-05 ***
## siteousPrisma  0.60847    0.30919   1.968  0.04911 *
## siteousSkyra   1.06897    0.07655  13.965  < 2e-16 ***
## siteUB         0.42902    0.15380   2.790  0.00529 **
## siteUCAM       0.22123    0.07946   2.784  0.00538 **
## siteUKB        0.72547    0.05853  12.395  < 2e-16 ***
## siteUmU        1.17851    0.09533  12.362  < 2e-16 ***
## ---
## Signif. codes:  0 '***' 0.001 '**' 0.01 '*' 0.05 '.' 0.1 ' ' 1
##
## Approximate significance of smooth terms:
##                      edf Ref.df      F p-value
## s(bl_age)          3.372  3.372 23.615 < 2e-16 ***
## s(bl_age):sleep_z 2.000  2.000  0.010   0.990
## s(bl_age):time    3.046  3.046  7.941 2.3e-05 ***
## s(sleep_z):time   1.000  1.000  0.129   0.719
## ---
## Signif. codes:  0 '***' 0.001 '**' 0.01 '*' 0.05 '.' 0.1 ' ' 1
##
## R-sq.(adj) =  0.138
## lmer.REML =  17386  Scale est. = 0.16139   n = 8176
```

Below are plots of a plot of `s(bl_age)`, `s(bl_age):sleep_z`, `s(bl_age):time`, and `s(sleep_z):time`. For `s(sleep_z):time`, roughly, if we cannot fit a straight line through the confidence bands, there is evidence that the effect of time depends on sleep duration. It is correct the that confidence interval is zero at one point along the axis; this happens because we do not include the main effect.

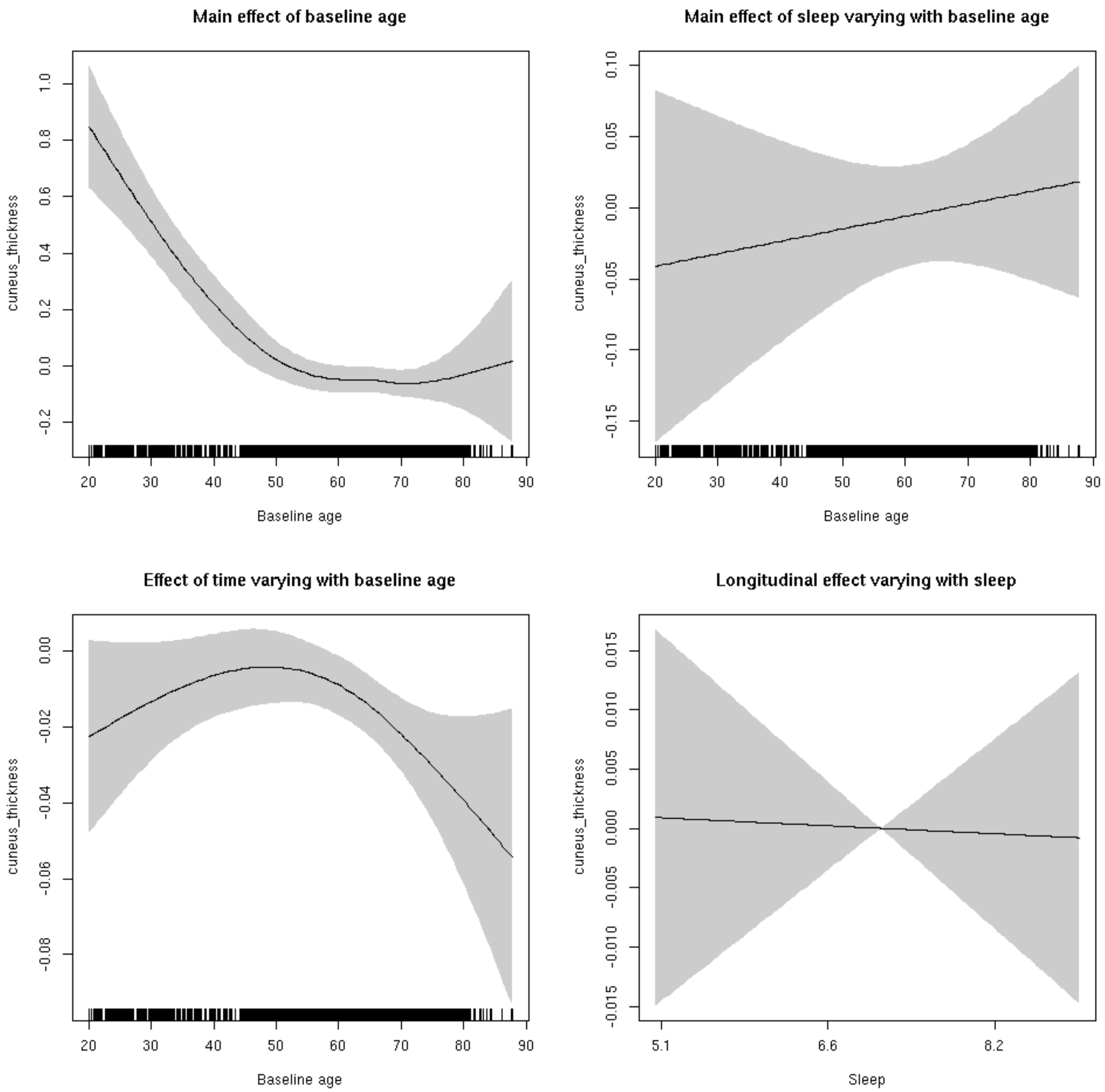

Restricted to sleep >= 5 and <= 9 hours Below is the row for `s(sleep_z):time` from the model output.

```
## s(sleep_z):time   1.000  1.000  0.013    0.909
```

Below are plots of a plot of `s(bl_age)`, `s(bl_age):sleep_z`, `s(bl_age):time`, and `s(sleep_z):time`. For `s(sleep_z):time`, roughly, if we cannot fit a straight line through the confidence bands, there is evidence that the effect of time depends on sleep duration. It is correct the that confidence interval is zero at one point along the axis; this happens because we do not include the main effect.

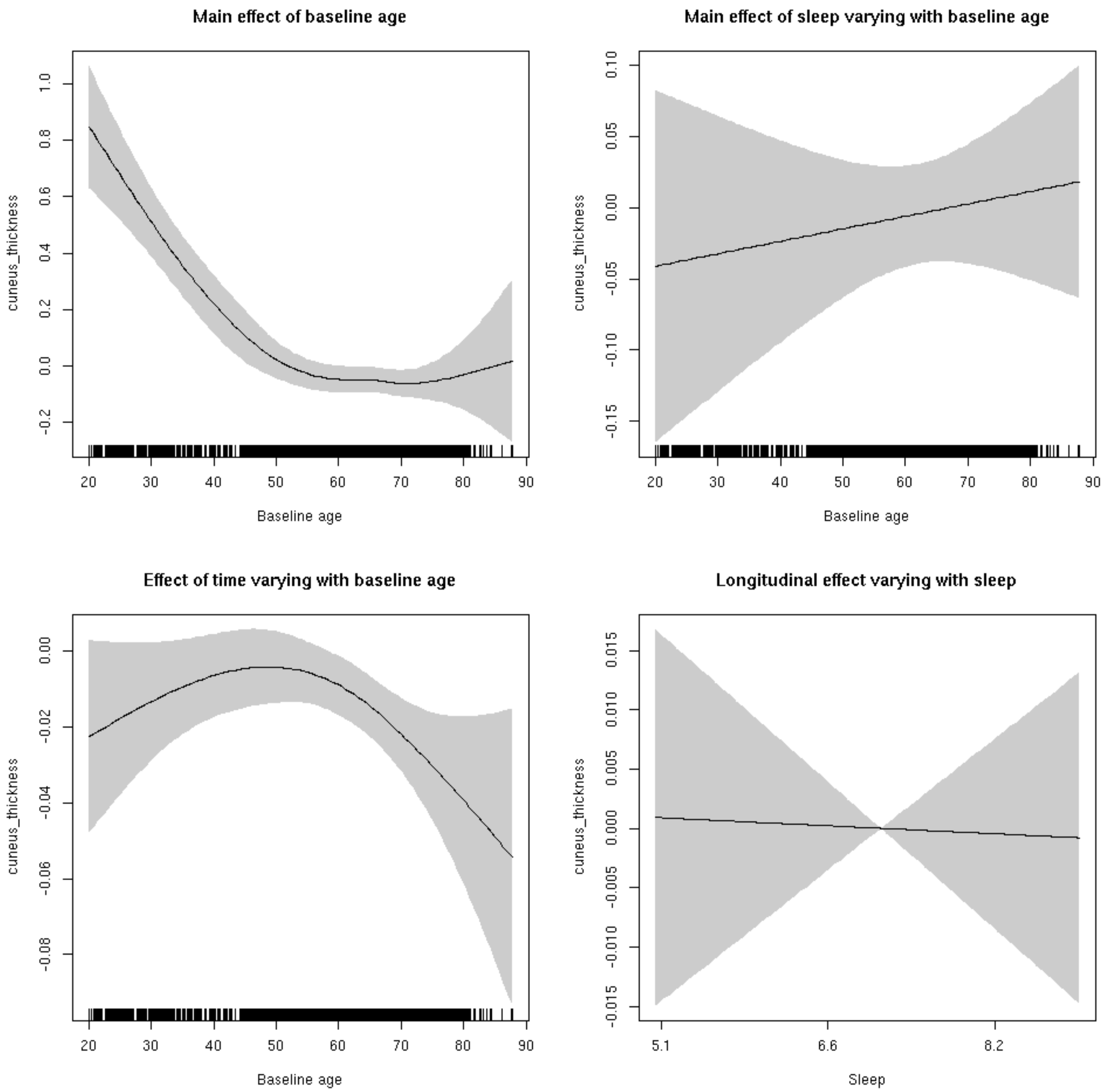

cuneus_volume: Stratified by sex

Female: Full data Below is the row for `s(sleep_z):time` from the model output.

```
## s(sleep_z):time   1.000  1.000  1.295   0.255
```

Below are plots of a plot of `s(bl_age)`, `s(bl_age):sleep_z`, `s(bl_age):time`, and `s(sleep_z):time`. For `s(sleep_z):time`, roughly, if we cannot fit a straight line through the confidence bands, there is evidence that the effect of time depends on sleep duration. It is correct the that confidence interval is zero at one point along the axis; this happens because we do not include the main effect.

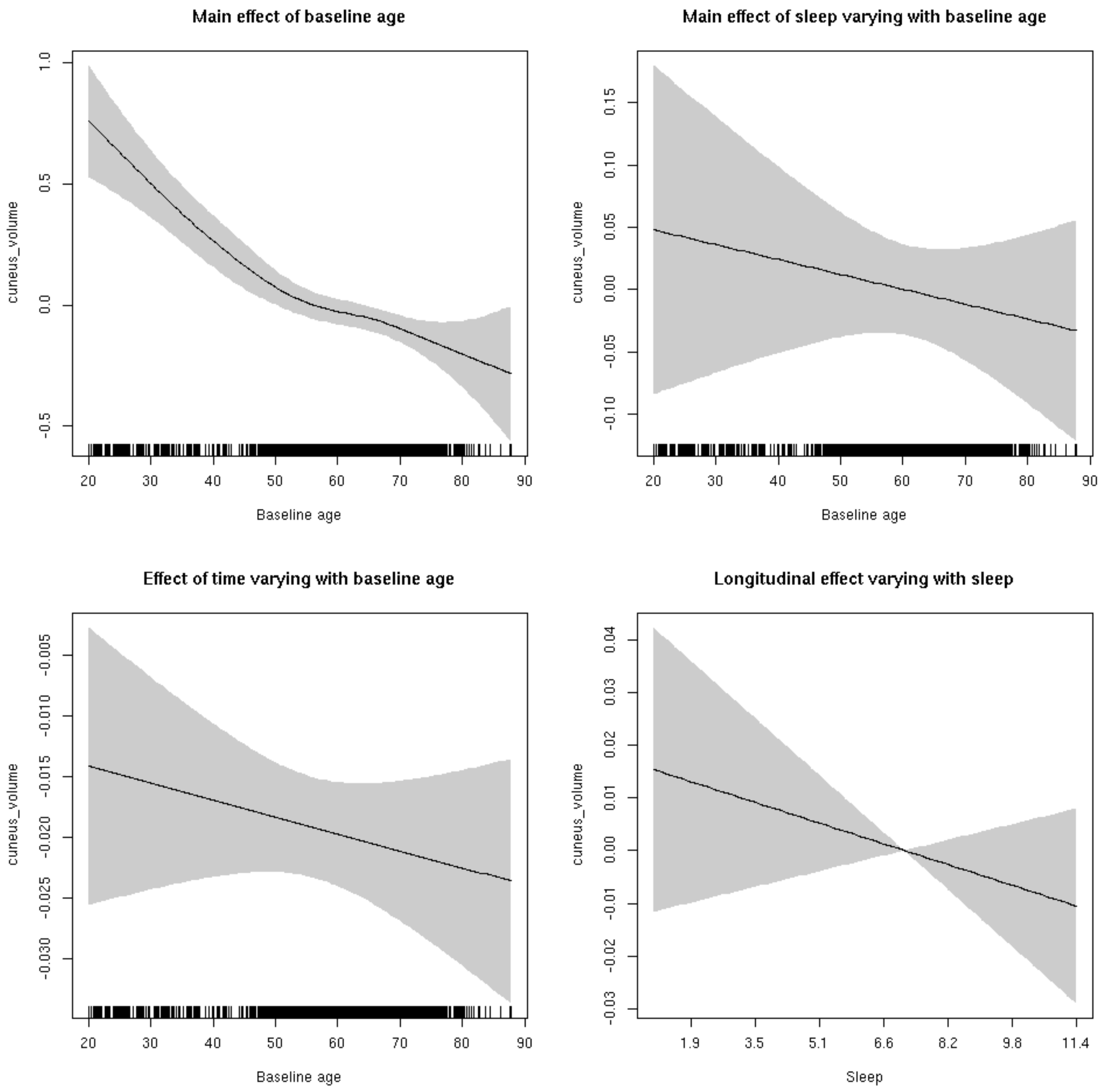

Male: Full data Below is the row for `s(sleep_z):time` from the model output.

```
## s(sleep_z):time   1.000  1.000  0.440   0.507
```

Below are plots of a plot of `s(bl_age)`, `s(bl_age):sleep_z`, `s(bl_age):time`, and `s(sleep_z):time`. For `s(sleep_z):time`, roughly, if we cannot fit a straight line through the confidence bands, there is evidence that the effect of time depends on sleep duration. It is correct the that confidence interval is zero at one point along the axis; this happens because we do not include the main effect.

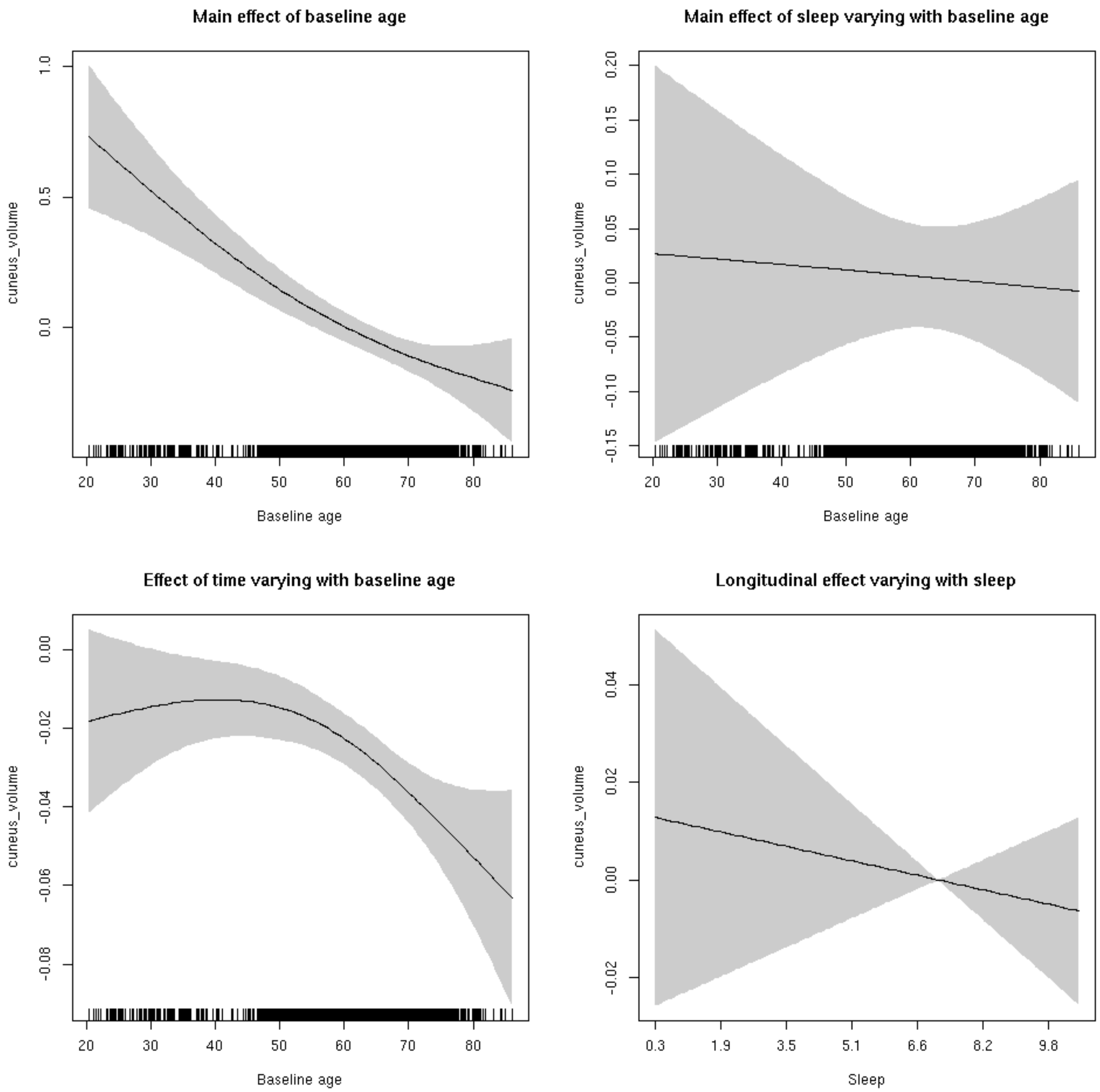

cuneus_volume

Full data Below is the model output. The term `s(sleep_z):time` is probably what we care most about.

```
##
## Family: gaussian
## Link function: identity
##
## Formula:
## value ~ sex + site + icv + s(bl_age, k = 10, bs = "cr") + s(bl_age,
##     by = sleep_z, bs = "cr") + +s(bl_age, by = time, k = 5, bs = "cr") +
##     s(sleep_z, by = time, k = 5, bs = "cr", pc = 0)
## <environment: 0x56300359ed80>
```

```
##
## Parametric coefficients:
##                Estimate Std. Error t value Pr(>|t|)
## (Intercept)   -0.125355   0.066544  -1.884   0.0596 .
## sexmale        0.384016   0.034206  11.227  < 2e-16 ***
## siteousAvanto -0.352107   0.081222  -4.335 1.47e-05 ***
## siteousPrisma  0.084941   0.181826   0.467   0.6404
## siteousSkyra   0.688462   0.078632   8.755  < 2e-16 ***
## siteUB        -0.104731   0.153440  -0.683   0.4949
## siteUCAM      -0.035666   0.082453  -0.433   0.6653
## siteUKB       -0.003912   0.063118  -0.062   0.9506
## siteUmU        0.524173   0.095037   5.515 3.59e-08 ***
## icv            0.308719   0.016802  18.374  < 2e-16 ***
## ---
## Signif. codes:  0 '***' 0.001 '**' 0.01 '*' 0.05 '.' 0.1 ' ' 1
##
## Approximate significance of smooth terms:
##                     edf Ref.df      F p-value
## s(bl_age)         2.732  2.732 39.541  <2e-16 ***
## s(bl_age):sleep_z 2.000  2.000  0.254   0.776
## s(bl_age):time    3.213  3.213 61.628  <2e-16 ***
## s(sleep_z):time   1.000  1.000  1.374   0.241
## ---
## Signif. codes:  0 '***' 0.001 '**' 0.01 '*' 0.05 '.' 0.1 ' ' 1
##
## R-sq.(adj) =  0.266
## lmer.REML =  11703  Scale est. = 0.044299  n = 8176
```

Below are plots of a plot of `s(bl_age)`, `s(bl_age):sleep_z`, `s(bl_age):time`, and `s(sleep_z):time`. For `s(sleep_z):time`, roughly, if we cannot fit a straight line through the confidence bands, there is evidence that the effect of time depends on sleep duration. It is correct the that confidence interval is zero at one point along the axis; this happens because we do not include the main effect.

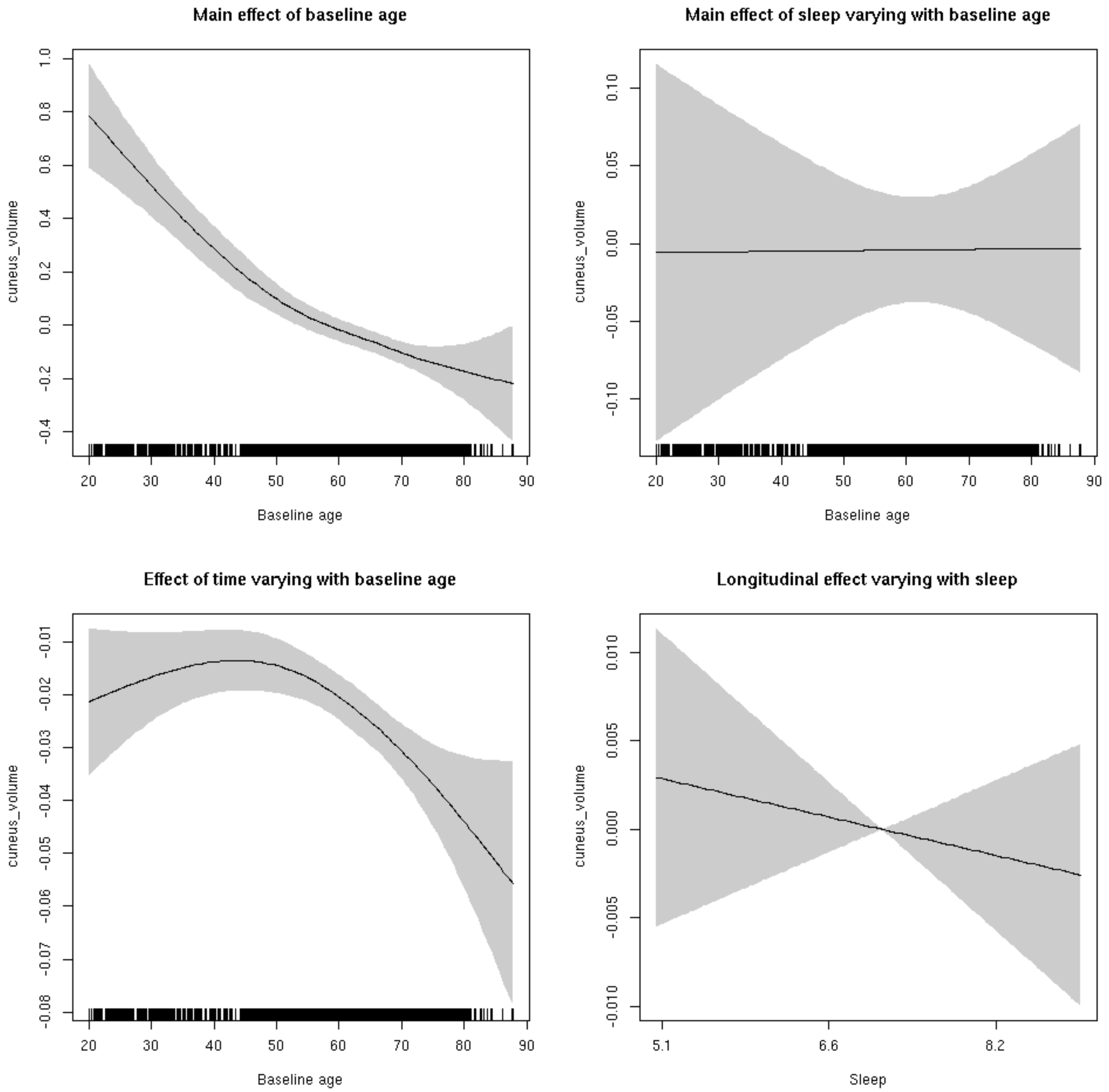

Restricted to sleep >= 5 and <= 9 hours Below is the row for `s(sleep_z):time` from the model output.

```
## s(sleep_z):time   1.000  1.000  0.484   0.487
```

Below are plots of a plot of `s(bl_age)`, `s(bl_age):sleep_z`, `s(bl_age):time`, and `s(sleep_z):time`. For `s(sleep_z):time`, roughly, if we cannot fit a straight line through the confidence bands, there is evidence that the effect of time depends on sleep duration. It is correct the that confidence interval is zero at one point along the axis; this happens because we do not include the main effect.

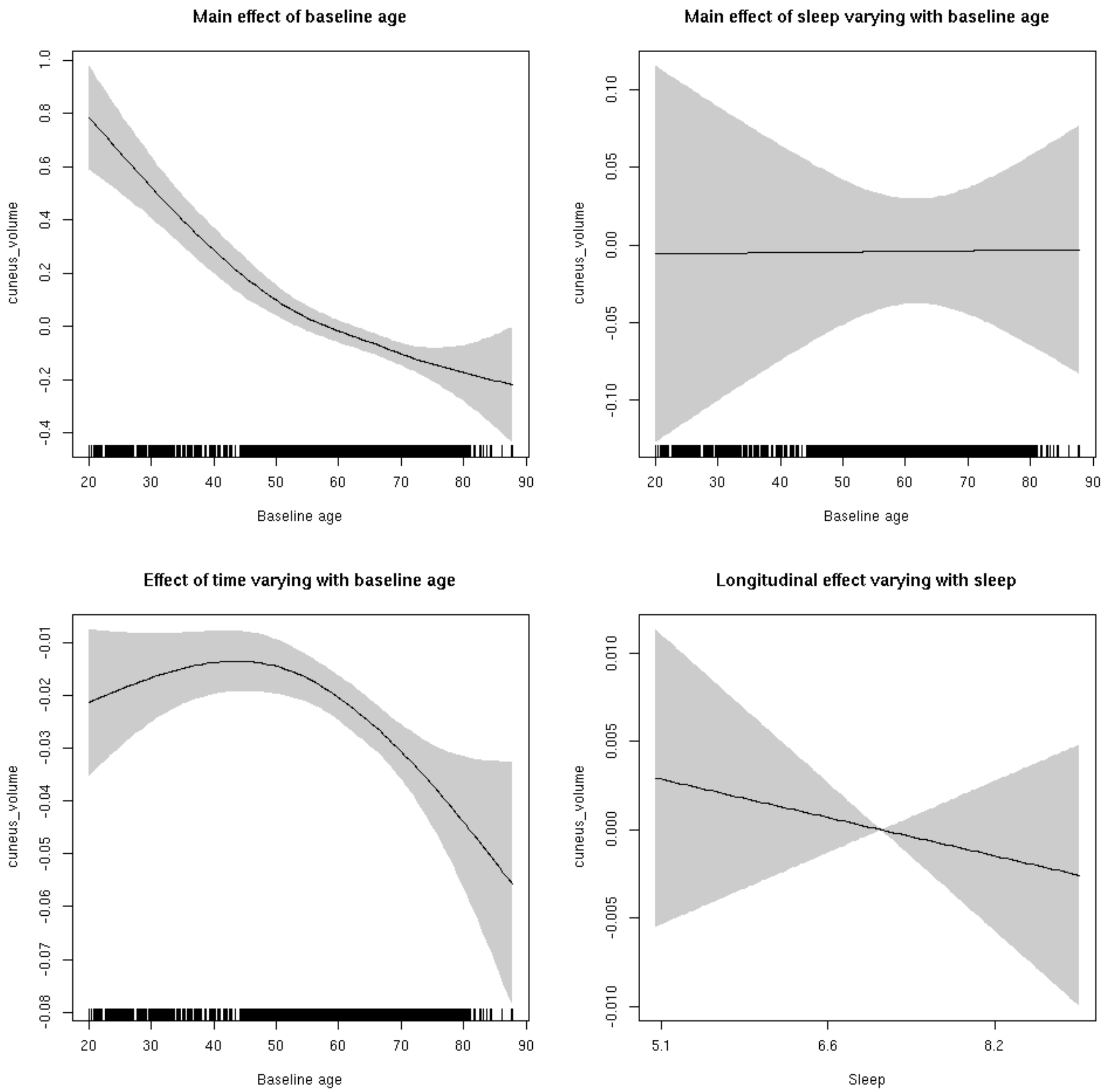

entorhinal_area: Stratified by sex

Female: Full data Below is the row for `s(sleep_z):time` from the model output.

```
## s(sleep_z):time     1       1  0.098    0.754
```

Below are plots of a plot of `s(bl_age)`, `s(bl_age):sleep_z`, `s(bl_age):time`, and `s(sleep_z):time`. For `s(sleep_z):time`, roughly, if we cannot fit a straight line through the confidence bands, there is evidence that the effect of time depends on sleep duration. It is correct the that confidence interval is zero at one point along the axis; this happens because we do not include the main effect.

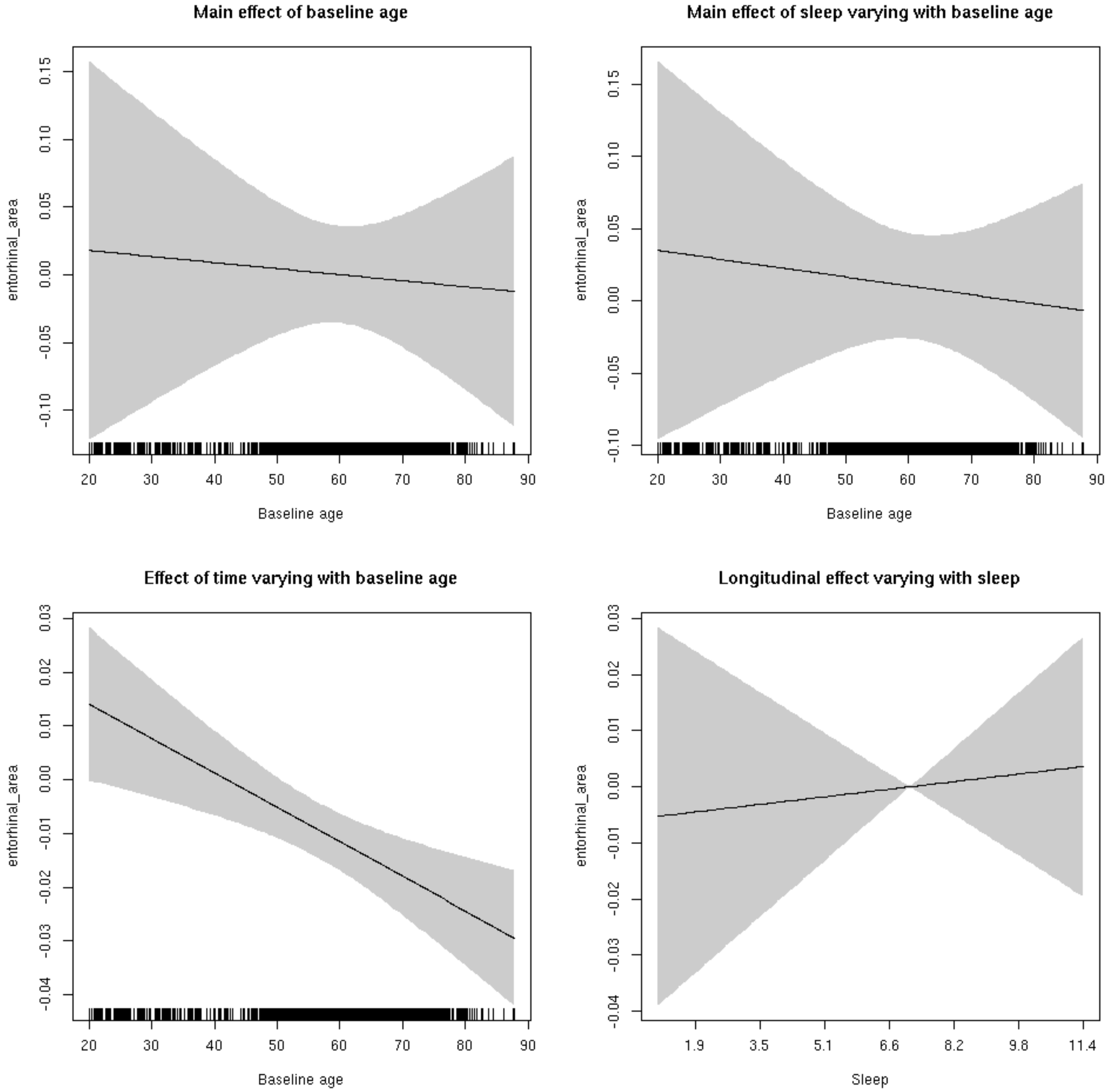

Male: Full data Below is the row for `s(sleep_z):time` from the model output.

```
## s(sleep_z):time     1      1 0.020  0.8863
```

Below are plots of a plot of `s(bl_age)`, `s(bl_age):sleep_z`, `s(bl_age):time`, and `s(sleep_z):time`. For `s(sleep_z):time`, roughly, if we cannot fit a straight line through the confidence bands, there is evidence that the effect of time depends on sleep duration. It is correct the that confidence interval is zero at one point along the axis; this happens because we do not include the main effect.

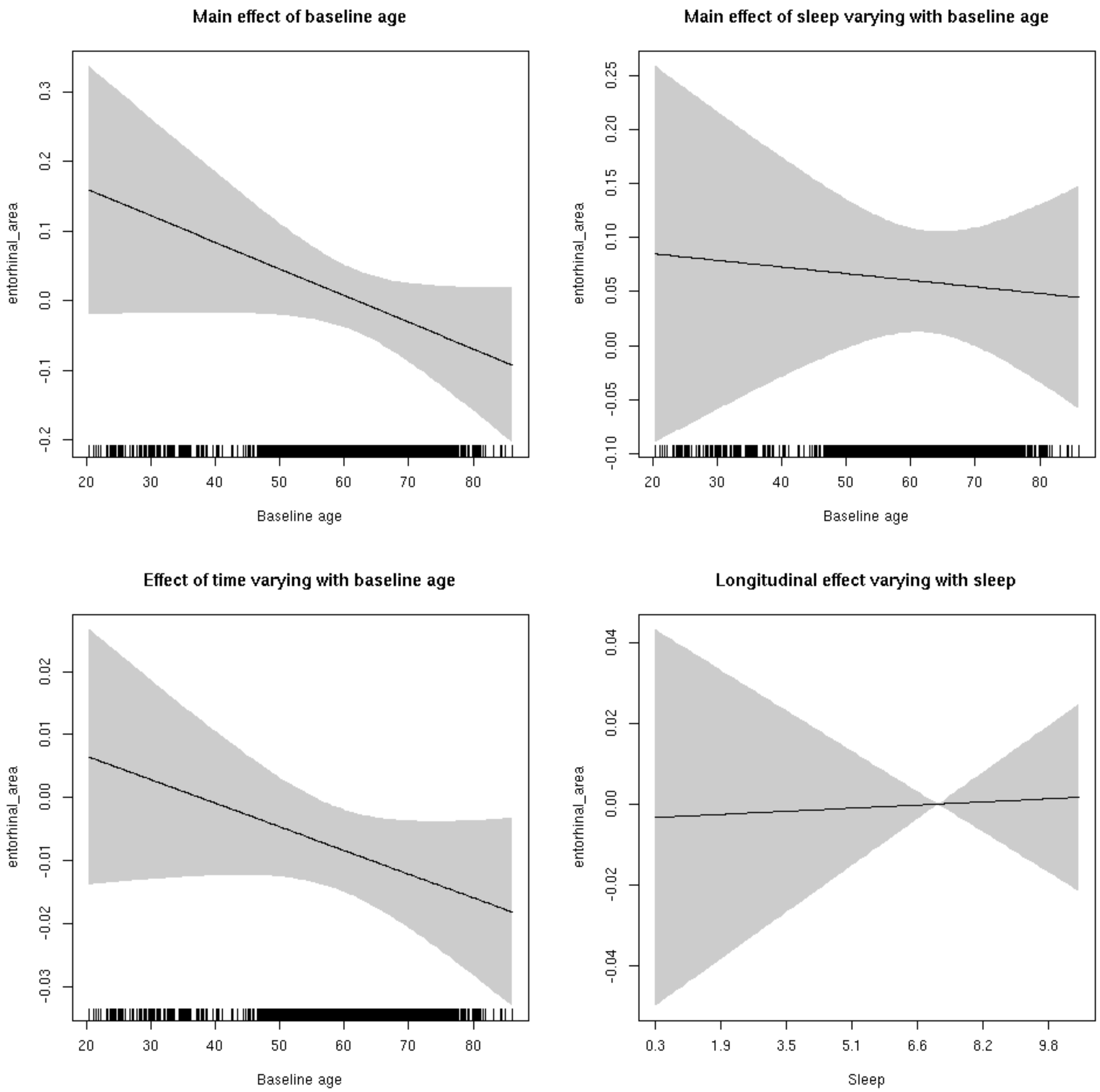

entorhinal_area

Full data Below is the model output. The term `s(sleep_z):time` is probably what we care most about.

```
##
## Family: gaussian
## Link function: identity
##
## Formula:
## value ~ sex + site + icv + s(bl_age, k = 10, bs = "cr") + s(bl_age,
##     by = sleep_z, bs = "cr") + +s(bl_age, by = time, k = 5, bs = "cr") +
##     s(sleep_z, by = time, k = 5, bs = "cr", pc = 0)
## <environment: 0x5630035aedb8>
```

```
##
## Parametric coefficients:
##                Estimate Std. Error t value Pr(>|t|)
## (Intercept)     0.34342    0.06622   5.186 2.20e-07 ***
## sexmale         0.17789    0.03439   5.173 2.36e-07 ***
## siteousAvanto -0.44460    0.08184  -5.433 5.71e-08 ***
## siteousPrisma -1.05135    0.21528  -4.884 1.06e-06 ***
## siteousSkyra  -0.61598    0.07914  -7.783 7.93e-15 ***
## siteUB        -0.67471    0.15356  -4.394 1.13e-05 ***
## siteUCAM      -0.56238    0.08237  -6.828 9.24e-12 ***
## siteUKB       -0.41371    0.06166  -6.710 2.08e-11 ***
## siteUmU       -0.81148    0.09498  -8.544  < 2e-16 ***
## icv            0.35021    0.01696  20.655  < 2e-16 ***
## ---
## Signif. codes:  0 ‘***’ 0.001 ‘**’ 0.01 ‘*’ 0.05 ‘.’ 0.1 ‘ ’ 1
##
## Approximate significance of smooth terms:
##                    edf Ref.df      F  p-value
## s(bl_age)            1      1  2.155    0.142
## s(bl_age):sleep_z    2      2  2.263    0.104
## s(bl_age):time       2      2 15.187 6.63e-07 ***
## s(sleep_z):time      1      1  0.140    0.708
## ---
## Signif. codes:  0 ‘***’ 0.001 ‘**’ 0.01 ‘*’ 0.05 ‘.’ 0.1 ‘ ’ 1
##
## R-sq.(adj) =  0.187
## lmer.REML =  13534  Scale est. = 0.067556  n = 8176
```

Below are plots of a plot of `s(bl_age)`, `s(bl_age):sleep_z`, `s(bl_age):time`, and `s(sleep_z):time`. For `s(sleep_z):time`, roughly, if we cannot fit a straight line through the confidence bands, there is evidence that the effect of time depends on sleep duration. It is correct the that confidence interval is zero at one point along the axis; this happens because we do not include the main effect.

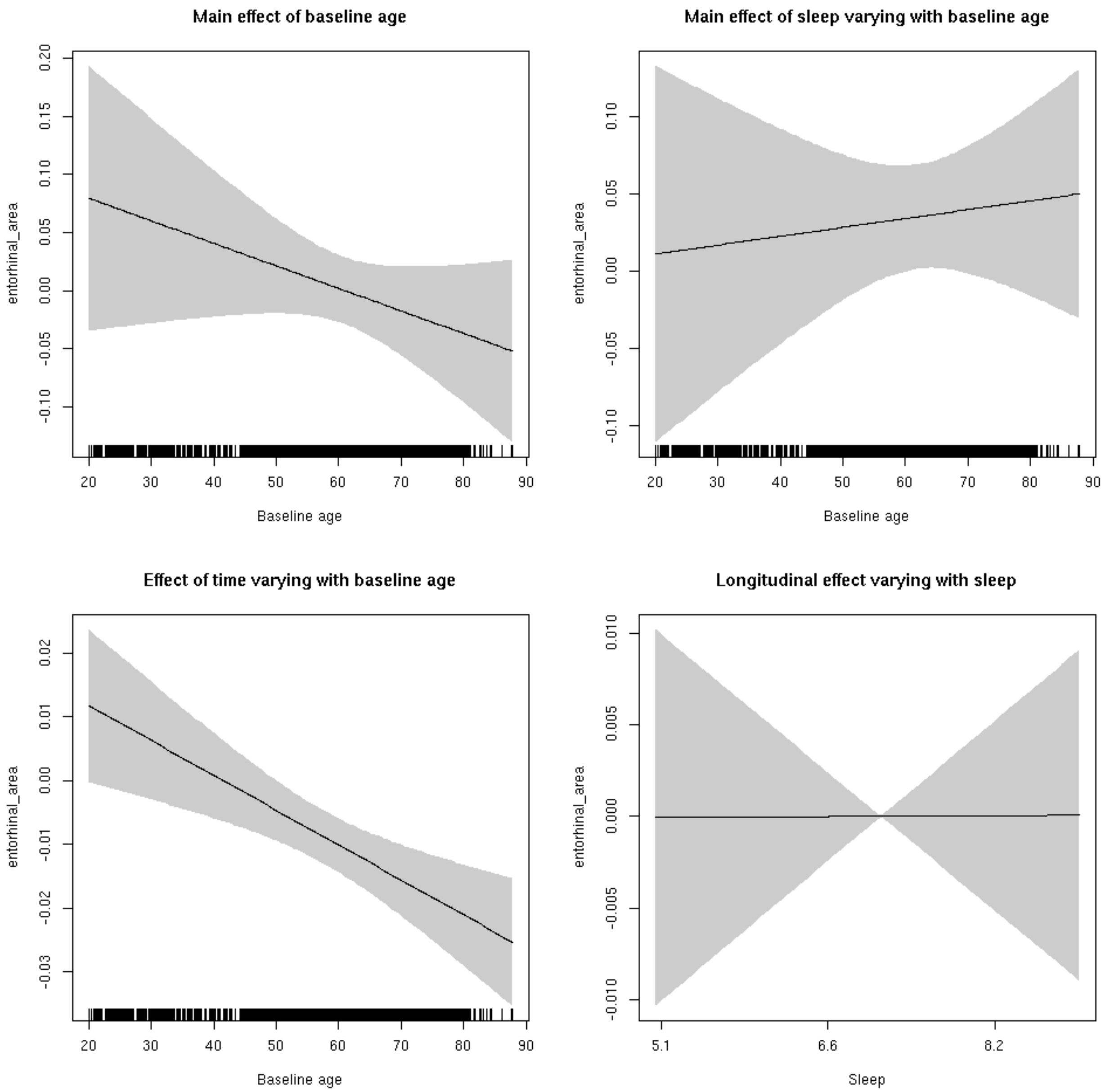

Restricted to sleep >= 5 and <= 9 hours Below is the row for `s(sleep_z):time` from the model output.

```
## s(sleep_z):time     1       1  0.000    0.990
```

Below are plots of a plot of `s(bl_age)`, `s(bl_age):sleep_z`, `s(bl_age):time`, and `s(sleep_z):time`. For `s(sleep_z):time`, roughly, if we cannot fit a straight line through the confidence bands, there is evidence that the effect of time depends on sleep duration. It is correct the that confidence interval is zero at one point along the axis; this happens because we do not include the main effect.

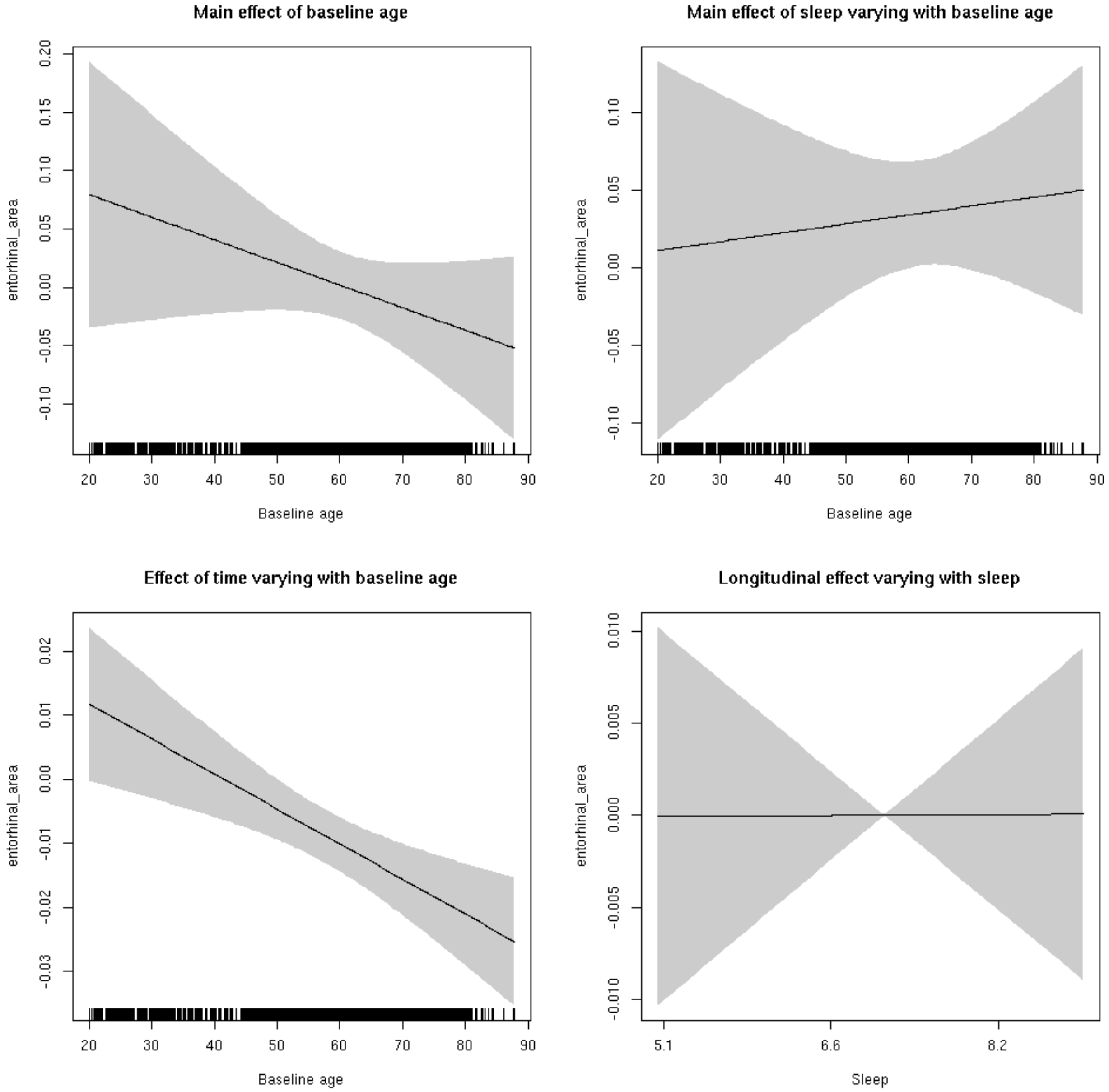

entorhinal_thickness: Stratified by sex

Female: Full data Below is the row for `s(sleep_z):time` from the model output.

```
## s(sleep_z):time   1.000  1.000  0.255   0.614
```

Below are plots of a plot of `s(bl_age)`, `s(bl_age):sleep_z`, `s(bl_age):time`, and `s(sleep_z):time`. For `s(sleep_z):time`, roughly, if we cannot fit a straight line through the confidence bands, there is evidence that the effect of time depends on sleep duration. It is correct the that confidence interval is zero at one point along the axis; this happens because we do not include the main effect.

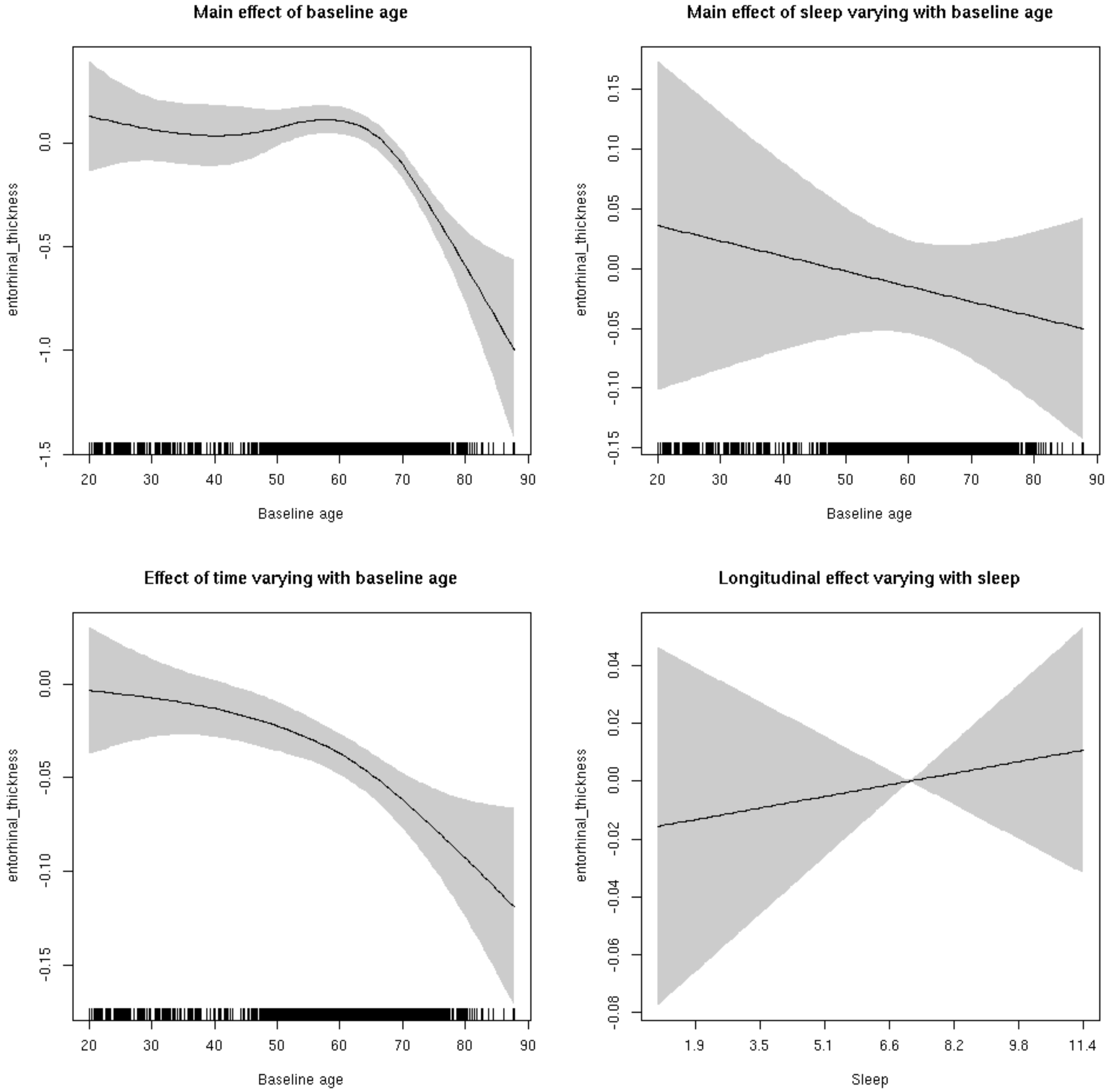

Male: Full data Below is the row for `s(sleep_z):time` from the model output.

```
## s(sleep_z):time   1.000  1.000  4.249  0.0393 *
```

Below are plots of a plot of `s(bl_age)`, `s(bl_age):sleep_z`, `s(bl_age):time`, and `s(sleep_z):time`. For `s(sleep_z):time`, roughly, if we cannot fit a straight line through the confidence bands, there is evidence that the effect of time depends on sleep duration. It is correct the that confidence interval is zero at one point along the axis; this happens because we do not include the main effect.

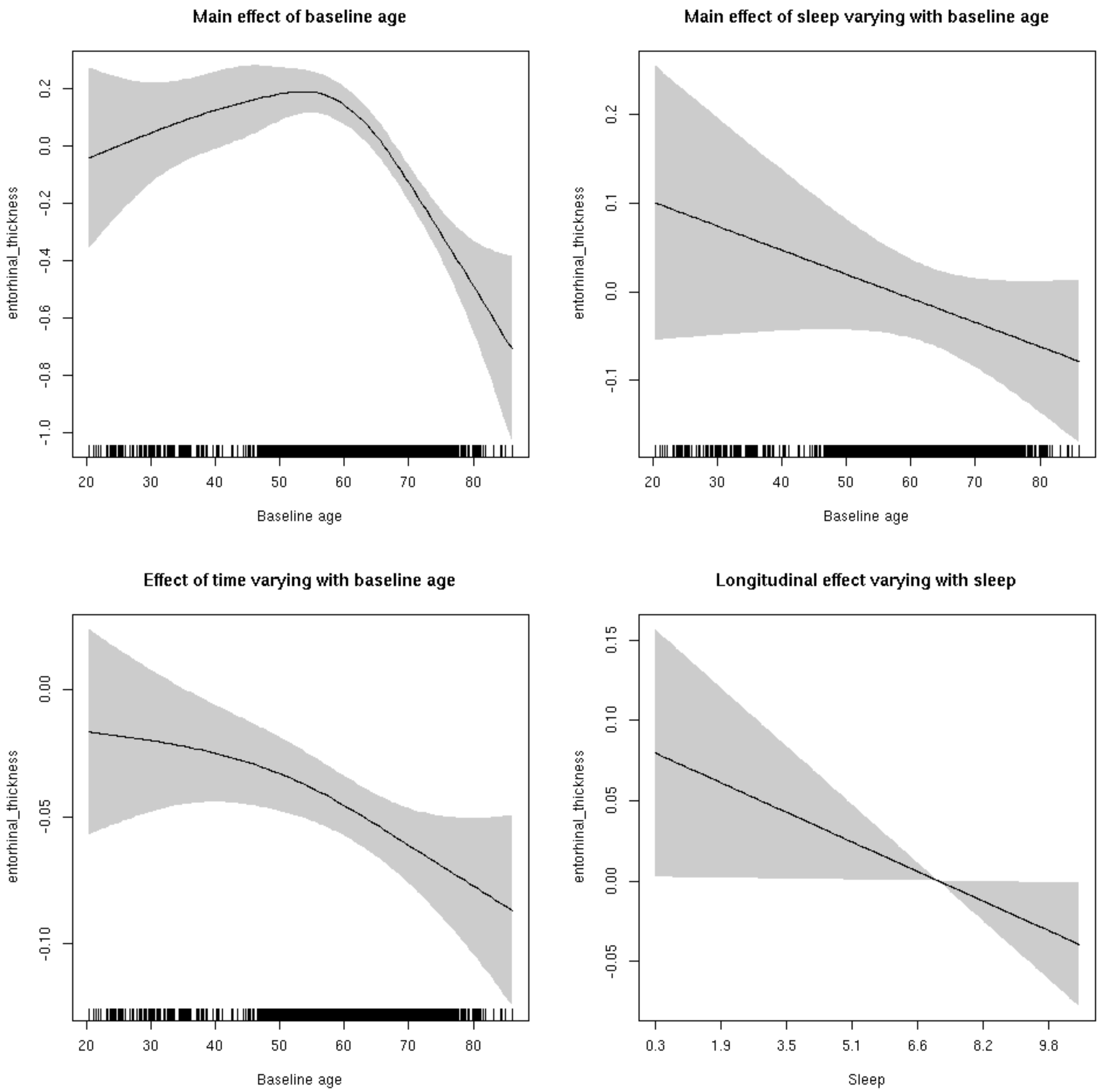

entorhinal_thickness

Full data Below is the model output. The term `s(sleep_z):time` is probably what we care most about.

```
##
## Family: gaussian
## Link function: identity
##
## Formula:
## value ~ sex + site + s(bl_age, k = 10, bs = "cr") + s(bl_age,
##     by = sleep_z, bs = "cr") + +s(bl_age, by = time, k = 5, bs = "cr") +
##     s(sleep_z, by = time, k = 5, bs = "cr", pc = 0)
## <environment: 0x5630035d31b8>
```

```
##
## Parametric coefficients:
##                Estimate Std. Error t value Pr(>|t|)
## (Intercept)   -0.21420    0.05431  -3.944 8.07e-05 ***
## sexmale       -0.01917    0.02717  -0.706  0.48035
## siteousAvanto  0.20849    0.07260   2.872  0.00409 **
## siteousPrisma -0.31946    0.34339  -0.930  0.35224
## siteousSkyra   0.19631    0.07257   2.705  0.00685 **
## siteUB         0.18476    0.14316   1.291  0.19689
## siteUCAM       0.01461    0.07486   0.195  0.84530
## siteUKB        0.35445    0.05507   6.436 1.29e-10 ***
## siteUmU       -0.15978    0.08940  -1.787  0.07395 .
## ---
## Signif. codes:  0 '***' 0.001 '**' 0.01 '*' 0.05 '.' 0.1 ' ' 1
##
## Approximate significance of smooth terms:
##                      edf Ref.df      F p-value
## s(bl_age)          4.241  4.241 33.476  <2e-16 ***
## s(bl_age):sleep_z 2.000  2.000  1.924   0.146
## s(bl_age):time    2.980  2.980 55.249  <2e-16 ***
## s(sleep_z):time   1.000  1.000  1.073   0.300
## ---
## Signif. codes:  0 '***' 0.001 '**' 0.01 '*' 0.05 '.' 0.1 ' ' 1
##
## R-sq.(adj) =  0.0789
## lmer.REML =  17963  Scale est. = 0.20939   n = 8166
```

Below are plots of a plot of `s(bl_age)`, `s(bl_age):sleep_z`, `s(bl_age):time`, and `s(sleep_z):time`. For `s(sleep_z):time`, roughly, if we cannot fit a straight line through the confidence bands, there is evidence that the effect of time depends on sleep duration. It is correct the that confidence interval is zero at one point along the axis; this happens because we do not include the main effect.

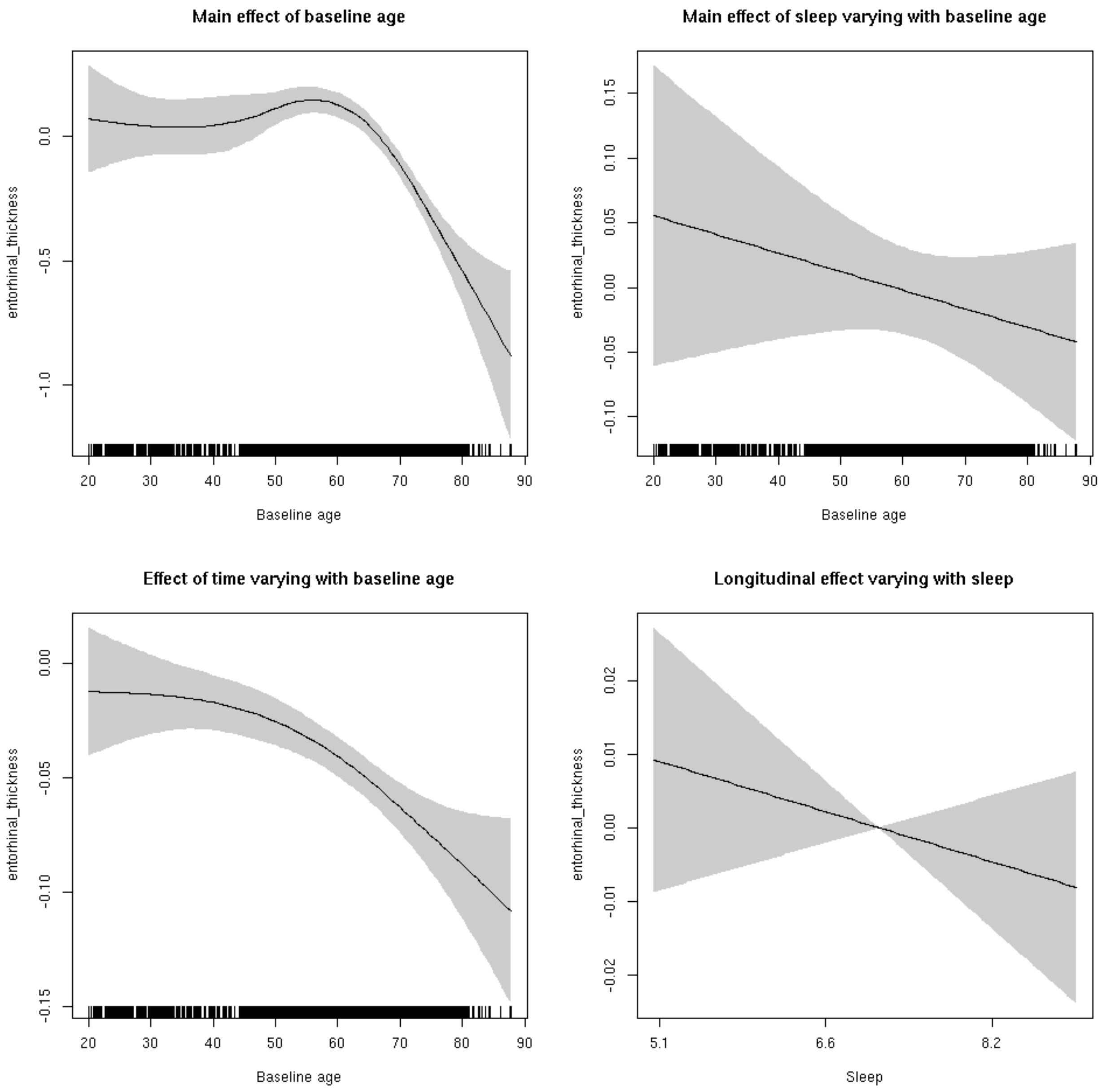

Restricted to sleep >= 5 and <= 9 hours Below is the row for `s(sleep_z):time` from the model output.

```
## s(sleep_z):time   1.000  1.000  1.056   0.304
```

Below are plots of a plot of `s(bl_age)`, `s(bl_age):sleep_z`, `s(bl_age):time`, and `s(sleep_z):time`. For `s(sleep_z):time`, roughly, if we cannot fit a straight line through the confidence bands, there is evidence that the effect of time depends on sleep duration. It is correct the that confidence interval is zero at one point along the axis; this happens because we do not include the main effect.

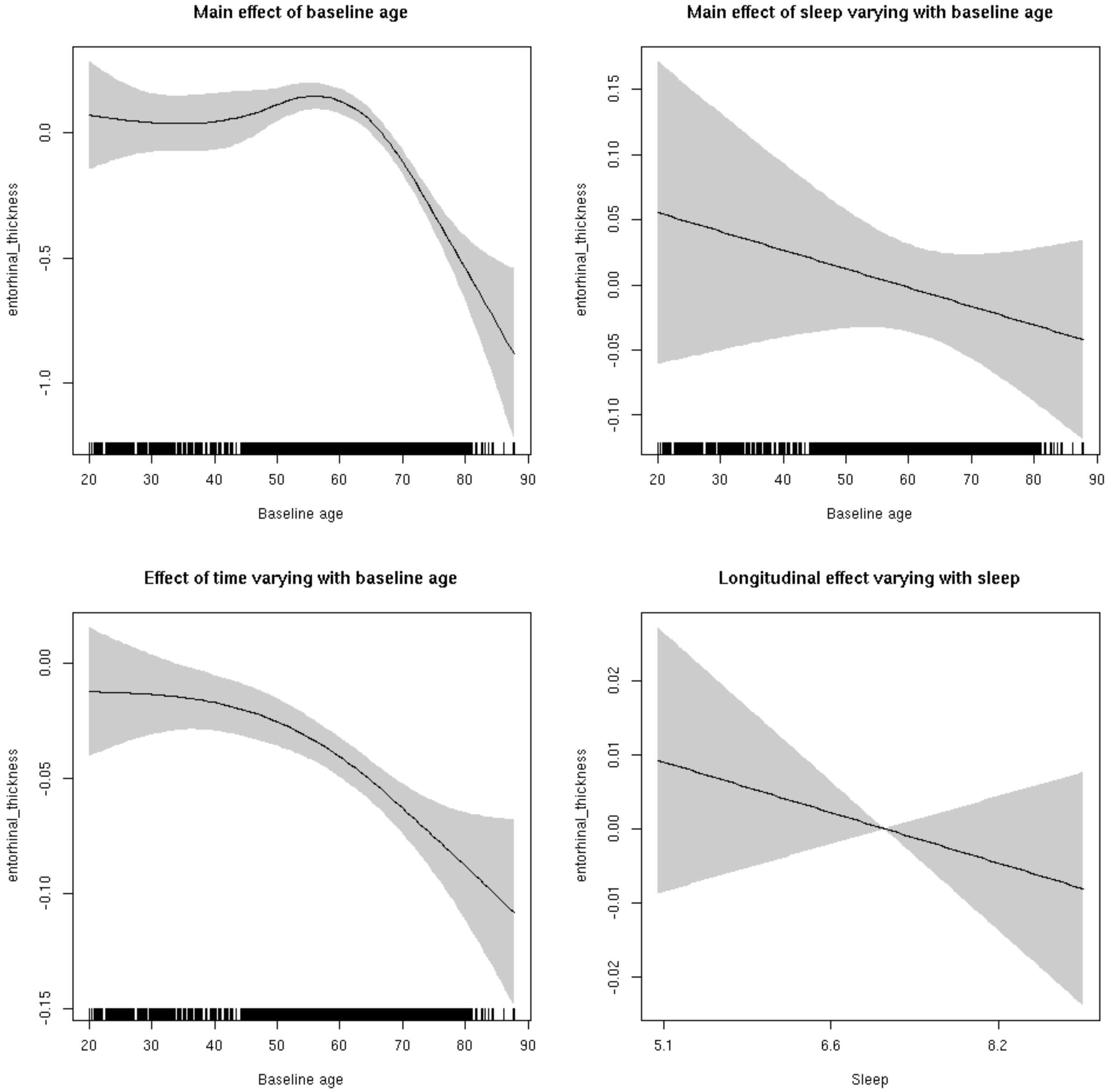

entorhinal_volume: Stratified by sex

Female: Full data Below is the row for `s(sleep_z):time` from the model output.

```
## s(sleep_z):time   1.000  1.000  0.084  0.7723
```

Below are plots of a plot of `s(bl_age)`, `s(bl_age):sleep_z`, `s(bl_age):time`, and `s(sleep_z):time`. For `s(sleep_z):time`, roughly, if we cannot fit a straight line through the confidence bands, there is evidence that the effect of time depends on sleep duration. It is correct the that confidence interval is zero at one point along the axis; this happens because we do not include the main effect.

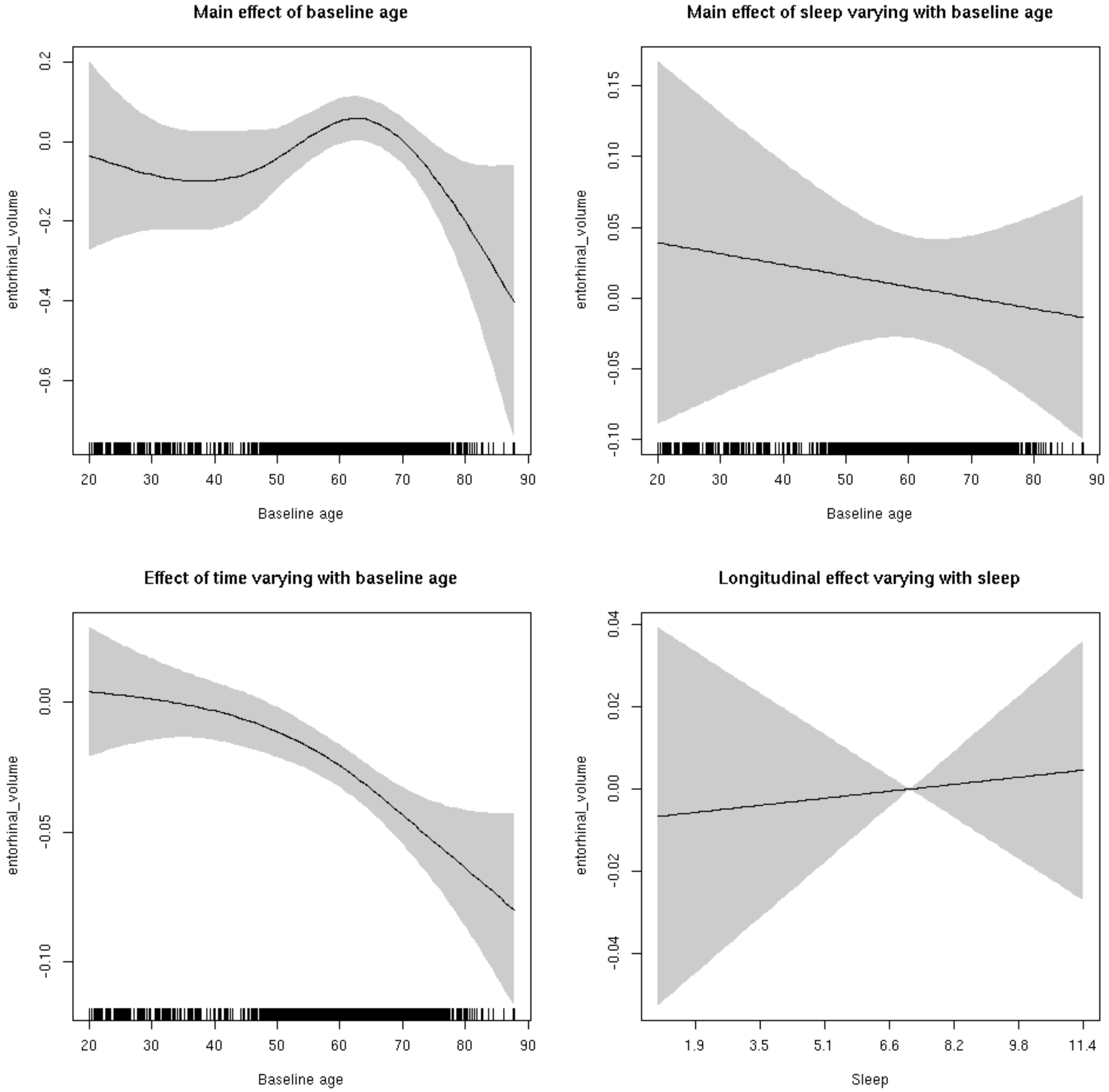

Male: Full data Below is the row for `s(sleep_z):time` from the model output.

```
## s(sleep_z):time   1.000  1.000  4.147 0.041786 *
```

Below are plots of a plot of `s(bl_age)`, `s(bl_age):sleep_z`, `s(bl_age):time`, and `s(sleep_z):time`. For `s(sleep_z):time`, roughly, if we cannot fit a straight line through the confidence bands, there is evidence that the effect of time depends on sleep duration. It is correct the that confidence interval is zero at one point along the axis; this happens because we do not include the main effect.

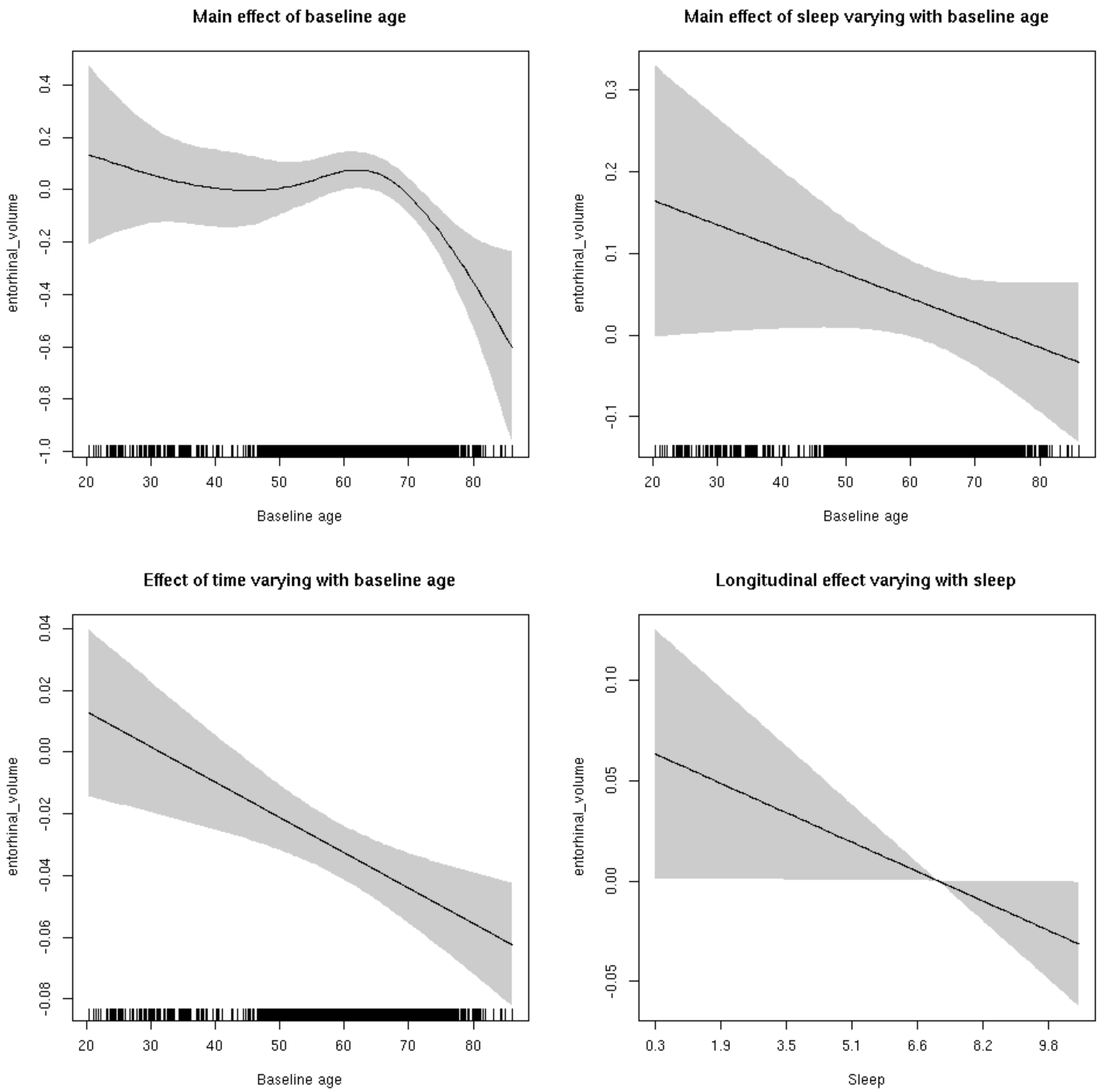

entorhinal_volume

Full data Below is the model output. The term `s(sleep_z):time` is probably what we care most about.

```
##
## Family: gaussian
## Link function: identity
##
## Formula:
## value ~ sex + site + icv + s(bl_age, k = 10, bs = "cr") + s(bl_age,
##     by = sleep_z, bs = "cr") + +s(bl_age, by = time, k = 5, bs = "cr") +
##     s(sleep_z, by = time, k = 5, bs = "cr", pc = 0)
## <environment: 0x5630035a4ed0>
```

```
##
## Parametric coefficients:
##                Estimate Std. Error t value Pr(>|t|)
## (Intercept)     0.54969    0.06459   8.511  < 2e-16 ***
## sexmale         0.18553    0.03311   5.604 2.17e-08 ***
## siteousAvanto -0.05028    0.07963  -0.631  0.52778
## siteousPrisma -0.88432    0.27736  -3.188  0.00144 **
## siteousSkyra  -0.38943    0.07708  -5.052 4.47e-07 ***
## siteUB        -0.36469    0.14694  -2.482  0.01309 *
## siteUCAM      -0.35548    0.08027  -4.429 9.60e-06 ***
## siteUKB       -0.59700    0.06135  -9.730  < 2e-16 ***
## siteUmU       -0.57627    0.09184  -6.275 3.68e-10 ***
## icv            0.30857    0.01640  18.819  < 2e-16 ***
## ---
## Signif. codes:  0 '***' 0.001 '**' 0.01 '*' 0.05 '.' 0.1 ' ' 1
##
## Approximate significance of smooth terms:
##                      edf Ref.df      F  p-value
## s(bl_age)          4.133  4.133  8.038 9.77e-07 ***
## s(bl_age):sleep_z 2.000  2.000  2.076    0.126
## s(bl_age):time    2.954  2.954 44.226  < 2e-16 ***
## s(sleep_z):time   1.000  1.000  1.291    0.256
## ---
## Signif. codes:  0 '***' 0.001 '**' 0.01 '*' 0.05 '.' 0.1 ' ' 1
##
## R-sq.(adj) =  0.177
## lmer.REML =  15844  Scale est. = 0.12487   n = 8176
```

Below are plots of a plot of `s(bl_age)`, `s(bl_age):sleep_z`, `s(bl_age):time`, and `s(sleep_z):time`. For `s(sleep_z):time`, roughly, if we cannot fit a straight line through the confidence bands, there is evidence that the effect of time depends on sleep duration. It is correct the that confidence interval is zero at one point along the axis; this happens because we do not include the main effect.

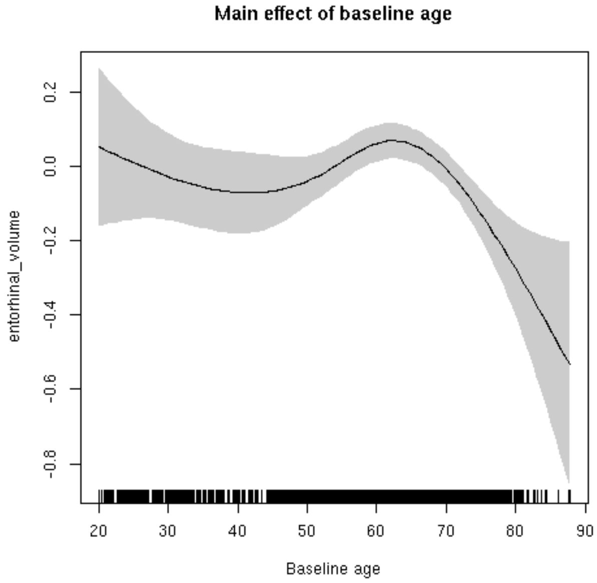

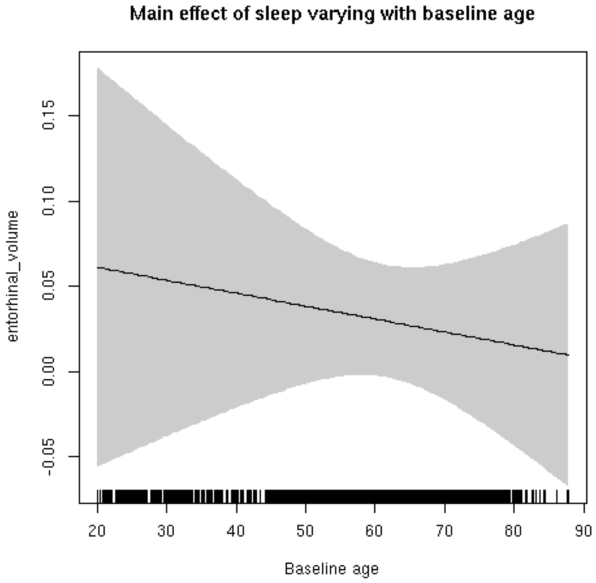

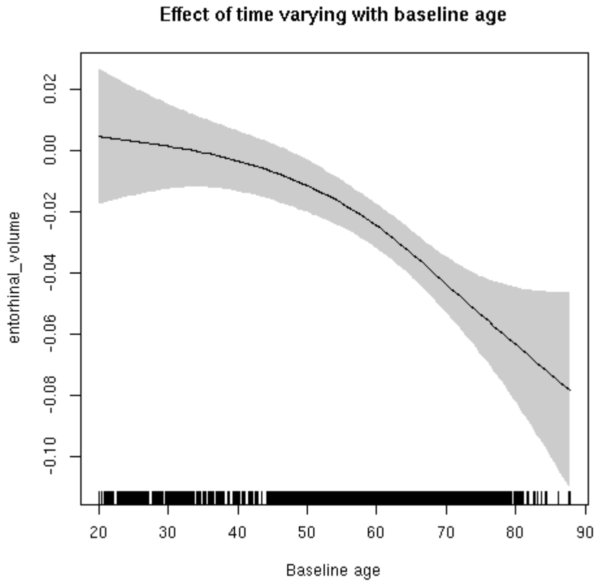

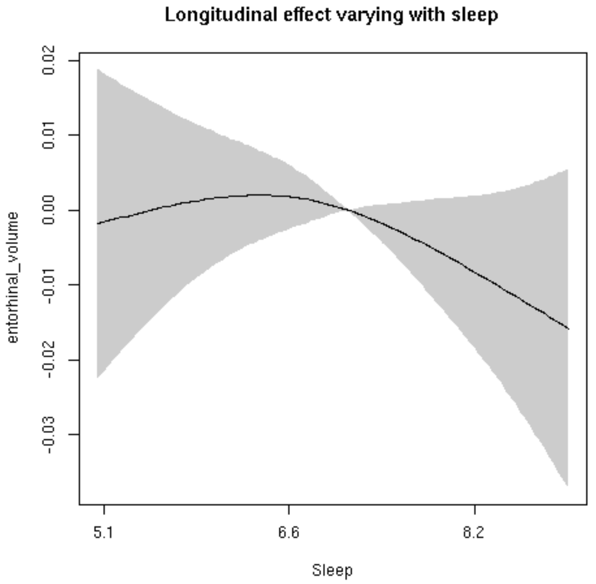

Restricted to sleep >= 5 and <= 9 hours Below is the row for `s(sleep_z):time` from the model output.

```
## s(sleep_z):time   1.648  1.648  0.754    0.303
```

Below are plots of a plot of `s(bl_age)`, `s(bl_age):sleep_z`, `s(bl_age):time`, and `s(sleep_z):time`. For `s(sleep_z):time`, roughly, if we cannot fit a straight line through the confidence bands, there is evidence that the effect of time depends on sleep duration. It is correct the that confidence interval is zero at one point along the axis; this happens because we do not include the main effect.

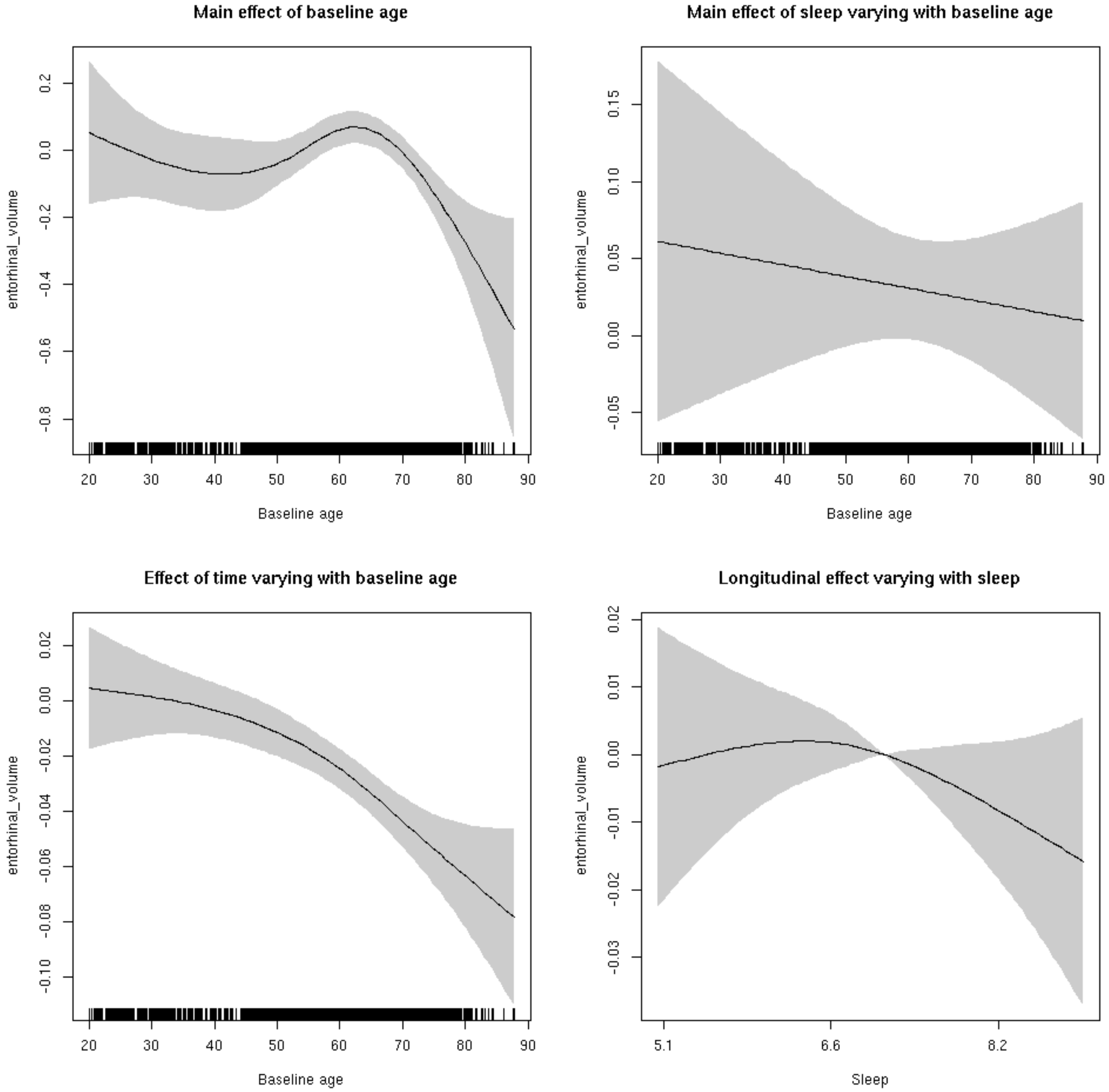

frontalpole_area: Stratified by sex

Female: Full data Below is the row for `s(sleep_z):time` from the model output.

```
## s(sleep_z):time   1.00   1.00  0.056   0.812
```

Below are plots of a plot of `s(bl_age)`, `s(bl_age):sleep_z`, `s(bl_age):time`, and `s(sleep_z):time`. For `s(sleep_z):time`, roughly, if we cannot fit a straight line through the confidence bands, there is evidence that the effect of time depends on sleep duration. It is correct the that confidence interval is zero at one point along the axis; this happens because we do not include the main effect.

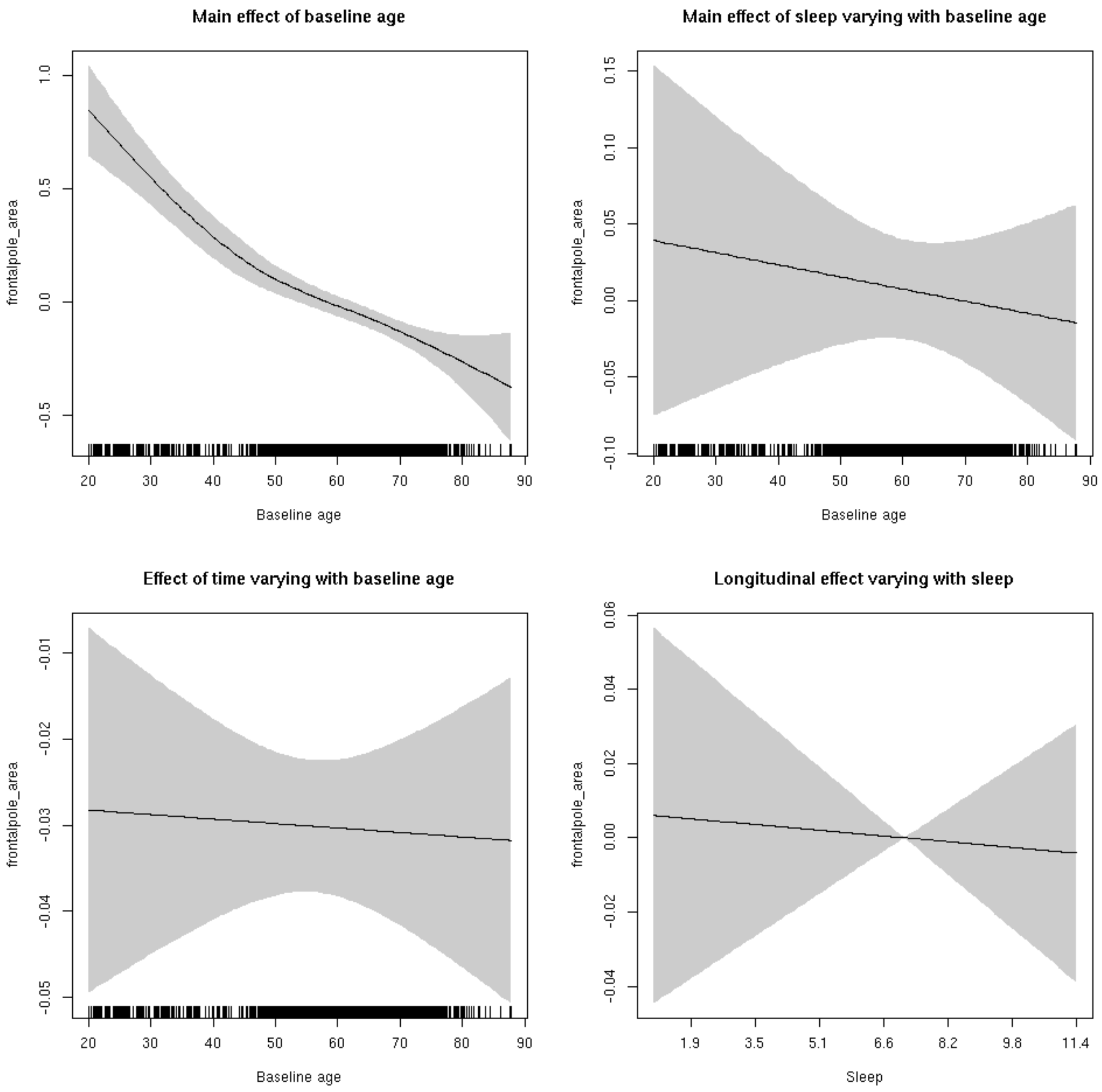

Male: Full data Below is the row for `s(sleep_z):time` from the model output.

```
## s(sleep_z):time   1.000  1.000  0.416  0.5189
```

Below are plots of a plot of `s(bl_age)`, `s(bl_age):sleep_z`, `s(bl_age):time`, and `s(sleep_z):time`. For `s(sleep_z):time`, roughly, if we cannot fit a straight line through the confidence bands, there is evidence that the effect of time depends on sleep duration. It is correct the that confidence interval is zero at one point along the axis; this happens because we do not include the main effect.

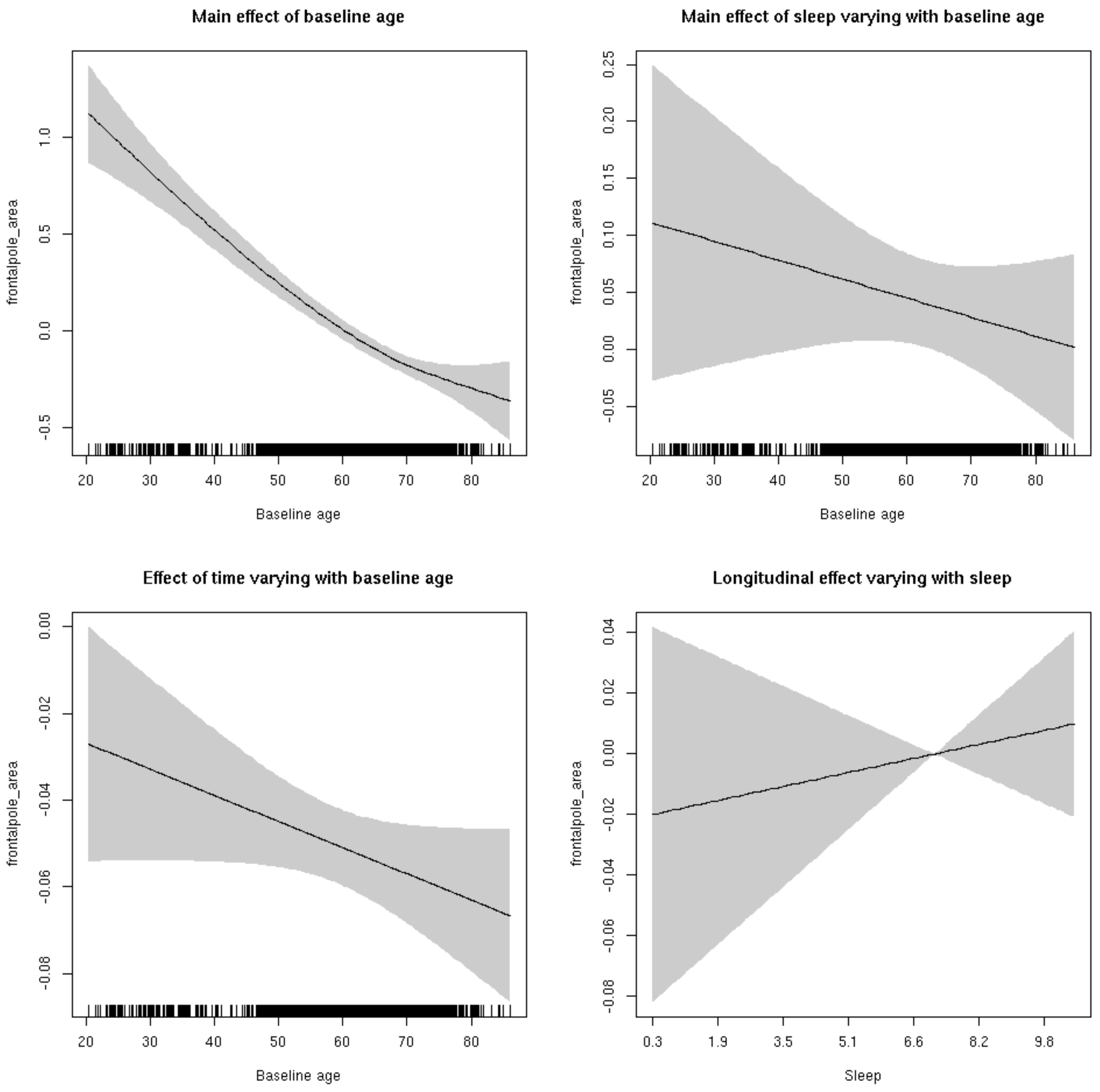

frontalpole_area

Full data Below is the model output. The term `s(sleep_z):time` is probably what we care most about.

```
##
## Family: gaussian
## Link function: identity
##
## Formula:
## value ~ sex + site + icv + s(bl_age, k = 10, bs = "cr") + s(bl_age,
##     by = sleep_z, bs = "cr") + +s(bl_age, by = time, k = 5, bs = "cr") +
##     s(sleep_z, by = time, k = 5, bs = "cr", pc = 0)
## <environment: 0x5630035a1ae8>
```

```
##
## Parametric coefficients:
##                Estimate Std. Error t value Pr(>|t|)
## (Intercept)     1.20705    0.05554  21.733  < 2e-16 ***
## sexmale         0.26855    0.02849   9.427  < 2e-16 ***
## siteousAvanto -0.27266    0.06908  -3.947 7.99e-05 ***
## siteousPrisma -0.34292    0.28339  -1.210    0.226
## siteousSkyra   0.47292    0.06685   7.074 1.62e-12 ***
## siteUB        -0.62984    0.12561  -5.014 5.44e-07 ***
## siteUCAM      -0.86885    0.06852 -12.680  < 2e-16 ***
## siteUKB       -1.32168    0.05249 -25.182  < 2e-16 ***
## siteUmU        0.32791    0.07893   4.154 3.30e-05 ***
## icv            0.41740    0.01412  29.560  < 2e-16 ***
## ---
## Signif. codes:  0 '***' 0.001 '**' 0.01 '*' 0.05 '.' 0.1 ' ' 1
##
## Approximate significance of smooth terms:
##                       edf Ref.df       F p-value
## s(bl_age)           2.559  2.559 107.131  <2e-16 ***
## s(bl_age):sleep_z 2.000  2.000   2.033   0.131
## s(bl_age):time    2.000  2.000  95.270  <2e-16 ***
## s(sleep_z):time   1.000  1.000   0.037   0.847
## ---
## Signif. codes:  0 '***' 0.001 '**' 0.01 '*' 0.05 '.' 0.1 ' ' 1
##
## R-sq.(adj) =  0.521
## lmer.REML =  15080  Scale est. = 0.138     n = 8172
```

Below are plots of a plot of `s(bl_age)`, `s(bl_age):sleep_z`, `s(bl_age):time`, and `s(sleep_z):time`. For `s(sleep_z):time`, roughly, if we cannot fit a straight line through the confidence bands, there is evidence that the effect of time depends on sleep duration. It is correct the that confidence interval is zero at one point along the axis; this happens because we do not include the main effect.

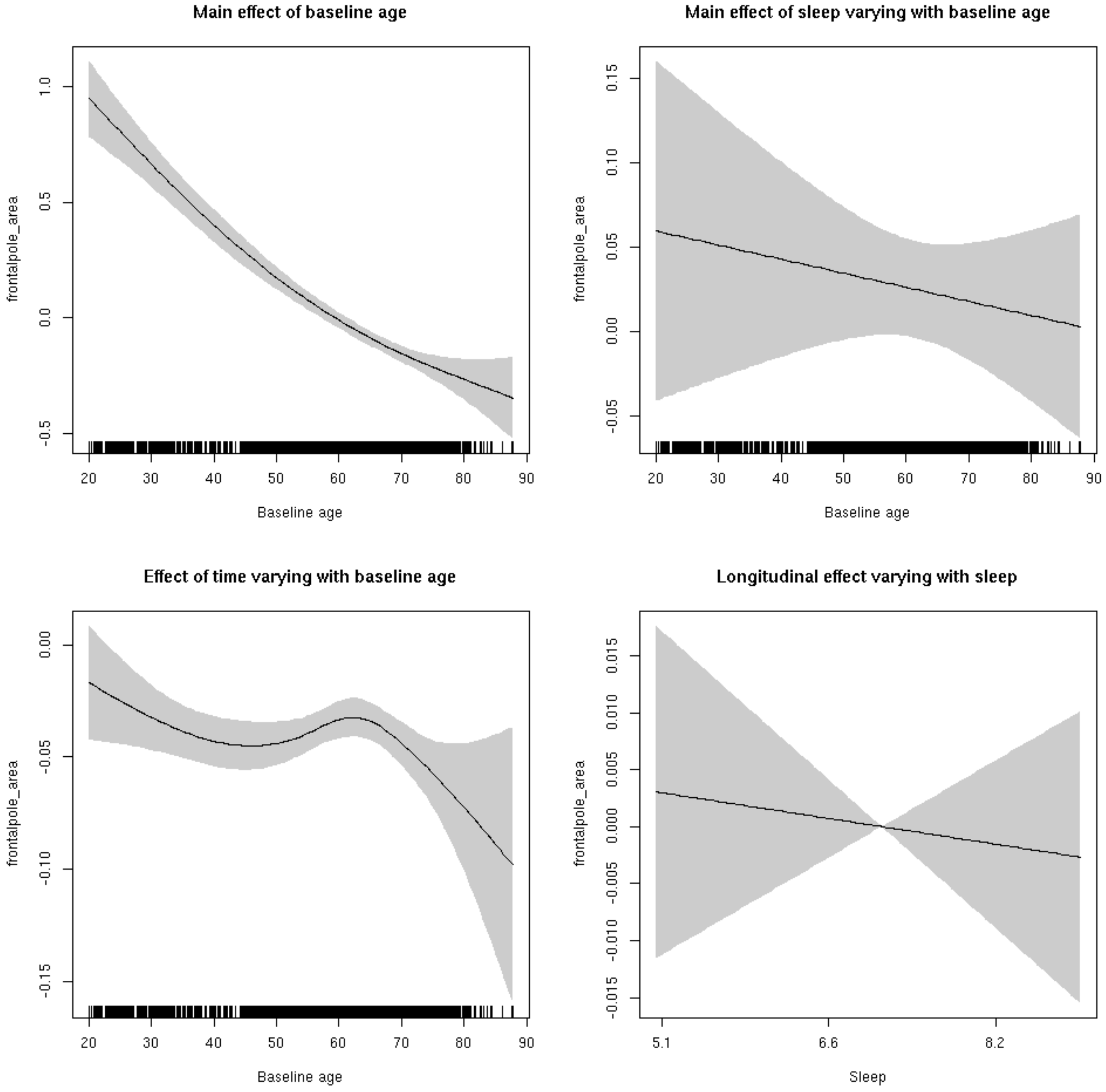

Restricted to sleep >= 5 and <= 9 hours Below is the row for `s(sleep_z):time` from the model output.

```
## s(sleep_z):time   1.000  1.000   0.175   0.676
```

Below are plots of a plot of `s(bl_age)`, `s(bl_age):sleep_z`, `s(bl_age):time`, and `s(sleep_z):time`. For `s(sleep_z):time`, roughly, if we cannot fit a straight line through the confidence bands, there is evidence that the effect of time depends on sleep duration. It is correct the that confidence interval is zero at one point along the axis; this happens because we do not include the main effect.

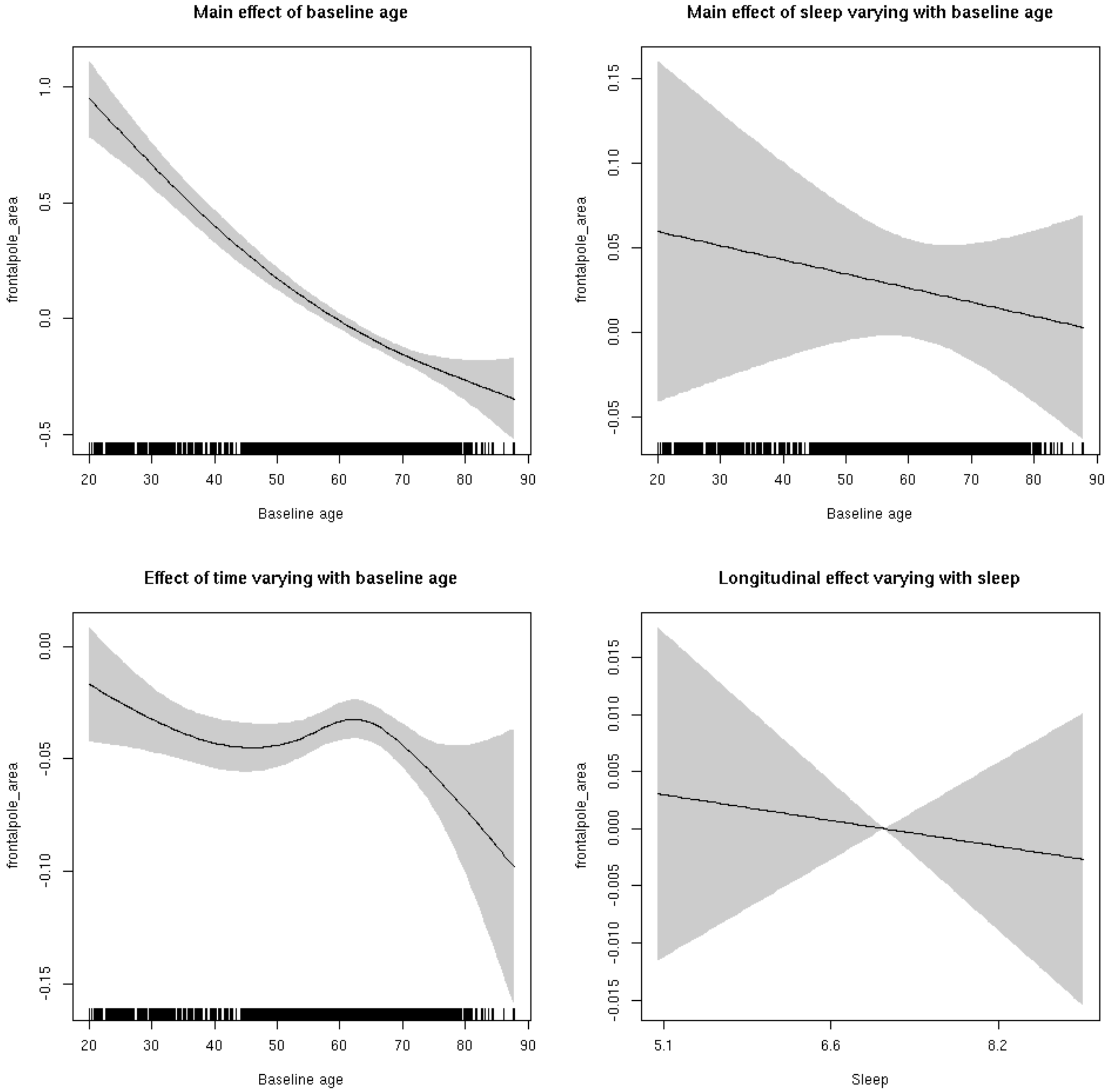

frontalpole_thickness: Stratified by sex

Female: Full data Below is the row for `s(sleep_z):time` from the model output.

```
## s(sleep_z):time   1.00   1.00  0.027 0.869333
```

Below are plots of a plot of `s(bl_age)`, `s(bl_age):sleep_z`, `s(bl_age):time`, and `s(sleep_z):time`. For `s(sleep_z):time`, roughly, if we cannot fit a straight line through the confidence bands, there is evidence that the effect of time depends on sleep duration. It is correct the that confidence interval is zero at one point along the axis; this happens because we do not include the main effect.

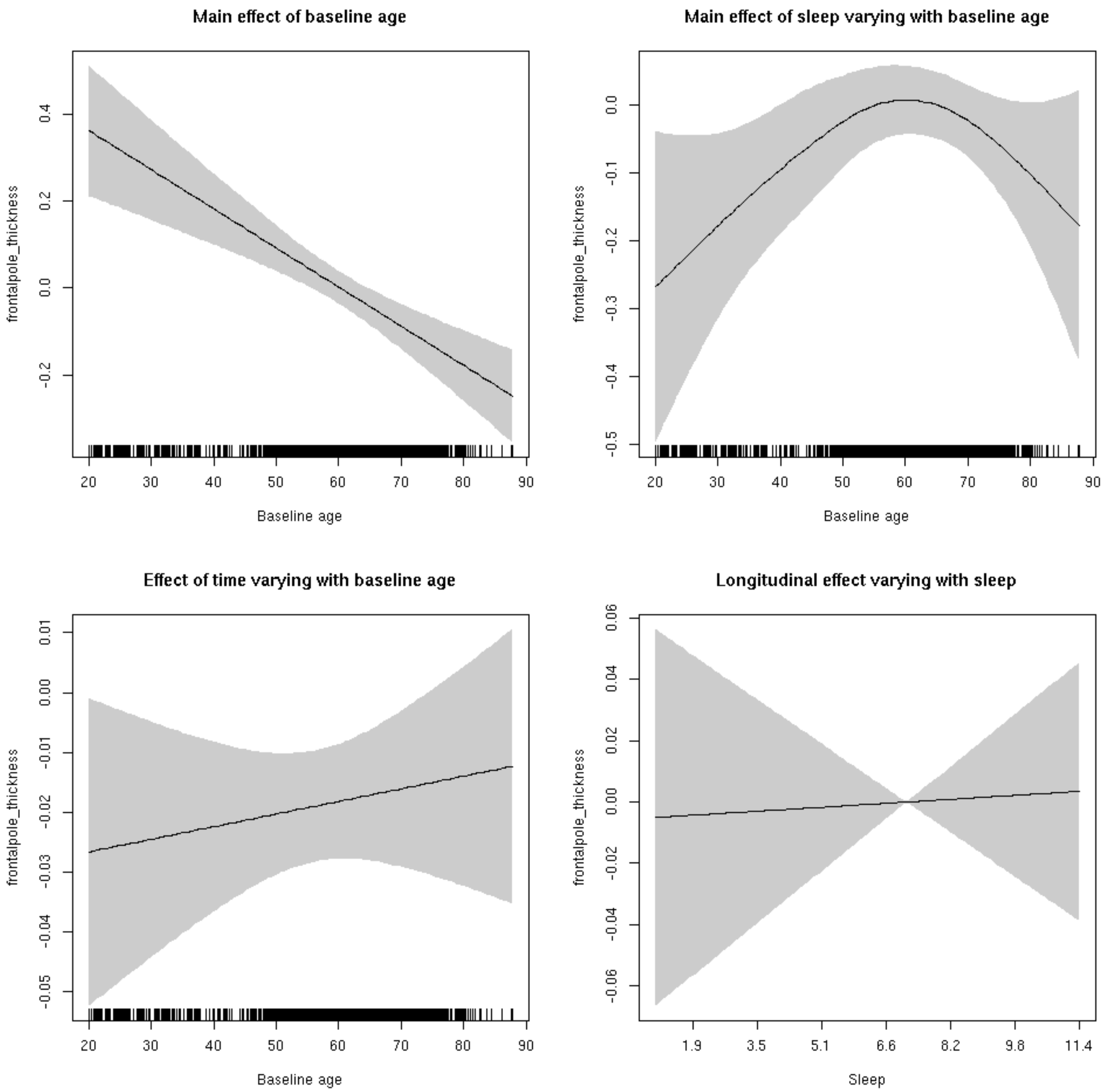

Male: Full data Below is the row for `s(sleep_z):time` from the model output.

```
## s(sleep_z):time   1.00   1.00  0.022    0.882
```

Below are plots of a plot of `s(bl_age)`, `s(bl_age):sleep_z`, `s(bl_age):time`, and `s(sleep_z):time`. For `s(sleep_z):time`, roughly, if we cannot fit a straight line through the confidence bands, there is evidence that the effect of time depends on sleep duration. It is correct the that confidence interval is zero at one point along the axis; this happens because we do not include the main effect.

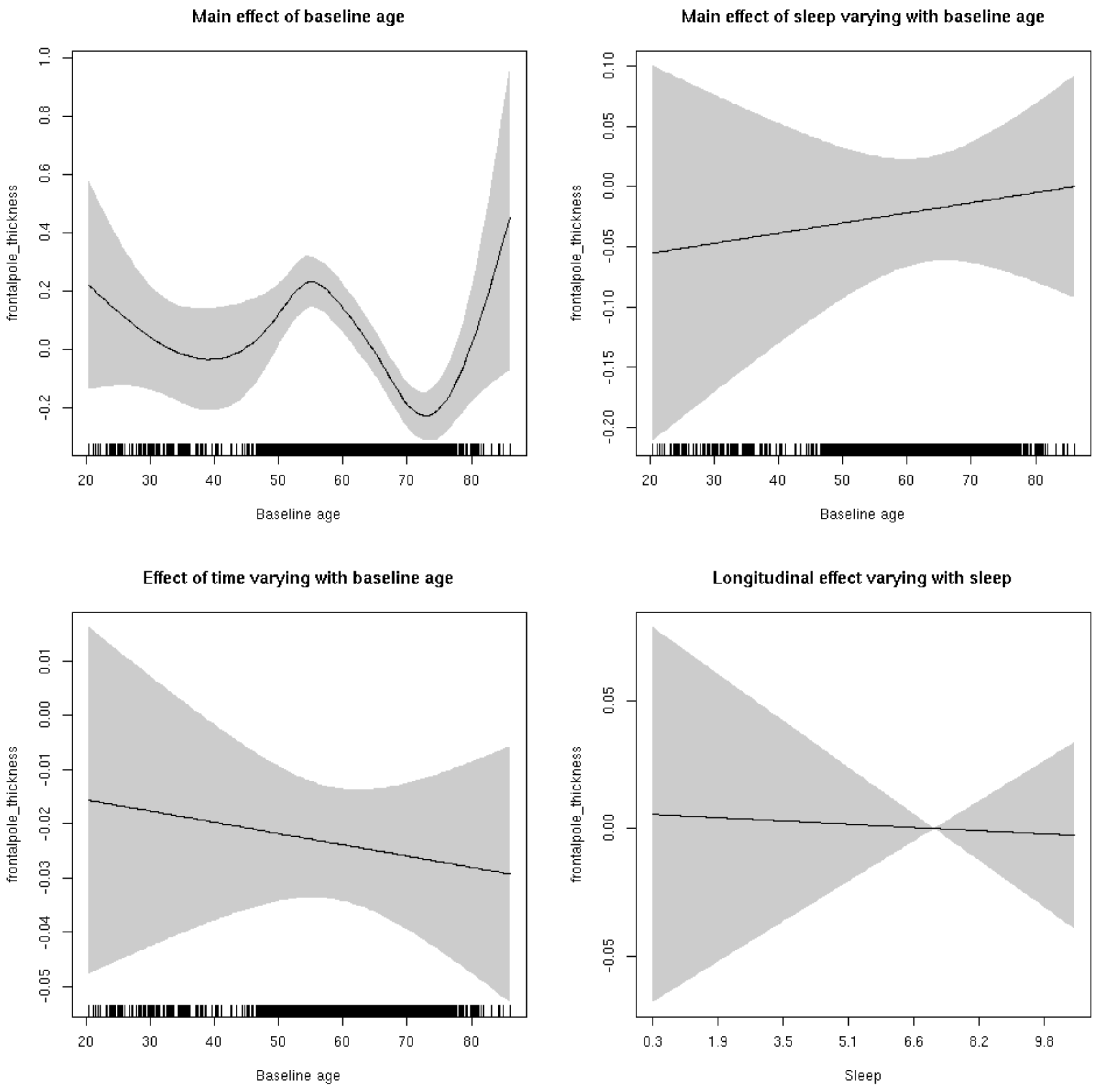

frontalpole_thickness

Full data Below is the model output. The term `s(sleep_z):time` is probably what we care most about.

```
##
## Family: gaussian
## Link function: identity
##
## Formula:
## value ~ sex + site + s(bl_age, k = 10, bs = "cr") + s(bl_age,
##     by = sleep_z, bs = "cr") + +s(bl_age, by = time, k = 5, bs = "cr") +
##     s(sleep_z, by = time, k = 5, bs = "cr", pc = 0)
## <environment: 0x5630035ca488>
```

```
##
## Parametric coefficients:
##                Estimate Std. Error t value Pr(>|t|)
## (Intercept)   -0.71136    0.05490 -12.956  < 2e-16 ***
## sexmale       -0.11631    0.02740  -4.244 2.22e-05 ***
## siteousAvanto  0.07407    0.07322   1.012    0.312
## siteousPrisma  0.39630    0.33654   1.178    0.239
## siteousSkyra  -0.54956    0.07322  -7.506 6.76e-14 ***
## siteUB         0.98731    0.14470   6.823 9.54e-12 ***
## siteUCAM       0.50947    0.07620   6.686 2.44e-11 ***
## siteUKB        0.90631    0.05581  16.238  < 2e-16 ***
## siteUmU       -0.55462    0.09027  -6.144 8.41e-10 ***
## ---
## Signif. codes:  0 '***' 0.001 '**' 0.01 '*' 0.05 '.' 0.1 ' ' 1
##
## Approximate significance of smooth terms:
##                      edf Ref.df      F p-value
## s(bl_age)          6.226  6.226 13.711  <2e-16 ***
## s(bl_age):sleep_z 2.213  2.213  1.866   0.176
## s(bl_age):time    2.000  2.000 18.193  <2e-16 ***
## s(sleep_z):time   1.000  1.000  0.019   0.889
## ---
## Signif. codes:  0 '***' 0.001 '**' 0.01 '*' 0.05 '.' 0.1 ' ' 1
##
## R-sq.(adj) =  0.244
## lmer.REML =  17837  Scale est. = 0.19929   n = 8171
```

Below are plots of a plot of `s(bl_age)`, `s(bl_age):sleep_z`, `s(bl_age):time`, and `s(sleep_z):time`. For `s(sleep_z):time`, roughly, if we cannot fit a straight line through the confidence bands, there is evidence that the effect of time depends on sleep duration. It is correct the that confidence interval is zero at one point along the axis; this happens because we do not include the main effect.

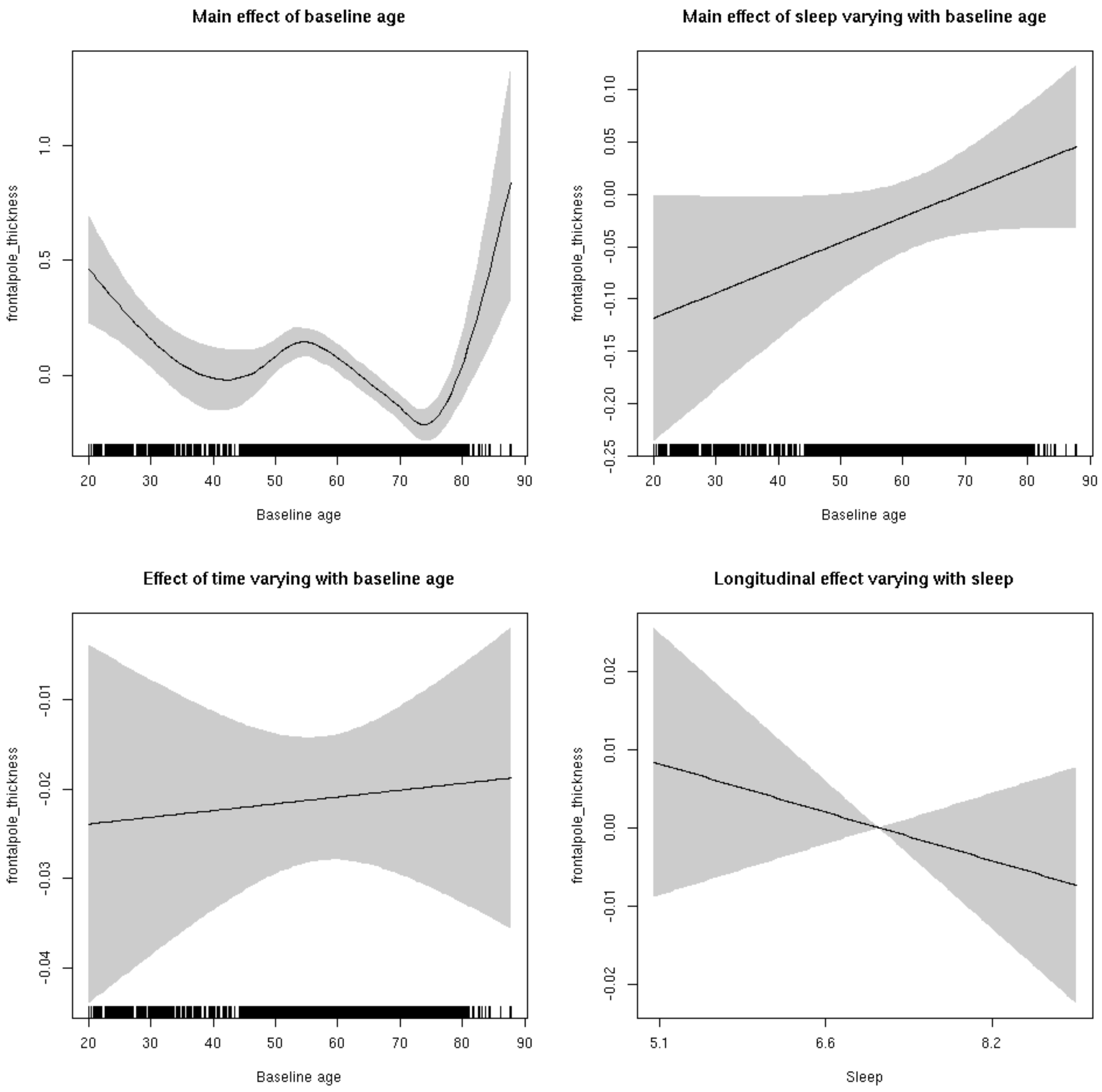

Restricted to sleep >= 5 and <= 9 hours Below is the row for `s(sleep_z):time` from the model output.

```
## s(sleep_z):time   1.000  1.000  0.946   0.331
```

Below are plots of a plot of `s(bl_age)`, `s(bl_age):sleep_z`, `s(bl_age):time`, and `s(sleep_z):time`. For `s(sleep_z):time`, roughly, if we cannot fit a straight line through the confidence bands, there is evidence that the effect of time depends on sleep duration. It is correct the that confidence interval is zero at one point along the axis; this happens because we do not include the main effect.

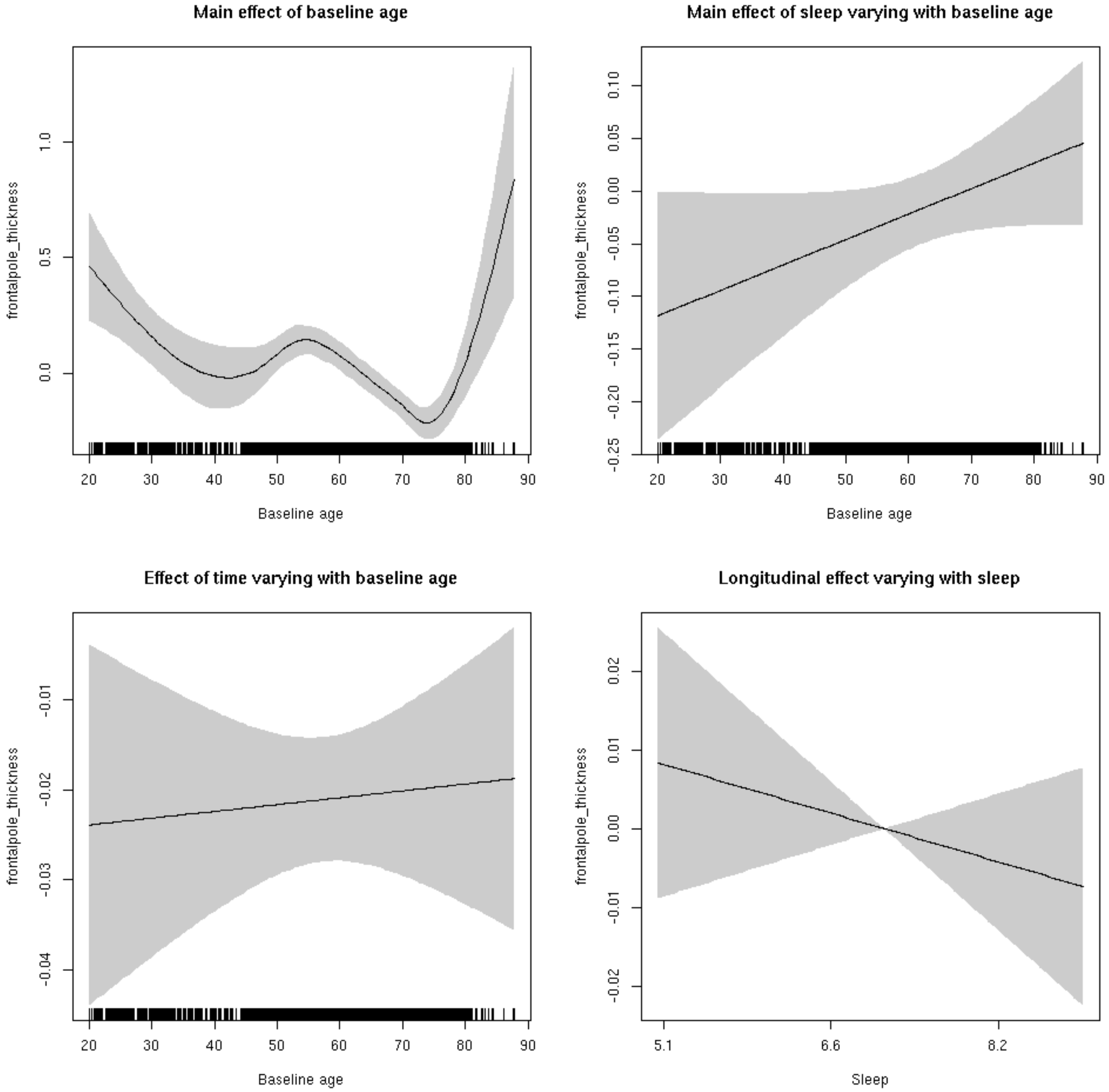

frontalpole_volume: Stratified by sex

Female: Full data Below is the row for `s(sleep_z):time` from the model output.

```
## s(sleep_z):time   1.000  1.000  0.031   0.861
```

Below are plots of a plot of `s(bl_age)`, `s(bl_age):sleep_z`, `s(bl_age):time`, and `s(sleep_z):time`. For `s(sleep_z):time`, roughly, if we cannot fit a straight line through the confidence bands, there is evidence that the effect of time depends on sleep duration. It is correct the that confidence interval is zero at one point along the axis; this happens because we do not include the main effect.

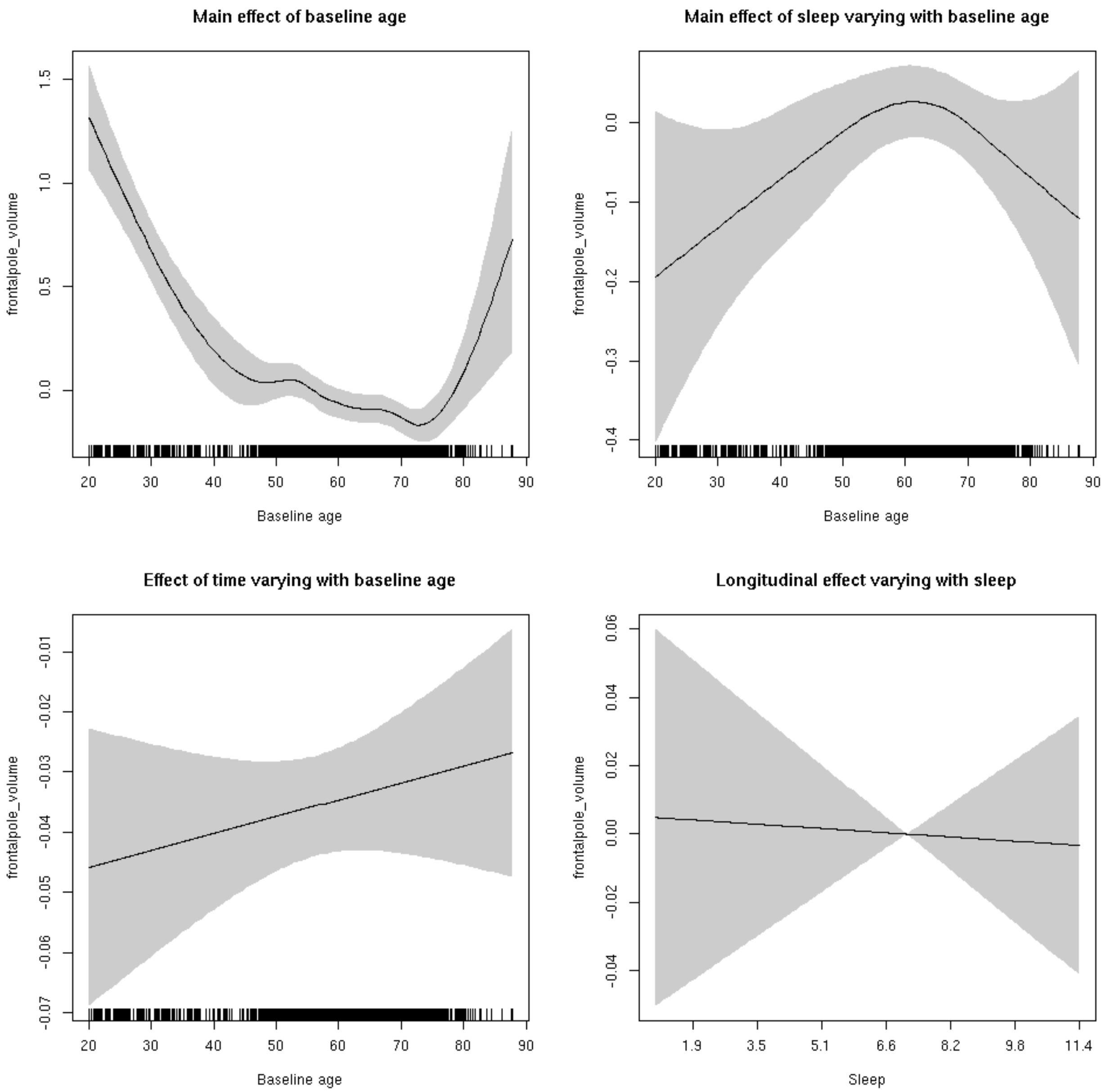

Male: Full data Below is the row for `s(sleep_z):time` from the model output.

```
## s(sleep_z):time   1.000  1.000  0.574   0.449
```

Below are plots of a plot of `s(bl_age)`, `s(bl_age):sleep_z`, `s(bl_age):time`, and `s(sleep_z):time`. For `s(sleep_z):time`, roughly, if we cannot fit a straight line through the confidence bands, there is evidence that the effect of time depends on sleep duration. It is correct the that confidence interval is zero at one point along the axis; this happens because we do not include the main effect.

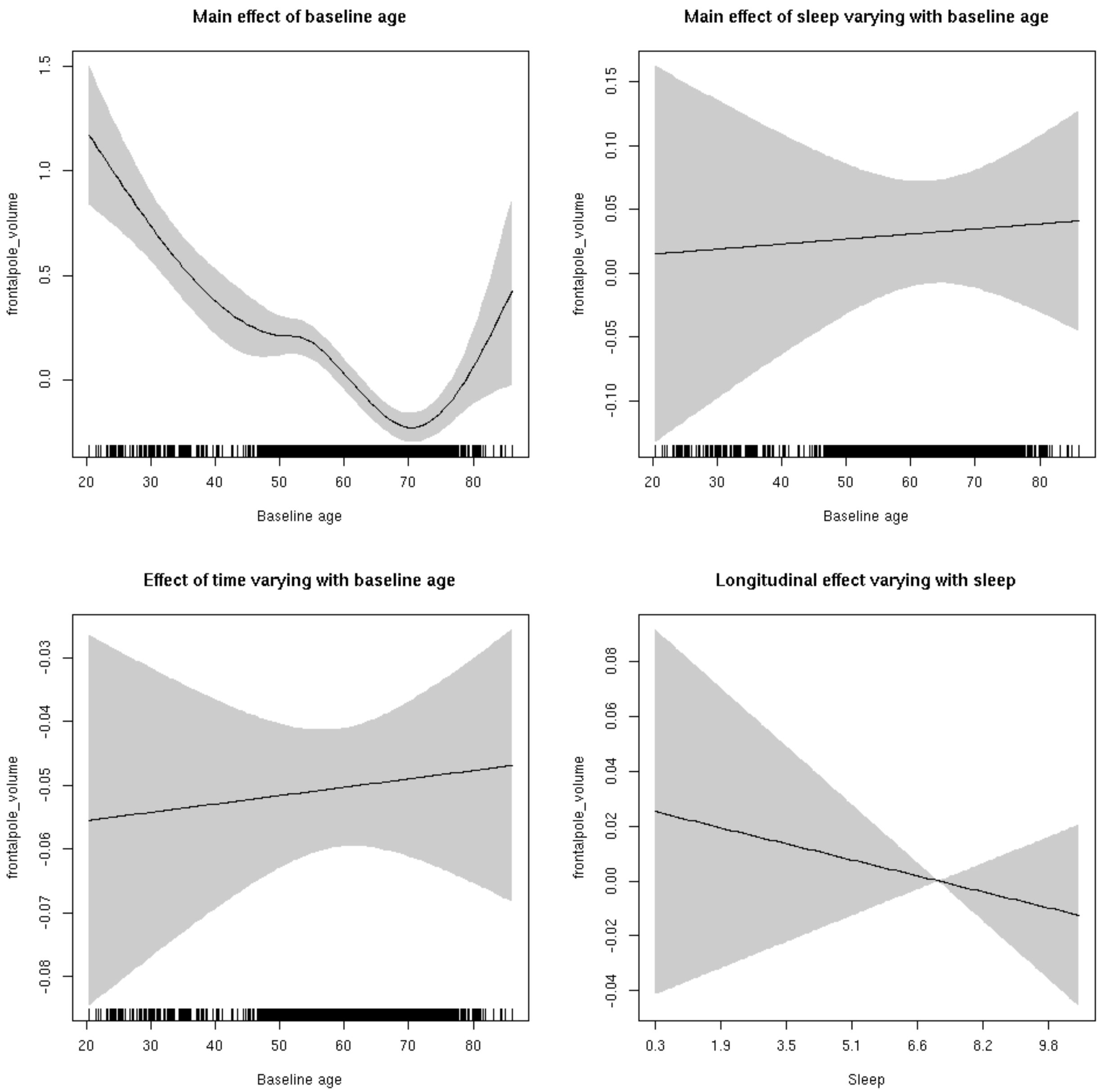

frontalpole_volume

Full data Below is the model output. The term `s(sleep_z):time` is probably what we care most about.

```
##
## Family: gaussian
## Link function: identity
##
## Formula:
## value ~ sex + site + icv + s(bl_age, k = 10, bs = "cr") + s(bl_age,
##     by = sleep_z, bs = "cr") + +s(bl_age, by = time, k = 5, bs = "cr") +
##     s(sleep_z, by = time, k = 5, bs = "cr", pc = 0)
## <environment: 0x5630035a29c8>
```

```
##
## Parametric coefficients:
##                Estimate Std. Error t value Pr(>|t|)
## (Intercept)     0.61850    0.05933  10.425  < 2e-16 ***
## sexmale         0.20154    0.03034   6.643 3.28e-11 ***
## siteousAvanto -0.38828    0.07373  -5.266 1.43e-07 ***
## siteousPrisma  0.08164    0.30503   0.268 0.788982
## siteousSkyra  -0.27516    0.07144  -3.852 0.000118 ***
## siteUB          0.22471    0.13377   1.680 0.093037 .
## siteUCAM       -0.21036    0.07423  -2.834 0.004610 **
## siteUKB        -0.59497    0.05644 -10.542  < 2e-16 ***
## siteUmU        -0.29136    0.08420  -3.460 0.000542 ***
## icv             0.25983    0.01504  17.273  < 2e-16 ***
## ---
## Signif. codes:  0 '***' 0.001 '**' 0.01 '*' 0.05 '.' 0.1 ' ' 1
##
## Approximate significance of smooth terms:
##                      edf Ref.df      F p-value
## s(bl_age)          6.318  6.318 44.322  <2e-16 ***
## s(bl_age):sleep_z 2.422  2.422  0.682   0.643
## s(bl_age):time    2.000  2.000 93.736  <2e-16 ***
## s(sleep_z):time   1.000  1.000  0.515   0.473
## ---
## Signif. codes:  0 '***' 0.001 '**' 0.01 '*' 0.05 '.' 0.1 ' ' 1
##
## R-sq.(adj) =  0.233
## lmer.REML =  16184  Scale est. = 0.16045   n = 8156
```

Below are plots of a plot of `s(bl_age)`, `s(bl_age):sleep_z`, `s(bl_age):time`, and `s(sleep_z):time`. For `s(sleep_z):time`, roughly, if we cannot fit a straight line through the confidence bands, there is evidence that the effect of time depends on sleep duration. It is correct the that confidence interval is zero at one point along the axis; this happens because we do not include the main effect.

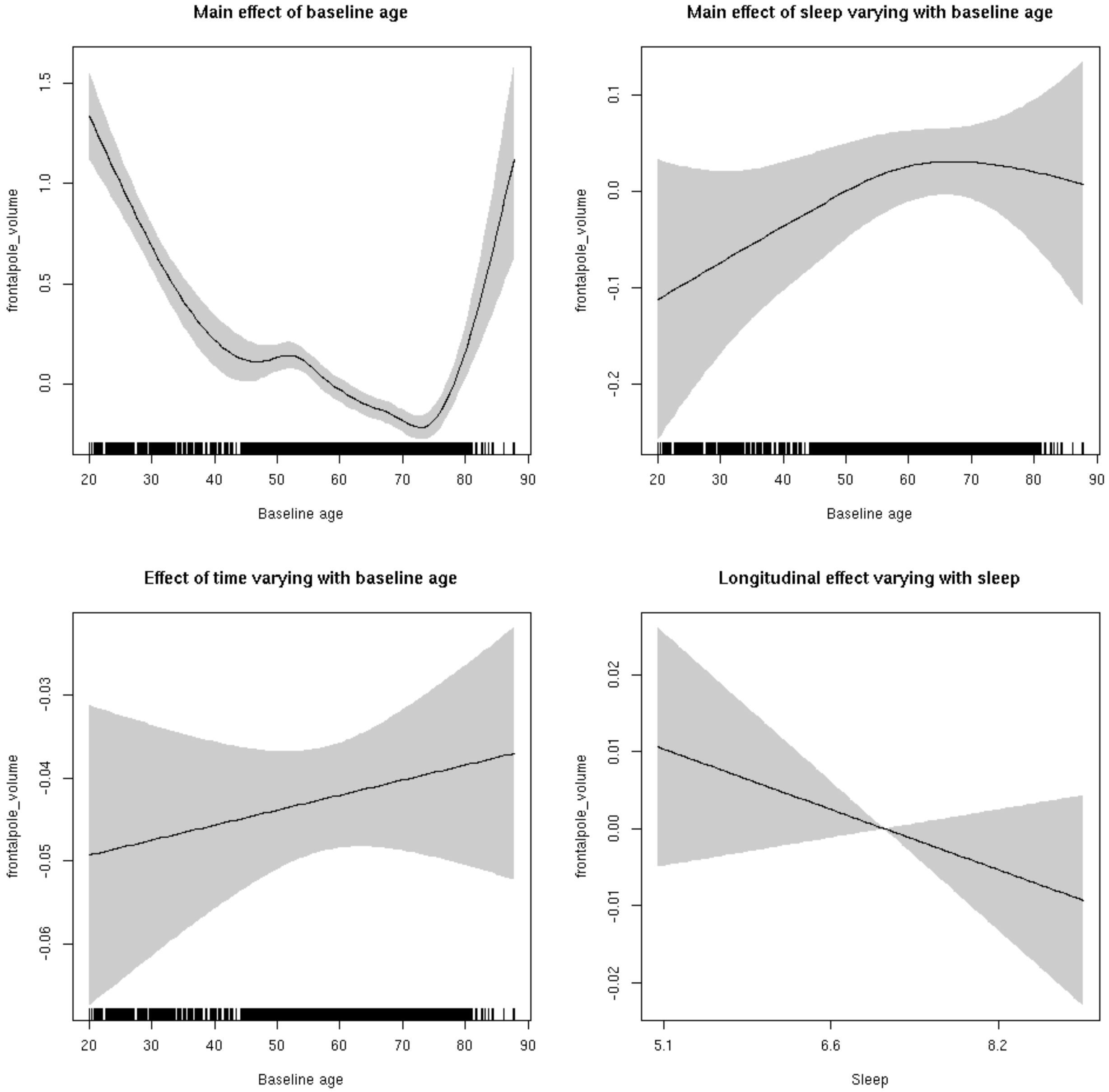

Restricted to sleep >= 5 and <= 9 hours Below is the row for `s(sleep_z):time` from the model output.

```
## s(sleep_z):time   1.000  1.000  1.855   0.173
```

Below are plots of a plot of `s(bl_age)`, `s(bl_age):sleep_z`, `s(bl_age):time`, and `s(sleep_z):time`. For `s(sleep_z):time`, roughly, if we cannot fit a straight line through the confidence bands, there is evidence that the effect of time depends on sleep duration. It is correct the that confidence interval is zero at one point along the axis; this happens because we do not include the main effect.

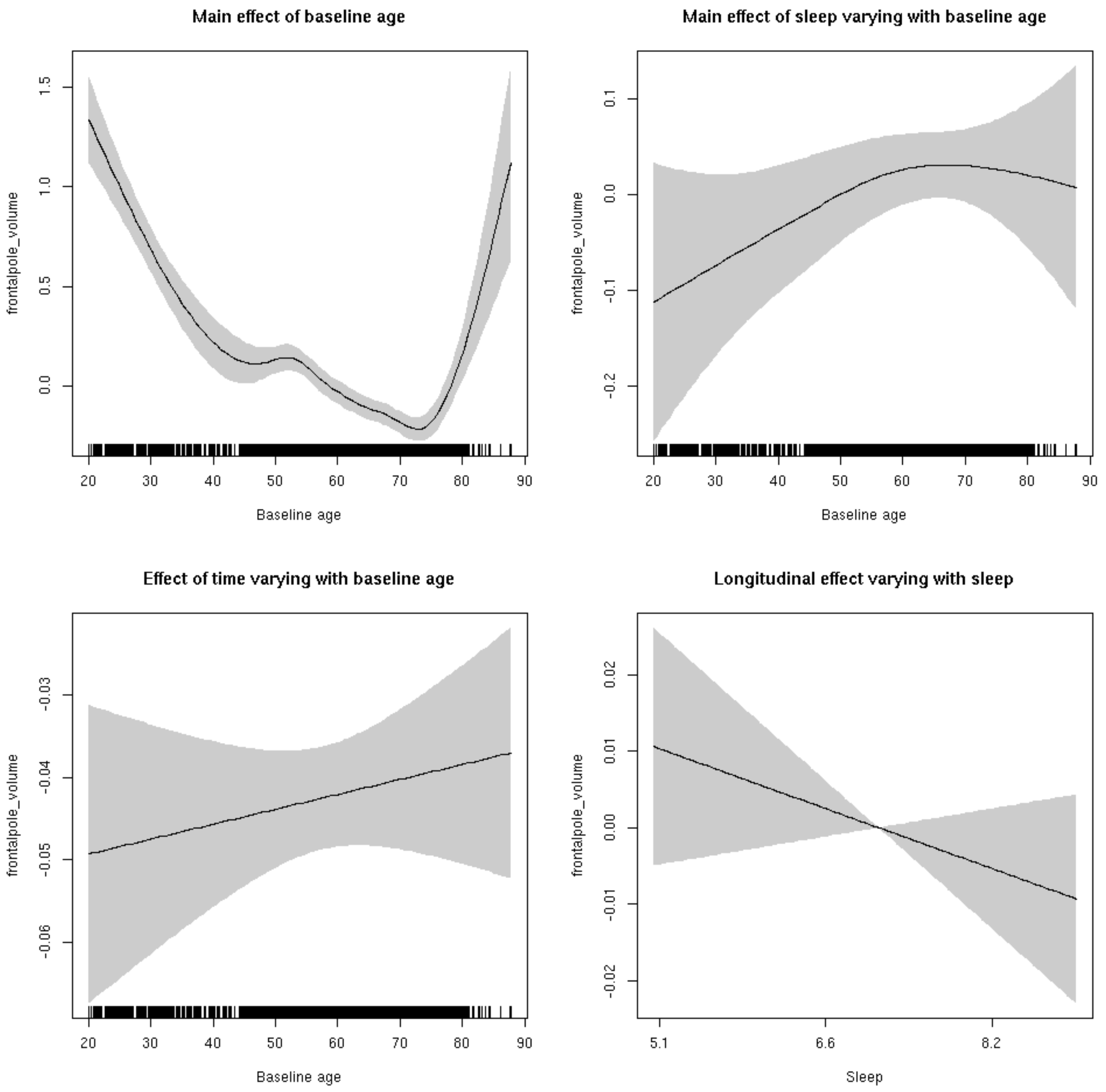

fusiform_area: Stratified by sex

Female: Full data Below is the row for `s(sleep_z):time` from the model output.

```
## s(sleep_z):time   1.000  1.000   2.055   0.152
```

Below are plots of a plot of `s(bl_age)`, `s(bl_age):sleep_z`, `s(bl_age):time`, and `s(sleep_z):time`. For `s(sleep_z):time`, roughly, if we cannot fit a straight line through the confidence bands, there is evidence that the effect of time depends on sleep duration. It is correct the that confidence interval is zero at one point along the axis; this happens because we do not include the main effect.

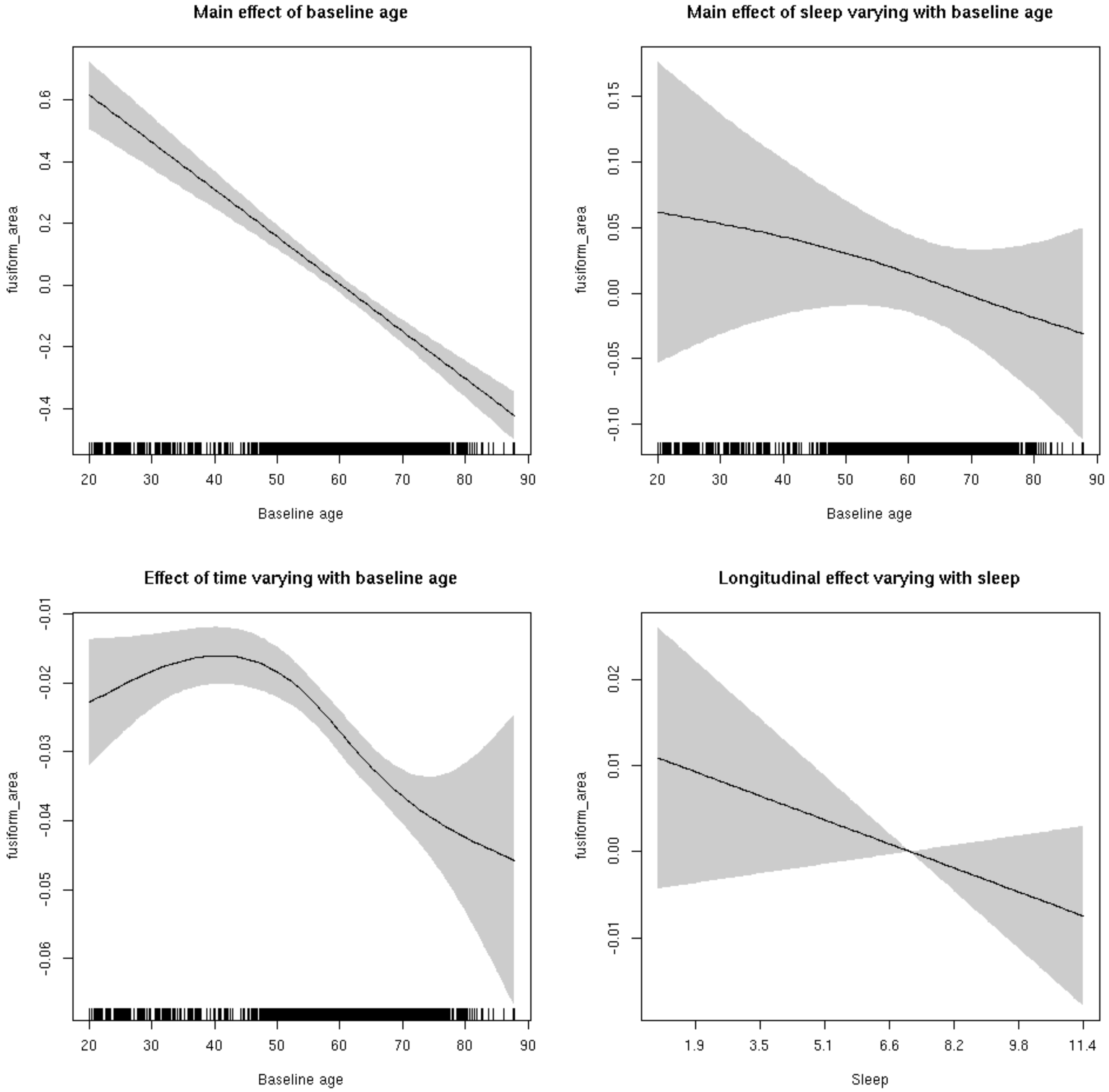

Male: Full data Below is the row for `s(sleep_z):time` from the model output.

```
## s(sleep_z):time   1.000  1.000   0.082   0.775
```

Below are plots of a plot of `s(bl_age)`, `s(bl_age):sleep_z`, `s(bl_age):time`, and `s(sleep_z):time`. For `s(sleep_z):time`, roughly, if we cannot fit a straight line through the confidence bands, there is evidence that the effect of time depends on sleep duration. It is correct the that confidence interval is zero at one point along the axis; this happens because we do not include the main effect.

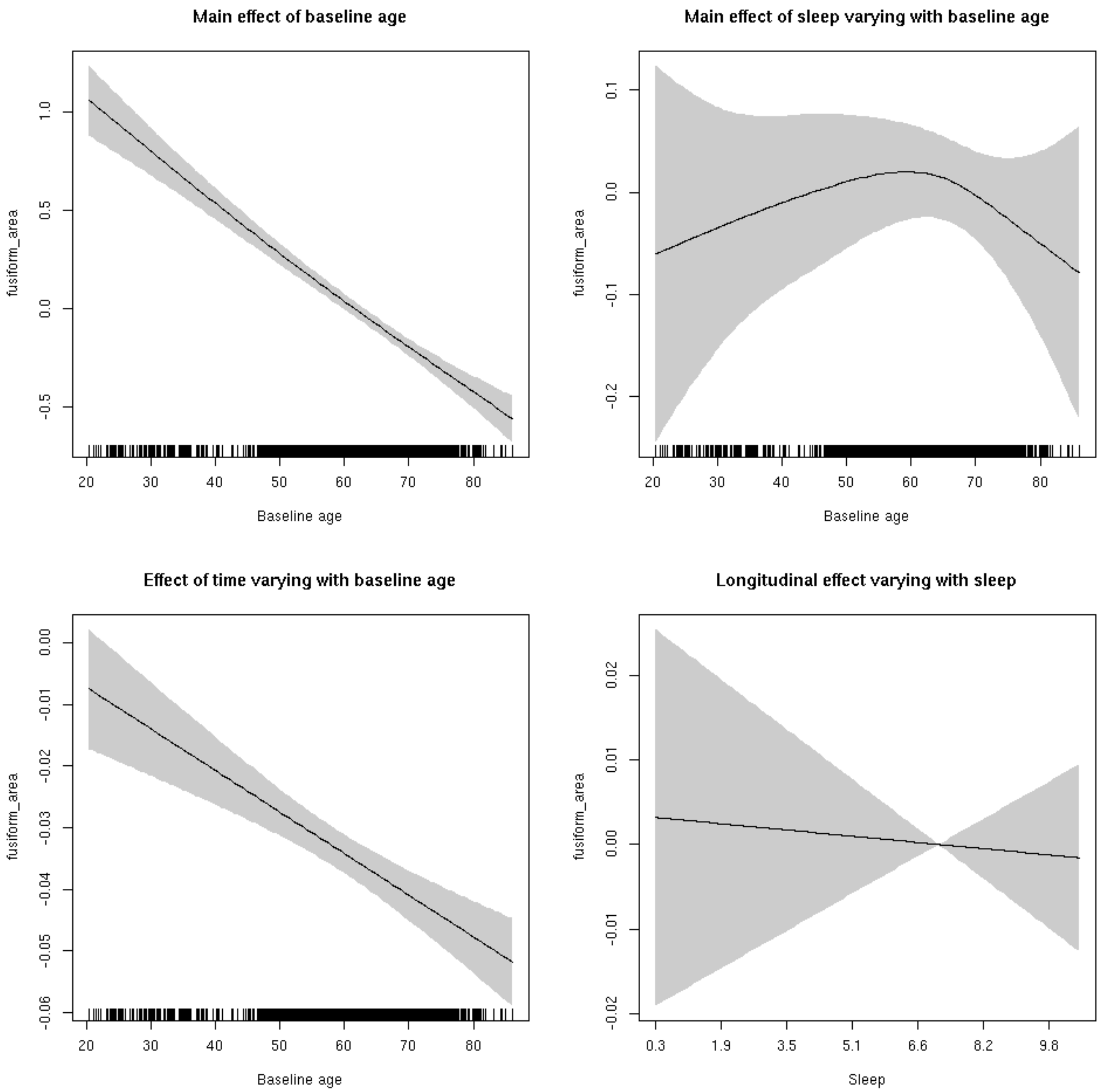

fusiform_area

Full data Below is the model output. The term `s(sleep_z):time` is probably what we care most about.

```
##
## Family: gaussian
## Link function: identity
##
## Formula:
## value ~ sex + site + icv + s(bl_age, k = 10, bs = "cr") + s(bl_age,
##     by = sleep_z, bs = "cr") + +s(bl_age, by = time, k = 5, bs = "cr") +
##     s(sleep_z, by = time, k = 5, bs = "cr", pc = 0)
## <environment: 0x5630035aae60>
```

```
##
## Parametric coefficients:
##                Estimate Std. Error t value Pr(>|t|)
## (Intercept)     0.52552    0.05062  10.381  < 2e-16 ***
## sexmale         0.40374    0.02632  15.340  < 2e-16 ***
## siteousAvanto -0.72947    0.06228 -11.713  < 2e-16 ***
## siteousPrisma -1.00984    0.11273  -8.958  < 2e-16 ***
## siteousSkyra  -0.30556    0.06032  -5.065 4.17e-07 ***
## siteUB        -0.78747    0.11853  -6.644 3.26e-11 ***
## siteUCAM      -0.63674    0.06322 -10.071  < 2e-16 ***
## siteUKB       -0.70737    0.04724 -14.973  < 2e-16 ***
## siteUmU       -0.54499    0.07298  -7.467 9.04e-14 ***
## icv            0.53246    0.01279  41.639  < 2e-16 ***
## ---
## Signif. codes:  0 '***' 0.001 '**' 0.01 '*' 0.05 '.' 0.1 ' ' 1
##
## Approximate significance of smooth terms:
##                      edf Ref.df       F p-value
## s(bl_age)          1.000  1.000 373.099  <2e-16 ***
## s(bl_age):sleep_z 3.172  3.172   1.693   0.174
## s(bl_age):time    3.988  3.988 265.645  <2e-16 ***
## s(sleep_z):time   1.000  1.000   0.983   0.322
## ---
## Signif. codes:  0 '***' 0.001 '**' 0.01 '*' 0.05 '.' 0.1 ' ' 1
##
## R-sq.(adj) =   0.54
## lmer.REML = 4890.2  Scale est. = 0.014406  n = 8164
```

Below are plots of a plot of `s(bl_age)`, `s(bl_age):sleep_z`, `s(bl_age):time`, and `s(sleep_z):time`. For `s(sleep_z):time`, roughly, if we cannot fit a straight line through the confidence bands, there is evidence that the effect of time depends on sleep duration. It is correct the that confidence interval is zero at one point along the axis; this happens because we do not include the main effect.

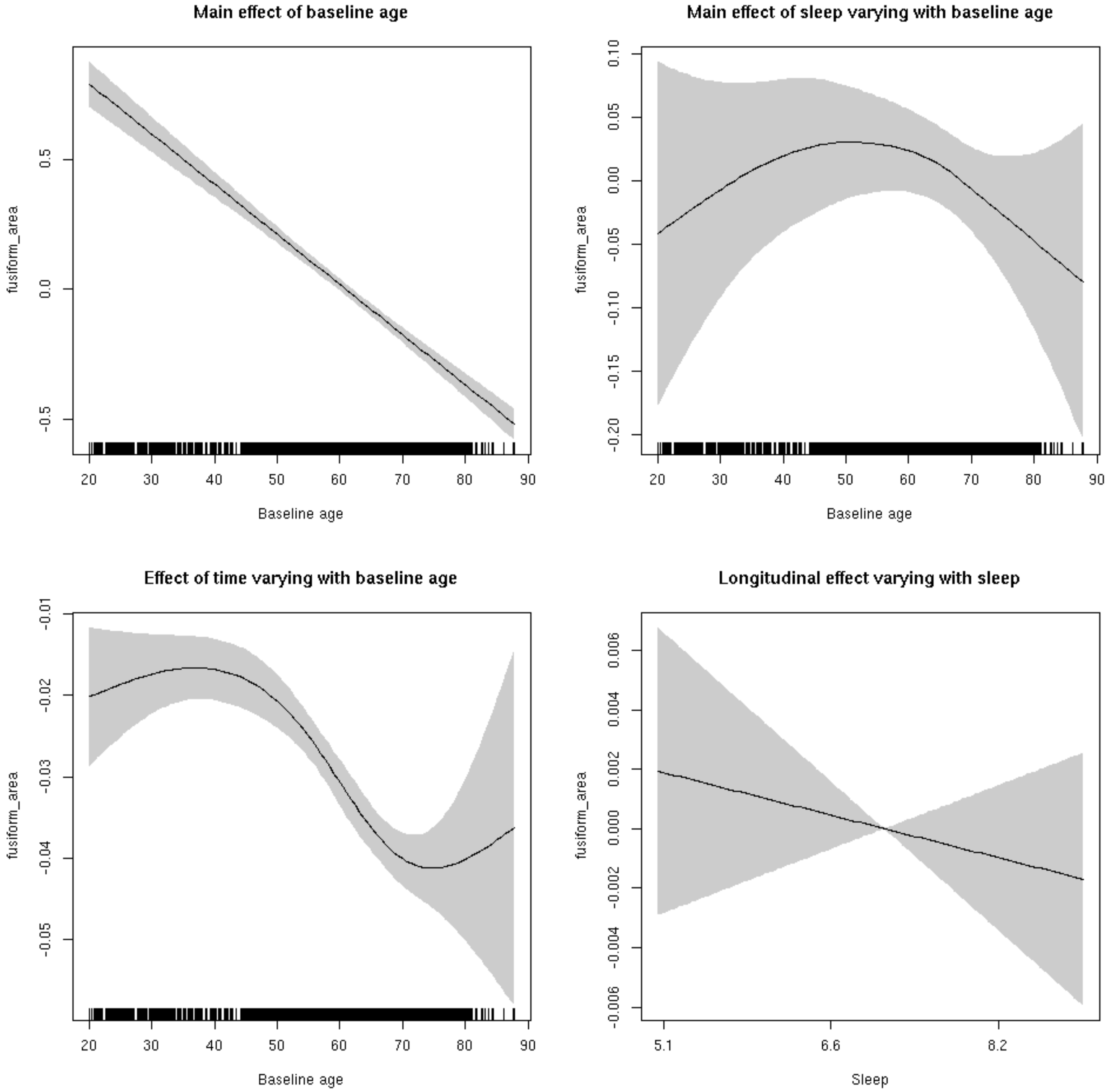

Restricted to sleep >= 5 and <= 9 hours Below is the row for `s(sleep_z):time` from the model output.

```
## s(sleep_z):time   1.000  1.000   0.639   0.424
```

Below are plots of a plot of `s(bl_age)`, `s(bl_age):sleep_z`, `s(bl_age):time`, and `s(sleep_z):time`. For `s(sleep_z):time`, roughly, if we cannot fit a straight line through the confidence bands, there is evidence that the effect of time depends on sleep duration. It is correct the that confidence interval is zero at one point along the axis; this happens because we do not include the main effect.

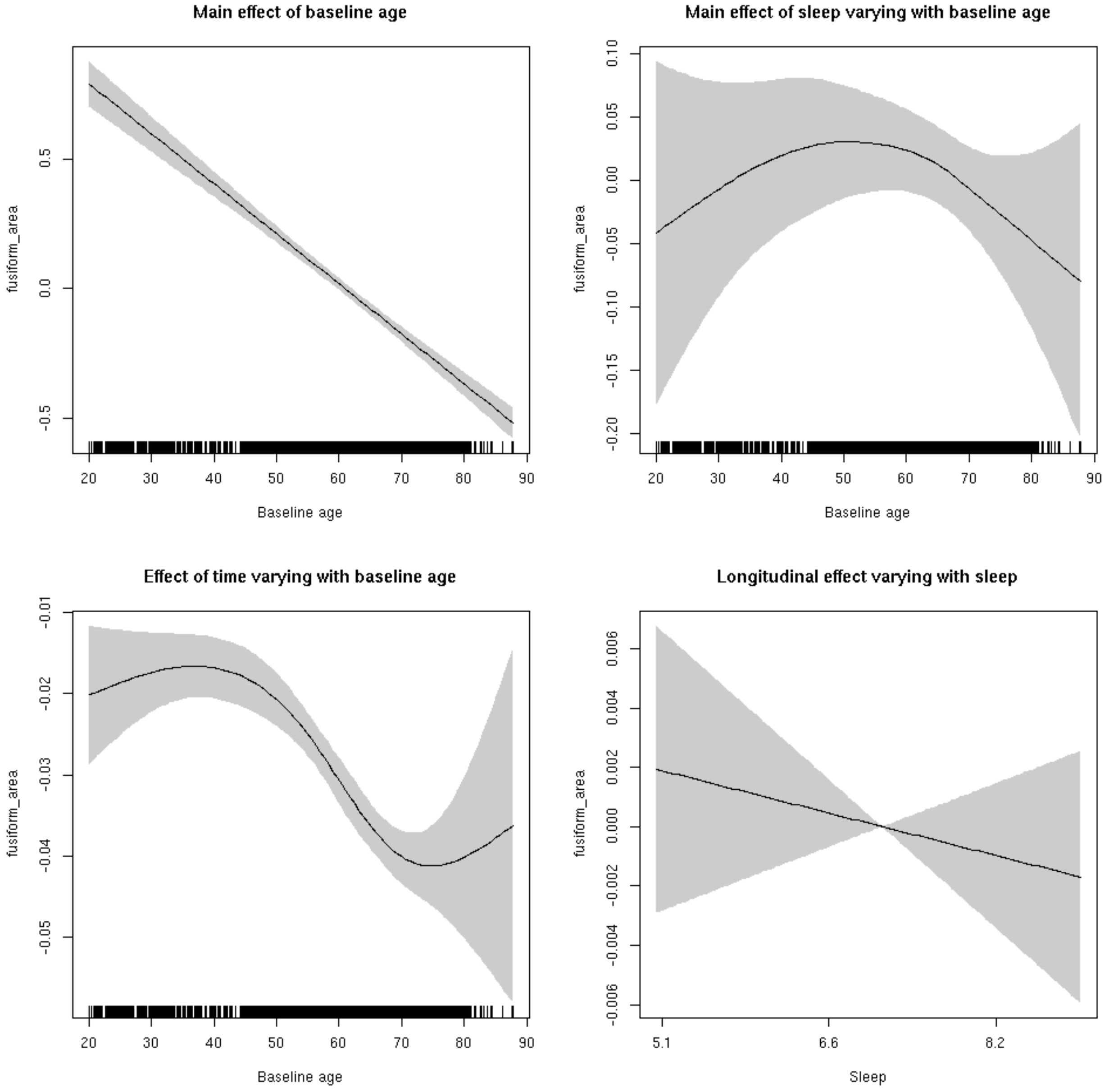

fusiform_thickness: Stratified by sex

Female: Full data Below is the row for `s(sleep_z):time` from the model output.

```
## s(sleep_z):time   1.000  1.000  2.275   0.132
```

Below are plots of a plot of `s(bl_age)`, `s(bl_age):sleep_z`, `s(bl_age):time`, and `s(sleep_z):time`. For `s(sleep_z):time`, roughly, if we cannot fit a straight line through the confidence bands, there is evidence that the effect of time depends on sleep duration. It is correct the that confidence interval is zero at one point along the axis; this happens because we do not include the main effect.

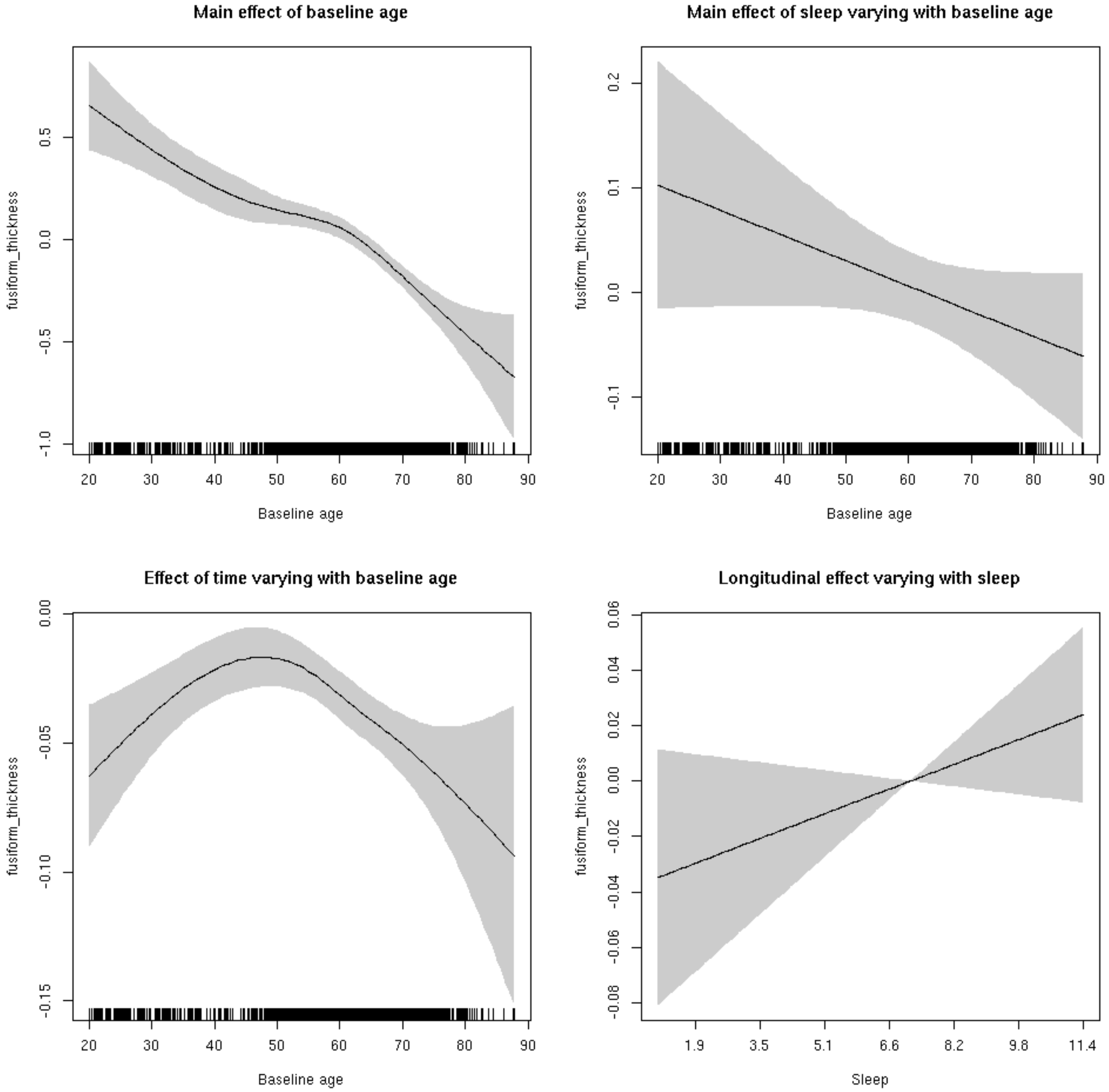

Male: Full data Below is the row for `s(sleep_z):time` from the model output.

```
## s(sleep_z):time   1.000  1.000  0.515   0.473
```

Below are plots of a plot of `s(bl_age)`, `s(bl_age):sleep_z`, `s(bl_age):time`, and `s(sleep_z):time`. For `s(sleep_z):time`, roughly, if we cannot fit a straight line through the confidence bands, there is evidence that the effect of time depends on sleep duration. It is correct the that confidence interval is zero at one point along the axis; this happens because we do not include the main effect.

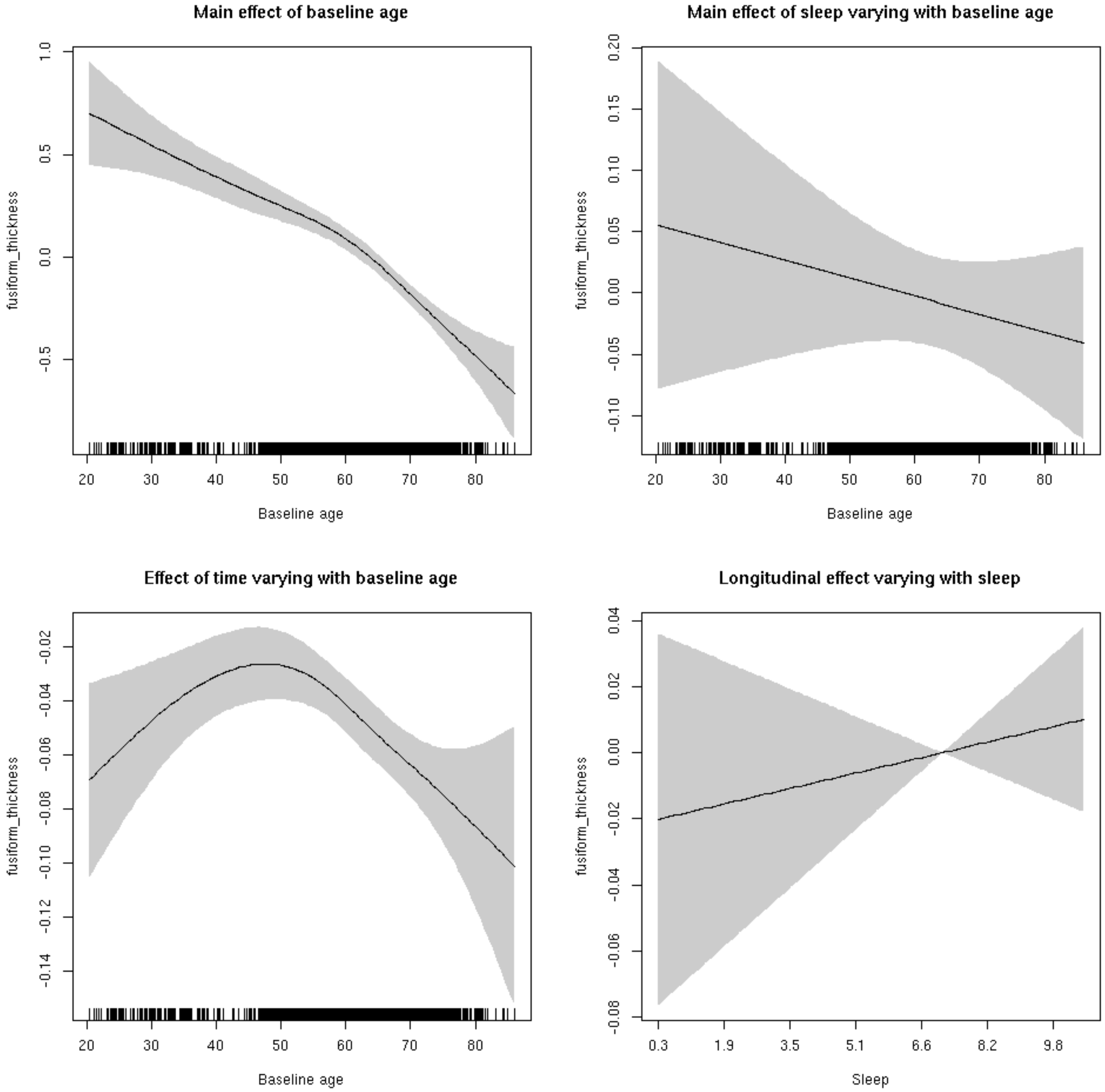

fusiform_thickness

Full data Below is the model output. The term `s(sleep_z):time` is probably what we care most about.

```
##
## Family: gaussian
## Link function: identity
##
## Formula:
## value ~ sex + site + s(bl_age, k = 10, bs = "cr") + s(bl_age,
##     by = sleep_z, bs = "cr") + +s(bl_age, by = time, k = 5, bs = "cr") +
##     s(sleep_z, by = time, k = 5, bs = "cr", pc = 0)
## <environment: 0x5630035ca300>
```

```
##
## Parametric coefficients:
##                Estimate Std. Error t value Pr(>|t|)    
## (Intercept)   -1.95267    0.04665 -41.861  < 2e-16 ***
## sexmale       -0.02020    0.02336  -0.864  0.38739    
## siteousAvanto  0.19443    0.06184   3.144  0.00167 ** 
## siteousPrisma  0.62291    0.25558   2.437  0.01482 *  
## siteousSkyra   0.85388    0.06182  13.813  < 2e-16 ***
## siteUB         0.42290    0.12382   3.415  0.00064 ***
## siteUCAM       0.70146    0.06431  10.908  < 2e-16 ***
## siteUKB        2.25496    0.04733  47.642  < 2e-16 ***
## siteUmU        0.34154    0.07685   4.444 8.93e-06 ***
## ---
## Signif. codes:  0 '***' 0.001 '**' 0.01 '*' 0.05 '.' 0.1 ' ' 1
##
## Approximate significance of smooth terms:
##                      edf Ref.df      F p-value    
## s(bl_age)          4.085  4.085 77.923  <2e-16 ***
## s(bl_age):sleep_z 2.000  2.000  2.024   0.132    
## s(bl_age):time     3.928  3.928 75.390  <2e-16 ***
## s(sleep_z):time    1.000  1.000  2.689   0.101    
## ---
## Signif. codes:  0 '***' 0.001 '**' 0.01 '*' 0.05 '.' 0.1 ' ' 1
##
## R-sq.(adj) =  0.581
## lmer.REML =  14094  Scale est. = 0.11103   n = 8170
```

Below are plots of a plot of `s(bl_age)`, `s(bl_age):sleep_z`, `s(bl_age):time`, and `s(sleep_z):time`. For `s(sleep_z):time`, roughly, if we cannot fit a straight line through the confidence bands, there is evidence that the effect of time depends on sleep duration. It is correct the that confidence interval is zero at one point along the axis; this happens because we do not include the main effect.

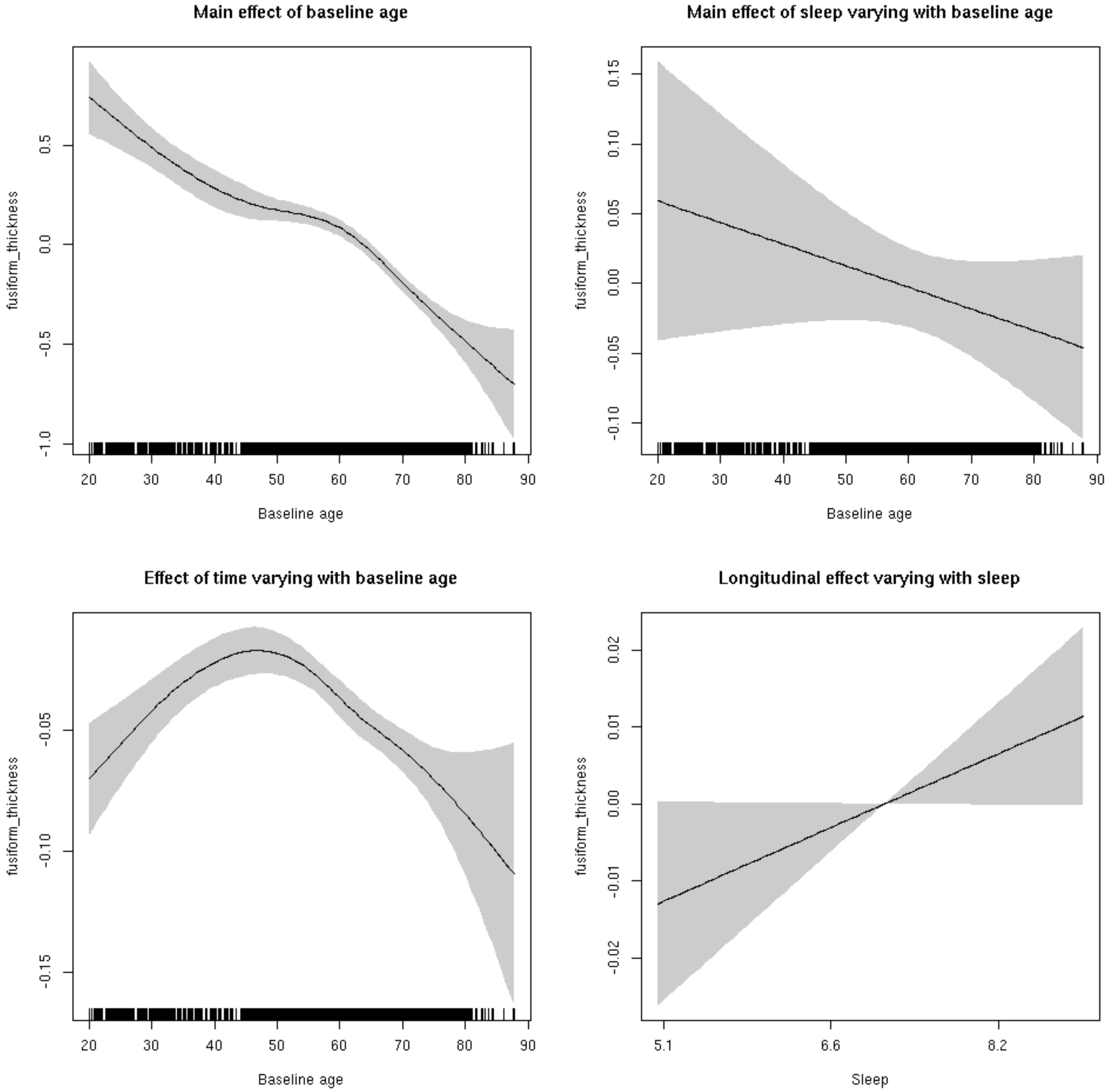

Restricted to sleep >= 5 and <= 9 hours Below is the row for `s(sleep_z):time` from the model output.

```
## s(sleep_z):time   1.000  1.000  3.842   0.050 .
```

Below are plots of a plot of `s(bl_age)`, `s(bl_age):sleep_z`, `s(bl_age):time`, and `s(sleep_z):time`. For `s(sleep_z):time`, roughly, if we cannot fit a straight line through the confidence bands, there is evidence that the effect of time depends on sleep duration. It is correct the that confidence interval is zero at one point along the axis; this happens because we do not include the main effect.

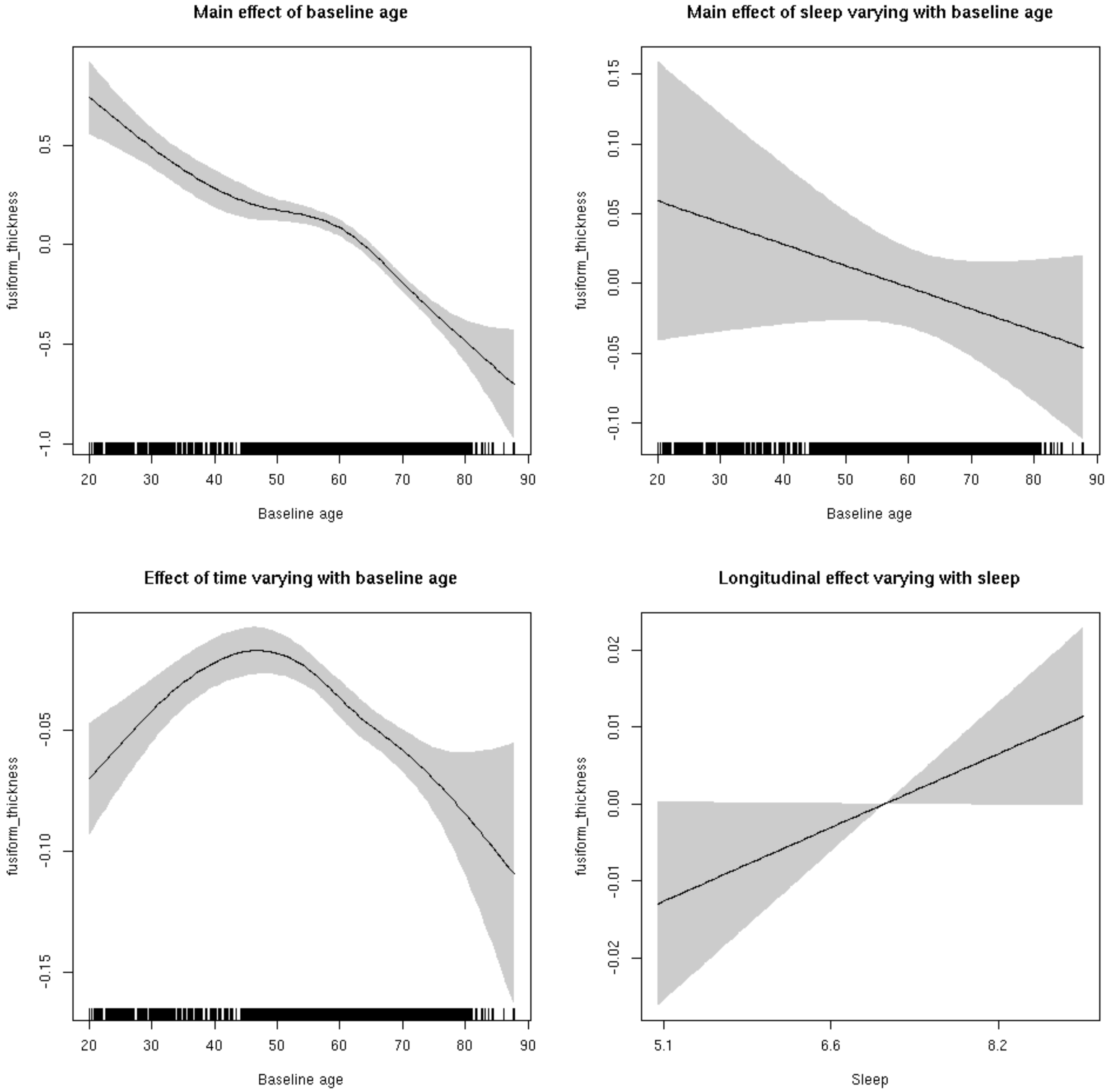

fusiform_volume: Stratified by sex

Female: Full data Below is the row for `s(sleep_z):time` from the model output.

```
## s(sleep_z):time   1.000  1.000   0.001  0.9709
```

Below are plots of a plot of `s(bl_age)`, `s(bl_age):sleep_z`, `s(bl_age):time`, and `s(sleep_z):time`. For `s(sleep_z):time`, roughly, if we cannot fit a straight line through the confidence bands, there is evidence that the effect of time depends on sleep duration. It is correct the that confidence interval is zero at one point along the axis; this happens because we do not include the main effect.

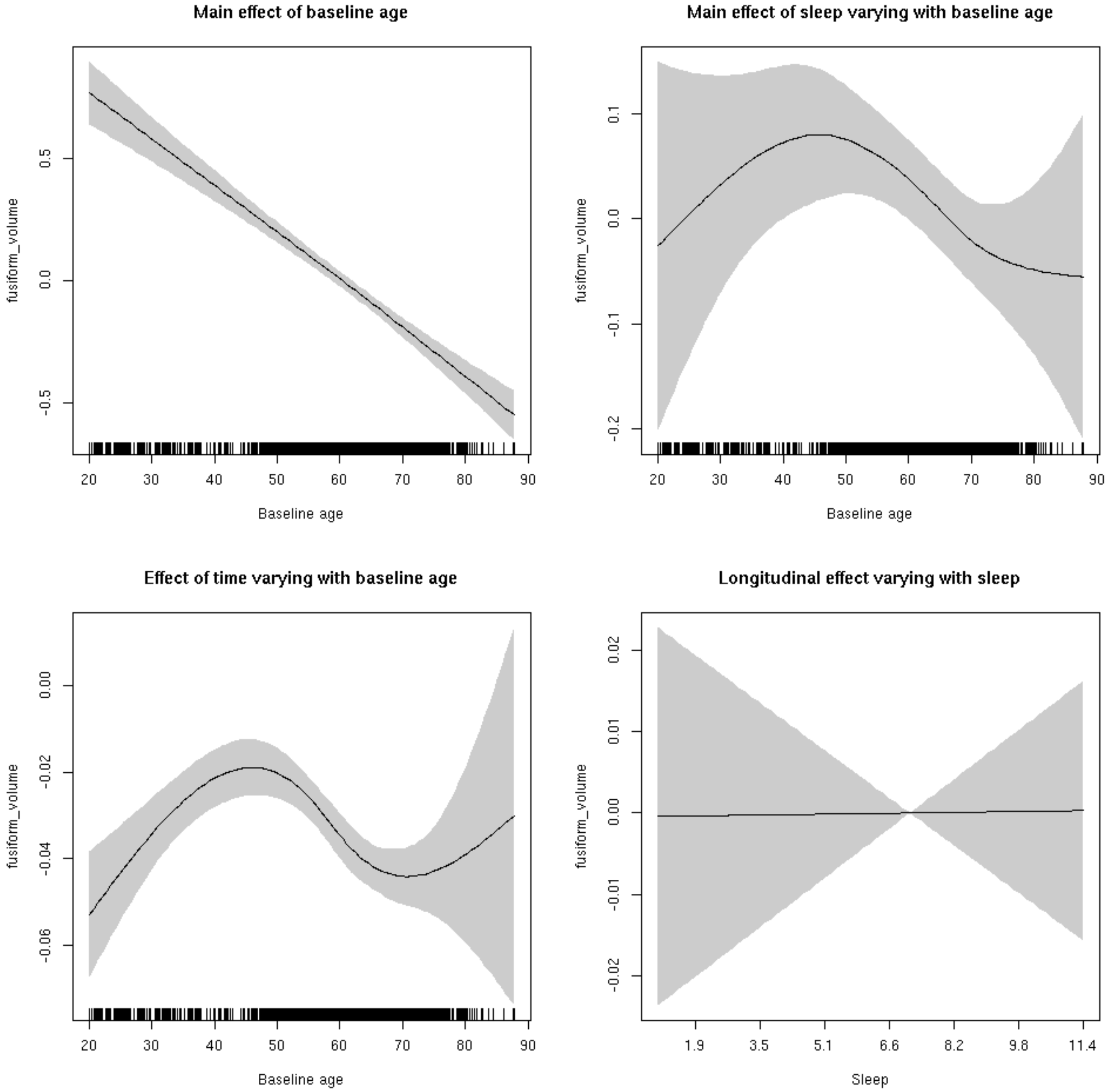

Male: Full data Below is the row for `s(sleep_z):time` from the model output.

```
## s(sleep_z):time   1.000  1.000   1.139   0.286
```

Below are plots of a plot of `s(bl_age)`, `s(bl_age):sleep_z`, `s(bl_age):time`, and `s(sleep_z):time`. For `s(sleep_z):time`, roughly, if we cannot fit a straight line through the confidence bands, there is evidence that the effect of time depends on sleep duration. It is correct the that confidence interval is zero at one point along the axis; this happens because we do not include the main effect.

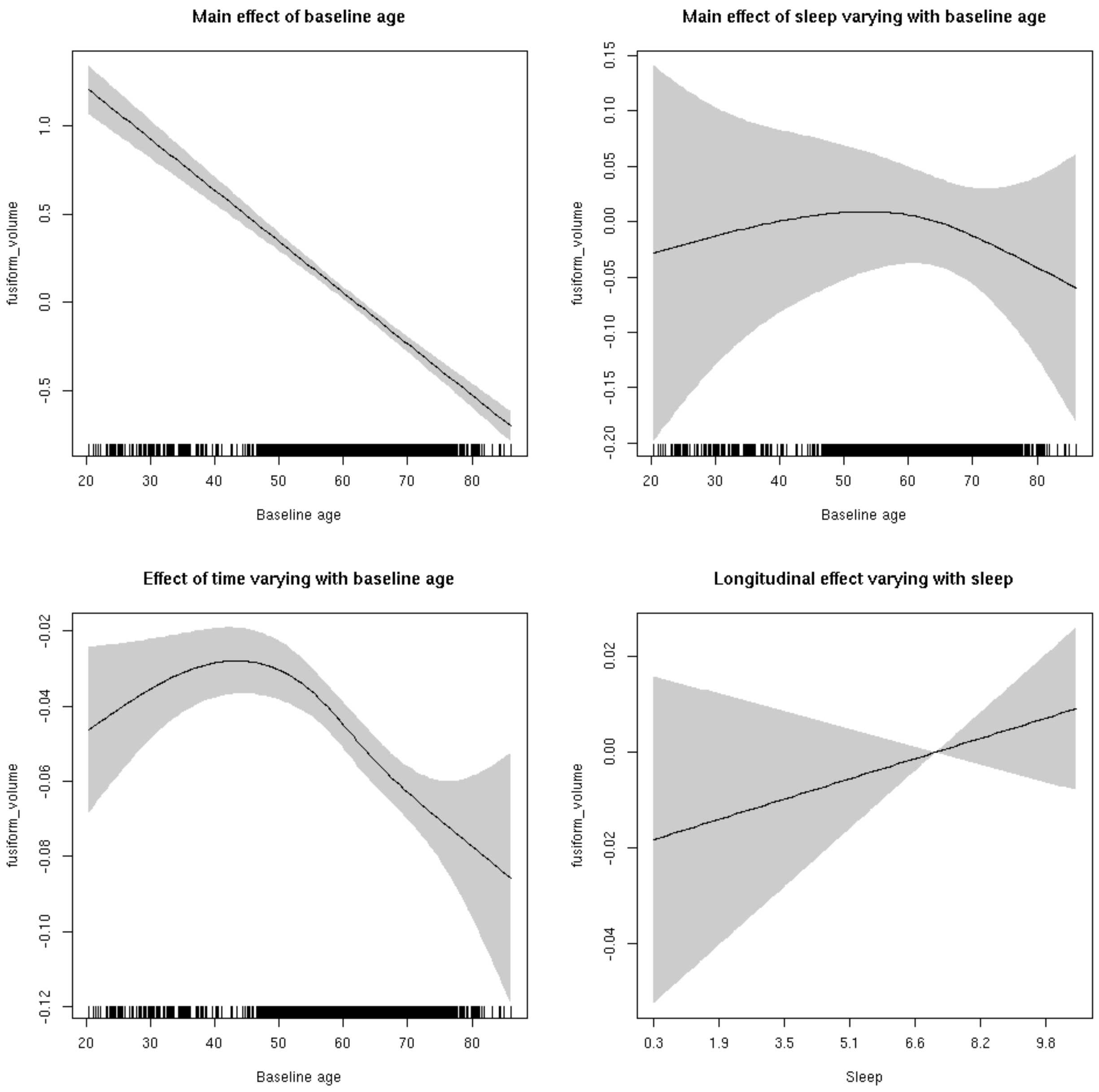

fusiform_volume

Full data Below is the model output. The term `s(sleep_z):time` is probably what we care most about.

```
##
## Family: gaussian
## Link function: identity
##
## Formula:
## value ~ sex + site + icv + s(bl_age, k = 10, bs = "cr") + s(bl_age,
##     by = sleep_z, bs = "cr") + +s(bl_age, by = time, k = 5, bs = "cr") +
##     s(sleep_z, by = time, k = 5, bs = "cr", pc = 0)
## <environment: 0x5630035a2060>
```

```
##
## Parametric coefficients:
##                Estimate Std. Error t value Pr(>|t|)
## (Intercept)   -0.48209    0.05203  -9.265  < 2e-16 ***
## sexmale        0.29929    0.02703  11.072  < 2e-16 ***
## siteousAvanto -0.45788    0.06421  -7.131 1.08e-12 ***
## siteousPrisma -0.44492    0.15543  -2.862  0.00421 **
## siteousSkyra   0.25646    0.06212   4.129 3.69e-05 ***
## siteUB        -0.37476    0.12092  -3.099  0.00195 **
## siteUCAM      -0.18123    0.06478  -2.797  0.00516 **
## siteUKB        0.51270    0.04848  10.576  < 2e-16 ***
## siteUmU       -0.11521    0.07468  -1.543  0.12297
## icv            0.51226    0.01330  38.515  < 2e-16 ***
## ---
## Signif. codes:  0 '***' 0.001 '**' 0.01 '*' 0.05 '.' 0.1 ' ' 1
##
## Approximate significance of smooth terms:
##                      edf Ref.df       F p-value
## s(bl_age)          1.000  1.000 534.375  <2e-16 ***
## s(bl_age):sleep_z 3.380  3.380   3.400  0.0208 *
## s(bl_age):time    4.109  4.109 205.216  <2e-16 ***
## s(sleep_z):time   1.000  1.000   0.613  0.4337
## ---
## Signif. codes:  0 '***' 0.001 '**' 0.01 '*' 0.05 '.' 0.1 ' ' 1
##
## R-sq.(adj) =  0.546
## lmer.REML = 8712.4  Scale est. = 0.033805  n = 8174
```

Below are plots of a plot of `s(bl_age)`, `s(bl_age):sleep_z`, `s(bl_age):time`, and `s(sleep_z):time`. For `s(sleep_z):time`, roughly, if we cannot fit a straight line through the confidence bands, there is evidence that the effect of time depends on sleep duration. It is correct the that confidence interval is zero at one point along the axis; this happens because we do not include the main effect.

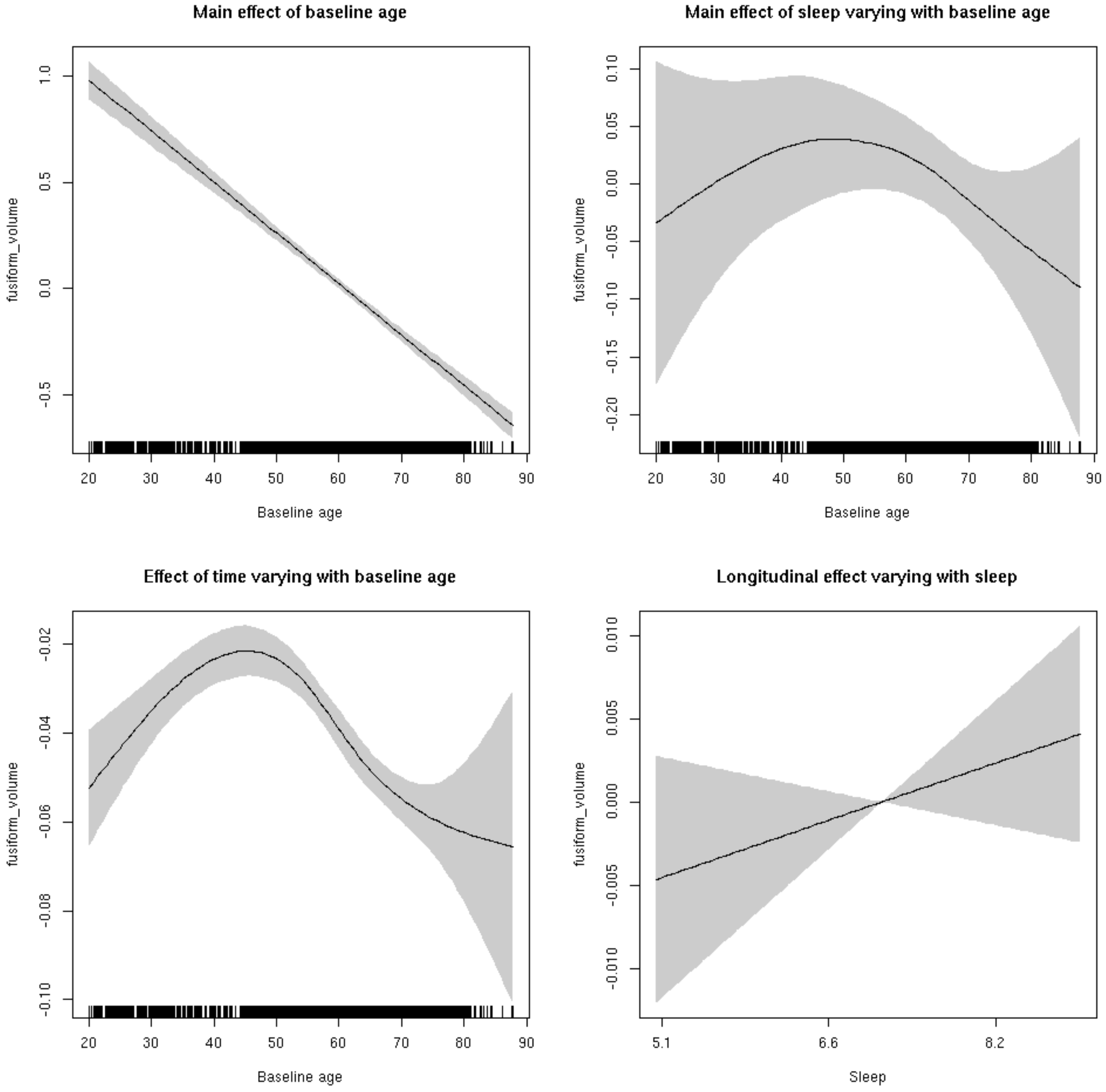

Restricted to sleep >= 5 and <= 9 hours Below is the row for `s(sleep_z):time` from the model output.

```
## s(sleep_z):time   1.000  1.000   1.571   0.210
```

Below are plots of a plot of `s(bl_age)`, `s(bl_age):sleep_z`, `s(bl_age):time`, and `s(sleep_z):time`. For `s(sleep_z):time`, roughly, if we cannot fit a straight line through the confidence bands, there is evidence that the effect of time depends on sleep duration. It is correct the that confidence interval is zero at one point along the axis; this happens because we do not include the main effect.

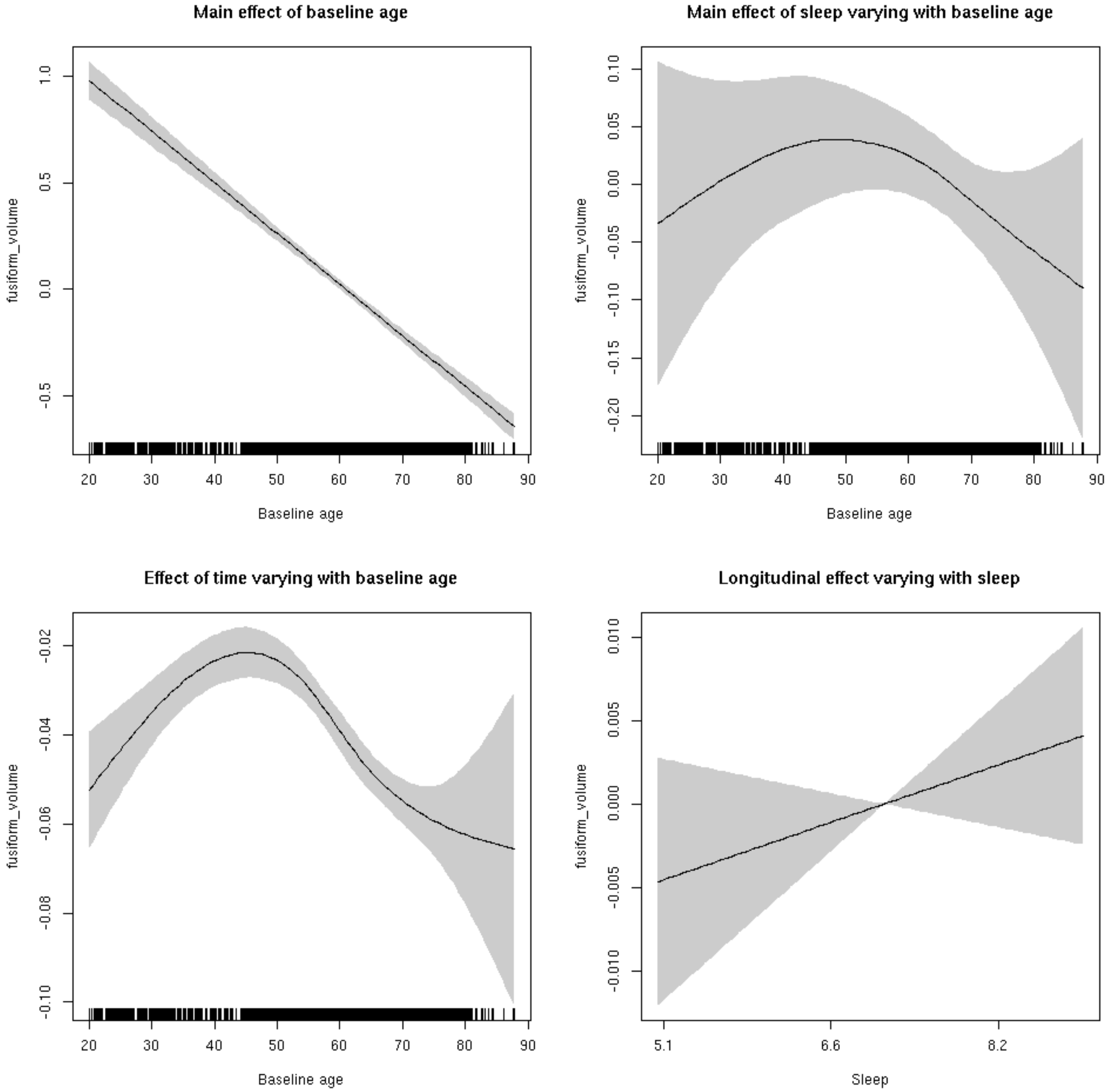

inferiorparietal_area: Stratified by sex

Female: Full data Below is the row for `s(sleep_z):time` from the model output.

```
## s(sleep_z):time   1.000  1.000   0.233   0.629
```

Below are plots of a plot of `s(bl_age)`, `s(bl_age):sleep_z`, `s(bl_age):time`, and `s(sleep_z):time`. For `s(sleep_z):time`, roughly, if we cannot fit a straight line through the confidence bands, there is evidence that the effect of time depends on sleep duration. It is correct the that confidence interval is zero at one point along the axis; this happens because we do not include the main effect.

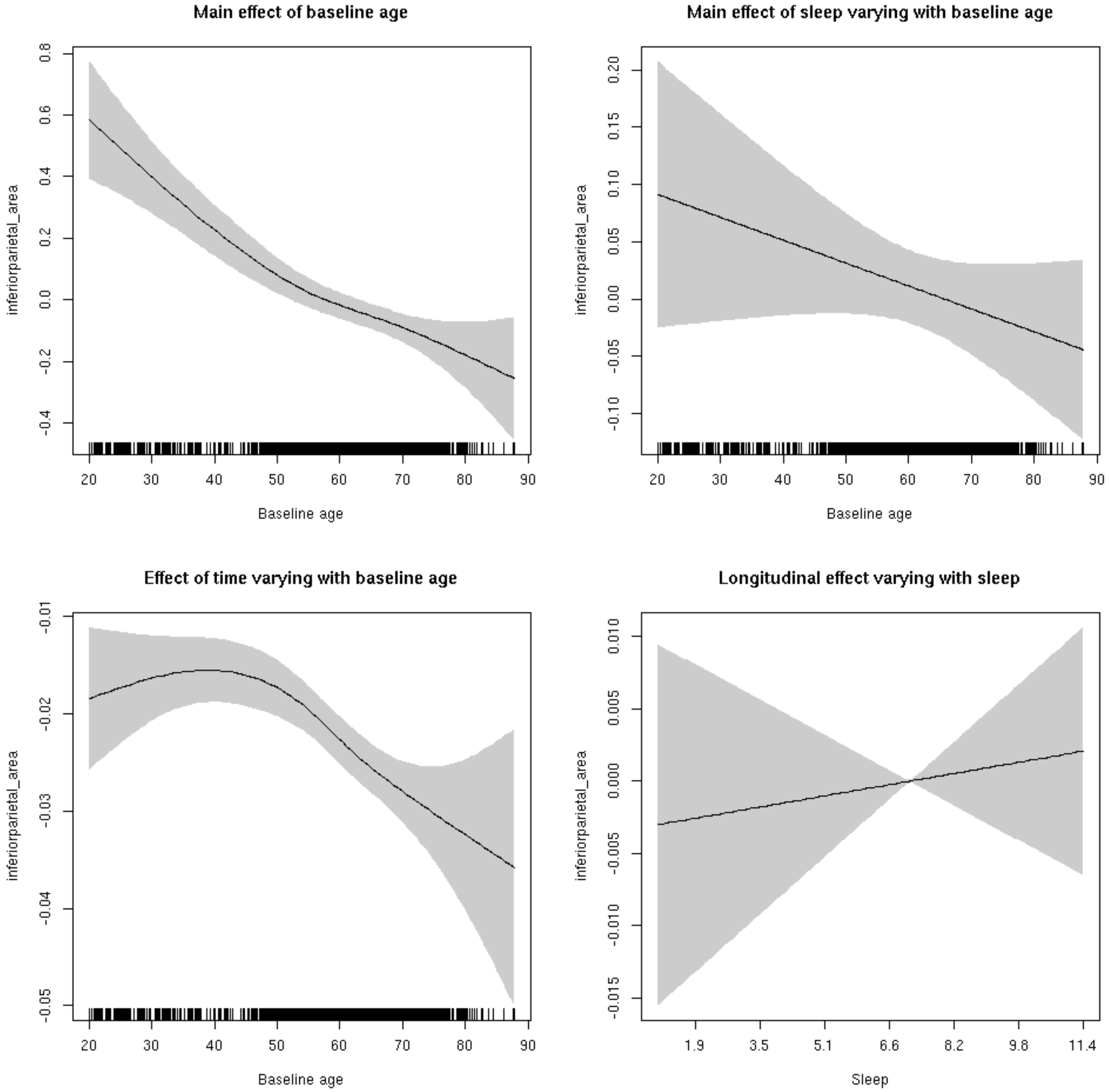

Male: Full data Below is the row for `s(sleep_z):time` from the model output.

```
## s(sleep_z):time   1.000  1.000   0.717   0.397
```

Below are plots of a plot of `s(bl_age)`, `s(bl_age):sleep_z`, `s(bl_age):time`, and `s(sleep_z):time`. For `s(sleep_z):time`, roughly, if we cannot fit a straight line through the confidence bands, there is evidence that the effect of time depends on sleep duration. It is correct the that confidence interval is zero at one point along the axis; this happens because we do not include the main effect.

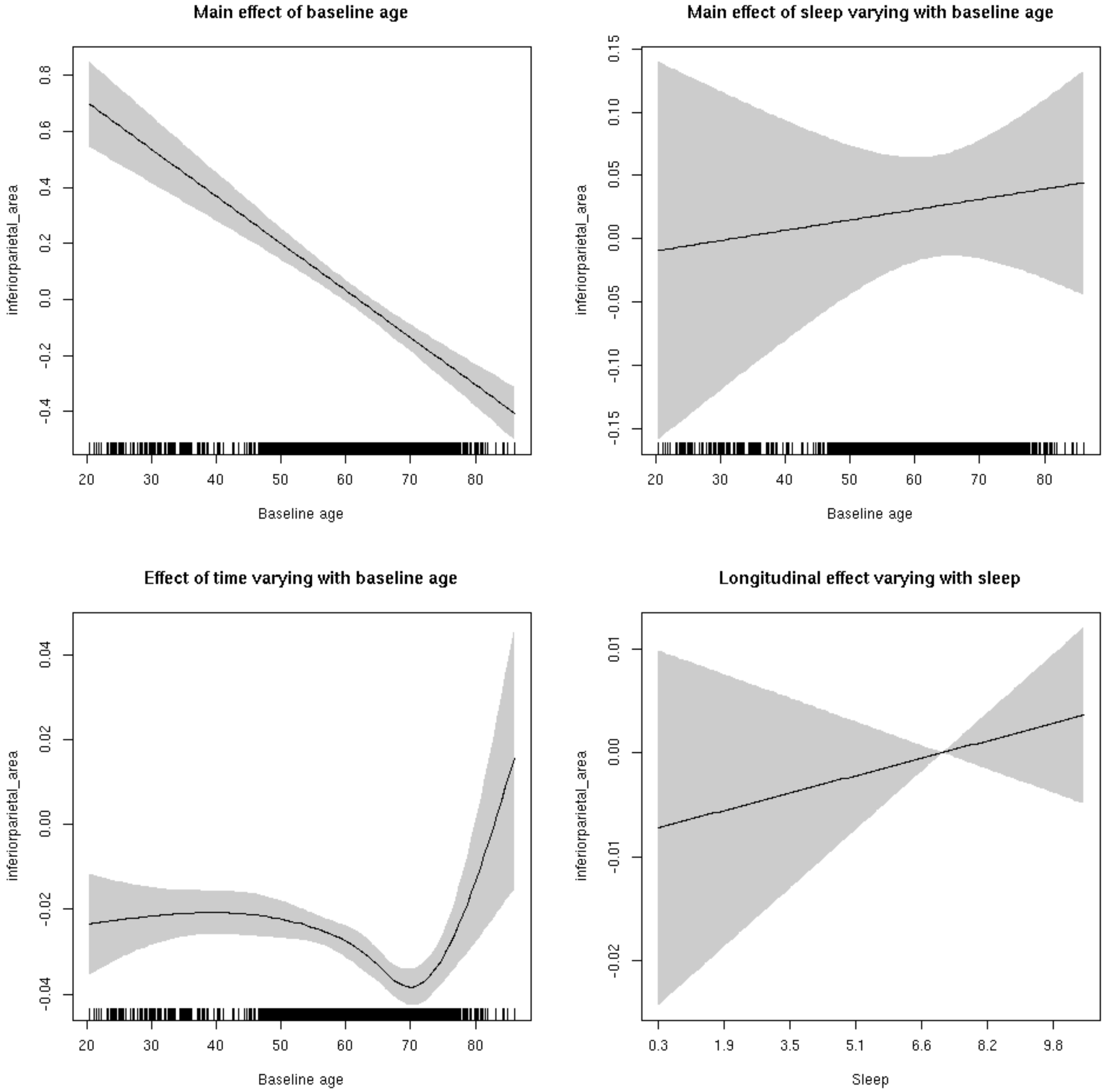

inferiorparietal_area

Full data Below is the model output. The term `s(sleep_z):time` is probably what we care most about.

```
##
## Family: gaussian
## Link function: identity
##
## Formula:
## value ~ sex + site + icv + s(bl_age, k = 10, bs = "cr") + s(bl_age,
##     by = sleep_z, bs = "cr") + +s(bl_age, by = time, k = 5, bs = "cr") +
##     s(sleep_z, by = time, k = 5, bs = "cr", pc = 0)
## <environment: 0x5630035a3768>
```

```
##
## Parametric coefficients:
##                Estimate Std. Error t value Pr(>|t|)
## (Intercept)     0.78687    0.05740  13.710  < 2e-16 ***
## sexmale         0.22658    0.02947   7.687 1.68e-14 ***
## siteousAvanto -0.75593    0.06991 -10.812  < 2e-16 ***
## siteousPrisma -0.76380    0.10282  -7.428 1.21e-13 ***
## siteousSkyra  -0.18162    0.06788  -2.676  0.00747 **
## siteUB        -1.02948    0.13409  -7.677 1.81e-14 ***
## siteUCAM      -0.87324    0.07195 -12.137  < 2e-16 ***
## siteUKB       -0.90011    0.05479 -16.430  < 2e-16 ***
## siteUmU       -0.05742    0.08277  -0.694  0.48786
## icv            0.51781    0.01403  36.899  < 2e-16 ***
## ---
## Signif. codes:  0 '***' 0.001 '**' 0.01 '*' 0.05 '.' 0.1 ' ' 1
##
## Approximate significance of smooth terms:
##                     edf Ref.df       F p-value
## s(bl_age)         3.296  3.296  45.108  <2e-16 ***
## s(bl_age):sleep_z 2.000  2.000   0.655   0.519
## s(bl_age):time    4.336  4.336 269.265  <2e-16 ***
## s(sleep_z):time   1.000  1.000   0.699   0.403
## ---
## Signif. codes:  0 '***' 0.001 '**' 0.01 '*' 0.05 '.' 0.1 ' ' 1
##
## R-sq.(adj) =  0.442
## lmer.REML = 3789.5  Scale est. = 0.008913  n = 8155
```

Below are plots of a plot of `s(bl_age)`, `s(bl_age):sleep_z`, `s(bl_age):time`, and `s(sleep_z):time`. For `s(sleep_z):time`, roughly, if we cannot fit a straight line through the confidence bands, there is evidence that the effect of time depends on sleep duration. It is correct the that confidence interval is zero at one point along the axis; this happens because we do not include the main effect.

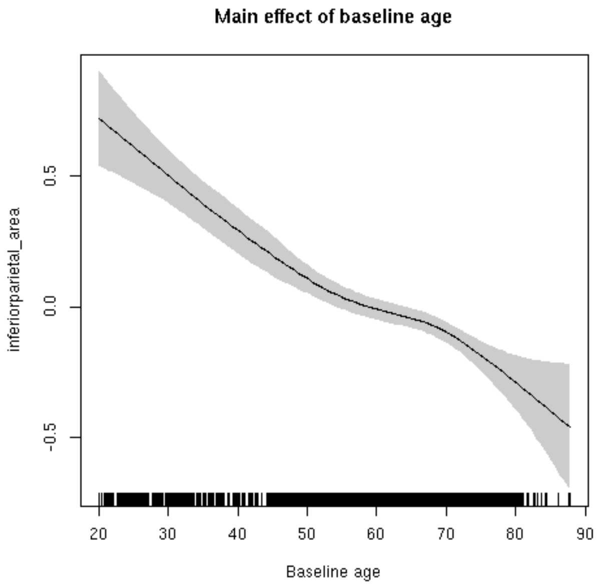

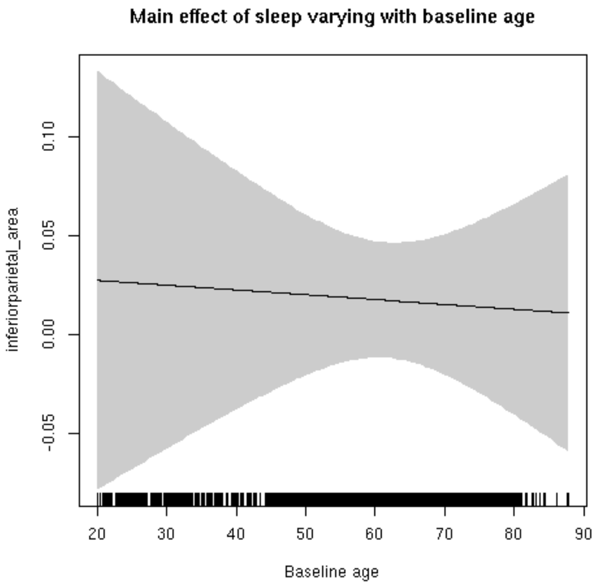

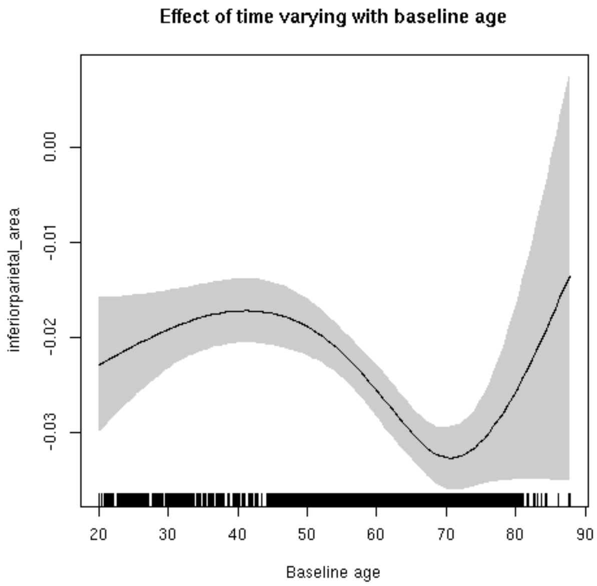

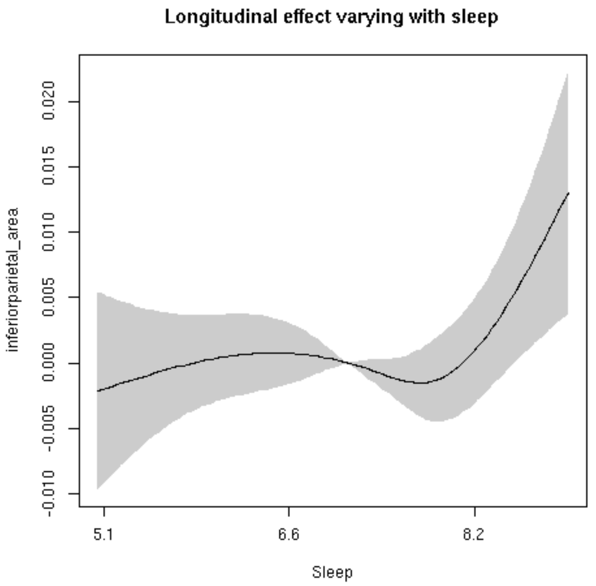

Restricted to sleep >= 5 and <= 9 hours Below is the row for `s(sleep_z):time` from the model output.

```
## s(sleep_z):time   3.185  3.185   1.103   0.199
```

Below are plots of a plot of `s(bl_age)`, `s(bl_age):sleep_z`, `s(bl_age):time`, and `s(sleep_z):time`. For `s(sleep_z):time`, roughly, if we cannot fit a straight line through the confidence bands, there is evidence that the effect of time depends on sleep duration. It is correct the that confidence interval is zero at one point along the axis; this happens because we do not include the main effect.

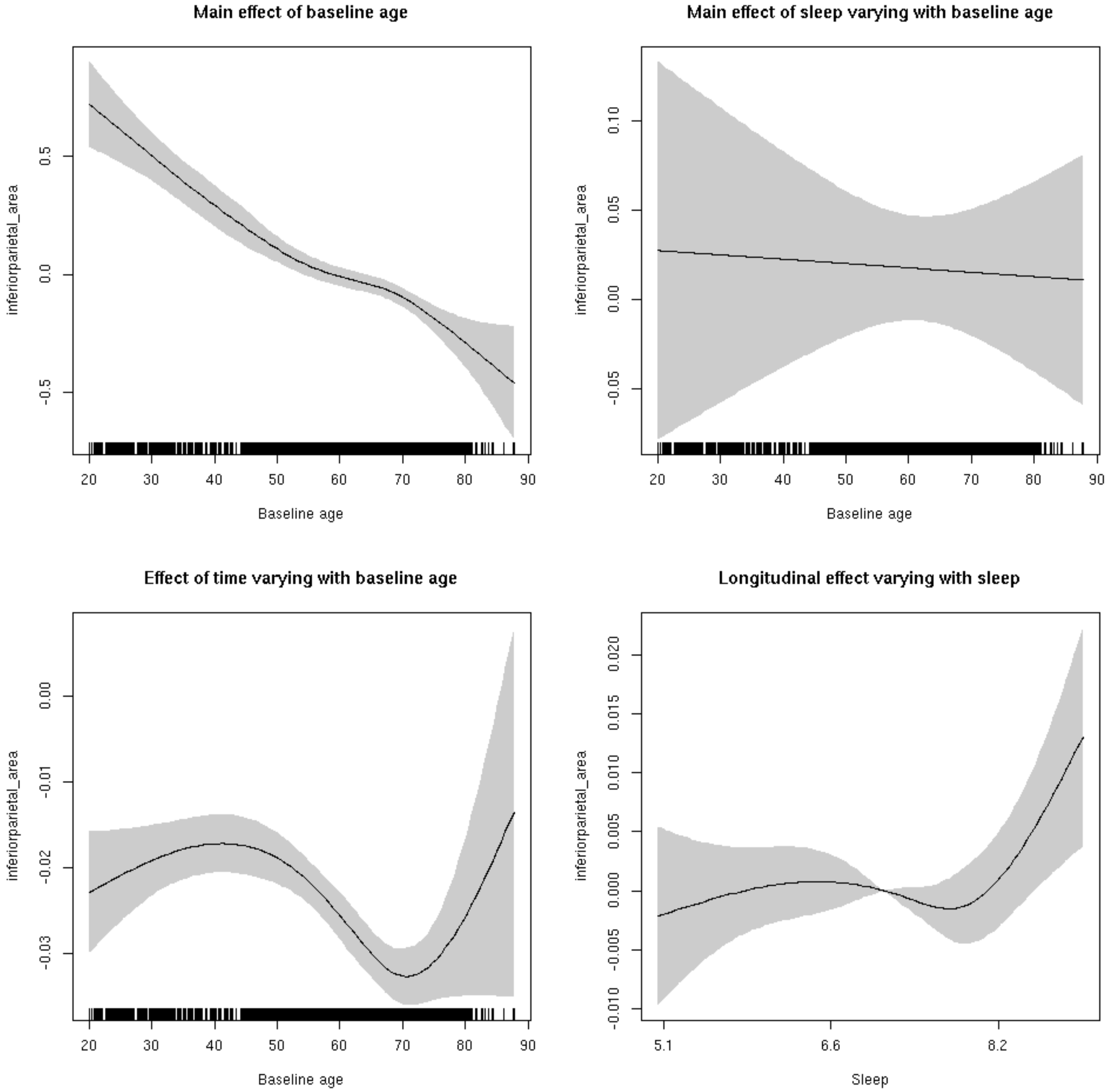

inferiorparietal_thickness: Stratified by sex

Female: Full data Below is the row for `s(sleep_z):time` from the model output.

```
## s(sleep_z):time   3.505  3.505   3.336 0.00894 **
```

Below are plots of a plot of `s(bl_age)`, `s(bl_age):sleep_z`, `s(bl_age):time`, and `s(sleep_z):time`. For `s(sleep_z):time`, roughly, if we cannot fit a straight line through the confidence bands, there is evidence that the effect of time depends on sleep duration. It is correct the that confidence interval is zero at one point along the axis; this happens because we do not include the main effect.

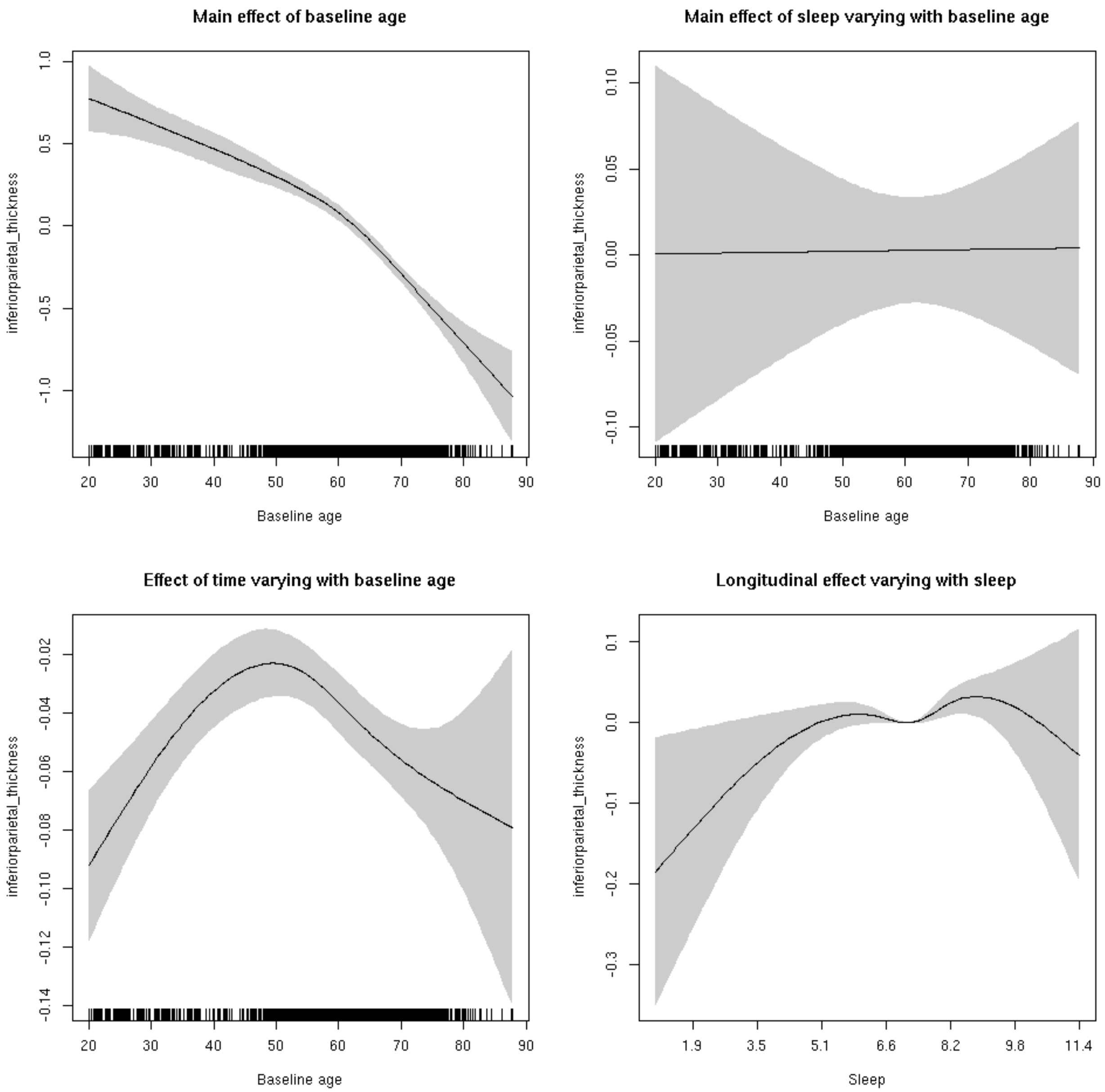

Male: Full data Below is the row for `s(sleep_z):time` from the model output.

```
## s(sleep_z):time   1.000  1.000   0.475   0.491
```

Below are plots of a plot of `s(bl_age)`, `s(bl_age):sleep_z`, `s(bl_age):time`, and `s(sleep_z):time`. For `s(sleep_z):time`, roughly, if we cannot fit a straight line through the confidence bands, there is evidence that the effect of time depends on sleep duration. It is correct the that confidence interval is zero at one point along the axis; this happens because we do not include the main effect.

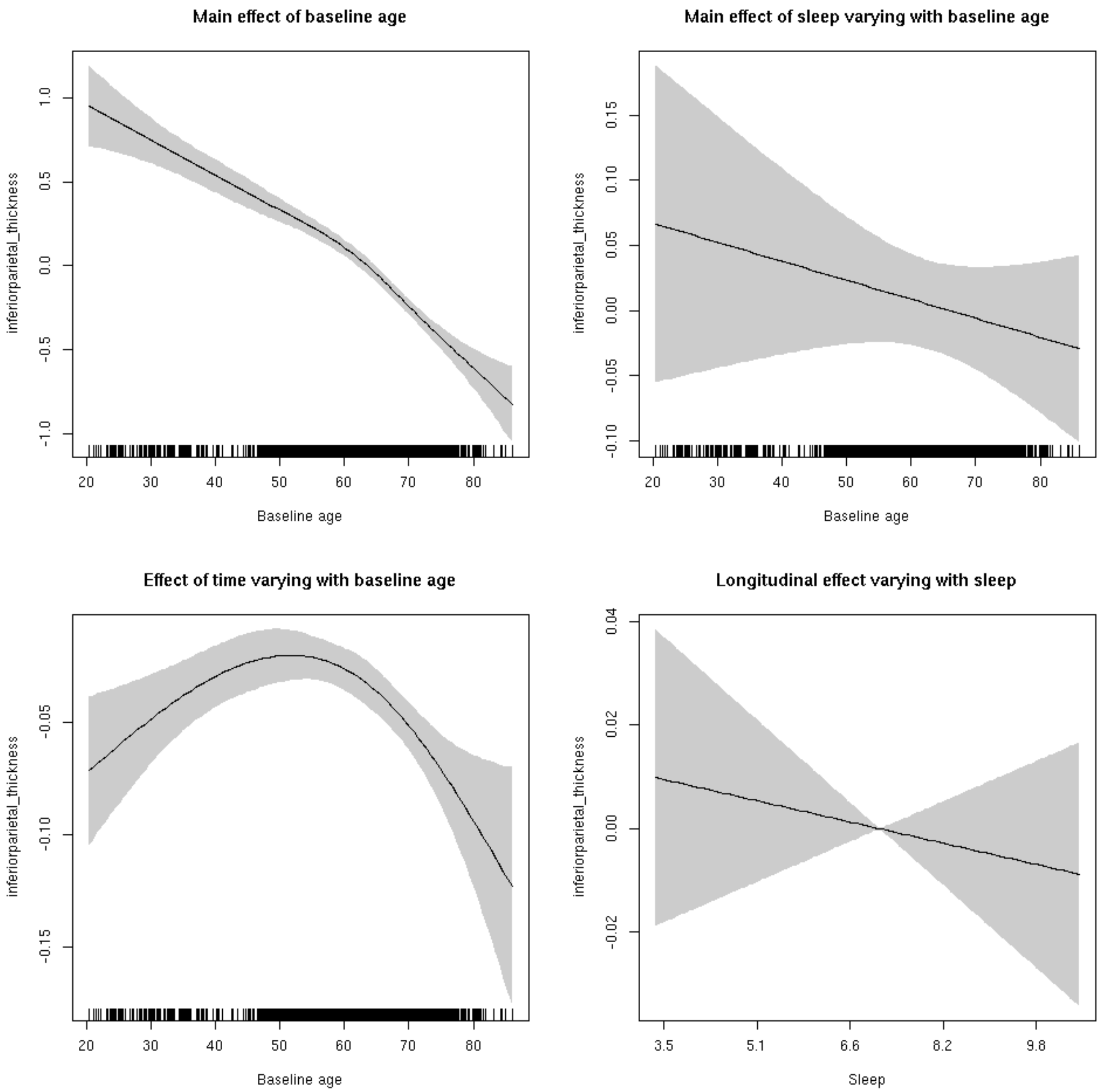

inferiorparietal_thickness

Full data Below is the model output. The term `s(sleep_z):time` is probably what we care most about.

```
##
## Family: gaussian
## Link function: identity
##
## Formula:
## value ~ sex + site + s(bl_age, k = 10, bs = "cr") + s(bl_age,
##     by = sleep_z, bs = "cr") + +s(bl_age, by = time, k = 5, bs = "cr") +
##     s(sleep_z, by = time, k = 5, bs = "cr", pc = 0)
## <environment: 0x5630035cb810>
```

```
##
## Parametric coefficients:
##                Estimate Std. Error t value Pr(>|t|)
## (Intercept)   -1.87946    0.04280 -43.911  < 2e-16 ***
## sexmale       -0.08125    0.02146  -3.786 0.000154 ***
## siteousAvanto  0.27077    0.05671   4.775 1.83e-06 ***
## siteousPrisma  0.74286    0.23042   3.224 0.001269 **
## siteousSkyra  -0.01915    0.05668  -0.338 0.735534
## siteUB         0.70319    0.11375   6.182 6.63e-10 ***
## siteUCAM       0.67956    0.05904  11.510  < 2e-16 ***
## siteUKB        2.21319    0.04342  50.972  < 2e-16 ***
## siteUmU       -0.24799    0.07055  -3.515 0.000442 ***
## ---
## Signif. codes:  0 '***' 0.001 '**' 0.01 '*' 0.05 '.' 0.1 ' ' 1
##
## Approximate significance of smooth terms:
##                      edf Ref.df       F p-value
## s(bl_age)          3.877  3.877 194.381  <2e-16 ***
## s(bl_age):sleep_z 2.000  2.000   0.310   0.733
## s(bl_age):time    3.888  3.888  75.048  <2e-16 ***
## s(sleep_z):time   1.000  1.000   0.571   0.450
## ---
## Signif. codes:  0 '***' 0.001 '**' 0.01 '*' 0.05 '.' 0.1 ' ' 1
##
## R-sq.(adj) =  0.663
## lmer.REML =  12514  Scale est. = 0.089784  n = 8165
```

Below are plots of a plot of `s(bl_age)`, `s(bl_age):sleep_z`, `s(bl_age):time`, and `s(sleep_z):time`. For `s(sleep_z):time`, roughly, if we cannot fit a straight line through the confidence bands, there is evidence that the effect of time depends on sleep duration. It is correct the that confidence interval is zero at one point along the axis; this happens because we do not include the main effect.

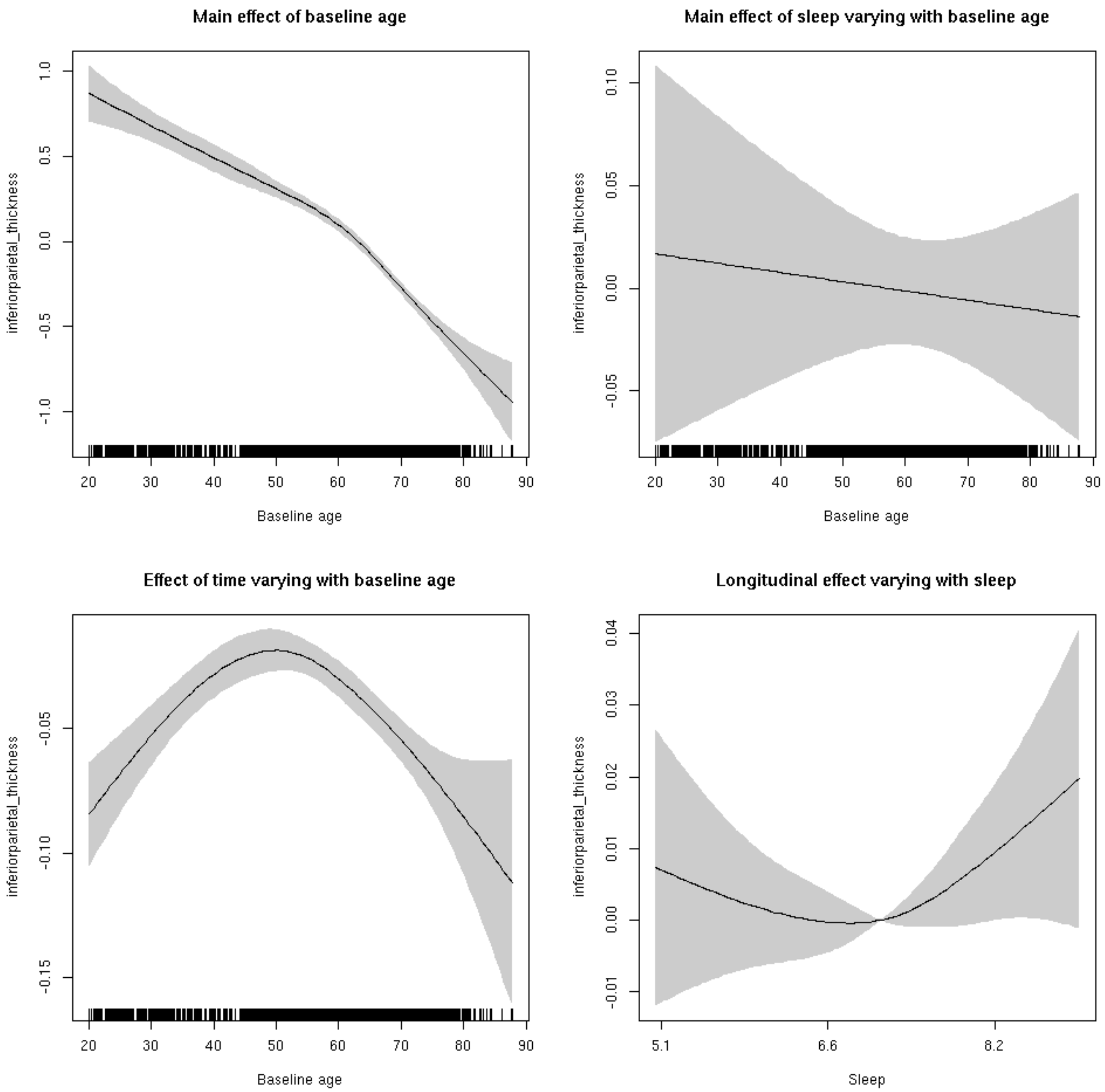

Restricted to sleep >= 5 and <= 9 hours Below is the row for `s(sleep_z):time` from the model output.

```
## s(sleep_z):time   1.992  1.992   2.072   0.112
```

Below are plots of a plot of `s(bl_age)`, `s(bl_age):sleep_z`, `s(bl_age):time`, and `s(sleep_z):time`. For `s(sleep_z):time`, roughly, if we cannot fit a straight line through the confidence bands, there is evidence that the effect of time depends on sleep duration. It is correct the that confidence interval is zero at one point along the axis; this happens because we do not include the main effect.

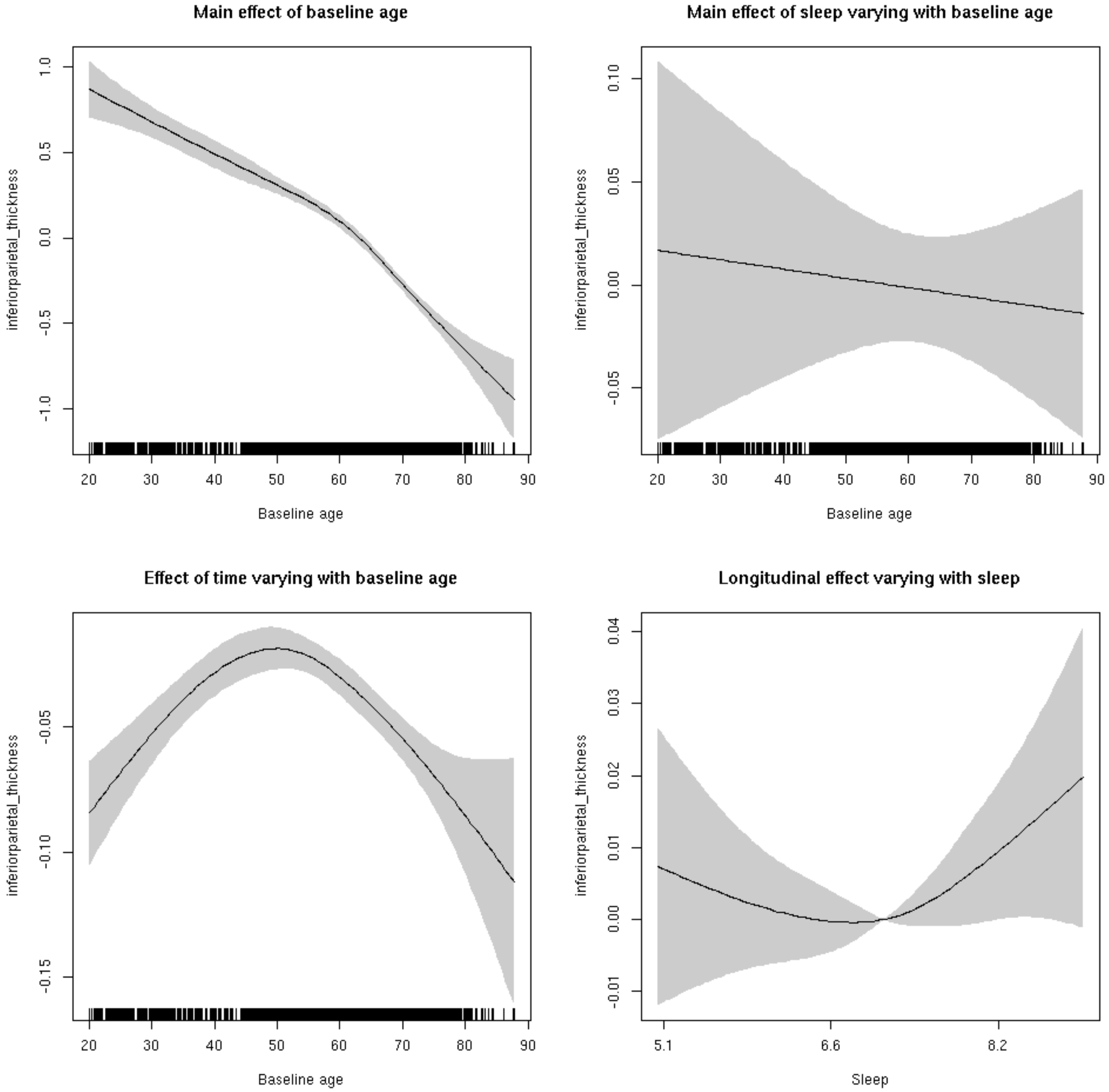

inferiorparietal_volume: Stratified by sex

Female: Full data Below is the row for `s(sleep_z):time` from the model output.

```
## s(sleep_z):time   1.000  1.000   1.069   0.301
```

Below are plots of a plot of `s(bl_age)`, `s(bl_age):sleep_z`, `s(bl_age):time`, and `s(sleep_z):time`. For `s(sleep_z):time`, roughly, if we cannot fit a straight line through the confidence bands, there is evidence that the effect of time depends on sleep duration. It is correct the that confidence interval is zero at one point along the axis; this happens because we do not include the main effect.

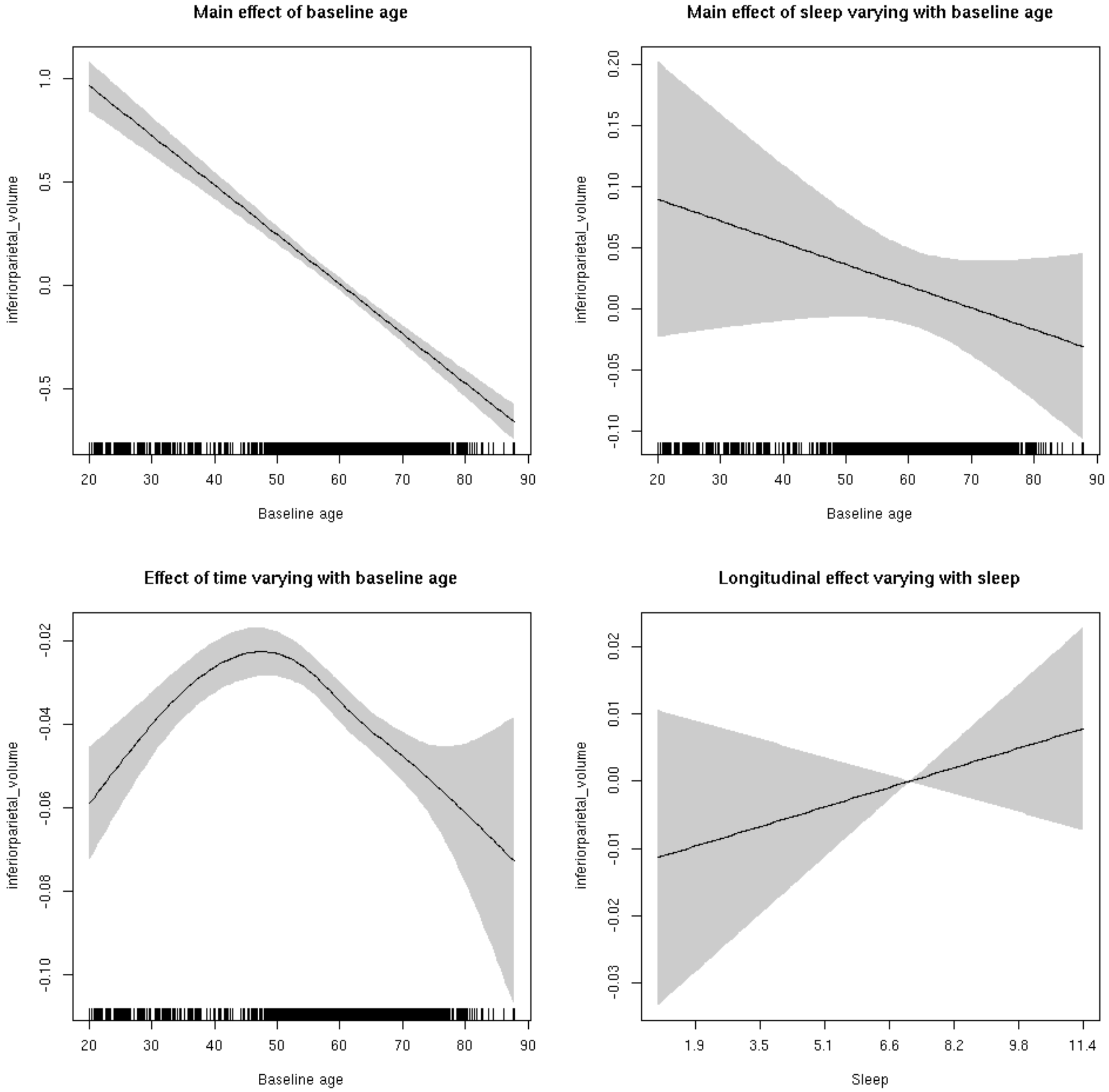

Male: Full data Below is the row for `s(sleep_z):time` from the model output.

```
## s(sleep_z):time   1.000  1.000   0.041   0.839
```

Below are plots of a plot of `s(bl_age)`, `s(bl_age):sleep_z`, `s(bl_age):time`, and `s(sleep_z):time`. For `s(sleep_z):time`, roughly, if we cannot fit a straight line through the confidence bands, there is evidence that the effect of time depends on sleep duration. It is correct the that confidence interval is zero at one point along the axis; this happens because we do not include the main effect.

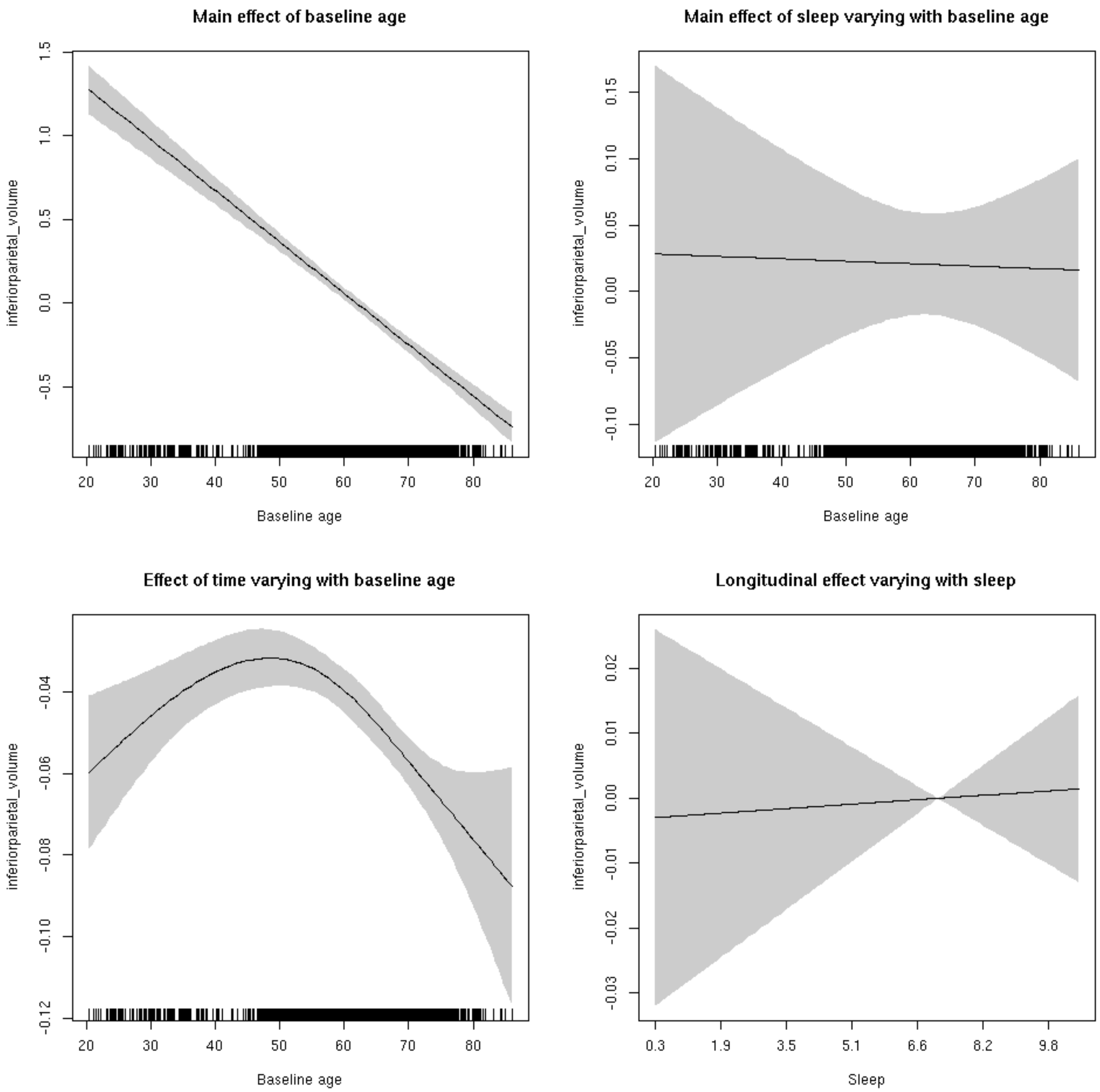

inferiorparietal_volume

Full data Below is the model output. The term `s(sleep_z):time` is probably what we care most about.

```
##
## Family: gaussian
## Link function: identity
##
## Formula:
## value ~ sex + site + icv + s(bl_age, k = 10, bs = "cr") + s(bl_age,
##     by = sleep_z, bs = "cr") + +s(bl_age, by = time, k = 5, bs = "cr") +
##     s(sleep_z, by = time, k = 5, bs = "cr", pc = 0)
## <environment: 0x5630035b8648>
```

```
##
## Parametric coefficients:
##                Estimate Std. Error t value Pr(>|t|)
## (Intercept)     0.06807    0.05589   1.218  0.22330
## sexmale         0.11930    0.02872   4.154 3.29e-05 ***
## siteousAvanto -0.69408    0.06811 -10.191  < 2e-16 ***
## siteousPrisma -0.44114    0.14361  -3.072  0.00214 **
## siteousSkyra  -0.18409    0.06599  -2.790  0.00529 **
## siteUB        -0.61675    0.12892  -4.784 1.75e-06 ***
## siteUCAM      -0.60526    0.06936  -8.726  < 2e-16 ***
## siteUKB        0.01147    0.05310   0.216  0.82894
## siteUmU       -0.21445    0.07982  -2.687  0.00723 **
## icv            0.54757    0.01409  38.854  < 2e-16 ***
## ---
## Signif. codes:  0 '***' 0.001 '**' 0.01 '*' 0.05 '.' 0.1 ' ' 1
##
## Approximate significance of smooth terms:
##                      edf Ref.df       F p-value
## s(bl_age)          3.130  3.130 194.646 < 2e-16 ***
## s(bl_age):sleep_z 2.000  2.000   1.170 0.31050
## s(bl_age):time    4.041  4.041 163.379 < 2e-16 ***
## s(sleep_z):time   3.667  3.667   4.093 0.00214 **
## ---
## Signif. codes:  0 '***' 0.001 '**' 0.01 '*' 0.05 '.' 0.1 ' ' 1
##
## R-sq.(adj) =  0.475
## lmer.REML = 8162.8  Scale est. = 0.026561  n = 8168
```

Below are plots of a plot of `s(bl_age)`, `s(bl_age):sleep_z`, `s(bl_age):time`, and `s(sleep_z):time`. For `s(sleep_z):time`, roughly, if we cannot fit a straight line through the confidence bands, there is evidence that the effect of time depends on sleep duration. It is correct the that confidence interval is zero at one point along the axis; this happens because we do not include the main effect.

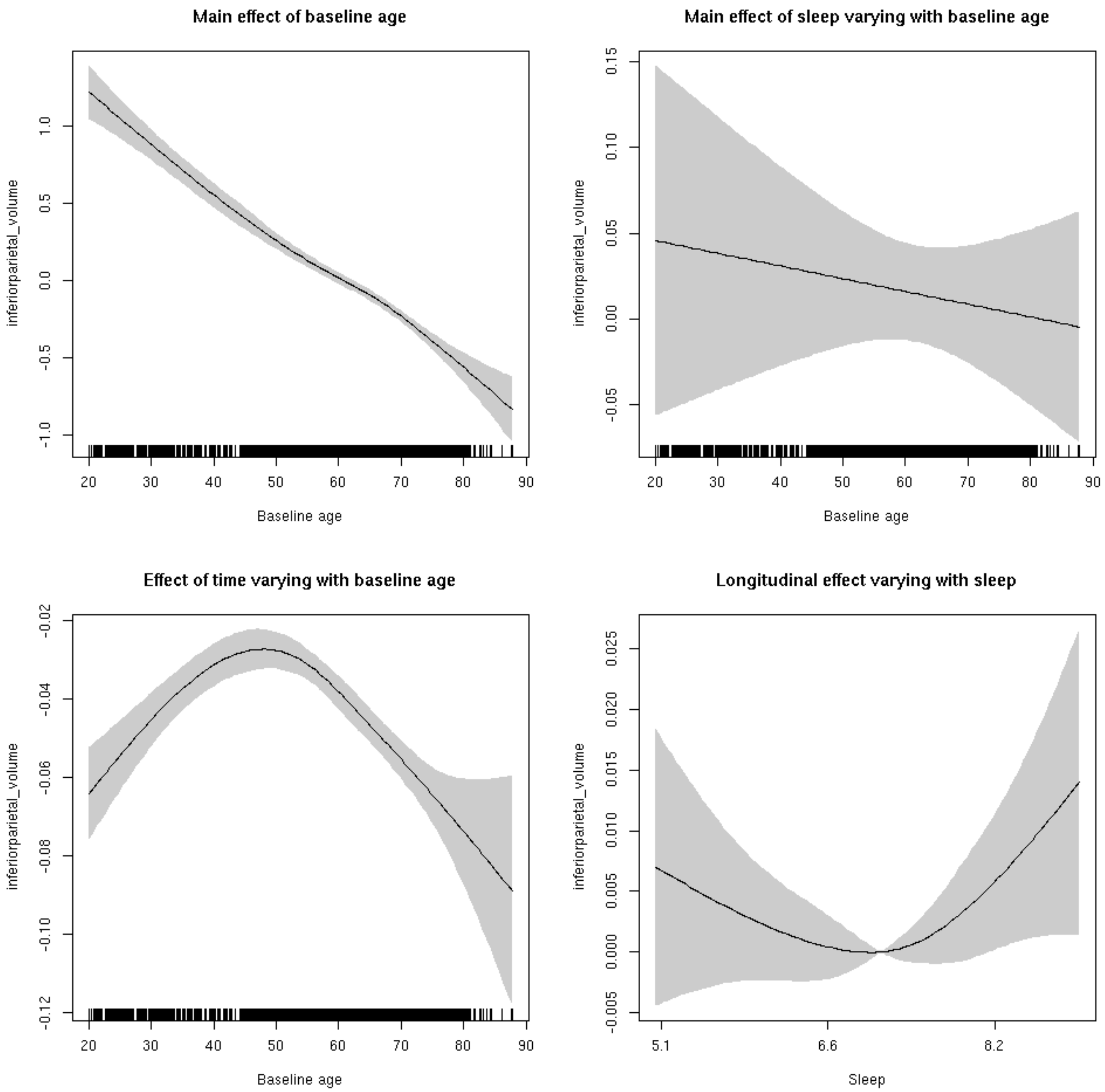

Restricted to sleep >= 5 and <= 9 hours Below is the row for `s(sleep_z):time` from the model output.

```
## s(sleep_z):time   2.215  2.215   2.454   0.073 .
```

Below are plots of a plot of `s(bl_age)`, `s(bl_age):sleep_z`, `s(bl_age):time`, and `s(sleep_z):time`. For `s(sleep_z):time`, roughly, if we cannot fit a straight line through the confidence bands, there is evidence that the effect of time depends on sleep duration. It is correct the that confidence interval is zero at one point along the axis; this happens because we do not include the main effect.

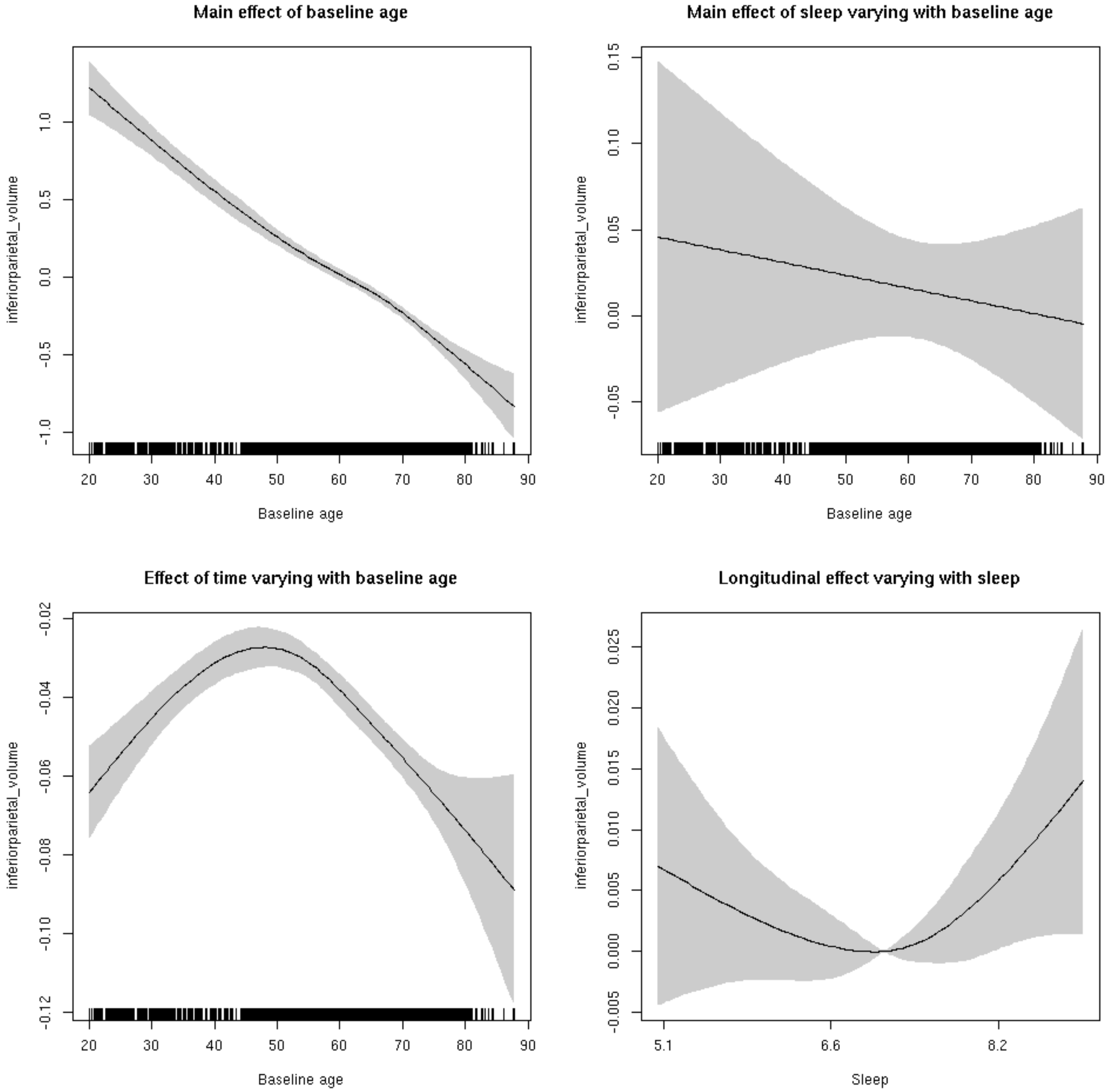

inferiortemporal_area: Stratified by sex

Female: Full data Below is the row for `s(sleep_z):time` from the model output.

```
## s(sleep_z):time   1.000  1.000   0.804   0.370
```

Below are plots of a plot of `s(bl_age)`, `s(bl_age):sleep_z`, `s(bl_age):time`, and `s(sleep_z):time`. For `s(sleep_z):time`, roughly, if we cannot fit a straight line through the confidence bands, there is evidence that the effect of time depends on sleep duration. It is correct the that confidence interval is zero at one point along the axis; this happens because we do not include the main effect.

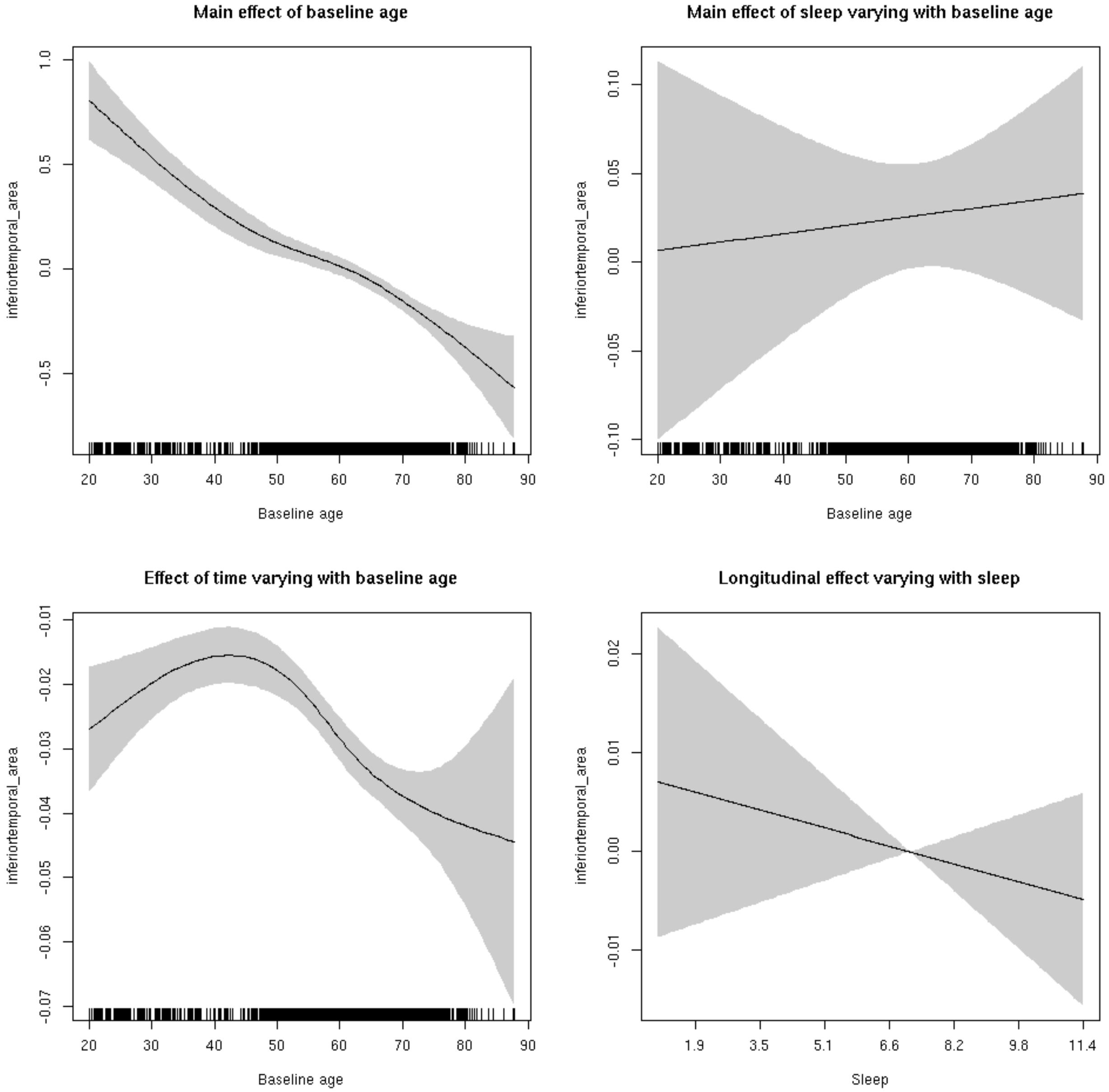

Male: Full data Below is the row for `s(sleep_z):time` from the model output.

```
## s(sleep_z):time   1.000  1.000   1.553   0.213
```

Below are plots of a plot of `s(bl_age)`, `s(bl_age):sleep_z`, `s(bl_age):time`, and `s(sleep_z):time`. For `s(sleep_z):time`, roughly, if we cannot fit a straight line through the confidence bands, there is evidence that the effect of time depends on sleep duration. It is correct the that confidence interval is zero at one point along the axis; this happens because we do not include the main effect.

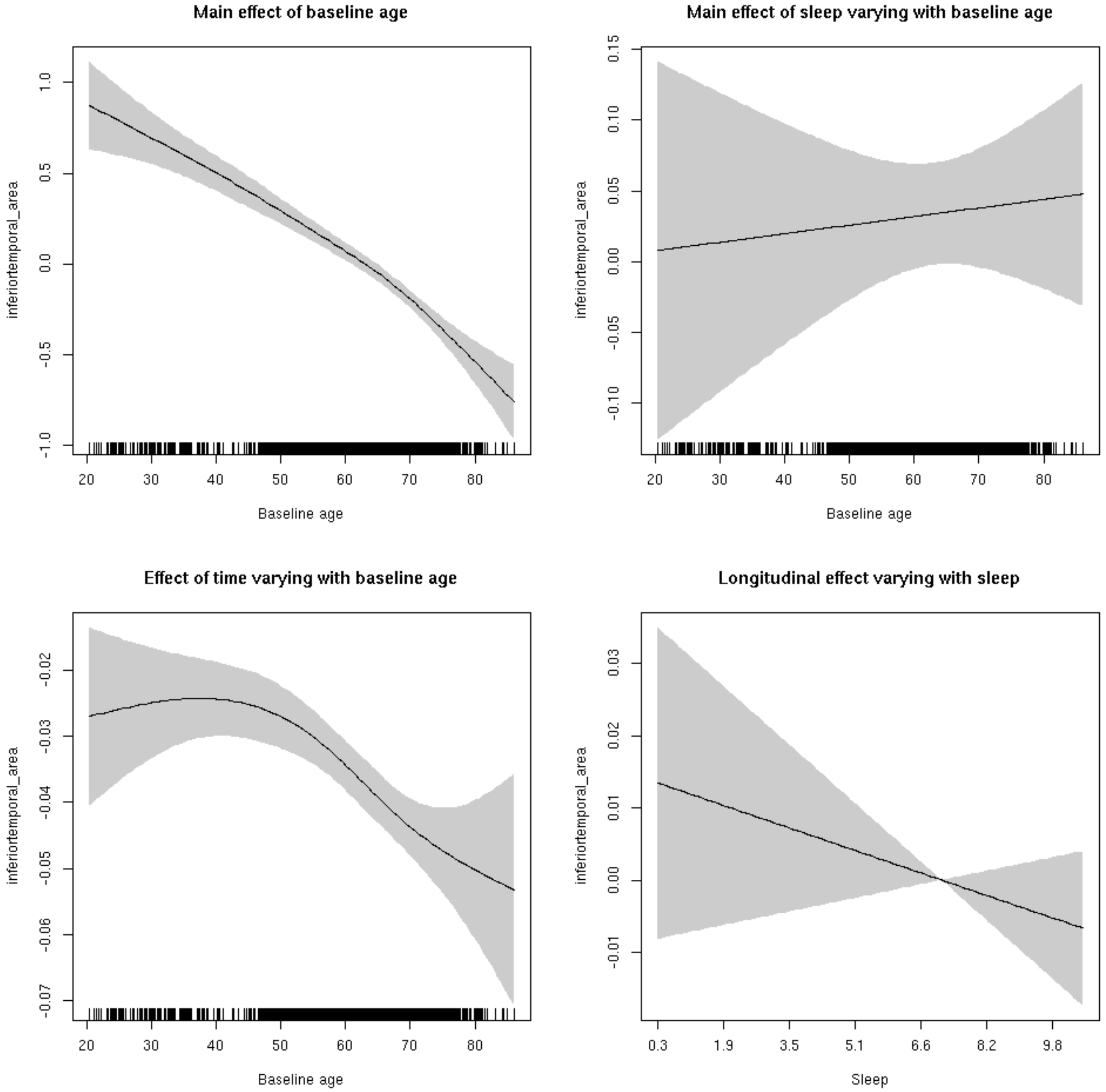

inferiortemporal_area

Full data Below is the model output. The term `s(sleep_z):time` is probably what we care most about.

```
##
## Family: gaussian
## Link function: identity
##
## Formula:
## value ~ sex + site + icv + s(bl_age, k = 10, bs = "cr") + s(bl_age,
##     by = sleep_z, bs = "cr") + +s(bl_age, by = time, k = 5, bs = "cr") +
##     s(sleep_z, by = time, k = 5, bs = "cr", pc = 0)
## <environment: 0x5630035b23a8>
```

```
##
## Parametric coefficients:
##                Estimate Std. Error t value Pr(>|t|)
## (Intercept)     0.73448    0.05236  14.027  < 2e-16 ***
## sexmale         0.31021    0.02689  11.535  < 2e-16 ***
## siteousAvanto -0.57154    0.06379  -8.960  < 2e-16 ***
## siteousPrisma -1.43967    0.11290 -12.752  < 2e-16 ***
## siteousSkyra  -0.17051    0.06182  -2.758  0.00582 **
## siteUB        -0.72259    0.12133  -5.955  2.7e-09 ***
## siteUCAM      -0.76953    0.06530 -11.785  < 2e-16 ***
## siteUKB       -0.85943    0.04987 -17.234  < 2e-16 ***
## siteUmU       -0.30512    0.07501  -4.068  4.8e-05 ***
## icv            0.54695    0.01306  41.868  < 2e-16 ***
## ---
## Signif. codes:  0 '***' 0.001 '**' 0.01 '*' 0.05 '.' 0.1 ' ' 1
##
## Approximate significance of smooth terms:
##                      edf Ref.df       F p-value
## s(bl_age)          3.460  3.460 113.707  <2e-16 ***
## s(bl_age):sleep_z 2.000  2.000   2.529  0.0798 .
## s(bl_age):time    4.063  4.063 287.520  <2e-16 ***
## s(sleep_z):time   1.000  1.000   2.099  0.1474
## ---
## Signif. codes:  0 '***' 0.001 '**' 0.01 '*' 0.05 '.' 0.1 ' ' 1
##
## R-sq.(adj) =  0.535
## lmer.REML = 4963.7  Scale est. = 0.014116  n = 8154
```

Below are plots of a plot of `s(bl_age)`, `s(bl_age):sleep_z`, `s(bl_age):time`, and `s(sleep_z):time`. For `s(sleep_z):time`, roughly, if we cannot fit a straight line through the confidence bands, there is evidence that the effect of time depends on sleep duration. It is correct the that confidence interval is zero at one point along the axis; this happens because we do not include the main effect.

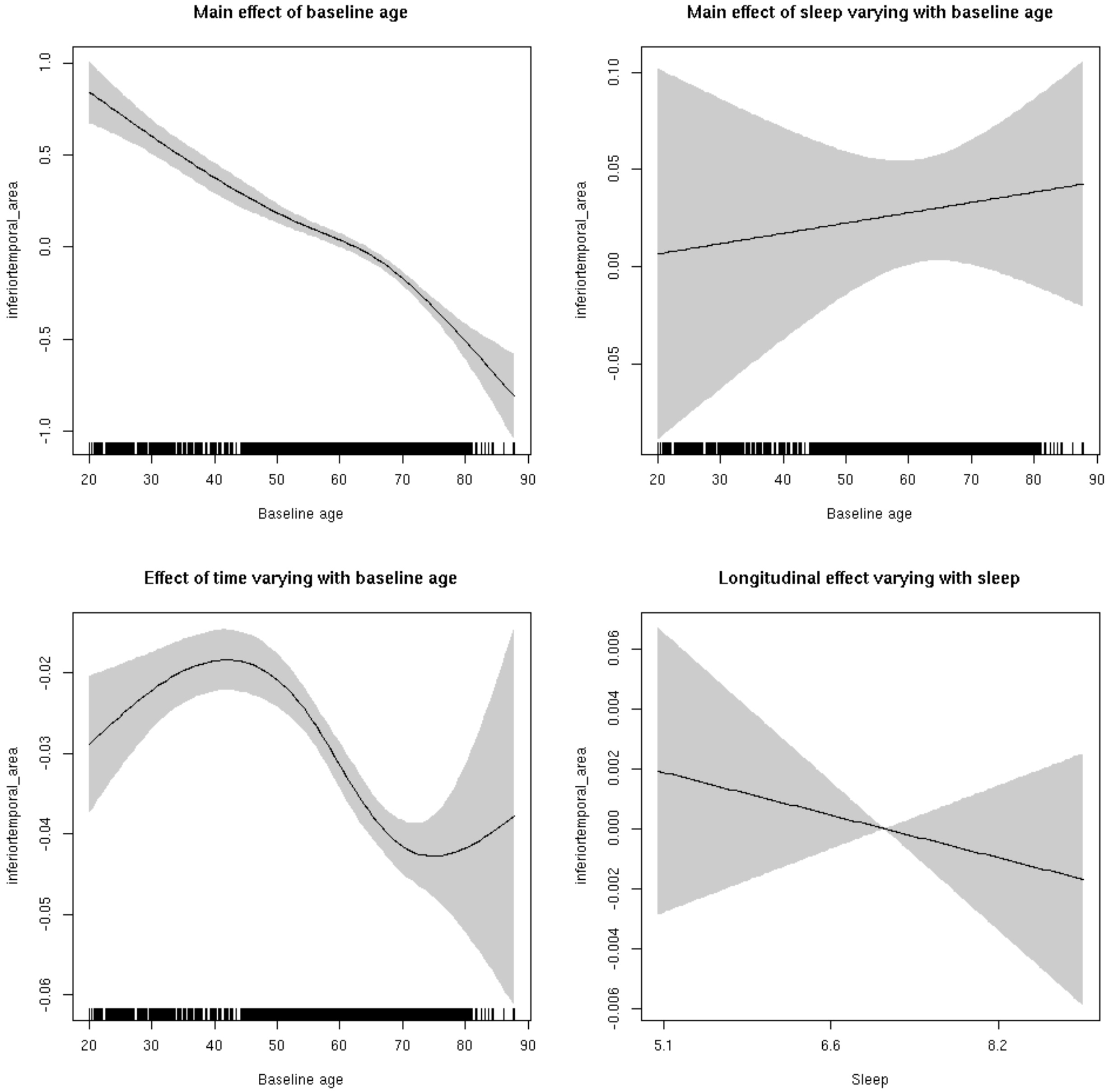

Restricted to sleep >= 5 and <= 9 hours Below is the row for `s(sleep_z):time` from the model output.

```
## s(sleep_z):time   1.000  1.000   0.646  0.4215
```

Below are plots of a plot of `s(bl_age)`, `s(bl_age):sleep_z`, `s(bl_age):time`, and `s(sleep_z):time`. For `s(sleep_z):time`, roughly, if we cannot fit a straight line through the confidence bands, there is evidence that the effect of time depends on sleep duration. It is correct the that confidence interval is zero at one point along the axis; this happens because we do not include the main effect.

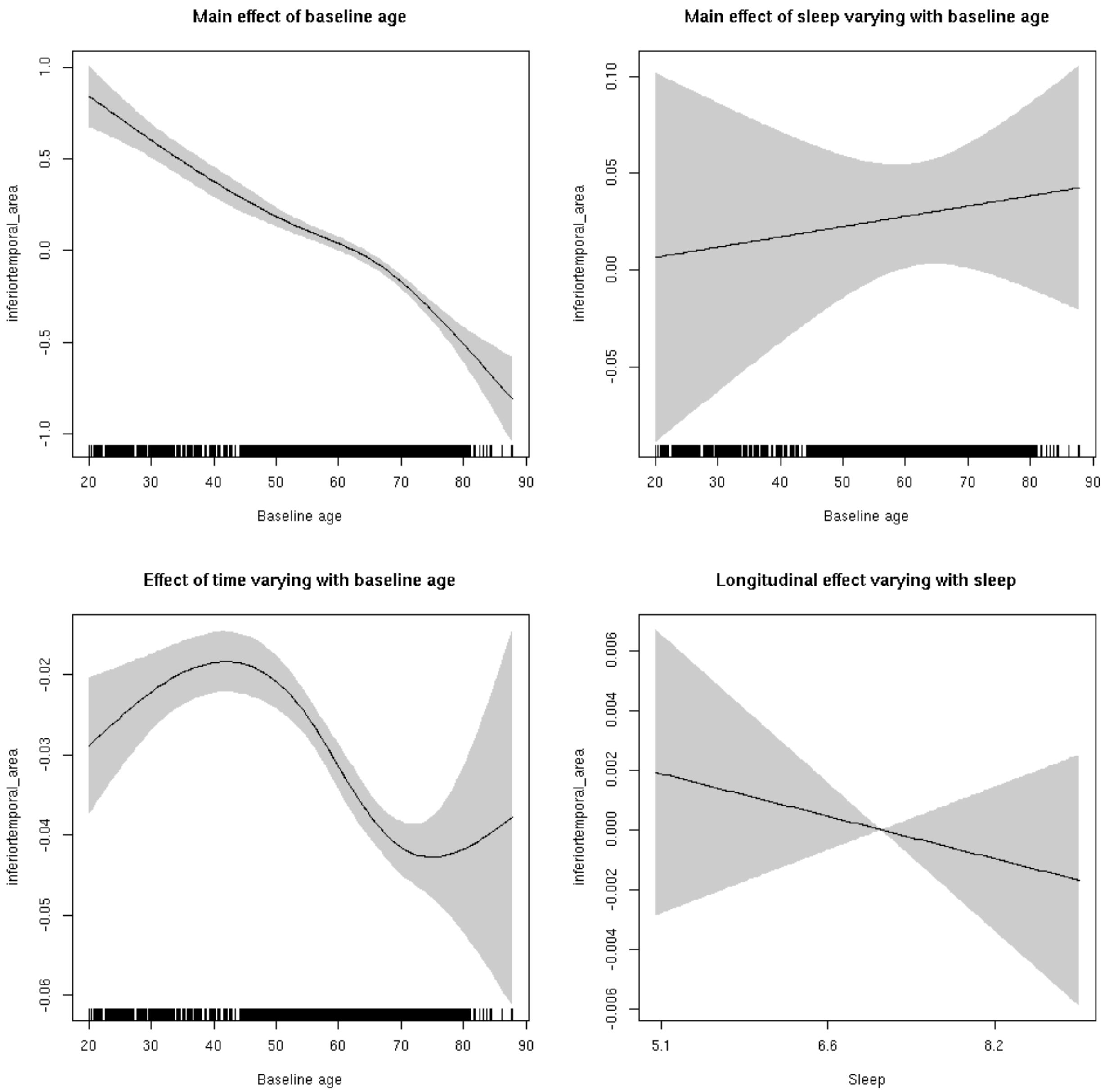

inferiortemporal_thickness: Stratified by sex

Female: Full data Below is the row for `s(sleep_z):time` from the model output.

```
## s(sleep_z):time   1.000  1.000  2.811  0.0937 .
```

Below are plots of a plot of `s(bl_age)`, `s(bl_age):sleep_z`, `s(bl_age):time`, and `s(sleep_z):time`. For `s(sleep_z):time`, roughly, if we cannot fit a straight line through the confidence bands, there is evidence that the effect of time depends on sleep duration. It is correct the that confidence interval is zero at one point along the axis; this happens because we do not include the main effect.

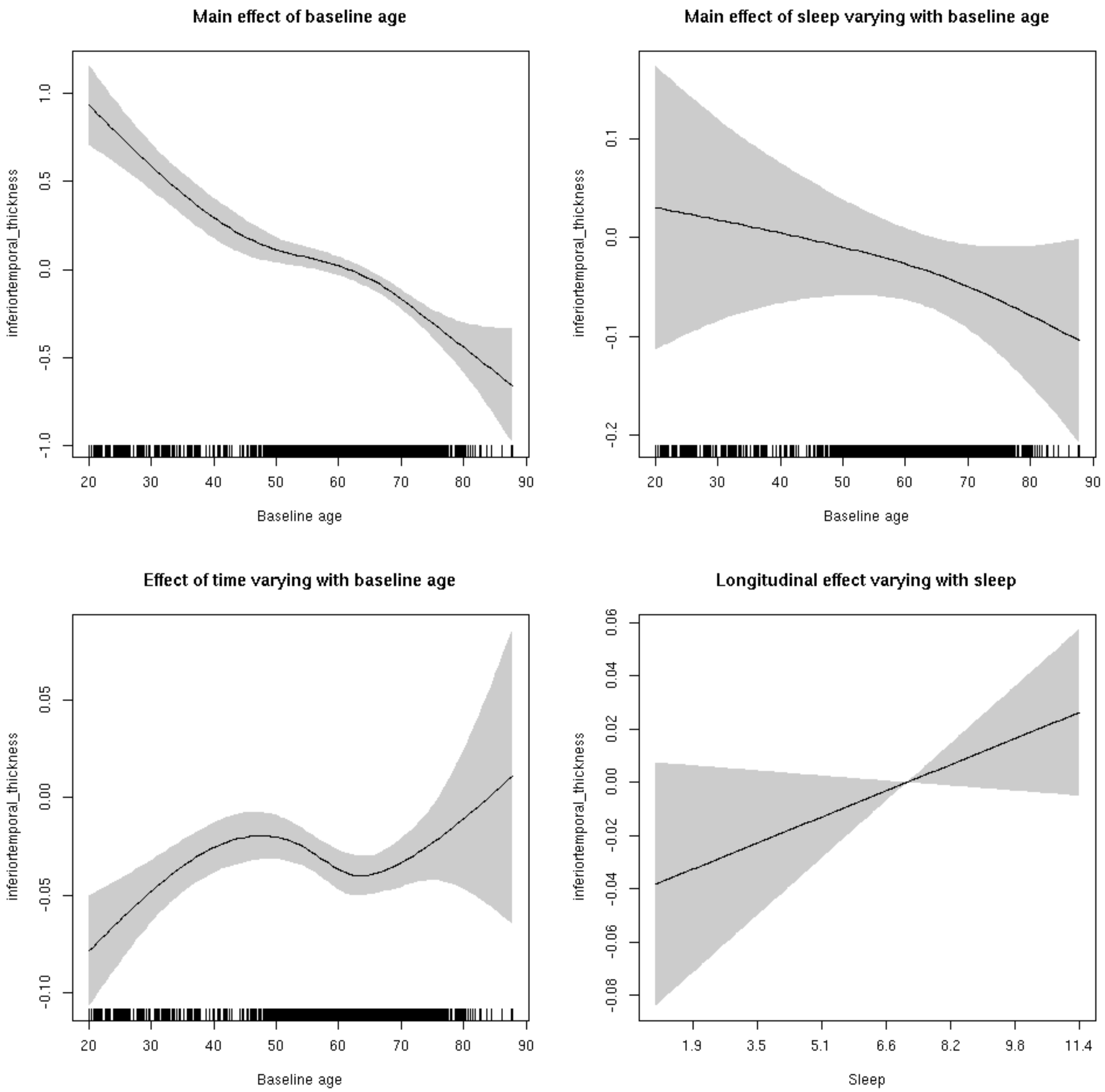

Male: Full data Below is the row for `s(sleep_z):time` from the model output.

```
## s(sleep_z):time   3.075  3.075   3.172  0.0422 *
```

Below are plots of a plot of `s(bl_age)`, `s(bl_age):sleep_z`, `s(bl_age):time`, and `s(sleep_z):time`. For `s(sleep_z):time`, roughly, if we cannot fit a straight line through the confidence bands, there is evidence that the effect of time depends on sleep duration. It is correct the that confidence interval is zero at one point along the axis; this happens because we do not include the main effect.

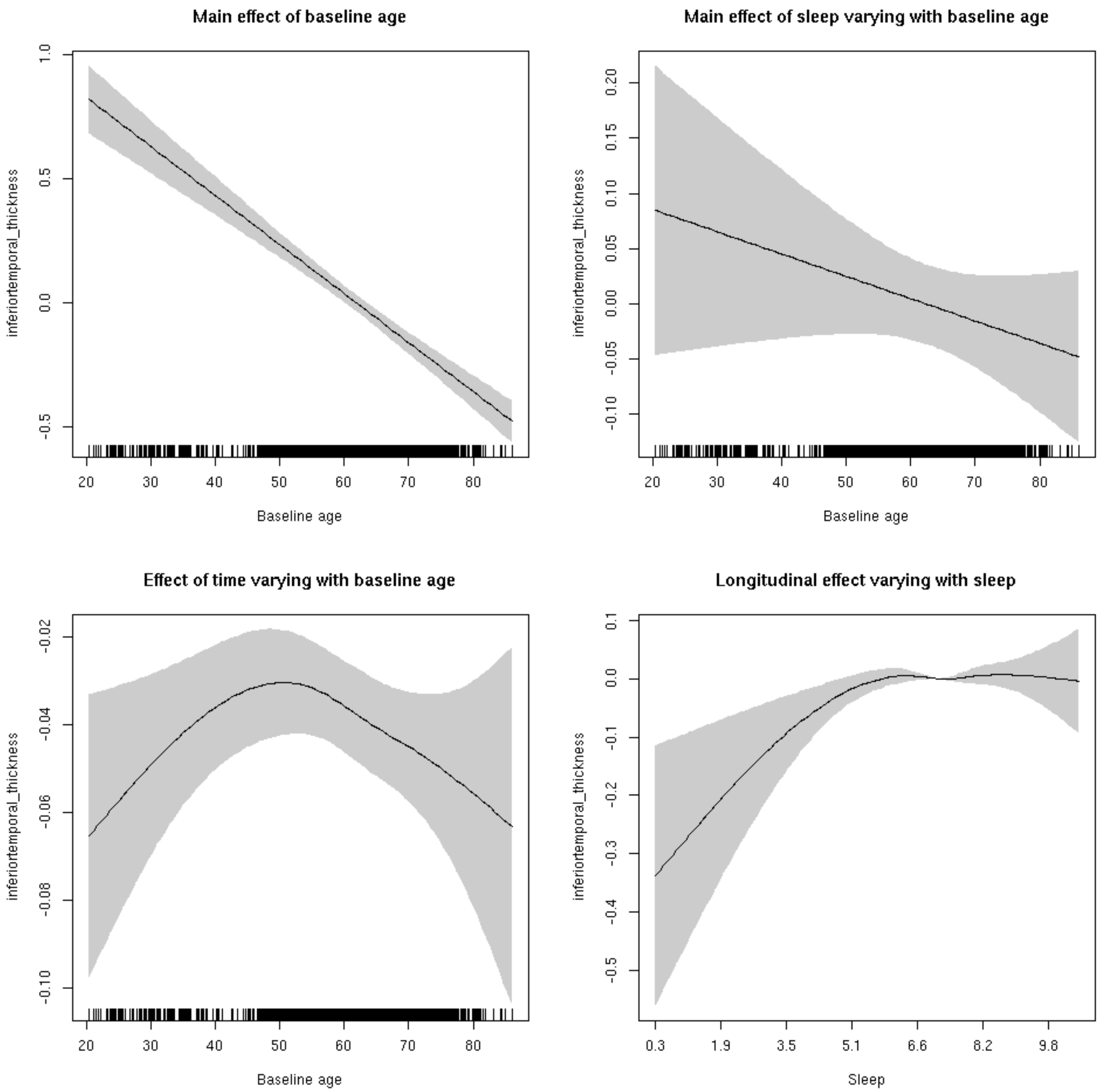

inferiortemporal_thickness

Full data Below is the model output. The term `s(sleep_z):time` is probably what we care most about.

```
##
## Family: gaussian
## Link function: identity
##
## Formula:
## value ~ sex + site + s(bl_age, k = 10, bs = "cr") + s(bl_age,
##     by = sleep_z, bs = "cr") + +s(bl_age, by = time, k = 5, bs = "cr") +
##     s(sleep_z, by = time, k = 5, bs = "cr", pc = 0)
## <environment: 0x5630035d4498>
```

```
##
## Parametric coefficients:
##                Estimate Std. Error t value Pr(>|t|)
## (Intercept)   -2.43490    0.04697 -51.835  < 2e-16 ***
## sexmale        0.14719    0.02358   6.241 4.56e-10 ***
## siteousAvanto  0.72305    0.06218  11.628  < 2e-16 ***
## siteousPrisma  1.26211    0.24513   5.149 2.68e-07 ***
## siteousSkyra   0.70438    0.06216  11.331  < 2e-16 ***
## siteUB         0.77904    0.12514   6.225 5.05e-10 ***
## siteUCAM       0.93968    0.06466  14.532  < 2e-16 ***
## siteUKB        2.66345    0.04759  55.967  < 2e-16 ***
## siteUmU        0.36558    0.07750   4.717 2.43e-06 ***
## ---
## Signif. codes:  0 '***' 0.001 '**' 0.01 '*' 0.05 '.' 0.1 ' ' 1
##
## Approximate significance of smooth terms:
##                      edf Ref.df      F p-value
## s(bl_age)          3.326  3.326 91.807  <2e-16 ***
## s(bl_age):sleep_z 2.000  2.000  3.120  0.0442 *
## s(bl_age):time    4.162  4.162 38.811  <2e-16 ***
## s(sleep_z):time   3.246  3.246  3.073  0.0141 *
## ---
## Signif. codes:  0 '***' 0.001 '**' 0.01 '*' 0.05 '.' 0.1 ' ' 1
##
## R-sq.(adj) =  0.624
## lmer.REML =  13760  Scale est. = 0.10066   n = 8172
```

Below are plots of a plot of `s(bl_age)`, `s(bl_age):sleep_z`, `s(bl_age):time`, and `s(sleep_z):time`. For `s(sleep_z):time`, roughly, if we cannot fit a straight line through the confidence bands, there is evidence that the effect of time depends on sleep duration. It is correct the that confidence interval is zero at one point along the axis; this happens because we do not include the main effect.

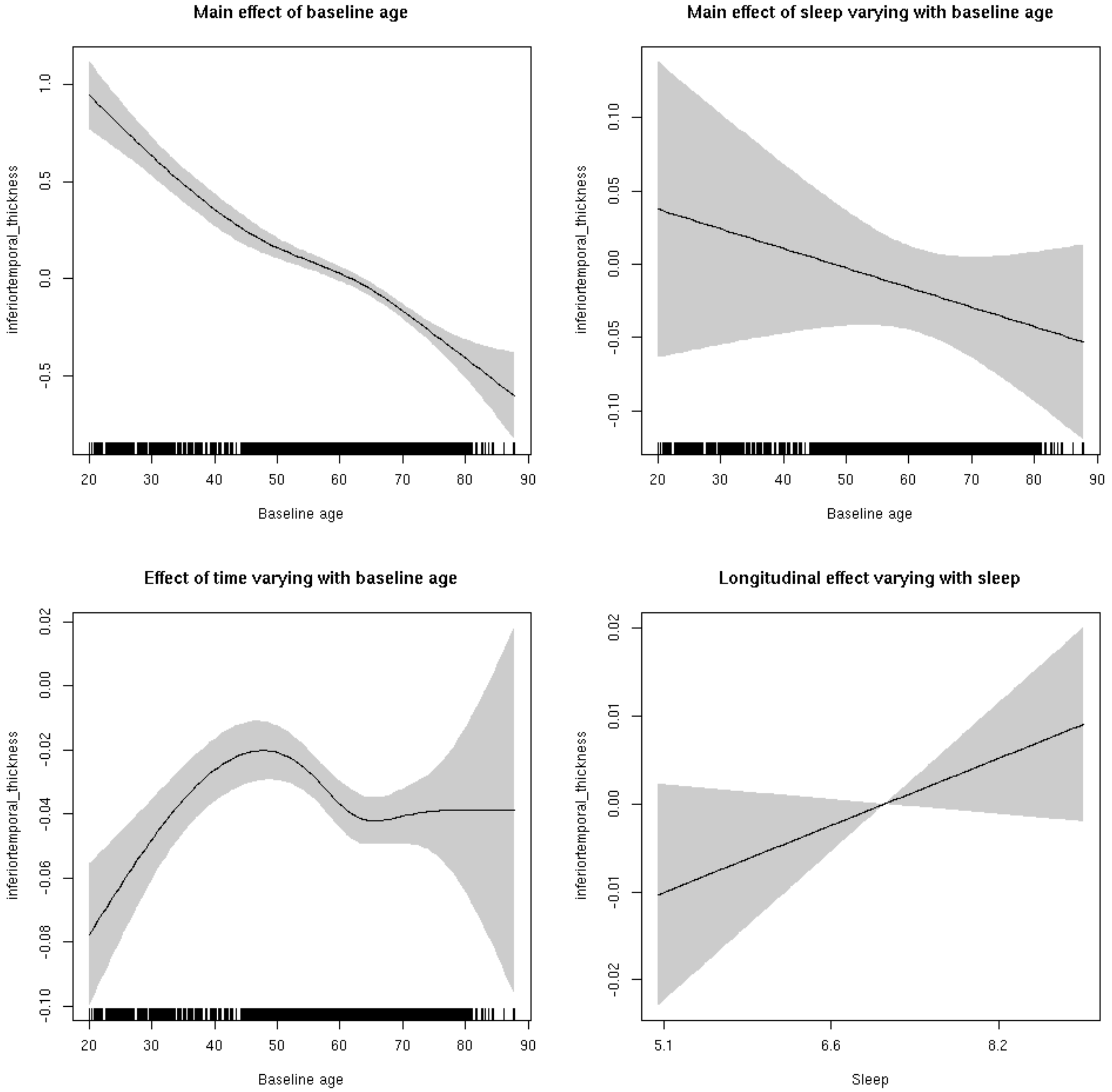

Restricted to sleep >= 5 and <= 9 hours Below is the row for `s(sleep_z):time` from the model output.

```
## s(sleep_z):time   1.000  1.000  2.683   0.101
```

Below are plots of a plot of `s(bl_age)`, `s(bl_age):sleep_z`, `s(bl_age):time`, and `s(sleep_z):time`. For `s(sleep_z):time`, roughly, if we cannot fit a straight line through the confidence bands, there is evidence that the effect of time depends on sleep duration. It is correct the that confidence interval is zero at one point along the axis; this happens because we do not include the main effect.

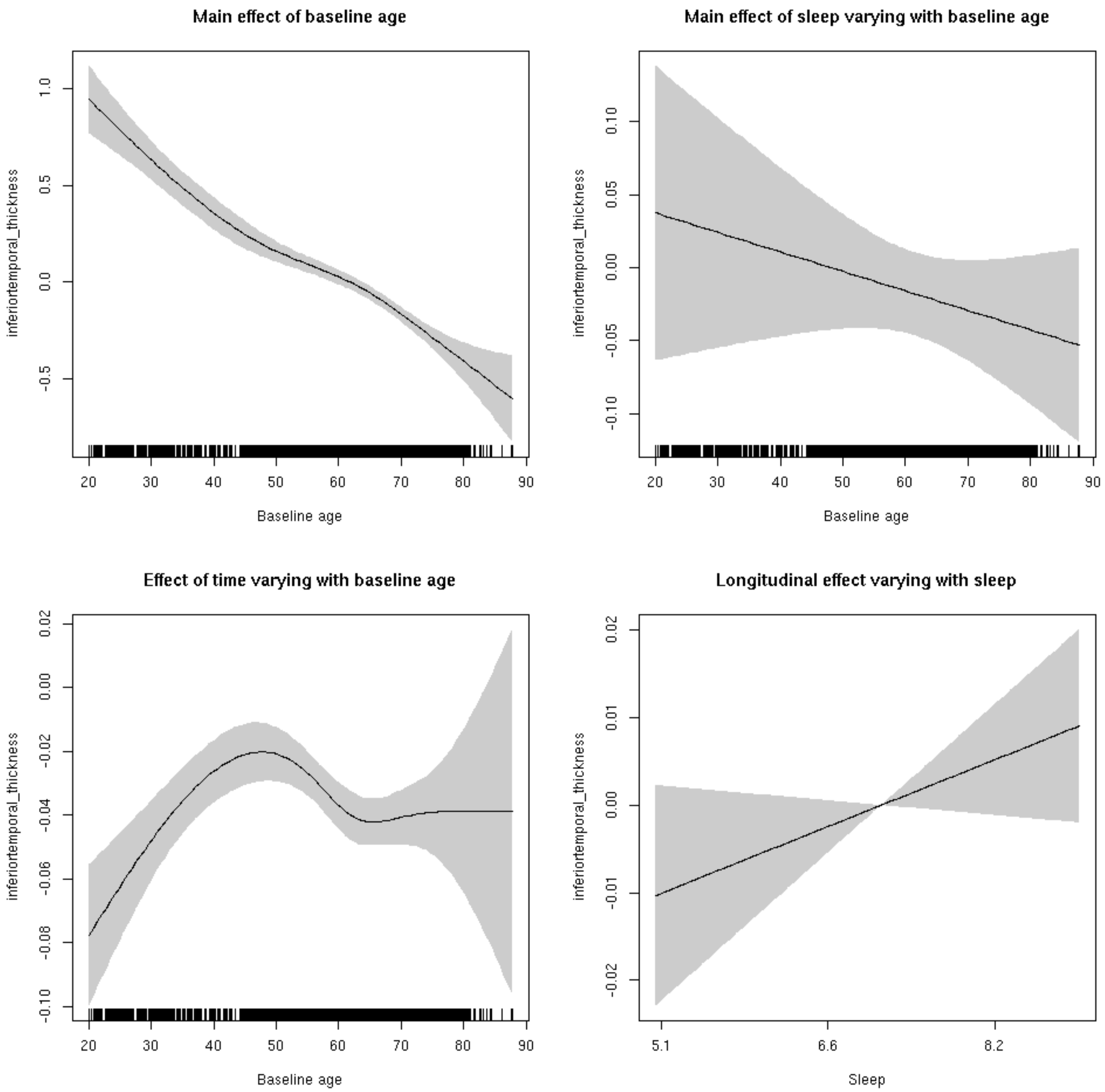

inferiortemporal_volume: Stratified by sex

Female: Full data Below is the row for `s(sleep_z):time` from the model output.

```
## s(sleep_z):time   1.000  1.000   0.041   0.840
```

Below are plots of a plot of `s(bl_age)`, `s(bl_age):sleep_z`, `s(bl_age):time`, and `s(sleep_z):time`. For `s(sleep_z):time`, roughly, if we cannot fit a straight line through the confidence bands, there is evidence that the effect of time depends on sleep duration. It is correct the that confidence interval is zero at one point along the axis; this happens because we do not include the main effect.

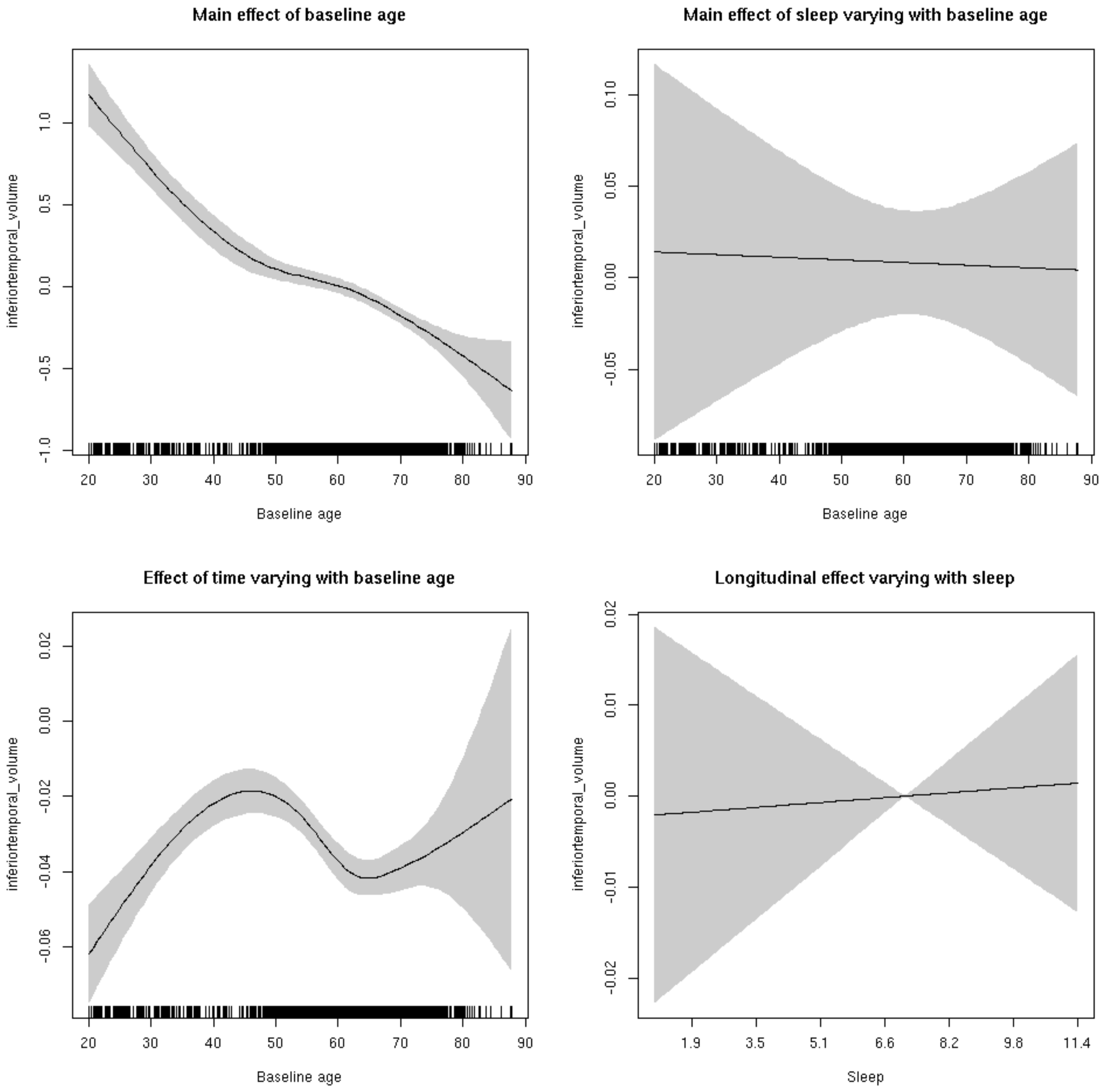

Male: Full data Below is the row for `s(sleep_z):time` from the model output.

```
## s(sleep_z):time   1.000  1.000   0.429   0.513
```

Below are plots of a plot of `s(bl_age)`, `s(bl_age):sleep_z`, `s(bl_age):time`, and `s(sleep_z):time`. For `s(sleep_z):time`, roughly, if we cannot fit a straight line through the confidence bands, there is evidence that the effect of time depends on sleep duration. It is correct the that confidence interval is zero at one point along the axis; this happens because we do not include the main effect.

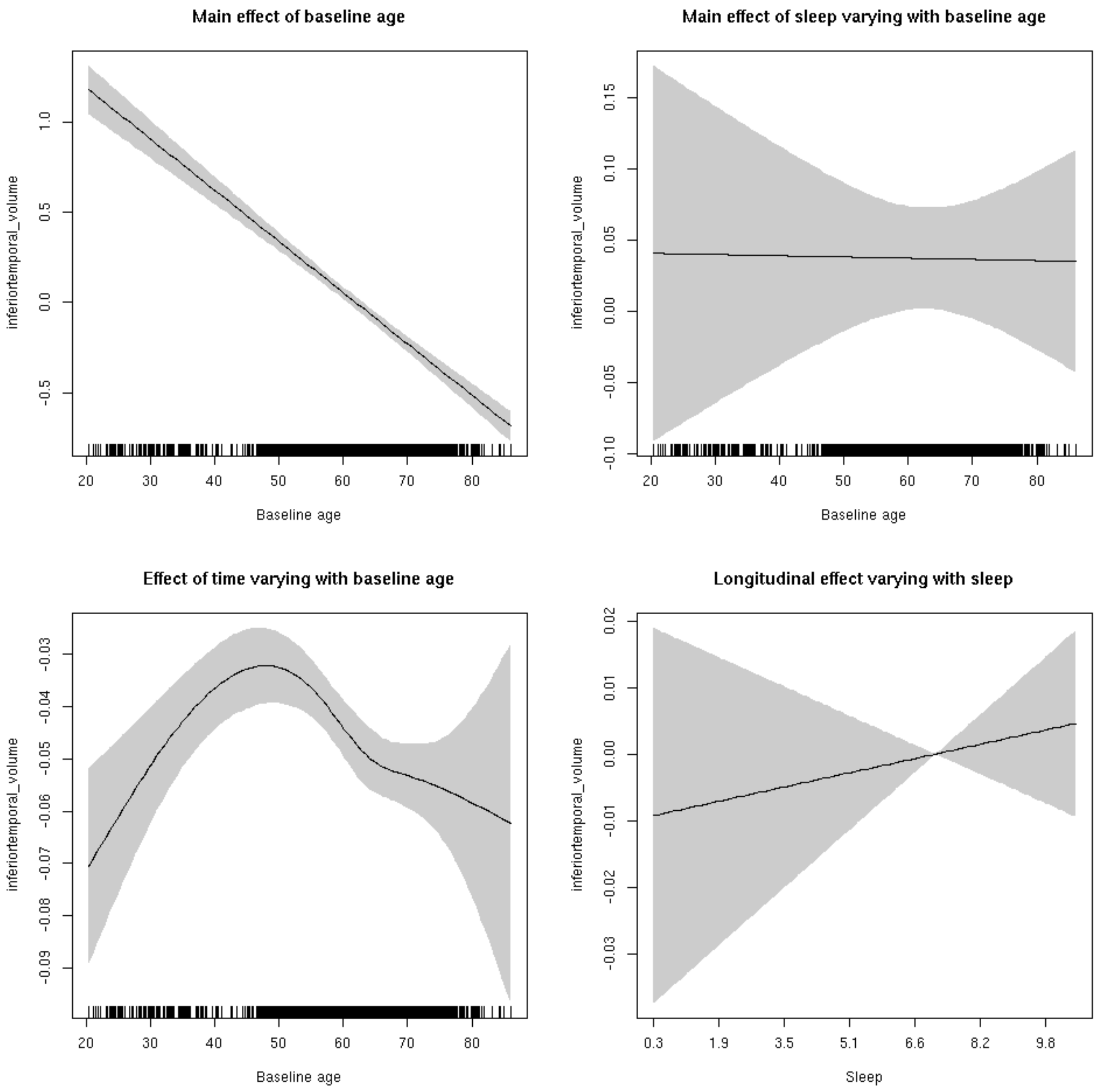

inferiortemporal_volume

Full data Below is the model output. The term `s(sleep_z):time` is probably what we care most about.

```
##
## Family: gaussian
## Link function: identity
##
## Formula:
## value ~ sex + site + icv + s(bl_age, k = 10, bs = "cr") + s(bl_age,
##     by = sleep_z, bs = "cr") + +s(bl_age, by = time, k = 5, bs = "cr") +
##     s(sleep_z, by = time, k = 5, bs = "cr", pc = 0)
## <environment: 0x5630035b2648>
```

```
##
## Parametric coefficients:
##                Estimate Std. Error t value Pr(>|t|)
## (Intercept)   -0.43134    0.05167  -8.347  < 2e-16 ***
## sexmale        0.30233    0.02652  11.399  < 2e-16 ***
## siteousAvanto -0.18416    0.06298  -2.924 0.003464 **
## siteousPrisma -0.56915    0.13632  -4.175 3.01e-05 ***
## siteousSkyra   0.19362    0.06099   3.174 0.001507 **
## siteUB        -0.17370    0.11906  -1.459 0.144625
## siteUCAM      -0.22099    0.06426  -3.439 0.000587 ***
## siteUKB        0.45927    0.04915   9.343  < 2e-16 ***
## siteUmU        0.15251    0.07375   2.068 0.038688 *
## icv            0.52774    0.01302  40.522  < 2e-16 ***
## ---
## Signif. codes:  0 '***' 0.001 '**' 0.01 '*' 0.05 '.' 0.1 ' ' 1
##
## Approximate significance of smooth terms:
##                     edf Ref.df       F p-value
## s(bl_age)         3.651  3.651 160.570  <2e-16 ***
## s(bl_age):sleep_z 2.000  2.000   1.191   0.304
## s(bl_age):time    4.486  4.486 241.131  <2e-16 ***
## s(sleep_z):time   1.000  1.000   0.457   0.499
## ---
## Signif. codes:  0 '***' 0.001 '**' 0.01 '*' 0.05 '.' 0.1 ' ' 1
##
## R-sq.(adj) =  0.555
## lmer.REML = 7162.2  Scale est. = 0.024358  n = 8164
```

Below are plots of a plot of `s(bl_age)`, `s(bl_age):sleep_z`, `s(bl_age):time`, and `s(sleep_z):time`. For `s(sleep_z):time`, roughly, if we cannot fit a straight line through the confidence bands, there is evidence that the effect of time depends on sleep duration. It is correct the that confidence interval is zero at one point along the axis; this happens because we do not include the main effect.

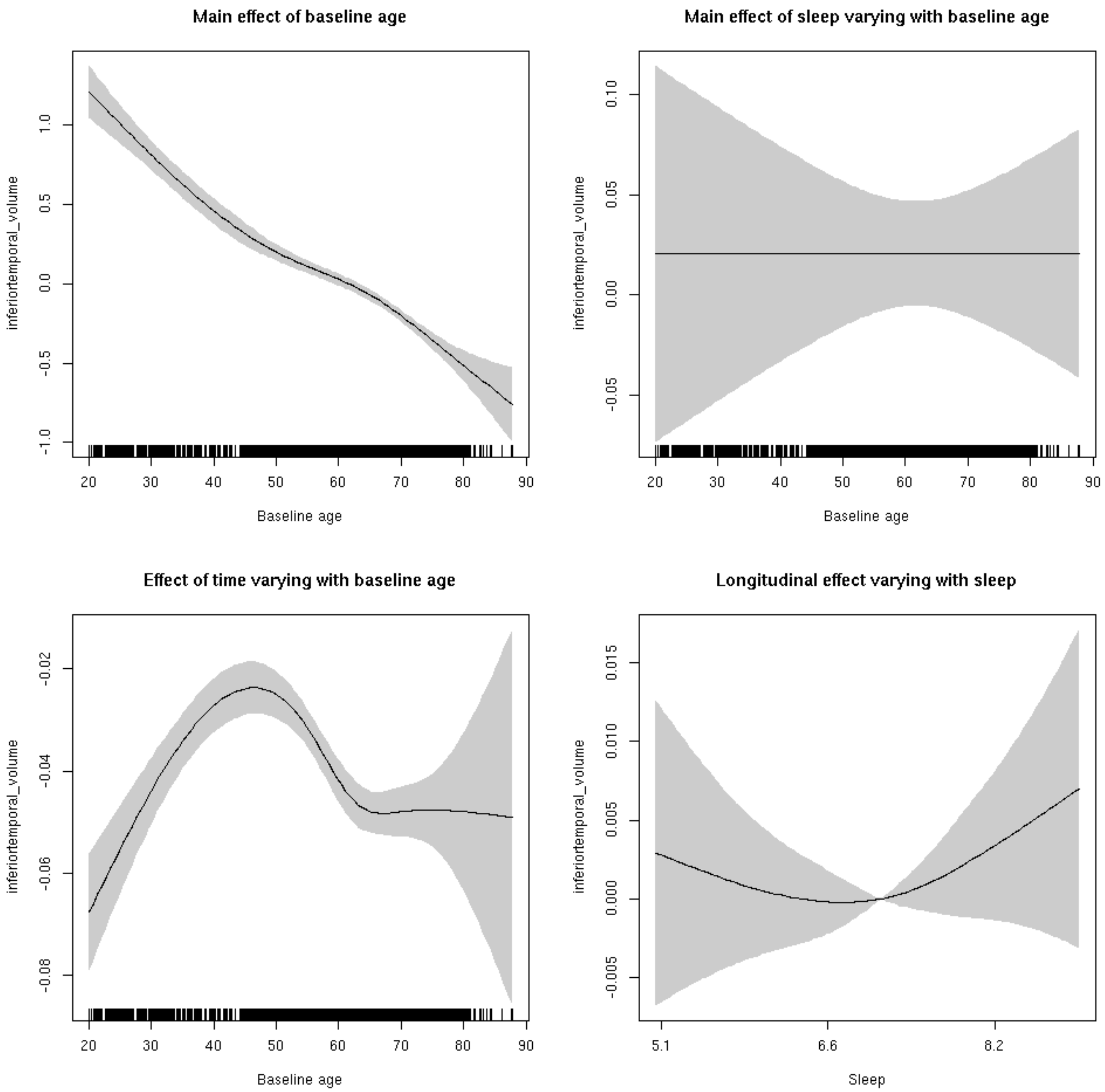

Restricted to sleep >= 5 and <= 9 hours Below is the row for `s(sleep_z):time` from the model output.

```
## s(sleep_z):time   1.739  1.739   0.876   0.354
```

Below are plots of a plot of `s(bl_age)`, `s(bl_age):sleep_z`, `s(bl_age):time`, and `s(sleep_z):time`. For `s(sleep_z):time`, roughly, if we cannot fit a straight line through the confidence bands, there is evidence that the effect of time depends on sleep duration. It is correct the that confidence interval is zero at one point along the axis; this happens because we do not include the main effect.

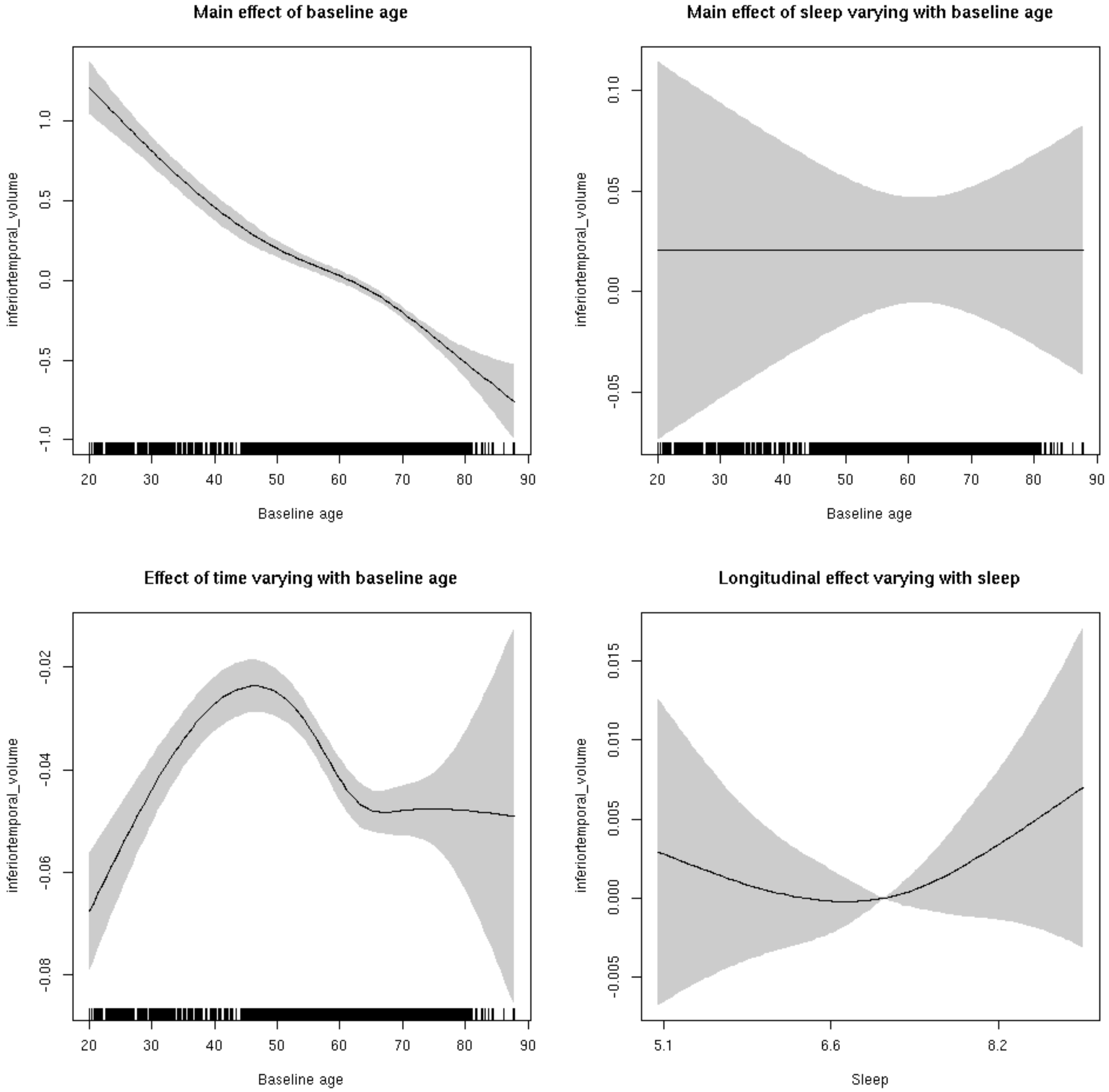

insula_area: Stratified by sex

Female: Full data Below is the row for `s(sleep_z):time` from the model output.

```
## s(sleep_z):time   1.000  1.000 0.013    0.910
```

Below are plots of a plot of `s(bl_age)`, `s(bl_age):sleep_z`, `s(bl_age):time`, and `s(sleep_z):time`. For `s(sleep_z):time`, roughly, if we cannot fit a straight line through the confidence bands, there is evidence that the effect of time depends on sleep duration. It is correct the that confidence interval is zero at one point along the axis; this happens because we do not include the main effect.

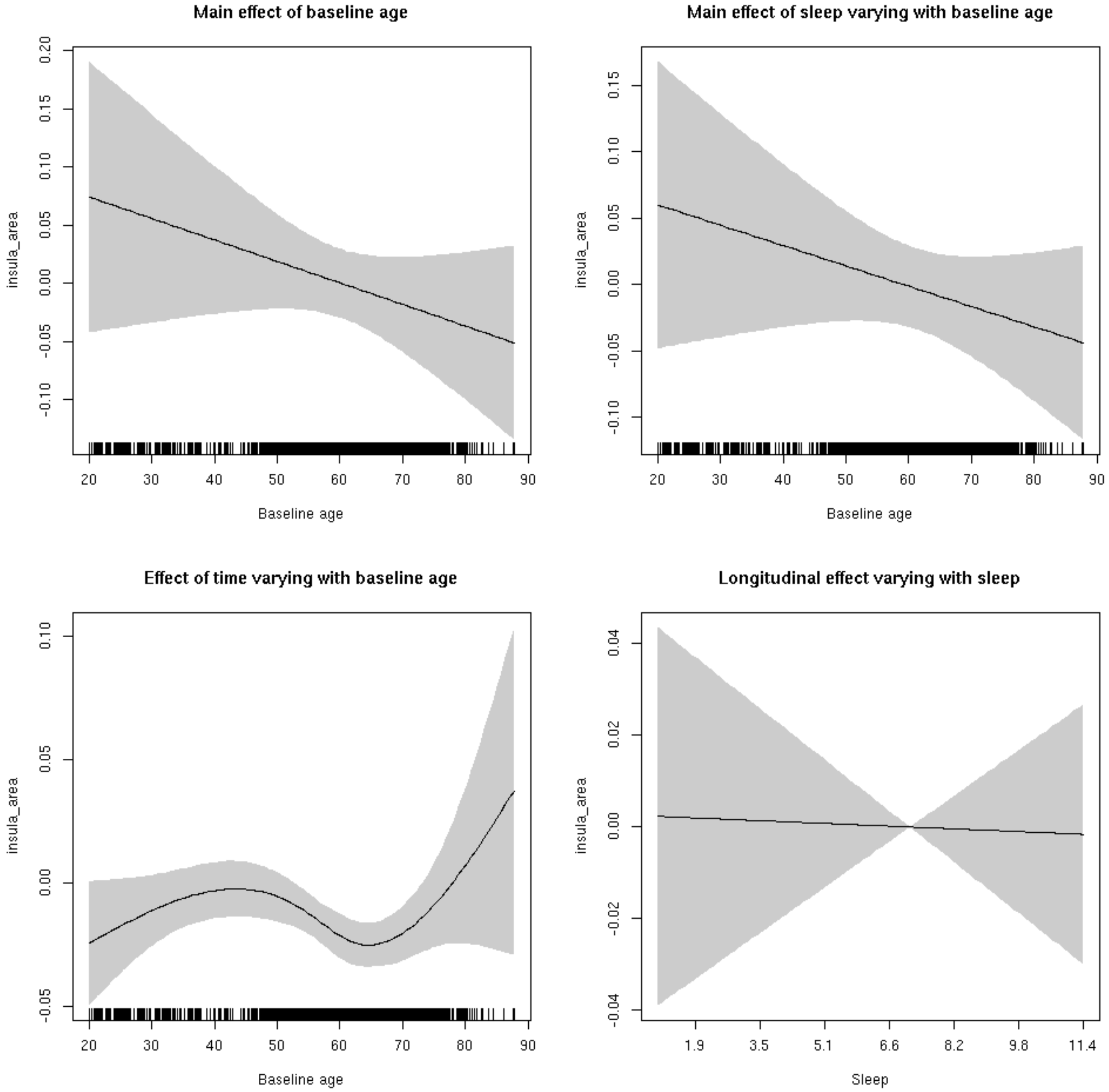

Male: Full data Below is the row for `s(sleep_z):time` from the model output.

```
## s(sleep_z):time     1       1  2.026   0.1547
```

Below are plots of a plot of `s(bl_age)`, `s(bl_age):sleep_z`, `s(bl_age):time`, and `s(sleep_z):time`. For `s(sleep_z):time`, roughly, if we cannot fit a straight line through the confidence bands, there is evidence that the effect of time depends on sleep duration. It is correct the that confidence interval is zero at one point along the axis; this happens because we do not include the main effect.

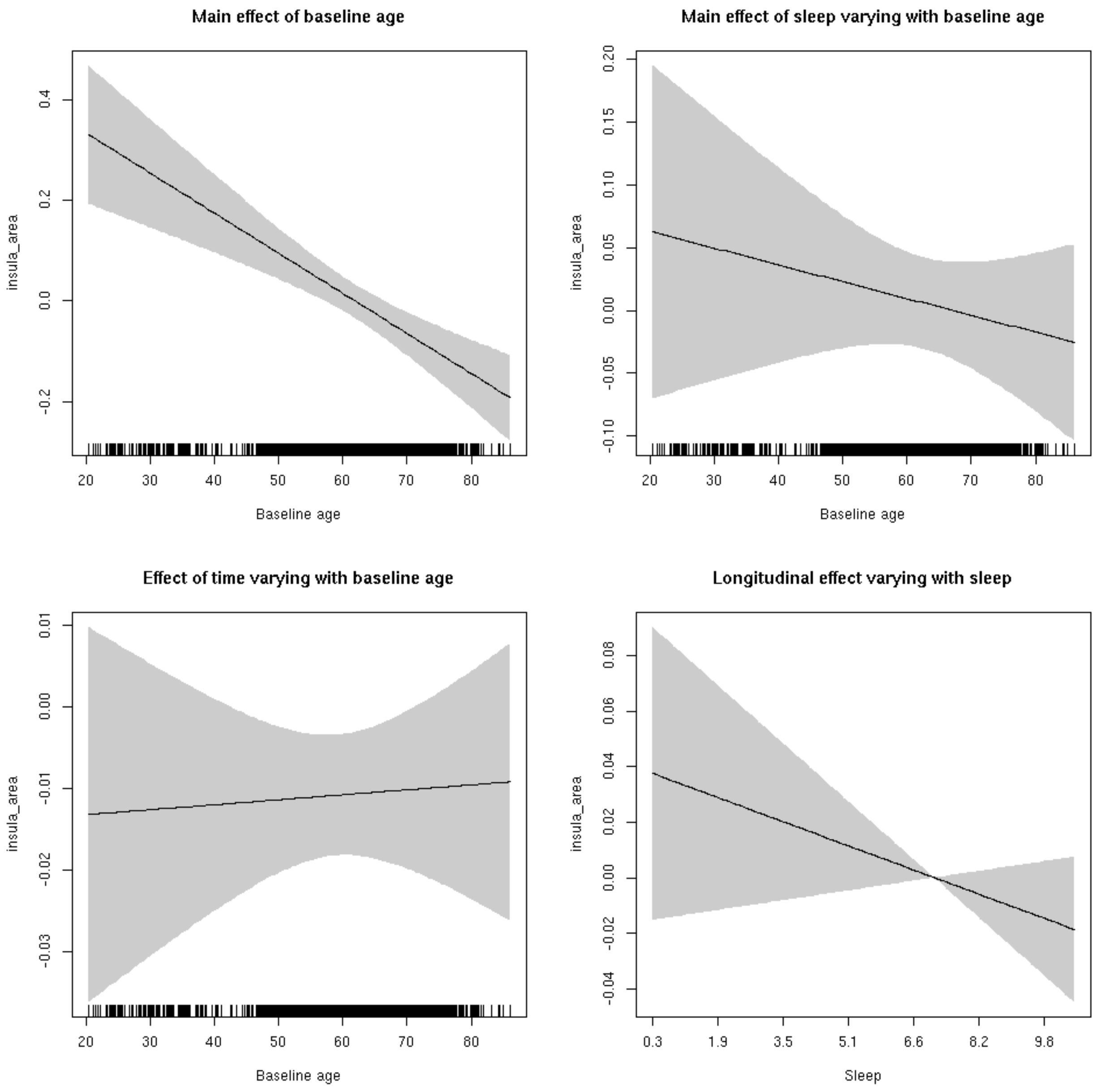

insula_area

Full data Below is the model output. The term `s(sleep_z):time` is probably what we care most about.

```
##
## Family: gaussian
## Link function: identity
##
## Formula:
## value ~ sex + site + icv + s(bl_age, k = 10, bs = "cr") + s(bl_age,
##     by = sleep_z, bs = "cr") + +s(bl_age, by = time, k = 5, bs = "cr") +
##     s(sleep_z, by = time, k = 5, bs = "cr", pc = 0)
## <environment: 0x5630035b39d0>
```

```
##
## Parametric coefficients:
##                 Estimate Std. Error t value Pr(>|t|)
## (Intercept)      0.56673    0.05234  10.827  < 2e-16 ***
## sexmale          0.28178    0.02715  10.377  < 2e-16 ***
## siteousAvanto -0.66267    0.06541 -10.131  < 2e-16 ***
## siteousPrisma -1.08530    0.23918  -4.538 5.77e-06 ***
## siteousSkyra  -0.57480    0.06319  -9.096  < 2e-16 ***
## siteUB        -0.83764    0.12023  -6.967 3.49e-12 ***
## siteUCAM      -0.76579    0.06501 -11.779  < 2e-16 ***
## siteUKB       -0.69504    0.04868 -14.276  < 2e-16 ***
## siteUmU       -0.37871    0.07494  -5.053 4.44e-07 ***
## icv            0.61672    0.01345  45.847  < 2e-16 ***
## ---
## Signif. codes:  0 '***' 0.001 '**' 0.01 '*' 0.05 '.' 0.1 ' ' 1
##
## Approximate significance of smooth terms:
##                     edf Ref.df      F  p-value
## s(bl_age)             1      1 20.632 5.80e-06 ***
## s(bl_age):sleep_z     2      2  1.250    0.287
## s(bl_age):time        2      2 15.845 2.27e-07 ***
## s(sleep_z):time       1      1  1.174    0.279
## ---
## Signif. codes:  0 '***' 0.001 '**' 0.01 '*' 0.05 '.' 0.1 ' ' 1
##
## R-sq.(adj) =  0.488
## lmer.REML =  13109  Scale est. = 0.094419  n = 8178
```

Below are plots of a plot of `s(bl_age)`, `s(bl_age):sleep_z`, `s(bl_age):time`, and `s(sleep_z):time`. For `s(sleep_z):time`, roughly, if we cannot fit a straight line through the confidence bands, there is evidence that the effect of time depends on sleep duration. It is correct the that confidence interval is zero at one point along the axis; this happens because we do not include the main effect.

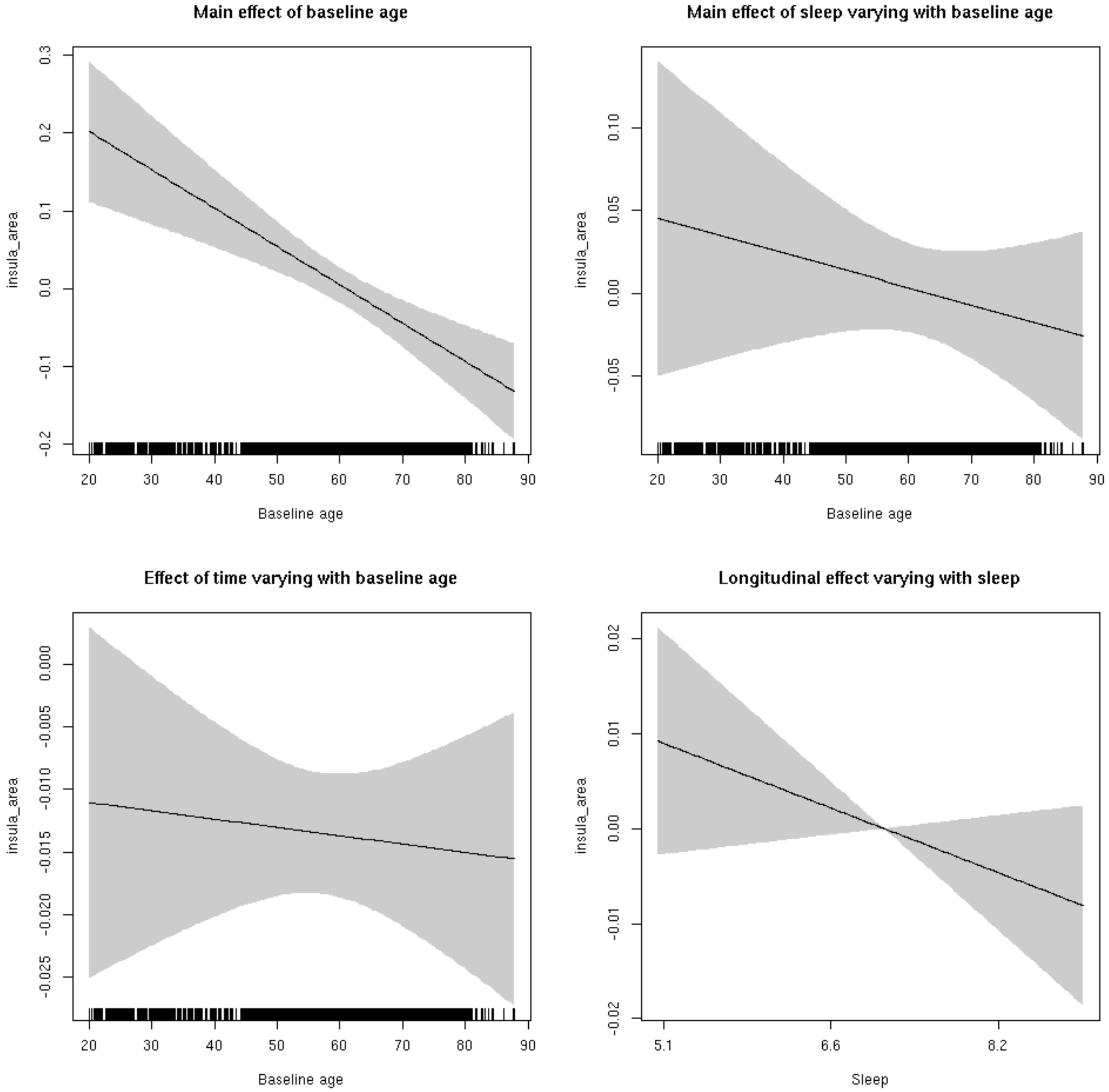

Restricted to sleep >= 5 and <= 9 hours Below is the row for `s(sleep_z):time` from the model output.

```
## s(sleep_z):time     1       1  2.362    0.124
```

Below are plots of a plot of `s(bl_age)`, `s(bl_age):sleep_z`, `s(bl_age):time`, and `s(sleep_z):time`. For `s(sleep_z):time`, roughly, if we cannot fit a straight line through the confidence bands, there is evidence that the effect of time depends on sleep duration. It is correct the that confidence interval is zero at one point along the axis; this happens because we do not include the main effect.

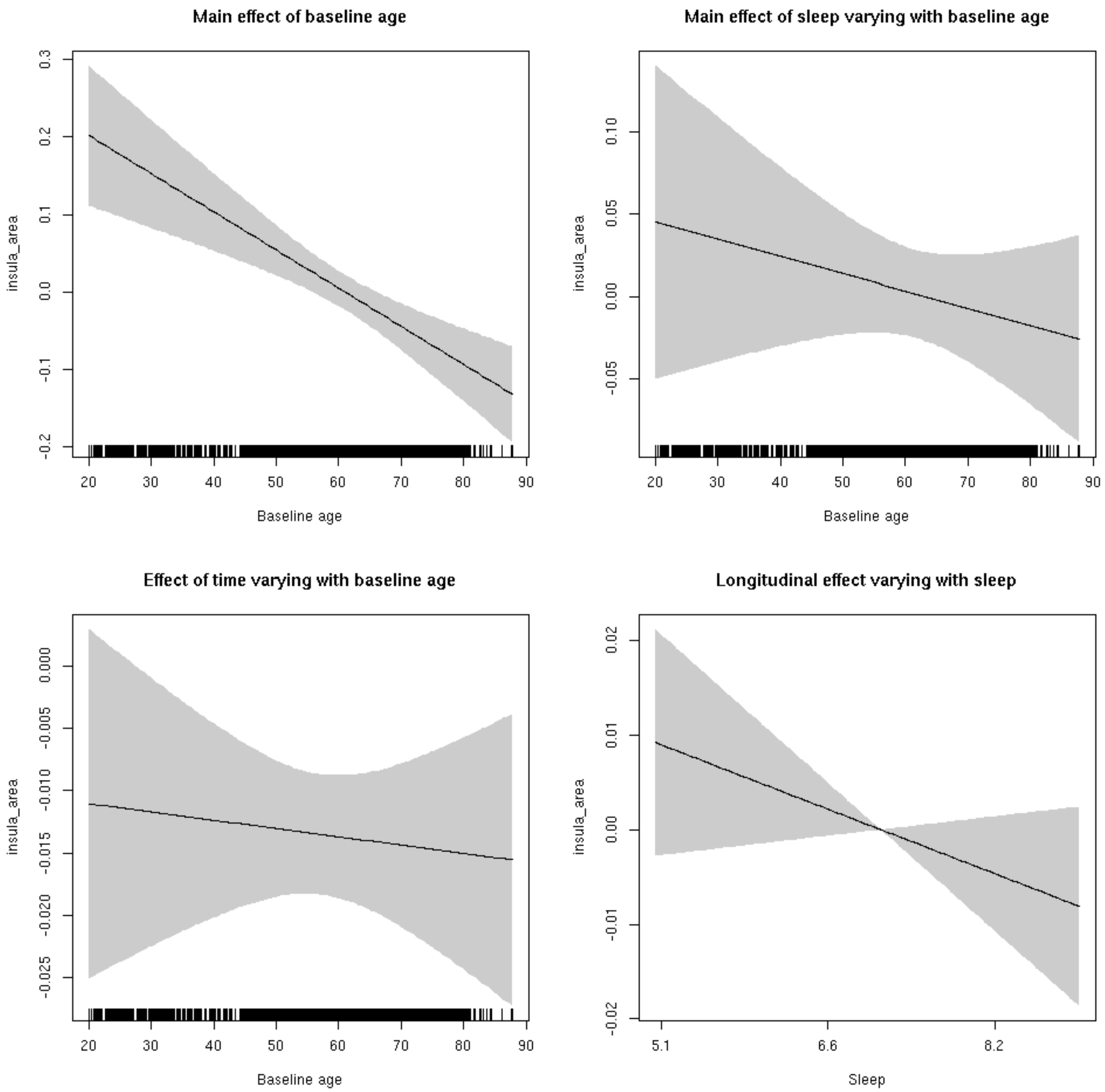

insula_thickness: Stratified by sex

Female: Full data Below is the row for `s(sleep_z):time` from the model output.

```
## s(sleep_z):time   1.948  1.948  1.638 0.210483
```

Below are plots of a plot of `s(bl_age)`, `s(bl_age):sleep_z`, `s(bl_age):time`, and `s(sleep_z):time`. For `s(sleep_z):time`, roughly, if we cannot fit a straight line through the confidence bands, there is evidence that the effect of time depends on sleep duration. It is correct the that confidence interval is zero at one point along the axis; this happens because we do not include the main effect.

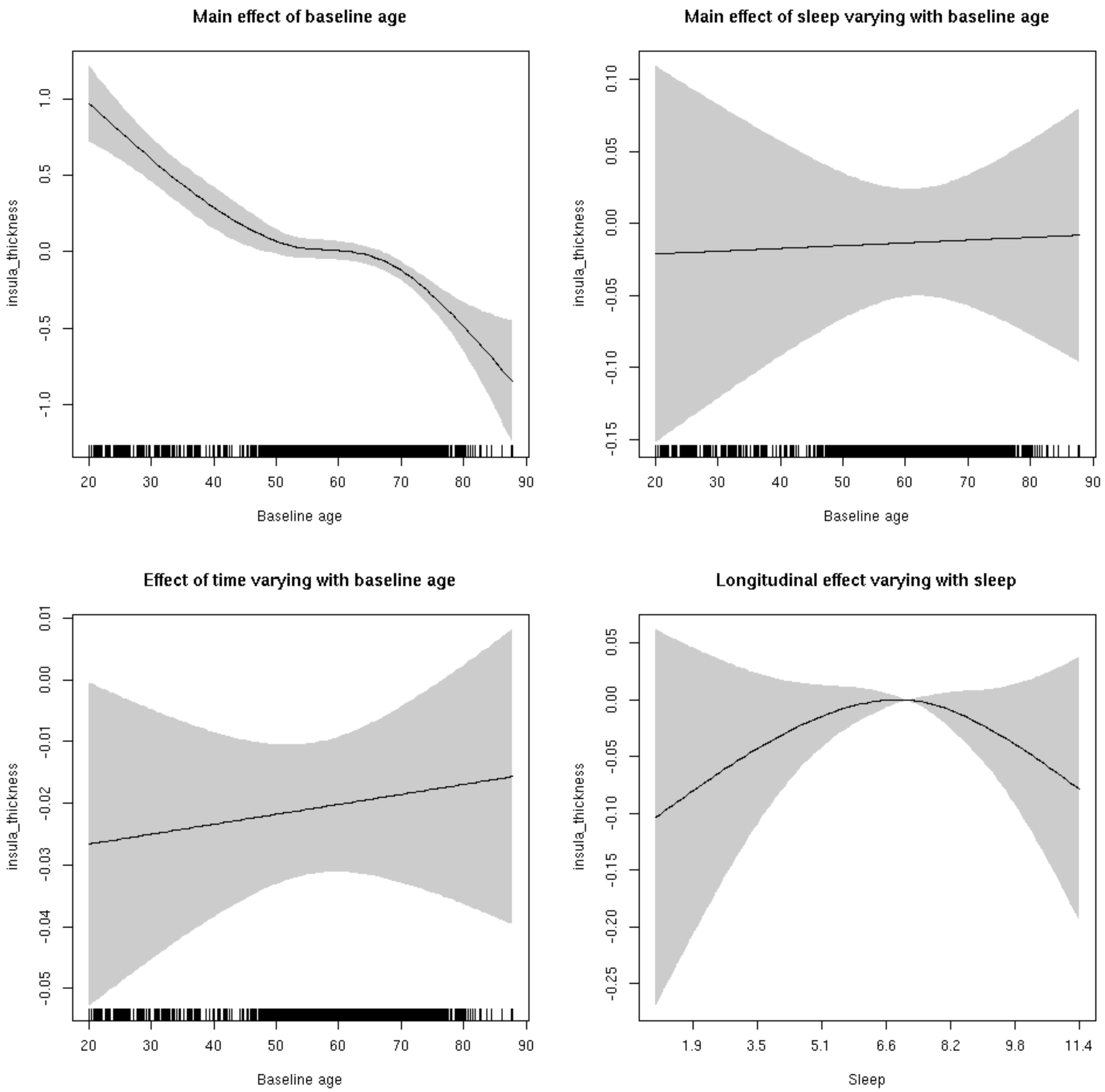

Male: Full data Below is the row for `s(sleep_z):time` from the model output.

```
## s(sleep_z):time   1.000  1.000   0.187   0.666
```

Below are plots of a plot of `s(bl_age)`, `s(bl_age):sleep_z`, `s(bl_age):time`, and `s(sleep_z):time`. For `s(sleep_z):time`, roughly, if we cannot fit a straight line through the confidence bands, there is evidence that the effect of time depends on sleep duration. It is correct the that confidence interval is zero at one point along the axis; this happens because we do not include the main effect.

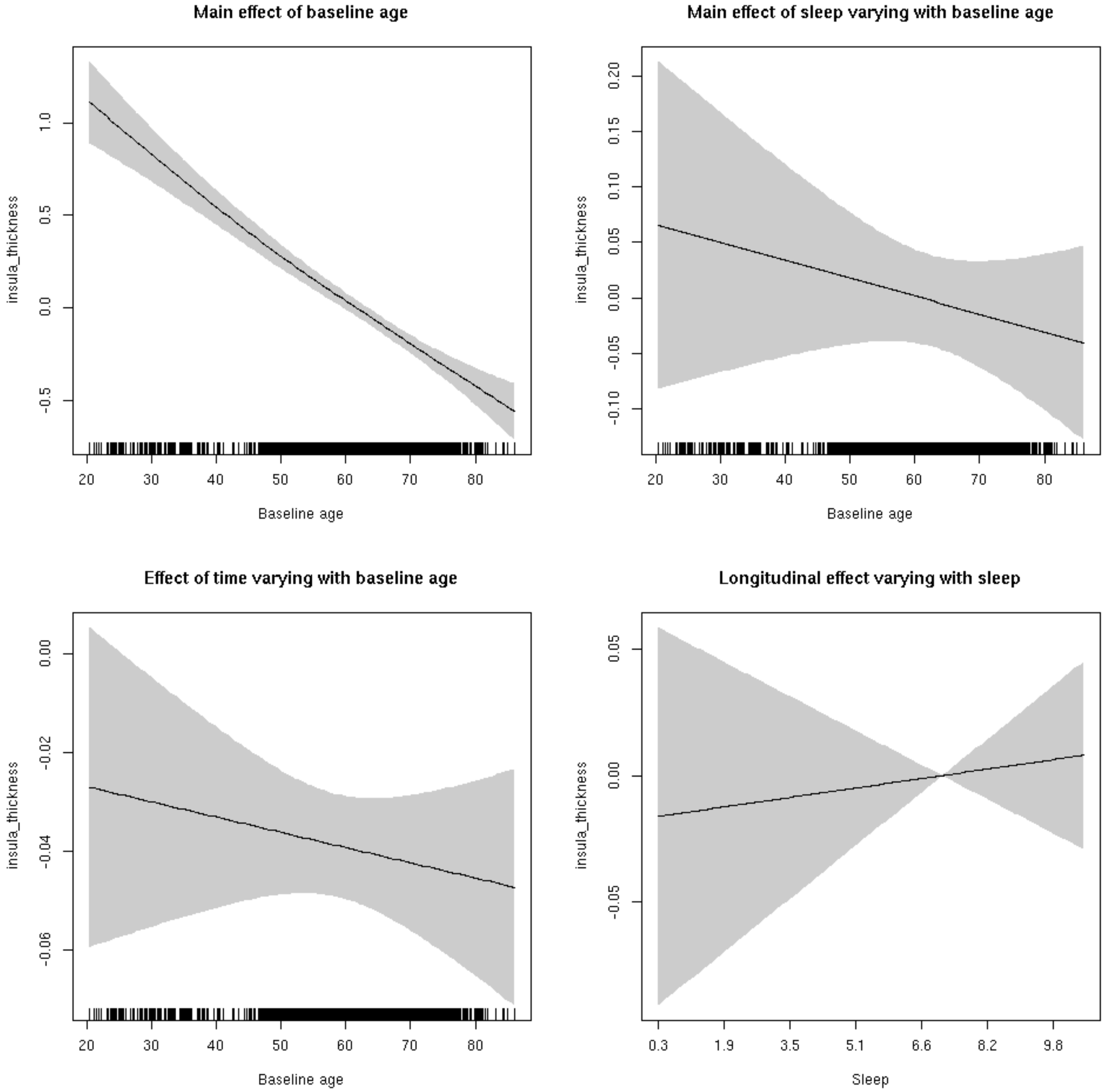

insula_thickness

Full data Below is the model output. The term `s(sleep_z):time` is probably what we care most about.

```
##
## Family: gaussian
## Link function: identity
##
## Formula:
## value ~ sex + site + s(bl_age, k = 10, bs = "cr") + s(bl_age,
##     by = sleep_z, bs = "cr") + +s(bl_age, by = time, k = 5, bs = "cr") +
##     s(sleep_z, by = time, k = 5, bs = "cr", pc = 0)
## <environment: 0x5630035d9688>
```

```
##
## Parametric coefficients:
##                Estimate Std. Error t value Pr(>|t|)
## (Intercept)   -1.28050    0.05173 -24.751  < 2e-16 ***
## sexmale        0.07028    0.02588   2.715  0.00664 **
## siteousAvanto  0.10193    0.06936   1.470  0.14169
## siteousPrisma  0.44448    0.33824   1.314  0.18885
## siteousSkyra   0.31652    0.06933   4.565 5.06e-06 ***
## siteUB         0.40202    0.13627   2.950  0.00319 **
## siteUCAM       0.23196    0.07116   3.260  0.00112 **
## siteUKB        1.46116    0.05240  27.887  < 2e-16 ***
## siteUmU       -0.05222    0.08522  -0.613  0.54006
## ---
## Signif. codes:  0 '***' 0.001 '**' 0.01 '*' 0.05 '.' 0.1 ' ' 1
##
## Approximate significance of smooth terms:
##                      edf Ref.df      F p-value
## s(bl_age)          3.732  3.732 81.121  <2e-16 ***
## s(bl_age):sleep_z 2.000  2.000  0.473   0.623
## s(bl_age):time    2.631  2.631 19.398  <2e-16 ***
## s(sleep_z):time   2.010  2.010  1.514   0.221
## ---
## Signif. codes:  0 '***' 0.001 '**' 0.01 '*' 0.05 '.' 0.1 ' ' 1
##
## R-sq.(adj) =  0.339
## lmer.REML =  17536  Scale est. = 0.20534   n = 8178
```

Below are plots of a plot of `s(bl_age)`, `s(bl_age):sleep_z`, `s(bl_age):time`, and `s(sleep_z):time`. For `s(sleep_z):time`, roughly, if we cannot fit a straight line through the confidence bands, there is evidence that the effect of time depends on sleep duration. It is correct the that confidence interval is zero at one point along the axis; this happens because we do not include the main effect.

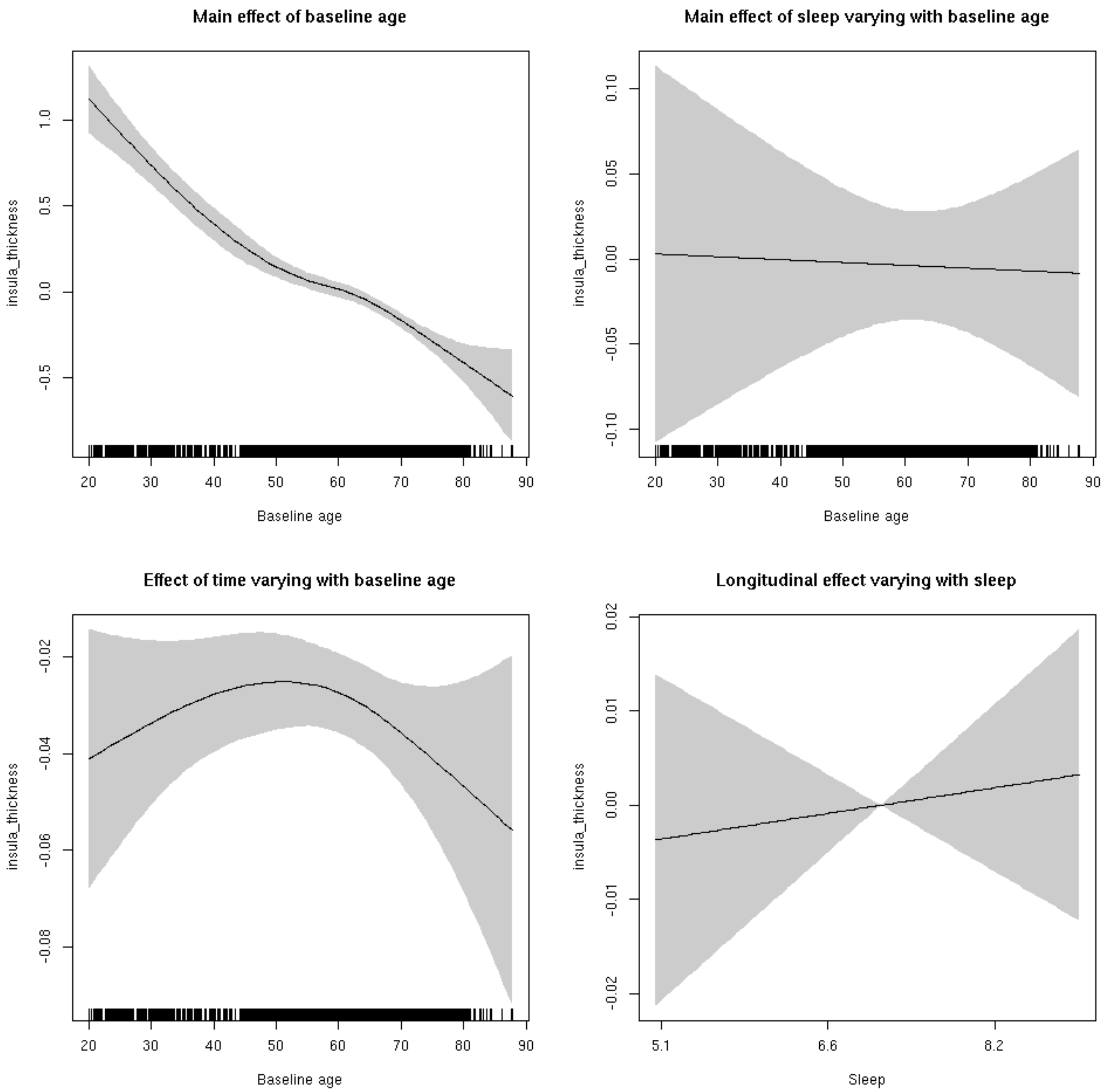

Restricted to sleep >= 5 and <= 9 hours Below is the row for `s(sleep_z):time` from the model output.

```
## s(sleep_z):time   1.000  1.000  0.175   0.676
```

Below are plots of a plot of `s(bl_age)`, `s(bl_age):sleep_z`, `s(bl_age):time`, and `s(sleep_z):time`. For `s(sleep_z):time`, roughly, if we cannot fit a straight line through the confidence bands, there is evidence that the effect of time depends on sleep duration. It is correct the that confidence interval is zero at one point along the axis; this happens because we do not include the main effect.

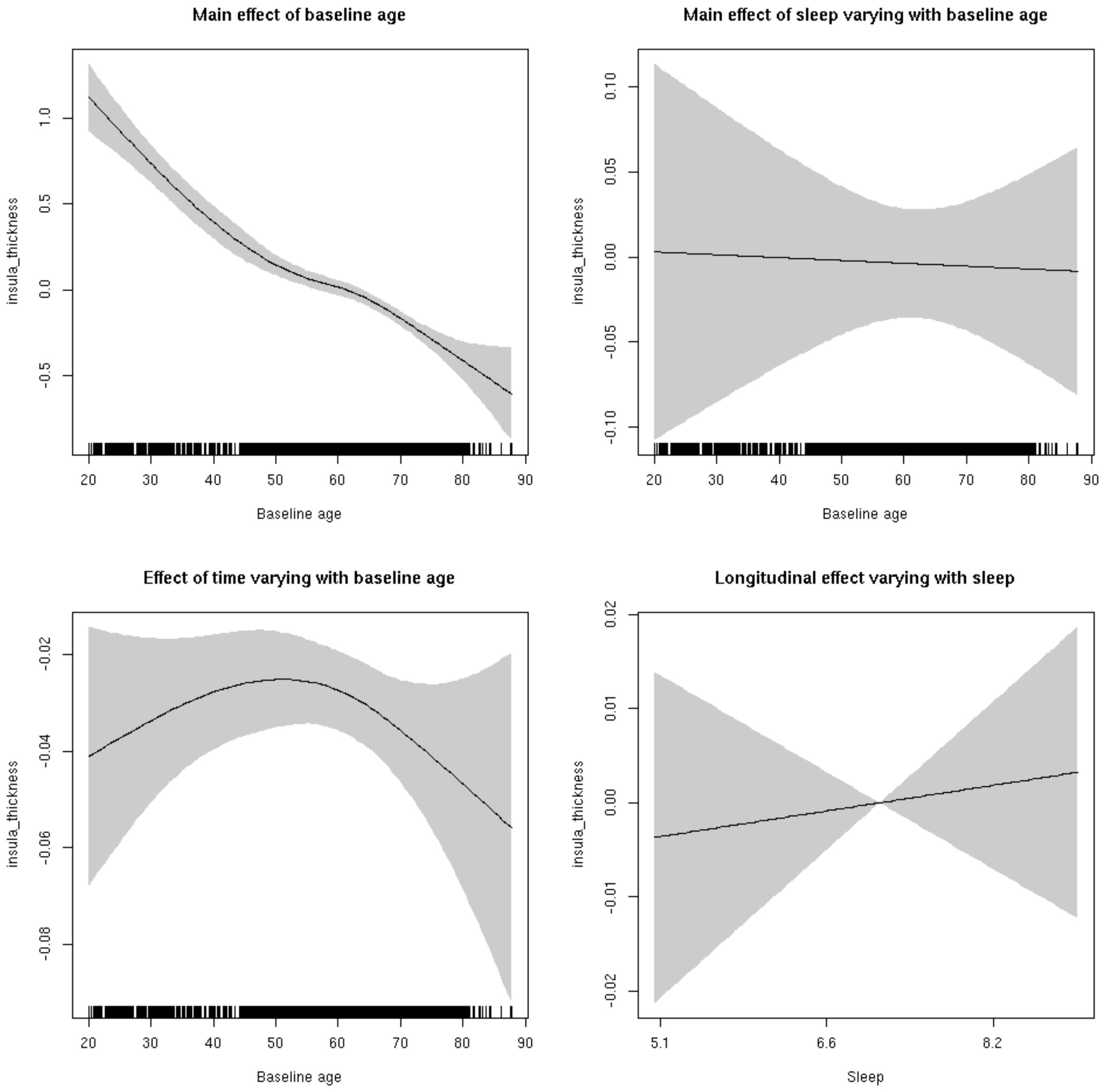

insula_volume: Stratified by sex

Female: Full data Below is the row for `s(sleep_z):time` from the model output.

```
## s(sleep_z):time   1.000  1.000  0.038   0.846
```

Below are plots of a plot of `s(bl_age)`, `s(bl_age):sleep_z`, `s(bl_age):time`, and `s(sleep_z):time`. For `s(sleep_z):time`, roughly, if we cannot fit a straight line through the confidence bands, there is evidence that the effect of time depends on sleep duration. It is correct the that confidence interval is zero at one point along the axis; this happens because we do not include the main effect.

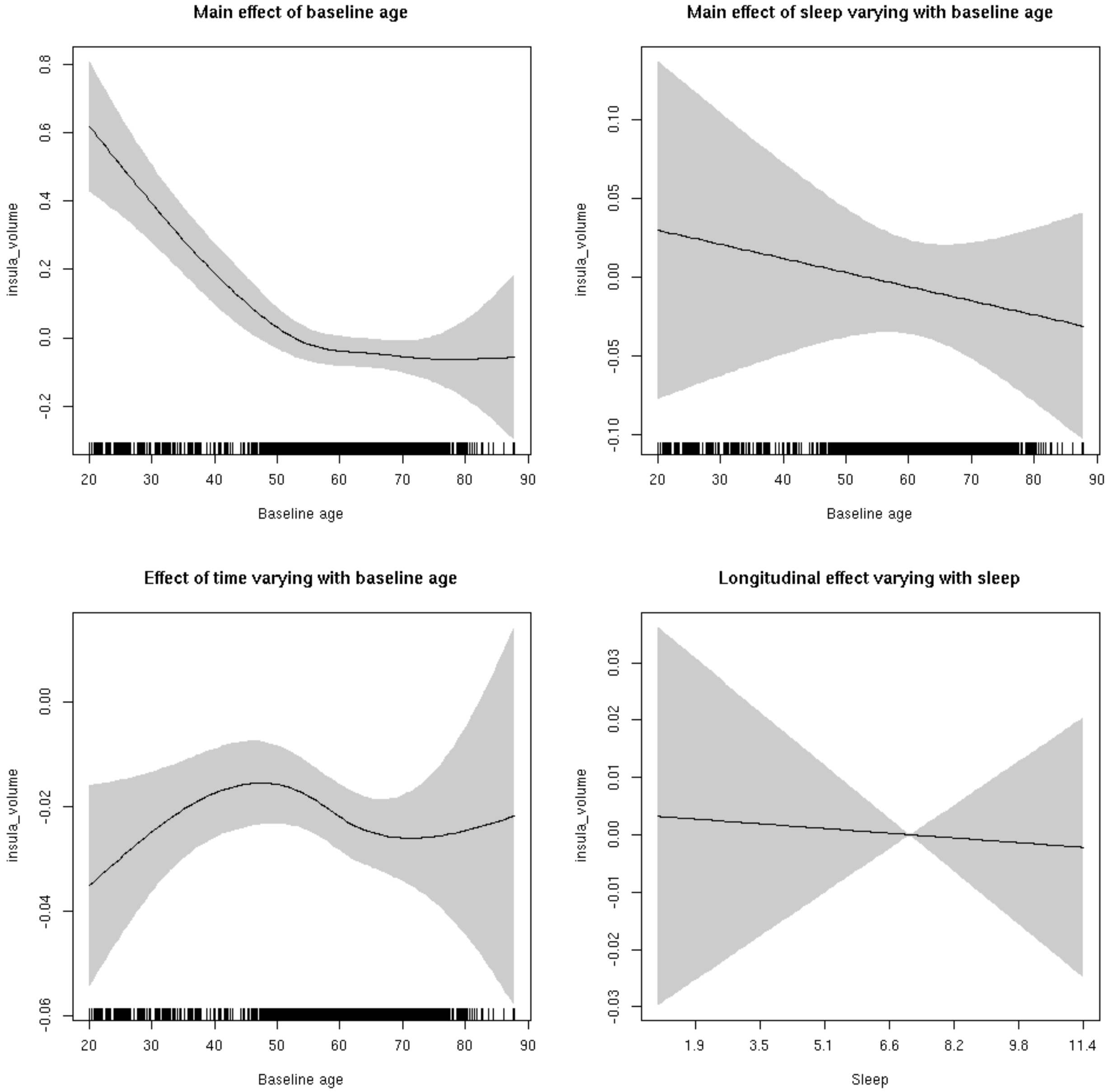

Male: Full data Below is the row for `s(sleep_z):time` from the model output.

```
## s(sleep_z):time   1.000  1.000  1.911   0.167
```

Below are plots of a plot of `s(bl_age)`, `s(bl_age):sleep_z`, `s(bl_age):time`, and `s(sleep_z):time`. For `s(sleep_z):time`, roughly, if we cannot fit a straight line through the confidence bands, there is evidence that the effect of time depends on sleep duration. It is correct the that confidence interval is zero at one point along the axis; this happens because we do not include the main effect.

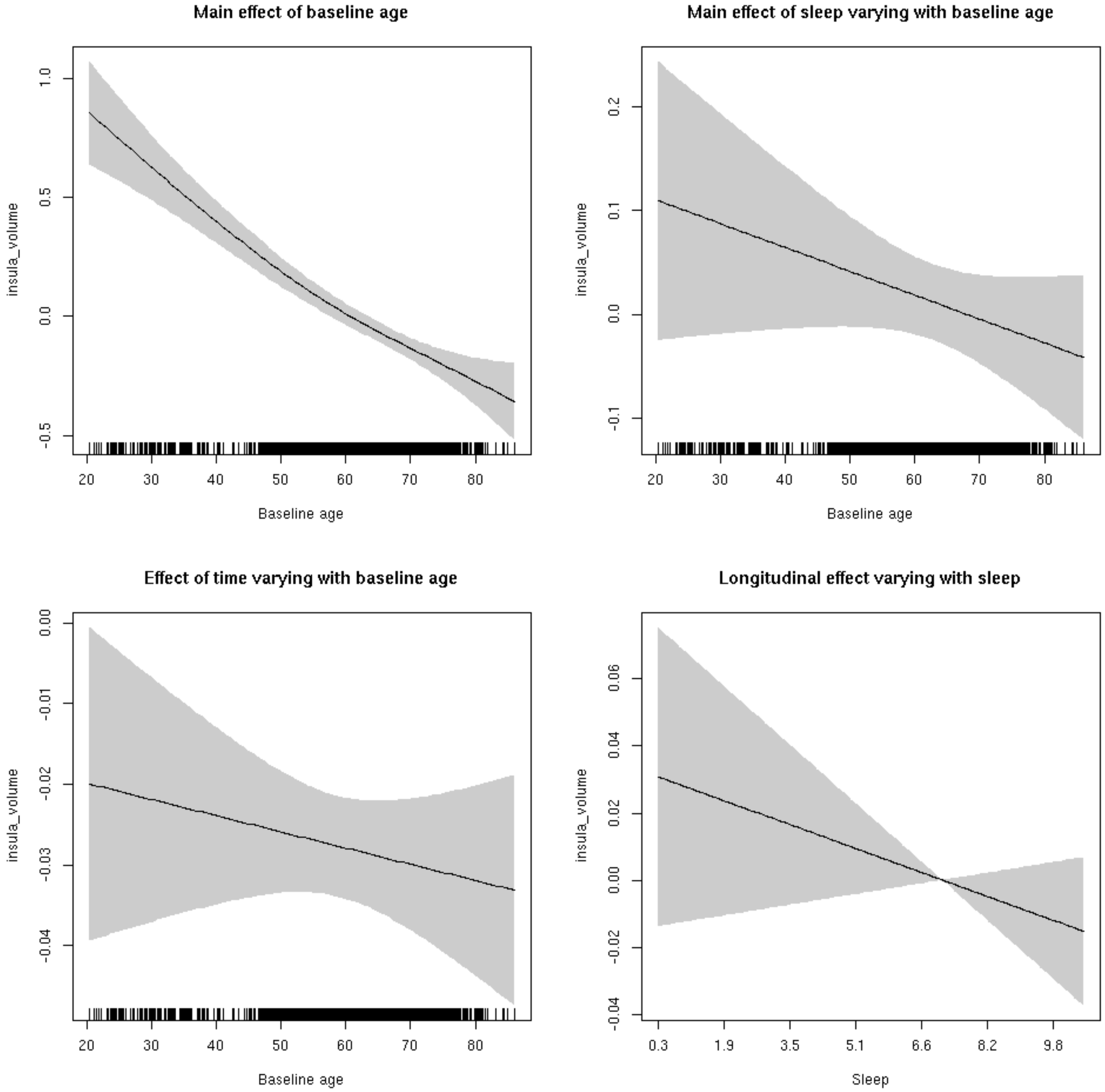

insula_volume

Full data Below is the model output. The term `s(sleep_z):time` is probably what we care most about.

```
##
## Family: gaussian
## Link function: identity
##
## Formula:
## value ~ sex + site + icv + s(bl_age, k = 10, bs = "cr") + s(bl_age,
##     by = sleep_z, bs = "cr") + +s(bl_age, by = time, k = 5, bs = "cr") +
##     s(sleep_z, by = time, k = 5, bs = "cr", pc = 0)
## <environment: 0x5630035ab250>
```

```
##
## Parametric coefficients:
##                Estimate Std. Error t value Pr(>|t|)
## (Intercept)   -0.07716    0.05313  -1.452 0.146430
## sexmale        0.33824    0.02728  12.398  < 2e-16 ***
## siteousAvanto -0.67056    0.06528 -10.272  < 2e-16 ***
## siteousPrisma -0.67770    0.20164  -3.361 0.000780 ***
## siteousSkyra  -0.28406    0.06317  -4.496 7.00e-06 ***
## siteUB        -0.63435    0.12149  -5.221 1.82e-07 ***
## siteUCAM      -0.69160    0.06573 -10.521  < 2e-16 ***
## siteUKB        0.01773    0.05035   0.352 0.724773
## siteUmU       -0.27019    0.07565  -3.571 0.000357 ***
## icv            0.56999    0.01349  42.245  < 2e-16 ***
## ---
## Signif. codes:  0 '***' 0.001 '**' 0.01 '*' 0.05 '.' 0.1 ' ' 1
##
## Approximate significance of smooth terms:
##                     edf Ref.df      F p-value
## s(bl_age)         2.922  2.922 53.653  <2e-16 ***
## s(bl_age):sleep_z 2.000  2.000  1.561   0.210
## s(bl_age):time    2.630  2.630 58.104  <2e-16 ***
## s(sleep_z):time   1.000  1.000  1.337   0.248
## ---
## Signif. codes:  0 '***' 0.001 '**' 0.01 '*' 0.05 '.' 0.1 ' ' 1
##
## R-sq.(adj) =  0.512
## lmer.REML =  11435  Scale est. = 0.063237  n = 8178
```

Below are plots of a plot of `s(bl_age)`, `s(bl_age):sleep_z`, `s(bl_age):time`, and `s(sleep_z):time`. For `s(sleep_z):time`, roughly, if we cannot fit a straight line through the confidence bands, there is evidence that the effect of time depends on sleep duration. It is correct the that confidence interval is zero at one point along the axis; this happens because we do not include the main effect.

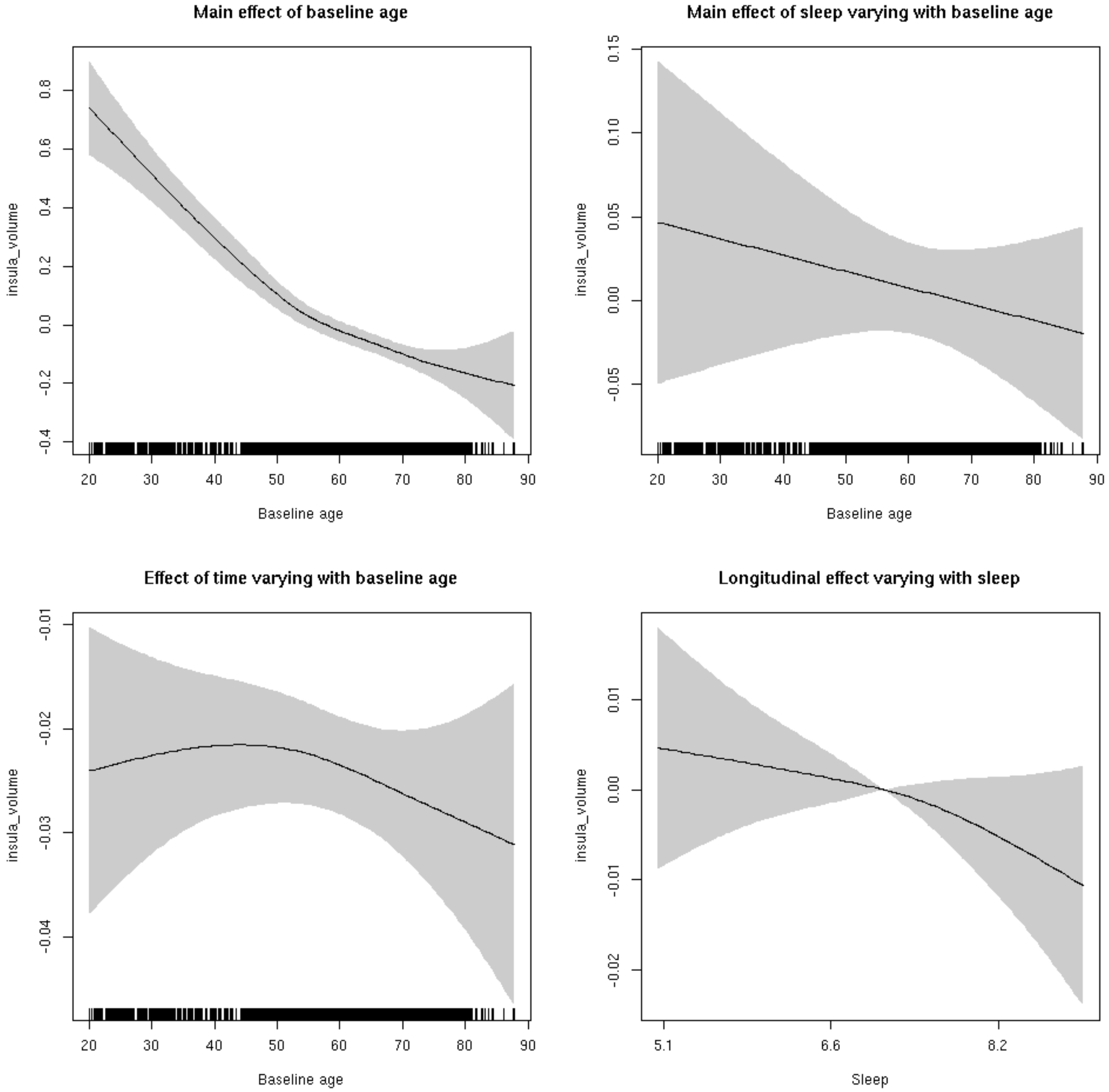

Restricted to sleep >= 5 and <= 9 hours Below is the row for `s(sleep_z):time` from the model output.

```
## s(sleep_z):time   1.421  1.421  1.001   0.229
```

Below are plots of a plot of `s(bl_age)`, `s(bl_age):sleep_z`, `s(bl_age):time`, and `s(sleep_z):time`. For `s(sleep_z):time`, roughly, if we cannot fit a straight line through the confidence bands, there is evidence that the effect of time depends on sleep duration. It is correct the that confidence interval is zero at one point along the axis; this happens because we do not include the main effect.

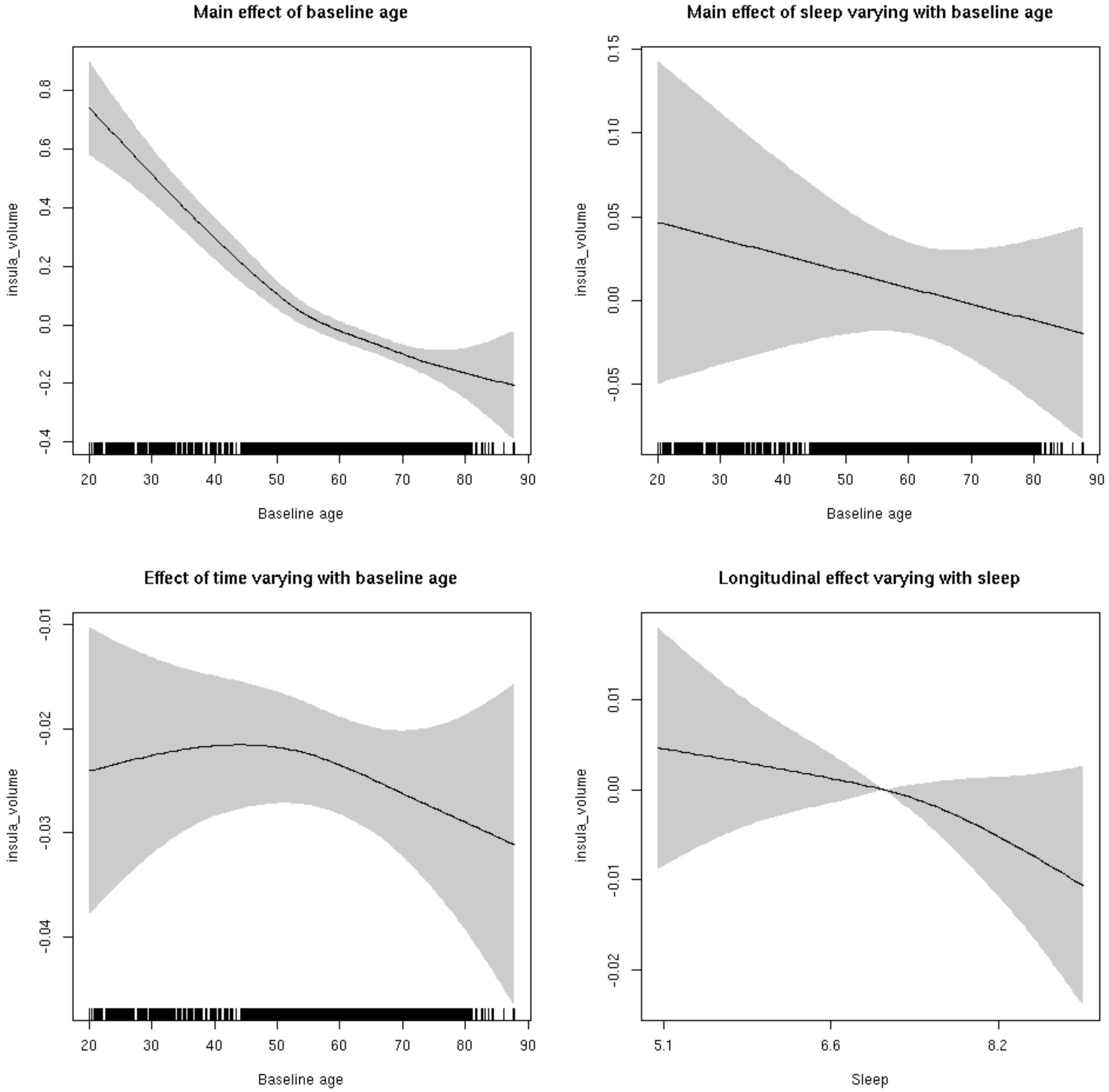

isthmuscingulate_area: Stratified by sex

Female: Full data Below is the row for `s(sleep_z):time` from the model output.

```
## s(sleep_z):time   1.000  1.000  1.246   0.264
```

Below are plots of a plot of `s(bl_age)`, `s(bl_age):sleep_z`, `s(bl_age):time`, and `s(sleep_z):time`. For `s(sleep_z):time`, roughly, if we cannot fit a straight line through the confidence bands, there is evidence that the effect of time depends on sleep duration. It is correct the that confidence interval is zero at one point along the axis; this happens because we do not include the main effect.

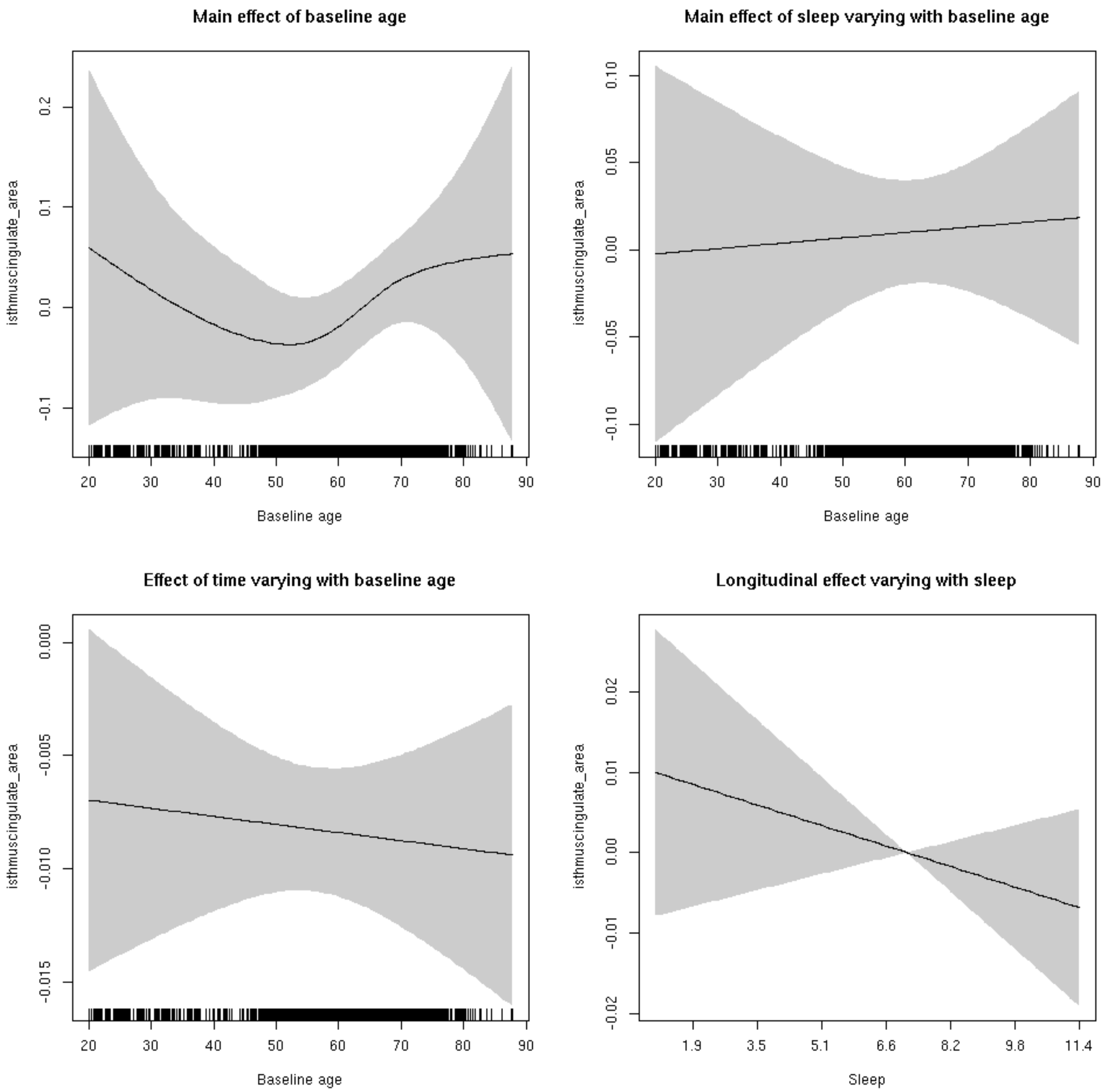

Male: Full data Below is the row for `s(sleep_z):time` from the model output.

```
## s(sleep_z):time     1      1  0.536  0.4642
```

Below are plots of a plot of `s(bl_age)`, `s(bl_age):sleep_z`, `s(bl_age):time`, and `s(sleep_z):time`. For `s(sleep_z):time`, roughly, if we cannot fit a straight line through the confidence bands, there is evidence that the effect of time depends on sleep duration. It is correct the that confidence interval is zero at one point along the axis; this happens because we do not include the main effect.

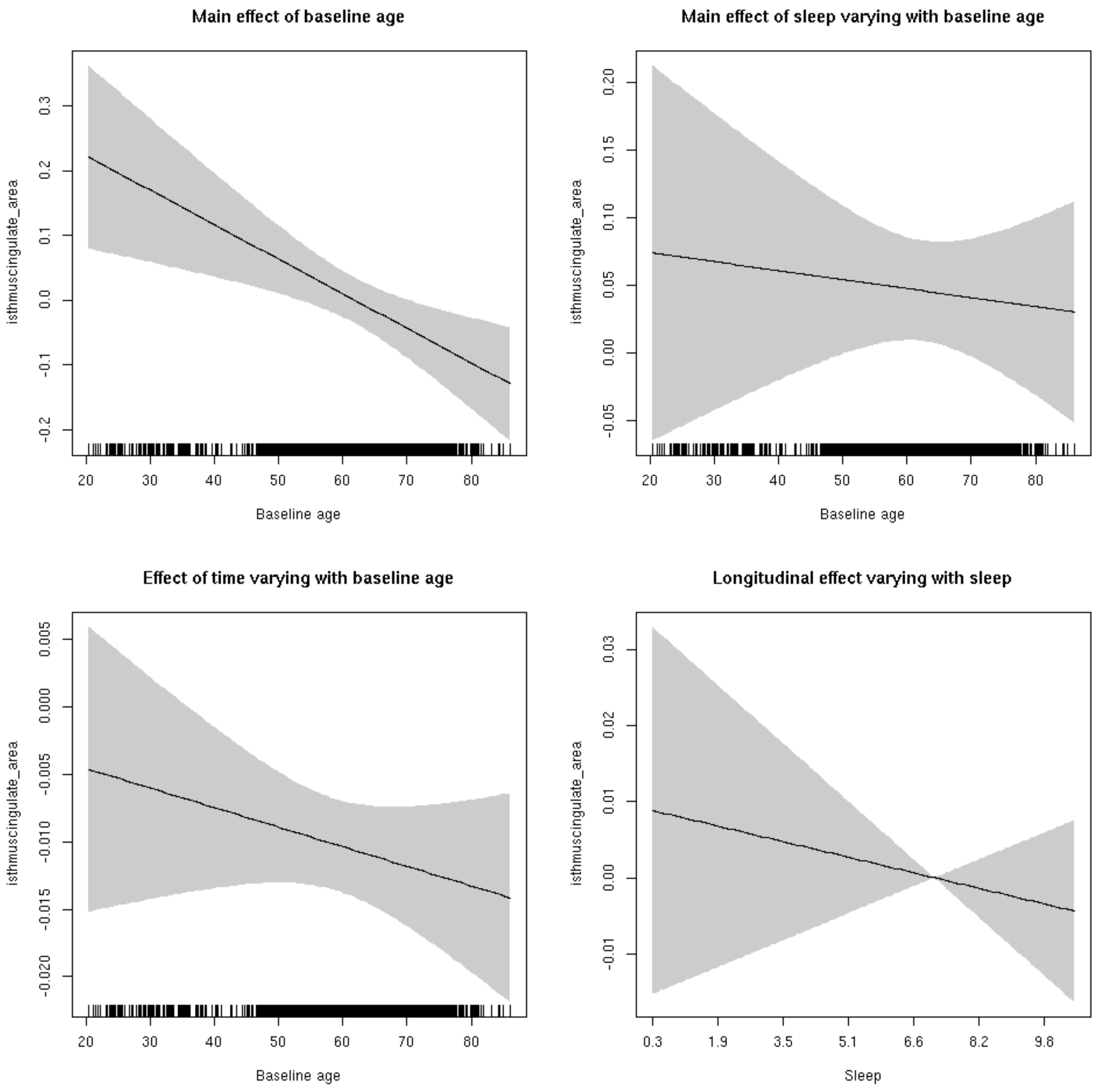

isthmuscingulate_area

Full data Below is the model output. The term `s(sleep_z):time` is probably what we care most about.

```
##
## Family: gaussian
## Link function: identity
##
## Formula:
## value ~ sex + site + icv + s(bl_age, k = 10, bs = "cr") + s(bl_age,
##     by = sleep_z, bs = "cr") + +s(bl_age, by = time, k = 5, bs = "cr") +
##     s(sleep_z, by = time, k = 5, bs = "cr", pc = 0)
## <environment: 0x5630035b5c70>
```

```
##
## Parametric coefficients:
##               Estimate Std. Error t value Pr(>|t|)
## (Intercept)    0.87704    0.05411  16.208  < 2e-16 ***
## sexmale        0.27963    0.02778  10.067  < 2e-16 ***
## siteousAvanto -0.83988    0.06586 -12.752  < 2e-16 ***
## siteousPrisma -0.77866    0.12463  -6.248 4.37e-10 ***
## siteousSkyra  -0.37954    0.06381  -5.948 2.83e-09 ***
## siteUB        -0.88226    0.12506  -7.054 1.87e-12 ***
## siteUCAM      -0.80035    0.06736 -11.882  < 2e-16 ***
## siteUKB       -1.06100    0.05150 -20.600  < 2e-16 ***
## siteUmU       -0.48229    0.07754  -6.220 5.22e-10 ***
## icv            0.54267    0.01357  39.984  < 2e-16 ***
## ---
## Signif. codes:  0 '***' 0.001 '**' 0.01 '*' 0.05 '.' 0.1 ' ' 1
##
## Approximate significance of smooth terms:
##                     edf Ref.df      F p-value
## s(bl_age)         3.609  3.609  1.584   0.101
## s(bl_age):sleep_z 2.000  2.000  2.036   0.131
## s(bl_age):time    2.000  2.000 36.929  <2e-16 ***
## s(sleep_z):time   1.000  1.000  1.875   0.171
## ---
## Signif. codes:  0 '***' 0.001 '**' 0.01 '*' 0.05 '.' 0.1 ' ' 1
##
## R-sq.(adj) =  0.463
## lmer.REML = 6331.7  Scale est. = 0.01838   n = 8170
```

Below are plots of a plot of `s(bl_age)`, `s(bl_age):sleep_z`, `s(bl_age):time`, and `s(sleep_z):time`. For `s(sleep_z):time`, roughly, if we cannot fit a straight line through the confidence bands, there is evidence that the effect of time depends on sleep duration. It is correct the that confidence interval is zero at one point along the axis; this happens because we do not include the main effect.

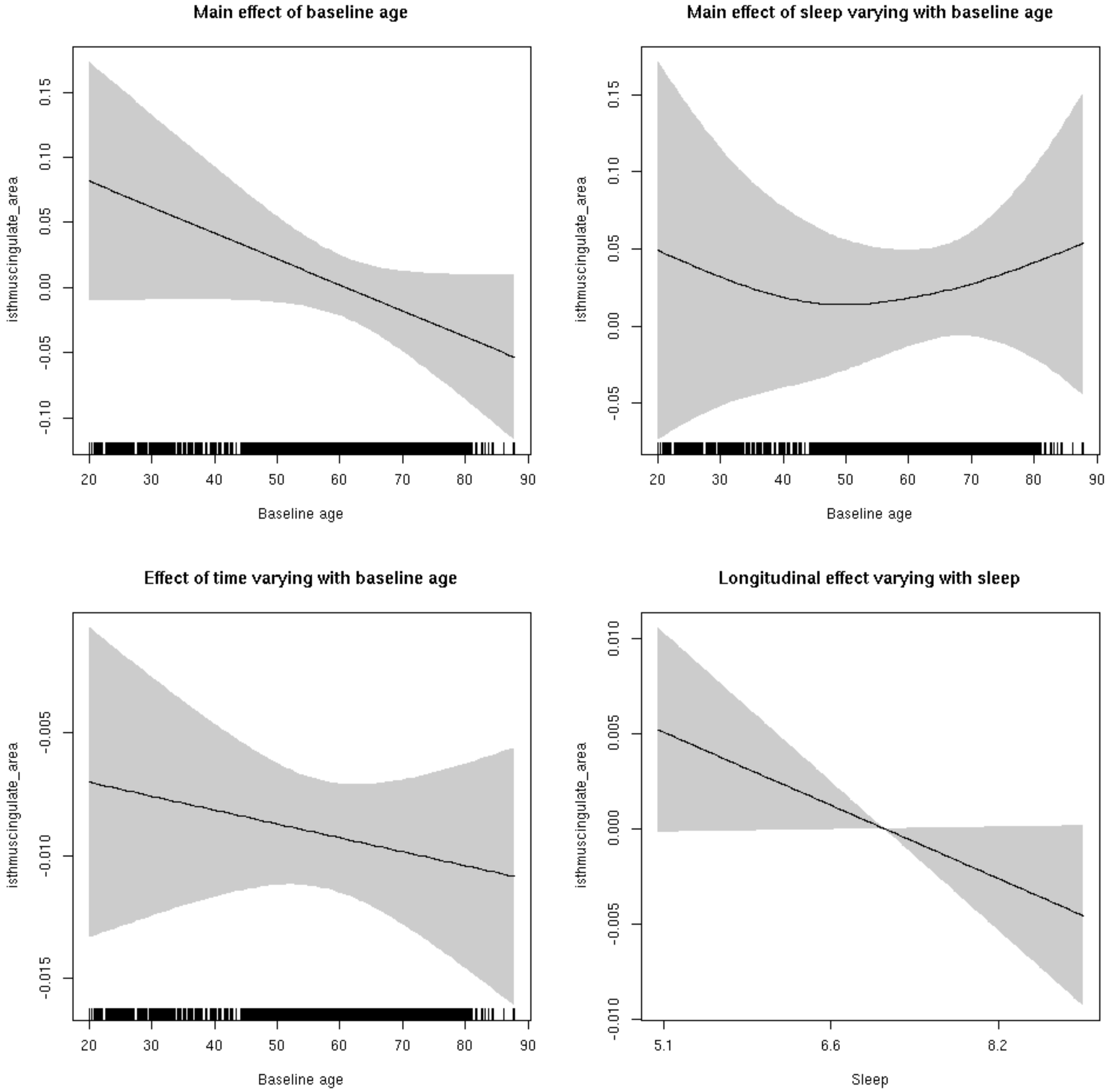

Restricted to sleep >= 5 and <= 9 hours Below is the row for `s(sleep_z):time` from the model output.

```
## s(sleep_z):time   1.000  1.000  3.735  0.0533 .
```

Below are plots of a plot of `s(bl_age)`, `s(bl_age):sleep_z`, `s(bl_age):time`, and `s(sleep_z):time`. For `s(sleep_z):time`, roughly, if we cannot fit a straight line through the confidence bands, there is evidence that the effect of time depends on sleep duration. It is correct the that confidence interval is zero at one point along the axis; this happens because we do not include the main effect.

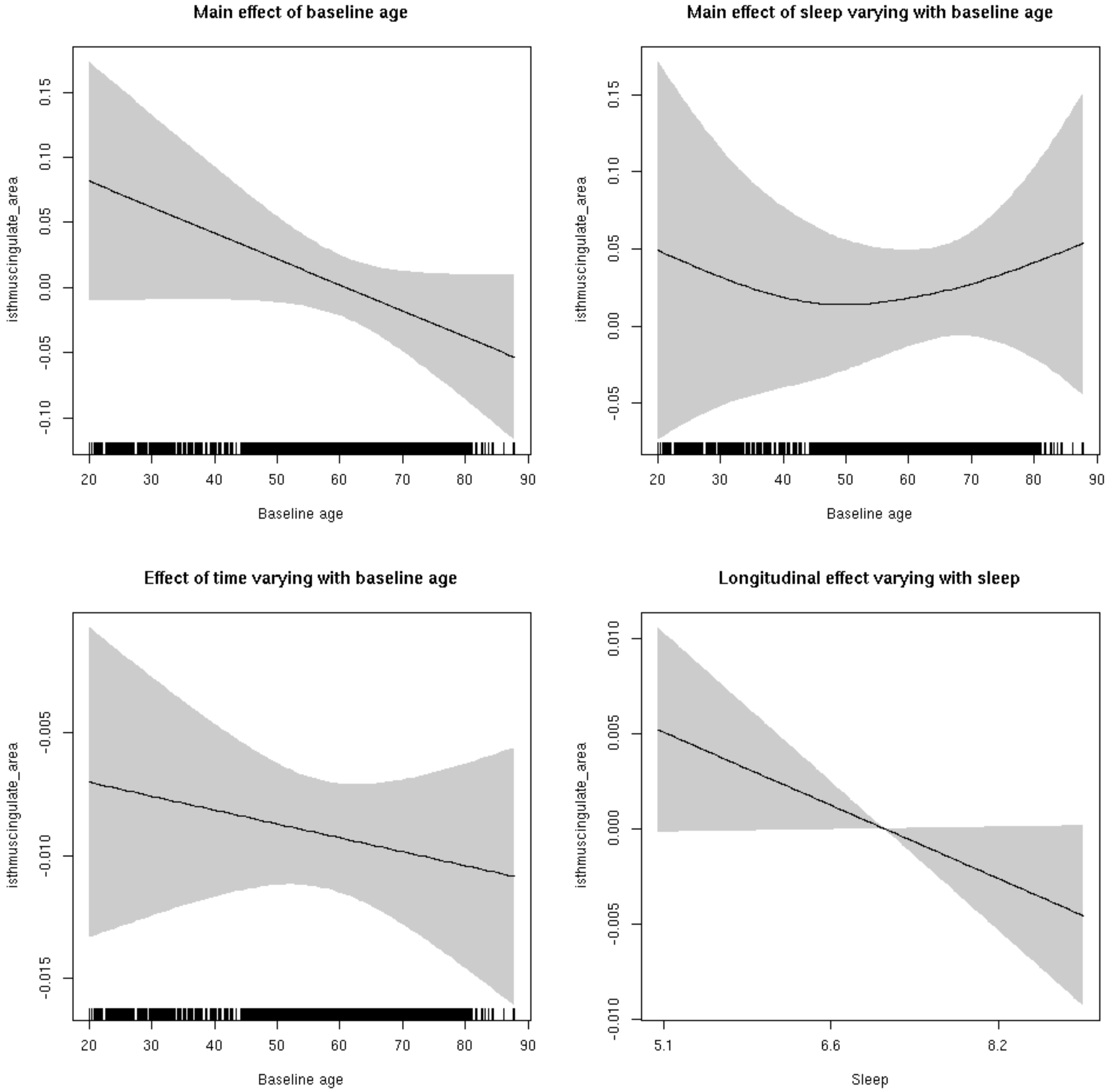

isthmuscingulate_thickness: Stratified by sex

Female: Full data Below is the row for `s(sleep_z):time` from the model output.

```
## s(sleep_z):time   1.000  1.000  0.001   0.969
```

Below are plots of a plot of `s(bl_age)`, `s(bl_age):sleep_z`, `s(bl_age):time`, and `s(sleep_z):time`. For `s(sleep_z):time`, roughly, if we cannot fit a straight line through the confidence bands, there is evidence that the effect of time depends on sleep duration. It is correct the that confidence interval is zero at one point along the axis; this happens because we do not include the main effect.

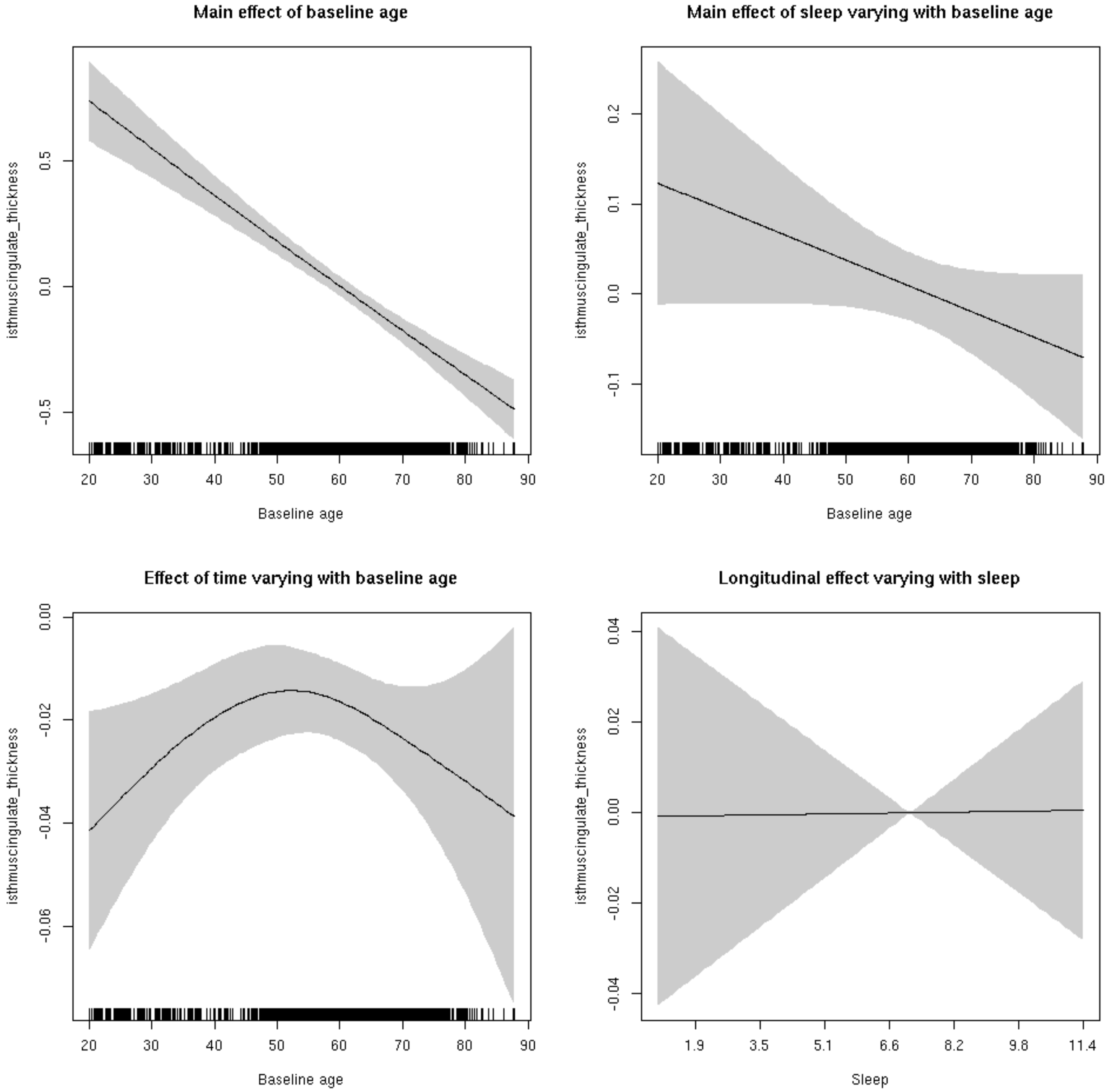

Male: Full data Below is the row for `s(sleep_z):time` from the model output.

```
## s(sleep_z):time      1       1   0.564   0.453
```

Below are plots of a plot of `s(bl_age)`, `s(bl_age):sleep_z`, `s(bl_age):time`, and `s(sleep_z):time`. For `s(sleep_z):time`, roughly, if we cannot fit a straight line through the confidence bands, there is evidence that the effect of time depends on sleep duration. It is correct the that confidence interval is zero at one point along the axis; this happens because we do not include the main effect.

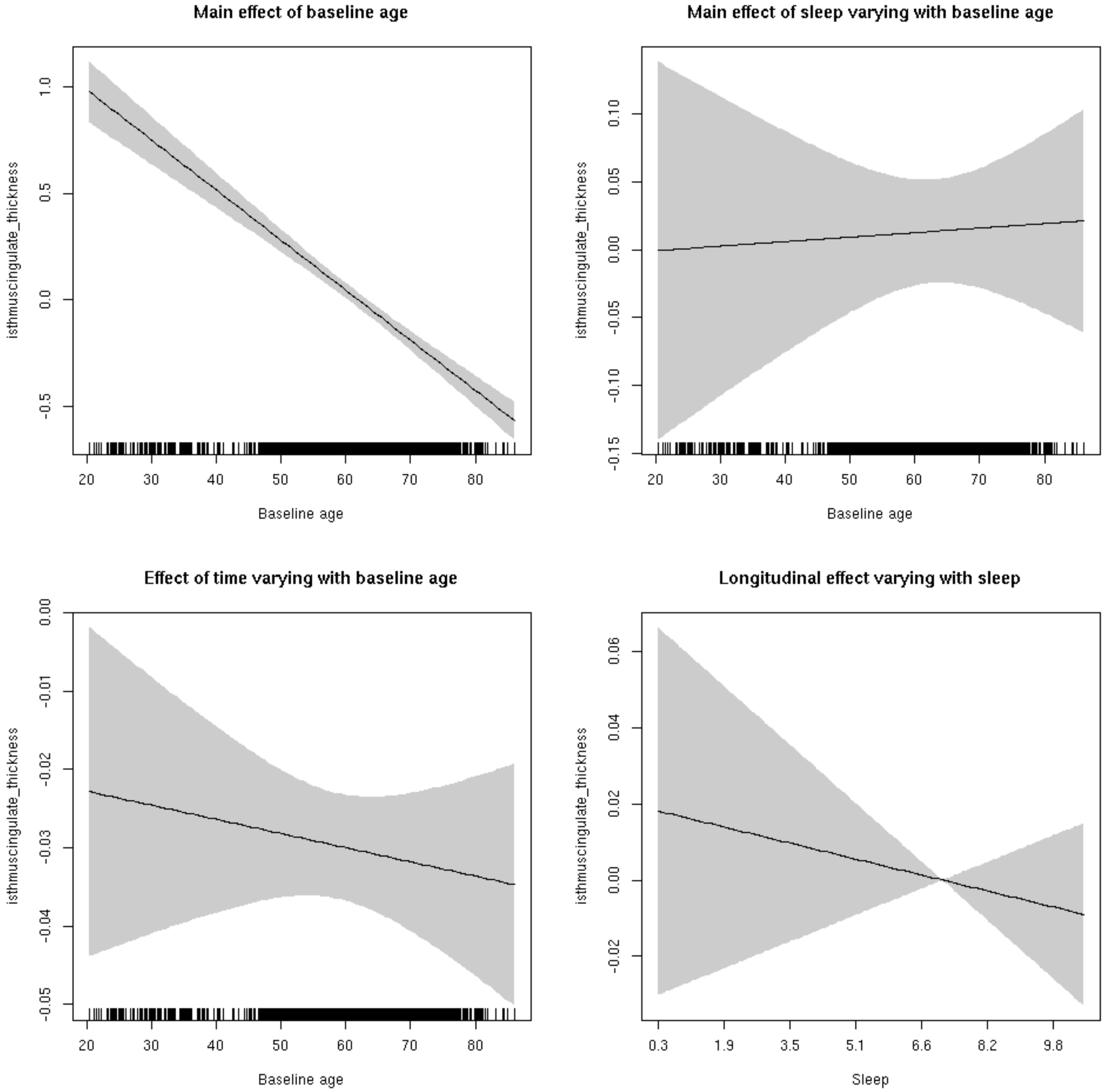

isthmuscingulate_thickness

Full data Below is the model output. The term `s(sleep_z):time` is probably what we care most about.

```
##
## Family: gaussian
## Link function: identity
##
## Formula:
## value ~ sex + site + s(bl_age, k = 10, bs = "cr") + s(bl_age,
##     by = sleep_z, bs = "cr") + +s(bl_age, by = time, k = 5, bs = "cr") +
##     s(sleep_z, by = time, k = 5, bs = "cr", pc = 0)
## <environment: 0x5630035d3810>
```

```
##
## Parametric coefficients:
##                Estimate Std. Error t value Pr(>|t|)
## (Intercept)   -1.64518    0.05137 -32.027  < 2e-16 ***
## sexmale       -0.13198    0.02573  -5.129 2.99e-07 ***
## siteousAvanto  0.23924    0.06749   3.545 0.000395 ***
## siteousPrisma  0.28800    0.23166   1.243 0.213829
## siteousSkyra   0.39181    0.06747   5.807 6.60e-09 ***
## siteUB         0.57565    0.13709   4.199 2.71e-05 ***
## siteUCAM       0.29750    0.07096   4.192 2.79e-05 ***
## siteUKB        1.96540    0.05219  37.658  < 2e-16 ***
## siteUmU        0.58615    0.08463   6.926 4.67e-12 ***
## ---
## Signif. codes:  0 ‘***’ 0.001 ‘**’ 0.01 ‘*’ 0.05 ‘.’ 0.1 ‘ ’ 1
##
## Approximate significance of smooth terms:
##                      edf Ref.df      F p-value
## s(bl_age)          4.482  4.482 66.315  <2e-16 ***
## s(bl_age):sleep_z 2.000  2.000  0.580   0.560
## s(bl_age):time    3.220  3.220 37.820  <2e-16 ***
## s(sleep_z):time   1.000  1.000  0.175   0.676
## ---
## Signif. codes:  0 ‘***’ 0.001 ‘**’ 0.01 ‘*’ 0.05 ‘.’ 0.1 ‘ ’ 1
##
## R-sq.(adj) =  0.473
## lmer.REML =  13784  Scale est. = 0.086341  n = 8176
```

Below are plots of a plot of `s(bl_age)`, `s(bl_age):sleep_z`, `s(bl_age):time`, and `s(sleep_z):time`. For `s(sleep_z):time`, roughly, if we cannot fit a straight line through the confidence bands, there is evidence that the effect of time depends on sleep duration. It is correct the that confidence interval is zero at one point along the axis; this happens because we do not include the main effect.

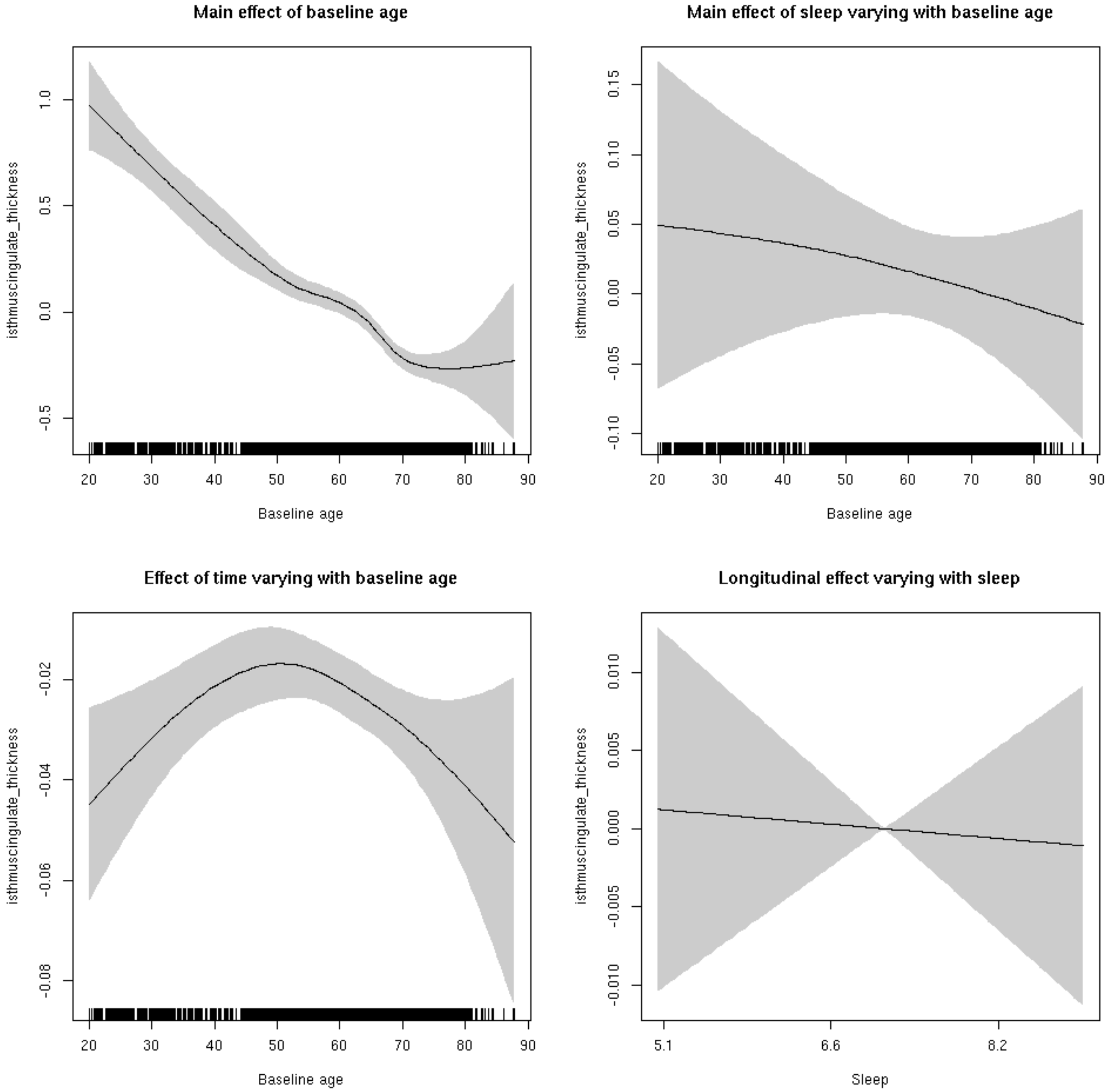

Restricted to sleep >= 5 and <= 9 hours Below is the row for `s(sleep_z):time` from the model output.

```
## s(sleep_z):time   1.000  1.000  0.045   0.831
```

Below are plots of a plot of `s(bl_age)`, `s(bl_age):sleep_z`, `s(bl_age):time`, and `s(sleep_z):time`. For `s(sleep_z):time`, roughly, if we cannot fit a straight line through the confidence bands, there is evidence that the effect of time depends on sleep duration. It is correct the that confidence interval is zero at one point along the axis; this happens because we do not include the main effect.

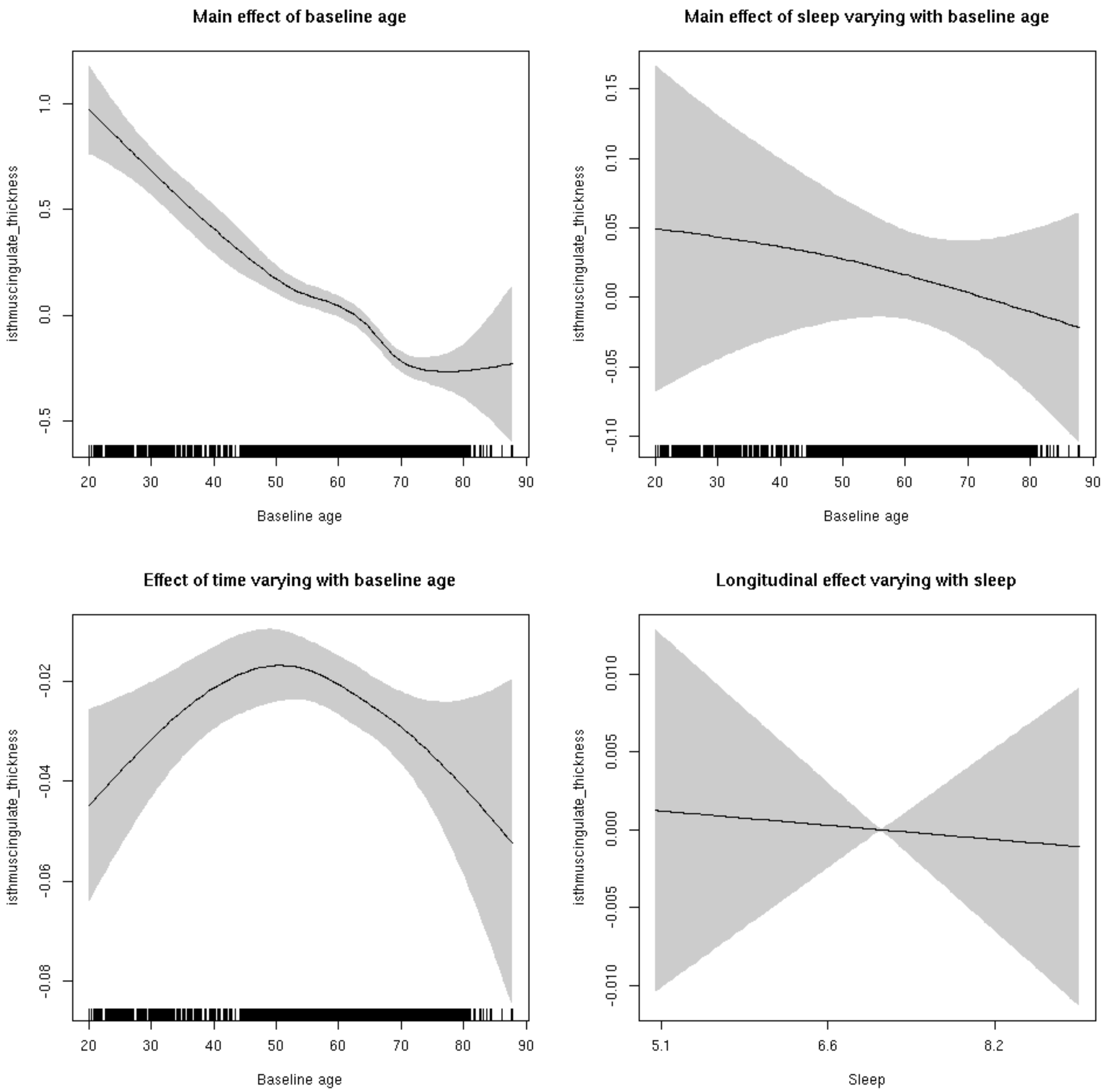

isthmuscingulate_volume: Stratified by sex

Female: Full data Below is the row for `s(sleep_z):time` from the model output.

```
## s(sleep_z):time   1.000  1.000  0.179   0.672
```

Below are plots of a plot of `s(bl_age)`, `s(bl_age):sleep_z`, `s(bl_age):time`, and `s(sleep_z):time`. For `s(sleep_z):time`, roughly, if we cannot fit a straight line through the confidence bands, there is evidence that the effect of time depends on sleep duration. It is correct the that confidence interval is zero at one point along the axis; this happens because we do not include the main effect.

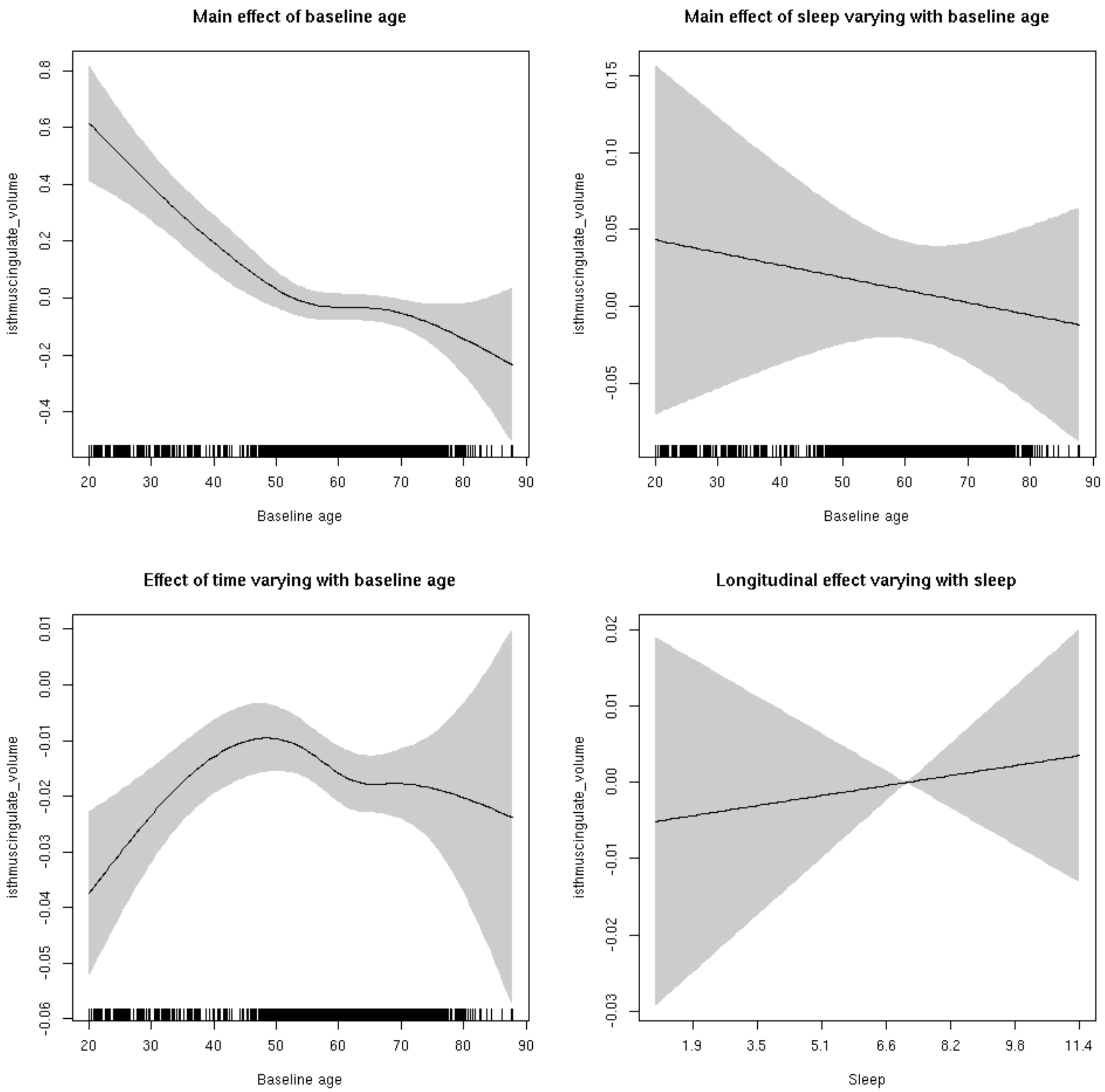

Male: Full data Below is the row for `s(sleep_z):time` from the model output.

```
## s(sleep_z):time   1.000  1.000   0.430   0.512
```

Below are plots of a plot of `s(bl_age)`, `s(bl_age):sleep_z`, `s(bl_age):time`, and `s(sleep_z):time`. For `s(sleep_z):time`, roughly, if we cannot fit a straight line through the confidence bands, there is evidence that the effect of time depends on sleep duration. It is correct the that confidence interval is zero at one point along the axis; this happens because we do not include the main effect.

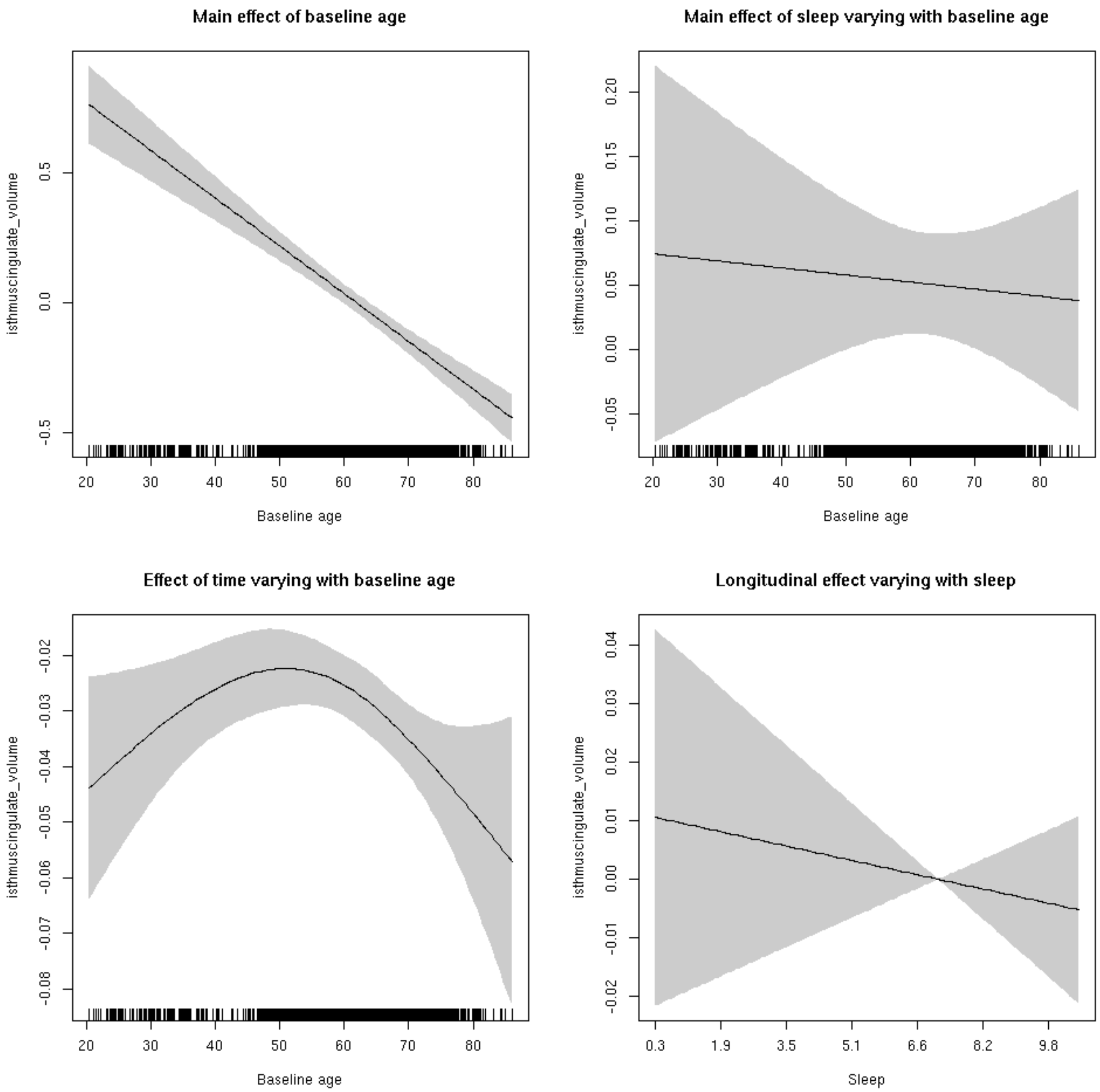

isthmuscingulate_volume

Full data Below is the model output. The term `s(sleep_z):time` is probably what we care most about.

```
##
## Family: gaussian
## Link function: identity
##
## Formula:
## value ~ sex + site + icv + s(bl_age, k = 10, bs = "cr") + s(bl_age,
##     by = sleep_z, bs = "cr") + +s(bl_age, by = time, k = 5, bs = "cr") +
##     s(sleep_z, by = time, k = 5, bs = "cr", pc = 0)
## <environment: 0x5630035ab650>
```

```
##
## Parametric coefficients:
##                Estimate Std. Error t value Pr(>|t|)
## (Intercept)   -0.20083    0.05708  -3.519 0.000436 ***
## sexmale        0.26042    0.02932   8.881  < 2e-16 ***
## siteousAvanto -0.61215    0.06964  -8.790  < 2e-16 ***
## siteousPrisma -0.45994    0.15615  -2.945 0.003234 **
## siteousSkyra   0.06533    0.06743   0.969 0.332639
## siteUB        -0.38888    0.13153  -2.956 0.003121 **
## siteUCAM      -0.41922    0.07078  -5.923 3.29e-09 ***
## siteUKB        0.18834    0.05419   3.475 0.000513 ***
## siteUmU       -0.07121    0.08148  -0.874 0.382216
## icv            0.49279    0.01441  34.188  < 2e-16 ***
## ---
## Signif. codes:  0 '***' 0.001 '**' 0.01 '*' 0.05 '.' 0.1 ' ' 1
##
## Approximate significance of smooth terms:
##                      edf Ref.df      F p-value
## s(bl_age)          2.999  2.999 49.309  <2e-16 ***
## s(bl_age):sleep_z 2.000  2.000  2.258   0.105
## s(bl_age):time    3.611  3.611 48.454  <2e-16 ***
## s(sleep_z):time   2.454  2.454  1.198   0.404
## ---
## Signif. codes:  0 '***' 0.001 '**' 0.01 '*' 0.05 '.' 0.1 ' ' 1
##
## R-sq.(adj) =   0.43
## lmer.REML = 9204.3  Scale est. = 0.032697  n = 8171
```

Below are plots of a plot of `s(bl_age)`, `s(bl_age):sleep_z`, `s(bl_age):time`, and `s(sleep_z):time`. For `s(sleep_z):time`, roughly, if we cannot fit a straight line through the confidence bands, there is evidence that the effect of time depends on sleep duration. It is correct the that confidence interval is zero at one point along the axis; this happens because we do not include the main effect.

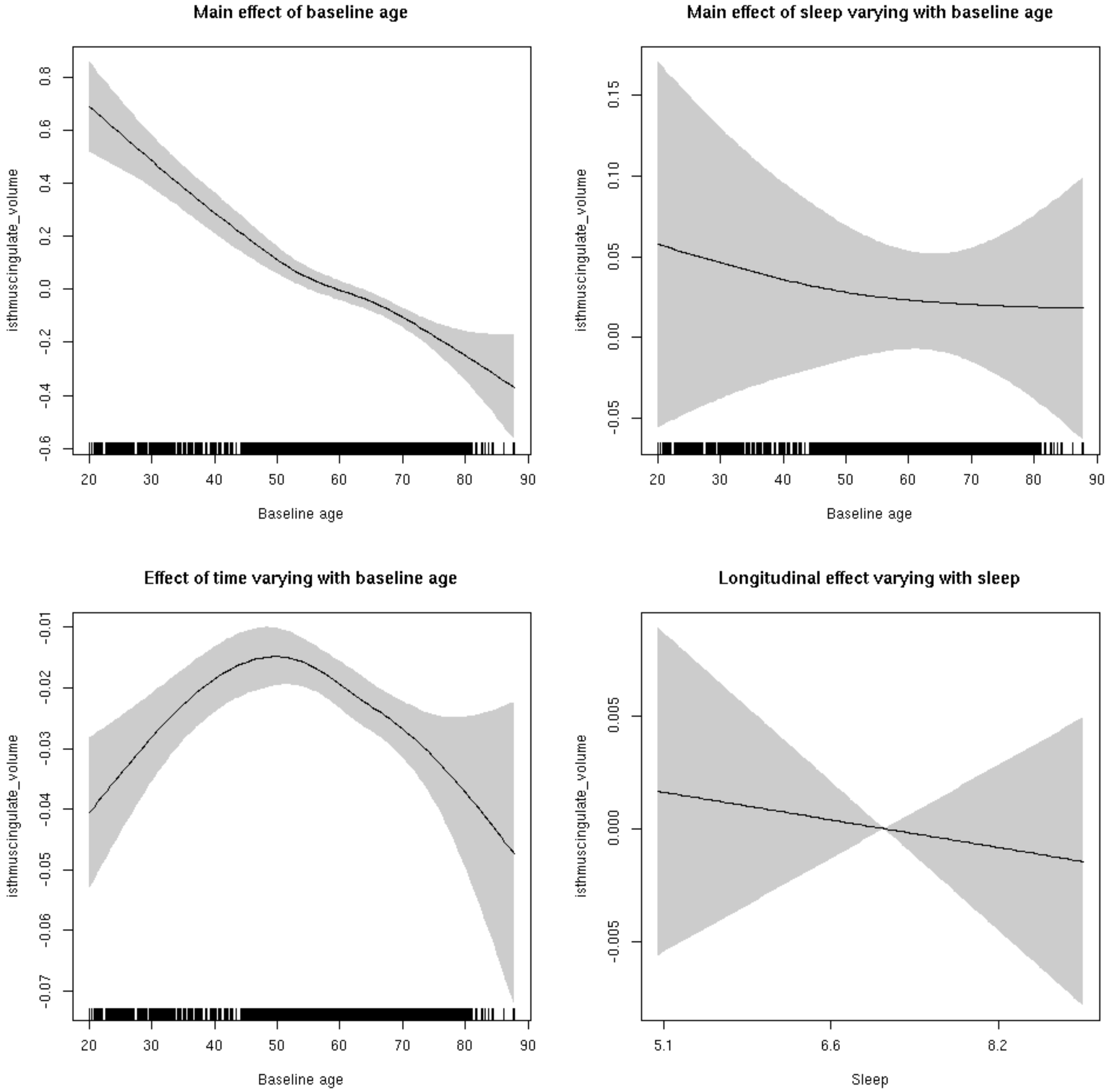

Restricted to sleep >= 5 and <= 9 hours Below is the row for `s(sleep_z):time` from the model output.

```
## s(sleep_z):time   1.000  1.000  0.208   0.648
```

Below are plots of a plot of `s(bl_age)`, `s(bl_age):sleep_z`, `s(bl_age):time`, and `s(sleep_z):time`. For `s(sleep_z):time`, roughly, if we cannot fit a straight line through the confidence bands, there is evidence that the effect of time depends on sleep duration. It is correct the that confidence interval is zero at one point along the axis; this happens because we do not include the main effect.

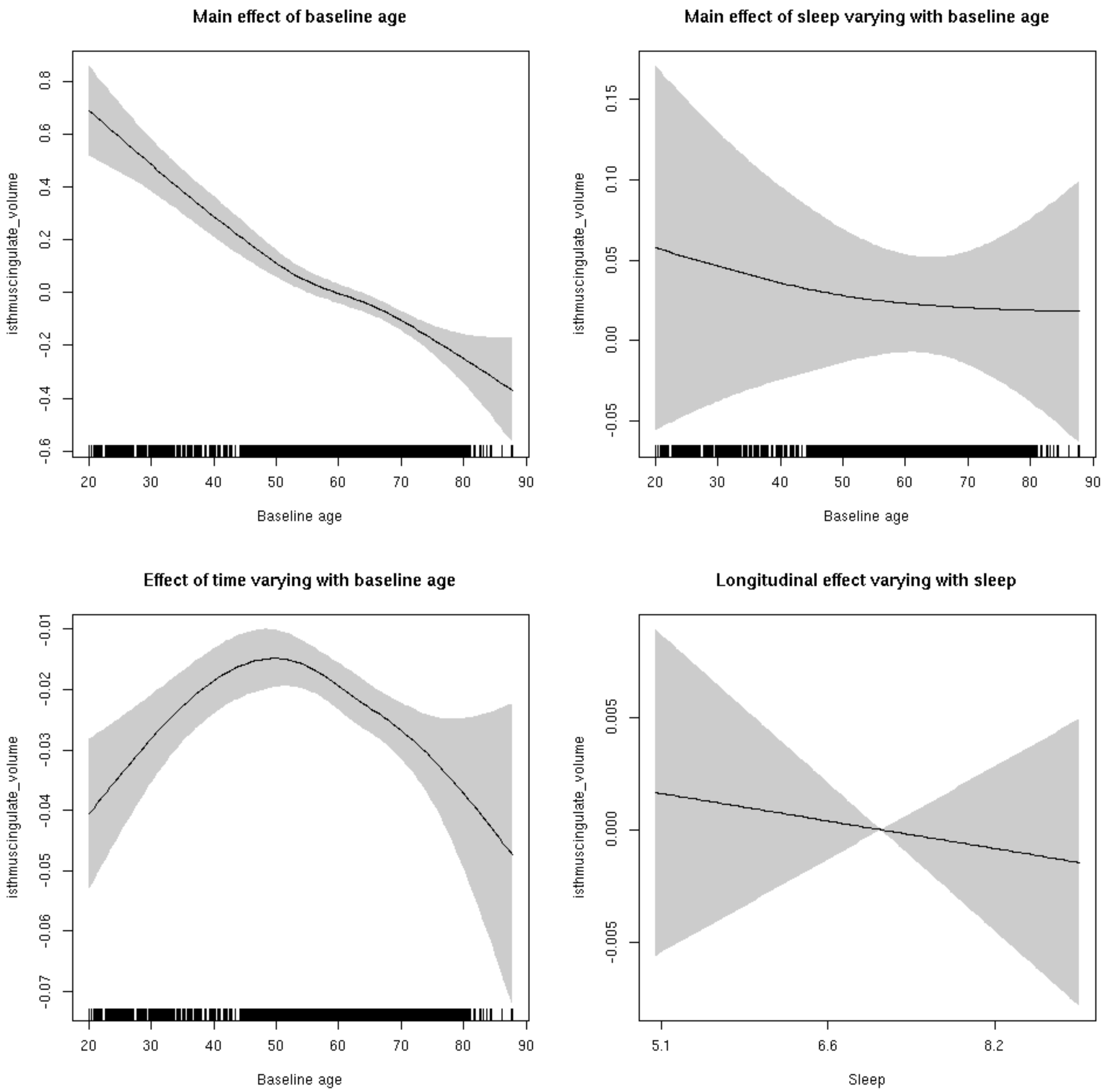

lateraloccipital_area: Stratified by sex

Female: Full data Below is the row for `s(sleep_z):time` from the model output.

```
## s(sleep_z):time   1.000  1.000   1.501   0.221
```

Below are plots of a plot of `s(bl_age)`, `s(bl_age):sleep_z`, `s(bl_age):time`, and `s(sleep_z):time`. For `s(sleep_z):time`, roughly, if we cannot fit a straight line through the confidence bands, there is evidence that the effect of time depends on sleep duration. It is correct the that confidence interval is zero at one point along the axis; this happens because we do not include the main effect.

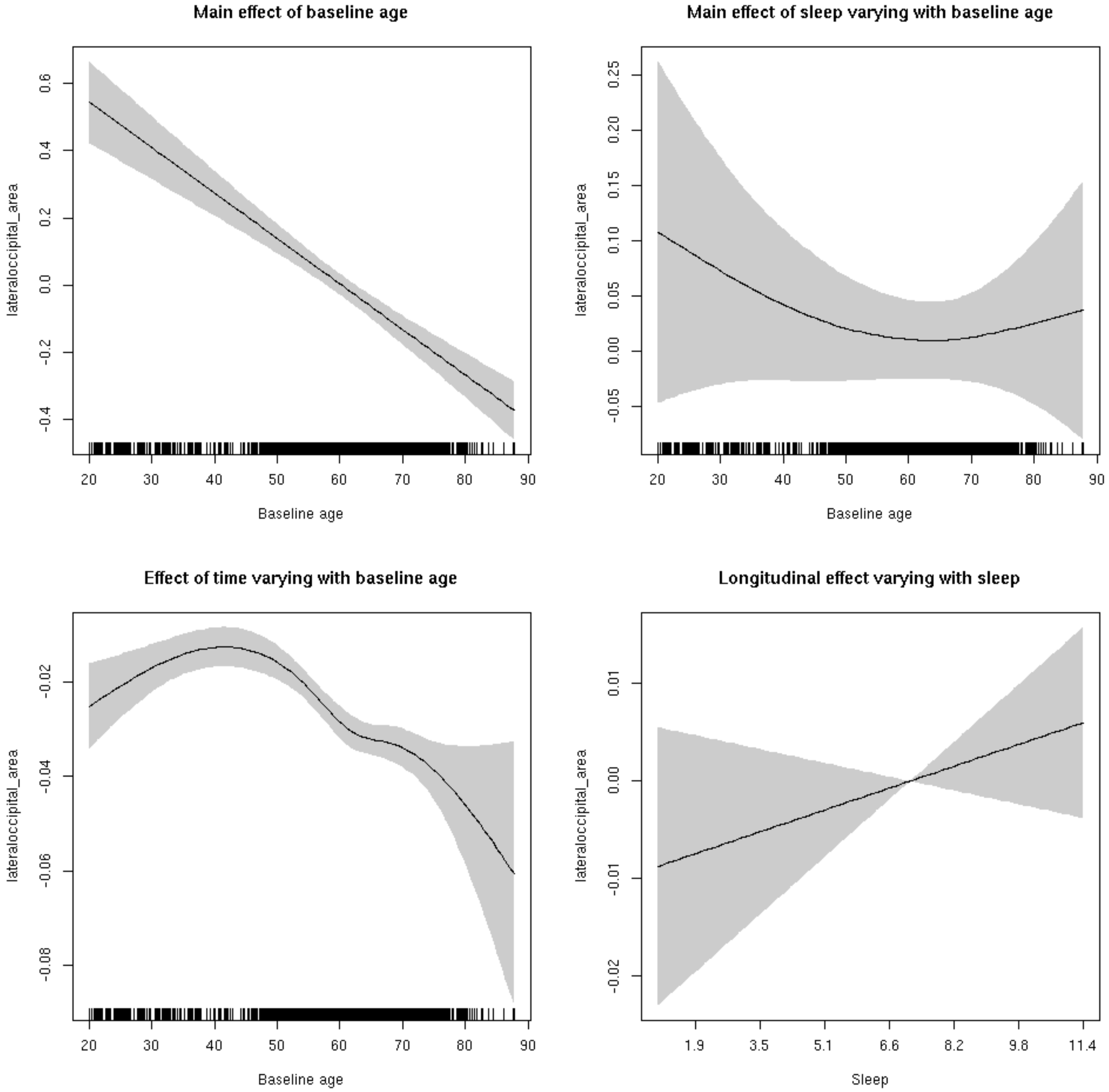

Male: Full data Below is the row for `s(sleep_z):time` from the model output.

```
## s(sleep_z):time     1       1   0.855   0.355
```

Below are plots of a plot of `s(bl_age)`, `s(bl_age):sleep_z`, `s(bl_age):time`, and `s(sleep_z):time`. For `s(sleep_z):time`, roughly, if we cannot fit a straight line through the confidence bands, there is evidence that the effect of time depends on sleep duration. It is correct the that confidence interval is zero at one point along the axis; this happens because we do not include the main effect.

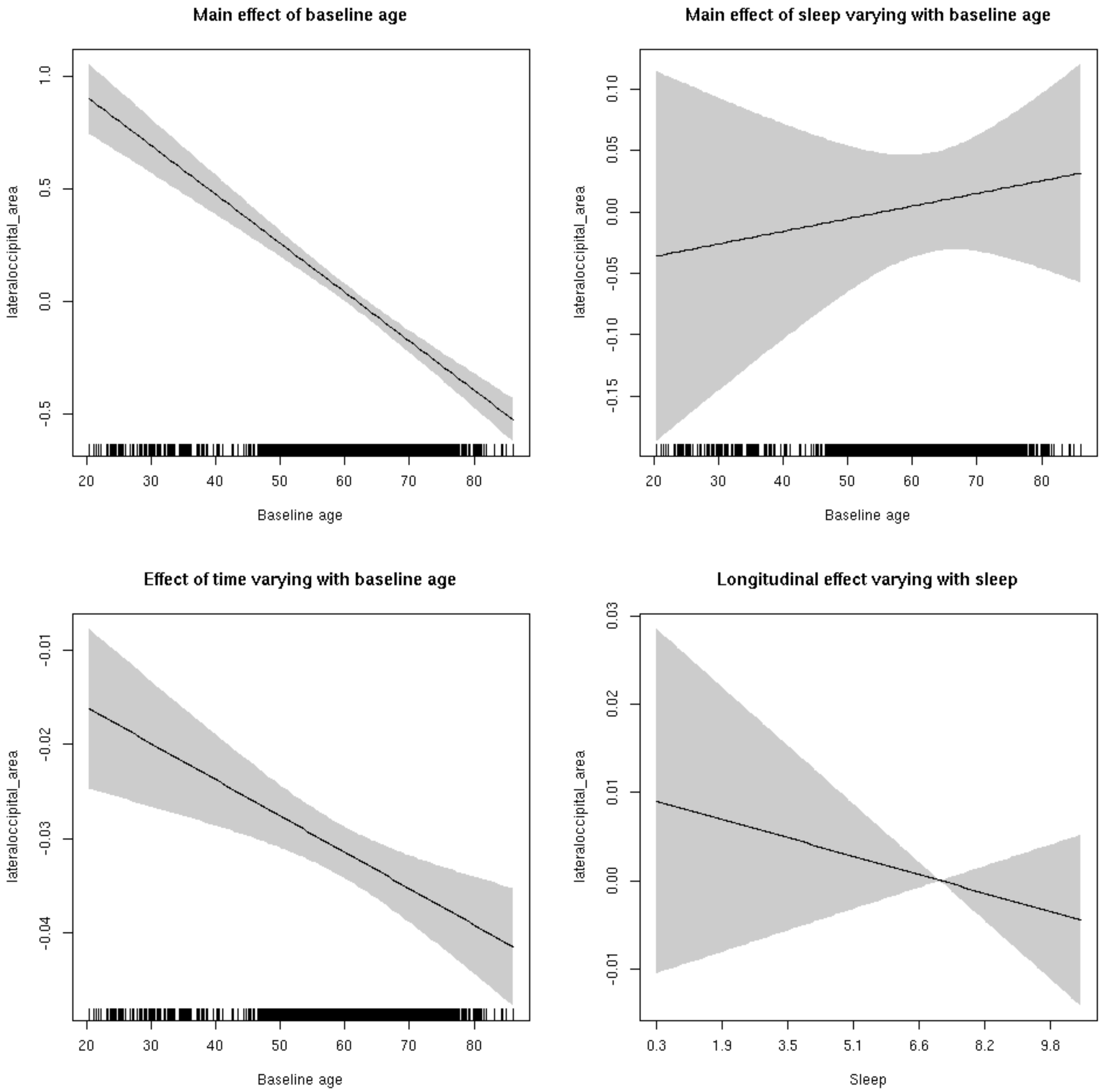

lateraloccipital_area

Full data Below is the model output. The term `s(sleep_z):time` is probably what we care most about.

```
##
## Family: gaussian
## Link function: identity
##
## Formula:
## value ~ sex + site + icv + s(bl_age, k = 10, bs = "cr") + s(bl_age,
##     by = sleep_z, bs = "cr") + +s(bl_age, by = time, k = 5, bs = "cr") +
##     s(sleep_z, by = time, k = 5, bs = "cr", pc = 0)
## <environment: 0x5630035b3260>
```

```
##
## Parametric coefficients:
##                Estimate Std. Error t value Pr(>|t|)
## (Intercept)     0.18924    0.05694   3.324 0.000892 ***
## sexmale         0.49916    0.02959  16.869  < 2e-16 ***
## siteousAvanto -0.43565    0.07007  -6.217 5.32e-10 ***
## siteousPrisma -0.71909    0.11077  -6.492 8.99e-11 ***
## siteousSkyra  -0.04920    0.06794  -0.724 0.468941
## siteUB        -0.76612    0.13404  -5.716 1.13e-08 ***
## siteUCAM      -0.42116    0.07142  -5.897 3.85e-09 ***
## siteUKB       -0.38282    0.05322  -7.193 6.92e-13 ***
## siteUmU        0.05486    0.08245   0.665 0.505814
## icv            0.41477    0.01421  29.194  < 2e-16 ***
## ---
## Signif. codes:  0 '***' 0.001 '**' 0.01 '*' 0.05 '.' 0.1 ' ' 1
##
## Approximate significance of smooth terms:
##                      edf Ref.df      F p-value
## s(bl_age)          1.000  1.000 232.40  <2e-16 ***
## s(bl_age):sleep_z 2.000  2.000   0.29   0.748
## s(bl_age):time    3.723  3.723 307.46  <2e-16 ***
## s(sleep_z):time   1.000  1.000   0.10   0.751
## ---
## Signif. codes:  0 '***' 0.001 '**' 0.01 '*' 0.05 '.' 0.1 ' ' 1
##
## R-sq.(adj) =  0.427
## lmer.REML = 4973.5  Scale est. = 0.011751  n = 8162
```

Below are plots of a plot of `s(bl_age)`, `s(bl_age):sleep_z`, `s(bl_age):time`, and `s(sleep_z):time`. For `s(sleep_z):time`, roughly, if we cannot fit a straight line through the confidence bands, there is evidence that the effect of time depends on sleep duration. It is correct the that confidence interval is zero at one point along the axis; this happens because we do not include the main effect.

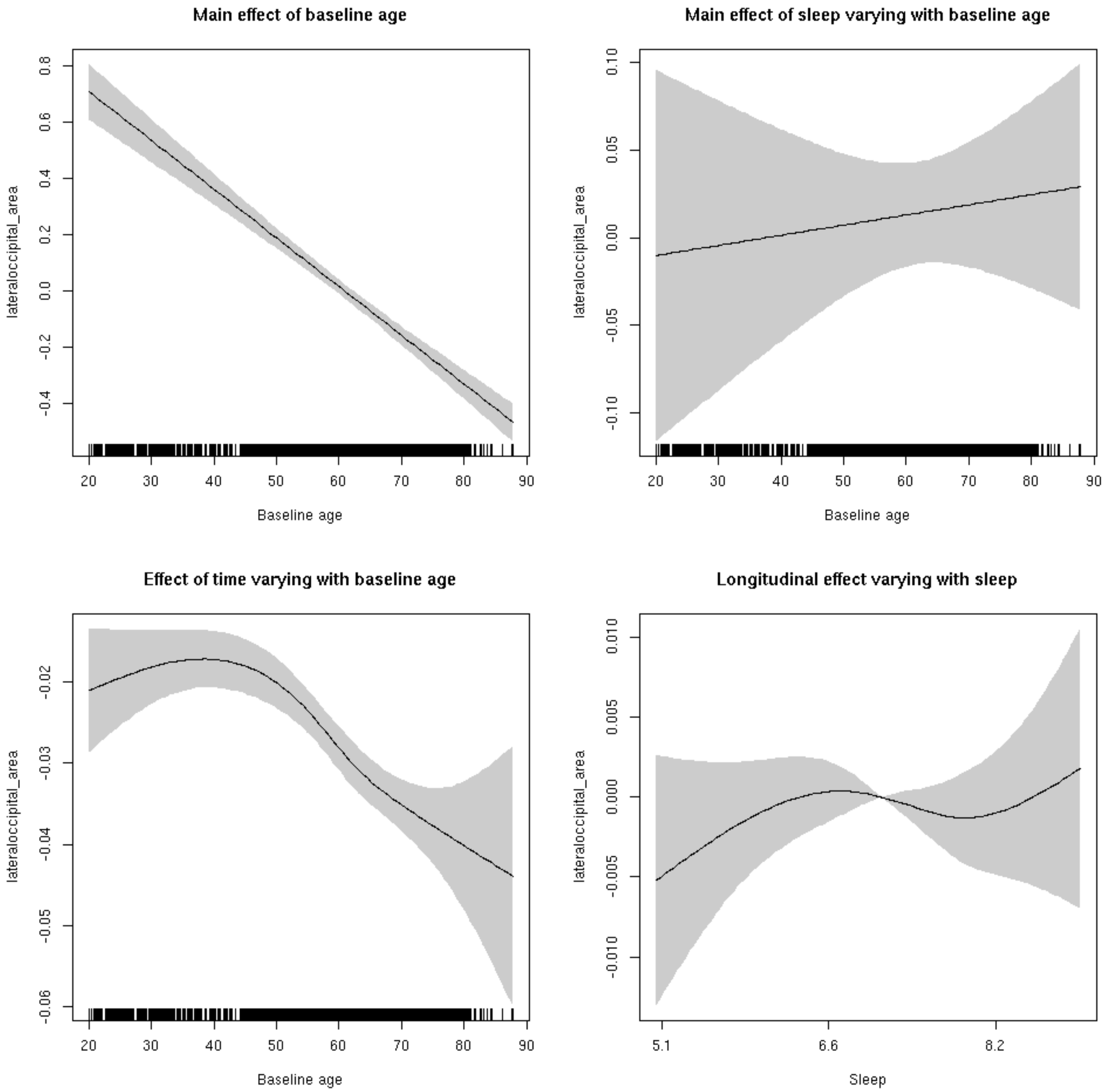

Restricted to sleep >= 5 and <= 9 hours Below is the row for `s(sleep_z):time` from the model output.

```
## s(sleep_z):time   2.343  2.343   0.545   0.537
```

Below are plots of a plot of `s(bl_age)`, `s(bl_age):sleep_z`, `s(bl_age):time`, and `s(sleep_z):time`. For `s(sleep_z):time`, roughly, if we cannot fit a straight line through the confidence bands, there is evidence that the effect of time depends on sleep duration. It is correct the that confidence interval is zero at one point along the axis; this happens because we do not include the main effect.

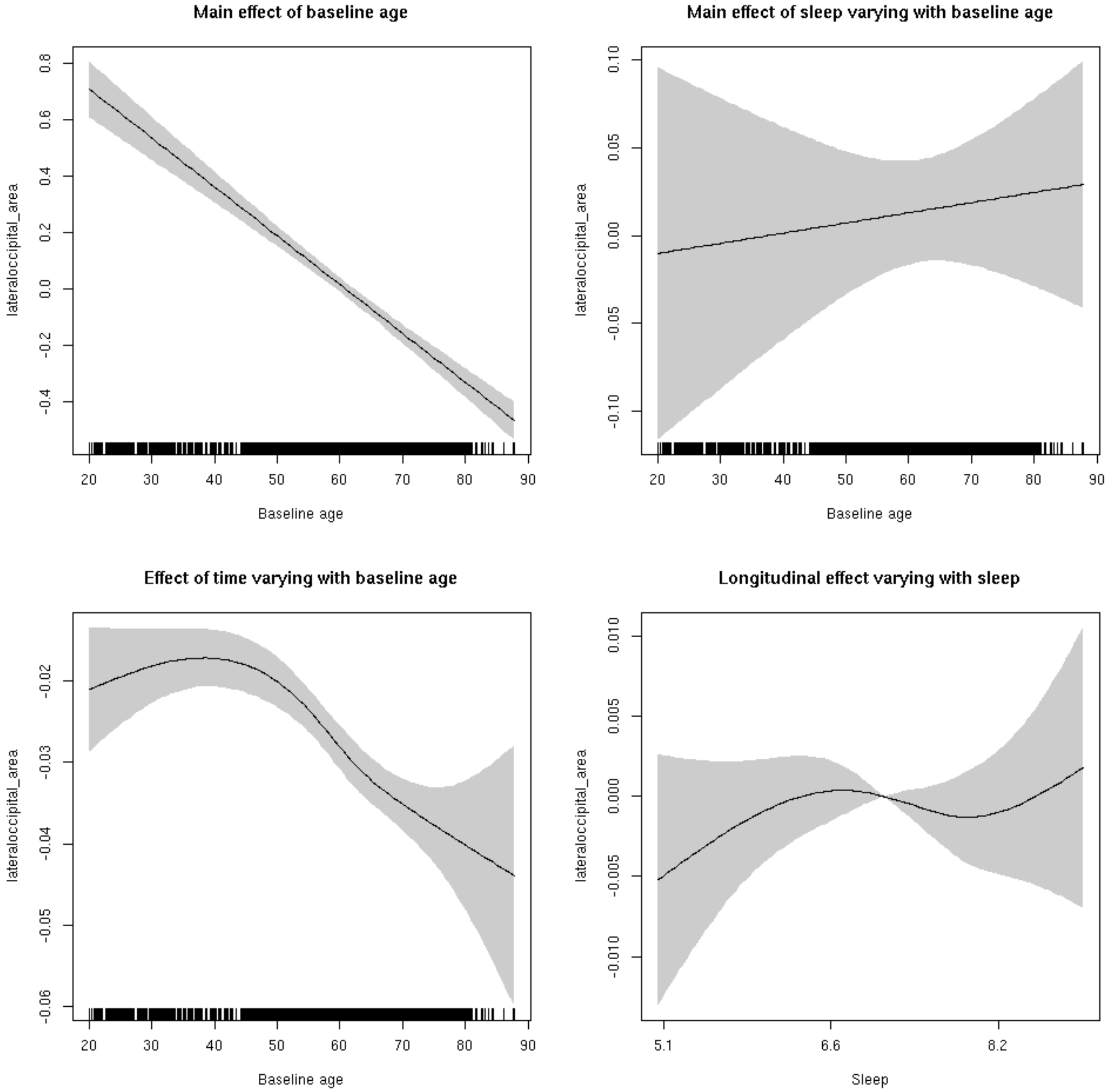

lateraloccipital_thickness: Stratified by sex

Female: Full data Below is the row for `s(sleep_z):time` from the model output.

```
## s(sleep_z):time   1.000  1.000  0.616   0.432
```

Below are plots of a plot of `s(bl_age)`, `s(bl_age):sleep_z`, `s(bl_age):time`, and `s(sleep_z):time`. For `s(sleep_z):time`, roughly, if we cannot fit a straight line through the confidence bands, there is evidence that the effect of time depends on sleep duration. It is correct the that confidence interval is zero at one point along the axis; this happens because we do not include the main effect.

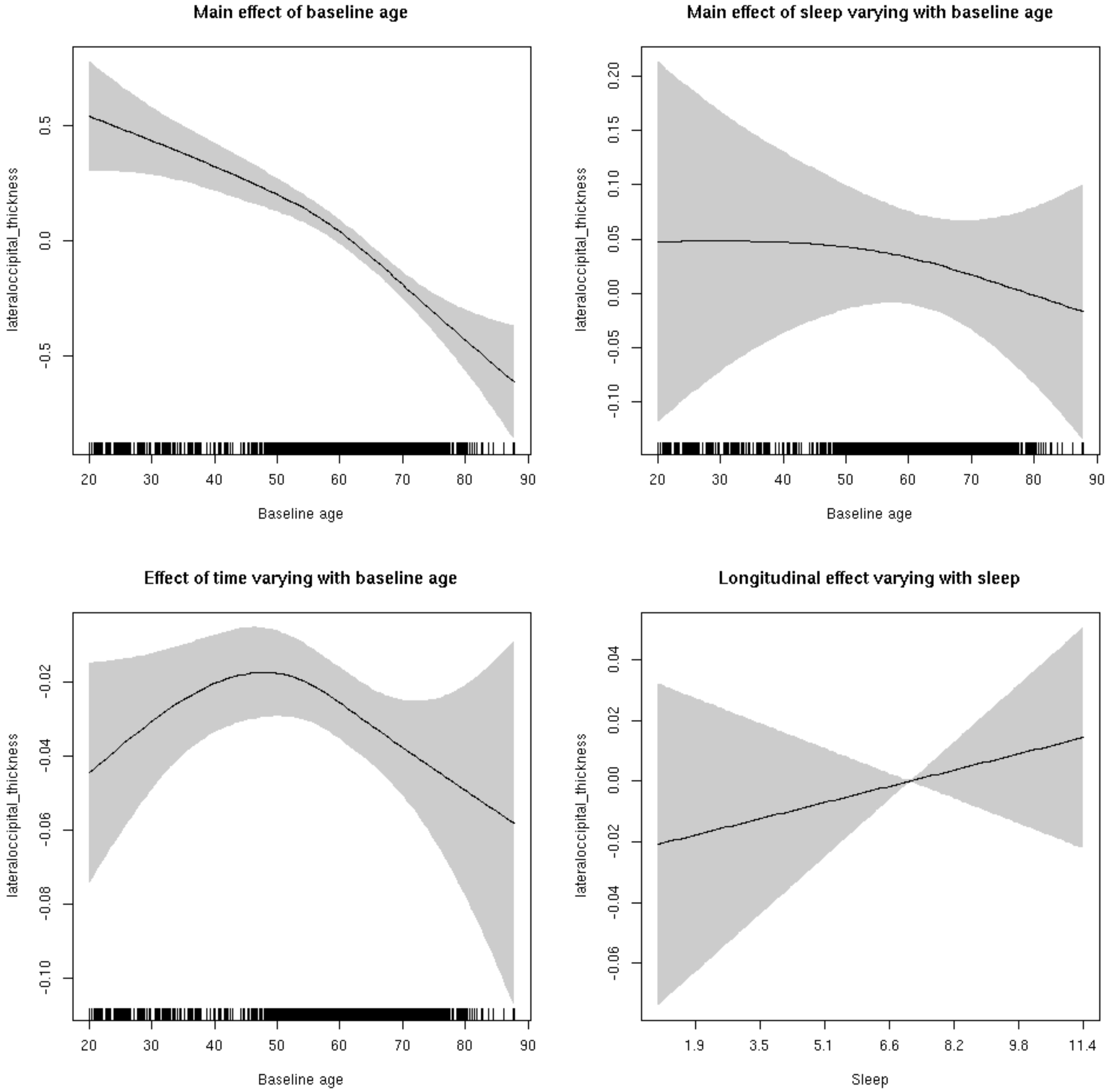

Male: Full data Below is the row for `s(sleep_z):time` from the model output.

```
## s(sleep_z):time   1.000  1.000  0.619   0.431
```

Below are plots of a plot of `s(bl_age)`, `s(bl_age):sleep_z`, `s(bl_age):time`, and `s(sleep_z):time`. For `s(sleep_z):time`, roughly, if we cannot fit a straight line through the confidence bands, there is evidence that the effect of time depends on sleep duration. It is correct the that confidence interval is zero at one point along the axis; this happens because we do not include the main effect.

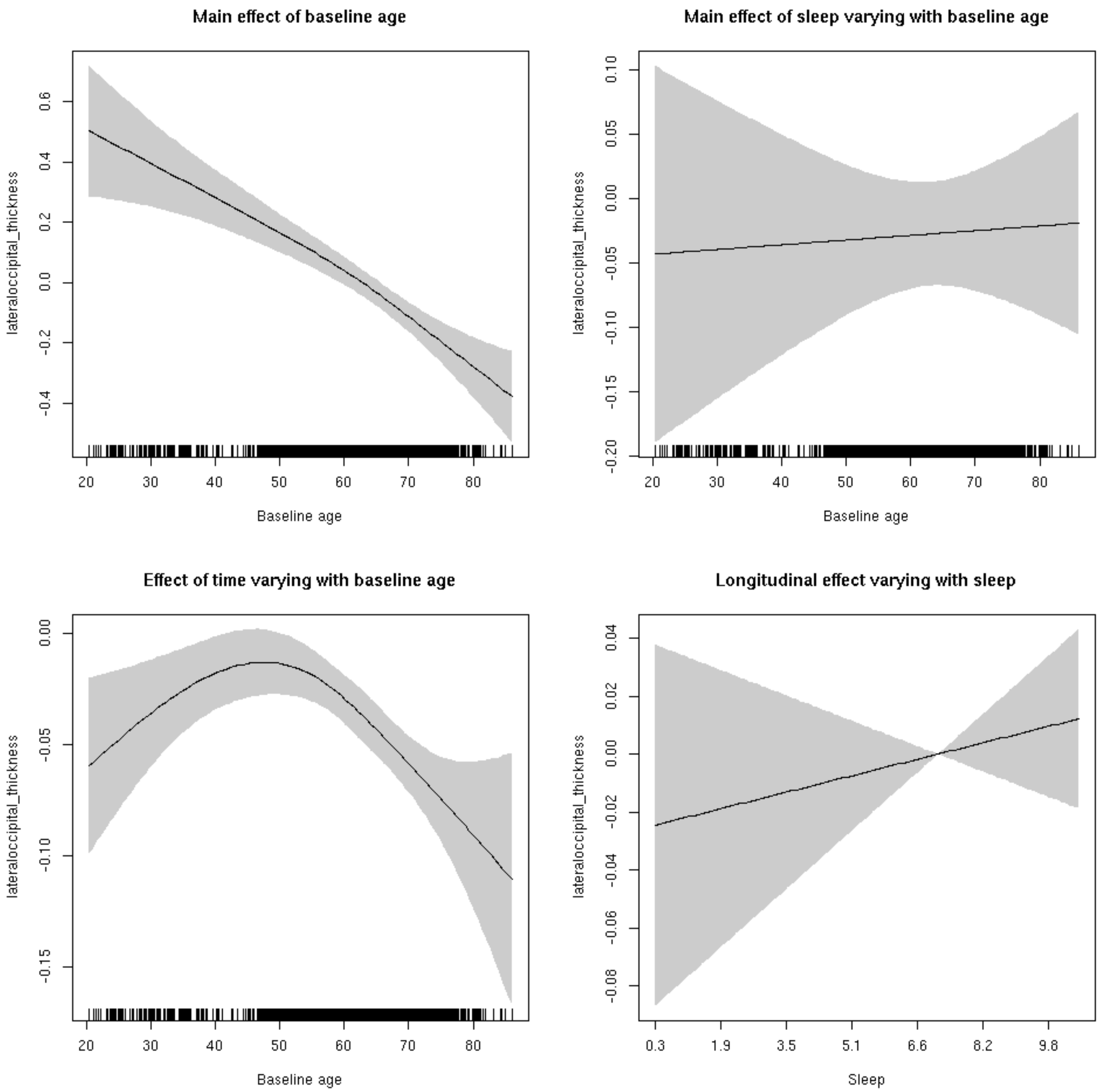

lateraloccipital_thickness

Full data Below is the model output. The term `s(sleep_z):time` is probably what we care most about.

```
##
## Family: gaussian
## Link function: identity
##
## Formula:
## value ~ sex + site + s(bl_age, k = 10, bs = "cr") + s(bl_age,
##     by = sleep_z, bs = "cr") + +s(bl_age, by = time, k = 5, bs = "cr") +
##     s(sleep_z, by = time, k = 5, bs = "cr", pc = 0)
## <environment: 0x5630035d58f0>
```

```
##
## Parametric coefficients:
##                Estimate Std. Error t value Pr(>|t|)
## (Intercept)   -1.63638    0.05417 -30.210  < 2e-16 ***
## sexmale        0.14835    0.02727   5.441 5.45e-08 ***
## siteousAvanto  0.23329    0.07191   3.244  0.00118 **
## siteousPrisma  1.14877    0.28975   3.965 7.41e-05 ***
## siteousSkyra   1.05764    0.07186  14.719  < 2e-16 ***
## siteUB         0.98103    0.14450   6.789 1.21e-11 ***
## siteUCAM       0.91554    0.07451  12.287  < 2e-16 ***
## siteUKB        1.77800    0.05476  32.471  < 2e-16 ***
## siteUmU        0.26648    0.08951   2.977  0.00292 **
## ---
## Signif. codes:  0 '***' 0.001 '**' 0.01 '*' 0.05 '.' 0.1 ' ' 1
##
## Approximate significance of smooth terms:
##                      edf Ref.df      F p-value
## s(bl_age)          2.747  2.747 52.092  <2e-16 ***
## s(bl_age):sleep_z 2.000  2.000  0.159   0.853
## s(bl_age):time    3.610  3.610 44.362  <2e-16 ***
## s(sleep_z):time   1.000  1.000  1.454   0.228
## ---
## Signif. codes:  0 '***' 0.001 '**' 0.01 '*' 0.05 '.' 0.1 ' ' 1
##
## R-sq.(adj) =   0.35
## lmer.REML =  16327  Scale est. = 0.14161   n = 8168
```

Below are plots of a plot of `s(bl_age)`, `s(bl_age):sleep_z`, `s(bl_age):time`, and `s(sleep_z):time`. For `s(sleep_z):time`, roughly, if we cannot fit a straight line through the confidence bands, there is evidence that the effect of time depends on sleep duration. It is correct the that confidence interval is zero at one point along the axis; this happens because we do not include the main effect.

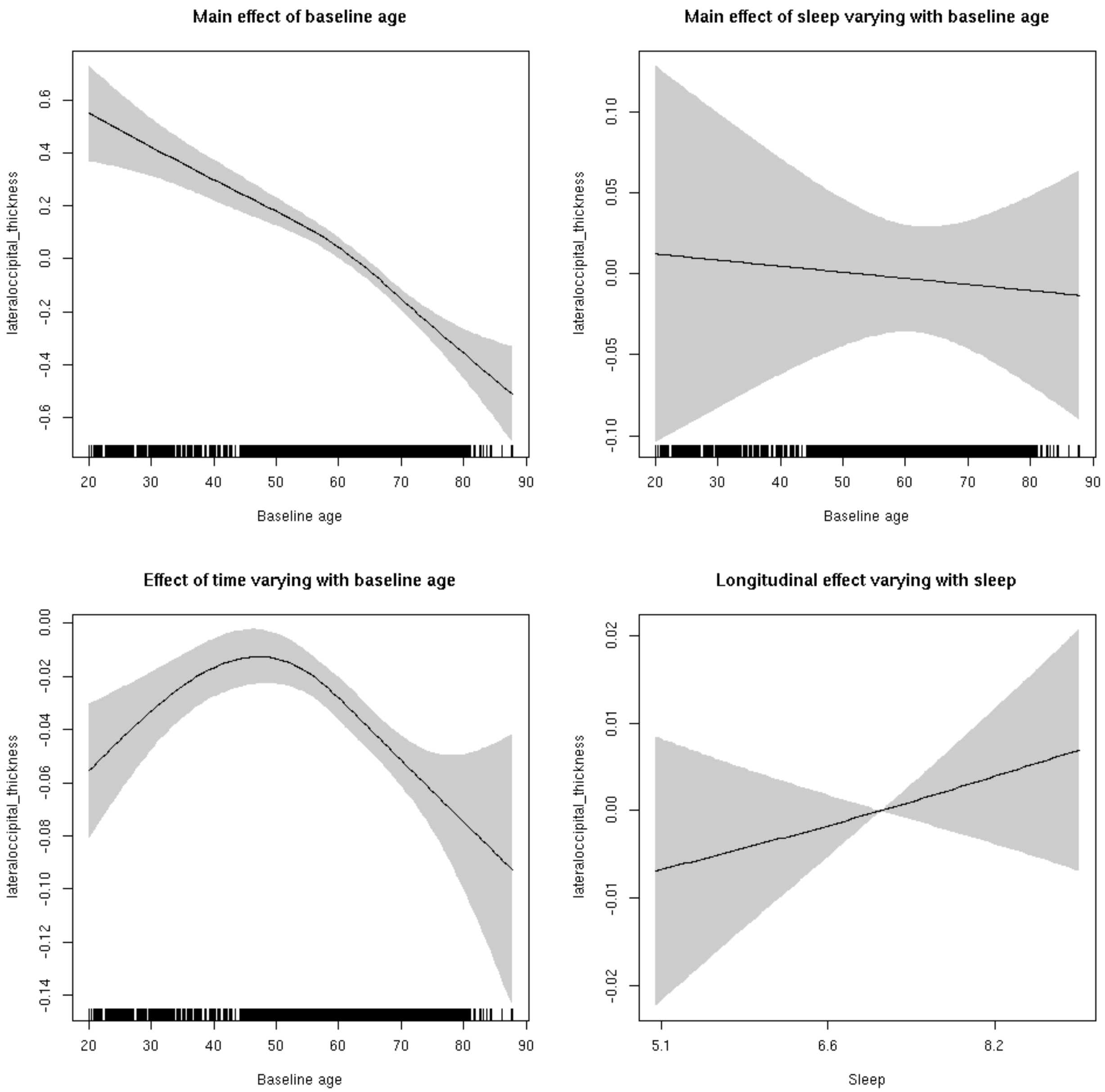

Restricted to sleep >= 5 and <= 9 hours Below is the row for `s(sleep_z):time` from the model output.

```
## s(sleep_z):time   1.039  1.039  0.879   0.333
```

Below are plots of a plot of `s(bl_age)`, `s(bl_age):sleep_z`, `s(bl_age):time`, and `s(sleep_z):time`. For `s(sleep_z):time`, roughly, if we cannot fit a straight line through the confidence bands, there is evidence that the effect of time depends on sleep duration. It is correct the that confidence interval is zero at one point along the axis; this happens because we do not include the main effect.

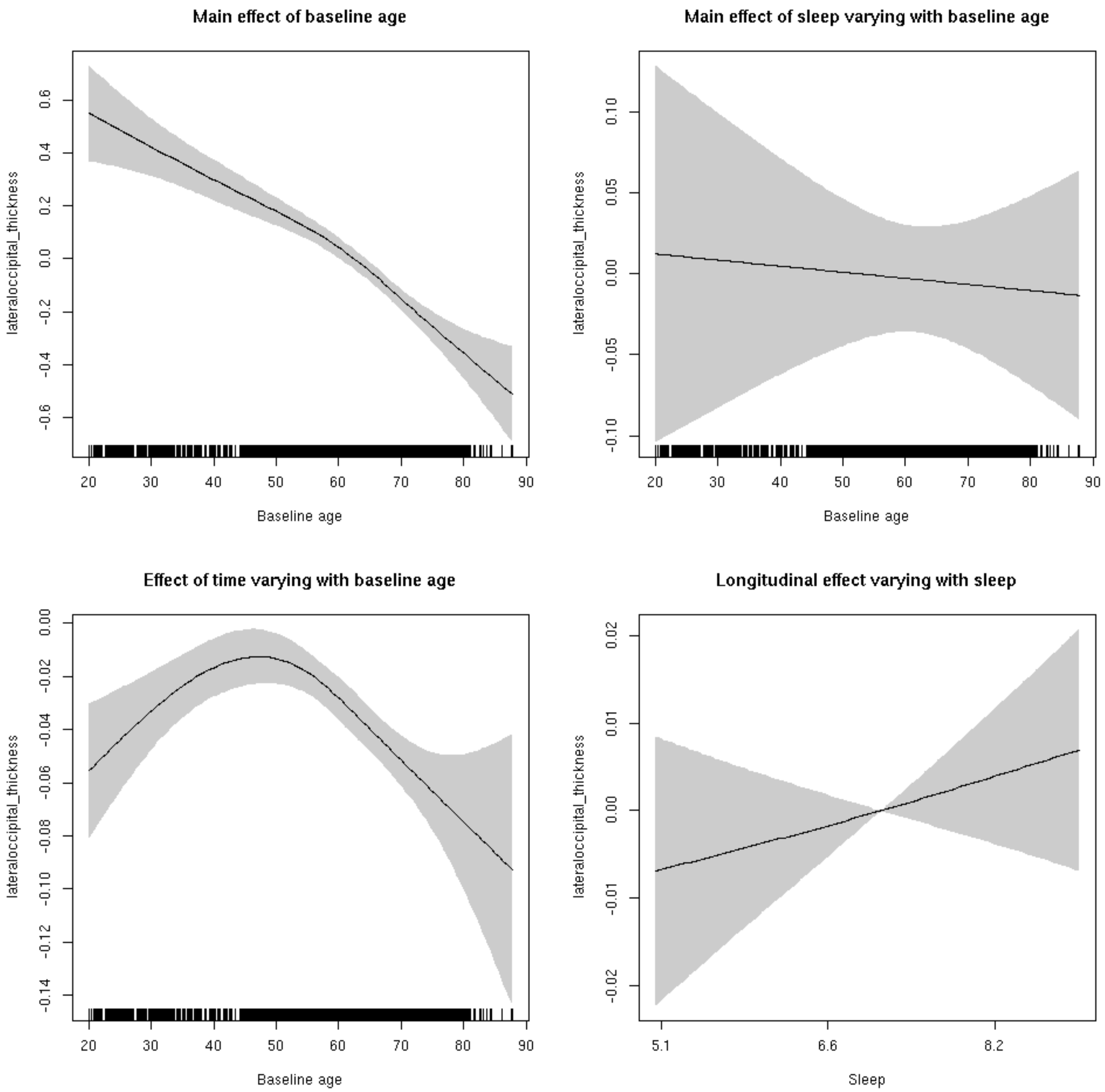

lateraloccipital_volume: Stratified by sex

Female: Full data Below is the row for `s(sleep_z):time` from the model output.

```
## s(sleep_z):time   1.000  1.000   0.055   0.814
```

Below are plots of a plot of `s(bl_age)`, `s(bl_age):sleep_z`, `s(bl_age):time`, and `s(sleep_z):time`. For `s(sleep_z):time`, roughly, if we cannot fit a straight line through the confidence bands, there is evidence that the effect of time depends on sleep duration. It is correct the that confidence interval is zero at one point along the axis; this happens because we do not include the main effect.

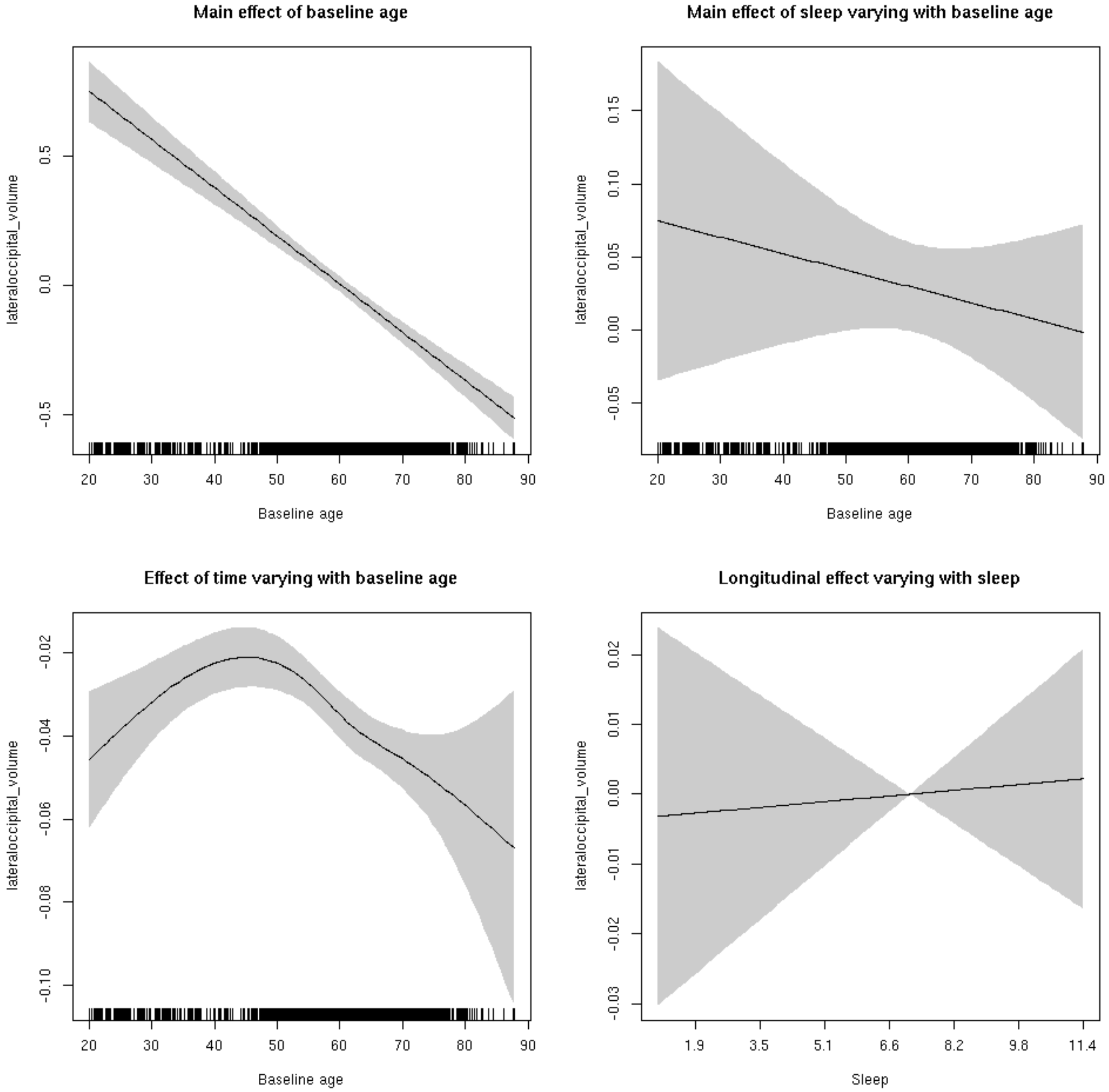

Male: Full data Below is the row for `s(sleep_z):time` from the model output.

```
## s(sleep_z):time   1.000  1.000   0.210   0.647
```

Below are plots of a plot of `s(bl_age)`, `s(bl_age):sleep_z`, `s(bl_age):time`, and `s(sleep_z):time`. For `s(sleep_z):time`, roughly, if we cannot fit a straight line through the confidence bands, there is evidence that the effect of time depends on sleep duration. It is correct the that confidence interval is zero at one point along the axis; this happens because we do not include the main effect.

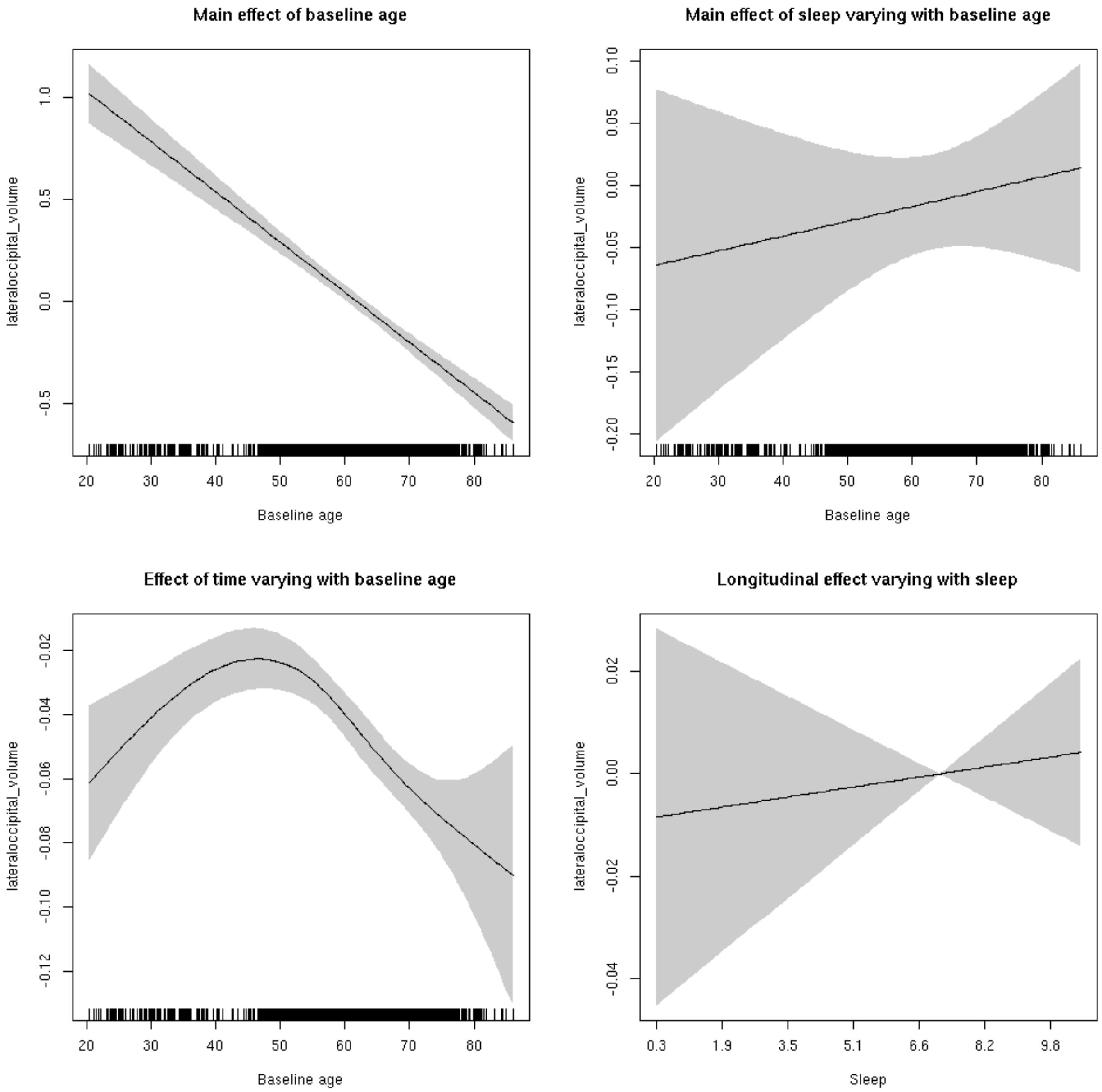

lateraloccipital_volume

Full data Below is the model output. The term `s(sleep_z):time` is probably what we care most about.

```
##
## Family: gaussian
## Link function: identity
##
## Formula:
## value ~ sex + site + icv + s(bl_age, k = 10, bs = "cr") + s(bl_age,
##     by = sleep_z, bs = "cr") + +s(bl_age, by = time, k = 5, bs = "cr") +
##     s(sleep_z, by = time, k = 5, bs = "cr", pc = 0)
## <environment: 0x5630035b3ed8>
```

```
##
## Parametric coefficients:
##                Estimate Std. Error t value Pr(>|t|)
## (Intercept)   -0.37705    0.05468  -6.896 5.76e-12 ***
## sexmale        0.44856    0.02839  15.799  < 2e-16 ***
## siteousAvanto -0.36565    0.06752  -5.416 6.29e-08 ***
## siteousPrisma -0.06669    0.17142  -0.389  0.69725
## siteousSkyra   0.58654    0.06530   8.982  < 2e-16 ***
## siteUB        -0.16388    0.12691  -1.291  0.19663
## siteUCAM       0.06757    0.06803   0.993  0.32061
## siteUKB        0.29244    0.05094   5.741 9.72e-09 ***
## siteUmU        0.22352    0.07845   2.849  0.00439 **
## icv            0.44478    0.01399  31.790  < 2e-16 ***
## ---
## Signif. codes:  0 '***' 0.001 '**' 0.01 '*' 0.05 '.' 0.1 ' ' 1
##
## Approximate significance of smooth terms:
##                      edf Ref.df       F p-value
## s(bl_age)          1.000  1.000 384.425  <2e-16 ***
## s(bl_age):sleep_z 2.000  2.000   0.151   0.859
## s(bl_age):time    4.003  4.003 169.333  <2e-16 ***
## s(sleep_z):time   1.000  1.000   0.284   0.594
## ---
## Signif. codes:  0 '***' 0.001 '**' 0.01 '*' 0.05 '.' 0.1 ' ' 1
##
## R-sq.(adj) =  0.477
## lmer.REML =  10027  Scale est. = 0.042103  n = 8174
```

Below are plots of a plot of `s(bl_age)`, `s(bl_age):sleep_z`, `s(bl_age):time`, and `s(sleep_z):time`. For `s(sleep_z):time`, roughly, if we cannot fit a straight line through the confidence bands, there is evidence that the effect of time depends on sleep duration. It is correct the that confidence interval is zero at one point along the axis; this happens because we do not include the main effect.

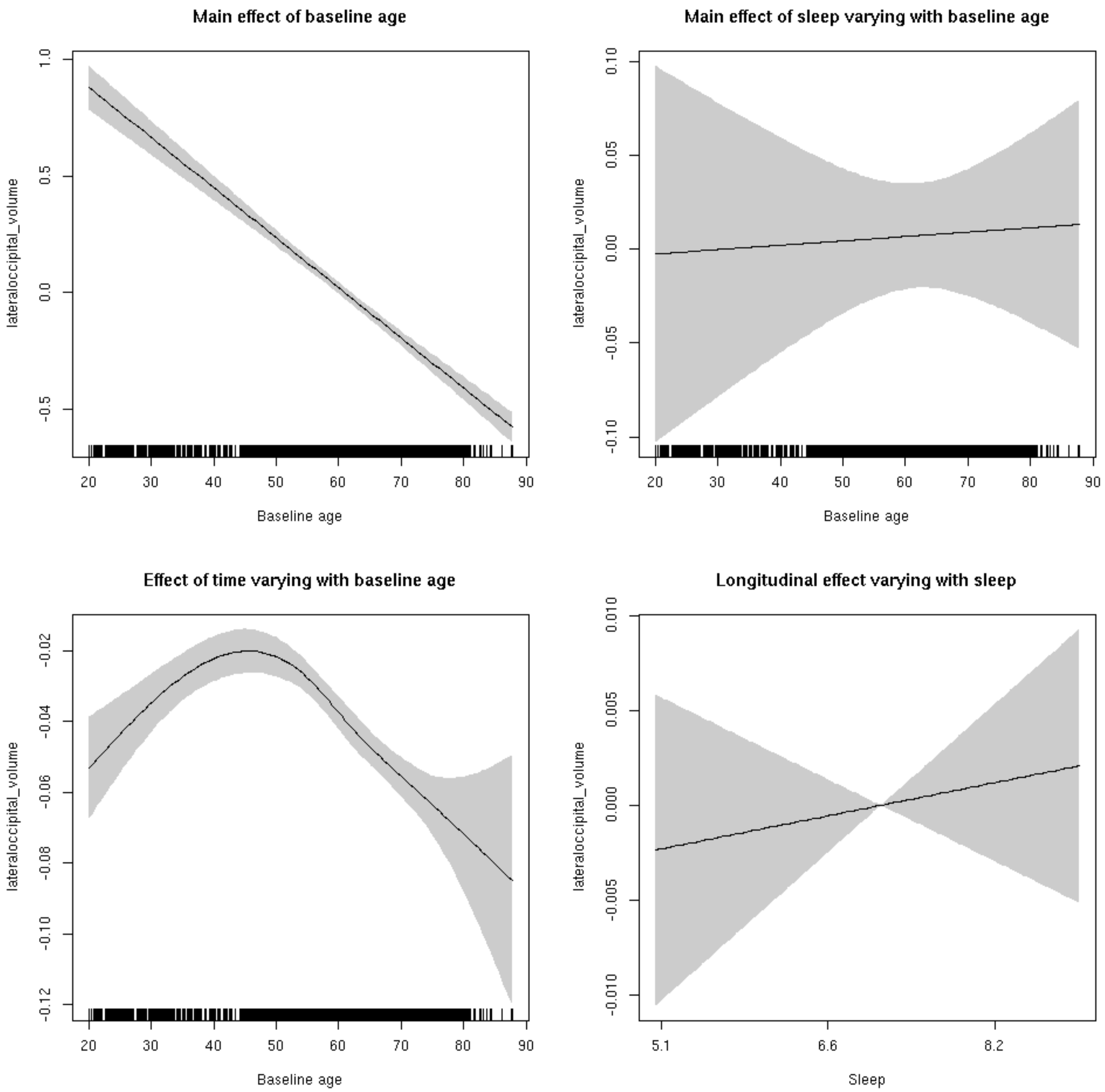

Restricted to sleep >= 5 and <= 9 hours Below is the row for `s(sleep_z):time` from the model output.

```
## s(sleep_z):time   1.00   1.00   0.332   0.565
```

Below are plots of a plot of `s(bl_age)`, `s(bl_age):sleep_z`, `s(bl_age):time`, and `s(sleep_z):time`. For `s(sleep_z):time`, roughly, if we cannot fit a straight line through the confidence bands, there is evidence that the effect of time depends on sleep duration. It is correct the that confidence interval is zero at one point along the axis; this happens because we do not include the main effect.

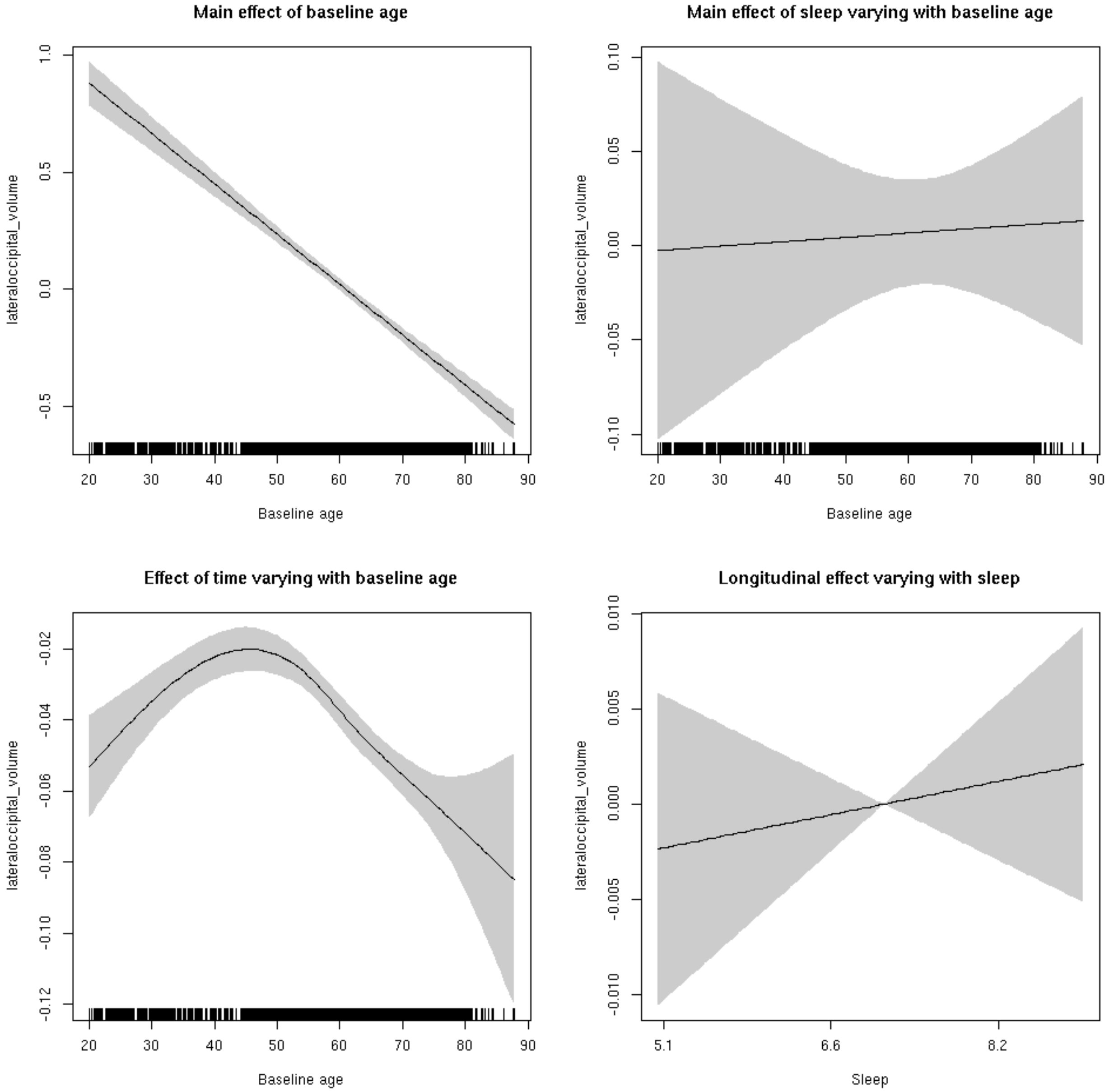

lateralorbitofrontal_area: Stratified by sex

Female: Full data Below is the row for `s(sleep_z):time` from the model output.

```
## s(sleep_z):time   1.000  1.000   1.059   0.303
```

Below are plots of a plot of `s(bl_age)`, `s(bl_age):sleep_z`, `s(bl_age):time`, and `s(sleep_z):time`. For `s(sleep_z):time`, roughly, if we cannot fit a straight line through the confidence bands, there is evidence that the effect of time depends on sleep duration. It is correct the that confidence interval is zero at one point along the axis; this happens because we do not include the main effect.

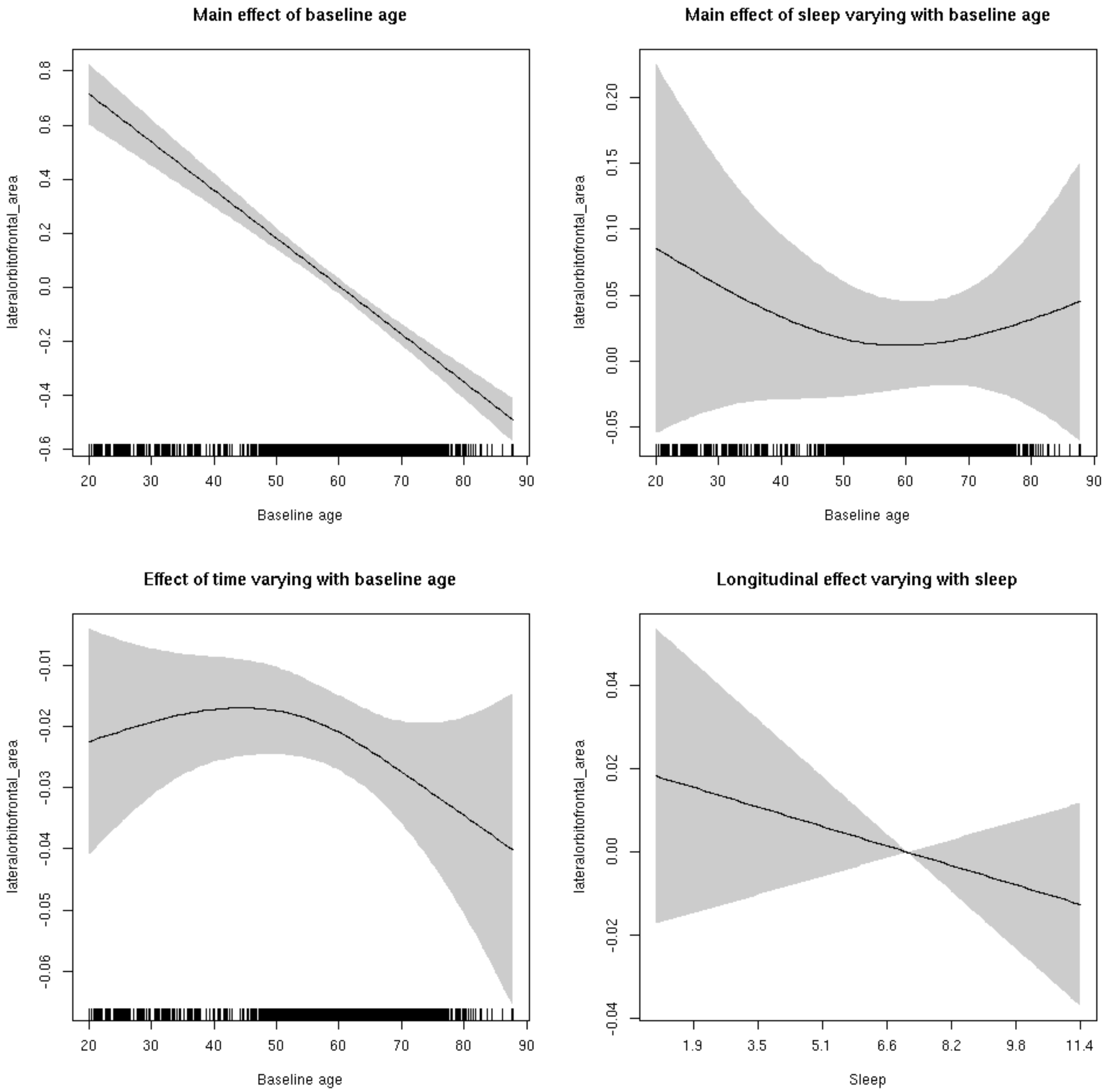

Male: Full data Below is the row for `s(sleep_z):time` from the model output.

```
## s(sleep_z):time   1.000  1.000   0.017  0.8967
```

Below are plots of a plot of `s(bl_age)`, `s(bl_age):sleep_z`, `s(bl_age):time`, and `s(sleep_z):time`. For `s(sleep_z):time`, roughly, if we cannot fit a straight line through the confidence bands, there is evidence that the effect of time depends on sleep duration. It is correct the that confidence interval is zero at one point along the axis; this happens because we do not include the main effect.

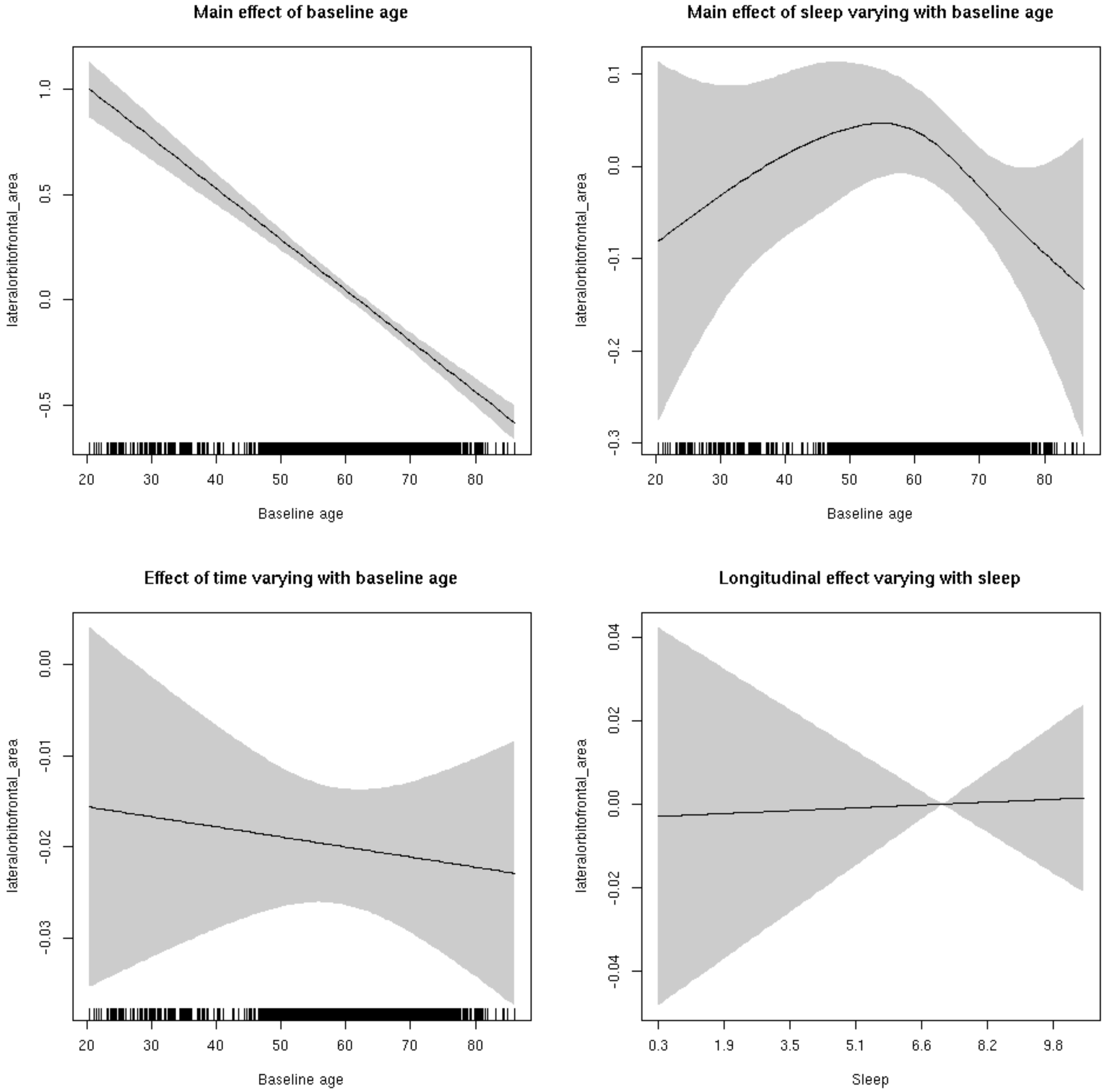

lateralorbitofrontal_area

Full data Below is the model output. The term `s(sleep_z):time` is probably what we care most about.

```
##
## Family: gaussian
## Link function: identity
##
## Formula:
## value ~ sex + site + icv + s(bl_age, k = 10, bs = "cr") + s(bl_age,
##     by = sleep_z, bs = "cr") + +s(bl_age, by = time, k = 5, bs = "cr") +
##     s(sleep_z, by = time, k = 5, bs = "cr", pc = 0)
## <environment: 0x5630035adbc8>
```

```
##
## Parametric coefficients:
##                Estimate Std. Error t value Pr(>|t|)
## (Intercept)     0.38950    0.05062   7.694 1.59e-14 ***
## sexmale         0.17683    0.02628   6.729 1.82e-11 ***
## siteousAvanto -0.24545    0.06297  -3.898 9.79e-05 ***
## siteousPrisma -1.26703    0.20825  -6.084 1.22e-09 ***
## siteousSkyra   0.10129    0.06086   1.664   0.0961 .
## siteUB        -0.67842    0.11664  -5.816 6.24e-09 ***
## siteUCAM      -0.60107    0.06288  -9.559  < 2e-16 ***
## siteUKB       -0.43469    0.04710  -9.228  < 2e-16 ***
## siteUmU        0.51990    0.07249   7.172 8.01e-13 ***
## icv            0.63664    0.01300  48.961  < 2e-16 ***
## ---
## Signif. codes:  0 '***' 0.001 '**' 0.01 '*' 0.05 '.' 0.1 ' ' 1
##
## Approximate significance of smooth terms:
##                      edf Ref.df       F p-value
## s(bl_age)          1.000  1.000 422.772  <2e-16 ***
## s(bl_age):sleep_z 2.000  2.000   0.838   0.433
## s(bl_age):time    2.849  2.849  35.880  <2e-16 ***
## s(sleep_z):time   1.000  1.000   0.280   0.597
## ---
## Signif. codes:  0 '***' 0.001 '**' 0.01 '*' 0.05 '.' 0.1 ' ' 1
##
## R-sq.(adj) =  0.563
## lmer.REML =  11512  Scale est. = 0.069118  n = 8171
```

Below are plots of a plot of `s(bl_age)`, `s(bl_age):sleep_z`, `s(bl_age):time`, and `s(sleep_z):time`. For `s(sleep_z):time`, roughly, if we cannot fit a straight line through the confidence bands, there is evidence that the effect of time depends on sleep duration. It is correct the that confidence interval is zero at one point along the axis; this happens because we do not include the main effect.

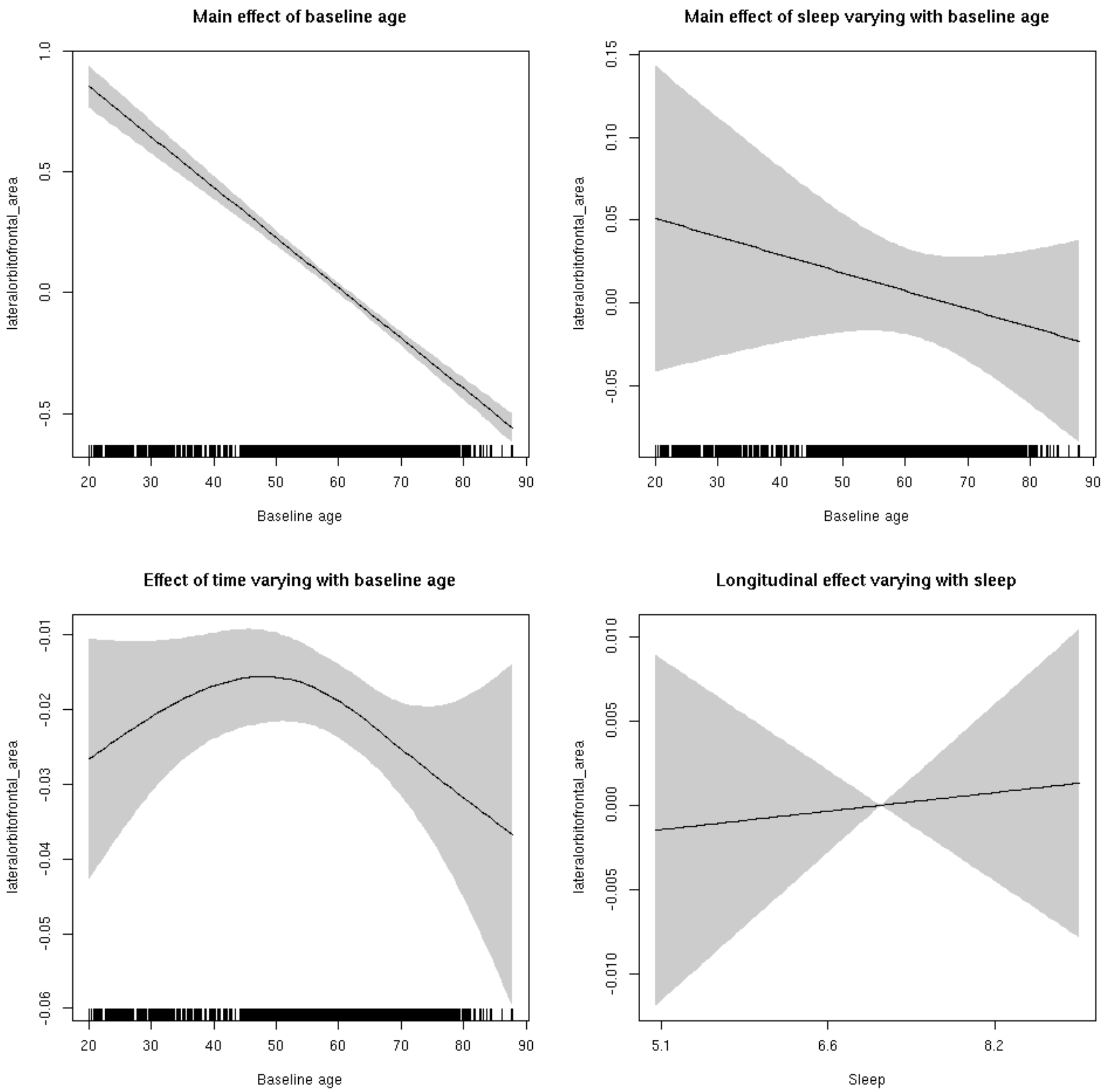

Restricted to sleep >= 5 and <= 9 hours Below is the row for `s(sleep_z):time` from the model output.

```
## s(sleep_z):time   1.000  1.000   0.082   0.775
```

Below are plots of a plot of `s(bl_age)`, `s(bl_age):sleep_z`, `s(bl_age):time`, and `s(sleep_z):time`. For `s(sleep_z):time`, roughly, if we cannot fit a straight line through the confidence bands, there is evidence that the effect of time depends on sleep duration. It is correct the that confidence interval is zero at one point along the axis; this happens because we do not include the main effect.

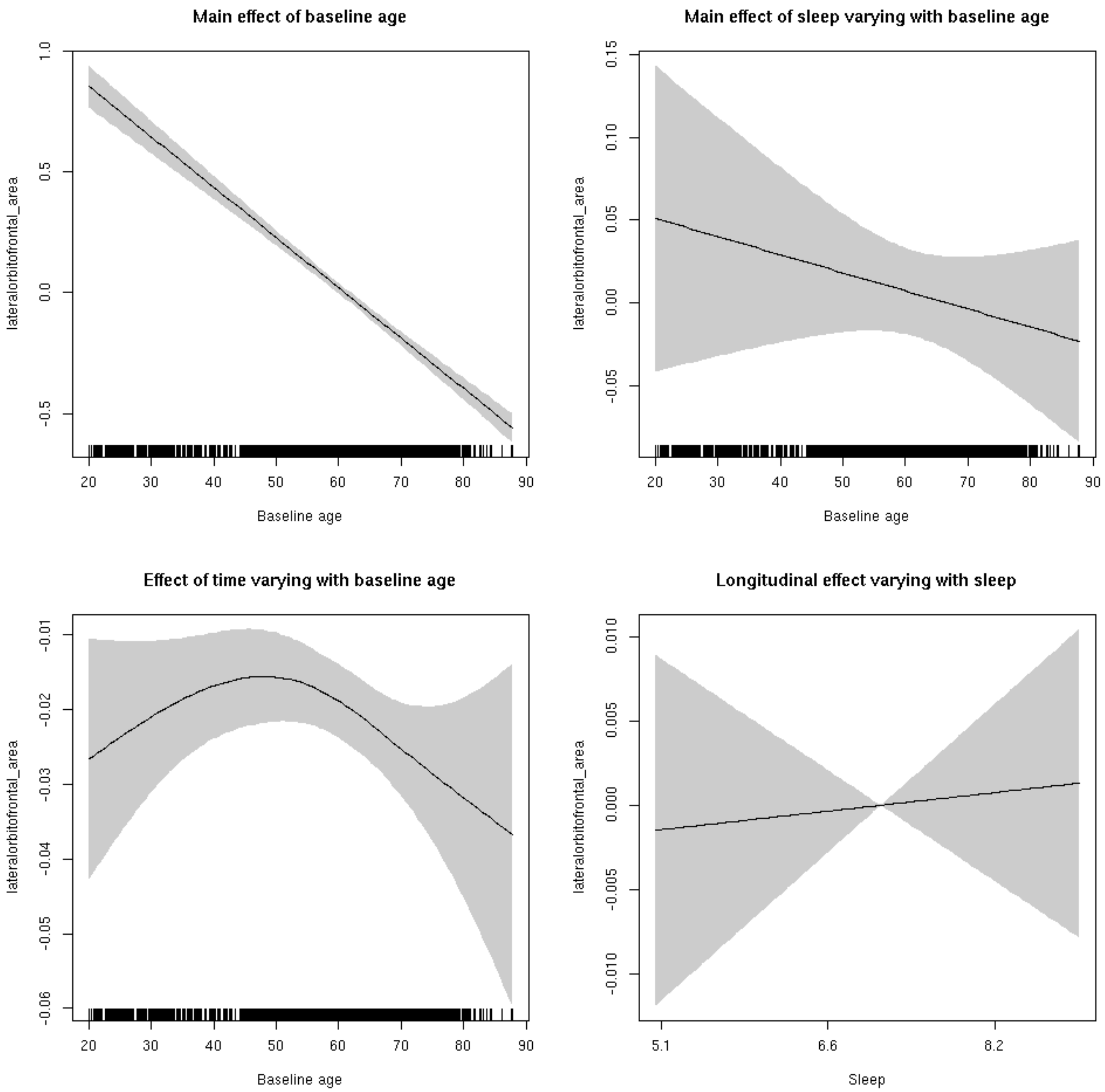

lateralorbitofrontal_thickness: Stratified by sex

Female: Full data Below is the row for `s(sleep_z):time` from the model output.

```
## s(sleep_z):time   1.000  1.000  1.377    0.241
```

Below are plots of a plot of `s(bl_age)`, `s(bl_age):sleep_z`, `s(bl_age):time`, and `s(sleep_z):time`. For `s(sleep_z):time`, roughly, if we cannot fit a straight line through the confidence bands, there is evidence that the effect of time depends on sleep duration. It is correct the that confidence interval is zero at one point along the axis; this happens because we do not include the main effect.

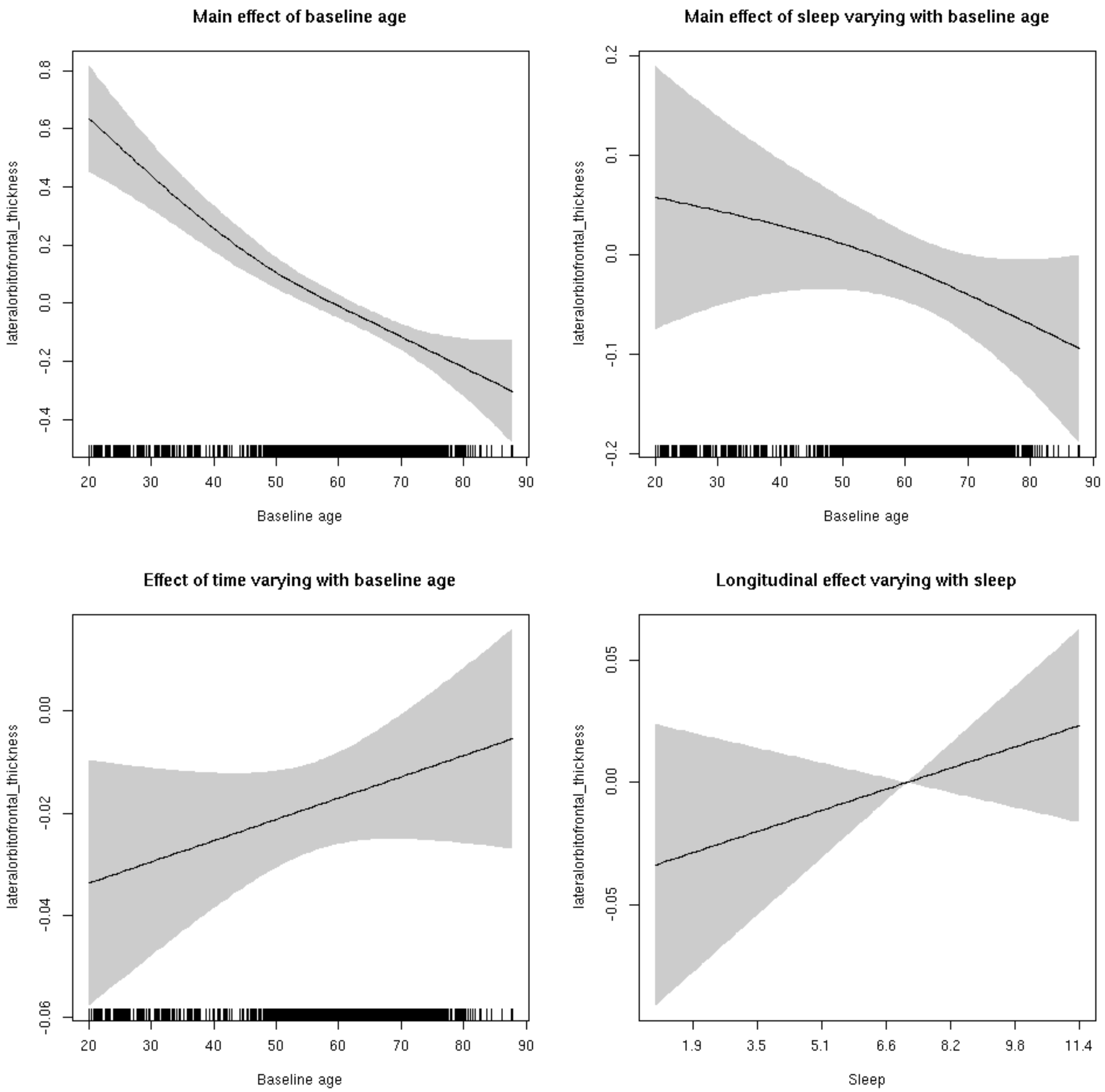

Male: Full data Below is the row for `s(sleep_z):time` from the model output.

```
## s(sleep_z):time   1.00   1.00  0.621    0.431
```

Below are plots of a plot of `s(bl_age)`, `s(bl_age):sleep_z`, `s(bl_age):time`, and `s(sleep_z):time`. For `s(sleep_z):time`, roughly, if we cannot fit a straight line through the confidence bands, there is evidence that the effect of time depends on sleep duration. It is correct the that confidence interval is zero at one point along the axis; this happens because we do not include the main effect.

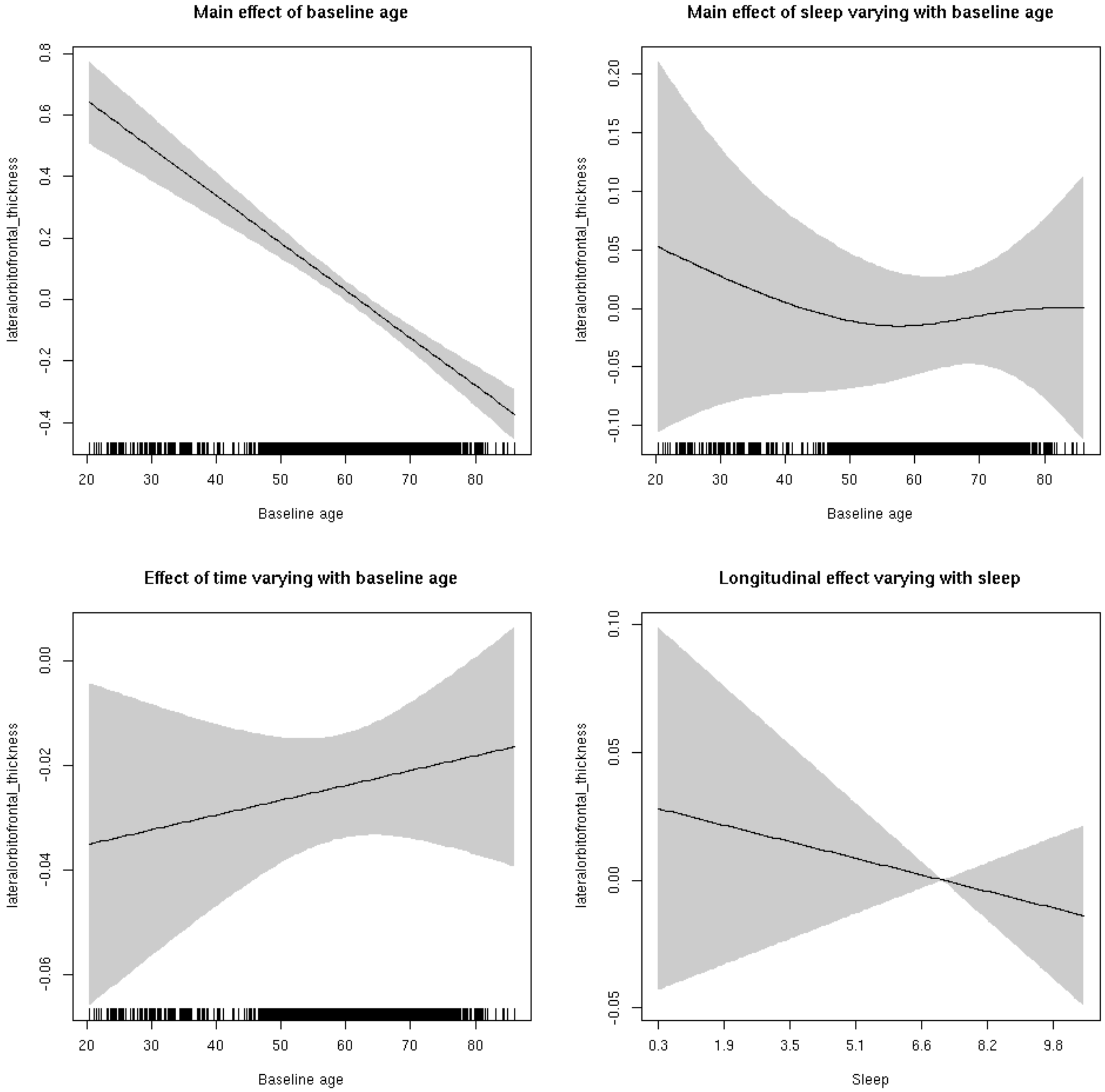

lateralorbitofrontal_thickness

Full data Below is the model output. The term `s(sleep_z):time` is probably what we care most about.

```
##
## Family: gaussian
## Link function: identity
##
## Formula:
## value ~ sex + site + s(bl_age, k = 10, bs = "cr") + s(bl_age,
##     by = sleep_z, bs = "cr") + +s(bl_age, by = time, k = 5, bs = "cr") +
##     s(sleep_z, by = time, k = 5, bs = "cr", pc = 0)
## <environment: 0x5630035ce460>
```

```
##
## Parametric coefficients:
##                Estimate Std. Error t value Pr(>|t|)
## (Intercept)   -1.84291    0.04369 -42.181  < 2e-16 ***
## sexmale        0.04122    0.02240   1.841   0.0657 .
## siteousAvanto  0.12068    0.06025   2.003   0.0452 *
## siteousPrisma  0.41598    0.31554   1.318   0.1874
## siteousSkyra  -0.09053    0.06008  -1.507   0.1319
## siteUB         0.61575    0.11713   5.257 1.50e-07 ***
## siteUCAM       0.47117    0.06069   7.763 9.27e-15 ***
## siteUKB        2.10444    0.04329  48.617  < 2e-16 ***
## siteUmU       -0.29050    0.07322  -3.967 7.33e-05 ***
## ---
## Signif. codes:  0 '***' 0.001 '**' 0.01 '*' 0.05 '.' 0.1 ' ' 1
##
## Approximate significance of smooth terms:
##                      edf Ref.df       F p-value
## s(bl_age)          1.056  1.056 160.363  <2e-16 ***
## s(bl_age):sleep_z 2.000  2.000   2.081   0.125
## s(bl_age):time    2.000  2.000  22.649  <2e-16 ***
## s(sleep_z):time   1.000  1.000   0.076   0.783
## ---
## Signif. codes:  0 '***' 0.001 '**' 0.01 '*' 0.05 '.' 0.1 ' ' 1
##
## R-sq.(adj) =  0.599
## lmer.REML =  15905  Scale est. = 0.18352   n = 8172
```

Below are plots of a plot of `s(bl_age)`, `s(bl_age):sleep_z`, `s(bl_age):time`, and `s(sleep_z):time`. For `s(sleep_z):time`, roughly, if we cannot fit a straight line through the confidence bands, there is evidence that the effect of time depends on sleep duration. It is correct the that confidence interval is zero at one point along the axis; this happens because we do not include the main effect.

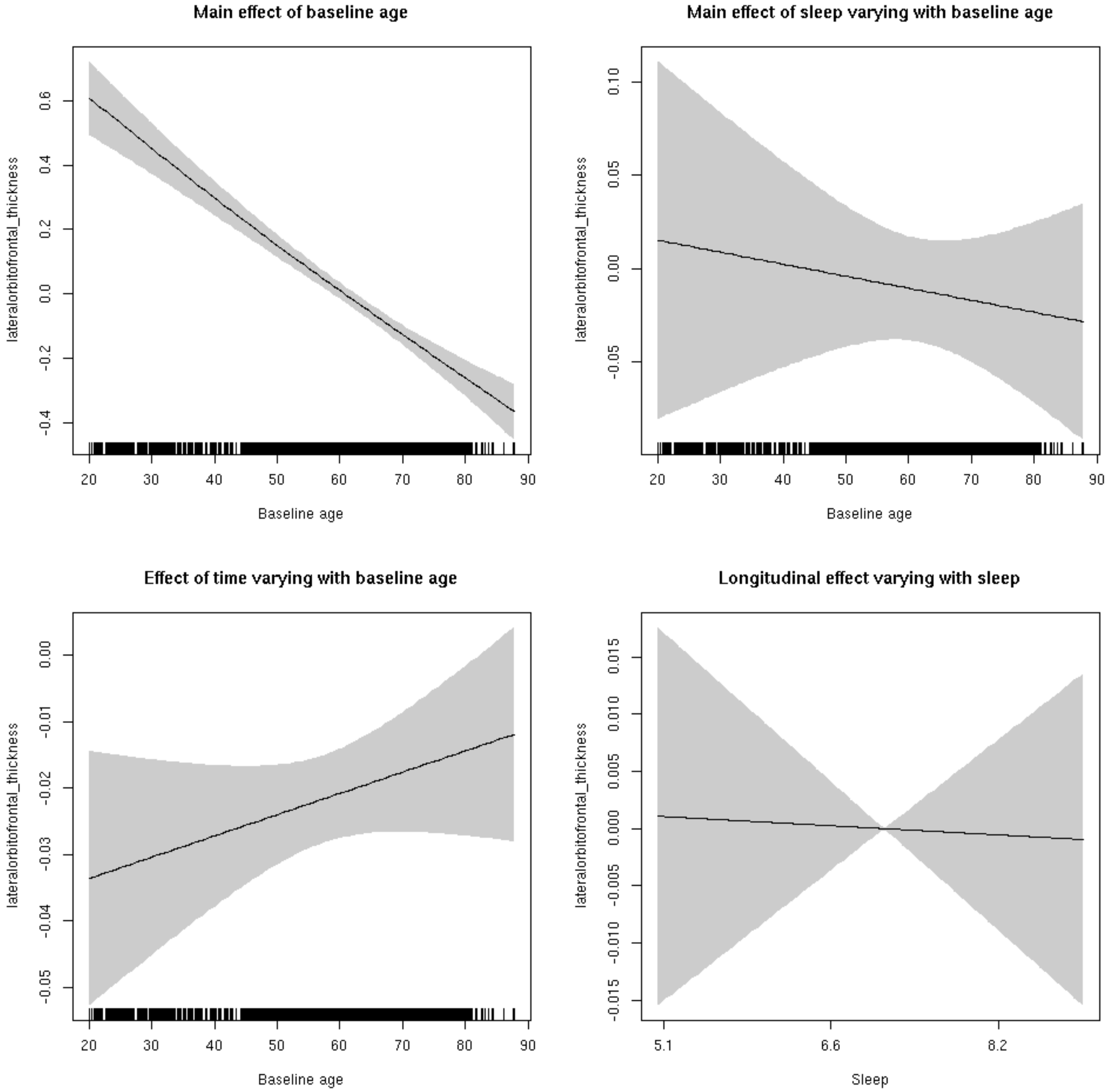

Restricted to sleep >= 5 and <= 9 hours Below is the row for `s(sleep_z):time` from the model output.

```
## s(sleep_z):time   1.000  1.000   0.017   0.897
```

Below are plots of a plot of `s(bl_age)`, `s(bl_age):sleep_z`, `s(bl_age):time`, and `s(sleep_z):time`. For `s(sleep_z):time`, roughly, if we cannot fit a straight line through the confidence bands, there is evidence that the effect of time depends on sleep duration. It is correct the that confidence interval is zero at one point along the axis; this happens because we do not include the main effect.

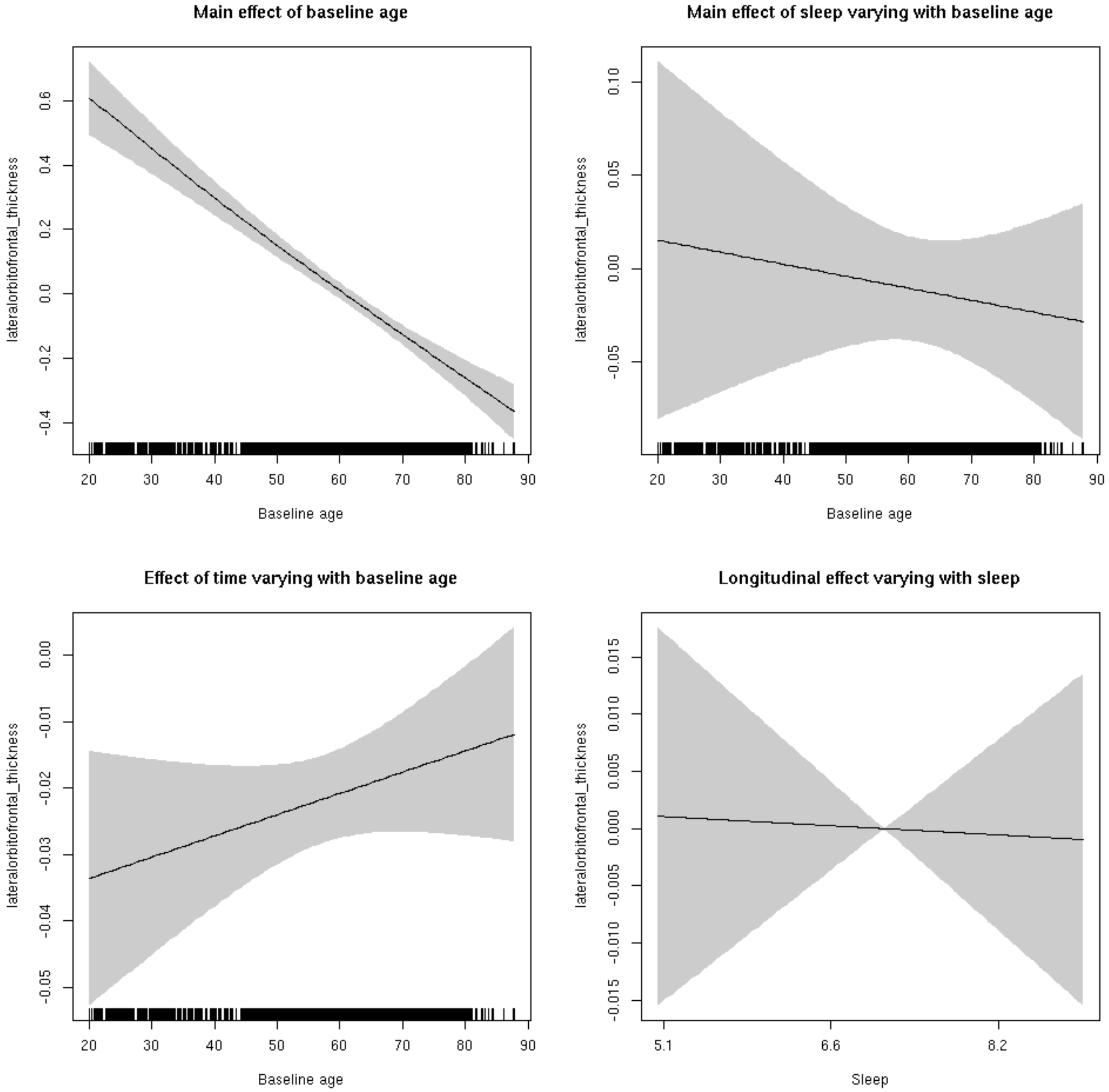

lateralorbitofrontal_volume: Stratified by sex

Female: Full data Below is the row for `s(sleep_z):time` from the model output.

```
## s(sleep_z):time   1.000  1.000   0.003   0.960
```

Below are plots of a plot of `s(bl_age)`, `s(bl_age):sleep_z`, `s(bl_age):time`, and `s(sleep_z):time`. For `s(sleep_z):time`, roughly, if we cannot fit a straight line through the confidence bands, there is evidence that the effect of time depends on sleep duration. It is correct the that confidence interval is zero at one point along the axis; this happens because we do not include the main effect.

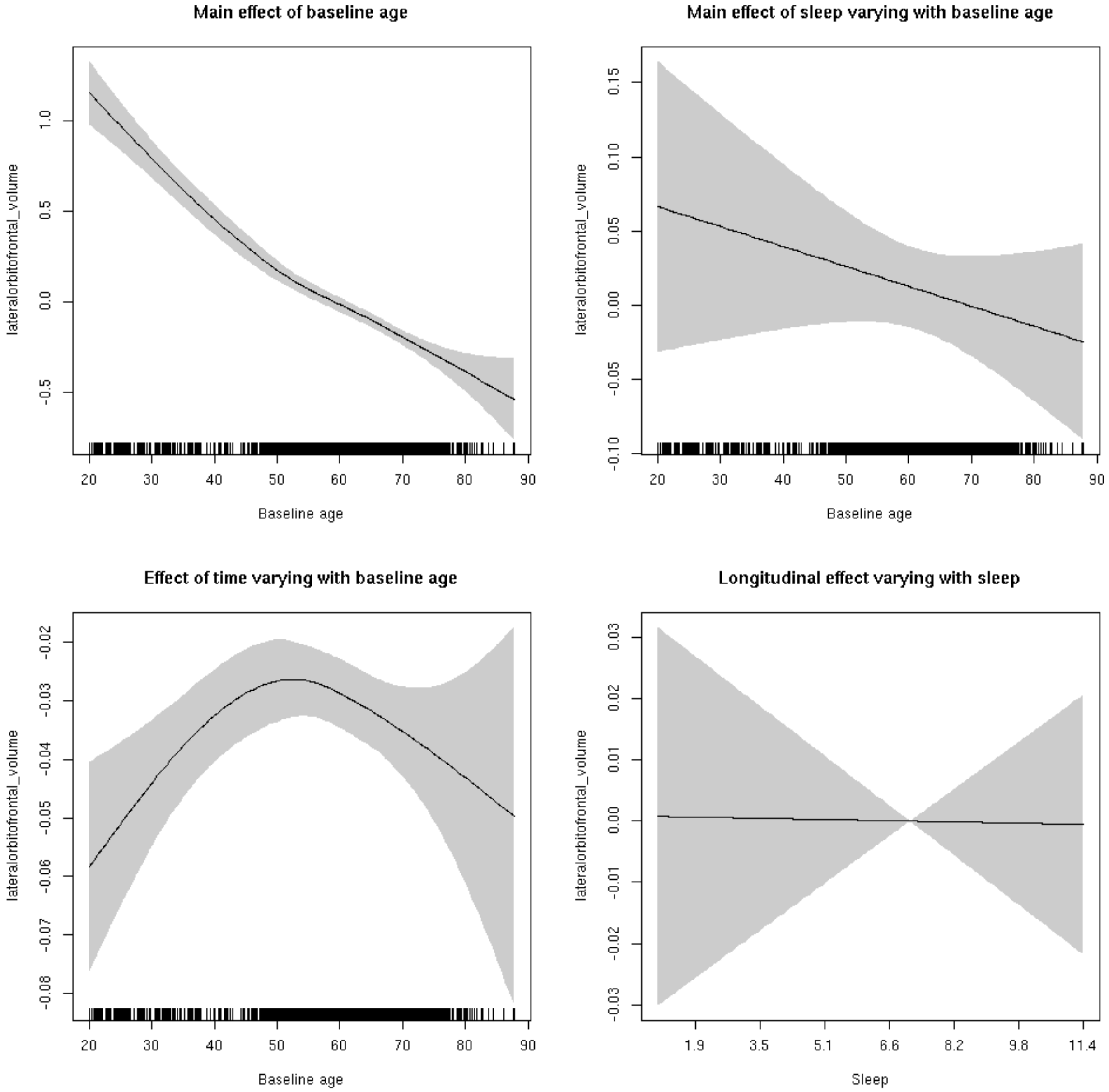

Male: Full data Below is the row for `s(sleep_z):time` from the model output.

```
## s(sleep_z):time   1.000  1.000   0.047   0.828
```

Below are plots of a plot of `s(bl_age)`, `s(bl_age):sleep_z`, `s(bl_age):time`, and `s(sleep_z):time`. For `s(sleep_z):time`, roughly, if we cannot fit a straight line through the confidence bands, there is evidence that the effect of time depends on sleep duration. It is correct the that confidence interval is zero at one point along the axis; this happens because we do not include the main effect.

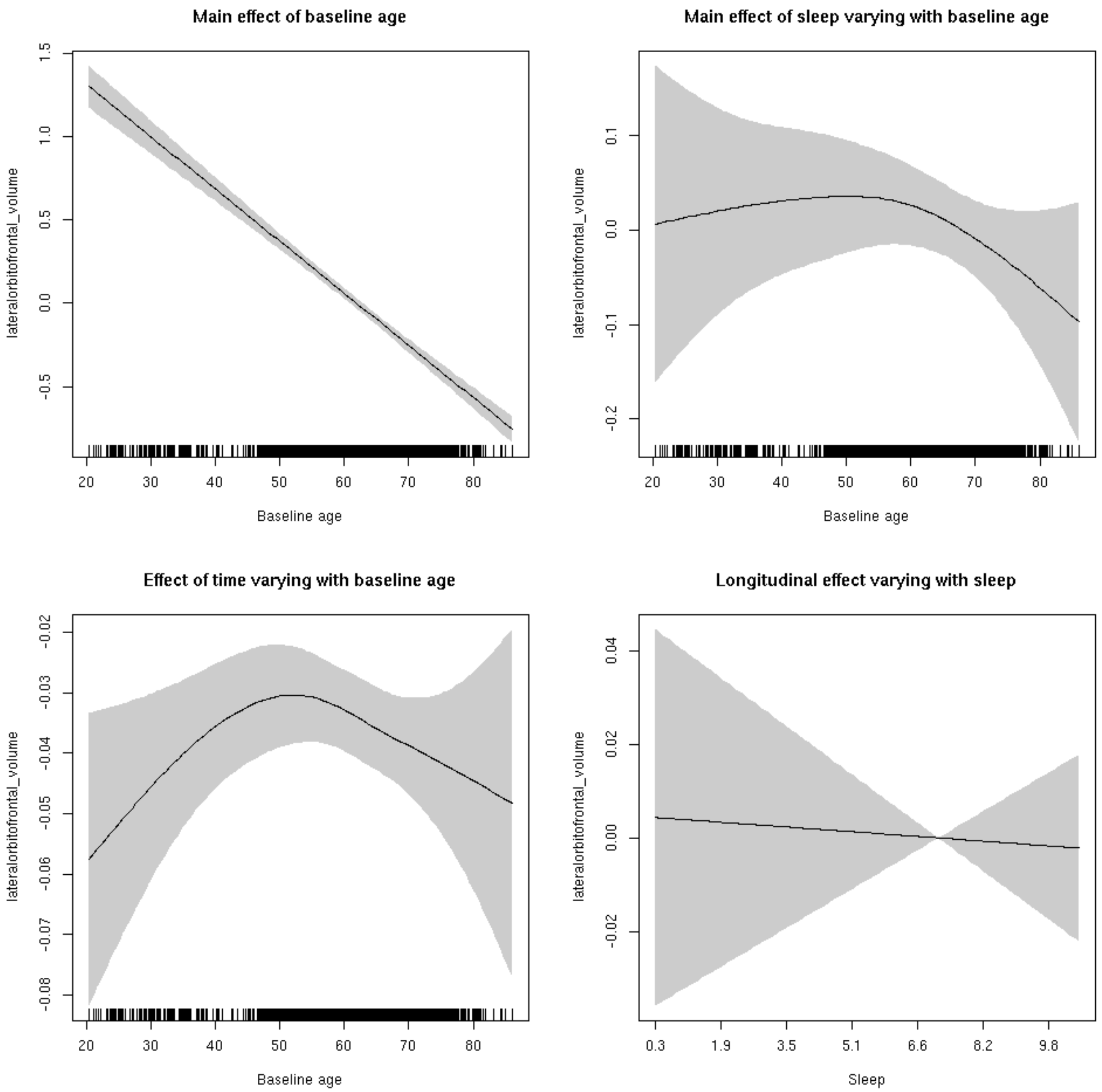

lateralorbitofrontal_volume

Full data Below is the model output. The term `s(sleep_z):time` is probably what we care most about.

```
##
## Family: gaussian
## Link function: identity
##
## Formula:
## value ~ sex + site + icv + s(bl_age, k = 10, bs = "cr") + s(bl_age,
##     by = sleep_z, bs = "cr") + +s(bl_age, by = time, k = 5, bs = "cr") +
##     s(sleep_z, by = time, k = 5, bs = "cr", pc = 0)
## <environment: 0x5630035aa1e8>
```

```
##
## Parametric coefficients:
##                Estimate Std. Error t value Pr(>|t|)
## (Intercept)   -0.46529    0.04864  -9.565  < 2e-16 ***
## sexmale        0.17028    0.02499   6.814 1.01e-11 ***
## siteousAvanto -0.41798    0.05975  -6.995 2.86e-12 ***
## siteousPrisma -0.82641    0.18421  -4.486 7.35e-06 ***
## siteousSkyra  -0.05862    0.05783  -1.014 0.310829
## siteUB        -0.31188    0.11121  -2.804 0.005054 **
## siteUCAM      -0.34102    0.06022  -5.663 1.54e-08 ***
## siteUKB        0.57940    0.04612  12.563  < 2e-16 ***
## siteUmU        0.24614    0.06925   3.554 0.000381 ***
## icv            0.61414    0.01236  49.699  < 2e-16 ***
## ---
## Signif. codes:  0 '***' 0.001 '**' 0.01 '*' 0.05 '.' 0.1 ' ' 1
##
## Approximate significance of smooth terms:
##                      edf Ref.df       F p-value
## s(bl_age)          3.051  3.051 256.162  <2e-16 ***
## s(bl_age):sleep_z 2.098  2.098   1.534   0.188
## s(bl_age):time     3.532  3.532 106.564  <2e-16 ***
## s(sleep_z):time    1.000  1.000   0.017   0.896
## ---
## Signif. codes:  0 '***' 0.001 '**' 0.01 '*' 0.05 '.' 0.1 ' ' 1
##
## R-sq.(adj) =  0.623
## lmer.REML = 9965.7  Scale est. = 0.052729  n = 8174
```

Below are plots of a plot of `s(bl_age)`, `s(bl_age):sleep_z`, `s(bl_age):time`, and `s(sleep_z):time`. For `s(sleep_z):time`, roughly, if we cannot fit a straight line through the confidence bands, there is evidence that the effect of time depends on sleep duration. It is correct the that confidence interval is zero at one point along the axis; this happens because we do not include the main effect.

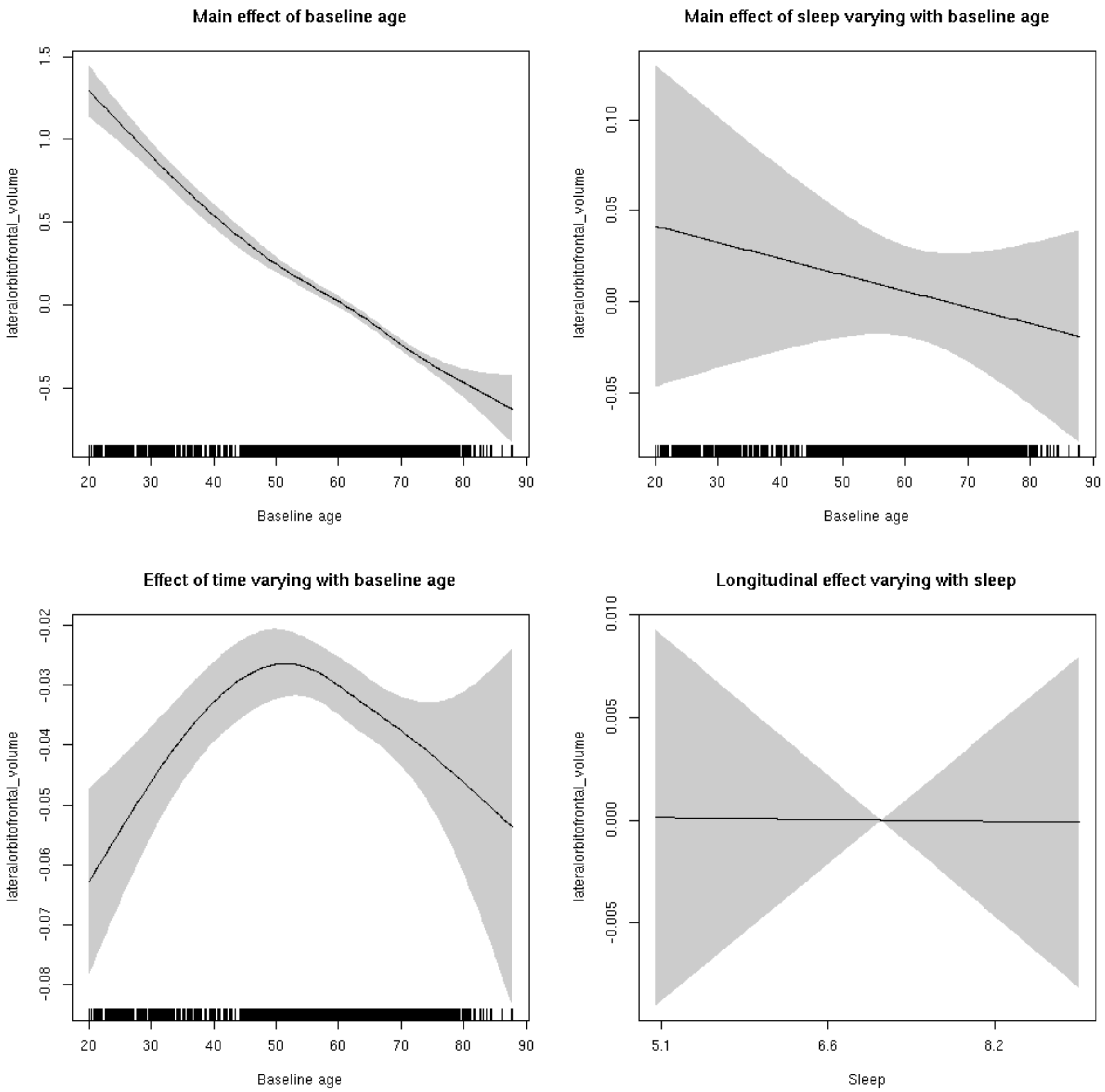

Restricted to sleep >= 5 and <= 9 hours Below is the row for `s(sleep_z):time` from the model output.

```
## s(sleep_z):time   1.000  1.000   0.001   0.978
```

Below are plots of a plot of `s(bl_age)`, `s(bl_age):sleep_z`, `s(bl_age):time`, and `s(sleep_z):time`. For `s(sleep_z):time`, roughly, if we cannot fit a straight line through the confidence bands, there is evidence that the effect of time depends on sleep duration. It is correct the that confidence interval is zero at one point along the axis; this happens because we do not include the main effect.

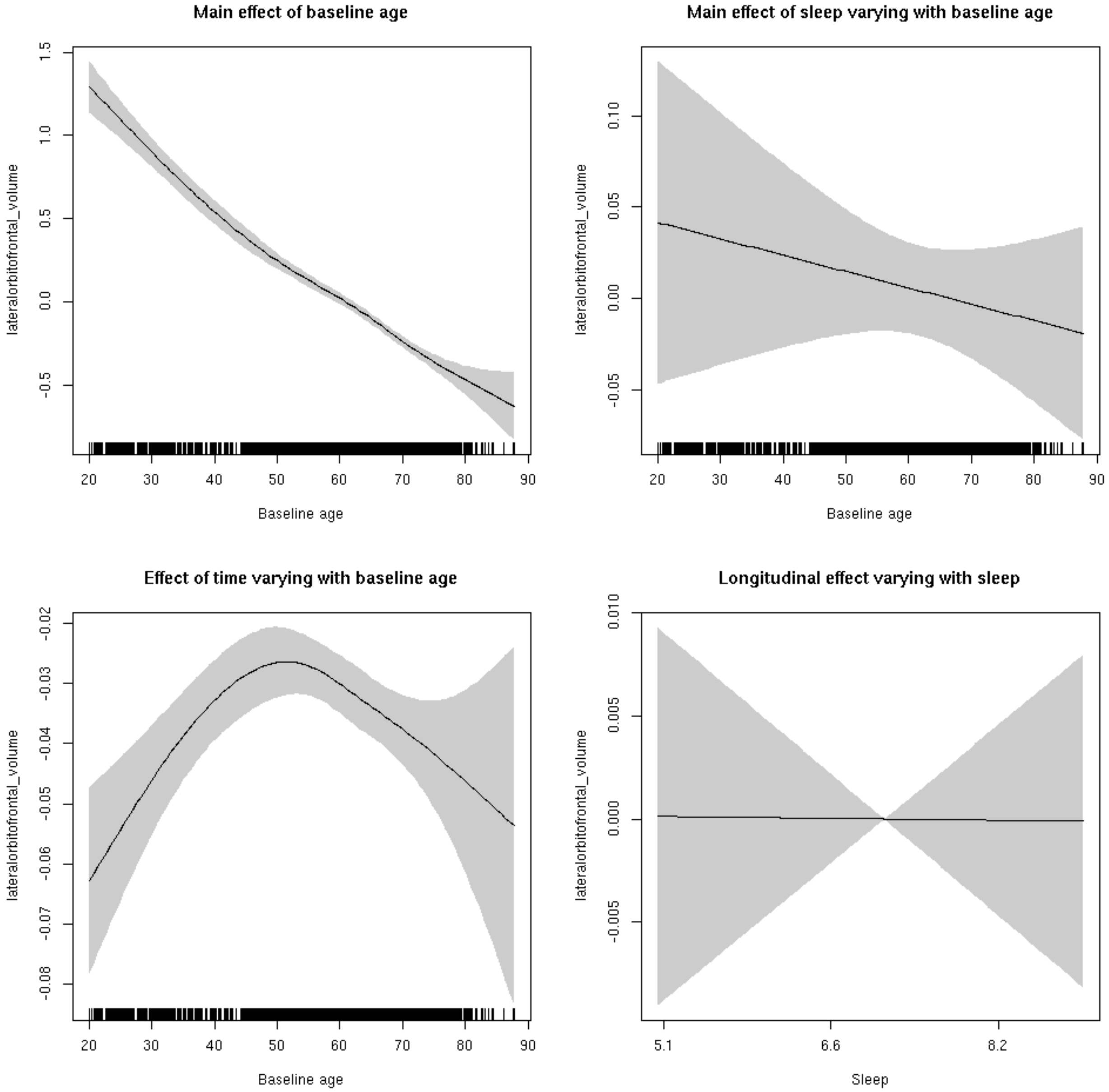

lingual_area: Stratified by sex

Female: Full data Below is the row for `s(sleep_z):time` from the model output.

```
## s(sleep_z):time   1.000  1.000   0.838   0.360
```

Below are plots of a plot of `s(bl_age)`, `s(bl_age):sleep_z`, `s(bl_age):time`, and `s(sleep_z):time`. For `s(sleep_z):time`, roughly, if we cannot fit a straight line through the confidence bands, there is evidence that the effect of time depends on sleep duration. It is correct the that confidence interval is zero at one point along the axis; this happens because we do not include the main effect.

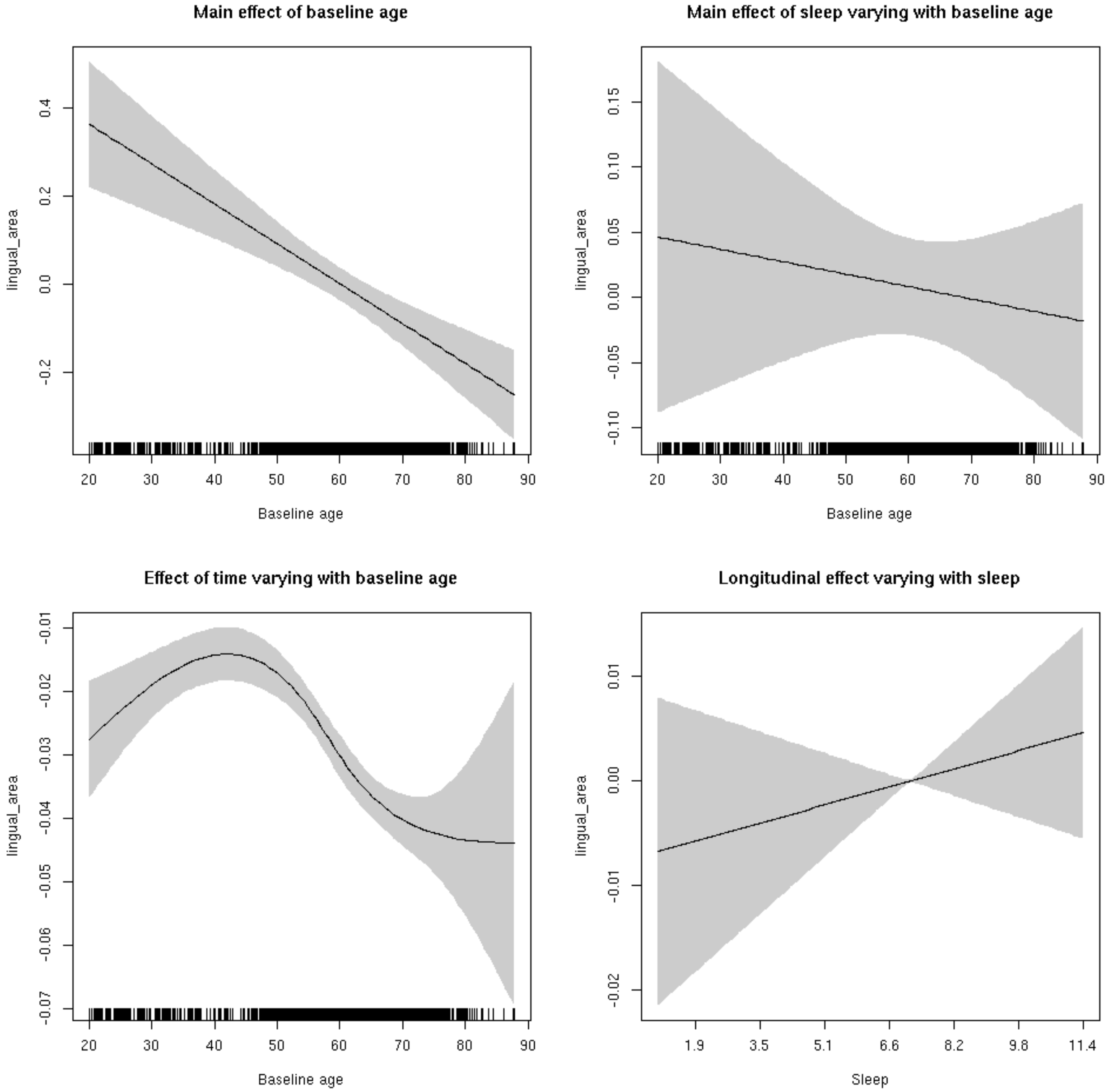

Male: Full data Below is the row for `s(sleep_z):time` from the model output.

```
## s(sleep_z):time   1.000  1.000   0.091   0.763
```

Below are plots of a plot of `s(bl_age)`, `s(bl_age):sleep_z`, `s(bl_age):time`, and `s(sleep_z):time`. For `s(sleep_z):time`, roughly, if we cannot fit a straight line through the confidence bands, there is evidence that the effect of time depends on sleep duration. It is correct the that confidence interval is zero at one point along the axis; this happens because we do not include the main effect.

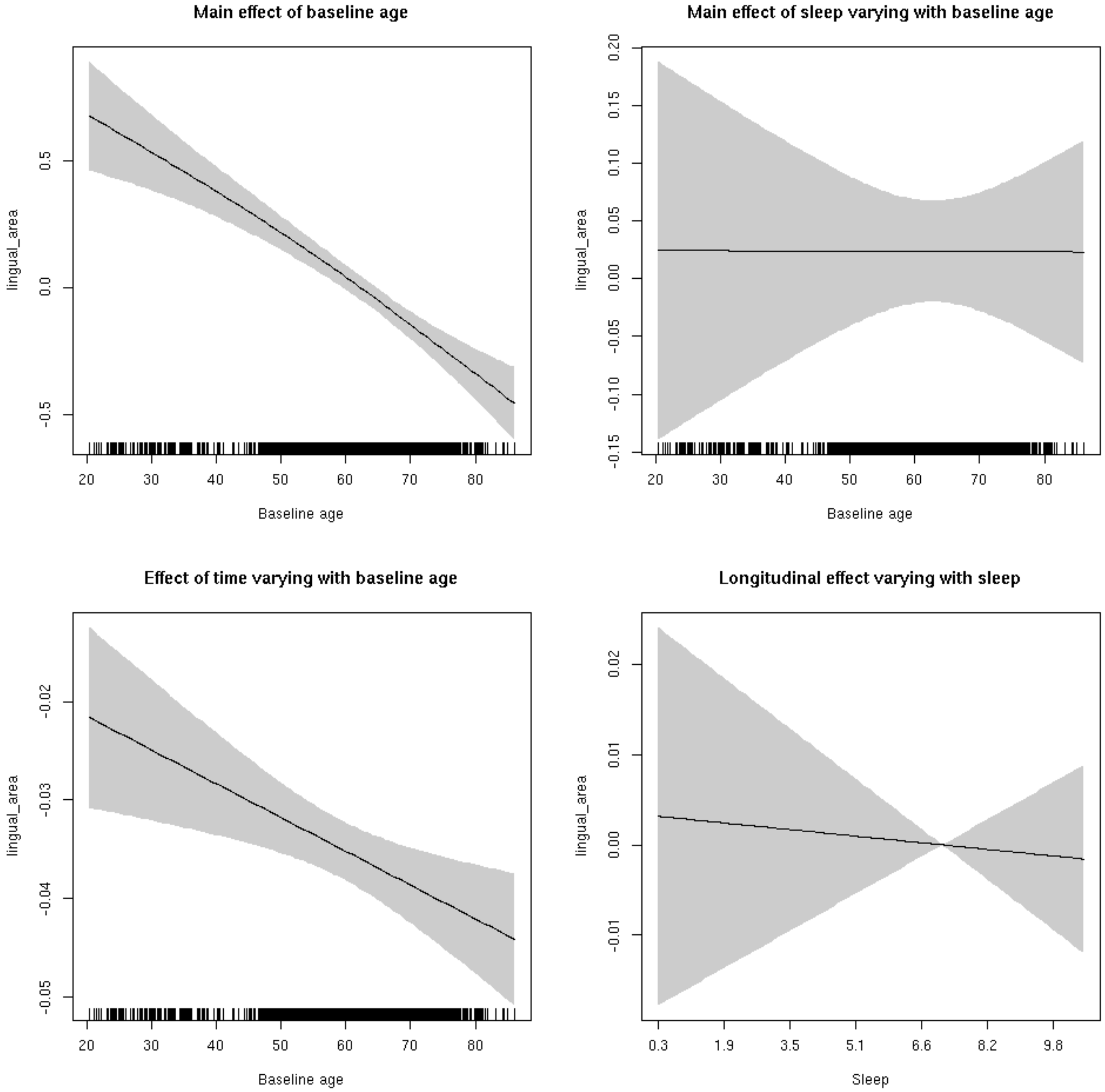

lingual_area

Full data Below is the model output. The term `s(sleep_z):time` is probably what we care most about.

```
##
## Family: gaussian
## Link function: identity
##
## Formula:
## value ~ sex + site + icv + s(bl_age, k = 10, bs = "cr") + s(bl_age,
##     by = sleep_z, bs = "cr") + +s(bl_age, by = time, k = 5, bs = "cr") +
##     s(sleep_z, by = time, k = 5, bs = "cr", pc = 0)
## <environment: 0x5630035b6258>
```

```
##
## Parametric coefficients:
##                 Estimate Std. Error t value Pr(>|t|)
## (Intercept)     0.286932   0.064589   4.442 9.01e-06 ***
## sexmale         0.259607   0.033294   7.797 7.10e-15 ***
## siteousAvanto -0.338114   0.078929  -4.284 1.86e-05 ***
## siteousPrisma -0.344497   0.120487  -2.859  0.00426 **
## siteousSkyra  -0.009223   0.076559  -0.120  0.90412
## siteUB        -0.419972   0.151198  -2.778  0.00549 **
## siteUCAM      -0.412015   0.080481  -5.119 3.13e-07 ***
## siteUKB       -0.372763   0.061223  -6.089 1.19e-09 ***
## siteUmU       -0.259305   0.093243  -2.781  0.00543 **
## icv            0.366247   0.015913  23.016  < 2e-16 ***
## ---
## Signif. codes:  0 '***' 0.001 '**' 0.01 '*' 0.05 '.' 0.1 ' ' 1
##
## Approximate significance of smooth terms:
##                       edf Ref.df       F p-value
## s(bl_age)           1.996  1.996  55.503  <2e-16 ***
## s(bl_age):sleep_z 2.000  2.000   0.613   0.542
## s(bl_age):time    4.161  4.161 303.749  <2e-16 ***
## s(sleep_z):time   1.000  1.000   0.402   0.526
## ---
## Signif. codes:  0 '***' 0.001 '**' 0.01 '*' 0.05 '.' 0.1 ' ' 1
##
## R-sq.(adj) =  0.263
## lmer.REML = 6378.5  Scale est. = 0.013129  n = 8166
```

Below are plots of a plot of `s(bl_age)`, `s(bl_age):sleep_z`, `s(bl_age):time`, and `s(sleep_z):time`. For `s(sleep_z):time`, roughly, if we cannot fit a straight line through the confidence bands, there is evidence that the effect of time depends on sleep duration. It is correct the that confidence interval is zero at one point along the axis; this happens because we do not include the main effect.

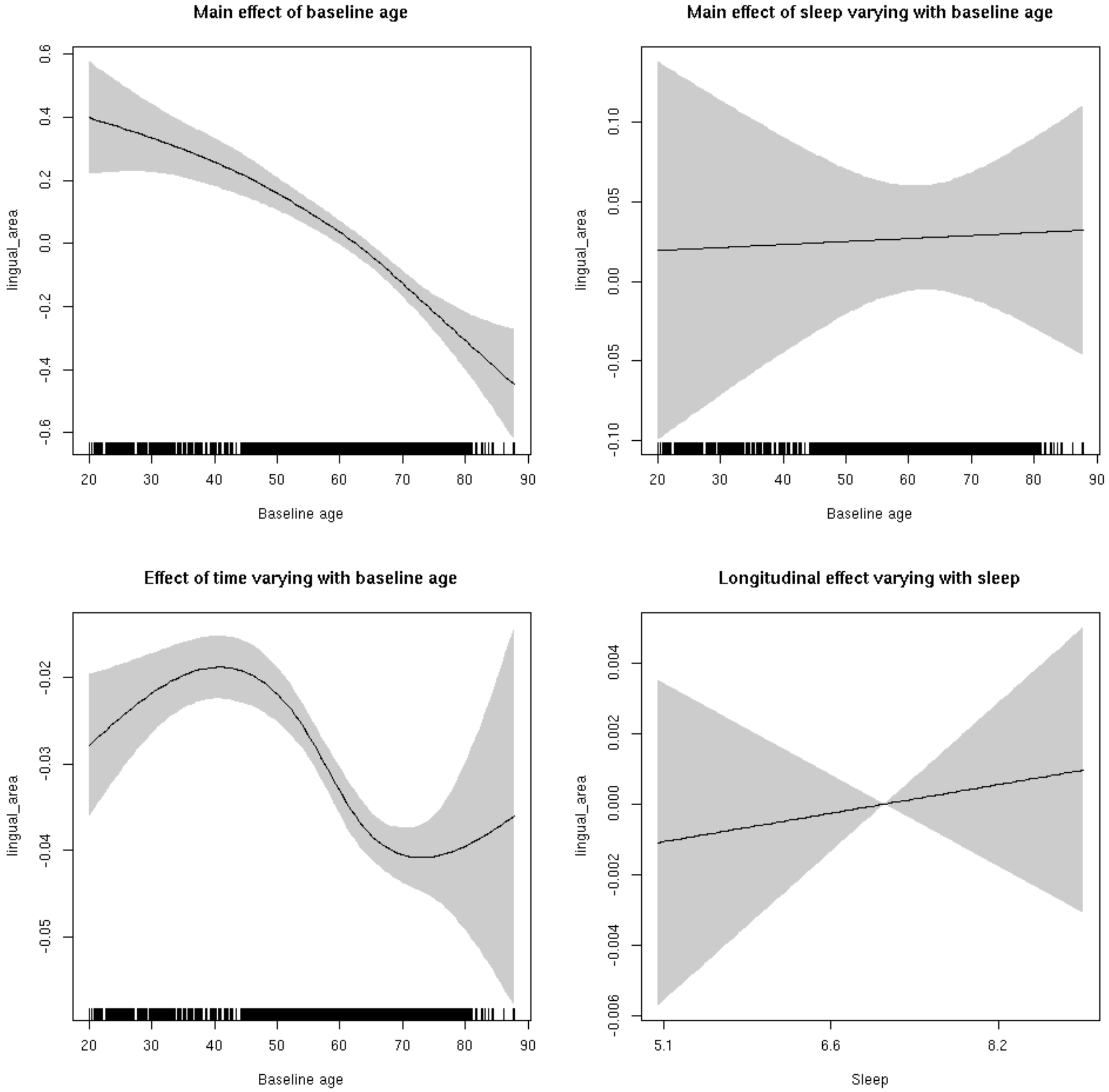

Restricted to sleep >= 5 and <= 9 hours Below is the row for `s(sleep_z):time` from the model output.

```
## s(sleep_z):time   1.000  1.000   0.227   0.634
```

Below are plots of a plot of `s(bl_age)`, `s(bl_age):sleep_z`, `s(bl_age):time`, and `s(sleep_z):time`. For `s(sleep_z):time`, roughly, if we cannot fit a straight line through the confidence bands, there is evidence that the effect of time depends on sleep duration. It is correct the that confidence interval is zero at one point along the axis; this happens because we do not include the main effect.

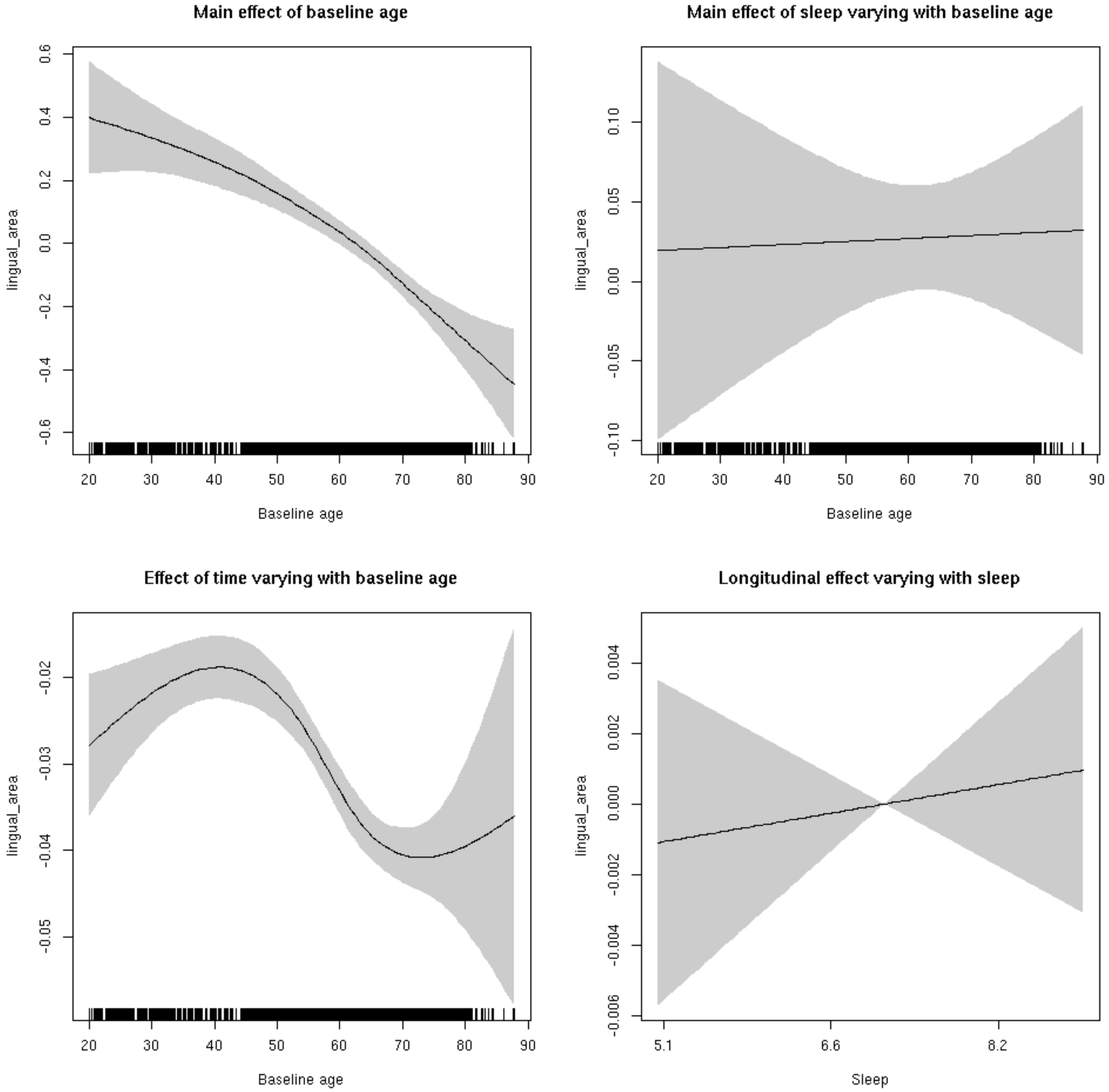

lingual_thickness: Stratified by sex

Female: Full data Below is the row for `s(sleep_z):time` from the model output.

```
## s(sleep_z):time   1.000  1.000  0.011  0.9155
```

Below are plots of a plot of `s(bl_age)`, `s(bl_age):sleep_z`, `s(bl_age):time`, and `s(sleep_z):time`. For `s(sleep_z):time`, roughly, if we cannot fit a straight line through the confidence bands, there is evidence that the effect of time depends on sleep duration. It is correct the that confidence interval is zero at one point along the axis; this happens because we do not include the main effect.

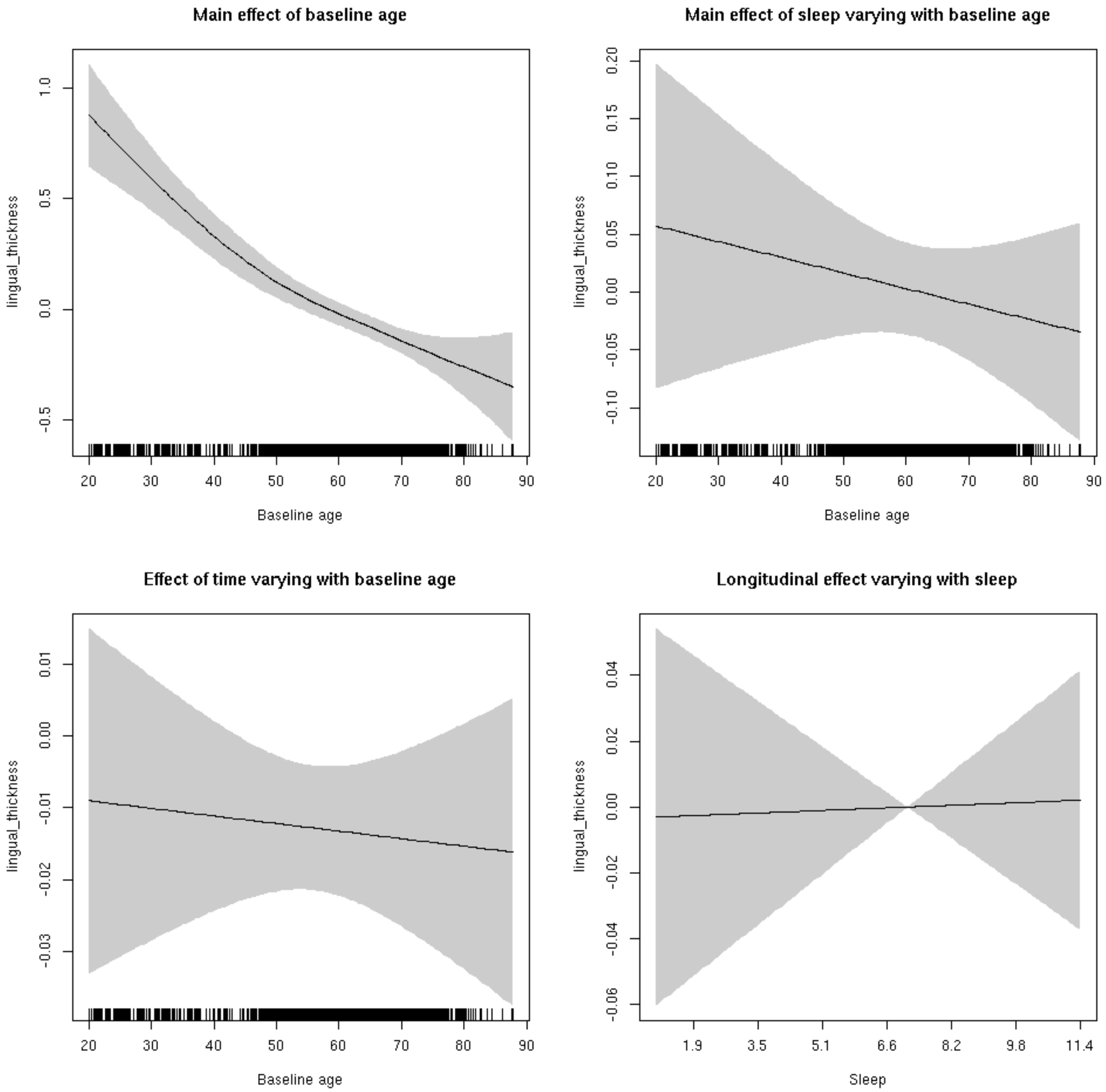

Male: Full data Below is the row for `s(sleep_z):time` from the model output.

```
## s(sleep_z):time   1.000  1.000  0.026    0.873
```

Below are plots of a plot of `s(bl_age)`, `s(bl_age):sleep_z`, `s(bl_age):time`, and `s(sleep_z):time`. For `s(sleep_z):time`, roughly, if we cannot fit a straight line through the confidence bands, there is evidence that the effect of time depends on sleep duration. It is correct the that confidence interval is zero at one point along the axis; this happens because we do not include the main effect.

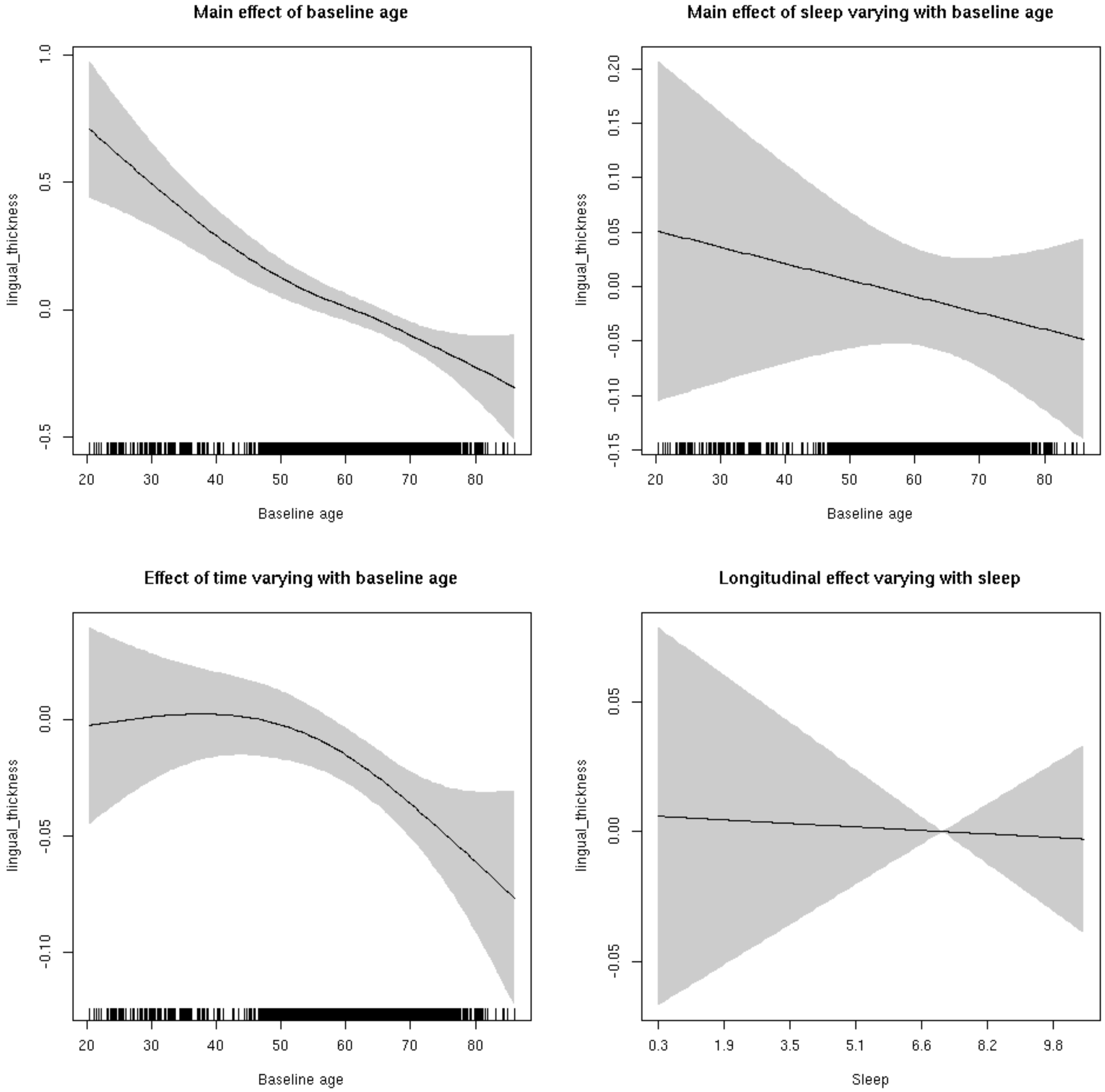

lingual_thickness

Full data Below is the model output. The term `s(sleep_z):time` is probably what we care most about.

```
##
## Family: gaussian
## Link function: identity
##
## Formula:
## value ~ sex + site + s(bl_age, k = 10, bs = "cr") + s(bl_age,
##     by = sleep_z, bs = "cr") + +s(bl_age, by = time, k = 5, bs = "cr") +
##     s(sleep_z, by = time, k = 5, bs = "cr", pc = 0)
## <environment: 0x5630035cc2c8>
```

```
##
## Parametric coefficients:
##                Estimate Std. Error t value Pr(>|t|)
## (Intercept)   -0.86427    0.05492 -15.736  < 2e-16 ***
## sexmale        0.09724    0.02755   3.530 0.000418 ***
## siteousAvanto  0.01162    0.07320   0.159 0.873844
## siteousPrisma  0.71563    0.32374   2.211 0.027097 *
## siteousSkyra   1.45433    0.07315  19.881  < 2e-16 ***
## siteUB         0.48510    0.14565   3.331 0.000870 ***
## siteUCAM       0.40078    0.07549   5.309 1.13e-07 ***
## siteUKB        0.94851    0.05560  17.059  < 2e-16 ***
## siteUmU        1.14592    0.09060  12.648  < 2e-16 ***
## ---
## Signif. codes:  0 '***' 0.001 '**' 0.01 '*' 0.05 '.' 0.1 ' ' 1
##
## Approximate significance of smooth terms:
##                      edf Ref.df      F p-value
## s(bl_age)          3.309  3.309 43.949  <2e-16 ***
## s(bl_age):sleep_z 2.000  2.000  0.916    0.40
## s(bl_age):time    3.160  3.160 11.426  <2e-16 ***
## s(sleep_z):time   1.000  1.000  0.006    0.94
## ---
## Signif. codes:  0 '***' 0.001 '**' 0.01 '*' 0.05 '.' 0.1 ' ' 1
##
## R-sq.(adj) =  0.178
## lmer.REML =  17503  Scale est. = 0.18197   n = 8178
```

Below are plots of a plot of `s(bl_age)`, `s(bl_age):sleep_z`, `s(bl_age):time`, and `s(sleep_z):time`. For `s(sleep_z):time`, roughly, if we cannot fit a straight line through the confidence bands, there is evidence that the effect of time depends on sleep duration. It is correct the that confidence interval is zero at one point along the axis; this happens because we do not include the main effect.

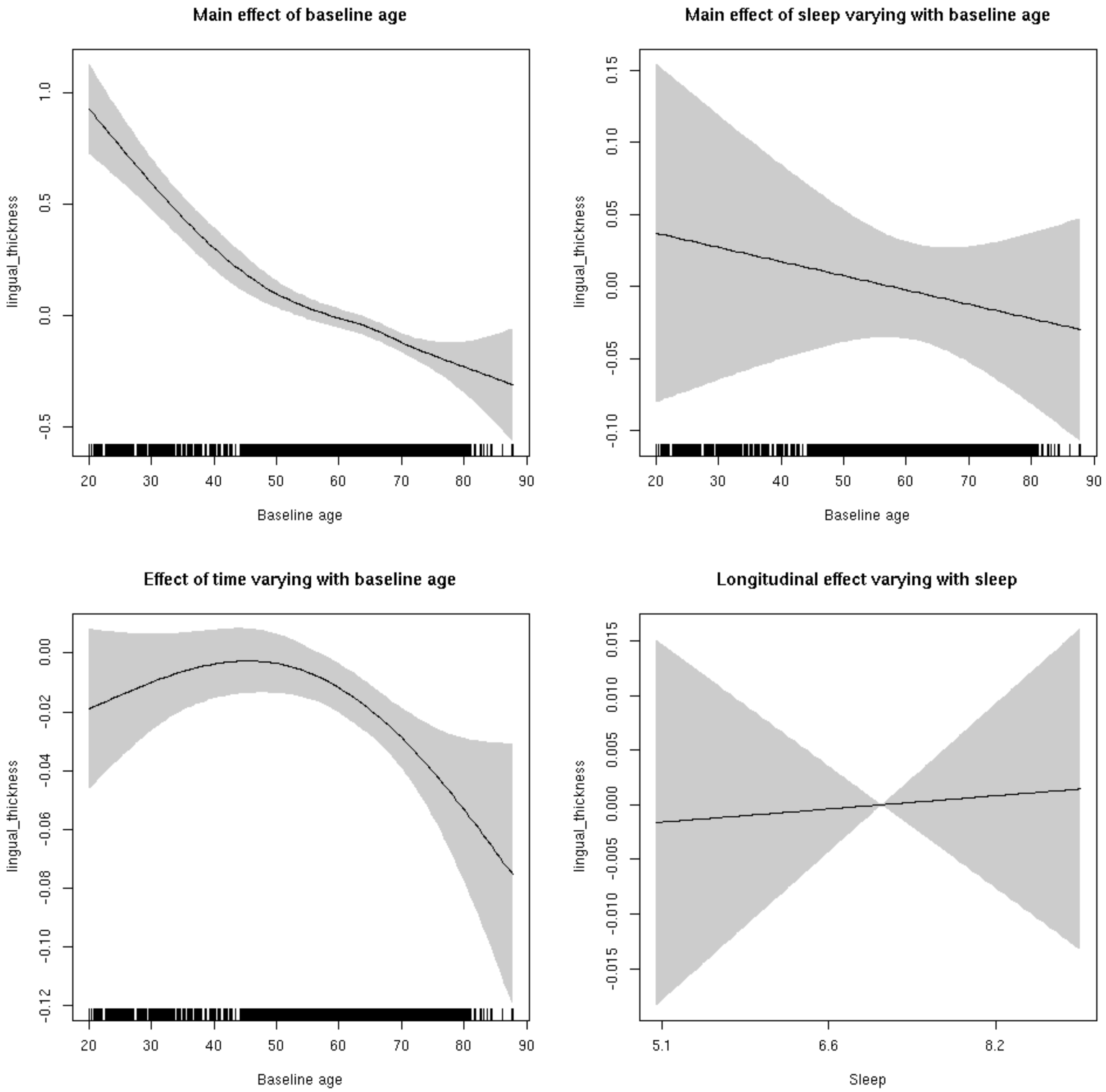

Restricted to sleep >= 5 and <= 9 hours Below is the row for `s(sleep_z):time` from the model output.

```
## s(sleep_z):time   1.000  1.000  0.038    0.845
```

Below are plots of a plot of `s(bl_age)`, `s(bl_age):sleep_z`, `s(bl_age):time`, and `s(sleep_z):time`. For `s(sleep_z):time`, roughly, if we cannot fit a straight line through the confidence bands, there is evidence that the effect of time depends on sleep duration. It is correct the that confidence interval is zero at one point along the axis; this happens because we do not include the main effect.

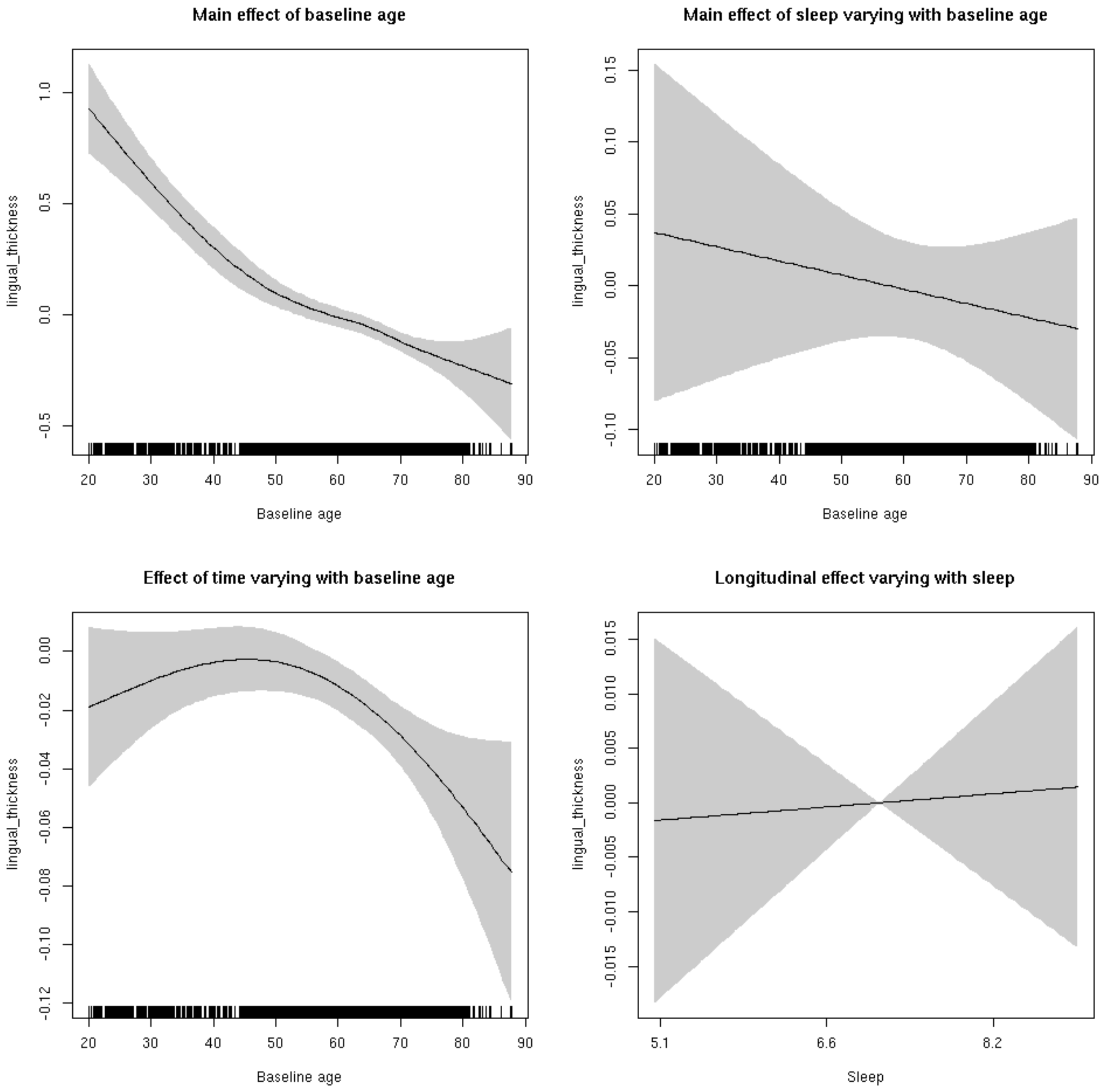

lingual_volume: Stratified by sex

Female: Full data Below is the row for `s(sleep_z):time` from the model output.

```
## s(sleep_z):time   1.000  1.000  0.047   0.828
```

Below are plots of a plot of `s(bl_age)`, `s(bl_age):sleep_z`, `s(bl_age):time`, and `s(sleep_z):time`. For `s(sleep_z):time`, roughly, if we cannot fit a straight line through the confidence bands, there is evidence that the effect of time depends on sleep duration. It is correct the that confidence interval is zero at one point along the axis; this happens because we do not include the main effect.

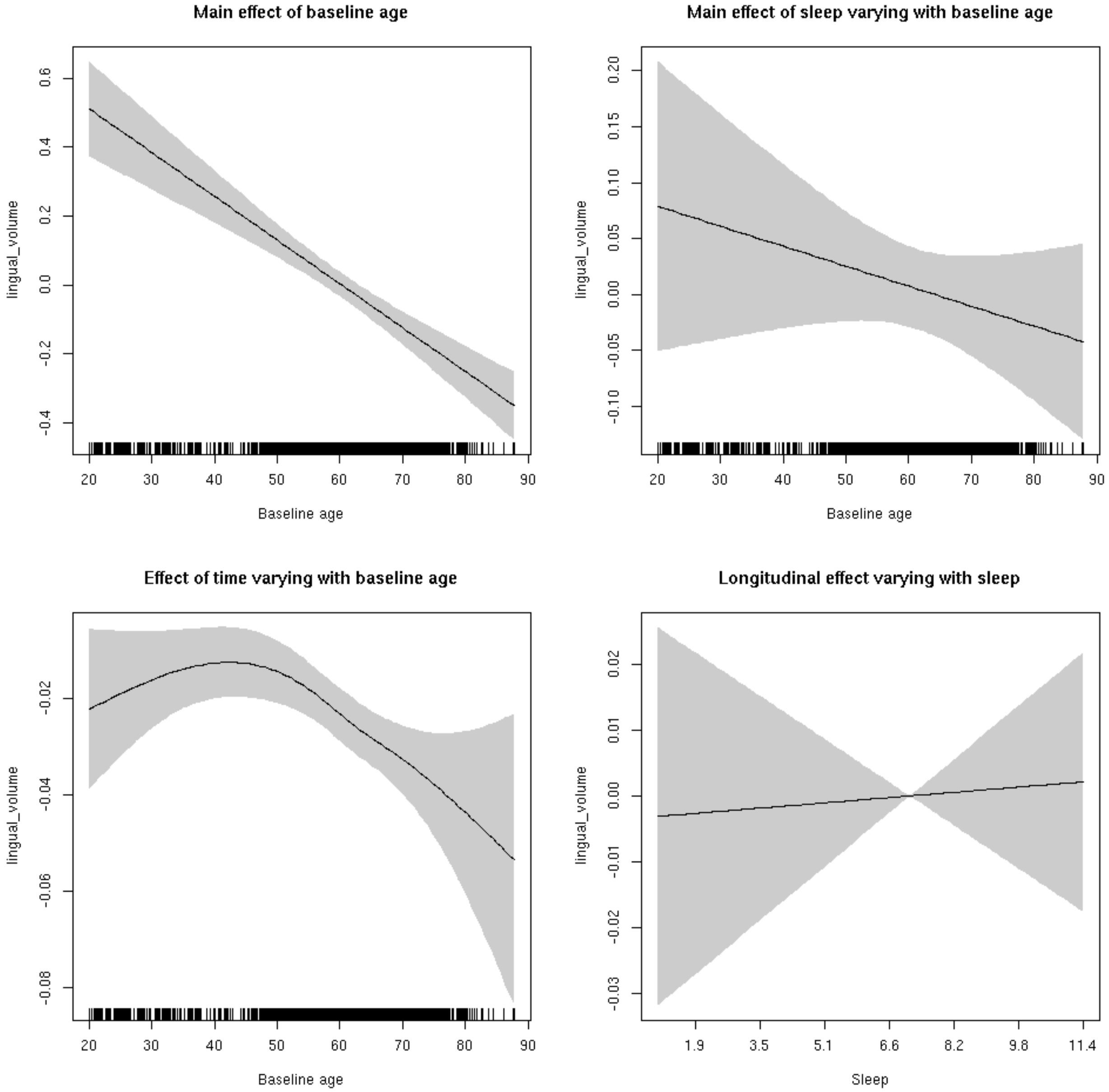

Male: Full data Below is the row for `s(sleep_z):time` from the model output.

```
## s(sleep_z):time   1.000  1.000  0.012   0.913
```

Below are plots of a plot of `s(bl_age)`, `s(bl_age):sleep_z`, `s(bl_age):time`, and `s(sleep_z):time`. For `s(sleep_z):time`, roughly, if we cannot fit a straight line through the confidence bands, there is evidence that the effect of time depends on sleep duration. It is correct the that confidence interval is zero at one point along the axis; this happens because we do not include the main effect.

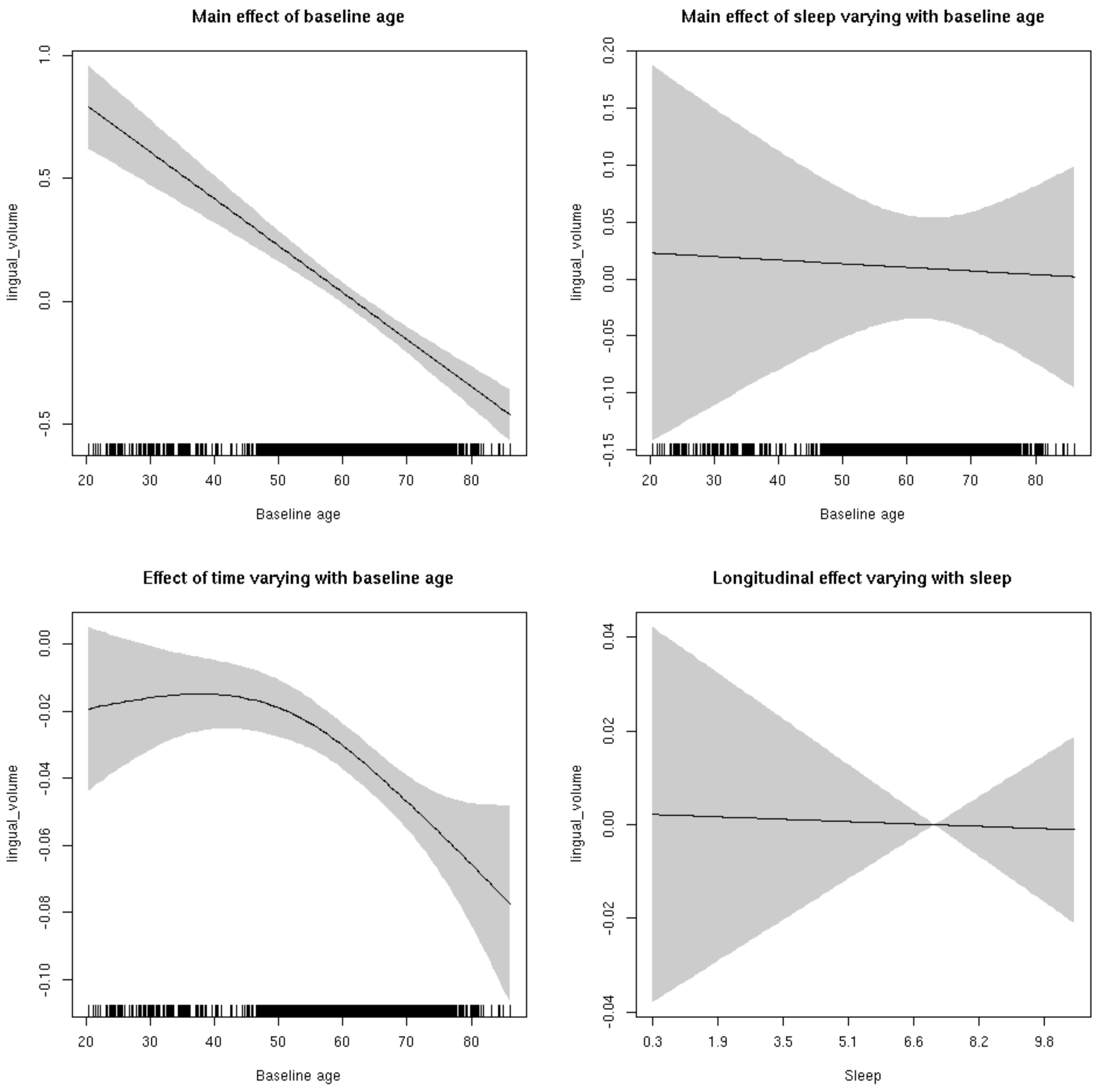

lingual_volume

Full data Below is the model output. The term `s(sleep_z):time` is probably what we care most about.

```
##
## Family: gaussian
## Link function: identity
##
## Formula:
## value ~ sex + site + icv + s(bl_age, k = 10, bs = "cr") + s(bl_age,
##     by = sleep_z, bs = "cr") + +s(bl_age, by = time, k = 5, bs = "cr") +
##     s(sleep_z, by = time, k = 5, bs = "cr", pc = 0)
## <environment: 0x5630035b1ce0>
```

```
##
## Parametric coefficients:
##                  Estimate Std. Error t value Pr(>|t|)
## (Intercept)       0.01533    0.06472   0.237   0.8128
## sexmale           0.19034    0.03318   5.737 9.95e-09 ***
## siteousAvanto -0.39819    0.07897  -5.043 4.69e-07 ***
## siteousPrisma  0.06932    0.18737   0.370   0.7114
## siteousSkyra   0.72423    0.07648   9.470  < 2e-16 ***
## siteUB        -0.09748    0.14871  -0.655   0.5122
## siteUCAM      -0.20378    0.08117  -2.510   0.0121 *
## siteUKB       -0.02567    0.06170  -0.416   0.6774
## siteUmU        0.41082    0.09226   4.453 8.59e-06 ***
## icv            0.36894    0.01633  22.596  < 2e-16 ***
## ---
## Signif. codes:  0 '***' 0.001 '**' 0.01 '*' 0.05 '.' 0.1 ' ' 1
##
## Approximate significance of smooth terms:
##                        edf Ref.df      F p-value
## s(bl_age)            5.920  5.920 27.386  <2e-16 ***
## s(bl_age):sleep_z 2.000  2.000  0.369   0.692
## s(bl_age):time      3.662  3.662 82.966  <2e-16 ***
## s(sleep_z):time     1.000  1.000  0.013   0.908
## ---
## Signif. codes:  0 '***' 0.001 '**' 0.01 '*' 0.05 '.' 0.1 ' ' 1
##
## R-sq.(adj) =  0.285
## lmer.REML =  11862  Scale est. = 0.048636  n = 8176
```

Below are plots of a plot of `s(bl_age)`, `s(bl_age):sleep_z`, `s(bl_age):time`, and `s(sleep_z):time`. For `s(sleep_z):time`, roughly, if we cannot fit a straight line through the confidence bands, there is evidence that the effect of time depends on sleep duration. It is correct the that confidence interval is zero at one point along the axis; this happens because we do not include the main effect.

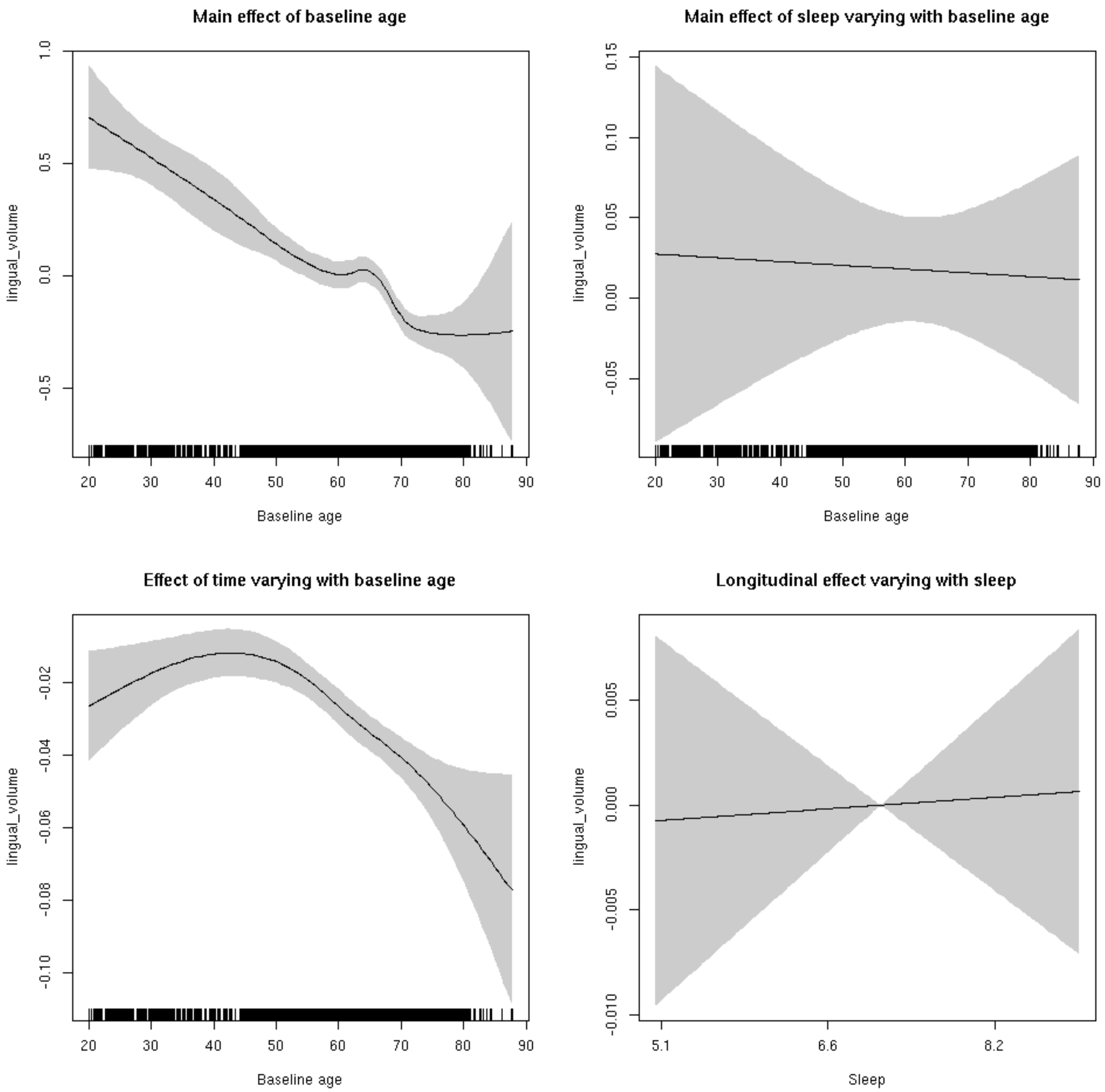

Restricted to sleep >= 5 and <= 9 hours Below is the row for `s(sleep_z):time` from the model output.

```
## s(sleep_z):time   1.000  1.000  0.028   0.867
```

Below are plots of a plot of `s(bl_age)`, `s(bl_age):sleep_z`, `s(bl_age):time`, and `s(sleep_z):time`. For `s(sleep_z):time`, roughly, if we cannot fit a straight line through the confidence bands, there is evidence that the effect of time depends on sleep duration. It is correct the that confidence interval is zero at one point along the axis; this happens because we do not include the main effect.

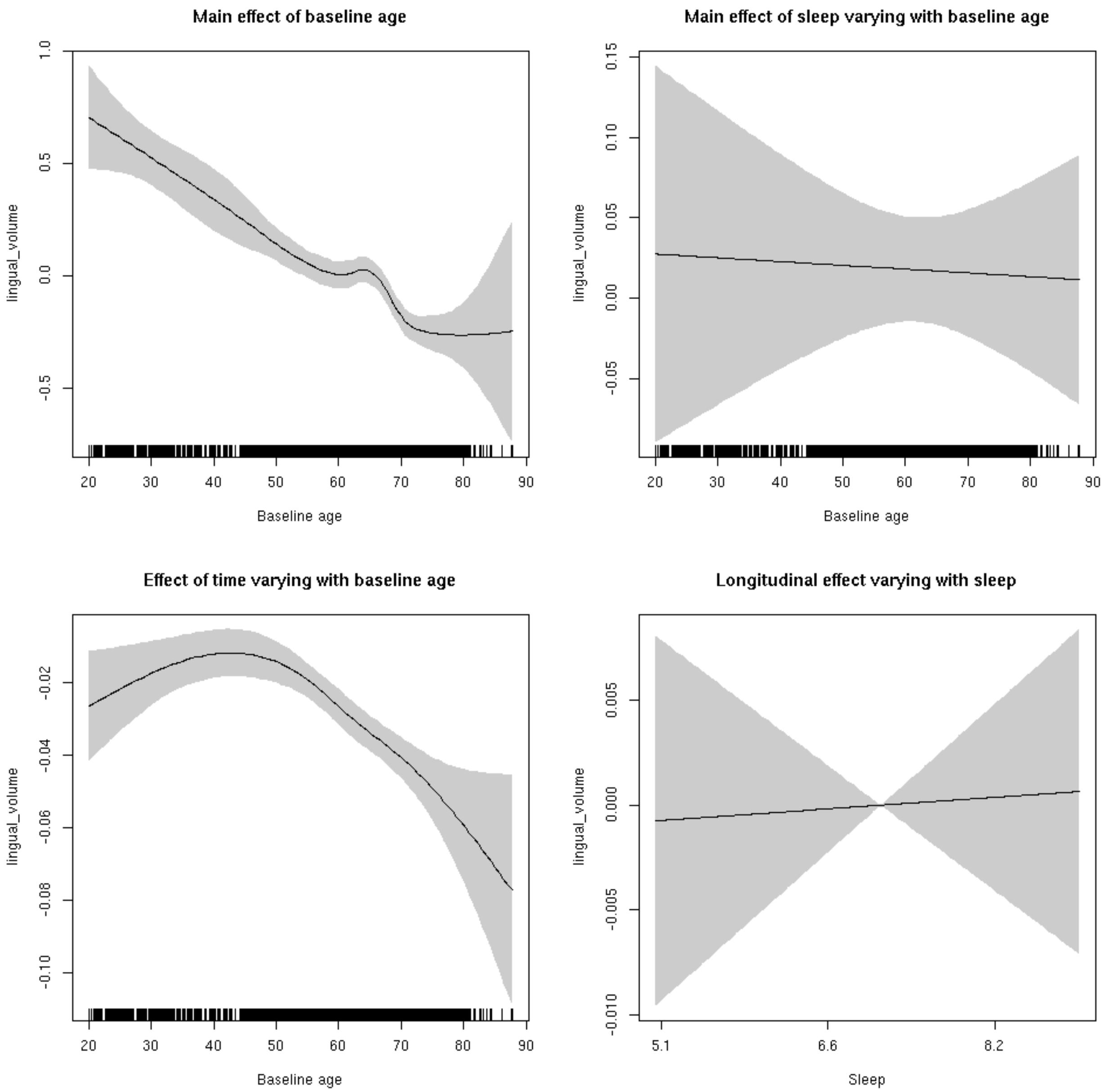

medialorbitofrontal_area: Stratified by sex

Female: Full data Below is the row for `s(sleep_z):time` from the model output.

```
## s(sleep_z):time   1.000  1.000  3.842  0.0501 .
```

Below are plots of a plot of `s(bl_age)`, `s(bl_age):sleep_z`, `s(bl_age):time`, and `s(sleep_z):time`. For `s(sleep_z):time`, roughly, if we cannot fit a straight line through the confidence bands, there is evidence that the effect of time depends on sleep duration. It is correct the that confidence interval is zero at one point along the axis; this happens because we do not include the main effect.

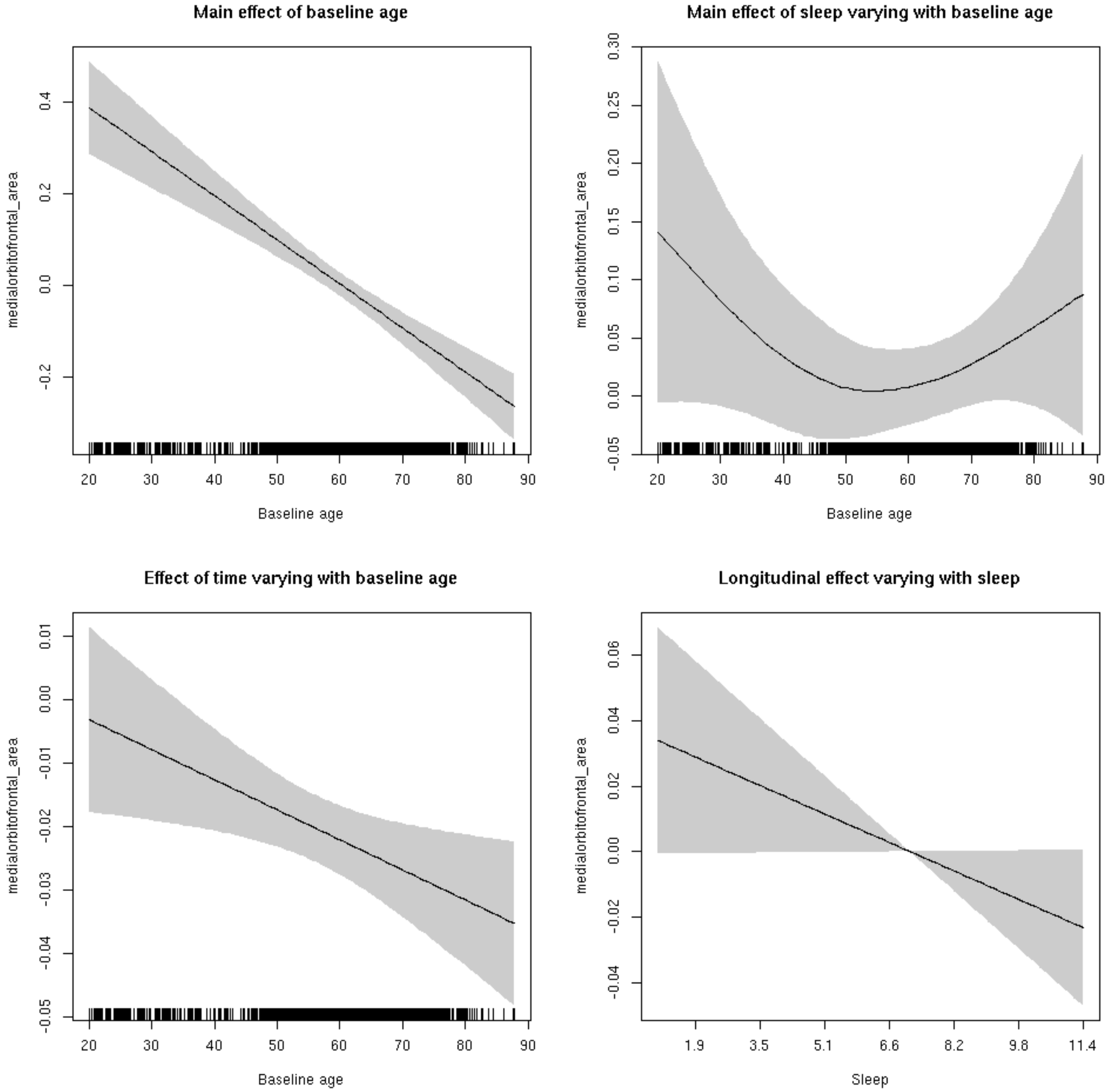

Male: Full data Below is the row for `s(sleep_z):time` from the model output.

```
## s(sleep_z):time   1.000  1.000  0.301  0.5834
```

Below are plots of a plot of `s(bl_age)`, `s(bl_age):sleep_z`, `s(bl_age):time`, and `s(sleep_z):time`. For `s(sleep_z):time`, roughly, if we cannot fit a straight line through the confidence bands, there is evidence that the effect of time depends on sleep duration. It is correct the that confidence interval is zero at one point along the axis; this happens because we do not include the main effect.

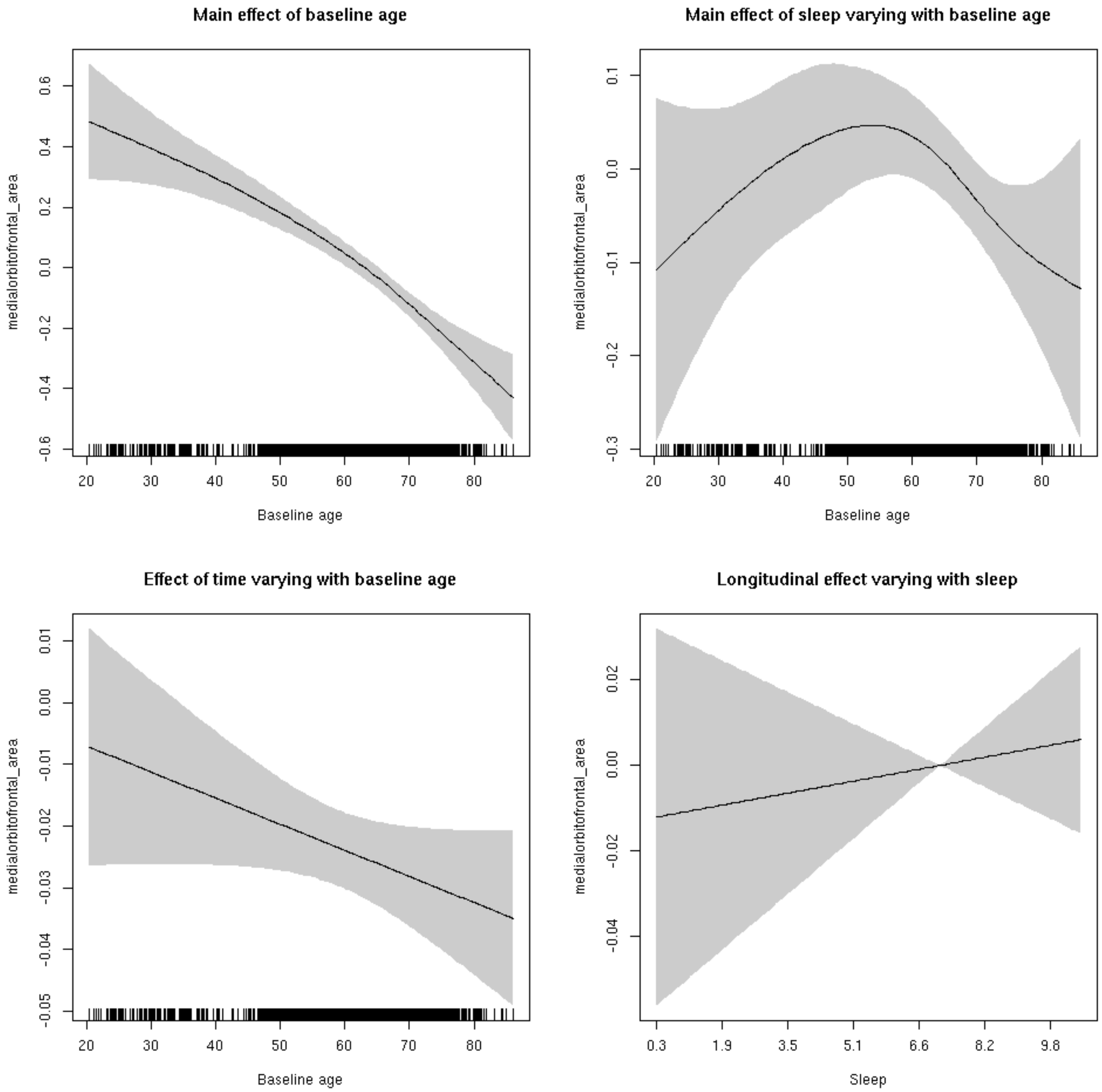

medialorbitofrontal_area

Full data Below is the model output. The term `s(sleep_z):time` is probably what we care most about.

```
##
## Family: gaussian
## Link function: identity
##
## Formula:
## value ~ sex + site + icv + s(bl_age, k = 10, bs = "cr") + s(bl_age,
##     by = sleep_z, bs = "cr") + +s(bl_age, by = time, k = 5, bs = "cr") +
##     s(sleep_z, by = time, k = 5, bs = "cr", pc = 0)
## <environment: 0x5630035ac840>
```

```
##
## Parametric coefficients:
##                Estimate Std. Error t value Pr(>|t|)
## (Intercept)     0.54567    0.04624  11.800  < 2e-16 ***
## sexmale         0.22432    0.02374   9.447  < 2e-16 ***
## siteousAvanto   0.09070    0.05712   1.588  0.11232
## siteousPrisma -0.86062    0.20192  -4.262 2.05e-05 ***
## siteousSkyra    0.50028    0.05526   9.053  < 2e-16 ***
## siteUB         -0.32091    0.10532  -3.047  0.00232 **
## siteUCAM       -0.40907    0.05715  -7.157 8.94e-13 ***
## siteUKB        -0.65163    0.04378 -14.885  < 2e-16 ***
## siteUmU         0.38995    0.06581   5.925 3.25e-09 ***
## icv             0.64373    0.01176  54.738  < 2e-16 ***
## ---
## Signif. codes:  0 '***' 0.001 '**' 0.01 '*' 0.05 '.' 0.1 ' ' 1
##
## Approximate significance of smooth terms:
##                       edf Ref.df      F p-value
## s(bl_age)           2.777  2.777 58.157  <2e-16 ***
## s(bl_age):sleep_z 2.000  2.000  0.929   0.395
## s(bl_age):time      2.000  2.000 62.084  <2e-16 ***
## s(sleep_z):time     1.000  1.000  0.997   0.318
## ---
## Signif. codes:  0 '***' 0.001 '**' 0.01 '*' 0.05 '.' 0.1 ' ' 1
##
## R-sq.(adj) =  0.632
## lmer.REML =  10559  Scale est. = 0.066523  n = 8178
```

Below are plots of a plot of `s(bl_age)`, `s(bl_age):sleep_z`, `s(bl_age):time`, and `s(sleep_z):time`. For `s(sleep_z):time`, roughly, if we cannot fit a straight line through the confidence bands, there is evidence that the effect of time depends on sleep duration. It is correct the that confidence interval is zero at one point along the axis; this happens because we do not include the main effect.

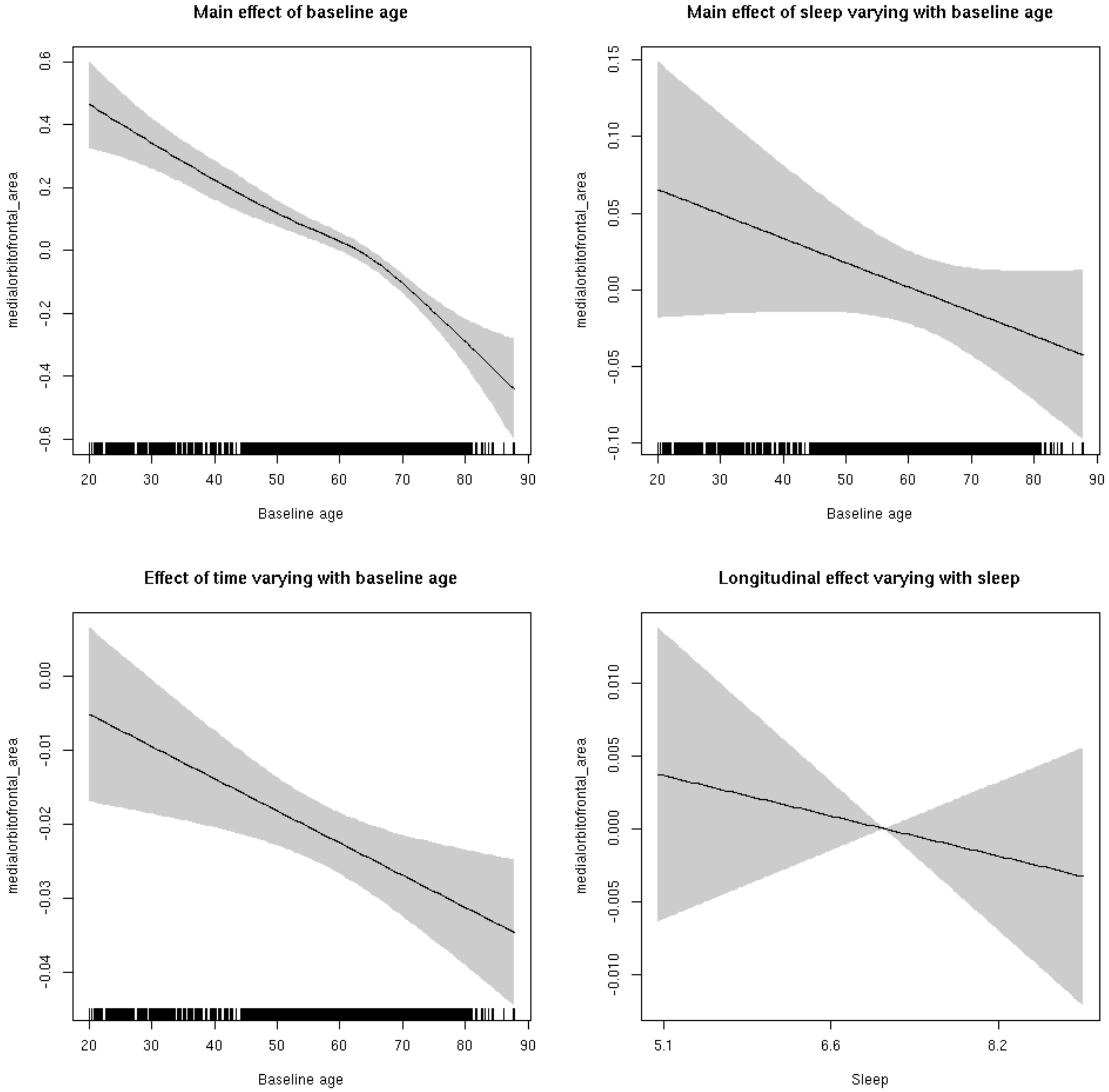

Restricted to sleep >= 5 and <= 9 hours Below is the row for `s(sleep_z):time` from the model output.

```
## s(sleep_z):time   1.000  1.000  0.547   0.460
```

Below are plots of a plot of `s(bl_age)`, `s(bl_age):sleep_z`, `s(bl_age):time`, and `s(sleep_z):time`. For `s(sleep_z):time`, roughly, if we cannot fit a straight line through the confidence bands, there is evidence that the effect of time depends on sleep duration. It is correct the that confidence interval is zero at one point along the axis; this happens because we do not include the main effect.

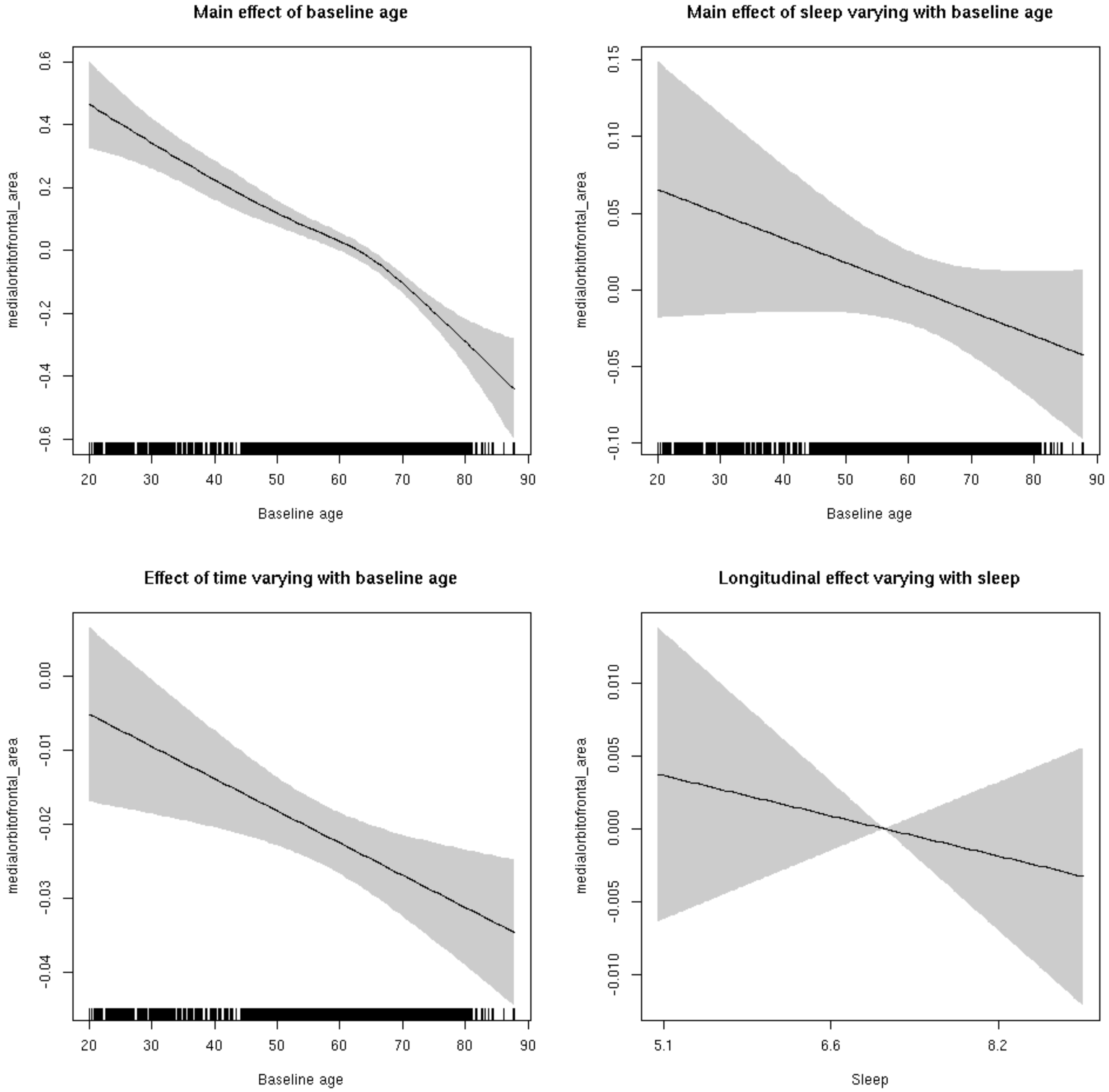

medialorbitofrontal_thickness: Stratified by sex

Female: Full data Below is the row for `s(sleep_z):time` from the model output.

```
## s(sleep_z):time   1.000  1.000  0.468   0.494
```

Below are plots of a plot of `s(bl_age)`, `s(bl_age):sleep_z`, `s(bl_age):time`, and `s(sleep_z):time`. For `s(sleep_z):time`, roughly, if we cannot fit a straight line through the confidence bands, there is evidence that the effect of time depends on sleep duration. It is correct the that confidence interval is zero at one point along the axis; this happens because we do not include the main effect.

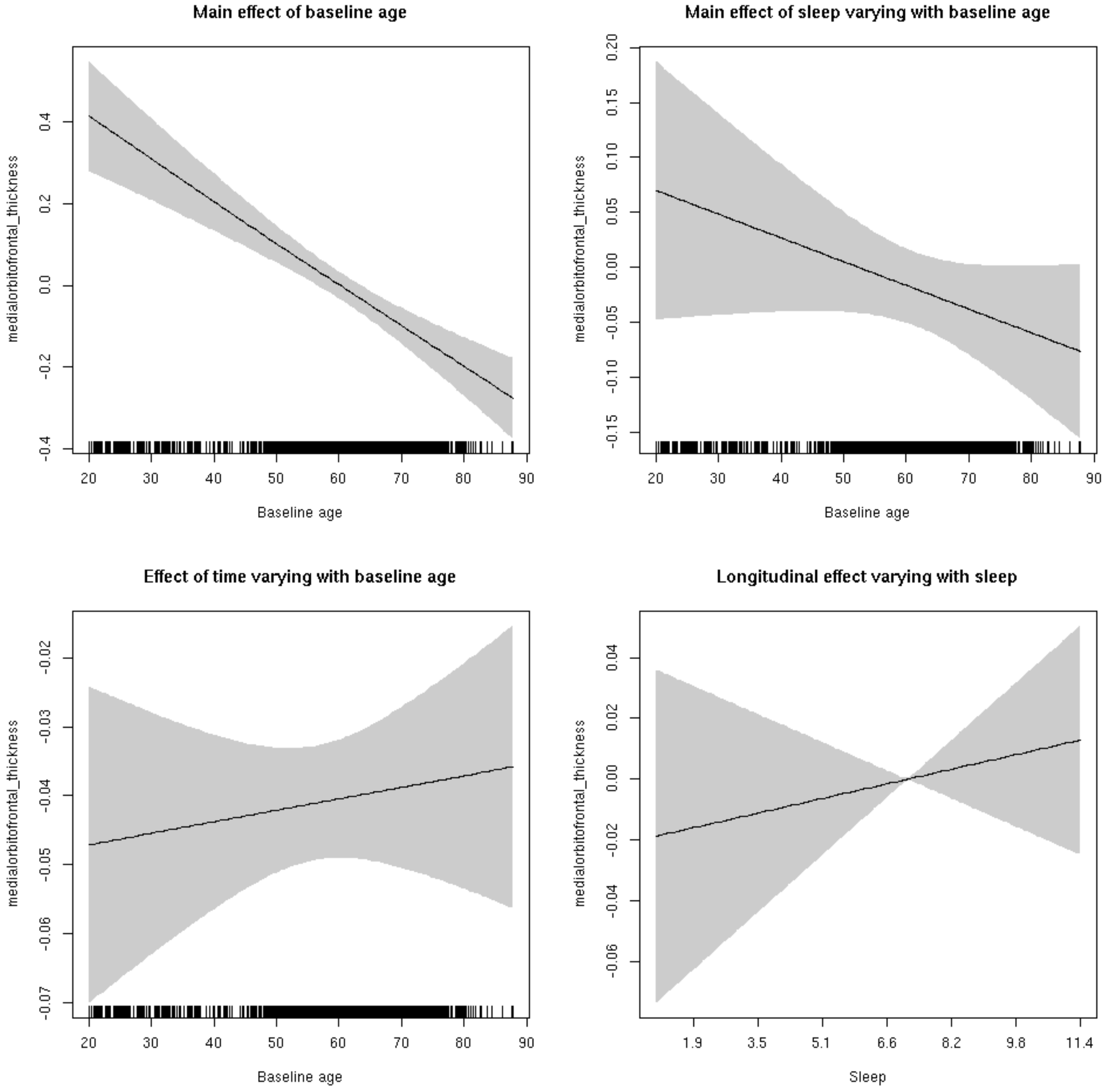

Male: Full data Below is the row for `s(sleep_z):time` from the model output.

```
## s(sleep_z):time   1.000  1.000  0.209   0.647
```

Below are plots of a plot of `s(bl_age)`, `s(bl_age):sleep_z`, `s(bl_age):time`, and `s(sleep_z):time`. For `s(sleep_z):time`, roughly, if we cannot fit a straight line through the confidence bands, there is evidence that the effect of time depends on sleep duration. It is correct the that confidence interval is zero at one point along the axis; this happens because we do not include the main effect.

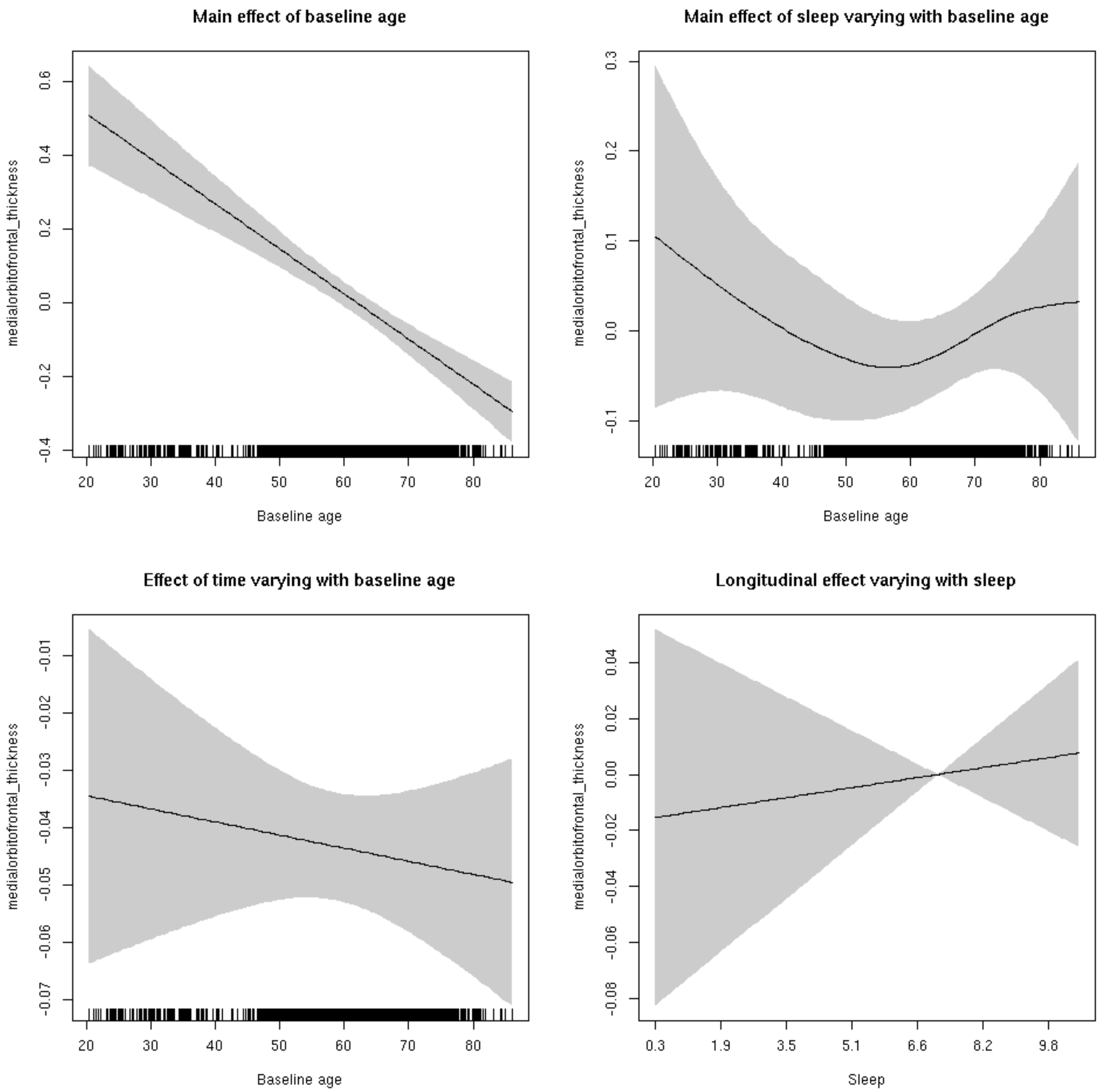

medialorbitofrontal_thickness

Full data Below is the model output. The term `s(sleep_z):time` is probably what we care most about.

```
##
## Family: gaussian
## Link function: identity
##
## Formula:
## value ~ sex + site + s(bl_age, k = 10, bs = "cr") + s(bl_age,
##     by = sleep_z, bs = "cr") + +s(bl_age, by = time, k = 5, bs = "cr") +
##     s(sleep_z, by = time, k = 5, bs = "cr", pc = 0)
## <environment: 0x5630035d88f8>
```

```
##
## Parametric coefficients:
##                Estimate Std. Error t value Pr(>|t|)
## (Intercept)   -1.87753    0.04471 -41.993  < 2e-16 ***
## sexmale        0.09978    0.02296   4.345 1.41e-05 ***
## siteousAvanto  0.08178    0.06140   1.332    0.183
## siteousPrisma  0.40723    0.30136   1.351    0.177
## siteousSkyra   0.06700    0.06124   1.094    0.274
## siteUB         0.49283    0.12054   4.088 4.39e-05 ***
## siteUCAM       0.28051    0.06219   4.510 6.56e-06 ***
## siteUKB        2.10031    0.04427  47.447  < 2e-16 ***
## siteUmU        0.44557    0.07501   5.940 2.96e-09 ***
## ---
## Signif. codes:  0 '***' 0.001 '**' 0.01 '*' 0.05 '.' 0.1 ' ' 1
##
## Approximate significance of smooth terms:
##                    edf Ref.df       F p-value
## s(bl_age)            1      1 102.183  <2e-16 ***
## s(bl_age):sleep_z    2      2   1.729   0.178
## s(bl_age):time       2      2  91.734  <2e-16 ***
## s(sleep_z):time      1      1   0.513   0.474
## ---
## Signif. codes:  0 '***' 0.001 '**' 0.01 '*' 0.05 '.' 0.1 ' ' 1
##
## R-sq.(adj) =   0.58
## lmer.REML =  15601  Scale est. = 0.16331   n = 8172
```

Below are plots of a plot of `s(bl_age)`, `s(bl_age):sleep_z`, `s(bl_age):time`, and `s(sleep_z):time`. For `s(sleep_z):time`, roughly, if we cannot fit a straight line through the confidence bands, there is evidence that the effect of time depends on sleep duration. It is correct the that confidence interval is zero at one point along the axis; this happens because we do not include the main effect.

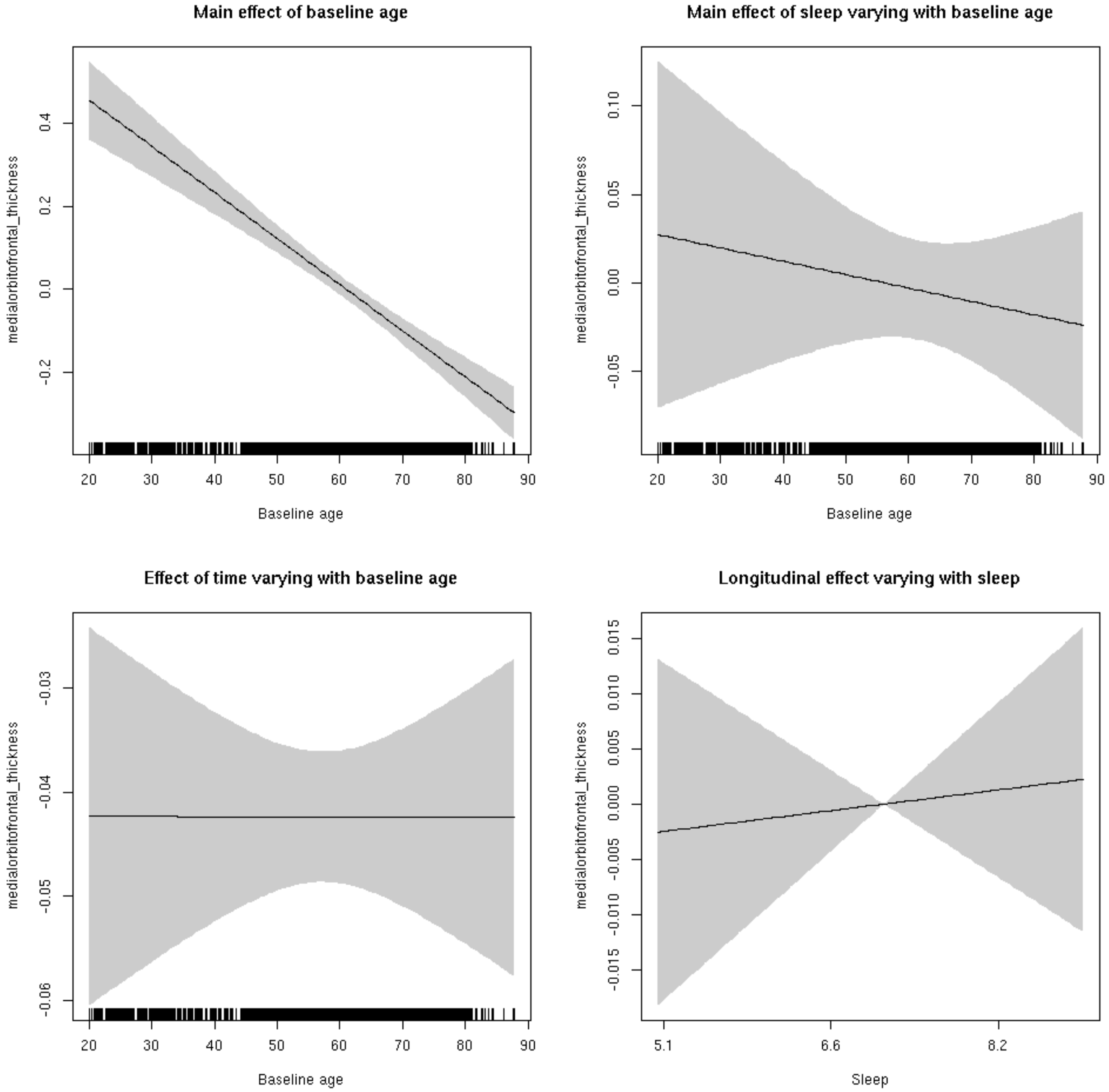

Restricted to sleep >= 5 and <= 9 hours Below is the row for `s(sleep_z):time` from the model output.

```
## s(sleep_z):time     1       1  0.105   0.746
```

Below are plots of a plot of `s(bl_age)`, `s(bl_age):sleep_z`, `s(bl_age):time`, and `s(sleep_z):time`. For `s(sleep_z):time`, roughly, if we cannot fit a straight line through the confidence bands, there is evidence that the effect of time depends on sleep duration. It is correct the that confidence interval is zero at one point along the axis; this happens because we do not include the main effect.

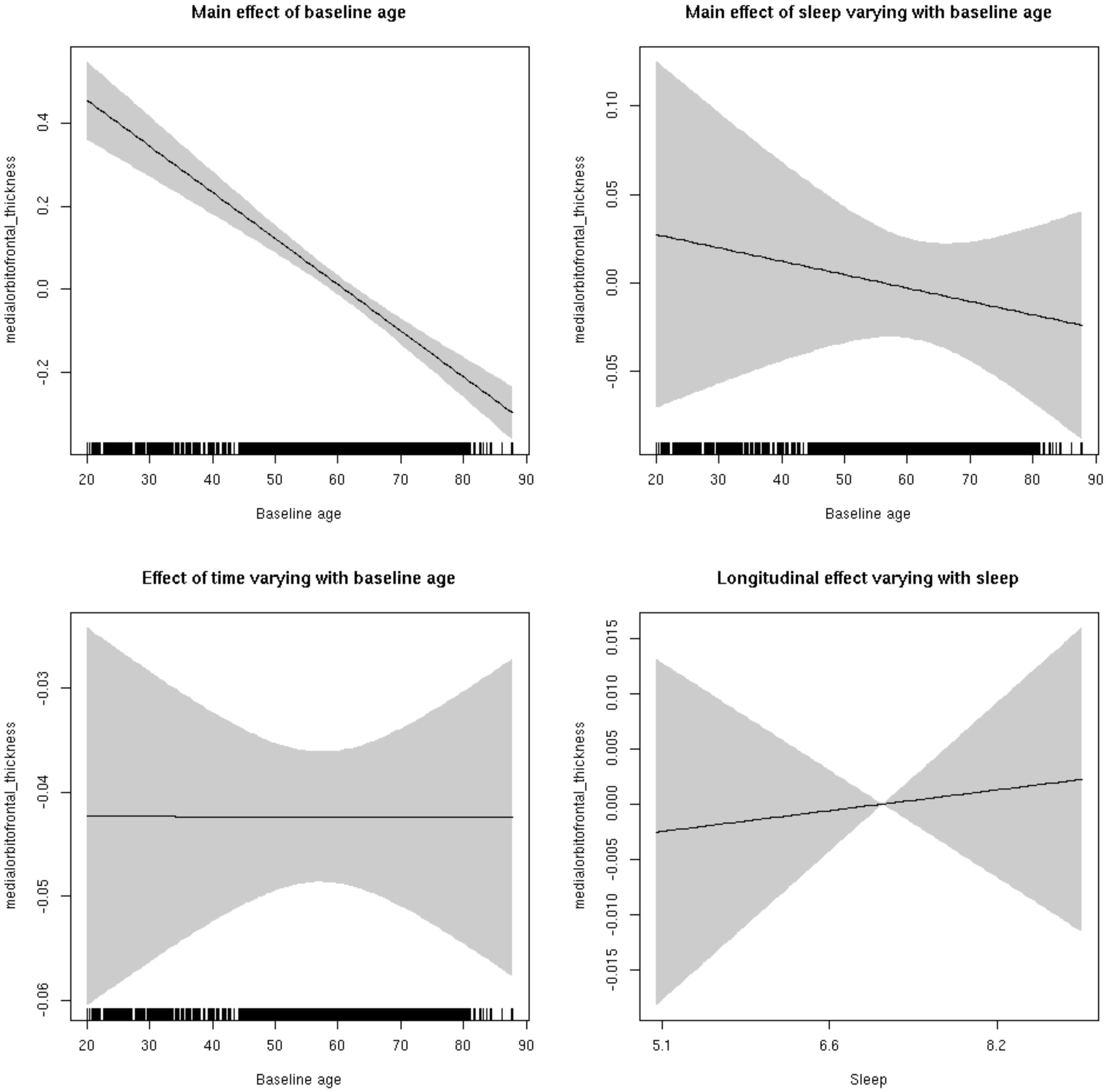

medialorbitofrontal_volume: Stratified by sex

Female: Full data Below is the row for `s(sleep_z):time` from the model output.

```
## s(sleep_z):time   1.000  1.000   0.487   0.485
```

Below are plots of a plot of `s(bl_age)`, `s(bl_age):sleep_z`, `s(bl_age):time`, and `s(sleep_z):time`. For `s(sleep_z):time`, roughly, if we cannot fit a straight line through the confidence bands, there is evidence that the effect of time depends on sleep duration. It is correct the that confidence interval is zero at one point along the axis; this happens because we do not include the main effect.

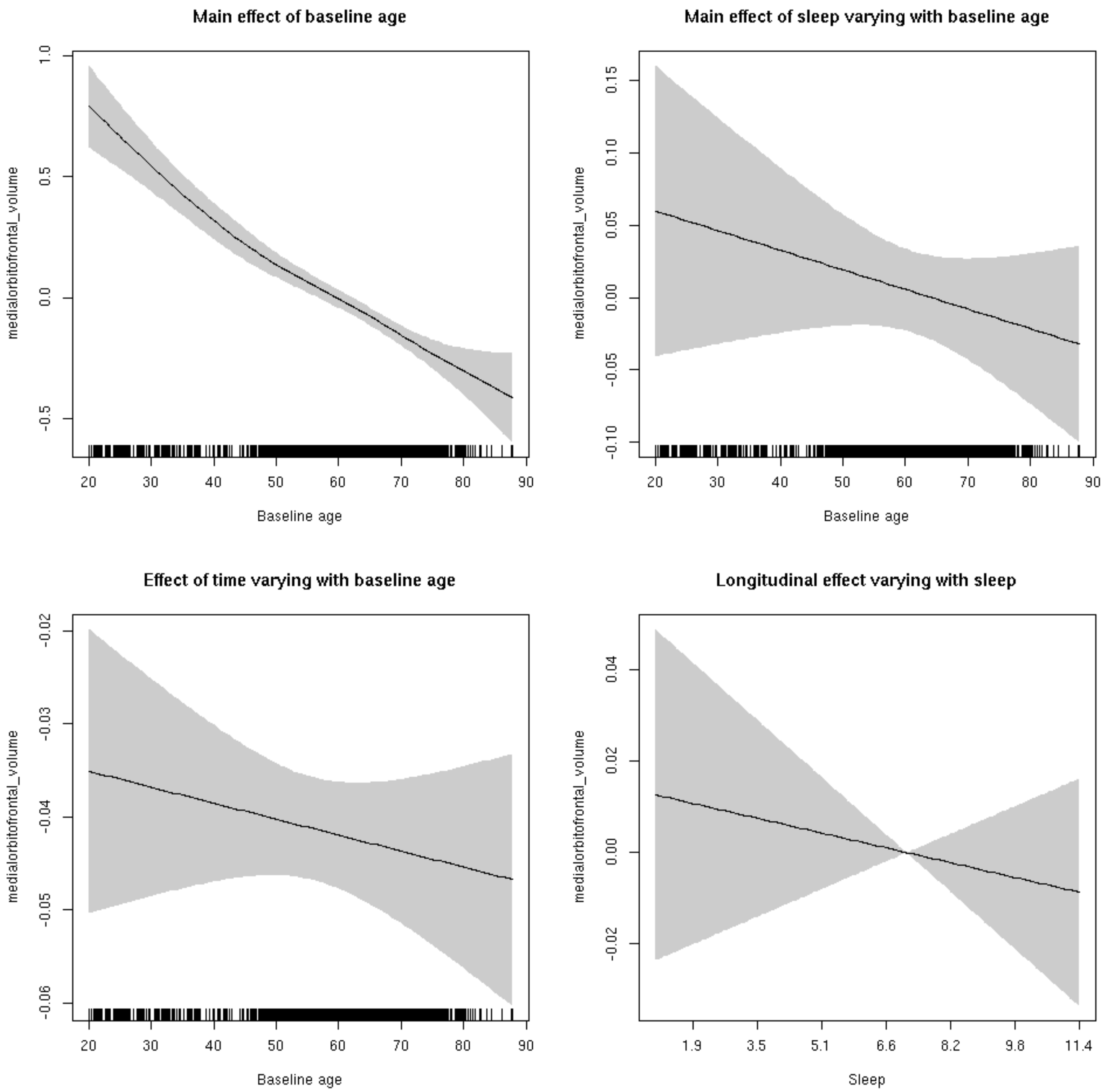

Male: Full data Below is the row for `s(sleep_z):time` from the model output.

```
## s(sleep_z):time   2.699  2.699   1.490   0.134
```

Below are plots of a plot of `s(bl_age)`, `s(bl_age):sleep_z`, `s(bl_age):time`, and `s(sleep_z):time`. For `s(sleep_z):time`, roughly, if we cannot fit a straight line through the confidence bands, there is evidence that the effect of time depends on sleep duration. It is correct the that confidence interval is zero at one point along the axis; this happens because we do not include the main effect.

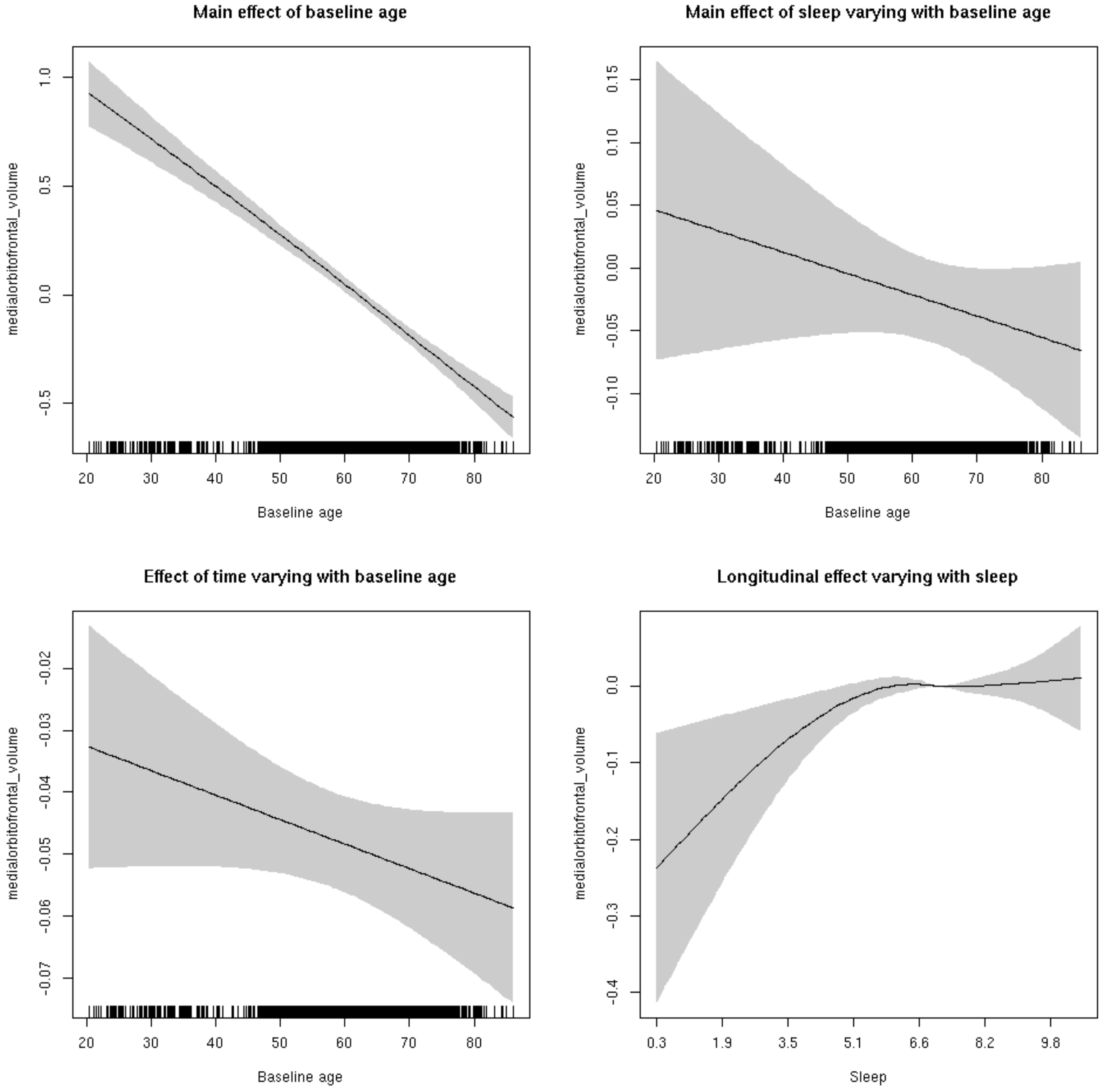

medialorbitofrontal__volume

Full data Below is the model output. The term `s(sleep_z):time` is probably what we care most about.

```
##
## Family: gaussian
## Link function: identity
##
## Formula:
## value ~ sex + site + icv + s(bl_age, k = 10, bs = "cr") + s(bl_age,
##     by = sleep_z, bs = "cr") + +s(bl_age, by = time, k = 5, bs = "cr") +
##     s(sleep_z, by = time, k = 5, bs = "cr", pc = 0)
## <environment: 0x5630035ac418>
```

```
##
## Parametric coefficients:
##                Estimate Std. Error t value Pr(>|t|)
## (Intercept)   -0.68288    0.04775 -14.302  < 2e-16 ***
## sexmale        0.25063    0.02475  10.127  < 2e-16 ***
## siteousAvanto -0.16002    0.05951  -2.689  0.00718 **
## siteousPrisma -0.39236    0.20761  -1.890  0.05881 .
## siteousSkyra   0.27655    0.05750   4.809 1.54e-06 ***
## siteUB        -0.07246    0.10976  -0.660  0.50916
## siteUCAM      -0.29295    0.05929  -4.941 7.93e-07 ***
## siteUKB        0.78923    0.04442  17.769  < 2e-16 ***
## siteUmU        0.56902    0.06832   8.329  < 2e-16 ***
## icv            0.54327    0.01225  44.336  < 2e-16 ***
## ---
## Signif. codes:  0 ‘***’ 0.001 ‘**’ 0.01 ‘*’ 0.05 ‘.’ 0.1 ‘ ’ 1
##
## Approximate significance of smooth terms:
##                    edf Ref.df       F p-value
## s(bl_age)            1      1 418.674  <2e-16 ***
## s(bl_age):sleep_z    2      2   2.846  0.0581 .
## s(bl_age):time       2      2 233.593  <2e-16 ***
## s(sleep_z):time      1      1   0.059  0.8085
## ---
## Signif. codes:  0 ‘***’ 0.001 ‘**’ 0.01 ‘*’ 0.05 ‘.’ 0.1 ‘ ’ 1
##
## R-sq.(adj) =  0.599
## lmer.REML =  11101  Scale est. = 0.070002  n = 8174
```

Below are plots of a plot of `s(bl_age)`, `s(bl_age):sleep_z`, `s(bl_age):time`, and `s(sleep_z):time`. For `s(sleep_z):time`, roughly, if we cannot fit a straight line through the confidence bands, there is evidence that the effect of time depends on sleep duration. It is correct the that confidence interval is zero at one point along the axis; this happens because we do not include the main effect.

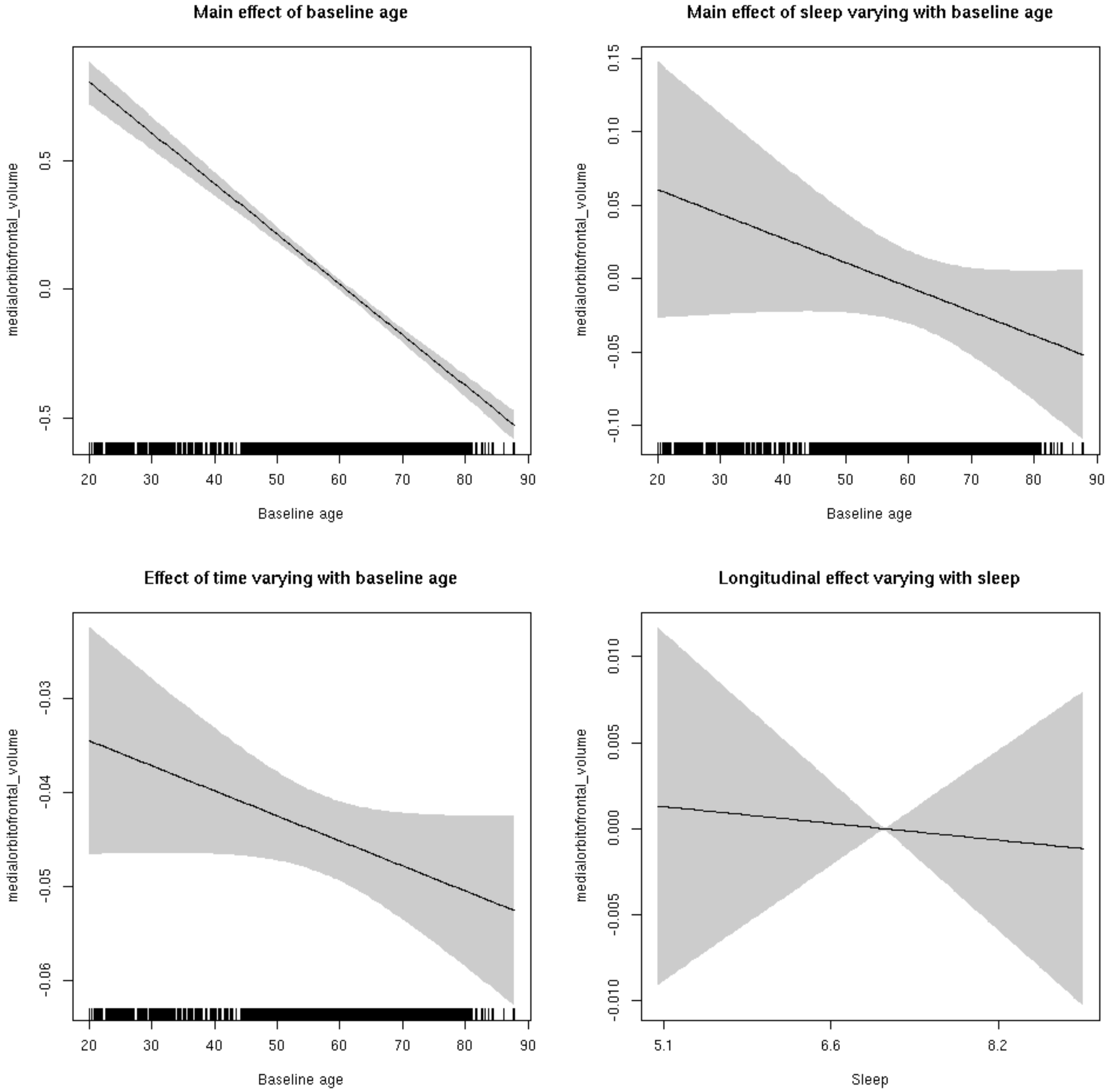

Restricted to sleep >= 5 and <= 9 hours Below is the row for `s(sleep_z):time` from the model output.

```
## s(sleep_z):time     1       1   0.064      0.8
```

Below are plots of a plot of `s(bl_age)`, `s(bl_age):sleep_z`, `s(bl_age):time`, and `s(sleep_z):time`. For `s(sleep_z):time`, roughly, if we cannot fit a straight line through the confidence bands, there is evidence that the effect of time depends on sleep duration. It is correct the that confidence interval is zero at one point along the axis; this happens because we do not include the main effect.

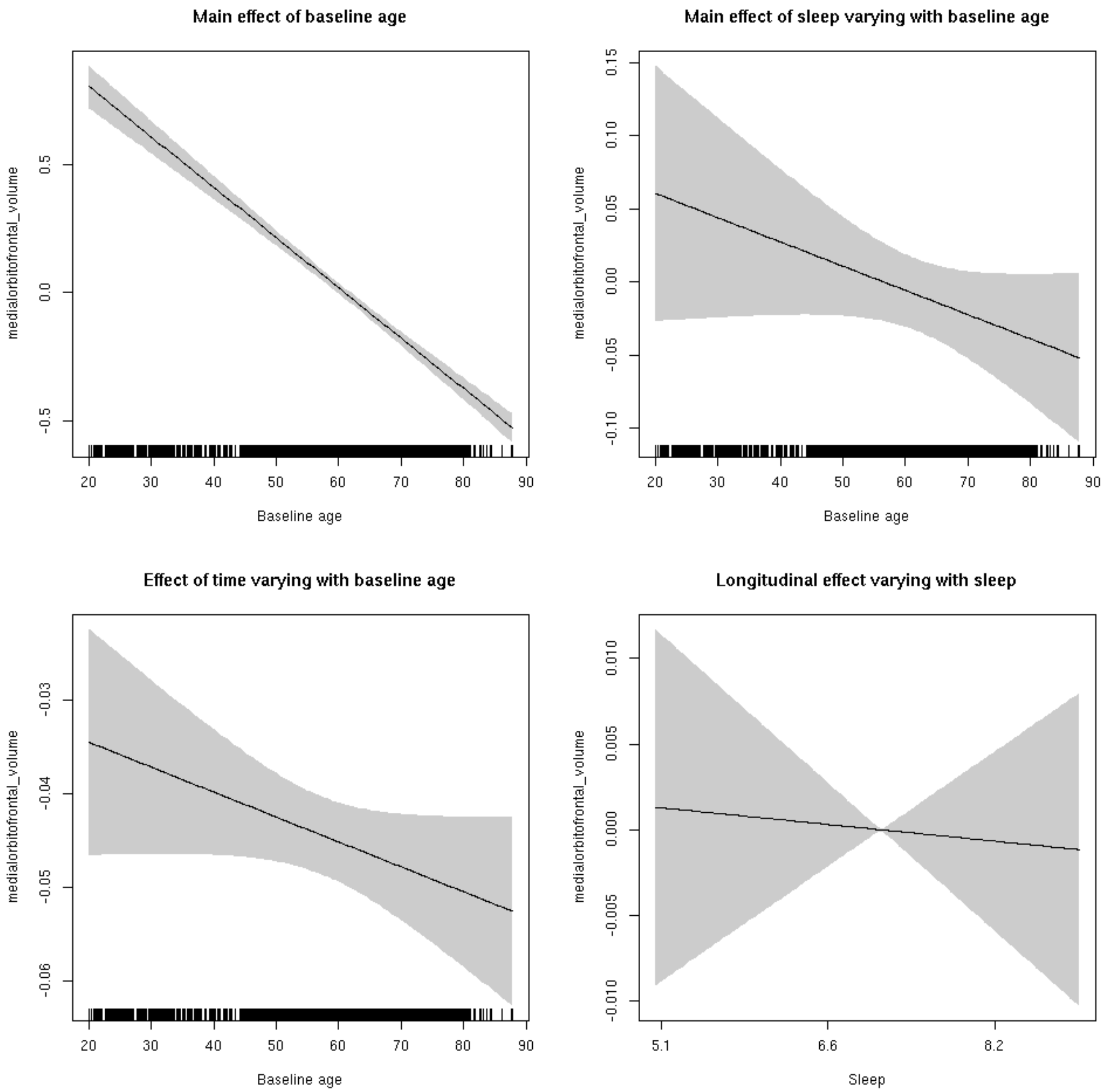

middletemporal_area: Stratified by sex

Female: Full data Below is the row for `s(sleep_z):time` from the model output.

```
## s(sleep_z):time   1.000  1.000   1.223   0.269
```

Below are plots of a plot of `s(bl_age)`, `s(bl_age):sleep_z`, `s(bl_age):time`, and `s(sleep_z):time`. For `s(sleep_z):time`, roughly, if we cannot fit a straight line through the confidence bands, there is evidence that the effect of time depends on sleep duration. It is correct the that confidence interval is zero at one point along the axis; this happens because we do not include the main effect.

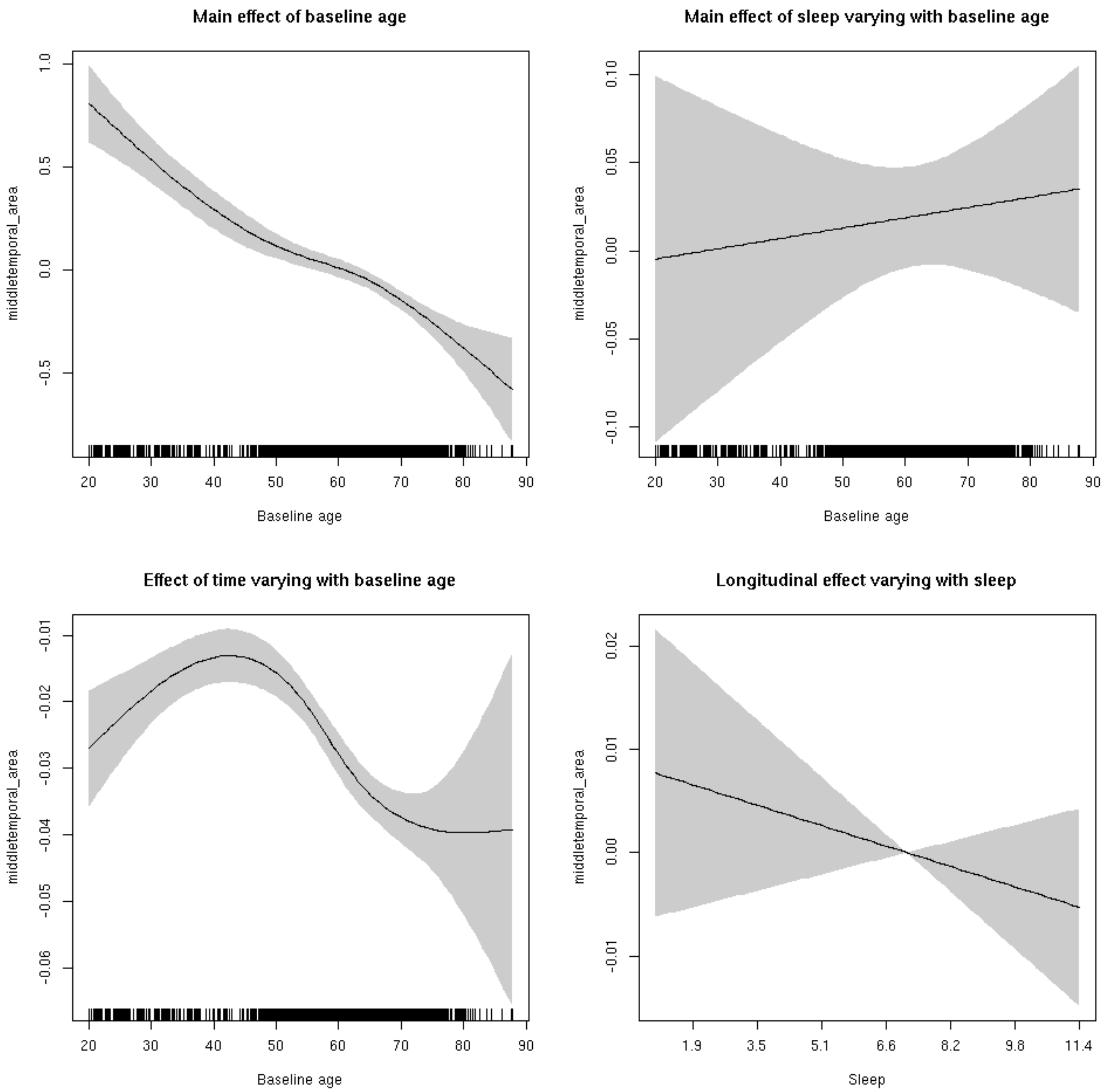

Male: Full data Below is the row for `s(sleep_z):time` from the model output.

```
## s(sleep_z):time   1.000  1.000   1.247   0.264
```

Below are plots of a plot of `s(bl_age)`, `s(bl_age):sleep_z`, `s(bl_age):time`, and `s(sleep_z):time`. For `s(sleep_z):time`, roughly, if we cannot fit a straight line through the confidence bands, there is evidence that the effect of time depends on sleep duration. It is correct the that confidence interval is zero at one point along the axis; this happens because we do not include the main effect.

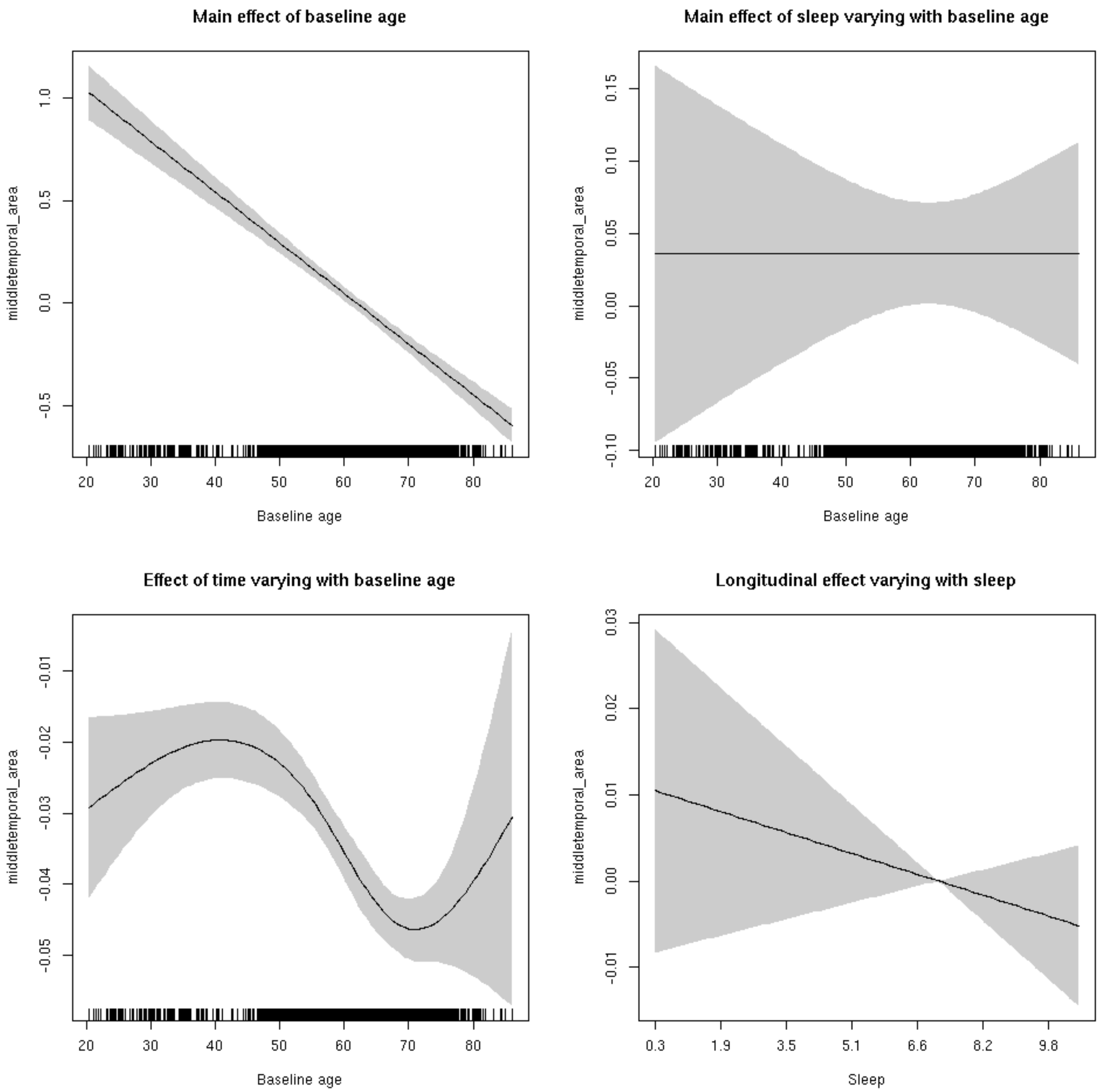

middletemporal_area

Full data Below is the model output. The term `s(sleep_z):time` is probably what we care most about.

```
##
## Family: gaussian
## Link function: identity
##
## Formula:
## value ~ sex + site + icv + s(bl_age, k = 10, bs = "cr") + s(bl_age,
##     by = sleep_z, bs = "cr") + +s(bl_age, by = time, k = 5, bs = "cr") +
##     s(sleep_z, by = time, k = 5, bs = "cr", pc = 0)
## <environment: 0x5630035abbc8>
```

```
##
## Parametric coefficients:
##                Estimate Std. Error t value Pr(>|t|)
## (Intercept)     0.83524    0.05104  16.363  < 2e-16 ***
## sexmale         0.21346    0.02621   8.144 4.40e-16 ***
## siteousAvanto -0.68254    0.06220 -10.974  < 2e-16 ***
## siteousPrisma -1.12136    0.10292 -10.896  < 2e-16 ***
## siteousSkyra  -0.17257    0.06030  -2.862  0.00422 **
## siteUB        -0.87933    0.11863  -7.412 1.37e-13 ***
## siteUCAM      -0.79598    0.06368 -12.500  < 2e-16 ***
## siteUKB       -0.91577    0.04863 -18.832  < 2e-16 ***
## siteUmU       -0.06206    0.07330  -0.847  0.39718
## icv            0.58613    0.01266  46.312  < 2e-16 ***
## ---
## Signif. codes:  0 ‘***’ 0.001 ‘**’ 0.01 ‘*’ 0.05 ‘.’ 0.1 ‘ ’ 1
##
## Approximate significance of smooth terms:
##                      edf Ref.df       F p-value
## s(bl_age)          3.225  3.225 128.564  <2e-16 ***
## s(bl_age):sleep_z 2.000  2.000   2.236   0.107
## s(bl_age):time    4.475  4.475 335.390  <2e-16 ***
## s(sleep_z):time   1.000  1.000   2.627   0.105
## ---
## Signif. codes:  0 ‘***’ 0.001 ‘**’ 0.01 ‘*’ 0.05 ‘.’ 0.1 ‘ ’ 1
##
## R-sq.(adj) =  0.563
## lmer.REML = 3663.1  Scale est. = 0.01082   n = 8164
```

Below are plots of a plot of `s(bl_age)`, `s(bl_age):sleep_z`, `s(bl_age):time`, and `s(sleep_z):time`. For `s(sleep_z):time`, roughly, if we cannot fit a straight line through the confidence bands, there is evidence that the effect of time depends on sleep duration. It is correct the that confidence interval is zero at one point along the axis; this happens because we do not include the main effect.

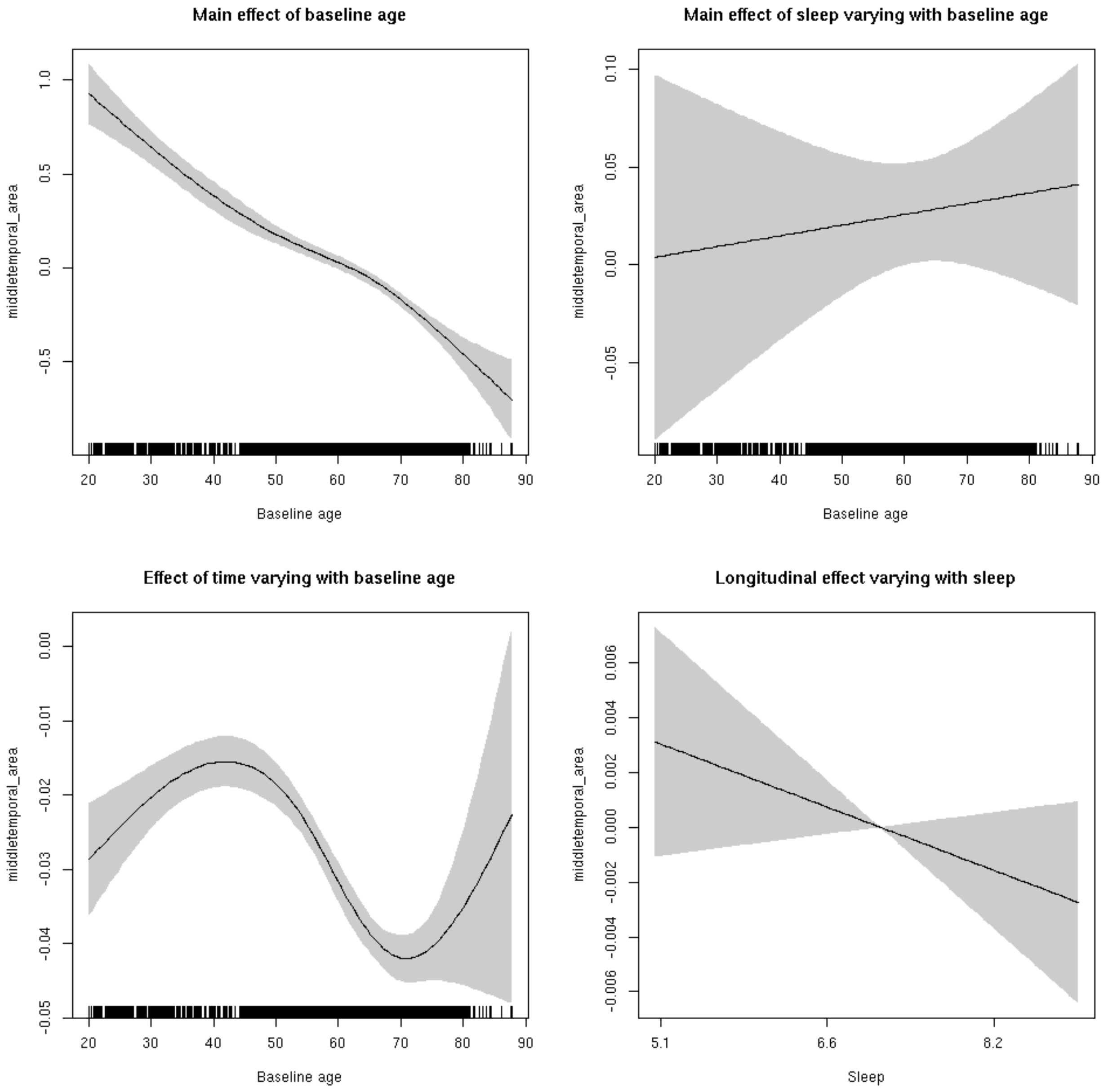

Restricted to sleep >= 5 and <= 9 hours Below is the row for `s(sleep_z):time` from the model output.

```
## s(sleep_z):time   1.000  1.000   2.209   0.137
```

Below are plots of a plot of `s(bl_age)`, `s(bl_age):sleep_z`, `s(bl_age):time`, and `s(sleep_z):time`. For `s(sleep_z):time`, roughly, if we cannot fit a straight line through the confidence bands, there is evidence that the effect of time depends on sleep duration. It is correct the that confidence interval is zero at one point along the axis; this happens because we do not include the main effect.

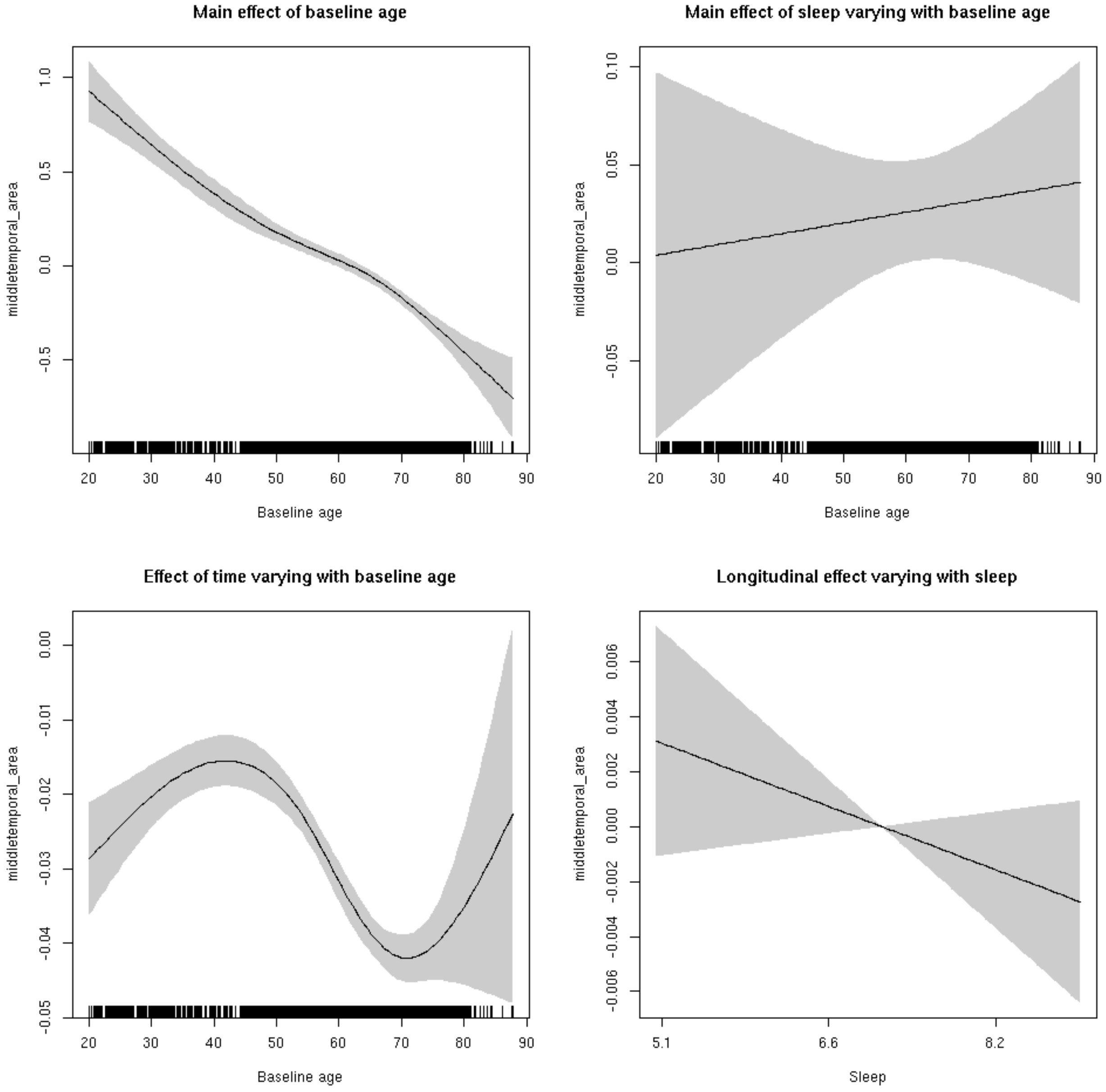

middletemporal_thickness: Stratified by sex

Female: Full data Below is the row for `s(sleep_z):time` from the model output.

```
## s(sleep_z):time   1.000  1.000  3.034  0.0816 .
```

Below are plots of a plot of `s(bl_age)`, `s(bl_age):sleep_z`, `s(bl_age):time`, and `s(sleep_z):time`. For `s(sleep_z):time`, roughly, if we cannot fit a straight line through the confidence bands, there is evidence that the effect of time depends on sleep duration. It is correct the that confidence interval is zero at one point along the axis; this happens because we do not include the main effect.

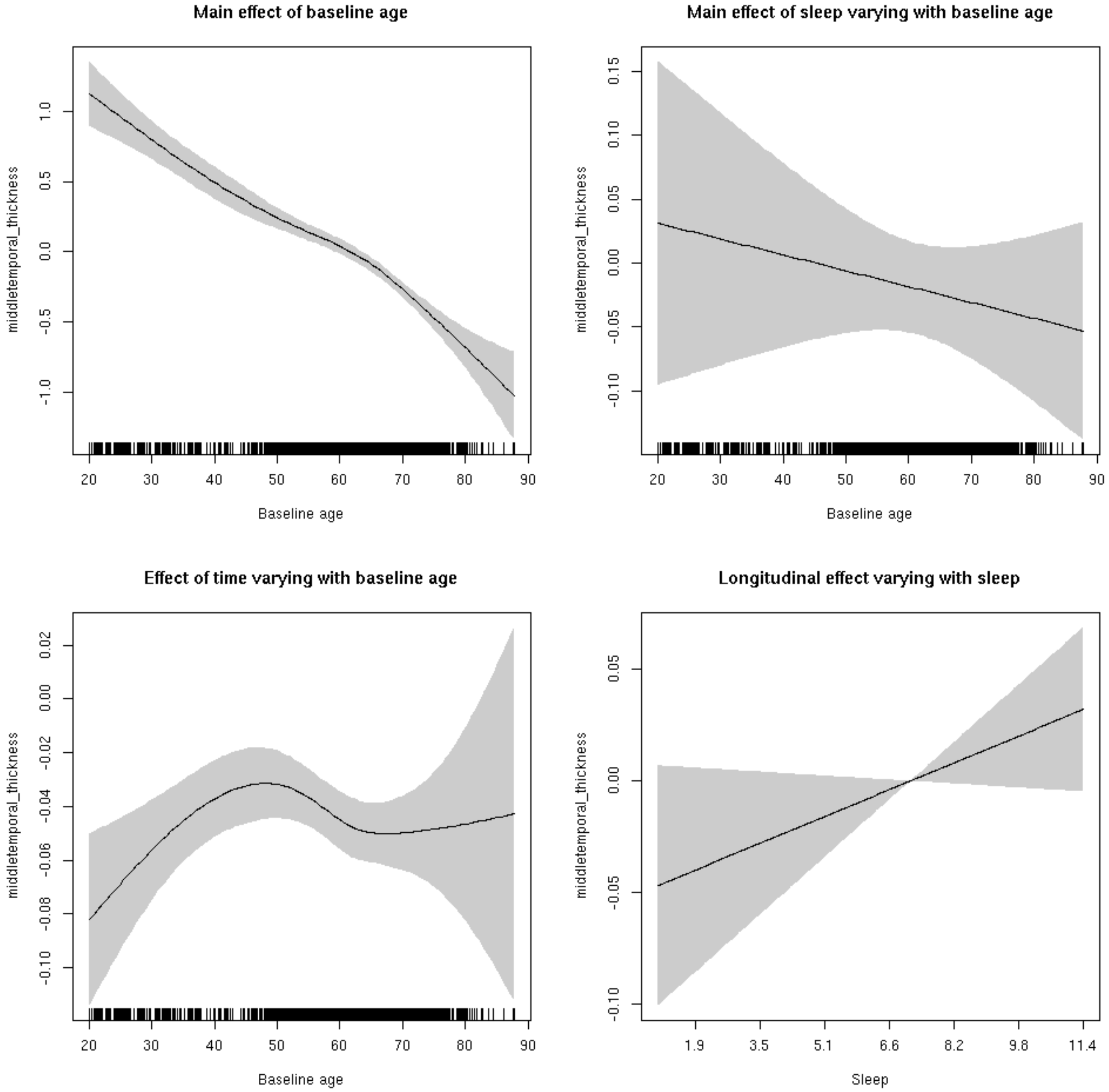

Male: Full data Below is the row for `s(sleep_z):time` from the model output.

```
## s(sleep_z):time   1.000  1.000   0.072   0.788
```

Below are plots of a plot of `s(bl_age)`, `s(bl_age):sleep_z`, `s(bl_age):time`, and `s(sleep_z):time`. For `s(sleep_z):time`, roughly, if we cannot fit a straight line through the confidence bands, there is evidence that the effect of time depends on sleep duration. It is correct the that confidence interval is zero at one point along the axis; this happens because we do not include the main effect.

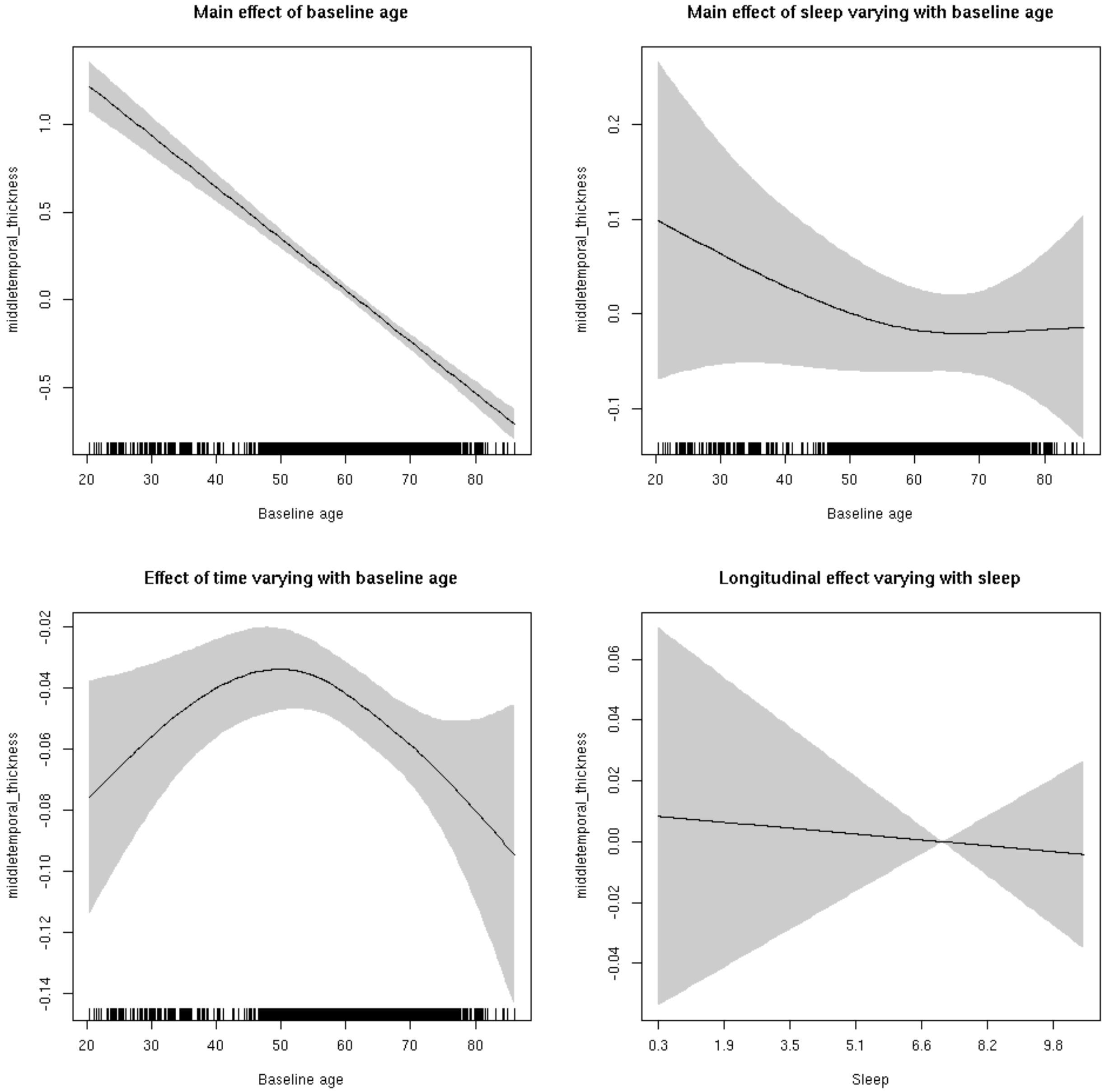

middletemporal_thickness

Full data Below is the model output. The term `s(sleep_z):time` is probably what we care most about.

```
##
## Family: gaussian
## Link function: identity
##
## Formula:
## value ~ sex + site + s(bl_age, k = 10, bs = "cr") + s(bl_age,
##     by = sleep_z, bs = "cr") + +s(bl_age, by = time, k = 5, bs = "cr") +
##     s(sleep_z, by = time, k = 5, bs = "cr", pc = 0)
## <environment: 0x5630035d29a0>
```

```
##
## Parametric coefficients:
##                Estimate Std. Error t value Pr(>|t|)
## (Intercept)   -1.68294    0.04873 -34.537  < 2e-16 ***
## sexmale        0.18960    0.02446   7.751 1.02e-14 ***
## siteousAvanto  0.27247    0.06496   4.195 2.76e-05 ***
## siteousPrisma  0.54618    0.28752   1.900  0.05752 .
## siteousSkyra   0.43431    0.06492   6.690 2.38e-11 ***
## siteUB         0.52805    0.12926   4.085 4.45e-05 ***
## siteUCAM       0.59762    0.06702   8.917  < 2e-16 ***
## siteUKB        1.84798    0.04932  37.473  < 2e-16 ***
## siteUmU       -0.25997    0.08040  -3.233  0.00123 **
## ---
## Signif. codes:  0 ‘***’ 0.001 ‘**’ 0.01 ‘*’ 0.05 ‘.’ 0.1 ‘ ’ 1
##
## Approximate significance of smooth terms:
##                      edf Ref.df       F p-value
## s(bl_age)          3.165  3.165 193.153  <2e-16 ***
## s(bl_age):sleep_z 2.000  2.000   1.807   0.164
## s(bl_age):time    3.797  3.797  72.400  <2e-16 ***
## s(sleep_z):time   1.000  1.000   1.437   0.231
## ---
## Signif. codes:  0 ‘***’ 0.001 ‘**’ 0.01 ‘*’ 0.05 ‘.’ 0.1 ‘ ’ 1
##
## R-sq.(adj) =  0.486
## lmer.REML =  15545  Scale est. = 0.14353   n = 8170
```

Below are plots of a plot of `s(bl_age)`, `s(bl_age):sleep_z`, `s(bl_age):time`, and `s(sleep_z):time`. For `s(sleep_z):time`, roughly, if we cannot fit a straight line through the confidence bands, there is evidence that the effect of time depends on sleep duration. It is correct the that confidence interval is zero at one point along the axis; this happens because we do not include the main effect.

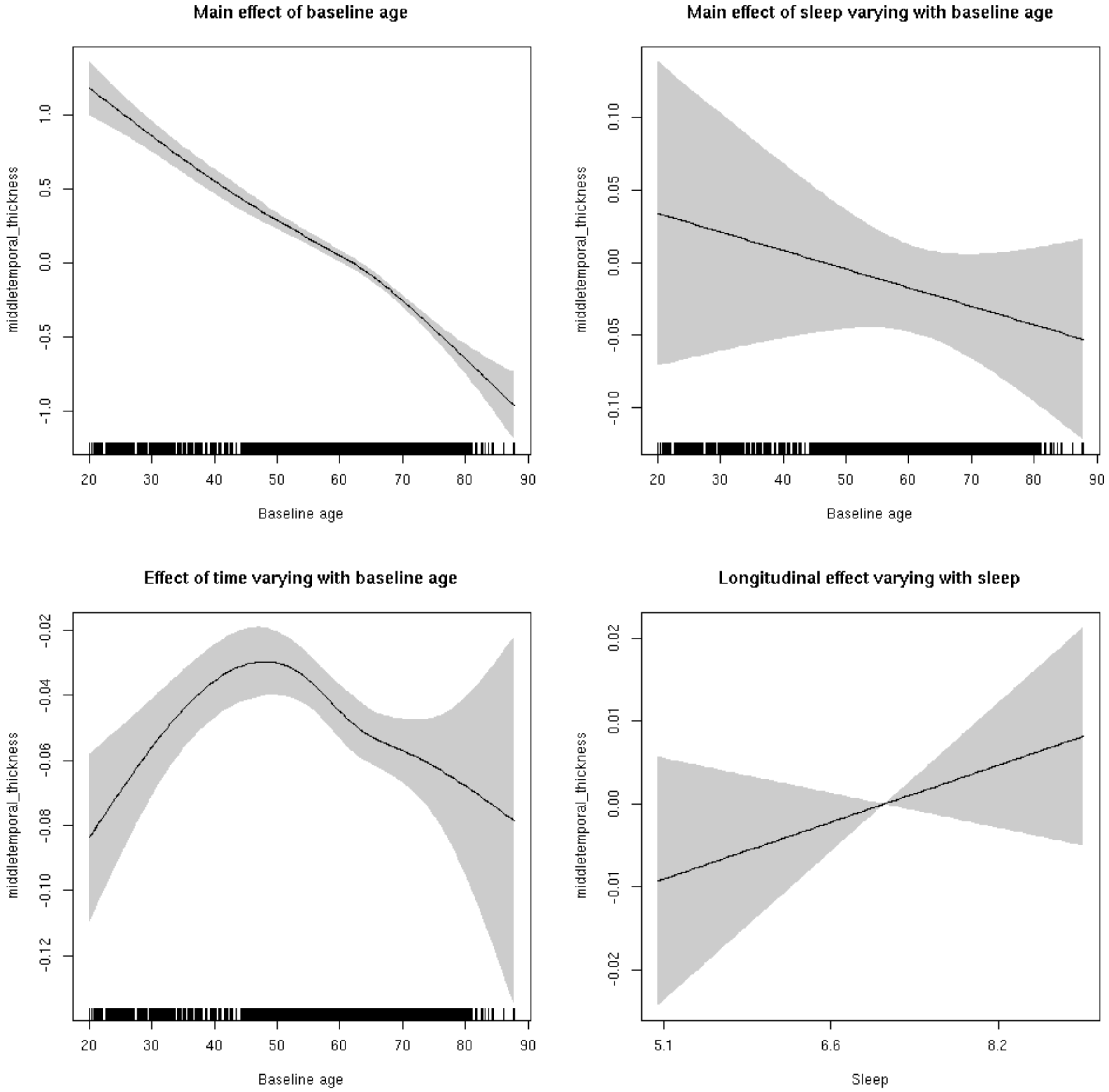

Restricted to sleep >= 5 and <= 9 hours Below is the row for `s(sleep_z):time` from the model output.

```
## s(sleep_z):time   1.000  1.000   1.535   0.215
```

Below are plots of a plot of `s(bl_age)`, `s(bl_age):sleep_z`, `s(bl_age):time`, and `s(sleep_z):time`. For `s(sleep_z):time`, roughly, if we cannot fit a straight line through the confidence bands, there is evidence that the effect of time depends on sleep duration. It is correct the that confidence interval is zero at one point along the axis; this happens because we do not include the main effect.

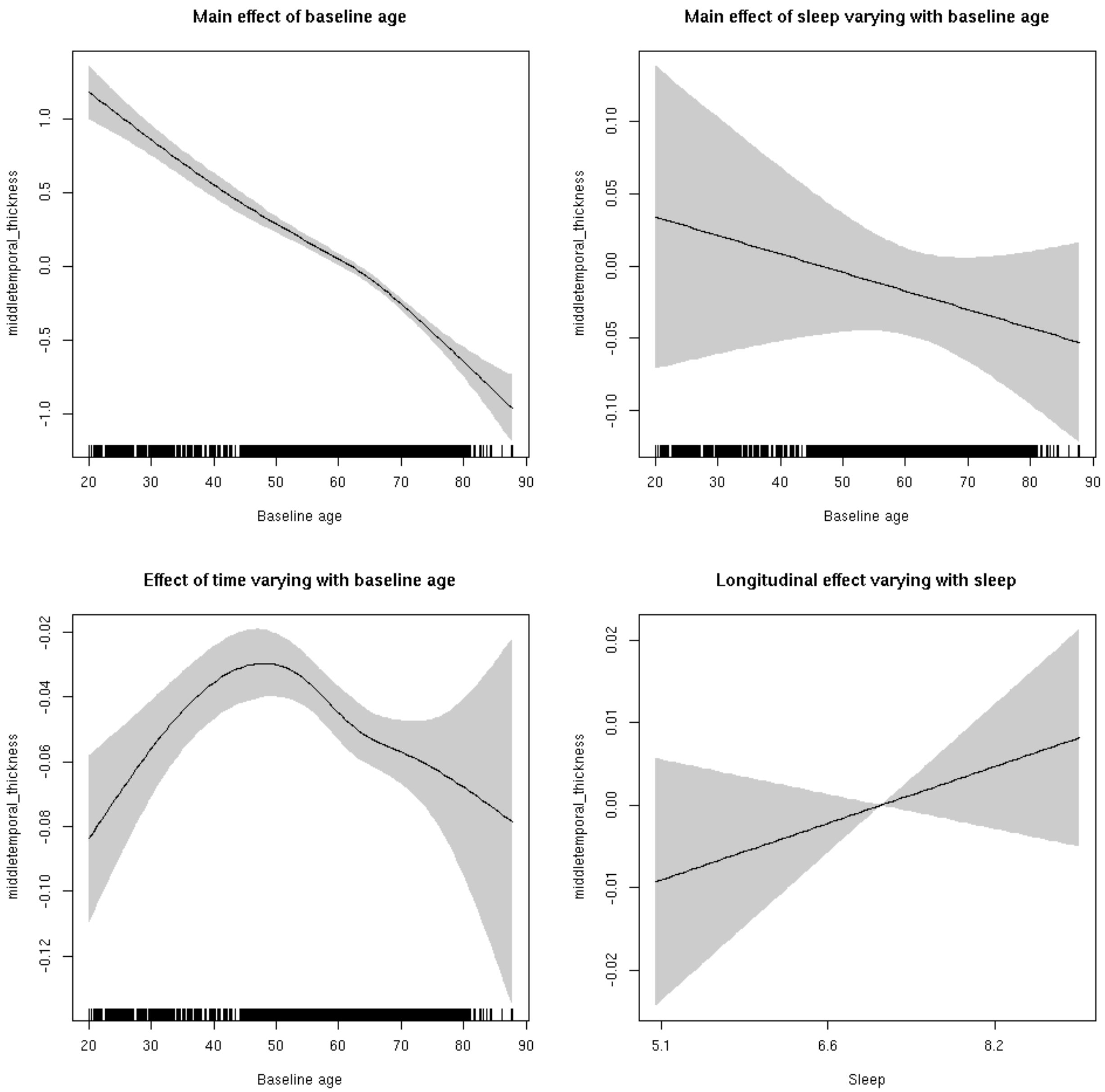

middletemporal_volume: Stratified by sex

Female: Full data Below is the row for `s(sleep_z):time` from the model output.

```
## s(sleep_z):time   1.000  1.000   0.006   0.939
```

Below are plots of a plot of `s(bl_age)`, `s(bl_age):sleep_z`, `s(bl_age):time`, and `s(sleep_z):time`. For `s(sleep_z):time`, roughly, if we cannot fit a straight line through the confidence bands, there is evidence that the effect of time depends on sleep duration. It is correct the that confidence interval is zero at one point along the axis; this happens because we do not include the main effect.

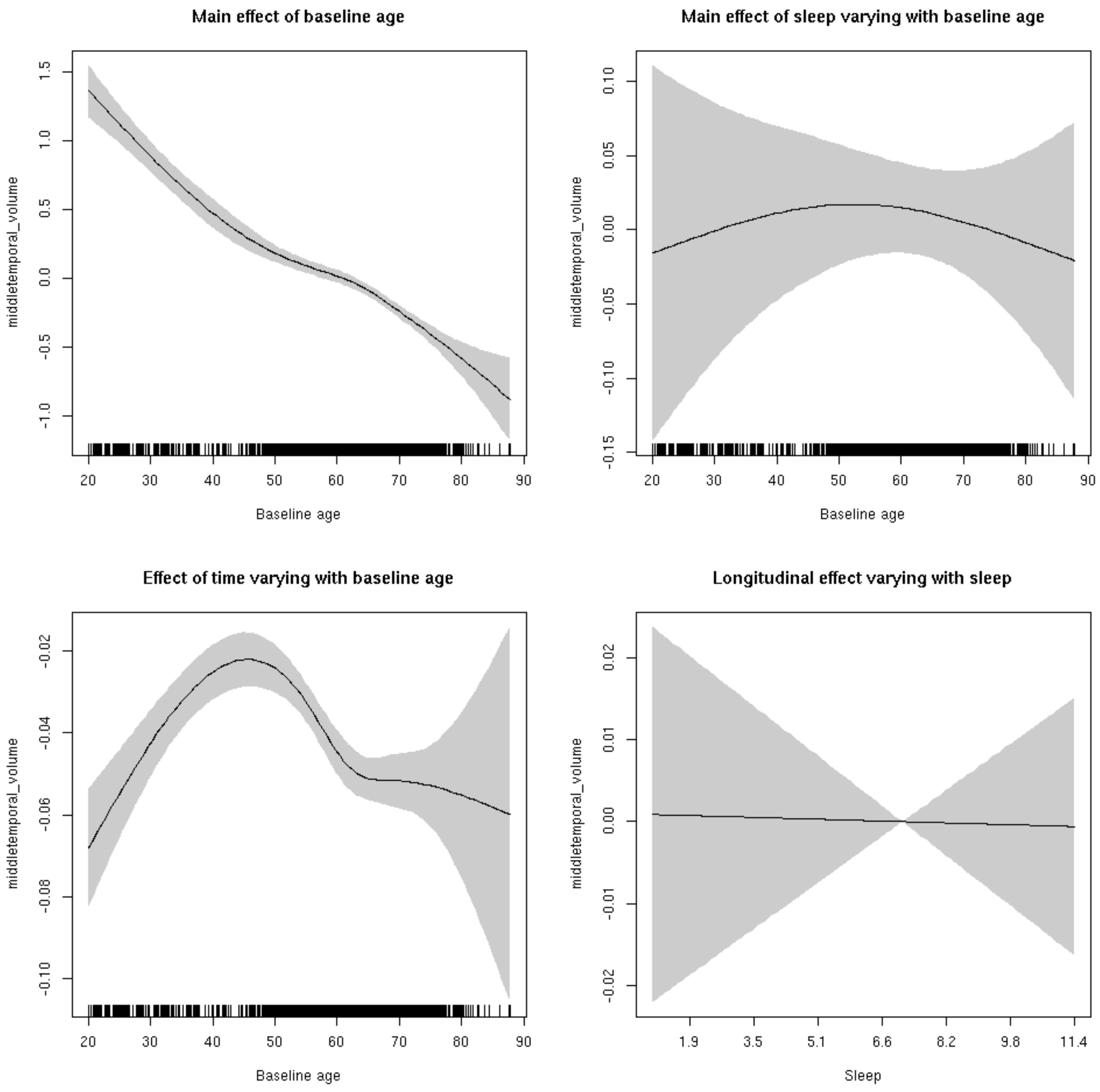

Male: Full data Below is the row for `s(sleep_z):time` from the model output.

```
## s(sleep_z):time   1.000  1.000   0.280   0.597
```

Below are plots of a plot of `s(bl_age)`, `s(bl_age):sleep_z`, `s(bl_age):time`, and `s(sleep_z):time`. For `s(sleep_z):time`, roughly, if we cannot fit a straight line through the confidence bands, there is evidence that the effect of time depends on sleep duration. It is correct the that confidence interval is zero at one point along the axis; this happens because we do not include the main effect.

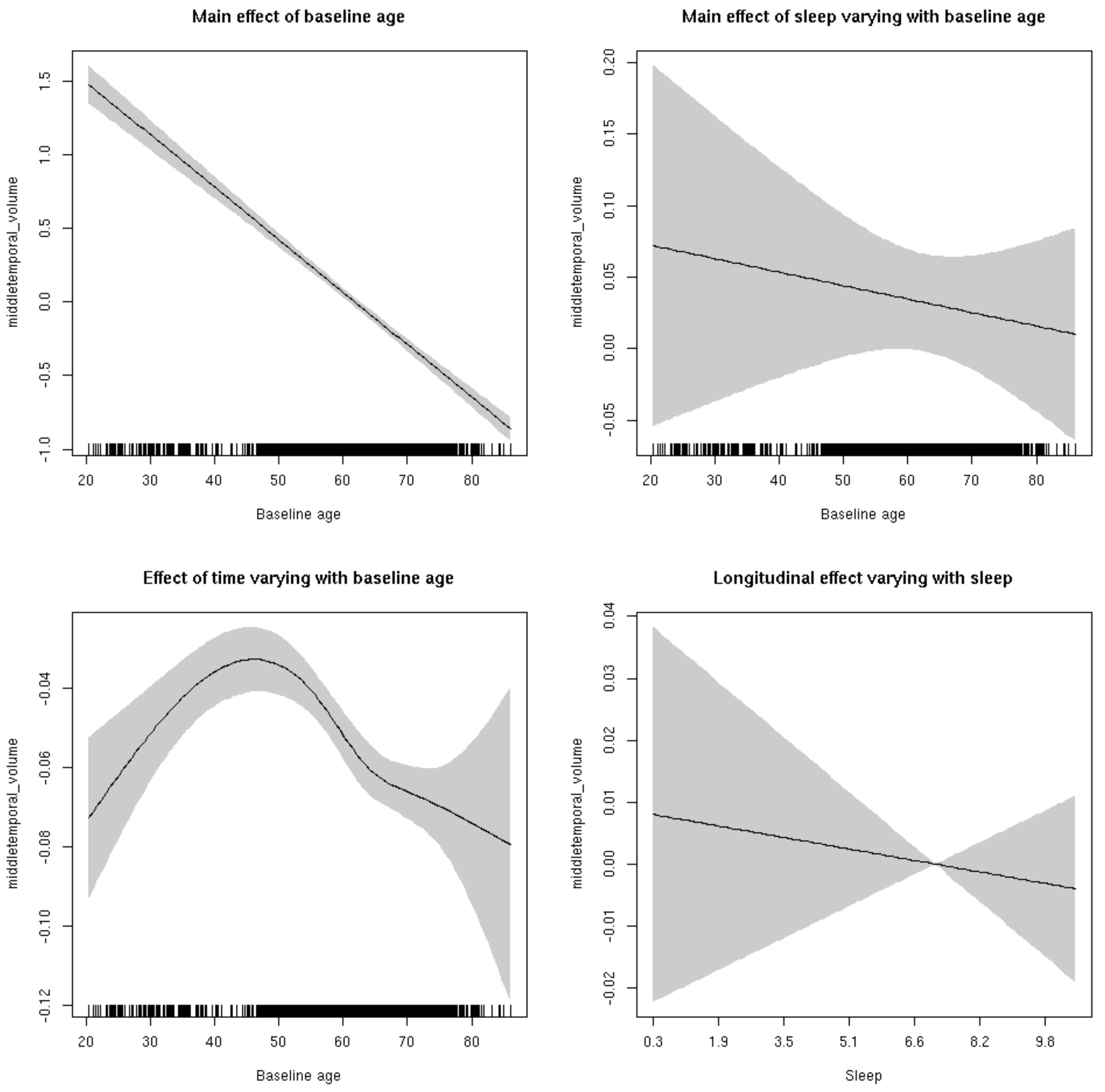

middletemporal_volume

Full data Below is the model output. The term `s(sleep_z):time` is probably what we care most about.

```
##
## Family: gaussian
## Link function: identity
##
## Formula:
## value ~ sex + site + icv + s(bl_age, k = 10, bs = "cr") + s(bl_age,
##     by = sleep_z, bs = "cr") + +s(bl_age, by = time, k = 5, bs = "cr") +
##     s(sleep_z, by = time, k = 5, bs = "cr", pc = 0)
## <environment: 0x5630035a6d80>
```

```
##
## Parametric coefficients:
##               Estimate Std. Error t value Pr(>|t|)
## (Intercept)   -0.04284    0.04985  -0.859   0.3902
## sexmale        0.25069    0.02558   9.800  < 2e-16 ***
## siteousAvanto -0.52696    0.06084  -8.662  < 2e-16 ***
## siteousPrisma -0.62904    0.14445  -4.355 1.35e-05 ***
## siteousSkyra   0.07473    0.05891   1.269   0.2046
## siteUB        -0.45708    0.11464  -3.987 6.75e-05 ***
## siteUCAM      -0.41830    0.06200  -6.747 1.61e-11 ***
## siteUKB        0.09437    0.04742   1.990   0.0466 *
## siteUmU       -0.04780    0.07109  -0.672   0.5014
## icv            0.54790    0.01259  43.513  < 2e-16 ***
## ---
## Signif. codes:  0 '***' 0.001 '**' 0.01 '*' 0.05 '.' 0.1 ' ' 1
##
## Approximate significance of smooth terms:
##                     edf Ref.df       F p-value
## s(bl_age)         3.804  3.804 266.772  <2e-16 ***
## s(bl_age):sleep_z 2.000  2.000   1.229   0.293
## s(bl_age):time    4.512  4.512 294.894  <2e-16 ***
## s(sleep_z):time   1.000  1.000   0.157   0.692
## ---
## Signif. codes:  0 '***' 0.001 '**' 0.01 '*' 0.05 '.' 0.1 ' ' 1
##
## R-sq.(adj) =  0.575
## lmer.REML = 7606.8  Scale est. = 0.028894  n = 8172
```

Below are plots of a plot of `s(bl_age)`, `s(bl_age):sleep_z`, `s(bl_age):time`, and `s(sleep_z):time`. For `s(sleep_z):time`, roughly, if we cannot fit a straight line through the confidence bands, there is evidence that the effect of time depends on sleep duration. It is correct the that confidence interval is zero at one point along the axis; this happens because we do not include the main effect.

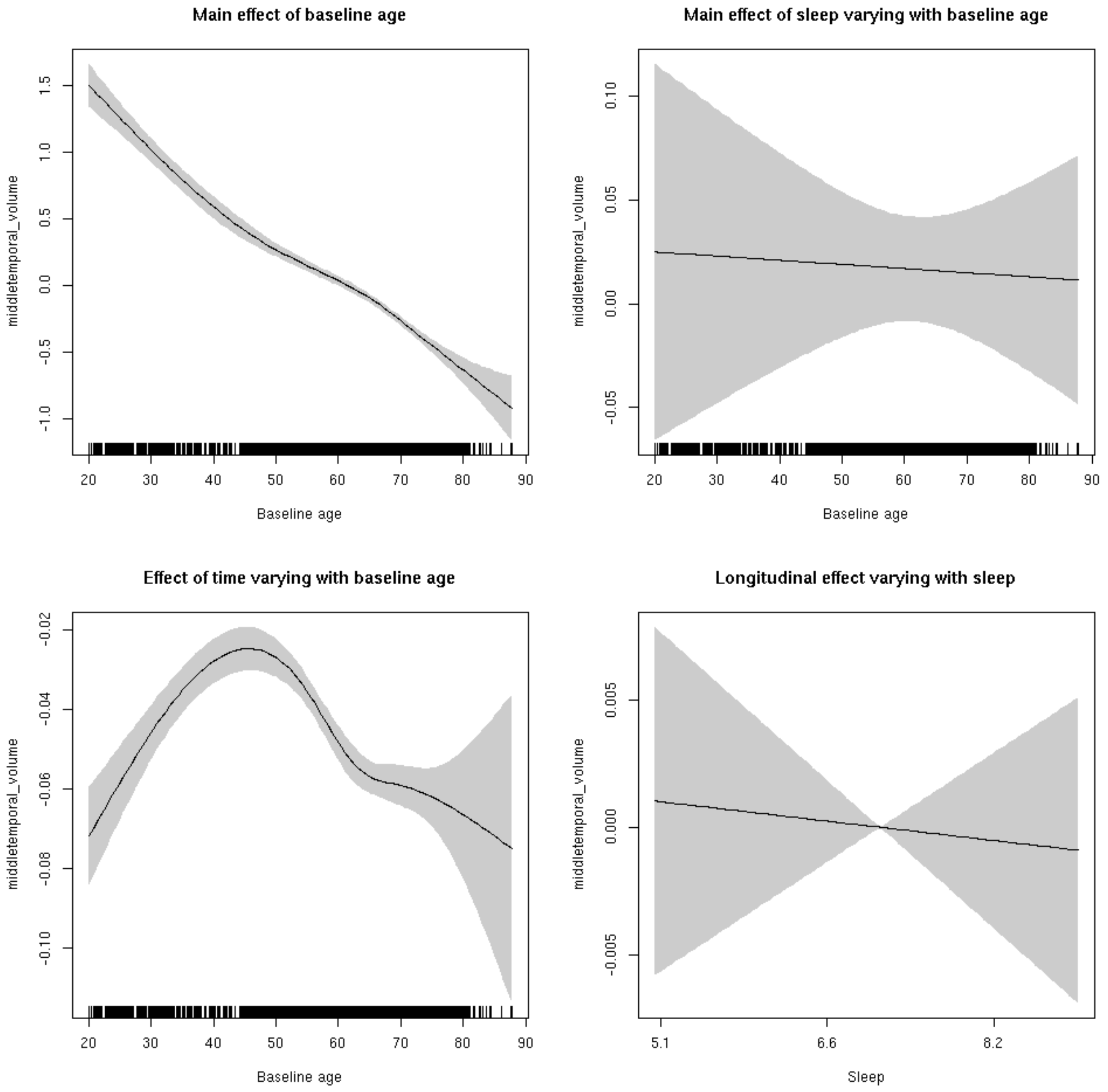

Restricted to sleep >= 5 and <= 9 hours Below is the row for `s(sleep_z):time` from the model output.

```
## s(sleep_z):time   1.000  1.000   0.090   0.764
```

Below are plots of a plot of `s(bl_age)`, `s(bl_age):sleep_z`, `s(bl_age):time`, and `s(sleep_z):time`. For `s(sleep_z):time`, roughly, if we cannot fit a straight line through the confidence bands, there is evidence that the effect of time depends on sleep duration. It is correct the that confidence interval is zero at one point along the axis; this happens because we do not include the main effect.

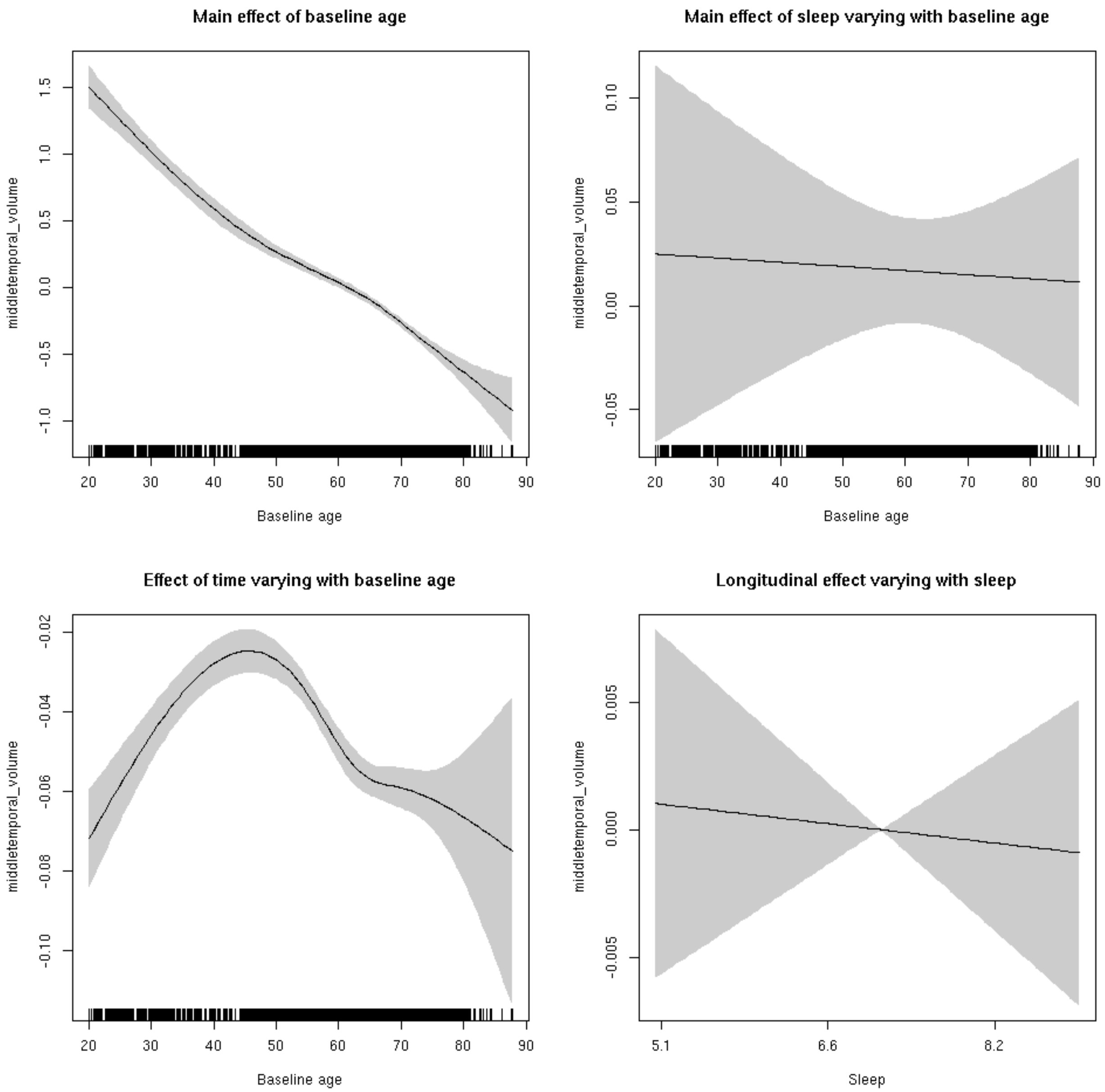

paracentral_area: Stratified by sex

Female: Full data Below is the row for `s(sleep_z):time` from the model output.

```
## s(sleep_z):time   1.000  1.000 0.158 0.69097
```

Below are plots of a plot of `s(bl_age)`, `s(bl_age):sleep_z`, `s(bl_age):time`, and `s(sleep_z):time`. For `s(sleep_z):time`, roughly, if we cannot fit a straight line through the confidence bands, there is evidence that the effect of time depends on sleep duration. It is correct the that confidence interval is zero at one point along the axis; this happens because we do not include the main effect.

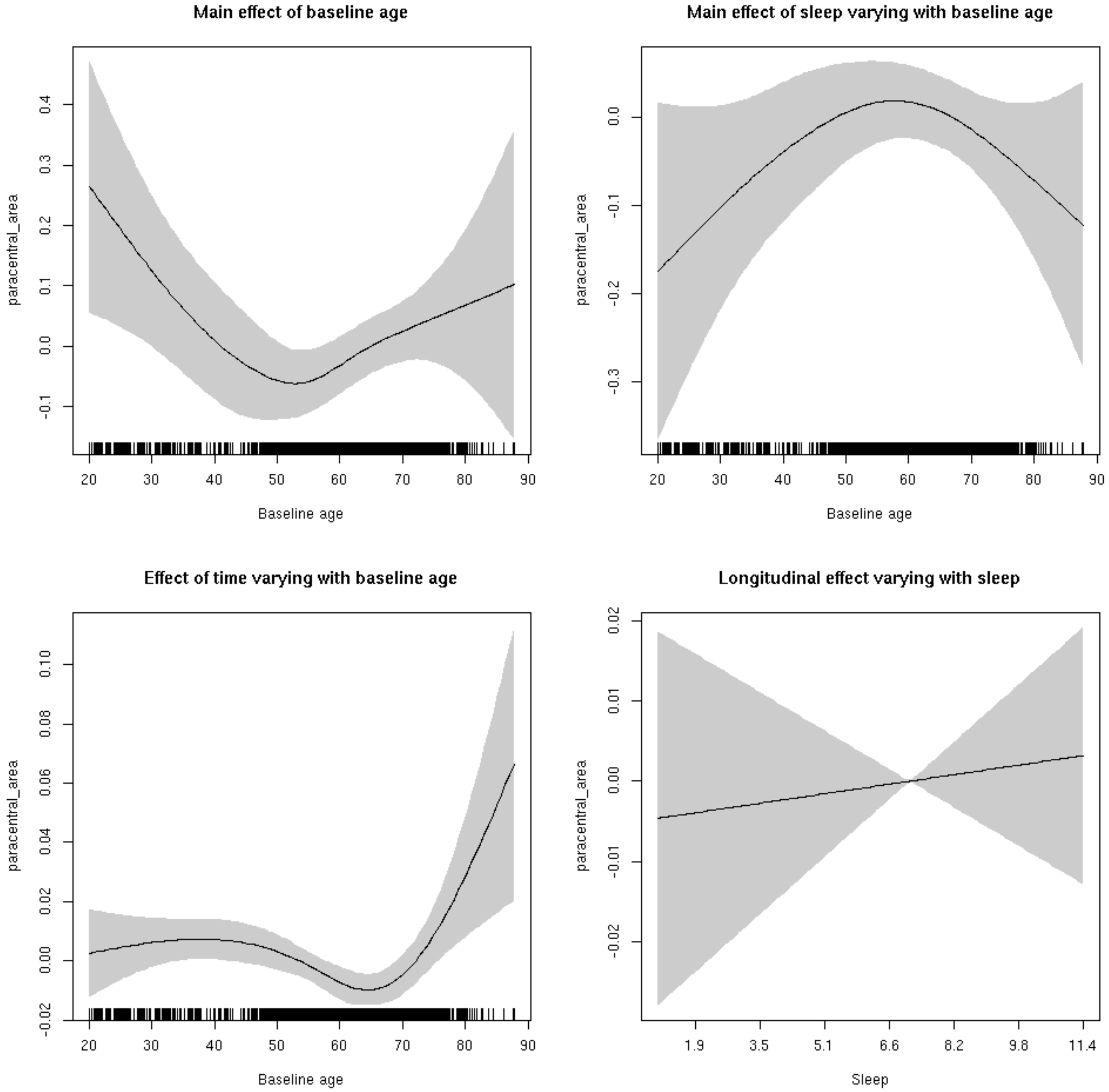

Male: Full data Below is the row for `s(sleep_z):time` from the model output.

```
## s(sleep_z):time     1       1 1.007  0.3156
```

Below are plots of a plot of `s(bl_age)`, `s(bl_age):sleep_z`, `s(bl_age):time`, and `s(sleep_z):time`. For `s(sleep_z):time`, roughly, if we cannot fit a straight line through the confidence bands, there is evidence that the effect of time depends on sleep duration. It is correct the that confidence interval is zero at one point along the axis; this happens because we do not include the main effect.

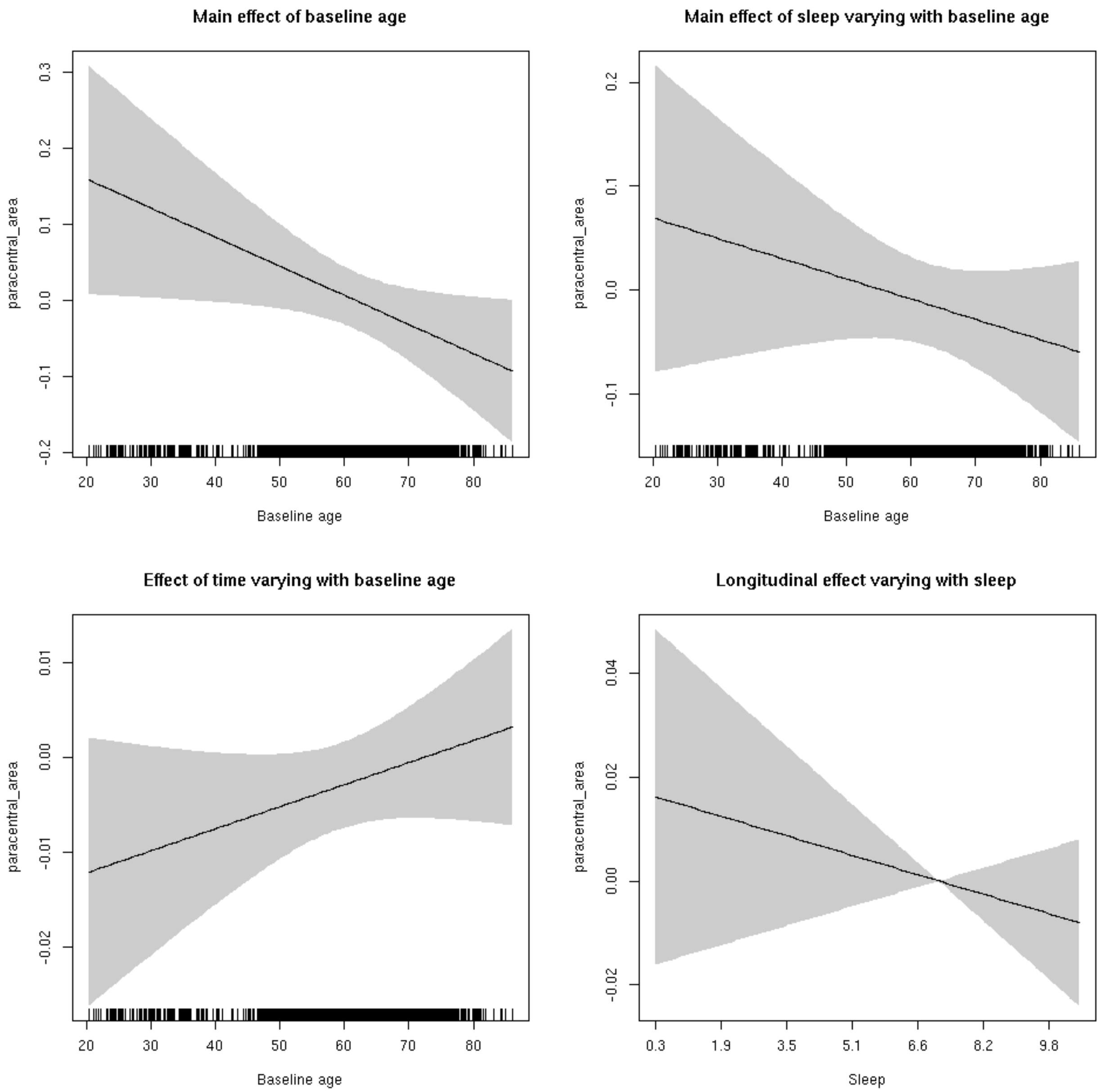

paracentral_area

Full data Below is the model output. The term `s(sleep_z):time` is probably what we care most about.

```
##
## Family: gaussian
## Link function: identity
##
## Formula:
## value ~ sex + site + icv + s(bl_age, k = 10, bs = "cr") + s(bl_age,
##     by = sleep_z, bs = "cr") + +s(bl_age, by = time, k = 5, bs = "cr") +
##     s(sleep_z, by = time, k = 5, bs = "cr", pc = 0)
## <environment: 0x5630035b6f88>
```

```
##
## Parametric coefficients:
##                Estimate Std. Error t value Pr(>|t|)
## (Intercept)     0.47336    0.05818   8.136 4.70e-16 ***
## sexmale         0.06377    0.02992   2.131   0.0331 *
## siteousAvanto -0.40734    0.07099  -5.738 9.91e-09 ***
## siteousPrisma -0.72013    0.15576  -4.623 3.84e-06 ***
## siteousSkyra  -0.59493    0.06875  -8.654  < 2e-16 ***
## siteUB        -0.57318    0.13416  -4.272 1.96e-05 ***
## siteUCAM      -0.47530    0.07211  -6.591 4.63e-11 ***
## siteUKB       -0.47642    0.05519  -8.633  < 2e-16 ***
## siteUmU       -0.15266    0.08308  -1.838   0.0662 .
## icv            0.58522    0.01470  39.798  < 2e-16 ***
## ---
## Signif. codes:  0 '***' 0.001 '**' 0.01 '*' 0.05 '.' 0.1 ' ' 1
##
## Approximate significance of smooth terms:
##                      edf Ref.df     F p-value
## s(bl_age)          2.762  2.762 4.450  0.0090 **
## s(bl_age):sleep_z 3.144  3.144 2.050  0.0882 .
## s(bl_age):time    2.000  2.000 1.365  0.2554
## s(sleep_z):time   1.000  1.000 0.087  0.7676
## ---
## Signif. codes:  0 '***' 0.001 '**' 0.01 '*' 0.05 '.' 0.1 ' ' 1
##
## R-sq.(adj) =  0.392
## lmer.REML =   9277  Scale est. = 0.032103  n = 8170
```

Below are plots of a plot of `s(bl_age)`, `s(bl_age):sleep_z`, `s(bl_age):time`, and `s(sleep_z):time`. For `s(sleep_z):time`, roughly, if we cannot fit a straight line through the confidence bands, there is evidence that the effect of time depends on sleep duration. It is correct the that confidence interval is zero at one point along the axis; this happens because we do not include the main effect.

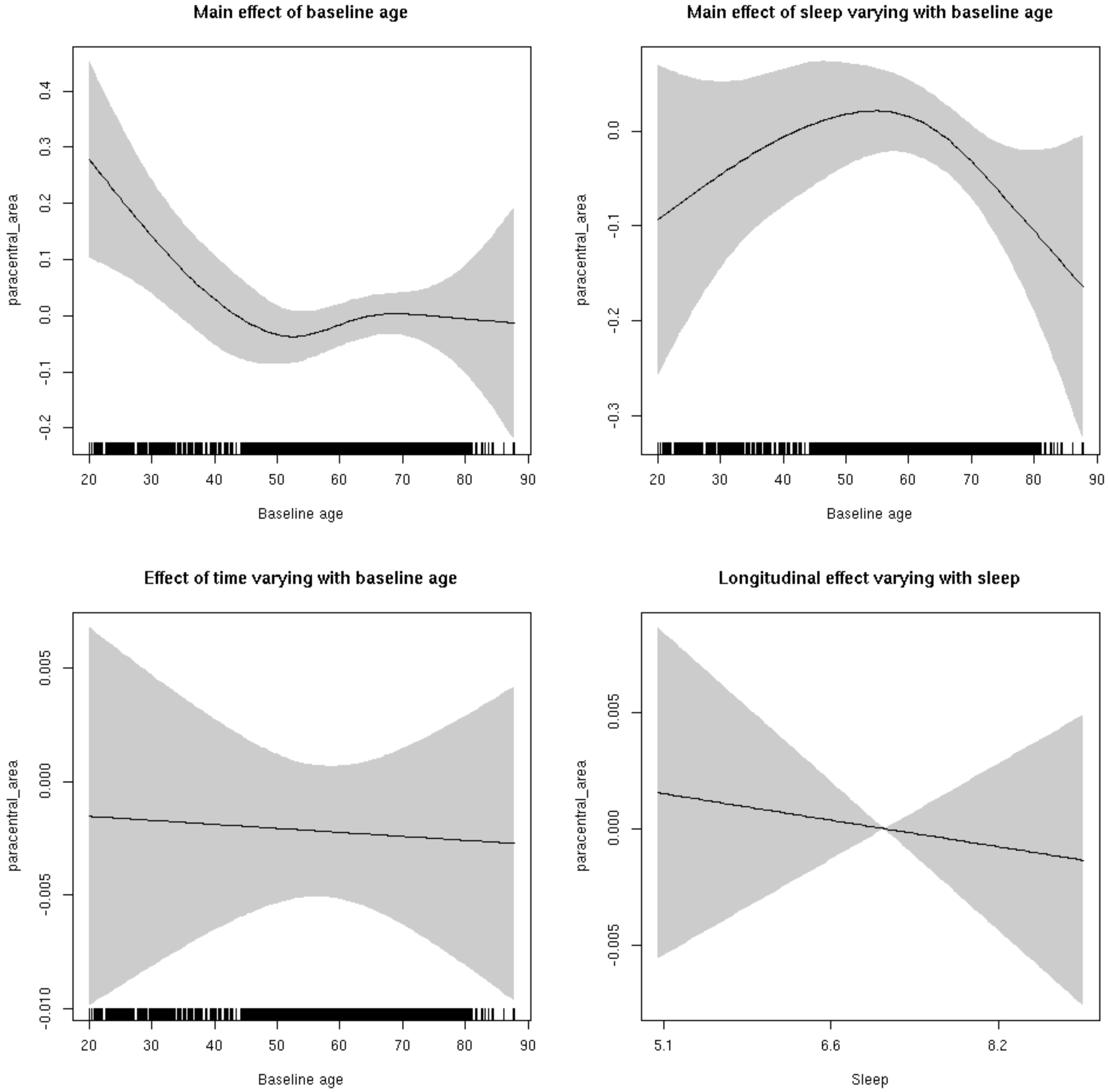

Restricted to sleep >= 5 and <= 9 hours Below is the row for `s(sleep_z):time` from the model output.

```
## s(sleep_z):time   1.000  1.000 0.188 0.66442
```

Below are plots of a plot of `s(bl_age)`, `s(bl_age):sleep_z`, `s(bl_age):time`, and `s(sleep_z):time`. For `s(sleep_z):time`, roughly, if we cannot fit a straight line through the confidence bands, there is evidence that the effect of time depends on sleep duration. It is correct the that confidence interval is zero at one point along the axis; this happens because we do not include the main effect.

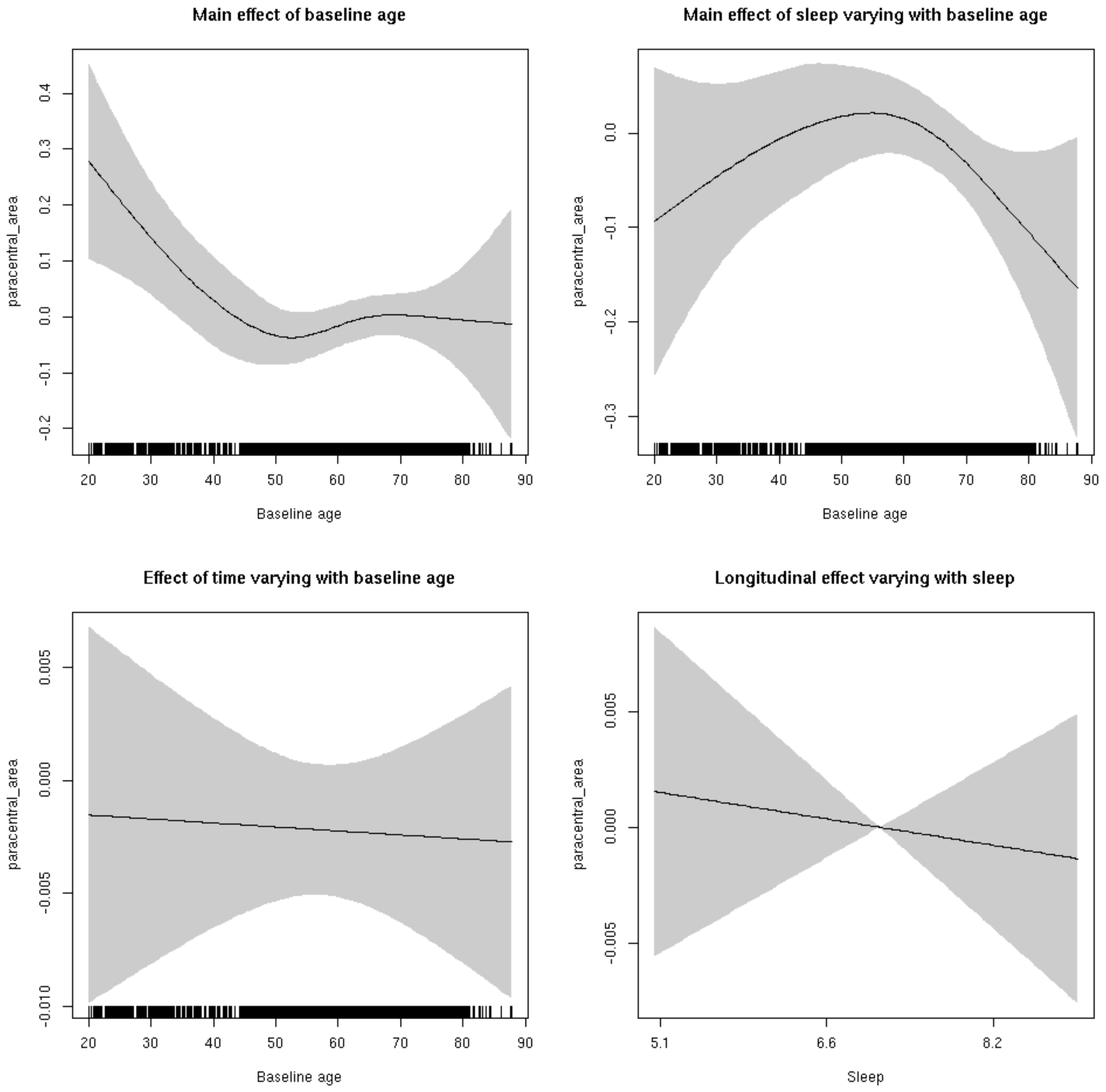

paracentral_thickness: Stratified by sex

Female: Full data Below is the row for `s(sleep_z):time` from the model output.

```
## s(sleep_z):time   1.000  1.000  0.222   0.637
```

Below are plots of a plot of `s(bl_age)`, `s(bl_age):sleep_z`, `s(bl_age):time`, and `s(sleep_z):time`. For `s(sleep_z):time`, roughly, if we cannot fit a straight line through the confidence bands, there is evidence that the effect of time depends on sleep duration. It is correct the that confidence interval is zero at one point along the axis; this happens because we do not include the main effect.

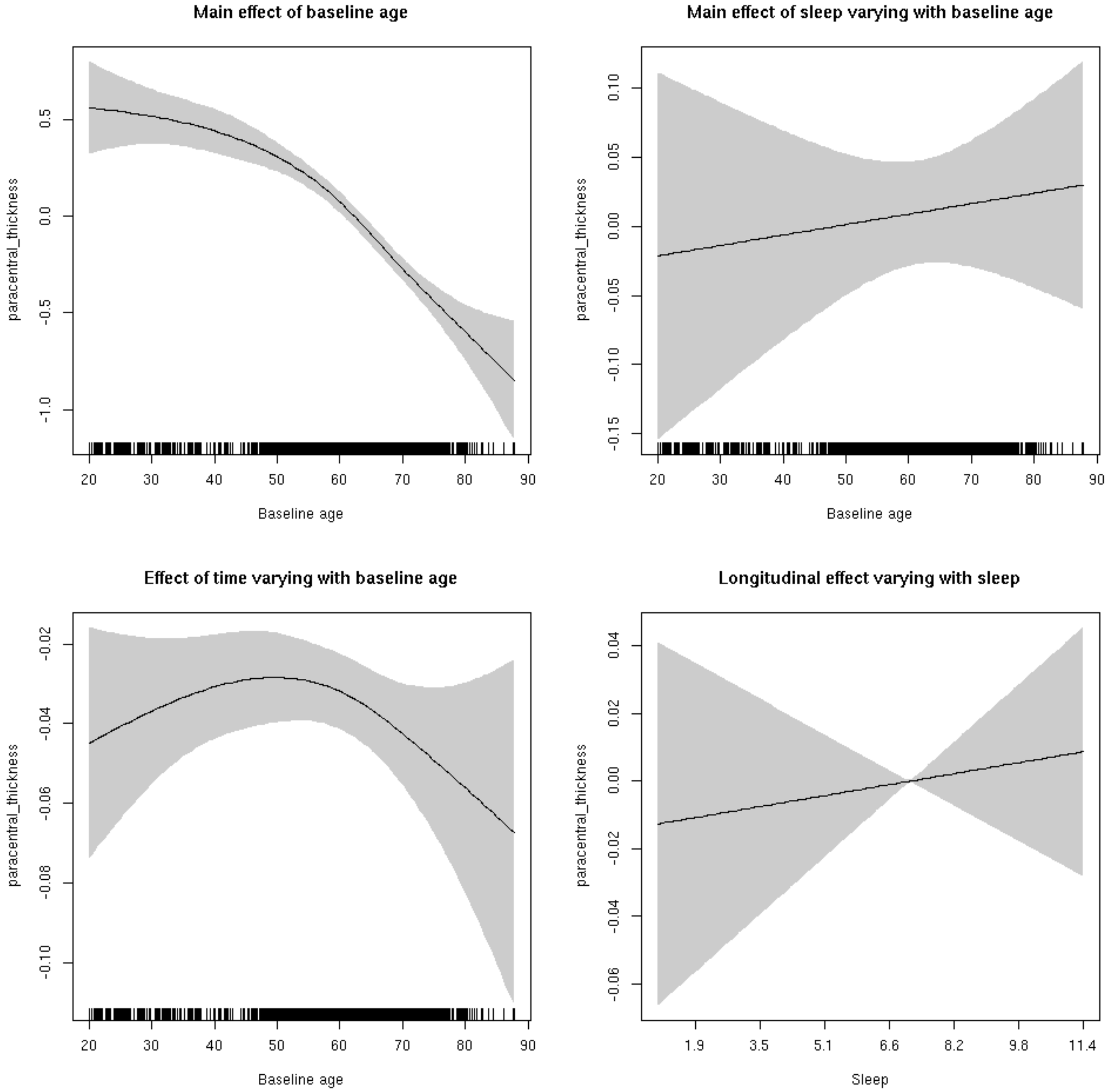

Male: Full data Below is the row for `s(sleep_z):time` from the model output.

```
## s(sleep_z):time   1.000  1.000  2.394   0.122
```

Below are plots of a plot of `s(bl_age)`, `s(bl_age):sleep_z`, `s(bl_age):time`, and `s(sleep_z):time`. For `s(sleep_z):time`, roughly, if we cannot fit a straight line through the confidence bands, there is evidence that the effect of time depends on sleep duration. It is correct the that confidence interval is zero at one point along the axis; this happens because we do not include the main effect.

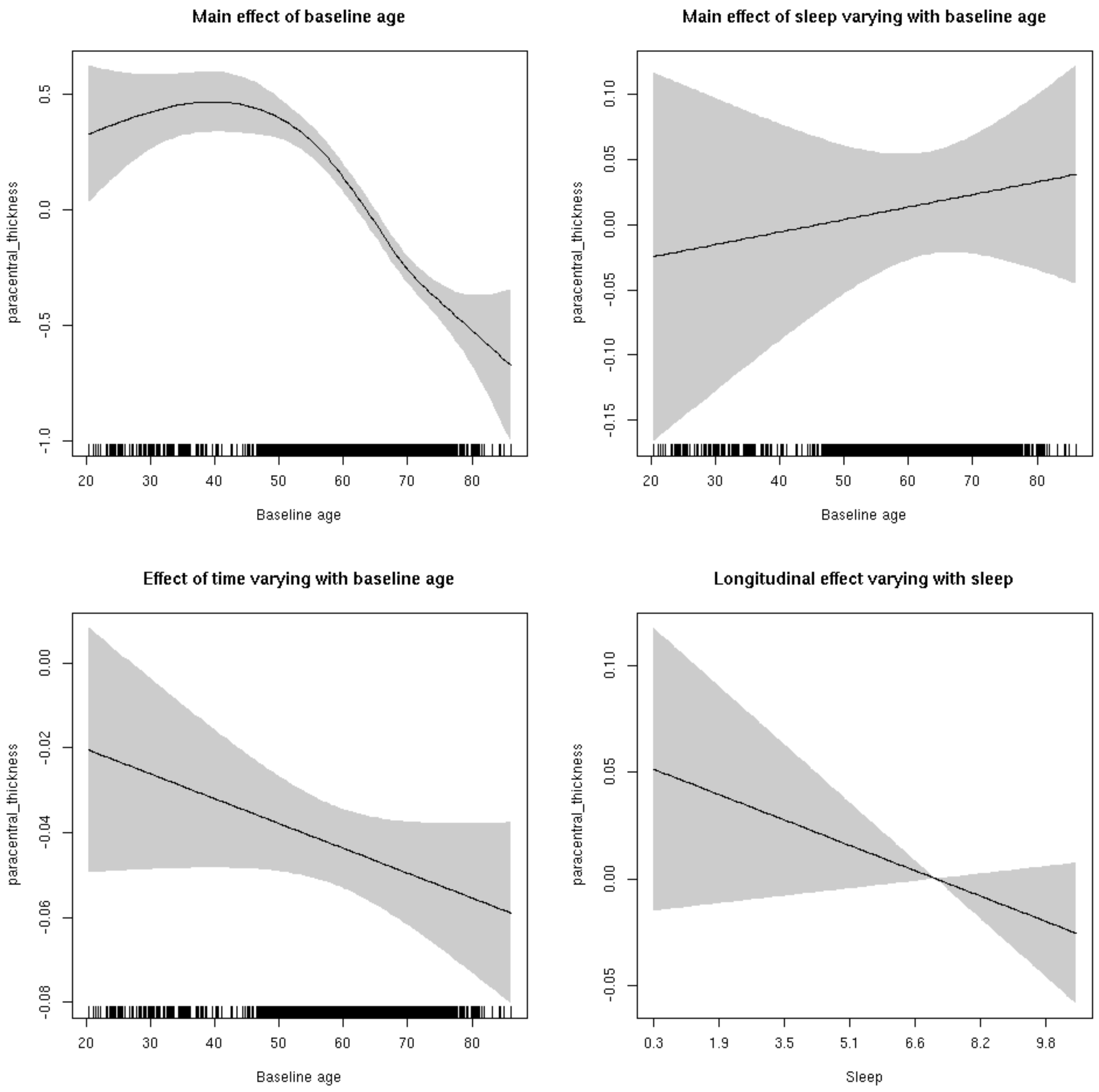

paracentral_thickness

Full data Below is the model output. The term `s(sleep_z):time` is probably what we care most about.

```
##
## Family: gaussian
## Link function: identity
##
## Formula:
## value ~ sex + site + s(bl_age, k = 10, bs = "cr") + s(bl_age,
##     by = sleep_z, bs = "cr") + +s(bl_age, by = time, k = 5, bs = "cr") +
##     s(sleep_z, by = time, k = 5, bs = "cr", pc = 0)
## <environment: 0x5630035c9228>
```

```
##
## Parametric coefficients:
##                Estimate Std. Error t value Pr(>|t|)
## (Intercept)   -0.80122    0.05101 -15.707  < 2e-16 ***
## sexmale       -0.08001    0.02554  -3.133 0.001737 **
## siteousAvanto -0.62007    0.06791  -9.131  < 2e-16 ***
## siteousPrisma  0.25221    0.29992   0.841 0.400423
## siteousSkyra  -0.23174    0.06788  -3.414 0.000643 ***
## siteUB         0.17647    0.13505   1.307 0.191357
## siteUCAM      -0.10217    0.07035  -1.452 0.146431
## siteUKB        0.99637    0.05172  19.263  < 2e-16 ***
## siteUmU        0.17245    0.08404   2.052 0.040209 *
## ---
## Signif. codes:  0 '***' 0.001 '**' 0.01 '*' 0.05 '.' 0.1 ' ' 1
##
## Approximate significance of smooth terms:
##                      edf Ref.df       F p-value
## s(bl_age)          3.964  3.964 108.772  <2e-16 ***
## s(bl_age):sleep_z 2.000  2.000   0.684   0.505
## s(bl_age):time     2.785  2.785  57.104  <2e-16 ***
## s(sleep_z):time    1.000  1.000   0.662   0.416
## ---
## Signif. codes:  0 '***' 0.001 '**' 0.01 '*' 0.05 '.' 0.1 ' ' 1
##
## R-sq.(adj) =  0.344
## lmer.REML =  16253  Scale est. = 0.15615   n = 8176
```

Below are plots of a plot of `s(bl_age)`, `s(bl_age):sleep_z`, `s(bl_age):time`, and `s(sleep_z):time`. For `s(sleep_z):time`, roughly, if we cannot fit a straight line through the confidence bands, there is evidence that the effect of time depends on sleep duration. It is correct the that confidence interval is zero at one point along the axis; this happens because we do not include the main effect.

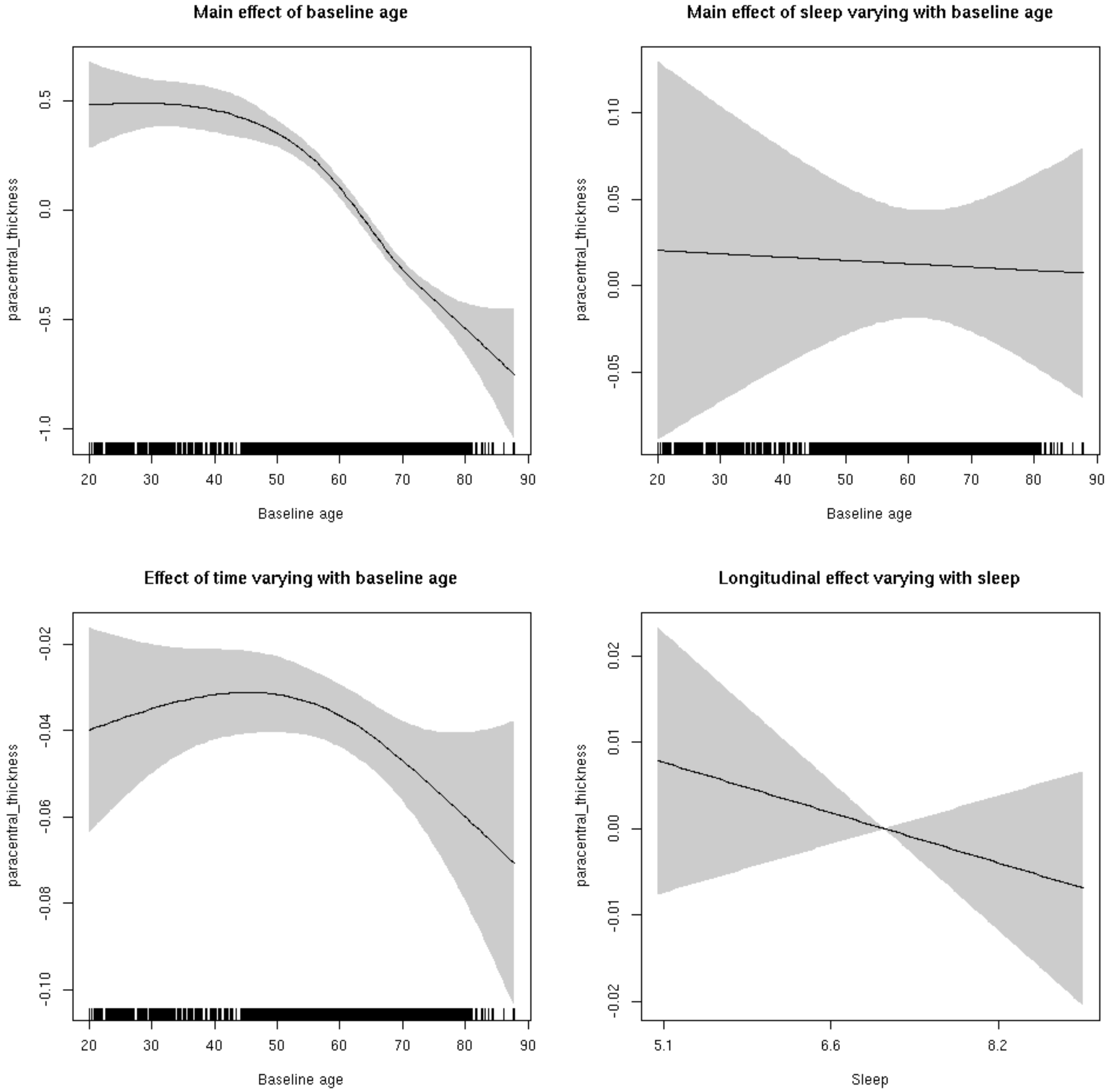

Restricted to sleep >= 5 and <= 9 hours Below is the row for `s(sleep_z):time` from the model output.

```
## s(sleep_z):time   1.000  1.000   1.033    0.31
```

Below are plots of a plot of `s(bl_age)`, `s(bl_age):sleep_z`, `s(bl_age):time`, and `s(sleep_z):time`. For `s(sleep_z):time`, roughly, if we cannot fit a straight line through the confidence bands, there is evidence that the effect of time depends on sleep duration. It is correct the that confidence interval is zero at one point along the axis; this happens because we do not include the main effect.

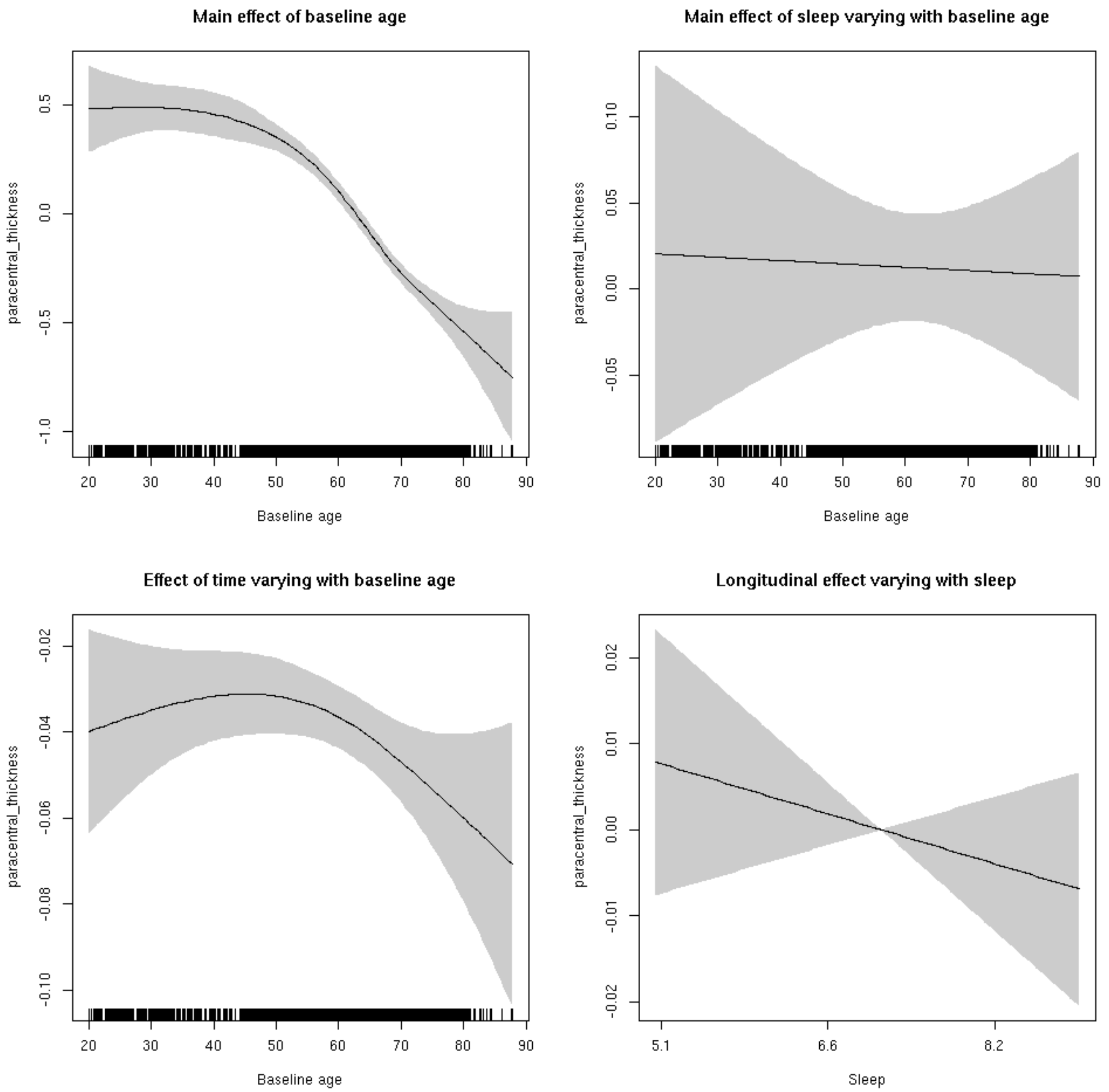

paracentral_volume: Stratified by sex

Female: Full data Below is the row for `s(sleep_z):time` from the model output.

```
## s(sleep_z):time   1.000  1.000   0.126   0.722
```

Below are plots of a plot of `s(bl_age)`, `s(bl_age):sleep_z`, `s(bl_age):time`, and `s(sleep_z):time`. For `s(sleep_z):time`, roughly, if we cannot fit a straight line through the confidence bands, there is evidence that the effect of time depends on sleep duration. It is correct the that confidence interval is zero at one point along the axis; this happens because we do not include the main effect.

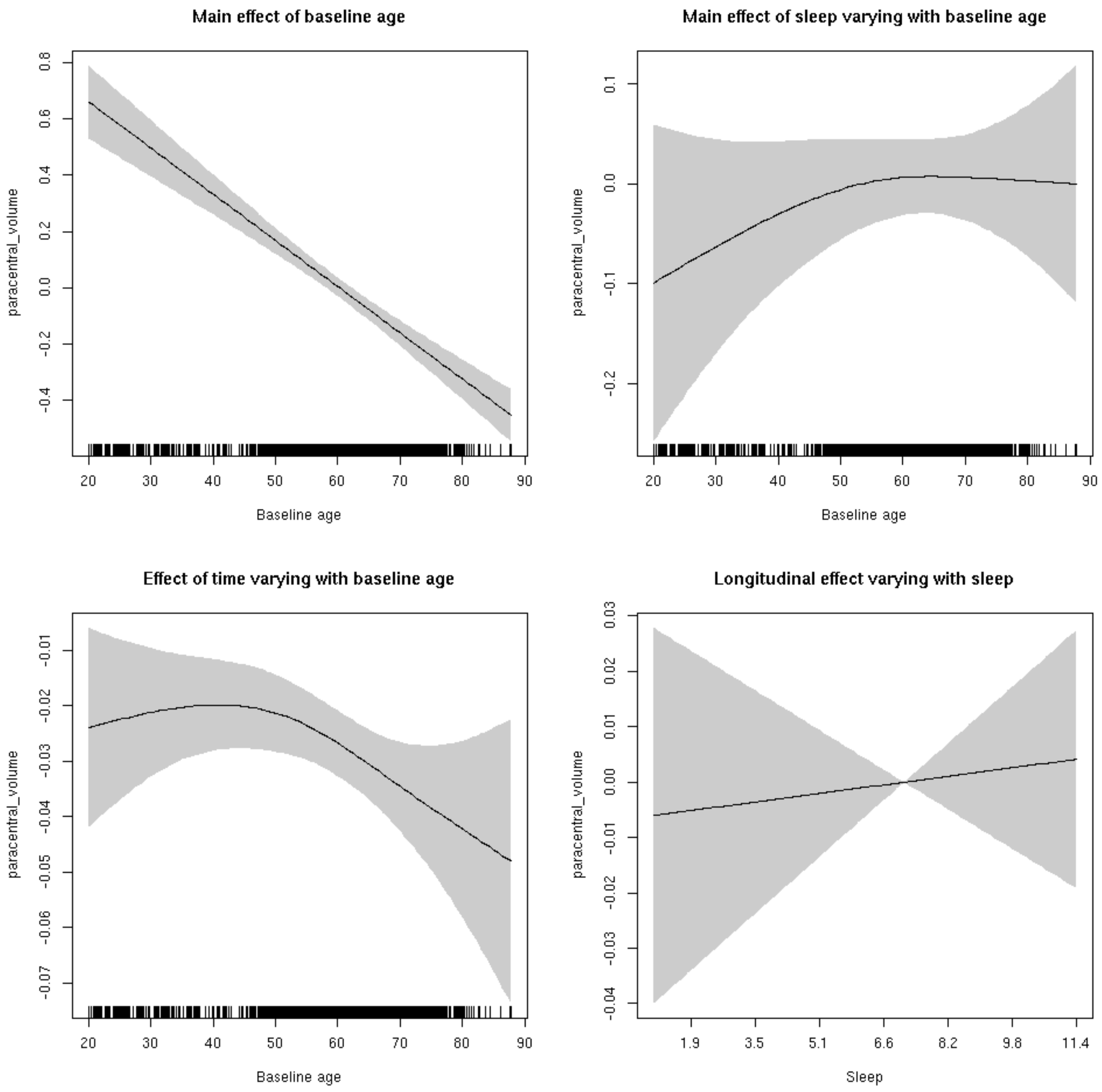

Male: Full data Below is the row for `s(sleep_z):time` from the model output.

```
## s(sleep_z):time   1.000  1.000  4.394  0.0361 *
```

Below are plots of a plot of `s(bl_age)`, `s(bl_age):sleep_z`, `s(bl_age):time`, and `s(sleep_z):time`. For `s(sleep_z):time`, roughly, if we cannot fit a straight line through the confidence bands, there is evidence that the effect of time depends on sleep duration. It is correct the that confidence interval is zero at one point along the axis; this happens because we do not include the main effect.

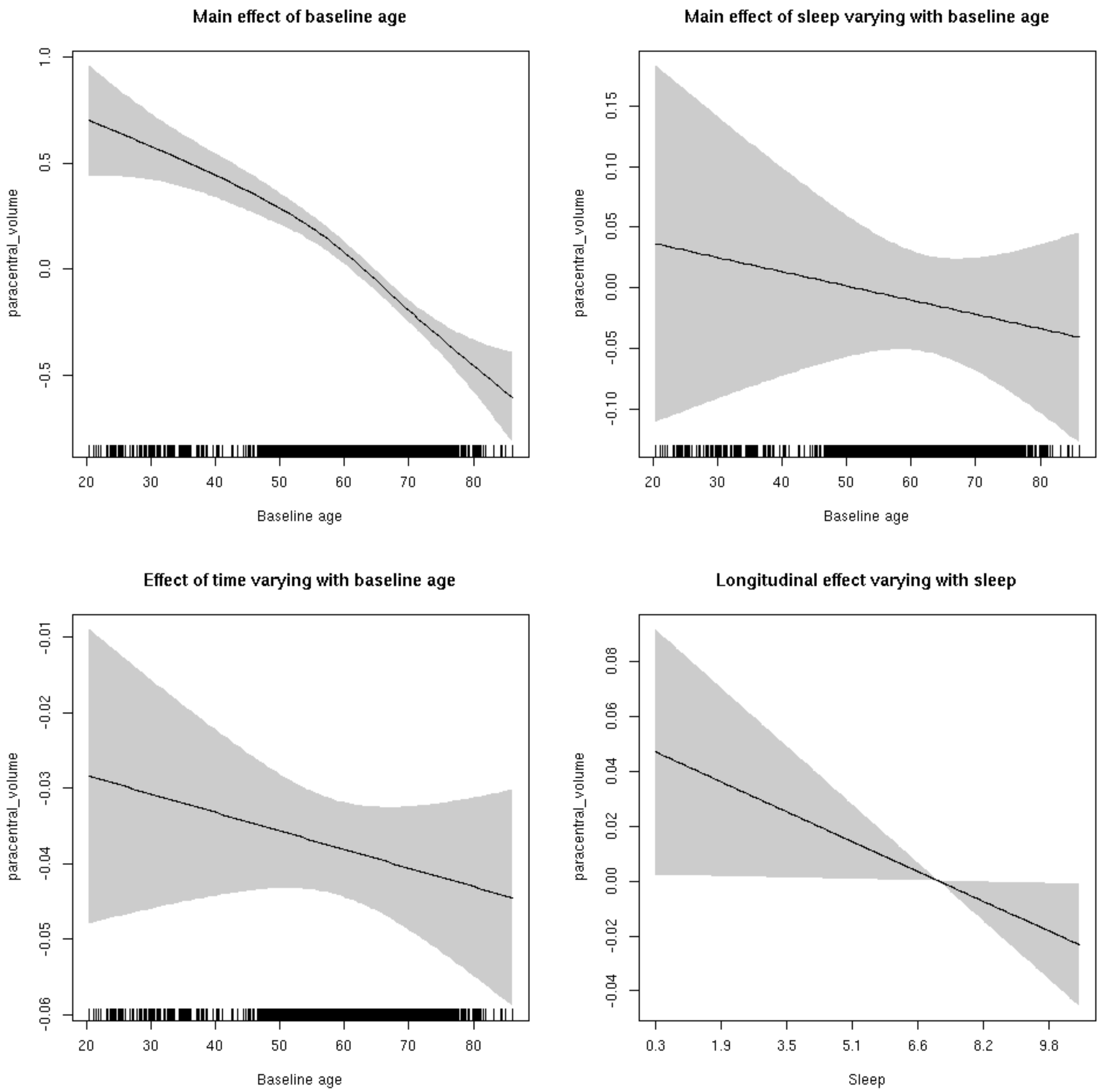

paracentral_volume

Full data Below is the model output. The term `s(sleep_z):time` is probably what we care most about.

```
##
## Family: gaussian
## Link function: identity
##
## Formula:
## value ~ sex + site + icv + s(bl_age, k = 10, bs = "cr") + s(bl_age,
##     by = sleep_z, bs = "cr") + +s(bl_age, by = time, k = 5, bs = "cr") +
##     s(sleep_z, by = time, k = 5, bs = "cr", pc = 0)
## <environment: 0x5630035ac8b0>
```

```
##
## Parametric coefficients:
##                 Estimate Std. Error t value Pr(>|t|)
## (Intercept)      0.02554    0.05886   0.434  0.66434
## sexmale         -0.03046    0.03032  -1.004  0.31520
## siteousAvanto -0.94501    0.07235 -13.062  < 2e-16 ***
## siteousPrisma -0.42821    0.20761  -2.063  0.03919 *
## siteousSkyra  -0.53940    0.06999  -7.707 1.44e-14 ***
## siteUB        -0.37346    0.13519  -2.762  0.00575 **
## siteUCAM      -0.56375    0.07275  -7.749 1.04e-14 ***
## siteUKB        0.11408    0.05553   2.054  0.03996 *
## siteUmU        0.09375    0.08398   1.116  0.26434
## icv            0.52594    0.01497  35.122  < 2e-16 ***
## ---
## Signif. codes:  0 '***' 0.001 '**' 0.01 '*' 0.05 '.' 0.1 ' ' 1
##
## Approximate significance of smooth terms:
##                       edf Ref.df       F p-value
## s(bl_age)           2.047  2.047 132.758  <2e-16 ***
## s(bl_age):sleep_z 2.400  2.400   0.248   0.798
## s(bl_age):time      2.000  2.000 129.088  <2e-16 ***
## s(sleep_z):time     1.000  1.000   2.262   0.133
## ---
## Signif. codes:  0 '***' 0.001 '**' 0.01 '*' 0.05 '.' 0.1 ' ' 1
##
## R-sq.(adj) =  0.393
## lmer.REML =  12391  Scale est. = 0.065177  n = 8178
```

Below are plots of a plot of `s(bl_age)`, `s(bl_age):sleep_z`, `s(bl_age):time`, and `s(sleep_z):time`. For `s(sleep_z):time`, roughly, if we cannot fit a straight line through the confidence bands, there is evidence that the effect of time depends on sleep duration. It is correct the that confidence interval is zero at one point along the axis; this happens because we do not include the main effect.

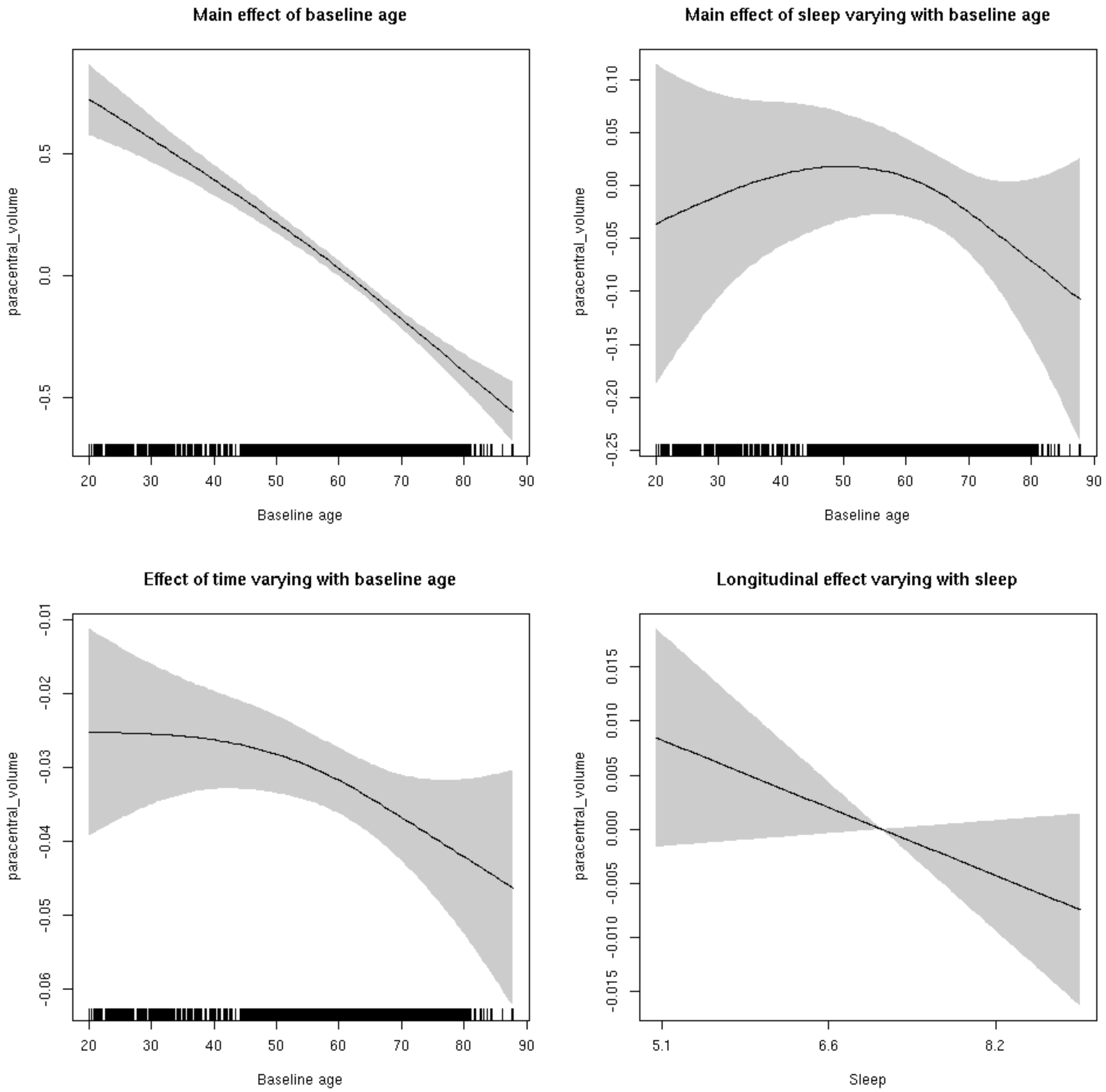

Restricted to sleep >= 5 and <= 9 hours Below is the row for `s(sleep_z):time` from the model output.

```
## s(sleep_z):time   1.000  1.000   2.815  0.0934 .
```

Below are plots of a plot of `s(bl_age)`, `s(bl_age):sleep_z`, `s(bl_age):time`, and `s(sleep_z):time`. For `s(sleep_z):time`, roughly, if we cannot fit a straight line through the confidence bands, there is evidence that the effect of time depends on sleep duration. It is correct the that confidence interval is zero at one point along the axis; this happens because we do not include the main effect.

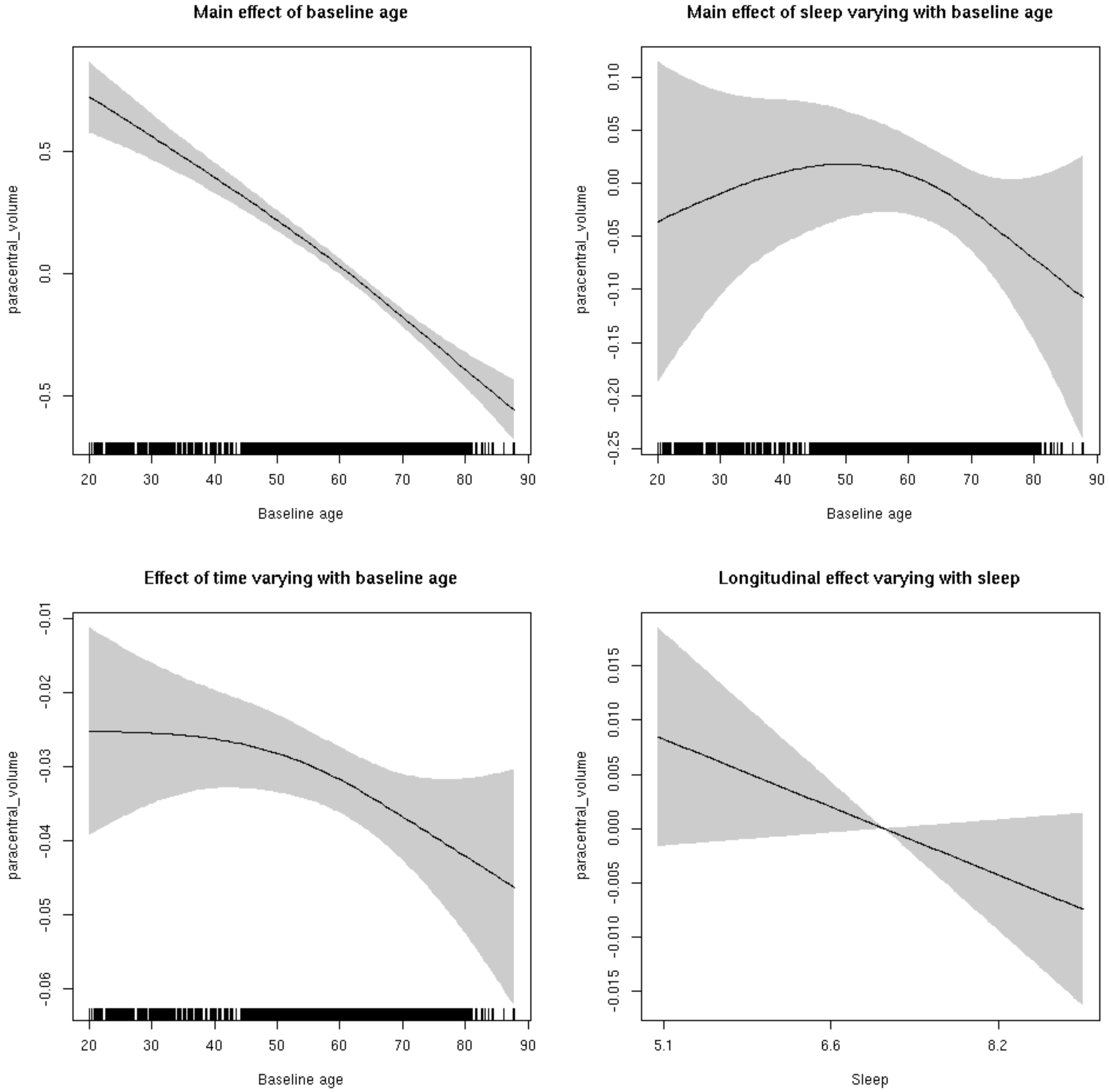

parahippocampal_area: Stratified by sex

Female: Full data Below is the row for `s(sleep_z):time` from the model output.

```
## s(sleep_z):time   1.000  1.000   0.073   0.787
```

Below are plots of a plot of `s(bl_age)`, `s(bl_age):sleep_z`, `s(bl_age):time`, and `s(sleep_z):time`. For `s(sleep_z):time`, roughly, if we cannot fit a straight line through the confidence bands, there is evidence that the effect of time depends on sleep duration. It is correct the that confidence interval is zero at one point along the axis; this happens because we do not include the main effect.

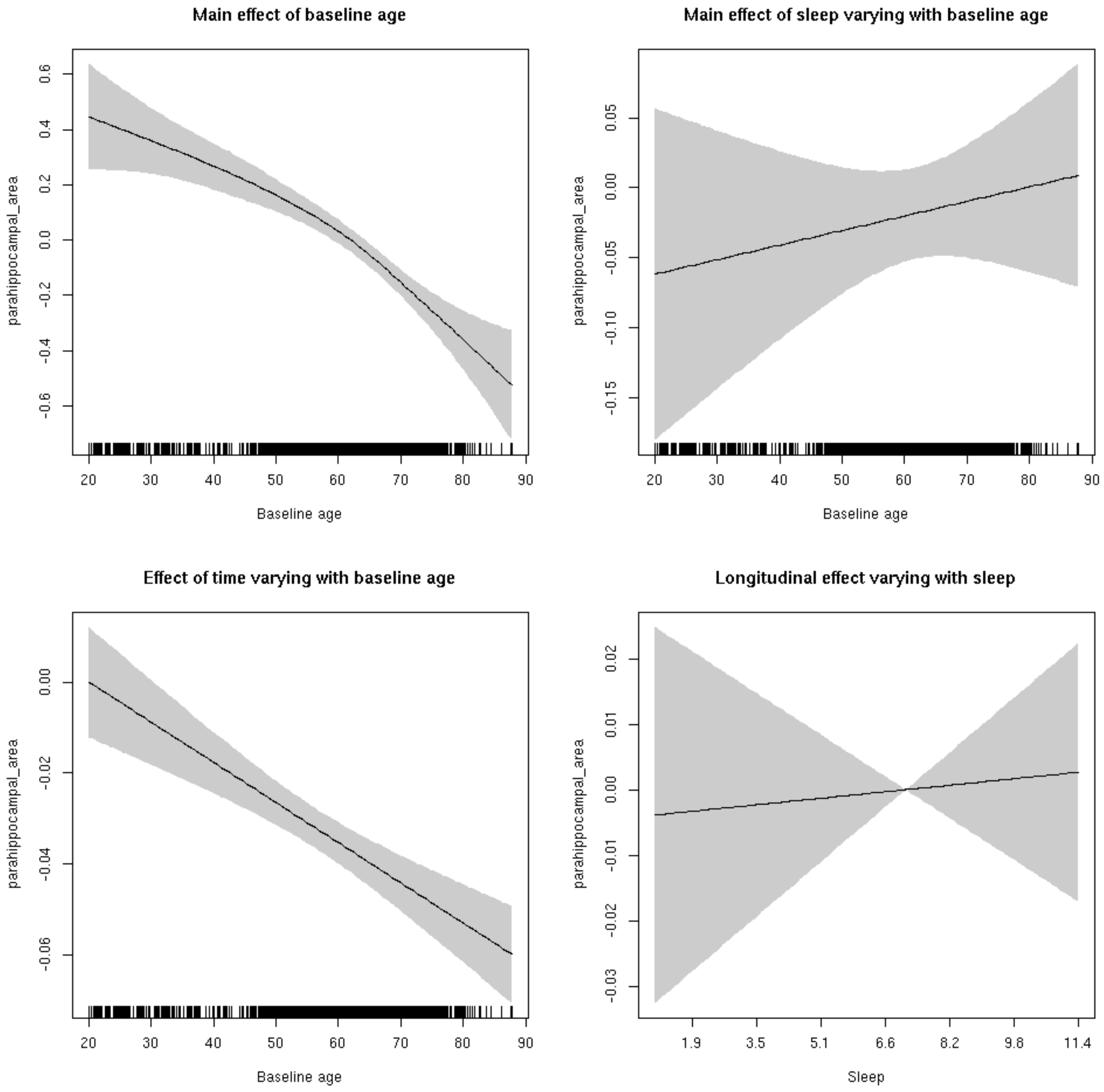

Male: Full data Below is the row for `s(sleep_z):time` from the model output.

```
## s(sleep_z):time   1.000  1.000   0.291   0.590
```

Below are plots of a plot of `s(bl_age)`, `s(bl_age):sleep_z`, `s(bl_age):time`, and `s(sleep_z):time`. For `s(sleep_z):time`, roughly, if we cannot fit a straight line through the confidence bands, there is evidence that the effect of time depends on sleep duration. It is correct the that confidence interval is zero at one point along the axis; this happens because we do not include the main effect.

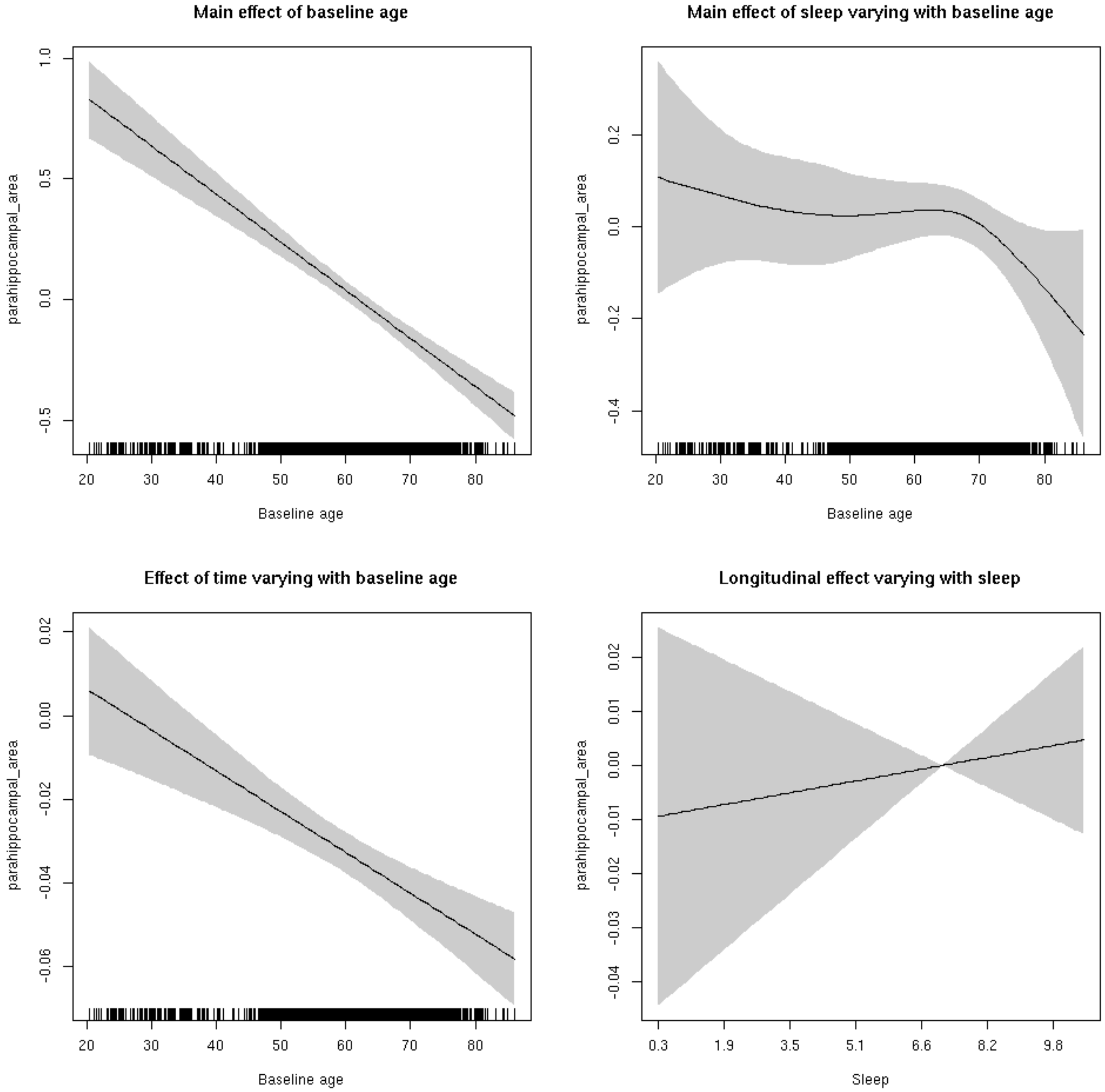

parahippocampal_area

Full data Below is the model output. The term `s(sleep_z):time` is probably what we care most about.

```
##
## Family: gaussian
## Link function: identity
##
## Formula:
## value ~ sex + site + icv + s(bl_age, k = 10, bs = "cr") + s(bl_age,
##     by = sleep_z, bs = "cr") + +s(bl_age, by = time, k = 5, bs = "cr") +
##     s(sleep_z, by = time, k = 5, bs = "cr", pc = 0)
## <environment: 0x5630035b1a08>
```

```
##
## Parametric coefficients:
##                Estimate Std. Error t value Pr(>|t|)
## (Intercept)     0.70912    0.05986  11.846  < 2e-16 ***
## sexmale         0.15942    0.03079   5.177 2.31e-07 ***
## siteousAvanto -0.88258    0.07318 -12.061  < 2e-16 ***
## siteousPrisma -1.21544    0.17518  -6.938 4.27e-12 ***
## siteousSkyra  -0.46729    0.07082  -6.598 4.41e-11 ***
## siteUB        -0.75145    0.13785  -5.451 5.15e-08 ***
## siteUCAM      -0.86386    0.07411 -11.656  < 2e-16 ***
## siteUKB       -0.76202    0.05672 -13.434  < 2e-16 ***
## siteUmU       -0.54912    0.08544  -6.427 1.38e-10 ***
## icv            0.49151    0.01515  32.442  < 2e-16 ***
## ---
## Signif. codes:  0 '***' 0.001 '**' 0.01 '*' 0.05 '.' 0.1 ' ' 1
##
## Approximate significance of smooth terms:
##                      edf Ref.df       F p-value
## s(bl_age)          2.598  2.598  77.274  <2e-16 ***
## s(bl_age):sleep_z 2.657  2.657   0.187   0.830
## s(bl_age):time    2.000  2.000 221.583  <2e-16 ***
## s(sleep_z):time   1.000  1.000   0.364   0.546
## ---
## Signif. codes:  0 '***' 0.001 '**' 0.01 '*' 0.05 '.' 0.1 ' ' 1
##
## R-sq.(adj) =  0.352
## lmer.REML =  10712  Scale est. = 0.042724  n = 8172
```

Below are plots of a plot of `s(bl_age)`, `s(bl_age):sleep_z`, `s(bl_age):time`, and `s(sleep_z):time`. For `s(sleep_z):time`, roughly, if we cannot fit a straight line through the confidence bands, there is evidence that the effect of time depends on sleep duration. It is correct the that confidence interval is zero at one point along the axis; this happens because we do not include the main effect.

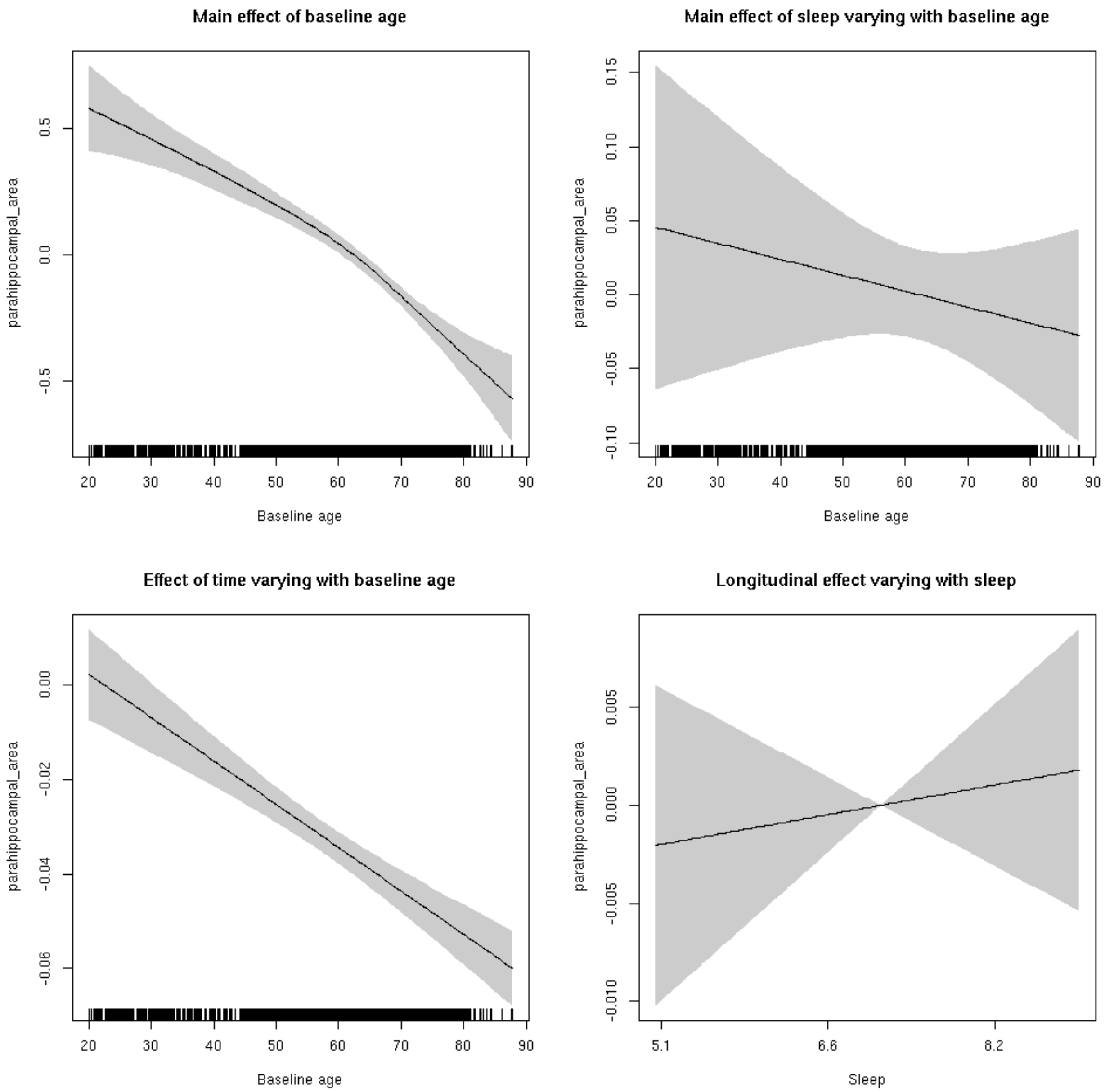

Restricted to sleep >= 5 and <= 9 hours Below is the row for `s(sleep_z):time` from the model output.

```
## s(sleep_z):time   1.000  1.000   0.251   0.617
```

Below are plots of a plot of `s(bl_age)`, `s(bl_age):sleep_z`, `s(bl_age):time`, and `s(sleep_z):time`. For `s(sleep_z):time`, roughly, if we cannot fit a straight line through the confidence bands, there is evidence that the effect of time depends on sleep duration. It is correct the that confidence interval is zero at one point along the axis; this happens because we do not include the main effect.

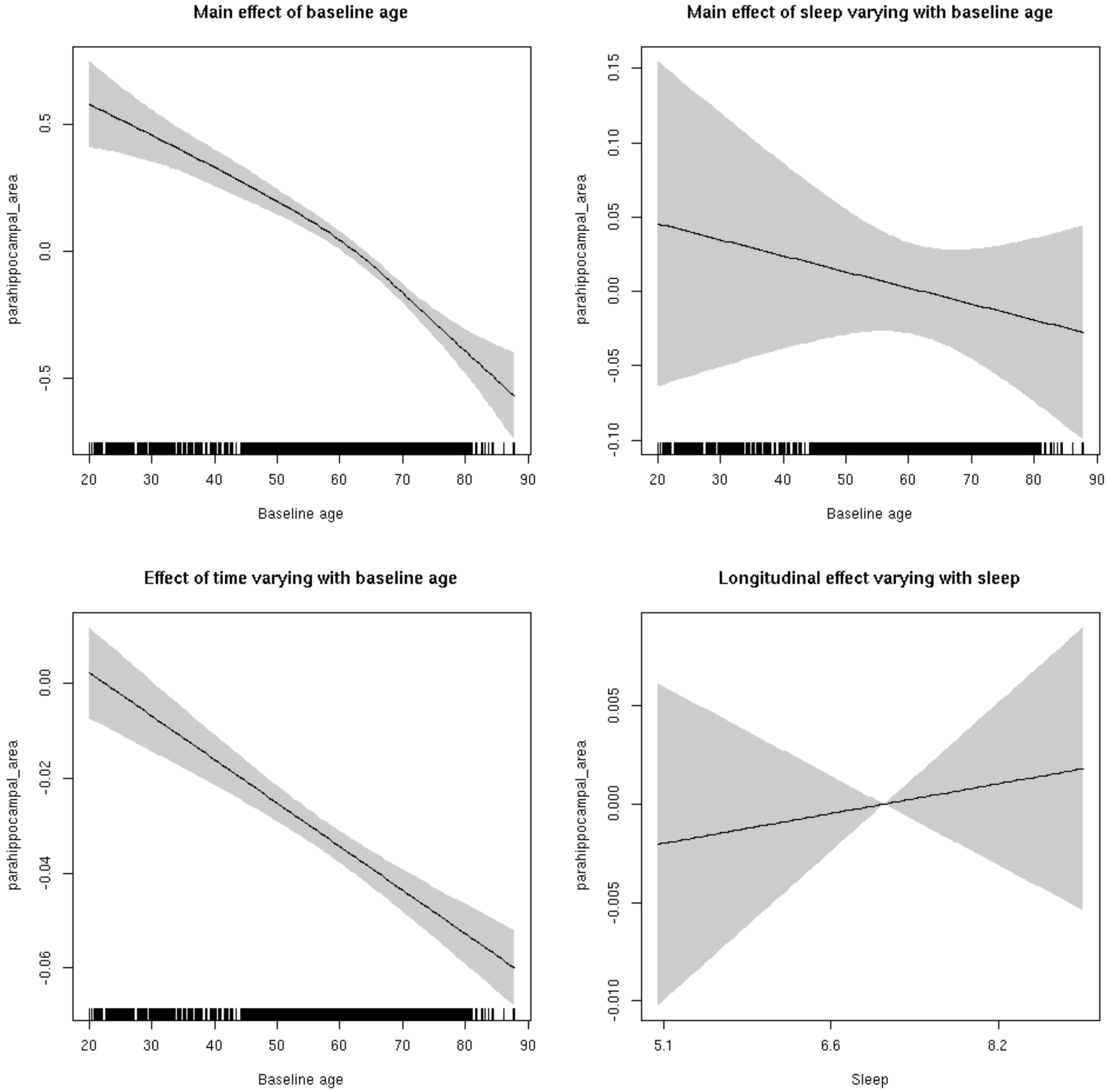

parahippocampal_thickness: Stratified by sex

Female: Full data Below is the row for `s(sleep_z):time` from the model output.

```
## s(sleep_z):time   1.000  1.000  0.887    0.346
```

Below are plots of a plot of `s(bl_age)`, `s(bl_age):sleep_z`, `s(bl_age):time`, and `s(sleep_z):time`. For `s(sleep_z):time`, roughly, if we cannot fit a straight line through the confidence bands, there is evidence that the effect of time depends on sleep duration. It is correct the that confidence interval is zero at one point along the axis; this happens because we do not include the main effect.

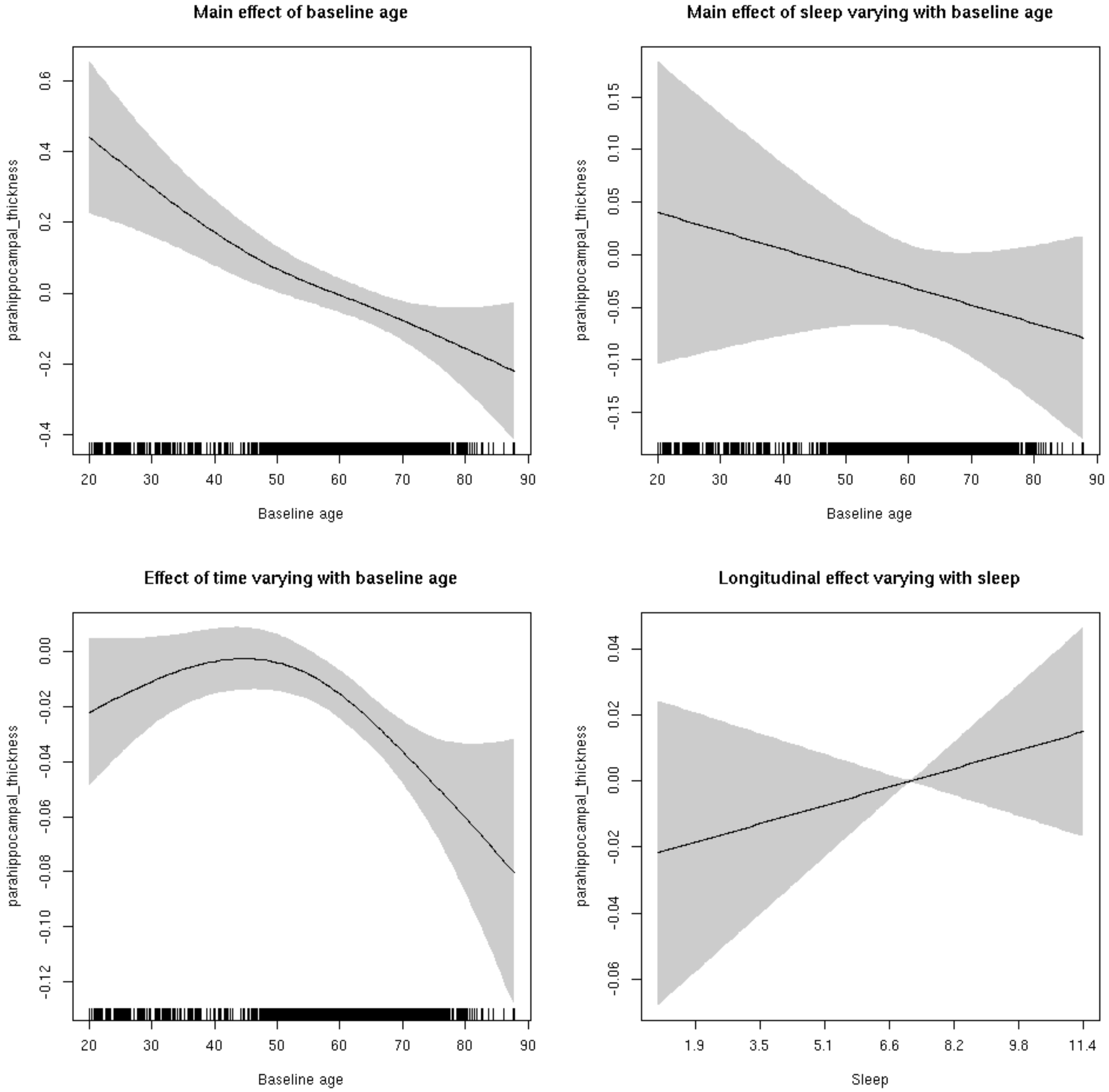

Male: Full data Below is the row for `s(sleep_z):time` from the model output.

```
## s(sleep_z):time   1.000  1.000  0.530  0.4666
```

Below are plots of a plot of `s(bl_age)`, `s(bl_age):sleep_z`, `s(bl_age):time`, and `s(sleep_z):time`. For `s(sleep_z):time`, roughly, if we cannot fit a straight line through the confidence bands, there is evidence that the effect of time depends on sleep duration. It is correct the that confidence interval is zero at one point along the axis; this happens because we do not include the main effect.

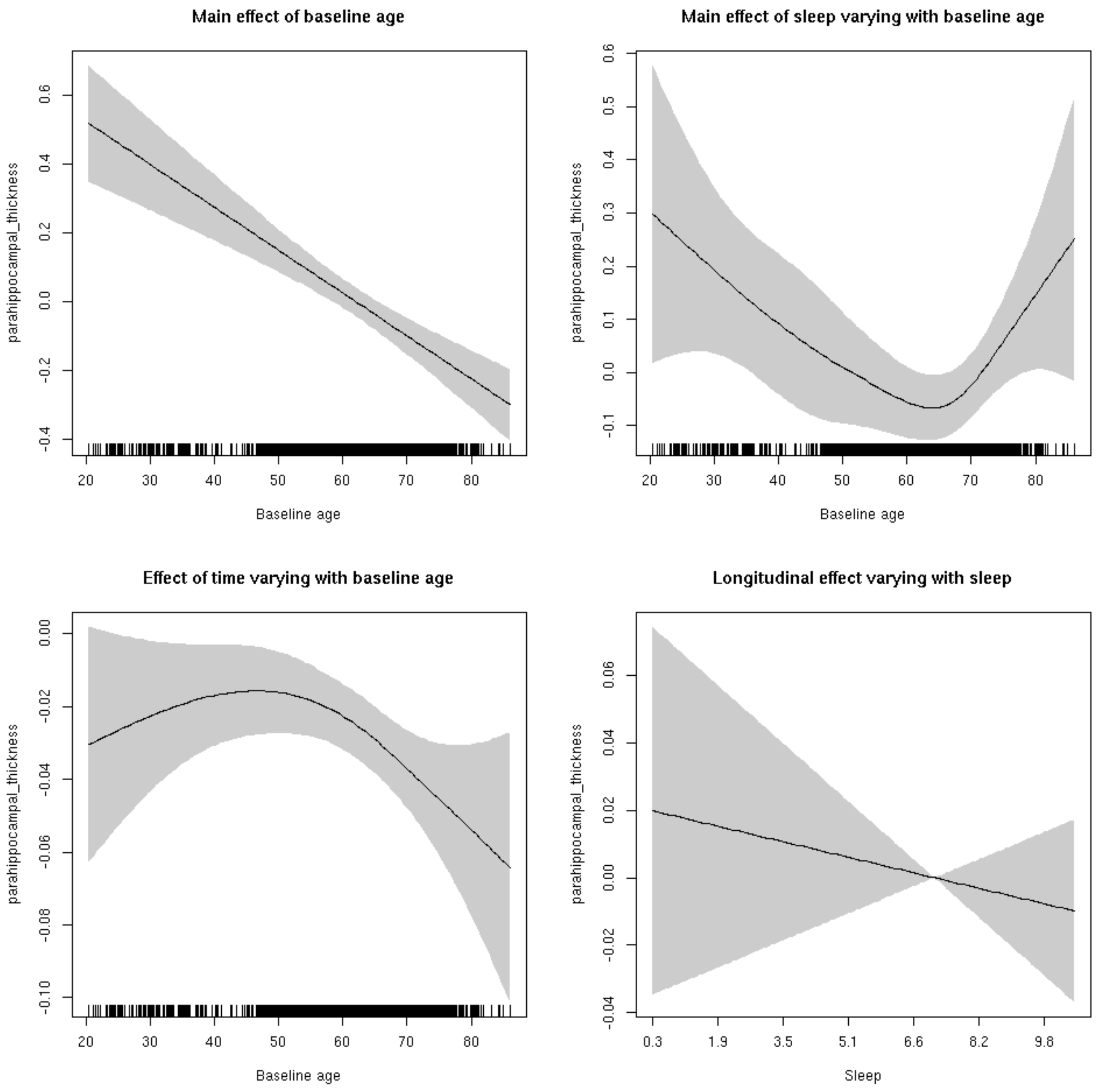

parahippocampal_thickness

Full data Below is the model output. The term `s(sleep_z):time` is probably what we care most about.

```
##
## Family: gaussian
## Link function: identity
##
## Formula:
## value ~ sex + site + s(bl_age, k = 10, bs = "cr") + s(bl_age,
##     by = sleep_z, bs = "cr") + +s(bl_age, by = time, k = 5, bs = "cr") +
##     s(sleep_z, by = time, k = 5, bs = "cr", pc = 0)
## <environment: 0x5630035d2ce8>
```

```
##
## Parametric coefficients:
##                Estimate Std. Error t value Pr(>|t|)
## (Intercept)   -0.303939   0.055674  -5.459 4.92e-08 ***
## sexmale       -0.309006   0.028672 -10.777  < 2e-16 ***
## siteousAvanto -0.071426   0.074869  -0.954   0.3401
## siteousPrisma  0.181506   0.257583   0.705   0.4810
## siteousSkyra   0.392405   0.074757   5.249 1.57e-07 ***
## siteUB         0.027298   0.152230   0.179   0.8577
## siteUCAM       0.178302   0.077556   2.299   0.0215 *
## siteUKB        0.557058   0.055205  10.091  < 2e-16 ***
## siteUmU       -0.009677   0.093423  -0.104   0.9175
## ---
## Signif. codes:  0 '***' 0.001 '**' 0.01 '*' 0.05 '.' 0.1 ' ' 1
##
## Approximate significance of smooth terms:
##                     edf Ref.df      F p-value
## s(bl_age)         1.000  1.000 57.308  <2e-16 ***
## s(bl_age):sleep_z 3.493  3.493  2.645   0.024 *
## s(bl_age):time    3.400  3.400 30.612  <2e-16 ***
## s(sleep_z):time   1.000  1.000  0.060   0.806
## ---
## Signif. codes:  0 '***' 0.001 '**' 0.01 '*' 0.05 '.' 0.1 ' ' 1
##
## R-sq.(adj) =  0.104
## lmer.REML =  15508  Scale est. = 0.10678   n = 8166
```

Below are plots of a plot of `s(bl_age)`, `s(bl_age):sleep_z`, `s(bl_age):time`, and `s(sleep_z):time`. For `s(sleep_z):time`, roughly, if we cannot fit a straight line through the confidence bands, there is evidence that the effect of time depends on sleep duration. It is correct the that confidence interval is zero at one point along the axis; this happens because we do not include the main effect.

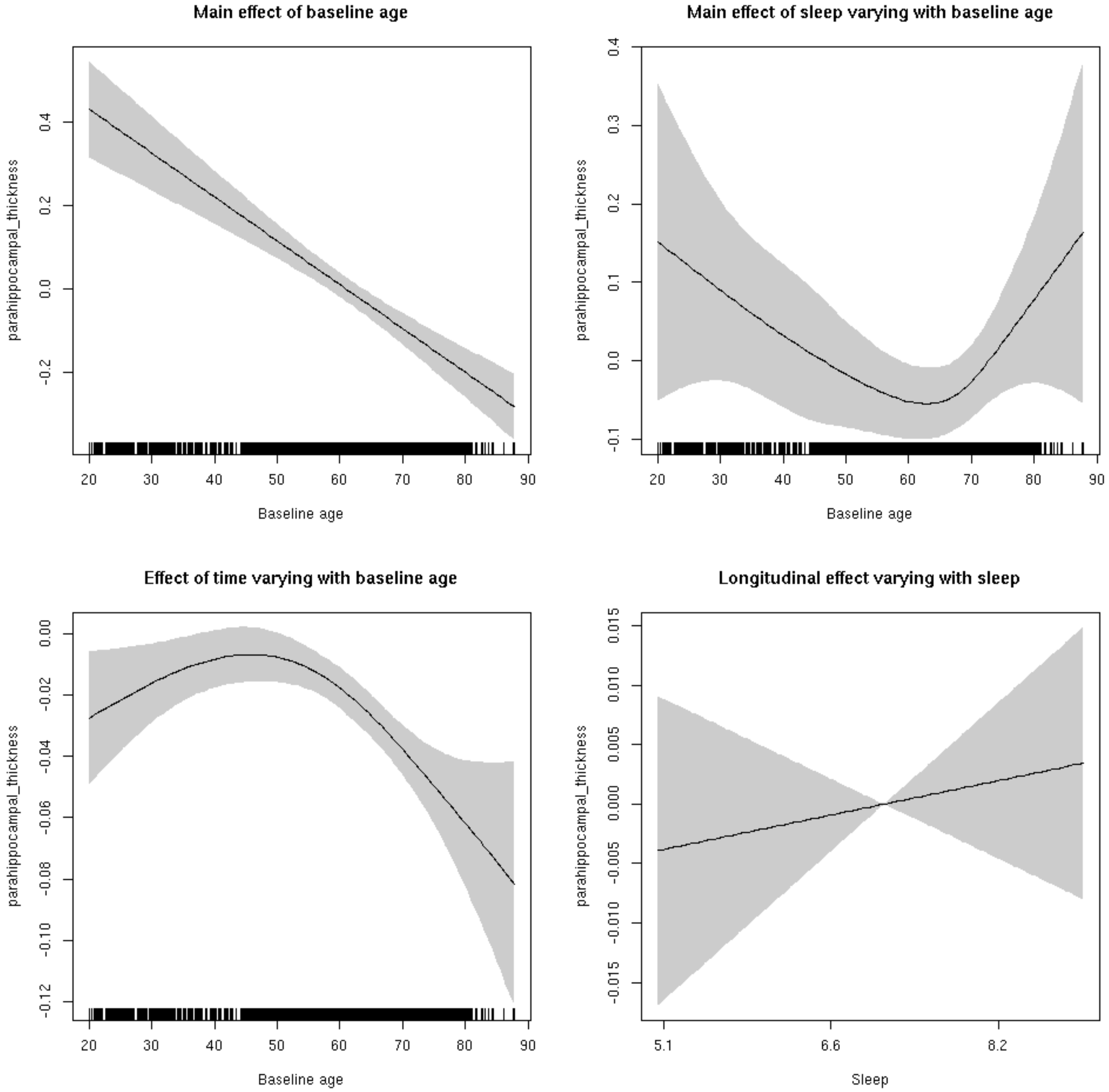

Restricted to sleep >= 5 and <= 9 hours Below is the row for `s(sleep_z):time` from the model output.

```
## s(sleep_z):time   1.000  1.000  0.363  0.5469
```

Below are plots of a plot of `s(bl_age)`, `s(bl_age):sleep_z`, `s(bl_age):time`, and `s(sleep_z):time`. For `s(sleep_z):time`, roughly, if we cannot fit a straight line through the confidence bands, there is evidence that the effect of time depends on sleep duration. It is correct the that confidence interval is zero at one point along the axis; this happens because we do not include the main effect.

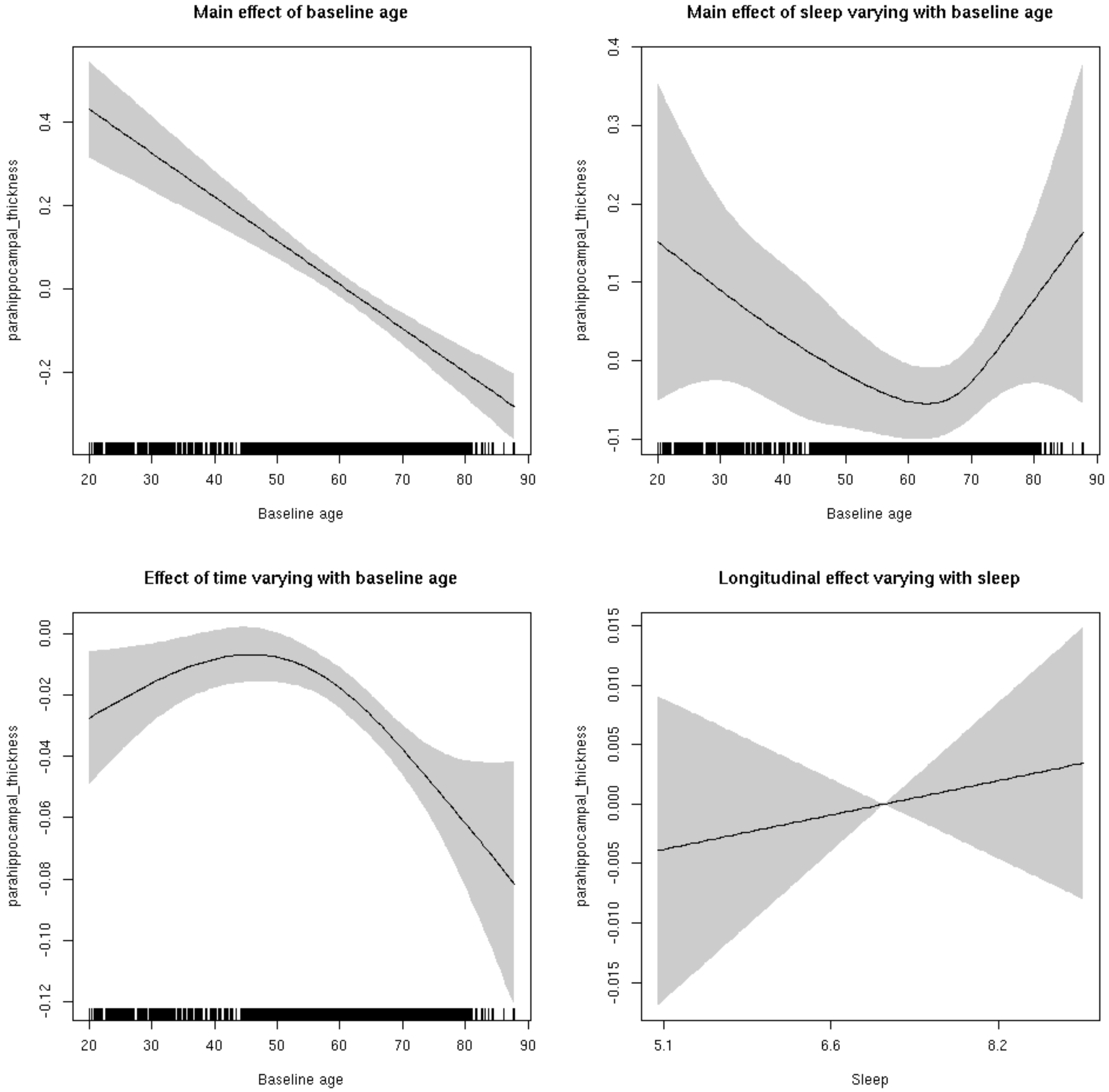

parahippocampal_volume: Stratified by sex

Female: Full data Below is the row for `s(sleep_z):time` from the model output.

```
## s(sleep_z):time   1.000  1.000  1.843  0.1747
```

Below are plots of a plot of `s(bl_age)`, `s(bl_age):sleep_z`, `s(bl_age):time`, and `s(sleep_z):time`. For `s(sleep_z):time`, roughly, if we cannot fit a straight line through the confidence bands, there is evidence that the effect of time depends on sleep duration. It is correct the that confidence interval is zero at one point along the axis; this happens because we do not include the main effect.

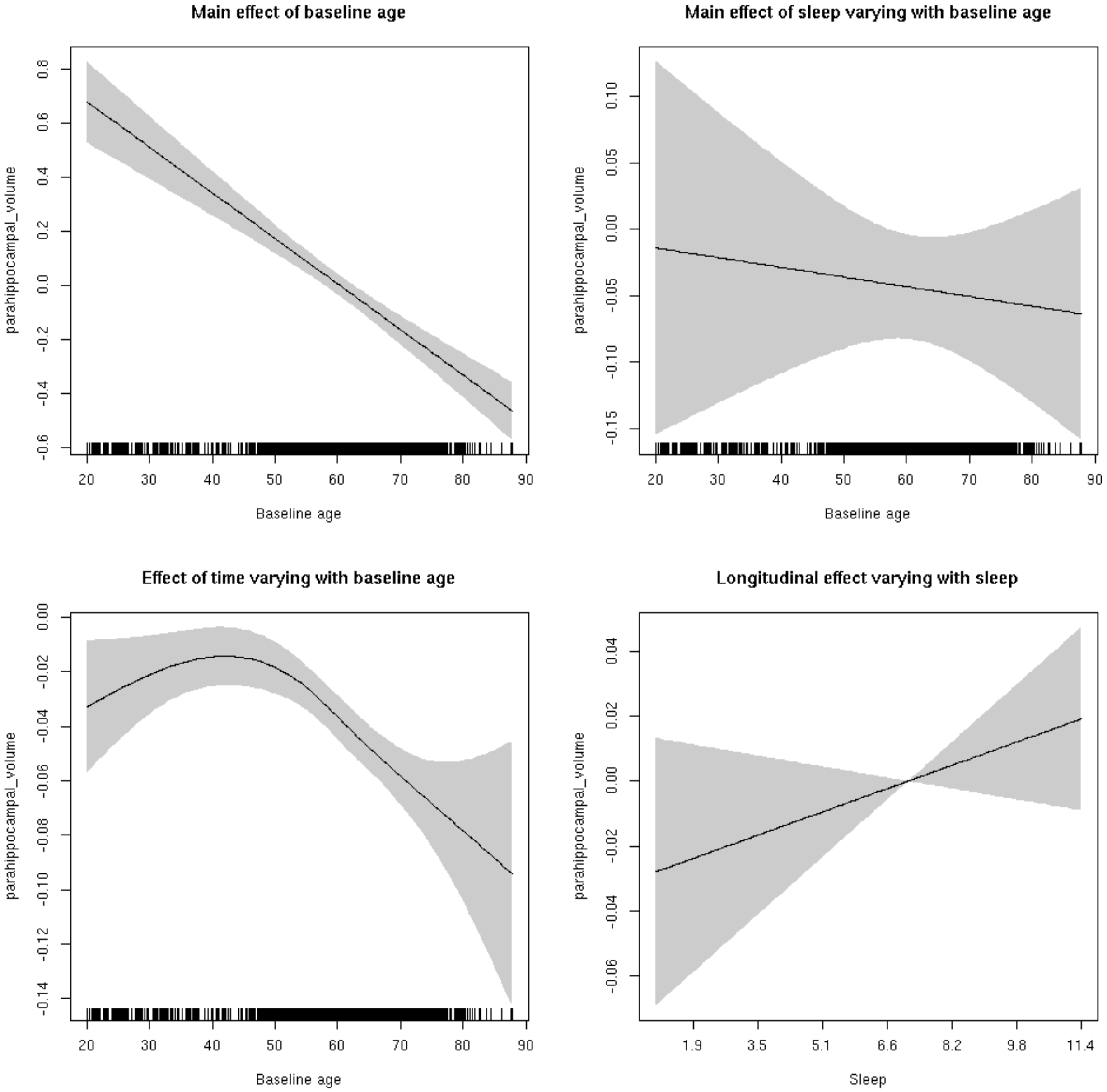

Male: Full data Below is the row for `s(sleep_z):time` from the model output.

```
## s(sleep_z):time   1.000  1.000   0.728  0.3934
```

Below are plots of a plot of `s(bl_age)`, `s(bl_age):sleep_z`, `s(bl_age):time`, and `s(sleep_z):time`. For `s(sleep_z):time`, roughly, if we cannot fit a straight line through the confidence bands, there is evidence that the effect of time depends on sleep duration. It is correct the that confidence interval is zero at one point along the axis; this happens because we do not include the main effect.

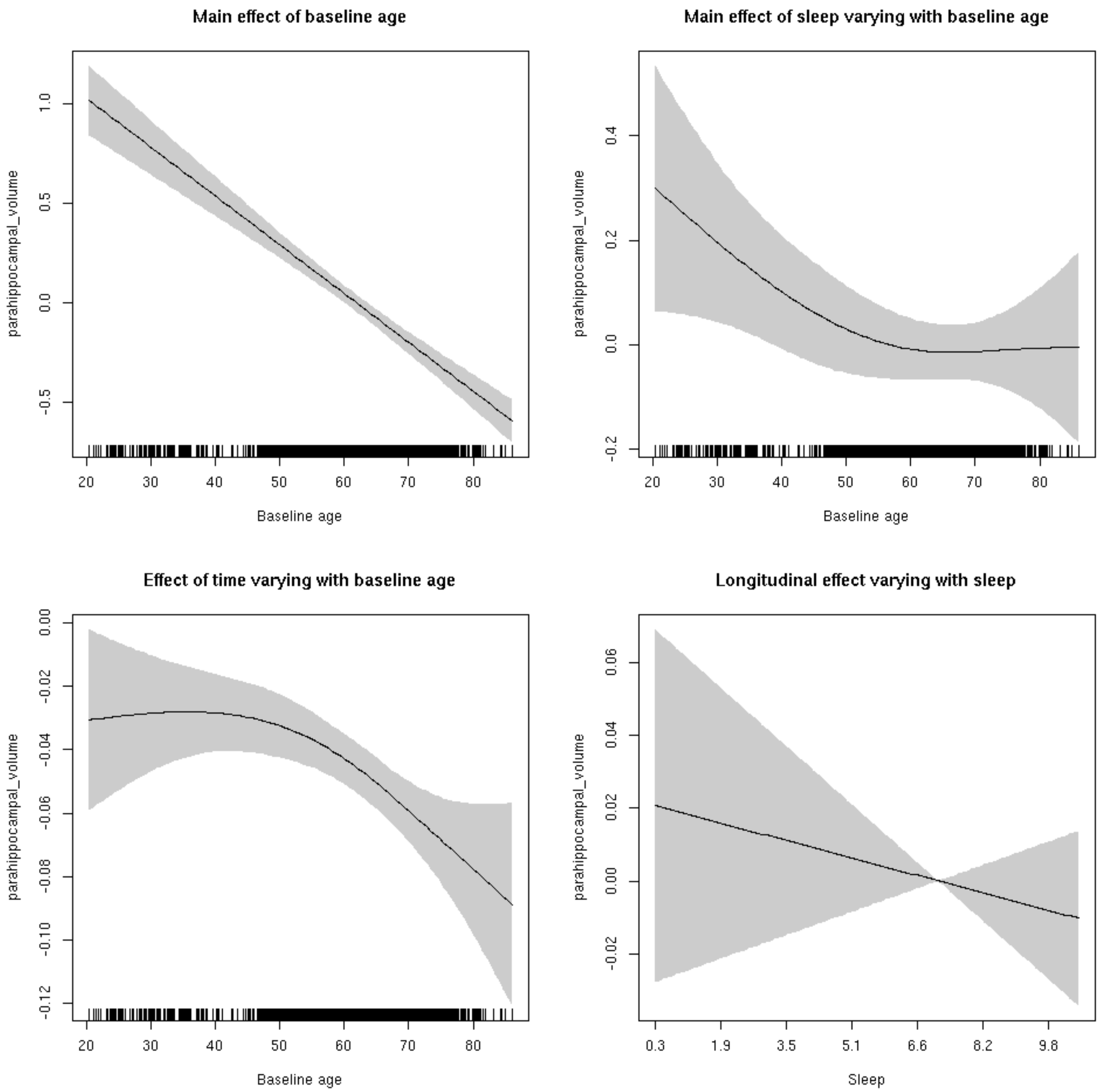

parahippocampal_volume

Full data Below is the model output. The term `s(sleep_z):time` is probably what we care most about.

```
##
## Family: gaussian
## Link function: identity
##
## Formula:
## value ~ sex + site + icv + s(bl_age, k = 10, bs = "cr") + s(bl_age,
##     by = sleep_z, bs = "cr") + +s(bl_age, by = time, k = 5, bs = "cr") +
##     s(sleep_z, by = time, k = 5, bs = "cr", pc = 0)
## <environment: 0x5630035aedb8>
```

```
##
## Parametric coefficients:
##                Estimate Std. Error t value Pr(>|t|)
## (Intercept)     0.45146    0.06731   6.707 2.12e-11 ***
## sexmale        -0.06012    0.03495  -1.720 0.085425 .
## siteousAvanto -0.41889    0.08335  -5.026 5.12e-07 ***
## siteousPrisma -0.35474    0.23658  -1.499 0.133796
## siteousSkyra   0.25745    0.08058   3.195 0.001403 **
## siteUB        -0.40486    0.15580  -2.599 0.009378 **
## siteUCAM      -0.31028    0.08369  -3.707 0.000211 ***
## siteUKB       -0.33158    0.06268  -5.290 1.25e-07 ***
## siteUmU       -0.18202    0.09649  -1.887 0.059262 .
## icv            0.29018    0.01726  16.815  < 2e-16 ***
## ---
## Signif. codes:  0 '***' 0.001 '**' 0.01 '*' 0.05 '.' 0.1 ' ' 1
##
## Approximate significance of smooth terms:
##                      edf Ref.df       F p-value
## s(bl_age)          1.000  1.000 225.322  <2e-16 ***
## s(bl_age):sleep_z 2.417  2.417   2.503   0.112
## s(bl_age):time    3.530  3.530 106.391  <2e-16 ***
## s(sleep_z):time   1.000  1.000   0.254   0.615
## ---
## Signif. codes:  0 '***' 0.001 '**' 0.01 '*' 0.05 '.' 0.1 ' ' 1
##
## R-sq.(adj) =  0.177
## lmer.REML =  14603  Scale est. = 0.084234  n = 8175
```

Below are plots of a plot of `s(bl_age)`, `s(bl_age):sleep_z`, `s(bl_age):time`, and `s(sleep_z):time`. For `s(sleep_z):time`, roughly, if we cannot fit a straight line through the confidence bands, there is evidence that the effect of time depends on sleep duration. It is correct the that confidence interval is zero at one point along the axis; this happens because we do not include the main effect.

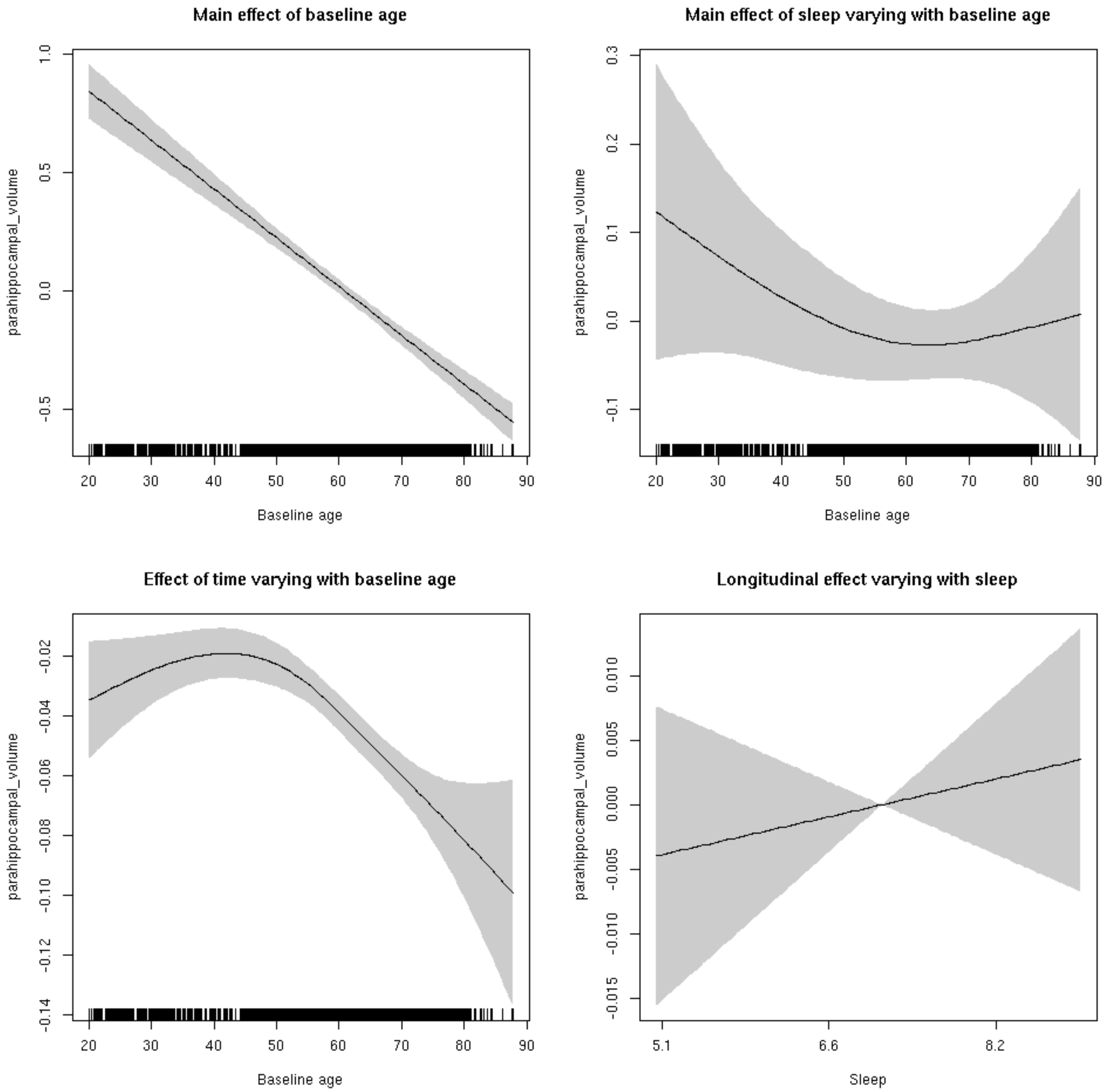

Restricted to sleep >= 5 and <= 9 hours Below is the row for `s(sleep_z):time` from the model output.

```
## s(sleep_z):time   1.000  1.000   0.467   0.494
```

Below are plots of a plot of `s(bl_age)`, `s(bl_age):sleep_z`, `s(bl_age):time`, and `s(sleep_z):time`. For `s(sleep_z):time`, roughly, if we cannot fit a straight line through the confidence bands, there is evidence that the effect of time depends on sleep duration. It is correct the that confidence interval is zero at one point along the axis; this happens because we do not include the main effect.

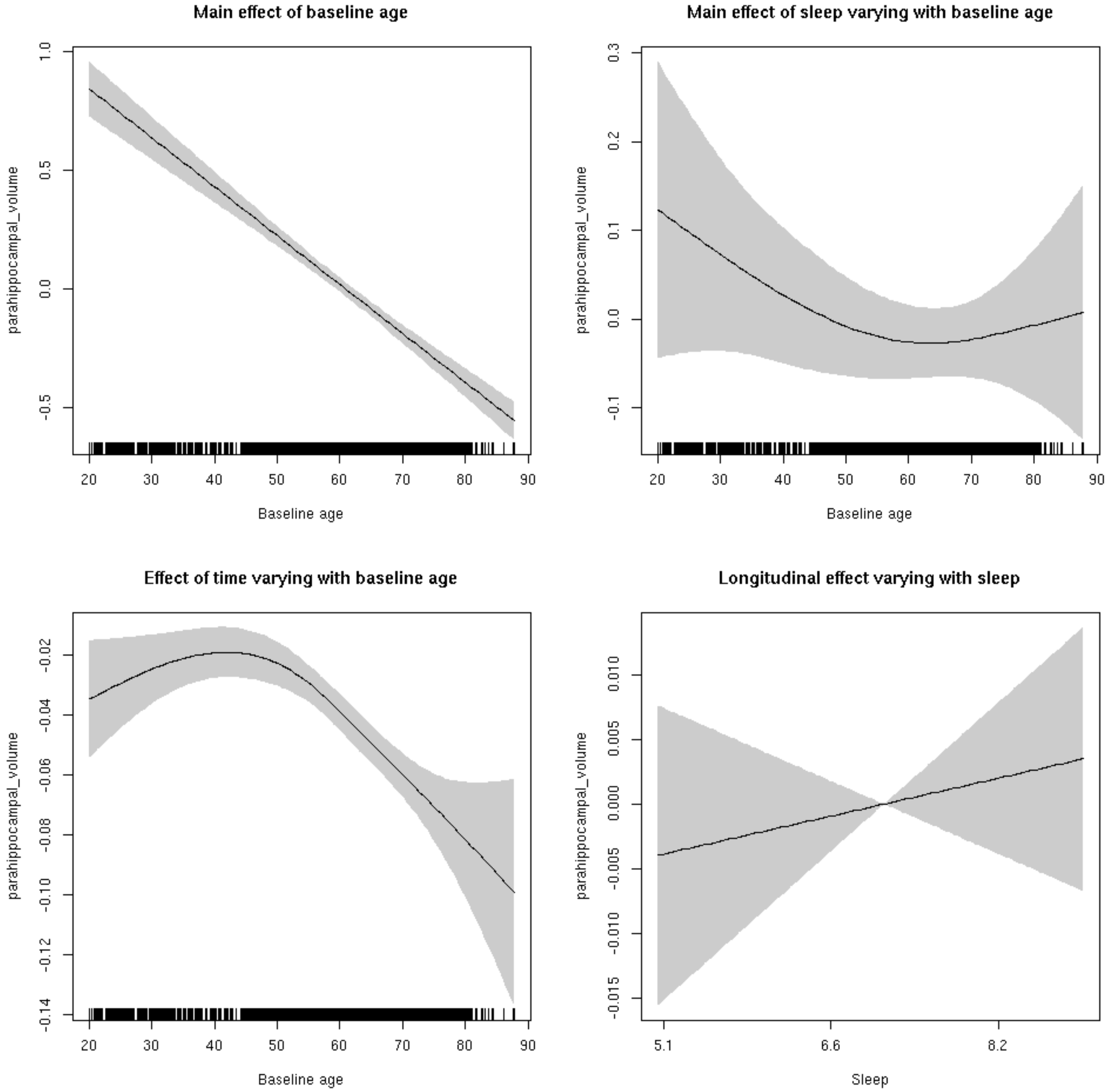

parsopercularis_area: Stratified by sex

Female: Full data Below is the row for `s(sleep_z):time` from the model output.

```
## s(sleep_z):time   3.13   3.13  1.935   0.304
```

Below are plots of a plot of `s(bl_age)`, `s(bl_age):sleep_z`, `s(bl_age):time`, and `s(sleep_z):time`. For `s(sleep_z):time`, roughly, if we cannot fit a straight line through the confidence bands, there is evidence that the effect of time depends on sleep duration. It is correct the that confidence interval is zero at one point along the axis; this happens because we do not include the main effect.

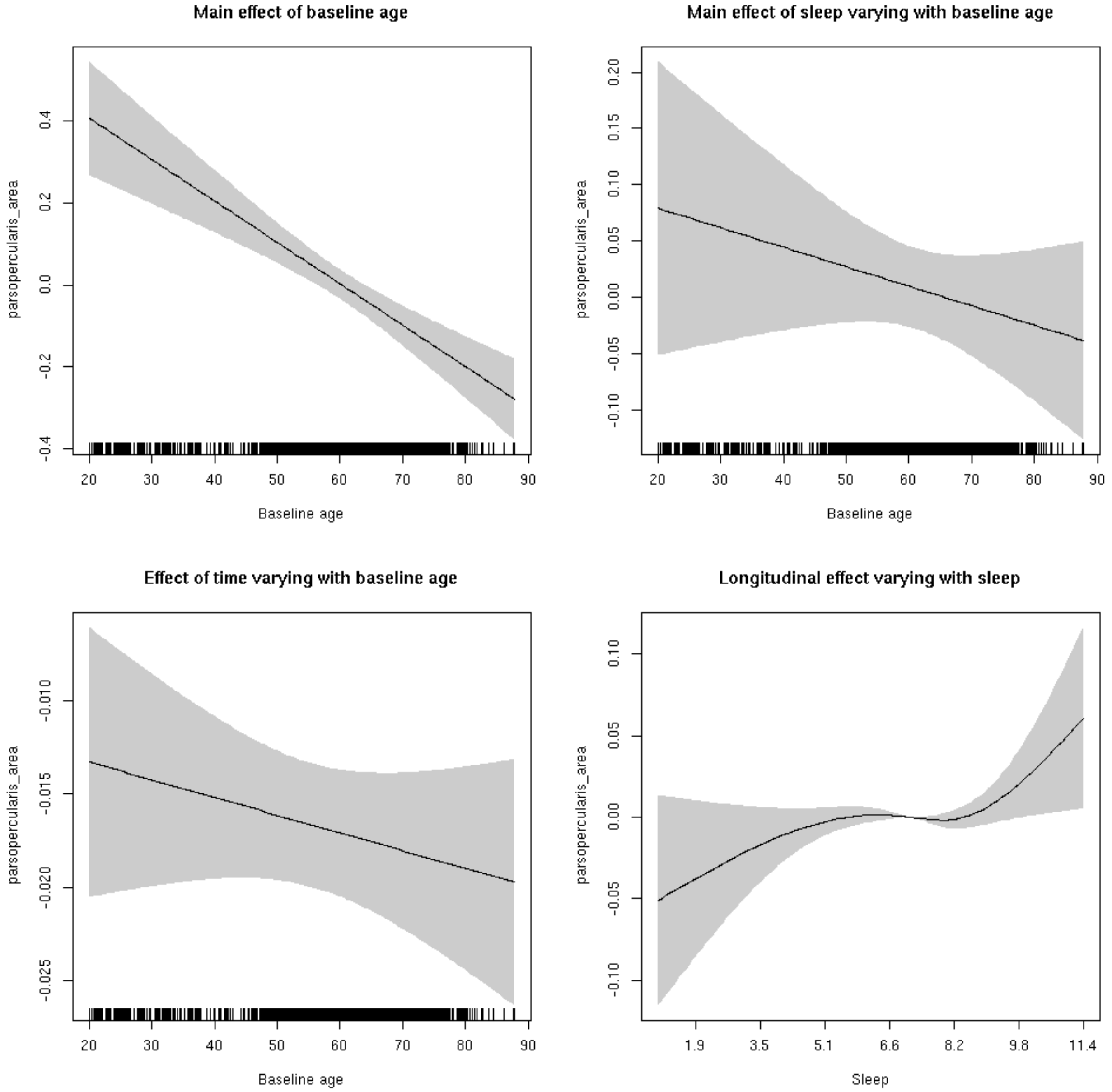

Male: Full data Below is the row for `s(sleep_z):time` from the model output.

```
## s(sleep_z):time   1.000  1.000  2.504   0.114
```

Below are plots of a plot of `s(bl_age)`, `s(bl_age):sleep_z`, `s(bl_age):time`, and `s(sleep_z):time`. For `s(sleep_z):time`, roughly, if we cannot fit a straight line through the confidence bands, there is evidence that the effect of time depends on sleep duration. It is correct the that confidence interval is zero at one point along the axis; this happens because we do not include the main effect.

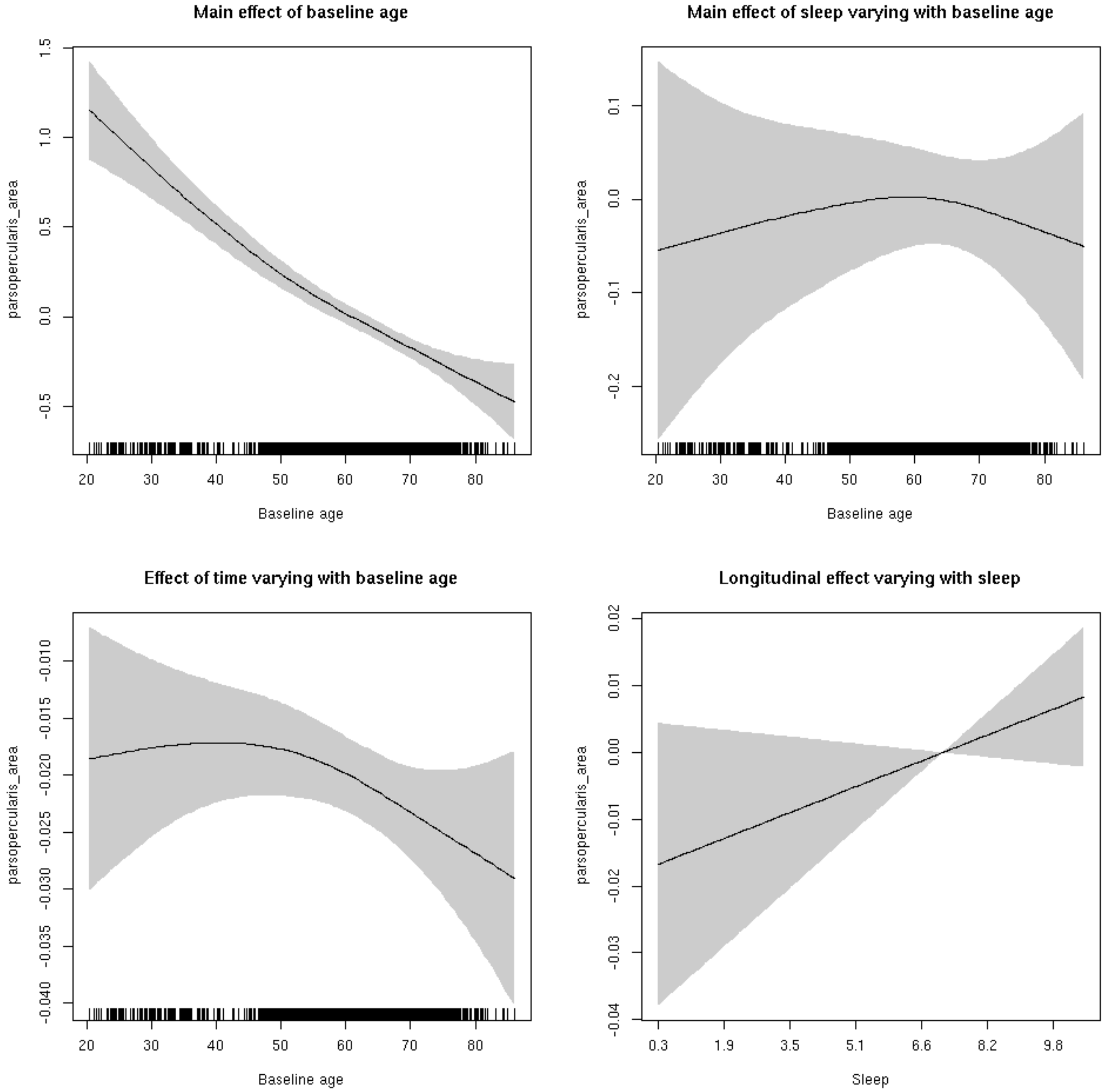

parsopercularis_area

Full data Below is the model output. The term `s(sleep_z):time` is probably what we care most about.

```
##
## Family: gaussian
## Link function: identity
##
## Formula:
## value ~ sex + site + icv + s(bl_age, k = 10, bs = "cr") + s(bl_age,
##     by = sleep_z, bs = "cr") + +s(bl_age, by = time, k = 5, bs = "cr") +
##     s(sleep_z, by = time, k = 5, bs = "cr", pc = 0)
## <environment: 0x5630035a5618>
```

```
##
## Parametric coefficients:
##               Estimate Std. Error t value Pr(>|t|)
## (Intercept)    0.68786    0.06331  10.866  < 2e-16 ***
## sexmale        0.10756    0.03291   3.268  0.00109 **
## siteousAvanto -0.62295    0.07790  -7.997 1.45e-15 ***
## siteousPrisma -0.35521    0.12334  -2.880  0.00399 **
## siteousSkyra  -0.30462    0.07555  -4.032 5.58e-05 ***
## siteUB        -0.77744    0.14888  -5.222 1.81e-07 ***
## siteUCAM      -0.80391    0.07933 -10.133  < 2e-16 ***
## siteUKB       -0.70175    0.05916 -11.861  < 2e-16 ***
## siteUmU       -0.13663    0.09158  -1.492  0.13575
## icv            0.46318    0.01584  29.238  < 2e-16 ***
## ---
## Signif. codes:  0 '***' 0.001 '**' 0.01 '*' 0.05 '.' 0.1 ' ' 1
##
## Approximate significance of smooth terms:
##                     edf Ref.df       F p-value
## s(bl_age)         1.000  1.000 166.142  <2e-16 ***
## s(bl_age):sleep_z 2.798  2.798   0.677   0.455
## s(bl_age):time    2.892  2.892 126.714  <2e-16 ***
## s(sleep_z):time   1.000  1.000   2.231   0.135
## ---
## Signif. codes:  0 '***' 0.001 '**' 0.01 '*' 0.05 '.' 0.1 ' ' 1
##
## R-sq.(adj) =  0.305
## lmer.REML =   6712  Scale est. = 0.014591  n = 8154
```

Below are plots of a plot of `s(bl_age)`, `s(bl_age):sleep_z`, `s(bl_age):time`, and `s(sleep_z):time`. For `s(sleep_z):time`, roughly, if we cannot fit a straight line through the confidence bands, there is evidence that the effect of time depends on sleep duration. It is correct the that confidence interval is zero at one point along the axis; this happens because we do not include the main effect.

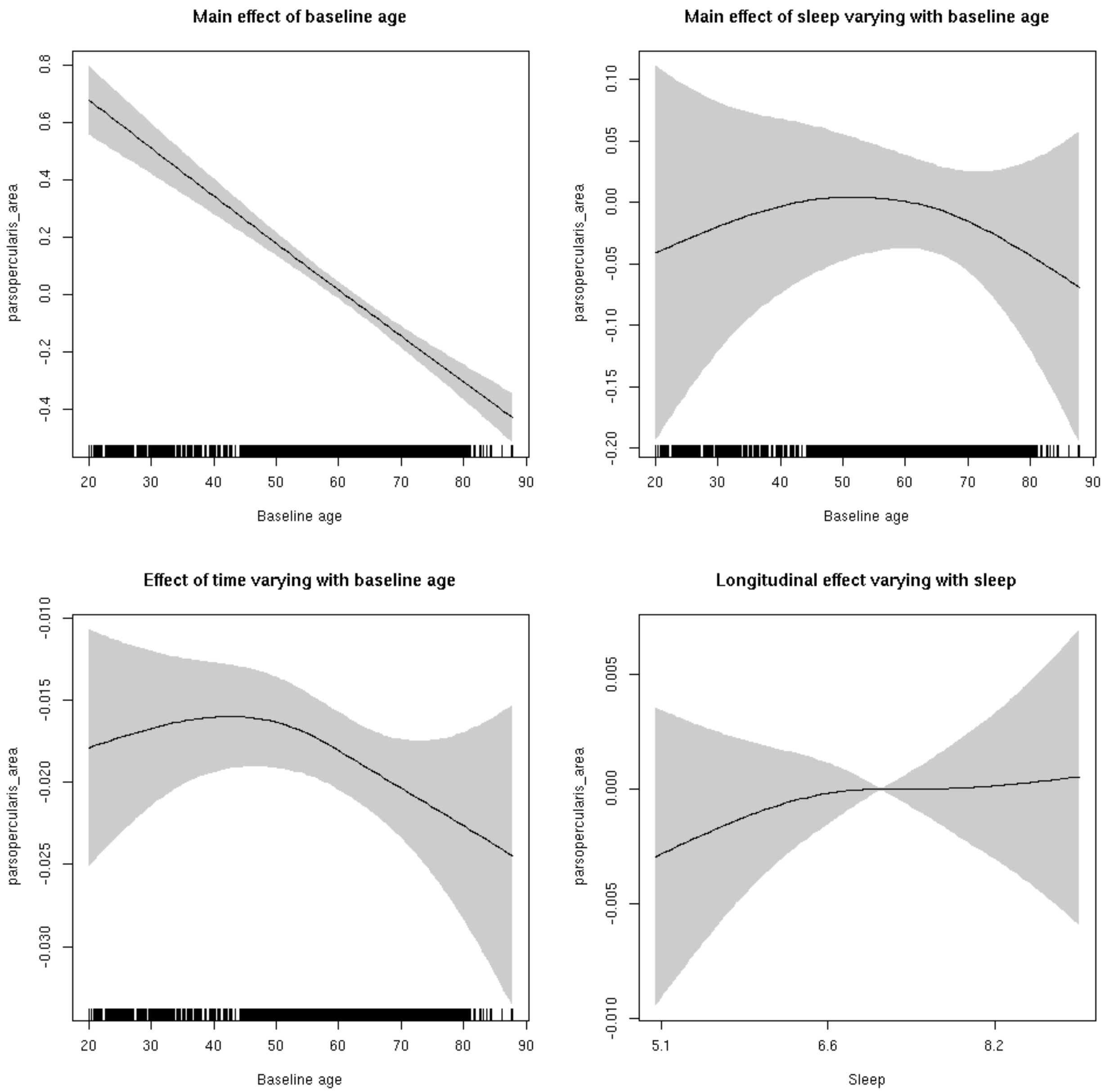

Restricted to sleep >= 5 and <= 9 hours Below is the row for `s(sleep_z):time` from the model output.

```
## s(sleep_z):time   1.407  1.407   0.478   0.681
```

Below are plots of a plot of `s(bl_age)`, `s(bl_age):sleep_z`, `s(bl_age):time`, and `s(sleep_z):time`. For `s(sleep_z):time`, roughly, if we cannot fit a straight line through the confidence bands, there is evidence that the effect of time depends on sleep duration. It is correct the that confidence interval is zero at one point along the axis; this happens because we do not include the main effect.

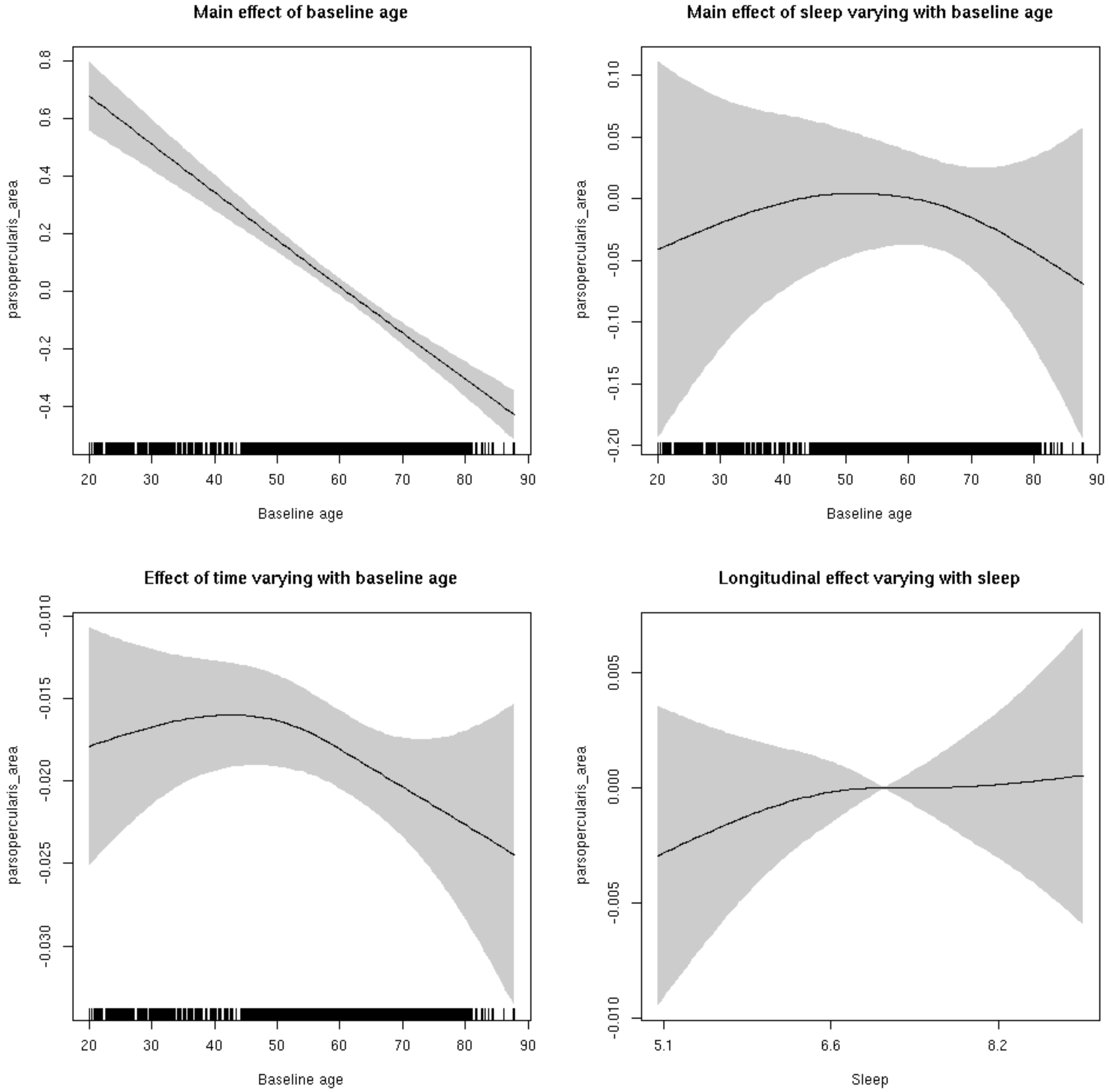

parsopercularis_thickness: Stratified by sex

Female: Full data Below is the row for `s(sleep_z):time` from the model output.

```
## s(sleep_z):time   1.000  1.000   0.001   0.980
```

Below are plots of a plot of `s(bl_age)`, `s(bl_age):sleep_z`, `s(bl_age):time`, and `s(sleep_z):time`. For `s(sleep_z):time`, roughly, if we cannot fit a straight line through the confidence bands, there is evidence that the effect of time depends on sleep duration. It is correct the that confidence interval is zero at one point along the axis; this happens because we do not include the main effect.

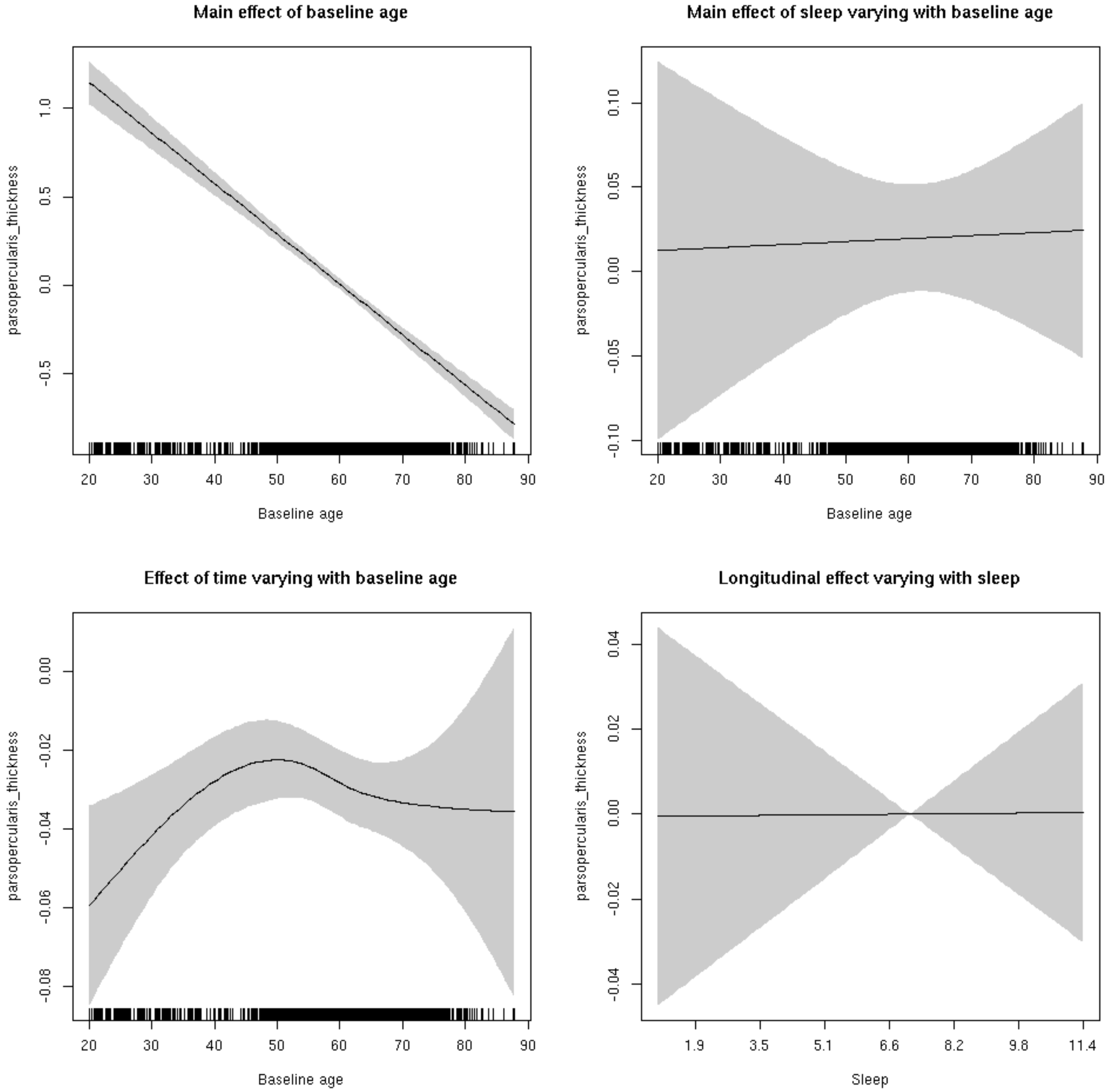

Male: Full data Below is the row for `s(sleep_z):time` from the model output.

```
## s(sleep_z):time   2.113  2.113   2.000   0.102
```

Below are plots of a plot of `s(bl_age)`, `s(bl_age):sleep_z`, `s(bl_age):time`, and `s(sleep_z):time`. For `s(sleep_z):time`, roughly, if we cannot fit a straight line through the confidence bands, there is evidence that the effect of time depends on sleep duration. It is correct the that confidence interval is zero at one point along the axis; this happens because we do not include the main effect.

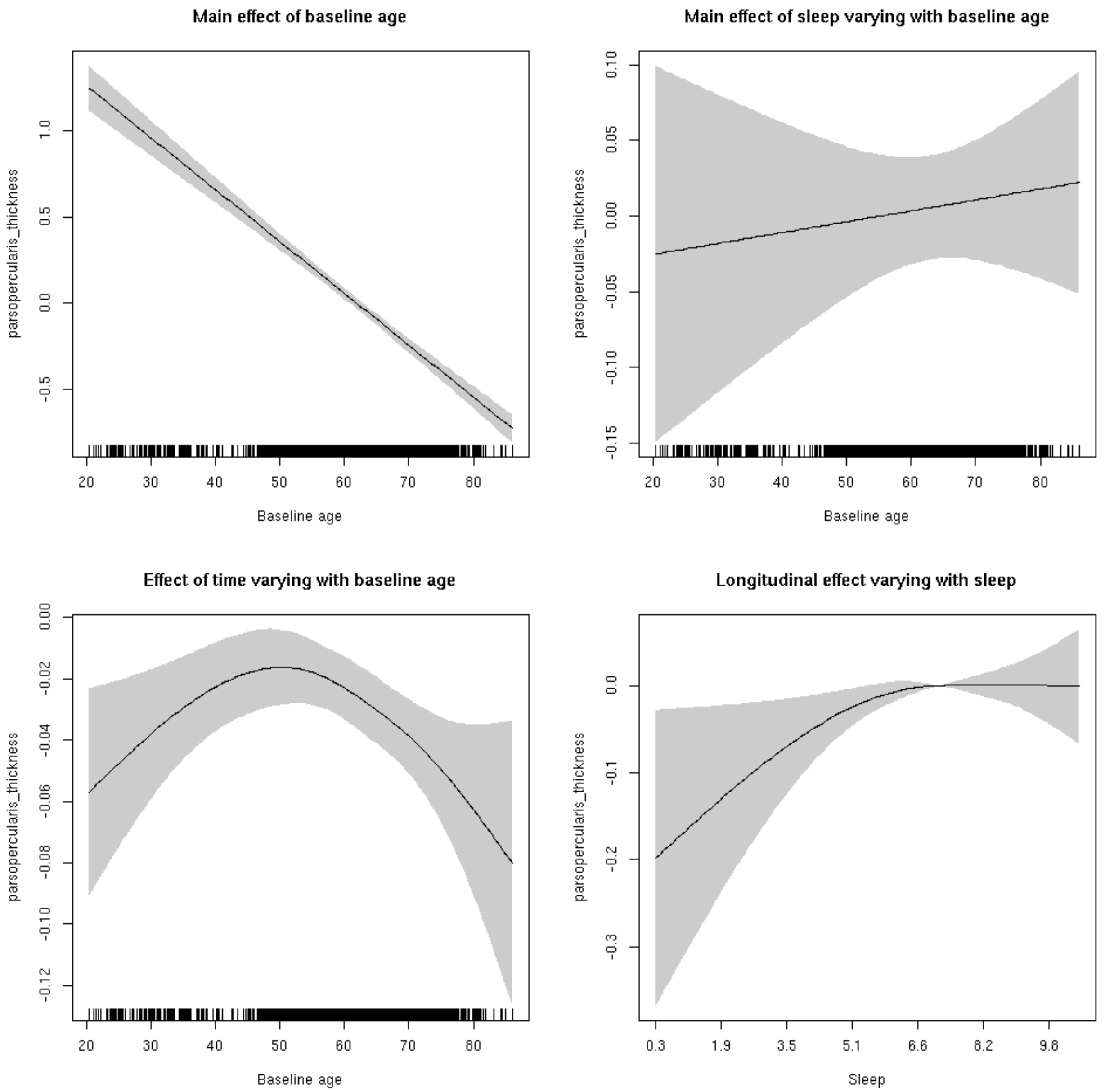

parsopercularis_thickness

Full data Below is the model output. The term `s(sleep_z):time` is probably what we care most about.

```
##
## Family: gaussian
## Link function: identity
##
## Formula:
## value ~ sex + site + s(bl_age, k = 10, bs = "cr") + s(bl_age,
##     by = sleep_z, bs = "cr") + +s(bl_age, by = time, k = 5, bs = "cr") +
##     s(sleep_z, by = time, k = 5, bs = "cr", pc = 0)
## <environment: 0x5630035da000>
```

```
##
## Parametric coefficients:
##                Estimate Std. Error t value Pr(>|t|)
## (Intercept)   -1.80233    0.04297 -41.946  < 2e-16 ***
## sexmale        0.09600    0.02208   4.347 1.40e-05 ***
## siteousAvanto  0.06309    0.05833   1.082 0.279477
## siteousPrisma  0.26362    0.24474   1.077 0.281443
## siteousSkyra  -0.05524    0.05820  -0.949 0.342611
## siteUB         0.38125    0.11668   3.267 0.001090 **
## siteUCAM       0.29054    0.05976   4.862 1.18e-06 ***
## siteUKB        2.07137    0.04259  48.631  < 2e-16 ***
## siteUmU       -0.26199    0.07206  -3.636 0.000279 ***
## ---
## Signif. codes:  0 '***' 0.001 '**' 0.01 '*' 0.05 '.' 0.1 ' ' 1
##
## Approximate significance of smooth terms:
##                       edf Ref.df       F p-value
## s(bl_age)         1.000  1.000 780.103  <2e-16 ***
## s(bl_age):sleep_z 2.000  2.000   0.602   0.548
## s(bl_age):time    3.707  3.707  47.344  <2e-16 ***
## s(sleep_z):time   1.000  1.000   1.595   0.207
## ---
## Signif. codes:  0 '***' 0.001 '**' 0.01 '*' 0.05 '.' 0.1 ' ' 1
##
## R-sq.(adj) =  0.627
## lmer.REML =  13294  Scale est. = 0.10224   n = 8168
```

Below are plots of a plot of `s(bl_age)`, `s(bl_age):sleep_z`, `s(bl_age):time`, and `s(sleep_z):time`. For `s(sleep_z):time`, roughly, if we cannot fit a straight line through the confidence bands, there is evidence that the effect of time depends on sleep duration. It is correct the that confidence interval is zero at one point along the axis; this happens because we do not include the main effect.

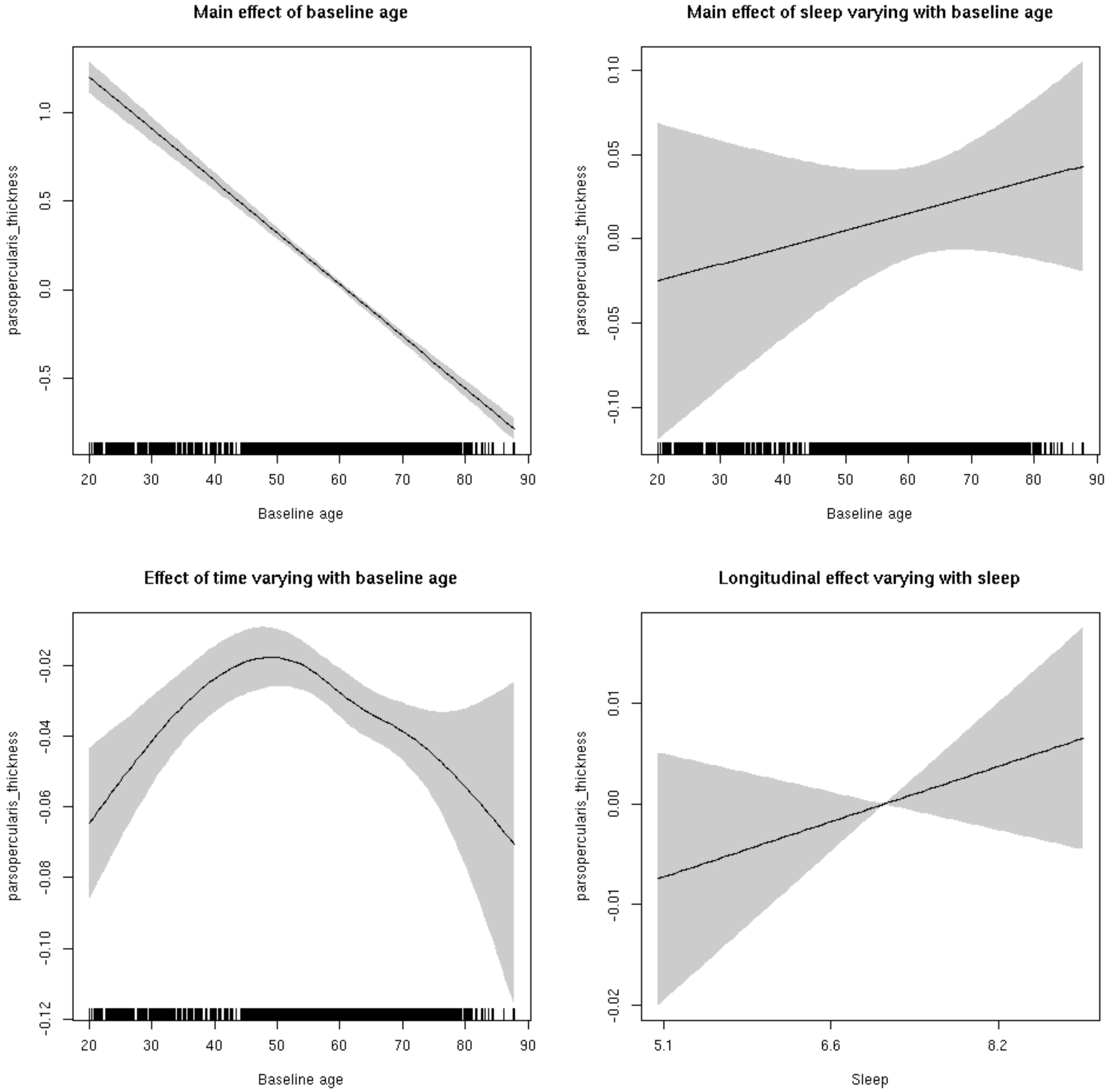

Restricted to sleep >= 5 and <= 9 hours Below is the row for `s(sleep_z):time` from the model output.

```
## s(sleep_z):time   1.000  1.000   1.396   0.237
```

Below are plots of a plot of `s(bl_age)`, `s(bl_age):sleep_z`, `s(bl_age):time`, and `s(sleep_z):time`. For `s(sleep_z):time`, roughly, if we cannot fit a straight line through the confidence bands, there is evidence that the effect of time depends on sleep duration. It is correct the that confidence interval is zero at one point along the axis; this happens because we do not include the main effect.

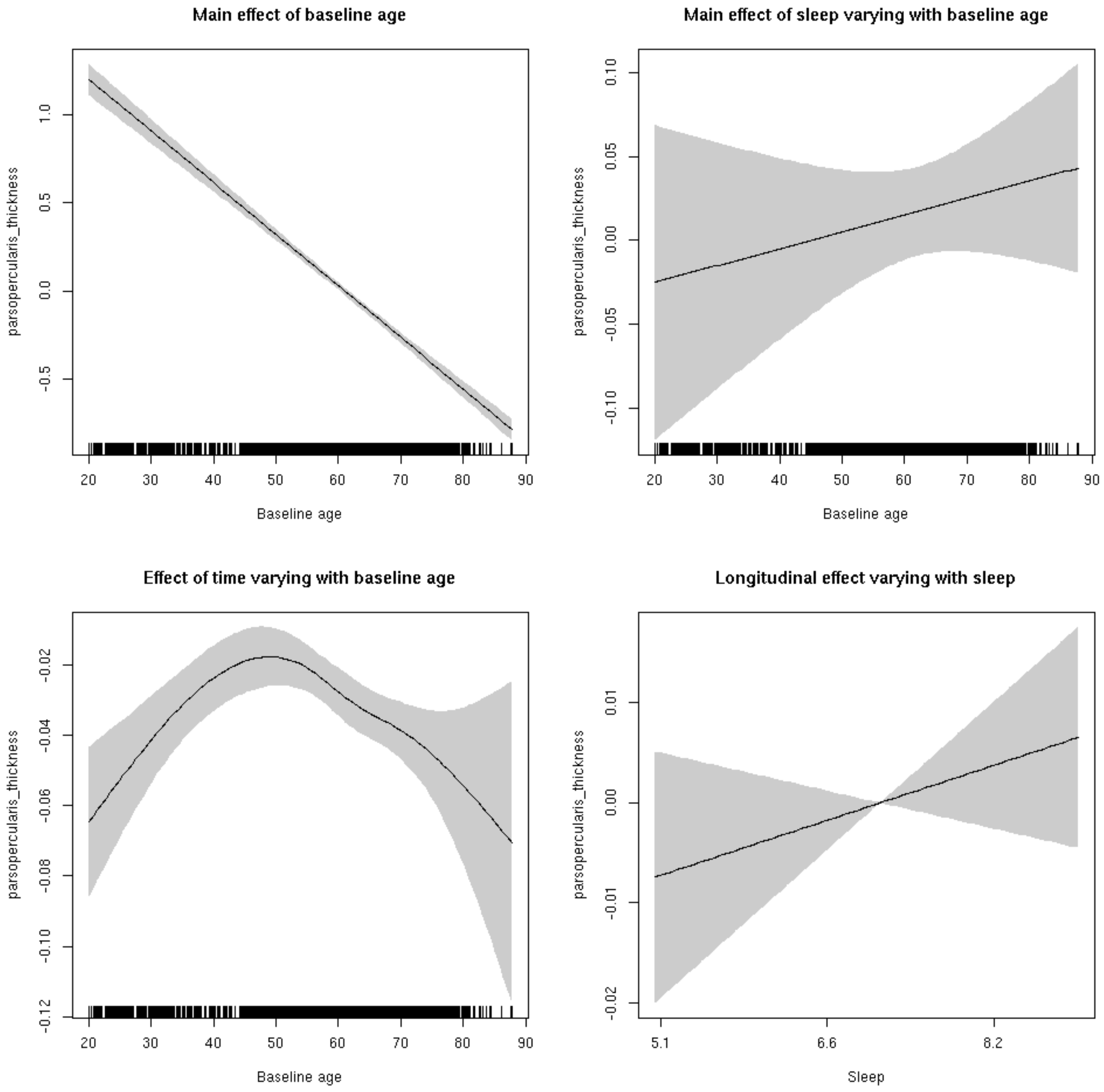

parsopercularis_volume: Stratified by sex

Female: Full data Below is the row for `s(sleep_z):time` from the model output.

```
## s(sleep_z):time   1.000  1.000   0.043   0.835
```

Below are plots of a plot of `s(bl_age)`, `s(bl_age):sleep_z`, `s(bl_age):time`, and `s(sleep_z):time`. For `s(sleep_z):time`, roughly, if we cannot fit a straight line through the confidence bands, there is evidence that the effect of time depends on sleep duration. It is correct the that confidence interval is zero at one point along the axis; this happens because we do not include the main effect.

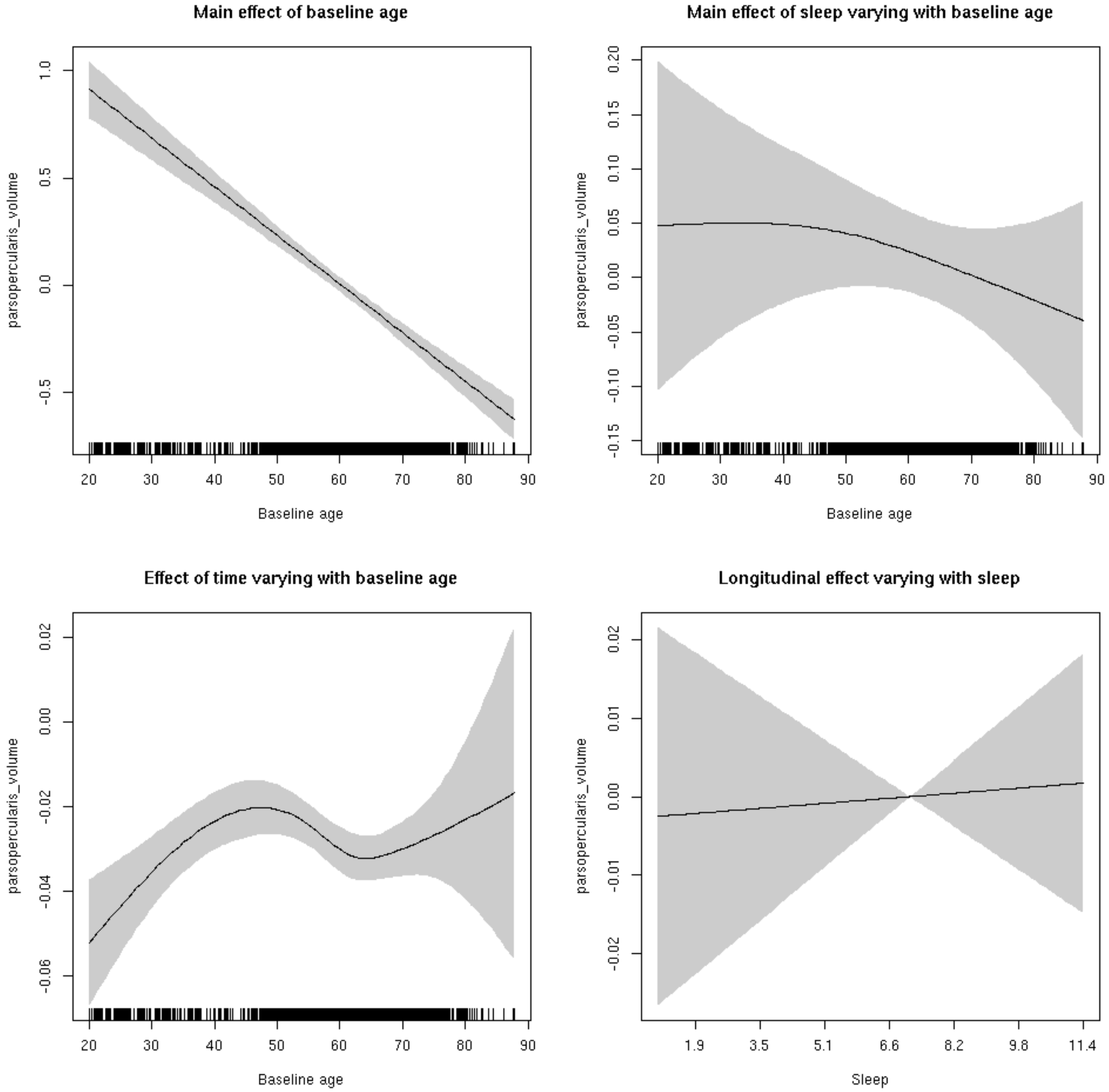

Male: Full data Below is the row for `s(sleep_z):time` from the model output.

```
## s(sleep_z):time   1.000  1.000   6.162  0.0131 *
```

Below are plots of a plot of `s(bl_age)`, `s(bl_age):sleep_z`, `s(bl_age):time`, and `s(sleep_z):time`. For `s(sleep_z):time`, roughly, if we cannot fit a straight line through the confidence bands, there is evidence that the effect of time depends on sleep duration. It is correct the that confidence interval is zero at one point along the axis; this happens because we do not include the main effect.

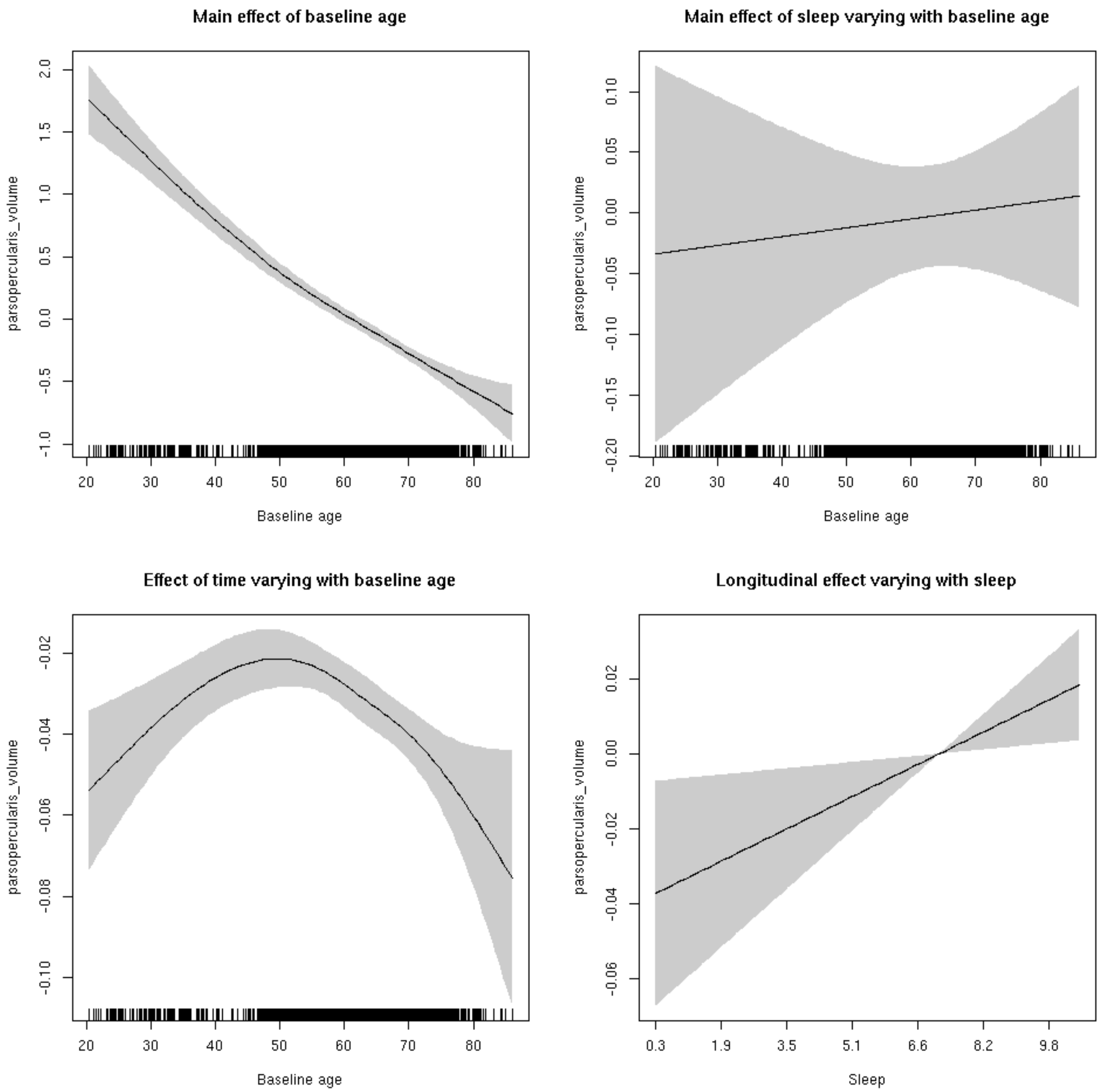

parsopercularis_volume

Full data Below is the model output. The term `s(sleep_z):time` is probably what we care most about.

```
##
## Family: gaussian
## Link function: identity
##
## Formula:
## value ~ sex + site + icv + s(bl_age, k = 10, bs = "cr") + s(bl_age,
##     by = sleep_z, bs = "cr") + +s(bl_age, by = time, k = 5, bs = "cr") +
##     s(sleep_z, by = time, k = 5, bs = "cr", pc = 0)
## <environment: 0x5630035a3dc0>
```

```
##
## Parametric coefficients:
##                Estimate Std. Error t value Pr(>|t|)
## (Intercept)   -0.04532    0.06100  -0.743 0.457569
## sexmale        0.06518    0.03150   2.069 0.038559 *
## siteousAvanto -0.66296    0.07460  -8.887  < 2e-16 ***
## siteousPrisma -0.34124    0.15376  -2.219 0.026493 *
## siteousSkyra  -0.37574    0.07224  -5.201 2.03e-07 ***
## siteUB        -0.56435    0.14131  -3.994 6.56e-05 ***
## siteUCAM      -0.64429    0.07560  -8.523  < 2e-16 ***
## siteUKB        0.20199    0.05753   3.511 0.000449 ***
## siteUmU       -0.27090    0.08735  -3.101 0.001933 **
## icv            0.45142    0.01543  29.256  < 2e-16 ***
## ---
## Signif. codes:  0 '***' 0.001 '**' 0.01 '*' 0.05 '.' 0.1 ' ' 1
##
## Approximate significance of smooth terms:
##                      edf Ref.df       F p-value
## s(bl_age)          1.841  1.841 274.821  <2e-16 ***
## s(bl_age):sleep_z 2.560  2.560   0.247  0.7341
## s(bl_age):time    4.198  4.198 123.651  <2e-16 ***
## s(sleep_z):time   1.000  1.000   3.697  0.0546 .
## ---
## Signif. codes:  0 '***' 0.001 '**' 0.01 '*' 0.05 '.' 0.1 ' ' 1
##
## R-sq.(adj) =  0.385
## lmer.REML = 9383.3  Scale est. = 0.029957  n = 8163
```

Below are plots of a plot of `s(bl_age)`, `s(bl_age):sleep_z`, `s(bl_age):time`, and `s(sleep_z):time`. For `s(sleep_z):time`, roughly, if we cannot fit a straight line through the confidence bands, there is evidence that the effect of time depends on sleep duration. It is correct the that confidence interval is zero at one point along the axis; this happens because we do not include the main effect.

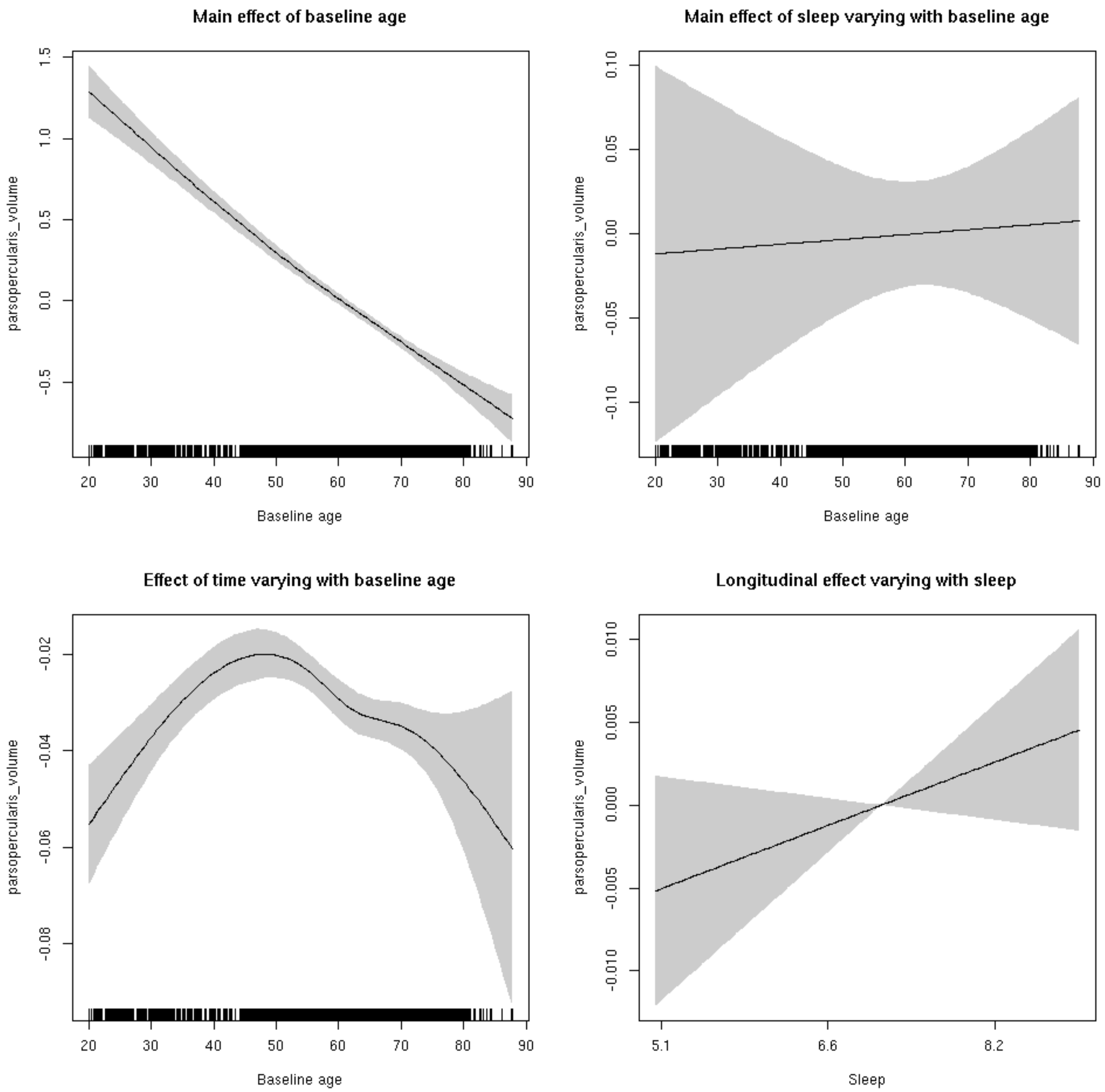

Restricted to sleep >= 5 and <= 9 hours Below is the row for `s(sleep_z):time` from the model output.

```
## s(sleep_z):time   1.000  1.000   2.221   0.136
```

Below are plots of a plot of `s(bl_age)`, `s(bl_age):sleep_z`, `s(bl_age):time`, and `s(sleep_z):time`. For `s(sleep_z):time`, roughly, if we cannot fit a straight line through the confidence bands, there is evidence that the effect of time depends on sleep duration. It is correct the that confidence interval is zero at one point along the axis; this happens because we do not include the main effect.

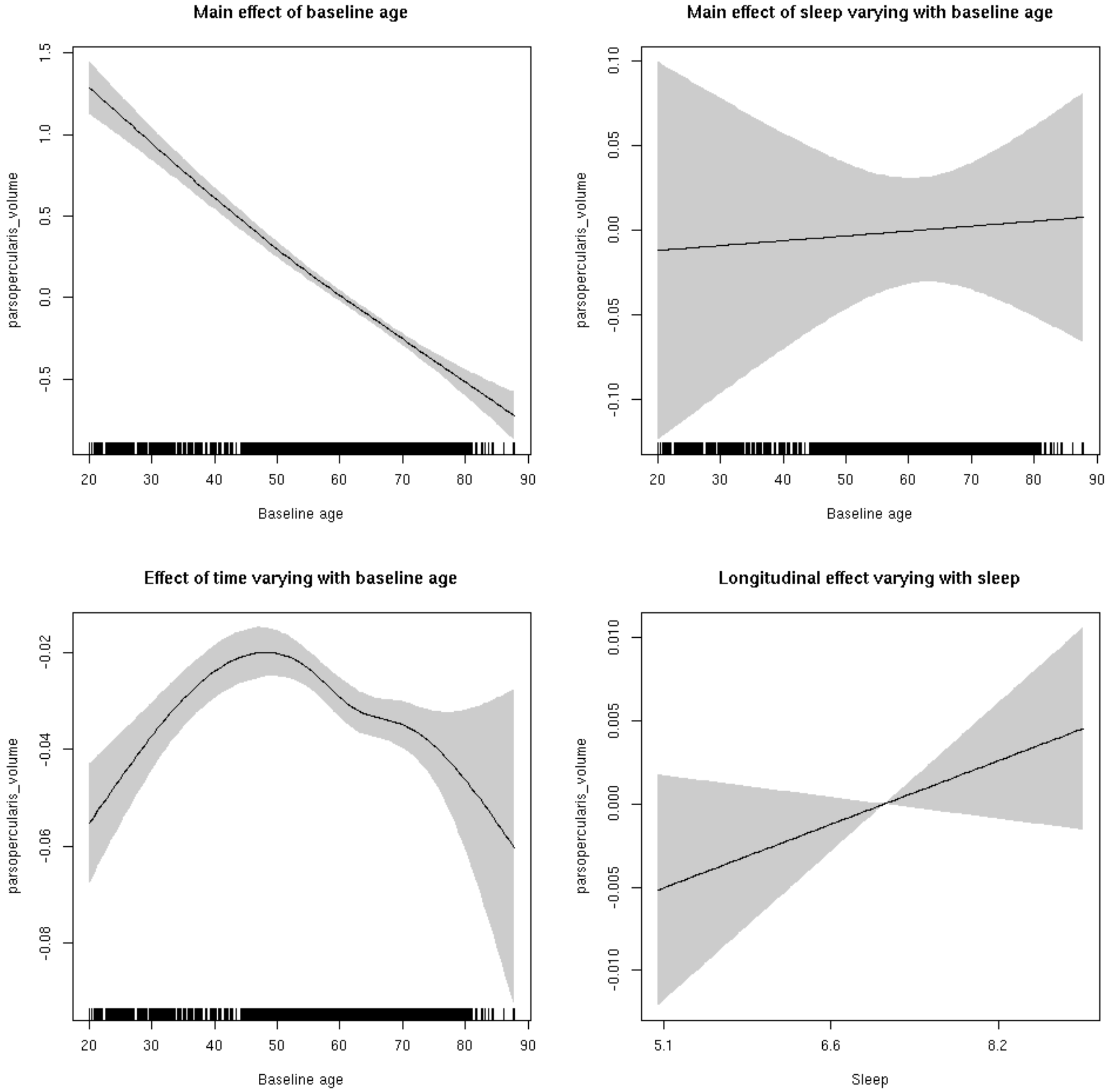

parsorbitalis_area: Stratified by sex

Female: Full data Below is the row for `s(sleep_z):time` from the model output.

```
## s(sleep_z):time   1.000  1.000  0.560  0.4542
```

Below are plots of a plot of `s(bl_age)`, `s(bl_age):sleep_z`, `s(bl_age):time`, and `s(sleep_z):time`. For `s(sleep_z):time`, roughly, if we cannot fit a straight line through the confidence bands, there is evidence that the effect of time depends on sleep duration. It is correct the that confidence interval is zero at one point along the axis; this happens because we do not include the main effect.

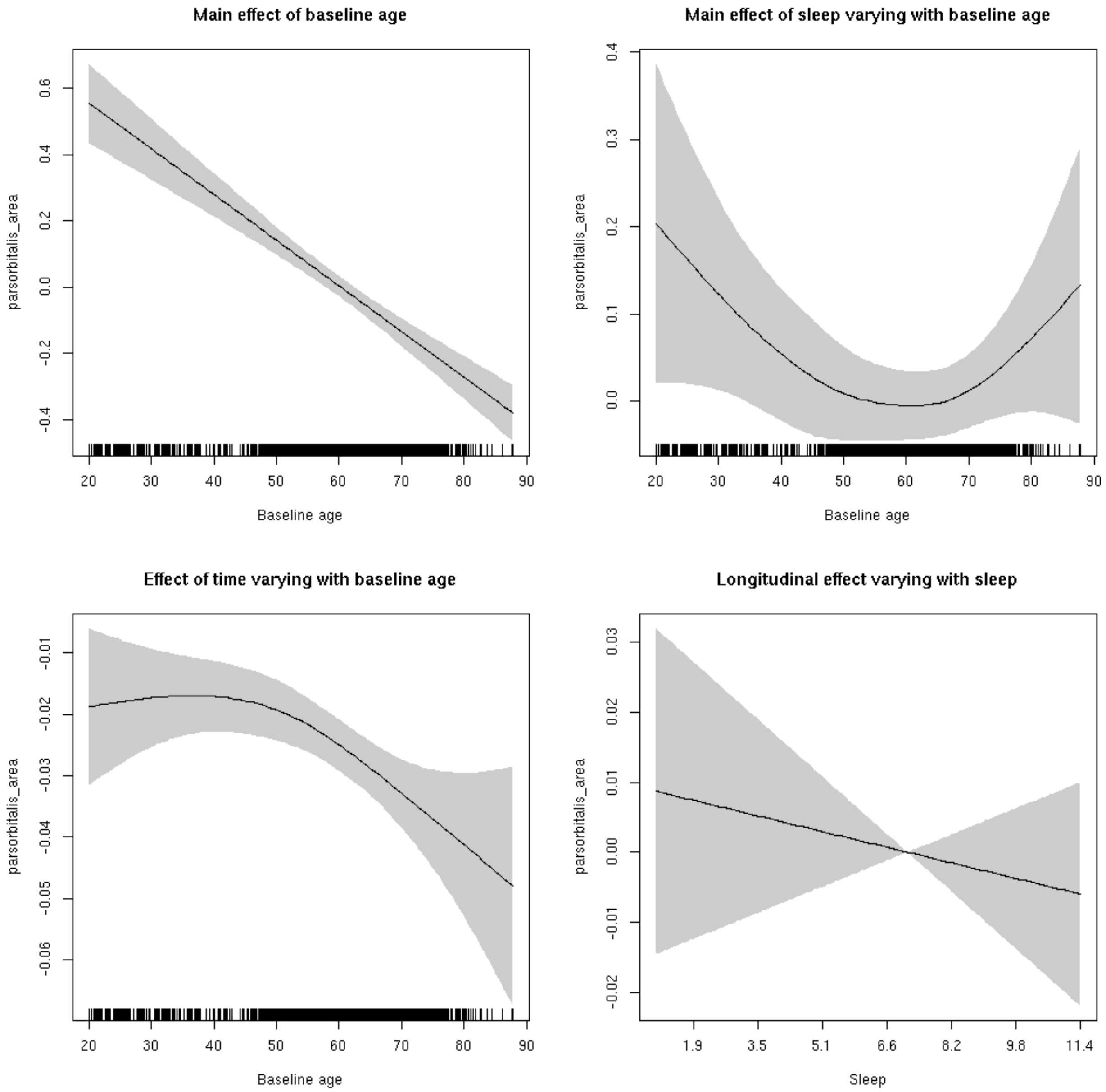

Male: Full data Below is the row for `s(sleep_z):time` from the model output.

```
## s(sleep_z):time   1.000  1.000   2.444   0.118
```

Below are plots of a plot of `s(bl_age)`, `s(bl_age):sleep_z`, `s(bl_age):time`, and `s(sleep_z):time`. For `s(sleep_z):time`, roughly, if we cannot fit a straight line through the confidence bands, there is evidence that the effect of time depends on sleep duration. It is correct the that confidence interval is zero at one point along the axis; this happens because we do not include the main effect.

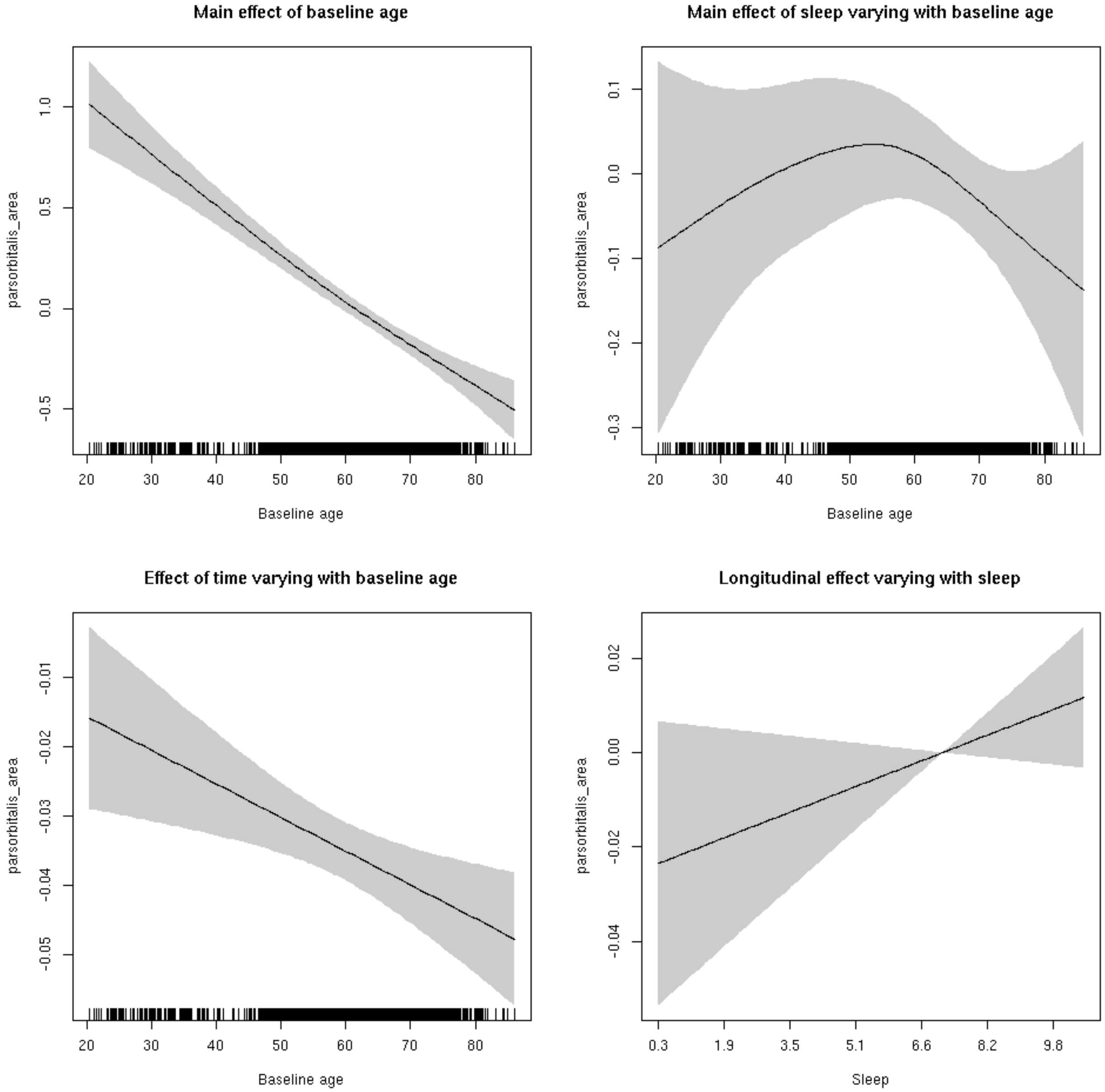

parsorbitalis_area

Full data Below is the model output. The term `s(sleep_z):time` is probably what we care most about.

```
##
## Family: gaussian
## Link function: identity
##
## Formula:
## value ~ sex + site + icv + s(bl_age, k = 10, bs = "cr") + s(bl_age,
##     by = sleep_z, bs = "cr") + +s(bl_age, by = time, k = 5, bs = "cr") +
##     s(sleep_z, by = time, k = 5, bs = "cr", pc = 0)
## <environment: 0x5630035b4648>
```

```
##
## Parametric coefficients:
##                 Estimate Std. Error t value Pr(>|t|)
## (Intercept)      0.78934    0.05713  13.816  < 2e-16 ***
## sexmale          0.41611    0.02969  14.017  < 2e-16 ***
## siteousAvanto -0.75204    0.07044 -10.677  < 2e-16 ***
## siteousPrisma -0.91877    0.15092  -6.088 1.20e-09 ***
## siteousSkyra  -0.19730    0.06817  -2.894  0.00381 **
## siteUB        -0.82621    0.13308  -6.208 5.62e-10 ***
## siteUCAM      -0.77470    0.07116 -10.887  < 2e-16 ***
## siteUKB       -0.96524    0.05324 -18.129  < 2e-16 ***
## siteUmU       -0.02261    0.08209  -0.275  0.78303
## icv            0.47658    0.01456  32.737  < 2e-16 ***
## ---
## Signif. codes:  0 '***' 0.001 '**' 0.01 '*' 0.05 '.' 0.1 ' ' 1
##
## Approximate significance of smooth terms:
##                       edf Ref.df       F p-value
## s(bl_age)           1.000  1.000 259.457  <2e-16 ***
## s(bl_age):sleep_z 2.000  2.000   0.724   0.485
## s(bl_age):time      2.831  2.831 165.820  <2e-16 ***
## s(sleep_z):time     1.000  1.000   0.330   0.566
## ---
## Signif. codes:  0 '***' 0.001 '**' 0.01 '*' 0.05 '.' 0.1 ' ' 1
##
## R-sq.(adj) =  0.458
## lmer.REML = 8887.6  Scale est. = 0.029685  n = 8173
```

Below are plots of a plot of `s(bl_age)`, `s(bl_age):sleep_z`, `s(bl_age):time`, and `s(sleep_z):time`. For `s(sleep_z):time`, roughly, if we cannot fit a straight line through the confidence bands, there is evidence that the effect of time depends on sleep duration. It is correct the that confidence interval is zero at one point along the axis; this happens because we do not include the main effect.

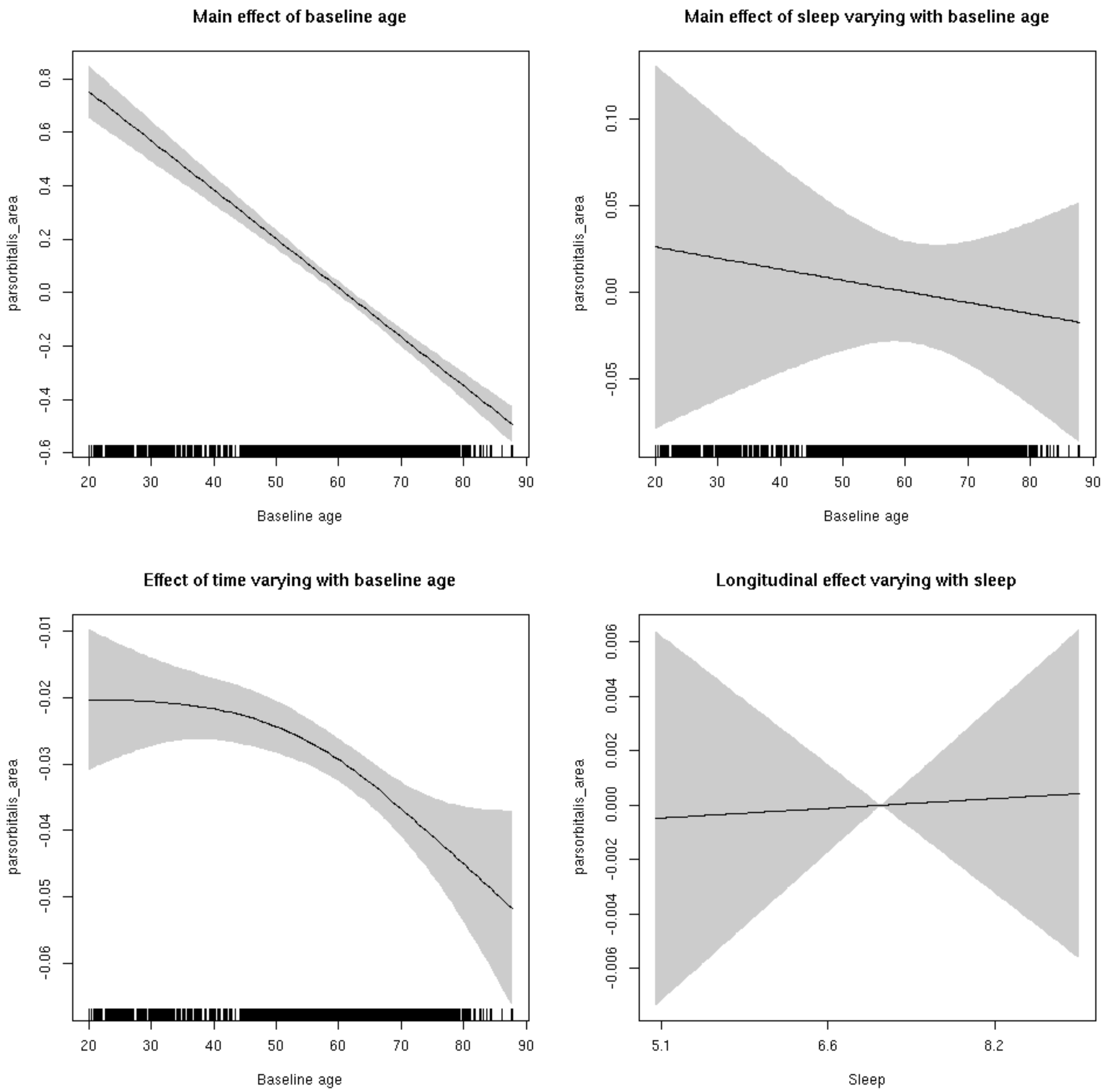

Restricted to sleep >= 5 and <= 9 hours Below is the row for `s(sleep_z):time` from the model output.

```
## s(sleep_z):time   1.000  1.000   0.020   0.888
```

Below are plots of a plot of `s(bl_age)`, `s(bl_age):sleep_z`, `s(bl_age):time`, and `s(sleep_z):time`. For `s(sleep_z):time`, roughly, if we cannot fit a straight line through the confidence bands, there is evidence that the effect of time depends on sleep duration. It is correct the that confidence interval is zero at one point along the axis; this happens because we do not include the main effect.

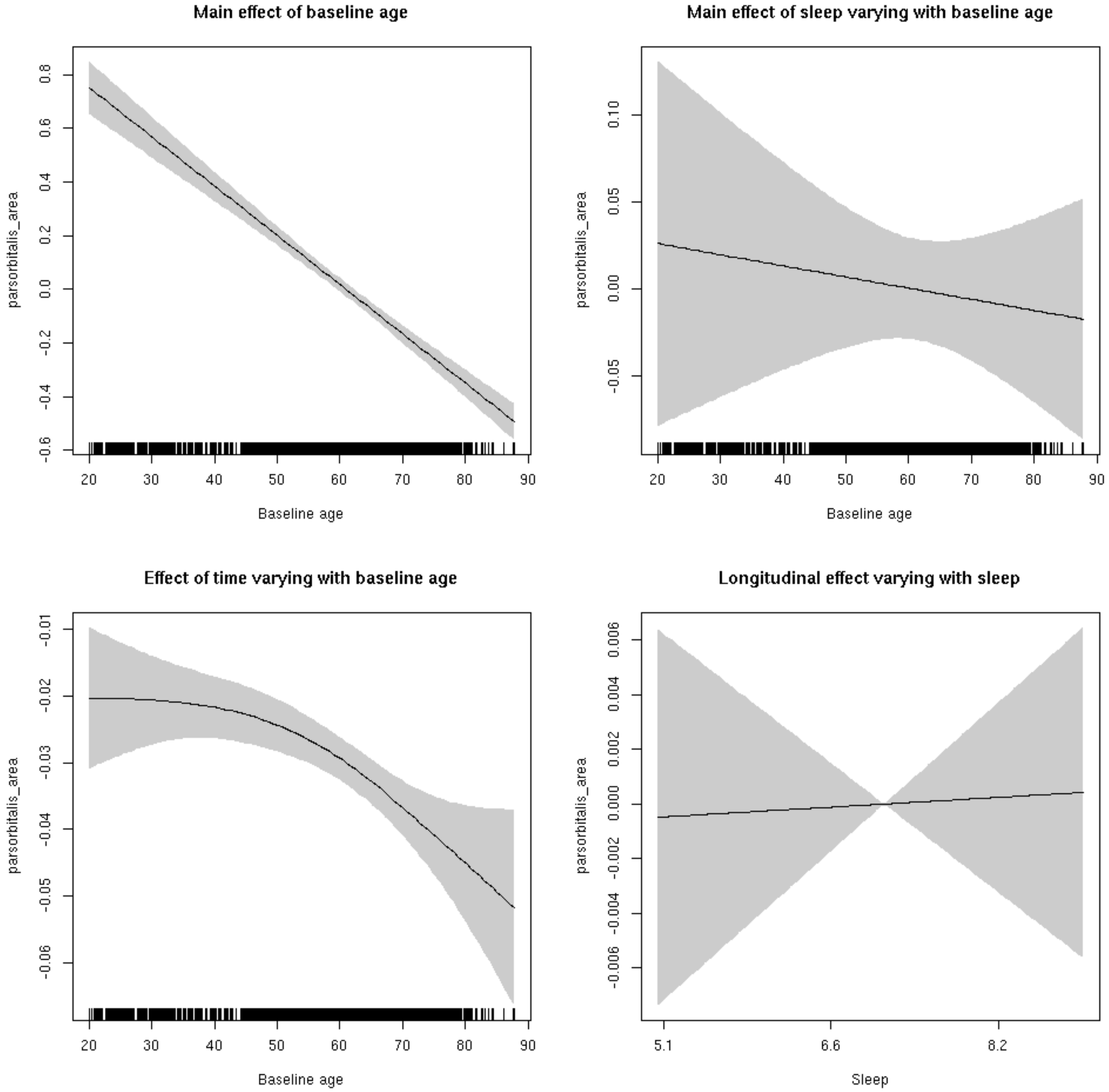

parsorbitalis_thickness: Stratified by sex

Female: Full data Below is the row for `s(sleep_z):time` from the model output.

```
## s(sleep_z):time   3.178  3.178   2.255   0.0410 *
```

Below are plots of a plot of `s(bl_age)`, `s(bl_age):sleep_z`, `s(bl_age):time`, and `s(sleep_z):time`. For `s(sleep_z):time`, roughly, if we cannot fit a straight line through the confidence bands, there is evidence that the effect of time depends on sleep duration. It is correct the that confidence interval is zero at one point along the axis; this happens because we do not include the main effect.

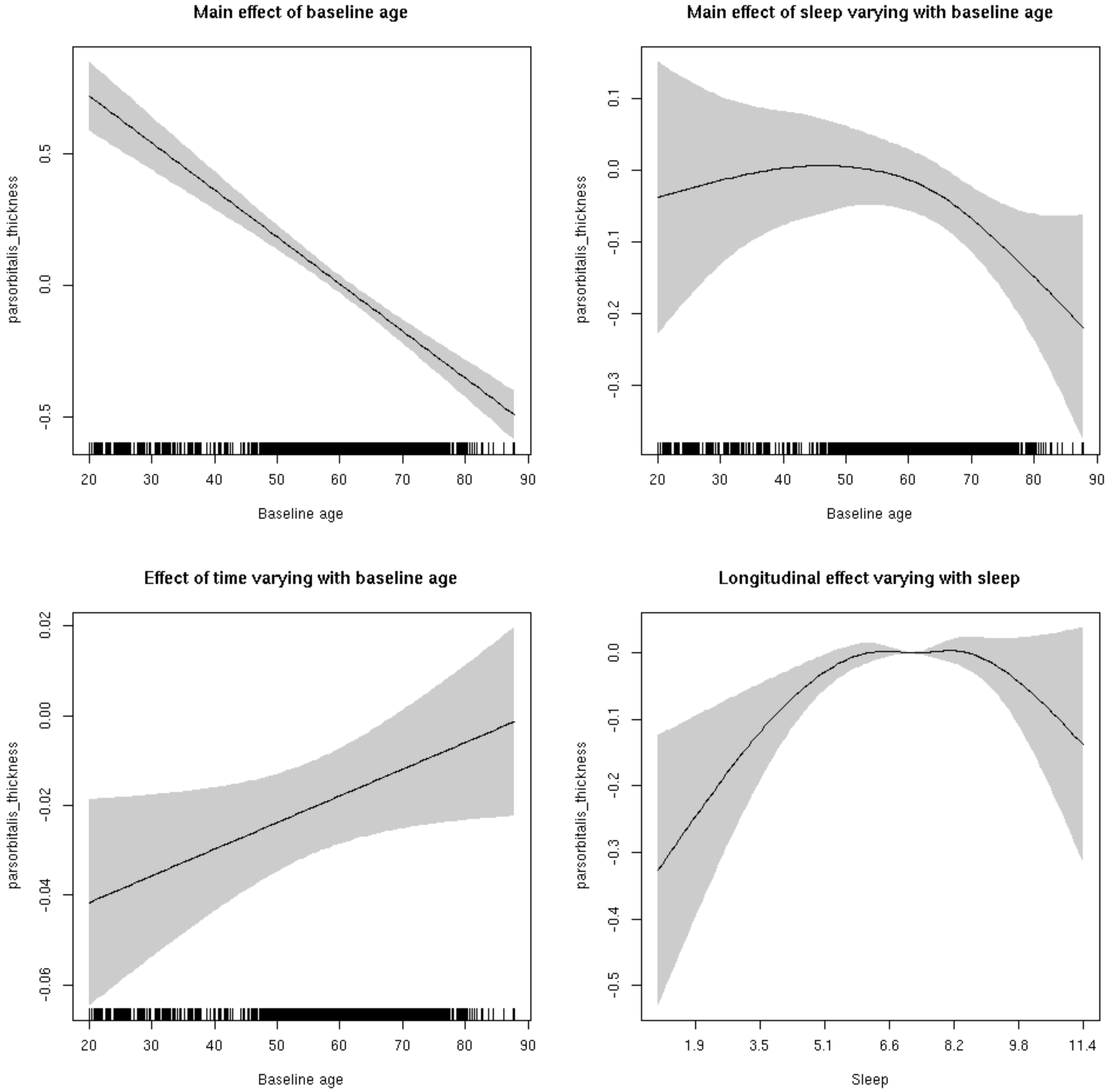

Male: Full data Below is the row for `s(sleep_z):time` from the model output.

```
## s(sleep_z):time   1.000  1.000  0.006 0.937602
```

Below are plots of a plot of `s(bl_age)`, `s(bl_age):sleep_z`, `s(bl_age):time`, and `s(sleep_z):time`. For `s(sleep_z):time`, roughly, if we cannot fit a straight line through the confidence bands, there is evidence that the effect of time depends on sleep duration. It is correct the that confidence interval is zero at one point along the axis; this happens because we do not include the main effect.

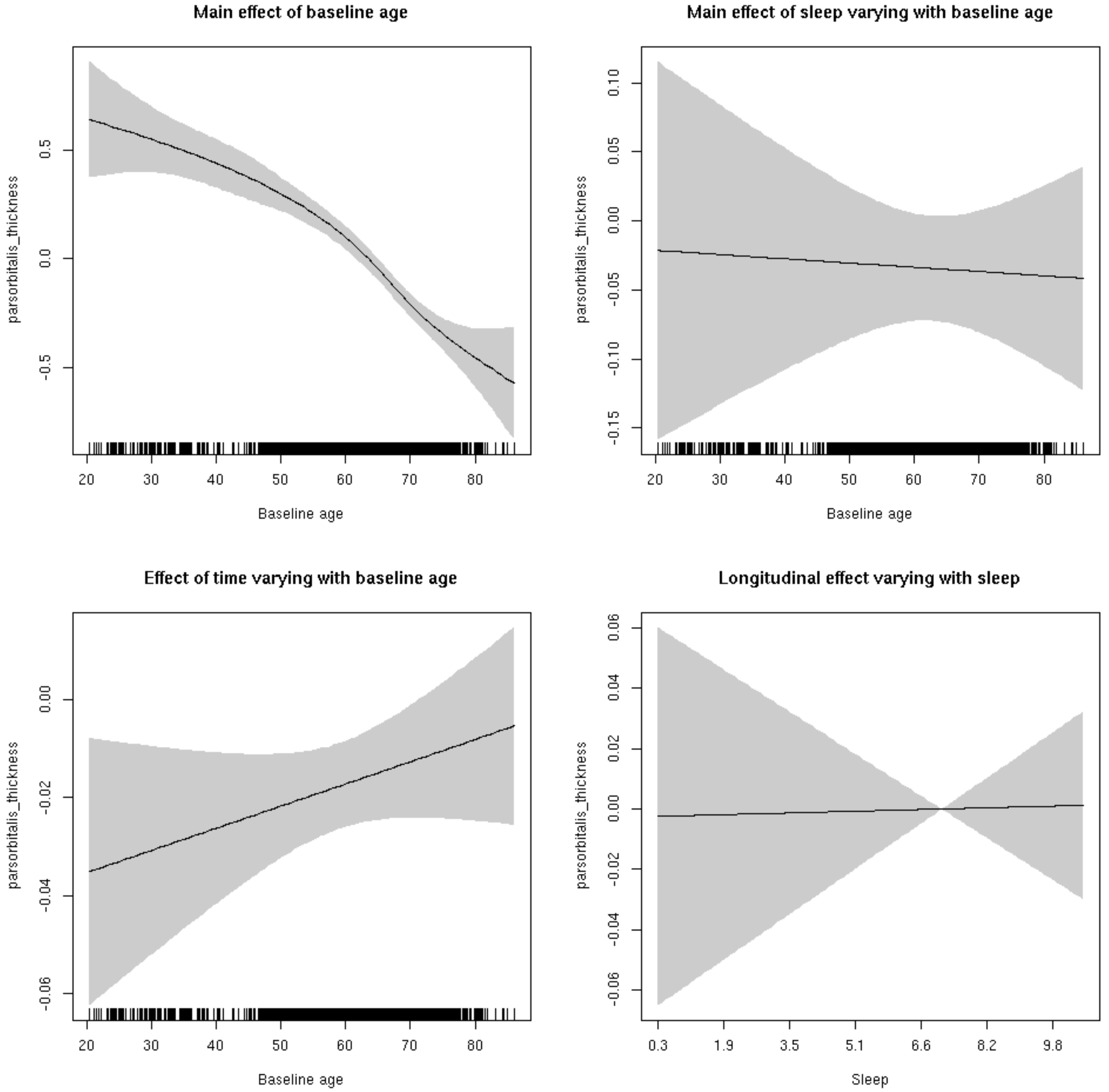

parsorbitalis__thickness

Full data Below is the model output. The term s(sleep_z):time is probably what we care most about.

```
##
## Family: gaussian
## Link function: identity
##
## Formula:
## value ~ sex + site + s(bl_age, k = 10, bs = "cr") + s(bl_age,
##     by = sleep_z, bs = "cr") + +s(bl_age, by = time, k = 5, bs = "cr") +
##     s(sleep_z, by = time, k = 5, bs = "cr", pc = 0)
## <environment: 0x5630035c7a08>
```

```
##
## Parametric coefficients:
##                 Estimate Std. Error t value Pr(>|t|)
## (Intercept)    -1.632236   0.048219 -33.851  < 2e-16 ***
## sexmale        -0.133169   0.024169  -5.510 3.70e-08 ***
## siteousAvanto   0.460040   0.064278   7.157 8.96e-13 ***
## siteousPrisma   0.212853   0.288786   0.737    0.461
## siteousSkyra    0.001281   0.064256   0.020    0.984
## siteUB          0.754028   0.127591   5.910 3.56e-09 ***
## siteUCAM        0.620259   0.066341   9.350  < 2e-16 ***
## siteUKB         1.958571   0.048855  40.090  < 2e-16 ***
## siteUmU        -0.333430   0.079694  -4.184 2.90e-05 ***
## ---
## Signif. codes:  0 '***' 0.001 '**' 0.01 '*' 0.05 '.' 0.1 ' ' 1
##
## Approximate significance of smooth terms:
##                      edf Ref.df      F  p-value
## s(bl_age)          3.694  3.694 83.607  < 2e-16 ***
## s(bl_age):sleep_z 2.874  2.874  4.181   0.0047 **
## s(bl_age):time     2.000  2.000 15.185 6.57e-07 ***
## s(sleep_z):time    2.865  2.865  1.777   0.1240
## ---
## Signif. codes:  0 '***' 0.001 '**' 0.01 '*' 0.05 '.' 0.1 ' ' 1
##
## R-sq.(adj) =  0.522
## lmer.REML =  15505  Scale est. = 0.14556   n = 8168
```

Below are plots of a plot of `s(bl_age)`, `s(bl_age):sleep_z`, `s(bl_age):time`, and `s(sleep_z):time`. For `s(sleep_z):time`, roughly, if we cannot fit a straight line through the confidence bands, there is evidence that the effect of time depends on sleep duration. It is correct the that confidence interval is zero at one point along the axis; this happens because we do not include the main effect.

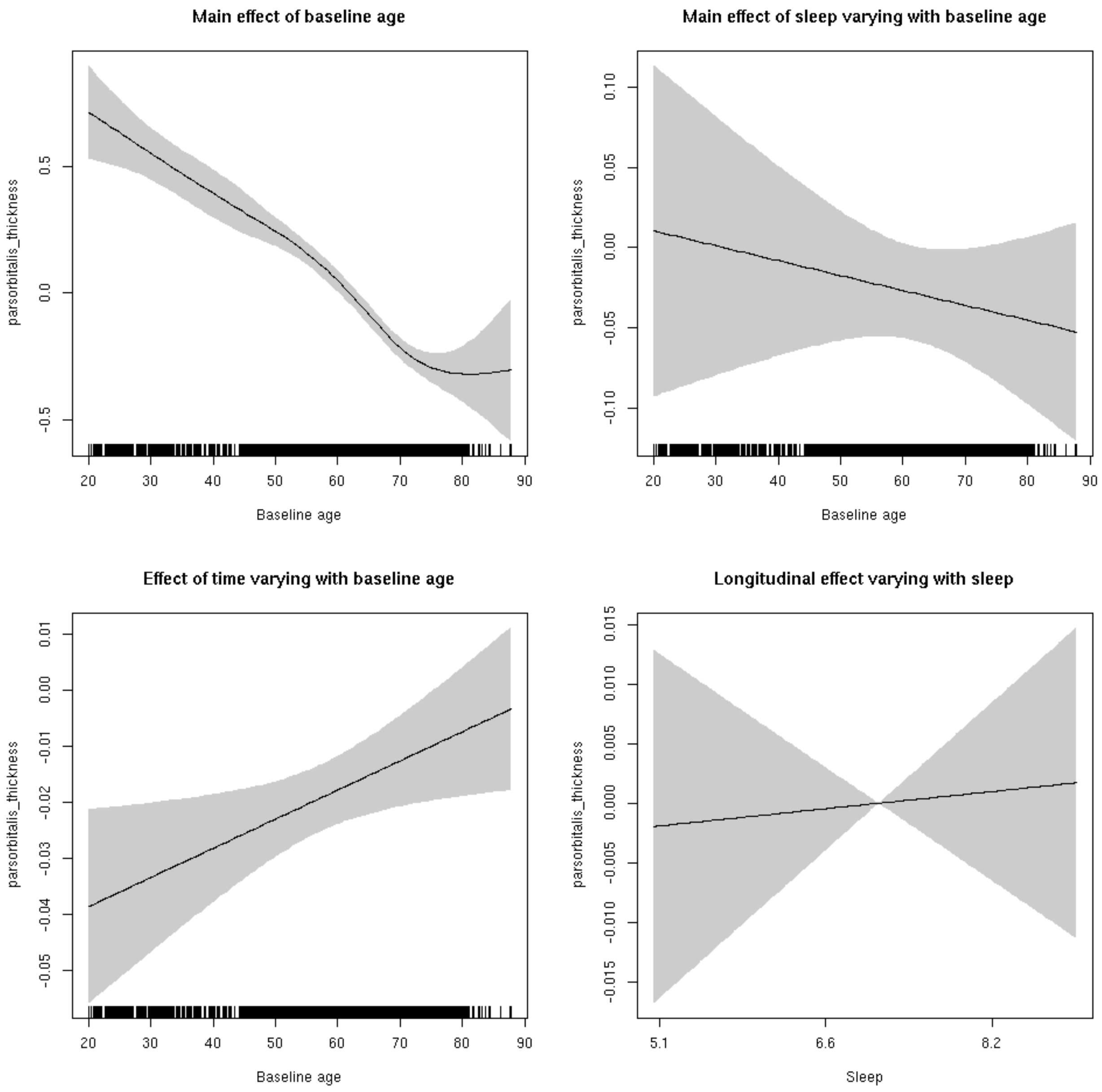

Restricted to sleep >= 5 and <= 9 hours Below is the row for `s(sleep_z):time` from the model output.

```
## s(sleep_z):time   1.000  1.000  0.069   0.793
```

Below are plots of a plot of `s(bl_age)`, `s(bl_age):sleep_z`, `s(bl_age):time`, and `s(sleep_z):time`. For `s(sleep_z):time`, roughly, if we cannot fit a straight line through the confidence bands, there is evidence that the effect of time depends on sleep duration. It is correct the that confidence interval is zero at one point along the axis; this happens because we do not include the main effect.

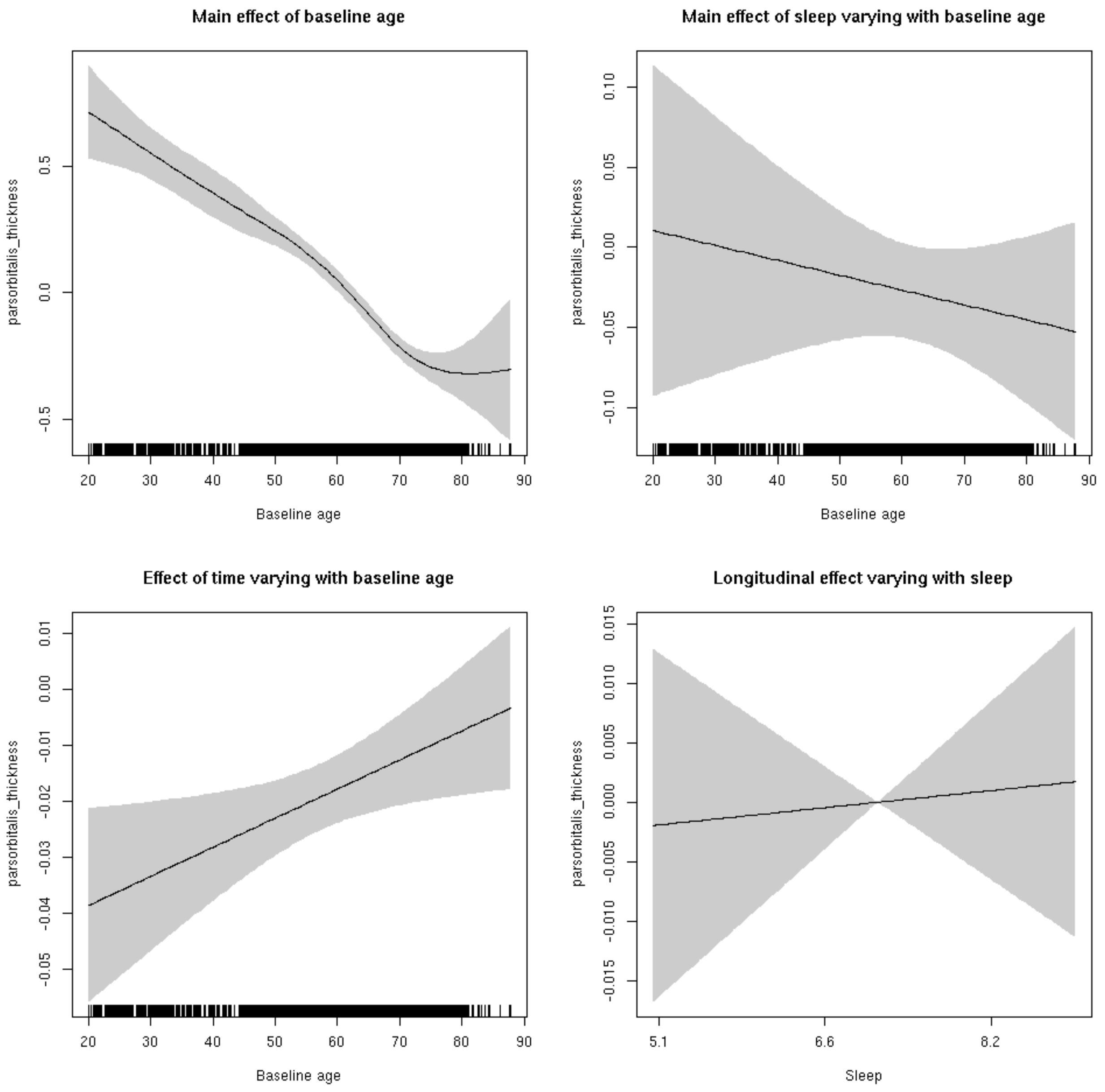

parsorbitalis_volume: Stratified by sex

Female: Full data Below is the row for `s(sleep_z):time` from the model output.

```
## s(sleep_z):time   1.000  1.000   0.062  0.8029
```

Below are plots of a plot of `s(bl_age)`, `s(bl_age):sleep_z`, `s(bl_age):time`, and `s(sleep_z):time`. For `s(sleep_z):time`, roughly, if we cannot fit a straight line through the confidence bands, there is evidence that the effect of time depends on sleep duration. It is correct the that confidence interval is zero at one point along the axis; this happens because we do not include the main effect.

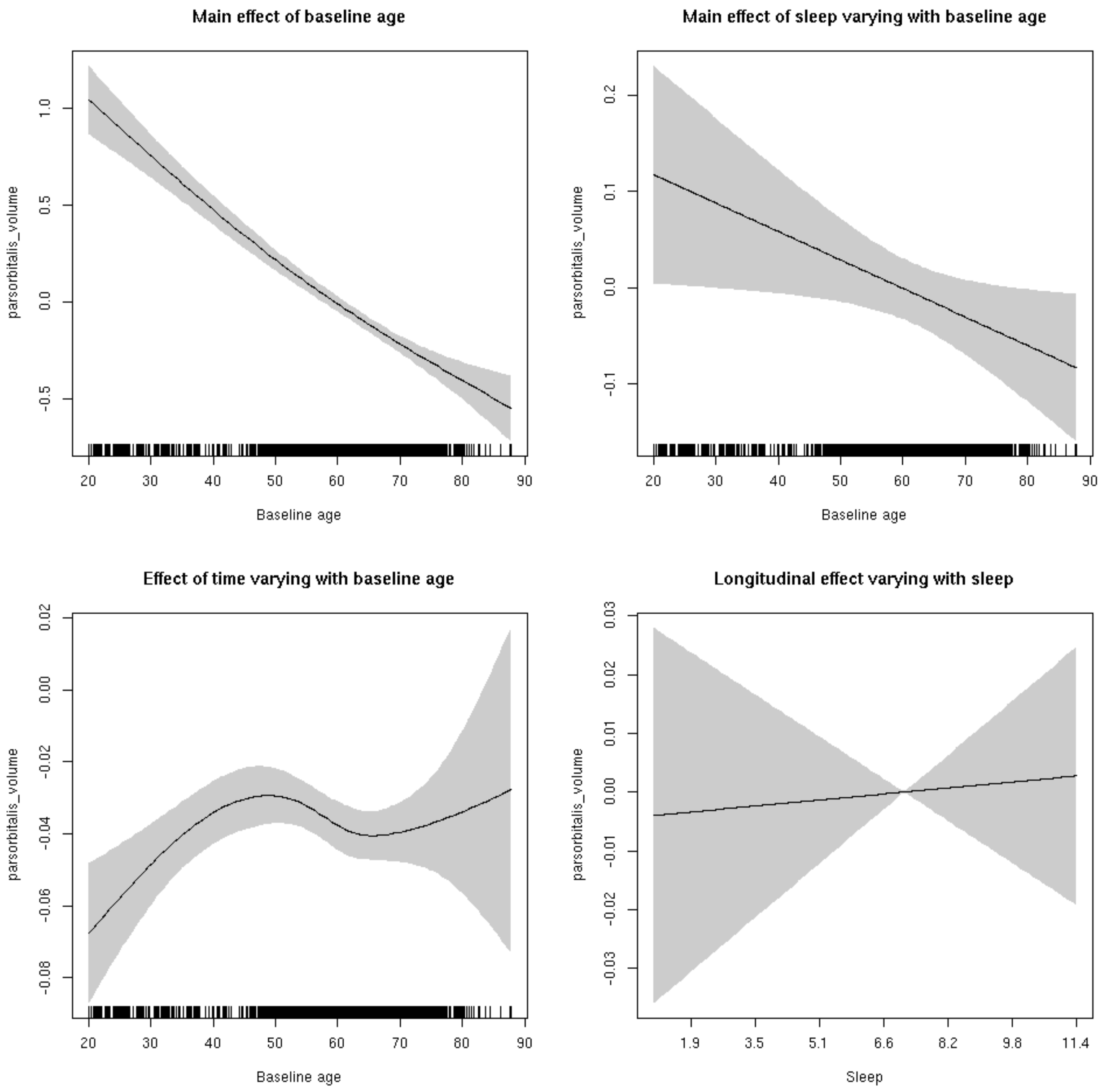

Male: Full data Below is the row for `s(sleep_z):time` from the model output.

```
## s(sleep_z):time   1.000  1.000   0.103   0.749
```

Below are plots of a plot of `s(bl_age)`, `s(bl_age):sleep_z`, `s(bl_age):time`, and `s(sleep_z):time`. For `s(sleep_z):time`, roughly, if we cannot fit a straight line through the confidence bands, there is evidence that the effect of time depends on sleep duration. It is correct the that confidence interval is zero at one point along the axis; this happens because we do not include the main effect.

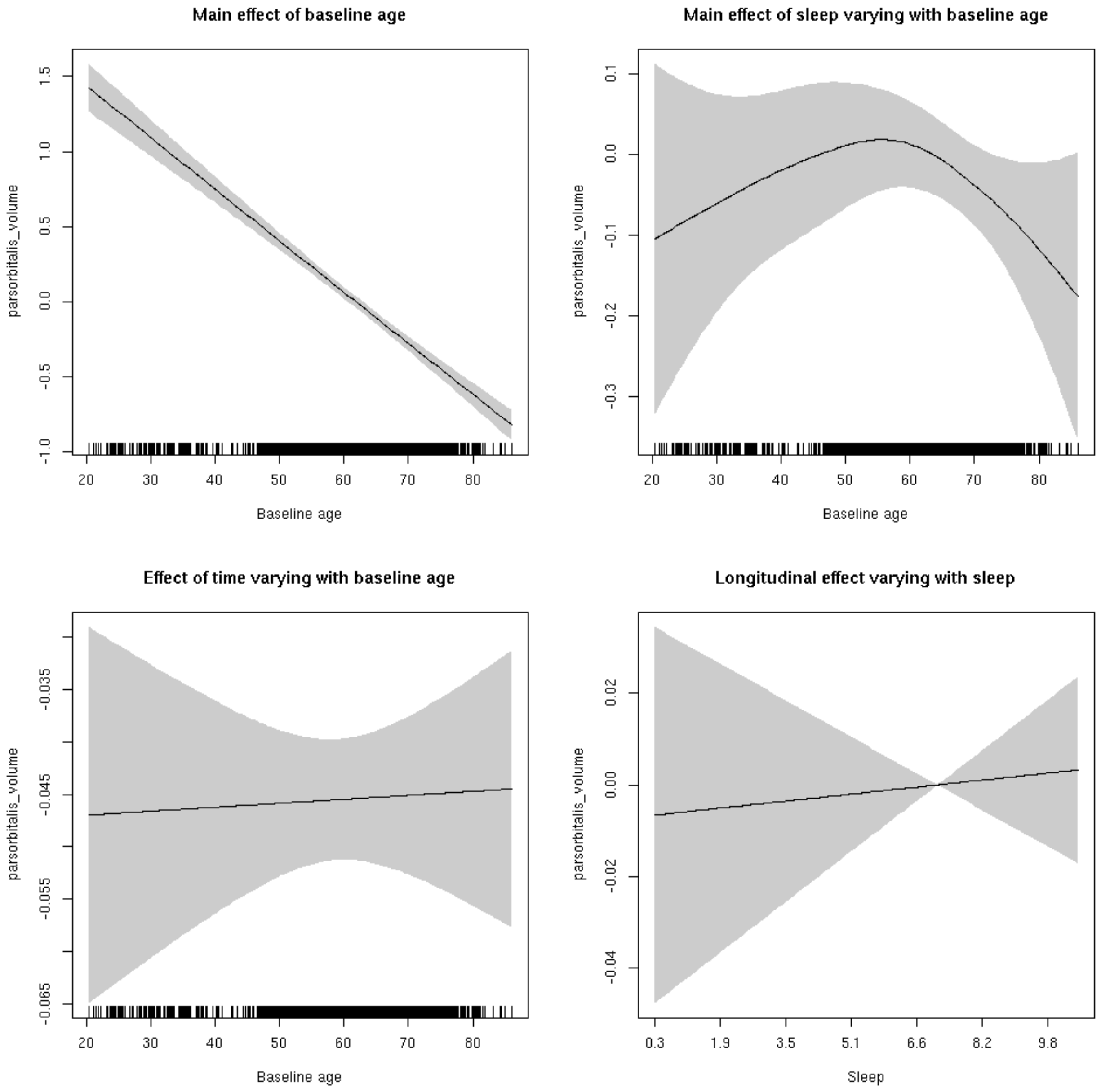

parsorbitalis_volume

Full data Below is the model output. The term `s(sleep_z):time` is probably what we care most about.

```
##
## Family: gaussian
## Link function: identity
##
## Formula:
## value ~ sex + site + icv + s(bl_age, k = 10, bs = "cr") + s(bl_age,
##     by = sleep_z, bs = "cr") + +s(bl_age, by = time, k = 5, bs = "cr") +
##     s(sleep_z, by = time, k = 5, bs = "cr", pc = 0)
## <environment: 0x5630035b15e0>
```

```
##
## Parametric coefficients:
##                 Estimate Std. Error t value Pr(>|t|)
## (Intercept)   -0.22652    0.05697  -3.976 7.07e-05 ***
## sexmale        0.31897    0.02935  10.867  < 2e-16 ***
## siteousAvanto -0.35545    0.06993  -5.083 3.81e-07 ***
## siteousPrisma -0.46387    0.19387  -2.393   0.0167 *
## siteousSkyra  -0.03700    0.06767  -0.547   0.5846
## siteUB        -0.15904    0.13092  -1.215   0.2245
## siteUCAM      -0.15687    0.07044  -2.227   0.0260 *
## siteUKB        0.26216    0.05381   4.872 1.13e-06 ***
## siteUmU       -0.12751    0.08152  -1.564   0.1178
## icv            0.39228    0.01448  27.087  < 2e-16 ***
## ---
## Signif. codes:  0 '***' 0.001 '**' 0.01 '*' 0.05 '.' 0.1 ' ' 1
##
## Approximate significance of smooth terms:
##                       edf Ref.df       F p-value
## s(bl_age)         2.148  2.148 296.840  <2e-16 ***
## s(bl_age):sleep_z 2.862  2.862   2.564  0.0347 *
## s(bl_age):time    3.899  3.899 129.166  <2e-16 ***
## s(sleep_z):time   1.000  1.000   0.308  0.5790
## ---
## Signif. codes:  0 '***' 0.001 '**' 0.01 '*' 0.05 '.' 0.1 ' ' 1
##
## R-sq.(adj) =  0.404
## lmer.REML =  11480  Scale est. = 0.05601   n = 8168
```

Below are plots of a plot of `s(bl_age)`, `s(bl_age):sleep_z`, `s(bl_age):time`, and `s(sleep_z):time`. For `s(sleep_z):time`, roughly, if we cannot fit a straight line through the confidence bands, there is evidence that the effect of time depends on sleep duration. It is correct the that confidence interval is zero at one point along the axis; this happens because we do not include the main effect.

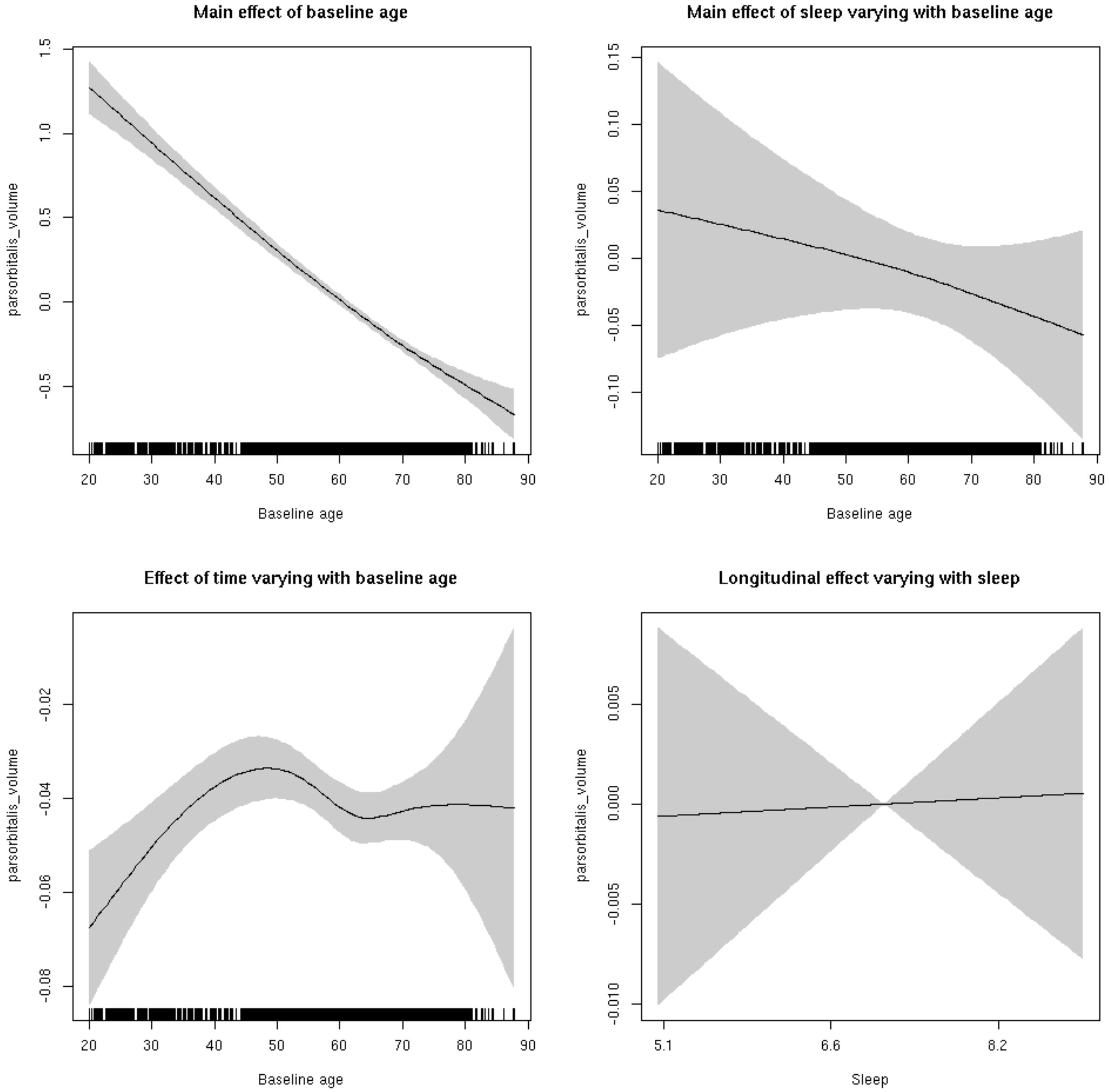

Restricted to sleep >= 5 and <= 9 hours Below is the row for `s(sleep_z):time` from the model output.

```
## s(sleep_z):time   1.000  1.000   0.017   0.897
```

Below are plots of a plot of `s(bl_age)`, `s(bl_age):sleep_z`, `s(bl_age):time`, and `s(sleep_z):time`. For `s(sleep_z):time`, roughly, if we cannot fit a straight line through the confidence bands, there is evidence that the effect of time depends on sleep duration. It is correct the that confidence interval is zero at one point along the axis; this happens because we do not include the main effect.

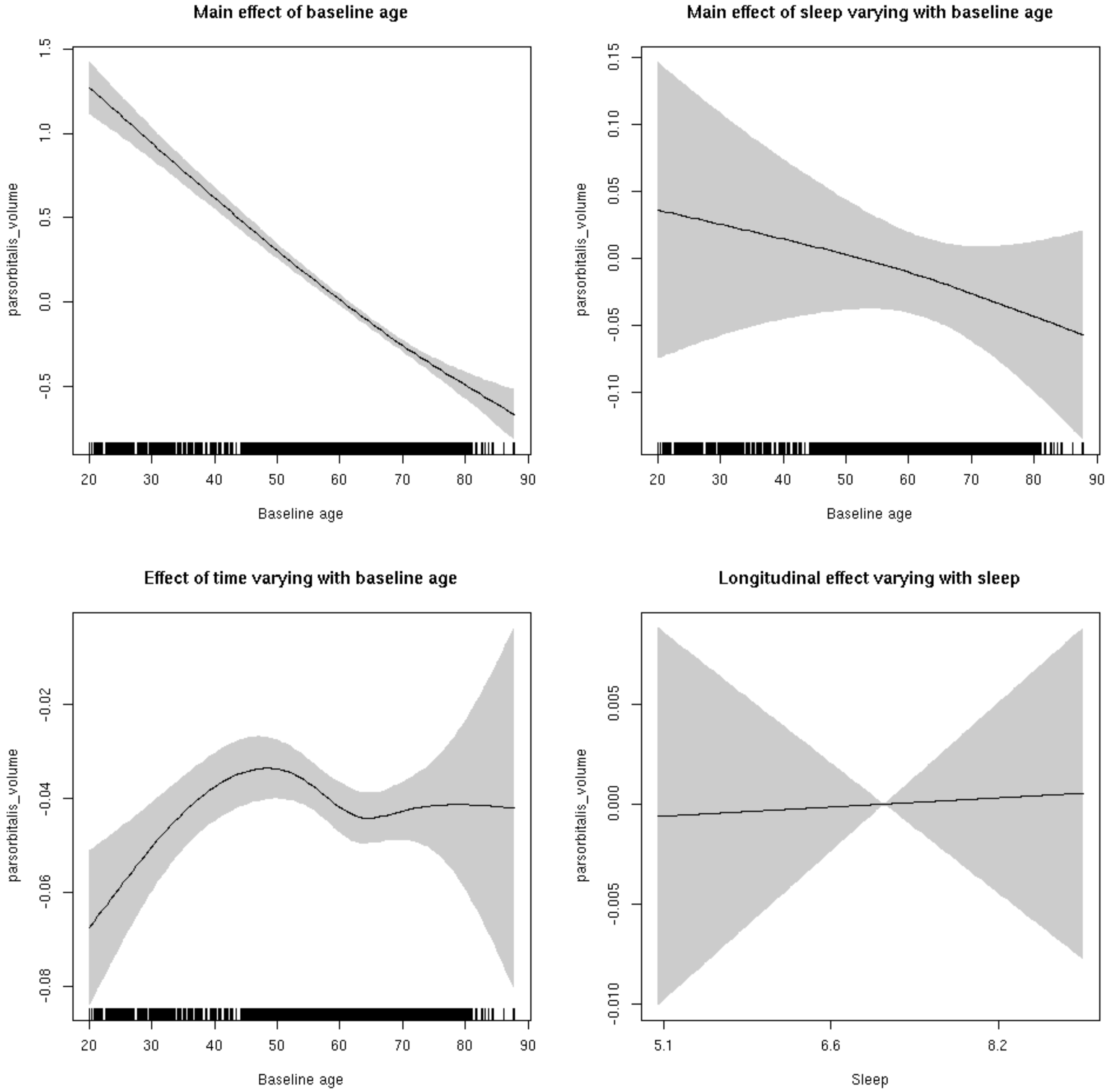

parstriangularis_area: Stratified by sex

Female: Full data Below is the row for `s(sleep_z):time` from the model output.

```
## s(sleep_z):time   1.000  1.000  0.073   0.787
```

Below are plots of a plot of `s(bl_age)`, `s(bl_age):sleep_z`, `s(bl_age):time`, and `s(sleep_z):time`. For `s(sleep_z):time`, roughly, if we cannot fit a straight line through the confidence bands, there is evidence that the effect of time depends on sleep duration. It is correct the that confidence interval is zero at one point along the axis; this happens because we do not include the main effect.

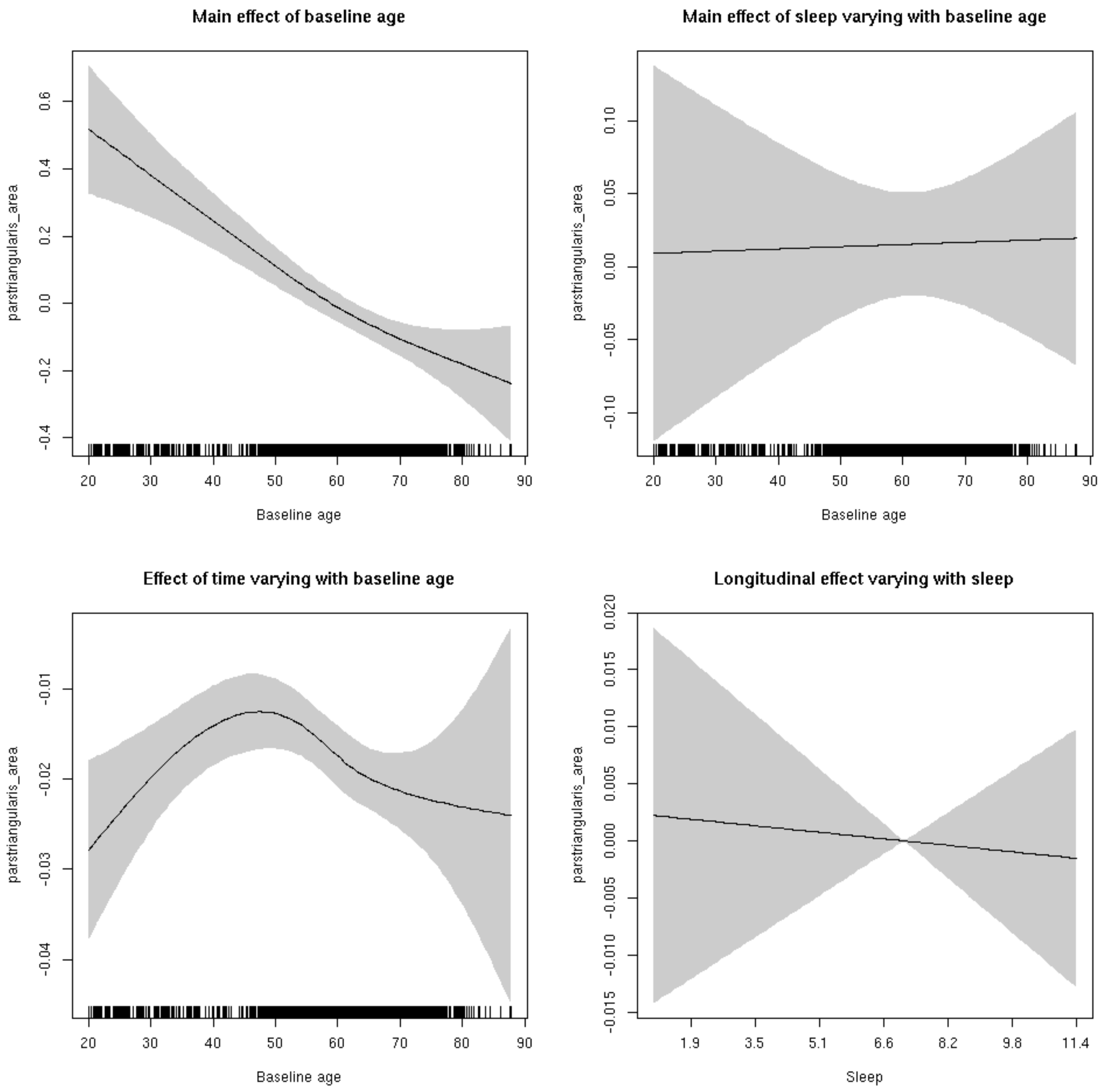

Male: Full data Below is the row for `s(sleep_z):time` from the model output.

```
## s(sleep_z):time   1.000  1.000   0.125   0.724
```

Below are plots of a plot of `s(bl_age)`, `s(bl_age):sleep_z`, `s(bl_age):time`, and `s(sleep_z):time`. For `s(sleep_z):time`, roughly, if we cannot fit a straight line through the confidence bands, there is evidence that the effect of time depends on sleep duration. It is correct the that confidence interval is zero at one point along the axis; this happens because we do not include the main effect.

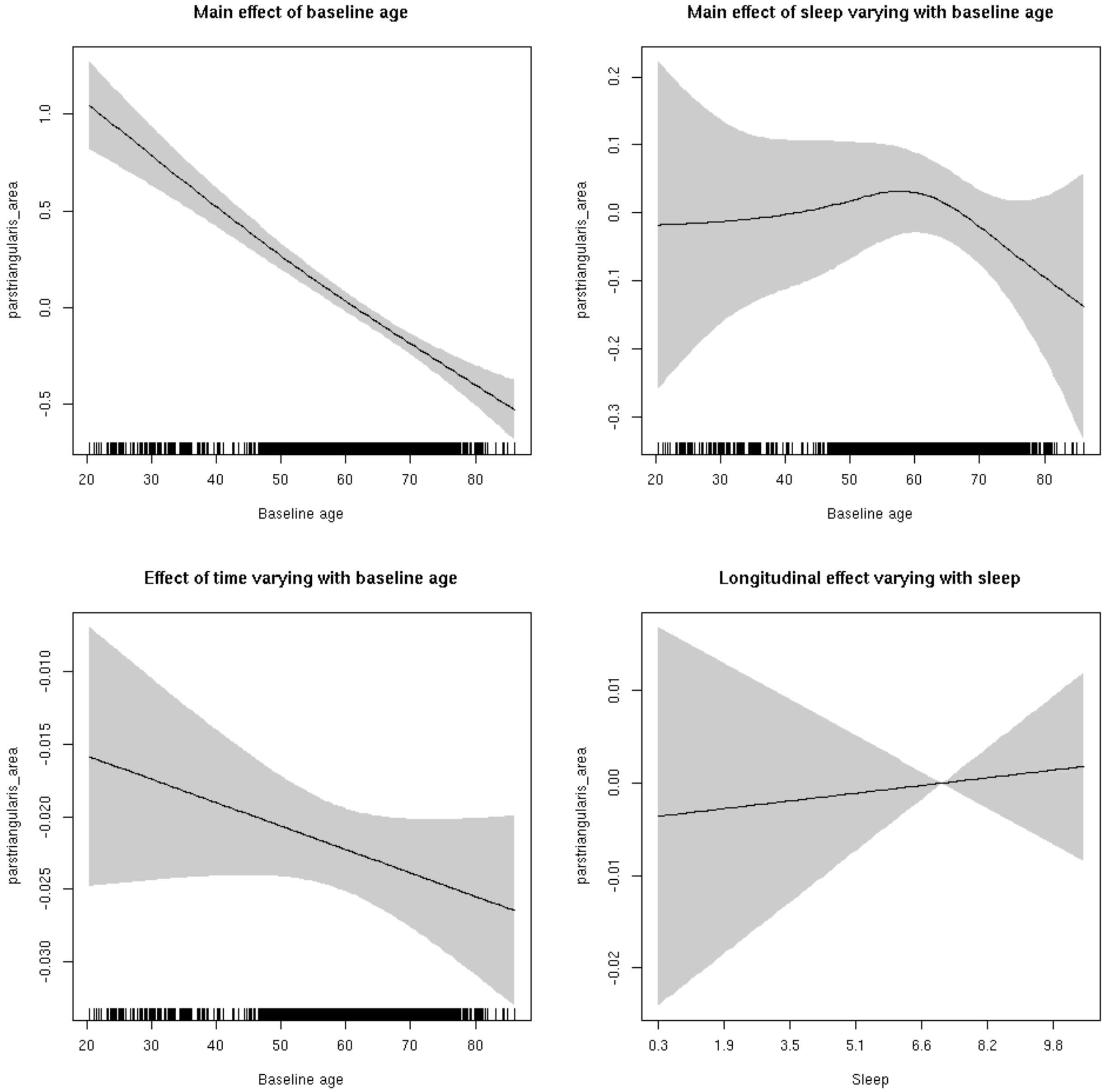

parstriangularis_area

Full data Below is the model output. The term `s(sleep_z):time` is probably what we care most about.

```
##
## Family: gaussian
## Link function: identity
##
## Formula:
## value ~ sex + site + icv + s(bl_age, k = 10, bs = "cr") + s(bl_age,
##     by = sleep_z, bs = "cr") + +s(bl_age, by = time, k = 5, bs = "cr") +
##     s(sleep_z, by = time, k = 5, bs = "cr", pc = 0)
## <environment: 0x5630035b28b0>
```

```
##
## Parametric coefficients:
##                Estimate Std. Error t value Pr(>|t|)
## (Intercept)     0.71083    0.06326  11.237  < 2e-16 ***
## sexmale         0.31697    0.03267   9.701  < 2e-16 ***
## siteousAvanto -0.72403    0.07740  -9.354  < 2e-16 ***
## siteousPrisma -0.76005    0.12182  -6.239 4.62e-10 ***
## siteousSkyra  -0.34084    0.07509  -4.539 5.73e-06 ***
## siteUB        -0.75211    0.14797  -5.083 3.80e-07 ***
## siteUCAM      -0.73406    0.07879  -9.316  < 2e-16 ***
## siteUKB       -0.84786    0.05975 -14.191  < 2e-16 ***
## siteUmU       -0.30254    0.09123  -3.316 0.000916 ***
## icv            0.41279    0.01572  26.264  < 2e-16 ***
## ---
## Signif. codes:  0 '***' 0.001 '**' 0.01 '*' 0.05 '.' 0.1 ' ' 1
##
## Approximate significance of smooth terms:
##                      edf Ref.df       F p-value
## s(bl_age)          1.721  1.721  99.971  <2e-16 ***
## s(bl_age):sleep_z 2.529  2.529   0.166   0.796
## s(bl_age):time    2.674  2.674 160.536  <2e-16 ***
## s(sleep_z):time   1.000  1.000   0.018   0.893
## ---
## Signif. codes:  0 '***' 0.001 '**' 0.01 '*' 0.05 '.' 0.1 ' ' 1
##
## R-sq.(adj) =  0.334
## lmer.REML = 6517.4  Scale est. = 0.014103  n = 8157
```

Below are plots of a plot of `s(bl_age)`, `s(bl_age):sleep_z`, `s(bl_age):time`, and `s(sleep_z):time`. For `s(sleep_z):time`, roughly, if we cannot fit a straight line through the confidence bands, there is evidence that the effect of time depends on sleep duration. It is correct the that confidence interval is zero at one point along the axis; this happens because we do not include the main effect.

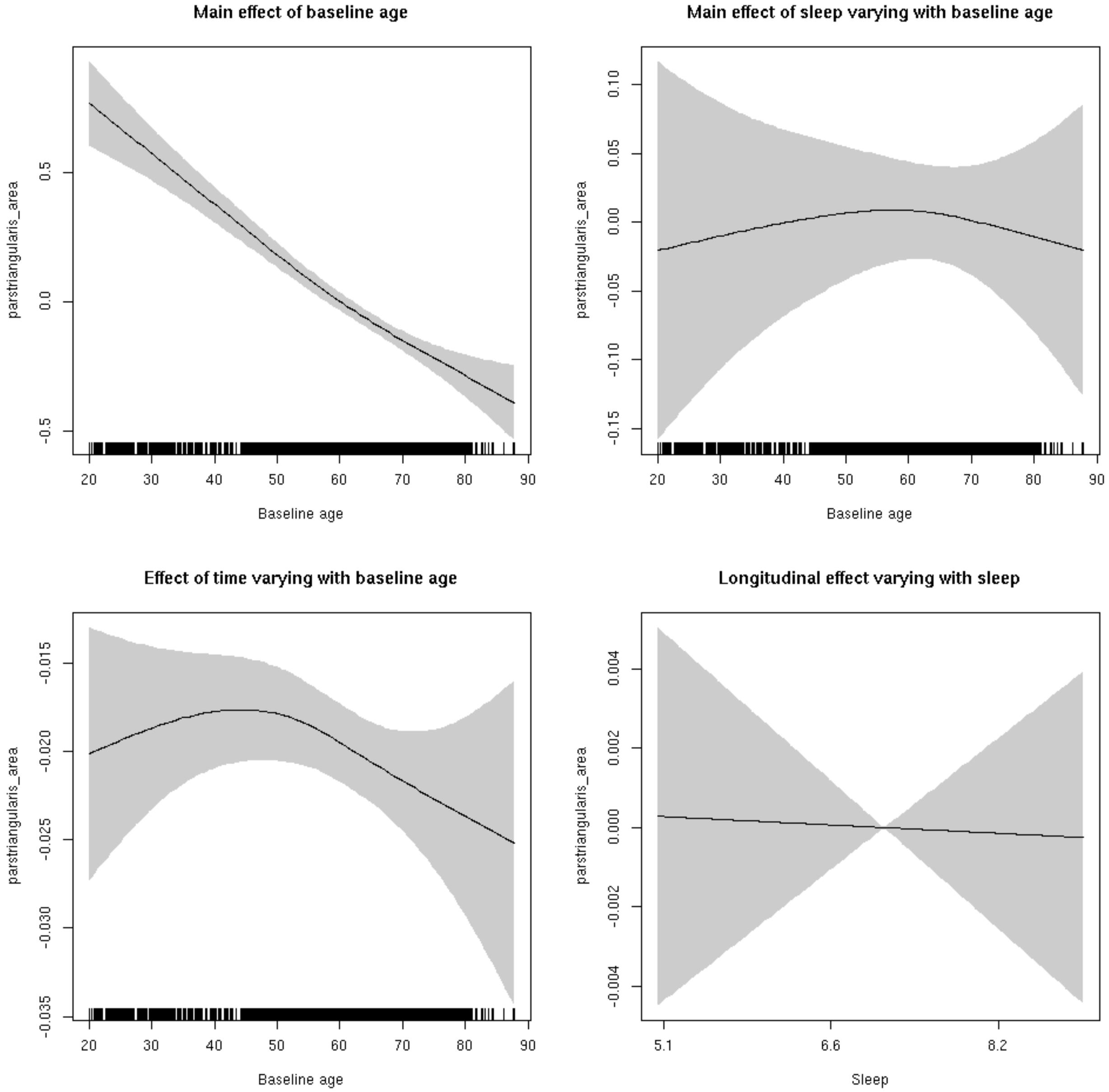

Restricted to sleep >= 5 and <= 9 hours Below is the row for `s(sleep_z):time` from the model output.

```
## s(sleep_z):time   1.000  1.000   0.014   0.905
```

Below are plots of a plot of `s(bl_age)`, `s(bl_age):sleep_z`, `s(bl_age):time`, and `s(sleep_z):time`. For `s(sleep_z):time`, roughly, if we cannot fit a straight line through the confidence bands, there is evidence that the effect of time depends on sleep duration. It is correct the that confidence interval is zero at one point along the axis; this happens because we do not include the main effect.

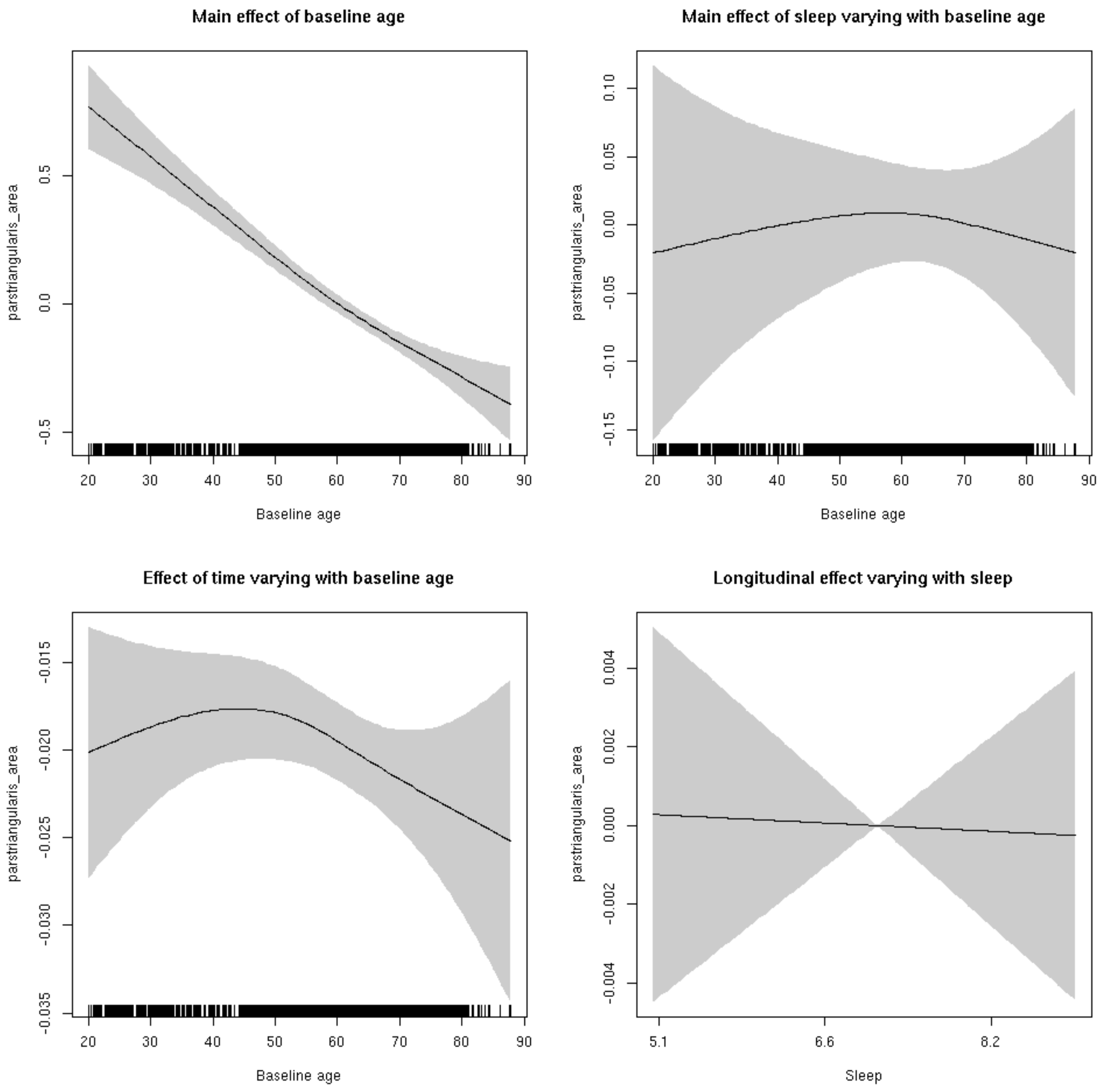

parstriangularis_thickness: Stratified by sex

Female: Full data Below is the row for `s(sleep_z):time` from the model output.

```
## s(sleep_z):time   1.000  1.000   0.139   0.709
```

Below are plots of a plot of `s(bl_age)`, `s(bl_age):sleep_z`, `s(bl_age):time`, and `s(sleep_z):time`. For `s(sleep_z):time`, roughly, if we cannot fit a straight line through the confidence bands, there is evidence that the effect of time depends on sleep duration. It is correct the that confidence interval is zero at one point along the axis; this happens because we do not include the main effect.

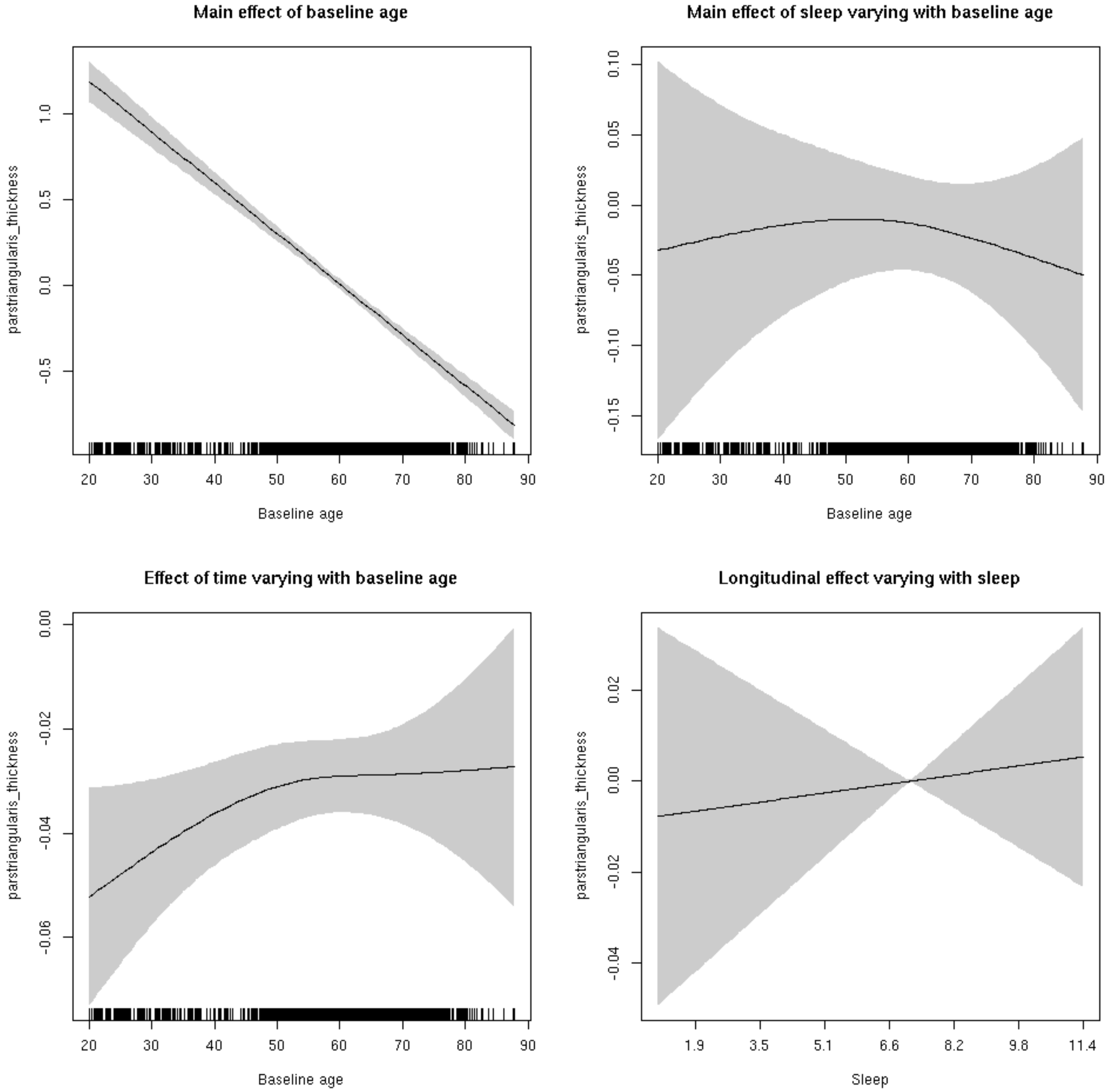

Male: Full data Below is the row for `s(sleep_z):time` from the model output.

```
## s(sleep_z):time   3.008  3.008   3.086  0.0291 *
```

Below are plots of a plot of `s(bl_age)`, `s(bl_age):sleep_z`, `s(bl_age):time`, and `s(sleep_z):time`. For `s(sleep_z):time`, roughly, if we cannot fit a straight line through the confidence bands, there is evidence that the effect of time depends on sleep duration. It is correct the that confidence interval is zero at one point along the axis; this happens because we do not include the main effect.

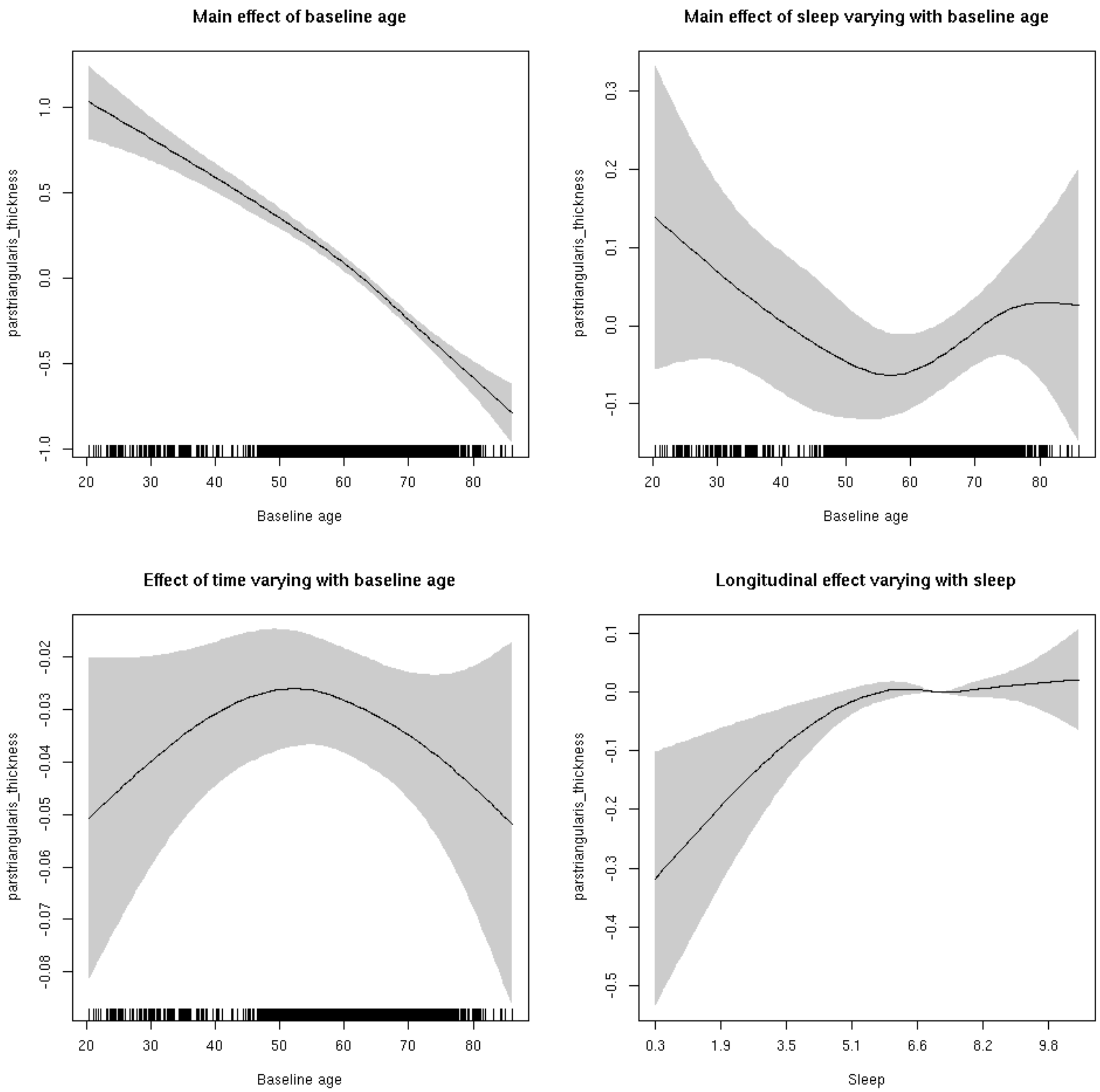

parstriangularis_thickness

Full data Below is the model output. The term `s(sleep_z):time` is probably what we care most about.

```
##
## Family: gaussian
## Link function: identity
##
## Formula:
## value ~ sex + site + s(bl_age, k = 10, bs = "cr") + s(bl_age,
##     by = sleep_z, bs = "cr") + +s(bl_age, by = time, k = 5, bs = "cr") +
##     s(sleep_z, by = time, k = 5, bs = "cr", pc = 0)
## <environment: 0x5630035d26c8>
```

```
##
## Parametric coefficients:
##                Estimate Std. Error t value Pr(>|t|)
## (Intercept)   -1.838519   0.042536 -43.223  < 2e-16 ***
## sexmale       -0.006378   0.021513  -0.296 0.766861
## siteousAvanto  0.208769   0.056730   3.680 0.000235 ***
## siteousPrisma  0.376239   0.232157   1.621 0.105138
## siteousSkyra  -0.021248   0.056690  -0.375 0.707803
## siteUB         0.394053   0.113851   3.461 0.000541 ***
## siteUCAM       0.337054   0.058403   5.771 8.16e-09 ***
## siteUKB        2.154408   0.042827  50.305  < 2e-16 ***
## siteUmU       -0.247174   0.070496  -3.506 0.000457 ***
## ---
## Signif. codes:  0 '***' 0.001 '**' 0.01 '*' 0.05 '.' 0.1 ' ' 1
##
## Approximate significance of smooth terms:
##                     edf Ref.df       F p-value
## s(bl_age)         2.113  2.113 382.702  <2e-16 ***
## s(bl_age):sleep_z 2.000  2.000   1.597  0.2026
## s(bl_age):time    3.166  3.166  39.527  <2e-16 ***
## s(sleep_z):time   3.280  3.280   2.580  0.0284 *
## ---
## Signif. codes:  0 '***' 0.001 '**' 0.01 '*' 0.05 '.' 0.1 ' ' 1
##
## R-sq.(adj) =  0.649
## lmer.REML =  12600  Scale est. = 0.09133   n = 8162
```

Below are plots of a plot of `s(bl_age)`, `s(bl_age):sleep_z`, `s(bl_age):time`, and `s(sleep_z):time`. For `s(sleep_z):time`, roughly, if we cannot fit a straight line through the confidence bands, there is evidence that the effect of time depends on sleep duration. It is correct the that confidence interval is zero at one point along the axis; this happens because we do not include the main effect.

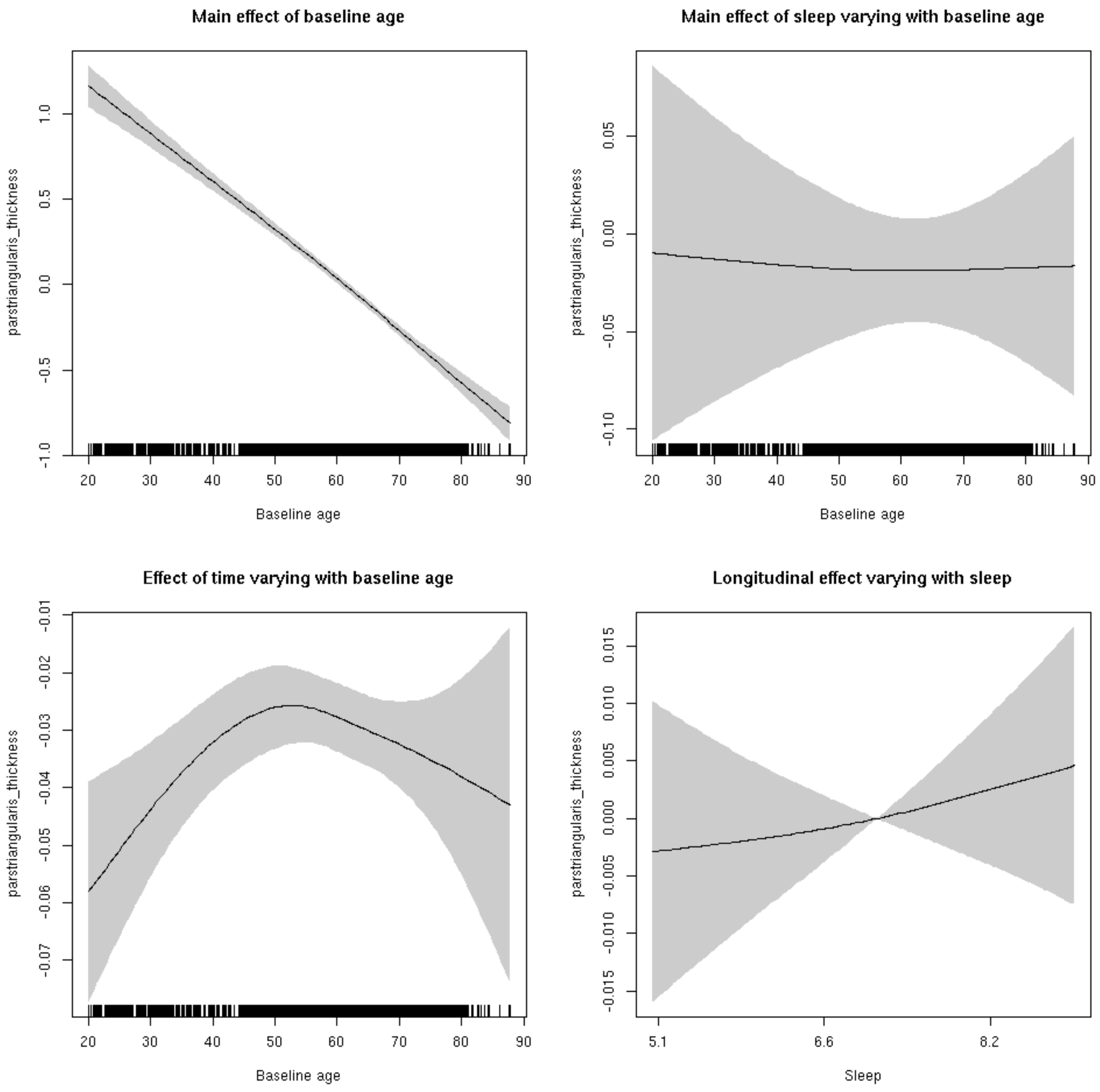

Restricted to sleep >= 5 and <= 9 hours Below is the row for `s(sleep_z):time` from the model output.

```
## s(sleep_z):time   1.108  1.108   0.299   0.552
```

Below are plots of a plot of `s(bl_age)`, `s(bl_age):sleep_z`, `s(bl_age):time`, and `s(sleep_z):time`. For `s(sleep_z):time`, roughly, if we cannot fit a straight line through the confidence bands, there is evidence that the effect of time depends on sleep duration. It is correct the that confidence interval is zero at one point along the axis; this happens because we do not include the main effect.

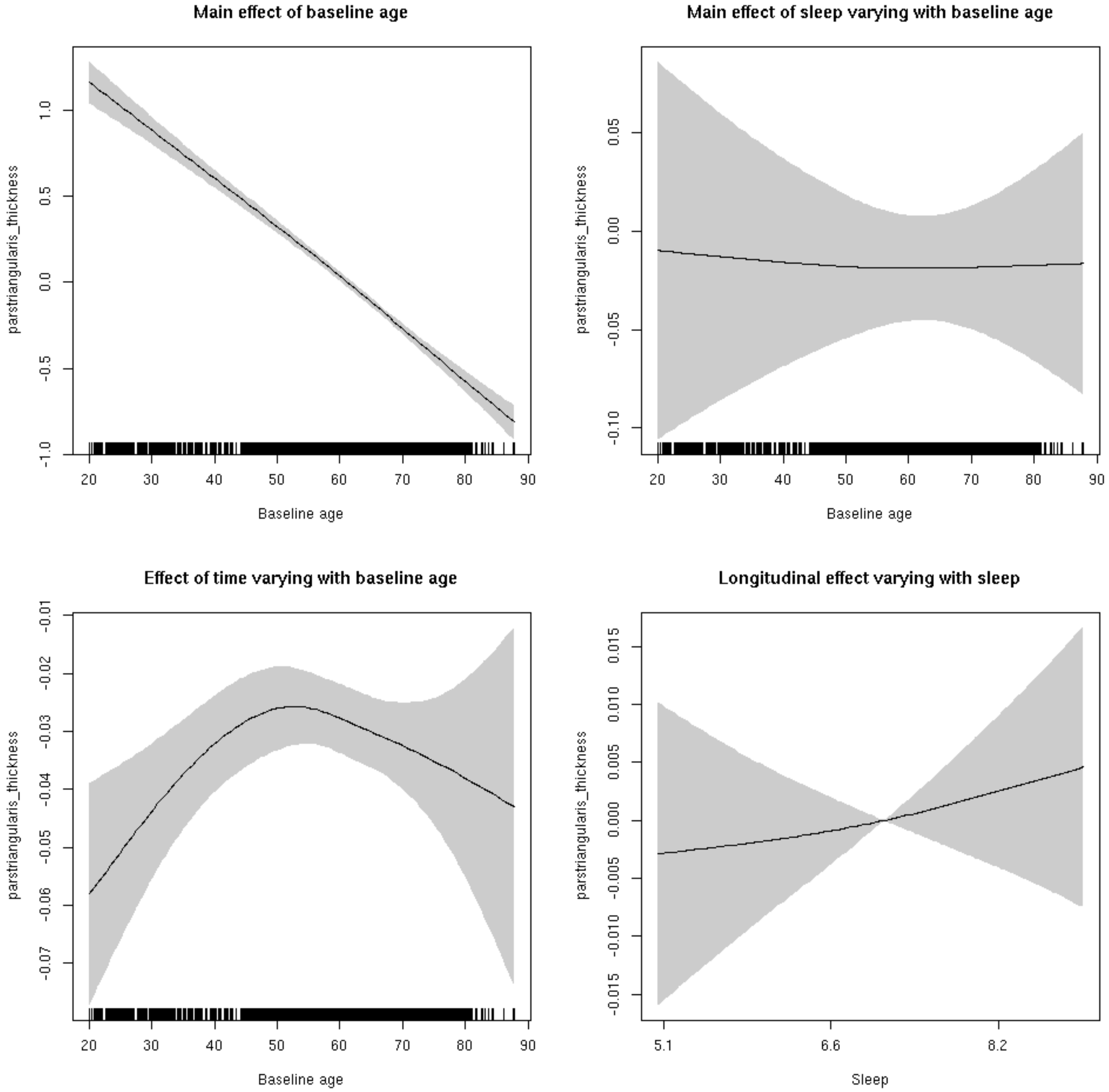

parstriangularis_volume: Stratified by sex

Female: Full data Below is the row for `s(sleep_z):time` from the model output.

```
## s(sleep_z):time   1.000  1.000   0.057   0.812
```

Below are plots of a plot of `s(bl_age)`, `s(bl_age):sleep_z`, `s(bl_age):time`, and `s(sleep_z):time`. For `s(sleep_z):time`, roughly, if we cannot fit a straight line through the confidence bands, there is evidence that the effect of time depends on sleep duration. It is correct the that confidence interval is zero at one point along the axis; this happens because we do not include the main effect.

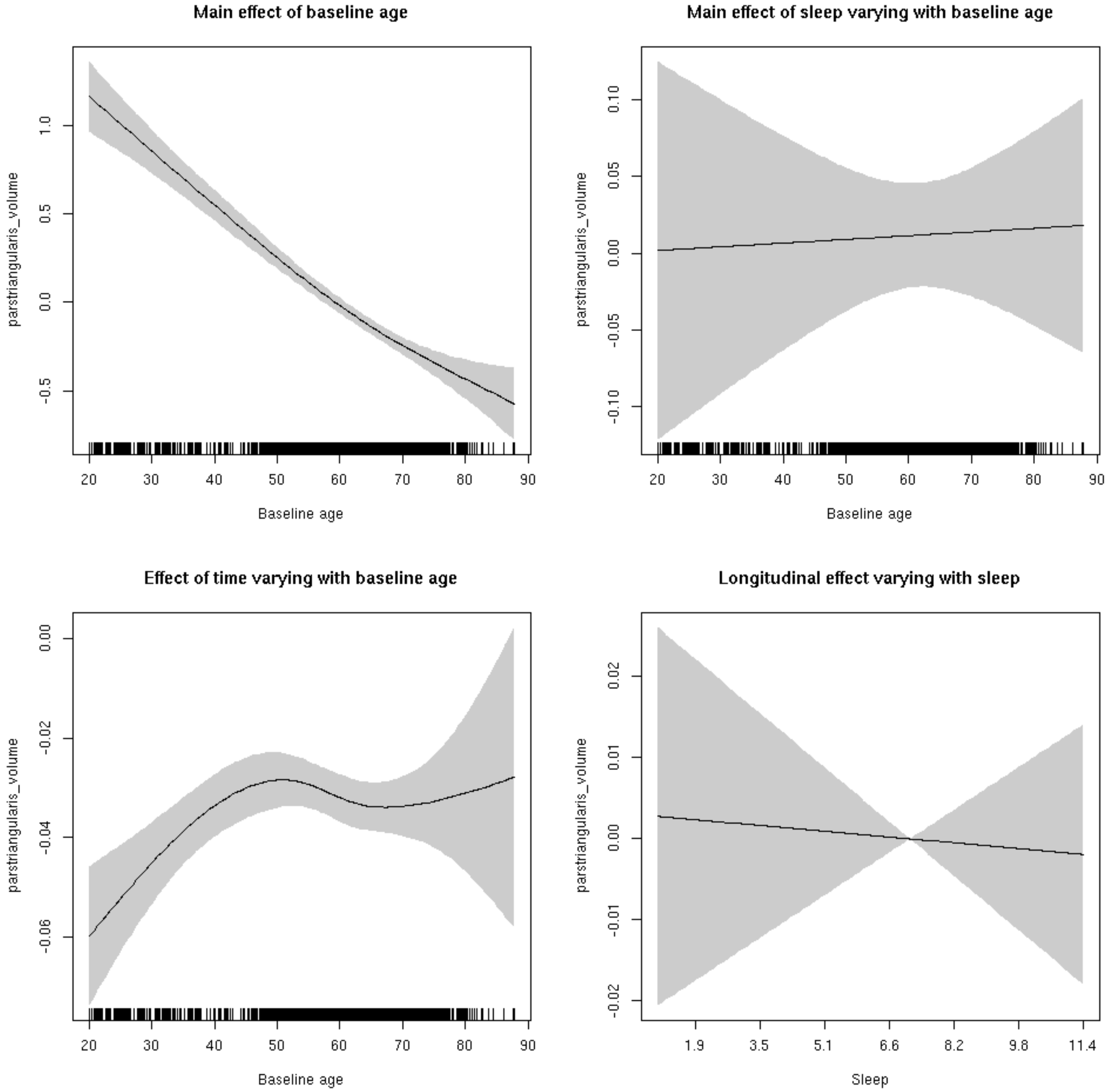

Male: Full data Below is the row for `s(sleep_z):time` from the model output.

```
## s(sleep_z):time   1.000  1.000   1.019   0.313
```

Below are plots of a plot of `s(bl_age)`, `s(bl_age):sleep_z`, `s(bl_age):time`, and `s(sleep_z):time`. For `s(sleep_z):time`, roughly, if we cannot fit a straight line through the confidence bands, there is evidence that the effect of time depends on sleep duration. It is correct the that confidence interval is zero at one point along the axis; this happens because we do not include the main effect.

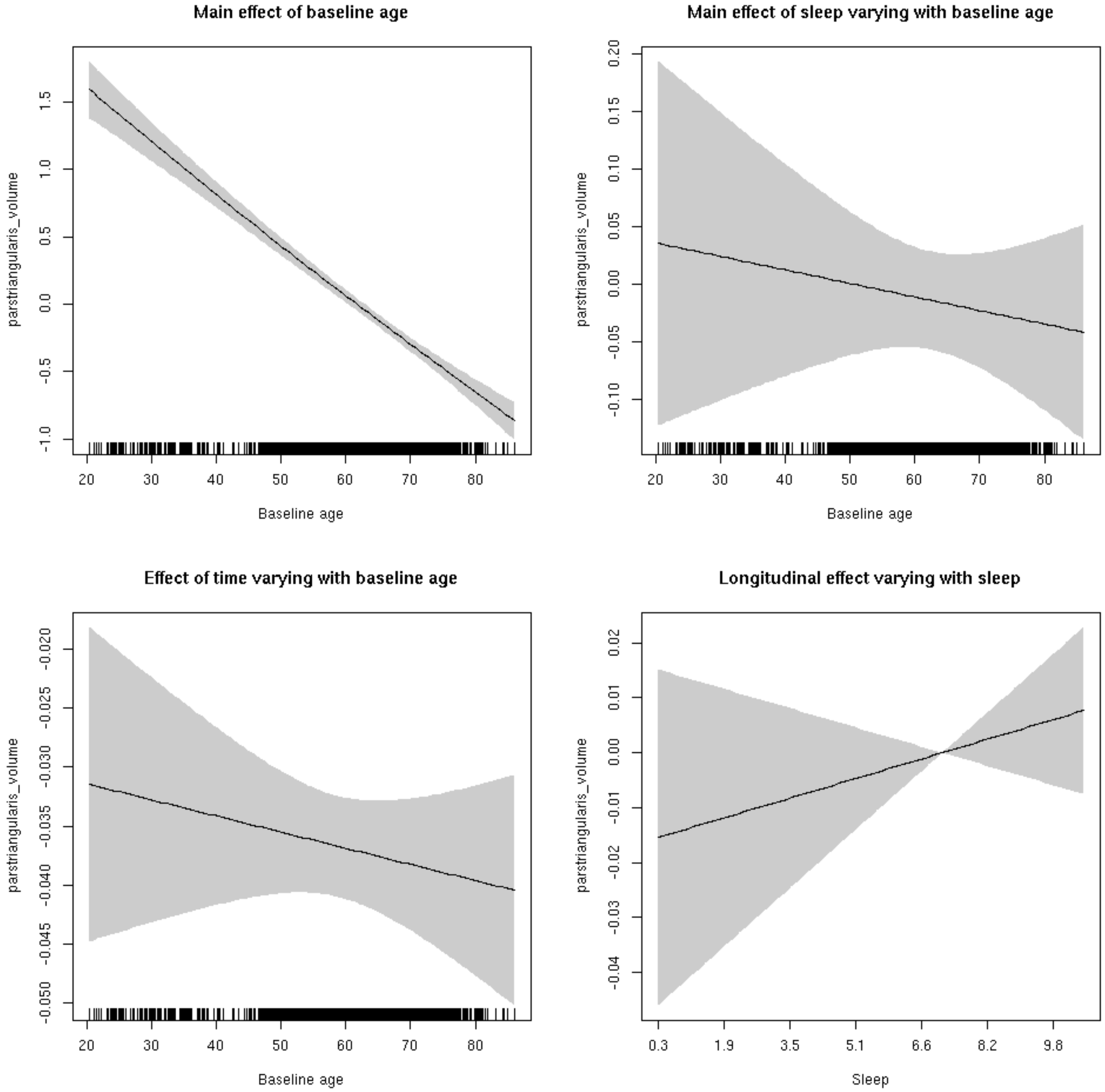

parstriangularis_volume

Full data Below is the model output. The term `s(sleep_z):time` is probably what we care most about.

```
##
## Family: gaussian
## Link function: identity
##
## Formula:
## value ~ sex + site + icv + s(bl_age, k = 10, bs = "cr") + s(bl_age,
##     by = sleep_z, bs = "cr") + +s(bl_age, by = time, k = 5, bs = "cr") +
##     s(sleep_z, by = time, k = 5, bs = "cr", pc = 0)
## <environment: 0x5630035a94c8>
```

```
##
## Parametric coefficients:
##                 Estimate Std. Error t value Pr(>|t|)
## (Intercept)   -0.05023    0.06141  -0.818 0.413473
## sexmale        0.25211    0.03166   7.964 1.89e-15 ***
## siteousAvanto -0.62402    0.07502  -8.318  < 2e-16 ***
## siteousPrisma -0.52966    0.15400  -3.439 0.000586 ***
## siteousSkyra  -0.34706    0.07267  -4.776 1.82e-06 ***
## siteUB        -0.46156    0.14214  -3.247 0.001171 **
## siteUCAM      -0.51042    0.07608  -6.709 2.09e-11 ***
## siteUKB        0.09208    0.05803   1.587 0.112566
## siteUmU       -0.38307    0.08789  -4.358 1.33e-05 ***
## icv            0.39802    0.01551  25.663  < 2e-16 ***
## ---
## Signif. codes:  0 '***' 0.001 '**' 0.01 '*' 0.05 '.' 0.1 ' ' 1
##
## Approximate significance of smooth terms:
##                     edf Ref.df       F p-value
## s(bl_age)         2.039  2.039 325.883  <2e-16 ***
## s(bl_age):sleep_z 2.201  2.201   0.159  0.8337
## s(bl_age):time    3.782  3.782 106.192  <2e-16 ***
## s(sleep_z):time   3.378  3.378   2.094  0.0491 *
## ---
## Signif. codes:  0 '***' 0.001 '**' 0.01 '*' 0.05 '.' 0.1 ' ' 1
##
## R-sq.(adj) =  0.371
## lmer.REML = 9442.5  Scale est. = 0.029965  n = 8168
```

Below are plots of a plot of `s(bl_age)`, `s(bl_age):sleep_z`, `s(bl_age):time`, and `s(sleep_z):time`. For `s(sleep_z):time`, roughly, if we cannot fit a straight line through the confidence bands, there is evidence that the effect of time depends on sleep duration. It is correct the that confidence interval is zero at one point along the axis; this happens because we do not include the main effect.

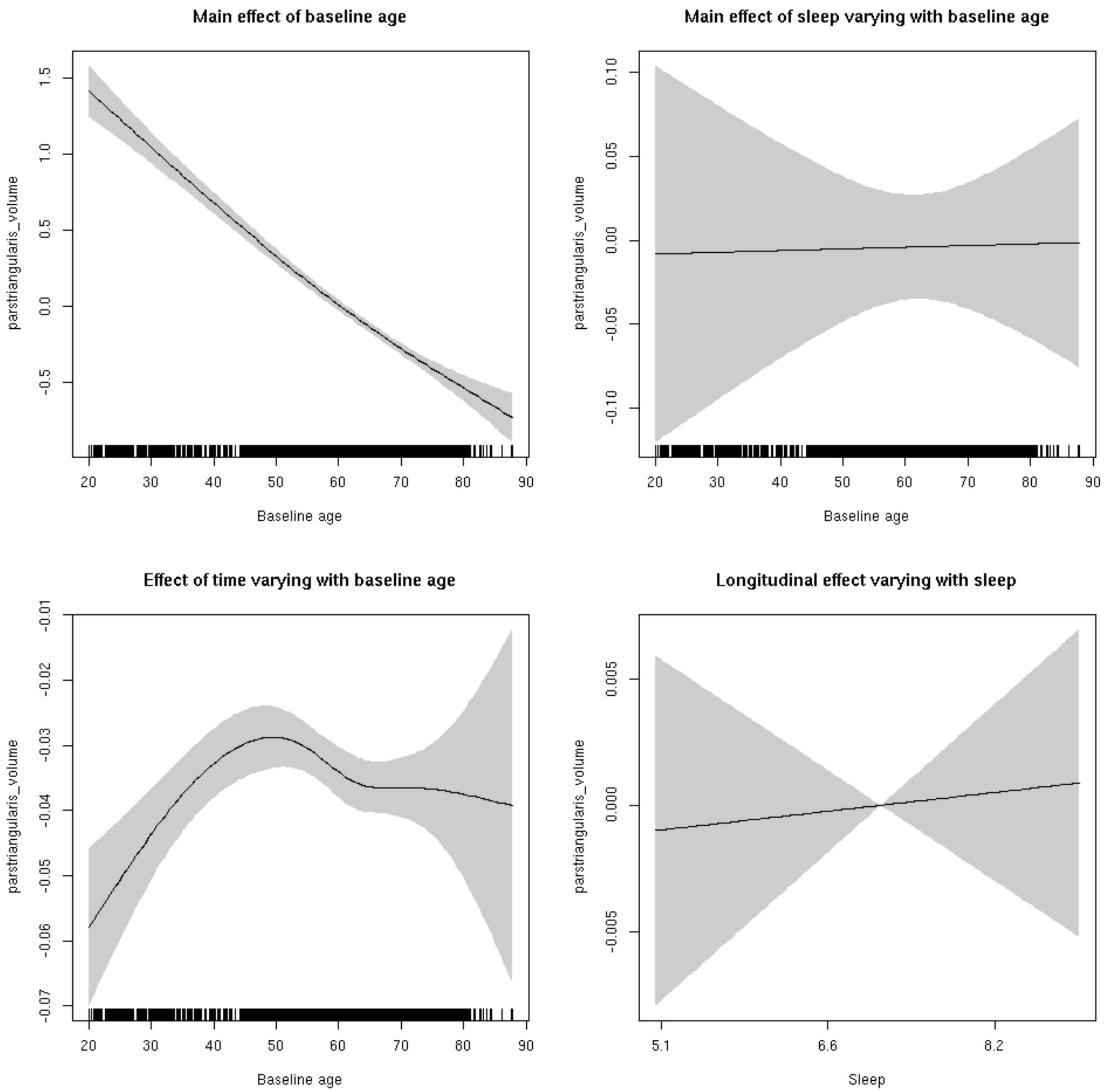

Restricted to sleep >= 5 and <= 9 hours Below is the row for `s(sleep_z):time` from the model output.

```
## s(sleep_z):time   1.000  1.000   0.084   0.772
```

Below are plots of a plot of `s(bl_age)`, `s(bl_age):sleep_z`, `s(bl_age):time`, and `s(sleep_z):time`. For `s(sleep_z):time`, roughly, if we cannot fit a straight line through the confidence bands, there is evidence that the effect of time depends on sleep duration. It is correct the that confidence interval is zero at one point along the axis; this happens because we do not include the main effect.

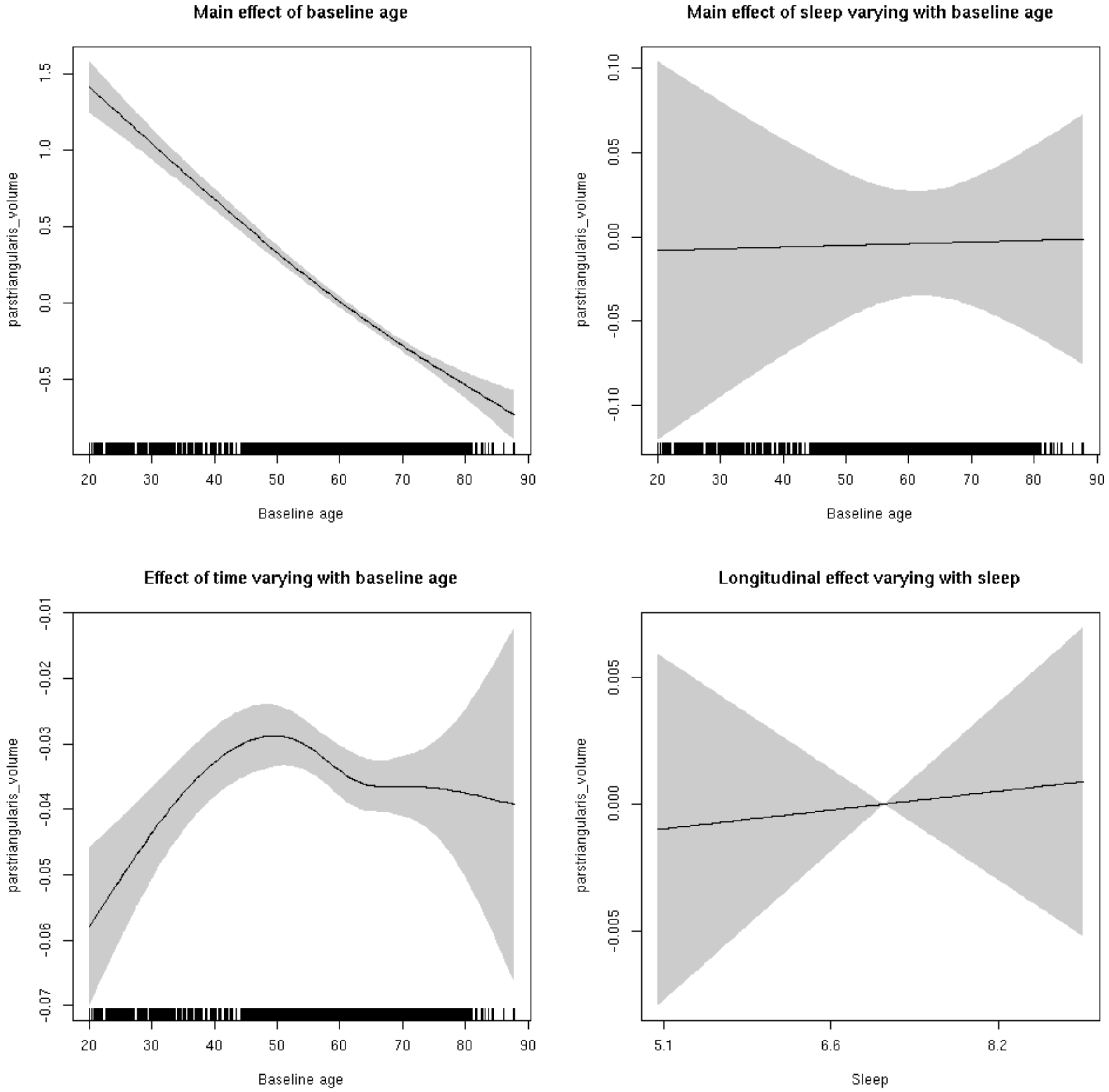

pericalcarine_area: Stratified by sex

Female: Full data Below is the row for `s(sleep_z):time` from the model output.

```
## s(sleep_z):time   1.000  1.000  0.476  0.4902
```

Below are plots of a plot of `s(bl_age)`, `s(bl_age):sleep_z`, `s(bl_age):time`, and `s(sleep_z):time`. For `s(sleep_z):time`, roughly, if we cannot fit a straight line through the confidence bands, there is evidence that the effect of time depends on sleep duration. It is correct the that confidence interval is zero at one point along the axis; this happens because we do not include the main effect.

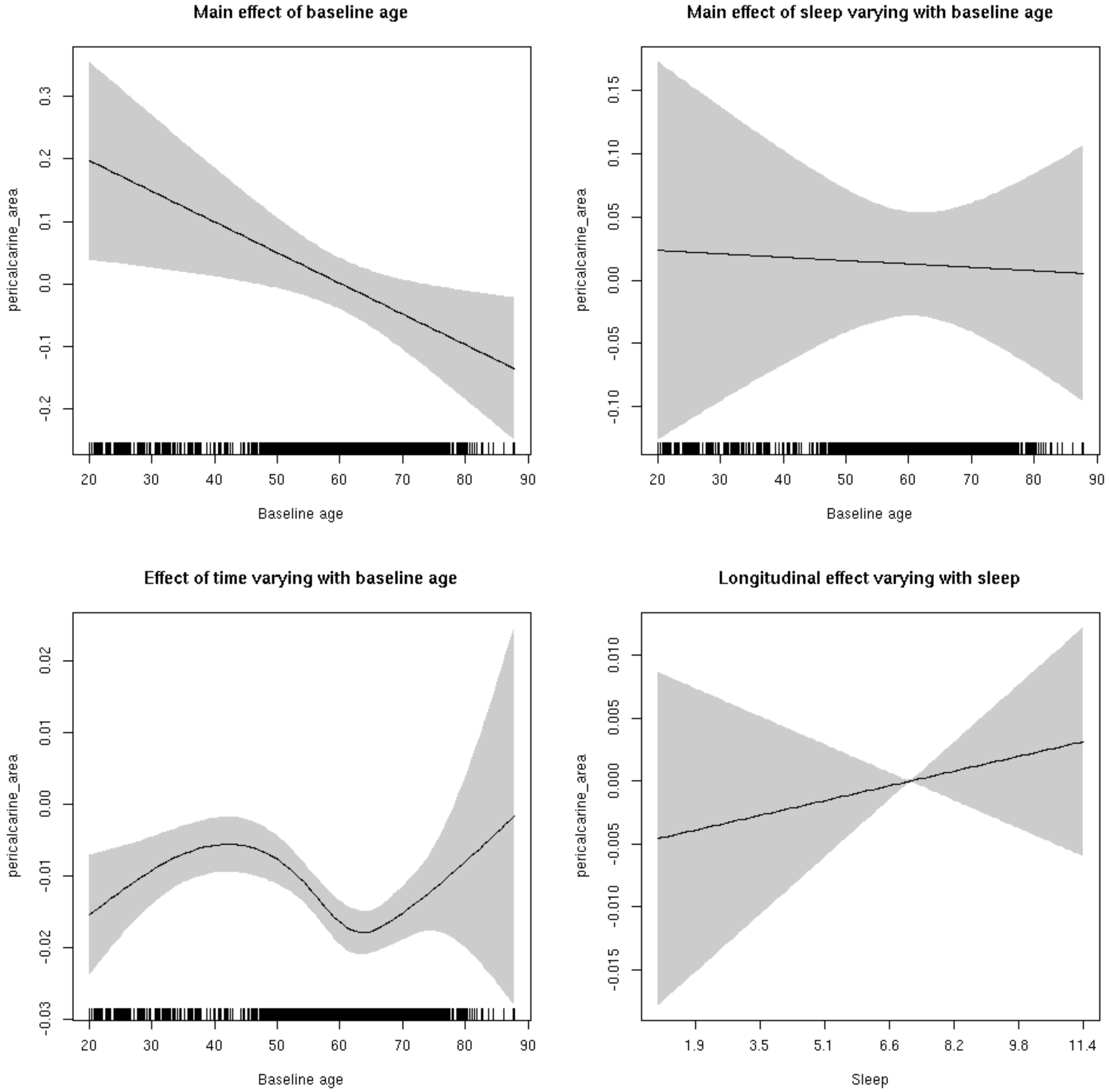

Male: Full data Below is the row for `s(sleep_z):time` from the model output.

```
## s(sleep_z):time     1       1  1.171    0.279
```

Below are plots of a plot of `s(bl_age)`, `s(bl_age):sleep_z`, `s(bl_age):time`, and `s(sleep_z):time`. For `s(sleep_z):time`, roughly, if we cannot fit a straight line through the confidence bands, there is evidence that the effect of time depends on sleep duration. It is correct the that confidence interval is zero at one point along the axis; this happens because we do not include the main effect.

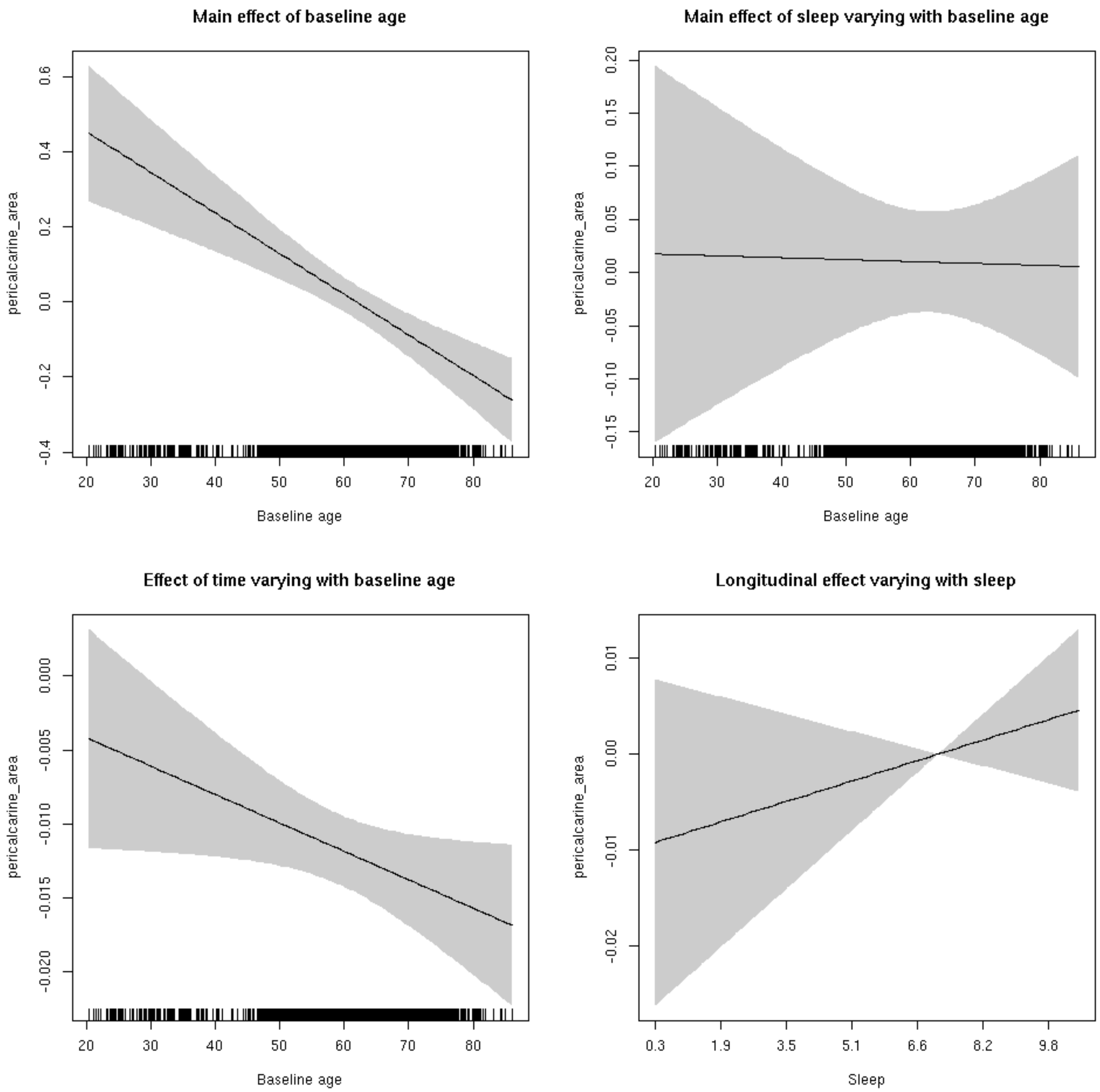

pericalcarine_area

Full data Below is the model output. The term `s(sleep_z):time` is probably what we care most about.

```
##
## Family: gaussian
## Link function: identity
##
## Formula:
## value ~ sex + site + icv + s(bl_age, k = 10, bs = "cr") + s(bl_age,
##     by = sleep_z, bs = "cr") + +s(bl_age, by = time, k = 5, bs = "cr") +
##     s(sleep_z, by = time, k = 5, bs = "cr", pc = 0)
## <environment: 0x5630035a87d0>
```

```
##
## Parametric coefficients:
##                Estimate Std. Error t value Pr(>|t|)
## (Intercept)     0.46287    0.06936   6.673 2.67e-11 ***
## sexmale         0.22109    0.03603   6.136 8.87e-10 ***
## siteousAvanto -0.24667    0.08563  -2.881  0.00398 **
## siteousPrisma -0.47009    0.11618  -4.046 5.25e-05 ***
## siteousSkyra  -0.16464    0.08320  -1.979  0.04788 *
## siteUB        -0.28019    0.16511  -1.697  0.08973 .
## siteUCAM      -0.41498    0.08745  -4.745 2.12e-06 ***
## siteUKB       -0.56913    0.06508  -8.745  < 2e-16 ***
## siteUmU       -0.71830    0.10141  -7.083 1.53e-12 ***
## icv            0.23280    0.01682  13.841  < 2e-16 ***
## ---
## Signif. codes:  0 '***' 0.001 '**' 0.01 '*' 0.05 '.' 0.1 ' ' 1
##
## Approximate significance of smooth terms:
##                      edf Ref.df      F p-value
## s(bl_age)          1.000  1.000 30.504  <2e-16 ***
## s(bl_age):sleep_z 2.000  2.000  0.261   0.771
## s(bl_age):time    4.057  4.057 57.407  <2e-16 ***
## s(sleep_z):time   2.104  2.104  2.779   0.088 .
## ---
## Signif. codes:  0 '***' 0.001 '**' 0.01 '*' 0.05 '.' 0.1 ' ' 1
##
## R-sq.(adj) =  0.138
## lmer.REML = 5666.8  Scale est. = 0.0094353  n = 8160
```

Below are plots of a plot of `s(bl_age)`, `s(bl_age):sleep_z`, `s(bl_age):time`, and `s(sleep_z):time`. For `s(sleep_z):time`, roughly, if we cannot fit a straight line through the confidence bands, there is evidence that the effect of time depends on sleep duration. It is correct the that confidence interval is zero at one point along the axis; this happens because we do not include the main effect.

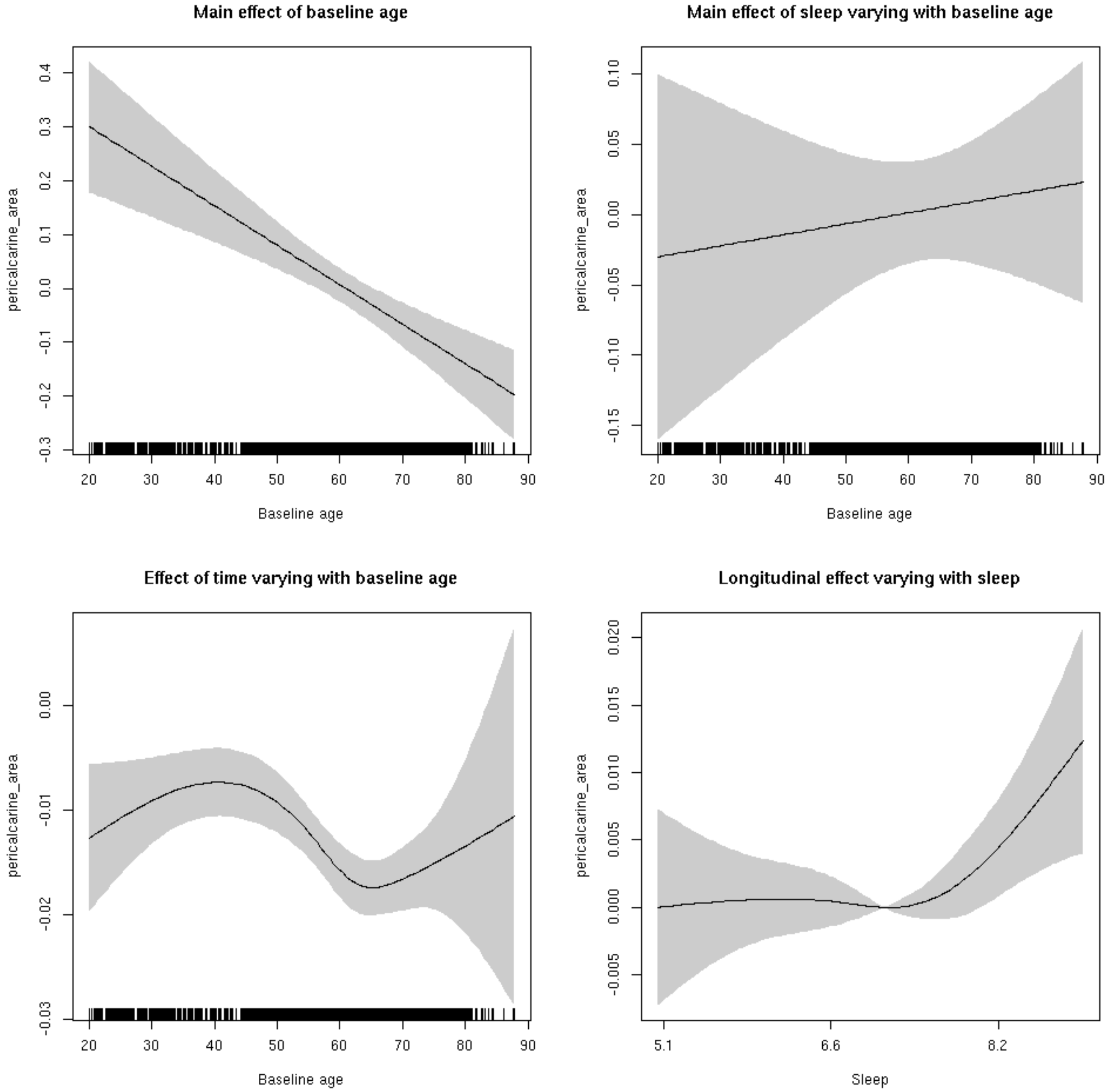

Restricted to sleep >= 5 and <= 9 hours Below is the row for `s(sleep_z):time` from the model output.

```
## s(sleep_z):time   2.569  2.569  2.470   0.0416 *
```

Below are plots of a plot of `s(bl_age)`, `s(bl_age):sleep_z`, `s(bl_age):time`, and `s(sleep_z):time`. For `s(sleep_z):time`, roughly, if we cannot fit a straight line through the confidence bands, there is evidence that the effect of time depends on sleep duration. It is correct the that confidence interval is zero at one point along the axis; this happens because we do not include the main effect.

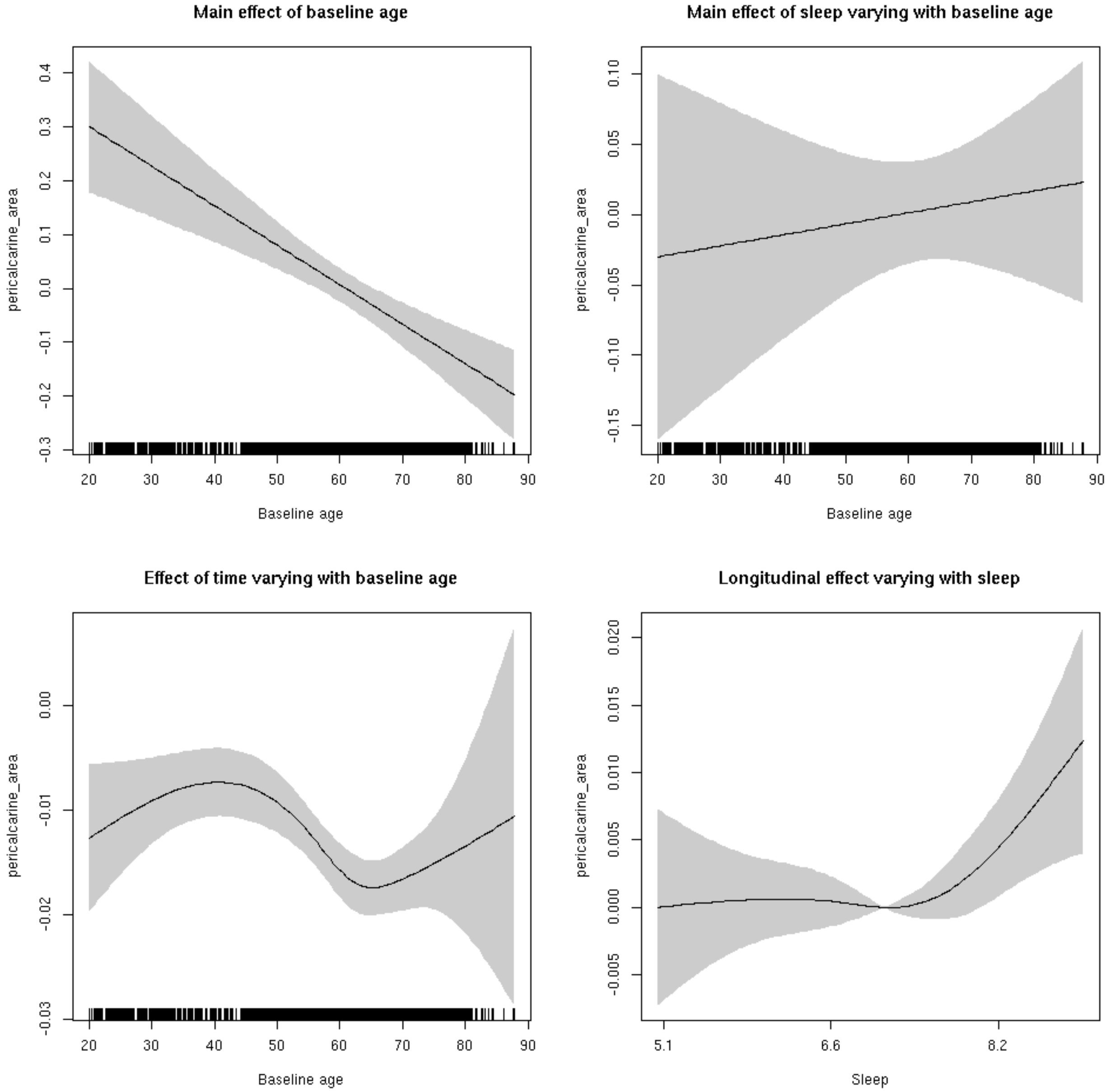

pericalcarine_thickness: Stratified by sex

Female: Full data Below is the row for `s(sleep_z):time` from the model output.

```
## s(sleep_z):time   1.000  1.000  0.402  0.5261
```

Below are plots of a plot of `s(bl_age)`, `s(bl_age):sleep_z`, `s(bl_age):time`, and `s(sleep_z):time`. For `s(sleep_z):time`, roughly, if we cannot fit a straight line through the confidence bands, there is evidence that the effect of time depends on sleep duration. It is correct the that confidence interval is zero at one point along the axis; this happens because we do not include the main effect.

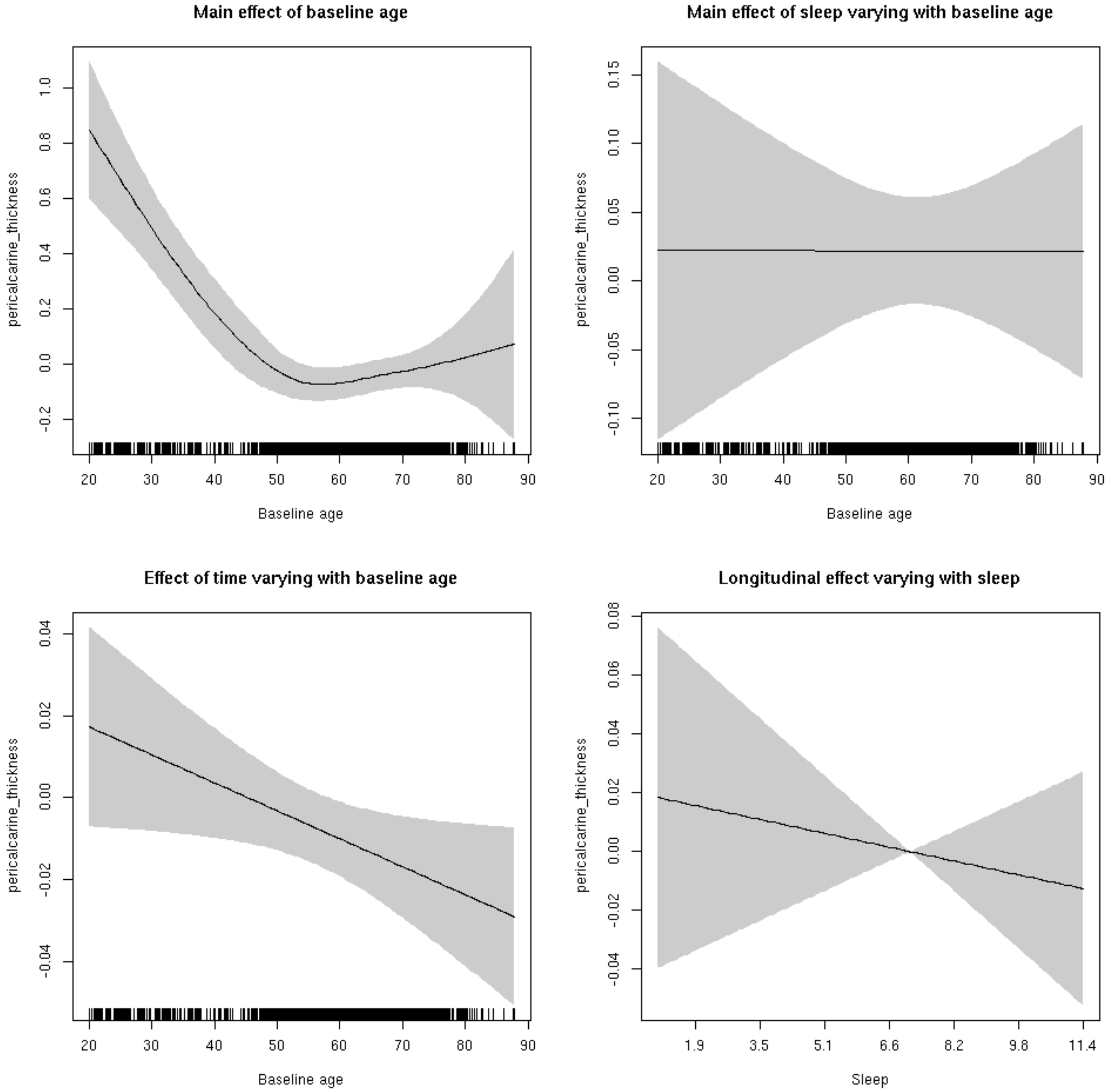

Male: Full data Below is the row for `s(sleep_z):time` from the model output.

```
## s(sleep_z):time   1.000  1.000  0.583 0.445230
```

Below are plots of a plot of `s(bl_age)`, `s(bl_age):sleep_z`, `s(bl_age):time`, and `s(sleep_z):time`. For `s(sleep_z):time`, roughly, if we cannot fit a straight line through the confidence bands, there is evidence that the effect of time depends on sleep duration. It is correct the that confidence interval is zero at one point along the axis; this happens because we do not include the main effect.

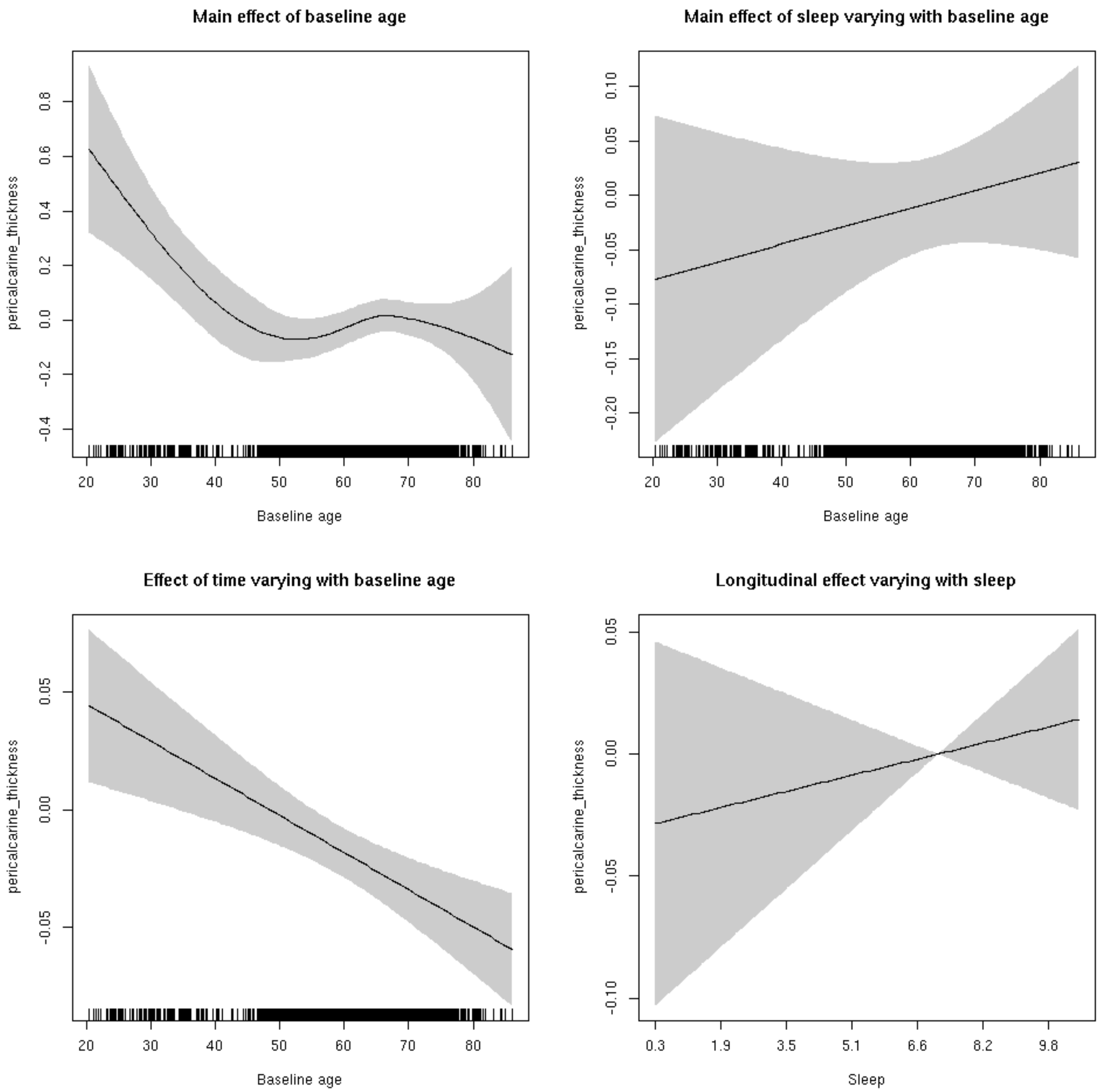

pericalcarine__thickness

Full data Below is the model output. The term `s(sleep_z):time` is probably what we care most about.

```
##
## Family: gaussian
## Link function: identity
##
## Formula:
## value ~ sex + site + s(bl_age, k = 10, bs = "cr") + s(bl_age,
##     by = sleep_z, bs = "cr") + +s(bl_age, by = time, k = 5, bs = "cr") +
##     s(sleep_z, by = time, k = 5, bs = "cr", pc = 0)
## <environment: 0x5630035cf7d8>
```

```
##
## Parametric coefficients:
##                Estimate Std. Error t value Pr(>|t|)
## (Intercept)   -0.79130    0.05345 -14.805  < 2e-16 ***
## sexmale        0.13391    0.02675   5.006 5.68e-07 ***
## siteousAvanto -0.70032    0.07138  -9.811  < 2e-16 ***
## siteousPrisma  0.65729    0.33003   1.992  0.04645 *
## siteousSkyra   1.30147    0.07133  18.245  < 2e-16 ***
## siteUB         0.52717    0.14118   3.734  0.00019 ***
## siteUCAM      -0.16864    0.07361  -2.291  0.02199 *
## siteUKB        0.81987    0.05417  15.135  < 2e-16 ***
## siteUmU        1.16304    0.08805  13.208  < 2e-16 ***
## ---
## Signif. codes:  0 '***' 0.001 '**' 0.01 '*' 0.05 '.' 0.1 ' ' 1
##
## Approximate significance of smooth terms:
##                      edf Ref.df      F p-value
## s(bl_age)          3.951  3.951 18.813  <2e-16 ***
## s(bl_age):sleep_z 2.000  2.000  0.277   0.758
## s(bl_age):time    2.000  2.000 16.440  <2e-16 ***
## s(sleep_z):time   1.000  1.000  0.018   0.892
## ---
## Signif. codes:  0 '***' 0.001 '**' 0.01 '*' 0.05 '.' 0.1 ' ' 1
##
## R-sq.(adj) =  0.245
## lmer.REML =  17500  Scale est. = 0.19195   n = 8178
```

Below are plots of a plot of `s(bl_age)`, `s(bl_age):sleep_z`, `s(bl_age):time`, and `s(sleep_z):time`. For `s(sleep_z):time`, roughly, if we cannot fit a straight line through the confidence bands, there is evidence that the effect of time depends on sleep duration. It is correct the that confidence interval is zero at one point along the axis; this happens because we do not include the main effect.

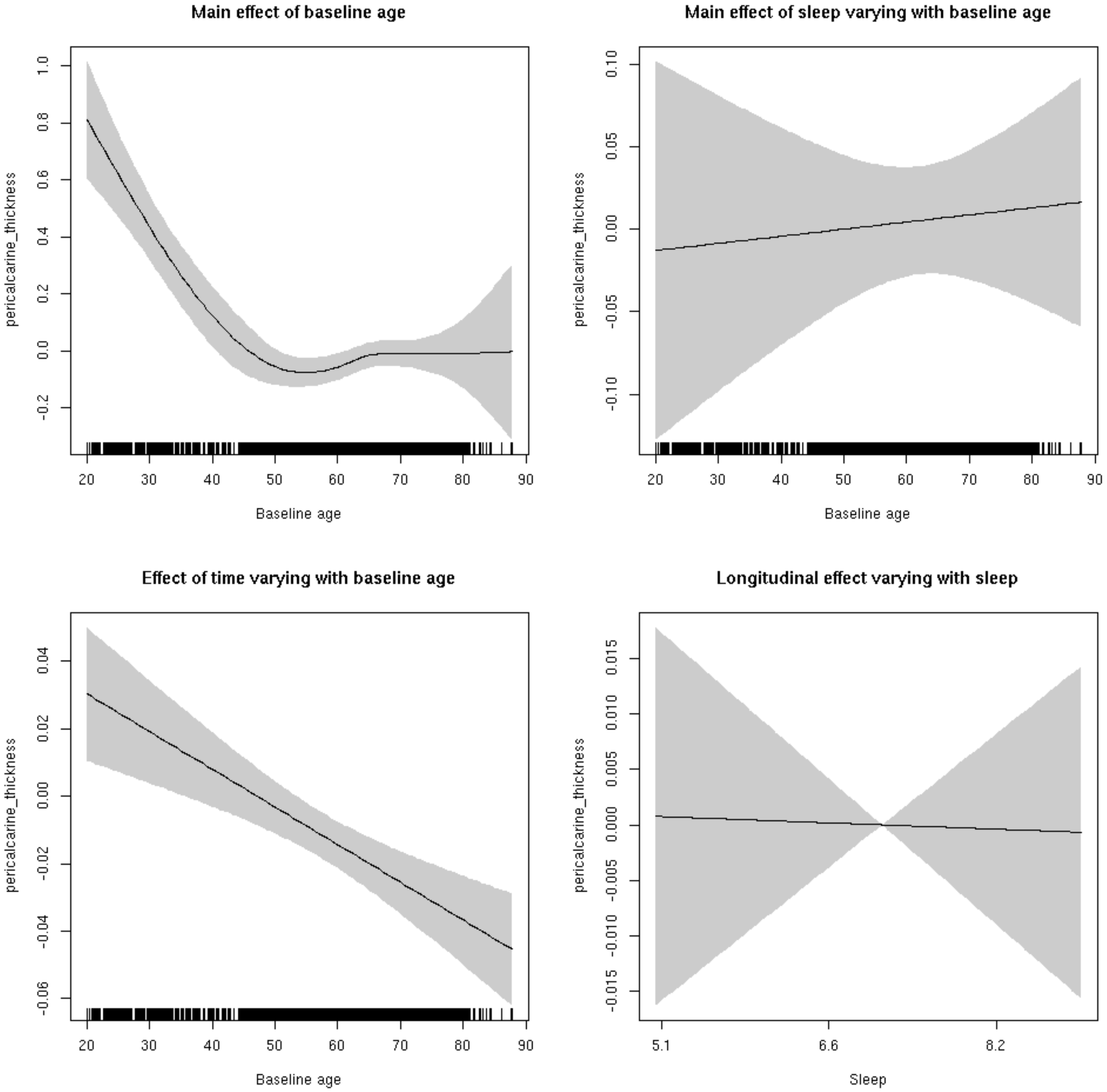

Restricted to sleep >= 5 and <= 9 hours Below is the row for `s(sleep_z):time` from the model output.

```
## s(sleep_z):time   1.000  1.000  0.008    0.928
```

Below are plots of a plot of `s(bl_age)`, `s(bl_age):sleep_z`, `s(bl_age):time`, and `s(sleep_z):time`. For `s(sleep_z):time`, roughly, if we cannot fit a straight line through the confidence bands, there is evidence that the effect of time depends on sleep duration. It is correct the that confidence interval is zero at one point along the axis; this happens because we do not include the main effect.

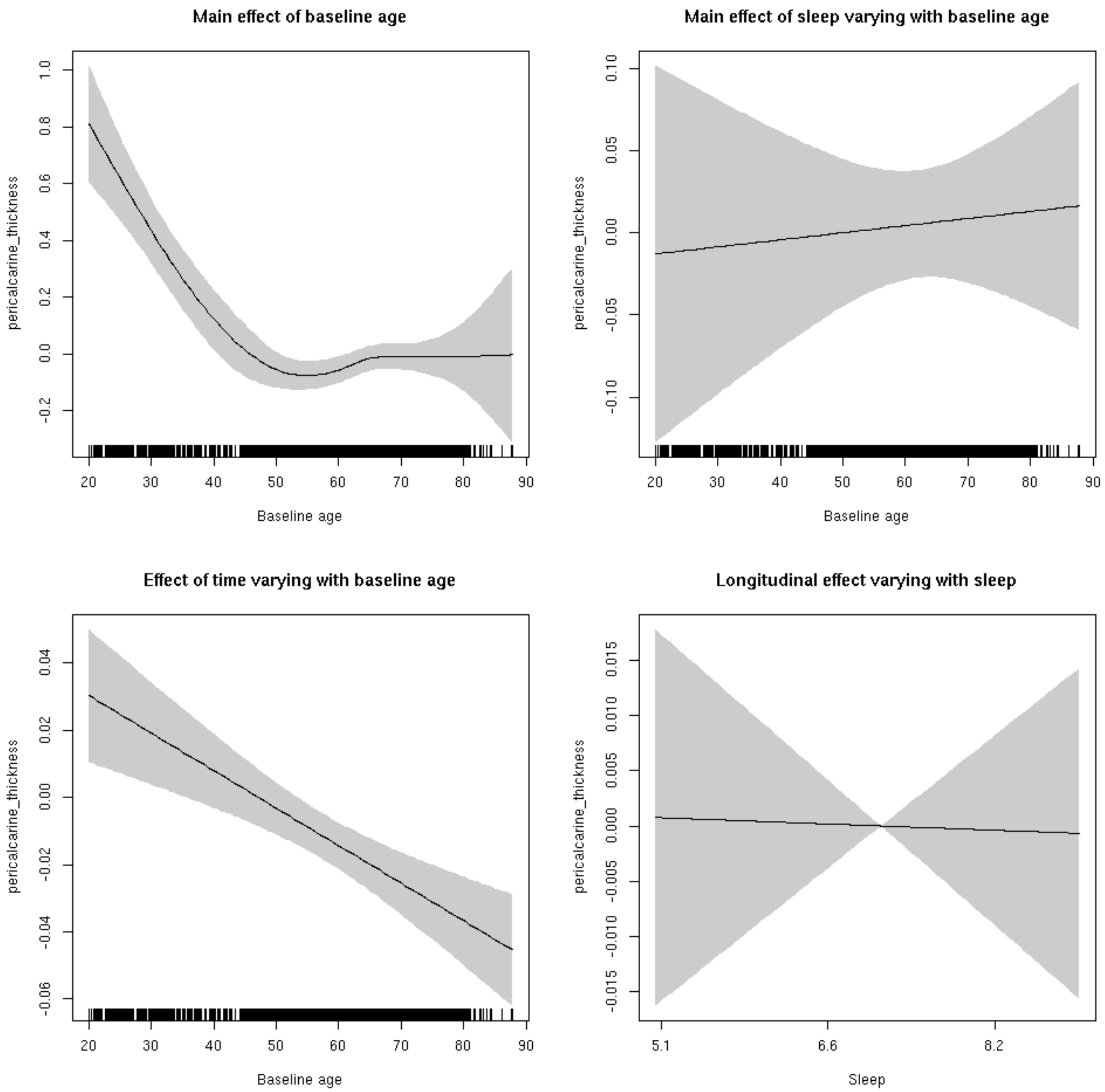

pericalcarine_volume: Stratified by sex

Female: Full data Below is the row for `s(sleep_z):time` from the model output.

```
## s(sleep_z):time   1.000  1.000  3.041   0.0812 .
```

Below are plots of a plot of `s(bl_age)`, `s(bl_age):sleep_z`, `s(bl_age):time`, and `s(sleep_z):time`. For `s(sleep_z):time`, roughly, if we cannot fit a straight line through the confidence bands, there is evidence that the effect of time depends on sleep duration. It is correct the that confidence interval is zero at one point along the axis; this happens because we do not include the main effect.

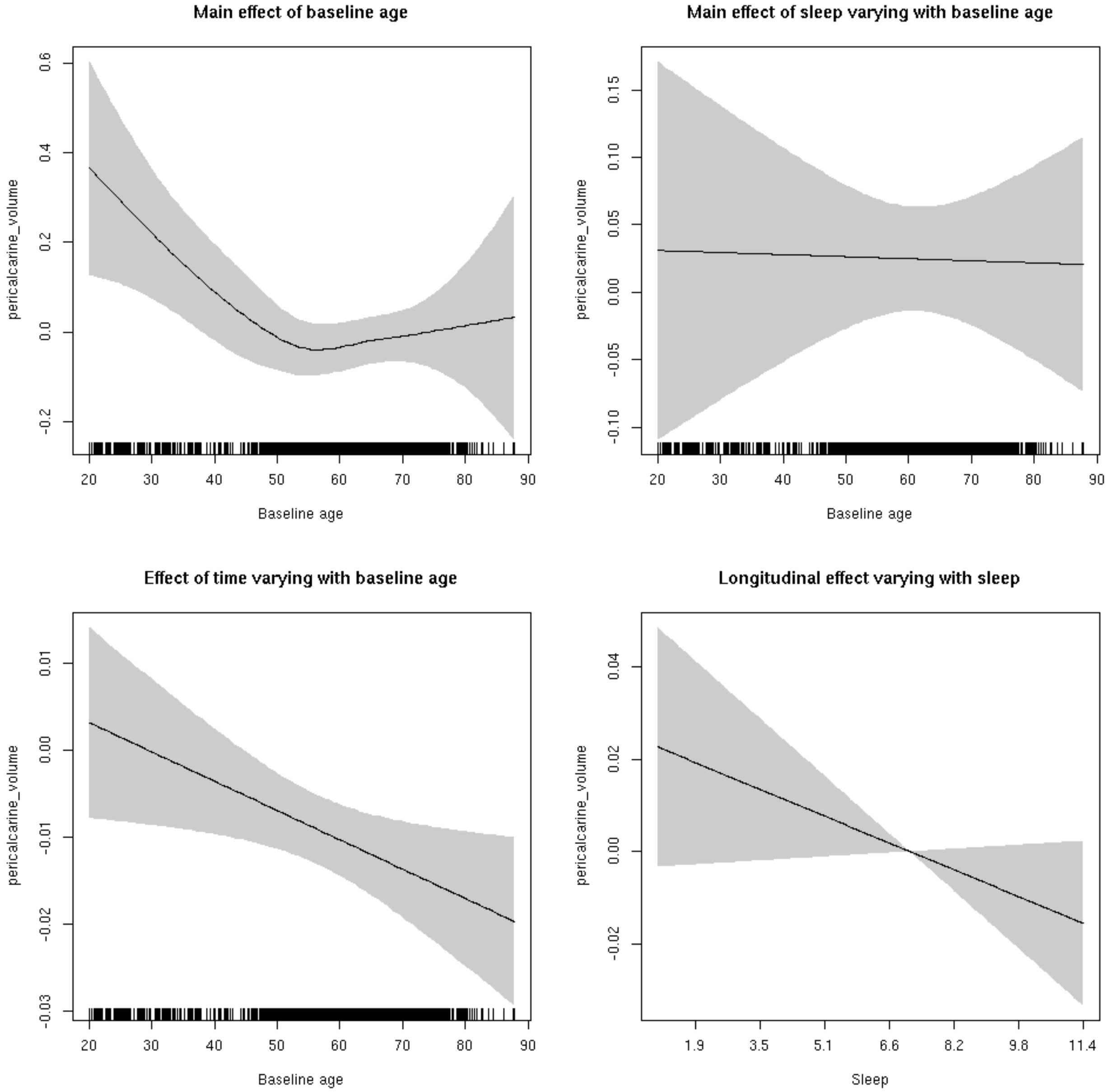

Male: Full data Below is the row for `s(sleep_z):time` from the model output.

```
## s(sleep_z):time   1.000  1.000  2.745 0.09765 .
```

Below are plots of a plot of `s(bl_age)`, `s(bl_age):sleep_z`, `s(bl_age):time`, and `s(sleep_z):time`. For `s(sleep_z):time`, roughly, if we cannot fit a straight line through the confidence bands, there is evidence that the effect of time depends on sleep duration. It is correct the that confidence interval is zero at one point along the axis; this happens because we do not include the main effect.

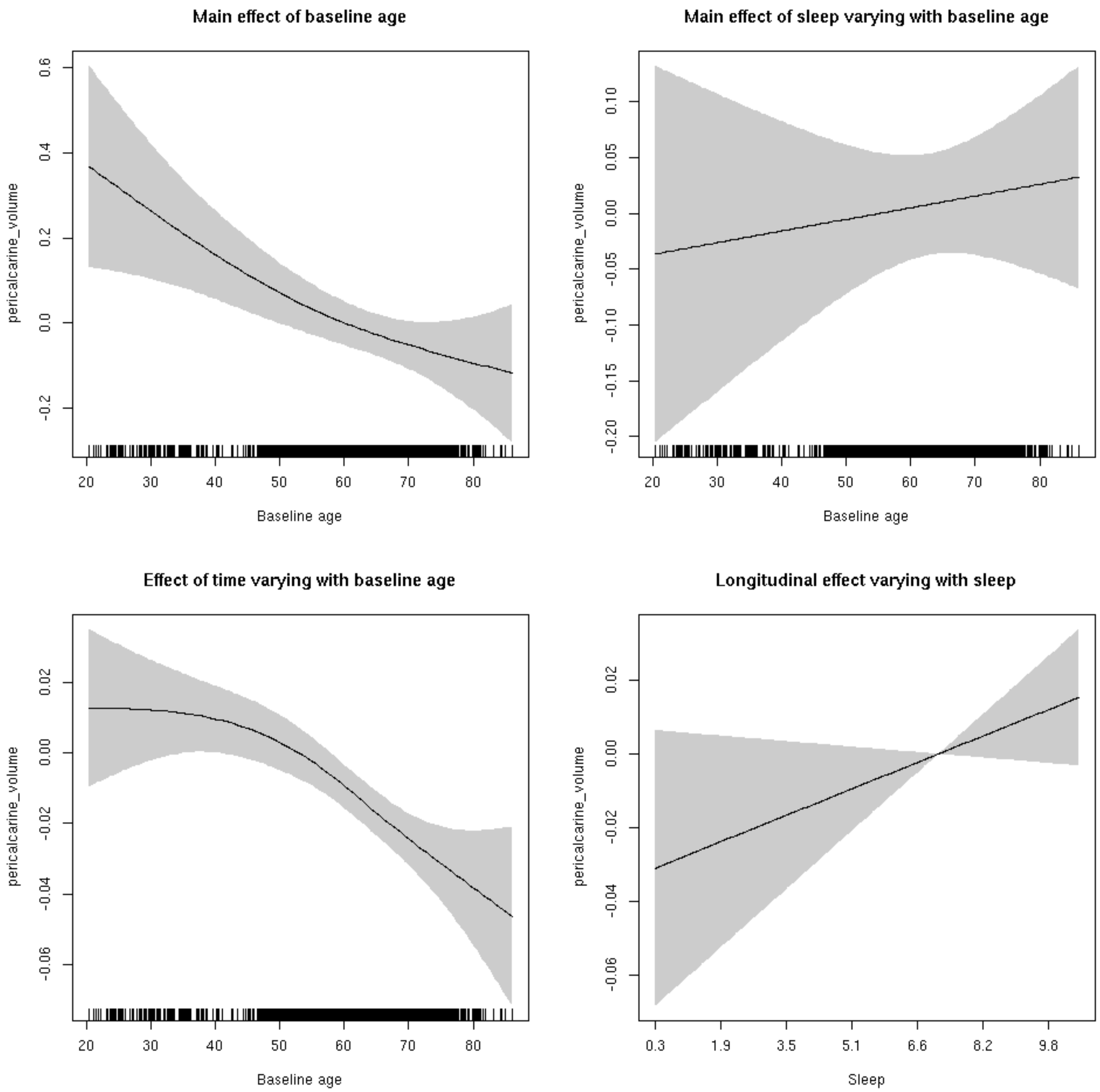

pericalcarine_volume

Full data Below is the model output. The term `s(sleep_z):time` is probably what we care most about.

```
##
## Family: gaussian
## Link function: identity
##
## Formula:
## value ~ sex + site + icv + s(bl_age, k = 10, bs = "cr") + s(bl_age,
##     by = sleep_z, bs = "cr") + +s(bl_age, by = time, k = 5, bs = "cr") +
##     s(sleep_z, by = time, k = 5, bs = "cr", pc = 0)
## <environment: 0x5630035adb90>
```

```
##
## Parametric coefficients:
##                Estimate Std. Error t value Pr(>|t|)
## (Intercept)     0.28404    0.06734   4.218 2.49e-05 ***
## sexmale         0.19690    0.03464   5.684 1.36e-08 ***
## siteousAvanto -0.75631    0.08222  -9.199  < 2e-16 ***
## siteousPrisma -0.19904    0.17802  -1.118    0.264
## siteousSkyra   0.36215    0.07959   4.550 5.44e-06 ***
## siteUB        -0.06300    0.15549  -0.405    0.685
## siteUCAM      -0.60946    0.08341  -7.307 3.00e-13 ***
## siteUKB       -0.38772    0.06380  -6.077 1.28e-09 ***
## siteUmU       -0.08121    0.09625  -0.844    0.399
## icv            0.26518    0.01700  15.598  < 2e-16 ***
## ---
## Signif. codes:  0 '***' 0.001 '**' 0.01 '*' 0.05 '.' 0.1 ' ' 1
##
## Approximate significance of smooth terms:
##                      edf Ref.df      F  p-value
## s(bl_age)          2.449  2.449  9.175 4.22e-05 ***
## s(bl_age):sleep_z 2.000  2.000  0.607    0.545
## s(bl_age):time    2.000  2.000 32.432  < 2e-16 ***
## s(sleep_z):time   1.000  1.000  0.027    0.869
## ---
## Signif. codes:  0 '***' 0.001 '**' 0.01 '*' 0.05 '.' 0.1 ' ' 1
##
## R-sq.(adj) =   0.17
## lmer.REML =  11528  Scale est. = 0.041593  n = 8172
```

Below are plots of a plot of `s(bl_age)`, `s(bl_age):sleep_z`, `s(bl_age):time`, and `s(sleep_z):time`. For `s(sleep_z):time`, roughly, if we cannot fit a straight line through the confidence bands, there is evidence that the effect of time depends on sleep duration. It is correct the that confidence interval is zero at one point along the axis; this happens because we do not include the main effect.

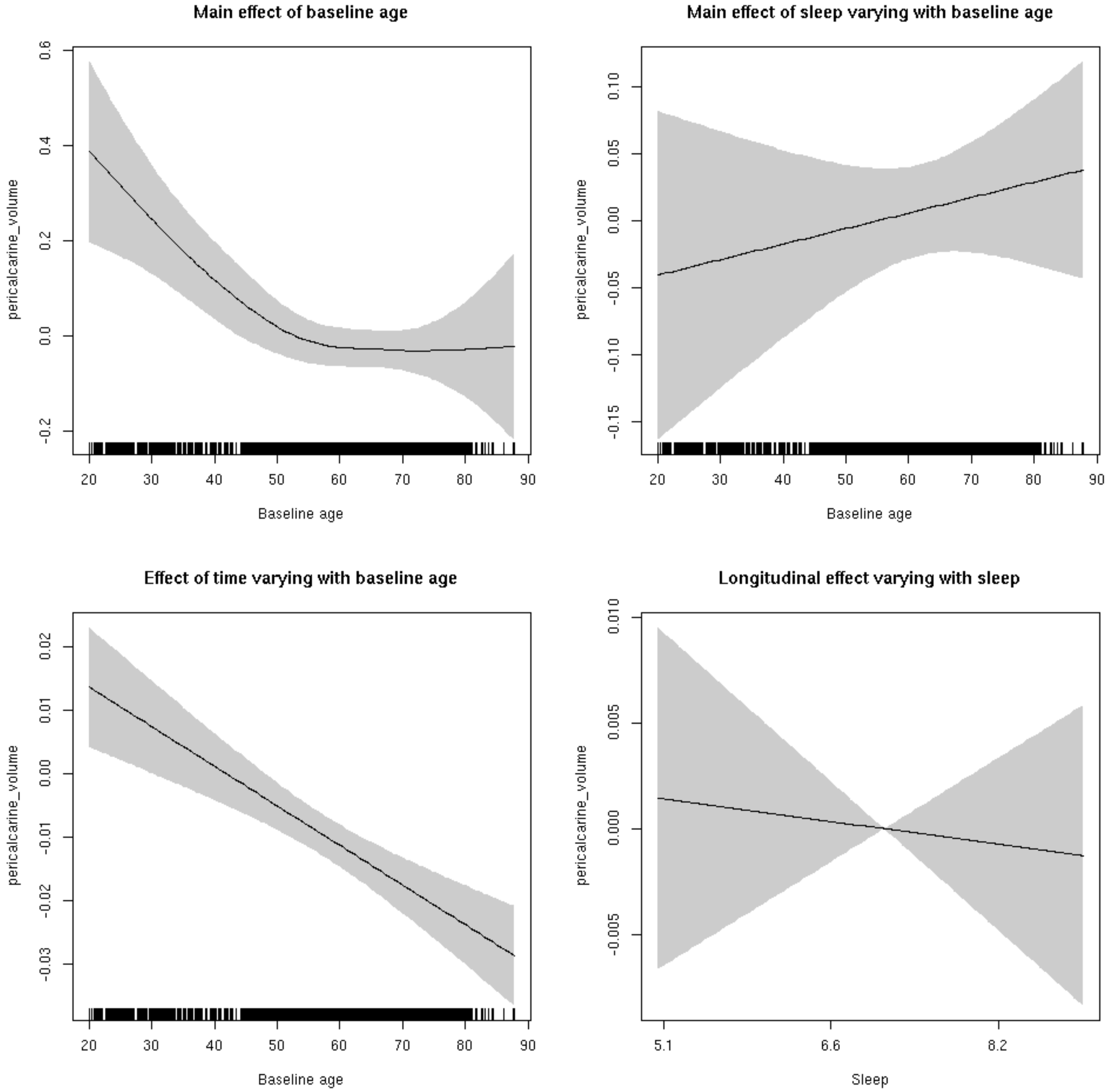

Restricted to sleep >= 5 and <= 9 hours Below is the row for `s(sleep_z):time` from the model output.

```
## s(sleep_z):time   1.000  1.000  0.126 0.722154
```

Below are plots of a plot of `s(bl_age)`, `s(bl_age):sleep_z`, `s(bl_age):time`, and `s(sleep_z):time`. For `s(sleep_z):time`, roughly, if we cannot fit a straight line through the confidence bands, there is evidence that the effect of time depends on sleep duration. It is correct the that confidence interval is zero at one point along the axis; this happens because we do not include the main effect.

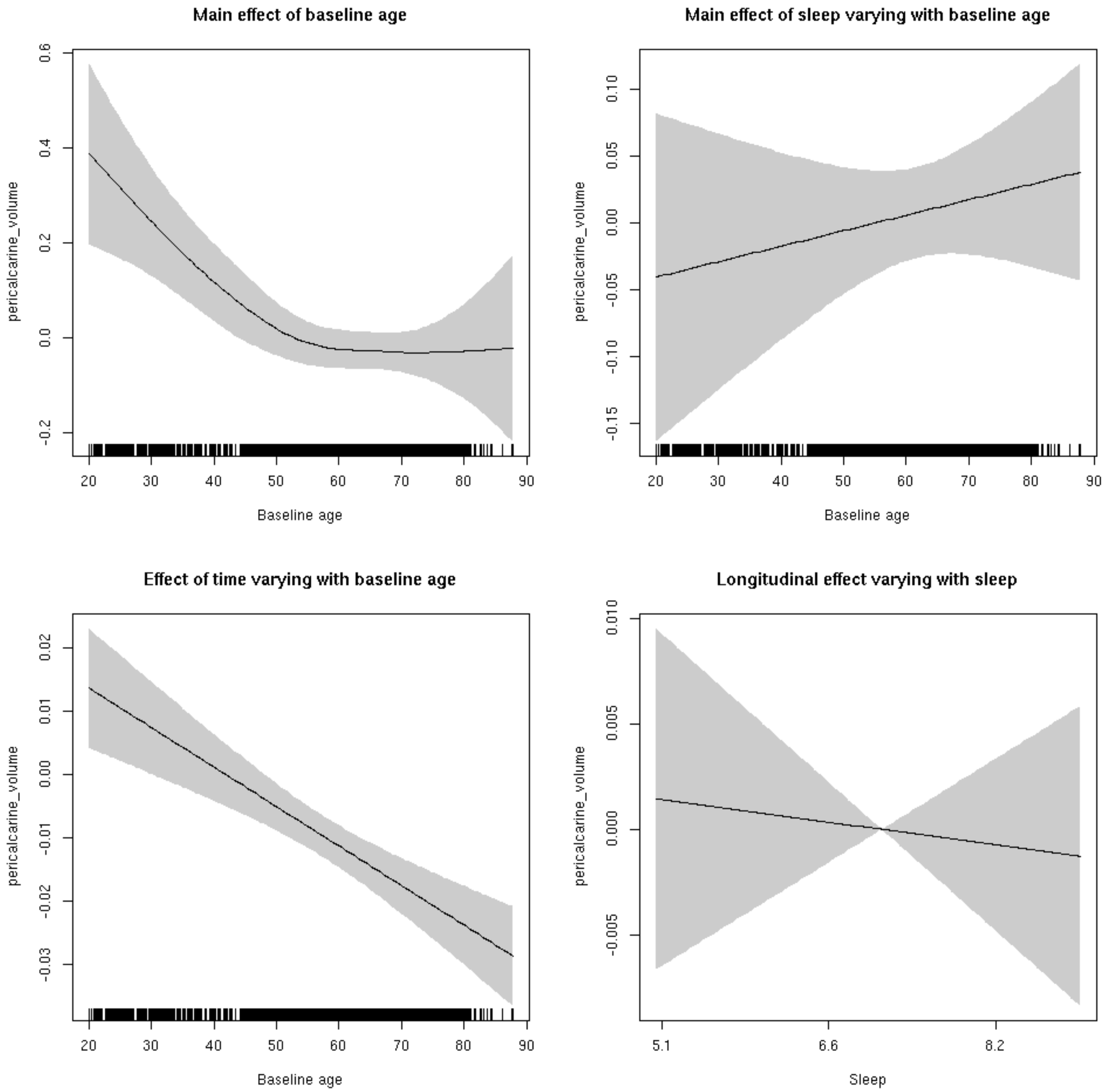

postcentral_area: Stratified by sex

Female: Full data Below is the row for `s(sleep_z):time` from the model output.

```
## s(sleep_z):time   1.000  1.000  0.012  0.9124
```

Below are plots of a plot of `s(bl_age)`, `s(bl_age):sleep_z`, `s(bl_age):time`, and `s(sleep_z):time`. For `s(sleep_z):time`, roughly, if we cannot fit a straight line through the confidence bands, there is evidence that the effect of time depends on sleep duration. It is correct the that confidence interval is zero at one point along the axis; this happens because we do not include the main effect.

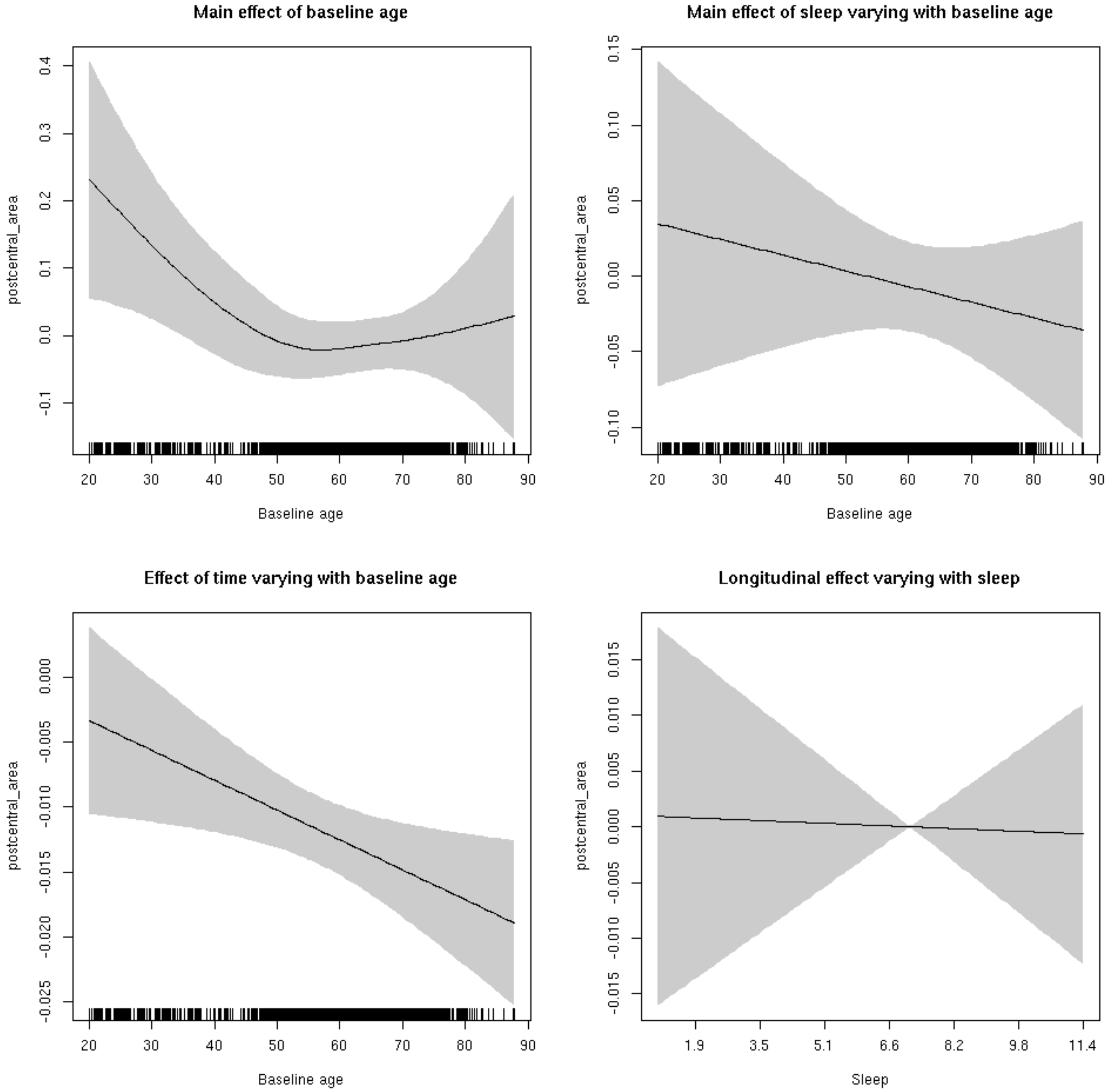

Male: Full data Below is the row for `s(sleep_z):time` from the model output.

```
## s(sleep_z):time   1.000  1.000  3.466  0.0627 .
```

Below are plots of a plot of `s(bl_age)`, `s(bl_age):sleep_z`, `s(bl_age):time`, and `s(sleep_z):time`. For `s(sleep_z):time`, roughly, if we cannot fit a straight line through the confidence bands, there is evidence that the effect of time depends on sleep duration. It is correct the that confidence interval is zero at one point along the axis; this happens because we do not include the main effect.

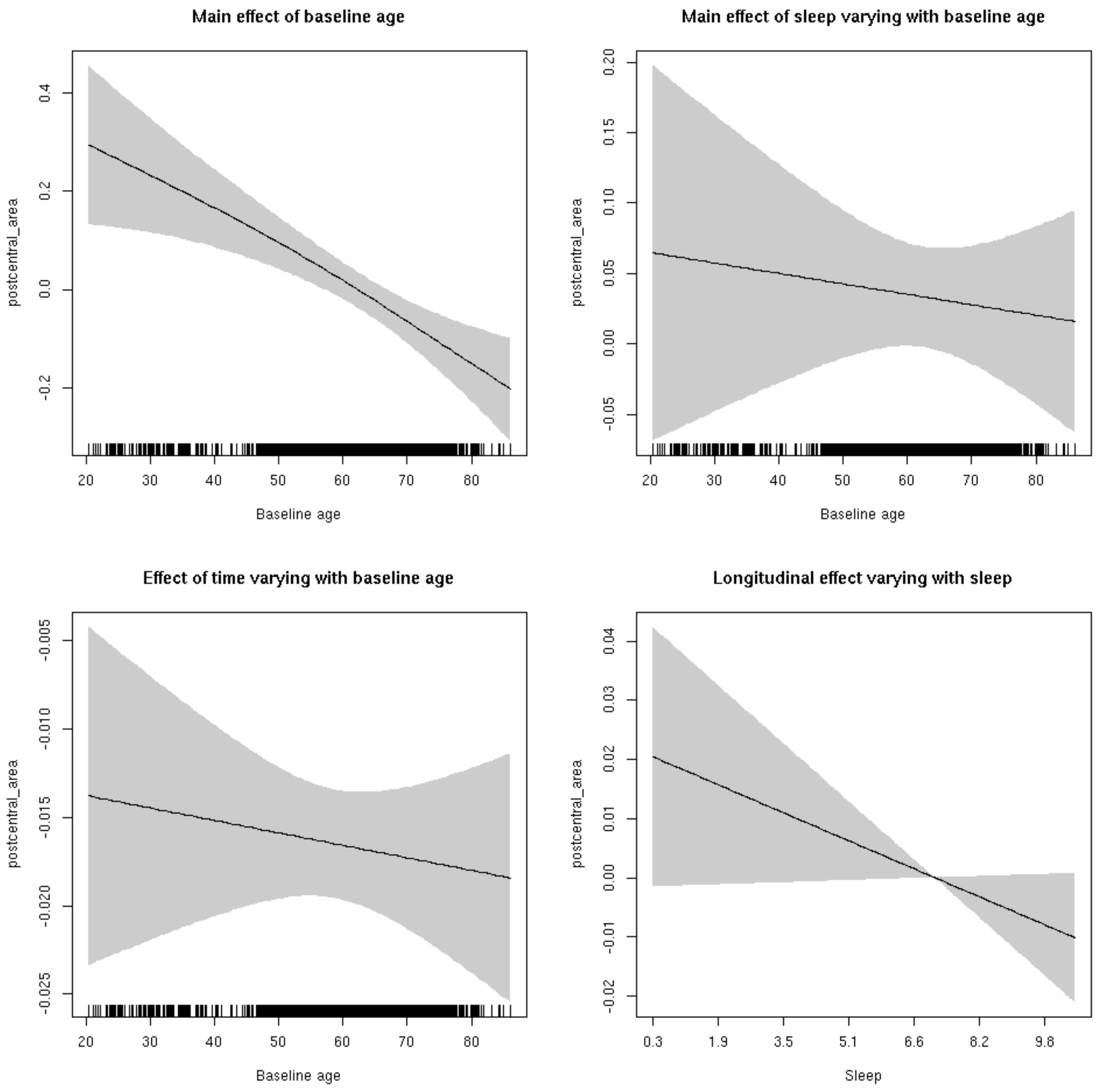

postcentral_area

Full data Below is the model output. The term `s(sleep_z):time` is probably what we care most about.

```
##
## Family: gaussian
## Link function: identity
##
## Formula:
## value ~ sex + site + icv + s(bl_age, k = 10, bs = "cr") + s(bl_age,
##     by = sleep_z, bs = "cr") + +s(bl_age, by = time, k = 5, bs = "cr") +
##     s(sleep_z, by = time, k = 5, bs = "cr", pc = 0)
## <environment: 0x5630035ad7d8>
```

```
##
## Parametric coefficients:
##                Estimate Std. Error t value Pr(>|t|)
## (Intercept)     0.65821    0.05200  12.658  < 2e-16 ***
## sexmale         0.21797    0.02703   8.063 8.54e-16 ***
## siteousAvanto -0.80195    0.06399 -12.533  < 2e-16 ***
## siteousPrisma -0.78346    0.11710  -6.691 2.37e-11 ***
## siteousSkyra  -0.58478    0.06202  -9.429  < 2e-16 ***
## siteUB        -0.72209    0.12163  -5.937 3.03e-09 ***
## siteUCAM      -0.68876    0.06493 -10.608  < 2e-16 ***
## siteUKB       -0.74537    0.04852 -15.362  < 2e-16 ***
## siteUmU        0.07430    0.07491   0.992    0.321
## icv            0.64144    0.01317  48.722  < 2e-16 ***
## ---
## Signif. codes:  0 '***' 0.001 '**' 0.01 '*' 0.05 '.' 0.1 ' ' 1
##
## Approximate significance of smooth terms:
##                    edf Ref.df       F p-value
## s(bl_age)            1      1  20.873 5.1e-06 ***
## s(bl_age):sleep_z    2      2   0.996   0.369
## s(bl_age):time       2      2 101.126 < 2e-16 ***
## s(sleep_z):time      1      1   1.908   0.167
## ---
## Signif. codes:  0 '***' 0.001 '**' 0.01 '*' 0.05 '.' 0.1 ' ' 1
##
## R-sq.(adj) =  0.518
## lmer.REML =   5458  Scale est. = 0.015719  n = 8144
```

Below are plots of a plot of `s(bl_age)`, `s(bl_age):sleep_z`, `s(bl_age):time`, and `s(sleep_z):time`. For `s(sleep_z):time`, roughly, if we cannot fit a straight line through the confidence bands, there is evidence that the effect of time depends on sleep duration. It is correct the that confidence interval is zero at one point along the axis; this happens because we do not include the main effect.

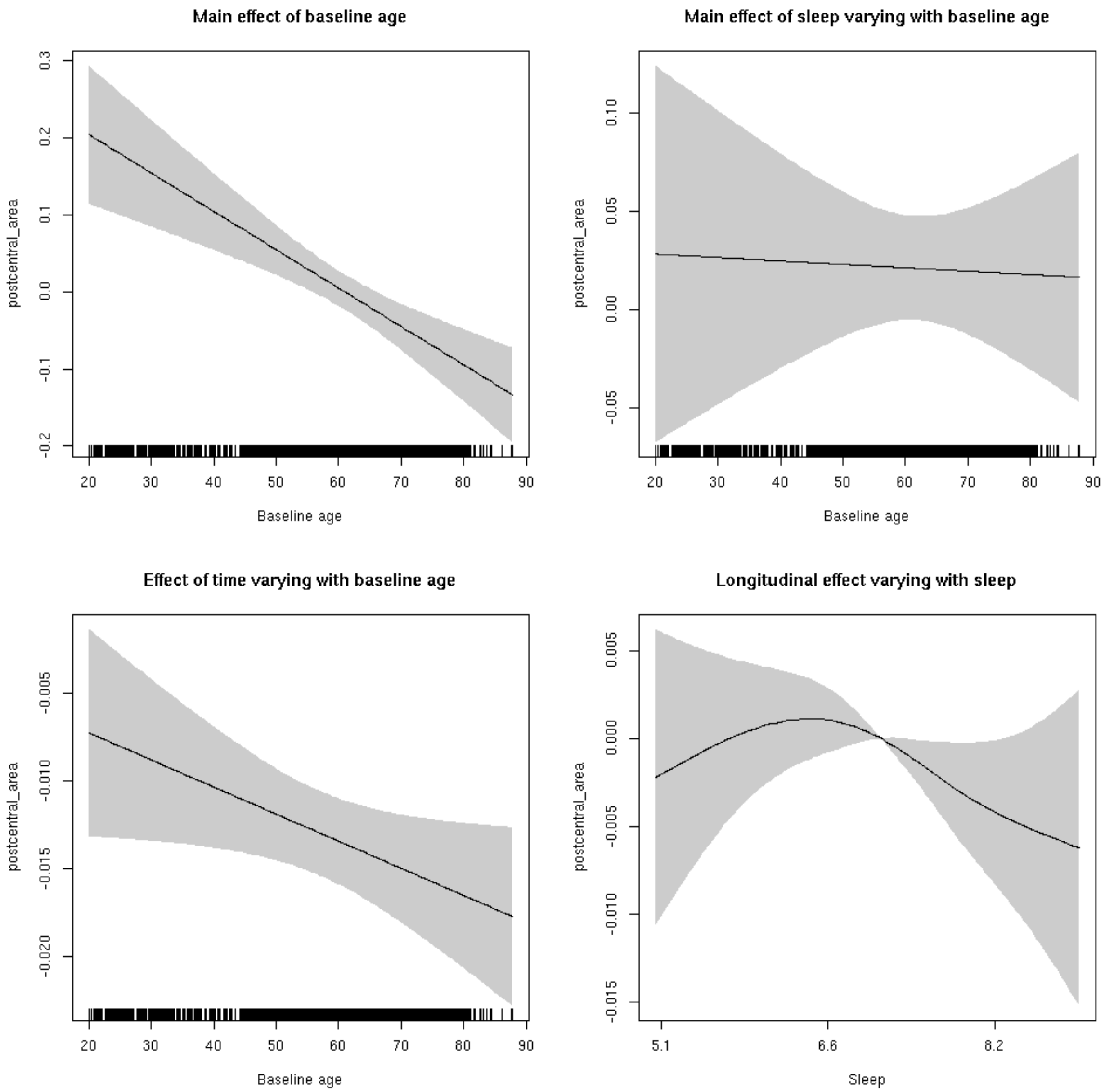

Restricted to sleep >= 5 and <= 9 hours Below is the row for `s(sleep_z):time` from the model output.

```
## s(sleep_z):time   2.007  2.007  2.265    0.115
```

Below are plots of a plot of `s(bl_age)`, `s(bl_age):sleep_z`, `s(bl_age):time`, and `s(sleep_z):time`. For `s(sleep_z):time`, roughly, if we cannot fit a straight line through the confidence bands, there is evidence that the effect of time depends on sleep duration. It is correct the that confidence interval is zero at one point along the axis; this happens because we do not include the main effect.

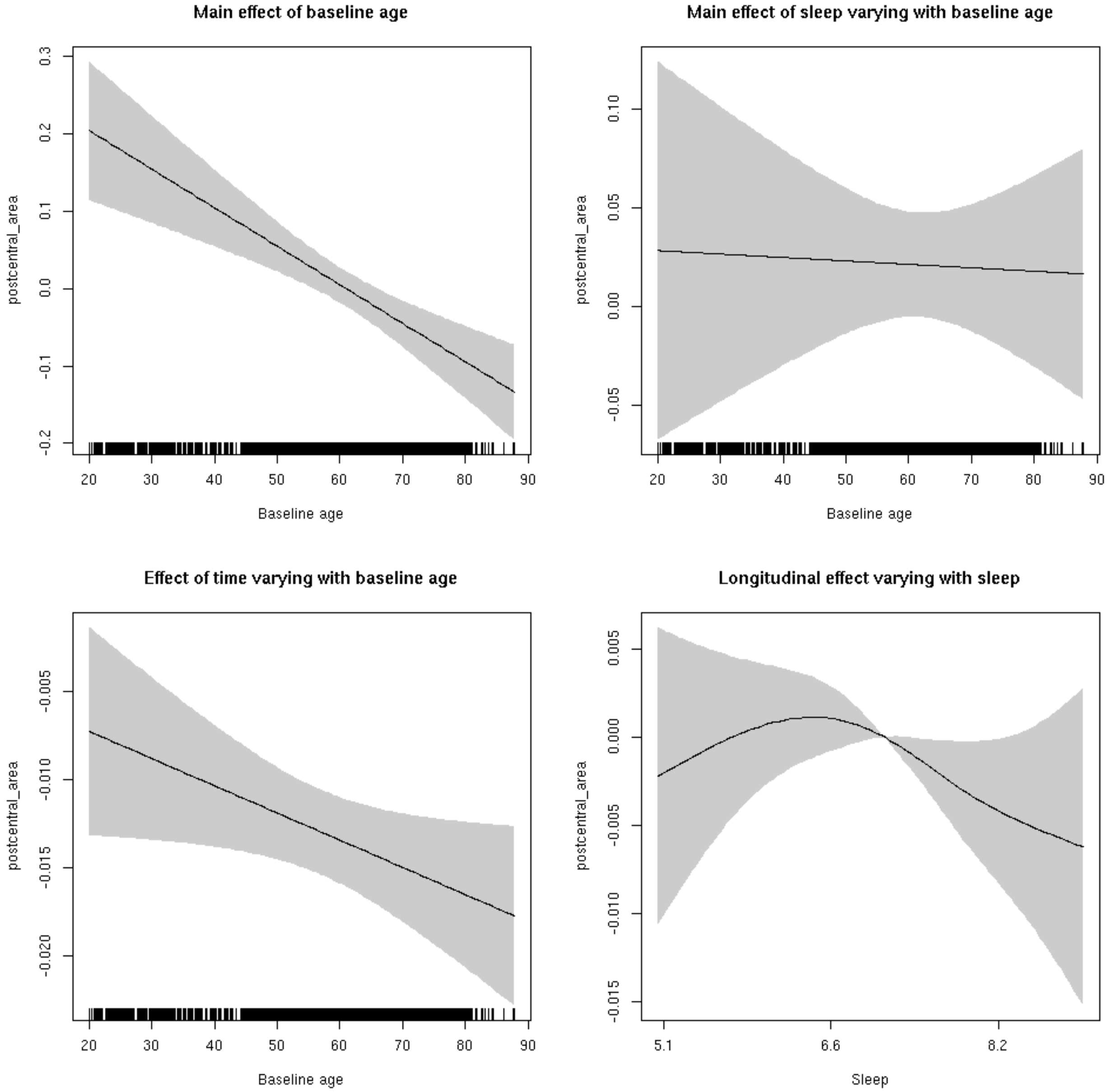

postcentral_thickness: Stratified by sex

Female: Full data Below is the row for `s(sleep_z):time` from the model output.

```
## s(sleep_z):time   1.000  1.000  0.585   0.445
```

Below are plots of a plot of `s(bl_age)`, `s(bl_age):sleep_z`, `s(bl_age):time`, and `s(sleep_z):time`. For `s(sleep_z):time`, roughly, if we cannot fit a straight line through the confidence bands, there is evidence that the effect of time depends on sleep duration. It is correct the that confidence interval is zero at one point along the axis; this happens because we do not include the main effect.

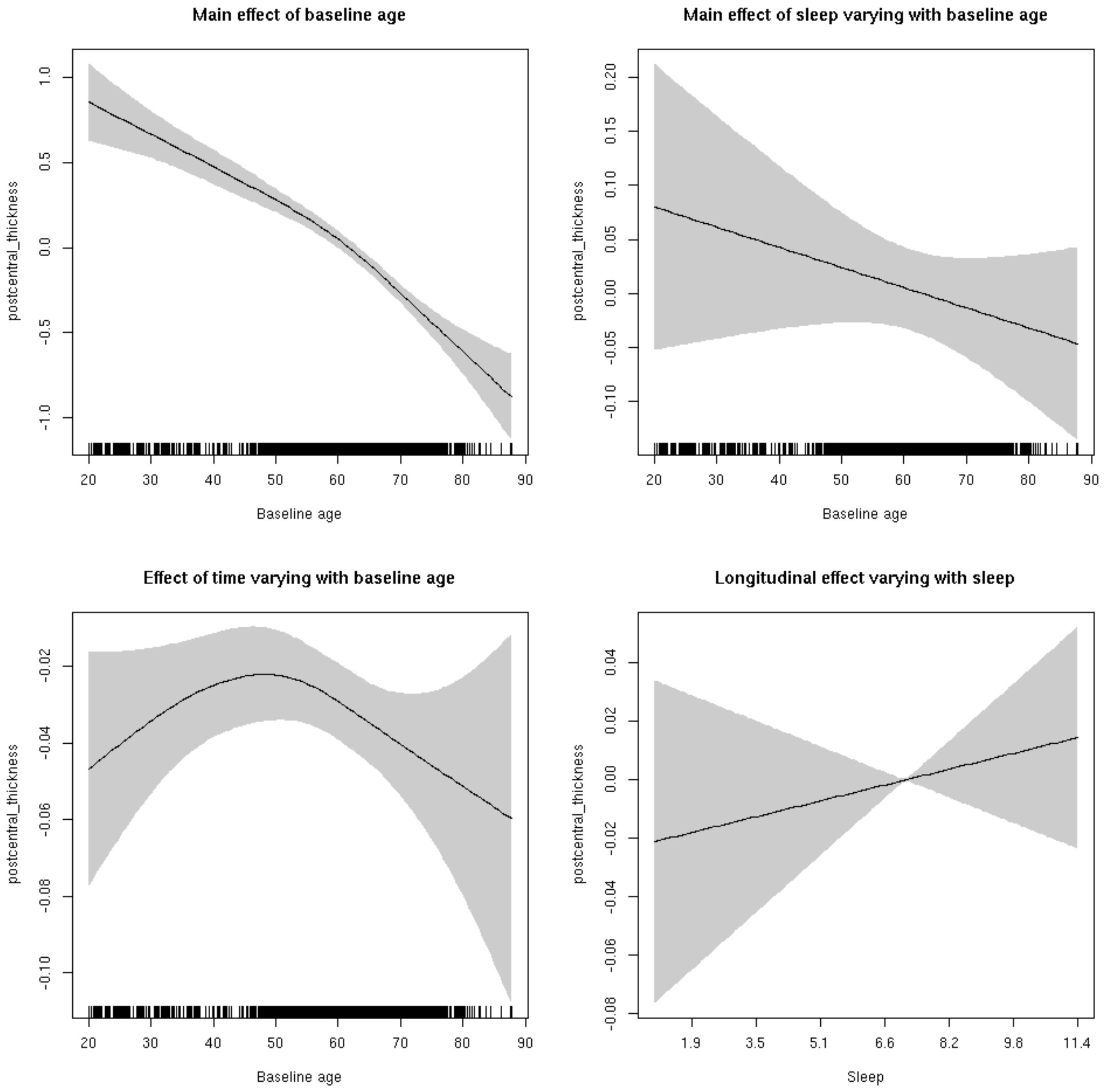

Male: Full data Below is the row for `s(sleep_z):time` from the model output.

```
## s(sleep_z):time   1.000  1.000  0.149   0.699
```

Below are plots of a plot of `s(bl_age)`, `s(bl_age):sleep_z`, `s(bl_age):time`, and `s(sleep_z):time`. For `s(sleep_z):time`, roughly, if we cannot fit a straight line through the confidence bands, there is evidence that the effect of time depends on sleep duration. It is correct the that confidence interval is zero at one point along the axis; this happens because we do not include the main effect.

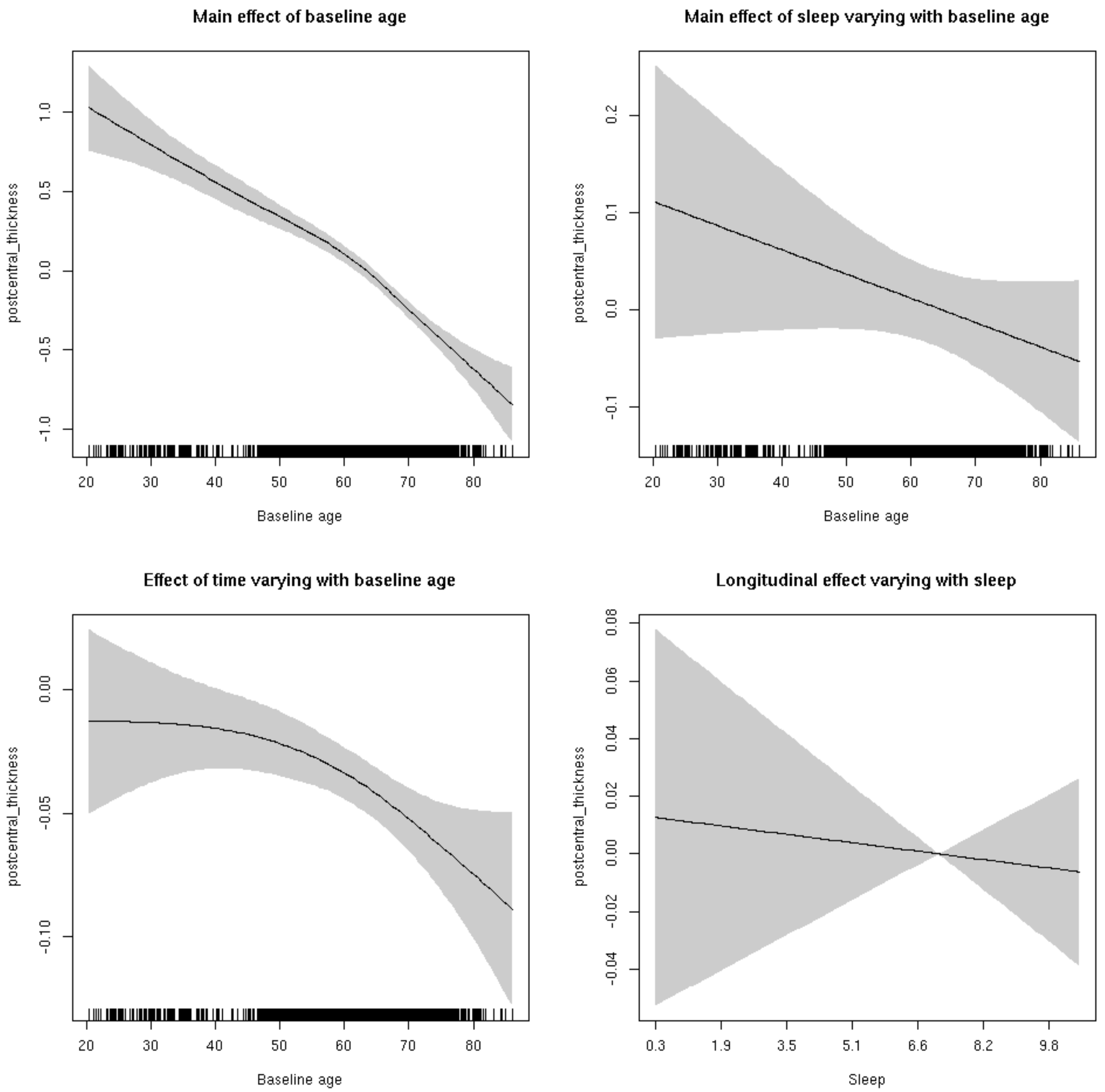

postcentral_thickness

Full data Below is the model output. The term `s(sleep_z):time` is probably what we care most about.

```
##
## Family: gaussian
## Link function: identity
##
## Formula:
## value ~ sex + site + s(bl_age, k = 10, bs = "cr") + s(bl_age,
##     by = sleep_z, bs = "cr") + +s(bl_age, by = time, k = 5, bs = "cr") +
##     s(sleep_z, by = time, k = 5, bs = "cr", pc = 0)
## <environment: 0x5630035da070>
```

```
##
## Parametric coefficients:
##                Estimate Std. Error t value Pr(>|t|)
## (Intercept)   -1.24775    0.05076 -24.581  < 2e-16 ***
## sexmale       -0.10689    0.02545  -4.200 2.70e-05 ***
## siteousAvanto -0.04822    0.06764  -0.713  0.47600
## siteousPrisma  0.68287    0.30023   2.274  0.02296 *
## siteousSkyra   0.21528    0.06760   3.184  0.00146 **
## siteUB         0.74864    0.13453   5.565 2.71e-08 ***
## siteUCAM       0.52603    0.06979   7.537 5.32e-14 ***
## siteUKB        1.51986    0.05141  29.565  < 2e-16 ***
## siteUmU        0.35430    0.08371   4.233 2.33e-05 ***
## ---
## Signif. codes:  0 '***' 0.001 '**' 0.01 '*' 0.05 '.' 0.1 ' ' 1
##
## Approximate significance of smooth terms:
##                      edf Ref.df       F p-value
## s(bl_age)          3.440  3.440 150.165  <2e-16 ***
## s(bl_age):sleep_z 2.000  2.000   2.067   0.127
## s(bl_age):time    3.218  3.218  43.800  <2e-16 ***
## s(sleep_z):time   1.000  1.000   0.109   0.741
## ---
## Signif. codes:  0 '***' 0.001 '**' 0.01 '*' 0.05 '.' 0.1 ' ' 1
##
## R-sq.(adj) =  0.389
## lmer.REML =  16241  Scale est. = 0.15668   n = 8176
```

Below are plots of a plot of `s(bl_age)`, `s(bl_age):sleep_z`, `s(bl_age):time`, and `s(sleep_z):time`. For `s(sleep_z):time`, roughly, if we cannot fit a straight line through the confidence bands, there is evidence that the effect of time depends on sleep duration. It is correct the that confidence interval is zero at one point along the axis; this happens because we do not include the main effect.

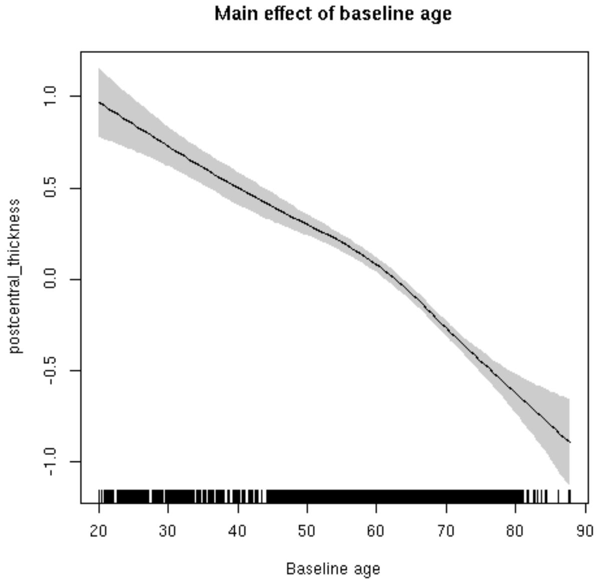

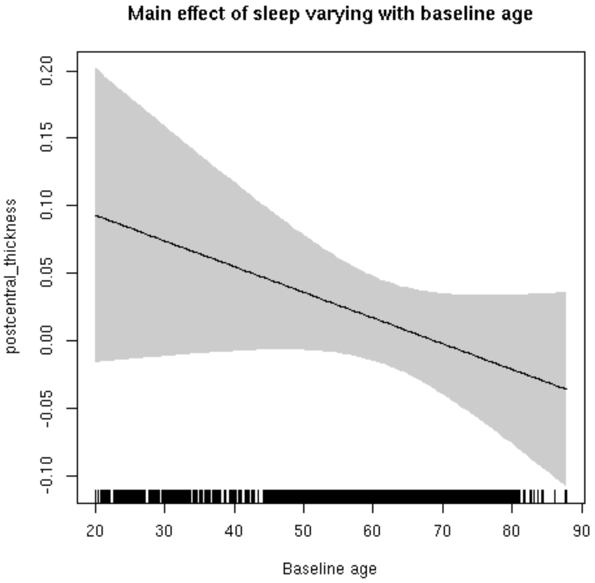

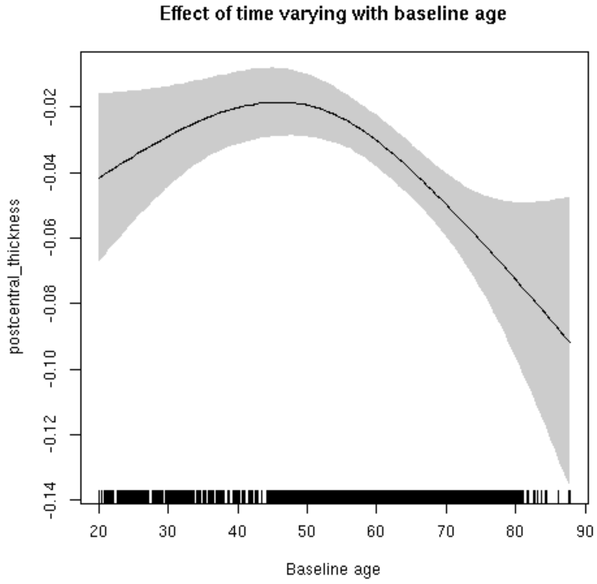

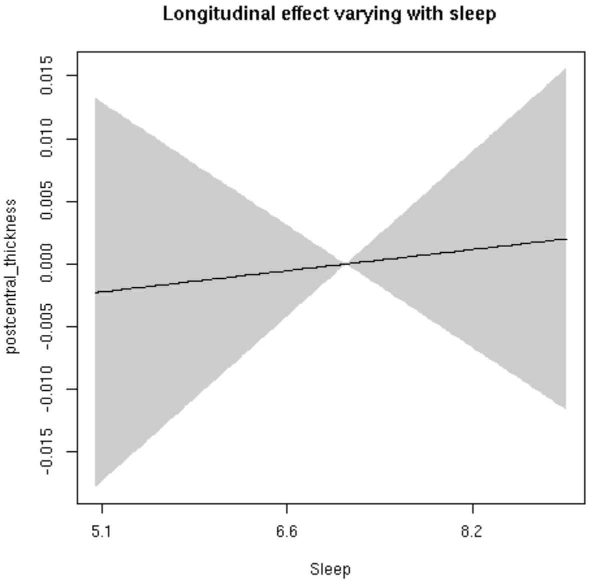

Restricted to sleep >= 5 and <= 9 hours Below is the row for `s(sleep_z):time` from the model output.

```
## s(sleep_z):time   1.000  1.000   0.086   0.769
```

Below are plots of a plot of `s(bl_age)`, `s(bl_age):sleep_z`, `s(bl_age):time`, and `s(sleep_z):time`. For `s(sleep_z):time`, roughly, if we cannot fit a straight line through the confidence bands, there is evidence that the effect of time depends on sleep duration. It is correct the that confidence interval is zero at one point along the axis; this happens because we do not include the main effect.

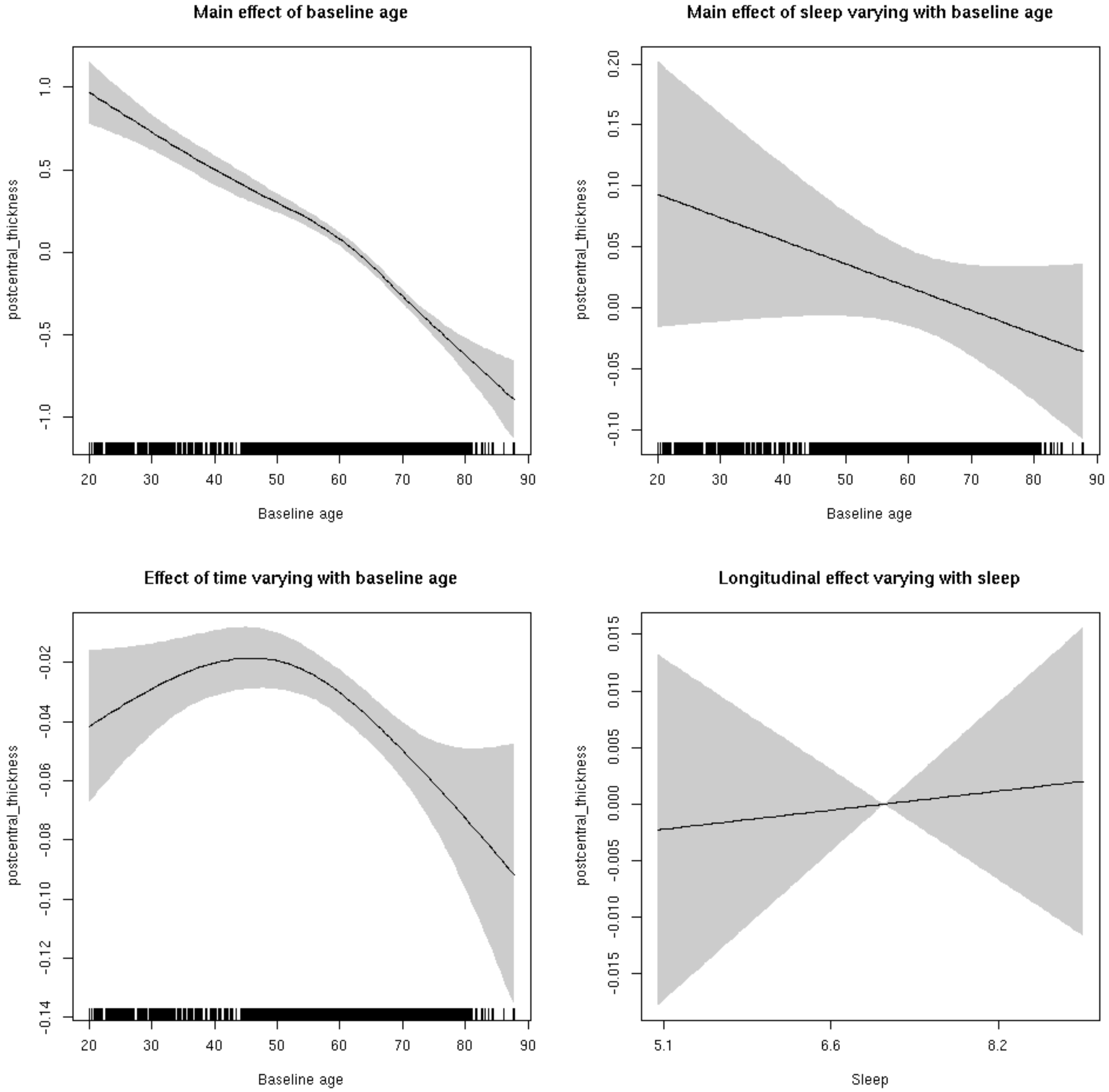

postcentral_volume: Stratified by sex

Female: Full data Below is the row for `s(sleep_z):time` from the model output.

```
## s(sleep_z):time   1.000  1.000   0.187   0.665
```

Below are plots of a plot of `s(bl_age)`, `s(bl_age):sleep_z`, `s(bl_age):time`, and `s(sleep_z):time`. For `s(sleep_z):time`, roughly, if we cannot fit a straight line through the confidence bands, there is evidence that the effect of time depends on sleep duration. It is correct the that confidence interval is zero at one point along the axis; this happens because we do not include the main effect.

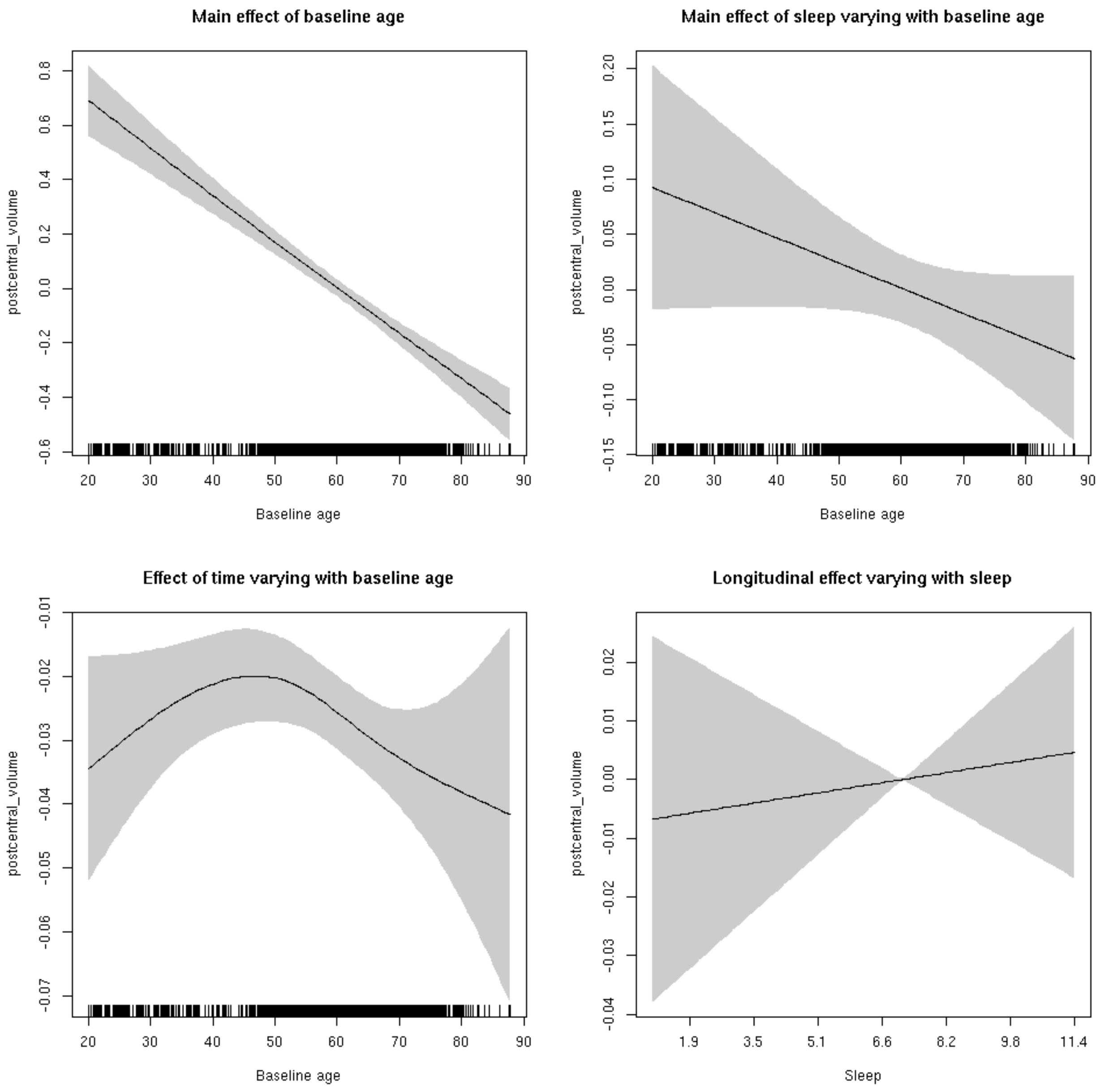

Male: Full data Below is the row for `s(sleep_z):time` from the model output.

```
## s(sleep_z):time   1.000  1.000   0.822   0.365
```

Below are plots of a plot of `s(bl_age)`, `s(bl_age):sleep_z`, `s(bl_age):time`, and `s(sleep_z):time`. For `s(sleep_z):time`, roughly, if we cannot fit a straight line through the confidence bands, there is evidence that the effect of time depends on sleep duration. It is correct the that confidence interval is zero at one point along the axis; this happens because we do not include the main effect.

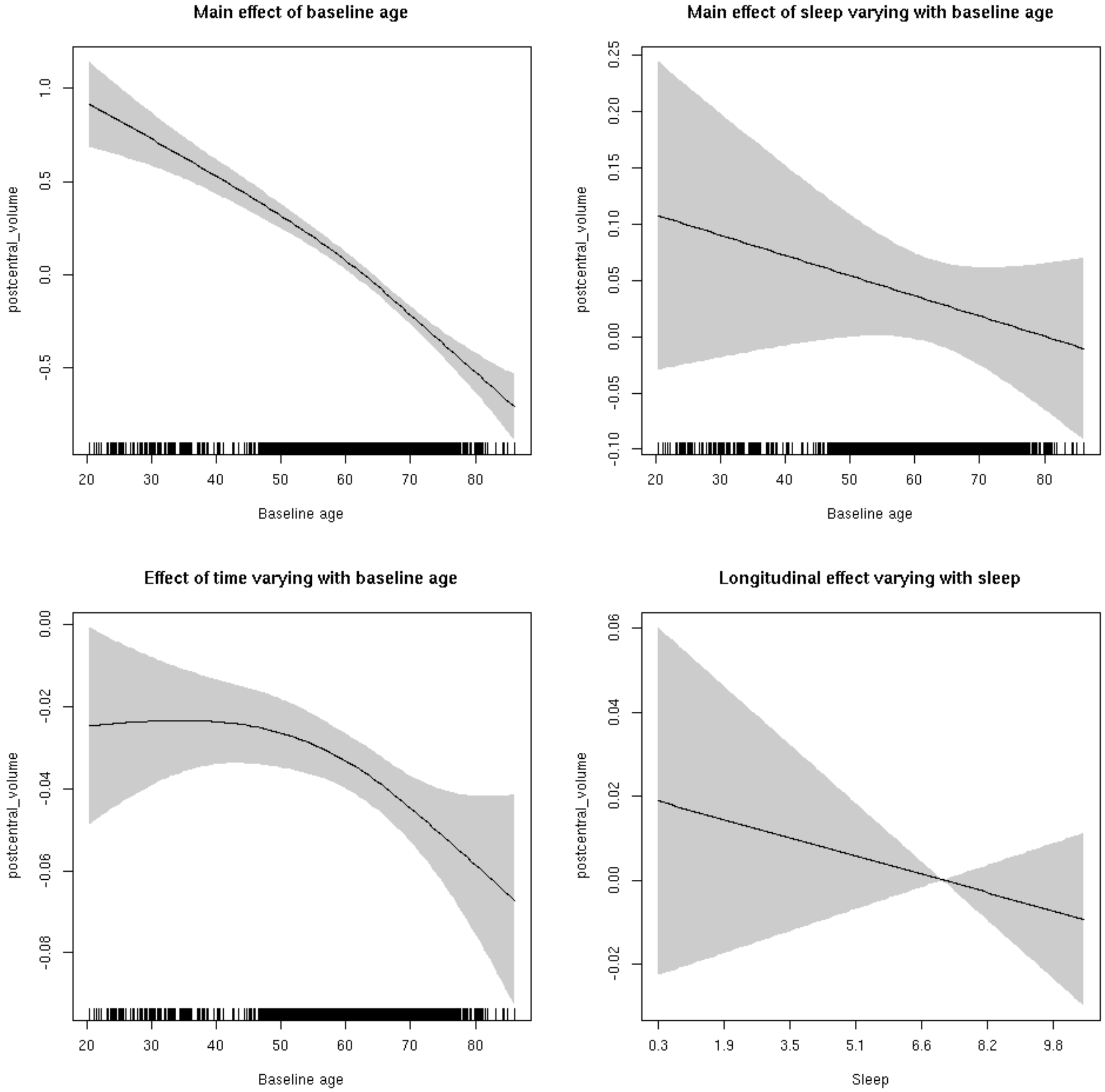

postcentral_volume

Full data Below is the model output. The term `s(sleep_z):time` is probably what we care most about.

```
##
## Family: gaussian
## Link function: identity
##
## Formula:
## value ~ sex + site + icv + s(bl_age, k = 10, bs = "cr") + s(bl_age,
##     by = sleep_z, bs = "cr") + +s(bl_age, by = time, k = 5, bs = "cr") +
##     s(sleep_z, by = time, k = 5, bs = "cr", pc = 0)
## <environment: 0x5630035ae3e0>
```

```
##
## Parametric coefficients:
##                 Estimate Std. Error t value Pr(>|t|)
## (Intercept)      0.08657    0.05425   1.596  0.11060
## sexmale          0.02551    0.02816   0.906  0.36514
## siteousAvanto   -0.94922    0.06716 -14.133  < 2e-16 ***
## siteousPrisma   -0.49833    0.19084  -2.611  0.00904 **
## siteousSkyra    -0.44382    0.06495  -6.833 8.92e-12 ***
## siteUB          -0.20985    0.12555  -1.671  0.09469 .
## siteUCAM        -0.41012    0.06744  -6.081 1.25e-09 ***
## siteUKB          0.05017    0.05051   0.993  0.32060
## siteUmU          0.18534    0.07776   2.384  0.01717 *
## icv              0.60402    0.01391  43.422  < 2e-16 ***
## ---
## Signif. codes:  0 '***' 0.001 '**' 0.01 '*' 0.05 '.' 0.1 ' ' 1
##
## Approximate significance of smooth terms:
##                       edf Ref.df       F p-value
## s(bl_age)           1.000  1.000 371.001  <2e-16 ***
## s(bl_age):sleep_z 2.000  2.000   2.722  0.0658 .
## s(bl_age):time      3.291  3.291  90.770  <2e-16 ***
## s(sleep_z):time     1.000  1.000   0.105  0.7460
## ---
## Signif. codes:  0 '***' 0.001 '**' 0.01 '*' 0.05 '.' 0.1 ' ' 1
##
## R-sq.(adj) =  0.481
## lmer.REML =  11085  Scale est. = 0.054832  n = 8176
```

Below are plots of a plot of `s(bl_age)`, `s(bl_age):sleep_z`, `s(bl_age):time`, and `s(sleep_z):time`. For `s(sleep_z):time`, roughly, if we cannot fit a straight line through the confidence bands, there is evidence that the effect of time depends on sleep duration. It is correct the that confidence interval is zero at one point along the axis; this happens because we do not include the main effect.

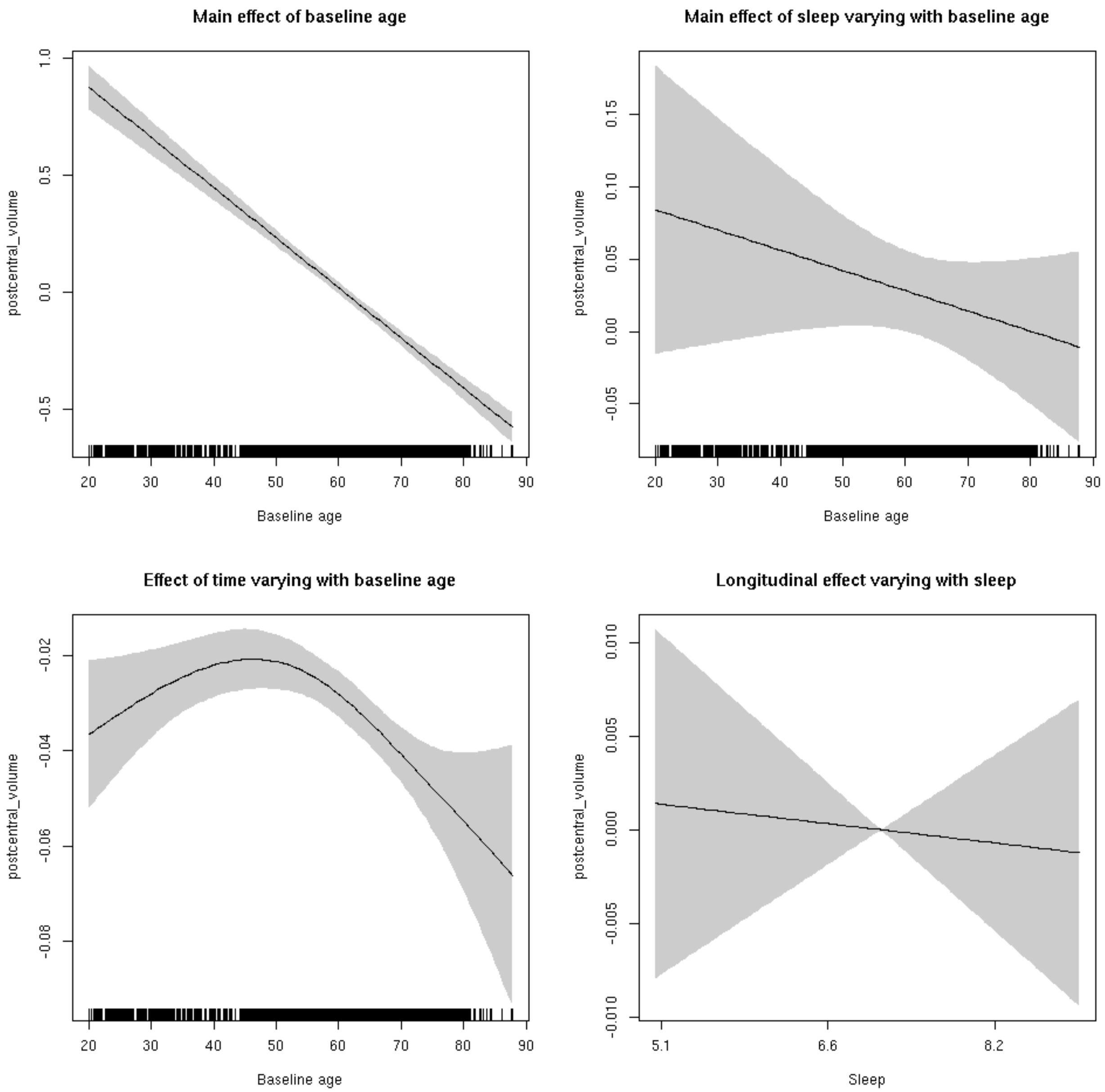

Restricted to sleep >= 5 and <= 9 hours Below is the row for `s(sleep_z):time` from the model output.

```
## s(sleep_z):time   1.000  1.000   0.090  0.7645
```

Below are plots of a plot of `s(bl_age)`, `s(bl_age):sleep_z`, `s(bl_age):time`, and `s(sleep_z):time`. For `s(sleep_z):time`, roughly, if we cannot fit a straight line through the confidence bands, there is evidence that the effect of time depends on sleep duration. It is correct the that confidence interval is zero at one point along the axis; this happens because we do not include the main effect.

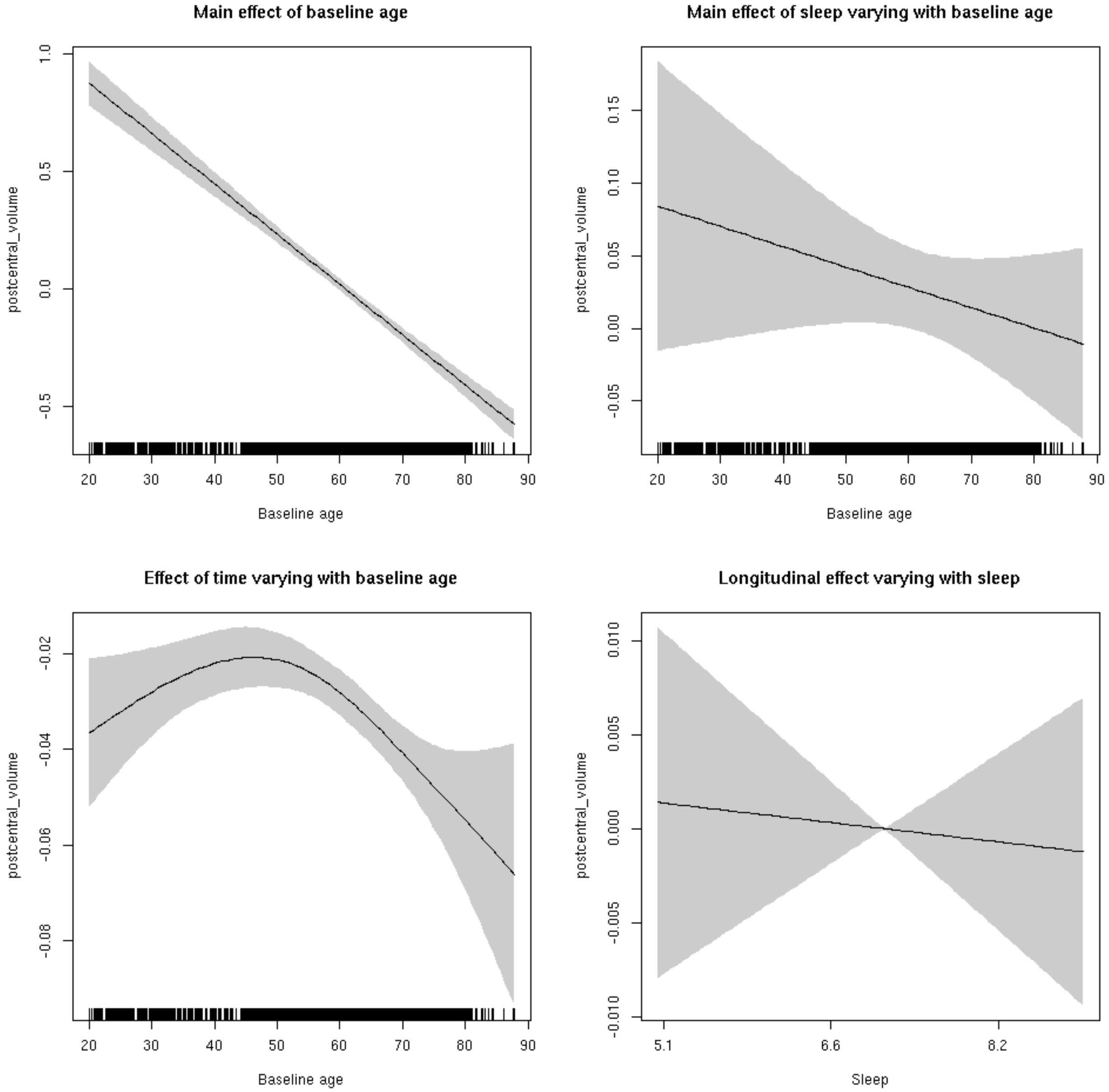

posteriorcingulate_area: Stratified by sex

Female: Full data Below is the row for `s(sleep_z):time` from the model output.

```
## s(sleep_z):time   1.000  1.000  0.378   0.538
```

Below are plots of a plot of `s(bl_age)`, `s(bl_age):sleep_z`, `s(bl_age):time`, and `s(sleep_z):time`. For `s(sleep_z):time`, roughly, if we cannot fit a straight line through the confidence bands, there is evidence that the effect of time depends on sleep duration. It is correct the that confidence interval is zero at one point along the axis; this happens because we do not include the main effect.

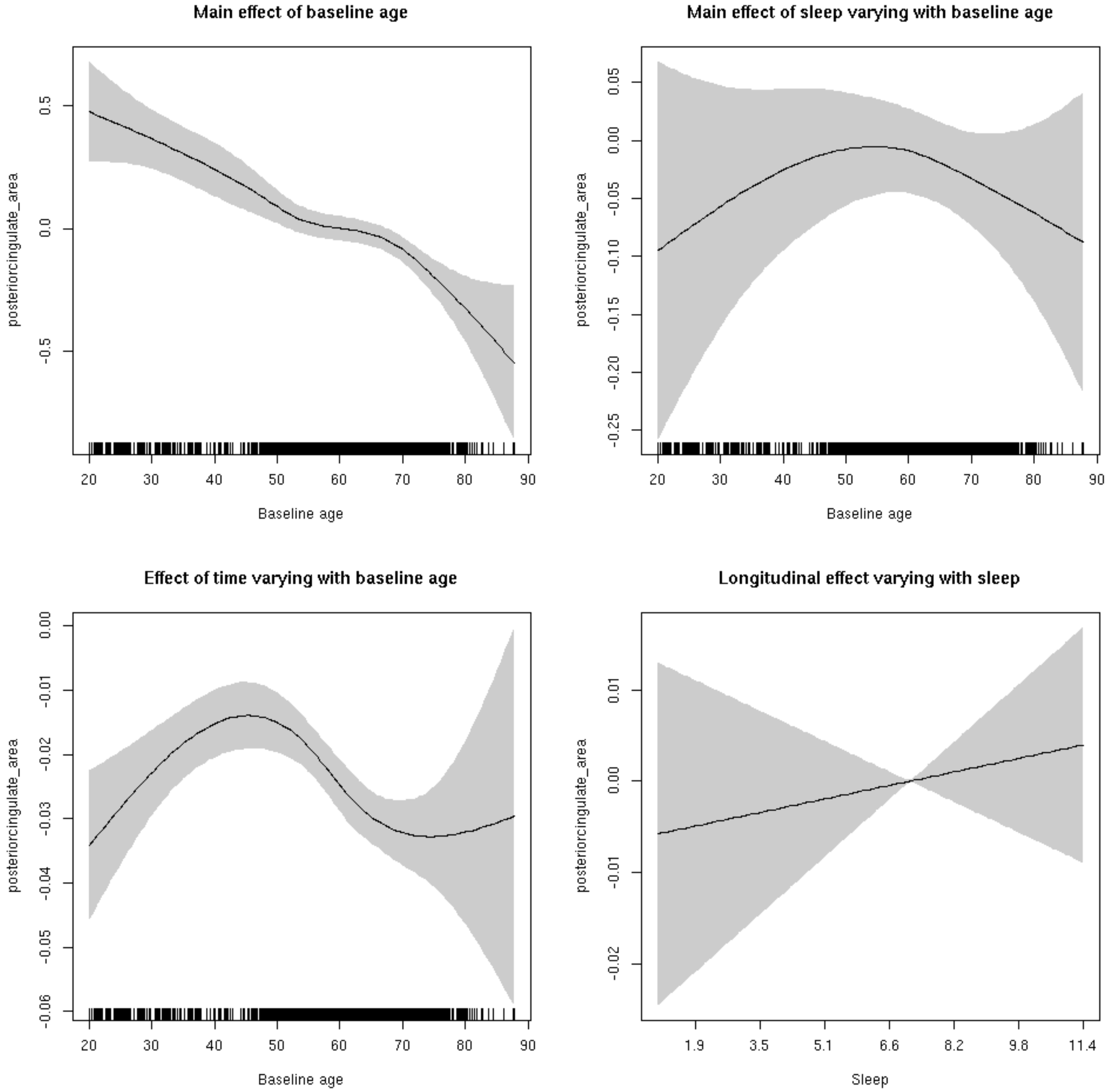

Male: Full data Below is the row for `s(sleep_z):time` from the model output.

```
## s(sleep_z):time   1.000  1.000   0.006   0.940
```

Below are plots of a plot of `s(bl_age)`, `s(bl_age):sleep_z`, `s(bl_age):time`, and `s(sleep_z):time`. For `s(sleep_z):time`, roughly, if we cannot fit a straight line through the confidence bands, there is evidence that the effect of time depends on sleep duration. It is correct the that confidence interval is zero at one point along the axis; this happens because we do not include the main effect.

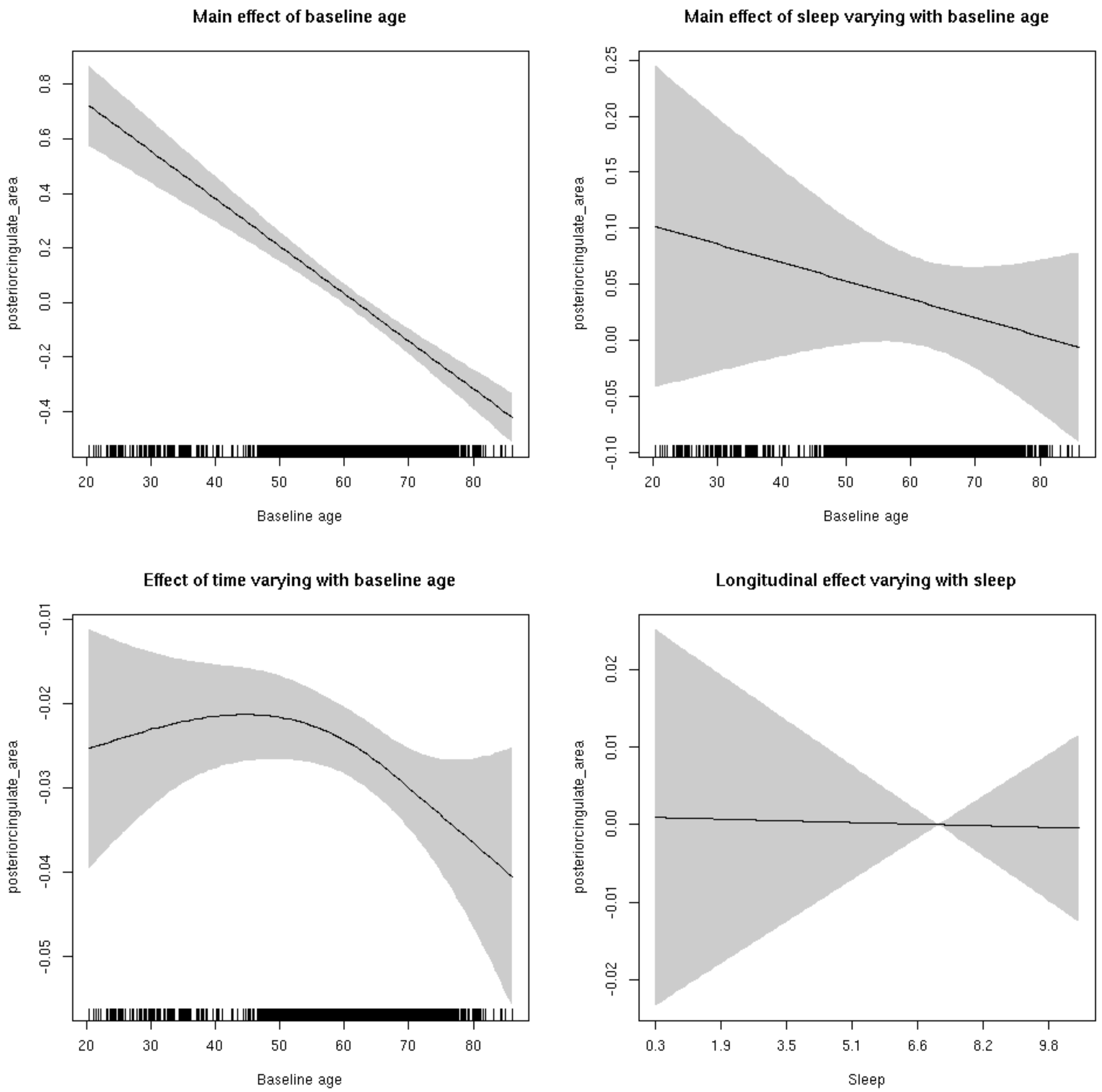

posteriorcingulate_area

Full data Below is the model output. The term `s(sleep_z):time` is probably what we care most about.

```
##
## Family: gaussian
## Link function: identity
##
## Formula:
## value ~ sex + site + icv + s(bl_age, k = 10, bs = "cr") + s(bl_age,
##     by = sleep_z, bs = "cr") + +s(bl_age, by = time, k = 5, bs = "cr") +
##     s(sleep_z, by = time, k = 5, bs = "cr", pc = 0)
## <environment: 0x5630035b3490>
```

```
##
## Parametric coefficients:
##                Estimate Std. Error t value Pr(>|t|)
## (Intercept)     1.16574    0.05585  20.872  < 2e-16 ***
## sexmale         0.09116    0.02864   3.184  0.00146 **
## siteousAvanto -0.93470    0.06790 -13.766  < 2e-16 ***
## siteousPrisma -0.65575    0.12748  -5.144 2.76e-07 ***
## siteousSkyra  -0.58272    0.06579  -8.857  < 2e-16 ***
## siteUB        -1.01103    0.12881  -7.849 4.72e-15 ***
## siteUCAM      -0.88418    0.06941 -12.738  < 2e-16 ***
## siteUKB       -1.20908    0.05315 -22.749  < 2e-16 ***
## siteUmU       -0.45663    0.07973  -5.727 1.06e-08 ***
## icv            0.61115    0.01401  43.629  < 2e-16 ***
## ---
## Signif. codes:  0 '***' 0.001 '**' 0.01 '*' 0.05 '.' 0.1 ' ' 1
##
## Approximate significance of smooth terms:
##                      edf Ref.df       F p-value
## s(bl_age)          3.556  3.556  46.041  <2e-16 ***
## s(bl_age):sleep_z 2.006  2.006   0.409   0.662
## s(bl_age):time    3.611  3.611 152.331  <2e-16 ***
## s(sleep_z):time   1.000  1.000   0.168   0.682
## ---
## Signif. codes:  0 '***' 0.001 '**' 0.01 '*' 0.05 '.' 0.1 ' ' 1
##
## R-sq.(adj) =  0.475
## lmer.REML = 6709.1  Scale est. = 0.019082  n = 8155
```

Below are plots of a plot of `s(bl_age)`, `s(bl_age):sleep_z`, `s(bl_age):time`, and `s(sleep_z):time`. For `s(sleep_z):time`, roughly, if we cannot fit a straight line through the confidence bands, there is evidence that the effect of time depends on sleep duration. It is correct the that confidence interval is zero at one point along the axis; this happens because we do not include the main effect.

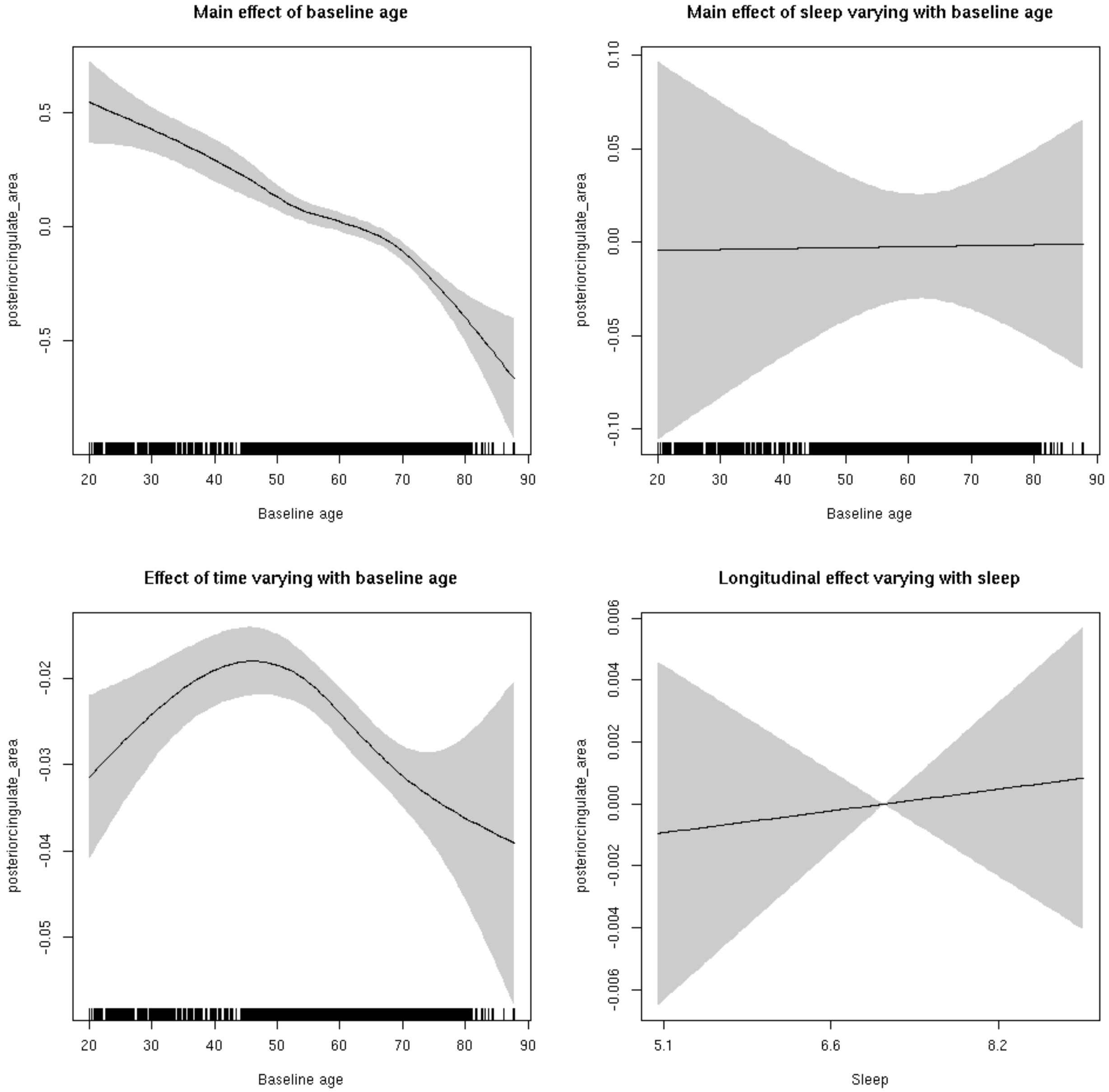

Restricted to sleep >= 5 and <= 9 hours Below is the row for `s(sleep_z):time` from the model output.

```
## s(sleep_z):time   1.000  1.000   0.117   0.732
```

Below are plots of a plot of `s(bl_age)`, `s(bl_age):sleep_z`, `s(bl_age):time`, and `s(sleep_z):time`. For `s(sleep_z):time`, roughly, if we cannot fit a straight line through the confidence bands, there is evidence that the effect of time depends on sleep duration. It is correct the that confidence interval is zero at one point along the axis; this happens because we do not include the main effect.

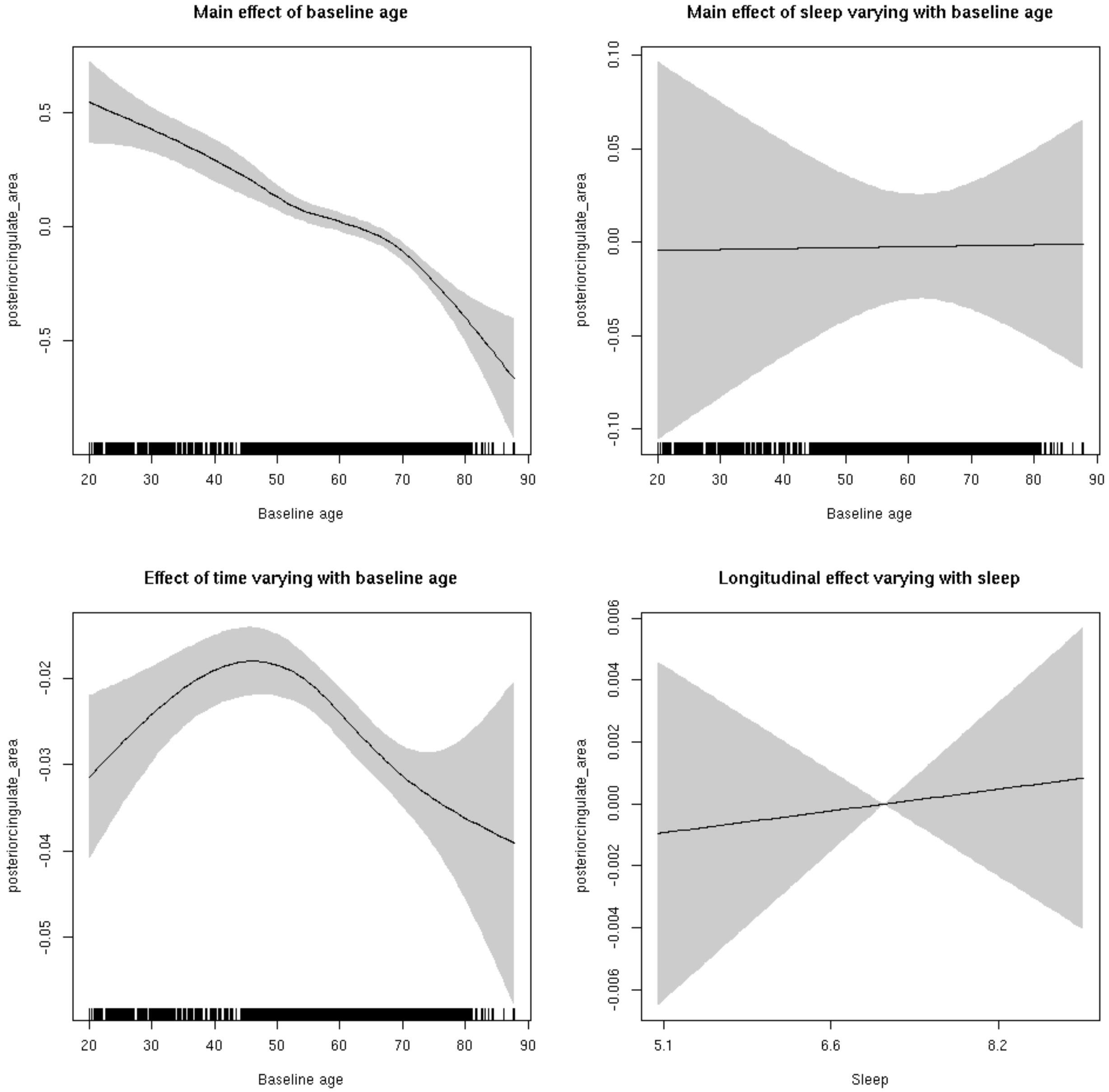

posteriorcingulate_thickness: Stratified by sex

Female: Full data Below is the row for `s(sleep_z):time` from the model output.

```
## s(sleep_z):time     1      1  0.147   0.701
```

Below are plots of a plot of `s(bl_age)`, `s(bl_age):sleep_z`, `s(bl_age):time`, and `s(sleep_z):time`. For `s(sleep_z):time`, roughly, if we cannot fit a straight line through the confidence bands, there is evidence that the effect of time depends on sleep duration. It is correct the that confidence interval is zero at one point along the axis; this happens because we do not include the main effect.

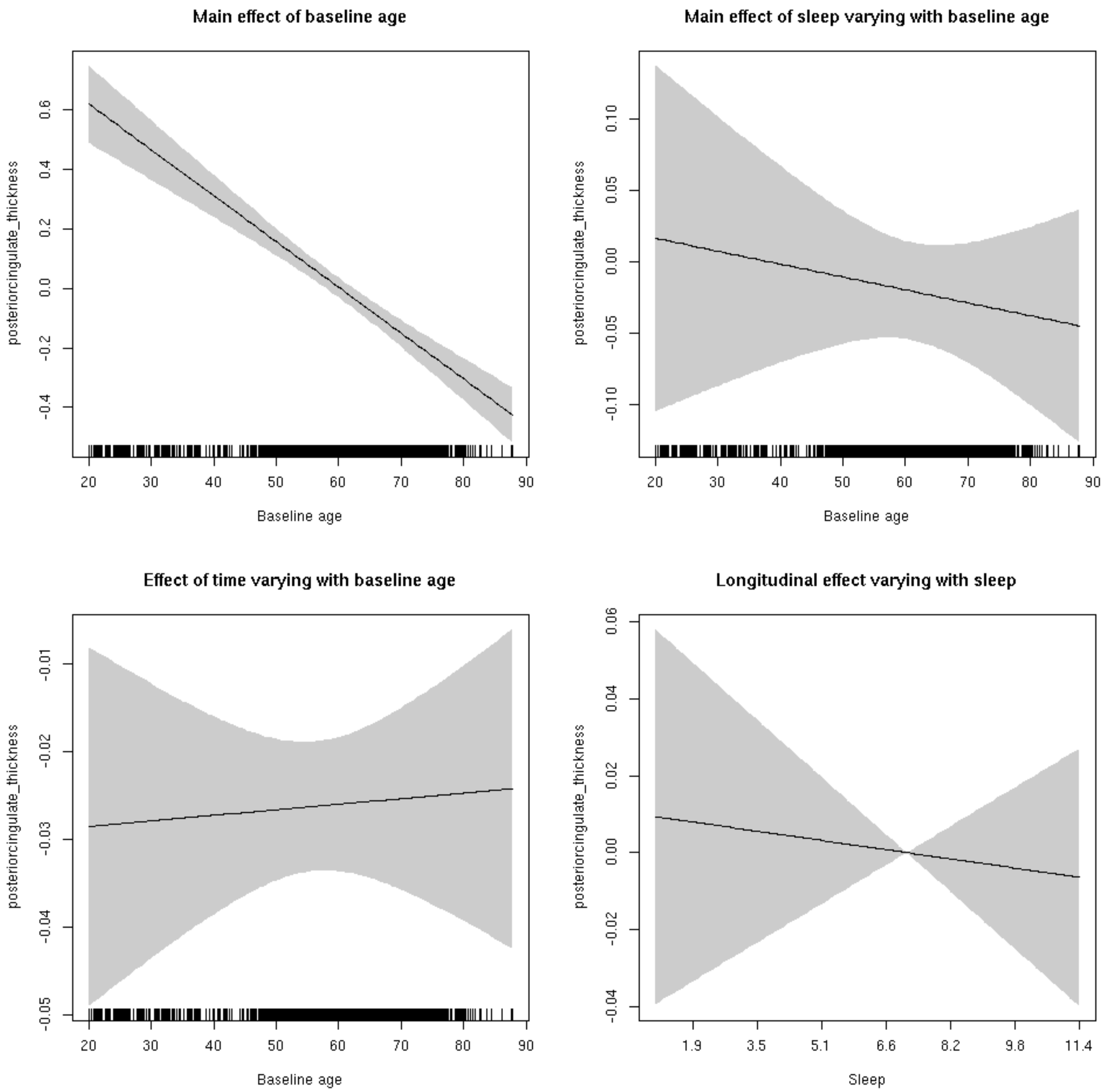

Male: Full data Below is the row for `s(sleep_z):time` from the model output.

```
## s(sleep_z):time     1       1  0.020   0.888
```

Below are plots of a plot of `s(bl_age)`, `s(bl_age):sleep_z`, `s(bl_age):time`, and `s(sleep_z):time`. For `s(sleep_z):time`, roughly, if we cannot fit a straight line through the confidence bands, there is evidence that the effect of time depends on sleep duration. It is correct the that confidence interval is zero at one point along the axis; this happens because we do not include the main effect.

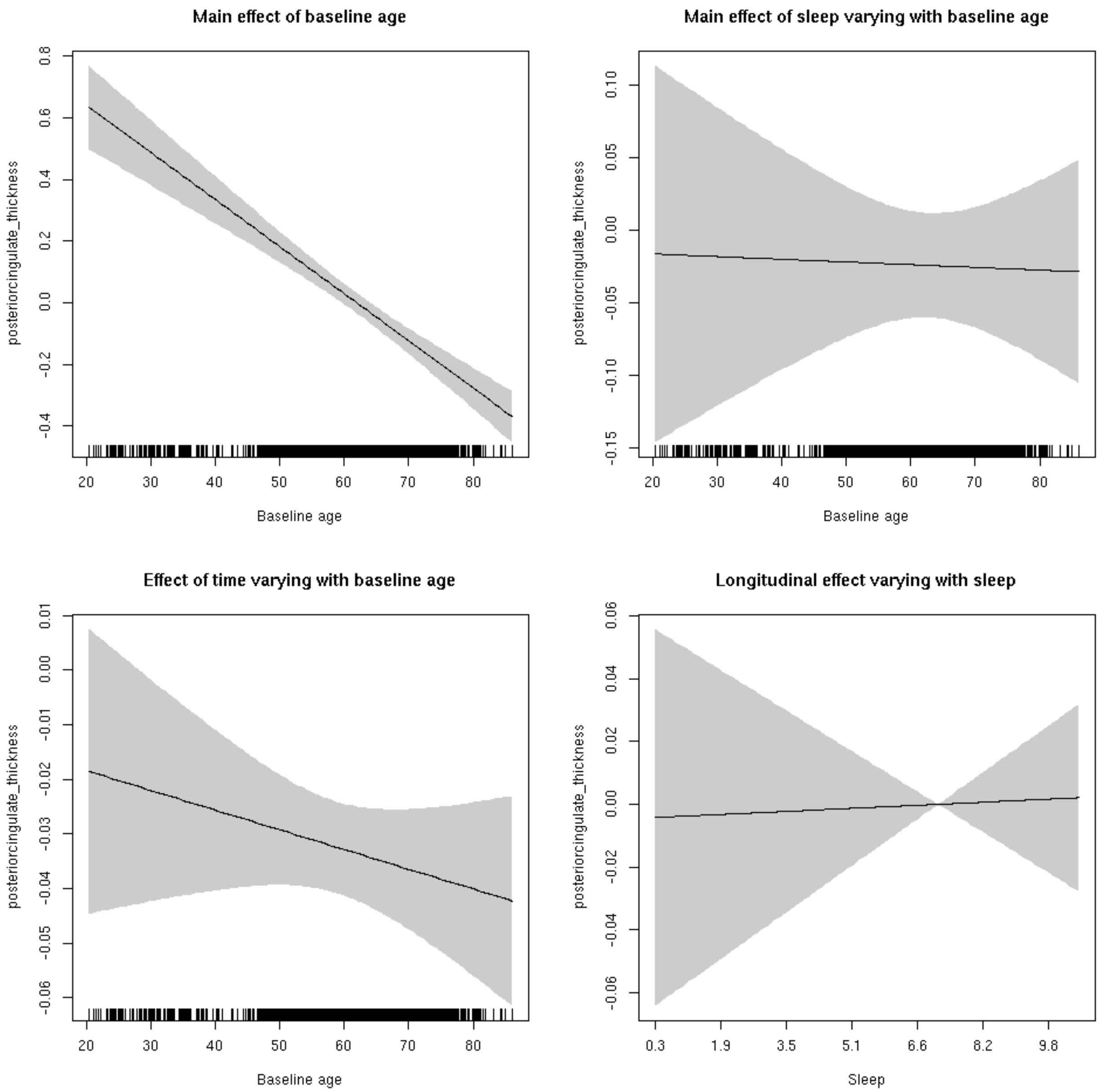

posteriorcingulate_thickness

Full data Below is the model output. The term `s(sleep_z):time` is probably what we care most about.

```
##
## Family: gaussian
## Link function: identity
##
## Formula:
## value ~ sex + site + s(bl_age, k = 10, bs = "cr") + s(bl_age,
##     by = sleep_z, bs = "cr") + +s(bl_age, by = time, k = 5, bs = "cr") +
##     s(sleep_z, by = time, k = 5, bs = "cr", pc = 0)
## <environment: 0x5630035ddfc8>
```

```
##
## Parametric coefficients:
##                Estimate Std. Error t value Pr(>|t|)
## (Intercept)   -1.57531    0.04672 -33.719  < 2e-16 ***
## sexmale       -0.08780    0.02338  -3.756 0.000174 ***
## siteousAvanto  0.04018    0.06215   0.646 0.518000
## siteousPrisma  0.03305    0.27224   0.121 0.903364
## siteousSkyra  -0.25572    0.06212  -4.117 3.88e-05 ***
## siteUB         0.25328    0.12367   2.048 0.040580 *
## siteUCAM       0.07527    0.06442   1.168 0.242682
## siteUKB        1.88770    0.04739  39.832  < 2e-16 ***
## siteUmU        0.27435    0.07694   3.566 0.000365 ***
## ---
## Signif. codes:  0 '***' 0.001 '**' 0.01 '*' 0.05 '.' 0.1 ' ' 1
##
## Approximate significance of smooth terms:
##                     edf Ref.df      F p-value
## s(bl_age)         4.242  4.242 46.869  <2e-16 ***
## s(bl_age):sleep_z 2.000  2.000  1.467   0.231
## s(bl_age):time    2.000  2.000 55.334  <2e-16 ***
## s(sleep_z):time   1.000  1.000  0.075   0.784
## ---
## Signif. codes:  0 '***' 0.001 '**' 0.01 '*' 0.05 '.' 0.1 ' ' 1
##
## R-sq.(adj) =  0.562
## lmer.REML =  14727  Scale est. = 0.12833   n = 8178
```

Below are plots of a plot of `s(bl_age)`, `s(bl_age):sleep_z`, `s(bl_age):time`, and `s(sleep_z):time`. For `s(sleep_z):time`, roughly, if we cannot fit a straight line through the confidence bands, there is evidence that the effect of time depends on sleep duration. It is correct the that confidence interval is zero at one point along the axis; this happens because we do not include the main effect.

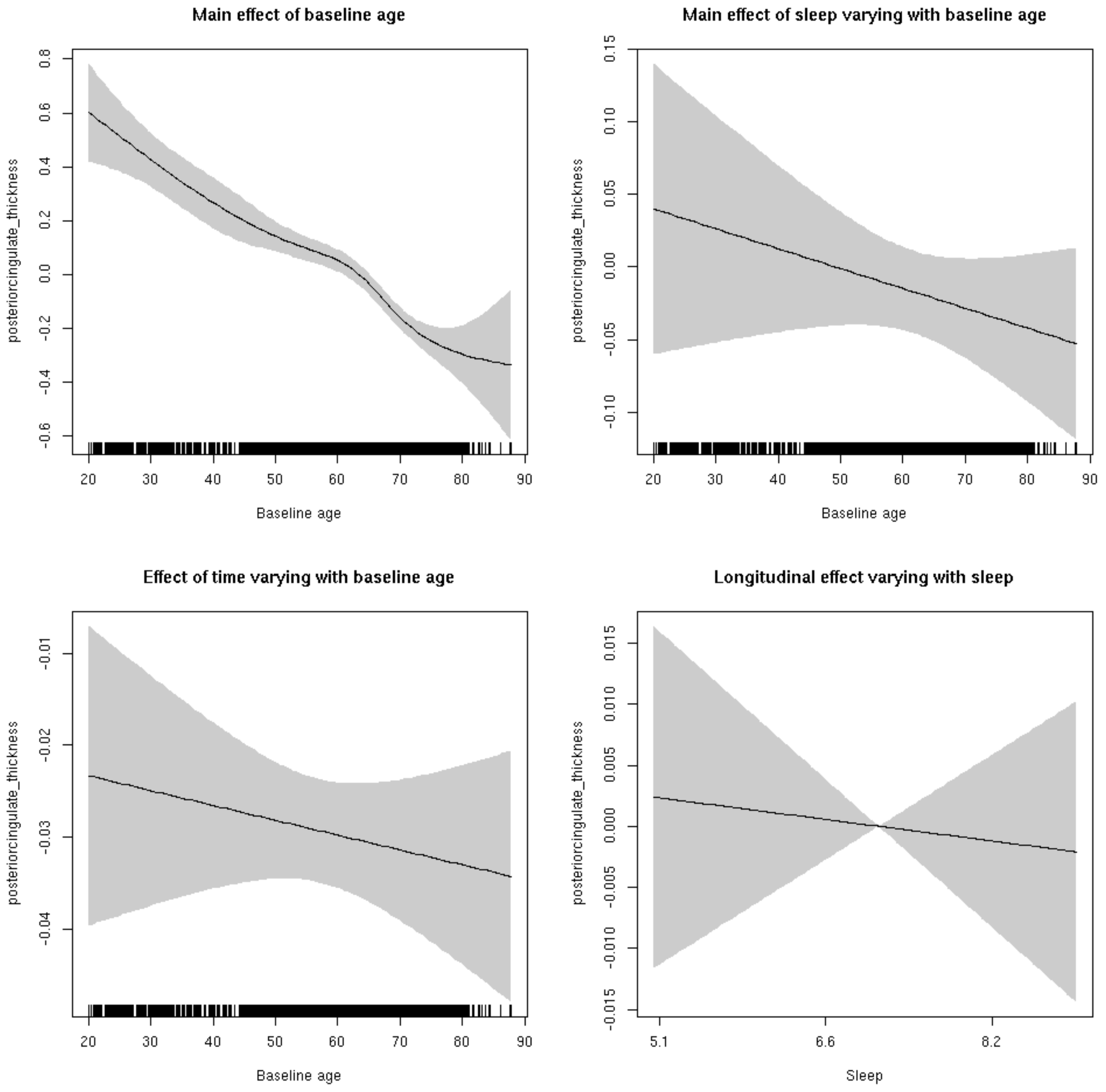

Restricted to sleep >= 5 and <= 9 hours Below is the row for `s(sleep_z):time` from the model output.

```
## s(sleep_z):time   1.000  1.000  0.116   0.733
```

Below are plots of a plot of `s(bl_age)`, `s(bl_age):sleep_z`, `s(bl_age):time`, and `s(sleep_z):time`. For `s(sleep_z):time`, roughly, if we cannot fit a straight line through the confidence bands, there is evidence that the effect of time depends on sleep duration. It is correct the that confidence interval is zero at one point along the axis; this happens because we do not include the main effect.

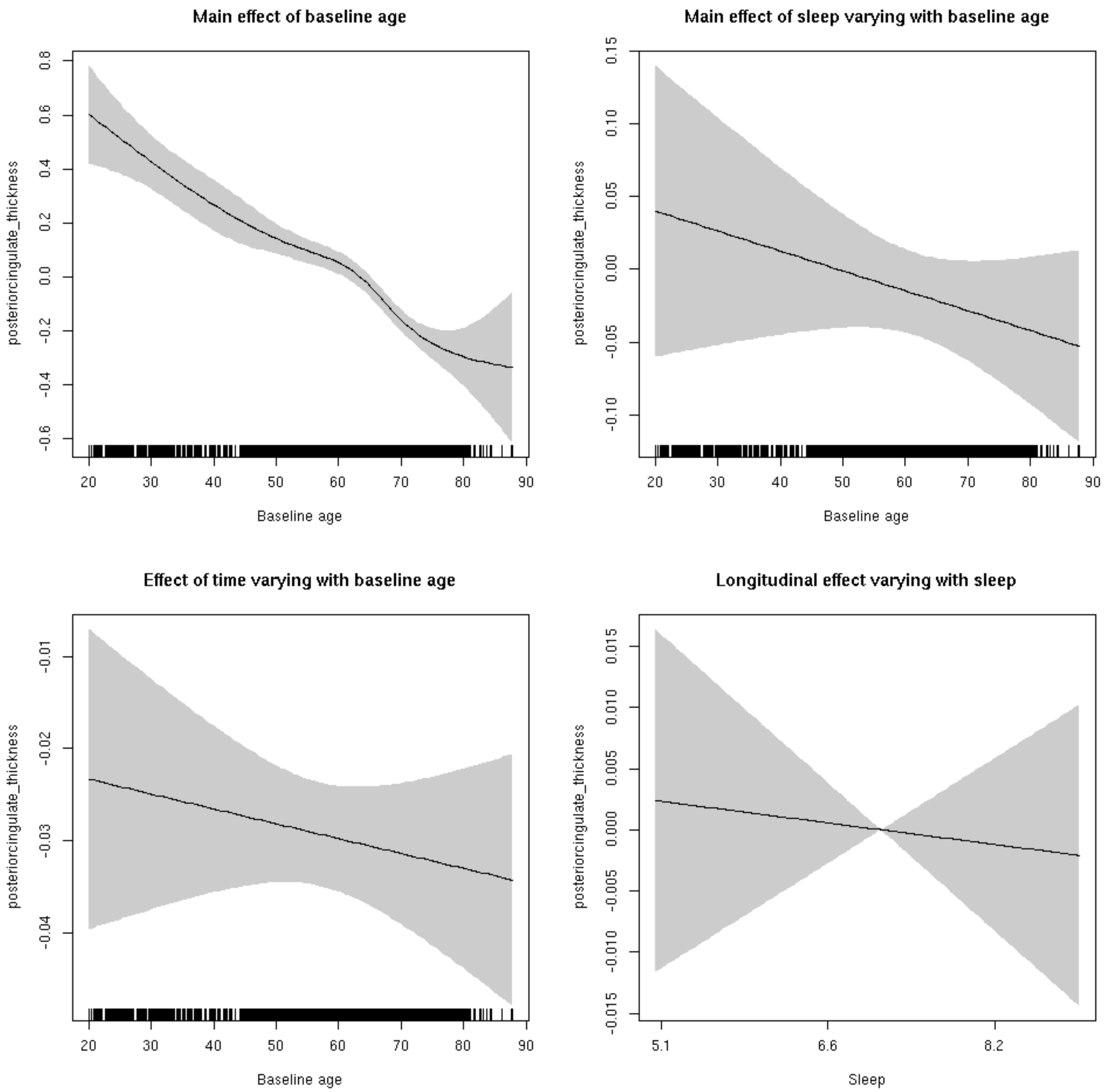

posteriorcingulate_volume: Stratified by sex

Female: Full data Below is the row for `s(sleep_z):time` from the model output.

```
## s(sleep_z):time   1.000  1.000  0.006  0.9374
```

Below are plots of a plot of `s(bl_age)`, `s(bl_age):sleep_z`, `s(bl_age):time`, and `s(sleep_z):time`. For `s(sleep_z):time`, roughly, if we cannot fit a straight line through the confidence bands, there is evidence that the effect of time depends on sleep duration. It is correct the that confidence interval is zero at one point along the axis; this happens because we do not include the main effect.

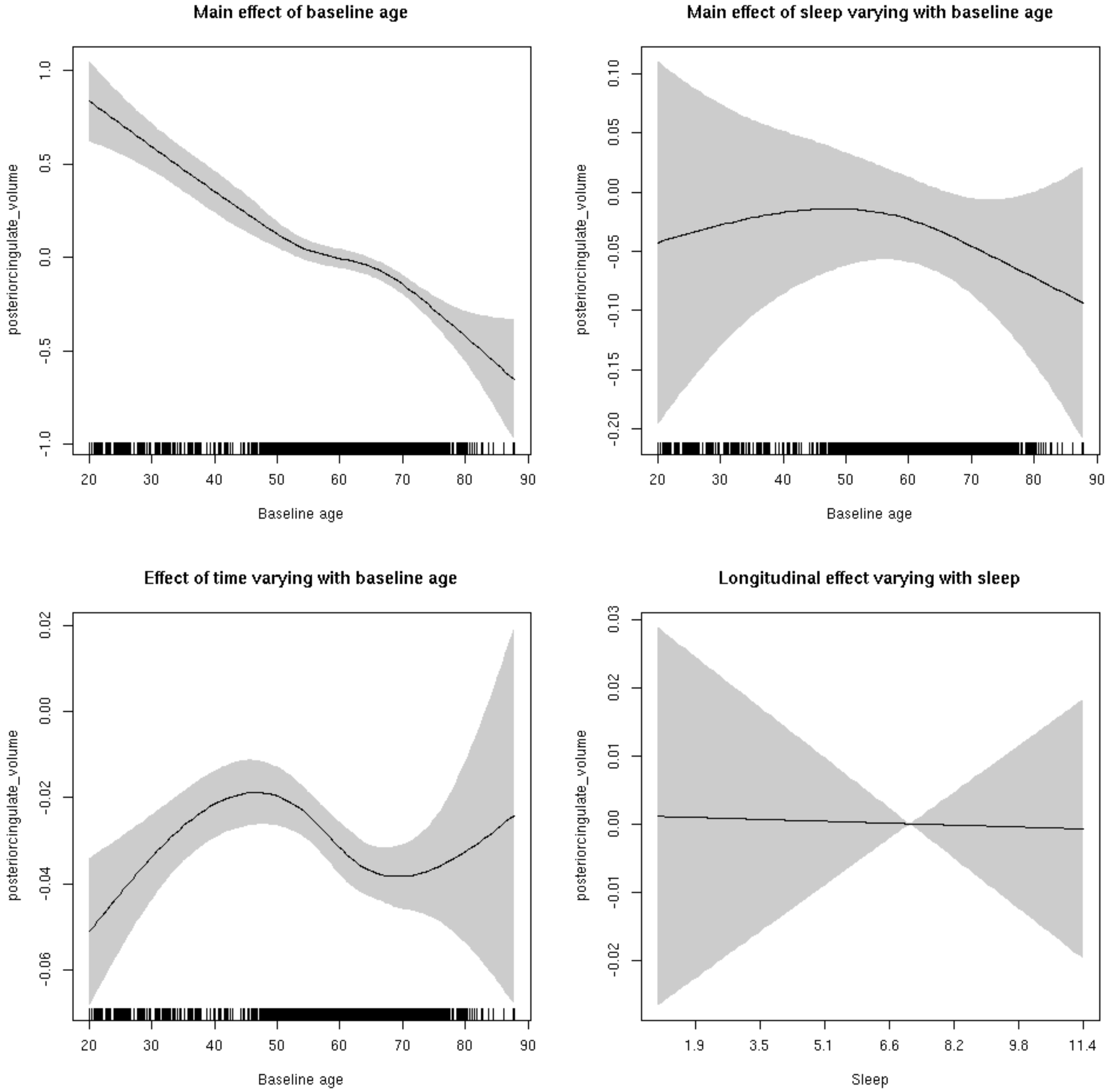

Male: Full data Below is the row for `s(sleep_z):time` from the model output.

```
## s(sleep_z):time   1.000  1.000   0.025   0.875
```

Below are plots of a plot of `s(bl_age)`, `s(bl_age):sleep_z`, `s(bl_age):time`, and `s(sleep_z):time`. For `s(sleep_z):time`, roughly, if we cannot fit a straight line through the confidence bands, there is evidence that the effect of time depends on sleep duration. It is correct the that confidence interval is zero at one point along the axis; this happens because we do not include the main effect.

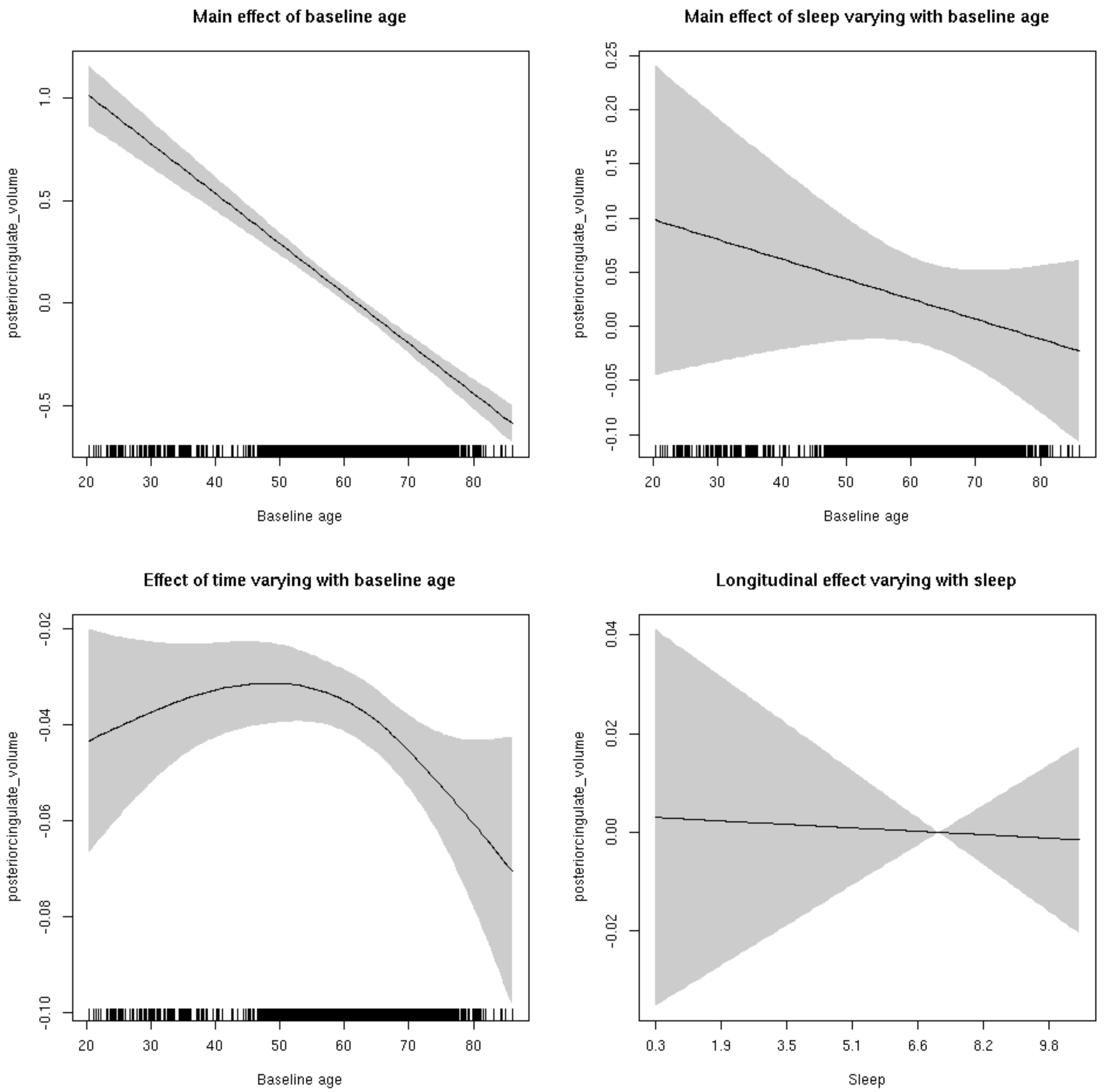

posteriorcingulate_volume

Full data Below is the model output. The term `s(sleep_z):time` is probably what we care most about.

```
##
## Family: gaussian
## Link function: identity
##
## Formula:
## value ~ sex + site + icv + s(bl_age, k = 10, bs = "cr") + s(bl_age,
##     by = sleep_z, bs = "cr") + +s(bl_age, by = time, k = 5, bs = "cr") +
##     s(sleep_z, by = time, k = 5, bs = "cr", pc = 0)
## <environment: 0x5630035ae418>
```

```
##
## Parametric coefficients:
##                Estimate Std. Error t value Pr(>|t|)
## (Intercept)   -0.07162    0.05655  -1.267 0.205357
## sexmale        0.11883    0.02907   4.088 4.39e-05 ***
## siteousAvanto -0.79560    0.06917 -11.502  < 2e-16 ***
## siteousPrisma -0.38630    0.17657  -2.188 0.028714 *
## siteousSkyra  -0.48033    0.06694  -7.175 7.86e-13 ***
## siteUB        -0.63739    0.12998  -4.904 9.58e-07 ***
## siteUCAM      -0.61191    0.06999  -8.743  < 2e-16 ***
## siteUKB        0.19725    0.05359   3.681 0.000234 ***
## siteUmU       -0.23604    0.08064  -2.927 0.003431 **
## icv            0.52138    0.01434  36.356  < 2e-16 ***
## ---
## Signif. codes:  0 '***' 0.001 '**' 0.01 '*' 0.05 '.' 0.1 ' ' 1
##
## Approximate significance of smooth terms:
##                     edf Ref.df       F p-value
## s(bl_age)         2.713  2.713 118.356  <2e-16 ***
## s(bl_age):sleep_z 2.000  2.000   1.235   0.291
## s(bl_age):time    3.490  3.490 128.634  <2e-16 ***
## s(sleep_z):time   1.000  1.000   0.031   0.861
## ---
## Signif. codes:  0 '***' 0.001 '**' 0.01 '*' 0.05 '.' 0.1 ' ' 1
##
## R-sq.(adj) =  0.452
## lmer.REML =  10454  Scale est. = 0.044804  n = 8166
```

Below are plots of a plot of `s(bl_age)`, `s(bl_age):sleep_z`, `s(bl_age):time`, and `s(sleep_z):time`. For `s(sleep_z):time`, roughly, if we cannot fit a straight line through the confidence bands, there is evidence that the effect of time depends on sleep duration. It is correct the that confidence interval is zero at one point along the axis; this happens because we do not include the main effect.

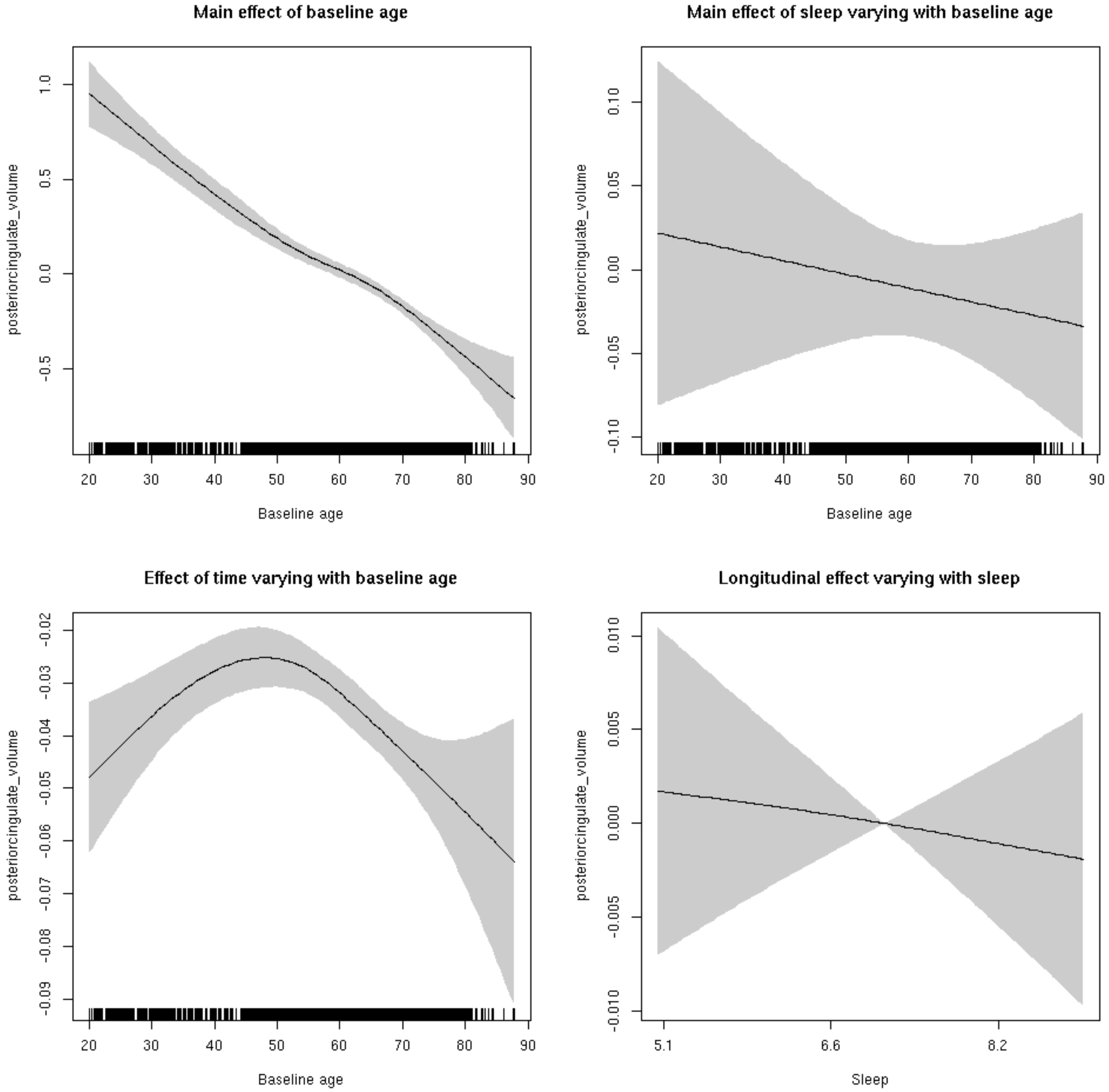

Restricted to sleep >= 5 and <= 9 hours Below is the row for `s(sleep_z):time` from the model output.

```
## s(sleep_z):time   1.032  1.032   0.182   0.663
```

Below are plots of a plot of `s(bl_age)`, `s(bl_age):sleep_z`, `s(bl_age):time`, and `s(sleep_z):time`. For `s(sleep_z):time`, roughly, if we cannot fit a straight line through the confidence bands, there is evidence that the effect of time depends on sleep duration. It is correct the that confidence interval is zero at one point along the axis; this happens because we do not include the main effect.

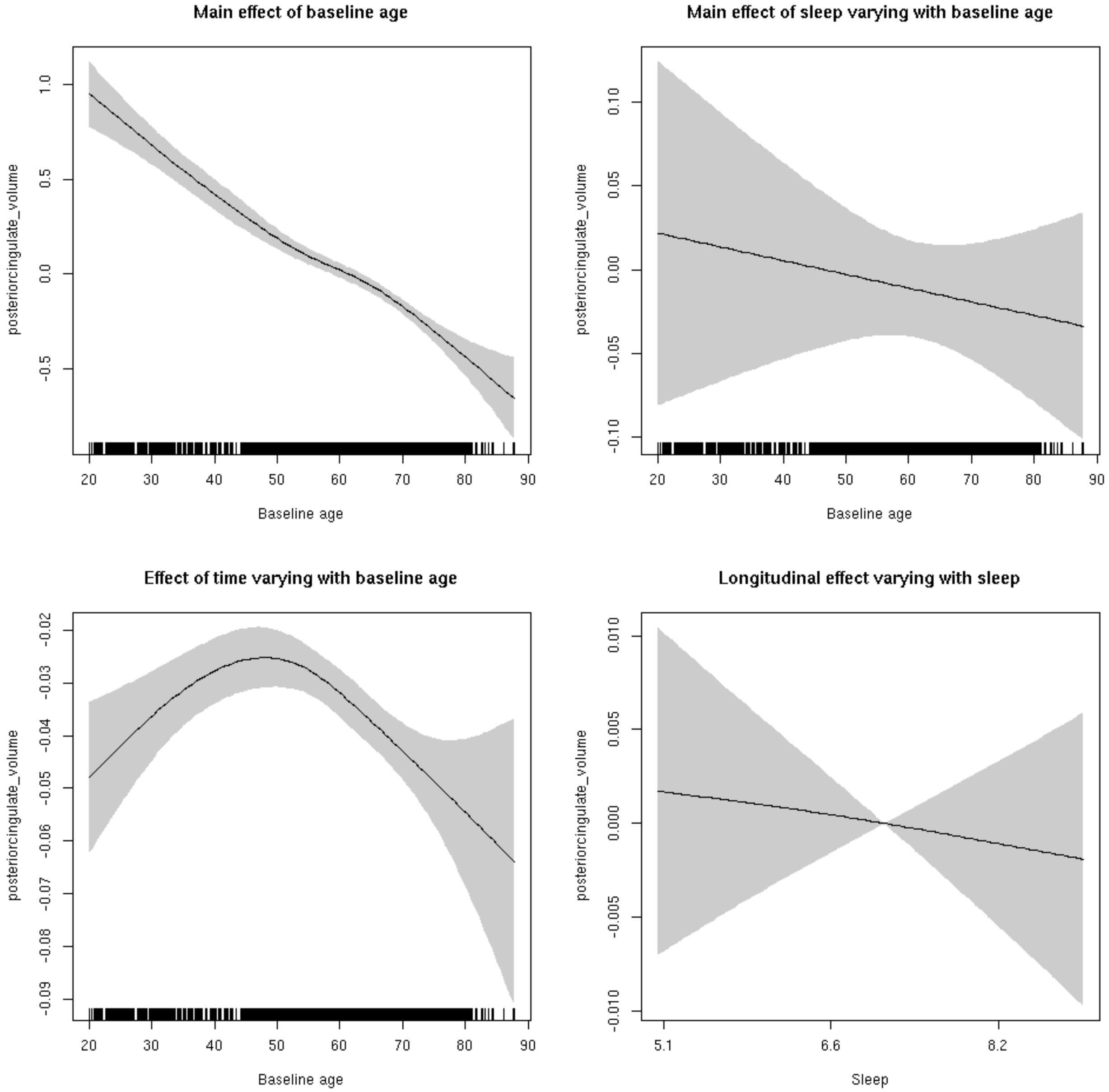

precentral_area: Stratified by sex

Female: Full data Below is the row for `s(sleep_z):time` from the model output.

```
## s(sleep_z):time   1.000  1.000 3.685 0.05497 .
```

Below are plots of a plot of `s(bl_age)`, `s(bl_age):sleep_z`, `s(bl_age):time`, and `s(sleep_z):time`. For `s(sleep_z):time`, roughly, if we cannot fit a straight line through the confidence bands, there is evidence that the effect of time depends on sleep duration. It is correct the that confidence interval is zero at one point along the axis; this happens because we do not include the main effect.

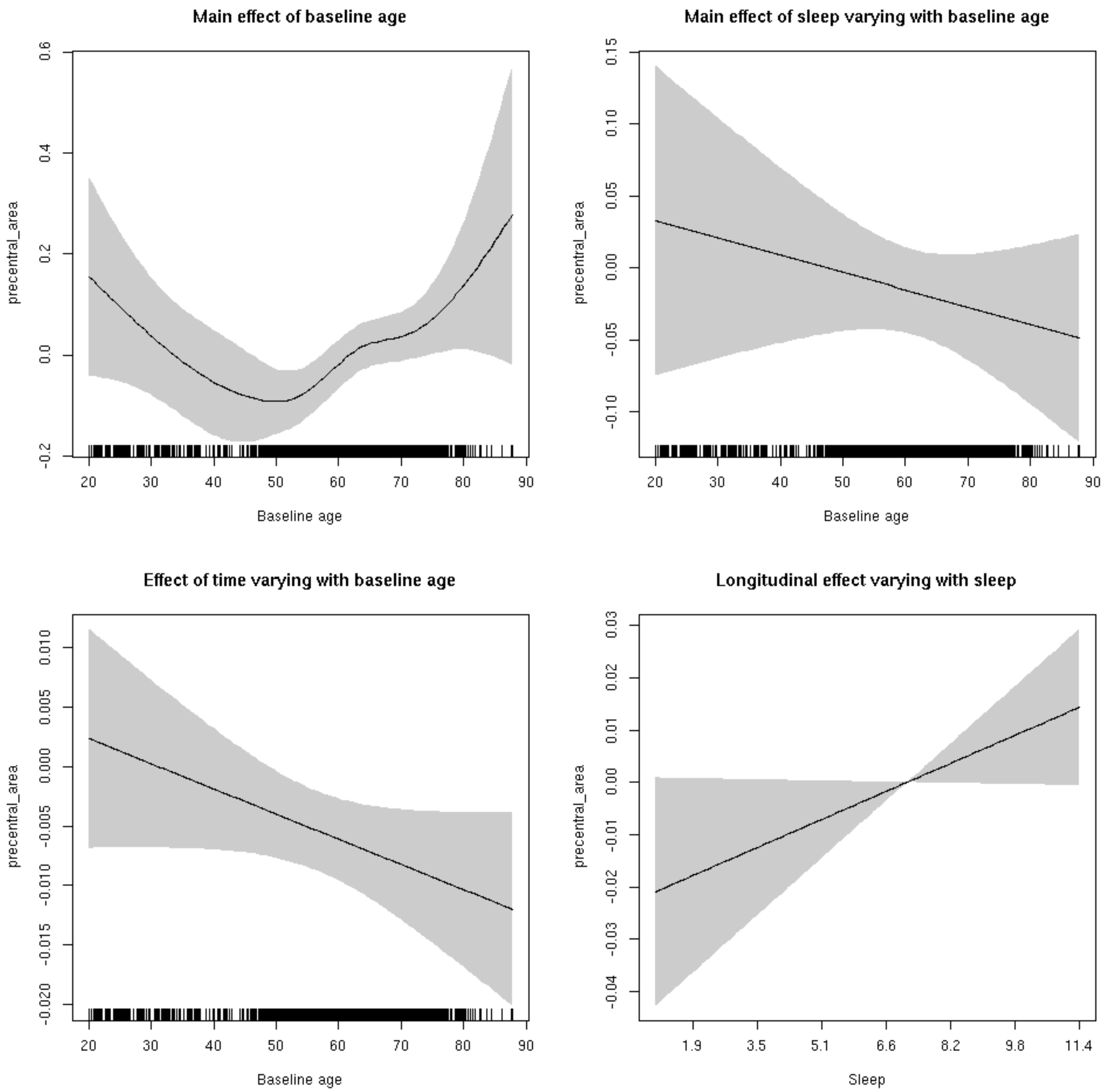

Male: Full data Below is the row for `s(sleep_z):time` from the model output.

```
## s(sleep_z):time     1       1  0.016   0.8979
```

Below are plots of a plot of `s(bl_age)`, `s(bl_age):sleep_z`, `s(bl_age):time`, and `s(sleep_z):time`. For `s(sleep_z):time`, roughly, if we cannot fit a straight line through the confidence bands, there is evidence that the effect of time depends on sleep duration. It is correct the that confidence interval is zero at one point along the axis; this happens because we do not include the main effect.

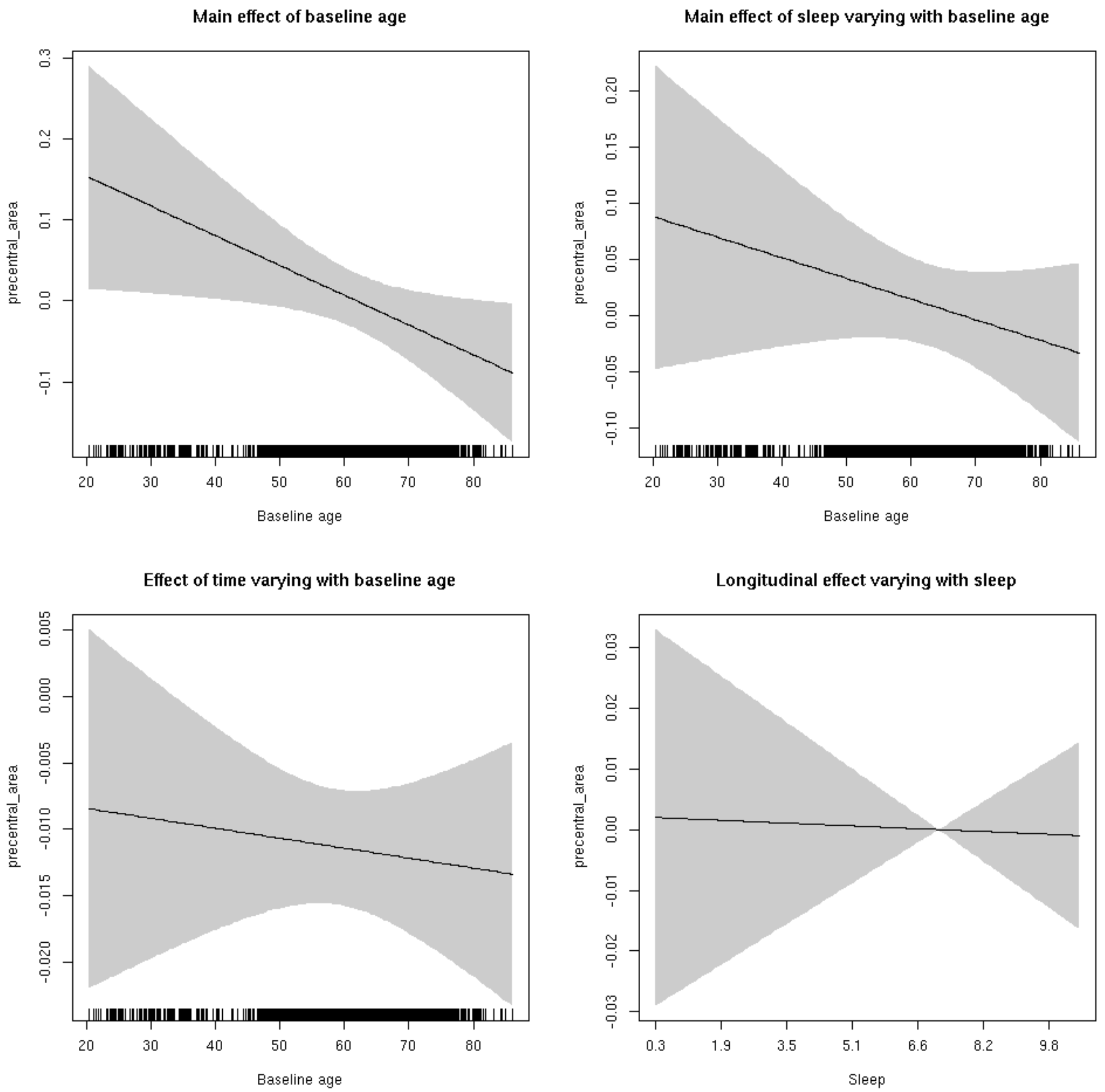

precentral_area

Full data Below is the model output. The term `s(sleep_z):time` is probably what we care most about.

```
##
## Family: gaussian
## Link function: identity
##
## Formula:
## value ~ sex + site + icv + s(bl_age, k = 10, bs = "cr") + s(bl_age,
##     by = sleep_z, bs = "cr") + +s(bl_age, by = time, k = 5, bs = "cr") +
##     s(sleep_z, by = time, k = 5, bs = "cr", pc = 0)
## <environment: 0x5630035aef78>
```

```
##
## Parametric coefficients:
##                Estimate Std. Error t value Pr(>|t|)
## (Intercept)     0.82064    0.05309  15.458  < 2e-16 ***
## sexmale         0.29179    0.02728  10.697  < 2e-16 ***
## siteousAvanto -0.59761    0.06477  -9.227  < 2e-16 ***
## siteousPrisma -0.75940    0.14656  -5.182 2.25e-07 ***
## siteousSkyra  -0.73987    0.06270 -11.800  < 2e-16 ***
## siteUB        -0.75414    0.12225  -6.169 7.20e-10 ***
## siteUCAM      -0.66237    0.06584 -10.060  < 2e-16 ***
## siteUKB       -0.98695    0.05040 -19.581  < 2e-16 ***
## siteUmU       -0.09258    0.07575  -1.222    0.222
## icv            0.60757    0.01341  45.317  < 2e-16 ***
## ---
## Signif. codes:  0 '***' 0.001 '**' 0.01 '*' 0.05 '.' 0.1 ' ' 1
##
## Approximate significance of smooth terms:
##                     edf Ref.df      F p-value
## s(bl_age)          3.13   3.13  2.931  0.0365 *
## s(bl_age):sleep_z 2.00   2.00  1.343  0.2611
## s(bl_age):time    2.00   2.00 19.633  <2e-16 ***
## s(sleep_z):time   1.00   1.00  1.319  0.2507
## ---
## Signif. codes:  0 '***' 0.001 '**' 0.01 '*' 0.05 '.' 0.1 ' ' 1
##
## R-sq.(adj) =  0.513
## lmer.REML = 8103.9  Scale est. = 0.028973  n = 8164
```

Below are plots of a plot of `s(bl_age)`, `s(bl_age):sleep_z`, `s(bl_age):time`, and `s(sleep_z):time`. For `s(sleep_z):time`, roughly, if we cannot fit a straight line through the confidence bands, there is evidence that the effect of time depends on sleep duration. It is correct the that confidence interval is zero at one point along the axis; this happens because we do not include the main effect.

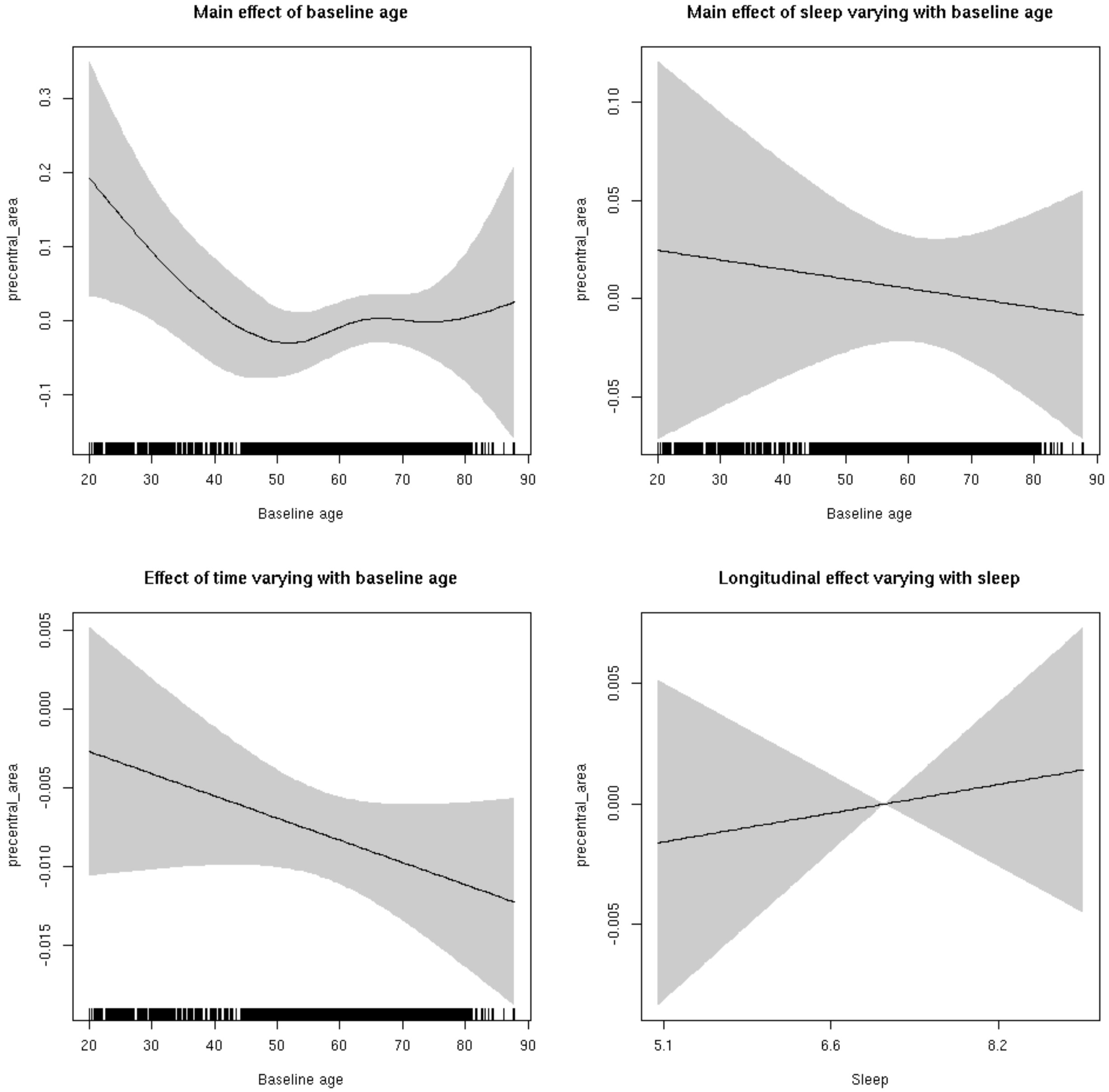

Restricted to sleep >= 5 and <= 9 hours Below is the row for `s(sleep_z):time` from the model output.

```
## s(sleep_z):time   1.000  1.000  0.228  0.6333
```

Below are plots of a plot of `s(bl_age)`, `s(bl_age):sleep_z`, `s(bl_age):time`, and `s(sleep_z):time`. For `s(sleep_z):time`, roughly, if we cannot fit a straight line through the confidence bands, there is evidence that the effect of time depends on sleep duration. It is correct the that confidence interval is zero at one point along the axis; this happens because we do not include the main effect.

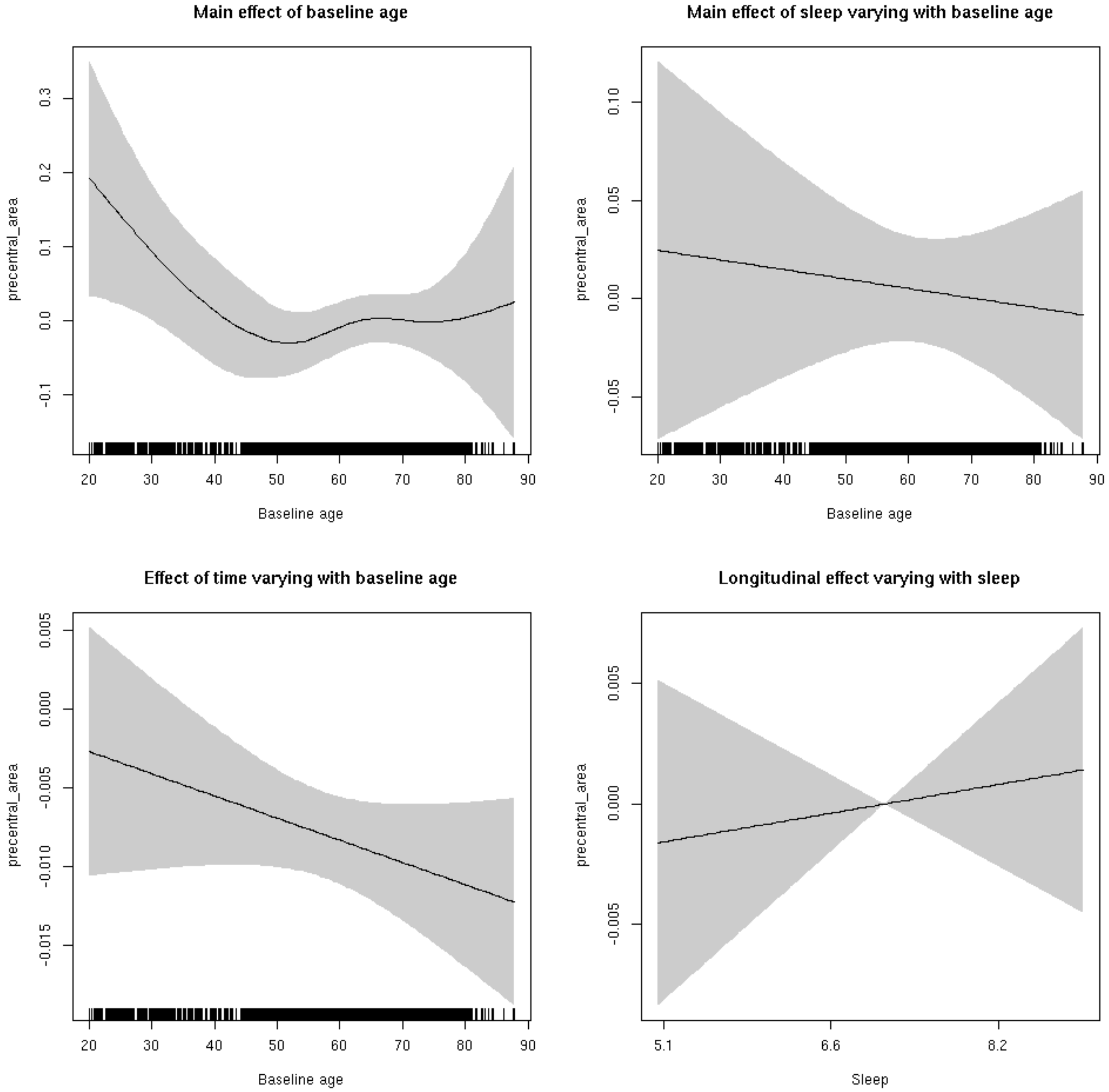

precentral_thickness: Stratified by sex

Female: Full data Below is the row for `s(sleep_z):time` from the model output.

```
## s(sleep_z):time   1.000  1.000   0.535   0.464
```

Below are plots of a plot of `s(bl_age)`, `s(bl_age):sleep_z`, `s(bl_age):time`, and `s(sleep_z):time`. For `s(sleep_z):time`, roughly, if we cannot fit a straight line through the confidence bands, there is evidence that the effect of time depends on sleep duration. It is correct the that confidence interval is zero at one point along the axis; this happens because we do not include the main effect.

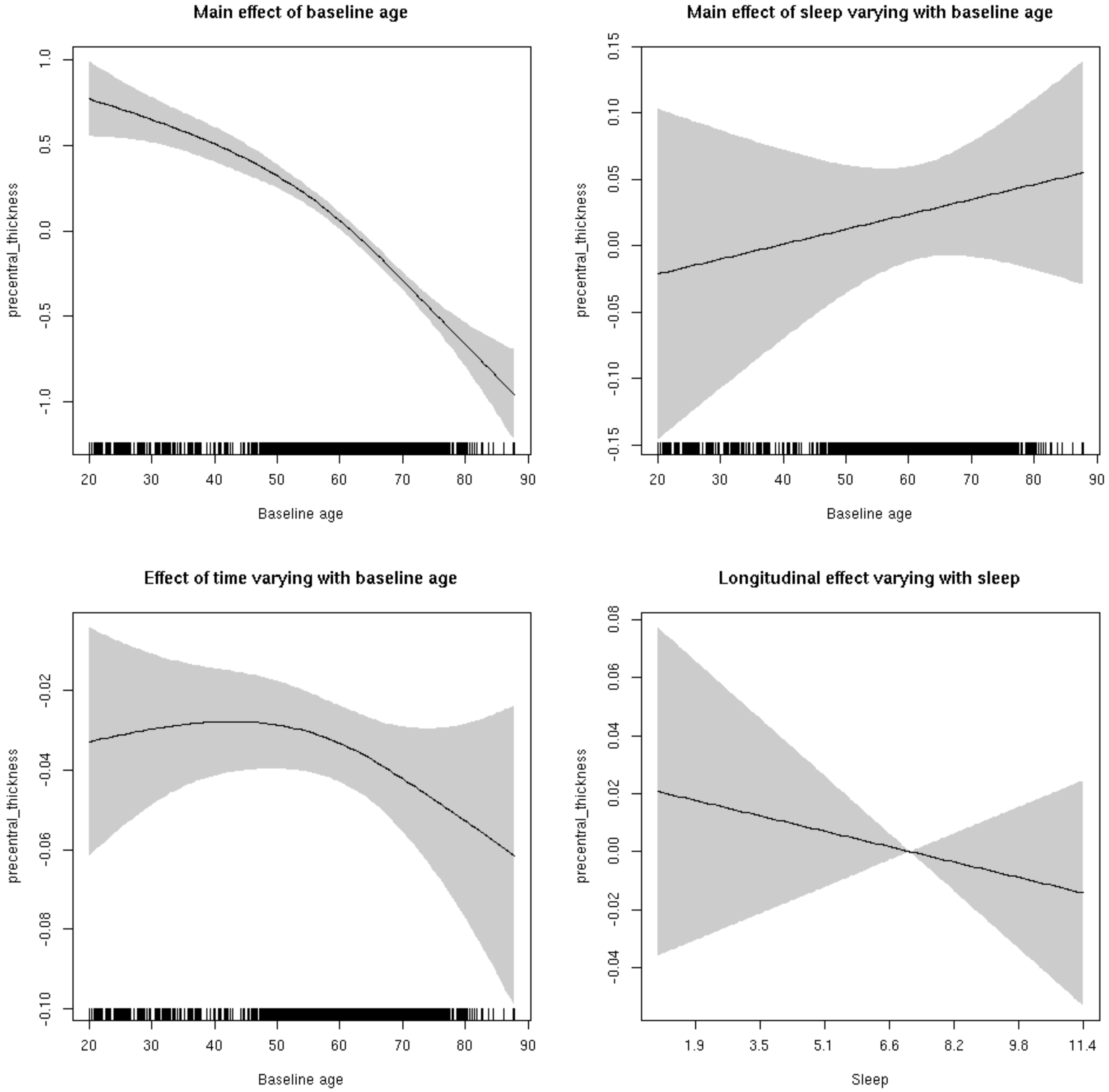

Male: Full data Below is the row for `s(sleep_z):time` from the model output.

```
## s(sleep_z):time   1.000  1.000   0.132   0.717
```

Below are plots of a plot of `s(bl_age)`, `s(bl_age):sleep_z`, `s(bl_age):time`, and `s(sleep_z):time`. For `s(sleep_z):time`, roughly, if we cannot fit a straight line through the confidence bands, there is evidence that the effect of time depends on sleep duration. It is correct the that confidence interval is zero at one point along the axis; this happens because we do not include the main effect.

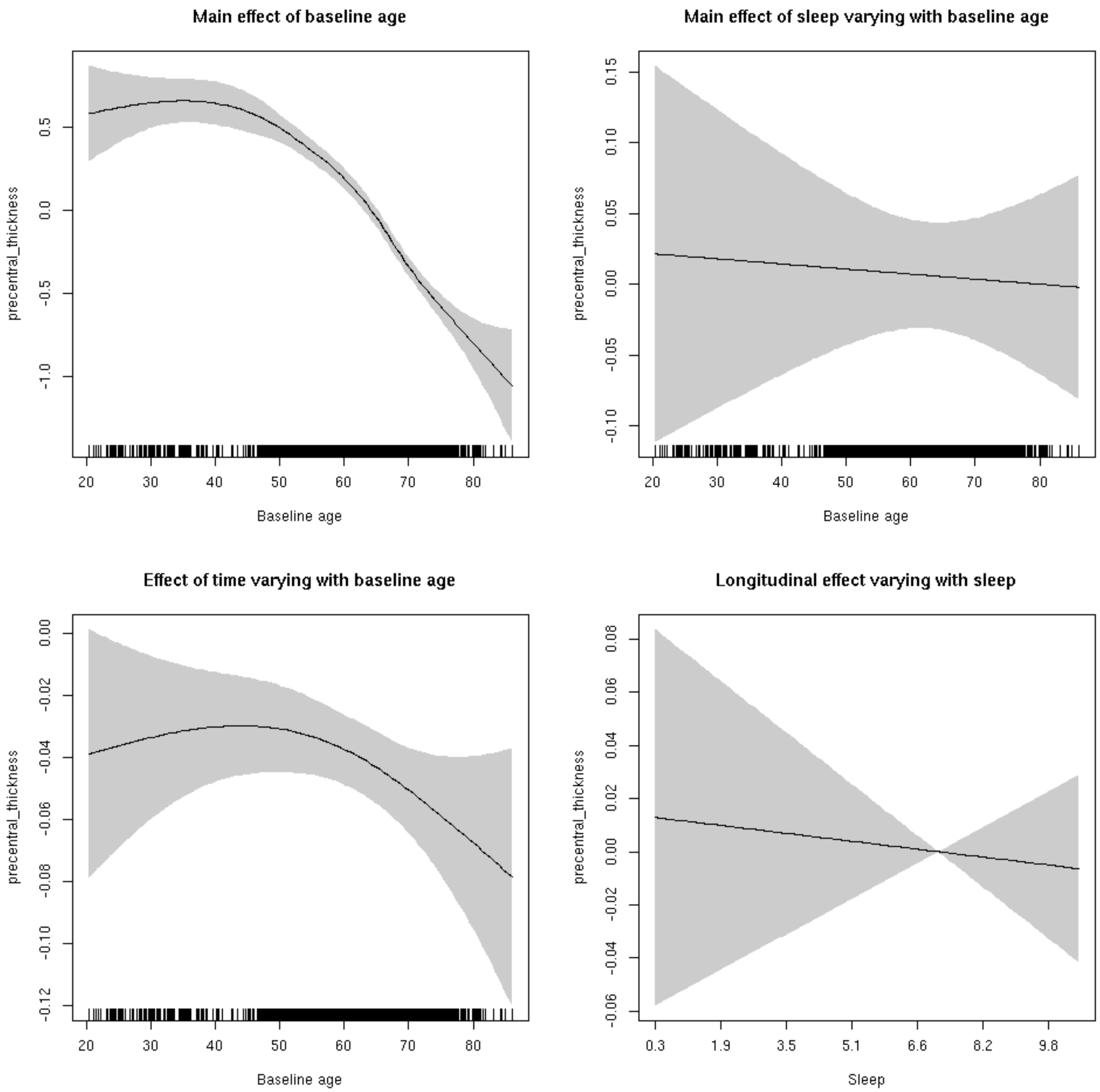

precentral_thickness

Full data Below is the model output. The term `s(sleep_z):time` is probably what we care most about.

```
##
## Family: gaussian
## Link function: identity
##
## Formula:
## value ~ sex + site + s(bl_age, k = 10, bs = "cr") + s(bl_age,
##     by = sleep_z, bs = "cr") + +s(bl_age, by = time, k = 5, bs = "cr") +
##     s(sleep_z, by = time, k = 5, bs = "cr", pc = 0)
## <environment: 0x5630035ce5b0>
```

```
##
## Parametric coefficients:
##                Estimate Std. Error t value Pr(>|t|)
## (Intercept)    -0.90170    0.04805 -18.765  < 2e-16 ***
## sexmale        -0.07366    0.02404  -3.064  0.00219 **
## siteousAvanto -0.60867    0.06440  -9.452  < 2e-16 ***
## siteousPrisma  0.10094    0.31365   0.322  0.74759
## siteousSkyra  -0.35788    0.06438  -5.559  2.8e-08 ***
## siteUB          0.29935    0.12653   2.366  0.01801 *
## siteUCAM       -0.01652    0.06623  -0.249  0.80302
## siteUKB         1.16066    0.04868  23.844  < 2e-16 ***
## siteUmU        -0.23623    0.07912  -2.986  0.00284 **
## ---
## Signif. codes:  0 ‘***’ 0.001 ‘**’ 0.01 ‘*’ 0.05 ‘.’ 0.1 ‘ ’ 1
##
## Approximate significance of smooth terms:
##                      edf Ref.df       F p-value
## s(bl_age)          3.802  3.802 192.699  <2e-16 ***
## s(bl_age):sleep_z 2.000  2.000   0.555   0.574
## s(bl_age):time     3.115  3.115  31.248  <2e-16 ***
## s(sleep_z):time    3.431  3.431   0.926   0.266
## ---
## Signif. codes:  0 ‘***’ 0.001 ‘**’ 0.01 ‘*’ 0.05 ‘.’ 0.1 ‘ ’ 1
##
## R-sq.(adj) =  0.439
## lmer.REML =  16297  Scale est. = 0.17647   n = 8169
```

Below are plots of a plot of `s(bl_age)`, `s(bl_age):sleep_z`, `s(bl_age):time`, and `s(sleep_z):time`. For `s(sleep_z):time`, roughly, if we cannot fit a straight line through the confidence bands, there is evidence that the effect of time depends on sleep duration. It is correct the that confidence interval is zero at one point along the axis; this happens because we do not include the main effect.

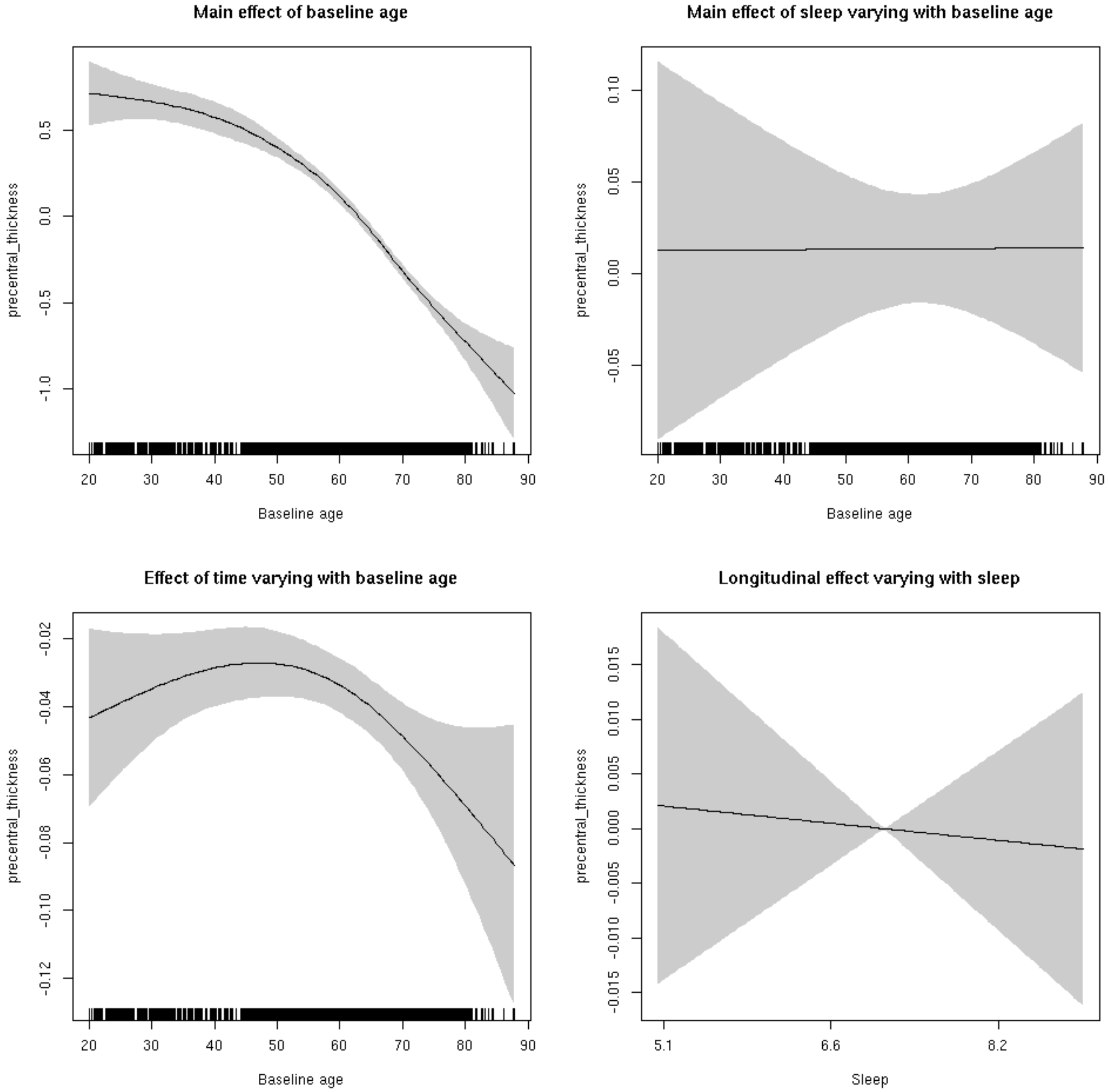

Restricted to sleep >= 5 and <= 9 hours Below is the row for `s(sleep_z):time` from the model output.

```
## s(sleep_z):time   1.000  1.000   0.067   0.795
```

Below are plots of a plot of `s(bl_age)`, `s(bl_age):sleep_z`, `s(bl_age):time`, and `s(sleep_z):time`. For `s(sleep_z):time`, roughly, if we cannot fit a straight line through the confidence bands, there is evidence that the effect of time depends on sleep duration. It is correct the that confidence interval is zero at one point along the axis; this happens because we do not include the main effect.

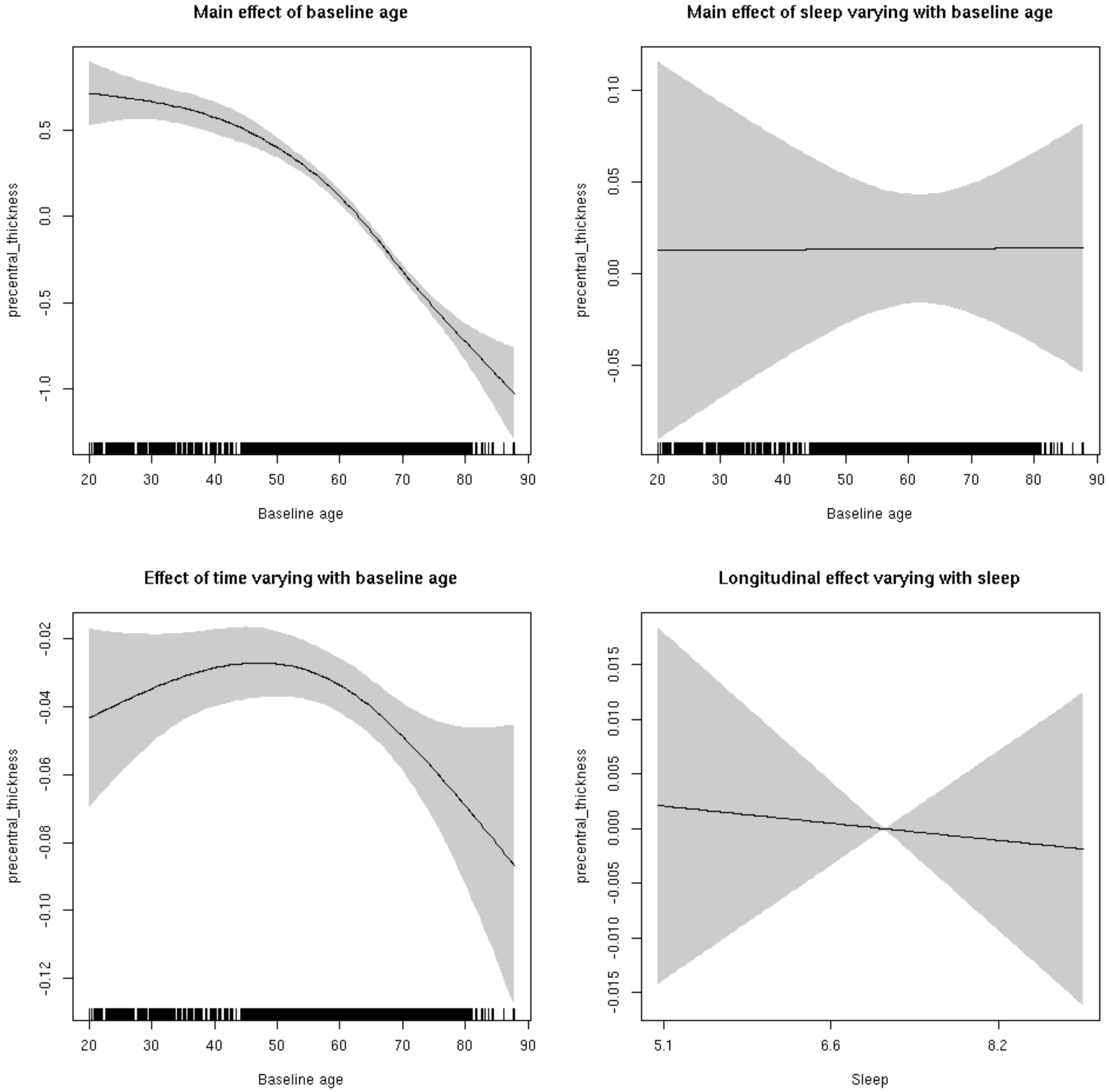

precentral_volume: Stratified by sex

Female: Full data Below is the row for `s(sleep_z):time` from the model output.

```
## s(sleep_z):time   3.778  3.778   3.450 0.00537 **
```

Below are plots of a plot of `s(bl_age)`, `s(bl_age):sleep_z`, `s(bl_age):time`, and `s(sleep_z):time`. For `s(sleep_z):time`, roughly, if we cannot fit a straight line through the confidence bands, there is evidence that the effect of time depends on sleep duration. It is correct the that confidence interval is zero at one point along the axis; this happens because we do not include the main effect.

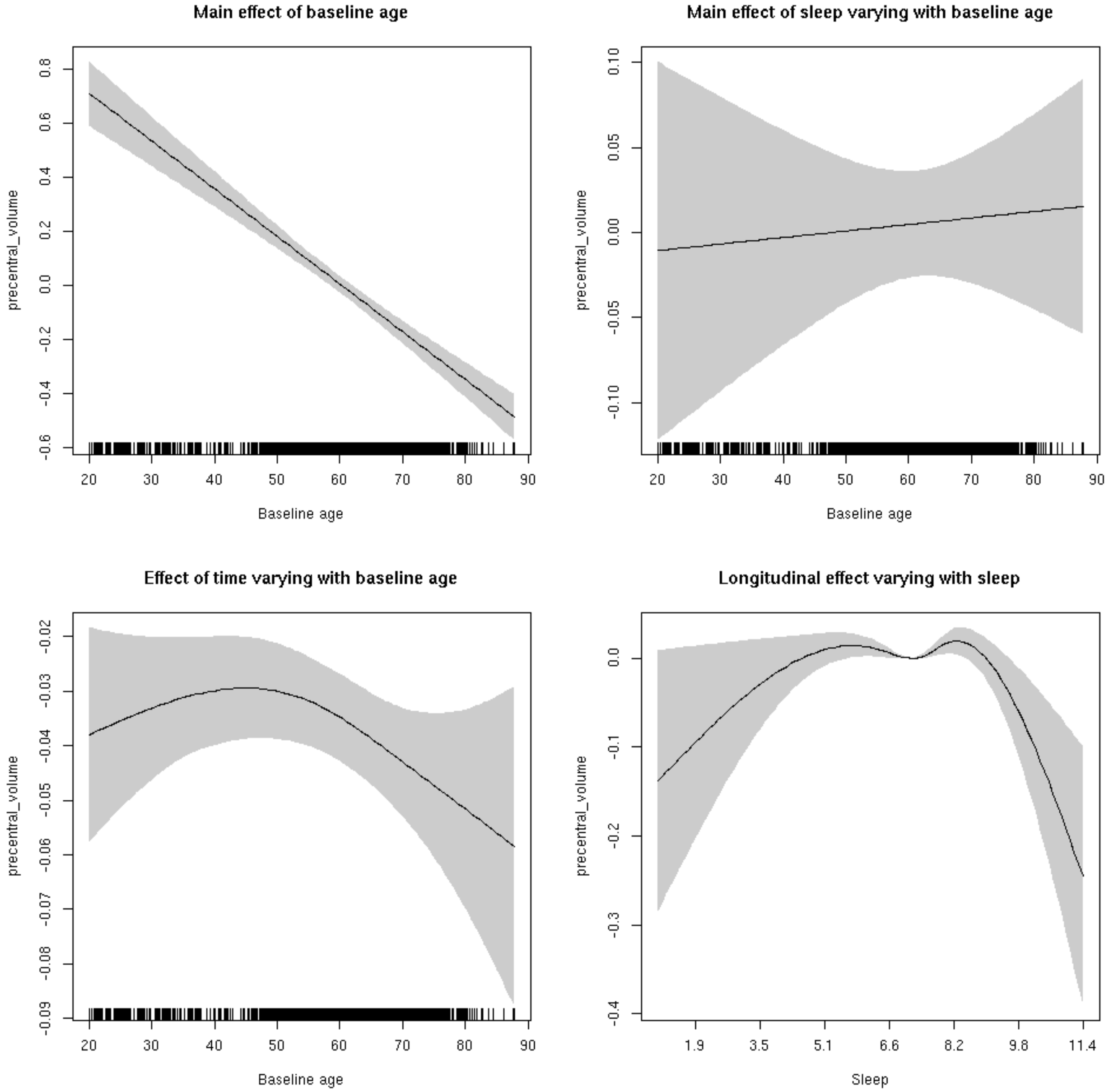

Male: Full data Below is the row for `s(sleep_z):time` from the model output.

```
## s(sleep_z):time   1.000  1.000   0.229   0.632
```

Below are plots of a plot of `s(bl_age)`, `s(bl_age):sleep_z`, `s(bl_age):time`, and `s(sleep_z):time`. For `s(sleep_z):time`, roughly, if we cannot fit a straight line through the confidence bands, there is evidence that the effect of time depends on sleep duration. It is correct the that confidence interval is zero at one point along the axis; this happens because we do not include the main effect.

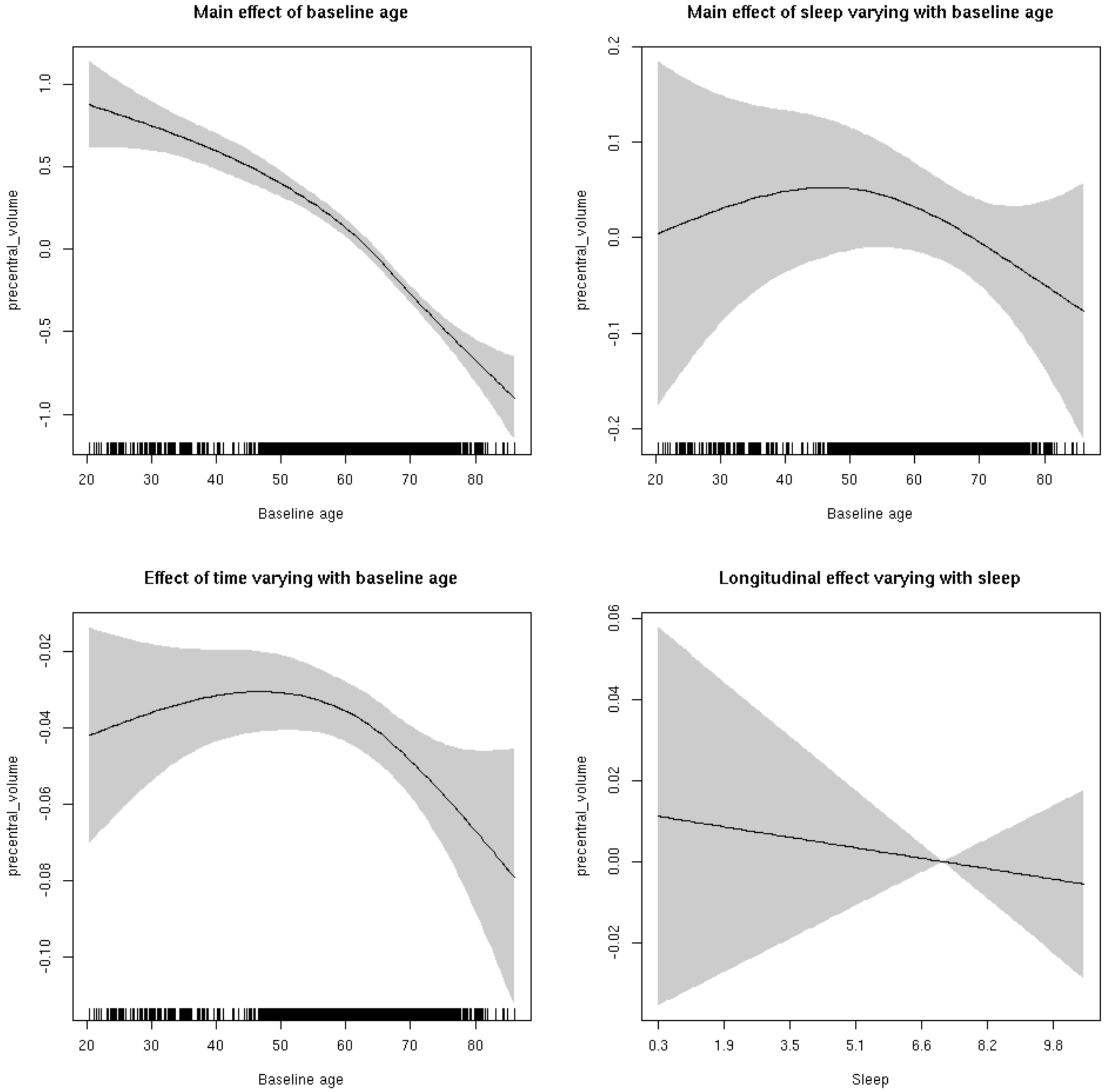

precentral_volume

Full data Below is the model output. The term `s(sleep_z):time` is probably what we care most about.

```
##
## Family: gaussian
## Link function: identity
##
## Formula:
## value ~ sex + site + icv + s(bl_age, k = 10, bs = "cr") + s(bl_age,
##     by = sleep_z, bs = "cr") + +s(bl_age, by = time, k = 5, bs = "cr") +
##     s(sleep_z, by = time, k = 5, bs = "cr", pc = 0)
## <environment: 0x5630035a74b8>
```

```
##
## Parametric coefficients:
##                Estimate Std. Error t value Pr(>|t|)
## (Intercept)     0.23678    0.05453   4.342 1.43e-05 ***
## sexmale         0.13572    0.02796   4.853 1.24e-06 ***
## siteousAvanto -1.17691    0.06698 -17.571  < 2e-16 ***
## siteousPrisma -0.57521    0.21078  -2.729 0.006368 **
## siteousSkyra  -0.79441    0.06485 -12.250  < 2e-16 ***
## siteUB        -0.44949    0.12447  -3.611 0.000306 ***
## siteUCAM      -0.67097    0.06779  -9.898  < 2e-16 ***
## siteUKB       -0.16369    0.05181  -3.159 0.001587 **
## siteUmU       -0.22556    0.07757  -2.908 0.003651 **
## icv            0.56513    0.01383  40.848  < 2e-16 ***
## ---
## Signif. codes:  0 ‘***’ 0.001 ‘**’ 0.01 ‘*’ 0.05 ‘.’ 0.1 ‘ ’ 1
##
## Approximate significance of smooth terms:
##                      edf Ref.df       F p-value
## s(bl_age)          4.015  4.015 122.003  <2e-16 ***
## s(bl_age):sleep_z 2.000  2.000   0.379   0.685
## s(bl_age):time     3.265  3.265  54.680  <2e-16 ***
## s(sleep_z):time    3.554  3.554   1.400   0.123
## ---
## Signif. codes:  0 ‘***’ 0.001 ‘**’ 0.01 ‘*’ 0.05 ‘.’ 0.1 ‘ ’ 1
##
## R-sq.(adj) =  0.478
## lmer.REML =  12032  Scale est. = 0.069567  n = 8170
```

Below are plots of a plot of `s(bl_age)`, `s(bl_age):sleep_z`, `s(bl_age):time`, and `s(sleep_z):time`. For `s(sleep_z):time`, roughly, if we cannot fit a straight line through the confidence bands, there is evidence that the effect of time depends on sleep duration. It is correct the that confidence interval is zero at one point along the axis; this happens because we do not include the main effect.

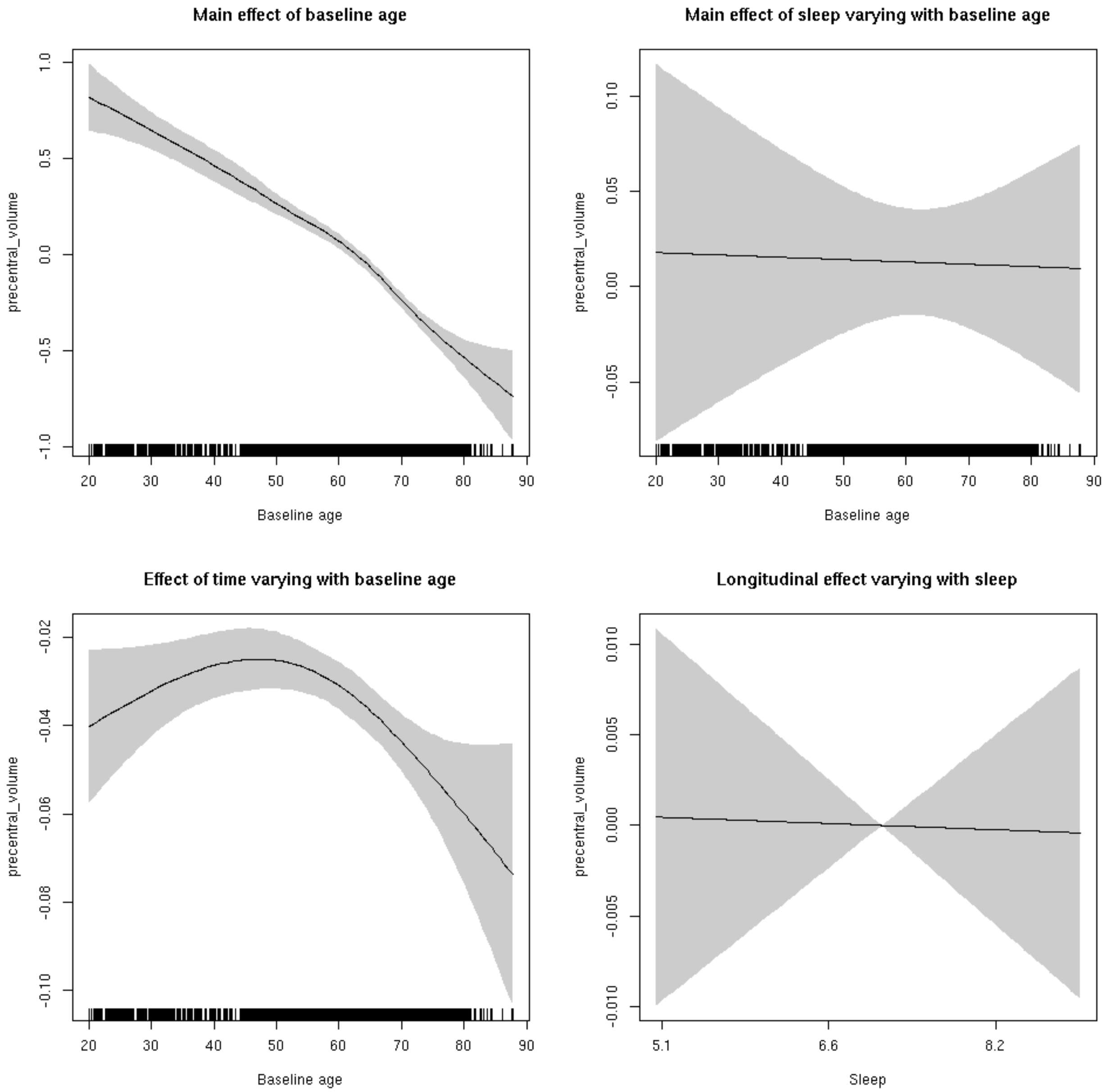

Restricted to sleep >= 5 and <= 9 hours Below is the row for `s(sleep_z):time` from the model output.

```
## s(sleep_z):time   1.000  1.000   0.008   0.929
```

Below are plots of a plot of `s(bl_age)`, `s(bl_age):sleep_z`, `s(bl_age):time`, and `s(sleep_z):time`. For `s(sleep_z):time`, roughly, if we cannot fit a straight line through the confidence bands, there is evidence that the effect of time depends on sleep duration. It is correct the that confidence interval is zero at one point along the axis; this happens because we do not include the main effect.

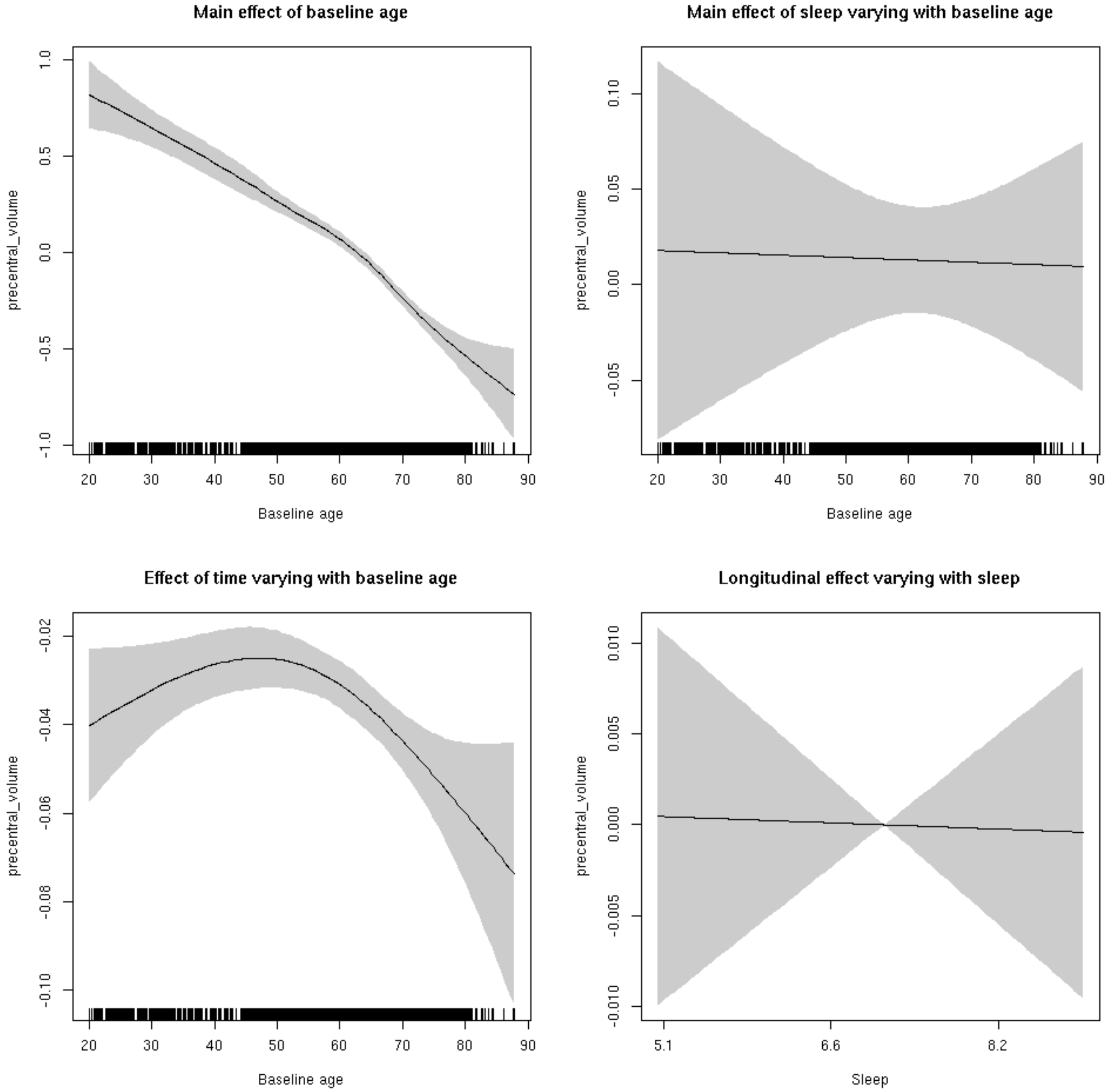

precuneus_area: Stratified by sex

Female: Full data Below is the row for `s(sleep_z):time` from the model output.

```
## s(sleep_z):time   1.000  1.000   0.002   0.962
```

Below are plots of a plot of `s(bl_age)`, `s(bl_age):sleep_z`, `s(bl_age):time`, and `s(sleep_z):time`. For `s(sleep_z):time`, roughly, if we cannot fit a straight line through the confidence bands, there is evidence that the effect of time depends on sleep duration. It is correct the that confidence interval is zero at one point along the axis; this happens because we do not include the main effect.

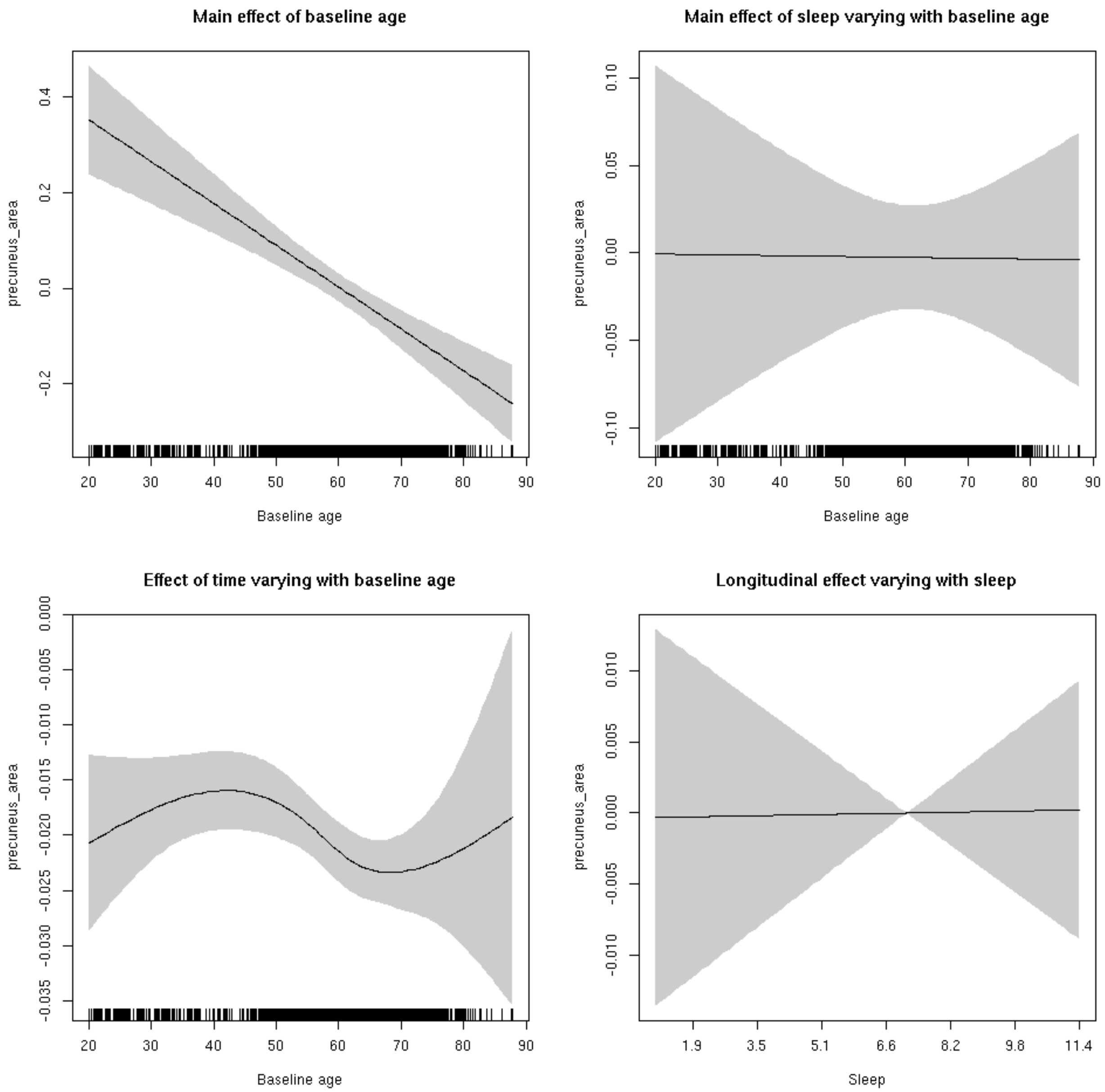

Male: Full data Below is the row for `s(sleep_z):time` from the model output.

```
## s(sleep_z):time   1.000  1.000   0.244   0.621
```

Below are plots of a plot of `s(bl_age)`, `s(bl_age):sleep_z`, `s(bl_age):time`, and `s(sleep_z):time`. For `s(sleep_z):time`, roughly, if we cannot fit a straight line through the confidence bands, there is evidence that the effect of time depends on sleep duration. It is correct the that confidence interval is zero at one point along the axis; this happens because we do not include the main effect.

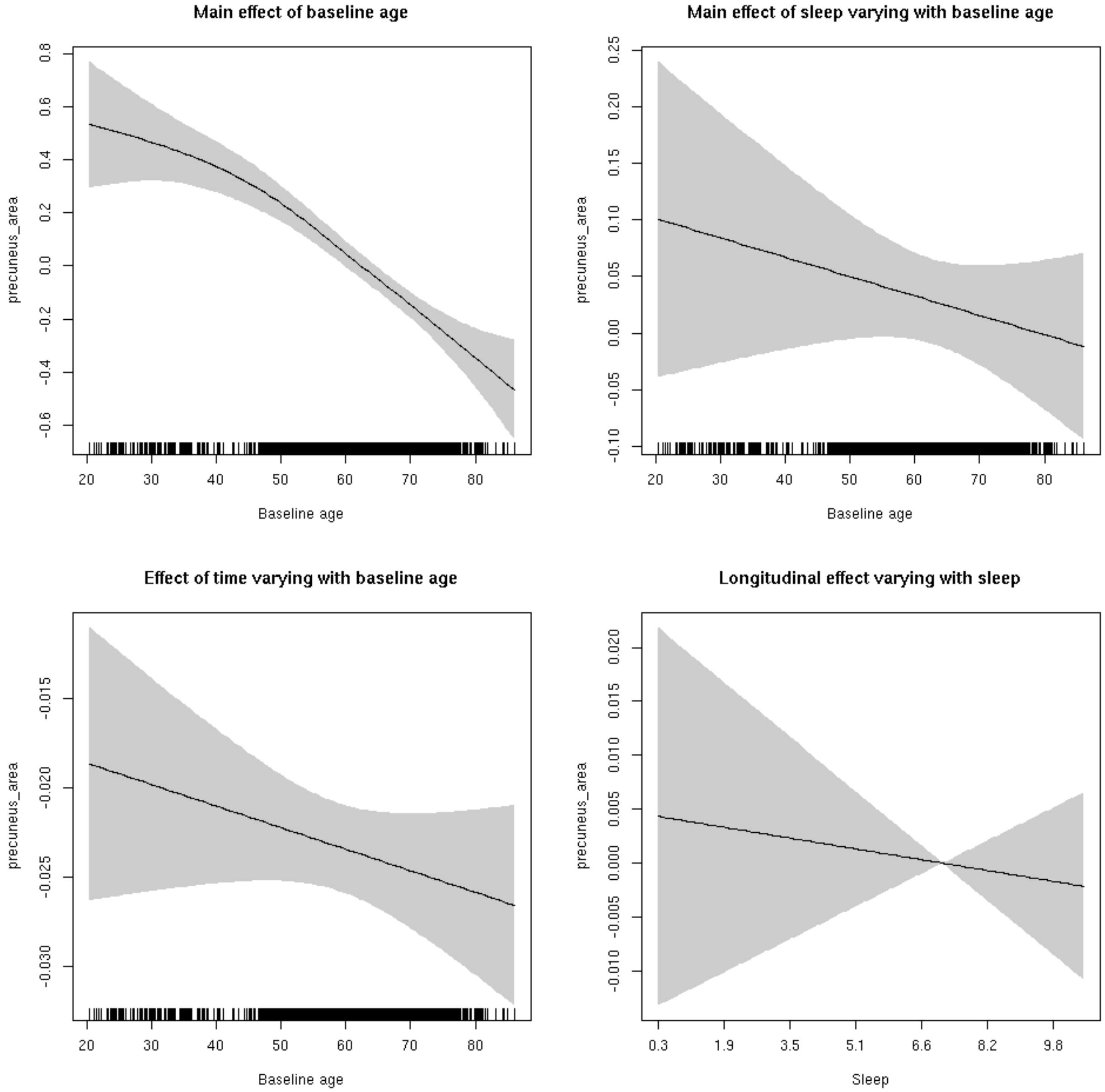

precuneus_area

Full data Below is the model output. The term `s(sleep_z):time` is probably what we care most about.

```
##
## Family: gaussian
## Link function: identity
##
## Formula:
## value ~ sex + site + icv + s(bl_age, k = 10, bs = "cr") + s(bl_age,
##     by = sleep_z, bs = "cr") + +s(bl_age, by = time, k = 5, bs = "cr") +
##     s(sleep_z, by = time, k = 5, bs = "cr", pc = 0)
## <environment: 0x5630035b2808>
```

```
##
## Parametric coefficients:
##                 Estimate Std. Error t value Pr(>|t|)
## (Intercept)      0.64311    0.05316  12.098  < 2e-16 ***
## sexmale          0.17077    0.02760   6.188 6.40e-10 ***
## siteousAvanto -0.81762    0.06534 -12.513  < 2e-16 ***
## siteousPrisma -0.76171    0.10210  -7.461 9.50e-14 ***
## siteousSkyra  -0.32531    0.06337  -5.133 2.91e-07 ***
## siteUB        -0.68707    0.12500  -5.497 3.99e-08 ***
## siteUCAM      -0.76422    0.06649 -11.494  < 2e-16 ***
## siteUKB       -0.69782    0.04979 -14.014  < 2e-16 ***
## siteUmU       -0.21071    0.07691  -2.740  0.00617 **
## icv            0.60946    0.01325  46.008  < 2e-16 ***
## ---
## Signif. codes:  0 ‘***’ 0.001 ‘**’ 0.01 ‘*’ 0.05 ‘.’ 0.1 ‘ ’ 1
##
## Approximate significance of smooth terms:
##                      edf Ref.df       F p-value
## s(bl_age)          1.128  1.128 121.930  <2e-16 ***
## s(bl_age):sleep_z 2.000  2.000   0.583   0.558
## s(bl_age):time    2.000  2.000 380.140  <2e-16 ***
## s(sleep_z):time   1.000  1.000   0.345   0.557
## ---
## Signif. codes:  0 ‘***’ 0.001 ‘**’ 0.01 ‘*’ 0.05 ‘.’ 0.1 ‘ ’ 1
##
## R-sq.(adj) =  0.491
## lmer.REML = 3650.2  Scale est. = 0.0098032  n = 8155
```

Below are plots of a plot of `s(bl_age)`, `s(bl_age):sleep_z`, `s(bl_age):time`, and `s(sleep_z):time`. For `s(sleep_z):time`, roughly, if we cannot fit a straight line through the confidence bands, there is evidence that the effect of time depends on sleep duration. It is correct the that confidence interval is zero at one point along the axis; this happens because we do not include the main effect.

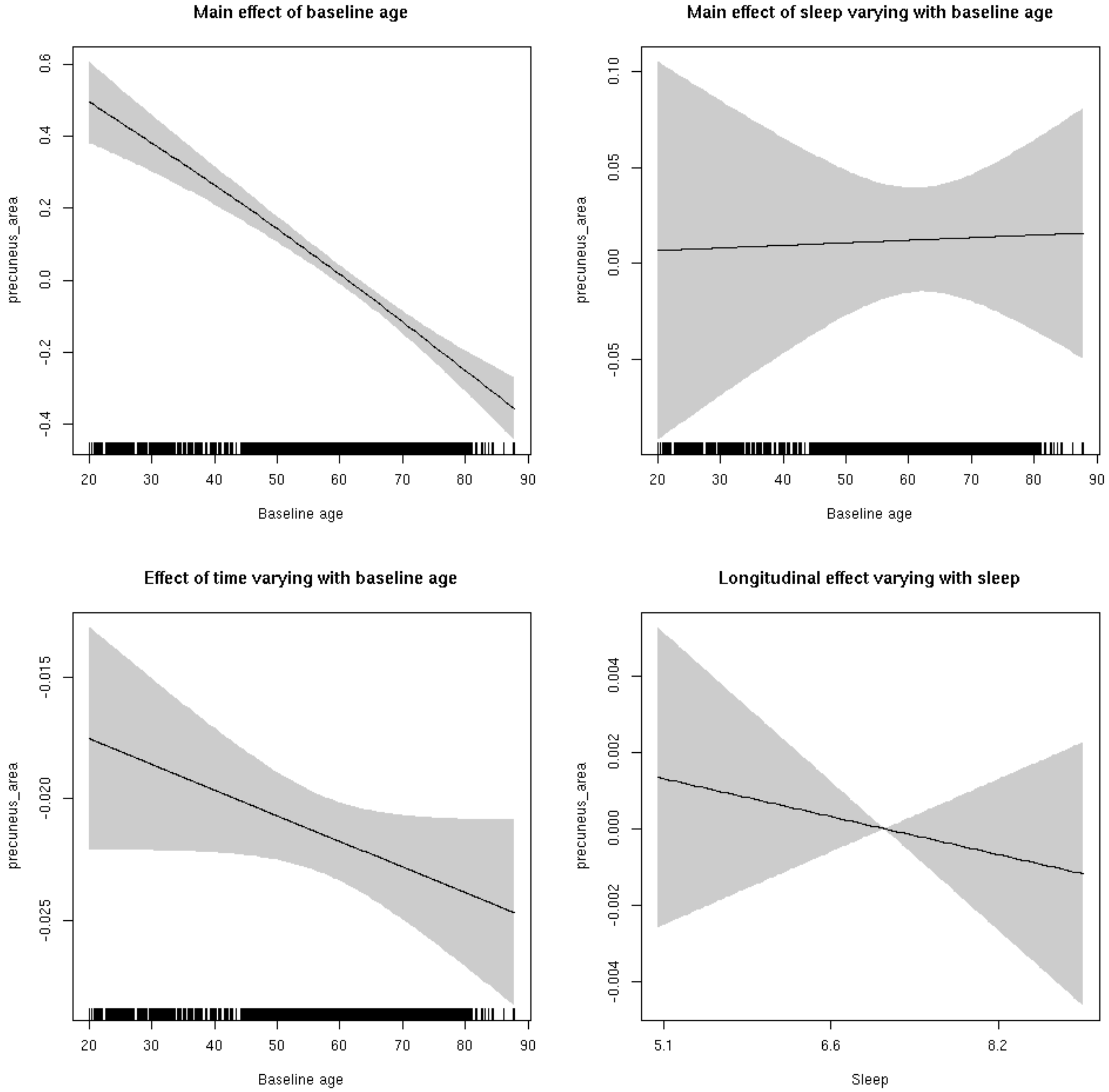

Restricted to sleep >= 5 and <= 9 hours Below is the row for `s(sleep_z):time` from the model output.

```
## s(sleep_z):time   1.000  1.000   0.470   0.493
```

Below are plots of a plot of `s(bl_age)`, `s(bl_age):sleep_z`, `s(bl_age):time`, and `s(sleep_z):time`. For `s(sleep_z):time`, roughly, if we cannot fit a straight line through the confidence bands, there is evidence that the effect of time depends on sleep duration. It is correct the that confidence interval is zero at one point along the axis; this happens because we do not include the main effect.

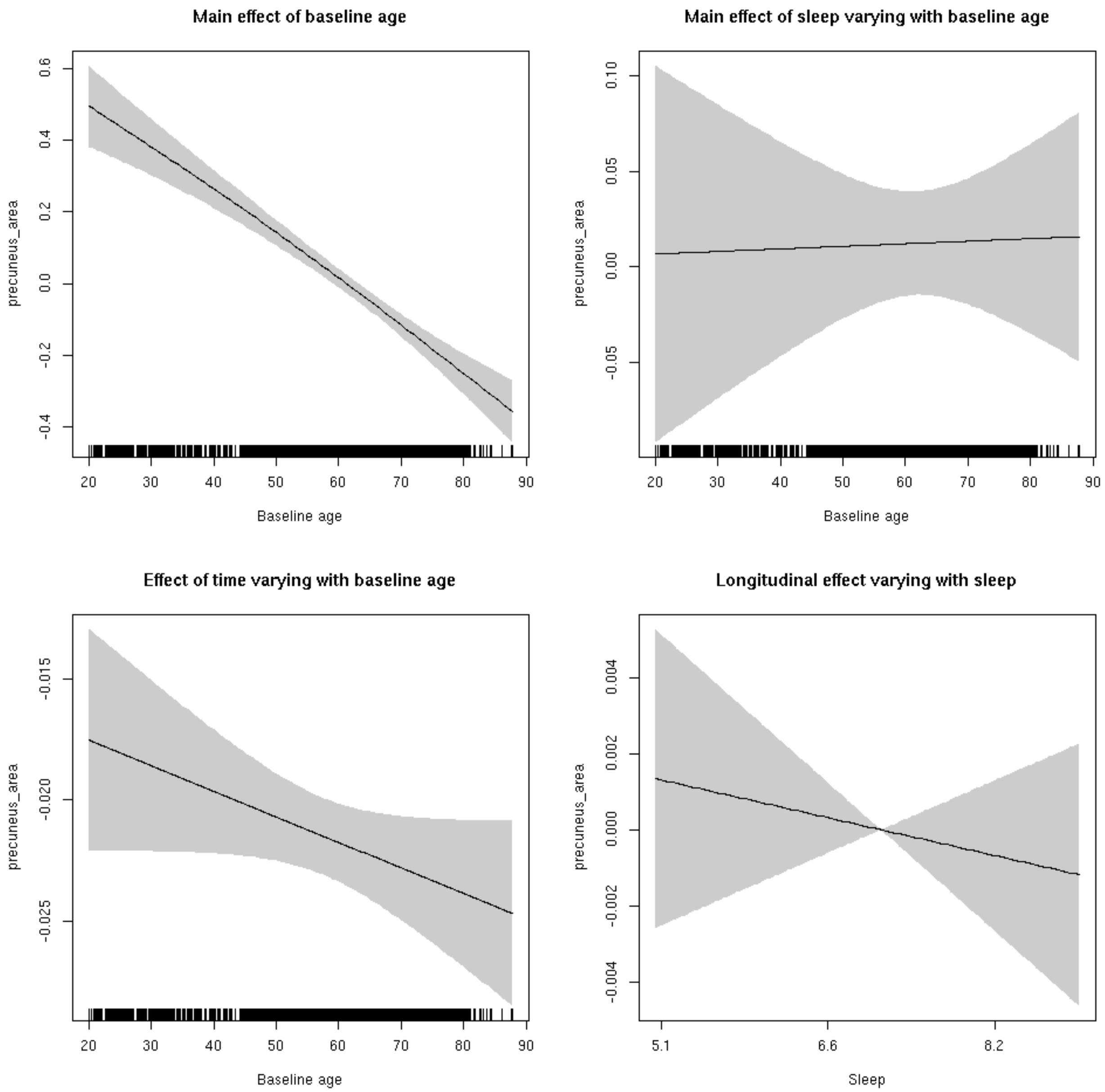

precuneus_thickness: Stratified by sex

Female: Full data Below is the row for `s(sleep_z):time` from the model output.

```
## s(sleep_z):time   1.000  1.000   0.01   0.919
```

Below are plots of a plot of `s(bl_age)`, `s(bl_age):sleep_z`, `s(bl_age):time`, and `s(sleep_z):time`. For `s(sleep_z):time`, roughly, if we cannot fit a straight line through the confidence bands, there is evidence that the effect of time depends on sleep duration. It is correct the that confidence interval is zero at one point along the axis; this happens because we do not include the main effect.

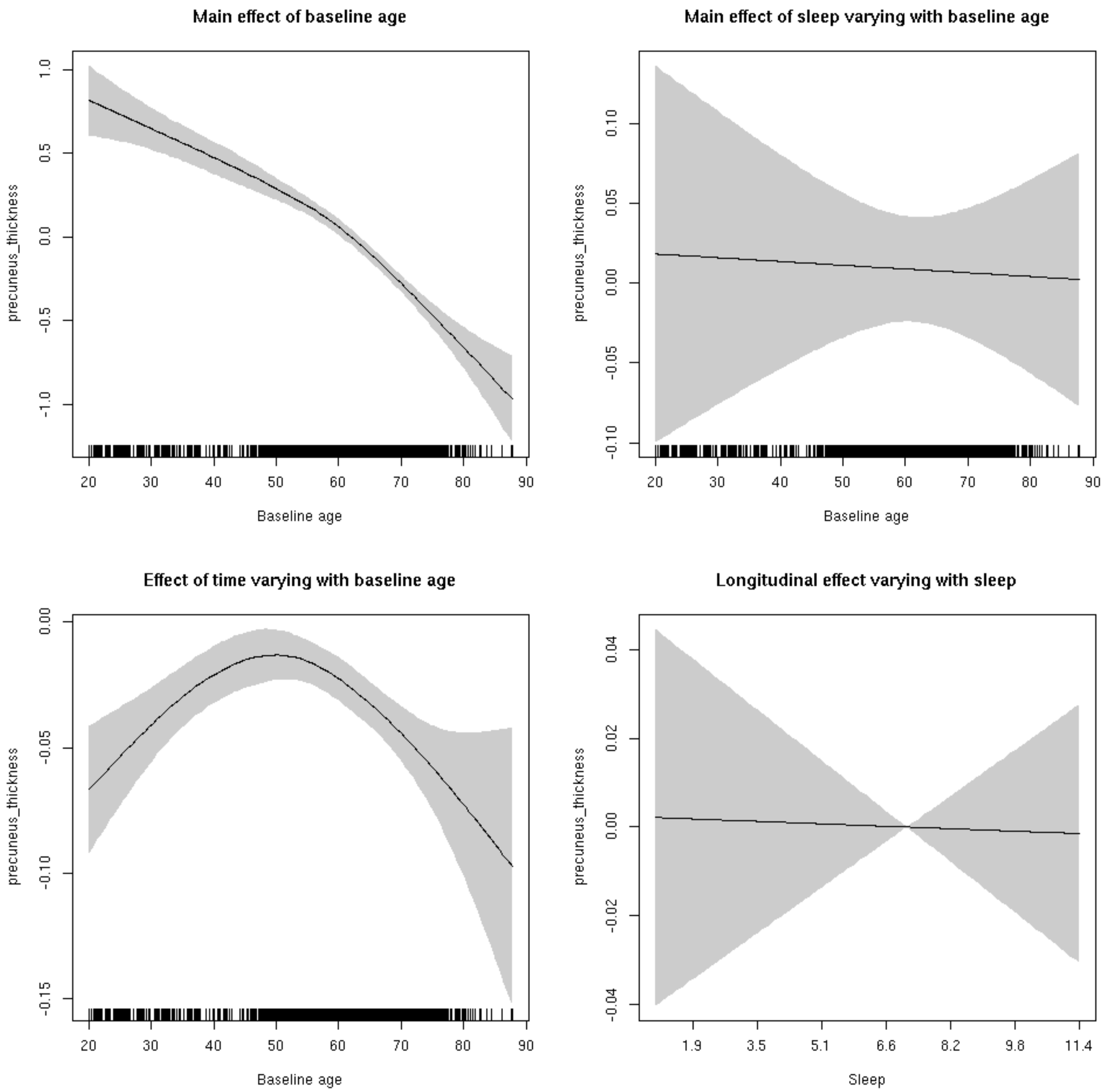

Male: Full data Below is the row for `s(sleep_z):time` from the model output.

```
## s(sleep_z):time   1.000  1.000  0.437   0.509
```

Below are plots of a plot of `s(bl_age)`, `s(bl_age):sleep_z`, `s(bl_age):time`, and `s(sleep_z):time`. For `s(sleep_z):time`, roughly, if we cannot fit a straight line through the confidence bands, there is evidence that the effect of time depends on sleep duration. It is correct the that confidence interval is zero at one point along the axis; this happens because we do not include the main effect.

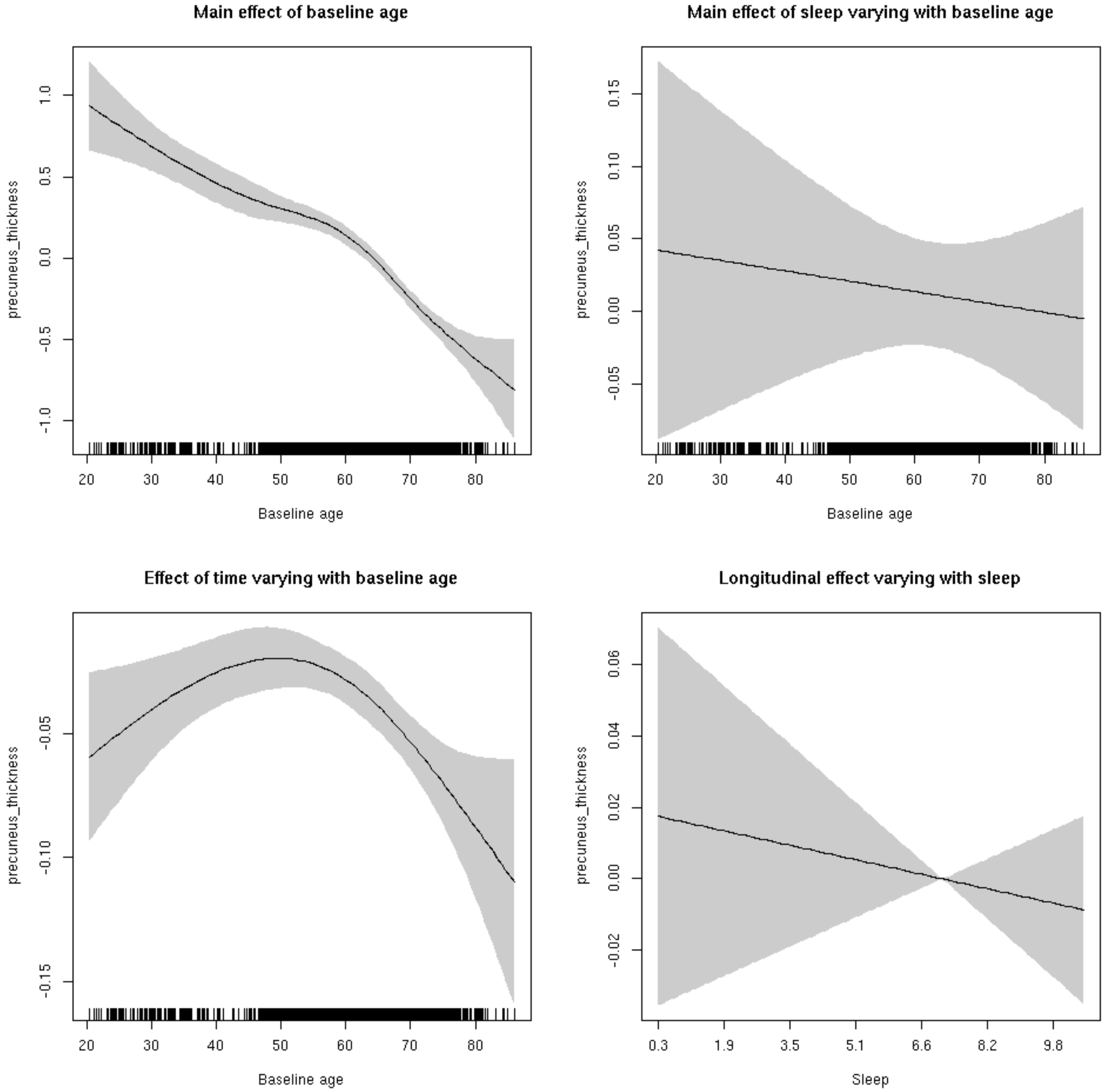

precuneus_thickness

Full data Below is the model output. The term `s(sleep_z):time` is probably what we care most about.

```
##
## Family: gaussian
## Link function: identity
##
## Formula:
## value ~ sex + site + s(bl_age, k = 10, bs = "cr") + s(bl_age,
##     by = sleep_z, bs = "cr") + +s(bl_age, by = time, k = 5, bs = "cr") +
##     s(sleep_z, by = time, k = 5, bs = "cr", pc = 0)
## <environment: 0x5630035dc850>
```

```
##
## Parametric coefficients:
##                Estimate Std. Error t value Pr(>|t|)
## (Intercept)   -1.65885    0.04605 -36.022  < 2e-16 ***
## sexmale       -0.01686    0.02309  -0.730 0.465350
## siteousAvanto -0.09729    0.06091  -1.597 0.110237
## siteousPrisma  0.35468    0.23909   1.483 0.137993
## siteousSkyra   0.04219    0.06089   0.693 0.488389
## siteUB         0.44062    0.12254   3.596 0.000325 ***
## siteUCAM       0.41549    0.06343   6.550  6.1e-11 ***
## siteUKB        1.93211    0.04672  41.359  < 2e-16 ***
## siteUmU        0.27052    0.07590   3.564 0.000367 ***
## ---
## Signif. codes:  0 '***' 0.001 '**' 0.01 '*' 0.05 '.' 0.1 ' ' 1
##
## Approximate significance of smooth terms:
##                      edf Ref.df       F p-value
## s(bl_age)          3.831  3.831 163.975  <2e-16 ***
## s(bl_age):sleep_z 2.000  2.000   0.429   0.651
## s(bl_age):time    3.835  3.835  42.729  <2e-16 ***
## s(sleep_z):time   2.887  2.887   2.269   0.116
## ---
## Signif. codes:  0 '***' 0.001 '**' 0.01 '*' 0.05 '.' 0.1 ' ' 1
##
## R-sq.(adj) =  0.579
## lmer.REML =  13373  Scale est. = 0.095679  n = 8170
```

Below are plots of a plot of `s(bl_age)`, `s(bl_age):sleep_z`, `s(bl_age):time`, and `s(sleep_z):time`. For `s(sleep_z):time`, roughly, if we cannot fit a straight line through the confidence bands, there is evidence that the effect of time depends on sleep duration. It is correct the that confidence interval is zero at one point along the axis; this happens because we do not include the main effect.

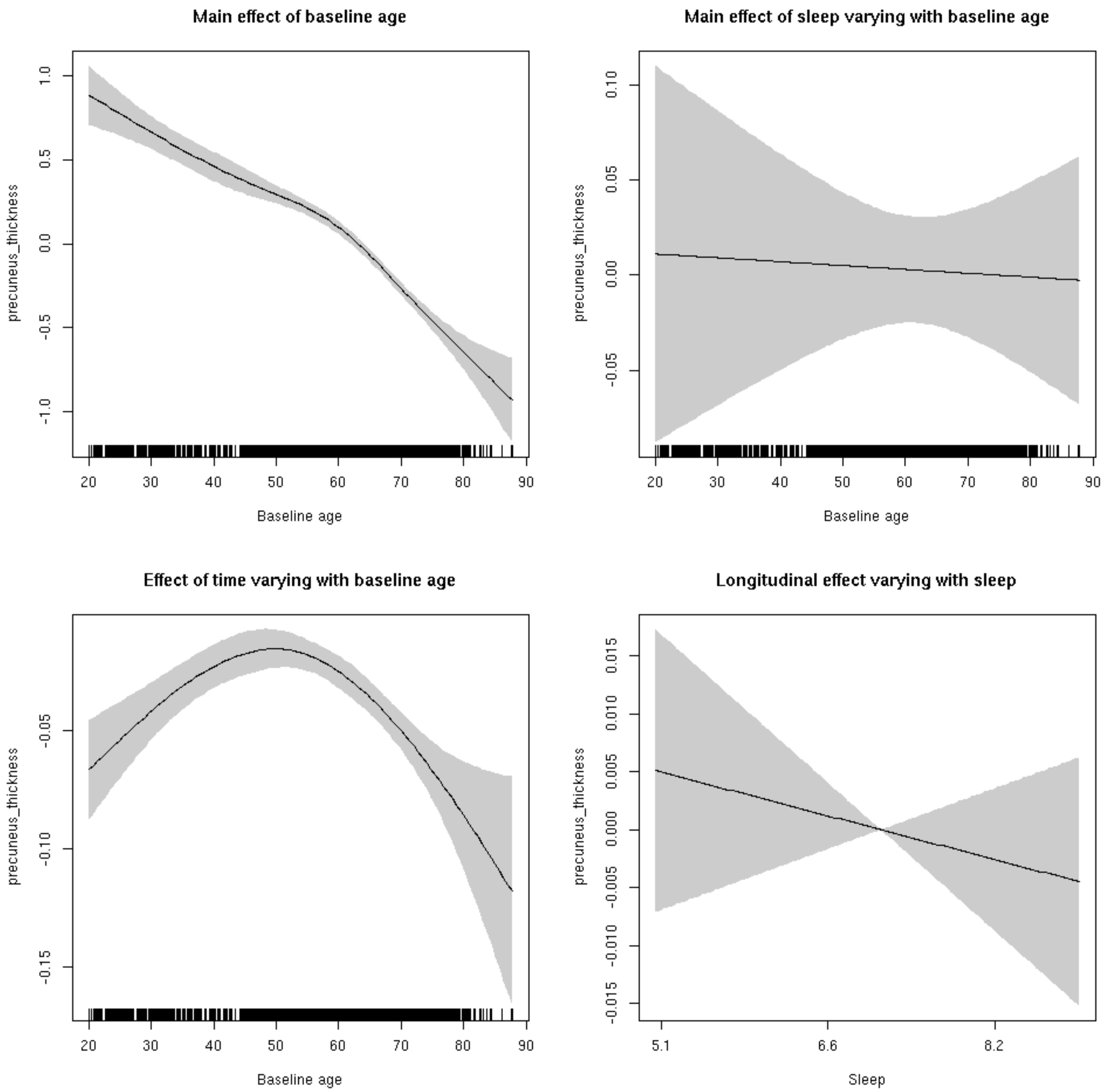

Restricted to sleep >= 5 and <= 9 hours Below is the row for `s(sleep_z):time` from the model output.

```
## s(sleep_z):time   1.000  1.000   0.701   0.403
```

Below are plots of a plot of `s(bl_age)`, `s(bl_age):sleep_z`, `s(bl_age):time`, and `s(sleep_z):time`. For `s(sleep_z):time`, roughly, if we cannot fit a straight line through the confidence bands, there is evidence that the effect of time depends on sleep duration. It is correct the that confidence interval is zero at one point along the axis; this happens because we do not include the main effect.

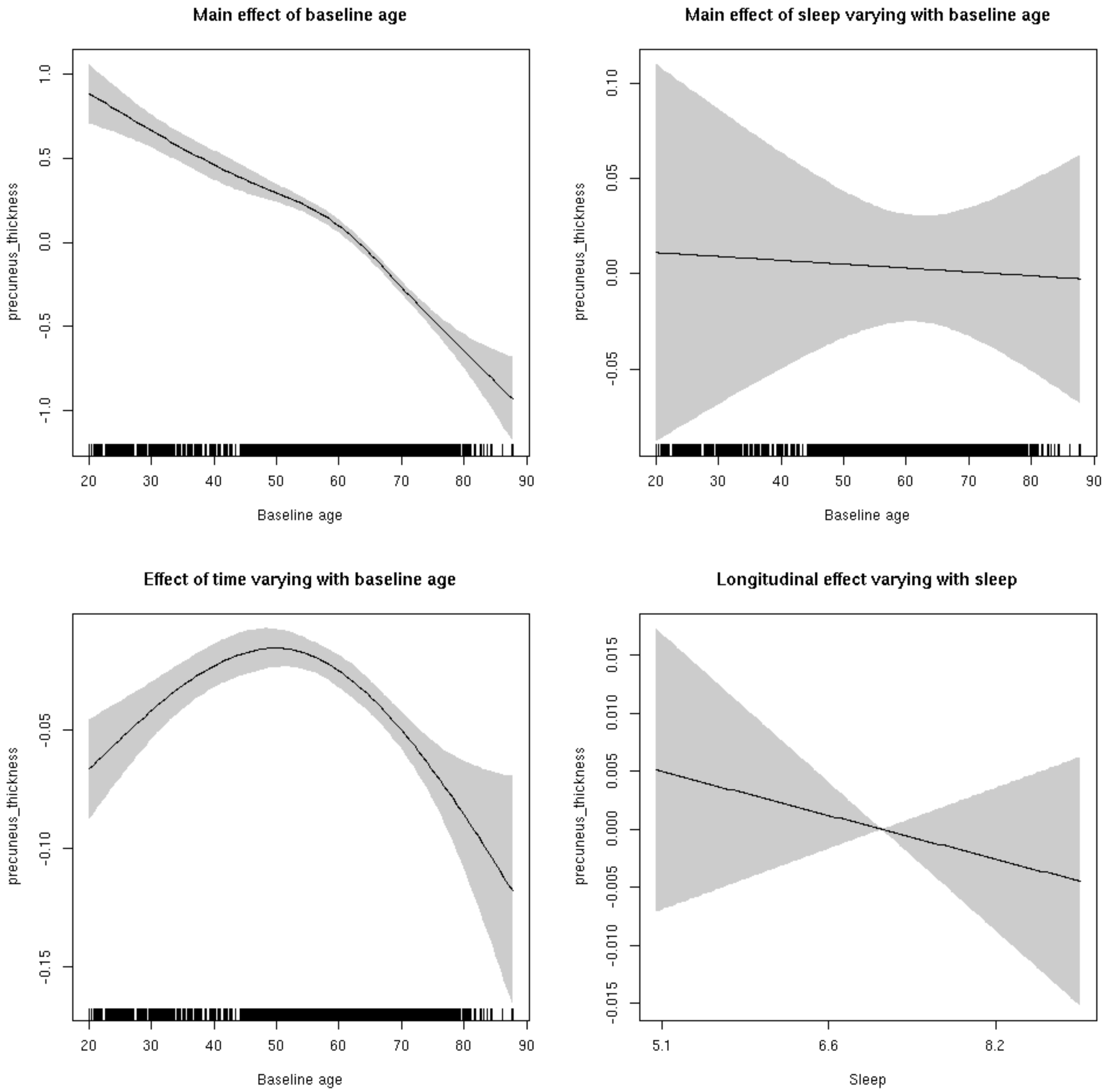

precuneus_volume: Stratified by sex

Female: Full data Below is the row for `s(sleep_z):time` from the model output.

```
## s(sleep_z):time   1.000  1.000   0.047   0.828
```

Below are plots of a plot of `s(bl_age)`, `s(bl_age):sleep_z`, `s(bl_age):time`, and `s(sleep_z):time`. For `s(sleep_z):time`, roughly, if we cannot fit a straight line through the confidence bands, there is evidence that the effect of time depends on sleep duration. It is correct the that confidence interval is zero at one point along the axis; this happens because we do not include the main effect.

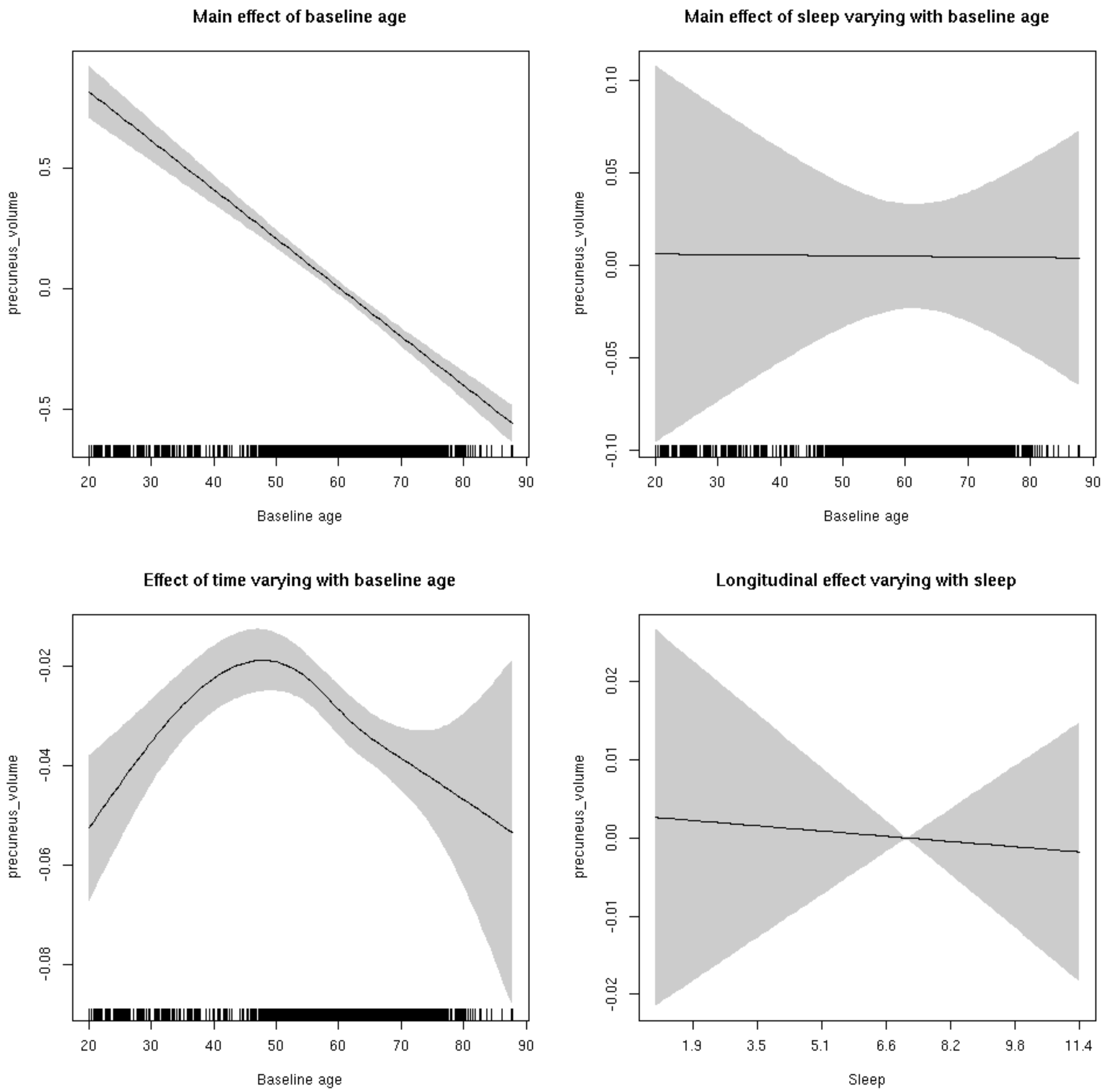

Male: Full data Below is the row for `s(sleep_z):time` from the model output.

```
## s(sleep_z):time   1.000  1.000   0.741  0.3894
```

Below are plots of a plot of `s(bl_age)`, `s(bl_age):sleep_z`, `s(bl_age):time`, and `s(sleep_z):time`. For `s(sleep_z):time`, roughly, if we cannot fit a straight line through the confidence bands, there is evidence that the effect of time depends on sleep duration. It is correct the that confidence interval is zero at one point along the axis; this happens because we do not include the main effect.

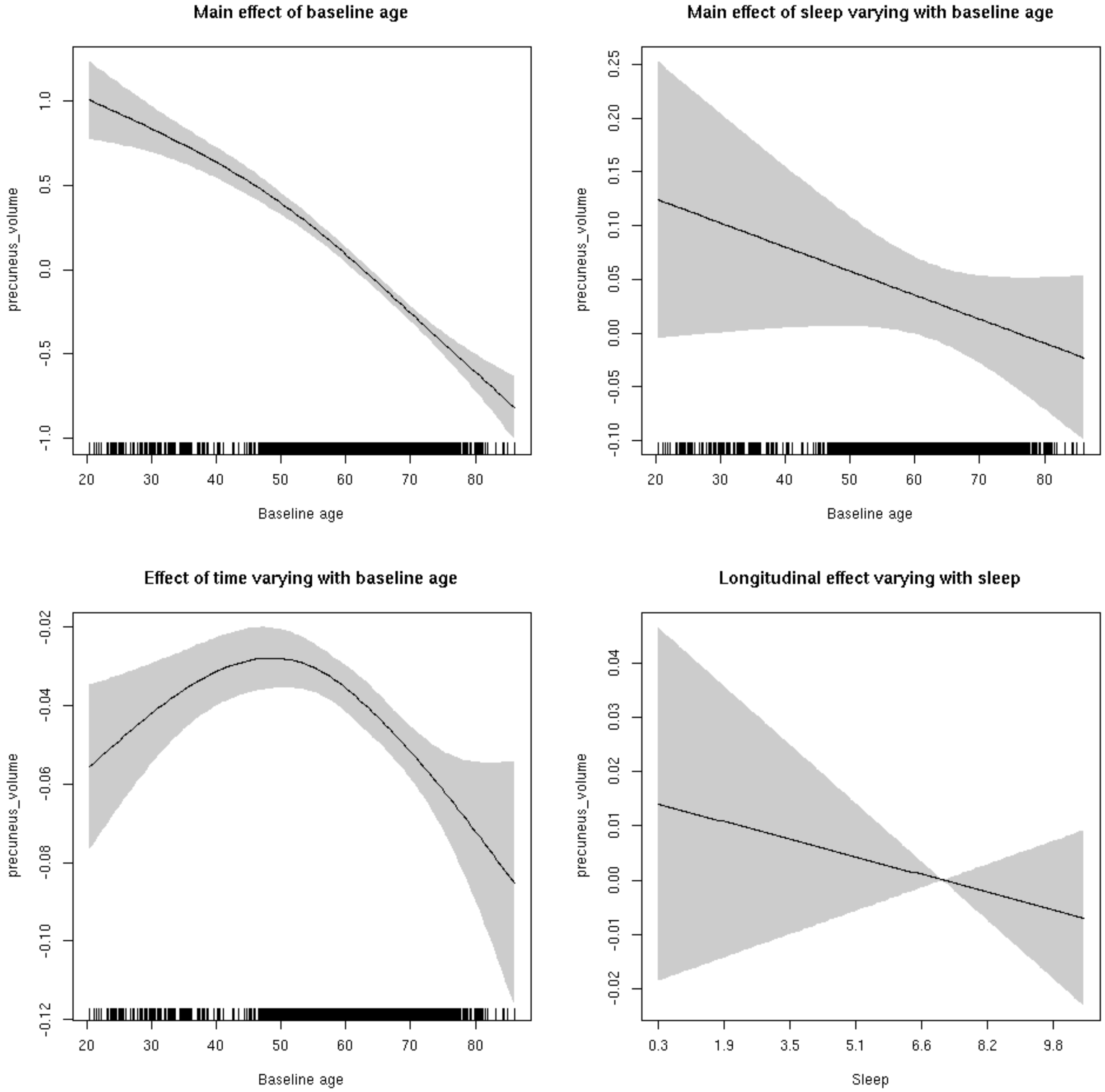

precuneus_volume

Full data Below is the model output. The term `s(sleep_z):time` is probably what we care most about.

```
##
## Family: gaussian
## Link function: identity
##
## Formula:
## value ~ sex + site + icv + s(bl_age, k = 10, bs = "cr") + s(bl_age,
##     by = sleep_z, bs = "cr") + +s(bl_age, by = time, k = 5, bs = "cr") +
##     s(sleep_z, by = time, k = 5, bs = "cr", pc = 0)
## <environment: 0x5630035b4d48>
```

```
##
## Parametric coefficients:
##                Estimate Std. Error t value Pr(>|t|)
## (Intercept)   -0.08801    0.05079  -1.733 0.083169 .
## sexmale        0.09852    0.02614   3.769 0.000165 ***
## siteousAvanto -0.93313    0.06211 -15.023  < 2e-16 ***
## siteousPrisma -0.63237    0.15308  -4.131 3.65e-05 ***
## siteousSkyra  -0.26754    0.06014  -4.449 8.75e-06 ***
## siteUB        -0.42637    0.11689  -3.648 0.000266 ***
## siteUCAM      -0.58089    0.06284  -9.244  < 2e-16 ***
## siteUKB        0.19784    0.04809   4.114 3.93e-05 ***
## siteUmU       -0.09230    0.07247  -1.274 0.202855
## icv            0.61257    0.01287  47.588  < 2e-16 ***
## ---
## Signif. codes:  0 '***' 0.001 '**' 0.01 '*' 0.05 '.' 0.1 ' ' 1
##
## Approximate significance of smooth terms:
##                      edf Ref.df       F p-value
## s(bl_age)          2.480  2.480 242.279  <2e-16 ***
## s(bl_age):sleep_z 2.000  2.000   1.590   0.204
## s(bl_age):time    3.917  3.917 168.874  <2e-16 ***
## s(sleep_z):time   1.000  1.000   0.717   0.397
## ---
## Signif. codes:  0 '***' 0.001 '**' 0.01 '*' 0.05 '.' 0.1 ' ' 1
##
## R-sq.(adj) =  0.575
## lmer.REML = 8338.9  Scale est. = 0.033091  n = 8167
```

Below are plots of a plot of `s(bl_age)`, `s(bl_age):sleep_z`, `s(bl_age):time`, and `s(sleep_z):time`. For `s(sleep_z):time`, roughly, if we cannot fit a straight line through the confidence bands, there is evidence that the effect of time depends on sleep duration. It is correct the that confidence interval is zero at one point along the axis; this happens because we do not include the main effect.

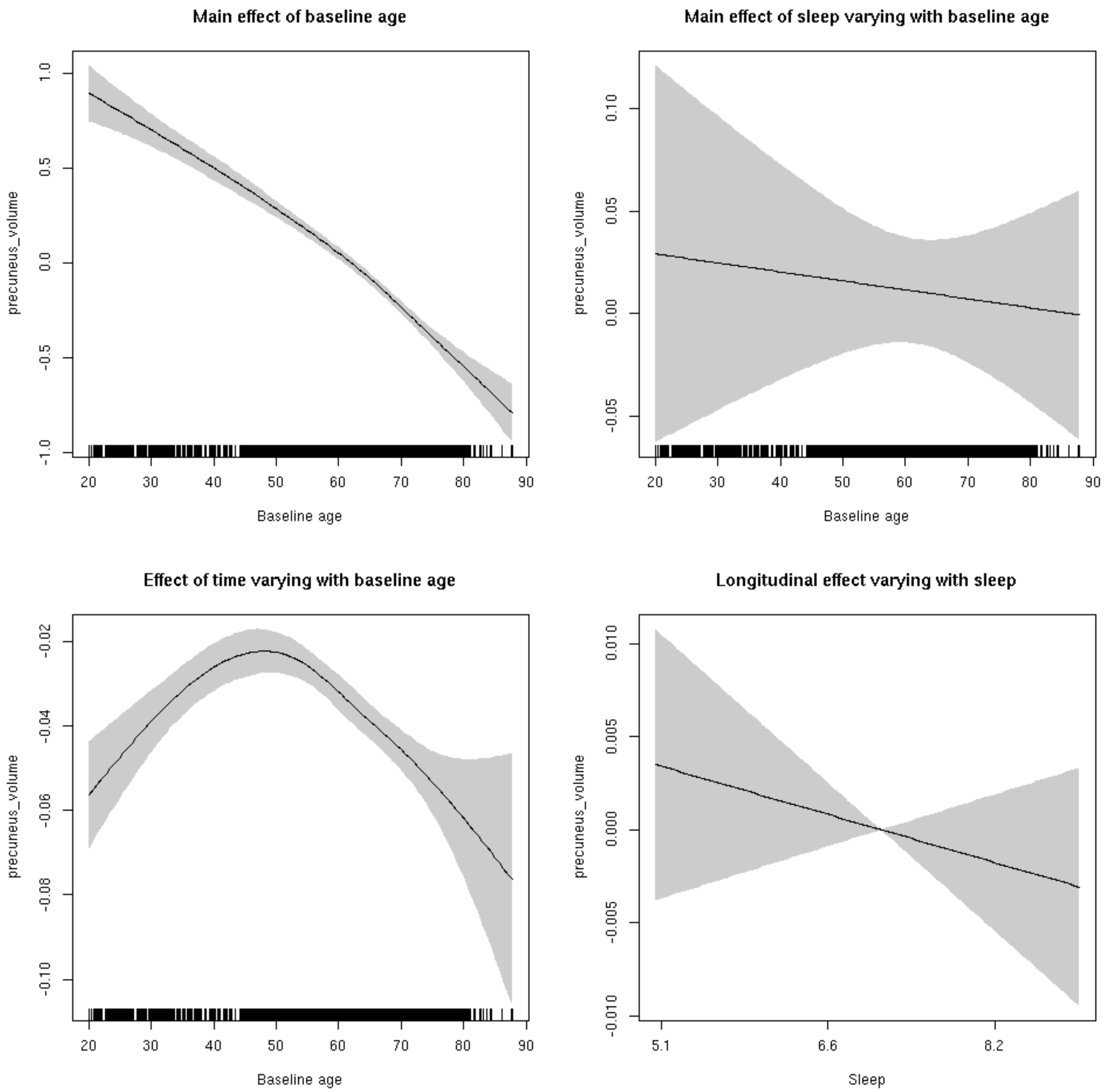

Restricted to sleep >= 5 and <= 9 hours Below is the row for `s(sleep_z):time` from the model output.

```
## s(sleep_z):time   1.000  1.000   0.923   0.337
```

Below are plots of a plot of `s(bl_age)`, `s(bl_age):sleep_z`, `s(bl_age):time`, and `s(sleep_z):time`. For `s(sleep_z):time`, roughly, if we cannot fit a straight line through the confidence bands, there is evidence that the effect of time depends on sleep duration. It is correct the that confidence interval is zero at one point along the axis; this happens because we do not include the main effect.

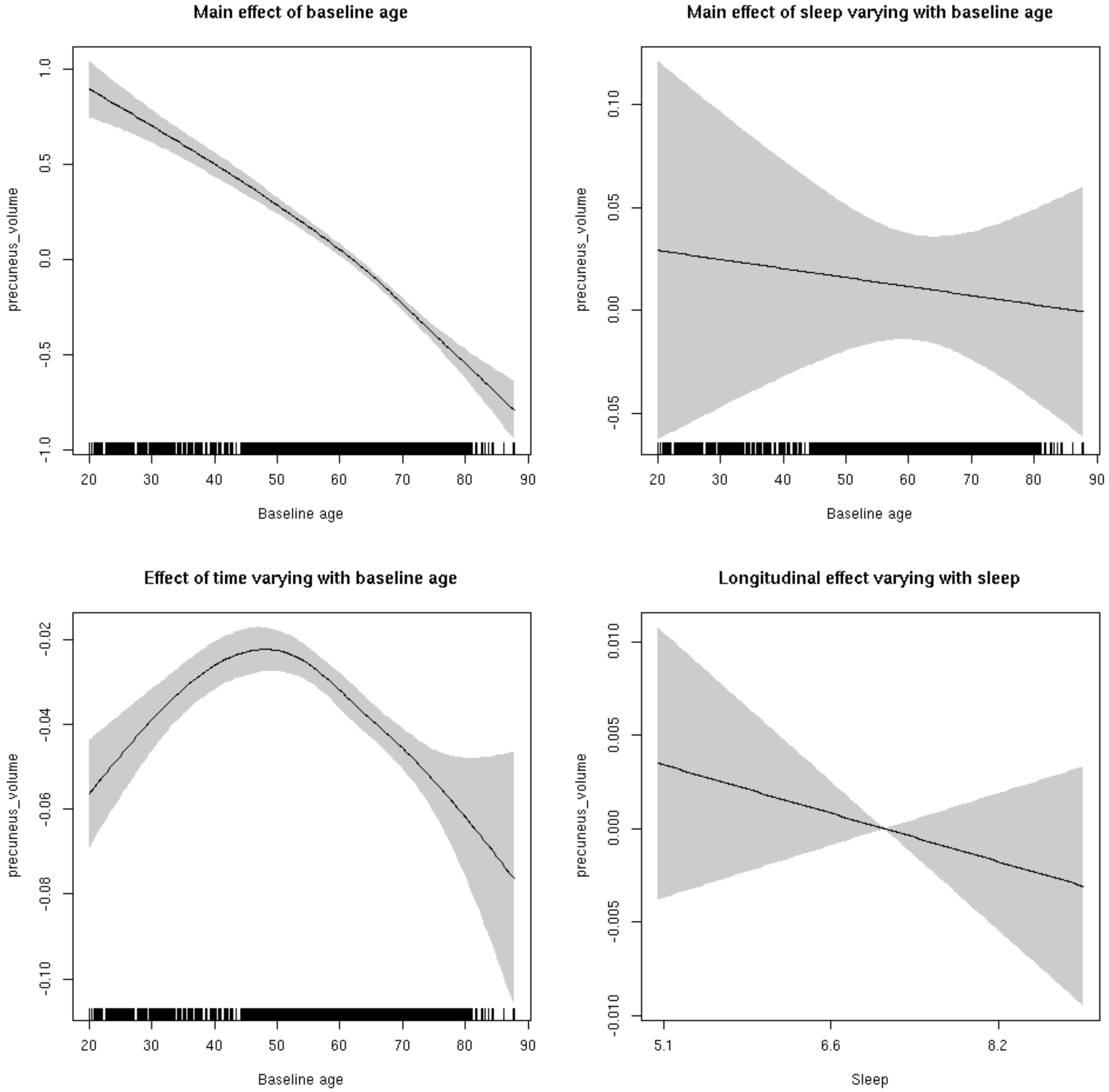

rostralanteriorcingulate_area: Stratified by sex

Female: Full data Below is the row for `s(sleep_z):time` from the model output.

```
## s(sleep_z):time   1.000  1.000  0.602 0.437819
```

Below are plots of a plot of `s(bl_age)`, `s(bl_age):sleep_z`, `s(bl_age):time`, and `s(sleep_z):time`. For `s(sleep_z):time`, roughly, if we cannot fit a straight line through the confidence bands, there is evidence that the effect of time depends on sleep duration. It is correct the that confidence interval is zero at one point along the axis; this happens because we do not include the main effect.

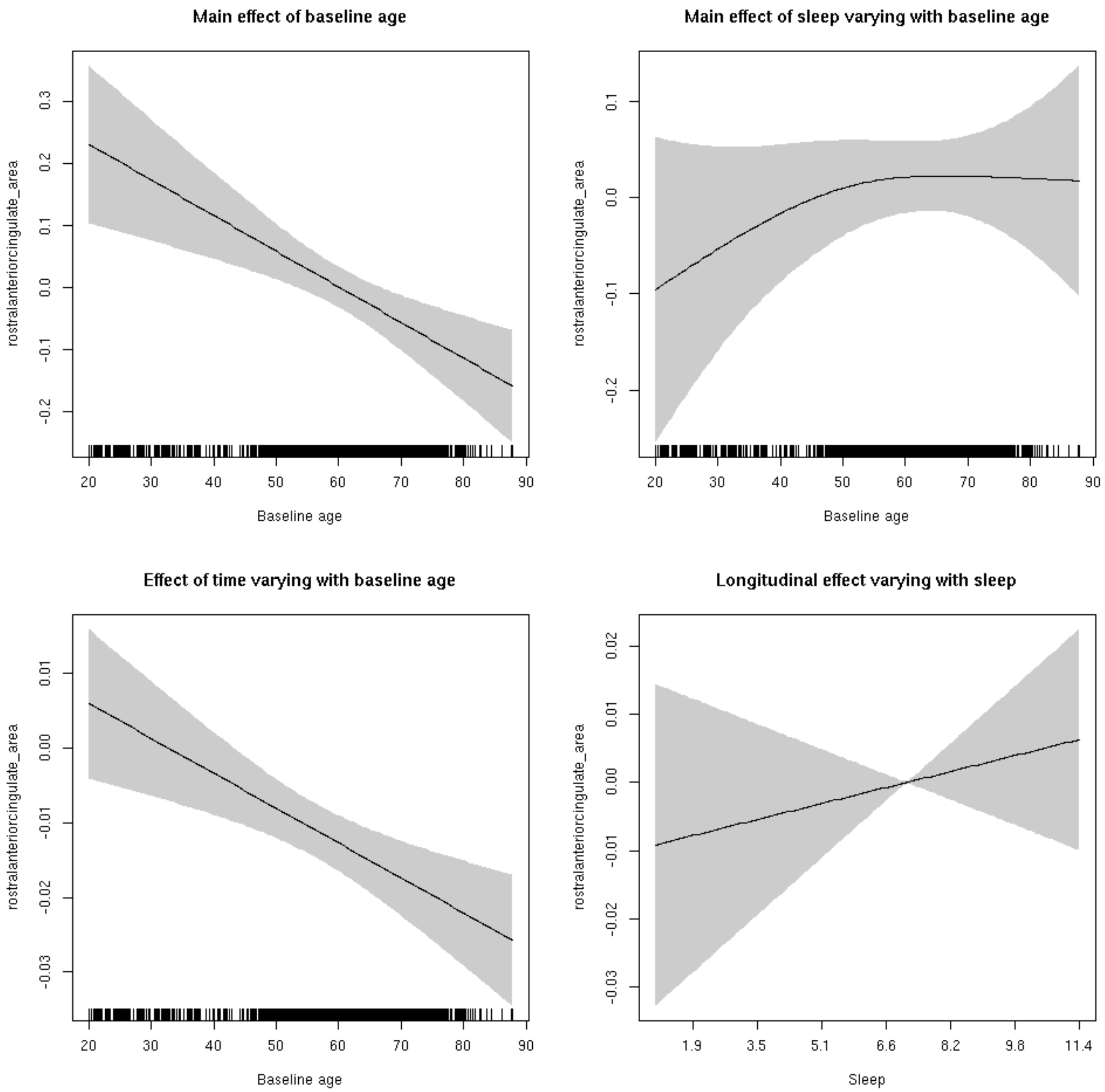

Male: Full data Below is the row for `s(sleep_z):time` from the model output.

```
## s(sleep_z):time   1.000  1.000  0.026    0.873
```

Below are plots of a plot of `s(bl_age)`, `s(bl_age):sleep_z`, `s(bl_age):time`, and `s(sleep_z):time`. For `s(sleep_z):time`, roughly, if we cannot fit a straight line through the confidence bands, there is evidence that the effect of time depends on sleep duration. It is correct the that confidence interval is zero at one point along the axis; this happens because we do not include the main effect.

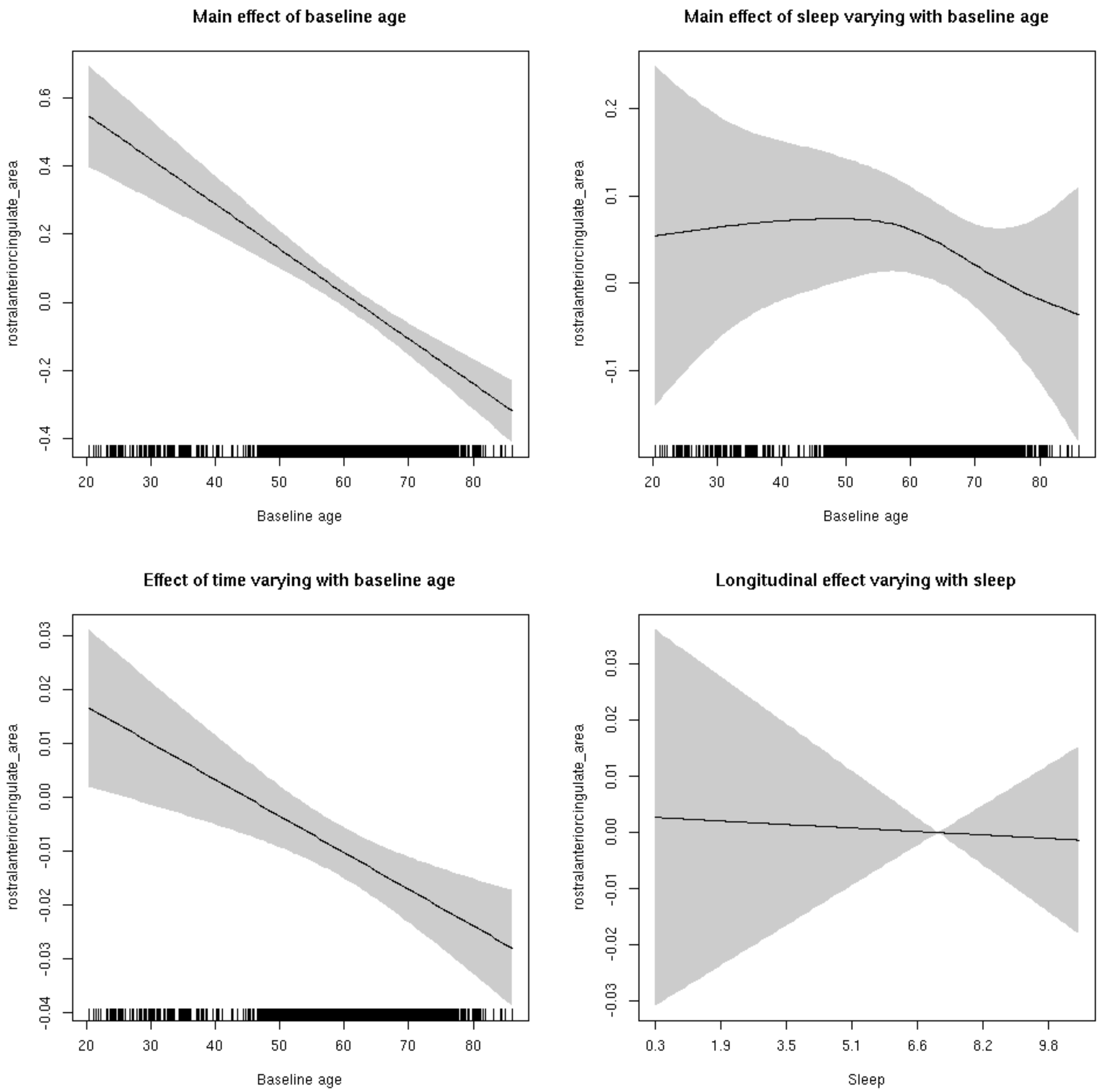

rostralanteriorcingulate_area

Full data Below is the model output. The term `s(sleep_z):time` is probably what we care most about.

```
##
## Family: gaussian
## Link function: identity
##
## Formula:
## value ~ sex + site + icv + s(bl_age, k = 10, bs = "cr") + s(bl_age,
##     by = sleep_z, bs = "cr") + +s(bl_age, by = time, k = 5, bs = "cr") +
##     s(sleep_z, by = time, k = 5, bs = "cr", pc = 0)
## <environment: 0x5630035b26b8>
```

```
##
## Parametric coefficients:
##               Estimate Std. Error t value Pr(>|t|)
## (Intercept)    0.69438    0.05781  12.012  < 2e-16 ***
## sexmale        0.14046    0.02974   4.724 2.35e-06 ***
## siteousAvanto -0.44227    0.07056  -6.268 3.83e-10 ***
## siteousPrisma -1.17272    0.15896  -7.377 1.77e-13 ***
## siteousSkyra  -0.16663    0.06830  -2.440   0.0147 *
## siteUB        -0.54250    0.13321  -4.073 4.69e-05 ***
## siteUCAM      -0.59118    0.07163  -8.253  < 2e-16 ***
## siteUKB       -0.80168    0.05483 -14.620  < 2e-16 ***
## siteUmU       -0.10728    0.08251  -1.300   0.1936
## icv            0.58310    0.01462  39.896  < 2e-16 ***
## ---
## Signif. codes:  0 '***' 0.001 '**' 0.01 '*' 0.05 '.' 0.1 ' ' 1
##
## Approximate significance of smooth terms:
##                     edf Ref.df      F p-value
## s(bl_age)         2.790  2.790 22.265  <2e-16 ***
## s(bl_age):sleep_z 3.123  3.123  2.320   0.075 .
## s(bl_age):time    2.000  2.000 38.697  <2e-16 ***
## s(sleep_z):time   1.000  1.000  0.132   0.717
## ---
## Signif. codes:  0 '***' 0.001 '**' 0.01 '*' 0.05 '.' 0.1 ' ' 1
##
## R-sq.(adj) =   0.44
## lmer.REML = 9445.7  Scale est. = 0.033994  n = 8160
```

Below are plots of a plot of `s(bl_age)`, `s(bl_age):sleep_z`, `s(bl_age):time`, and `s(sleep_z):time`. For `s(sleep_z):time`, roughly, if we cannot fit a straight line through the confidence bands, there is evidence that the effect of time depends on sleep duration. It is correct the that confidence interval is zero at one point along the axis; this happens because we do not include the main effect.

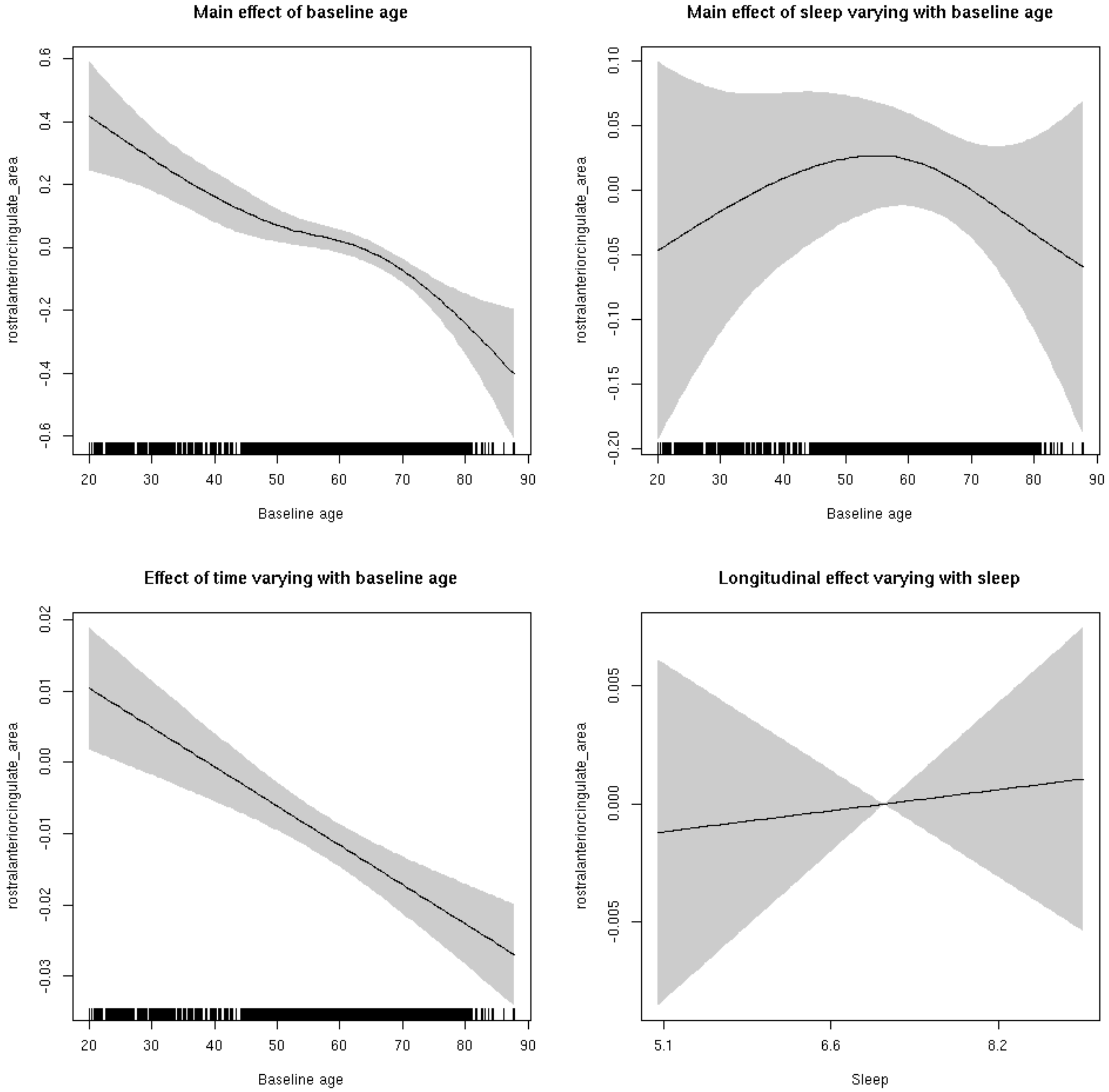

Restricted to sleep >= 5 and <= 9 hours Below is the row for `s(sleep_z):time` from the model output.

```
## s(sleep_z):time   1.000  1.000  0.108   0.742
```

Below are plots of a plot of `s(bl_age)`, `s(bl_age):sleep_z`, `s(bl_age):time`, and `s(sleep_z):time`. For `s(sleep_z):time`, roughly, if we cannot fit a straight line through the confidence bands, there is evidence that the effect of time depends on sleep duration. It is correct the that confidence interval is zero at one point along the axis; this happens because we do not include the main effect.

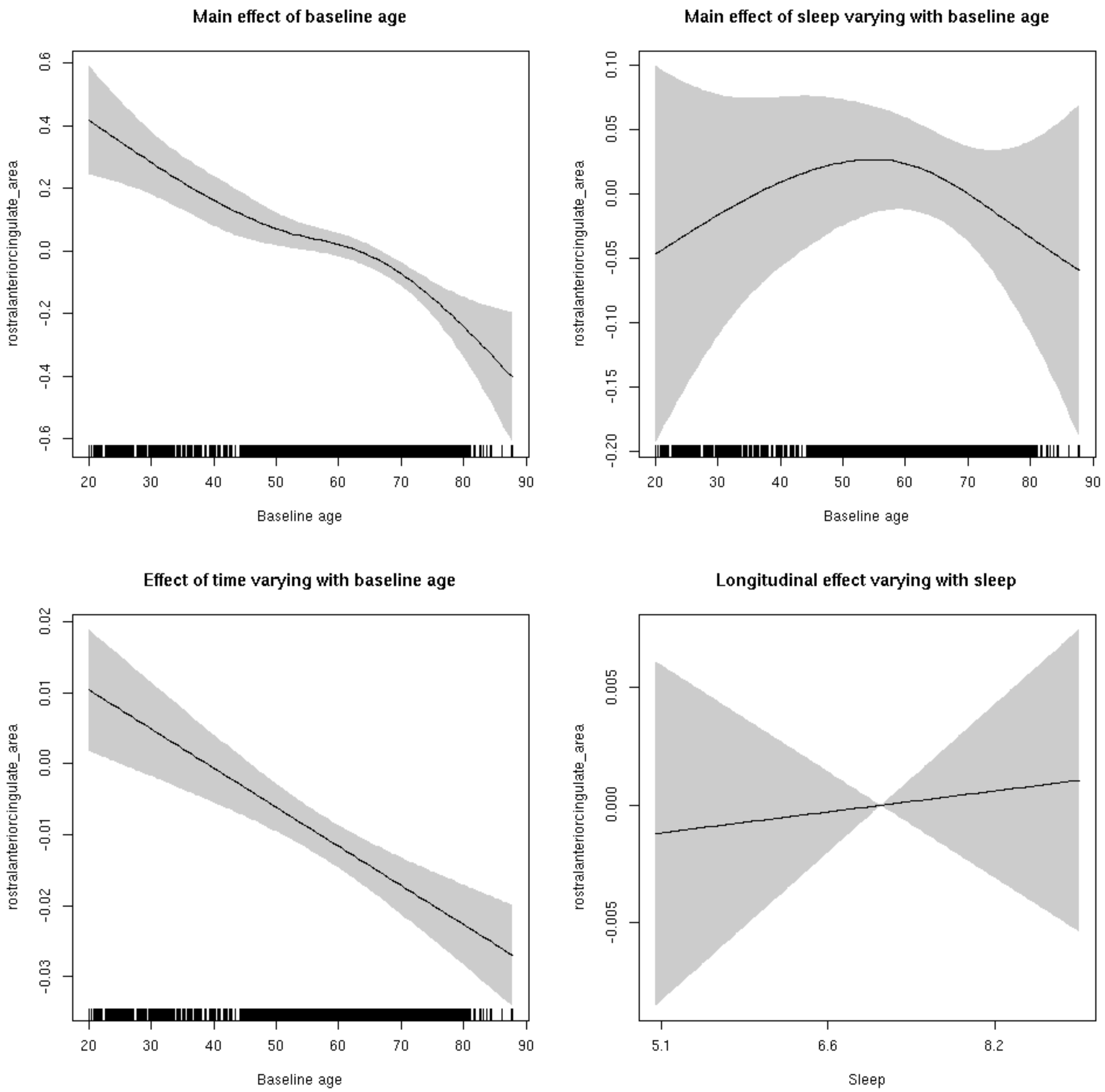

rostralanteriorcingulate_thickness: Stratified by sex

Female: Full data Below is the row for `s(sleep_z):time` from the model output.

```
## s(sleep_z):time   1.000  1.000  1.638   0.201
```

Below are plots of a plot of `s(bl_age)`, `s(bl_age):sleep_z`, `s(bl_age):time`, and `s(sleep_z):time`. For `s(sleep_z):time`, roughly, if we cannot fit a straight line through the confidence bands, there is evidence that the effect of time depends on sleep duration. It is correct the that confidence interval is zero at one point along the axis; this happens because we do not include the main effect.

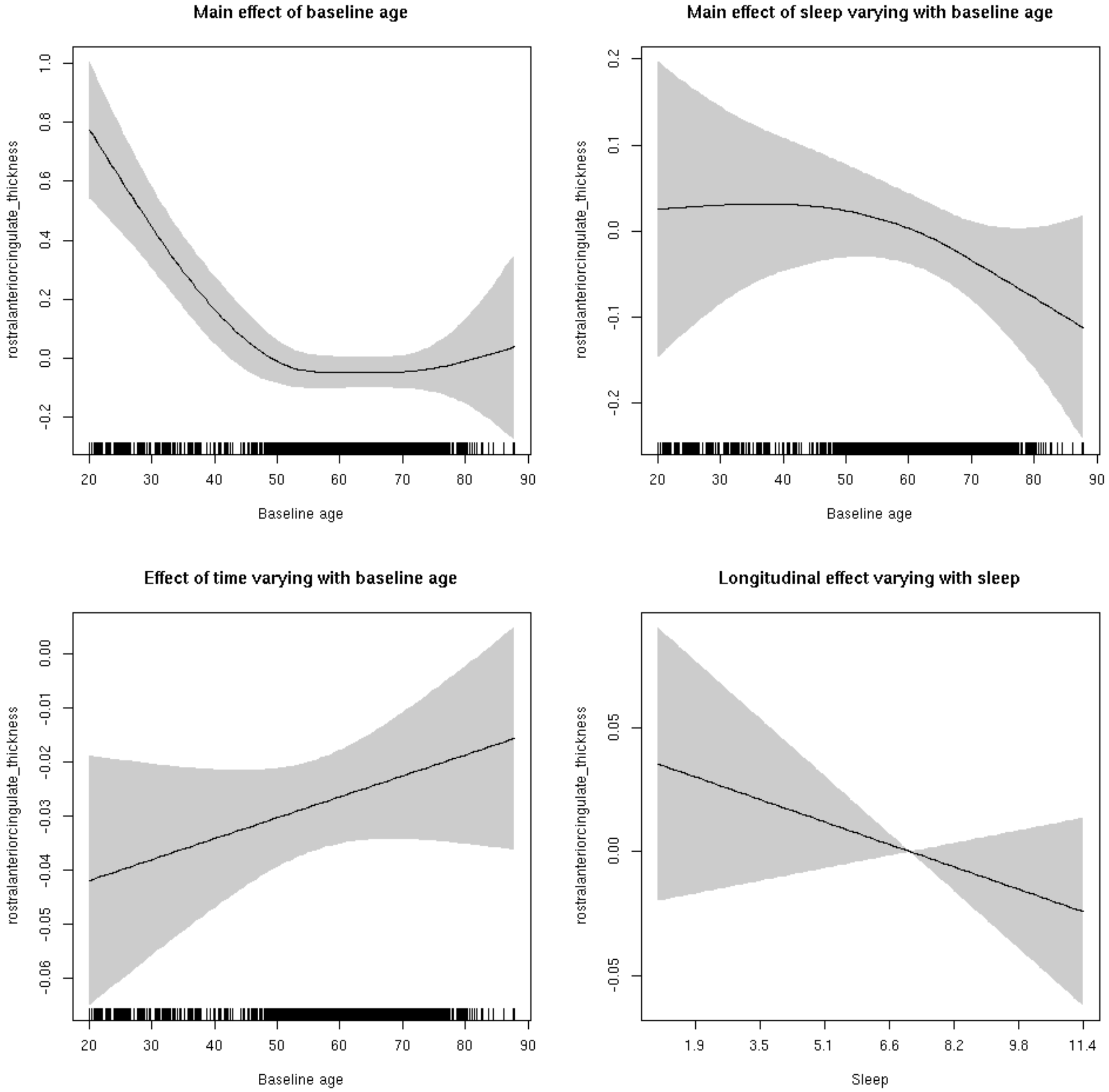

Male: Full data Below is the row for `s(sleep_z):time` from the model output.

```
## s(sleep_z):time   1.000  1.000  0.003   0.956
```

Below are plots of a plot of `s(bl_age)`, `s(bl_age):sleep_z`, `s(bl_age):time`, and `s(sleep_z):time`. For `s(sleep_z):time`, roughly, if we cannot fit a straight line through the confidence bands, there is evidence that the effect of time depends on sleep duration. It is correct the that confidence interval is zero at one point along the axis; this happens because we do not include the main effect.

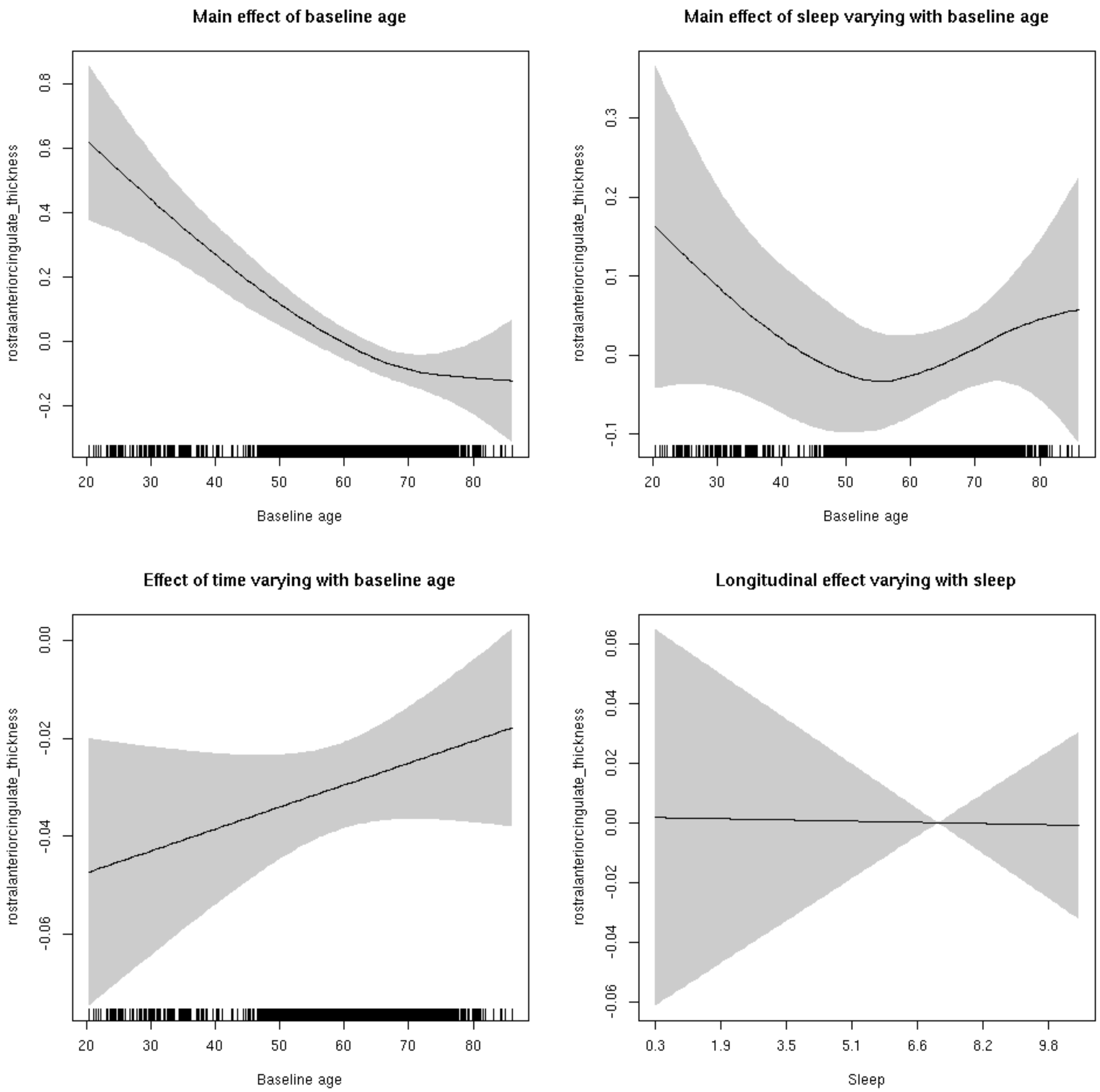

rostralanteriorcingulate__thickness

Full data Below is the model output. The term `s(sleep_z):time` is probably what we care most about.

```
##
## Family: gaussian
## Link function: identity
##
## Formula:
## value ~ sex + site + s(bl_age, k = 10, bs = "cr") + s(bl_age,
##     by = sleep_z, bs = "cr") + +s(bl_age, by = time, k = 5, bs = "cr") +
##     s(sleep_z, by = time, k = 5, bs = "cr", pc = 0)
## <environment: 0x5630035da1f8>
```

```
##
## Parametric coefficients:
##                Estimate Std. Error t value Pr(>|t|)
## (Intercept)   -1.72064    0.04935 -34.867  < 2e-16 ***
## sexmale       -0.06089    0.02476  -2.460 0.013925 *
## siteousAvanto  0.08987    0.06584   1.365 0.172308
## siteousPrisma  0.63264    0.29624   2.136 0.032747 *
## siteousSkyra   0.06030    0.06579   0.917 0.359425
## siteUB         0.32773    0.13073   2.507 0.012202 *
## siteUCAM       0.18488    0.06782   2.726 0.006423 **
## siteUKB        1.98391    0.04995  39.717  < 2e-16 ***
## siteUmU        0.30606    0.08140   3.760 0.000171 ***
## ---
## Signif. codes:  0 '***' 0.001 '**' 0.01 '*' 0.05 '.' 0.1 ' ' 1
##
## Approximate significance of smooth terms:
##                      edf Ref.df      F p-value
## s(bl_age)          3.325  3.325 27.462  <2e-16 ***
## s(bl_age):sleep_z 2.000  2.000  1.190   0.304
## s(bl_age):time    2.000  2.000 46.665  <2e-16 ***
## s(sleep_z):time   1.000  1.000  1.029   0.310
## ---
## Signif. codes:  0 '***' 0.001 '**' 0.01 '*' 0.05 '.' 0.1 ' ' 1
##
## R-sq.(adj) =  0.515
## lmer.REML =  15917  Scale est. = 0.15325   n = 8172
```

Below are plots of a plot of `s(bl_age)`, `s(bl_age):sleep_z`, `s(bl_age):time`, and `s(sleep_z):time`. For `s(sleep_z):time`, roughly, if we cannot fit a straight line through the confidence bands, there is evidence that the effect of time depends on sleep duration. It is correct the that confidence interval is zero at one point along the axis; this happens because we do not include the main effect.

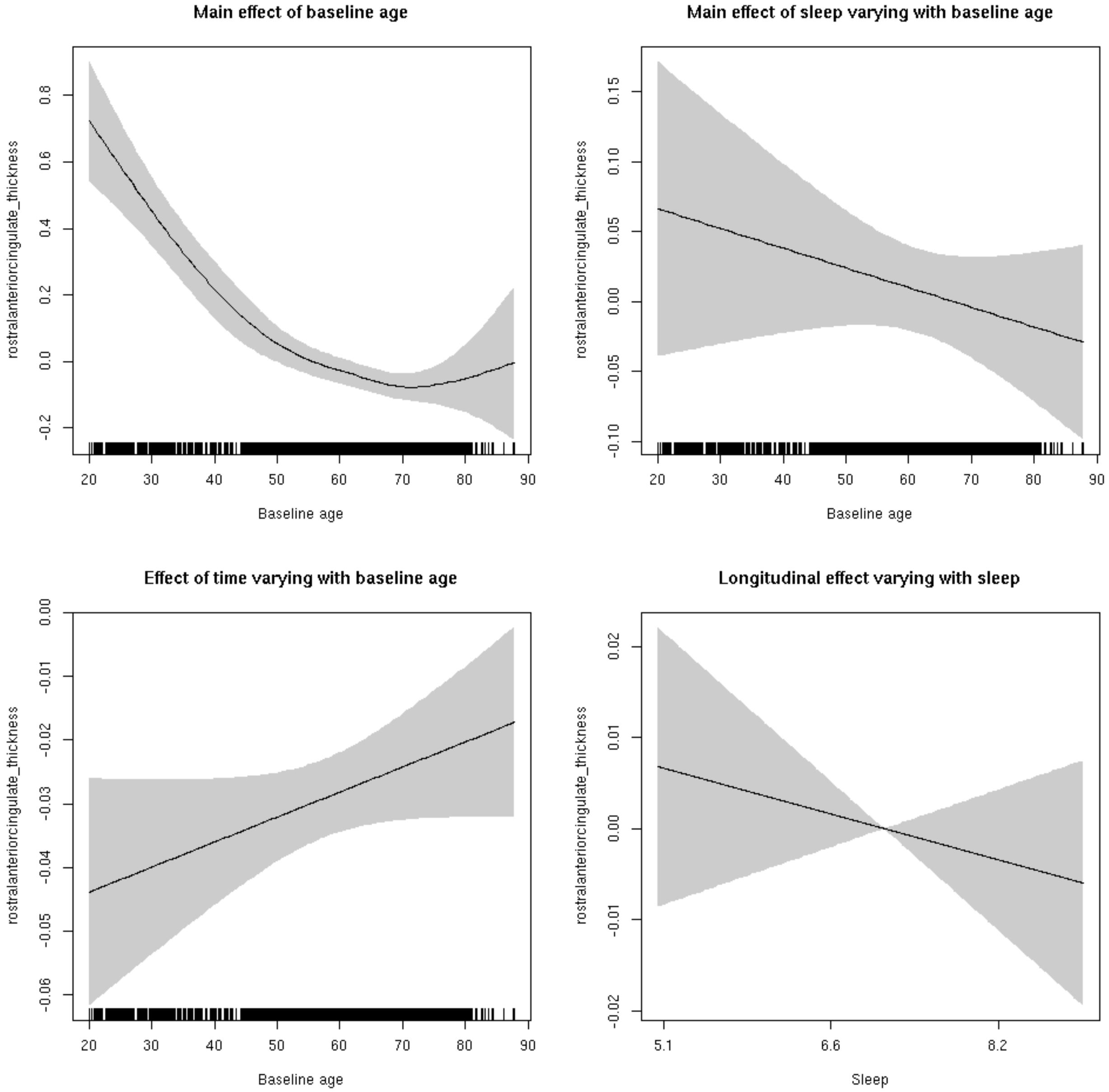

Restricted to sleep >= 5 and <= 9 hours Below is the row for `s(sleep_z):time` from the model output.

```
## s(sleep_z):time   1.000  1.000  0.782   0.376
```

Below are plots of a plot of `s(bl_age)`, `s(bl_age):sleep_z`, `s(bl_age):time`, and `s(sleep_z):time`. For `s(sleep_z):time`, roughly, if we cannot fit a straight line through the confidence bands, there is evidence that the effect of time depends on sleep duration. It is correct the that confidence interval is zero at one point along the axis; this happens because we do not include the main effect.

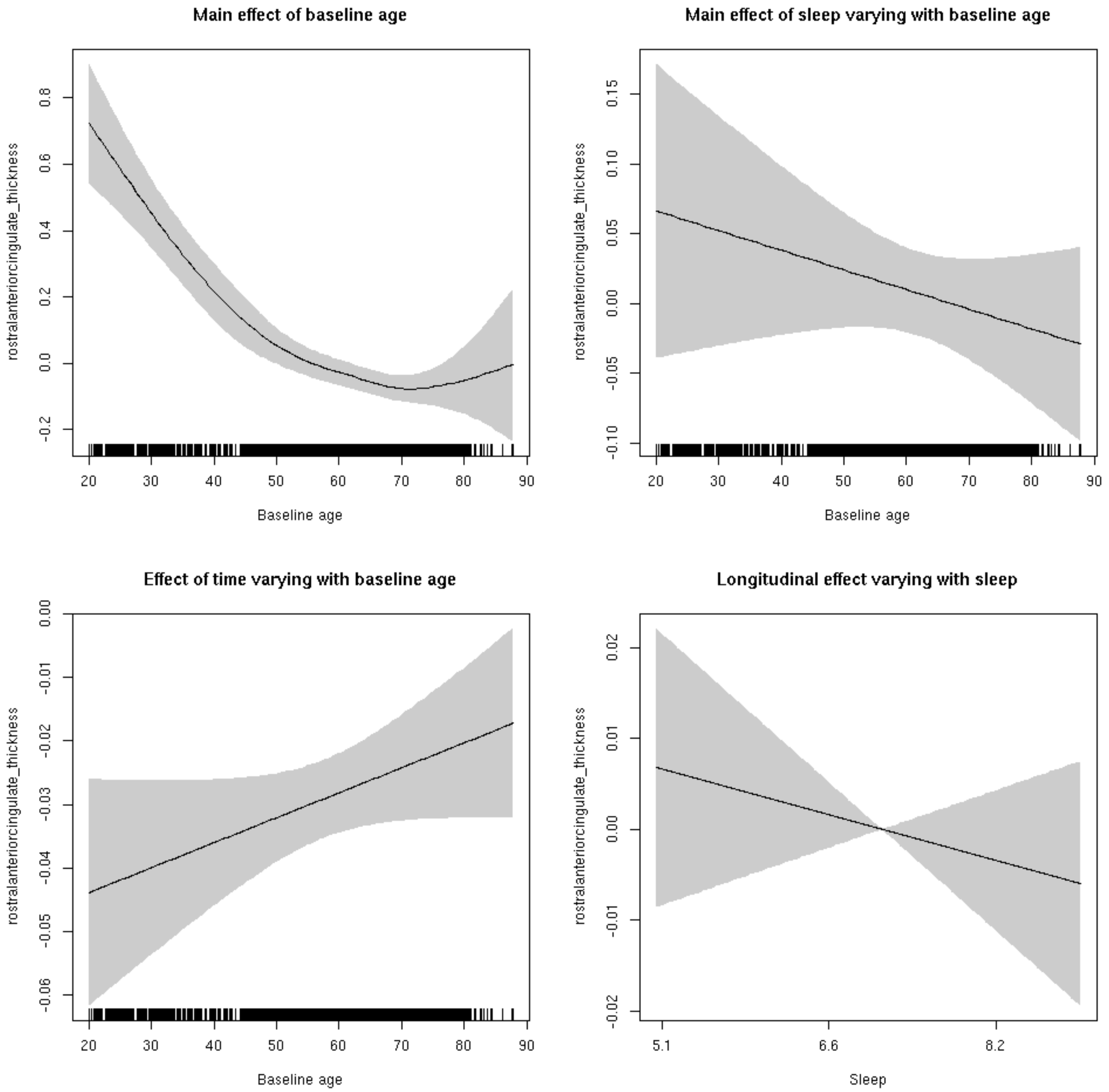

rostralanteriorcingulate_volume: Stratified by sex

Female: Full data Below is the row for `s(sleep_z):time` from the model output.

```
## s(sleep_z):time   1.000  1.000  0.538   0.463
```

Below are plots of a plot of `s(bl_age)`, `s(bl_age):sleep_z`, `s(bl_age):time`, and `s(sleep_z):time`. For `s(sleep_z):time`, roughly, if we cannot fit a straight line through the confidence bands, there is evidence that the effect of time depends on sleep duration. It is correct the that confidence interval is zero at one point along the axis; this happens because we do not include the main effect.

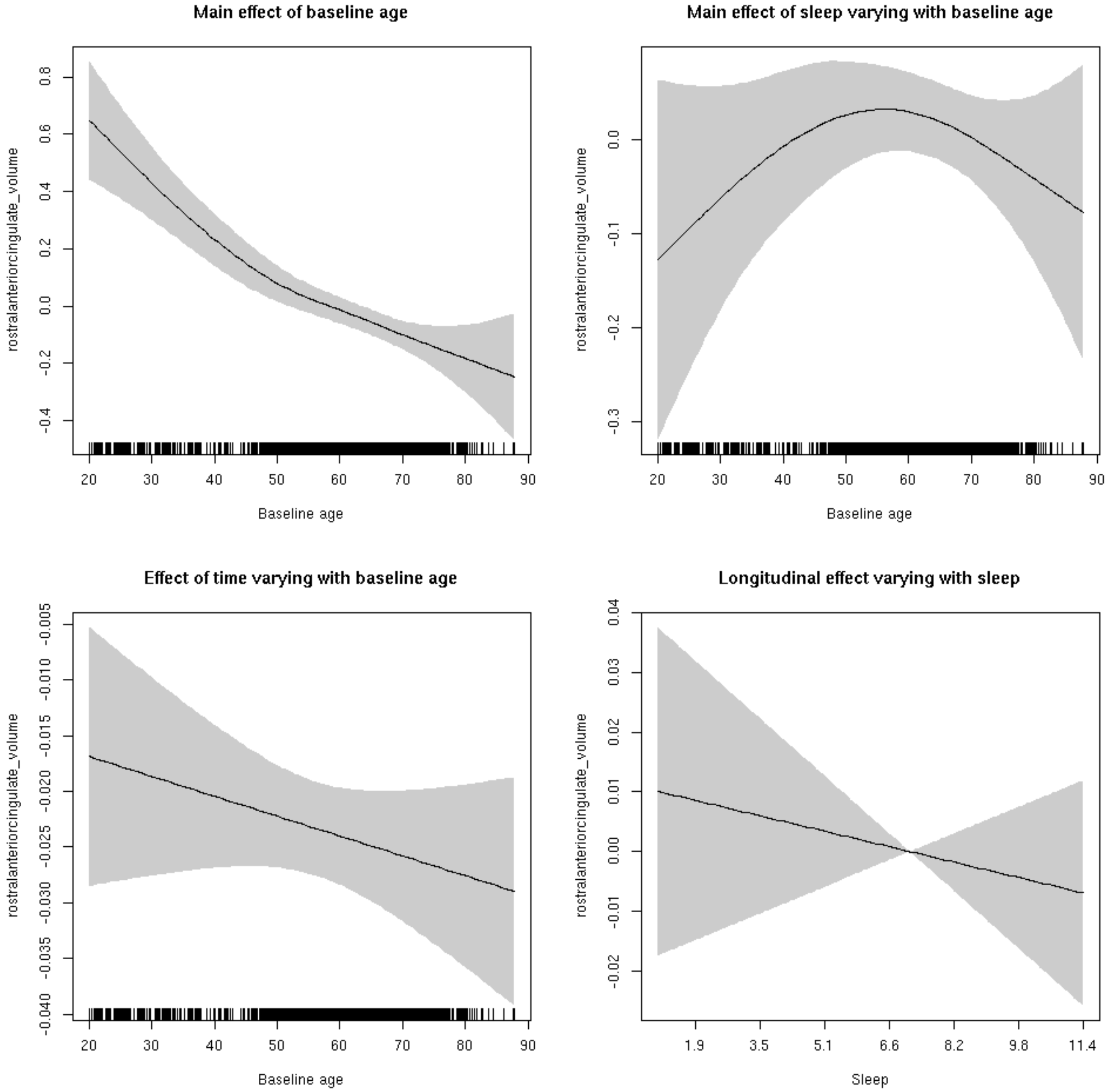

Male: Full data Below is the row for `s(sleep_z):time` from the model output.

```
## s(sleep_z):time     1      1   0.120  0.7292
```

Below are plots of a plot of `s(bl_age)`, `s(bl_age):sleep_z`, `s(bl_age):time`, and `s(sleep_z):time`. For `s(sleep_z):time`, roughly, if we cannot fit a straight line through the confidence bands, there is evidence that the effect of time depends on sleep duration. It is correct the that confidence interval is zero at one point along the axis; this happens because we do not include the main effect.

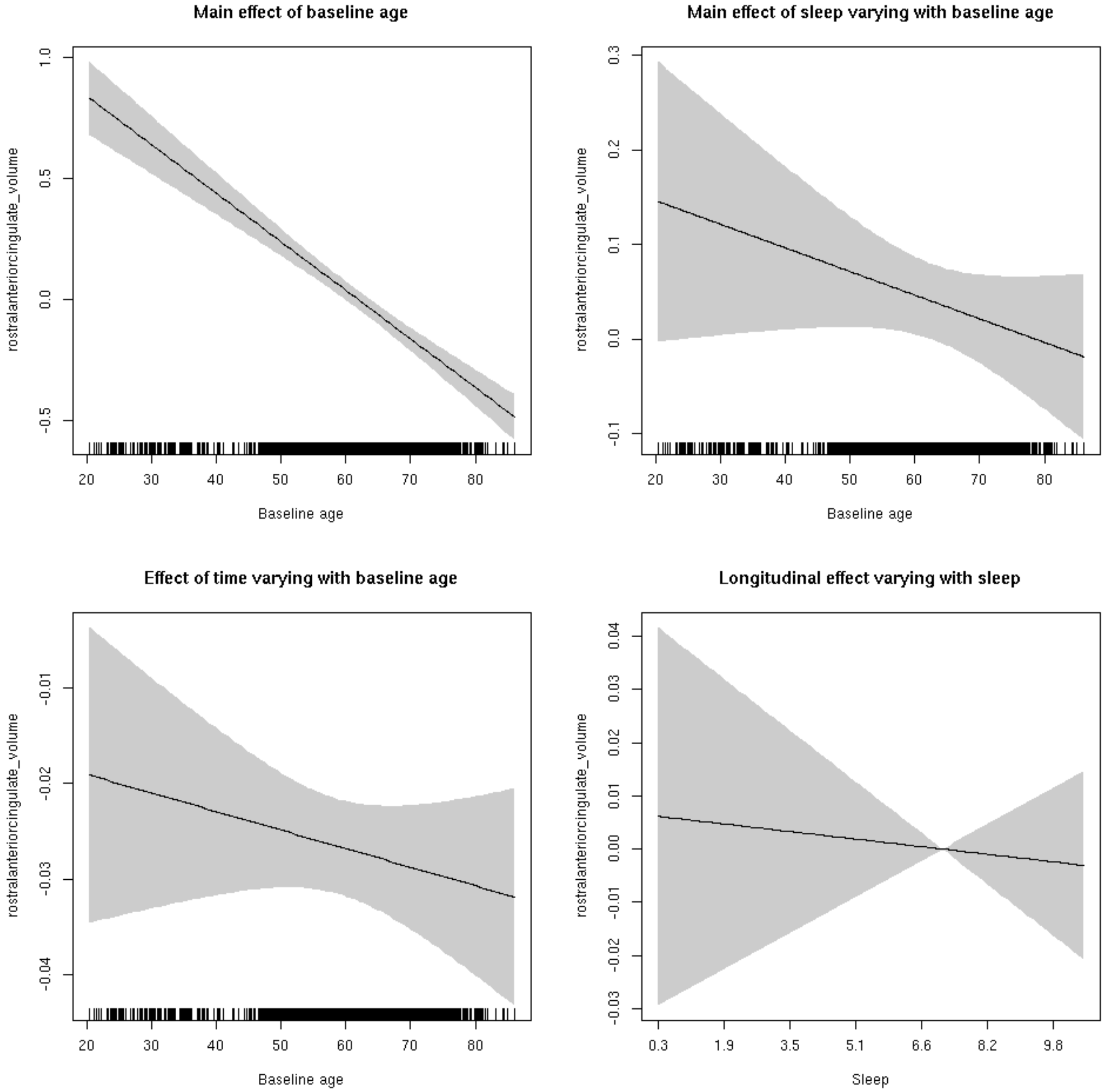

rostralanteriorcingulate_volume

Full data Below is the model output. The term `s(sleep_z):time` is probably what we care most about.

```
##
## Family: gaussian
## Link function: identity
##
## Formula:
## value ~ sex + site + icv + s(bl_age, k = 10, bs = "cr") + s(bl_age,
##     by = sleep_z, bs = "cr") + +s(bl_age, by = time, k = 5, bs = "cr") +
##     s(sleep_z, by = time, k = 5, bs = "cr", pc = 0)
## <environment: 0x5630035ad458>
```

```
##
## Parametric coefficients:
##                Estimate Std. Error t value Pr(>|t|)
## (Intercept)   -0.20390    0.05966  -3.417 0.000635 ***
## sexmale        0.12978    0.03070   4.227 2.39e-05 ***
## siteousAvanto -0.39602    0.07296  -5.428 5.85e-08 ***
## siteousPrisma -0.60293    0.17324  -3.480 0.000503 ***
## siteousSkyra  -0.13968    0.07060  -1.978 0.047915 *
## siteUB        -0.28287    0.13744  -2.058 0.039604 *
## siteUCAM      -0.40126    0.07383  -5.435 5.64e-08 ***
## siteUKB        0.24295    0.05644   4.304 1.69e-05 ***
## siteUmU        0.10220    0.08516   1.200 0.230176
## icv            0.51333    0.01514  33.906  < 2e-16 ***
## ---
## Signif. codes:  0 '***' 0.001 '**' 0.01 '*' 0.05 '.' 0.1 ' ' 1
##
## Approximate significance of smooth terms:
##                      edf Ref.df       F p-value
## s(bl_age)          2.346  2.346  74.389  <2e-16 ***
## s(bl_age):sleep_z 2.938  2.938   1.735   0.122
## s(bl_age):time    2.000  2.000 119.618  <2e-16 ***
## s(sleep_z):time   1.000  1.000   0.614   0.433
## ---
## Signif. codes:  0 '***' 0.001 '**' 0.01 '*' 0.05 '.' 0.1 ' ' 1
##
## R-sq.(adj) =  0.396
## lmer.REML =  10575  Scale est. = 0.041594  n = 8174
```

Below are plots of a plot of `s(bl_age)`, `s(bl_age):sleep_z`, `s(bl_age):time`, and `s(sleep_z):time`. For `s(sleep_z):time`, roughly, if we cannot fit a straight line through the confidence bands, there is evidence that the effect of time depends on sleep duration. It is correct the that confidence interval is zero at one point along the axis; this happens because we do not include the main effect.

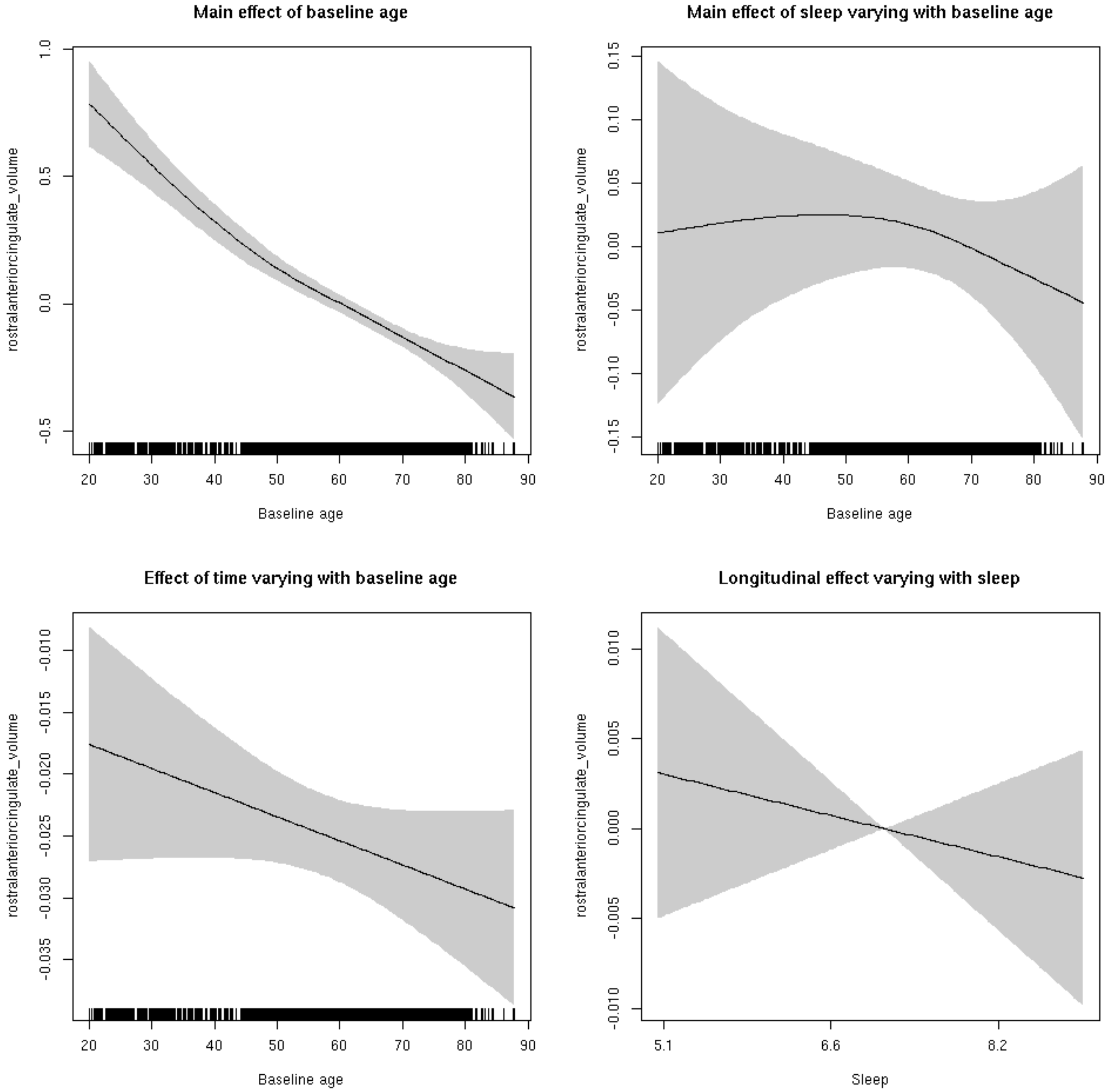

Restricted to sleep >= 5 and <= 9 hours Below is the row for `s(sleep_z):time` from the model output.

```
## s(sleep_z):time   1.000  1.000   0.590   0.442
```

Below are plots of a plot of `s(bl_age)`, `s(bl_age):sleep_z`, `s(bl_age):time`, and `s(sleep_z):time`. For `s(sleep_z):time`, roughly, if we cannot fit a straight line through the confidence bands, there is evidence that the effect of time depends on sleep duration. It is correct the that confidence interval is zero at one point along the axis; this happens because we do not include the main effect.

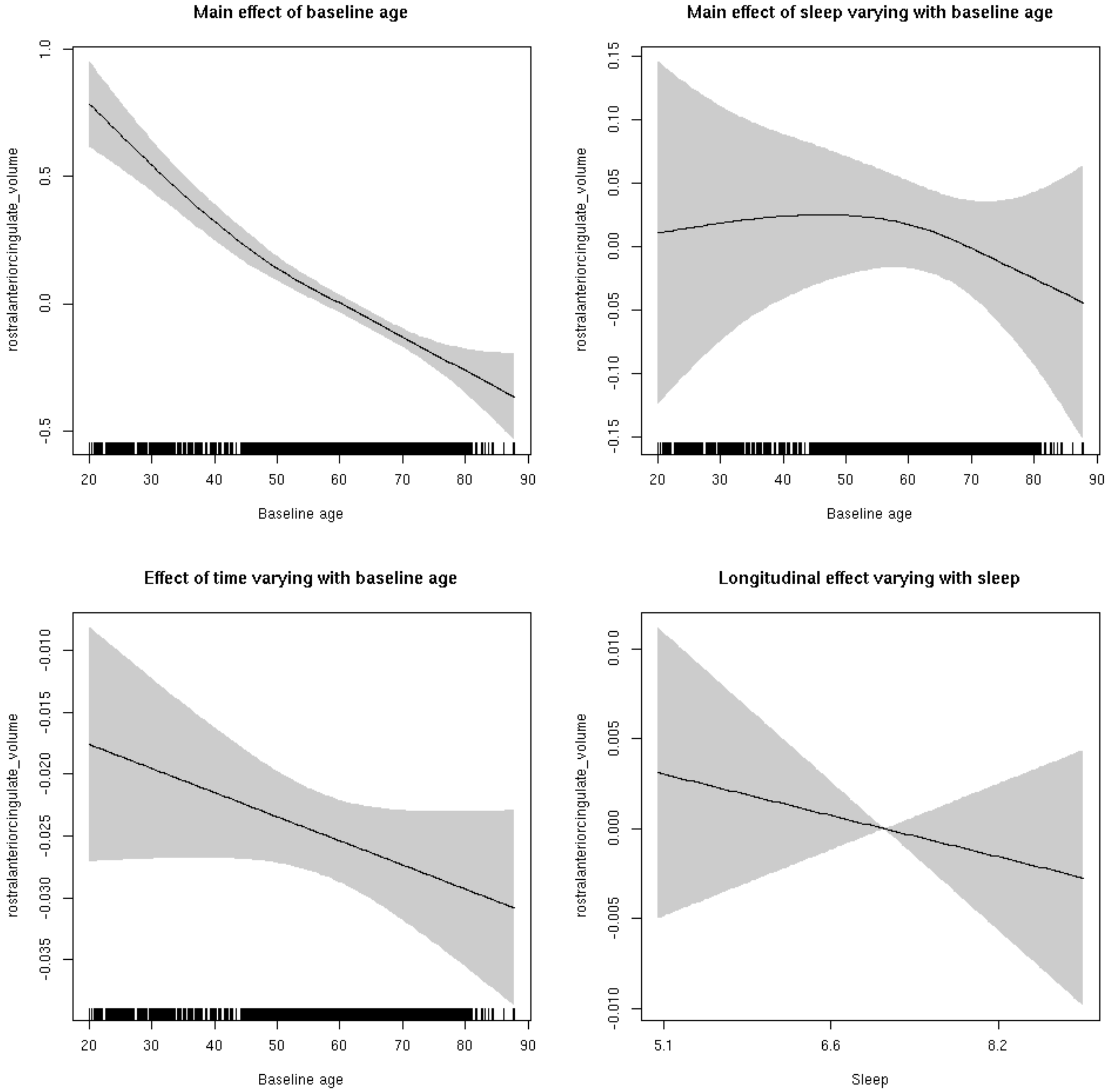

rostralmiddlefrontal_area: Stratified by sex

Female: Full data Below is the row for `s(sleep_z):time` from the model output.

```
## s(sleep_z):time   1.000  1.000  0.007   0.931
```

Below are plots of a plot of `s(bl_age)`, `s(bl_age):sleep_z`, `s(bl_age):time`, and `s(sleep_z):time`. For `s(sleep_z):time`, roughly, if we cannot fit a straight line through the confidence bands, there is evidence that the effect of time depends on sleep duration. It is correct the that confidence interval is zero at one point along the axis; this happens because we do not include the main effect.

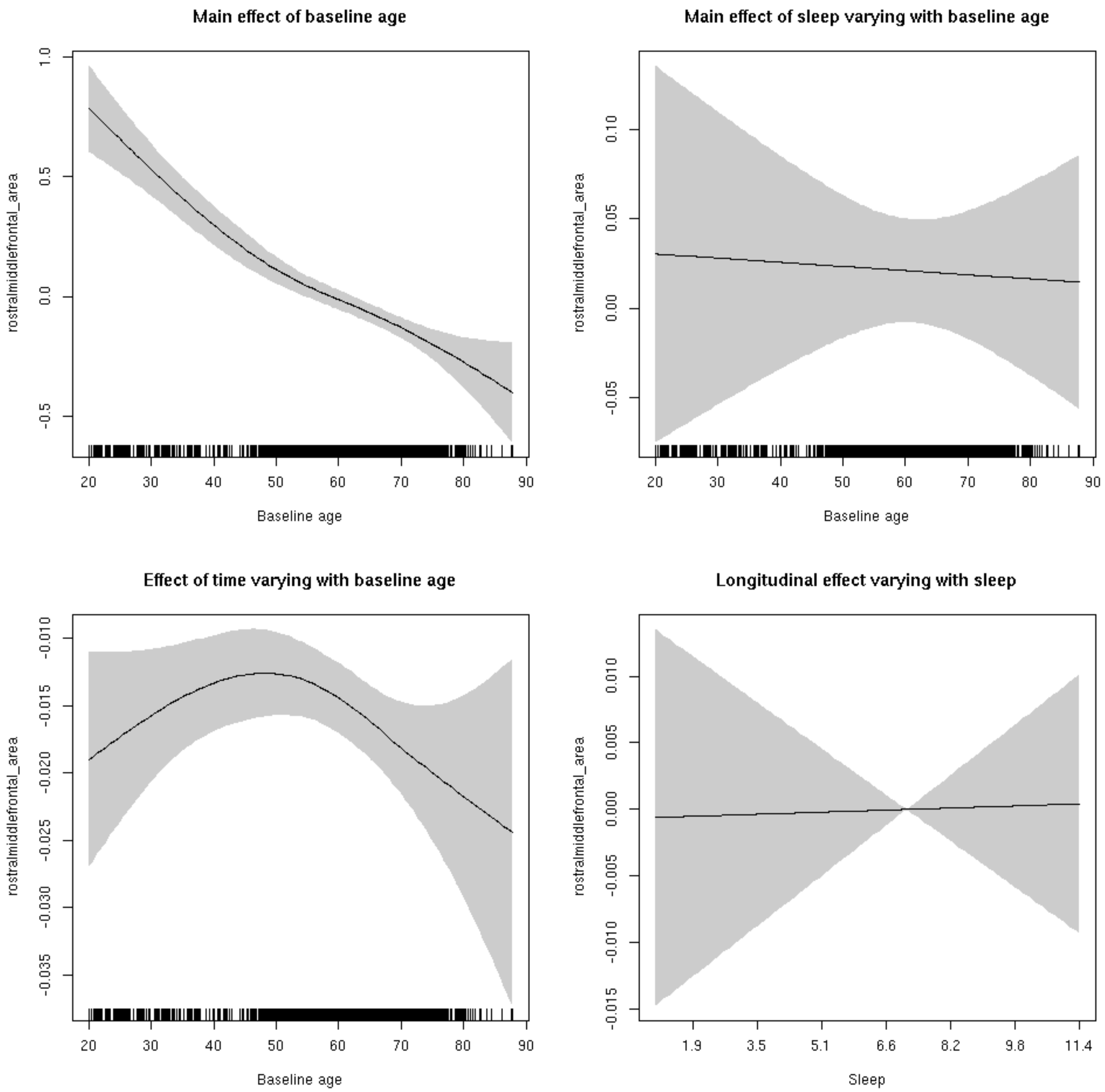

Male: Full data Below is the row for `s(sleep_z):time` from the model output.

```
## s(sleep_z):time   1.000  1.000   0.294   0.588
```

Below are plots of a plot of `s(bl_age)`, `s(bl_age):sleep_z`, `s(bl_age):time`, and `s(sleep_z):time`. For `s(sleep_z):time`, roughly, if we cannot fit a straight line through the confidence bands, there is evidence that the effect of time depends on sleep duration. It is correct the that confidence interval is zero at one point along the axis; this happens because we do not include the main effect.

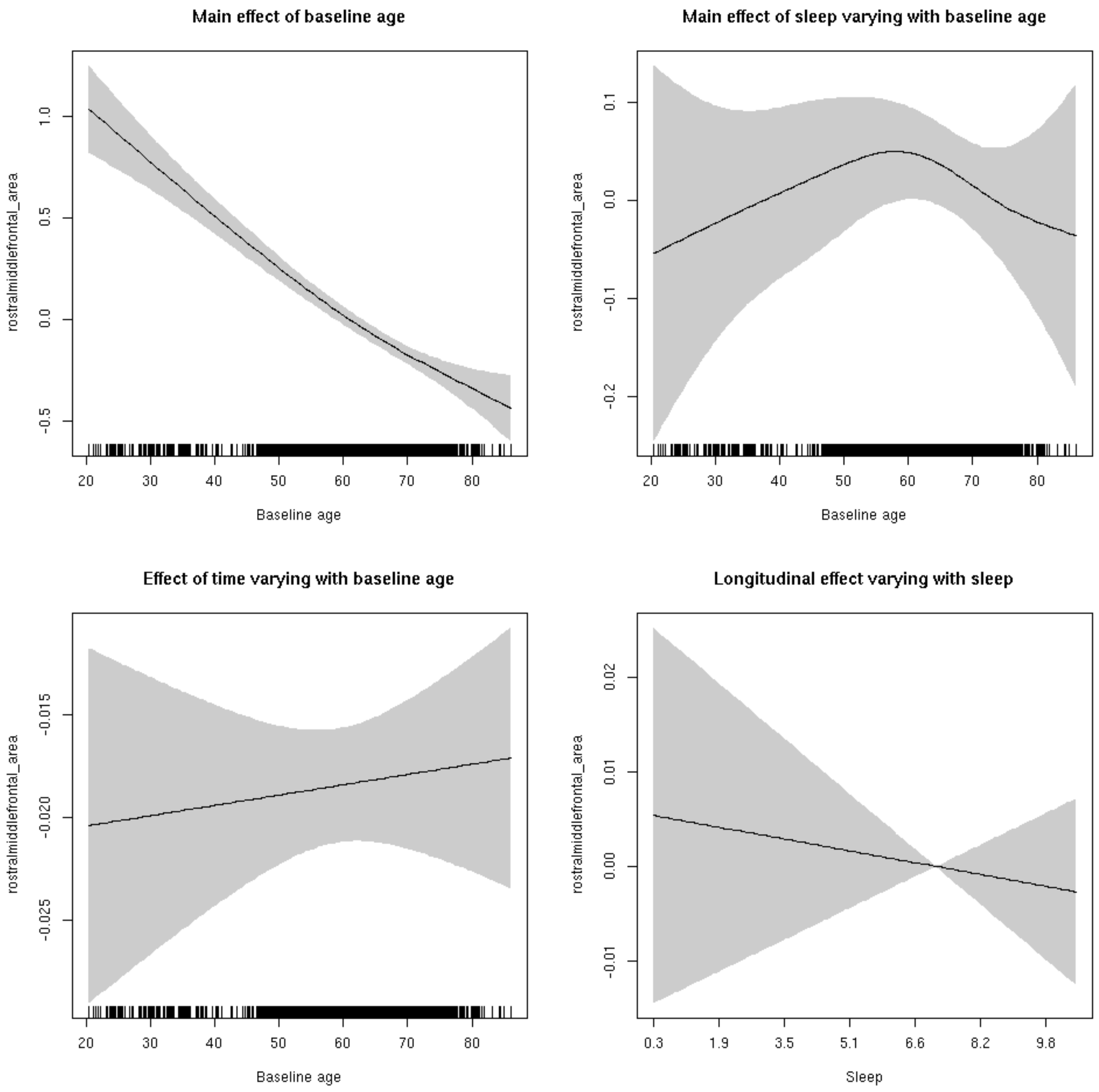

rostralmiddlefrontal_area

Full data Below is the model output. The term `s(sleep_z):time` is probably what we care most about.

```
##
## Family: gaussian
## Link function: identity
##
## Formula:
## value ~ sex + site + icv + s(bl_age, k = 10, bs = "cr") + s(bl_age,
##     by = sleep_z, bs = "cr") + +s(bl_age, by = time, k = 5, bs = "cr") +
##     s(sleep_z, by = time, k = 5, bs = "cr", pc = 0)
## <environment: 0x5630035b5768>
```

```
##
## Parametric coefficients:
##                Estimate Std. Error t value Pr(>|t|)
## (Intercept)     0.96791    0.05148  18.800  < 2e-16 ***
## sexmale         0.24640    0.02648   9.304  < 2e-16 ***
## siteousAvanto -0.88720    0.06280 -14.127  < 2e-16 ***
## siteousPrisma -1.09739    0.10632 -10.321  < 2e-16 ***
## siteousSkyra  -0.67450    0.06089 -11.078  < 2e-16 ***
## siteUB        -0.98657    0.11948  -8.257  < 2e-16 ***
## siteUCAM      -1.07879    0.06393 -16.875  < 2e-16 ***
## siteUKB       -1.09294    0.04882 -22.385  < 2e-16 ***
## siteUmU       -0.27848    0.07383  -3.772 0.000163 ***
## icv            0.60907    0.01283  47.459  < 2e-16 ***
## ---
## Signif. codes:  0 '***' 0.001 '**' 0.01 '*' 0.05 '.' 0.1 ' ' 1
##
## Approximate significance of smooth terms:
##                      edf Ref.df       F p-value
## s(bl_age)          2.330  2.330 140.416  <2e-16 ***
## s(bl_age):sleep_z 2.661  2.661   1.008   0.311
## s(bl_age):time     2.000  2.000 185.890  <2e-16 ***
## s(sleep_z):time    1.000  1.000   0.225   0.636
## ---
## Signif. codes:  0 '***' 0.001 '**' 0.01 '*' 0.05 '.' 0.1 ' ' 1
##
## R-sq.(adj) =   0.55
## lmer.REML = 4117.5  Scale est. = 0.01189   n = 8154
```

Below are plots of a plot of `s(bl_age)`, `s(bl_age):sleep_z`, `s(bl_age):time`, and `s(sleep_z):time`. For `s(sleep_z):time`, roughly, if we cannot fit a straight line through the confidence bands, there is evidence that the effect of time depends on sleep duration. It is correct the that confidence interval is zero at one point along the axis; this happens because we do not include the main effect.

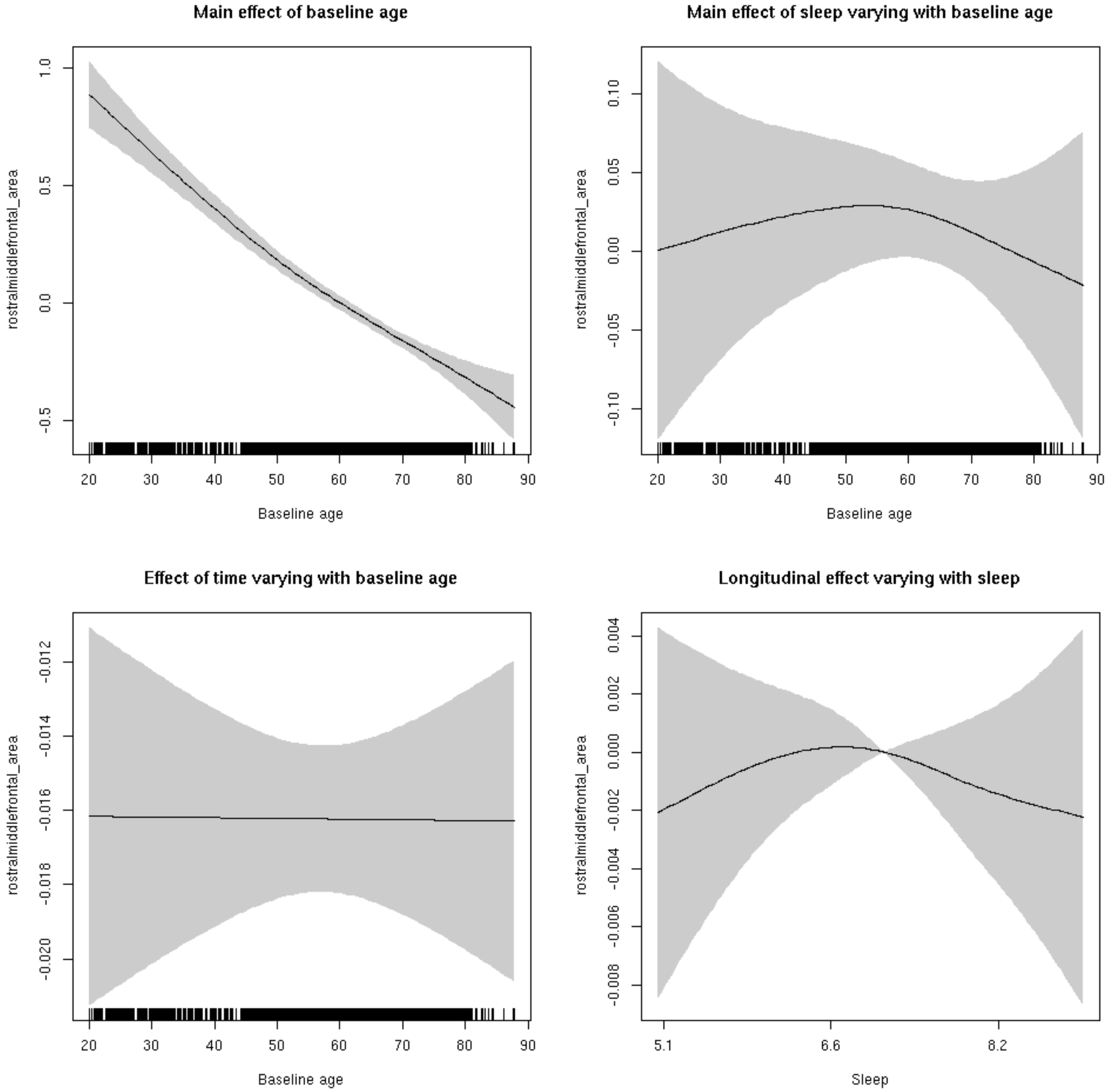

Restricted to sleep >= 5 and <= 9 hours Below is the row for `s(sleep_z):time` from the model output.

```
## s(sleep_z):time   1.603  1.603   0.396   0.614
```

Below are plots of a plot of `s(bl_age)`, `s(bl_age):sleep_z`, `s(bl_age):time`, and `s(sleep_z):time`. For `s(sleep_z):time`, roughly, if we cannot fit a straight line through the confidence bands, there is evidence that the effect of time depends on sleep duration. It is correct the that confidence interval is zero at one point along the axis; this happens because we do not include the main effect.

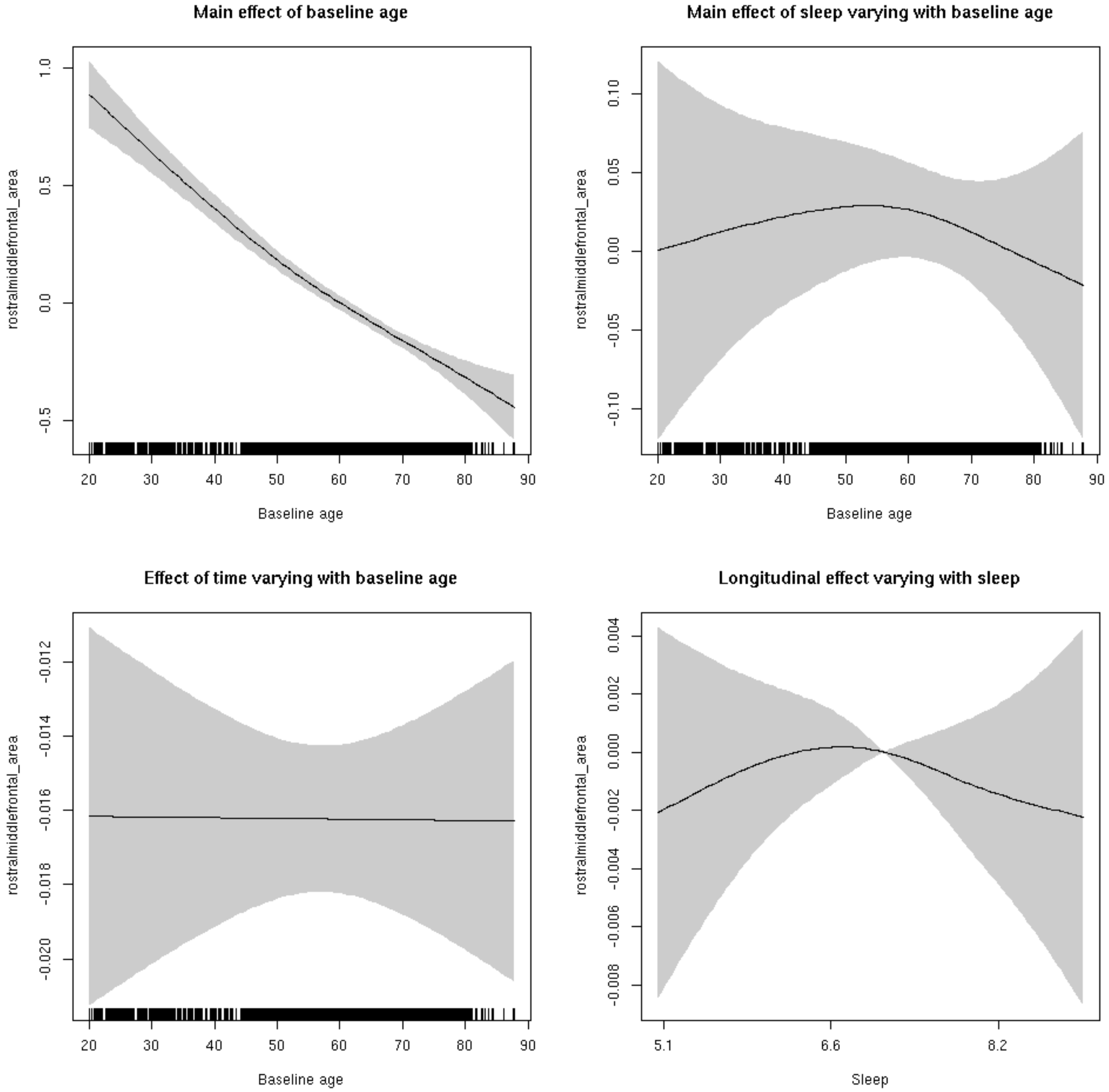

rostralmiddlefrontal_thickness: Stratified by sex

Female: Full data Below is the row for `s(sleep_z):time` from the model output.

```
## s(sleep_z):time   1.000  1.000  0.239   0.625
```

Below are plots of a plot of `s(bl_age)`, `s(bl_age):sleep_z`, `s(bl_age):time`, and `s(sleep_z):time`. For `s(sleep_z):time`, roughly, if we cannot fit a straight line through the confidence bands, there is evidence that the effect of time depends on sleep duration. It is correct the that confidence interval is zero at one point along the axis; this happens because we do not include the main effect.

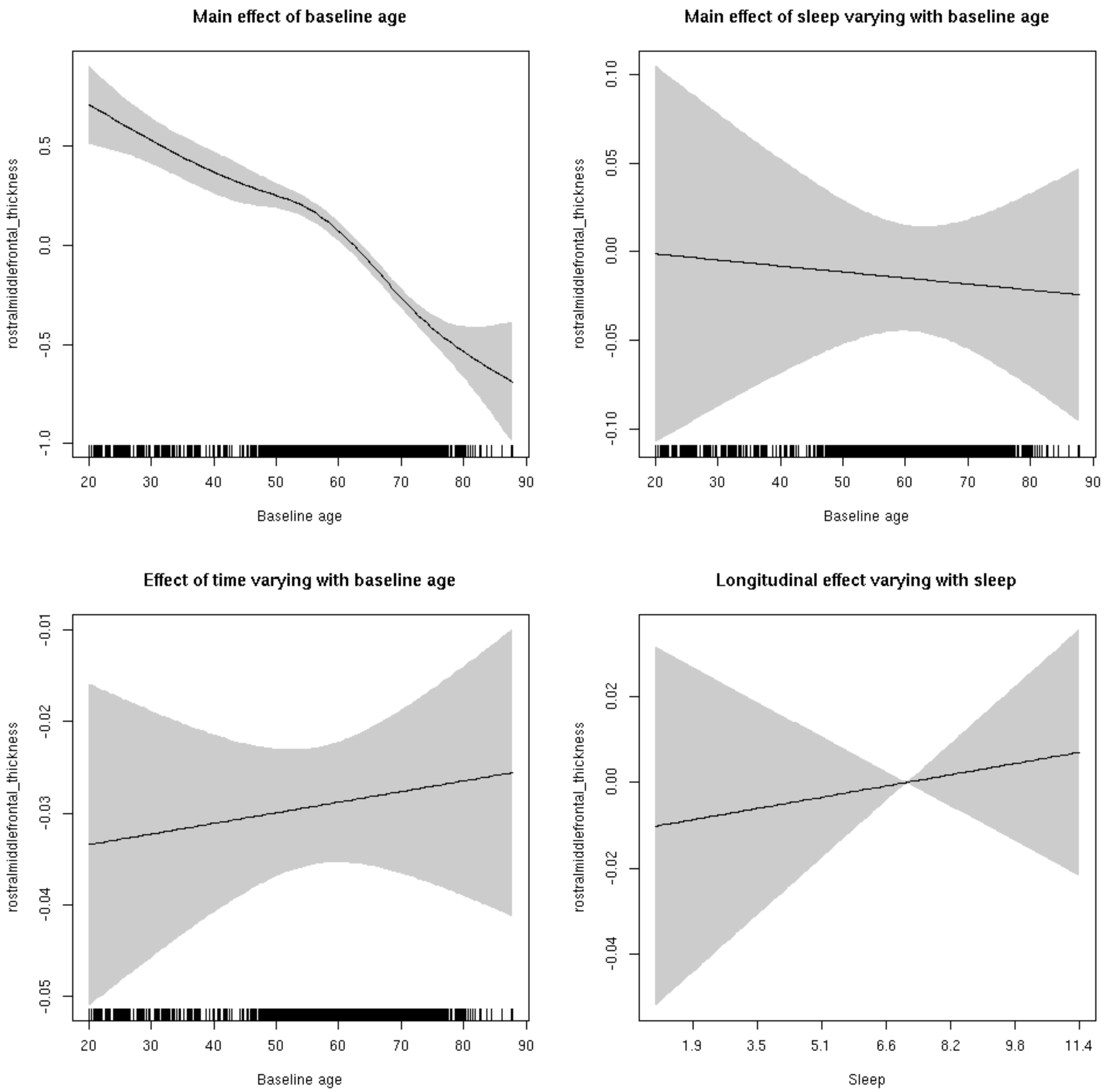

Male: Full data Below is the row for `s(sleep_z):time` from the model output.

```
## s(sleep_z):time   2.940  2.940  2.505  0.0451 *
```

Below are plots of a plot of `s(bl_age)`, `s(bl_age):sleep_z`, `s(bl_age):time`, and `s(sleep_z):time`. For `s(sleep_z):time`, roughly, if we cannot fit a straight line through the confidence bands, there is evidence that the effect of time depends on sleep duration. It is correct the that confidence interval is zero at one point along the axis; this happens because we do not include the main effect.

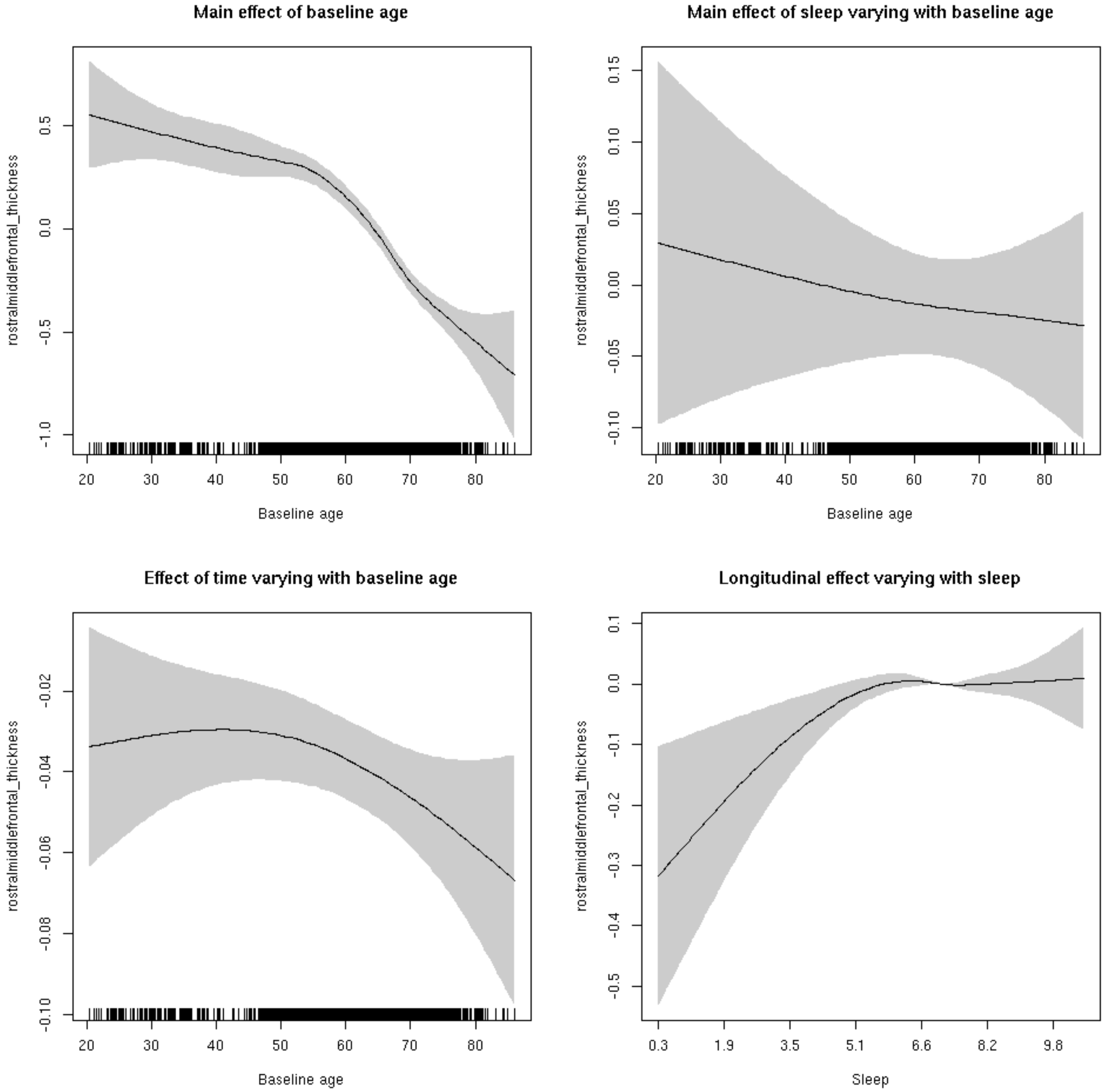

rostralmiddlefrontal_thickness

Full data Below is the model output. The term `s(sleep_z):time` is probably what we care most about.

```
##
## Family: gaussian
## Link function: identity
##
## Formula:
## value ~ sex + site + s(bl_age, k = 10, bs = "cr") + s(bl_age,
##     by = sleep_z, bs = "cr") + +s(bl_age, by = time, k = 5, bs = "cr") +
##     s(sleep_z, by = time, k = 5, bs = "cr", pc = 0)
## <environment: 0x5630035d3880>
```

```
##
## Parametric coefficients:
##                Estimate Std. Error t value Pr(>|t|)
## (Intercept)   -1.89118    0.04196 -45.076  < 2e-16 ***
## sexmale        0.05932    0.02098   2.828  0.00470 **
## siteousAvanto  0.18758    0.05563   3.372  0.00075 ***
## siteousPrisma  0.49185    0.23390   2.103  0.03551 *
## siteousSkyra  -0.36322    0.05564  -6.529 7.03e-11 ***
## siteUB         0.61863    0.11110   5.568 2.65e-08 ***
## siteUCAM       0.40727    0.05805   7.016 2.47e-12 ***
## siteUKB        2.17533    0.04263  51.027  < 2e-16 ***
## siteUmU       -0.18132    0.06902  -2.627  0.00863 **
## ---
## Signif. codes:  0 '***' 0.001 '**' 0.01 '*' 0.05 '.' 0.1 ' ' 1
##
## Approximate significance of smooth terms:
##                      edf Ref.df       F p-value
## s(bl_age)          5.192  5.192 119.589  <2e-16 ***
## s(bl_age):sleep_z 2.000  2.000   1.268  0.2816
## s(bl_age):time    2.462  2.462  51.087  <2e-16 ***
## s(sleep_z):time   3.309  3.309   3.063  0.0131 *
## ---
## Signif. codes:  0 '***' 0.001 '**' 0.01 '*' 0.05 '.' 0.1 ' ' 1
##
## R-sq.(adj) =  0.689
## lmer.REML =  12513  Scale est. = 0.093526  n = 8164
```

Below are plots of a plot of `s(bl_age)`, `s(bl_age):sleep_z`, `s(bl_age):time`, and `s(sleep_z):time`. For `s(sleep_z):time`, roughly, if we cannot fit a straight line through the confidence bands, there is evidence that the effect of time depends on sleep duration. It is correct the that confidence interval is zero at one point along the axis; this happens because we do not include the main effect.

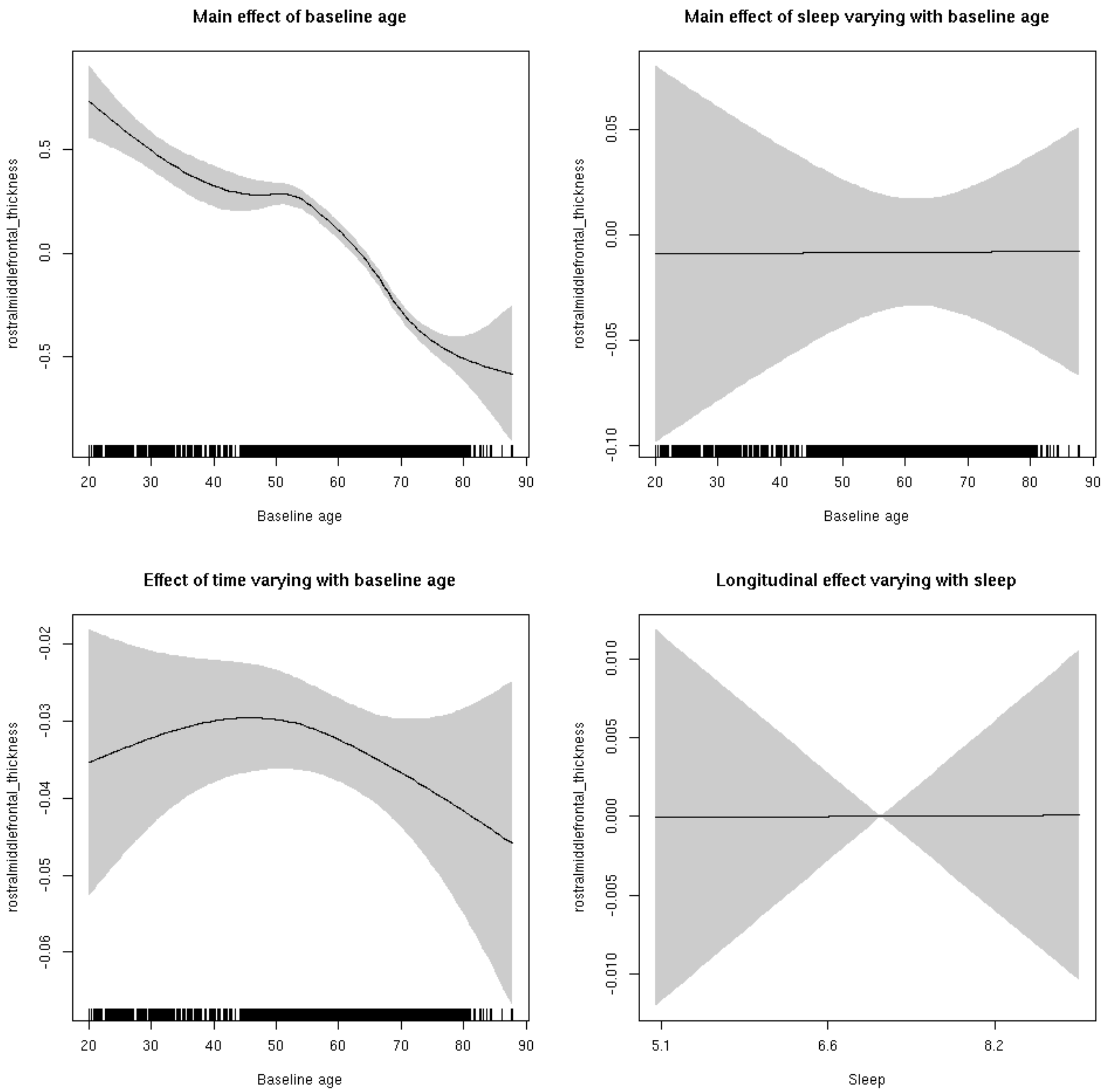

Restricted to sleep >= 5 and <= 9 hours Below is the row for `s(sleep_z):time` from the model output.

```
## s(sleep_z):time   1.000  1.000   0.000   0.990
```

Below are plots of a plot of `s(bl_age)`, `s(bl_age):sleep_z`, `s(bl_age):time`, and `s(sleep_z):time`. For `s(sleep_z):time`, roughly, if we cannot fit a straight line through the confidence bands, there is evidence that the effect of time depends on sleep duration. It is correct the that confidence interval is zero at one point along the axis; this happens because we do not include the main effect.

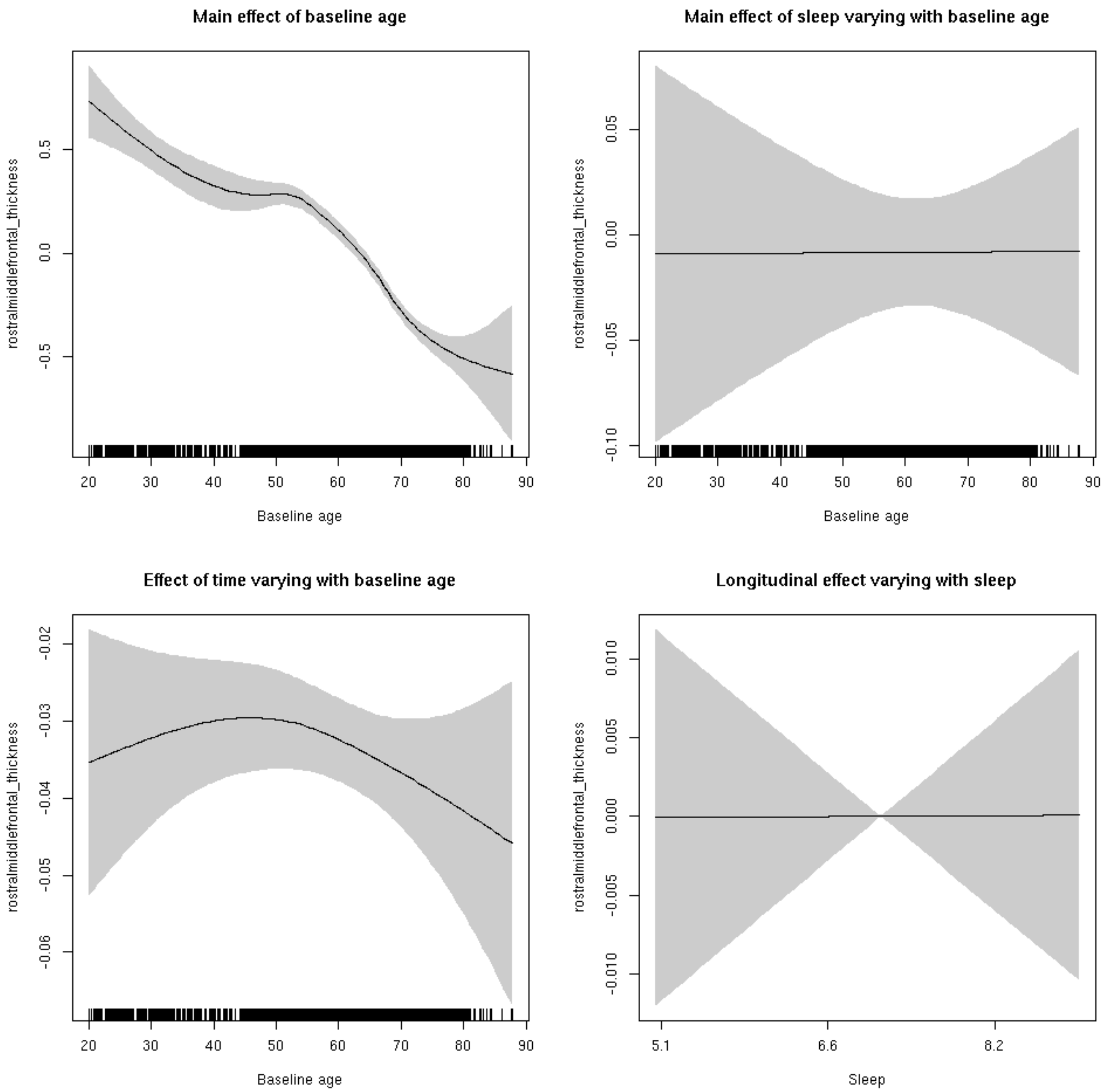

rostralmiddlefrontal_volume: Stratified by sex

Female: Full data Below is the row for `s(sleep_z):time` from the model output.

```
## s(sleep_z):time   1.000  1.000   0.011   0.917
```

Below are plots of a plot of `s(bl_age)`, `s(bl_age):sleep_z`, `s(bl_age):time`, and `s(sleep_z):time`. For `s(sleep_z):time`, roughly, if we cannot fit a straight line through the confidence bands, there is evidence that the effect of time depends on sleep duration. It is correct the that confidence interval is zero at one point along the axis; this happens because we do not include the main effect.

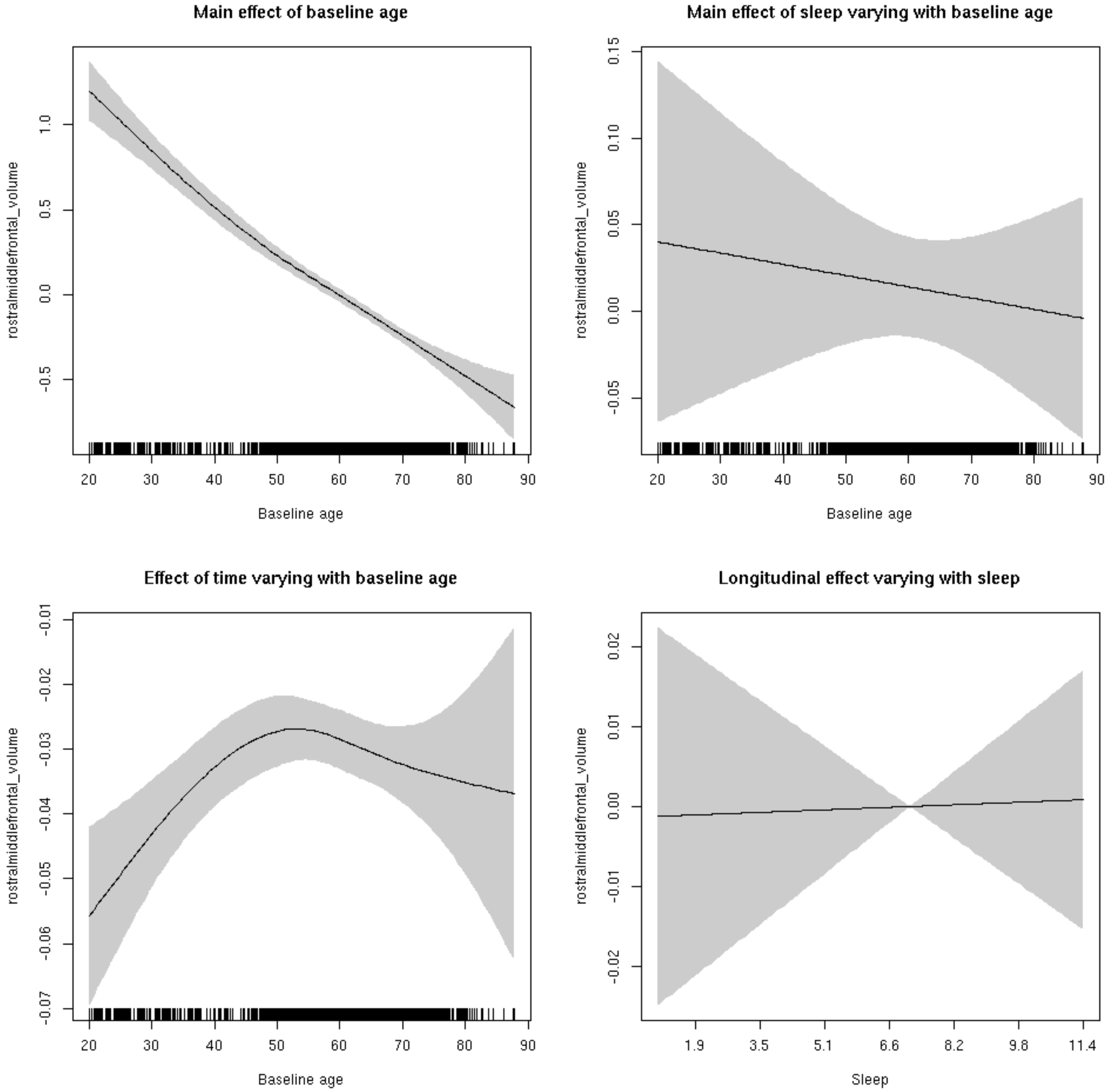

Male: Full data Below is the row for `s(sleep_z):time` from the model output.

```
## s(sleep_z):time   1.000  1.000   1.041   0.308
```

Below are plots of a plot of `s(bl_age)`, `s(bl_age):sleep_z`, `s(bl_age):time`, and `s(sleep_z):time`. For `s(sleep_z):time`, roughly, if we cannot fit a straight line through the confidence bands, there is evidence that the effect of time depends on sleep duration. It is correct the that confidence interval is zero at one point along the axis; this happens because we do not include the main effect.

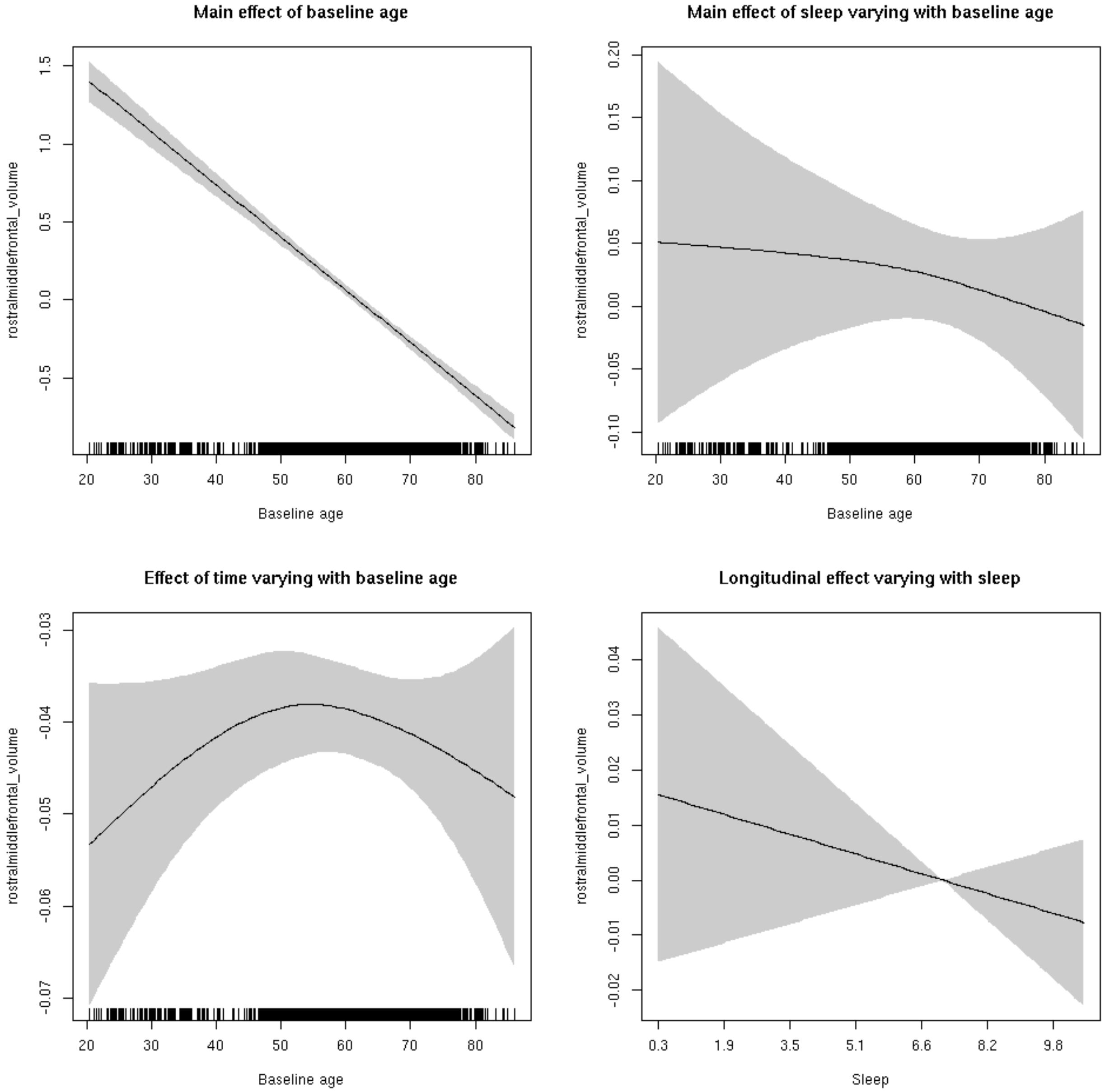

rostralmiddlefrontal_volume

Full data Below is the model output. The term `s(sleep_z):time` is probably what we care most about.

```
##
## Family: gaussian
## Link function: identity
##
## Formula:
## value ~ sex + site + icv + s(bl_age, k = 10, bs = "cr") + s(bl_age,
##     by = sleep_z, bs = "cr") + +s(bl_age, by = time, k = 5, bs = "cr") +
##     s(sleep_z, by = time, k = 5, bs = "cr", pc = 0)
## <environment: 0x5630035ac108>
```

```
##
## Parametric coefficients:
##                Estimate Std. Error t value Pr(>|t|)
## (Intercept)     0.24198    0.05062   4.780 1.78e-06 ***
## sexmale         0.23556    0.02610   9.025  < 2e-16 ***
## siteousAvanto -0.90866    0.06197 -14.664  < 2e-16 ***
## siteousPrisma -0.71842    0.14718  -4.881 1.07e-06 ***
## siteousSkyra  -0.83979    0.06000 -13.996  < 2e-16 ***
## siteUB        -0.60093    0.11671  -5.149 2.68e-07 ***
## siteUCAM      -0.87276    0.06262 -13.938  < 2e-16 ***
## siteUKB       -0.20719    0.04783  -4.332 1.49e-05 ***
## siteUmU       -0.36971    0.07229  -5.114 3.22e-07 ***
## icv            0.58973    0.01284  45.930  < 2e-16 ***
## ---
## Signif. codes:  0 '***' 0.001 '**' 0.01 '*' 0.05 '.' 0.1 ' ' 1
##
## Approximate significance of smooth terms:
##                      edf Ref.df       F p-value
## s(bl_age)          2.123  2.123 407.921  <2e-16 ***
## s(bl_age):sleep_z 2.673  2.673   0.936  0.2897
## s(bl_age):time    3.425  3.425 116.222  <2e-16 ***
## s(sleep_z):time   3.256  3.256   2.159  0.0452 *
## ---
## Signif. codes:  0 '***' 0.001 '**' 0.01 '*' 0.05 '.' 0.1 ' ' 1
##
## R-sq.(adj) =  0.565
## lmer.REML = 7913.8  Scale est. = 0.030008  n = 8168
```

Below are plots of a plot of `s(bl_age)`, `s(bl_age):sleep_z`, `s(bl_age):time`, and `s(sleep_z):time`. For `s(sleep_z):time`, roughly, if we cannot fit a straight line through the confidence bands, there is evidence that the effect of time depends on sleep duration. It is correct the that confidence interval is zero at one point along the axis; this happens because we do not include the main effect.

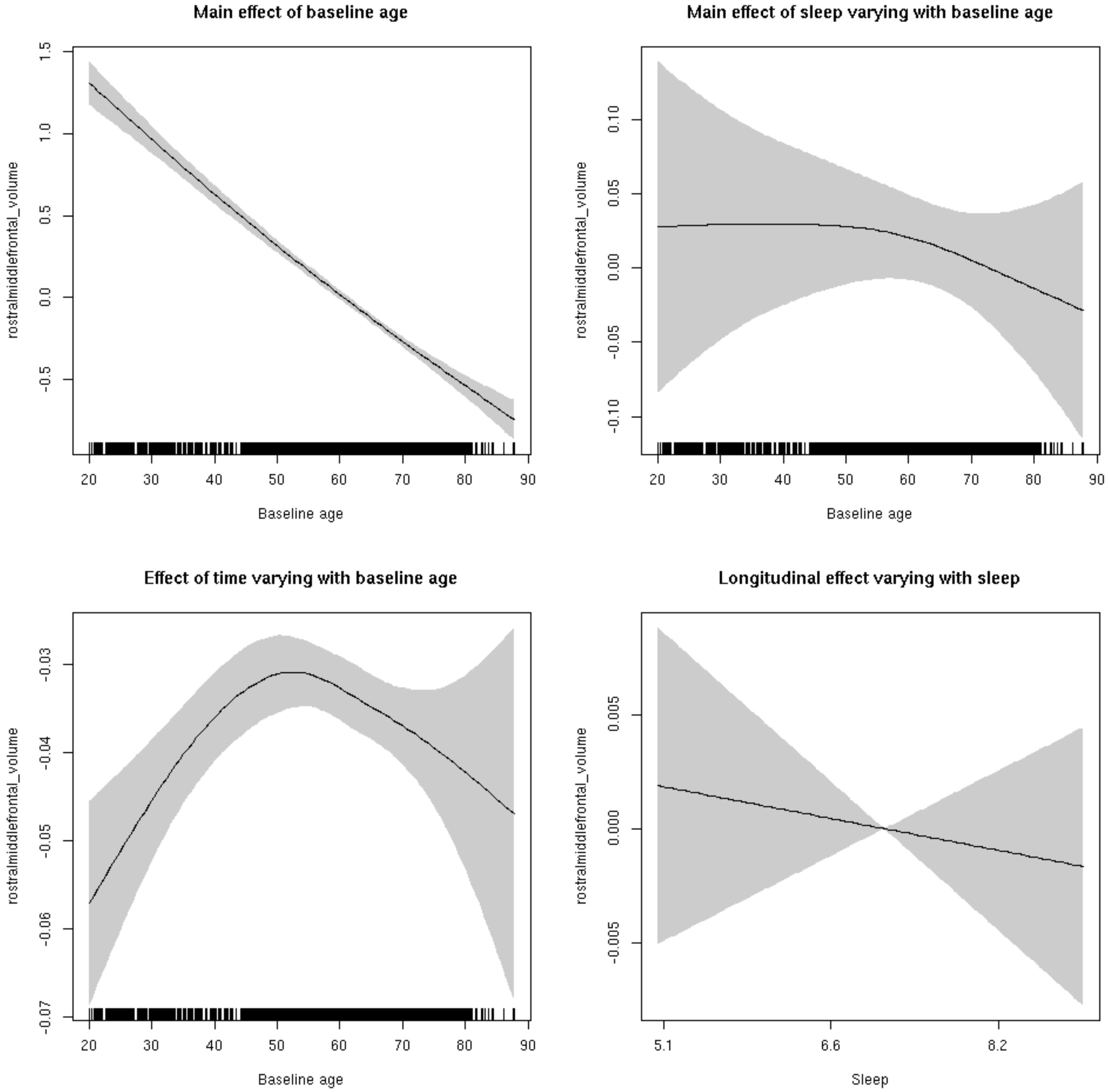

Restricted to sleep >= 5 and <= 9 hours Below is the row for `s(sleep_z):time` from the model output.

```
## s(sleep_z):time   1.000  1.000   0.295   0.587
```

Below are plots of a plot of `s(bl_age)`, `s(bl_age):sleep_z`, `s(bl_age):time`, and `s(sleep_z):time`. For `s(sleep_z):time`, roughly, if we cannot fit a straight line through the confidence bands, there is evidence that the effect of time depends on sleep duration. It is correct the that confidence interval is zero at one point along the axis; this happens because we do not include the main effect.

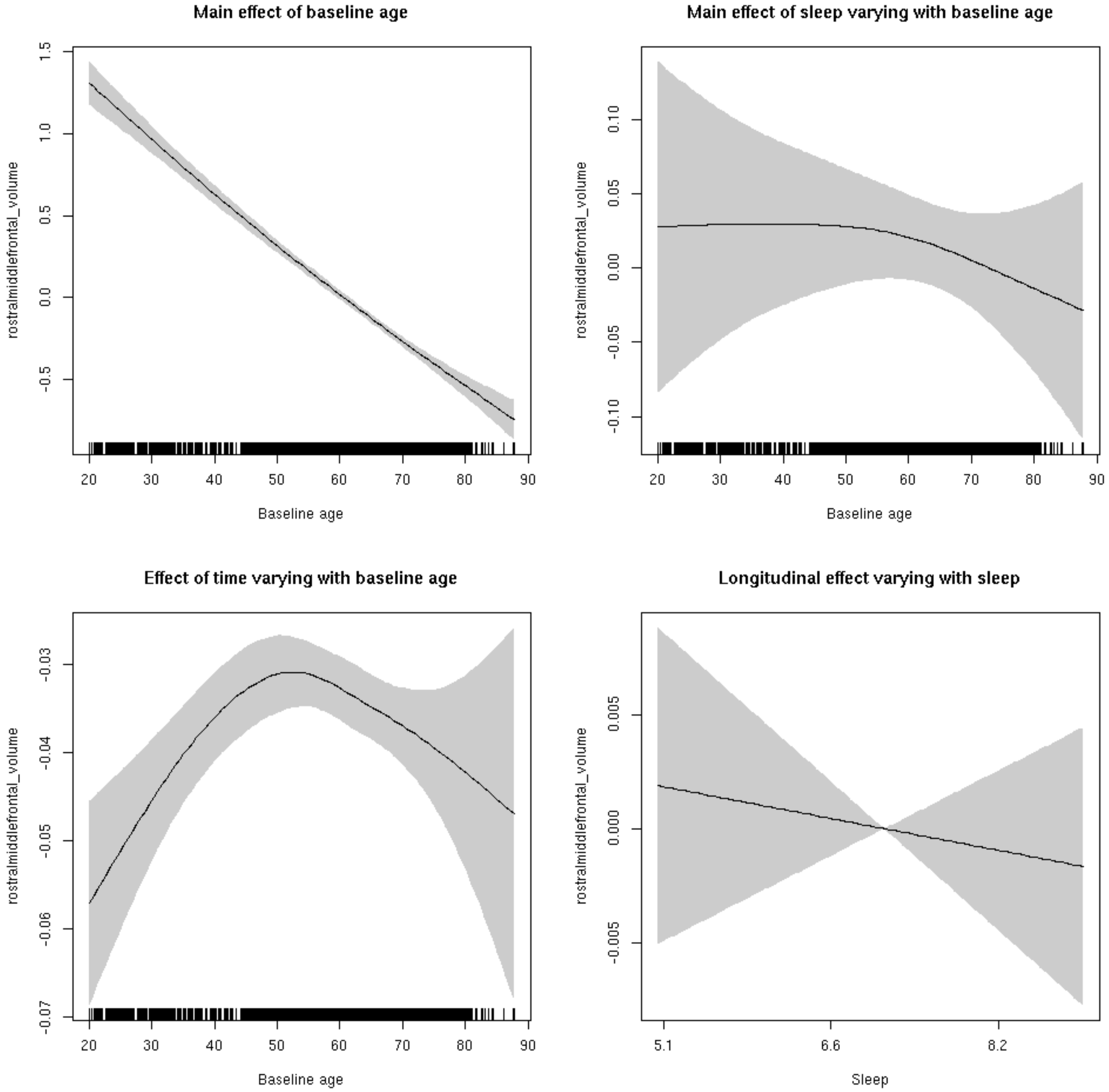

superiorfrontal_area: Stratified by sex

Female: Full data Below is the row for `s(sleep_z):time` from the model output.

```
## s(sleep_z):time   1.000  1.000  0.053   0.817
```

Below are plots of a plot of `s(bl_age)`, `s(bl_age):sleep_z`, `s(bl_age):time`, and `s(sleep_z):time`. For `s(sleep_z):time`, roughly, if we cannot fit a straight line through the confidence bands, there is evidence that the effect of time depends on sleep duration. It is correct the that confidence interval is zero at one point along the axis; this happens because we do not include the main effect.

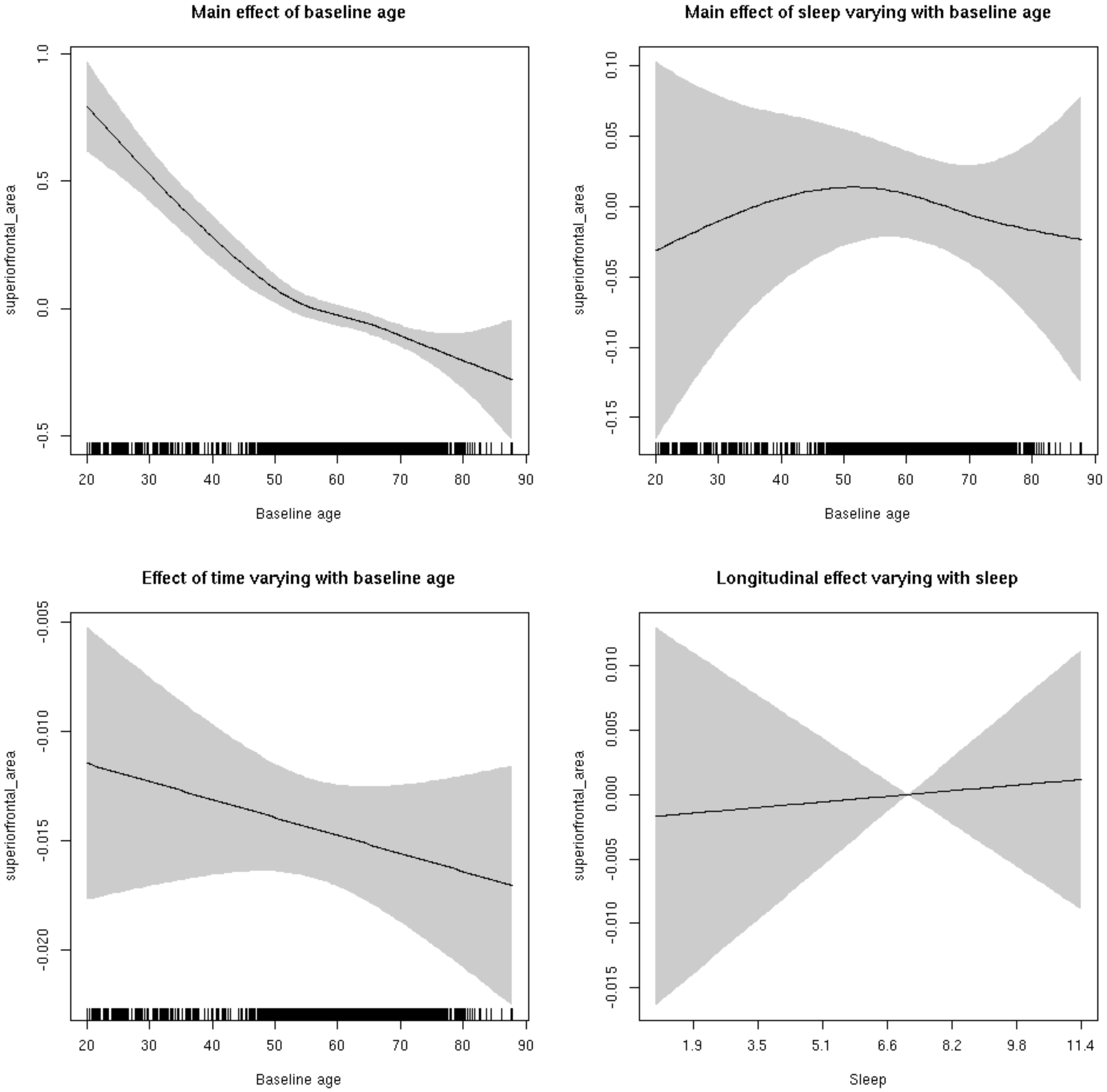

Male: Full data Below is the row for `s(sleep_z):time` from the model output.

```
## s(sleep_z):time   1.000  1.000  0.029   0.865
```

Below are plots of a plot of `s(bl_age)`, `s(bl_age):sleep_z`, `s(bl_age):time`, and `s(sleep_z):time`. For `s(sleep_z):time`, roughly, if we cannot fit a straight line through the confidence bands, there is evidence that the effect of time depends on sleep duration. It is correct the that confidence interval is zero at one point along the axis; this happens because we do not include the main effect.

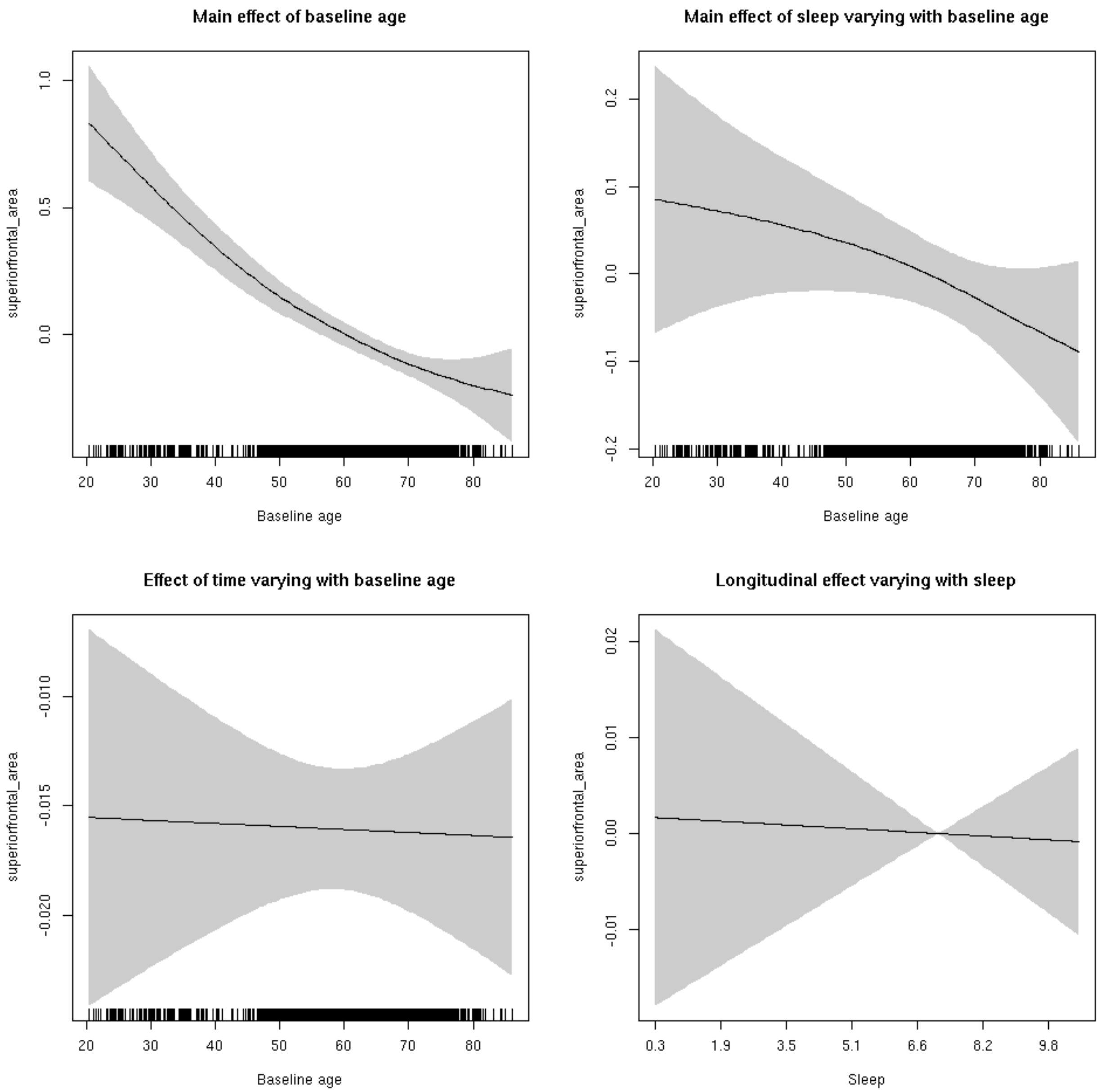

superiorfrontal_area

Full data Below is the model output. The term `s(sleep_z):time` is probably what we care most about.

```
##
## Family: gaussian
## Link function: identity
##
## Formula:
## value ~ sex + site + icv + s(bl_age, k = 10, bs = "cr") + s(bl_age,
##     by = sleep_z, bs = "cr") + +s(bl_age, by = time, k = 5, bs = "cr") +
##     s(sleep_z, by = time, k = 5, bs = "cr", pc = 0)
## <environment: 0x5630035b3ae8>
```

```
##
## Parametric coefficients:
##                Estimate Std. Error t value Pr(>|t|)
## (Intercept)     0.90008    0.04992  18.032  < 2e-16 ***
## sexmale         0.20567    0.02568   8.010 1.30e-15 ***
## siteousAvanto -0.86455    0.06076 -14.229  < 2e-16 ***
## siteousPrisma -0.97681    0.10577  -9.236  < 2e-16 ***
## siteousSkyra  -0.73426    0.05891 -12.465  < 2e-16 ***
## siteUB        -0.81673    0.11569  -7.060 1.81e-12 ***
## siteUCAM      -0.74582    0.06212 -12.007  < 2e-16 ***
## siteUKB       -0.98916    0.04748 -20.834  < 2e-16 ***
## siteUmU       -0.23033    0.07150  -3.221  0.00128 **
## icv            0.67458    0.01247  54.077  < 2e-16 ***
## ---
## Signif. codes:  0 '***' 0.001 '**' 0.01 '*' 0.05 '.' 0.1 ' ' 1
##
## Approximate significance of smooth terms:
##                       edf Ref.df       F p-value
## s(bl_age)           3.095  3.095  72.539  <2e-16 ***
## s(bl_age):sleep_z 2.992  2.992   1.767   0.133
## s(bl_age):time    2.000  2.000 149.712  <2e-16 ***
## s(sleep_z):time   1.000  1.000   0.010   0.921
## ---
## Signif. codes:  0 '***' 0.001 '**' 0.01 '*' 0.05 '.' 0.1 ' ' 1
##
## R-sq.(adj) =  0.585
## lmer.REML = 3962.1  Scale est. = 0.012158  n = 8144
```

Below are plots of a plot of `s(bl_age)`, `s(bl_age):sleep_z`, `s(bl_age):time`, and `s(sleep_z):time`. For `s(sleep_z):time`, roughly, if we cannot fit a straight line through the confidence bands, there is evidence that the effect of time depends on sleep duration. It is correct the that confidence interval is zero at one point along the axis; this happens because we do not include the main effect.

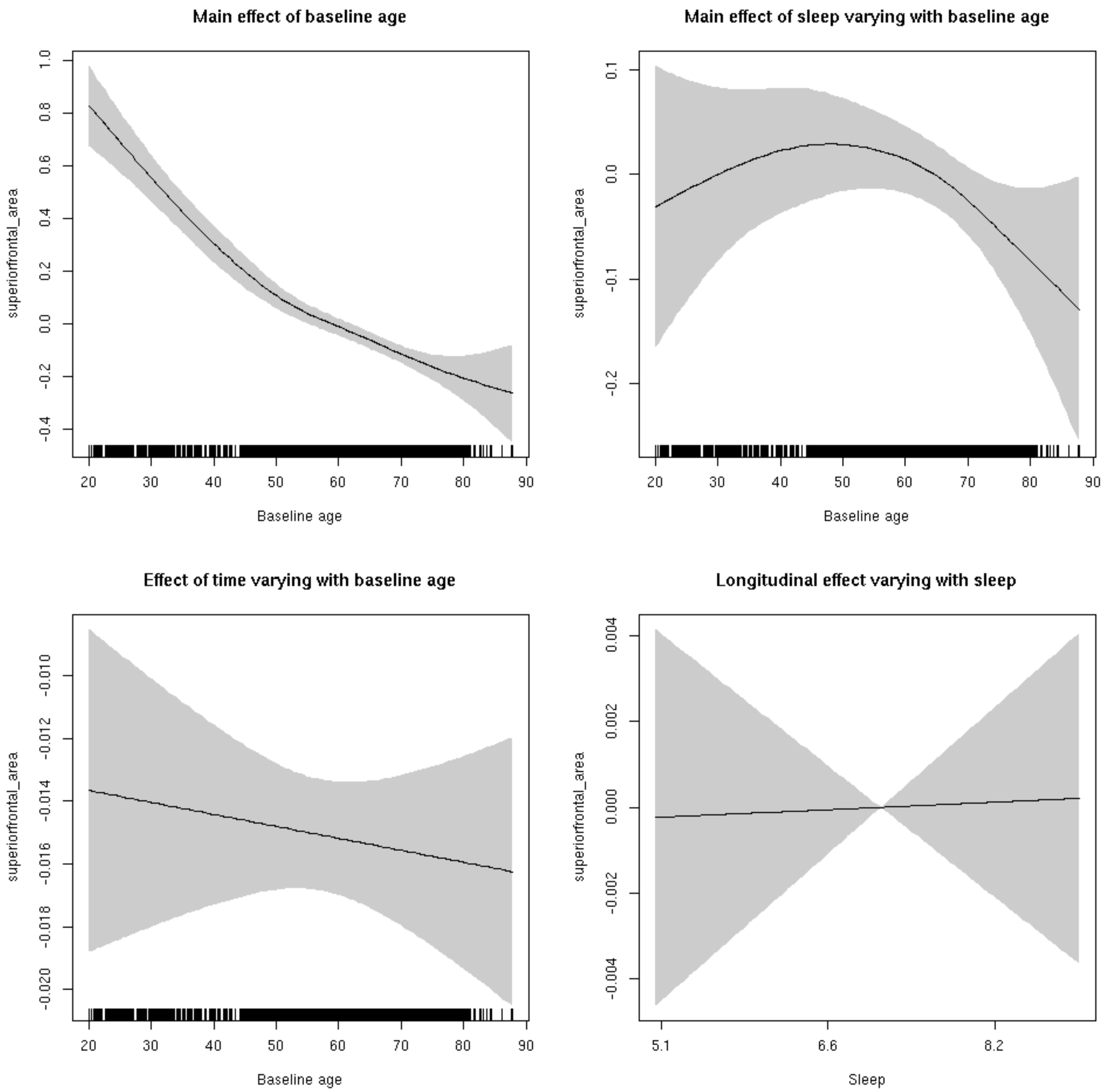

Restricted to sleep >= 5 and <= 9 hours Below is the row for `s(sleep_z):time` from the model output.

```
## s(sleep_z):time   1.000  1.000   0.012  0.9137
```

Below are plots of a plot of `s(bl_age)`, `s(bl_age):sleep_z`, `s(bl_age):time`, and `s(sleep_z):time`. For `s(sleep_z):time`, roughly, if we cannot fit a straight line through the confidence bands, there is evidence that the effect of time depends on sleep duration. It is correct the that confidence interval is zero at one point along the axis; this happens because we do not include the main effect.

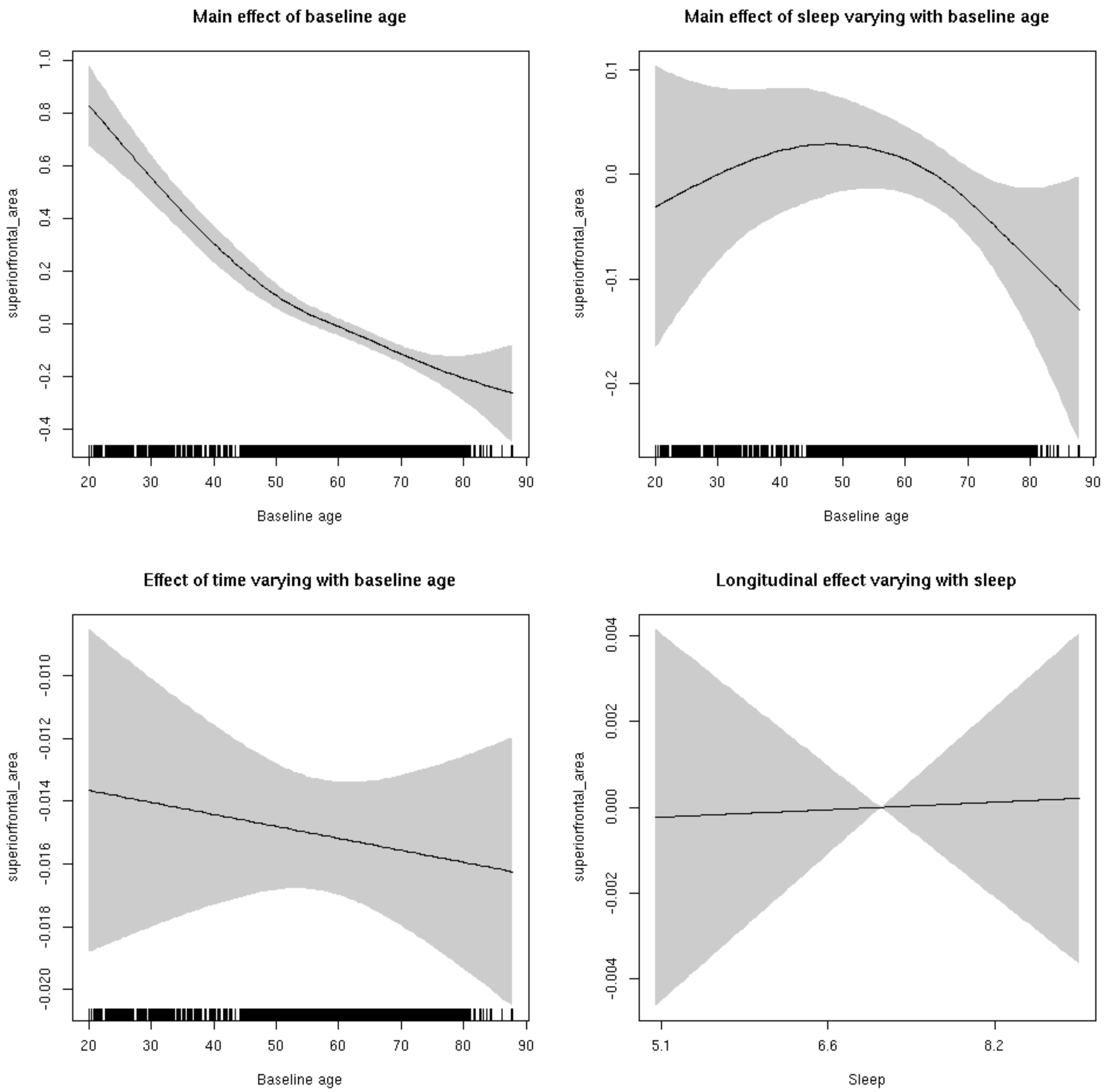

superiorfrontal_thickness: Stratified by sex

Female: Full data Below is the row for `s(sleep_z):time` from the model output.

```
## s(sleep_z):time   1.000  1.000   0.210   0.647
```

Below are plots of a plot of `s(bl_age)`, `s(bl_age):sleep_z`, `s(bl_age):time`, and `s(sleep_z):time`. For `s(sleep_z):time`, roughly, if we cannot fit a straight line through the confidence bands, there is evidence that the effect of time depends on sleep duration. It is correct the that confidence interval is zero at one point along the axis; this happens because we do not include the main effect.

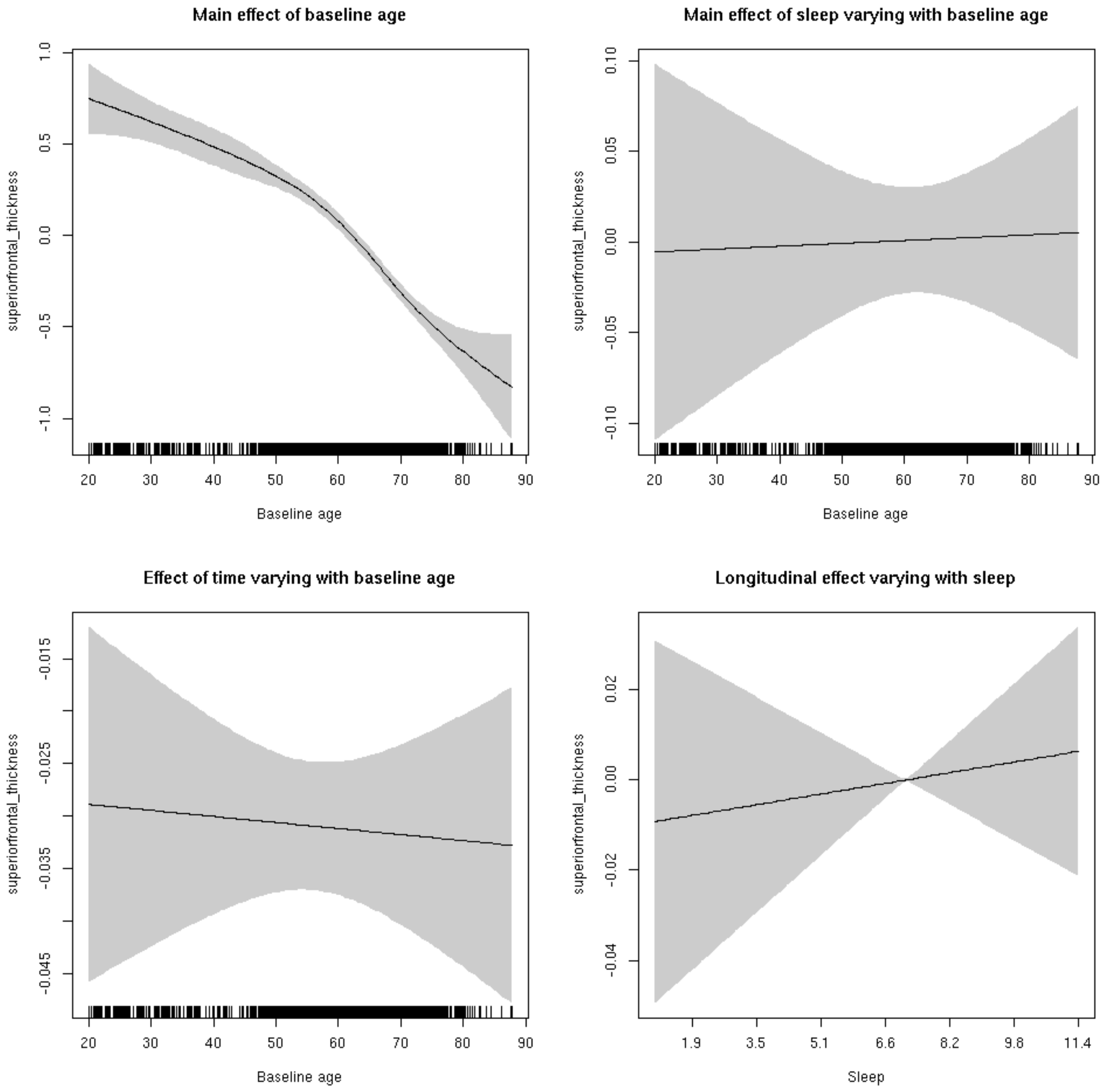

Male: Full data Below is the row for `s(sleep_z):time` from the model output.

```
## s(sleep_z):time   1.000  1.000   0.787   0.375
```

Below are plots of a plot of `s(bl_age)`, `s(bl_age):sleep_z`, `s(bl_age):time`, and `s(sleep_z):time`. For `s(sleep_z):time`, roughly, if we cannot fit a straight line through the confidence bands, there is evidence that the effect of time depends on sleep duration. It is correct the that confidence interval is zero at one point along the axis; this happens because we do not include the main effect.

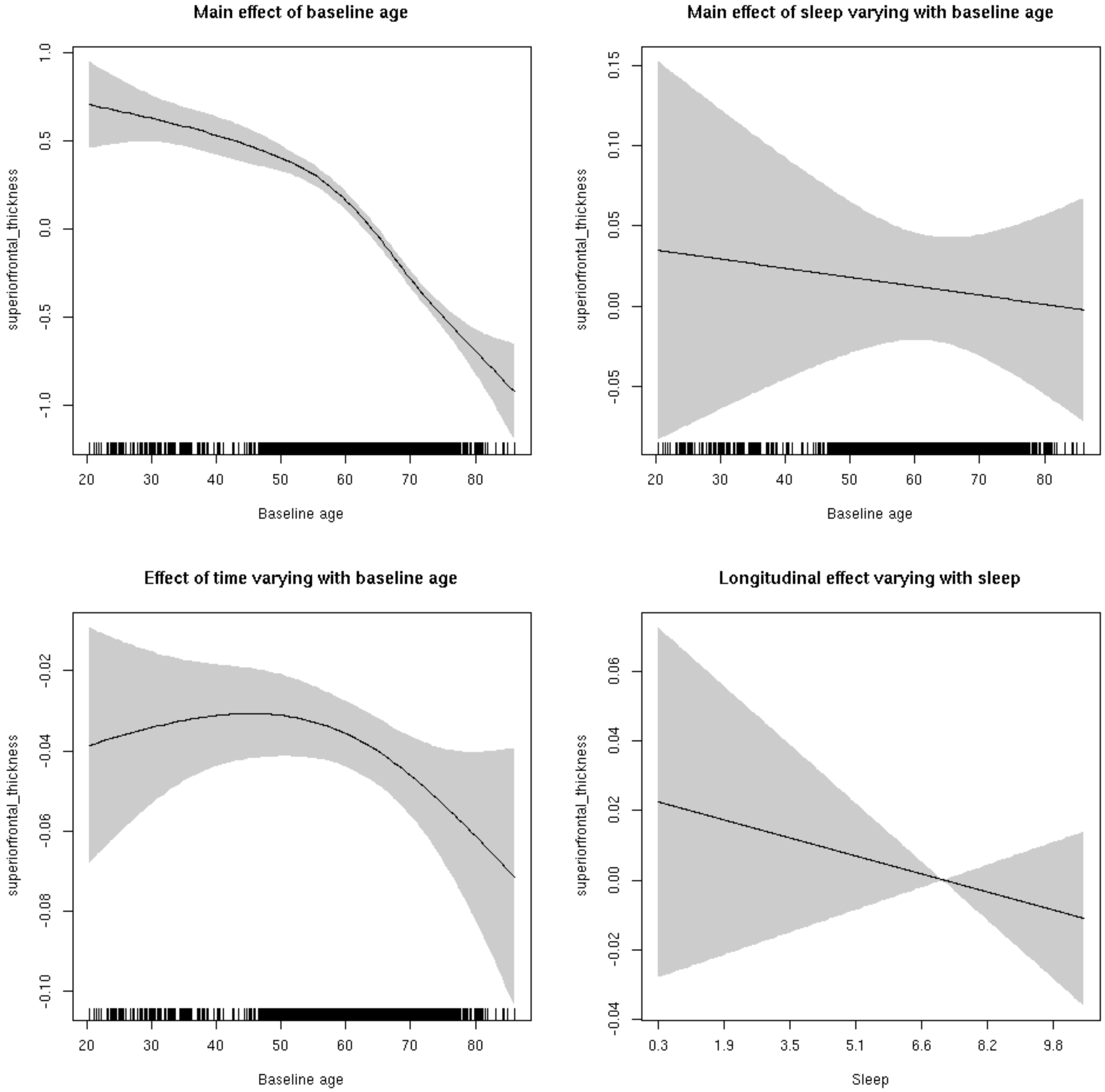

superiorfrontal_thickness

Full data Below is the model output. The term `s(sleep_z):time` is probably what we care most about.

```
##
## Family: gaussian
## Link function: identity
##
## Formula:
## value ~ sex + site + s(bl_age, k = 10, bs = "cr") + s(bl_age,
##     by = sleep_z, bs = "cr") + +s(bl_age, by = time, k = 5, bs = "cr") +
##     s(sleep_z, by = time, k = 5, bs = "cr", pc = 0)
## <environment: 0x5630035df768>
```

```
##
## Parametric coefficients:
##                 Estimate Std. Error t value Pr(>|t|)
## (Intercept)    -1.505786   0.041171 -36.574  < 2e-16 ***
## sexmale        -0.085143   0.020599  -4.133 3.61e-05 ***
## siteousAvanto   0.004912   0.054579   0.090 0.928289
## siteousPrisma   0.220276   0.227106   0.970 0.332111
## siteousSkyra   -1.039787   0.054573 -19.053  < 2e-16 ***
## siteUB          0.370319   0.109160   3.392 0.000696 ***
## siteUCAM        0.217076   0.056837   3.819 0.000135 ***
## siteUKB         1.893367   0.041802  45.294  < 2e-16 ***
## siteUmU        -0.282255   0.067778  -4.164 3.15e-05 ***
## ---
## Signif. codes:  0 '***' 0.001 '**' 0.01 '*' 0.05 '.' 0.1 ' ' 1
##
## Approximate significance of smooth terms:
##                      edf Ref.df       F p-value
## s(bl_age)          4.536  4.536 198.433  <2e-16 ***
## s(bl_age):sleep_z 2.000  2.000   0.153   0.859
## s(bl_age):time    2.841  2.841  52.271  <2e-16 ***
## s(sleep_z):time   2.308  2.308   1.886   0.280
## ---
## Signif. codes:  0 '***' 0.001 '**' 0.01 '*' 0.05 '.' 0.1 ' ' 1
##
## R-sq.(adj) =  0.699
## lmer.REML =  12117  Scale est. = 0.087886  n = 8172
```

Below are plots of a plot of `s(bl_age)`, `s(bl_age):sleep_z`, `s(bl_age):time`, and `s(sleep_z):time`. For `s(sleep_z):time`, roughly, if we cannot fit a straight line through the confidence bands, there is evidence that the effect of time depends on sleep duration. It is correct the that confidence interval is zero at one point along the axis; this happens because we do not include the main effect.

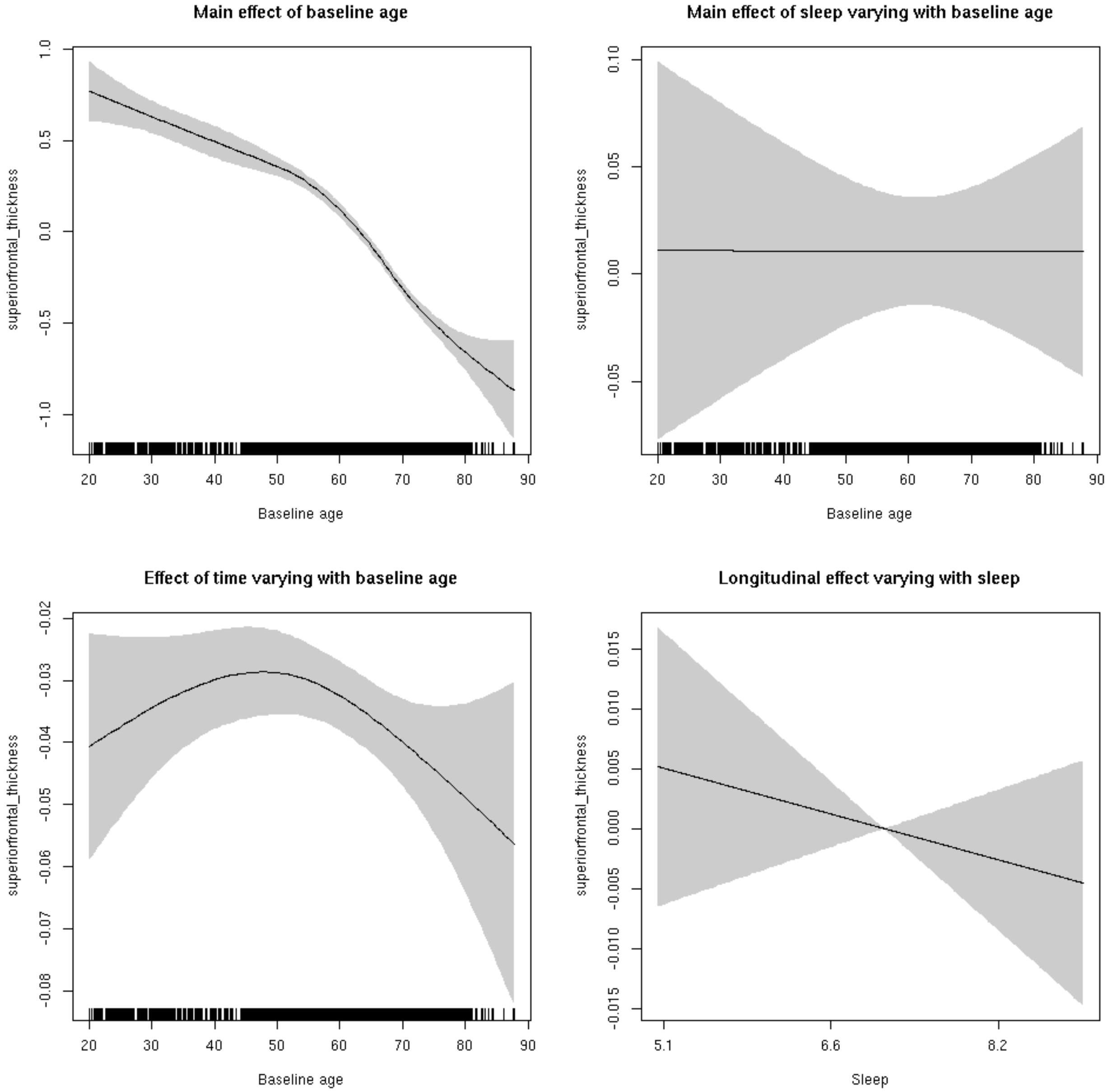

Restricted to sleep >= 5 and <= 9 hours Below is the row for `s(sleep_z):time` from the model output.

```
## s(sleep_z):time   1.000  1.000   0.784   0.376
```

Below are plots of a plot of `s(bl_age)`, `s(bl_age):sleep_z`, `s(bl_age):time`, and `s(sleep_z):time`. For `s(sleep_z):time`, roughly, if we cannot fit a straight line through the confidence bands, there is evidence that the effect of time depends on sleep duration. It is correct the that confidence interval is zero at one point along the axis; this happens because we do not include the main effect.

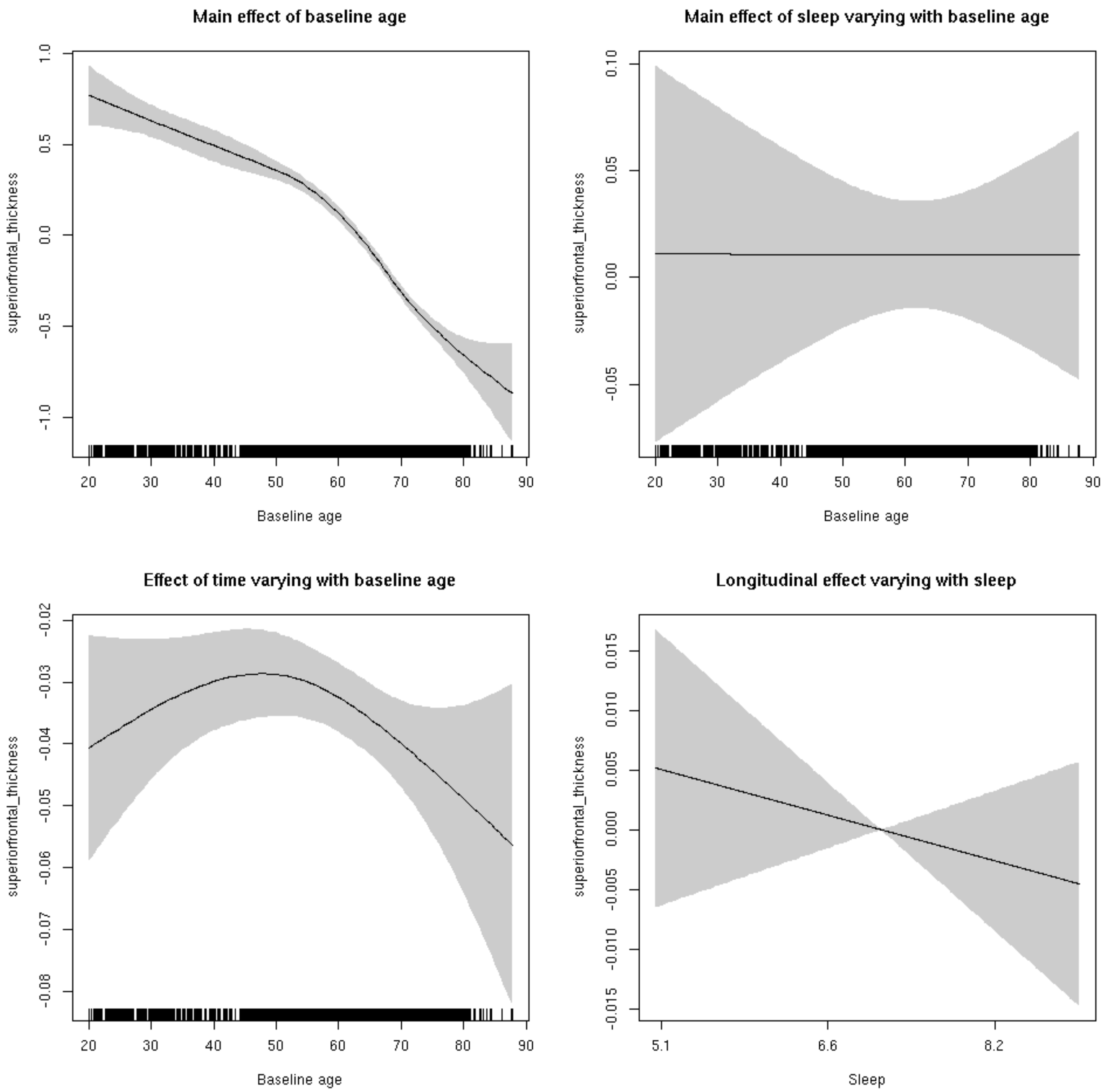

superiorfrontal_volume: Stratified by sex

Female: Full data Below is the row for `s(sleep_z):time` from the model output.

```
## s(sleep_z):time   2.599  2.599   1.382   0.284
```

Below are plots of a plot of `s(bl_age)`, `s(bl_age):sleep_z`, `s(bl_age):time`, and `s(sleep_z):time`. For `s(sleep_z):time`, roughly, if we cannot fit a straight line through the confidence bands, there is evidence that the effect of time depends on sleep duration. It is correct the that confidence interval is zero at one point along the axis; this happens because we do not include the main effect.

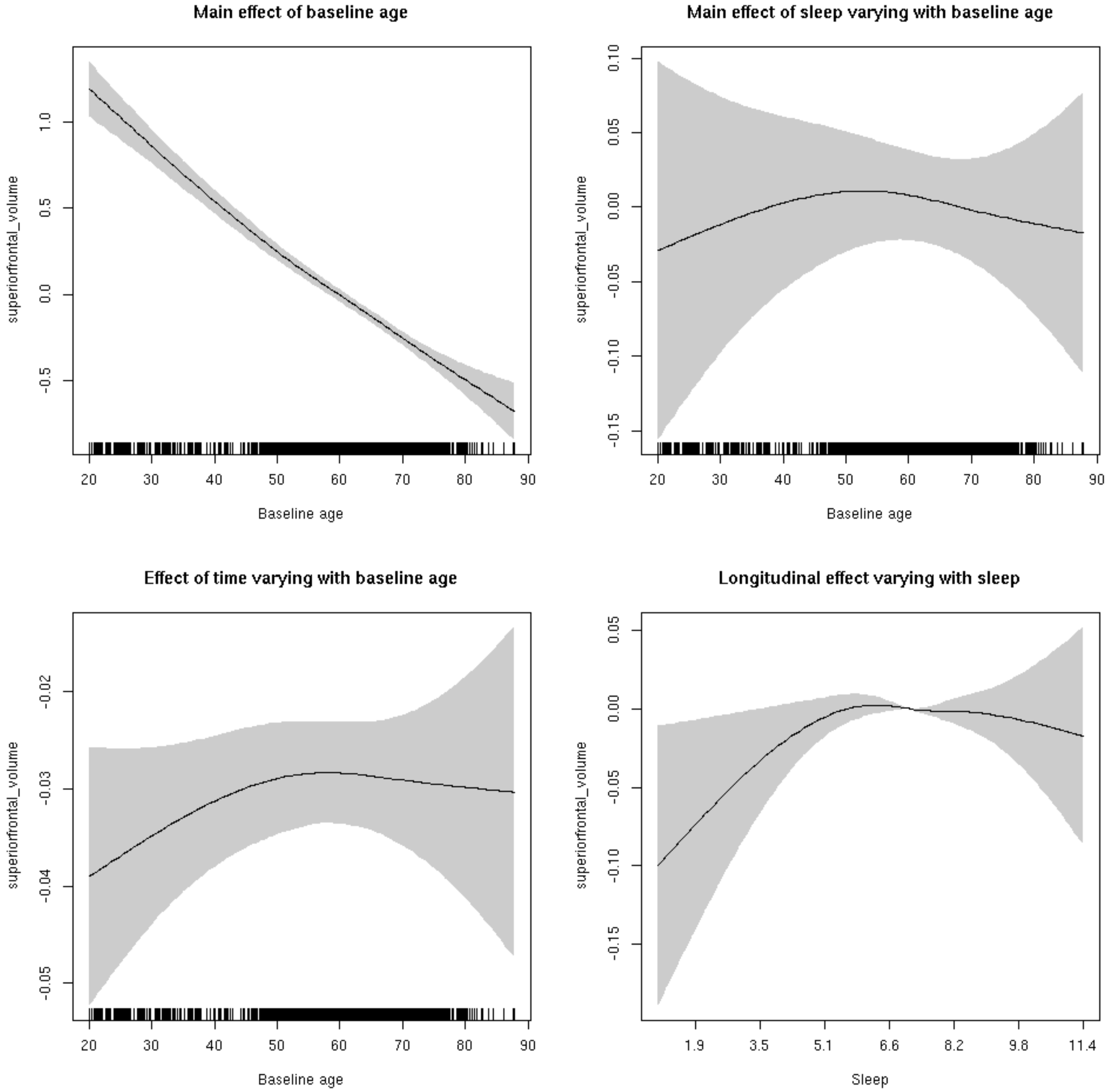

Male: Full data Below is the row for `s(sleep_z):time` from the model output.

```
## s(sleep_z):time   1.000  1.000   0.476  0.4905
```

Below are plots of a plot of `s(bl_age)`, `s(bl_age):sleep_z`, `s(bl_age):time`, and `s(sleep_z):time`. For `s(sleep_z):time`, roughly, if we cannot fit a straight line through the confidence bands, there is evidence that the effect of time depends on sleep duration. It is correct the that confidence interval is zero at one point along the axis; this happens because we do not include the main effect.

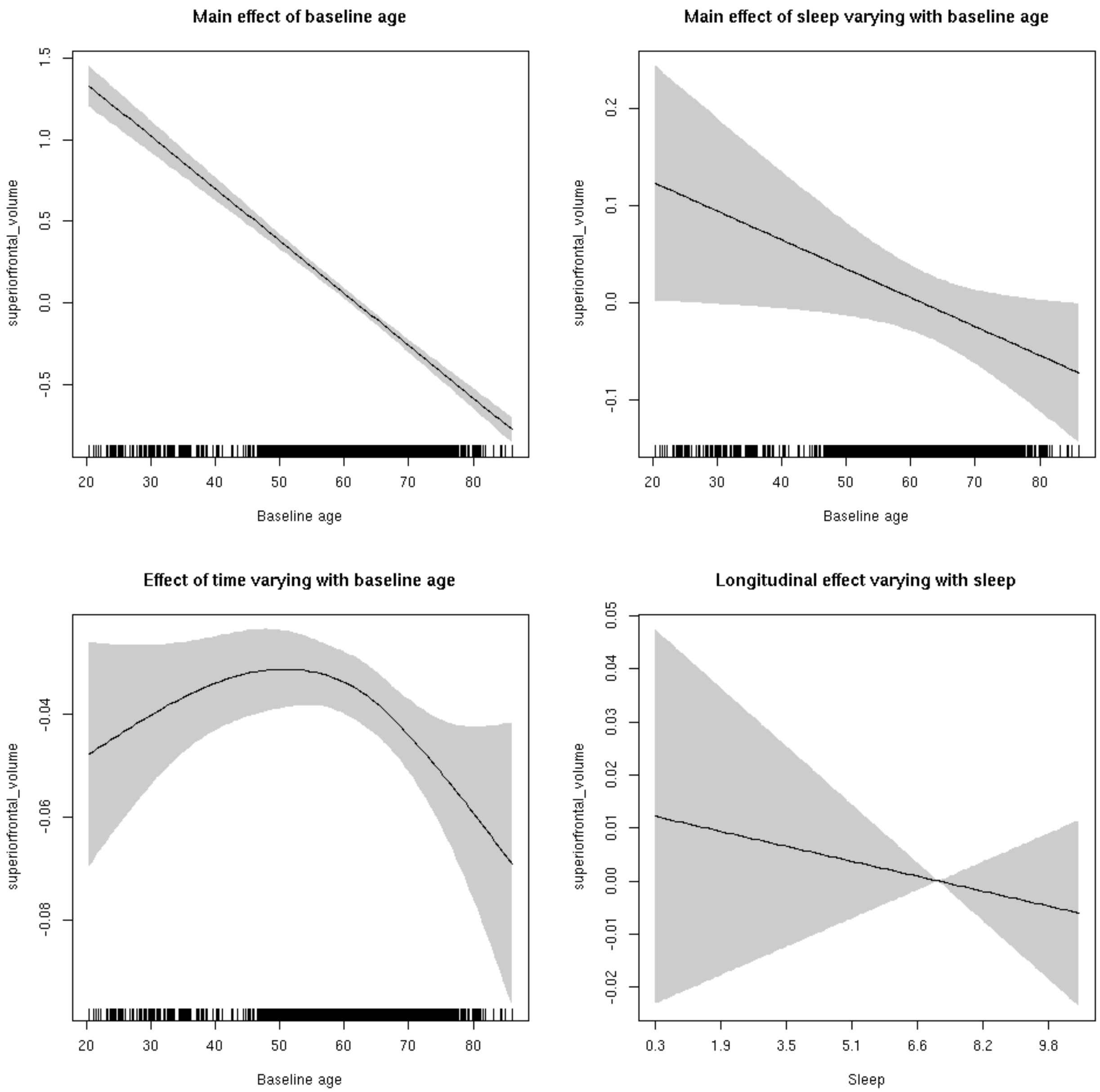

superiorfrontal_volume

Full data Below is the model output. The term `s(sleep_z):time` is probably what we care most about.

```
##
## Family: gaussian
## Link function: identity
##
## Formula:
## value ~ sex + site + icv + s(bl_age, k = 10, bs = "cr") + s(bl_age,
##     by = sleep_z, bs = "cr") + +s(bl_age, by = time, k = 5, bs = "cr") +
##     s(sleep_z, by = time, k = 5, bs = "cr", pc = 0)
## <environment: 0x5630035b4098>
```

```
##
## Parametric coefficients:
##                 Estimate Std. Error t value Pr(>|t|)
## (Intercept)      0.140875   0.048253   2.920  0.00352 **
## sexmale          0.158561   0.024767   6.402 1.62e-10 ***
## siteousAvanto -0.970298   0.059063 -16.428  < 2e-16 ***
## siteousPrisma -0.676788   0.161652  -4.187 2.86e-05 ***
## siteousSkyra  -1.256849   0.057178 -21.981  < 2e-16 ***
## siteUB        -0.555481   0.110616  -5.022 5.23e-07 ***
## siteUCAM      -0.623602   0.059877 -10.415  < 2e-16 ***
## siteUKB       -0.008757   0.045833  -0.191  0.84849
## siteUmU       -0.279725   0.068735  -4.070 4.75e-05 ***
## icv            0.637480   0.012225  52.144  < 2e-16 ***
## ---
## Signif. codes:  0 '***' 0.001 '**' 0.01 '*' 0.05 '.' 0.1 ' ' 1
##
## Approximate significance of smooth terms:
##                       edf Ref.df       F p-value
## s(bl_age)           3.509  3.509 268.553  <2e-16 ***
## s(bl_age):sleep_z 2.476  2.476   1.099   0.220
## s(bl_age):time    3.291  3.291  92.473  <2e-16 ***
## s(sleep_z):time   2.585  2.585   2.093   0.194
## ---
## Signif. codes:  0 '***' 0.001 '**' 0.01 '*' 0.05 '.' 0.1 ' ' 1
##
## R-sq.(adj) =  0.624
## lmer.REML = 8597.4  Scale est. = 0.038744  n = 8176
```

Below are plots of a plot of `s(bl_age)`, `s(bl_age):sleep_z`, `s(bl_age):time`, and `s(sleep_z):time`. For `s(sleep_z):time`, roughly, if we cannot fit a straight line through the confidence bands, there is evidence that the effect of time depends on sleep duration. It is correct the that confidence interval is zero at one point along the axis; this happens because we do not include the main effect.

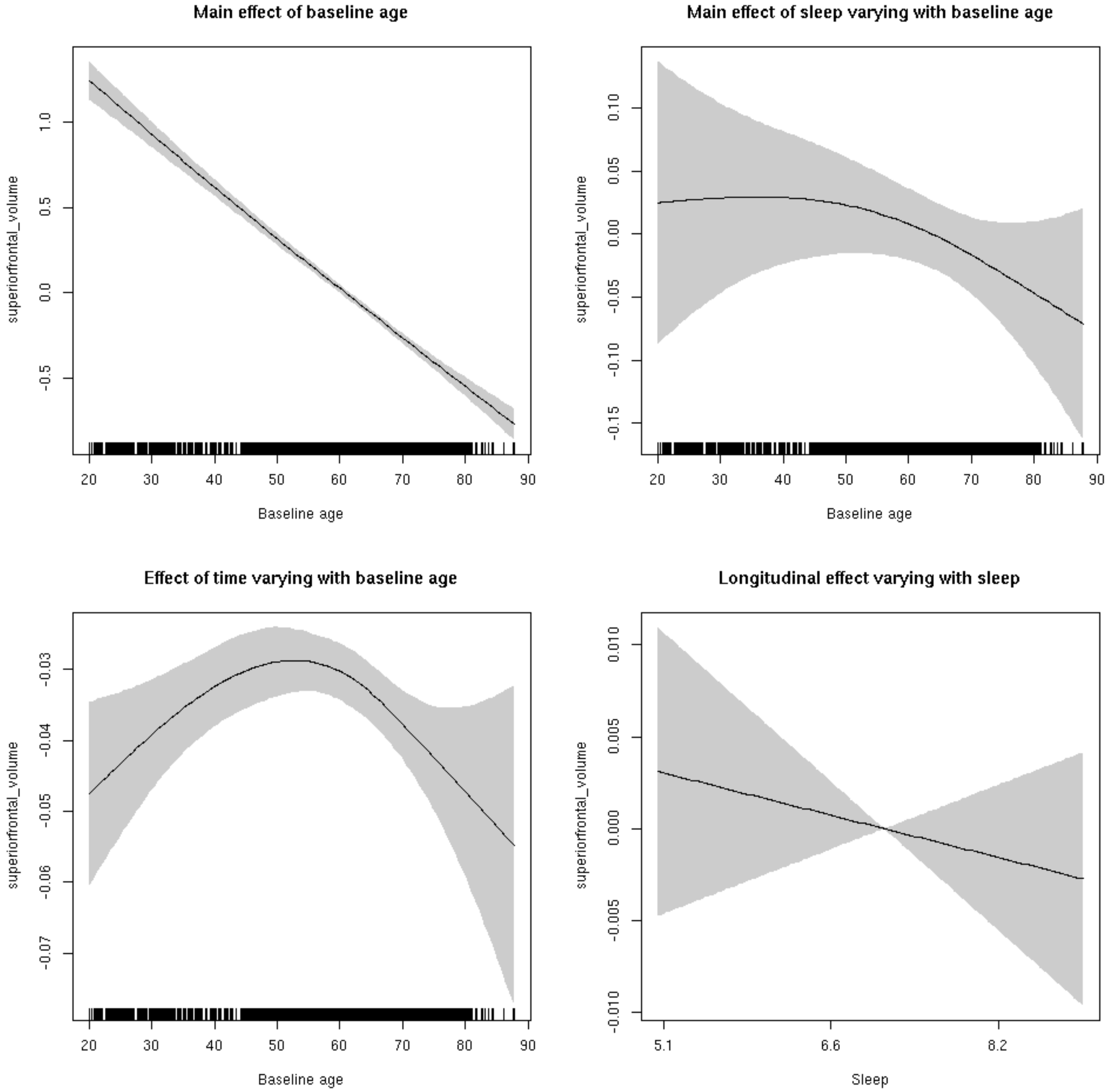

Restricted to sleep >= 5 and <= 9 hours Below is the row for `s(sleep_z):time` from the model output.

```
## s(sleep_z):time   1.000  1.000   0.628   0.428
```

Below are plots of a plot of `s(bl_age)`, `s(bl_age):sleep_z`, `s(bl_age):time`, and `s(sleep_z):time`. For `s(sleep_z):time`, roughly, if we cannot fit a straight line through the confidence bands, there is evidence that the effect of time depends on sleep duration. It is correct the that confidence interval is zero at one point along the axis; this happens because we do not include the main effect.

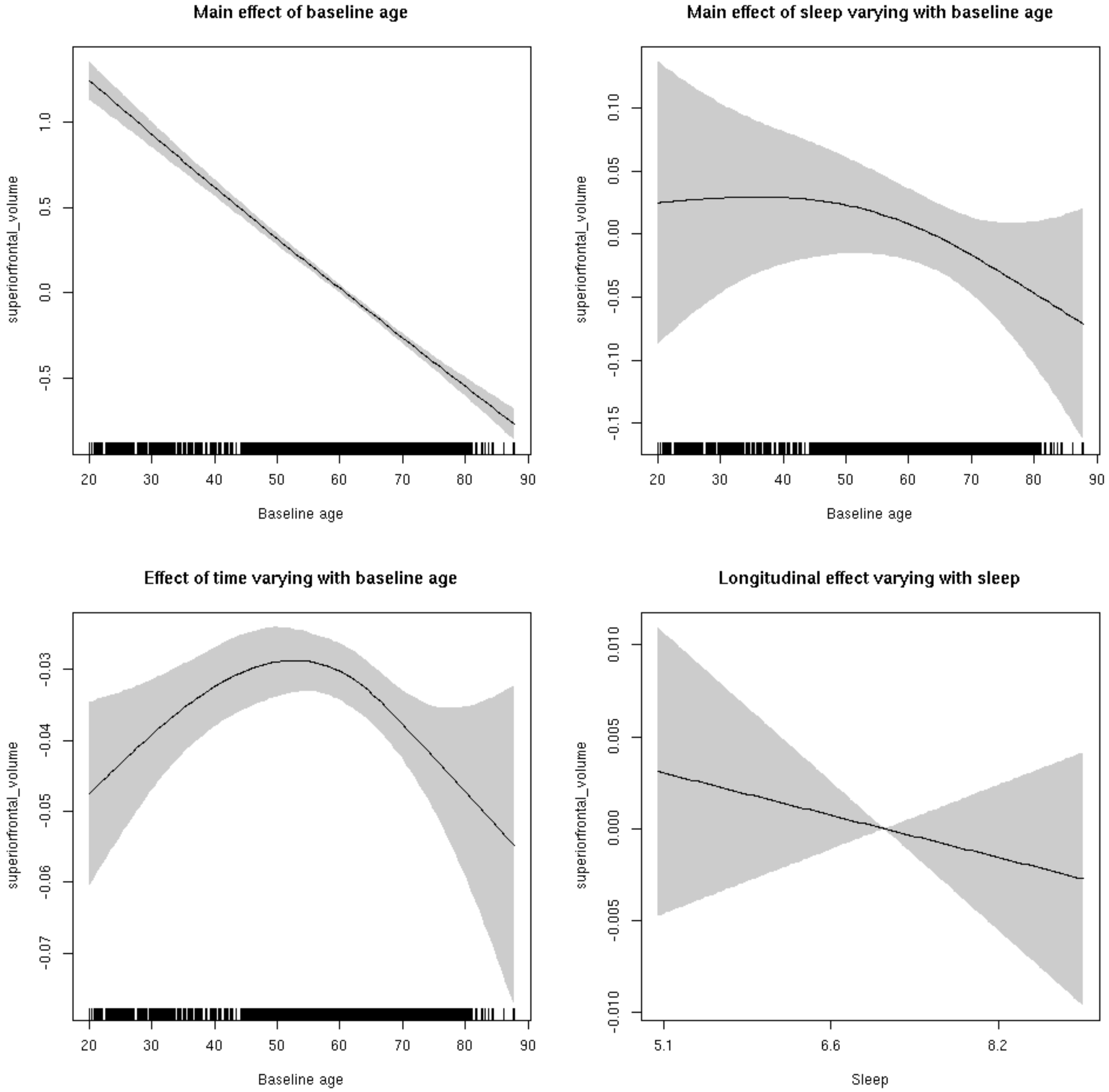

superiorparietal_area: Stratified by sex

Female: Full data Below is the row for `s(sleep_z):time` from the model output.

```
## s(sleep_z):time     1       1   0.494    0.482
```

Below are plots of a plot of `s(bl_age)`, `s(bl_age):sleep_z`, `s(bl_age):time`, and `s(sleep_z):time`. For `s(sleep_z):time`, roughly, if we cannot fit a straight line through the confidence bands, there is evidence that the effect of time depends on sleep duration. It is correct the that confidence interval is zero at one point along the axis; this happens because we do not include the main effect.

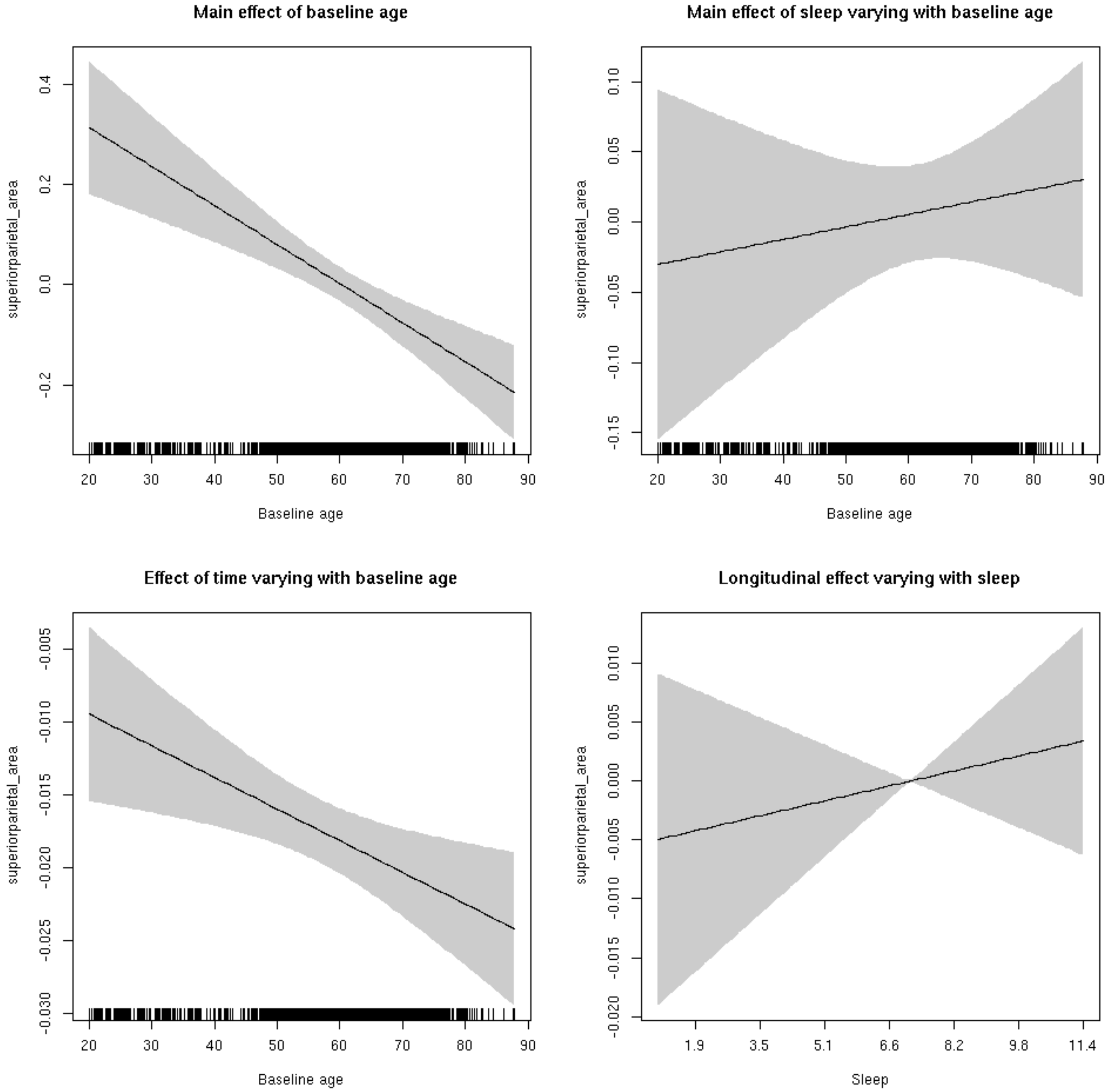

Male: Full data Below is the row for `s(sleep_z):time` from the model output.

```
## s(sleep_z):time     1       1   0.171   0.679
```

Below are plots of a plot of `s(bl_age)`, `s(bl_age):sleep_z`, `s(bl_age):time`, and `s(sleep_z):time`. For `s(sleep_z):time`, roughly, if we cannot fit a straight line through the confidence bands, there is evidence that the effect of time depends on sleep duration. It is correct the that confidence interval is zero at one point along the axis; this happens because we do not include the main effect.

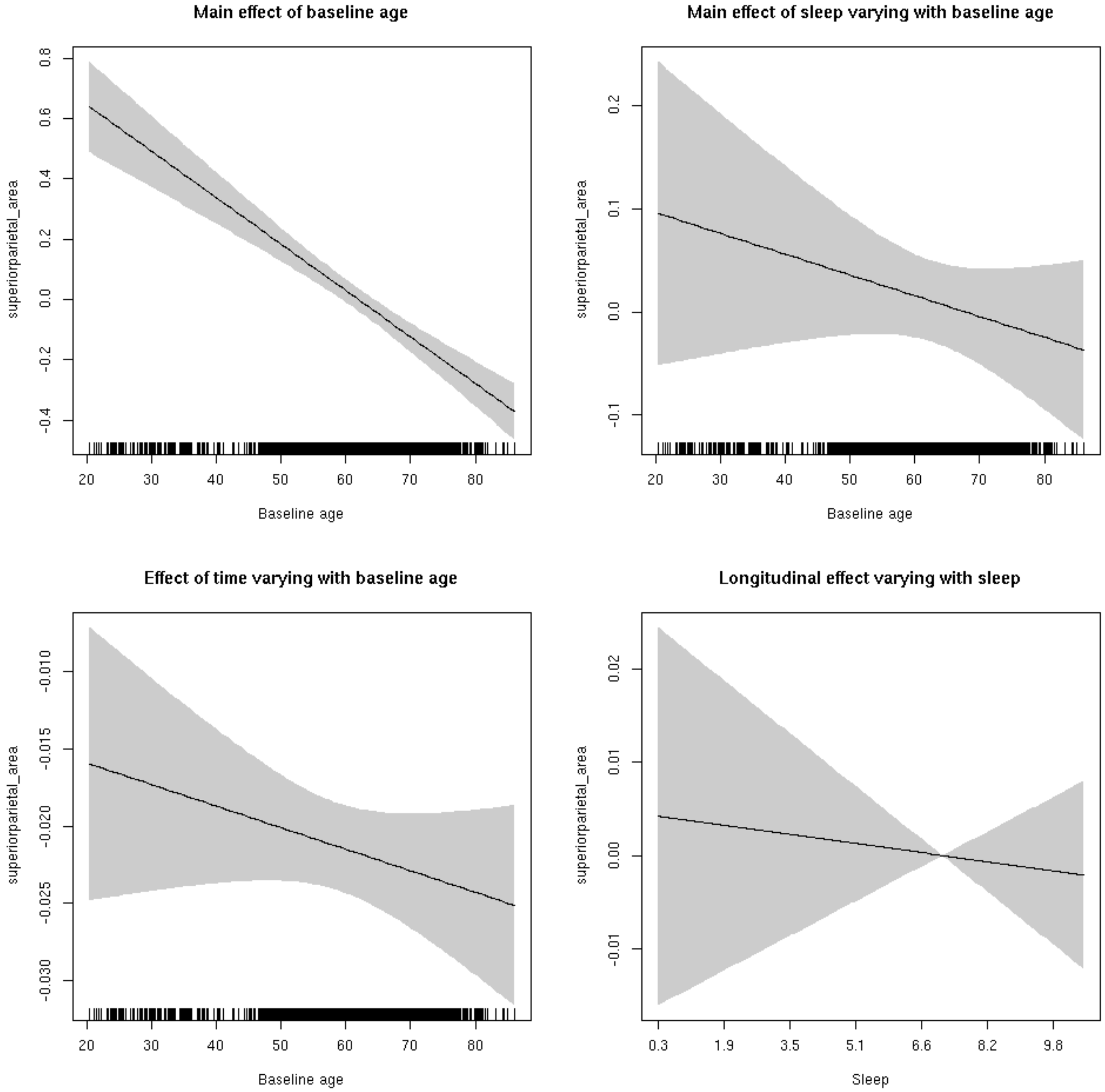

superiorparietal_area

Full data Below is the model output. The term `s(sleep_z):time` is probably what we care most about.

```
##
## Family: gaussian
## Link function: identity
##
## Formula:
## value ~ sex + site + icv + s(bl_age, k = 10, bs = "cr") + s(bl_age,
##     by = sleep_z, bs = "cr") + +s(bl_age, by = time, k = 5, bs = "cr") +
##     s(sleep_z, by = time, k = 5, bs = "cr", pc = 0)
## <environment: 0x5630035b24c0>
```

```
##
## Parametric coefficients:
##                Estimate Std. Error t value Pr(>|t|)
## (Intercept)     0.60498    0.05858  10.328  < 2e-16 ***
## sexmale         0.04323    0.03047   1.419   0.1560
## siteousAvanto -0.76435    0.07209 -10.603  < 2e-16 ***
## siteousPrisma -0.71614    0.11334  -6.319 2.78e-10 ***
## siteousSkyra  -0.28177    0.06993  -4.029 5.64e-05 ***
## siteUB        -0.69727    0.13779  -5.060 4.28e-07 ***
## siteUCAM      -0.77930    0.07331 -10.630  < 2e-16 ***
## siteUKB       -0.59222    0.05474 -10.819  < 2e-16 ***
## siteUmU        0.14541    0.08476   1.716   0.0863 .
## icv            0.57437    0.01464  39.233  < 2e-16 ***
## ---
## Signif. codes:  0 '***' 0.001 '**' 0.01 '*' 0.05 '.' 0.1 ' ' 1
##
## Approximate significance of smooth terms:
##                    edf Ref.df       F p-value
## s(bl_age)            1      1  97.816  <2e-16 ***
## s(bl_age):sleep_z    2      2   0.211   0.809
## s(bl_age):time       2      2 241.460  <2e-16 ***
## s(sleep_z):time      1      1   0.005   0.941
## ---
## Signif. codes:  0 '***' 0.001 '**' 0.01 '*' 0.05 '.' 0.1 ' ' 1
##
## R-sq.(adj) =  0.392
## lmer.REML = 5341.5  Scale est. = 0.012193  n = 8141
```

Below are plots of a plot of `s(bl_age)`, `s(bl_age):sleep_z`, `s(bl_age):time`, and `s(sleep_z):time`. For `s(sleep_z):time`, roughly, if we cannot fit a straight line through the confidence bands, there is evidence that the effect of time depends on sleep duration. It is correct the that confidence interval is zero at one point along the axis; this happens because we do not include the main effect.

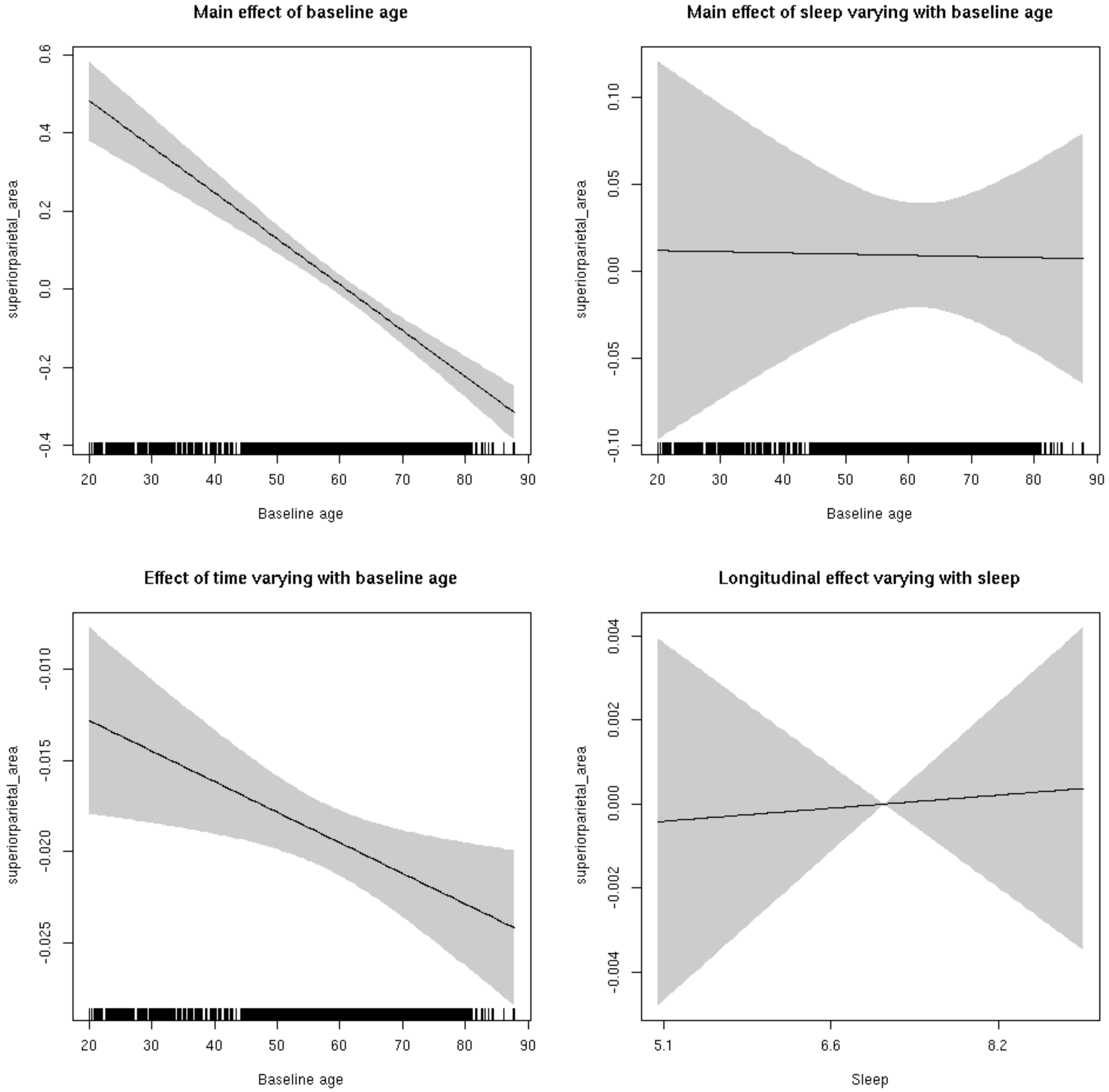

Restricted to sleep >= 5 and <= 9 hours Below is the row for `s(sleep_z):time` from the model output.

```
## s(sleep_z):time     1       1   0.038   0.846
```

Below are plots of a plot of `s(bl_age)`, `s(bl_age):sleep_z`, `s(bl_age):time`, and `s(sleep_z):time`. For `s(sleep_z):time`, roughly, if we cannot fit a straight line through the confidence bands, there is evidence that the effect of time depends on sleep duration. It is correct the that confidence interval is zero at one point along the axis; this happens because we do not include the main effect.

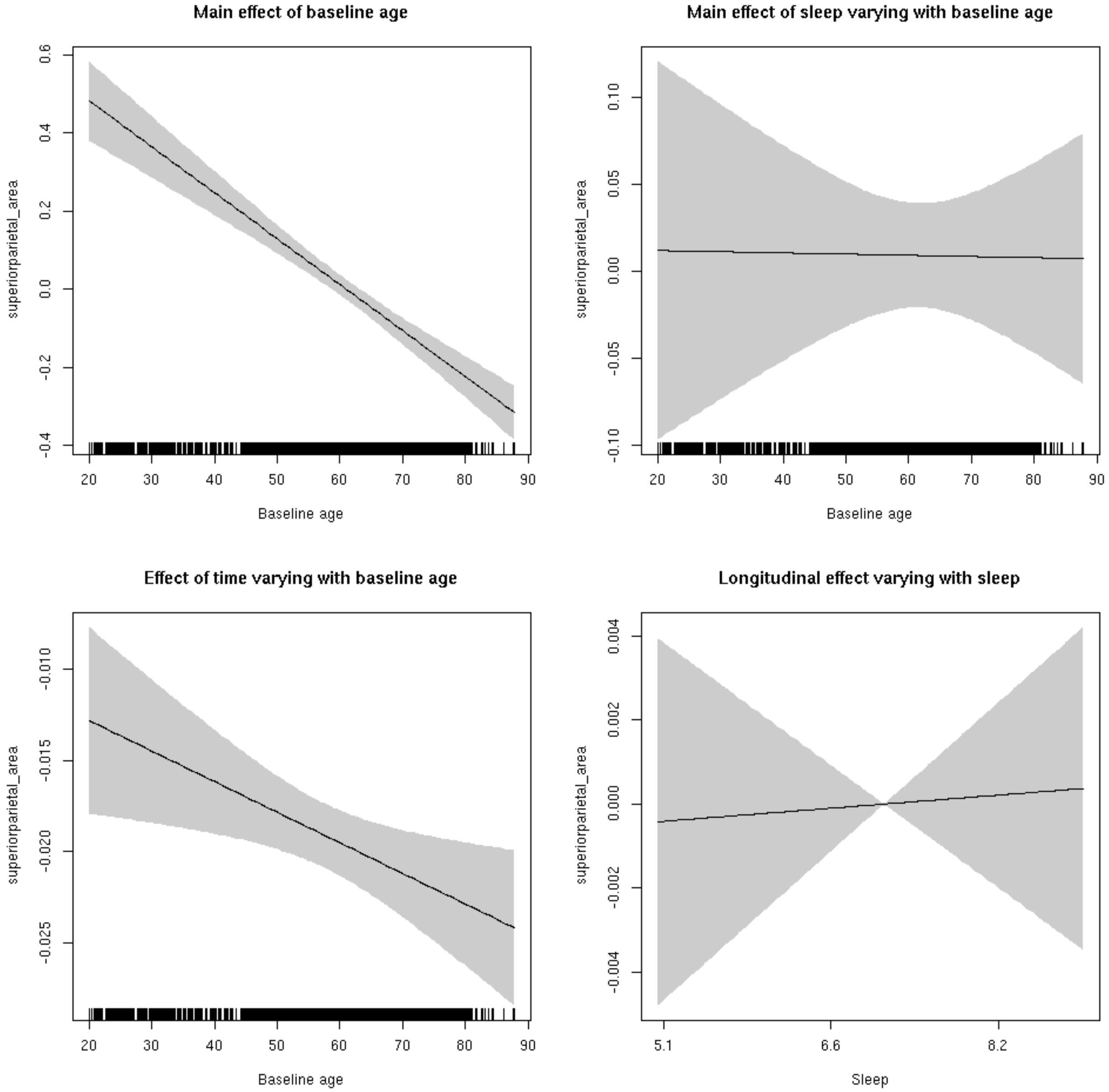

superiorparietal_thickness: Stratified by sex

Female: Full data Below is the row for `s(sleep_z):time` from the model output.

```
## s(sleep_z):time   1.000  1.000  2.136   0.144
```

Below are plots of a plot of `s(bl_age)`, `s(bl_age):sleep_z`, `s(bl_age):time`, and `s(sleep_z):time`. For `s(sleep_z):time`, roughly, if we cannot fit a straight line through the confidence bands, there is evidence that the effect of time depends on sleep duration. It is correct the that confidence interval is zero at one point along the axis; this happens because we do not include the main effect.

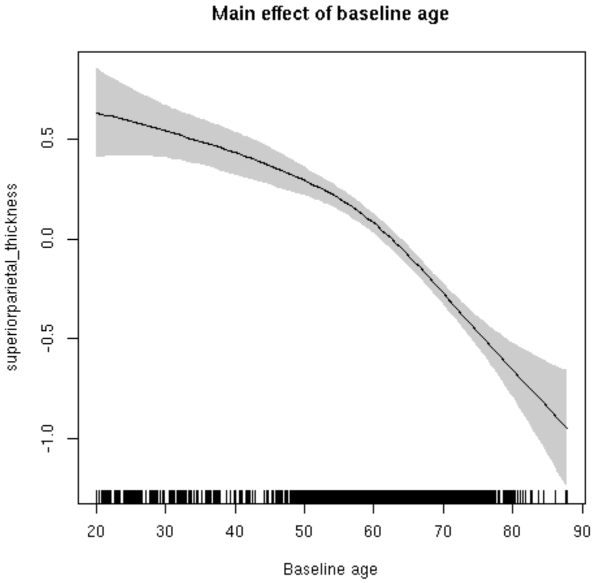

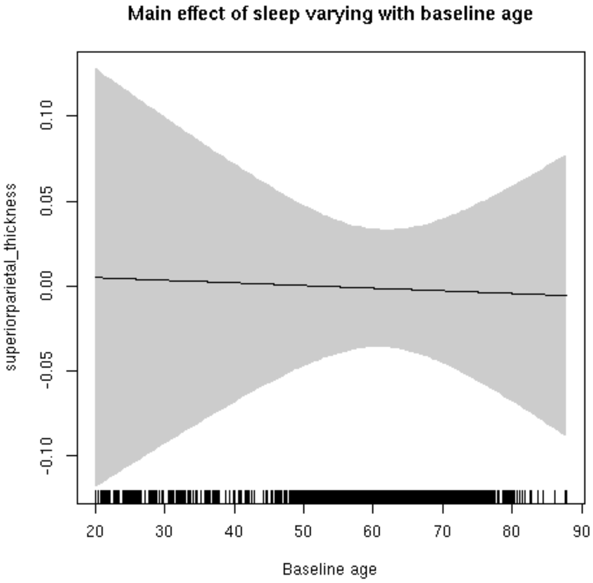

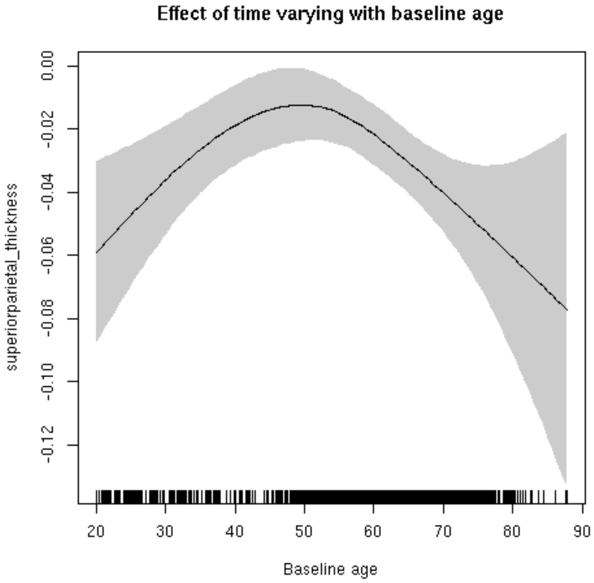

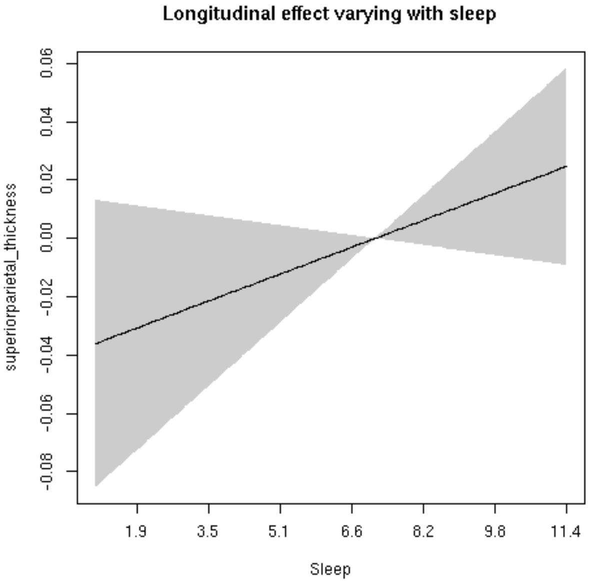

Male: Full data Below is the row for `s(sleep_z):time` from the model output.

```
## s(sleep_z):time   3.314  3.314  1.708  0.0849 .
```

Below are plots of a plot of `s(bl_age)`, `s(bl_age):sleep_z`, `s(bl_age):time`, and `s(sleep_z):time`. For `s(sleep_z):time`, roughly, if we cannot fit a straight line through the confidence bands, there is evidence that the effect of time depends on sleep duration. It is correct the that confidence interval is zero at one point along the axis; this happens because we do not include the main effect.

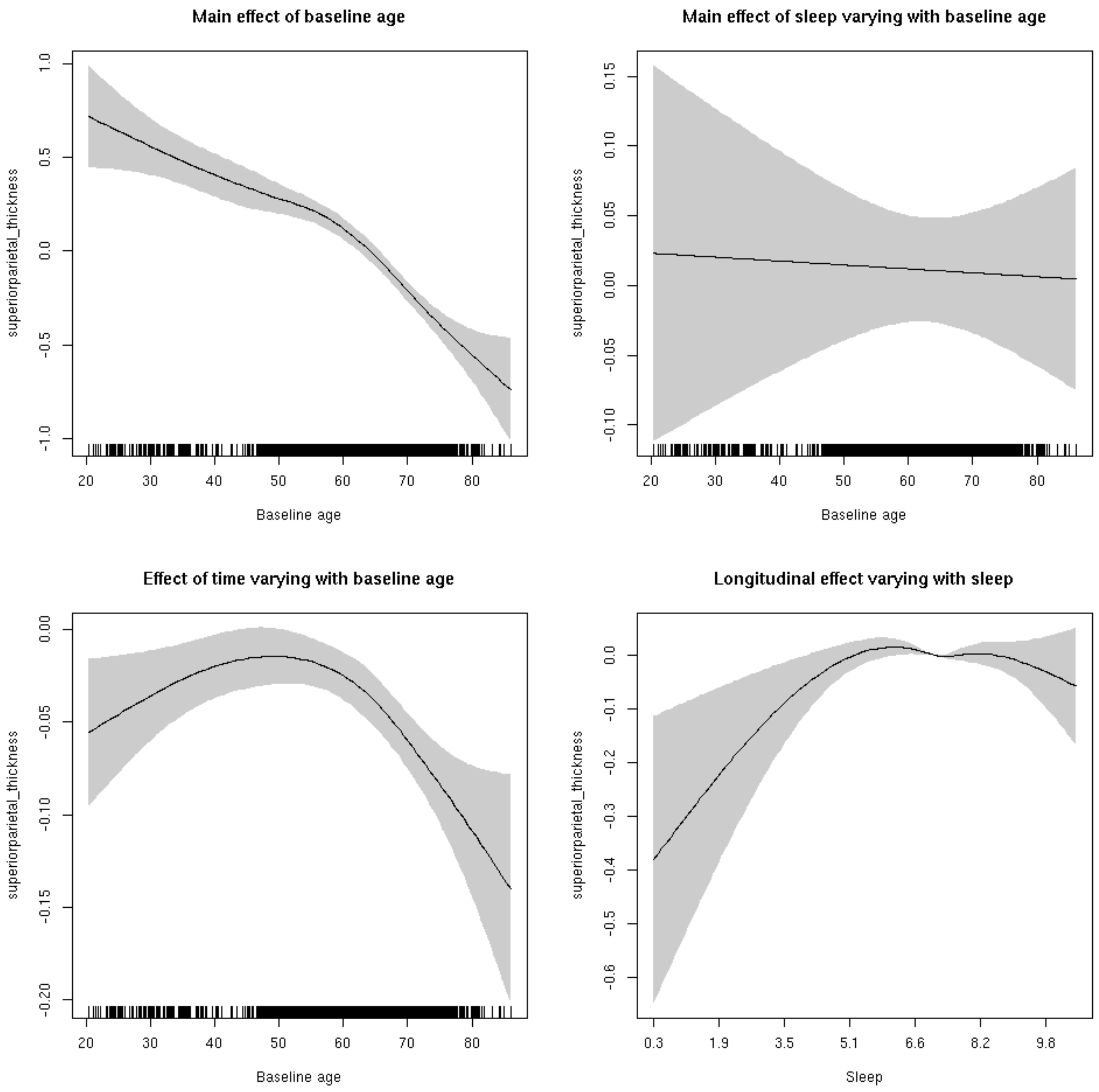

superiorparietal_thickness

Full data Below is the model output. The term `s(sleep_z):time` is probably what we care most about.

```
##
## Family: gaussian
## Link function: identity
##
## Formula:
## value ~ sex + site + s(bl_age, k = 10, bs = "cr") + s(bl_age,
##     by = sleep_z, bs = "cr") + +s(bl_age, by = time, k = 5, bs = "cr") +
##     s(sleep_z, by = time, k = 5, bs = "cr", pc = 0)
## <environment: 0x5630035cf1b8>
```

```
##
## Parametric coefficients:
##                Estimate Std. Error t value Pr(>|t|)
## (Intercept)   -1.40582    0.04785 -29.380  < 2e-16 ***
## sexmale       -0.07850    0.02398  -3.274 0.001065 **
## siteousAvanto  0.01922    0.06357   0.302 0.762446
## siteousPrisma  0.83445    0.27091   3.080 0.002076 **
## siteousSkyra  -0.23995    0.06356  -3.775 0.000161 ***
## siteUB         0.66094    0.12693   5.207 1.96e-07 ***
## siteUCAM       0.61170    0.06588   9.286  < 2e-16 ***
## siteUKB        1.70408    0.04851  35.128  < 2e-16 ***
## siteUmU        0.04644    0.07885   0.589 0.555929
## ---
## Signif. codes:  0 '***' 0.001 '**' 0.01 '*' 0.05 '.' 0.1 ' ' 1
##
## Approximate significance of smooth terms:
##                      edf Ref.df       F p-value
## s(bl_age)          3.765  3.765 126.289  <2e-16 ***
## s(bl_age):sleep_z 2.000  2.000   0.095  0.9092
## s(bl_age):time    3.751  3.751  33.148  <2e-16 ***
## s(sleep_z):time   3.533  3.533   2.969  0.0126 *
## ---
## Signif. codes:  0 '***' 0.001 '**' 0.01 '*' 0.05 '.' 0.1 ' ' 1
##
## R-sq.(adj) =  0.504
## lmer.REML =  14845  Scale est. = 0.12593   n = 8172
```

Below are plots of a plot of `s(bl_age)`, `s(bl_age):sleep_z`, `s(bl_age):time`, and `s(sleep_z):time`. For `s(sleep_z):time`, roughly, if we cannot fit a straight line through the confidence bands, there is evidence that the effect of time depends on sleep duration. It is correct the that confidence interval is zero at one point along the axis; this happens because we do not include the main effect.

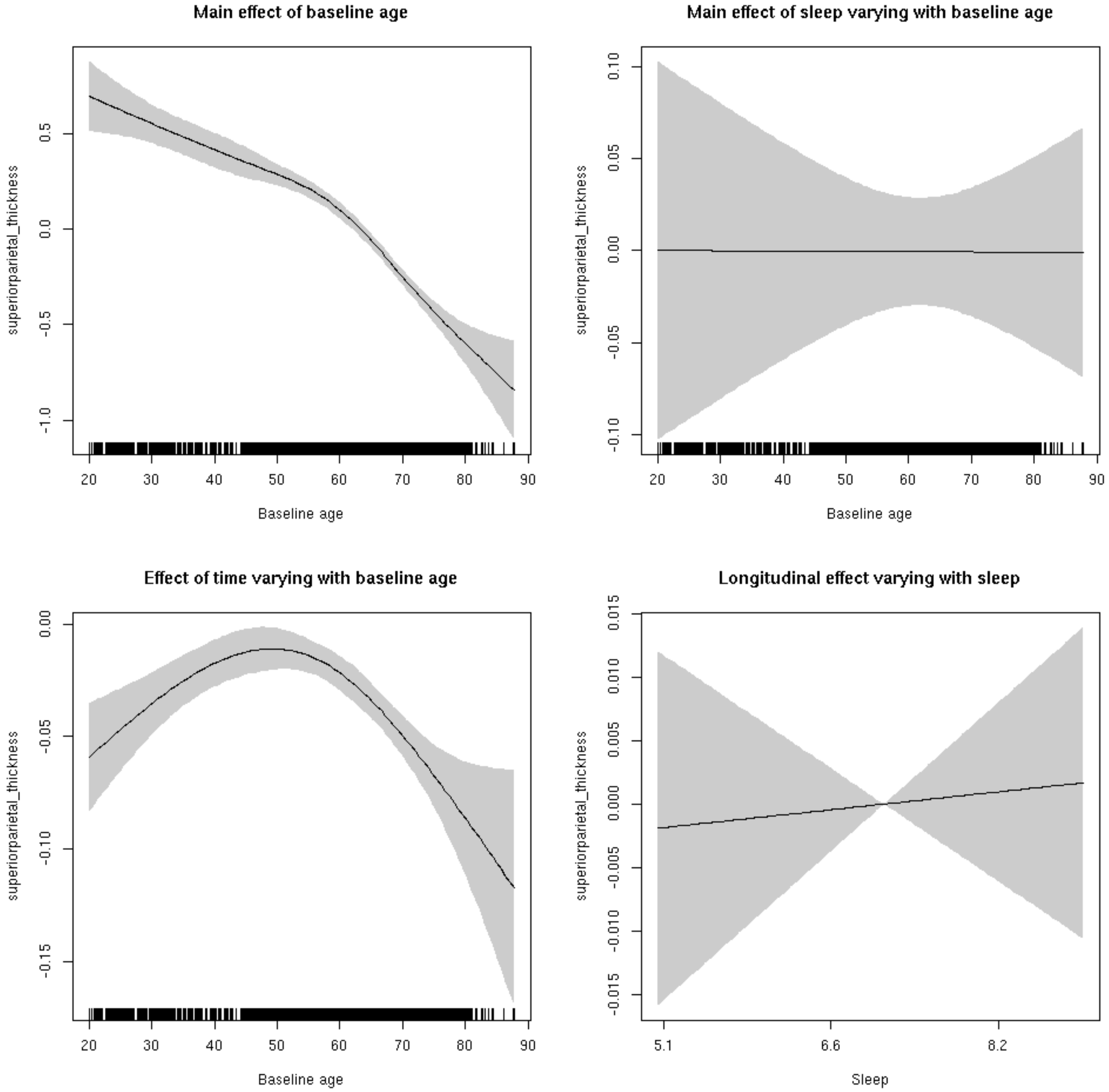

Restricted to sleep >= 5 and <= 9 hours Below is the row for `s(sleep_z):time` from the model output.

```
## s(sleep_z):time   1.000  1.000   0.075   0.785
```

Below are plots of a plot of `s(bl_age)`, `s(bl_age):sleep_z`, `s(bl_age):time`, and `s(sleep_z):time`. For `s(sleep_z):time`, roughly, if we cannot fit a straight line through the confidence bands, there is evidence that the effect of time depends on sleep duration. It is correct the that confidence interval is zero at one point along the axis; this happens because we do not include the main effect.

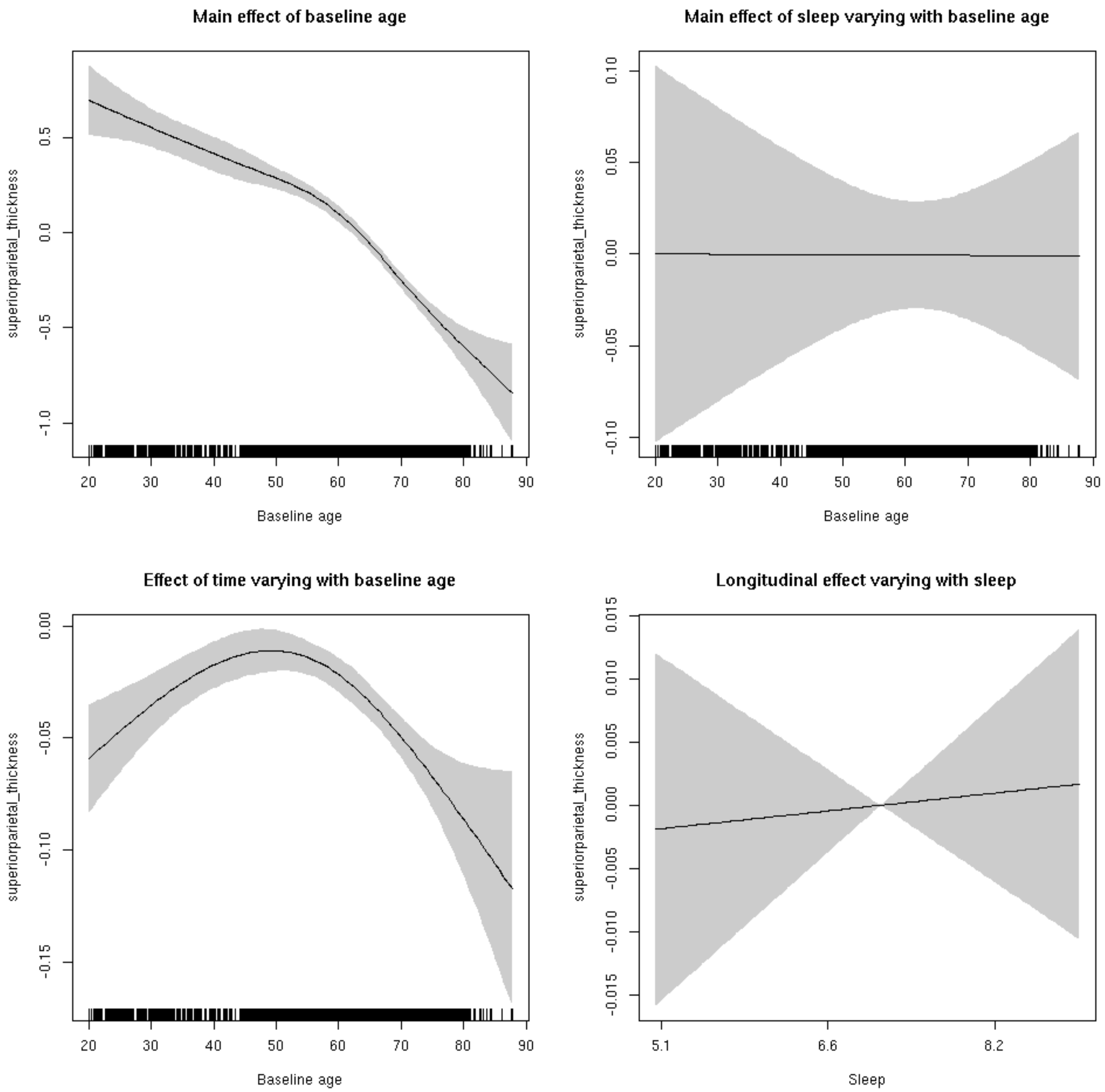

superiorparietal_volume: Stratified by sex

Female: Full data Below is the row for `s(sleep_z):time` from the model output.

```
## s(sleep_z):time   1.000  1.000   0.867   0.352
```

Below are plots of a plot of `s(bl_age)`, `s(bl_age):sleep_z`, `s(bl_age):time`, and `s(sleep_z):time`. For `s(sleep_z):time`, roughly, if we cannot fit a straight line through the confidence bands, there is evidence that the effect of time depends on sleep duration. It is correct the that confidence interval is zero at one point along the axis; this happens because we do not include the main effect.

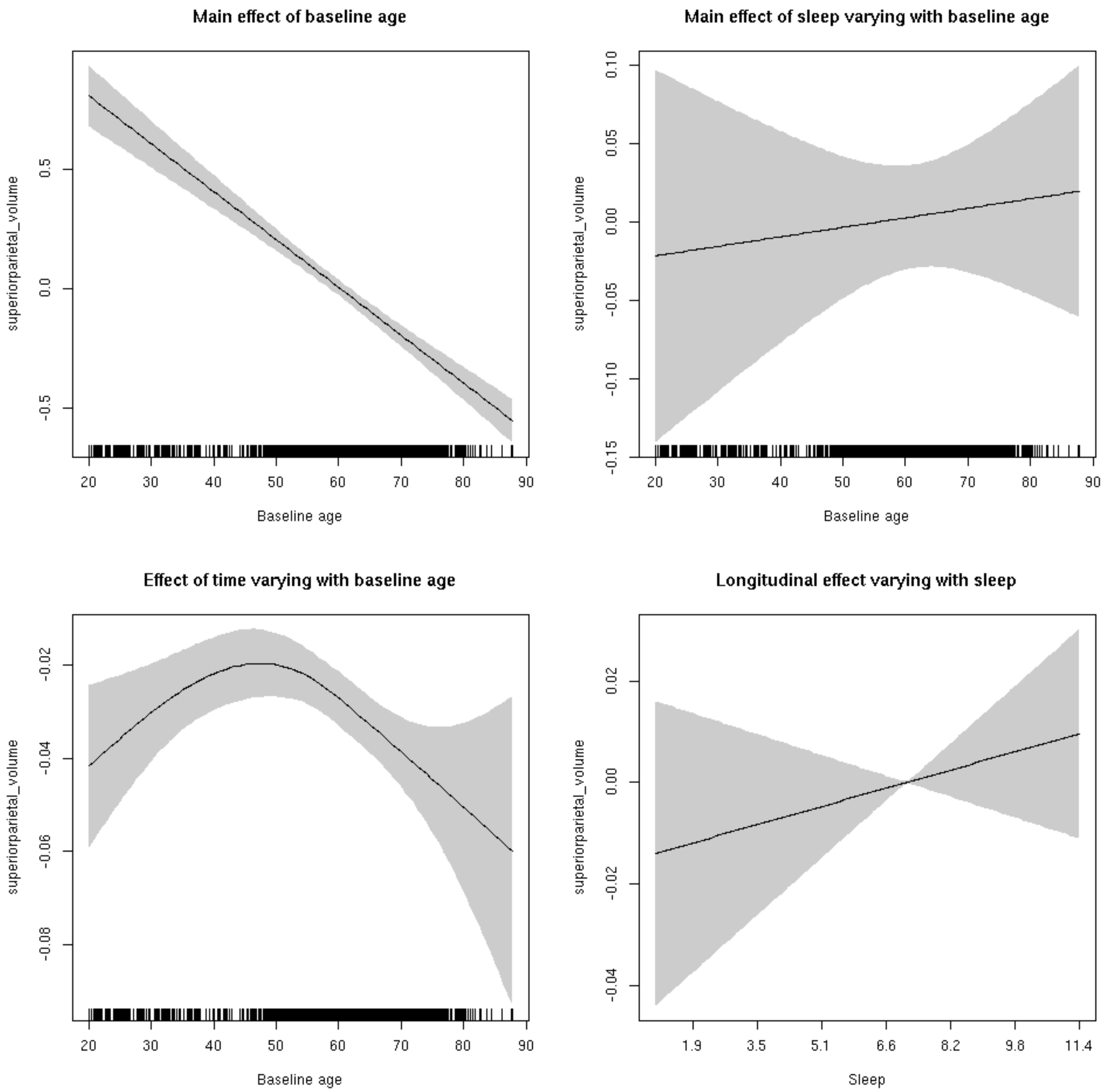

Male: Full data Below is the row for `s(sleep_z):time` from the model output.

```
## s(sleep_z):time   1.000  1.000   0.236   0.627
```

Below are plots of a plot of `s(bl_age)`, `s(bl_age):sleep_z`, `s(bl_age):time`, and `s(sleep_z):time`. For `s(sleep_z):time`, roughly, if we cannot fit a straight line through the confidence bands, there is evidence that the effect of time depends on sleep duration. It is correct the that confidence interval is zero at one point along the axis; this happens because we do not include the main effect.

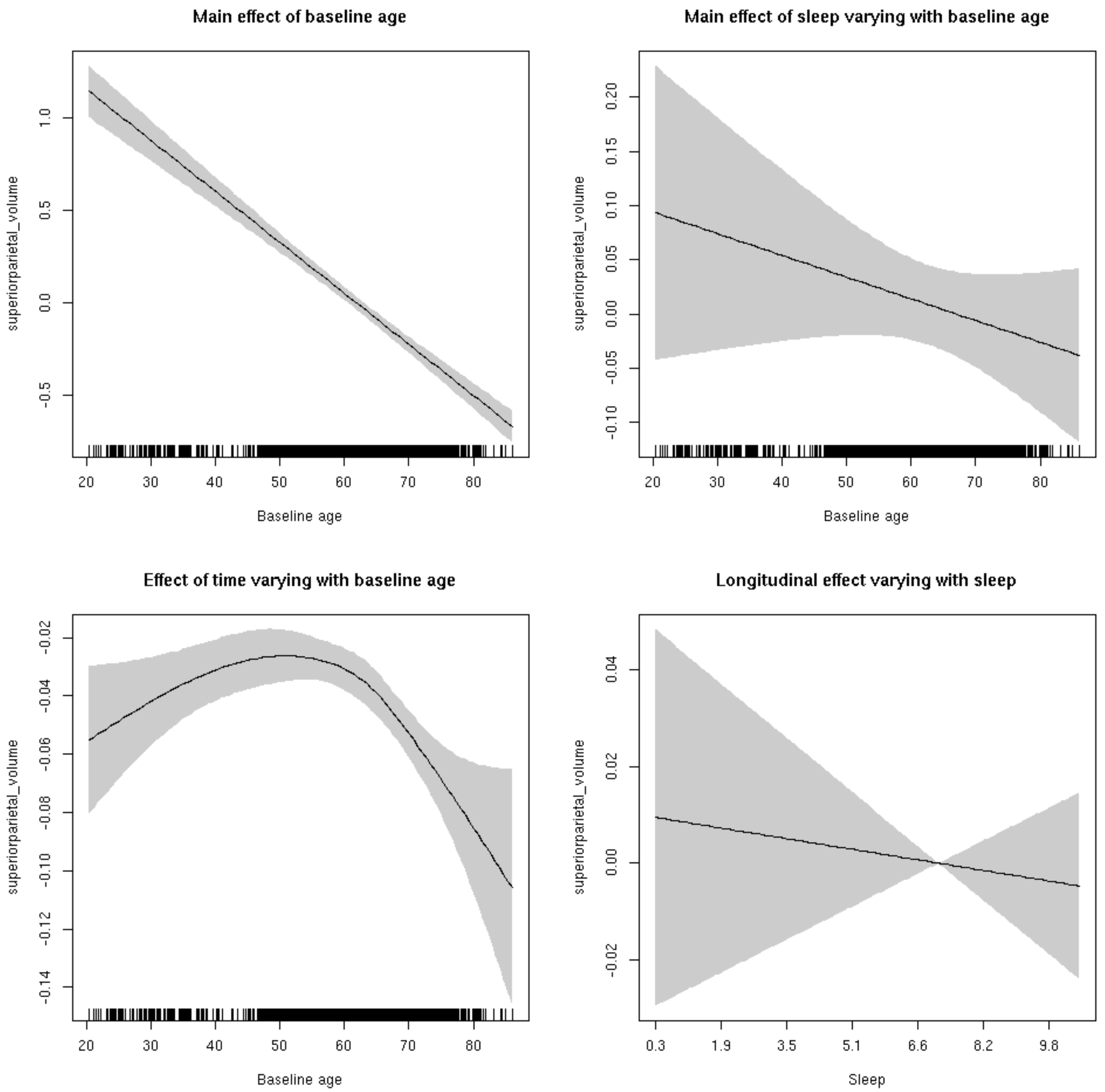

superiorparietal_volume

Full data Below is the model output. The term `s(sleep_z):time` is probably what we care most about.

```
##
## Family: gaussian
## Link function: identity
##
## Formula:
## value ~ sex + site + icv + s(bl_age, k = 10, bs = "cr") + s(bl_age,
##     by = sleep_z, bs = "cr") + +s(bl_age, by = time, k = 5, bs = "cr") +
##     s(sleep_z, by = time, k = 5, bs = "cr", pc = 0)
## <environment: 0x5630035b43e0>
```

```
##
## Parametric coefficients:
##                 Estimate Std. Error t value Pr(>|t|)
## (Intercept)      0.02135    0.05595   0.382  0.70275
## sexmale         -0.07591    0.02886  -2.630  0.00855 **
## siteousAvanto -0.83228    0.06872 -12.112  < 2e-16 ***
## siteousPrisma -0.25604    0.18295  -1.400  0.16169
## siteousSkyra  -0.51168    0.06652  -7.692 1.62e-14 ***
## siteUB        -0.31690    0.12887  -2.459  0.01395 *
## siteUCAM      -0.46708    0.06921  -6.749 1.59e-11 ***
## siteUKB        0.19260    0.05271   3.654  0.00026 ***
## siteUmU        0.03787    0.07992   0.474  0.63563
## icv            0.58180    0.01424  40.851  < 2e-16 ***
## ---
## Signif. codes:  0 '***' 0.001 '**' 0.01 '*' 0.05 '.' 0.1 ' ' 1
##
## Approximate significance of smooth terms:
##                       edf Ref.df       F p-value
## s(bl_age)           1.845  1.845 204.779  <2e-16 ***
## s(bl_age):sleep_z 2.000  2.000   0.369   0.691
## s(bl_age):time    3.703  3.703 114.901  <2e-16 ***
## s(sleep_z):time   1.000  1.000   0.043   0.836
## ---
## Signif. codes:  0 '***' 0.001 '**' 0.01 '*' 0.05 '.' 0.1 ' ' 1
##
## R-sq.(adj) =  0.471
## lmer.REML =  10789  Scale est. = 0.049017  n = 8166
```

Below are plots of a plot of `s(bl_age)`, `s(bl_age):sleep_z`, `s(bl_age):time`, and `s(sleep_z):time`. For `s(sleep_z):time`, roughly, if we cannot fit a straight line through the confidence bands, there is evidence that the effect of time depends on sleep duration. It is correct the that confidence interval is zero at one point along the axis; this happens because we do not include the main effect.

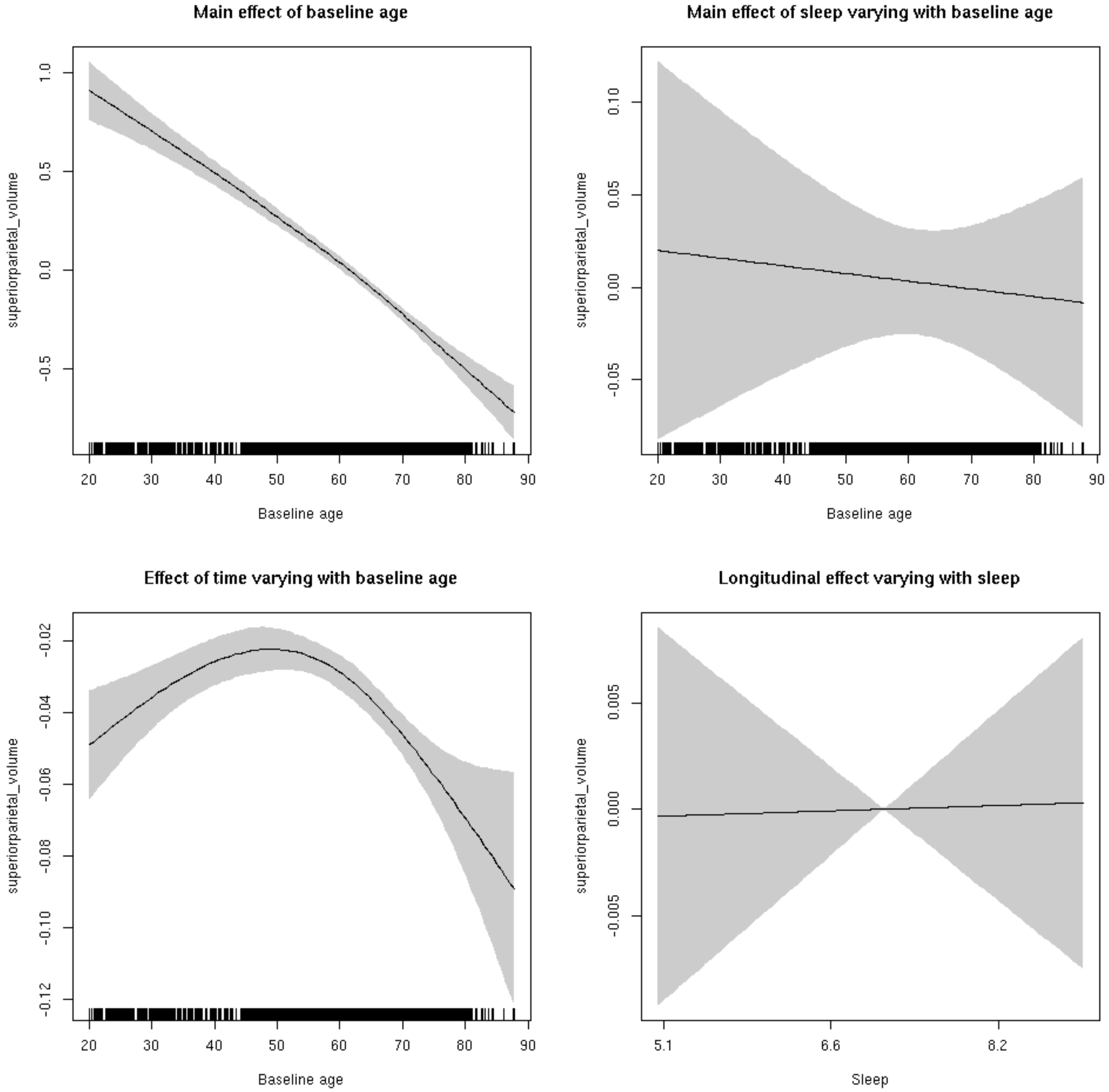

Restricted to sleep >= 5 and <= 9 hours Below is the row for `s(sleep_z):time` from the model output.

```
## s(sleep_z):time   1.000  1.000   0.006   0.939
```

Below are plots of a plot of `s(bl_age)`, `s(bl_age):sleep_z`, `s(bl_age):time`, and `s(sleep_z):time`. For `s(sleep_z):time`, roughly, if we cannot fit a straight line through the confidence bands, there is evidence that the effect of time depends on sleep duration. It is correct the that confidence interval is zero at one point along the axis; this happens because we do not include the main effect.

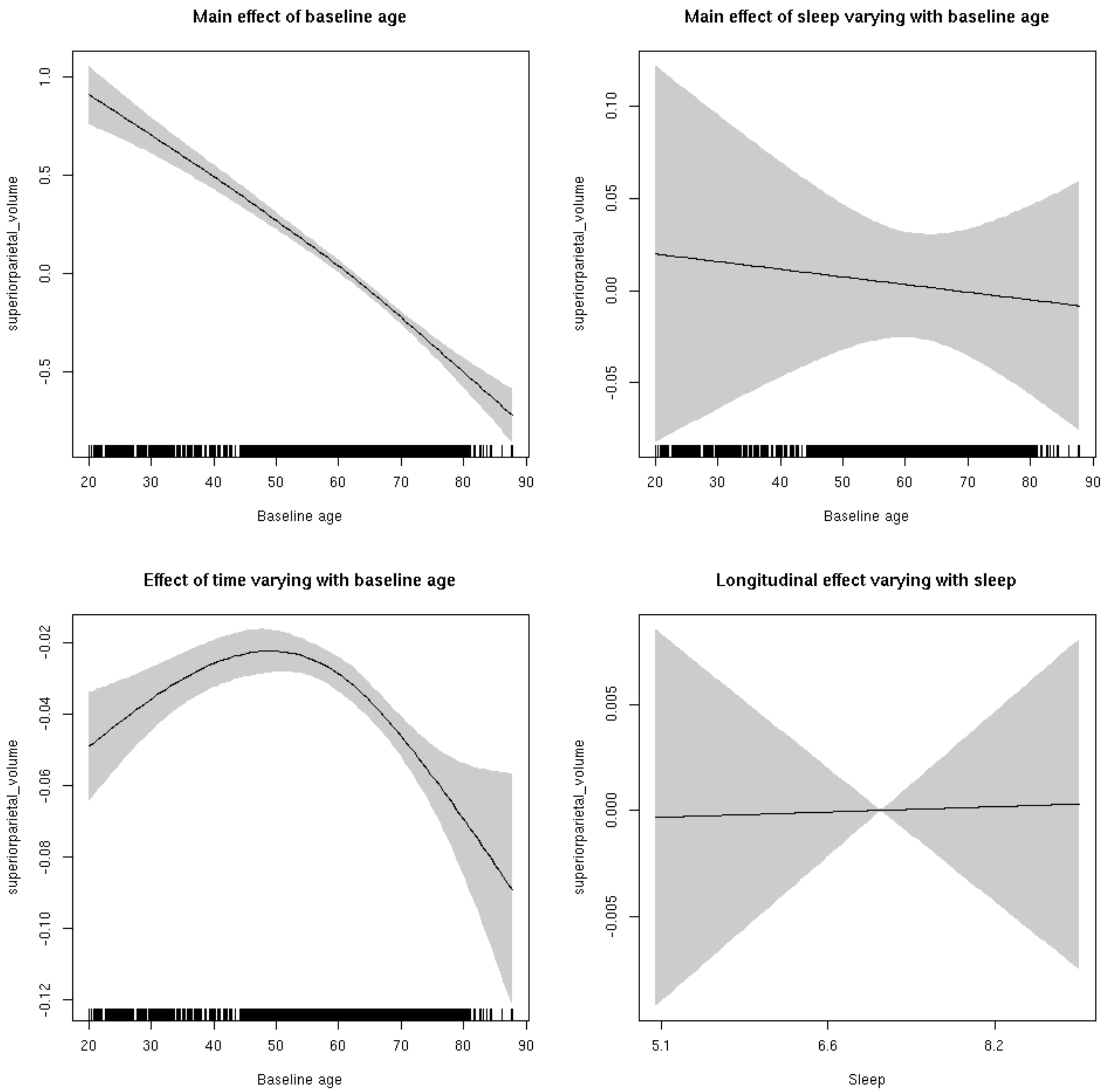

superiortemporal_area: Stratified by sex

Female: Full data Below is the row for `s(sleep_z):time` from the model output.

```
## s(sleep_z):time   1.000  1.000  0.238   0.626
```

Below are plots of a plot of `s(bl_age)`, `s(bl_age):sleep_z`, `s(bl_age):time`, and `s(sleep_z):time`. For `s(sleep_z):time`, roughly, if we cannot fit a straight line through the confidence bands, there is evidence that the effect of time depends on sleep duration. It is correct the that confidence interval is zero at one point along the axis; this happens because we do not include the main effect.

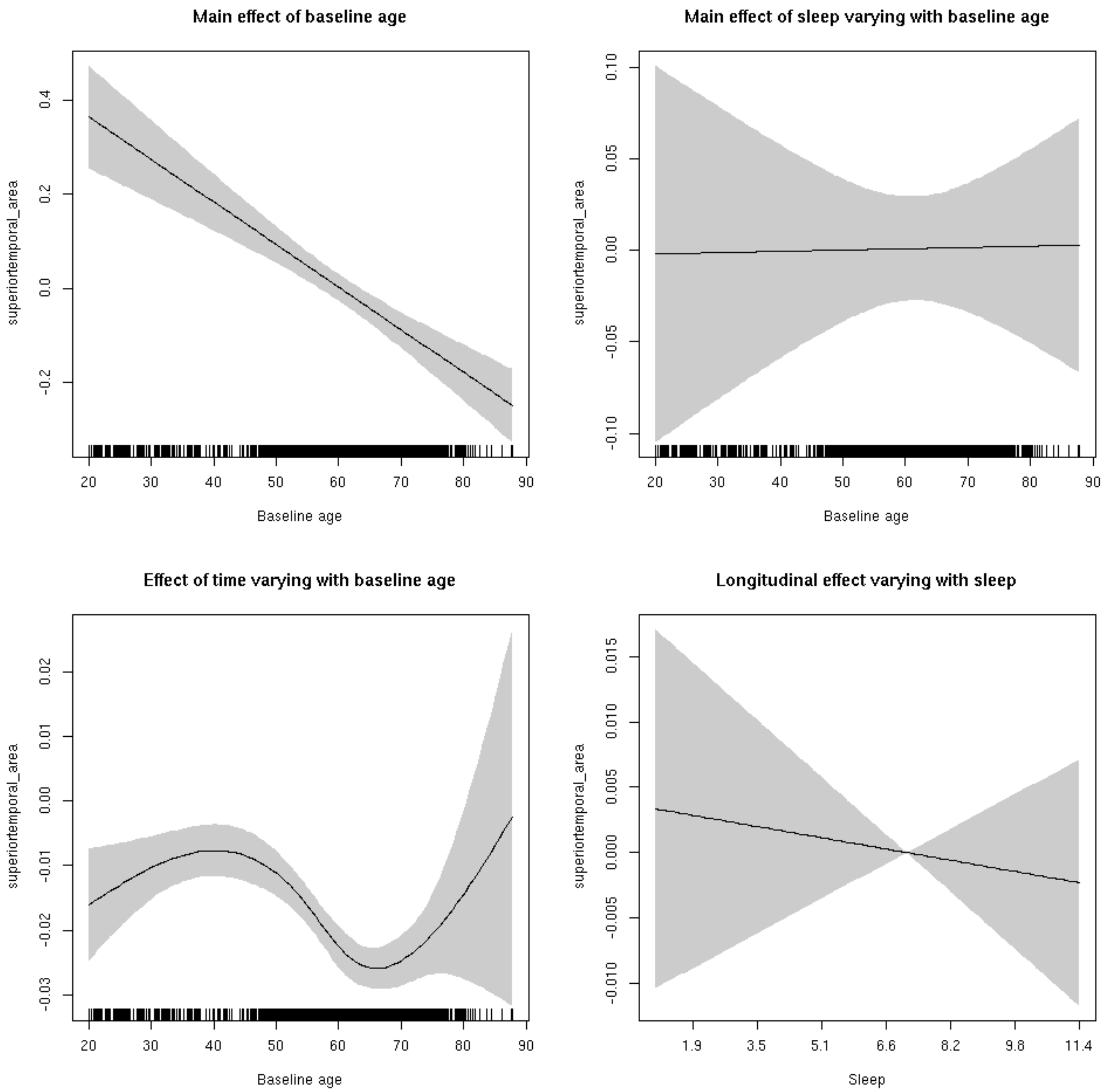

Male: Full data Below is the row for `s(sleep_z):time` from the model output.

```
## s(sleep_z):time   1.000  1.000   0.188   0.665
```

Below are plots of a plot of `s(bl_age)`, `s(bl_age):sleep_z`, `s(bl_age):time`, and `s(sleep_z):time`. For `s(sleep_z):time`, roughly, if we cannot fit a straight line through the confidence bands, there is evidence that the effect of time depends on sleep duration. It is correct the that confidence interval is zero at one point along the axis; this happens because we do not include the main effect.

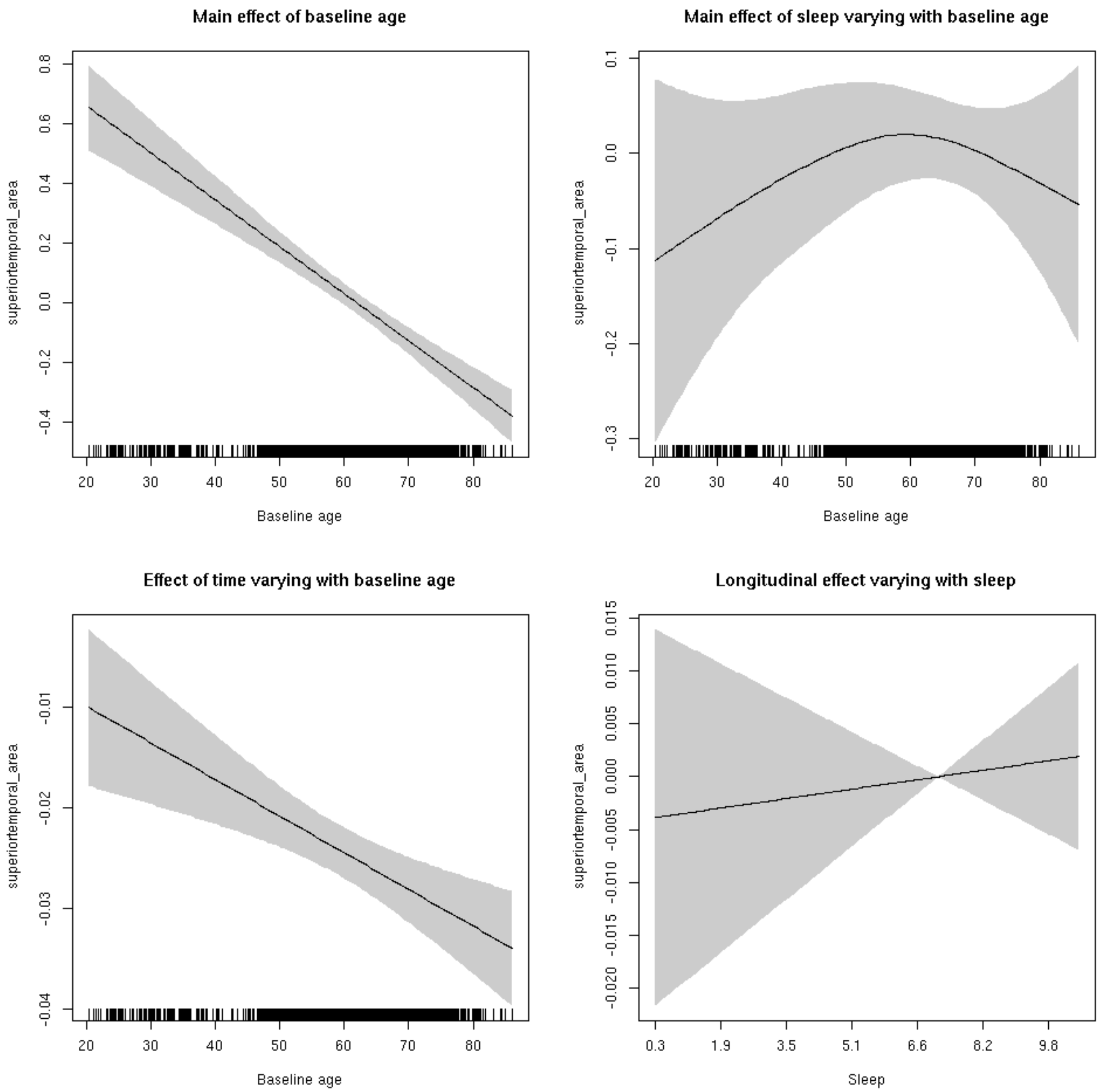

superiortemporal_area

Full data Below is the model output. The term `s(sleep_z):time` is probably what we care most about.

```
##
## Family: gaussian
## Link function: identity
##
## Formula:
## value ~ sex + site + icv + s(bl_age, k = 10, bs = "cr") + s(bl_age,
##     by = sleep_z, bs = "cr") + +s(bl_age, by = time, k = 5, bs = "cr") +
##     s(sleep_z, by = time, k = 5, bs = "cr", pc = 0)
## <environment: 0x5630035aed10>
```

```
##
## Parametric coefficients:
##                Estimate Std. Error t value Pr(>|t|)
## (Intercept)     0.68797    0.05213  13.197  < 2e-16 ***
## sexmale         0.25289    0.02708   9.339  < 2e-16 ***
## siteousAvanto -0.74913    0.06421 -11.667  < 2e-16 ***
## siteousPrisma -0.90811    0.10273  -8.840  < 2e-16 ***
## siteousSkyra  -0.31745    0.06225  -5.099 3.48e-07 ***
## siteUB        -0.82574    0.12266  -6.732 1.79e-11 ***
## siteUCAM      -0.69508    0.06533 -10.639  < 2e-16 ***
## siteUKB       -0.79773    0.04872 -16.373  < 2e-16 ***
## siteUmU       -0.15480    0.07546  -2.052   0.0402 *
## icv            0.61215    0.01302  46.999  < 2e-16 ***
## ---
## Signif. codes:  0 '***' 0.001 '**' 0.01 '*' 0.05 '.' 0.1 ' ' 1
##
## Approximate significance of smooth terms:
##                      edf Ref.df       F p-value
## s(bl_age)          1.000  1.000 135.412  <2e-16 ***
## s(bl_age):sleep_z 2.000  2.000   0.045   0.956
## s(bl_age):time    4.295  4.295 173.329  <2e-16 ***
## s(sleep_z):time   1.000  1.000   0.003   0.957
## ---
## Signif. codes:  0 '***' 0.001 '**' 0.01 '*' 0.05 '.' 0.1 ' ' 1
##
## R-sq.(adj) =  0.536
## lmer.REML = 3721.1  Scale est. = 0.010287  n = 8171
```

Below are plots of a plot of `s(bl_age)`, `s(bl_age):sleep_z`, `s(bl_age):time`, and `s(sleep_z):time`. For `s(sleep_z):time`, roughly, if we cannot fit a straight line through the confidence bands, there is evidence that the effect of time depends on sleep duration. It is correct the that confidence interval is zero at one point along the axis; this happens because we do not include the main effect.

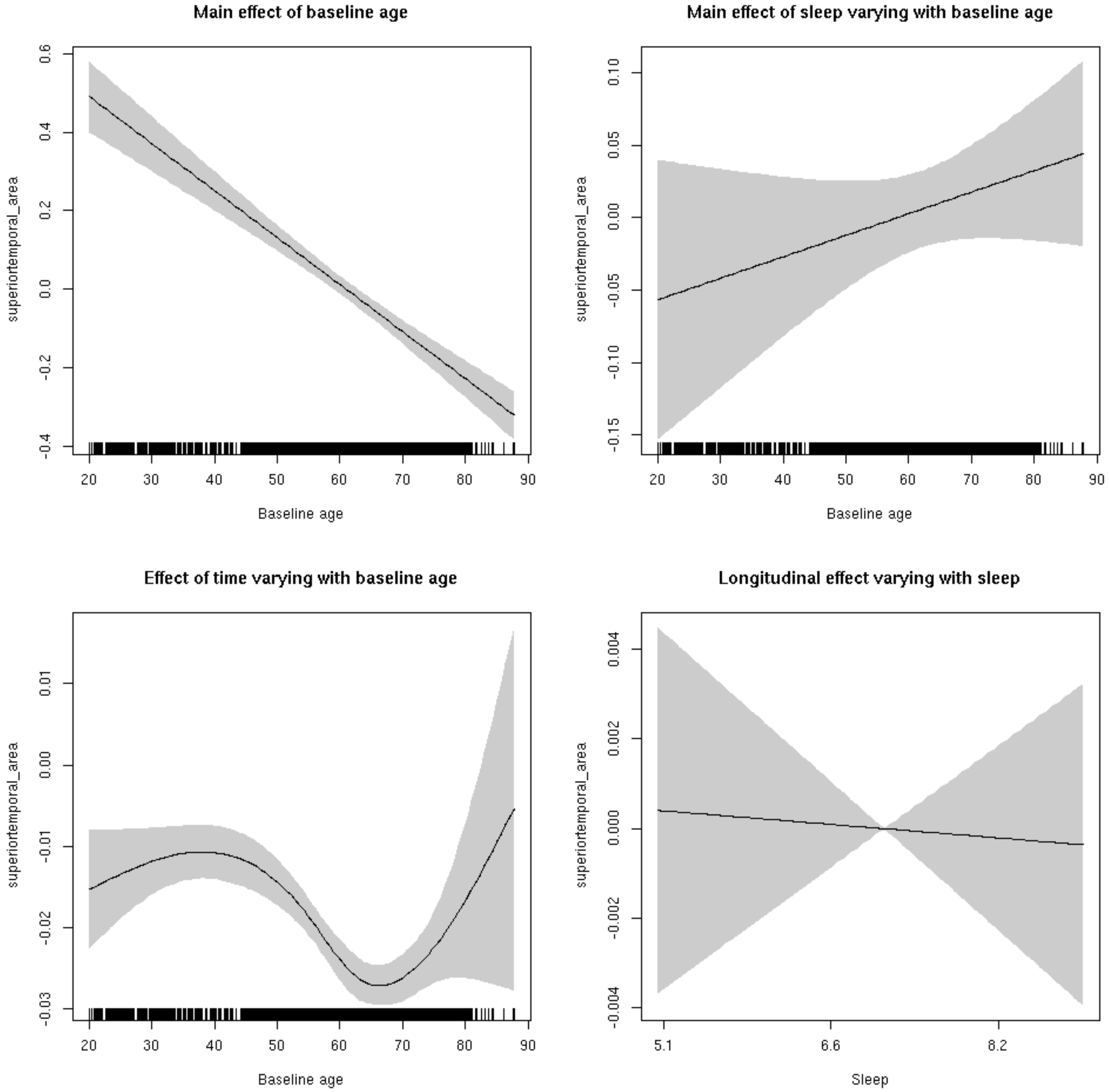

Restricted to sleep >= 5 and <= 9 hours Below is the row for `s(sleep_z):time` from the model output.

```
## s(sleep_z):time   1.0    1.0   0.040   0.842
```

Below are plots of a plot of `s(bl_age)`, `s(bl_age):sleep_z`, `s(bl_age):time`, and `s(sleep_z):time`. For `s(sleep_z):time`, roughly, if we cannot fit a straight line through the confidence bands, there is evidence that the effect of time depends on sleep duration. It is correct the that confidence interval is zero at one point along the axis; this happens because we do not include the main effect.

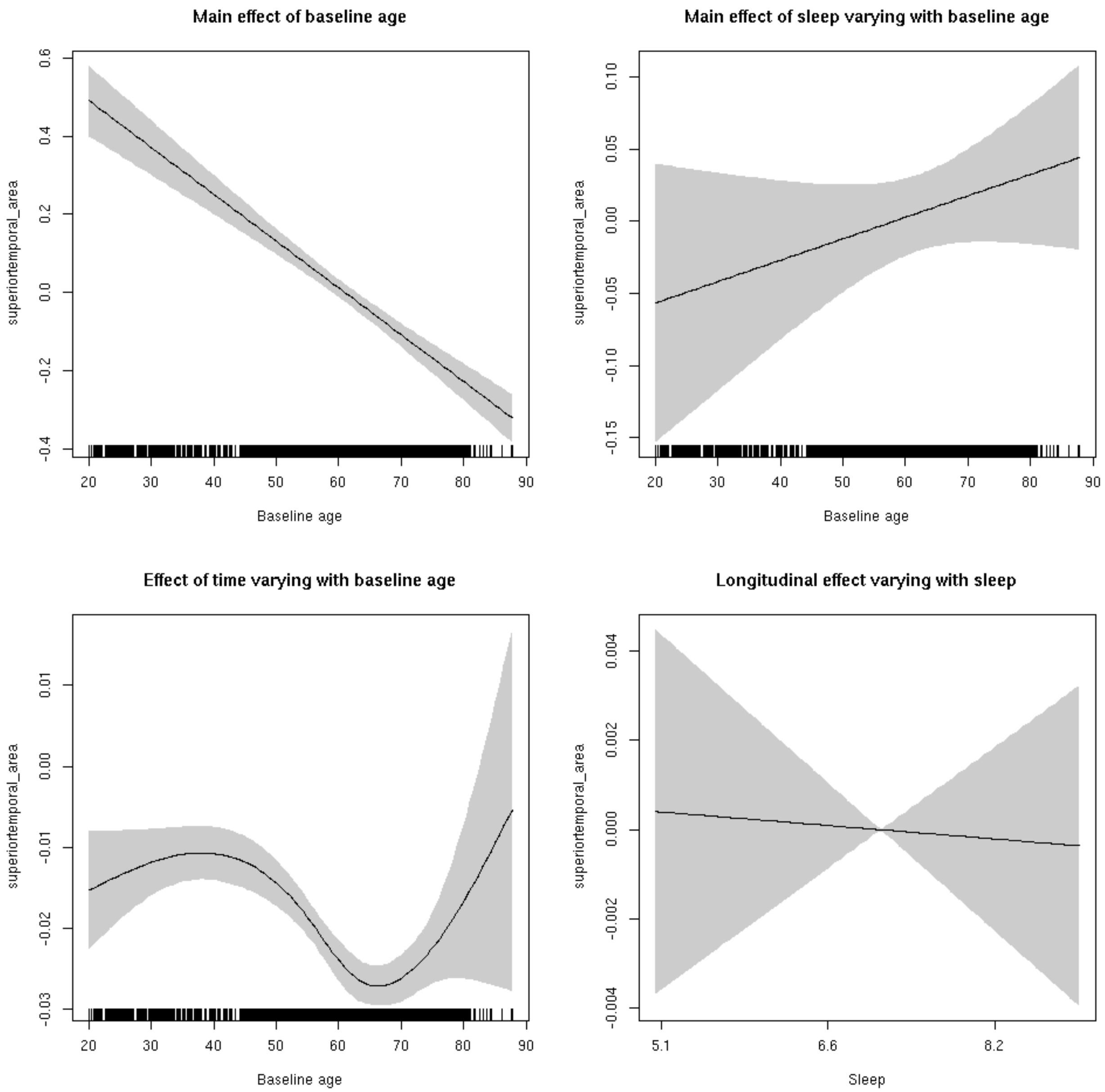

superiortemporal_thickness: Stratified by sex

Female: Full data Below is the row for `s(sleep_z):time` from the model output.

```
## s(sleep_z):time   1.000  1.000   0.634   0.426
```

Below are plots of a plot of `s(bl_age)`, `s(bl_age):sleep_z`, `s(bl_age):time`, and `s(sleep_z):time`. For `s(sleep_z):time`, roughly, if we cannot fit a straight line through the confidence bands, there is evidence that the effect of time depends on sleep duration. It is correct the that confidence interval is zero at one point along the axis; this happens because we do not include the main effect.

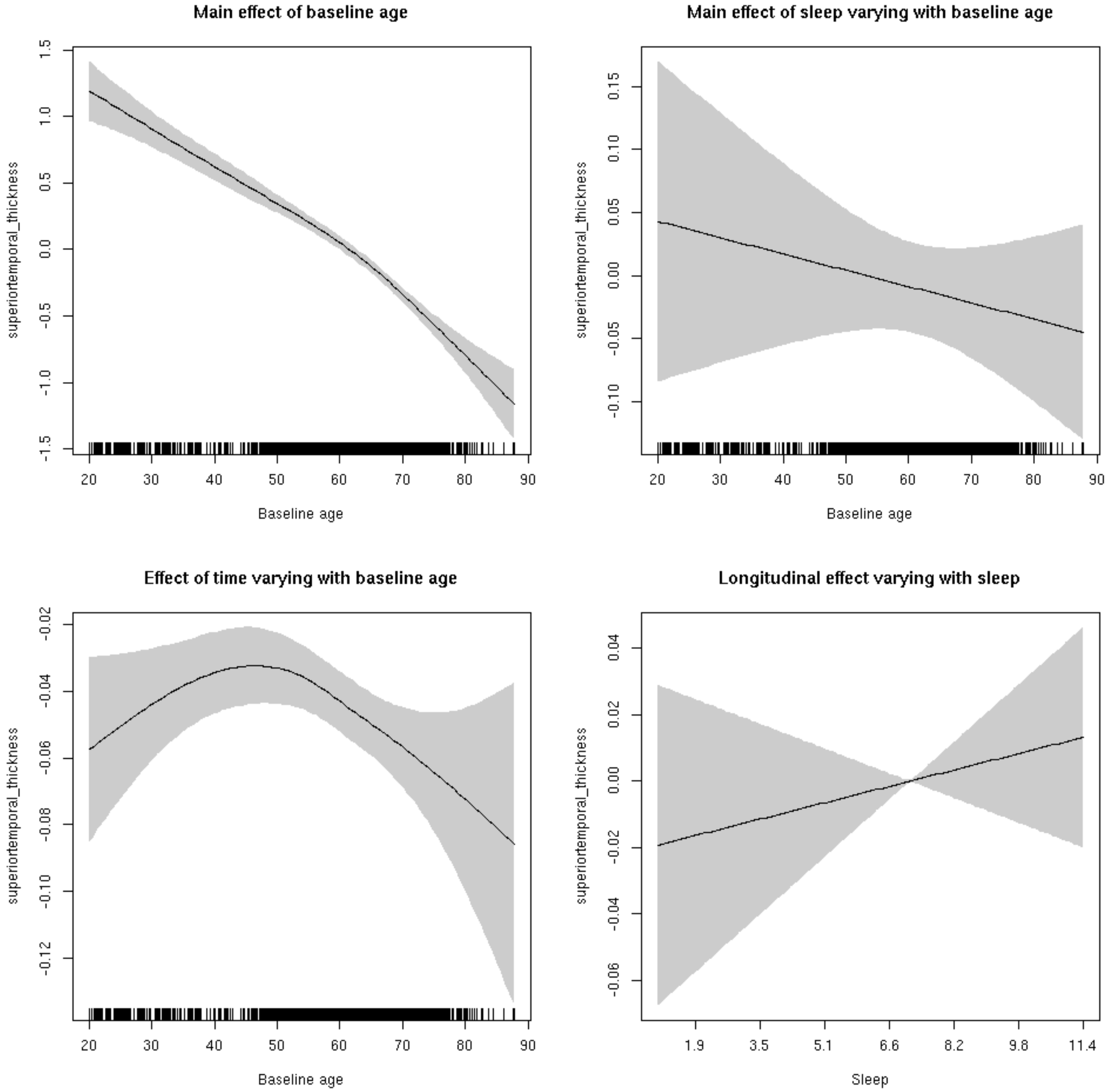

Male: Full data Below is the row for `s(sleep_z):time` from the model output.

```
## s(sleep_z):time   1.000  1.000   0.240   0.625
```

Below are plots of a plot of `s(bl_age)`, `s(bl_age):sleep_z`, `s(bl_age):time`, and `s(sleep_z):time`. For `s(sleep_z):time`, roughly, if we cannot fit a straight line through the confidence bands, there is evidence that the effect of time depends on sleep duration. It is correct the that confidence interval is zero at one point along the axis; this happens because we do not include the main effect.

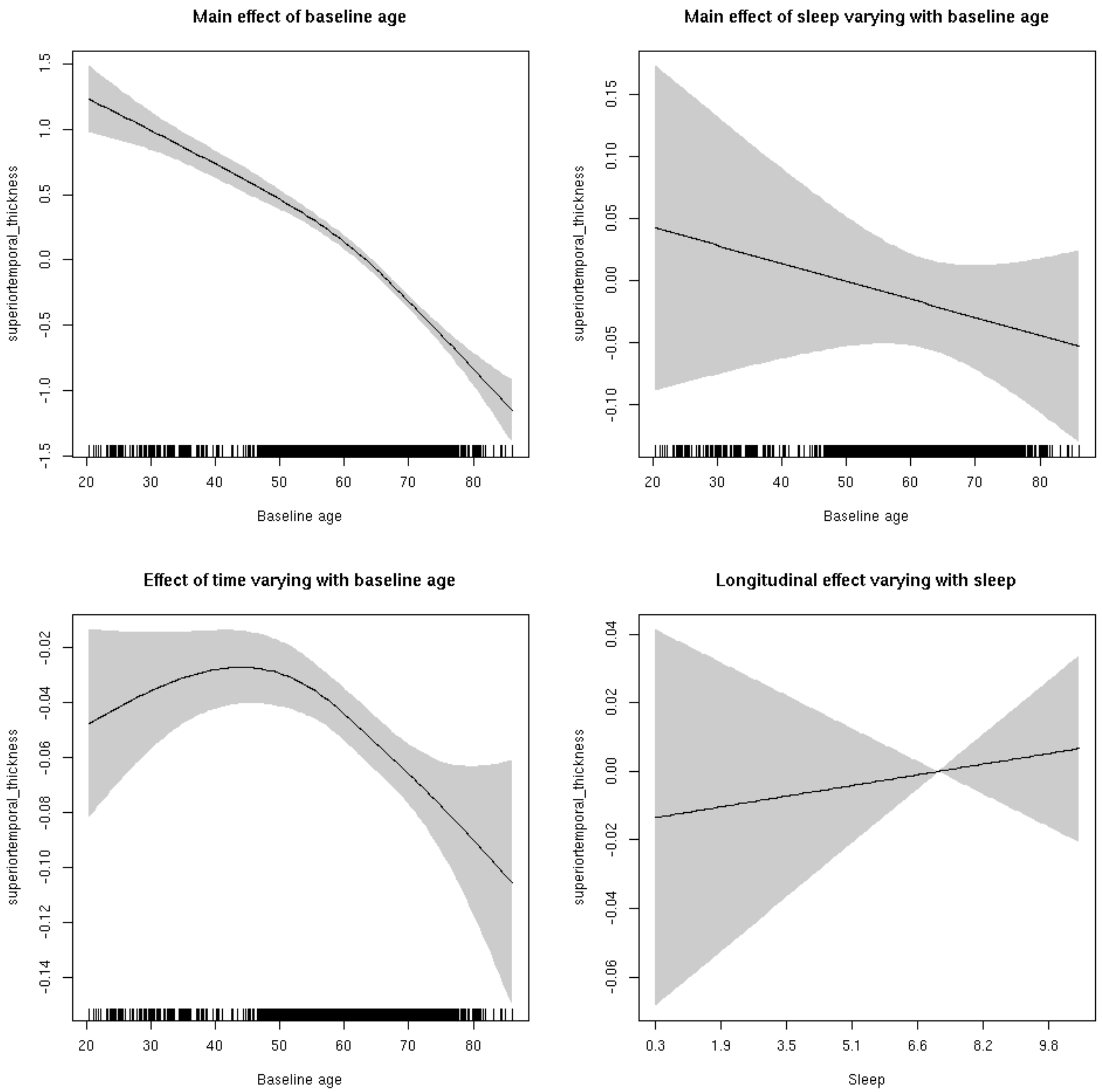

superiortemporal_thickness

Full data Below is the model output. The term `s(sleep_z):time` is probably what we care most about.

```
##
## Family: gaussian
## Link function: identity
##
## Formula:
## value ~ sex + site + s(bl_age, k = 10, bs = "cr") + s(bl_age,
##     by = sleep_z, bs = "cr") + +s(bl_age, by = time, k = 5, bs = "cr") +
##     s(sleep_z, by = time, k = 5, bs = "cr", pc = 0)
## <environment: 0x5630035d1d88>
```

```
##
## Parametric coefficients:
##                Estimate Std. Error t value Pr(>|t|)
## (Intercept)   -1.27183    0.04806 -26.464  < 2e-16 ***
## sexmale        0.08185    0.02411   3.396 0.000688 ***
## siteousAvanto  0.07115    0.06371   1.117 0.264081
## siteousPrisma  0.27432    0.25948   1.057 0.290454
## siteousSkyra   0.31245    0.06368   4.907 9.43e-07 ***
## siteUB         0.17430    0.12780   1.364 0.172660
## siteUCAM       0.23506    0.06614   3.554 0.000382 ***
## siteUKB        1.49102    0.04872  30.607  < 2e-16 ***
## siteUmU       -0.42296    0.07925  -5.337 9.72e-08 ***
## ---
## Signif. codes:  0 '***' 0.001 '**' 0.01 '*' 0.05 '.' 0.1 ' ' 1
##
## Approximate significance of smooth terms:
##                      edf Ref.df       F p-value
## s(bl_age)          3.575  3.575 273.627  <2e-16 ***
## s(bl_age):sleep_z 2.000  2.000   2.001   0.135
## s(bl_age):time    3.677  3.677  95.771  <2e-16 ***
## s(sleep_z):time   1.000  1.000   1.045   0.307
## ---
## Signif. codes:  0 '***' 0.001 '**' 0.01 '*' 0.05 '.' 0.1 ' ' 1
##
## R-sq.(adj) =  0.473
## lmer.REML =  14454  Scale est. = 0.11395   n = 8174
```

Below are plots of a plot of `s(bl_age)`, `s(bl_age):sleep_z`, `s(bl_age):time`, and `s(sleep_z):time`. For `s(sleep_z):time`, roughly, if we cannot fit a straight line through the confidence bands, there is evidence that the effect of time depends on sleep duration. It is correct the that confidence interval is zero at one point along the axis; this happens because we do not include the main effect.

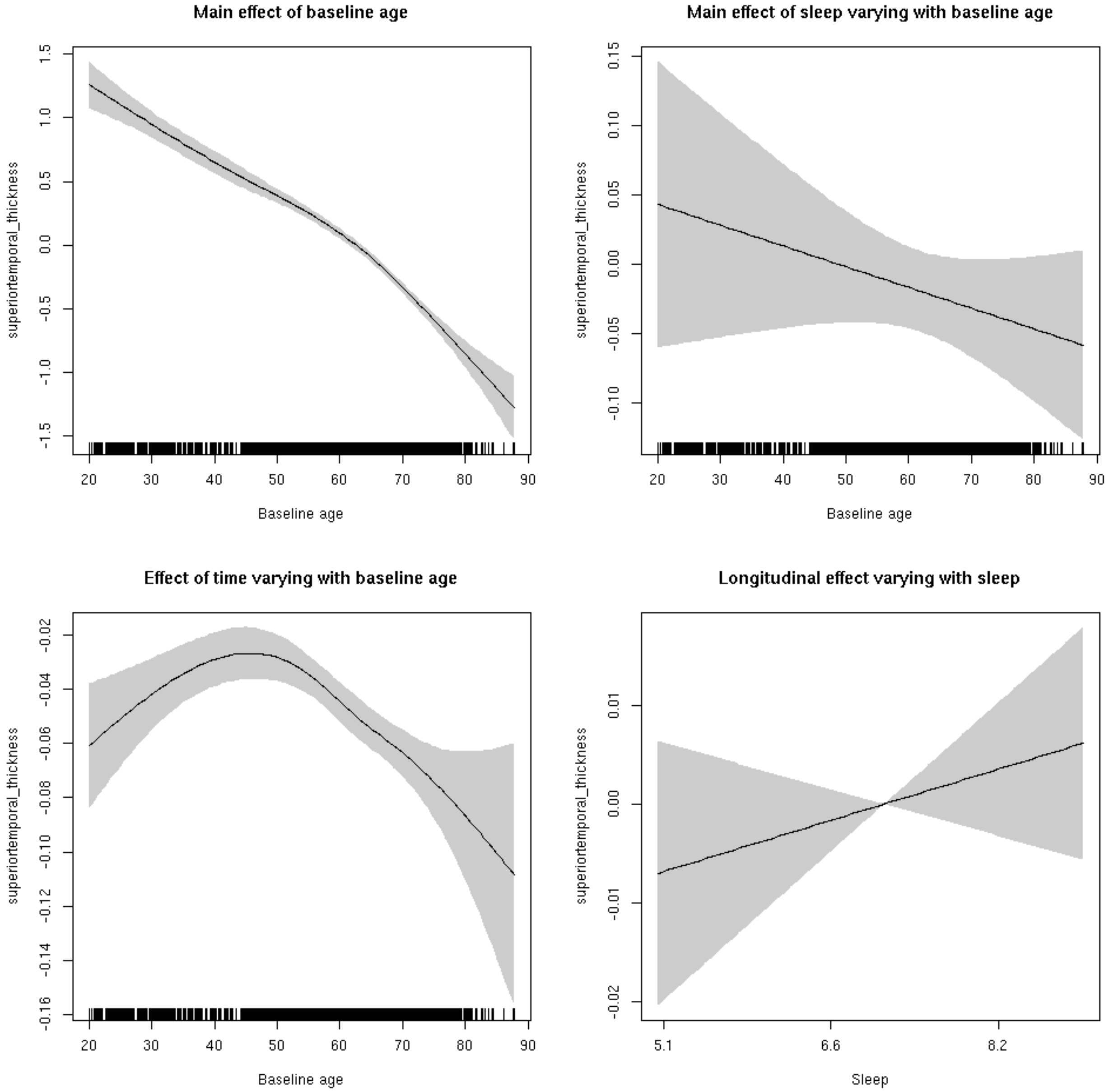

Restricted to sleep >= 5 and <= 9 hours Below is the row for `s(sleep_z):time` from the model output.

```
## s(sleep_z):time   1.000  1.000   1.096   0.295
```

Below are plots of a plot of `s(bl_age)`, `s(bl_age):sleep_z`, `s(bl_age):time`, and `s(sleep_z):time`. For `s(sleep_z):time`, roughly, if we cannot fit a straight line through the confidence bands, there is evidence that the effect of time depends on sleep duration. It is correct the that confidence interval is zero at one point along the axis; this happens because we do not include the main effect.

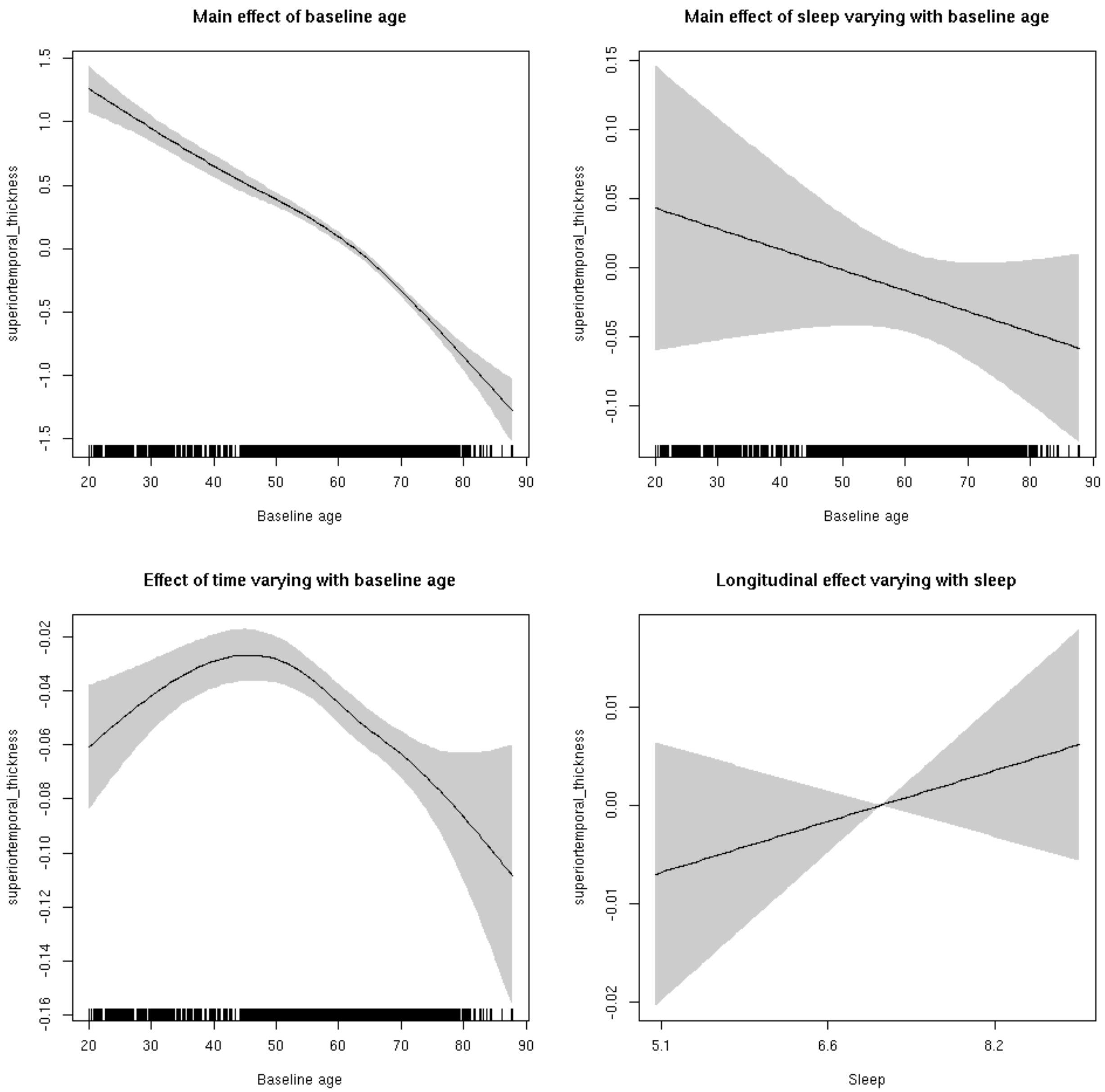

superiortemporal_volume: Stratified by sex

Female: Full data Below is the row for `s(sleep_z):time` from the model output.

```
## s(sleep_z):time   1.000  1.000   0.037   0.847
```

Below are plots of a plot of `s(bl_age)`, `s(bl_age):sleep_z`, `s(bl_age):time`, and `s(sleep_z):time`. For `s(sleep_z):time`, roughly, if we cannot fit a straight line through the confidence bands, there is evidence that the effect of time depends on sleep duration. It is correct the that confidence interval is zero at one point along the axis; this happens because we do not include the main effect.

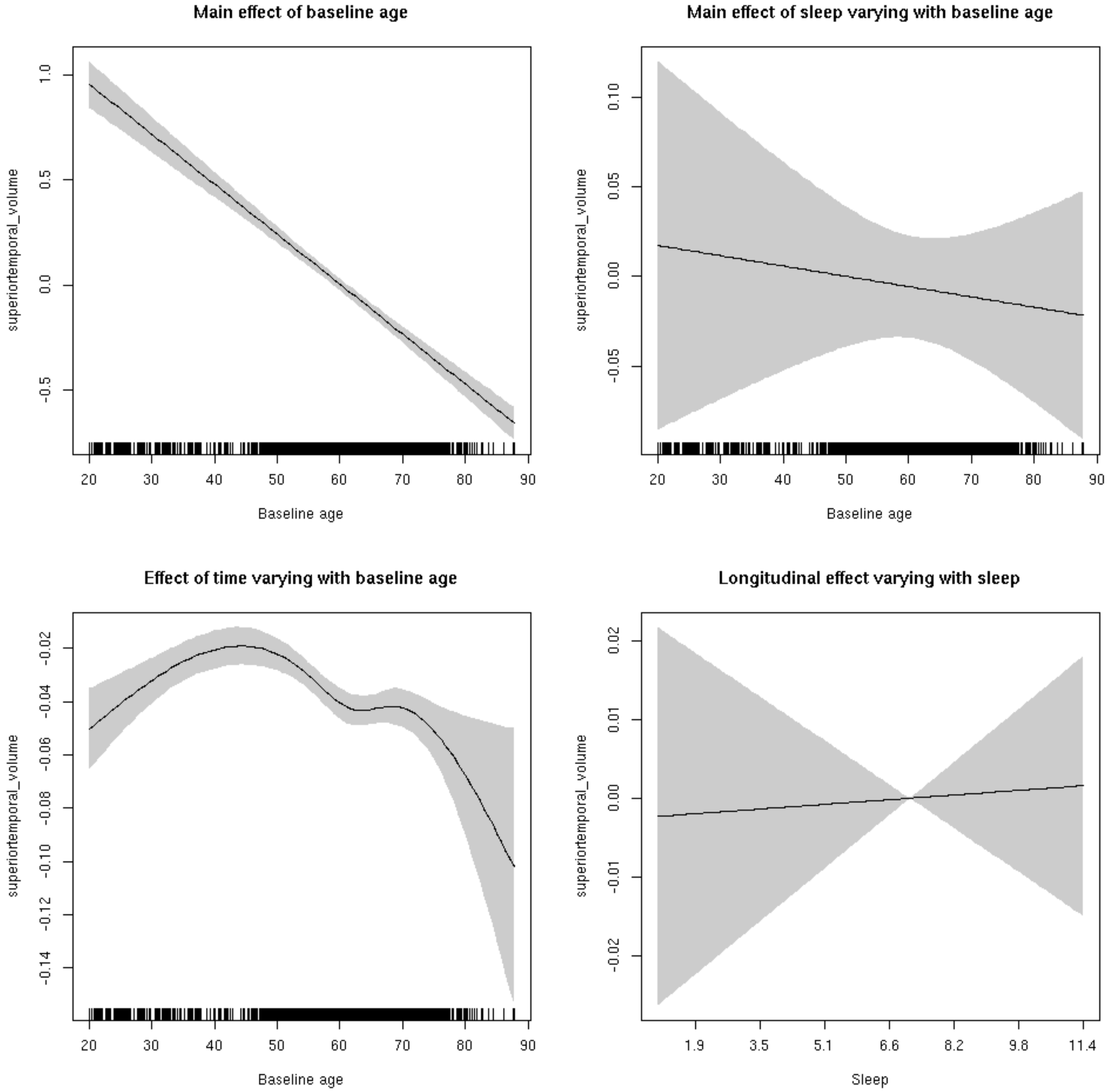

Male: Full data Below is the row for `s(sleep_z):time` from the model output.

```
## s(sleep_z):time   1.000  1.000   0.354   0.552
```

Below are plots of a plot of `s(bl_age)`, `s(bl_age):sleep_z`, `s(bl_age):time`, and `s(sleep_z):time`. For `s(sleep_z):time`, roughly, if we cannot fit a straight line through the confidence bands, there is evidence that the effect of time depends on sleep duration. It is correct the that confidence interval is zero at one point along the axis; this happens because we do not include the main effect.

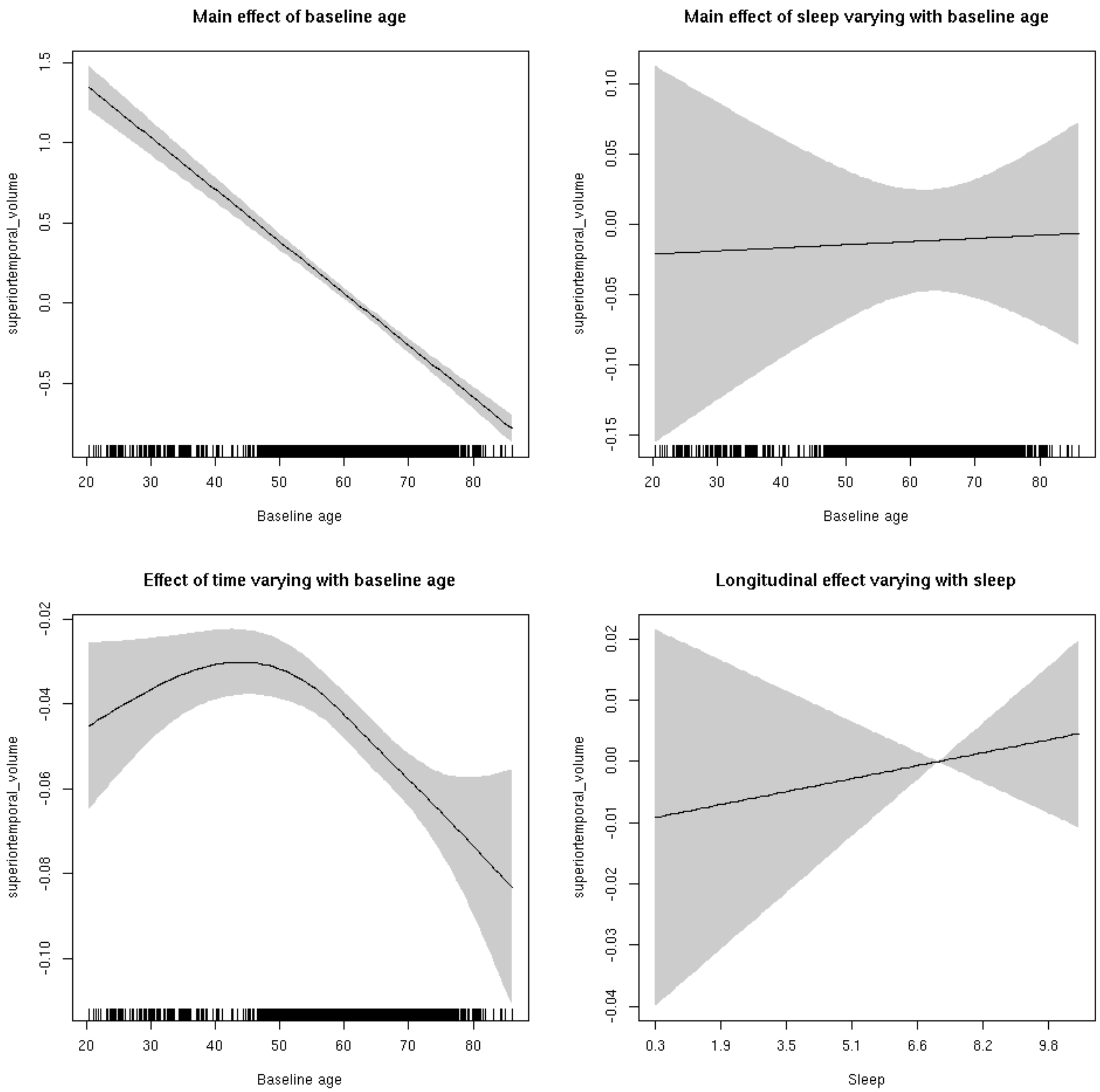

superiortemporal__volume

Full data Below is the model output. The term `s(sleep_z):time` is probably what we care most about.

```
##
## Family: gaussian
## Link function: identity
##
## Formula:
## value ~ sex + site + icv + s(bl_age, k = 10, bs = "cr") + s(bl_age,
##     by = sleep_z, bs = "cr") + +s(bl_age, by = time, k = 5, bs = "cr") +
##     s(sleep_z, by = time, k = 5, bs = "cr", pc = 0)
## <environment: 0x5630035aebc0>
```

```
##
## Parametric coefficients:
##                Estimate Std. Error t value Pr(>|t|)
## (Intercept)     0.04753    0.05168   0.920 0.357749
## sexmale         0.18112    0.02684   6.748 1.61e-11 ***
## siteousAvanto -0.72815    0.06372 -11.428  < 2e-16 ***
## siteousPrisma -0.57276    0.14994  -3.820 0.000135 ***
## siteousSkyra  -0.11954    0.06164  -1.939 0.052507 .
## siteUB        -0.58926    0.12014  -4.905 9.54e-07 ***
## siteUCAM      -0.53897    0.06432  -8.380  < 2e-16 ***
## siteUKB        0.02466    0.04815   0.512 0.608482
## siteUmU       -0.31502    0.07419  -4.246 2.20e-05 ***
## icv            0.56570    0.01320  42.855  < 2e-16 ***
## ---
## Signif. codes:  0 '***' 0.001 '**' 0.01 '*' 0.05 '.' 0.1 ' ' 1
##
## Approximate significance of smooth terms:
##                     edf Ref.df       F p-value
## s(bl_age)          1.00   1.00 723.883  <2e-16 ***
## s(bl_age):sleep_z 2.00   2.00   0.631   0.532
## s(bl_age):time    4.47   4.47 205.403  <2e-16 ***
## s(sleep_z):time   1.00   1.00   0.322   0.570
## ---
## Signif. codes:  0 '***' 0.001 '**' 0.01 '*' 0.05 '.' 0.1 ' ' 1
##
## R-sq.(adj) =  0.538
## lmer.REML = 8283.2  Scale est. = 0.030977  n = 8172
```

Below are plots of a plot of `s(bl_age)`, `s(bl_age):sleep_z`, `s(bl_age):time`, and `s(sleep_z):time`. For `s(sleep_z):time`, roughly, if we cannot fit a straight line through the confidence bands, there is evidence that the effect of time depends on sleep duration. It is correct the that confidence interval is zero at one point along the axis; this happens because we do not include the main effect.

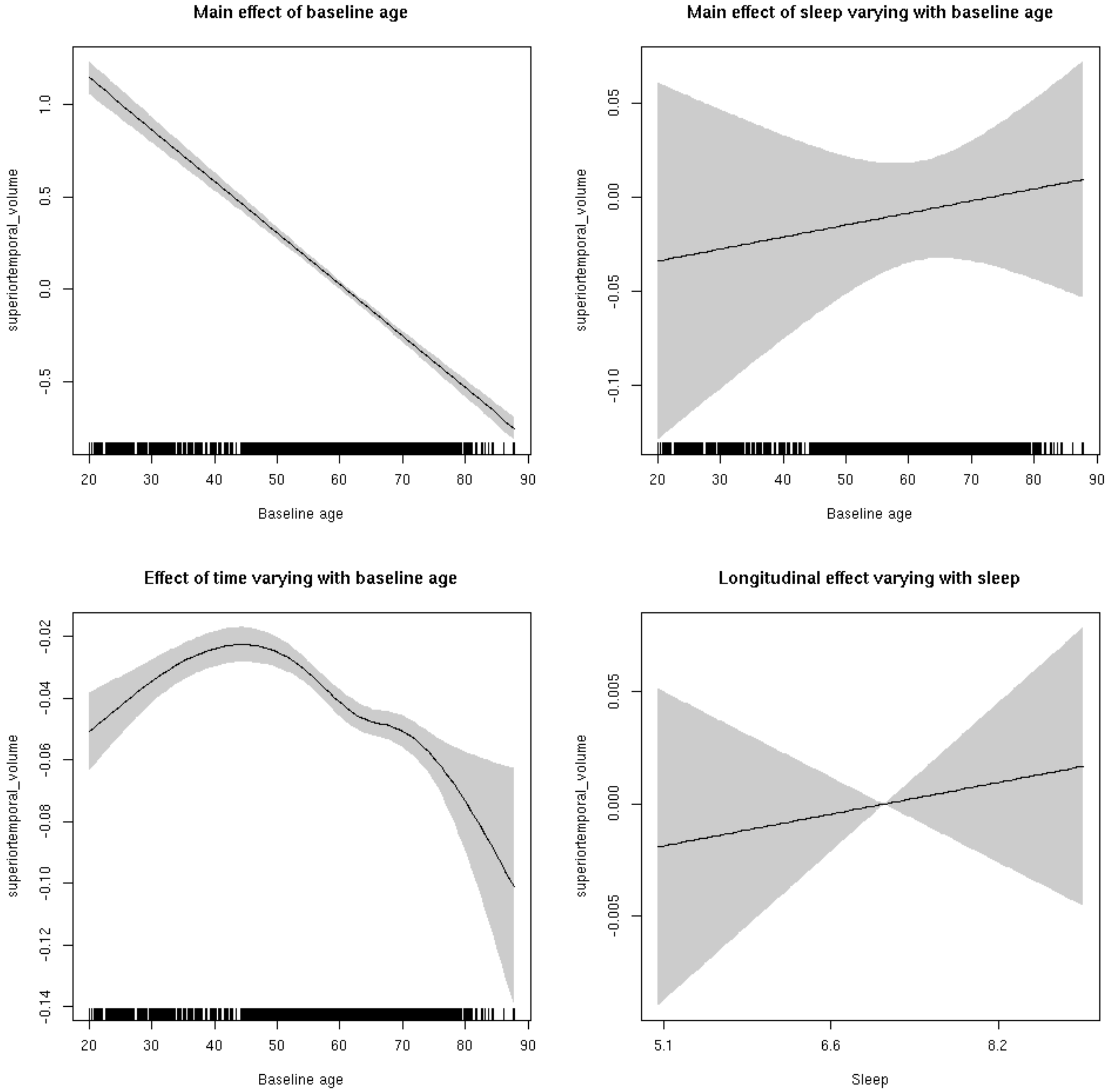

Restricted to sleep >= 5 and <= 9 hours Below is the row for `s(sleep_z):time` from the model output.

```
## s(sleep_z):time   1.000  1.000   0.293   0.588
```

Below are plots of a plot of `s(bl_age)`, `s(bl_age):sleep_z`, `s(bl_age):time`, and `s(sleep_z):time`. For `s(sleep_z):time`, roughly, if we cannot fit a straight line through the confidence bands, there is evidence that the effect of time depends on sleep duration. It is correct the that confidence interval is zero at one point along the axis; this happens because we do not include the main effect.

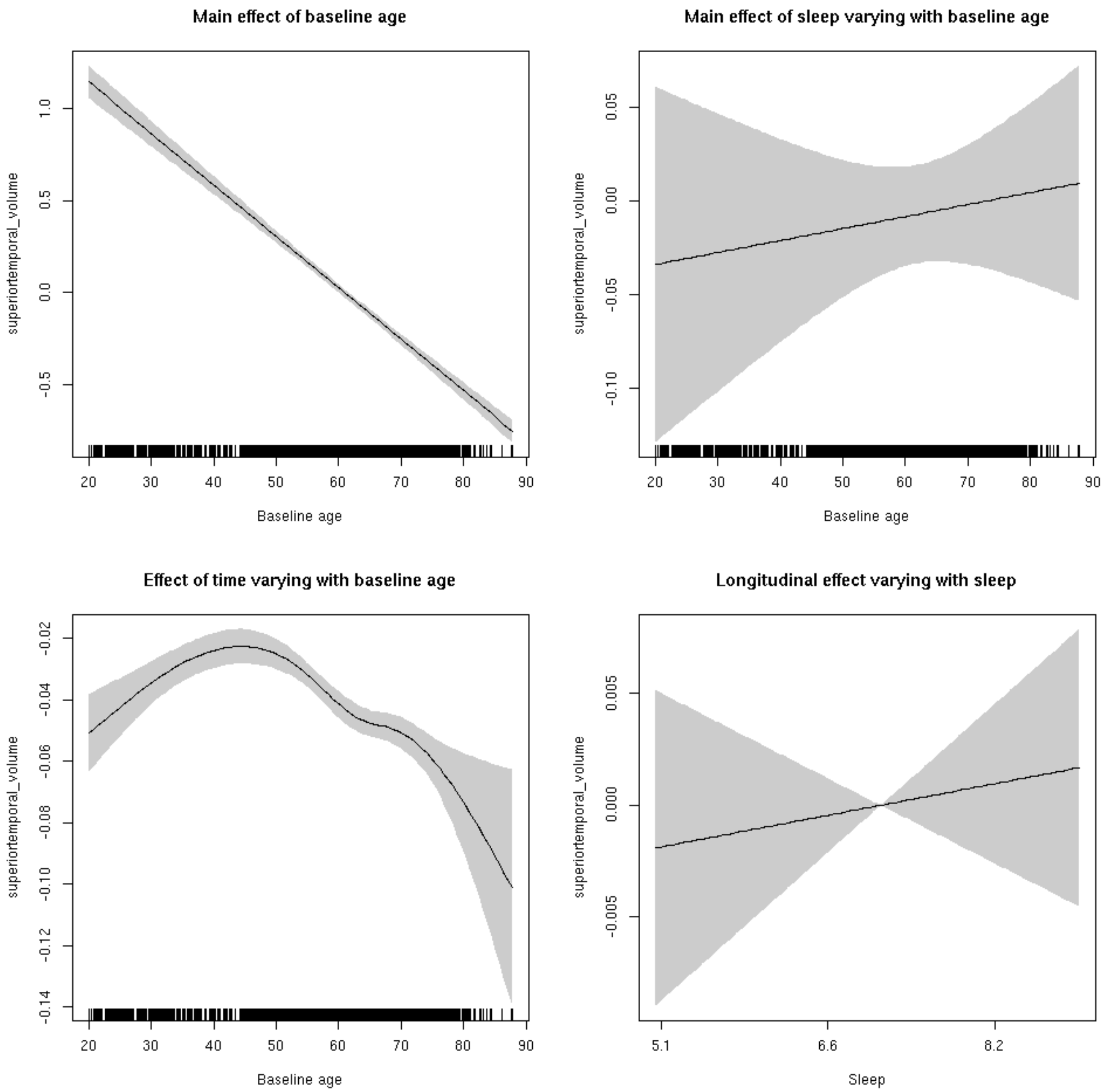

supramarginal_area: Stratified by sex

Female: Full data Below is the row for `s(sleep_z):time` from the model output.

```
## s(sleep_z):time   1.000  1.000  2.337    0.126
```

Below are plots of a plot of `s(bl_age)`, `s(bl_age):sleep_z`, `s(bl_age):time`, and `s(sleep_z):time`. For `s(sleep_z):time`, roughly, if we cannot fit a straight line through the confidence bands, there is evidence that the effect of time depends on sleep duration. It is correct the that confidence interval is zero at one point along the axis; this happens because we do not include the main effect.

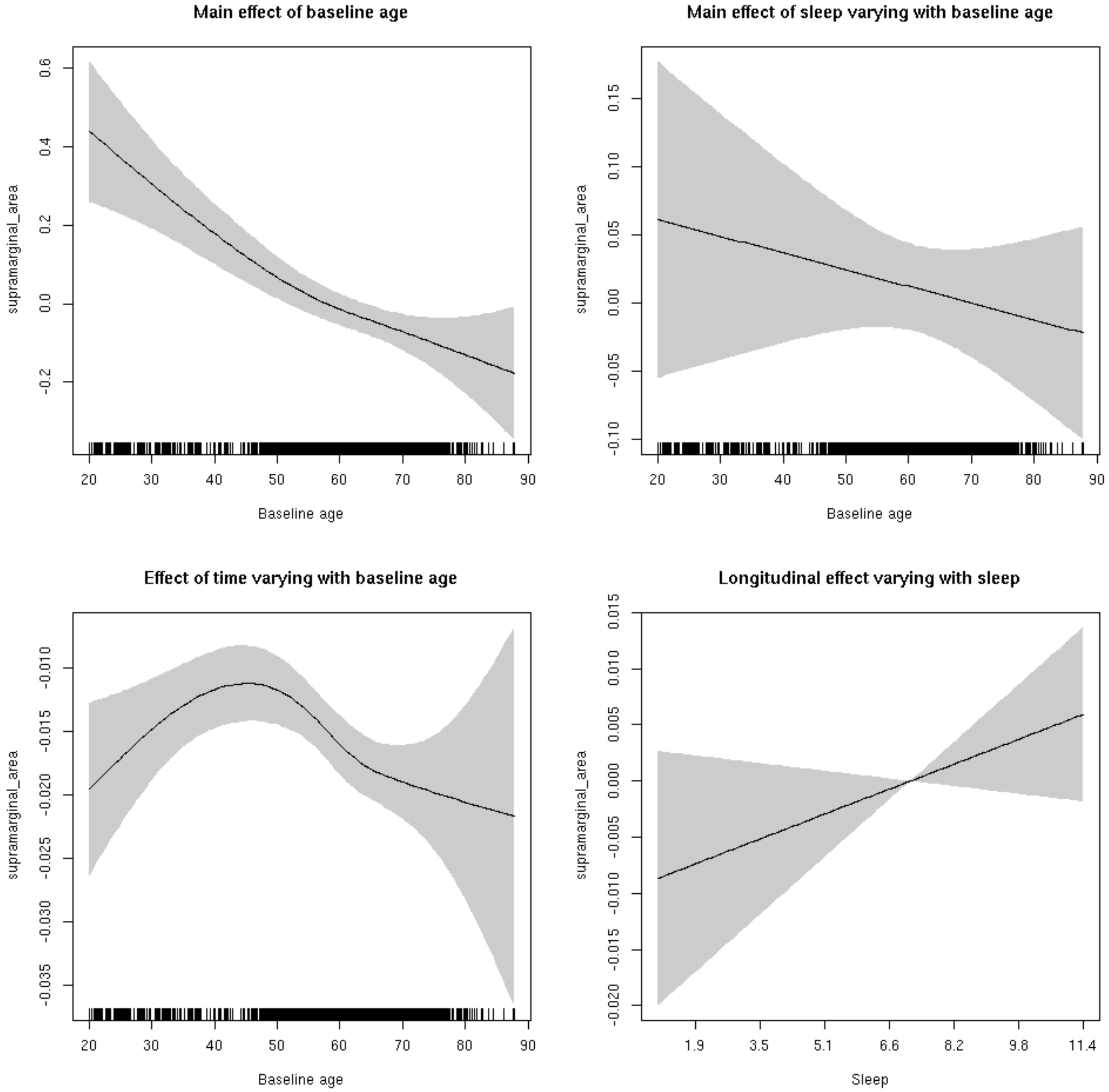

Male: Full data Below is the row for `s(sleep_z):time` from the model output.

```
## s(sleep_z):time   3.137  3.137  1.942  0.0759 .
```

Below are plots of a plot of `s(bl_age)`, `s(bl_age):sleep_z`, `s(bl_age):time`, and `s(sleep_z):time`. For `s(sleep_z):time`, roughly, if we cannot fit a straight line through the confidence bands, there is evidence that the effect of time depends on sleep duration. It is correct the that confidence interval is zero at one point along the axis; this happens because we do not include the main effect.

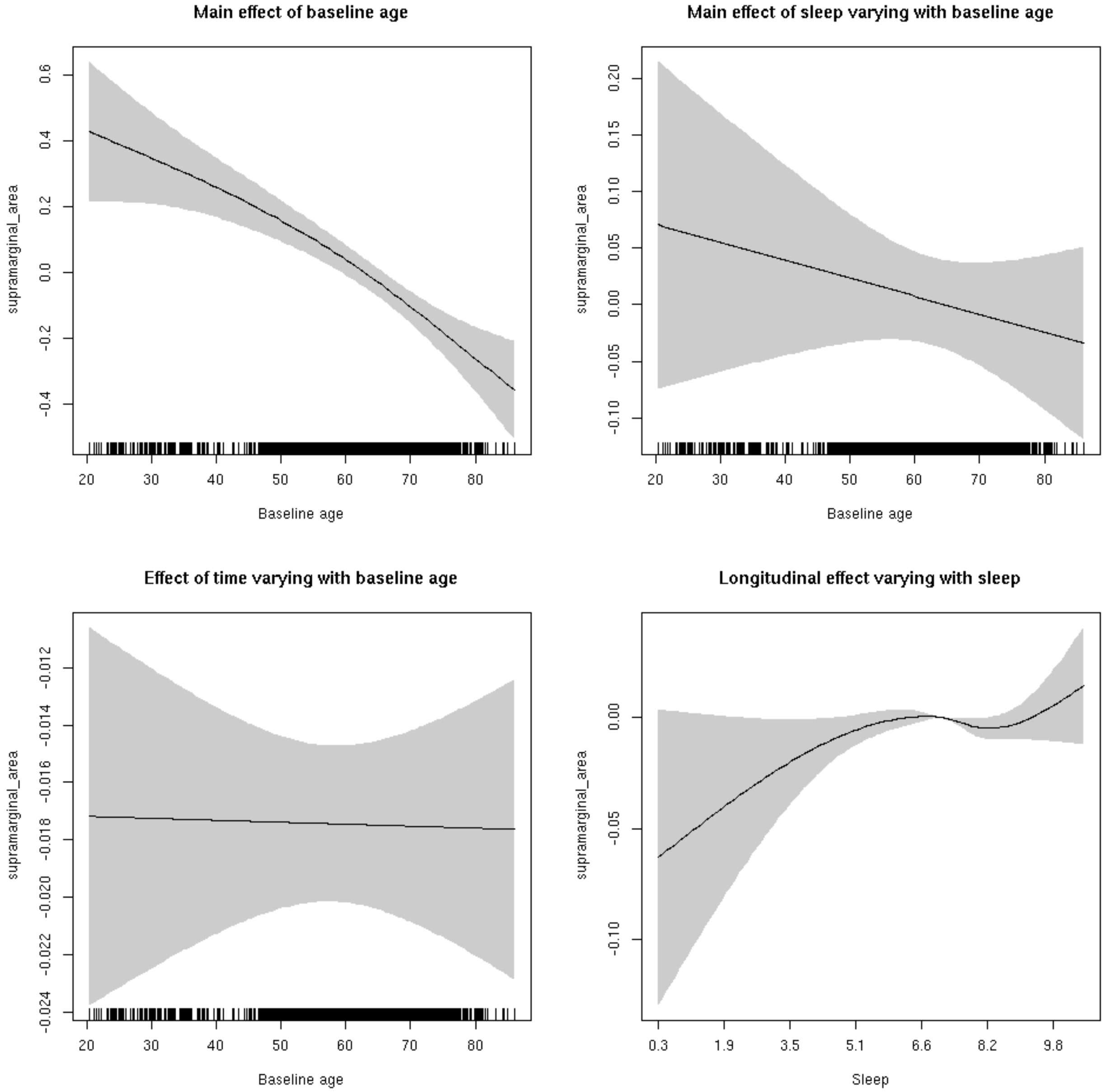

supramarginal_area

Full data Below is the model output. The term `s(sleep_z):time` is probably what we care most about.

```
##
## Family: gaussian
## Link function: identity
##
## Formula:
## value ~ sex + site + icv + s(bl_age, k = 10, bs = "cr") + s(bl_age,
##     by = sleep_z, bs = "cr") + +s(bl_age, by = time, k = 5, bs = "cr") +
##     s(sleep_z, by = time, k = 5, bs = "cr", pc = 0)
## <environment: 0x5630035aca00>
```

```
##
## Parametric coefficients:
##                Estimate Std. Error t value Pr(>|t|)
## (Intercept)     0.54140    0.05551   9.754  < 2e-16 ***
## sexmale         0.27559    0.02887   9.545  < 2e-16 ***
## siteousAvanto -0.79208    0.06842 -11.576  < 2e-16 ***
## siteousPrisma -0.90982    0.09599  -9.478  < 2e-16 ***
## siteousSkyra  -0.39428    0.06647  -5.932 3.12e-09 ***
## siteUB        -0.66046    0.13150  -5.022 5.21e-07 ***
## siteUCAM      -0.56823    0.06976  -8.145 4.34e-16 ***
## siteUKB       -0.64236    0.05200 -12.354  < 2e-16 ***
## siteUmU       -0.04162    0.08080  -0.515    0.606
## icv            0.53952    0.01362  39.603  < 2e-16 ***
## ---
## Signif. codes:  0 ‘***’ 0.001 ‘**’ 0.01 ‘*’ 0.05 ‘.’ 0.1 ‘ ’ 1
##
## Approximate significance of smooth terms:
##                      edf Ref.df       F p-value
## s(bl_age)          1.000  1.000  85.047  <2e-16 ***
## s(bl_age):sleep_z 2.000  2.000   1.427   0.240
## s(bl_age):time    3.129  3.129 214.130  <2e-16 ***
## s(sleep_z):time   1.000  1.000   1.149   0.284
## ---
## Signif. codes:  0 ‘***’ 0.001 ‘**’ 0.01 ‘*’ 0.05 ‘.’ 0.1 ‘ ’ 1
##
## R-sq.(adj) =   0.44
## lmer.REML = 2626.1  Scale est. = 0.007011  n = 8133
```

Below are plots of a plot of `s(bl_age)`, `s(bl_age):sleep_z`, `s(bl_age):time`, and `s(sleep_z):time`. For `s(sleep_z):time`, roughly, if we cannot fit a straight line through the confidence bands, there is evidence that the effect of time depends on sleep duration. It is correct the that confidence interval is zero at one point along the axis; this happens because we do not include the main effect.

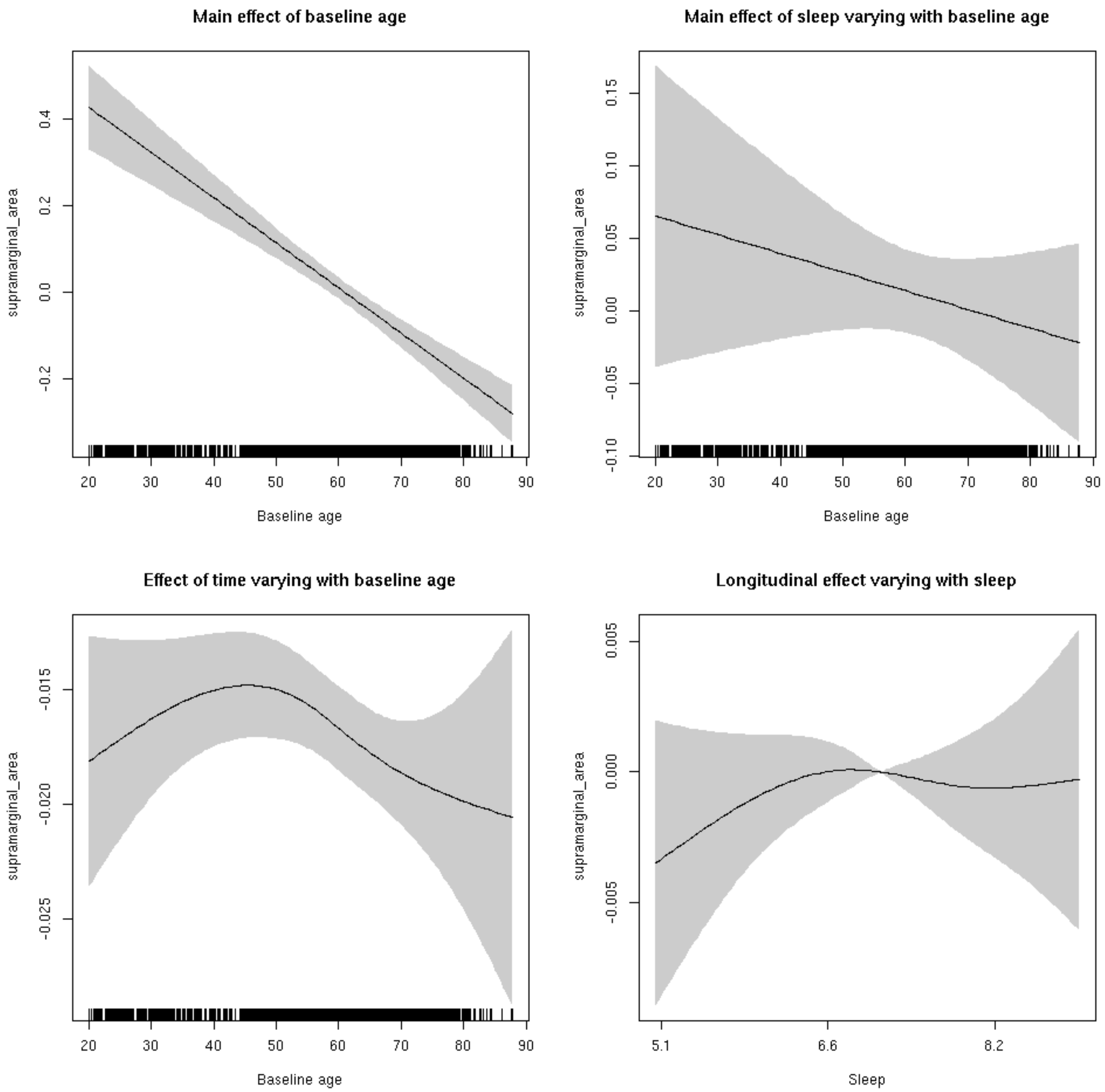

Restricted to sleep >= 5 and <= 9 hours Below is the row for `s(sleep_z):time` from the model output.

```
## s(sleep_z):time   1.862  1.862   0.524   0.530
```

Below are plots of a plot of `s(bl_age)`, `s(bl_age):sleep_z`, `s(bl_age):time`, and `s(sleep_z):time`. For `s(sleep_z):time`, roughly, if we cannot fit a straight line through the confidence bands, there is evidence that the effect of time depends on sleep duration. It is correct the that confidence interval is zero at one point along the axis; this happens because we do not include the main effect.

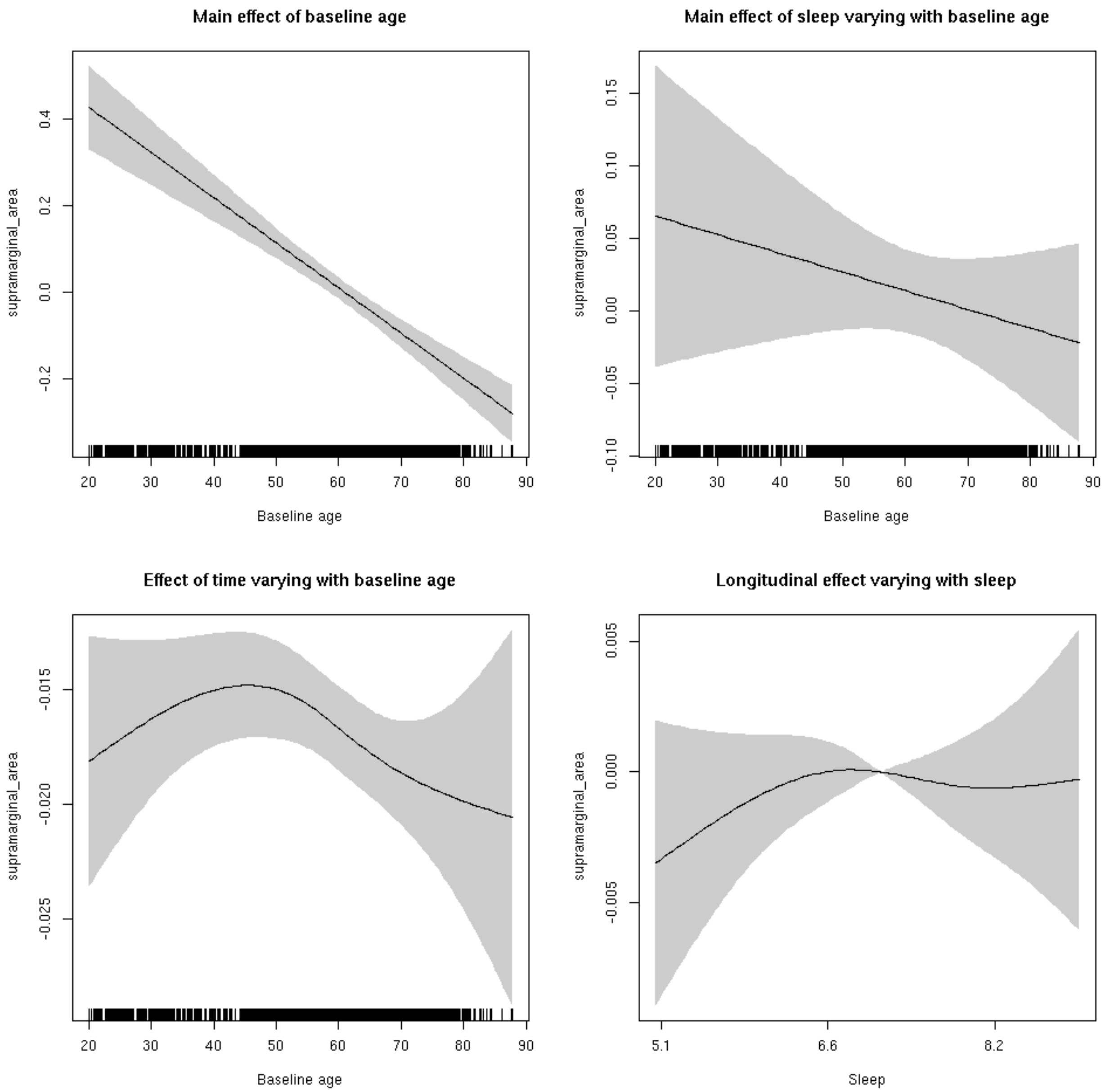

supramarginal_thickness: Stratified by sex

Female: Full data Below is the row for `s(sleep_z):time` from the model output.

```
## s(sleep_z):time   3.612  3.612   1.977  0.0569 .
```

Below are plots of a plot of `s(bl_age)`, `s(bl_age):sleep_z`, `s(bl_age):time`, and `s(sleep_z):time`. For `s(sleep_z):time`, roughly, if we cannot fit a straight line through the confidence bands, there is evidence that the effect of time depends on sleep duration. It is correct the that confidence interval is zero at one point along the axis; this happens because we do not include the main effect.

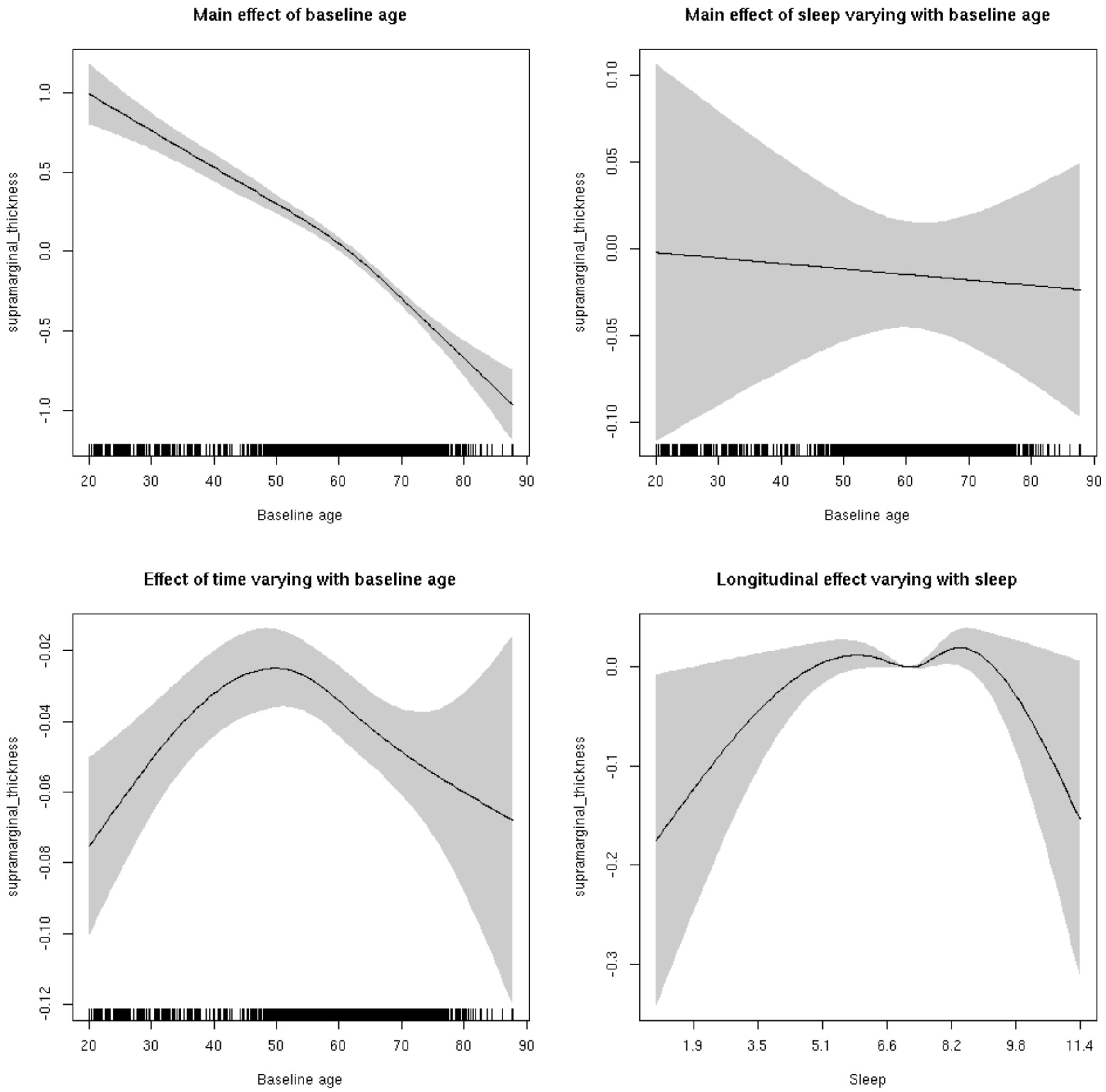

Male: Full data Below is the row for `s(sleep_z):time` from the model output.

```
## s(sleep_z):time   1.000  1.000   0.193   0.661
```

Below are plots of a plot of `s(bl_age)`, `s(bl_age):sleep_z`, `s(bl_age):time`, and `s(sleep_z):time`. For `s(sleep_z):time`, roughly, if we cannot fit a straight line through the confidence bands, there is evidence that the effect of time depends on sleep duration. It is correct the that confidence interval is zero at one point along the axis; this happens because we do not include the main effect.

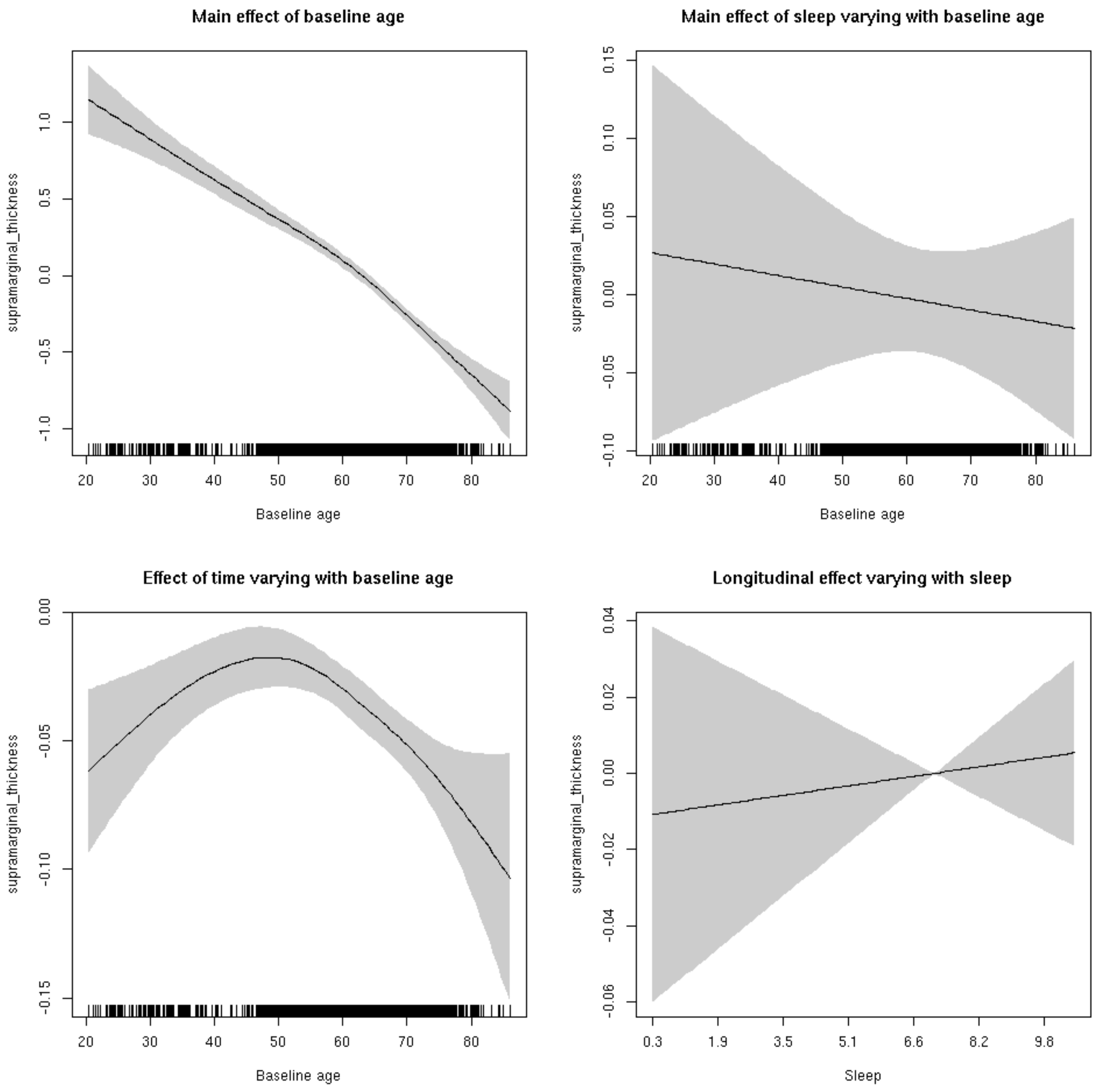

supramarginal_thickness

Full data Below is the model output. The term `s(sleep_z):time` is probably what we care most about.

```
##
## Family: gaussian
## Link function: identity
##
## Formula:
## value ~ sex + site + s(bl_age, k = 10, bs = "cr") + s(bl_age,
##     by = sleep_z, bs = "cr") + +s(bl_age, by = time, k = 5, bs = "cr") +
##     s(sleep_z, by = time, k = 5, bs = "cr", pc = 0)
## <environment: 0x5630035d3848>
```

```
##
## Parametric coefficients:
##                Estimate Std. Error t value Pr(>|t|)
## (Intercept)   -1.81400    0.04248 -42.702  < 2e-16 ***
## sexmale       -0.09958    0.02132  -4.671 3.04e-06 ***
## siteousAvanto  0.16063    0.05626   2.855  0.00432 **
## siteousPrisma  0.59838    0.22586   2.649  0.00808 **
## siteousSkyra  -0.15519    0.05625  -2.759  0.00581 **
## siteUB         0.58606    0.11303   5.185 2.21e-07 ***
## siteUCAM       0.51529    0.05844   8.817  < 2e-16 ***
## siteUKB        2.17801    0.04306  50.582  < 2e-16 ***
## siteUmU       -0.21081    0.07005  -3.009  0.00263 **
## ---
## Signif. codes:  0 '***' 0.001 '**' 0.01 '*' 0.05 '.' 0.1 ' ' 1
##
## Approximate significance of smooth terms:
##                       edf Ref.df       F p-value
## s(bl_age)           3.510  3.510 252.609  <2e-16 ***
## s(bl_age):sleep_z 2.000  2.000   0.624  0.5357
## s(bl_age):time    3.865  3.865  49.261  <2e-16 ***
## s(sleep_z):time   3.639  3.639   2.551  0.0225 *
## ---
## Signif. codes:  0 '***' 0.001 '**' 0.01 '*' 0.05 '.' 0.1 ' ' 1
##
## R-sq.(adj) =   0.67
## lmer.REML =  12289  Scale est. = 0.085939  n = 8168
```

Below are plots of a plot of `s(bl_age)`, `s(bl_age):sleep_z`, `s(bl_age):time`, and `s(sleep_z):time`. For `s(sleep_z):time`, roughly, if we cannot fit a straight line through the confidence bands, there is evidence that the effect of time depends on sleep duration. It is correct the that confidence interval is zero at one point along the axis; this happens because we do not include the main effect.

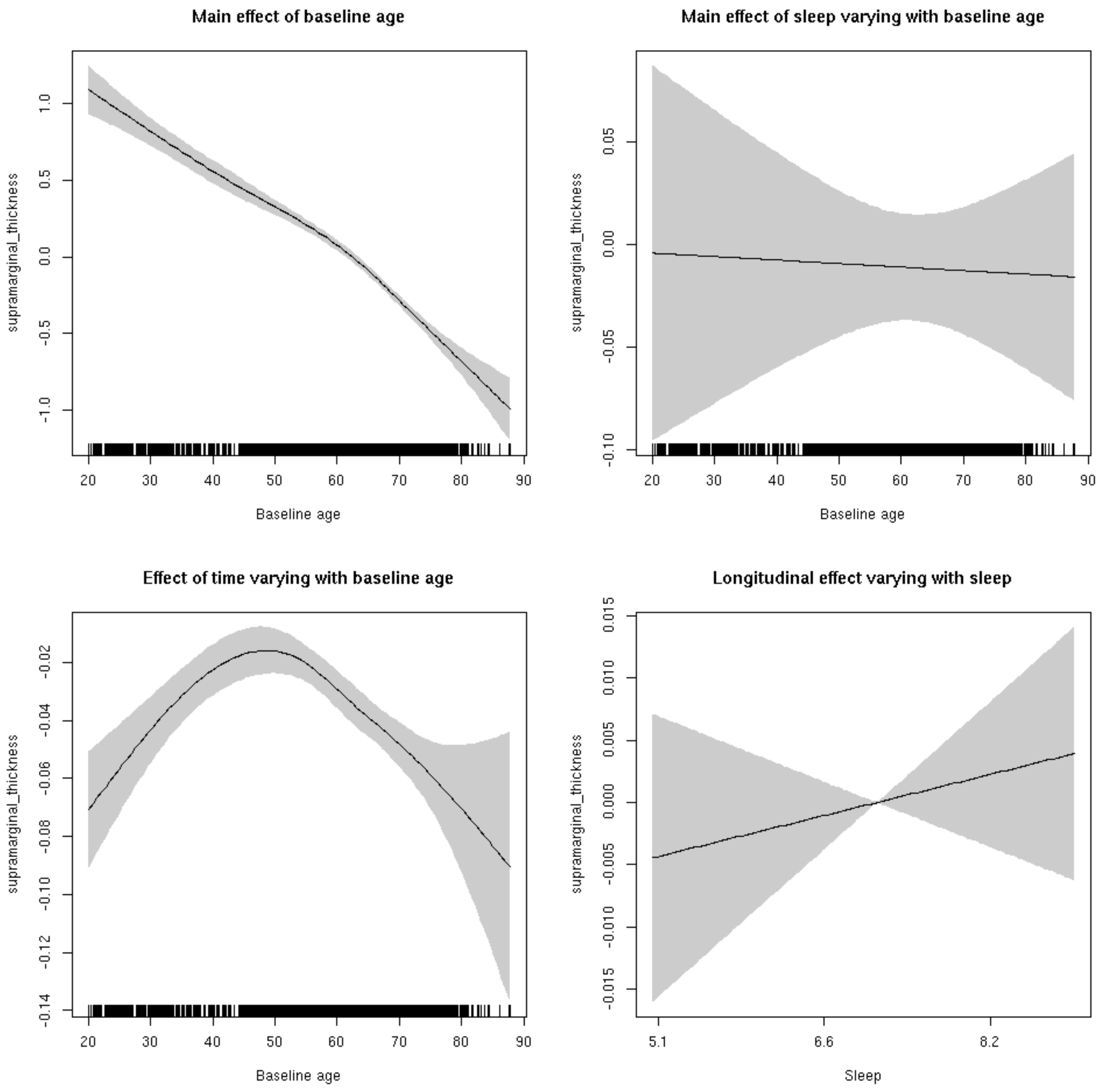

Restricted to sleep >= 5 and <= 9 hours Below is the row for `s(sleep_z):time` from the model output.

```
## s(sleep_z):time   1.001  1.001   0.592   0.441
```

Below are plots of a plot of `s(bl_age)`, `s(bl_age):sleep_z`, `s(bl_age):time`, and `s(sleep_z):time`. For `s(sleep_z):time`, roughly, if we cannot fit a straight line through the confidence bands, there is evidence that the effect of time depends on sleep duration. It is correct the that confidence interval is zero at one point along the axis; this happens because we do not include the main effect.

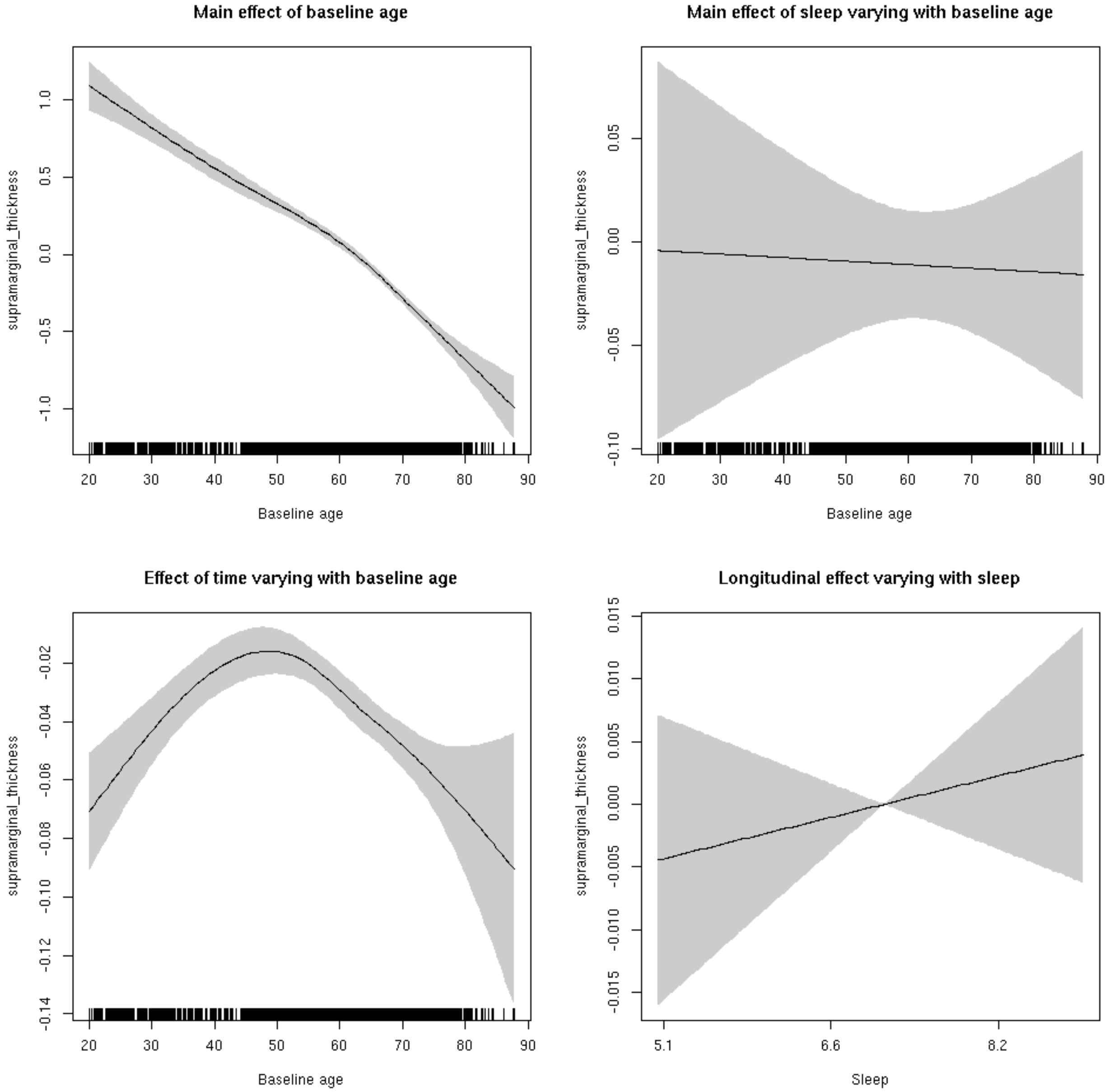

supramarginal_volume: Stratified by sex

Female: Full data Below is the row for `s(sleep_z):time` from the model output.

```
## s(sleep_z):time   3.665  3.665  1.991  0.0596 .
```

Below are plots of a plot of `s(bl_age)`, `s(bl_age):sleep_z`, `s(bl_age):time`, and `s(sleep_z):time`. For `s(sleep_z):time`, roughly, if we cannot fit a straight line through the confidence bands, there is evidence that the effect of time depends on sleep duration. It is correct the that confidence interval is zero at one point along the axis; this happens because we do not include the main effect.

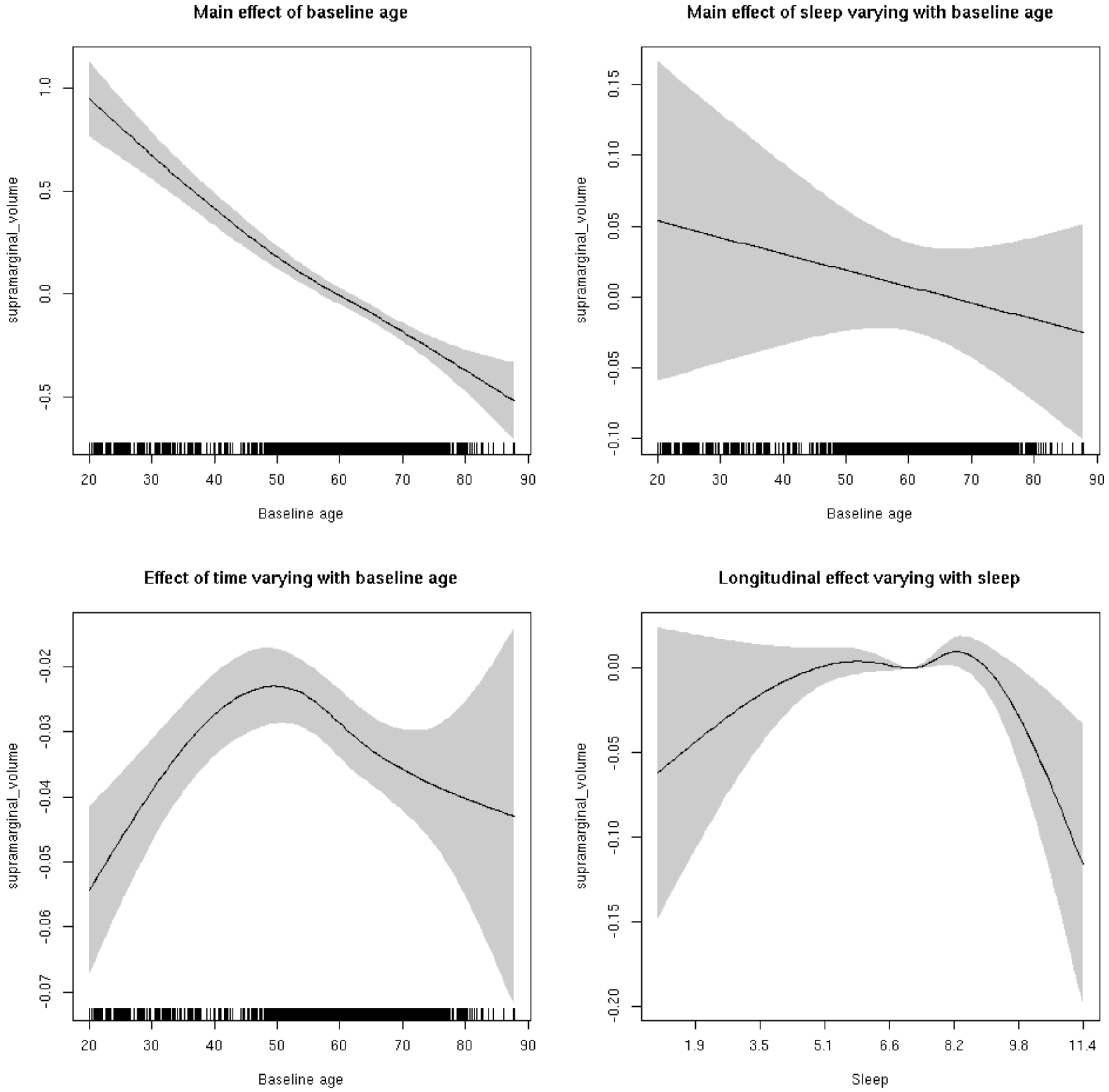

Male: Full data Below is the row for `s(sleep_z):time` from the model output.

```
## s(sleep_z):time   2.975  2.975   2.628  0.0455 *
```

Below are plots of a plot of `s(bl_age)`, `s(bl_age):sleep_z`, `s(bl_age):time`, and `s(sleep_z):time`. For `s(sleep_z):time`, roughly, if we cannot fit a straight line through the confidence bands, there is evidence that the effect of time depends on sleep duration. It is correct the that confidence interval is zero at one point along the axis; this happens because we do not include the main effect.

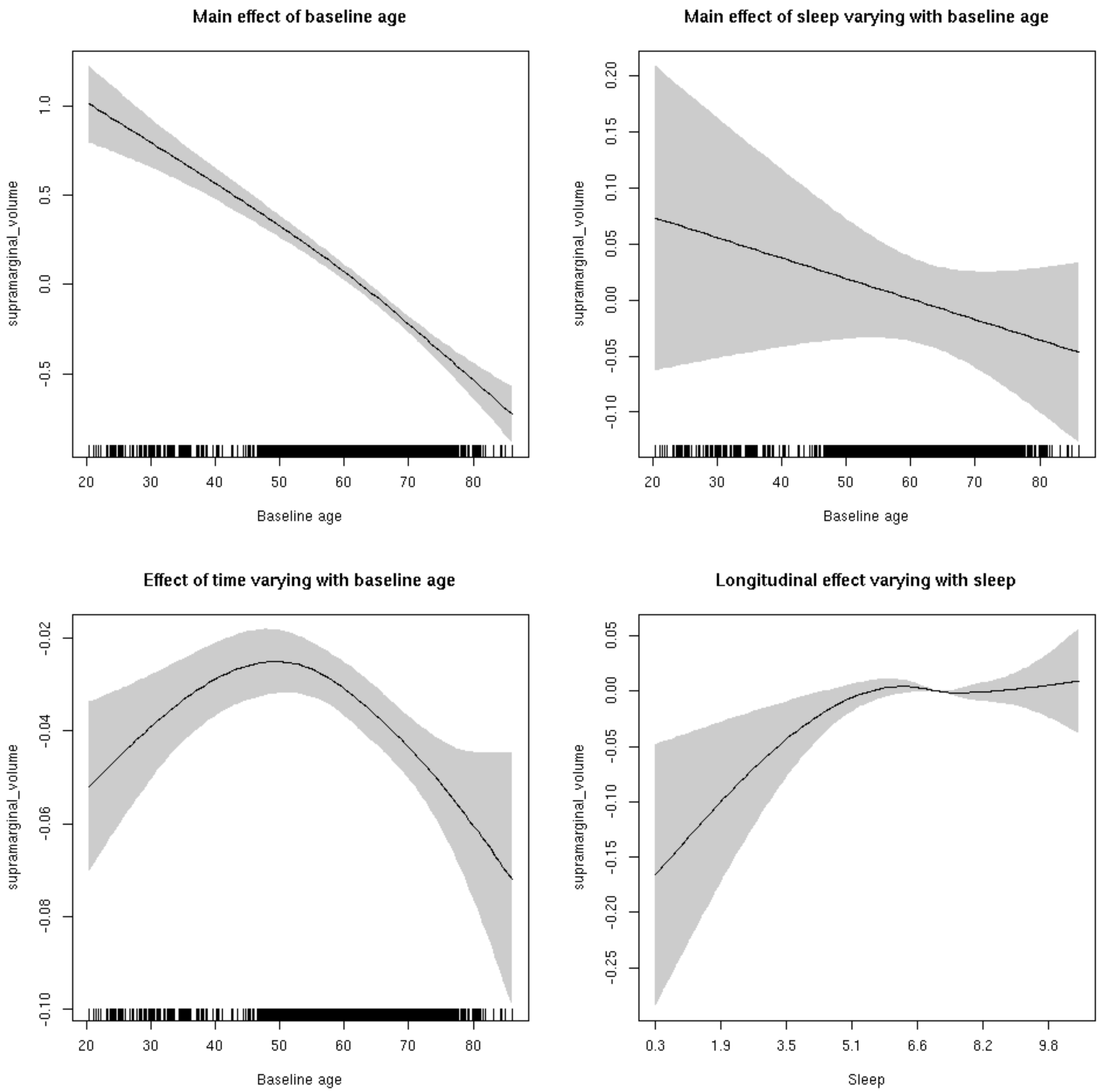

supramarginal_volume

Full data Below is the model output. The term `s(sleep_z):time` is probably what we care most about.

```
##
## Family: gaussian
## Link function: identity
##
## Formula:
## value ~ sex + site + icv + s(bl_age, k = 10, bs = "cr") + s(bl_age,
##     by = sleep_z, bs = "cr") + +s(bl_age, by = time, k = 5, bs = "cr") +
##     s(sleep_z, by = time, k = 5, bs = "cr", pc = 0)
## <environment: 0x5630035aaa00>
```

```
##
## Parametric coefficients:
##                Estimate Std. Error t value Pr(>|t|)
## (Intercept)   -0.16470    0.05401  -3.049  0.00230 **
## sexmale        0.18369    0.02809   6.540 6.51e-11 ***
## siteousAvanto -0.77497    0.06646 -11.660  < 2e-16 ***
## siteousPrisma -0.57321    0.13667  -4.194 2.77e-05 ***
## siteousSkyra  -0.45577    0.06435  -7.083 1.53e-12 ***
## siteUB        -0.40996    0.12589  -3.257  0.00113 **
## siteUCAM      -0.41263    0.06727  -6.134 8.99e-10 ***
## siteUKB        0.25149    0.05035   4.994 6.02e-07 ***
## siteUmU       -0.17016    0.07762  -2.192  0.02838 *
## icv            0.54139    0.01375  39.367  < 2e-16 ***
## ---
## Signif. codes:  0 '***' 0.001 '**' 0.01 '*' 0.05 '.' 0.1 ' ' 1
##
## Approximate significance of smooth terms:
##                      edf Ref.df       F p-value
## s(bl_age)          1.000  1.000 474.443  <2e-16 ***
## s(bl_age):sleep_z 2.000  2.000   1.558   0.211
## s(bl_age):time     3.861  3.861 184.606  <2e-16 ***
## s(sleep_z):time    1.000  1.000   0.459   0.498
## ---
## Signif. codes:  0 '***' 0.001 '**' 0.01 '*' 0.05 '.' 0.1 ' ' 1
##
## R-sq.(adj) =  0.507
## lmer.REML = 7464.5  Scale est. = 0.023634  n = 8144
```

Below are plots of a plot of `s(bl_age)`, `s(bl_age):sleep_z`, `s(bl_age):time`, and `s(sleep_z):time`. For `s(sleep_z):time`, roughly, if we cannot fit a straight line through the confidence bands, there is evidence that the effect of time depends on sleep duration. It is correct the that confidence interval is zero at one point along the axis; this happens because we do not include the main effect.

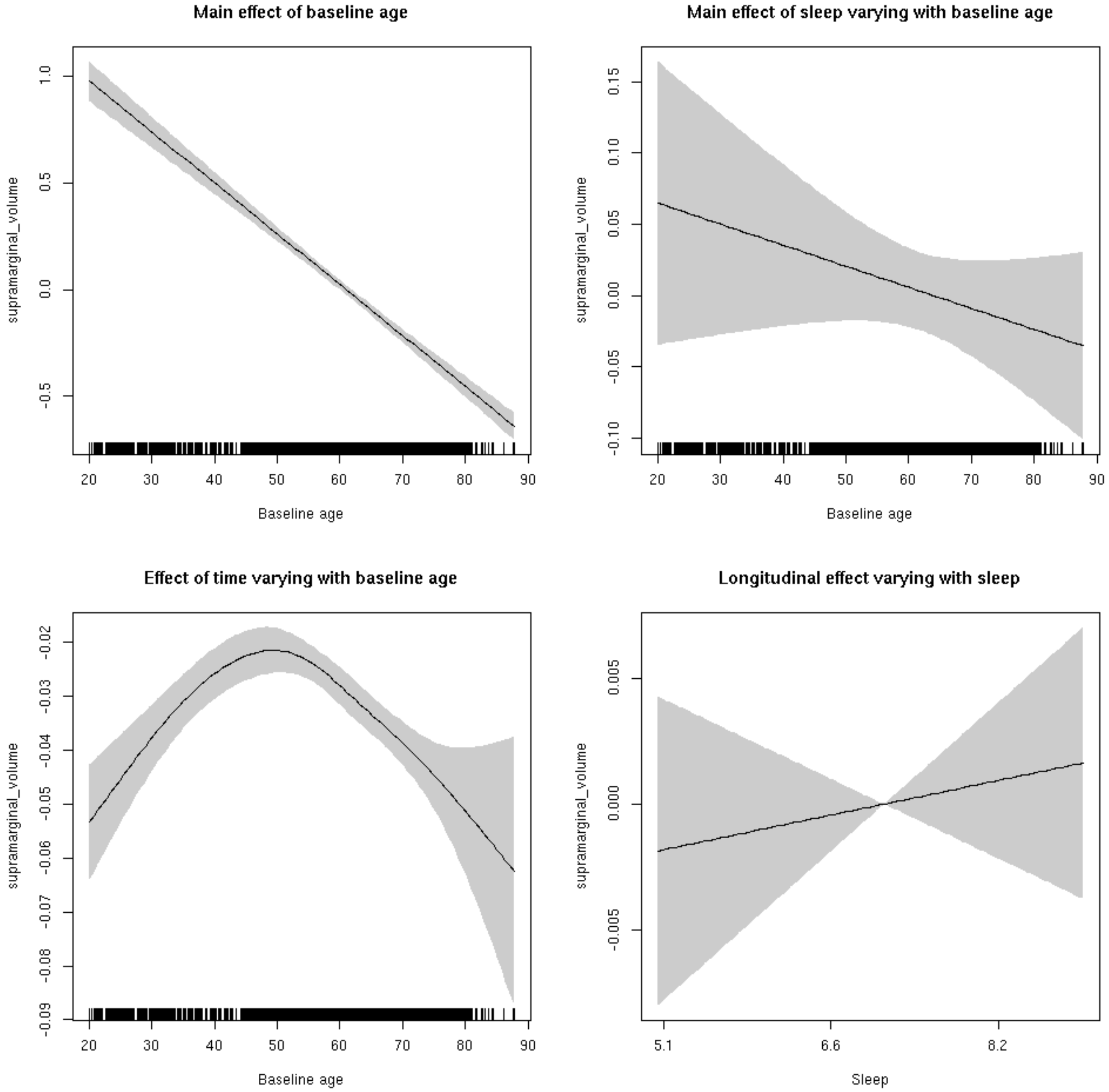

Restricted to sleep >= 5 and <= 9 hours Below is the row for `s(sleep_z):time` from the model output.

```
## s(sleep_z):time   1.000  1.000   0.366   0.545
```

Below are plots of a plot of `s(bl_age)`, `s(bl_age):sleep_z`, `s(bl_age):time`, and `s(sleep_z):time`. For `s(sleep_z):time`, roughly, if we cannot fit a straight line through the confidence bands, there is evidence that the effect of time depends on sleep duration. It is correct the that confidence interval is zero at one point along the axis; this happens because we do not include the main effect.

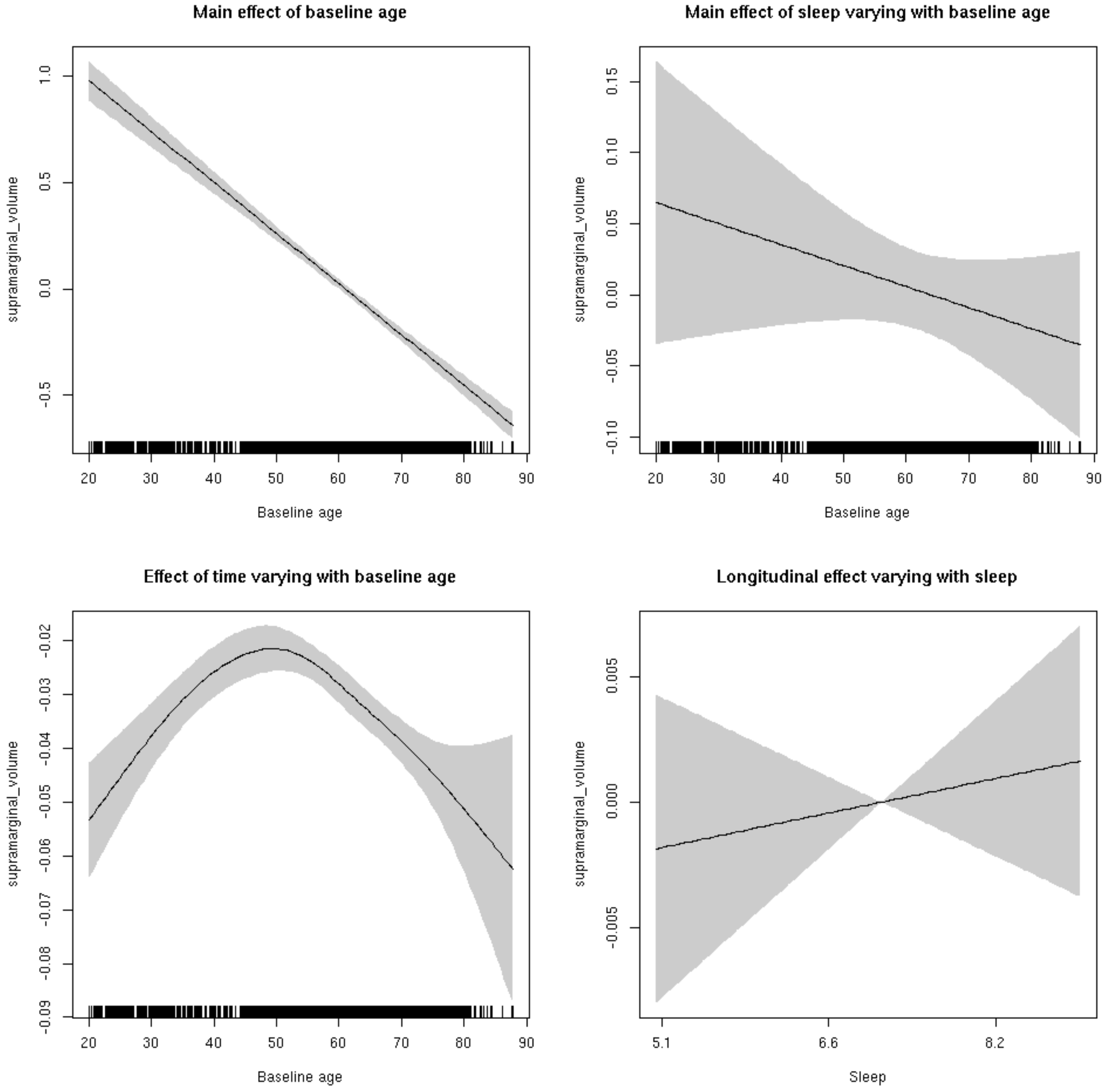

transversetemporal_area: Stratified by sex

Female: Full data Below is the row for `s(sleep_z):time` from the model output.

```
## s(sleep_z):time     1      1  5.423  0.0199 *
```

Below are plots of a plot of `s(bl_age)`, `s(bl_age):sleep_z`, `s(bl_age):time`, and `s(sleep_z):time`. For `s(sleep_z):time`, roughly, if we cannot fit a straight line through the confidence bands, there is evidence that the effect of time depends on sleep duration. It is correct the that confidence interval is zero at one point along the axis; this happens because we do not include the main effect.

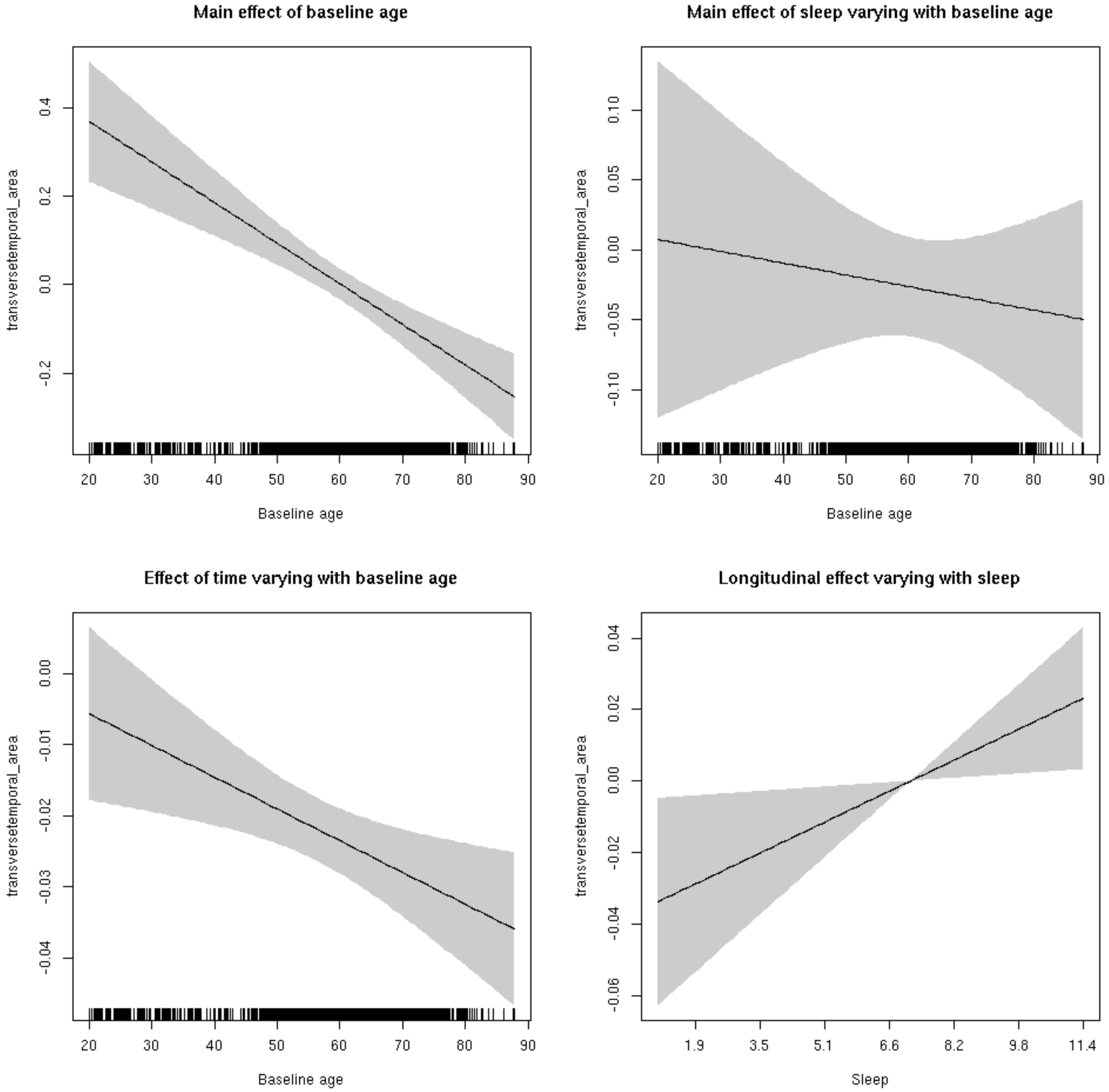

Male: Full data Below is the row for `s(sleep_z):time` from the model output.

```
## s(sleep_z):time   2.231  2.231  3.279    0.022 *
```

Below are plots of a plot of `s(bl_age)`, `s(bl_age):sleep_z`, `s(bl_age):time`, and `s(sleep_z):time`. For `s(sleep_z):time`, roughly, if we cannot fit a straight line through the confidence bands, there is evidence that the effect of time depends on sleep duration. It is correct the that confidence interval is zero at one point along the axis; this happens because we do not include the main effect.

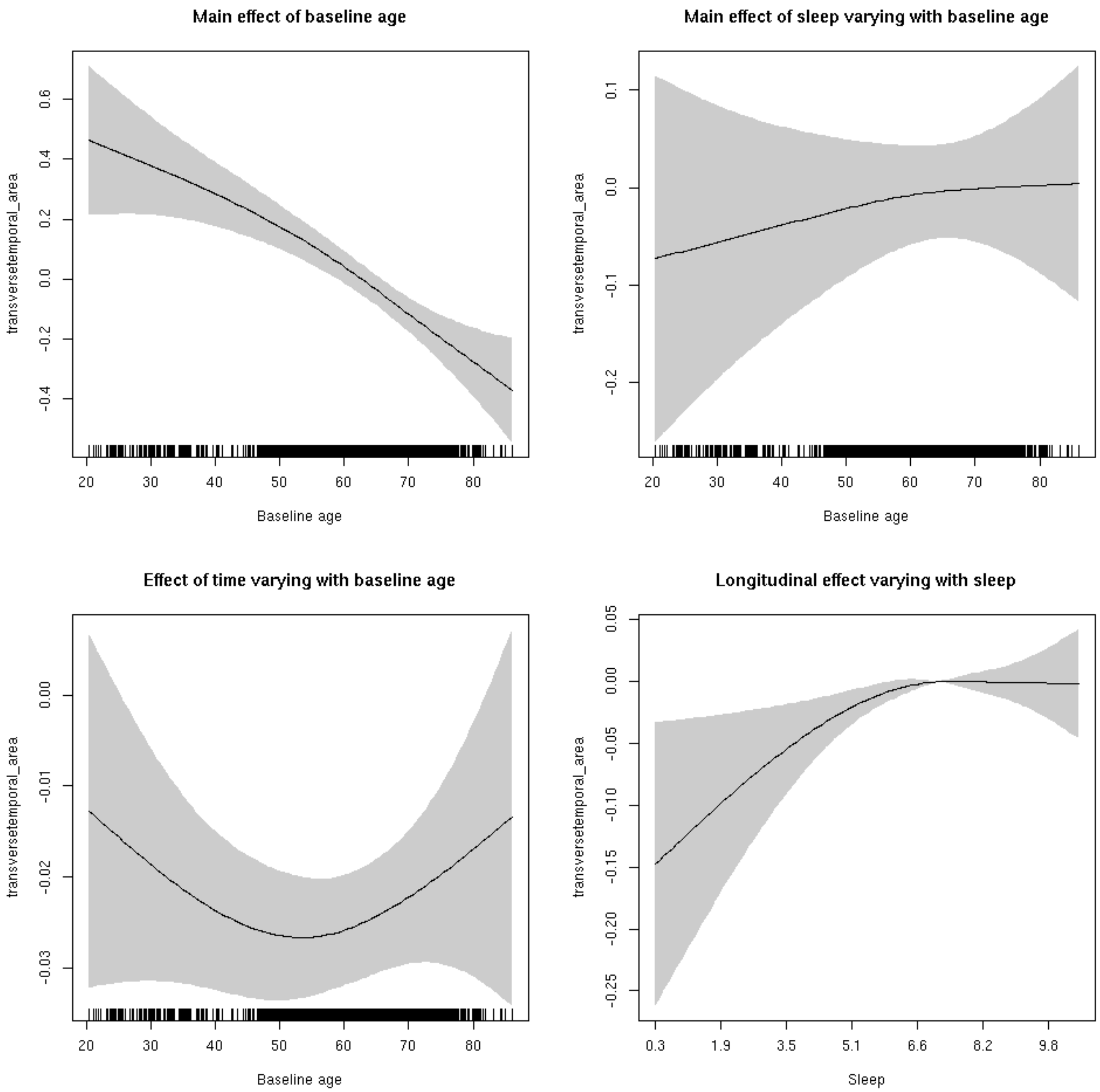

transversetemporal_area

Full data Below is the model output. The term `s(sleep_z):time` is probably what we care most about.

```
##
## Family: gaussian
## Link function: identity
##
## Formula:
## value ~ sex + site + icv + s(bl_age, k = 10, bs = "cr") + s(bl_age,
##     by = sleep_z, bs = "cr") + +s(bl_age, by = time, k = 5, bs = "cr") +
##     s(sleep_z, by = time, k = 5, bs = "cr", pc = 0)
## <environment: 0x5630035b1c00>
```

```
##
## Parametric coefficients:
##                 Estimate Std. Error t value Pr(>|t|)
## (Intercept)      0.22267    0.06522   3.414 0.000643 ***
## sexmale          0.03791    0.03364   1.127 0.259792
## siteousAvanto -0.56594    0.07983  -7.089 1.46e-12 ***
## siteousPrisma -0.41755    0.17521  -2.383 0.017188 *
## siteousSkyra  -0.17776    0.07727  -2.300 0.021447 *
## siteUB        -0.35461    0.15091  -2.350 0.018810 *
## siteUCAM      -0.25649    0.08079  -3.175 0.001505 **
## siteUKB       -0.17758    0.06149  -2.888 0.003886 **
## siteUmU       -0.04399    0.09335  -0.471 0.637484
## icv            0.47755    0.01651  28.922  < 2e-16 ***
## ---
## Signif. codes:  0 '***' 0.001 '**' 0.01 '*' 0.05 '.' 0.1 ' ' 1
##
## Approximate significance of smooth terms:
##                       edf Ref.df      F  p-value
## s(bl_age)           1.843  1.843 32.084  < 2e-16 ***
## s(bl_age):sleep_z 2.000  2.000  0.898 0.407349
## s(bl_age):time    2.000  2.000 65.937  < 2e-16 ***
## s(sleep_z):time   2.467  2.467  5.698 0.000997 ***
## ---
## Signif. codes:  0 '***' 0.001 '**' 0.01 '*' 0.05 '.' 0.1 ' ' 1
##
## R-sq.(adj) =  0.271
## lmer.REML =  11214  Scale est. = 0.04064   n = 8175
```

Below are plots of a plot of `s(bl_age)`, `s(bl_age):sleep_z`, `s(bl_age):time`, and `s(sleep_z):time`. For `s(sleep_z):time`, roughly, if we cannot fit a straight line through the confidence bands, there is evidence that the effect of time depends on sleep duration. It is correct the that confidence interval is zero at one point along the axis; this happens because we do not include the main effect.

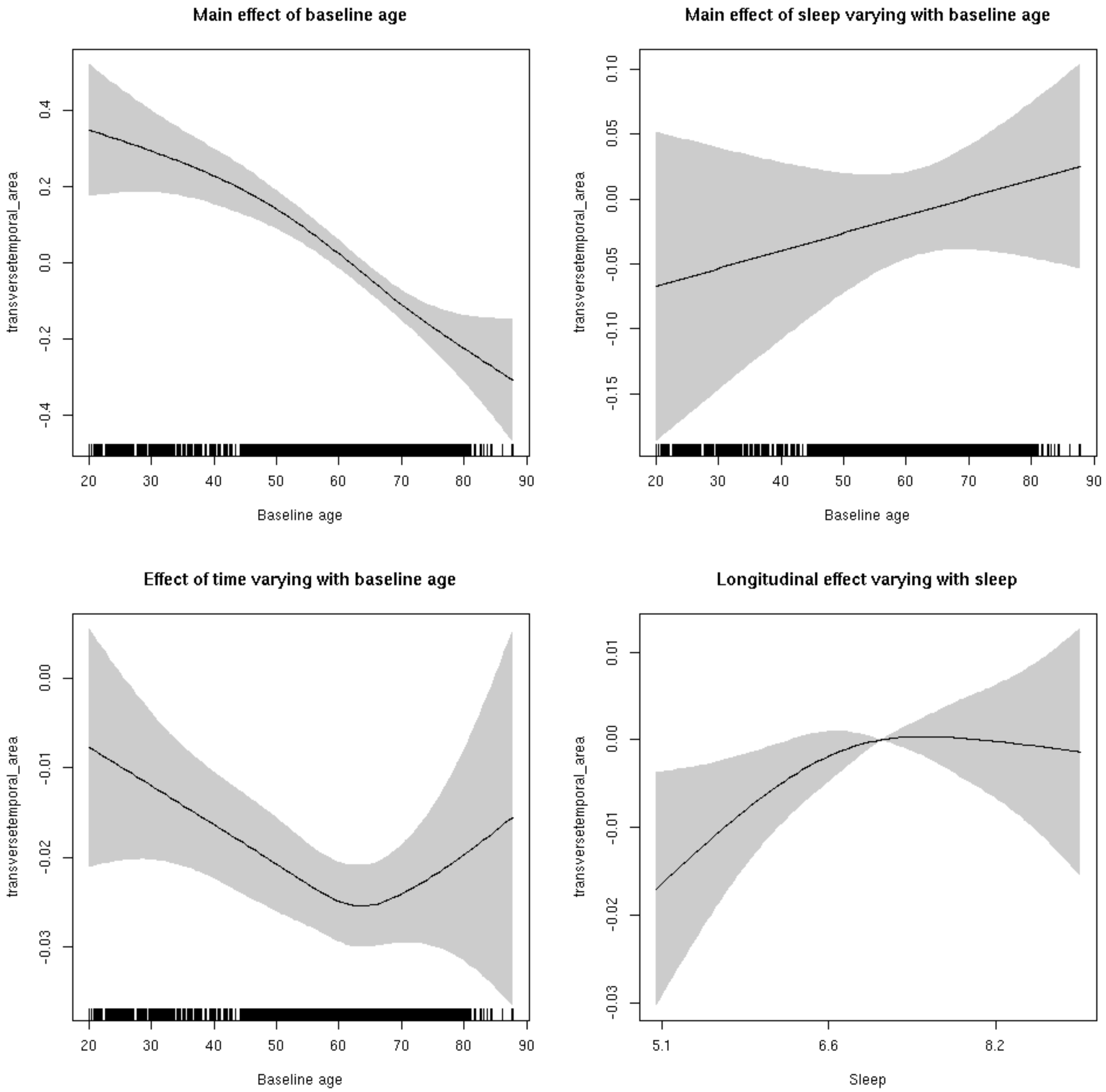

Restricted to sleep >= 5 and <= 9 hours Below is the row for `s(sleep_z):time` from the model output.

```
## s(sleep_z):time   1.922  1.922  3.738  0.0372 *
```

Below are plots of a plot of `s(bl_age)`, `s(bl_age):sleep_z`, `s(bl_age):time`, and `s(sleep_z):time`. For `s(sleep_z):time`, roughly, if we cannot fit a straight line through the confidence bands, there is evidence that the effect of time depends on sleep duration. It is correct the that confidence interval is zero at one point along the axis; this happens because we do not include the main effect.

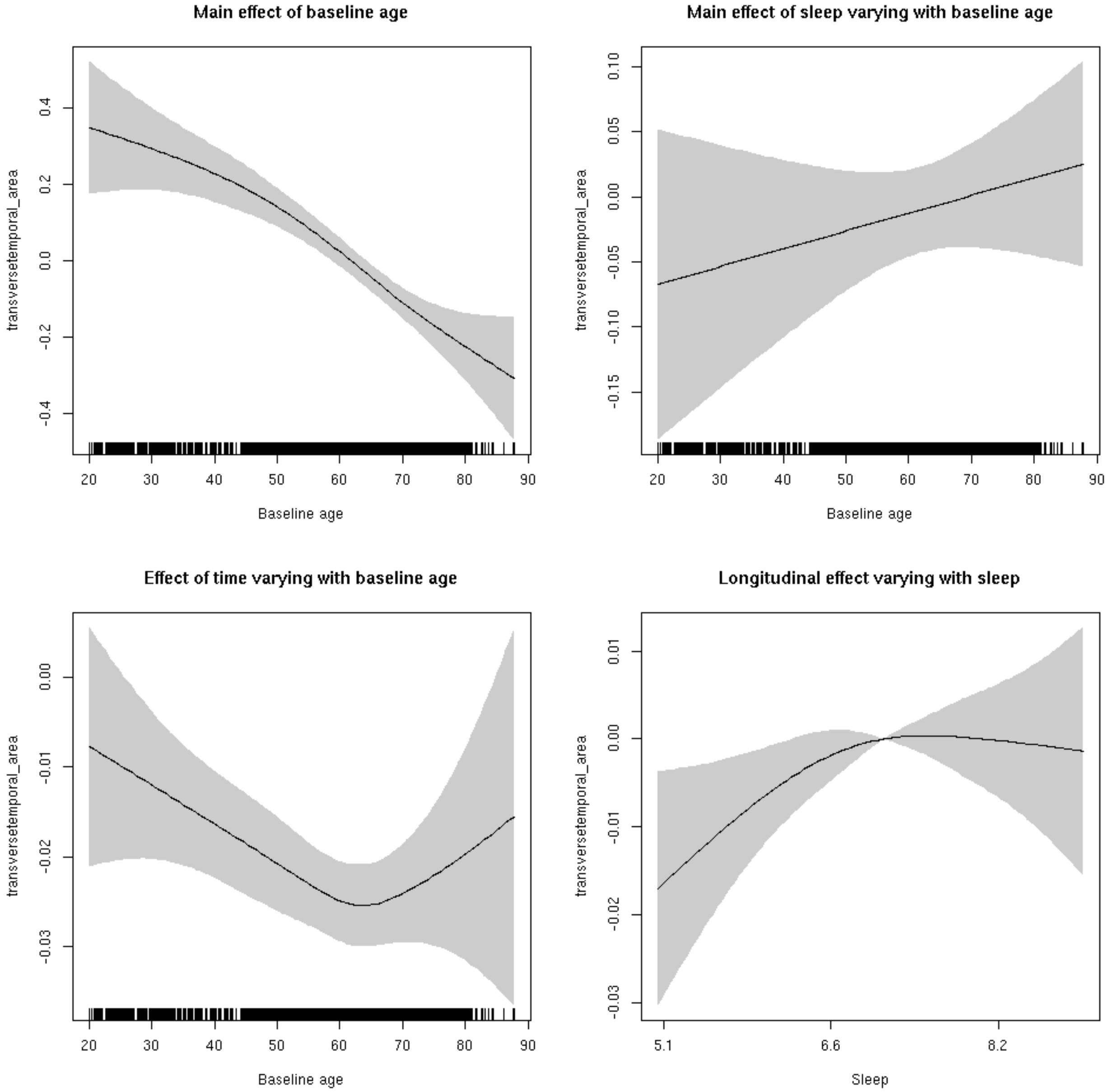

transversetemporal_thickness: Stratified by sex

Female: Full data Below is the row for `s(sleep_z):time` from the model output.

```
## s(sleep_z):time   1.000  1.000  0.105  0.7462
```

Below are plots of a plot of `s(bl_age)`, `s(bl_age):sleep_z`, `s(bl_age):time`, and `s(sleep_z):time`. For `s(sleep_z):time`, roughly, if we cannot fit a straight line through the confidence bands, there is evidence that the effect of time depends on sleep duration. It is correct the that confidence interval is zero at one point along the axis; this happens because we do not include the main effect.

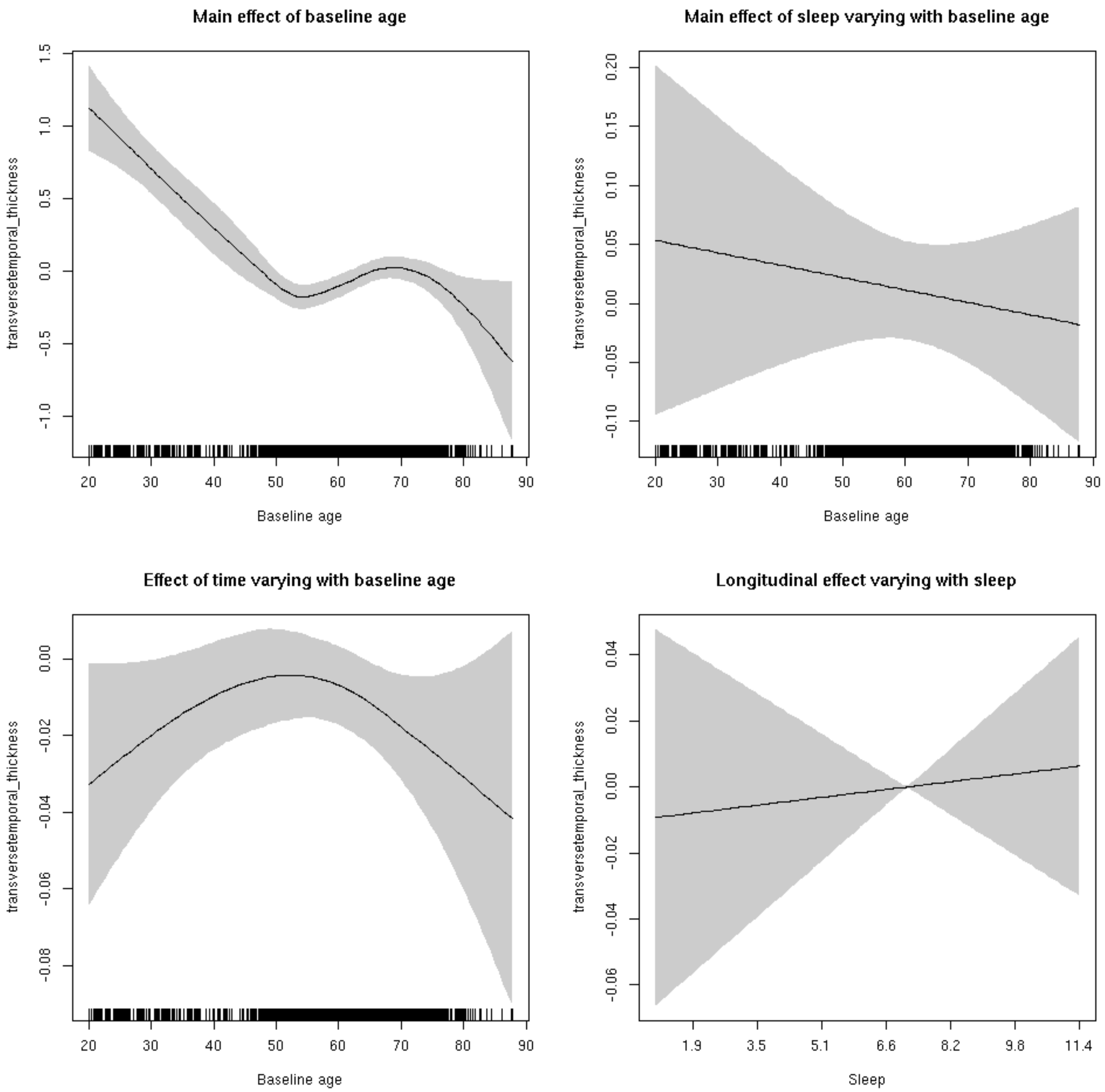

Male: Full data Below is the row for `s(sleep_z):time` from the model output.

```
## s(sleep_z):time   1.000  1.000  0.523   0.470
```

Below are plots of a plot of `s(bl_age)`, `s(bl_age):sleep_z`, `s(bl_age):time`, and `s(sleep_z):time`. For `s(sleep_z):time`, roughly, if we cannot fit a straight line through the confidence bands, there is evidence that the effect of time depends on sleep duration. It is correct the that confidence interval is zero at one point along the axis; this happens because we do not include the main effect.

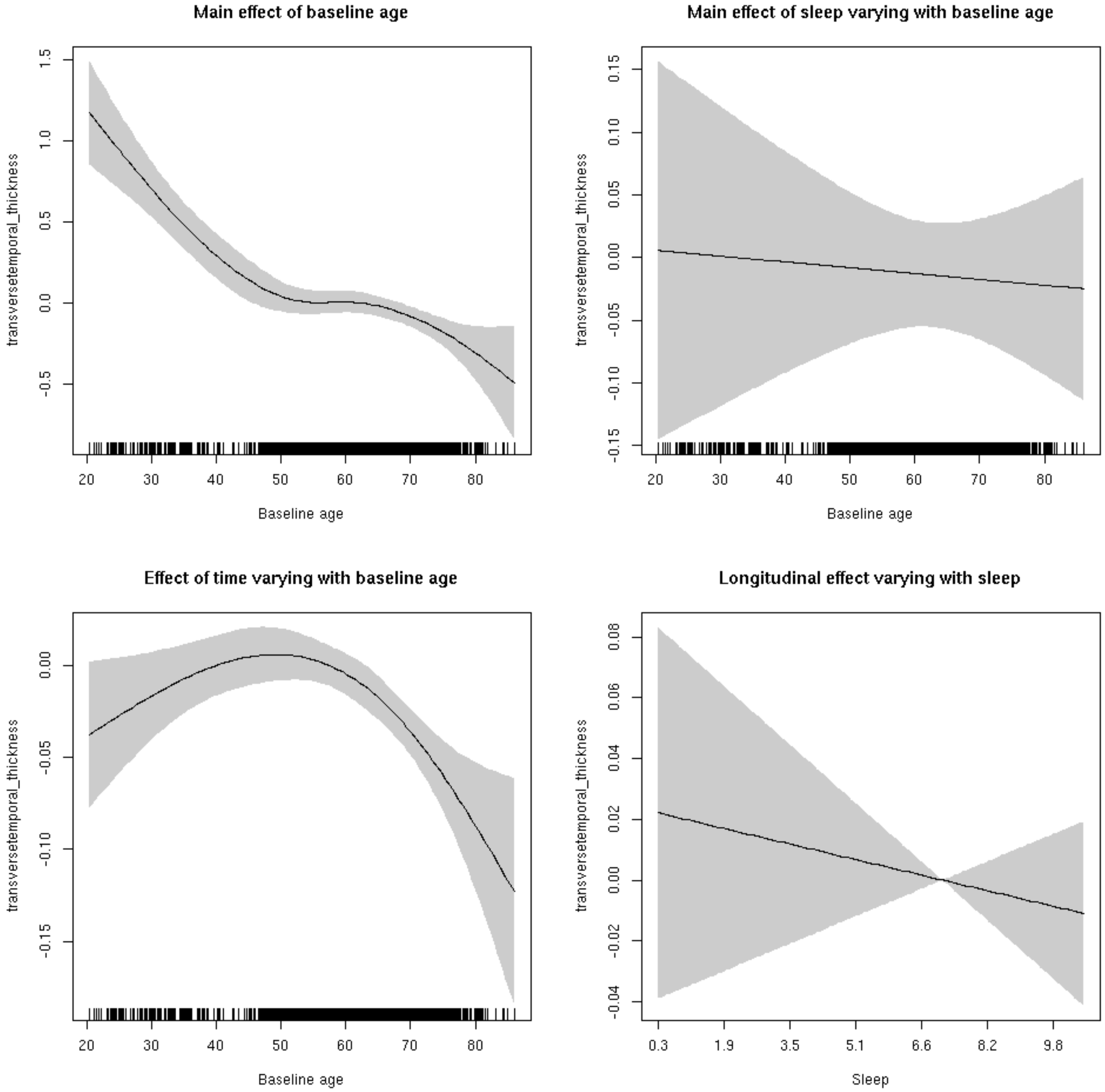

transversetemporal_thickness

Full data Below is the model output. The term `s(sleep_z):time` is probably what we care most about.

```
##
## Family: gaussian
## Link function: identity
##
## Formula:
## value ~ sex + site + s(bl_age, k = 10, bs = "cr") + s(bl_age,
##     by = sleep_z, bs = "cr") + +s(bl_age, by = time, k = 5, bs = "cr") +
##     s(sleep_z, by = time, k = 5, bs = "cr", pc = 0)
## <environment: 0x5630035d10d8>
```

```
##
## Parametric coefficients:
##                Estimate Std. Error t value Pr(>|t|)
## (Intercept)   -0.87845    0.05588 -15.720  < 2e-16 ***
## sexmale       -0.02949    0.02794  -1.056 0.291214
## siteousAvanto -0.04714    0.07396  -0.637 0.523887
## siteousPrisma  0.44534    0.29850   1.492 0.135754
## siteousSkyra   0.51062    0.07394   6.906 5.37e-12 ***
## siteUB         0.48881    0.14830   3.296 0.000984 ***
## siteUCAM       0.32313    0.07736   4.177 2.98e-05 ***
## siteUKB        1.00056    0.05679  17.619  < 2e-16 ***
## siteUmU        0.29509    0.09199   3.208 0.001342 **
## ---
## Signif. codes:  0 '***' 0.001 '**' 0.01 '*' 0.05 '.' 0.1 ' ' 1
##
## Approximate significance of smooth terms:
##                      edf Ref.df      F p-value
## s(bl_age)          5.107  5.107 36.588  <2e-16 ***
## s(bl_age):sleep_z 2.000  2.000  0.438   0.645
## s(bl_age):time    3.635  3.635 13.006  <2e-16 ***
## s(sleep_z):time   1.000  1.000  0.041   0.839
## ---
## Signif. codes:  0 '***' 0.001 '**' 0.01 '*' 0.05 '.' 0.1 ' ' 1
##
## R-sq.(adj) =  0.153
## lmer.REML =  16811  Scale est. = 0.15041   n = 8180
```

Below are plots of a plot of `s(bl_age)`, `s(bl_age):sleep_z`, `s(bl_age):time`, and `s(sleep_z):time`. For `s(sleep_z):time`, roughly, if we cannot fit a straight line through the confidence bands, there is evidence that the effect of time depends on sleep duration. It is correct the that confidence interval is zero at one point along the axis; this happens because we do not include the main effect.

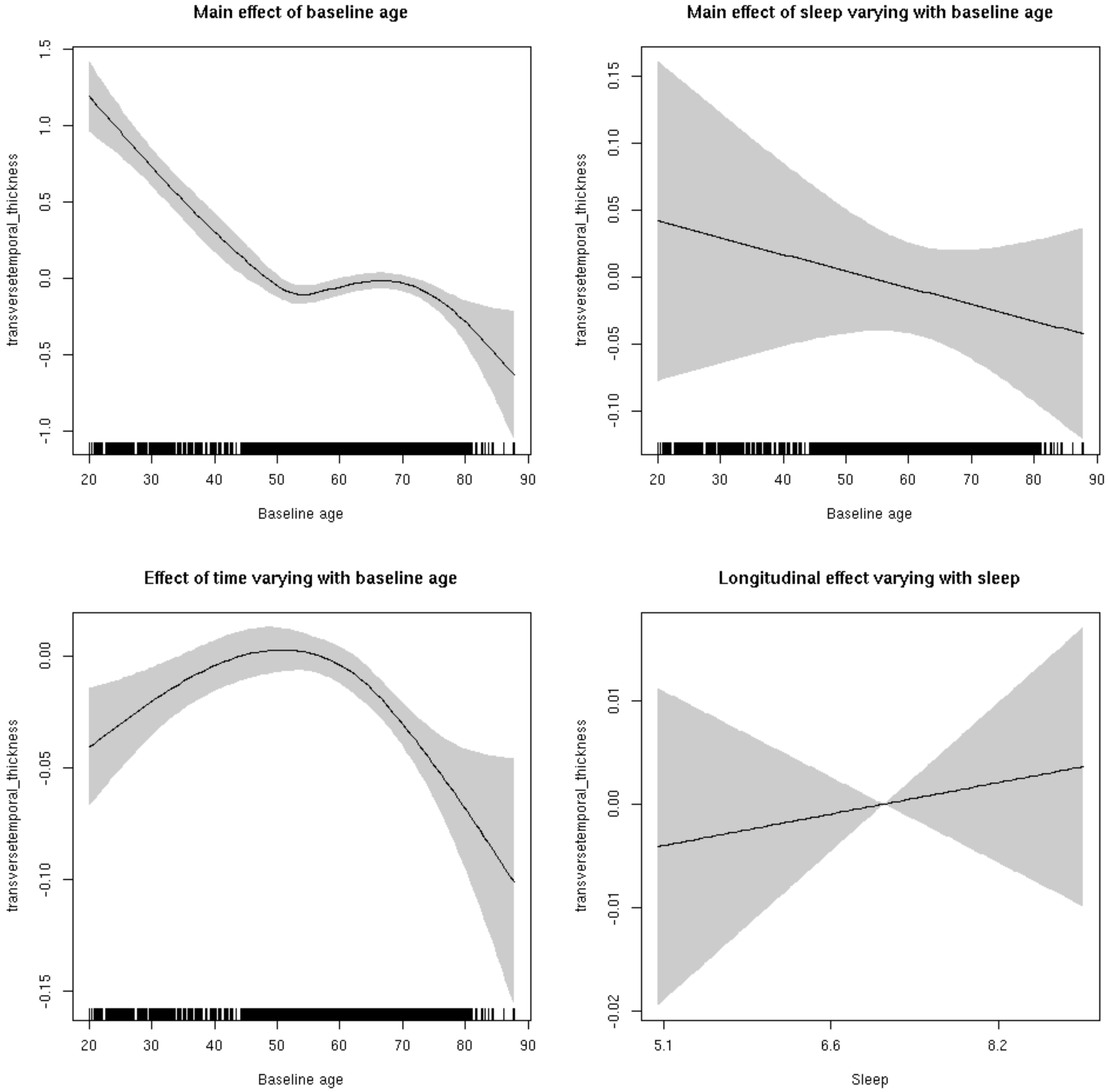

Restricted to sleep >= 5 and <= 9 hours Below is the row for `s(sleep_z):time` from the model output.

```
## s(sleep_z):time   1.000  1.000  0.287   0.592
```

Below are plots of a plot of `s(bl_age)`, `s(bl_age):sleep_z`, `s(bl_age):time`, and `s(sleep_z):time`. For `s(sleep_z):time`, roughly, if we cannot fit a straight line through the confidence bands, there is evidence that the effect of time depends on sleep duration. It is correct the that confidence interval is zero at one point along the axis; this happens because we do not include the main effect.

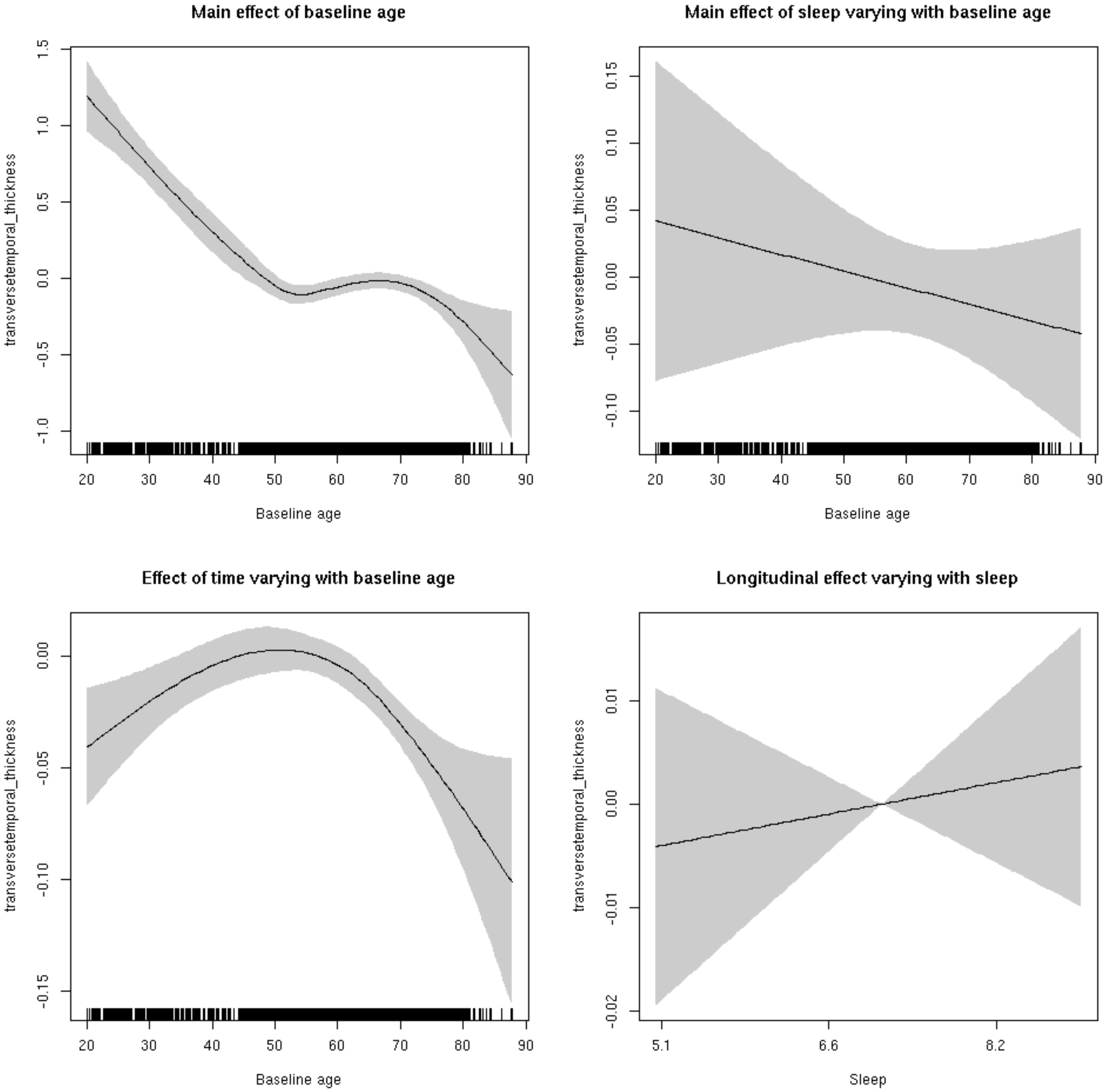

transversetemporal_volume: Stratified by sex

Female: Full data Below is the row for `s(sleep_z):time` from the model output.

```
## s(sleep_z):time   1.000  1.000  1.066   0.302
```

Below are plots of a plot of `s(bl_age)`, `s(bl_age):sleep_z`, `s(bl_age):time`, and `s(sleep_z):time`. For `s(sleep_z):time`, roughly, if we cannot fit a straight line through the confidence bands, there is evidence that the effect of time depends on sleep duration. It is correct the that confidence interval is zero at one point along the axis; this happens because we do not include the main effect.

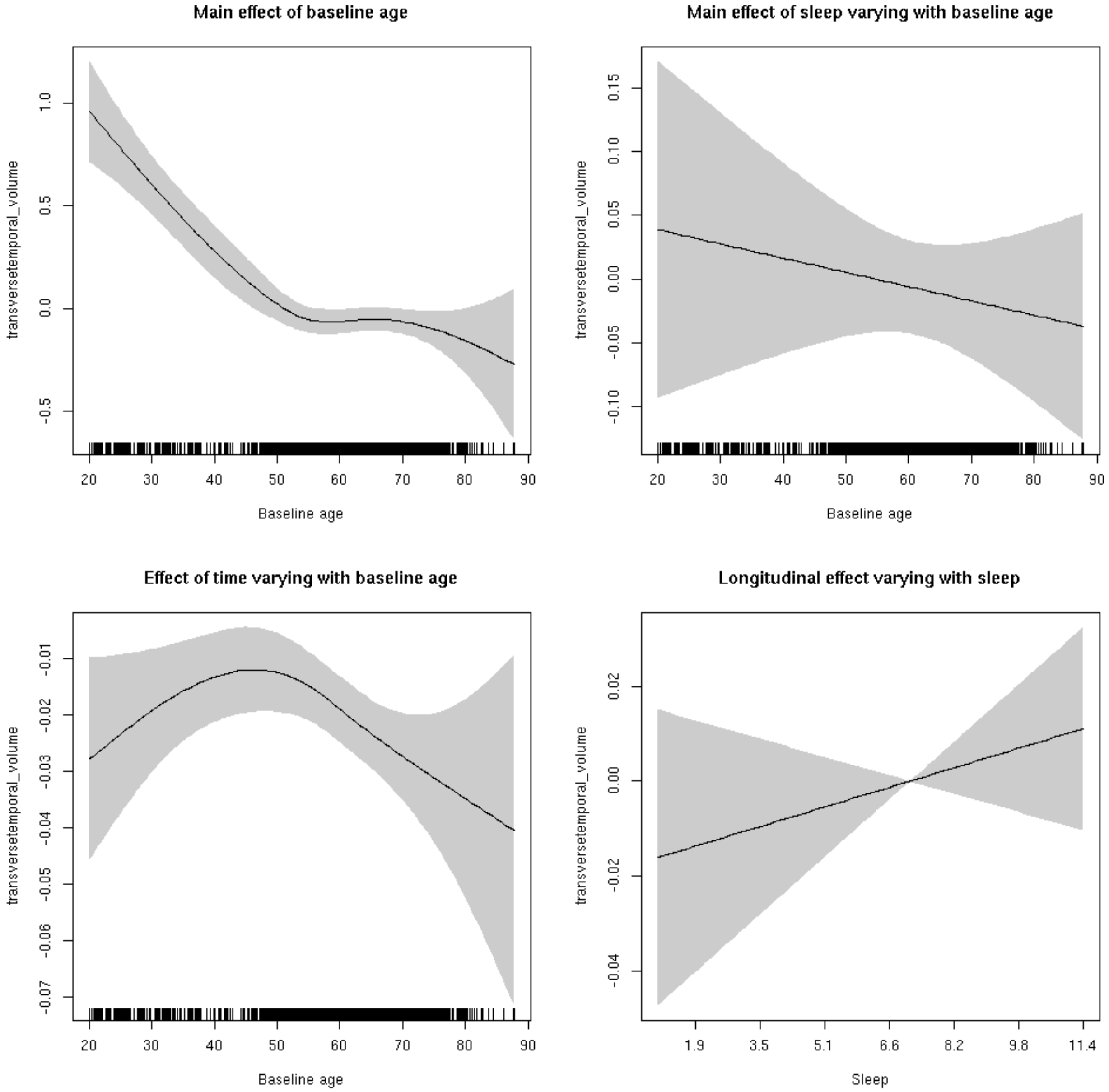

Male: Full data Below is the row for `s(sleep_z):time` from the model output.

```
## s(sleep_z):time   1.000  1.000   0.029   0.865
```

Below are plots of a plot of `s(bl_age)`, `s(bl_age):sleep_z`, `s(bl_age):time`, and `s(sleep_z):time`. For `s(sleep_z):time`, roughly, if we cannot fit a straight line through the confidence bands, there is evidence that the effect of time depends on sleep duration. It is correct the that confidence interval is zero at one point along the axis; this happens because we do not include the main effect.

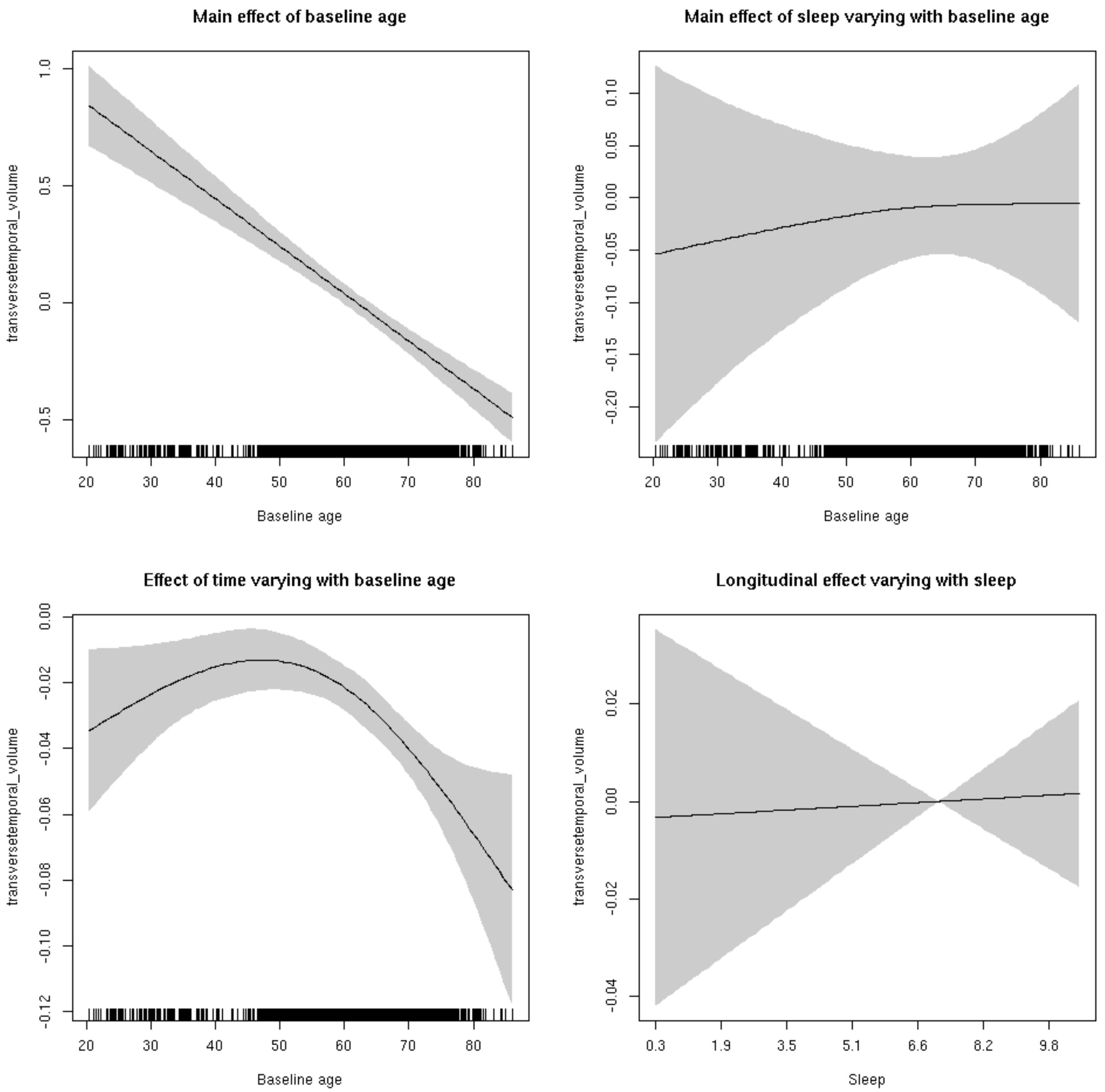

transversetemporal_volume

Full data Below is the model output. The term `s(sleep_z):time` is probably what we care most about.

```
##
## Family: gaussian
## Link function: identity
##
## Formula:
## value ~ sex + site + icv + s(bl_age, k = 10, bs = "cr") + s(bl_age,
##     by = sleep_z, bs = "cr") + +s(bl_age, by = time, k = 5, bs = "cr") +
##     s(sleep_z, by = time, k = 5, bs = "cr", pc = 0)
## <environment: 0x5630035ad538>
```

```
##
## Parametric coefficients:
##                 Estimate Std. Error t value Pr(>|t|)
## (Intercept)   -0.22865    0.06570  -3.480 0.000504 ***
## sexmale        0.05148    0.03372   1.527 0.126899
## siteousAvanto -0.63418    0.08018  -7.910 2.92e-15 ***
## siteousPrisma -0.20768    0.19094  -1.088 0.276768
## siteousSkyra   0.08348    0.07762   1.076 0.282176
## siteUB        -0.06620    0.15101  -0.438 0.661134
## siteUCAM      -0.10443    0.08160  -1.280 0.200673
## siteUKB        0.31928    0.06244   5.114 3.23e-07 ***
## siteUmU        0.08869    0.09365   0.947 0.343653
## icv            0.40486    0.01662  24.357  < 2e-16 ***
## ---
## Signif. codes:  0 '***' 0.001 '**' 0.01 '*' 0.05 '.' 0.1 ' ' 1
##
## Approximate significance of smooth terms:
##                       edf Ref.df      F p-value
## s(bl_age)           3.586  3.586 49.773  <2e-16 ***
## s(bl_age):sleep_z 2.000  2.000  0.593   0.553
## s(bl_age):time    3.578  3.578 59.046  <2e-16 ***
## s(sleep_z):time   1.000  1.000  0.721   0.396
## ---
## Signif. codes:  0 '***' 0.001 '**' 0.01 '*' 0.05 '.' 0.1 ' ' 1
##
## R-sq.(adj) =  0.269
## lmer.REML =  12147  Scale est. = 0.050593  n = 8174
```

Below are plots of a plot of `s(bl_age)`, `s(bl_age):sleep_z`, `s(bl_age):time`, and `s(sleep_z):time`. For `s(sleep_z):time`, roughly, if we cannot fit a straight line through the confidence bands, there is evidence that the effect of time depends on sleep duration. It is correct the that confidence interval is zero at one point along the axis; this happens because we do not include the main effect.

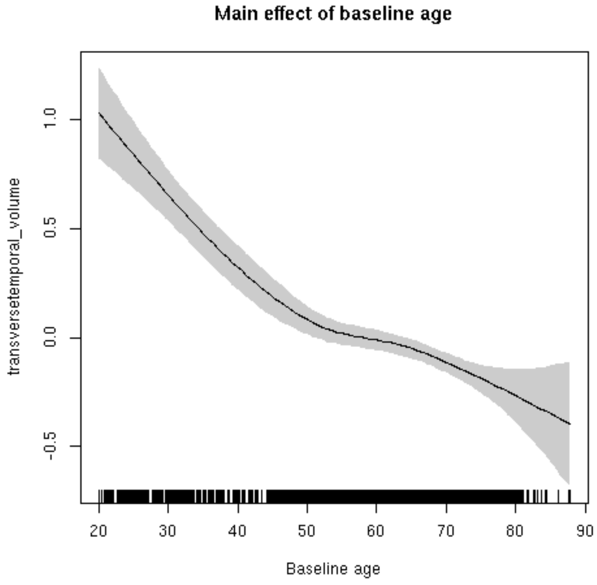

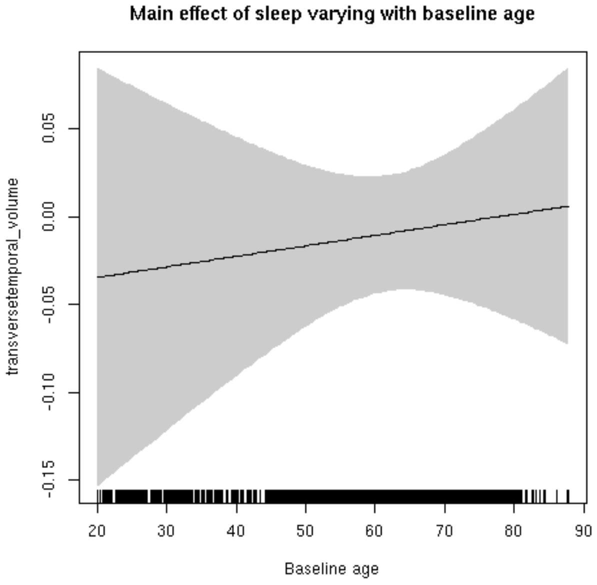

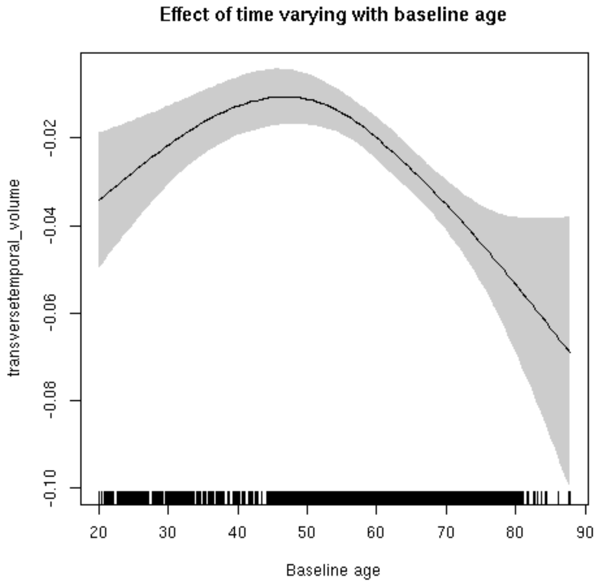

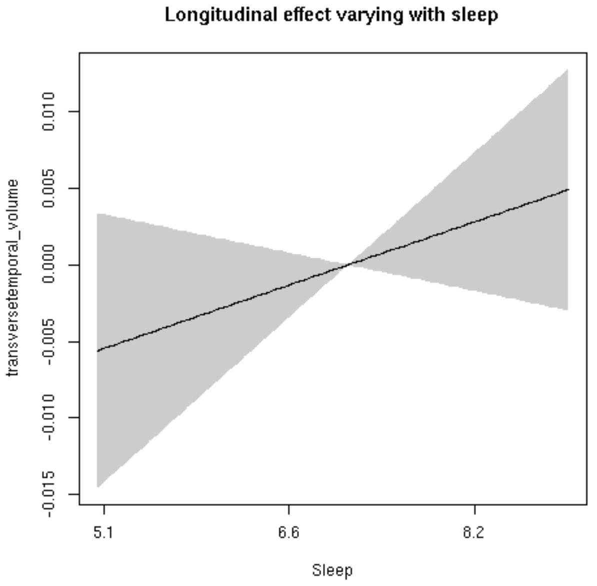

Restricted to sleep >= 5 and <= 9 hours Below is the row for `s(sleep_z):time` from the model output.

```
## s(sleep_z):time   1.000  1.000  1.549   0.213
```

Below are plots of a plot of `s(bl_age)`, `s(bl_age):sleep_z`, `s(bl_age):time`, and `s(sleep_z):time`. For `s(sleep_z):time`, roughly, if we cannot fit a straight line through the confidence bands, there is evidence that the effect of time depends on sleep duration. It is correct the that confidence interval is zero at one point along the axis; this happens because we do not include the main effect.

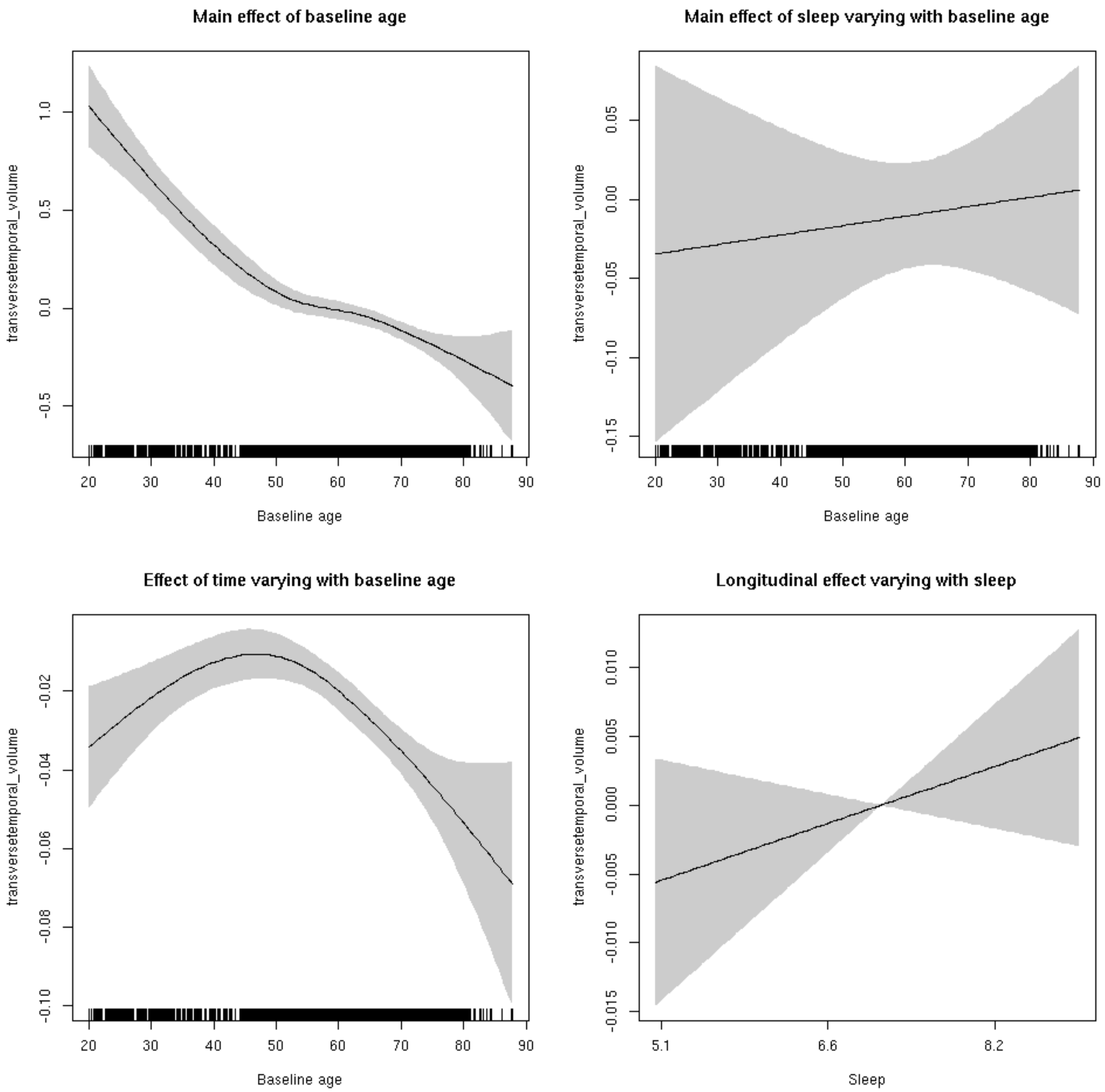

P-values corrected for multiple testing

Below is a table showing the p-values for `s(sleep_z, by = time)` adjusted for multiple testing using the Benjamini-Hochberg procedure for the main models not controling for SES, BMI, or depression, and using only subjects with two or more observations.

region	type	pval	adjusted_pval
bankssts_area	Full data	0.0142	0.6476
caudalanteriorcingulate_area	Full data	0.9621	0.9907
caudalmiddlefrontal_area	Full data	0.2890	0.9805
cuneus_area	Full data	0.9557	0.9907
entorhinal_area	Full data	0.7084	0.9896

region	type	pval	adjusted_pval
frontalpole_area	Full data	0.8474	0.9896
fusiform_area	Full data	0.3215	0.9805
inferiorparietal_area	Full data	0.4033	0.9885
inferiortemporal_area	Full data	0.1474	0.8769
insula_area	Full data	0.2785	0.9805
isthmuscingulate_area	Full data	0.1709	0.9315
lateraloccipital_area	Full data	0.7515	0.9896
lateralorbitofrontal_area	Full data	0.5970	0.9896
lingual_area	Full data	0.5260	0.9896
medialorbitofrontal_area	Full data	0.3181	0.9805
middletemporal_area	Full data	0.1051	0.8416
paracentral_area	Full data	0.7676	0.9896
parahippocampal_area	Full data	0.5461	0.9896
parsopercularis_area	Full data	0.1353	0.8581
parsorbitalis_area	Full data	0.5659	0.9896
parstriangularis_area	Full data	0.8927	0.9896
pericalcarine_area	Full data	0.0880	0.8296
postcentral_area	Full data	0.1672	0.9197
posteriorcingulate_area	Full data	0.6817	0.9896
precentral_area	Full data	0.2507	0.9805
precuneus_area	Full data	0.5567	0.9896
rostralanteriorcingulate_area	Full data	0.7168	0.9896
rostralmiddlefrontal_area	Full data	0.6356	0.9896
superiorfrontal_area	Full data	0.9209	0.9896
superiorparietal_area	Full data	0.9414	0.9896
superiortemporal_area	Full data	0.9568	0.9907
supramarginal_area	Full data	0.2838	0.9805
transversetemporal_area	Full data	0.0010	0.2960
bankssts_area	Restricted to sleep >= 5 and <= 9 hours	0.0677	0.8080
caudalanteriorcingulate_area	Restricted to sleep >= 5 and <= 9 hours	0.7225	0.9896
caudalmiddlefrontal_area	Restricted to sleep >= 5 and <= 9 hours	0.7788	0.9896
cuneus_area	Restricted to sleep >= 5 and <= 9 hours	0.8673	0.9896
entorhinal_area	Restricted to sleep >= 5 and <= 9 hours	0.9897	0.9947
frontalpole_area	Restricted to sleep >= 5 and <= 9 hours	0.6758	0.9896
fusiform_area	Restricted to sleep >= 5 and <= 9 hours	0.4240	0.9885
inferiorparietal_area	Restricted to sleep >= 5 and <= 9 hours	0.1985	0.9805
inferiortemporal_area	Restricted to sleep >= 5 and <= 9 hours	0.4215	0.9885
insula_area	Restricted to sleep >= 5 and <= 9 hours	0.1243	0.8581
isthmuscingulate_area	Restricted to sleep >= 5 and <= 9 hours	0.0533	0.7420
lateraloccipital_area	Restricted to sleep >= 5 and <= 9 hours	0.5367	0.9896
lateralorbitofrontal_area	Restricted to sleep >= 5 and <= 9 hours	0.7750	0.9896
lingual_area	Restricted to sleep >= 5 and <= 9 hours	0.6341	0.9896
medialorbitofrontal_area	Restricted to sleep >= 5 and <= 9 hours	0.4597	0.9885
middletemporal_area	Restricted to sleep >= 5 and <= 9 hours	0.1372	0.8581
paracentral_area	Restricted to sleep >= 5 and <= 9 hours	0.6644	0.9896
parahippocampal_area	Restricted to sleep >= 5 and <= 9 hours	0.6167	0.9896
parsopercularis_area	Restricted to sleep >= 5 and <= 9 hours	0.6814	0.9896
parsorbitalis_area	Restricted to sleep >= 5 and <= 9 hours	0.8884	0.9896
parstriangularis_area	Restricted to sleep >= 5 and <= 9 hours	0.9052	0.9896
pericalcarine_area	Restricted to sleep >= 5 and <= 9 hours	0.0416	0.7207
postcentral_area	Restricted to sleep >= 5 and <= 9 hours	0.1153	0.8581
posteriorcingulate_area	Restricted to sleep >= 5 and <= 9 hours	0.7323	0.9896

region	type	pval	adjusted_pval
precentral_area	Restricted to sleep >= 5 and <= 9 hours	0.6333	0.9896
precuneus_area	Restricted to sleep >= 5 and <= 9 hours	0.4931	0.9885
rostralanteriorcingulate_area	Restricted to sleep >= 5 and <= 9 hours	0.7422	0.9896
rostralmiddlefrontal_area	Restricted to sleep >= 5 and <= 9 hours	0.6140	0.9896
superiorfrontal_area	Restricted to sleep >= 5 and <= 9 hours	0.9137	0.9896
superiorparietal_area	Restricted to sleep >= 5 and <= 9 hours	0.8464	0.9896
superiortemporal_area	Restricted to sleep >= 5 and <= 9 hours	0.8416	0.9896
supramarginal_area	Restricted to sleep >= 5 and <= 9 hours	0.5299	0.9896
transversetemporal_area	Restricted to sleep >= 5 and <= 9 hours	0.0372	0.7207

region	type	pval	adjusted_pval
bankssts_thickness	Full data	0.5303	0.9896
caudalanteriorcingulate_thickness	Full data	0.3036	0.9805
caudalmiddlefrontal_thickness	Full data	0.3517	0.9885
cuneus_thickness	Full data	0.7195	0.9896
entorhinal_thickness	Full data	0.3002	0.9805
frontalpole_thickness	Full data	0.8891	0.9896
fusiform_thickness	Full data	0.1011	0.8416
inferiorparietal_thickness	Full data	0.4500	0.9885
inferiortemporal_thickness	Full data	0.0141	0.6476
insula_thickness	Full data	0.2205	0.9805
isthmuscingulate_thickness	Full data	0.6761	0.9896
lateraloccipital_thickness	Full data	0.2279	0.9805
lateralorbitofrontal_thickness	Full data	0.7831	0.9896
lingual_thickness	Full data	0.9405	0.9896
medialorbitofrontal_thickness	Full data	0.4737	0.9885
middletemporal_thickness	Full data	0.2306	0.9805
paracentral_thickness	Full data	0.4159	0.9885
parahippocampal_thickness	Full data	0.8064	0.9896
parsopercularis_thickness	Full data	0.2067	0.9805
parsorbitalis_thickness	Full data	0.1240	0.8581
parstriangularis_thickness	Full data	0.0284	0.7207
pericalcarine_thickness	Full data	0.8919	0.9896
postcentral_thickness	Full data	0.7411	0.9896
posteriorcingulate_thickness	Full data	0.7845	0.9896
precentral_thickness	Full data	0.2662	0.9805
precuneus_thickness	Full data	0.1163	0.8581
rostralanteriorcingulate_thickness	Full data	0.3104	0.9805
rostralmiddlefrontal_thickness	Full data	0.0131	0.6476
superiorfrontal_thickness	Full data	0.2804	0.9805
superiorparietal_thickness	Full data	0.0126	0.6476
superiortemporal_thickness	Full data	0.3068	0.9805
supramarginal_thickness	Full data	0.0225	0.7207
transversetemporal_thickness	Full data	0.8394	0.9896
bankssts_thickness	Restricted to sleep >= 5 and <= 9 hours	0.6085	0.9896
caudalanteriorcingulate_thickness	Restricted to sleep >= 5 and <= 9 hours	0.4786	0.9885
caudalmiddlefrontal_thickness	Restricted to sleep >= 5 and <= 9 hours	0.7160	0.9896
cuneus_thickness	Restricted to sleep >= 5 and <= 9 hours	0.9086	0.9896
entorhinal_thickness	Restricted to sleep >= 5 and <= 9 hours	0.3042	0.9805
frontalpole_thickness	Restricted to sleep >= 5 and <= 9 hours	0.3307	0.9822
fusiform_thickness	Restricted to sleep >= 5 and <= 9 hours	0.0500	0.7253

region	type	pval	adjusted_pval
inferiorparietal__thickness	Restricted to sleep >= 5 and <= 9 hours	0.1119	0.8581
inferiortemporal__thickness	Restricted to sleep >= 5 and <= 9 hours	0.1015	0.8416
insula__thickness	Restricted to sleep >= 5 and <= 9 hours	0.6758	0.9896
isthmuscingulate__thickness	Restricted to sleep >= 5 and <= 9 hours	0.8314	0.9896
lateraloccipital__thickness	Restricted to sleep >= 5 and <= 9 hours	0.3327	0.9832
lateralorbitofrontal__thickness	Restricted to sleep >= 5 and <= 9 hours	0.8967	0.9896
lingual__thickness	Restricted to sleep >= 5 and <= 9 hours	0.8450	0.9896
medialorbitofrontal__thickness	Restricted to sleep >= 5 and <= 9 hours	0.7460	0.9896
middletemporal__thickness	Restricted to sleep >= 5 and <= 9 hours	0.2154	0.9805
paracentral__thickness	Restricted to sleep >= 5 and <= 9 hours	0.3096	0.9805
parahippocampal__thickness	Restricted to sleep >= 5 and <= 9 hours	0.5469	0.9896
parsopercularis__thickness	Restricted to sleep >= 5 and <= 9 hours	0.2374	0.9805
parsorbitalis__thickness	Restricted to sleep >= 5 and <= 9 hours	0.7928	0.9896
parstriangularis__thickness	Restricted to sleep >= 5 and <= 9 hours	0.5524	0.9896
pericalcarine__thickness	Restricted to sleep >= 5 and <= 9 hours	0.9284	0.9896
postcentral__thickness	Restricted to sleep >= 5 and <= 9 hours	0.7689	0.9896
posteriorcingulate__thickness	Restricted to sleep >= 5 and <= 9 hours	0.7335	0.9896
precentral__thickness	Restricted to sleep >= 5 and <= 9 hours	0.7951	0.9896
precuneus__thickness	Restricted to sleep >= 5 and <= 9 hours	0.4026	0.9885
rostralanteriorcingulate__thickness	Restricted to sleep >= 5 and <= 9 hours	0.3764	0.9885
rostralmiddlefrontal__thickness	Restricted to sleep >= 5 and <= 9 hours	0.9896	0.9947
superiorfrontal__thickness	Restricted to sleep >= 5 and <= 9 hours	0.3761	0.9885
superiorparietal__thickness	Restricted to sleep >= 5 and <= 9 hours	0.7847	0.9896
superiortemporal__thickness	Restricted to sleep >= 5 and <= 9 hours	0.2952	0.9805
supramarginal__thickness	Restricted to sleep >= 5 and <= 9 hours	0.4413	0.9885
transversetemporal__thickness	Restricted to sleep >= 5 and <= 9 hours	0.5919	0.9896

region	type	pval	adjusted_pval
bankssts__volume	Full data	0.4166	0.9885
caudalanteriorcingulate__volume	Full data	0.2375	0.9805
caudalmiddlefrontal__volume	Full data	0.1050	0.8416
cuneus__volume	Full data	0.2412	0.9805
entorhinal__volume	Full data	0.2558	0.9805
frontalpole__volume	Full data	0.4731	0.9885
fusiform__volume	Full data	0.4337	0.9885
inferiorparietal__volume	Full data	0.0021	0.4245
inferiortemporal__volume	Full data	0.4990	0.9896
insula__volume	Full data	0.2475	0.9805
isthmuscingulate__volume	Full data	0.4044	0.9885
lateraloccipital__volume	Full data	0.5943	0.9896
lateralorbitofrontal__volume	Full data	0.8956	0.9896
lingual__volume	Full data	0.9075	0.9896
medialorbitofrontal__volume	Full data	0.8085	0.9896
middletemporal__volume	Full data	0.6923	0.9896
paracentral__volume	Full data	0.1326	0.8581
parahippocampal__volume	Full data	0.6146	0.9896
parsopercularis__volume	Full data	0.0546	0.7420
parsorbitalis__volume	Full data	0.5790	0.9896
parstriangularis__volume	Full data	0.0491	0.7253
pericalcarine__volume	Full data	0.8694	0.9896
postcentral__volume	Full data	0.7460	0.9896

region	type	pval	adjusted_pval
posteriorcingulate_volume	Full data	0.8607	0.9896
precentral_volume	Full data	0.1230	0.8581
precuneus_volume	Full data	0.3973	0.9885
rostralanteriorcingulate_volume	Full data	0.4334	0.9885
rostralmiddlefrontal_volume	Full data	0.0452	0.7207
superiorfrontal_volume	Full data	0.1940	0.9805
superiorparietal_volume	Full data	0.8357	0.9896
superiortemporal_volume	Full data	0.5701	0.9896
supramarginal_volume	Full data	0.4983	0.9896
transversetemporal_volume	Full data	0.3959	0.9885
bankssts_volume	Restricted to sleep >= 5 and <= 9 hours	0.2429	0.9805
caudalanteriorcingulate_volume	Restricted to sleep >= 5 and <= 9 hours	0.2999	0.9805
caudalmiddlefrontal_volume	Restricted to sleep >= 5 and <= 9 hours	0.9692	0.9907
cuneus_volume	Restricted to sleep >= 5 and <= 9 hours	0.4868	0.9885
entorhinal_volume	Restricted to sleep >= 5 and <= 9 hours	0.3033	0.9805
frontalpole_volume	Restricted to sleep >= 5 and <= 9 hours	0.1732	0.9349
fusiform_volume	Restricted to sleep >= 5 and <= 9 hours	0.2101	0.9805
inferiorparietal_volume	Restricted to sleep >= 5 and <= 9 hours	0.0730	0.8080
inferiortemporal_volume	Restricted to sleep >= 5 and <= 9 hours	0.3542	0.9885
insula_volume	Restricted to sleep >= 5 and <= 9 hours	0.2292	0.9805
isthmuscingulate_volume	Restricted to sleep >= 5 and <= 9 hours	0.6482	0.9896
lateraloccipital_volume	Restricted to sleep >= 5 and <= 9 hours	0.5646	0.9896
lateralorbitofrontal_volume	Restricted to sleep >= 5 and <= 9 hours	0.9782	0.9932
lingual_volume	Restricted to sleep >= 5 and <= 9 hours	0.8670	0.9896
medialorbitofrontal_volume	Restricted to sleep >= 5 and <= 9 hours	0.7999	0.9896
middletemporal_volume	Restricted to sleep >= 5 and <= 9 hours	0.7636	0.9896
paracentral_volume	Restricted to sleep >= 5 and <= 9 hours	0.0934	0.8416
parahippocampal_volume	Restricted to sleep >= 5 and <= 9 hours	0.4942	0.9885
parsopercularis_volume	Restricted to sleep >= 5 and <= 9 hours	0.1362	0.8581
parsorbitalis_volume	Restricted to sleep >= 5 and <= 9 hours	0.8966	0.9896
parstriangularis_volume	Restricted to sleep >= 5 and <= 9 hours	0.7719	0.9896
pericalcarine_volume	Restricted to sleep >= 5 and <= 9 hours	0.7222	0.9896
postcentral_volume	Restricted to sleep >= 5 and <= 9 hours	0.7645	0.9896
posteriorcingulate_volume	Restricted to sleep >= 5 and <= 9 hours	0.6627	0.9896
precentral_volume	Restricted to sleep >= 5 and <= 9 hours	0.9288	0.9896
precuneus_volume	Restricted to sleep >= 5 and <= 9 hours	0.3367	0.9853
rostralanteriorcingulate_volume	Restricted to sleep >= 5 and <= 9 hours	0.4423	0.9885
rostralmiddlefrontal_volume	Restricted to sleep >= 5 and <= 9 hours	0.5873	0.9896
superiorfrontal_volume	Restricted to sleep >= 5 and <= 9 hours	0.4283	0.9885
superiorparietal_volume	Restricted to sleep >= 5 and <= 9 hours	0.9393	0.9896
superiortemporal_volume	Restricted to sleep >= 5 and <= 9 hours	0.5884	0.9896
supramarginal_volume	Restricted to sleep >= 5 and <= 9 hours	0.5451	0.9896
transversetemporal_volume	Restricted to sleep >= 5 and <= 9 hours	0.2133	0.9805

Below is a plot of corrected and raw p-values for the case of using all data with two or more timepoints.

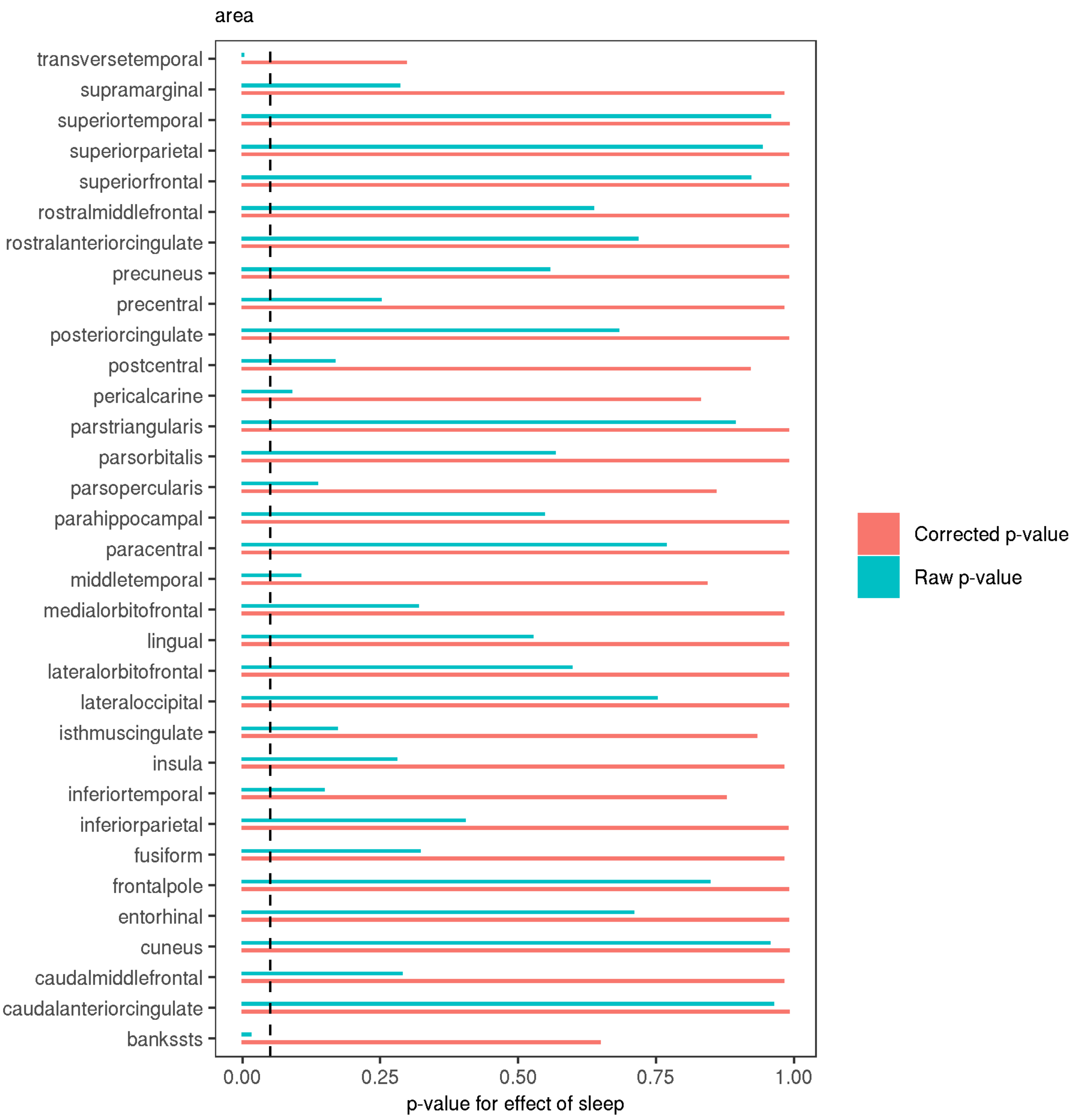

Below is a comparison of corrected p-values for the various cases.

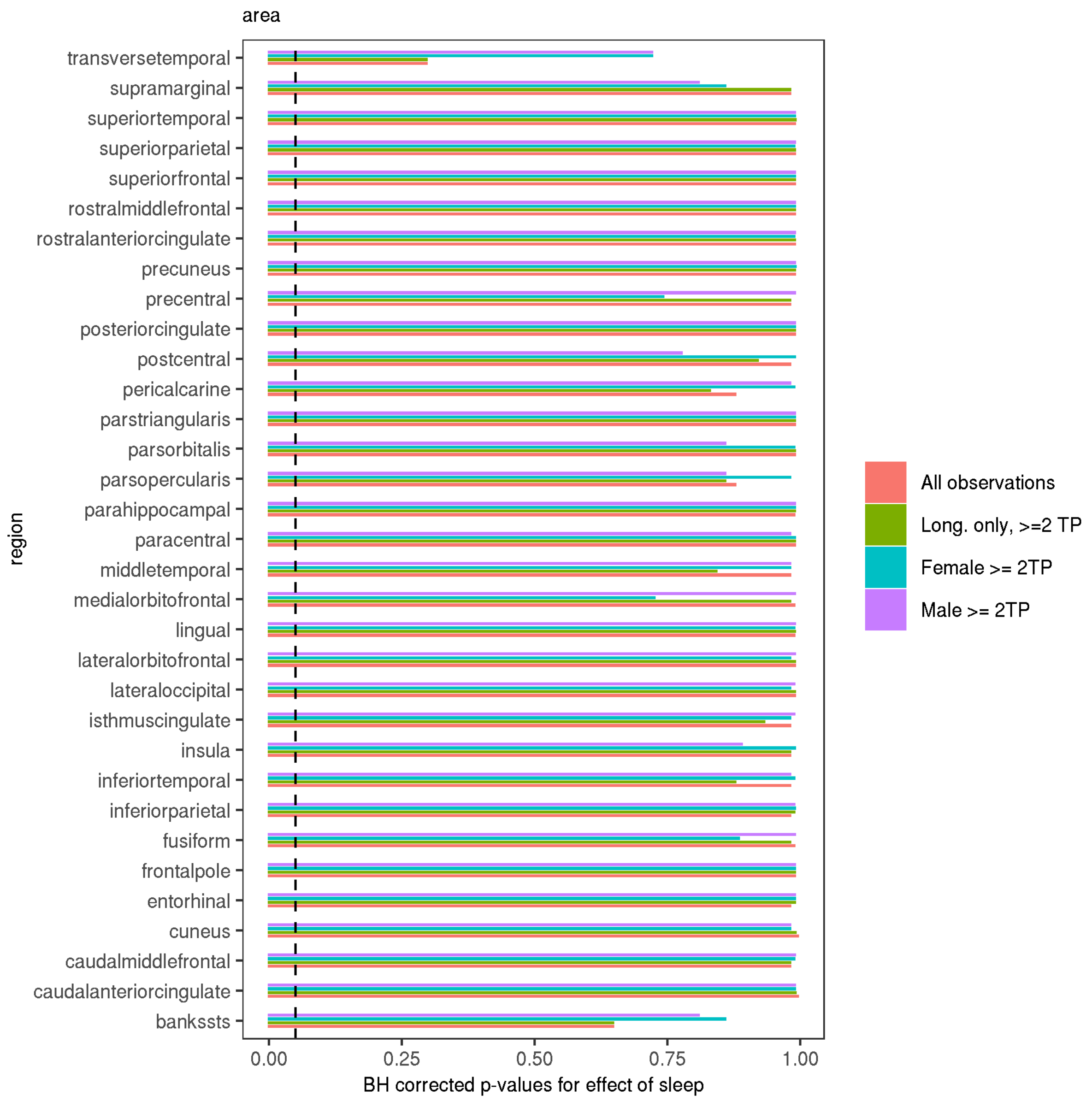

Below is a plot of corrected and raw p-values for the case of using all data with two or more timepoints.

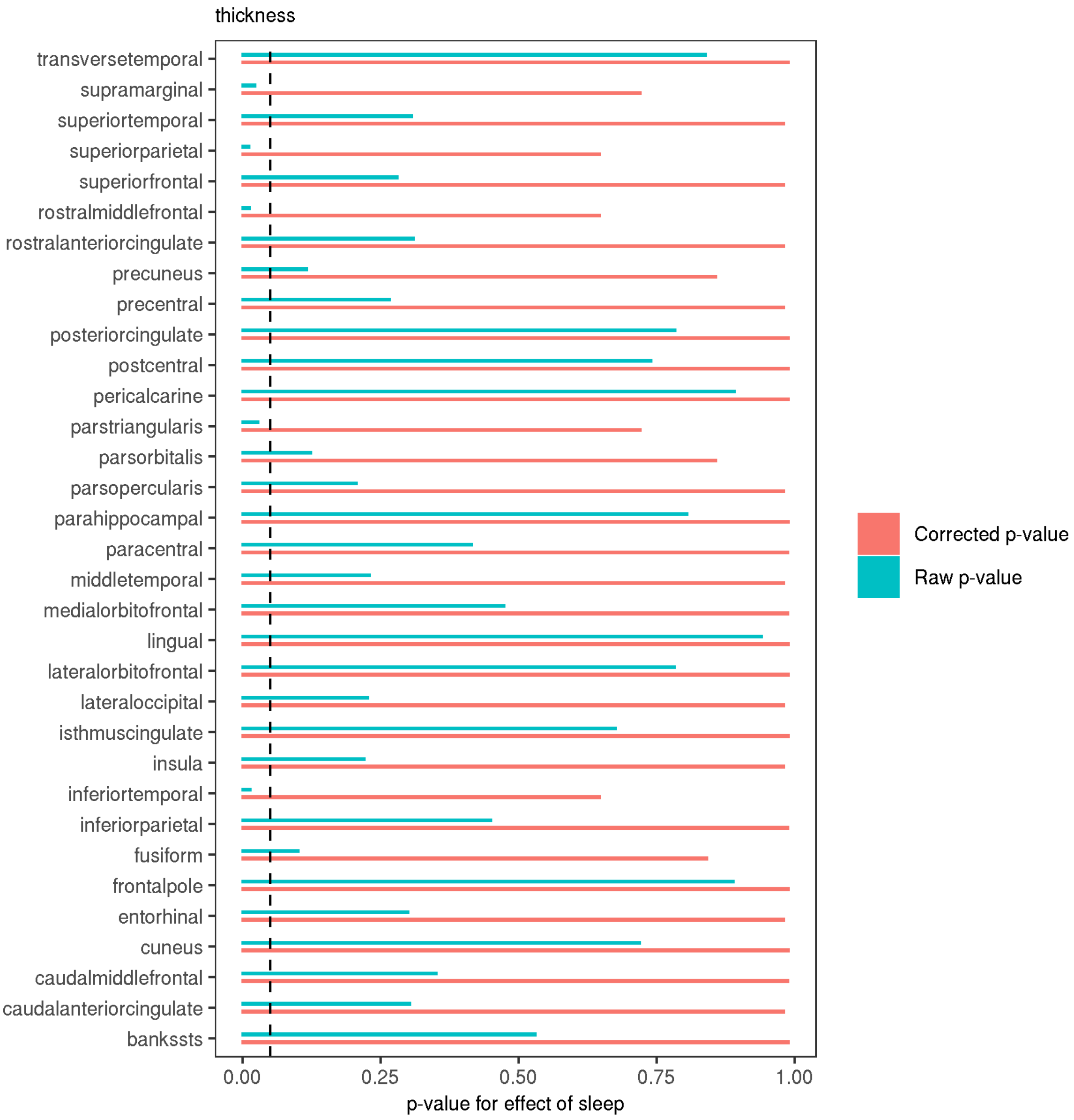

Below is a comparison of corrected p-values for the various cases.

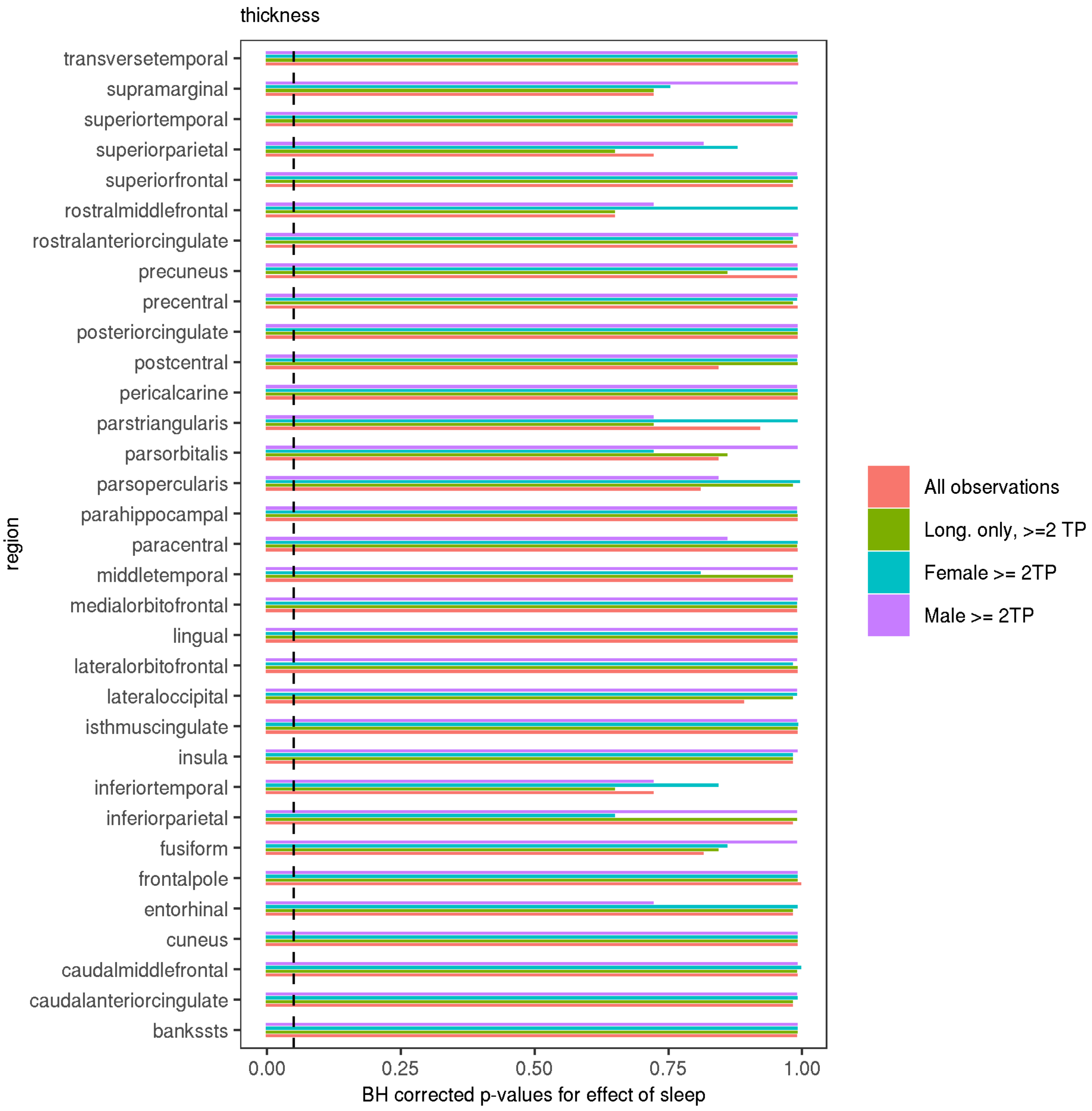

Below is a plot of corrected and raw p-values for the case of using all data with two or more timepoints.

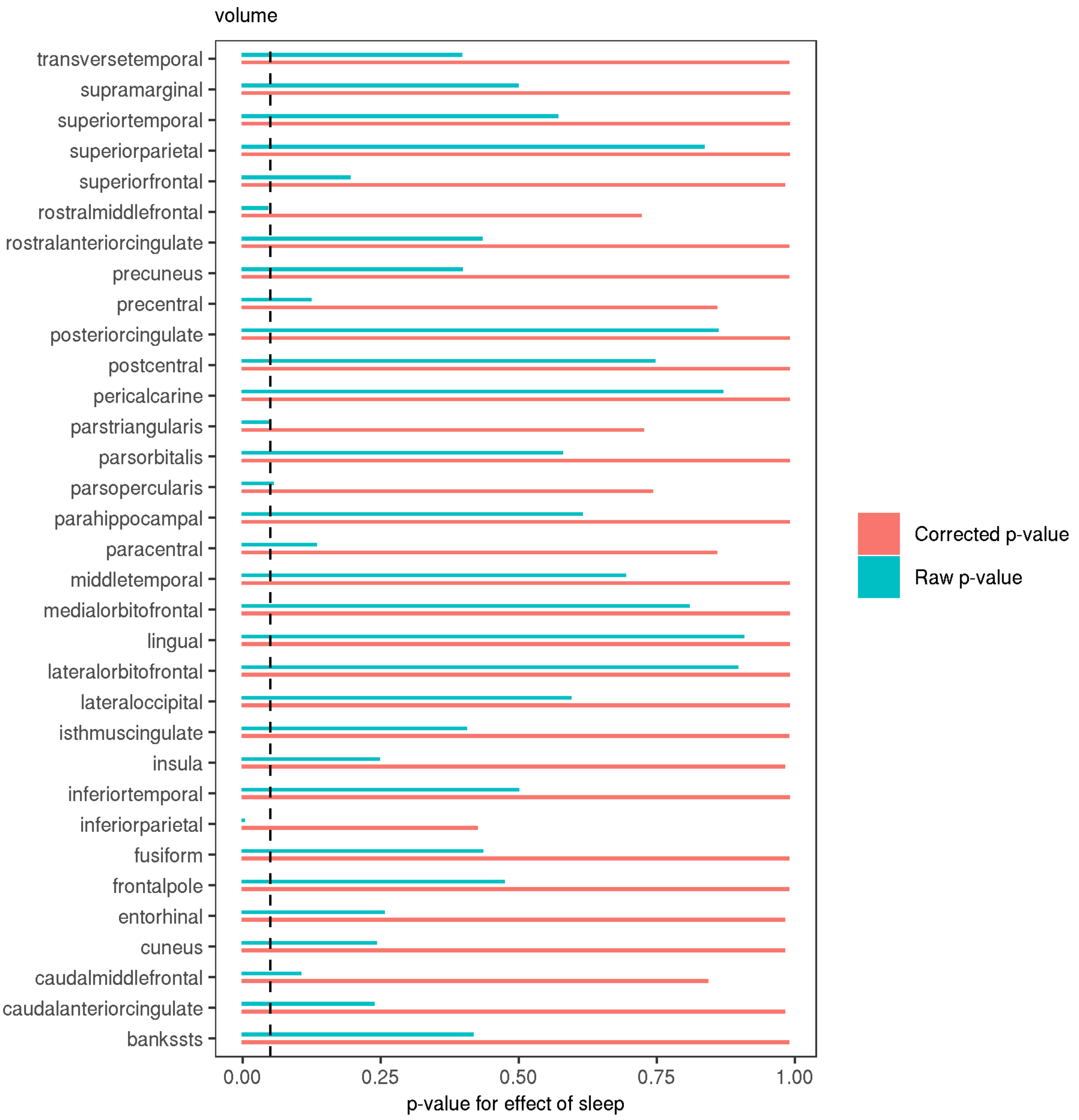

Below is a comparison of corrected p-values for the various cases.

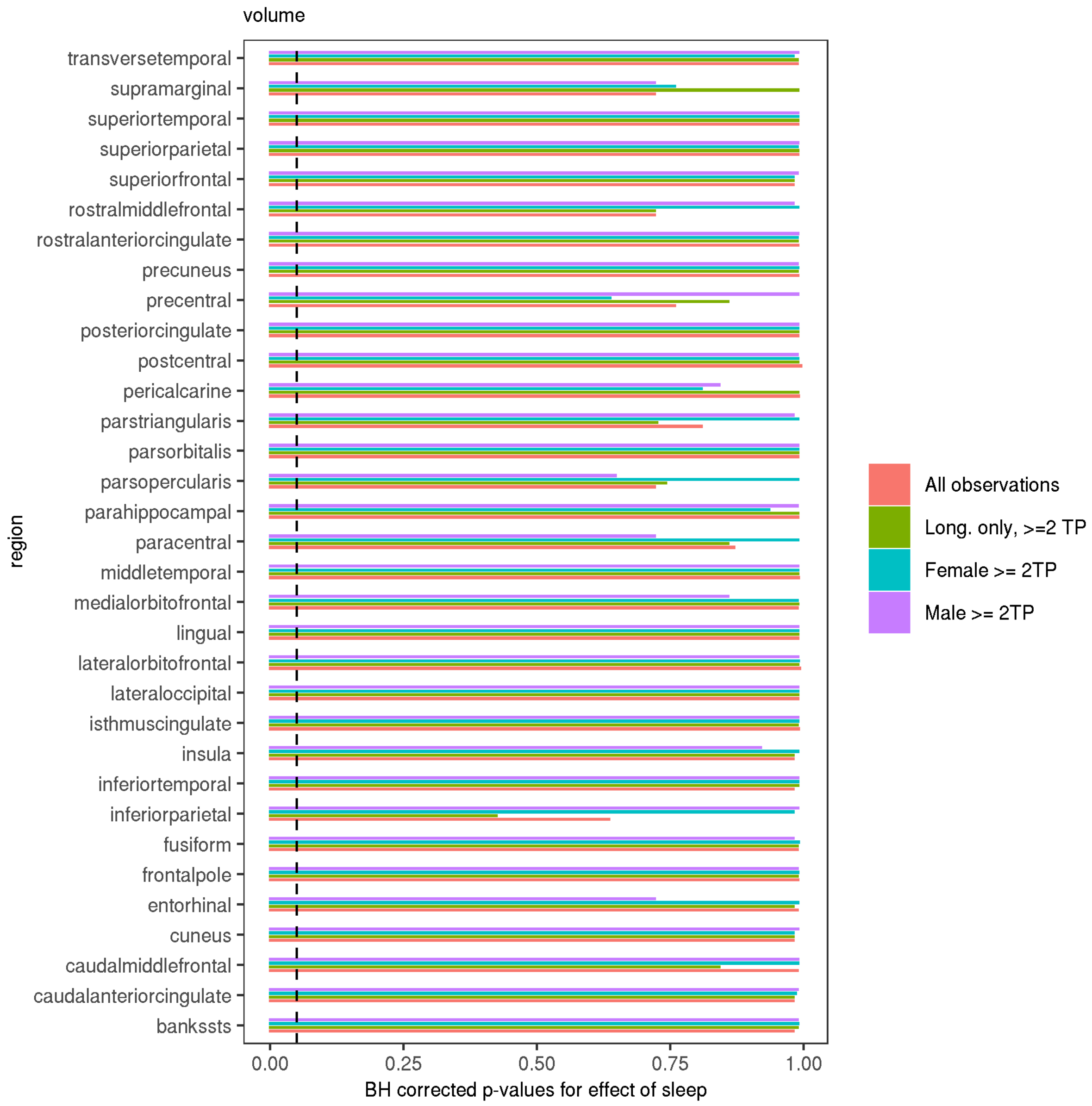

Models per cluster

Area cluster 1

All longitudinal data

```
##
## Family: gaussian
## Link function: identity
##
## Formula:
## value ~ sex + site + s(bl_age, k = 10, bs = "cr") + s(bl_age,
##     by = sleep_z, bs = "cr") + +s(bl_age, by = time, k = 5, bs = "cr") +
```

```
##     s(sleep_z, by = time, k = 5, bs = "cr", pc = 0)
## <environment: 0x563034c82f38>
##
## Parametric coefficients:
##               Estimate Std. Error t value Pr(>|t|)
## (Intercept)    40068.0      235.4 170.211  < 2e-16 ***
## sexmale         4109.4      121.3  33.882  < 2e-16 ***
## siteousAvanto   2277.6      312.3   7.293 3.32e-13 ***
## siteousPrisma   2225.1      648.6   3.431 0.000605 ***
## siteousSkyra    2849.2      312.2   9.127  < 2e-16 ***
## siteUB          -709.5      648.5  -1.094 0.273952
## siteUCAM         681.8      327.9   2.079 0.037650 *
## siteUKB          885.1      233.5   3.790 0.000152 ***
## siteUmU         2183.0      395.3   5.523 3.44e-08 ***
## ---
## Signif. codes:  0 '***' 0.001 '**' 0.01 '*' 0.05 '.' 0.1 ' ' 1
##
## Approximate significance of smooth terms:
##                     edf Ref.df       F p-value
## s(bl_age)         1.000  1.000 221.669  <2e-16 ***
## s(bl_age):sleep_z 2.000  2.000   1.773   0.170
## s(bl_age):time    3.346  3.346 150.081  <2e-16 ***
## s(sleep_z):time   1.000  1.000   0.008   0.929
## ---
## Signif. codes:  0 '***' 0.001 '**' 0.01 '*' 0.05 '.' 0.1 ' ' 1
##
## R-sq.(adj) =  0.277
## lmer.REML = 1.4678e+05  Scale est. = 5.4843e+05  n = 8178
```

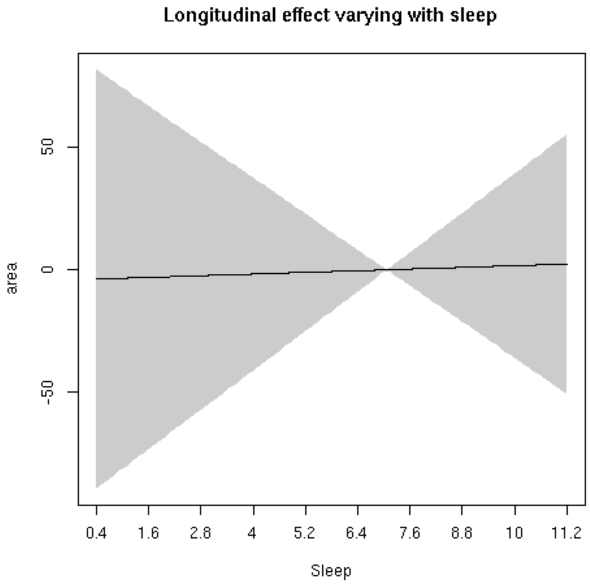

Participants sleeping 5-9 hours

```
##
## Family: gaussian
```

```
## Link function: identity
##
## Formula:
## value ~ sex + site + s(bl_age, k = 10, bs = "cr") + s(bl_age,
##     by = sleep_z, bs = "cr") + +s(bl_age, by = time, k = 5, bs = "cr") +
##     s(sleep_z, by = time, k = 5, bs = "cr", pc = 0)
## <environment: 0x563034c82f38>
##
## Parametric coefficients:
##               Estimate Std. Error t value Pr(>|t|)
## (Intercept)    40068.0      235.4 170.211  < 2e-16 ***
## sexmale         4109.4      121.3  33.882  < 2e-16 ***
## siteousAvanto   2277.6      312.3   7.293 3.32e-13 ***
## siteousPrisma   2225.1      648.6   3.431 0.000605 ***
## siteousSkyra    2849.2      312.2   9.127  < 2e-16 ***
## siteUB          -709.5      648.5  -1.094 0.273952
## siteUCAM         681.8      327.9   2.079 0.037650 *
## siteUKB          885.1      233.5   3.790 0.000152 ***
## siteUmU         2183.0      395.3   5.523 3.44e-08 ***
## ---
## Signif. codes:  0 '***' 0.001 '**' 0.01 '*' 0.05 '.' 0.1 ' ' 1
##
## Approximate significance of smooth terms:
##                     edf Ref.df       F p-value
## s(bl_age)         1.000  1.000 221.669  <2e-16 ***
## s(bl_age):sleep_z 2.000  2.000   1.773   0.170
## s(bl_age):time    3.346  3.346 150.081  <2e-16 ***
## s(sleep_z):time   1.000  1.000   0.008   0.929
## ---
## Signif. codes:  0 '***' 0.001 '**' 0.01 '*' 0.05 '.' 0.1 ' ' 1
##
## R-sq.(adj) =  0.277
## lmer.REML = 1.4678e+05  Scale est. = 5.4843e+05  n = 8178
```

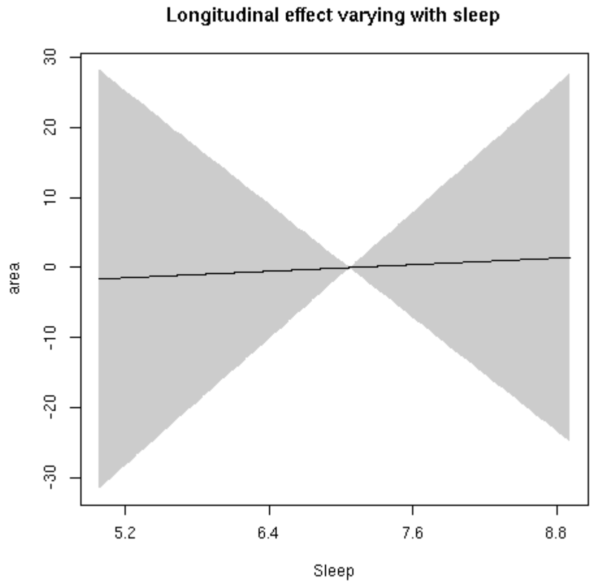

Thickness cluster 1

All longitudinal data

```
##
## Family: gaussian
## Link function: identity
##
## Formula:
## value ~ sex + site + s(bl_age, k = 10, bs = "cr") + s(bl_age,
##     by = sleep_z, bs = "cr") + +s(bl_age, by = time, k = 5, bs = "cr") +
##     s(sleep_z, by = time, k = 5, bs = "cr", pc = 0)
## <environment: 0x563005f9cdf8>
##
## Parametric coefficients:
##                  Estimate Std. Error t value Pr(>|t|)
## (Intercept)     2.1062223  0.0056688 371.543  < 2e-16 ***
## sexmale         0.0037645  0.0028417   1.325  0.18529
## siteousAvanto -0.0006973  0.0075599  -0.092  0.92651
## siteousPrisma   0.1030956  0.0338983   3.041  0.00236 **
## siteousSkyra    0.0756025  0.0075556  10.006  < 2e-16 ***
## siteUB          0.0893057  0.0150182   5.947 2.85e-09 ***
## siteUCAM        0.0717592  0.0077948   9.206  < 2e-16 ***
## siteUKB         0.2241622  0.0057408  39.047  < 2e-16 ***
## siteUmU         0.0689326  0.0093485   7.374 1.82e-13 ***
## ---
## Signif. codes:  0 ‘***’ 0.001 ‘**’ 0.01 ‘*’ 0.05 ‘.’ 0.1 ‘ ’ 1
##
## Approximate significance of smooth terms:
##                      edf Ref.df       F p-value
## s(bl_age)          3.429  3.429 122.718  <2e-16 ***
## s(bl_age):sleep_z 2.000  2.000   0.224   0.800
## s(bl_age):time    3.527  3.527  41.841  <2e-16 ***
## s(sleep_z):time   1.000  1.000   0.040   0.841
## ---
## Signif. codes:  0 ‘***’ 0.001 ‘**’ 0.01 ‘*’ 0.05 ‘.’ 0.1 ‘ ’ 1
##
## R-sq.(adj) =  0.475
## lmer.REML = -19501  Scale est. = 0.0020038  n = 8178
```

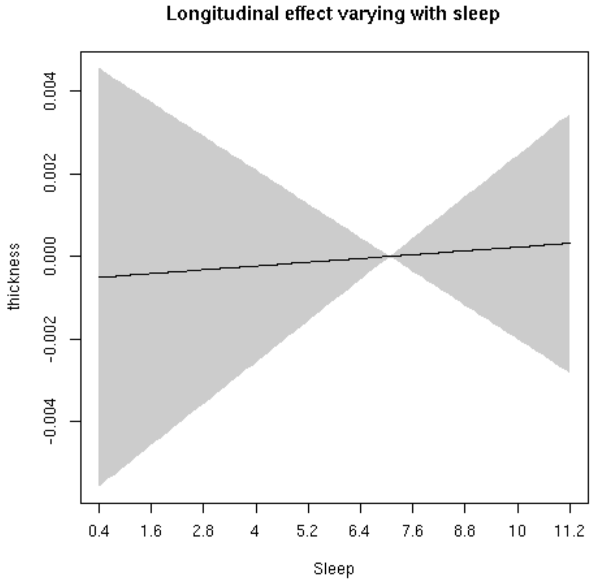

Participants sleeping 5-9 hours

```
##
## Family: gaussian
## Link function: identity
##
## Formula:
## value ~ sex + site + s(bl_age, k = 10, bs = "cr") + s(bl_age,
##     by = sleep_z, bs = "cr") + +s(bl_age, by = time, k = 5, bs = "cr") +
##     s(sleep_z, by = time, k = 5, bs = "cr", pc = 0)
## <environment: 0x563005f9cdf8>
##
## Parametric coefficients:
##                 Estimate Std. Error t value Pr(>|t|)
## (Intercept)    2.1062223  0.0056688 371.543  < 2e-16 ***
## sexmale        0.0037645  0.0028417   1.325  0.18529
## siteousAvanto -0.0006973  0.0075599  -0.092  0.92651
## siteousPrisma  0.1030956  0.0338983   3.041  0.00236 **
## siteousSkyra   0.0756025  0.0075556  10.006  < 2e-16 ***
## siteUB         0.0893057  0.0150182   5.947 2.85e-09 ***
## siteUCAM       0.0717592  0.0077948   9.206  < 2e-16 ***
## siteUKB        0.2241622  0.0057408  39.047  < 2e-16 ***
## siteUmU        0.0689326  0.0093485   7.374 1.82e-13 ***
## ---
## Signif. codes:  0 ‘***’ 0.001 ‘**’ 0.01 ‘*’ 0.05 ‘.’ 0.1 ‘ ’ 1
##
## Approximate significance of smooth terms:
##                     edf Ref.df       F p-value
## s(bl_age)         3.429  3.429 122.718  <2e-16 ***
## s(bl_age):sleep_z 2.000  2.000   0.224   0.800
## s(bl_age):time    3.527  3.527  41.841  <2e-16 ***
## s(sleep_z):time   1.000  1.000   0.040   0.841
```

```
## ---
## Signif. codes:  0 ‘***’ 0.001 ‘**’ 0.01 ‘*’ 0.05 ‘.’ 0.1 ‘ ’ 1
##
## R-sq.(adj) =  0.475
## lmer.REML = -19501  Scale est. = 0.0020038  n = 8178
```

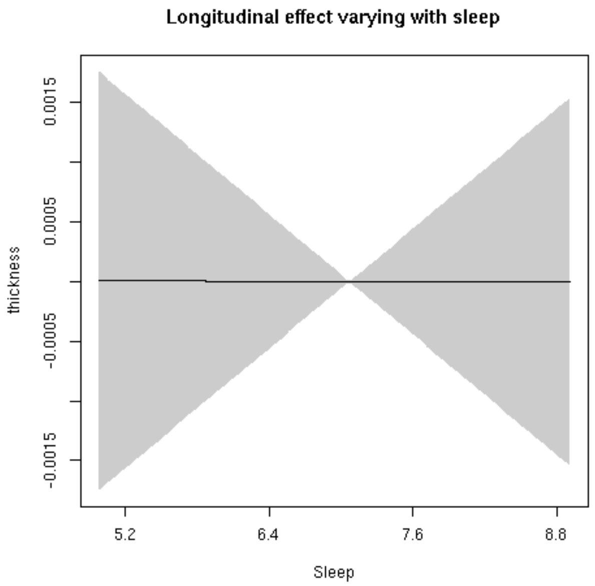

Volume cluster 1

All longitudinal data

```
##
## Family: gaussian
## Link function: identity
##
## Formula:
## value ~ sex + site + s(bl_age, k = 10, bs = "cr") + s(bl_age,
##     by = sleep_z, bs = "cr") + +s(bl_age, by = time, k = 5, bs = "cr") +
##     s(sleep_z, by = time, k = 5, bs = "cr", pc = 0)
## <environment: 0x563005e93210>
##
## Parametric coefficients:
##                Estimate Std. Error t value Pr(>|t|)
## (Intercept)    207103.9     1262.5 164.042  < 2e-16 ***
## sexmale         25397.8      641.8  39.575  < 2e-16 ***
## siteousAvanto   15482.5     1660.9   9.322  < 2e-16 ***
## siteousPrisma   27545.8     4292.3   6.417 1.46e-10 ***
## siteousSkyra    18875.1     1660.1  11.370  < 2e-16 ***
## siteUB          10358.6     3428.0   3.022  0.00252 **
## siteUCAM        16949.3     1739.4   9.744  < 2e-16 ***
## siteUKB         34700.7     1268.5  27.356  < 2e-16 ***
## siteUmU         18233.2     2100.0   8.682  < 2e-16 ***
## ---
## Signif. codes:  0 ‘***’ 0.001 ‘**’ 0.01 ‘*’ 0.05 ‘.’ 0.1 ‘ ’ 1
```

```
##
## Approximate significance of smooth terms:
##                      edf Ref.df       F p-value
## s(bl_age)          1.797  1.797 371.314  <2e-16 ***
## s(bl_age):sleep_z 2.413  2.413   1.296   0.199
## s(bl_age):time    3.997  3.997 202.442  <2e-16 ***
## s(sleep_z):time   1.000  1.000   0.044   0.835
## ---
## Signif. codes:  0 '***' 0.001 '**' 0.01 '*' 0.05 '.' 0.1 ' ' 1
##
## R-sq.(adj) =  0.448
## lmer.REML = 1.7642e+05  Scale est. = 2.6866e+07  n = 8178
```

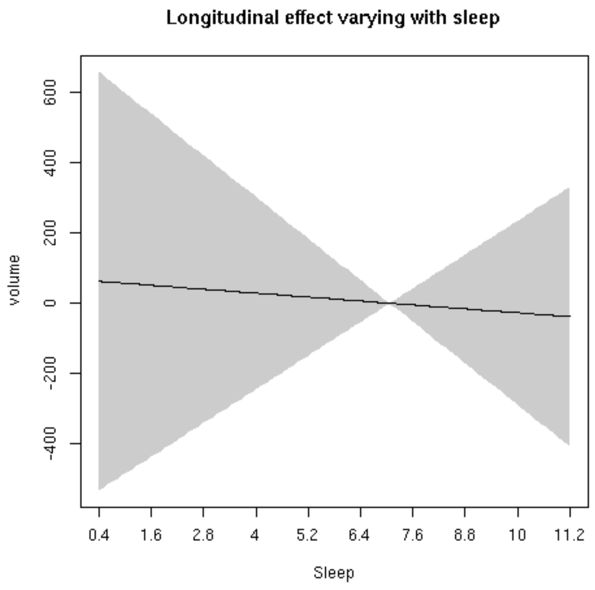

Participants sleeping 5-9 hours

```
##
## Family: gaussian
## Link function: identity
##
## Formula:
## value ~ sex + site + s(bl_age, k = 10, bs = "cr") + s(bl_age,
##     by = sleep_z, bs = "cr") + +s(bl_age, by = time, k = 5, bs = "cr") +
##     s(sleep_z, by = time, k = 5, bs = "cr", pc = 0)
## <environment: 0x563005e93210>
##
## Parametric coefficients:
##                Estimate Std. Error t value Pr(>|t|)
## (Intercept)    207103.9     1262.5 164.042  < 2e-16 ***
## sexmale         25397.8      641.8  39.575  < 2e-16 ***
## siteousAvanto   15482.5     1660.9   9.322  < 2e-16 ***
## siteousPrisma   27545.8     4292.3   6.417 1.46e-10 ***
## siteousSkyra    18875.1     1660.1  11.370  < 2e-16 ***
## siteUB          10358.6     3428.0   3.022  0.00252 **
```

```
## siteUCAM       16949.3     1739.4   9.744  < 2e-16 ***
## siteUKB        34700.7     1268.5  27.356  < 2e-16 ***
## siteUmU        18233.2     2100.0   8.682  < 2e-16 ***
## ---
## Signif. codes:  0 ‘***’ 0.001 ‘**’ 0.01 ‘*’ 0.05 ‘.’ 0.1 ‘ ’ 1
##
## Approximate significance of smooth terms:
##                     edf Ref.df       F p-value
## s(bl_age)         1.797  1.797 371.314  <2e-16 ***
## s(bl_age):sleep_z 2.413  2.413   1.296   0.199
## s(bl_age):time    3.997  3.997 202.442  <2e-16 ***
## s(sleep_z):time   1.000  1.000   0.044   0.835
## ---
## Signif. codes:  0 ‘***’ 0.001 ‘**’ 0.01 ‘*’ 0.05 ‘.’ 0.1 ‘ ’ 1
##
## R-sq.(adj) =  0.448
## lmer.REML = 1.7642e+05  Scale est. = 2.6866e+07  n = 8178
```

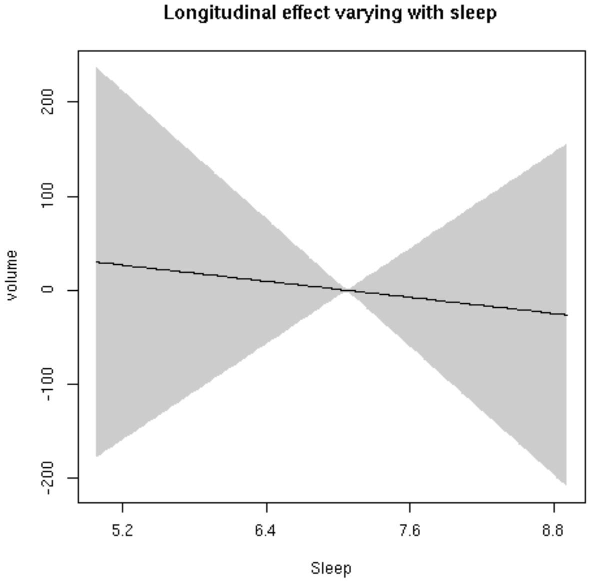

Area cluster 2

All longitudinal data

```
##
## Family: gaussian
## Link function: identity
##
## Formula:
## value ~ sex + site + s(bl_age, k = 10, bs = "cr") + s(bl_age,
##     by = sleep_z, bs = "cr") + +s(bl_age, by = time, k = 5, bs = "cr") +
##     s(sleep_z, by = time, k = 5, bs = "cr", pc = 0)
## <environment: 0x563004d659a8>
##
## Parametric coefficients:
```

```
##                Estimate Std. Error t value Pr(>|t|)
## (Intercept)   16483.39      98.57 167.220  < 2e-16 ***
## sexmale        1805.53      50.79  35.549  < 2e-16 ***
## siteousAvanto  1481.92     130.85  11.325  < 2e-16 ***
## siteousPrisma   790.78     280.95   2.815  0.00489 **
## siteousSkyra   1400.82     130.79  10.711  < 2e-16 ***
## siteUB         -118.91     271.49  -0.438  0.66141
## siteUCAM        546.27     137.33   3.978 7.02e-05 ***
## siteUKB         413.25      97.79   4.226 2.41e-05 ***
## siteUmU        1537.00     165.52   9.286  < 2e-16 ***
## ---
## Signif. codes:  0 '***' 0.001 '**' 0.01 '*' 0.05 '.' 0.1 ' ' 1
##
## Approximate significance of smooth terms:
##                      edf Ref.df       F p-value
## s(bl_age)          1.000  1.000 334.383  <2e-16 ***
## s(bl_age):sleep_z 2.302  2.302   2.000  0.0986 .
## s(bl_age):time    4.698  4.698  88.586  <2e-16 ***
## s(sleep_z):time   1.000  1.000   0.624  0.4294
## ---
## Signif. codes:  0 '***' 0.001 '**' 0.01 '*' 0.05 '.' 0.1 ' ' 1
##
## R-sq.(adj) =  0.329
## lmer.REML = 1.3292e+05  Scale est. = 1.0494e+05  n = 8178
```

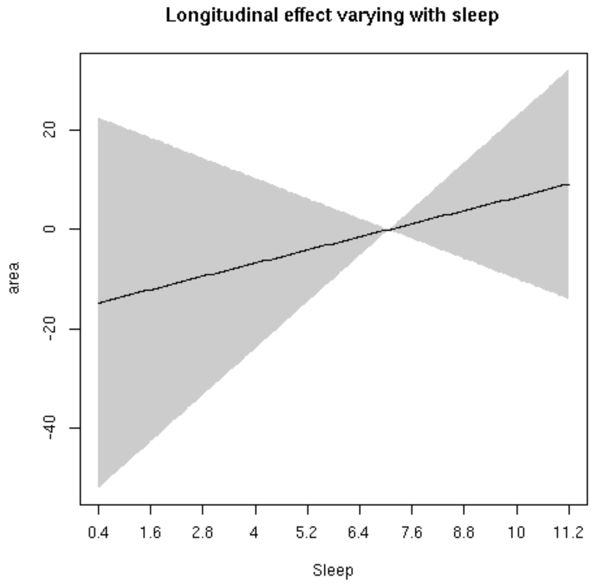

Participants sleeping 5-9 hours

```
##
## Family: gaussian
## Link function: identity
##
## Formula:
## value ~ sex + site + s(bl_age, k = 10, bs = "cr") + s(bl_age,
```

```
##     by = sleep_z, bs = "cr") + +s(bl_age, by = time, k = 5, bs = "cr") +
##     s(sleep_z, by = time, k = 5, bs = "cr", pc = 0)
## <environment: 0x563004d659a8>
##
## Parametric coefficients:
##               Estimate Std. Error t value Pr(>|t|)
## (Intercept)   16483.39      98.57 167.220  < 2e-16 ***
## sexmale        1805.53      50.79  35.549  < 2e-16 ***
## siteousAvanto  1481.92     130.85  11.325  < 2e-16 ***
## siteousPrisma   790.78     280.95   2.815  0.00489 **
## siteousSkyra   1400.82     130.79  10.711  < 2e-16 ***
## siteUB         -118.91     271.49  -0.438  0.66141
## siteUCAM        546.27     137.33   3.978 7.02e-05 ***
## siteUKB         413.25      97.79   4.226 2.41e-05 ***
## siteUmU        1537.00     165.52   9.286  < 2e-16 ***
## ---
## Signif. codes:  0 ‘***’ 0.001 ‘**’ 0.01 ‘*’ 0.05 ‘.’ 0.1 ‘ ’ 1
##
## Approximate significance of smooth terms:
##                       edf Ref.df       F p-value
## s(bl_age)           1.000  1.000 334.383  <2e-16 ***
## s(bl_age):sleep_z 2.302  2.302   2.000  0.0986 .
## s(bl_age):time    4.698  4.698  88.586  <2e-16 ***
## s(sleep_z):time   1.000  1.000   0.624  0.4294
## ---
## Signif. codes:  0 ‘***’ 0.001 ‘**’ 0.01 ‘*’ 0.05 ‘.’ 0.1 ‘ ’ 1
##
## R-sq.(adj) =  0.329
## lmer.REML = 1.3292e+05  Scale est. = 1.0494e+05  n = 8178
```

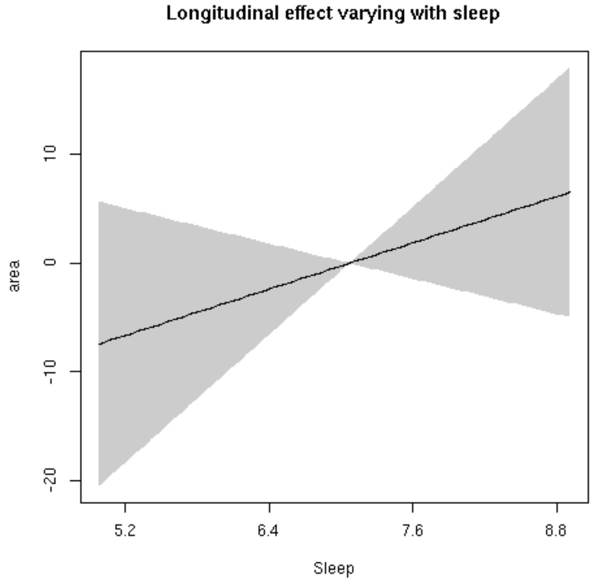

Thickness cluster 2

All longitudinal data

```
##
## Family: gaussian
## Link function: identity
##
## Formula:
## value ~ sex + site + s(bl_age, k = 10, bs = "cr") + s(bl_age,
##     by = sleep_z, bs = "cr") + +s(bl_age, by = time, k = 5, bs = "cr") +
##     s(sleep_z, by = time, k = 5, bs = "cr", pc = 0)
## <environment: 0x56300a2a08d0>
##
## Parametric coefficients:
##                Estimate Std. Error t value Pr(>|t|)
## (Intercept)    2.503990   0.005808 431.145  < 2e-16 ***
## sexmale       -0.002010   0.002907  -0.691   0.4893
## siteousAvanto  0.012177   0.007730   1.575   0.1152
## siteousPrisma  0.075641   0.034028   2.223   0.0262 *
## siteousSkyra  -0.015444   0.007727  -1.999   0.0457 *
## siteUB         0.081258   0.015380   5.283  1.3e-07 ***
## siteUCAM       0.067074   0.008002   8.382  < 2e-16 ***
## siteUKB        0.307295   0.005889  52.179  < 2e-16 ***
## siteUmU       -0.024603   0.009569  -2.571   0.0102 *
## ---
## Signif. codes:  0 ‘***’ 0.001 ‘**’ 0.01 ‘*’ 0.05 ‘.’ 0.1 ‘ ’ 1
##
## Approximate significance of smooth terms:
##                     edf Ref.df       F p-value
## s(bl_age)         3.961  3.961 255.420  <2e-16 ***
## s(bl_age):sleep_z 2.000  2.000   0.799   0.450
## s(bl_age):time    3.678  3.678  86.913  <2e-16 ***
## s(sleep_z):time   1.000  1.000   0.425   0.515
## ---
## Signif. codes:  0 ‘***’ 0.001 ‘**’ 0.01 ‘*’ 0.05 ‘.’ 0.1 ‘ ’ 1
##
## R-sq.(adj) =  0.686
## lmer.REML = -19313  Scale est. = 0.0020073  n = 8178
```

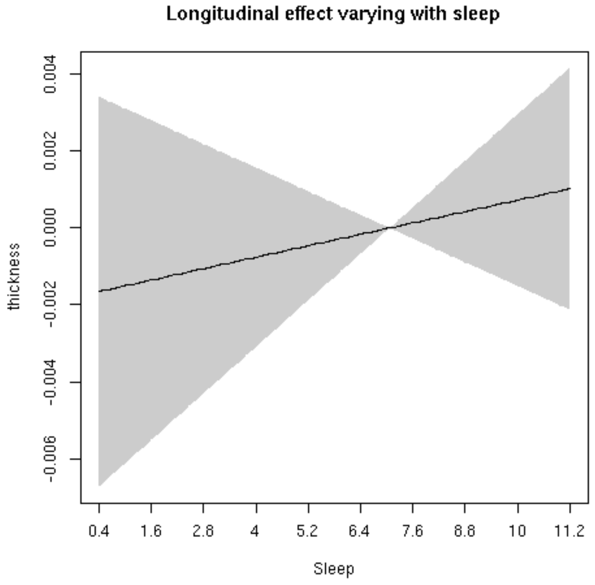

Participants sleeping 5-9 hours

```
##
## Family: gaussian
## Link function: identity
##
## Formula:
## value ~ sex + site + s(bl_age, k = 10, bs = "cr") + s(bl_age,
##     by = sleep_z, bs = "cr") + +s(bl_age, by = time, k = 5, bs = "cr") +
##     s(sleep_z, by = time, k = 5, bs = "cr", pc = 0)
## <environment: 0x56300a2a08d0>
##
## Parametric coefficients:
##                Estimate Std. Error t value Pr(>|t|)
## (Intercept)    2.503990   0.005808 431.145  < 2e-16 ***
## sexmale       -0.002010   0.002907  -0.691   0.4893
## siteousAvanto  0.012177   0.007730   1.575   0.1152
## siteousPrisma  0.075641   0.034028   2.223   0.0262 *
## siteousSkyra  -0.015444   0.007727  -1.999   0.0457 *
## siteUB         0.081258   0.015380   5.283  1.3e-07 ***
## siteUCAM       0.067074   0.008002   8.382  < 2e-16 ***
## siteUKB        0.307295   0.005889  52.179  < 2e-16 ***
## siteUmU       -0.024603   0.009569  -2.571   0.0102 *
## ---
## Signif. codes:  0 ‘***’ 0.001 ‘**’ 0.01 ‘*’ 0.05 ‘.’ 0.1 ‘ ’ 1
##
## Approximate significance of smooth terms:
##                     edf Ref.df       F p-value
## s(bl_age)         3.961  3.961 255.420  <2e-16 ***
## s(bl_age):sleep_z 2.000  2.000   0.799   0.450
## s(bl_age):time    3.678  3.678  86.913  <2e-16 ***
## s(sleep_z):time   1.000  1.000   0.425   0.515
```

```
## ---
## Signif. codes:  0 ‘***’ 0.001 ‘**’ 0.01 ‘*’ 0.05 ‘.’ 0.1 ‘ ’ 1
##
## R-sq.(adj) =  0.686
## lmer.REML = -19313  Scale est. = 0.0020073  n = 8178
```

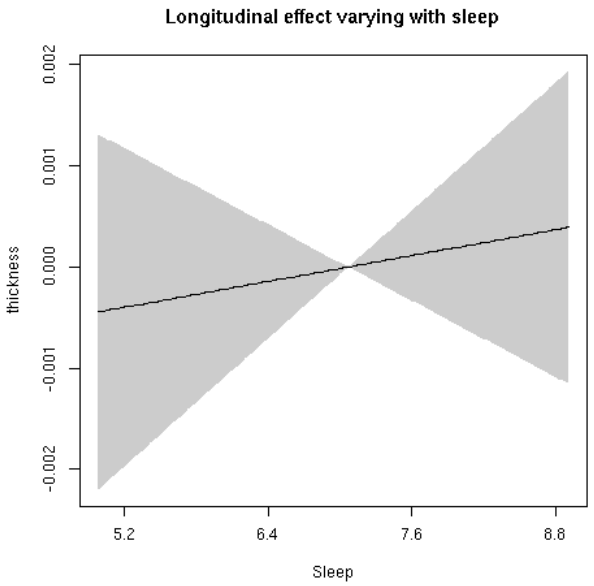

Volume cluster 2

All longitudinal data

```
##
## Family: gaussian
## Link function: identity
##
## Formula:
## value ~ sex + site + s(bl_age, k = 10, bs = "cr") + s(bl_age,
##     by = sleep_z, bs = "cr") + +s(bl_age, by = time, k = 5, bs = "cr") +
##     s(sleep_z, by = time, k = 5, bs = "cr", pc = 0)
## <environment: 0x563006cee588>
##
## Parametric coefficients:
##                Estimate Std. Error t value Pr(>|t|)
## (Intercept)    115801.2      712.2 162.585  < 2e-16 ***
## sexmale         13835.3      366.9  37.707  < 2e-16 ***
## siteousAvanto    7487.2      949.1   7.889 3.45e-15 ***
## siteousPrisma   13287.7     2443.6   5.438 5.55e-08 ***
## siteousSkyra    11115.2      948.3  11.721  < 2e-16 ***
## siteUB           4556.7     1958.1   2.327     0.02 *
## siteUCAM         6448.6      992.7   6.496 8.72e-11 ***
## siteUKB         15442.9      706.5  21.859  < 2e-16 ***
## siteUmU          8974.2     1195.8   7.504 6.82e-14 ***
## ---
## Signif. codes:  0 ‘***’ 0.001 ‘**’ 0.01 ‘*’ 0.05 ‘.’ 0.1 ‘ ’ 1
```

```
##
## Approximate significance of smooth terms:
##                       edf Ref.df       F p-value
## s(bl_age)           1.000  1.000 759.346  <2e-16 ***
## s(bl_age):sleep_z 3.237  3.237   1.829  0.0953 .
## s(bl_age):time    3.555  3.555 195.093  <2e-16 ***
## s(sleep_z):time   1.000  1.000   0.227  0.6338
## ---
## Signif. codes:  0 ‘***’ 0.001 ‘**’ 0.01 ‘*’ 0.05 ‘.’ 0.1 ‘ ’ 1
##
## R-sq.(adj) =  0.397
## lmer.REML = 1.6727e+05  Scale est. = 8.696e+06  n = 8180
```

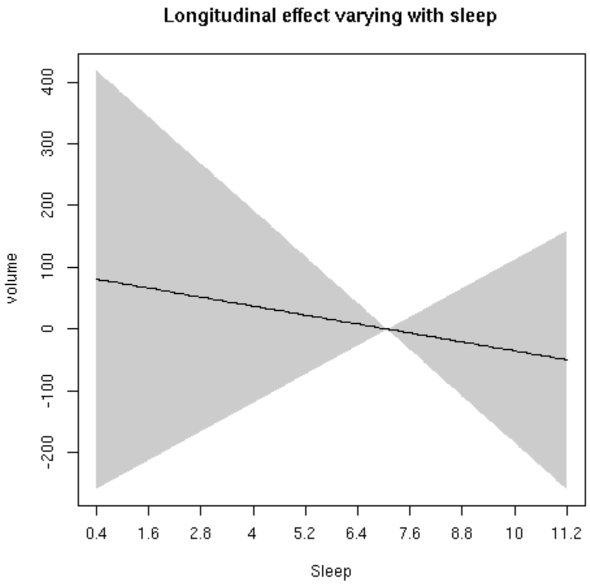

Participants sleeping 5-9 hours

```
##
## Family: gaussian
## Link function: identity
##
## Formula:
## value ~ sex + site + s(bl_age, k = 10, bs = "cr") + s(bl_age,
##     by = sleep_z, bs = "cr") + +s(bl_age, by = time, k = 5, bs = "cr") +
##     s(sleep_z, by = time, k = 5, bs = "cr", pc = 0)
## <environment: 0x563006cee588>
##
## Parametric coefficients:
##               Estimate Std. Error t value Pr(>|t|)
## (Intercept)   115801.2      712.2 162.585  < 2e-16 ***
## sexmale        13835.3      366.9  37.707  < 2e-16 ***
## siteousAvanto   7487.2      949.1   7.889 3.45e-15 ***
## siteousPrisma  13287.7     2443.6   5.438 5.55e-08 ***
## siteousSkyra   11115.2      948.3  11.721  < 2e-16 ***
## siteUB          4556.7     1958.1   2.327     0.02 *
```

```
## siteUCAM        6448.6      992.7   6.496 8.72e-11 ***
## siteUKB        15442.9      706.5  21.859  < 2e-16 ***
## siteUmU         8974.2     1195.8   7.504 6.82e-14 ***
## ---
## Signif. codes:  0 '***' 0.001 '**' 0.01 '*' 0.05 '.' 0.1 ' ' 1
##
## Approximate significance of smooth terms:
##                       edf Ref.df       F p-value
## s(bl_age)           1.000  1.000 759.346  <2e-16 ***
## s(bl_age):sleep_z 3.237  3.237   1.829  0.0953 .
## s(bl_age):time    3.555  3.555 195.093  <2e-16 ***
## s(sleep_z):time   1.000  1.000   0.227  0.6338
## ---
## Signif. codes:  0 '***' 0.001 '**' 0.01 '*' 0.05 '.' 0.1 ' ' 1
##
## R-sq.(adj) =  0.397
## lmer.REML = 1.6727e+05  Scale est. = 8.696e+06  n = 8180
```

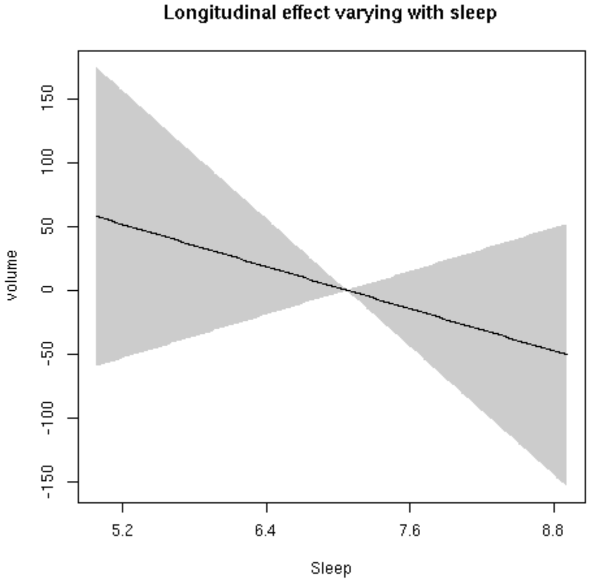

Area cluster 3

All longitudinal data

```
##
## Family: gaussian
## Link function: identity
##
## Formula:
## value ~ sex + site + s(bl_age, k = 10, bs = "cr") + s(bl_age,
##     by = sleep_z, bs = "cr") + +s(bl_age, by = time, k = 5, bs = "cr") +
##     s(sleep_z, by = time, k = 5, bs = "cr", pc = 0)
## <environment: 0x563034333450>
##
## Parametric coefficients:
```

```
##                Estimate Std. Error t value Pr(>|t|)    
## (Intercept)   101688.9      554.9 183.271  < 2e-16 ***
## sexmale        11622.1      286.0  40.639  < 2e-16 ***
## siteousAvanto   7079.8      736.5   9.612  < 2e-16 ***
## siteousPrisma   5695.3     1554.0   3.665 0.000249 ***
## siteousSkyra    5441.0      736.2   7.391 1.60e-13 ***
## siteUB          -328.8     1528.8  -0.215 0.829736    
## siteUCAM        3201.0      773.4   4.139 3.52e-05 ***
## siteUKB         1263.7      550.4   2.296 0.021698 *  
## siteUmU         5965.9      931.9   6.402 1.62e-10 ***
## ---
## Signif. codes:  0 '***' 0.001 '**' 0.01 '*' 0.05 '.' 0.1 ' ' 1
## 
## Approximate significance of smooth terms:
##                     edf Ref.df       F p-value    
## s(bl_age)         1.000  1.000 221.086  <2e-16 ***
## s(bl_age):sleep_z 2.805  2.805   1.622   0.120    
## s(bl_age):time    2.000  2.000 146.794  <2e-16 ***
## s(sleep_z):time   1.000  1.000   0.827   0.363    
## ---
## Signif. codes:  0 '***' 0.001 '**' 0.01 '*' 0.05 '.' 0.1 ' ' 1
## 
## R-sq.(adj) =  0.343   
## lmer.REML = 1.6102e+05  Scale est. = 3.1807e+06  n = 8180
```

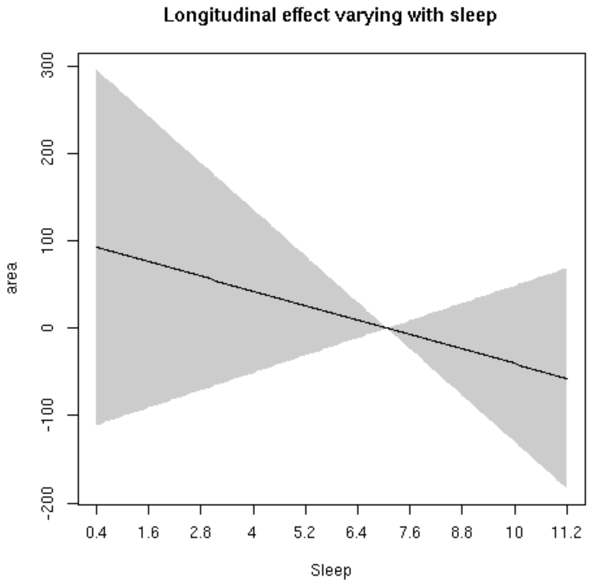

Participants sleeping 5-9 hours

```
## 
## Family: gaussian 
## Link function: identity 
## 
## Formula:
## value ~ sex + site + s(bl_age, k = 10, bs = "cr") + s(bl_age, 
```

```
##     by = sleep_z, bs = "cr") + +s(bl_age, by = time, k = 5, bs = "cr") +
##     s(sleep_z, by = time, k = 5, bs = "cr", pc = 0)
## <environment: 0x563034333450>
##
## Parametric coefficients:
##               Estimate Std. Error t value Pr(>|t|)
## (Intercept)   101688.9      554.9 183.271  < 2e-16 ***
## sexmale        11622.1      286.0  40.639  < 2e-16 ***
## siteousAvanto   7079.8      736.5   9.612  < 2e-16 ***
## siteousPrisma   5695.3     1554.0   3.665 0.000249 ***
## siteousSkyra    5441.0      736.2   7.391 1.60e-13 ***
## siteUB          -328.8     1528.8  -0.215 0.829736
## siteUCAM        3201.0      773.4   4.139 3.52e-05 ***
## siteUKB         1263.7      550.4   2.296 0.021698 *
## siteUmU         5965.9      931.9   6.402 1.62e-10 ***
## ---
## Signif. codes:  0 '***' 0.001 '**' 0.01 '*' 0.05 '.' 0.1 ' ' 1
##
## Approximate significance of smooth terms:
##                       edf Ref.df       F p-value
## s(bl_age)           1.000  1.000 221.086  <2e-16 ***
## s(bl_age):sleep_z 2.805  2.805   1.622   0.120
## s(bl_age):time    2.000  2.000 146.794  <2e-16 ***
## s(sleep_z):time   1.000  1.000   0.827   0.363
## ---
## Signif. codes:  0 '***' 0.001 '**' 0.01 '*' 0.05 '.' 0.1 ' ' 1
##
## R-sq.(adj) =  0.343
## lmer.REML = 1.6102e+05  Scale est. = 3.1807e+06  n = 8180
```

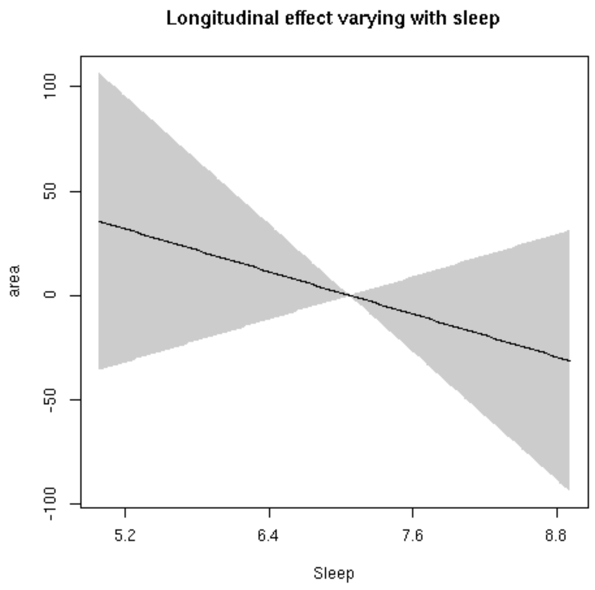

Thickness cluster 3

All longitudinal data

```
##
## Family: gaussian
## Link function: identity
##
## Formula:
## value ~ sex + site + s(bl_age, k = 10, bs = "cr") + s(bl_age,
##     by = sleep_z, bs = "cr") + +s(bl_age, by = time, k = 5, bs = "cr") +
##     s(sleep_z, by = time, k = 5, bs = "cr", pc = 0)
## <environment: 0x5630074621f0>
##
## Parametric coefficients:
##                Estimate Std. Error t value Pr(>|t|)
## (Intercept)    2.542424   0.005926 429.016  < 2e-16 ***
## sexmale       -0.001732   0.003042  -0.569 0.569054
## siteousAvanto -0.009717   0.008089  -1.201 0.229727
## siteousPrisma  0.054343   0.036952   1.471 0.141424
## siteousSkyra   0.001682   0.008069   0.208 0.834898
## siteUB         0.062181   0.016031   3.879 0.000106 ***
## siteUCAM       0.029428   0.008240   3.571 0.000357 ***
## siteUKB        0.284573   0.005872  48.463  < 2e-16 ***
## siteUmU        0.015502   0.009937   1.560 0.118806
## ---
## Signif. codes:  0 '***' 0.001 '**' 0.01 '*' 0.05 '.' 0.1 ' ' 1
##
## Approximate significance of smooth terms:
##                      edf Ref.df       F p-value
## s(bl_age)          1.000  1.000 482.425  <2e-16 ***
## s(bl_age):sleep_z 2.000  2.000   0.655   0.520
## s(bl_age):time    3.052  3.052  81.066  <2e-16 ***
## s(sleep_z):time   1.000  1.000   0.131   0.718
## ---
## Signif. codes:  0 '***' 0.001 '**' 0.01 '*' 0.05 '.' 0.1 ' ' 1
##
## R-sq.(adj) =  0.616
## lmer.REML = -18210  Scale est. = 0.0023948  n = 8178
```

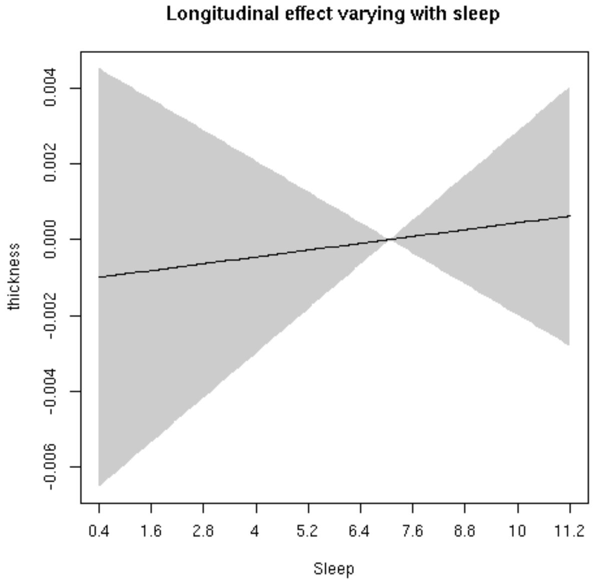

Participants sleeping 5-9 hours

```
##
## Family: gaussian
## Link function: identity
##
## Formula:
## value ~ sex + site + s(bl_age, k = 10, bs = "cr") + s(bl_age,
##     by = sleep_z, bs = "cr") + +s(bl_age, by = time, k = 5, bs = "cr") +
##     s(sleep_z, by = time, k = 5, bs = "cr", pc = 0)
## <environment: 0x5630074621f0>
##
## Parametric coefficients:
##                Estimate Std. Error t value Pr(>|t|)
## (Intercept)    2.542424   0.005926 429.016  < 2e-16 ***
## sexmale       -0.001732   0.003042  -0.569 0.569054
## siteousAvanto -0.009717   0.008089  -1.201 0.229727
## siteousPrisma  0.054343   0.036952   1.471 0.141424
## siteousSkyra   0.001682   0.008069   0.208 0.834898
## siteUB         0.062181   0.016031   3.879 0.000106 ***
## siteUCAM       0.029428   0.008240   3.571 0.000357 ***
## siteUKB        0.284573   0.005872  48.463  < 2e-16 ***
## siteUmU        0.015502   0.009937   1.560 0.118806
## ---
## Signif. codes:  0 ‘***’ 0.001 ‘**’ 0.01 ‘*’ 0.05 ‘.’ 0.1 ‘ ’ 1
##
## Approximate significance of smooth terms:
##                     edf Ref.df       F p-value
## s(bl_age)         1.000  1.000 482.425  <2e-16 ***
## s(bl_age):sleep_z 2.000  2.000   0.655   0.520
## s(bl_age):time    3.052  3.052  81.066  <2e-16 ***
## s(sleep_z):time   1.000  1.000   0.131   0.718
```

```
## ---
## Signif. codes:  0 '***' 0.001 '**' 0.01 '*' 0.05 '.' 0.1 ' ' 1
##
## R-sq.(adj) =  0.616
## lmer.REML = -18210  Scale est. = 0.0023948  n = 8178
```

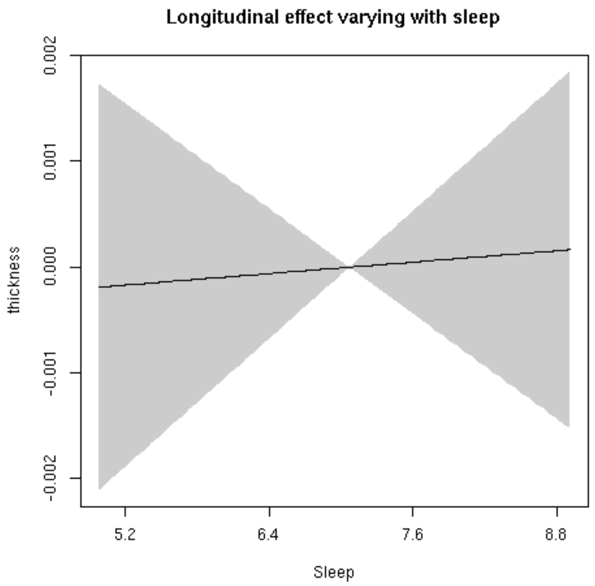

Volume cluster 3

All longitudinal data

```
##
## Family: gaussian
## Link function: identity
##
## Formula:
## value ~ sex + site + s(bl_age, k = 10, bs = "cr") + s(bl_age,
##     by = sleep_z, bs = "cr") + +s(bl_age, by = time, k = 5, bs = "cr") +
##     s(sleep_z, by = time, k = 5, bs = "cr", pc = 0)
## <environment: 0x56300419f738>
##
## Parametric coefficients:
##                Estimate Std. Error t value Pr(>|t|)
## (Intercept)     91602.8      584.4 156.755  < 2e-16 ***
## sexmale         10693.6      298.6  35.809  < 2e-16 ***
## siteousAvanto    5076.1      772.5   6.571 5.30e-11 ***
## siteousPrisma   11108.1     1971.6   5.634 1.82e-08 ***
## siteousSkyra     6876.6      772.0   8.907  < 2e-16 ***
## siteUB           2323.4     1594.8   1.457    0.145
## siteUCAM         4655.6      808.3   5.759 8.74e-09 ***
## siteUKB         12056.3      584.1  20.639  < 2e-16 ***
## siteUmU          5853.0      975.7   5.999 2.07e-09 ***
## ---
## Signif. codes:  0 '***' 0.001 '**' 0.01 '*' 0.05 '.' 0.1 ' ' 1
```

```
##
## Approximate significance of smooth terms:
##                       edf Ref.df       F p-value
## s(bl_age)           1.434  1.434 437.154  <2e-16 ***
## s(bl_age):sleep_z 2.243  2.243   2.005   0.108
## s(bl_age):time    3.729  3.729 197.411  <2e-16 ***
## s(sleep_z):time   1.000  1.000   0.067   0.796
## ---
## Signif. codes:  0 '***' 0.001 '**' 0.01 '*' 0.05 '.' 0.1 ' ' 1
##
## R-sq.(adj) =  0.381
## lmer.REML = 1.6377e+05  Scale est. = 5.6397e+06  n = 8178
```

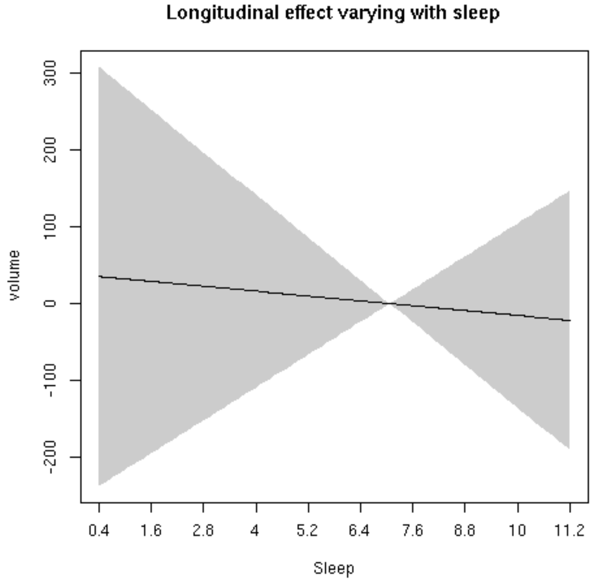

Participants sleeping 5-9 hours

```
##
## Family: gaussian
## Link function: identity
##
## Formula:
## value ~ sex + site + s(bl_age, k = 10, bs = "cr") + s(bl_age,
##     by = sleep_z, bs = "cr") + +s(bl_age, by = time, k = 5, bs = "cr") +
##     s(sleep_z, by = time, k = 5, bs = "cr", pc = 0)
## <environment: 0x56300419f738>
##
## Parametric coefficients:
##               Estimate Std. Error t value Pr(>|t|)
## (Intercept)    91602.8      584.4 156.755  < 2e-16 ***
## sexmale        10693.6      298.6  35.809  < 2e-16 ***
## siteousAvanto   5076.1      772.5   6.571 5.30e-11 ***
## siteousPrisma  11108.1     1971.6   5.634 1.82e-08 ***
## siteousSkyra    6876.6      772.0   8.907  < 2e-16 ***
## siteUB          2323.4     1594.8   1.457    0.145
```

```
## siteUCAM        4655.6      808.3   5.759 8.74e-09 ***
## siteUKB        12056.3      584.1  20.639  < 2e-16 ***
## siteUmU         5853.0      975.7   5.999 2.07e-09 ***
## ---
## Signif. codes:  0 '***' 0.001 '**' 0.01 '*' 0.05 '.' 0.1 ' ' 1
##
## Approximate significance of smooth terms:
##                       edf Ref.df       F p-value
## s(bl_age)           1.434  1.434 437.154  <2e-16 ***
## s(bl_age):sleep_z 2.243  2.243   2.005   0.108
## s(bl_age):time      3.729  3.729 197.411  <2e-16 ***
## s(sleep_z):time     1.000  1.000   0.067   0.796
## ---
## Signif. codes:  0 '***' 0.001 '**' 0.01 '*' 0.05 '.' 0.1 ' ' 1
##
## R-sq.(adj) =  0.381
## lmer.REML = 1.6377e+05  Scale est. = 5.6397e+06  n = 8178
```

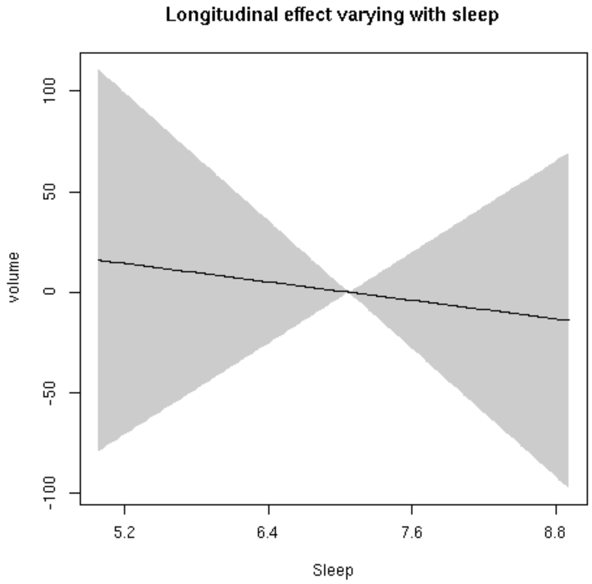

Subcortical volumes longitudinal

Contents

Introduction **7**
- Data . . . 7
- Models . . . 7

Results per region **8**
- Accumbens-area . . . 8
  - Main analysis . . . 8
    - Full data . . . 8
    - Restricted to sleep >= 5 and <= 9 hours . . . 9
  - Controling for socioeconomic status . . . 10
    - Full data . . . 10
    - Restricted to sleep >= 5 and <= 9 hours . . . 12
  - Controling for BMI . . . 13
    - Full data . . . 13
    - Restricted to sleep >= 5 and <= 9 hours . . . 15
  - Controling for depression . . . 16
    - Full data . . . 16
    - Restricted to sleep >= 5 and <= 9 hours . . . 18
  - Controling for sleep quality . . . 19
    - Full data . . . 19
    - Restricted to sleep >= 5 and <= 9 hours . . . 21
- Amygdala . . . 22
  - Main analysis . . . 22
    - Full data . . . 22
    - Restricted to sleep >= 5 and <= 9 hours . . . 24
  - Controling for socioeconomic status . . . 25
    - Full data . . . 25
    - Restricted to sleep >= 5 and <= 9 hours . . . 27
  - Controling for BMI . . . 28
    - Full data . . . 28
    - Restricted to sleep >= 5 and <= 9 hours . . . 30
  - Controling for depression . . . 31
    - Full data . . . 31
    - Restricted to sleep >= 5 and <= 9 hours . . . 33
  - Controling for sleep quality . . . 34
    - Full data . . . 34
    - Restricted to sleep >= 5 and <= 9 hours . . . 36
- Brain-Stem . . . 37
  - Main analysis . . . 37
    - Full data . . . 37
    - Restricted to sleep >= 5 and <= 9 hours . . . 39
  - Controling for socioeconomic status . . . 40
    - Full data . . . 40
    - Restricted to sleep >= 5 and <= 9 hours . . . 42

Controling for BMI . . . 43
Full data . . . 43
Restricted to sleep >= 5 and <= 9 hours . . . 45
Controling for depression . . . 46
Full data . . . 46
Restricted to sleep >= 5 and <= 9 hours . . . 48
Controling for sleep quality . . . 49
Full data . . . 49
Restricted to sleep >= 5 and <= 9 hours . . . 51
Caudate . . . 52
Main analysis . . . 52
Full data . . . 52
Restricted to sleep >= 5 and <= 9 hours . . . 54
Controling for socioeconomic status . . . 55
Full data . . . 55
Restricted to sleep >= 5 and <= 9 hours . . . 57
Controling for BMI . . . 58
Full data . . . 58
Restricted to sleep >= 5 and <= 9 hours . . . 60
Controling for depression . . . 61
Full data . . . 61
Restricted to sleep >= 5 and <= 9 hours . . . 63
Controling for sleep quality . . . 64
Full data . . . 64
Restricted to sleep >= 5 and <= 9 hours . . . 66
CC_Anterior . . . 67
Main analysis . . . 67
Full data . . . 67
Restricted to sleep >= 5 and <= 9 hours . . . 69
Controling for socioeconomic status . . . 70
Full data . . . 70
Restricted to sleep >= 5 and <= 9 hours . . . 72
Controling for BMI . . . 73
Full data . . . 73
Restricted to sleep >= 5 and <= 9 hours . . . 75
Controling for depression . . . 76
Full data . . . 76
Restricted to sleep >= 5 and <= 9 hours . . . 78
Controling for sleep quality . . . 79
Full data . . . 79
Restricted to sleep >= 5 and <= 9 hours . . . 81
CC_Central . . . 82
Main analysis . . . 82
Full data . . . 82
Restricted to sleep >= 5 and <= 9 hours . . . 84
Controling for socioeconomic status . . . 85
Full data . . . 85
Restricted to sleep >= 5 and <= 9 hours . . . 87
Controling for BMI . . . 88
Full data . . . 88
Restricted to sleep >= 5 and <= 9 hours . . . 90
Controling for depression . . . 91
Full data . . . 91
Restricted to sleep >= 5 and <= 9 hours . . . 93

Controling for sleep quality 94
Full data 94
Restricted to sleep >= 5 and <= 9 hours 96
CC_Mid_Anterior 97
Main analysis 97
Full data 97
Restricted to sleep >= 5 and <= 9 hours 99
Controling for socioeconomic status 100
Full data 100
Restricted to sleep >= 5 and <= 9 hours 102
Controling for BMI 103
Full data 103
Restricted to sleep >= 5 and <= 9 hours 105
Controling for depression 106
Full data 106
Restricted to sleep >= 5 and <= 9 hours 108
Controling for sleep quality 109
Full data 109
Restricted to sleep >= 5 and <= 9 hours 111
CC_Mid_Posterior 112
Main analysis 112
Full data 112
Restricted to sleep >= 5 and <= 9 hours 114
Controling for socioeconomic status 115
Full data 115
Restricted to sleep >= 5 and <= 9 hours 117
Controling for BMI 118
Full data 118
Restricted to sleep >= 5 and <= 9 hours 120
Controling for depression 121
Full data 121
Restricted to sleep >= 5 and <= 9 hours 123
Controling for sleep quality 124
Full data 124
Restricted to sleep >= 5 and <= 9 hours 126
CC_Posterior 127
Main analysis 127
Full data 127
Restricted to sleep >= 5 and <= 9 hours 129
Controling for socioeconomic status 130
Full data 130
Restricted to sleep >= 5 and <= 9 hours 132
Controling for BMI 133
Full data 133
Restricted to sleep >= 5 and <= 9 hours 135
Controling for depression 136
Full data 136
Restricted to sleep >= 5 and <= 9 hours 138
Controling for sleep quality 139
Full data 139
Restricted to sleep >= 5 and <= 9 hours 141
Cerebellum-Cortex 142
Main analysis 142
Full data 142

Restricted to sleep >= 5 and <= 9 hours . . . 144
Controling for socioeconomic status . . . 145
Full data . . . 145
Restricted to sleep >= 5 and <= 9 hours . . . 147
Controling for BMI . . . 148
Full data . . . 148
Restricted to sleep >= 5 and <= 9 hours . . . 150
Controling for depression . . . 151
Full data . . . 151
Restricted to sleep >= 5 and <= 9 hours . . . 153
Controling for sleep quality . . . 154
Full data . . . 154
Restricted to sleep >= 5 and <= 9 hours . . . 156
Cerebellum-White-Matter . . . 157
Main analysis . . . 157
Full data . . . 157
Restricted to sleep >= 5 and <= 9 hours . . . 159
Controling for socioeconomic status . . . 160
Full data . . . 160
Restricted to sleep >= 5 and <= 9 hours . . . 162
Controling for BMI . . . 163
Full data . . . 163
Restricted to sleep >= 5 and <= 9 hours . . . 165
Controling for depression . . . 166
Full data . . . 166
Restricted to sleep >= 5 and <= 9 hours . . . 168
Controling for sleep quality . . . 169
Full data . . . 169
Restricted to sleep >= 5 and <= 9 hours . . . 171
CerebralWhiteMatterVol . . . 172
Main analysis . . . 172
Full data . . . 172
Restricted to sleep >= 5 and <= 9 hours . . . 174
Controling for socioeconomic status . . . 175
Full data . . . 175
Restricted to sleep >= 5 and <= 9 hours . . . 177
Controling for BMI . . . 178
Full data . . . 178
Restricted to sleep >= 5 and <= 9 hours . . . 180
Controling for depression . . . 181
Full data . . . 181
Restricted to sleep >= 5 and <= 9 hours . . . 183
Controling for sleep quality . . . 184
Full data . . . 184
Restricted to sleep >= 5 and <= 9 hours . . . 186
EstimatedTotalIntraCranialVol . . . 187
Main analysis . . . 187
Full data . . . 187
Restricted to sleep >= 5 and <= 9 hours . . . 189
Controling for socioeconomic status . . . 190
Full data . . . 190
Restricted to sleep >= 5 and <= 9 hours . . . 192
Controling for BMI . . . 193
Full data . . . 193

Restricted to sleep >= 5 and <= 9 hours . . . 195
Controling for depression . . . 196
Full data . . . 196
Restricted to sleep >= 5 and <= 9 hours . . . 198
Controling for sleep quality . . . 199
Full data . . . 199
Restricted to sleep >= 5 and <= 9 hours . . . 201
Hippocampus . . . 202
Main analysis . . . 202
Full data . . . 202
Restricted to sleep >= 5 and <= 9 hours . . . 204
Controling for socioeconomic status . . . 205
Full data . . . 205
Restricted to sleep >= 5 and <= 9 hours . . . 207
Controling for BMI . . . 208
Full data . . . 208
Restricted to sleep >= 5 and <= 9 hours . . . 210
Controling for depression . . . 211
Full data . . . 211
Restricted to sleep >= 5 and <= 9 hours . . . 213
Controling for sleep quality . . . 214
Full data . . . 214
Restricted to sleep >= 5 and <= 9 hours . . . 216
Pallidum . . . 217
Main analysis . . . 217
Full data . . . 217
Restricted to sleep >= 5 and <= 9 hours . . . 219
Controling for socioeconomic status . . . 220
Full data . . . 220
Restricted to sleep >= 5 and <= 9 hours . . . 222
Controling for BMI . . . 223
Full data . . . 223
Restricted to sleep >= 5 and <= 9 hours . . . 225
Controling for depression . . . 226
Full data . . . 226
Restricted to sleep >= 5 and <= 9 hours . . . 228
Controling for sleep quality . . . 229
Full data . . . 229
Restricted to sleep >= 5 and <= 9 hours . . . 231
Putamen . . . 232
Main analysis . . . 232
Full data . . . 232
Restricted to sleep >= 5 and <= 9 hours . . . 234
Controling for socioeconomic status . . . 235
Full data . . . 235
Restricted to sleep >= 5 and <= 9 hours . . . 237
Controling for BMI . . . 238
Full data . . . 238
Restricted to sleep >= 5 and <= 9 hours . . . 240
Controling for depression . . . 241
Full data . . . 241
Restricted to sleep >= 5 and <= 9 hours . . . 243
Controling for sleep quality . . . 244
Full data . . . 244

Restricted to sleep >= 5 and <= 9 hours . . . 246
Thalamus . . . 247
Main analysis . . . 247
Full data . . . 247
Restricted to sleep >= 5 and <= 9 hours . . . 249
Controling for socioeconomic status . . . 250
Full data . . . 250
Restricted to sleep >= 5 and <= 9 hours . . . 252
Controling for BMI . . . 253
Full data . . . 253
Restricted to sleep >= 5 and <= 9 hours . . . 255
Controling for depression . . . 256
Full data . . . 256
Restricted to sleep >= 5 and <= 9 hours . . . 258
Controling for sleep quality . . . 259
Full data . . . 259
Restricted to sleep >= 5 and <= 9 hours . . . 261
TotalGrayVol . . . 262
Main analysis . . . 262
Full data . . . 262
Restricted to sleep >= 5 and <= 9 hours . . . 264
Controling for socioeconomic status . . . 265
Full data . . . 265
Restricted to sleep >= 5 and <= 9 hours . . . 267
Controling for BMI . . . 268
Full data . . . 268
Restricted to sleep >= 5 and <= 9 hours . . . 270
Controling for depression . . . 271
Full data . . . 271
Restricted to sleep >= 5 and <= 9 hours . . . 273
Controling for sleep quality . . . 274
Full data . . . 274
Restricted to sleep >= 5 and <= 9 hours . . . 276
Ventricles . . . 277
Main analysis . . . 277
Full data . . . 277
Restricted to sleep >= 5 and <= 9 hours . . . 279
Controling for socioeconomic status . . . 280
Full data . . . 280
Restricted to sleep >= 5 and <= 9 hours . . . 282
Controling for BMI . . . 283
Full data . . . 283
Restricted to sleep >= 5 and <= 9 hours . . . 285
Controling for depression . . . 286
Full data . . . 286
Restricted to sleep >= 5 and <= 9 hours . . . 288
Controling for sleep quality . . . 289
Full data . . . 289
Restricted to sleep >= 5 and <= 9 hours . . . 291

P-values corrected for multiple testing 292
Controling for quality . . . 294
P-values controling for BMI . . . 295
P-values controling for SES . . . 296

P-values controling for Depression 297

```
library(tidyverse)
```

Introduction

Data

All participants with two or more timepoints were included. A count is shown below.

study	Participants	Observations
MPIB	284	568
UB	38	112
UCAM	252	504
UiO	340	1011
UKB	2846	5692
UmU	133	266

Max. follow-up	Mean follow-up	SD follow-up
11.22656	2.510435	1.448605

Models

The following GAMM was fitted:

```
mod <- gamm4(
  value ~ sex + site + icv + s(bl_age, k = 10, bs = 'cr') +
    s(bl_age, by = sleep_z, bs = 'cr') +
    s(bl_age, by = time, k = 5, bs = 'cr') +
    s(sleep_z, by = time, k = 5, bs = 'cr', pc = 0),
  random = ~(1|id), data = long_dat, REML = FALSE
)
```

Here is an explanation of each of the smooth terms. The number of "knots" was chosen relatively low for the sake of computational speed, but increasing this would have minimal impact on the results, as the estimated degrees of freedom is well below the maximum provided by the `k`.

- `s(bl_age, k = 10, bs = 'cr')` models the main effect of baseline age. In a linear model, the equivalent would be `bl_age`.
- `s(bl_age, by = sleep_z, bs = 'cr')` models the effect of sleep and how this effect varies with baseline age. In a linear model, the equivalent to this term would be `sleep_z + bl_age:sleep_z`.
- `s(bl_age, by = time, k = 5, bs = 'cr')` models how the effect of time depends on baseline age. In a linear model, the equivalent to this term would be `time + bl_age:time`.
- `s(sleep_z, by = time, k = 5, bs = 'cr', pc = 0)` models the interaction between time and sleep. The argument `pc = 0` constrains this term to not include a main effect of `time`, as this is covered by the previous term. In a linear model, the equivalent to this term would be `sleep_z:time`. This is the term of main interest.

Results per region

Accumbens-area

Main analysis

Full data Below is the model output. The term `s(sleep_z):time` is probably what we care most about.

```
##
## Family: gaussian
## Link function: identity
##
## Formula:
## value ~ sex + site + icv + s(bl_age, k = 10, bs = "cr") + s(bl_age,
##     by = sleep_z, bs = "cr") + +s(bl_age, by = time, k = 5, bs = "cr") +
##     s(sleep_z, by = time, k = 5, bs = "cr", pc = 0)
## <environment: 0x56391542fa28>
##
## Parametric coefficients:
##                 Estimate Std. Error t value Pr(>|t|)
## (Intercept)      925.987     10.605  87.319  < 2e-16 ***
## sexmale           14.029      5.443   2.577  0.00998 **
## siteousAvanto    114.136     13.087   8.722  < 2e-16 ***
## siteousPrisma    -66.977     42.893  -1.561  0.11845
## siteousSkyra     274.062     12.714  21.557  < 2e-16 ***
## siteUB           -24.983     24.241  -1.031  0.30274
## siteUCAM        -100.142     13.242  -7.562 4.39e-14 ***
## siteUKB          -10.489     10.089  -1.040  0.29852
## siteUmU          289.790     15.136  19.145  < 2e-16 ***
## icv               57.456      2.656  21.632  < 2e-16 ***
## ---
## Signif. codes:  0 ‘***’ 0.001 ‘**’ 0.01 ‘*’ 0.05 ‘.’ 0.1 ‘ ’ 1
##
## Approximate significance of smooth terms:
##                     edf Ref.df       F p-value
## s(bl_age)          4.34   4.34 490.591  <2e-16 ***
## s(bl_age):sleep_z 2.00   2.00   0.522   0.593
## s(bl_age):time    2.00   2.00 275.339  <2e-16 ***
## s(sleep_z):time   1.00   1.00   0.070   0.791
## ---
## Signif. codes:  0 ‘***’ 0.001 ‘**’ 0.01 ‘*’ 0.05 ‘.’ 0.1 ‘ ’ 1
##
## R-sq.(adj) =   0.58
## lmer.REML =  98394  Scale est. = 2920.6    n = 8153
```

Below are plots of a plot of `s(bl_age)`, `s(bl_age):sleep_z`, `s(bl_age):time`, and `s(sleep_z):time`. For `s(sleep_z):time`, roughly, if we cannot fit a straight line through the confidence bands, there is evidence that the effect of time depends on sleep duration. It is correct the that confidence interval is zero at one point along the axis; this happens because we do not include the main effect.

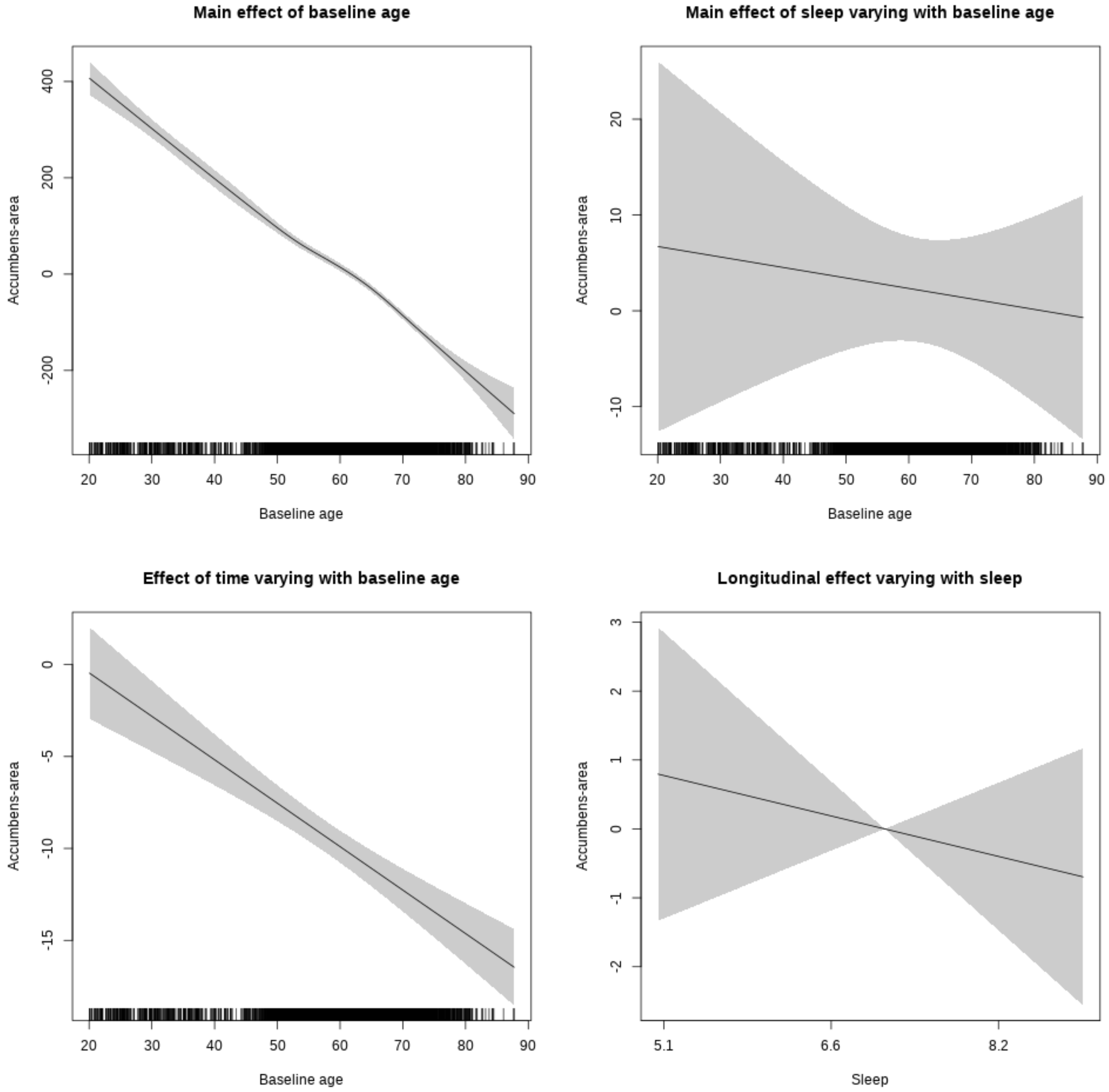

Restricted to sleep >= 5 and <= 9 hours Below is the row for `s(sleep_z):time` from the model output.

```
## s(sleep_z):time   1.000  1.000   0.557   0.455
```

Below are plots of a plot of `s(bl_age)`, `s(bl_age):sleep_z`, `s(bl_age):time`, and `s(sleep_z):time`. For `s(sleep_z):time`, roughly, if we cannot fit a straight line through the confidence bands, there is evidence that the effect of time depends on sleep duration. It is correct the that confidence interval is zero at one point along the axis; this happens because we do not include the main effect.

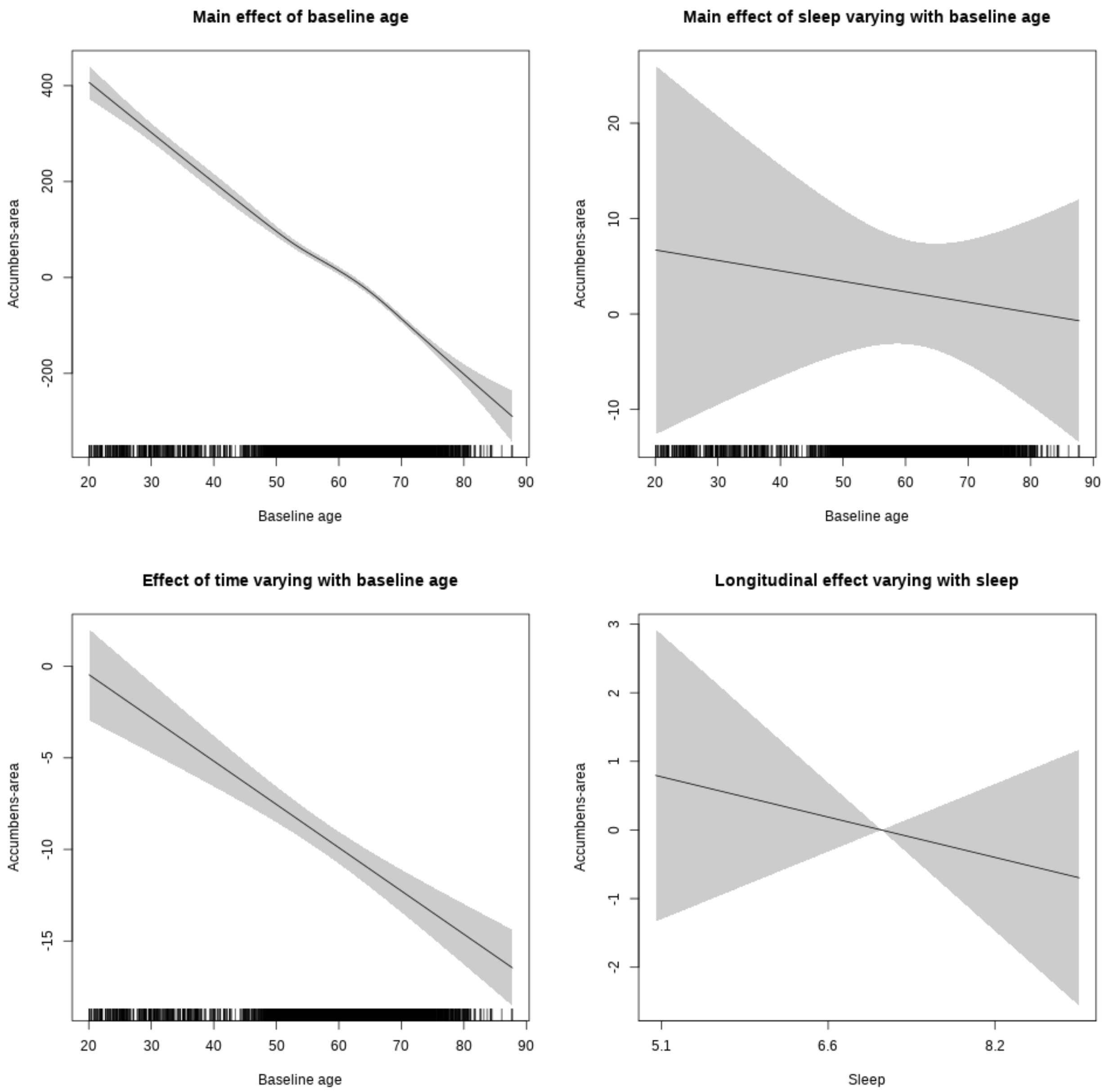

Controling for socioeconomic status

Full data Below is the model output. The term `s(sleep_z):time` is probably what we care most about.

```
##
## Family: gaussian
## Link function: identity
##
## Formula:
## value ~ sex + site + income_scaled + education_scaled + icv +
##     s(bl_age, k = 10, bs = "cr") + s(bl_age, by = sleep_z, bs = "cr") +
##     +s(bl_age, by = time, k = 5, bs = "cr") + s(sleep_z, by = time,
##     k = 5, bs = "cr", pc = 0)
```

```
## <environment: 0x563911288808>
##
## Parametric coefficients:
##                  Estimate Std. Error t value Pr(>|t|)
## (Intercept)       963.363     23.978  40.176  < 2e-16 ***
## sexmale            18.001      6.722   2.678  0.00744 **
## siteousAvanto      64.443     27.362   2.355  0.01855 *
## siteousPrisma    -115.147     69.742  -1.651  0.09879 .
## siteousSkyra      257.089     24.708  10.405  < 2e-16 ***
## siteUKB           -60.927     22.548  -2.702  0.00692 **
## income_scaled      -3.563      8.180  -0.436  0.66313
## education_scaled   10.484      9.512   1.102  0.27043
## icv                64.532      3.390  19.038  < 2e-16 ***
## ---
## Signif. codes:  0 ‘***’ 0.001 ‘**’ 0.01 ‘*’ 0.05 ‘.’ 0.1 ‘ ’ 1
##
## Approximate significance of smooth terms:
##                   edf Ref.df       F p-value
## s(bl_age)           1      1 916.159  <2e-16 ***
## s(bl_age):sleep_z   2      2   1.195  0.3029
## s(bl_age):time      2      2 116.109  <2e-16 ***
## s(sleep_z):time     1      1   2.728  0.0986 .
## ---
## Signif. codes:  0 ‘***’ 0.001 ‘**’ 0.01 ‘*’ 0.05 ‘.’ 0.1 ‘ ’ 1
##
## R-sq.(adj) =  0.589
## lmer.REML =  56008  Scale est. = 2889.7    n = 4654
```

Below are plots of a plot of `s(bl_age)`, `s(bl_age):sleep_z`, `s(bl_age):time`, and `s(sleep_z):time`. For `s(sleep_z):time`, roughly, if we cannot fit a straight line through the confidence bands, there is evidence that the effect of time depends on sleep duration. It is correct the that confidence interval is zero at one point along the axis; this happens because we do not include the main effect.

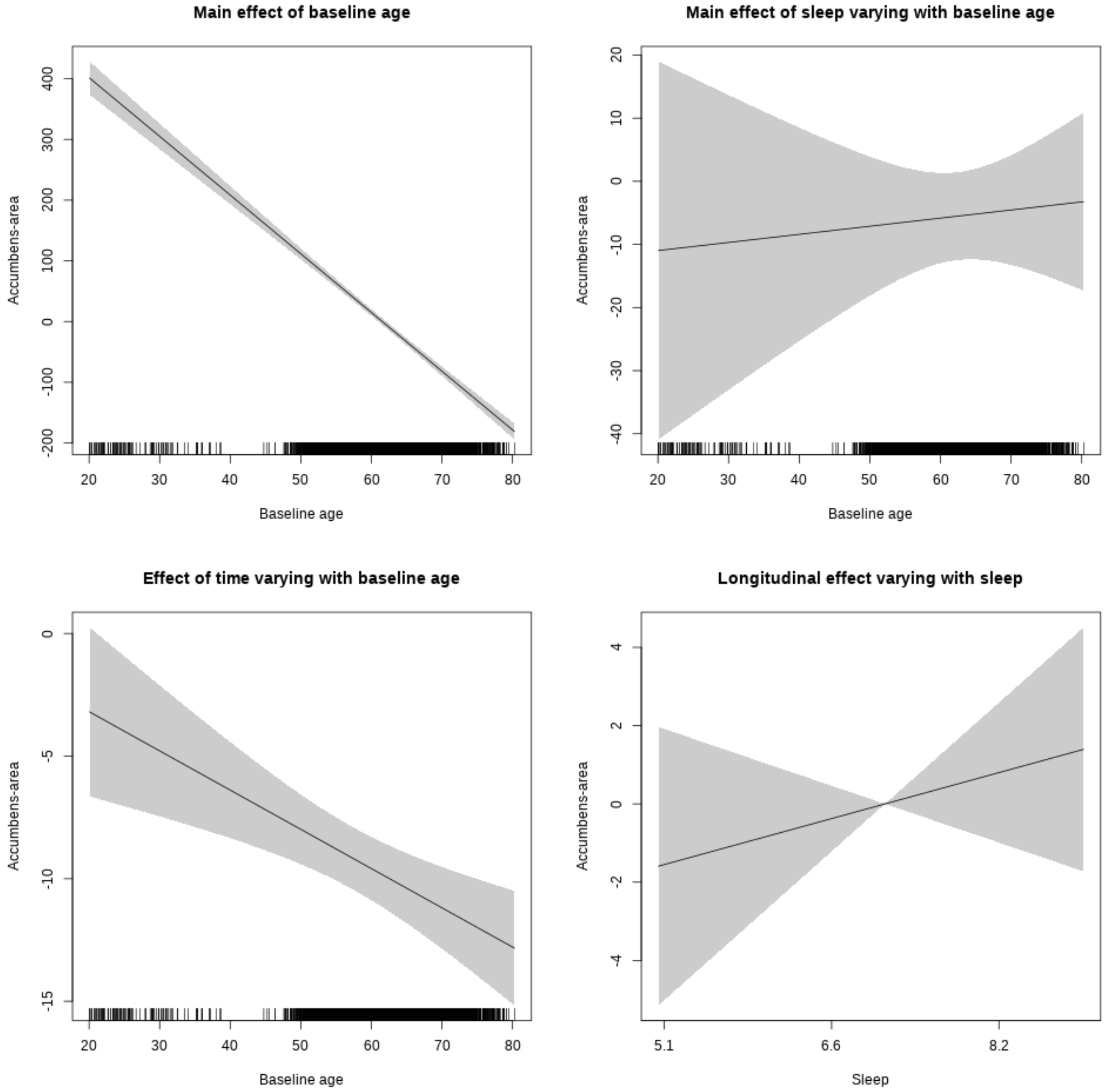

Restricted to sleep >= 5 and <= 9 hours Below is the row for `s(sleep_z):time` from the model output.

```
## s(sleep_z):time     1      1   0.800   0.371
```

Below are plots of a plot of `s(bl_age)`, `s(bl_age):sleep_z`, `s(bl_age):time`, and `s(sleep_z):time`. For `s(sleep_z):time`, roughly, if we cannot fit a straight line through the confidence bands, there is evidence that the effect of time depends on sleep duration. It is correct the that confidence interval is zero at one point along the axis; this happens because we do not include the main effect.

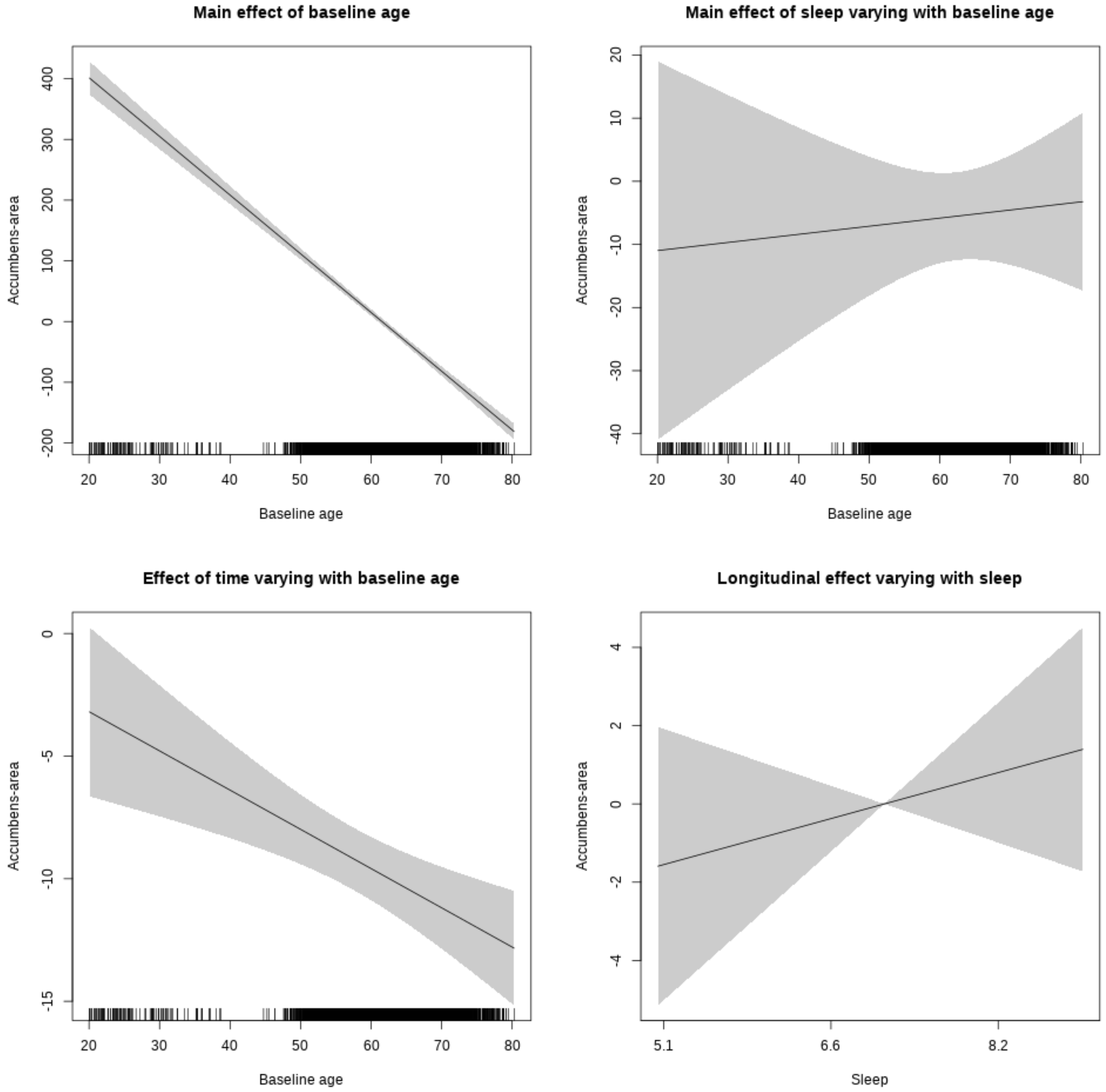

Controling for BMI

Full data Below is the model output. The term `s(sleep_z):time` is probably what we care most about.

```
##
## Family: gaussian
## Link function: identity
##
## Formula:
## value ~ sex + site + bmi + icv + s(bl_age, k = 10, bs = "cr") +
##     s(bl_age, by = sleep_z, bs = "cr") + +s(bl_age, by = time,
##     k = 5, bs = "cr") + s(sleep_z, by = time, k = 5, bs = "cr",
##     pc = 0)
```

```
## <environment: 0x563916cde2d8>
##
## Parametric coefficients:
##                  Estimate Std. Error t value Pr(>|t|)
## (Intercept)     1023.6533    22.6285  45.237  < 2e-16 ***
## sexmale           17.5180     6.2884   2.786 0.005359 **
## siteousPrisma   -153.3027    43.0596  -3.560 0.000374 ***
## siteousSkyra     188.7443    13.4537  14.029  < 2e-16 ***
## siteUCAM        -217.4941    18.2012 -11.949  < 2e-16 ***
## siteUKB         -122.4731    16.5913  -7.382  1.8e-13 ***
## siteUmU          185.1536    20.3144   9.114  < 2e-16 ***
## bmi                0.4684     0.6467   0.724 0.468902
## icv               63.1016     3.1474  20.049  < 2e-16 ***
## ---
## Signif. codes:  0 '***' 0.001 '**' 0.01 '*' 0.05 '.' 0.1 ' ' 1
##
## Approximate significance of smooth terms:
##                       edf Ref.df        F p-value
## s(bl_age)           1.000  1.000 1432.161  <2e-16 ***
## s(bl_age):sleep_z 2.000  2.000    0.003   0.997
## s(bl_age):time    2.000  2.000  137.493  <2e-16 ***
## s(sleep_z):time   2.089  2.089    1.012   0.271
## ---
## Signif. codes:  0 '***' 0.001 '**' 0.01 '*' 0.05 '.' 0.1 ' ' 1
##
## R-sq.(adj) =  0.613
## lmer.REML =  65309  Scale est. = 2929.1    n = 5416
```

Below are plots of a plot of `s(bl_age)`, `s(bl_age):sleep_z`, `s(bl_age):time`, and `s(sleep_z):time`. For `s(sleep_z):time`, roughly, if we cannot fit a straight line through the confidence bands, there is evidence that the effect of time depends on sleep duration. It is correct the that confidence interval is zero at one point along the axis; this happens because we do not include the main effect.

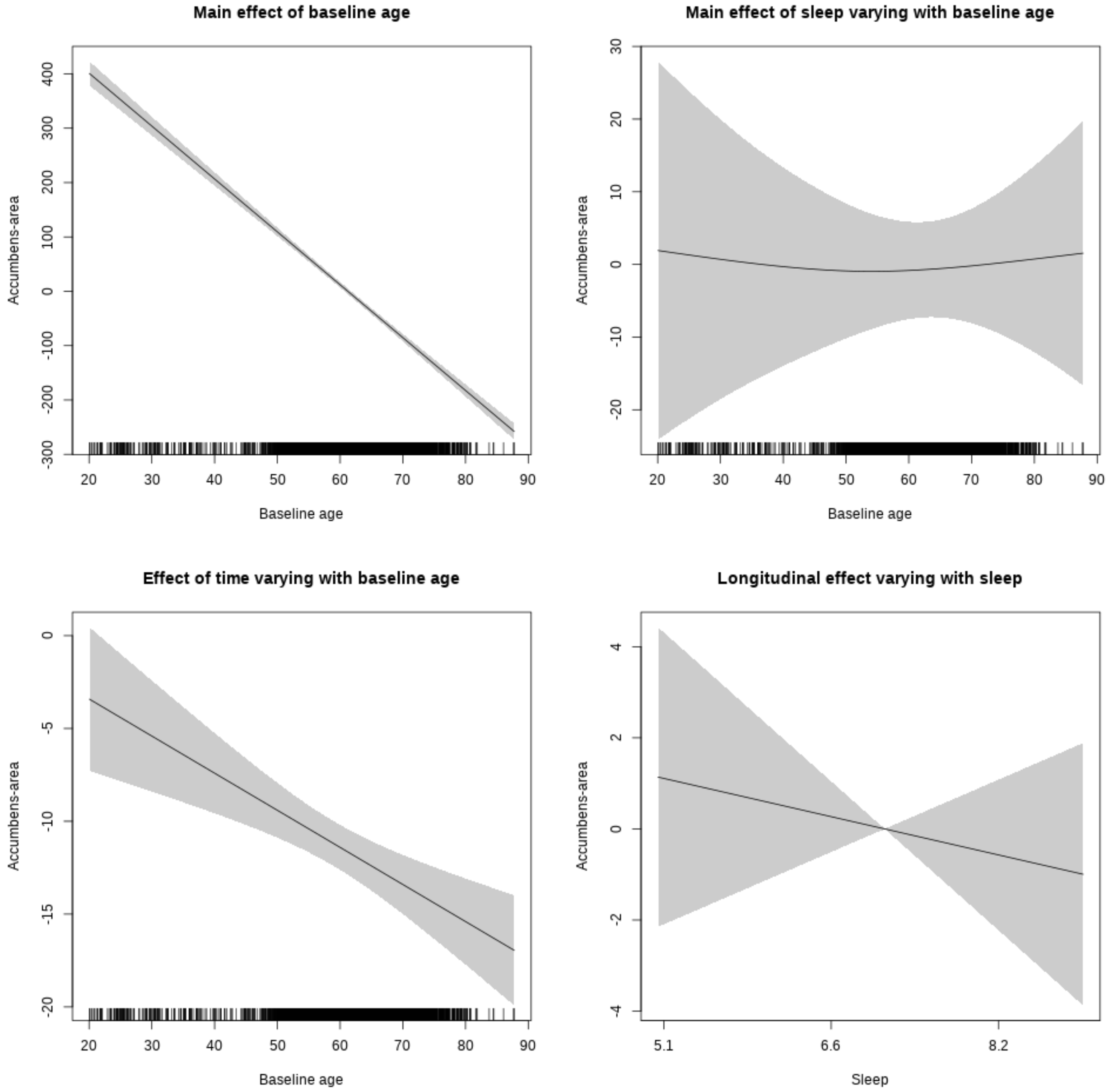

Restricted to sleep >= 5 and <= 9 hours Below is the row for `s(sleep_z):time` from the model output.

```
## s(sleep_z):time   1.000  1.000    0.478   0.489
```

Below are plots of a plot of `s(bl_age)`, `s(bl_age):sleep_z`, `s(bl_age):time`, and `s(sleep_z):time`. For `s(sleep_z):time`, roughly, if we cannot fit a straight line through the confidence bands, there is evidence that the effect of time depends on sleep duration. It is correct the that confidence interval is zero at one point along the axis; this happens because we do not include the main effect.

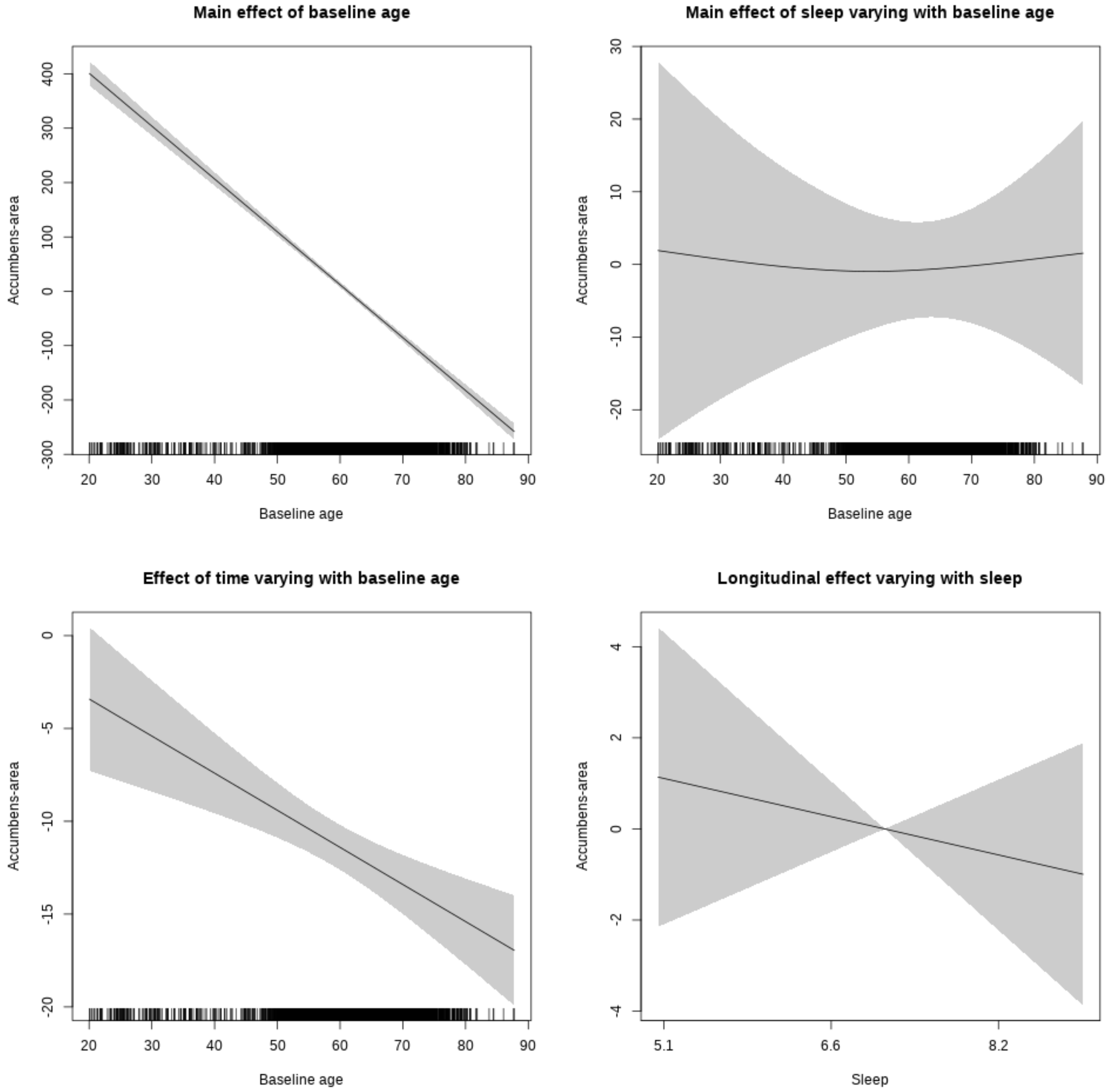

Controling for depression

Full data Below is the model output. The term `s(sleep_z):time` is probably what we care most about.

```
##
## Family: gaussian
## Link function: identity
##
## Formula:
## value ~ sex + site + depression + icv + s(bl_age, k = 10, bs = "cr") +
##     s(bl_age, by = sleep_z, bs = "cr") + +s(bl_age, by = time,
##     k = 5, bs = "cr") + s(sleep_z, by = time, k = 5, bs = "cr",
##     pc = 0)
```

```
## <environment: 0x563910d56fa8>
##
## Parametric coefficients:
##                Estimate Std. Error t value Pr(>|t|)
## (Intercept)     910.565     12.024  75.731  < 2e-16 ***
## sexmale          22.564      6.286   3.590 0.000334 ***
## siteousAvanto   215.784     27.752   7.775 8.88e-15 ***
## siteousPrisma    45.699     55.725   0.820 0.412204
## siteousSkyra    382.181     21.953  17.409  < 2e-16 ***
## siteUCAM        -83.393     14.426  -5.781 7.83e-09 ***
## siteUKB          -6.496     11.030  -0.589 0.555912
## siteUmU         319.973     17.538  18.244  < 2e-16 ***
## depression      -42.505     21.958  -1.936 0.052948 .
## icv              54.438      3.071  17.728  < 2e-16 ***
## ---
## Signif. codes:  0 '***' 0.001 '**' 0.01 '*' 0.05 '.' 0.1 ' ' 1
##
## Approximate significance of smooth terms:
##                      edf Ref.df       F p-value
## s(bl_age)          3.670  3.670 310.427  <2e-16 ***
## s(bl_age):sleep_z 2.000  2.000   0.195   0.823
## s(bl_age):time    2.000  2.000 141.044  <2e-16 ***
## s(sleep_z):time   2.423  2.423   1.941   0.147
## ---
## Signif. codes:  0 '***' 0.001 '**' 0.01 '*' 0.05 '.' 0.1 ' ' 1
##
## R-sq.(adj) =  0.575
## lmer.REML =  68640  Scale est. = 2880      n = 5683
```

Below are plots of a plot of `s(bl_age)`, `s(bl_age):sleep_z`, `s(bl_age):time`, and `s(sleep_z):time`. For `s(sleep_z):time`, roughly, if we cannot fit a straight line through the confidence bands, there is evidence that the effect of time depends on sleep duration. It is correct the that confidence interval is zero at one point along the axis; this happens because we do not include the main effect.

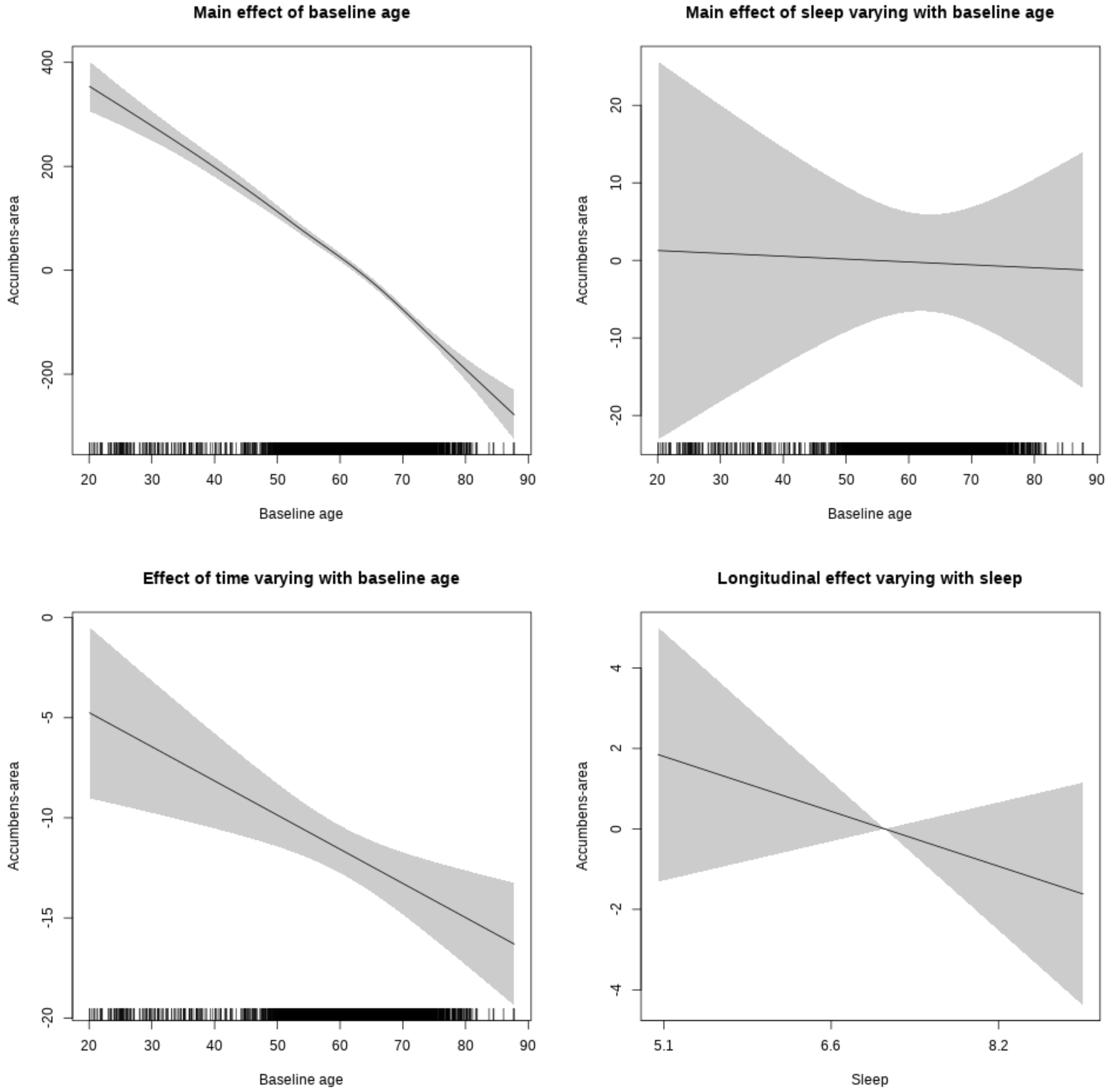

Restricted to sleep >= 5 and <= 9 hours Below is the row for `s(sleep_z):time` from the model output.

```
## s(sleep_z):time   1.000  1.000   1.366   0.242
```

Below are plots of a plot of `s(bl_age)`, `s(bl_age):sleep_z`, `s(bl_age):time`, and `s(sleep_z):time`. For `s(sleep_z):time`, roughly, if we cannot fit a straight line through the confidence bands, there is evidence that the effect of time depends on sleep duration. It is correct the that confidence interval is zero at one point along the axis; this happens because we do not include the main effect.

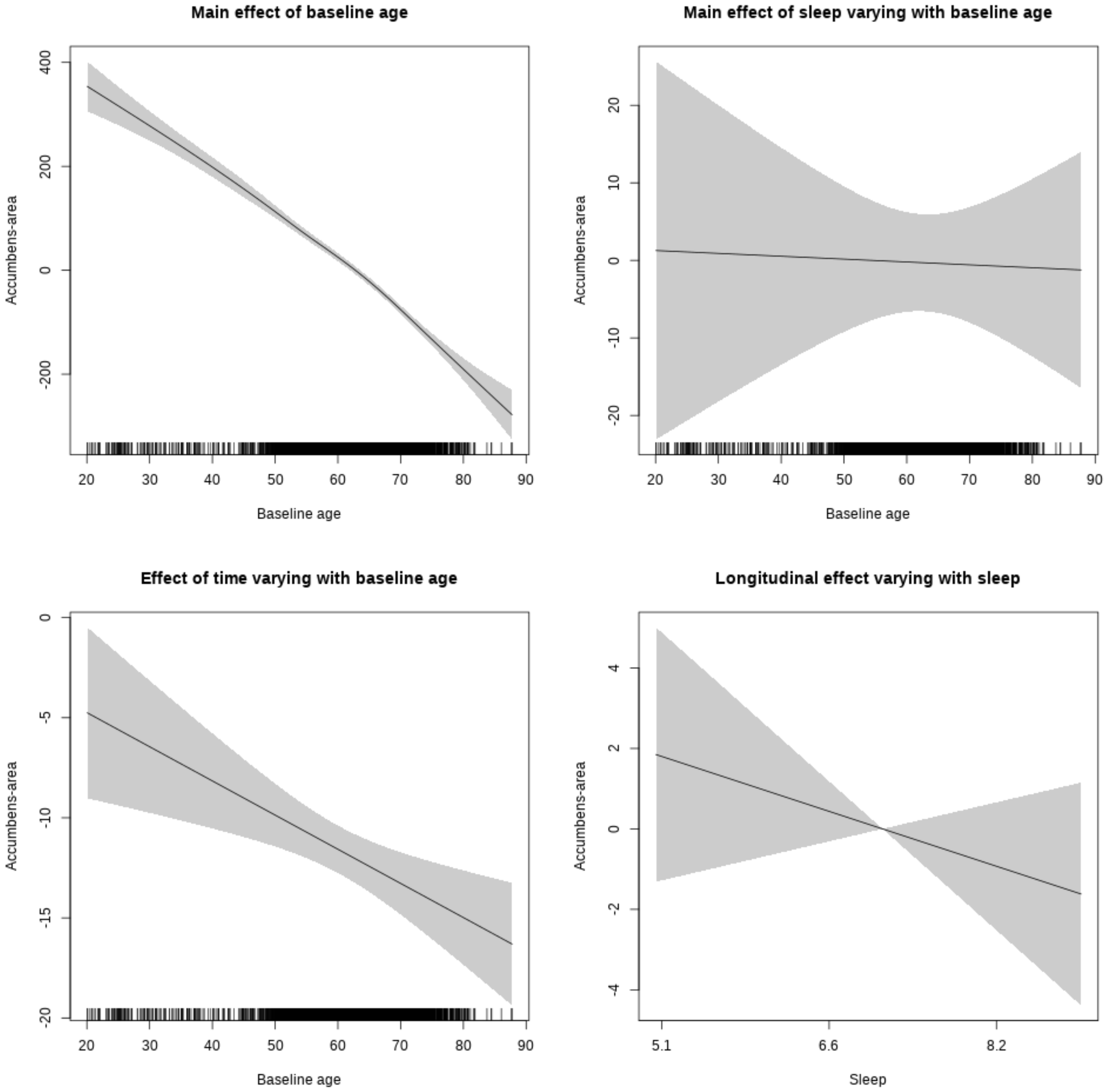

Controling for sleep quality

Full data Below is the model output. The term `s(sleep_z):time` is probably what we care most about.

```
##
## Family: gaussian
## Link function: identity
##
## Formula:
## value ~ X + s(bl_age, k = 10, bs = "cr") + s(bl_age, by = sleep_z,
##     bs = "cr") + s(bl_age, by = time, k = 5, bs = "cr") + s(sleep_z,
##     by = time, k = 5, bs = "cr", pc = 0)
## <environment: 0x56391126a060>
```

```
##
## Parametric coefficients:
##                          Estimate Std. Error t value Pr(>|t|)
## (Intercept)               848.422     19.544  43.411   <2e-16 ***
## Xsexmale                   76.748      7.308  10.502   <2e-16 ***
## XsiteousAvanto            223.256     18.733  11.918   <2e-16 ***
## XsiteousPrisma             63.225     44.850   1.410   0.1587
## XsiteousSkyra             360.755     18.854  19.134   <2e-16 ***
## XsiteUB                    37.339     29.599   1.262   0.2072
## XsiteUCAM                 -16.653     18.955  -0.879   0.3797
## XsiteUKB                   55.497     59.243   0.937   0.3489
## XsiteUmU                  354.424     21.222  16.701   <2e-16 ***
## Xukb_dummy:sleep_accel      8.210      4.596   1.786   0.0741 .
## Xukb_dummy:chronotype      -4.546      6.119  -0.743   0.4576
## Xukb_dummy:dozing           7.403     12.646   0.585   0.5583
## Xukb_dummy:sleeplessness  -13.387      7.858  -1.704   0.0885 .
## Xukb_dummy:snoring        -10.245     11.164  -0.918   0.3589
## Xukb_dummy:gettingup       -3.582      8.523  -0.420   0.6743
## Xnotukb_dummy:PSQI_Global  -4.236      2.411  -1.757   0.0790 .
## ---
## Signif. codes:  0 ‘***’ 0.001 ‘**’ 0.01 ‘*’ 0.05 ‘.’ 0.1 ‘ ’ 1
##
## Approximate significance of smooth terms:
##                     edf Ref.df       F p-value
## s(bl_age)         1.594  1.594 613.737  <2e-16 ***
## s(bl_age):sleep_z 2.000  2.000   1.011  0.3641
## s(bl_age):time    2.000  2.000 191.074  <2e-16 ***
## s(sleep_z):time   1.000  1.000   3.179  0.0747 .
## ---
## Signif. codes:  0 ‘***’ 0.001 ‘**’ 0.01 ‘*’ 0.05 ‘.’ 0.1 ‘ ’ 1
##
## R-sq.(adj) =  0.586
## lmer.REML =  51913  Scale est. = 2896.5    n = 4287
```

Below are plots of a plot of `s(bl_age)`, `s(bl_age):sleep_z`, `s(bl_age):time`, and `s(sleep_z):time`. For `s(sleep_z):time`, roughly, if we cannot fit a straight line through the confidence bands, there is evidence that the effect of time depends on sleep duration. It is correct the that confidence interval is zero at one point along the axis; this happens because we do not include the main effect.

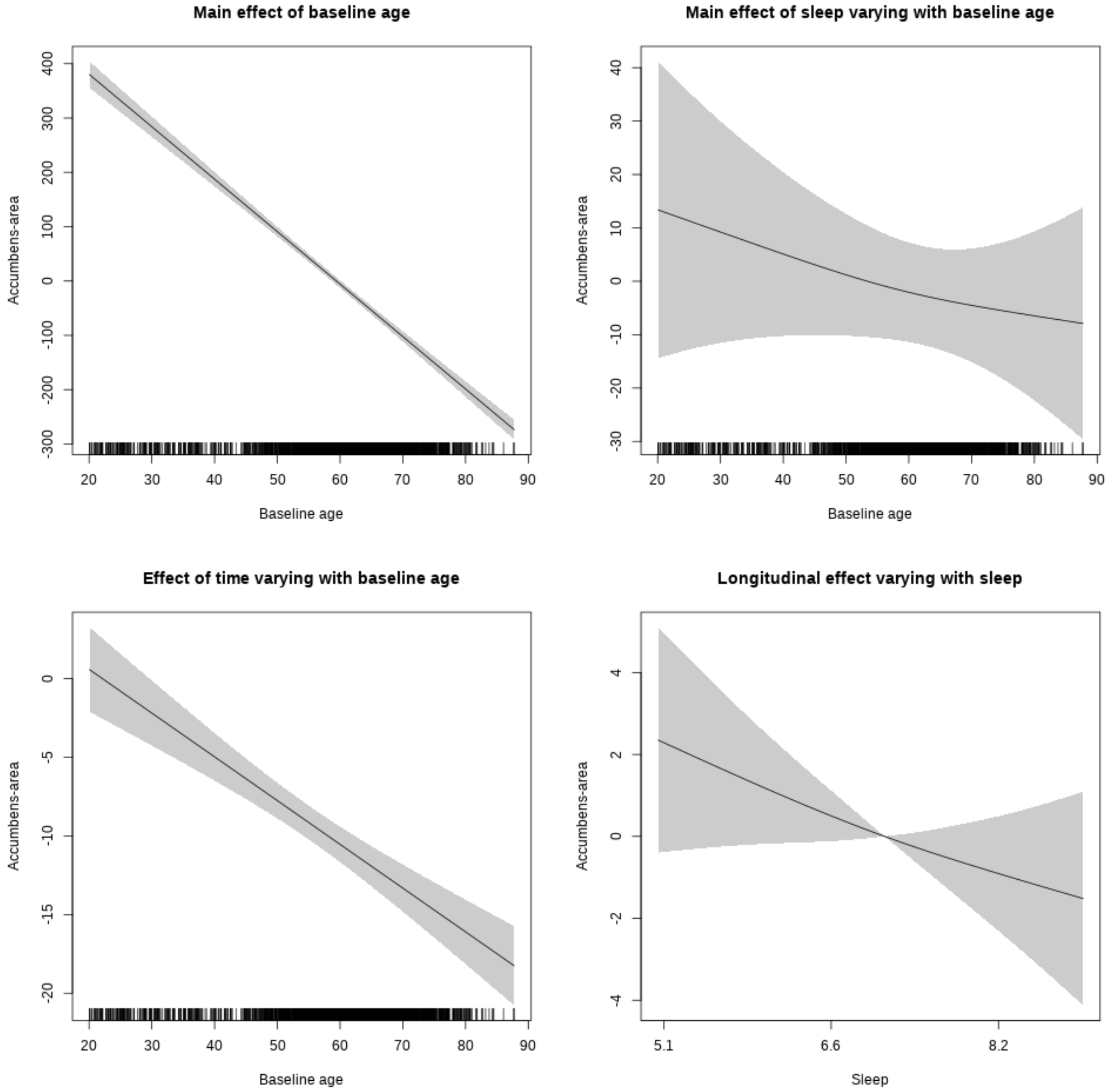

Restricted to sleep >= 5 and <= 9 hours Below is the row for `s(sleep_z):time` from the model output.

```
## s(sleep_z):time   1.120  1.120    2.859   0.104
```

Below are plots of a plot of `s(bl_age)`, `s(bl_age):sleep_z`, `s(bl_age):time`, and `s(sleep_z):time`. For `s(sleep_z):time`, roughly, if we cannot fit a straight line through the confidence bands, there is evidence that the effect of time depends on sleep duration. It is correct the that confidence interval is zero at one point along the axis; this happens because we do not include the main effect.

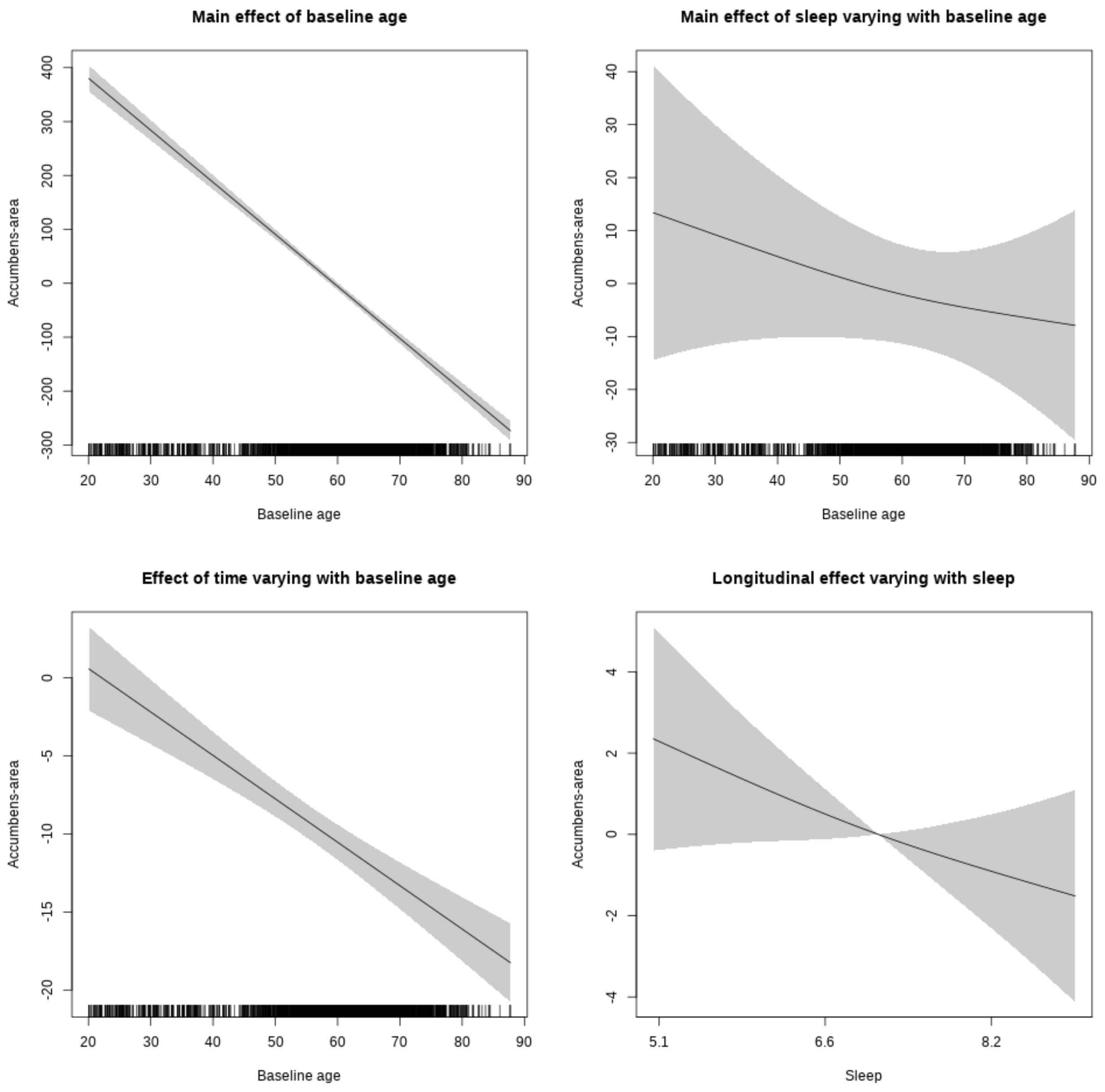

Amygdala

Main analysis

Full data Below is the model output. The term `s(sleep_z):time` is probably what we care most about.

```
##
## Family: gaussian
## Link function: identity
##
## Formula:
## value ~ sex + site + icv + s(bl_age, k = 10, bs = "cr") + s(bl_age,
##     by = sleep_z, bs = "cr") + +s(bl_age, by = time, k = 5, bs = "cr") +
```

```
##     s(sleep_z, by = time, k = 5, bs = "cr", pc = 0)
## <environment: 0x563917dfbe60>
##
## Parametric coefficients:
##               Estimate Std. Error t value Pr(>|t|)
## (Intercept)   3397.660     24.121 140.857  < 2e-16 ***
## sexmale        133.497     12.419  10.750  < 2e-16 ***
## siteousAvanto -464.628     29.667 -15.661  < 2e-16 ***
## siteousPrisma -346.387     87.177  -3.973 7.15e-05 ***
## siteousSkyra   117.770     28.837   4.084 4.47e-05 ***
## siteUB        -241.039     55.553  -4.339 1.45e-05 ***
## siteUCAM      -235.264     30.127  -7.809 6.48e-15 ***
## siteUKB       -126.316     22.973  -5.498 3.95e-08 ***
## siteUmU       -171.752     34.558  -4.970 6.83e-07 ***
## icv            197.508      6.015  32.838  < 2e-16 ***
## ---
## Signif. codes:  0 '***' 0.001 '**' 0.01 '*' 0.05 '.' 0.1 ' ' 1
##
## Approximate significance of smooth terms:
##                     edf Ref.df       F p-value
## s(bl_age)         4.015  4.015 250.654  <2e-16 ***
## s(bl_age):sleep_z 2.000  2.000   0.836  0.4335
## s(bl_age):time    3.647  3.647 112.471  <2e-16 ***
## s(sleep_z):time   1.000  1.000   4.151  0.0416 *
## ---
## Signif. codes:  0 '***' 0.001 '**' 0.01 '*' 0.05 '.' 0.1 ' ' 1
##
## R-sq.(adj) =  0.494
## lmer.REML = 1.1069e+05  Scale est. = 11576     n = 8151
```

Below are plots of a plot of `s(bl_age)`, `s(bl_age):sleep_z`, `s(bl_age):time`, and `s(sleep_z):time`. For `s(sleep_z):time`, roughly, if we cannot fit a straight line through the confidence bands, there is evidence that the effect of time depends on sleep duration. It is correct the that confidence interval is zero at one point along the axis; this happens because we do not include the main effect.

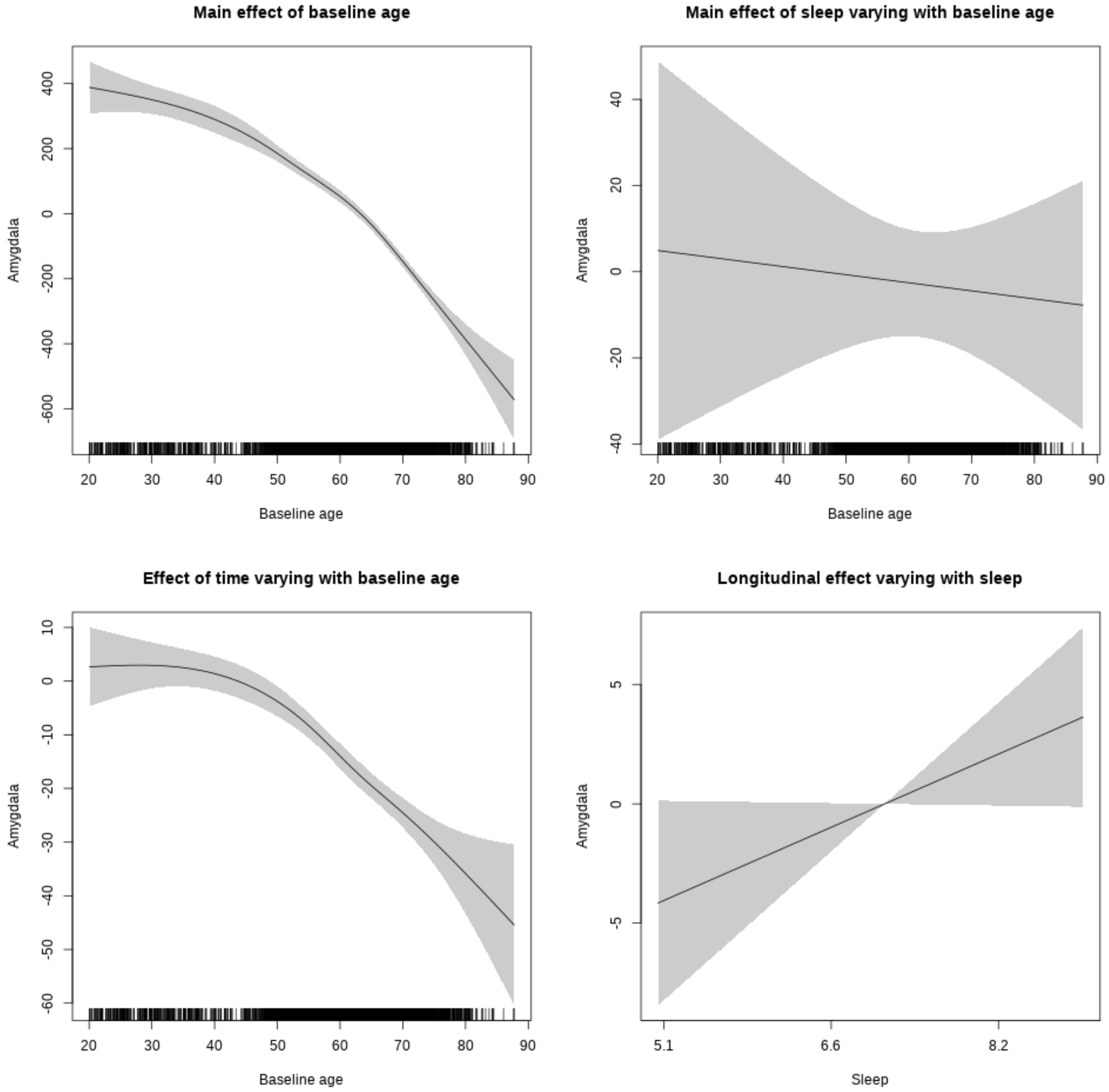

Restricted to sleep >= 5 and <= 9 hours Below is the row for `s(sleep_z):time` from the model output.

```
## s(sleep_z):time   1.000  1.000   3.745   0.053 .
```

Below are plots of a plot of `s(bl_age)`, `s(bl_age):sleep_z`, `s(bl_age):time`, and `s(sleep_z):time`. For `s(sleep_z):time`, roughly, if we cannot fit a straight line through the confidence bands, there is evidence that the effect of time depends on sleep duration. It is correct the that confidence interval is zero at one point along the axis; this happens because we do not include the main effect.

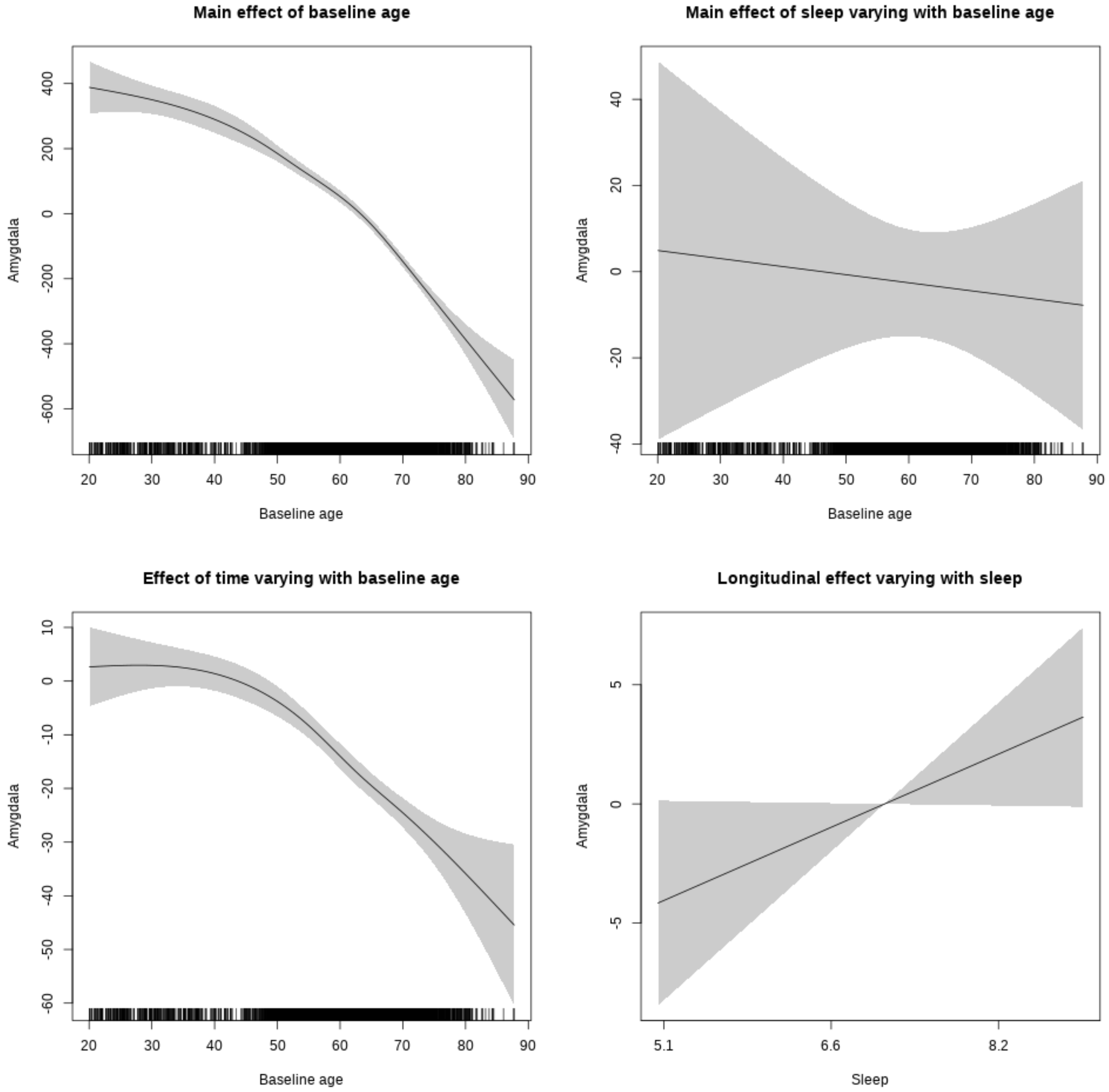

Controling for socioeconomic status

Full data Below is the model output. The term `s(sleep_z):time` is probably what we care most about.

```
##
## Family: gaussian
## Link function: identity
##
## Formula:
## value ~ sex + site + income_scaled + education_scaled + icv +
##     s(bl_age, k = 10, bs = "cr") + s(bl_age, by = sleep_z, bs = "cr") +
##     +s(bl_age, by = time, k = 5, bs = "cr") + s(sleep_z, by = time,
##     k = 5, bs = "cr", pc = 0)
```

```
## <environment: 0x563911093fb8>
##
## Parametric coefficients:
##                  Estimate Std. Error t value Pr(>|t|)
## (Intercept)      3443.033     58.769  58.585  < 2e-16 ***
## sexmale           128.538     16.183   7.943 2.47e-15 ***
## siteousAvanto    -469.250     65.240  -7.193 7.37e-13 ***
## siteousPrisma    -183.765    146.483  -1.255  0.20972
## siteousSkyra      134.123     60.032   2.234  0.02552 *
## siteUKB          -160.793     55.595  -2.892  0.00384 **
## income_scaled      -9.097     20.659  -0.440  0.65970
## education_scaled  -16.944     22.856  -0.741  0.45853
## icv               224.618      8.055  27.886  < 2e-16 ***
## ---
## Signif. codes:  0 '***' 0.001 '**' 0.01 '*' 0.05 '.' 0.1 ' ' 1
##
## Approximate significance of smooth terms:
##                     edf Ref.df       F p-value
## s(bl_age)         3.454  3.454 116.883  <2e-16 ***
## s(bl_age):sleep_z 5.428  5.428   1.364   0.147
## s(bl_age):time    2.359  2.359  51.834  <2e-16 ***
## s(sleep_z):time   2.657  2.657   1.840   0.104
## ---
## Signif. codes:  0 '***' 0.001 '**' 0.01 '*' 0.05 '.' 0.1 ' ' 1
##
## R-sq.(adj) =  0.489
## lmer.REML =  63373  Scale est. = 11975     n = 4652
```

Below are plots of a plot of `s(bl_age)`, `s(bl_age):sleep_z`, `s(bl_age):time`, and `s(sleep_z):time`. For `s(sleep_z):time`, roughly, if we cannot fit a straight line through the confidence bands, there is evidence that the effect of time depends on sleep duration. It is correct the that confidence interval is zero at one point along the axis; this happens because we do not include the main effect.

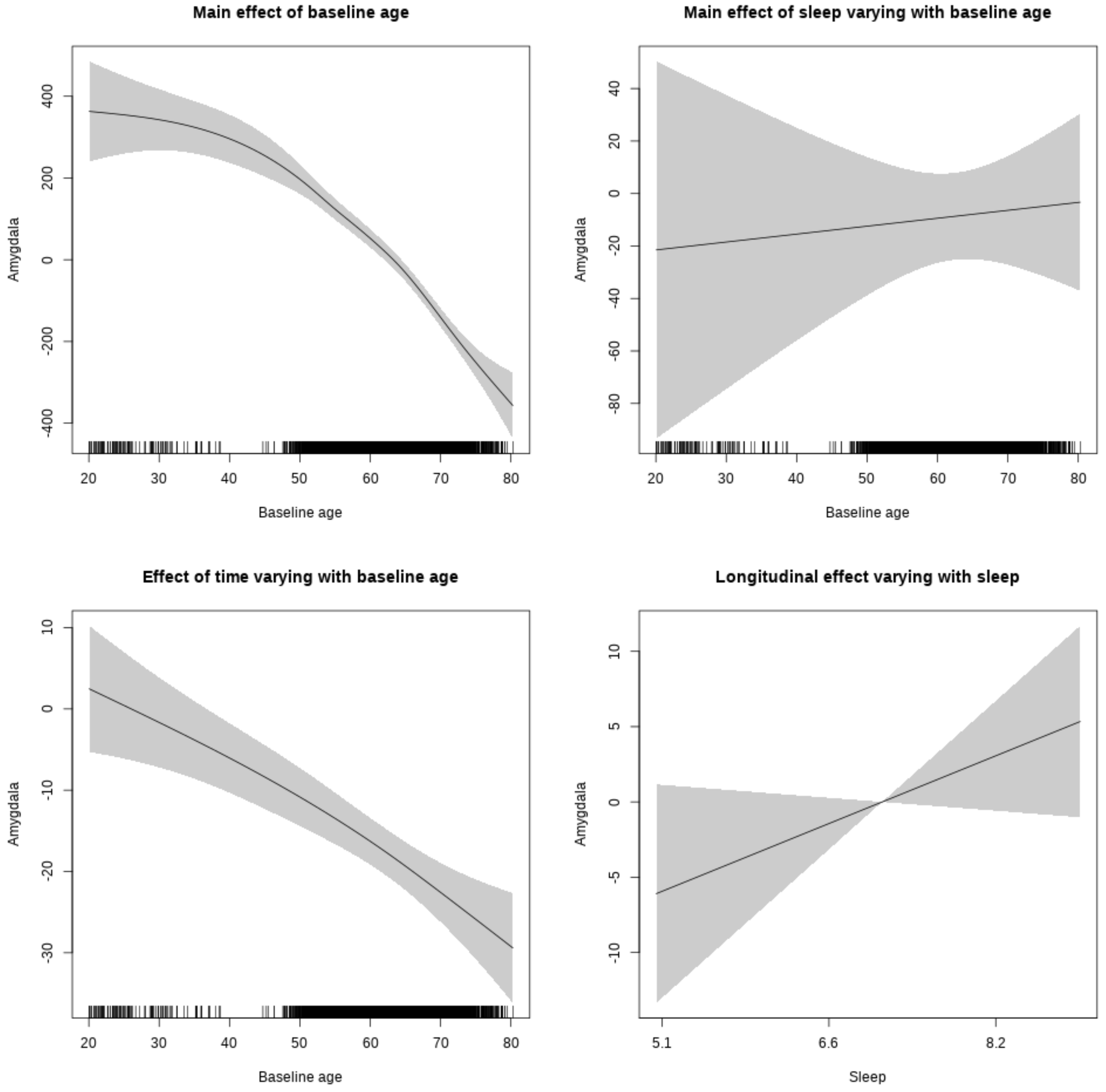

Restricted to sleep >= 5 and <= 9 hours Below is the row for `s(sleep_z):time` from the model output.

```
## s(sleep_z):time   1.000  1.000   2.819  0.0932 .
```

Below are plots of a plot of `s(bl_age)`, `s(bl_age):sleep_z`, `s(bl_age):time`, and `s(sleep_z):time`. For `s(sleep_z):time`, roughly, if we cannot fit a straight line through the confidence bands, there is evidence that the effect of time depends on sleep duration. It is correct the that confidence interval is zero at one point along the axis; this happens because we do not include the main effect.

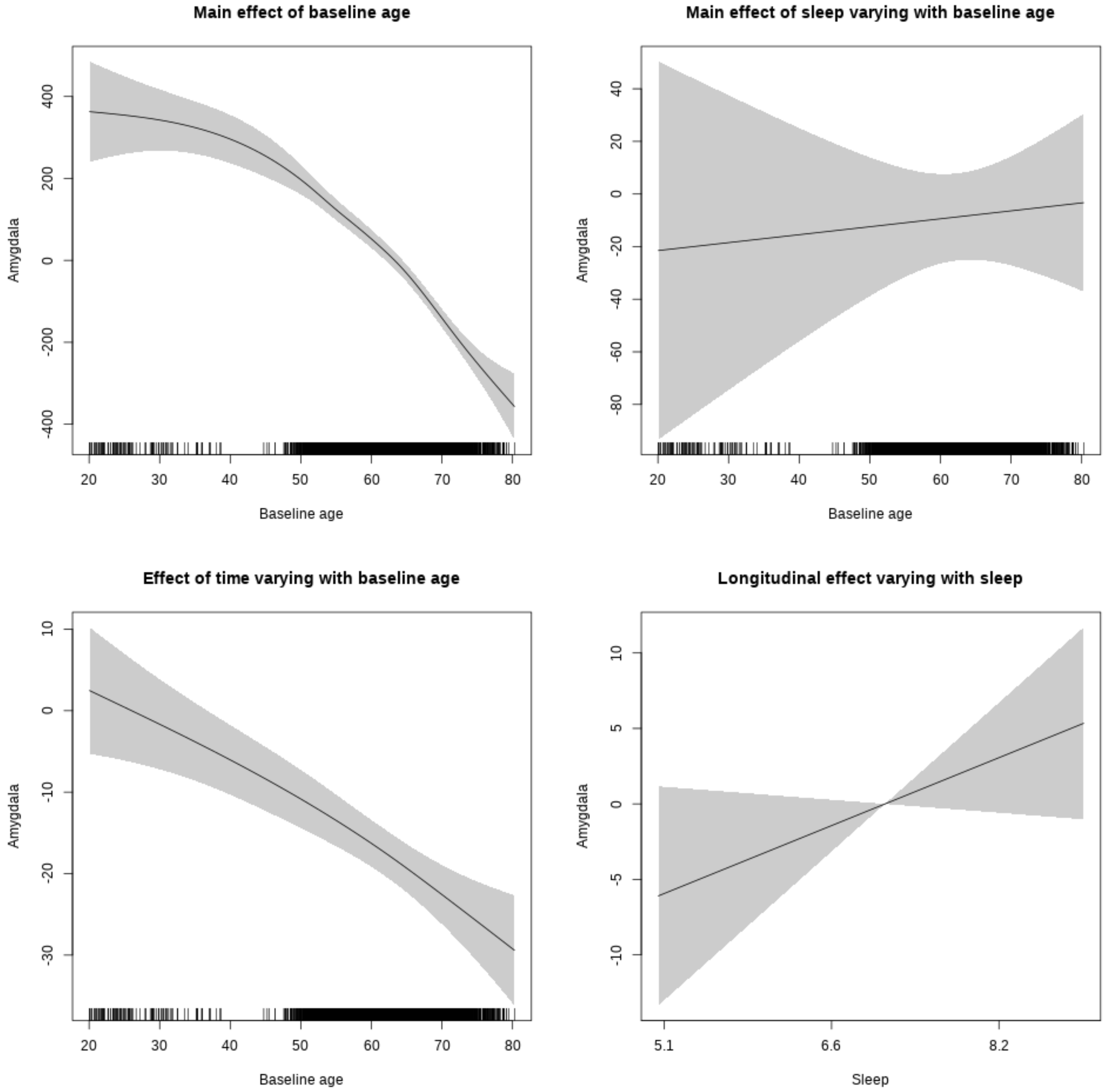

Controling for BMI

Full data Below is the model output. The term `s(sleep_z):time` is probably what we care most about.

```
##
## Family: gaussian
## Link function: identity
##
## Formula:
## value ~ sex + site + bmi + icv + s(bl_age, k = 10, bs = "cr") +
##     s(bl_age, by = sleep_z, bs = "cr") + +s(bl_age, by = time,
##     k = 5, bs = "cr") + s(sleep_z, by = time, k = 5, bs = "cr",
##     pc = 0)
```

```
## <environment: 0x5639154898e8>
##
## Parametric coefficients:
##                Estimate Std. Error t value Pr(>|t|)
## (Intercept)    2889.729     53.352  54.164  < 2e-16 ***
## sexmale         127.089     14.813   8.580  < 2e-16 ***
## siteousPrisma   130.493     85.097   1.533   0.1252
## siteousSkyra    606.348     26.867  22.569  < 2e-16 ***
## siteUCAM        201.688     41.169   4.899 9.92e-07 ***
## siteUKB         293.716     38.610   7.607 3.28e-14 ***
## siteUmU         288.470     46.835   6.159 7.83e-10 ***
## bmi               3.067      1.530   2.004   0.0451 *
## icv             217.316      7.349  29.571  < 2e-16 ***
## ---
## Signif. codes:  0 '***' 0.001 '**' 0.01 '*' 0.05 '.' 0.1 ' ' 1
##
## Approximate significance of smooth terms:
##                      edf Ref.df       F p-value
## s(bl_age)          3.532  3.532 172.542 < 2e-16 ***
## s(bl_age):sleep_z 2.000  2.000   0.040 0.96052
## s(bl_age):time    2.303  2.303  54.497 < 2e-16 ***
## s(sleep_z):time   3.078  3.078   4.501 0.00348 **
## ---
## Signif. codes:  0 '***' 0.001 '**' 0.01 '*' 0.05 '.' 0.1 ' ' 1
##
## R-sq.(adj) =  0.492
## lmer.REML =  73562  Scale est. = 11191     n = 5414
```

Below are plots of a plot of `s(bl_age)`, `s(bl_age):sleep_z`, `s(bl_age):time`, and `s(sleep_z):time`. For `s(sleep_z):time`, roughly, if we cannot fit a straight line through the confidence bands, there is evidence that the effect of time depends on sleep duration. It is correct the that confidence interval is zero at one point along the axis; this happens because we do not include the main effect.

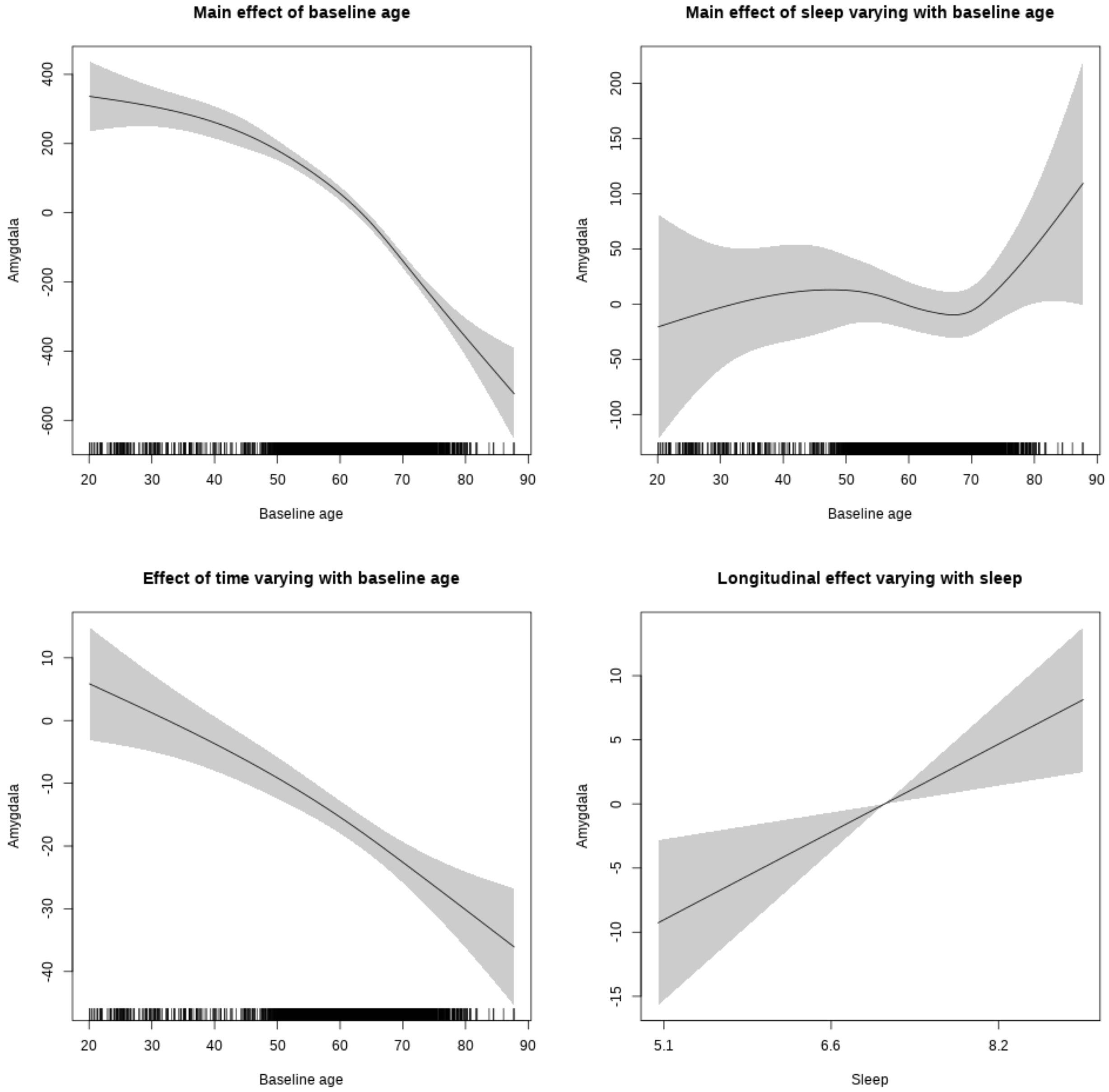

Restricted to sleep >= 5 and <= 9 hours Below is the row for `s(sleep_z):time` from the model output.

```
## s(sleep_z):time   1.000  1.000   8.286 0.00401 **
```

Below are plots of a plot of `s(bl_age)`, `s(bl_age):sleep_z`, `s(bl_age):time`, and `s(sleep_z):time`. For `s(sleep_z):time`, roughly, if we cannot fit a straight line through the confidence bands, there is evidence that the effect of time depends on sleep duration. It is correct the that confidence interval is zero at one point along the axis; this happens because we do not include the main effect.

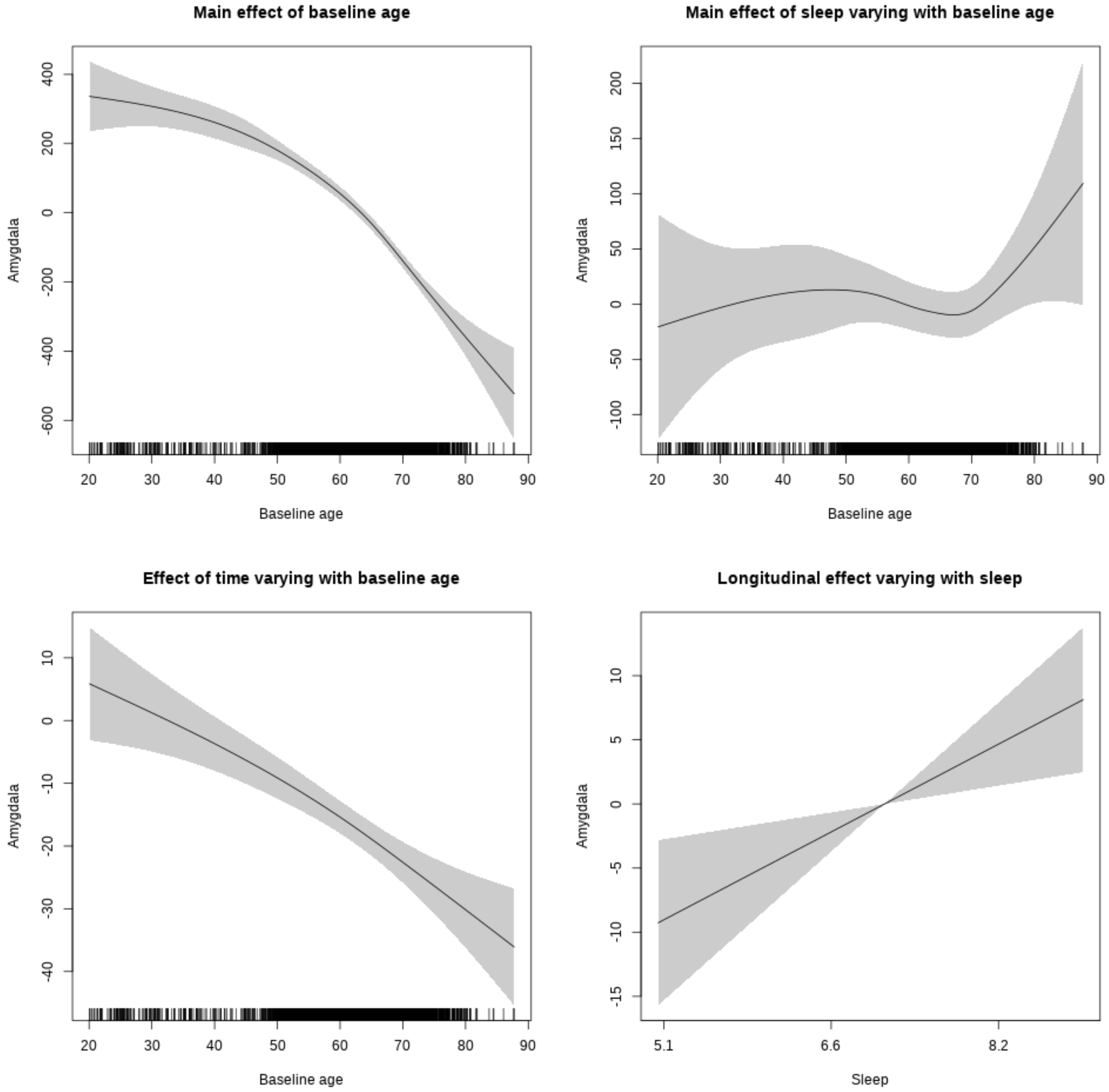

Controling for depression

Full data Below is the model output. The term `s(sleep_z):time` is probably what we care most about.

```
##
## Family: gaussian
## Link function: identity
##
## Formula:
## value ~ sex + site + depression + icv + s(bl_age, k = 10, bs = "cr") +
##     s(bl_age, by = sleep_z, bs = "cr") + +s(bl_age, by = time,
##     k = 5, bs = "cr") + s(sleep_z, by = time, k = 5, bs = "cr",
##     pc = 0)
```

```
## <environment: 0x56390ec80b80>
##
## Parametric coefficients:
##                Estimate Std. Error t value Pr(>|t|)
## (Intercept)    3390.991     28.548 118.783  < 2e-16 ***
## sexmale         126.203     14.925   8.456  < 2e-16 ***
## siteousAvanto -431.053     62.185  -6.932 4.62e-12 ***
## siteousPrisma -367.633    113.563  -3.237  0.00121 **
## siteousSkyra   227.021     52.127   4.355 1.35e-05 ***
## siteUCAM      -213.701     34.327  -6.225 5.15e-10 ***
## siteUKB       -130.707     26.226  -4.984 6.42e-07 ***
## siteUmU        -99.948     41.779  -2.392  0.01677 *
## depression     -97.924     52.322  -1.872  0.06132 .
## icv            199.938      7.229  27.659  < 2e-16 ***
## ---
## Signif. codes:  0 '***' 0.001 '**' 0.01 '*' 0.05 '.' 0.1 ' ' 1
##
## Approximate significance of smooth terms:
##                       edf Ref.df       F p-value
## s(bl_age)           3.639  3.639 151.891  <2e-16 ***
## s(bl_age):sleep_z 2.000  2.000   0.684  0.5046
## s(bl_age):time    2.333  2.333 101.515  <2e-16 ***
## s(sleep_z):time   1.000  1.000   3.830  0.0504 .
## ---
## Signif. codes:  0 '***' 0.001 '**' 0.01 '*' 0.05 '.' 0.1 ' ' 1
##
## R-sq.(adj) =   0.46
## lmer.REML =  77317  Scale est. = 10874     n = 5681
```

Below are plots of a plot of `s(bl_age)`, `s(bl_age):sleep_z`, `s(bl_age):time`, and `s(sleep_z):time`. For `s(sleep_z):time`, roughly, if we cannot fit a straight line through the confidence bands, there is evidence that the effect of time depends on sleep duration. It is correct the that confidence interval is zero at one point along the axis; this happens because we do not include the main effect.

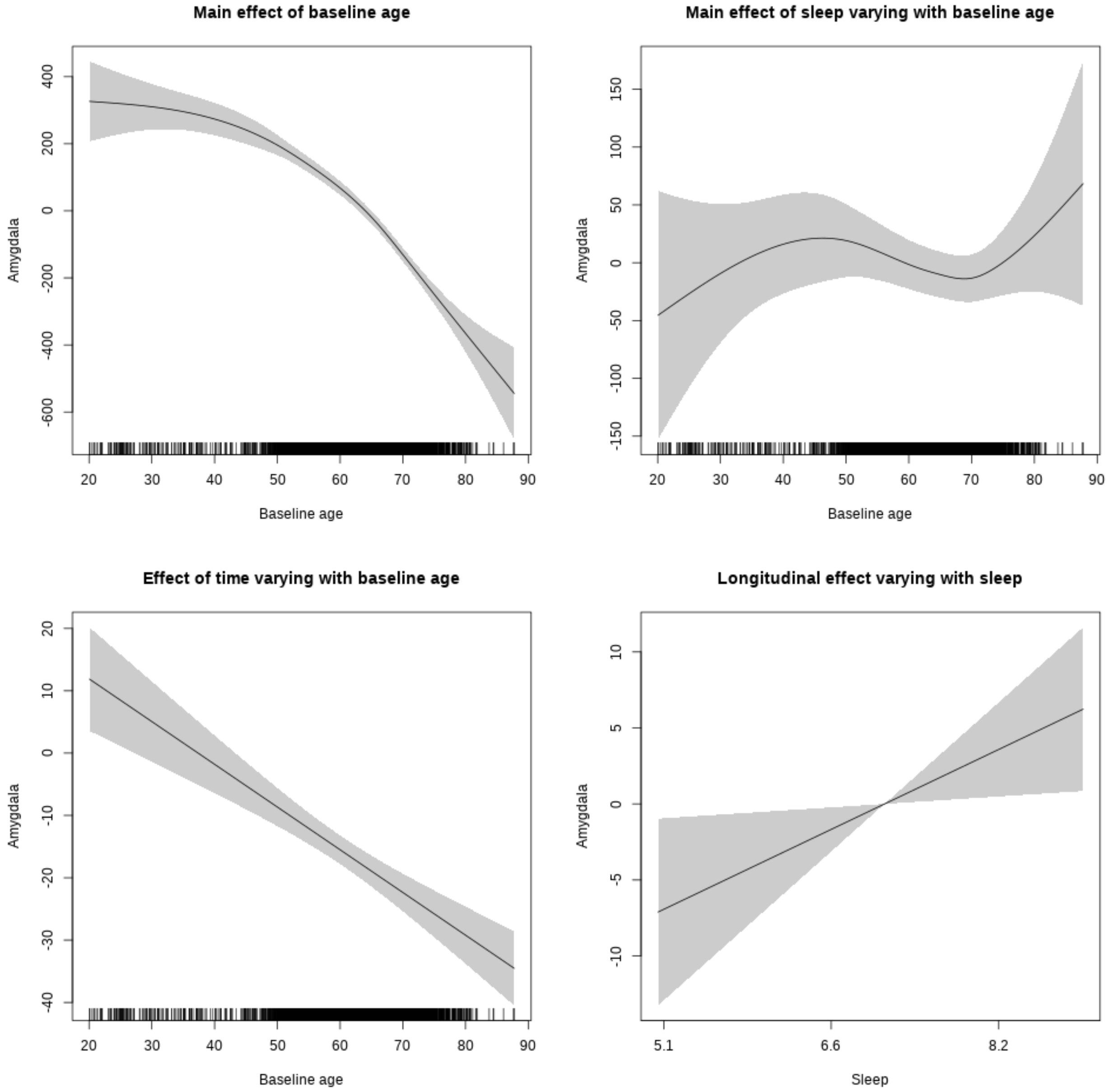

Restricted to sleep >= 5 and <= 9 hours Below is the row for `s(sleep_z):time` from the model output.

```
## s(sleep_z):time   1.000  1.000   5.361  0.0206 *
```

Below are plots of a plot of `s(bl_age)`, `s(bl_age):sleep_z`, `s(bl_age):time`, and `s(sleep_z):time`. For `s(sleep_z):time`, roughly, if we cannot fit a straight line through the confidence bands, there is evidence that the effect of time depends on sleep duration. It is correct the that confidence interval is zero at one point along the axis; this happens because we do not include the main effect.

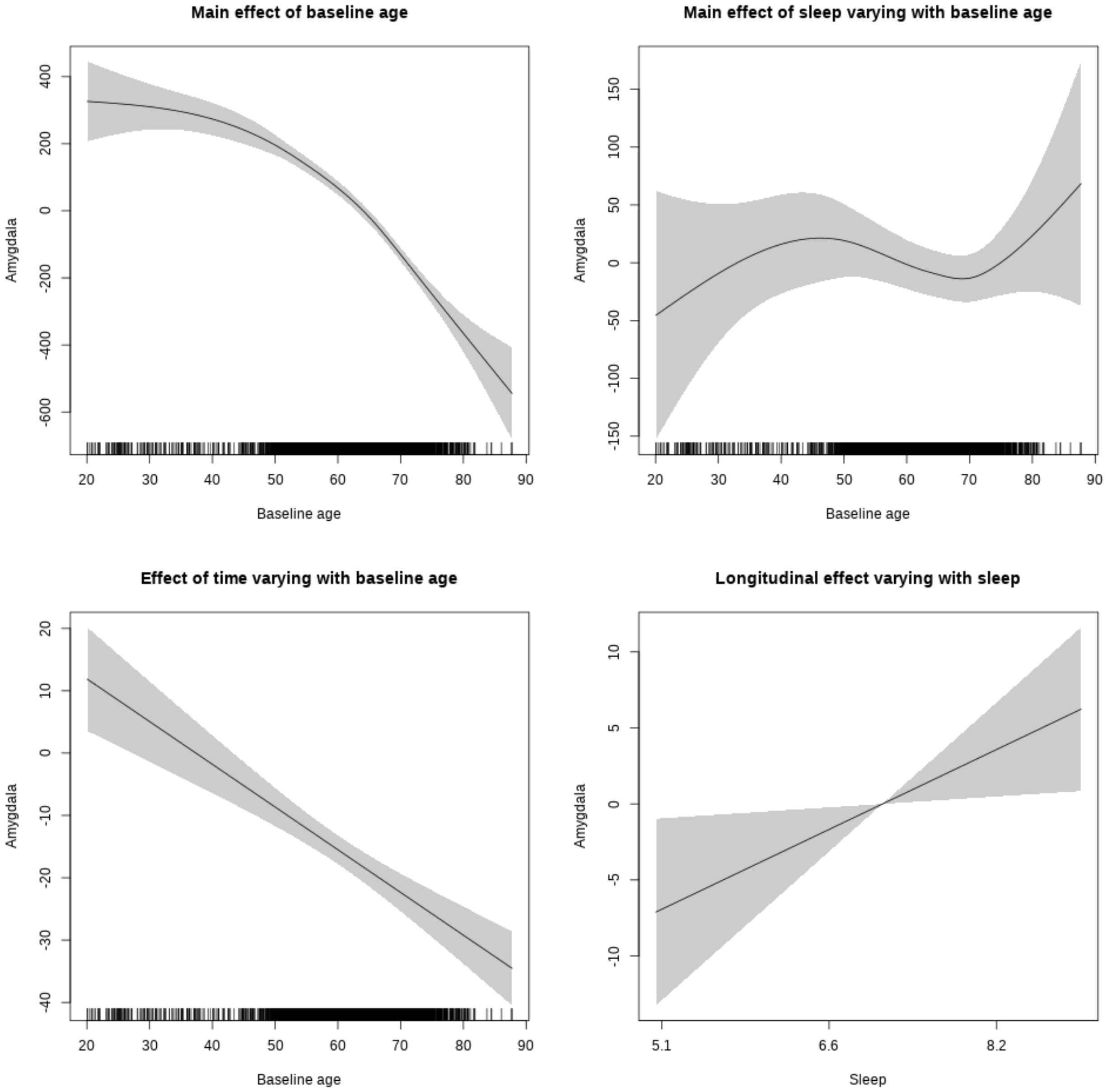

Controling for sleep quality

Full data Below is the model output. The term `s(sleep_z):time` is probably what we care most about.

```
##
## Family: gaussian
## Link function: identity
##
## Formula:
## value ~ X + s(bl_age, k = 10, bs = "cr") + s(bl_age, by = sleep_z,
##     bs = "cr") + s(bl_age, by = time, k = 5, bs = "cr") + s(sleep_z,
##     by = time, k = 5, bs = "cr", pc = 0)
## <environment: 0x563910fc7f88>
```

```
##
## Parametric coefficients:
##                            Estimate Std. Error t value Pr(>|t|)
## (Intercept)                2965.788     46.349  63.989  < 2e-16 ***
## Xsexmale                    352.404     17.334  20.330  < 2e-16 ***
## XsiteousAvanto              -20.540     44.344  -0.463  0.64324
## XsiteousPrisma              176.043     91.136   1.932  0.05347 .
## XsiteousSkyra               481.125     44.534  10.804  < 2e-16 ***
## XsiteUB                      41.765     70.331   0.594  0.55265
## XsiteUCAM                   136.176     45.382   3.001  0.00271 **
## XsiteUKB                    367.194    141.125   2.602  0.00930 **
## XsiteUmU                    119.840     50.481   2.374  0.01764 *
## Xukb_dummy:sleep_accel        9.487     10.902   0.870  0.38423
## Xukb_dummy:chronotype        -7.087     14.516  -0.488  0.62540
## Xukb_dummy:dozing            44.390     29.997   1.480  0.13900
## Xukb_dummy:sleeplessness    -37.512     18.690  -2.007  0.04481 *
## Xukb_dummy:snoring          -17.975     26.476  -0.679  0.49723
## Xukb_dummy:gettingup        -27.031     20.313  -1.331  0.18335
## Xnotukb_dummy:PSQI_Global    -1.912      5.736  -0.333  0.73894
## ---
## Signif. codes:  0 ‘***’ 0.001 ‘**’ 0.01 ‘*’ 0.05 ‘.’ 0.1 ‘ ’ 1
##
## Approximate significance of smooth terms:
##                      edf Ref.df       F p-value
## s(bl_age)          3.269  3.269 140.995  <2e-16 ***
## s(bl_age):sleep_z 2.000  2.000   0.877   0.416
## s(bl_age):time    4.112  4.112  69.529  <2e-16 ***
## s(sleep_z):time   1.000  1.000   0.831   0.362
## ---
## Signif. codes:  0 ‘***’ 0.001 ‘**’ 0.01 ‘*’ 0.05 ‘.’ 0.1 ‘ ’ 1
##
## R-sq.(adj) =  0.385
## lmer.REML =  58397  Scale est. = 10884     n = 4287
```

Below are plots of a plot of `s(bl_age)`, `s(bl_age):sleep_z`, `s(bl_age):time`, and `s(sleep_z):time`. For `s(sleep_z):time`, roughly, if we cannot fit a straight line through the confidence bands, there is evidence that the effect of time depends on sleep duration. It is correct the that confidence interval is zero at one point along the axis; this happens because we do not include the main effect.

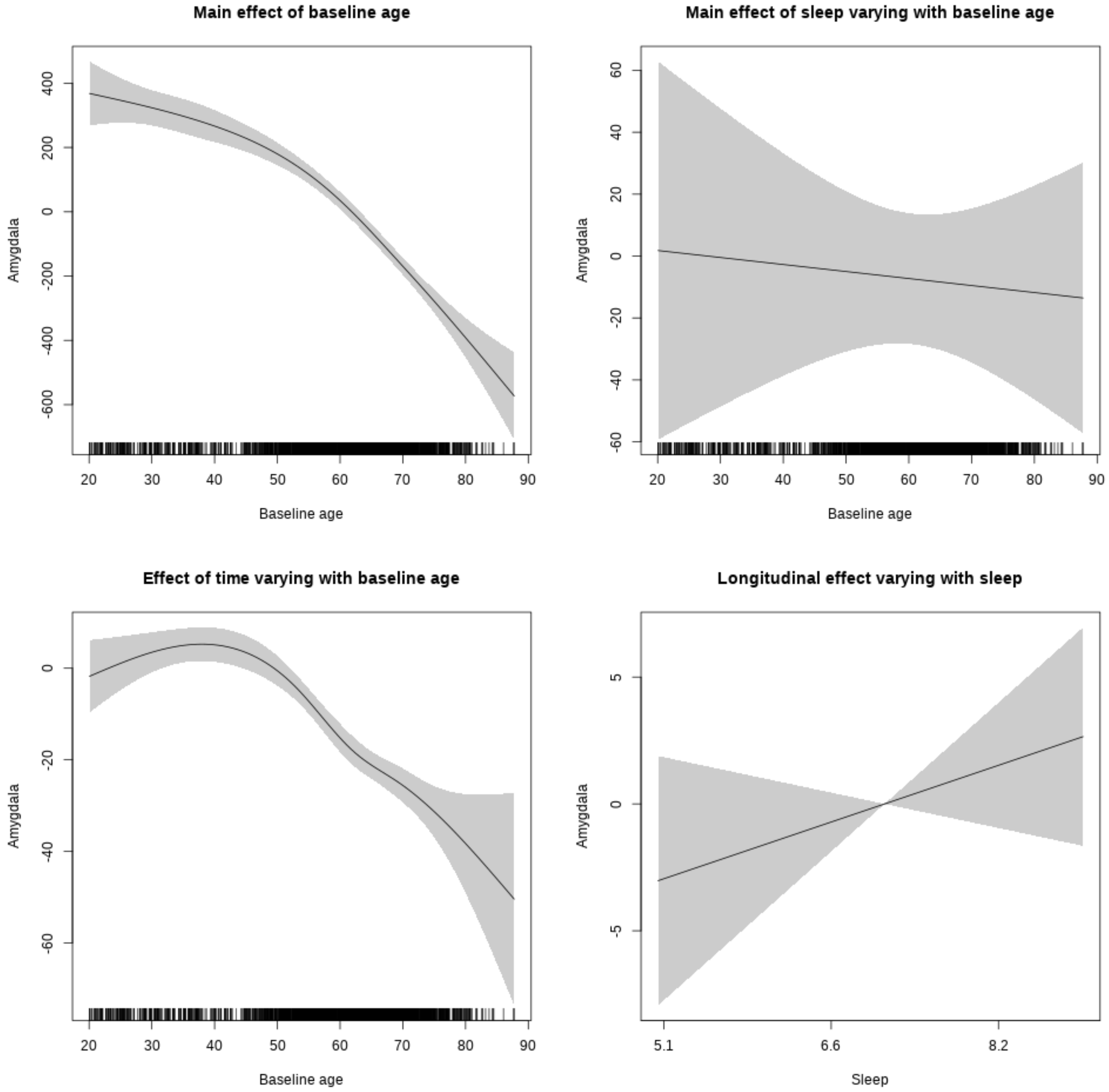

Restricted to sleep >= 5 and <= 9 hours Below is the row for `s(sleep_z):time` from the model output.

```
## s(sleep_z):time   1.000  1.000   1.516   0.218
```

Below are plots of a plot of `s(bl_age)`, `s(bl_age):sleep_z`, `s(bl_age):time`, and `s(sleep_z):time`. For `s(sleep_z):time`, roughly, if we cannot fit a straight line through the confidence bands, there is evidence that the effect of time depends on sleep duration. It is correct the that confidence interval is zero at one point along the axis; this happens because we do not include the main effect.

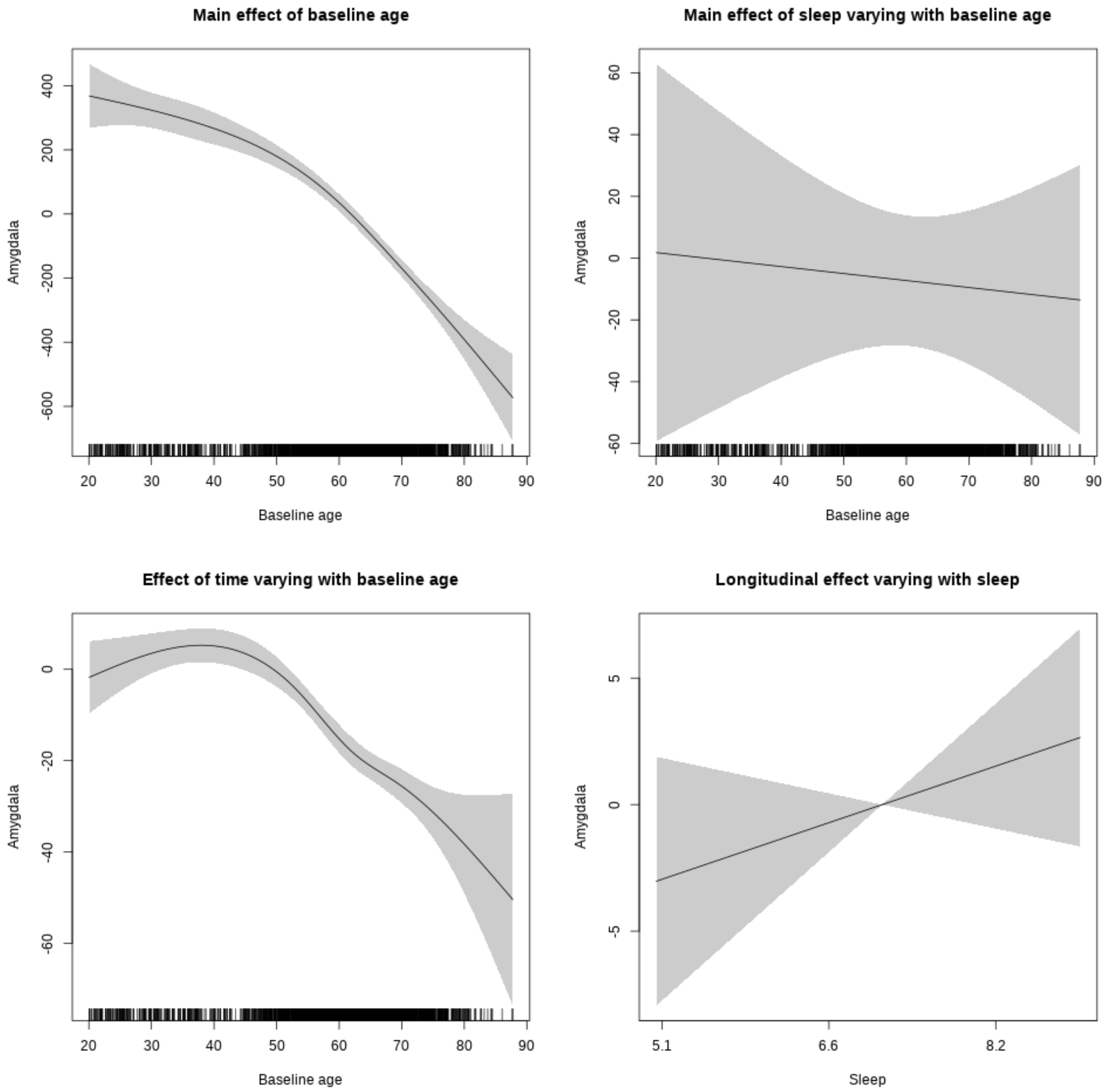

Brain-Stem

Main analysis

Full data Below is the model output. The term `s(sleep_z):time` is probably what we care most about.

```
##
## Family: gaussian
## Link function: identity
##
## Formula:
## value ~ sex + site + icv + s(bl_age, k = 10, bs = "cr") + s(bl_age,
##     by = sleep_z, bs = "cr") + +s(bl_age, by = time, k = 5, bs = "cr") +
```

```
##     s(sleep_z, by = time, k = 5, bs = "cr", pc = 0)
## <environment: 0x5639116bca58>
##
## Parametric coefficients:
##                Estimate Std. Error t value Pr(>|t|)
## (Intercept)   23182.25     131.24 176.643  < 2e-16 ***
## sexmale         806.65      67.38  11.971  < 2e-16 ***
## siteousAvanto -2480.44     161.10 -15.397  < 2e-16 ***
## siteousPrisma -3068.28     294.53 -10.418  < 2e-16 ***
## siteousSkyra  -1801.27     157.24 -11.456  < 2e-16 ***
## siteUB        -2539.85     310.26  -8.186 3.11e-16 ***
## siteUCAM      -2668.75     167.22 -15.960  < 2e-16 ***
## siteUKB       -1520.46     126.37 -12.032  < 2e-16 ***
## siteUmU       -3606.40     191.70 -18.812  < 2e-16 ***
## icv            1370.90      30.66  44.706  < 2e-16 ***
## ---
## Signif. codes:  0 '***' 0.001 '**' 0.01 '*' 0.05 '.' 0.1 ' ' 1
##
## Approximate significance of smooth terms:
##                      edf Ref.df      F p-value
## s(bl_age)          5.538  5.538 16.738  <2e-16 ***
## s(bl_age):sleep_z 2.000  2.000  2.081  0.1249
## s(bl_age):time    3.339  3.339 81.431  <2e-16 ***
## s(sleep_z):time   2.269  2.269  4.223  0.0122 *
## ---
## Signif. codes:  0 '***' 0.001 '**' 0.01 '*' 0.05 '.' 0.1 ' ' 1
##
## R-sq.(adj) =  0.496
## lmer.REML = 1.3297e+05  Scale est. = 99287     n = 8137
```

Below are plots of a plot of `s(bl_age)`, `s(bl_age):sleep_z`, `s(bl_age):time`, and `s(sleep_z):time`. For `s(sleep_z):time`, roughly, if we cannot fit a straight line through the confidence bands, there is evidence that the effect of time depends on sleep duration. It is correct the that confidence interval is zero at one point along the axis; this happens because we do not include the main effect.

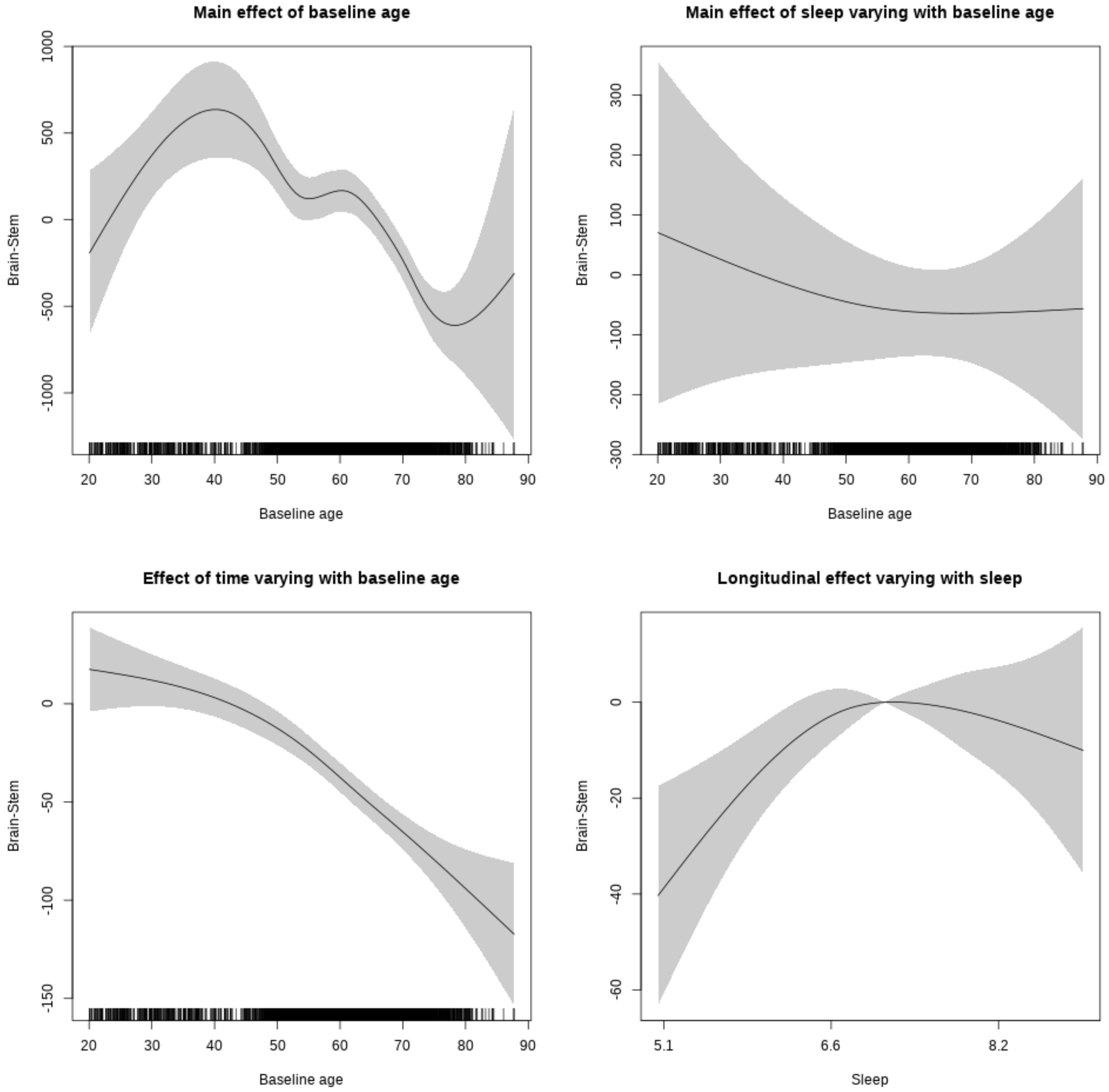

Restricted to sleep >= 5 and <= 9 hours Below is the row for `s(sleep_z):time` from the model output.

```
## s(sleep_z):time   2.346  2.346  4.525  0.0054 **
```

Below are plots of a plot of `s(bl_age)`, `s(bl_age):sleep_z`, `s(bl_age):time`, and `s(sleep_z):time`. For `s(sleep_z):time`, roughly, if we cannot fit a straight line through the confidence bands, there is evidence that the effect of time depends on sleep duration. It is correct the that confidence interval is zero at one point along the axis; this happens because we do not include the main effect.

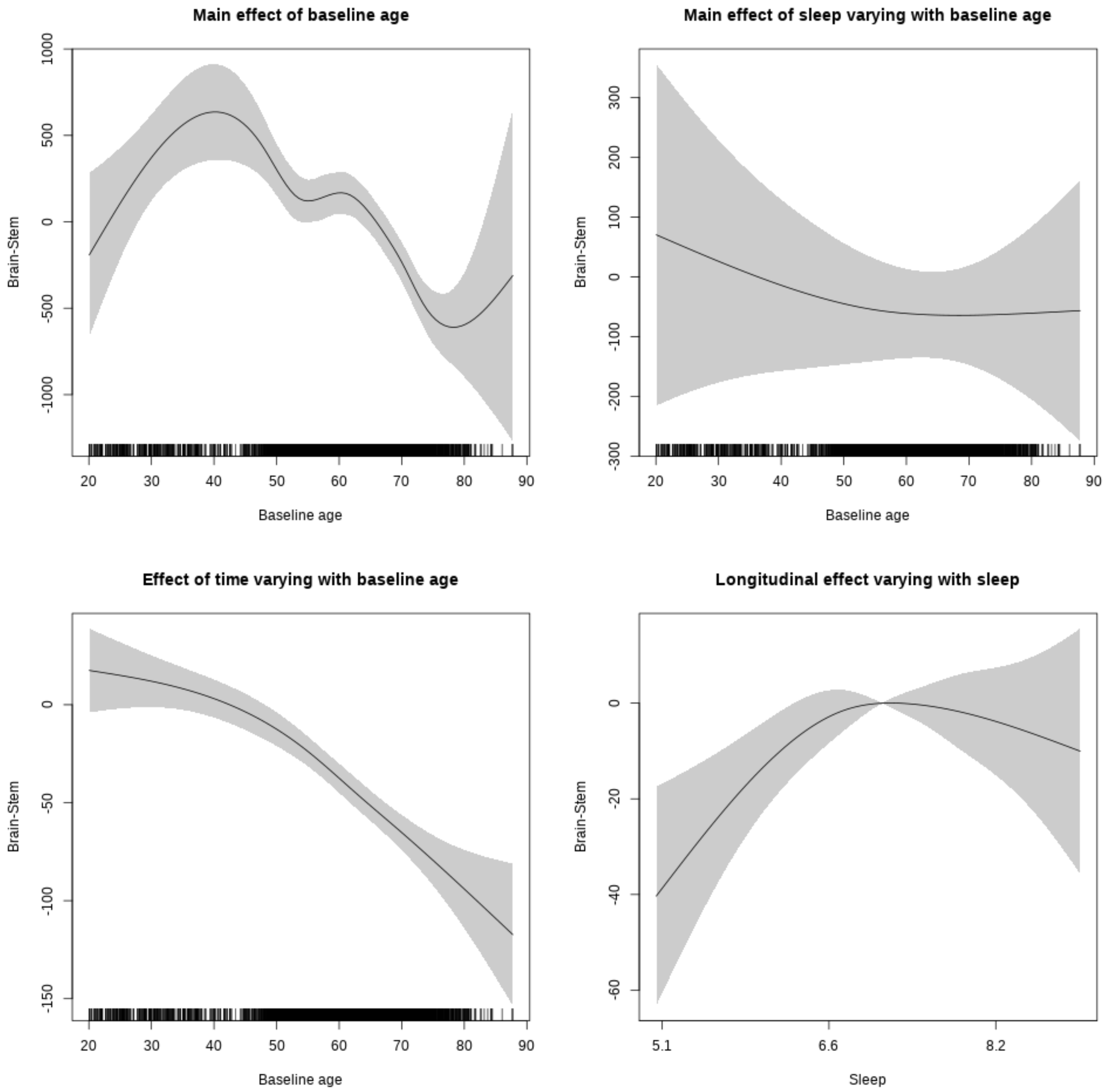

Controling for socioeconomic status

Full data Below is the model output. The term `s(sleep_z):time` is probably what we care most about.

```
##
## Family: gaussian
## Link function: identity
##
## Formula:
## value ~ sex + site + income_scaled + education_scaled + icv +
##     s(bl_age, k = 10, bs = "cr") + s(bl_age, by = sleep_z, bs = "cr") +
##     +s(bl_age, by = time, k = 5, bs = "cr") + s(sleep_z, by = time,
##     k = 5, bs = "cr", pc = 0)
```

```
## <environment: 0x563914e762e0>
##
## Parametric coefficients:
##                  Estimate Std. Error t value Pr(>|t|)
## (Intercept)      23439.91     321.39  72.933  < 2e-16 ***
## sexmale            693.90      87.25   7.953 2.28e-15 ***
## siteousAvanto    -3113.29     341.20  -9.125  < 2e-16 ***
## siteousPrisma    -3801.22     518.06  -7.337 2.56e-13 ***
## siteousSkyra     -2338.99     329.62  -7.096 1.48e-12 ***
## siteUKB          -2056.22     305.31  -6.735 1.84e-11 ***
## income_scaled       74.69     114.18   0.654    0.513
## education_scaled   307.89     126.48   2.434    0.015 *
## icv               1576.61      40.89  38.555  < 2e-16 ***
## ---
## Signif. codes:  0 ‘***’ 0.001 ‘**’ 0.01 ‘*’ 0.05 ‘.’ 0.1 ‘ ’ 1
##
## Approximate significance of smooth terms:
##                     edf Ref.df      F  p-value
## s(bl_age)         3.092  3.092  8.732 6.36e-06 ***
## s(bl_age):sleep_z 2.000  2.000  0.022    0.978
## s(bl_age):time    3.169  3.169 22.780  < 2e-16 ***
## s(sleep_z):time   1.000  1.000  2.254    0.133
## ---
## Signif. codes:  0 ‘***’ 0.001 ‘**’ 0.01 ‘*’ 0.05 ‘.’ 0.1 ‘ ’ 1
##
## R-sq.(adj) =  0.502
## lmer.REML =  76010  Scale est. = 1.0221e+05  n = 4636
```

Below are plots of a plot of `s(bl_age)`, `s(bl_age):sleep_z`, `s(bl_age):time`, and `s(sleep_z):time`. For `s(sleep_z):time`, roughly, if we cannot fit a straight line through the confidence bands, there is evidence that the effect of time depends on sleep duration. It is correct the that confidence interval is zero at one point along the axis; this happens because we do not include the main effect.

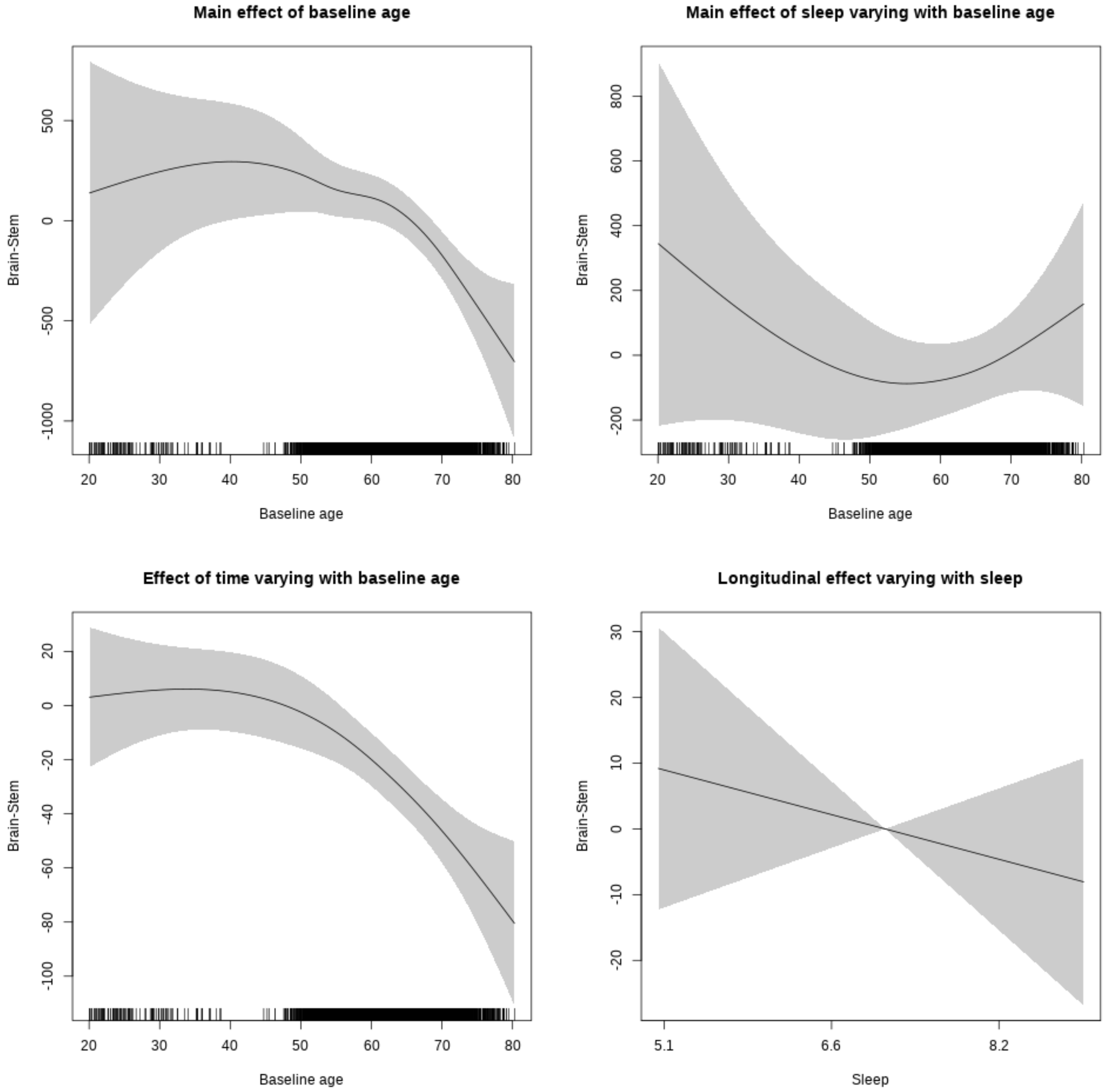

Restricted to sleep >= 5 and <= 9 hours Below is the row for `s(sleep_z):time` from the model output.

```
## s(sleep_z):time   1.000  1.000  0.734    0.392
```

Below are plots of a plot of `s(bl_age)`, `s(bl_age):sleep_z`, `s(bl_age):time`, and `s(sleep_z):time`. For `s(sleep_z):time`, roughly, if we cannot fit a straight line through the confidence bands, there is evidence that the effect of time depends on sleep duration. It is correct the that confidence interval is zero at one point along the axis; this happens because we do not include the main effect.

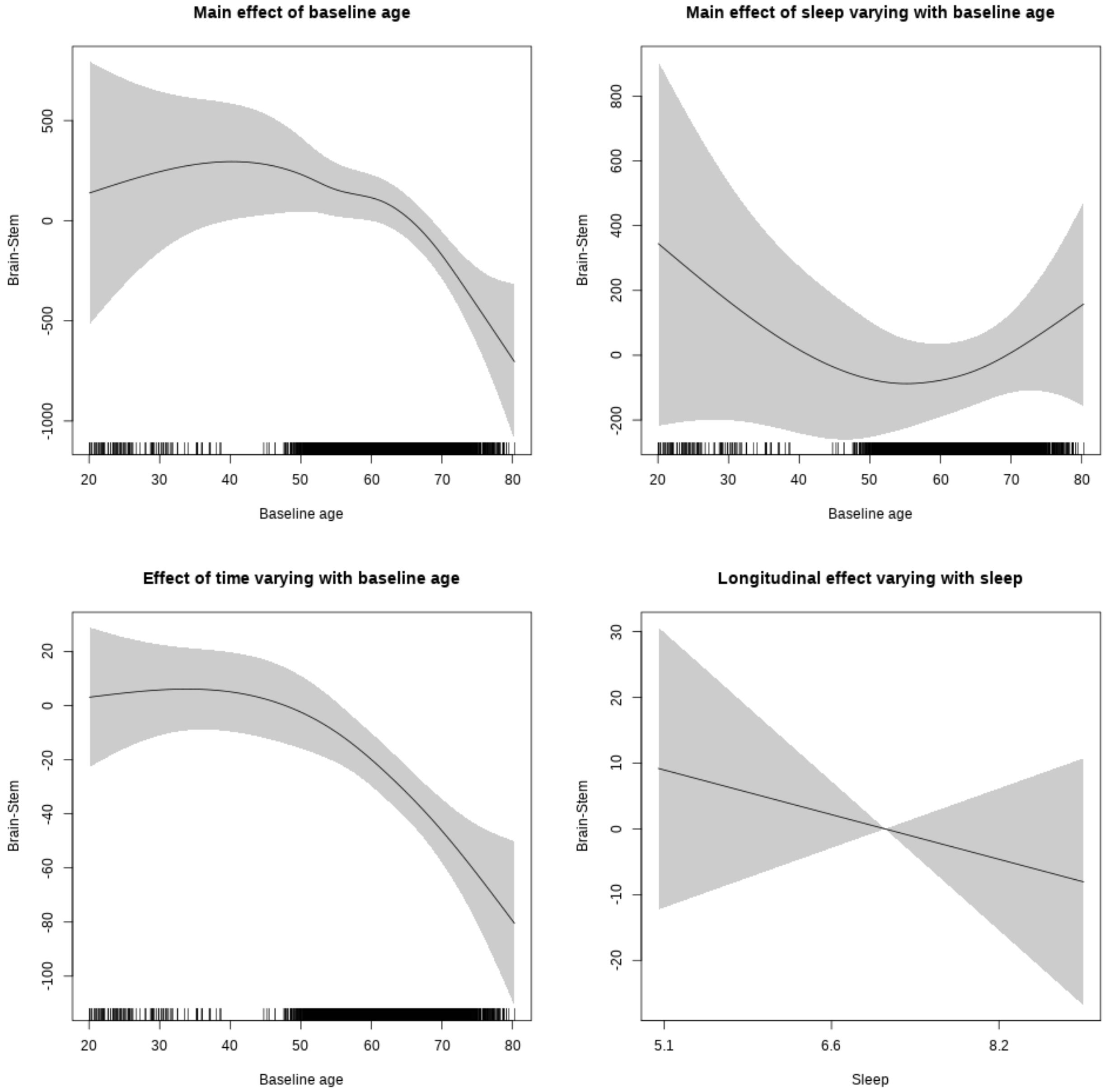

Controling for BMI

Full data Below is the model output. The term `s(sleep_z):time` is probably what we care most about.

```
##
## Family: gaussian
## Link function: identity
##
## Formula:
## value ~ sex + site + bmi + icv + s(bl_age, k = 10, bs = "cr") +
##     s(bl_age, by = sleep_z, bs = "cr") + +s(bl_age, by = time,
##     k = 5, bs = "cr") + s(sleep_z, by = time, k = 5, bs = "cr",
##     pc = 0)
```

```
## <environment: 0x563910d47f30>
##
## Parametric coefficients:
##                Estimate Std. Error t value Pr(>|t|)
## (Intercept)   21243.187    276.915  76.714  < 2e-16 ***
## sexmale         695.147     80.404   8.646  < 2e-16 ***
## siteousPrisma  -715.468    256.576  -2.789  0.00531 **
## siteousSkyra    763.039     82.789   9.217  < 2e-16 ***
## siteUCAM       -181.974    203.157  -0.896  0.37044
## siteUKB         885.031    184.853   4.788 1.73e-06 ***
## siteUmU       -1019.589    235.504  -4.329 1.52e-05 ***
## bmi             -17.571      8.505  -2.066  0.03888 *
## icv            1540.970     37.754  40.816  < 2e-16 ***
## ---
## Signif. codes:  0 '***' 0.001 '**' 0.01 '*' 0.05 '.' 0.1 ' ' 1
##
## Approximate significance of smooth terms:
##                     edf Ref.df      F p-value
## s(bl_age)         5.143  5.143 10.661  <2e-16 ***
## s(bl_age):sleep_z 2.000  2.000  0.962   0.382
## s(bl_age):time    3.276  3.276 44.097  <2e-16 ***
## s(sleep_z):time   1.000  1.000  1.078   0.299
## ---
## Signif. codes:  0 '***' 0.001 '**' 0.01 '*' 0.05 '.' 0.1 ' ' 1
##
## R-sq.(adj) =  0.516
## lmer.REML =  88421  Scale est. = 97997     n = 5398
```

Below are plots of a plot of `s(bl_age)`, `s(bl_age):sleep_z`, `s(bl_age):time`, and `s(sleep_z):time`. For `s(sleep_z):time`, roughly, if we cannot fit a straight line through the confidence bands, there is evidence that the effect of time depends on sleep duration. It is correct the that confidence interval is zero at one point along the axis; this happens because we do not include the main effect.

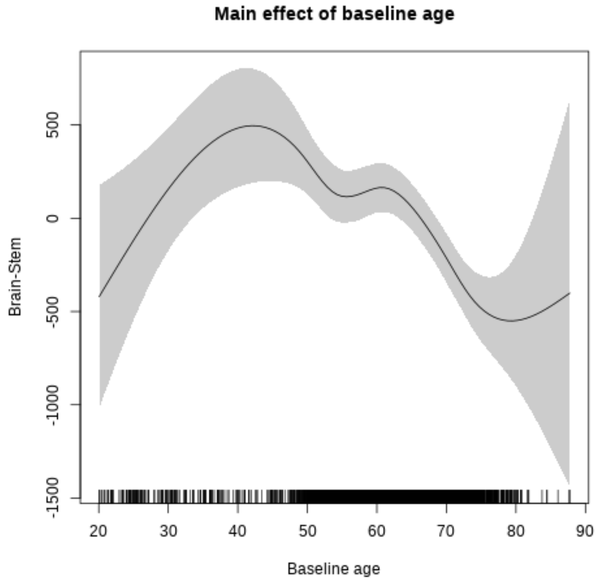

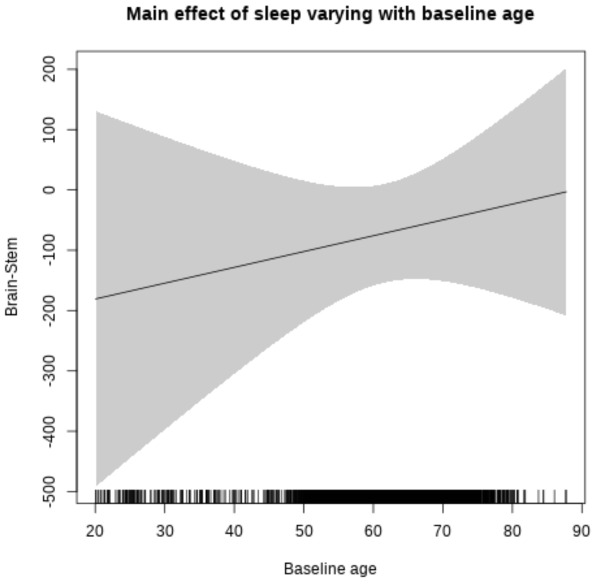

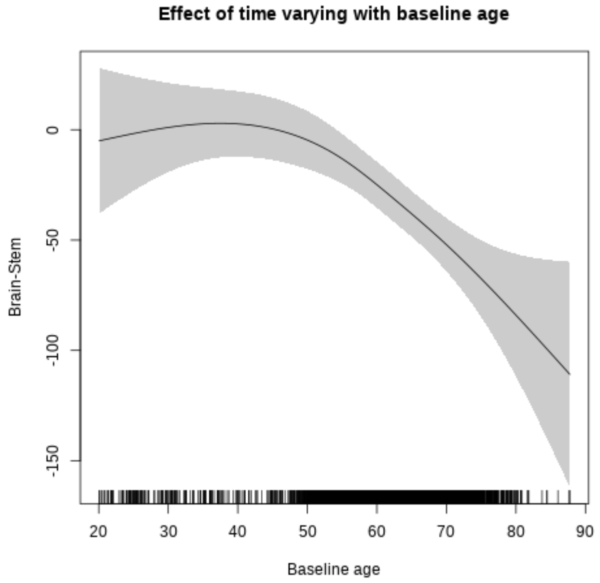

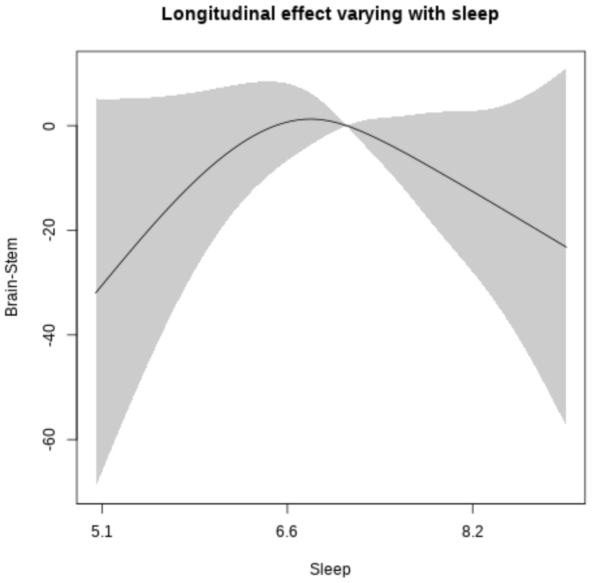

Restricted to sleep >= 5 and <= 9 hours Below is the row for `s(sleep_z):time` from the model output.

```
## s(sleep_z):time   2.189  2.189  1.998   0.134
```

Below are plots of a plot of `s(bl_age)`, `s(bl_age):sleep_z`, `s(bl_age):time`, and `s(sleep_z):time`. For `s(sleep_z):time`, roughly, if we cannot fit a straight line through the confidence bands, there is evidence that the effect of time depends on sleep duration. It is correct the that confidence interval is zero at one point along the axis; this happens because we do not include the main effect.

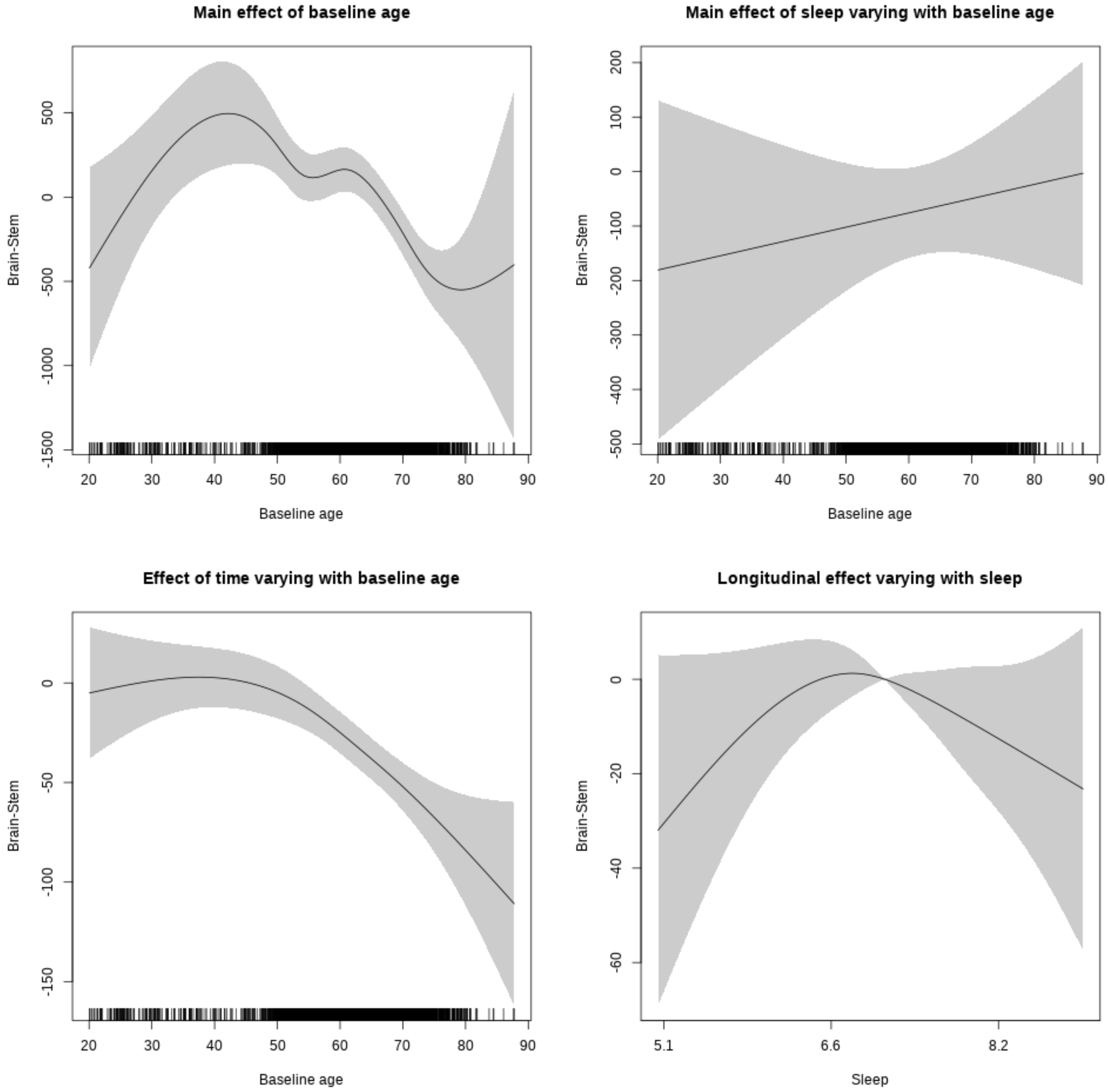

Controling for depression

Full data Below is the model output. The term `s(sleep_z):time` is probably what we care most about.

```
##
## Family: gaussian
## Link function: identity
##
## Formula:
## value ~ sex + site + depression + icv + s(bl_age, k = 10, bs = "cr") +
##     s(bl_age, by = sleep_z, bs = "cr") + +s(bl_age, by = time,
##     k = 5, bs = "cr") + s(sleep_z, by = time, k = 5, bs = "cr",
##     pc = 0)
```

```
## <environment: 0x5639141b54e0>
##
## Parametric coefficients:
##                Estimate Std. Error t value Pr(>|t|)
## (Intercept)    23229.29     153.31 151.517  < 2e-16 ***
## sexmale          693.60      79.87   8.684  < 2e-16 ***
## siteousAvanto -2209.19     304.66  -7.251 4.68e-13 ***
## siteousPrisma -2684.20     421.29  -6.371 2.02e-10 ***
## siteousSkyra  -1233.83     284.56  -4.336 1.48e-05 ***
## siteUCAM      -2509.35     188.97 -13.279  < 2e-16 ***
## siteUKB       -1531.88     141.97 -10.790  < 2e-16 ***
## siteUmU       -3223.73     228.40 -14.115  < 2e-16 ***
## depression     -582.50     286.66  -2.032   0.0422 *
## icv            1446.86      36.76  39.363  < 2e-16 ***
## ---
## Signif. codes:  0 '***' 0.001 '**' 0.01 '*' 0.05 '.' 0.1 ' ' 1
##
## Approximate significance of smooth terms:
##                      edf Ref.df      F p-value
## s(bl_age)          5.456  5.456 12.681  <2e-16 ***
## s(bl_age):sleep_z 2.000  2.000  0.874   0.417
## s(bl_age):time    3.119  3.119 46.150  <2e-16 ***
## s(sleep_z):time   1.688  1.688  1.421   0.142
## ---
## Signif. codes:  0 '***' 0.001 '**' 0.01 '*' 0.05 '.' 0.1 ' ' 1
##
## R-sq.(adj) =  0.491
## lmer.REML =  92925  Scale est. = 97038     n = 5665
```

Below are plots of a plot of `s(bl_age)`, `s(bl_age):sleep_z`, `s(bl_age):time`, and `s(sleep_z):time`. For `s(sleep_z):time`, roughly, if we cannot fit a straight line through the confidence bands, there is evidence that the effect of time depends on sleep duration. It is correct the that confidence interval is zero at one point along the axis; this happens because we do not include the main effect.

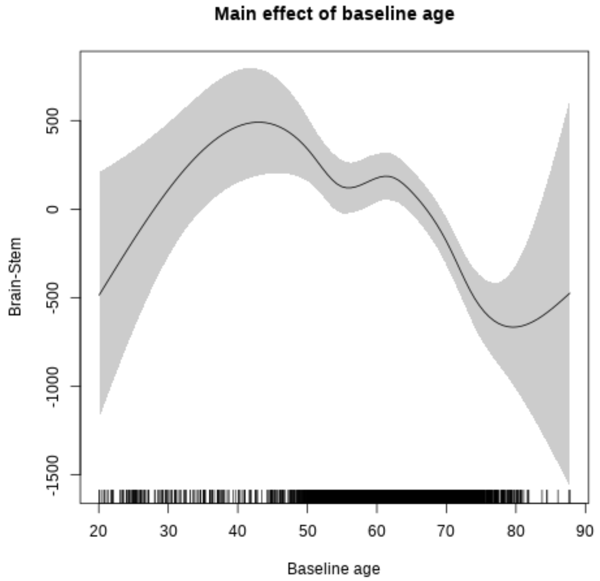

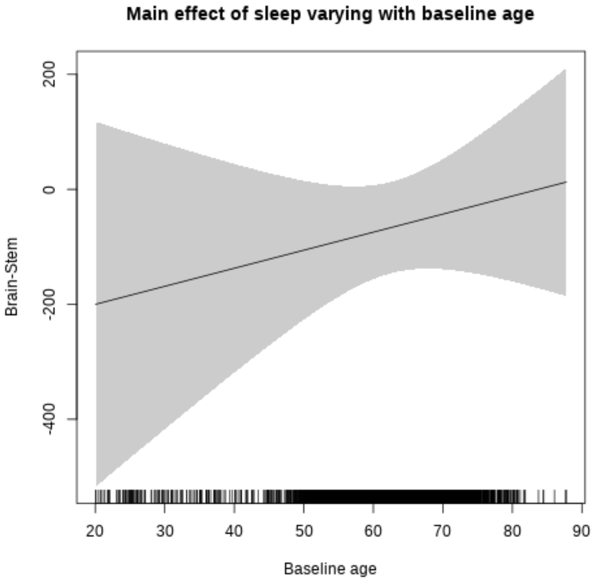

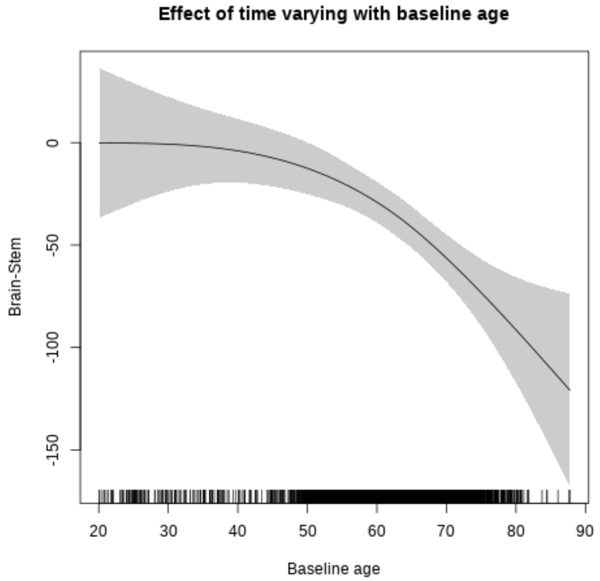

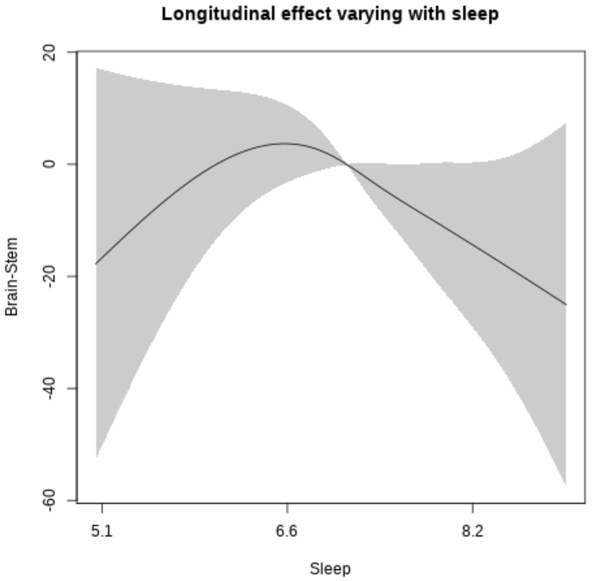

Restricted to sleep >= 5 and <= 9 hours Below is the row for `s(sleep_z):time` from the model output.

```
## s(sleep_z):time   2.172  2.172  2.289   0.123
```

Below are plots of a plot of `s(bl_age)`, `s(bl_age):sleep_z`, `s(bl_age):time`, and `s(sleep_z):time`. For `s(sleep_z):time`, roughly, if we cannot fit a straight line through the confidence bands, there is evidence that the effect of time depends on sleep duration. It is correct the that confidence interval is zero at one point along the axis; this happens because we do not include the main effect.

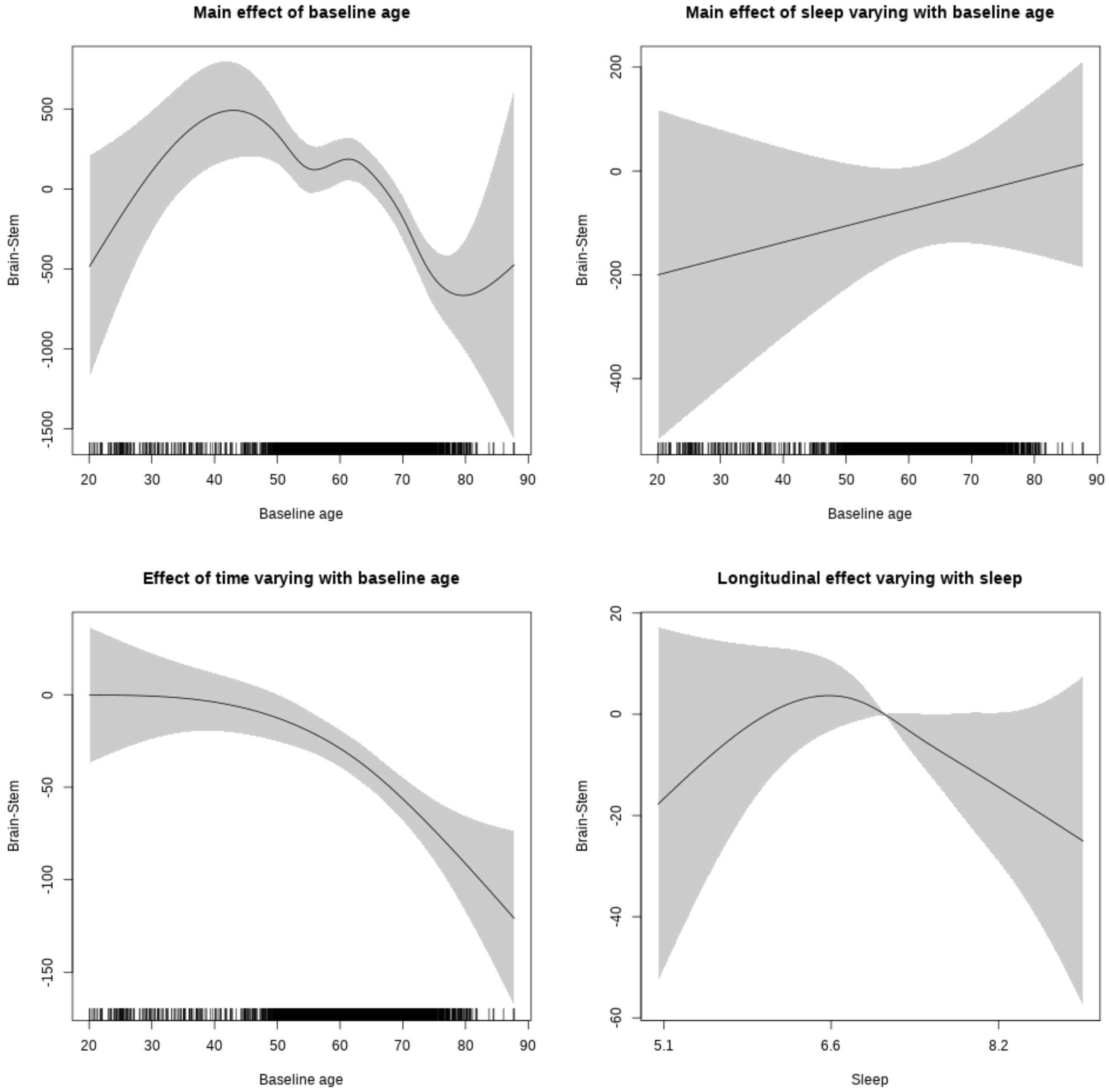

Controling for sleep quality

Full data Below is the model output. The term `s(sleep_z):time` is probably what we care most about.

```
##
## Family: gaussian
## Link function: identity
##
## Formula:
## value ~ X + s(bl_age, k = 10, bs = "cr") + s(bl_age, by = sleep_z,
##     bs = "cr") + s(bl_age, by = time, k = 5, bs = "cr") + s(sleep_z,
##     by = time, k = 5, bs = "cr", pc = 0)
## <environment: 0x5639110937a0>
```

```
##
## Parametric coefficients:
##                            Estimate Std. Error t value Pr(>|t|)
## (Intercept)                20466.09     259.53  78.858  < 2e-16 ***
## Xsexmale                    2315.65      97.31  23.797  < 2e-16 ***
## XsiteousAvanto               506.72     247.38   2.048  0.04059 *
## XsiteousPrisma               588.28     350.97   1.676  0.09378 .
## XsiteousSkyra                710.96     247.71   2.870  0.00412 **
## XsiteUB                     -729.86     394.94  -1.848  0.06467 .
## XsiteUCAM                   -199.06     253.86  -0.784  0.43300
## XsiteUKB                     858.54     794.90   1.080  0.28018
## XsiteUmU                   -1839.66     282.54  -6.511 8.32e-11 ***
## Xukb_dummy:sleep_accel        30.69      61.55   0.499  0.61801
## Xukb_dummy:chronotype         11.93      81.52   0.146  0.88370
## Xukb_dummy:dozing             70.58     168.19   0.420  0.67477
## Xukb_dummy:sleeplessness    -285.45     104.83  -2.723  0.00649 **
## Xukb_dummy:snoring           148.52     148.57   1.000  0.31754
## Xukb_dummy:gettingup         -86.35     114.04  -0.757  0.44900
## Xnotukb_dummy:PSQI_Global    -53.65      32.16  -1.668  0.09530 .
## ---
## Signif. codes:  0 '***' 0.001 '**' 0.01 '*' 0.05 '.' 0.1 ' ' 1
##
## Approximate significance of smooth terms:
##                      edf Ref.df      F p-value
## s(bl_age)          3.147  3.147 14.890 < 2e-16 ***
## s(bl_age):sleep_z 2.000  2.000  2.101 0.12252
## s(bl_age):time    3.502  3.502 92.190 < 2e-16 ***
## s(sleep_z):time   1.000  1.000  7.834 0.00515 **
## ---
## Signif. codes:  0 '***' 0.001 '**' 0.01 '*' 0.05 '.' 0.1 ' ' 1
##
## R-sq.(adj) =  0.304
## lmer.REML =  70279  Scale est. = 1.0422e+05  n = 4279
```

Below are plots of a plot of `s(bl_age)`, `s(bl_age):sleep_z`, `s(bl_age):time`, and `s(sleep_z):time`. For `s(sleep_z):time`, roughly, if we cannot fit a straight line through the confidence bands, there is evidence that the effect of time depends on sleep duration. It is correct the that confidence interval is zero at one point along the axis; this happens because we do not include the main effect.

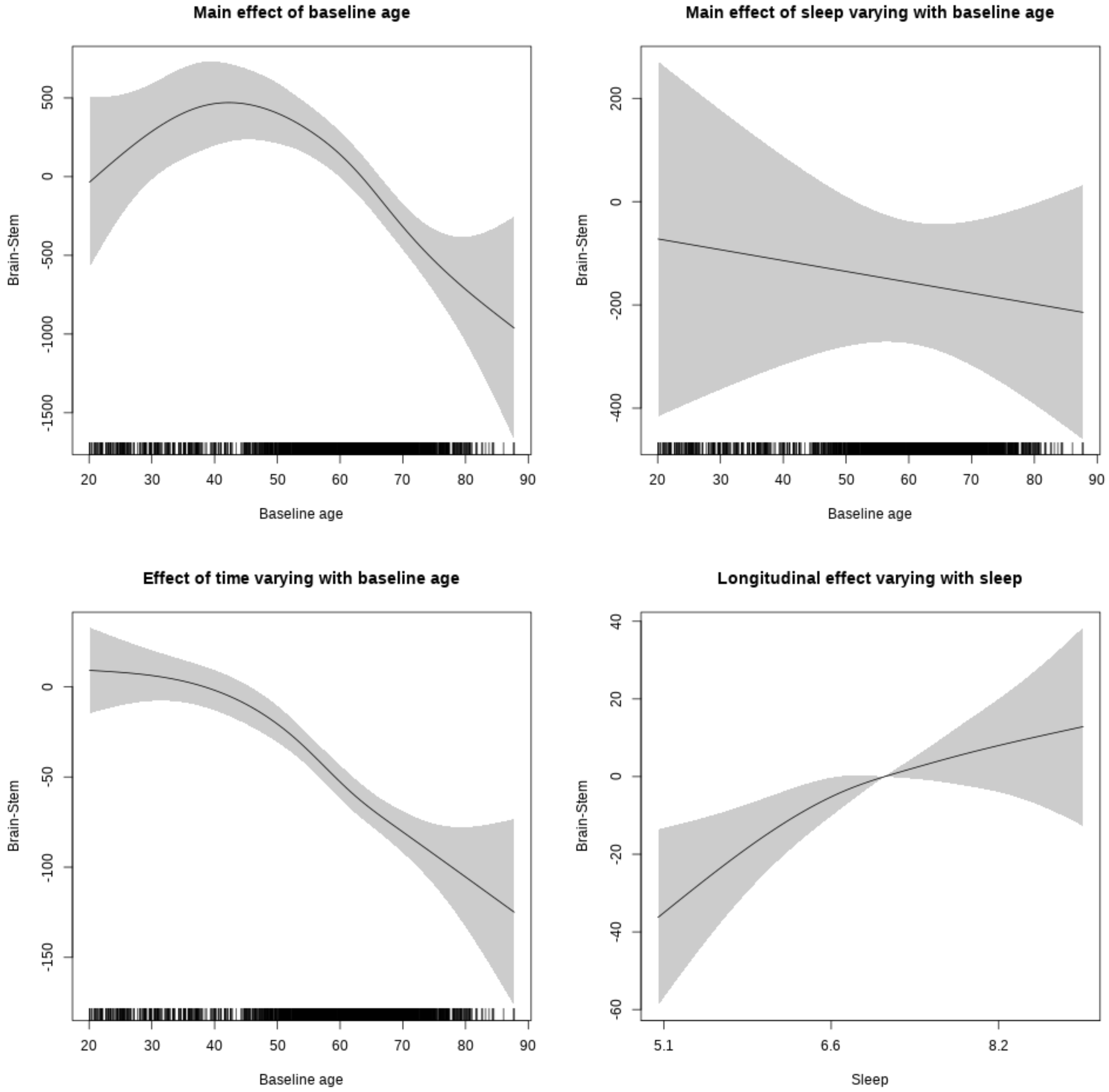

Restricted to sleep >= 5 and <= 9 hours Below is the row for `s(sleep_z):time` from the model output.

```
## s(sleep_z):time   1.713  1.713  7.007 0.00217 **
```

Below are plots of a plot of `s(bl_age)`, `s(bl_age):sleep_z`, `s(bl_age):time`, and `s(sleep_z):time`. For `s(sleep_z):time`, roughly, if we cannot fit a straight line through the confidence bands, there is evidence that the effect of time depends on sleep duration. It is correct the that confidence interval is zero at one point along the axis; this happens because we do not include the main effect.

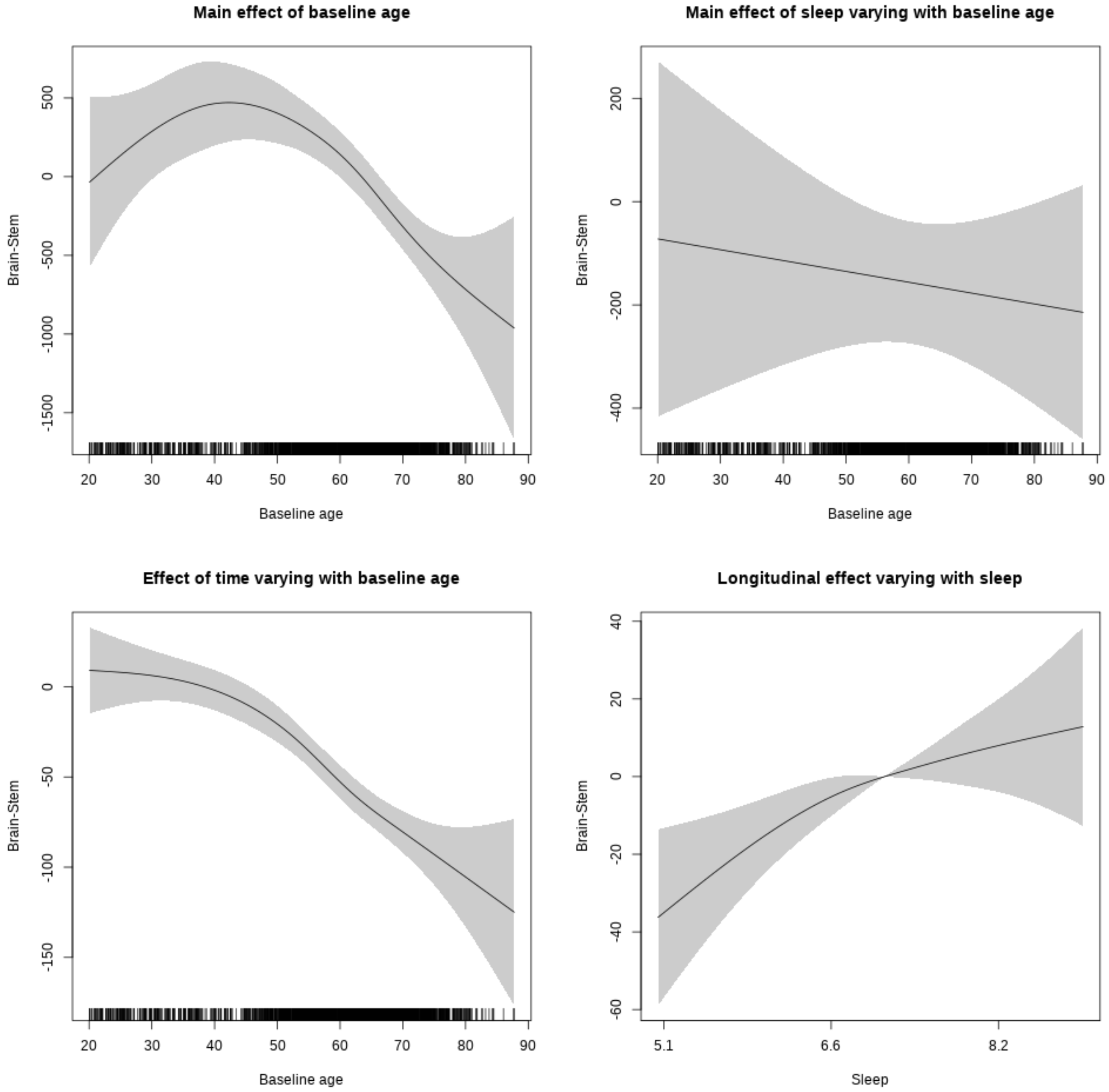

Caudate

Main analysis

Full data Below is the model output. The term `s(sleep_z):time` is probably what we care most about.

```
##
## Family: gaussian
## Link function: identity
##
## Formula:
## value ~ sex + site + icv + s(bl_age, k = 10, bs = "cr") + s(bl_age,
##     by = sleep_z, bs = "cr") + +s(bl_age, by = time, k = 5, bs = "cr") +
```

```
##     s(sleep_z, by = time, k = 5, bs = "cr", pc = 0)
## <environment: 0x563917994a50>
##
## Parametric coefficients:
##               Estimate Std. Error t value Pr(>|t|)
## (Intercept)    7795.85      54.17 143.925  < 2e-16 ***
## sexmale          57.96      27.80   2.085   0.0371 *
## siteousAvanto  -760.65      66.46 -11.445  < 2e-16 ***
## siteousPrisma  -326.14     134.62  -2.423   0.0154 *
## siteousSkyra   -379.47      64.79  -5.857 4.89e-09 ***
## siteUB         -651.78     127.04  -5.131 2.96e-07 ***
## siteUCAM       -660.32      68.55  -9.632  < 2e-16 ***
## siteUKB       -1099.51      51.96 -21.160  < 2e-16 ***
## siteUmU        -429.26      78.62  -5.460 4.90e-08 ***
## icv             451.20      12.91  34.951  < 2e-16 ***
## ---
## Signif. codes:  0 '***' 0.001 '**' 0.01 '*' 0.05 '.' 0.1 ' ' 1
##
## Approximate significance of smooth terms:
##                     edf Ref.df      F p-value
## s(bl_age)         4.831  4.831 46.082  <2e-16 ***
## s(bl_age):sleep_z 2.235  2.235  1.272  0.2880
## s(bl_age):time    2.623  2.623 79.946  <2e-16 ***
## s(sleep_z):time   1.000  1.000  8.479  0.0036 **
## ---
## Signif. codes:  0 '***' 0.001 '**' 0.01 '*' 0.05 '.' 0.1 ' ' 1
##
## R-sq.(adj) =  0.408
## lmer.REML = 1.1988e+05  Scale est. = 22652     n = 8146
```

Below are plots of a plot of `s(bl_age)`, `s(bl_age):sleep_z`, `s(bl_age):time`, and `s(sleep_z):time`. For `s(sleep_z):time`, roughly, if we cannot fit a straight line through the confidence bands, there is evidence that the effect of time depends on sleep duration. It is correct the that confidence interval is zero at one point along the axis; this happens because we do not include the main effect.

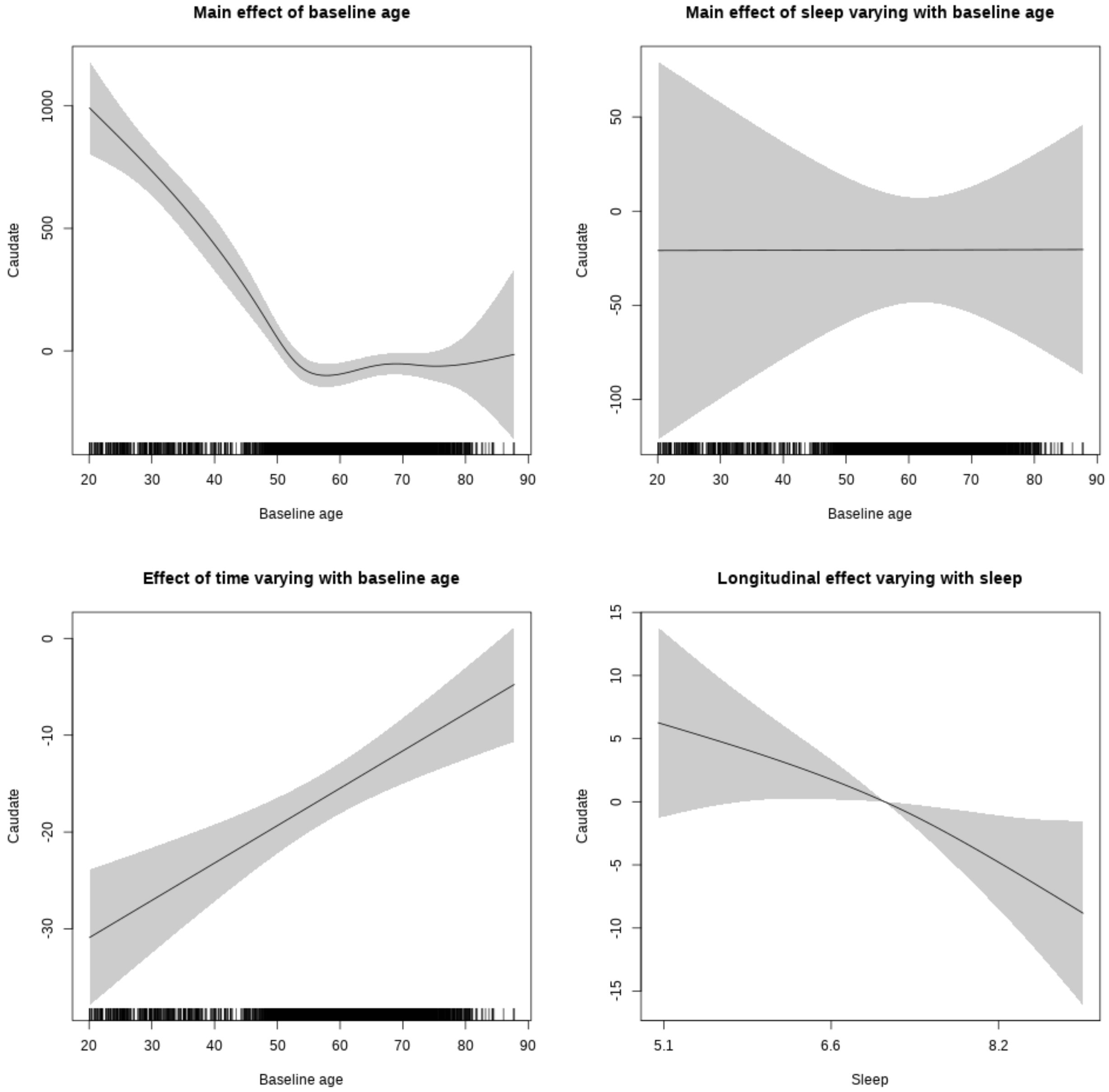

Restricted to sleep >= 5 and <= 9 hours Below is the row for `s(sleep_z):time` from the model output.

```
## s(sleep_z):time   1.298  1.298  4.260  0.0179 *
```

Below are plots of a plot of `s(bl_age)`, `s(bl_age):sleep_z`, `s(bl_age):time`, and `s(sleep_z):time`. For `s(sleep_z):time`, roughly, if we cannot fit a straight line through the confidence bands, there is evidence that the effect of time depends on sleep duration. It is correct the that confidence interval is zero at one point along the axis; this happens because we do not include the main effect.

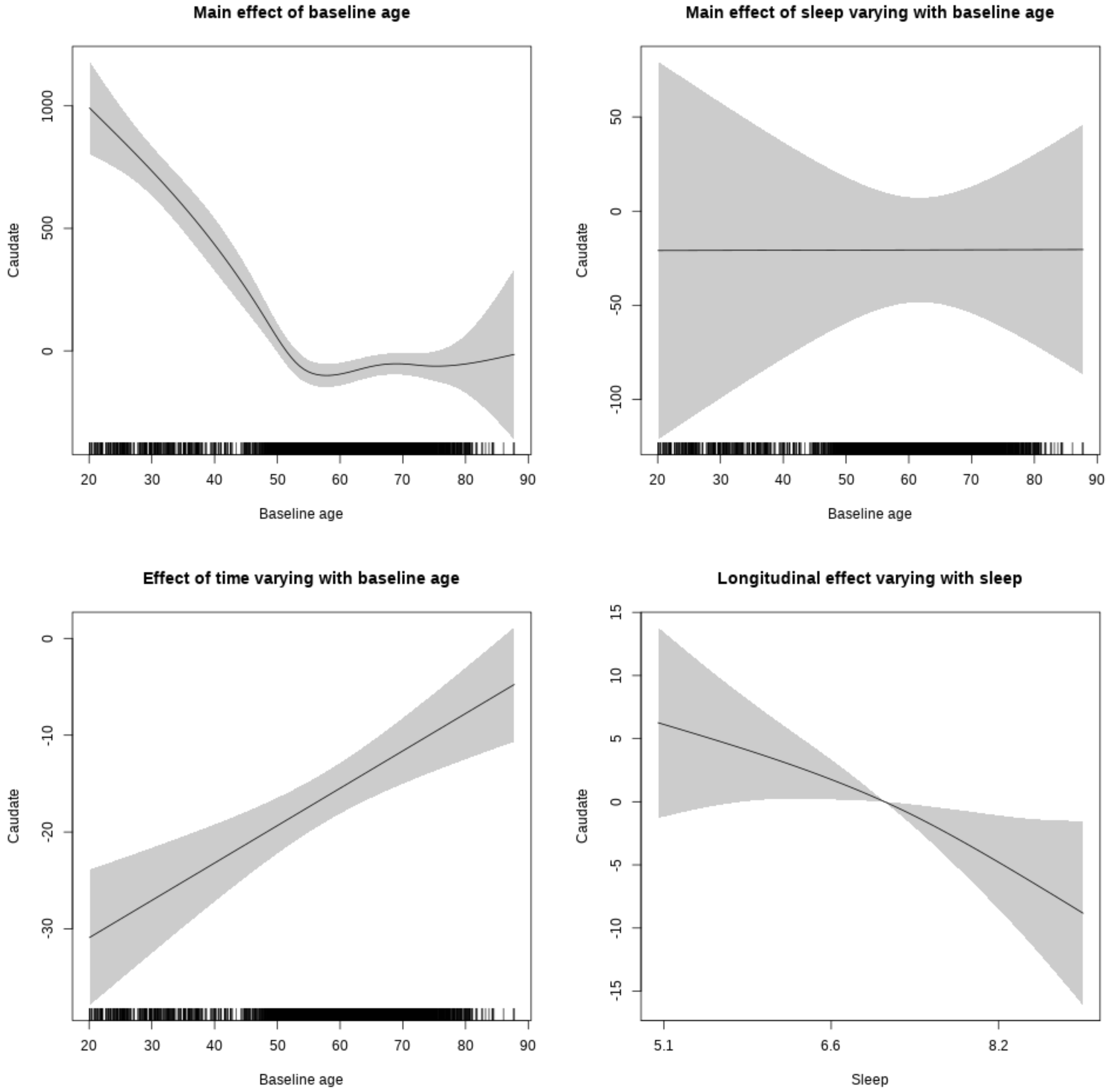

Controling for socioeconomic status

Full data Below is the model output. The term `s(sleep_z):time` is probably what we care most about.

```
##
## Family: gaussian
## Link function: identity
##
## Formula:
## value ~ sex + site + income_scaled + education_scaled + icv +
##     s(bl_age, k = 10, bs = "cr") + s(bl_age, by = sleep_z, bs = "cr") +
##     +s(bl_age, by = time, k = 5, bs = "cr") + s(sleep_z, by = time,
##     k = 5, bs = "cr", pc = 0)
```

```
## <environment: 0x5639112dce10>
##
## Parametric coefficients:
##                   Estimate Std. Error t value Pr(>|t|)
## (Intercept)       8223.112    131.530  62.519  < 2e-16 ***
## sexmale             -7.659     35.725  -0.214 0.830264
## siteousAvanto    -1348.246    140.224  -9.615  < 2e-16 ***
## siteousPrisma     -776.484    227.743  -3.409 0.000656 ***
## siteousSkyra      -934.800    134.651  -6.942 4.39e-12 ***
## siteUKB          -1609.641    124.935 -12.884  < 2e-16 ***
## income_scaled       44.750     46.594   0.960 0.336897
## education_scaled    79.499     51.480   1.544 0.122587
## icv                482.107     16.960  28.426  < 2e-16 ***
## ---
## Signif. codes:  0 '***' 0.001 '**' 0.01 '*' 0.05 '.' 0.1 ' ' 1
##
## Approximate significance of smooth terms:
##                     edf Ref.df      F p-value
## s(bl_age)         3.664  3.664 17.255  <2e-16 ***
## s(bl_age):sleep_z 2.000  2.000  0.891   0.410
## s(bl_age):time    2.332  2.332 24.916  <2e-16 ***
## s(sleep_z):time   1.000  1.000  0.995   0.318
## ---
## Signif. codes:  0 '***' 0.001 '**' 0.01 '*' 0.05 '.' 0.1 ' ' 1
##
## R-sq.(adj) =  0.389
## lmer.REML =  68485  Scale est. = 21861     n = 4650
```

Below are plots of a plot of `s(bl_age)`, `s(bl_age):sleep_z`, `s(bl_age):time`, and `s(sleep_z):time`. For `s(sleep_z):time`, roughly, if we cannot fit a straight line through the confidence bands, there is evidence that the effect of time depends on sleep duration. It is correct the that confidence interval is zero at one point along the axis; this happens because we do not include the main effect.

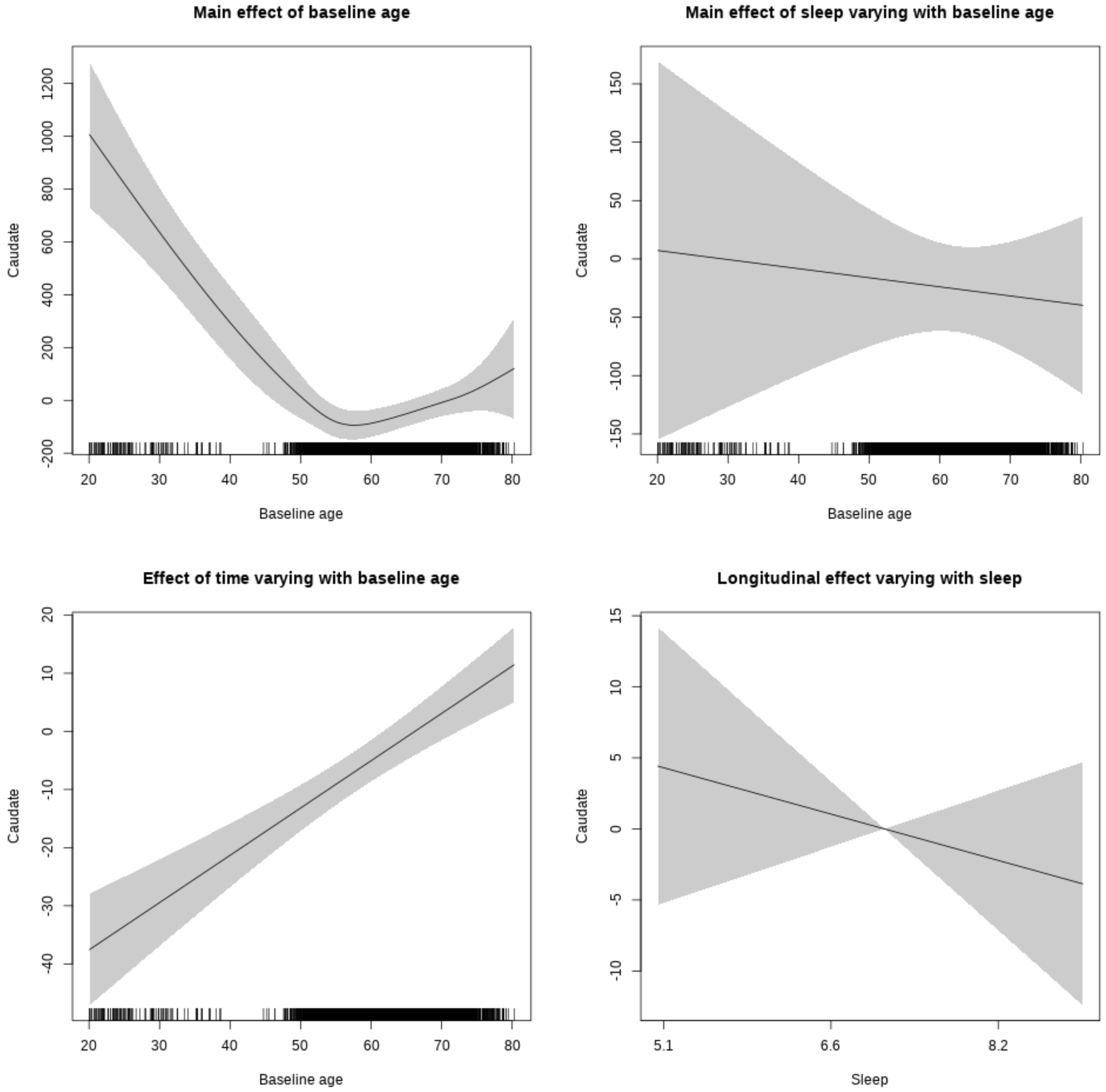

Restricted to sleep >= 5 and <= 9 hours Below is the row for `s(sleep_z):time` from the model output.

```
## s(sleep_z):time   1.000  1.000  0.817   0.366
```

Below are plots of a plot of `s(bl_age)`, `s(bl_age):sleep_z`, `s(bl_age):time`, and `s(sleep_z):time`. For `s(sleep_z):time`, roughly, if we cannot fit a straight line through the confidence bands, there is evidence that the effect of time depends on sleep duration. It is correct the that confidence interval is zero at one point along the axis; this happens because we do not include the main effect.

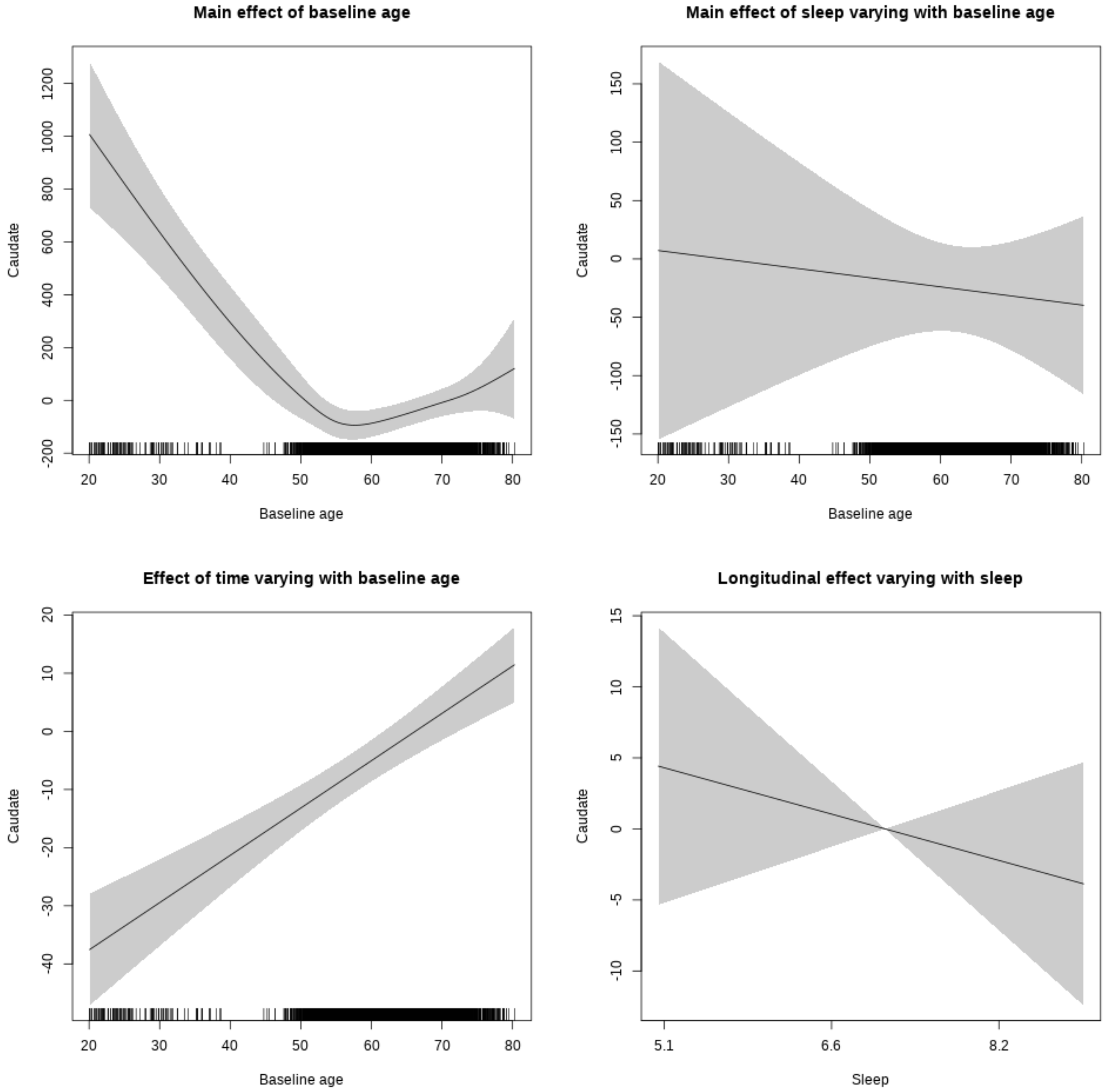

Controling for BMI

Full data Below is the model output. The term `s(sleep_z):time` is probably what we care most about.

```
##
## Family: gaussian
## Link function: identity
##
## Formula:
## value ~ sex + site + bmi + icv + s(bl_age, k = 10, bs = "cr") +
##     s(bl_age, by = sleep_z, bs = "cr") + +s(bl_age, by = time,
##     k = 5, bs = "cr") + s(sleep_z, by = time, k = 5, bs = "cr",
##     pc = 0)
```

```
## <environment: 0x56390aaf10d8>
##
## Parametric coefficients:
##               Estimate Std. Error t value Pr(>|t|)
## (Intercept)   6764.562    114.848  58.900  < 2e-16 ***
## sexmale          5.097     33.200   0.154  0.87800
## siteousPrisma  361.350    122.929   2.940  0.00330 **
## siteousSkyra   356.842     39.424   9.051  < 2e-16 ***
## siteUCAM       169.074     84.745   1.995  0.04608 *
## siteUKB       -292.756     77.822  -3.762  0.00017 ***
## siteUmU        390.952     98.225   3.980 6.98e-05 ***
## bmi              9.065      3.488   2.599  0.00937 **
## icv            482.201     15.838  30.445  < 2e-16 ***
## ---
## Signif. codes:  0 '***' 0.001 '**' 0.01 '*' 0.05 '.' 0.1 ' ' 1
##
## Approximate significance of smooth terms:
##                     edf Ref.df      F p-value
## s(bl_age)         4.336  4.336 29.985 < 2e-16 ***
## s(bl_age):sleep_z 2.000  2.000  1.978 0.13847
## s(bl_age):time    2.000  2.000 47.244 < 2e-16 ***
## s(sleep_z):time   1.000  1.000 10.391 0.00127 **
## ---
## Signif. codes:  0 '***' 0.001 '**' 0.01 '*' 0.05 '.' 0.1 ' ' 1
##
## R-sq.(adj) =   0.41
## lmer.REML =  79869  Scale est. = 22658     n = 5412
```

Below are plots of a plot of `s(bl_age)`, `s(bl_age):sleep_z`, `s(bl_age):time`, and `s(sleep_z):time`. For `s(sleep_z):time`, roughly, if we cannot fit a straight line through the confidence bands, there is evidence that the effect of time depends on sleep duration. It is correct the that confidence interval is zero at one point along the axis; this happens because we do not include the main effect.

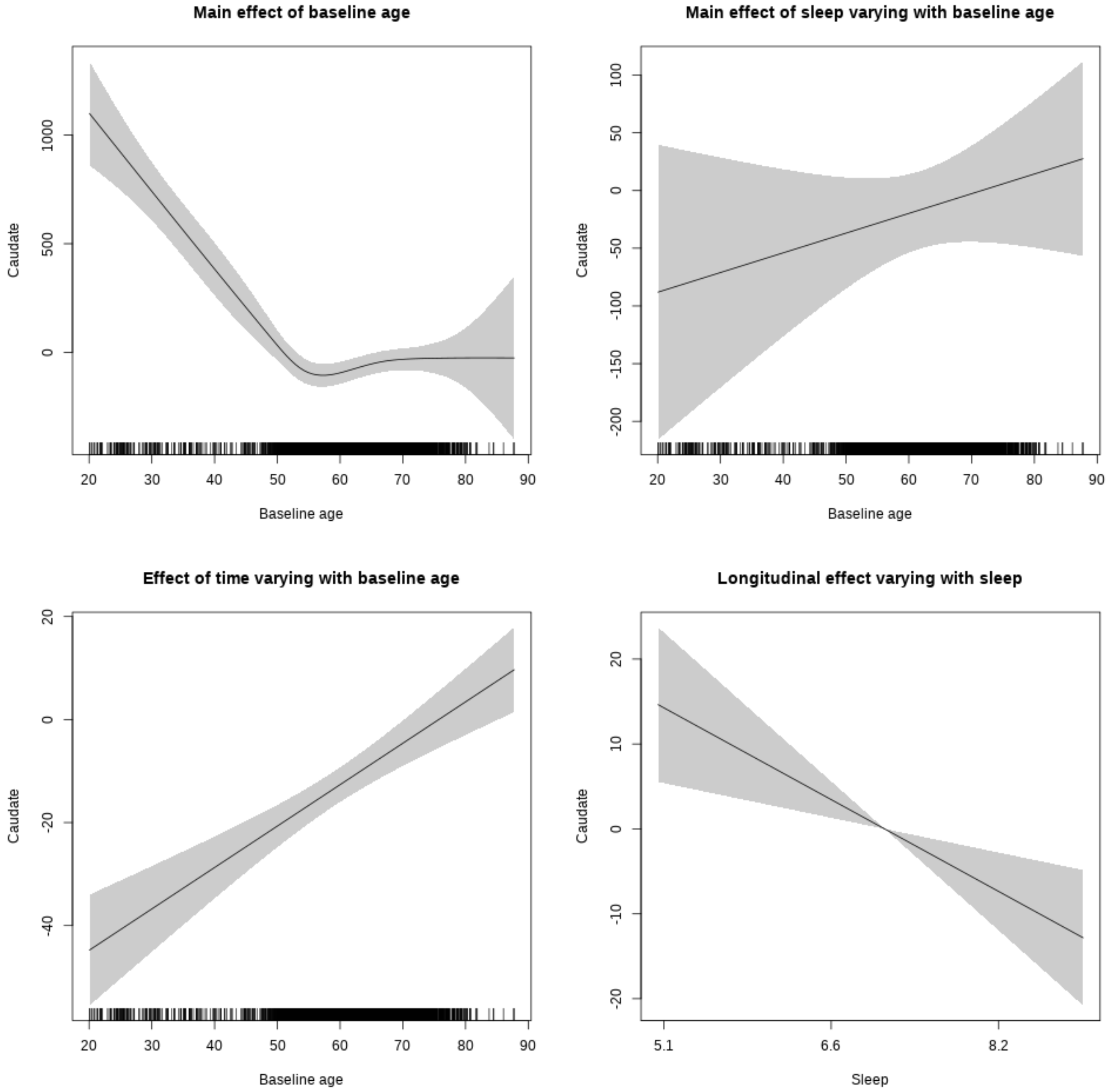

Restricted to sleep >= 5 and <= 9 hours Below is the row for `s(sleep_z):time` from the model output.

```
## s(sleep_z):time   1.000  1.000 10.343 0.00131 **
```

Below are plots of a plot of `s(bl_age)`, `s(bl_age):sleep_z`, `s(bl_age):time`, and `s(sleep_z):time`. For `s(sleep_z):time`, roughly, if we cannot fit a straight line through the confidence bands, there is evidence that the effect of time depends on sleep duration. It is correct the that confidence interval is zero at one point along the axis; this happens because we do not include the main effect.

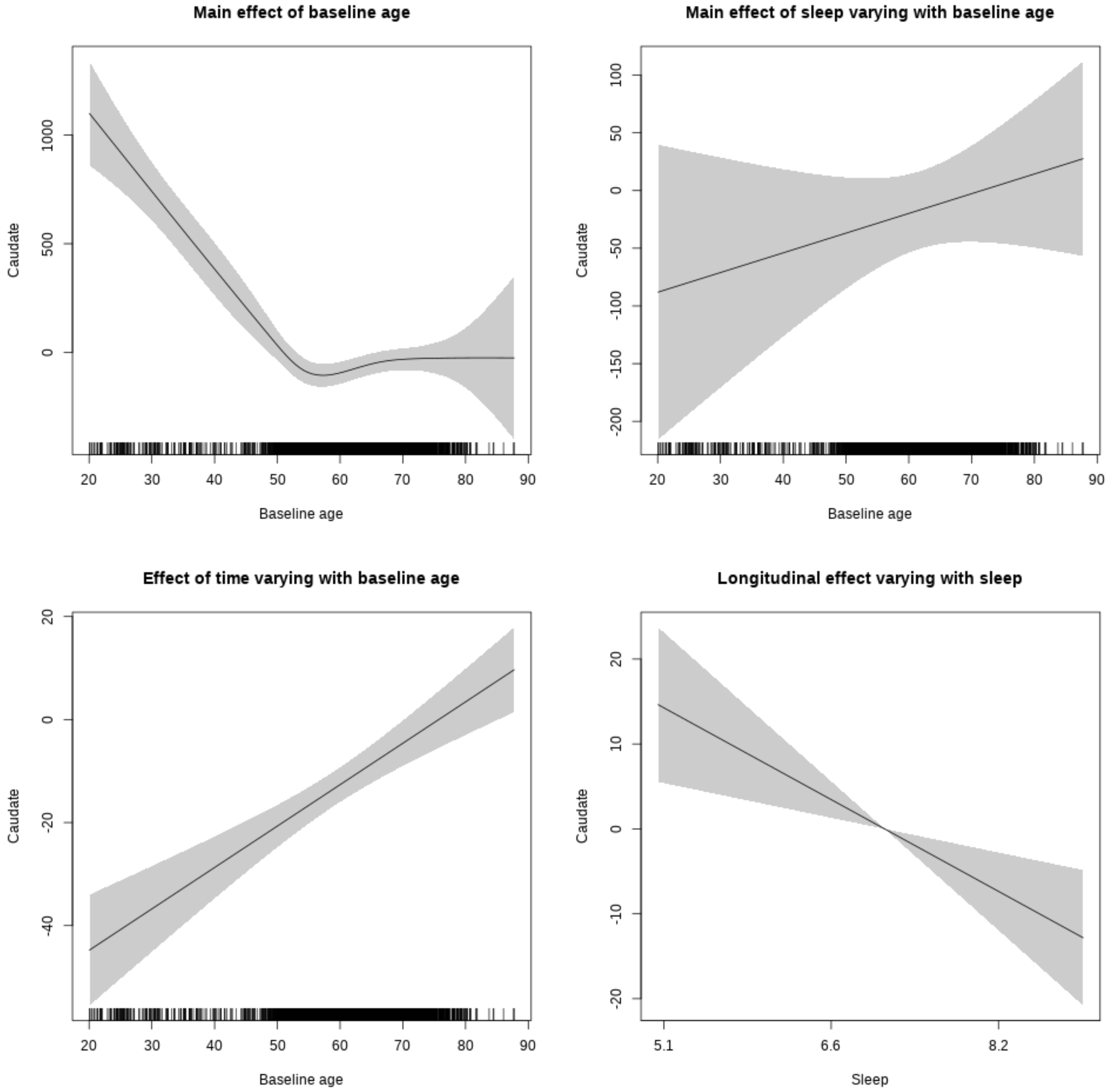

Controling for depression

Full data Below is the model output. The term `s(sleep_z):time` is probably what we care most about.

```
##
## Family: gaussian
## Link function: identity
##
## Formula:
## value ~ sex + site + depression + icv + s(bl_age, k = 10, bs = "cr") +
##     s(bl_age, by = sleep_z, bs = "cr") + +s(bl_age, by = time,
##     k = 5, bs = "cr") + s(sleep_z, by = time, k = 5, bs = "cr",
##     pc = 0)
```

```
## <environment: 0x563910f8b5a0>
##
## Parametric coefficients:
##                Estimate Std. Error t value Pr(>|t|)
## (Intercept)    7718.61      62.44 123.615  < 2e-16 ***
## sexmale           5.03      32.60   0.154   0.8774
## siteousAvanto  -652.73     126.33  -5.167 2.46e-07 ***
## siteousPrisma  -315.51     189.11  -1.668   0.0953 .
## siteousSkyra   -188.65     115.28  -1.636   0.1018
## siteUCAM       -580.74      76.31  -7.611 3.17e-14 ***
## siteUKB       -1034.91      57.67 -17.946  < 2e-16 ***
## siteUmU        -415.77      92.45  -4.497 7.02e-06 ***
## depression      201.67     115.91   1.740   0.0819 .
## icv             473.95      15.27  31.036  < 2e-16 ***
## ---
## Signif. codes:  0 '***' 0.001 '**' 0.01 '*' 0.05 '.' 0.1 ' ' 1
##
## Approximate significance of smooth terms:
##                      edf Ref.df      F  p-value
## s(bl_age)          4.759  4.759 17.656  < 2e-16 ***
## s(bl_age):sleep_z 2.780  2.780  2.494    0.118
## s(bl_age):time    2.000  2.000 44.080  < 2e-16 ***
## s(sleep_z):time   1.000  1.000 18.514 1.75e-05 ***
## ---
## Signif. codes:  0 '***' 0.001 '**' 0.01 '*' 0.05 '.' 0.1 ' ' 1
##
## R-sq.(adj) =  0.395
## lmer.REML =  83929  Scale est. = 23006     n = 5679
```

Below are plots of a plot of `s(bl_age)`, `s(bl_age):sleep_z`, `s(bl_age):time`, and `s(sleep_z):time`. For `s(sleep_z):time`, roughly, if we cannot fit a straight line through the confidence bands, there is evidence that the effect of time depends on sleep duration. It is correct the that confidence interval is zero at one point along the axis; this happens because we do not include the main effect.

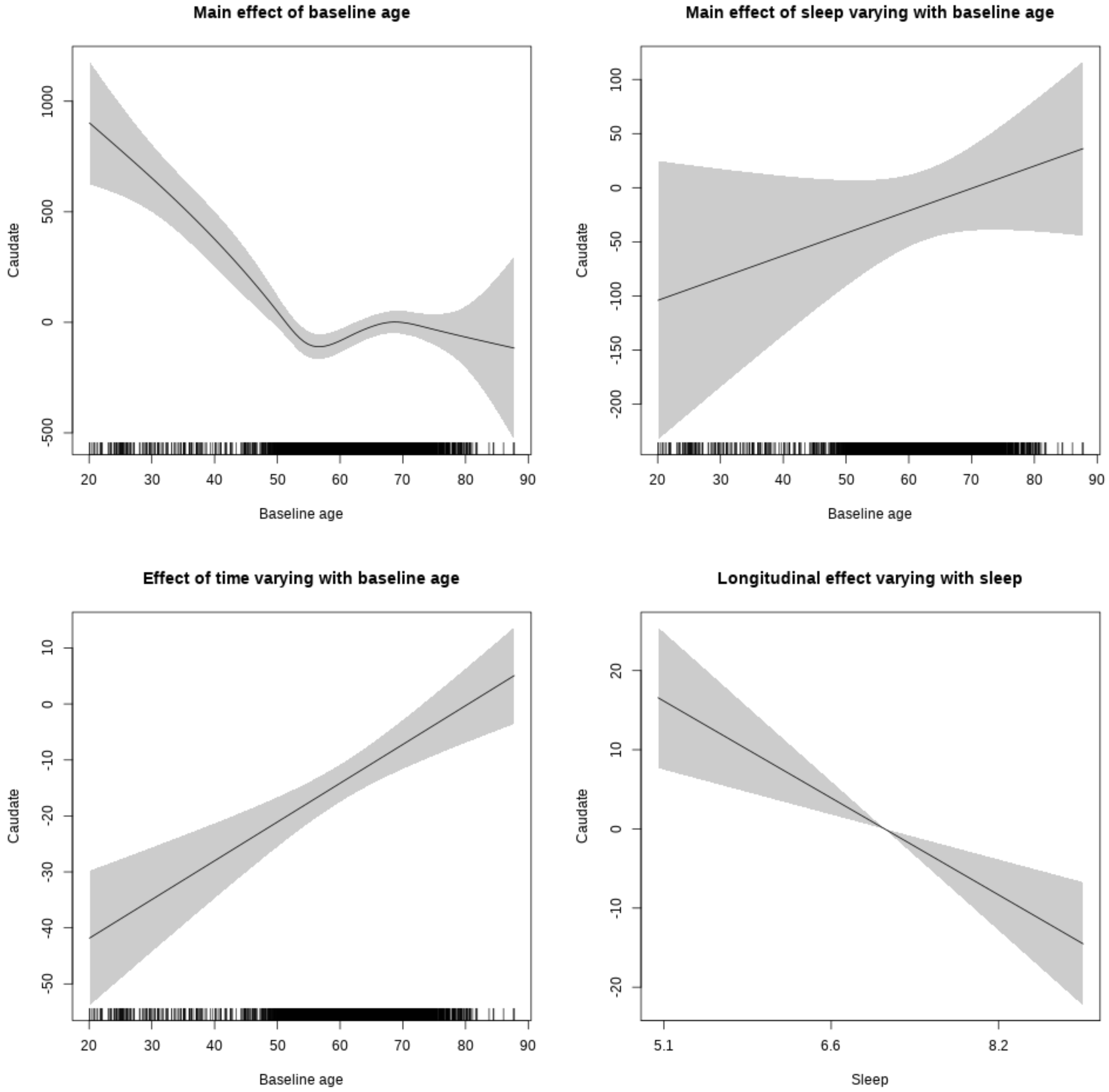

Restricted to sleep >= 5 and <= 9 hours Below is the row for `s(sleep_z):time` from the model output.

```
## s(sleep_z):time   1.000  1.000 13.899 0.000195 ***
```

Below are plots of a plot of `s(bl_age)`, `s(bl_age):sleep_z`, `s(bl_age):time`, and `s(sleep_z):time`. For `s(sleep_z):time`, roughly, if we cannot fit a straight line through the confidence bands, there is evidence that the effect of time depends on sleep duration. It is correct the that confidence interval is zero at one point along the axis; this happens because we do not include the main effect.

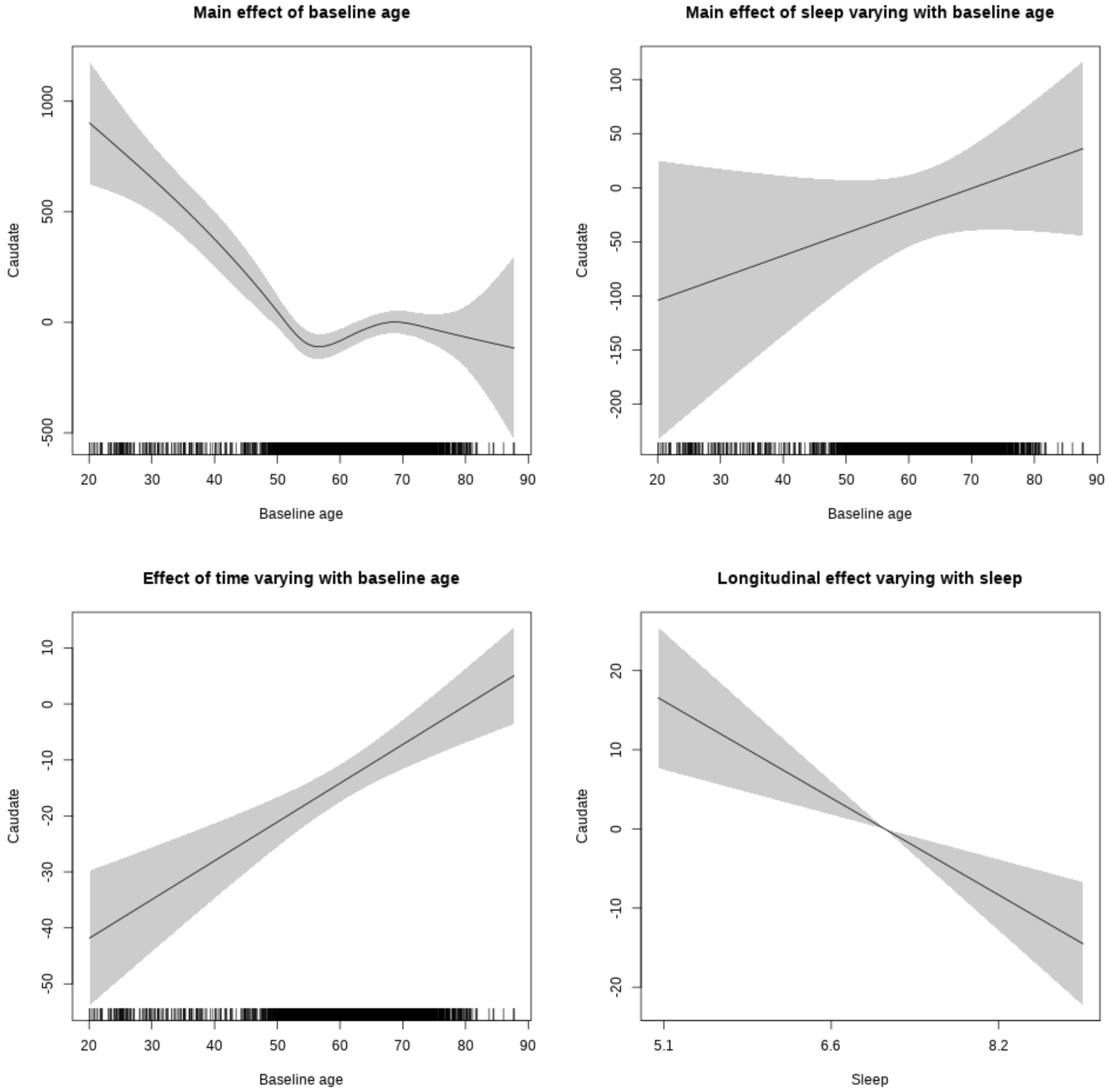

Controling for sleep quality

Full data Below is the model output. The term `s(sleep_z):time` is probably what we care most about.

```
##
## Family: gaussian
## Link function: identity
##
## Formula:
## value ~ X + s(bl_age, k = 10, bs = "cr") + s(bl_age, by = sleep_z,
##     bs = "cr") + s(bl_age, by = time, k = 5, bs = "cr") + s(sleep_z,
##     by = time, k = 5, bs = "cr", pc = 0)
## <environment: 0x563911572570>
```

```
##
## Parametric coefficients:
##                          Estimate Std. Error t value Pr(>|t|)
## (Intercept)              6962.122    108.003  64.462  < 2e-16 ***
## Xsexmale                  579.793     40.401  14.351  < 2e-16 ***
## XsiteousAvanto            101.771    103.047   0.988  0.32339
## XsiteousPrisma            766.477    157.255   4.874 1.13e-06 ***
## XsiteousSkyra             332.058    103.229   3.217  0.00131 **
## XsiteUB                  -146.993    164.238  -0.895  0.37084
## XsiteUCAM                  41.829    105.820   0.395  0.69265
## XsiteUKB                 -162.218    328.893  -0.493  0.62188
## XsiteUmU                   20.577    117.599   0.175  0.86111
## Xukb_dummy:sleep_accel     -8.372     25.409  -0.329  0.74180
## Xukb_dummy:chronotype      -2.542     33.827  -0.075  0.94010
## Xukb_dummy:dozing         126.505     70.015   1.807  0.07086 .
## Xukb_dummy:sleeplessness  -65.834     43.556  -1.511  0.13074
## Xukb_dummy:snoring         38.082     61.689   0.617  0.53705
## Xukb_dummy:gettingup      -47.924     47.347  -1.012  0.31150
## Xnotukb_dummy:PSQI_Global  -4.013     13.378  -0.300  0.76424
## ---
## Signif. codes:  0 '***' 0.001 '**' 0.01 '*' 0.05 '.' 0.1 ' ' 1
##
## Approximate significance of smooth terms:
##                     edf Ref.df       F p-value
## s(bl_age)          3.42   3.42  33.732 < 2e-16 ***
## s(bl_age):sleep_z 2.00   2.00   1.122 0.32589
## s(bl_age):time    2.00   2.00 110.983 < 2e-16 ***
## s(sleep_z):time   1.00   1.00   8.544 0.00348 **
## ---
## Signif. codes:  0 '***' 0.001 '**' 0.01 '*' 0.05 '.' 0.1 ' ' 1
##
## R-sq.(adj) =   0.26
## lmer.REML =  63518  Scale est. = 23812     n = 4286
```

Below are plots of a plot of `s(bl_age)`, `s(bl_age):sleep_z`, `s(bl_age):time`, and `s(sleep_z):time`. For `s(sleep_z):time`, roughly, if we cannot fit a straight line through the confidence bands, there is evidence that the effect of time depends on sleep duration. It is correct the that confidence interval is zero at one point along the axis; this happens because we do not include the main effect.

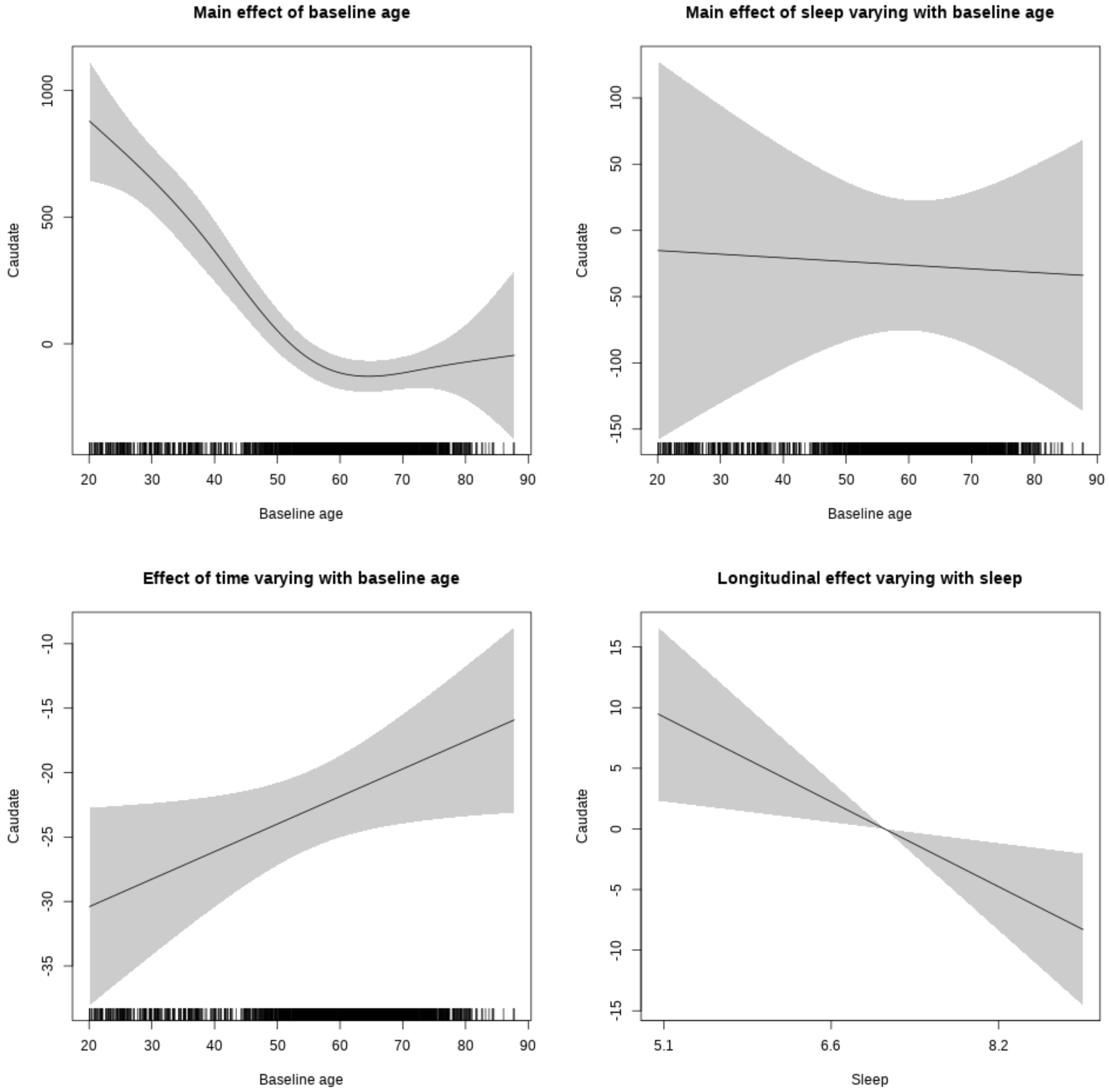

Restricted to sleep >= 5 and <= 9 hours Below is the row for `s(sleep_z):time` from the model output.

```
## s(sleep_z):time   1.000  1.000   7.031 0.00804 **
```

Below are plots of a plot of `s(bl_age)`, `s(bl_age):sleep_z`, `s(bl_age):time`, and `s(sleep_z):time`. For `s(sleep_z):time`, roughly, if we cannot fit a straight line through the confidence bands, there is evidence that the effect of time depends on sleep duration. It is correct the that confidence interval is zero at one point along the axis; this happens because we do not include the main effect.

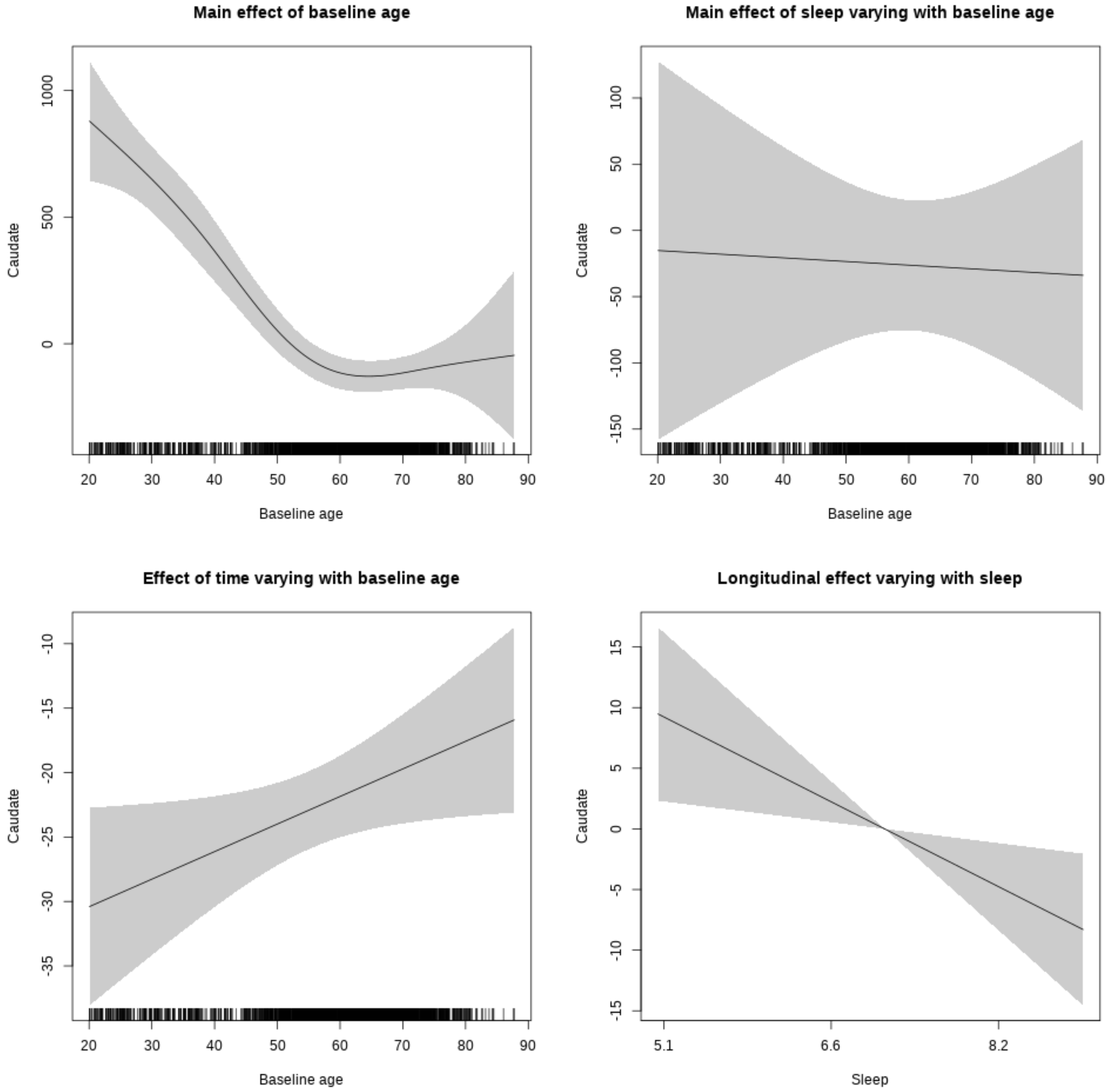

CC_Anterior

Main analysis

Full data Below is the model output. The term `s(sleep_z):time` is probably what we care most about.

```
##
## Family: gaussian
## Link function: identity
##
## Formula:
## value ~ sex + site + icv + s(bl_age, k = 10, bs = "cr") + s(bl_age,
##     by = sleep_z, bs = "cr") + +s(bl_age, by = time, k = 5, bs = "cr") +
```

```
##     s(sleep_z, by = time, k = 5, bs = "cr", pc = 0)
## <environment: 0x563910f11468>
##
## Parametric coefficients:
##                Estimate Std. Error t value Pr(>|t|)
## (Intercept)    1041.729     10.716  97.211  < 2e-16 ***
## sexmale         -48.920      5.522  -8.859  < 2e-16 ***
## siteousAvanto -183.159     13.155 -13.923  < 2e-16 ***
## siteousPrisma -180.910     34.397  -5.259 1.48e-07 ***
## siteousSkyra  -149.870     12.792 -11.716  < 2e-16 ***
## siteUB        -154.162     24.824  -6.210 5.55e-10 ***
## siteUCAM      -164.357     13.384 -12.280  < 2e-16 ***
## siteUKB        -65.716     10.210  -6.437 1.29e-10 ***
## siteUmU       -113.667     15.405  -7.379 1.76e-13 ***
## icv             84.617      2.650  31.936  < 2e-16 ***
## ---
## Signif. codes:  0 ‘***’ 0.001 ‘**’ 0.01 ‘*’ 0.05 ‘.’ 0.1 ‘ ’ 1
##
## Approximate significance of smooth terms:
##                      edf Ref.df      F p-value
## s(bl_age)          3.514  3.514 40.379 < 2e-16 ***
## s(bl_age):sleep_z 2.000  2.000  1.567   0.209
## s(bl_age):time    2.514  2.514 12.844 6.1e-07 ***
## s(sleep_z):time   1.000  1.000  0.009   0.925
## ---
## Signif. codes:  0 ‘***’ 0.001 ‘**’ 0.01 ‘*’ 0.05 ‘.’ 0.1 ‘ ’ 1
##
## R-sq.(adj) =  0.249
## lmer.REML =  96135  Scale est. = 1716.1    n = 8140
```

Below are plots of a plot of `s(bl_age)`, `s(bl_age):sleep_z`, `s(bl_age):time`, and `s(sleep_z):time`. For `s(sleep_z):time`, roughly, if we cannot fit a straight line through the confidence bands, there is evidence that the effect of time depends on sleep duration. It is correct the that confidence interval is zero at one point along the axis; this happens because we do not include the main effect.

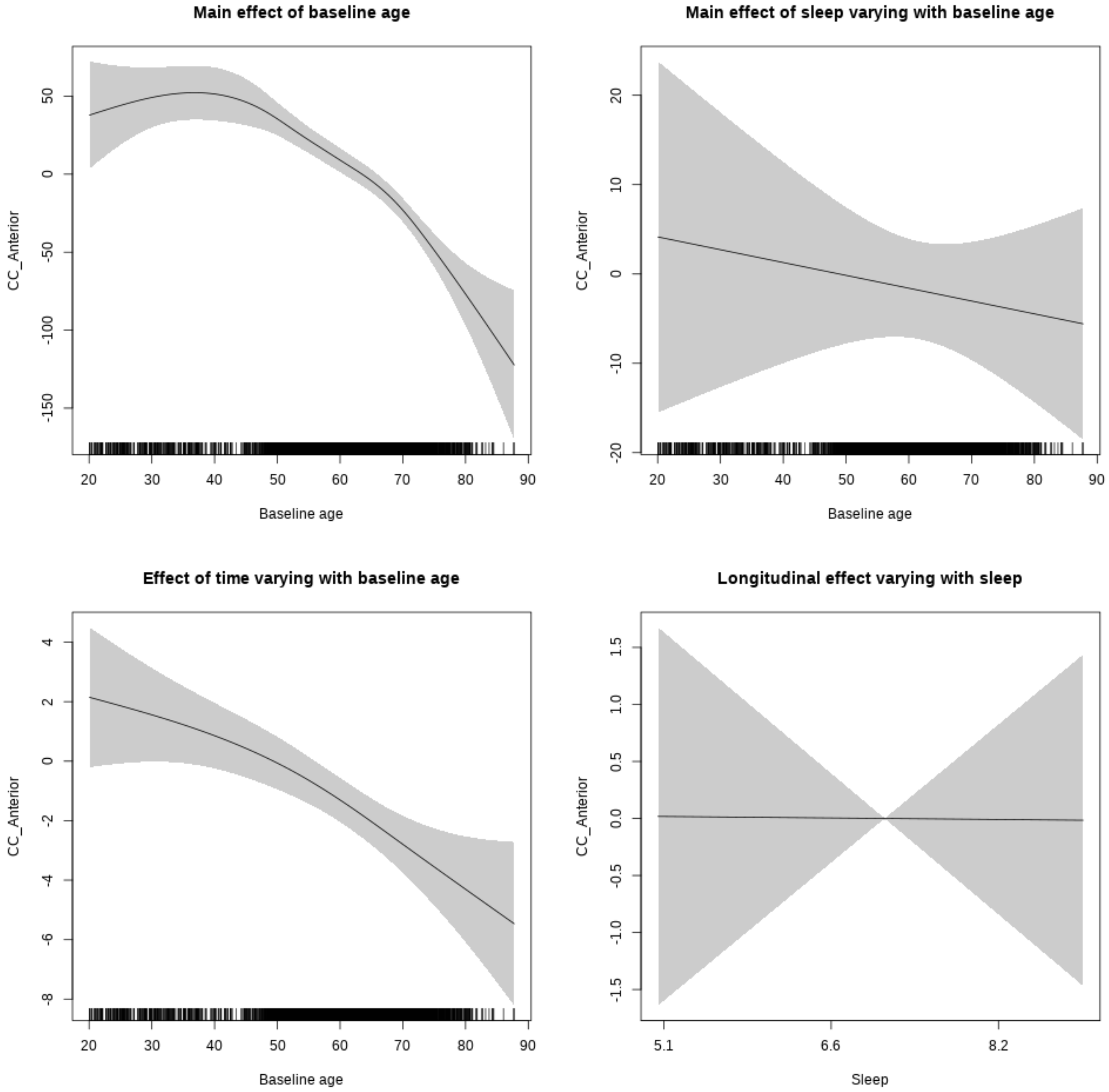

Restricted to sleep >= 5 and <= 9 hours Below is the row for `s(sleep_z):time` from the model output.

```
## s(sleep_z):time   1.000  1.000  0.000    0.983
```

Below are plots of a plot of `s(bl_age)`, `s(bl_age):sleep_z`, `s(bl_age):time`, and `s(sleep_z):time`. For `s(sleep_z):time`, roughly, if we cannot fit a straight line through the confidence bands, there is evidence that the effect of time depends on sleep duration. It is correct the that confidence interval is zero at one point along the axis; this happens because we do not include the main effect.

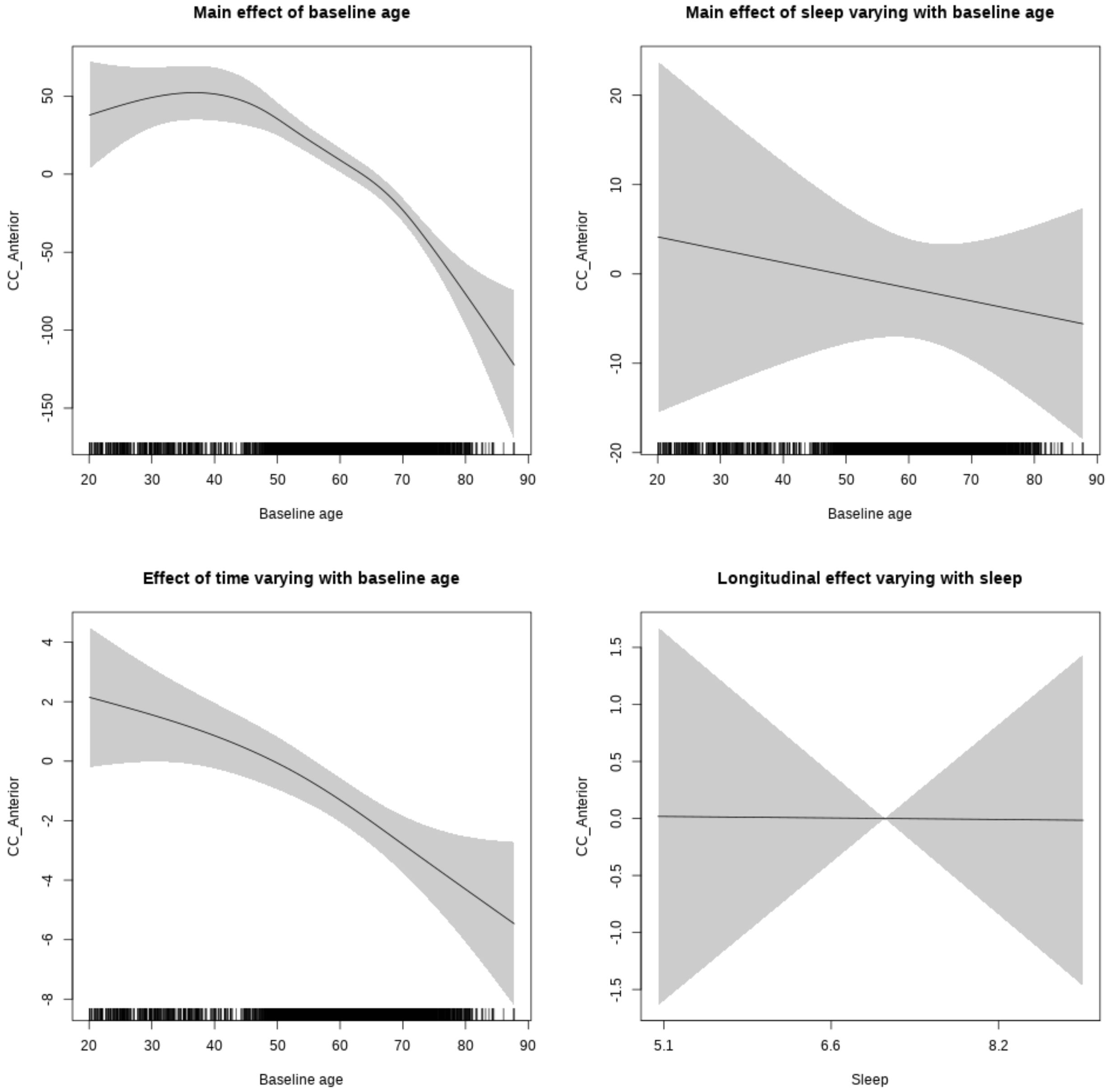

Controling for socioeconomic status

Full data Below is the model output. The term `s(sleep_z):time` is probably what we care most about.

```
##
## Family: gaussian
## Link function: identity
##
## Formula:
## value ~ sex + site + income_scaled + education_scaled + icv +
##     s(bl_age, k = 10, bs = "cr") + s(bl_age, by = sleep_z, bs = "cr") +
##     +s(bl_age, by = time, k = 5, bs = "cr") + s(sleep_z, by = time,
##     k = 5, bs = "cr", pc = 0)
```

```
## <environment: 0x563914d94f00>
##
## Parametric coefficients:
##                  Estimate Std. Error t value Pr(>|t|)
## (Intercept)      1091.214     26.127  41.765  < 2e-16 ***
## sexmale           -68.151      7.214  -9.447  < 2e-16 ***
## siteousAvanto    -229.025     28.838  -7.942 2.49e-15 ***
## siteousPrisma    -227.859     62.088  -3.670 0.000245 ***
## siteousSkyra     -177.742     26.733  -6.649 3.30e-11 ***
## siteUKB          -111.670     24.679  -4.525 6.19e-06 ***
## income_scaled     -10.445      9.162  -1.140 0.254322
## education_scaled    6.501     10.243   0.635 0.525636
## icv               100.231      3.581  27.992  < 2e-16 ***
## ---
## Signif. codes:  0 '***' 0.001 '**' 0.01 '*' 0.05 '.' 0.1 ' ' 1
##
## Approximate significance of smooth terms:
##                     edf Ref.df      F  p-value
## s(bl_age)         2.289  2.289 12.581 1.56e-06 ***
## s(bl_age):sleep_z 2.000  2.000  1.406  0.24513
## s(bl_age):time    3.144  3.144  4.588  0.00515 **
## s(sleep_z):time   1.000  1.000  0.445  0.50494
## ---
## Signif. codes:  0 '***' 0.001 '**' 0.01 '*' 0.05 '.' 0.1 ' ' 1
##
## R-sq.(adj) =  0.244
## lmer.REML =  55412  Scale est. = 2091.1    n = 4640
```

Below are plots of a plot of `s(bl_age)`, `s(bl_age):sleep_z`, `s(bl_age):time`, and `s(sleep_z):time`. For `s(sleep_z):time`, roughly, if we cannot fit a straight line through the confidence bands, there is evidence that the effect of time depends on sleep duration. It is correct the that confidence interval is zero at one point along the axis; this happens because we do not include the main effect.

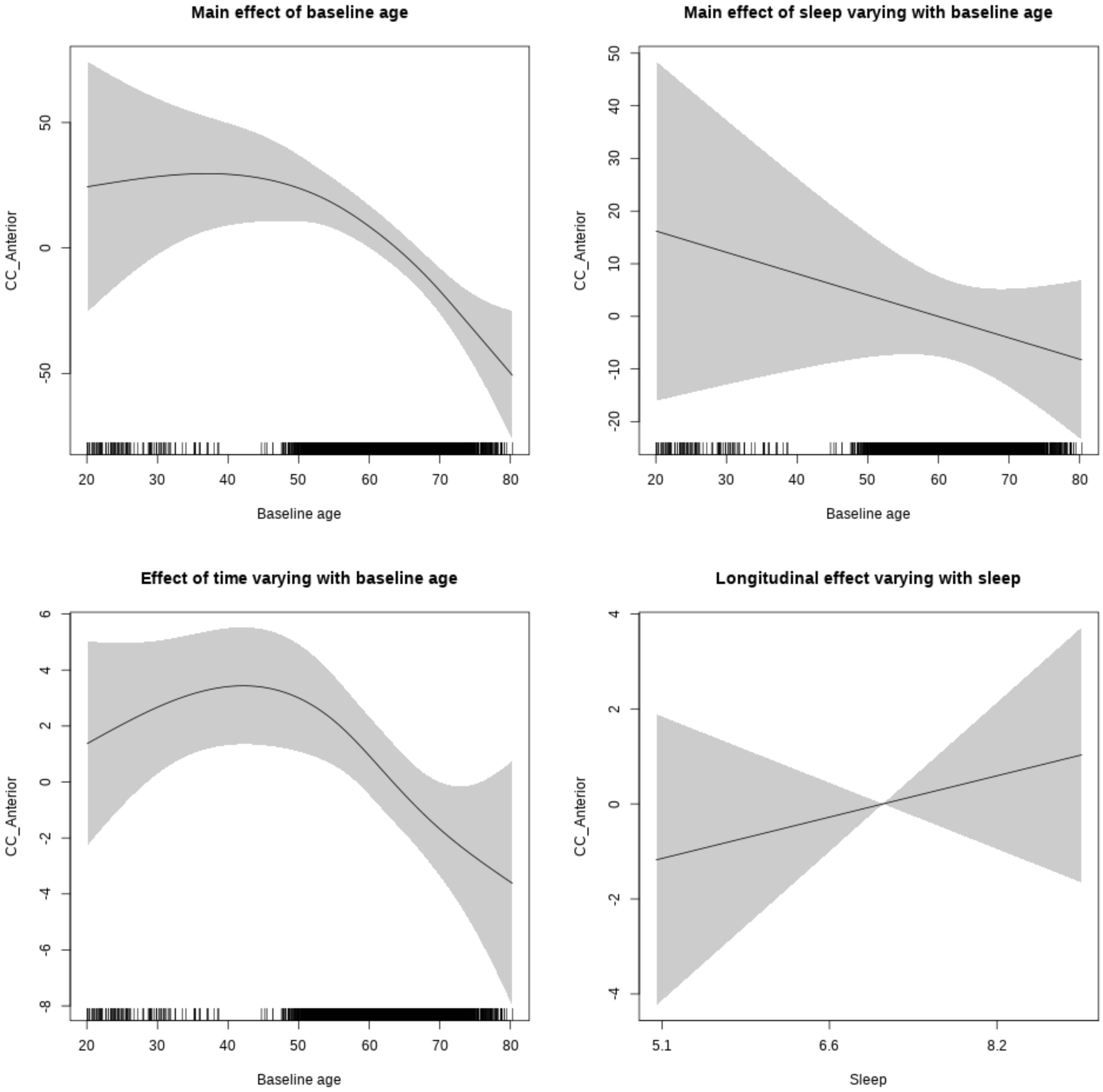

Restricted to sleep >= 5 and <= 9 hours Below is the row for `s(sleep_z):time` from the model output.

```
## s(sleep_z):time   1.000  1.000  0.591   0.4422
```

Below are plots of a plot of `s(bl_age)`, `s(bl_age):sleep_z`, `s(bl_age):time`, and `s(sleep_z):time`. For `s(sleep_z):time`, roughly, if we cannot fit a straight line through the confidence bands, there is evidence that the effect of time depends on sleep duration. It is correct the that confidence interval is zero at one point along the axis; this happens because we do not include the main effect.

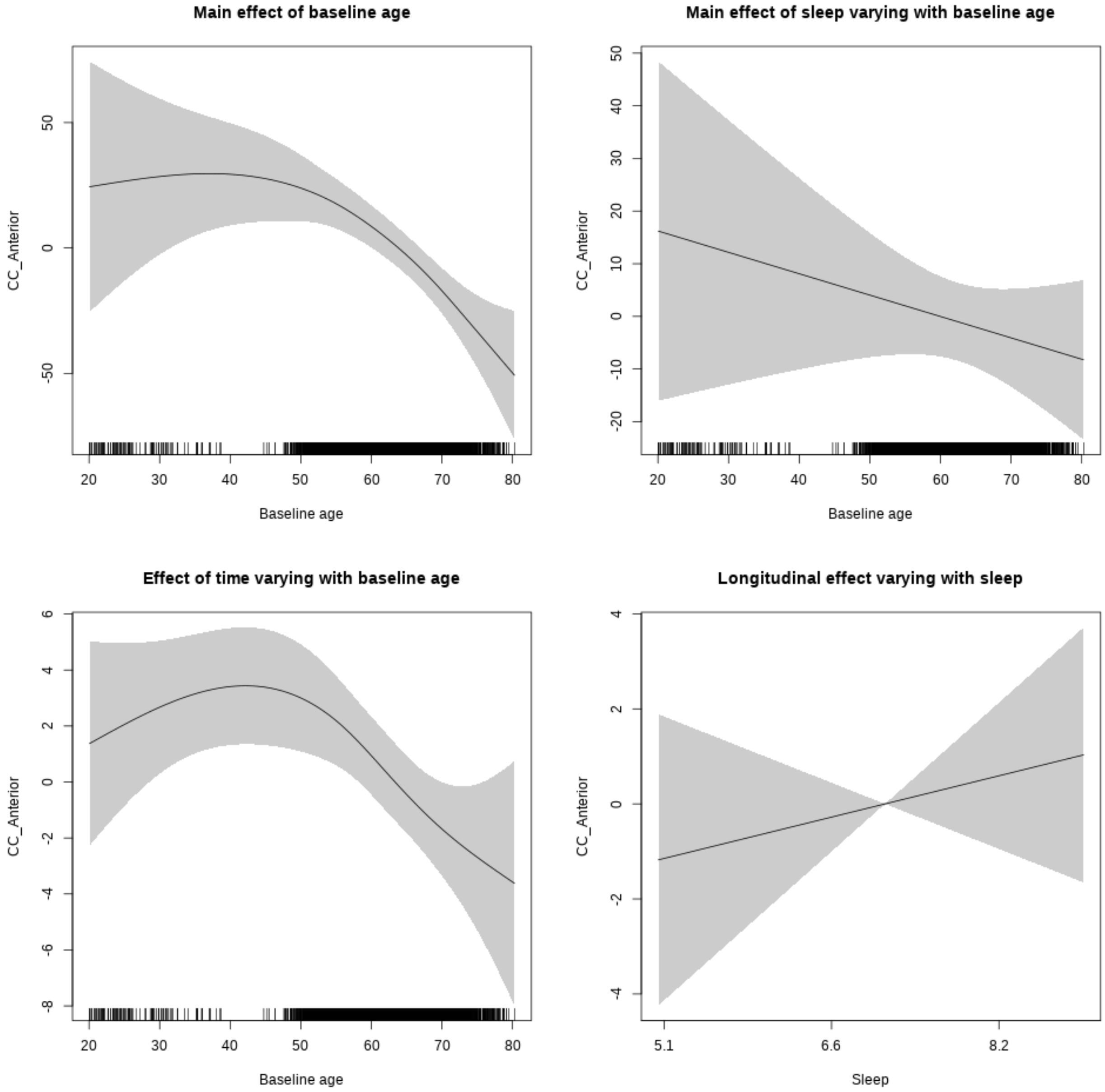

Controling for BMI

Full data Below is the model output. The term `s(sleep_z):time` is probably what we care most about.

```
##
## Family: gaussian
## Link function: identity
##
## Formula:
## value ~ sex + site + bmi + icv + s(bl_age, k = 10, bs = "cr") +
##     s(bl_age, by = sleep_z, bs = "cr") + +s(bl_age, by = time,
##     k = 5, bs = "cr") + s(sleep_z, by = time, k = 5, bs = "cr",
##     pc = 0)
```

```
## <environment: 0x5639110b8150>
##
## Parametric coefficients:
##                Estimate Std. Error t value Pr(>|t|)
## (Intercept)    913.0309    23.5848  38.713  < 2e-16 ***
## sexmale        -55.3715     6.6246  -8.358  < 2e-16 ***
## siteousPrisma   -0.2780    35.7937  -0.008 0.993804
## siteousSkyra    41.9660    11.3363   3.702 0.000216 ***
## siteUCAM        17.1302    17.9869   0.952 0.340951
## siteUKB        108.5606    16.8330   6.449 1.22e-10 ***
## siteUmU         71.4450    20.6060   3.467 0.000530 ***
## bmi             -1.7878     0.6862  -2.606 0.009198 **
## icv             93.2012     3.2743  28.465  < 2e-16 ***
## ---
## Signif. codes:  0 '***' 0.001 '**' 0.01 '*' 0.05 '.' 0.1 ' ' 1
##
## Approximate significance of smooth terms:
##                      edf Ref.df      F p-value
## s(bl_age)          2.799  2.799 20.977 < 2e-16 ***
## s(bl_age):sleep_z 2.000  2.000  0.418 0.65817
## s(bl_age):time    2.888  2.888  4.984 0.00127 **
## s(sleep_z):time   1.000  1.000  0.155 0.69341
## ---
## Signif. codes:  0 '***' 0.001 '**' 0.01 '*' 0.05 '.' 0.1 ' ' 1
##
## R-sq.(adj) =  0.249
## lmer.REML =  64354  Scale est. = 1968.3    n = 5402
```

Below are plots of a plot of `s(bl_age)`, `s(bl_age):sleep_z`, `s(bl_age):time`, and `s(sleep_z):time`. For `s(sleep_z):time`, roughly, if we cannot fit a straight line through the confidence bands, there is evidence that the effect of time depends on sleep duration. It is correct the that confidence interval is zero at one point along the axis; this happens because we do not include the main effect.

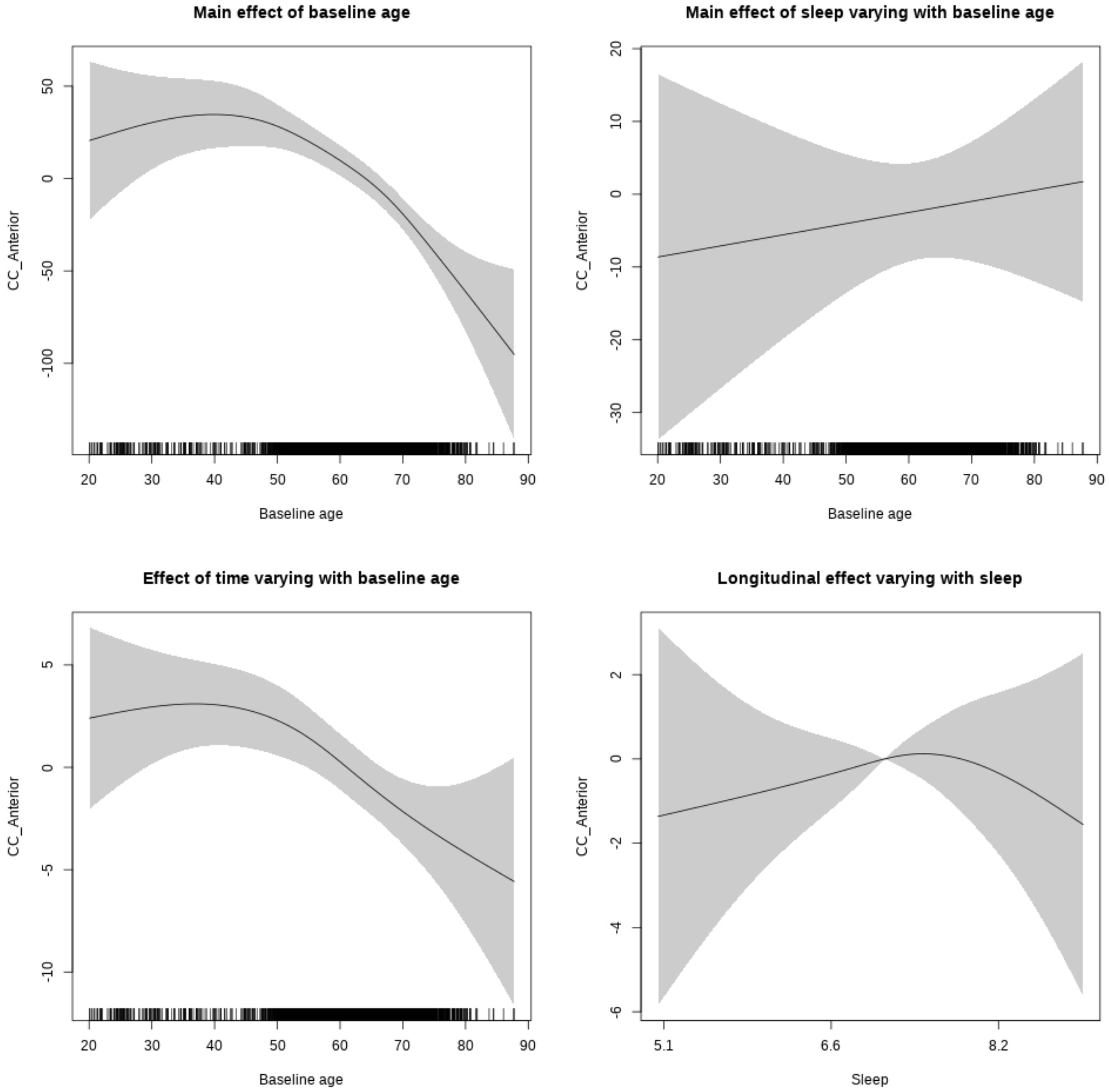

Restricted to sleep >= 5 and <= 9 hours Below is the row for `s(sleep_z):time` from the model output.

```
## s(sleep_z):time   1.725  1.725  0.447 0.67040
```

Below are plots of a plot of `s(bl_age)`, `s(bl_age):sleep_z`, `s(bl_age):time`, and `s(sleep_z):time`. For `s(sleep_z):time`, roughly, if we cannot fit a straight line through the confidence bands, there is evidence that the effect of time depends on sleep duration. It is correct the that confidence interval is zero at one point along the axis; this happens because we do not include the main effect.

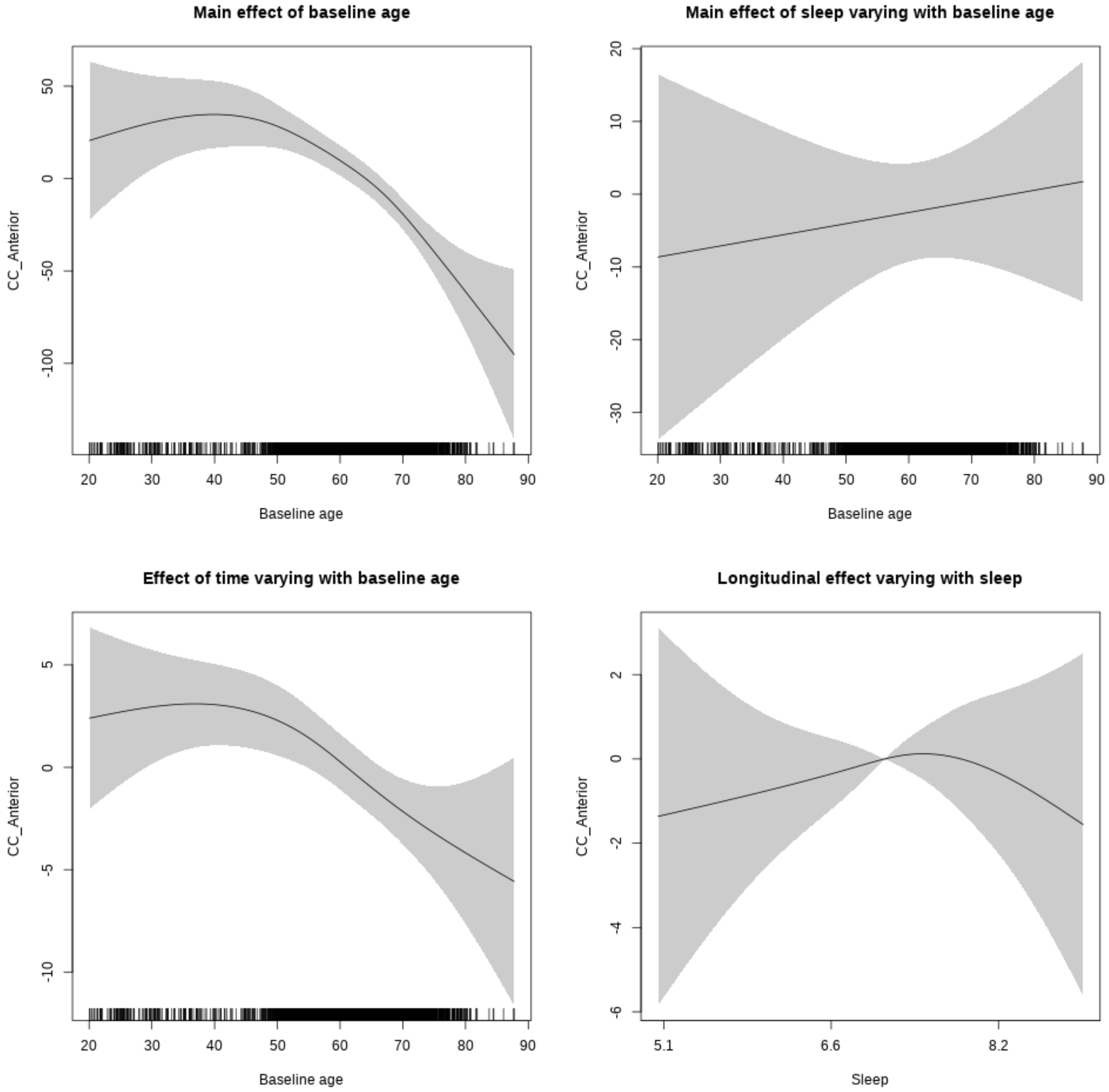

Controling for depression

Full data Below is the model output. The term `s(sleep_z):time` is probably what we care most about.

```
##
## Family: gaussian
## Link function: identity
##
## Formula:
## value ~ sex + site + depression + icv + s(bl_age, k = 10, bs = "cr") +
##     s(bl_age, by = sleep_z, bs = "cr") + +s(bl_age, by = time,
##     k = 5, bs = "cr") + s(sleep_z, by = time, k = 5, bs = "cr",
##     pc = 0)
```

```
## <environment: 0x563914f44da8>
##
## Parametric coefficients:
##                 Estimate Std. Error t value Pr(>|t|)
## (Intercept)     1029.829     12.580  81.860  < 2e-16 ***
## sexmale          -54.666      6.583  -8.304  < 2e-16 ***
## siteousAvanto -156.425     27.039  -5.785 7.63e-09 ***
## siteousPrisma -152.857     48.347  -3.162  0.00158 **
## siteousSkyra  -114.034     22.887  -4.983 6.46e-07 ***
## siteUCAM      -142.953     15.074  -9.484  < 2e-16 ***
## siteUKB        -55.391     11.553  -4.794 1.67e-06 ***
## siteUmU        -86.171     18.440  -4.673 3.04e-06 ***
## depression     -17.759     23.106  -0.769  0.44218
## icv             87.739      3.179  27.597  < 2e-16 ***
## ---
## Signif. codes:  0 '***' 0.001 '**' 0.01 '*' 0.05 '.' 0.1 ' ' 1
##
## Approximate significance of smooth terms:
##                      edf Ref.df      F p-value
## s(bl_age)          2.987  2.987 20.000 < 2e-16 ***
## s(bl_age):sleep_z 2.000  2.000  1.760 0.17209
## s(bl_age):time    2.655  2.655  4.266 0.00414 **
## s(sleep_z):time   1.000  1.000  0.045 0.83189
## ---
## Signif. codes:  0 '***' 0.001 '**' 0.01 '*' 0.05 '.' 0.1 ' ' 1
##
## R-sq.(adj) =  0.242
## lmer.REML =  67610  Scale est. = 1925.6    n = 5669
```

Below are plots of a plot of `s(bl_age)`, `s(bl_age):sleep_z`, `s(bl_age):time`, and `s(sleep_z):time`. For `s(sleep_z):time`, roughly, if we cannot fit a straight line through the confidence bands, there is evidence that the effect of time depends on sleep duration. It is correct the that confidence interval is zero at one point along the axis; this happens because we do not include the main effect.

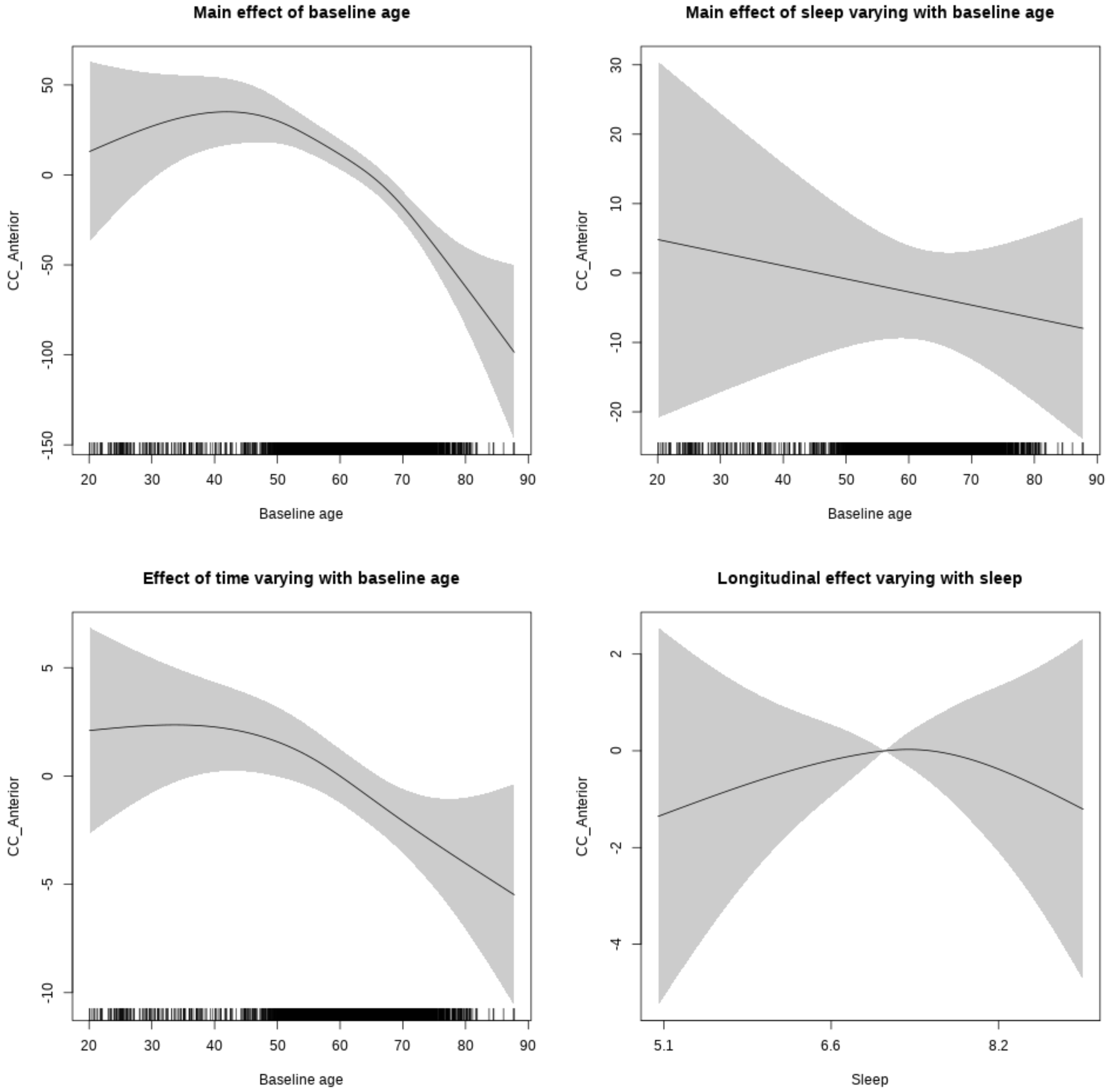

Restricted to sleep >= 5 and <= 9 hours Below is the row for `s(sleep_z):time` from the model output.

```
## s(sleep_z):time   1.534  1.534  0.374  0.6866
```

Below are plots of a plot of `s(bl_age)`, `s(bl_age):sleep_z`, `s(bl_age):time`, and `s(sleep_z):time`. For `s(sleep_z):time`, roughly, if we cannot fit a straight line through the confidence bands, there is evidence that the effect of time depends on sleep duration. It is correct the that confidence interval is zero at one point along the axis; this happens because we do not include the main effect.

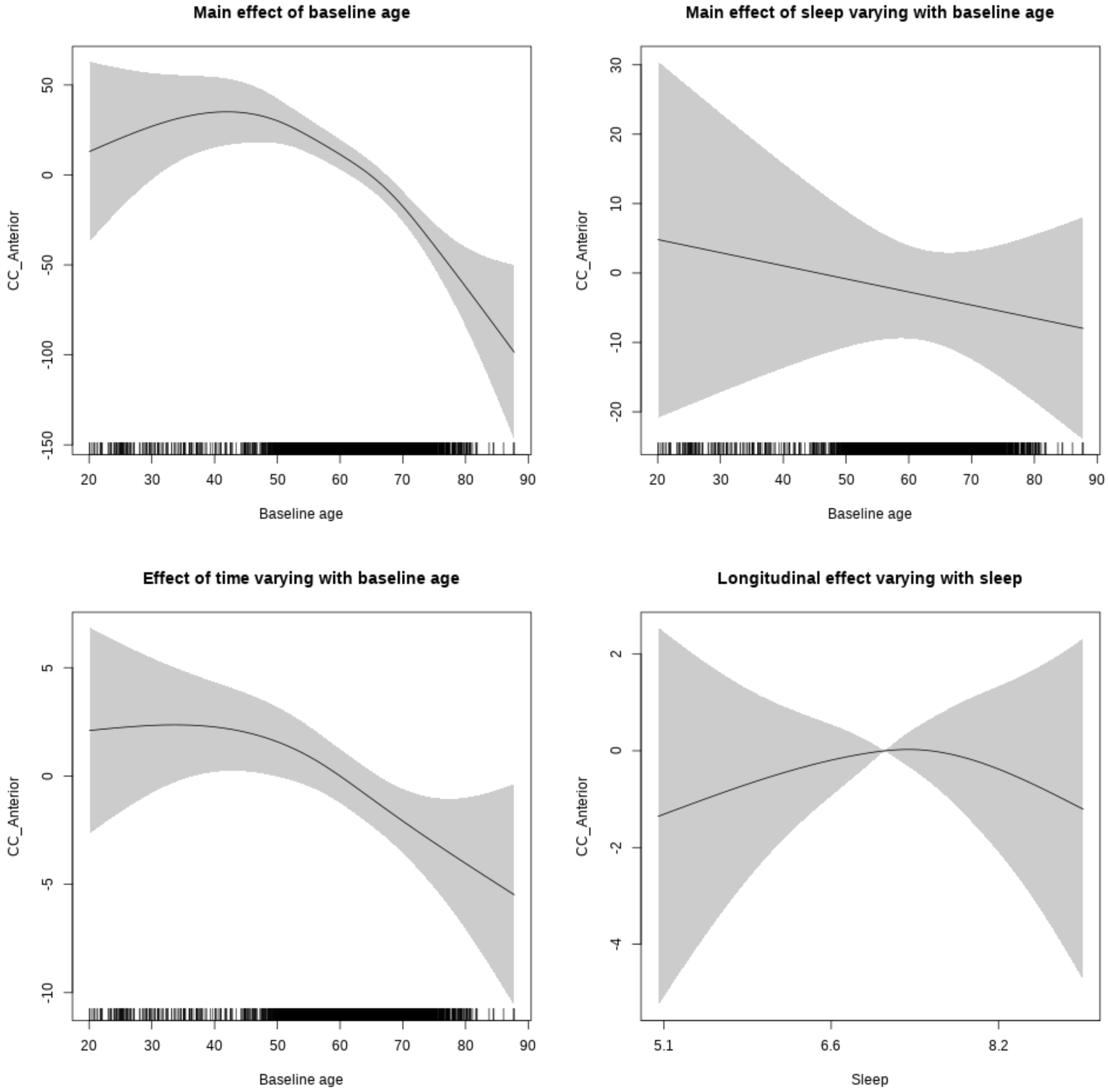

Controling for sleep quality

Full data Below is the model output. The term `s(sleep_z):time` is probably what we care most about.

```
##
## Family: gaussian
## Link function: identity
##
## Formula:
## value ~ X + s(bl_age, k = 10, bs = "cr") + s(bl_age, by = sleep_z,
##     bs = "cr") + s(bl_age, by = time, k = 5, bs = "cr") + s(sleep_z,
##     by = time, k = 5, bs = "cr", pc = 0)
## <environment: 0x563910056020>
```

```
##
## Parametric coefficients:
##                            Estimate Std. Error t value Pr(>|t|)
## (Intercept)                859.0983    19.2650  44.594  < 2e-16 ***
## Xsexmale                    49.2199     7.2099   6.827 9.92e-12 ***
## XsiteousAvanto               0.8138    18.4176   0.044   0.9648
## XsiteousPrisma              51.8573    34.7785   1.491   0.1360
## XsiteousSkyra                7.7569    18.4791   0.420   0.6747
## XsiteUB                    -31.3840    29.2477  -1.073   0.2833
## XsiteUCAM                   -8.1021    18.8804  -0.429   0.6679
## XsiteUKB                   -97.4471    58.8306  -1.656   0.0977 .
## XsiteUmU                    -7.3338    20.9808  -0.350   0.7267
## Xukb_dummy:sleep_accel       8.5035     4.5310   1.877   0.0606 .
## Xukb_dummy:chronotype        6.5705     6.0671   1.083   0.2789
## Xukb_dummy:dozing            9.5405    12.4861   0.764   0.4449
## Xukb_dummy:sleeplessness     6.0568     7.7865   0.778   0.4367
## Xukb_dummy:snoring          11.7254    11.0357   1.062   0.2881
## Xukb_dummy:gettingup        20.8107     8.4829   2.453   0.0142 *
## Xnotukb_dummy:PSQI_Global   -0.7046     2.3844  -0.295   0.7676
## ---
## Signif. codes:  0 '***' 0.001 '**' 0.01 '*' 0.05 '.' 0.1 ' ' 1
##
## Approximate significance of smooth terms:
##                      edf Ref.df      F p-value
## s(bl_age)          3.452  3.452 31.110  <2e-16 ***
## s(bl_age):sleep_z  2.000  2.000  0.205   0.814
## s(bl_age):time     2.000  2.000 37.724  <2e-16 ***
## s(sleep_z):time    1.000  1.000  0.001   0.980
## ---
## Signif. codes:  0 '***' 0.001 '**' 0.01 '*' 0.05 '.' 0.1 ' ' 1
##
## R-sq.(adj) =  0.131
## lmer.REML =  50229  Scale est. = 1486.4    n = 4280
```

Below are plots of a plot of `s(bl_age)`, `s(bl_age):sleep_z`, `s(bl_age):time`, and `s(sleep_z):time`. For `s(sleep_z):time`, roughly, if we cannot fit a straight line through the confidence bands, there is evidence that the effect of time depends on sleep duration. It is correct the that confidence interval is zero at one point along the axis; this happens because we do not include the main effect.

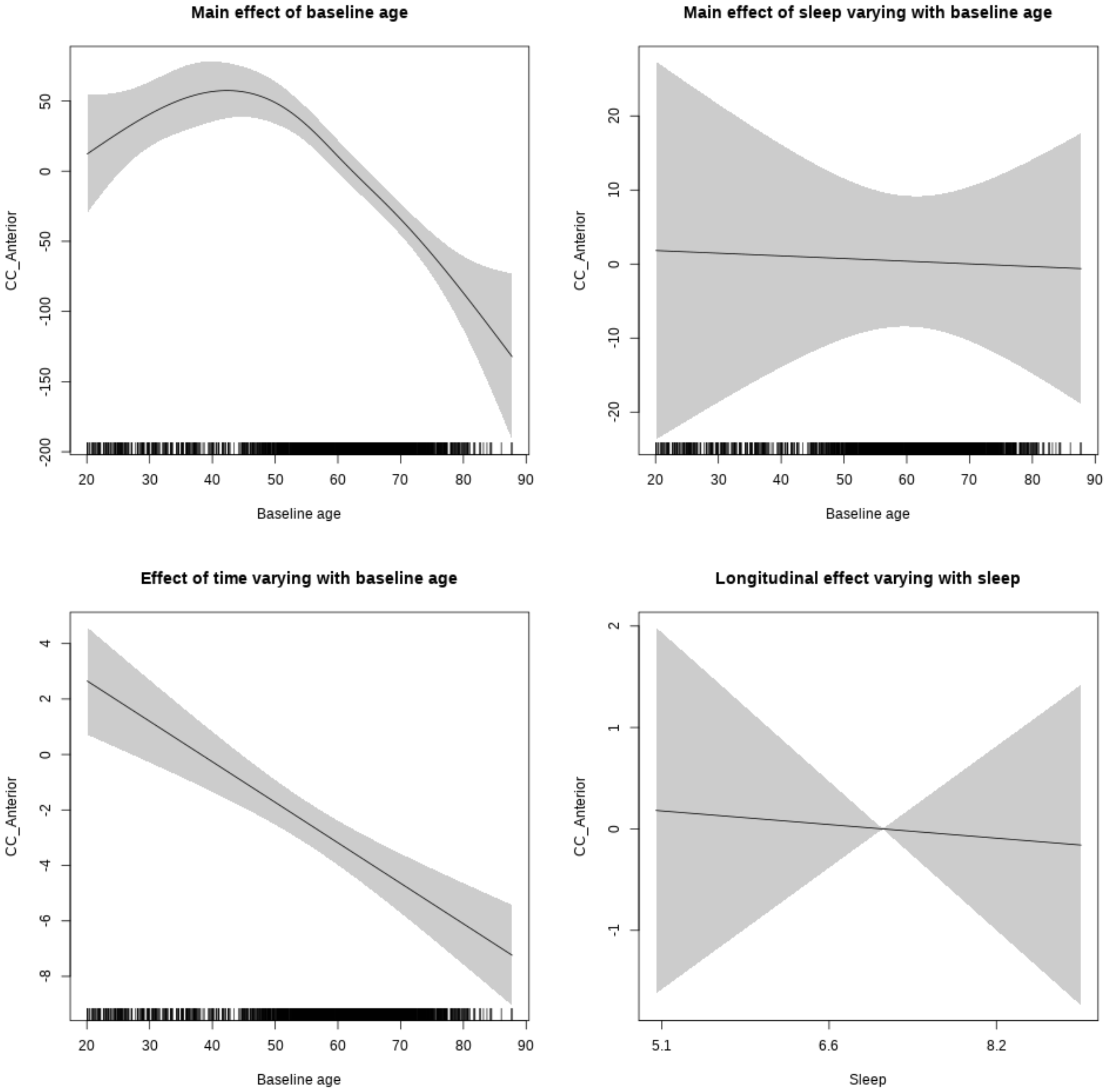

Restricted to sleep >= 5 and <= 9 hours Below is the row for `s(sleep_z):time` from the model output.

```
## s(sleep_z):time   1.000  1.000  0.041   0.840
```

Below are plots of a plot of `s(bl_age)`, `s(bl_age):sleep_z`, `s(bl_age):time`, and `s(sleep_z):time`. For `s(sleep_z):time`, roughly, if we cannot fit a straight line through the confidence bands, there is evidence that the effect of time depends on sleep duration. It is correct the that confidence interval is zero at one point along the axis; this happens because we do not include the main effect.

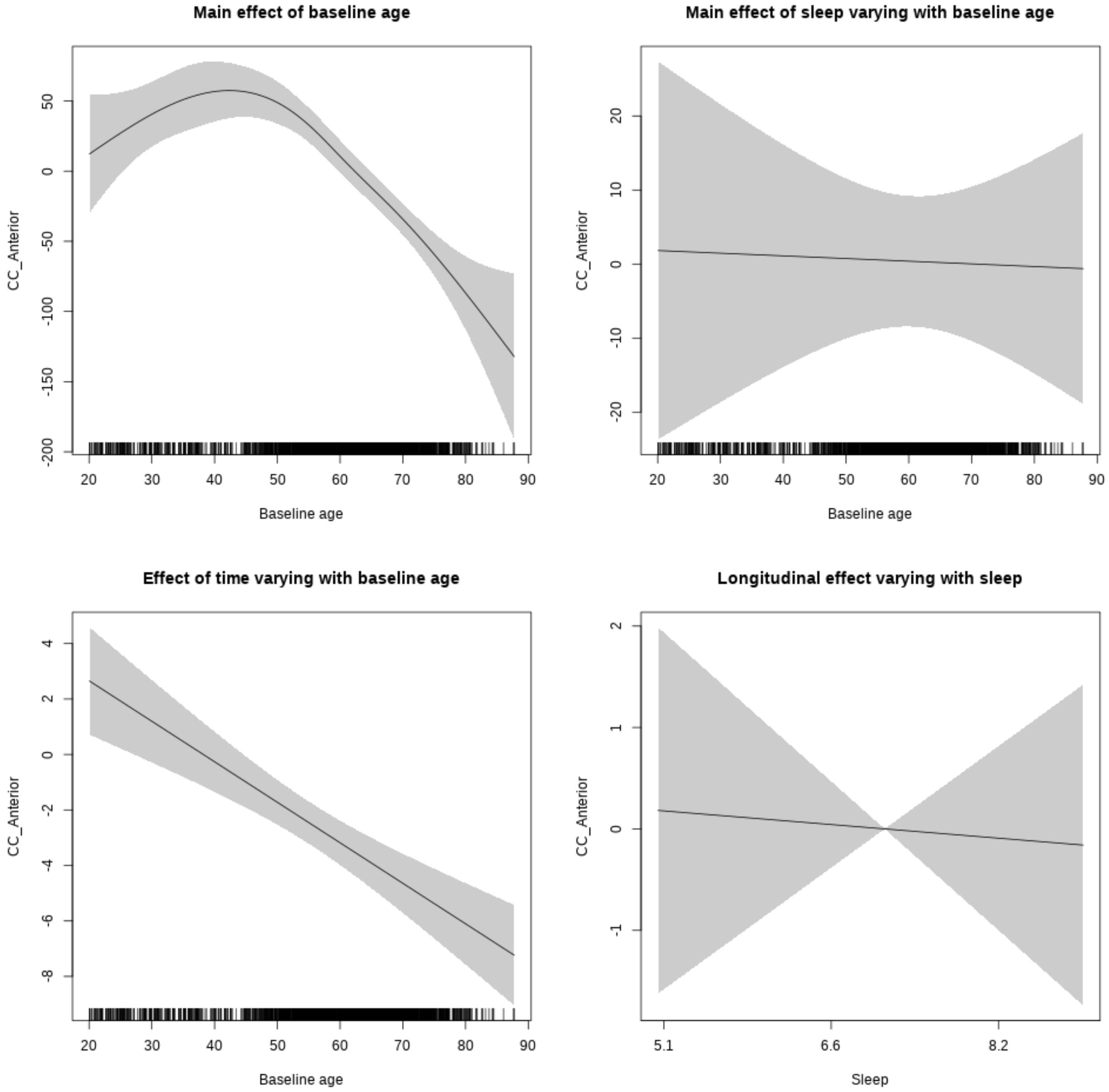

CC_Central

Main analysis

Full data Below is the model output. The term `s(sleep_z):time` is probably what we care most about.

```
##
## Family: gaussian
## Link function: identity
##
## Formula:
## value ~ sex + site + icv + s(bl_age, k = 10, bs = "cr") + s(bl_age,
##     by = sleep_z, bs = "cr") + +s(bl_age, by = time, k = 5, bs = "cr") +
```

```
##     s(sleep_z, by = time, k = 5, bs = "cr", pc = 0)
## <environment: 0x56391119f7e0>
##
## Parametric coefficients:
##               Estimate Std. Error t value Pr(>|t|)
## (Intercept)    536.885      8.292  64.750  < 2e-16 ***
## sexmale        -20.795      4.275  -4.864 1.17e-06 ***
## siteousAvanto  -80.679     10.191  -7.917 2.76e-15 ***
## siteousPrisma  -50.566     28.378  -1.782  0.07480 .
## siteousSkyra   -86.897      9.907  -8.771  < 2e-16 ***
## siteUB         -50.600     19.156  -2.642  0.00827 **
## siteUCAM       -26.178     10.351  -2.529  0.01146 *
## siteUKB          8.208      7.893   1.040  0.29839
## siteUmU         -8.750     11.900  -0.735  0.46218
## icv             22.677      2.061  11.002  < 2e-16 ***
## ---
## Signif. codes:  0 '***' 0.001 '**' 0.01 '*' 0.05 '.' 0.1 ' ' 1
##
## Approximate significance of smooth terms:
##                     edf Ref.df       F p-value
## s(bl_age)         3.481  3.481 142.841  <2e-16 ***
## s(bl_age):sleep_z 3.294  3.294   3.069  0.0452 *
## s(bl_age):time    3.125  3.125  65.424  <2e-16 ***
## s(sleep_z):time   1.000  1.000   0.405  0.5245
## ---
## Signif. codes:  0 '***' 0.001 '**' 0.01 '*' 0.05 '.' 0.1 ' ' 1
##
## R-sq.(adj) =  0.171
## lmer.REML =  92655  Scale est. = 1200.4    n = 8144
```

Below are plots of a plot of `s(bl_age)`, `s(bl_age):sleep_z`, `s(bl_age):time`, and `s(sleep_z):time`. For `s(sleep_z):time`, roughly, if we cannot fit a straight line through the confidence bands, there is evidence that the effect of time depends on sleep duration. It is correct the that confidence interval is zero at one point along the axis; this happens because we do not include the main effect.

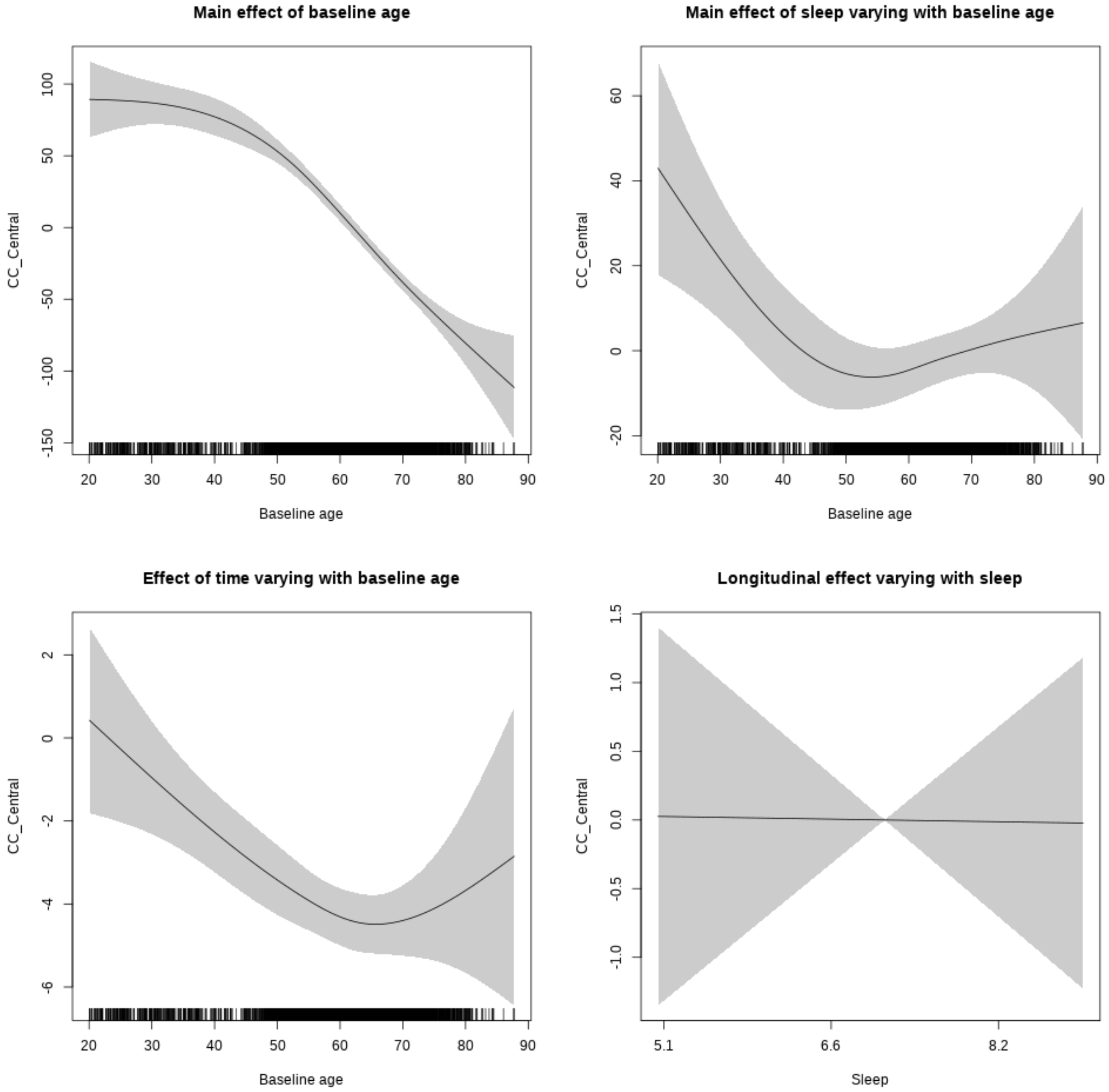

Restricted to sleep >= 5 and <= 9 hours Below is the row for `s(sleep_z):time` from the model output.

```
## s(sleep_z):time   1.000  1.000   0.001 0.97009
```

Below are plots of a plot of `s(bl_age)`, `s(bl_age):sleep_z`, `s(bl_age):time`, and `s(sleep_z):time`. For `s(sleep_z):time`, roughly, if we cannot fit a straight line through the confidence bands, there is evidence that the effect of time depends on sleep duration. It is correct the that confidence interval is zero at one point along the axis; this happens because we do not include the main effect.

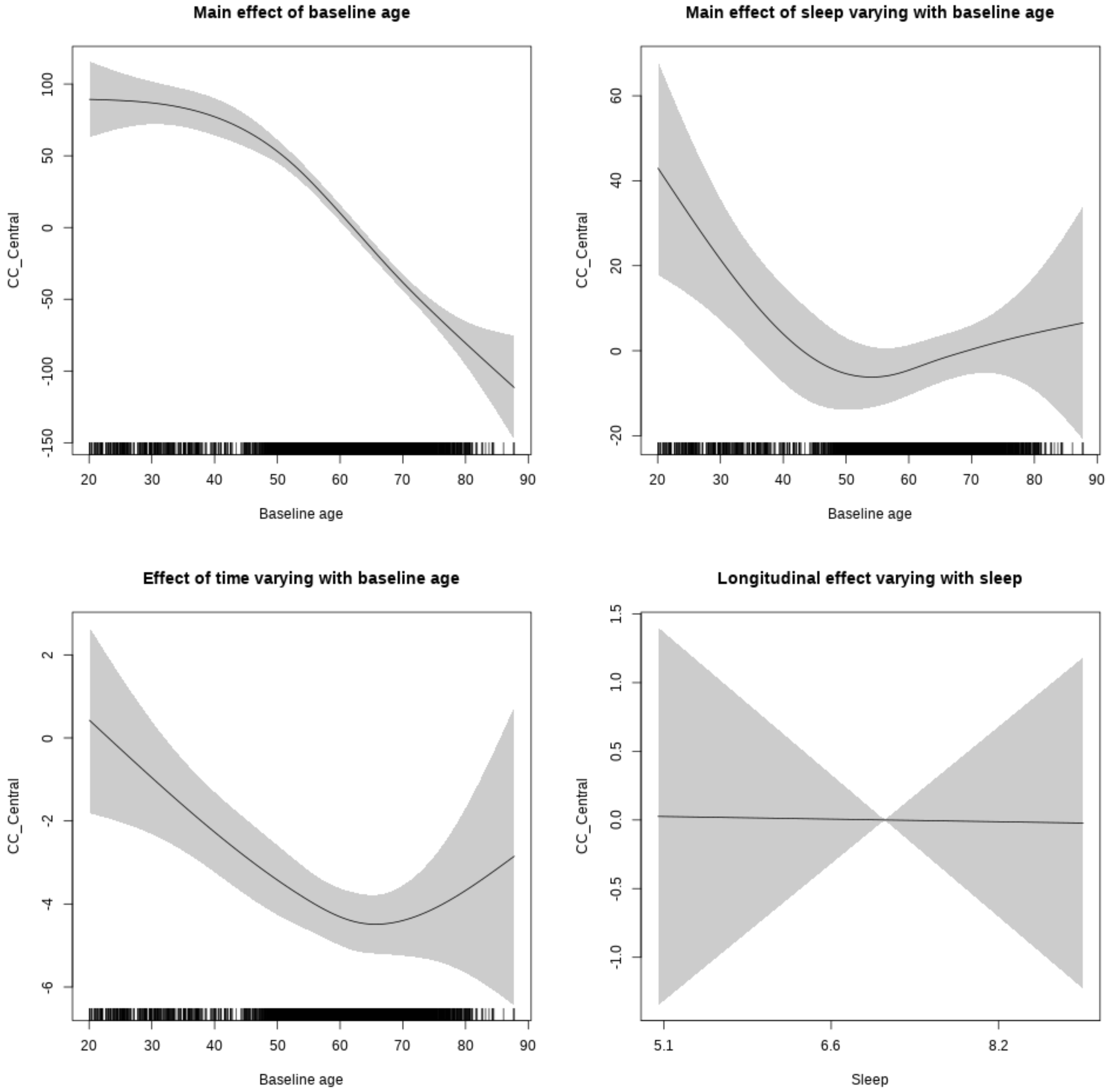

Controling for socioeconomic status

Full data Below is the model output. The term `s(sleep_z):time` is probably what we care most about.

```
##
## Family: gaussian
## Link function: identity
##
## Formula:
## value ~ sex + site + income_scaled + education_scaled + icv +
##     s(bl_age, k = 10, bs = "cr") + s(bl_age, by = sleep_z, bs = "cr") +
##     +s(bl_age, by = time, k = 5, bs = "cr") + s(sleep_z, by = time,
##     k = 5, bs = "cr", pc = 0)
```

```
## <environment: 0x563914d942f8>
##
## Parametric coefficients:
##                   Estimate Std. Error t value Pr(>|t|)
## (Intercept)        543.530     20.660  26.308  < 2e-16 ***
## sexmale            -24.010      5.684  -4.224 2.45e-05 ***
## siteousAvanto      -86.377     22.865  -3.778  0.00016 ***
## siteousPrisma        2.220     50.546   0.044  0.96497
## siteousSkyra       -85.065     21.092  -4.033 5.60e-05 ***
## siteUKB             17.325     19.538   0.887  0.37526
## income_scaled       -3.531      7.270  -0.486  0.62718
## education_scaled   -18.296      8.040  -2.276  0.02291 *
## icv                 28.530      2.826  10.095  < 2e-16 ***
## ---
## Signif. codes:  0 '***' 0.001 '**' 0.01 '*' 0.05 '.' 0.1 ' ' 1
##
## Approximate significance of smooth terms:
##                       edf Ref.df      F p-value
## s(bl_age)           3.208  3.208 73.587  <2e-16 ***
## s(bl_age):sleep_z 2.000  2.000  0.263   0.769
## s(bl_age):time    2.882  2.882 35.084  <2e-16 ***
## s(sleep_z):time   1.000  1.000  0.024   0.878
## ---
## Signif. codes:  0 '***' 0.001 '**' 0.01 '*' 0.05 '.' 0.1 ' ' 1
##
## R-sq.(adj) =  0.145
## lmer.REML =  53457  Scale est. = 1410.7    n = 4646
```

Below are plots of a plot of `s(bl_age)`, `s(bl_age):sleep_z`, `s(bl_age):time`, and `s(sleep_z):time`. For `s(sleep_z):time`, roughly, if we cannot fit a straight line through the confidence bands, there is evidence that the effect of time depends on sleep duration. It is correct the that confidence interval is zero at one point along the axis; this happens because we do not include the main effect.

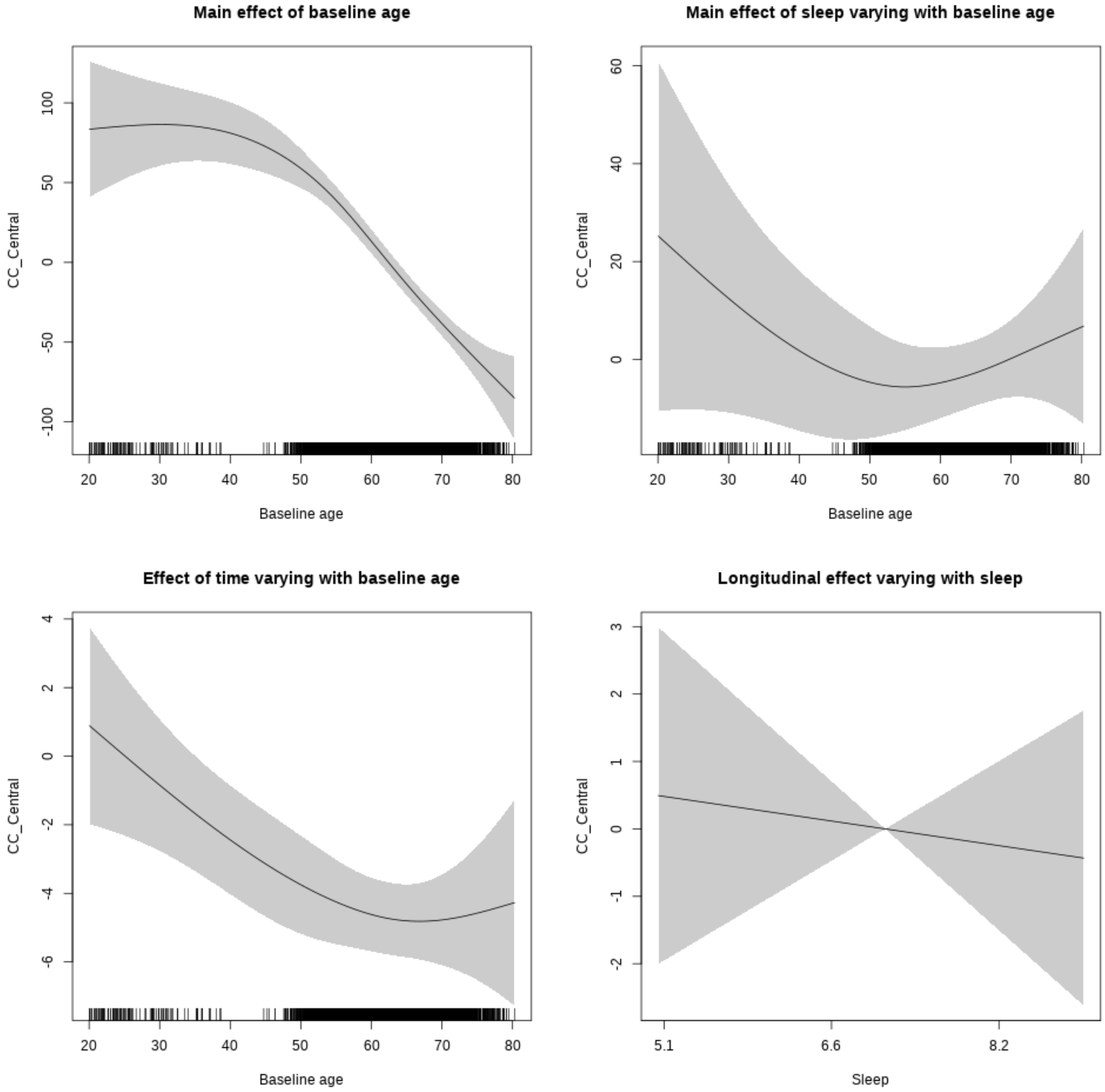

Restricted to sleep >= 5 and <= 9 hours Below is the row for `s(sleep_z):time` from the model output.

```
## s(sleep_z):time   1.000  1.000  0.157   0.692
```

Below are plots of a plot of `s(bl_age)`, `s(bl_age):sleep_z`, `s(bl_age):time`, and `s(sleep_z):time`. For `s(sleep_z):time`, roughly, if we cannot fit a straight line through the confidence bands, there is evidence that the effect of time depends on sleep duration. It is correct the that confidence interval is zero at one point along the axis; this happens because we do not include the main effect.

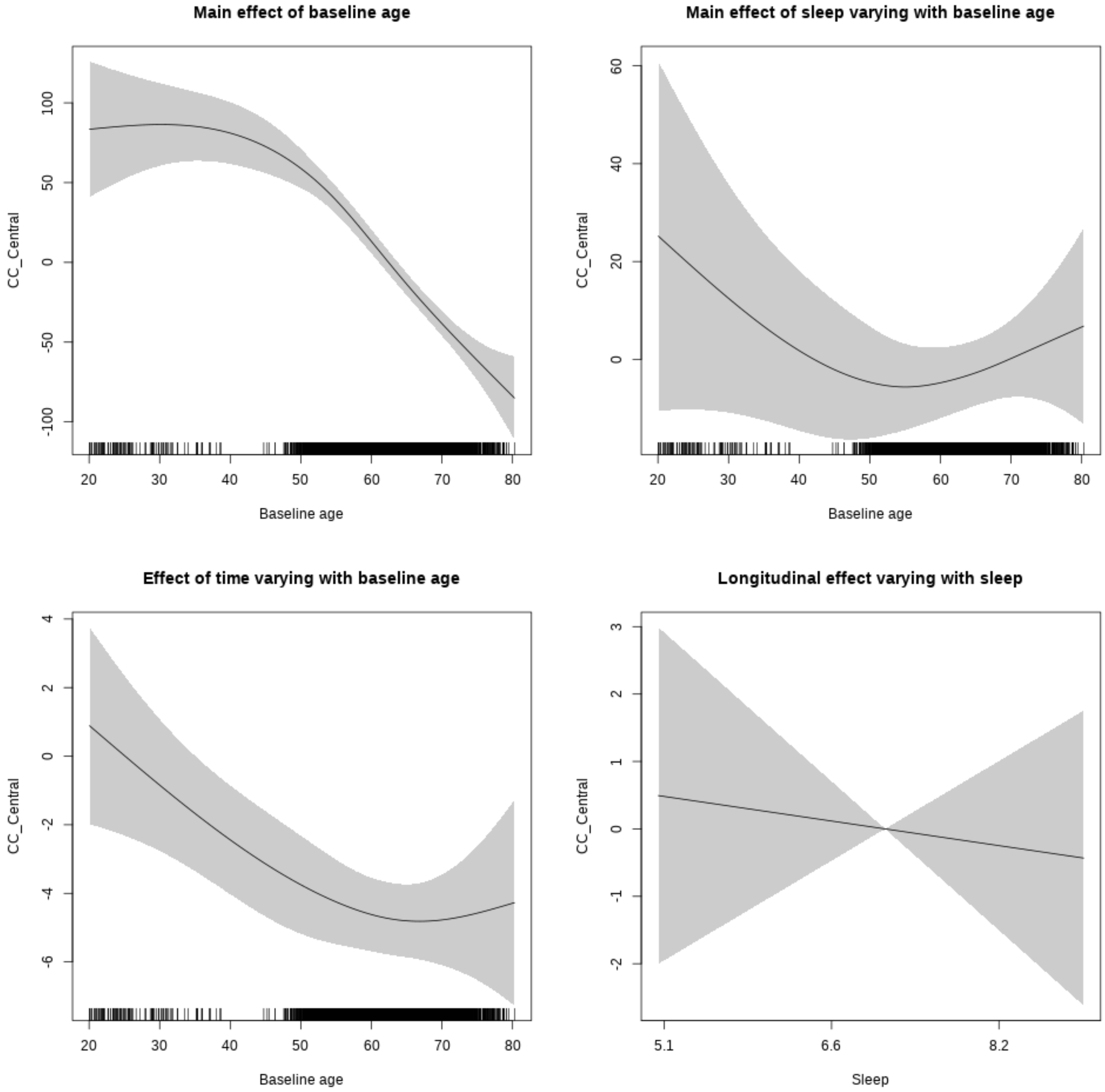

Controling for BMI

Full data Below is the model output. The term `s(sleep_z):time` is probably what we care most about.

```
##
## Family: gaussian
## Link function: identity
##
## Formula:
## value ~ sex + site + bmi + icv + s(bl_age, k = 10, bs = "cr") +
##     s(bl_age, by = sleep_z, bs = "cr") + +s(bl_age, by = time,
##     k = 5, bs = "cr") + s(sleep_z, by = time, k = 5, bs = "cr",
##     pc = 0)
```

```
## <environment: 0x563910f9aa40>
##
## Parametric coefficients:
##                Estimate Std. Error t value Pr(>|t|)
## (Intercept)    464.8117    18.8440  24.666  < 2e-16 ***
## sexmale        -19.0298     5.2496  -3.625 0.000292 ***
## siteousPrisma   36.8842    29.5311   1.249 0.211720
## siteousSkyra     4.5738     9.3437   0.490 0.624504
## siteUCAM        64.0764    14.5031   4.418 1.02e-05 ***
## siteUKB         96.9971    13.5902   7.137 1.08e-12 ***
## siteUmU         82.9643    16.5188   5.022 5.27e-07 ***
## bmi             -0.7186     0.5424  -1.325 0.185267
## icv             25.6322     2.6000   9.859  < 2e-16 ***
## ---
## Signif. codes:  0 '***' 0.001 '**' 0.01 '*' 0.05 '.' 0.1 ' ' 1
##
## Approximate significance of smooth terms:
##                      edf Ref.df      F p-value
## s(bl_age)          3.582  3.582 91.914  <2e-16 ***
## s(bl_age):sleep_z 2.000  2.000  1.409   0.245
## s(bl_age):time     3.784  3.784 25.408  <2e-16 ***
## s(sleep_z):time    1.000  1.000  1.086   0.297
## ---
## Signif. codes:  0 '***' 0.001 '**' 0.01 '*' 0.05 '.' 0.1 ' ' 1
##
## R-sq.(adj) =  0.151
## lmer.REML =  62138  Scale est. = 1344.3    n = 5408
```

Below are plots of a plot of `s(bl_age)`, `s(bl_age):sleep_z`, `s(bl_age):time`, and `s(sleep_z):time`. For `s(sleep_z):time`, roughly, if we cannot fit a straight line through the confidence bands, there is evidence that the effect of time depends on sleep duration. It is correct the that confidence interval is zero at one point along the axis; this happens because we do not include the main effect.

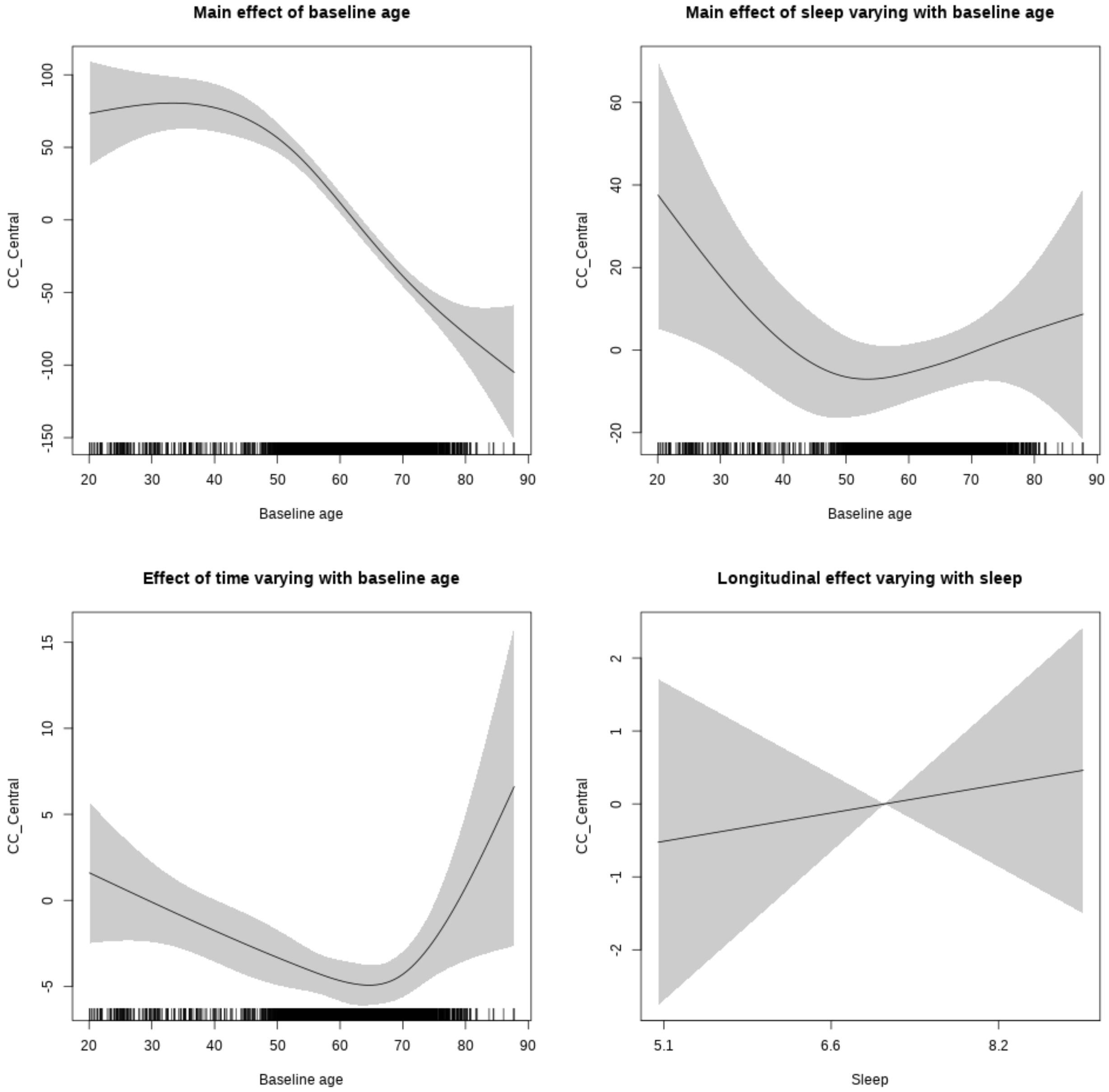

Restricted to sleep >= 5 and <= 9 hours Below is the row for `s(sleep_z):time` from the model output.

```
## s(sleep_z):time   1.000  1.000  0.221  0.6384
```

Below are plots of a plot of `s(bl_age)`, `s(bl_age):sleep_z`, `s(bl_age):time`, and `s(sleep_z):time`. For `s(sleep_z):time`, roughly, if we cannot fit a straight line through the confidence bands, there is evidence that the effect of time depends on sleep duration. It is correct the that confidence interval is zero at one point along the axis; this happens because we do not include the main effect.

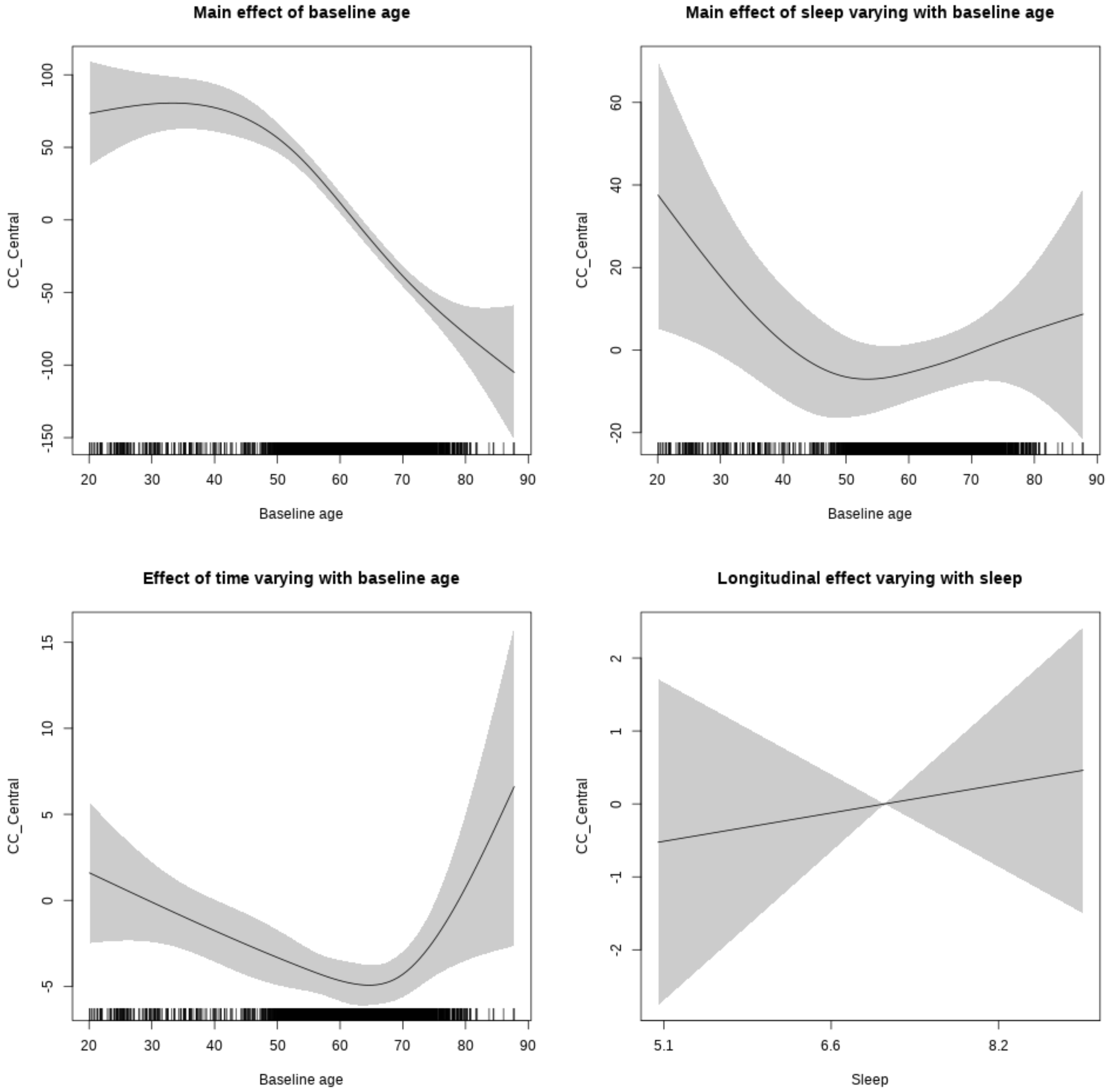

Controling for depression

Full data Below is the model output. The term `s(sleep_z):time` is probably what we care most about.

```
##
## Family: gaussian
## Link function: identity
##
## Formula:
## value ~ sex + site + depression + icv + s(bl_age, k = 10, bs = "cr") +
##     s(bl_age, by = sleep_z, bs = "cr") + +s(bl_age, by = time,
##     k = 5, bs = "cr") + s(sleep_z, by = time, k = 5, bs = "cr",
##     pc = 0)
```

```
## <environment: 0x563914fe5af8>
##
## Parametric coefficients:
##                Estimate Std. Error t value Pr(>|t|)
## (Intercept)    529.3237     9.7801  54.122  < 2e-16 ***
## sexmale        -16.6018     5.1161  -3.245  0.00118 **
## siteousAvanto  -98.1983    21.3945  -4.590 4.53e-06 ***
## siteousPrisma -100.2944    39.3846  -2.547  0.01091 *
## siteousSkyra  -105.7618    17.8411  -5.928 3.25e-09 ***
## siteUCAM       -23.8927    11.7466  -2.034  0.04200 *
## siteUKB         11.7027     8.9815   1.303  0.19263
## siteUmU         -0.1954    14.3080  -0.014  0.98911
## depression     -14.6110    17.9214  -0.815  0.41495
## icv             21.5778     2.4787   8.705  < 2e-16 ***
## ---
## Signif. codes:  0 '***' 0.001 '**' 0.01 '*' 0.05 '.' 0.1 ' ' 1
##
## Approximate significance of smooth terms:
##                     edf Ref.df      F p-value
## s(bl_age)         3.472  3.472 97.430  <2e-16 ***
## s(bl_age):sleep_z 2.000  2.000  2.030   0.131
## s(bl_age):time    3.311  3.311 32.908  <2e-16 ***
## s(sleep_z):time   1.000  1.000  0.554   0.457
## ---
## Signif. codes:  0 '***' 0.001 '**' 0.01 '*' 0.05 '.' 0.1 ' ' 1
##
## R-sq.(adj) =  0.153
## lmer.REML =  65173  Scale est. = 1319.3    n = 5675
```

Below are plots of a plot of `s(bl_age)`, `s(bl_age):sleep_z`, `s(bl_age):time`, and `s(sleep_z):time`. For `s(sleep_z):time`, roughly, if we cannot fit a straight line through the confidence bands, there is evidence that the effect of time depends on sleep duration. It is correct the that confidence interval is zero at one point along the axis; this happens because we do not include the main effect.

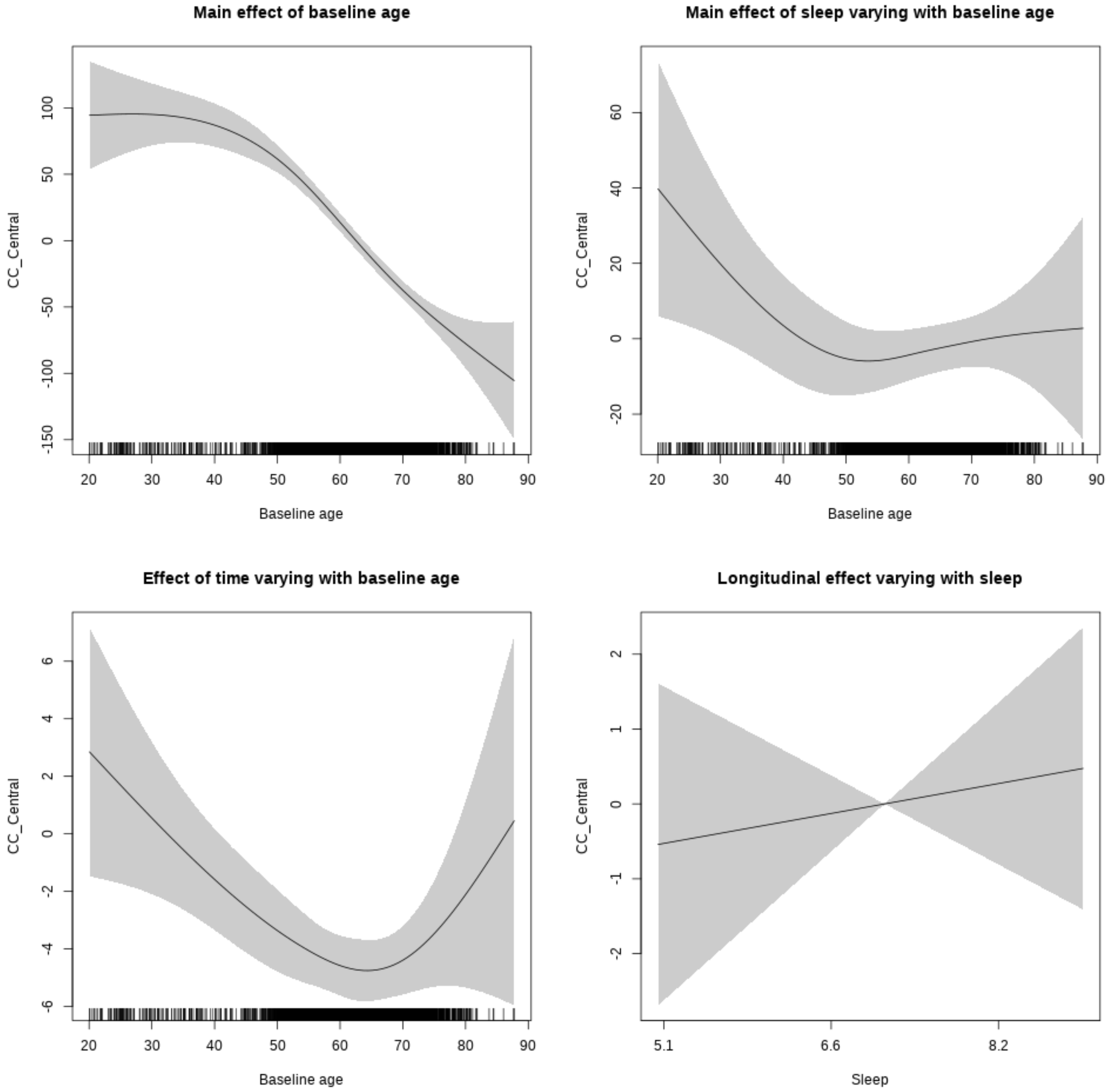

Restricted to sleep >= 5 and <= 9 hours Below is the row for `s(sleep_z):time` from the model output.

```
## s(sleep_z):time   1.000  1.000  0.253   0.615
```

Below are plots of a plot of `s(bl_age)`, `s(bl_age):sleep_z`, `s(bl_age):time`, and `s(sleep_z):time`. For `s(sleep_z):time`, roughly, if we cannot fit a straight line through the confidence bands, there is evidence that the effect of time depends on sleep duration. It is correct the that confidence interval is zero at one point along the axis; this happens because we do not include the main effect.

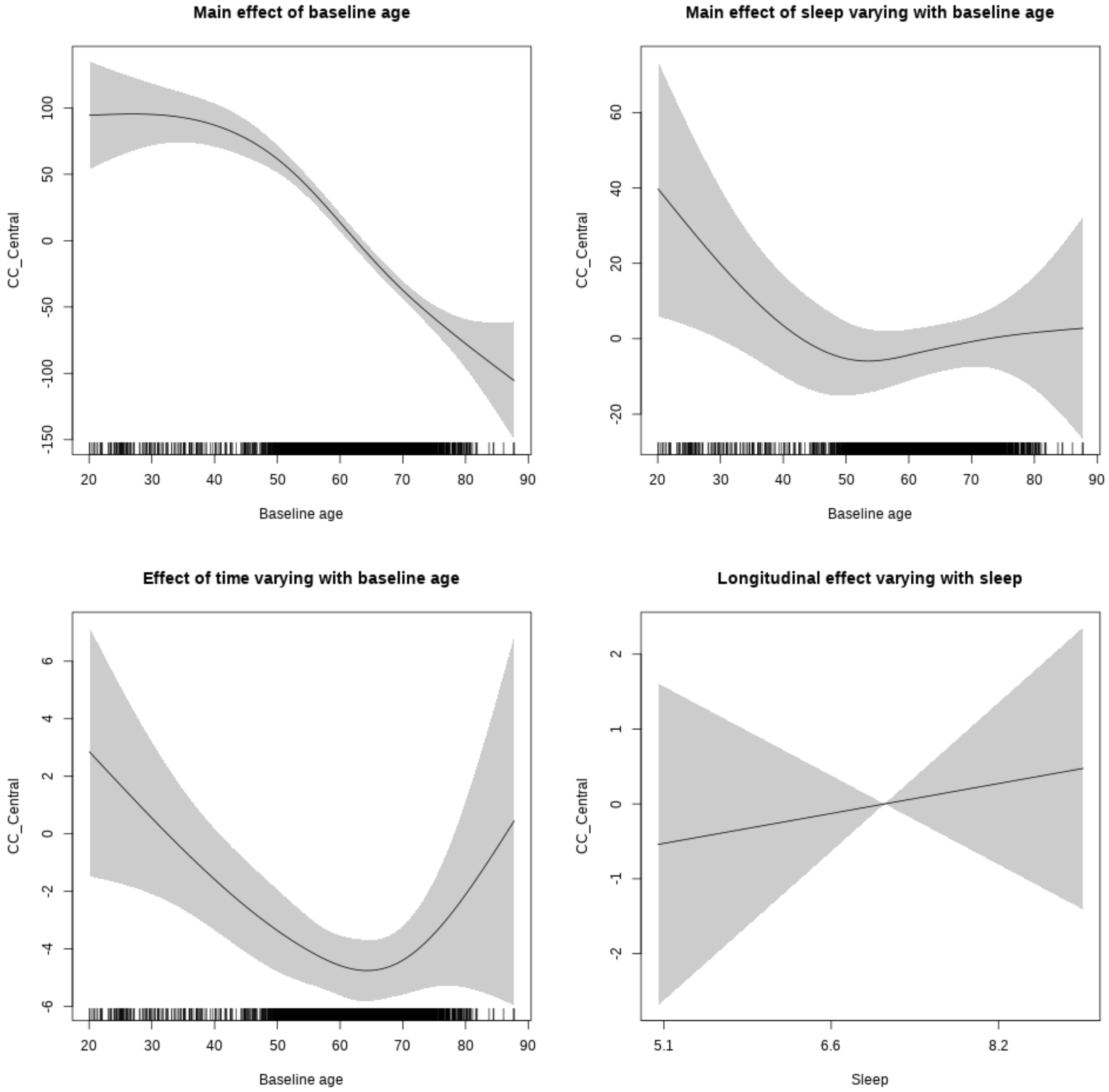

Controling for sleep quality

Full data Below is the model output. The term `s(sleep_z):time` is probably what we care most about.

```
##
## Family: gaussian
## Link function: identity
##
## Formula:
## value ~ X + s(bl_age, k = 10, bs = "cr") + s(bl_age, by = sleep_z,
##     bs = "cr") + s(bl_age, by = time, k = 5, bs = "cr") + s(sleep_z,
##     by = time, k = 5, bs = "cr", pc = 0)
## <environment: 0x563911192378>
```

```
##
## Parametric coefficients:
##                            Estimate Std. Error t value Pr(>|t|)
## (Intercept)                 497.153     14.092  35.279  < 2e-16 ***
## Xsexmale                     13.146      5.275   2.492  0.01273 *
## XsiteousAvanto              -19.372     13.482  -1.437  0.15083
## XsiteousPrisma               16.132     27.844   0.579  0.56236
## XsiteousSkyra               -36.422     13.539  -2.690  0.00717 **
## XsiteUB                     -16.255     21.382  -0.760  0.44716
## XsiteUCAM                    21.772     13.806   1.577  0.11486
## XsiteUKB                    -41.501     42.968  -0.966  0.33417
## XsiteUmU                     29.728     15.349   1.937  0.05283 .
## Xukb_dummy:sleep_accel        8.649      3.319   2.606  0.00918 **
## Xukb_dummy:chronotype         1.396      4.422   0.316  0.75224
## Xukb_dummy:dozing            13.242      9.128   1.451  0.14692
## Xukb_dummy:sleeplessness     -7.124      5.684  -1.253  0.21014
## Xukb_dummy:snoring            2.179      8.056   0.270  0.78680
## Xukb_dummy:gettingup          5.877      6.187   0.950  0.34222
## Xnotukb_dummy:PSQI_Global    -4.025      1.745  -2.307  0.02111 *
## ---
## Signif. codes:  0 '***' 0.001 '**' 0.01 '*' 0.05 '.' 0.1 ' ' 1
##
## Approximate significance of smooth terms:
##                      edf Ref.df      F p-value
## s(bl_age)          3.272  3.272 80.335  <2e-16 ***
## s(bl_age):sleep_z 2.000  2.000  2.594  0.0748 .
## s(bl_age):time     3.338  3.338 30.081  <2e-16 ***
## s(sleep_z):time    1.000  1.000  1.850  0.1738
## ---
## Signif. codes:  0 '***' 0.001 '**' 0.01 '*' 0.05 '.' 0.1 ' ' 1
##
## R-sq.(adj) =  0.168
## lmer.REML =  48179  Scale est. = 1020      n = 4284
```

Below are plots of a plot of `s(bl_age)`, `s(bl_age):sleep_z`, `s(bl_age):time`, and `s(sleep_z):time`. For `s(sleep_z):time`, roughly, if we cannot fit a straight line through the confidence bands, there is evidence that the effect of time depends on sleep duration. It is correct the that confidence interval is zero at one point along the axis; this happens because we do not include the main effect.

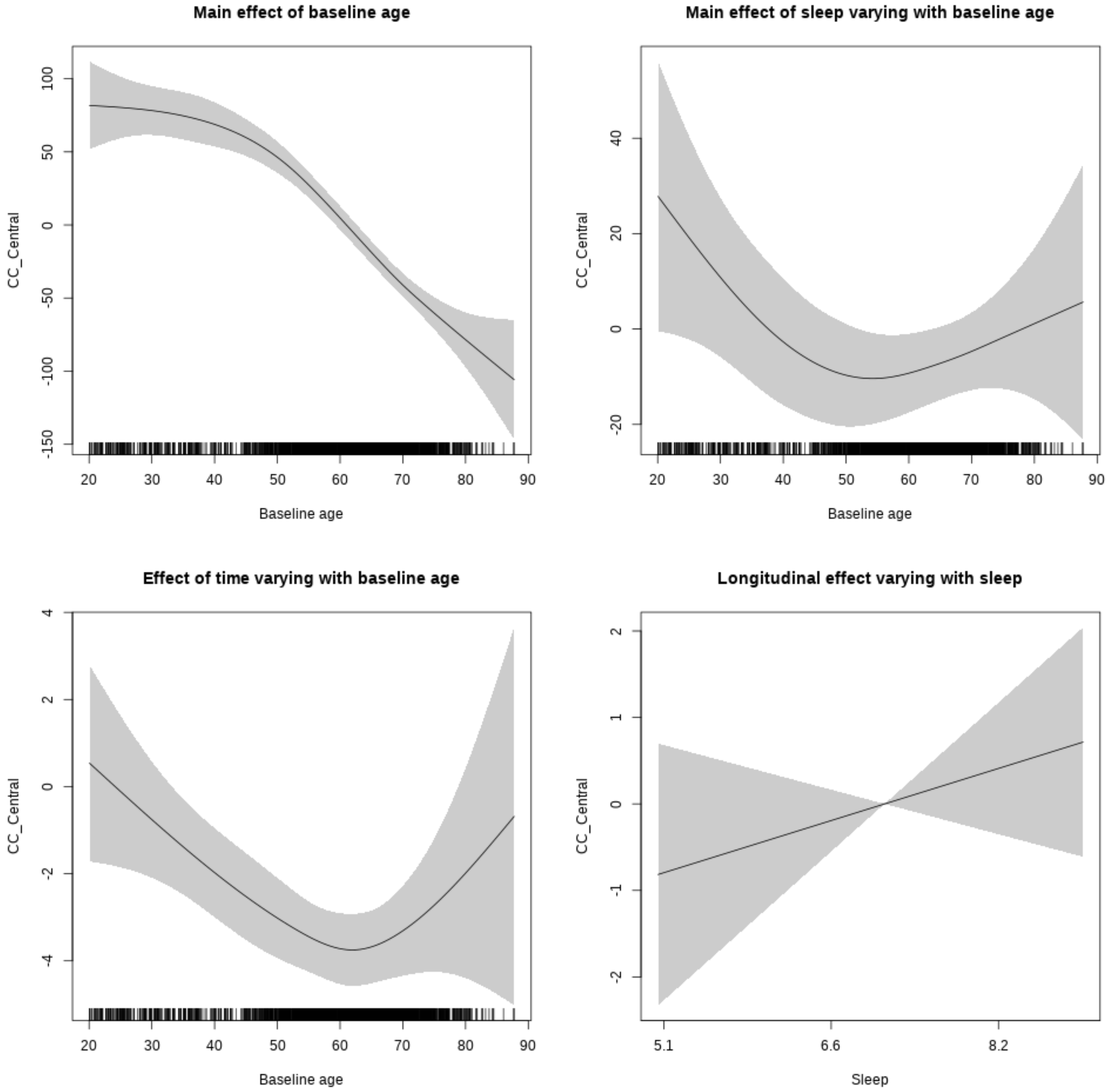

Restricted to sleep >= 5 and <= 9 hours Below is the row for `s(sleep_z):time` from the model output.

```
## s(sleep_z):time   1.000  1.000  1.162  0.2812
```

Below are plots of a plot of `s(bl_age)`, `s(bl_age):sleep_z`, `s(bl_age):time`, and `s(sleep_z):time`. For `s(sleep_z):time`, roughly, if we cannot fit a straight line through the confidence bands, there is evidence that the effect of time depends on sleep duration. It is correct the that confidence interval is zero at one point along the axis; this happens because we do not include the main effect.

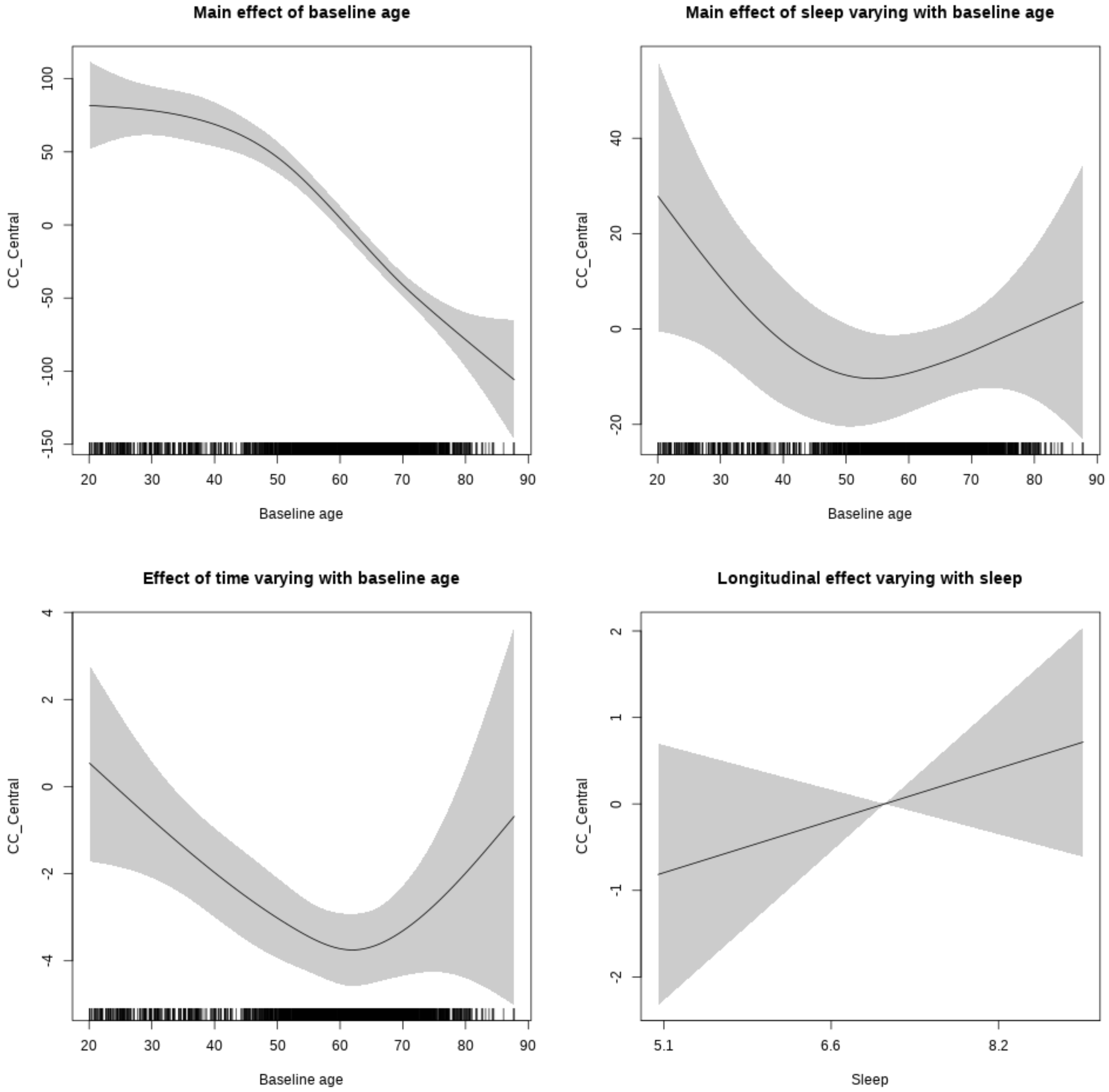

CC_Mid_Anterior

Main analysis

Full data Below is the model output. The term `s(sleep_z):time` is probably what we care most about.

```
##
## Family: gaussian
## Link function: identity
##
## Formula:
## value ~ sex + site + icv + s(bl_age, k = 10, bs = "cr") + s(bl_age,
##     by = sleep_z, bs = "cr") + +s(bl_age, by = time, k = 5, bs = "cr") +
```

```
##     s(sleep_z, by = time, k = 5, bs = "cr", pc = 0)
## <environment: 0x56391e8b1180>
##
## Parametric coefficients:
##               Estimate Std. Error t value Pr(>|t|)
## (Intercept)    563.829      8.595  65.599  < 2e-16 ***
## sexmale        -20.603      4.427  -4.654 3.31e-06 ***
## siteousAvanto -107.935     10.557 -10.224  < 2e-16 ***
## siteousPrisma  -97.186     29.640  -3.279 0.001046 **
## siteousSkyra  -104.598     10.261 -10.194  < 2e-16 ***
## siteUB         -73.856     19.814  -3.727 0.000195 ***
## siteUCAM       -60.014     10.746  -5.585 2.42e-08 ***
## siteUKB        -14.247      8.187  -1.740 0.081872 .
## siteUmU        -38.067     12.316  -3.091 0.002003 **
## icv             34.402      2.143  16.056  < 2e-16 ***
## ---
## Signif. codes:  0 '***' 0.001 '**' 0.01 '*' 0.05 '.' 0.1 ' ' 1
##
## Approximate significance of smooth terms:
##                     edf Ref.df       F p-value
## s(bl_age)         4.024  4.024 122.979  <2e-16 ***
## s(bl_age):sleep_z 2.410  2.410   3.213  0.0625 .
## s(bl_age):time    2.598  2.598  86.249  <2e-16 ***
## s(sleep_z):time   1.000  1.000   1.403  0.2362
## ---
## Signif. codes:  0 '***' 0.001 '**' 0.01 '*' 0.05 '.' 0.1 ' ' 1
##
## R-sq.(adj) =  0.195
## lmer.REML =  93162  Scale est. = 1314.1    n = 8132
```

Below are plots of a plot of `s(bl_age)`, `s(bl_age):sleep_z`, `s(bl_age):time`, and `s(sleep_z):time`. For `s(sleep_z):time`, roughly, if we cannot fit a straight line through the confidence bands, there is evidence that the effect of time depends on sleep duration. It is correct the that confidence interval is zero at one point along the axis; this happens because we do not include the main effect.

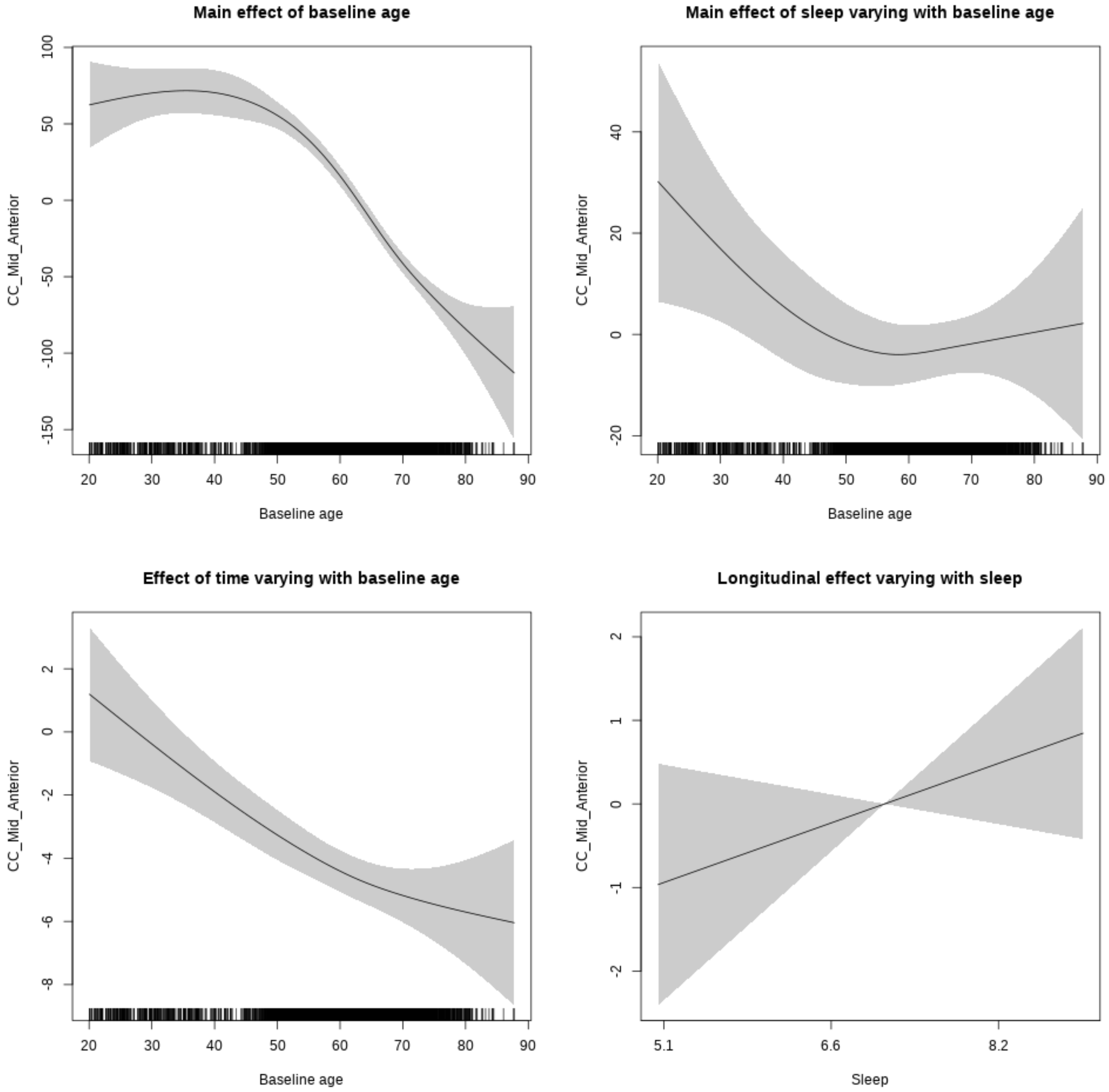

Restricted to sleep >= 5 and <= 9 hours Below is the row for `s(sleep_z):time` from the model output.

```
## s(sleep_z):time   1.000  1.000   1.781  0.1820
```

Below are plots of a plot of `s(bl_age)`, `s(bl_age):sleep_z`, `s(bl_age):time`, and `s(sleep_z):time`. For `s(sleep_z):time`, roughly, if we cannot fit a straight line through the confidence bands, there is evidence that the effect of time depends on sleep duration. It is correct the that confidence interval is zero at one point along the axis; this happens because we do not include the main effect.

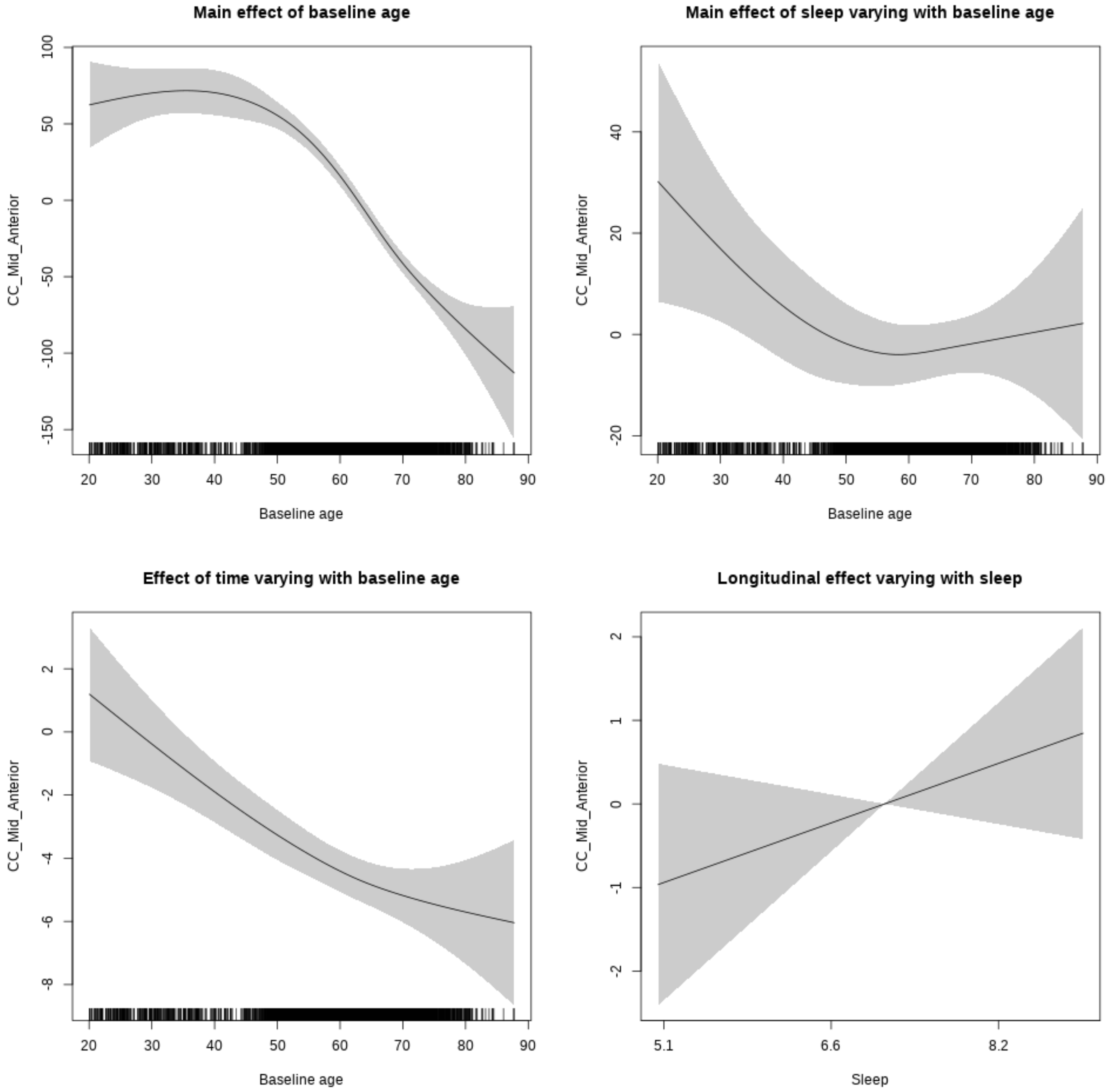

Controling for socioeconomic status

Full data Below is the model output. The term `s(sleep_z):time` is probably what we care most about.

```
##
## Family: gaussian
## Link function: identity
##
## Formula:
## value ~ sex + site + income_scaled + education_scaled + icv +
##     s(bl_age, k = 10, bs = "cr") + s(bl_age, by = sleep_z, bs = "cr") +
##     +s(bl_age, by = time, k = 5, bs = "cr") + s(sleep_z, by = time,
##     k = 5, bs = "cr", pc = 0)
```

```
## <environment: 0x5639115261a8>
##
## Parametric coefficients:
##                  Estimate Std. Error t value Pr(>|t|)
## (Intercept)       580.538     21.276  27.286  < 2e-16 ***
## sexmale           -26.812      5.857  -4.578 4.82e-06 ***
## siteousAvanto    -120.463     23.590  -5.106 3.41e-07 ***
## siteousPrisma    -101.101     53.155  -1.902   0.0572 .
## siteousSkyra     -111.115     21.700  -5.121 3.17e-07 ***
## siteUKB           -13.996     20.115  -0.696   0.4866
## income_scaled      -4.692      7.485  -0.627   0.5308
## education_scaled  -17.436      8.275  -2.107   0.0352 *
## icv                40.273      2.927  13.760  < 2e-16 ***
## ---
## Signif. codes:  0 '***' 0.001 '**' 0.01 '*' 0.05 '.' 0.1 ' ' 1
##
## Approximate significance of smooth terms:
##                     edf Ref.df      F p-value
## s(bl_age)         3.444  3.444 72.604  <2e-16 ***
## s(bl_age):sleep_z 2.001  2.001  0.582   0.559
## s(bl_age):time    2.000  2.000 59.063  <2e-16 ***
## s(sleep_z):time   1.000  1.000  0.044   0.834
## ---
## Signif. codes:  0 '***' 0.001 '**' 0.01 '*' 0.05 '.' 0.1 ' ' 1
##
## R-sq.(adj) =  0.177
## lmer.REML =  53756  Scale est. = 1581.3    n = 4638
```

Below are plots of a plot of `s(bl_age)`, `s(bl_age):sleep_z`, `s(bl_age):time`, and `s(sleep_z):time`. For `s(sleep_z):time`, roughly, if we cannot fit a straight line through the confidence bands, there is evidence that the effect of time depends on sleep duration. It is correct the that confidence interval is zero at one point along the axis; this happens because we do not include the main effect.

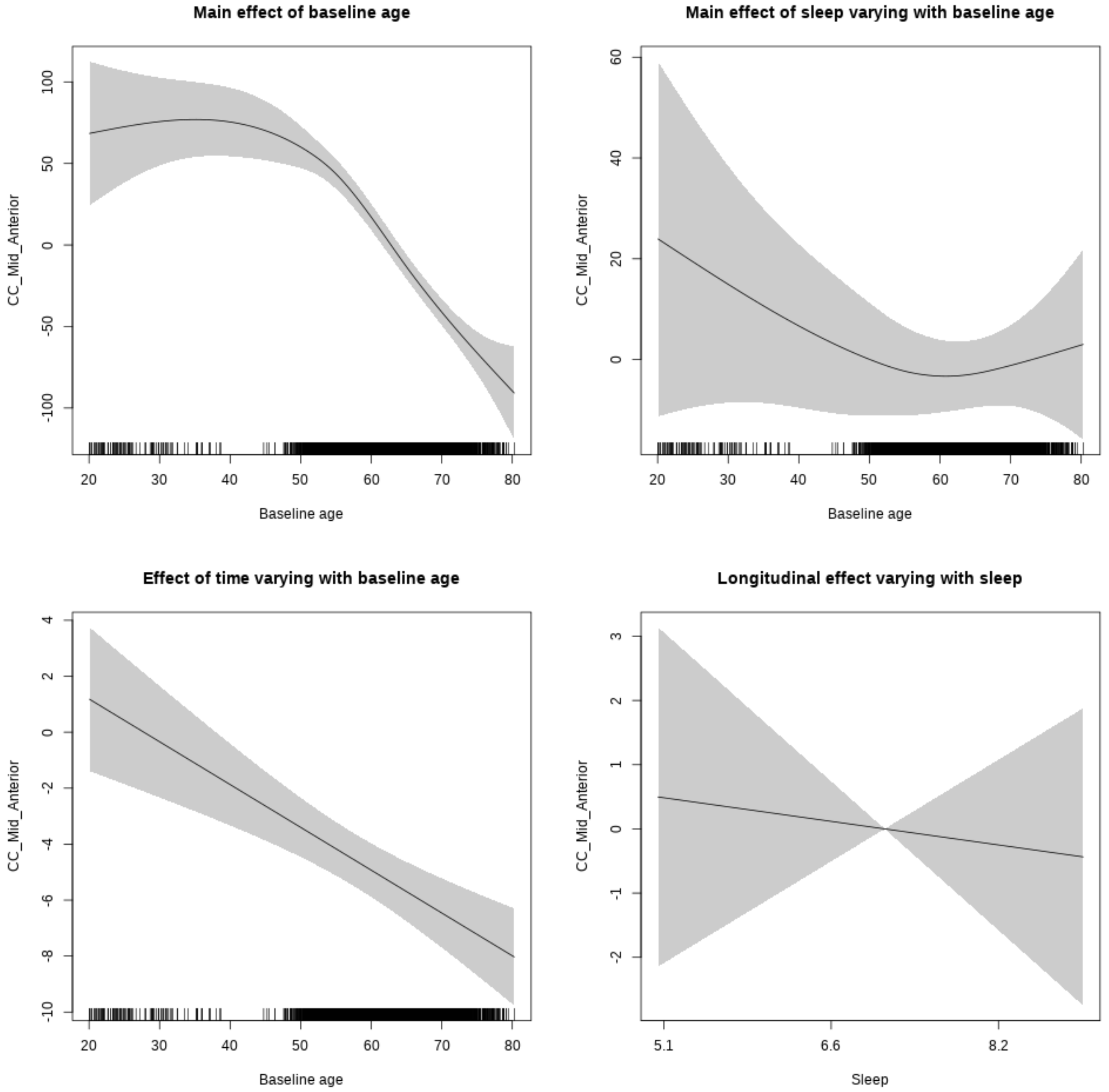

Restricted to sleep >= 5 and <= 9 hours Below is the row for `s(sleep_z):time` from the model output.

```
## s(sleep_z):time   1.000  1.000  0.142   0.706
```

Below are plots of a plot of `s(bl_age)`, `s(bl_age):sleep_z`, `s(bl_age):time`, and `s(sleep_z):time`. For `s(sleep_z):time`, roughly, if we cannot fit a straight line through the confidence bands, there is evidence that the effect of time depends on sleep duration. It is correct the that confidence interval is zero at one point along the axis; this happens because we do not include the main effect.

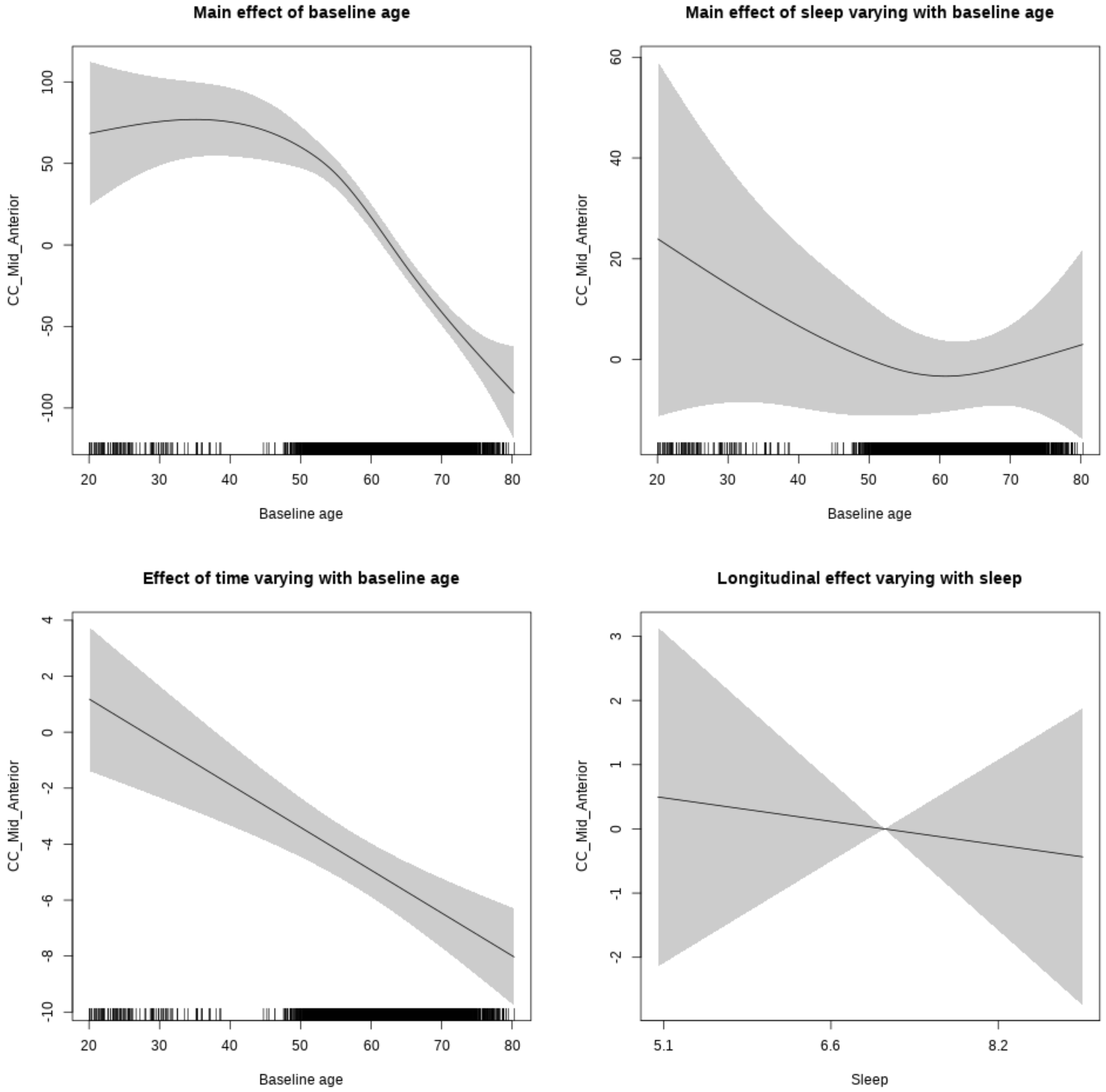

Controling for BMI

Full data Below is the model output. The term `s(sleep_z):time` is probably what we care most about.

```
##
## Family: gaussian
## Link function: identity
##
## Formula:
## value ~ sex + site + bmi + icv + s(bl_age, k = 10, bs = "cr") +
##     s(bl_age, by = sleep_z, bs = "cr") + +s(bl_age, by = time,
##     k = 5, bs = "cr") + s(sleep_z, by = time, k = 5, bs = "cr",
##     pc = 0)
```

```
## <environment: 0x56390d705bb0>
##
## Parametric coefficients:
##                Estimate Std. Error t value Pr(>|t|)
## (Intercept)    479.3422    19.1897  24.979  < 2e-16 ***
## sexmale        -20.2357     5.3221  -3.802 0.000145 ***
## siteousPrisma   10.5392    30.8568   0.342 0.732702
## siteousSkyra     6.4182     9.7384   0.659 0.509888
## siteUCAM        46.0625    14.8742   3.097 0.001966 **
## siteUKB         90.0374    13.9221   6.467 1.09e-10 ***
## siteUmU         74.7093    16.8524   4.433 9.47e-06 ***
## bmi             -0.8638     0.5488  -1.574 0.115524
## icv             38.0056     2.6529  14.326  < 2e-16 ***
## ---
## Signif. codes:  0 '***' 0.001 '**' 0.01 '*' 0.05 '.' 0.1 ' ' 1
##
## Approximate significance of smooth terms:
##                      edf Ref.df      F p-value
## s(bl_age)          4.154  4.154 81.724  <2e-16 ***
## s(bl_age):sleep_z 2.000  2.000  2.172   0.114
## s(bl_age):time    2.000  2.000 54.759  <2e-16 ***
## s(sleep_z):time   1.000  1.000  1.093   0.296
## ---
## Signif. codes:  0 '***' 0.001 '**' 0.01 '*' 0.05 '.' 0.1 ' ' 1
##
## R-sq.(adj) =  0.186
## lmer.REML =  62329  Scale est. = 1474.1    n = 5398
```

Below are plots of a plot of `s(bl_age)`, `s(bl_age):sleep_z`, `s(bl_age):time`, and `s(sleep_z):time`. For `s(sleep_z):time`, roughly, if we cannot fit a straight line through the confidence bands, there is evidence that the effect of time depends on sleep duration. It is correct the that confidence interval is zero at one point along the axis; this happens because we do not include the main effect.

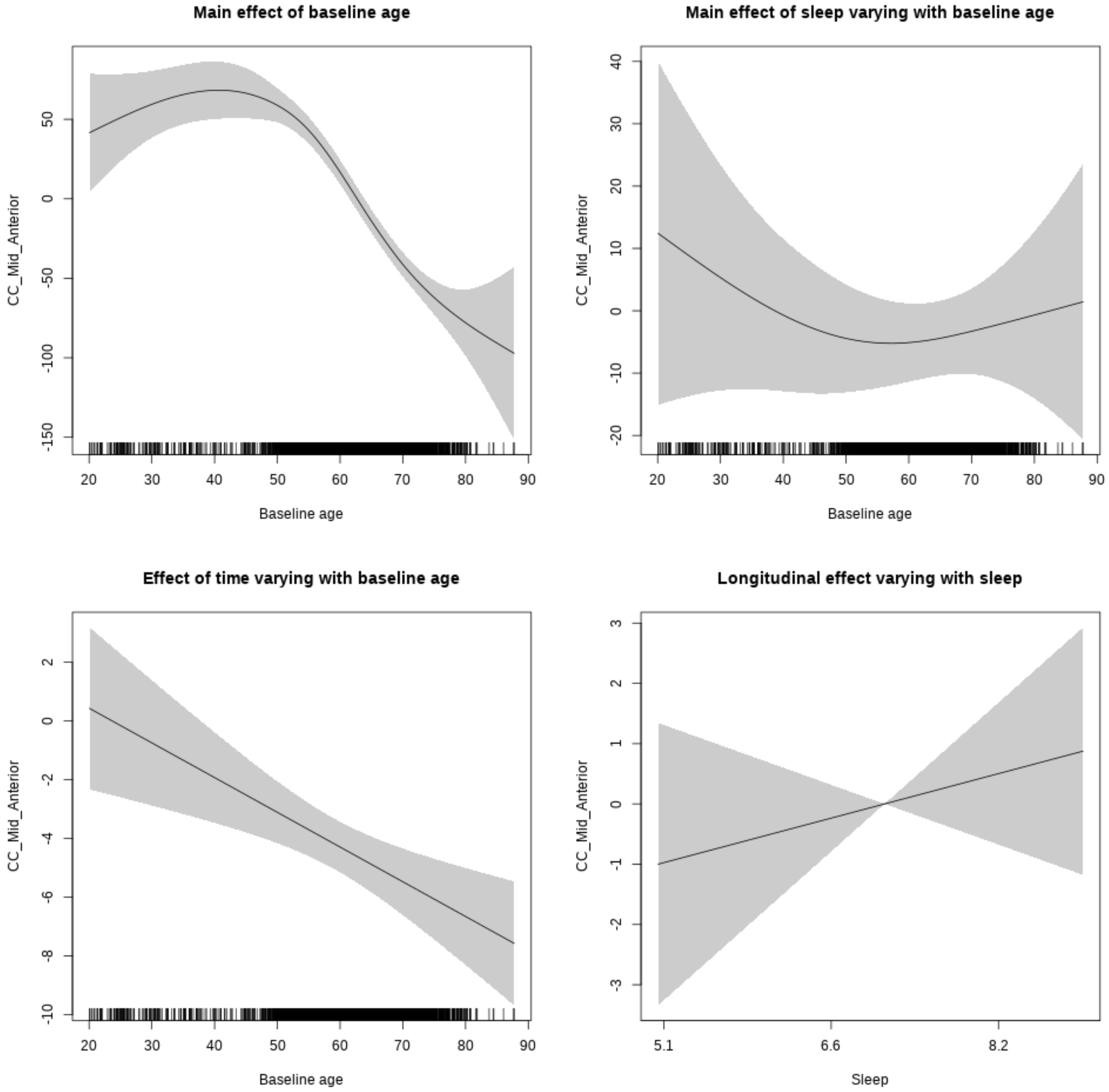

Restricted to sleep >= 5 and <= 9 hours Below is the row for `s(sleep_z):time` from the model output.

```
## s(sleep_z):time   1.000  1.000  0.728   0.394
```

Below are plots of a plot of `s(bl_age)`, `s(bl_age):sleep_z`, `s(bl_age):time`, and `s(sleep_z):time`. For `s(sleep_z):time`, roughly, if we cannot fit a straight line through the confidence bands, there is evidence that the effect of time depends on sleep duration. It is correct the that confidence interval is zero at one point along the axis; this happens because we do not include the main effect.

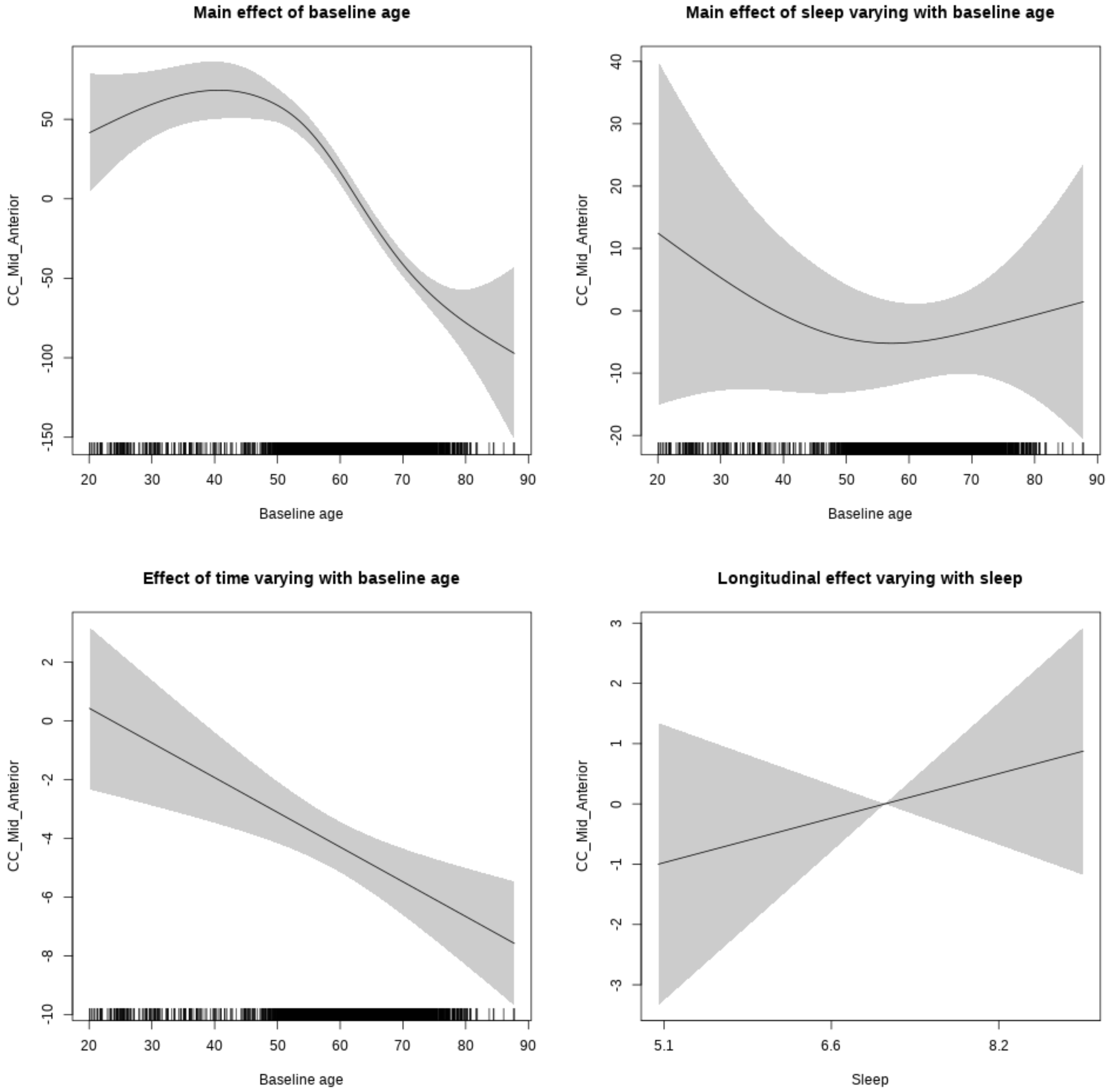

Controling for depression

Full data Below is the model output. The term `s(sleep_z):time` is probably what we care most about.

```
##
## Family: gaussian
## Link function: identity
##
## Formula:
## value ~ sex + site + depression + icv + s(bl_age, k = 10, bs = "cr") +
##     s(bl_age, by = sleep_z, bs = "cr") + +s(bl_age, by = time,
##     k = 5, bs = "cr") + s(sleep_z, by = time, k = 5, bs = "cr",
##     pc = 0)
```

```
## <environment: 0x5639111c6b30>
##
## Parametric coefficients:
##                Estimate Std. Error t value Pr(>|t|)
## (Intercept)     550.026     10.015  54.918  < 2e-16 ***
## sexmale         -17.877      5.235  -3.415 0.000643 ***
## siteousAvanto -112.018     22.002  -5.091 3.67e-07 ***
## siteousPrisma -103.846     40.877  -2.540 0.011097 *
## siteousSkyra  -112.105     18.279  -6.133 9.21e-10 ***
## siteUCAM       -54.934     12.057  -4.556 5.32e-06 ***
## siteUKB         -8.115      9.195  -0.883 0.377510
## siteUmU        -38.185     14.617  -2.612 0.009014 **
## depression      27.609     18.307   1.508 0.131580
## icv             33.062      2.550  12.966  < 2e-16 ***
## ---
## Signif. codes:  0 '***' 0.001 '**' 0.01 '*' 0.05 '.' 0.1 ' ' 1
##
## Approximate significance of smooth terms:
##                      edf Ref.df      F p-value
## s(bl_age)          4.004  4.004 82.817  <2e-16 ***
## s(bl_age):sleep_z 2.000  2.000  1.895   0.150
## s(bl_age):time    2.000  2.000 63.518  <2e-16 ***
## s(sleep_z):time   1.000  1.000  0.729   0.393
## ---
## Signif. codes:  0 '***' 0.001 '**' 0.01 '*' 0.05 '.' 0.1 ' ' 1
##
## R-sq.(adj) =  0.177
## lmer.REML =  65411  Scale est. = 1434.8    n = 5665
```

Below are plots of a plot of `s(bl_age)`, `s(bl_age):sleep_z`, `s(bl_age):time`, and `s(sleep_z):time`. For `s(sleep_z):time`, roughly, if we cannot fit a straight line through the confidence bands, there is evidence that the effect of time depends on sleep duration. It is correct the that confidence interval is zero at one point along the axis; this happens because we do not include the main effect.

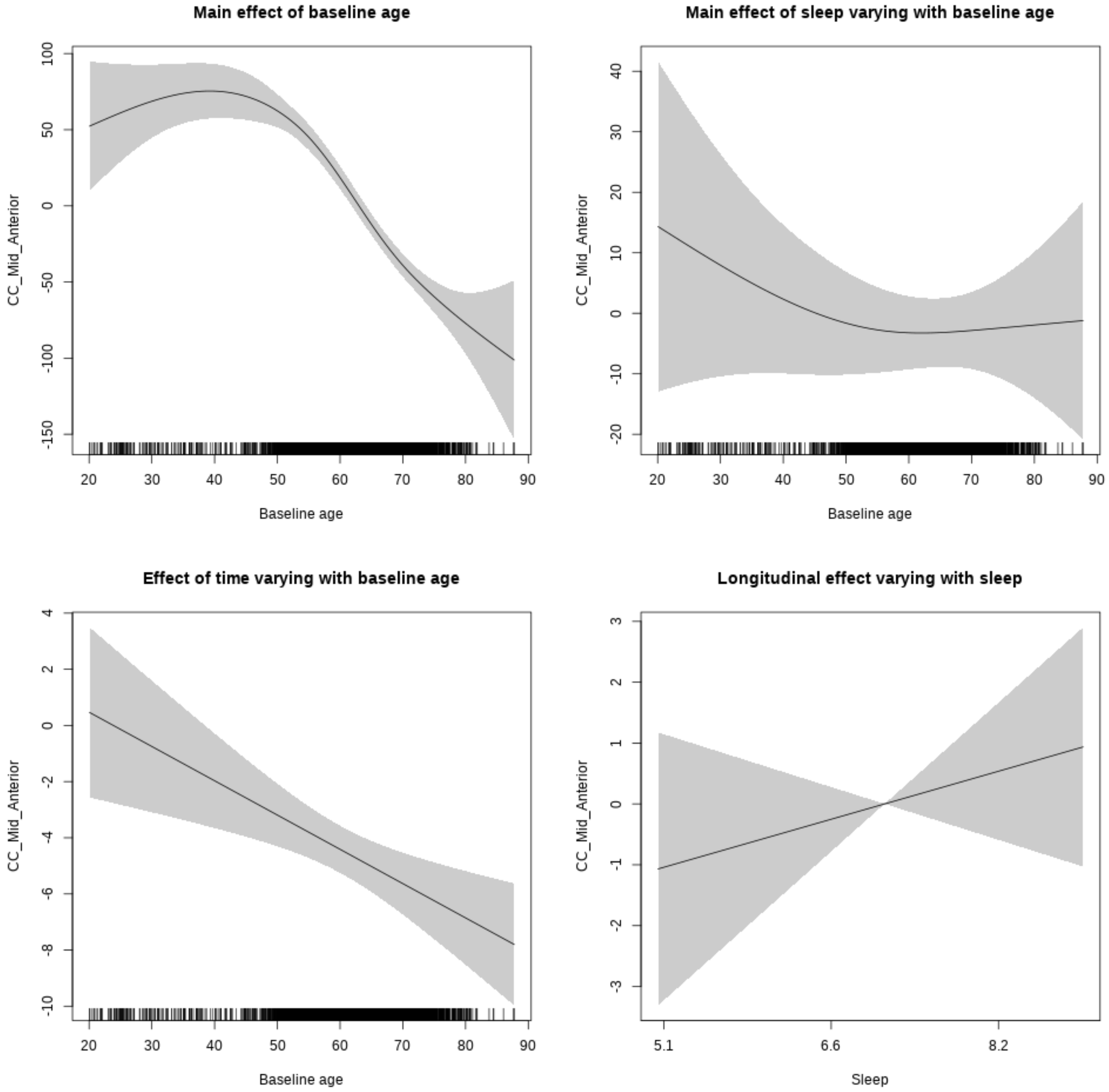

Restricted to sleep >= 5 and <= 9 hours Below is the row for `s(sleep_z):time` from the model output.

```
## s(sleep_z):time   1.000  1.000  0.907   0.341
```

Below are plots of a plot of `s(bl_age)`, `s(bl_age):sleep_z`, `s(bl_age):time`, and `s(sleep_z):time`. For `s(sleep_z):time`, roughly, if we cannot fit a straight line through the confidence bands, there is evidence that the effect of time depends on sleep duration. It is correct the that confidence interval is zero at one point along the axis; this happens because we do not include the main effect.

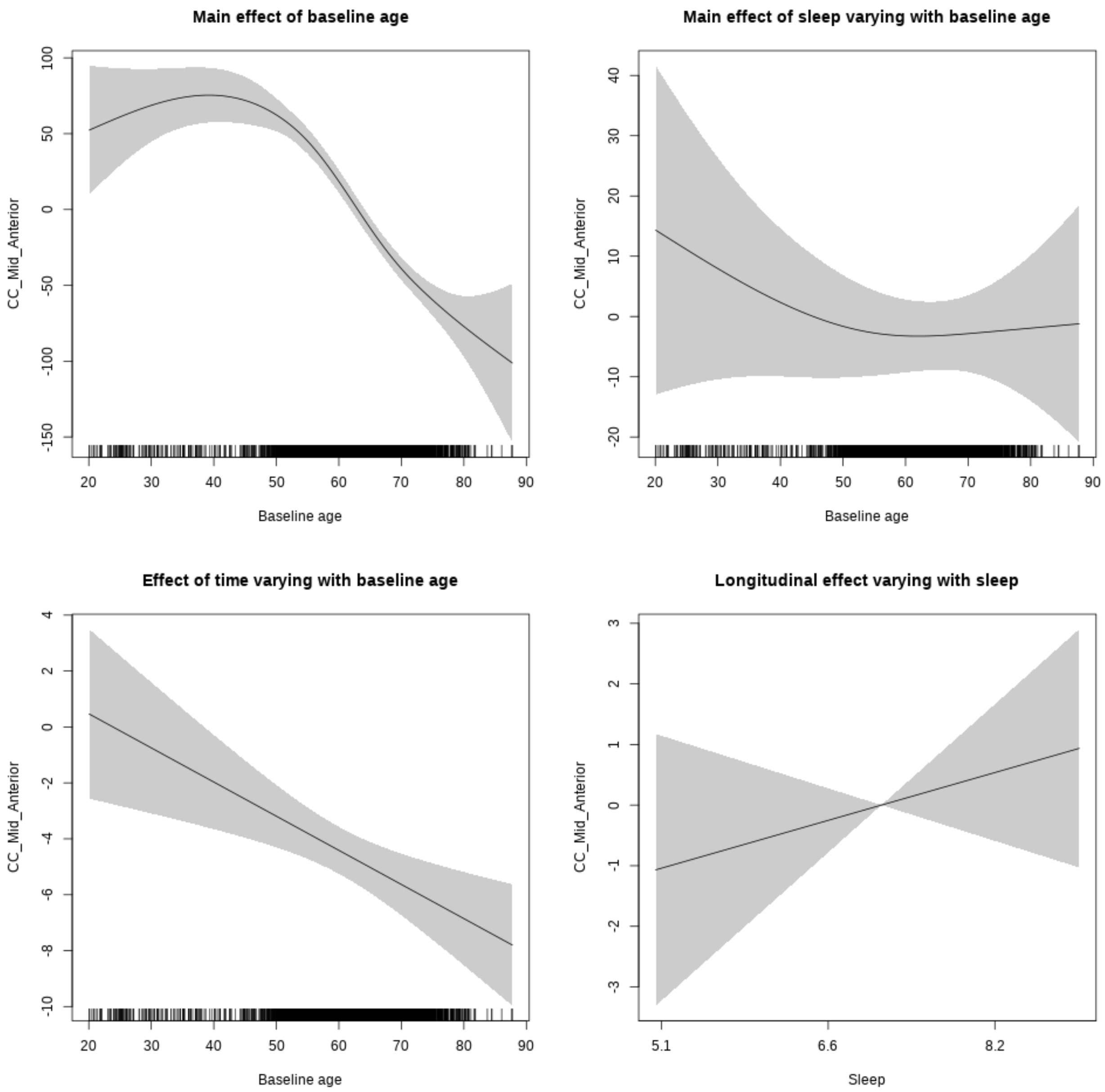

Controling for sleep quality

Full data Below is the model output. The term `s(sleep_z):time` is probably what we care most about.

```
##
## Family: gaussian
## Link function: identity
##
## Formula:
## value ~ X + s(bl_age, k = 10, bs = "cr") + s(bl_age, by = sleep_z,
##     bs = "cr") + s(bl_age, by = time, k = 5, bs = "cr") + s(sleep_z,
##     by = time, k = 5, bs = "cr", pc = 0)
## <environment: 0x563911801560>
```

```
##
## Parametric coefficients:
##                           Estimate Std. Error t value Pr(>|t|)
## (Intercept)                489.698     14.571  33.609  < 2e-16 ***
## Xsexmale                    31.303      5.454   5.740 1.01e-08 ***
## XsiteousAvanto             -22.994     13.951  -1.648  0.09939 .
## XsiteousPrisma               2.215     28.529   0.078  0.93812
## XsiteousSkyra              -34.114     14.009  -2.435  0.01493 *
## XsiteUB                    -21.003     22.083  -0.951  0.34161
## XsiteUCAM                    9.105     14.330   0.635  0.52521
## XsiteUKB                   -60.490     44.567  -1.357  0.17476
## XsiteUmU                    15.138     15.865   0.954  0.34005
## Xukb_dummy:sleep_accel       9.752      3.425   2.847  0.00444 **
## Xukb_dummy:chronotype        6.213      4.580   1.356  0.17506
## Xukb_dummy:dozing           20.565      9.435   2.180  0.02935 *
## Xukb_dummy:sleeplessness    -5.898      5.885  -1.002  0.31637
## Xukb_dummy:snoring           8.624      8.333   1.035  0.30077
## Xukb_dummy:gettingup         2.445      6.428   0.380  0.70368
## Xnotukb_dummy:PSQI_Global   -2.608      1.803  -1.447  0.14811
## ---
## Signif. codes:  0 '***' 0.001 '**' 0.01 '*' 0.05 '.' 0.1 ' ' 1
##
## Approximate significance of smooth terms:
##                     edf Ref.df      F p-value
## s(bl_age)         4.043  4.043 64.549  <2e-16 ***
## s(bl_age):sleep_z 2.000  2.000  2.434  0.0878 .
## s(bl_age):time    2.000  2.000 68.358  <2e-16 ***
## s(sleep_z):time   1.000  1.000  5.157  0.0232 *
## ---
## Signif. codes:  0 '***' 0.001 '**' 0.01 '*' 0.05 '.' 0.1 ' ' 1
##
## R-sq.(adj) =   0.18
## lmer.REML =  48263  Scale est. = 1064.1    n = 4272
```

Below are plots of a plot of `s(bl_age)`, `s(bl_age):sleep_z`, `s(bl_age):time`, and `s(sleep_z):time`. For `s(sleep_z):time`, roughly, if we cannot fit a straight line through the confidence bands, there is evidence that the effect of time depends on sleep duration. It is correct the that confidence interval is zero at one point along the axis; this happens because we do not include the main effect.

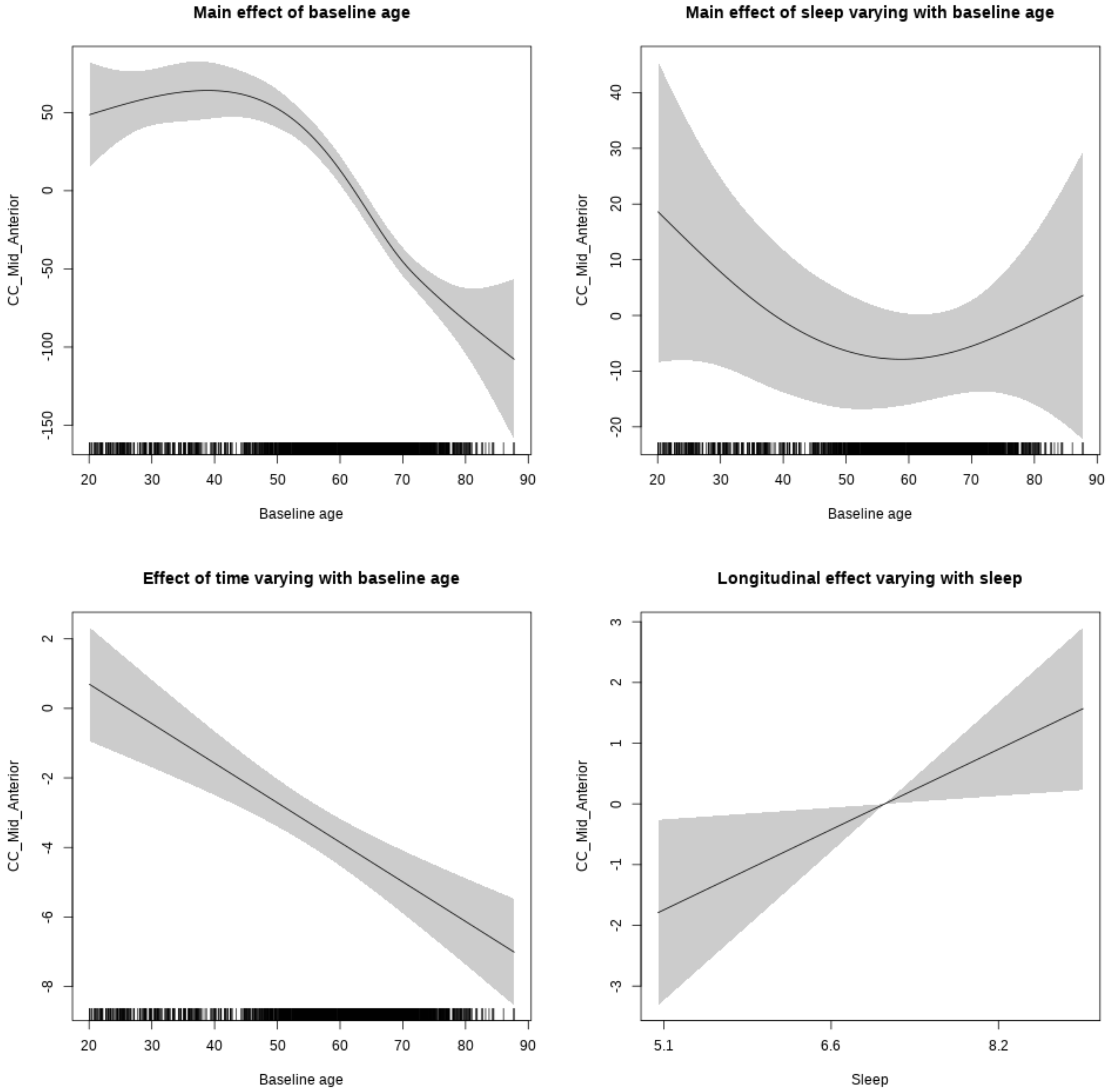

Restricted to sleep >= 5 and <= 9 hours Below is the row for `s(sleep_z):time` from the model output.

```
## s(sleep_z):time   1.000  1.000  5.494  0.0191 *
```

Below are plots of a plot of `s(bl_age)`, `s(bl_age):sleep_z`, `s(bl_age):time`, and `s(sleep_z):time`. For `s(sleep_z):time`, roughly, if we cannot fit a straight line through the confidence bands, there is evidence that the effect of time depends on sleep duration. It is correct the that confidence interval is zero at one point along the axis; this happens because we do not include the main effect.

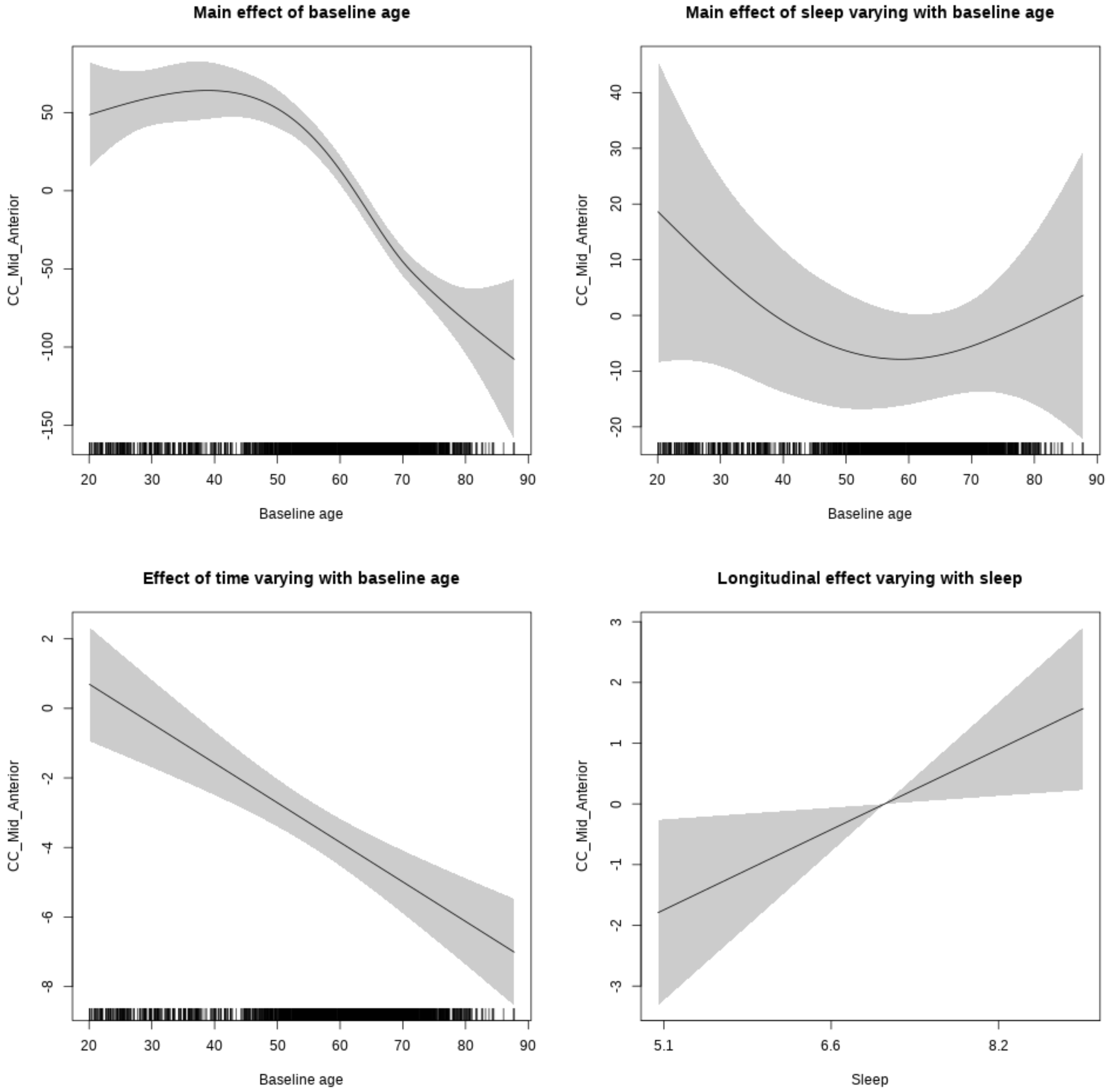

CC_Mid_Posterior

Main analysis

Full data Below is the model output. The term `s(sleep_z):time` is probably what we care most about.

```
##
## Family: gaussian
## Link function: identity
##
## Formula:
## value ~ sex + site + icv + s(bl_age, k = 10, bs = "cr") + s(bl_age,
##     by = sleep_z, bs = "cr") + +s(bl_age, by = time, k = 5, bs = "cr") +
```

```
##     s(sleep_z, by = time, k = 5, bs = "cr", pc = 0)
## <environment: 0x563910f11008>
##
## Parametric coefficients:
##               Estimate Std. Error t value Pr(>|t|)
## (Intercept)    543.492      7.370  73.741  < 2e-16 ***
## sexmale        -18.605      3.806  -4.888 1.04e-06 ***
## siteousAvanto  -76.370      9.050  -8.439  < 2e-16 ***
## siteousPrisma  -16.092     22.740  -0.708  0.47919
## siteousSkyra   -91.132      8.800 -10.356  < 2e-16 ***
## siteUB         -51.131     17.117  -2.987  0.00282 **
## siteUCAM       -45.675      9.186  -4.973 6.74e-07 ***
## siteUKB         14.291      7.010   2.039  0.04152 *
## siteUmU        -17.164     10.611  -1.618  0.10581
## icv             26.335      1.820  14.471  < 2e-16 ***
## ---
## Signif. codes:  0 ‘***’ 0.001 ‘**’ 0.01 ‘*’ 0.05 ‘.’ 0.1 ‘ ’ 1
##
## Approximate significance of smooth terms:
##                      edf Ref.df       F p-value
## s(bl_age)          2.788  2.788 144.535  <2e-16 ***
## s(bl_age):sleep_z 2.000  2.000   0.421   0.657
## s(bl_age):time    2.000  2.000 146.645  <2e-16 ***
## s(sleep_z):time   1.000  1.000   0.028   0.868
## ---
## Signif. codes:  0 ‘***’ 0.001 ‘**’ 0.01 ‘*’ 0.05 ‘.’ 0.1 ‘ ’ 1
##
## R-sq.(adj) =  0.196
## lmer.REML =  89355  Scale est. = 736.13    n = 8114
```

Below are plots of a plot of `s(bl_age)`, `s(bl_age):sleep_z`, `s(bl_age):time`, and `s(sleep_z):time`. For `s(sleep_z):time`, roughly, if we cannot fit a straight line through the confidence bands, there is evidence that the effect of time depends on sleep duration. It is correct the that confidence interval is zero at one point along the axis; this happens because we do not include the main effect.

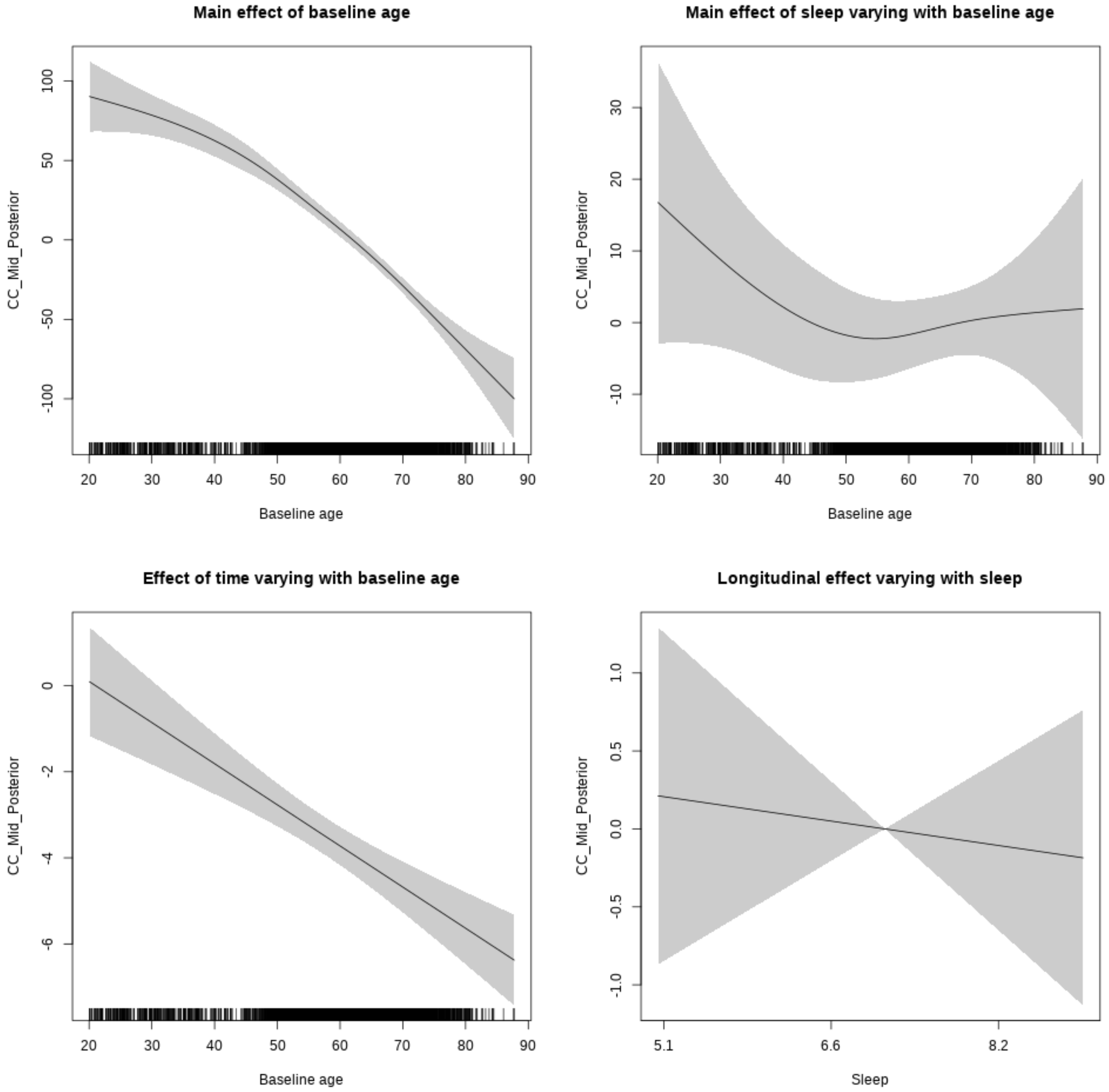

Restricted to sleep >= 5 and <= 9 hours Below is the row for `s(sleep_z):time` from the model output.

```
## s(sleep_z):time   1.000  1.000   0.154   0.695
```

Below are plots of a plot of `s(bl_age)`, `s(bl_age):sleep_z`, `s(bl_age):time`, and `s(sleep_z):time`. For `s(sleep_z):time`, roughly, if we cannot fit a straight line through the confidence bands, there is evidence that the effect of time depends on sleep duration. It is correct the that confidence interval is zero at one point along the axis; this happens because we do not include the main effect.

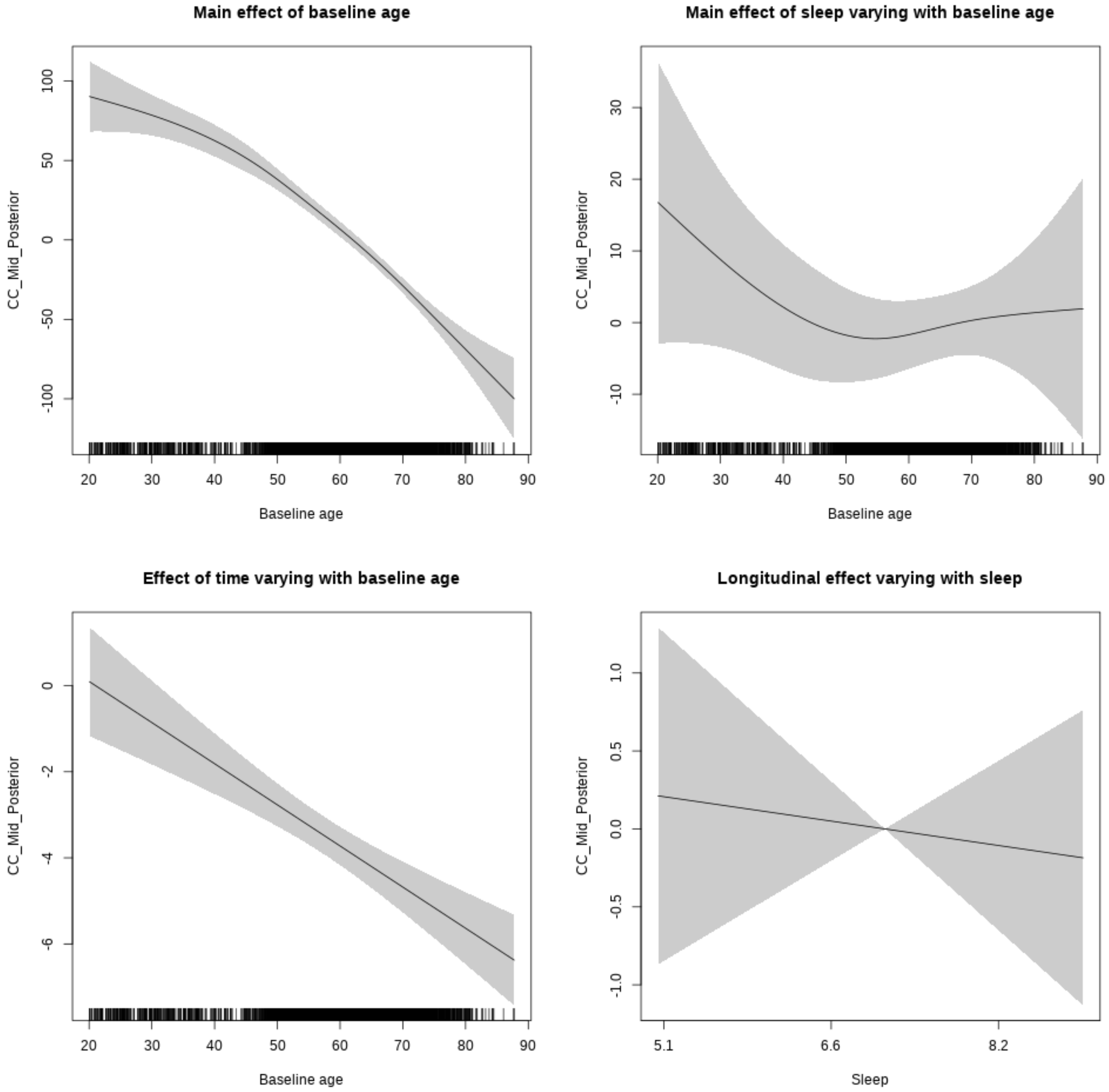

Controling for socioeconomic status

Full data Below is the model output. The term `s(sleep_z):time` is probably what we care most about.

```
##
## Family: gaussian
## Link function: identity
##
## Formula:
## value ~ sex + site + income_scaled + education_scaled + icv +
##     s(bl_age, k = 10, bs = "cr") + s(bl_age, by = sleep_z, bs = "cr") +
##     +s(bl_age, by = time, k = 5, bs = "cr") + s(sleep_z, by = time,
##     k = 5, bs = "cr", pc = 0)
```

```
## <environment: 0x56391e7cac68>
##
## Parametric coefficients:
##                  Estimate Std. Error t value Pr(>|t|)
## (Intercept)      528.3024    18.3165  28.843  < 2e-16 ***
## sexmale          -25.0482     5.0420  -4.968 7.01e-07 ***
## siteousAvanto    -59.7970    19.9856  -2.992 0.002786 **
## siteousPrisma    -13.9781    40.0209  -0.349 0.726902
## siteousSkyra     -67.0974    18.7186  -3.585 0.000341 ***
## siteUKB           31.0684    17.3241   1.793 0.072980 .
## income_scaled      0.1082     6.4638   0.017 0.986646
## education_scaled  -1.9616     7.1690  -0.274 0.784390
## icv               31.7309     2.4826  12.781  < 2e-16 ***
## ---
## Signif. codes:  0 '***' 0.001 '**' 0.01 '*' 0.05 '.' 0.1 ' ' 1
##
## Approximate significance of smooth terms:
##                     edf Ref.df      F p-value
## s(bl_age)         2.874  2.874 50.297  <2e-16 ***
## s(bl_age):sleep_z 2.000  2.000  0.704   0.494
## s(bl_age):time    4.685  4.685 27.758  <2e-16 ***
## s(sleep_z):time   1.000  1.000  1.319   0.251
## ---
## Signif. codes:  0 '***' 0.001 '**' 0.01 '*' 0.05 '.' 0.1 ' ' 1
##
## R-sq.(adj) =  0.141
## lmer.REML =  51433  Scale est. = 821.32    n = 4626
```

Below are plots of a plot of `s(bl_age)`, `s(bl_age):sleep_z`, `s(bl_age):time`, and `s(sleep_z):time`. For `s(sleep_z):time`, roughly, if we cannot fit a straight line through the confidence bands, there is evidence that the effect of time depends on sleep duration. It is correct the that confidence interval is zero at one point along the axis; this happens because we do not include the main effect.

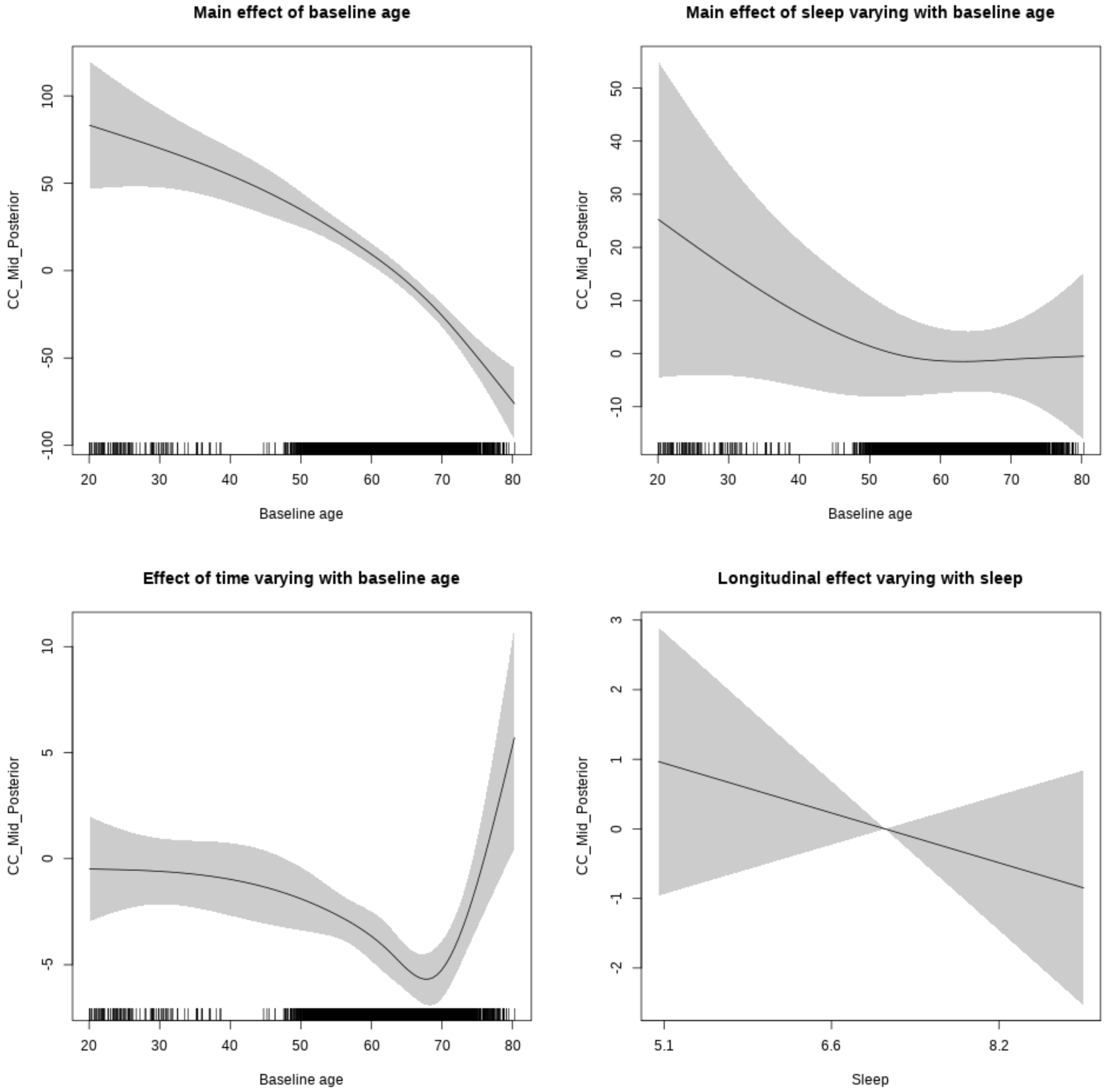

Restricted to sleep >= 5 and <= 9 hours Below is the row for `s(sleep_z):time` from the model output.

```
## s(sleep_z):time   1.000  1.000  1.009   0.315
```

Below are plots of a plot of `s(bl_age)`, `s(bl_age):sleep_z`, `s(bl_age):time`, and `s(sleep_z):time`. For `s(sleep_z):time`, roughly, if we cannot fit a straight line through the confidence bands, there is evidence that the effect of time depends on sleep duration. It is correct the that confidence interval is zero at one point along the axis; this happens because we do not include the main effect.

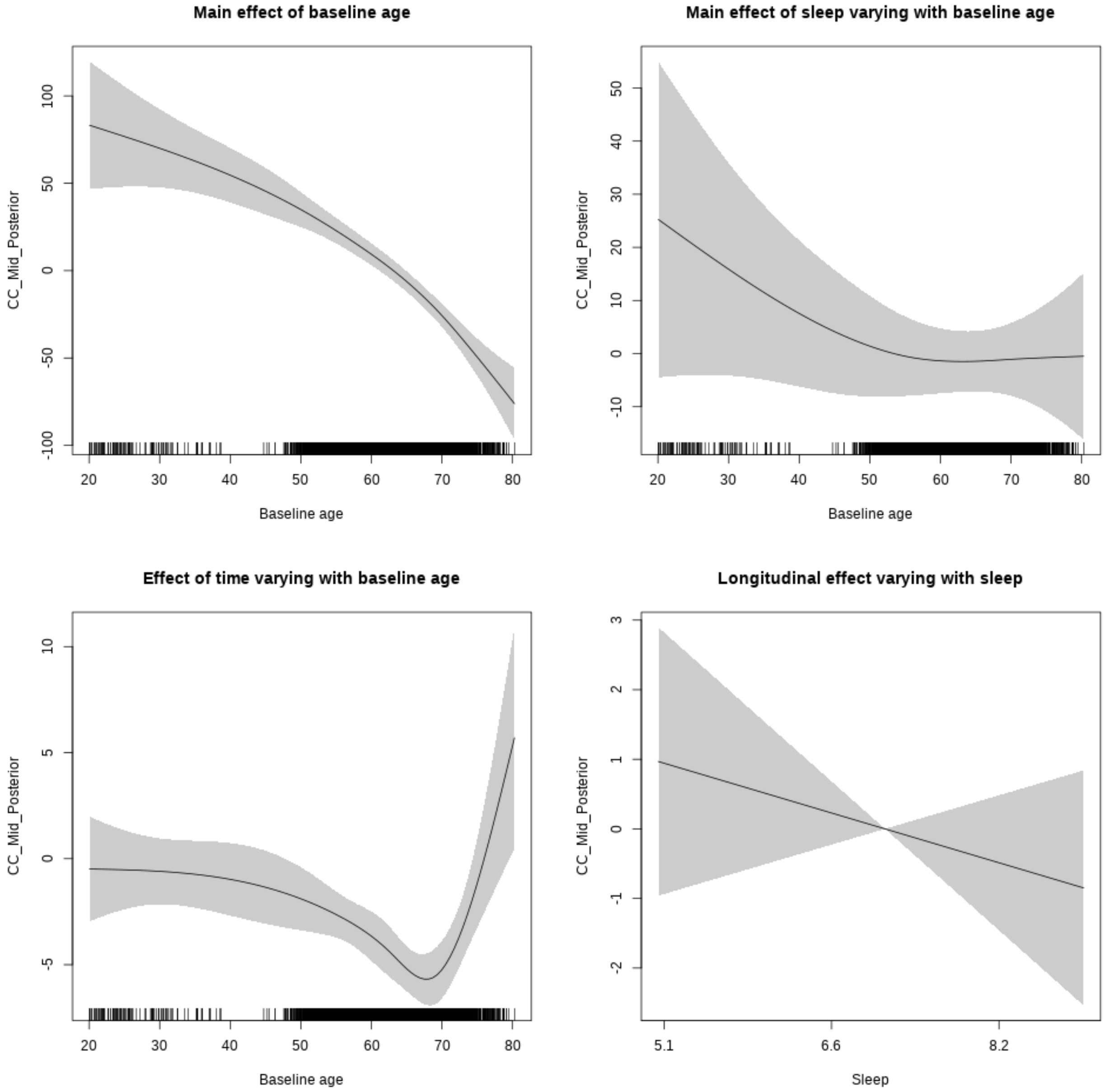

Controling for BMI

Full data Below is the model output. The term `s(sleep_z):time` is probably what we care most about.

```
##
## Family: gaussian
## Link function: identity
##
## Formula:
## value ~ sex + site + bmi + icv + s(bl_age, k = 10, bs = "cr") +
##     s(bl_age, by = sleep_z, bs = "cr") + +s(bl_age, by = time,
##     k = 5, bs = "cr") + s(sleep_z, by = time, k = 5, bs = "cr",
##     pc = 0)
```

```
## <environment: 0x5639112f1120>
##
## Parametric coefficients:
##               Estimate Std. Error t value Pr(>|t|)
## (Intercept)   499.6173    16.2194  30.804  < 2e-16 ***
## sexmale       -20.1870     4.6125  -4.377 1.23e-05 ***
## siteousPrisma  57.6551    22.7232   2.537 0.011200 *
## siteousSkyra   -6.1750     7.2402  -0.853 0.393772
## siteUCAM       38.4410    12.1840   3.155 0.001614 **
## siteUKB        98.5972    11.3445   8.691  < 2e-16 ***
## siteUmU        71.0525    14.0517   5.056 4.41e-07 ***
## bmi            -1.5974     0.4809  -3.321 0.000901 ***
## icv            30.5484     2.2670  13.475  < 2e-16 ***
## ---
## Signif. codes:  0 ‘***’ 0.001 ‘**’ 0.01 ‘*’ 0.05 ‘.’ 0.1 ‘ ’ 1
##
## Approximate significance of smooth terms:
##                     edf Ref.df       F p-value
## s(bl_age)         2.501  2.501 101.546  <2e-16 ***
## s(bl_age):sleep_z 2.000  2.000   1.376   0.253
## s(bl_age):time    4.784  4.784  26.405  <2e-16 ***
## s(sleep_z):time   1.000  1.000   0.224   0.636
## ---
## Signif. codes:  0 ‘***’ 0.001 ‘**’ 0.01 ‘*’ 0.05 ‘.’ 0.1 ‘ ’ 1
##
## R-sq.(adj) =   0.17
## lmer.REML =  59778  Scale est. = 786.39    n = 5388
```

Below are plots of a plot of `s(bl_age)`, `s(bl_age):sleep_z`, `s(bl_age):time`, and `s(sleep_z):time`. For `s(sleep_z):time`, roughly, if we cannot fit a straight line through the confidence bands, there is evidence that the effect of time depends on sleep duration. It is correct the that confidence interval is zero at one point along the axis; this happens because we do not include the main effect.

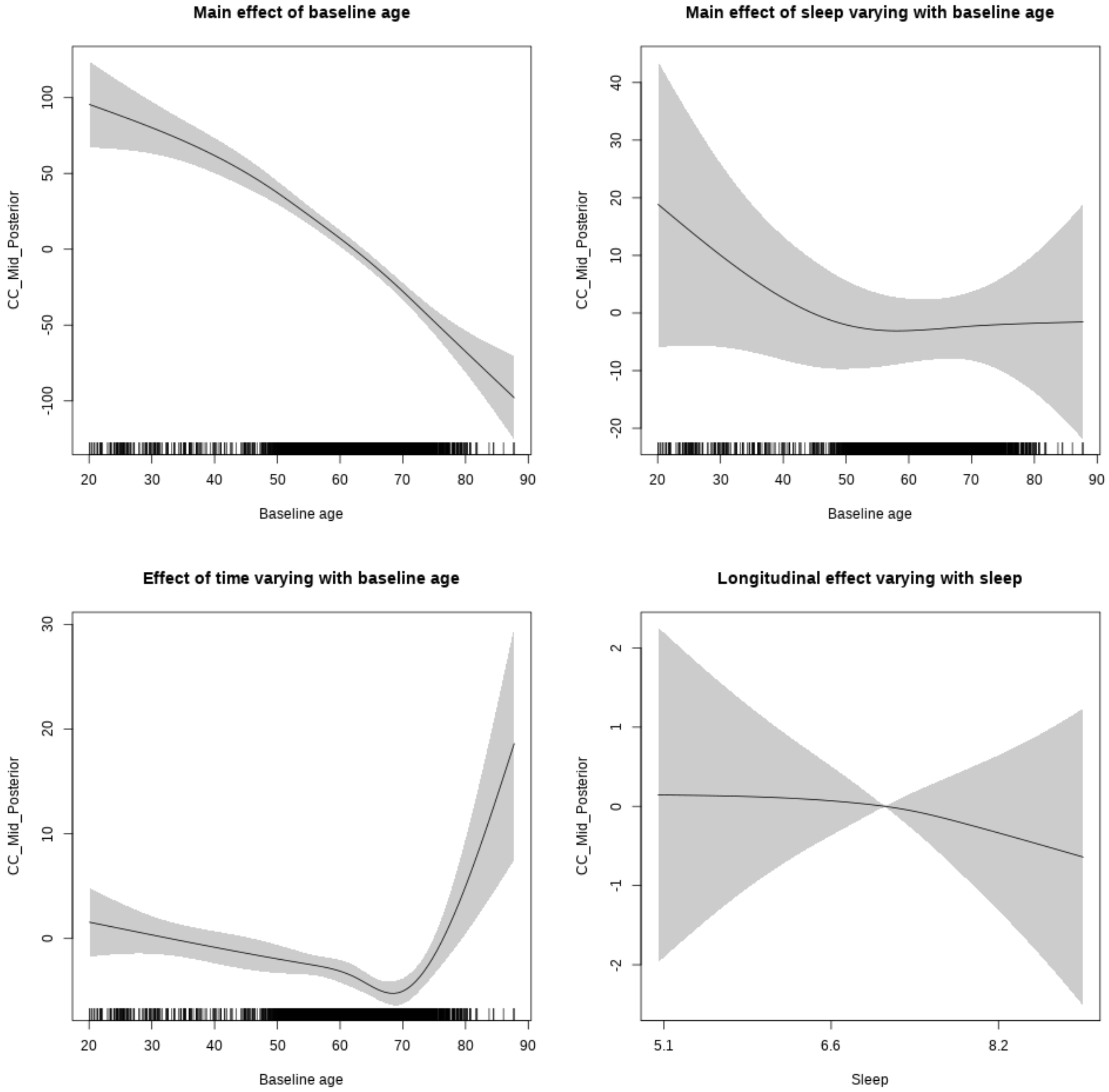

Restricted to sleep >= 5 and <= 9 hours Below is the row for `s(sleep_z):time` from the model output.

```
## s(sleep_z):time   1.199  1.199   0.128   0.702
```

Below are plots of a plot of `s(bl_age)`, `s(bl_age):sleep_z`, `s(bl_age):time`, and `s(sleep_z):time`. For `s(sleep_z):time`, roughly, if we cannot fit a straight line through the confidence bands, there is evidence that the effect of time depends on sleep duration. It is correct the that confidence interval is zero at one point along the axis; this happens because we do not include the main effect.

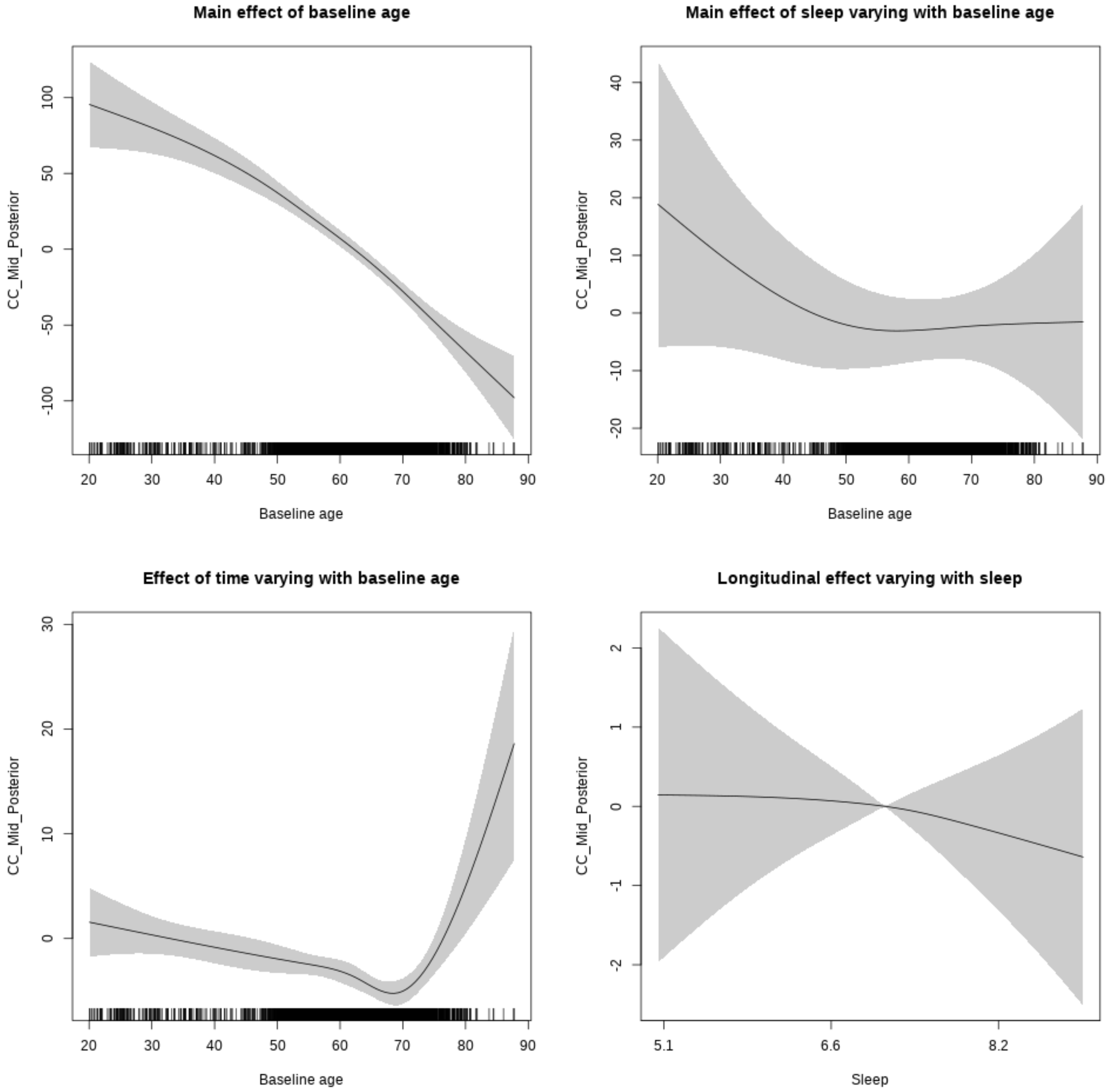

Controling for depression

Full data Below is the model output. The term `s(sleep_z):time` is probably what we care most about.

```
##
## Family: gaussian
## Link function: identity
##
## Formula:
## value ~ sex + site + depression + icv + s(bl_age, k = 10, bs = "cr") +
##     s(bl_age, by = sleep_z, bs = "cr") + +s(bl_age, by = time,
##     k = 5, bs = "cr") + s(sleep_z, by = time, k = 5, bs = "cr",
##     pc = 0)
```

```
## <environment: 0x5639159473f8>
##
## Parametric coefficients:
##                Estimate Std. Error t value Pr(>|t|)
## (Intercept)     539.563      8.673  62.215  < 2e-16 ***
## sexmale         -18.902      4.542  -4.161 3.21e-05 ***
## siteousAvanto   -58.042     18.265  -3.178  0.00149 **
## siteousPrisma   -12.774     31.373  -0.407  0.68390
## siteousSkyra    -85.673     15.737  -5.444 5.43e-08 ***
## siteUCAM        -43.444     10.380  -4.185 2.89e-05 ***
## siteUKB          14.498      7.965   1.820  0.06878 .
## siteUmU          -9.861     12.733  -0.774  0.43868
## depression      -11.417     15.962  -0.715  0.47448
## icv              27.347      2.183  12.528  < 2e-16 ***
## ---
## Signif. codes:  0 '***' 0.001 '**' 0.01 '*' 0.05 '.' 0.1 ' ' 1
##
## Approximate significance of smooth terms:
##                      edf Ref.df      F p-value
## s(bl_age)          2.687  2.687 83.438  <2e-16 ***
## s(bl_age):sleep_z 2.000  2.000  0.868   0.420
## s(bl_age):time    4.588  4.588 29.142  <2e-16 ***
## s(sleep_z):time   1.000  1.000  0.362   0.547
## ---
## Signif. codes:  0 '***' 0.001 '**' 0.01 '*' 0.05 '.' 0.1 ' ' 1
##
## R-sq.(adj) =  0.156
## lmer.REML =  62780  Scale est. = 773.82    n = 5655
```

Below are plots of a plot of `s(bl_age)`, `s(bl_age):sleep_z`, `s(bl_age):time`, and `s(sleep_z):time`. For `s(sleep_z):time`, roughly, if we cannot fit a straight line through the confidence bands, there is evidence that the effect of time depends on sleep duration. It is correct the that confidence interval is zero at one point along the axis; this happens because we do not include the main effect.

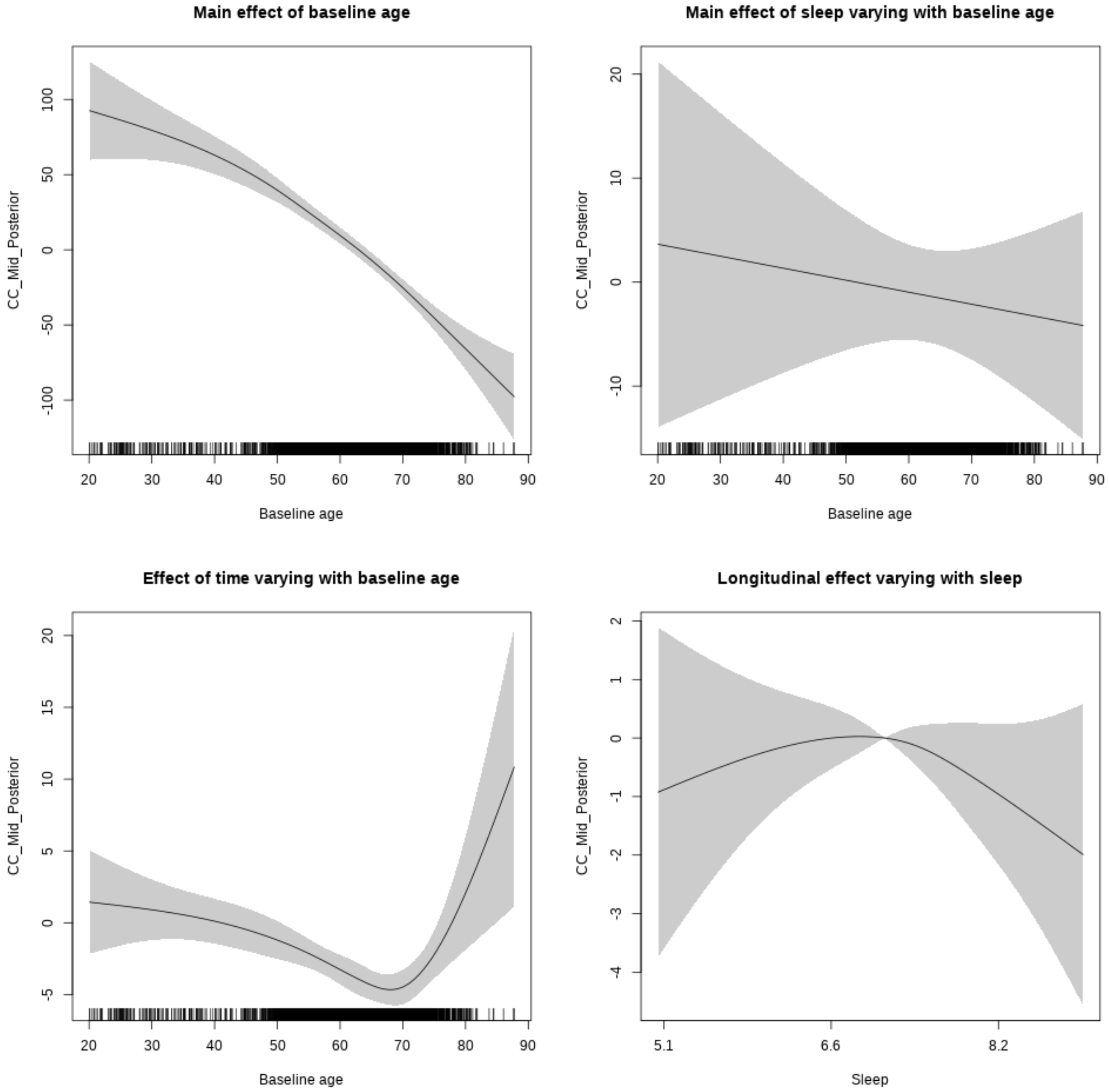

Restricted to sleep >= 5 and <= 9 hours Below is the row for `s(sleep_z):time` from the model output.

```
## s(sleep_z):time   1.840  1.840  1.114   0.235
```

Below are plots of a plot of `s(bl_age)`, `s(bl_age):sleep_z`, `s(bl_age):time`, and `s(sleep_z):time`. For `s(sleep_z):time`, roughly, if we cannot fit a straight line through the confidence bands, there is evidence that the effect of time depends on sleep duration. It is correct the that confidence interval is zero at one point along the axis; this happens because we do not include the main effect.

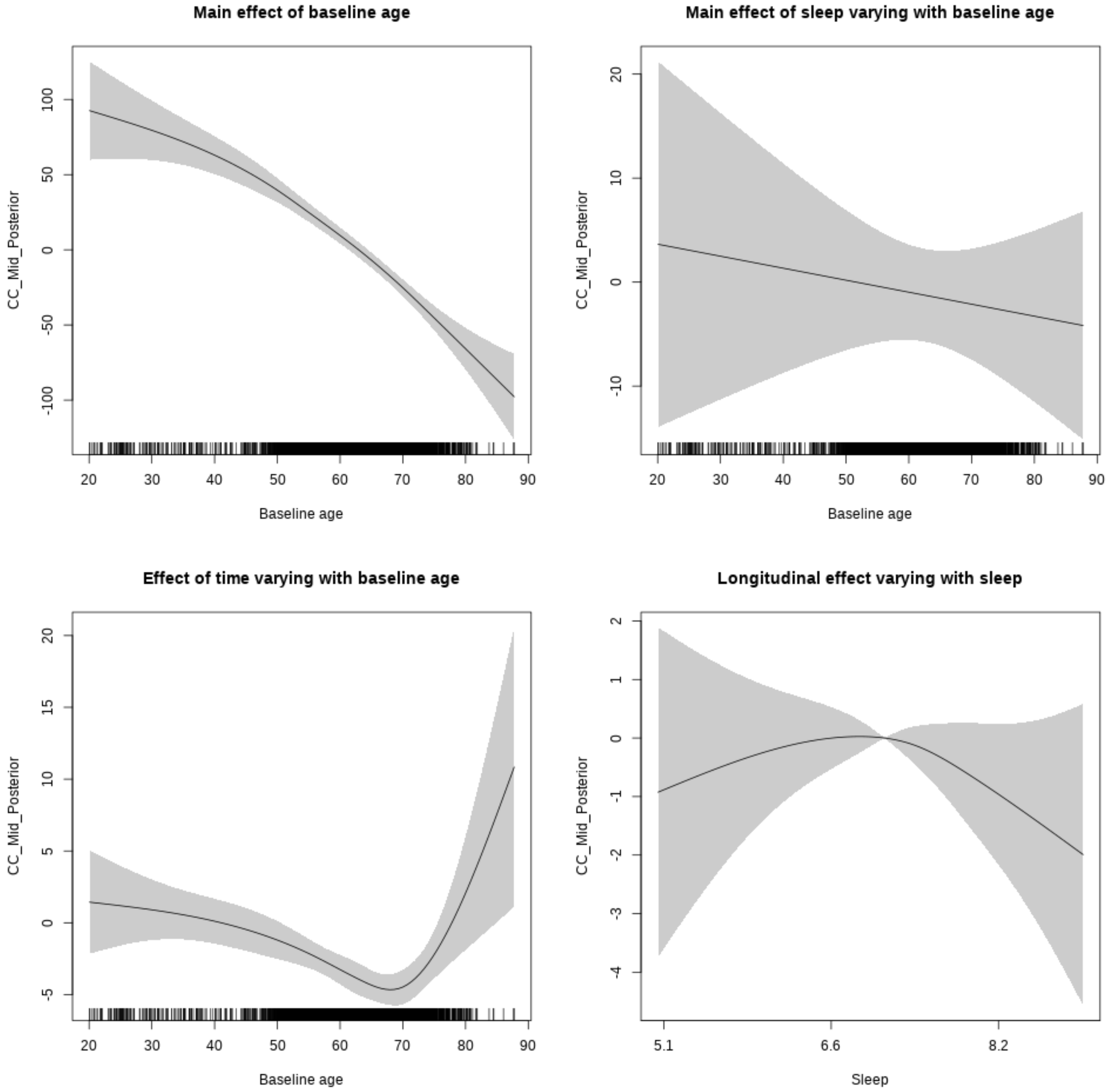

Controling for sleep quality

Full data Below is the model output. The term `s(sleep_z):time` is probably what we care most about.

```
##
## Family: gaussian
## Link function: identity
##
## Formula:
## value ~ X + s(bl_age, k = 10, bs = "cr") + s(bl_age, by = sleep_z,
##     bs = "cr") + s(bl_age, by = time, k = 5, bs = "cr") + s(sleep_z,
##     by = time, k = 5, bs = "cr", pc = 0)
## <environment: 0x563910f8bb18>
```

```
##
## Parametric coefficients:
##                          Estimate Std. Error t value Pr(>|t|)
## (Intercept)              489.8766    12.5975  38.887  < 2e-16 ***
## Xsexmale                  18.5635     4.7194   3.933 8.51e-05 ***
## XsiteousAvanto           -22.1210    12.0377  -1.838  0.06618 .
## XsiteousPrisma            49.0018    23.7239   2.066  0.03894 *
## XsiteousSkyra            -47.6088    12.0832  -3.940 8.28e-05 ***
## XsiteUB                  -21.8010    19.1308  -1.140  0.25453
## XsiteUCAM                 -3.8704    12.3002  -0.315  0.75303
## XsiteUKB                 -20.6948    38.3934  -0.539  0.58990
## XsiteUmU                   9.9853    13.7160   0.728  0.46665
## Xukb_dummy:sleep_accel     8.9601     2.9701   3.017  0.00257 **
## Xukb_dummy:chronotype      0.8177     3.9600   0.206  0.83642
## Xukb_dummy:dozing          9.0564     8.1680   1.109  0.26760
## Xukb_dummy:sleeplessness  -6.2274     5.0921  -1.223  0.22141
## Xukb_dummy:snoring        12.2063     7.2147   1.692  0.09074 .
## Xukb_dummy:gettingup      -0.6289     5.5342  -0.114  0.90953
## Xnotukb_dummy:PSQI_Global  0.1219     1.5599   0.078  0.93770
## ---
## Signif. codes:  0 '***' 0.001 '**' 0.01 '*' 0.05 '.' 0.1 ' ' 1
##
## Approximate significance of smooth terms:
##                      edf Ref.df      F p-value
## s(bl_age)          2.852  2.852 84.132  <2e-16 ***
## s(bl_age):sleep_z 2.000  2.000  1.462  0.2319
## s(bl_age):time    2.000  2.000 71.900  <2e-16 ***
## s(sleep_z):time   1.000  1.000  4.234  0.0397 *
## ---
## Signif. codes:  0 '***' 0.001 '**' 0.01 '*' 0.05 '.' 0.1 ' ' 1
##
## R-sq.(adj) =  0.193
## lmer.REML =  46848  Scale est. = 715.83    n = 4278
```

Below are plots of a plot of `s(bl_age)`, `s(bl_age):sleep_z`, `s(bl_age):time`, and `s(sleep_z):time`. For `s(sleep_z):time`, roughly, if we cannot fit a straight line through the confidence bands, there is evidence that the effect of time depends on sleep duration. It is correct the that confidence interval is zero at one point along the axis; this happens because we do not include the main effect.

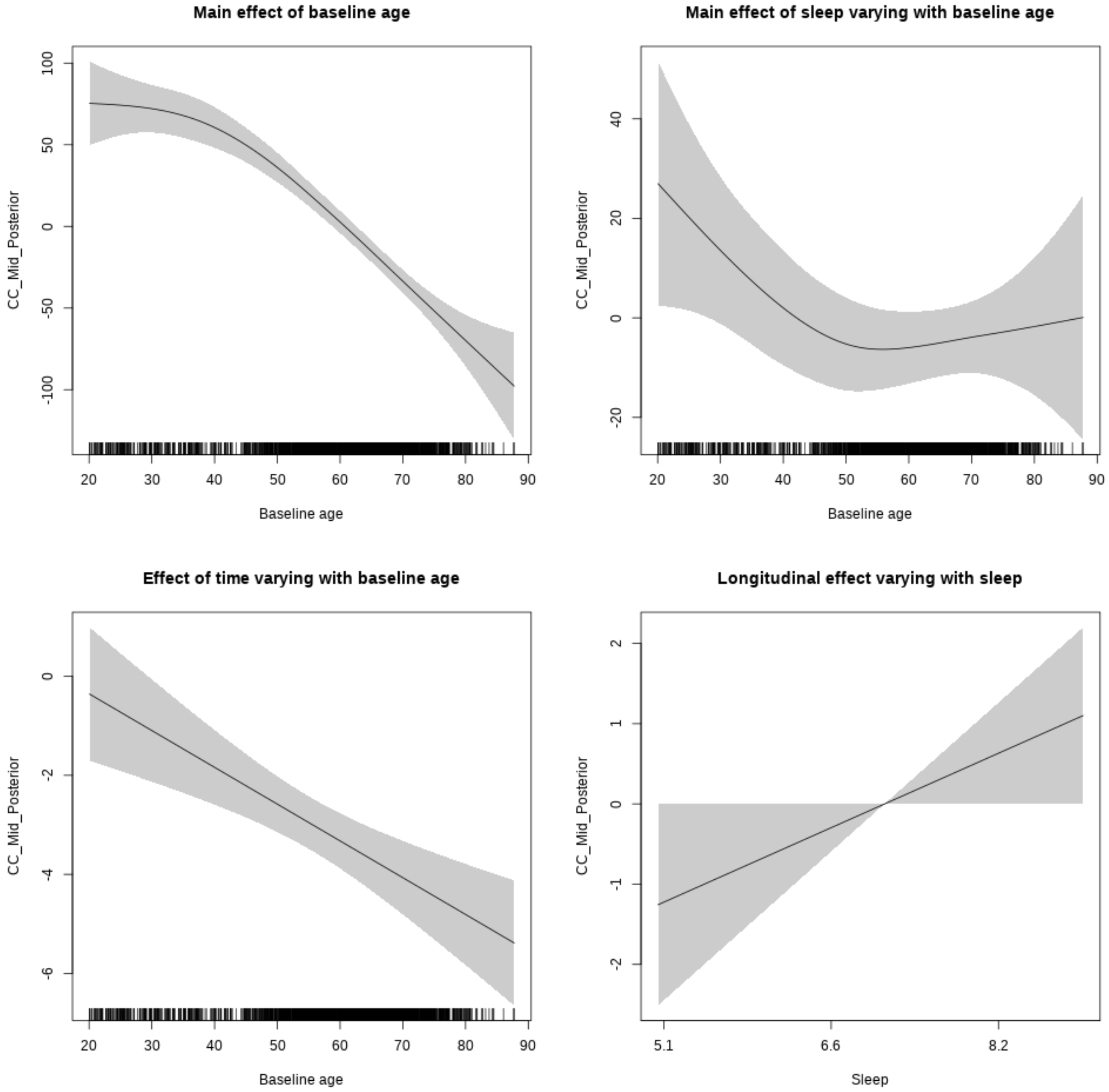

Restricted to sleep >= 5 and <= 9 hours Below is the row for `s(sleep_z):time` from the model output.

```
## s(sleep_z):time   1.000  1.000  4.007  0.0454 *
```

Below are plots of a plot of `s(bl_age)`, `s(bl_age):sleep_z`, `s(bl_age):time`, and `s(sleep_z):time`. For `s(sleep_z):time`, roughly, if we cannot fit a straight line through the confidence bands, there is evidence that the effect of time depends on sleep duration. It is correct the that confidence interval is zero at one point along the axis; this happens because we do not include the main effect.

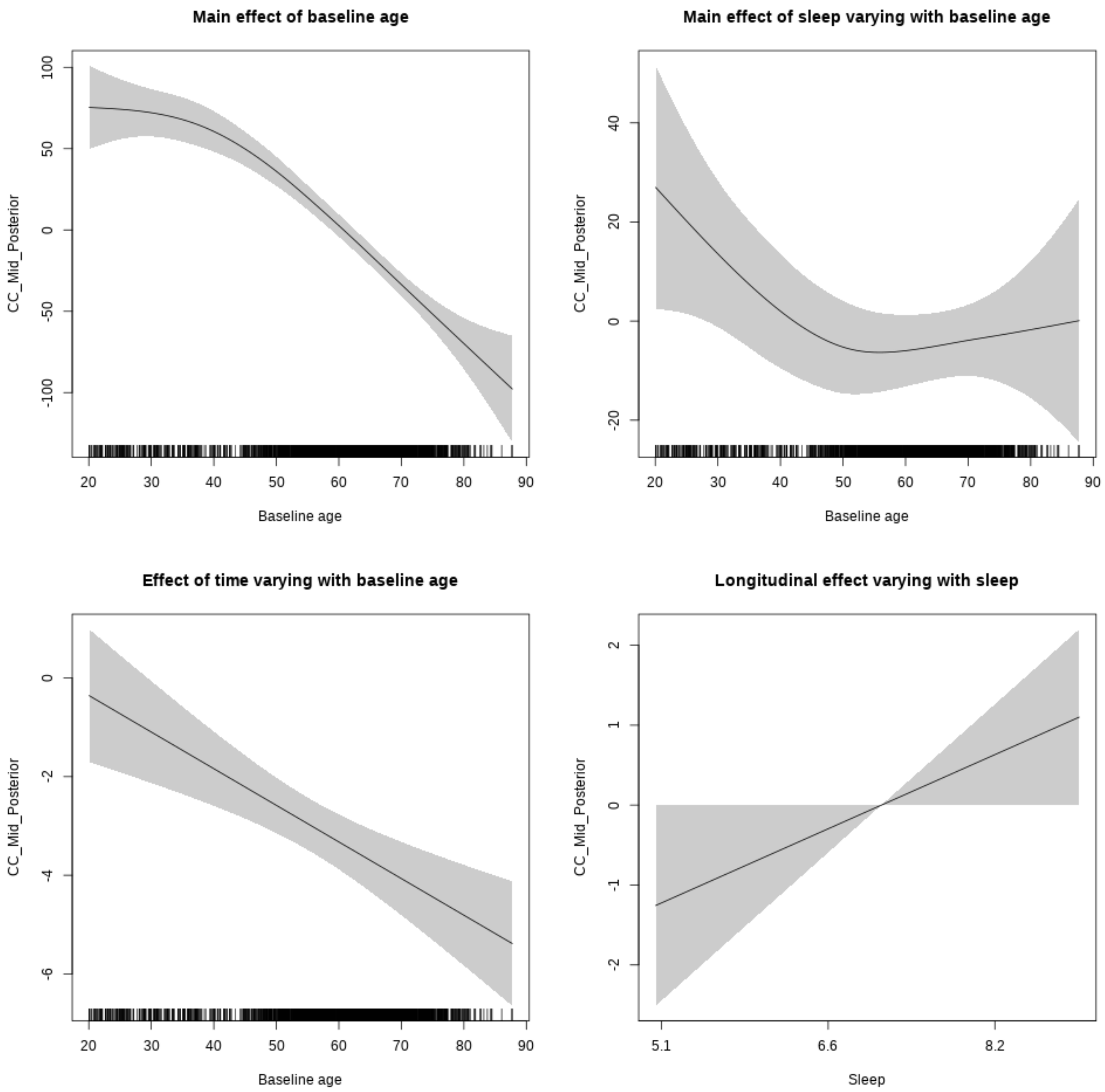

CC_Posterior

Main analysis

Full data Below is the model output. The term `s(sleep_z):time` is probably what we care most about.

```
##
## Family: gaussian
## Link function: identity
##
## Formula:
## value ~ sex + site + icv + s(bl_age, k = 10, bs = "cr") + s(bl_age,
##     by = sleep_z, bs = "cr") + +s(bl_age, by = time, k = 5, bs = "cr") +
```

```
##     s(sleep_z, by = time, k = 5, bs = "cr", pc = 0)
## <environment: 0x56391e8b18b8>
##
## Parametric coefficients:
##                Estimate Std. Error t value Pr(>|t|)
## (Intercept)    1066.718     10.725  99.460  < 2e-16 ***
## sexmale         -33.472      5.595  -5.983 2.29e-09 ***
## siteousAvanto   -95.527     13.286  -7.190 7.04e-13 ***
## siteousPrisma -101.803     28.703  -3.547 0.000392 ***
## siteousSkyra    -81.073     12.929  -6.271 3.78e-10 ***
## siteUB          -84.105     25.343  -3.319 0.000908 ***
## siteUCAM        -66.165     13.527  -4.891 1.02e-06 ***
## siteUKB          -1.886     10.044  -0.188 0.851090
## siteUmU         -14.624     15.653  -0.934 0.350226
## icv              65.278      2.626  24.862  < 2e-16 ***
## ---
## Signif. codes:  0 '***' 0.001 '**' 0.01 '*' 0.05 '.' 0.1 ' ' 1
##
## Approximate significance of smooth terms:
##                    edf Ref.df      F p-value
## s(bl_age)            1      1  7.734 0.00543 **
## s(bl_age):sleep_z    2      2  0.362 0.69643
## s(bl_age):time       2      2 31.778 < 2e-16 ***
## s(sleep_z):time      1      1  2.723 0.09896 .
## ---
## Signif. codes:  0 '***' 0.001 '**' 0.01 '*' 0.05 '.' 0.1 ' ' 1
##
## R-sq.(adj) =  0.167
## lmer.REML =  93848  Scale est. = 1077.4    n = 8100
```

Below are plots of a plot of `s(bl_age)`, `s(bl_age):sleep_z`, `s(bl_age):time`, and `s(sleep_z):time`. For `s(sleep_z):time`, roughly, if we cannot fit a straight line through the confidence bands, there is evidence that the effect of time depends on sleep duration. It is correct the that confidence interval is zero at one point along the axis; this happens because we do not include the main effect.

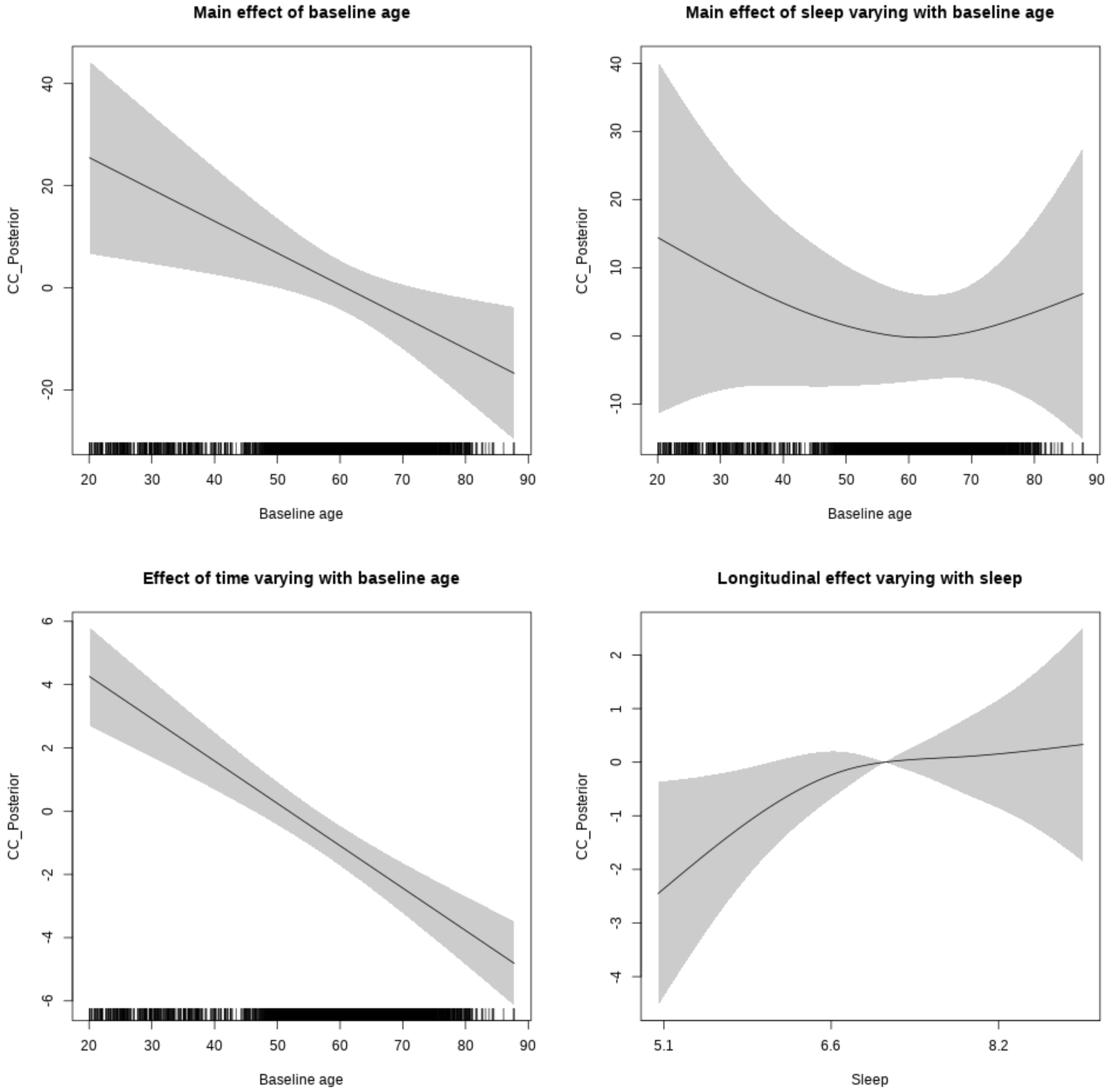

Restricted to sleep >= 5 and <= 9 hours Below is the row for `s(sleep_z):time` from the model output.

```
## s(sleep_z):time   1.810  1.810  2.953 0.06042 .
```

Below are plots of a plot of `s(bl_age)`, `s(bl_age):sleep_z`, `s(bl_age):time`, and `s(sleep_z):time`. For `s(sleep_z):time`, roughly, if we cannot fit a straight line through the confidence bands, there is evidence that the effect of time depends on sleep duration. It is correct the that confidence interval is zero at one point along the axis; this happens because we do not include the main effect.

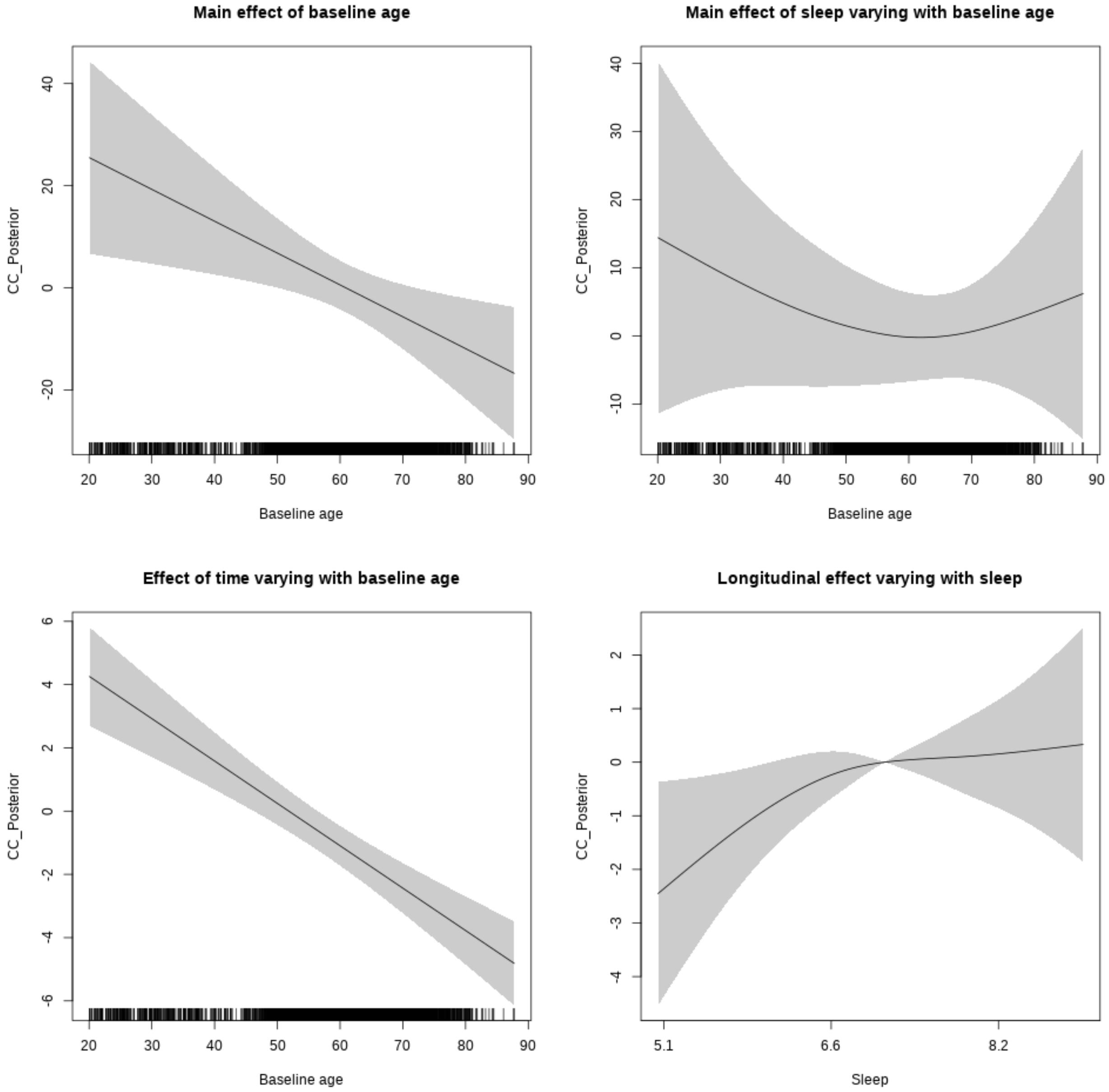

Controling for socioeconomic status

Full data Below is the model output. The term `s(sleep_z):time` is probably what we care most about.

```
##
## Family: gaussian
## Link function: identity
##
## Formula:
## value ~ sex + site + income_scaled + education_scaled + icv +
##     s(bl_age, k = 10, bs = "cr") + s(bl_age, by = sleep_z, bs = "cr") +
##     +s(bl_age, by = time, k = 5, bs = "cr") + s(sleep_z, by = time,
##     k = 5, bs = "cr", pc = 0)
```

```
## <environment: 0x56391152c058>
##
## Parametric coefficients:
##                   Estimate Std. Error t value Pr(>|t|)
## (Intercept)       1109.758     25.376  43.733  < 2e-16 ***
## sexmale            -50.304      7.087  -7.098 1.46e-12 ***
## siteousAvanto     -127.404     27.537  -4.627 3.82e-06 ***
## siteousPrisma     -141.671     49.920  -2.838  0.00456 **
## siteousSkyra      -109.322     26.172  -4.177 3.01e-05 ***
## siteUKB            -39.090     23.920  -1.634  0.10228
## income_scaled      -13.112      8.735  -1.501  0.13341
## education_scaled     5.778     10.154   0.569  0.56939
## icv                 76.809      3.448  22.279  < 2e-16 ***
## ---
## Signif. codes:  0 '***' 0.001 '**' 0.01 '*' 0.05 '.' 0.1 ' ' 1
##
## Approximate significance of smooth terms:
##                     edf Ref.df      F  p-value
## s(bl_age)         1.000  1.000  0.043    0.836
## s(bl_age):sleep_z 3.092  3.092  1.400    0.194
## s(bl_age):time    2.000  2.000 13.206 2.15e-06 ***
## s(sleep_z):time   1.000  1.000  0.708    0.400
## ---
## Signif. codes:  0 '***' 0.001 '**' 0.01 '*' 0.05 '.' 0.1 ' ' 1
##
## R-sq.(adj) =  0.177
## lmer.REML =  53779  Scale est. = 1185.4    n = 4618
```

Below are plots of a plot of `s(bl_age)`, `s(bl_age):sleep_z`, `s(bl_age):time`, and `s(sleep_z):time`. For `s(sleep_z):time`, roughly, if we cannot fit a straight line through the confidence bands, there is evidence that the effect of time depends on sleep duration. It is correct the that confidence interval is zero at one point along the axis; this happens because we do not include the main effect.

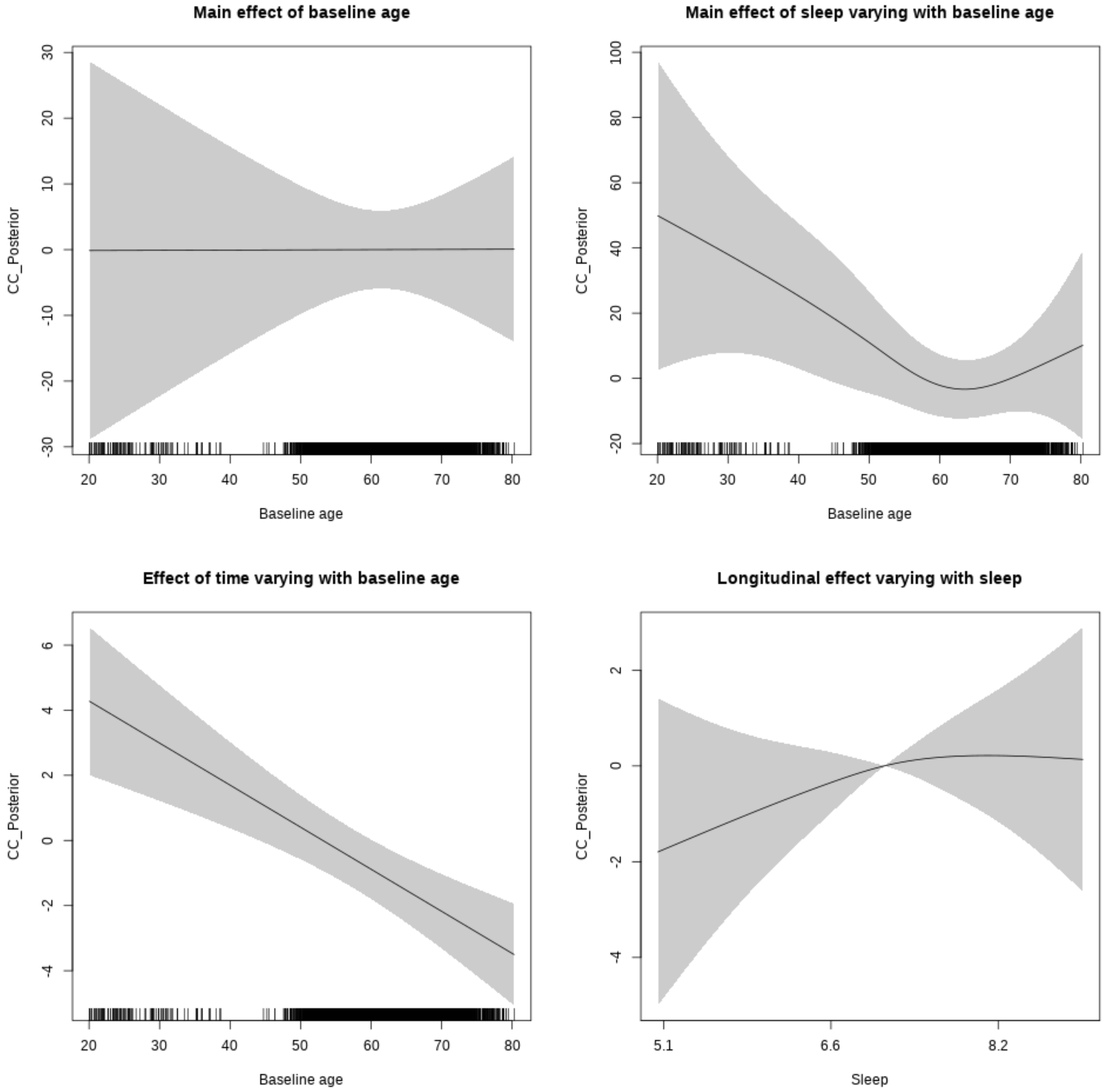

Restricted to sleep >= 5 and <= 9 hours Below is the row for `s(sleep_z):time` from the model output.

```
## s(sleep_z):time   1.344  1.344  1.171    0.440
```

Below are plots of a plot of `s(bl_age)`, `s(bl_age):sleep_z`, `s(bl_age):time`, and `s(sleep_z):time`. For `s(sleep_z):time`, roughly, if we cannot fit a straight line through the confidence bands, there is evidence that the effect of time depends on sleep duration. It is correct the that confidence interval is zero at one point along the axis; this happens because we do not include the main effect.

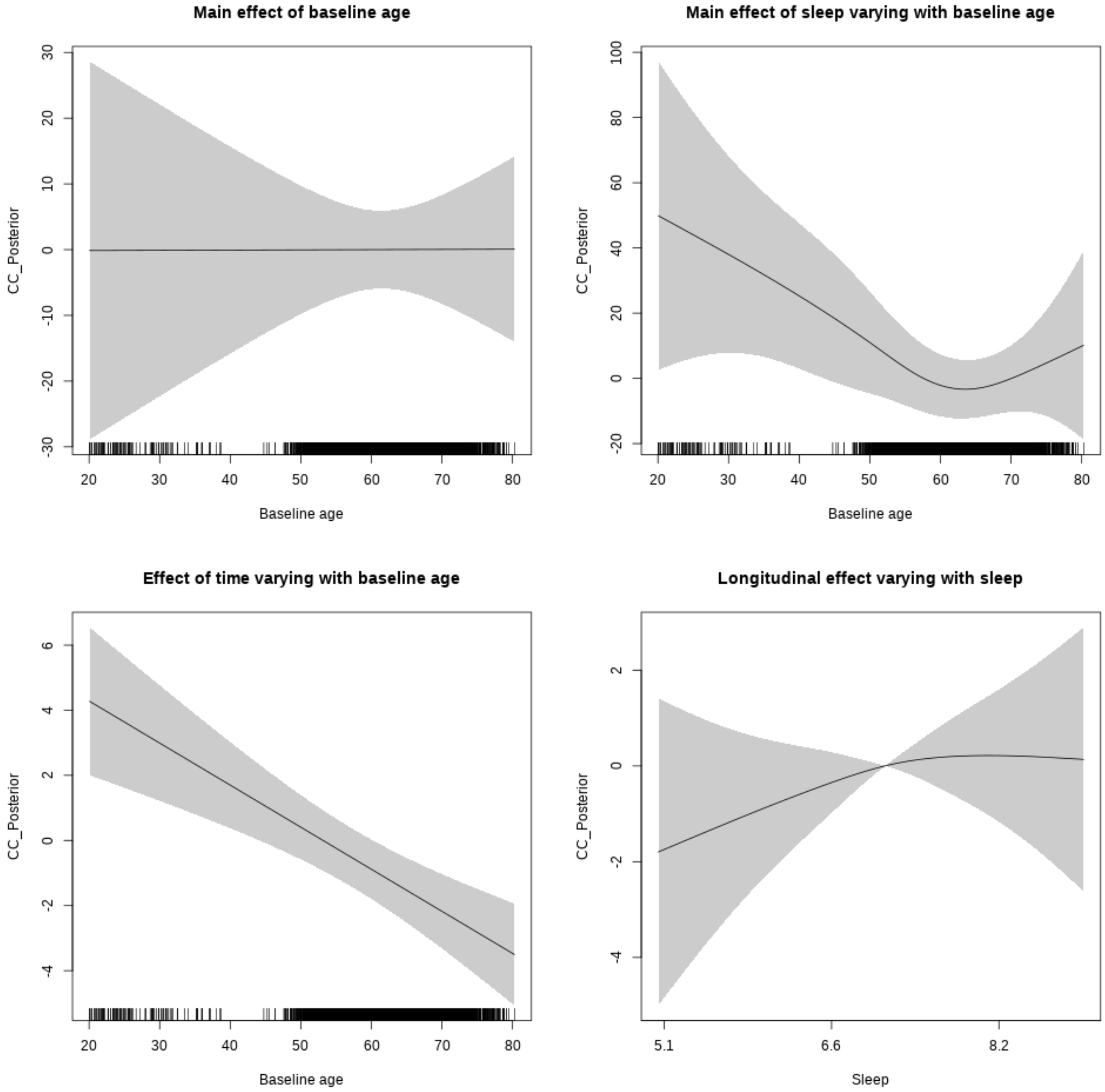

Controling for BMI

Full data Below is the model output. The term `s(sleep_z):time` is probably what we care most about.

```
##
## Family: gaussian
## Link function: identity
##
## Formula:
## value ~ sex + site + bmi + icv + s(bl_age, k = 10, bs = "cr") +
##     s(bl_age, by = sleep_z, bs = "cr") + +s(bl_age, by = time,
##     k = 5, bs = "cr") + s(sleep_z, by = time, k = 5, bs = "cr",
##     pc = 0)
```

```
## <environment: 0x56390d716a20>
##
## Parametric coefficients:
##                  Estimate Std. Error t value Pr(>|t|)
## (Intercept)   1013.7167    21.8739  46.344  < 2e-16 ***
## sexmale        -39.2040     6.6064  -5.934 3.14e-09 ***
## siteousPrisma  -14.7426    27.1087  -0.544   0.5866
## siteousSkyra    12.2445     8.6634   1.413   0.1576
## siteUCAM        36.4606    16.4621   2.215   0.0268 *
## siteUKB         92.9192    14.3513   6.475 1.04e-10 ***
## siteUmU         91.0937    18.9511   4.807 1.58e-06 ***
## bmi             -1.6126     0.6886  -2.342   0.0192 *
## icv             72.9317     3.1990  22.799  < 2e-16 ***
## ---
## Signif. codes:  0 '***' 0.001 '**' 0.01 '*' 0.05 '.' 0.1 ' ' 1
##
## Approximate significance of smooth terms:
##                       edf Ref.df      F  p-value
## s(bl_age)           1.000  1.000  0.381    0.537
## s(bl_age):sleep_z 2.511  2.511  1.046    0.522
## s(bl_age):time    2.000  2.000 15.496 1.89e-07 ***
## s(sleep_z):time   1.000  1.000  2.390    0.122
## ---
## Signif. codes:  0 '***' 0.001 '**' 0.01 '*' 0.05 '.' 0.1 ' ' 1
##
## R-sq.(adj) =  0.168
## lmer.REML =  62452  Scale est. = 1107.9    n = 5372
```

Below are plots of a plot of `s(bl_age)`, `s(bl_age):sleep_z`, `s(bl_age):time`, and `s(sleep_z):time`. For `s(sleep_z):time`, roughly, if we cannot fit a straight line through the confidence bands, there is evidence that the effect of time depends on sleep duration. It is correct the that confidence interval is zero at one point along the axis; this happens because we do not include the main effect.

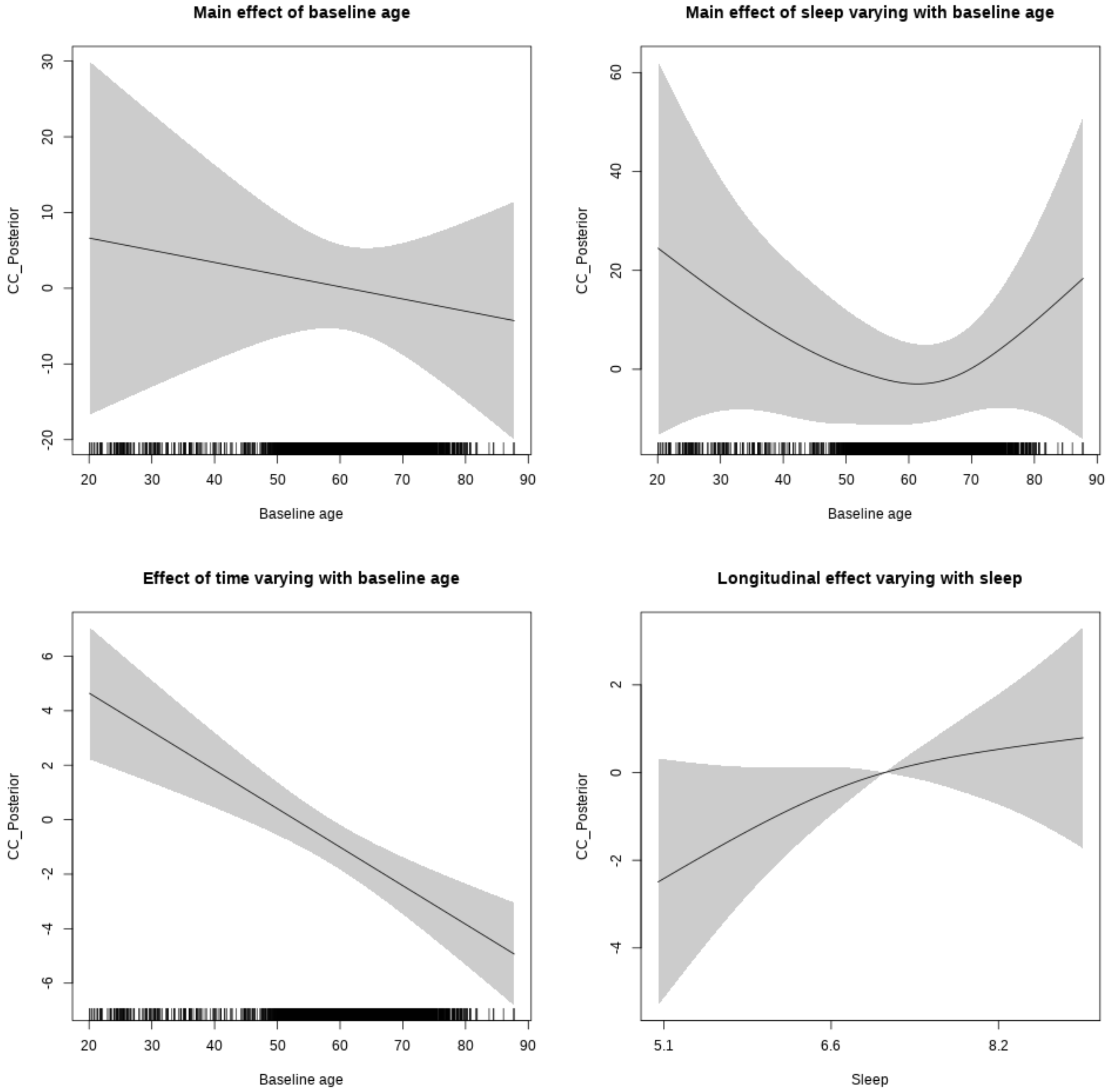

Restricted to sleep >= 5 and <= 9 hours Below is the row for `s(sleep_z):time` from the model output.

```
## s(sleep_z):time   1.358  1.358  2.815    0.155
```

Below are plots of a plot of `s(bl_age)`, `s(bl_age):sleep_z`, `s(bl_age):time`, and `s(sleep_z):time`. For `s(sleep_z):time`, roughly, if we cannot fit a straight line through the confidence bands, there is evidence that the effect of time depends on sleep duration. It is correct the that confidence interval is zero at one point along the axis; this happens because we do not include the main effect.

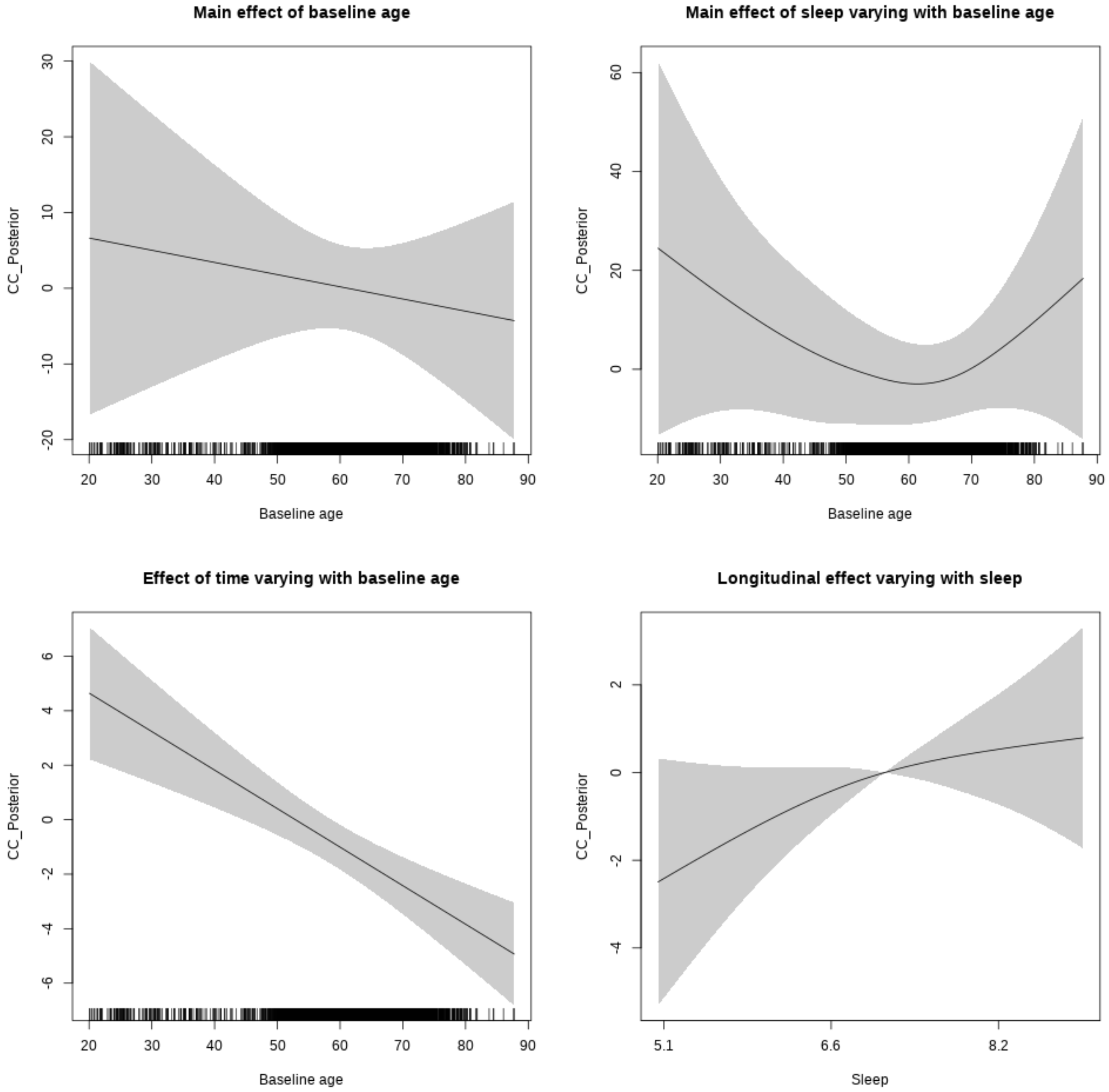

Controling for depression

Full data Below is the model output. The term `s(sleep_z):time` is probably what we care most about.

```
##
## Family: gaussian
## Link function: identity
##
## Formula:
## value ~ sex + site + depression + icv + s(bl_age, k = 10, bs = "cr") +
##     s(bl_age, by = sleep_z, bs = "cr") + +s(bl_age, by = time,
##     k = 5, bs = "cr") + s(sleep_z, by = time, k = 5, bs = "cr",
##     pc = 0)
```

```
## <environment: 0x5639111d2bd8>
##
## Parametric coefficients:
##                Estimate Std. Error t value Pr(>|t|)
## (Intercept)    1053.862     12.711  82.911  < 2e-16 ***
## sexmale         -34.266      6.676  -5.133 2.95e-07 ***
## siteousAvanto   -75.012     24.940  -3.008  0.00264 **
## siteousPrisma   -73.859     39.307  -1.879  0.06030 .
## siteousSkyra    -50.827     22.164  -2.293  0.02187 *
## siteUCAM        -45.245     15.104  -2.996  0.00275 **
## siteUKB           8.813     11.598   0.760  0.44733
## siteUmU           8.814     18.860   0.467  0.64026
## depression      -10.843     23.579  -0.460  0.64564
## icv              66.226      3.153  21.005  < 2e-16 ***
## ---
## Signif. codes:  0 '***' 0.001 '**' 0.01 '*' 0.05 '.' 0.1 ' ' 1
##
## Approximate significance of smooth terms:
##                    edf Ref.df      F  p-value
## s(bl_age)            1      1  0.150    0.699
## s(bl_age):sleep_z    2      2  0.678    0.508
## s(bl_age):time       2      2 12.592 3.92e-06 ***
## s(sleep_z):time      1      1  1.258    0.262
## ---
## Signif. codes:  0 '***' 0.001 '**' 0.01 '*' 0.05 '.' 0.1 ' ' 1
##
## R-sq.(adj) =  0.158
## lmer.REML =  65703  Scale est. = 1078.5    n = 5637
```

Below are plots of a plot of `s(bl_age)`, `s(bl_age):sleep_z`, `s(bl_age):time`, and `s(sleep_z):time`. For `s(sleep_z):time`, roughly, if we cannot fit a straight line through the confidence bands, there is evidence that the effect of time depends on sleep duration. It is correct the that confidence interval is zero at one point along the axis; this happens because we do not include the main effect.

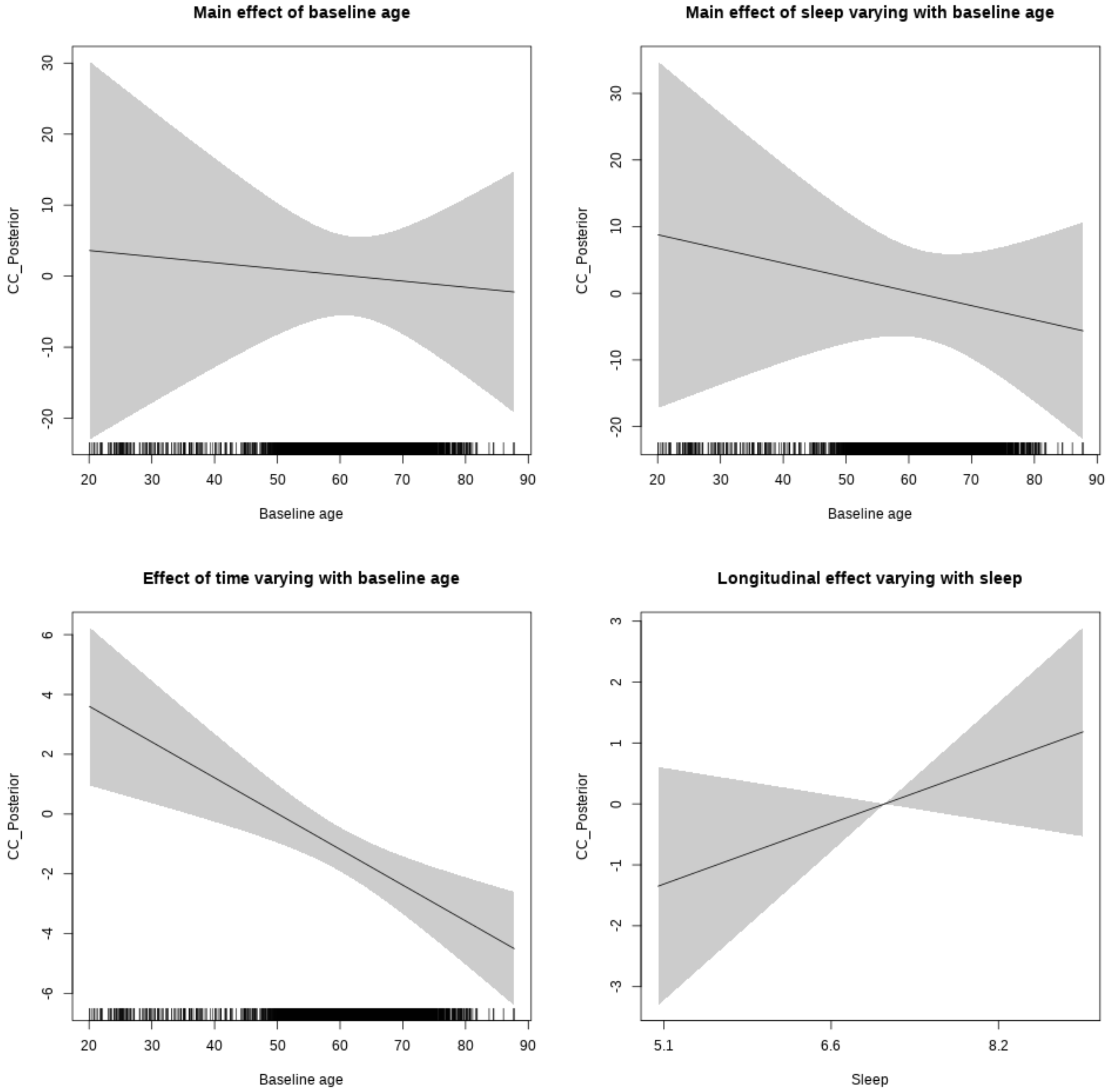

Restricted to sleep >= 5 and <= 9 hours Below is the row for `s(sleep_z):time` from the model output.

```
## s(sleep_z):time     1       1  1.903    0.168
```

Below are plots of a plot of `s(bl_age)`, `s(bl_age):sleep_z`, `s(bl_age):time`, and `s(sleep_z):time`. For `s(sleep_z):time`, roughly, if we cannot fit a straight line through the confidence bands, there is evidence that the effect of time depends on sleep duration. It is correct the that confidence interval is zero at one point along the axis; this happens because we do not include the main effect.

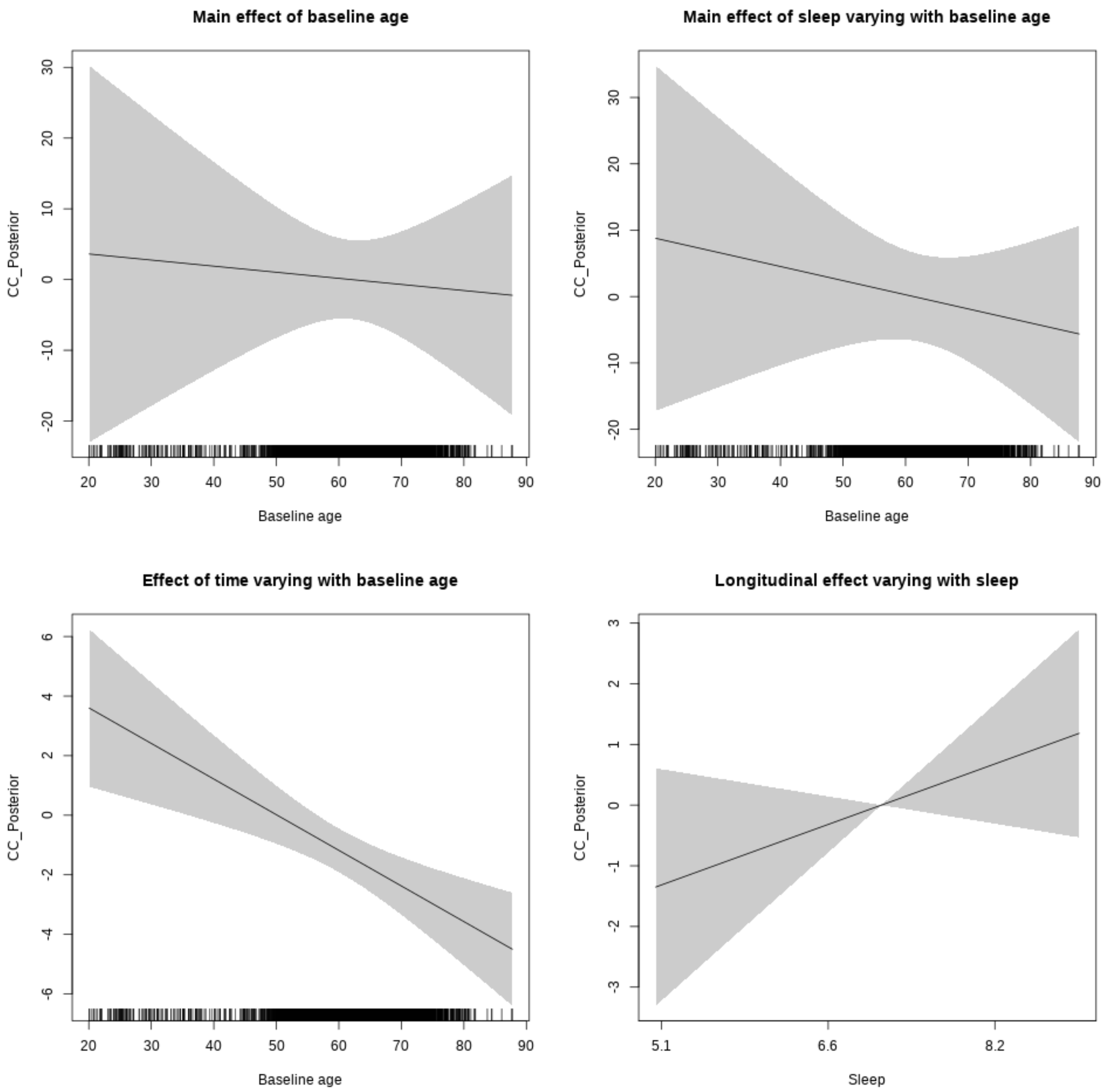

Controling for sleep quality

Full data Below is the model output. The term `s(sleep_z):time` is probably what we care most about.

```
##
## Family: gaussian
## Link function: identity
##
## Formula:
## value ~ X + s(bl_age, k = 10, bs = "cr") + s(bl_age, by = sleep_z,
##     bs = "cr") + s(bl_age, by = time, k = 5, bs = "cr") + s(sleep_z,
##     by = time, k = 5, bs = "cr", pc = 0)
## <environment: 0x563911573b28>
```

```
##
## Parametric coefficients:
##                           Estimate Std. Error t value Pr(>|t|)
## (Intercept)                914.592     19.109  47.861  < 2e-16 ***
## Xsexmale                    43.397      7.202   6.026 1.83e-09 ***
## XsiteousAvanto              44.940     18.219   2.467 0.013677 *
## XsiteousPrisma              70.428     29.478   2.389 0.016929 *
## XsiteousSkyra               36.002     18.261   1.971 0.048737 *
## XsiteUB                      4.047     29.114   0.139 0.889452
## XsiteUCAM                   53.475     18.507   2.889 0.003879 **
## XsiteUKB                   -73.213     58.114  -1.260 0.207807
## XsiteUmU                    66.190     20.695   3.198 0.001392 **
## Xukb_dummy:sleep_accel      15.549      4.523   3.438 0.000593 ***
## Xukb_dummy:chronotype        9.648      6.041   1.597 0.110367
## Xukb_dummy:dozing            9.642     12.440   0.775 0.438353
## Xukb_dummy:sleeplessness     2.217      7.725   0.287 0.774084
## Xukb_dummy:snoring           6.672     10.991   0.607 0.543834
## Xukb_dummy:gettingup        11.762      8.378   1.404 0.160457
## Xnotukb_dummy:PSQI_Global    1.613      2.379   0.678 0.497744
## ---
## Signif. codes:  0 '***' 0.001 '**' 0.01 '*' 0.05 '.' 0.1 ' ' 1
##
## Approximate significance of smooth terms:
##                      edf Ref.df      F p-value
## s(bl_age)          1.000  1.000  7.159 0.00749 **
## s(bl_age):sleep_z 2.745  2.745  0.623 0.61359
## s(bl_age):time     2.000  2.000 33.512 < 2e-16 ***
## s(sleep_z):time    1.000  1.000  2.010 0.15634
## ---
## Signif. codes:  0 '***' 0.001 '**' 0.01 '*' 0.05 '.' 0.1 ' ' 1
##
## R-sq.(adj) =  0.0717
## lmer.REML =  48811  Scale est. = 908.09    n = 4258
```

Below are plots of a plot of `s(bl_age)`, `s(bl_age):sleep_z`, `s(bl_age):time`, and `s(sleep_z):time`. For `s(sleep_z):time`, roughly, if we cannot fit a straight line through the confidence bands, there is evidence that the effect of time depends on sleep duration. It is correct the that confidence interval is zero at one point along the axis; this happens because we do not include the main effect.

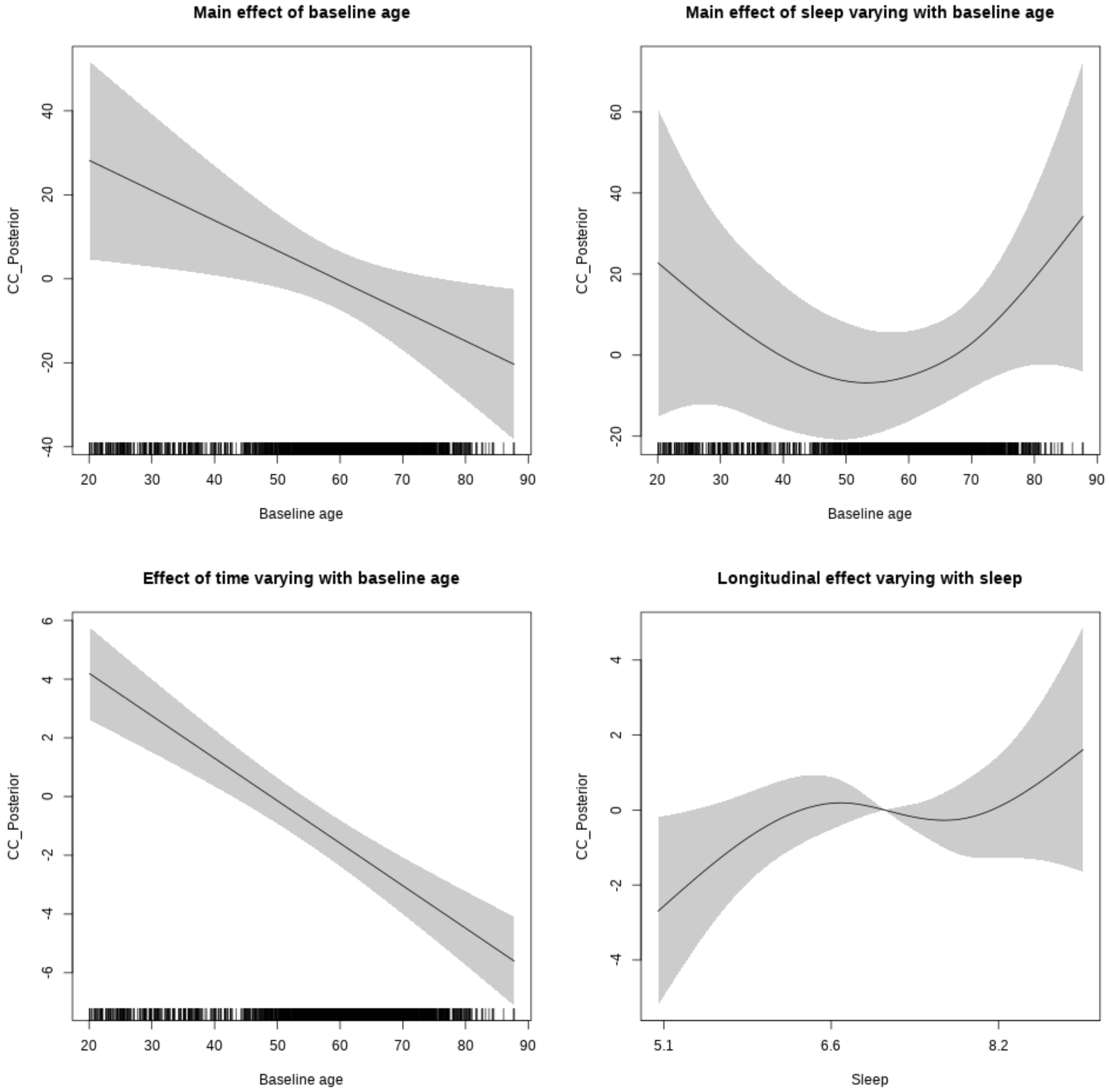

Restricted to sleep >= 5 and <= 9 hours Below is the row for `s(sleep_z):time` from the model output.

```
## s(sleep_z):time   2.554  2.554  1.156  0.1942
```

Below are plots of a plot of `s(bl_age)`, `s(bl_age):sleep_z`, `s(bl_age):time`, and `s(sleep_z):time`. For `s(sleep_z):time`, roughly, if we cannot fit a straight line through the confidence bands, there is evidence that the effect of time depends on sleep duration. It is correct the that confidence interval is zero at one point along the axis; this happens because we do not include the main effect.

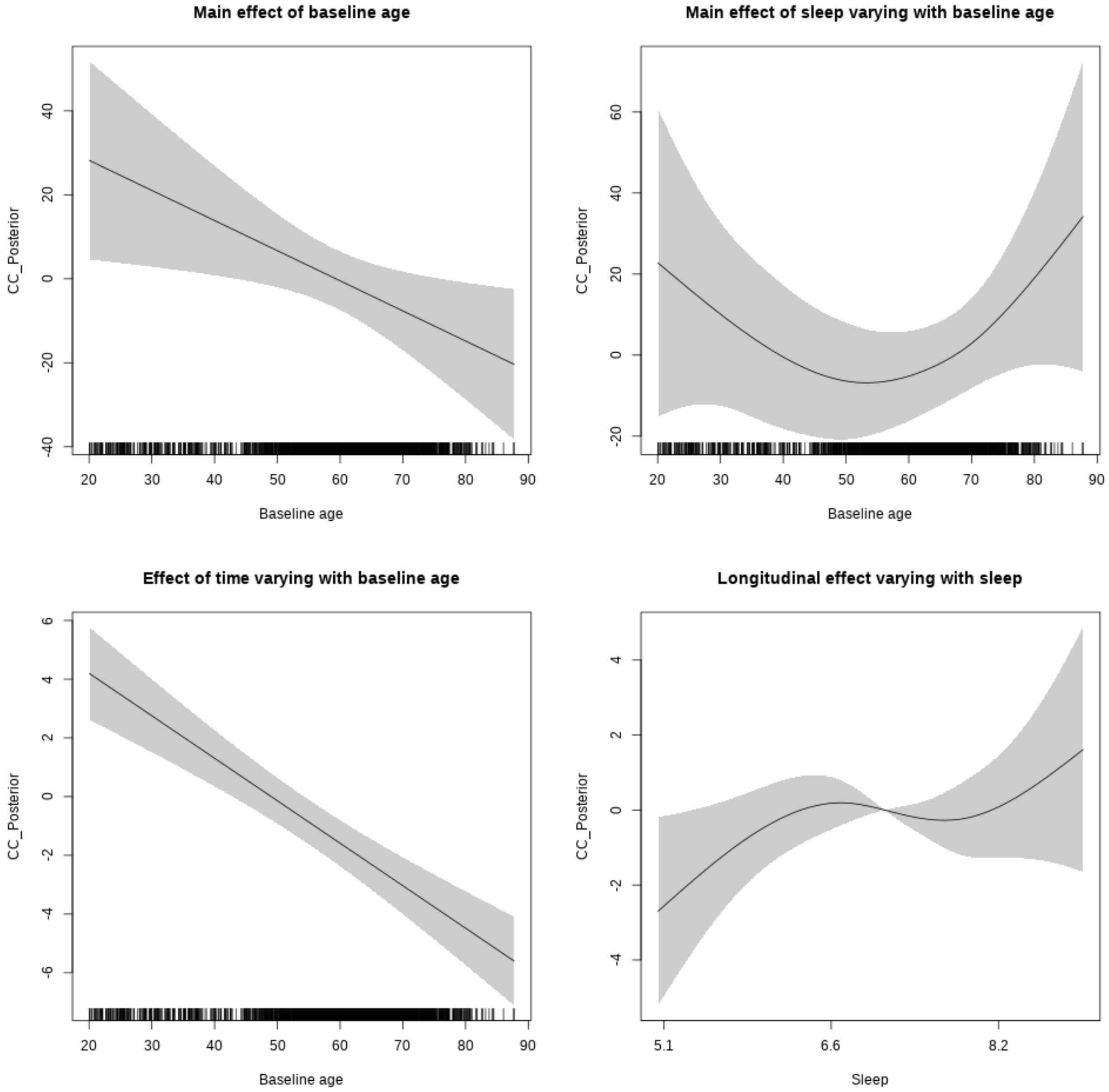

Cerebellum-Cortex

Main analysis

Full data Below is the model output. The term `s(sleep_z):time` is probably what we care most about.

```
##
## Family: gaussian
## Link function: identity
##
## Formula:
## value ~ sex + site + icv + s(bl_age, k = 10, bs = "cr") + s(bl_age,
##     by = sleep_z, bs = "cr") + +s(bl_age, by = time, k = 5, bs = "cr") +
```

```
##     s(sleep_z, by = time, k = 5, bs = "cr", pc = 0)
## <environment: 0x56391e79b038>
##
## Parametric coefficients:
##                Estimate Std. Error t value Pr(>|t|)
## (Intercept)    108107.6      681.0 158.742  < 2e-16 ***
## sexmale          5093.1      350.8  14.519  < 2e-16 ***
## siteousAvanto   -8330.3      835.0  -9.977  < 2e-16 ***
## siteousPrisma   -7046.9     1750.2  -4.026 5.72e-05 ***
## siteousSkyra   -10734.5      813.2 -13.201  < 2e-16 ***
## siteUB          -7610.1     1595.1  -4.771 1.86e-06 ***
## siteUCAM        -3445.1      855.2  -4.028 5.67e-05 ***
## siteUKB          2319.4      651.3   3.561 0.000371 ***
## siteUmU        -13029.2      995.8 -13.084  < 2e-16 ***
## icv              5414.1      164.3  32.950  < 2e-16 ***
## ---
## Signif. codes:  0 '***' 0.001 '**' 0.01 '*' 0.05 '.' 0.1 ' ' 1
##
## Approximate significance of smooth terms:
##                      edf Ref.df       F p-value
## s(bl_age)          3.497  3.497 143.431  <2e-16 ***
## s(bl_age):sleep_z 2.000  2.000   1.743   0.175
## s(bl_age):time     4.030  4.030  78.208  <2e-16 ***
## s(sleep_z):time    1.000  1.000   0.395   0.530
## ---
## Signif. codes:  0 '***' 0.001 '**' 0.01 '*' 0.05 '.' 0.1 ' ' 1
##
## R-sq.(adj) =   0.49
## lmer.REML = 1.6117e+05  Scale est. = 3.9226e+06  n = 8129
```

Below are plots of a plot of `s(bl_age)`, `s(bl_age):sleep_z`, `s(bl_age):time`, and `s(sleep_z):time`. For `s(sleep_z):time`, roughly, if we cannot fit a straight line through the confidence bands, there is evidence that the effect of time depends on sleep duration. It is correct the that confidence interval is zero at one point along the axis; this happens because we do not include the main effect.

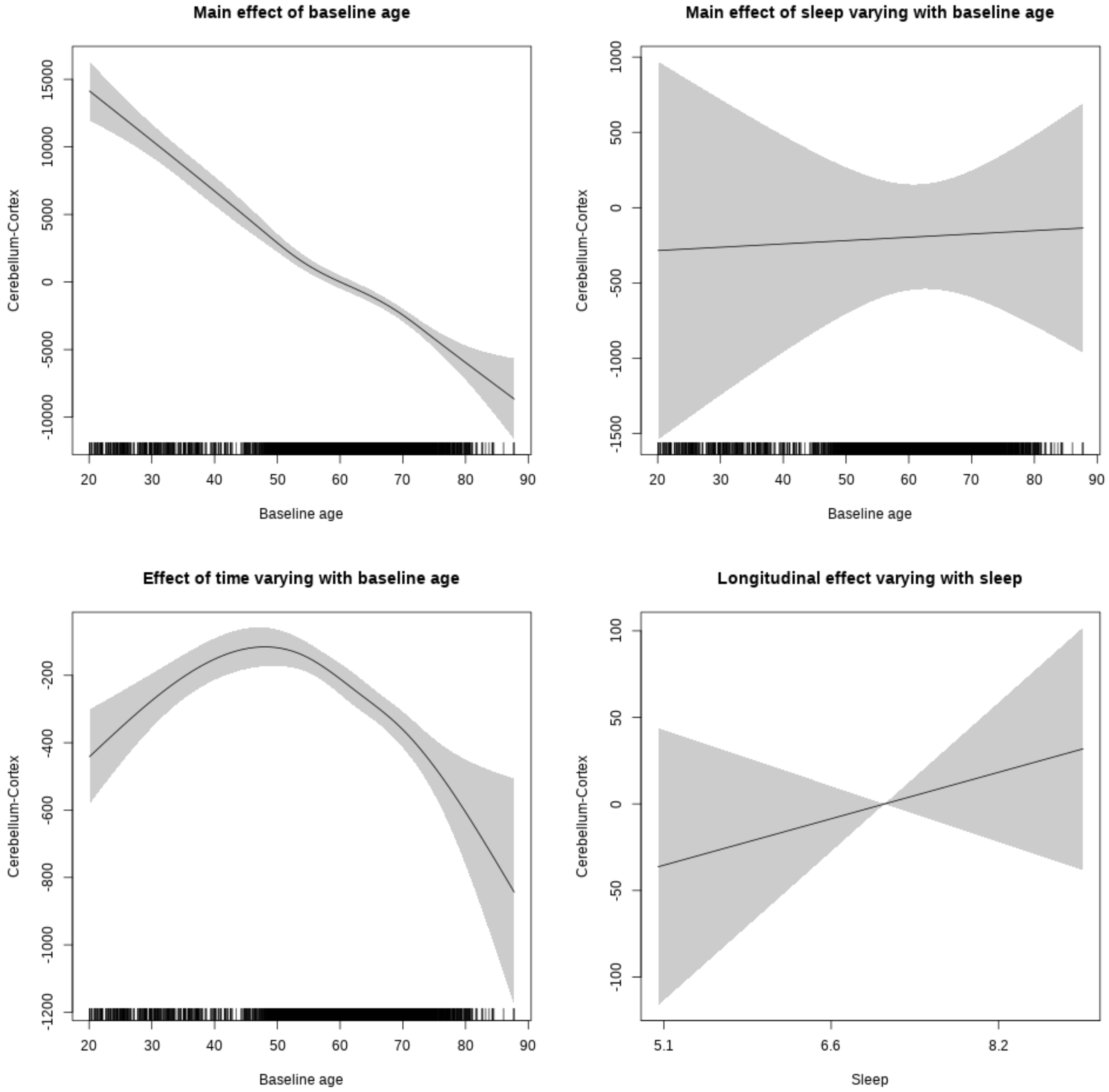

Restricted to sleep >= 5 and <= 9 hours Below is the row for `s(sleep_z):time` from the model output.

```
## s(sleep_z):time   1.000  1.000   0.823   0.364
```

Below are plots of a plot of `s(bl_age)`, `s(bl_age):sleep_z`, `s(bl_age):time`, and `s(sleep_z):time`. For `s(sleep_z):time`, roughly, if we cannot fit a straight line through the confidence bands, there is evidence that the effect of time depends on sleep duration. It is correct the that confidence interval is zero at one point along the axis; this happens because we do not include the main effect.

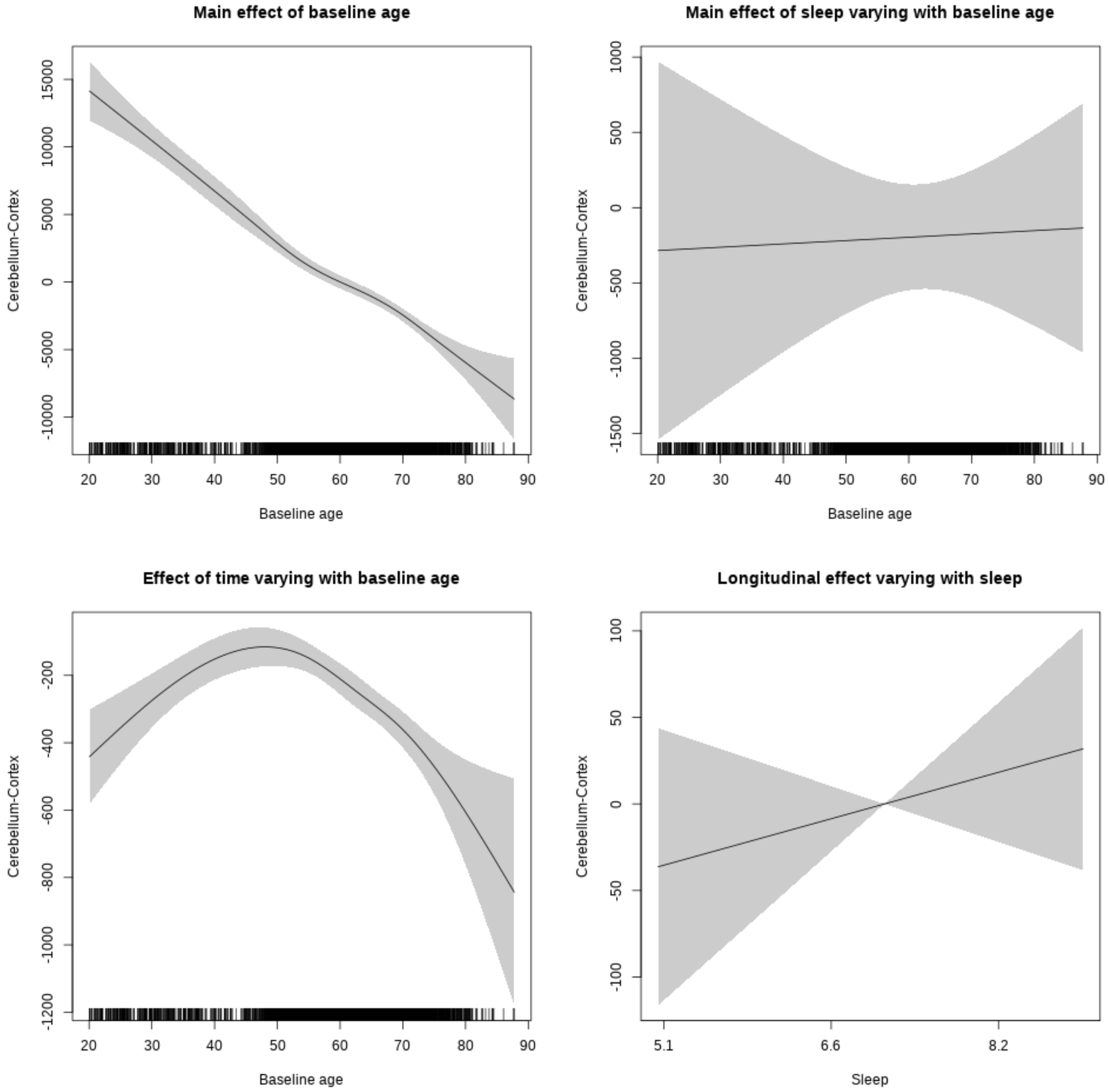

Controling for socioeconomic status

Full data Below is the model output. The term `s(sleep_z):time` is probably what we care most about.

```
##
## Family: gaussian
## Link function: identity
##
## Formula:
## value ~ sex + site + income_scaled + education_scaled + icv +
##     s(bl_age, k = 10, bs = "cr") + s(bl_age, by = sleep_z, bs = "cr") +
##     +s(bl_age, by = time, k = 5, bs = "cr") + s(sleep_z, by = time,
##     k = 5, bs = "cr", pc = 0)
```

```
## <environment: 0x56391139afc8>
##
## Parametric coefficients:
##                   Estimate Std. Error t value Pr(>|t|)
## (Intercept)       110333.5     1705.3  64.699  < 2e-16 ***
## sexmale             4471.6      465.4   9.609  < 2e-16 ***
## siteousAvanto     -12686.7     1821.3  -6.966 3.73e-12 ***
## siteousPrisma      -8616.3     2993.0  -2.879  0.00401 **
## siteousSkyra      -13831.2     1746.0  -7.922 2.92e-15 ***
## siteUKB             -713.8     1617.3  -0.441  0.65900
## income_scaled        544.0      603.8   0.901  0.36771
## education_scaled     549.4      669.1   0.821  0.41164
## icv                 6319.7      223.1  28.331  < 2e-16 ***
## ---
## Signif. codes:  0 '***' 0.001 '**' 0.01 '*' 0.05 '.' 0.1 ' ' 1
##
## Approximate significance of smooth terms:
##                      edf Ref.df      F p-value
## s(bl_age)          3.087  3.087 46.803  <2e-16 ***
## s(bl_age):sleep_z 2.000  2.000  0.352   0.704
## s(bl_age):time    3.586  3.586 32.520  <2e-16 ***
## s(sleep_z):time   1.000  1.000  0.000   0.995
## ---
## Signif. codes:  0 '***' 0.001 '**' 0.01 '*' 0.05 '.' 0.1 ' ' 1
##
## R-sq.(adj) =  0.426
## lmer.REML =  92132  Scale est. = 3.8173e+06  n = 4636
```

Below are plots of a plot of `s(bl_age)`, `s(bl_age):sleep_z`, `s(bl_age):time`, and `s(sleep_z):time`. For `s(sleep_z):time`, roughly, if we cannot fit a straight line through the confidence bands, there is evidence that the effect of time depends on sleep duration. It is correct the that confidence interval is zero at one point along the axis; this happens because we do not include the main effect.

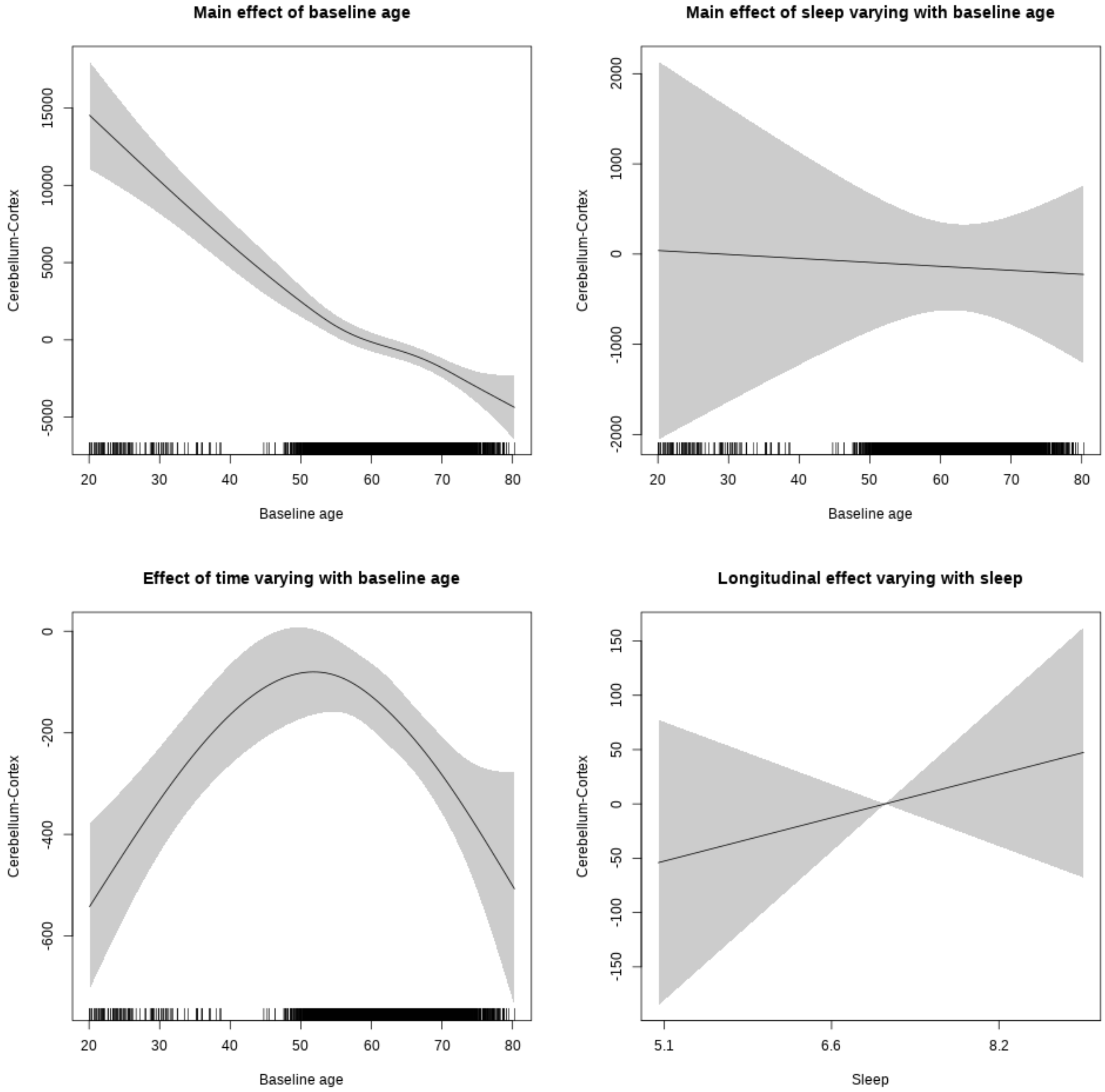

Restricted to sleep >= 5 and <= 9 hours Below is the row for `s(sleep_z):time` from the model output.

```
## s(sleep_z):time   1.000  1.000  0.676   0.411
```

Below are plots of a plot of `s(bl_age)`, `s(bl_age):sleep_z`, `s(bl_age):time`, and `s(sleep_z):time`. For `s(sleep_z):time`, roughly, if we cannot fit a straight line through the confidence bands, there is evidence that the effect of time depends on sleep duration. It is correct the that confidence interval is zero at one point along the axis; this happens because we do not include the main effect.

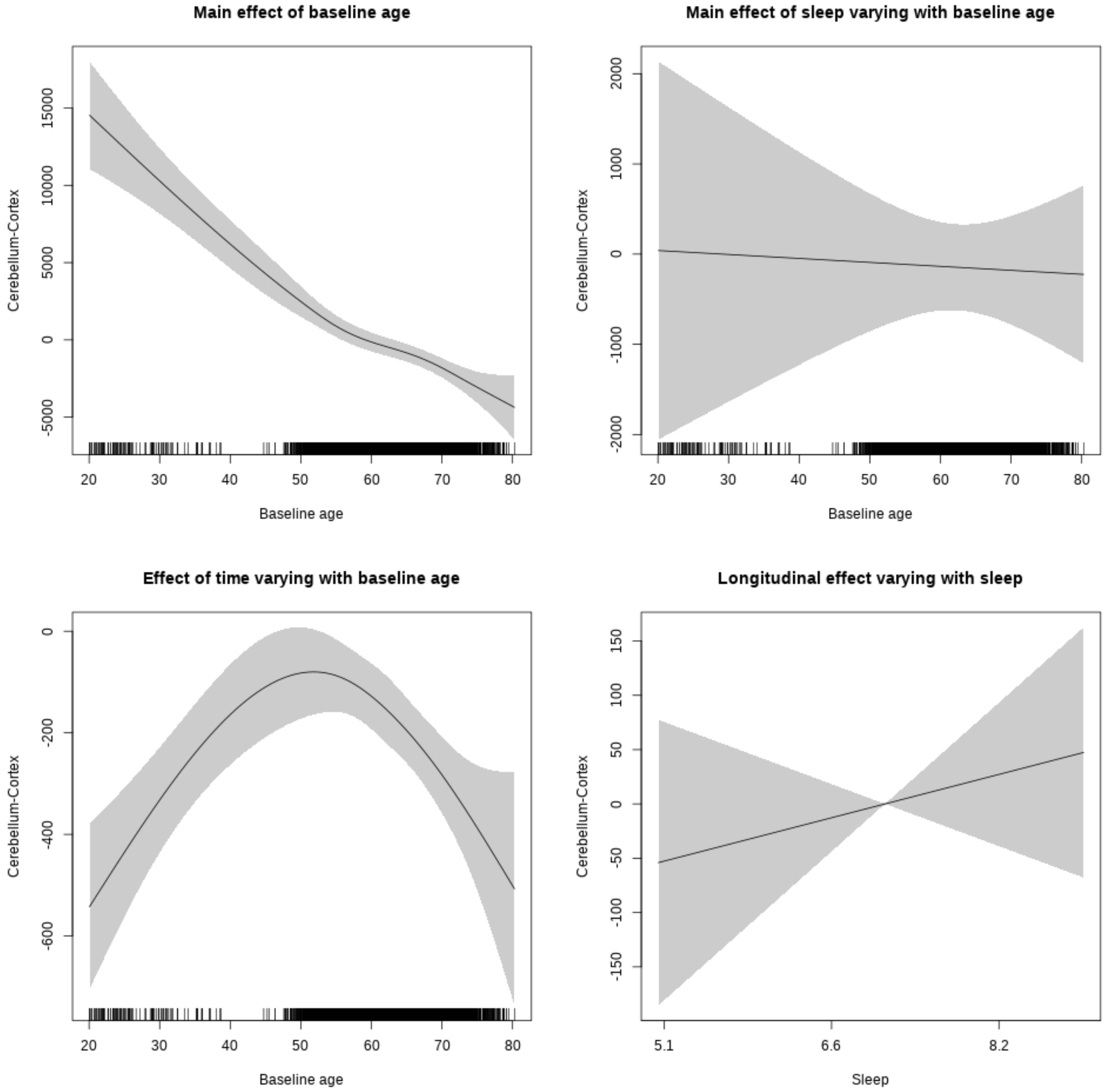

Controling for BMI

Full data Below is the model output. The term `s(sleep_z):time` is probably what we care most about.

```
##
## Family: gaussian
## Link function: identity
##
## Formula:
## value ~ sex + site + bmi + icv + s(bl_age, k = 10, bs = "cr") +
##     s(bl_age, by = sleep_z, bs = "cr") + +s(bl_age, by = time,
##     k = 5, bs = "cr") + s(sleep_z, by = time, k = 5, bs = "cr",
##     pc = 0)
```

```
## <environment: 0x5639111f2bd8>
##
## Parametric coefficients:
##                 Estimate Std. Error t value Pr(>|t|)
## (Intercept)    101906.54    1466.12  69.508  < 2e-16 ***
## sexmale          4657.12     425.22  10.952  < 2e-16 ***
## siteousPrisma    1720.32    1621.21   1.061  0.28868
## siteousSkyra    -1032.52     520.49  -1.984  0.04734 *
## siteUCAM         5774.28    1073.54   5.379 7.82e-08 ***
## siteUKB         11675.73     991.89  11.771  < 2e-16 ***
## siteUmU         -2982.40    1261.21  -2.365  0.01808 *
## bmi              -124.20      44.62  -2.784  0.00539 **
## icv              6203.12     205.08  30.248  < 2e-16 ***
## ---
## Signif. codes:  0 ‘***’ 0.001 ‘**’ 0.01 ‘*’ 0.05 ‘.’ 0.1 ‘ ’ 1
##
## Approximate significance of smooth terms:
##                      edf Ref.df      F p-value
## s(bl_age)          2.917  2.917 98.322  <2e-16 ***
## s(bl_age):sleep_z 2.000  2.000  2.454  0.0861 .
## s(bl_age):time     3.871  3.871 41.461  <2e-16 ***
## s(sleep_z):time    1.000  1.000  0.018  0.8936
## ---
## Signif. codes:  0 ‘***’ 0.001 ‘**’ 0.01 ‘*’ 0.05 ‘.’ 0.1 ‘ ’ 1
##
## R-sq.(adj) =   0.48
## lmer.REML = 1.0717e+05  Scale est. = 3.9421e+06  n = 5390
```

Below are plots of a plot of `s(bl_age)`, `s(bl_age):sleep_z`, `s(bl_age):time`, and `s(sleep_z):time`. For `s(sleep_z):time`, roughly, if we cannot fit a straight line through the confidence bands, there is evidence that the effect of time depends on sleep duration. It is correct the that confidence interval is zero at one point along the axis; this happens because we do not include the main effect.

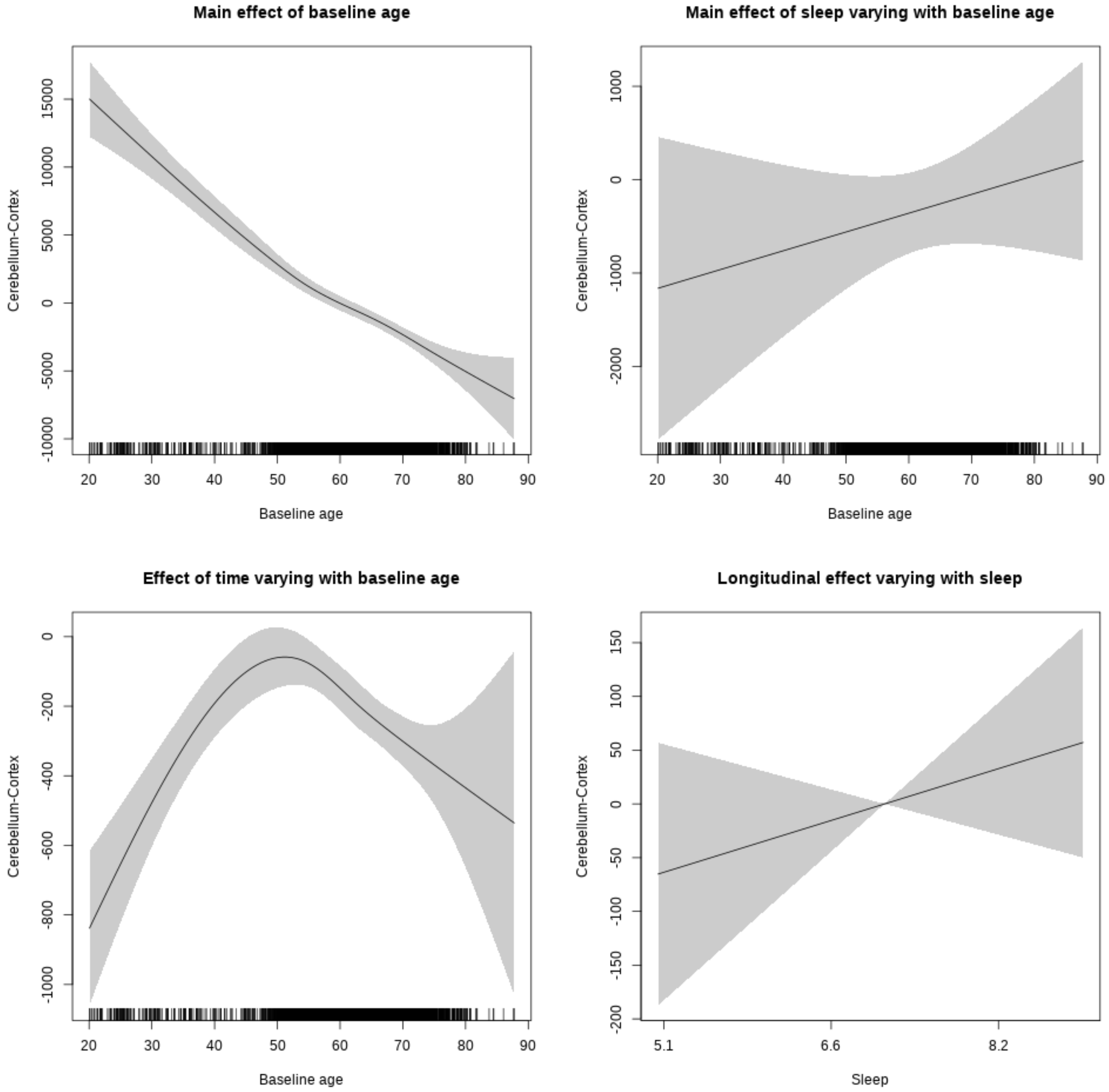

Restricted to sleep >= 5 and <= 9 hours Below is the row for `s(sleep_z):time` from the model output.

```
## s(sleep_z):time   1.000  1.000  1.140   0.286
```

Below are plots of a plot of `s(bl_age)`, `s(bl_age):sleep_z`, `s(bl_age):time`, and `s(sleep_z):time`. For `s(sleep_z):time`, roughly, if we cannot fit a straight line through the confidence bands, there is evidence that the effect of time depends on sleep duration. It is correct the that confidence interval is zero at one point along the axis; this happens because we do not include the main effect.

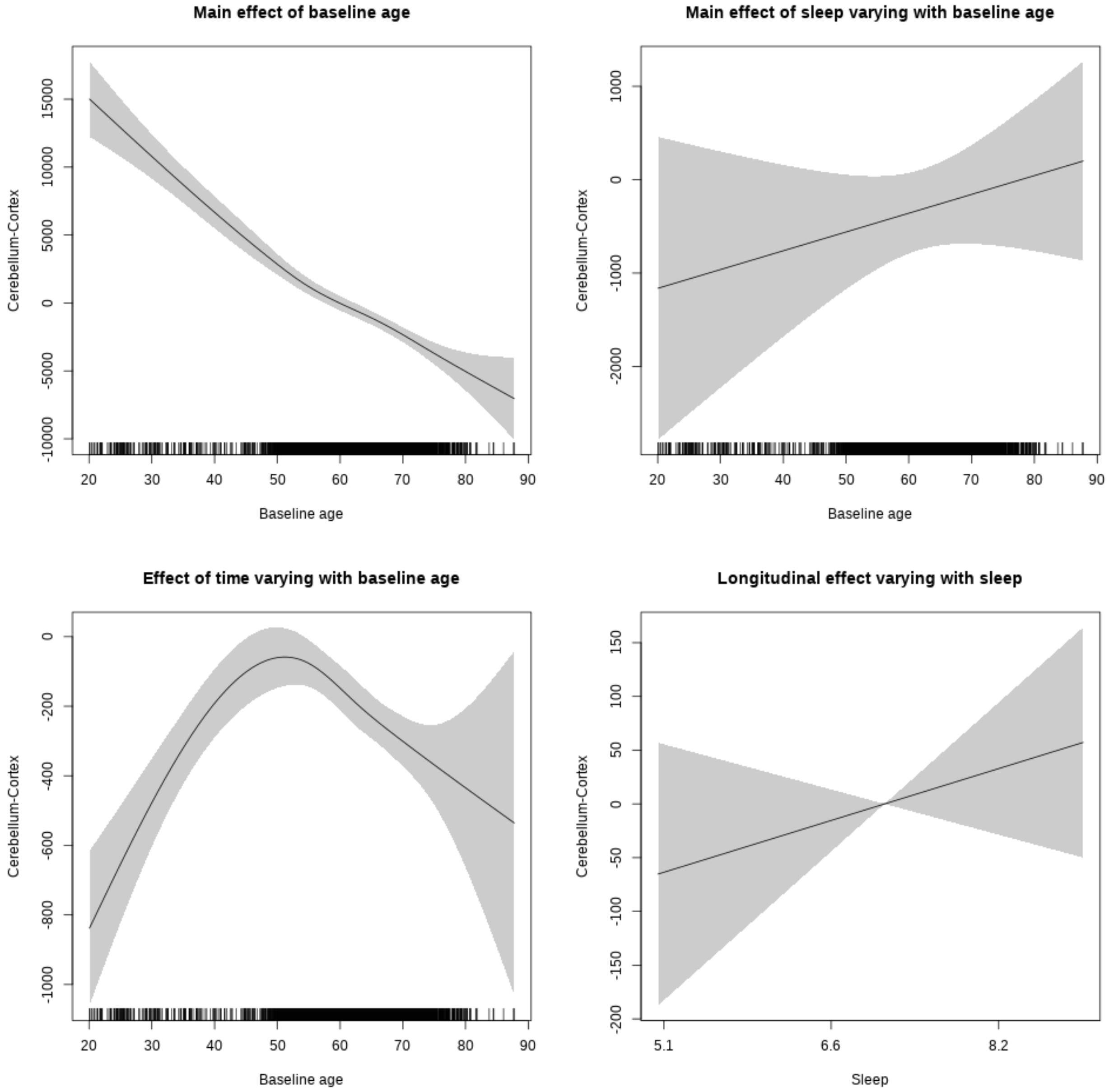

Controling for depression

Full data Below is the model output. The term `s(sleep_z):time` is probably what we care most about.

```
##
## Family: gaussian
## Link function: identity
##
## Formula:
## value ~ sex + site + depression + icv + s(bl_age, k = 10, bs = "cr") +
##     s(bl_age, by = sleep_z, bs = "cr") + +s(bl_age, by = time,
##     k = 5, bs = "cr") + s(sleep_z, by = time, k = 5, bs = "cr",
##     pc = 0)
```

```
## <environment: 0x56391587d9f8>
##
## Parametric coefficients:
##                Estimate Std. Error t value Pr(>|t|)
## (Intercept)    108144.1      800.4 135.120  < 2e-16 ***
## sexmale          4718.7      418.2  11.283  < 2e-16 ***
## siteousAvanto   -7569.8     1608.8  -4.705 2.60e-06 ***
## siteousPrisma   -8467.5     2406.8  -3.518 0.000438 ***
## siteousSkyra    -9772.0     1465.8  -6.667 2.86e-11 ***
## siteUCAM        -3658.5      966.0  -3.787 0.000154 ***
## siteUKB          1989.3      737.8   2.696 0.007033 **
## siteUmU        -12413.1     1194.3 -10.393  < 2e-16 ***
## depression      -1014.5     1488.4  -0.682 0.495522
## icv              5834.8      197.1  29.611  < 2e-16 ***
## ---
## Signif. codes:  0 '***' 0.001 '**' 0.01 '*' 0.05 '.' 0.1 ' ' 1
##
## Approximate significance of smooth terms:
##                       edf Ref.df      F p-value
## s(bl_age)           3.204  3.204 78.342  <2e-16 ***
## s(bl_age):sleep_z 2.000  2.000  1.178   0.308
## s(bl_age):time      3.693  3.693 42.813  <2e-16 ***
## s(sleep_z):time     1.000  1.000  0.014   0.906
## ---
## Signif. codes:  0 '***' 0.001 '**' 0.01 '*' 0.05 '.' 0.1 ' ' 1
##
## R-sq.(adj) =  0.469
## lmer.REML = 1.1241e+05  Scale est. = 3.723e+06  n = 5657
```

Below are plots of a plot of `s(bl_age)`, `s(bl_age):sleep_z`, `s(bl_age):time`, and `s(sleep_z):time`. For `s(sleep_z):time`, roughly, if we cannot fit a straight line through the confidence bands, there is evidence that the effect of time depends on sleep duration. It is correct the that confidence interval is zero at one point along the axis; this happens because we do not include the main effect.

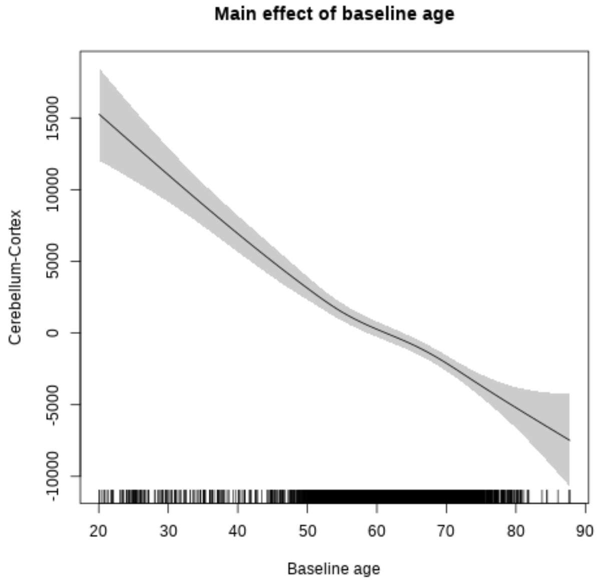

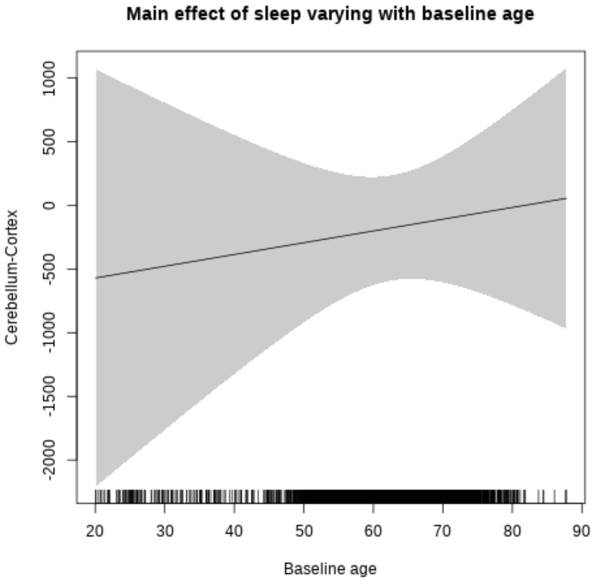

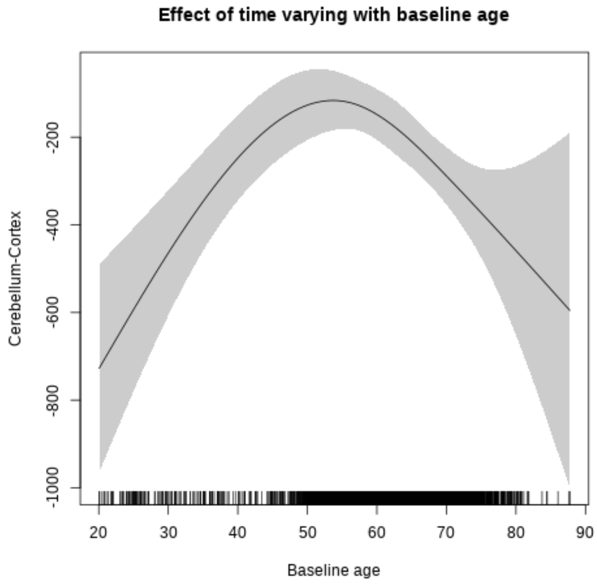

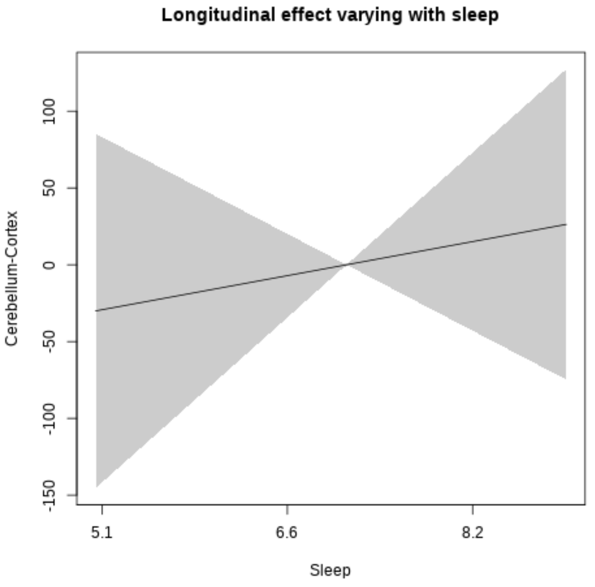

Restricted to sleep >= 5 and <= 9 hours Below is the row for `s(sleep_z):time` from the model output.

```
## s(sleep_z):time   1.000  1.000  0.271   0.603
```

Below are plots of a plot of `s(bl_age)`, `s(bl_age):sleep_z`, `s(bl_age):time`, and `s(sleep_z):time`. For `s(sleep_z):time`, roughly, if we cannot fit a straight line through the confidence bands, there is evidence that the effect of time depends on sleep duration. It is correct the that confidence interval is zero at one point along the axis; this happens because we do not include the main effect.

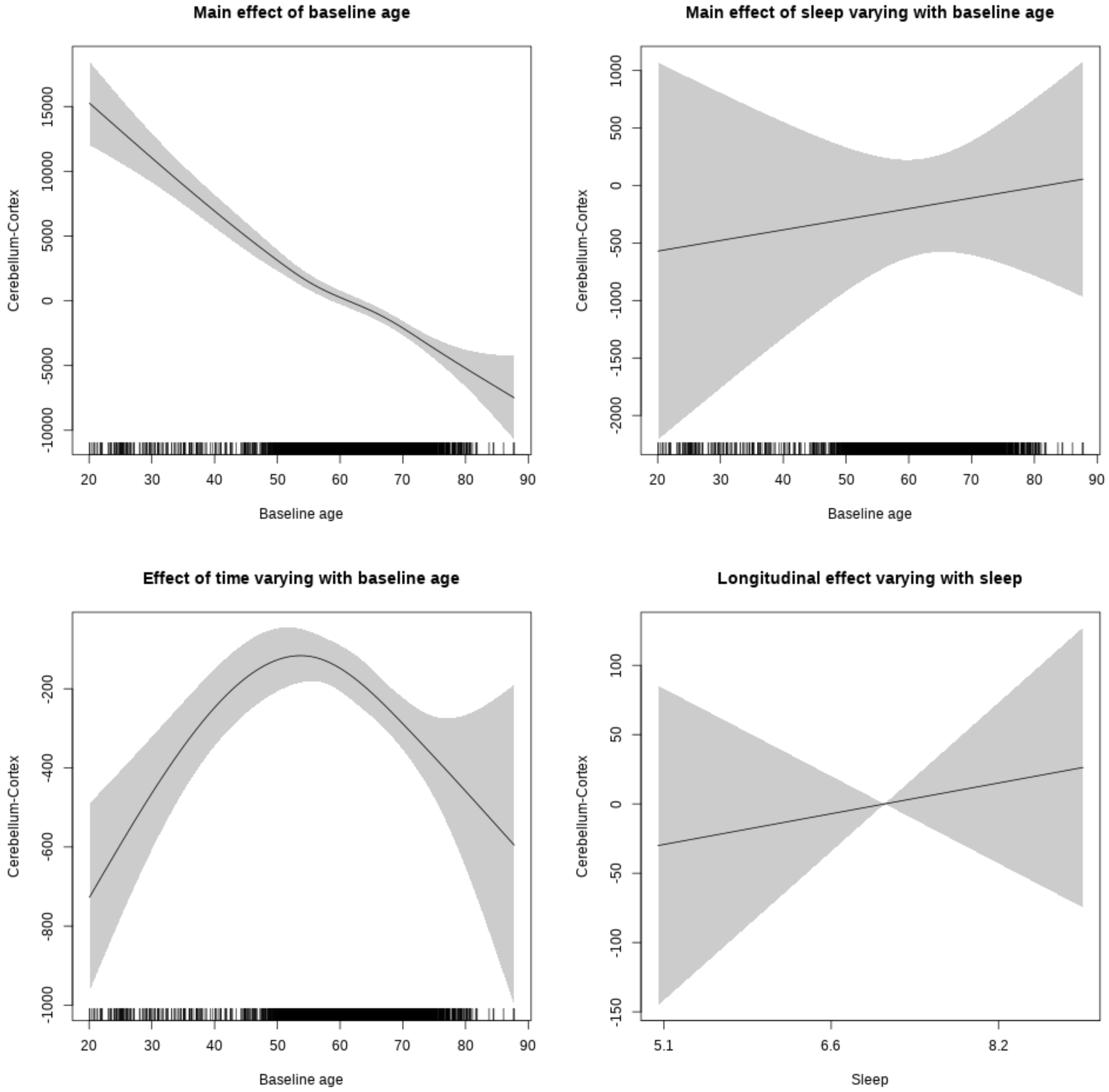

Controling for sleep quality

Full data Below is the model output. The term `s(sleep_z):time` is probably what we care most about.

```
##
## Family: gaussian
## Link function: identity
##
## Formula:
## value ~ X + s(bl_age, k = 10, bs = "cr") + s(bl_age, by = sleep_z,
##     bs = "cr") + s(bl_age, by = time, k = 5, bs = "cr") + s(sleep_z,
##     by = time, k = 5, bs = "cr", pc = 0)
## <environment: 0x563910f8b370>
```

```
##
## Parametric coefficients:
##                          Estimate Std. Error t value Pr(>|t|)
## (Intercept)              97015.78    1251.13  77.543  < 2e-16 ***
## Xsexmale                 11360.56     471.19  24.110  < 2e-16 ***
## XsiteousAvanto            3193.09    1192.64   2.677 0.007450 **
## XsiteousPrisma            7053.04    1968.94   3.582 0.000345 ***
## XsiteousSkyra            -1139.77    1195.57  -0.953 0.340480
## XsiteUB                   -165.93    1906.94  -0.087 0.930666
## XsiteUCAM                 5944.13    1210.21   4.912 9.37e-07 ***
## XsiteUKB                 10142.99    3829.99   2.648 0.008120 **
## XsiteUmU                 -6615.07    1362.98  -4.853 1.26e-06 ***
## Xukb_dummy:sleep_accel      -67.56     299.88  -0.225 0.821756
## Xukb_dummy:chronotype       -72.97     394.30  -0.185 0.853183
## Xukb_dummy:dozing            69.90     813.35   0.086 0.931519
## Xukb_dummy:sleeplessness   -616.65     504.52  -1.222 0.221680
## Xukb_dummy:snoring         1131.61     718.63   1.575 0.115408
## Xukb_dummy:gettingup        291.26     547.61   0.532 0.594844
## Xnotukb_dummy:PSQI_Global   -32.31     155.02  -0.208 0.834902
## ---
## Signif. codes:  0 ‘***’ 0.001 ‘**’ 0.01 ‘*’ 0.05 ‘.’ 0.1 ‘ ’ 1
##
## Approximate significance of smooth terms:
##                      edf Ref.df       F p-value
## s(bl_age)          1.000  1.000 358.406  <2e-16 ***
## s(bl_age):sleep_z  2.000  2.000   0.872   0.418
## s(bl_age):time     3.777  3.777  60.575  <2e-16 ***
## s(sleep_z):time    1.000  1.000   0.338   0.561
## ---
## Signif. codes:  0 ‘***’ 0.001 ‘**’ 0.01 ‘*’ 0.05 ‘.’ 0.1 ‘ ’ 1
##
## R-sq.(adj) =  0.415
## lmer.REML =  84841  Scale est. = 4.1522e+06  n = 4271
```

Below are plots of a plot of `s(bl_age)`, `s(bl_age):sleep_z`, `s(bl_age):time`, and `s(sleep_z):time`. For `s(sleep_z):time`, roughly, if we cannot fit a straight line through the confidence bands, there is evidence that the effect of time depends on sleep duration. It is correct the that confidence interval is zero at one point along the axis; this happens because we do not include the main effect.

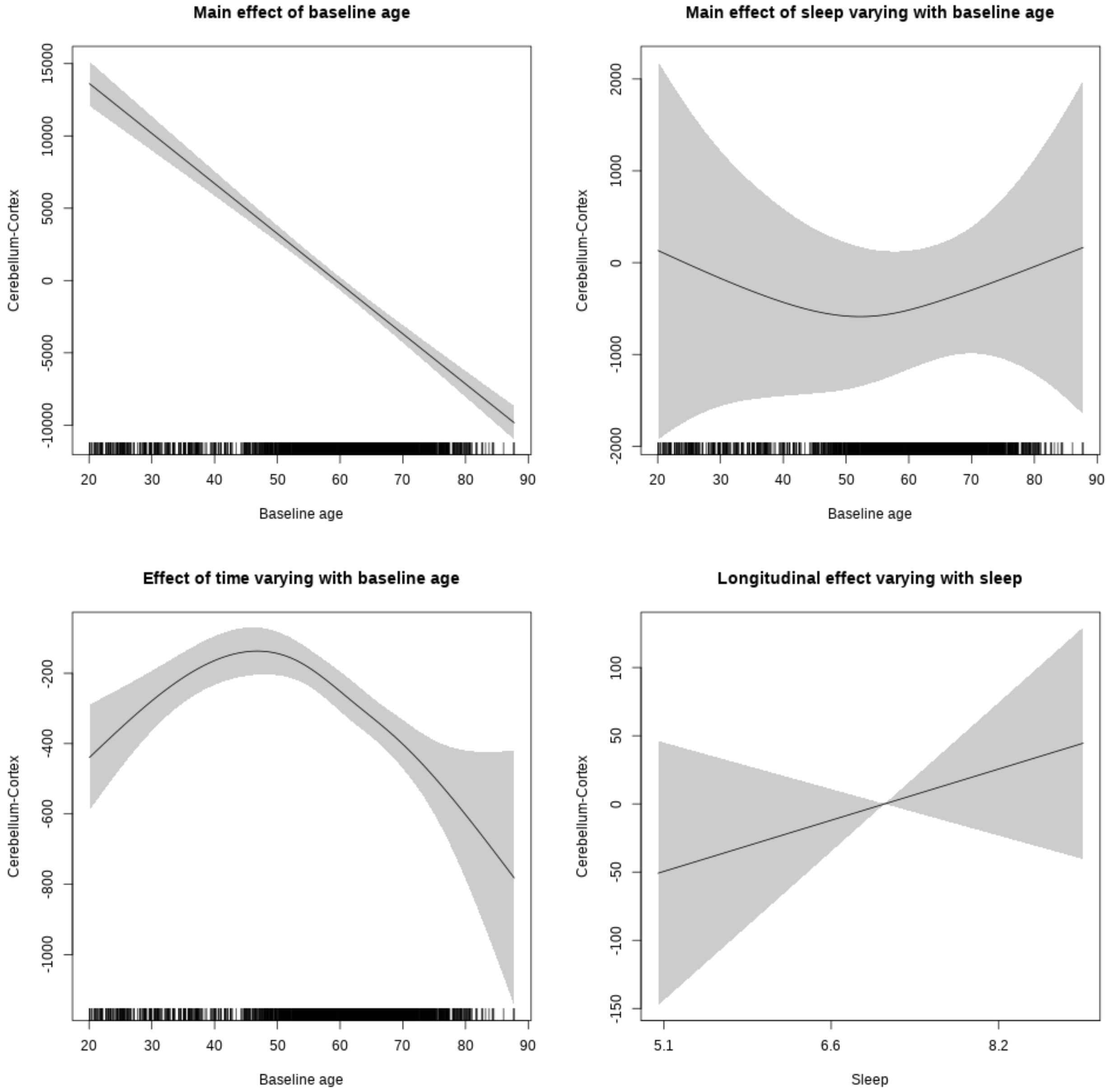

Restricted to sleep >= 5 and <= 9 hours Below is the row for `s(sleep_z):time` from the model output.

```
## s(sleep_z):time   1.000  1.000   1.097   0.295
```

Below are plots of a plot of `s(bl_age)`, `s(bl_age):sleep_z`, `s(bl_age):time`, and `s(sleep_z):time`. For `s(sleep_z):time`, roughly, if we cannot fit a straight line through the confidence bands, there is evidence that the effect of time depends on sleep duration. It is correct the that confidence interval is zero at one point along the axis; this happens because we do not include the main effect.

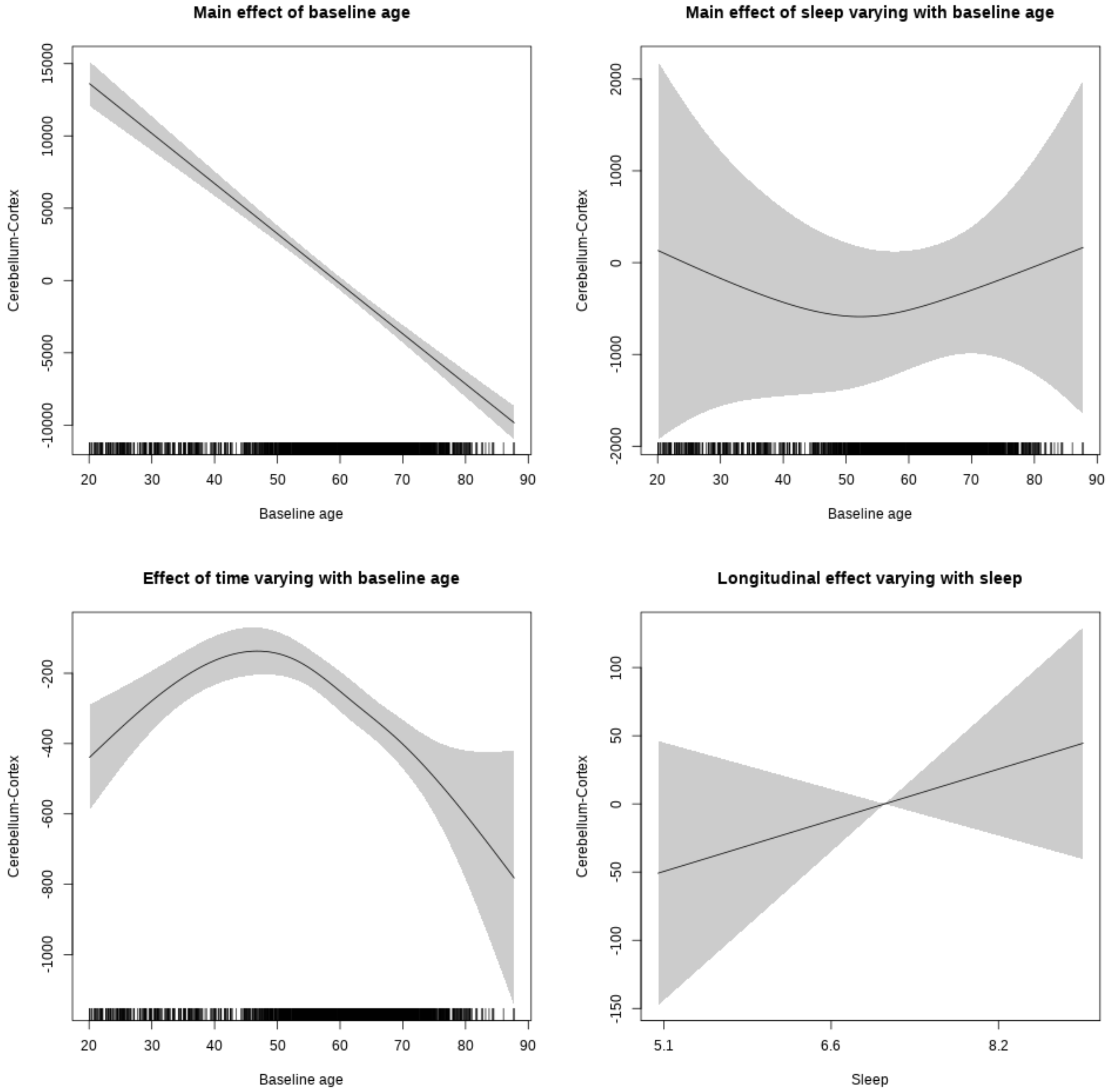

Cerebellum-White-Matter

Main analysis

Full data Below is the model output. The term `s(sleep_z):time` is probably what we care most about.

```
##
## Family: gaussian
## Link function: identity
##
## Formula:
## value ~ sex + site + icv + s(bl_age, k = 10, bs = "cr") + s(bl_age,
##     by = sleep_z, bs = "cr") + +s(bl_age, by = time, k = 5, bs = "cr") +
```

```
##     s(sleep_z, by = time, k = 5, bs = "cr", pc = 0)
## <environment: 0x56391e8b20d0>
##
## Parametric coefficients:
##                Estimate Std. Error t value Pr(>|t|)
## (Intercept)    30087.46     271.40 110.860  < 2e-16 ***
## sexmale         -273.13     139.60  -1.956 0.050444 .
## siteousAvanto -1395.55     333.91  -4.179 2.95e-05 ***
## siteousPrisma -6666.95    1020.59  -6.532 6.86e-11 ***
## siteousSkyra   1110.30     324.51   3.421 0.000626 ***
## siteUB        -2289.24     621.85  -3.681 0.000234 ***
## siteUCAM      -1771.92     340.99  -5.196 2.08e-07 ***
## siteUKB        1361.36     258.77   5.261 1.47e-07 ***
## siteUmU        8270.55     390.80  21.163  < 2e-16 ***
## icv            2003.82      67.87  29.525  < 2e-16 ***
## ---
## Signif. codes:  0 ‘***’ 0.001 ‘**’ 0.01 ‘*’ 0.05 ‘.’ 0.1 ‘ ’ 1
##
## Approximate significance of smooth terms:
##                       edf Ref.df      F  p-value
## s(bl_age)           5.814  5.814 64.811  < 2e-16 ***
## s(bl_age):sleep_z 2.000  2.000  0.058 0.943578
## s(bl_age):time    4.565  4.565 23.237  < 2e-16 ***
## s(sleep_z):time   1.000  1.000 13.463 0.000245 ***
## ---
## Signif. codes:  0 ‘***’ 0.001 ‘**’ 0.01 ‘*’ 0.05 ‘.’ 0.1 ‘ ’ 1
##
## R-sq.(adj) =  0.401
## lmer.REML = 1.4986e+05  Scale est. = 1.6102e+06  n = 8116
```

Below are plots of a plot of `s(bl_age)`, `s(bl_age):sleep_z`, `s(bl_age):time`, and `s(sleep_z):time`. For `s(sleep_z):time`, roughly, if we cannot fit a straight line through the confidence bands, there is evidence that the effect of time depends on sleep duration. It is correct the that confidence interval is zero at one point along the axis; this happens because we do not include the main effect.

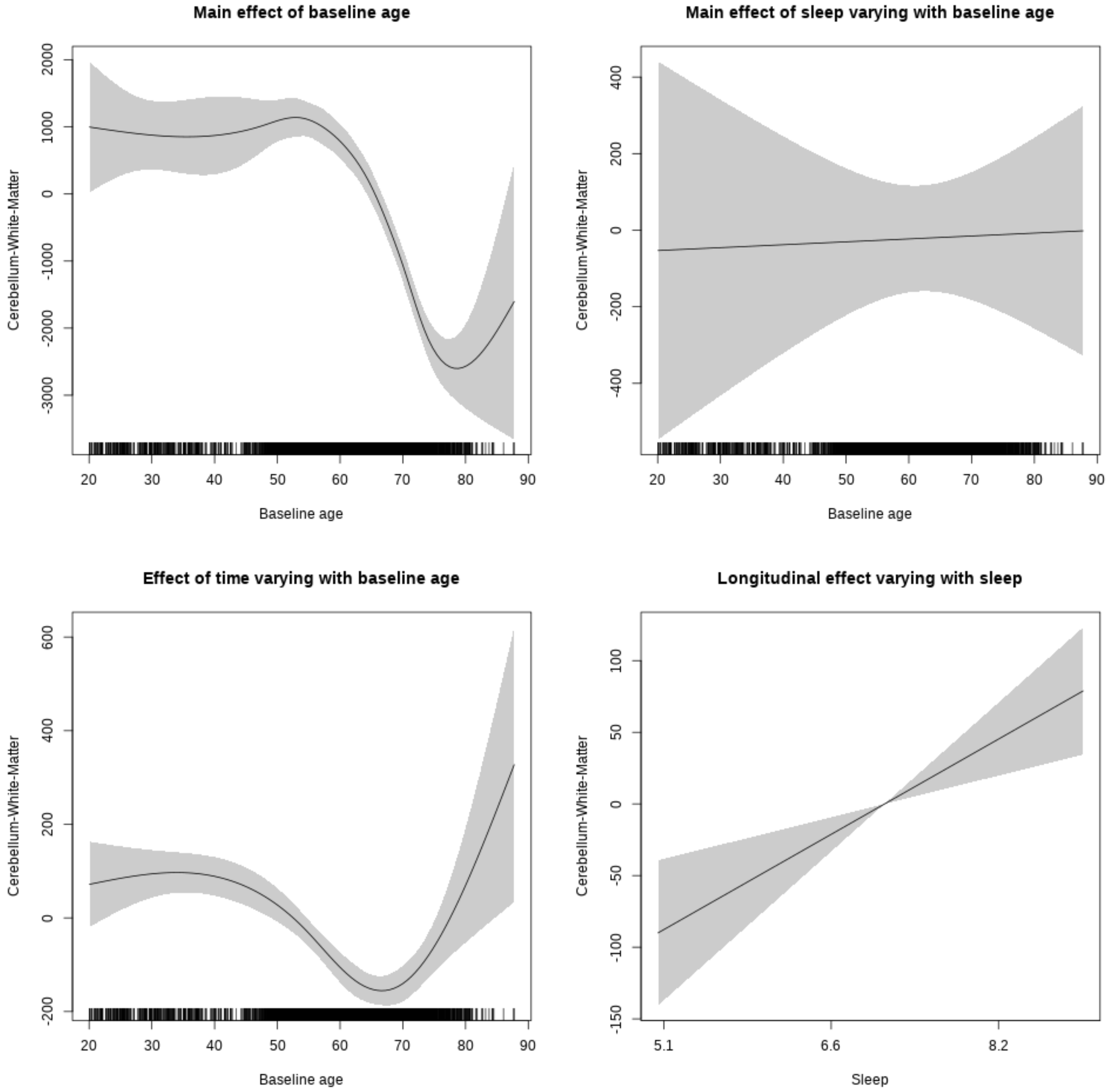

Restricted to sleep >= 5 and <= 9 hours Below is the row for `s(sleep_z):time` from the model output.

```
## s(sleep_z):time   1.000  1.000 12.630 0.000382 ***
```

Below are plots of a plot of `s(bl_age)`, `s(bl_age):sleep_z`, `s(bl_age):time`, and `s(sleep_z):time`. For `s(sleep_z):time`, roughly, if we cannot fit a straight line through the confidence bands, there is evidence that the effect of time depends on sleep duration. It is correct the that confidence interval is zero at one point along the axis; this happens because we do not include the main effect.

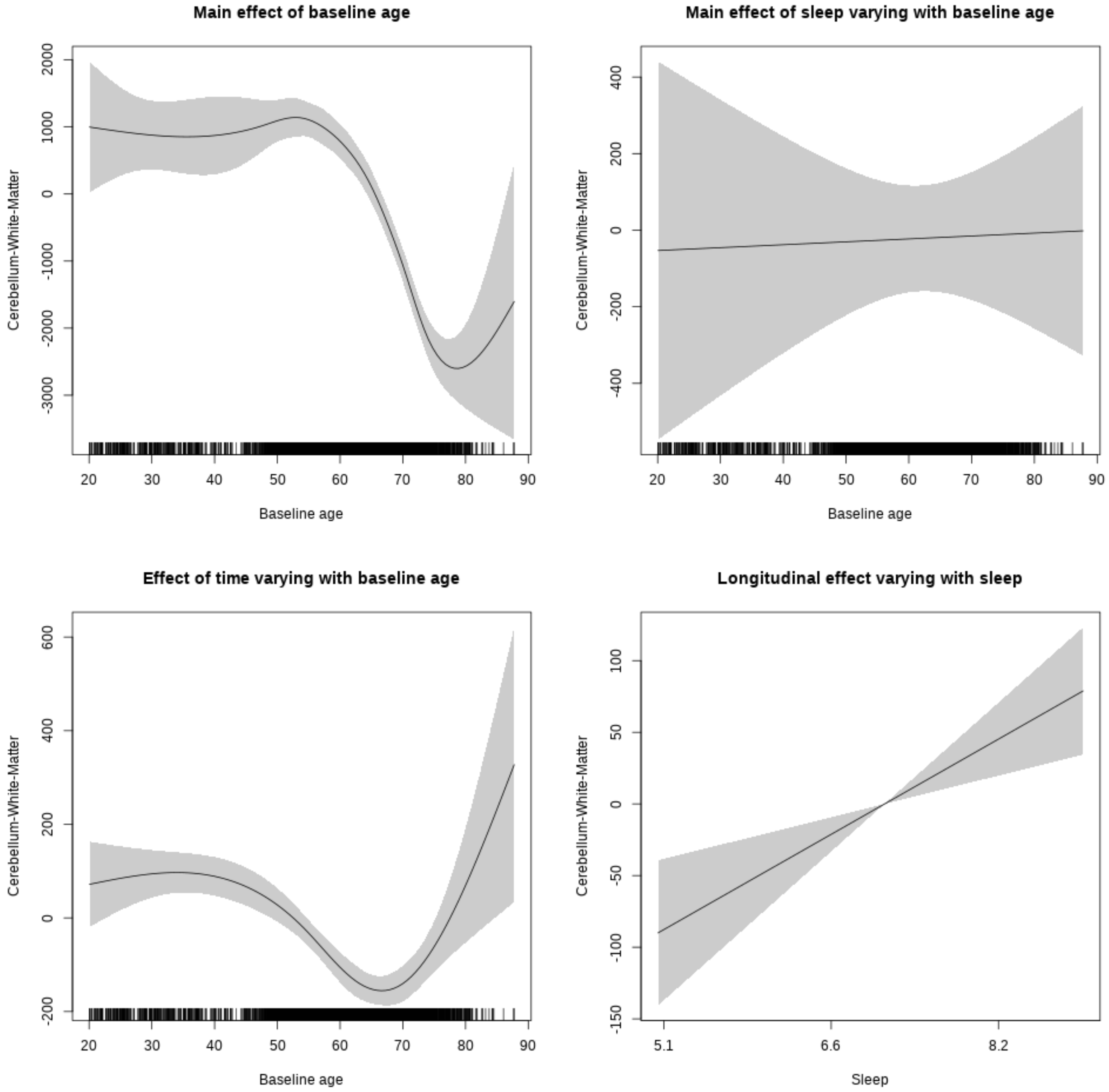

Controling for socioeconomic status

Full data Below is the model output. The term `s(sleep_z):time` is probably what we care most about.

```
##
## Family: gaussian
## Link function: identity
##
## Formula:
## value ~ sex + site + income_scaled + education_scaled + icv +
##     s(bl_age, k = 10, bs = "cr") + s(bl_age, by = sleep_z, bs = "cr") +
##     +s(bl_age, by = time, k = 5, bs = "cr") + s(sleep_z, by = time,
##     k = 5, bs = "cr", pc = 0)
```

```
## <environment: 0x563911525f08>
##
## Parametric coefficients:
##                  Estimate Std. Error t value Pr(>|t|)
## (Intercept)      30332.06     605.57  50.089  < 2e-16 ***
## sexmale           -386.45     166.73  -2.318   0.0205 *
## siteousAvanto    -2685.15     683.28  -3.930 8.63e-05 ***
## siteousPrisma    -7902.84    1698.00  -4.654 3.34e-06 ***
## siteousSkyra       824.24     617.15   1.336   0.1818
## siteUKB            852.65     572.87   1.488   0.1367
## income_scaled      112.78     212.85   0.530   0.5962
## education_scaled   401.28     234.96   1.708   0.0877 .
## icv               2208.52      83.62  26.411  < 2e-16 ***
## ---
## Signif. codes:  0 '***' 0.001 '**' 0.01 '*' 0.05 '.' 0.1 ' ' 1
##
## Approximate significance of smooth terms:
##                     edf Ref.df      F p-value
## s(bl_age)         3.978  3.978 57.970  <2e-16 ***
## s(bl_age):sleep_z 2.000  2.000  0.189   0.828
## s(bl_age):time    4.282  4.282 19.429  <2e-16 ***
## s(sleep_z):time   1.000  1.000  0.853   0.356
## ---
## Signif. codes:  0 '***' 0.001 '**' 0.01 '*' 0.05 '.' 0.1 ' ' 1
##
## R-sq.(adj) =   0.35
## lmer.REML =  85307  Scale est. = 1.6828e+06  n = 4630
```

Below are plots of a plot of `s(bl_age)`, `s(bl_age):sleep_z`, `s(bl_age):time`, and `s(sleep_z):time`. For `s(sleep_z):time`, roughly, if we cannot fit a straight line through the confidence bands, there is evidence that the effect of time depends on sleep duration. It is correct the that confidence interval is zero at one point along the axis; this happens because we do not include the main effect.

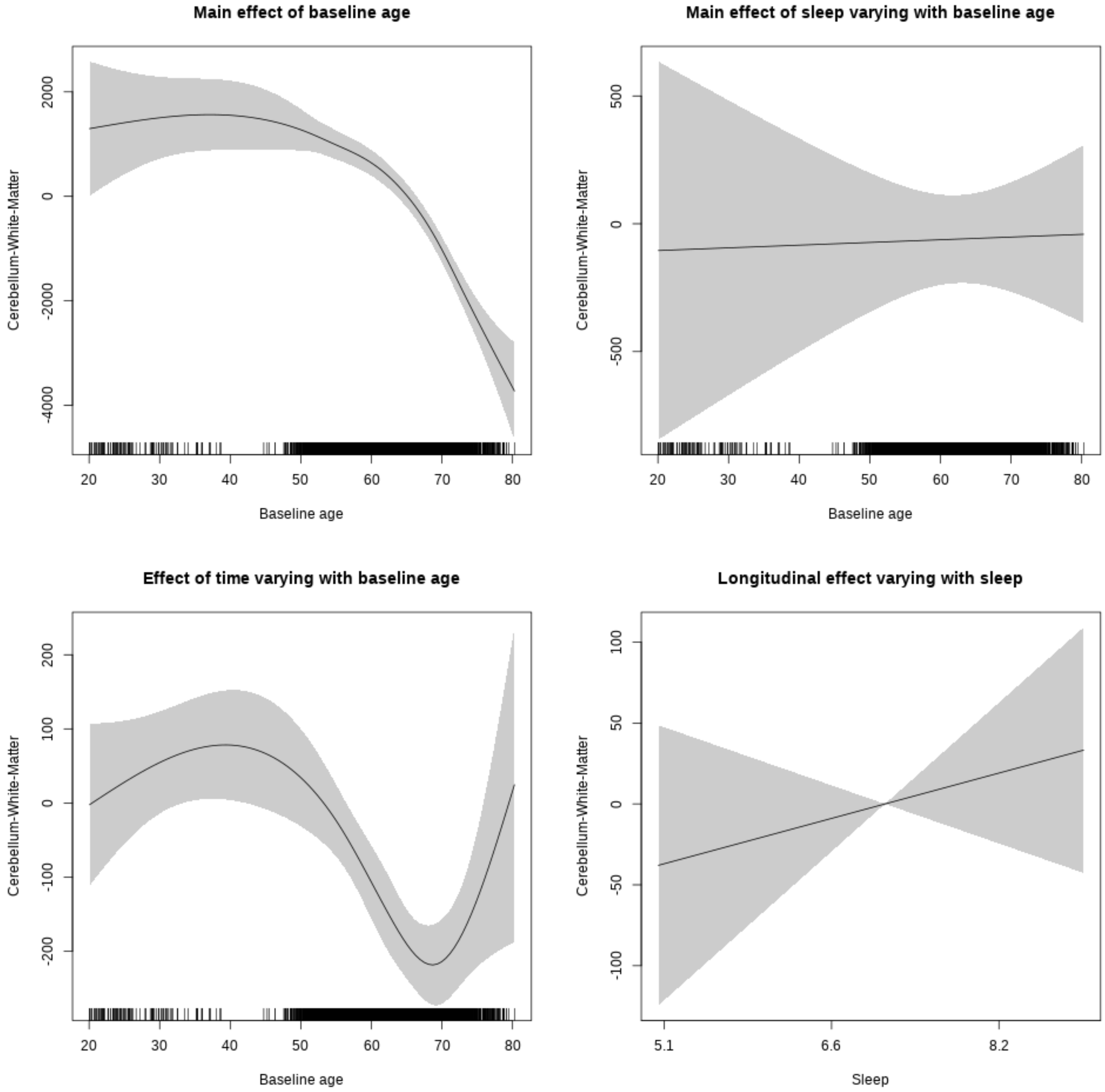

Restricted to sleep >= 5 and <= 9 hours Below is the row for `s(sleep_z):time` from the model output.

```
## s(sleep_z):time   1.000  1.000  0.768   0.381
```

Below are plots of a plot of `s(bl_age)`, `s(bl_age):sleep_z`, `s(bl_age):time`, and `s(sleep_z):time`. For `s(sleep_z):time`, roughly, if we cannot fit a straight line through the confidence bands, there is evidence that the effect of time depends on sleep duration. It is correct the that confidence interval is zero at one point along the axis; this happens because we do not include the main effect.

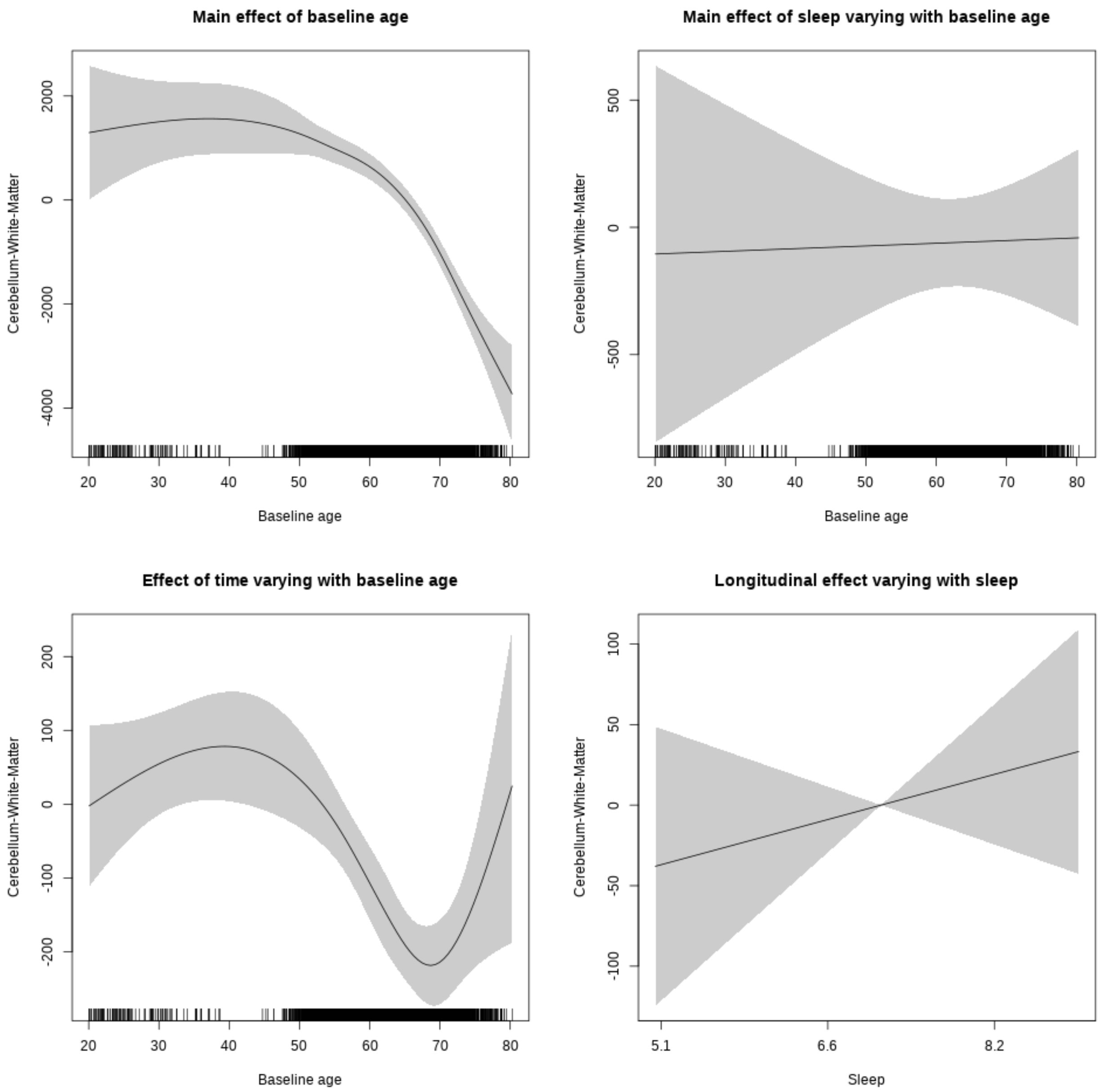

Controling for BMI

Full data Below is the model output. The term `s(sleep_z):time` is probably what we care most about.

```
##
## Family: gaussian
## Link function: identity
##
## Formula:
## value ~ sex + site + bmi + icv + s(bl_age, k = 10, bs = "cr") +
##     s(bl_age, by = sleep_z, bs = "cr") + +s(bl_age, by = time,
##     k = 5, bs = "cr") + s(sleep_z, by = time, k = 5, bs = "cr",
##     pc = 0)
```

```
## <environment: 0x56390d7065c0>
##
## Parametric coefficients:
##                Estimate Std. Error t value Pr(>|t|)
## (Intercept)    30043.95     633.80  47.403  < 2e-16 ***
## sexmale         -398.24     174.09  -2.288   0.0222 *
## siteousPrisma -5470.07    1054.99  -5.185 2.24e-07 ***
## siteousSkyra   2674.14     333.26   8.024 1.25e-15 ***
## siteUCAM       -492.43     495.50  -0.994   0.3204
## siteUKB        2219.33     463.32   4.790 1.71e-06 ***
## siteUmU        9124.39     561.20  16.259  < 2e-16 ***
## bmi             -34.31      17.98  -1.908   0.0564 .
## icv            2226.66      86.68  25.689  < 2e-16 ***
## ---
## Signif. codes:  0 '***' 0.001 '**' 0.01 '*' 0.05 '.' 0.1 ' ' 1
##
## Approximate significance of smooth terms:
##                       edf Ref.df      F  p-value
## s(bl_age)           5.409  5.409 43.748  < 2e-16 ***
## s(bl_age):sleep_z 2.000  2.000  0.890 0.410711
## s(bl_age):time      4.664  4.664  9.238  < 2e-16 ***
## s(sleep_z):time     1.000  1.000 12.661 0.000377 ***
## ---
## Signif. codes:  0 '***' 0.001 '**' 0.01 '*' 0.05 '.' 0.1 ' ' 1
##
## R-sq.(adj) =  0.353
## lmer.REML =  99975  Scale est. = 1.7288e+06  n = 5384
```

Below are plots of a plot of `s(bl_age)`, `s(bl_age):sleep_z`, `s(bl_age):time`, and `s(sleep_z):time`. For `s(sleep_z):time`, roughly, if we cannot fit a straight line through the confidence bands, there is evidence that the effect of time depends on sleep duration. It is correct the that confidence interval is zero at one point along the axis; this happens because we do not include the main effect.

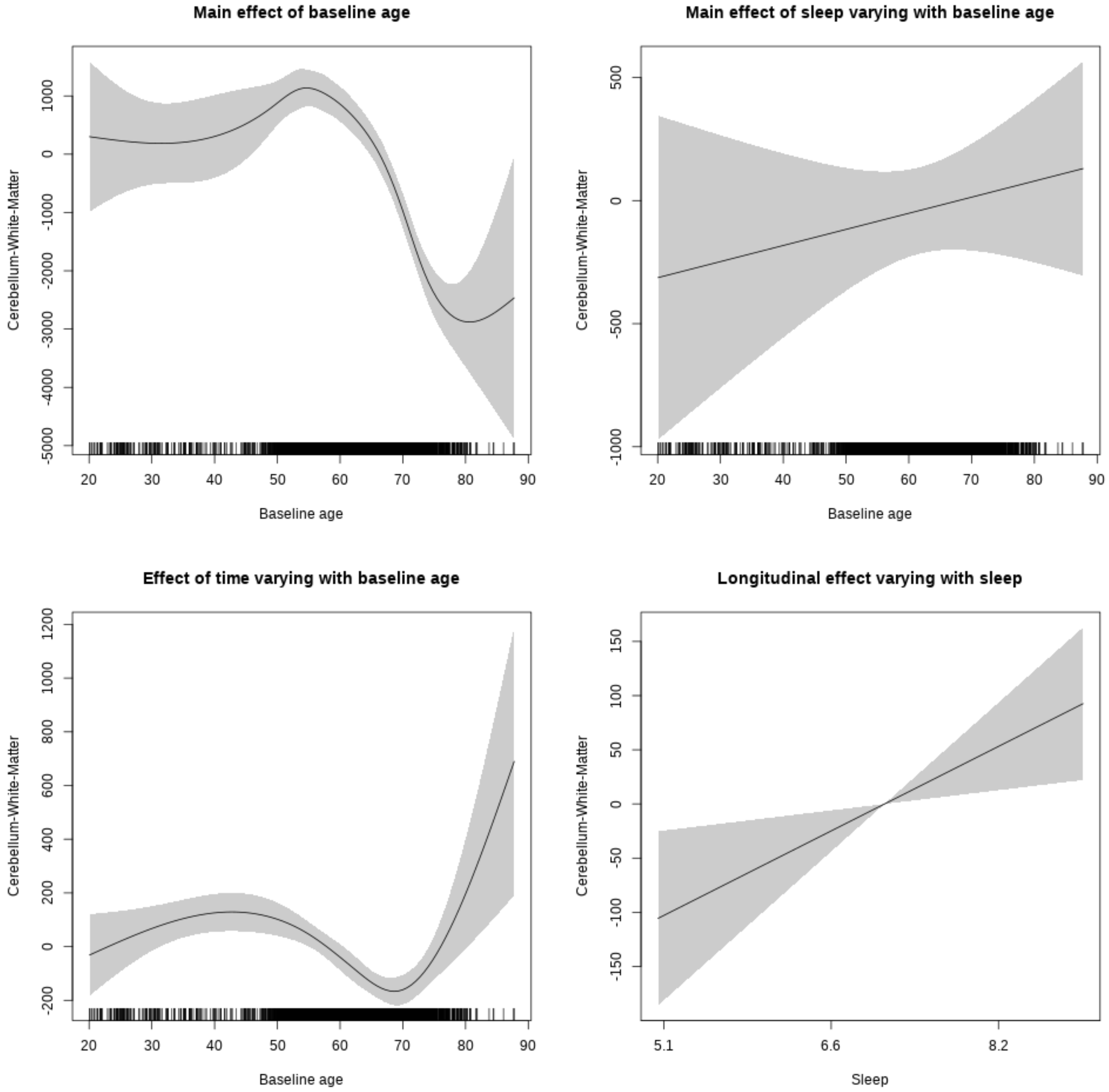

Restricted to sleep >= 5 and <= 9 hours Below is the row for `s(sleep_z):time` from the model output.

```
## s(sleep_z):time   1.000  1.000  6.906 0.00862 **
```

Below are plots of a plot of `s(bl_age)`, `s(bl_age):sleep_z`, `s(bl_age):time`, and `s(sleep_z):time`. For `s(sleep_z):time`, roughly, if we cannot fit a straight line through the confidence bands, there is evidence that the effect of time depends on sleep duration. It is correct the that confidence interval is zero at one point along the axis; this happens because we do not include the main effect.

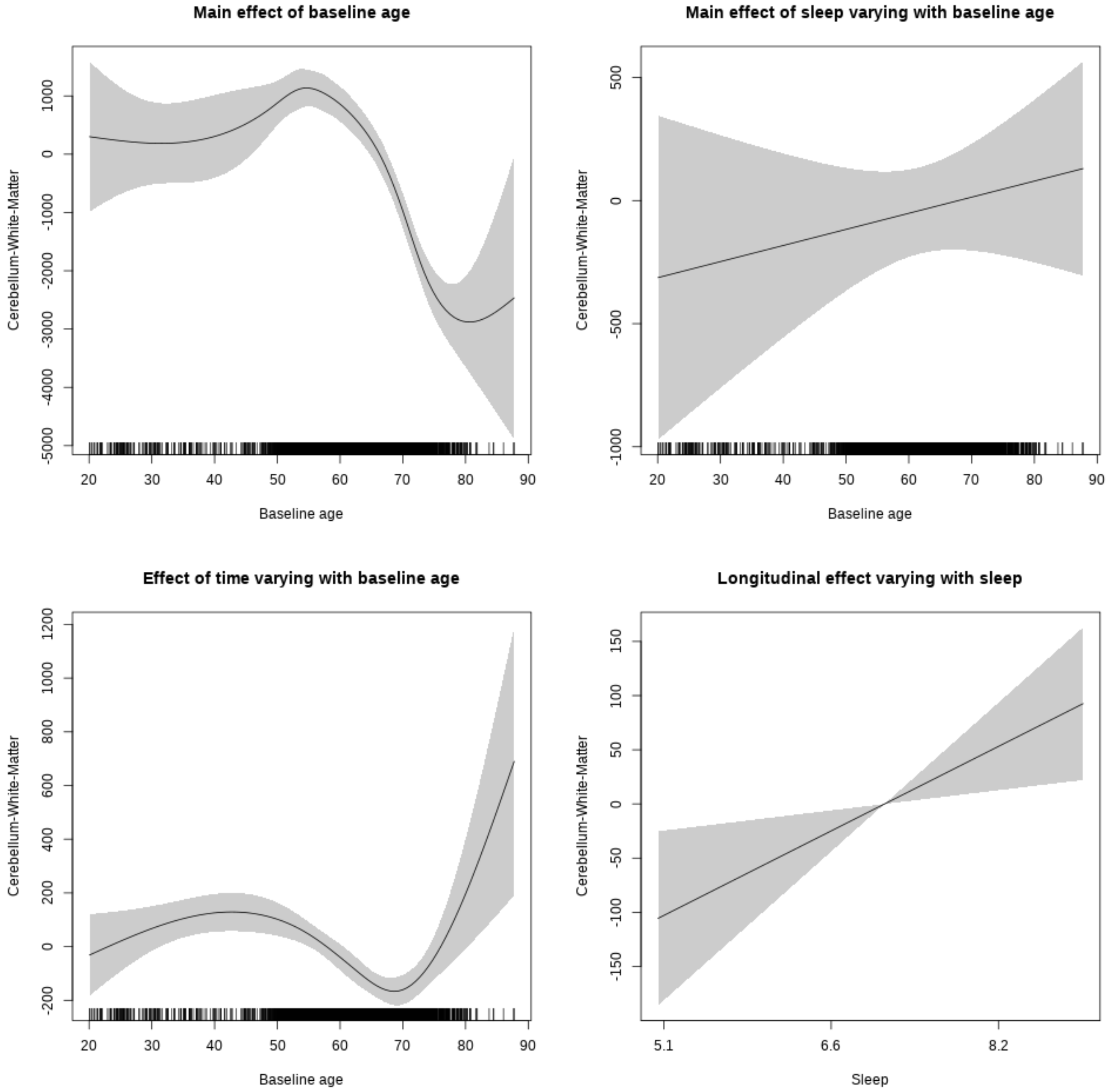

Controling for depression

Full data Below is the model output. The term `s(sleep_z):time` is probably what we care most about.

```
##
## Family: gaussian
## Link function: identity
##
## Formula:
## value ~ sex + site + depression + icv + s(bl_age, k = 10, bs = "cr") +
##     s(bl_age, by = sleep_z, bs = "cr") + +s(bl_age, by = time,
##     k = 5, bs = "cr") + s(sleep_z, by = time, k = 5, bs = "cr",
##     pc = 0)
```

```
## <environment: 0x5639111c77a8>
##
## Parametric coefficients:
##                 Estimate Std. Error t value Pr(>|t|)
## (Intercept)     29741.57     326.25  91.163  < 2e-16 ***
## sexmale          -390.13     170.74  -2.285 0.022355 *
## siteousAvanto    -257.15     729.01  -0.353 0.724293
## siteousPrisma  -4734.86    1386.69  -3.415 0.000643 ***
## siteousSkyra    2624.65     597.43   4.393 1.14e-05 ***
## siteUCAM         -992.44     396.38  -2.504 0.012316 *
## siteUKB          1604.80     299.59   5.357 8.82e-08 ***
## siteUmU          8679.86     479.58  18.099  < 2e-16 ***
## depression       -531.54     597.29  -0.890 0.373545
## icv              2037.15      83.11  24.512  < 2e-16 ***
## ---
## Signif. codes:  0 '***' 0.001 '**' 0.01 '*' 0.05 '.' 0.1 ' ' 1
##
## Approximate significance of smooth terms:
##                       edf Ref.df      F p-value
## s(bl_age)           5.338  5.338 43.680 < 2e-16 ***
## s(bl_age):sleep_z 2.000  2.000  0.014 0.98619
## s(bl_age):time      4.639  4.639  9.912 < 2e-16 ***
## s(sleep_z):time     1.000  1.000  8.763 0.00309 **
## ---
## Signif. codes:  0 '***' 0.001 '**' 0.01 '*' 0.05 '.' 0.1 ' ' 1
##
## R-sq.(adj) =  0.396
## lmer.REML = 1.0493e+05  Scale est. = 1.6926e+06  n = 5651
```

Below are plots of a plot of `s(bl_age)`, `s(bl_age):sleep_z`, `s(bl_age):time`, and `s(sleep_z):time`. For `s(sleep_z):time`, roughly, if we cannot fit a straight line through the confidence bands, there is evidence that the effect of time depends on sleep duration. It is correct the that confidence interval is zero at one point along the axis; this happens because we do not include the main effect.

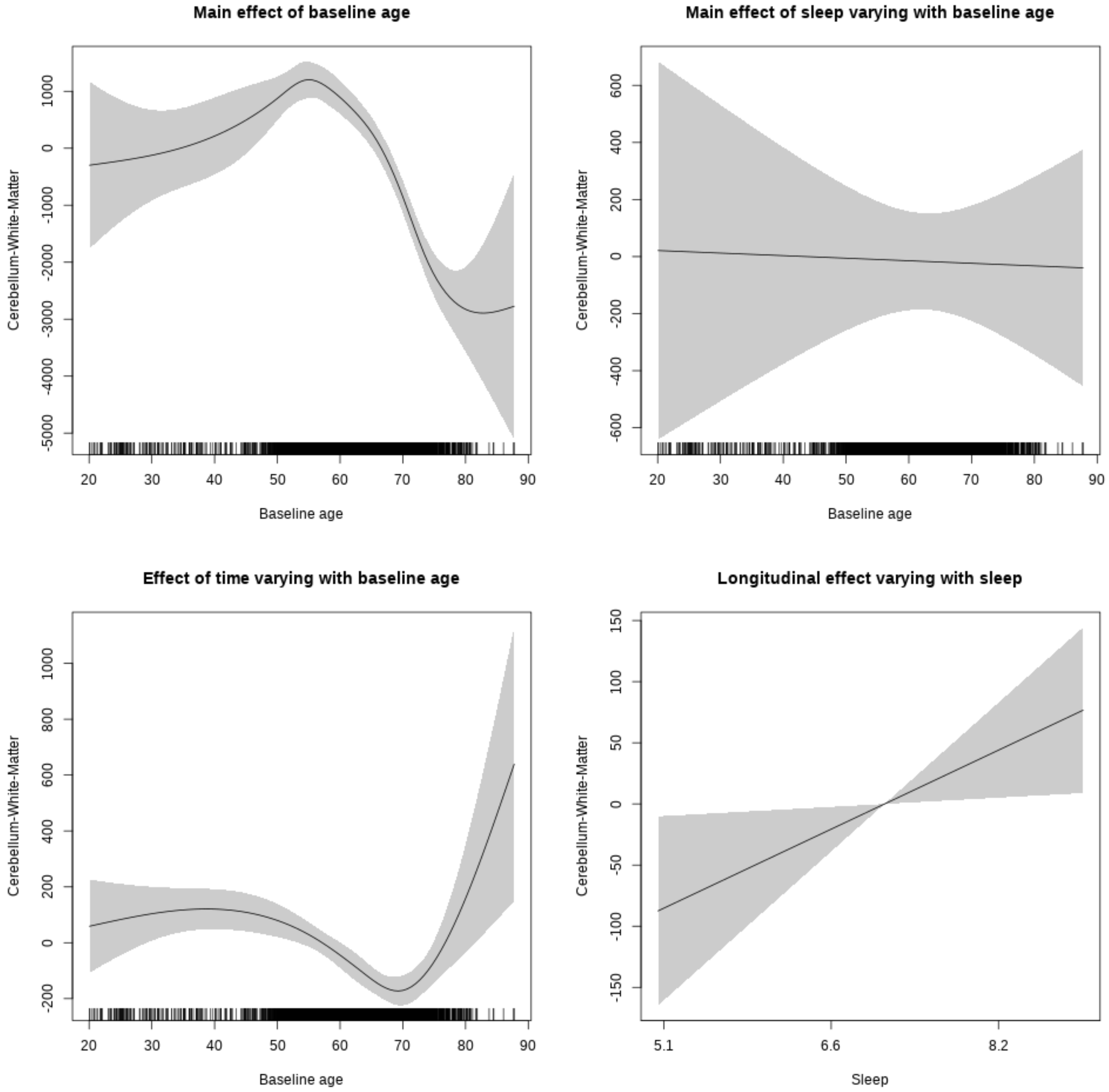

Restricted to sleep >= 5 and <= 9 hours Below is the row for `s(sleep_z):time` from the model output.

```
## s(sleep_z):time   1.000  1.000  5.120  0.0237 *
```

Below are plots of a plot of `s(bl_age)`, `s(bl_age):sleep_z`, `s(bl_age):time`, and `s(sleep_z):time`. For `s(sleep_z):time`, roughly, if we cannot fit a straight line through the confidence bands, there is evidence that the effect of time depends on sleep duration. It is correct the that confidence interval is zero at one point along the axis; this happens because we do not include the main effect.

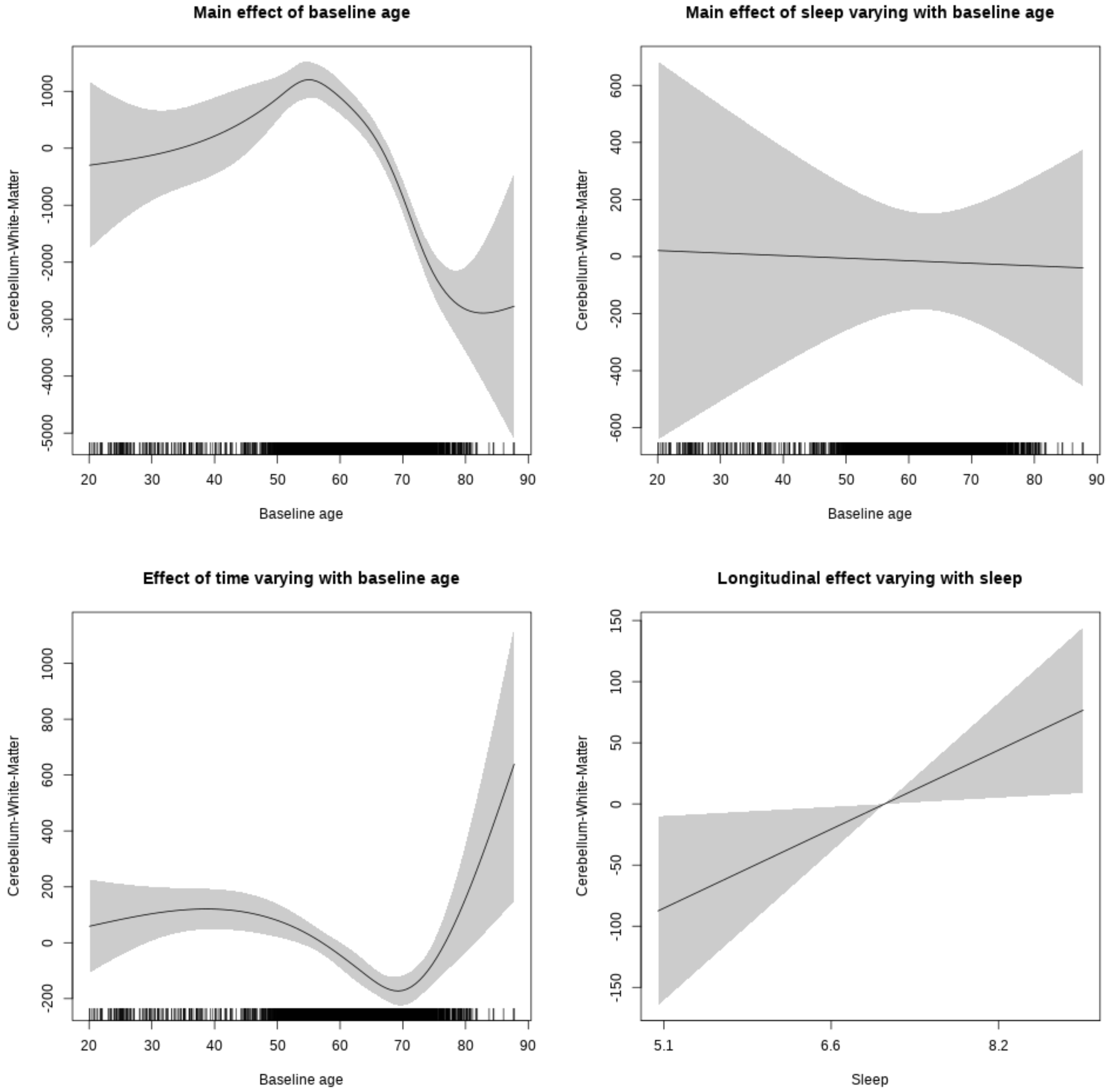

Controling for sleep quality

Full data Below is the model output. The term `s(sleep_z):time` is probably what we care most about.

```
##
## Family: gaussian
## Link function: identity
##
## Formula:
## value ~ X + s(bl_age, k = 10, bs = "cr") + s(bl_age, by = sleep_z,
##     bs = "cr") + s(bl_age, by = time, k = 5, bs = "cr") + s(sleep_z,
##     by = time, k = 5, bs = "cr", pc = 0)
## <environment: 0x56391180b598>
```

```
##
## Parametric coefficients:
##                            Estimate Std. Error t value Pr(>|t|)
## (Intercept)                26425.79     521.22  50.700  < 2e-16 ***
## Xsexmale                    2129.31     194.80  10.931  < 2e-16 ***
## XsiteousAvanto              2802.77     499.67   5.609 2.16e-08 ***
## XsiteousPrisma             -1585.63    1072.29  -1.479  0.13929
## XsiteousSkyra               4563.87     502.02   9.091  < 2e-16 ***
## XsiteUB                      197.05     788.90   0.250  0.80277
## XsiteUCAM                   1609.57     513.07   3.137  0.00172 **
## XsiteUKB                    4394.63    1594.29   2.756  0.00587 **
## XsiteUmU                   10234.97     570.72  17.934  < 2e-16 ***
## Xukb_dummy:sleep_accel        56.19     123.41   0.455  0.64888
## Xukb_dummy:chronotype       -112.67     162.93  -0.692  0.48928
## Xukb_dummy:dozing             44.23     337.31   0.131  0.89568
## Xukb_dummy:sleeplessness    -313.78     210.25  -1.492  0.13567
## Xukb_dummy:snoring           393.56     297.49   1.323  0.18592
## Xukb_dummy:gettingup        -196.69     228.58  -0.860  0.38958
## Xnotukb_dummy:PSQI_Global  -131.36      64.45  -2.038  0.04158 *
## ---
## Signif. codes:  0 ‘***’ 0.001 ‘**’ 0.01 ‘*’ 0.05 ‘.’ 0.1 ‘ ’ 1
##
## Approximate significance of smooth terms:
##                      edf Ref.df      F p-value
## s(bl_age)          4.837  4.837 36.438 < 2e-16 ***
## s(bl_age):sleep_z 2.000  2.000  1.121   0.326
## s(bl_age):time    4.354  4.354 16.400 < 2e-16 ***
## s(sleep_z):time   1.000  1.000 27.319 8.2e-07 ***
## ---
## Signif. codes:  0 ‘***’ 0.001 ‘**’ 0.01 ‘*’ 0.05 ‘.’ 0.1 ‘ ’ 1
##
## R-sq.(adj) =  0.298
## lmer.REML =  79135  Scale est. = 1.5516e+06  n = 4272
```

Below are plots of a plot of `s(bl_age)`, `s(bl_age):sleep_z`, `s(bl_age):time`, and `s(sleep_z):time`. For `s(sleep_z):time`, roughly, if we cannot fit a straight line through the confidence bands, there is evidence that the effect of time depends on sleep duration. It is correct the that confidence interval is zero at one point along the axis; this happens because we do not include the main effect.

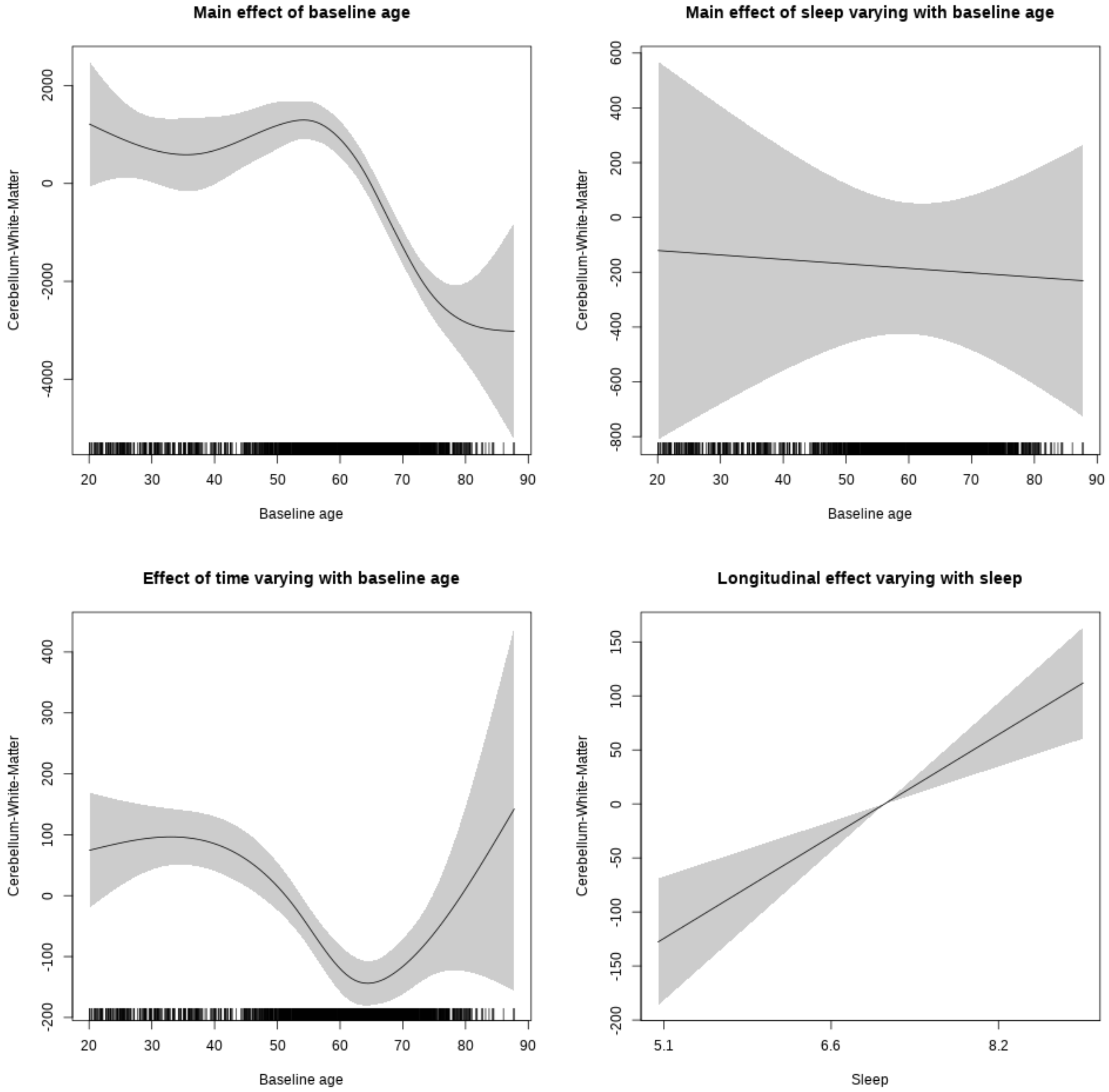

Restricted to sleep >= 5 and <= 9 hours Below is the row for `s(sleep_z):time` from the model output.

```
## s(sleep_z):time   1.000  1.000 18.905 1.42e-05 ***
```

Below are plots of a plot of `s(bl_age)`, `s(bl_age):sleep_z`, `s(bl_age):time`, and `s(sleep_z):time`. For `s(sleep_z):time`, roughly, if we cannot fit a straight line through the confidence bands, there is evidence that the effect of time depends on sleep duration. It is correct the that confidence interval is zero at one point along the axis; this happens because we do not include the main effect.

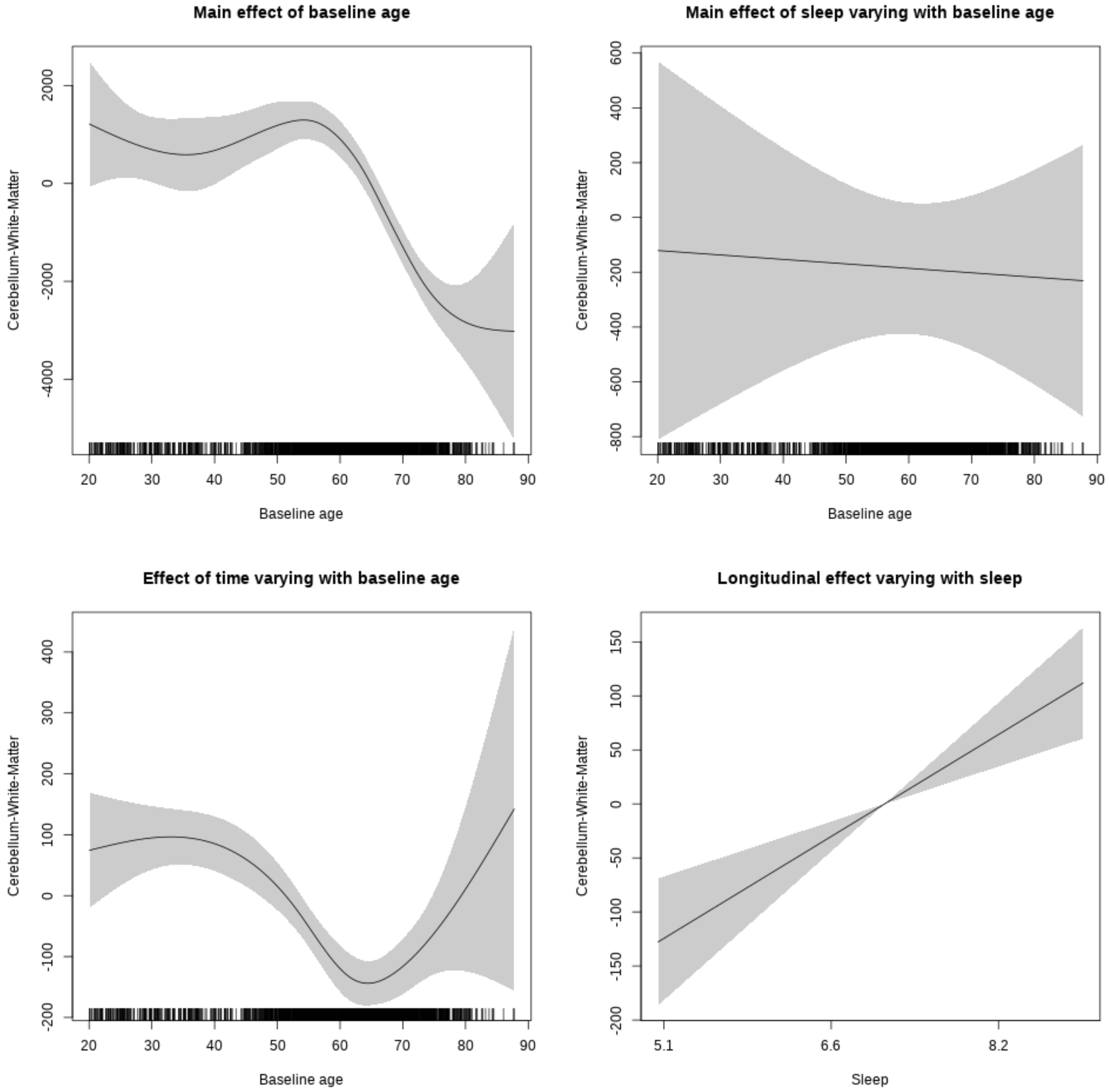

CerebralWhiteMatterVol

Main analysis

Full data Below is the model output. The term `s(sleep_z):time` is probably what we care most about.

```
##
## Family: gaussian
## Link function: identity
##
## Formula:
## value ~ sex + site + icv + s(bl_age, k = 10, bs = "cr") + s(bl_age,
##     by = sleep_z, bs = "cr") + +s(bl_age, by = time, k = 5, bs = "cr") +
```

```
##     s(sleep_z, by = time, k = 5, bs = "cr", pc = 0)
## <environment: 0x563910f09c80>
##
## Parametric coefficients:
##                Estimate Std. Error t value Pr(>|t|)
## (Intercept)   500685.2     2298.4  217.84   <2e-16 ***
## sexmale        12007.5     1180.8   10.17   <2e-16 ***
## siteousAvanto -51917.3     2818.7  -18.42   <2e-16 ***
## siteousPrisma -85972.2     5802.6  -14.82   <2e-16 ***
## siteousSkyra  -57172.6     2747.6  -20.81   <2e-16 ***
## siteUB        -62997.0     5392.4  -11.68   <2e-16 ***
## siteUCAM      -61480.7     2921.8  -21.04   <2e-16 ***
## siteUKB       -25078.6     2206.5  -11.37   <2e-16 ***
## siteUmU       -36533.6     3336.2  -10.95   <2e-16 ***
## icv            41090.0      549.3   74.81   <2e-16 ***
## ---
## Signif. codes:  0 ‘***’ 0.001 ‘**’ 0.01 ‘*’ 0.05 ‘.’ 0.1 ‘ ’ 1
##
## Approximate significance of smooth terms:
##                     edf Ref.df       F p-value
## s(bl_age)         5.621  5.621 119.799  <2e-16 ***
## s(bl_age):sleep_z 2.000  2.000   3.165  0.0422 *
## s(bl_age):time    4.787  4.787 188.311  <2e-16 ***
## s(sleep_z):time   3.387  3.387   1.841  0.1046
## ---
## Signif. codes:  0 ‘***’ 0.001 ‘**’ 0.01 ‘*’ 0.05 ‘.’ 0.1 ‘ ’ 1
##
## R-sq.(adj) =  0.734
## lmer.REML = 1.8123e+05  Scale est. = 4.2549e+07  n = 8150
```

Below are plots of a plot of `s(bl_age)`, `s(bl_age):sleep_z`, `s(bl_age):time`, and `s(sleep_z):time`. For `s(sleep_z):time`, roughly, if we cannot fit a straight line through the confidence bands, there is evidence that the effect of time depends on sleep duration. It is correct the that confidence interval is zero at one point along the axis; this happens because we do not include the main effect.

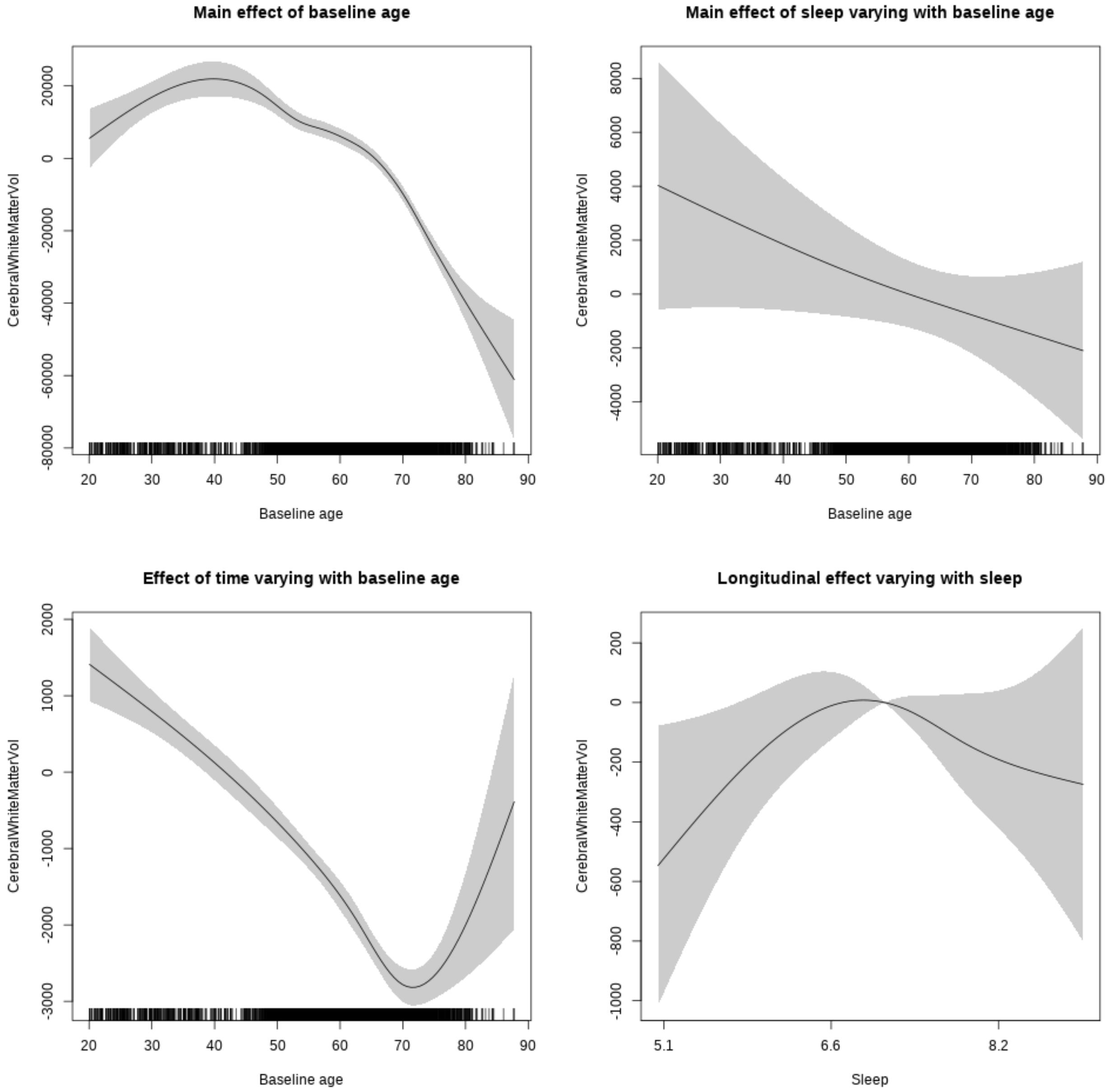

Restricted to sleep >= 5 and <= 9 hours Below is the row for `s(sleep_z):time` from the model output.

```
## s(sleep_z):time   2.354  2.354   2.626  0.0605 .
```

Below are plots of a plot of `s(bl_age)`, `s(bl_age):sleep_z`, `s(bl_age):time`, and `s(sleep_z):time`. For `s(sleep_z):time`, roughly, if we cannot fit a straight line through the confidence bands, there is evidence that the effect of time depends on sleep duration. It is correct the that confidence interval is zero at one point along the axis; this happens because we do not include the main effect.

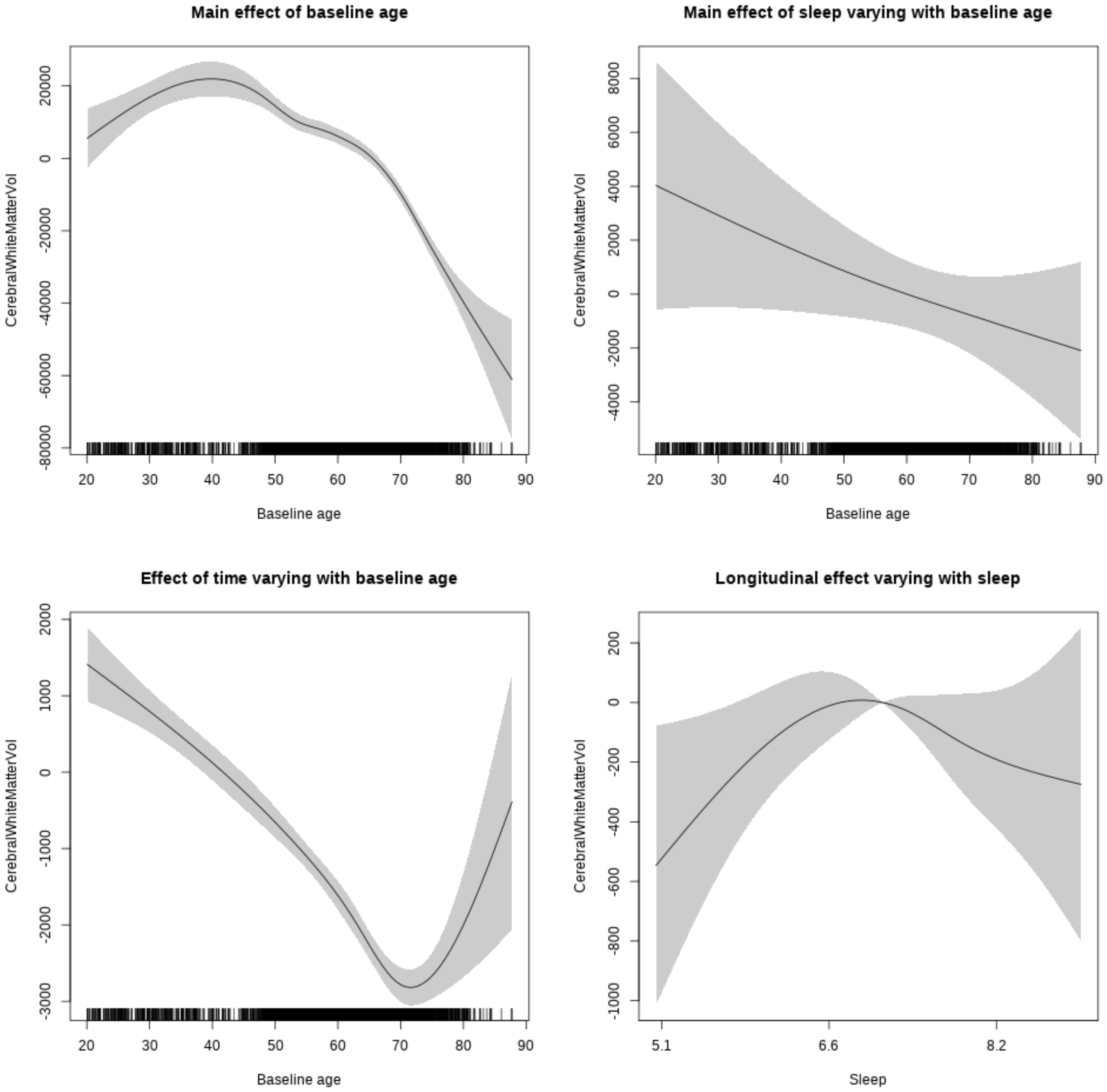

Controling for socioeconomic status

Full data Below is the model output. The term `s(sleep_z):time` is probably what we care most about.

```
##
## Family: gaussian
## Link function: identity
##
## Formula:
## value ~ sex + site + income_scaled + education_scaled + icv +
##     s(bl_age, k = 10, bs = "cr") + s(bl_age, by = sleep_z, bs = "cr") +
##     +s(bl_age, by = time, k = 5, bs = "cr") + s(sleep_z, by = time,
##     k = 5, bs = "cr", pc = 0)
```

```
## <environment: 0x5639172bd690>
##
## Parametric coefficients:
##                   Estimate Std. Error t value Pr(>|t|)
## (Intercept)       511313.0     5410.8  94.499  < 2e-16 ***
## sexmale             9969.7     1469.4   6.785 1.31e-11 ***
## siteousAvanto     -70555.3     5787.2 -12.192  < 2e-16 ***
## siteousPrisma    -116932.2     9854.9 -11.865  < 2e-16 ***
## siteousSkyra      -70122.7     5528.5 -12.684  < 2e-16 ***
## siteUKB           -38206.1     5140.9  -7.432 1.27e-13 ***
## income_scaled       3937.9     1911.8   2.060   0.0395 *
## education_scaled     730.7     2109.2   0.346   0.7290
## icv                44060.8      705.3  62.474  < 2e-16 ***
## ---
## Signif. codes:  0 ‘***’ 0.001 ‘**’ 0.01 ‘*’ 0.05 ‘.’ 0.1 ‘ ’ 1
##
## Approximate significance of smooth terms:
##                     edf Ref.df      F p-value
## s(bl_age)         4.383  4.383 54.307  <2e-16 ***
## s(bl_age):sleep_z 2.000  2.000  1.247   0.287
## s(bl_age):time    4.696  4.696 73.072  <2e-16 ***
## s(sleep_z):time   3.495  3.495  1.080   0.221
## ---
## Signif. codes:  0 ‘***’ 0.001 ‘**’ 0.01 ‘*’ 0.05 ‘.’ 0.1 ‘ ’ 1
##
## R-sq.(adj) =  0.748
## lmer.REML = 1.0345e+05  Scale est. = 4.2974e+07  n = 4652
```

Below are plots of a plot of `s(bl_age)`, `s(bl_age):sleep_z`, `s(bl_age):time`, and `s(sleep_z):time`. For `s(sleep_z):time`, roughly, if we cannot fit a straight line through the confidence bands, there is evidence that the effect of time depends on sleep duration. It is correct the that confidence interval is zero at one point along the axis; this happens because we do not include the main effect.

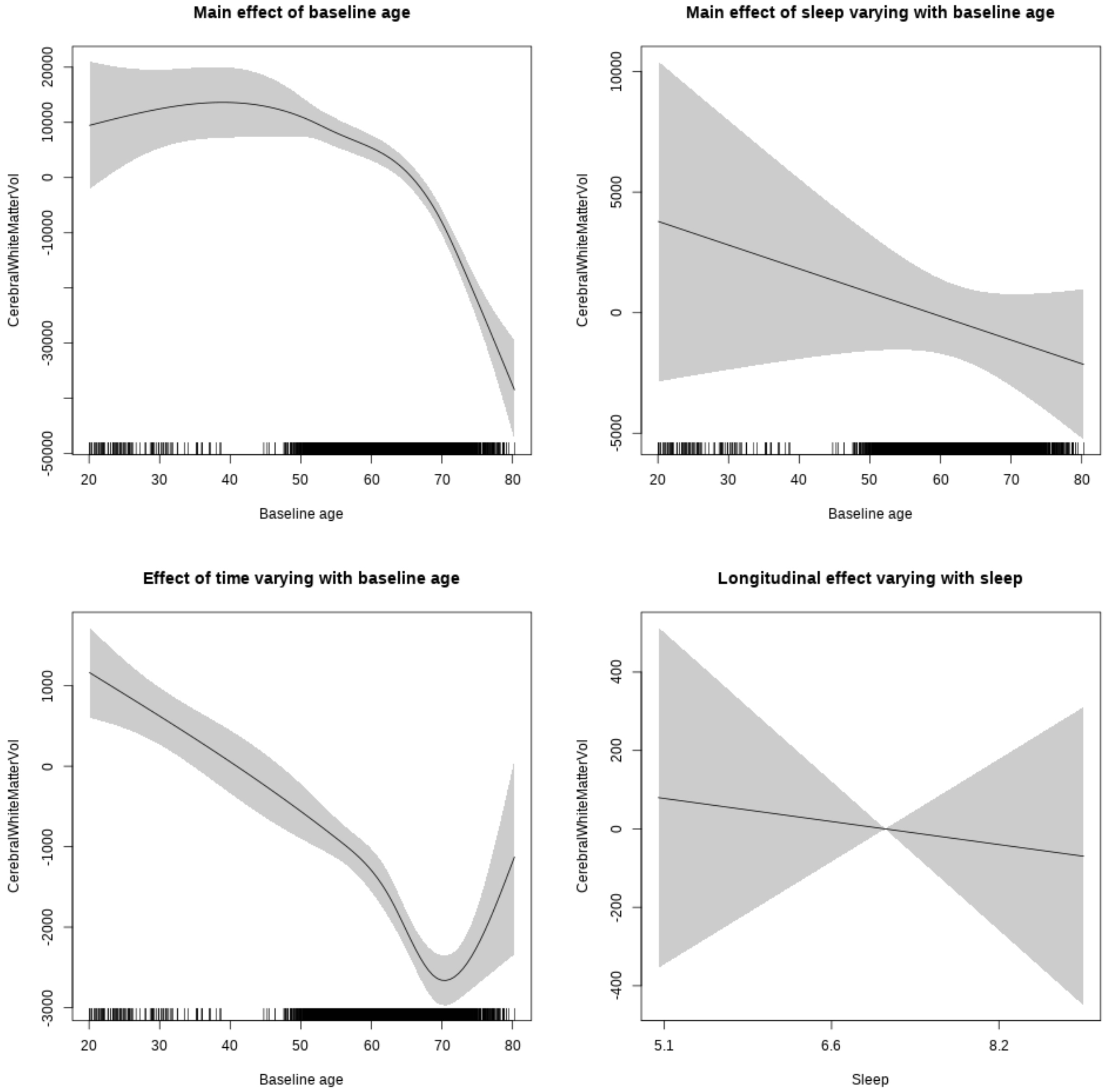

Restricted to sleep >= 5 and <= 9 hours Below is the row for `s(sleep_z):time` from the model output.

```
## s(sleep_z):time   1.000  1.000   0.135   0.714
```

Below are plots of a plot of `s(bl_age)`, `s(bl_age):sleep_z`, `s(bl_age):time`, and `s(sleep_z):time`. For `s(sleep_z):time`, roughly, if we cannot fit a straight line through the confidence bands, there is evidence that the effect of time depends on sleep duration. It is correct the that confidence interval is zero at one point along the axis; this happens because we do not include the main effect.

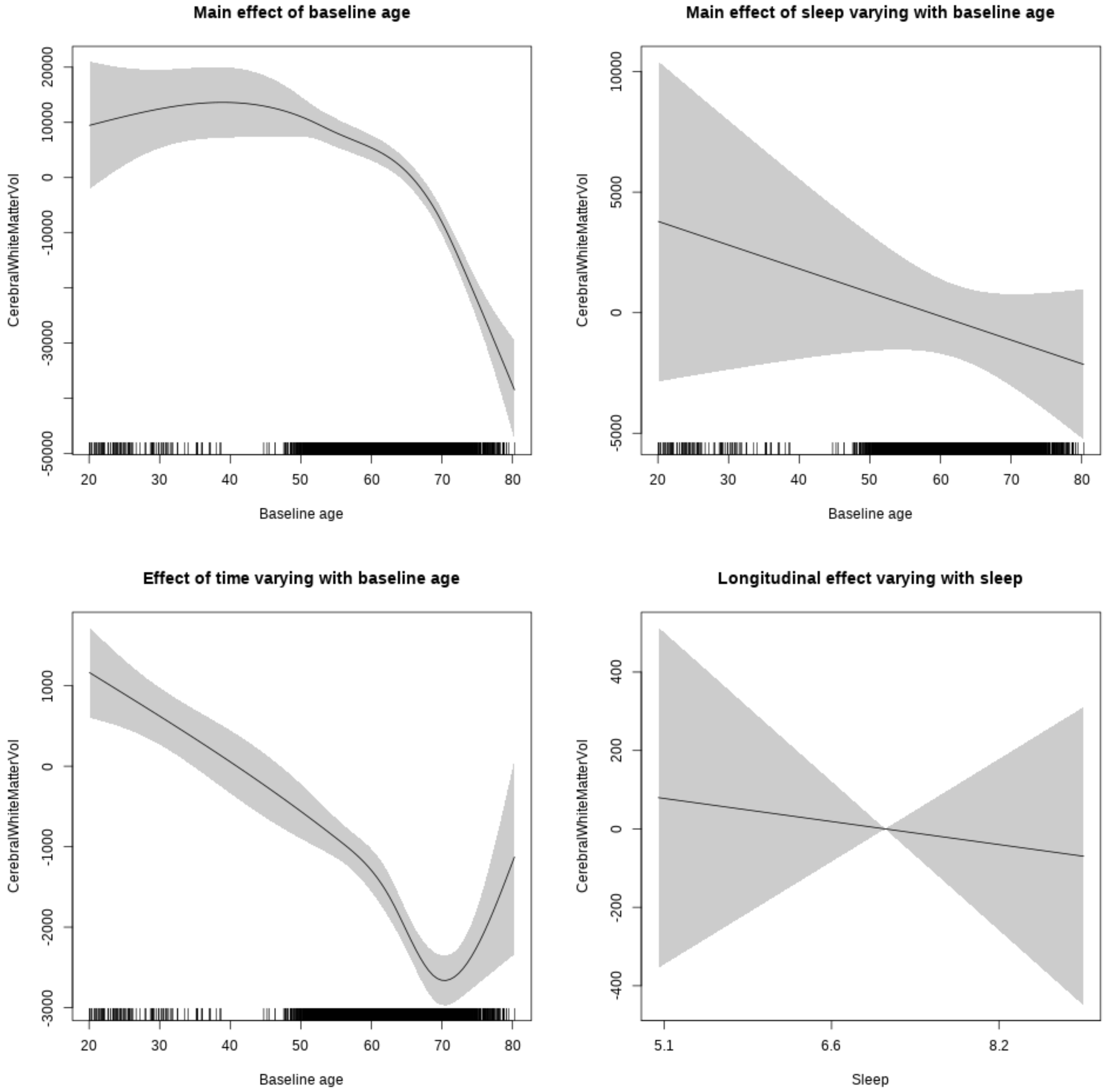

Controling for BMI

Full data Below is the model output. The term `s(sleep_z):time` is probably what we care most about.

```
##
## Family: gaussian
## Link function: identity
##
## Formula:
## value ~ sex + site + bmi + icv + s(bl_age, k = 10, bs = "cr") +
##     s(bl_age, by = sleep_z, bs = "cr") + +s(bl_age, by = time,
##     k = 5, bs = "cr") + s(sleep_z, by = time, k = 5, bs = "cr",
##     pc = 0)
```

```
## <environment: 0x5639112ecfd0>
##
## Parametric coefficients:
##                Estimate Std. Error t value Pr(>|t|)
## (Intercept)   464975.4     4691.2  99.118  < 2e-16 ***
## sexmale        11118.2     1352.9   8.218 2.57e-16 ***
## siteousPrisma -34840.0     5244.5  -6.643 3.37e-11 ***
## siteousSkyra   -2191.6     1686.2  -1.300   0.1938
## siteUCAM       -7163.1     3494.2  -2.050   0.0404 *
## siteUKB        25523.6     3200.6   7.975 1.85e-15 ***
## siteUmU        19006.5     4024.6   4.723 2.39e-06 ***
## bmi             -610.6      141.7  -4.309 1.67e-05 ***
## icv            43913.1      649.3  67.635  < 2e-16 ***
## ---
## Signif. codes:  0 '***' 0.001 '**' 0.01 '*' 0.05 '.' 0.1 ' ' 1
##
## Approximate significance of smooth terms:
##                      edf Ref.df       F p-value
## s(bl_age)          5.032  5.032  82.126  <2e-16 ***
## s(bl_age):sleep_z 2.000  2.000   0.315   0.730
## s(bl_age):time    4.791  4.791 139.462  <2e-16 ***
## s(sleep_z):time   1.000  1.000   0.245   0.621
## ---
## Signif. codes:  0 '***' 0.001 '**' 0.01 '*' 0.05 '.' 0.1 ' ' 1
##
## R-sq.(adj) =  0.753
## lmer.REML = 1.2026e+05  Scale est. = 4.1264e+07  n = 5412
```

Below are plots of a plot of `s(bl_age)`, `s(bl_age):sleep_z`, `s(bl_age):time`, and `s(sleep_z):time`. For `s(sleep_z):time`, roughly, if we cannot fit a straight line through the confidence bands, there is evidence that the effect of time depends on sleep duration. It is correct the that confidence interval is zero at one point along the axis; this happens because we do not include the main effect.

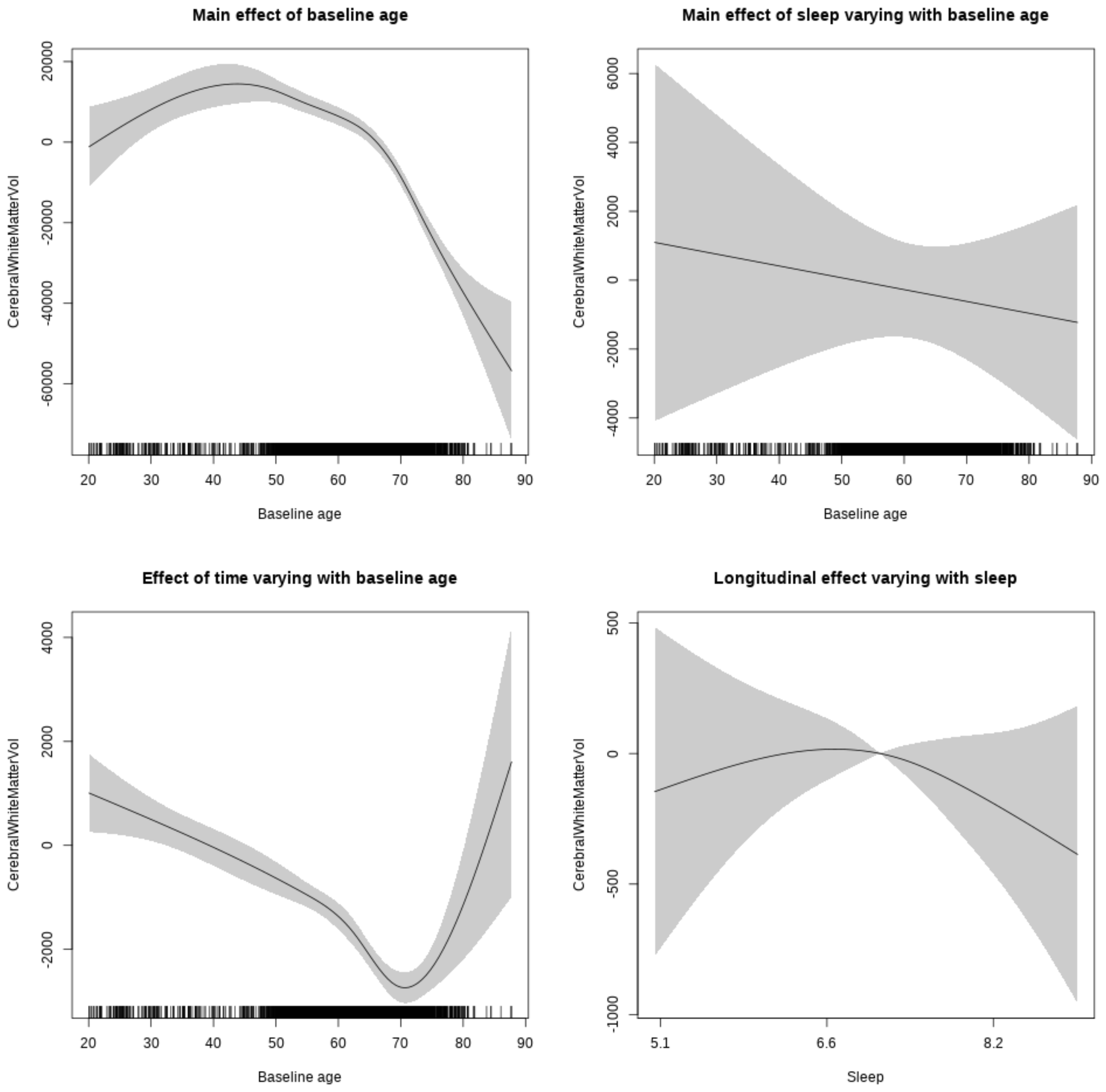

Restricted to sleep >= 5 and <= 9 hours Below is the row for `s(sleep_z):time` from the model output.

```
## s(sleep_z):time   1.668  1.668   0.626   0.367
```

Below are plots of a plot of `s(bl_age)`, `s(bl_age):sleep_z`, `s(bl_age):time`, and `s(sleep_z):time`. For `s(sleep_z):time`, roughly, if we cannot fit a straight line through the confidence bands, there is evidence that the effect of time depends on sleep duration. It is correct the that confidence interval is zero at one point along the axis; this happens because we do not include the main effect.

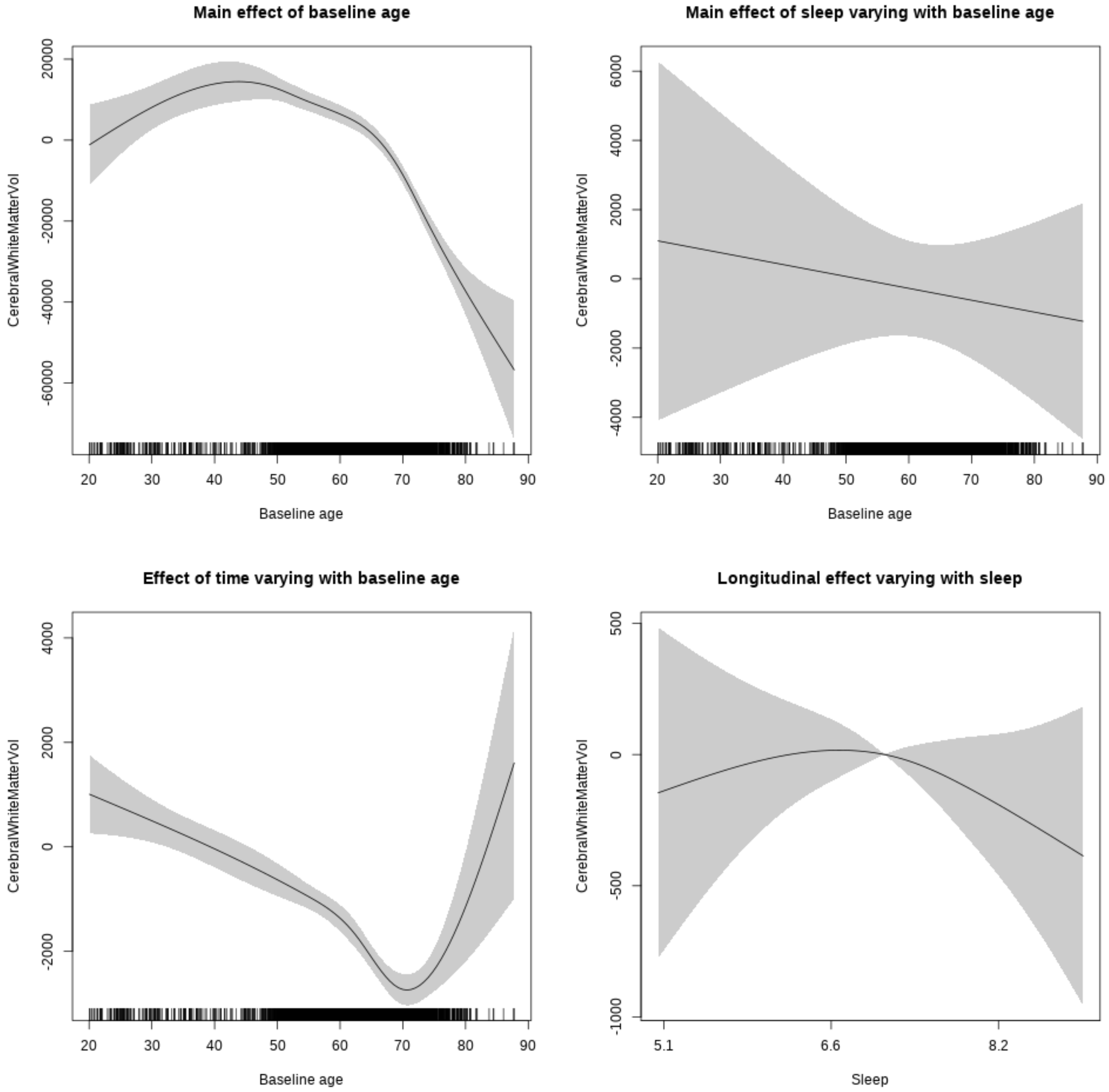

Controling for depression

Full data Below is the model output. The term `s(sleep_z):time` is probably what we care most about.

```
##
## Family: gaussian
## Link function: identity
##
## Formula:
## value ~ sex + site + depression + icv + s(bl_age, k = 10, bs = "cr") +
##     s(bl_age, by = sleep_z, bs = "cr") + +s(bl_age, by = time,
##     k = 5, bs = "cr") + s(sleep_z, by = time, k = 5, bs = "cr",
##     pc = 0)
```

```
## <environment: 0x563914fe9468>
##
## Parametric coefficients:
##                Estimate Std. Error t value Pr(>|t|)
## (Intercept)    495686.9     2706.5 183.148  < 2e-16 ***
## sexmale         11631.2     1411.3   8.241  < 2e-16 ***
## siteousAvanto -41709.9     5460.9  -7.638 2.58e-14 ***
## siteousPrisma -67067.8     8050.7  -8.331  < 2e-16 ***
## siteousSkyra  -46157.1     5004.7  -9.223  < 2e-16 ***
## siteUCAM      -52841.7     3326.4 -15.886  < 2e-16 ***
## siteUKB       -20828.3     2501.1  -8.328  < 2e-16 ***
## siteUmU       -23545.0     4012.7  -5.868 4.67e-09 ***
## depression    -13258.7     5030.9  -2.635  0.00842 **
## icv            41007.2      659.1  62.217  < 2e-16 ***
## ---
## Signif. codes:  0 '***' 0.001 '**' 0.01 '*' 0.05 '.' 0.1 ' ' 1
##
## Approximate significance of smooth terms:
##                      edf Ref.df       F p-value
## s(bl_age)          5.249  5.249  79.734  <2e-16 ***
## s(bl_age):sleep_z 2.000  2.000   1.388    0.25
## s(bl_age):time    4.721  4.721 165.981  <2e-16 ***
## s(sleep_z):time   1.000  1.000   0.219    0.64
## ---
## Signif. codes:  0 '***' 0.001 '**' 0.01 '*' 0.05 '.' 0.1 ' ' 1
##
## R-sq.(adj) =  0.722
## lmer.REML = 1.2658e+05  Scale est. = 4.056e+07  n = 5679
```

Below are plots of a plot of `s(bl_age)`, `s(bl_age):sleep_z`, `s(bl_age):time`, and `s(sleep_z):time`. For `s(sleep_z):time`, roughly, if we cannot fit a straight line through the confidence bands, there is evidence that the effect of time depends on sleep duration. It is correct the that confidence interval is zero at one point along the axis; this happens because we do not include the main effect.

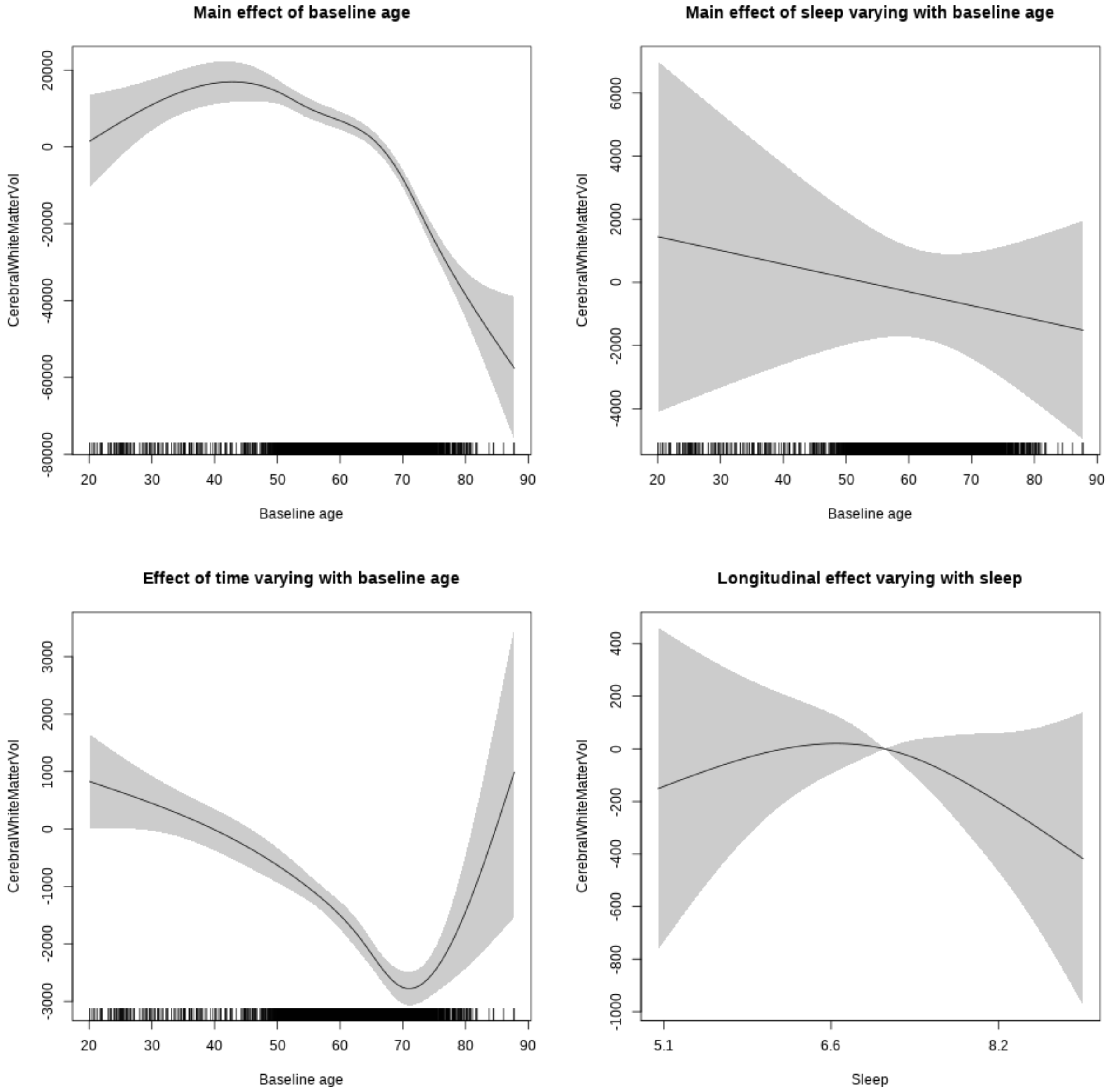

Restricted to sleep >= 5 and <= 9 hours Below is the row for `s(sleep_z):time` from the model output.

```
## s(sleep_z):time   1.725  1.725   0.828   0.292
```

Below are plots of a plot of `s(bl_age)`, `s(bl_age):sleep_z`, `s(bl_age):time`, and `s(sleep_z):time`. For `s(sleep_z):time`, roughly, if we cannot fit a straight line through the confidence bands, there is evidence that the effect of time depends on sleep duration. It is correct the that confidence interval is zero at one point along the axis; this happens because we do not include the main effect.

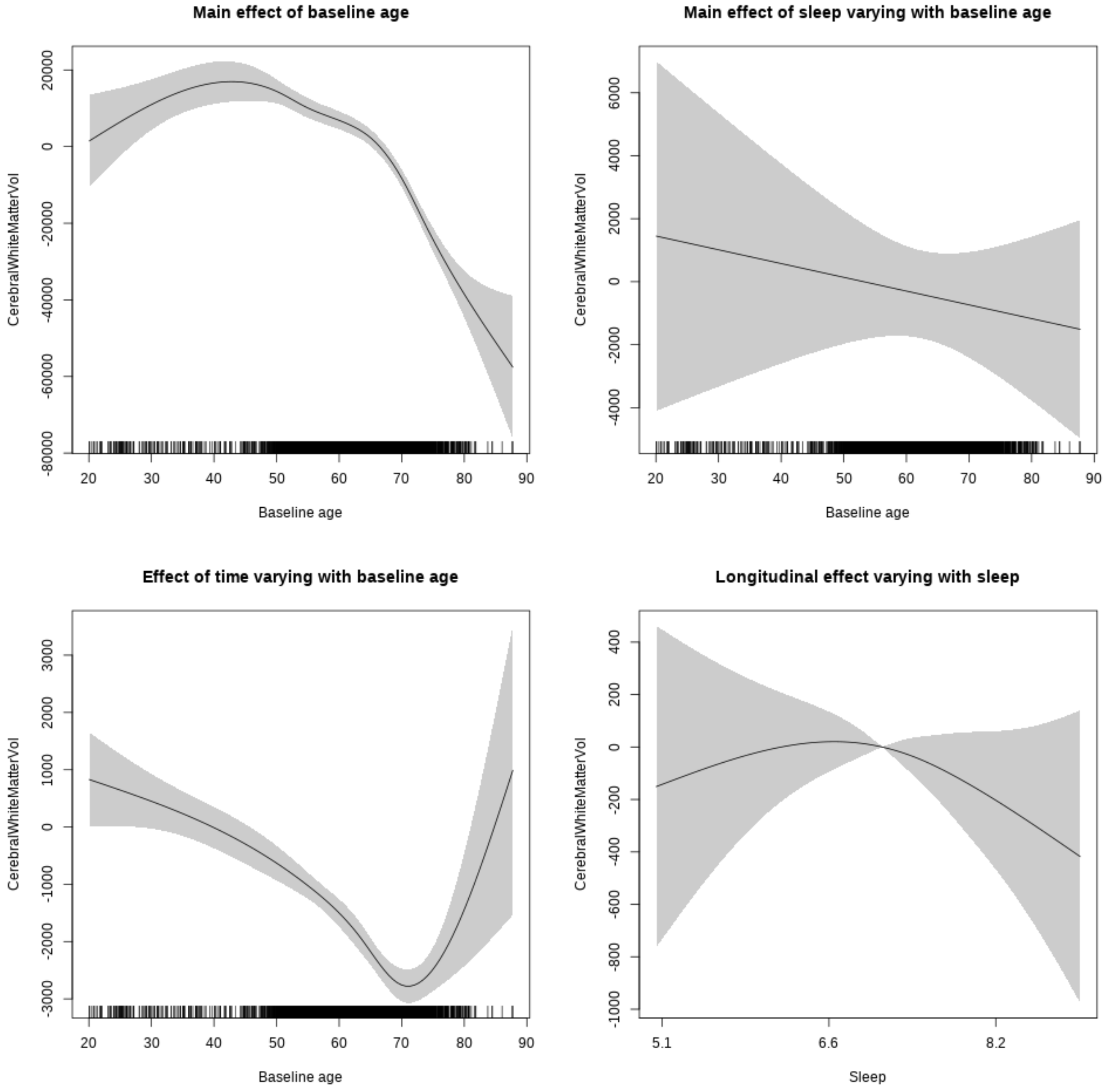

Controling for sleep quality

Full data Below is the model output. The term `s(sleep_z):time` is probably what we care most about.

```
##
## Family: gaussian
## Link function: identity
##
## Formula:
## value ~ X + s(bl_age, k = 10, bs = "cr") + s(bl_age, by = sleep_z,
##     bs = "cr") + s(bl_age, by = time, k = 5, bs = "cr") + s(sleep_z,
##     by = time, k = 5, bs = "cr", pc = 0)
## <environment: 0x563910f87cd8>
```

```
##
## Parametric coefficients:
##                            Estimate Std. Error t value Pr(>|t|)
## (Intercept)                418071.7     6006.6  69.602  < 2e-16 ***
## Xsexmale                    58565.1     2246.7  26.067  < 2e-16 ***
## XsiteousAvanto              35350.7     5727.5   6.172 7.37e-10 ***
## XsiteousPrisma              21091.3     7609.7   2.772  0.00560 **
## XsiteousSkyra               15606.1     5733.3   2.722  0.00651 **
## XsiteUB                     -8796.7     9136.3  -0.963  0.33569
## XsiteUCAM                   10377.3     5900.2   1.759  0.07868 .
## XsiteUKB                     6181.6    18289.0   0.338  0.73538
## XsiteUmU                    14898.6     6538.8   2.279  0.02275 *
## Xukb_dummy:sleep_accel       1601.4     1411.3   1.135  0.25655
## Xukb_dummy:chronotype        2423.4     1879.2   1.290  0.19726
## Xukb_dummy:dozing            2443.3     3883.3   0.629  0.52925
## Xukb_dummy:sleeplessness    -4725.1     2421.0  -1.952  0.05104 .
## Xukb_dummy:snoring           3731.6     3427.5   1.089  0.27634
## Xukb_dummy:gettingup         5389.2     2632.0   2.048  0.04067 *
## Xnotukb_dummy:PSQI_Global   -1245.3      744.3  -1.673  0.09436 .
## ---
## Signif. codes:  0 '***' 0.001 '**' 0.01 '*' 0.05 '.' 0.1 ' ' 1
##
## Approximate significance of smooth terms:
##                      edf Ref.df       F p-value
## s(bl_age)          3.741  3.741  52.982  <2e-16 ***
## s(bl_age):sleep_z  2.000  2.000   2.270   0.103
## s(bl_age):time     4.655  4.655 239.556  <2e-16 ***
## s(sleep_z):time    1.000  1.000   0.908   0.341
## ---
## Signif. codes:  0 '***' 0.001 '**' 0.01 '*' 0.05 '.' 0.1 ' ' 1
##
## R-sq.(adj) =  0.371
## lmer.REML =  96673  Scale est. = 4.207e+07  n = 4286
```

Below are plots of a plot of `s(bl_age)`, `s(bl_age):sleep_z`, `s(bl_age):time`, and `s(sleep_z):time`. For `s(sleep_z):time`, roughly, if we cannot fit a straight line through the confidence bands, there is evidence that the effect of time depends on sleep duration. It is correct the that confidence interval is zero at one point along the axis; this happens because we do not include the main effect.

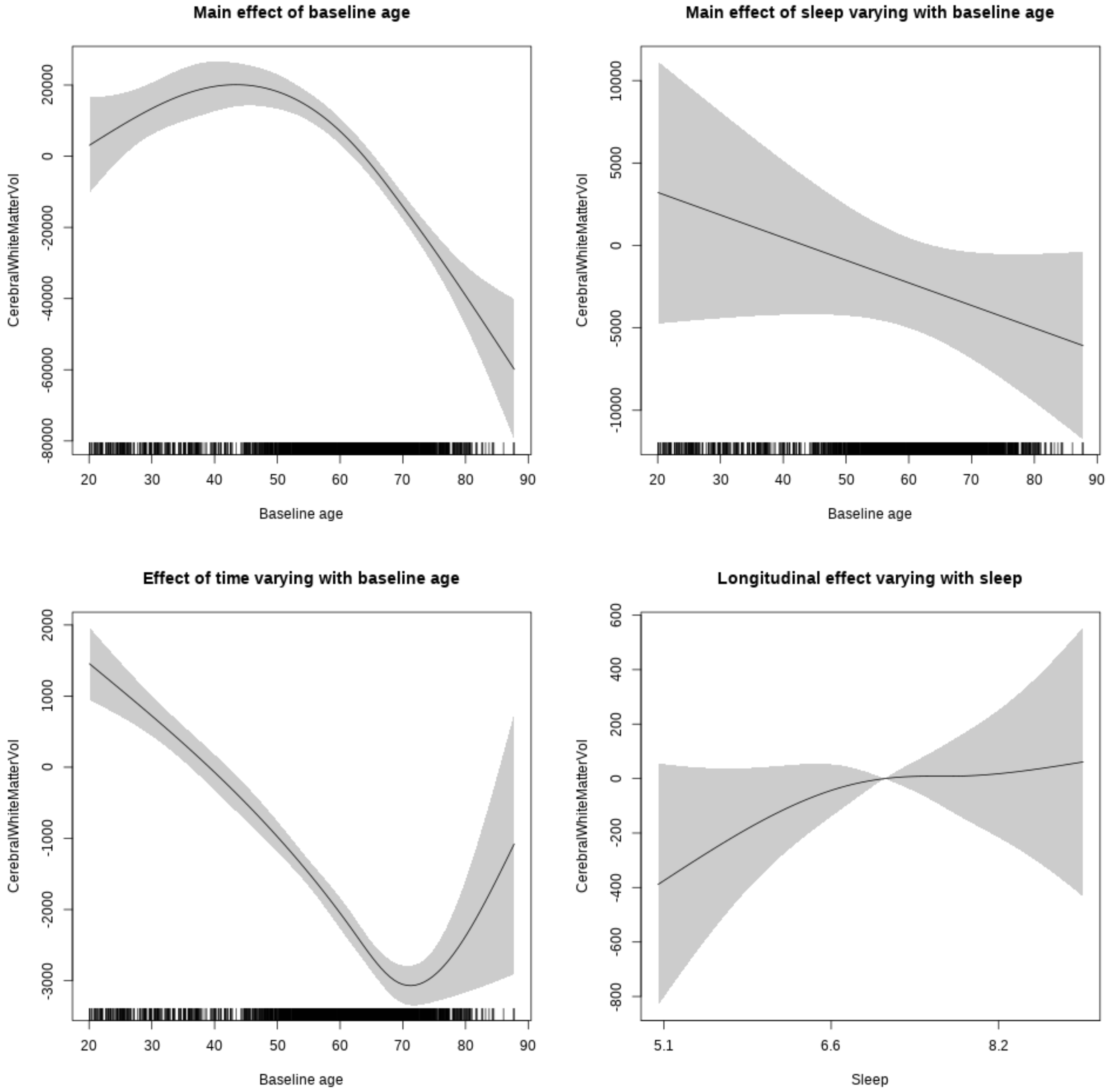

Restricted to sleep >= 5 and <= 9 hours Below is the row for `s(sleep_z):time` from the model output.

```
## s(sleep_z):time   1.647  1.647   2.094   0.200
```

Below are plots of a plot of `s(bl_age)`, `s(bl_age):sleep_z`, `s(bl_age):time`, and `s(sleep_z):time`. For `s(sleep_z):time`, roughly, if we cannot fit a straight line through the confidence bands, there is evidence that the effect of time depends on sleep duration. It is correct the that confidence interval is zero at one point along the axis; this happens because we do not include the main effect.

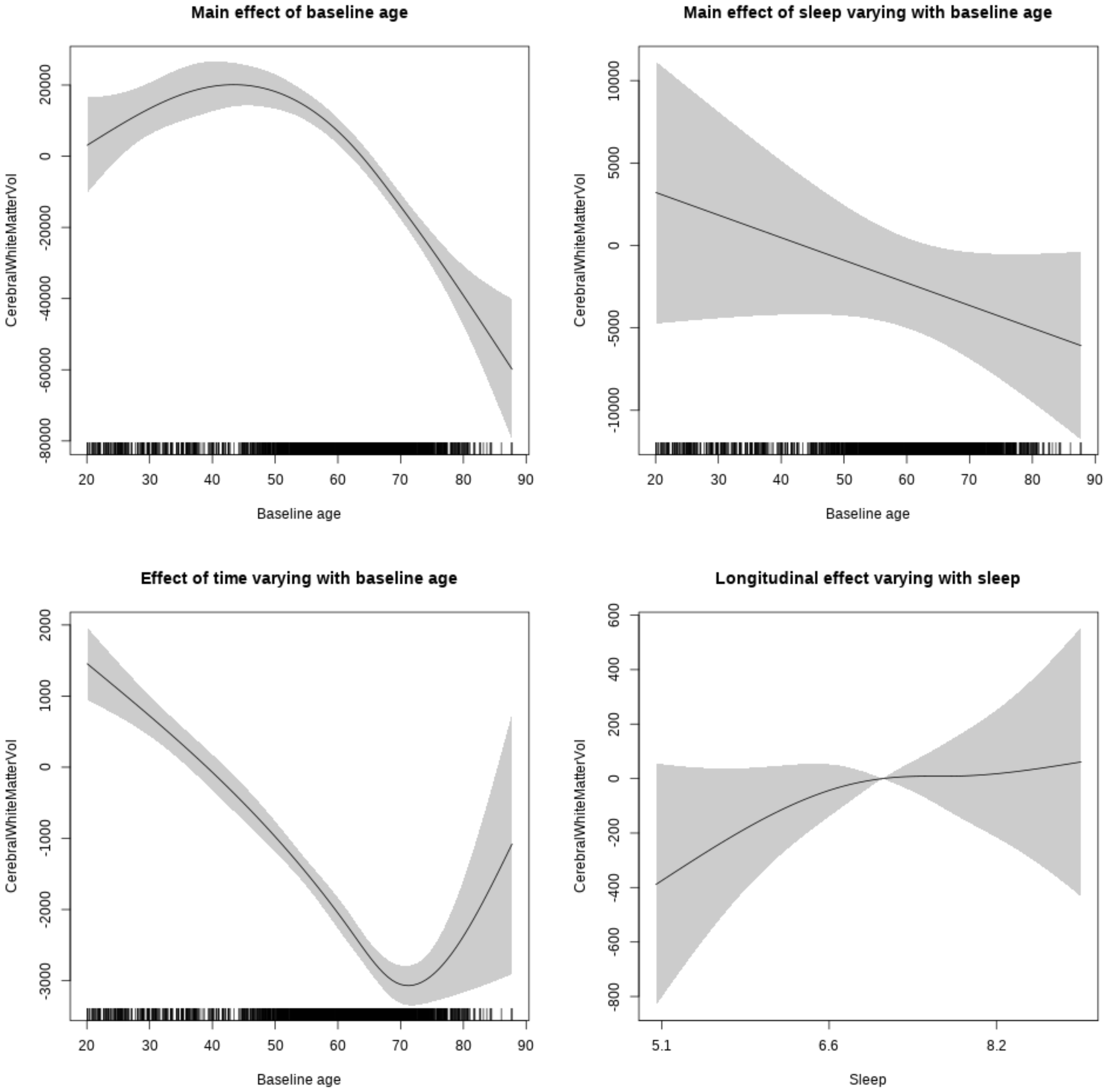

EstimatedTotalIntraCranialVol

Main analysis

Full data Below is the model output. The term `s(sleep_z):time` is probably what we care most about.

```
##
## Family: gaussian
## Link function: identity
##
## Formula:
## value ~ sex + site + s(bl_age, k = 10, bs = "cr") + s(bl_age,
##     by = sleep_z, bs = "cr") + +s(bl_age, by = time, k = 5, bs = "cr") +
```

```
##     s(sleep_z, by = time, k = 5, bs = "cr", pc = 0)
## <environment: 0x56391e8b1490>
##
## Parametric coefficients:
##               Estimate Std. Error t value Pr(>|t|)
## (Intercept)    1212478       8353 145.158   <2e-16 ***
## sexmale         182182       4199  43.389   <2e-16 ***
## siteousAvanto   325968      10789  30.212   <2e-16 ***
## siteousPrisma   405176      14379  28.179   <2e-16 ***
## siteousSkyra    270576      10789  25.079   <2e-16 ***
## siteUB          200552      22522   8.905   <2e-16 ***
## siteUCAM        267882      11544  23.205   <2e-16 ***
## siteUKB         252457       8495  29.718   <2e-16 ***
## siteUmU         175339      13779  12.725   <2e-16 ***
## ---
## Signif. codes:  0 '***' 0.001 '**' 0.01 '*' 0.05 '.' 0.1 ' ' 1
##
## Approximate significance of smooth terms:
##                     edf Ref.df      F  p-value
## s(bl_age)         4.309  4.309  7.227 1.48e-05 ***
## s(bl_age):sleep_z 2.000  2.000  1.488    0.226
## s(bl_age):time    2.000  2.000 87.786  < 2e-16 ***
## s(sleep_z):time   1.000  1.000  0.344    0.557
## ---
## Signif. codes:  0 '***' 0.001 '**' 0.01 '*' 0.05 '.' 0.1 ' ' 1
##
## R-sq.(adj) =  0.425
## lmer.REML = 1.9804e+05  Scale est. = 1.5165e+08  n = 8160
```

Below are plots of a plot of `s(bl_age)`, `s(bl_age):sleep_z`, `s(bl_age):time`, and `s(sleep_z):time`. For `s(sleep_z):time`, roughly, if we cannot fit a straight line through the confidence bands, there is evidence that the effect of time depends on sleep duration. It is correct the that confidence interval is zero at one point along the axis; this happens because we do not include the main effect.

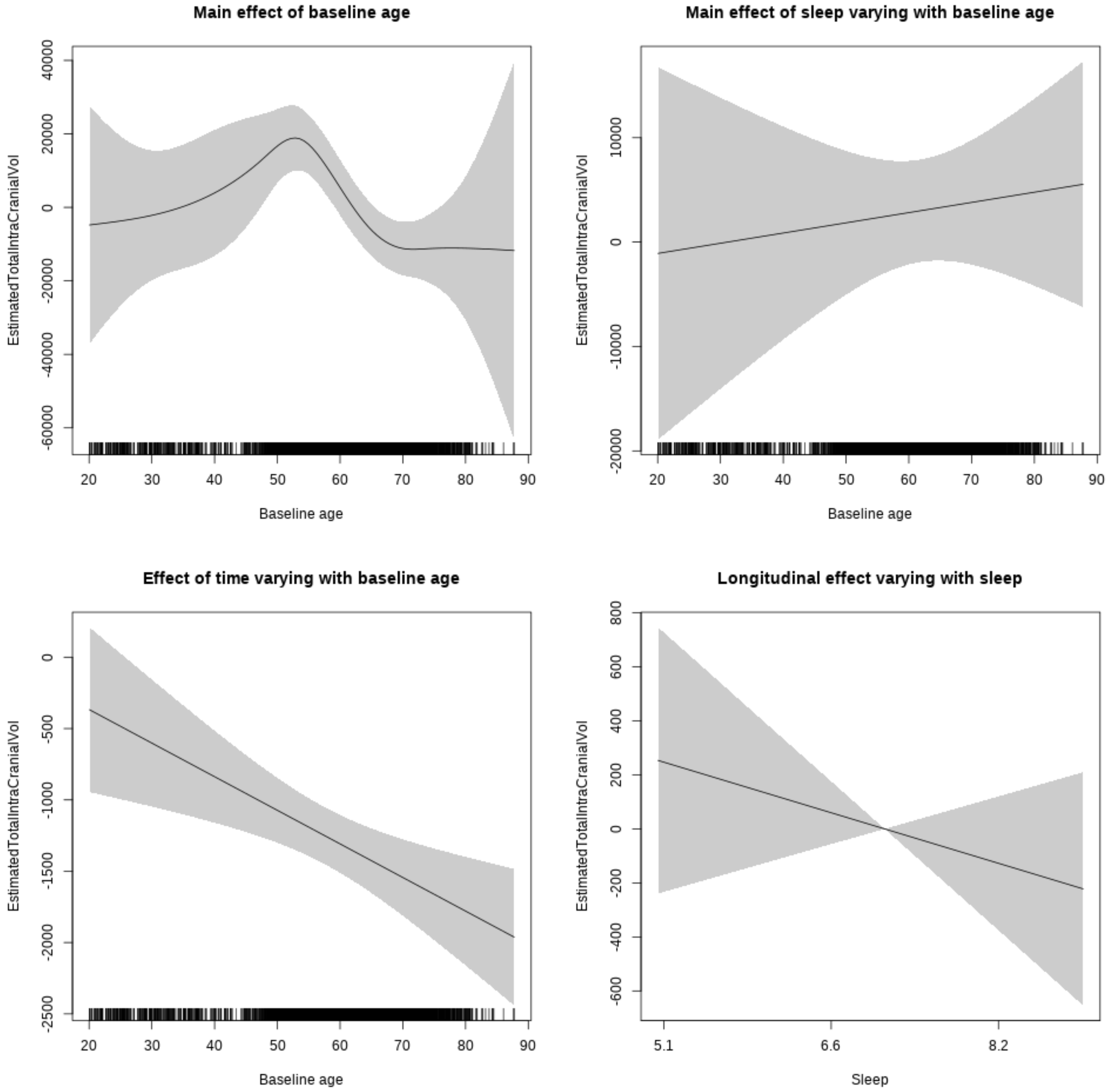

Restricted to sleep >= 5 and <= 9 hours Below is the row for `s(sleep_z):time` from the model output.

```
## s(sleep_z):time   1.000  1.000  1.058    0.304
```

Below are plots of a plot of `s(bl_age)`, `s(bl_age):sleep_z`, `s(bl_age):time`, and `s(sleep_z):time`. For `s(sleep_z):time`, roughly, if we cannot fit a straight line through the confidence bands, there is evidence that the effect of time depends on sleep duration. It is correct the that confidence interval is zero at one point along the axis; this happens because we do not include the main effect.

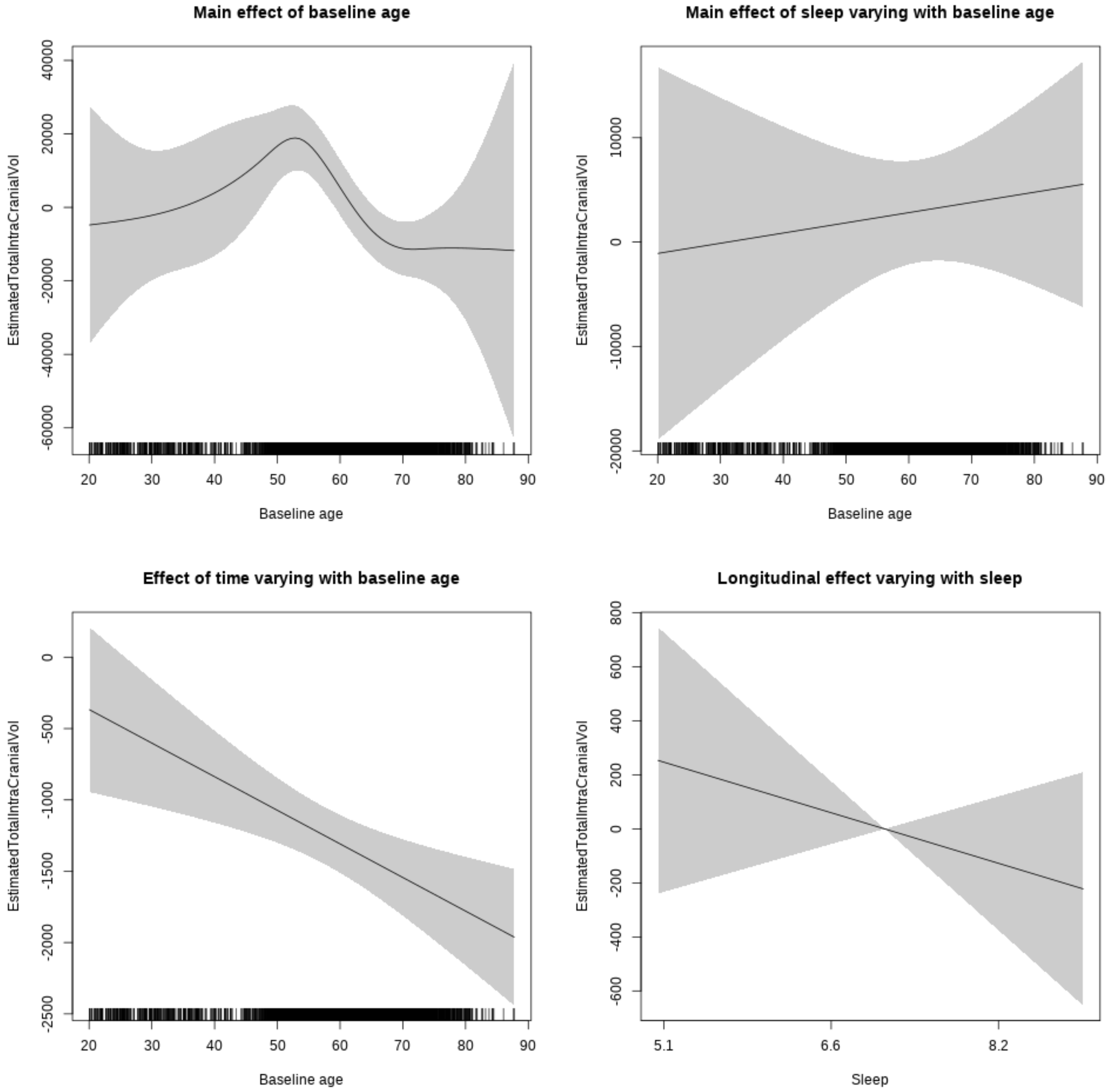

Controling for socioeconomic status

Full data Below is the model output. The term `s(sleep_z):time` is probably what we care most about.

```
##
## Family: gaussian
## Link function: identity
##
## Formula:
## value ~ sex + site + income_scaled + education_scaled + s(bl_age,
##     k = 10, bs = "cr") + s(bl_age, by = sleep_z, bs = "cr") +
##     +s(bl_age, by = time, k = 5, bs = "cr") + s(sleep_z, by = time,
##     k = 5, bs = "cr", pc = 0)
```

```
## <environment: 0x5639115506e8>
##
## Parametric coefficients:
##                   Estimate Std. Error t value Pr(>|t|)
## (Intercept)       1125783      21078  53.409  < 2e-16 ***
## sexmale            176576       5285  33.408  < 2e-16 ***
## siteousAvanto      346349      22789  15.198  < 2e-16 ***
## siteousPrisma      435828      27502  15.847  < 2e-16 ***
## siteousSkyra       286279      22611  12.661  < 2e-16 ***
## siteUKB            294233      20525  14.336  < 2e-16 ***
## income_scaled       39628       7708   5.141 2.85e-07 ***
## education_scaled    42911       8972   4.783 1.78e-06 ***
## ---
## Signif. codes:  0 '***' 0.001 '**' 0.01 '*' 0.05 '.' 0.1 ' ' 1
##
## Approximate significance of smooth terms:
##                     edf Ref.df      F p-value
## s(bl_age)         1.000  1.000  6.238  0.0125 *
## s(bl_age):sleep_z 2.935  2.935  0.571  0.6109
## s(bl_age):time    3.304  3.304 46.095  <2e-16 ***
## s(sleep_z):time   1.709  1.709  0.875  0.3876
## ---
## Signif. codes:  0 '***' 0.001 '**' 0.01 '*' 0.05 '.' 0.1 ' ' 1
##
## R-sq.(adj) =   0.38
## lmer.REML = 1.133e+05  Scale est. = 1.6337e+08  n = 4654
```

Below are plots of a plot of `s(bl_age)`, `s(bl_age):sleep_z`, `s(bl_age):time`, and `s(sleep_z):time`. For `s(sleep_z):time`, roughly, if we cannot fit a straight line through the confidence bands, there is evidence that the effect of time depends on sleep duration. It is correct the that confidence interval is zero at one point along the axis; this happens because we do not include the main effect.

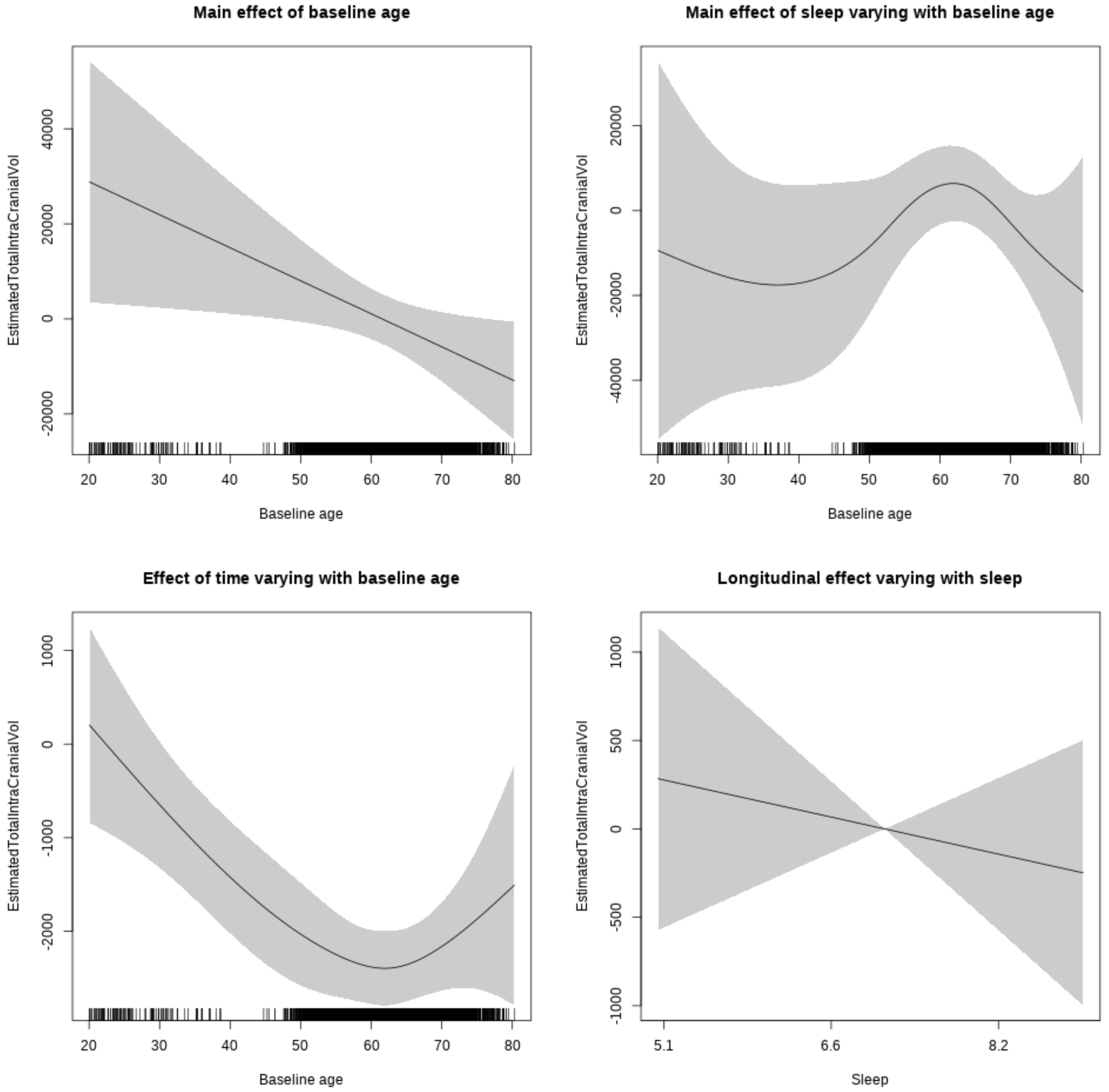

Restricted to sleep >= 5 and <= 9 hours Below is the row for `s(sleep_z):time` from the model output.

```
## s(sleep_z):time   1.000  1.000  0.440  0.5073
```

Below are plots of a plot of `s(bl_age)`, `s(bl_age):sleep_z`, `s(bl_age):time`, and `s(sleep_z):time`. For `s(sleep_z):time`, roughly, if we cannot fit a straight line through the confidence bands, there is evidence that the effect of time depends on sleep duration. It is correct the that confidence interval is zero at one point along the axis; this happens because we do not include the main effect.

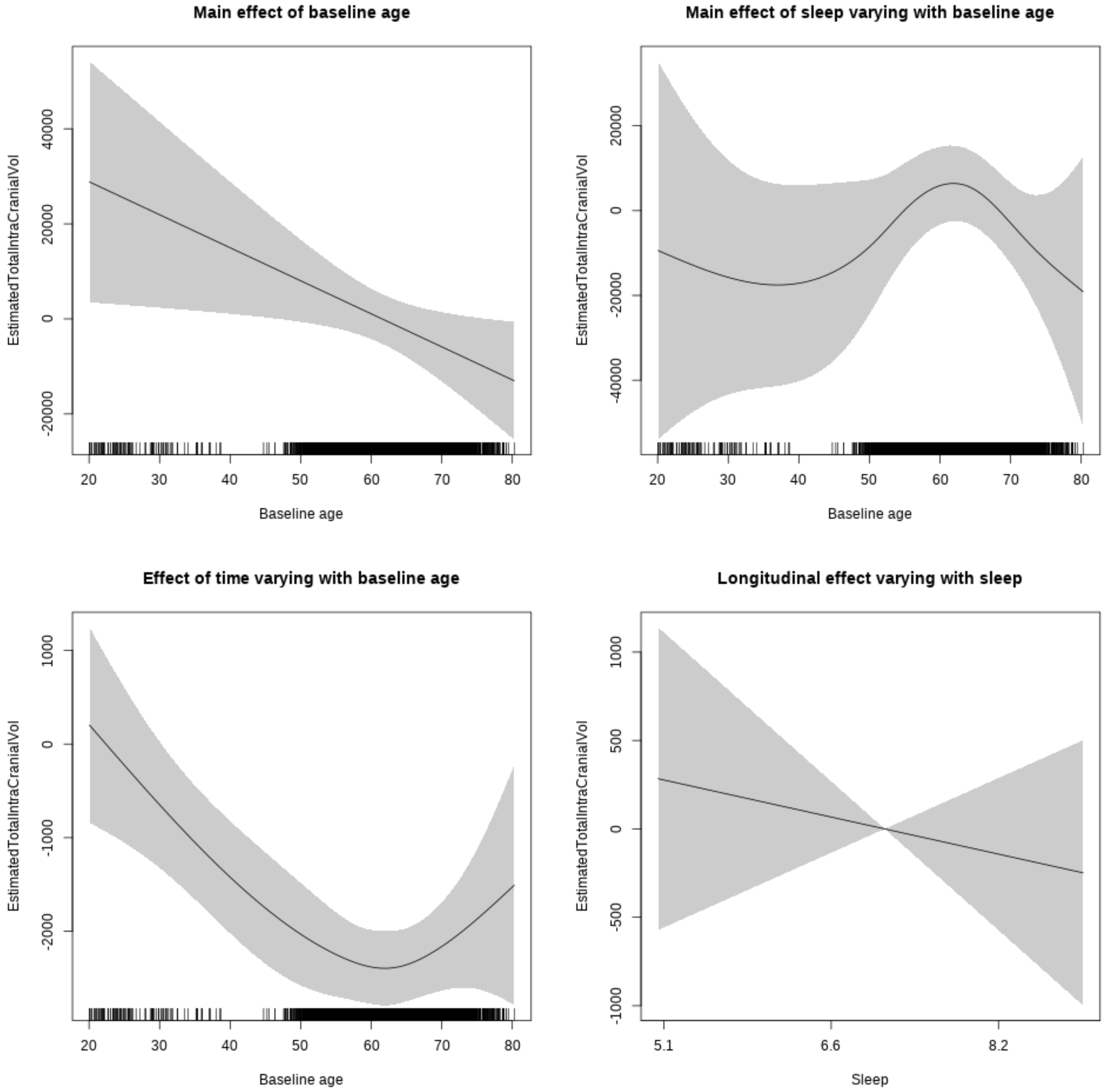

Controling for BMI

Full data Below is the model output. The term `s(sleep_z):time` is probably what we care most about.

```
##
## Family: gaussian
## Link function: identity
##
## Formula:
## value ~ sex + site + bmi + s(bl_age, k = 10, bs = "cr") + s(bl_age,
##     by = sleep_z, bs = "cr") + +s(bl_age, by = time, k = 5, bs = "cr") +
##     s(sleep_z, by = time, k = 5, bs = "cr", pc = 0)
## <environment: 0x56390d7cfaf0>
```

```
##
## Parametric coefficients:
##                 Estimate Std. Error t value Pr(>|t|)
## (Intercept)   1474290.4    19351.0  76.187  < 2e-16 ***
## sexmale        178569.1     4940.6  36.143  < 2e-16 ***
## siteousPrisma   78841.5     9927.4   7.942 2.41e-15 ***
## siteousSkyra   -57629.2     3177.7 -18.136  < 2e-16 ***
## siteUCAM       -46813.8    13636.5  -3.433 0.000601 ***
## siteUKB        -57810.2    12358.7  -4.678 2.97e-06 ***
## siteUmU       -141293.9    16117.9  -8.766  < 2e-16 ***
## bmi              2054.0      613.6   3.348 0.000821 ***
## ---
## Signif. codes:  0 '***' 0.001 '**' 0.01 '*' 0.05 '.' 0.1 ' ' 1
##
## Approximate significance of smooth terms:
##                      edf Ref.df      F  p-value
## s(bl_age)          2.509  2.509 11.078 7.02e-05 ***
## s(bl_age):sleep_z  2.000  2.000  0.973    0.378
## s(bl_age):time     3.301  3.301 60.002  < 2e-16 ***
## s(sleep_z):time    1.000  1.000  0.284    0.594
## ---
## Signif. codes:  0 '***' 0.001 '**' 0.01 '*' 0.05 '.' 0.1 ' ' 1
##
## R-sq.(adj) =  0.341
## lmer.REML = 1.3166e+05  Scale est. = 1.4603e+08  n = 5416
```

Below are plots of a plot of `s(bl_age)`, `s(bl_age):sleep_z`, `s(bl_age):time`, and `s(sleep_z):time`. For `s(sleep_z):time`, roughly, if we cannot fit a straight line through the confidence bands, there is evidence that the effect of time depends on sleep duration. It is correct the that confidence interval is zero at one point along the axis; this happens because we do not include the main effect.

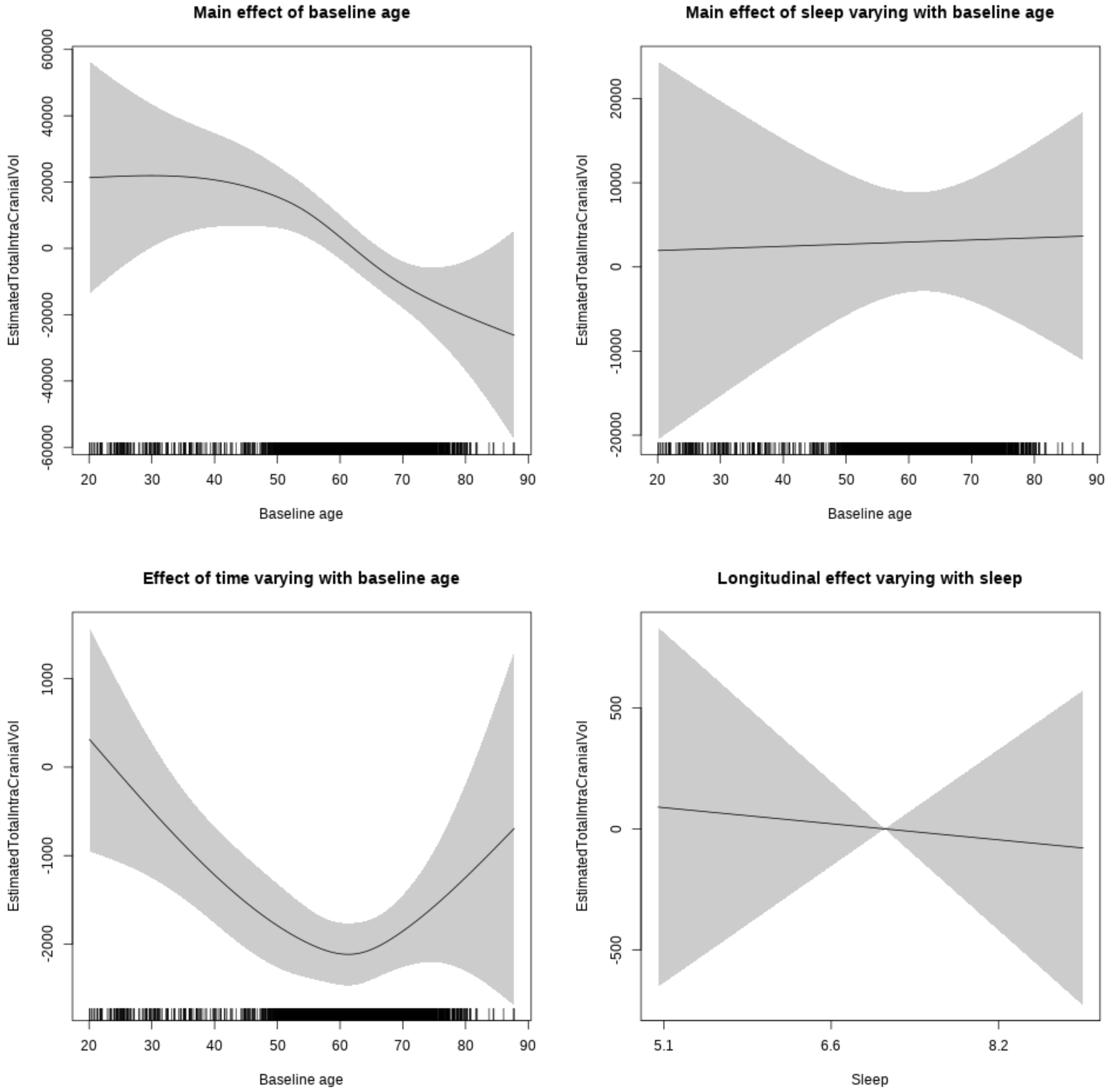

Restricted to sleep >= 5 and <= 9 hours Below is the row for `s(sleep_z):time` from the model output.

```
## s(sleep_z):time   1.000  1.000  0.059 0.808435
```

Below are plots of a plot of `s(bl_age)`, `s(bl_age):sleep_z`, `s(bl_age):time`, and `s(sleep_z):time`. For `s(sleep_z):time`, roughly, if we cannot fit a straight line through the confidence bands, there is evidence that the effect of time depends on sleep duration. It is correct the that confidence interval is zero at one point along the axis; this happens because we do not include the main effect.

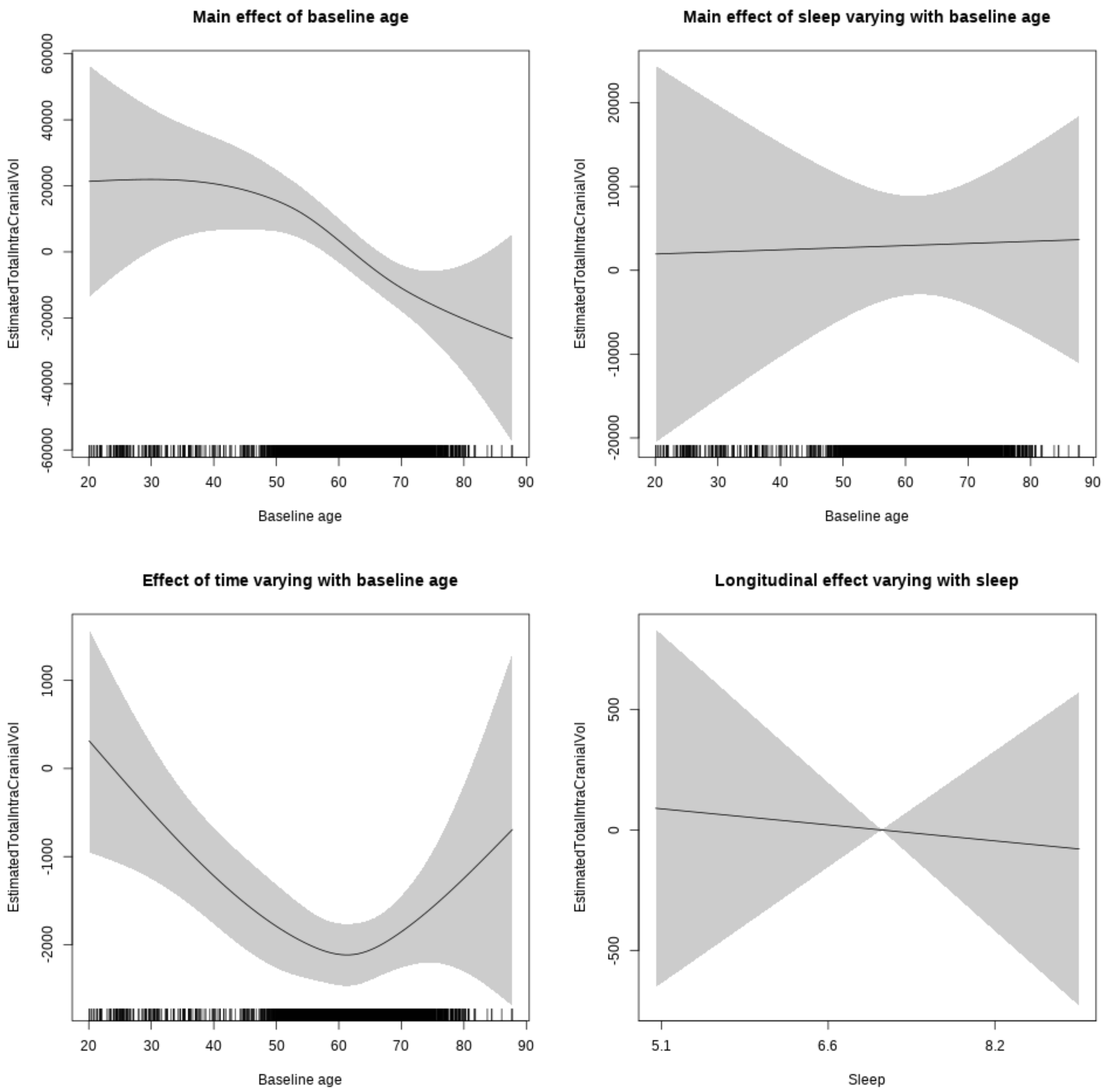

Controling for depression

Full data Below is the model output. The term `s(sleep_z):time` is probably what we care most about.

```
##
## Family: gaussian
## Link function: identity
##
## Formula:
## value ~ sex + site + depression + s(bl_age, k = 10, bs = "cr") +
##     s(bl_age, by = sleep_z, bs = "cr") + +s(bl_age, by = time,
##     k = 5, bs = "cr") + s(sleep_z, by = time, k = 5, bs = "cr",
##     pc = 0)
```

```
## <environment: 0x563911fef868>
##
## Parametric coefficients:
##                Estimate Std. Error t value Pr(>|t|)
## (Intercept)     1214420       9627 126.149  < 2e-16 ***
## sexmale          185634       4890  37.962  < 2e-16 ***
## siteousAvanto    278056      20538  13.539  < 2e-16 ***
## siteousPrisma    345121      23302  14.811  < 2e-16 ***
## siteousSkyra     216900      20160  10.759  < 2e-16 ***
## siteUCAM         245980      12856  19.133  < 2e-16 ***
## siteUKB          248363       9397  26.429  < 2e-16 ***
## siteUmU          140552      16396   8.572  < 2e-16 ***
## depression        58524      20758   2.819  0.00483 **
## ---
## Signif. codes:  0 '***' 0.001 '**' 0.01 '*' 0.05 '.' 0.1 ' ' 1
##
## Approximate significance of smooth terms:
##                      edf Ref.df      F  p-value
## s(bl_age)          3.315  3.315  5.010 0.000832 ***
## s(bl_age):sleep_z 2.000  2.000  1.278 0.278550
## s(bl_age):time    3.648  3.648 53.345  < 2e-16 ***
## s(sleep_z):time   1.000  1.000  0.073 0.786631
## ---
## Signif. codes:  0 '***' 0.001 '**' 0.01 '*' 0.05 '.' 0.1 ' ' 1
##
## R-sq.(adj) =  0.426
## lmer.REML = 1.3826e+05  Scale est. = 1.4137e+08  n = 5683
```

Below are plots of a plot of `s(bl_age)`, `s(bl_age):sleep_z`, `s(bl_age):time`, and `s(sleep_z):time`. For `s(sleep_z):time`, roughly, if we cannot fit a straight line through the confidence bands, there is evidence that the effect of time depends on sleep duration. It is correct the that confidence interval is zero at one point along the axis; this happens because we do not include the main effect.

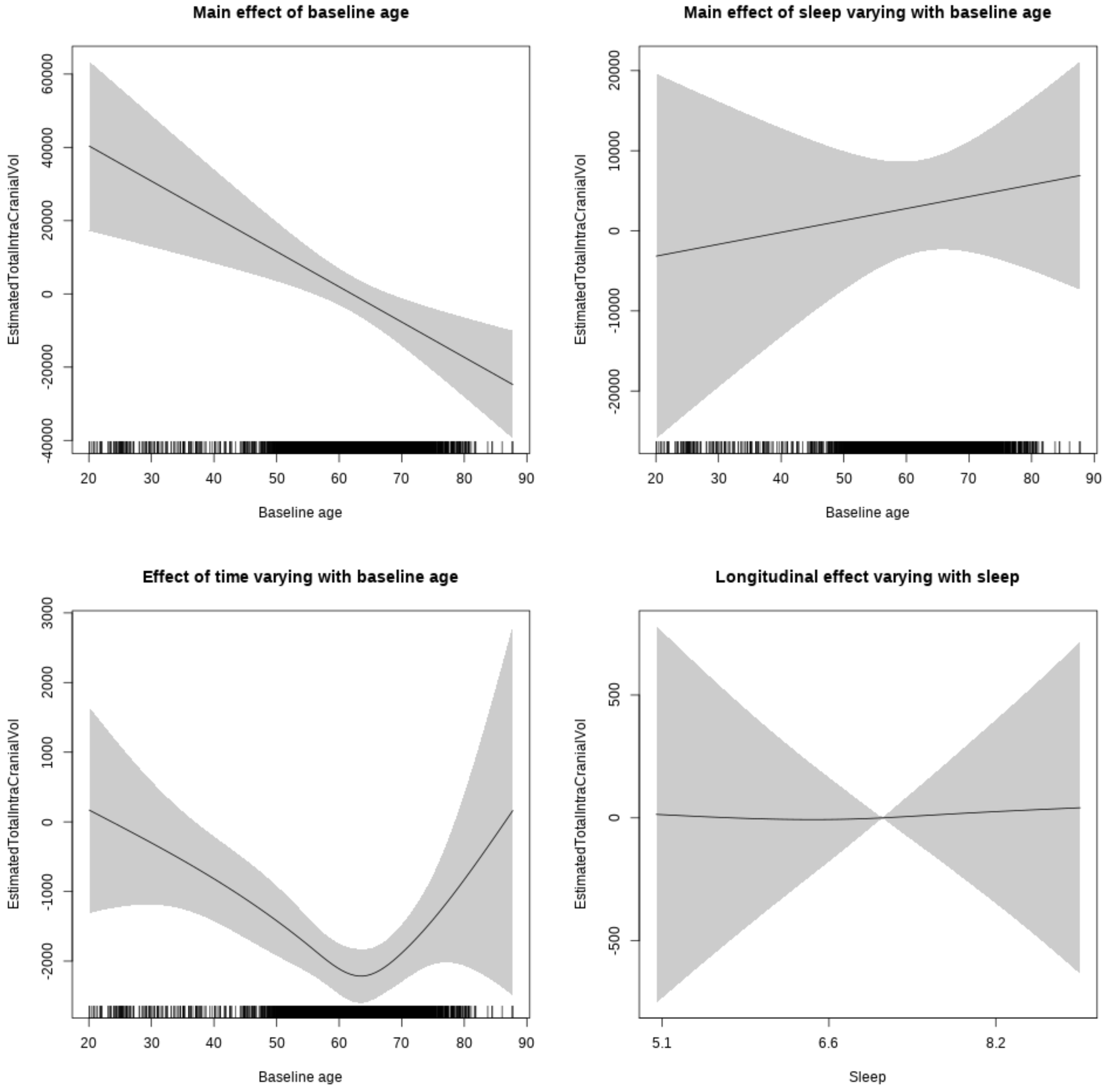

Restricted to sleep >= 5 and <= 9 hours Below is the row for `s(sleep_z):time` from the model output.

```
## s(sleep_z):time   1.067  1.067  0.003 0.977572
```

Below are plots of a plot of `s(bl_age)`, `s(bl_age):sleep_z`, `s(bl_age):time`, and `s(sleep_z):time`. For `s(sleep_z):time`, roughly, if we cannot fit a straight line through the confidence bands, there is evidence that the effect of time depends on sleep duration. It is correct the that confidence interval is zero at one point along the axis; this happens because we do not include the main effect.

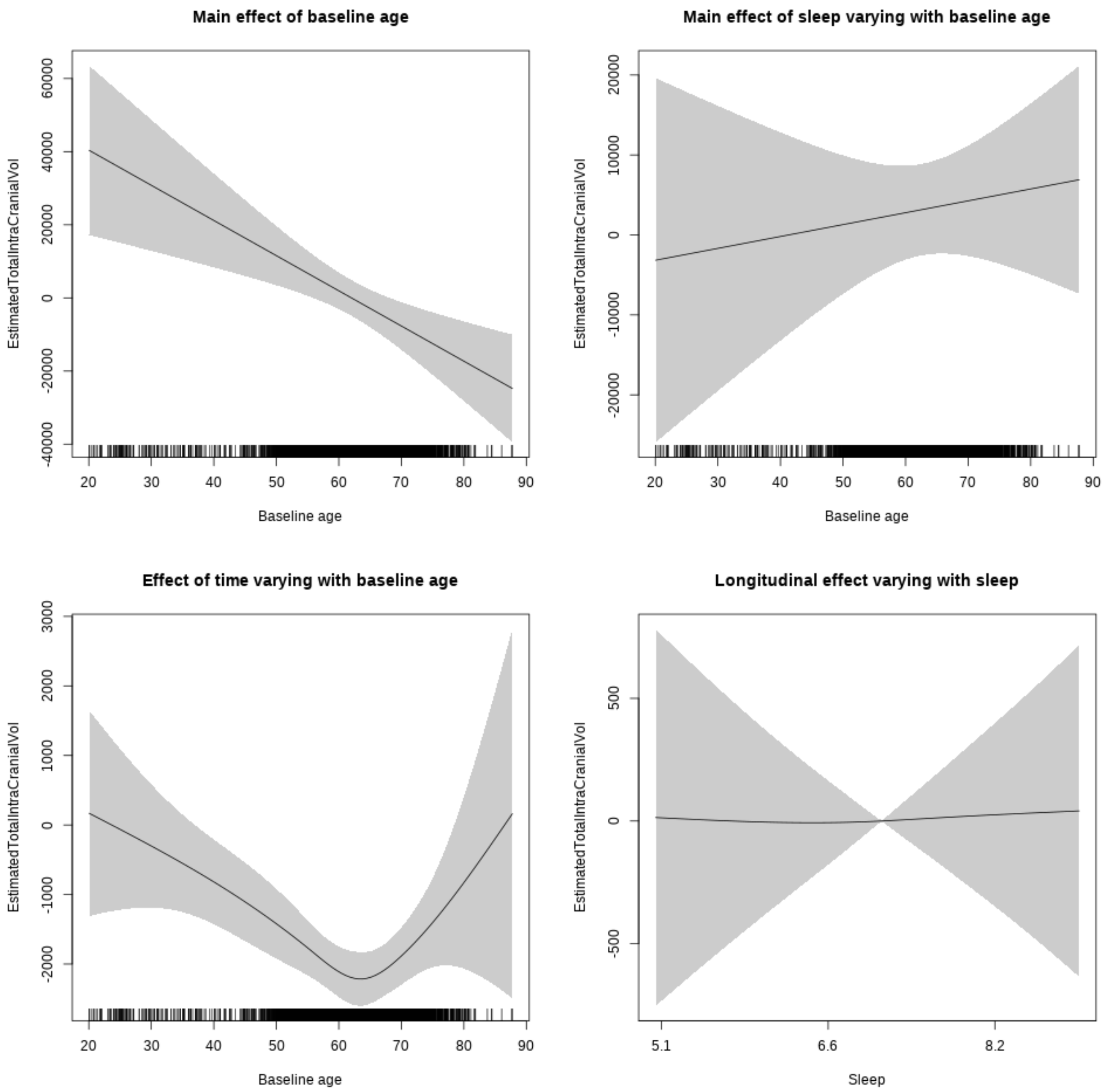

Controling for sleep quality

Full data Below is the model output. The term `s(sleep_z):time` is probably what we care most about.

```
##
## Family: gaussian
## Link function: identity
##
## Formula:
## value ~ X + s(bl_age, k = 10, bs = "cr") + s(bl_age, by = sleep_z,
##     bs = "cr") + s(bl_age, by = time, k = 5, bs = "cr") + s(sleep_z,
##     by = time, k = 5, bs = "cr", pc = 0)
## <environment: 0x563915389488>
```

```
##
## Parametric coefficients:
##                             Estimate Std. Error t value Pr(>|t|)
## (Intercept)                1218247.2    15741.4  77.391  < 2e-16 ***
## Xsexmale                    177387.2     5903.9  30.046  < 2e-16 ***
## XsiteousAvanto              338458.7    14973.4  22.604  < 2e-16 ***
## XsiteousPrisma              413974.1    17488.3  23.671  < 2e-16 ***
## XsiteousSkyra               281067.8    14980.8  18.762  < 2e-16 ***
## XsiteUB                     213377.4    24011.4   8.887  < 2e-16 ***
## XsiteUCAM                   284547.2    15293.9  18.605  < 2e-16 ***
## XsiteUKB                    252347.0    47881.6   5.270 1.43e-07 ***
## XsiteUmU                    196492.9    17118.9  11.478  < 2e-16 ***
## Xukb_dummy:sleep_accel        -759.6     3709.7  -0.205   0.8378
## Xukb_dummy:chronotype         5779.3     4939.0   1.170   0.2420
## Xukb_dummy:dozing            14355.5    10207.1   1.406   0.1597
## Xukb_dummy:sleeplessness    -12091.0     6349.0  -1.904   0.0569 .
## Xukb_dummy:snoring            9478.5     9009.8   1.052   0.2929
## Xukb_dummy:gettingup          -485.3     6892.1  -0.070   0.9439
## Xnotukb_dummy:PSQI_Global    -4948.4     1953.2  -2.533   0.0113 *
## ---
## Signif. codes:  0 ‘***’ 0.001 ‘**’ 0.01 ‘*’ 0.05 ‘.’ 0.1 ‘ ’ 1
##
## Approximate significance of smooth terms:
##                      edf Ref.df      F p-value
## s(bl_age)          2.157  2.157  4.793 0.00536 **
## s(bl_age):sleep_z 2.000  2.000  0.460 0.63143
## s(bl_age):time    2.000  2.000 22.682 < 2e-16 ***
## s(sleep_z):time   1.000  1.000  0.000 0.98501
## ---
## Signif. codes:  0 ‘***’ 0.001 ‘**’ 0.01 ‘*’ 0.05 ‘.’ 0.1 ‘ ’ 1
##
## R-sq.(adj) =  0.427
## lmer.REML = 1.0329e+05  Scale est. = 1.365e+08  n = 4290
```

Below are plots of a plot of `s(bl_age)`, `s(bl_age):sleep_z`, `s(bl_age):time`, and `s(sleep_z):time`. For `s(sleep_z):time`, roughly, if we cannot fit a straight line through the confidence bands, there is evidence that the effect of time depends on sleep duration. It is correct the that confidence interval is zero at one point along the axis; this happens because we do not include the main effect.

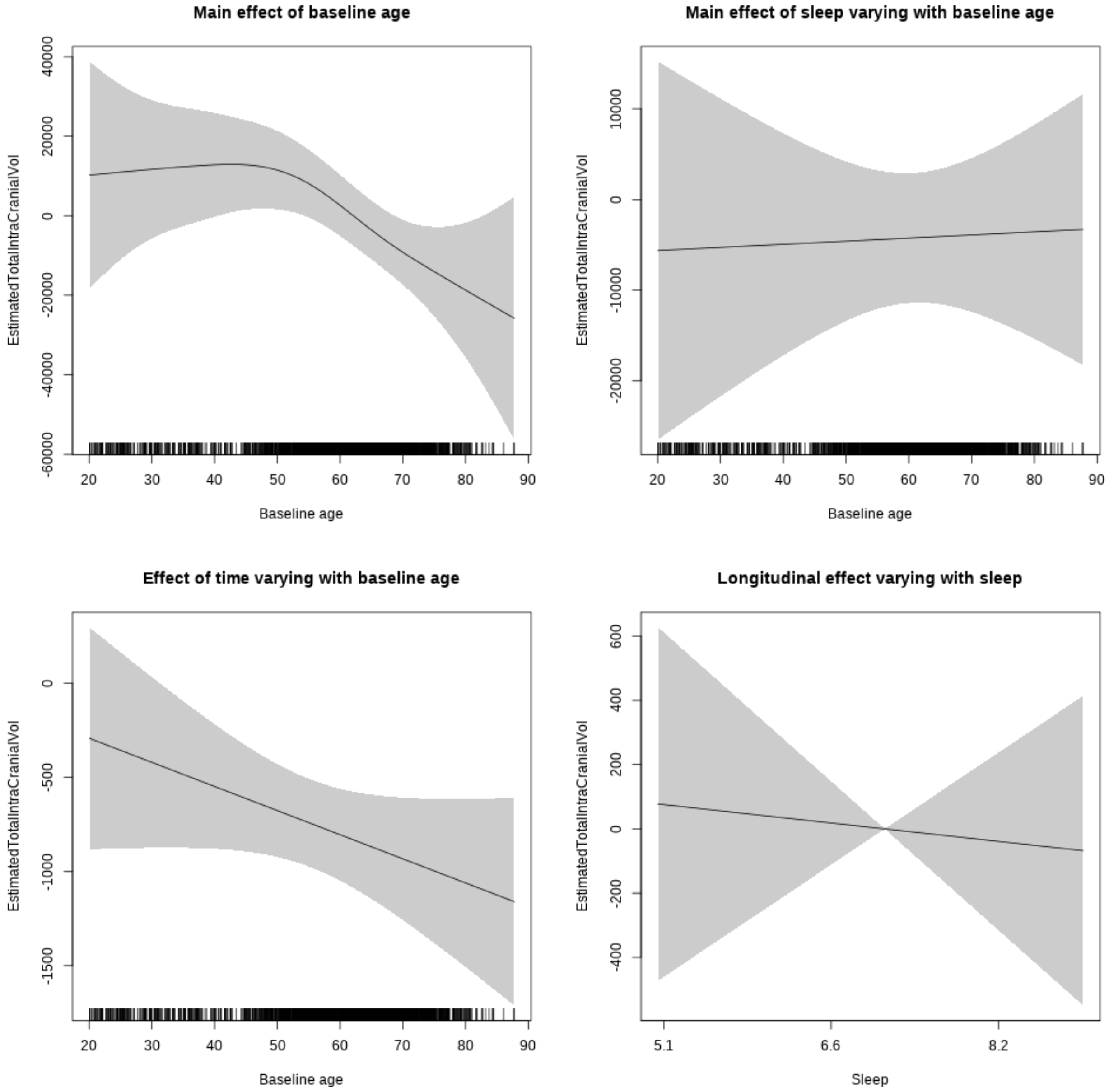

Restricted to sleep >= 5 and <= 9 hours Below is the row for `s(sleep_z):time` from the model output.

```
## s(sleep_z):time   1.000  1.000  0.079 0.77828
```

Below are plots of a plot of `s(bl_age)`, `s(bl_age):sleep_z`, `s(bl_age):time`, and `s(sleep_z):time`. For `s(sleep_z):time`, roughly, if we cannot fit a straight line through the confidence bands, there is evidence that the effect of time depends on sleep duration. It is correct the that confidence interval is zero at one point along the axis; this happens because we do not include the main effect.

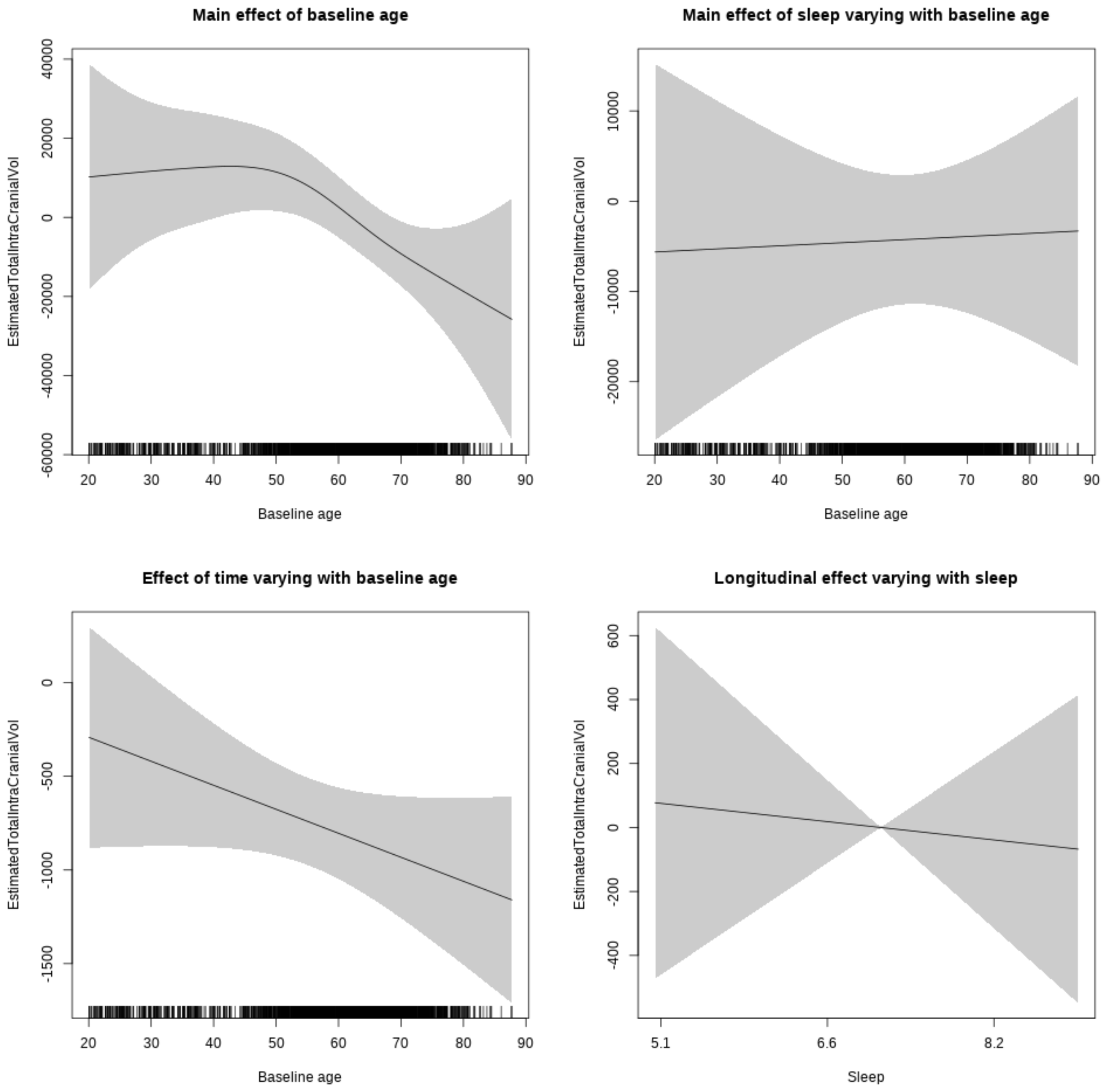

Hippocampus

Main analysis

Full data Below is the model output. The term `s(sleep_z):time` is probably what we care most about.

```
##
## Family: gaussian
## Link function: identity
##
## Formula:
## value ~ sex + site + icv + s(bl_age, k = 10, bs = "cr") + s(bl_age,
##     by = sleep_z, bs = "cr") + +s(bl_age, by = time, k = 5, bs = "cr") +
```

```
##     s(sleep_z, by = time, k = 5, bs = "cr", pc = 0)
## <environment: 0x56390da03b10>
##
## Parametric coefficients:
##                Estimate Std. Error t value Pr(>|t|)
## (Intercept)     8494.38      47.48 178.899  < 2e-16 ***
## sexmale           71.91      24.42   2.945 0.003240 **
## siteousAvanto   -843.25      58.21 -14.486  < 2e-16 ***
## siteousPrisma   -873.64     129.01  -6.772 1.36e-11 ***
## siteousSkyra    -552.16      56.69  -9.740  < 2e-16 ***
## siteUB          -417.88     110.86  -3.769 0.000165 ***
## siteUCAM        -481.02      59.91  -8.028 1.13e-15 ***
## siteUKB         -363.10      45.47  -7.986 1.59e-15 ***
## siteUmU         -305.70      68.65  -4.453 8.58e-06 ***
## icv              397.84      11.50  34.584  < 2e-16 ***
## ---
## Signif. codes:  0 '***' 0.001 '**' 0.01 '*' 0.05 '.' 0.1 ' ' 1
##
## Approximate significance of smooth terms:
##                      edf Ref.df       F p-value
## s(bl_age)          4.863  4.863 229.766 < 2e-16 ***
## s(bl_age):sleep_z  2.000  2.000   4.914 0.00736 **
## s(bl_age):time     4.166  4.166 298.865 < 2e-16 ***
## s(sleep_z):time    1.800  1.800   1.997 0.23218
## ---
## Signif. codes:  0 '***' 0.001 '**' 0.01 '*' 0.05 '.' 0.1 ' ' 1
##
## R-sq.(adj) =  0.443
## lmer.REML = 1.1878e+05  Scale est. = 22110     n = 8150
```

Below are plots of a plot of `s(bl_age)`, `s(bl_age):sleep_z`, `s(bl_age):time`, and `s(sleep_z):time`. For `s(sleep_z):time`, roughly, if we cannot fit a straight line through the confidence bands, there is evidence that the effect of time depends on sleep duration. It is correct the that confidence interval is zero at one point along the axis; this happens because we do not include the main effect.

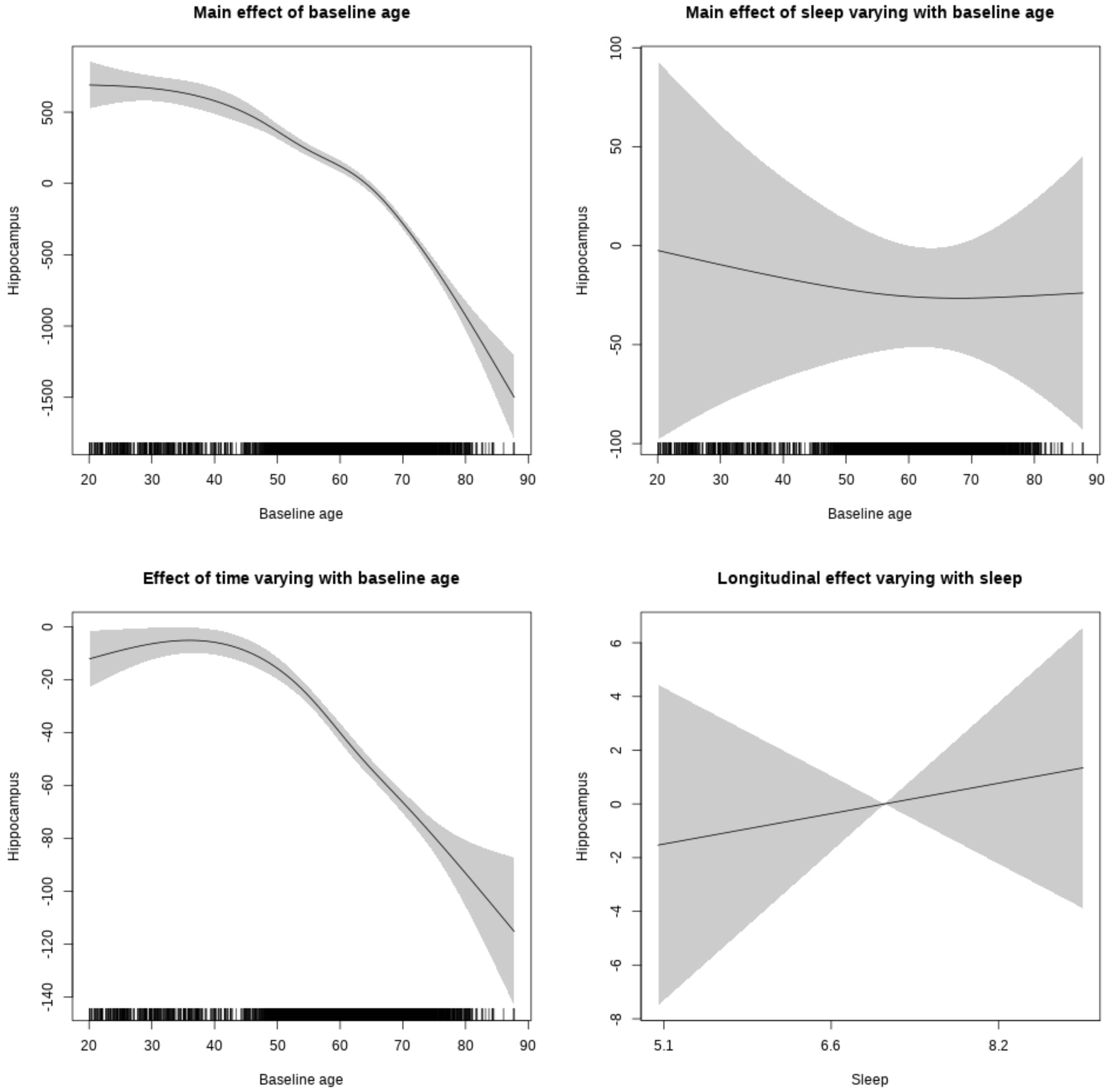

Restricted to sleep >= 5 and <= 9 hours Below is the row for `s(sleep_z):time` from the model output.

```
## s(sleep_z):time   1.000  1.000   0.264   0.608
```

Below are plots of a plot of `s(bl_age)`, `s(bl_age):sleep_z`, `s(bl_age):time`, and `s(sleep_z):time`. For `s(sleep_z):time`, roughly, if we cannot fit a straight line through the confidence bands, there is evidence that the effect of time depends on sleep duration. It is correct the that confidence interval is zero at one point along the axis; this happens because we do not include the main effect.

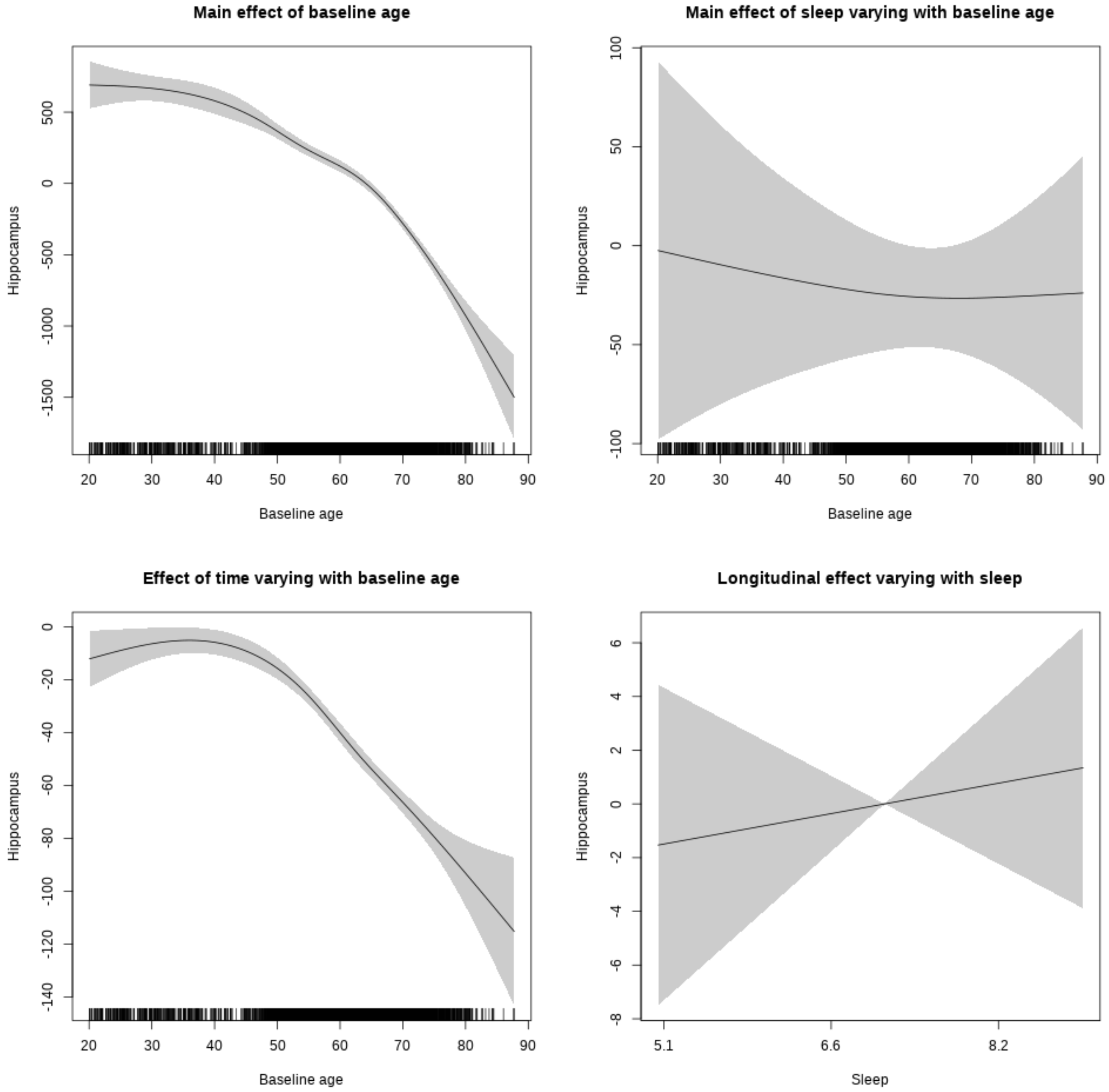

Controling for socioeconomic status

Full data Below is the model output. The term `s(sleep_z):time` is probably what we care most about.

```
##
## Family: gaussian
## Link function: identity
##
## Formula:
## value ~ sex + site + income_scaled + education_scaled + icv +
##     s(bl_age, k = 10, bs = "cr") + s(bl_age, by = sleep_z, bs = "cr") +
##     +s(bl_age, by = time, k = 5, bs = "cr") + s(sleep_z, by = time,
##     k = 5, bs = "cr", pc = 0)
```

```
## <environment: 0x56391158aa40>
##
## Parametric coefficients:
##                  Estimate Std. Error t value Pr(>|t|)
## (Intercept)       8615.66     112.85  76.348  < 2e-16 ***
## sexmale             33.10      30.83   1.074    0.283
## siteousAvanto     -979.54     121.58  -8.056 9.91e-16 ***
## siteousPrisma    -1186.51     220.66  -5.377 7.94e-08 ***
## siteousSkyra      -625.47     115.29  -5.425 6.08e-08 ***
## siteUKB           -491.63     106.98  -4.596 4.43e-06 ***
## income_scaled       45.06      39.85   1.131    0.258
## education_scaled   -24.10      44.02  -0.548    0.584
## icv                452.86      14.99  30.213  < 2e-16 ***
## ---
## Signif. codes:  0 '***' 0.001 '**' 0.01 '*' 0.05 '.' 0.1 ' ' 1
##
## Approximate significance of smooth terms:
##                     edf Ref.df       F p-value
## s(bl_age)         3.676  3.676 100.186  <2e-16 ***
## s(bl_age):sleep_z 2.000  2.000   1.245   0.288
## s(bl_age):time    3.728  3.728 131.022  <2e-16 ***
## s(sleep_z):time   2.326  2.326   1.883   0.151
## ---
## Signif. codes:  0 '***' 0.001 '**' 0.01 '*' 0.05 '.' 0.1 ' ' 1
##
## R-sq.(adj) =  0.426
## lmer.REML =  67844  Scale est. = 23122     n = 4646
```

Below are plots of a plot of `s(bl_age)`, `s(bl_age):sleep_z`, `s(bl_age):time`, and `s(sleep_z):time`. For `s(sleep_z):time`, roughly, if we cannot fit a straight line through the confidence bands, there is evidence that the effect of time depends on sleep duration. It is correct the that confidence interval is zero at one point along the axis; this happens because we do not include the main effect.

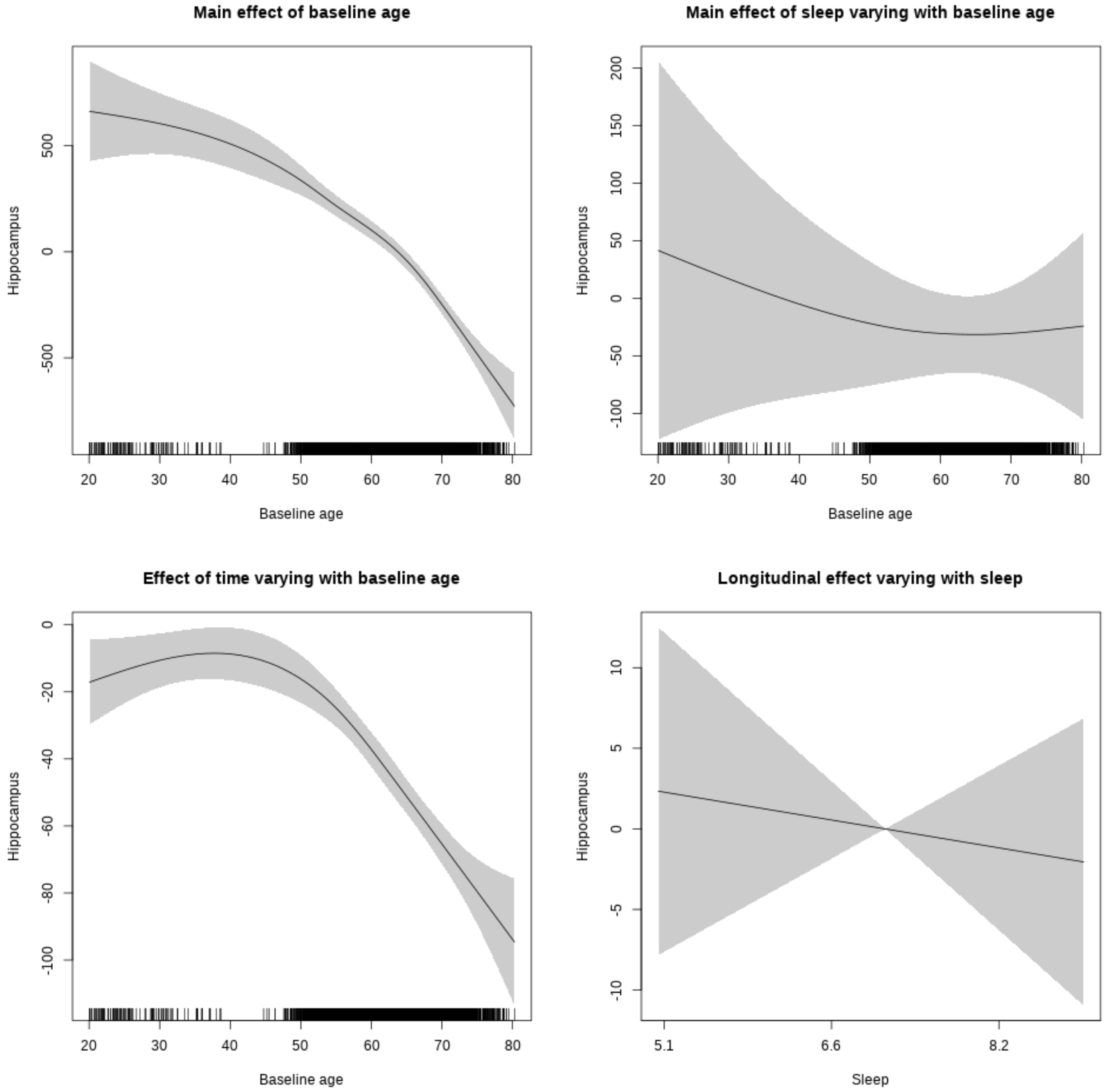

Restricted to sleep >= 5 and <= 9 hours Below is the row for `s(sleep_z):time` from the model output.

```
## s(sleep_z):time   1.000  1.000   0.212   0.645
```

Below are plots of a plot of `s(bl_age)`, `s(bl_age):sleep_z`, `s(bl_age):time`, and `s(sleep_z):time`. For `s(sleep_z):time`, roughly, if we cannot fit a straight line through the confidence bands, there is evidence that the effect of time depends on sleep duration. It is correct the that confidence interval is zero at one point along the axis; this happens because we do not include the main effect.

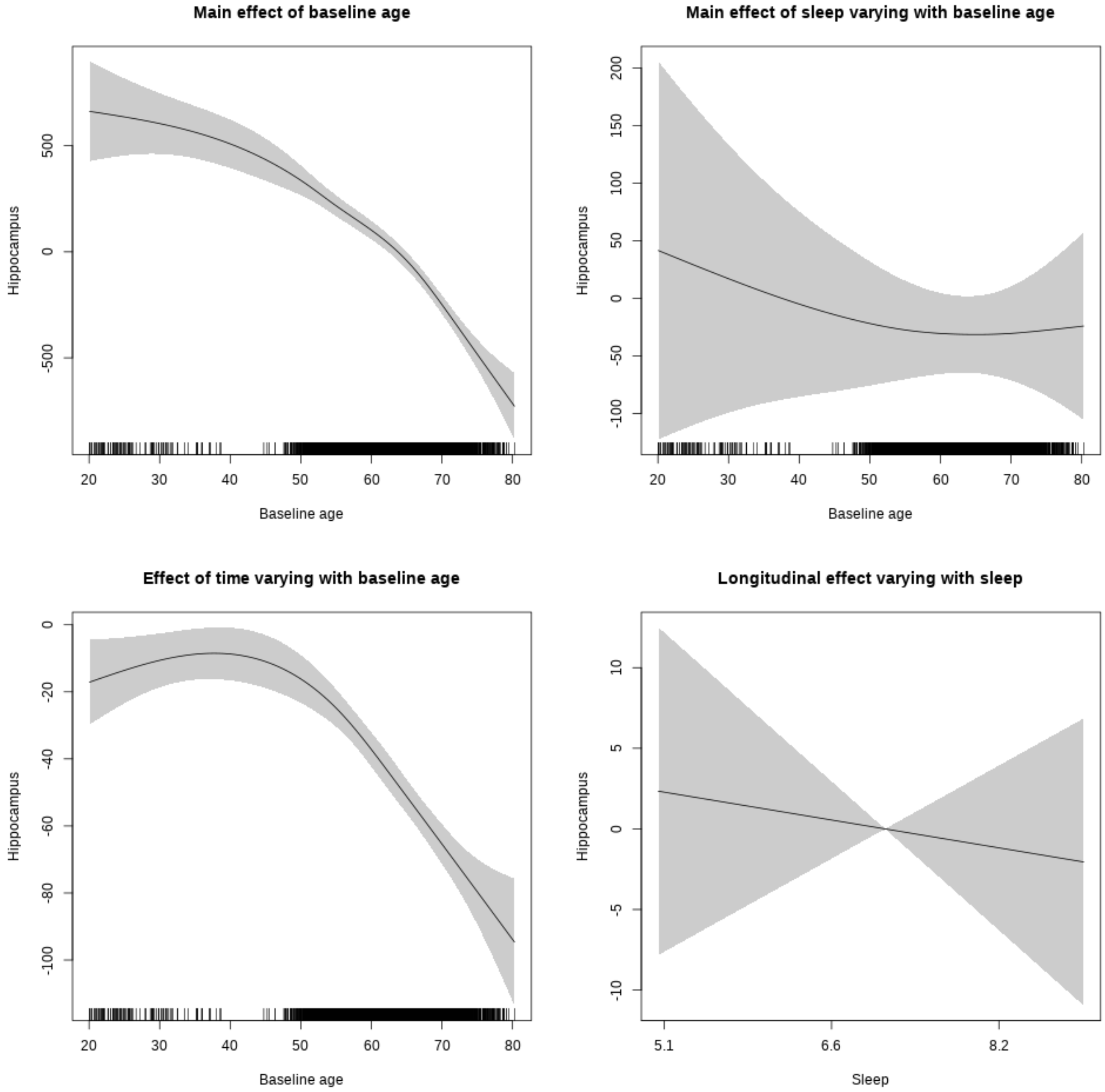

Controling for BMI

Full data Below is the model output. The term `s(sleep_z):time` is probably what we care most about.

```
##
## Family: gaussian
## Link function: identity
##
## Formula:
## value ~ sex + site + bmi + icv + s(bl_age, k = 10, bs = "cr") +
##     s(bl_age, by = sleep_z, bs = "cr") + +s(bl_age, by = time,
##     k = 5, bs = "cr") + s(sleep_z, by = time, k = 5, bs = "cr",
##     pc = 0)
```

```
## <environment: 0x563916b210c0>
##
## Parametric coefficients:
##                Estimate Std. Error t value Pr(>|t|)
## (Intercept)   7626.4513   101.6712  75.011  < 2e-16 ***
## sexmale         46.1591    29.2421   1.579    0.115
## siteousPrisma   -0.9683   121.2842  -0.008    0.994
## siteousSkyra   377.2601    38.8418   9.713  < 2e-16 ***
## siteUCAM       384.7857    75.7643   5.079 3.93e-07 ***
## siteUKB        458.8471    69.9005   6.564 5.72e-11 ***
## siteUmU        578.6973    87.4513   6.617 4.01e-11 ***
## bmi              0.9308     3.0534   0.305    0.761
## icv            442.0685    14.1570  31.226  < 2e-16 ***
## ---
## Signif. codes:  0 '***' 0.001 '**' 0.01 '*' 0.05 '.' 0.1 ' ' 1
##
## Approximate significance of smooth terms:
##                     edf Ref.df       F p-value
## s(bl_age)         4.269  4.269 144.147  <2e-16 ***
## s(bl_age):sleep_z 2.000  2.000   0.927   0.396
## s(bl_age):time    4.037  4.037 143.559  <2e-16 ***
## s(sleep_z):time   1.863  1.863   1.331   0.283
## ---
## Signif. codes:  0 '***' 0.001 '**' 0.01 '*' 0.05 '.' 0.1 ' ' 1
##
## R-sq.(adj) =   0.43
## lmer.REML =  79045  Scale est. = 22153     n = 5408
```

Below are plots of a plot of `s(bl_age)`, `s(bl_age):sleep_z`, `s(bl_age):time`, and `s(sleep_z):time`. For `s(sleep_z):time`, roughly, if we cannot fit a straight line through the confidence bands, there is evidence that the effect of time depends on sleep duration. It is correct the that confidence interval is zero at one point along the axis; this happens because we do not include the main effect.

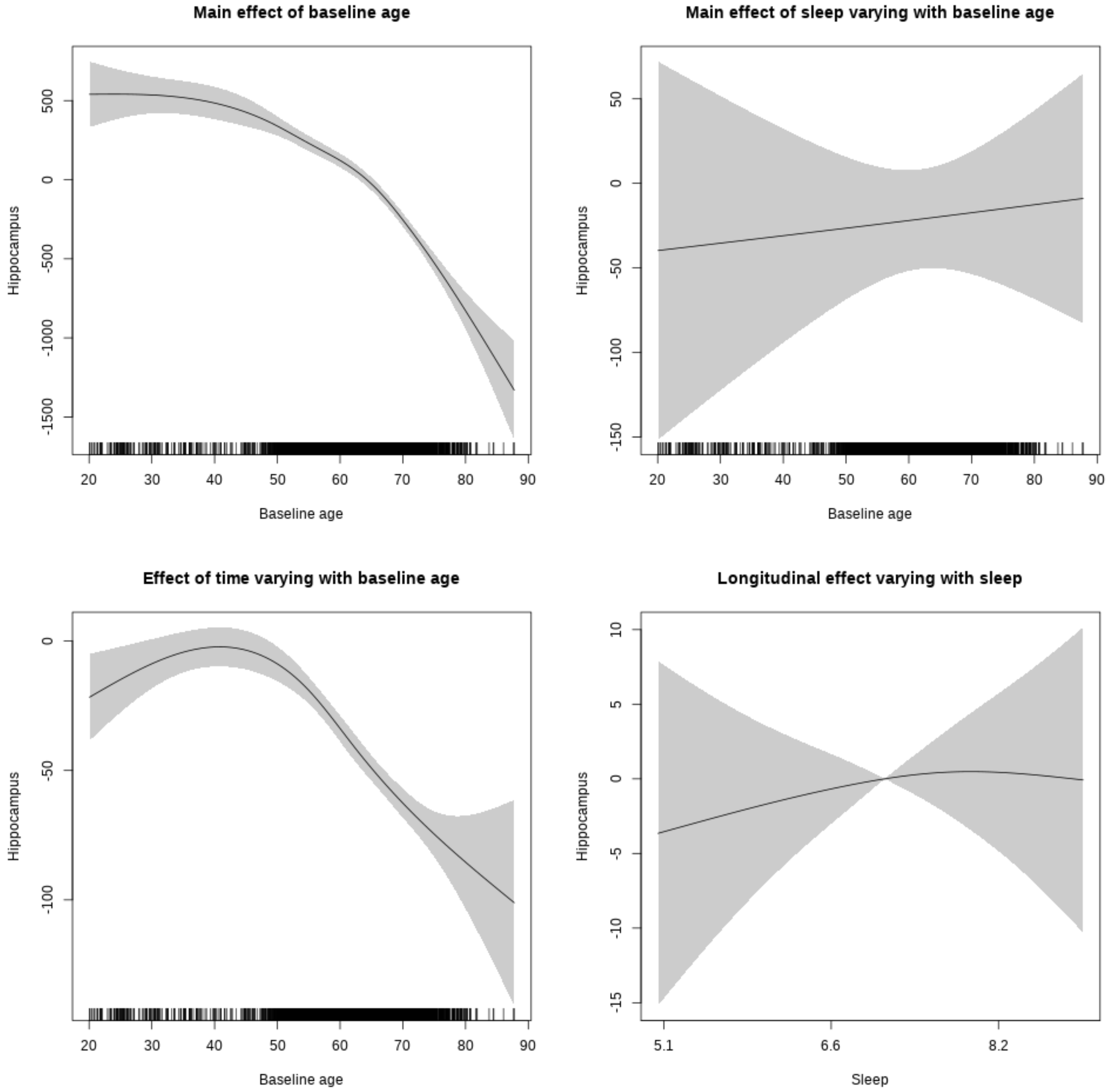

Restricted to sleep >= 5 and <= 9 hours Below is the row for `s(sleep_z):time` from the model output.

```
## s(sleep_z):time   1.236  1.236   0.341   0.737
```

Below are plots of a plot of `s(bl_age)`, `s(bl_age):sleep_z`, `s(bl_age):time`, and `s(sleep_z):time`. For `s(sleep_z):time`, roughly, if we cannot fit a straight line through the confidence bands, there is evidence that the effect of time depends on sleep duration. It is correct the that confidence interval is zero at one point along the axis; this happens because we do not include the main effect.

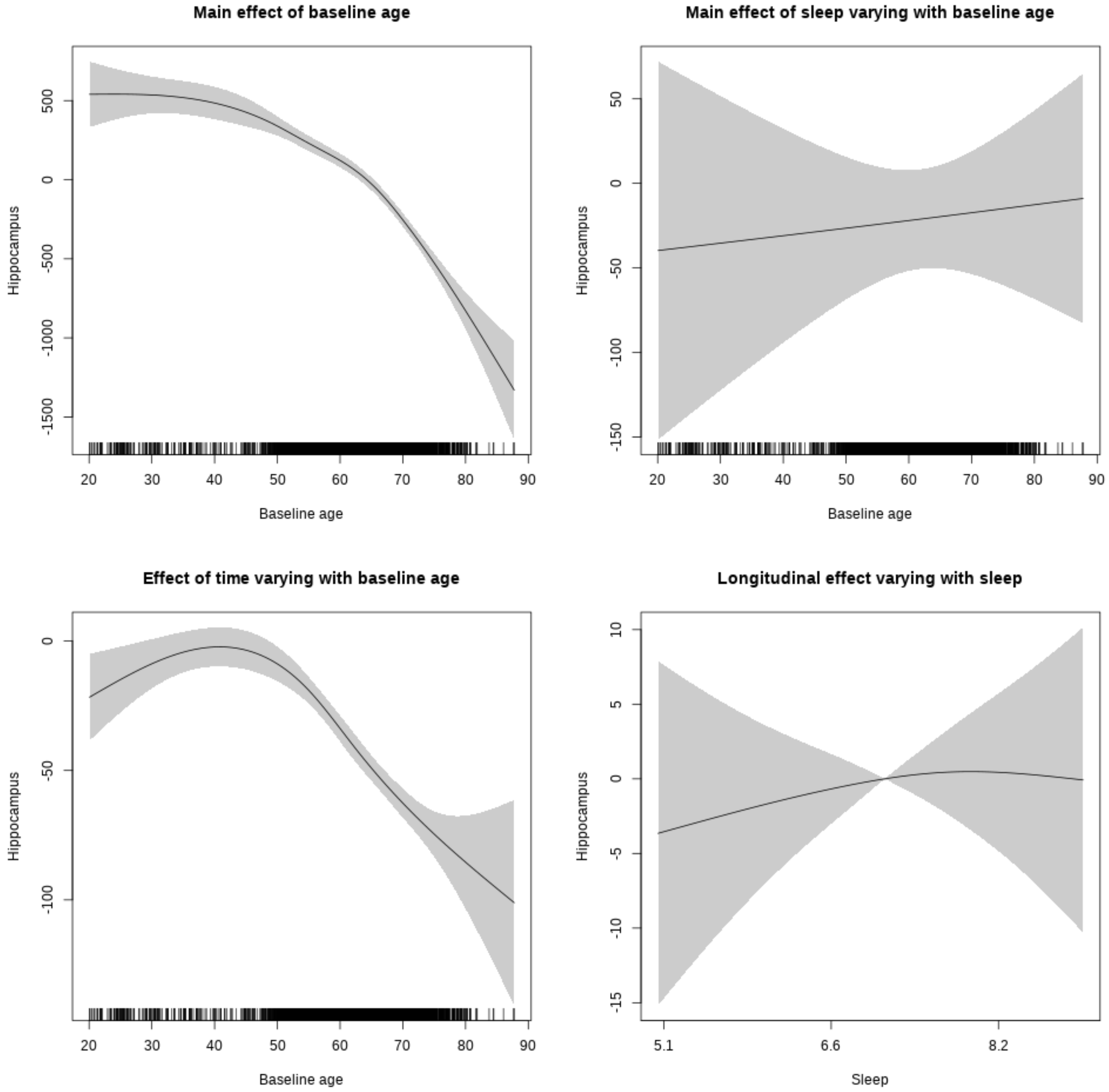

Controling for depression

Full data Below is the model output. The term `s(sleep_z):time` is probably what we care most about.

```
##
## Family: gaussian
## Link function: identity
##
## Formula:
## value ~ sex + site + depression + icv + s(bl_age, k = 10, bs = "cr") +
##     s(bl_age, by = sleep_z, bs = "cr") + +s(bl_age, by = time,
##     k = 5, bs = "cr") + s(sleep_z, by = time, k = 5, bs = "cr",
##     pc = 0)
```

```
## <environment: 0x563910fa6960>
##
## Parametric coefficients:
##                Estimate Std. Error t value Pr(>|t|)
## (Intercept)    8495.52      56.08 151.484  < 2e-16 ***
## sexmale          40.26      29.28   1.375 0.169184
## siteousAvanto  -828.88     114.71  -7.226 5.63e-13 ***
## siteousPrisma  -656.52     178.58  -3.676 0.000239 ***
## siteousSkyra   -428.16     103.20  -4.149 3.39e-05 ***
## siteUCAM       -418.75      68.34  -6.127 9.56e-10 ***
## siteUKB        -376.04      51.72  -7.271 4.06e-13 ***
## siteUmU        -124.89      82.73  -1.510 0.131214
## depression     -358.71     103.78  -3.456 0.000552 ***
## icv             411.32      13.86  29.676  < 2e-16 ***
## ---
## Signif. codes:  0 '***' 0.001 '**' 0.01 '*' 0.05 '.' 0.1 ' ' 1
##
## Approximate significance of smooth terms:
##                      edf Ref.df       F p-value
## s(bl_age)          4.724  4.724 138.834  <2e-16 ***
## s(bl_age):sleep_z 2.000  2.000   3.771  0.0231 *
## s(bl_age):time    3.835  3.835 159.842  <2e-16 ***
## s(sleep_z):time   1.974  1.974   1.755  0.1822
## ---
## Signif. codes:  0 '***' 0.001 '**' 0.01 '*' 0.05 '.' 0.1 ' ' 1
##
## R-sq.(adj) =  0.415
## lmer.REML =  83105  Scale est. = 21886     n = 5675
```

Below are plots of a plot of `s(bl_age)`, `s(bl_age):sleep_z`, `s(bl_age):time`, and `s(sleep_z):time`. For `s(sleep_z):time`, roughly, if we cannot fit a straight line through the confidence bands, there is evidence that the effect of time depends on sleep duration. It is correct the that confidence interval is zero at one point along the axis; this happens because we do not include the main effect.

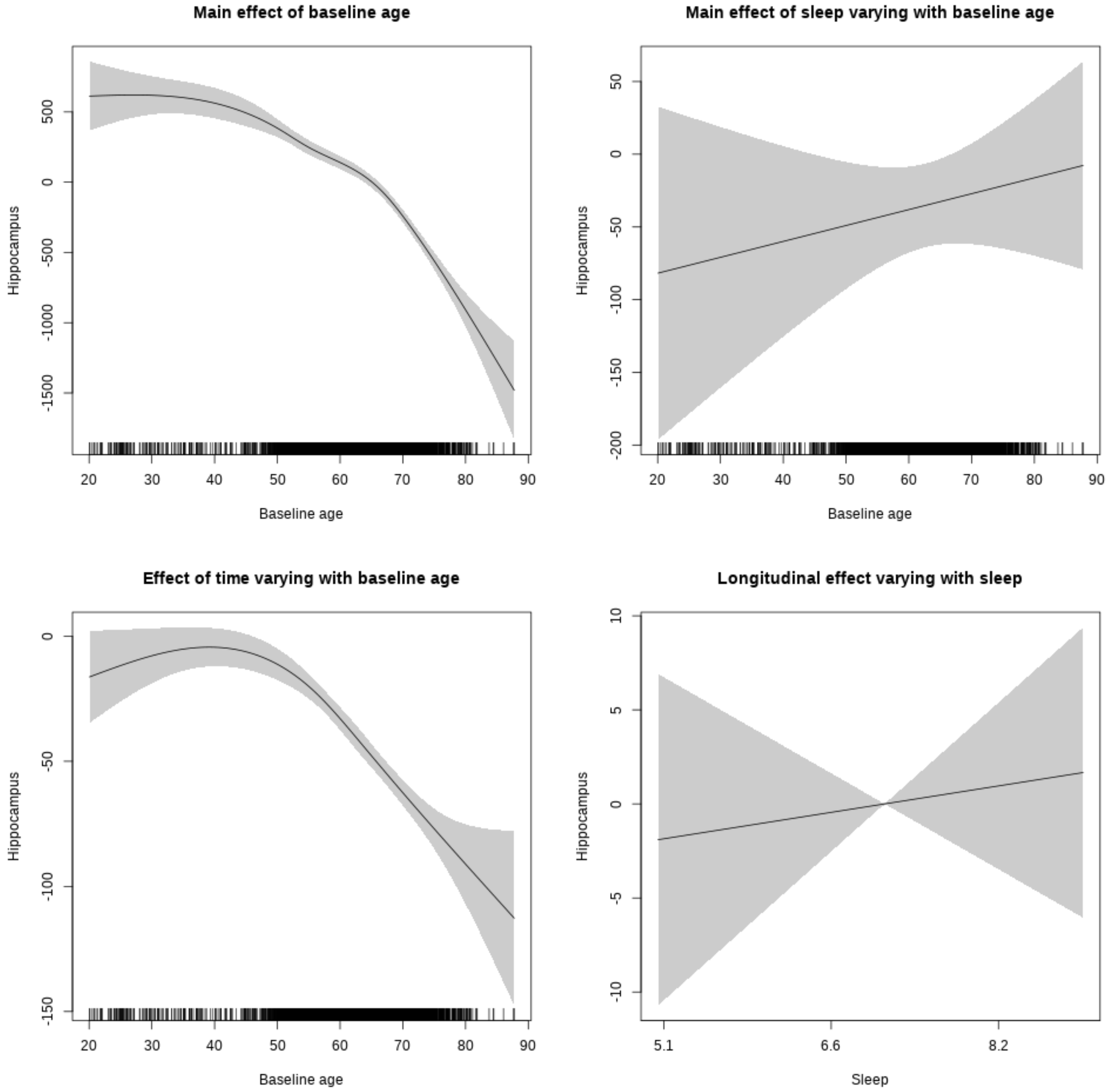

Restricted to sleep >= 5 and <= 9 hours Below is the row for `s(sleep_z):time` from the model output.

```
## s(sleep_z):time   1.000  1.000   0.186  0.6660
```

Below are plots of a plot of `s(bl_age)`, `s(bl_age):sleep_z`, `s(bl_age):time`, and `s(sleep_z):time`. For `s(sleep_z):time`, roughly, if we cannot fit a straight line through the confidence bands, there is evidence that the effect of time depends on sleep duration. It is correct the that confidence interval is zero at one point along the axis; this happens because we do not include the main effect.

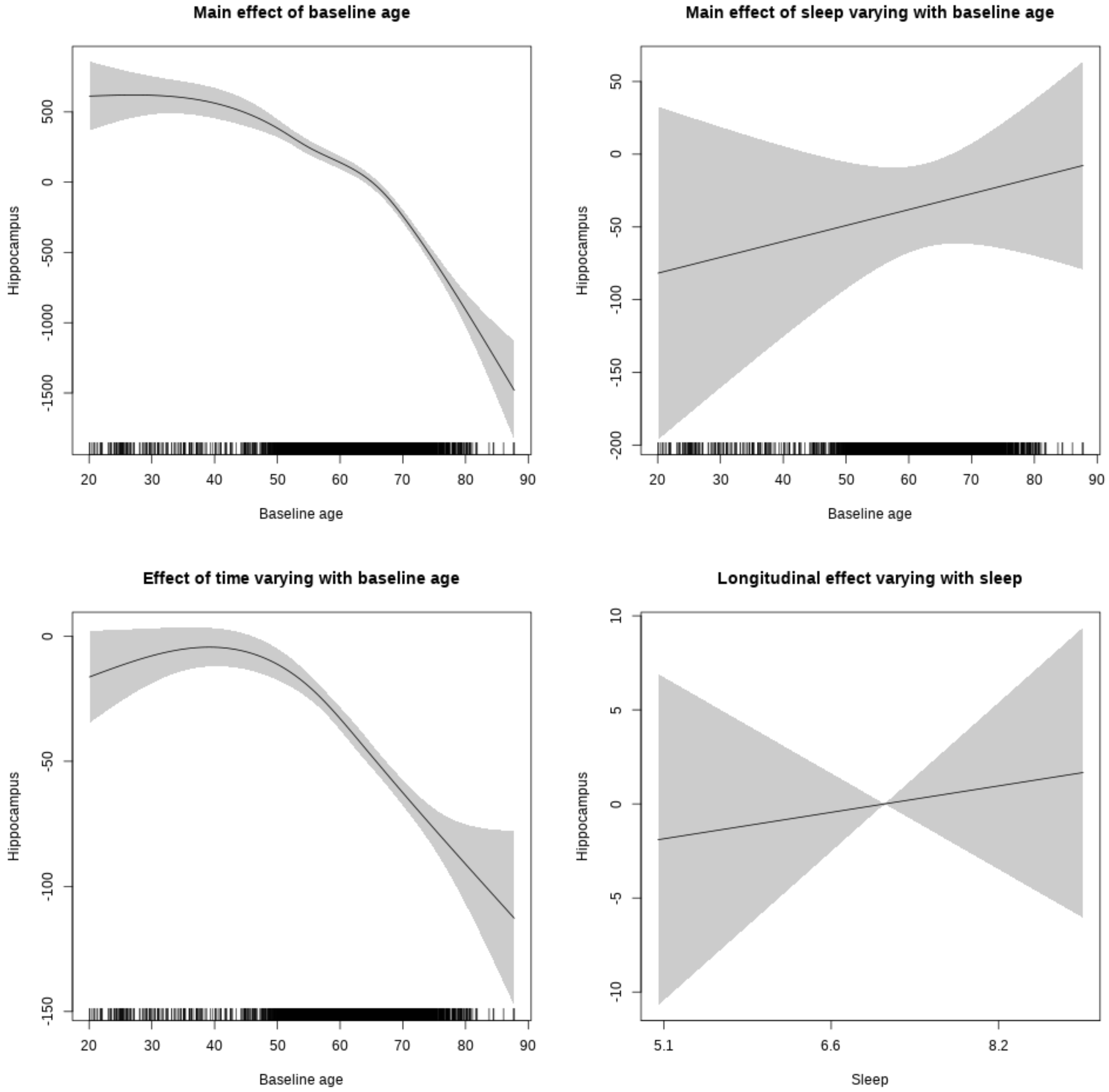

Controling for sleep quality

Full data Below is the model output. The term `s(sleep_z):time` is probably what we care most about.

```
##
## Family: gaussian
## Link function: identity
##
## Formula:
## value ~ X + s(bl_age, k = 10, bs = "cr") + s(bl_age, by = sleep_z,
##     bs = "cr") + s(bl_age, by = time, k = 5, bs = "cr") + s(sleep_z,
##     by = time, k = 5, bs = "cr", pc = 0)
## <environment: 0x563914d995c8>
```

```
##
## Parametric coefficients:
##                           Estimate Std. Error t value Pr(>|t|)
## (Intercept)               7711.273     91.404  84.364  < 2e-16 ***
## Xsexmale                   504.237     34.169  14.757  < 2e-16 ***
## XsiteousAvanto              32.488     87.323   0.372  0.70988
## XsiteousPrisma             183.249    142.988   1.282  0.20006
## XsiteousSkyra              179.076     87.515   2.046  0.04080 *
## XsiteUB                    124.095    138.851   0.894  0.37152
## XsiteUCAM                  233.726     89.777   2.603  0.00926 **
## XsiteUKB                   340.495    278.427   1.223  0.22143
## XsiteUmU                   220.617     99.523   2.217  0.02669 *
## Xukb_dummy:sleep_accel       3.659     21.472   0.170  0.86471
## Xukb_dummy:chronotype       -5.275     28.615  -0.184  0.85376
## Xukb_dummy:dozing           64.470     59.089   1.091  0.27531
## Xukb_dummy:sleeplessness   -60.274     36.842  -1.636  0.10191
## Xukb_dummy:snoring          15.768     52.172   0.302  0.76249
## Xukb_dummy:gettingup        -2.671     40.079  -0.067  0.94687
## Xnotukb_dummy:PSQI_Global  -14.485     11.312  -1.281  0.20041
## ---
## Signif. codes:  0 '***' 0.001 '**' 0.01 '*' 0.05 '.' 0.1 ' ' 1
##
## Approximate significance of smooth terms:
##                     edf Ref.df       F p-value
## s(bl_age)         3.982  3.982 135.628  <2e-16 ***
## s(bl_age):sleep_z 2.000  2.000   3.135  0.0436 *
## s(bl_age):time    4.197  4.197 242.666  <2e-16 ***
## s(sleep_z):time   1.000  1.000   0.829  0.3625
## ---
## Signif. codes:  0 '***' 0.001 '**' 0.01 '*' 0.05 '.' 0.1 ' ' 1
##
## R-sq.(adj) =  0.314
## lmer.REML =  62678  Scale est. = 21687     n = 4288
```

Below are plots of a plot of `s(bl_age)`, `s(bl_age):sleep_z`, `s(bl_age):time`, and `s(sleep_z):time`. For `s(sleep_z):time`, roughly, if we cannot fit a straight line through the confidence bands, there is evidence that the effect of time depends on sleep duration. It is correct the that confidence interval is zero at one point along the axis; this happens because we do not include the main effect.

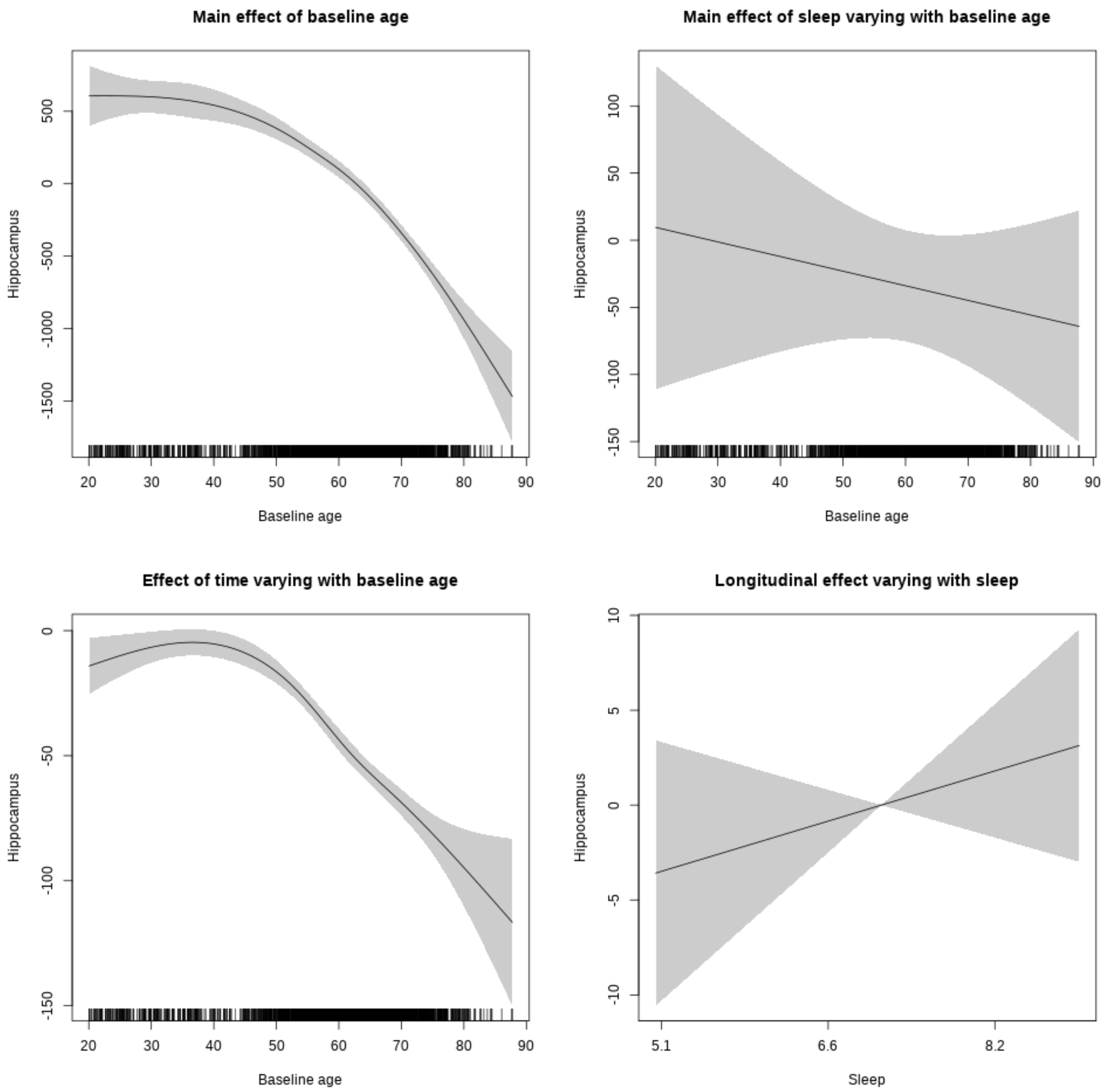

Restricted to sleep >= 5 and <= 9 hours Below is the row for `s(sleep_z):time` from the model output.

```
## s(sleep_z):time   1.000  1.000   1.049   0.306
```

Below are plots of a plot of `s(bl_age)`, `s(bl_age):sleep_z`, `s(bl_age):time`, and `s(sleep_z):time`. For `s(sleep_z):time`, roughly, if we cannot fit a straight line through the confidence bands, there is evidence that the effect of time depends on sleep duration. It is correct the that confidence interval is zero at one point along the axis; this happens because we do not include the main effect.

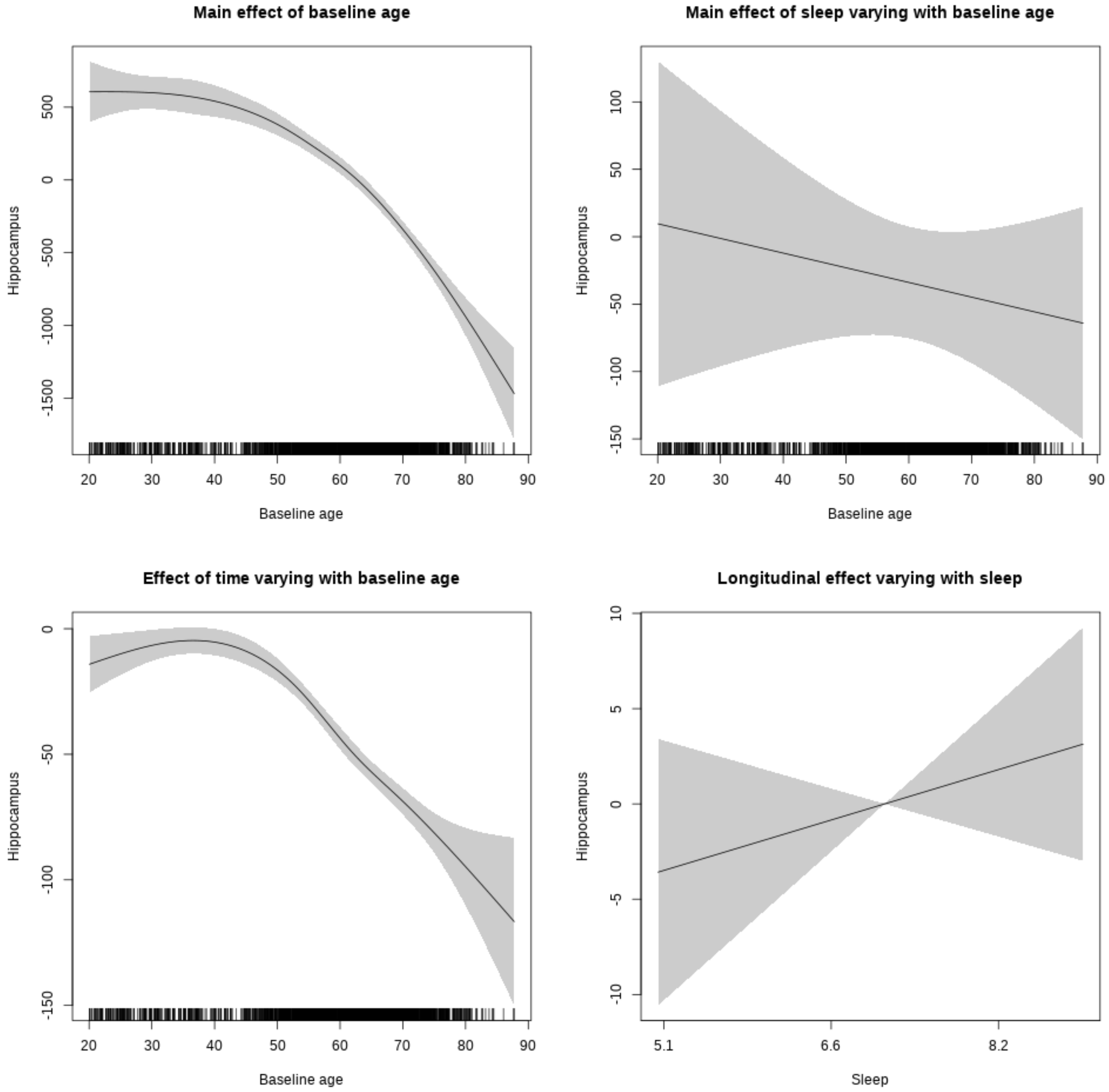

Pallidum

Main analysis

Full data Below is the model output. The term `s(sleep_z):time` is probably what we care most about.

```
##
## Family: gaussian
## Link function: identity
##
## Formula:
## value ~ sex + site + icv + s(bl_age, k = 10, bs = "cr") + s(bl_age,
##     by = sleep_z, bs = "cr") + +s(bl_age, by = time, k = 5, bs = "cr") +
```

```
##     s(sleep_z, by = time, k = 5, bs = "cr", pc = 0)
## <environment: 0x563911250488>
##
## Parametric coefficients:
##                Estimate Std. Error t value Pr(>|t|)
## (Intercept)    4118.887     25.109 164.038  < 2e-16 ***
## sexmale          95.577     13.079   7.308 2.97e-13 ***
## siteousAvanto  -410.669     31.179 -13.171  < 2e-16 ***
## siteousPrisma  -552.765     87.023  -6.352 2.24e-10 ***
## siteousSkyra   -315.404     30.276 -10.417  < 2e-16 ***
## siteUB         -381.617     58.618  -6.510 7.95e-11 ***
## siteUCAM       -284.678     31.384  -9.071  < 2e-16 ***
## siteUKB        -129.191     23.436  -5.512 3.65e-08 ***
## siteUmU        -319.398     36.273  -8.805  < 2e-16 ***
## icv             257.020      6.305  40.762  < 2e-16 ***
## ---
## Signif. codes:  0 '***' 0.001 '**' 0.01 '*' 0.05 '.' 0.1 ' ' 1
##
## Approximate significance of smooth terms:
##                     edf Ref.df       F p-value
## s(bl_age)         1.000  1.000 264.764  <2e-16 ***
## s(bl_age):sleep_z 2.000  2.000   1.734  0.1766
## s(bl_age):time    3.686  3.686  20.472  <2e-16 ***
## s(sleep_z):time   1.000  1.000   5.464  0.0194 *
## ---
## Signif. codes:  0 '***' 0.001 '**' 0.01 '*' 0.05 '.' 0.1 ' ' 1
##
## R-sq.(adj) =  0.456
## lmer.REML = 1.1109e+05  Scale est. = 11296     n = 8157
```

Below are plots of a plot of `s(bl_age)`, `s(bl_age):sleep_z`, `s(bl_age):time`, and `s(sleep_z):time`. For `s(sleep_z):time`, roughly, if we cannot fit a straight line through the confidence bands, there is evidence that the effect of time depends on sleep duration. It is correct the that confidence interval is zero at one point along the axis; this happens because we do not include the main effect.

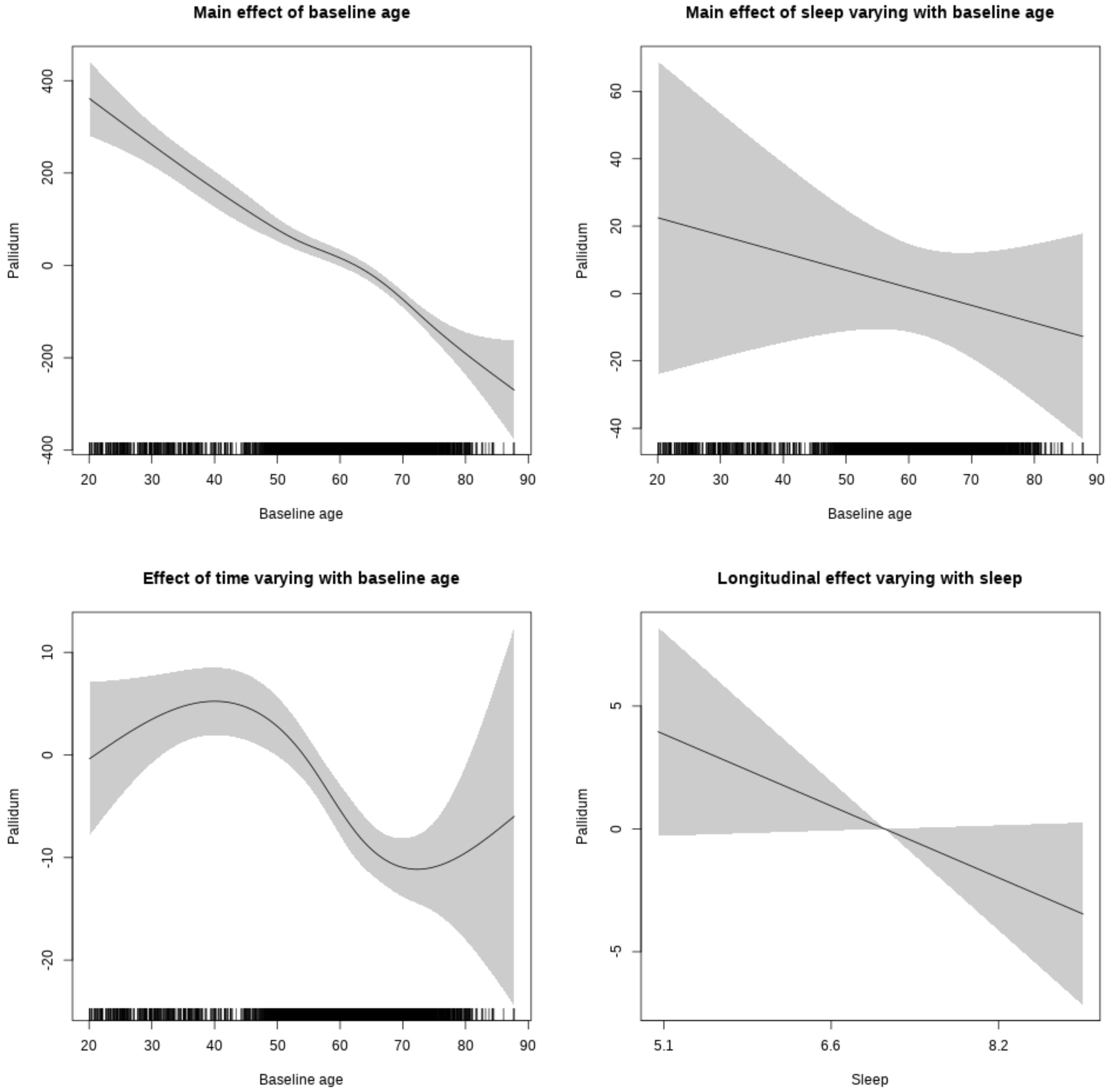

Restricted to sleep >= 5 and <= 9 hours Below is the row for `s(sleep_z):time` from the model output.

```
## s(sleep_z):time   1.000  1.000  3.470  0.0625 .
```

Below are plots of a plot of `s(bl_age)`, `s(bl_age):sleep_z`, `s(bl_age):time`, and `s(sleep_z):time`. For `s(sleep_z):time`, roughly, if we cannot fit a straight line through the confidence bands, there is evidence that the effect of time depends on sleep duration. It is correct the that confidence interval is zero at one point along the axis; this happens because we do not include the main effect.

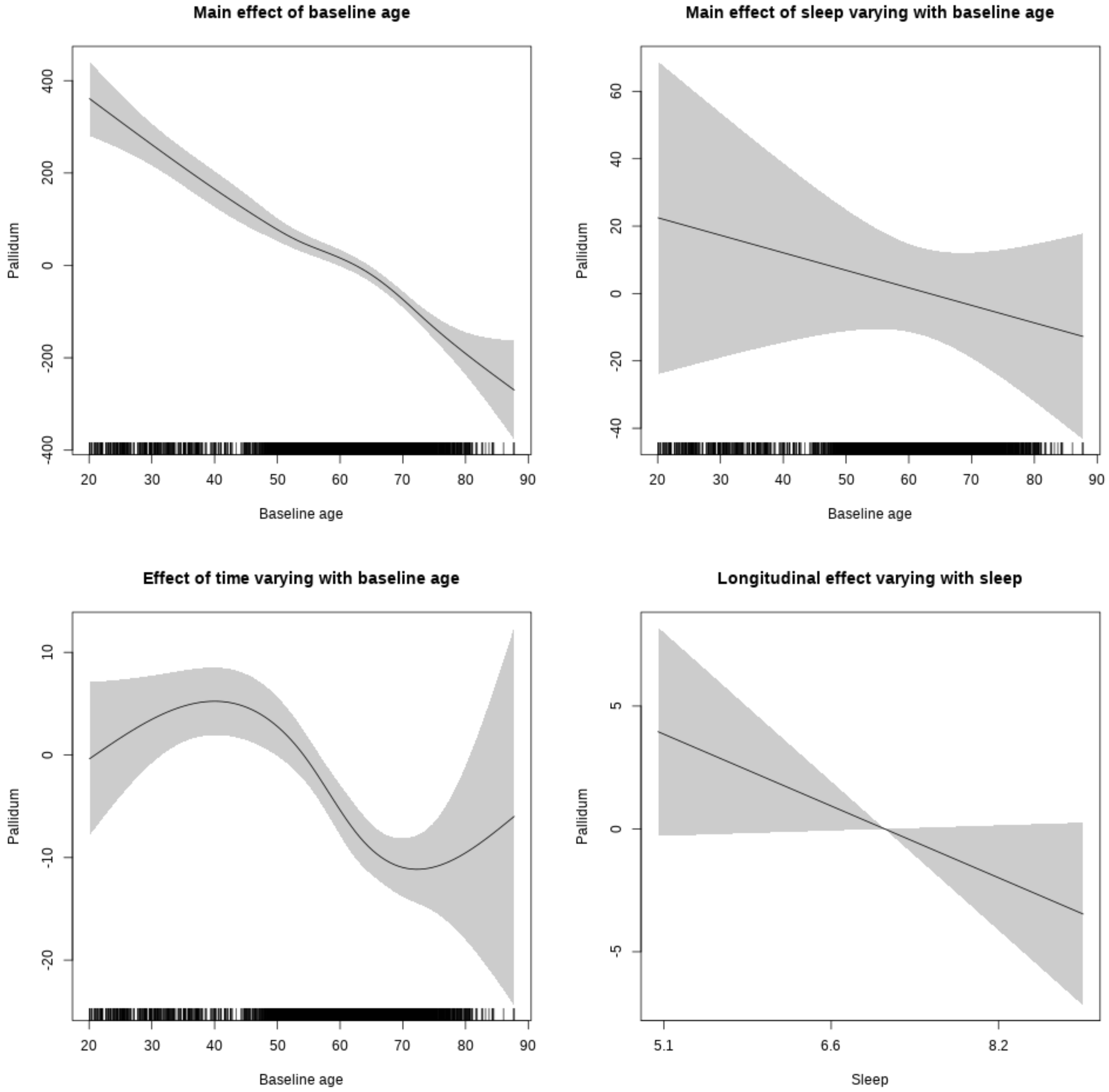

Controling for socioeconomic status

Full data Below is the model output. The term `s(sleep_z):time` is probably what we care most about.

```
##
## Family: gaussian
## Link function: identity
##
## Formula:
## value ~ sex + site + income_scaled + education_scaled + icv +
##     s(bl_age, k = 10, bs = "cr") + s(bl_age, by = sleep_z, bs = "cr") +
##     +s(bl_age, by = time, k = 5, bs = "cr") + s(sleep_z, by = time,
##     k = 5, bs = "cr", pc = 0)
```

```
## <environment: 0x56391159b7a8>
##
## Parametric coefficients:
##                   Estimate Std. Error t value Pr(>|t|)    
## (Intercept)       4137.759     57.358  72.139  < 2e-16 ***
## sexmale             81.481     16.046   5.078 3.97e-07 ***
## siteousAvanto     -429.395     64.157  -6.693 2.45e-11 ***
## siteousPrisma     -837.935    146.328  -5.726 1.09e-08 ***
## siteousSkyra      -324.768     59.114  -5.494 4.14e-08 ***
## siteUKB           -190.549     53.980  -3.530  0.00042 ***
## income_scaled        8.774     19.619   0.447  0.65473    
## education_scaled    51.554     22.783   2.263  0.02369 *  
## icv                271.256      8.028  33.787  < 2e-16 ***
## ---
## Signif. codes:  0 ‘***’ 0.001 ‘**’ 0.01 ‘*’ 0.05 ‘.’ 0.1 ‘ ’ 1
##
## Approximate significance of smooth terms:
##                      edf Ref.df       F  p-value    
## s(bl_age)          1.000  1.000 103.154  < 2e-16 ***
## s(bl_age):sleep_z 2.000  2.000   0.858   0.4243    
## s(bl_age):time    2.834  2.834   9.683 4.18e-06 ***
## s(sleep_z):time   2.240  2.240   3.408   0.0176 *  
## ---
## Signif. codes:  0 ‘***’ 0.001 ‘**’ 0.01 ‘*’ 0.05 ‘.’ 0.1 ‘ ’ 1
##
## R-sq.(adj) =  0.465   
## lmer.REML =  63382  Scale est. = 11989     n = 4654
```

Below are plots of a plot of `s(bl_age)`, `s(bl_age):sleep_z`, `s(bl_age):time`, and `s(sleep_z):time`. For `s(sleep_z):time`, roughly, if we cannot fit a straight line through the confidence bands, there is evidence that the effect of time depends on sleep duration. It is correct the that confidence interval is zero at one point along the axis; this happens because we do not include the main effect.

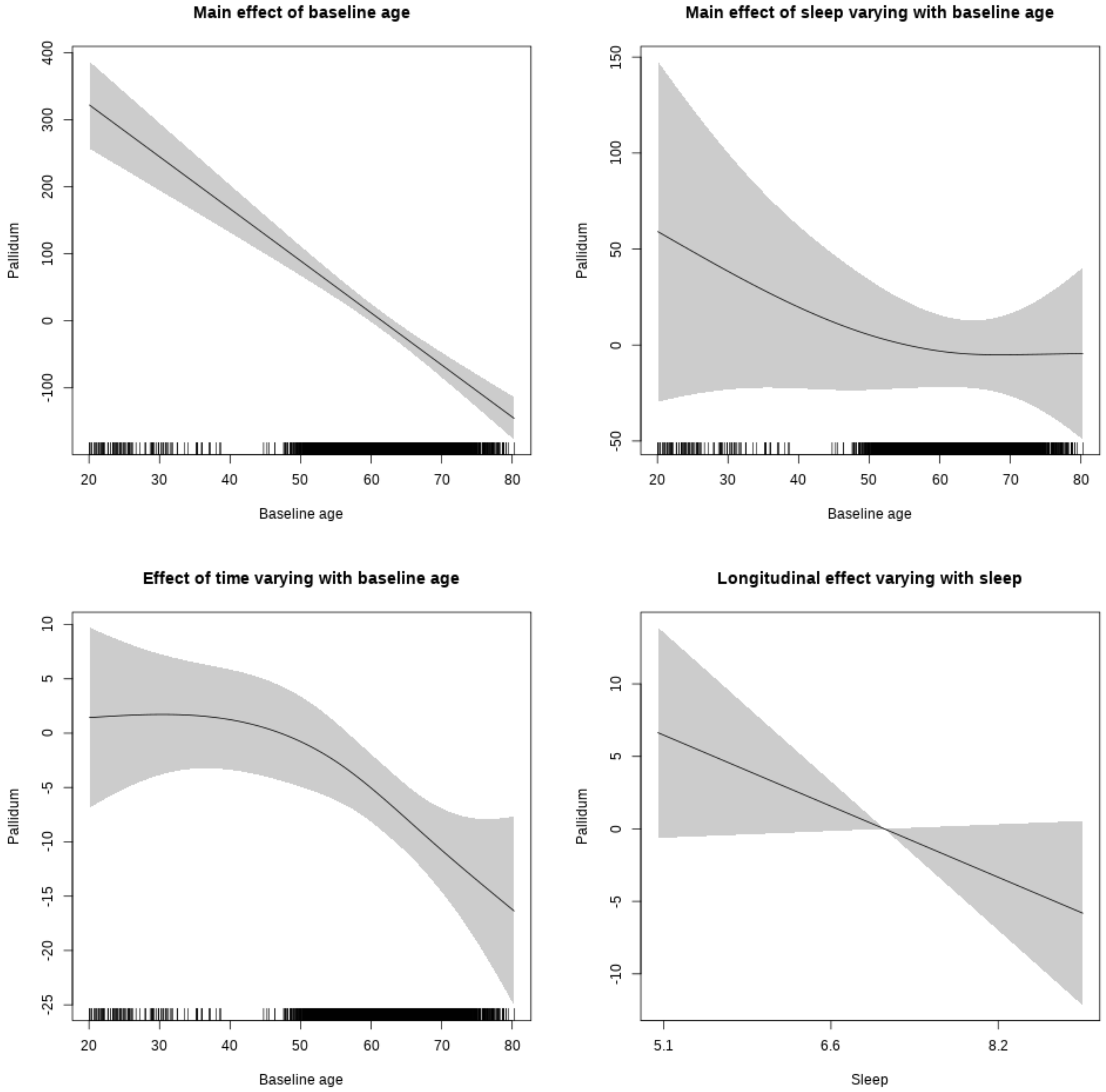

Restricted to sleep >= 5 and <= 9 hours Below is the row for `s(sleep_z):time` from the model output.

```
## s(sleep_z):time   1.000  1.000   3.340   0.0677 .
```

Below are plots of a plot of `s(bl_age)`, `s(bl_age):sleep_z`, `s(bl_age):time`, and `s(sleep_z):time`. For `s(sleep_z):time`, roughly, if we cannot fit a straight line through the confidence bands, there is evidence that the effect of time depends on sleep duration. It is correct the that confidence interval is zero at one point along the axis; this happens because we do not include the main effect.

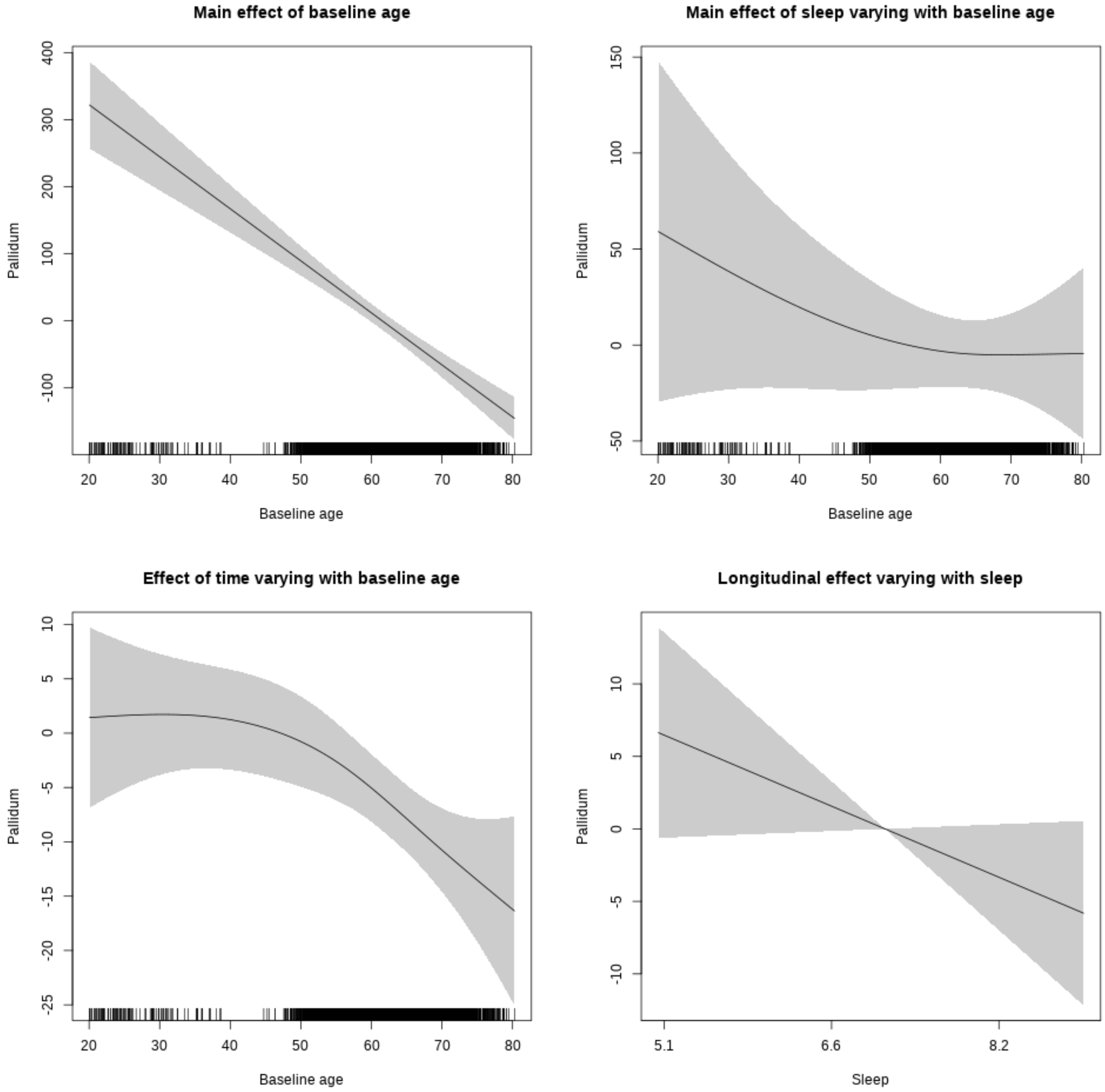

Controling for BMI

Full data Below is the model output. The term `s(sleep_z):time` is probably what we care most about.

```
##
## Family: gaussian
## Link function: identity
##
## Formula:
## value ~ sex + site + bmi + icv + s(bl_age, k = 10, bs = "cr") +
##     s(bl_age, by = sleep_z, bs = "cr") + +s(bl_age, by = time,
##     k = 5, bs = "cr") + s(sleep_z, by = time, k = 5, bs = "cr",
##     pc = 0)
```

```
## <environment: 0x563916b23638>
##
## Parametric coefficients:
##                Estimate Std. Error t value Pr(>|t|)
## (Intercept)    3829.254     54.745  69.947  < 2e-16 ***
## sexmale          81.895     15.286   5.358 8.79e-08 ***
## siteousPrisma -111.371     86.956  -1.281 0.200331
## siteousSkyra    147.877     27.455   5.386 7.50e-08 ***
## siteUCAM        140.532     42.071   3.340 0.000843 ***
## siteUKB         273.815     39.411   6.948 4.15e-12 ***
## siteUmU         111.823     48.052   2.327 0.019996 *
## bmi              -4.360      1.580  -2.760 0.005807 **
## icv             277.932      7.578  36.677  < 2e-16 ***
## ---
## Signif. codes:  0 '***' 0.001 '**' 0.01 '*' 0.05 '.' 0.1 ' ' 1
##
## Approximate significance of smooth terms:
##                      edf Ref.df      F p-value
## s(bl_age)          2.569  2.569 52.411  <2e-16 ***
## s(bl_age):sleep_z 2.000  2.000  0.909  0.4030
## s(bl_age):time    2.000  2.000 20.131  <2e-16 ***
## s(sleep_z):time   1.000  1.000  5.829  0.0158 *
## ---
## Signif. codes:  0 '***' 0.001 '**' 0.01 '*' 0.05 '.' 0.1 ' ' 1
##
## R-sq.(adj) =  0.467
## lmer.REML =  73870  Scale est. = 11682     n = 5416
```

Below are plots of a plot of `s(bl_age)`, `s(bl_age):sleep_z`, `s(bl_age):time`, and `s(sleep_z):time`. For `s(sleep_z):time`, roughly, if we cannot fit a straight line through the confidence bands, there is evidence that the effect of time depends on sleep duration. It is correct the that confidence interval is zero at one point along the axis; this happens because we do not include the main effect.

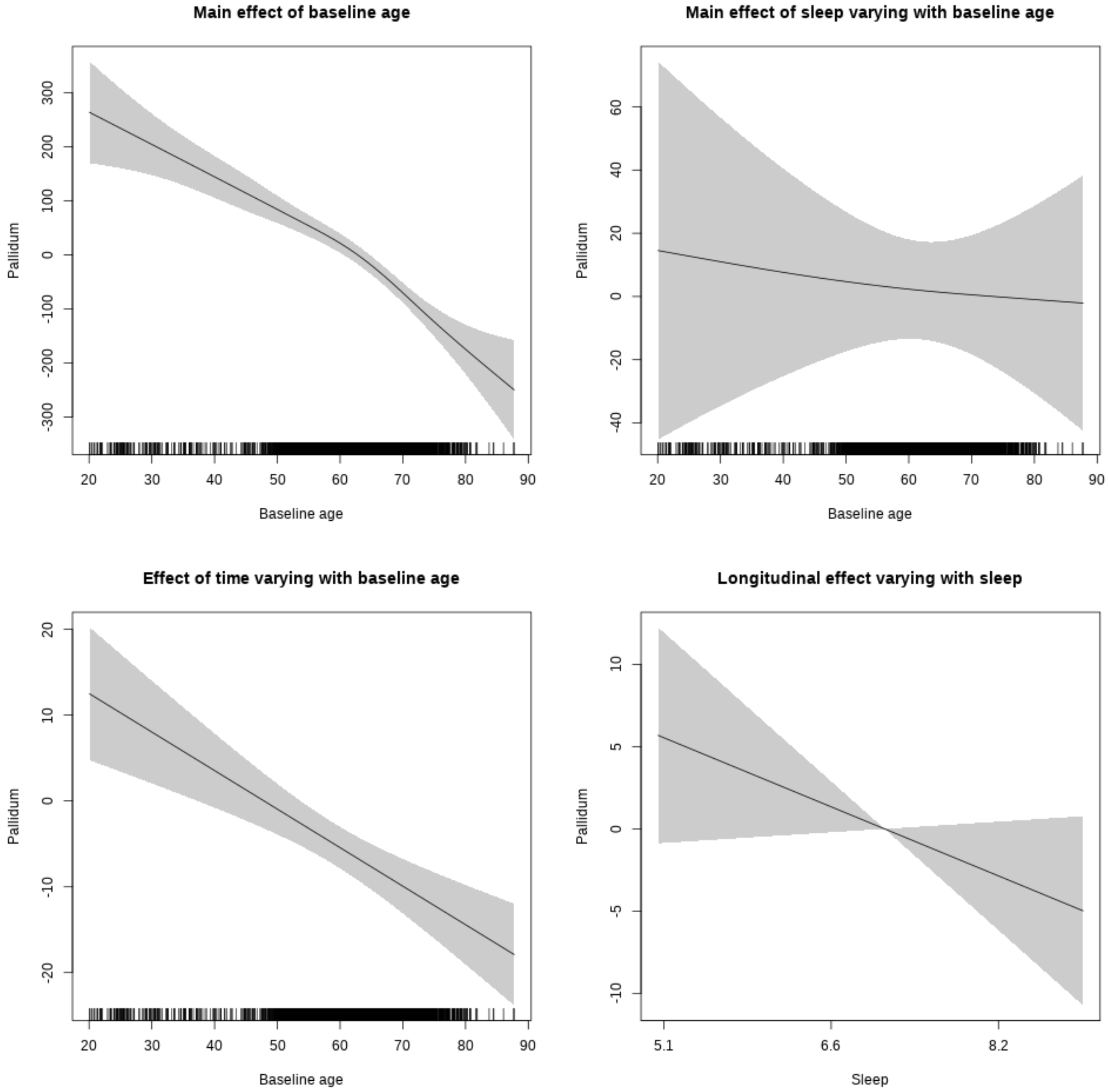

Restricted to sleep >= 5 and <= 9 hours Below is the row for `s(sleep_z):time` from the model output.

```
## s(sleep_z):time   1.000  1.000  3.001  0.0833 .
```

Below are plots of a plot of `s(bl_age)`, `s(bl_age):sleep_z`, `s(bl_age):time`, and `s(sleep_z):time`. For `s(sleep_z):time`, roughly, if we cannot fit a straight line through the confidence bands, there is evidence that the effect of time depends on sleep duration. It is correct the that confidence interval is zero at one point along the axis; this happens because we do not include the main effect.

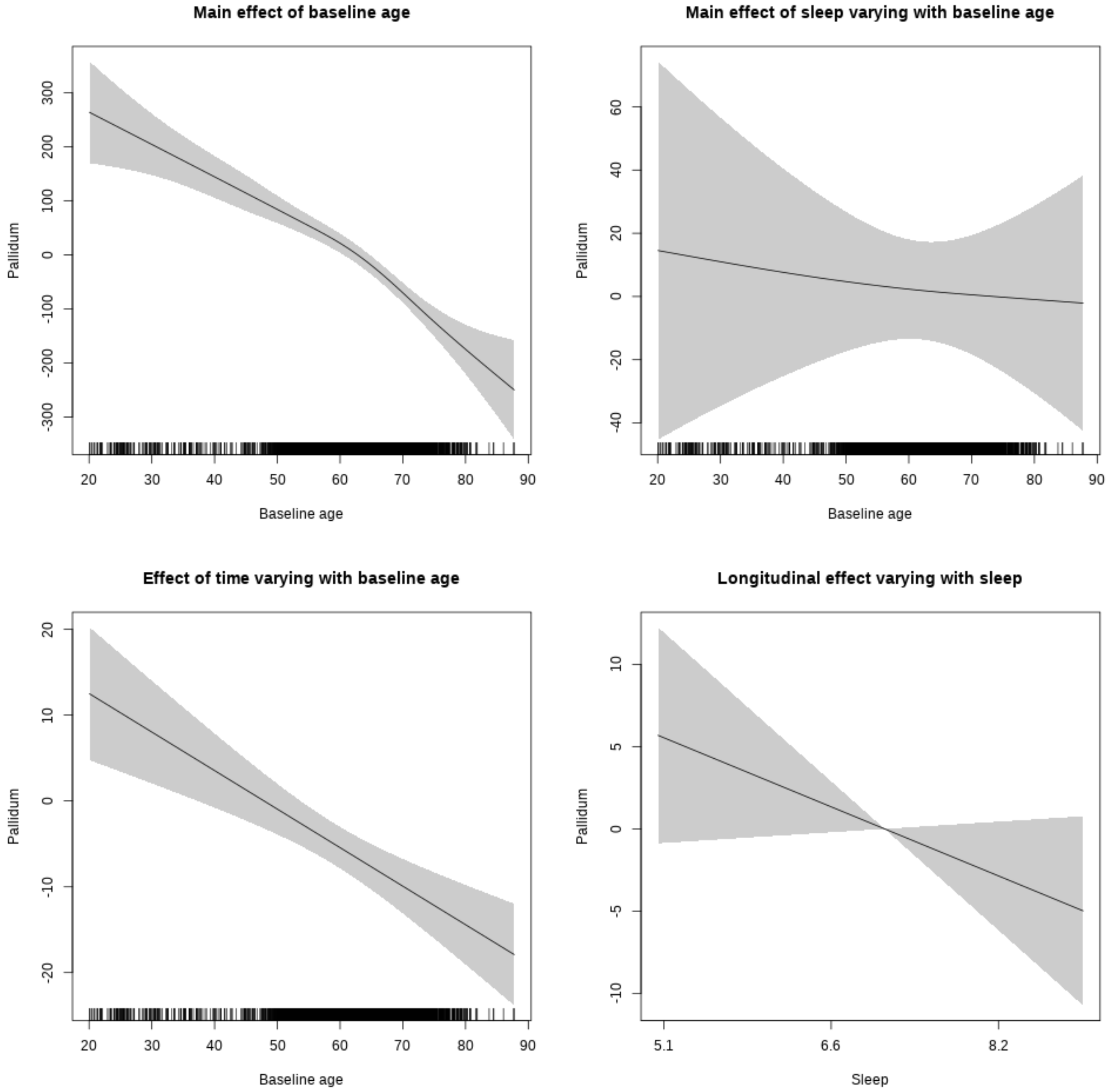

Controling for depression

Full data Below is the model output. The term `s(sleep_z):time` is probably what we care most about.

```
##
## Family: gaussian
## Link function: identity
##
## Formula:
## value ~ sex + site + depression + icv + s(bl_age, k = 10, bs = "cr") +
##     s(bl_age, by = sleep_z, bs = "cr") + +s(bl_age, by = time,
##     k = 5, bs = "cr") + s(sleep_z, by = time, k = 5, bs = "cr",
##     pc = 0)
```

```
## <environment: 0x563910fa3ea8>
##
## Parametric coefficients:
##                Estimate Std. Error t value Pr(>|t|)
## (Intercept)    4101.058     29.794 137.646  < 2e-16 ***
## sexmale          85.660     15.566   5.503 3.90e-08 ***
## siteousAvanto -229.444     64.669  -3.548 0.000391 ***
## siteousPrisma -260.829    116.872  -2.232 0.025671 *
## siteousSkyra  -189.398     54.566  -3.471 0.000522 ***
## siteUCAM      -250.953     36.045  -6.962 3.73e-12 ***
## siteUKB       -119.263     27.391  -4.354 1.36e-05 ***
## siteUmU       -283.708     43.619  -6.504 8.48e-11 ***
## depression      -8.407     54.627  -0.154 0.877693
## icv            258.907      7.530  34.385  < 2e-16 ***
## ---
## Signif. codes:  0 ‘***’ 0.001 ‘**’ 0.01 ‘*’ 0.05 ‘.’ 0.1 ‘ ’ 1
##
## Approximate significance of smooth terms:
##                     edf Ref.df      F p-value
## s(bl_age)         4.452  4.452 30.062  <2e-16 ***
## s(bl_age):sleep_z 2.000  2.000  0.778  0.4593
## s(bl_age):time    2.000  2.000 24.324  <2e-16 ***
## s(sleep_z):time   1.000  1.000  4.300  0.0382 *
## ---
## Signif. codes:  0 ‘***’ 0.001 ‘**’ 0.01 ‘*’ 0.05 ‘.’ 0.1 ‘ ’ 1
##
## R-sq.(adj) =  0.442
## lmer.REML =  77719  Scale est. = 11387     n = 5683
```

Below are plots of a plot of `s(bl_age)`, `s(bl_age):sleep_z`, `s(bl_age):time`, and `s(sleep_z):time`. For `s(sleep_z):time`, roughly, if we cannot fit a straight line through the confidence bands, there is evidence that the effect of time depends on sleep duration. It is correct the that confidence interval is zero at one point along the axis; this happens because we do not include the main effect.

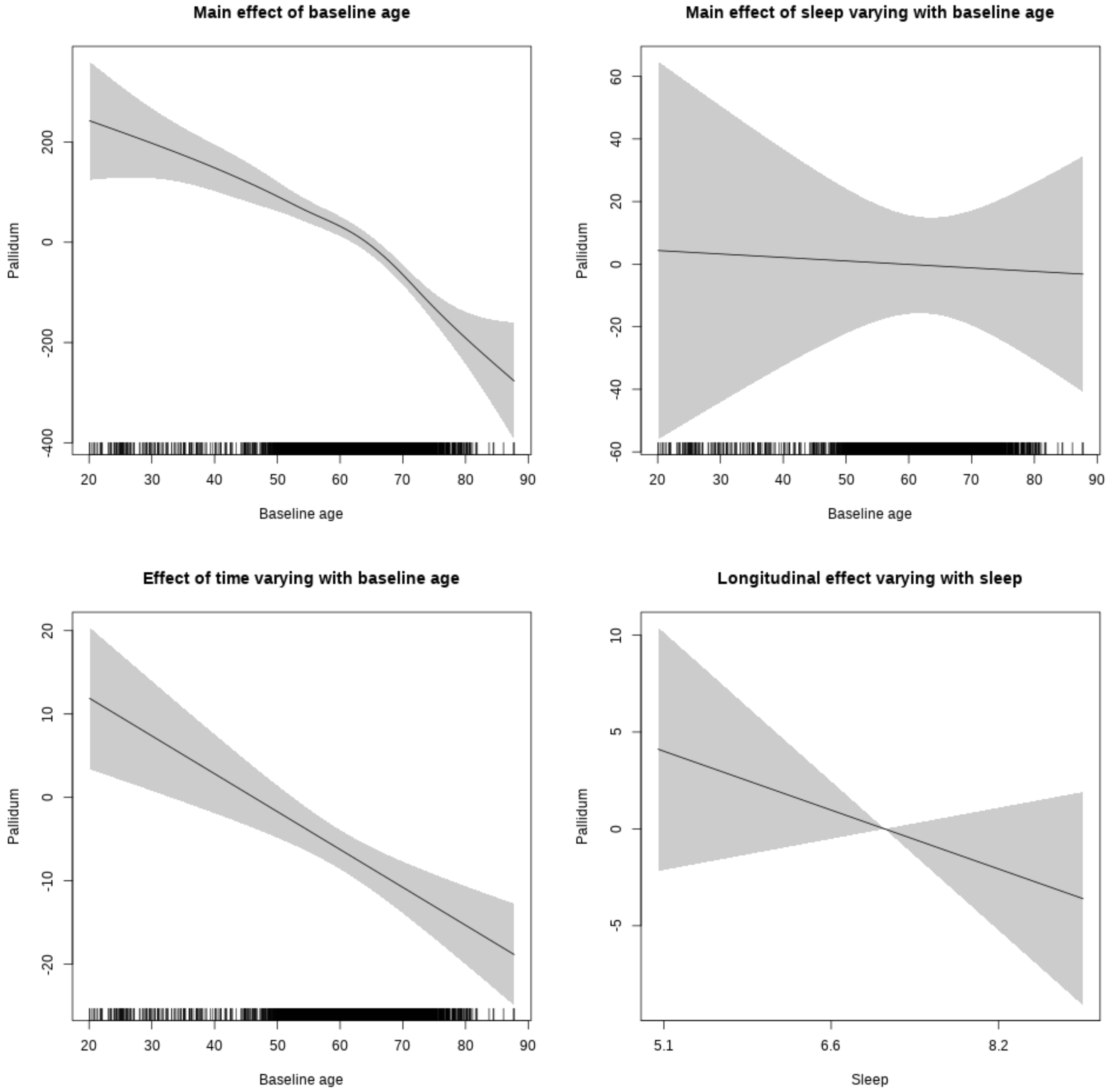

Restricted to sleep >= 5 and <= 9 hours Below is the row for `s(sleep_z):time` from the model output.

```
## s(sleep_z):time   1.00   1.00  1.711   0.191
```

Below are plots of a plot of `s(bl_age)`, `s(bl_age):sleep_z`, `s(bl_age):time`, and `s(sleep_z):time`. For `s(sleep_z):time`, roughly, if we cannot fit a straight line through the confidence bands, there is evidence that the effect of time depends on sleep duration. It is correct the that confidence interval is zero at one point along the axis; this happens because we do not include the main effect.

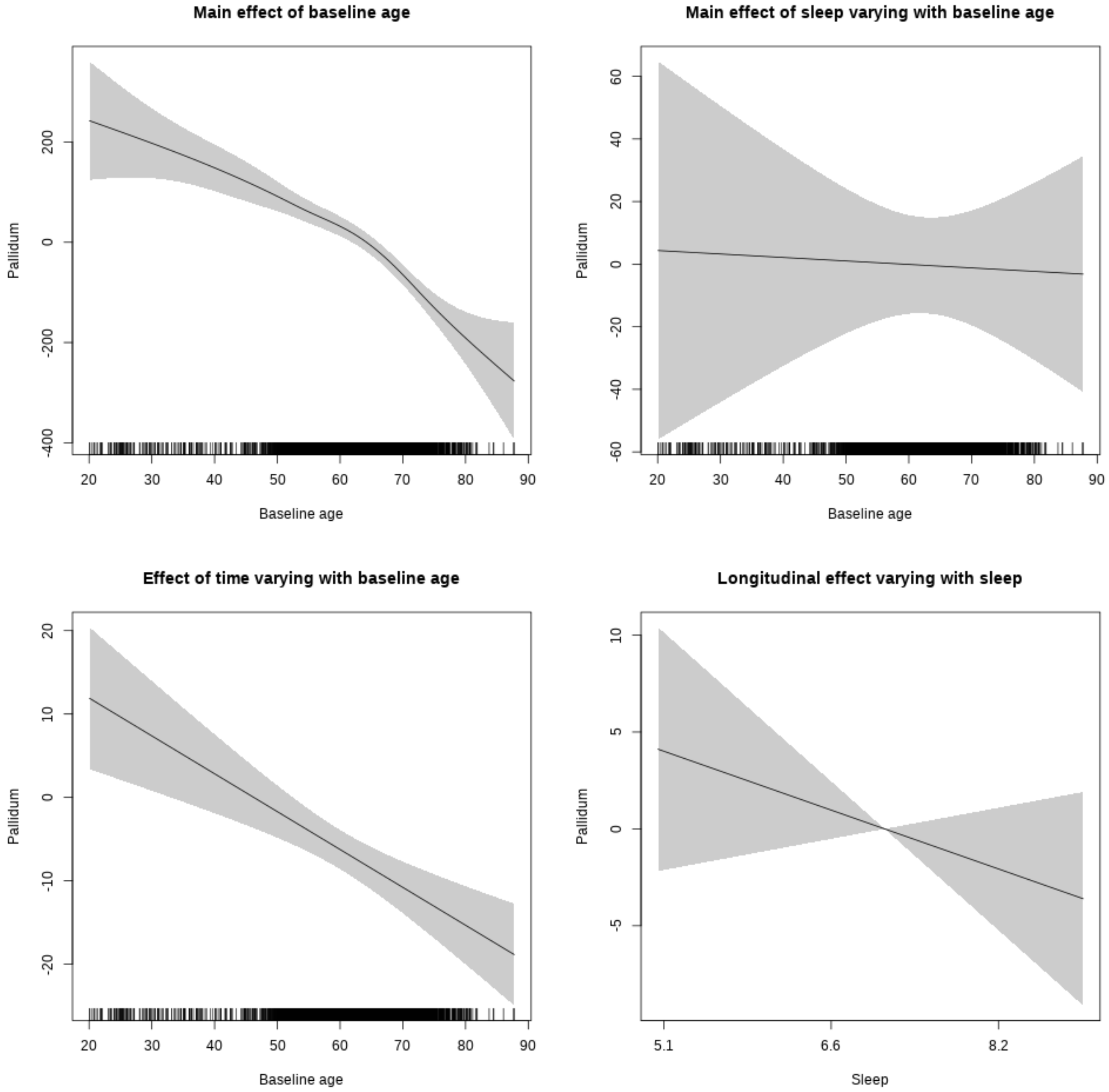

Controling for sleep quality

Full data Below is the model output. The term `s(sleep_z):time` is probably what we care most about.

```
##
## Family: gaussian
## Link function: identity
##
## Formula:
## value ~ X + s(bl_age, k = 10, bs = "cr") + s(bl_age, by = sleep_z,
##     bs = "cr") + s(bl_age, by = time, k = 5, bs = "cr") + s(sleep_z,
##     by = time, k = 5, bs = "cr", pc = 0)
## <environment: 0x563914d995c8>
```

```
##
## Parametric coefficients:
##                          Estimate Std. Error t value Pr(>|t|)
## (Intercept)              3611.635     51.121  70.649  < 2e-16 ***
## Xsexmale                  393.918     19.204  20.512  < 2e-16 ***
## XsiteousAvanto            120.731     48.807   2.474  0.01341 *
## XsiteousPrisma             78.227     91.740   0.853  0.39387
## XsiteousSkyra             117.125     48.982   2.391  0.01684 *
## XsiteUB                   -74.068     77.868  -0.951  0.34156
## XsiteUCAM                 150.377     49.470   3.040  0.00238 **
## XsiteUKB                  410.632    155.183   2.646  0.00817 **
## XsiteUmU                  -20.461     55.348  -0.370  0.71164
## Xukb_dummy:sleep_accel      8.188     12.065   0.679  0.49740
## Xukb_dummy:chronotype     -11.165     16.068  -0.695  0.48716
## Xukb_dummy:dozing          -7.024     33.203  -0.212  0.83247
## Xukb_dummy:sleeplessness  -43.394     20.611  -2.105  0.03532 *
## Xukb_dummy:snoring         11.179     29.322   0.381  0.70304
## Xukb_dummy:gettingup      -32.246     22.328  -1.444  0.14876
## Xnotukb_dummy:PSQI_Global  -5.466      6.330  -0.864  0.38790
## ---
## Signif. codes:  0 '***' 0.001 '**' 0.01 '*' 0.05 '.' 0.1 ' ' 1
##
## Approximate significance of smooth terms:
##                      edf Ref.df       F p-value
## s(bl_age)          1.000  1.000 126.721  <2e-16 ***
## s(bl_age):sleep_z 2.000  2.000   0.903  0.4053
## s(bl_age):time     4.134  4.134  13.766  <2e-16 ***
## s(sleep_z):time    1.000  1.000   4.013  0.0452 *
## ---
## Signif. codes:  0 '***' 0.001 '**' 0.01 '*' 0.05 '.' 0.1 ' ' 1
##
## R-sq.(adj) =  0.257
## lmer.REML =  58690  Scale est. = 10284     n = 4289
```

Below are plots of a plot of `s(bl_age)`, `s(bl_age):sleep_z`, `s(bl_age):time`, and `s(sleep_z):time`. For `s(sleep_z):time`, roughly, if we cannot fit a straight line through the confidence bands, there is evidence that the effect of time depends on sleep duration. It is correct the that confidence interval is zero at one point along the axis; this happens because we do not include the main effect.

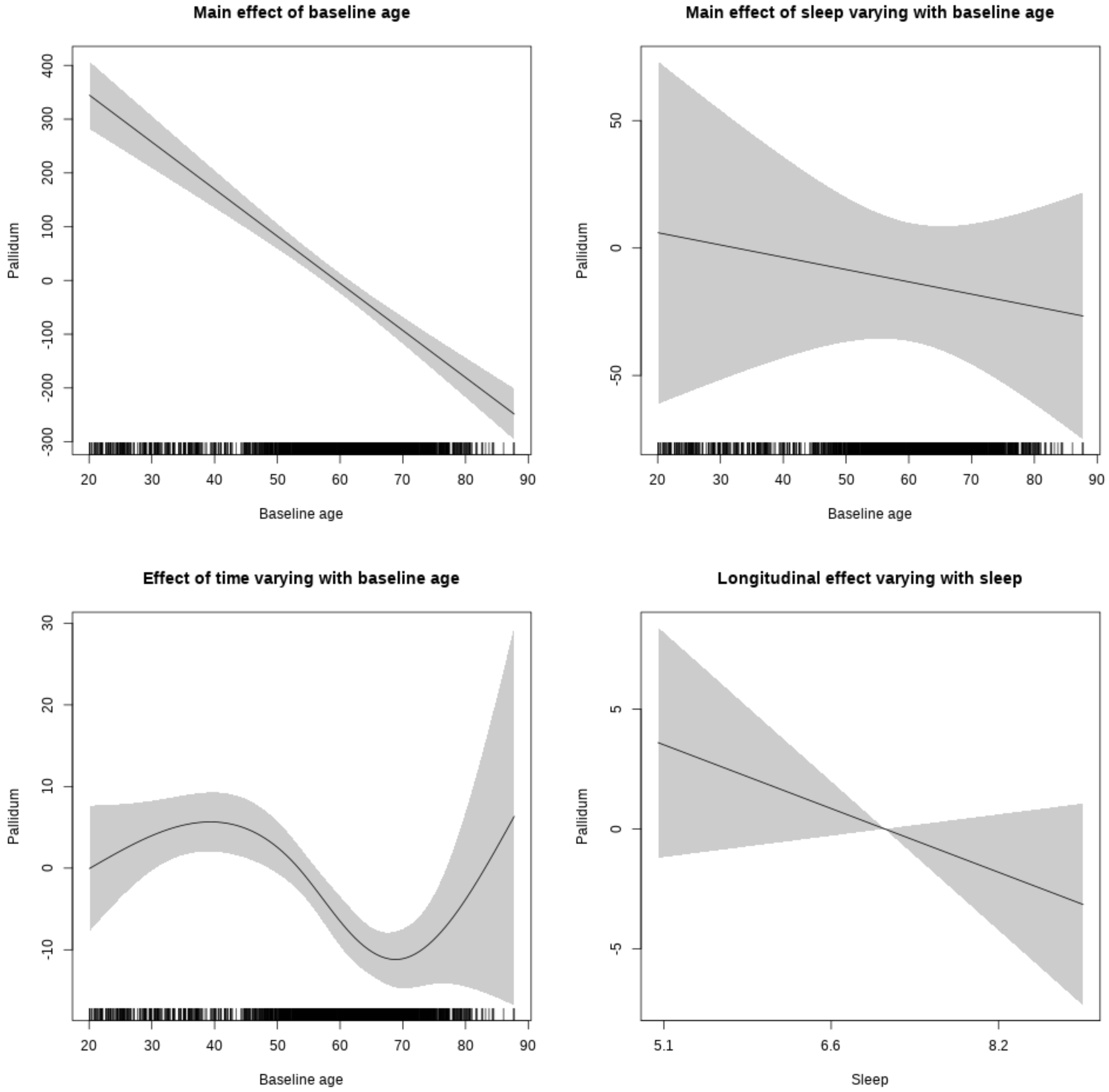

Restricted to sleep >= 5 and <= 9 hours Below is the row for `s(sleep_z):time` from the model output.

```
## s(sleep_z):time   1.000  1.000   2.242   0.134
```

Below are plots of a plot of `s(bl_age)`, `s(bl_age):sleep_z`, `s(bl_age):time`, and `s(sleep_z):time`. For `s(sleep_z):time`, roughly, if we cannot fit a straight line through the confidence bands, there is evidence that the effect of time depends on sleep duration. It is correct the that confidence interval is zero at one point along the axis; this happens because we do not include the main effect.

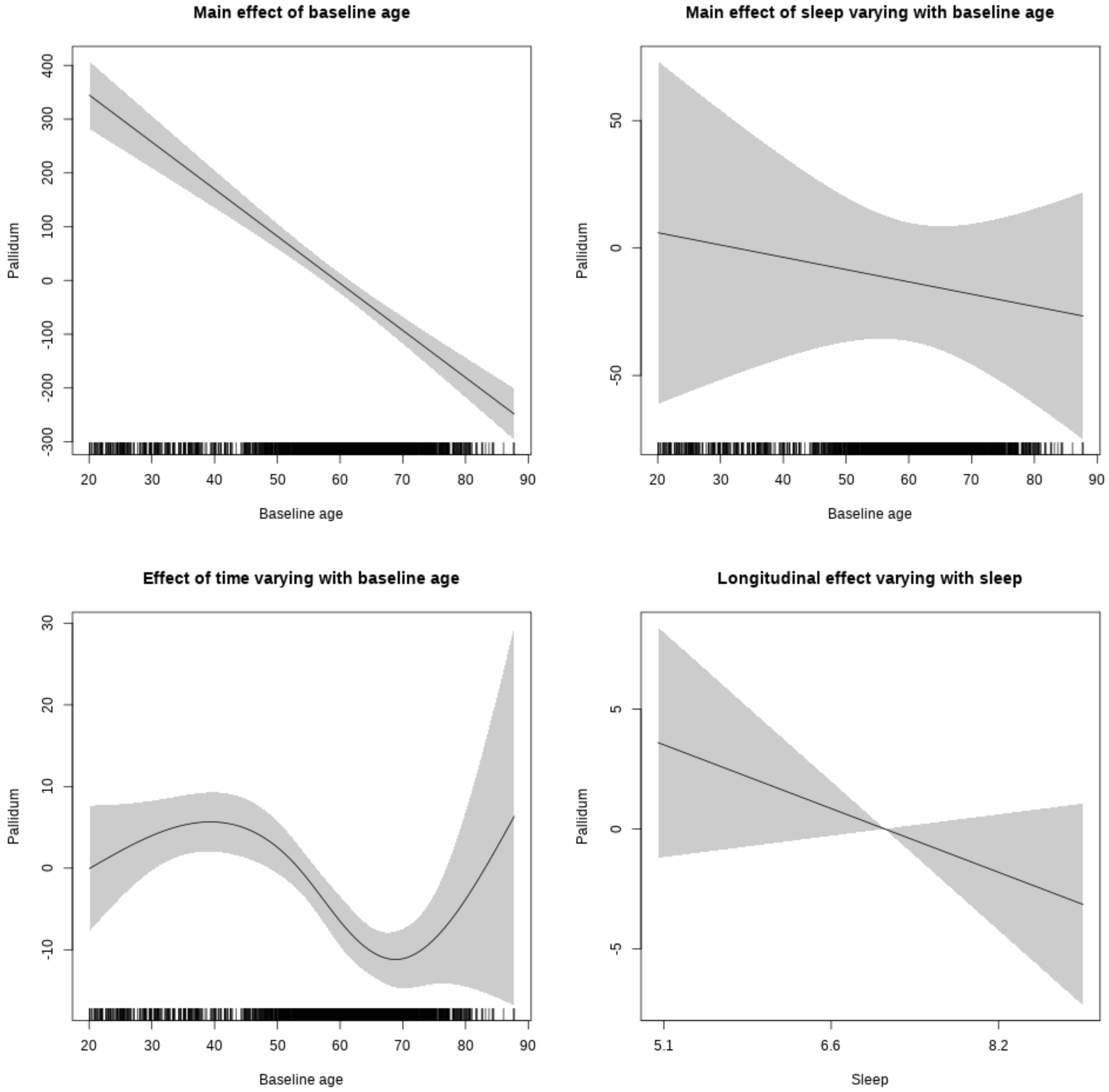

Putamen

Main analysis

Full data Below is the model output. The term `s(sleep_z):time` is probably what we care most about.

```
##
## Family: gaussian
## Link function: identity
##
## Formula:
## value ~ sex + site + icv + s(bl_age, k = 10, bs = "cr") + s(bl_age,
##     by = sleep_z, bs = "cr") + +s(bl_age, by = time, k = 5, bs = "cr") +
```

```
##     s(sleep_z, by = time, k = 5, bs = "cr", pc = 0)
## <environment: 0x5639112565a0>
##
## Parametric coefficients:
##                Estimate Std. Error t value Pr(>|t|)
## (Intercept)    10036.64      63.37 158.373  < 2e-16 ***
## sexmale          342.33      32.69  10.470  < 2e-16 ***
## siteousAvanto   -807.60      77.86 -10.372  < 2e-16 ***
## siteousPrisma   -931.38     165.29  -5.635 1.81e-08 ***
## siteousSkyra    -147.00      75.83  -1.939  0.05257 .
## siteUB          -437.94     148.68  -2.946  0.00323 **
## siteUCAM        -633.92      79.38  -7.986 1.58e-15 ***
## siteUKB         -932.52      60.46 -15.424  < 2e-16 ***
## siteUmU         -378.20      91.94  -4.114 3.93e-05 ***
## icv              505.61      15.28  33.083  < 2e-16 ***
## ---
## Signif. codes:  0 ‘***’ 0.001 ‘**’ 0.01 ‘*’ 0.05 ‘.’ 0.1 ‘ ’ 1
##
## Approximate significance of smooth terms:
##                     edf Ref.df       F p-value
## s(bl_age)         2.737  2.737 342.208  <2e-16 ***
## s(bl_age):sleep_z 2.000  2.000   0.750   0.473
## s(bl_age):time    2.000  2.000 357.290  <2e-16 ***
## s(sleep_z):time   1.000  1.000   0.866   0.352
## ---
## Signif. codes:  0 ‘***’ 0.001 ‘**’ 0.01 ‘*’ 0.05 ‘.’ 0.1 ‘ ’ 1
##
## R-sq.(adj) =  0.474
## lmer.REML = 1.2288e+05  Scale est. = 35312     n = 8138
```

Below are plots of a plot of `s(bl_age)`, `s(bl_age):sleep_z`, `s(bl_age):time`, and `s(sleep_z):time`. For `s(sleep_z):time`, roughly, if we cannot fit a straight line through the confidence bands, there is evidence that the effect of time depends on sleep duration. It is correct the that confidence interval is zero at one point along the axis; this happens because we do not include the main effect.

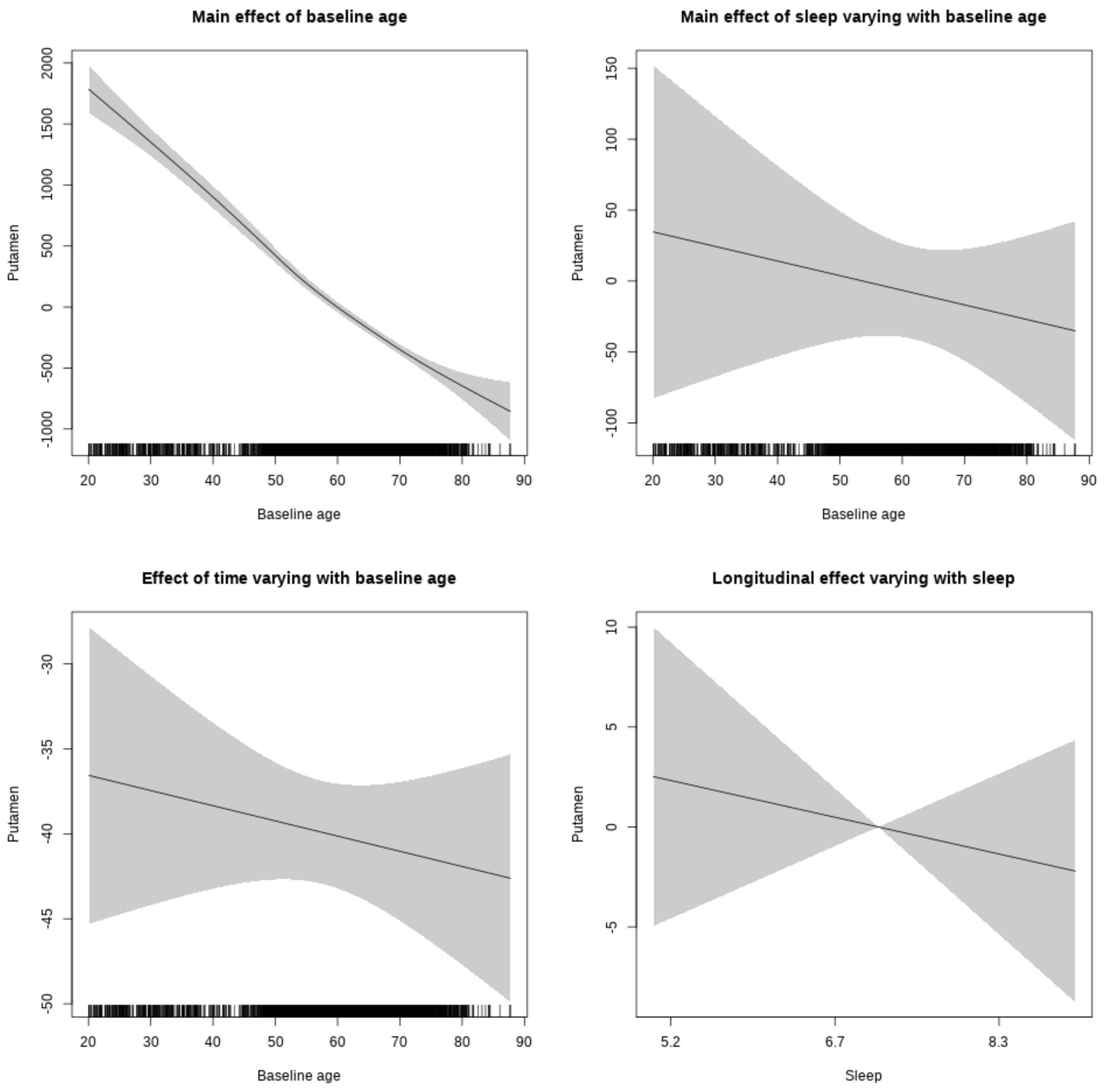

Restricted to sleep >= 5 and <= 9 hours Below is the row for `s(sleep_z):time` from the model output.

```
## s(sleep_z):time   1.000  1.000   0.454   0.501
```

Below are plots of a plot of `s(bl_age)`, `s(bl_age):sleep_z`, `s(bl_age):time`, and `s(sleep_z):time`. For `s(sleep_z):time`, roughly, if we cannot fit a straight line through the confidence bands, there is evidence that the effect of time depends on sleep duration. It is correct the that confidence interval is zero at one point along the axis; this happens because we do not include the main effect.

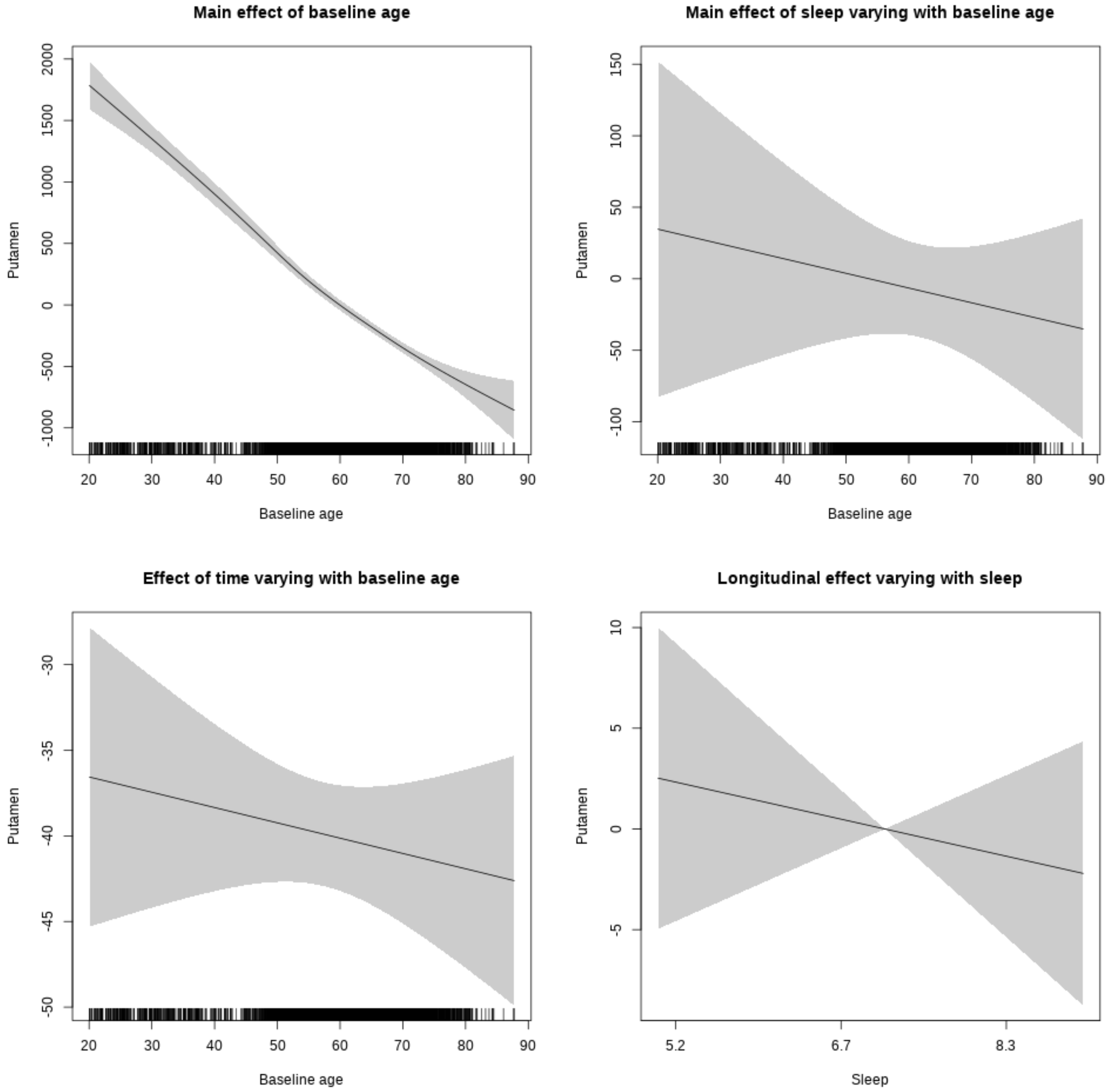

Controling for socioeconomic status

Full data Below is the model output. The term `s(sleep_z):time` is probably what we care most about.

```
##
## Family: gaussian
## Link function: identity
##
## Formula:
## value ~ sex + site + income_scaled + education_scaled + icv +
##     s(bl_age, k = 10, bs = "cr") + s(bl_age, by = sleep_z, bs = "cr") +
##     +s(bl_age, by = time, k = 5, bs = "cr") + s(sleep_z, by = time,
##     k = 5, bs = "cr", pc = 0)
```

```
## <environment: 0x563911591ea8>
##
## Parametric coefficients:
##                  Estimate Std. Error t value Pr(>|t|)
## (Intercept)      10108.80     147.55  68.509  < 2e-16 ***
## sexmale            343.99      40.91   8.408  < 2e-16 ***
## siteousAvanto    -1013.74     159.28  -6.365 2.15e-10 ***
## siteousPrisma    -1146.25     274.55  -4.175 3.03e-05 ***
## siteousSkyra      -185.49     152.19  -1.219   0.2230
## siteUKB          -1118.78     139.34  -8.029 1.24e-15 ***
## income_scaled      -20.12      51.02  -0.394   0.6933
## education_scaled   106.93      58.83   1.818   0.0692 .
## icv                533.52      19.73  27.038  < 2e-16 ***
## ---
## Signif. codes:  0 '***' 0.001 '**' 0.01 '*' 0.05 '.' 0.1 ' ' 1
##
## Approximate significance of smooth terms:
##                     edf Ref.df       F p-value
## s(bl_age)         1.198  1.198 309.447  <2e-16 ***
## s(bl_age):sleep_z 2.000  2.000   1.101   0.332
## s(bl_age):time    2.000  2.000 185.898  <2e-16 ***
## s(sleep_z):time   1.000  1.000   0.573   0.449
## ---
## Signif. codes:  0 '***' 0.001 '**' 0.01 '*' 0.05 '.' 0.1 ' ' 1
##
## R-sq.(adj) =  0.485
## lmer.REML =  70015  Scale est. = 34065     n = 4642
```

Below are plots of a plot of `s(bl_age)`, `s(bl_age):sleep_z`, `s(bl_age):time`, and `s(sleep_z):time`. For `s(sleep_z):time`, roughly, if we cannot fit a straight line through the confidence bands, there is evidence that the effect of time depends on sleep duration. It is correct the that confidence interval is zero at one point along the axis; this happens because we do not include the main effect.

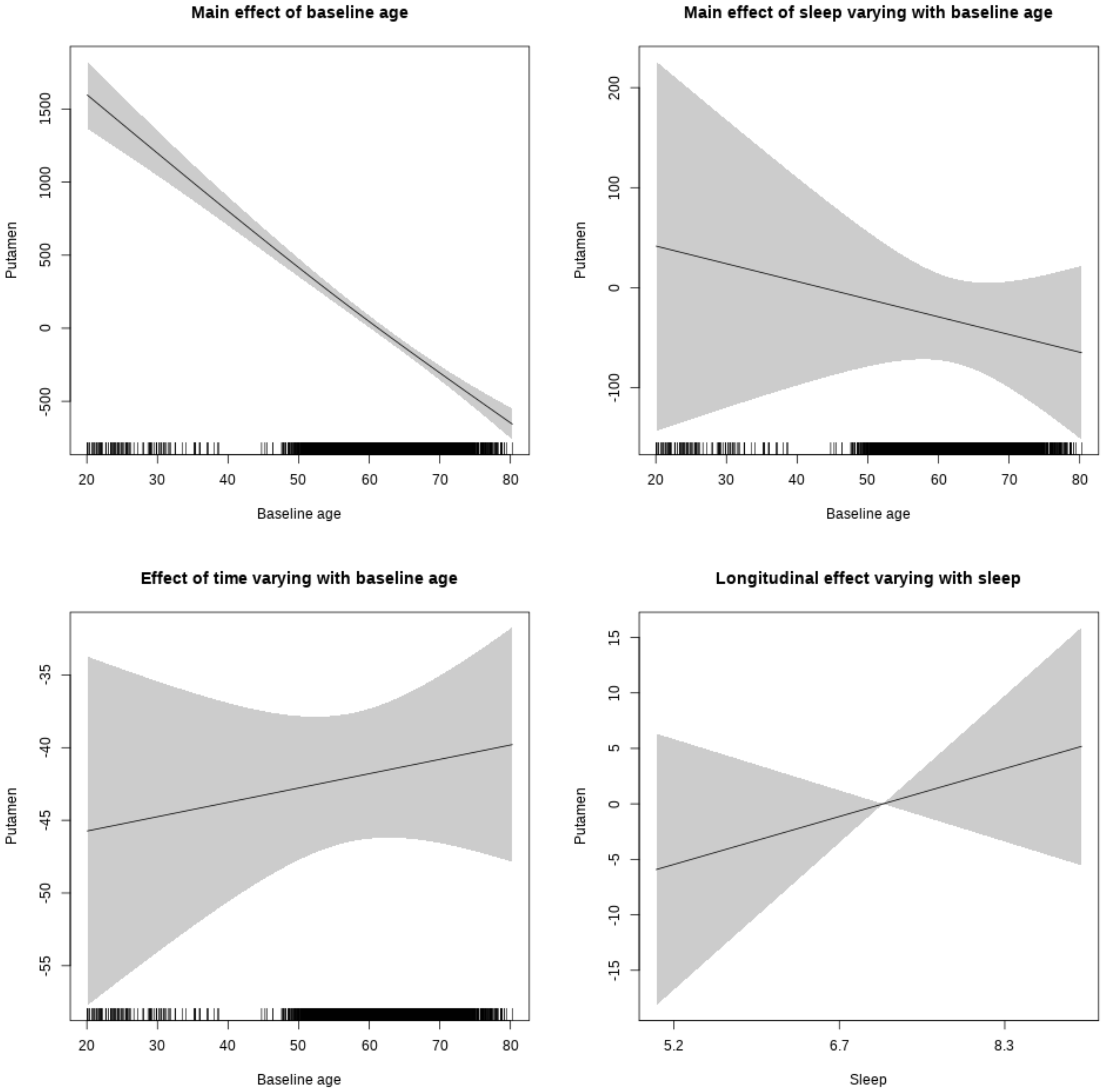

Restricted to sleep >= 5 and <= 9 hours Below is the row for `s(sleep_z):time` from the model output.

```
## s(sleep_z):time   1.000  1.000   0.934   0.334
```

Below are plots of a plot of `s(bl_age)`, `s(bl_age):sleep_z`, `s(bl_age):time`, and `s(sleep_z):time`. For `s(sleep_z):time`, roughly, if we cannot fit a straight line through the confidence bands, there is evidence that the effect of time depends on sleep duration. It is correct the that confidence interval is zero at one point along the axis; this happens because we do not include the main effect.

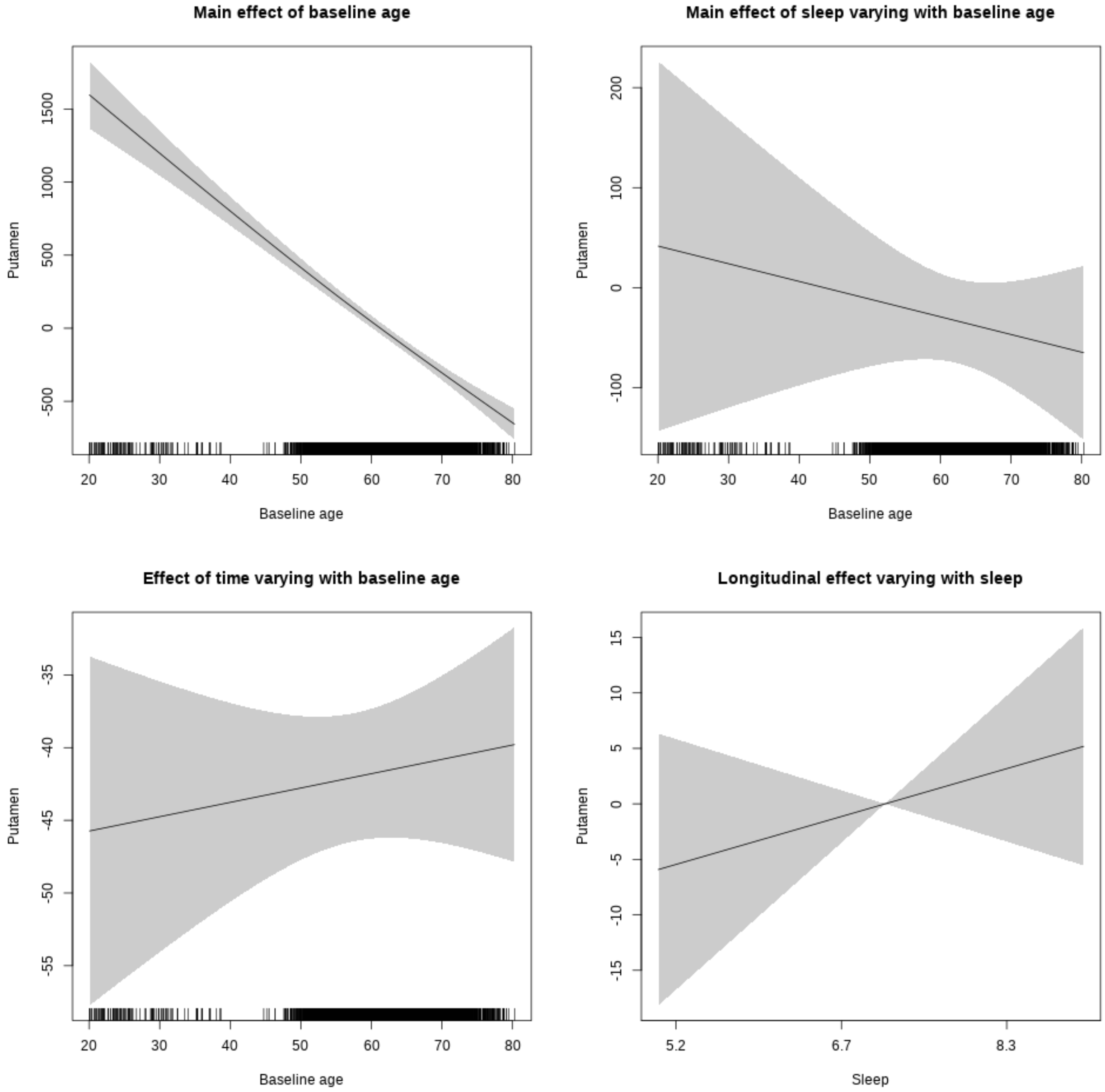

Controling for BMI

Full data Below is the model output. The term `s(sleep_z):time` is probably what we care most about.

```
##
## Family: gaussian
## Link function: identity
##
## Formula:
## value ~ sex + site + bmi + icv + s(bl_age, k = 10, bs = "cr") +
##     s(bl_age, by = sleep_z, bs = "cr") + +s(bl_age, by = time,
##     k = 5, bs = "cr") + s(sleep_z, by = time, k = 5, bs = "cr",
##     pc = 0)
```

```
## <environment: 0x563916ea8cc8>
##
## Parametric coefficients:
##                Estimate Std. Error t value Pr(>|t|)
## (Intercept)    8837.967    130.471  67.739  < 2e-16 ***
## sexmale         343.302     38.212   8.984  < 2e-16 ***
## siteousPrisma    -6.474    148.880  -0.043   0.9653
## siteousSkyra    763.741     47.703  16.010  < 2e-16 ***
## siteUCAM        278.635     95.937   2.904   0.0037 **
## siteUKB         -51.824     87.875  -0.590   0.5554
## siteUmU         551.764    112.060   4.924 8.74e-07 ***
## bmi              11.475      3.996   2.872   0.0041 **
## icv             520.040     18.361  28.323  < 2e-16 ***
## ---
## Signif. codes:  0 '***' 0.001 '**' 0.01 '*' 0.05 '.' 0.1 ' ' 1
##
## Approximate significance of smooth terms:
##                       edf Ref.df       F p-value
## s(bl_age)           2.069  2.069 288.833  <2e-16 ***
## s(bl_age):sleep_z 2.000  2.000   0.952   0.386
## s(bl_age):time    2.711  2.711 201.808  <2e-16 ***
## s(sleep_z):time   1.000  1.000   0.013   0.911
## ---
## Signif. codes:  0 '***' 0.001 '**' 0.01 '*' 0.05 '.' 0.1 ' ' 1
##
## R-sq.(adj) =  0.498
## lmer.REML =  81532  Scale est. = 33294     n = 5404
```

Below are plots of a plot of `s(bl_age)`, `s(bl_age):sleep_z`, `s(bl_age):time`, and `s(sleep_z):time`. For `s(sleep_z):time`, roughly, if we cannot fit a straight line through the confidence bands, there is evidence that the effect of time depends on sleep duration. It is correct the that confidence interval is zero at one point along the axis; this happens because we do not include the main effect.

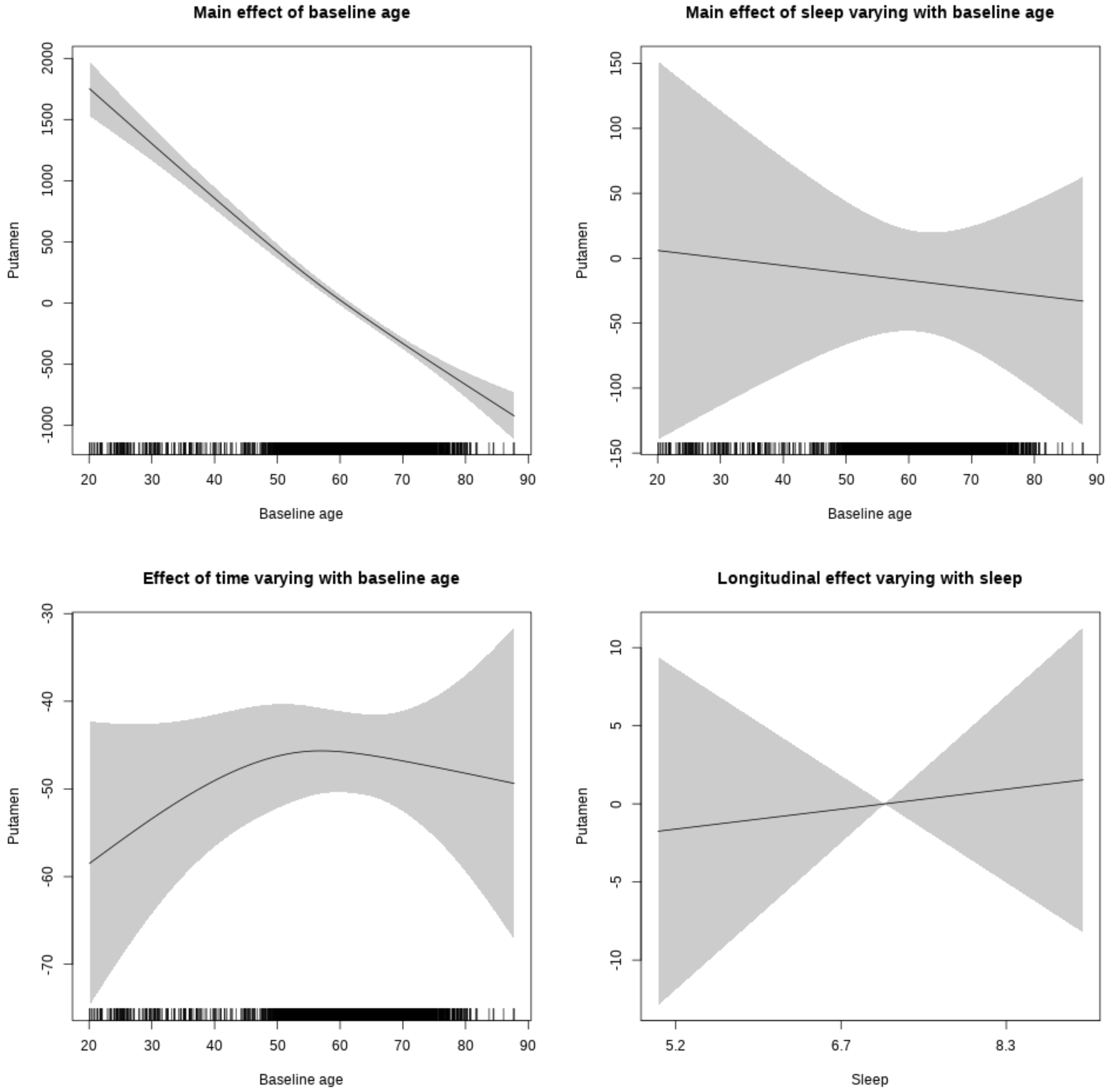

Restricted to sleep >= 5 and <= 9 hours Below is the row for `s(sleep_z):time` from the model output.

```
## s(sleep_z):time   1.000  1.000   0.099   0.753
```

Below are plots of a plot of `s(bl_age)`, `s(bl_age):sleep_z`, `s(bl_age):time`, and `s(sleep_z):time`. For `s(sleep_z):time`, roughly, if we cannot fit a straight line through the confidence bands, there is evidence that the effect of time depends on sleep duration. It is correct the that confidence interval is zero at one point along the axis; this happens because we do not include the main effect.

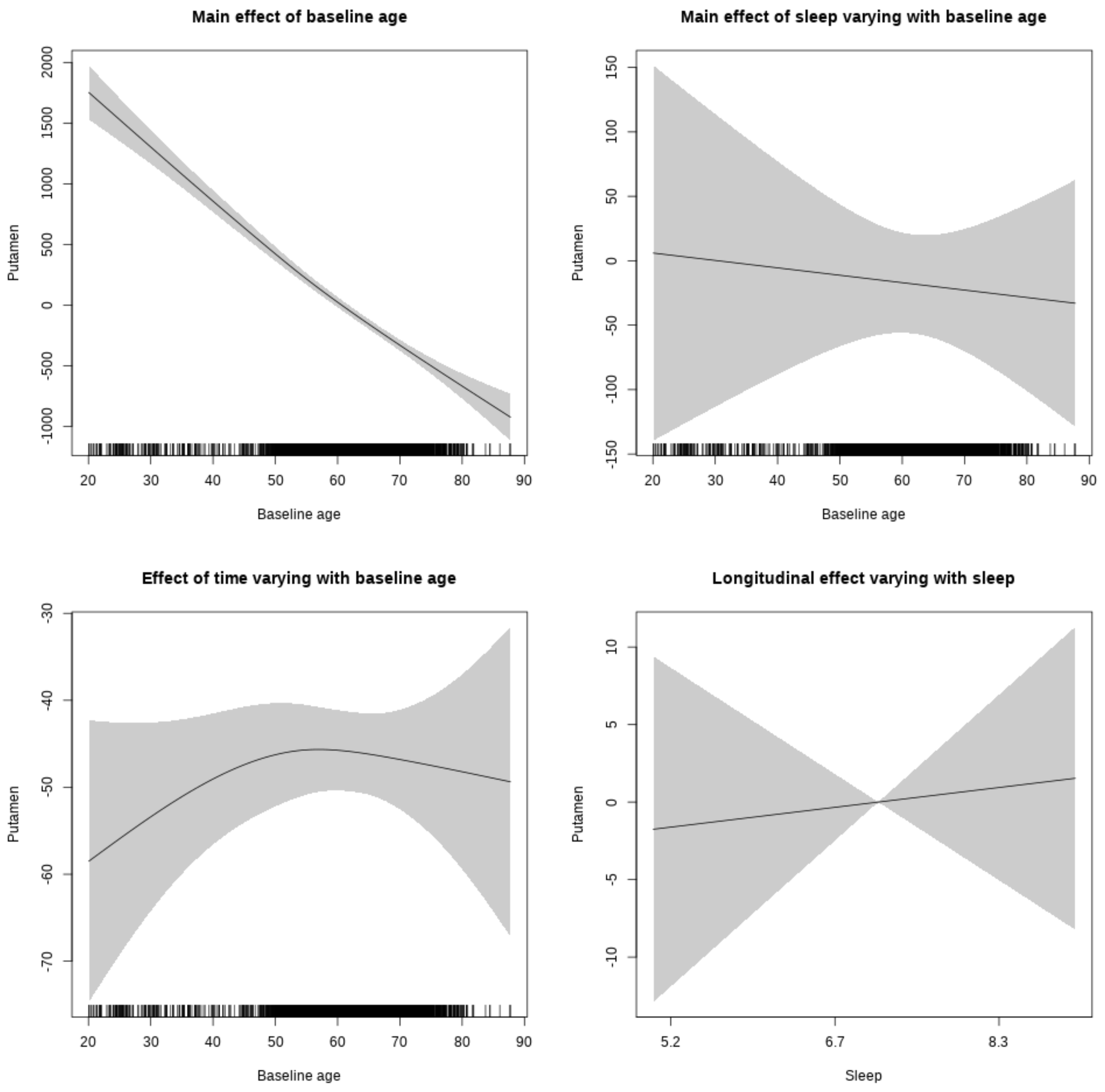

Controling for depression

Full data Below is the model output. The term `s(sleep_z):time` is probably what we care most about.

```
##
## Family: gaussian
## Link function: identity
##
## Formula:
## value ~ sex + site + depression + icv + s(bl_age, k = 10, bs = "cr") +
##     s(bl_age, by = sleep_z, bs = "cr") + +s(bl_age, by = time,
##     k = 5, bs = "cr") + s(sleep_z, by = time, k = 5, bs = "cr",
##     pc = 0)
```

```
## <environment: 0x563910fa49d0>
##
## Parametric coefficients:
##                Estimate Std. Error t value Pr(>|t|)
## (Intercept)     9922.28      71.47 138.823  < 2e-16 ***
## sexmale          352.63      37.43   9.421  < 2e-16 ***
## siteousAvanto  -668.08     143.32  -4.661 3.21e-06 ***
## siteousPrisma  -529.59     222.43  -2.381  0.01730 *
## siteousSkyra    159.28     128.64   1.238  0.21570
## siteUCAM       -546.69      85.46  -6.397 1.71e-10 ***
## siteUKB        -873.40      65.63 -13.307  < 2e-16 ***
## siteUmU        -281.37     105.79  -2.660  0.00784 **
## depression       12.86     132.58   0.097  0.92274
## icv             496.79      17.65  28.148  < 2e-16 ***
## ---
## Signif. codes:  0 '***' 0.001 '**' 0.01 '*' 0.05 '.' 0.1 ' ' 1
##
## Approximate significance of smooth terms:
##                      edf Ref.df       F p-value
## s(bl_age)          1.918  1.918 291.900  <2e-16 ***
## s(bl_age):sleep_z 2.000  2.000   0.599   0.549
## s(bl_age):time    2.753  2.753 185.716  <2e-16 ***
## s(sleep_z):time   1.000  1.000   1.494   0.222
## ---
## Signif. codes:  0 '***' 0.001 '**' 0.01 '*' 0.05 '.' 0.1 ' ' 1
##
## R-sq.(adj) =  0.477
## lmer.REML =  85664  Scale est. = 33794     n = 5671
```

Below are plots of a plot of `s(bl_age)`, `s(bl_age):sleep_z`, `s(bl_age):time`, and `s(sleep_z):time`. For `s(sleep_z):time`, roughly, if we cannot fit a straight line through the confidence bands, there is evidence that the effect of time depends on sleep duration. It is correct the that confidence interval is zero at one point along the axis; this happens because we do not include the main effect.

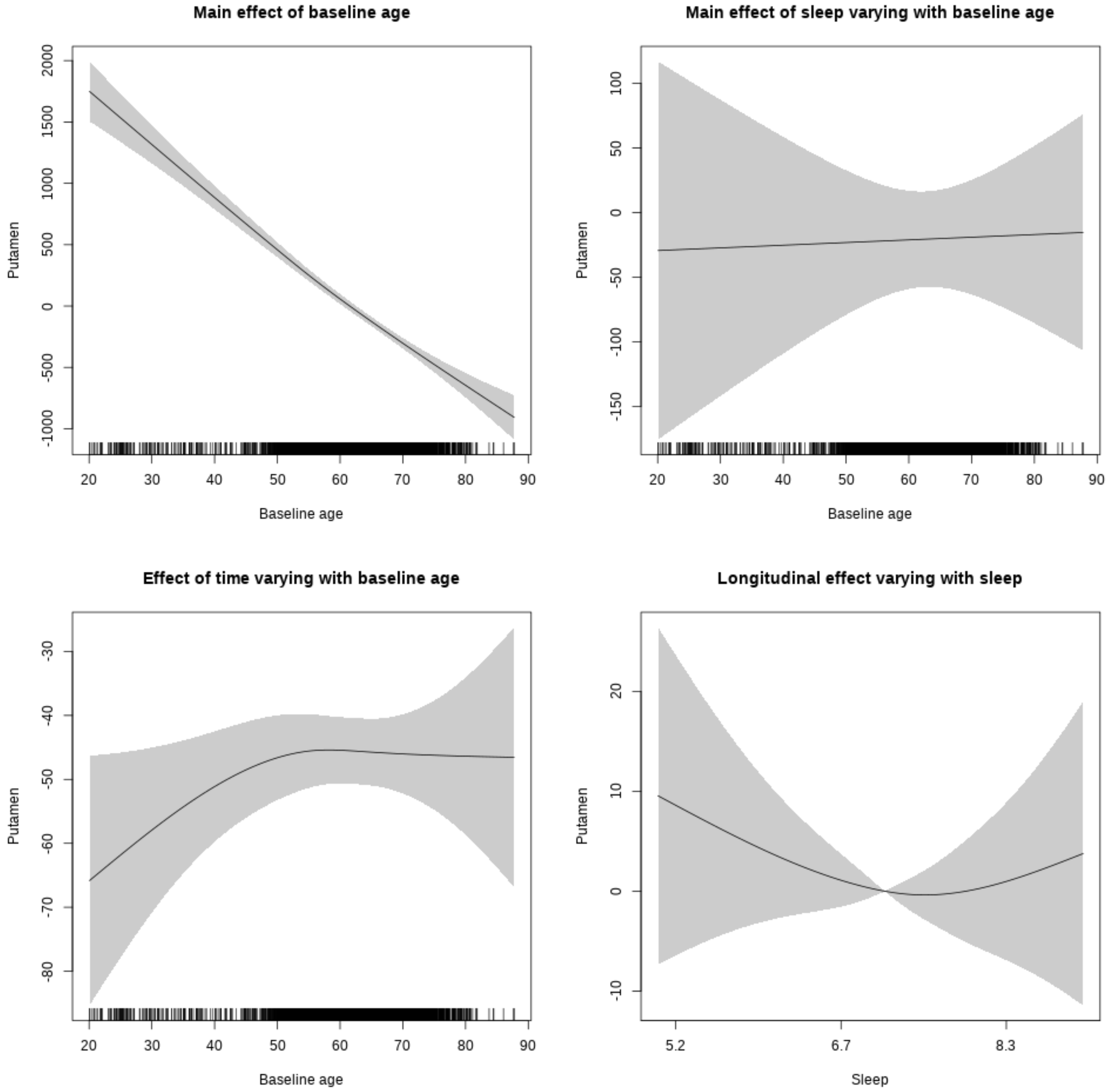

Restricted to sleep >= 5 and <= 9 hours Below is the row for `s(sleep_z):time` from the model output.

```
## s(sleep_z):time   1.590  1.590   0.931   0.505
```

Below are plots of a plot of `s(bl_age)`, `s(bl_age):sleep_z`, `s(bl_age):time`, and `s(sleep_z):time`. For `s(sleep_z):time`, roughly, if we cannot fit a straight line through the confidence bands, there is evidence that the effect of time depends on sleep duration. It is correct the that confidence interval is zero at one point along the axis; this happens because we do not include the main effect.

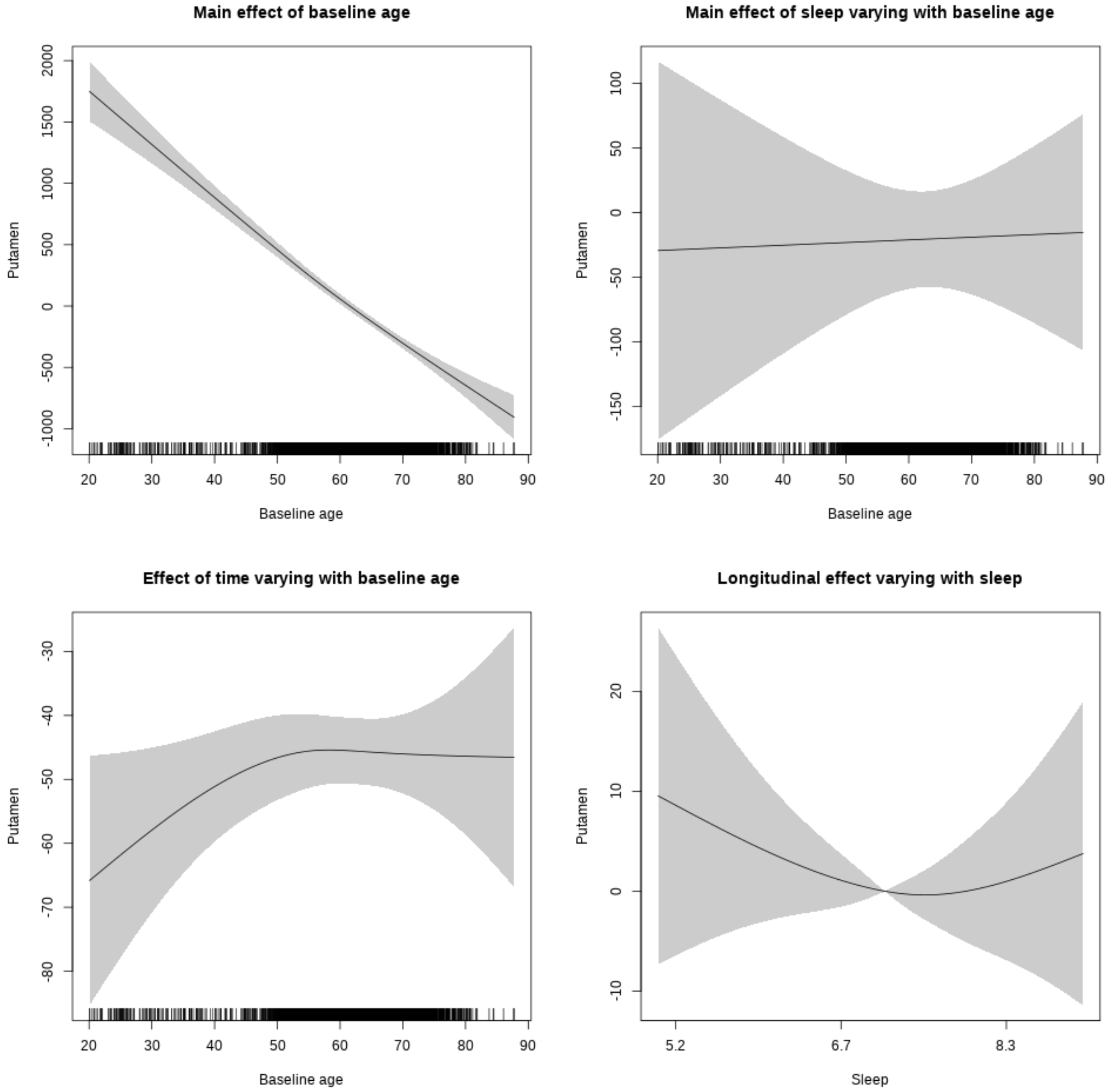

Controling for sleep quality

Full data Below is the model output. The term `s(sleep_z):time` is probably what we care most about.

```
##
## Family: gaussian
## Link function: identity
##
## Formula:
## value ~ X + s(bl_age, k = 10, bs = "cr") + s(bl_age, by = sleep_z,
##     bs = "cr") + s(bl_age, by = time, k = 5, bs = "cr") + s(sleep_z,
##     by = time, k = 5, bs = "cr", pc = 0)
## <environment: 0x563914d995c8>
```

```
##
## Parametric coefficients:
##                           Estimate Std. Error t value Pr(>|t|)
## (Intercept)               8916.543    119.965  74.326  < 2e-16 ***
## Xsexmale                   878.383     45.062  19.493  < 2e-16 ***
## XsiteousAvanto             404.481    114.284   3.539 0.000406 ***
## XsiteousPrisma             496.952    183.650   2.706 0.006838 **
## XsiteousSkyra              877.905    114.532   7.665  2.2e-14 ***
## XsiteUB                    286.924    182.756   1.570 0.116493
## XsiteUCAM                  381.125    116.003   3.285 0.001026 **
## XsiteUKB                   604.876    364.256   1.661 0.096871 .
## XsiteUmU                   347.787    129.881   2.678 0.007441 **
## Xukb_dummy:sleep_accel     -14.825     28.289  -0.524 0.600268
## Xukb_dummy:chronotype       -8.904     37.725  -0.236 0.813433
## Xukb_dummy:dozing           81.039     77.851   1.041 0.297960
## Xukb_dummy:sleeplessness  -119.550     48.367  -2.472 0.013484 *
## Xukb_dummy:snoring         -77.248     68.762  -1.123 0.261324
## Xukb_dummy:gettingup       -30.098     52.434  -0.574 0.565988
## Xnotukb_dummy:PSQI_Global    2.456     14.906   0.165 0.869147
## ---
## Signif. codes:  0 '***' 0.001 '**' 0.01 '*' 0.05 '.' 0.1 ' ' 1
##
## Approximate significance of smooth terms:
##                       edf Ref.df       F p-value
## s(bl_age)           1.178  1.178 431.280  <2e-16 ***
## s(bl_age):sleep_z 2.000  2.000   1.790  0.1671
## s(bl_age):time      2.000  2.000 258.458  <2e-16 ***
## s(sleep_z):time     1.000  1.000   6.278  0.0123 *
## ---
## Signif. codes:  0 '***' 0.001 '**' 0.01 '*' 0.05 '.' 0.1 ' ' 1
##
## R-sq.(adj) =  0.392
## lmer.REML =  64712  Scale est. = 34945     n = 4278
```

Below are plots of a plot of `s(bl_age)`, `s(bl_age):sleep_z`, `s(bl_age):time`, and `s(sleep_z):time`. For `s(sleep_z):time`, roughly, if we cannot fit a straight line through the confidence bands, there is evidence that the effect of time depends on sleep duration. It is correct the that confidence interval is zero at one point along the axis; this happens because we do not include the main effect.

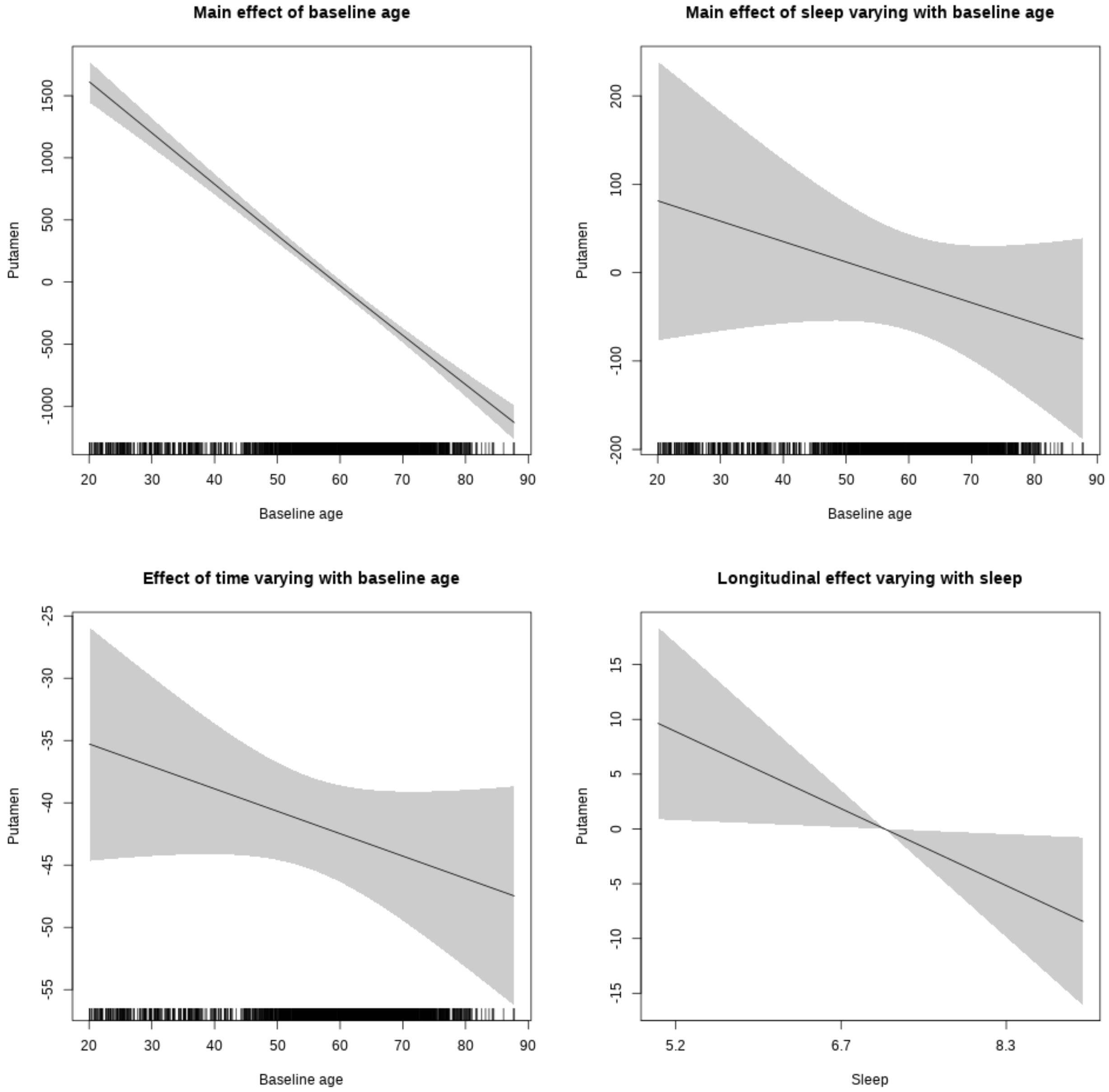

Restricted to sleep >= 5 and <= 9 hours Below is the row for `s(sleep_z):time` from the model output.

```
## s(sleep_z):time   1.000  1.000   4.843  0.0278 *
```

Below are plots of a plot of `s(bl_age)`, `s(bl_age):sleep_z`, `s(bl_age):time`, and `s(sleep_z):time`. For `s(sleep_z):time`, roughly, if we cannot fit a straight line through the confidence bands, there is evidence that the effect of time depends on sleep duration. It is correct the that confidence interval is zero at one point along the axis; this happens because we do not include the main effect.

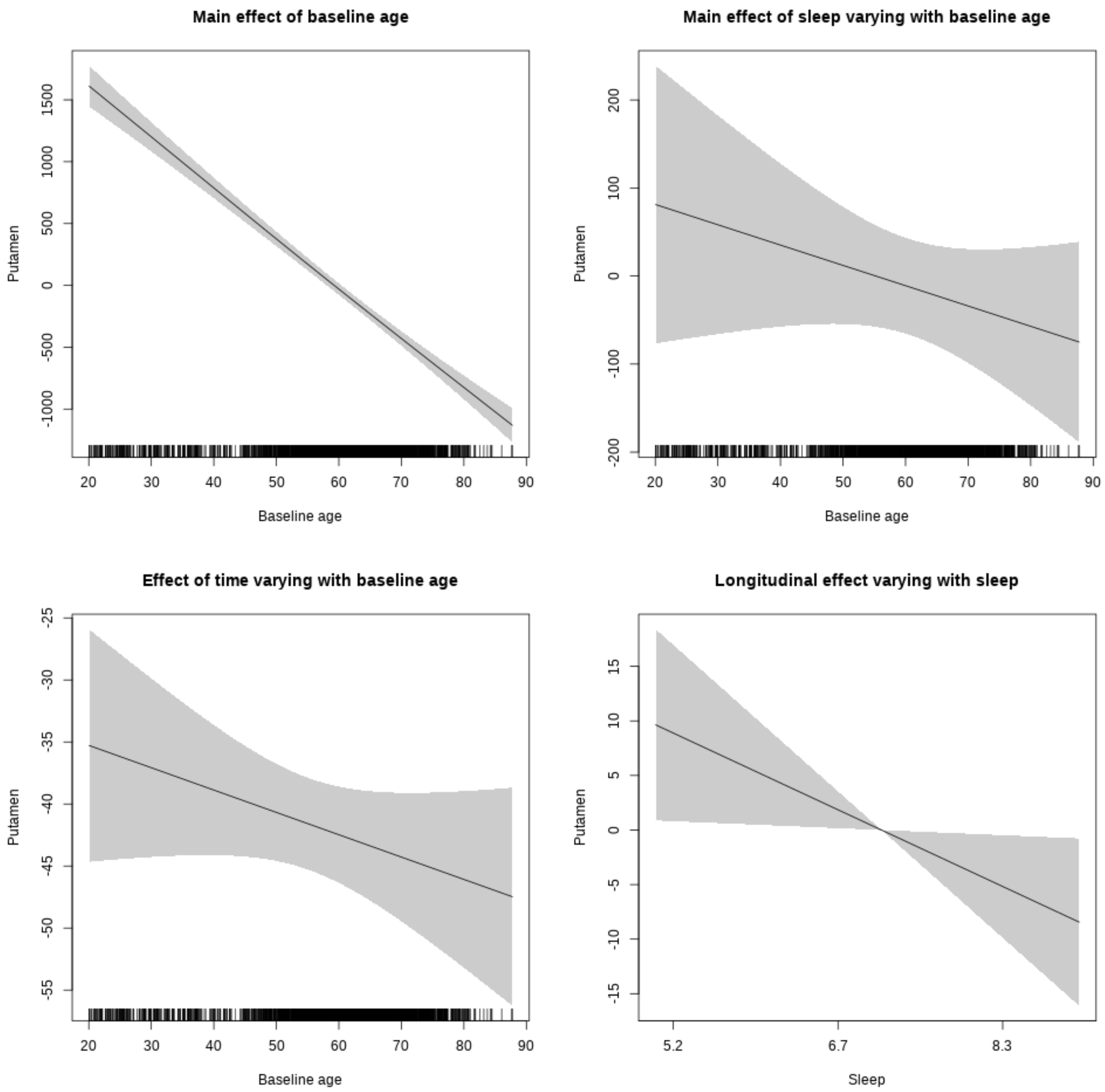

Thalamus

Main analysis

Full data Below is the model output. The term `s(sleep_z):time` is probably what we care most about.

```
##
## Family: gaussian
## Link function: identity
##
## Formula:
## value ~ sex + site + icv + s(bl_age, k = 10, bs = "cr") + s(bl_age,
##     by = sleep_z, bs = "cr") + +s(bl_age, by = time, k = 5, bs = "cr") +
```

```
##     s(sleep_z, by = time, k = 5, bs = "cr", pc = 0)
## <environment: 0x563911250648>
##
## Parametric coefficients:
##                Estimate Std. Error t value Pr(>|t|)
## (Intercept)   15114.94      71.51 211.376  < 2e-16 ***
## sexmale         191.81      36.80   5.212 1.92e-07 ***
## siteousAvanto -1484.08      87.75 -16.913  < 2e-16 ***
## siteousPrisma -1016.87     227.67  -4.466 8.06e-06 ***
## siteousSkyra  -1321.26      85.36 -15.479  < 2e-16 ***
## siteUB         -479.61     165.60  -2.896  0.00379 **
## siteUCAM       -600.07      89.81  -6.682 2.52e-11 ***
## siteUKB       -1343.41      68.28 -19.675  < 2e-16 ***
## siteUmU       -1481.90     102.79 -14.417  < 2e-16 ***
## icv             875.66      17.65  49.611  < 2e-16 ***
## ---
## Signif. codes:  0 ‘***’ 0.001 ‘**’ 0.01 ‘*’ 0.05 ‘.’ 0.1 ‘ ’ 1
##
## Approximate significance of smooth terms:
##                      edf Ref.df       F  p-value
## s(bl_age)          4.690  4.690 390.659  < 2e-16 ***
## s(bl_age):sleep_z 2.000  2.000   4.306 0.013511 *
## s(bl_age):time    4.152  4.152 345.105  < 2e-16 ***
## s(sleep_z):time   1.000  1.000  12.385 0.000435 ***
## ---
## Signif. codes:  0 ‘***’ 0.001 ‘**’ 0.01 ‘*’ 0.05 ‘.’ 0.1 ‘ ’ 1
##
## R-sq.(adj) =  0.618
## lmer.REML = 1.2711e+05  Scale est. = 74884     n = 8150
```

Below are plots of a plot of `s(bl_age)`, `s(bl_age):sleep_z`, `s(bl_age):time`, and `s(sleep_z):time`. For `s(sleep_z):time`, roughly, if we cannot fit a straight line through the confidence bands, there is evidence that the effect of time depends on sleep duration. It is correct the that confidence interval is zero at one point along the axis; this happens because we do not include the main effect.

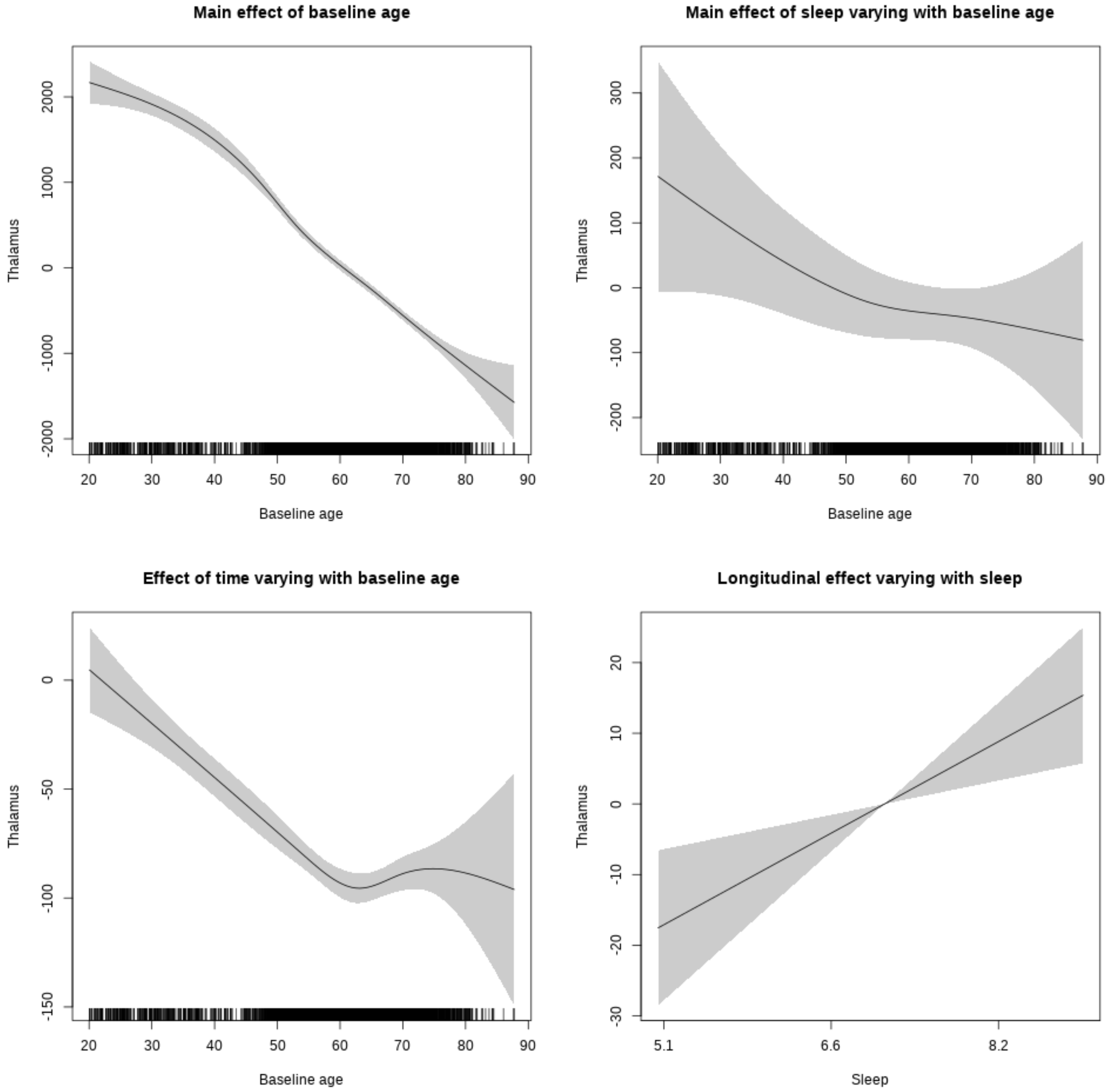

Restricted to sleep >= 5 and <= 9 hours Below is the row for `s(sleep_z):time` from the model output.

```
## s(sleep_z):time   1.000  1.000  10.227 0.00139 **
```

Below are plots of a plot of `s(bl_age)`, `s(bl_age):sleep_z`, `s(bl_age):time`, and `s(sleep_z):time`. For `s(sleep_z):time`, roughly, if we cannot fit a straight line through the confidence bands, there is evidence that the effect of time depends on sleep duration. It is correct the that confidence interval is zero at one point along the axis; this happens because we do not include the main effect.

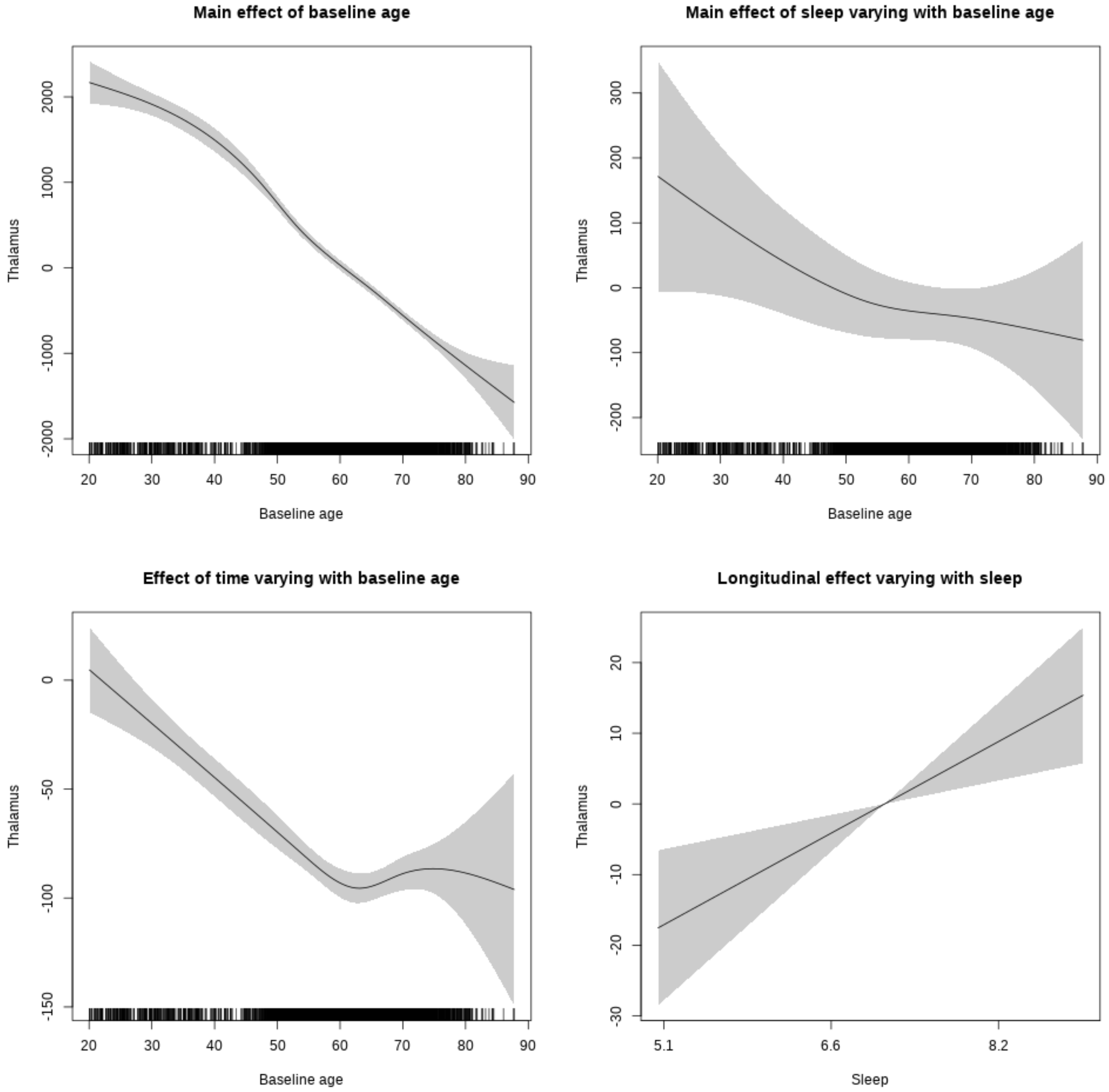

Controling for socioeconomic status

Full data Below is the model output. The term `s(sleep_z):time` is probably what we care most about.

```
##
## Family: gaussian
## Link function: identity
##
## Formula:
## value ~ sex + site + income_scaled + education_scaled + icv +
##     s(bl_age, k = 10, bs = "cr") + s(bl_age, by = sleep_z, bs = "cr") +
##     +s(bl_age, by = time, k = 5, bs = "cr") + s(sleep_z, by = time,
##     k = 5, bs = "cr", pc = 0)
```

```
## <environment: 0x563911590e40>
##
## Parametric coefficients:
##                  Estimate Std. Error t value Pr(>|t|)
## (Intercept)      15164.94     165.44  91.665  < 2e-16 ***
## sexmale            154.68      46.21   3.348 0.000822 ***
## siteousAvanto    -1945.43     182.91 -10.636  < 2e-16 ***
## siteousPrisma     -767.44     386.82  -1.984 0.047320 *
## siteousSkyra     -1499.75     170.54  -8.794  < 2e-16 ***
## siteUKB          -1576.56     155.79 -10.120  < 2e-16 ***
## income_scaled       36.64      56.70   0.646 0.518194
## education_scaled   135.05      65.86   2.051 0.040355 *
## icv                955.59      22.96  41.622  < 2e-16 ***
## ---
## Signif. codes:  0 '***' 0.001 '**' 0.01 '*' 0.05 '.' 0.1 ' ' 1
##
## Approximate significance of smooth terms:
##                     edf Ref.df       F p-value
## s(bl_age)         1.000  1.000 691.854  <2e-16 ***
## s(bl_age):sleep_z 2.000  2.000   0.546  0.5794
## s(bl_age):time    3.977  3.977 114.185  <2e-16 ***
## s(sleep_z):time   1.000  1.000   3.280  0.0702 .
## ---
## Signif. codes:  0 '***' 0.001 '**' 0.01 '*' 0.05 '.' 0.1 ' ' 1
##
## R-sq.(adj) =   0.61
## lmer.REML =  72657  Scale est. = 79667     n = 4650
```

Below are plots of a plot of `s(bl_age)`, `s(bl_age):sleep_z`, `s(bl_age):time`, and `s(sleep_z):time`. For `s(sleep_z):time`, roughly, if we cannot fit a straight line through the confidence bands, there is evidence that the effect of time depends on sleep duration. It is correct the that confidence interval is zero at one point along the axis; this happens because we do not include the main effect.

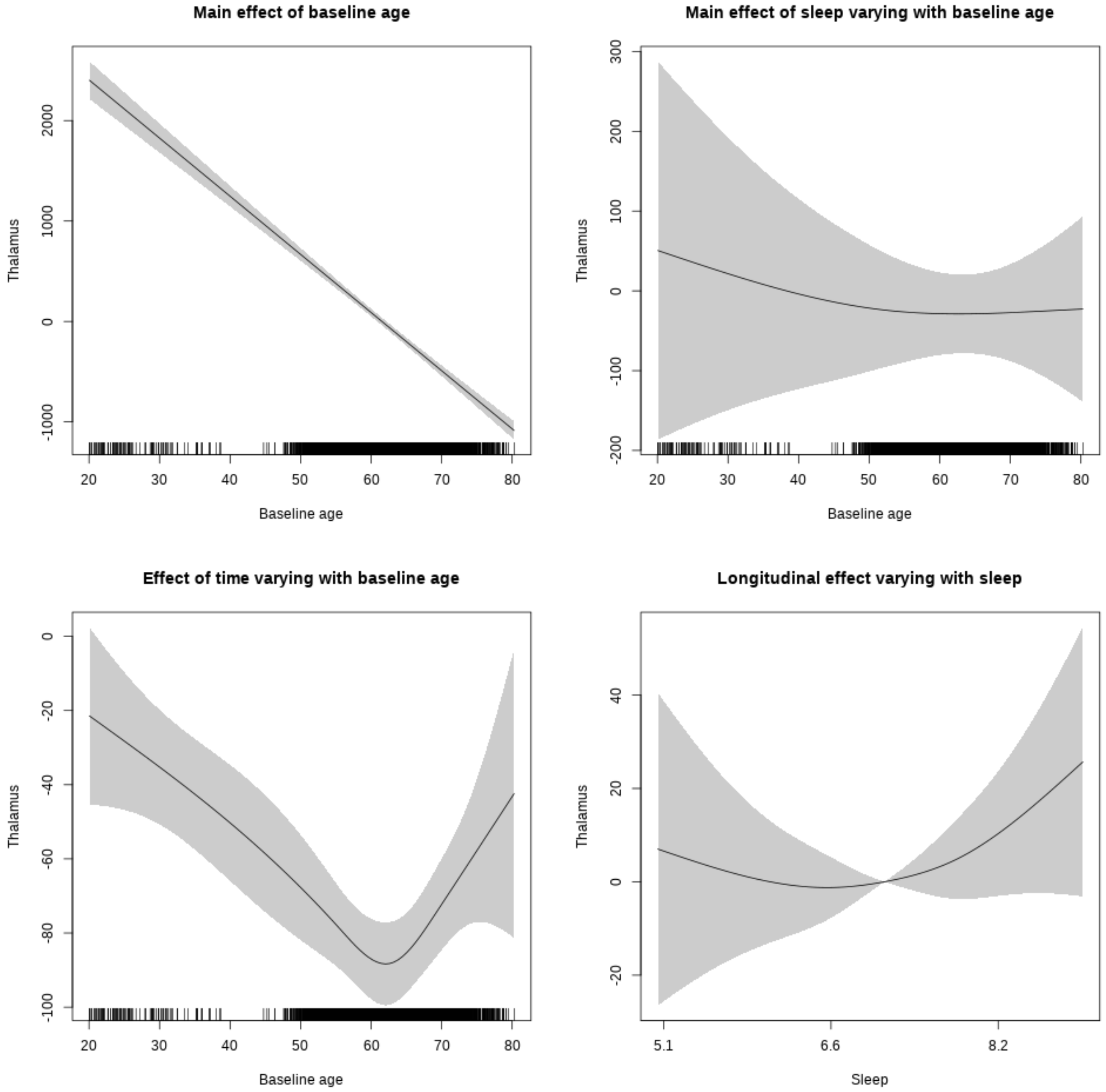

Restricted to sleep >= 5 and <= 9 hours Below is the row for `s(sleep_z):time` from the model output.

```
## s(sleep_z):time   1.842  1.842   1.030   0.256
```

Below are plots of a plot of `s(bl_age)`, `s(bl_age):sleep_z`, `s(bl_age):time`, and `s(sleep_z):time`. For `s(sleep_z):time`, roughly, if we cannot fit a straight line through the confidence bands, there is evidence that the effect of time depends on sleep duration. It is correct the that confidence interval is zero at one point along the axis; this happens because we do not include the main effect.

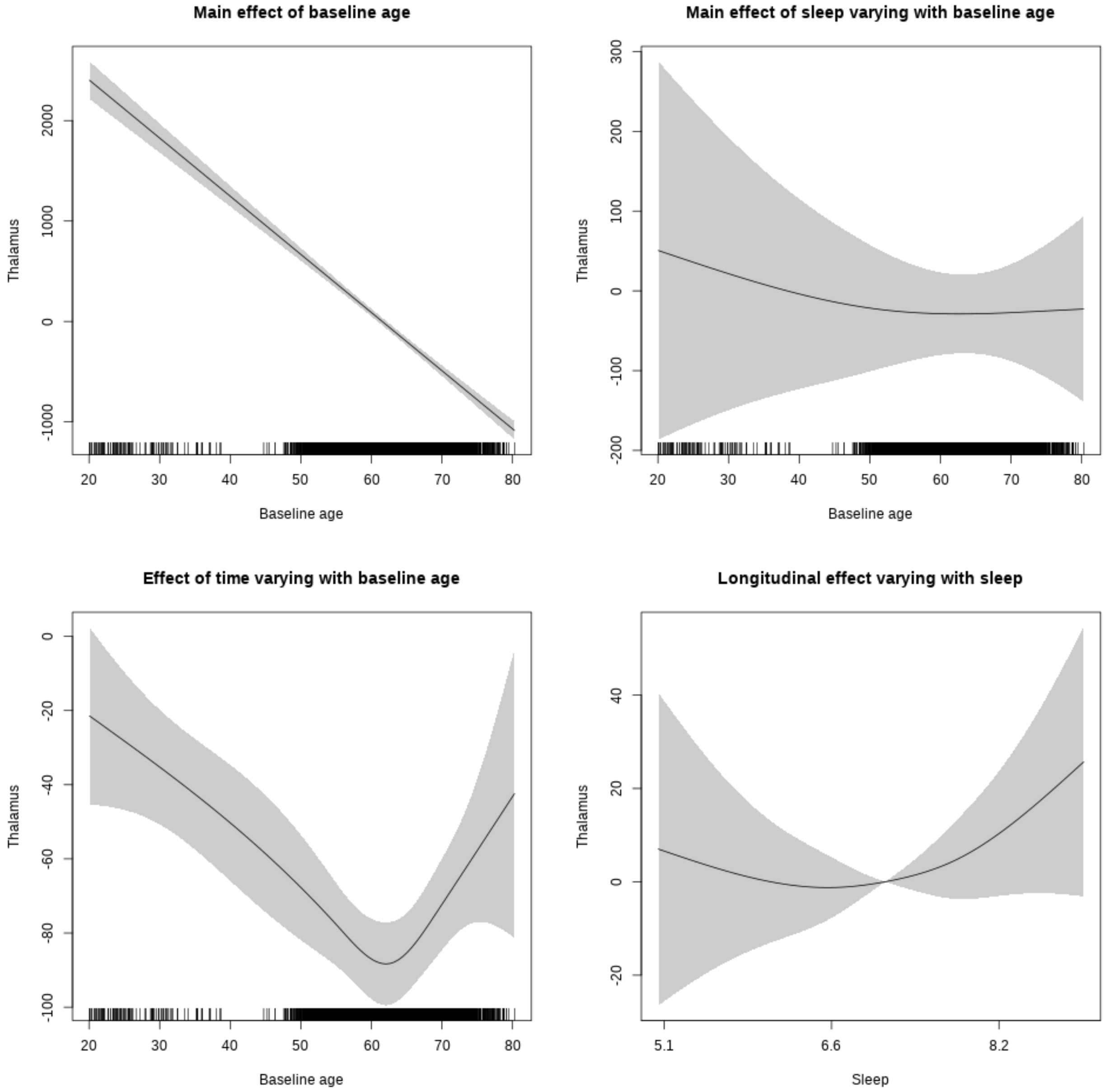

Controling for BMI

Full data Below is the model output. The term `s(sleep_z):time` is probably what we care most about.

```
##
## Family: gaussian
## Link function: identity
##
## Formula:
## value ~ sex + site + bmi + icv + s(bl_age, k = 10, bs = "cr") +
##     s(bl_age, by = sleep_z, bs = "cr") + +s(bl_age, by = time,
##     k = 5, bs = "cr") + s(sleep_z, by = time, k = 5, bs = "cr",
##     pc = 0)
```

```
## <environment: 0x563916b2ebb8>
##
## Parametric coefficients:
##                Estimate Std. Error t value Pr(>|t|)
## (Intercept)   13546.691    146.724  92.328  < 2e-16 ***
## sexmale         153.588     43.090   3.564 0.000368 ***
## siteousPrisma   475.689    219.412   2.168 0.030201 *
## siteousSkyra    266.377     69.724   3.820 0.000135 ***
## siteUCAM       1095.049    112.932   9.696  < 2e-16 ***
## siteUKB         236.783    100.339   2.360 0.018319 *
## siteUmU         193.892    128.602   1.508 0.131692
## bmi              -1.855      4.473  -0.415 0.678411
## icv             939.193     21.225  44.250  < 2e-16 ***
## ---
## Signif. codes:  0 '***' 0.001 '**' 0.01 '*' 0.05 '.' 0.1 ' ' 1
##
## Approximate significance of smooth terms:
##                     edf Ref.df        F p-value
## s(bl_age)         1.000  1.000 1192.516 < 2e-16 ***
## s(bl_age):sleep_z 2.000  2.000    1.364 0.25576
## s(bl_age):time    4.113  4.113  143.055 < 2e-16 ***
## s(sleep_z):time   1.000  1.000    8.973 0.00275 **
## ---
## Signif. codes:  0 '***' 0.001 '**' 0.01 '*' 0.05 '.' 0.1 ' ' 1
##
## R-sq.(adj) =  0.644
## lmer.REML =  84425  Scale est. = 73529     n = 5412
```

Below are plots of a plot of `s(bl_age)`, `s(bl_age):sleep_z`, `s(bl_age):time`, and `s(sleep_z):time`. For `s(sleep_z):time`, roughly, if we cannot fit a straight line through the confidence bands, there is evidence that the effect of time depends on sleep duration. It is correct the that confidence interval is zero at one point along the axis; this happens because we do not include the main effect.

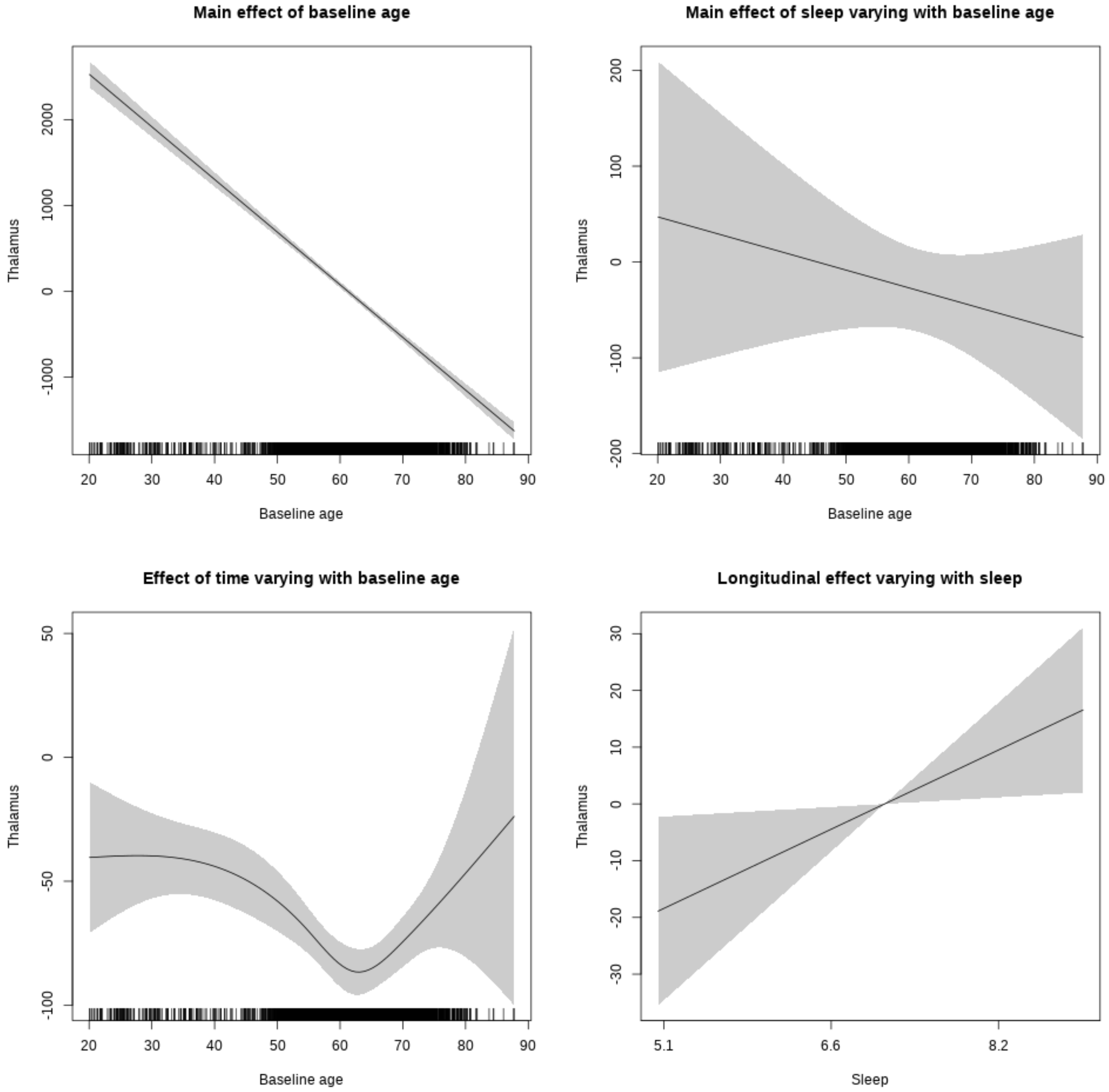

Restricted to sleep >= 5 and <= 9 hours Below is the row for `s(sleep_z):time` from the model output.

```
## s(sleep_z):time   1.000  1.000    5.175   0.023 *
```

Below are plots of a plot of `s(bl_age)`, `s(bl_age):sleep_z`, `s(bl_age):time`, and `s(sleep_z):time`. For `s(sleep_z):time`, roughly, if we cannot fit a straight line through the confidence bands, there is evidence that the effect of time depends on sleep duration. It is correct the that confidence interval is zero at one point along the axis; this happens because we do not include the main effect.

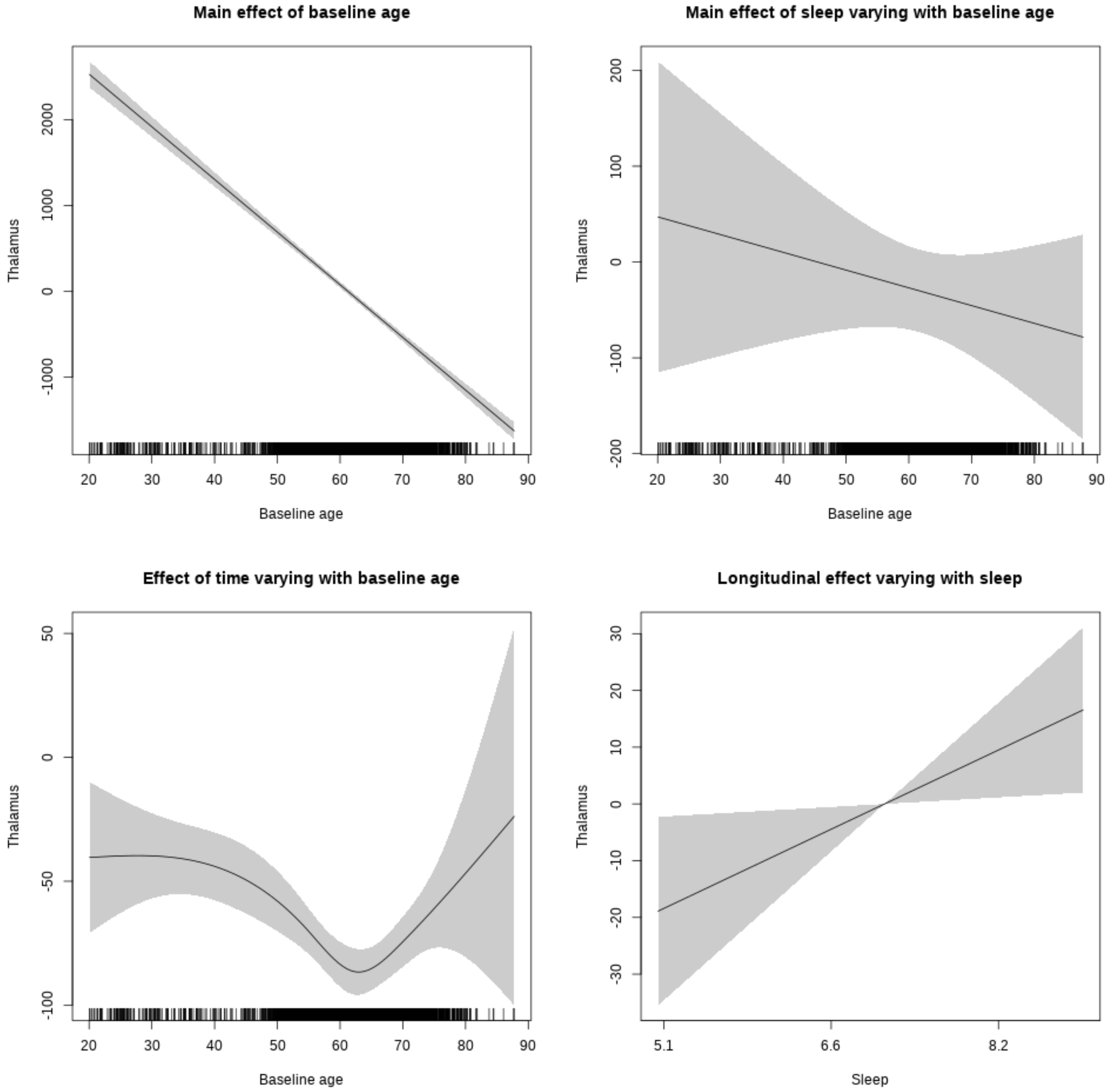

Controling for depression

Full data Below is the model output. The term `s(sleep_z):time` is probably what we care most about.

```
##
## Family: gaussian
## Link function: identity
##
## Formula:
## value ~ sex + site + depression + icv + s(bl_age, k = 10, bs = "cr") +
##     s(bl_age, by = sleep_z, bs = "cr") + +s(bl_age, by = time,
##     k = 5, bs = "cr") + s(sleep_z, by = time, k = 5, bs = "cr",
##     pc = 0)
```

```
## <environment: 0x563910fb0378>
##
## Parametric coefficients:
##                Estimate Std. Error t value Pr(>|t|)
## (Intercept)    15029.29      82.09 183.084  < 2e-16 ***
## sexmale          172.68      42.88   4.027 5.71e-05 ***
## siteousAvanto -1391.76     174.36  -7.982 1.73e-15 ***
## siteousPrisma -1173.10     300.27  -3.907 9.46e-05 ***
## siteousSkyra  -1012.24     150.65  -6.719 2.01e-11 ***
## siteUCAM       -532.94      99.92  -5.334 9.98e-08 ***
## siteUKB       -1329.19      75.55 -17.593  < 2e-16 ***
## siteUmU       -1300.74     120.45 -10.799  < 2e-16 ***
## depression     -258.94     150.84  -1.717   0.0861 .
## icv             882.67      20.62  42.808  < 2e-16 ***
## ---
## Signif. codes:  0 ‘***’ 0.001 ‘**’ 0.01 ‘*’ 0.05 ‘.’ 0.1 ‘ ’ 1
##
## Approximate significance of smooth terms:
##                       edf Ref.df       F p-value
## s(bl_age)           5.091  5.091 200.242  <2e-16 ***
## s(bl_age):sleep_z 2.000  2.000   1.626  0.1968
## s(bl_age):time    2.891  2.891 237.956  <2e-16 ***
## s(sleep_z):time   1.000  1.000   4.606  0.0319 *
## ---
## Signif. codes:  0 ‘***’ 0.001 ‘**’ 0.01 ‘*’ 0.05 ‘.’ 0.1 ‘ ’ 1
##
## R-sq.(adj) =  0.618
## lmer.REML =  88636  Scale est. = 71111     n = 5679
```

Below are plots of a plot of `s(bl_age)`, `s(bl_age):sleep_z`, `s(bl_age):time`, and `s(sleep_z):time`. For `s(sleep_z):time`, roughly, if we cannot fit a straight line through the confidence bands, there is evidence that the effect of time depends on sleep duration. It is correct the that confidence interval is zero at one point along the axis; this happens because we do not include the main effect.

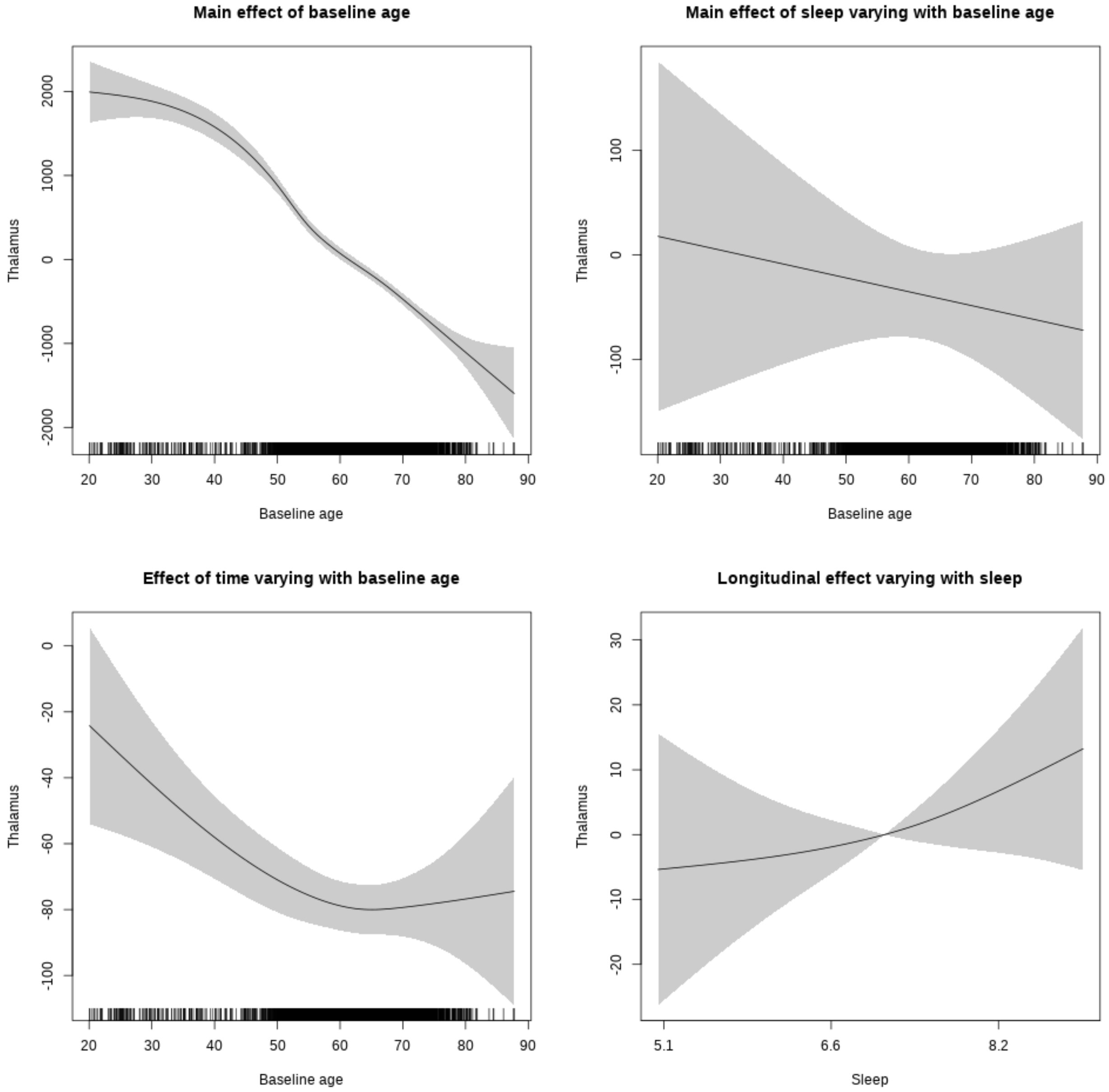

Restricted to sleep >= 5 and <= 9 hours Below is the row for `s(sleep_z):time` from the model output.

```
## s(sleep_z):time   1.317  1.317   0.886   0.269
```

Below are plots of a plot of `s(bl_age)`, `s(bl_age):sleep_z`, `s(bl_age):time`, and `s(sleep_z):time`. For `s(sleep_z):time`, roughly, if we cannot fit a straight line through the confidence bands, there is evidence that the effect of time depends on sleep duration. It is correct the that confidence interval is zero at one point along the axis; this happens because we do not include the main effect.

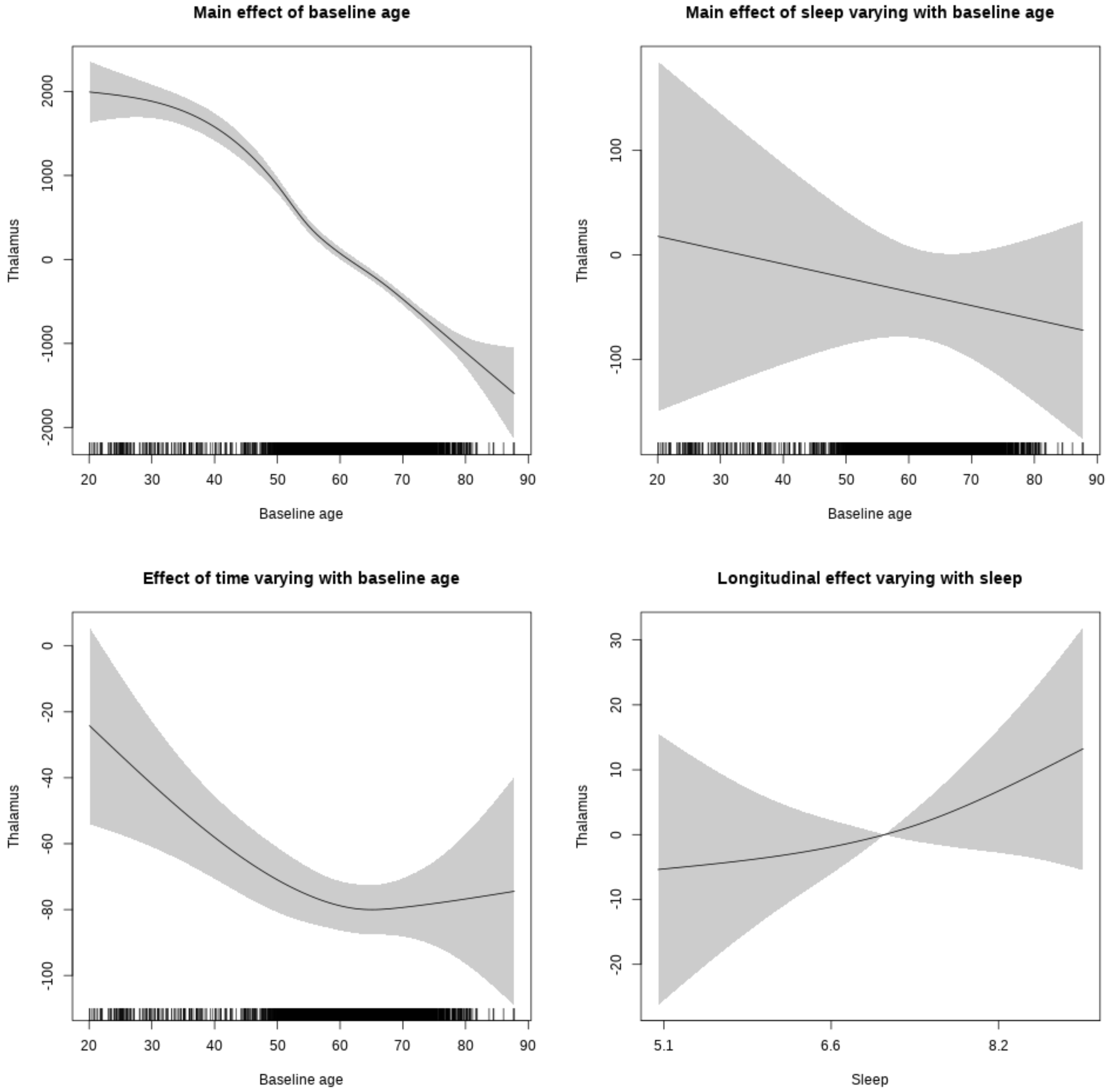

Controling for sleep quality

Full data Below is the model output. The term `s(sleep_z):time` is probably what we care most about.

```
##
## Family: gaussian
## Link function: identity
##
## Formula:
## value ~ X + s(bl_age, k = 10, bs = "cr") + s(bl_age, by = sleep_z,
##     bs = "cr") + s(bl_age, by = time, k = 5, bs = "cr") + s(sleep_z,
##     by = time, k = 5, bs = "cr", pc = 0)
## <environment: 0x563914d995c8>
```

```
##
## Parametric coefficients:
##                            Estimate Std. Error t value Pr(>|t|)
## (Intercept)                13395.43     148.41  90.263  < 2e-16 ***
## Xsexmale                    1139.87      55.51  20.534  < 2e-16 ***
## XsiteousAvanto               414.63     141.84   2.923  0.00348 **
## XsiteousPrisma              1294.26     248.77   5.203 2.06e-07 ***
## XsiteousSkyra                259.72     142.22   1.826  0.06790 .
## XsiteUB                      709.72     225.35   3.149  0.00165 **
## XsiteUCAM                    981.53     145.69   6.737 1.83e-11 ***
## XsiteUKB                      55.23     453.67   0.122  0.90311
## XsiteUmU                    -354.61     161.60  -2.194  0.02826 *
## Xukb_dummy:sleep_accel        35.87      35.13   1.021  0.30725
## Xukb_dummy:chronotype         40.01      46.51   0.860  0.38965
## Xukb_dummy:dozing             42.45      95.97   0.442  0.65829
## Xukb_dummy:sleeplessness    -188.93      59.86  -3.156  0.00161 **
## Xukb_dummy:snoring           -14.40      84.71  -0.170  0.86501
## Xukb_dummy:gettingup          19.62      65.11   0.301  0.76318
## Xnotukb_dummy:PSQI_Global    -20.75      18.36  -1.130  0.25862
## ---
## Signif. codes:  0 '***' 0.001 '**' 0.01 '*' 0.05 '.' 0.1 ' ' 1
##
## Approximate significance of smooth terms:
##                     edf Ref.df       F  p-value
## s(bl_age)         3.842  3.842 214.494  < 2e-16 ***
## s(bl_age):sleep_z 2.000  2.000   2.594 0.074851 .
## s(bl_age):time    3.804  3.804 285.641  < 2e-16 ***
## s(sleep_z):time   1.000  1.000  14.047 0.000181 ***
## ---
## Signif. codes:  0 '***' 0.001 '**' 0.01 '*' 0.05 '.' 0.1 ' ' 1
##
## R-sq.(adj) =  0.463
## lmer.REML =  67270  Scale est. = 70944     n = 4284
```

Below are plots of a plot of `s(bl_age)`, `s(bl_age):sleep_z`, `s(bl_age):time`, and `s(sleep_z):time`. For `s(sleep_z):time`, roughly, if we cannot fit a straight line through the confidence bands, there is evidence that the effect of time depends on sleep duration. It is correct the that confidence interval is zero at one point along the axis; this happens because we do not include the main effect.

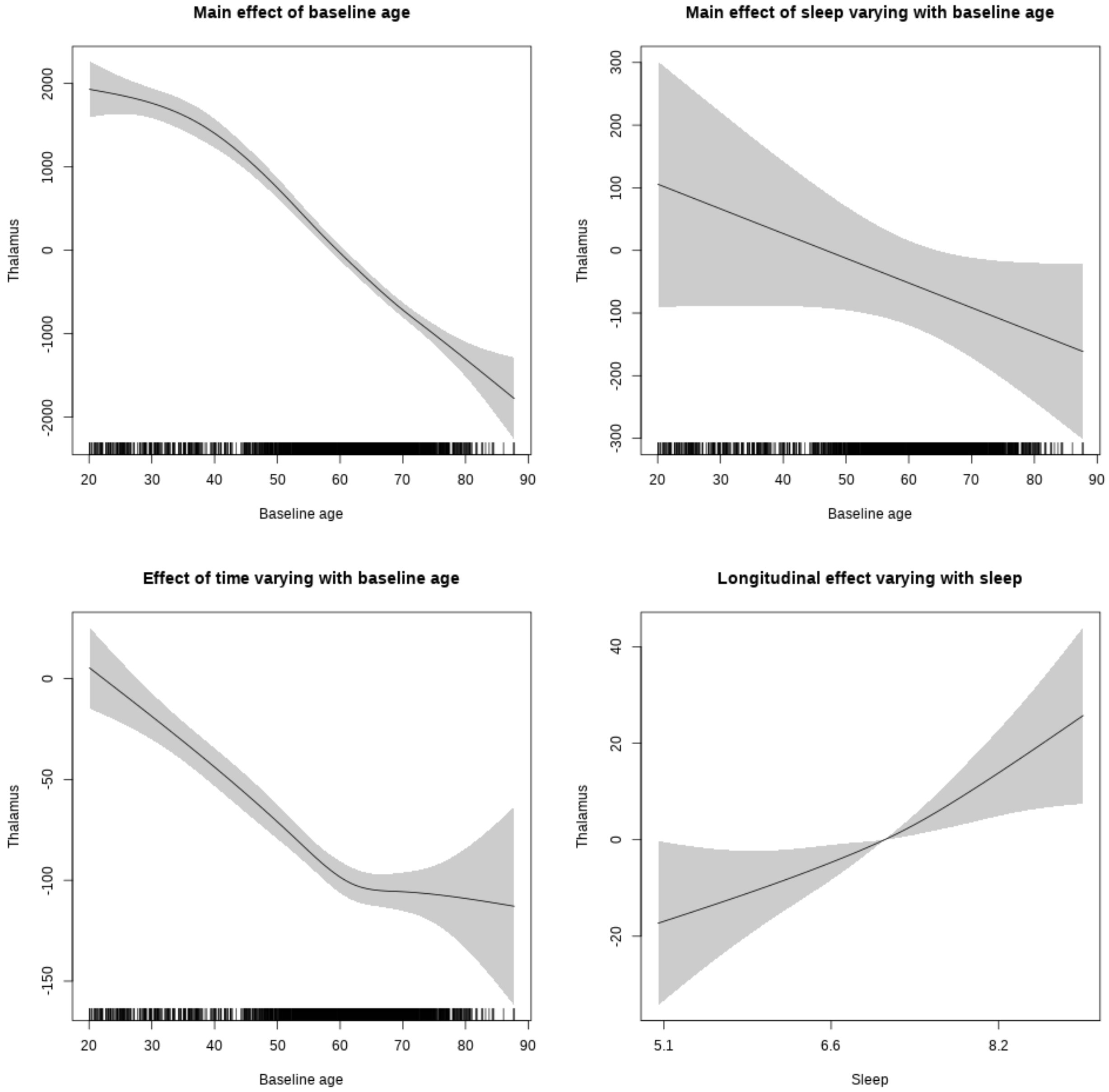

Restricted to sleep >= 5 and <= 9 hours Below is the row for `s(sleep_z):time` from the model output.

```
## s(sleep_z):time   1.473  1.473   5.768 0.00451 **
```

Below are plots of a plot of `s(bl_age)`, `s(bl_age):sleep_z`, `s(bl_age):time`, and `s(sleep_z):time`. For `s(sleep_z):time`, roughly, if we cannot fit a straight line through the confidence bands, there is evidence that the effect of time depends on sleep duration. It is correct the that confidence interval is zero at one point along the axis; this happens because we do not include the main effect.

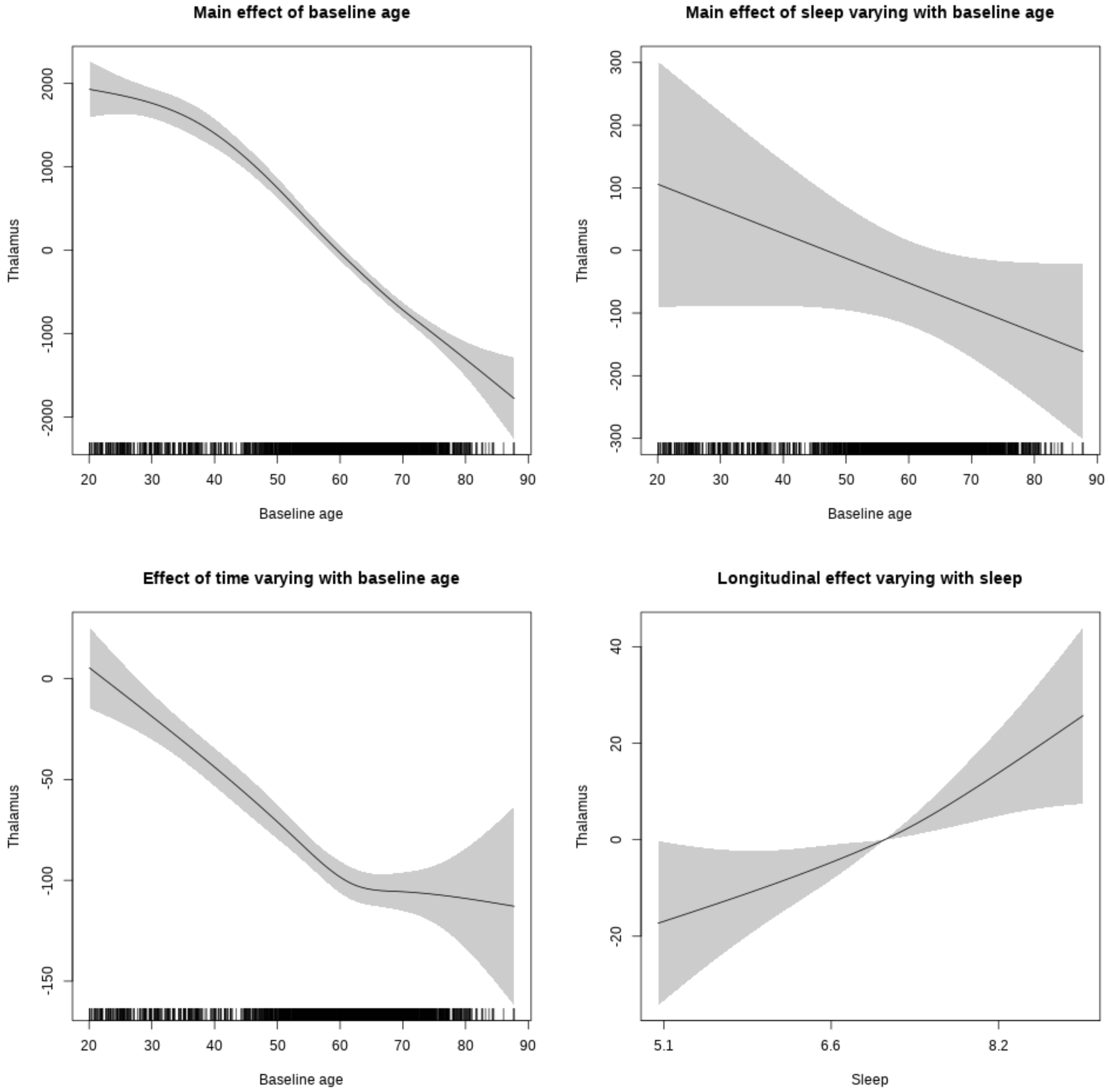

TotalGrayVol

Main analysis

Full data Below is the model output. The term `s(sleep_z):time` is probably what we care most about.

```
##
## Family: gaussian
## Link function: identity
##
## Formula:
## value ~ sex + site + icv + s(bl_age, k = 10, bs = "cr") + s(bl_age,
##     by = sleep_z, bs = "cr") + +s(bl_age, by = time, k = 5, bs = "cr") +
```

```
##     s(sleep_z, by = time, k = 5, bs = "cr", pc = 0)
## <environment: 0x563911248ae0>
##
## Parametric coefficients:
##               Estimate Std. Error t value Pr(>|t|)
## (Intercept)   663161.6     2098.3 316.049  < 2e-16 ***
## sexmale        16218.2     1079.8  15.020  < 2e-16 ***
## siteousAvanto -60084.4     2584.1 -23.251  < 2e-16 ***
## siteousPrisma -46885.4     8104.3  -5.785 7.51e-09 ***
## siteousSkyra  -34623.0     2511.2 -13.787  < 2e-16 ***
## siteUB        -38331.3     4816.6  -7.958 1.98e-15 ***
## siteUCAM      -38678.3     2626.4 -14.727  < 2e-16 ***
## siteUKB         2069.2     1998.9   1.035    0.301
## siteUmU       -23067.1     3001.3  -7.686 1.70e-14 ***
## icv            43972.4      525.3  83.705  < 2e-16 ***
## ---
## Signif. codes:  0 '***' 0.001 '**' 0.01 '*' 0.05 '.' 0.1 ' ' 1
##
## Approximate significance of smooth terms:
##                     edf Ref.df       F p-value
## s(bl_age)         4.701  4.701 510.142  <2e-16 ***
## s(bl_age):sleep_z 2.000  2.000   1.666   0.189
## s(bl_age):time    4.062  4.062 256.529  <2e-16 ***
## s(sleep_z):time   1.000  1.000   0.316   0.574
## ---
## Signif. codes:  0 '***' 0.001 '**' 0.01 '*' 0.05 '.' 0.1 ' ' 1
##
## R-sq.(adj) =  0.805
## lmer.REML = 1.8416e+05  Scale est. = 1.0252e+08  n = 8152
```

Below are plots of a plot of `s(bl_age)`, `s(bl_age):sleep_z`, `s(bl_age):time`, and `s(sleep_z):time`. For `s(sleep_z):time`, roughly, if we cannot fit a straight line through the confidence bands, there is evidence that the effect of time depends on sleep duration. It is correct the that confidence interval is zero at one point along the axis; this happens because we do not include the main effect.

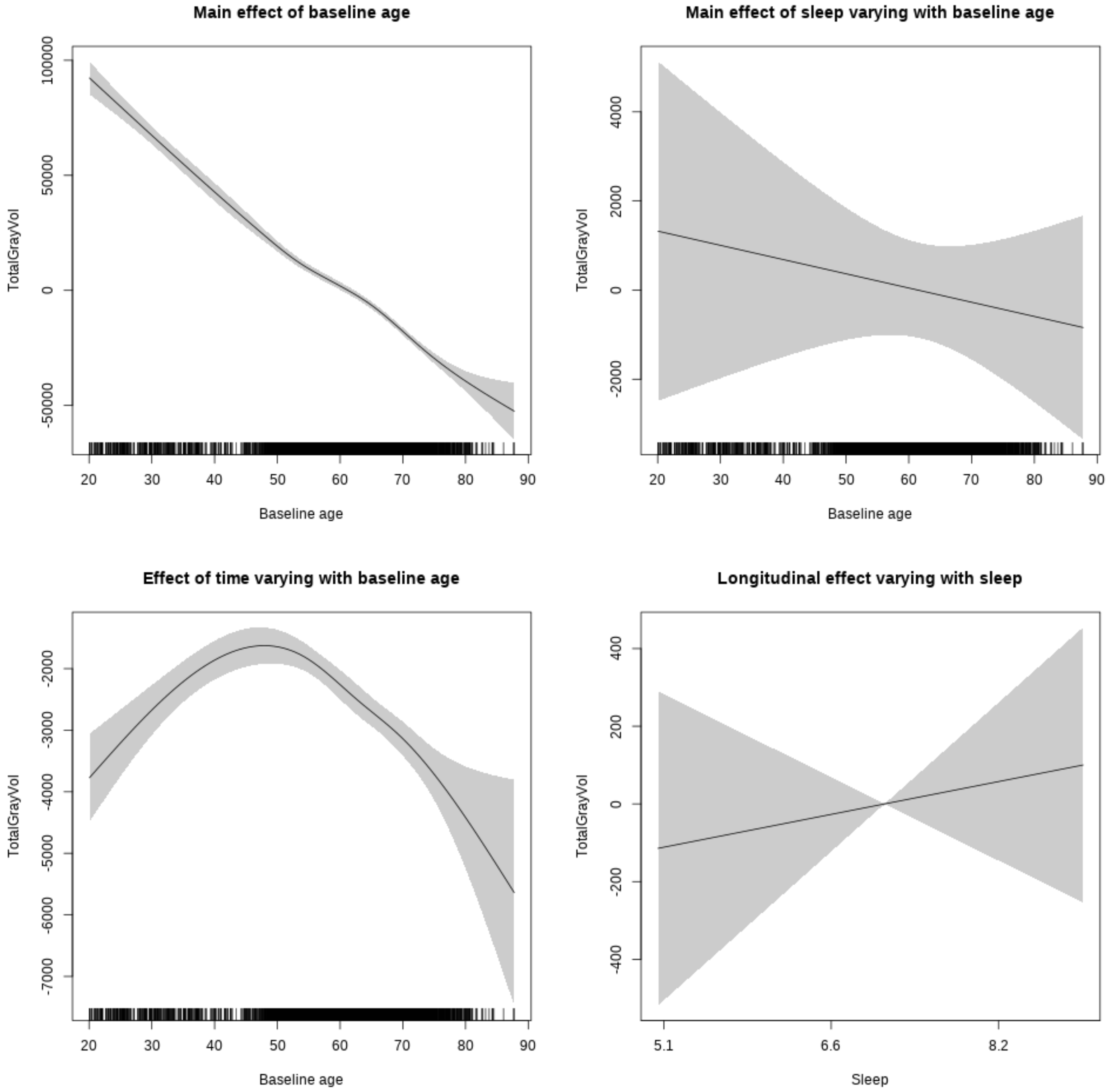

Restricted to sleep >= 5 and <= 9 hours Below is the row for `s(sleep_z):time` from the model output.

```
## s(sleep_z):time   1.000  1.000   0.320   0.572
```

Below are plots of a plot of `s(bl_age)`, `s(bl_age):sleep_z`, `s(bl_age):time`, and `s(sleep_z):time`. For `s(sleep_z):time`, roughly, if we cannot fit a straight line through the confidence bands, there is evidence that the effect of time depends on sleep duration. It is correct the that confidence interval is zero at one point along the axis; this happens because we do not include the main effect.

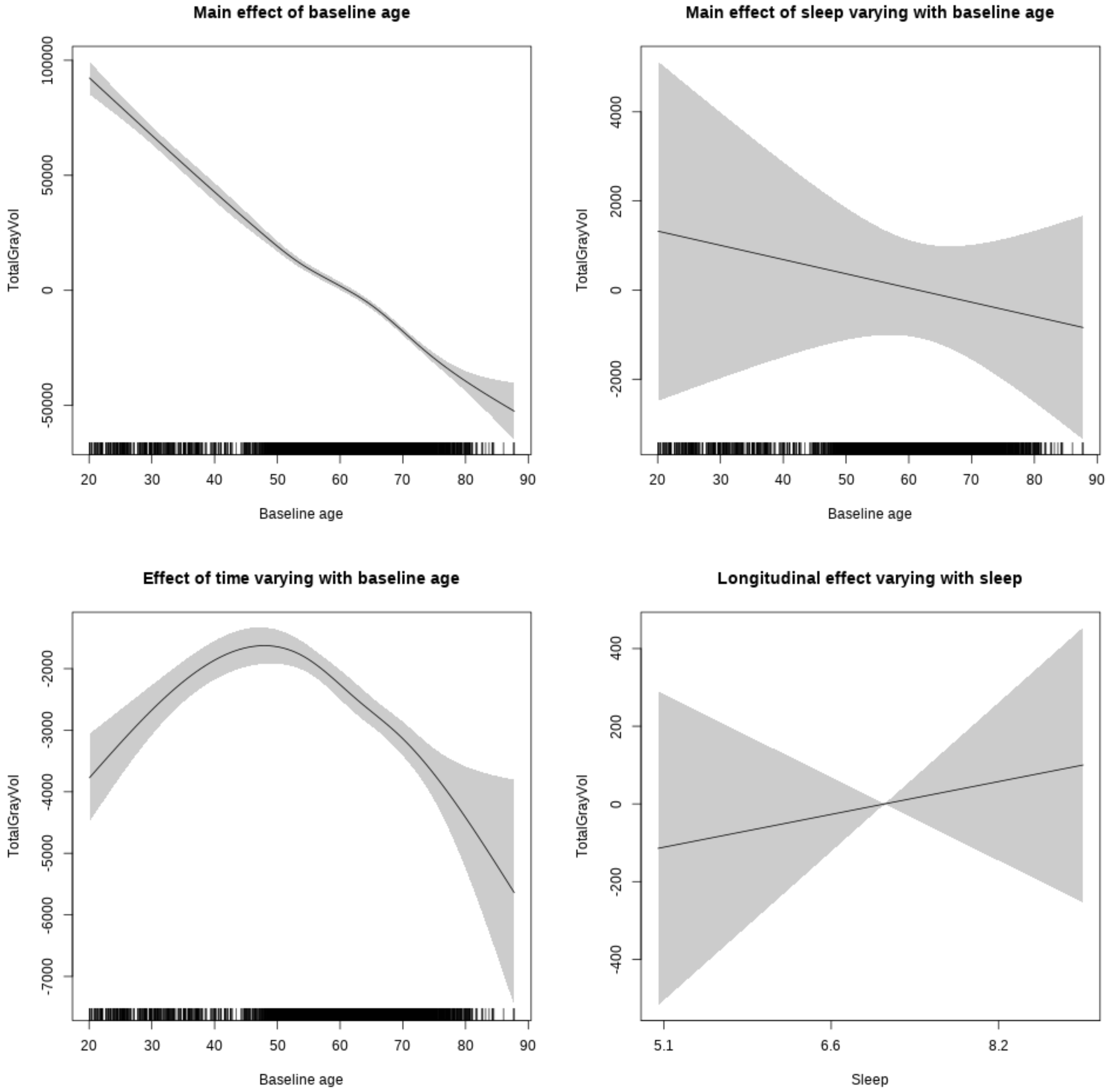

Controling for socioeconomic status

Full data Below is the model output. The term `s(sleep_z):time` is probably what we care most about.

```
##
## Family: gaussian
## Link function: identity
##
## Formula:
## value ~ sex + site + income_scaled + education_scaled + icv +
##     s(bl_age, k = 10, bs = "cr") + s(bl_age, by = sleep_z, bs = "cr") +
##     +s(bl_age, by = time, k = 5, bs = "cr") + s(sleep_z, by = time,
##     k = 5, bs = "cr", pc = 0)
```

```
## <environment: 0x563911590e78>
##
## Parametric coefficients:
##                  Estimate Std. Error t value Pr(>|t|)
## (Intercept)      678565.2     4973.5 136.436  < 2e-16 ***
## sexmale           14964.2     1366.3  10.953  < 2e-16 ***
## siteousAvanto    -82081.7     5627.5 -14.586  < 2e-16 ***
## siteousPrisma    -61767.0    14204.3  -4.348  1.4e-05 ***
## siteousSkyra     -50177.8     5066.8  -9.903  < 2e-16 ***
## siteUKB          -14979.3     4707.1  -3.182  0.00147 **
## income_scaled      2213.5     1744.5   1.269  0.20457
## education_scaled  -1300.8     1925.2  -0.676  0.49928
## icv               47185.8      686.4  68.747  < 2e-16 ***
## ---
## Signif. codes:  0 ‘***’ 0.001 ‘**’ 0.01 ‘*’ 0.05 ‘.’ 0.1 ‘ ’ 1
##
## Approximate significance of smooth terms:
##                     edf Ref.df       F p-value
## s(bl_age)         4.310  4.310 200.061  <2e-16 ***
## s(bl_age):sleep_z 2.000  2.000   1.089   0.337
## s(bl_age):time    3.781  3.781  71.819  <2e-16 ***
## s(sleep_z):time   3.289  3.289   1.536   0.109
## ---
## Signif. codes:  0 ‘***’ 0.001 ‘**’ 0.01 ‘*’ 0.05 ‘.’ 0.1 ‘ ’ 1
##
## R-sq.(adj) =  0.794
## lmer.REML = 1.0532e+05  Scale est. = 1.1889e+08  n = 4648
```

Below are plots of a plot of `s(bl_age)`, `s(bl_age):sleep_z`, `s(bl_age):time`, and `s(sleep_z):time`. For `s(sleep_z):time`, roughly, if we cannot fit a straight line through the confidence bands, there is evidence that the effect of time depends on sleep duration. It is correct the that confidence interval is zero at one point along the axis; this happens because we do not include the main effect.

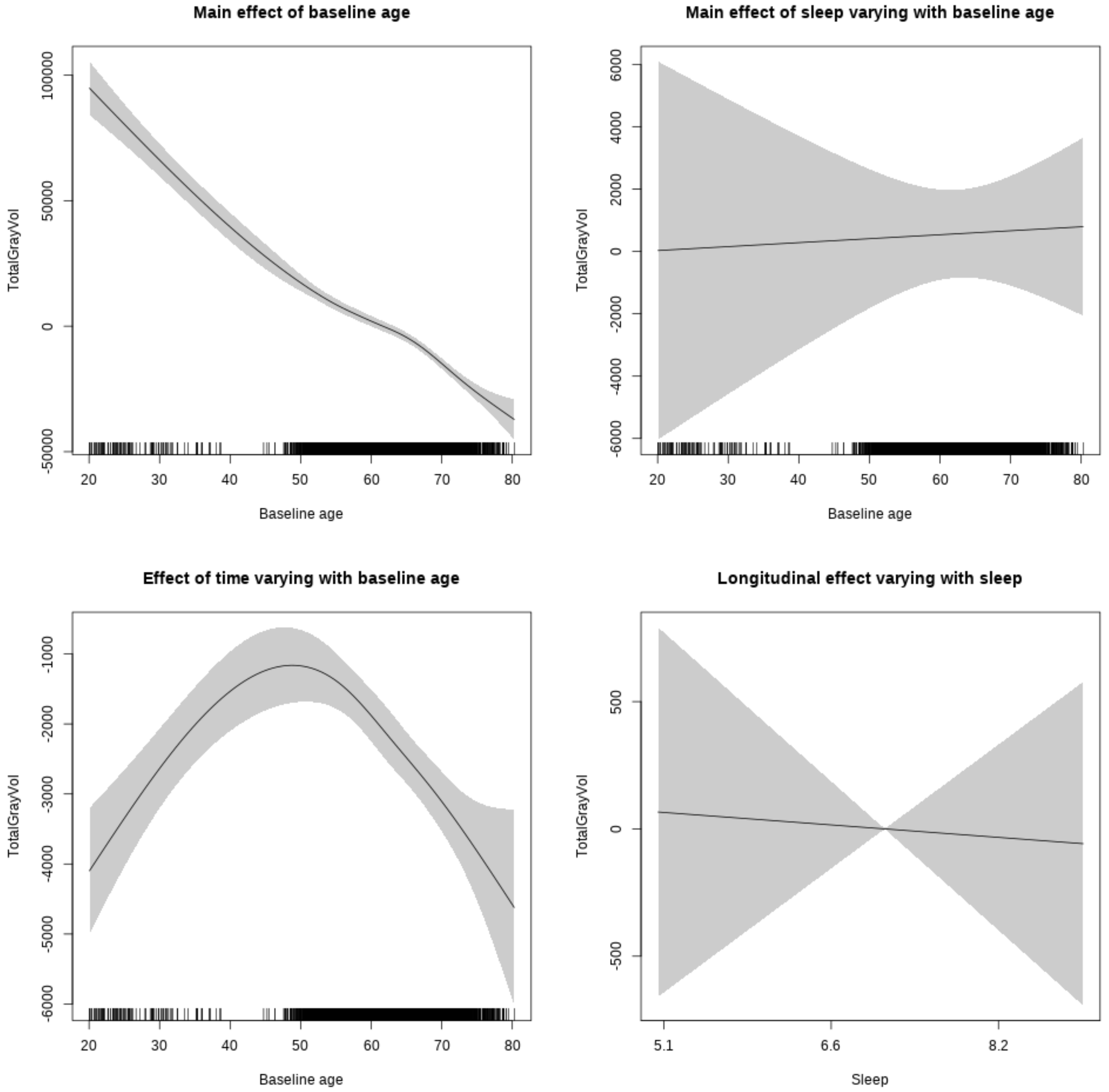

Restricted to sleep >= 5 and <= 9 hours Below is the row for `s(sleep_z):time` from the model output.

```
## s(sleep_z):time   1.000  1.000   0.033   0.855
```

Below are plots of a plot of `s(bl_age)`, `s(bl_age):sleep_z`, `s(bl_age):time`, and `s(sleep_z):time`. For `s(sleep_z):time`, roughly, if we cannot fit a straight line through the confidence bands, there is evidence that the effect of time depends on sleep duration. It is correct the that confidence interval is zero at one point along the axis; this happens because we do not include the main effect.

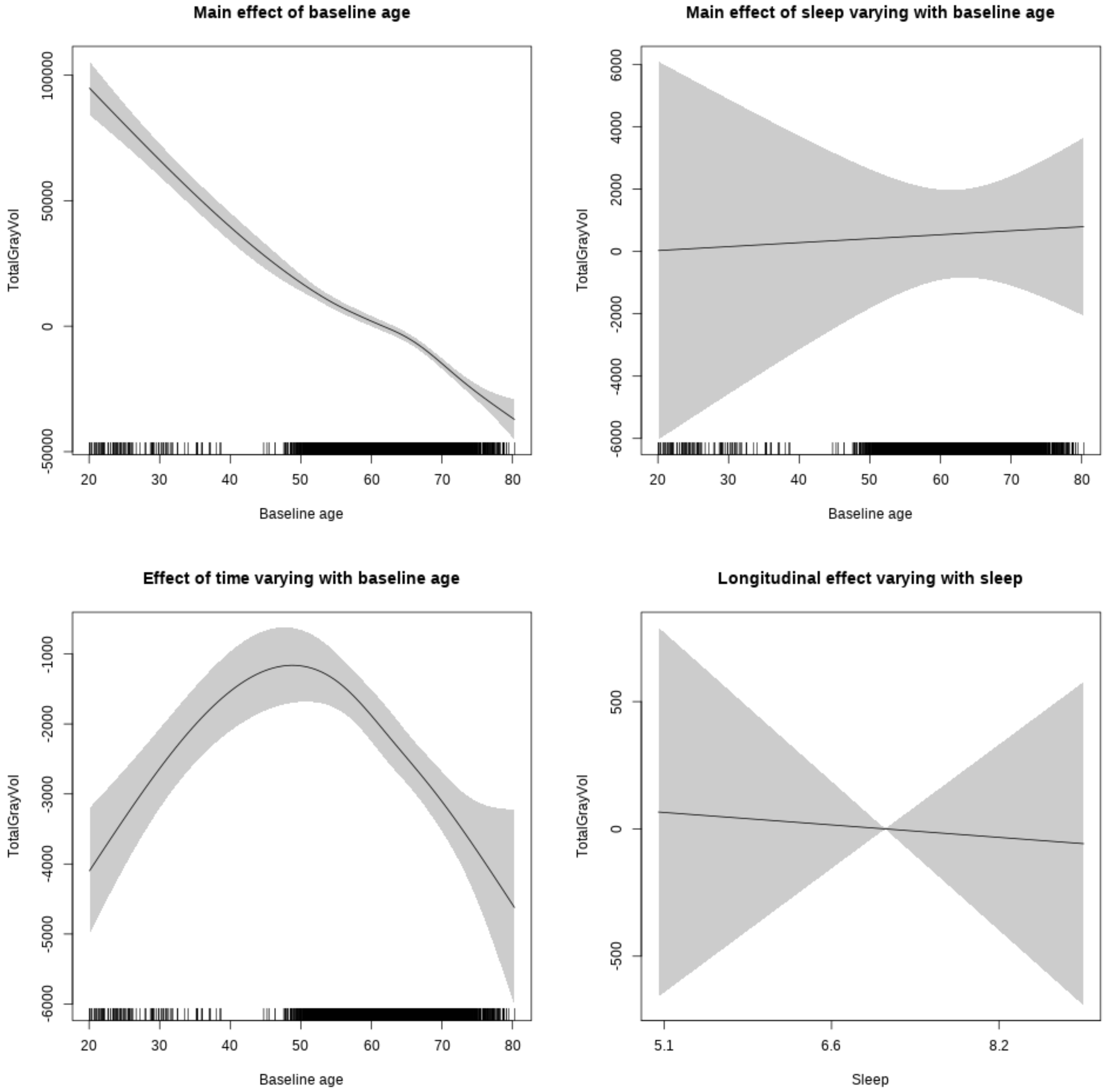

Controling for BMI

Full data Below is the model output. The term `s(sleep_z):time` is probably what we care most about.

```
##
## Family: gaussian
## Link function: identity
##
## Formula:
## value ~ sex + site + bmi + icv + s(bl_age, k = 10, bs = "cr") +
##     s(bl_age, by = sleep_z, bs = "cr") + +s(bl_age, by = time,
##     k = 5, bs = "cr") + s(sleep_z, by = time, k = 5, bs = "cr",
##     pc = 0)
```

```
## <environment: 0x563916b28678>
##
## Parametric coefficients:
##                Estimate Std. Error t value Pr(>|t|)
## (Intercept)    602881.9     4609.0 130.805  < 2e-16 ***
## sexmale         15186.1     1238.3  12.263  < 2e-16 ***
## siteousPrisma   15280.9     8384.7   1.822   0.0684 .
## siteousSkyra    30008.1     2627.8  11.419  < 2e-16 ***
## siteUCAM        25136.5     3649.5   6.888 6.32e-12 ***
## siteUKB         63166.5     3442.1  18.351  < 2e-16 ***
## siteUmU         41713.5     4091.9  10.194  < 2e-16 ***
## bmi               -81.9      127.8  -0.641   0.5216
## icv             46731.1      619.3  75.462  < 2e-16 ***
## ---
## Signif. codes:  0 '***' 0.001 '**' 0.01 '*' 0.05 '.' 0.1 ' ' 1
##
## Approximate significance of smooth terms:
##                       edf Ref.df       F p-value
## s(bl_age)           4.335  4.335 329.496  <2e-16 ***
## s(bl_age):sleep_z 2.209  2.209   0.567   0.641
## s(bl_age):time    3.787  3.787  82.965  <2e-16 ***
## s(sleep_z):time   3.196  3.196   2.813   0.146
## ---
## Signif. codes:  0 '***' 0.001 '**' 0.01 '*' 0.05 '.' 0.1 ' ' 1
##
## R-sq.(adj) =  0.806
## lmer.REML = 1.2233e+05  Scale est. = 1.1075e+08  n = 5410
```

Below are plots of a plot of `s(bl_age)`, `s(bl_age):sleep_z`, `s(bl_age):time`, and `s(sleep_z):time`. For `s(sleep_z):time`, roughly, if we cannot fit a straight line through the confidence bands, there is evidence that the effect of time depends on sleep duration. It is correct the that confidence interval is zero at one point along the axis; this happens because we do not include the main effect.

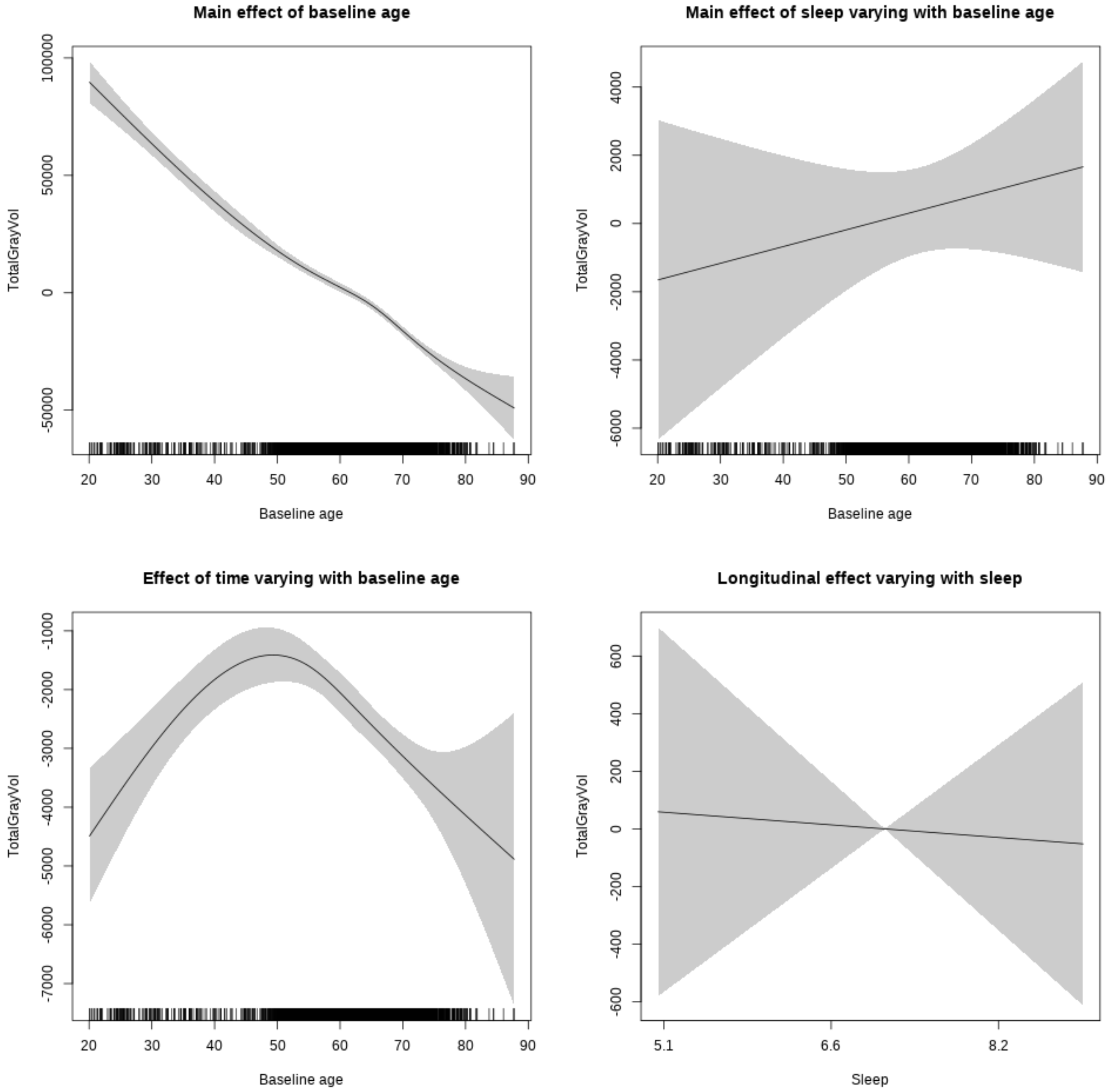

Restricted to sleep >= 5 and <= 9 hours Below is the row for `s(sleep_z):time` from the model output.

```
## s(sleep_z):time   1.000  1.000   0.034   0.853
```

Below are plots of a plot of `s(bl_age)`, `s(bl_age):sleep_z`, `s(bl_age):time`, and `s(sleep_z):time`. For `s(sleep_z):time`, roughly, if we cannot fit a straight line through the confidence bands, there is evidence that the effect of time depends on sleep duration. It is correct the that confidence interval is zero at one point along the axis; this happens because we do not include the main effect.

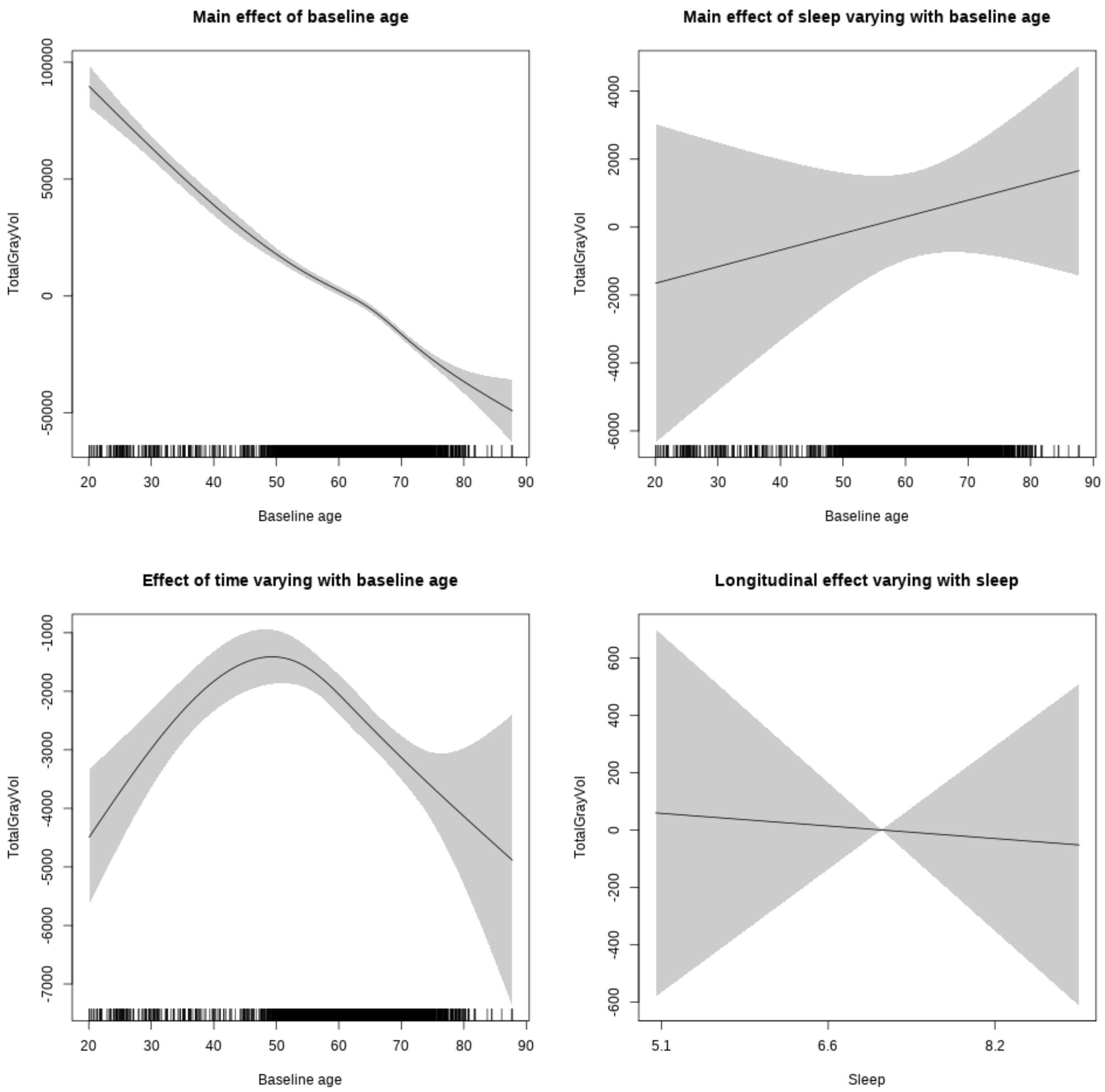

Controling for depression

Full data Below is the model output. The term `s(sleep_z):time` is probably what we care most about.

```
##
## Family: gaussian
## Link function: identity
##
## Formula:
## value ~ sex + site + depression + icv + s(bl_age, k = 10, bs = "cr") +
##     s(bl_age, by = sleep_z, bs = "cr") + +s(bl_age, by = time,
##     k = 5, bs = "cr") + s(sleep_z, by = time, k = 5, bs = "cr",
##     pc = 0)
```

```
## <environment: 0x563910fa3450>
##
## Parametric coefficients:
##                Estimate Std. Error t value Pr(>|t|)
## (Intercept)    659071.4     2476.4 266.145  < 2e-16 ***
## sexmale         15623.3     1295.4  12.060  < 2e-16 ***
## siteousAvanto -48465.5     5605.9  -8.645  < 2e-16 ***
## siteousPrisma -38577.3    10906.2  -3.537 0.000408 ***
## siteousSkyra  -22441.6     4528.6  -4.956 7.42e-07 ***
## siteUCAM      -33190.3     2984.0 -11.123  < 2e-16 ***
## siteUKB         3963.8     2273.3   1.744 0.081282 .
## siteUmU       -13366.1     3614.4  -3.698 0.000219 ***
## depression    -11909.4     4526.2  -2.631 0.008532 **
## icv            44061.0      631.2  69.804  < 2e-16 ***
## ---
## Signif. codes:  0 '***' 0.001 '**' 0.01 '*' 0.05 '.' 0.1 ' ' 1
##
## Approximate significance of smooth terms:
##                      edf Ref.df       F p-value
## s(bl_age)          4.129  4.129 263.850  <2e-16 ***
## s(bl_age):sleep_z 2.887  2.887   0.957   0.338
## s(bl_age):time    3.681  3.681  89.125  <2e-16 ***
## s(sleep_z):time   3.216  3.216   2.671   0.211
## ---
## Signif. codes:  0 '***' 0.001 '**' 0.01 '*' 0.05 '.' 0.1 ' ' 1
##
## R-sq.(adj) =  0.792
## lmer.REML = 1.2869e+05  Scale est. = 1.0687e+08  n = 5677
```

Below are plots of a plot of `s(bl_age)`, `s(bl_age):sleep_z`, `s(bl_age):time`, and `s(sleep_z):time`. For `s(sleep_z):time`, roughly, if we cannot fit a straight line through the confidence bands, there is evidence that the effect of time depends on sleep duration. It is correct the that confidence interval is zero at one point along the axis; this happens because we do not include the main effect.

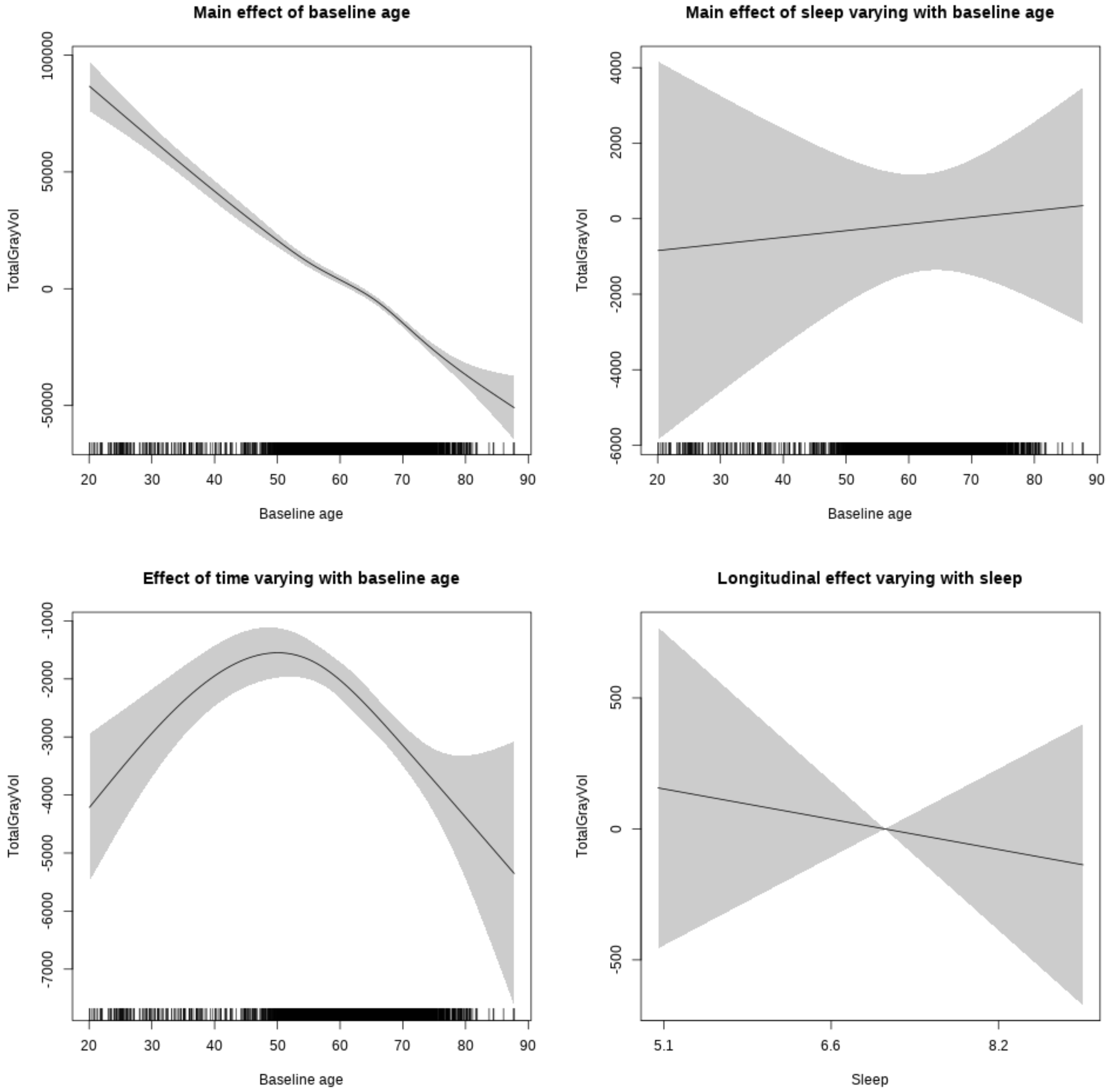

Restricted to sleep >= 5 and <= 9 hours Below is the row for `s(sleep_z):time` from the model output.

```
## s(sleep_z):time   1.000  1.000   0.261   0.610
```

Below are plots of a plot of `s(bl_age)`, `s(bl_age):sleep_z`, `s(bl_age):time`, and `s(sleep_z):time`. For `s(sleep_z):time`, roughly, if we cannot fit a straight line through the confidence bands, there is evidence that the effect of time depends on sleep duration. It is correct the that confidence interval is zero at one point along the axis; this happens because we do not include the main effect.

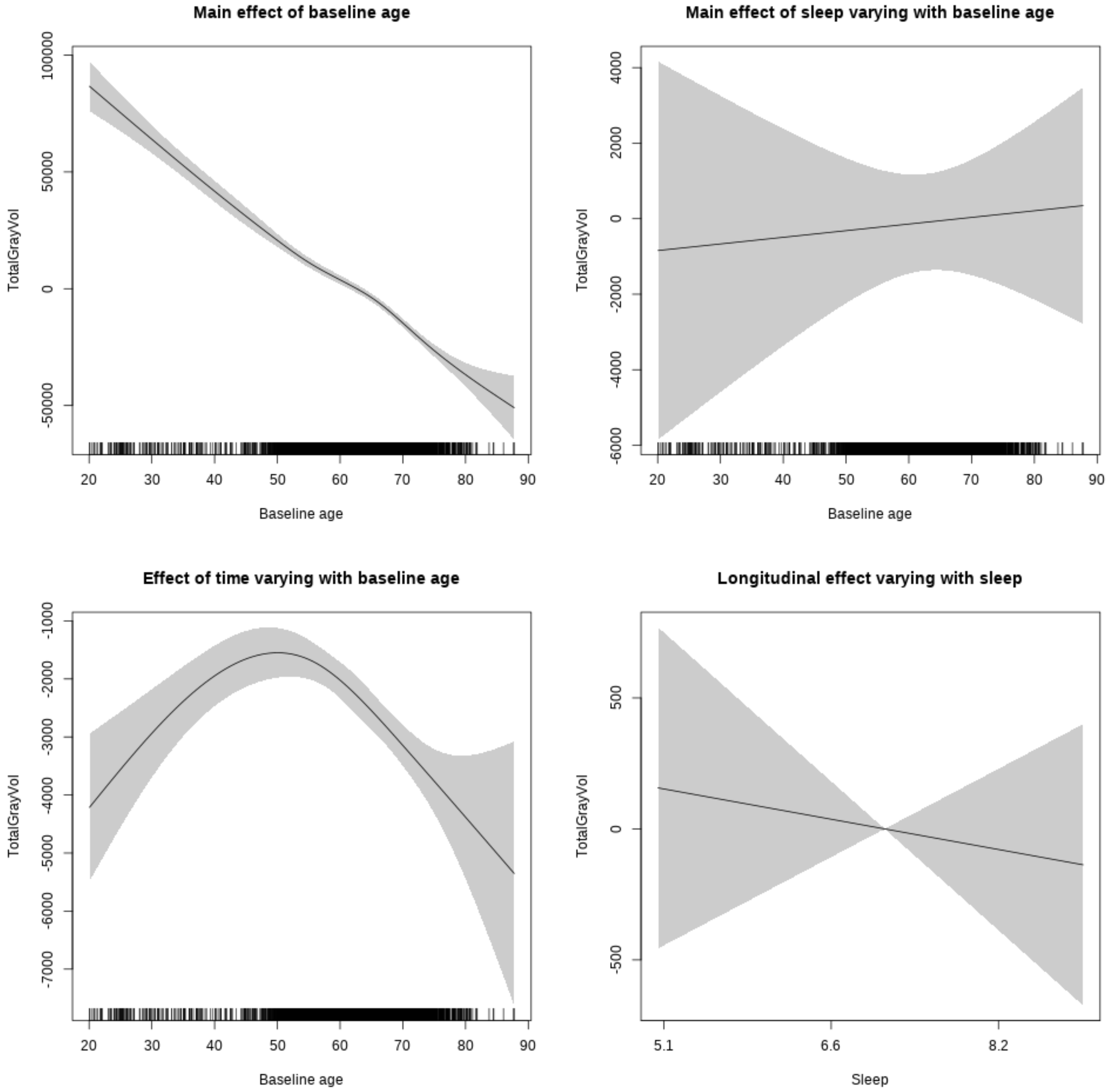

Controling for sleep quality

Full data Below is the model output. The term `s(sleep_z):time` is probably what we care most about.

```
##
## Family: gaussian
## Link function: identity
##
## Formula:
## value ~ X + s(bl_age, k = 10, bs = "cr") + s(bl_age, by = sleep_z,
##     bs = "cr") + s(bl_age, by = time, k = 5, bs = "cr") + s(sleep_z,
##     by = time, k = 5, bs = "cr", pc = 0)
## <environment: 0x563914d995c8>
```

```
##
## Parametric coefficients:
##                           Estimate Std. Error t value Pr(>|t|)
## (Intercept)               576234.1     5556.7 103.702  < 2e-16 ***
## Xsexmale                   63767.5     2088.8  30.528  < 2e-16 ***
## XsiteousAvanto             32772.7     5301.9   6.181 6.95e-10 ***
## XsiteousPrisma             65401.2     9397.3   6.960 3.93e-12 ***
## XsiteousSkyra              42428.2     5317.9   7.978 1.89e-15 ***
## XsiteUB                    19527.3     8467.9   2.306   0.0212 *
## XsiteUCAM                  39294.5     5376.9   7.308 3.22e-13 ***
## XsiteUKB                   72094.5    16926.3   4.259 2.09e-05 ***
## XsiteUmU                   31453.3     6015.6   5.229 1.79e-07 ***
## Xukb_dummy:sleep_accel      -294.4     1321.4  -0.223   0.8237
## Xukb_dummy:chronotype        887.4     1748.3   0.508   0.6118
## Xukb_dummy:dozing           3365.8     3610.1   0.932   0.3512
## Xukb_dummy:sleeplessness   -4502.9     2241.2  -2.009   0.0446 *
## Xukb_dummy:snoring          3232.6     3188.5   1.014   0.3107
## Xukb_dummy:gettingup         866.7     2428.9   0.357   0.7212
## Xnotukb_dummy:PSQI_Global   -991.4      688.2  -1.441   0.1498
## ---
## Signif. codes:  0 '***' 0.001 '**' 0.01 '*' 0.05 '.' 0.1 ' ' 1
##
## Approximate significance of smooth terms:
##                      edf Ref.df       F p-value
## s(bl_age)          1.000  1.000 721.468  <2e-16 ***
## s(bl_age):sleep_z 2.000  2.000   0.573   0.564
## s(bl_age):time    3.818  3.818 207.783  <2e-16 ***
## s(sleep_z):time   1.000  1.000   0.070   0.792
## ---
## Signif. codes:  0 '***' 0.001 '**' 0.01 '*' 0.05 '.' 0.1 ' ' 1
##
## R-sq.(adj) =  0.502
## lmer.REML =  98436  Scale est. = 1.0225e+08  n = 4286
```

Below are plots of a plot of `s(bl_age)`, `s(bl_age):sleep_z`, `s(bl_age):time`, and `s(sleep_z):time`. For `s(sleep_z):time`, roughly, if we cannot fit a straight line through the confidence bands, there is evidence that the effect of time depends on sleep duration. It is correct the that confidence interval is zero at one point along the axis; this happens because we do not include the main effect.

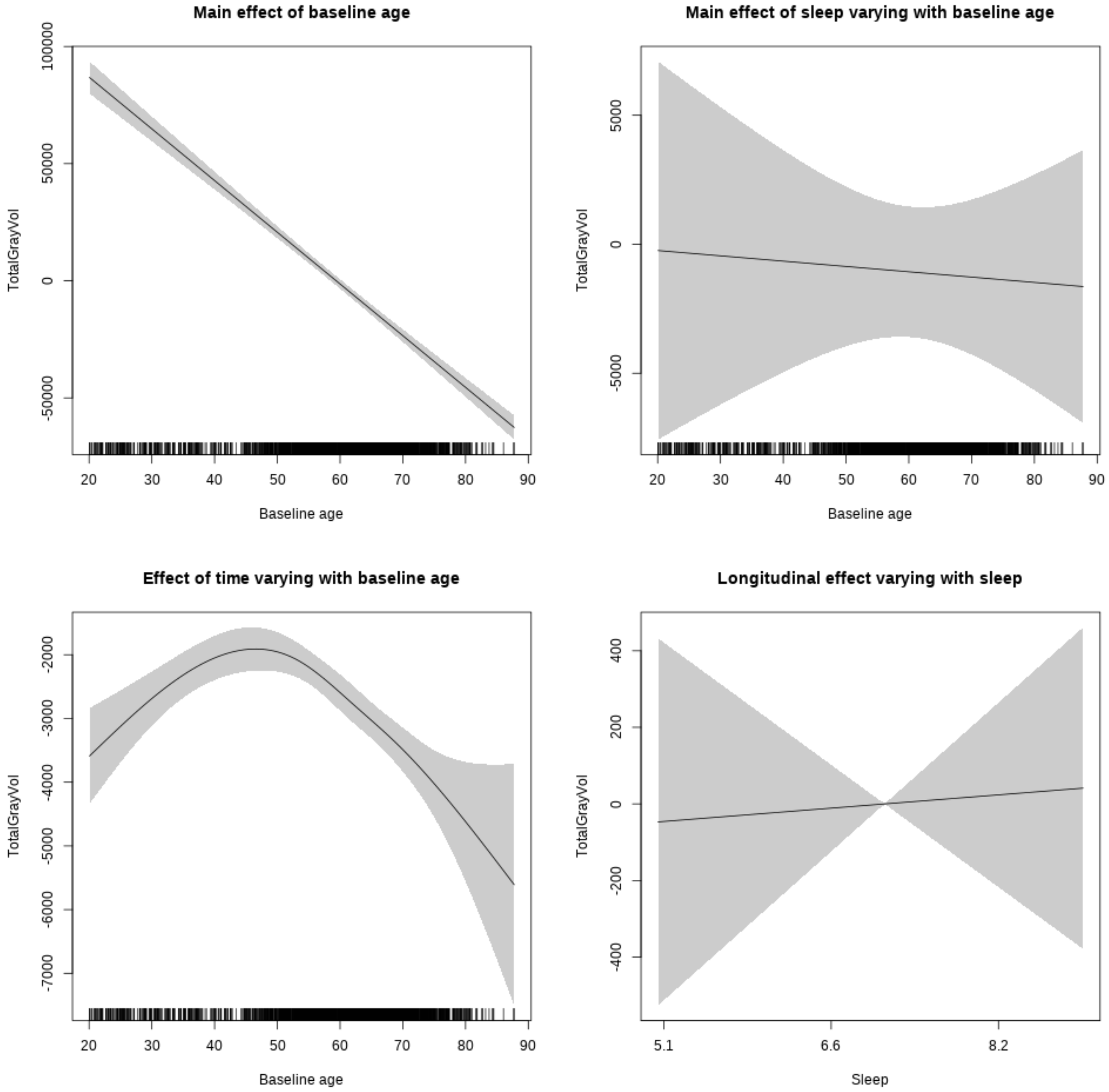

Restricted to sleep >= 5 and <= 9 hours Below is the row for `s(sleep_z):time` from the model output.

```
## s(sleep_z):time   1.000  1.000   0.038   0.845
```

Below are plots of a plot of `s(bl_age)`, `s(bl_age):sleep_z`, `s(bl_age):time`, and `s(sleep_z):time`. For `s(sleep_z):time`, roughly, if we cannot fit a straight line through the confidence bands, there is evidence that the effect of time depends on sleep duration. It is correct the that confidence interval is zero at one point along the axis; this happens because we do not include the main effect.

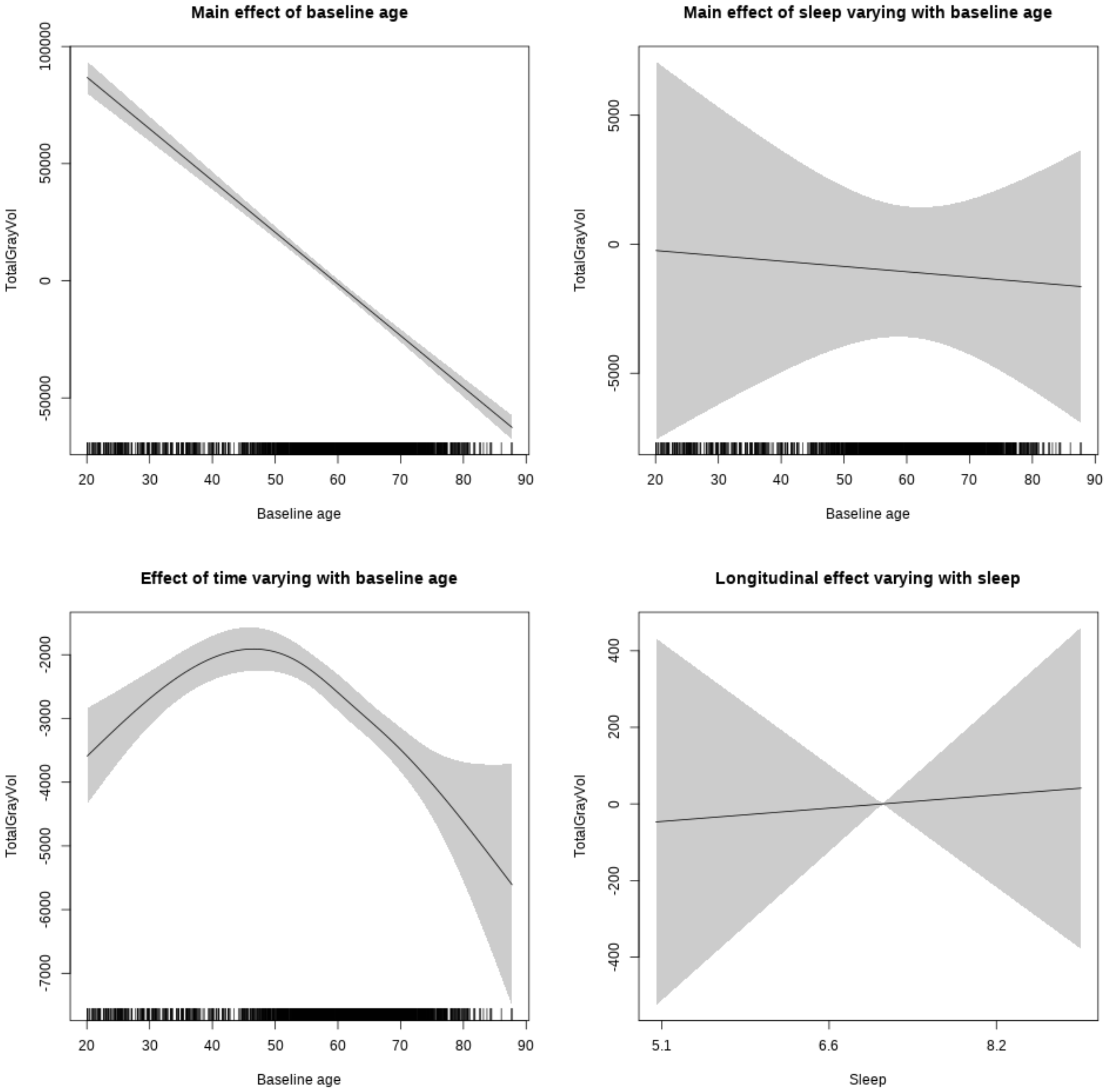

Ventricles

Main analysis

Full data Below is the model output. The term `s(sleep_z):time` is probably what we care most about.

```
##
## Family: gaussian
## Link function: identity
##
## Formula:
## value ~ sex + site + icv + s(bl_age, k = 10, bs = "cr") + s(bl_age,
##     by = sleep_z, bs = "cr") + +s(bl_age, by = time, k = 5, bs = "cr") +
```

```
##     s(sleep_z, by = time, k = 5, bs = "cr", pc = 0)
## <environment: 0x563911250b18>
##
## Parametric coefficients:
##               Estimate Std. Error t value Pr(>|t|)
## (Intercept)    38884.8      877.9  44.291  < 2e-16 ***
## sexmale         3005.7      449.0   6.694 2.31e-11 ***
## siteousAvanto  -8466.6     1087.4  -7.786 7.77e-15 ***
## siteousPrisma -10556.7     1564.9  -6.746 1.63e-11 ***
## siteousSkyra   -5150.1     1066.8  -4.828 1.41e-06 ***
## siteUB        -11340.7     2141.1  -5.297 1.21e-07 ***
## siteUCAM       -7919.5     1135.8  -6.972 3.36e-12 ***
## siteUKB       -10873.6      854.8 -12.720  < 2e-16 ***
## siteUmU        -6191.8     1318.3  -4.697 2.69e-06 ***
## icv             5214.9      183.2  28.470  < 2e-16 ***
## ---
## Signif. codes:  0 ‘***’ 0.001 ‘**’ 0.01 ‘*’ 0.05 ‘.’ 0.1 ‘ ’ 1
##
## Approximate significance of smooth terms:
##                     edf Ref.df        F p-value
## s(bl_age)         4.590  4.590  284.669 < 2e-16 ***
## s(bl_age):sleep_z 2.000  2.000    2.107 0.12170
## s(bl_age):time    4.393  4.393 1654.046 < 2e-16 ***
## s(sleep_z):time   1.000  1.000    9.241 0.00237 **
## ---
## Signif. codes:  0 ‘***’ 0.001 ‘**’ 0.01 ‘*’ 0.05 ‘.’ 0.1 ‘ ’ 1
##
## R-sq.(adj) =  0.429
## lmer.REML = 1.6083e+05  Scale est. = 2.0002e+06  n = 8139
```

Below are plots of a plot of `s(bl_age)`, `s(bl_age):sleep_z`, `s(bl_age):time`, and `s(sleep_z):time`. For `s(sleep_z):time`, roughly, if we cannot fit a straight line through the confidence bands, there is evidence that the effect of time depends on sleep duration. It is correct the that confidence interval is zero at one point along the axis; this happens because we do not include the main effect.

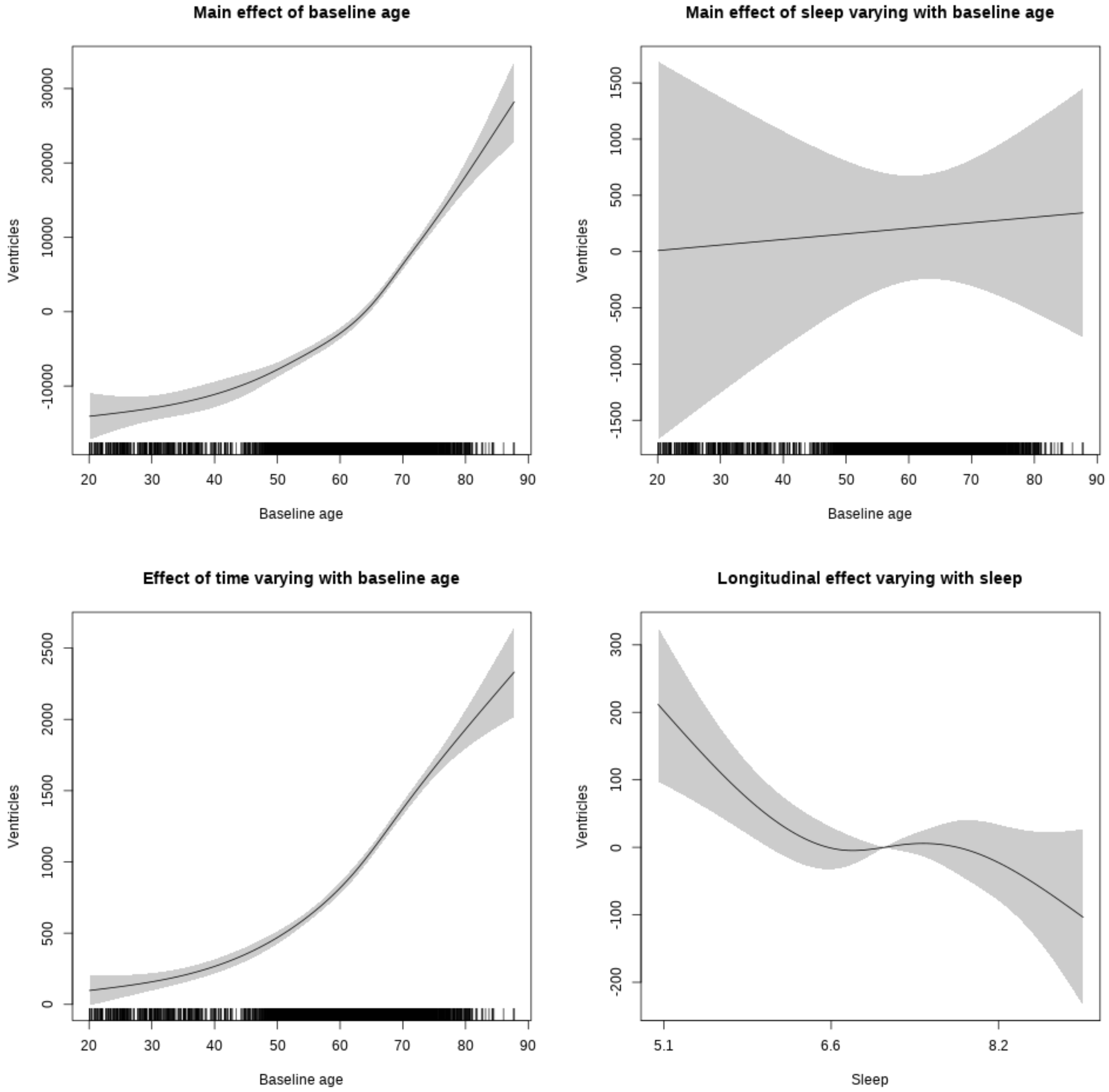

Restricted to sleep >= 5 and <= 9 hours Below is the row for `s(sleep_z):time` from the model output.

```
## s(sleep_z):time   2.860  2.860    4.558  0.0022 **
```

Below are plots of a plot of `s(bl_age)`, `s(bl_age):sleep_z`, `s(bl_age):time`, and `s(sleep_z):time`. For `s(sleep_z):time`, roughly, if we cannot fit a straight line through the confidence bands, there is evidence that the effect of time depends on sleep duration. It is correct the that confidence interval is zero at one point along the axis; this happens because we do not include the main effect.

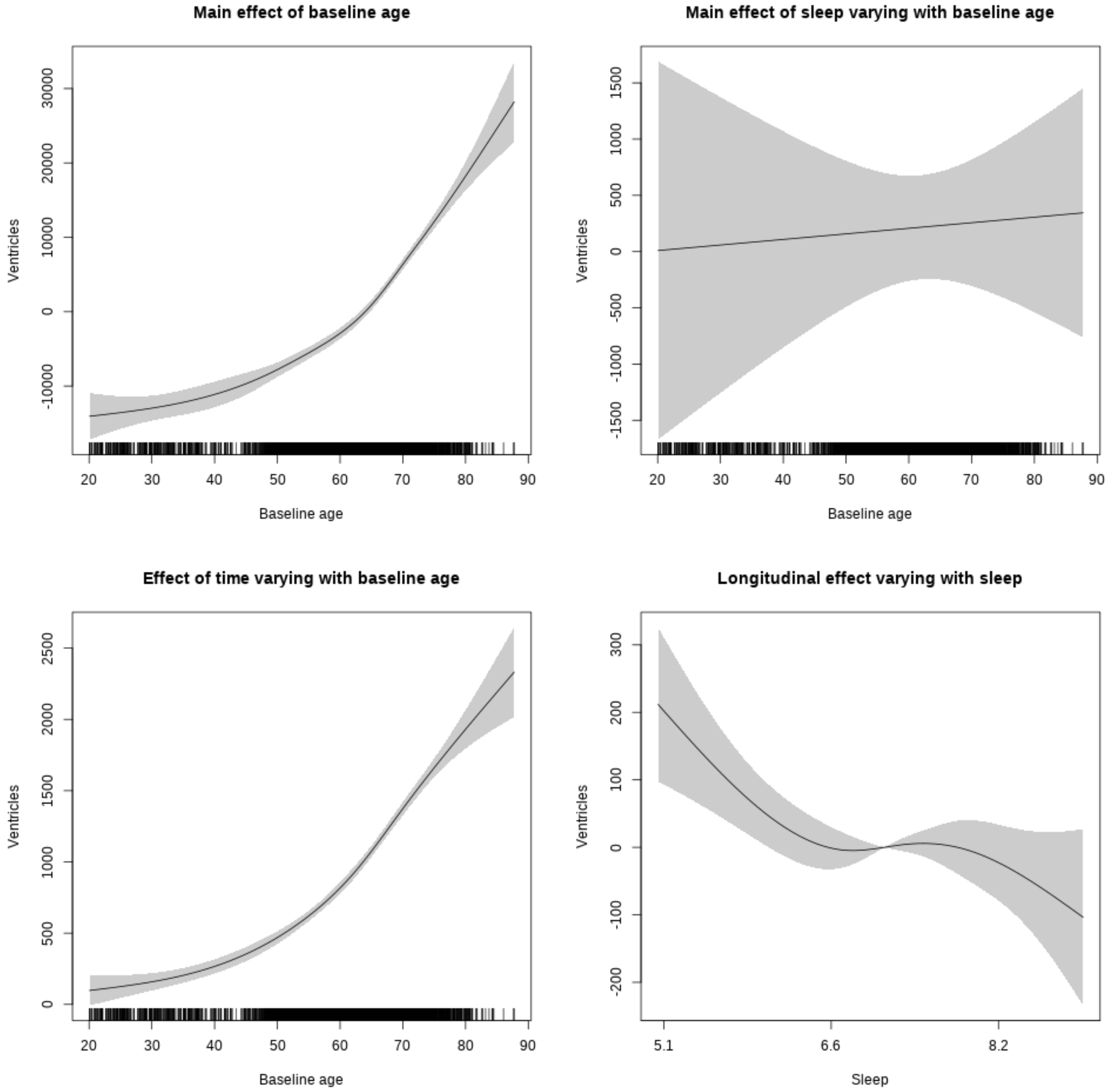

Controling for socioeconomic status

Full data Below is the model output. The term `s(sleep_z):time` is probably what we care most about.

```
##
## Family: gaussian
## Link function: identity
##
## Formula:
## value ~ sex + site + income_scaled + education_scaled + icv +
##     s(bl_age, k = 10, bs = "cr") + s(bl_age, by = sleep_z, bs = "cr") +
##     +s(bl_age, by = time, k = 5, bs = "cr") + s(sleep_z, by = time,
##     k = 5, bs = "cr", pc = 0)
```

```
## <environment: 0x563911595d20>
##
## Parametric coefficients:
##                   Estimate Std. Error t value Pr(>|t|)
## (Intercept)        37660.0     2151.5  17.504  < 2e-16 ***
## sexmale             2900.2      566.1   5.123 3.13e-07 ***
## siteousAvanto     -10760.5     2260.7  -4.760 2.00e-06 ***
## siteousPrisma     -15399.4     2778.2  -5.543 3.14e-08 ***
## siteousSkyra       -6998.7     2223.0  -3.148  0.00165 **
## siteUKB           -10198.7     2063.0  -4.944 7.94e-07 ***
## income_scaled      -1400.6      775.7  -1.806  0.07105 .
## education_scaled    2117.1      856.4   2.472  0.01347 *
## icv                 4895.2      230.1  21.273  < 2e-16 ***
## ---
## Signif. codes:  0 '***' 0.001 '**' 0.01 '*' 0.05 '.' 0.1 ' ' 1
##
## Approximate significance of smooth terms:
##                      edf Ref.df       F p-value
## s(bl_age)          4.006  4.006 137.442  <2e-16 ***
## s(bl_age):sleep_z 2.000  2.000   0.126   0.882
## s(bl_age):time     4.521  4.521 908.512  <2e-16 ***
## s(sleep_z):time    1.000  1.000   0.077   0.781
## ---
## Signif. codes:  0 '***' 0.001 '**' 0.01 '*' 0.05 '.' 0.1 ' ' 1
##
## R-sq.(adj) =  0.392
## lmer.REML =  91653  Scale est. = 1.6932e+06  n = 4650
```

Below are plots of a plot of `s(bl_age)`, `s(bl_age):sleep_z`, `s(bl_age):time`, and `s(sleep_z):time`. For `s(sleep_z):time`, roughly, if we cannot fit a straight line through the confidence bands, there is evidence that the effect of time depends on sleep duration. It is correct the that confidence interval is zero at one point along the axis; this happens because we do not include the main effect.

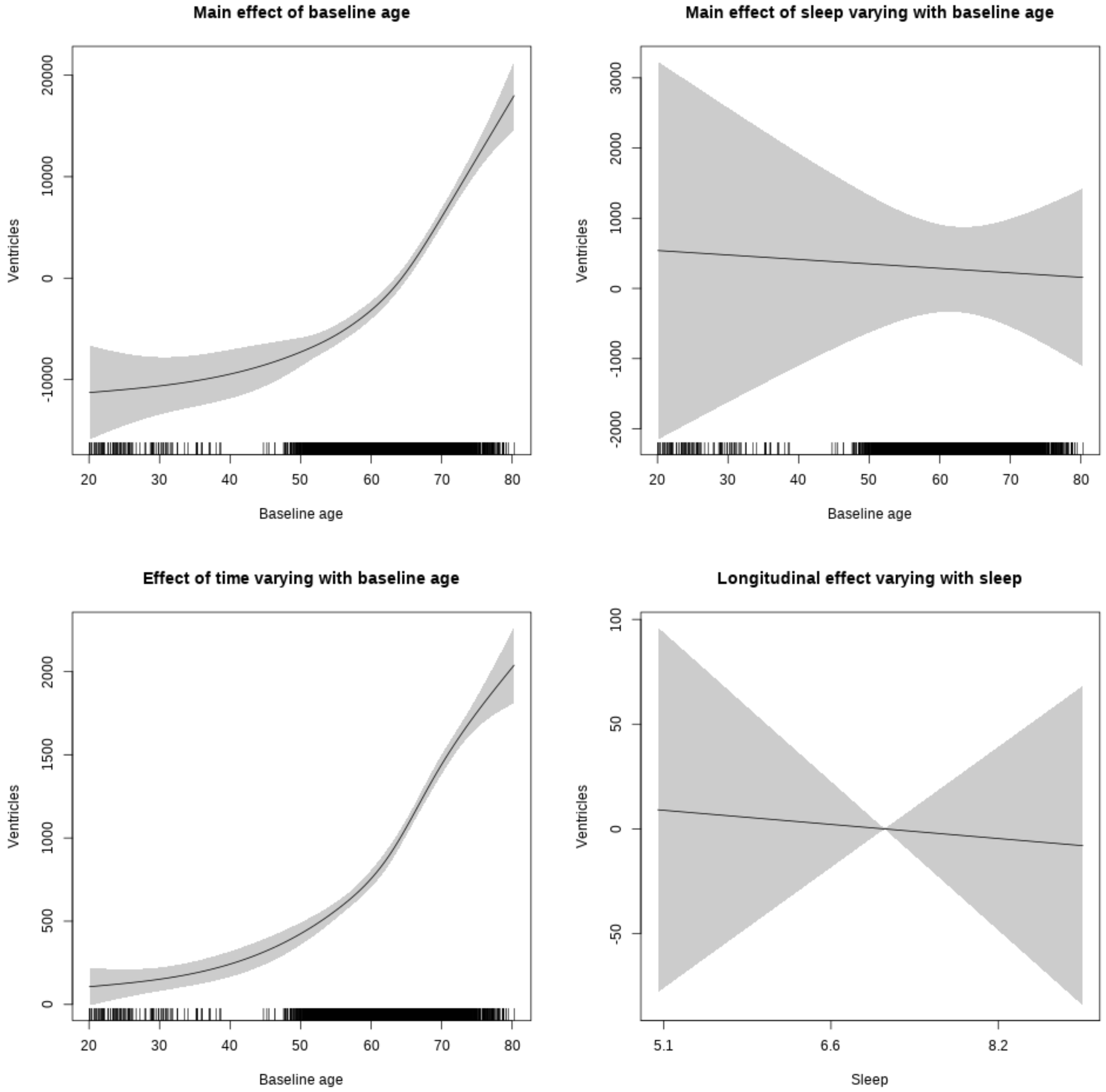

Restricted to sleep >= 5 and <= 9 hours Below is the row for `s(sleep_z):time` from the model output.

```
## s(sleep_z):time   1.000  1.000   0.044   0.834
```

Below are plots of a plot of `s(bl_age)`, `s(bl_age):sleep_z`, `s(bl_age):time`, and `s(sleep_z):time`. For `s(sleep_z):time`, roughly, if we cannot fit a straight line through the confidence bands, there is evidence that the effect of time depends on sleep duration. It is correct the that confidence interval is zero at one point along the axis; this happens because we do not include the main effect.

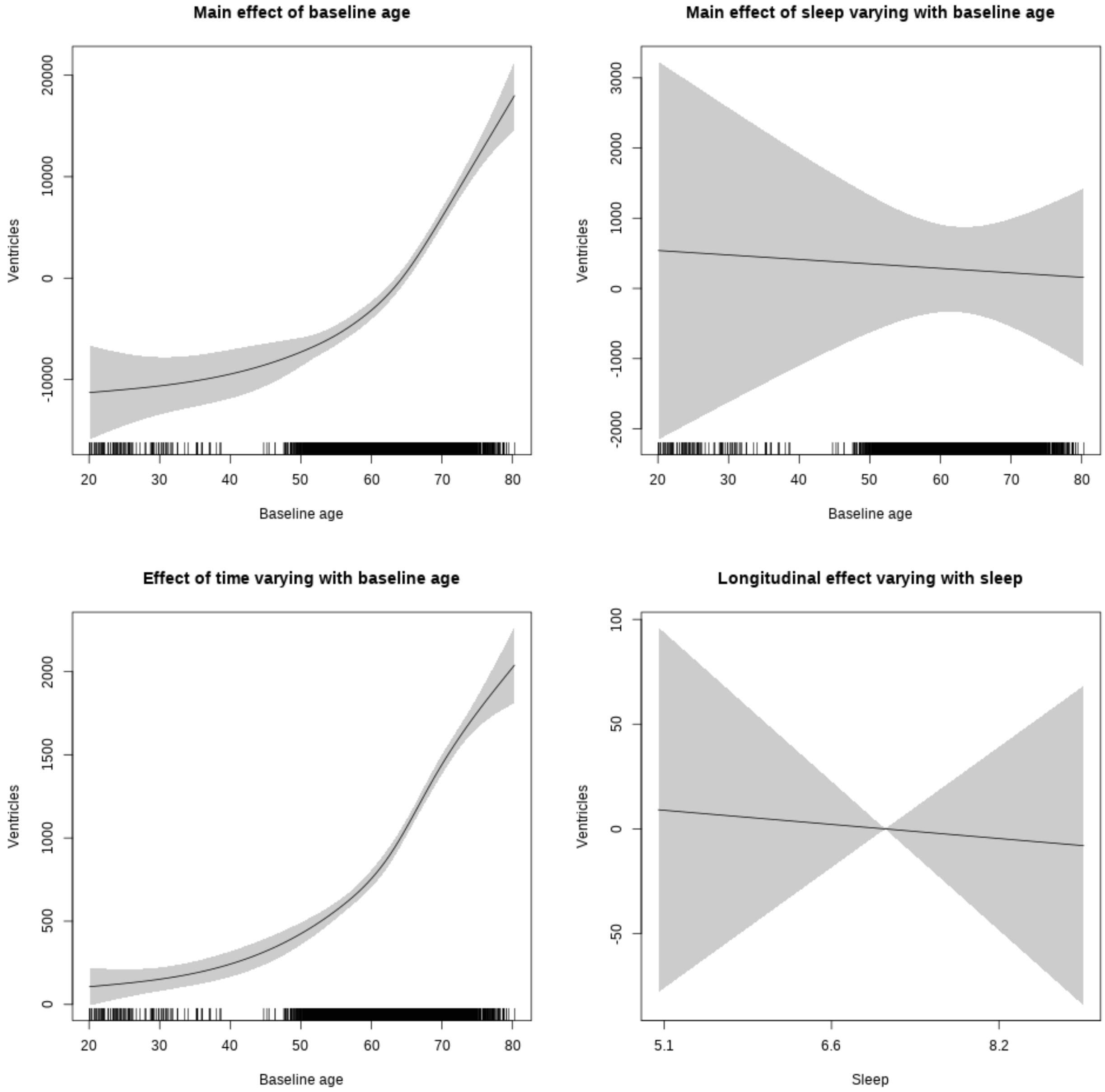

Controling for BMI

Full data Below is the model output. The term `s(sleep_z):time` is probably what we care most about.

```
##
## Family: gaussian
## Link function: identity
##
## Formula:
## value ~ sex + site + bmi + icv + s(bl_age, k = 10, bs = "cr") +
##     s(bl_age, by = sleep_z, bs = "cr") + +s(bl_age, by = time,
##     k = 5, bs = "cr") + s(sleep_z, by = time, k = 5, bs = "cr",
##     pc = 0)
```

```
## <environment: 0x563916ce02c0>
##
## Parametric coefficients:
##                Estimate Std. Error t value Pr(>|t|)
## (Intercept)    26395.94    1875.38  14.075  < 2e-16 ***
## sexmale         2792.68     533.99   5.230 1.76e-07 ***
## siteousPrisma  -1374.82    1101.48  -1.248  0.21203
## siteousSkyra    3693.64     367.30  10.056  < 2e-16 ***
## siteUCAM         178.37    1346.88   0.132  0.89465
## siteUKB        -2210.57    1218.09  -1.815  0.06961 .
## siteUmU         1905.16    1576.79   1.208  0.22700
## bmi              158.03      58.71   2.691  0.00714 **
## icv             5013.14     221.89  22.593  < 2e-16 ***
## ---
## Signif. codes:  0 '***' 0.001 '**' 0.01 '*' 0.05 '.' 0.1 ' ' 1
##
## Approximate significance of smooth terms:
##                      edf Ref.df       F p-value
## s(bl_age)          4.735  4.735 187.008  <2e-16 ***
## s(bl_age):sleep_z 2.000  2.000   0.537   0.585
## s(bl_age):time    4.588  4.588 973.972  <2e-16 ***
## s(sleep_z):time   1.000  1.000   1.842   0.175
## ---
## Signif. codes:  0 '***' 0.001 '**' 0.01 '*' 0.05 '.' 0.1 ' ' 1
##
## R-sq.(adj) =  0.405
## lmer.REML = 1.0689e+05  Scale est. = 1.7767e+06  n = 5409
```

Below are plots of a plot of `s(bl_age)`, `s(bl_age):sleep_z`, `s(bl_age):time`, and `s(sleep_z):time`. For `s(sleep_z):time`, roughly, if we cannot fit a straight line through the confidence bands, there is evidence that the effect of time depends on sleep duration. It is correct the that confidence interval is zero at one point along the axis; this happens because we do not include the main effect.

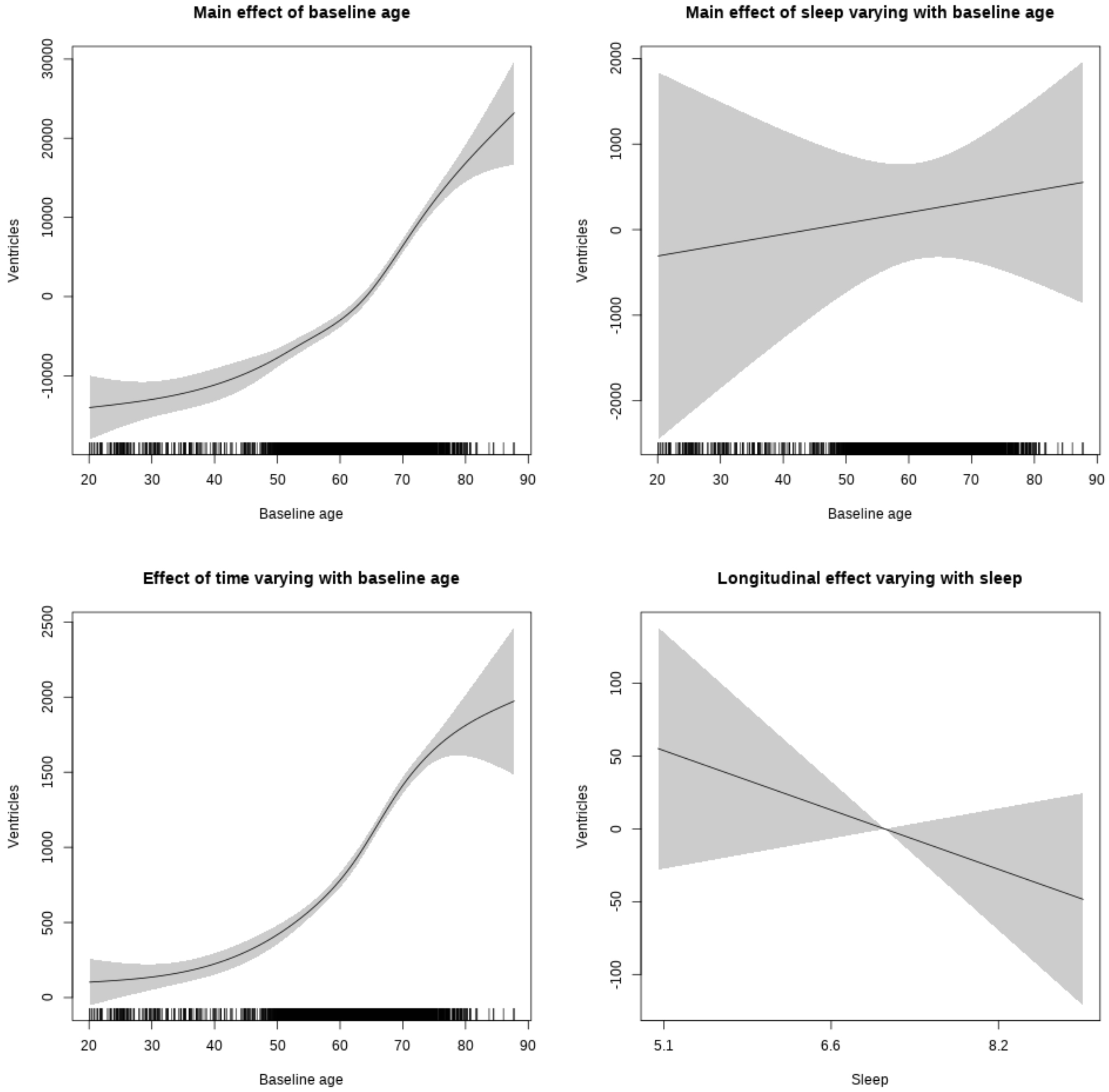

Restricted to sleep >= 5 and <= 9 hours Below is the row for `s(sleep_z):time` from the model output.

```
## s(sleep_z):time   1.000  1.000   1.759   0.185
```

Below are plots of a plot of `s(bl_age)`, `s(bl_age):sleep_z`, `s(bl_age):time`, and `s(sleep_z):time`. For `s(sleep_z):time`, roughly, if we cannot fit a straight line through the confidence bands, there is evidence that the effect of time depends on sleep duration. It is correct the that confidence interval is zero at one point along the axis; this happens because we do not include the main effect.

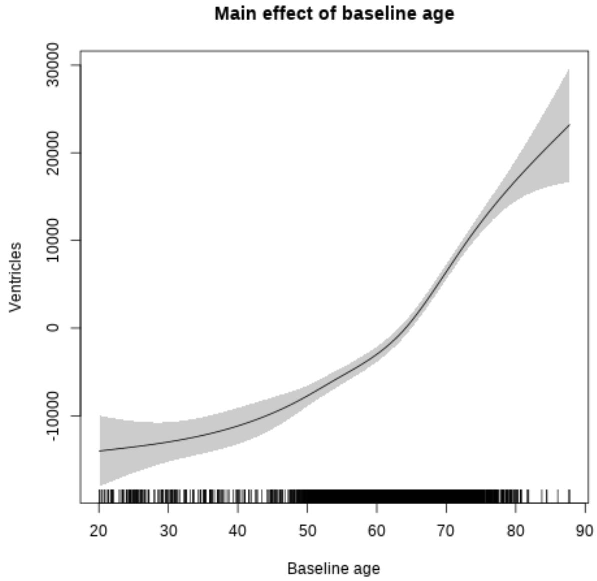

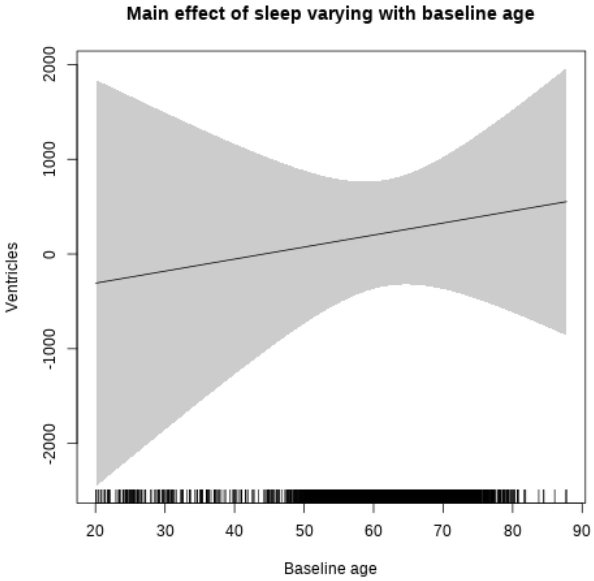

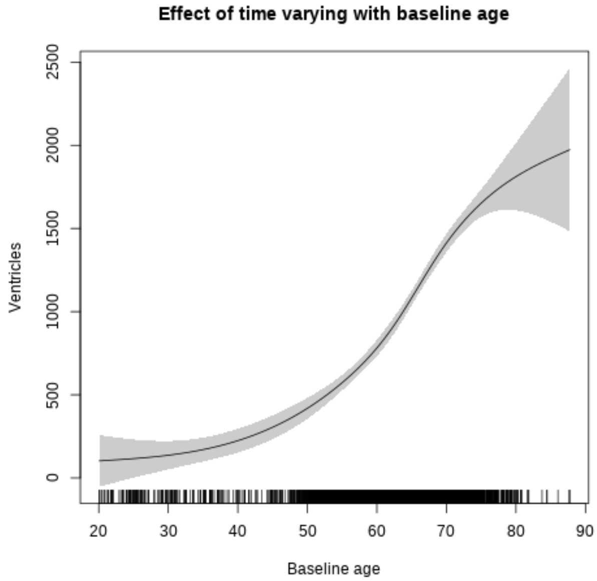

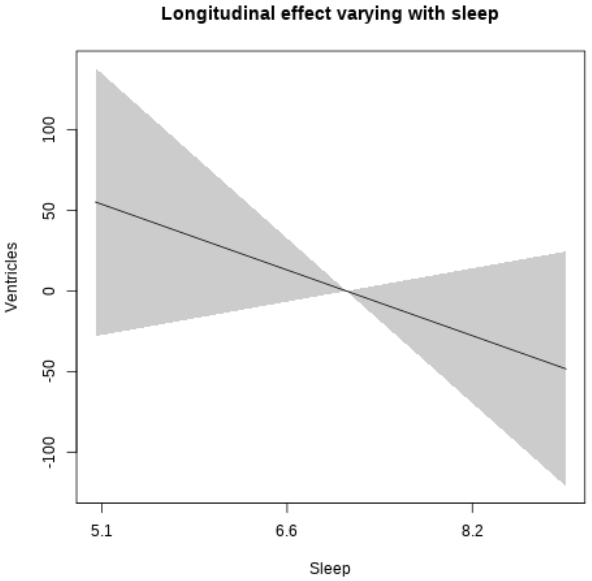

Controling for depression

Full data Below is the model output. The term `s(sleep_z):time` is probably what we care most about.

```
##
## Family: gaussian
## Link function: identity
##
## Formula:
## value ~ sex + site + depression + icv + s(bl_age, k = 10, bs = "cr") +
##     s(bl_age, by = sleep_z, bs = "cr") + +s(bl_age, by = time,
##     k = 5, bs = "cr") + s(sleep_z, by = time, k = 5, bs = "cr",
##     pc = 0)
```

```
## <environment: 0x563910fa56f0>
##
## Parametric coefficients:
##                Estimate Std. Error t value Pr(>|t|)
## (Intercept)     38524.3     1020.9  37.734  < 2e-16 ***
## sexmale          2348.2      529.2   4.438 9.27e-06 ***
## siteousAvanto   -8998.2     2009.3  -4.478 7.68e-06 ***
## siteousPrisma   -8631.2     2385.1  -3.619 0.000299 ***
## siteousSkyra    -5194.1     1944.9  -2.671 0.007592 **
## siteUCAM        -8390.3     1276.8  -6.572 5.42e-11 ***
## siteUKB        -10154.5      956.2 -10.620  < 2e-16 ***
## siteUmU         -9558.2     1567.3  -6.098 1.14e-09 ***
## depression       9042.6     1971.2   4.587 4.59e-06 ***
## icv              5401.5      218.3  24.739  < 2e-16 ***
## ---
## Signif. codes:  0 '***' 0.001 '**' 0.01 '*' 0.05 '.' 0.1 ' ' 1
##
## Approximate significance of smooth terms:
##                      edf Ref.df        F p-value
## s(bl_age)          4.465  4.465  183.530  <2e-16 ***
## s(bl_age):sleep_z 2.000  2.000    3.315  0.0364 *
## s(bl_age):time    4.448  4.448 1067.363  <2e-16 ***
## s(sleep_z):time   1.000  1.000    2.211  0.1371
## ---
## Signif. codes:  0 '***' 0.001 '**' 0.01 '*' 0.05 '.' 0.1 ' ' 1
##
## R-sq.(adj) =  0.419
## lmer.REML = 1.1244e+05  Scale est. = 1.8371e+06  n = 5678
```

Below are plots of a plot of `s(bl_age)`, `s(bl_age):sleep_z`, `s(bl_age):time`, and `s(sleep_z):time`. For `s(sleep_z):time`, roughly, if we cannot fit a straight line through the confidence bands, there is evidence that the effect of time depends on sleep duration. It is correct the that confidence interval is zero at one point along the axis; this happens because we do not include the main effect.

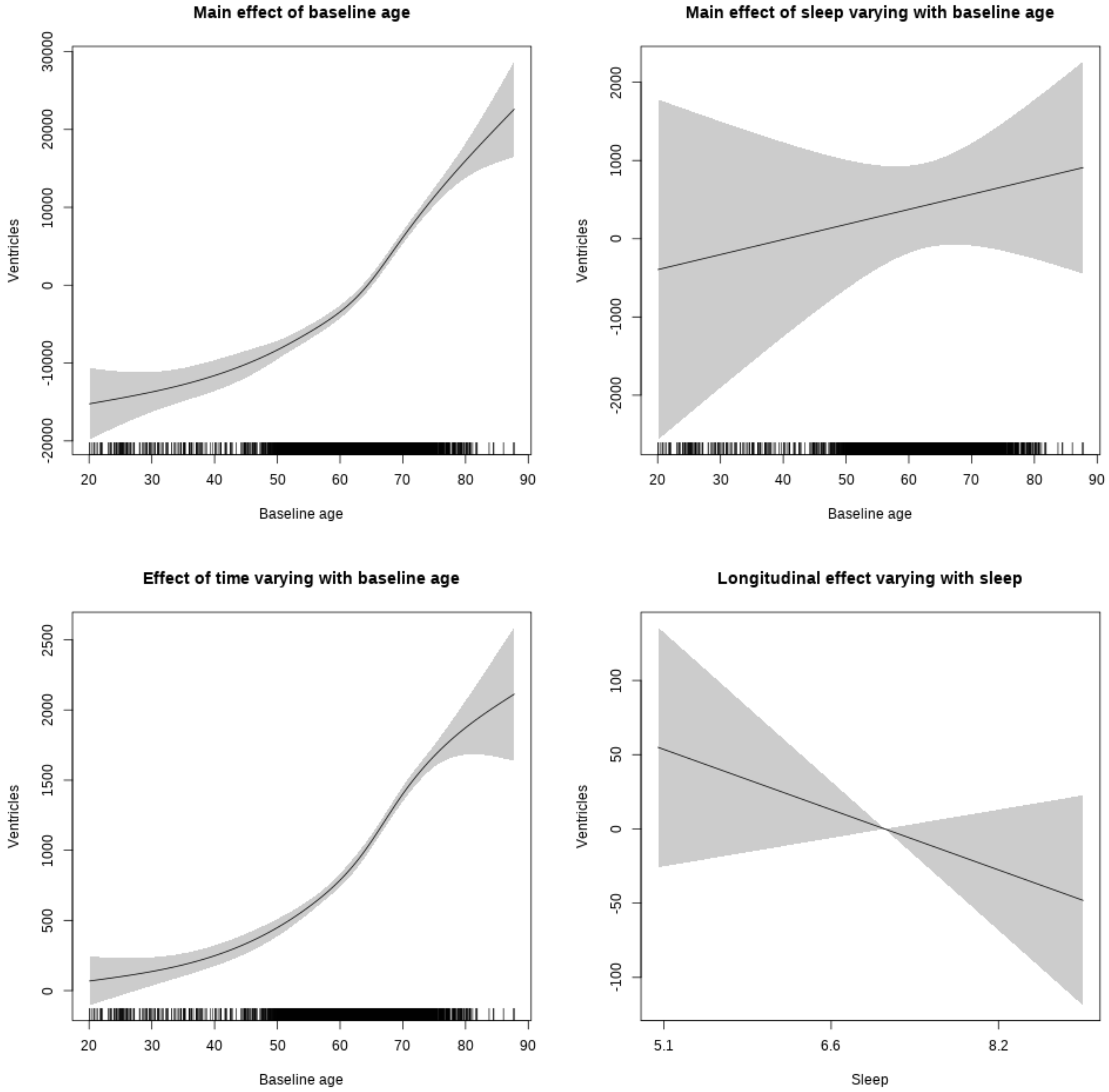

Restricted to sleep >= 5 and <= 9 hours Below is the row for `s(sleep_z):time` from the model output.

```
## s(sleep_z):time   1.000  1.000    1.850   0.174
```

Below are plots of a plot of `s(bl_age)`, `s(bl_age):sleep_z`, `s(bl_age):time`, and `s(sleep_z):time`. For `s(sleep_z):time`, roughly, if we cannot fit a straight line through the confidence bands, there is evidence that the effect of time depends on sleep duration. It is correct the that confidence interval is zero at one point along the axis; this happens because we do not include the main effect.

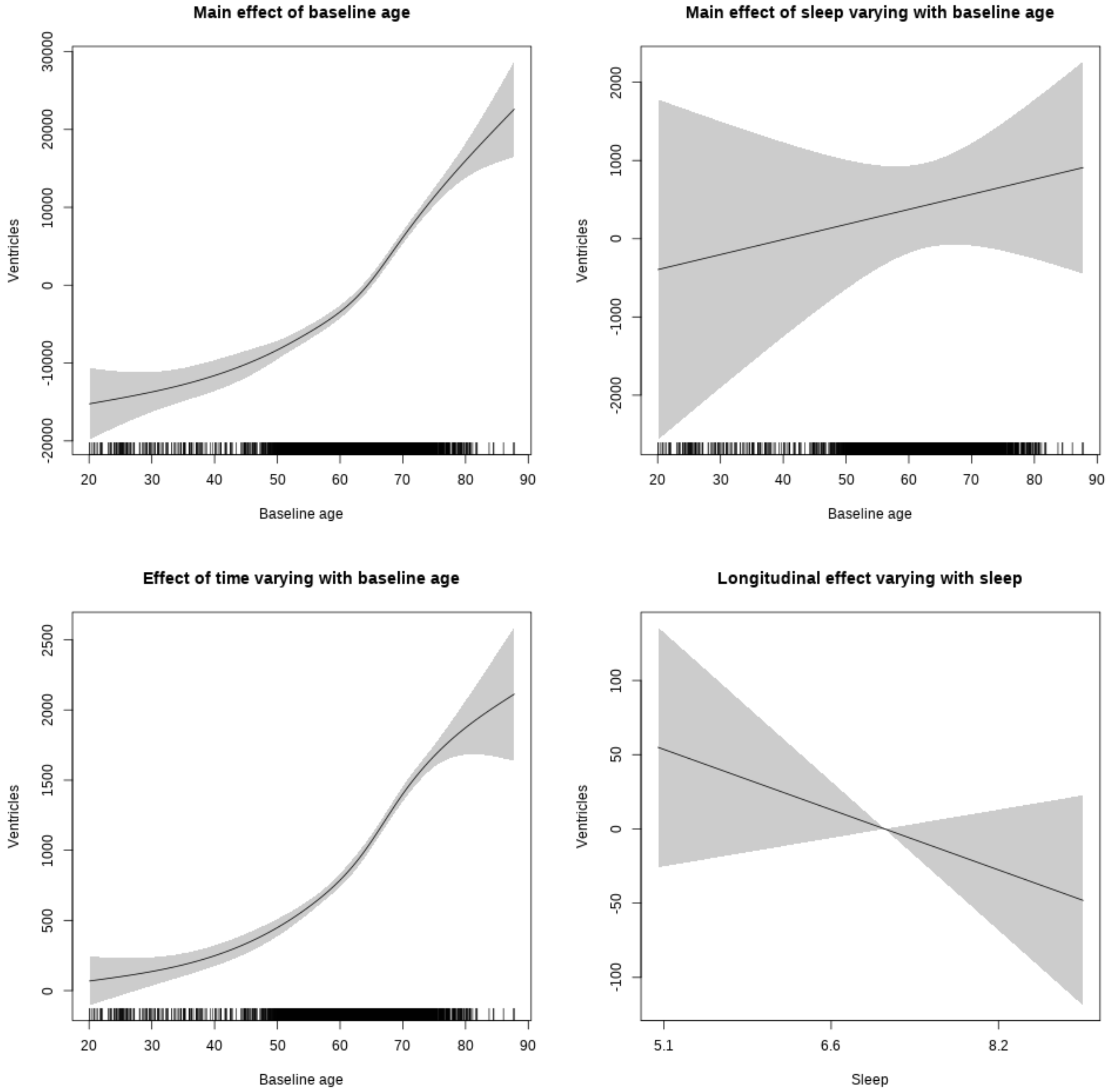

Controling for sleep quality

Full data Below is the model output. The term `s(sleep_z):time` is probably what we care most about.

```
##
## Family: gaussian
## Link function: identity
##
## Formula:
## value ~ X + s(bl_age, k = 10, bs = "cr") + s(bl_age, by = sleep_z,
##     bs = "cr") + s(bl_age, by = time, k = 5, bs = "cr") + s(sleep_z,
##     by = time, k = 5, bs = "cr", pc = 0)
## <environment: 0x563914d995c8>
```

```
##
## Parametric coefficients:
##                          Estimate Std. Error t value Pr(>|t|)
## (Intercept)               28955.47    1695.82  17.075   <2e-16 ***
## Xsexmale                   8317.05     633.09  13.137   <2e-16 ***
## XsiteousAvanto              727.83    1618.52   0.450   0.6530
## XsiteousPrisma             1033.03    1994.08   0.518   0.6045
## XsiteousSkyra              2102.25    1619.52   1.298   0.1943
## XsiteUB                   -5556.93    2577.96  -2.156   0.0312 *
## XsiteUCAM                  -417.92    1669.57  -0.250   0.8024
## XsiteUKB                   2040.06    5165.61   0.395   0.6929
## XsiteUmU                  -1872.19    1845.78  -1.014   0.3105
## Xukb_dummy:sleep_accel     -720.26     397.80  -1.811   0.0703 .
## Xukb_dummy:chronotype      -188.99     529.84  -0.357   0.7213
## Xukb_dummy:dozing           573.38    1094.76   0.524   0.6005
## Xukb_dummy:sleeplessness     27.97     683.09   0.041   0.9673
## Xukb_dummy:snoring          250.64     966.33   0.259   0.7954
## Xukb_dummy:gettingup       -201.57     743.07  -0.271   0.7862
## Xnotukb_dummy:PSQI_Global   -71.70     209.81  -0.342   0.7326
## ---
## Signif. codes:  0 ‘***’ 0.001 ‘**’ 0.01 ‘*’ 0.05 ‘.’ 0.1 ‘ ’ 1
##
## Approximate significance of smooth terms:
##                     edf Ref.df       F  p-value
## s(bl_age)         4.933  4.933 120.356  < 2e-16 ***
## s(bl_age):sleep_z 2.000  2.000   1.561 0.210086
## s(bl_age):time    4.122  4.122 860.189  < 2e-16 ***
## s(sleep_z):time   1.000  1.000  12.709 0.000368 ***
## ---
## Signif. codes:  0 ‘***’ 0.001 ‘**’ 0.01 ‘*’ 0.05 ‘.’ 0.1 ‘ ’ 1
##
## R-sq.(adj) =  0.334
## lmer.REML =  84735  Scale est. = 2.2708e+06  n = 4275
```

Below are plots of a plot of `s(bl_age)`, `s(bl_age):sleep_z`, `s(bl_age):time`, and `s(sleep_z):time`. For `s(sleep_z):time`, roughly, if we cannot fit a straight line through the confidence bands, there is evidence that the effect of time depends on sleep duration. It is correct the that confidence interval is zero at one point along the axis; this happens because we do not include the main effect.

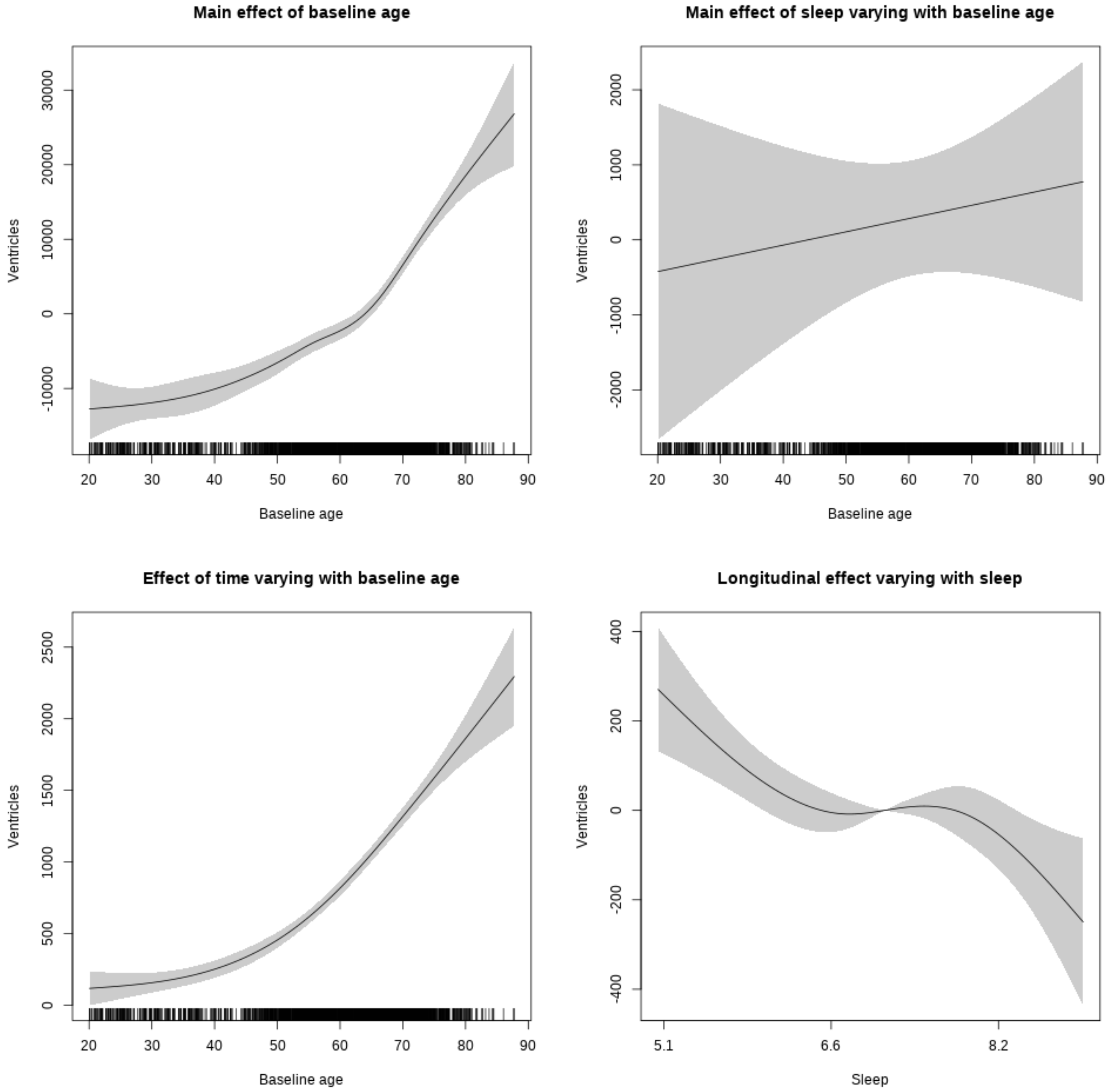

Restricted to sleep >= 5 and <= 9 hours Below is the row for `s(sleep_z):time` from the model output.

```
## s(sleep_z):time   3.090  3.090   7.705 2.6e-05 ***
```

Below are plots of a plot of `s(bl_age)`, `s(bl_age):sleep_z`, `s(bl_age):time`, and `s(sleep_z):time`. For `s(sleep_z):time`, roughly, if we cannot fit a straight line through the confidence bands, there is evidence that the effect of time depends on sleep duration. It is correct the that confidence interval is zero at one point along the axis; this happens because we do not include the main effect.

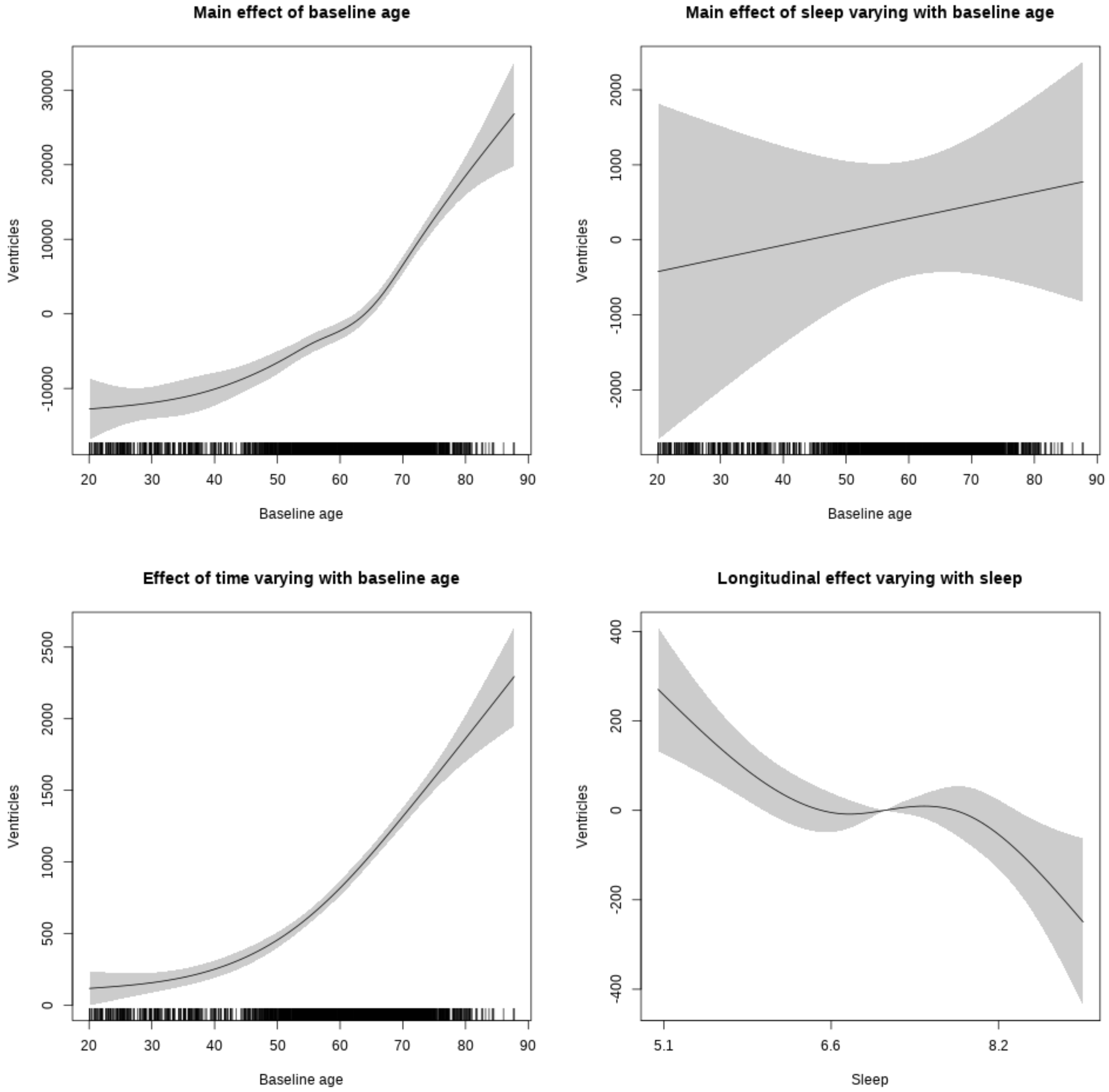

# P-values corrected for multiple testing

Below is a table showing the p-values for `s(sleep_z, by = time)` adjusted for multiple testing using the Benjamini-Hochberg procedure for the main models not controling for SES, BMI, or depression.

controlvar	region	type	pval	adjusted_pval
default	Accumbens-area	Full data	0.7914	0.8845
default	Amygdala	Full data	0.0416	0.1318
default	Brain-Stem	Full data	0.0122	0.0514
default	Caudate	Full data	0.0036	0.0196
default	CC_Anterior	Full data	0.9246	0.9760
default	CC_Central	Full data	0.5245	0.7033

controlvar	region	type	pval	adjusted_pval
default	CC_Mid_Anterior	Full data	0.2362	0.4275
default	CC_Mid_Posterior	Full data	0.8680	0.9425
default	CC_Posterior	Full data	0.0990	0.2208
default	Cerebellum-Cortex	Full data	0.5299	0.7033
default	Cerebellum-White-Matter	Full data	0.0002	0.0055
default	CerebralWhiteMatterVol	Full data	0.1046	0.2208
default	EstimatedTotalIntraCranialVol	Full data	0.5573	0.7033
default	Hippocampus	Full data	0.2322	0.4275
default	Pallidum	Full data	0.0194	0.0671
default	Putamen	Full data	0.3522	0.5771
default	Thalamus	Full data	0.0004	0.0055
default	TotalGrayVol	Full data	0.5737	0.7033
default	Ventricles	Full data	0.0024	0.0150
default	Accumbens-area	Restricted to sleep >= 5 and <= 9 hours	0.4554	0.6922
default	Amygdala	Restricted to sleep >= 5 and <= 9 hours	0.0530	0.1485
default	Brain-Stem	Restricted to sleep >= 5 and <= 9 hours	0.0054	0.0256
default	Caudate	Restricted to sleep >= 5 and <= 9 hours	0.0179	0.0671
default	CC_Anterior	Restricted to sleep >= 5 and <= 9 hours	0.9828	0.9828
default	CC_Central	Restricted to sleep >= 5 and <= 9 hours	0.9701	0.9828
default	CC_Mid_Anterior	Restricted to sleep >= 5 and <= 9 hours	0.1820	0.3640
default	CC_Mid_Posterior	Restricted to sleep >= 5 and <= 9 hours	0.6951	0.8004
default	CC_Posterior	Restricted to sleep >= 5 and <= 9 hours	0.0604	0.1485
default	Cerebellum-Cortex	Restricted to sleep >= 5 and <= 9 hours	0.3645	0.5771
default	Cerebellum-White-Matter	Restricted to sleep >= 5 and <= 9 hours	0.0004	0.0055
default	CerebralWhiteMatterVol	Restricted to sleep >= 5 and <= 9 hours	0.0605	0.1485
default	EstimatedTotalIntraCranialVol	Restricted to sleep >= 5 and <= 9 hours	0.3036	0.5244
default	Hippocampus	Restricted to sleep >= 5 and <= 9 hours	0.6076	0.7215
default	Pallidum	Restricted to sleep >= 5 and <= 9 hours	0.0625	0.1485
default	Putamen	Restricted to sleep >= 5 and <= 9 hours	0.5006	0.7033
default	Thalamus	Restricted to sleep >= 5 and <= 9 hours	0.0014	0.0132
default	TotalGrayVol	Restricted to sleep >= 5 and <= 9 hours	0.5718	0.7033
default	Ventricles	Restricted to sleep >= 5 and <= 9 hours	0.0022	0.0150

Controling for quality

Below is a plot comparing the p-values for `s(sleep_z, by = time)` adjusted for multiple testing using the Benjamini-Hochberg procedure for the the model controling for sleep quality and the default model.

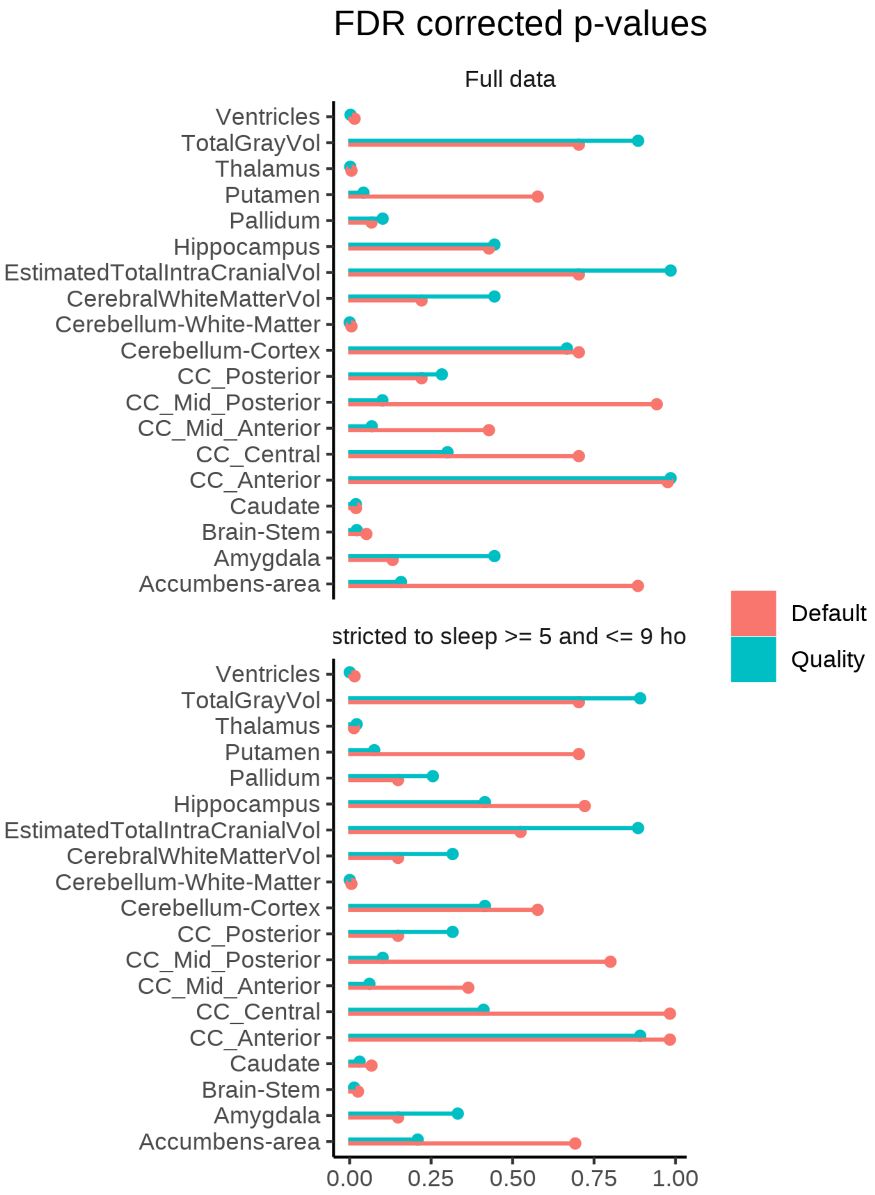

Next is a table of all the p-values for this model controling for sleep quality.

region	type	pval	adjusted_pval
Accumbens-area	Full data	0.0747	0.1577
Amygdala	Full data	0.3619	0.4444
Brain-Stem	Full data	0.0051	0.0217
Caudate	Full data	0.0035	0.0189
CC_Anterior	Full data	0.9799	0.9850
CC_Central	Full data	0.1738	0.3002
CC_Mid_Anterior	Full data	0.0232	0.0678

region	type	pval	adjusted_pval
CC_Mid_Posterior	Full data	0.0397	0.1005
CC_Posterior	Full data	0.1563	0.2829
Cerebellum-Cortex	Full data	0.5612	0.6664
Cerebellum-White-Matter	Full data	0.0000	0.0000
CerebralWhiteMatterVol	Full data	0.3408	0.4444
EstimatedTotalIntraCranialVol	Full data	0.9850	0.9850
Hippocampus	Full data	0.3625	0.4444
Pallidum	Full data	0.0452	0.1014
Putamen	Full data	0.0123	0.0424
Thalamus	Full data	0.0002	0.0017
TotalGrayVol	Full data	0.7920	0.8851
Ventricles	Full data	0.0004	0.0028
Accumbens-area	Restricted to sleep >= 5 and <= 9 hours	0.1044	0.2088
Amygdala	Restricted to sleep >= 5 and <= 9 hours	0.2183	0.3318
Brain-Stem	Restricted to sleep >= 5 and <= 9 hours	0.0022	0.0137
Caudate	Restricted to sleep >= 5 and <= 9 hours	0.0080	0.0305
CC_Anterior	Restricted to sleep >= 5 and <= 9 hours	0.8402	0.8915
CC_Central	Restricted to sleep >= 5 and <= 9 hours	0.2812	0.4109
CC_Mid_Anterior	Restricted to sleep >= 5 and <= 9 hours	0.0191	0.0606
CC_Mid_Posterior	Restricted to sleep >= 5 and <= 9 hours	0.0454	0.1014
CC_Posterior	Restricted to sleep >= 5 and <= 9 hours	0.1942	0.3159
Cerebellum-Cortex	Restricted to sleep >= 5 and <= 9 hours	0.2950	0.4150
Cerebellum-White-Matter	Restricted to sleep >= 5 and <= 9 hours	0.0000	0.0003
CerebralWhiteMatterVol	Restricted to sleep >= 5 and <= 9 hours	0.1995	0.3159
EstimatedTotalIntraCranialVol	Restricted to sleep >= 5 and <= 9 hours	0.7783	0.8851
Hippocampus	Restricted to sleep >= 5 and <= 9 hours	0.3058	0.4150
Pallidum	Restricted to sleep >= 5 and <= 9 hours	0.1344	0.2553
Putamen	Restricted to sleep >= 5 and <= 9 hours	0.0278	0.0755
Thalamus	Restricted to sleep >= 5 and <= 9 hours	0.0045	0.0214
TotalGrayVol	Restricted to sleep >= 5 and <= 9 hours	0.8446	0.8915
Ventricles	Restricted to sleep >= 5 and <= 9 hours	0.0000	0.0003

P-values controling for BMI

region	type	pval	adjusted_pval
Accumbens-area	Full data	0.2710	0.5169
Amygdala	Full data	0.0035	0.0254
Brain-Stem	Full data	0.2992	0.5169
Caudate	Full data	0.0013	0.0166
CC_Anterior	Full data	0.6934	0.8341
CC_Central	Full data	0.2973	0.5169
CC_Mid_Anterior	Full data	0.2958	0.5169
CC_Mid_Posterior	Full data	0.6361	0.8341
CC_Posterior	Full data	0.1222	0.4204
Cerebellum-Cortex	Full data	0.8936	0.9108
Cerebellum-White-Matter	Full data	0.0004	0.0143
CerebralWhiteMatterVol	Full data	0.6206	0.8341
EstimatedTotalIntraCranialVol	Full data	0.5942	0.8341
Hippocampus	Full data	0.2834	0.5169
Pallidum	Full data	0.0158	0.0750

region	type	pval	adjusted_pval
Putamen	Full data	0.9108	0.9108
Thalamus	Full data	0.0028	0.0254
TotalGrayVol	Full data	0.1461	0.4204
Ventricles	Full data	0.1747	0.4388
Accumbens-area	Restricted to sleep >= 5 and <= 9 hours	0.4891	0.7435
Amygdala	Restricted to sleep >= 5 and <= 9 hours	0.0040	0.0254
Brain-Stem	Restricted to sleep >= 5 and <= 9 hours	0.1338	0.4204
Caudate	Restricted to sleep >= 5 and <= 9 hours	0.0013	0.0166
CC_Anterior	Restricted to sleep >= 5 and <= 9 hours	0.6704	0.8341
CC_Central	Restricted to sleep >= 5 and <= 9 hours	0.6384	0.8341
CC_Mid_Anterior	Restricted to sleep >= 5 and <= 9 hours	0.3937	0.6233
CC_Mid_Posterior	Restricted to sleep >= 5 and <= 9 hours	0.7024	0.8341
CC_Posterior	Restricted to sleep >= 5 and <= 9 hours	0.1549	0.4204
Cerebellum-Cortex	Restricted to sleep >= 5 and <= 9 hours	0.2856	0.5169
Cerebellum-White-Matter	Restricted to sleep >= 5 and <= 9 hours	0.0086	0.0468
CerebralWhiteMatterVol	Restricted to sleep >= 5 and <= 9 hours	0.3666	0.6057
EstimatedTotalIntraCranialVol	Restricted to sleep >= 5 and <= 9 hours	0.8084	0.8777
Hippocampus	Restricted to sleep >= 5 and <= 9 hours	0.7366	0.8419
Pallidum	Restricted to sleep >= 5 and <= 9 hours	0.0833	0.3165
Putamen	Restricted to sleep >= 5 and <= 9 hours	0.7533	0.8419
Thalamus	Restricted to sleep >= 5 and <= 9 hours	0.0230	0.0969
TotalGrayVol	Restricted to sleep >= 5 and <= 9 hours	0.8528	0.9002
Ventricles	Restricted to sleep >= 5 and <= 9 hours	0.1848	0.4388

P-values controling for SES

region	type	pval	adjusted_pval
Accumbens-area	Full data	0.0986	0.5908
Amygdala	Full data	0.1039	0.5908
Brain-Stem	Full data	0.1334	0.6335
Caudate	Full data	0.3185	0.6562
CC_Anterior	Full data	0.5049	0.6885
CC_Central	Full data	0.8780	0.9018
CC_Mid_Anterior	Full data	0.8340	0.9018
CC_Mid_Posterior	Full data	0.2508	0.6562
CC_Posterior	Full data	0.4001	0.6562
Cerebellum-Cortex	Full data	0.9947	0.9947
Cerebellum-White-Matter	Full data	0.3558	0.6562
CerebralWhiteMatterVol	Full data	0.2209	0.6562
EstimatedTotalIntraCranialVol	Full data	0.3876	0.6562
Hippocampus	Full data	0.1515	0.6397
Pallidum	Full data	0.0176	0.5908
Putamen	Full data	0.4490	0.6562
Thalamus	Full data	0.0702	0.5908
TotalGrayVol	Full data	0.1088	0.5908
Ventricles	Full data	0.7810	0.8993
Accumbens-area	Restricted to sleep >= 5 and <= 9 hours	0.3712	0.6562
Amygdala	Restricted to sleep >= 5 and <= 9 hours	0.0932	0.5908
Brain-Stem	Restricted to sleep >= 5 and <= 9 hours	0.3915	0.6562
Caudate	Restricted to sleep >= 5 and <= 9 hours	0.3660	0.6562

region	type	pval	adjusted_pval
CC_Anterior	Restricted to sleep >= 5 and <= 9 hours	0.4422	0.6562
CC_Central	Restricted to sleep >= 5 and <= 9 hours	0.6923	0.8476
CC_Mid_Anterior	Restricted to sleep >= 5 and <= 9 hours	0.7064	0.8476
CC_Mid_Posterior	Restricted to sleep >= 5 and <= 9 hours	0.3153	0.6562
CC_Posterior	Restricted to sleep >= 5 and <= 9 hours	0.4395	0.6562
Cerebellum-Cortex	Restricted to sleep >= 5 and <= 9 hours	0.4110	0.6562
Cerebellum-White-Matter	Restricted to sleep >= 5 and <= 9 hours	0.3810	0.6562
CerebralWhiteMatterVol	Restricted to sleep >= 5 and <= 9 hours	0.7137	0.8476
EstimatedTotalIntraCranialVol	Restricted to sleep >= 5 and <= 9 hours	0.5073	0.6885
Hippocampus	Restricted to sleep >= 5 and <= 9 hours	0.6452	0.8454
Pallidum	Restricted to sleep >= 5 and <= 9 hours	0.0677	0.5908
Putamen	Restricted to sleep >= 5 and <= 9 hours	0.3340	0.6562
Thalamus	Restricted to sleep >= 5 and <= 9 hours	0.2561	0.6562
TotalGrayVol	Restricted to sleep >= 5 and <= 9 hours	0.8550	0.9018
Ventricles	Restricted to sleep >= 5 and <= 9 hours	0.8340	0.9018

P-values controling for Depression

region	type	pval	adjusted_pval
Accumbens-area	Full data	0.1468	0.4534
Amygdala	Full data	0.0504	0.2394
Brain-Stem	Full data	0.1416	0.4534
Caudate	Full data	0.0000	0.0007
CC_Anterior	Full data	0.8319	0.8781
CC_Central	Full data	0.4567	0.6675
CC_Mid_Anterior	Full data	0.3933	0.5979
CC_Mid_Posterior	Full data	0.5472	0.7426
CC_Posterior	Full data	0.2621	0.4643
Cerebellum-Cortex	Full data	0.9059	0.9304
Cerebellum-White-Matter	Full data	0.0031	0.0391
CerebralWhiteMatterVol	Full data	0.6399	0.7598
EstimatedTotalIntraCranialVol	Full data	0.7866	0.8541
Hippocampus	Full data	0.1822	0.4534
Pallidum	Full data	0.0382	0.2072
Putamen	Full data	0.2216	0.4607
Thalamus	Full data	0.0319	0.2021
TotalGrayVol	Full data	0.2111	0.4607
Ventricles	Full data	0.1371	0.4534
Accumbens-area	Restricted to sleep >= 5 and <= 9 hours	0.2425	0.4607
Amygdala	Restricted to sleep >= 5 and <= 9 hours	0.0206	0.1800
Brain-Stem	Restricted to sleep >= 5 and <= 9 hours	0.1232	0.4534
Caudate	Restricted to sleep >= 5 and <= 9 hours	0.0002	0.0037
CC_Anterior	Restricted to sleep >= 5 and <= 9 hours	0.6866	0.7674
CC_Central	Restricted to sleep >= 5 and <= 9 hours	0.6148	0.7536
CC_Mid_Anterior	Restricted to sleep >= 5 and <= 9 hours	0.3409	0.5398
CC_Mid_Posterior	Restricted to sleep >= 5 and <= 9 hours	0.2351	0.4607
CC_Posterior	Restricted to sleep >= 5 and <= 9 hours	0.1678	0.4534
Cerebellum-Cortex	Restricted to sleep >= 5 and <= 9 hours	0.6028	0.7536
Cerebellum-White-Matter	Restricted to sleep >= 5 and <= 9 hours	0.0237	0.1800
CerebralWhiteMatterVol	Restricted to sleep >= 5 and <= 9 hours	0.2919	0.4823

region	type	pval	adjusted_pval
EstimatedTotalIntraCranialVol	Restricted to sleep >= 5 and <= 9 hours	0.9776	0.9776
Hippocampus	Restricted to sleep >= 5 and <= 9 hours	0.6660	0.7669
Pallidum	Restricted to sleep >= 5 and <= 9 hours	0.1909	0.4534
Putamen	Restricted to sleep >= 5 and <= 9 hours	0.5049	0.7107
Thalamus	Restricted to sleep >= 5 and <= 9 hours	0.2688	0.4643
TotalGrayVol	Restricted to sleep >= 5 and <= 9 hours	0.6096	0.7536
Ventricles	Restricted to sleep >= 5 and <= 9 hours	0.1739	0.4534

Clustering of cortical regions

Contents

Area 8
Description of clusters . . . 8
GAMM fits in each cluster . . . 11
Meta analysis . . . 12

Thickness 13
Description of clusters . . . 13
GAMM fits in each cluster . . . 16
Meta analysis . . . 17

Volume 18
Description of clusters . . . 18
GAMM fits in each cluster . . . 21
Meta analysis . . . 22

References 23

Data preparation, including outlier removal, was done exactly as for the subcortical volumes, and won't be repeated here. Analyses were conducted in R version 4.0.0 (R Core Team 2020). The `tidyverse` (Wickham et al. 2019) was used for data manipulation and visualization, and `ggseg` (Mowinckel and Vidal-Piñeiro 2020) was used for plotting of brain regions. The `metgam` package was used for meta analysis of GAMM fits, using the methods described in Sørensen et al. (2021).

For each measure (area, thickness, volume), the following GAMMs was fitted to each of 33 cortical ROIs, using `gamm4` (Wood and Scheipl 2020). When the measure was thickness, the term `icv` was not included.

```
mod <- gamm4(
  value ~ sex + site + icv + s(age, k = 10, bs = "cr") + s(sleep, k = 5, bs = "cr"),
  random =~(1|id), data = dat,
  REML = FALSE,
  knots = list(age_z = age_knots, sleep_z = sleep_knots)
  )
```

The additional arguments `age_knots` and `sleep_knots` are knot locations which make sure that the basis functions used are identical for all regions. This allows us to perform functional cluster using only the estimated spline coefficients.

The plots below show raw p-values and p-values corrected for multiple testing with the Benjamini-Hochberg procedure, for the effect of sleep and the given measure.The estimated false discovery rate is written in the plot titles. The `sgof` package(Conde and Una Alvarez 2020) was used for this.

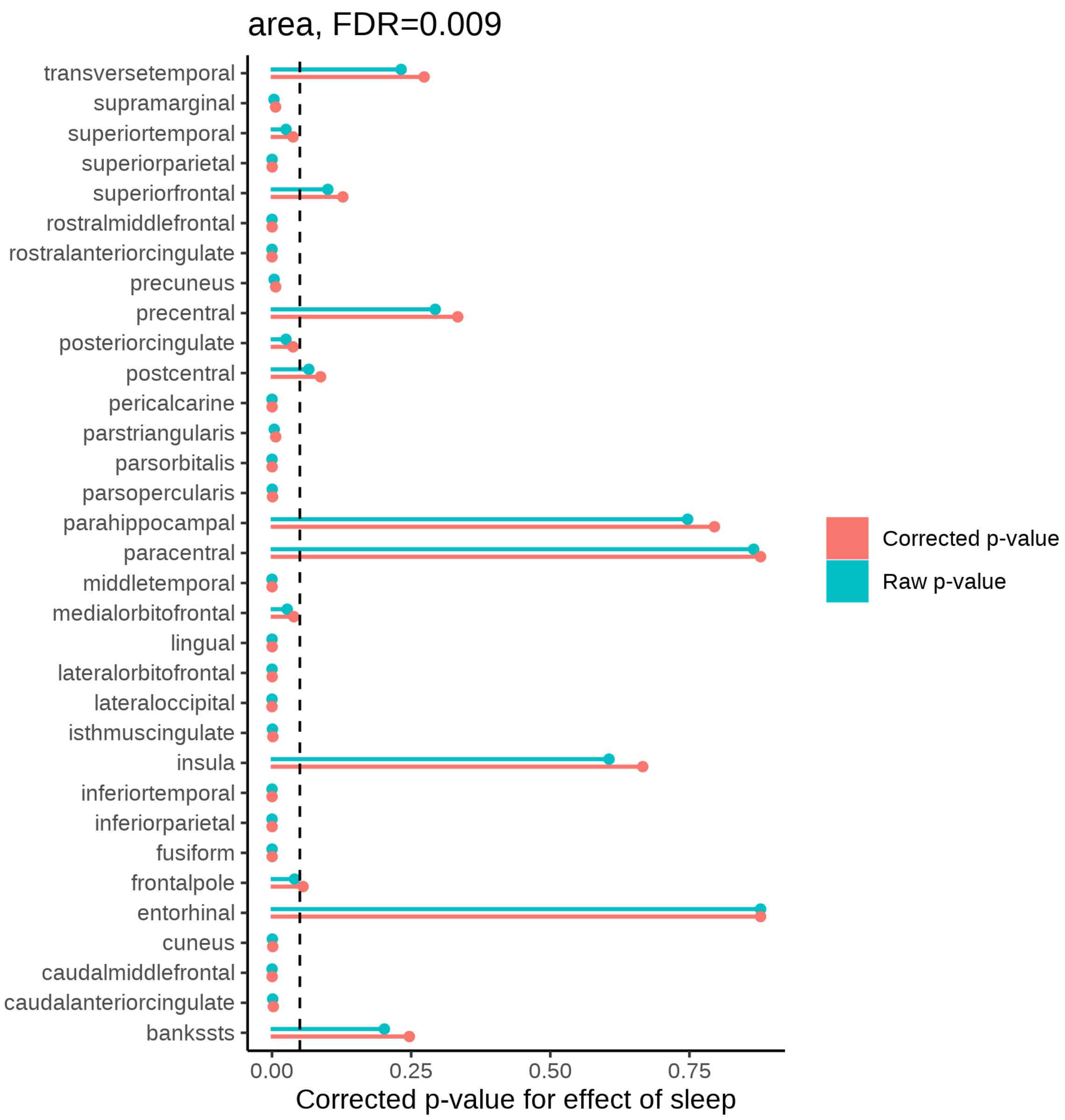
area, FDR=0.009
transversetemporal
supramarginal
superiortemporal
superiorparietal
superiorfrontal
rostralmiddlefrontal
rostralanteriorcingulate
precuneus
precentral
posteriorcingulate
postcentral
pericalcarine
parstriangularis
parsorbitalis
parsopercularis
parahippocampal
paracentral
middletemporal
medialorbitofrontal
lingual
lateralorbitofrontal
lateraloccipital
isthmuscingulate
insula
inferiortemporal
inferiorparietal
fusiform
frontalpole
entorhinal
cuneus
caudalmiddlefrontal
caudalanteriorcingulate
bankssts
0.00
0.25
0.50
0.75
Corrected p-value for effect of sleep
Corrected p-value
Raw p-value

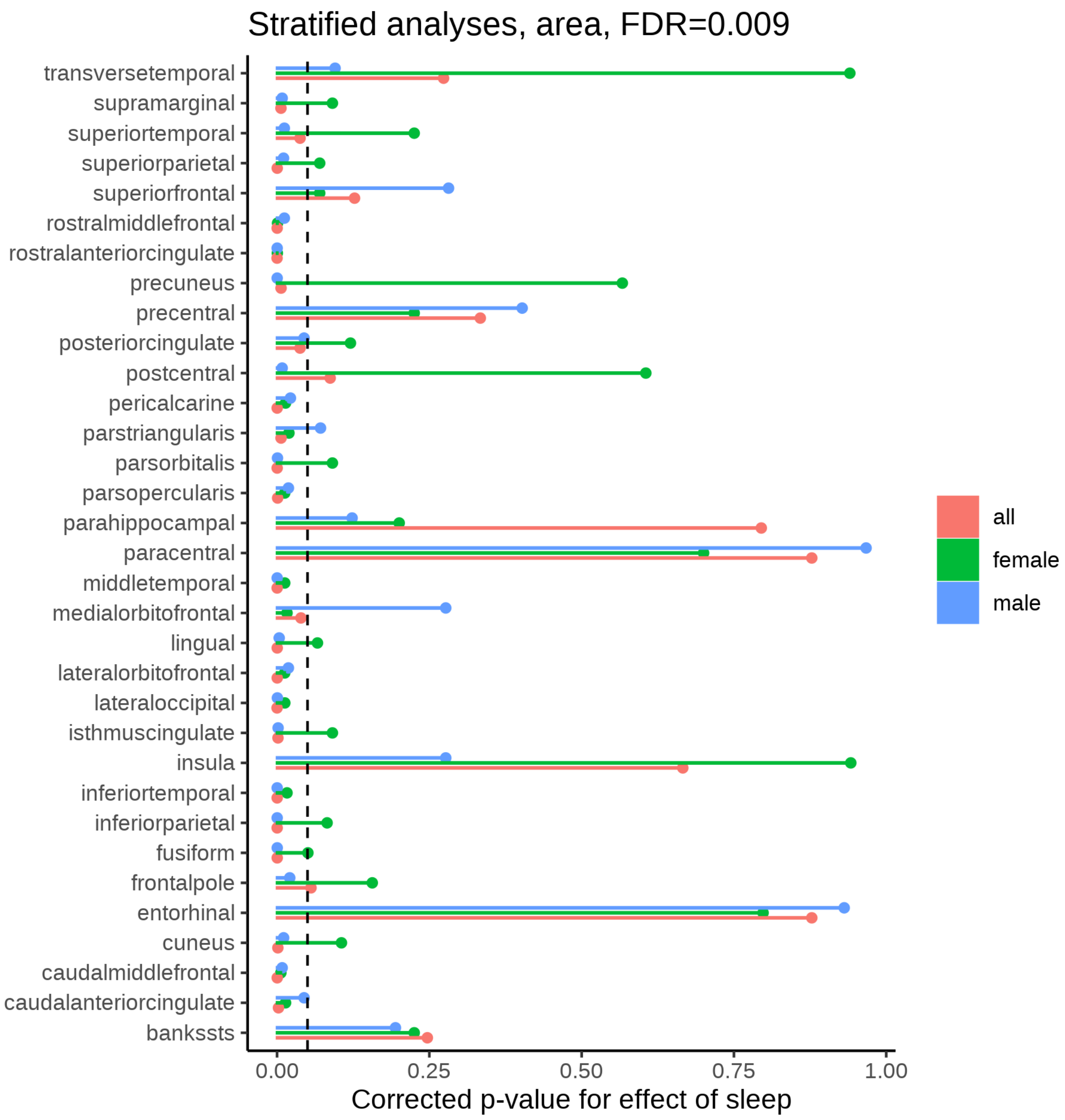
Stratified analyses, area, FDR=0.009
transversetemporal
supramarginal
superiortemporal
superiorparietal
superiorfrontal
rostralmiddlefrontal
rostralanteriorcingulate
precuneus
precentral
posteriorcingulate
postcentral
pericalcarine
parstriangularis
parsorbitalis
parsopercularis
parahippocampal
paracentral
middletemporal
medialorbitofrontal
lingual
lateralorbitofrontal
lateraloccipital
isthmuscingulate
insula
inferiortemporal
inferiorparietal
fusiform
frontalpole
entorhinal
cuneus
caudalmiddlefrontal
caudalanteriorcingulate
bankssts
0.00
0.25
0.50
0.75
1.00
Corrected p-value for effect of sleep
all
female
male

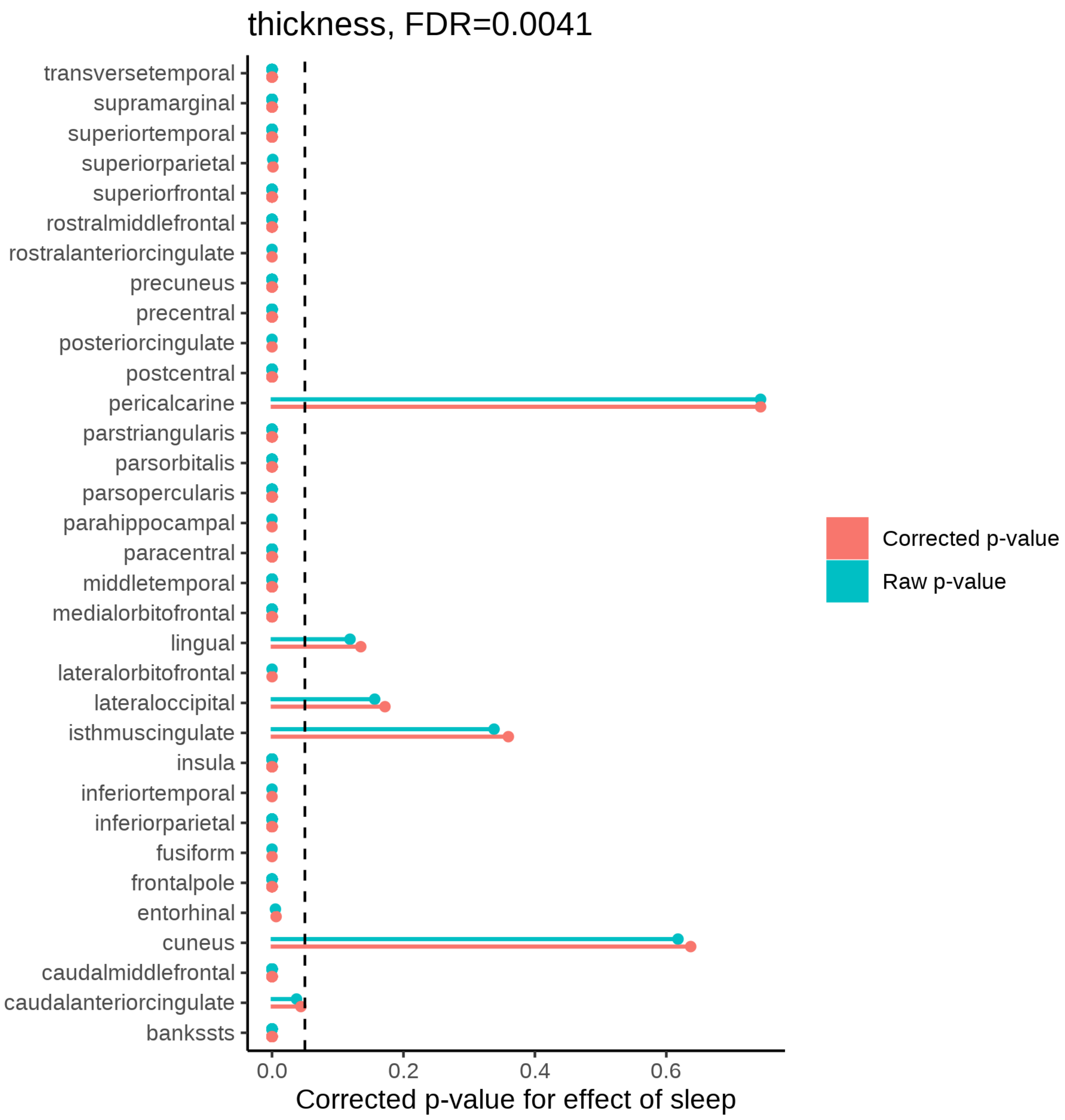
thickness, FDR=0.0041
transversetemporal
supramarginal
superiortemporal
superiorparietal
superiorfrontal
rostralmiddlefrontal
rostralanteriorcingulate
precuneus
precentral
posteriorcingulate
postcentral
pericalcarine
parstriangularis
parsorbitalis
parsopercularis
parahippocampal
paracentral
middletemporal
medialorbitofrontal
lingual
lateralorbitofrontal
lateraloccipital
isthmuscingulate
insula
inferiortemporal
inferiorparietal
fusiform
frontalpole
entorhinal
cuneus
caudalmiddlefrontal
caudalanteriorcingulate
bankssts
0.0
0.2
0.4
0.6
Corrected p-value for effect of sleep
Corrected p-value
Raw p-value

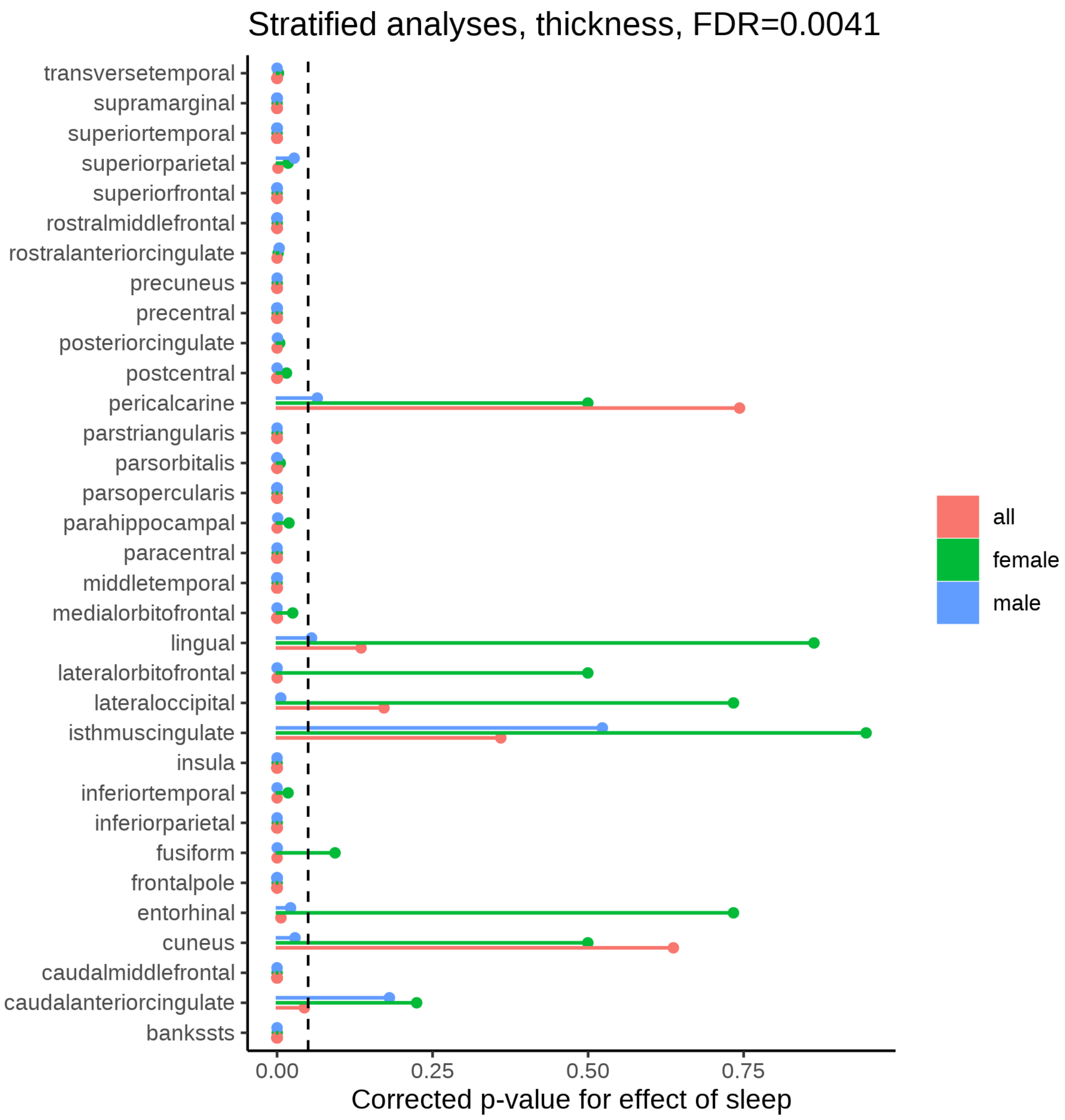

Stratified analyses, thickness, FDR=0.0041
transversetemporal
supramarginal
superiortemporal
superiorparietal
superiorfrontal
rostralmiddlefrontal
rostralanteriorcingulate
precuneus
precentral
posteriorcingulate
postcentral
pericalcarine
parstriangularis
parsorbitalis
parsopercularis
parahippocampal
paracentral
middletemporal
medialorbitofrontal
lingual
lateralorbitofrontal
lateraloccipital
isthmuscingulate
insula
inferiortemporal
inferiorparietal
fusiform
frontalpole
entorhinal
cuneus
caudalmiddlefrontal
caudalanteriorcingulate
bankssts
0.00
0.25
0.50
0.75
Corrected p-value for effect of sleep
all
female
male

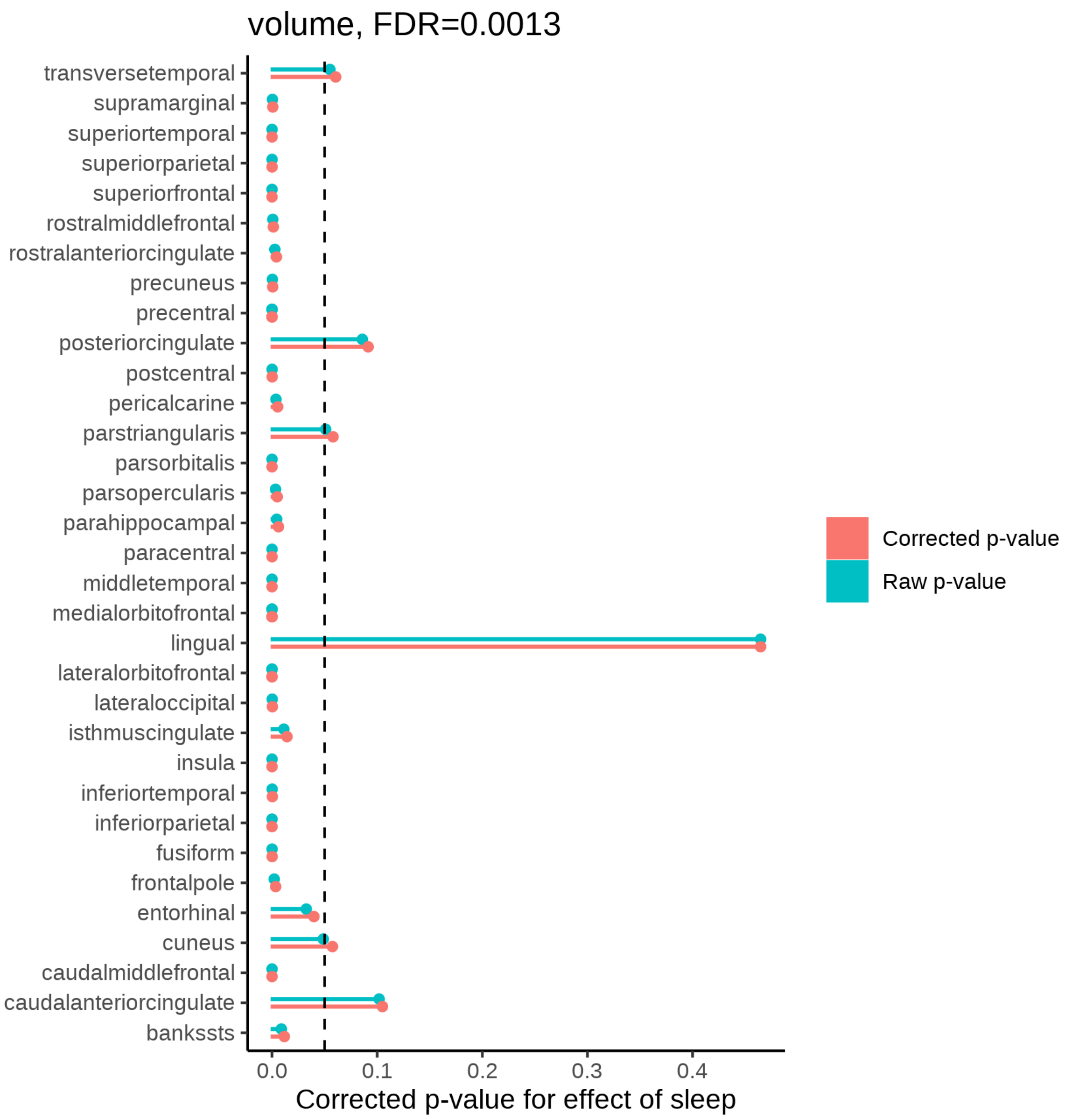

volume, FDR=0.0013
transversetemporal
supramarginal
superiortemporal
superiorparietal
superiorfrontal
rostralmiddlefrontal
rostralanteriorcingulate
precuneus
precentral
posteriorcingulate
postcentral
pericalcarine
parstriangularis
parsorbitalis
parsopercularis
parahippocampal
paracentral
middletemporal
medialorbitofrontal
lingual
lateralorbitofrontal
lateraloccipital
isthmuscingulate
insula
inferiortemporal
inferiorparietal
fusiform
frontalpole
entorhinal
cuneus
caudalmiddlefrontal
caudalanteriorcingulate
bankssts
0.0
0.1
0.2
0.3
0.4
Corrected p-value for effect of sleep
Corrected p-value
Raw p-value

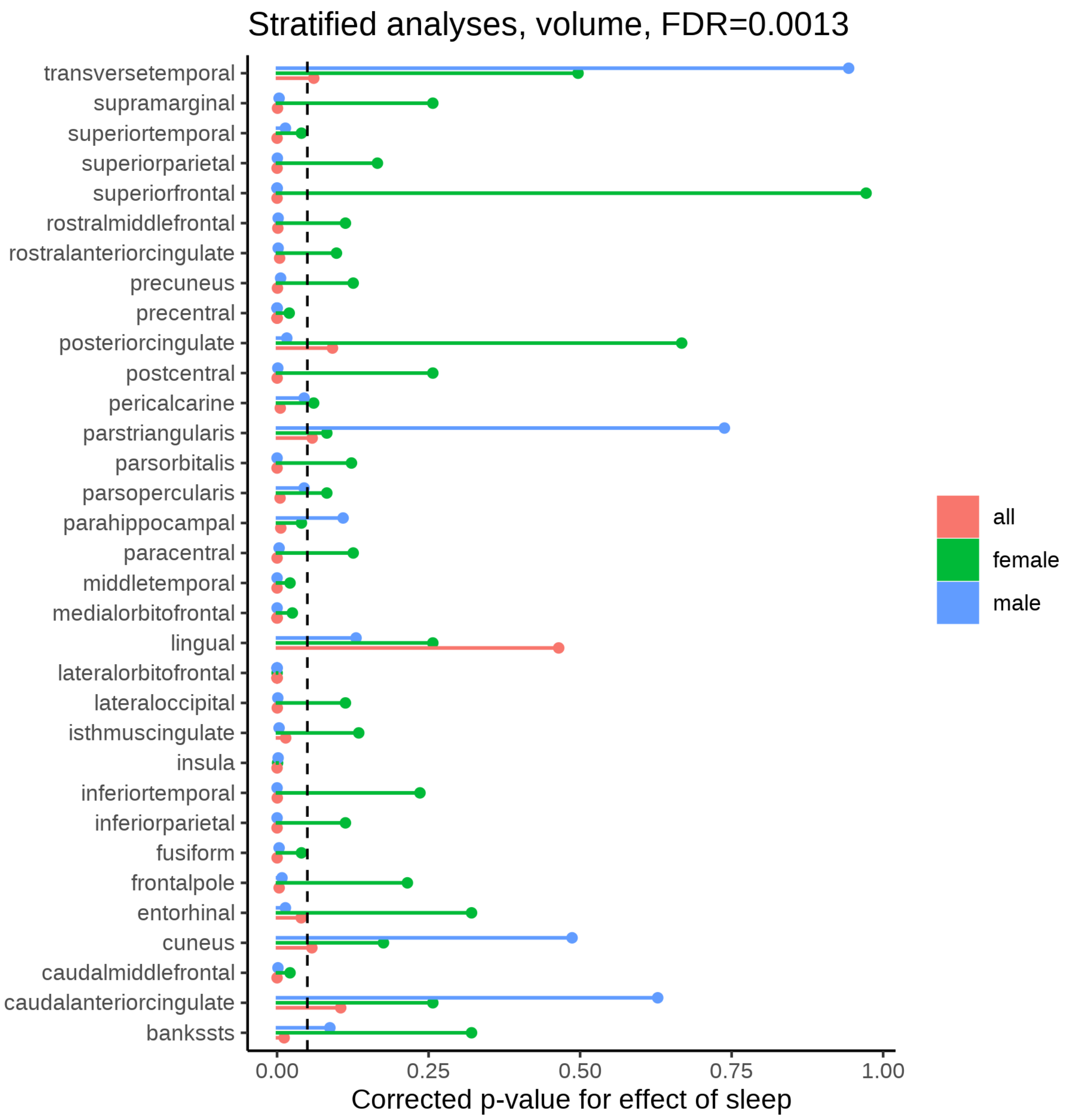

K-means clustering was run for each measure separately, dividing the regions based on the similarity of the sleep-brain curves described by the term `s(age)`. The plots below show how the total within-cluster sum-of-squares depends on the number of clusters. As usual, the plots are not very conclusive, but three clusters were chosen for each measure.

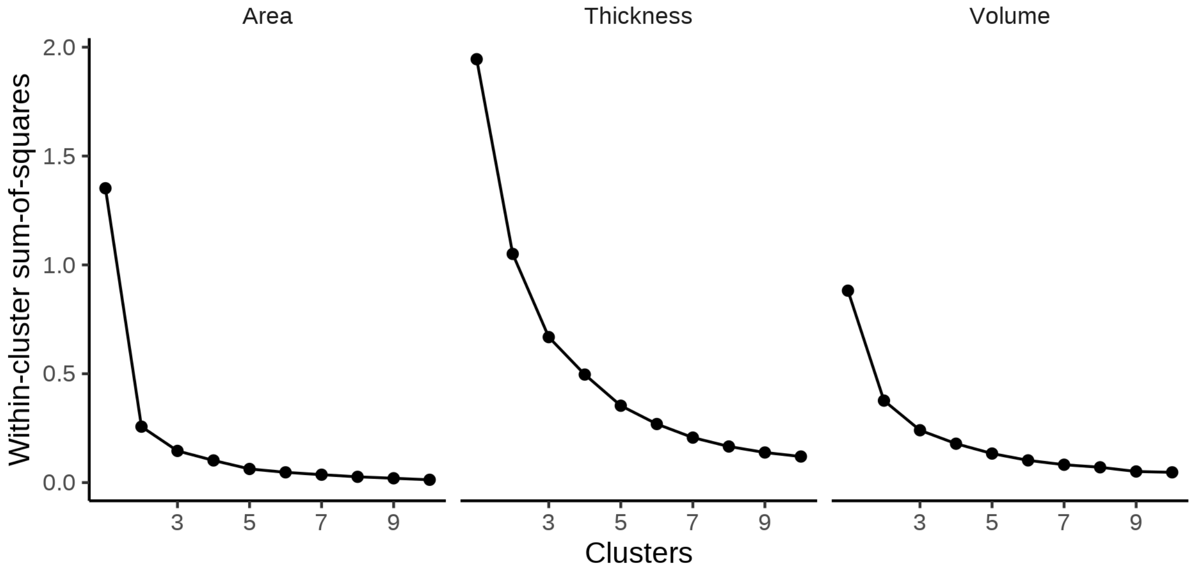

Area

The following regions go in each cluster:

- Cluster 1: cuneus, inferiorparietal, lateraloccipital, lingual, pericalcarine, superiorparietal
- Cluster 2: caudalmiddlefrontal, lateralorbitofrontal, middletemporal, parsorbitalis
- Cluster 3: bankssts, caudalanteriorcingulate, entorhinal, frontalpole, fusiform, inferiortemporal, insula, isthmuscingulate, medialorbitofrontal, paracentral, parahippocampal, parsopercularis, parstriangularis, postcentral, posteriorcingulate, precentral, precuneus, rostralanteriorcingulate, rostralmiddlefrontal, superiorfrontal, superiortemporal, supramarginal, transversetemporal

Description of clusters

First are spaghetti plots for the three clusters.

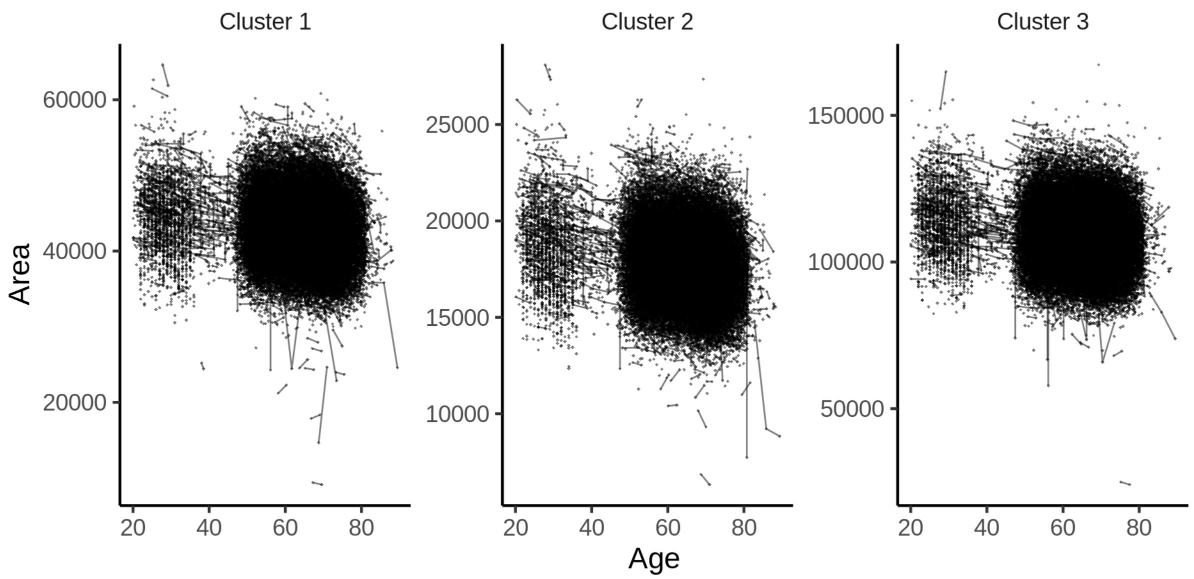

The plot below shows the sleep-volume curves for the three clusters.

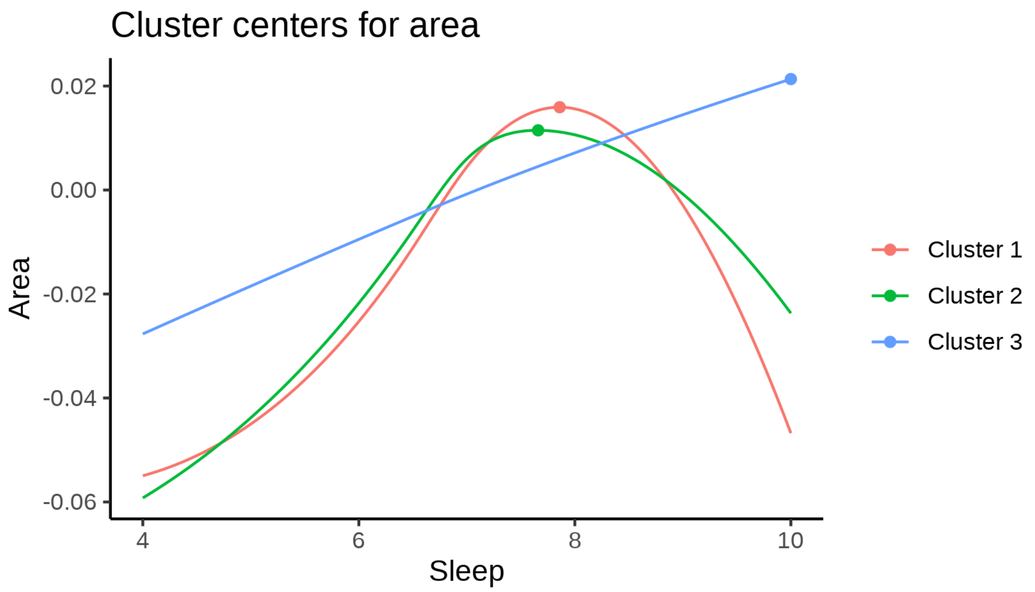

The maximum values for each cluster occur at the following sleep durations:

Cluster	Sleep
Cluster 1	7.86
Cluster 2	7.66
Cluster 3	10.00

The next plot shows the effect of varying the sleep duration as a percentage of the maximum value, obtained at the top of the curve. We have to scrutinize these percentages a bit, because actually defining the mean and standard deviations for each cluster turned out to be not that straightforward, but I believe that what I did makes sense. The problem is that the original clustering is done on a Z-transformed scale, since we are interested in differences in shapes of the curves, and not differences in absolute values of area/thickness/volume

between the regions. Then I had to define conversion factors by weighting the mean and variances of each region in each cluster, and try to scale it back.

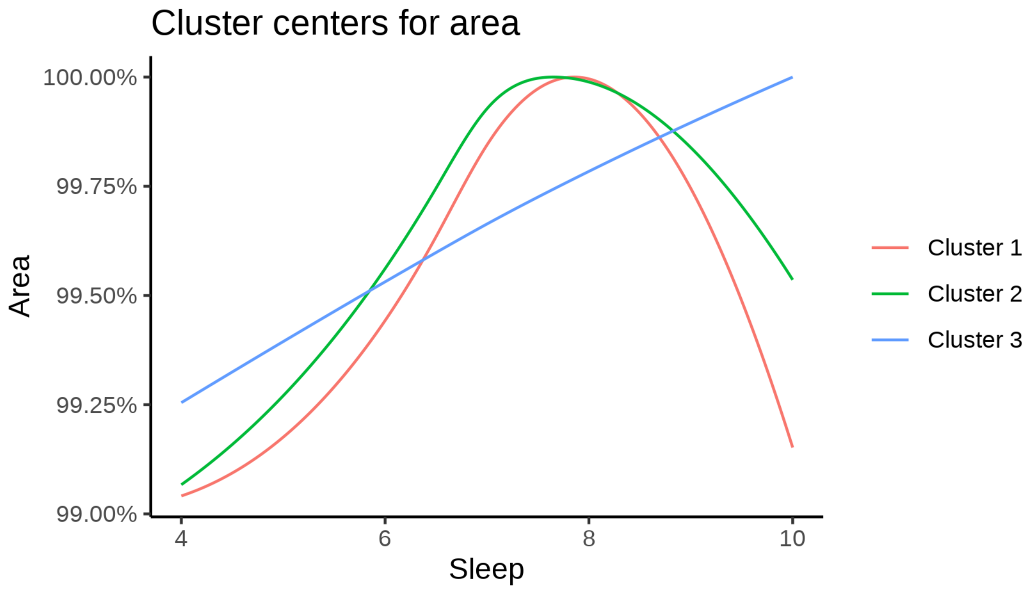

For completeness, we also show the cluster curves upon which the percentage plot above was based. Note that the values differ between clusters, since their sizes are different.

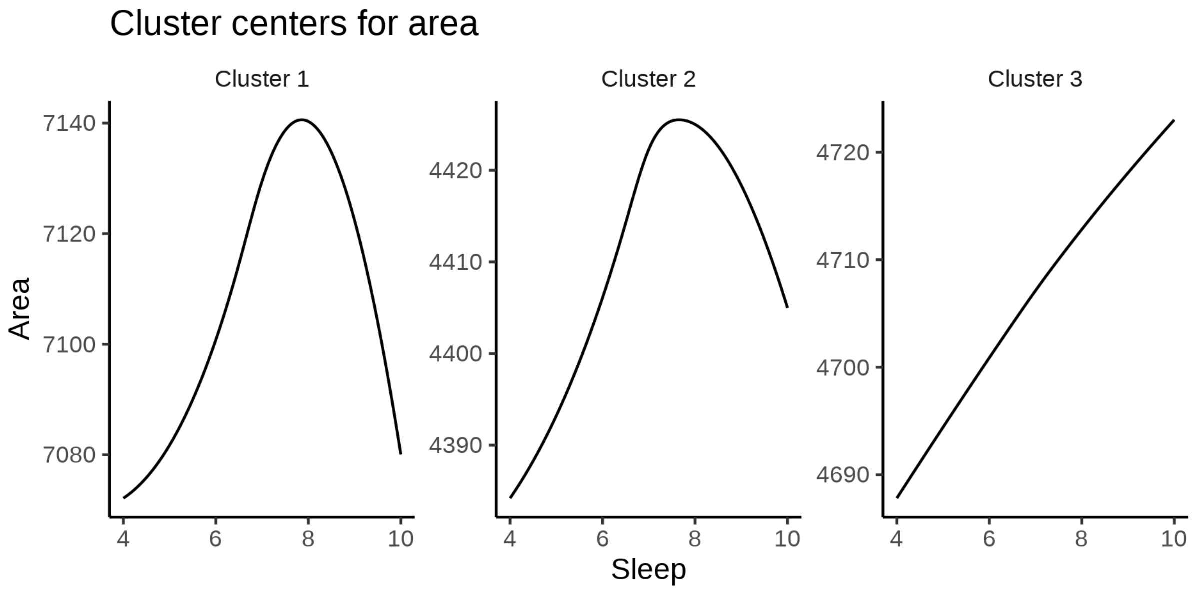

The next plot shows the location of the clusters.

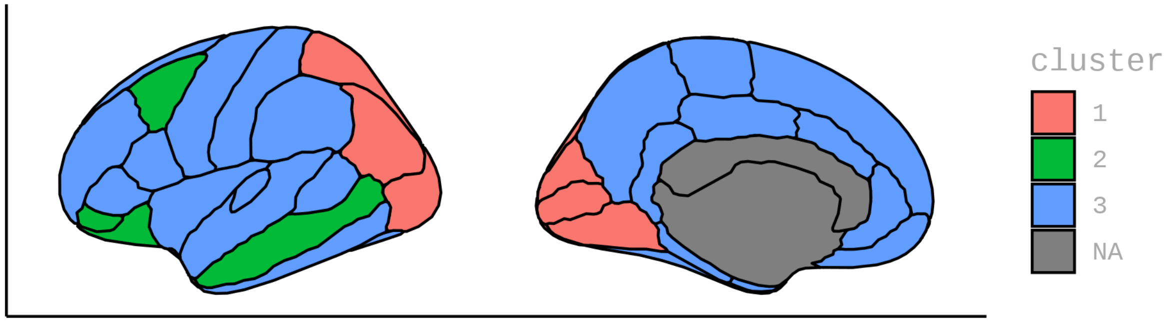

GAMM fits in each cluster

The plot below shows the results of fitting GAMMs to the (weighted) average in each cluster.

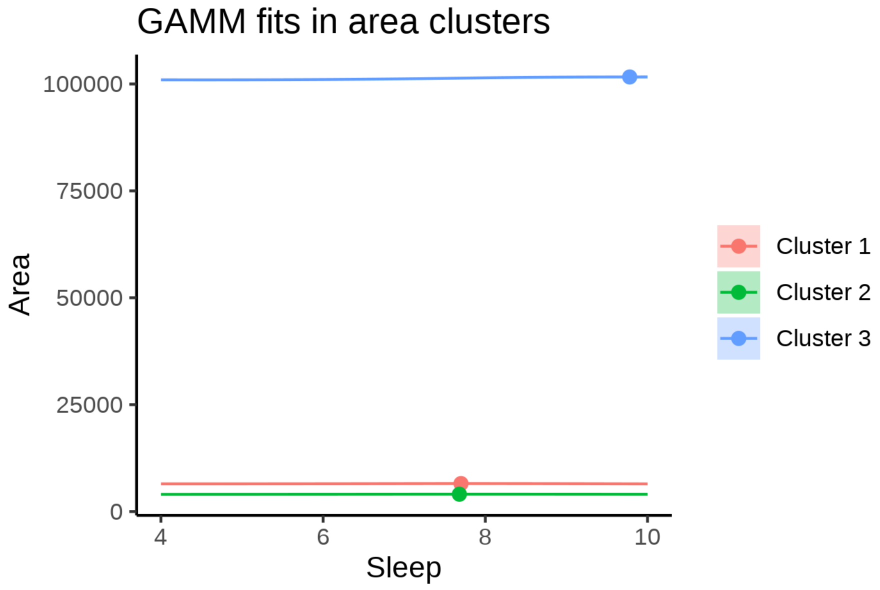

The next plot shows the same thing with percentages of cluster maximum.

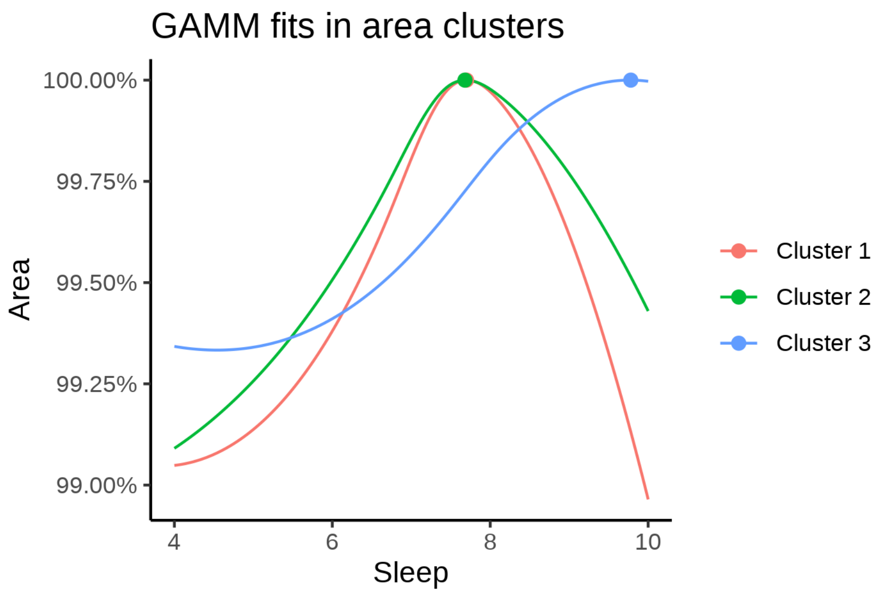

The table below shows the sleep duration associated with maximum value in each cluster, with 95% CIs.

Cluster	Sleep at max. (95% CI)
Cluster 1	7.7 (7.5, 8.1)
Cluster 2	7.7 (7.5, 8.2)
Cluster 3	9.8 (8.7, 10)

Meta analysis

The same GAMMs were estimated separately for each scanner and combined using meta analysis (metagam). The plot compares the resulting curves to the ones obtained by fitting a model to the whole data.

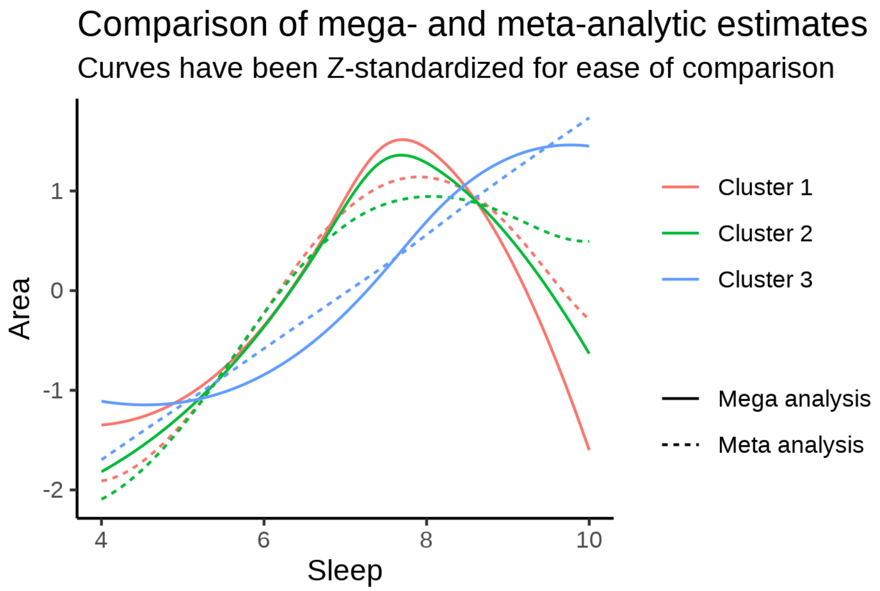

The table below shows the sleep duration associated with maximum value in each cluster, using this meta

analysis. Confidence statements are not easy to obtain when meta analyzing GAMMs, so only point estimates are shown.

Cluster	Sleep at max.
Cluster 1	7.9
Cluster 2	8.1
Cluster 3	10

Thickness

The following regions go in each cluster:

- Cluster 1: caudalanteriorcingulate, cuneus, isthmuscingulate, lateraloccipital, lingual, parsorbitalis, pericalcarine, precuneus, superiorparietal
- Cluster 2: bankssts, caudalmiddlefrontal, entorhinal, fusiform, inferiorparietal, inferiortemporal, lateralorbitofrontal, middletemporal, parstriangularis, postcentral, precentral, rostralmiddlefrontal, superiorfrontal, superiortemporal, supramarginal, transversetemporal
- Cluster 3: frontalpole, insula, medialorbitofrontal, paracentral, parahippocampal, parsopercularis, posteriorcingulate, rostralanteriorcingulate

Description of clusters

First are spaghetti plots for the three clusters.

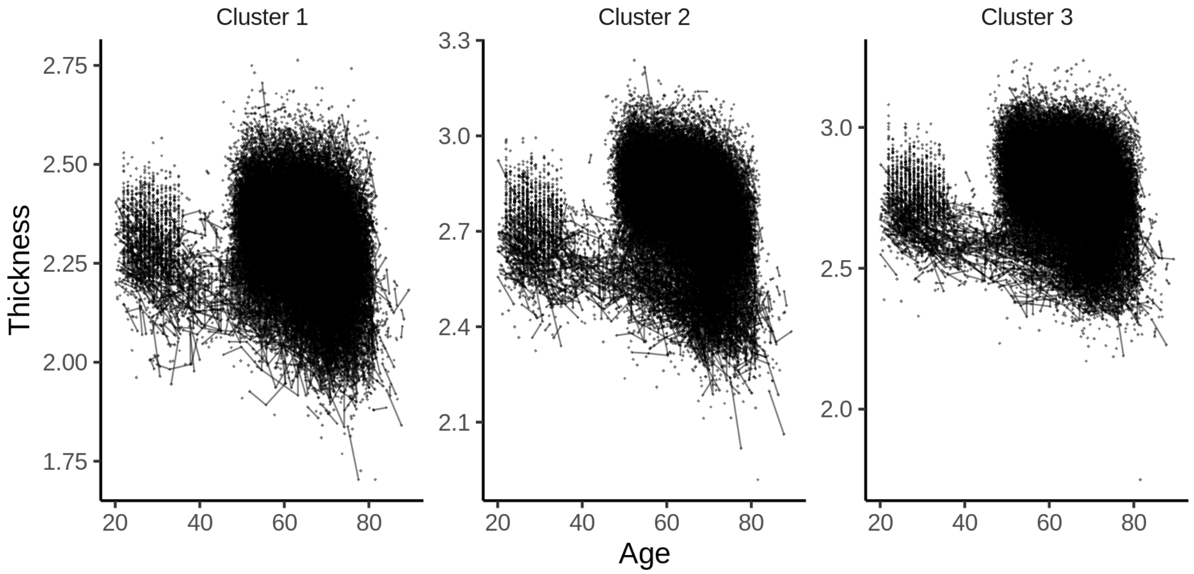

The plot below shows the sleep-volume curves for the three clusters.

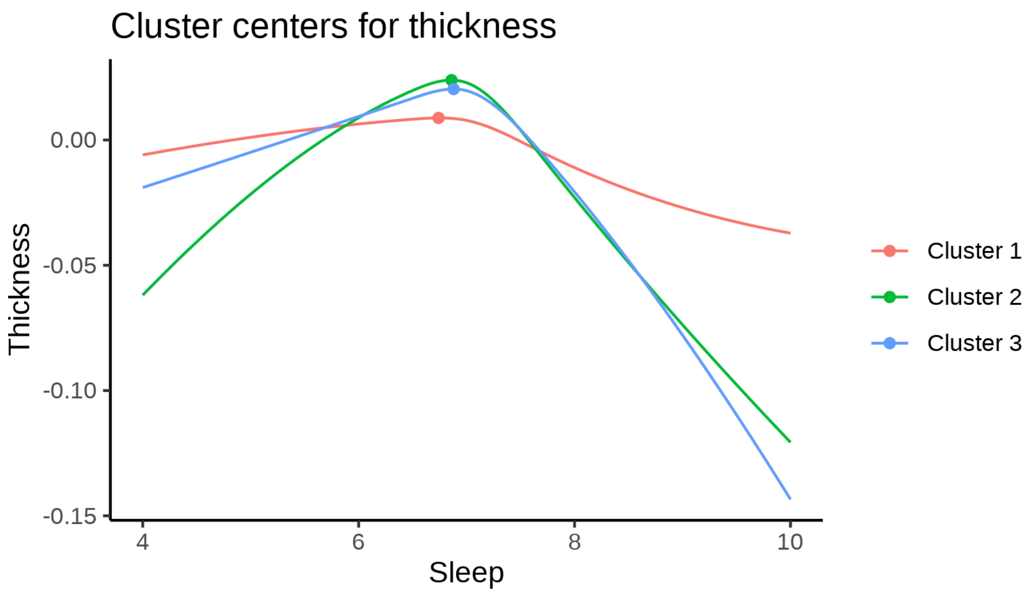

The maximum values for each cluster occur at the following sleep durations:

Cluster	Sleep
Cluster 1	6.74
Cluster 2	6.86
Cluster 3	6.88

The next plot shows the effect of varying the sleep duration as a percentage of the maximum value, obtained at the top of the curve. We have to scrutinize these percentages a bit, because actually defining the mean and standard deviations for each cluster turned out to be not that straightforward, but I believe that what I did makes sense. The problem is that the original clustering is done on a Z-transformed scale, since we are interested in differences in shapes of the curves, and not differences in absolute values of area/thickness/volume between the regions. Then I had to define conversion factors by weighting the mean and variances of each region in each cluster, and try to scale it back.

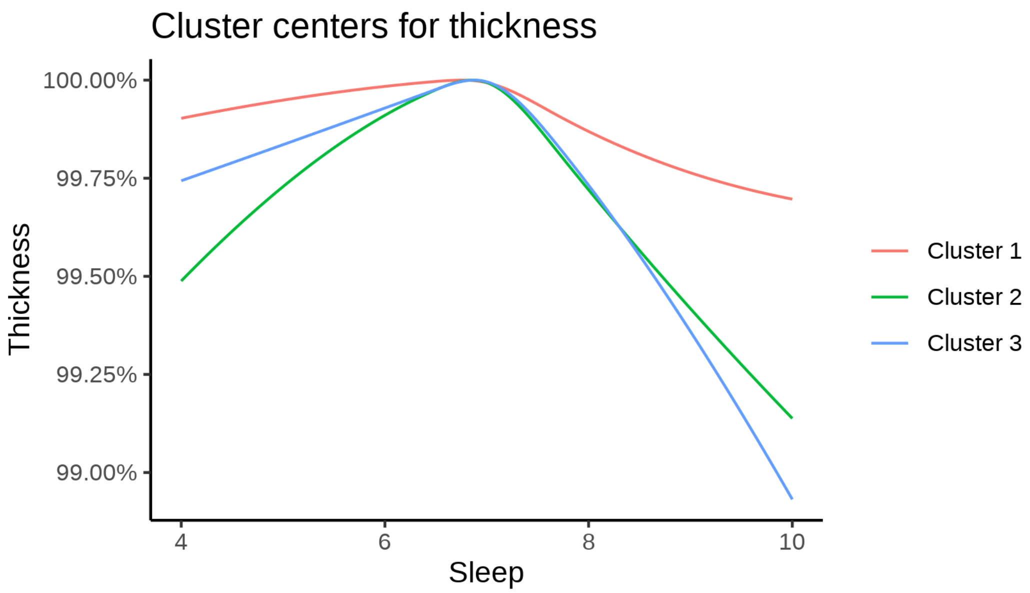

For completeness, we also show the cluster curves upon which the percentage plot above was based.

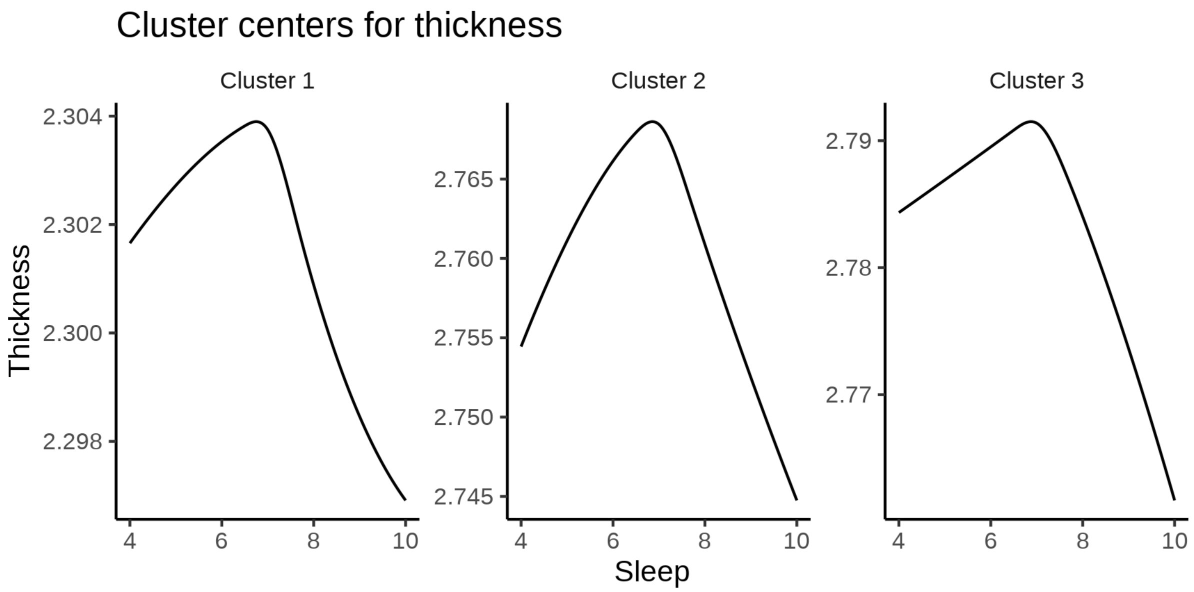

The next plot shows the location of the clusters.

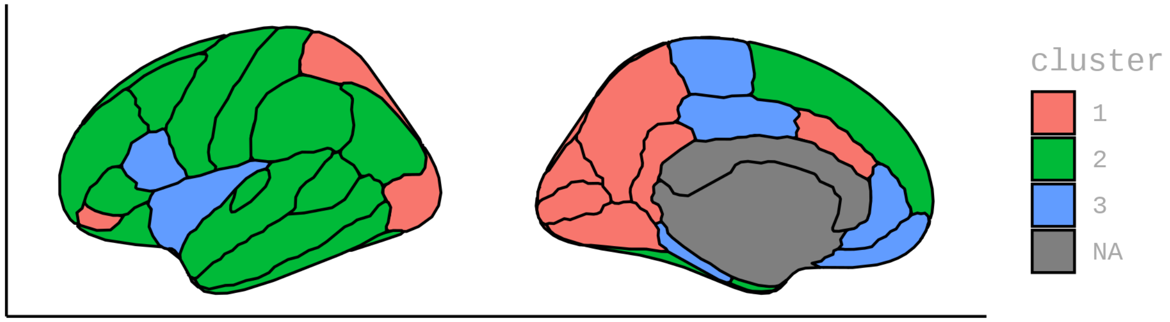

GAMM fits in each cluster

The plot below shows the results of fitting GAMMs to the (weighted) average in each cluster.

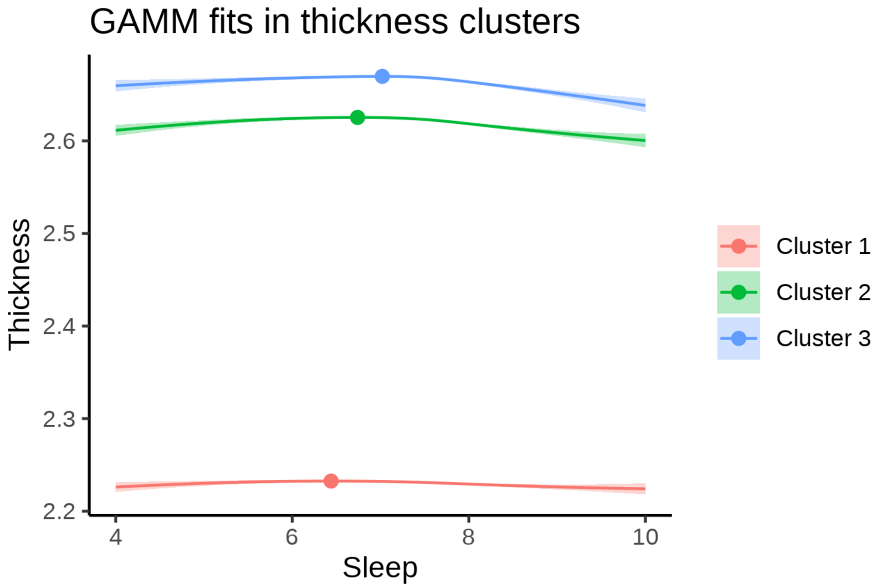

The next plot shows the same thing with percentages of cluster maximum.

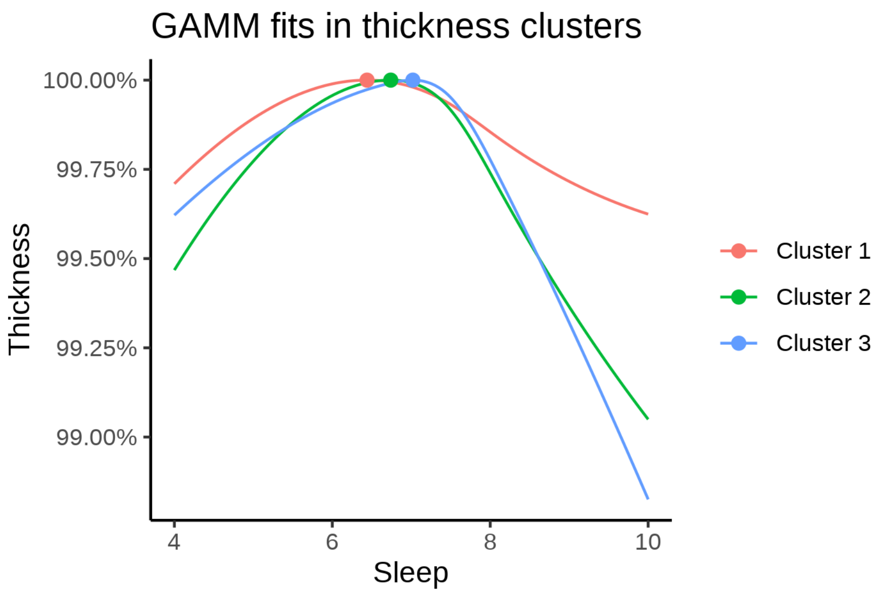

The table below shows the sleep duration associated with maximum value in each cluster, with 95% CIs.

Cluster	Sleep at max. (95% CI)
Cluster 1	6.4 (5.7, 7.1)
Cluster 2	6.7 (6.3, 7.1)
Cluster 3	7 (6.4, 7.2)

Meta analysis

The same GAMMs were estimated separately for each scanner and combined using meta analysis (metagam). The plot compares the resulting curves to the ones obtained by fitting a model to the whole data.

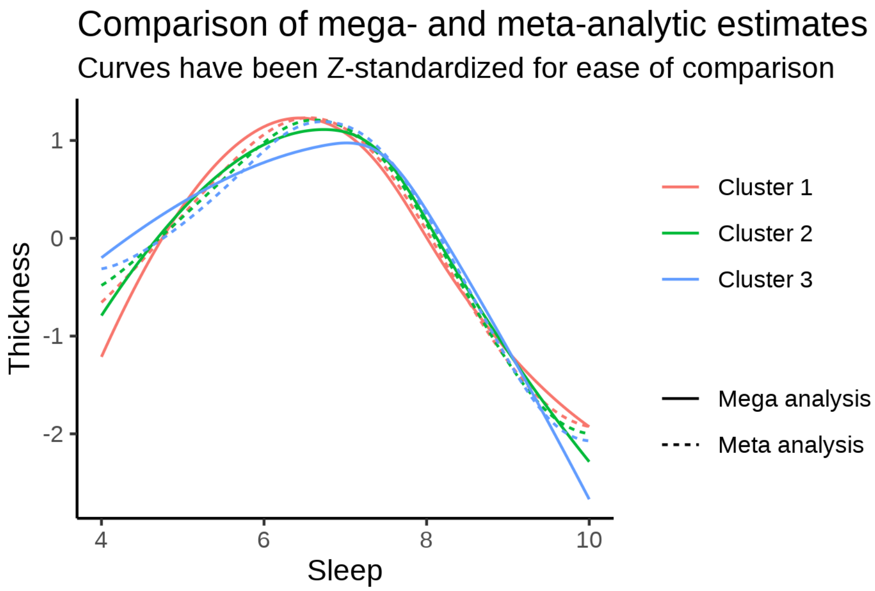

The table below shows the sleep duration associated with maximum value in each cluster, using this meta

analysis. Confidence statements are not easy to obtain when meta analyzing GAMMs, so only point estimates are shown.

Cluster	Sleep at max.
Cluster 1	6.6
Cluster 2	6.6
Cluster 3	6.7

Volume

The following regions go in each cluster:

- Cluster 1: bankssts, fusiform, inferiortemporal, lateraloccipital, medialorbitofrontal, paracentral, parsopercularis, pericalcarine, postcentral, superiorfrontal, superiorparietal, superiortemporal, supramarginal
- Cluster 2: caudalanteriorcingulate, cuneus, entorhinal, frontalpole, isthmuscingulate, lateralorbitofrontal, lingual, parahippocampal, parsorbitalis, parstriangularis, posteriorcingulate, precuneus, rostralanteriorcingulate, rostralmiddlefrontal, transversetemporal
- Cluster 3: caudalmiddlefrontal, inferiorparietal, insula, middletemporal, precentral

Description of clusters

First are spaghetti plots for the three clusters.

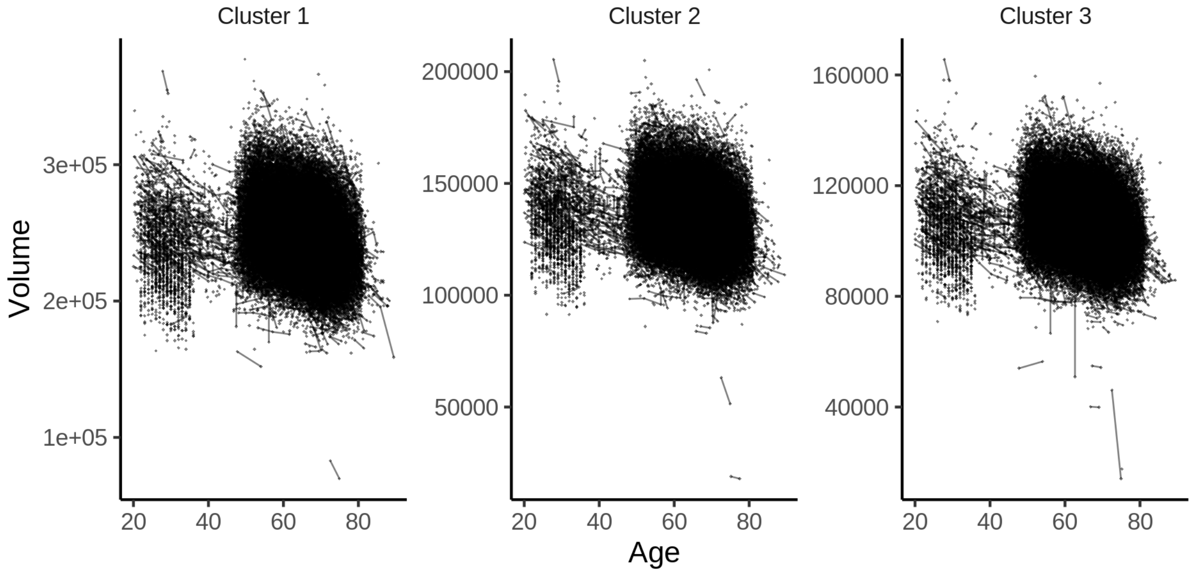

The plot below shows the sleep-volume curves for the three clusters.

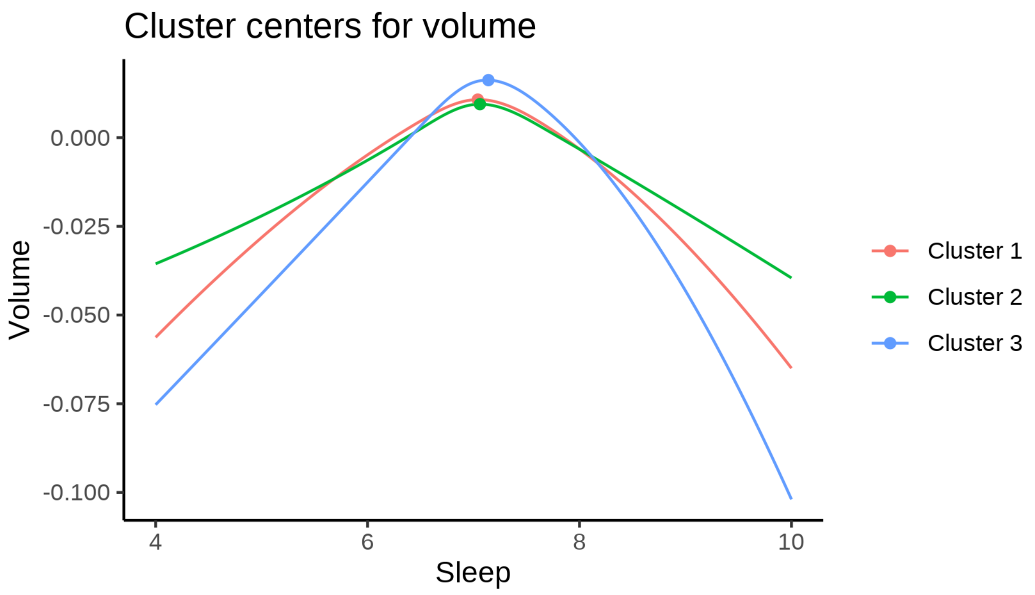

The maximum values for each cluster occur at the following sleep durations:

Cluster	Sleep
Cluster 1	7.04
Cluster 2	7.06
Cluster 3	7.14

The next plot shows the effect of varying the sleep duration as a percentage of the maximum value, obtained at the top of the curve. We have to scrutinize these percentages a bit, because actually defining the mean and standard deviations for each cluster turned out to be not that straightforward, but I believe that what I did makes sense. The problem is that the original clustering is done on a Z-transformed scale, since we are interested in differences in shapes of the curves, and not differences in absolute values of area/thickness/volume between the regions. Then I had to define conversion factors by weighting the mean and variances of each region in each cluster, and try to scale it back.

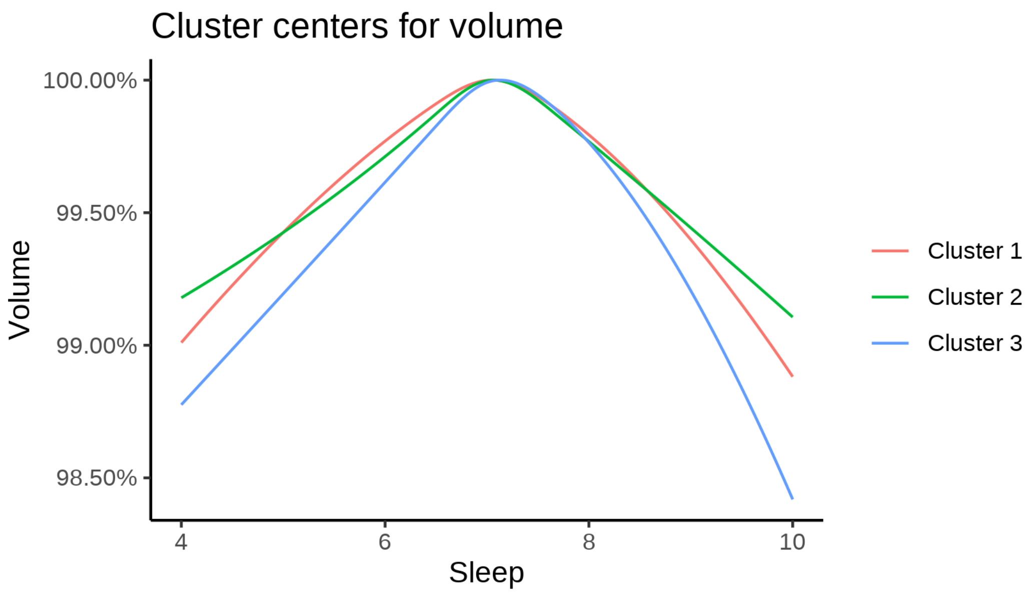

For completeness, we also show the cluster curves upon which the percentage plot above was based. Note that the values differ between clusters, since their sizes are different.

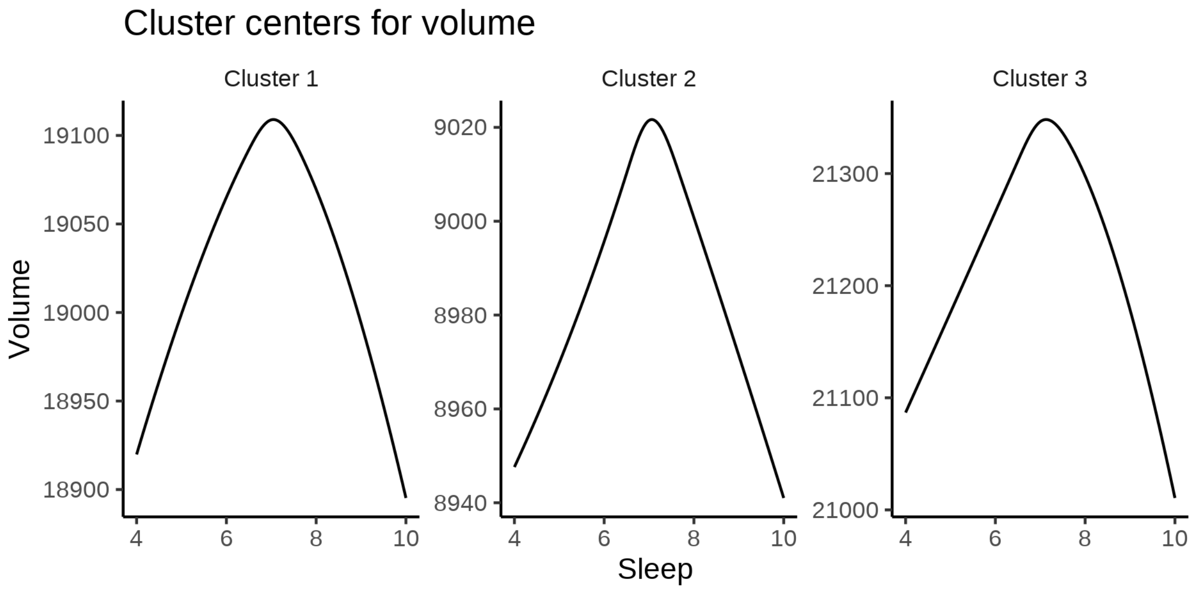

The next plot shows the location of the clusters.

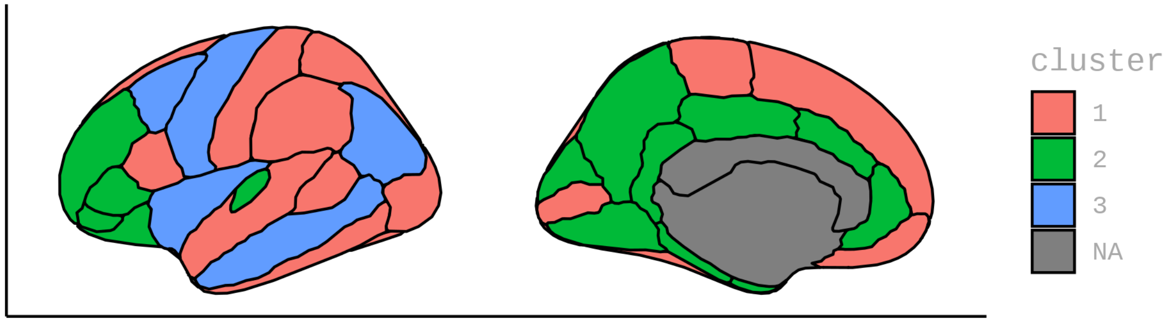

GAMM fits in each cluster

The plot below shows the results of fitting GAMMs to the (weighted) average in each cluster.

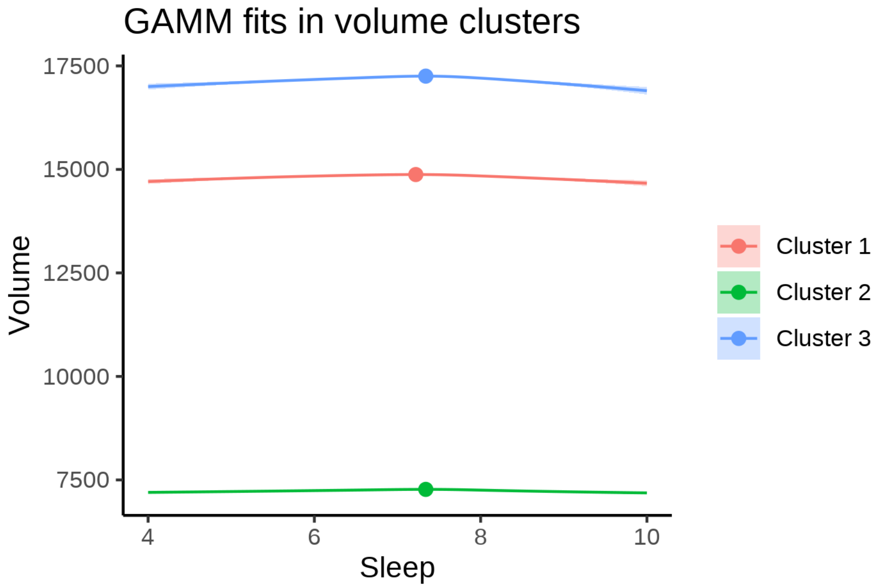

The next plot shows the same thing with percentages of cluster maximum.

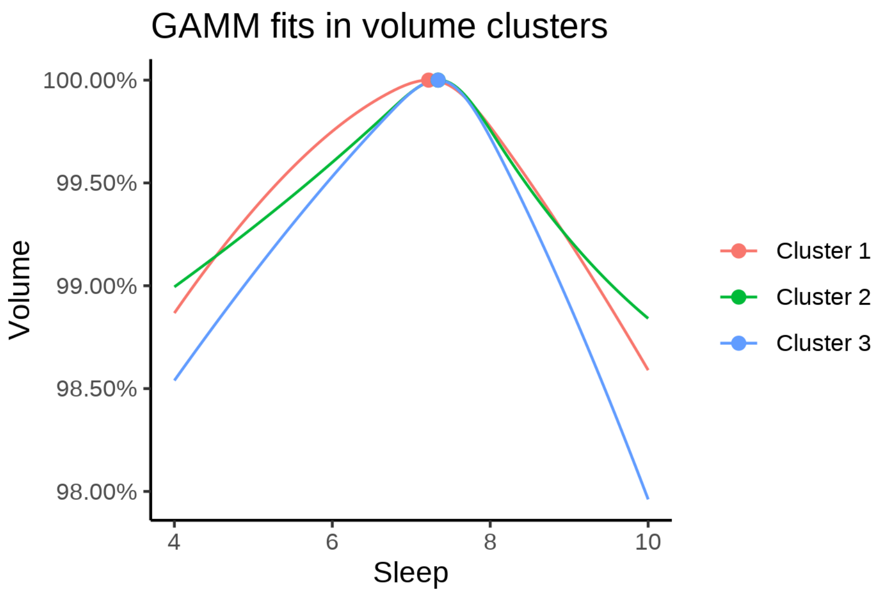

The table below shows the sleep duration associated with maximum value in each cluster, with 95% CIs.

Cluster	Sleep at max. (95% CI)
Cluster 1	7.2 (6.9, 7.4)
Cluster 2	7.3 (7.2, 7.4)
Cluster 3	7.3 (7.2, 7.4)

Meta analysis

The same GAMMs were estimated separately for each scanner and combined using meta analysis (metagam). The plot compares the resulting curves to the ones obtained by fitting a model to the whole data.

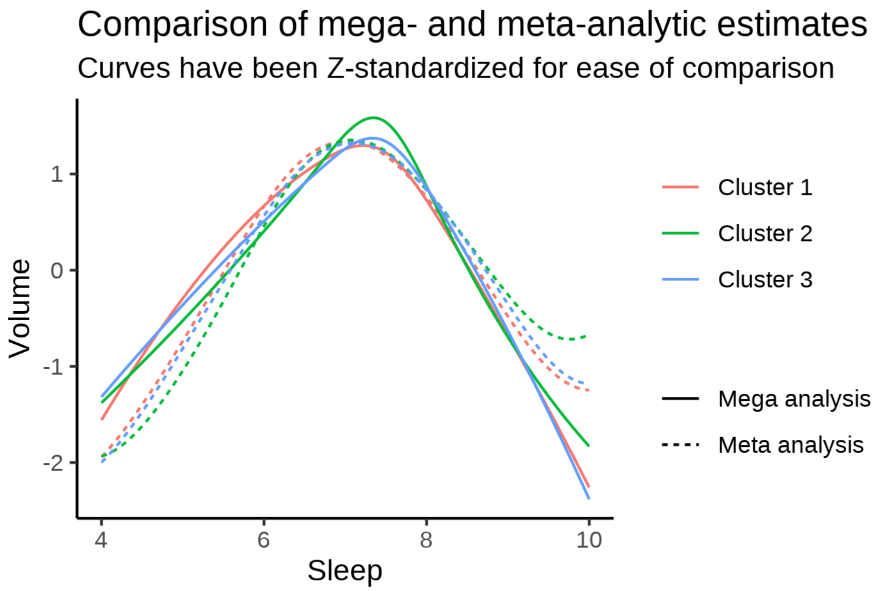

The table below shows the sleep duration associated with maximum value in each cluster, using this meta

analysis. Confidence statements are not easy to obtain when meta analyzing GAMMs, so only point estimates are shown.

Cluster	Sleep at max.
Cluster 1	7
Cluster 2	7.1
Cluster 3	7.1

References

Conde, Irene Castro, and Jacobo de Una Alvarez. 2020. *Sgof: Multiple Hypothesis Testing*. https://CRAN.R-project.org/package=sgof.

Mowinckel, Athanasia M., and Didac Vidal-Piñeiro. 2020. "Visualization of Brain Statistics with r Packages Ggseg and Ggseg3d." *Advances in Methods and Practices in Psychological Science* 3 (4): 466–83. https://doi.org/10.1177/2515245920928009.

R Core Team. 2020. *R: A Language and Environment for Statistical Computing*. Vienna, Austria: R Foundation for Statistical Computing. https://www.R-project.org/.

Sørensen, Øystein, Andreas M. Brandmaier, Didac Macia, Klaus Ebmeier, Paolo Ghisletta, Rogier A. Kievit, Athanasia M. Mowinckel, Kristine B. Walhovd, Rene Westerhausen, and Anders Fjell. 2021. "Meta-Analysis of Generalized Additive Models in Neuroimaging Studies." *NeuroImage* 224: 117416. https://doi.org/10.1016/j.neuroimage.2020.117416.

Wickham, Hadley, Mara Averick, Jennifer Bryan, Winston Chang, Lucy D'Agostino McGowan, Romain François, Garrett Grolemund, et al. 2019. "Welcome to the tidyverse." *Journal of Open Source Software* 4 (43): 1686. https://doi.org/10.21105/joss.01686.

Wood, Simon, and Fabian Scheipl. 2020. *Gamm4: Generalized Additive Mixed Models Using 'Mgcv' and 'Lme4'*. https://CRAN.R-project.org/package=gamm4.

Subcortical volumes

Overview of software and methods

All analyses were run in R version 4.0.0 (R Core Team 2020). The set of R packages provided by `tidyverse` (Wickham et al. 2019) were invaluable for data manipulation and visualization, in particular `ggplot2` (Wickham 2016), `dplyr` (Wickham et al. 2021), `tidyr` (Wickham 2021) and `purrr` (Henry and Wickham 2020). Furthermore, `gamm4` (S. Wood and Scheipl 2020) and `mgcv` (S. N. Wood 2017) were used for fitting generalized additive models. Posterior intervals for the sleep duration associated with maximum volume were computed with a method described in Sørensen, Walhovd, and Fjell (2021), based on Ruppert, Wand, and Carroll (2003). The `HDInterval` package was used to compute highest posterior density intervals, and `sgof` was used to correct for multiple comparisons using the Benjamini-Hochberg procedure (Benjamini and Hochberg 1995). Finally, `glue` (Hester 2020) and `stringr` (Wickham 2019) were used extensively for text manipulation.

Data overview

The total number of observations was 51309, from 47034 unique participants. The age range was from 20 to 89.4. The sex count is shown below.

sex	n
female	26805
male	24504

Follow-up intervals are shown below.

Participants with follow-up	Mean follow-up	Max follow-up	Min follow-up
3904	2.509695	11.22656	0.0054795

In order to remove outliers, the following model was fit to each region:

```
mod <- gamm4(value ~ s(age, bs = "cr") + sex + site,
             random = ~(1|id), data = data)
```

Outliers were defined by having a residual more than four times the magnitude of the residuals standard error. Technically, all values which returned `TRUE` on the following test were retained, all others were removed as outliers.

```
abs(residuals(mod$mer)) < 4 * sigma(mod$mer)
```

The table below shows the number of outliers removed per region.

region	initial_rows	retained_rows	outliers_removed
Accumbens-area	51309	51304	5

region	initial_rows	retained_rows	outliers_removed
Amygdala	51309	51303	6
Brain-Stem	51309	51286	23
Caudate	51309	51295	14
CC_Anterior	51309	51293	16
CC_Central	51309	51296	13
CC_Mid_Anterior	51309	51287	22
CC_Mid_Posterior	51309	51268	41
CC_Posterior	51309	51255	54
Cerebellum-Cortex	51309	51280	29
Cerebellum-White-Matter	51309	51275	34
CerebralWhiteMatterVol	51309	51300	9
EstimatedTotalIntraCranialVol	51309	51289	20
Hippocampus	51309	51299	10
Pallidum	51309	51300	9
Putamen	51309	51287	22
Thalamus	51308	51288	20
TotalGrayVol	51309	51302	7
Ventricles	51309	51288	21

The plot below shows the average sleep duration as a function of age, fit with a GAM. This model will be used for comparisons later. All sleep durations in this document are confined to lie between 4 and 10 hours, to avoid nonsensical answers.

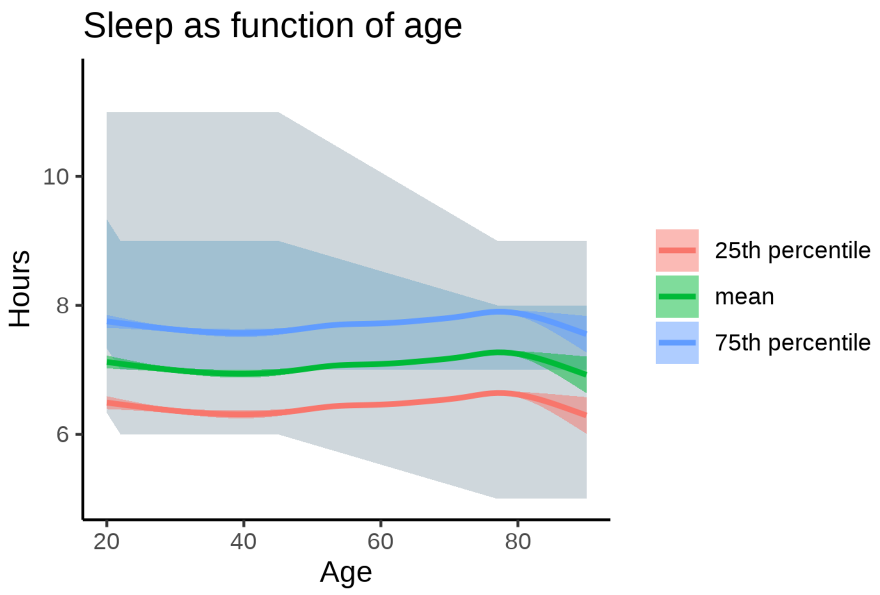

The model statistics is shown next.

```
##
## Family: gaussian
## Link function: identity
##
## Formula:
## sleep ~ s(age, bs = "cr")
##
## Parametric coefficients:
```

```
##             Estimate Std. Error t value Pr(>|t|)
## (Intercept) 7.134088   0.004306    1657   <2e-16 ***
## ---
## Signif. codes:  0 '***' 0.001 '**' 0.01 '*' 0.05 '.' 0.1 ' ' 1
##
## Approximate significance of smooth terms:
##          edf Ref.df     F p-value
## s(age) 6.866  7.865 33.11  <2e-16 ***
## ---
## Signif. codes:  0 '***' 0.001 '**' 0.01 '*' 0.05 '.' 0.1 ' ' 1
##
## R-sq.(adj) =  0.00555   Deviance explained = 0.57%
## -REML =  63539  Scale est. = 0.87222   n = 47034
```

Accumbens-area

Descriptive statistics

Study	Observations	Unique IDs	Mean age	Age range
HCP	974	974	28.8	22 - 37
MPIB	675	391	63.2	24 - 83
UB	113	39	70.9	64 - 81
UCAM	884	632	55.1	20 - 88
UiO	1474	803	49.4	20 - 89
UKB	45983	43137	64.5	45 - 83
UmU	412	279	62.2	25 - 85
UOXF	769	769	69.8	60 - 85

Spaghetti plot

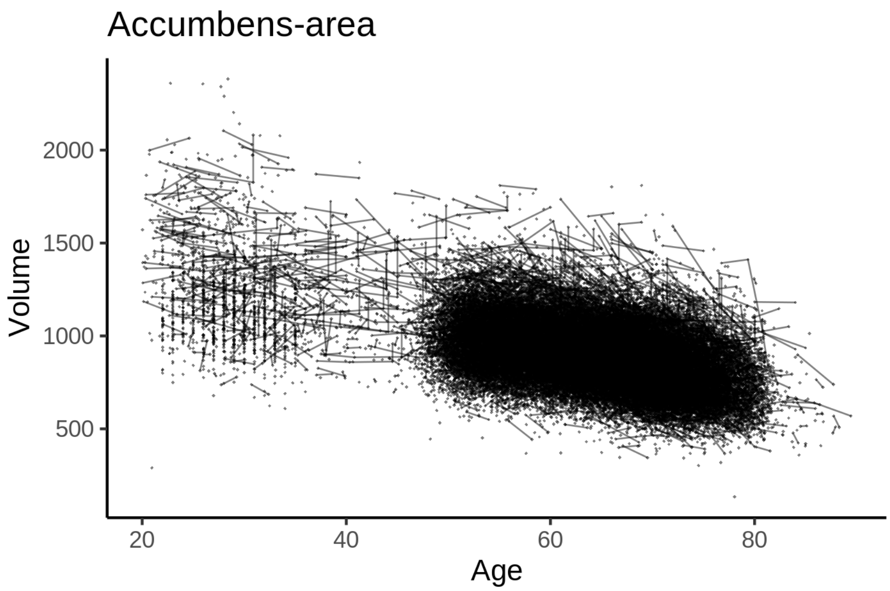

Models Controling for ICV

Model outputs

Model without sleep term

```
##
## Family: gaussian
## Link function: identity
##
## Formula:
## value ~ sex + site + icv + s(age_z, k = 10, bs = "cr")
## <environment: 0x55895ffe2630>
##
## Parametric coefficients:
##               Estimate Std. Error t value Pr(>|t|)
## (Intercept)   804.8127     7.7589 103.728  < 2e-16 ***
## sexmale        19.6585     1.5399  12.766  < 2e-16 ***
## siteMPIB       93.4406     9.6688   9.664  < 2e-16 ***
## siteousAvanto 238.5581     8.2505  28.914  < 2e-16 ***
## siteousPrisma 101.7601    13.6246   7.469 8.22e-14 ***
## siteousSkyra  407.8635     7.4140  55.013  < 2e-16 ***
## siteUB         64.9295    22.1399   2.933  0.00336 **
## siteUCAM        4.7150     8.4310   0.559  0.57599
## siteUKB        82.5578     7.9644  10.366  < 2e-16 ***
## siteUmU       370.4055    10.8585  34.112  < 2e-16 ***
## siteUOXF       59.9033     9.3475   6.408 1.48e-10 ***
## icv            59.6320     0.7755  76.891  < 2e-16 ***
## ---
## Signif. codes:  0 ‘***’ 0.001 ‘**’ 0.01 ‘*’ 0.05 ‘.’ 0.1 ‘ ’ 1
##
## Approximate significance of smooth terms:
##            edf Ref.df    F p-value
## s(age_z) 8.146  8.146 1886  <2e-16 ***
## ---
## Signif. codes:  0 ‘***’ 0.001 ‘**’ 0.01 ‘*’ 0.05 ‘.’ 0.1 ‘ ’ 1
##
## R-sq.(adj) =  0.469
## lmer.REML = 6.4415e+05  Scale est. = 2924      n = 51284
```

Model with only main effects of age and sleep

```
##
## Family: gaussian
## Link function: identity
##
## Formula:
## value ~ sex + site + icv + s(age_z, k = 10, bs = "cr") + s(sleep_z,
##     k = 5, bs = "cr")
## <environment: 0x55895ffe2630>
##
## Parametric coefficients:
##               Estimate Std. Error t value Pr(>|t|)
## (Intercept)   804.8190     7.7665 103.626  < 2e-16 ***
## sexmale        19.6587     1.5399  12.766  < 2e-16 ***
```

```
## siteMPIB       93.4336     9.6760   9.656  < 2e-16 ***
## siteousAvanto 238.5549     8.2525  28.907  < 2e-16 ***
## siteousPrisma 101.7580    13.6251   7.468 8.25e-14 ***
## siteousSkyra  407.8601     7.4164  54.994  < 2e-16 ***
## siteUB         64.9277    22.1402   2.933  0.00336 **
## siteUCAM        4.7118     8.4330   0.559  0.57634
## siteUKB        82.5511     7.9728  10.354  < 2e-16 ***
## siteUmU       370.3912    10.8845  34.029  < 2e-16 ***
## siteUOXF       59.9001     9.3493   6.407 1.50e-10 ***
## icv            59.6315     0.7759  76.855  < 2e-16 ***
## ---
## Signif. codes:  0 '***' 0.001 '**' 0.01 '*' 0.05 '.' 0.1 ' ' 1
##
## Approximate significance of smooth terms:
##              edf Ref.df    F p-value
## s(age_z)   8.146  8.146 1881  <2e-16 ***
## s(sleep_z) 1.000  1.000    0   0.984
## ---
## Signif. codes:  0 '***' 0.001 '**' 0.01 '*' 0.05 '.' 0.1 ' ' 1
##
## R-sq.(adj) =  0.469
## lmer.REML = 6.4415e+05  Scale est. = 2924      n = 51284
```

Model with full interaction between age and sleep

```
##
## Family: gaussian
## Link function: identity
##
## Formula:
## value ~ sex + site + icv + t2(age_z, sleep_z, k = c(10, 4), bs = "cr")
## <environment: 0x55895ffe2630>
##
## Parametric coefficients:
##               Estimate Std. Error t value Pr(>|t|)
## (Intercept)   805.9358     7.8163 103.110  < 2e-16 ***
## sexmale        19.6516     1.5433  12.733  < 2e-16 ***
## siteMPIB       92.5734     9.7397   9.505  < 2e-16 ***
## siteousAvanto 237.6040     8.3004  28.626  < 2e-16 ***
## siteousPrisma 100.7575    13.6575   7.377 1.64e-13 ***
## siteousSkyra  406.9034     7.4726  54.453  < 2e-16 ***
## siteUB         63.8693    22.1645   2.882  0.00396 **
## siteUCAM        3.5306     8.4923   0.416  0.67760
## siteUKB        81.4073     8.0235  10.146  < 2e-16 ***
## siteUmU       369.4282    10.9343  33.786  < 2e-16 ***
## siteUOXF       58.7589     9.4007   6.251 4.12e-10 ***
## icv            59.6143     0.7761  76.811  < 2e-16 ***
## ---
## Signif. codes:  0 '***' 0.001 '**' 0.01 '*' 0.05 '.' 0.1 ' ' 1
##
## Approximate significance of smooth terms:
##                     edf Ref.df     F p-value
## t2(age_z,sleep_z) 13.57  13.57 17.89  <2e-16 ***
## ---
## Signif. codes:  0 '***' 0.001 '**' 0.01 '*' 0.05 '.' 0.1 ' ' 1
```

```
##
## R-sq.(adj) =  0.469
## lmer.REML = 6.4417e+05  Scale est. = 2923.4    n = 51284
```

Model comparison

`mod_no_sleep` refers to model without sleep term, `mod_no_interaction` refers to model with only main effect of sleep, and `mod_full` refers to model with a full interaction between age and sleep. This is a nested model comparison, and the p-value at a given line refers to comparing the model at the line to the model on the line above. Hence, significance implies that the more complicated model is supported on statistical grounds.

To be even more specific, the p-value on the second row tests whether there is an association between sleep and volume. The p-value on the third row tests whether this association depends on age.

```
## Data: NULL
## Models:
## ml$mod_no_sleep$mer: NULL
## ml$mod_no_interaction$mer: NULL
## ml$mod_full$mer: NULL
##                           npar    AIC    BIC  logLik deviance Chisq Df Pr(>Chisq)
## ml$mod_no_sleep$mer         16 644185 644327 -322077   644153
## ml$mod_no_interaction$mer   18 644189 644349 -322077   644153 4e-04  2     0.9998
## ml$mod_full$mer             20 644206 644383 -322083   644166 0e+00  2     1.0000
```

We chose the model based on the likelihood ratio test with 5 % significance level, which was `mod_no_sleep`.

Lifespan brain trajectory

The trajectory shown is from the chosen model `mod_no_sleep`.

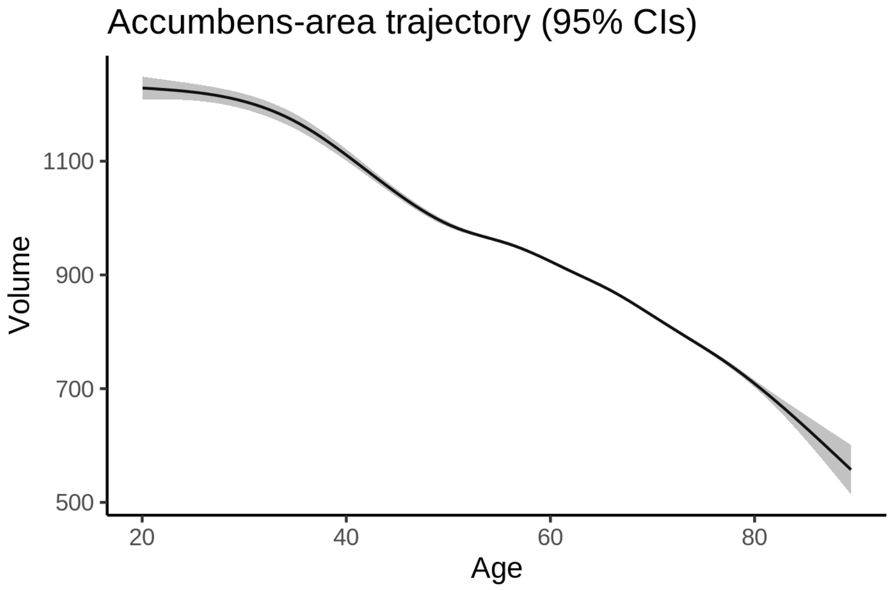

Effect of sleep

The chosen model did not include a sleep term, and hence we don't have any estimated effect of sleep.

We show the full interaction model for completeness, although it was not selected.

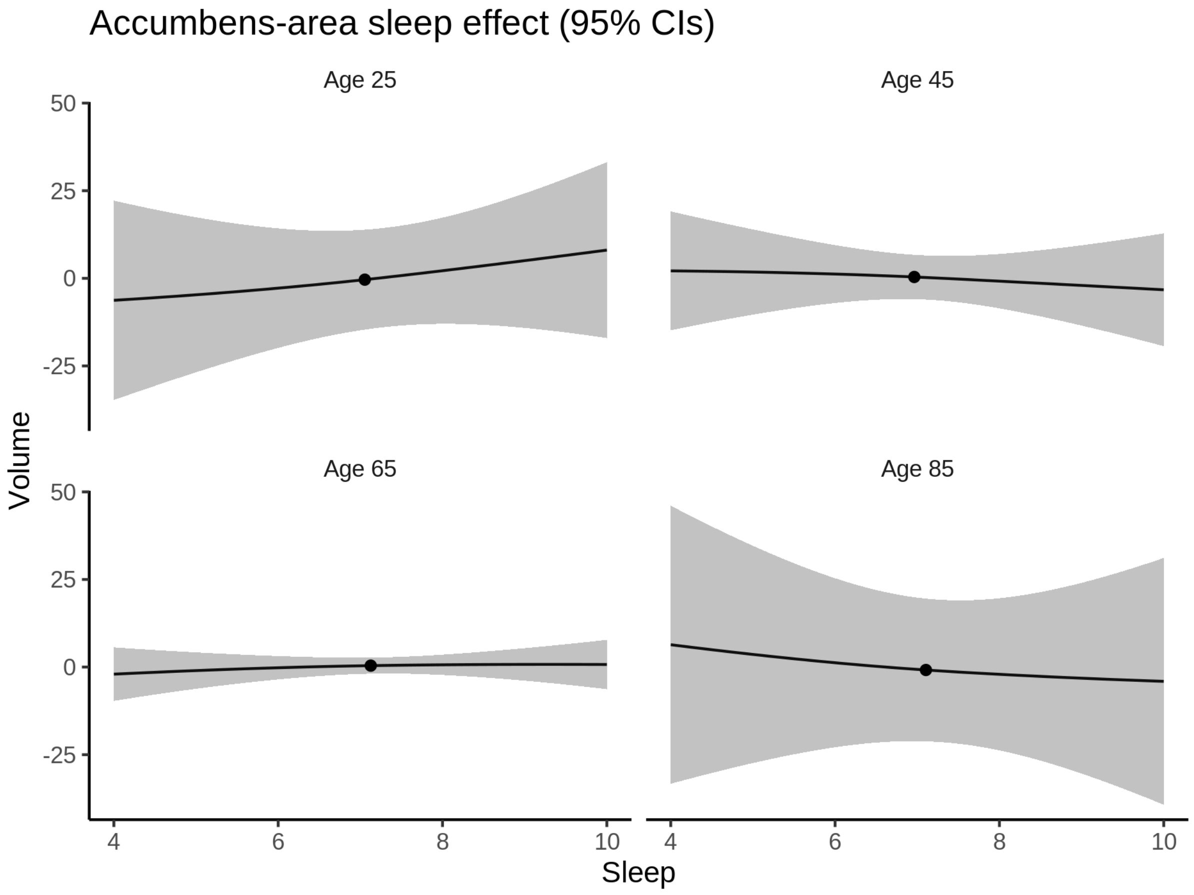

Deviation from sleep associated with maximal volume

Model with no sleep term was selected. No plots to show. (Although we can of course dig up the plots, which will be pretty flat).

Comparison of mean sleep and sleep associated with maximum volume

Nothing to show, as we did not find an association between sleep and volume.

Controling for covariates

Below is the output for a model in which we only include data with income and education.

```
##
## Family: gaussian
## Link function: identity
##
## Formula:
## value ~ sex + site + s(age_z, k = 10, bs = "cr") + s(sleep_z,
##     k = 5, bs = "cr") + icv
## <environment: 0x55895e488040>
```

```
##
## Parametric coefficients:
##               Estimate Std. Error t value Pr(>|t|)
## (Intercept)   938.3691    17.3056  54.223  < 2e-16 ***
## sexmale        19.0636     1.9217   9.920  < 2e-16 ***
## siteousAvanto 102.1468    22.2358   4.594 4.37e-06 ***
## siteousPrisma  -3.9207    21.5069  -0.182 0.855348
## siteousSkyra  297.1959    18.7414  15.858  < 2e-16 ***
## siteUKB       -57.7167    17.2759  -3.341 0.000836 ***
## siteUOXF      -52.5807    19.4907  -2.698 0.006985 **
## icv            61.5419     0.9881  62.283  < 2e-16 ***
## ---
## Signif. codes:  0 ‘***’ 0.001 ‘**’ 0.01 ‘*’ 0.05 ‘.’ 0.1 ‘ ’ 1
##
## Approximate significance of smooth terms:
##              edf Ref.df        F p-value
## s(age_z)   5.584  5.584 1484.432  <2e-16 ***
## s(sleep_z) 1.000  1.000    0.007   0.936
## ---
## Signif. codes:  0 ‘***’ 0.001 ‘**’ 0.01 ‘*’ 0.05 ‘.’ 0.1 ‘ ’ 1
##
## R-sq.(adj) =   0.42
## lmer.REML = 3.9053e+05  Scale est. = 2896.3    n = 31198
```

Below is the output for a model in which we control for the main effects of income and education.

```
##
## Family: gaussian
## Link function: identity
##
## Formula:
## value ~ sex + site + s(age_z, k = 10, bs = "cr") + s(sleep_z,
##     k = 5, bs = "cr") + icv + income_scaled + education_scaled
## <environment: 0x55895e488040>
##
## Parametric coefficients:
##                  Estimate Std. Error t value Pr(>|t|)
## (Intercept)      938.5088    17.4209  53.873  < 2e-16 ***
## sexmale           19.0807     1.9274   9.900  < 2e-16 ***
## siteousAvanto    102.0966    22.2413   4.590 4.44e-06 ***
## siteousPrisma     -3.9060    21.5093  -0.182  0.85590
## siteousSkyra     297.1522    18.7469  15.851  < 2e-16 ***
## siteUKB          -57.7938    17.2878  -3.343  0.00083 ***
## siteUOXF         -52.6609    19.5034  -2.700  0.00694 **
## icv               61.5526     0.9970  61.741  < 2e-16 ***
## income_scaled     -0.3286     2.2887  -0.144  0.88585
## education_scaled   0.1071     2.7026   0.040  0.96838
## ---
## Signif. codes:  0 ‘***’ 0.001 ‘**’ 0.01 ‘*’ 0.05 ‘.’ 0.1 ‘ ’ 1
##
## Approximate significance of smooth terms:
##              edf Ref.df        F p-value
## s(age_z)   5.582  5.582 1369.990  <2e-16 ***
## s(sleep_z) 1.000  1.000    0.007   0.934
## ---
```

```
## Signif. codes:  0 ‘***’ 0.001 ‘**’ 0.01 ‘*’ 0.05 ‘.’ 0.1 ‘ ’ 1
##
## R-sq.(adj) =   0.42
## lmer.REML = 3.9052e+05  Scale est. = 2896.2    n = 31198
```

We also included interaction effects between sleep duration and education and income, in another model. The output is shown below, and the interaction terms are `income_scaled:sleep_z` and `education_scaled:sleep_z`.

```
##
## Family: gaussian
## Link function: identity
##
## Formula:
## value ~ sex + site + s(age_z, k = 10, bs = "cr") + s(sleep_z,
##     k = 5, bs = "cr") + icv + income_scaled + education_scaled +
##     income_scaled:sleep_z + education_scaled:sleep_z
## <environment: 0x55895e488040>
##
## Parametric coefficients:
##                           Estimate Std. Error t value Pr(>|t|)
## (Intercept)              938.32418   17.42226  53.858  < 2e-16 ***
## sexmale                   18.97004    1.92879   9.835  < 2e-16 ***
## siteousAvanto            102.05287   22.24188   4.588 4.49e-06 ***
## siteousPrisma             -4.25676   21.51145  -0.198 0.843137
## siteousSkyra             297.08491   18.74720  15.847  < 2e-16 ***
## siteUKB                  -57.56930   17.28989  -3.330 0.000871 ***
## siteUOXF                 -52.19445   19.50916  -2.675 0.007468 **
## icv                       61.55597    0.99705  61.738  < 2e-16 ***
## income_scaled             -0.21103    2.29004  -0.092 0.926577
## education_scaled           0.05215    2.70291   0.019 0.984607
## income_scaled:sleep_z     -3.84086    2.26352  -1.697 0.089734 .
## education_scaled:sleep_z   0.64169    2.61657   0.245 0.806271
## ---
## Signif. codes:  0 ‘***’ 0.001 ‘**’ 0.01 ‘*’ 0.05 ‘.’ 0.1 ‘ ’ 1
##
## Approximate significance of smooth terms:
##              edf Ref.df        F p-value
## s(age_z)   5.592  5.592 1366.359  <2e-16 ***
## s(sleep_z) 1.000  1.000    0.238   0.626
## ---
## Signif. codes:  0 ‘***’ 0.001 ‘**’ 0.01 ‘*’ 0.05 ‘.’ 0.1 ‘ ’ 1
##
## R-sq.(adj) =   0.42
## lmer.REML = 3.9051e+05  Scale est. = 2896.4    n = 31198
```

We did the same controling for BMI. Below is the model with no covariates but only keeping data with BMI.

```
##
## Family: gaussian
## Link function: identity
##
## Formula:
## value ~ sex + site + s(age_z, k = 10, bs = "cr") + s(sleep_z,
##     k = 5, bs = "cr") + icv
## <environment: 0x55895cab6808>
```

```
##
## Parametric coefficients:
##                Estimate Std. Error t value Pr(>|t|)
## (Intercept)   1037.137     14.861  69.788  < 2e-16 ***
## sexmale         15.633      1.877   8.330  < 2e-16 ***
## siteousPrisma -123.716     18.711  -6.612 3.85e-11 ***
## siteousSkyra   198.225     13.111  15.118  < 2e-16 ***
## siteUCAM      -226.695     15.369 -14.750  < 2e-16 ***
## siteUKB       -151.901     14.946 -10.163  < 2e-16 ***
## siteUmU        144.024     16.752   8.598  < 2e-16 ***
## icv             64.181      0.968  66.301  < 2e-16 ***
## ---
## Signif. codes:  0 '***' 0.001 '**' 0.01 '*' 0.05 '.' 0.1 ' ' 1
##
## Approximate significance of smooth terms:
##              edf Ref.df        F p-value
## s(age_z)   7.459  7.459 1368.800  <2e-16 ***
## s(sleep_z) 1.000  1.000    0.001    0.97
## ---
## Signif. codes:  0 '***' 0.001 '**' 0.01 '*' 0.05 '.' 0.1 ' ' 1
##
## R-sq.(adj) =  0.489
## lmer.REML = 4.1878e+05  Scale est. = 2945      n = 33442
```

Below is the model output with main effect.

```
##
## Family: gaussian
## Link function: identity
##
## Formula:
## value ~ sex + site + s(age_z, k = 10, bs = "cr") + s(sleep_z,
##     k = 5, bs = "cr") + icv + bmi
## <environment: 0x55895cab6808>
##
## Parametric coefficients:
##                  Estimate Std. Error t value Pr(>|t|)
## (Intercept)   1038.54882   15.55427  66.769  < 2e-16 ***
## sexmale         15.67761    1.88225   8.329  < 2e-16 ***
## siteousPrisma -123.64785   18.71229  -6.608 3.96e-11 ***
## siteousSkyra   198.23852   13.11150  15.119  < 2e-16 ***
## siteUCAM      -226.65415   15.36997 -14.747  < 2e-16 ***
## siteUKB       -151.85605   14.94697 -10.160  < 2e-16 ***
## siteUmU        144.08791   16.75299   8.601  < 2e-16 ***
## icv             64.18900    0.96840  66.284  < 2e-16 ***
## bmi             -0.05605    0.18247  -0.307    0.759
## ---
## Signif. codes:  0 '***' 0.001 '**' 0.01 '*' 0.05 '.' 0.1 ' ' 1
##
## Approximate significance of smooth terms:
##              edf Ref.df        F p-value
## s(age_z)   7.459  7.459 1368.808  <2e-16 ***
## s(sleep_z) 1.000  1.000    0.001   0.981
## ---
## Signif. codes:  0 '***' 0.001 '**' 0.01 '*' 0.05 '.' 0.1 ' ' 1
```

```
##
## R-sq.(adj) =  0.489
## lmer.REML = 4.1878e+05  Scale est. = 2945      n = 33442
```

Next is the model with BMI-sleep interaction.

```
##
## Family: gaussian
## Link function: identity
##
## Formula:
## value ~ sex + site + s(age_z, k = 10, bs = "cr") + s(sleep_z,
##     k = 5, bs = "cr") + icv + bmi + bmi:sleep_z
## <environment: 0x55895cab6808>
##
## Parametric coefficients:
##                 Estimate Std. Error t value Pr(>|t|)
## (Intercept)   1038.81270   15.56014  66.761  < 2e-16 ***
## sexmale         15.67329    1.88228   8.327  < 2e-16 ***
## siteousPrisma -123.66484   18.71242  -6.609 3.94e-11 ***
## siteousSkyra   198.19674   13.11171  15.116  < 2e-16 ***
## siteUCAM      -226.76771   15.37113 -14.753  < 2e-16 ***
## siteUKB       -151.96657   14.94810 -10.166  < 2e-16 ***
## siteUmU        143.98302   16.75393   8.594  < 2e-16 ***
## icv             64.18484    0.96843  66.277  < 2e-16 ***
## bmi             -0.05987    0.18258  -0.328    0.743
## bmi:sleep_z     -0.10713    0.17278  -0.620    0.535
## ---
## Signif. codes:  0 ‘***’ 0.001 ‘**’ 0.01 ‘*’ 0.05 ‘.’ 0.1 ‘ ’ 1
##
## Approximate significance of smooth terms:
##              edf Ref.df        F p-value
## s(age_z)   7.459  7.459 1368.038  <2e-16 ***
## s(sleep_z) 1.000  1.000    0.379   0.538
## ---
## Signif. codes:  0 ‘***’ 0.001 ‘**’ 0.01 ‘*’ 0.05 ‘.’ 0.1 ‘ ’ 1
##
## R-sq.(adj) =  0.489
## lmer.REML = 4.1878e+05  Scale est. = 2945      n = 33442
```

We did the same controling for depression. Below is the model with no covariates but only keeping data with depression.

```
##
## Family: gaussian
## Link function: identity
##
## Formula:
## value ~ sex + site + s(age_z, k = 10, bs = "cr") + s(sleep_z,
##     k = 5, bs = "cr") + icv
## <environment: 0x55895c125240>
##
## Parametric coefficients:
##               Estimate Std. Error t value Pr(>|t|)
## (Intercept)   896.1686     8.7617 102.282  < 2e-16 ***
## sexmale        16.2695     1.8846   8.633  < 2e-16 ***
```

```
## siteousAvanto 201.9051    23.0072   8.776  < 2e-16 ***
## siteousPrisma  34.5885    17.5391   1.972   0.0486 *
## siteousSkyra  366.4358    13.5550  27.033  < 2e-16 ***
## siteUCAM      -89.7385    12.0883  -7.424 1.17e-13 ***
## siteUKB       -13.3478     8.6607  -1.541   0.1233
## siteUmU       283.6285    11.8626  23.909  < 2e-16 ***
## icv            63.3724     0.9707  65.289  < 2e-16 ***
## ---
## Signif. codes:  0 ‘***’ 0.001 ‘**’ 0.01 ‘*’ 0.05 ‘.’ 0.1 ‘ ’ 1
##
## Approximate significance of smooth terms:
##              edf Ref.df        F p-value
## s(age_z)   7.877  7.877 1158.899  <2e-16 ***
## s(sleep_z) 1.000  1.000    0.014   0.907
## ---
## Signif. codes:  0 ‘***’ 0.001 ‘**’ 0.01 ‘*’ 0.05 ‘.’ 0.1 ‘ ’ 1
##
## R-sq.(adj) =  0.471
## lmer.REML = 4.1751e+05  Scale est. = 2868.6    n = 33354
```

Below is the model output with main effect.

```
##
## Family: gaussian
## Link function: identity
##
## Formula:
## value ~ sex + site + s(age_z, k = 10, bs = "cr") + s(sleep_z,
##     k = 5, bs = "cr") + icv + depression
## <environment: 0x55895c125240>
##
## Parametric coefficients:
##               Estimate Std. Error t value Pr(>|t|)
## (Intercept)   898.6620     8.8014 102.104  < 2e-16 ***
## sexmale        16.0252     1.8862   8.496  < 2e-16 ***
## siteousAvanto 203.1304    23.0090   8.828  < 2e-16 ***
## siteousPrisma  35.6586    17.5408   2.033  0.04207 *
## siteousSkyra  367.8310    13.5616  27.123  < 2e-16 ***
## siteUCAM      -89.1712    12.0884  -7.377 1.66e-13 ***
## siteUKB       -14.4307     8.6674  -1.665  0.09594 .
## siteUmU       290.8900    12.1146  24.011  < 2e-16 ***
## icv            63.3940     0.9706  65.317  < 2e-16 ***
## depression    -19.6724     6.6789  -2.945  0.00323 **
## ---
## Signif. codes:  0 ‘***’ 0.001 ‘**’ 0.01 ‘*’ 0.05 ‘.’ 0.1 ‘ ’ 1
##
## Approximate significance of smooth terms:
##              edf Ref.df        F p-value
## s(age_z)   7.877  7.877 1148.886  <2e-16 ***
## s(sleep_z) 1.000  1.000    0.008   0.928
## ---
## Signif. codes:  0 ‘***’ 0.001 ‘**’ 0.01 ‘*’ 0.05 ‘.’ 0.1 ‘ ’ 1
##
## R-sq.(adj) =  0.471
## lmer.REML = 4.1749e+05  Scale est. = 2868.3    n = 33354
```

Next is the model with depression-sleep interaction.

```
##
## Family: gaussian
## Link function: identity
##
## Formula:
## value ~ sex + site + s(age_z, k = 10, bs = "cr") + s(sleep_z,
##     k = 5, bs = "cr") + icv + depression + depression:sleep_z
## <environment: 0x55895c125240>
##
## Parametric coefficients:
##                     Estimate Std. Error t value Pr(>|t|)
## (Intercept)         898.6614     8.8016 102.102  < 2e-16 ***
## sexmale              16.0145     1.8865   8.489  < 2e-16 ***
## siteousAvanto       203.0555    23.0103   8.825  < 2e-16 ***
## siteousPrisma        35.5880    17.5425   2.029  0.04250 *
## siteousSkyra        367.7589    13.5638  27.113  < 2e-16 ***
## siteUCAM            -89.2462    12.0908  -7.381  1.6e-13 ***
## siteUKB             -14.4267     8.6676  -1.664  0.09603 .
## siteUmU             291.4032    12.2158  23.855  < 2e-16 ***
## icv                  63.3952     0.9706  65.317  < 2e-16 ***
## depression          -19.7945     6.6894  -2.959  0.00309 **
## depression:sleep_z  -1.5940     4.8795  -0.327  0.74392
## ---
## Signif. codes:  0 ‘***’ 0.001 ‘**’ 0.01 ‘*’ 0.05 ‘.’ 0.1 ‘ ’ 1
##
## Approximate significance of smooth terms:
##               edf Ref.df       F p-value
## s(age_z)    7.875  7.875 1149.08  <2e-16 ***
## s(sleep_z) 1.000  1.000    0.01   0.921
## ---
## Signif. codes:  0 ‘***’ 0.001 ‘**’ 0.01 ‘*’ 0.05 ‘.’ 0.1 ‘ ’ 1
##
## R-sq.(adj) =  0.471
## lmer.REML = 4.1749e+05  Scale est. = 2868.2    n = 33354
```

The plot below shows the sleep-volume curve for the original model and for the model with main effects of SES.

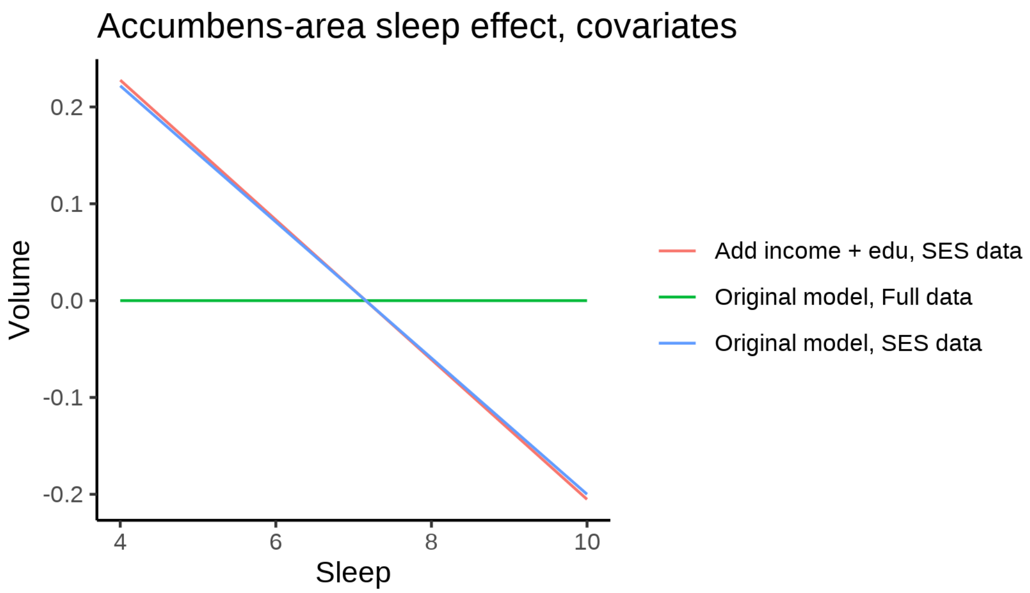

The plot below shows the sleep-volume curve for the original model and for the model with main effects of BMI.

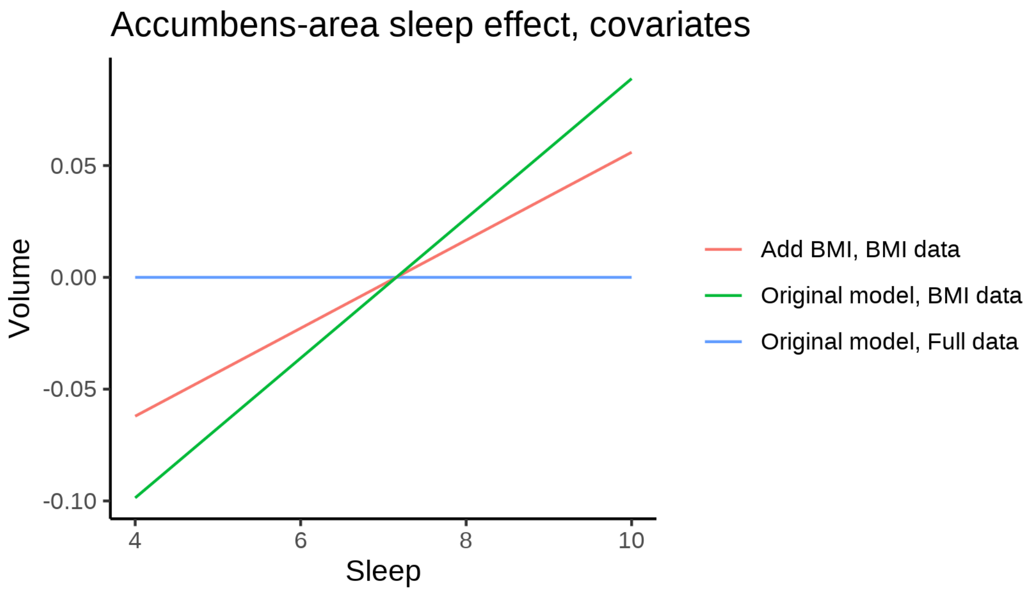

The plot below shows the sleep-volume curve for the original model and for the model with main effects of depression.

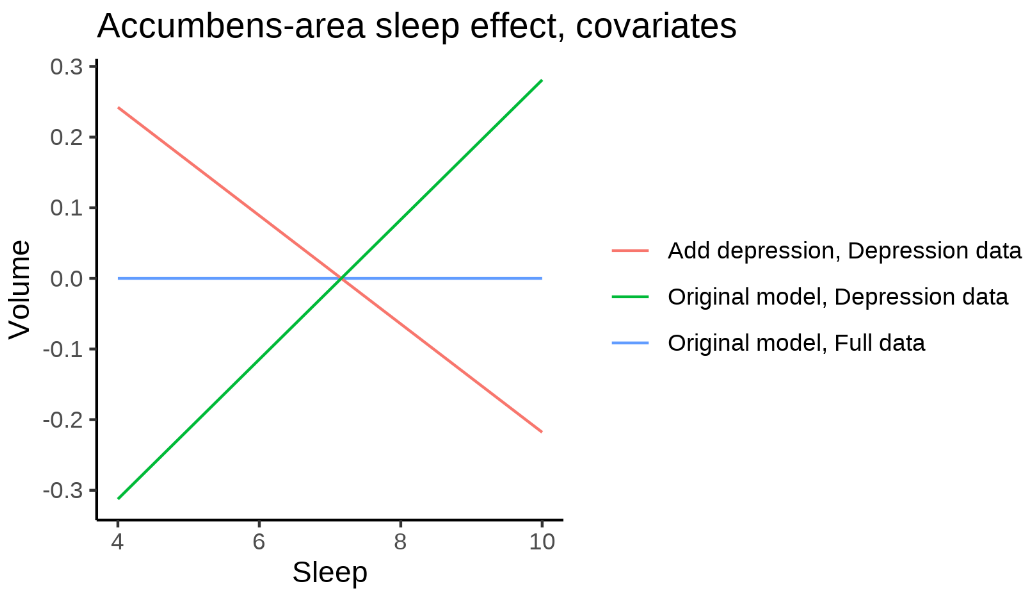

Models Not controling for ICV

Model outputs

Model without sleep term

```
##
## Family: gaussian
## Link function: identity
##
## Formula:
## value ~ sex + site + s(age_z, k = 10, bs = "cr")
## <environment: 0x558963cc0500>
##
## Parametric coefficients:
##                Estimate Std. Error t value Pr(>|t|)
## (Intercept)     773.606      8.179  94.585  < 2e-16 ***
## sexmale          87.321      1.341  65.138  < 2e-16 ***
## siteMPIB         -3.296     10.168  -0.324   0.7458
## siteousAvanto   265.157      8.640  30.688  < 2e-16 ***
## siteousPrisma   110.575     14.379   7.690 1.50e-14 ***
## siteousSkyra    411.381      7.848  52.422  < 2e-16 ***
## siteUB           45.689     23.584   1.937   0.0527 .
## siteUCAM          6.989      8.928   0.783   0.4337
## siteUKB          82.731      8.408   9.840  < 2e-16 ***
## siteUmU         344.760     11.503  29.970  < 2e-16 ***
## siteUOXF         44.497      9.880   4.504 6.68e-06 ***
## ---
## Signif. codes:  0 '***' 0.001 '**' 0.01 '*' 0.05 '.' 0.1 ' ' 1
##
## Approximate significance of smooth terms:
##             edf Ref.df    F p-value
## s(age_z) 7.81   7.81 1939  <2e-16 ***
```

```
## ---
## Signif. codes:  0 ‘***’ 0.001 ‘**’ 0.01 ‘*’ 0.05 ‘.’ 0.1 ‘ ’ 1
##
## R-sq.(adj) =  0.403
## lmer.REML = 6.4972e+05  Scale est. = 2940.8    n = 51284
```

Model with only main effects of age and sleep

```
##
## Family: gaussian
## Link function: identity
##
## Formula:
## value ~ sex + site + s(age_z, k = 10, bs = "cr") + s(sleep_z,
##     k = 5, bs = "cr")
## <environment: 0x558963cc0500>
##
## Parametric coefficients:
##               Estimate Std. Error t value Pr(>|t|)
## (Intercept)    774.946      8.187  94.654  < 2e-16 ***
## sexmale         87.148      1.341  64.985  < 2e-16 ***
## siteMPIB        -3.925     10.172  -0.386   0.6996
## siteousAvanto  264.233      8.642  30.574  < 2e-16 ***
## siteousPrisma  109.984     14.377   7.650 2.05e-14 ***
## siteousSkyra   410.433      7.850  52.286  < 2e-16 ***
## siteUB          45.881     23.580   1.946   0.0517 .
## siteUCAM         6.585      8.928   0.738   0.4608
## siteUKB         81.409      8.416   9.673  < 2e-16 ***
## siteUmU        343.134     11.529  29.764  < 2e-16 ***
## siteUOXF        43.850      9.879   4.439 9.08e-06 ***
## ---
## Signif. codes:  0 ‘***’ 0.001 ‘**’ 0.01 ‘*’ 0.05 ‘.’ 0.1 ‘ ’ 1
##
## Approximate significance of smooth terms:
##              edf Ref.df        F  p-value
## s(age_z)   7.802  7.802 1930.227  < 2e-16 ***
## s(sleep_z) 2.669  2.669    5.875 0.000697 ***
## ---
## Signif. codes:  0 ‘***’ 0.001 ‘**’ 0.01 ‘*’ 0.05 ‘.’ 0.1 ‘ ’ 1
##
## R-sq.(adj) =  0.404
## lmer.REML = 6.4971e+05  Scale est. = 2940.8    n = 51284
```

Model with full interaction between age and sleep

```
##
## Family: gaussian
## Link function: identity
##
## Formula:
## value ~ sex + site + t2(age_z, sleep_z, k = c(10, 4), bs = "cr")
## <environment: 0x558963cc0500>
##
## Parametric coefficients:
##               Estimate Std. Error t value Pr(>|t|)
## (Intercept)    778.540      8.107  96.036  < 2e-16 ***
```

```
## sexmale         87.250      1.344  64.922  < 2e-16 ***
## siteMPIB        -7.443     10.164  -0.732   0.4640    
## siteousAvanto  261.837      8.647  30.281  < 2e-16 ***
## siteousPrisma  107.793     14.404   7.483 7.35e-14 ***
## siteousSkyra   408.062      7.862  51.906  < 2e-16 ***
## siteUB          41.606     23.557   1.766   0.0774 .  
## siteUCAM         3.015      8.920   0.338   0.7353    
## siteUKB         77.658      8.326   9.327  < 2e-16 ***
## siteUmU        339.591     11.496  29.540  < 2e-16 ***
## siteUOXF        39.858      9.820   4.059 4.94e-05 ***
## ---
## Signif. codes:  0 ‘***’ 0.001 ‘**’ 0.01 ‘*’ 0.05 ‘.’ 0.1 ‘ ’ 1
##
## Approximate significance of smooth terms:
##                     edf Ref.df     F p-value    
## t2(age_z,sleep_z) 12.58  12.58 255.5  <2e-16 ***
## ---
## Signif. codes:  0 ‘***’ 0.001 ‘**’ 0.01 ‘*’ 0.05 ‘.’ 0.1 ‘ ’ 1
##
## R-sq.(adj) =  0.403   
## lmer.REML = 6.4972e+05  Scale est. = 2939.6    n = 51284
```

Model comparison

`mod_no_sleep` refers to model without sleep term, `mod_no_interaction` refers to model with only main effect of sleep, and `mod_full` refers to model with a full interaction between age and sleep. This is a nested model comparison, and the p-value at a given line refers to comparing the model at the line to the model on the line above. Hence, significance implies that the more complicated model is supported on statistical grounds.

To be even more specific, the p-value on the second row tests whether there is an association between sleep and volume. The p-value on the third row tests whether this association depends on age.

```
## Data: NULL
## Models:
## ml$mod_no_sleep$mer: NULL
## ml$mod_no_interaction$mer: NULL
## ml$mod_full$mer: NULL
##                           npar    AIC    BIC  logLik deviance Chisq Df Pr(>Chisq)   
## ml$mod_no_sleep$mer         15 649754 649887 -324862   649724                       
## ml$mod_no_interaction$mer   17 649746 649896 -324856   649712 12.31  2   0.002123 **
## ml$mod_full$mer             19 649758 649926 -324860   649720  0.00  2   1.000000   
## ---
## Signif. codes:  0 ‘***’ 0.001 ‘**’ 0.01 ‘*’ 0.05 ‘.’ 0.1 ‘ ’ 1
```

We chose the model based on the likelihood ratio test with 5 % significance level, which was `mod_no_interaction`.

Lifespan brain trajectory

The trajectory shown is from the chosen model `mod_no_interaction_no_icv`.

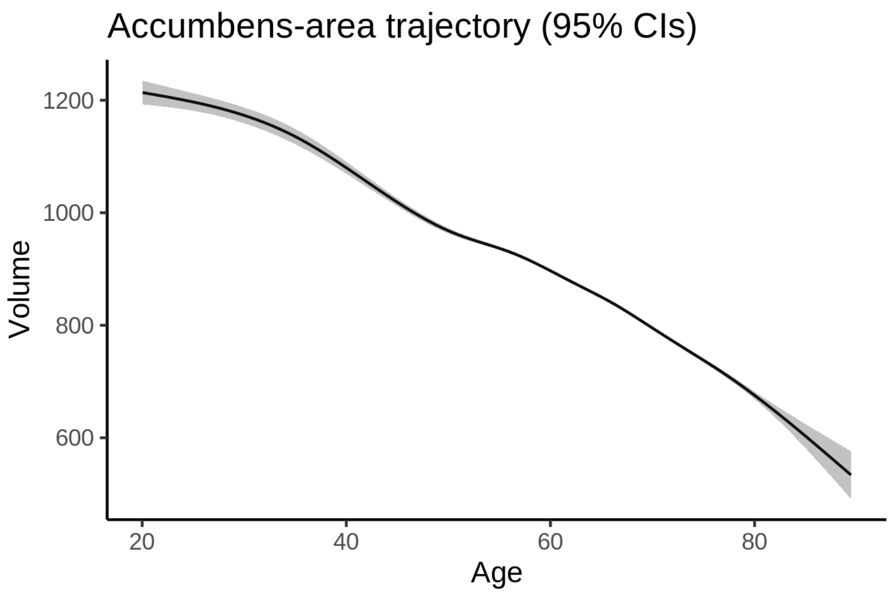

Effect of sleep

The chosen model only included the main effect of sleep, and hence the effect does not vary with age. The black dot shows the average sleep duration across all ages in the sample.

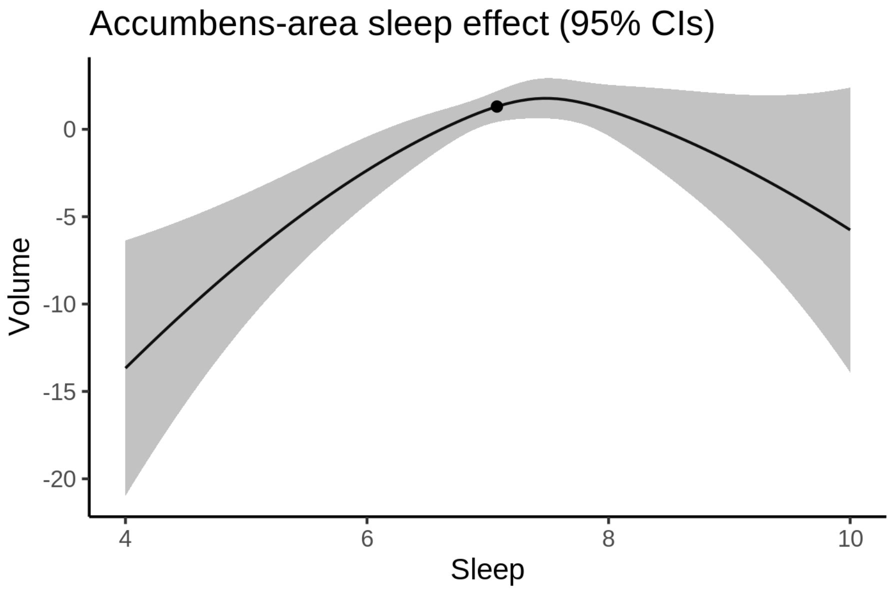

We also show the full interaction model for completeness, although it was not selected.

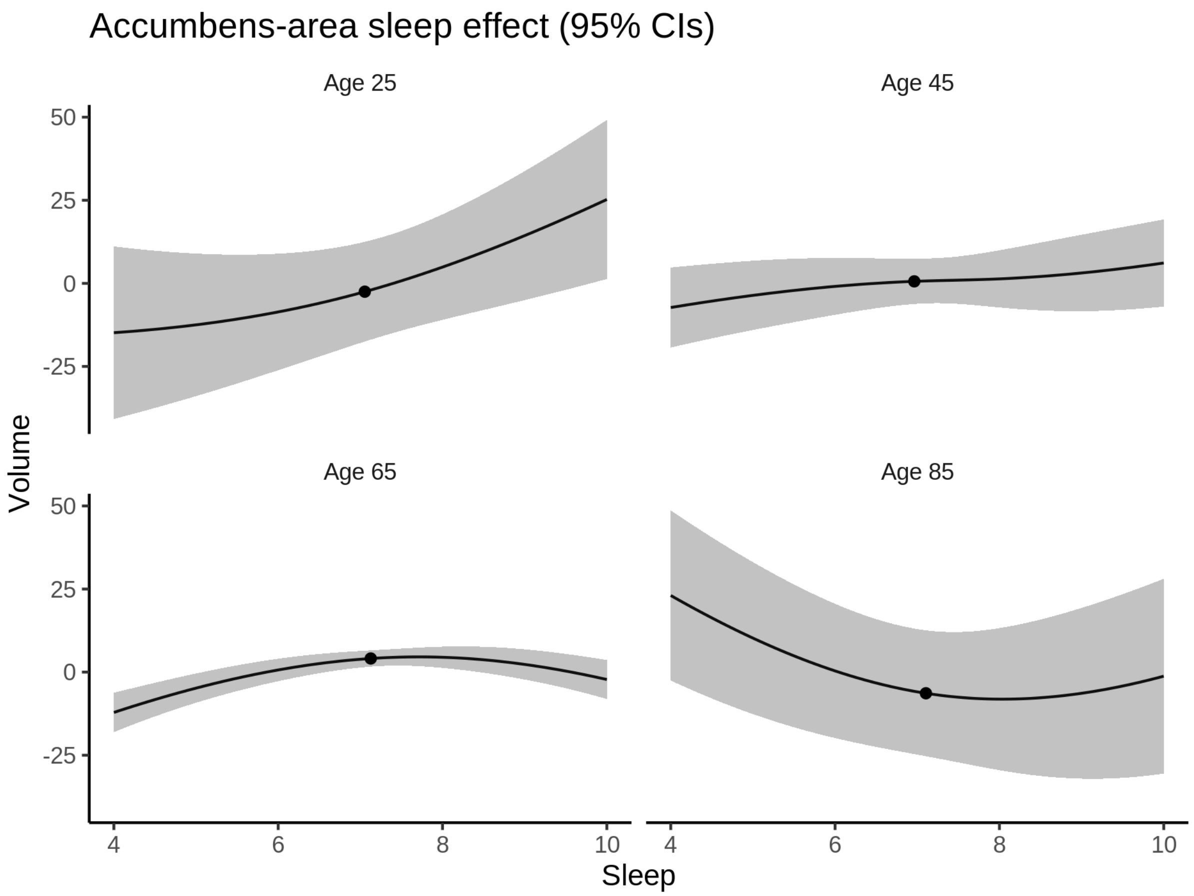

Deviation from sleep associated with maximal volume

Model with only main effect of sleep was chosen, so we show it for all ages at once. Maximum volume is attained at 7.5 hours of sleep. The percentage values in the plot are calculated as follows: The maximum at 100 % refers to a person at an arbitrary age with a sleep duration associated with maximum volume. For a female, this volume is 924 and for a male it is 1011. The other percentage values show how large the expected volume is for someone with other sleep durations. For example, 99 % implies a 1 % reduction.

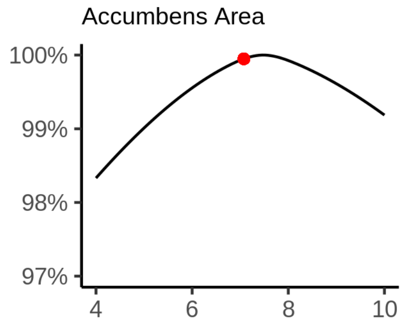

Comparison of mean sleep and sleep associated with maximum volume

A 95 % confidence interval for the sleep associated with maximum volume is [6.67, 8.48].

The plot below compares average sleep to the sleep associated with maximum volume.

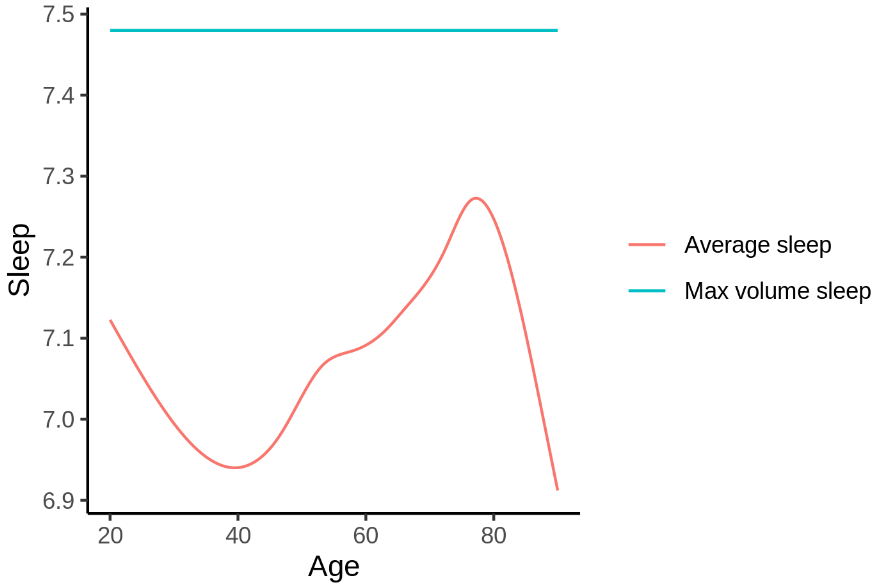

The next plot shows the difference between average sleep and sleep associated with maximum volume. The shaded region is a 95 % confidence interval.

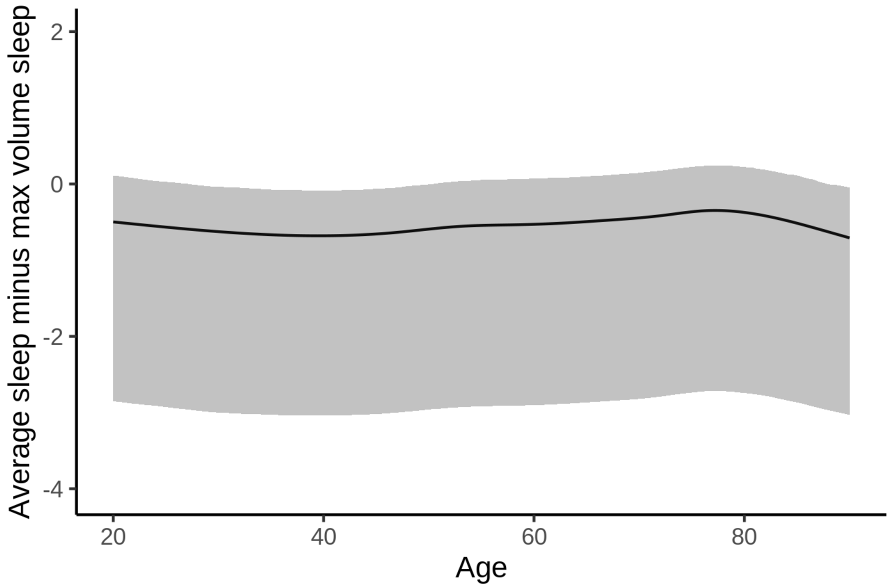

The next plot shows the probability that the sleep duration associated with maximum volume is longer than the average sleep duration, as a function of age. Probability below .05 can be interpreted as evidence that the sleep associated with maximum volume is shorter than the mean sleep, and probability above .95 can be interpreted the opposite way.

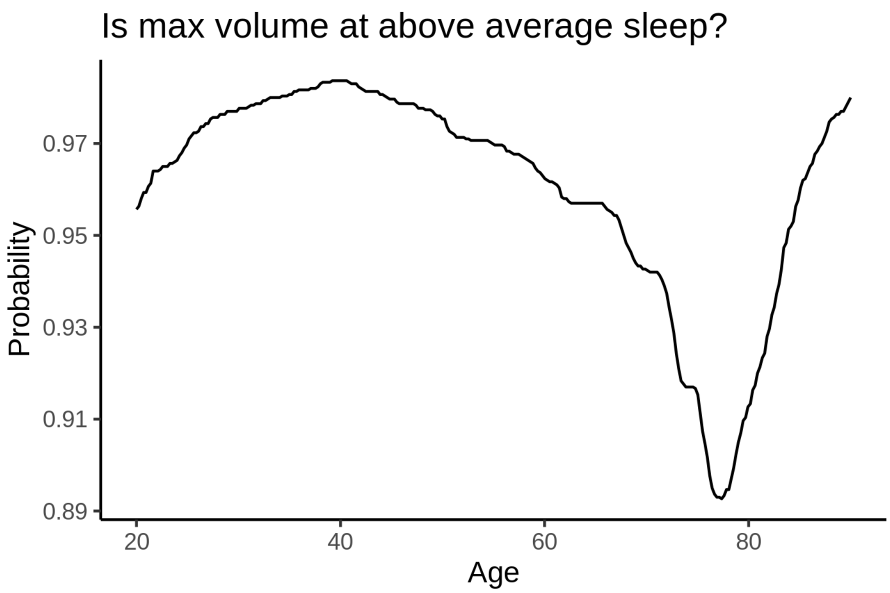

Amygdala

Descriptive statistics

Study	Observations	Unique IDs	Mean age	Age range
HCP	974	974	28.8	22 - 37
MPIB	677	391	63.1	24 - 83
UB	113	39	70.9	64 - 81
UCAM	884	632	55.1	20 - 88
UiO	1472	803	49.3	20 - 89
UKB	45982	43138	64.5	45 - 83
UmU	412	279	62.2	25 - 85
UOXF	769	769	69.8	60 - 85

Spaghetti plot

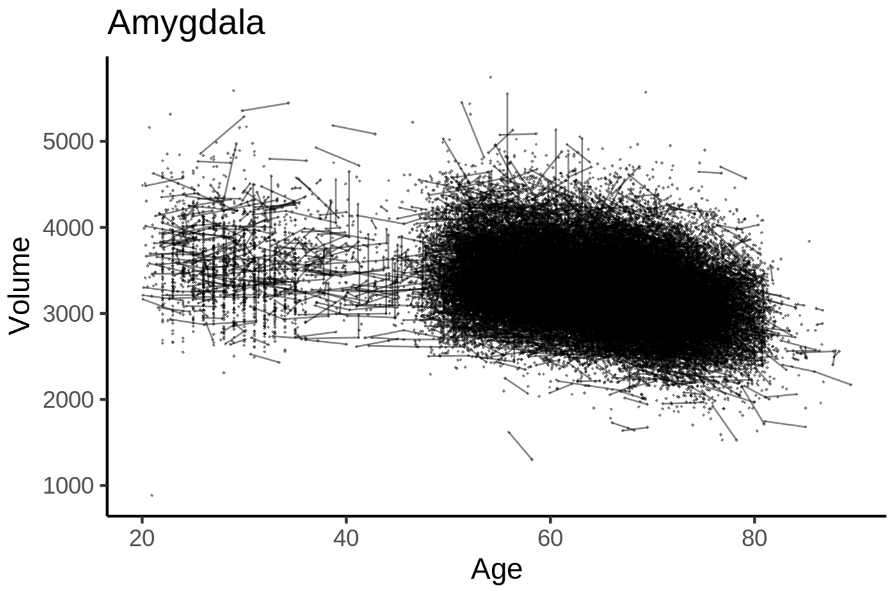

Models Controling for ICV

Model outputs

Model without sleep term

```
##
## Family: gaussian
## Link function: identity
##
## Formula:
## value ~ sex + site + icv + s(age_z, k = 10, bs = "cr")
## <environment: 0x558a46c11a78>
##
## Parametric coefficients:
##                Estimate Std. Error t value Pr(>|t|)
## (Intercept)    3033.328     18.123 167.373  < 2e-16 ***
## sexmale         147.429      3.645  40.442  < 2e-16 ***
## siteMPIB        342.212     22.853  14.974  < 2e-16 ***
## siteousAvanto -101.209     18.892  -5.357 8.49e-08 ***
## siteousPrisma  188.489     31.725   5.941 2.85e-09 ***
## siteousSkyra   508.459     17.451  29.136  < 2e-16 ***
## siteUB          81.508     53.036   1.537   0.1243
## siteUCAM       102.306     19.864   5.150 2.61e-07 ***
## siteUKB        190.359     18.596  10.237  < 2e-16 ***
## siteUmU        161.778     25.640   6.310 2.82e-10 ***
## siteUOXF        45.667     21.912   2.084   0.0372 *
## icv            202.684      1.835 110.473  < 2e-16 ***
## ---
## Signif. codes:  0 ‘***’ 0.001 ‘**’ 0.01 ‘*’ 0.05 ‘.’ 0.1 ‘ ’ 1
##
```

```
## Approximate significance of smooth terms:
##            edf Ref.df    F p-value
## s(age_z) 7.493  7.493 1303  <2e-16 ***
## ---
## Signif. codes:  0 '***' 0.001 '**' 0.01 '*' 0.05 '.' 0.1 ' ' 1
##
## R-sq.(adj) =  0.457
## lmer.REML = 7.311e+05  Scale est. = 11621     n = 51283
```

Model with only main effects of age and sleep

```
##
## Family: gaussian
## Link function: identity
##
## Formula:
## value ~ sex + site + icv + s(age_z, k = 10, bs = "cr") + s(sleep_z,
##     k = 5, bs = "cr")
## <environment: 0x558a46c11a78>
##
## Parametric coefficients:
##                Estimate Std. Error t value Pr(>|t|)
## (Intercept)    3031.788     18.141 167.125  < 2e-16 ***
## sexmale         147.375      3.645  40.427  < 2e-16 ***
## siteMPIB        343.882     22.869  15.037  < 2e-16 ***
## siteousAvanto  -100.394     18.897  -5.313 1.08e-07 ***
## siteousPrisma   189.034     31.726   5.958 2.56e-09 ***
## siteousSkyra    509.306     17.456  29.176  < 2e-16 ***
## siteUB           82.000     53.034   1.546   0.1221
## siteUCAM        103.109     19.867   5.190 2.11e-07 ***
## siteUKB         191.995     18.615  10.314  < 2e-16 ***
## siteUmU         165.156     25.700   6.426 1.32e-10 ***
## siteUOXF         46.478     21.916   2.121   0.0339 *
## icv             202.791      1.835 110.485  < 2e-16 ***
## ---
## Signif. codes:  0 '***' 0.001 '**' 0.01 '*' 0.05 '.' 0.1 ' ' 1
##
## Approximate significance of smooth terms:
##              edf Ref.df        F p-value
## s(age_z)   7.496  7.496 1294.978  <2e-16 ***
## s(sleep_z) 1.000  1.000    3.633  0.0567 .
## ---
## Signif. codes:  0 '***' 0.001 '**' 0.01 '*' 0.05 '.' 0.1 ' ' 1
##
## R-sq.(adj) =  0.457
## lmer.REML = 7.3109e+05  Scale est. = 11621     n = 51283
```

Model with full interaction between age and sleep

```
##
## Family: gaussian
## Link function: identity
##
## Formula:
## value ~ sex + site + icv + t2(age_z, sleep_z, k = c(10, 4), bs = "cr")
## <environment: 0x558a46c11a78>
```

```
##
## Parametric coefficients:
##                Estimate Std. Error t value Pr(>|t|)
## (Intercept)   3034.734     18.219 166.569  < 2e-16 ***
## sexmale        147.468      3.653  40.364  < 2e-16 ***
## siteMPIB       341.058     22.998  14.830  < 2e-16 ***
## siteousAvanto -102.931     18.997  -5.418 6.05e-08 ***
## siteousPrisma  186.332     31.795   5.860 4.64e-09 ***
## siteousSkyra   506.615     17.572  28.830  < 2e-16 ***
## siteUB          79.017     53.073   1.489   0.1365
## siteUCAM        99.982     19.985   5.003 5.67e-07 ***
## siteUKB        188.939     18.694  10.107  < 2e-16 ***
## siteUmU        162.233     25.792   6.290 3.20e-10 ***
## siteUOXF        43.300     22.002   1.968   0.0491 *
## icv            202.701      1.836 110.398  < 2e-16 ***
## ---
## Signif. codes:  0 ‘***’ 0.001 ‘**’ 0.01 ‘*’ 0.05 ‘.’ 0.1 ‘ ’ 1
##
## Approximate significance of smooth terms:
##                      edf Ref.df     F p-value
## t2(age_z,sleep_z) 12.86  12.86 29.32  <2e-16 ***
## ---
## Signif. codes:  0 ‘***’ 0.001 ‘**’ 0.01 ‘*’ 0.05 ‘.’ 0.1 ‘ ’ 1
##
## R-sq.(adj) =  0.457
## lmer.REML = 7.311e+05  Scale est. = 11620     n = 51283
```

Model comparison

`mod_no_sleep` refers to model without sleep term, `mod_no_interaction` refers to model with only main effect of sleep, and `mod_full` refers to model with a full interaction between age and sleep. This is a nested model comparison, and the p-value at a given line refers to comparing the model at the line to the model on the line above. Hence, significance implies that the more complicated model is supported on statistical grounds.

To be even more specific, the p-value on the second row tests whether there is an association between sleep and volume. The p-value on the third row tests whether this association depends on age.

```
## Data: NULL
## Models:
## ml$mod_no_sleep$mer: NULL
## ml$mod_no_interaction$mer: NULL
## ml$mod_full$mer: NULL
##                           npar    AIC    BIC  logLik deviance  Chisq Df Pr(>Chisq)
## ml$mod_no_sleep$mer         16 731129 731270 -365548   731097
## ml$mod_no_interaction$mer   18 731129 731288 -365547   731093 3.6326  2     0.1626
## ml$mod_full$mer             20 731141 731317 -365550   731101 0.0000  2     1.0000
```

We chose the model based on the likelihood ratio test with 5 % significance level, which was `mod_no_sleep`.

Lifespan brain trajectory

The trajectory shown is from the chosen model `mod_no_sleep`.

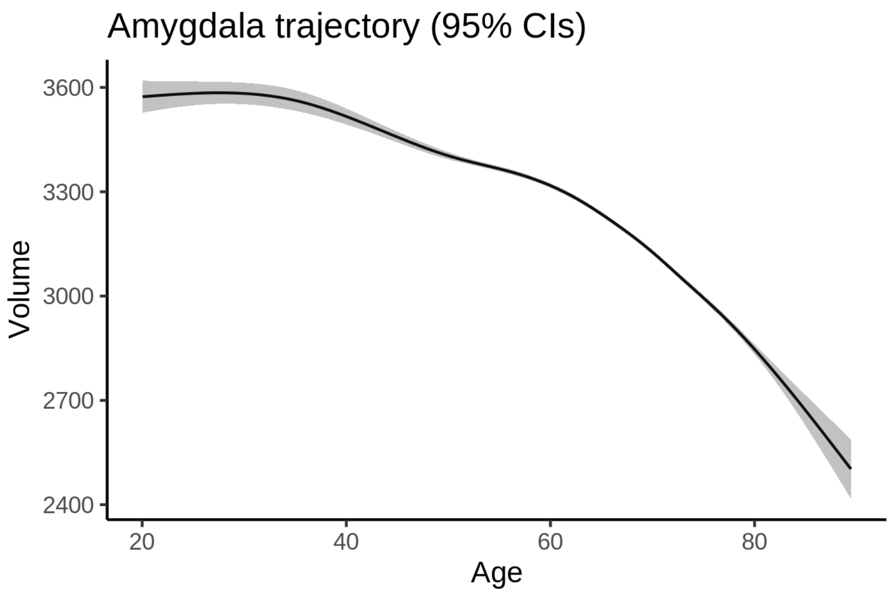

Effect of sleep

The chosen model did not include a sleep term, and hence we don't have any estimated effect of sleep. We show the full interaction model for completeness, although it was not selected.

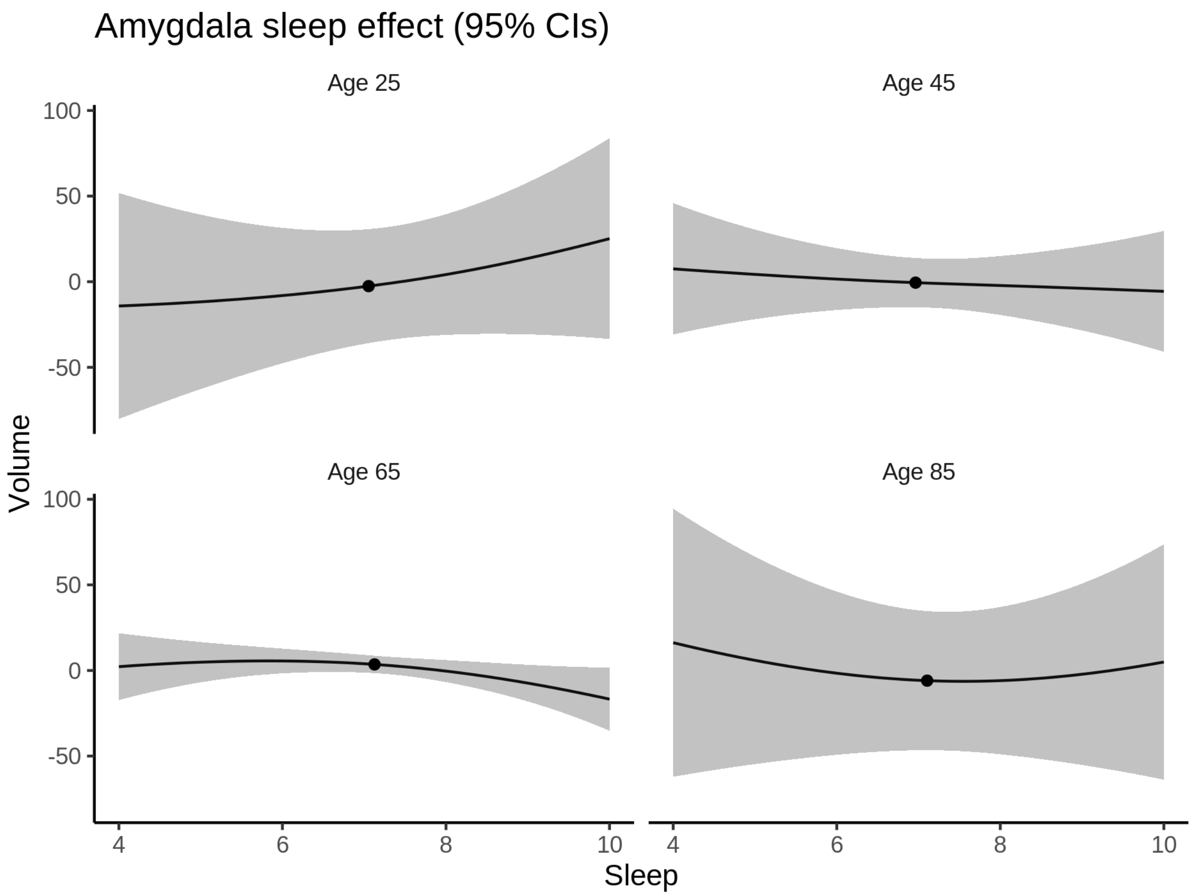

Deviation from sleep associated with maximal volume

Model with no sleep term was selected. No plots to show. (Although we can of course dig up the plots, which will be pretty flat).

Comparison of mean sleep and sleep associated with maximum volume

Nothing to show, as we did not find an association between sleep and volume.

Controling for covariates

Below is the output for a model in which we only include data with income and education.

```
##
## Family: gaussian
## Link function: identity
##
## Formula:
## value ~ sex + site + s(age_z, k = 10, bs = "cr") + s(sleep_z,
##     k = 5, bs = "cr") + icv
## <environment: 0x558a47c64150>
##
## Parametric coefficients:
```

```
##                Estimate Std. Error t value Pr(>|t|)
## (Intercept)   3402.860     42.153  80.726  < 2e-16 ***
## sexmale        139.850      4.645  30.108  < 2e-16 ***
## siteousAvanto -458.663     52.202  -8.786  < 2e-16 ***
## siteousPrisma -125.733     52.018  -2.417   0.0157 *
## siteousSkyra   146.530     45.568   3.216   0.0013 **
## siteUKB       -182.379     42.083  -4.334 1.47e-05 ***
## siteUOXF      -294.745     47.394  -6.219 5.07e-10 ***
## icv            210.327      2.387  88.121  < 2e-16 ***
## ---
## Signif. codes:  0 '***' 0.001 '**' 0.01 '*' 0.05 '.' 0.1 ' ' 1
##
## Approximate significance of smooth terms:
##              edf Ref.df       F p-value
## s(age_z)   6.126  6.126 855.383  <2e-16 ***
## s(sleep_z) 1.000  1.000   3.973  0.0462 *
## ---
## Signif. codes:  0 '***' 0.001 '**' 0.01 '*' 0.05 '.' 0.1 ' ' 1
##
## R-sq.(adj) =  0.442
## lmer.REML = 4.4478e+05  Scale est. = 12026     n = 31197
```

Below is the output for a model in which we control for the main effects of income and education.

```
##
## Family: gaussian
## Link function: identity
##
## Formula:
## value ~ sex + site + s(age_z, k = 10, bs = "cr") + s(sleep_z,
##     k = 5, bs = "cr") + icv + income_scaled + education_scaled
## <environment: 0x558a47c64150>
##
## Parametric coefficients:
##                   Estimate Std. Error t value Pr(>|t|)
## (Intercept)       3402.466     42.426  80.198  < 2e-16 ***
## sexmale            140.395      4.659  30.137  < 2e-16 ***
## siteousAvanto     -460.369     52.213  -8.817  < 2e-16 ***
## siteousPrisma     -125.966     52.021  -2.421  0.01547 *
## siteousSkyra       144.943     45.580   3.180  0.00147 **
## siteUKB           -184.690     42.109  -4.386 1.16e-05 ***
## siteUOXF          -295.666     47.422  -6.235 4.58e-10 ***
## icv                210.348      2.408  87.356  < 2e-16 ***
## income_scaled       -7.131      5.530  -1.289  0.19725
## education_scaled     7.429      6.534   1.137  0.25557
## ---
## Signif. codes:  0 '***' 0.001 '**' 0.01 '*' 0.05 '.' 0.1 ' ' 1
##
## Approximate significance of smooth terms:
##              edf Ref.df       F p-value
## s(age_z)   6.123  6.123 792.701  <2e-16 ***
## s(sleep_z) 1.000  1.000   4.125  0.0423 *
## ---
## Signif. codes:  0 '***' 0.001 '**' 0.01 '*' 0.05 '.' 0.1 ' ' 1
##
```

```
## R-sq.(adj) =  0.442
## lmer.REML = 4.4477e+05  Scale est. = 12025     n = 31197
```

We also included interaction effects between sleep duration and education and income, in another model. The output is shown below, and the interaction terms are `income_scaled:sleep_z` and `education_scaled:sleep_z`.

```
##
## Family: gaussian
## Link function: identity
##
## Formula:
## value ~ sex + site + s(age_z, k = 10, bs = "cr") + s(sleep_z,
##     k = 5, bs = "cr") + icv + income_scaled + education_scaled +
##     income_scaled:sleep_z + education_scaled:sleep_z
## <environment: 0x558a47c64150>
##
## Parametric coefficients:
##                          Estimate Std. Error t value Pr(>|t|)
## (Intercept)              3402.156     42.429  80.185  < 2e-16 ***
## sexmale                   140.160      4.662  30.065  < 2e-16 ***
## siteousAvanto            -460.517     52.215  -8.820  < 2e-16 ***
## siteousPrisma            -126.726     52.027  -2.436  0.01487 *
## siteousSkyra              144.770     45.582   3.176  0.00149 **
## siteUKB                  -184.310     42.114  -4.376 1.21e-05 ***
## siteUOXF                 -294.825     47.436  -6.215 5.19e-10 ***
## icv                       210.358      2.408  87.350  < 2e-16 ***
## income_scaled              -6.882      5.533  -1.244  0.21361
## education_scaled            7.311      6.535   1.119  0.26328
## income_scaled:sleep_z      -7.959      5.472  -1.454  0.14582
## education_scaled:sleep_z    1.899      6.326   0.300  0.76400
## ---
## Signif. codes:  0 ‘***’ 0.001 ‘**’ 0.01 ‘*’ 0.05 ‘.’ 0.1 ‘ ’ 1
##
## Approximate significance of smooth terms:
##              edf Ref.df       F p-value
## s(age_z)   6.127  6.127 791.504  <2e-16 ***
## s(sleep_z) 1.000  1.000   0.189   0.664
## ---
## Signif. codes:  0 ‘***’ 0.001 ‘**’ 0.01 ‘*’ 0.05 ‘.’ 0.1 ‘ ’ 1
##
## R-sq.(adj) =  0.442
## lmer.REML = 4.4475e+05  Scale est. = 12025     n = 31197
```

We did the same controling for BMI. Below is the model with no covariates but only keeping data with BMI.

```
##
## Family: gaussian
## Link function: identity
##
## Formula:
## value ~ sex + site + s(age_z, k = 10, bs = "cr") + s(sleep_z,
##     k = 5, bs = "cr") + icv
## <environment: 0x558a477aef48>
##
## Parametric coefficients:
```

```
##               Estimate Std. Error t value Pr(>|t|)
## (Intercept)   2949.354     31.673  93.120  < 2e-16 ***
## sexmale        137.756      4.508  30.558  < 2e-16 ***
## siteousPrisma  287.825     41.029   7.015 2.34e-12 ***
## siteousSkyra   623.686     26.292  23.721  < 2e-16 ***
## siteUCAM       187.890     33.054   5.684 1.32e-08 ***
## siteUKB        274.790     31.899   8.614  < 2e-16 ***
## siteUmU        277.804     36.788   7.552 4.41e-14 ***
## icv            212.295      2.324  91.363  < 2e-16 ***
## ---
## Signif. codes:  0 '***' 0.001 '**' 0.01 '*' 0.05 '.' 0.1 ' ' 1
##
## Approximate significance of smooth terms:
##             edf Ref.df       F p-value
## s(age_z)   6.798  6.798 884.250  <2e-16 ***
## s(sleep_z) 1.000  1.000   3.664  0.0556 .
## ---
## Signif. codes:  0 '***' 0.001 '**' 0.01 '*' 0.05 '.' 0.1 ' ' 1
##
## R-sq.(adj) =  0.456
## lmer.REML = 4.7627e+05  Scale est. = 11299     n = 33441
```

Below is the model output with main effect.

```
##
## Family: gaussian
## Link function: identity
##
## Formula:
## value ~ sex + site + s(age_z, k = 10, bs = "cr") + s(sleep_z,
##     k = 5, bs = "cr") + icv + bmi
## <environment: 0x558a477aef48>
##
## Parametric coefficients:
##                Estimate Std. Error t value Pr(>|t|)
## (Intercept)   2888.4783    33.5410  86.118  < 2e-16 ***
## sexmale        135.8299     4.5195  30.054  < 2e-16 ***
## siteousPrisma  285.2852    41.0214   6.955 3.60e-12 ***
## siteousSkyra   623.3041    26.2915  23.707  < 2e-16 ***
## siteUCAM       186.3332    33.0480   5.638 1.73e-08 ***
## siteUKB        272.9746    31.8951   8.559  < 2e-16 ***
## siteUmU        275.1780    36.7809   7.482 7.53e-14 ***
## icv            211.9529     2.3234  91.227  < 2e-16 ***
## bmi              2.4120     0.4383   5.503 3.75e-08 ***
## ---
## Signif. codes:  0 '***' 0.001 '**' 0.01 '*' 0.05 '.' 0.1 ' ' 1
##
## Approximate significance of smooth terms:
##             edf Ref.df       F p-value
## s(age_z)   6.802  6.802 883.768  <2e-16 ***
## s(sleep_z) 1.000  1.000   2.775  0.0957 .
## ---
## Signif. codes:  0 '***' 0.001 '**' 0.01 '*' 0.05 '.' 0.1 ' ' 1
##
## R-sq.(adj) =  0.457
```

```
## lmer.REML = 4.7624e+05  Scale est. = 11299     n = 33441
```

Next is the model with BMI-sleep interaction.

```
##
## Family: gaussian
## Link function: identity
##
## Formula:
## value ~ sex + site + s(age_z, k = 10, bs = "cr") + s(sleep_z,
##     k = 5, bs = "cr") + icv + bmi + bmi:sleep_z
## <environment: 0x558a477aef48>
##
## Parametric coefficients:
##                Estimate Std. Error t value Pr(>|t|)
## (Intercept)   2.888e+03  3.356e+01  86.076  < 2e-16 ***
## sexmale       1.358e+02  4.520e+00  30.054  < 2e-16 ***
## siteousPrisma 2.853e+02  4.102e+01   6.955 3.60e-12 ***
## siteousSkyra  6.233e+02  2.629e+01  23.708  < 2e-16 ***
## siteUCAM      1.864e+02  3.305e+01   5.640 1.71e-08 ***
## siteUKB       2.730e+02  3.190e+01   8.560  < 2e-16 ***
## siteUmU       2.752e+02  3.678e+01   7.483 7.45e-14 ***
## icv           2.120e+02  2.323e+00  91.225  < 2e-16 ***
## bmi           2.415e+00  4.385e-01   5.506 3.69e-08 ***
## bmi:sleep_z   7.574e-02  4.150e-01   0.183    0.855
## ---
## Signif. codes:  0 '***' 0.001 '**' 0.01 '*' 0.05 '.' 0.1 ' ' 1
##
## Approximate significance of smooth terms:
##              edf Ref.df      F p-value
## s(age_z)   6.802  6.802 883.64  <2e-16 ***
## s(sleep_z) 1.000  1.000   0.21   0.647
## ---
## Signif. codes:  0 '***' 0.001 '**' 0.01 '*' 0.05 '.' 0.1 ' ' 1
##
## R-sq.(adj) =  0.457
## lmer.REML = 4.7624e+05  Scale est. = 11299     n = 33441
```

We did the same controling for depression. Below is the model with no covariates but only keeping data with depression.

```
##
## Family: gaussian
## Link function: identity
##
## Formula:
## value ~ sex + site + s(age_z, k = 10, bs = "cr") + s(sleep_z,
##     k = 5, bs = "cr") + icv
## <environment: 0x558a460bdb78>
##
## Parametric coefficients:
##               Estimate Std. Error t value Pr(>|t|)
## (Intercept)   3356.346     21.378 156.997  < 2e-16 ***
## sexmale        137.015      4.539  30.184  < 2e-16 ***
## siteousAvanto -406.134     49.620  -8.185 2.82e-16 ***
## siteousPrisma -101.198     41.796  -2.421   0.0155 *
```

```
## siteousSkyra   262.626     32.559   8.066 7.50e-16 ***
## siteUCAM      -205.903     29.356  -7.014 2.36e-12 ***
## siteUKB       -134.556     21.137  -6.366 1.97e-10 ***
## siteUmU       -127.785     28.849  -4.429 9.48e-06 ***
## icv            210.505      2.336  90.107  < 2e-16 ***
## ---
## Signif. codes:  0 '***' 0.001 '**' 0.01 '*' 0.05 '.' 0.1 ' ' 1
##
## Approximate significance of smooth terms:
##              edf Ref.df       F p-value
## s(age_z)   6.701  6.701 842.666  <2e-16 ***
## s(sleep_z) 1.000  1.000   5.179  0.0229 *
## ---
## Signif. codes:  0 '***' 0.001 '**' 0.01 '*' 0.05 '.' 0.1 ' ' 1
##
## R-sq.(adj) =  0.448
## lmer.REML = 4.7492e+05  Scale est. = 10881     n = 33353
```

Below is the model output with main effect.

```
##
## Family: gaussian
## Link function: identity
##
## Formula:
## value ~ sex + site + s(age_z, k = 10, bs = "cr") + s(sleep_z,
##     k = 5, bs = "cr") + icv + depression
## <environment: 0x558a460bdb78>
##
## Parametric coefficients:
##                Estimate Std. Error t value Pr(>|t|)
## (Intercept)   3359.404     21.475 156.434  < 2e-16 ***
## sexmale        136.714      4.544  30.089  < 2e-16 ***
## siteousAvanto -404.624     49.630  -8.153 3.68e-16 ***
## siteousPrisma  -99.875     41.804  -2.389   0.0169 *
## siteousSkyra   264.328     32.578   8.114 5.07e-16 ***
## siteUCAM      -205.203     29.359  -6.989 2.81e-12 ***
## siteUKB       -135.881     21.155  -6.423 1.35e-10 ***
## siteUmU       -118.856     29.455  -4.035 5.47e-05 ***
## icv            210.531      2.336  90.117  < 2e-16 ***
## depression     -24.152     16.096  -1.501   0.1335
## ---
## Signif. codes:  0 '***' 0.001 '**' 0.01 '*' 0.05 '.' 0.1 ' ' 1
##
## Approximate significance of smooth terms:
##              edf Ref.df       F p-value
## s(age_z)   6.698  6.698 833.761  <2e-16 ***
## s(sleep_z) 1.000  1.000   5.645  0.0175 *
## ---
## Signif. codes:  0 '***' 0.001 '**' 0.01 '*' 0.05 '.' 0.1 ' ' 1
##
## R-sq.(adj) =  0.448
## lmer.REML = 4.7491e+05  Scale est. = 10882     n = 33353
```

Next is the model with depression-sleep interaction.

```
##
## Family: gaussian
## Link function: identity
##
## Formula:
## value ~ sex + site + s(age_z, k = 10, bs = "cr") + s(sleep_z,
##     k = 5, bs = "cr") + icv + depression + depression:sleep_z
## <environment: 0x558a460bdb78>
##
## Parametric coefficients:
##                    Estimate Std. Error t value Pr(>|t|)
## (Intercept)        3359.401     21.475 156.432  < 2e-16 ***
## sexmale             136.692      4.544  30.079  < 2e-16 ***
## siteousAvanto      -404.788     49.633  -8.156 3.60e-16 ***
## siteousPrisma      -100.032     41.809  -2.393   0.0167 *
## siteousSkyra        264.171     32.583   8.108 5.34e-16 ***
## siteUCAM           -205.365     29.365  -6.993 2.73e-12 ***
## siteUKB            -135.872     21.156  -6.423 1.36e-10 ***
## siteUmU            -117.801     29.698  -3.967 7.31e-05 ***
## icv                 210.534      2.336  90.116  < 2e-16 ***
## depression          -24.402     16.121  -1.514   0.1301
## depression:sleep_z   -3.272     11.762  -0.278   0.7809
## ---
## Signif. codes:  0 ‘***’ 0.001 ‘**’ 0.01 ‘*’ 0.05 ‘.’ 0.1 ‘ ’ 1
##
## Approximate significance of smooth terms:
##              edf Ref.df       F p-value
## s(age_z)   6.696  6.696 834.004  <2e-16 ***
## s(sleep_z) 1.000  1.000   3.448  0.0633 .
## ---
## Signif. codes:  0 ‘***’ 0.001 ‘**’ 0.01 ‘*’ 0.05 ‘.’ 0.1 ‘ ’ 1
##
## R-sq.(adj) =  0.448
## lmer.REML = 4.7491e+05  Scale est. = 10882     n = 33353
```

The plot below shows the sleep-volume curve for the original model and for the model with main effects of SES.

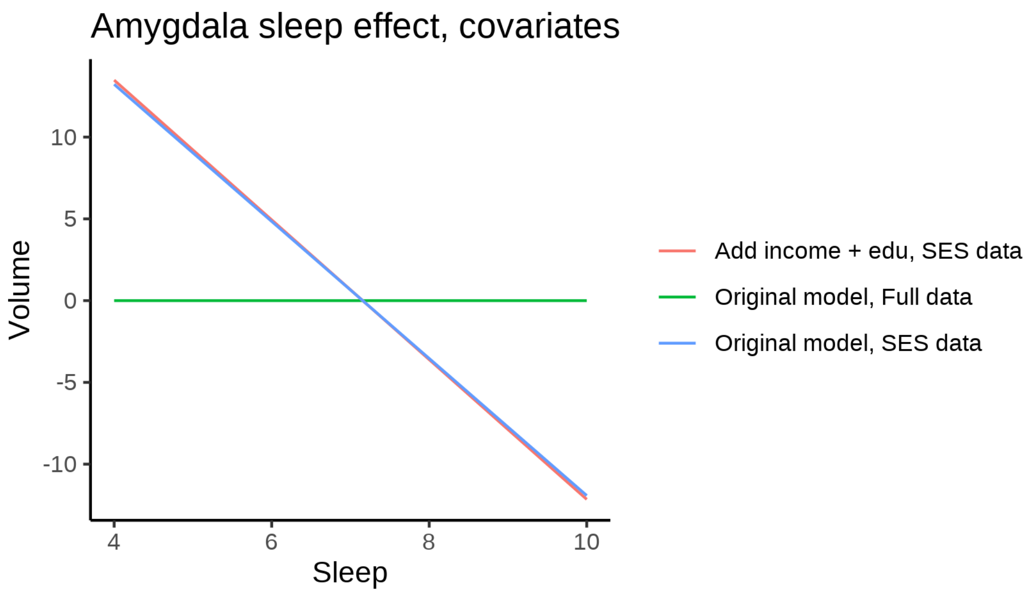

The plot below shows the sleep-volume curve for the original model and for the model with main effects of BMI.

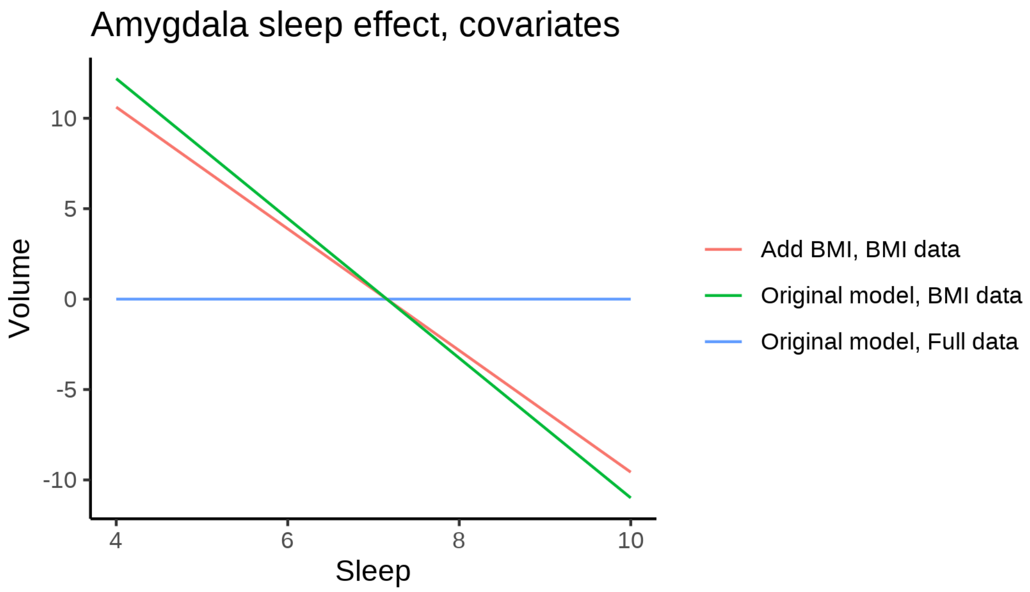

The plot below shows the sleep-volume curve for the original model and for the model with main effects of depression.

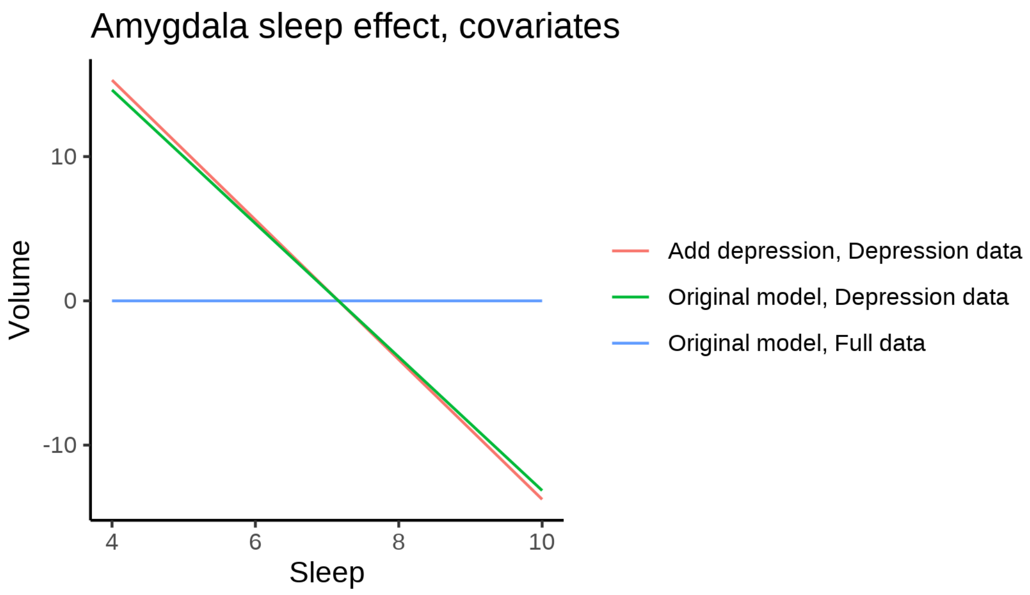

Models Not controling for ICV

Model outputs

Model without sleep term

```
##
## Family: gaussian
## Link function: identity
##
## Formula:
## value ~ sex + site + s(age_z, k = 10, bs = "cr")
## <environment: 0x558adb36df98>
##
## Parametric coefficients:
##                Estimate Std. Error t value Pr(>|t|)
## (Intercept)    2922.189     19.996 146.140  < 2e-16 ***
## sexmale         377.390      3.356 112.436  < 2e-16 ***
## siteMPIB         16.879     25.324   0.667  0.50507
## siteousAvanto   -11.356     20.740  -0.548  0.58401
## siteousPrisma   225.766     35.123   6.428 1.30e-10 ***
## siteousSkyra    522.979     19.454  26.883  < 2e-16 ***
## siteUB           21.173     59.737   0.354  0.72301
## siteUCAM        113.525     22.141   5.127 2.95e-07 ***
## siteUKB         196.236     20.543   9.552  < 2e-16 ***
## siteUmU          79.507     28.615   2.779  0.00546 **
## siteUOXF         -1.751     24.312  -0.072  0.94259
## ---
## Signif. codes:  0 '***' 0.001 '**' 0.01 '*' 0.05 '.' 0.1 ' ' 1
##
## Approximate significance of smooth terms:
##            edf Ref.df    F p-value
## s(age_z) 6.795  6.795 1373  <2e-16 ***
```

```
## ---
## Signif. codes:  0 ‘***’ 0.001 ‘**’ 0.01 ‘*’ 0.05 ‘.’ 0.1 ‘ ’ 1
##
## R-sq.(adj) =  0.318
## lmer.REML = 7.4196e+05  Scale est. = 11821     n = 51283
```

Model with only main effects of age and sleep

```
##
## Family: gaussian
## Link function: identity
##
## Formula:
## value ~ sex + site + s(age_z, k = 10, bs = "cr") + s(sleep_z,
##     k = 5, bs = "cr")
## <environment: 0x558adb36df98>
##
## Parametric coefficients:
##               Estimate Std. Error t value Pr(>|t|)
## (Intercept)   2924.680     20.015 146.125  < 2e-16 ***
## sexmale        376.926      3.358 112.252  < 2e-16 ***
## siteMPIB        16.319     25.334   0.644  0.51947
## siteousAvanto  -13.174     20.744  -0.635  0.52539
## siteousPrisma  224.457     35.119   6.391 1.66e-10 ***
## siteousSkyra   521.067     19.459  26.778  < 2e-16 ***
## siteUB          22.213     59.726   0.372  0.70995
## siteUCAM       113.249     22.141   5.115 3.15e-07 ***
## siteUKB        193.840     20.562   9.427  < 2e-16 ***
## siteUmU         77.714     28.676   2.710  0.00673 **
## siteUOXF        -2.816     24.310  -0.116  0.90778
## ---
## Signif. codes:  0 ‘***’ 0.001 ‘**’ 0.01 ‘*’ 0.05 ‘.’ 0.1 ‘ ’ 1
##
## Approximate significance of smooth terms:
##              edf Ref.df       F  p-value
## s(age_z)   6.784  6.784 1361.89  < 2e-16 ***
## s(sleep_z) 2.945  2.945    5.76 0.000779 ***
## ---
## Signif. codes:  0 ‘***’ 0.001 ‘**’ 0.01 ‘*’ 0.05 ‘.’ 0.1 ‘ ’ 1
##
## R-sq.(adj) =  0.318
## lmer.REML = 7.4195e+05  Scale est. = 11821     n = 51283
```

Model with full interaction between age and sleep

```
##
## Family: gaussian
## Link function: identity
##
## Formula:
## value ~ sex + site + t2(age_z, sleep_z, k = c(10, 4), bs = "cr")
## <environment: 0x558adb36df98>
##
## Parametric coefficients:
##               Estimate Std. Error t value Pr(>|t|)
## (Intercept)   2926.587     20.045 146.003  < 2e-16 ***
```

```
## sexmale        377.422      3.365 112.171  < 2e-16 ***
## siteMPIB        11.945     25.438   0.470  0.63867
## siteousAvanto  -14.814     20.836  -0.711  0.47711
## siteousPrisma  221.806     35.190   6.303 2.94e-10 ***
## siteousSkyra   519.150     19.564  26.536  < 2e-16 ***
## siteUB          17.479     59.747   0.293  0.76987
## siteUCAM       109.066     22.235   4.905 9.36e-07 ***
## siteUKB        191.738     20.587   9.313  < 2e-16 ***
## siteUmU         74.459     28.735   2.591  0.00957 **
## siteUOXF        -6.238     24.358  -0.256  0.79789
## ---
## Signif. codes:  0 '***' 0.001 '**' 0.01 '*' 0.05 '.' 0.1 ' ' 1
##
## Approximate significance of smooth terms:
##                     edf Ref.df     F p-value
## t2(age_z,sleep_z) 11.71  11.71 45.58  <2e-16 ***
## ---
## Signif. codes:  0 '***' 0.001 '**' 0.01 '*' 0.05 '.' 0.1 ' ' 1
##
## R-sq.(adj) =  0.318
## lmer.REML = 7.4195e+05  Scale est. = 11821     n = 51283
```

Model comparison

`mod_no_sleep` refers to model without sleep term, `mod_no_interaction` refers to model with only main effect of sleep, and `mod_full` refers to model with a full interaction between age and sleep. This is a nested model comparison, and the p-value at a given line refers to comparing the model at the line to the model on the line above. Hence, significance implies that the more complicated model is supported on statistical grounds.

To be even more specific, the p-value on the second row tests whether there is an association between sleep and volume. The p-value on the third row tests whether this association depends on age.

```
## Data: NULL
## Models:
## ml$mod_no_sleep$mer: NULL
## ml$mod_no_interaction$mer: NULL
## ml$mod_full$mer: NULL
##                           npar    AIC    BIC  logLik deviance   Chisq Df Pr(>Chisq)
## ml$mod_no_sleep$mer         15 741990 742123 -370980   741960
## ml$mod_no_interaction$mer   17 741982 742133 -370974   741948 11.6152  2   0.003005 **
## ml$mod_full$mer             19 741985 742153 -370974   741947  0.9682  2   0.616263
## ---
## Signif. codes:  0 '***' 0.001 '**' 0.01 '*' 0.05 '.' 0.1 ' ' 1
```

We chose the model based on the likelihood ratio test with 5 % significance level, which was `mod_no_interaction`.

Lifespan brain trajectory

The trajectory shown is from the chosen model `mod_no_interaction_no_icv`.

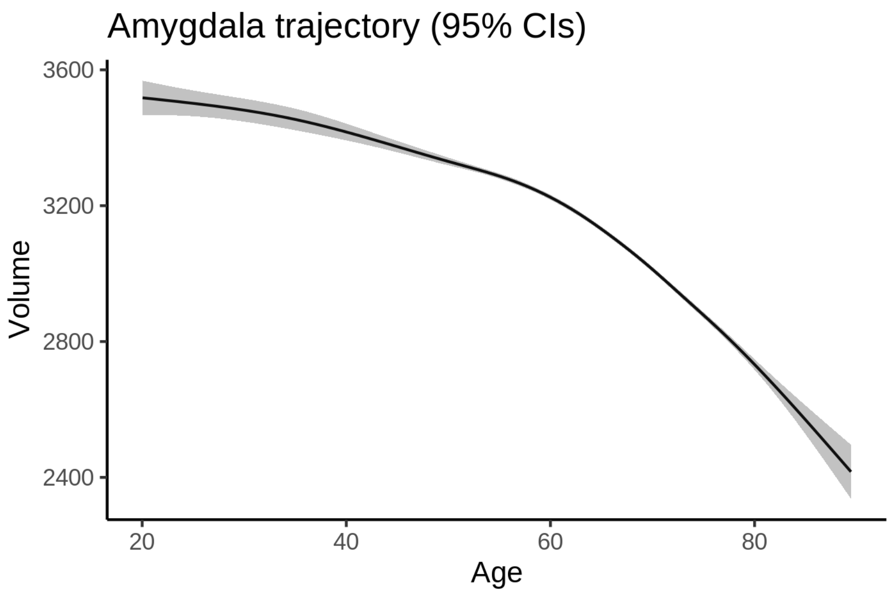

Effect of sleep

The chosen model only included the main effect of sleep, and hence the effect does not vary with age. The black dot shows the average sleep duration across all ages in the sample.

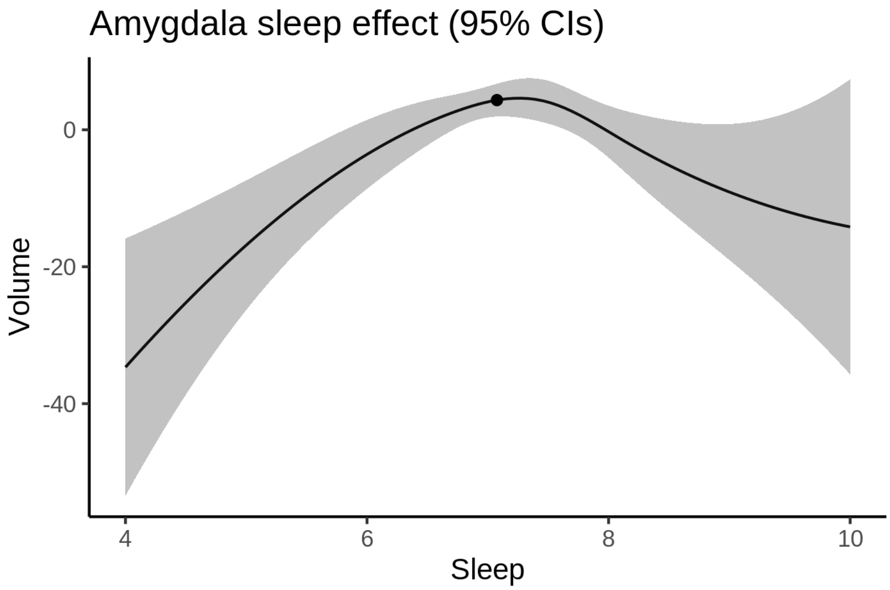

We also show the full interaction model for completeness, although it was not selected.

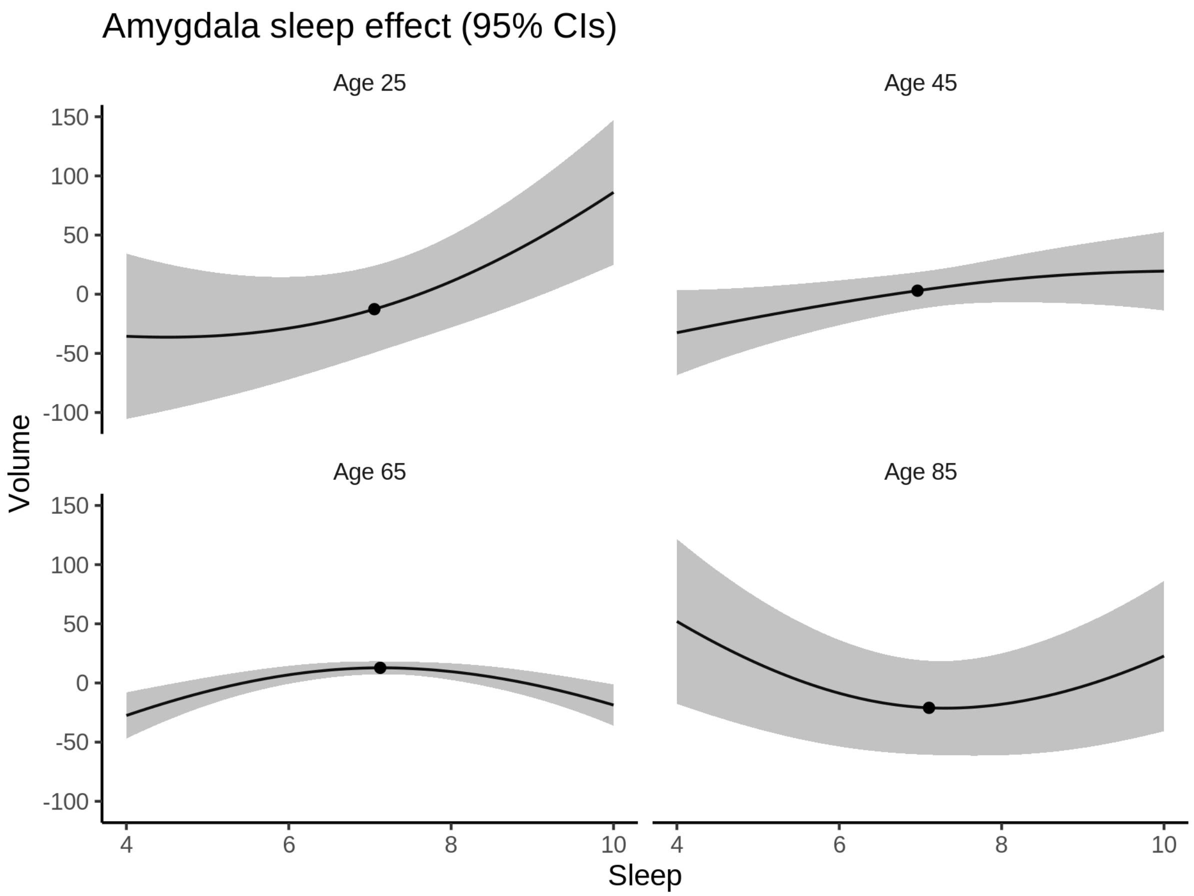

Deviation from sleep associated with maximal volume

Model with only main effect of sleep was chosen, so we show it for all ages at once. Maximum volume is attained at 7.3 hours of sleep. The percentage values in the plot are calculated as follows: The maximum at 100 % refers to a person at an arbitrary age with a sleep duration associated with maximum volume. For a female, this volume is 3166 and for a male it is 3543. The other percentage values show how large the expected volume is for someone with other sleep durations. For example, 99 % implies a 1 % reduction.

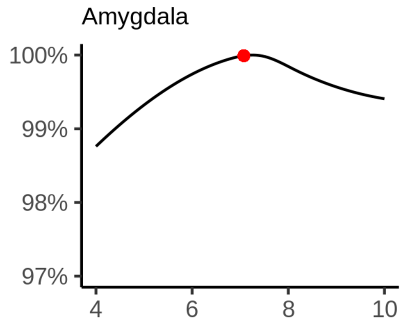

Comparison of mean sleep and sleep associated with maximum volume

A 95 % confidence interval for the sleep associated with maximum volume is [6.85, 10].

The plot below compares average sleep to the sleep associated with maximum volume.

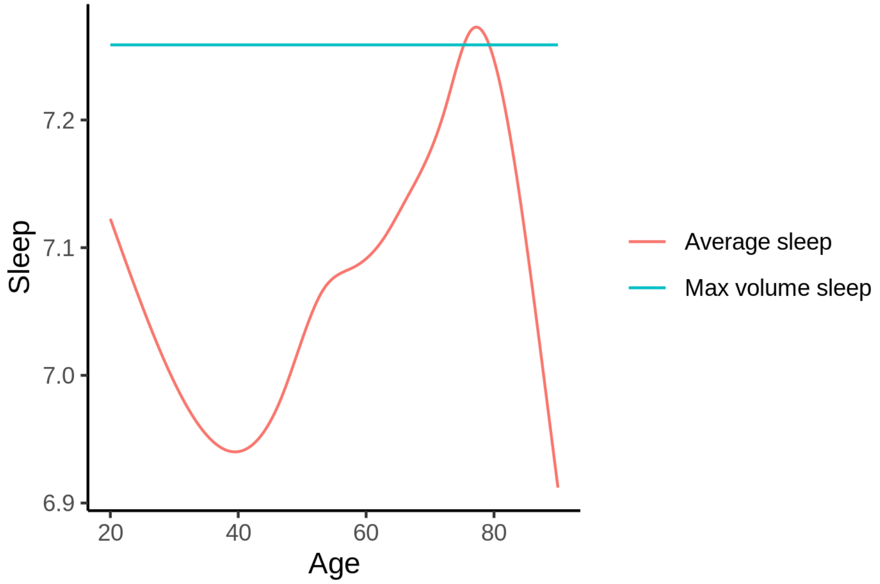

The next plot shows the difference between average sleep and sleep associated with maximum volume. The shaded region is a 95 % confidence interval.

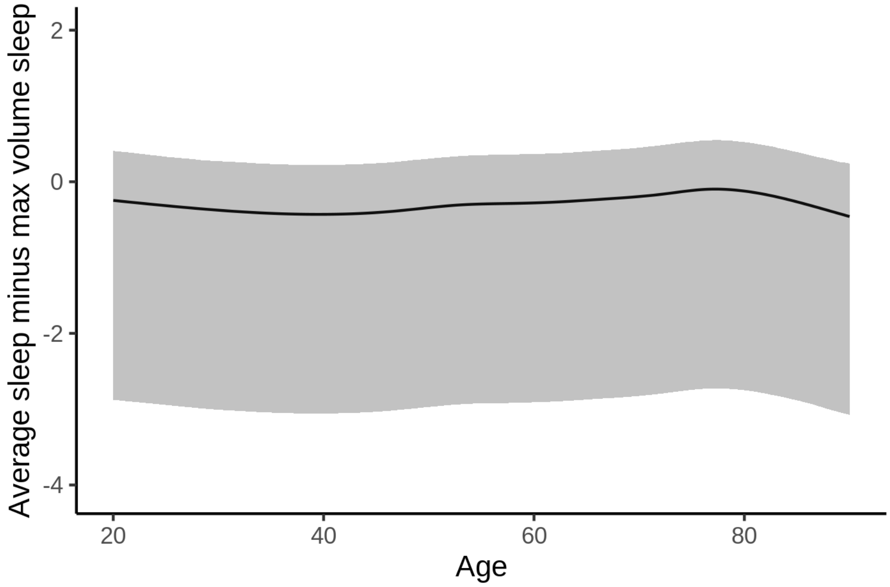

The next plot shows the probability that the sleep duration associated with maximum volume is longer than the average sleep duration, as a function of age. Probability below .05 can be interpreted as evidence that the sleep associated with maximum volume is shorter than the mean sleep, and probability above .95 can be interpreted the opposite way.

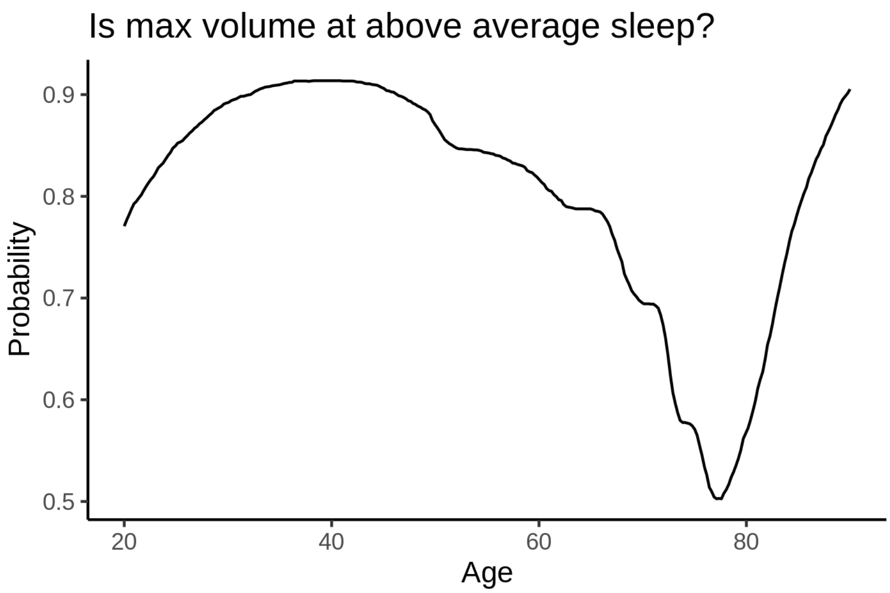

Brain-Stem

Descriptive statistics

Study	Observations	Unique IDs	Mean age	Age range
HCP	974	974	28.8	22 - 37
MPIB	677	391	63.1	24 - 83
UB	113	39	70.9	64 - 81
UCAM	884	632	55.1	20 - 88
UiO	1474	803	49.3	20 - 88
UKB	45963	43127	64.5	45 - 83
UmU	412	279	62.2	25 - 85
UOXF	769	769	69.8	60 - 85

Spaghetti plot

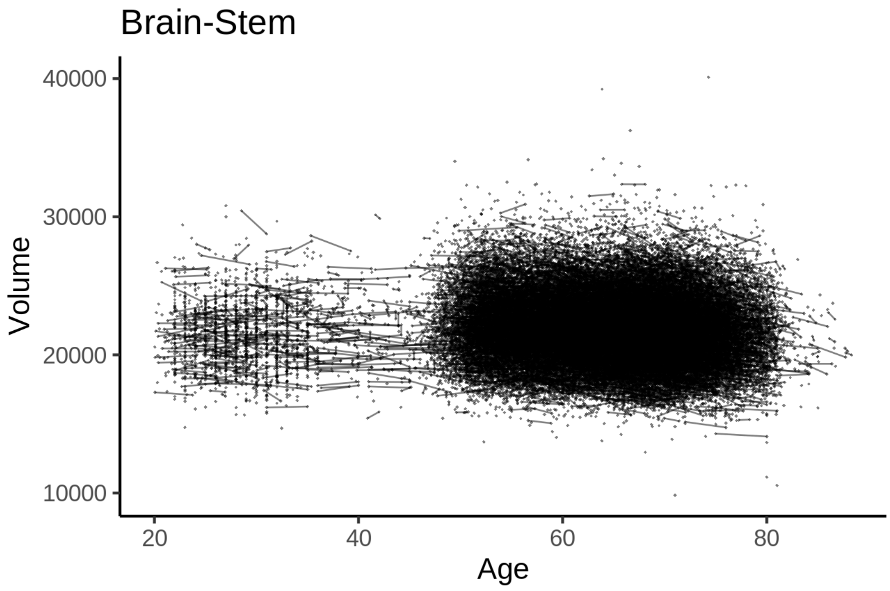

Models Controling for ICV

Model outputs

Model without sleep term

```
##
## Family: gaussian
## Link function: identity
##
## Formula:
## value ~ sex + site + icv + s(age_z, k = 10, bs = "cr")
## <environment: 0x5589630b6ab8>
##
## Parametric coefficients:
##                Estimate Std. Error t value Pr(>|t|)
## (Intercept)   20854.571     93.022 224.191  < 2e-16 ***
## sexmale         797.839     20.273  39.355  < 2e-16 ***
## siteMPIB       2478.732    124.147  19.966  < 2e-16 ***
## siteousAvanto    -8.274     96.082  -0.086  0.93138
## siteousPrisma   255.398    158.133   1.615  0.10630
## siteousSkyra    669.272     94.265   7.100 1.26e-12 ***
## siteUB         -295.703    299.758  -0.986  0.32391
## siteUCAM       -118.871    106.878  -1.112  0.26605
## siteUKB         747.541     95.171   7.855 4.09e-15 ***
## siteUmU       -1560.356    138.989 -11.226  < 2e-16 ***
## siteUOXF        314.785    115.166   2.733  0.00627 **
## icv            1481.073     10.144 146.008  < 2e-16 ***
## ---
## Signif. codes:  0 ‘***’ 0.001 ‘**’ 0.01 ‘*’ 0.05 ‘.’ 0.1 ‘ ’ 1
##
```

```
## Approximate significance of smooth terms:
##            edf Ref.df     F p-value
## s(age_z) 6.779  6.779 129.5  <2e-16 ***
## ---
## Signif. codes:  0 ‘***’ 0.001 ‘**’ 0.01 ‘*’ 0.05 ‘.’ 0.1 ‘ ’ 1
##
## R-sq.(adj) =  0.486
## lmer.REML = 9.0169e+05  Scale est. = 1.0143e+05  n = 51266
```

Model with only main effects of age and sleep

```
##
## Family: gaussian
## Link function: identity
##
## Formula:
## value ~ sex + site + icv + s(age_z, k = 10, bs = "cr") + s(sleep_z,
##     k = 5, bs = "cr")
## <environment: 0x5589630b6ab8>
##
## Parametric coefficients:
##                Estimate Std. Error t value Pr(>|t|)
## (Intercept)    20846.75      93.09 223.946  < 2e-16 ***
## sexmale          797.25      20.27  39.339  < 2e-16 ***
## siteMPIB        2496.24     124.17  20.103  < 2e-16 ***
## siteousAvanto     -5.36      96.08  -0.056   0.9555
## siteousPrisma    257.07     158.10   1.626   0.1040
## siteousSkyra     670.88      94.27   7.117 1.12e-12 ***
## siteUB          -284.42     299.63  -0.949   0.3425
## siteUCAM        -109.80     106.85  -1.028   0.3041
## siteUKB          755.87      95.25   7.936 2.13e-15 ***
## siteUmU        -1518.94     139.25 -10.908  < 2e-16 ***
## siteUOXF         318.32     115.14   2.765   0.0057 **
## icv             1480.70      10.15 145.865  < 2e-16 ***
## ---
## Signif. codes:  0 ‘***’ 0.001 ‘**’ 0.01 ‘*’ 0.05 ‘.’ 0.1 ‘ ’ 1
##
## Approximate significance of smooth terms:
##              edf Ref.df       F  p-value
## s(age_z)   6.774  6.774 124.362  < 2e-16 ***
## s(sleep_z) 3.579  3.579   9.923 1.64e-07 ***
## ---
## Signif. codes:  0 ‘***’ 0.001 ‘**’ 0.01 ‘*’ 0.05 ‘.’ 0.1 ‘ ’ 1
##
## R-sq.(adj) =  0.487
## lmer.REML = 9.0166e+05  Scale est. = 1.015e+05  n = 51266
```

Model with full interaction between age and sleep

```
##
## Family: gaussian
## Link function: identity
##
## Formula:
## value ~ sex + site + icv + t2(age_z, sleep_z, k = c(10, 4), bs = "cr")
## <environment: 0x5589630b6ab8>
```

```
##
## Parametric coefficients:
##                Estimate Std. Error t value Pr(>|t|)
## (Intercept)   20822.86      92.63 224.797  < 2e-16 ***
## sexmale         796.46      20.31  39.222  < 2e-16 ***
## siteMPIB       2517.89     124.32  20.253  < 2e-16 ***
## siteousAvanto    15.23      96.37   0.158  0.87446
## siteousPrisma   276.12     158.42   1.743  0.08135 .
## siteousSkyra    694.07      94.57   7.339 2.18e-13 ***
## siteUB         -270.91     299.56  -0.904  0.36582
## siteUCAM        -91.77     106.94  -0.858  0.39084
## siteUKB         780.82      94.73   8.242  < 2e-16 ***
## siteUmU       -1501.23     139.13 -10.790  < 2e-16 ***
## siteUOXF        340.22     114.89   2.961  0.00307 **
## icv            1481.79      10.15 146.036  < 2e-16 ***
## ---
## Signif. codes:  0 ‘***’ 0.001 ‘**’ 0.01 ‘*’ 0.05 ‘.’ 0.1 ‘ ’ 1
##
## Approximate significance of smooth terms:
##                       edf Ref.df     F p-value
## t2(age_z,sleep_z) 10.97  10.97 14.77  <2e-16 ***
## ---
## Signif. codes:  0 ‘***’ 0.001 ‘**’ 0.01 ‘*’ 0.05 ‘.’ 0.1 ‘ ’ 1
##
## R-sq.(adj) =  0.487
## lmer.REML = 9.0167e+05  Scale est. = 1.0149e+05  n = 51266
```

Model comparison

`mod_no_sleep` refers to model without sleep term, `mod_no_interaction` refers to model with only main effect of sleep, and `mod_full` refers to model with a full interaction between age and sleep. This is a nested model comparison, and the p-value at a given line refers to comparing the model at the line to the model on the line above. Hence, significance implies that the more complicated model is supported on statistical grounds.

To be even more specific, the p-value on the second row tests whether there is an association between sleep and volume. The p-value on the third row tests whether this association depends on age.

```
## Data: NULL
## Models:
## ml$mod_no_sleep$mer: NULL
## ml$mod_no_interaction$mer: NULL
## ml$mod_full$mer: NULL
##                           npar    AIC    BIC  logLik deviance  Chisq Df Pr(>Chisq)
## ml$mod_no_sleep$mer         16 901725 901866 -450846   901693
## ml$mod_no_interaction$mer   18 901699 901858 -450831   901663 29.727  2  3.506e-07 ***
## ml$mod_full$mer             20 901707 901884 -450833   901667  0.000  2          1
## ---
## Signif. codes:  0 ‘***’ 0.001 ‘**’ 0.01 ‘*’ 0.05 ‘.’ 0.1 ‘ ’ 1
```

We chose the model based on the likelihood ratio test with 5 % significance level, which was `mod_no_interaction`.

Lifespan brain trajectory

The trajectory shown is from the chosen model `mod_no_interaction`.

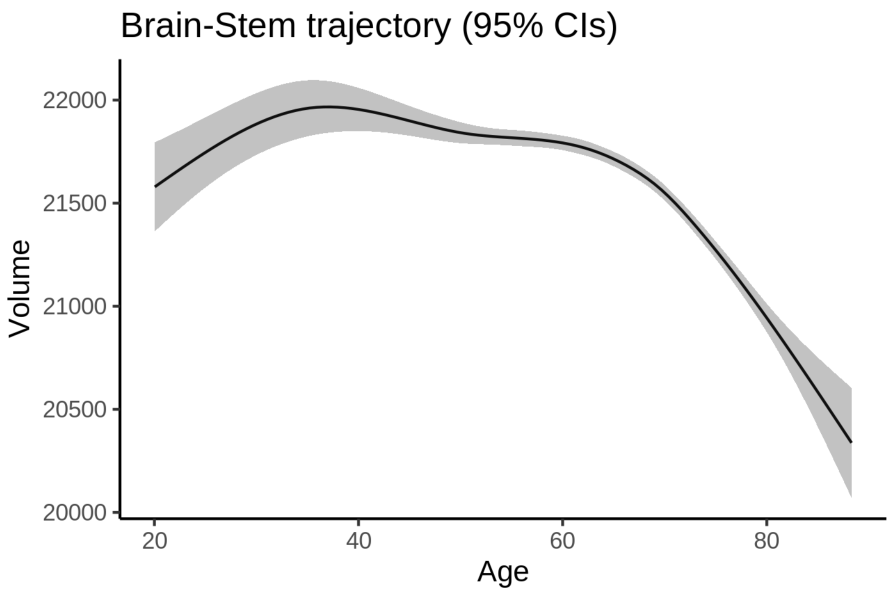

Effect of sleep

The chosen model only included the main effect of sleep, and hence the effect does not vary with age. The black dot shows the average sleep duration across all ages in the sample.

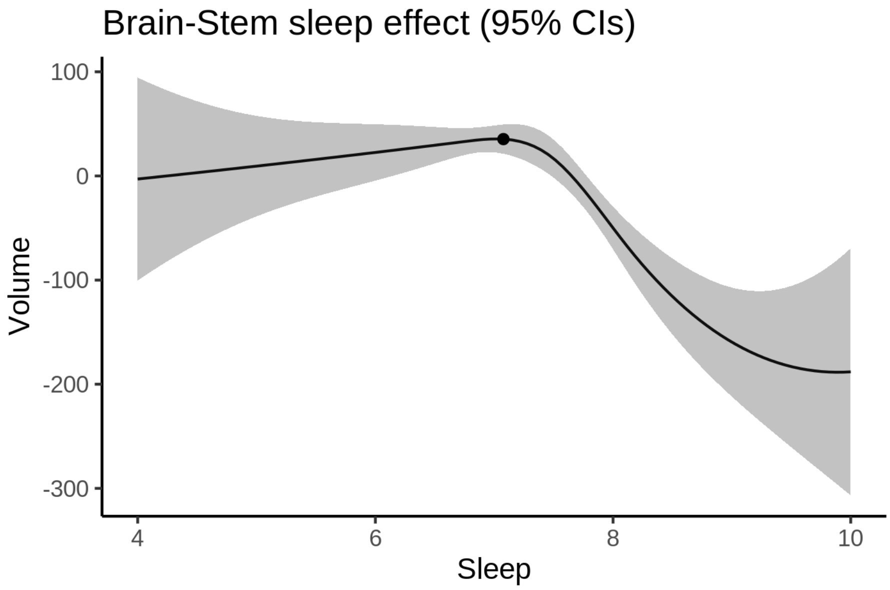

We also show the full interaction model for completeness, although it was not selected.

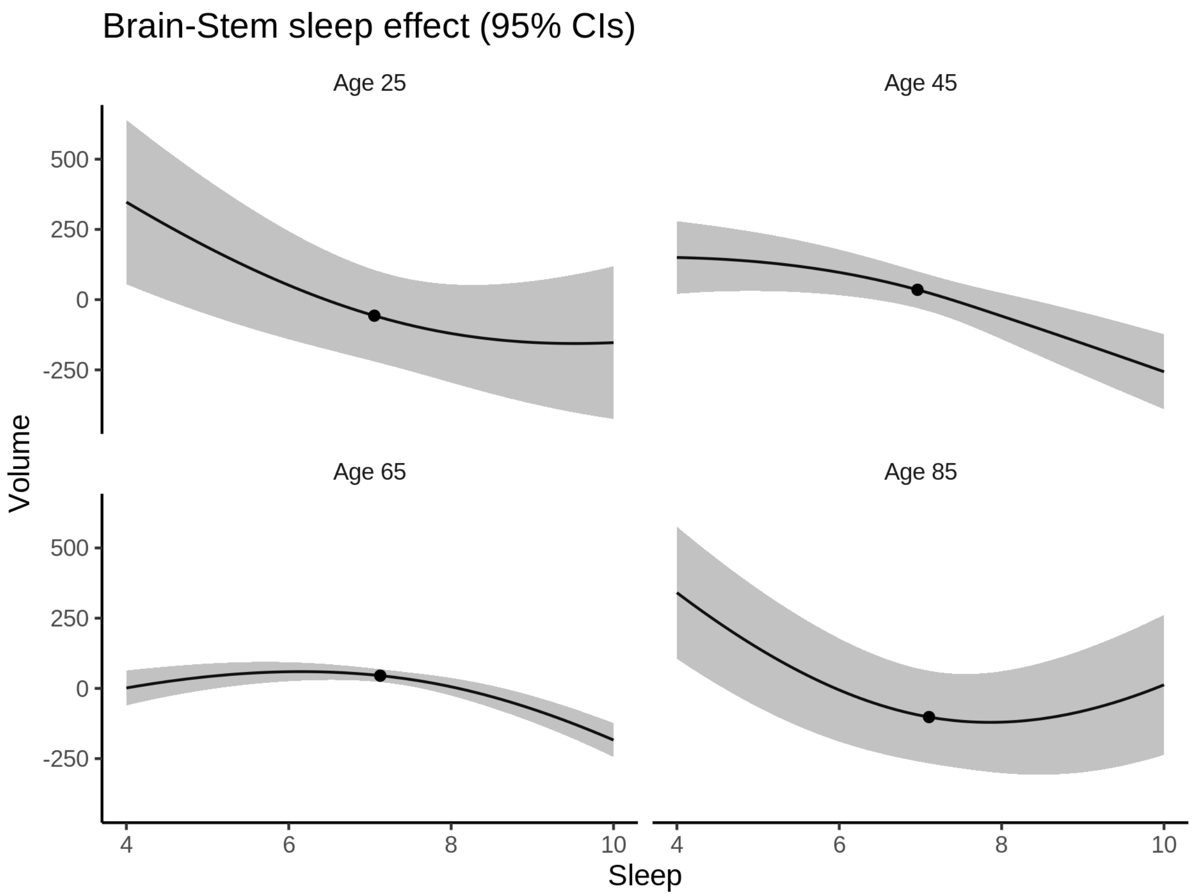

Deviation from sleep associated with maximal volume

Model with only main effect of sleep was chosen, so we show it for all ages at once. Maximum volume is attained at 7 hours of sleep. The percentage values in the plot are calculated as follows: The maximum at 100 % refers to a person at an arbitrary age with a sleep duration associated with maximum volume. For a female, this volume is 21555 and for a male it is 22352. The other percentage values show how large the expected volume is for someone with other sleep durations. For example, 99 % implies a 1 % reduction.

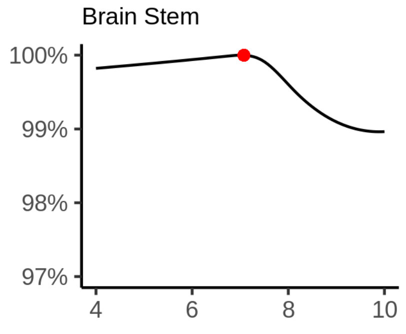

Comparison of mean sleep and sleep associated with maximum volume

A 95 % confidence interval for the sleep associated with maximum volume is [4, 7.21].

The plot below compares average sleep to the sleep associated with maximum volume.

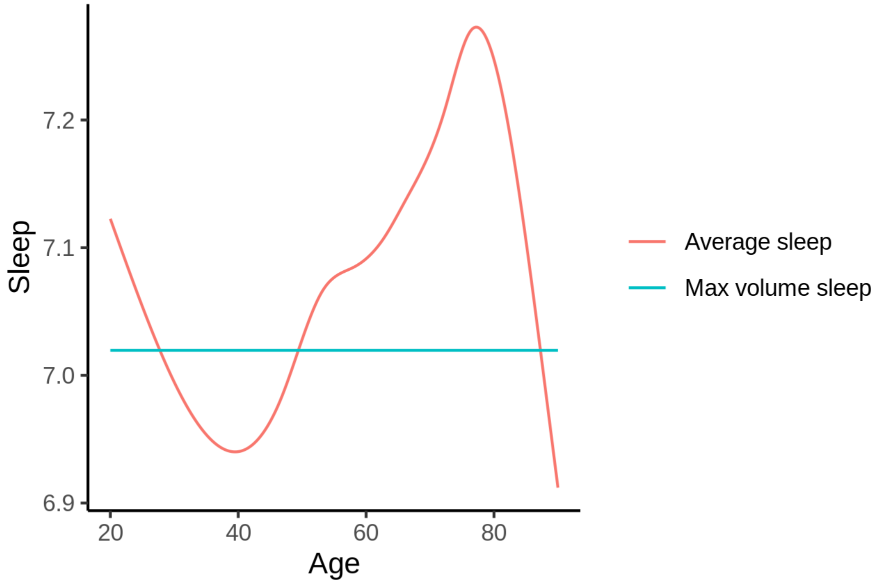

The next plot shows the difference between average sleep and sleep associated with maximum volume. The shaded region is a 95 % confidence interval.

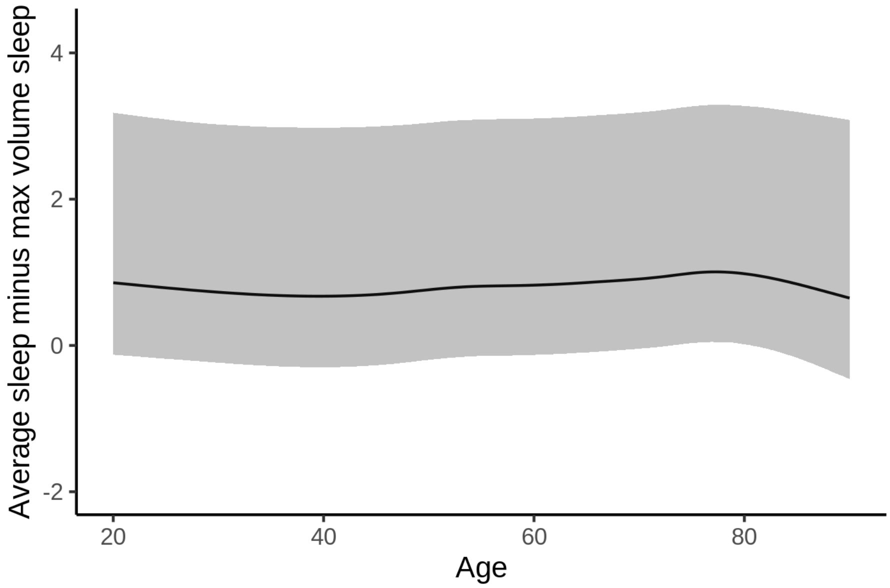

The next plot shows the probability that the sleep duration associated with maximum volume is longer than the average sleep duration, as a function of age. Probability below .05 can be interpreted as evidence that the sleep associated with maximum volume is shorter than the mean sleep, and probability above .95 can be interpreted the opposite way.

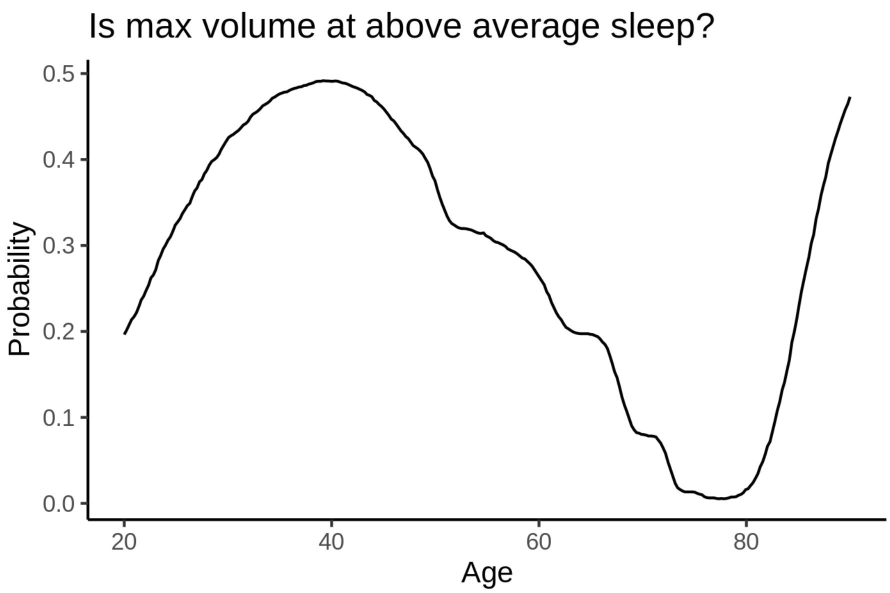

Controling for covariates

Below is the output for a model in which we only include data with income and education.

```
##
## Family: gaussian
## Link function: identity
##
## Formula:
## value ~ sex + site + s(age_z, k = 10, bs = "cr") + s(sleep_z,
##     k = 5, bs = "cr") + icv
## <environment: 0x55895b00c6b0>
##
## Parametric coefficients:
##                Estimate Std. Error t value Pr(>|t|)
## (Intercept)    23651.71     235.72 100.339  < 2e-16 ***
## sexmale          745.12      25.82  28.856  < 2e-16 ***
## siteousAvanto -3029.84     268.46 -11.286  < 2e-16 ***
## siteousPrisma -2324.03     284.19  -8.178 3.00e-16 ***
## siteousSkyra  -2235.92     254.91  -8.772  < 2e-16 ***
## siteUKB       -2024.86     235.21  -8.609  < 2e-16 ***
## siteUOXF      -2140.69     264.66  -8.088 6.26e-16 ***
## icv            1557.91      13.21 117.978  < 2e-16 ***
## ---
## Signif. codes:  0 ‘***’ 0.001 ‘**’ 0.01 ‘*’ 0.05 ‘.’ 0.1 ‘ ’ 1
##
## Approximate significance of smooth terms:
##              edf Ref.df      F  p-value
## s(age_z)   6.340  6.340 54.431  < 2e-16 ***
## s(sleep_z) 3.126  3.126  8.593 7.44e-06 ***
## ---
## Signif. codes:  0 ‘***’ 0.001 ‘**’ 0.01 ‘*’ 0.05 ‘.’ 0.1 ‘ ’ 1
##
```

```
## R-sq.(adj) =  0.489
## lmer.REML = 5.4865e+05  Scale est. = 1.0298e+05  n = 31180
```

Below is the output for a model in which we control for the main effects of income and education.

```
##
## Family: gaussian
## Link function: identity
##
## Formula:
## value ~ sex + site + s(age_z, k = 10, bs = "cr") + s(sleep_z,
##     k = 5, bs = "cr") + icv + income_scaled + education_scaled
## <environment: 0x55895b00c6b0>
##
## Parametric coefficients:
##                  Estimate Std. Error t value Pr(>|t|)
## (Intercept)      23423.16     236.97  98.845  < 2e-16 ***
## sexmale            745.41      25.86  28.823  < 2e-16 ***
## siteousAvanto    -3025.99     268.24 -11.281  < 2e-16 ***
## siteousPrisma    -2339.71     283.89  -8.242  < 2e-16 ***
## siteousSkyra     -2237.19     254.66  -8.785  < 2e-16 ***
## siteUKB          -2025.90     235.14  -8.616  < 2e-16 ***
## siteUOXF         -2069.35     264.54  -7.822 5.35e-15 ***
## icv               1543.44      13.30 116.082  < 2e-16 ***
## income_scaled      154.01      30.72   5.014 5.36e-07 ***
## education_scaled   200.91      36.41   5.518 3.46e-08 ***
## ---
## Signif. codes:  0 '***' 0.001 '**' 0.01 '*' 0.05 '.' 0.1 ' ' 1
##
## Approximate significance of smooth terms:
##              edf Ref.df      F  p-value
## s(age_z)   6.485  6.485 43.182  < 2e-16 ***
## s(sleep_z) 2.866  2.866  7.319 0.000102 ***
## ---
## Signif. codes:  0 '***' 0.001 '**' 0.01 '*' 0.05 '.' 0.1 ' ' 1
##
## R-sq.(adj) =   0.49
## lmer.REML = 5.4856e+05  Scale est. = 1.0308e+05  n = 31180
```

We also included interaction effects between sleep duration and education and income, in another model. The output is shown below, and the interaction terms are `income_scaled:sleep_z` and `education_scaled:sleep_z`.

```
##
## Family: gaussian
## Link function: identity
##
## Formula:
## value ~ sex + site + s(age_z, k = 10, bs = "cr") + s(sleep_z,
##     k = 5, bs = "cr") + icv + income_scaled + education_scaled +
##     income_scaled:sleep_z + education_scaled:sleep_z
## <environment: 0x55895b00c6b0>
##
## Parametric coefficients:
##                          Estimate Std. Error t value Pr(>|t|)
## (Intercept)              23425.11     236.98  98.848  < 2e-16 ***
```

```
## sexmale                     743.13      25.88  28.717  < 2e-16 ***
## siteousAvanto            -3030.57     268.24 -11.298  < 2e-16 ***
## siteousPrisma            -2348.82     283.90  -8.273  < 2e-16 ***
## siteousSkyra             -2241.56     254.65  -8.802  < 2e-16 ***
## siteUKB                  -2028.05     235.16  -8.624  < 2e-16 ***
## siteUOXF                 -2070.95     264.61  -7.826 5.18e-15 ***
## icv                       1543.75      13.30 116.102  < 2e-16 ***
## income_scaled              156.23      30.73   5.084 3.72e-07 ***
## education_scaled           199.45      36.42   5.477 4.36e-08 ***
## income_scaled:sleep_z      -62.14      30.71  -2.024   0.0430 *
## education_scaled:sleep_z    60.43      35.34   1.710   0.0873 .
## ---
## Signif. codes:  0 ‘***’ 0.001 ‘**’ 0.01 ‘*’ 0.05 ‘.’ 0.1 ‘ ’ 1
##
## Approximate significance of smooth terms:
##              edf Ref.df      F p-value
## s(age_z)   6.498  6.498 42.990 < 2e-16 ***
## s(sleep_z) 2.850  2.850  4.669 0.00385 **
## ---
## Signif. codes:  0 ‘***’ 0.001 ‘**’ 0.01 ‘*’ 0.05 ‘.’ 0.1 ‘ ’ 1
##
## R-sq.(adj) =   0.49
## lmer.REML = 5.4853e+05  Scale est. = 1.0307e+05  n = 31180
```

We did the same controling for BMI. Below is the model with no covariates but only keeping data with BMI.

```
##
## Family: gaussian
## Link function: identity
##
## Formula:
## value ~ sex + site + s(age_z, k = 10, bs = "cr") + s(sleep_z,
##     k = 5, bs = "cr") + icv
## <environment: 0x558ad6ffe670>
##
## Parametric coefficients:
##               Estimate Std. Error t value Pr(>|t|)
## (Intercept)   20970.70     129.46 161.986  < 2e-16 ***
## sexmale         732.00      25.06  29.215  < 2e-16 ***
## siteousPrisma   259.72     171.31   1.516     0.13
## siteousSkyra    759.64      81.02   9.375  < 2e-16 ***
## siteUCAM       -152.42     140.96  -1.081     0.28
## siteUKB         658.14     130.96   5.025 5.05e-07 ***
## siteUmU       -1464.17     167.22  -8.756  < 2e-16 ***
## icv            1561.97      12.85 121.525  < 2e-16 ***
## ---
## Signif. codes:  0 ‘***’ 0.001 ‘**’ 0.01 ‘*’ 0.05 ‘.’ 0.1 ‘ ’ 1
##
## Approximate significance of smooth terms:
##              edf Ref.df      F p-value
## s(age_z)   6.261  6.261 69.073 < 2e-16 ***
## s(sleep_z) 2.762  2.762  8.828 3.7e-05 ***
## ---
## Signif. codes:  0 ‘***’ 0.001 ‘**’ 0.01 ‘*’ 0.05 ‘.’ 0.1 ‘ ’ 1
##
```

```
## R-sq.(adj) =  0.496
## lmer.REML = 5.8743e+05  Scale est. = 99310     n = 33424
```

Below is the model output with main effect.

```
##
## Family: gaussian
## Link function: identity
##
## Formula:
## value ~ sex + site + s(age_z, k = 10, bs = "cr") + s(sleep_z,
##     k = 5, bs = "cr") + icv + bmi
## <environment: 0x558ad6ffe670>
##
## Parametric coefficients:
##                 Estimate Std. Error t value Pr(>|t|)
## (Intercept)   21424.611    143.309 149.500  < 2e-16 ***
## sexmale         746.400     25.110  29.725  < 2e-16 ***
## siteousPrisma   271.762    171.247   1.587    0.113
## siteousSkyra    760.386     81.026   9.384  < 2e-16 ***
## siteUCAM       -142.537    140.892  -1.012    0.312
## siteUKB         673.668    130.931   5.145 2.69e-07 ***
## siteUmU       -1444.849    167.143  -8.644  < 2e-16 ***
## icv            1564.756     12.847 121.798  < 2e-16 ***
## bmi             -18.054      2.447  -7.377 1.66e-13 ***
## ---
## Signif. codes:  0 ‘***’ 0.001 ‘**’ 0.01 ‘*’ 0.05 ‘.’ 0.1 ‘ ’ 1
##
## Approximate significance of smooth terms:
##              edf Ref.df      F  p-value
## s(age_z)   6.281  6.281 69.935  < 2e-16 ***
## s(sleep_z) 2.613  2.613  9.489 3.26e-05 ***
## ---
## Signif. codes:  0 ‘***’ 0.001 ‘**’ 0.01 ‘*’ 0.05 ‘.’ 0.1 ‘ ’ 1
##
## R-sq.(adj) =  0.496
## lmer.REML = 5.8737e+05  Scale est. = 99316     n = 33424
```

Next is the model with BMI-sleep interaction.

```
##
## Family: gaussian
## Link function: identity
##
## Formula:
## value ~ sex + site + s(age_z, k = 10, bs = "cr") + s(sleep_z,
##     k = 5, bs = "cr") + icv + bmi + bmi:sleep_z
## <environment: 0x558ad6ffe670>
##
## Parametric coefficients:
##                 Estimate Std. Error t value Pr(>|t|)
## (Intercept)   21420.428    143.392 149.383  < 2e-16 ***
## sexmale         746.477     25.110  29.728  < 2e-16 ***
## siteousPrisma   271.737    171.246   1.587    0.113
## siteousSkyra    760.655     81.025   9.388  < 2e-16 ***
## siteUCAM       -141.206    140.900  -1.002    0.316
```

```
## siteUKB         675.112    130.937   5.156 2.54e-07 ***
## siteUmU       -1443.701    167.147  -8.637  < 2e-16 ***
## icv           1564.859     12.847 121.803  < 2e-16 ***
## bmi            -17.987      2.449  -7.345 2.10e-13 ***
## bmi:sleep_z      1.965      2.322   0.846    0.397
## ---
## Signif. codes:  0 '***' 0.001 '**' 0.01 '*' 0.05 '.' 0.1 ' ' 1
##
## Approximate significance of smooth terms:
##              edf Ref.df      F p-value
## s(age_z)   6.277  6.277 70.050  <2e-16 ***
## s(sleep_z) 2.565  2.565  3.789  0.0273 *
## ---
## Signif. codes:  0 '***' 0.001 '**' 0.01 '*' 0.05 '.' 0.1 ' ' 1
##
## R-sq.(adj) =  0.496
## lmer.REML = 5.8737e+05  Scale est. = 99312     n = 33424
```

We did the same controling for depression. Below is the model with no covariates but only keeping data with depression.

```
##
## Family: gaussian
## Link function: identity
##
## Formula:
## value ~ sex + site + s(age_z, k = 10, bs = "cr") + s(sleep_z,
##     k = 5, bs = "cr") + icv
## <environment: 0x558ad8b83828>
##
## Parametric coefficients:
##               Estimate Std. Error t value Pr(>|t|)
## (Intercept)   23164.51     120.83 191.705  < 2e-16 ***
## sexmale         737.13      25.17  29.281  < 2e-16 ***
## siteousAvanto -2311.23     214.51 -10.774  < 2e-16 ***
## siteousPrisma -1768.20     218.91  -8.077 6.85e-16 ***
## siteousSkyra  -1338.79     176.52  -7.584 3.43e-14 ***
## siteUCAM      -2487.83     164.86 -15.091  < 2e-16 ***
## siteUKB       -1538.00     119.52 -12.868  < 2e-16 ***
## siteUmU       -3663.47     162.37 -22.562  < 2e-16 ***
## icv            1552.52      12.90 120.390  < 2e-16 ***
## ---
## Signif. codes:  0 '***' 0.001 '**' 0.01 '*' 0.05 '.' 0.1 ' ' 1
##
## Approximate significance of smooth terms:
##              edf Ref.df      F  p-value
## s(age_z)   6.483  6.483 71.750  < 2e-16 ***
## s(sleep_z) 2.708  2.708  8.008 6.92e-05 ***
## ---
## Signif. codes:  0 '***' 0.001 '**' 0.01 '*' 0.05 '.' 0.1 ' ' 1
##
## R-sq.(adj) =  0.491
## lmer.REML = 5.8558e+05  Scale est. = 98487     n = 33336
```

Below is the model output with main effect.

```
##
## Family: gaussian
## Link function: identity
##
## Formula:
## value ~ sex + site + s(age_z, k = 10, bs = "cr") + s(sleep_z,
##     k = 5, bs = "cr") + icv + depression
## <environment: 0x558ad8b83828>
##
## Parametric coefficients:
##                Estimate Std. Error t value Pr(>|t|)
## (Intercept)    23211.94     121.31 191.346  < 2e-16 ***
## sexmale          732.13      25.19  29.059  < 2e-16 ***
## siteousAvanto -2286.15     214.50 -10.658  < 2e-16 ***
## siteousPrisma -1744.78     218.89  -7.971 1.63e-15 ***
## siteousSkyra  -1310.90     176.56  -7.425 1.16e-13 ***
## siteUCAM      -2476.21     164.83 -15.023  < 2e-16 ***
## siteUKB       -1557.36     119.58 -13.024  < 2e-16 ***
## siteUmU       -3518.91     165.75 -21.230  < 2e-16 ***
## icv            1553.23      12.89 120.472  < 2e-16 ***
## depression     -389.37      90.16  -4.319 1.57e-05 ***
## ---
## Signif. codes:  0 '***' 0.001 '**' 0.01 '*' 0.05 '.' 0.1 ' ' 1
##
## Approximate significance of smooth terms:
##              edf Ref.df     F  p-value
## s(age_z)   6.459  6.459 74.26  < 2e-16 ***
## s(sleep_z) 2.546  2.546  8.15 7.71e-05 ***
## ---
## Signif. codes:  0 '***' 0.001 '**' 0.01 '*' 0.05 '.' 0.1 ' ' 1
##
## R-sq.(adj) =  0.491
## lmer.REML = 5.8556e+05  Scale est. = 98413     n = 33336
```

Next is the model with depression-sleep interaction.

```
##
## Family: gaussian
## Link function: identity
##
## Formula:
## value ~ sex + site + s(age_z, k = 10, bs = "cr") + s(sleep_z,
##     k = 5, bs = "cr") + icv + depression + depression:sleep_z
## <environment: 0x558ad8b83828>
##
## Parametric coefficients:
##                     Estimate Std. Error t value Pr(>|t|)
## (Intercept)         23211.92     121.31 191.343  < 2e-16 ***
## sexmale               732.27      25.20  29.060  < 2e-16 ***
## siteousAvanto       -2285.17     214.52 -10.652  < 2e-16 ***
## siteousPrisma       -1743.77     218.92  -7.965 1.70e-15 ***
## siteousSkyra        -1309.94     176.59  -7.418 1.22e-13 ***
## siteUCAM            -2475.15     164.87 -15.013  < 2e-16 ***
## siteUKB             -1557.38     119.58 -13.024  < 2e-16 ***
## siteUmU             -3526.03     167.18 -21.091  < 2e-16 ***
```

```
## icv                  1553.23      12.89 120.470  < 2e-16 ***
## depression           -387.68      90.32  -4.292 1.78e-05 ***
## depression:sleep_z    21.77      66.45   0.328    0.743
## ---
## Signif. codes:  0 '***' 0.001 '**' 0.01 '*' 0.05 '.' 0.1 ' ' 1
##
## Approximate significance of smooth terms:
##               edf Ref.df      F  p-value
## s(age_z)    6.461  6.461 74.252  < 2e-16 ***
## s(sleep_z) 2.509  2.509  8.024 0.000369 ***
## ---
## Signif. codes:  0 '***' 0.001 '**' 0.01 '*' 0.05 '.' 0.1 ' ' 1
##
## R-sq.(adj) =  0.491
## lmer.REML = 5.8555e+05  Scale est. = 98414     n = 33336
```

The plot below shows the sleep-volume curve for the original model and for the model with main effects of SES.

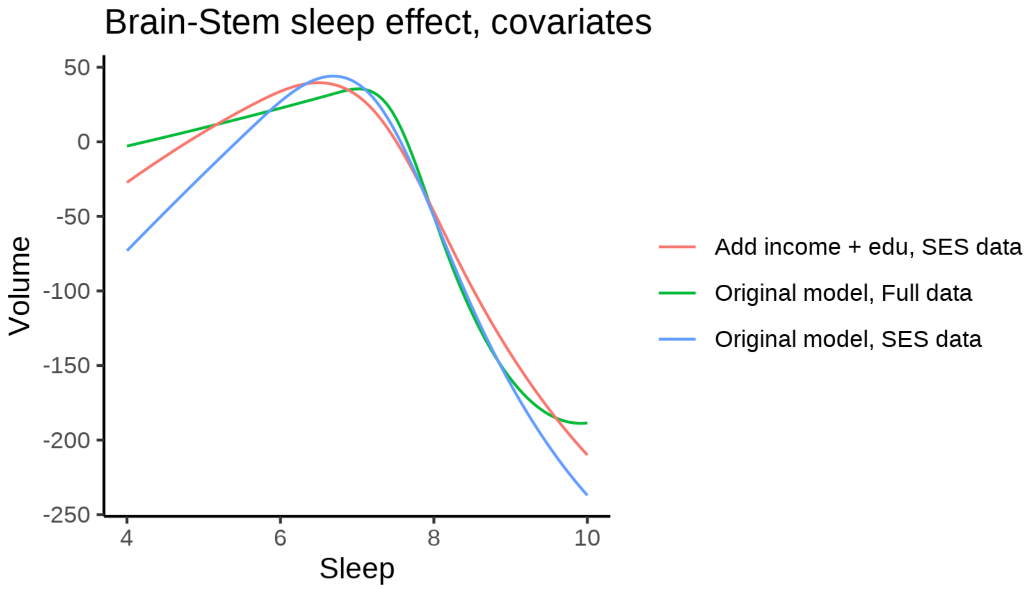

The plot below shows the sleep-volume curve for the original model and for the model with main effects of BMI.

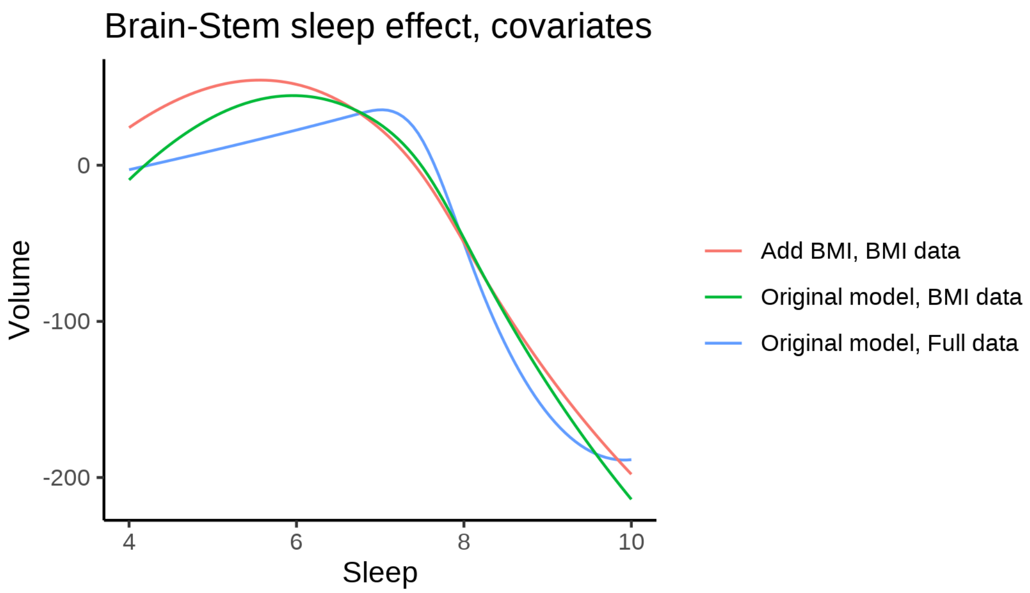

The plot below shows the sleep-volume curve for the original model and for the model with main effects of depression.

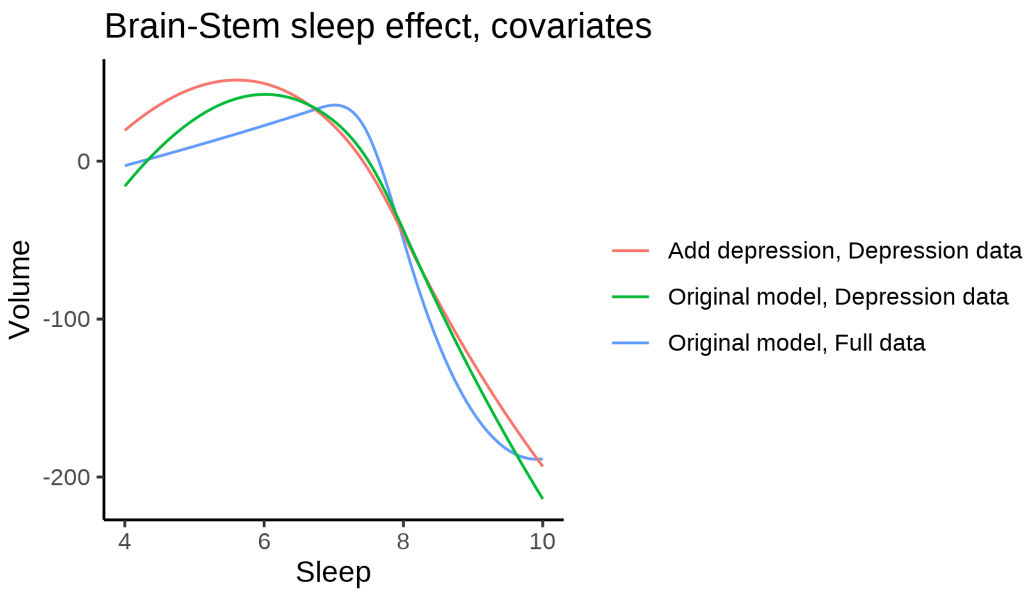

Models Not controling for ICV

Model outputs

Model without sleep term

```
##
## Family: gaussian
## Link function: identity
##
## Formula:
```

```
## value ~ sex + site + s(age_z, k = 10, bs = "cr")
## <environment: 0x558961d9b4f8>
##
## Parametric coefficients:
##                Estimate Std. Error t value Pr(>|t|)
## (Intercept)   19947.34     107.26 185.972  < 2e-16 ***
## sexmale        2479.60      20.07 123.552  < 2e-16 ***
## siteMPIB        174.49     146.05   1.195   0.2322
## siteousAvanto   643.83     113.28   5.683 1.33e-08 ***
## siteousPrisma   789.30     182.41   4.327 1.51e-05 ***
## siteousSkyra    796.62     111.94   7.117 1.12e-12 ***
## siteUB         -634.70     359.94  -1.763   0.0778 .
## siteUCAM         34.99     126.40   0.277   0.7819
## siteUKB         888.61     109.83   8.091 6.05e-16 ***
## siteUmU       -2072.41     164.75 -12.579  < 2e-16 ***
## siteUOXF         70.38     134.77   0.522   0.6015
## ---
## Signif. codes:  0 ‘***’ 0.001 ‘**’ 0.01 ‘*’ 0.05 ‘.’ 0.1 ‘ ’ 1
##
## Approximate significance of smooth terms:
##             edf Ref.df     F p-value
## s(age_z) 5.918  5.918 211.5  <2e-16 ***
## ---
## Signif. codes:  0 ‘***’ 0.001 ‘**’ 0.01 ‘*’ 0.05 ‘.’ 0.1 ‘ ’ 1
##
## R-sq.(adj) =  0.258
## lmer.REML = 9.1936e+05  Scale est. = 1.0895e+05  n = 51266
```

Model with only main effects of age and sleep

```
##
## Family: gaussian
## Link function: identity
##
## Formula:
## value ~ sex + site + s(age_z, k = 10, bs = "cr") + s(sleep_z,
##     k = 5, bs = "cr")
## <environment: 0x558961d9b4f8>
##
## Parametric coefficients:
##                Estimate Std. Error t value Pr(>|t|)
## (Intercept)   19970.23     107.30 186.121  < 2e-16 ***
## sexmale        2475.01      20.07 123.342  < 2e-16 ***
## siteMPIB        178.11     145.99   1.220   0.2225
## siteousAvanto   627.20     113.25   5.538 3.07e-08 ***
## siteousPrisma   774.82     182.34   4.249 2.15e-05 ***
## siteousSkyra    778.21     111.90   6.955 3.58e-12 ***
## siteUB         -616.25     359.65  -1.713   0.0866 .
## siteUCAM         36.77     126.31   0.291   0.7710
## siteUKB         866.56     109.86   7.888 3.14e-15 ***
## siteUmU       -2068.68     164.98 -12.539  < 2e-16 ***
## siteUOXF         61.42     134.67   0.456   0.6483
## ---
## Signif. codes:  0 ‘***’ 0.001 ‘**’ 0.01 ‘*’ 0.05 ‘.’ 0.1 ‘ ’ 1
##
```

```
## Approximate significance of smooth terms:
##               edf Ref.df      F p-value
## s(age_z)   5.896  5.896 204.97  <2e-16 ***
## s(sleep_z) 3.793  3.793  17.59  <2e-16 ***
## ---
## Signif. codes:  0 '***' 0.001 '**' 0.01 '*' 0.05 '.' 0.1 ' ' 1
##
## R-sq.(adj) =  0.259
## lmer.REML = 9.193e+05  Scale est. = 1.0903e+05  n = 51266
```

Model with full interaction between age and sleep

```
##
## Family: gaussian
## Link function: identity
##
## Formula:
## value ~ sex + site + t2(age_z, sleep_z, k = c(10, 4), bs = "cr")
## <environment: 0x558961d9b4f8>
##
## Parametric coefficients:
##                Estimate Std. Error t value Pr(>|t|)
## (Intercept)    19968.49     107.08 186.488  < 2e-16 ***
## sexmale         2480.29      20.10 123.368  < 2e-16 ***
## siteMPIB         156.64     146.28   1.071   0.2843
## siteousAvanto    636.75     113.67   5.602 2.14e-08 ***
## siteousPrisma    783.46     182.78   4.286 1.82e-05 ***
## siteousSkyra     788.70     112.35   7.020 2.24e-12 ***
## siteUB          -657.57     359.81  -1.828   0.0676 .
## siteUCAM          21.84     126.57   0.173   0.8630
## siteUKB          866.17     109.59   7.904 2.75e-15 ***
## siteUmU        -2086.19     165.02 -12.642  < 2e-16 ***
## siteUOXF          46.68     134.67   0.347   0.7289
## ---
## Signif. codes:  0 '***' 0.001 '**' 0.01 '*' 0.05 '.' 0.1 ' ' 1
##
## Approximate significance of smooth terms:
##                     edf Ref.df     F p-value
## t2(age_z,sleep_z) 9.64   9.64 18.51  <2e-16 ***
## ---
## Signif. codes:  0 '***' 0.001 '**' 0.01 '*' 0.05 '.' 0.1 ' ' 1
##
## R-sq.(adj) =  0.258
## lmer.REML = 9.1934e+05  Scale est. = 1.0906e+05  n = 51266
```

Model comparison

`mod_no_sleep` refers to model without sleep term, `mod_no_interaction` refers to model with only main effect of sleep, and `mod_full` refers to model with a full interaction between age and sleep. This is a nested model comparison, and the p-value at a given line refers to comparing the model at the line to the model on the line above. Hence, significance implies that the more complicated model is supported on statistical grounds.

To be even more specific, the p-value on the second row tests whether there is an association between sleep and volume. The p-value on the third row tests whether this association depends on age.

```
## Data: NULL
## Models:
## ml$mod_no_sleep$mer: NULL
## ml$mod_no_interaction$mer: NULL
## ml$mod_full$mer: NULL
##                           npar    AIC    BIC  logLik deviance  Chisq Df Pr(>Chisq)
## ml$mod_no_sleep$mer         15 919389 919521 -459679   919359
## ml$mod_no_interaction$mer   17 919333 919483 -459649   919299 59.565  2  1.163e-13 ***
## ml$mod_full$mer             19 919377 919545 -459669   919339  0.000  2          1
## ---
## Signif. codes:  0 '***' 0.001 '**' 0.01 '*' 0.05 '.' 0.1 ' ' 1
```

We chose the model based on the likelihood ratio test with 5 % significance level, which was `mod_no_interaction`.

Lifespan brain trajectory

The trajectory shown is from the chosen model `mod_no_interaction_no_icv`.

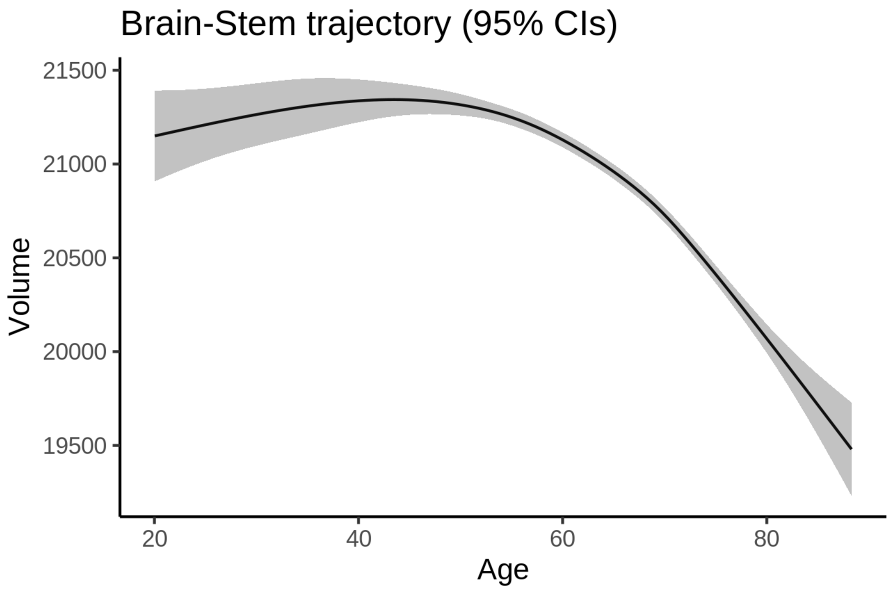

Effect of sleep

The chosen model only included the main effect of sleep, and hence the effect does not vary with age. The black dot shows the average sleep duration across all ages in the sample.

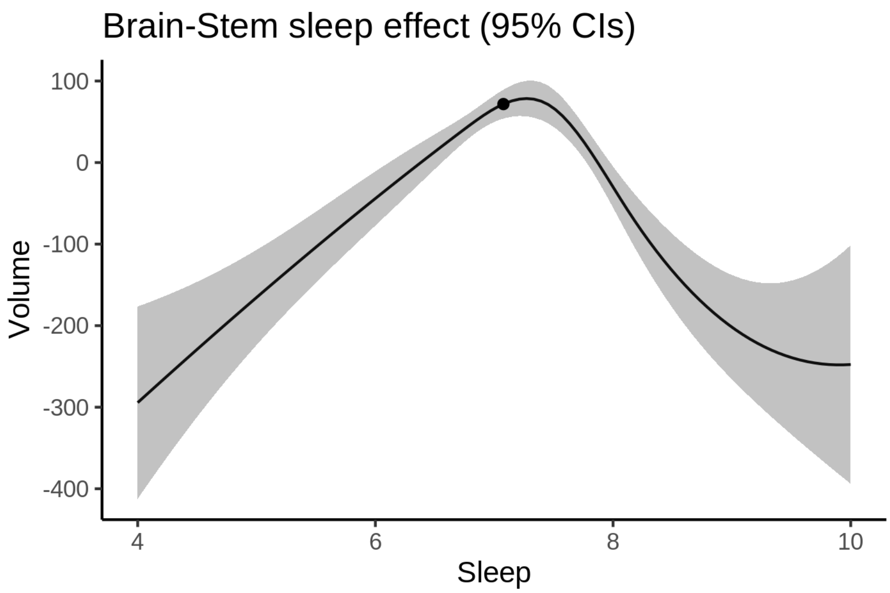

We also show the full interaction model for completeness, although it was not selected.

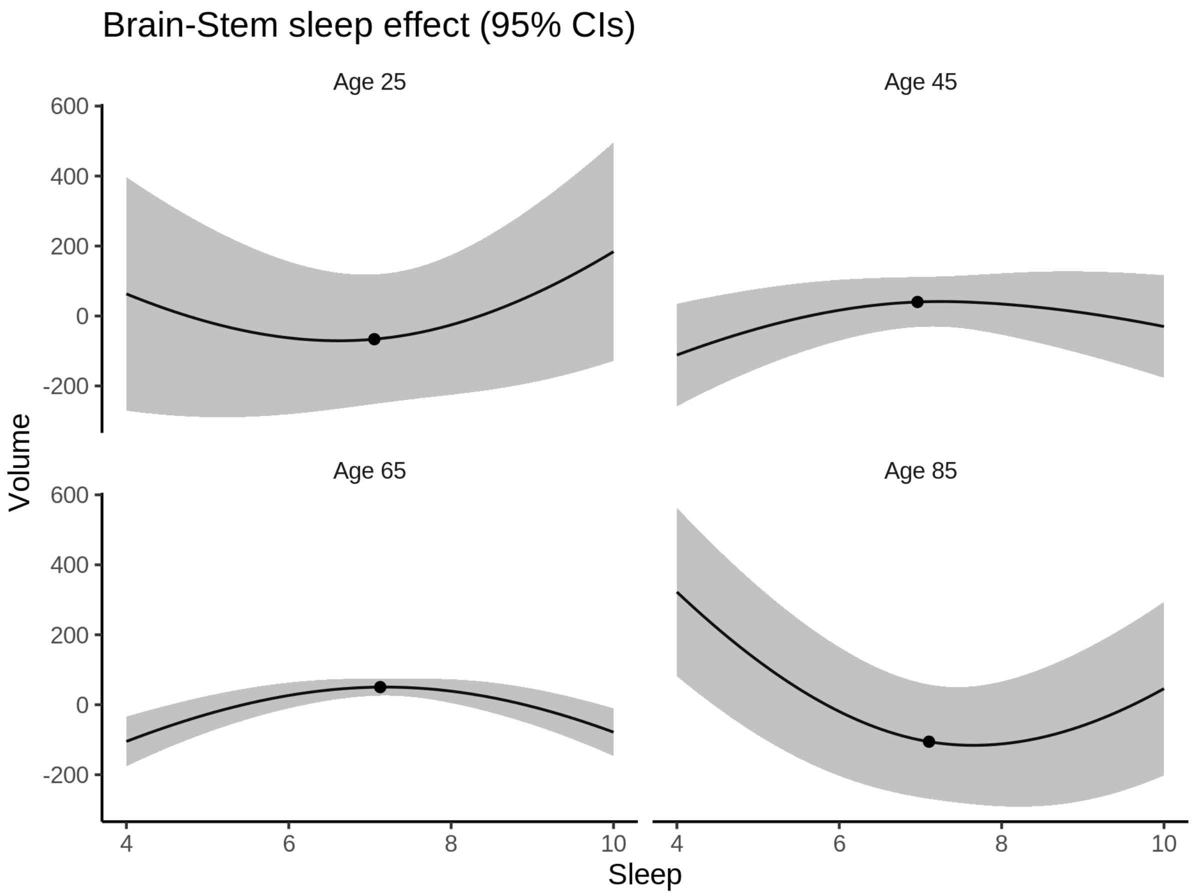

Deviation from sleep associated with maximal volume

Model with only main effect of sleep was chosen, so we show it for all ages at once. Maximum volume is attained at 7.3 hours of sleep. The percentage values in the plot are calculated as follows: The maximum at 100 % refers to a person at an arbitrary age with a sleep duration associated with maximum volume. For a female, this volume is 20872 and for a male it is 23347. The other percentage values show how large the expected volume is for someone with other sleep durations. For example, 99 % implies a 1 % reduction.

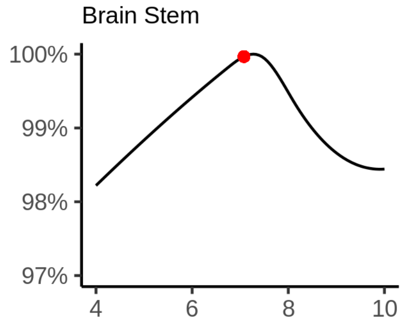

Comparison of mean sleep and sleep associated with maximum volume

A 95 % confidence interval for the sleep associated with maximum volume is [7.15, 7.33].

The plot below compares average sleep to the sleep associated with maximum volume.

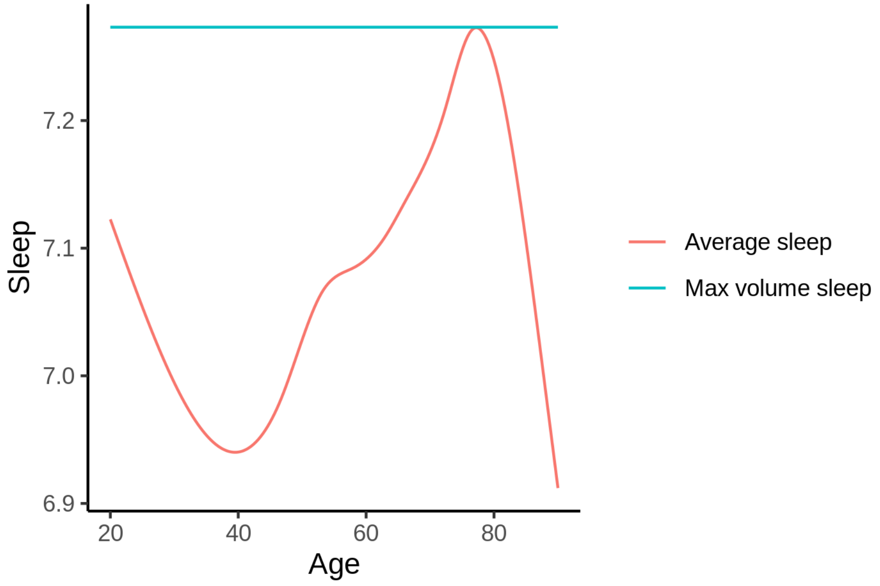

The next plot shows the difference between average sleep and sleep associated with maximum volume. The shaded region is a 95 % confidence interval.

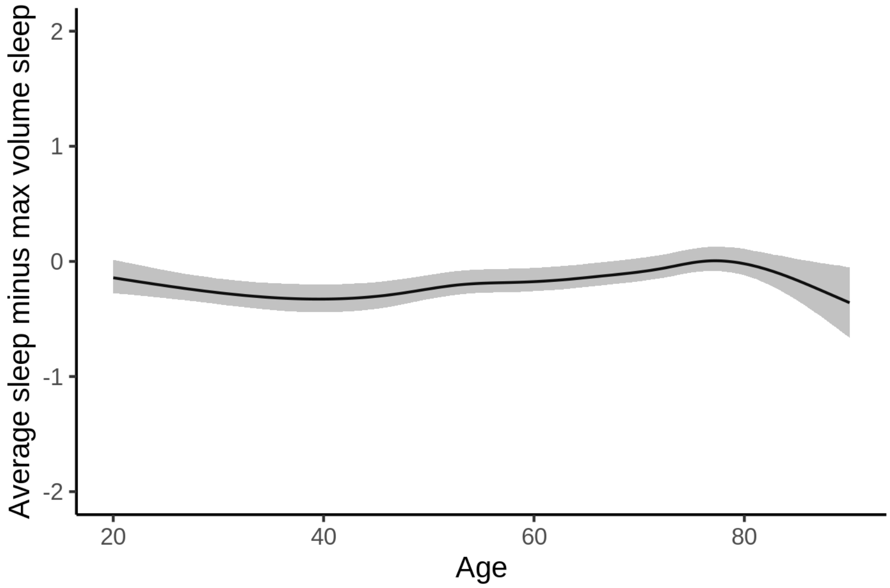

The next plot shows the probability that the sleep duration associated with maximum volume is longer than the average sleep duration, as a function of age. Probability below .05 can be interpreted as evidence that the sleep associated with maximum volume is shorter than the mean sleep, and probability above .95 can be interpreted the opposite way.

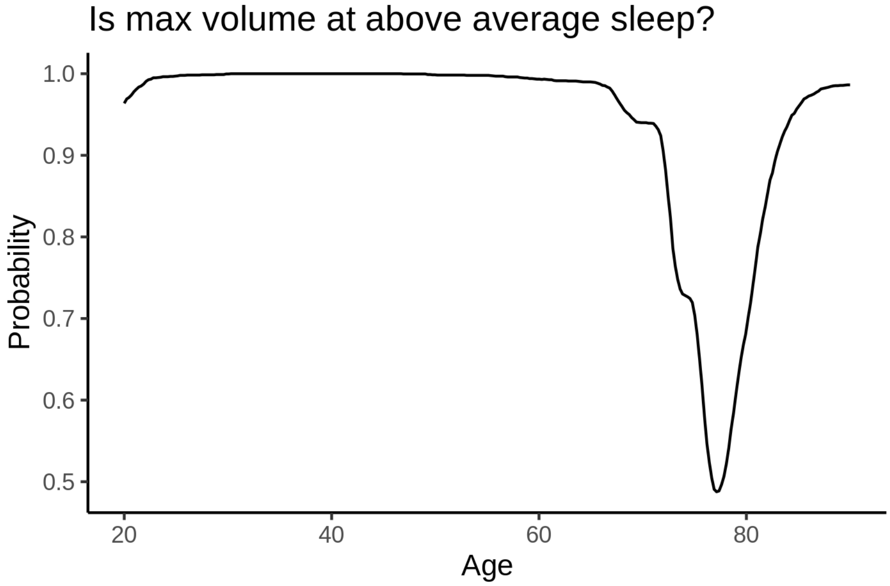

Caudate

Descriptive statistics

Study	Observations	Unique IDs	Mean age	Age range
HCP	974	974	28.8	22 - 37
MPIB	675	390	63.2	24 - 83

Study	Observations	Unique IDs	Mean age	Age range
UB	113	39	70.9	64 - 81
UCAM	884	632	55.1	20 - 88
UiO	1473	803	49.3	20 - 88
UKB	45976	43134	64.5	45 - 83
UmU	412	279	62.2	25 - 85
UOXF	769	769	69.8	60 - 85

Spaghetti plot

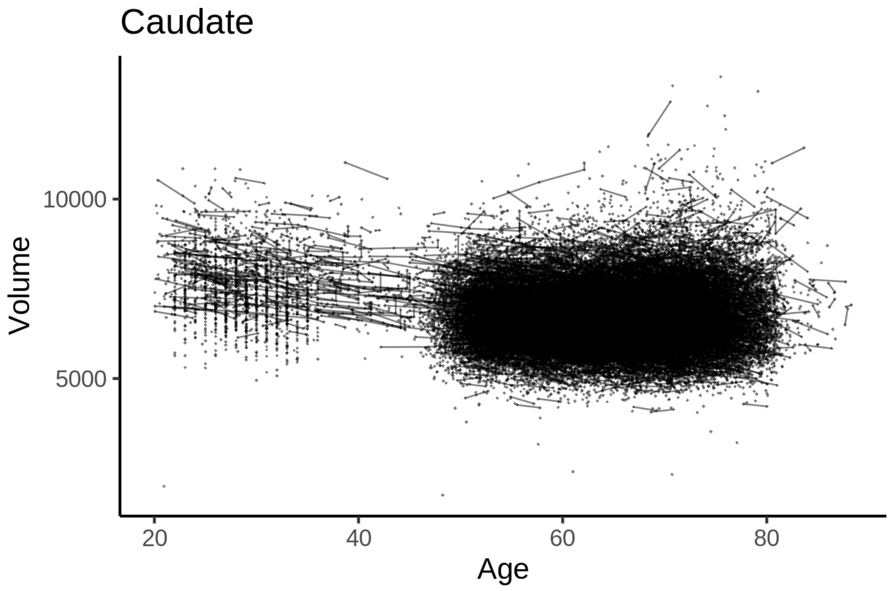

Models Controling for ICV

Model outputs

Model without sleep term

```
##
## Family: gaussian
## Link function: identity
##
## Formula:
## value ~ sex + site + icv + s(age_z, k = 10, bs = "cr")
## <environment: 0x558adafc2c50>
##
## Parametric coefficients:
##                Estimate Std. Error t value Pr(>|t|)
## (Intercept)    6550.991     39.662 165.169  < 2e-16 ***
## sexmale          18.827      8.357   2.253 0.024273 *
## siteMPIB       1253.514     51.829  24.185  < 2e-16 ***
## siteousAvanto   497.301     40.406  12.308  < 2e-16 ***
## siteousPrisma   830.539     67.549  12.295  < 2e-16 ***
## siteousSkyra    836.760     39.297  21.293  < 2e-16 ***
```

```
## siteUB         554.409    123.512   4.489 7.18e-06 ***
## siteUCAM       618.009     44.688  13.829  < 2e-16 ***
## siteUKB        149.850     40.630   3.688 0.000226 ***
## siteUmU        795.984     57.972  13.731  < 2e-16 ***
## siteUOXF       631.897     48.600  13.002  < 2e-16 ***
## icv            462.192      4.190 110.312  < 2e-16 ***
## ---
## Signif. codes:  0 '***' 0.001 '**' 0.01 '*' 0.05 '.' 0.1 ' ' 1
##
## Approximate significance of smooth terms:
##            edf Ref.df     F p-value
## s(age_z) 7.779  7.779 77.05  <2e-16 ***
## ---
## Signif. codes:  0 '***' 0.001 '**' 0.01 '*' 0.05 '.' 0.1 ' ' 1
##
## R-sq.(adj) =   0.33
## lmer.REML = 8.1219e+05  Scale est. = 23405     n = 51276
```

Model with only main effects of age and sleep

```
##
## Family: gaussian
## Link function: identity
##
## Formula:
## value ~ sex + site + icv + s(age_z, k = 10, bs = "cr") + s(sleep_z,
##     k = 5, bs = "cr")
## <environment: 0x558adafc2c50>
##
## Parametric coefficients:
##               Estimate Std. Error t value Pr(>|t|)
## (Intercept)   6554.418     39.694 165.125  < 2e-16 ***
## sexmale         18.950      8.357   2.268 0.023358 *
## siteMPIB      1249.743     51.859  24.099  < 2e-16 ***
## siteousAvanto  495.434     40.414  12.259  < 2e-16 ***
## siteousPrisma  829.229     67.548  12.276  < 2e-16 ***
## siteousSkyra   834.859     39.306  21.240  < 2e-16 ***
## siteUB         553.494    123.508   4.481 7.43e-06 ***
## siteUCAM       616.315     44.693  13.790  < 2e-16 ***
## siteUKB        146.206     40.665   3.595 0.000324 ***
## siteUmU        788.079     58.100  13.564  < 2e-16 ***
## siteUOXF       630.235     48.603  12.967  < 2e-16 ***
## icv            461.937      4.192 110.205  < 2e-16 ***
## ---
## Signif. codes:  0 '***' 0.001 '**' 0.01 '*' 0.05 '.' 0.1 ' ' 1
##
## Approximate significance of smooth terms:
##              edf Ref.df      F p-value
## s(age_z)   7.772  7.772 76.587  <2e-16 ***
## s(sleep_z) 1.000  1.000  4.033  0.0446 *
## ---
## Signif. codes:  0 '***' 0.001 '**' 0.01 '*' 0.05 '.' 0.1 ' ' 1
##
## R-sq.(adj) =   0.33
## lmer.REML = 8.1219e+05  Scale est. = 23399     n = 51276
```

Model with full interaction between age and sleep

```
##
## Family: gaussian
## Link function: identity
##
## Formula:
## value ~ sex + site + icv + t2(age_z, sleep_z, k = c(10, 4), bs = "cr")
## <environment: 0x558adafc2c50>
##
## Parametric coefficients:
##                Estimate Std. Error t value Pr(>|t|)
## (Intercept)    6557.739     39.932 164.223  < 2e-16 ***
## sexmale          19.843      8.374   2.370 0.017807 *
## siteMPIB       1246.007     52.154  23.891  < 2e-16 ***
## siteousAvanto   493.916     40.680  12.142  < 2e-16 ***
## siteousPrisma   827.163     67.709  12.216  < 2e-16 ***
## siteousSkyra    832.530     39.593  21.027  < 2e-16 ***
## siteUB          548.289    123.615   4.435 9.21e-06 ***
## siteUCAM        610.422     44.978  13.572  < 2e-16 ***
## siteUKB         142.411     40.908   3.481 0.000499 ***
## siteUmU         787.049     58.325  13.494  < 2e-16 ***
## siteUOXF        624.092     48.851  12.775  < 2e-16 ***
## icv             461.792      4.192 110.152  < 2e-16 ***
## ---
## Signif. codes:  0 ‘***’ 0.001 ‘**’ 0.01 ‘*’ 0.05 ‘.’ 0.1 ‘ ’ 1
##
## Approximate significance of smooth terms:
##                      edf Ref.df     F p-value
## t2(age_z,sleep_z) 12.28  12.28 10.52  <2e-16 ***
## ---
## Signif. codes:  0 ‘***’ 0.001 ‘**’ 0.01 ‘*’ 0.05 ‘.’ 0.1 ‘ ’ 1
##
## R-sq.(adj) =   0.33
## lmer.REML = 8.1219e+05  Scale est. = 23381     n = 51276
```

Model comparison

`mod_no_sleep` refers to model without sleep term, `mod_no_interaction` refers to model with only main effect of sleep, and `mod_full` refers to model with a full interaction between age and sleep. This is a nested model comparison, and the p-value at a given line refers to comparing the model at the line to the model on the line above. Hence, significance implies that the more complicated model is supported on statistical grounds.

To be even more specific, the p-value on the second row tests whether there is an association between sleep and volume. The p-value on the third row tests whether this association depends on age.

```
## Data: NULL
## Models:
## ml$mod_no_sleep$mer: NULL
## ml$mod_no_interaction$mer: NULL
## ml$mod_full$mer: NULL
##                           npar    AIC    BIC  logLik deviance  Chisq Df Pr(>Chisq)
## ml$mod_no_sleep$mer         16 812224 812365 -406096   812192
## ml$mod_no_interaction$mer   18 812224 812383 -406094   812188 4.0326  2     0.1331
```

```
## ml$mod_full$mer             20 812233 812410 -406096   812193 0.0000  2     1.0000
```

We chose the model based on the likelihood ratio test with 5 % significance level, which was `mod_no_sleep`.

Lifespan brain trajectory

The trajectory shown is from the chosen model `mod_no_sleep`.

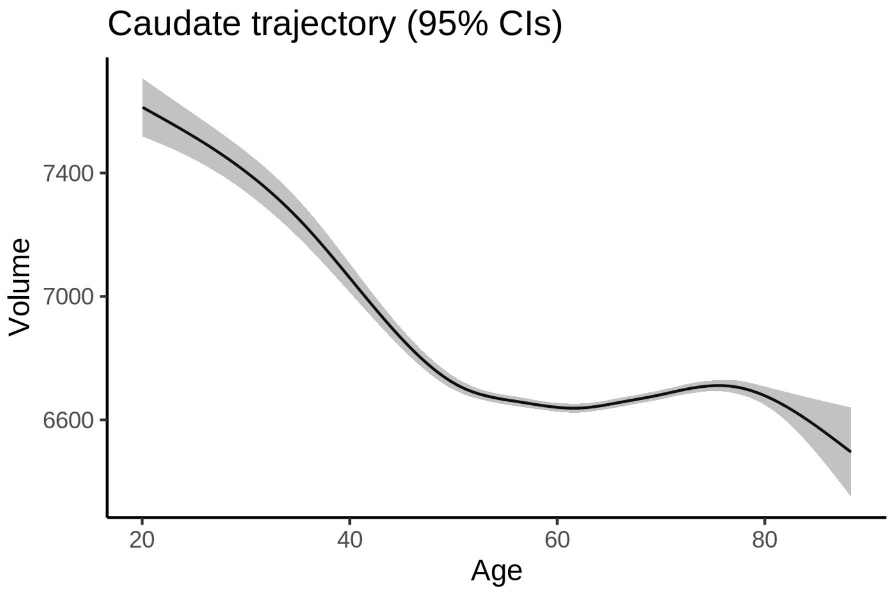

Effect of sleep

The chosen model did not include a sleep term, and hence we don't have any estimated effect of sleep.

We show the full interaction model for completeness, although it was not selected.

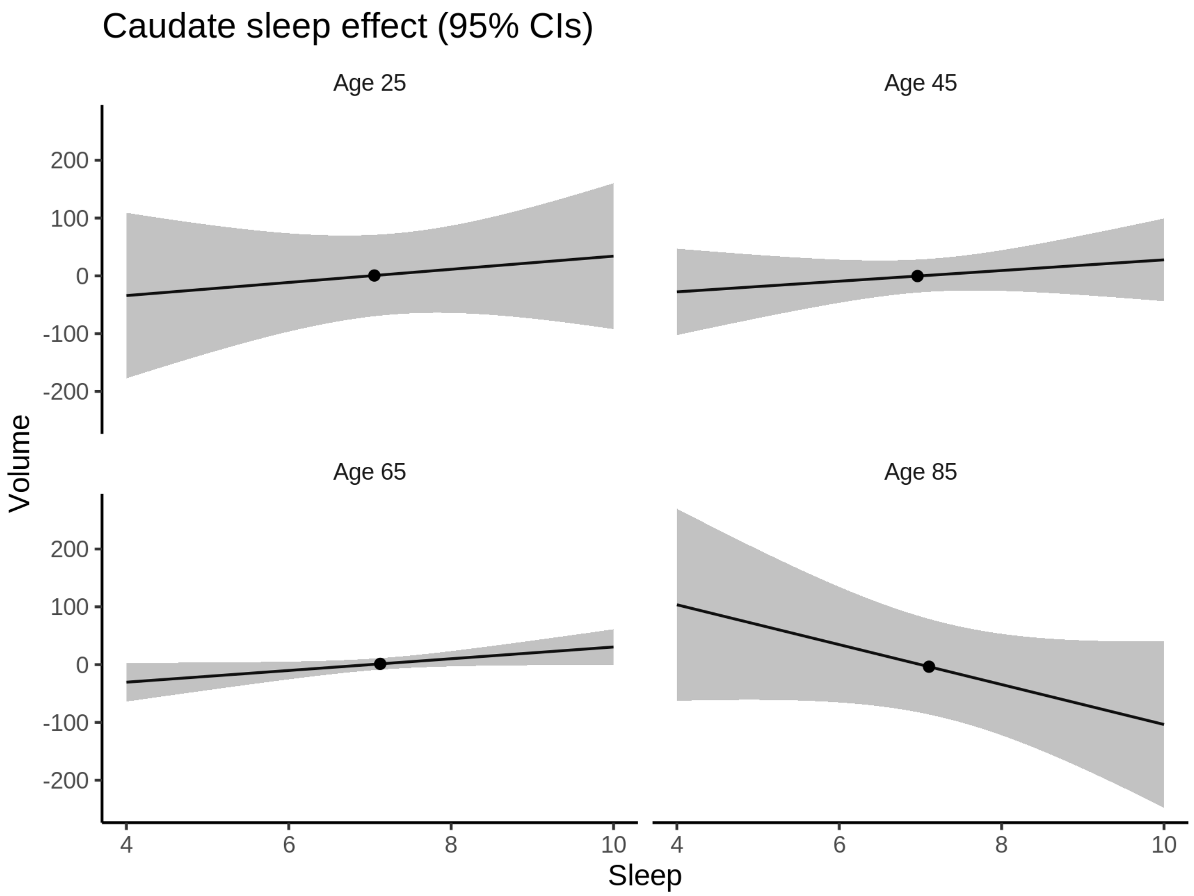

Deviation from sleep associated with maximal volume

Model with no sleep term was selected. No plots to show. (Although we can of course dig up the plots, which will be pretty flat).

Comparison of mean sleep and sleep associated with maximum volume

Nothing to show, as we did not find an association between sleep and volume.

Controling for covariates

Below is the output for a model in which we only include data with income and education.

```
##
## Family: gaussian
## Link function: identity
##
## Formula:
## value ~ sex + site + s(age_z, k = 10, bs = "cr") + s(sleep_z,
##     k = 5, bs = "cr") + icv
## <environment: 0x558ada722128>
##
## Parametric coefficients:
```

```
##                Estimate Std. Error t value Pr(>|t|)
## (Intercept)    8195.179     96.415  84.999  < 2e-16 ***
## sexmale           3.556     10.583   0.336    0.737
## siteousAvanto -1132.854    111.188 -10.189  < 2e-16 ***
## siteousPrisma  -657.472    117.070  -5.616 1.97e-08 ***
## siteousSkyra   -747.335    104.471  -7.154 8.64e-13 ***
## siteUKB       -1512.887     96.206 -15.725  < 2e-16 ***
## siteUOXF       -930.264    108.296  -8.590  < 2e-16 ***
## icv             468.475      5.414  86.526  < 2e-16 ***
## ---
## Signif. codes:  0 ‘***’ 0.001 ‘**’ 0.01 ‘*’ 0.05 ‘.’ 0.1 ‘ ’ 1
##
## Approximate significance of smooth terms:
##              edf Ref.df      F p-value
## s(age_z)   5.543  5.543 35.010  <2e-16 ***
## s(sleep_z) 1.000  1.000  2.432   0.119
## ---
## Signif. codes:  0 ‘***’ 0.001 ‘**’ 0.01 ‘*’ 0.05 ‘.’ 0.1 ‘ ’ 1
##
## R-sq.(adj) =  0.305
## lmer.REML = 4.9378e+05  Scale est. = 22017     n = 31194
```

Below is the output for a model in which we control for the main effects of income and education.

```
##
## Family: gaussian
## Link function: identity
##
## Formula:
## value ~ sex + site + s(age_z, k = 10, bs = "cr") + s(sleep_z,
##     k = 5, bs = "cr") + icv + income_scaled + education_scaled
## <environment: 0x558ada722128>
##
## Parametric coefficients:
##                   Estimate Std. Error t value Pr(>|t|)
## (Intercept)       8163.561     96.992  84.167  < 2e-16 ***
## sexmale              4.769     10.612   0.449  0.65314
## siteousAvanto    -1136.526    111.207 -10.220  < 2e-16 ***
## siteousPrisma     -661.245    117.063  -5.649 1.63e-08 ***
## siteousSkyra      -751.455    104.486  -7.192 6.53e-13 ***
## siteUKB          -1518.066     96.250 -15.772  < 2e-16 ***
## siteUOXF          -922.490    108.337  -8.515  < 2e-16 ***
## icv                466.476      5.460  85.430  < 2e-16 ***
## income_scaled        5.535     12.579   0.440  0.65993
## education_scaled    43.054     14.919   2.886  0.00391 **
## ---
## Signif. codes:  0 ‘***’ 0.001 ‘**’ 0.01 ‘*’ 0.05 ‘.’ 0.1 ‘ ’ 1
##
## Approximate significance of smooth terms:
##              edf Ref.df     F p-value
## s(age_z)   5.566  5.566 34.89  <2e-16 ***
## s(sleep_z) 1.000  1.000  2.23   0.135
## ---
## Signif. codes:  0 ‘***’ 0.001 ‘**’ 0.01 ‘*’ 0.05 ‘.’ 0.1 ‘ ’ 1
##
```

```
## R-sq.(adj) =  0.305
## lmer.REML = 4.9375e+05  Scale est. = 22023     n = 31194
```

We also included interaction effects between sleep duration and education and income, in another model. The output is shown below, and the interaction terms are `income_scaled:sleep_z` and `education_scaled:sleep_z`.

```
##
## Family: gaussian
## Link function: identity
##
## Formula:
## value ~ sex + site + s(age_z, k = 10, bs = "cr") + s(sleep_z,
##     k = 5, bs = "cr") + icv + income_scaled + education_scaled +
##     income_scaled:sleep_z + education_scaled:sleep_z
## <environment: 0x558ada722128>
##
## Parametric coefficients:
##                           Estimate Std. Error t value Pr(>|t|)
## (Intercept)               8160.728     96.983  84.146  < 2e-16 ***
## sexmale                      4.147     10.618   0.391  0.69608
## siteousAvanto            -1135.808    111.195 -10.215  < 2e-16 ***
## siteousPrisma             -661.555    117.057  -5.652 1.60e-08 ***
## siteousSkyra              -750.758    104.473  -7.186 6.81e-13 ***
## siteUKB                  -1514.383     96.244 -15.735  < 2e-16 ***
## siteUOXF                  -914.964    108.350  -8.445  < 2e-16 ***
## icv                        466.353      5.460  85.411  < 2e-16 ***
## income_scaled                6.161     12.584   0.490  0.62444
## education_scaled            42.857     14.919   2.873  0.00407 **
## income_scaled:sleep_z      -30.836     12.490  -2.469  0.01356 *
## education_scaled:sleep_z   -20.726     14.440  -1.435  0.15123
## ---
## Signif. codes:  0 ‘***’ 0.001 ‘**’ 0.01 ‘*’ 0.05 ‘.’ 0.1 ‘ ’ 1
##
## Approximate significance of smooth terms:
##              edf Ref.df      F p-value
## s(age_z)   5.568  5.568 34.942 < 2e-16 ***
## s(sleep_z) 1.000  1.000  9.089 0.00257 **
## ---
## Signif. codes:  0 ‘***’ 0.001 ‘**’ 0.01 ‘*’ 0.05 ‘.’ 0.1 ‘ ’ 1
##
## R-sq.(adj) =  0.305
## lmer.REML = 4.9373e+05  Scale est. = 22024     n = 31194
```

We did the same controling for BMI. Below is the model with no covariates but only keeping data with BMI.

```
##
## Family: gaussian
## Link function: identity
##
## Formula:
## value ~ sex + site + s(age_z, k = 10, bs = "cr") + s(sleep_z,
##     k = 5, bs = "cr") + icv
## <environment: 0x558a4a136af8>
##
## Parametric coefficients:
```

```
##                Estimate Std. Error t value Pr(>|t|)
## (Intercept)   7032.760     57.252 122.838  < 2e-16 ***
## sexmale         -3.318     10.285  -0.323    0.747
## siteousPrisma  346.047     76.074   4.549 5.41e-06 ***
## siteousSkyra   345.832     38.865   8.898  < 2e-16 ***
## siteUCAM       115.784     61.317   1.888    0.059 .
## siteUKB       -337.430     57.876  -5.830 5.59e-09 ***
## siteUmU        303.460     71.683   4.233 2.31e-05 ***
## icv            476.551      5.281  90.238  < 2e-16 ***
## ---
## Signif. codes:  0 '***' 0.001 '**' 0.01 '*' 0.05 '.' 0.1 ' ' 1
##
## Approximate significance of smooth terms:
##              edf Ref.df     F p-value
## s(age_z)   7.633  7.633 51.37  <2e-16 ***
## s(sleep_z) 1.000  1.000  1.92   0.166
## ---
## Signif. codes:  0 '***' 0.001 '**' 0.01 '*' 0.05 '.' 0.1 ' ' 1
##
## R-sq.(adj) =  0.328
## lmer.REML = 5.2896e+05  Scale est. = 23120     n = 33438
```

Below is the model output with main effect.

```
##
## Family: gaussian
## Link function: identity
##
## Formula:
## value ~ sex + site + s(age_z, k = 10, bs = "cr") + s(sleep_z,
##     k = 5, bs = "cr") + icv + bmi
## <environment: 0x558a4a136af8>
##
## Parametric coefficients:
##                Estimate Std. Error t value Pr(>|t|)
## (Intercept)   6969.977     62.597 111.347  < 2e-16 ***
## sexmale         -5.312     10.316  -0.515   0.6066
## siteousPrisma  344.232     76.076   4.525 6.06e-06 ***
## siteousSkyra   345.758     38.867   8.896  < 2e-16 ***
## siteUCAM       114.409     61.318   1.866   0.0621 .
## siteUKB       -339.208     57.880  -5.861 4.66e-09 ***
## siteUmU        300.903     71.687   4.197 2.71e-05 ***
## icv            476.207      5.282  90.149  < 2e-16 ***
## bmi              2.484      1.002   2.478   0.0132 *
## ---
## Signif. codes:  0 '***' 0.001 '**' 0.01 '*' 0.05 '.' 0.1 ' ' 1
##
## Approximate significance of smooth terms:
##              edf Ref.df      F p-value
## s(age_z)   7.639  7.639 51.811  <2e-16 ***
## s(sleep_z) 1.000  1.000  2.238   0.135
## ---
## Signif. codes:  0 '***' 0.001 '**' 0.01 '*' 0.05 '.' 0.1 ' ' 1
##
## R-sq.(adj) =  0.328
```

```
## lmer.REML = 5.2895e+05  Scale est. = 23122     n = 33438
```

Next is the model with BMI-sleep interaction.

```
##
## Family: gaussian
## Link function: identity
##
## Formula:
## value ~ sex + site + s(age_z, k = 10, bs = "cr") + s(sleep_z,
##     k = 5, bs = "cr") + icv + bmi + bmi:sleep_z
## <environment: 0x558a4a136af8>
##
## Parametric coefficients:
##               Estimate Std. Error t value Pr(>|t|)
## (Intercept)   6966.212     62.629 111.229  < 2e-16 ***
## sexmale         -5.239     10.316  -0.508   0.6116
## siteousPrisma  344.195     76.074   4.524 6.08e-06 ***
## siteousSkyra   346.018     38.867   8.903  < 2e-16 ***
## siteUCAM       115.771     61.321   1.888   0.0590 .
## siteUKB       -337.909     57.883  -5.838 5.34e-09 ***
## siteUmU        302.102     71.688   4.214 2.51e-05 ***
## icv            476.272      5.282  90.163  < 2e-16 ***
## bmi              2.545      1.003   2.538   0.0112 *
## bmi:sleep_z      1.725      0.949   1.817   0.0692 .
## ---
## Signif. codes:  0 '***' 0.001 '**' 0.01 '*' 0.05 '.' 0.1 ' ' 1
##
## Approximate significance of smooth terms:
##              edf Ref.df      F p-value
## s(age_z)   7.639  7.639 51.935  <2e-16 ***
## s(sleep_z) 1.000  1.000  2.379   0.123
## ---
## Signif. codes:  0 '***' 0.001 '**' 0.01 '*' 0.05 '.' 0.1 ' ' 1
##
## R-sq.(adj) =  0.328
## lmer.REML = 5.2894e+05  Scale est. = 23122     n = 33438
```

We did the same controling for depression. Below is the model with no covariates but only keeping data with depression.

```
##
## Family: gaussian
## Link function: identity
##
## Formula:
## value ~ sex + site + s(age_z, k = 10, bs = "cr") + s(sleep_z,
##     k = 5, bs = "cr") + icv
## <environment: 0x558ad9361bd0>
##
## Parametric coefficients:
##                Estimate Std. Error t value Pr(>|t|)
## (Intercept)    7701.734     49.303 156.213  < 2e-16 ***
## sexmale          -1.979     10.313  -0.192  0.84781
## siteousAvanto  -630.865     93.833  -6.723 1.81e-11 ***
## siteousPrisma  -288.132     92.159  -3.126  0.00177 **
```

```
## siteousSkyra   -187.936     73.512  -2.557  0.01058 *
## siteUCAM       -525.589     67.566  -7.779 7.53e-15 ***
## siteUKB       -1012.006     48.763 -20.753  < 2e-16 ***
## siteUmU        -359.475     66.335  -5.419 6.03e-08 ***
## icv             474.353      5.289  89.687  < 2e-16 ***
## ---
## Signif. codes:  0 ‘***’ 0.001 ‘**’ 0.01 ‘*’ 0.05 ‘.’ 0.1 ‘ ’ 1
##
## Approximate significance of smooth terms:
##              edf Ref.df      F p-value
## s(age_z)   7.609  7.609 28.478  <2e-16 ***
## s(sleep_z) 1.000  1.000  1.959   0.162
## ---
## Signif. codes:  0 ‘***’ 0.001 ‘**’ 0.01 ‘*’ 0.05 ‘.’ 0.1 ‘ ’ 1
##
## R-sq.(adj) =   0.32
## lmer.REML = 5.2725e+05  Scale est. = 23572     n = 33350
```

Below is the model output with main effect.

```
##
## Family: gaussian
## Link function: identity
##
## Formula:
## value ~ sex + site + s(age_z, k = 10, bs = "cr") + s(sleep_z,
##     k = 5, bs = "cr") + icv + depression
## <environment: 0x558ad9361bd0>
##
## Parametric coefficients:
##                 Estimate Std. Error t value Pr(>|t|)
## (Intercept)    7695.519     49.517 155.412  < 2e-16 ***
## sexmale          -1.360     10.323  -0.132  0.89520
## siteousAvanto  -633.999     93.865  -6.754 1.46e-11 ***
## siteousPrisma  -290.976     92.183  -3.157  0.00160 **
## siteousSkyra   -191.419     73.556  -2.602  0.00926 **
## siteUCAM       -527.067     67.573  -7.800 6.37e-15 ***
## siteUKB       -1009.331     48.802 -20.682  < 2e-16 ***
## siteUmU        -377.754     67.708  -5.579 2.44e-08 ***
## icv             474.301      5.289  89.677  < 2e-16 ***
## depression       49.299     36.623   1.346  0.17827
## ---
## Signif. codes:  0 ‘***’ 0.001 ‘**’ 0.01 ‘*’ 0.05 ‘.’ 0.1 ‘ ’ 1
##
## Approximate significance of smooth terms:
##              edf Ref.df      F p-value
## s(age_z)   7.611  7.611 28.602  <2e-16 ***
## s(sleep_z) 1.000  1.000  2.224   0.136
## ---
## Signif. codes:  0 ‘***’ 0.001 ‘**’ 0.01 ‘*’ 0.05 ‘.’ 0.1 ‘ ’ 1
##
## R-sq.(adj) =   0.32
## lmer.REML = 5.2724e+05  Scale est. = 23579     n = 33350
```

Next is the model with depression-sleep interaction.

```
##
## Family: gaussian
## Link function: identity
##
## Formula:
## value ~ sex + site + s(age_z, k = 10, bs = "cr") + s(sleep_z,
##     k = 5, bs = "cr") + icv + depression + depression:sleep_z
## <environment: 0x558ad9361bd0>
##
## Parametric coefficients:
##                      Estimate Std. Error t value Pr(>|t|)
## (Intercept)          7695.525     49.518 155.410  < 2e-16 ***
## sexmale                -1.310     10.325  -0.127  0.89903
## siteousAvanto        -633.666     93.875  -6.750 1.50e-11 ***
## siteousPrisma        -290.658     92.193  -3.153  0.00162 **
## siteousSkyra         -191.101     73.568  -2.598  0.00939 **
## siteUCAM             -526.703     67.588  -7.793 6.74e-15 ***
## siteUKB             -1009.353     48.803 -20.682  < 2e-16 ***
## siteUmU              -380.150     68.262  -5.569 2.58e-08 ***
## icv                   474.296      5.289  89.674  < 2e-16 ***
## depression             49.862     36.680   1.359  0.17404
## depression:sleep_z      7.392     26.778   0.276  0.78250
## ---
## Signif. codes:  0 '***' 0.001 '**' 0.01 '*' 0.05 '.' 0.1 ' ' 1
##
## Approximate significance of smooth terms:
##              edf Ref.df      F p-value
## s(age_z)   7.613  7.613 28.579  <2e-16 ***
## s(sleep_z) 1.000  1.000  1.235   0.266
## ---
## Signif. codes:  0 '***' 0.001 '**' 0.01 '*' 0.05 '.' 0.1 ' ' 1
##
## R-sq.(adj) =   0.32
## lmer.REML = 5.2723e+05  Scale est. = 23579     n = 33350
```

The plot below shows the sleep-volume curve for the original model and for the model with main effects of SES.

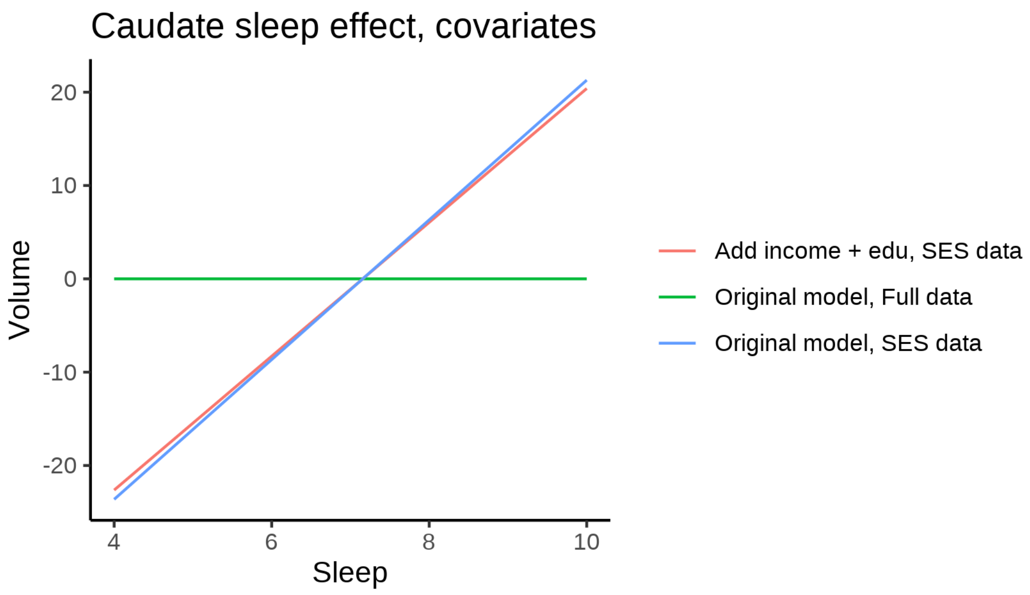

The plot below shows the sleep-volume curve for the original model and for the model with main effects of BMI.

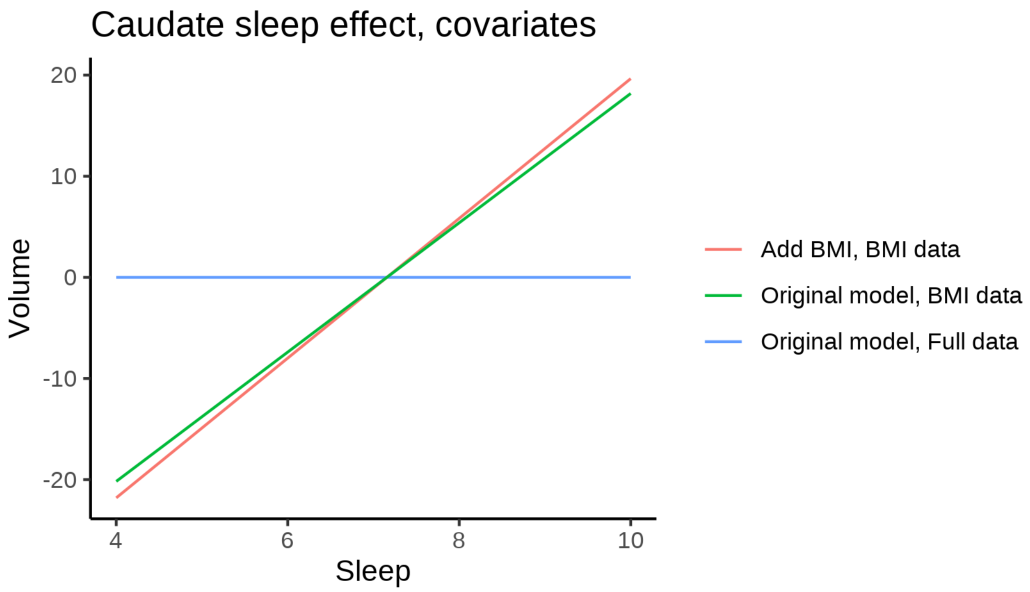

The plot below shows the sleep-volume curve for the original model and for the model with main effects of depression.

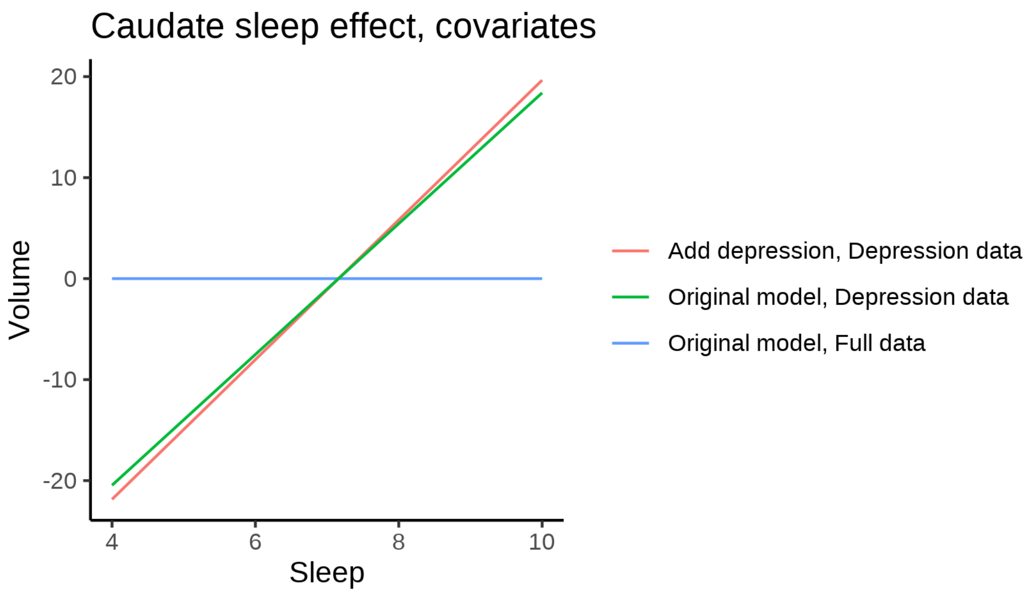

Models Not controling for ICV

Model outputs

Model without sleep term

```
##
## Family: gaussian
## Link function: identity
##
## Formula:
## value ~ sex + site + s(age_z, k = 10, bs = "cr")
## <environment: 0x5589766c7e80>
##
## Parametric coefficients:
##                Estimate Std. Error t value Pr(>|t|)
## (Intercept)    6293.736     43.361 145.148  < 2e-16 ***
## sexmale         543.806      7.705  70.574  < 2e-16 ***
## siteMPIB        512.017     57.161   8.957  < 2e-16 ***
## siteousAvanto   687.685     44.604  15.418  < 2e-16 ***
## siteousPrisma   988.985     73.901  13.382  < 2e-16 ***
## siteousSkyra    862.553     43.694  19.741  < 2e-16 ***
## siteUB          422.673    138.445   3.053 0.002267 **
## siteUCAM        645.614     49.594  13.018  < 2e-16 ***
## siteUKB         166.870     44.471   3.752 0.000175 ***
## siteUmU         612.482     64.424   9.507  < 2e-16 ***
## siteUOXF        528.850     53.600   9.867  < 2e-16 ***
## ---
## Signif. codes:  0 ‘***’ 0.001 ‘**’ 0.01 ‘*’ 0.05 ‘.’ 0.1 ‘ ’ 1
##
## Approximate significance of smooth terms:
##             edf Ref.df     F p-value
## s(age_z) 7.028  7.028 83.63  <2e-16 ***
```

```
## ---
## Signif. codes:  0 ‘***’ 0.001 ‘**’ 0.01 ‘*’ 0.05 ‘.’ 0.1 ‘ ’ 1
##
## R-sq.(adj) =  0.156
## lmer.REML = 8.2303e+05  Scale est. = 23853     n = 51276
```

Model with only main effects of age and sleep

```
##
## Family: gaussian
## Link function: identity
##
## Formula:
## value ~ sex + site + s(age_z, k = 10, bs = "cr") + s(sleep_z,
##     k = 5, bs = "cr")
## <environment: 0x5589766c7e80>
##
## Parametric coefficients:
##               Estimate Std. Error t value Pr(>|t|)
## (Intercept)   6304.577     43.384 145.320  < 2e-16 ***
## sexmale        542.914      7.707  70.447  < 2e-16 ***
## siteMPIB       503.657     57.168   8.810  < 2e-16 ***
## siteousAvanto  681.187     44.605  15.272  < 2e-16 ***
## siteousPrisma  984.177     73.886  13.320  < 2e-16 ***
## siteousSkyra   856.110     43.695  19.593  < 2e-16 ***
## siteUB         421.902    138.404   3.048 0.002302 **
## siteUCAM       641.354     49.583  12.935  < 2e-16 ***
## siteUKB        155.948     44.493   3.505 0.000457 ***
## siteUmU        592.211     64.542   9.176  < 2e-16 ***
## siteUOXF       524.069     53.581   9.781  < 2e-16 ***
## ---
## Signif. codes:  0 ‘***’ 0.001 ‘**’ 0.01 ‘*’ 0.05 ‘.’ 0.1 ‘ ’ 1
##
## Approximate significance of smooth terms:
##              edf Ref.df     F  p-value
## s(age_z)   6.999  6.999 83.71  < 2e-16 ***
## s(sleep_z) 2.054  2.054 13.57 1.02e-06 ***
## ---
## Signif. codes:  0 ‘***’ 0.001 ‘**’ 0.01 ‘*’ 0.05 ‘.’ 0.1 ‘ ’ 1
##
## R-sq.(adj) =  0.157
## lmer.REML = 8.23e+05  Scale est. = 23843     n = 51276
```

Model with full interaction between age and sleep

```
##
## Family: gaussian
## Link function: identity
##
## Formula:
## value ~ sex + site + t2(age_z, sleep_z, k = c(10, 4), bs = "cr")
## <environment: 0x5589766c7e80>
##
## Parametric coefficients:
##               Estimate Std. Error t value Pr(>|t|)
## (Intercept)    6312.93      43.56 144.908  < 2e-16 ***
```

```
## sexmale         544.92       7.72  70.588  < 2e-16 ***
## siteMPIB        491.50      57.44   8.556  < 2e-16 ***
## siteousAvanto   676.10      44.87  15.069  < 2e-16 ***
## siteousPrisma   977.64      74.05  13.203  < 2e-16 ***
## siteousSkyra    849.50      43.98  19.317  < 2e-16 ***
## siteUB          406.48     138.49   2.935  0.00334 **
## siteUCAM        627.46      49.85  12.586  < 2e-16 ***
## siteUKB         146.56      44.67   3.281  0.00104 **
## siteUmU         585.09      64.74   9.038  < 2e-16 ***
## siteUOXF        509.67      53.78   9.476  < 2e-16 ***
## ---
## Signif. codes:  0 ‘***’ 0.001 ‘**’ 0.01 ‘*’ 0.05 ‘.’ 0.1 ‘ ’ 1
##
## Approximate significance of smooth terms:
##                      edf Ref.df     F p-value
## t2(age_z,sleep_z) 10.66  10.66 9.943  <2e-16 ***
## ---
## Signif. codes:  0 ‘***’ 0.001 ‘**’ 0.01 ‘*’ 0.05 ‘.’ 0.1 ‘ ’ 1
##
## R-sq.(adj) =  0.157
## lmer.REML = 8.23e+05  Scale est. = 23811     n = 51276
```

Model comparison

`mod_no_sleep` refers to model without sleep term, `mod_no_interaction` refers to model with only main effect of sleep, and `mod_full` refers to model with a full interaction between age and sleep. This is a nested model comparison, and the p-value at a given line refers to comparing the model at the line to the model on the line above. Hence, significance implies that the more complicated model is supported on statistical grounds.

To be even more specific, the p-value on the second row tests whether there is an association between sleep and volume. The p-value on the third row tests whether this association depends on age.

```
## Data: NULL
## Models:
## ml$mod_no_sleep$mer: NULL
## ml$mod_no_interaction$mer: NULL
## ml$mod_full$mer: NULL
##                           npar    AIC    BIC  logLik deviance   Chisq Df Pr(>Chisq)
## ml$mod_no_sleep$mer         15 823059 823191 -411514   823029
## ml$mod_no_interaction$mer   17 823038 823188 -411502   823004 24.7369  2  4.251e-06 ***
## ml$mod_full$mer             19 823039 823207 -411500   823001  3.2705  2     0.1949
## ---
## Signif. codes:  0 ‘***’ 0.001 ‘**’ 0.01 ‘*’ 0.05 ‘.’ 0.1 ‘ ’ 1
```

We chose the model based on the likelihood ratio test with 5 % significance level, which was `mod_no_interaction`.

Lifespan brain trajectory

The trajectory shown is from the chosen model `mod_no_interaction_no_icv`.

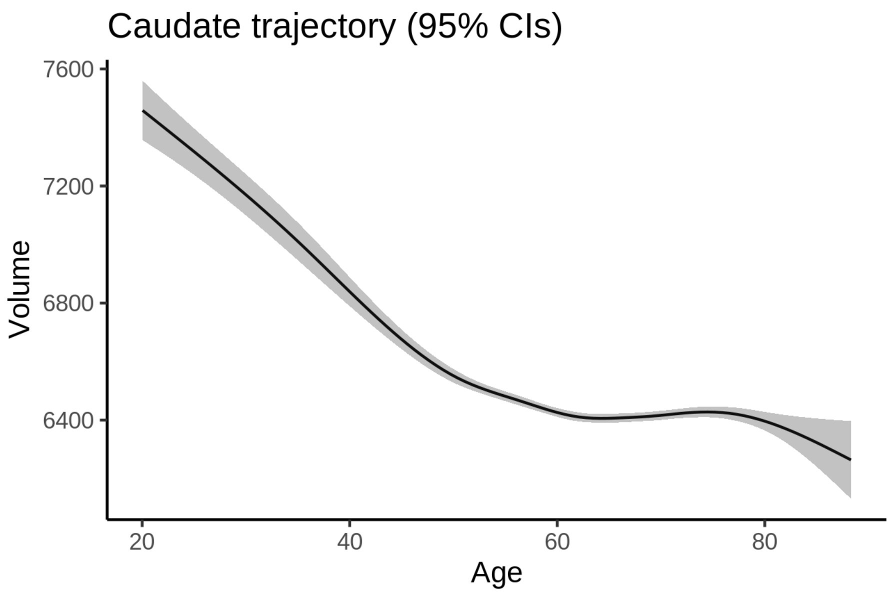

Effect of sleep

The chosen model only included the main effect of sleep, and hence the effect does not vary with age. The black dot shows the average sleep duration across all ages in the sample.

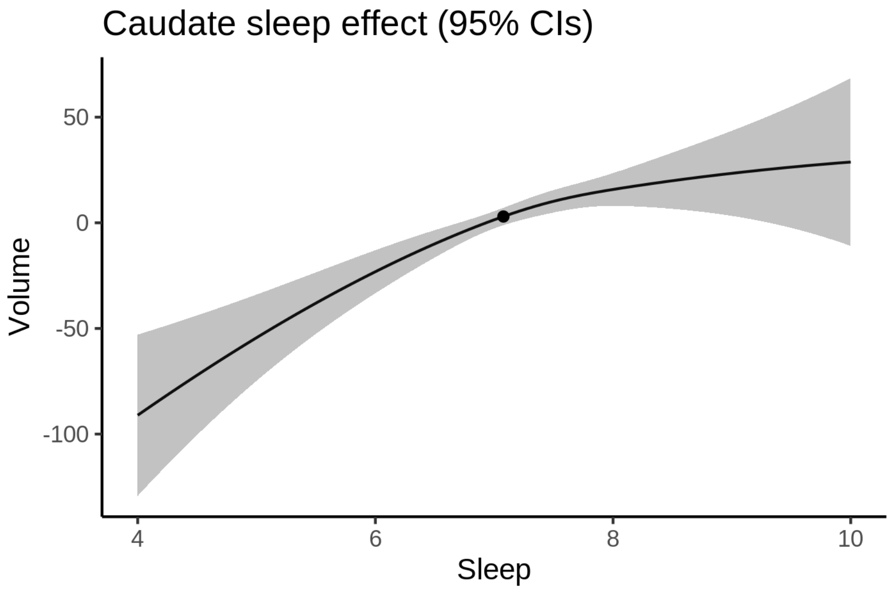

We also show the full interaction model for completeness, although it was not selected.

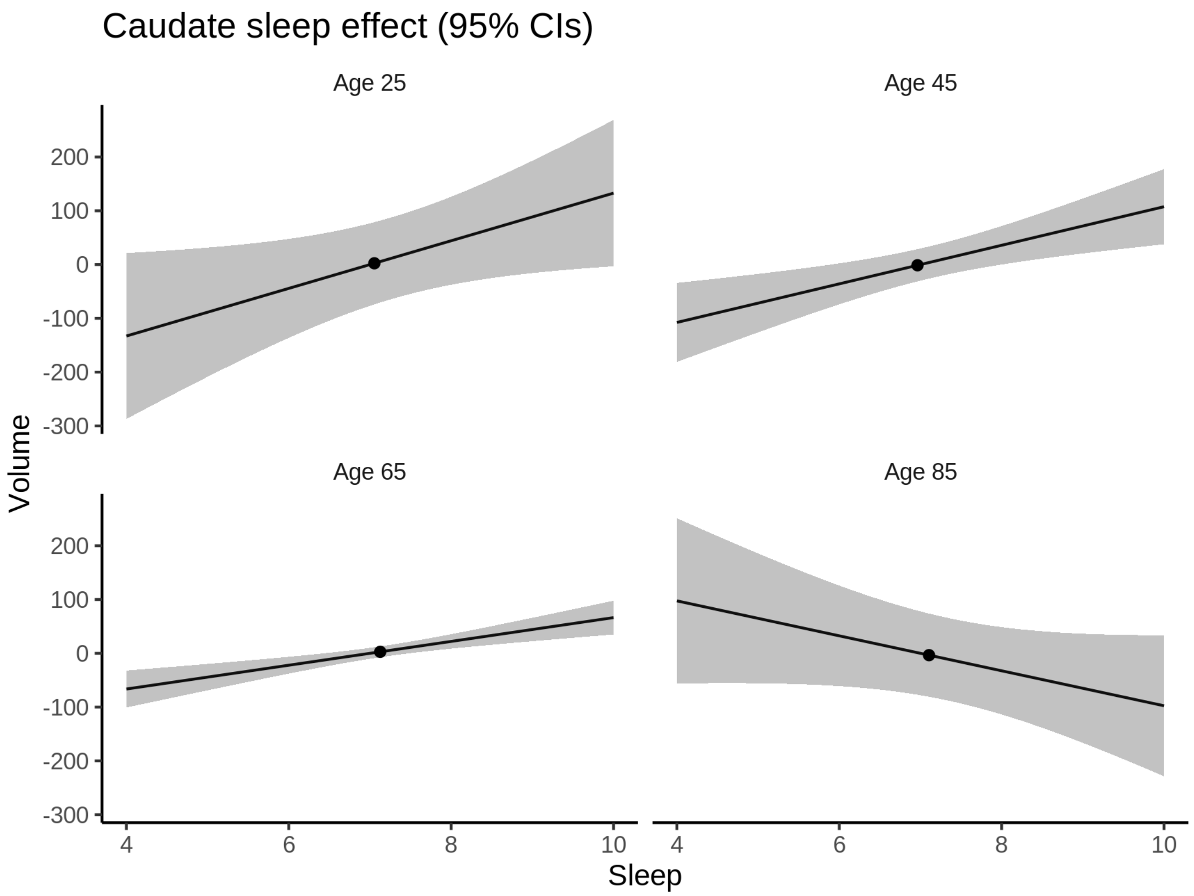

Deviation from sleep associated with maximal volume

Model with only main effect of sleep was chosen, so we show it for all ages at once. Maximum volume is attained at 10 hours of sleep. The percentage values in the plot are calculated as follows: The maximum at 100 % refers to a person at an arbitrary age with a sleep duration associated with maximum volume. For a female, this volume is 6667 and for a male it is 7210. The other percentage values show how large the expected volume is for someone with other sleep durations. For example, 99 % implies a 1 % reduction.

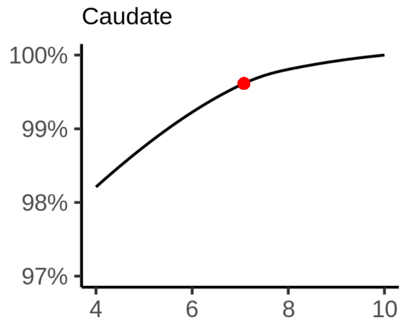

Comparison of mean sleep and sleep associated with maximum volume

A 95 % confidence interval for the sleep associated with maximum volume is [7.82, 10].

The plot below compares average sleep to the sleep associated with maximum volume.

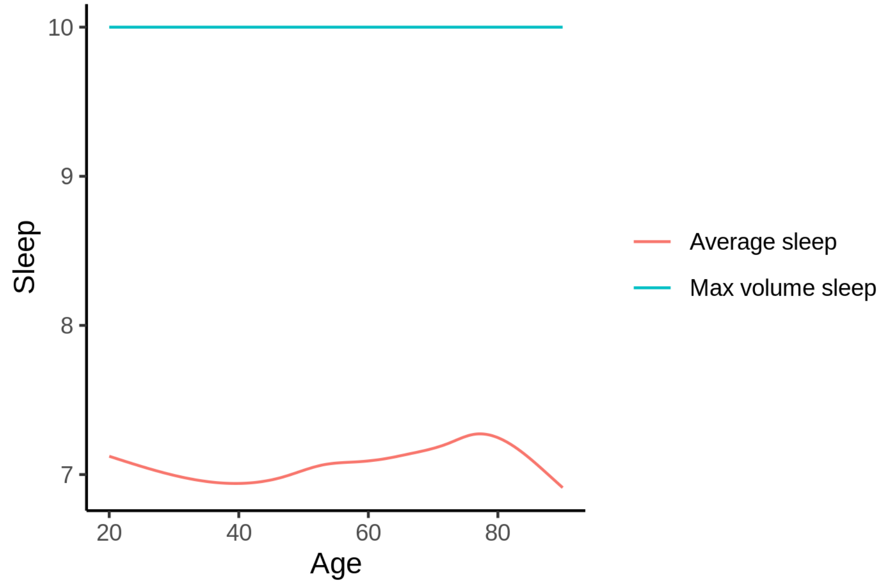

The next plot shows the difference between average sleep and sleep associated with maximum volume. The shaded region is a 95 % confidence interval.

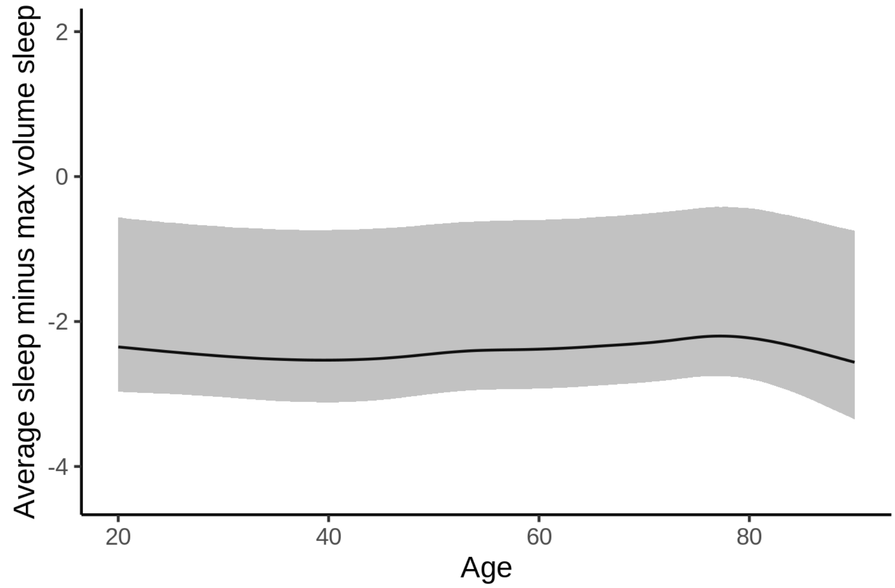

The next plot shows the probability that the sleep duration associated with maximum volume is longer than the average sleep duration, as a function of age. Probability below .05 can be interpreted as evidence that the sleep associated with maximum volume is shorter than the mean sleep, and probability above .95 can be interpreted the opposite way.

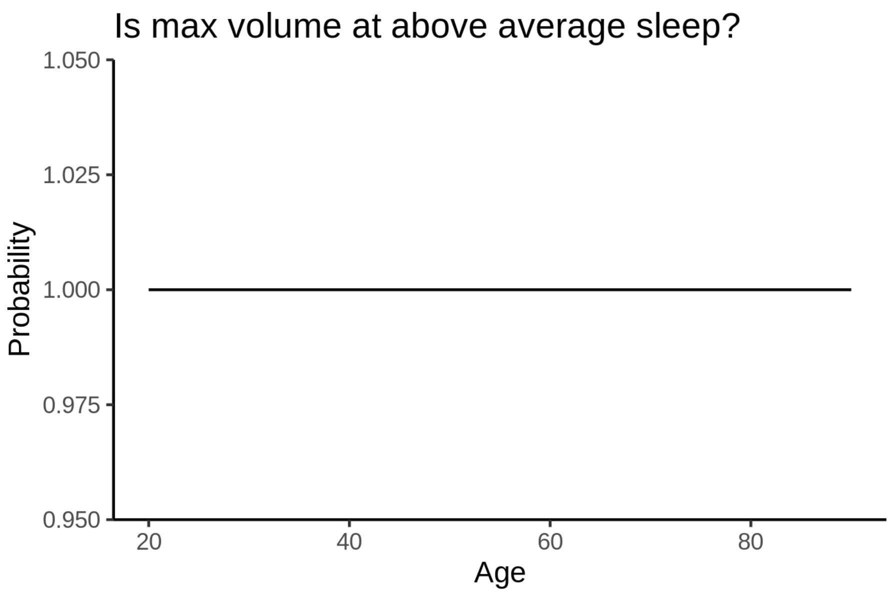

CC_Anterior

Descriptive statistics

Study	Observations	Unique IDs	Mean age	Age range
HCP	974	974	28.8	22 - 37
MPIB	677	391	63.1	24 - 83
UB	113	39	70.9	64 - 81
UCAM	884	632	55.1	20 - 88
UiO	1475	803	49.4	20 - 89
UKB	45969	43132	64.5	45 - 83
UmU	412	279	62.2	25 - 85
UOXF	769	769	69.8	60 - 85

Spaghetti plot

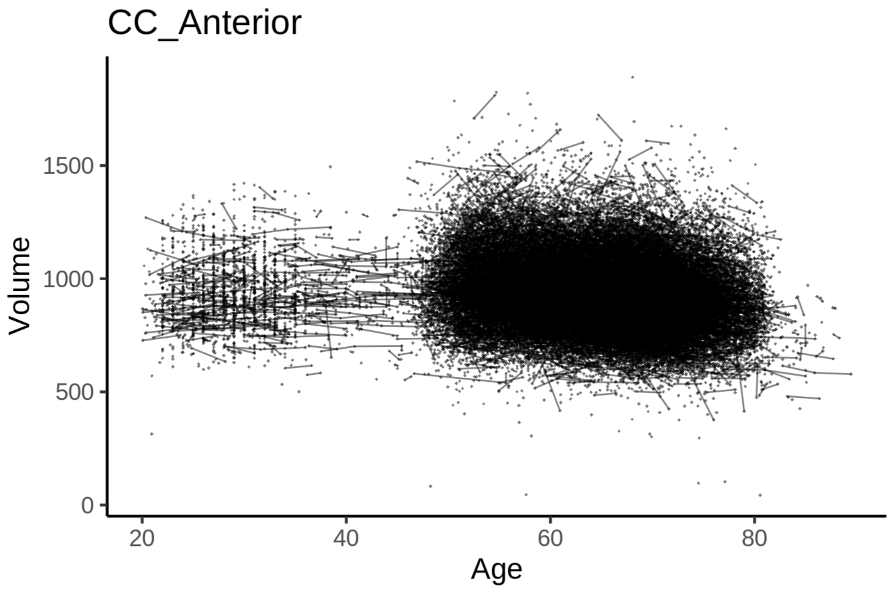

Models Controling for ICV

Model outputs

Model without sleep term

```
##
## Family: gaussian
## Link function: identity
##
## Formula:
## value ~ sex + site + icv + s(age_z, k = 10, bs = "cr")
## <environment: 0x558ad7ccd240>
##
## Parametric coefficients:
##                Estimate Std. Error t value Pr(>|t|)
## (Intercept)    938.2982     8.0511 116.542  < 2e-16 ***
## sexmale        -40.6634     1.6409 -24.781  < 2e-16 ***
## siteMPIB        94.4094    10.2592   9.202  < 2e-16 ***
## siteousAvanto -71.2842     8.2739  -8.616  < 2e-16 ***
## siteousPrisma -31.7765    14.0095  -2.268 0.023321 *
## siteousSkyra  -32.7960     7.8127  -4.198 2.70e-05 ***
## siteUB        -59.2729    24.0473  -2.465 0.013710 *
## siteUCAM      -62.0527     8.8953  -6.976 3.08e-12 ***
## siteUKB        22.4239     8.2575   2.716 0.006618 **
## siteUmU       -10.9414    11.5018  -0.951 0.341469
## siteUOXF      -32.4647     9.7697  -3.323 0.000891 ***
## icv            81.0000     0.8251  98.172  < 2e-16 ***
## ---
## Signif. codes:  0 ‘***’ 0.001 ‘**’ 0.01 ‘*’ 0.05 ‘.’ 0.1 ‘ ’ 1
##
```

```
## Approximate significance of smooth terms:
##            edf Ref.df     F p-value
## s(age_z) 7.379  7.379 130.4  <2e-16 ***
## ---
## Signif. codes:  0 ‘***’ 0.001 ‘**’ 0.01 ‘*’ 0.05 ‘.’ 0.1 ‘ ’ 1
##
## R-sq.(adj) =  0.215
## lmer.REML = 6.478e+05  Scale est. = 1718.9    n = 51273
```

Model with only main effects of age and sleep

```
##
## Family: gaussian
## Link function: identity
##
## Formula:
## value ~ sex + site + icv + s(age_z, k = 10, bs = "cr") + s(sleep_z,
##     k = 5, bs = "cr")
## <environment: 0x558ad7ccd240>
##
## Parametric coefficients:
##                Estimate Std. Error t value Pr(>|t|)
## (Intercept)    937.3624     8.0590 116.312  < 2e-16 ***
## sexmale        -40.7085     1.6408 -24.810  < 2e-16 ***
## siteMPIB        95.5652    10.2655   9.309  < 2e-16 ***
## siteousAvanto -70.8321     8.2758  -8.559  < 2e-16 ***
## siteousPrisma -31.4785    14.0088  -2.247  0.02464 *
## siteousSkyra  -32.3471     7.8151  -4.139 3.49e-05 ***
## siteUB        -58.8205    24.0450  -2.446  0.01444 *
## siteUCAM      -61.4874     8.8964  -6.911 4.85e-12 ***
## siteUKB        23.4231     8.2662   2.834  0.00460 **
## siteUmU        -8.5511    11.5279  -0.742  0.45823
## siteUOXF      -31.9830     9.7709  -3.273  0.00106 **
## icv            81.0448     0.8256  98.163  < 2e-16 ***
## ---
## Signif. codes:  0 ‘***’ 0.001 ‘**’ 0.01 ‘*’ 0.05 ‘.’ 0.1 ‘ ’ 1
##
## Approximate significance of smooth terms:
##              edf Ref.df       F p-value
## s(age_z)   7.386  7.386 127.637  <2e-16 ***
## s(sleep_z) 1.649  1.649   7.825  0.0101 *
## ---
## Signif. codes:  0 ‘***’ 0.001 ‘**’ 0.01 ‘*’ 0.05 ‘.’ 0.1 ‘ ’ 1
##
## R-sq.(adj) =  0.215
## lmer.REML = 6.4779e+05  Scale est. = 1718.8    n = 51273
```

Model with full interaction between age and sleep

```
##
## Family: gaussian
## Link function: identity
##
## Formula:
## value ~ sex + site + icv + t2(age_z, sleep_z, k = c(10, 4), bs = "cr")
## <environment: 0x558ad7ccd240>
```

```
##
## Parametric coefficients:
##                Estimate Std. Error t value Pr(>|t|)
## (Intercept)    937.1574     7.9760 117.497  < 2e-16 ***
## sexmale        -40.6707     1.6443 -24.734  < 2e-16 ***
## siteMPIB        95.8570    10.2584   9.344  < 2e-16 ***
## siteousAvanto -70.6390     8.2826  -8.529  < 2e-16 ***
## siteousPrisma -31.6091    14.0325  -2.253  0.02429 *
## siteousSkyra  -32.1757     7.8260  -4.111 3.94e-05 ***
## siteUB        -58.8333    24.0205  -2.449  0.01432 *
## siteUCAM      -61.1803     8.8841  -6.886 5.78e-12 ***
## siteUKB        23.6120     8.1755   2.888  0.00388 **
## siteUmU        -8.2187    11.4935  -0.715  0.47456
## siteUOXF      -31.9516     9.7105  -3.290  0.00100 **
## icv            81.0074     0.8255  98.128  < 2e-16 ***
## ---
## Signif. codes:  0 '***' 0.001 '**' 0.01 '*' 0.05 '.' 0.1 ' ' 1
##
## Approximate significance of smooth terms:
##                     edf Ref.df     F p-value
## t2(age_z,sleep_z) 11.58  11.58 10.95  <2e-16 ***
## ---
## Signif. codes:  0 '***' 0.001 '**' 0.01 '*' 0.05 '.' 0.1 ' ' 1
##
## R-sq.(adj) =  0.215
## lmer.REML = 6.478e+05  Scale est. = 1718.9    n = 51273
```

Model comparison

`mod_no_sleep` refers to model without sleep term, `mod_no_interaction` refers to model with only main effect of sleep, and `mod_full` refers to model with a full interaction between age and sleep. This is a nested model comparison, and the p-value at a given line refers to comparing the model at the line to the model on the line above. Hence, significance implies that the more complicated model is supported on statistical grounds.

To be even more specific, the p-value on the second row tests whether there is an association between sleep and volume. The p-value on the third row tests whether this association depends on age.

```
## Data: NULL
## Models:
## ml$mod_no_sleep$mer: NULL
## ml$mod_no_interaction$mer: NULL
## ml$mod_full$mer: NULL
##                           npar    AIC    BIC  logLik deviance  Chisq Df Pr(>Chisq)
## ml$mod_no_sleep$mer         16 647834 647975 -323901   647802
## ml$mod_no_interaction$mer   18 647829 647988 -323896   647793 8.8945  2    0.01171 *
## ml$mod_full$mer             20 647835 648012 -323898   647795 0.0000  2    1.00000
## ---
## Signif. codes:  0 '***' 0.001 '**' 0.01 '*' 0.05 '.' 0.1 ' ' 1
```

We chose the model based on the likelihood ratio test with 5 % significance level, which was `mod_no_interaction`.

Lifespan brain trajectory

The trajectory shown is from the chosen model `mod_no_interaction`.

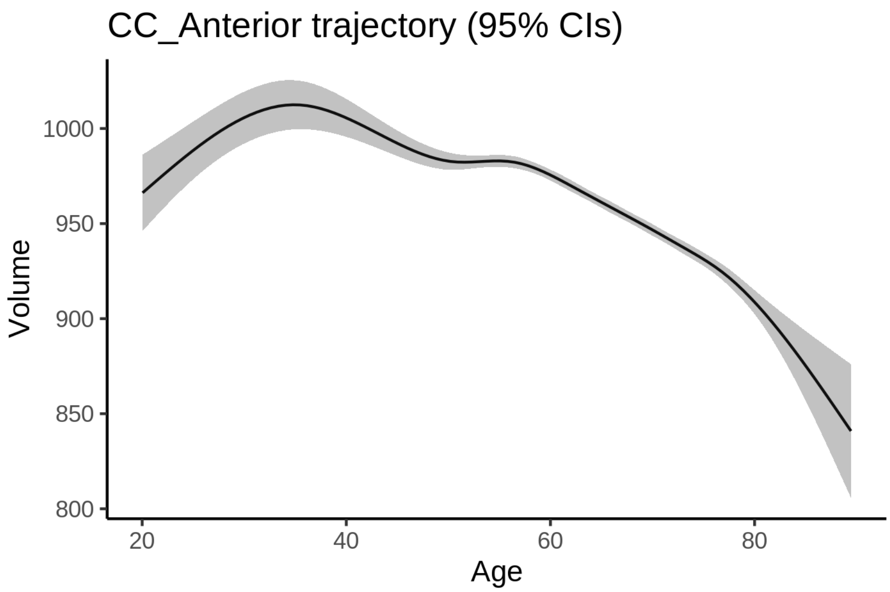

Effect of sleep

The chosen model only included the main effect of sleep, and hence the effect does not vary with age. The black dot shows the average sleep duration across all ages in the sample.

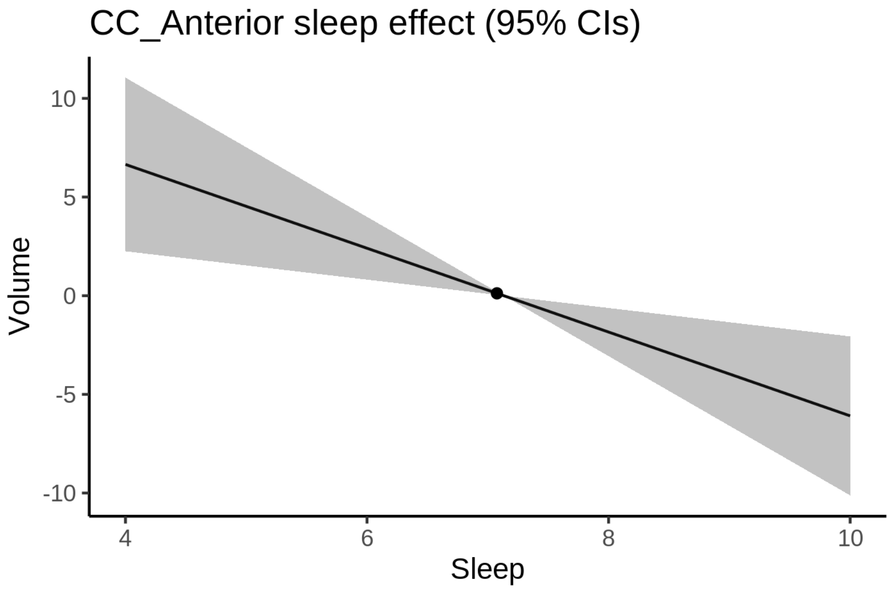

We also show the full interaction model for completeness, although it was not selected.

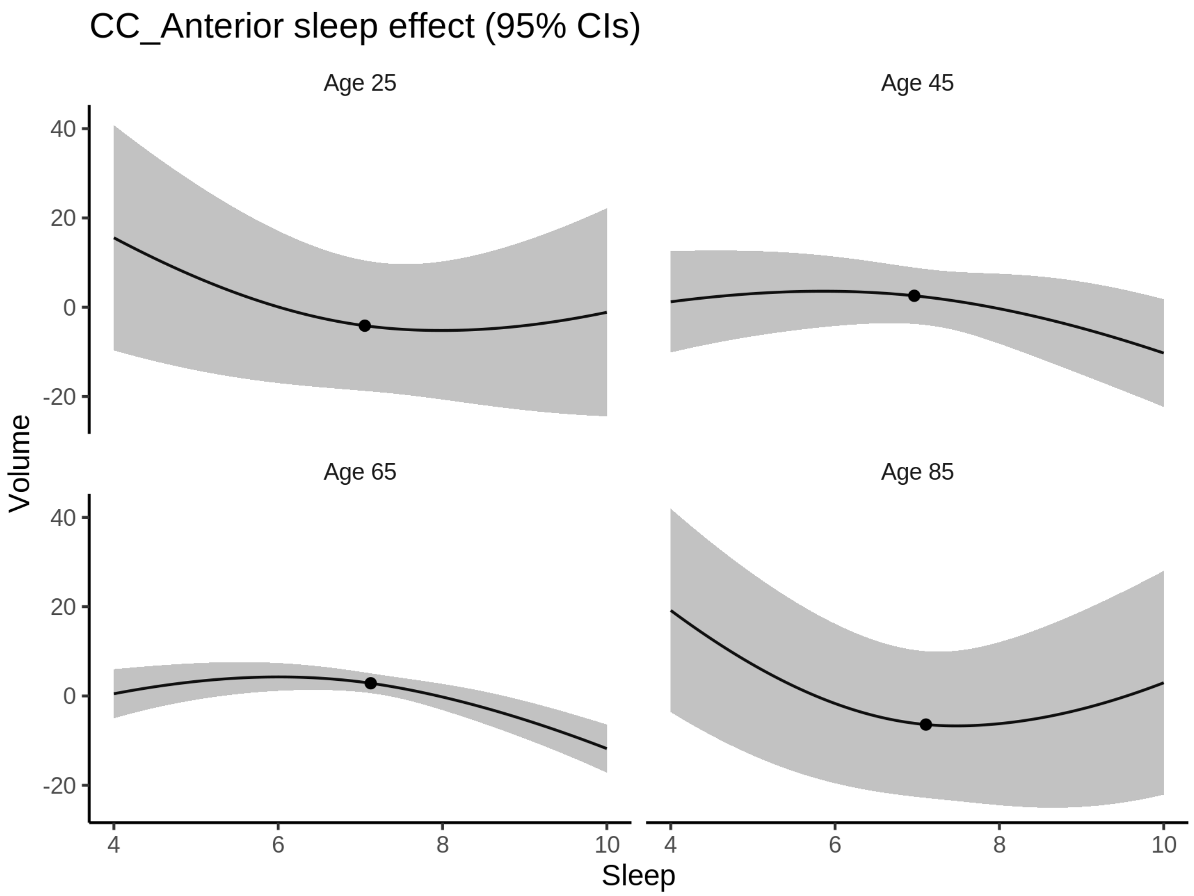

Deviation from sleep associated with maximal volume

Model with only main effect of sleep was chosen, so we show it for all ages at once. Maximum volume is attained at 4 hours of sleep. The percentage values in the plot are calculated as follows: The maximum at 100 % refers to a person at an arbitrary age with a sleep duration associated with maximum volume. For a female, this volume is 962 and for a male it is 921. The other percentage values show how large the expected volume is for someone with other sleep durations. For example, 99 % implies a 1 % reduction.

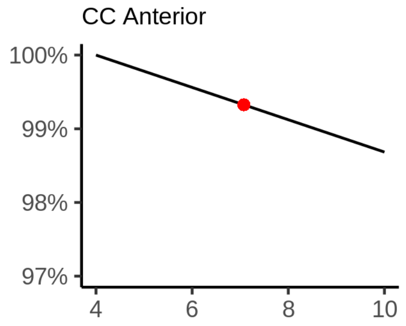

Comparison of mean sleep and sleep associated with maximum volume

A 95 % confidence interval for the sleep associated with maximum volume is [4, 6.24].

The plot below compares average sleep to the sleep associated with maximum volume.

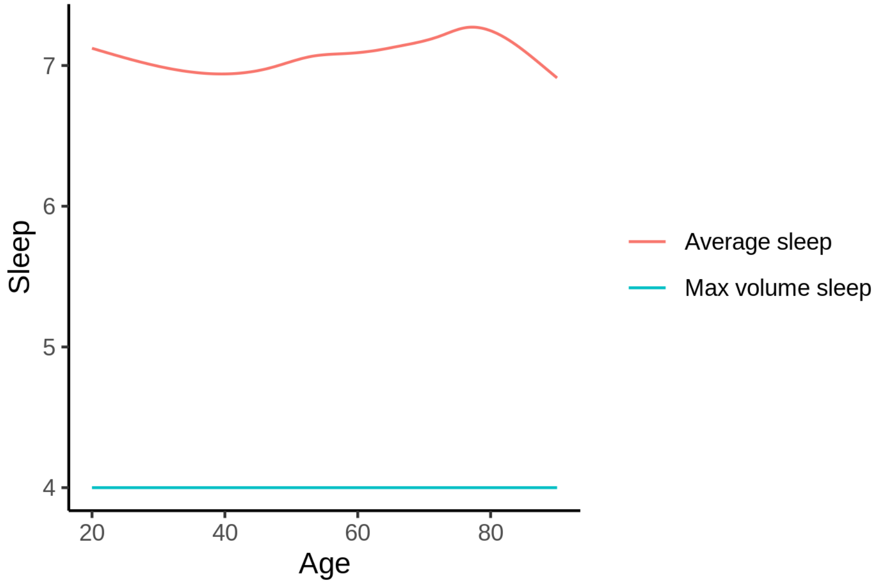

The next plot shows the difference between average sleep and sleep associated with maximum volume. The shaded region is a 95 % confidence interval.

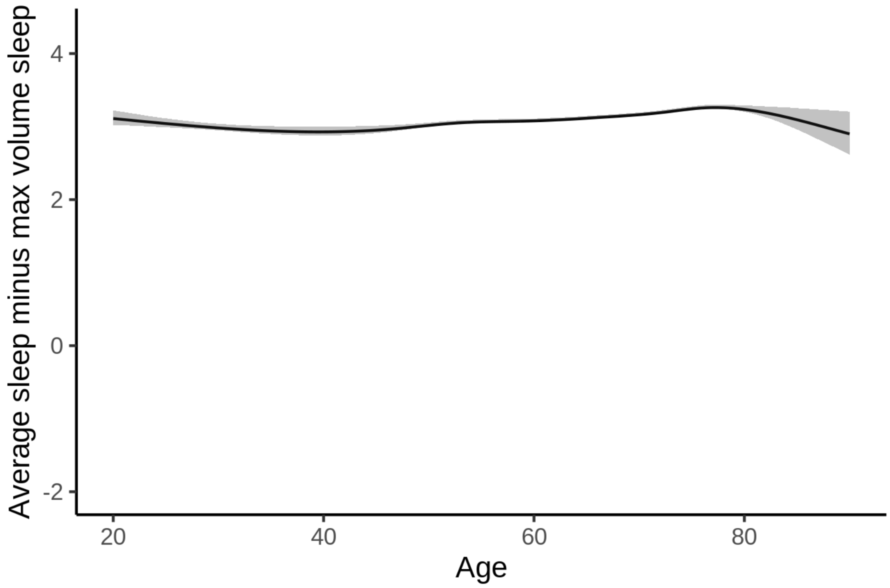

The next plot shows the probability that the sleep duration associated with maximum volume is longer than the average sleep duration, as a function of age. Probability below .05 can be interpreted as evidence that the sleep associated with maximum volume is shorter than the mean sleep, and probability above .95 can be interpreted the opposite way.

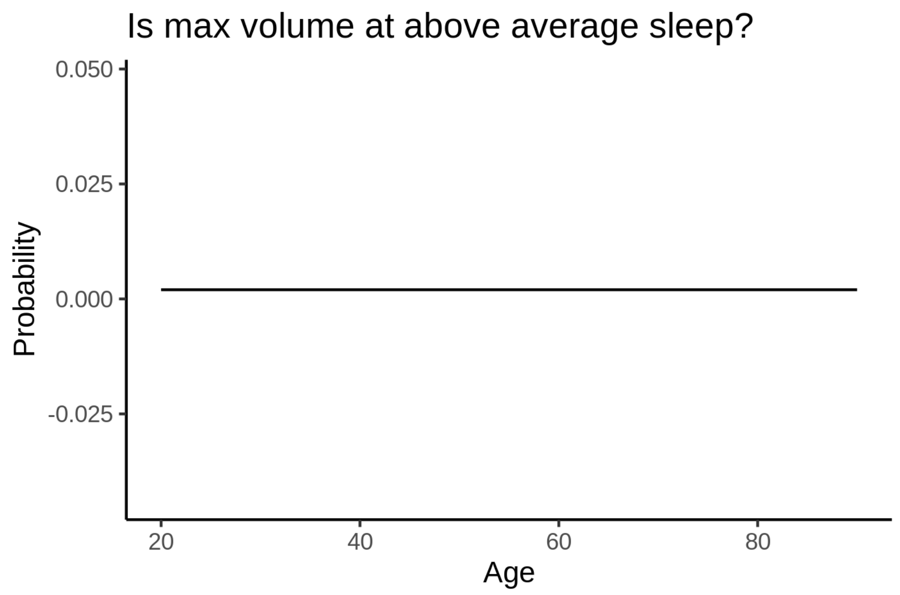

Controling for covariates

Below is the output for a model in which we only include data with income and education.

```
##
## Family: gaussian
## Link function: identity
##
## Formula:
## value ~ sex + site + s(age_z, k = 10, bs = "cr") + s(sleep_z,
##     k = 5, bs = "cr") + icv
## <environment: 0x558ada8f8bd8>
##
## Parametric coefficients:
##                Estimate Std. Error t value Pr(>|t|)
## (Intercept)    1070.007     19.035  56.214  < 2e-16 ***
## sexmale         -43.547      2.102 -20.719  < 2e-16 ***
## siteousAvanto -202.615     23.314  -8.691  < 2e-16 ***
## siteousPrisma -143.408     23.506  -6.101 1.07e-09 ***
## siteousSkyra  -147.718     20.647  -7.154 8.58e-13 ***
## siteUKB       -109.953     18.998  -5.788 7.21e-09 ***
## siteUOXF      -149.823     21.405  -7.000 2.62e-12 ***
## icv             84.895      1.080  78.609  < 2e-16 ***
## ---
## Signif. codes:  0 ‘***’ 0.001 ‘**’ 0.01 ‘*’ 0.05 ‘.’ 0.1 ‘ ’ 1
##
## Approximate significance of smooth terms:
##              edf Ref.df     F p-value
## s(age_z)   4.989  4.989 83.70  <2e-16 ***
## s(sleep_z) 1.705  1.705  2.23  0.0679 .
## ---
## Signif. codes:  0 ‘***’ 0.001 ‘**’ 0.01 ‘*’ 0.05 ‘.’ 0.1 ‘ ’ 1
##
```

```
## R-sq.(adj) =  0.211
## lmer.REML = 3.9487e+05  Scale est. = 2119.2    n = 31188
```

Below is the output for a model in which we control for the main effects of income and education.

```
##
## Family: gaussian
## Link function: identity
##
## Formula:
## value ~ sex + site + s(age_z, k = 10, bs = "cr") + s(sleep_z,
##     k = 5, bs = "cr") + icv + income_scaled + education_scaled
## <environment: 0x558ada8f8bd8>
##
## Parametric coefficients:
##                   Estimate Std. Error t value Pr(>|t|)
## (Intercept)      1069.8153    19.1589  55.839  < 2e-16 ***
## sexmale           -43.5166     2.1080 -20.643  < 2e-16 ***
## siteousAvanto    -202.7119    23.3200  -8.693  < 2e-16 ***
## siteousPrisma    -143.4438    23.5081  -6.102 1.06e-09 ***
## siteousSkyra     -147.8118    20.6533  -7.157 8.44e-13 ***
## siteUKB          -110.0777    19.0100  -5.791 7.08e-09 ***
## siteUOXF         -149.8169    21.4174  -6.995 2.70e-12 ***
## icv                84.8856     1.0894  77.922  < 2e-16 ***
## income_scaled      -0.2847     2.5045  -0.114    0.909
## education_scaled    0.5576     2.9587   0.188    0.851
## ---
## Signif. codes:  0 '***' 0.001 '**' 0.01 '*' 0.05 '.' 0.1 ' ' 1
##
## Approximate significance of smooth terms:
##              edf Ref.df      F p-value
## s(age_z)   4.992  4.992 76.609  <2e-16 ***
## s(sleep_z) 1.703  1.703  2.239  0.0674 .
## ---
## Signif. codes:  0 '***' 0.001 '**' 0.01 '*' 0.05 '.' 0.1 ' ' 1
##
## R-sq.(adj) =  0.211
## lmer.REML = 3.9486e+05  Scale est. = 2119.2    n = 31188
```

We also included interaction effects between sleep duration and education and income, in another model. The output is shown below, and the interaction terms are `income_scaled:sleep_z` and `education_scaled:sleep_z`.

```
##
## Family: gaussian
## Link function: identity
##
## Formula:
## value ~ sex + site + s(age_z, k = 10, bs = "cr") + s(sleep_z,
##     k = 5, bs = "cr") + icv + income_scaled + education_scaled +
##     income_scaled:sleep_z + education_scaled:sleep_z
## <environment: 0x558ada8f8bd8>
##
## Parametric coefficients:
##                           Estimate Std. Error t value Pr(>|t|)
## (Intercept)              1069.7661    19.1603  55.832  < 2e-16 ***
```

```
## sexmale                  -43.4054     2.1095 -20.576  < 2e-16 ***
## siteousAvanto           -202.5312    23.3209  -8.685  < 2e-16 ***
## siteousPrisma           -142.9873    23.5108  -6.082 1.20e-09 ***
## siteousSkyra            -147.6294    20.6540  -7.148 9.01e-13 ***
## siteUKB                 -110.0328    19.0123  -5.787 7.21e-09 ***
## siteUOXF                -149.8447    21.4237  -6.994 2.72e-12 ***
## icv                       84.8737     1.0895  77.904  < 2e-16 ***
## income_scaled             -0.3927     2.5056  -0.157    0.875
## education_scaled           0.6298     2.9591   0.213    0.831
## income_scaled:sleep_z      3.2233     2.4815   1.299    0.194
## education_scaled:sleep_z  -2.5041     2.8678  -0.873    0.383
## ---
## Signif. codes:  0 ‘***’ 0.001 ‘**’ 0.01 ‘*’ 0.05 ‘.’ 0.1 ‘ ’ 1
##
## Approximate significance of smooth terms:
##              edf Ref.df      F p-value
## s(age_z)   4.997  4.997 76.708  <2e-16 ***
## s(sleep_z) 1.644  1.644  0.355   0.544
## ---
## Signif. codes:  0 ‘***’ 0.001 ‘**’ 0.01 ‘*’ 0.05 ‘.’ 0.1 ‘ ’ 1
##
## R-sq.(adj) =  0.211
## lmer.REML = 3.9485e+05  Scale est. = 2119.2    n = 31188
```

We did the same controling for BMI. Below is the model with no covariates but only keeping data with BMI.

```
##
## Family: gaussian
## Link function: identity
##
## Formula:
## value ~ sex + site + s(age_z, k = 10, bs = "cr") + s(sleep_z,
##     k = 5, bs = "cr") + icv
## <environment: 0x558adb36c900>
##
## Parametric coefficients:
##               Estimate Std. Error t value Pr(>|t|)
## (Intercept)    876.934     13.626  64.360  < 2e-16 ***
## sexmale        -43.283      2.035 -21.269  < 2e-16 ***
## siteousPrisma   42.906     17.919   2.394 0.016654 *
## siteousSkyra    46.658     11.074   4.213 2.52e-05 ***
## siteUCAM         7.328     14.327   0.511 0.609011
## siteUKB         83.057     13.726   6.051 1.45e-09 ***
## siteUmU         56.345     16.065   3.507 0.000453 ***
## icv             84.939      1.049  80.992  < 2e-16 ***
## ---
## Signif. codes:  0 ‘***’ 0.001 ‘**’ 0.01 ‘*’ 0.05 ‘.’ 0.1 ‘ ’ 1
##
## Approximate significance of smooth terms:
##              edf Ref.df      F p-value
## s(age_z)   5.019  5.019 98.572  <2e-16 ***
## s(sleep_z) 1.290  1.290  4.145  0.0528 .
## ---
## Signif. codes:  0 ‘***’ 0.001 ‘**’ 0.01 ‘*’ 0.05 ‘.’ 0.1 ‘ ’ 1
##
```

```
## R-sq.(adj) =  0.214
## lmer.REML = 4.2259e+05  Scale est. = 1983.1    n = 33432
```

Below is the model output with main effect.

```
##
## Family: gaussian
## Link function: identity
##
## Formula:
## value ~ sex + site + s(age_z, k = 10, bs = "cr") + s(sleep_z,
##     k = 5, bs = "cr") + icv + bmi
## <environment: 0x558adb36c900>
##
## Parametric coefficients:
##                Estimate Std. Error t value Pr(>|t|)
## (Intercept)    926.3514    14.5003  63.885  < 2e-16 ***
## sexmale        -41.7136     2.0380 -20.468  < 2e-16 ***
## siteousPrisma   44.9219    17.9037   2.509 0.012109 *
## siteousSkyra    46.9869    11.0718   4.244 2.20e-05 ***
## siteUCAM         8.5913    14.3167   0.600 0.548448
## siteUKB         84.6928    13.7200   6.173 6.78e-10 ***
## siteUmU         58.5390    16.0525   3.647 0.000266 ***
## icv             85.2279     1.0473  81.380  < 2e-16 ***
## bmi             -1.9640     0.1977  -9.935  < 2e-16 ***
## ---
## Signif. codes:  0 ‘***’ 0.001 ‘**’ 0.01 ‘*’ 0.05 ‘.’ 0.1 ‘ ’ 1
##
## Approximate significance of smooth terms:
##              edf Ref.df      F p-value
## s(age_z)   5.079  5.079 98.899  <2e-16 ***
## s(sleep_z) 1.000  1.000  6.561  0.0104 *
## ---
## Signif. codes:  0 ‘***’ 0.001 ‘**’ 0.01 ‘*’ 0.05 ‘.’ 0.1 ‘ ’ 1
##
## R-sq.(adj) =  0.217
## lmer.REML = 4.225e+05  Scale est. = 1982.5    n = 33432
```

Next is the model with BMI-sleep interaction.

```
##
## Family: gaussian
## Link function: identity
##
## Formula:
## value ~ sex + site + s(age_z, k = 10, bs = "cr") + s(sleep_z,
##     k = 5, bs = "cr") + icv + bmi + bmi:sleep_z
## <environment: 0x558adb36c900>
##
## Parametric coefficients:
##                Estimate Std. Error t value Pr(>|t|)
## (Intercept)    926.9231    14.5068  63.896  < 2e-16 ***
## sexmale        -41.7235     2.0380 -20.473  < 2e-16 ***
## siteousPrisma   44.9119    17.9035   2.509  0.01213 *
## siteousSkyra    46.9251    11.0717   4.238 2.26e-05 ***
## siteUCAM         8.3675    14.3176   0.584  0.55894
```

```
## siteUKB        84.4741    13.7210   6.157 7.52e-10 ***
## siteUmU        58.3350    16.0531   3.634  0.00028 ***
## icv            85.2184     1.0473  81.369  < 2e-16 ***
## bmi            -1.9728     0.1978  -9.974  < 2e-16 ***
## bmi:sleep_z    -0.2464     0.1872  -1.317  0.18799
## ---
## Signif. codes:  0 ‘***’ 0.001 ‘**’ 0.01 ‘*’ 0.05 ‘.’ 0.1 ‘ ’ 1
##
## Approximate significance of smooth terms:
##             edf Ref.df      F p-value
## s(age_z)   5.08   5.08 98.689  <2e-16 ***
## s(sleep_z) 1.00   1.00  0.758   0.384
## ---
## Signif. codes:  0 ‘***’ 0.001 ‘**’ 0.01 ‘*’ 0.05 ‘.’ 0.1 ‘ ’ 1
##
## R-sq.(adj) =  0.217
## lmer.REML = 4.225e+05  Scale est. = 1982.5    n = 33432
```

We did the same controling for depression. Below is the model with no covariates but only keeping data with depression.

```
##
## Family: gaussian
## Link function: identity
##
## Formula:
## value ~ sex + site + s(age_z, k = 10, bs = "cr") + s(sleep_z,
##     k = 5, bs = "cr") + icv
## <environment: 0x558a48df5cd0>
##
## Parametric coefficients:
##               Estimate Std. Error t value Pr(>|t|)
## (Intercept)   1012.299      9.677 104.611  < 2e-16 ***
## sexmale        -43.504      2.048 -21.243  < 2e-16 ***
## siteousAvanto -129.369     21.558  -6.001 1.98e-09 ***
## siteousPrisma  -85.761     18.654  -4.597 4.29e-06 ***
## siteousSkyra   -78.864     14.536  -5.425 5.82e-08 ***
## siteUCAM      -129.663     13.223  -9.806  < 2e-16 ***
## siteUKB        -52.529      9.568  -5.490 4.04e-08 ***
## siteUmU        -79.822     13.040  -6.122 9.37e-10 ***
## icv             84.721      1.054  80.385  < 2e-16 ***
## ---
## Signif. codes:  0 ‘***’ 0.001 ‘**’ 0.01 ‘*’ 0.05 ‘.’ 0.1 ‘ ’ 1
##
## Approximate significance of smooth terms:
##              edf Ref.df      F p-value
## s(age_z)   5.189  5.189 88.936  <2e-16 ***
## s(sleep_z) 1.568  1.568  4.952  0.0371 *
## ---
## Signif. codes:  0 ‘***’ 0.001 ‘**’ 0.01 ‘*’ 0.05 ‘.’ 0.1 ‘ ’ 1
##
## R-sq.(adj) =  0.212
## lmer.REML = 4.2136e+05  Scale est. = 1930      n = 33344
```

Below is the model output with main effect.

```
##
## Family: gaussian
## Link function: identity
##
## Formula:
## value ~ sex + site + s(age_z, k = 10, bs = "cr") + s(sleep_z,
##     k = 5, bs = "cr") + icv + depression
## <environment: 0x558a48df5cd0>
##
## Parametric coefficients:
##                Estimate Std. Error t value Pr(>|t|)
## (Intercept)    1011.215      9.720 104.038  < 2e-16 ***
## sexmale         -43.392      2.050 -21.167  < 2e-16 ***
## siteousAvanto -129.911     21.560  -6.025 1.70e-09 ***
## siteousPrisma  -86.240     18.656  -4.623 3.80e-06 ***
## siteousSkyra   -79.485     14.542  -5.466 4.64e-08 ***
## siteUCAM      -129.902     13.224  -9.824  < 2e-16 ***
## siteUKB        -52.090      9.576  -5.440 5.37e-08 ***
## siteUmU        -83.123     13.315  -6.243 4.35e-10 ***
## icv             84.701      1.054  80.355  < 2e-16 ***
## depression       8.945      7.295   1.226     0.22
## ---
## Signif. codes:  0 ‘***’ 0.001 ‘**’ 0.01 ‘*’ 0.05 ‘.’ 0.1 ‘ ’ 1
##
## Approximate significance of smooth terms:
##              edf Ref.df     F p-value
## s(age_z)   5.172  5.172 86.19  <2e-16 ***
## s(sleep_z) 1.750  1.750  4.54  0.0445 *
## ---
## Signif. codes:  0 ‘***’ 0.001 ‘**’ 0.01 ‘*’ 0.05 ‘.’ 0.1 ‘ ’ 1
##
## R-sq.(adj) =  0.212
## lmer.REML = 4.2135e+05  Scale est. = 1929.8    n = 33344
```

Next is the model with depression-sleep interaction.

```
##
## Family: gaussian
## Link function: identity
##
## Formula:
## value ~ sex + site + s(age_z, k = 10, bs = "cr") + s(sleep_z,
##     k = 5, bs = "cr") + icv + depression + depression:sleep_z
## <environment: 0x558a48df5cd0>
##
## Parametric coefficients:
##                     Estimate Std. Error t value Pr(>|t|)
## (Intercept)         1011.213      9.720 104.038  < 2e-16 ***
## sexmale              -43.424      2.050 -21.179  < 2e-16 ***
## siteousAvanto       -130.159     21.562  -6.037 1.59e-09 ***
## siteousPrisma        -86.484     18.657  -4.635 3.58e-06 ***
## siteousSkyra         -79.723     14.544  -5.482 4.24e-08 ***
## siteUCAM            -130.140     13.226  -9.840  < 2e-16 ***
## siteUKB              -52.077      9.576  -5.438 5.41e-08 ***
## siteUmU              -81.607     13.427  -6.078 1.23e-09 ***
```

```
## icv                   84.704      1.054  80.357  < 2e-16 ***
## depression             8.582      7.308   1.174     0.24
## depression:sleep_z    -4.690      5.339  -0.878     0.38
## ---
## Signif. codes:  0 '***' 0.001 '**' 0.01 '*' 0.05 '.' 0.1 ' ' 1
##
## Approximate significance of smooth terms:
##               edf Ref.df      F p-value
## s(age_z)    5.162  5.162 86.387  <2e-16 ***
## s(sleep_z) 1.744  1.744  2.466   0.187
## ---
## Signif. codes:  0 '***' 0.001 '**' 0.01 '*' 0.05 '.' 0.1 ' ' 1
##
## R-sq.(adj) =  0.212
## lmer.REML = 4.2135e+05  Scale est. = 1929.9    n = 33344
```

The plot below shows the sleep-volume curve for the original model and for the model with main effects of SES.

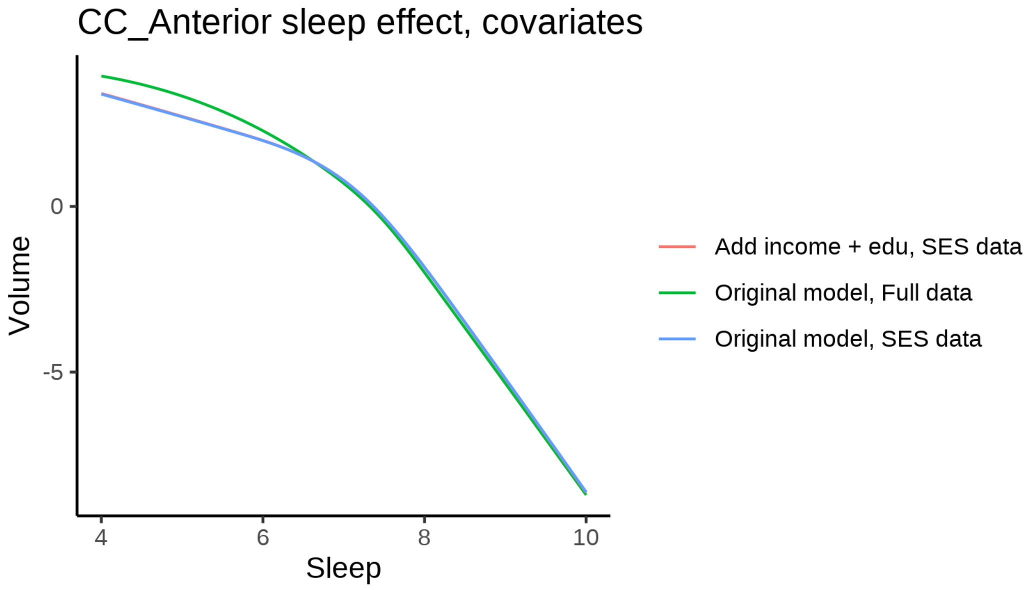

The plot below shows the sleep-volume curve for the original model and for the model with main effects of BMI.

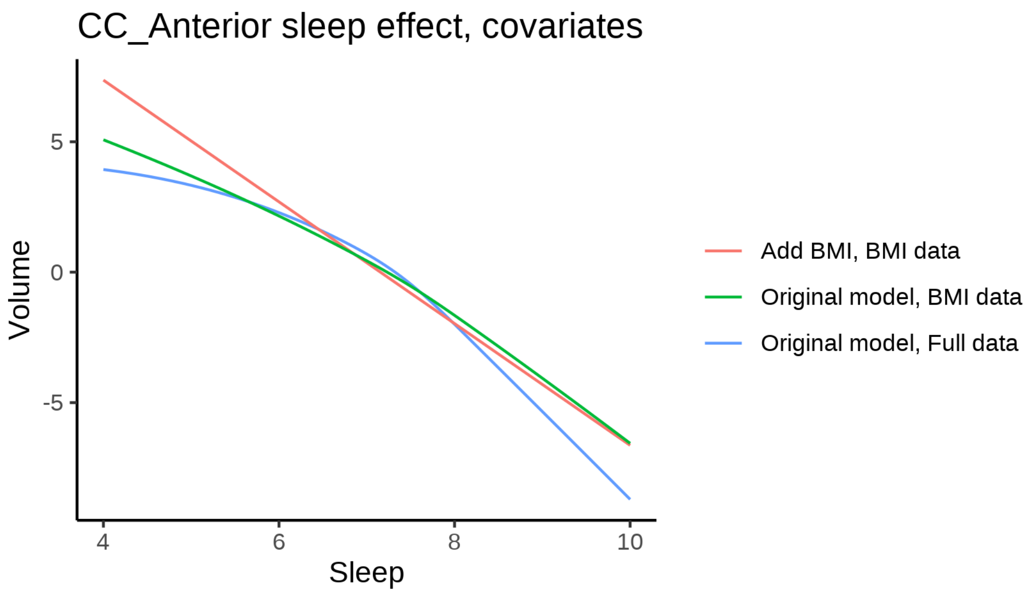

The plot below shows the sleep-volume curve for the original model and for the model with main effects of depression.

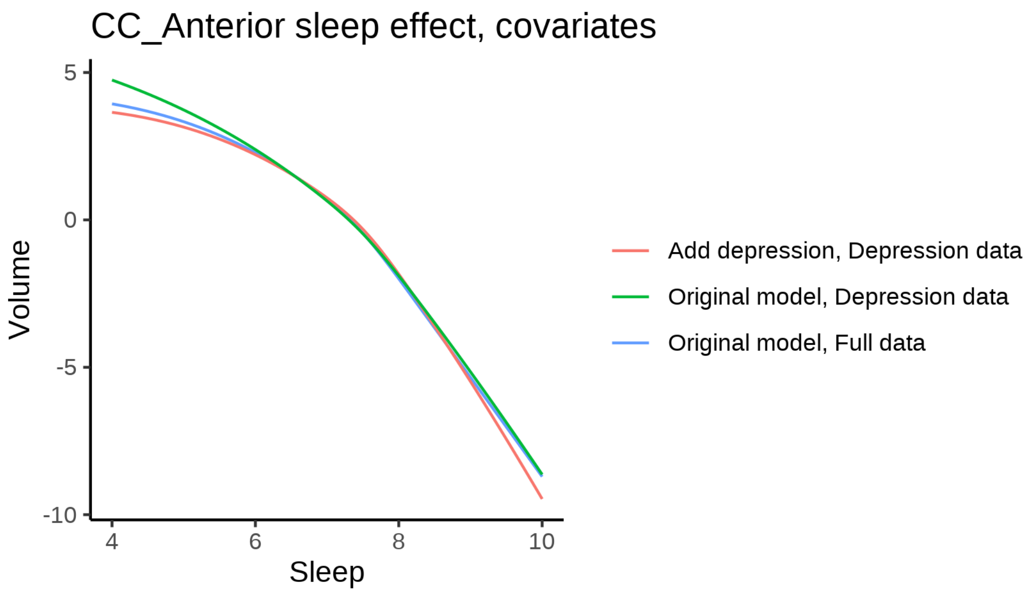

Models Not controling for ICV

Model outputs

Model without sleep term

```
##
## Family: gaussian
## Link function: identity
##
## Formula:
```

```
## value ~ sex + site + s(age_z, k = 10, bs = "cr")
## <environment: 0x558ad80a4f08>
##
## Parametric coefficients:
##                Estimate Std. Error t value Pr(>|t|)
## (Intercept)     893.135      8.646 103.303  < 2e-16 ***
## sexmale          51.234      1.477  34.699  < 2e-16 ***
## siteMPIB        -34.977     11.083  -3.156 0.001602 **
## siteousAvanto   -36.043      8.930  -4.036 5.44e-05 ***
## siteousPrisma   -13.435     15.191  -0.884 0.376461
## siteousSkyra    -27.290      8.500  -3.211 0.001326 **
## siteUB          -82.619     26.367  -3.133 0.001729 **
## siteUCAM        -57.205      9.669  -5.916 3.31e-09 ***
## siteUKB          25.552      8.878   2.878 0.004004 **
## siteUmU         -43.101     12.516  -3.444 0.000574 ***
## siteUOXF        -50.780     10.564  -4.807 1.54e-06 ***
## ---
## Signif. codes:  0 '***' 0.001 '**' 0.01 '*' 0.05 '.' 0.1 ' ' 1
##
## Approximate significance of smooth terms:
##             edf Ref.df     F p-value
## s(age_z) 6.313  6.313 200.3  <2e-16 ***
## ---
## Signif. codes:  0 '***' 0.001 '**' 0.01 '*' 0.05 '.' 0.1 ' ' 1
##
## R-sq.(adj) =  0.0585
## lmer.REML = 6.566e+05  Scale est. = 1804.2    n = 51273
```

Model with only main effects of age and sleep

```
##
## Family: gaussian
## Link function: identity
##
## Formula:
## value ~ sex + site + s(age_z, k = 10, bs = "cr") + s(sleep_z,
##     k = 5, bs = "cr")
## <environment: 0x558ad80a4f08>
##
## Parametric coefficients:
##                Estimate Std. Error t value Pr(>|t|)
## (Intercept)     894.111      8.652 103.342  < 2e-16 ***
## sexmale          51.001      1.477  34.530  < 2e-16 ***
## siteMPIB        -34.718     11.085  -3.132 0.001738 **
## siteousAvanto   -36.847      8.931  -4.126 3.70e-05 ***
## siteousPrisma   -14.028     15.187  -0.924 0.355677
## siteousSkyra    -28.169      8.501  -3.314 0.000921 ***
## siteUB          -81.746     26.358  -3.101 0.001927 **
## siteUCAM        -57.105      9.667  -5.907 3.50e-09 ***
## siteUKB          24.632      8.884   2.773 0.005565 **
## siteUmU         -42.831     12.540  -3.416 0.000637 ***
## siteUOXF        -51.176     10.560  -4.846 1.26e-06 ***
## ---
## Signif. codes:  0 '***' 0.001 '**' 0.01 '*' 0.05 '.' 0.1 ' ' 1
##
```

```
## Approximate significance of smooth terms:
##              edf Ref.df       F  p-value
## s(age_z)   6.292  6.292 195.855  < 2e-16 ***
## s(sleep_z) 3.407  3.407   8.035 8.74e-06 ***
## ---
## Signif. codes:  0 ‘***’ 0.001 ‘**’ 0.01 ‘*’ 0.05 ‘.’ 0.1 ‘ ’ 1
##
## R-sq.(adj) =  0.0591
## lmer.REML = 6.5658e+05  Scale est. = 1804.2    n = 51273
```

Model with full interaction between age and sleep

```
##
## Family: gaussian
## Link function: identity
##
## Formula:
## value ~ sex + site + t2(age_z, sleep_z, k = c(10, 4), bs = "cr")
## <environment: 0x558ad80a4f08>
##
## Parametric coefficients:
##               Estimate Std. Error t value Pr(>|t|)
## (Intercept)    895.300      8.600 104.107  < 2e-16 ***
## sexmale         51.332      1.480  34.689  < 2e-16 ***
## siteMPIB       -37.092     11.090  -3.345 0.000824 ***
## siteousAvanto  -37.252      8.952  -4.161 3.17e-05 ***
## siteousPrisma  -14.550     15.216  -0.956 0.338963
## siteousSkyra   -28.553      8.526  -3.349 0.000811 ***
## siteUB         -85.278     26.348  -3.237 0.001210 **
## siteUCAM       -58.606      9.671  -6.060 1.37e-09 ***
## siteUKB         23.258      8.825   2.636 0.008403 **
## siteUmU        -44.655     12.522  -3.566 0.000363 ***
## siteUOXF       -53.305     10.528  -5.063 4.13e-07 ***
## ---
## Signif. codes:  0 ‘***’ 0.001 ‘**’ 0.01 ‘*’ 0.05 ‘.’ 0.1 ‘ ’ 1
##
## Approximate significance of smooth terms:
##                     edf Ref.df     F p-value
## t2(age_z,sleep_z) 10.32  10.32 11.74  <2e-16 ***
## ---
## Signif. codes:  0 ‘***’ 0.001 ‘**’ 0.01 ‘*’ 0.05 ‘.’ 0.1 ‘ ’ 1
##
## R-sq.(adj) =  0.0589
## lmer.REML = 6.5659e+05  Scale est. = 1804.5    n = 51273
```

Model comparison

`mod_no_sleep` refers to model without sleep term, `mod_no_interaction` refers to model with only main effect of sleep, and `mod_full` refers to model with a full interaction between age and sleep. This is a nested model comparison, and the p-value at a given line refers to comparing the model at the line to the model on the line above. Hence, significance implies that the more complicated model is supported on statistical grounds.

To be even more specific, the p-value on the second row tests whether there is an association between sleep and volume. The p-value on the third row tests whether this association depends on age.

```
## Data: NULL
## Models:
## ml$mod_no_sleep$mer: NULL
## ml$mod_no_interaction$mer: NULL
## ml$mod_full$mer: NULL
##                           npar    AIC    BIC  logLik deviance  Chisq Df Pr(>Chisq)
## ml$mod_no_sleep$mer         15 656628 656760 -328299   656598
## ml$mod_no_interaction$mer   17 656610 656760 -328288   656576 21.778  2  1.867e-05 ***
## ml$mod_full$mer             19 656623 656791 -328293   656585  0.000  2          1
## ---
## Signif. codes:  0 '***' 0.001 '**' 0.01 '*' 0.05 '.' 0.1 ' ' 1
```

We chose the model based on the likelihood ratio test with 5 % significance level, which was `mod_no_interaction`.

Lifespan brain trajectory

The trajectory shown is from the chosen model `mod_no_interaction_no_icv`.

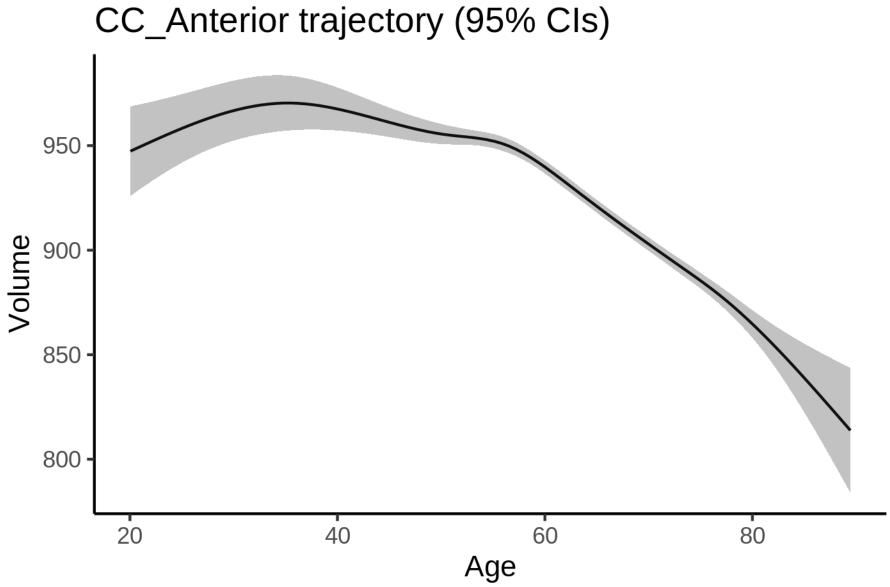

Effect of sleep

The chosen model only included the main effect of sleep, and hence the effect does not vary with age. The black dot shows the average sleep duration across all ages in the sample.

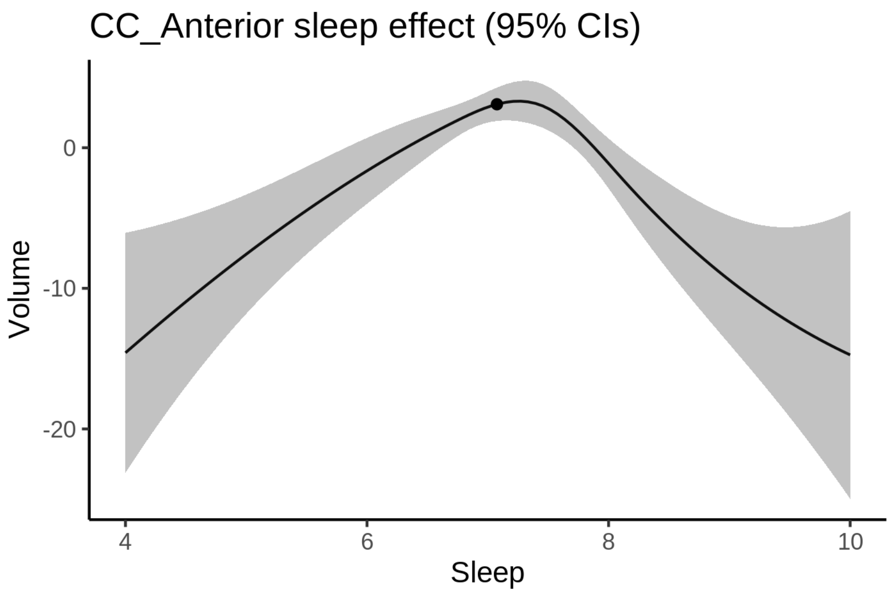

We also show the full interaction model for completeness, although it was not selected.

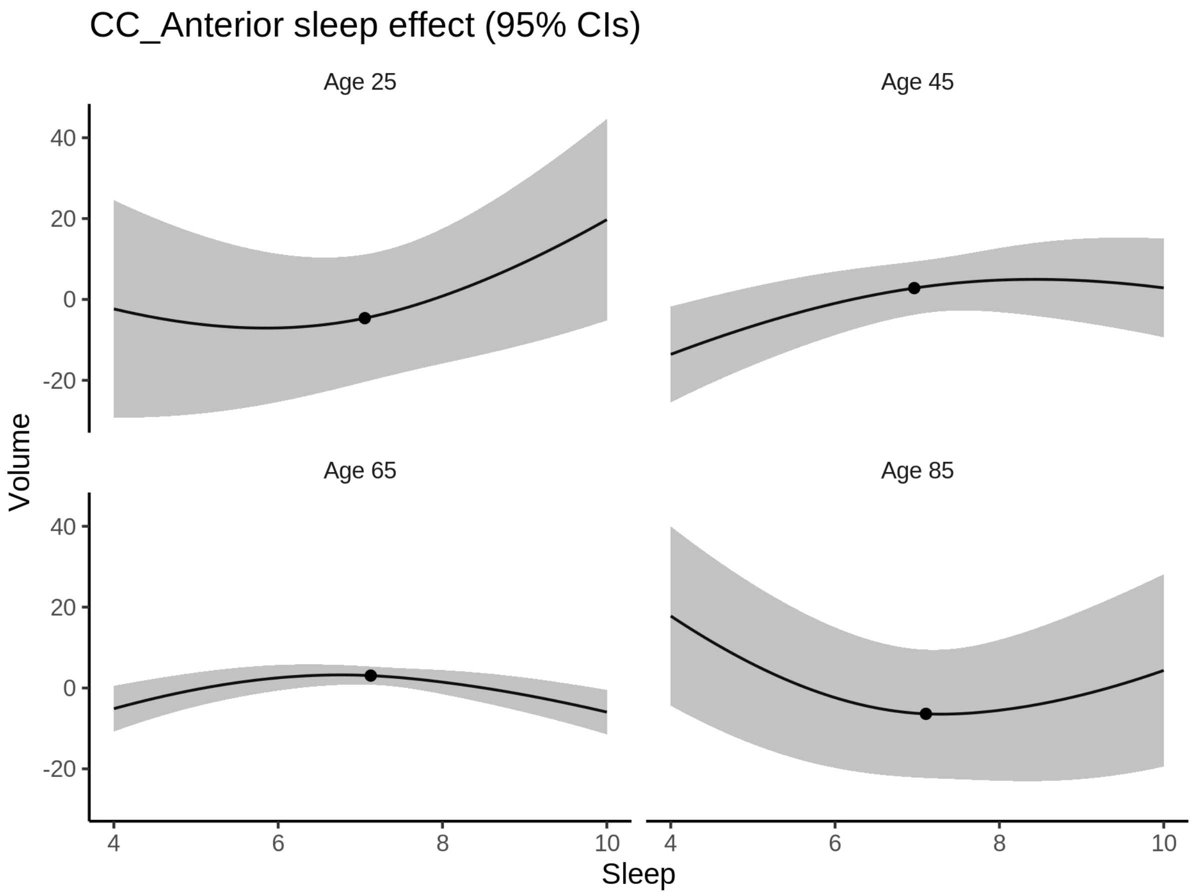

Deviation from sleep associated with maximal volume

Model with only main effect of sleep was chosen, so we show it for all ages at once. Maximum volume is attained at 7.3 hours of sleep. The percentage values in the plot are calculated as follows: The maximum at 100 % refers to a person at an arbitrary age with a sleep duration associated with maximum volume. For a female, this volume is 926 and for a male it is 977. The other percentage values show how large the expected volume is for someone with other sleep durations. For example, 99 % implies a 1 % reduction.

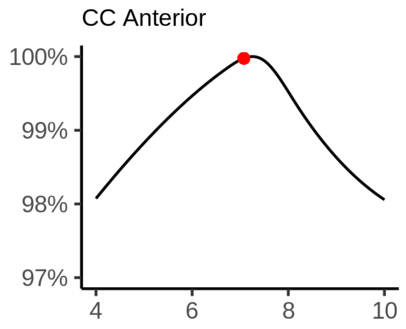

Comparison of mean sleep and sleep associated with maximum volume

A 95 % confidence interval for the sleep associated with maximum volume is [7.03, 7.39].

The plot below compares average sleep to the sleep associated with maximum volume.

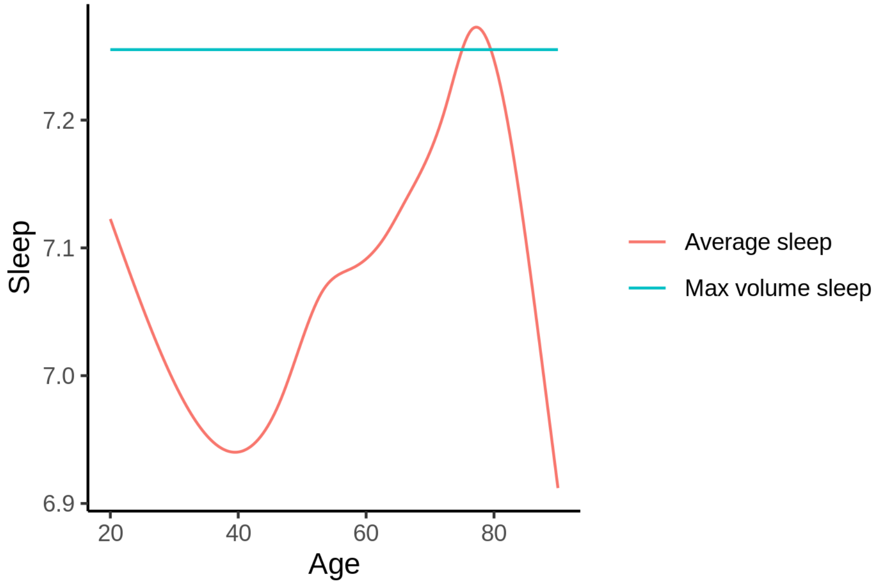

The next plot shows the difference between average sleep and sleep associated with maximum volume. The shaded region is a 95 % confidence interval.

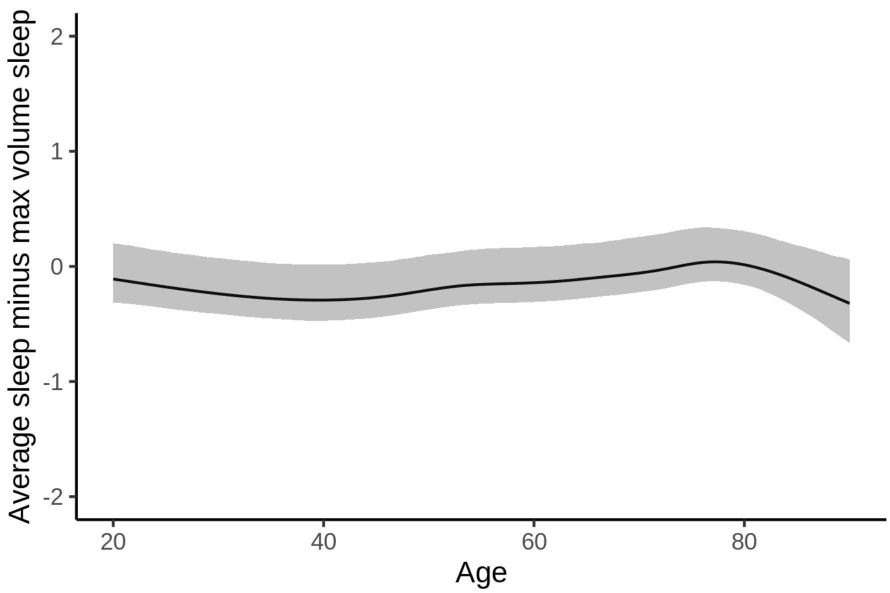

The next plot shows the probability that the sleep duration associated with maximum volume is longer than the average sleep duration, as a function of age. Probability below .05 can be interpreted as evidence that the sleep associated with maximum volume is shorter than the mean sleep, and probability above .95 can be interpreted the opposite way.

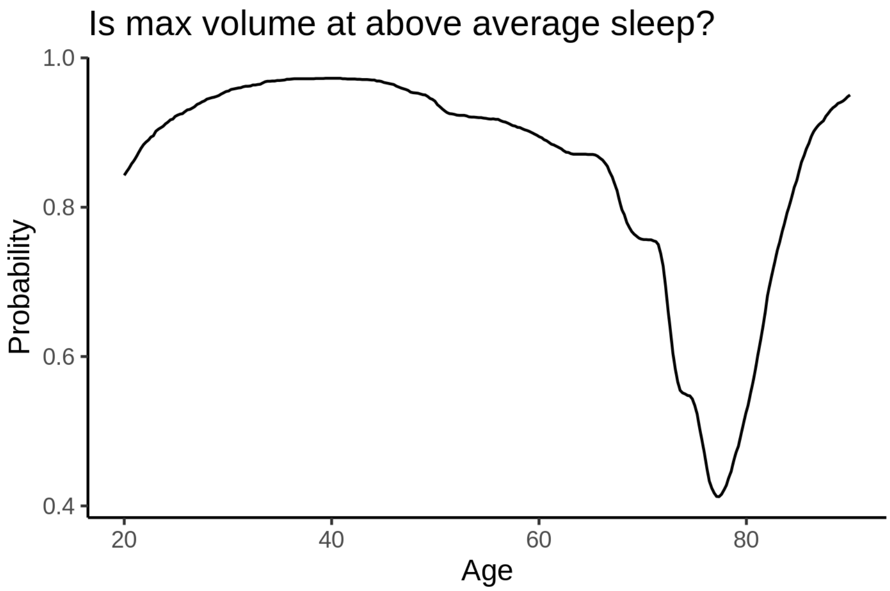

CC_Central

Descriptive statistics

Study	Observations	Unique IDs	Mean age	Age range
HCP	974	974	28.8	22 - 37
MPIB	677	391	63.1	24 - 83

Study	Observations	Unique IDs	Mean age	Age range
UB	113	39	70.9	64 - 81
UCAM	883	632	55.1	20 - 88
UiO	1475	803	49.4	20 - 89
UKB	45975	43135	64.5	45 - 83
UmU	412	279	62.2	25 - 85
UOXF	769	769	69.8	60 - 85

Spaghetti plot

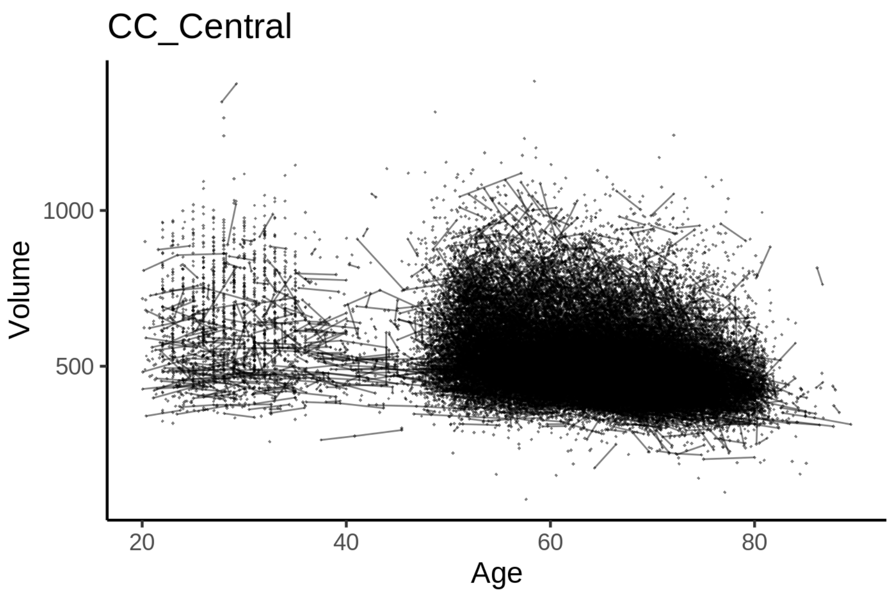

Models Controling for ICV

Model outputs

Model without sleep term

```
##
## Family: gaussian
## Link function: identity
##
## Formula:
## value ~ sex + site + icv + s(age_z, k = 10, bs = "cr")
## <environment: 0x558a47d30568>
##
## Parametric coefficients:
##                 Estimate Std. Error t value Pr(>|t|)
## (Intercept)     591.2557     6.1119  96.739  < 2e-16 ***
## sexmale         -18.8030     1.2657 -14.856  < 2e-16 ***
## siteMPIB        -66.5837     7.8616  -8.470  < 2e-16 ***
## siteousAvanto -149.3246     6.4166 -23.272  < 2e-16 ***
## siteousPrisma  -68.2283    10.9082  -6.255 4.01e-10 ***
## siteousSkyra  -156.5680     5.9919 -26.130  < 2e-16 ***
```

```
## siteUB        -115.5241    18.4358  -6.266 3.73e-10 ***
## siteUCAM       -84.2211     6.8119 -12.364  < 2e-16 ***
## siteUKB        -58.6568     6.2648  -9.363  < 2e-16 ***
## siteUmU        -79.5667     8.8112  -9.030  < 2e-16 ***
## siteUOXF       -45.5595     7.4501  -6.115 9.71e-10 ***
## icv             20.4541     0.6367  32.126  < 2e-16 ***
## ---
## Signif. codes:  0 ‘***’ 0.001 ‘**’ 0.01 ‘*’ 0.05 ‘.’ 0.1 ‘ ’ 1
##
## Approximate significance of smooth terms:
##            edf Ref.df     F p-value
## s(age_z) 5.608  5.608 811.4  <2e-16 ***
## ---
## Signif. codes:  0 ‘***’ 0.001 ‘**’ 0.01 ‘*’ 0.05 ‘.’ 0.1 ‘ ’ 1
##
## R-sq.(adj) =  0.146
## lmer.REML = 6.219e+05  Scale est. = 1202.3    n = 51278
```

Model with only main effects of age and sleep

```
##
## Family: gaussian
## Link function: identity
##
## Formula:
## value ~ sex + site + icv + s(age_z, k = 10, bs = "cr") + s(sleep_z,
##     k = 5, bs = "cr")
## <environment: 0x558a47d30568>
##
## Parametric coefficients:
##                Estimate Std. Error t value Pr(>|t|)
## (Intercept)    590.1206     6.1140  96.519  < 2e-16 ***
## sexmale        -18.8428     1.2655 -14.889  < 2e-16 ***
## siteMPIB       -65.3460     7.8645  -8.309  < 2e-16 ***
## siteousAvanto -148.7233     6.4163 -23.179  < 2e-16 ***
## siteousPrisma  -67.8263    10.9067  -6.219 5.05e-10 ***
## siteousSkyra  -155.9481     5.9917 -26.027  < 2e-16 ***
## siteUB        -115.1788    18.4315  -6.249 4.16e-10 ***
## siteUCAM       -83.6424     6.8107 -12.281  < 2e-16 ***
## siteUKB        -57.4503     6.2674  -9.167  < 2e-16 ***
## siteUmU        -77.0457     8.8289  -8.727  < 2e-16 ***
## siteUOXF       -44.9763     7.4477  -6.039 1.56e-09 ***
## icv             20.5337     0.6369  32.242  < 2e-16 ***
## ---
## Signif. codes:  0 ‘***’ 0.001 ‘**’ 0.01 ‘*’ 0.05 ‘.’ 0.1 ‘ ’ 1
##
## Approximate significance of smooth terms:
##              edf Ref.df      F  p-value
## s(age_z)   5.582  5.582 807.19  < 2e-16 ***
## s(sleep_z) 1.000  1.000  17.06 3.62e-05 ***
## ---
## Signif. codes:  0 ‘***’ 0.001 ‘**’ 0.01 ‘*’ 0.05 ‘.’ 0.1 ‘ ’ 1
##
## R-sq.(adj) =  0.146
## lmer.REML = 6.2188e+05  Scale est. = 1202.2    n = 51278
```

Model with full interaction between age and sleep

```
##
## Family: gaussian
## Link function: identity
##
## Formula:
## value ~ sex + site + icv + t2(age_z, sleep_z, k = c(10, 4), bs = "cr")
## <environment: 0x558a47d30568>
##
## Parametric coefficients:
##                Estimate Std. Error t value Pr(>|t|)
## (Intercept)     590.011      6.140  96.096  < 2e-16 ***
## sexmale         -19.068      1.268 -15.039  < 2e-16 ***
## siteMPIB        -64.909      7.906  -8.210 2.25e-16 ***
## siteousAvanto -148.806      6.449 -23.073  < 2e-16 ***
## siteousPrisma  -67.938     10.927  -6.217 5.10e-10 ***
## siteousSkyra  -155.865      6.030 -25.848  < 2e-16 ***
## siteUB        -114.489     18.443  -6.208 5.41e-10 ***
## siteUCAM       -83.145      6.849 -12.140  < 2e-16 ***
## siteUKB        -57.239      6.293  -9.095  < 2e-16 ***
## siteUmU        -77.175      8.857  -8.713  < 2e-16 ***
## siteUOXF       -44.297      7.477  -5.925 3.15e-09 ***
## icv             20.546      0.637  32.257  < 2e-16 ***
## ---
## Signif. codes:  0 ‘***’ 0.001 ‘**’ 0.01 ‘*’ 0.05 ‘.’ 0.1 ‘ ’ 1
##
## Approximate significance of smooth terms:
##                      edf Ref.df     F p-value
## t2(age_z,sleep_z) 9.226  9.226 27.07  <2e-16 ***
## ---
## Signif. codes:  0 ‘***’ 0.001 ‘**’ 0.01 ‘*’ 0.05 ‘.’ 0.1 ‘ ’ 1
##
## R-sq.(adj) =  0.146
## lmer.REML = 6.2188e+05  Scale est. = 1202.1    n = 51278
```

Model comparison

`mod_no_sleep` refers to model without sleep term, `mod_no_interaction` refers to model with only main effect of sleep, and `mod_full` refers to model with a full interaction between age and sleep. This is a nested model comparison, and the p-value at a given line refers to comparing the model at the line to the model on the line above. Hence, significance implies that the more complicated model is supported on statistical grounds.

To be even more specific, the p-value on the second row tests whether there is an association between sleep and volume. The p-value on the third row tests whether this association depends on age.

```
## Data: NULL
## Models:
## ml$mod_no_sleep$mer: NULL
## ml$mod_no_interaction$mer: NULL
## ml$mod_full$mer: NULL
##                           npar    AIC    BIC  logLik deviance   Chisq Df Pr(>Chisq)
## ml$mod_no_sleep$mer         16 621932 622074 -310950   621900
## ml$mod_no_interaction$mer   18 621919 622078 -310942   621883 17.0577  2  0.0001977 ***
```

```
## ml$mod_full$mer            20 621919 622096 -310939   621879  4.3163  2  0.1155375
## ---
## Signif. codes:  0 '***' 0.001 '**' 0.01 '*' 0.05 '.' 0.1 ' ' 1
```

We chose the model based on the likelihood ratio test with 5 % significance level, which was `mod_no_interaction`.

Lifespan brain trajectory

The trajectory shown is from the chosen model `mod_no_interaction`.

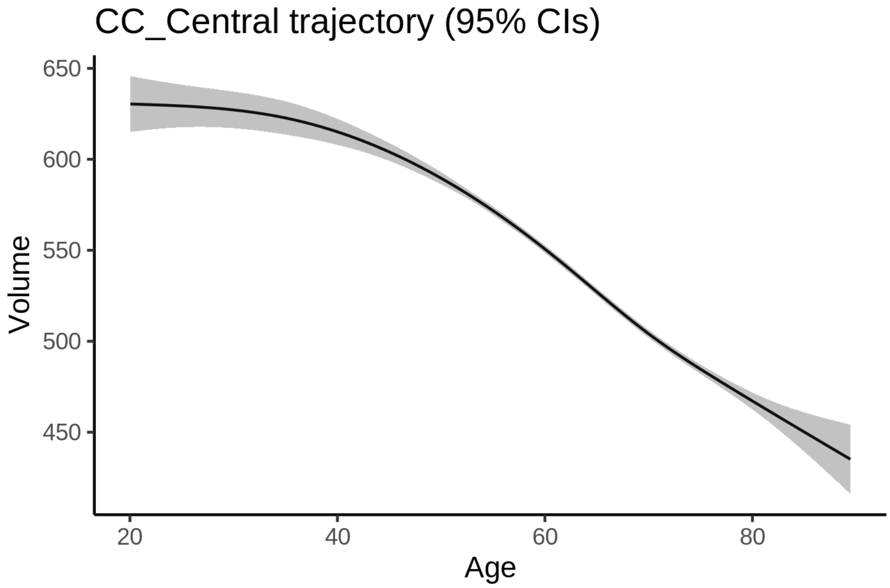

Effect of sleep

The chosen model only included the main effect of sleep, and hence the effect does not vary with age. The black dot shows the average sleep duration across all ages in the sample.

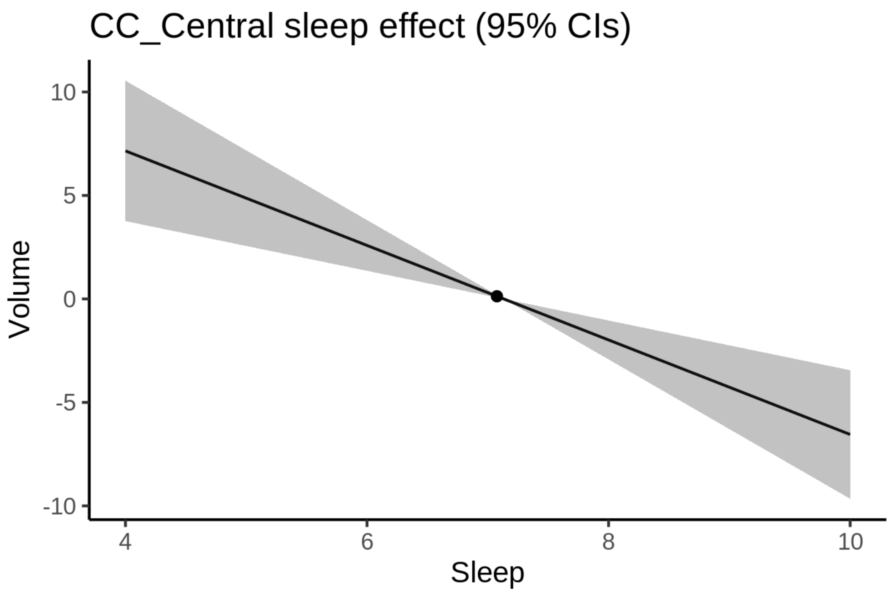

We also show the full interaction model for completeness, although it was not selected.

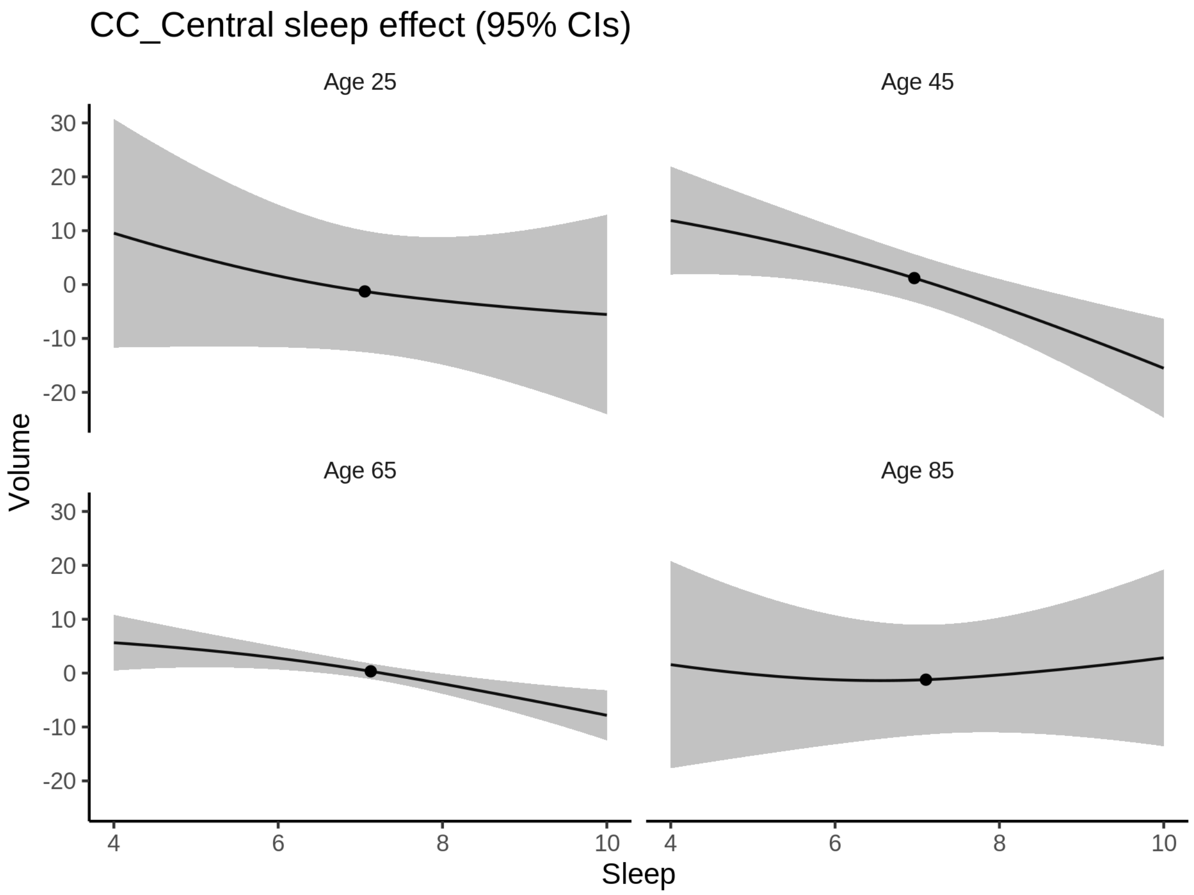

Deviation from sleep associated with maximal volume

Model with only main effect of sleep was chosen, so we show it for all ages at once. Maximum volume is attained at 4 hours of sleep. The percentage values in the plot are calculated as follows: The maximum at 100 % refers to a person at an arbitrary age with a sleep duration associated with maximum volume. For a female, this volume is 555 and for a male it is 536. The other percentage values show how large the expected volume is for someone with other sleep durations. For example, 99 % implies a 1 % reduction.

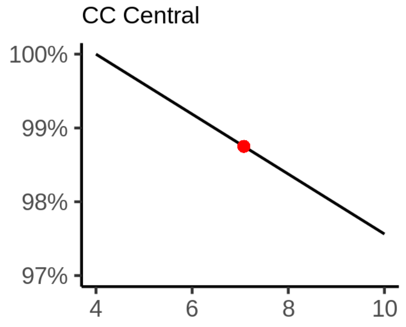

Comparison of mean sleep and sleep associated with maximum volume

A 95 % confidence interval for the sleep associated with maximum volume is [4, 4].

The plot below compares average sleep to the sleep associated with maximum volume.

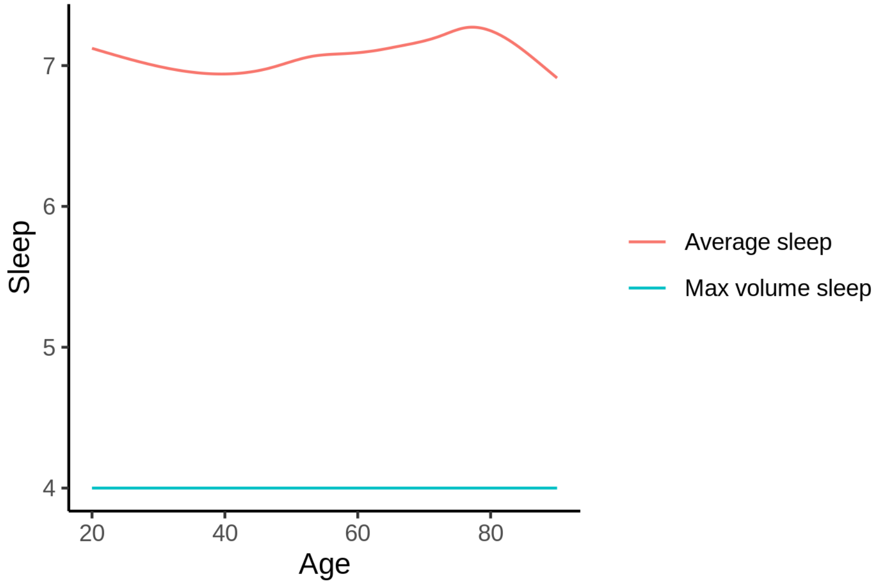

The next plot shows the difference between average sleep and sleep associated with maximum volume. The shaded region is a 95 % confidence interval.

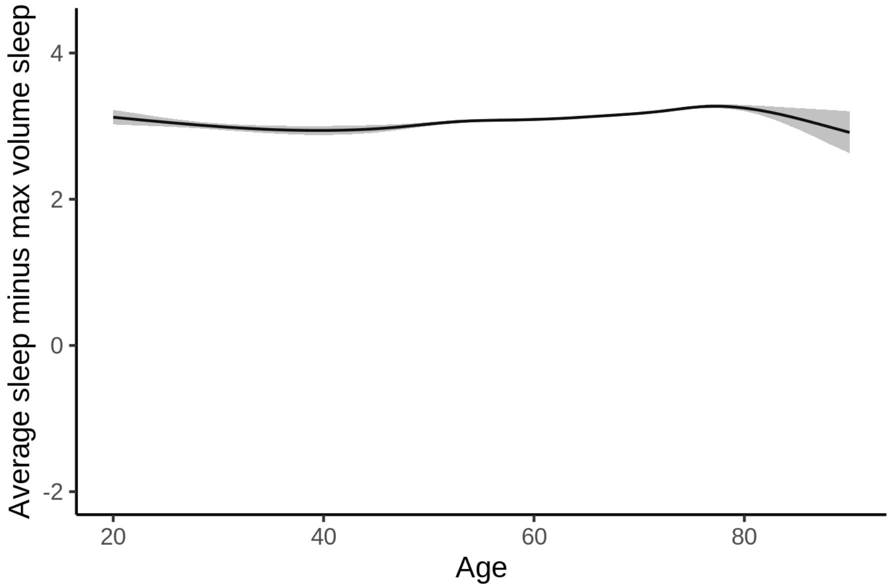

The next plot shows the probability that the sleep duration associated with maximum volume is longer than the average sleep duration, as a function of age. Probability below .05 can be interpreted as evidence that the sleep associated with maximum volume is shorter than the mean sleep, and probability above .95 can be interpreted the opposite way.

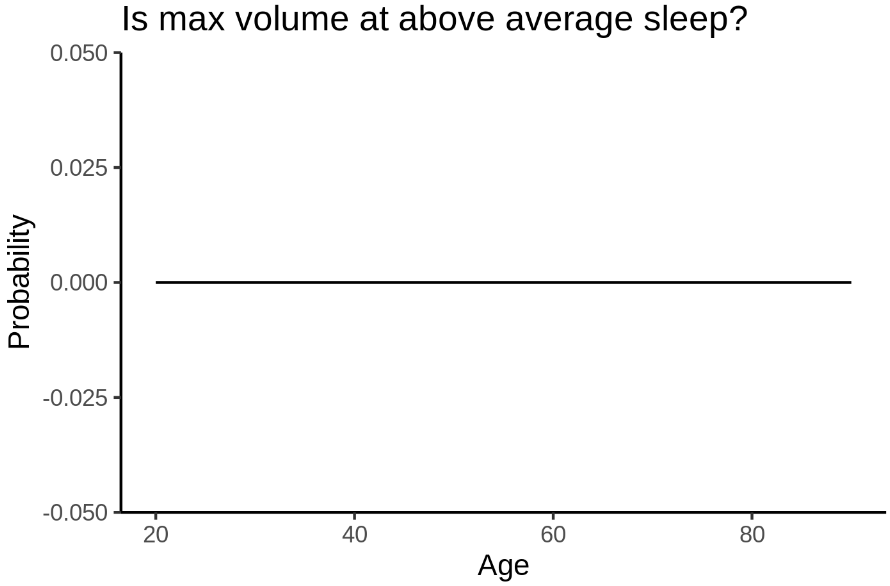

Controling for covariates

Below is the output for a model in which we only include data with income and education.

```
##
## Family: gaussian
## Link function: identity
##
## Formula:
```

```
## value ~ sex + site + s(age_z, k = 10, bs = "cr") + s(sleep_z,
##     k = 5, bs = "cr") + icv
## <environment: 0x558ad81d0018>
##
## Parametric coefficients:
##              Estimate Std. Error t value Pr(>|t|)
## (Intercept)   516.2388    14.4730  35.669  < 2e-16 ***
## sexmale       -18.7158     1.6012 -11.689  < 2e-16 ***
## siteousAvanto -88.3558    17.9518  -4.922 8.62e-07 ***
## siteousPrisma  12.4867    17.9160   0.697    0.486
## siteousSkyra  -89.0267    15.7033  -5.669 1.45e-08 ***
## siteUKB        14.0811    14.4453   0.975    0.330
## siteUOXF       22.1866    16.2830   1.363    0.173
## icv            21.1410     0.8227  25.697  < 2e-16 ***
## ---
## Signif. codes:  0 ‘***’ 0.001 ‘**’ 0.01 ‘*’ 0.05 ‘.’ 0.1 ‘ ’ 1
##
## Approximate significance of smooth terms:
##              edf Ref.df       F p-value
## s(age_z)   4.899  4.899 548.764  <2e-16 ***
## s(sleep_z) 1.000  1.000   5.131  0.0235 *
## ---
## Signif. codes:  0 ‘***’ 0.001 ‘**’ 0.01 ‘*’ 0.05 ‘.’ 0.1 ‘ ’ 1
##
## R-sq.(adj) =  0.114
## lmer.REML = 3.7828e+05  Scale est. = 1410.2    n = 31193
```

Below is the output for a model in which we control for the main effects of income and education.

```
##
## Family: gaussian
## Link function: identity
##
## Formula:
## value ~ sex + site + s(age_z, k = 10, bs = "cr") + s(sleep_z,
##     k = 5, bs = "cr") + icv + income_scaled + education_scaled
## <environment: 0x558ad81d0018>
##
## Parametric coefficients:
##                  Estimate Std. Error t value Pr(>|t|)
## (Intercept)      521.4263    14.5683  35.792  < 2e-16 ***
## sexmale          -18.7668     1.6057 -11.688  < 2e-16 ***
## siteousAvanto    -88.0801    17.9557  -4.905 9.37e-07 ***
## siteousPrisma     13.1710    17.9162   0.735   0.4623
## siteousSkyra     -88.7254    15.7063  -5.649 1.63e-08 ***
## siteUKB           14.1600    14.4559   0.980   0.3273
## siteUOXF          20.5483    16.2934   1.261   0.2073
## icv               21.4757     0.8299  25.876  < 2e-16 ***
## income_scaled     -2.8554     1.9058  -1.498   0.1341
## education_scaled  -4.9985     2.2525  -2.219   0.0265 *
## ---
## Signif. codes:  0 ‘***’ 0.001 ‘**’ 0.01 ‘*’ 0.05 ‘.’ 0.1 ‘ ’ 1
##
## Approximate significance of smooth terms:
##              edf Ref.df       F p-value
```

```
## s(age_z)   4.965  4.965 507.278  <2e-16 ***
## s(sleep_z) 1.075  1.075   4.386  0.0298 *
## ---
## Signif. codes:  0 ‘***’ 0.001 ‘**’ 0.01 ‘*’ 0.05 ‘.’ 0.1 ‘ ’ 1
##
## R-sq.(adj) =  0.115
## lmer.REML = 3.7826e+05  Scale est. = 1409.9    n = 31193
```

We also included interaction effects between sleep duration and education and income, in another model. The output is shown below, and the interaction terms are `income_scaled:sleep_z` and `education_scaled:sleep_z`.

```
##
## Family: gaussian
## Link function: identity
##
## Formula:
## value ~ sex + site + s(age_z, k = 10, bs = "cr") + s(sleep_z,
##     k = 5, bs = "cr") + icv + income_scaled + education_scaled +
##     income_scaled:sleep_z + education_scaled:sleep_z
## <environment: 0x558ad81d0018>
##
## Parametric coefficients:
##                          Estimate Std. Error t value Pr(>|t|)
## (Intercept)               521.303     14.569  35.782  < 2e-16 ***
## sexmale                   -18.651      1.607 -11.607  < 2e-16 ***
## siteousAvanto             -87.833     17.956  -4.892 1.00e-06 ***
## siteousPrisma              13.723     17.918   0.766   0.4438
## siteousSkyra              -88.486     15.706  -5.634 1.78e-08 ***
## siteUKB                    14.311     14.457   0.990   0.3222
## siteUOXF                   20.741     16.298   1.273   0.2032
## icv                        21.456      0.830  25.851  < 2e-16 ***
## income_scaled              -2.974      1.907  -1.560   0.1189
## education_scaled           -4.922      2.253  -2.185   0.0289 *
## income_scaled:sleep_z       2.915      1.886   1.545   0.1223
## education_scaled:sleep_z   -3.701      2.181  -1.697   0.0897 .
## ---
## Signif. codes:  0 ‘***’ 0.001 ‘**’ 0.01 ‘*’ 0.05 ‘.’ 0.1 ‘ ’ 1
##
## Approximate significance of smooth terms:
##              edf Ref.df       F p-value
## s(age_z)   4.976  4.976 506.826  <2e-16 ***
## s(sleep_z) 1.000  1.000   0.001   0.977
## ---
## Signif. codes:  0 ‘***’ 0.001 ‘**’ 0.01 ‘*’ 0.05 ‘.’ 0.1 ‘ ’ 1
##
## R-sq.(adj) =  0.115
## lmer.REML = 3.7825e+05  Scale est. = 1409.8    n = 31193
```

We did the same controling for BMI. Below is the model with no covariates but only keeping data with BMI.

```
##
## Family: gaussian
## Link function: identity
##
## Formula:
```

```
## value ~ sex + site + s(age_z, k = 10, bs = "cr") + s(sleep_z,
##     k = 5, bs = "cr") + icv
## <environment: 0x558a45f6e9a0>
##
## Parametric coefficients:
##               Estimate Std. Error t value Pr(>|t|)
## (Intercept)   429.8584    10.8786  39.514  < 2e-16 ***
## sexmale       -18.2469     1.5547 -11.737  < 2e-16 ***
## siteousPrisma  91.7384    14.1515   6.483 9.14e-11 ***
## siteousSkyra    0.4134     9.0635   0.046    0.964
## siteUCAM       77.0922    11.3823   6.773 1.28e-11 ***
## siteUKB       101.2798    10.9536   9.246  < 2e-16 ***
## siteUmU        84.1167    12.6626   6.643 3.12e-11 ***
## icv            20.9390     0.8013  26.130  < 2e-16 ***
## ---
## Signif. codes:  0 '***' 0.001 '**' 0.01 '*' 0.05 '.' 0.1 ' ' 1
##
## Approximate significance of smooth terms:
##              edf Ref.df       F p-value
## s(age_z)   5.412  5.412 557.193  <2e-16 ***
## s(sleep_z) 1.000  1.000   5.654  0.0174 *
## ---
## Signif. codes:  0 '***' 0.001 '**' 0.01 '*' 0.05 '.' 0.1 ' ' 1
##
## R-sq.(adj) =  0.119
## lmer.REML = 4.0505e+05  Scale est. = 1345.4    n = 33437
```

Below is the model output with main effect.

```
##
## Family: gaussian
## Link function: identity
##
## Formula:
## value ~ sex + site + s(age_z, k = 10, bs = "cr") + s(sleep_z,
##     k = 5, bs = "cr") + icv + bmi
## <environment: 0x558a45f6e9a0>
##
## Parametric coefficients:
##               Estimate Std. Error t value Pr(>|t|)
## (Intercept)   461.3772    11.5205  40.048  < 2e-16 ***
## sexmale       -17.2469     1.5577 -11.072  < 2e-16 ***
## siteousPrisma  93.0875    14.1432   6.582 4.72e-11 ***
## siteousSkyra    0.6295     9.0619   0.069    0.945
## siteUCAM       77.9095    11.3767   6.848 7.61e-12 ***
## siteUKB       102.2671    10.9497   9.340  < 2e-16 ***
## siteUmU        85.5083    12.6559   6.756 1.44e-11 ***
## icv            21.1154     0.8008  26.369  < 2e-16 ***
## bmi            -1.2506     0.1511  -8.279  < 2e-16 ***
## ---
## Signif. codes:  0 '***' 0.001 '**' 0.01 '*' 0.05 '.' 0.1 ' ' 1
##
## Approximate significance of smooth terms:
##              edf Ref.df      F p-value
## s(age_z)   5.442  5.442 555.33 < 2e-16 ***
```

```
## s(sleep_z) 1.000  1.000   7.56 0.00597 **
## ---
## Signif. codes:  0 '***' 0.001 '**' 0.01 '*' 0.05 '.' 0.1 ' ' 1
##
## R-sq.(adj) =  0.121
## lmer.REML = 4.0498e+05  Scale est. = 1345.1    n = 33437
```

Next is the model with BMI-sleep interaction.

```
##
## Family: gaussian
## Link function: identity
##
## Formula:
## value ~ sex + site + s(age_z, k = 10, bs = "cr") + s(sleep_z,
##     k = 5, bs = "cr") + icv + bmi + bmi:sleep_z
## <environment: 0x558a45f6e9a0>
##
## Parametric coefficients:
##               Estimate Std. Error t value Pr(>|t|)
## (Intercept)   461.1069    11.5255  40.008  < 2e-16 ***
## sexmale       -17.2422     1.5577 -11.069  < 2e-16 ***
## siteousPrisma  93.0947    14.1432   6.582 4.70e-11 ***
## siteousSkyra    0.6619     9.0619   0.073    0.942
## siteUCAM       78.0172    11.3775   6.857 7.15e-12 ***
## siteUKB       102.3724    10.9506   9.349  < 2e-16 ***
## siteUmU        85.6067    12.6565   6.764 1.37e-11 ***
## icv            21.1199     0.8008  26.374  < 2e-16 ***
## bmi            -1.2465     0.1511  -8.247  < 2e-16 ***
## bmi:sleep_z     0.1154     0.1430   0.806    0.420
## ---
## Signif. codes:  0 '***' 0.001 '**' 0.01 '*' 0.05 '.' 0.1 ' ' 1
##
## Approximate significance of smooth terms:
##              edf Ref.df       F p-value
## s(age_z)   5.443  5.443 555.329  <2e-16 ***
## s(sleep_z) 1.000  1.000   1.572    0.21
## ---
## Signif. codes:  0 '***' 0.001 '**' 0.01 '*' 0.05 '.' 0.1 ' ' 1
##
## R-sq.(adj) =  0.121
## lmer.REML = 4.0499e+05  Scale est. = 1345.1    n = 33437
```

We did the same controling for depression. Below is the model with no covariates but only keeping data with depression.

```
##
## Family: gaussian
## Link function: identity
##
## Formula:
## value ~ sex + site + s(age_z, k = 10, bs = "cr") + s(sleep_z,
##     k = 5, bs = "cr") + icv
## <environment: 0x558a48036f40>
##
## Parametric coefficients:
```

```
##                Estimate Std. Error t value Pr(>|t|)
## (Intercept)   517.8638     7.3368  70.584  < 2e-16 ***
## sexmale       -18.0327     1.5588 -11.568  < 2e-16 ***
## siteousAvanto -91.4470    17.0927  -5.350 8.85e-08 ***
## siteousPrisma  -1.0022    14.2674  -0.070   0.9440
## siteousSkyra  -97.4031    11.0807  -8.790  < 2e-16 ***
## siteUCAM      -18.0590    10.0338  -1.800   0.0719 .
## siteUKB        12.5073     7.2535   1.724   0.0847 .
## siteUmU        -6.0696     9.8959  -0.613   0.5397
## icv            20.5088     0.8023  25.563  < 2e-16 ***
## ---
## Signif. codes:  0 ‘***’ 0.001 ‘**’ 0.01 ‘*’ 0.05 ‘.’ 0.1 ‘ ’ 1
##
## Approximate significance of smooth terms:
##             edf Ref.df       F p-value
## s(age_z)   5.28   5.28 548.477  <2e-16 ***
## s(sleep_z) 1.00   1.00   5.371  0.0205 *
## ---
## Signif. codes:  0 ‘***’ 0.001 ‘**’ 0.01 ‘*’ 0.05 ‘.’ 0.1 ‘ ’ 1
##
## R-sq.(adj) =  0.117
## lmer.REML = 4.0368e+05  Scale est. = 1319      n = 33349
```

Below is the model output with main effect.

```
##
## Family: gaussian
## Link function: identity
##
## Formula:
## value ~ sex + site + s(age_z, k = 10, bs = "cr") + s(sleep_z,
##     k = 5, bs = "cr") + icv + depression
## <environment: 0x558a48036f40>
##
## Parametric coefficients:
##                Estimate Std. Error t value Pr(>|t|)
## (Intercept)   519.1124     7.3698  70.438  < 2e-16 ***
## sexmale       -18.1551     1.5603 -11.636  < 2e-16 ***
## siteousAvanto -90.8188    17.0971  -5.312 1.09e-07 ***
## siteousPrisma  -0.4505    14.2721  -0.032   0.9748
## siteousSkyra  -96.6939    11.0893  -8.720  < 2e-16 ***
## siteUCAM      -17.7827    10.0355  -1.772   0.0764 .
## siteUKB        11.9659     7.2596   1.648   0.0993 .
## siteUmU        -2.4288    10.1042  -0.240   0.8100
## icv            20.5194     0.8023  25.576  < 2e-16 ***
## depression     -9.8610     5.5272  -1.784   0.0744 .
## ---
## Signif. codes:  0 ‘***’ 0.001 ‘**’ 0.01 ‘*’ 0.05 ‘.’ 0.1 ‘ ’ 1
##
## Approximate significance of smooth terms:
##              edf Ref.df       F p-value
## s(age_z)   5.307  5.307 540.209  <2e-16 ***
## s(sleep_z) 1.000  1.000   5.941  0.0148 *
## ---
## Signif. codes:  0 ‘***’ 0.001 ‘**’ 0.01 ‘*’ 0.05 ‘.’ 0.1 ‘ ’ 1
```

```
##
## R-sq.(adj) =  0.117
## lmer.REML = 4.0367e+05  Scale est. = 1318.9    n = 33349
```

Next is the model with depression-sleep interaction.

```
##
## Family: gaussian
## Link function: identity
##
## Formula:
## value ~ sex + site + s(age_z, k = 10, bs = "cr") + s(sleep_z,
##     k = 5, bs = "cr") + icv + depression + depression:sleep_z
## <environment: 0x558a48036f40>
##
## Parametric coefficients:
##                     Estimate Std. Error t value Pr(>|t|)
## (Intercept)        519.10625    7.36948  70.440  < 2e-16 ***
## sexmale            -18.20764    1.56044 -11.668  < 2e-16 ***
## siteousAvanto      -91.24448   17.09763  -5.337 9.53e-08 ***
## siteousPrisma       -0.86233   14.27220  -0.060   0.9518
## siteousSkyra       -97.10237   11.08979  -8.756  < 2e-16 ***
## siteUCAM           -18.17753   10.03688  -1.811   0.0701 .
## siteUKB             11.99039    7.25925   1.652   0.0986 .
## siteUmU              0.03934   10.18674   0.004   0.9969
## icv                 20.52535    0.80225  25.585  < 2e-16 ***
## depression         -10.44598    5.53554  -1.887   0.0592 .
## depression:sleep_z  -7.67600    4.03881  -1.901   0.0574 .
## ---
## Signif. codes:  0 ‘***’ 0.001 ‘**’ 0.01 ‘*’ 0.05 ‘.’ 0.1 ‘ ’ 1
##
## Approximate significance of smooth terms:
##              edf Ref.df       F p-value
## s(age_z)   5.296  5.296 541.702  <2e-16 ***
## s(sleep_z) 1.000  1.000   1.081   0.299
## ---
## Signif. codes:  0 ‘***’ 0.001 ‘**’ 0.01 ‘*’ 0.05 ‘.’ 0.1 ‘ ’ 1
##
## R-sq.(adj) =  0.117
## lmer.REML = 4.0366e+05  Scale est. = 1319      n = 33349
```

The plot below shows the sleep-volume curve for the original model and for the model with main effects of SES.

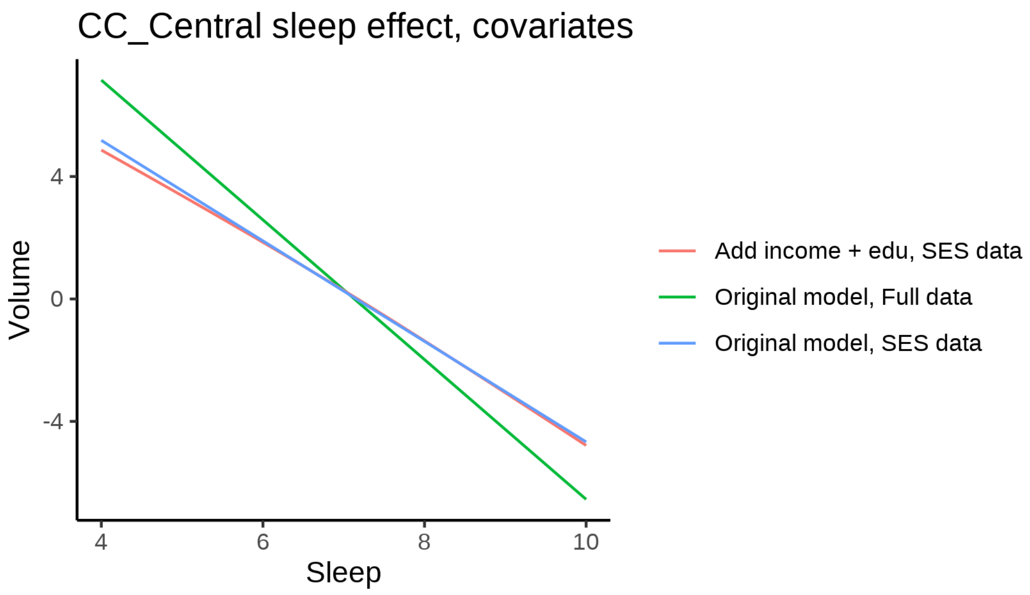

The plot below shows the sleep-volume curve for the original model and for the model with main effects of BMI.

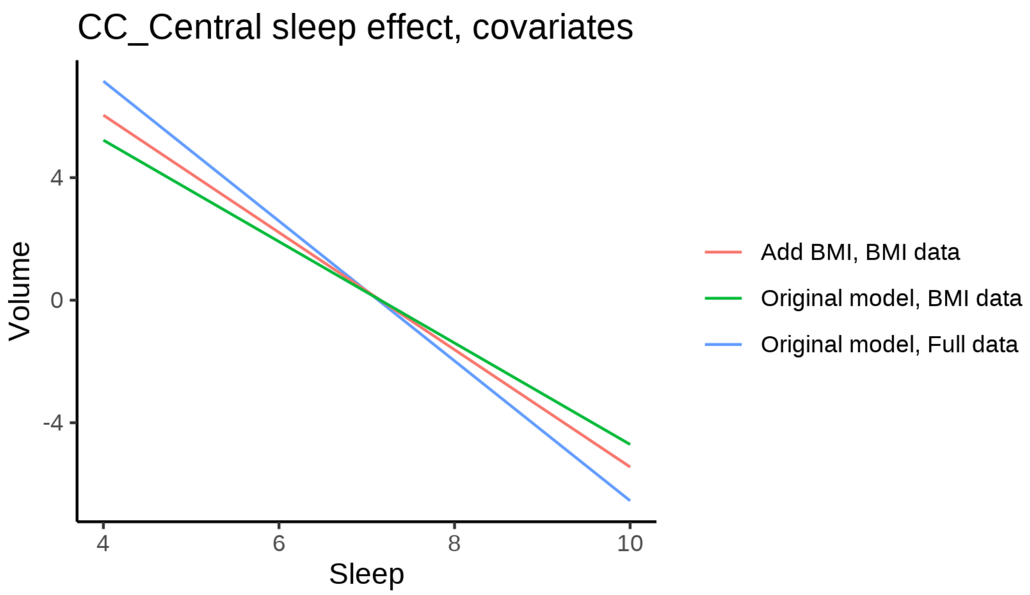

The plot below shows the sleep-volume curve for the original model and for the model with main effects of depression.

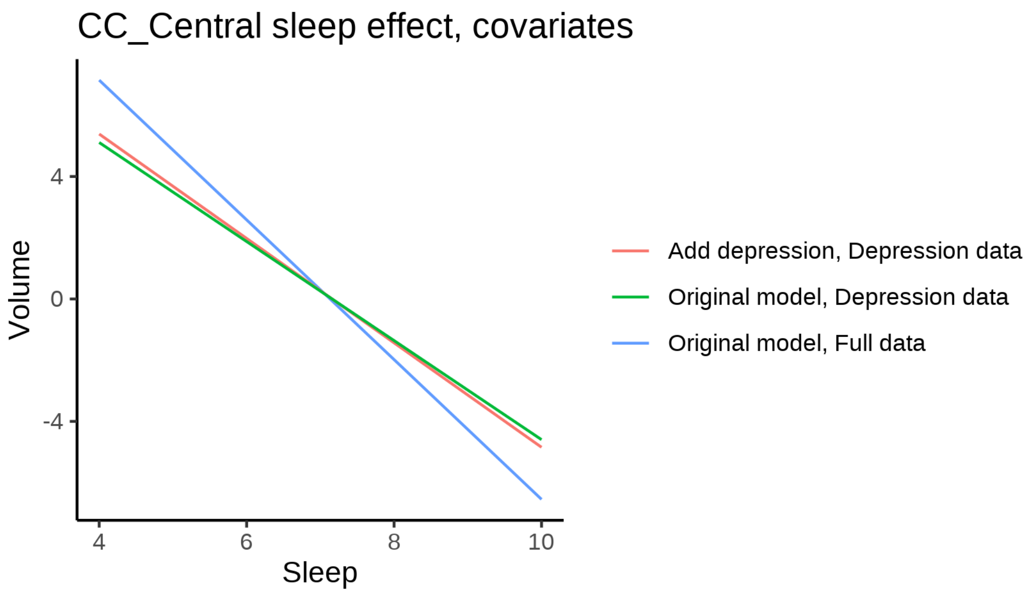

Models Not controling for ICV

Model outputs

Model without sleep term

```
##
## Family: gaussian
## Link function: identity
##
## Formula:
## value ~ sex + site + s(age_z, k = 10, bs = "cr")
## <environment: 0x55895ab3e980>
##
## Parametric coefficients:
##                 Estimate Std. Error t value Pr(>|t|)
## (Intercept)      579.107      6.174  93.796  < 2e-16 ***
## sexmale            4.407      1.050   4.197 2.71e-05 ***
## siteMPIB         -98.715      7.884 -12.520  < 2e-16 ***
## siteousAvanto   -139.881      6.476 -21.601  < 2e-16 ***
## siteousPrisma    -63.834     11.015  -5.795 6.87e-09 ***
## siteousSkyra    -154.597      6.057 -25.522  < 2e-16 ***
## siteUB          -120.631     18.635  -6.473 9.67e-11 ***
## siteUCAM         -82.423      6.888 -11.967  < 2e-16 ***
## siteUKB          -57.095      6.341  -9.004  < 2e-16 ***
## siteUmU          -87.045      8.906  -9.774  < 2e-16 ***
## siteUOXF         -49.369      7.536  -6.551 5.76e-11 ***
## ---
## Signif. codes:  0 '***' 0.001 '**' 0.01 '*' 0.05 '.' 0.1 ' ' 1
##
## Approximate significance of smooth terms:
##            edf Ref.df     F p-value
## s(age_z) 5.732  5.732 849.7  <2e-16 ***
```

```
## ---
## Signif. codes:  0 ‘***’ 0.001 ‘**’ 0.01 ‘*’ 0.05 ‘.’ 0.1 ‘ ’ 1
##
## R-sq.(adj) =  0.128
## lmer.REML = 6.2292e+05  Scale est. = 1216.3    n = 51278
```

Model with only main effects of age and sleep

```
##
## Family: gaussian
## Link function: identity
##
## Formula:
## value ~ sex + site + s(age_z, k = 10, bs = "cr") + s(sleep_z,
##     k = 5, bs = "cr")
## <environment: 0x55895ab3e980>
##
## Parametric coefficients:
##               Estimate Std. Error t value Pr(>|t|)
## (Intercept)    578.362      6.178  93.611  < 2e-16 ***
## sexmale          4.398      1.050   4.188 2.82e-05 ***
## siteMPIB       -97.800      7.887 -12.400  < 2e-16 ***
## siteousAvanto -139.544      6.477 -21.546  < 2e-16 ***
## siteousPrisma  -63.615     11.014  -5.776 7.71e-09 ***
## siteousSkyra  -154.279      6.058 -25.465  < 2e-16 ***
## siteUB        -120.217     18.632  -6.452 1.11e-10 ***
## siteUCAM       -81.956      6.887 -11.900  < 2e-16 ***
## siteUKB        -56.313      6.345  -8.875  < 2e-16 ***
## siteUmU        -85.087      8.924  -9.534  < 2e-16 ***
## siteUOXF       -49.001      7.534  -6.504 7.90e-11 ***
## ---
## Signif. codes:  0 ‘***’ 0.001 ‘**’ 0.01 ‘*’ 0.05 ‘.’ 0.1 ‘ ’ 1
##
## Approximate significance of smooth terms:
##              edf Ref.df      F p-value
## s(age_z)   5.710  5.710 843.24 < 2e-16 ***
## s(sleep_z) 1.938  1.938   7.66 0.00236 **
## ---
## Signif. codes:  0 ‘***’ 0.001 ‘**’ 0.01 ‘*’ 0.05 ‘.’ 0.1 ‘ ’ 1
##
## R-sq.(adj) =  0.129
## lmer.REML = 6.2291e+05  Scale est. = 1216.4    n = 51278
```

Model with full interaction between age and sleep

```
##
## Family: gaussian
## Link function: identity
##
## Formula:
## value ~ sex + site + t2(age_z, sleep_z, k = c(10, 4), bs = "cr")
## <environment: 0x55895ab3e980>
##
## Parametric coefficients:
##               Estimate Std. Error t value Pr(>|t|)
## (Intercept)    578.548      6.214  93.101  < 2e-16 ***
```

```
## sexmale          4.252      1.052   4.041 5.34e-05 ***
## siteMPIB       -97.966      7.932 -12.351  < 2e-16 ***
## siteousAvanto -139.768      6.514 -21.458  < 2e-16 ***
## siteousPrisma  -63.901     11.037  -5.790 7.09e-09 ***
## siteousSkyra  -154.350      6.100 -25.302  < 2e-16 ***
## siteUB        -120.238     18.647  -6.448 1.14e-10 ***
## siteUCAM       -81.943      6.930 -11.824  < 2e-16 ***
## siteUKB        -56.437      6.381  -8.844  < 2e-16 ***
## siteUmU        -85.690      8.958  -9.566  < 2e-16 ***
## siteUOXF       -48.793      7.572  -6.444 1.17e-10 ***
## ---
## Signif. codes:  0 '***' 0.001 '**' 0.01 '*' 0.05 '.' 0.1 ' ' 1
##
## Approximate significance of smooth terms:
##                      edf Ref.df     F p-value
## t2(age_z,sleep_z) 9.787  9.787 23.04  <2e-16 ***
## ---
## Signif. codes:  0 '***' 0.001 '**' 0.01 '*' 0.05 '.' 0.1 ' ' 1
##
## R-sq.(adj) =  0.129
## lmer.REML = 6.2291e+05  Scale est. = 1216.4    n = 51278
```

Model comparison

`mod_no_sleep` refers to model without sleep term, `mod_no_interaction` refers to model with only main effect of sleep, and `mod_full` refers to model with a full interaction between age and sleep. This is a nested model comparison, and the p-value at a given line refers to comparing the model at the line to the model on the line above. Hence, significance implies that the more complicated model is supported on statistical grounds.

To be even more specific, the p-value on the second row tests whether there is an association between sleep and volume. The p-value on the third row tests whether this association depends on age.

```
## Data: NULL
## Models:
## ml$mod_no_sleep$mer: NULL
## ml$mod_no_interaction$mer: NULL
## ml$mod_full$mer: NULL
##                           npar    AIC    BIC  logLik deviance   Chisq Df Pr(>Chisq)
## ml$mod_no_sleep$mer         15 622952 623084 -311461   622922
## ml$mod_no_interaction$mer   17 622945 623096 -311456   622911 10.4719  2   0.005322 **
## ml$mod_full$mer             19 622946 623115 -311454   622908  2.8984  2   0.234760
## ---
## Signif. codes:  0 '***' 0.001 '**' 0.01 '*' 0.05 '.' 0.1 ' ' 1
```

We chose the model based on the likelihood ratio test with 5 % significance level, which was `mod_no_interaction`.

Lifespan brain trajectory

The trajectory shown is from the chosen model `mod_no_interaction_no_icv`.

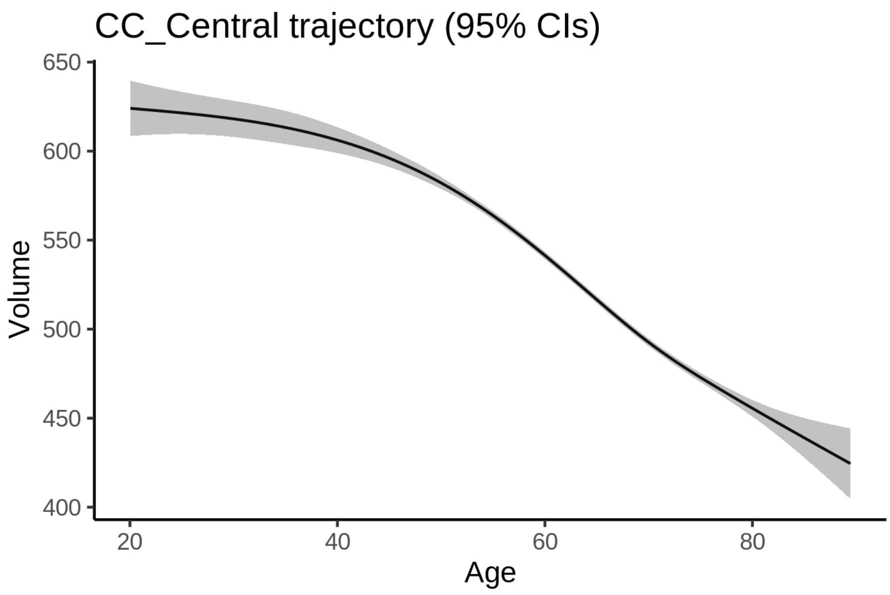

Effect of sleep

The chosen model only included the main effect of sleep, and hence the effect does not vary with age. The black dot shows the average sleep duration across all ages in the sample.

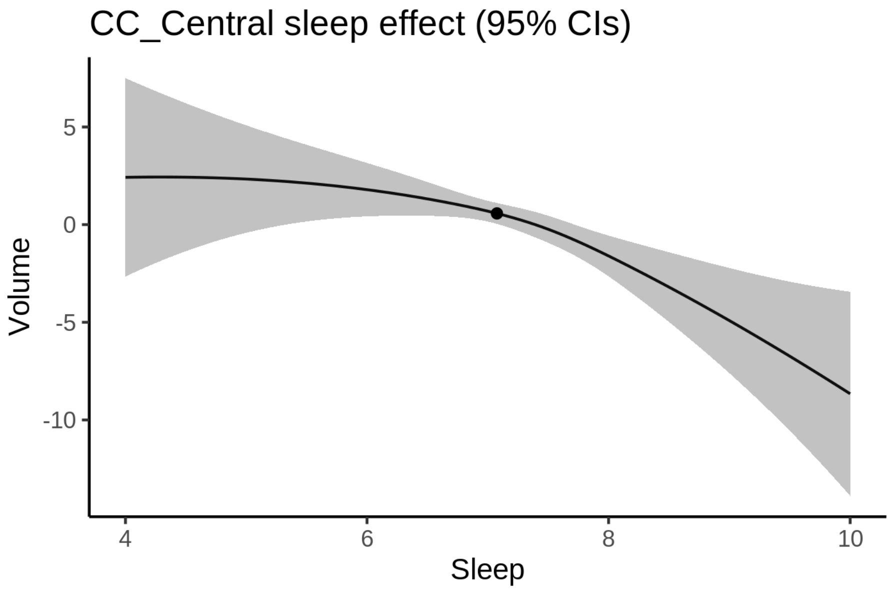

We also show the full interaction model for completeness, although it was not selected.

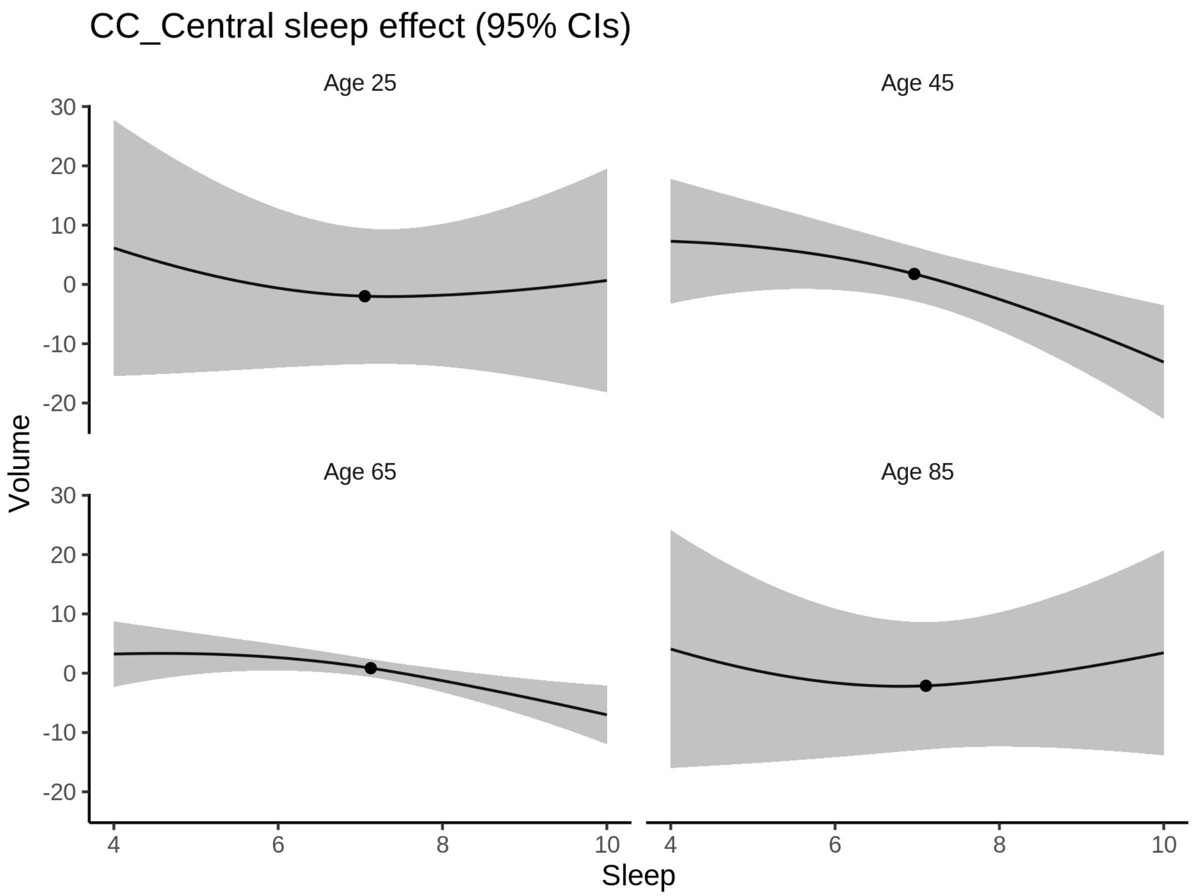

Deviation from sleep associated with maximal volume

Model with only main effect of sleep was chosen, so we show it for all ages at once. Maximum volume is attained at 4.3 hours of sleep. The percentage values in the plot are calculated as follows: The maximum at 100 % refers to a person at an arbitrary age with a sleep duration associated with maximum volume. For a female, this volume is 546 and for a male it is 550. The other percentage values show how large the expected volume is for someone with other sleep durations. For example, 99 % implies a 1 % reduction.

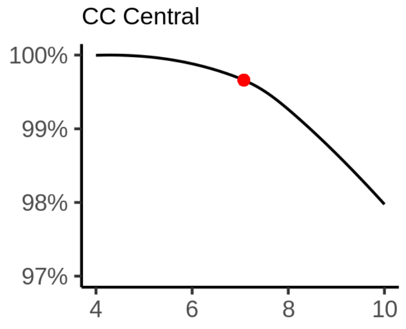

Comparison of mean sleep and sleep associated with maximum volume

A 95 % confidence interval for the sleep associated with maximum volume is [4, 6.55].

The plot below compares average sleep to the sleep associated with maximum volume.

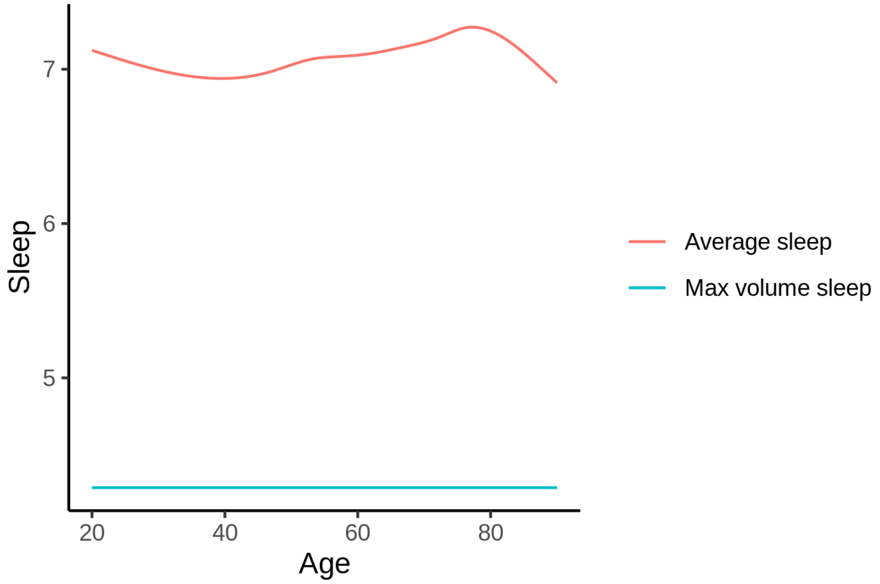

The next plot shows the difference between average sleep and sleep associated with maximum volume. The shaded region is a 95 % confidence interval.

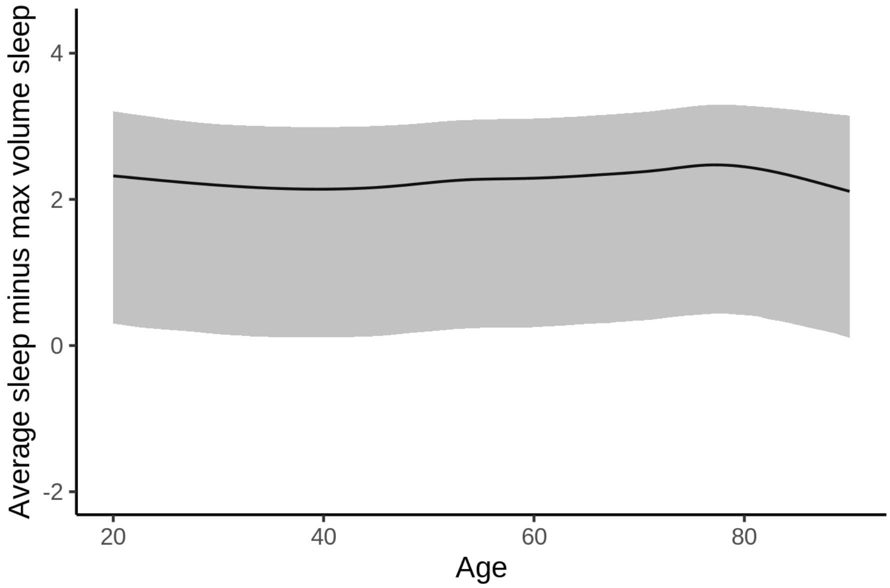

The next plot shows the probability that the sleep duration associated with maximum volume is longer than the average sleep duration, as a function of age. Probability below .05 can be interpreted as evidence that the sleep associated with maximum volume is shorter than the mean sleep, and probability above .95 can be interpreted the opposite way.

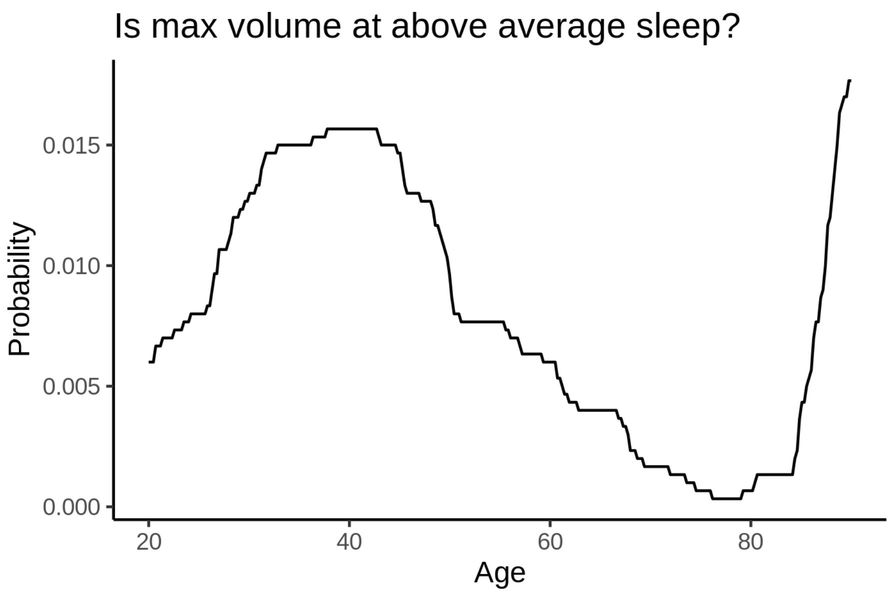

CC_Mid_Anterior

Descriptive statistics

Study	Observations	Unique IDs	Mean age	Age range
HCP	974	974	28.8	22 - 37
MPIB	677	391	63.1	24 - 83
UB	113	39	70.9	64 - 81
UCAM	881	631	55.1	20 - 88
UiO	1475	803	49.4	20 - 89
UKB	45966	43131	64.5	45 - 83
UmU	412	279	62.2	25 - 85
UOXF	769	769	69.8	60 - 85

Spaghetti plot

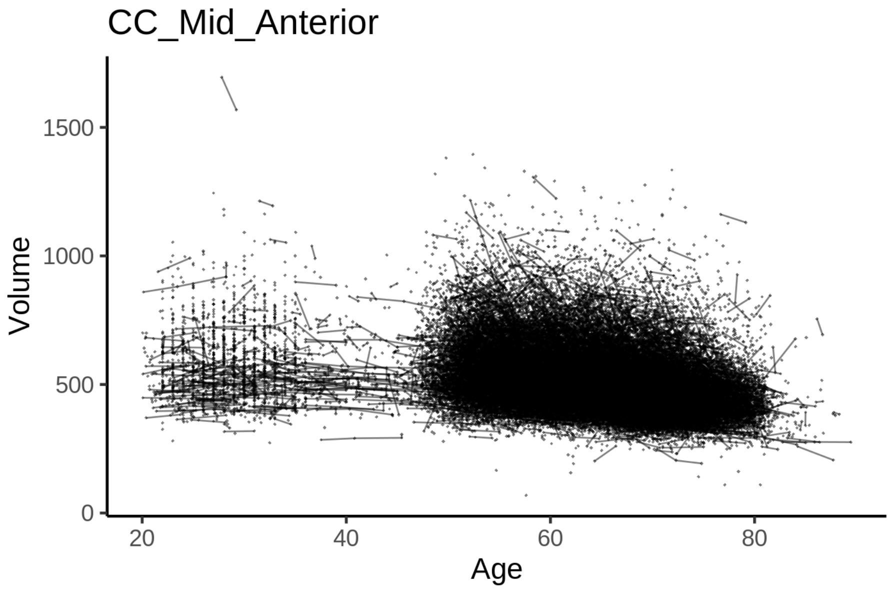

Models Controling for ICV

Model outputs

Model without sleep term

```
##
## Family: gaussian
## Link function: identity
##
## Formula:
## value ~ sex + site + icv + s(age_z, k = 10, bs = "cr")
## <environment: 0x558a46b71da8>
##
## Parametric coefficients:
##                 Estimate Std. Error t value Pr(>|t|)
## (Intercept)    552.4539     6.3902  86.453  < 2e-16 ***
## sexmale        -17.1079     1.3104 -13.056  < 2e-16 ***
## siteMPIB        -0.8416     8.1661  -0.103  0.91791
## siteousAvanto -104.8390     6.6735 -15.710  < 2e-16 ***
## siteousPrisma  -38.5730    11.3110  -3.410  0.00065 ***
## siteousSkyra  -101.7833     6.2253 -16.350  < 2e-16 ***
## siteUB         -75.3639    19.0957  -3.947 7.94e-05 ***
## siteUCAM       -53.0345     7.0829  -7.488 7.12e-14 ***
## siteUKB        -18.4993     6.5525  -2.823  0.00476 **
## siteUmU        -38.7947     9.1550  -4.238 2.26e-05 ***
## siteUOXF        -8.7402     7.7673  -1.125  0.26049
## icv             30.8475     0.6593  46.788  < 2e-16 ***
## ---
## Signif. codes:  0 ‘***’ 0.001 ‘**’ 0.01 ‘*’ 0.05 ‘.’ 0.1 ‘ ’ 1
##
```

```
## Approximate significance of smooth terms:
##             edf Ref.df     F p-value
## s(age_z) 6.186  6.186 844.3  <2e-16 ***
## ---
## Signif. codes:  0 ‘***’ 0.001 ‘**’ 0.01 ‘*’ 0.05 ‘.’ 0.1 ‘ ’ 1
##
## R-sq.(adj) =   0.16
## lmer.REML = 6.2541e+05  Scale est. = 1316.3    n = 51267
```

Model with only main effects of age and sleep

```
##
## Family: gaussian
## Link function: identity
##
## Formula:
## value ~ sex + site + icv + s(age_z, k = 10, bs = "cr") + s(sleep_z,
##     k = 5, bs = "cr")
## <environment: 0x558a46b71da8>
##
## Parametric coefficients:
##                 Estimate Std. Error t value Pr(>|t|)
## (Intercept)    551.1118     6.3930  86.205  < 2e-16 ***
## sexmale        -17.1553     1.3101 -13.095  < 2e-16 ***
## siteMPIB         0.6217     8.1690   0.076 0.939338
## siteousAvanto -104.1294     6.6732 -15.604  < 2e-16 ***
## siteousPrisma  -38.0963    11.3090  -3.369 0.000756 ***
## siteousSkyra  -101.0504     6.2251 -16.233  < 2e-16 ***
## siteUB         -74.9556    19.0902  -3.926 8.63e-05 ***
## siteUCAM       -52.3477     7.0817  -7.392 1.47e-13 ***
## siteUKB        -17.0724     6.5558  -2.604 0.009212 **
## siteUmU        -35.8133     9.1734  -3.904 9.47e-05 ***
## siteUOXF        -8.0508     7.7651  -1.037 0.299842
## icv             30.9418     0.6595  46.921  < 2e-16 ***
## ---
## Signif. codes:  0 ‘***’ 0.001 ‘**’ 0.01 ‘*’ 0.05 ‘.’ 0.1 ‘ ’ 1
##
## Approximate significance of smooth terms:
##               edf Ref.df     F  p-value
## s(age_z)   6.163  6.163 837.7  < 2e-16 ***
## s(sleep_z) 1.000  1.000  22.3 2.36e-06 ***
## ---
## Signif. codes:  0 ‘***’ 0.001 ‘**’ 0.01 ‘*’ 0.05 ‘.’ 0.1 ‘ ’ 1
##
## R-sq.(adj) =   0.16
## lmer.REML = 6.2539e+05  Scale est. = 1316.5    n = 51267
```

Model with full interaction between age and sleep

```
##
## Family: gaussian
## Link function: identity
##
## Formula:
## value ~ sex + site + icv + t2(age_z, sleep_z, k = c(10, 4), bs = "cr")
## <environment: 0x558a46b71da8>
```

```
##
## Parametric coefficients:
##                Estimate Std. Error t value Pr(>|t|)
## (Intercept)    550.8009     6.4207  85.785  < 2e-16 ***
## sexmale        -17.3358     1.3127 -13.207  < 2e-16 ***
## siteMPIB         1.1641     8.2135   0.142 0.887294
## siteousAvanto -104.0985     6.7088 -15.517  < 2e-16 ***
## siteousPrisma  -38.1260    11.3312  -3.365 0.000767 ***
## siteousSkyra  -100.9104     6.2664 -16.104  < 2e-16 ***
## siteUB         -74.1469    19.1034  -3.881 0.000104 ***
## siteUCAM       -51.8033     7.1235  -7.272 3.59e-13 ***
## siteUKB        -16.6696     6.5836  -2.532 0.011344 *
## siteUmU        -35.7616     9.2044  -3.885 0.000102 ***
## siteUOXF        -7.2821     7.7960  -0.934 0.350268
## icv             30.9627     0.6595  46.947  < 2e-16 ***
## ---
## Signif. codes:  0 ‘***’ 0.001 ‘**’ 0.01 ‘*’ 0.05 ‘.’ 0.1 ‘ ’ 1
##
## Approximate significance of smooth terms:
##                      edf Ref.df     F p-value
## t2(age_z,sleep_z) 9.537  9.537 19.29  <2e-16 ***
## ---
## Signif. codes:  0 ‘***’ 0.001 ‘**’ 0.01 ‘*’ 0.05 ‘.’ 0.1 ‘ ’ 1
##
## R-sq.(adj) =   0.16
## lmer.REML = 6.2539e+05  Scale est. = 1315.9    n = 51267
```

Model comparison

`mod_no_sleep` refers to model without sleep term, `mod_no_interaction` refers to model with only main effect of sleep, and `mod_full` refers to model with a full interaction between age and sleep. This is a nested model comparison, and the p-value at a given line refers to comparing the model at the line to the model on the line above. Hence, significance implies that the more complicated model is supported on statistical grounds.

To be even more specific, the p-value on the second row tests whether there is an association between sleep and volume. The p-value on the third row tests whether this association depends on age.

```
## Data: NULL
## Models:
## ml$mod_no_sleep$mer: NULL
## ml$mod_no_interaction$mer: NULL
## ml$mod_full$mer: NULL
##                           npar    AIC    BIC  logLik deviance  Chisq Df Pr(>Chisq)
## ml$mod_no_sleep$mer         16 625445 625587 -312707   625413
## ml$mod_no_interaction$mer   18 625427 625586 -312695   625391 22.296  2   1.44e-05 ***
## ml$mod_full$mer             20 625432 625609 -312696   625392  0.000  2          1
## ---
## Signif. codes:  0 ‘***’ 0.001 ‘**’ 0.01 ‘*’ 0.05 ‘.’ 0.1 ‘ ’ 1
```

We chose the model based on the likelihood ratio test with 5 % significance level, which was `mod_no_interaction`.

Lifespan brain trajectory

The trajectory shown is from the chosen model `mod_no_interaction`.

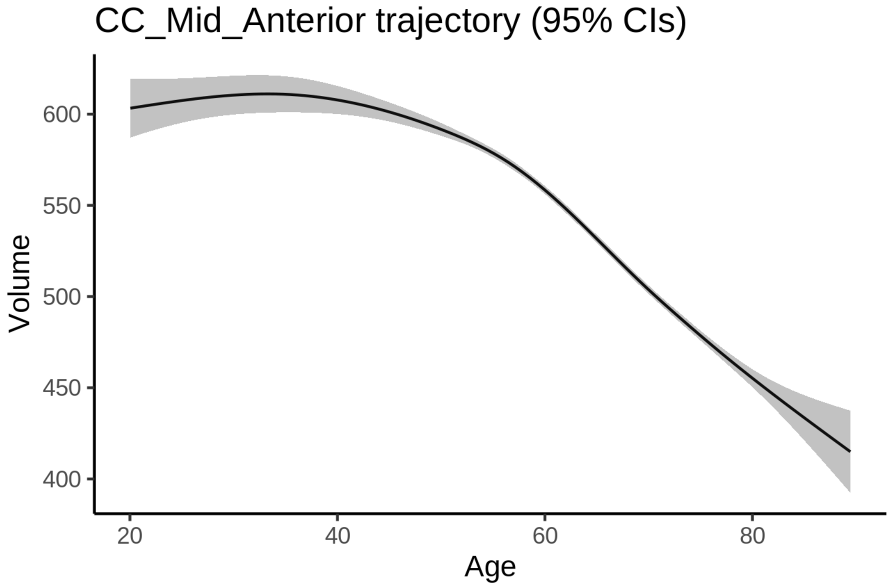

Effect of sleep

The chosen model only included the main effect of sleep, and hence the effect does not vary with age. The black dot shows the average sleep duration across all ages in the sample.

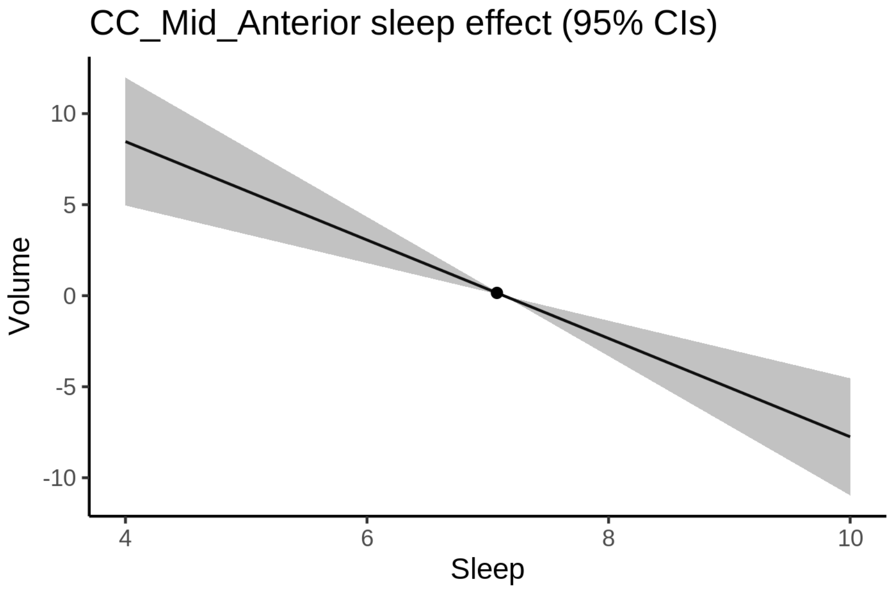

We also show the full interaction model for completeness, although it was not selected.

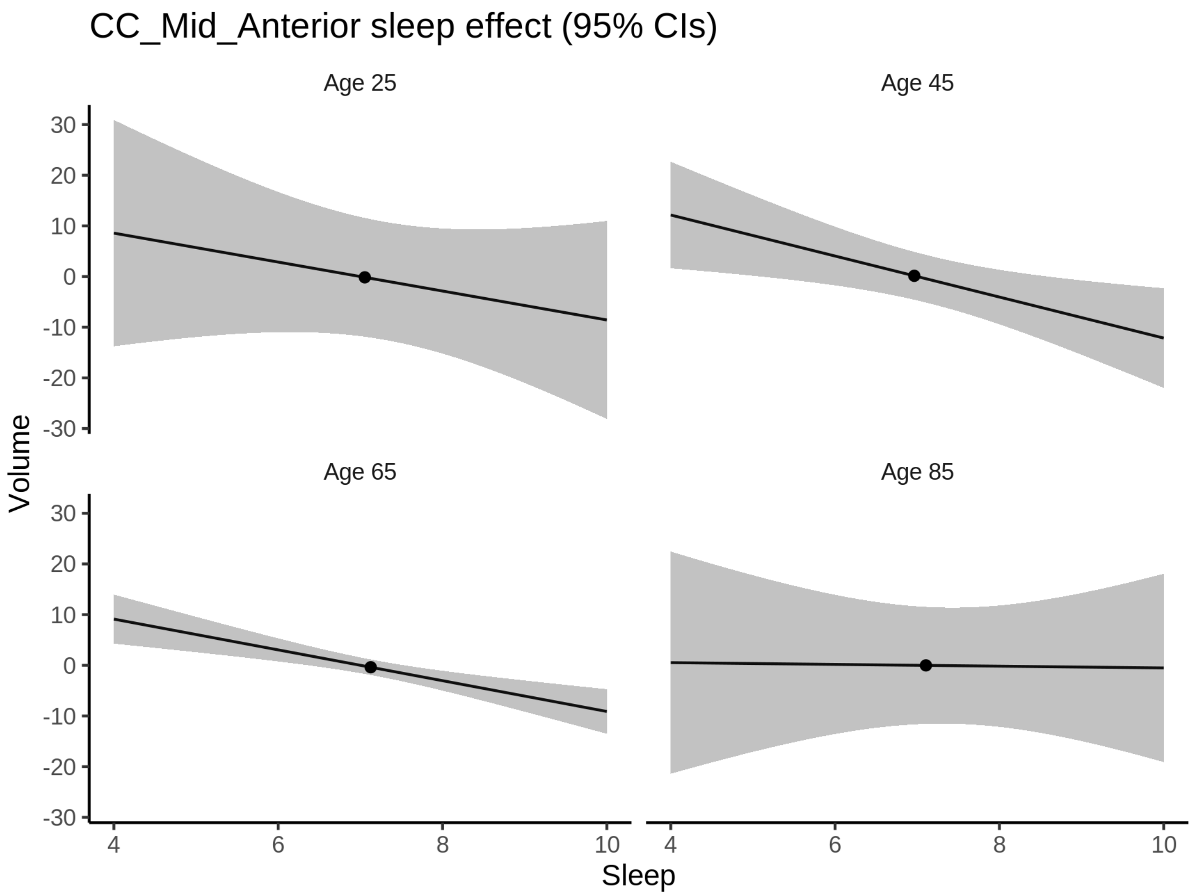

Deviation from sleep associated with maximal volume

Model with only main effect of sleep was chosen, so we show it for all ages at once. Maximum volume is attained at 4 hours of sleep. The percentage values in the plot are calculated as follows: The maximum at 100 % refers to a person at an arbitrary age with a sleep duration associated with maximum volume. For a female, this volume is 548 and for a male it is 531. The other percentage values show how large the expected volume is for someone with other sleep durations. For example, 99 % implies a 1 % reduction.

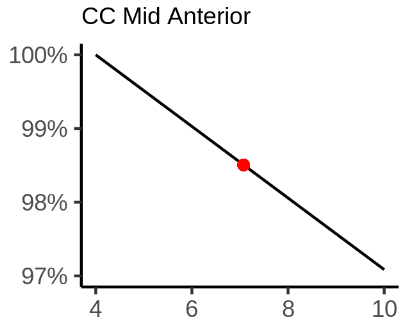

Comparison of mean sleep and sleep associated with maximum volume

A 95 % confidence interval for the sleep associated with maximum volume is [4, 4].

The plot below compares average sleep to the sleep associated with maximum volume.

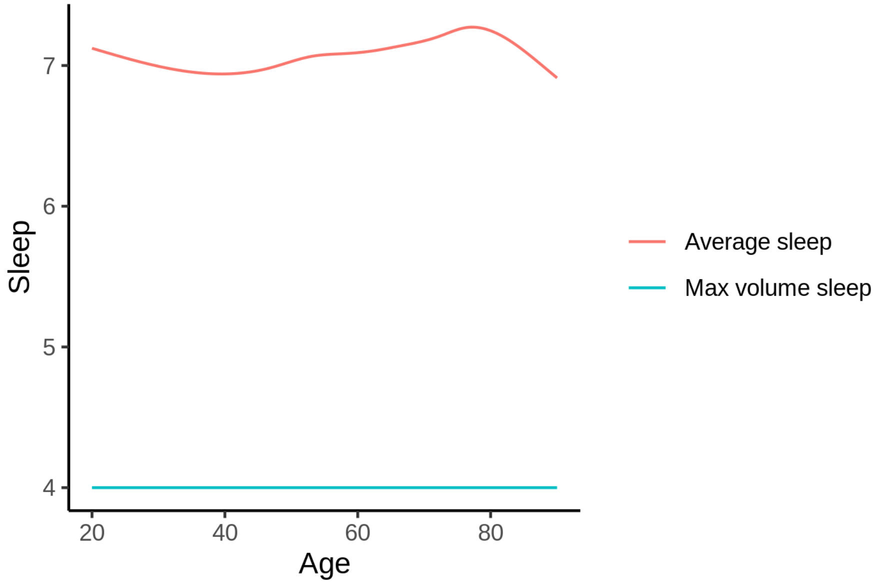

The next plot shows the difference between average sleep and sleep associated with maximum volume. The shaded region is a 95 % confidence interval.

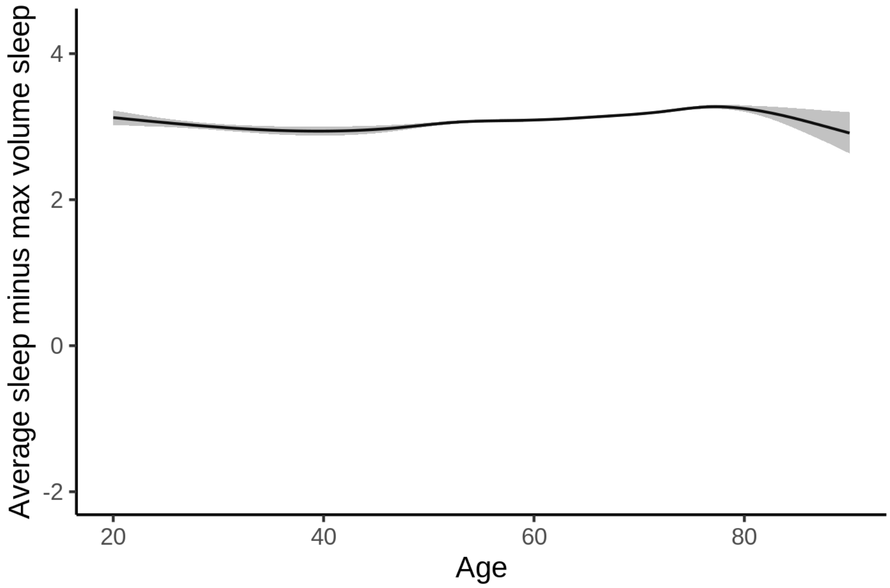

The next plot shows the probability that the sleep duration associated with maximum volume is longer than the average sleep duration, as a function of age. Probability below .05 can be interpreted as evidence that the sleep associated with maximum volume is shorter than the mean sleep, and probability above .95 can be interpreted the opposite way.

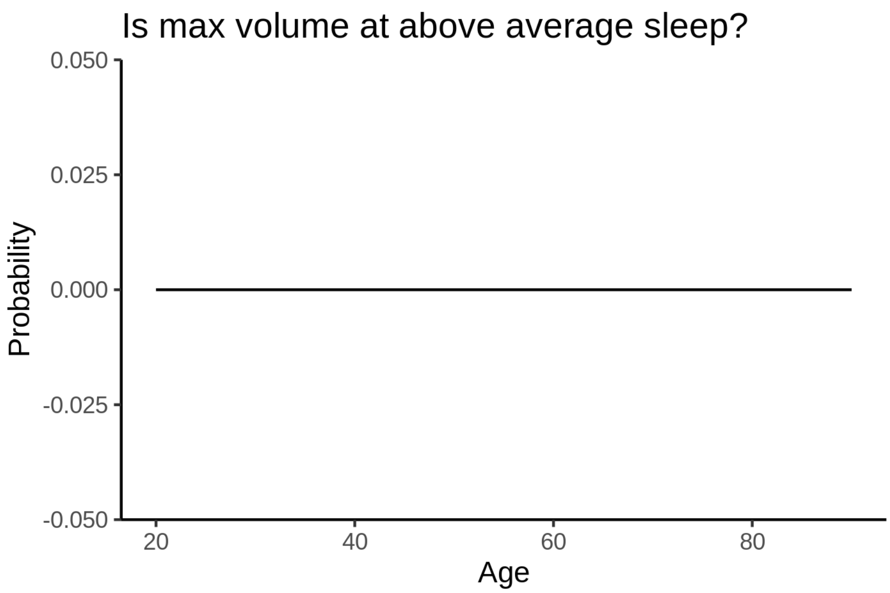

Controling for covariates

Below is the output for a model in which we only include data with income and education.

```
##
## Family: gaussian
## Link function: identity
##
## Formula:
## value ~ sex + site + s(age_z, k = 10, bs = "cr") + s(sleep_z,
##     k = 5, bs = "cr") + icv
## <environment: 0x558adb4f17f0>
##
## Parametric coefficients:
##                Estimate Std. Error t value Pr(>|t|)
## (Intercept)    555.8013    14.9399  37.203  < 2e-16 ***
## sexmale        -18.4100     1.6524 -11.141  < 2e-16 ***
## siteousAvanto -119.1874    18.6147  -6.403 1.55e-10 ***
## siteousPrisma  -41.8377    18.4940  -2.262   0.0237 *
## siteousSkyra  -111.3992    16.1943  -6.879 6.15e-12 ***
## siteUKB        -23.5825    14.9125  -1.581   0.1138
## siteUOXF       -15.0957    16.8079  -0.898   0.3691
## icv             32.3772     0.8492  38.128  < 2e-16 ***
## ---
## Signif. codes:  0 ‘***’ 0.001 ‘**’ 0.01 ‘*’ 0.05 ‘.’ 0.1 ‘ ’ 1
##
## Approximate significance of smooth terms:
##              edf Ref.df       F p-value
## s(age_z)   5.218  5.218 608.285 < 2e-16 ***
## s(sleep_z) 1.000  1.000   9.265 0.00234 **
## ---
## Signif. codes:  0 ‘***’ 0.001 ‘**’ 0.01 ‘*’ 0.05 ‘.’ 0.1 ‘ ’ 1
##
```

```
## R-sq.(adj) =   0.15
## lmer.REML = 3.8026e+05  Scale est. = 1585.3    n = 31185
```

Below is the output for a model in which we control for the main effects of income and education.

```
##
## Family: gaussian
## Link function: identity
##
## Formula:
## value ~ sex + site + s(age_z, k = 10, bs = "cr") + s(sleep_z,
##     k = 5, bs = "cr") + icv + income_scaled + education_scaled
## <environment: 0x558adb4f17f0>
##
## Parametric coefficients:
##                   Estimate Std. Error t value Pr(>|t|)
## (Intercept)       561.7266    15.0377  37.354  < 2e-16 ***
## sexmale           -18.4897     1.6570 -11.159  < 2e-16 ***
## siteousAvanto    -118.8309    18.6176  -6.383 1.76e-10 ***
## siteousPrisma     -41.0324    18.4934  -2.219   0.0265 *
## siteousSkyra     -110.9974    16.1967  -6.853 7.36e-12 ***
## siteUKB           -23.4352    14.9228  -1.570   0.1163
## siteUOXF          -16.9538    16.8180  -1.008   0.3134
## icv                32.7587     0.8566  38.243  < 2e-16 ***
## income_scaled      -2.9597     1.9667  -1.505   0.1324
## education_scaled   -5.9524     2.3242  -2.561   0.0104 *
## ---
## Signif. codes:  0 ‘***’ 0.001 ‘**’ 0.01 ‘*’ 0.05 ‘.’ 0.1 ‘ ’ 1
##
## Approximate significance of smooth terms:
##              edf Ref.df       F p-value
## s(age_z)   5.279  5.279 561.241 < 2e-16 ***
## s(sleep_z) 1.000  1.000   8.956 0.00277 **
## ---
## Signif. codes:  0 ‘***’ 0.001 ‘**’ 0.01 ‘*’ 0.05 ‘.’ 0.1 ‘ ’ 1
##
## R-sq.(adj) =  0.151
## lmer.REML = 3.8024e+05  Scale est. = 1584.9    n = 31185
```

We also included interaction effects between sleep duration and education and income, in another model. The output is shown below, and the interaction terms are `income_scaled:sleep_z` and `education_scaled:sleep_z`.

```
##
## Family: gaussian
## Link function: identity
##
## Formula:
## value ~ sex + site + s(age_z, k = 10, bs = "cr") + s(sleep_z,
##     k = 5, bs = "cr") + icv + income_scaled + education_scaled +
##     income_scaled:sleep_z + education_scaled:sleep_z
## <environment: 0x558adb4f17f0>
##
## Parametric coefficients:
##                           Estimate Std. Error t value Pr(>|t|)
## (Intercept)               561.8600    15.0390  37.360  < 2e-16 ***
```

```
## sexmale                   -18.3803     1.6581 -11.085  < 2e-16 ***
## siteousAvanto            -118.7398    18.6180  -6.378 1.82e-10 ***
## siteousPrisma             -40.6528    18.4951  -2.198   0.0280 *
## siteousSkyra             -110.8987    16.1968  -6.847 7.68e-12 ***
## siteUKB                   -23.5952    14.9246  -1.581   0.1139
## siteUOXF                  -17.3015    16.8229  -1.028   0.3037
## icv                        32.7523     0.8567  38.232  < 2e-16 ***
## income_scaled              -3.0764     1.9678  -1.563   0.1180
## education_scaled           -5.8955     2.3244  -2.536   0.0112 *
## income_scaled:sleep_z       3.5910     1.9462   1.845   0.0650 .
## education_scaled:sleep_z   -1.1372     2.2498  -0.505   0.6132
## ---
## Signif. codes:  0 '***' 0.001 '**' 0.01 '*' 0.05 '.' 0.1 ' ' 1
##
## Approximate significance of smooth terms:
##              edf Ref.df       F p-value
## s(age_z)   5.293  5.293 560.334  <2e-16 ***
## s(sleep_z) 1.000  1.000   2.303   0.129
## ---
## Signif. codes:  0 '***' 0.001 '**' 0.01 '*' 0.05 '.' 0.1 ' ' 1
##
## R-sq.(adj) =  0.151
## lmer.REML = 3.8023e+05  Scale est. = 1584.9    n = 31185
```

We did the same controling for BMI. Below is the model with no covariates but only keeping data with BMI.

```
##
## Family: gaussian
## Link function: identity
##
## Formula:
## value ~ sex + site + s(age_z, k = 10, bs = "cr") + s(sleep_z,
##     k = 5, bs = "cr") + icv
## <environment: 0x558a48640ae0>
##
## Parametric coefficients:
##               Estimate Std. Error t value Pr(>|t|)
## (Intercept)   449.3776    11.3326  39.653  < 2e-16 ***
## sexmale       -17.4913     1.5998 -10.933  < 2e-16 ***
## siteousPrisma  64.7979    14.6793   4.414 1.02e-05 ***
## siteousSkyra    1.8439     9.4843   0.194    0.846
## siteUCAM       51.1513    11.8342   4.322 1.55e-05 ***
## siteUKB        83.1626    11.4105   7.288 3.21e-13 ***
## siteUmU        70.3292    13.1408   5.352 8.76e-08 ***
## icv            32.1600     0.8249  38.987  < 2e-16 ***
## ---
## Signif. codes:  0 '***' 0.001 '**' 0.01 '*' 0.05 '.' 0.1 ' ' 1
##
## Approximate significance of smooth terms:
##              edf Ref.df       F p-value
## s(age_z)   5.961  5.961 574.971 < 2e-16 ***
## s(sleep_z) 1.053  1.053   8.751 0.00252 **
## ---
## Signif. codes:  0 '***' 0.001 '**' 0.01 '*' 0.05 '.' 0.1 ' ' 1
##
```

```
## R-sq.(adj) =  0.153
## lmer.REML = 4.0694e+05  Scale est. = 1477.4    n = 33427
```

Below is the model output with main effect.

```
##
## Family: gaussian
## Link function: identity
##
## Formula:
## value ~ sex + site + s(age_z, k = 10, bs = "cr") + s(sleep_z,
##     k = 5, bs = "cr") + icv + bmi
## <environment: 0x558a48640ae0>
##
## Parametric coefficients:
##               Estimate Std. Error t value Pr(>|t|)
## (Intercept)   470.8131    11.9908  39.264  < 2e-16 ***
## sexmale       -16.8099     1.6039 -10.481  < 2e-16 ***
## siteousPrisma  65.7366    14.6763   4.479 7.52e-06 ***
## siteousSkyra    2.0169     9.4839   0.213    0.832
## siteUCAM       51.6584    11.8325   4.366 1.27e-05 ***
## siteUKB        83.8436    11.4100   7.348 2.05e-13 ***
## siteUmU        71.2178    13.1390   5.420 5.99e-08 ***
## icv            32.2968     0.8251  39.143  < 2e-16 ***
## bmi            -0.8509     0.1557  -5.466 4.64e-08 ***
## ---
## Signif. codes:  0 '***' 0.001 '**' 0.01 '*' 0.05 '.' 0.1 ' ' 1
##
## Approximate significance of smooth terms:
##              edf Ref.df      F p-value
## s(age_z)   5.994  5.994 572.68 < 2e-16 ***
## s(sleep_z) 1.583  1.583   5.96 0.00368 **
## ---
## Signif. codes:  0 '***' 0.001 '**' 0.01 '*' 0.05 '.' 0.1 ' ' 1
##
## R-sq.(adj) =  0.154
## lmer.REML = 4.0691e+05  Scale est. = 1477.3    n = 33427
```

Next is the model with BMI-sleep interaction.

```
##
## Family: gaussian
## Link function: identity
##
## Formula:
## value ~ sex + site + s(age_z, k = 10, bs = "cr") + s(sleep_z,
##     k = 5, bs = "cr") + icv + bmi + bmi:sleep_z
## <environment: 0x558a48640ae0>
##
## Parametric coefficients:
##                Estimate Std. Error t value Pr(>|t|)
## (Intercept)   470.88875   11.99589  39.254  < 2e-16 ***
## sexmale       -16.81142    1.60392 -10.481  < 2e-16 ***
## siteousPrisma  65.73333   14.67645   4.479 7.53e-06 ***
## siteousSkyra    2.00678    9.48402   0.212    0.832
## siteUCAM       51.62851   11.83340   4.363 1.29e-05 ***
```

```
## siteUKB        83.81257   11.41090   7.345 2.11e-13 ***
## siteUmU        71.19076   13.13968   5.418 6.07e-08 ***
## icv            32.29504    0.82512  39.140  < 2e-16 ***
## bmi            -0.85196    0.15576  -5.470 4.54e-08 ***
## bmi:sleep_z    -0.03294    0.14741  -0.223    0.823
## ---
## Signif. codes:  0 ‘***’ 0.001 ‘**’ 0.01 ‘*’ 0.05 ‘.’ 0.1 ‘ ’ 1
##
## Approximate significance of smooth terms:
##              edf Ref.df       F p-value
## s(age_z)   5.994  5.994 572.449  <2e-16 ***
## s(sleep_z) 1.567  1.567   0.186   0.713
## ---
## Signif. codes:  0 ‘***’ 0.001 ‘**’ 0.01 ‘*’ 0.05 ‘.’ 0.1 ‘ ’ 1
##
## R-sq.(adj) =  0.154
## lmer.REML = 4.0691e+05  Scale est. = 1477.3    n = 33427
```

We did the same controling for depression. Below is the model with no covariates but only keeping data with depression.

```
##
## Family: gaussian
## Link function: identity
##
## Formula:
## value ~ sex + site + s(age_z, k = 10, bs = "cr") + s(sleep_z,
##     k = 5, bs = "cr") + icv
## <environment: 0x558a470629c8>
##
## Parametric coefficients:
##                Estimate Std. Error t value Pr(>|t|)
## (Intercept)    542.1358     7.5572  71.737  < 2e-16 ***
## sexmale        -17.5721     1.6067 -10.936  < 2e-16 ***
## siteousAvanto -101.3186    17.7317  -5.714 1.11e-08 ***
## siteousPrisma  -30.0581    14.7351  -2.040   0.0414 *
## siteousSkyra  -101.5079    11.4459  -8.869  < 2e-16 ***
## siteUCAM       -47.6812    10.3543  -4.605 4.14e-06 ***
## siteUKB        -10.0733     7.4714  -1.348   0.1776
## siteUmU        -23.6996    10.1956  -2.324   0.0201 *
## icv             31.8265     0.8272  38.475  < 2e-16 ***
## ---
## Signif. codes:  0 ‘***’ 0.001 ‘**’ 0.01 ‘*’ 0.05 ‘.’ 0.1 ‘ ’ 1
##
## Approximate significance of smooth terms:
##             edf Ref.df      F p-value
## s(age_z)   5.56   5.56 600.81 < 2e-16 ***
## s(sleep_z) 1.00   1.00   9.16 0.00248 **
## ---
## Signif. codes:  0 ‘***’ 0.001 ‘**’ 0.01 ‘*’ 0.05 ‘.’ 0.1 ‘ ’ 1
##
## R-sq.(adj) =   0.15
## lmer.REML = 4.0564e+05  Scale est. = 1433      n = 33339
```

Below is the model output with main effect.

```
##
## Family: gaussian
## Link function: identity
##
## Formula:
## value ~ sex + site + s(age_z, k = 10, bs = "cr") + s(sleep_z,
##     k = 5, bs = "cr") + icv + depression
## <environment: 0x558a470629c8>
##
## Parametric coefficients:
##                Estimate Std. Error t value Pr(>|t|)
## (Intercept)    542.4867     7.5917  71.458  < 2e-16 ***
## sexmale        -17.6065     1.6083 -10.947  < 2e-16 ***
## siteousAvanto -101.1405    17.7354  -5.703 1.19e-08 ***
## siteousPrisma  -29.9018    14.7389  -2.029   0.0425 *
## siteousSkyra  -101.3074    11.4533  -8.845  < 2e-16 ***
## siteUCAM       -47.6006    10.3559  -4.596 4.31e-06 ***
## siteUKB        -10.2256     7.4780  -1.367   0.1715
## siteUmU        -22.6760    10.4108  -2.178   0.0294 *
## icv             31.8295     0.8272  38.477  < 2e-16 ***
## depression      -2.7696     5.6967  -0.486   0.6269
## ---
## Signif. codes:  0 ‘***’ 0.001 ‘**’ 0.01 ‘*’ 0.05 ‘.’ 0.1 ‘ ’ 1
##
## Approximate significance of smooth terms:
##              edf Ref.df       F p-value
## s(age_z)   5.565  5.565 590.232 < 2e-16 ***
## s(sleep_z) 1.000  1.000   9.322 0.00227 **
## ---
## Signif. codes:  0 ‘***’ 0.001 ‘**’ 0.01 ‘*’ 0.05 ‘.’ 0.1 ‘ ’ 1
##
## R-sq.(adj) =   0.15
## lmer.REML = 4.0563e+05  Scale est. = 1433      n = 33339
```

Next is the model with depression-sleep interaction.

```
##
## Family: gaussian
## Link function: identity
##
## Formula:
## value ~ sex + site + s(age_z, k = 10, bs = "cr") + s(sleep_z,
##     k = 5, bs = "cr") + icv + depression + depression:sleep_z
## <environment: 0x558a470629c8>
##
## Parametric coefficients:
##                     Estimate Std. Error t value Pr(>|t|)
## (Intercept)         542.4812     7.5914  71.460  < 2e-16 ***
## sexmale             -17.6582     1.6085 -10.978  < 2e-16 ***
## siteousAvanto      -101.5506    17.7365  -5.726 1.04e-08 ***
## siteousPrisma       -30.2976    14.7397  -2.056   0.0398 *
## siteousSkyra       -101.6999    11.4546  -8.878  < 2e-16 ***
## siteUCAM            -47.9891    10.3576  -4.633 3.61e-06 ***
## siteUKB             -10.2022     7.4777  -1.364   0.1725
## siteUmU             -20.2439    10.4960  -1.929   0.0538 .
```

```
## icv                  31.8355     0.8272  38.486  < 2e-16 ***
## depression           -3.3475     5.7053  -0.587   0.5574
## depression:sleep_z   -7.5644     4.1621  -1.817   0.0692 .
## ---
## Signif. codes:  0 '***' 0.001 '**' 0.01 '*' 0.05 '.' 0.1 ' ' 1
##
## Approximate significance of smooth terms:
##              edf Ref.df       F p-value
## s(age_z)   5.563  5.563 590.870  <2e-16 ***
## s(sleep_z) 1.000  1.000   2.573   0.109
## ---
## Signif. codes:  0 '***' 0.001 '**' 0.01 '*' 0.05 '.' 0.1 ' ' 1
##
## R-sq.(adj) =   0.15
## lmer.REML = 4.0563e+05  Scale est. = 1433      n = 33339
```

The plot below shows the sleep-volume curve for the original model and for the model with main effects of SES.

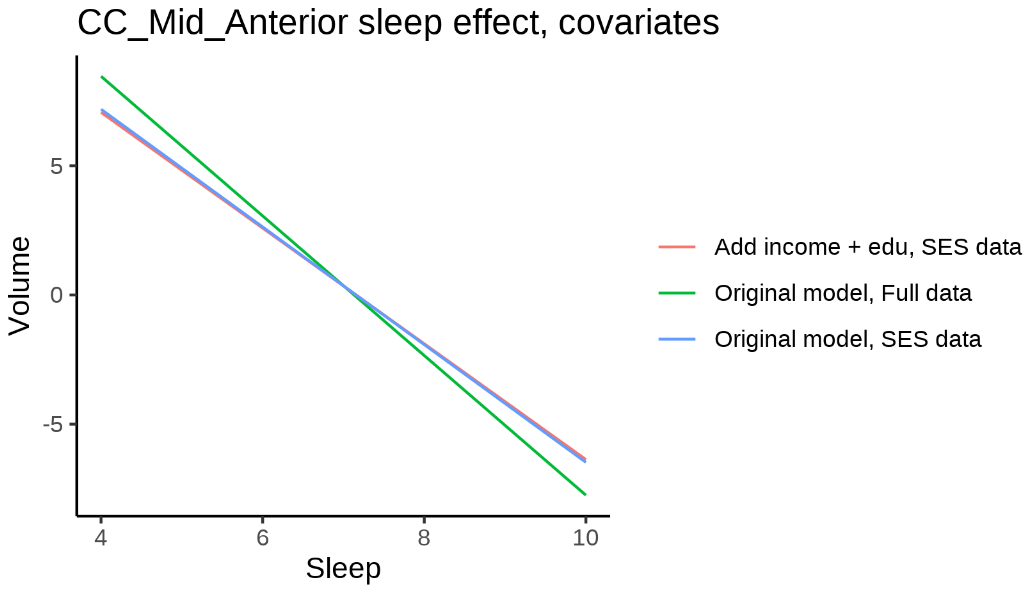

The plot below shows the sleep-volume curve for the original model and for the model with main effects of BMI.

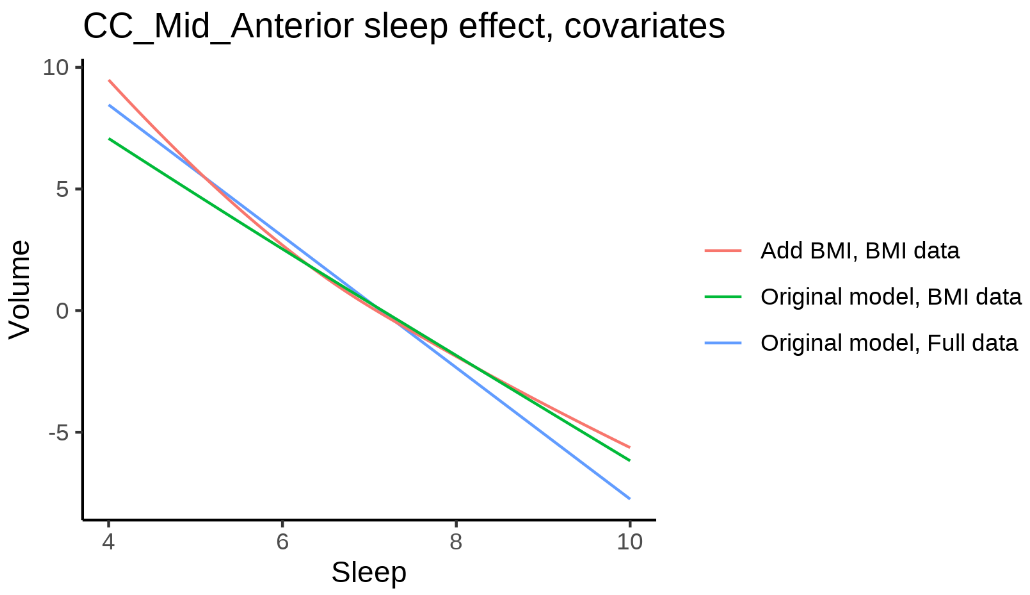

The plot below shows the sleep-volume curve for the original model and for the model with main effects of depression.

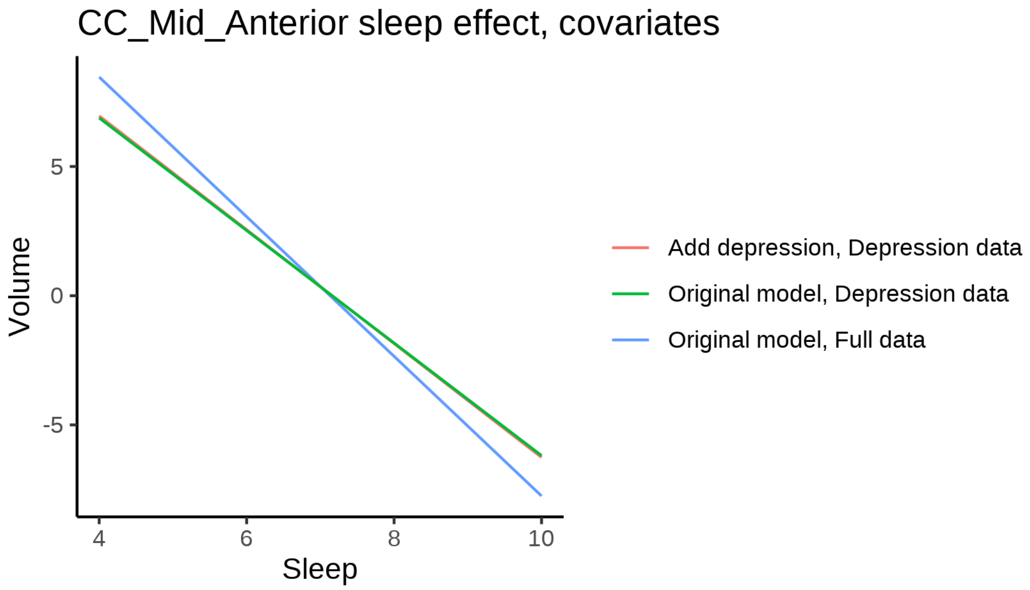

Models Not controling for ICV

Model outputs

Model without sleep term

```
##
## Family: gaussian
## Link function: identity
##
## Formula:
```

```
## value ~ sex + site + s(age_z, k = 10, bs = "cr")
## <environment: 0x558b6057dab8>
##
## Parametric coefficients:
##                Estimate Std. Error t value Pr(>|t|)
## (Intercept)    534.311      6.525  81.892  < 2e-16 ***
## sexmale         17.891      1.100  16.269  < 2e-16 ***
## siteMPIB       -49.407      8.282  -5.965 2.46e-09 ***
## siteousAvanto  -90.688      6.805 -13.328  < 2e-16 ***
## siteousPrisma  -32.112     11.544  -2.782  0.00541 **
## siteousSkyra   -98.919      6.364 -15.543  < 2e-16 ***
## siteUB         -83.260     19.531  -4.263 2.02e-05 ***
## siteUCAM       -50.546      7.242  -6.980 2.99e-12 ***
## siteUKB        -16.331      6.703  -2.436  0.01484 *
## siteUmU        -50.212      9.358  -5.366 8.10e-08 ***
## siteUOXF       -14.669      7.942  -1.847  0.06476 .
## ---
## Signif. codes:  0 '***' 0.001 '**' 0.01 '*' 0.05 '.' 0.1 ' ' 1
##
## Approximate significance of smooth terms:
##            edf Ref.df     F p-value
## s(age_z) 6.307  6.307 889.9  <2e-16 ***
## ---
## Signif. codes:  0 '***' 0.001 '**' 0.01 '*' 0.05 '.' 0.1 ' ' 1
##
## R-sq.(adj) =  0.123
## lmer.REML = 6.2756e+05  Scale est. = 1343.2    n = 51267
```

Model with only main effects of age and sleep

```
##
## Family: gaussian
## Link function: identity
##
## Formula:
## value ~ sex + site + s(age_z, k = 10, bs = "cr") + s(sleep_z,
##     k = 5, bs = "cr")
## <environment: 0x558b6057dab8>
##
## Parametric coefficients:
##                Estimate Std. Error t value Pr(>|t|)
## (Intercept)    533.516      6.530  81.707  < 2e-16 ***
## sexmale         17.883      1.100  16.257  < 2e-16 ***
## siteMPIB       -48.422      8.285  -5.844 5.12e-09 ***
## siteousAvanto  -90.325      6.806 -13.272  < 2e-16 ***
## siteousPrisma  -31.877     11.543  -2.761  0.00576 **
## siteousSkyra   -98.577      6.365 -15.486  < 2e-16 ***
## siteUB         -82.805     19.528  -4.240 2.24e-05 ***
## siteUCAM       -50.037      7.242  -6.910 4.91e-12 ***
## siteUKB        -15.497      6.708  -2.310  0.02087 *
## siteUmU        -48.094      9.377  -5.129 2.93e-07 ***
## siteUOXF       -14.271      7.941  -1.797  0.07233 .
## ---
## Signif. codes:  0 '***' 0.001 '**' 0.01 '*' 0.05 '.' 0.1 ' ' 1
##
```

```
## Approximate significance of smooth terms:
##               edf Ref.df       F p-value
## s(age_z)   6.287  6.287 881.579 < 2e-16 ***
## s(sleep_z) 2.049  2.049   6.463 0.00127 **
## ---
## Signif. codes:  0 ‘***’ 0.001 ‘**’ 0.01 ‘*’ 0.05 ‘.’ 0.1 ‘ ’ 1
##
## R-sq.(adj) =  0.123
## lmer.REML = 6.2754e+05  Scale est. = 1343.3    n = 51267
```

Model with full interaction between age and sleep

```
##
## Family: gaussian
## Link function: identity
##
## Formula:
## value ~ sex + site + t2(age_z, sleep_z, k = c(10, 4), bs = "cr")
## <environment: 0x558b6057dab8>
##
## Parametric coefficients:
##                Estimate Std. Error t value Pr(>|t|)
## (Intercept)     533.358      6.556  81.354  < 2e-16 ***
## sexmale          17.806      1.102  16.155  < 2e-16 ***
## siteMPIB        -48.478      8.329  -5.820 5.91e-09 ***
## siteousAvanto   -90.386      6.841 -13.212  < 2e-16 ***
## siteousPrisma   -32.109     11.567  -2.776  0.00551 **
## siteousSkyra    -98.556      6.406 -15.384  < 2e-16 ***
## siteUB          -82.677     19.541  -4.231 2.33e-05 ***
## siteUCAM        -49.968      7.283  -6.861 6.93e-12 ***
## siteUKB         -15.289      6.734  -2.271  0.02318 *
## siteUmU         -48.420      9.408  -5.147 2.66e-07 ***
## siteUOXF        -13.904      7.971  -1.744  0.08111 .
## ---
## Signif. codes:  0 ‘***’ 0.001 ‘**’ 0.01 ‘*’ 0.05 ‘.’ 0.1 ‘ ’ 1
##
## Approximate significance of smooth terms:
##                     edf Ref.df     F p-value
## t2(age_z,sleep_z) 10.23  10.23 14.77  <2e-16 ***
## ---
## Signif. codes:  0 ‘***’ 0.001 ‘**’ 0.01 ‘*’ 0.05 ‘.’ 0.1 ‘ ’ 1
##
## R-sq.(adj) =  0.123
## lmer.REML = 6.2755e+05  Scale est. = 1342.9    n = 51267
```

Model comparison

`mod_no_sleep` refers to model without sleep term, `mod_no_interaction` refers to model with only main effect of sleep, and `mod_full` refers to model with a full interaction between age and sleep. This is a nested model comparison, and the p-value at a given line refers to comparing the model at the line to the model on the line above. Hence, significance implies that the more complicated model is supported on statistical grounds.

To be even more specific, the p-value on the second row tests whether there is an association between sleep and volume. The p-value on the third row tests whether this association depends on age.

```
## Data: NULL
## Models:
## ml$mod_no_sleep$mer: NULL
## ml$mod_no_interaction$mer: NULL
## ml$mod_full$mer: NULL
##                            npar    AIC    BIC  logLik deviance  Chisq Df Pr(>Chisq)
## ml$mod_no_sleep$mer          15 627586 627718 -313778   627556
## ml$mod_no_interaction$mer    17 627579 627729 -313772   627545 10.976  2   0.004136 **
## ml$mod_full$mer              19 627586 627754 -313774   627548  0.000  2   1.000000
## ---
## Signif. codes:  0 ‘***’ 0.001 ‘**’ 0.01 ‘*’ 0.05 ‘.’ 0.1 ‘ ’ 1
```

We chose the model based on the likelihood ratio test with 5 % significance level, which was `mod_no_interaction`.

Lifespan brain trajectory

The trajectory shown is from the chosen model `mod_no_interaction_no_icv`.

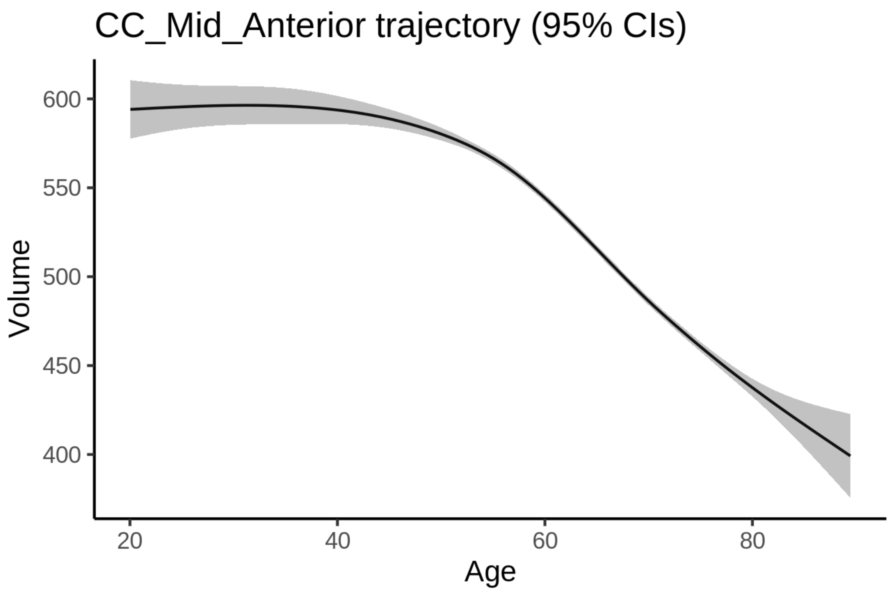

Effect of sleep

The chosen model only included the main effect of sleep, and hence the effect does not vary with age. The black dot shows the average sleep duration across all ages in the sample.

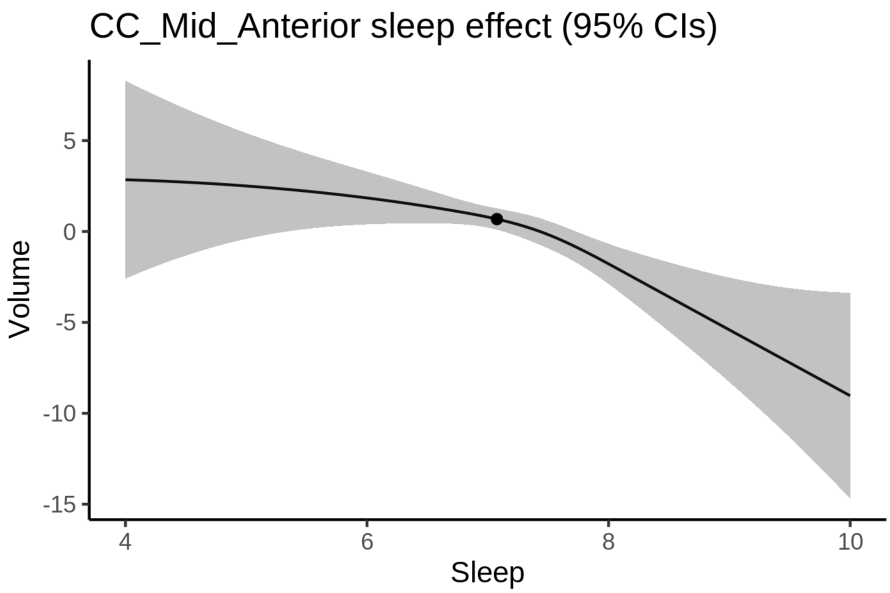

We also show the full interaction model for completeness, although it was not selected.

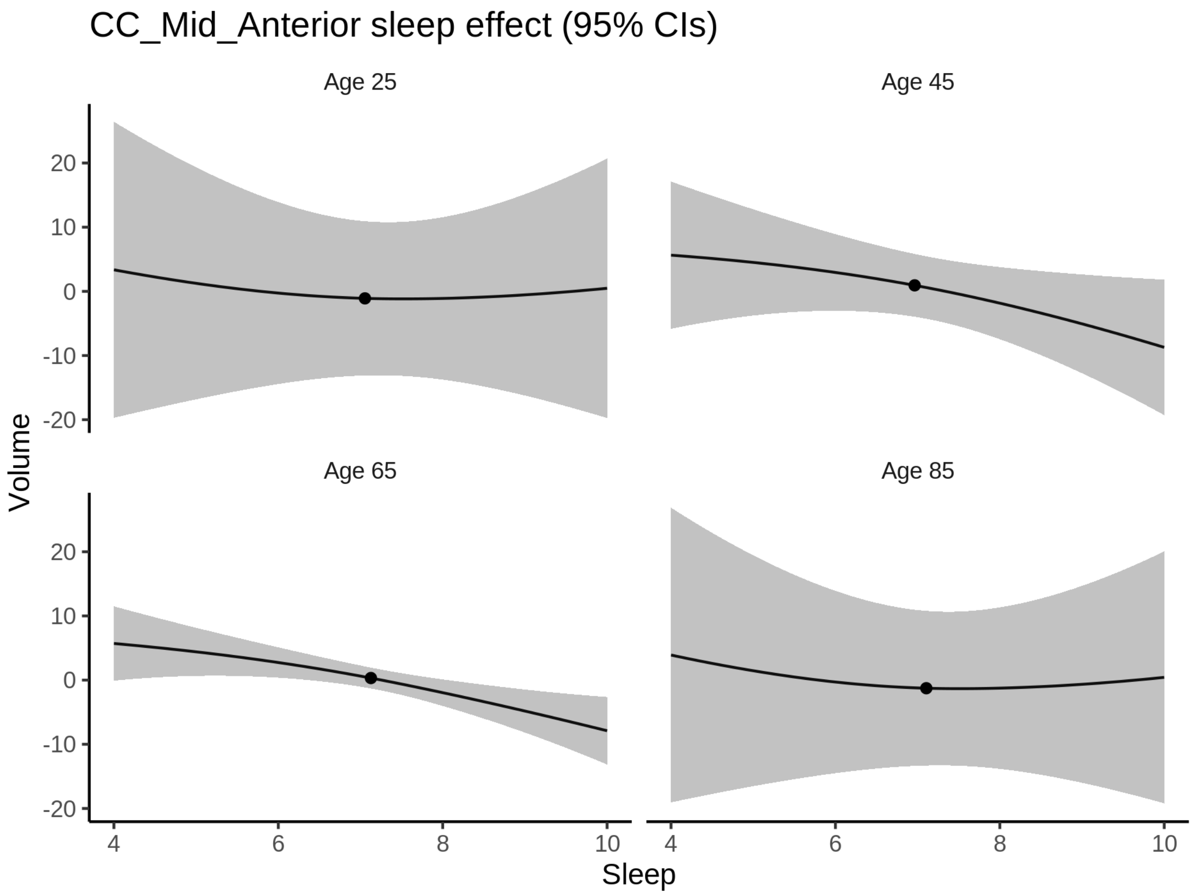

Deviation from sleep associated with maximal volume

Model with only main effect of sleep was chosen, so we show it for all ages at once. Maximum volume is attained at 4 hours of sleep. The percentage values in the plot are calculated as follows: The maximum at 100 % refers to a person at an arbitrary age with a sleep duration associated with maximum volume. For a female, this volume is 533 and for a male it is 551. The other percentage values show how large the expected volume is for someone with other sleep durations. For example, 99 % implies a 1 % reduction.

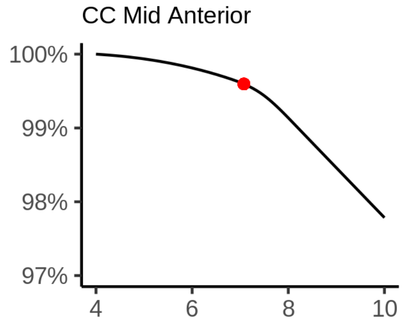

Comparison of mean sleep and sleep associated with maximum volume

A 95 % confidence interval for the sleep associated with maximum volume is [4, 6.73].

The plot below compares average sleep to the sleep associated with maximum volume.

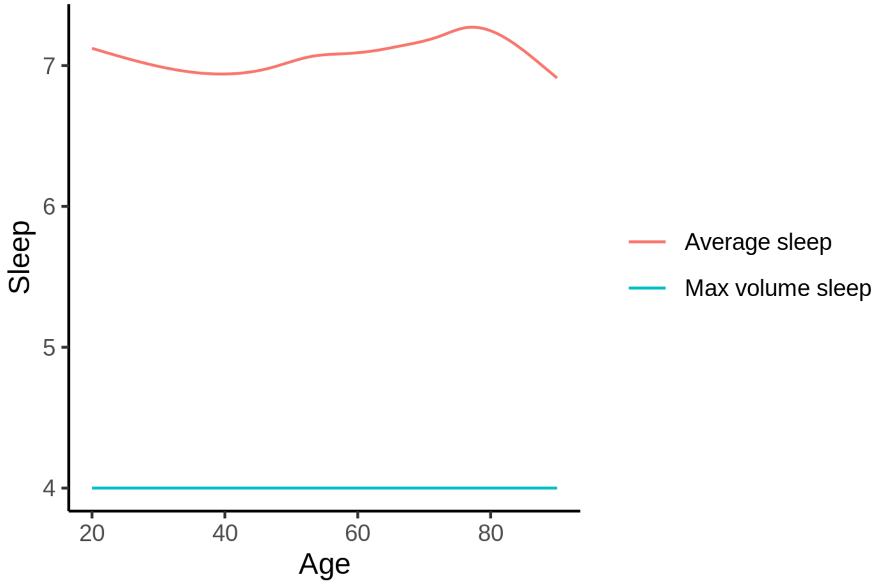

The next plot shows the difference between average sleep and sleep associated with maximum volume. The shaded region is a 95 % confidence interval.

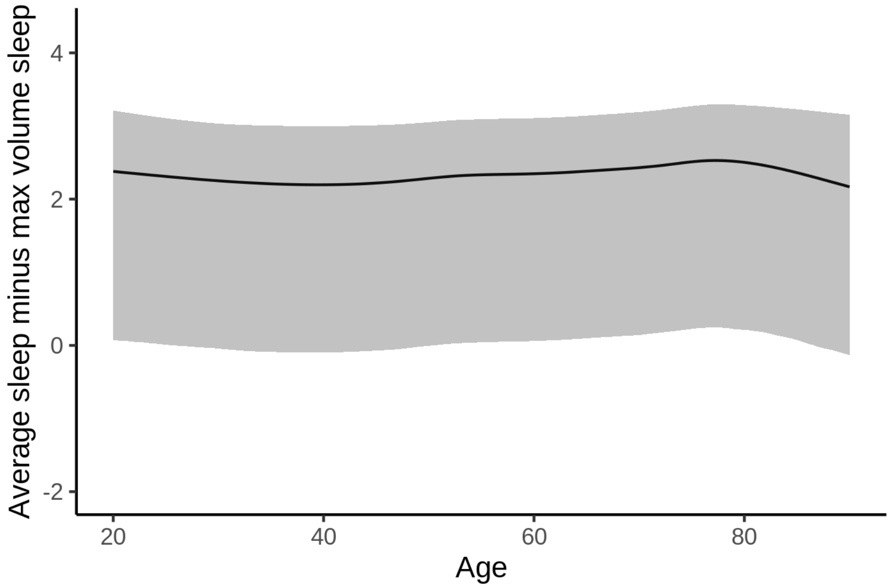

The next plot shows the probability that the sleep duration associated with maximum volume is longer than the average sleep duration, as a function of age. Probability below .05 can be interpreted as evidence that the sleep associated with maximum volume is shorter than the mean sleep, and probability above .95 can be interpreted the opposite way.

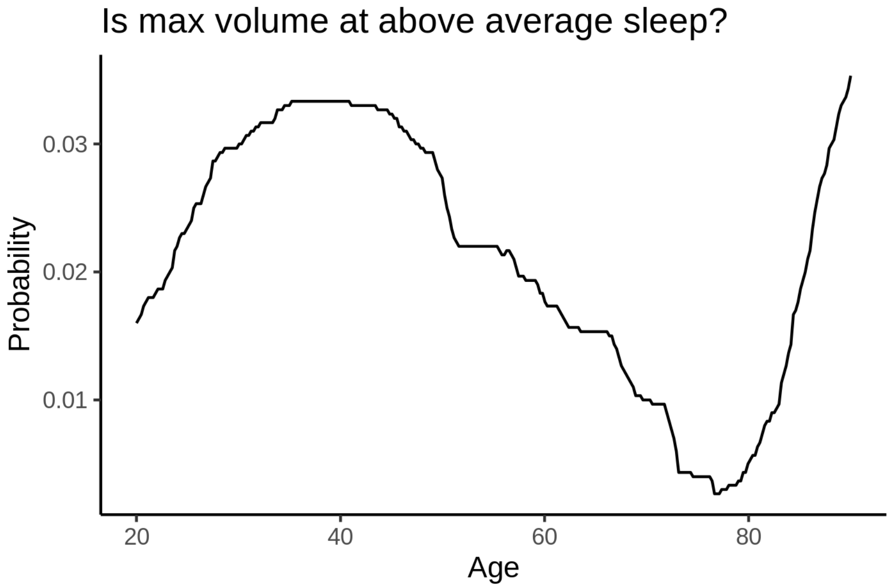

CC_Mid_Posterior

Descriptive statistics

Study	Observations	Unique IDs	Mean age	Age range
HCP	974	974	28.8	22 - 37
MPIB	677	391	63.1	24 - 83

Study	Observations	Unique IDs	Mean age	Age range
UB	113	39	70.9	64 - 81
UCAM	884	632	55.1	20 - 88
UiO	1475	803	49.4	20 - 89
UKB	45944	43120	64.5	45 - 83
UmU	412	279	62.2	25 - 85
UOXF	769	769	69.8	60 - 85

Spaghetti plot

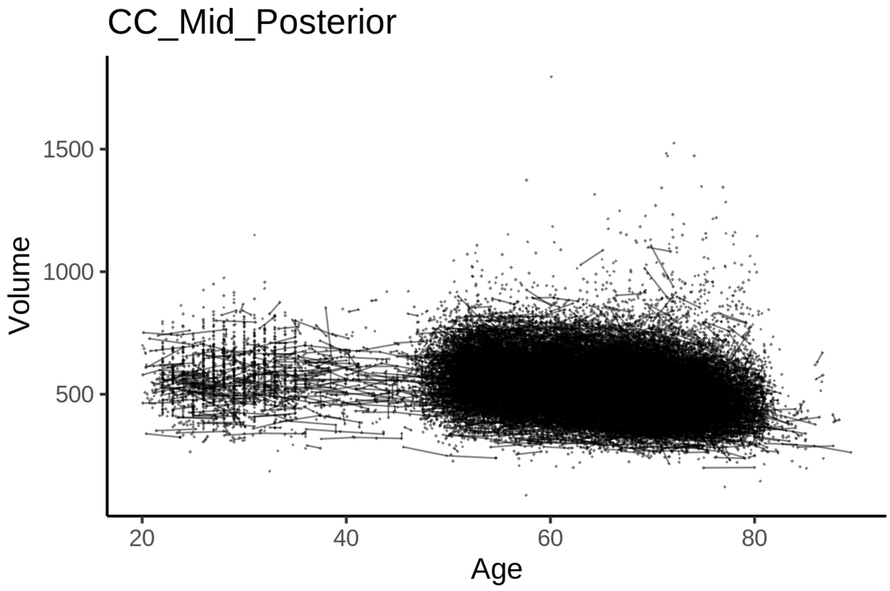

Models Controling for ICV

Model outputs

Model without sleep term

```
##
## Family: gaussian
## Link function: identity
##
## Formula:
## value ~ sex + site + icv + s(age_z, k = 10, bs = "cr")
## <environment: 0x558963829d10>
##
## Parametric coefficients:
##                Estimate Std. Error t value Pr(>|t|)
## (Intercept)   526.4579     5.6779  92.721  < 2e-16 ***
## sexmale       -18.4762     1.1774 -15.692  < 2e-16 ***
## siteMPIB       14.6888     7.3224   2.006   0.0449 *
## siteousAvanto -60.7891     5.8413 -10.407  < 2e-16 ***
## siteousPrisma -21.6652     9.9162  -2.185   0.0289 *
## siteousSkyra  -76.2296     5.5690 -13.688  < 2e-16 ***
```

```
## siteUB        -44.2387    17.2900  -2.559   0.0105 *
## siteUCAM      -29.2563     6.3359  -4.618 3.89e-06 ***
## siteUKB        23.6146     5.8199   4.058 4.97e-05 ***
## siteUmU       -13.9926     8.2045  -1.705   0.0881 .
## siteUOXF       -6.6890     6.9237  -0.966   0.3340
## icv            27.3397     0.5917  46.207  < 2e-16 ***
## ---
## Signif. codes:  0 '***' 0.001 '**' 0.01 '*' 0.05 '.' 0.1 ' ' 1
##
## Approximate significance of smooth terms:
##            edf Ref.df     F p-value
## s(age_z) 6.555  6.555 460.9  <2e-16 ***
## ---
## Signif. codes:  0 '***' 0.001 '**' 0.01 '*' 0.05 '.' 0.1 ' ' 1
##
## R-sq.(adj) =  0.124
## lmer.REML = 6.1274e+05  Scale est. = 737.91    n = 51248
```

Model with only main effects of age and sleep

```
##
## Family: gaussian
## Link function: identity
##
## Formula:
## value ~ sex + site + icv + s(age_z, k = 10, bs = "cr") + s(sleep_z,
##     k = 5, bs = "cr")
## <environment: 0x558963829d10>
##
## Parametric coefficients:
##               Estimate Std. Error t value Pr(>|t|)
## (Intercept)   526.2690     5.6829  92.606  < 2e-16 ***
## sexmale       -18.4917     1.1775 -15.705  < 2e-16 ***
## siteMPIB       15.0131     7.3269   2.049   0.0405 *
## siteousAvanto -60.7267     5.8427 -10.394  < 2e-16 ***
## siteousPrisma -21.6266     9.9163  -2.181   0.0292 *
## siteousSkyra  -76.1794     5.5707 -13.675  < 2e-16 ***
## siteUB        -44.0650    17.2893  -2.549   0.0108 *
## siteUCAM      -29.1036     6.3366  -4.593 4.38e-06 ***
## siteUKB        23.8191     5.8255   4.089 4.34e-05 ***
## siteUmU       -13.2967     8.2231  -1.617   0.1059
## siteUOXF       -6.6060     6.9242  -0.954   0.3401
## icv            27.3386     0.5921  46.170  < 2e-16 ***
## ---
## Signif. codes:  0 '***' 0.001 '**' 0.01 '*' 0.05 '.' 0.1 ' ' 1
##
## Approximate significance of smooth terms:
##              edf Ref.df       F p-value
## s(age_z)   6.547  6.547 457.357  <2e-16 ***
## s(sleep_z) 1.721  1.721   2.398   0.208
## ---
## Signif. codes:  0 '***' 0.001 '**' 0.01 '*' 0.05 '.' 0.1 ' ' 1
##
## R-sq.(adj) =  0.124
## lmer.REML = 6.1274e+05  Scale est. = 737.9     n = 51248
```

Model with full interaction between age and sleep

```
##
## Family: gaussian
## Link function: identity
##
## Formula:
## value ~ sex + site + icv + t2(age_z, sleep_z, k = c(10, 4), bs = "cr")
## <environment: 0x558963829d10>
##
## Parametric coefficients:
##                Estimate Std. Error t value Pr(>|t|)
## (Intercept)   526.7810     5.6978  92.453  < 2e-16 ***
## sexmale       -18.5333     1.1799 -15.708  < 2e-16 ***
## siteMPIB       14.5936     7.3615   1.982   0.0474 *
## siteousAvanto -61.1165     5.8720 -10.408  < 2e-16 ***
## siteousPrisma -21.9813     9.9366  -2.212   0.0270 *
## siteousSkyra  -76.5169     5.6036 -13.655  < 2e-16 ***
## siteUB        -44.6157    17.2979  -2.579   0.0099 **
## siteUCAM      -29.5530     6.3676  -4.641 3.47e-06 ***
## siteUKB        23.3101     5.8400   3.991 6.58e-05 ***
## siteUmU       -13.8046     8.2448  -1.674   0.0941 .
## siteUOXF       -7.0923     6.9435  -1.021   0.3071
## icv            27.3435     0.5921  46.180  < 2e-16 ***
## ---
## Signif. codes:  0 ‘***’ 0.001 ‘**’ 0.01 ‘*’ 0.05 ‘.’ 0.1 ‘ ’ 1
##
## Approximate significance of smooth terms:
##                      edf Ref.df     F p-value
## t2(age_z,sleep_z) 10.93  10.93 10.15  <2e-16 ***
## ---
## Signif. codes:  0 ‘***’ 0.001 ‘**’ 0.01 ‘*’ 0.05 ‘.’ 0.1 ‘ ’ 1
##
## R-sq.(adj) =  0.124
## lmer.REML = 6.1274e+05  Scale est. = 737.76    n = 51248
```

Model comparison

`mod_no_sleep` refers to model without sleep term, `mod_no_interaction` refers to model with only main effect of sleep, and `mod_full` refers to model with a full interaction between age and sleep. This is a nested model comparison, and the p-value at a given line refers to comparing the model at the line to the model on the line above. Hence, significance implies that the more complicated model is supported on statistical grounds.

To be even more specific, the p-value on the second row tests whether there is an association between sleep and volume. The p-value on the third row tests whether this association depends on age.

```
## Data: NULL
## Models:
## ml$mod_no_sleep$mer: NULL
## ml$mod_no_interaction$mer: NULL
## ml$mod_full$mer: NULL
##                           npar    AIC    BIC  logLik deviance  Chisq Df Pr(>Chisq)
## ml$mod_no_sleep$mer         16 612769 612911 -306369   612737
## ml$mod_no_interaction$mer   18 612772 612931 -306368   612736 1.6528  2     0.4376
```

```
## ml$mod_full$mer            20 612779 612955 -306369   612739 0.0000  2     1.0000
```

We chose the model based on the likelihood ratio test with 5 % significance level, which was `mod_no_sleep`.

Lifespan brain trajectory

The trajectory shown is from the chosen model `mod_no_sleep`.

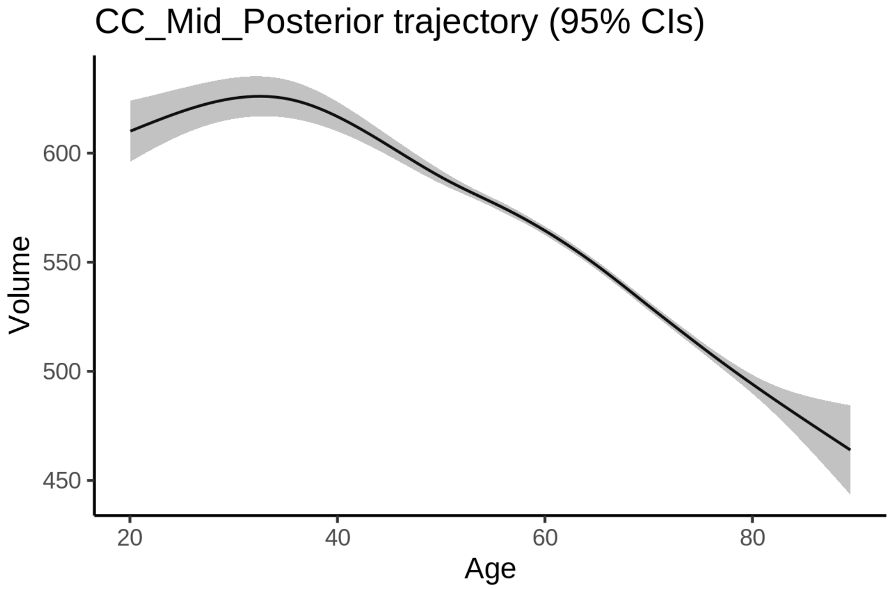

Effect of sleep

The chosen model did not include a sleep term, and hence we don't have any estimated effect of sleep.

We show the full interaction model for completeness, although it was not selected.

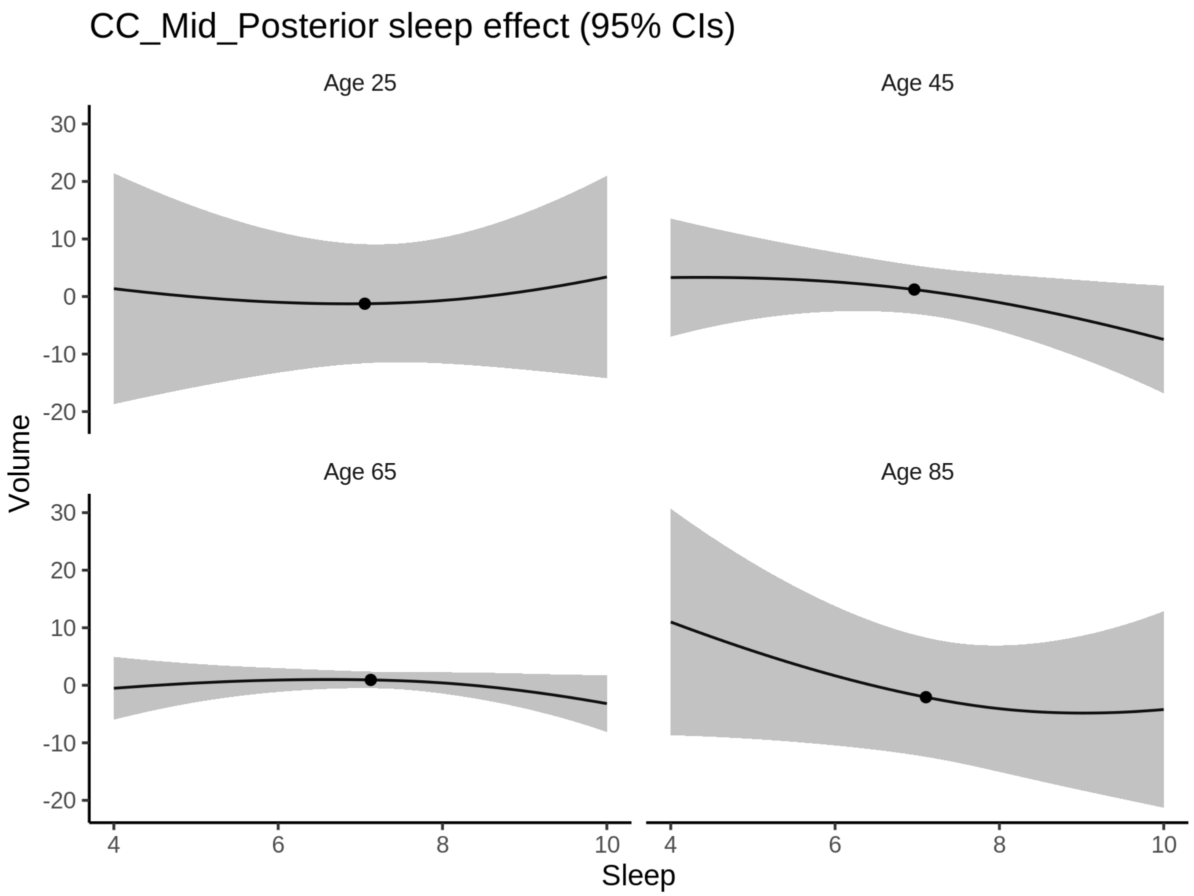

Deviation from sleep associated with maximal volume

Model with no sleep term was selected. No plots to show. (Although we can of course dig up the plots, which will be pretty flat).

Comparison of mean sleep and sleep associated with maximum volume

Nothing to show, as we did not find an association between sleep and volume.

Controling for covariates

Below is the output for a model in which we only include data with income and education.

```
##
## Family: gaussian
## Link function: identity
##
## Formula:
## value ~ sex + site + s(age_z, k = 10, bs = "cr") + s(sleep_z,
##     k = 5, bs = "cr") + icv
## <environment: 0x558a4ab49d80>
##
## Parametric coefficients:
```

```
##                Estimate Std. Error t value Pr(>|t|)
## (Intercept)    531.399     13.646  38.943  < 2e-16 ***
## sexmale        -18.919      1.508 -12.548  < 2e-16 ***
## siteousAvanto  -72.440     16.379  -4.423 9.78e-06 ***
## siteousPrisma  -29.602     16.810  -1.761   0.0782 .
## siteousSkyra   -81.703     14.836  -5.507 3.68e-08 ***
## siteUKB         17.200     13.616   1.263   0.2065
## siteUOXF       -14.930     15.345  -0.973   0.3306
## icv             28.695      0.774  37.072  < 2e-16 ***
## ---
## Signif. codes:  0 ‘***’ 0.001 ‘**’ 0.01 ‘*’ 0.05 ‘.’ 0.1 ‘ ’ 1
##
## Approximate significance of smooth terms:
##               edf Ref.df       F p-value
## s(age_z)   4.220  4.220 385.916  <2e-16 ***
## s(sleep_z) 1.628  1.628   0.305   0.592
## ---
## Signif. codes:  0 ‘***’ 0.001 ‘**’ 0.01 ‘*’ 0.05 ‘.’ 0.1 ‘ ’ 1
##
## R-sq.(adj) =  0.106
## lmer.REML = 3.7337e+05  Scale est. = 832.95    n = 31173
```

Below is the output for a model in which we control for the main effects of income and education.

```
##
## Family: gaussian
## Link function: identity
##
## Formula:
## value ~ sex + site + s(age_z, k = 10, bs = "cr") + s(sleep_z,
##     k = 5, bs = "cr") + icv + income_scaled + education_scaled
## <environment: 0x558a4ab49d80>
##
## Parametric coefficients:
##                     Estimate Std. Error t value Pr(>|t|)
## (Intercept)      528.740982  13.723365  38.529  < 2e-16 ***
## sexmale          -19.093018   1.511886 -12.629  < 2e-16 ***
## siteousAvanto    -71.899113  16.378732  -4.390 1.14e-05 ***
## siteousPrisma    -29.770209  16.806516  -1.771   0.0765 .
## siteousSkyra     -81.212463  14.837373  -5.474 4.45e-08 ***
## siteUKB           17.954970  13.614773   1.319   0.1873
## siteUOXF         -13.766604  15.344999  -0.897   0.3697
## icv               28.511600   0.780628  36.524  < 2e-16 ***
## income_scaled      4.206282   1.793564   2.345   0.0190 *
## education_scaled   0.007938   2.122795   0.004   0.9970
## ---
## Signif. codes:  0 ‘***’ 0.001 ‘**’ 0.01 ‘*’ 0.05 ‘.’ 0.1 ‘ ’ 1
##
## Approximate significance of smooth terms:
##               edf Ref.df       F p-value
## s(age_z)   4.104  4.104 352.244  <2e-16 ***
## s(sleep_z) 1.359  1.359   0.118   0.725
## ---
## Signif. codes:  0 ‘***’ 0.001 ‘**’ 0.01 ‘*’ 0.05 ‘.’ 0.1 ‘ ’ 1
##
```

```
## R-sq.(adj) =  0.107
## lmer.REML = 3.7336e+05  Scale est. = 833.44    n = 31173
```

We also included interaction effects between sleep duration and education and income, in another model. The output is shown below, and the interaction terms are `income_scaled:sleep_z` and `education_scaled:sleep_z`.

```
##
## Family: gaussian
## Link function: identity
##
## Formula:
## value ~ sex + site + s(age_z, k = 10, bs = "cr") + s(sleep_z,
##     k = 5, bs = "cr") + icv + income_scaled + education_scaled +
##     income_scaled:sleep_z + education_scaled:sleep_z
## <environment: 0x558a4ab49d80>
##
## Parametric coefficients:
##                           Estimate Std. Error t value Pr(>|t|)
## (Intercept)              528.48501   13.72341  38.510  < 2e-16 ***
## sexmale                  -18.99006    1.51289 -12.552  < 2e-16 ***
## siteousAvanto            -71.60894   16.37879  -4.372 1.23e-05 ***
## siteousPrisma            -29.23597   16.80780  -1.739   0.0820 .
## siteousSkyra             -80.93085   14.83713  -5.455 4.95e-08 ***
## siteUKB                   18.23289   13.61581   1.339   0.1805
## siteUOXF                 -13.39657   15.34894  -0.873   0.3828
## icv                       28.49346    0.78063  36.500  < 2e-16 ***
## income_scaled              4.12342    1.79370   2.299   0.0215 *
## education_scaled           0.09451    2.12265   0.045   0.9645
## income_scaled:sleep_z      2.35731    1.77758   1.326   0.1848
## education_scaled:sleep_z  -4.03540    2.05515  -1.964   0.0496 *
## ---
## Signif. codes:  0 ‘***’ 0.001 ‘**’ 0.01 ‘*’ 0.05 ‘.’ 0.1 ‘ ’ 1
##
## Approximate significance of smooth terms:
##              edf Ref.df       F p-value
## s(age_z)   4.114  4.114 352.036  <2e-16 ***
## s(sleep_z) 1.111  1.111   1.181   0.313
## ---
## Signif. codes:  0 ‘***’ 0.001 ‘**’ 0.01 ‘*’ 0.05 ‘.’ 0.1 ‘ ’ 1
##
## R-sq.(adj) =  0.107
## lmer.REML = 3.7335e+05  Scale est. = 833.42    n = 31173
```

We did the same controling for BMI. Below is the model with no covariates but only keeping data with BMI.

```
##
## Family: gaussian
## Link function: identity
##
## Formula:
## value ~ sex + site + s(age_z, k = 10, bs = "cr") + s(sleep_z,
##     k = 5, bs = "cr") + icv
## <environment: 0x5589628f4758>
##
## Parametric coefficients:
```

```
##                Estimate Std. Error t value Pr(>|t|)
## (Intercept)   461.1165     9.1717  50.276  < 2e-16 ***
## sexmale       -18.3813     1.4622 -12.571  < 2e-16 ***
## siteousPrisma  43.5868    12.1782   3.579 0.000345 ***
## siteousSkyra   -7.1751     7.0792  -1.014 0.310811
## siteUCAM       36.6438     9.6964   3.779 0.000158 ***
## siteUKB        87.9018     9.2499   9.503  < 2e-16 ***
## siteUmU        53.5881    11.0254   4.860 1.18e-06 ***
## icv            28.7018     0.7529  38.120  < 2e-16 ***
## ---
## Signif. codes:  0 '***' 0.001 '**' 0.01 '*' 0.05 '.' 0.1 ' ' 1
##
## Approximate significance of smooth terms:
##              edf Ref.df       F p-value
## s(age_z)   5.692  5.692 330.447  <2e-16 ***
## s(sleep_z) 1.268  1.268   0.105   0.904
## ---
## Signif. codes:  0 '***' 0.001 '**' 0.01 '*' 0.05 '.' 0.1 ' ' 1
##
## R-sq.(adj) =  0.114
## lmer.REML = 3.9965e+05  Scale est. = 794.26    n = 33417
```

Below is the model output with main effect.

```
##
## Family: gaussian
## Link function: identity
##
## Formula:
## value ~ sex + site + s(age_z, k = 10, bs = "cr") + s(sleep_z,
##     k = 5, bs = "cr") + icv + bmi
## <environment: 0x5589628f4758>
##
## Parametric coefficients:
##                Estimate Std. Error t value Pr(>|t|)
## (Intercept)   499.5209     9.8384  50.773  < 2e-16 ***
## sexmale       -17.1654     1.4640 -11.725  < 2e-16 ***
## siteousPrisma  44.9821    12.1659   3.697 0.000218 ***
## siteousSkyra   -7.0110     7.0776  -0.991 0.321896
## siteUCAM       37.5731     9.6864   3.879 0.000105 ***
## siteUKB        89.0859     9.2410   9.640  < 2e-16 ***
## siteUmU        55.2298    11.0125   5.015 5.33e-07 ***
## icv            28.9227     0.7517  38.476  < 2e-16 ***
## bmi            -1.5230     0.1421 -10.719  < 2e-16 ***
## ---
## Signif. codes:  0 '***' 0.001 '**' 0.01 '*' 0.05 '.' 0.1 ' ' 1
##
## Approximate significance of smooth terms:
##              edf Ref.df     F p-value
## s(age_z)   5.675  5.675 333.2  <2e-16 ***
## s(sleep_z) 1.000  1.000   0.4   0.527
## ---
## Signif. codes:  0 '***' 0.001 '**' 0.01 '*' 0.05 '.' 0.1 ' ' 1
##
## R-sq.(adj) =  0.117
```

```
## lmer.REML = 3.9954e+05  Scale est. = 794.11    n = 33417
```

Next is the model with BMI-sleep interaction.

```
##
## Family: gaussian
## Link function: identity
##
## Formula:
## value ~ sex + site + s(age_z, k = 10, bs = "cr") + s(sleep_z,
##     k = 5, bs = "cr") + icv + bmi + bmi:sleep_z
## <environment: 0x5589628f4758>
##
## Parametric coefficients:
##               Estimate Std. Error t value Pr(>|t|)
## (Intercept)   499.1361     9.8431  50.709  < 2e-16 ***
## sexmale       -17.1584     1.4640 -11.721  < 2e-16 ***
## siteousPrisma  44.9838    12.1658   3.698 0.000218 ***
## siteousSkyra   -6.9758     7.0776  -0.986 0.324329
## siteUCAM       37.7190     9.6870   3.894 9.89e-05 ***
## siteUKB        89.2268     9.2416   9.655  < 2e-16 ***
## siteUmU        55.3608    11.0129   5.027 5.01e-07 ***
## icv            28.9292     0.7517  38.484  < 2e-16 ***
## bmi            -1.5169     0.1422 -10.670  < 2e-16 ***
## bmi:sleep_z     0.1689     0.1345   1.256 0.209213
## ---
## Signif. codes:  0 ‘***’ 0.001 ‘**’ 0.01 ‘*’ 0.05 ‘.’ 0.1 ‘ ’ 1
##
## Approximate significance of smooth terms:
##              edf Ref.df       F p-value
## s(age_z)   5.674  5.674 333.525  <2e-16 ***
## s(sleep_z) 1.000  1.000   1.805   0.179
## ---
## Signif. codes:  0 ‘***’ 0.001 ‘**’ 0.01 ‘*’ 0.05 ‘.’ 0.1 ‘ ’ 1
##
## R-sq.(adj) =  0.117
## lmer.REML = 3.9954e+05  Scale est. = 794.1     n = 33417
```

We did the same controling for depression. Below is the model with no covariates but only keeping data with depression.

```
##
## Family: gaussian
## Link function: identity
##
## Formula:
## value ~ sex + site + s(age_z, k = 10, bs = "cr") + s(sleep_z,
##     k = 5, bs = "cr") + icv
## <environment: 0x558ada4d57f8>
##
## Parametric coefficients:
##               Estimate Std. Error t value Pr(>|t|)
## (Intercept)   532.8544     6.9724  76.424  < 2e-16 ***
## sexmale       -18.1468     1.4689 -12.354  < 2e-16 ***
## siteousAvanto -54.5723    14.6336  -3.729 0.000192 ***
## siteousPrisma -27.4611    13.2918  -2.066 0.038835 *
```

```
## siteousSkyra  -77.5324    10.4213  -7.440 1.03e-13 ***
## siteUCAM      -39.7849     9.5266  -4.176 2.97e-05 ***
## siteUKB        15.5185     6.8946   2.251 0.024403 *
## siteUmU       -19.9476     9.3871  -2.125 0.033594 *
## icv            28.3413     0.7553  37.522  < 2e-16 ***
## ---
## Signif. codes:  0 ‘***’ 0.001 ‘**’ 0.01 ‘*’ 0.05 ‘.’ 0.1 ‘ ’ 1
##
## Approximate significance of smooth terms:
##              edf Ref.df       F p-value
## s(age_z)   5.436  5.436 325.992  <2e-16 ***
## s(sleep_z) 1.380  1.380   0.223   0.833
## ---
## Signif. codes:  0 ‘***’ 0.001 ‘**’ 0.01 ‘*’ 0.05 ‘.’ 0.1 ‘ ’ 1
##
## R-sq.(adj) =   0.11
## lmer.REML = 3.9837e+05  Scale est. = 779.46    n = 33329
```

Below is the model output with main effect.

```
##
## Family: gaussian
## Link function: identity
##
## Formula:
## value ~ sex + site + s(age_z, k = 10, bs = "cr") + s(sleep_z,
##     k = 5, bs = "cr") + icv + depression
## <environment: 0x558ada4d57f8>
##
## Parametric coefficients:
##               Estimate Std. Error t value Pr(>|t|)
## (Intercept)   534.3633     7.0026  76.309  < 2e-16 ***
## sexmale       -18.3019     1.4703 -12.448  < 2e-16 ***
## siteousAvanto -53.8246    14.6375  -3.677 0.000236 ***
## siteousPrisma -26.8027    13.2955  -2.016 0.043816 *
## siteousSkyra  -76.6840    10.4285  -7.353 1.98e-13 ***
## siteUCAM      -39.4575     9.5277  -4.141 3.46e-05 ***
## siteUKB        14.8959     6.8996   2.159 0.030861 *
## siteUmU       -15.4031     9.5825  -1.607 0.107973
## icv            28.3640     0.7551  37.561  < 2e-16 ***
## depression    -12.2990     5.2109  -2.360 0.018269 *
## ---
## Signif. codes:  0 ‘***’ 0.001 ‘**’ 0.01 ‘*’ 0.05 ‘.’ 0.1 ‘ ’ 1
##
## Approximate significance of smooth terms:
##              edf Ref.df       F p-value
## s(age_z)   5.457  5.457 324.371  <2e-16 ***
## s(sleep_z) 1.000  1.000   0.195   0.659
## ---
## Signif. codes:  0 ‘***’ 0.001 ‘**’ 0.01 ‘*’ 0.05 ‘.’ 0.1 ‘ ’ 1
##
## R-sq.(adj) =   0.11
## lmer.REML = 3.9836e+05  Scale est. = 779.38    n = 33329
```

Next is the model with depression-sleep interaction.

```
##
## Family: gaussian
## Link function: identity
##
## Formula:
## value ~ sex + site + s(age_z, k = 10, bs = "cr") + s(sleep_z,
##     k = 5, bs = "cr") + icv + depression + depression:sleep_z
## <environment: 0x558ada4d57f8>
##
## Parametric coefficients:
##                    Estimate Std. Error t value Pr(>|t|)
## (Intercept)        534.3569     7.0023  76.312  < 2e-16 ***
## sexmale            -18.3507     1.4704 -12.480  < 2e-16 ***
## siteousAvanto      -54.1876    14.6370  -3.702 0.000214 ***
## siteousPrisma      -27.1496    13.2942  -2.042 0.041138 *
## siteousSkyra       -77.0308    10.4274  -7.387 1.53e-13 ***
## siteUCAM           -39.8012     9.5286  -4.177 2.96e-05 ***
## siteUKB             14.9182     6.8993   2.162 0.030603 *
## siteUmU            -13.0927     9.6604  -1.355 0.175332
## icv                 28.3693     0.7551  37.570  < 2e-16 ***
## depression         -12.8423     5.2188  -2.461 0.013869 *
## depression:sleep_z  -7.1334     3.8088  -1.873 0.061092 .
## ---
## Signif. codes:  0 ‘***’ 0.001 ‘**’ 0.01 ‘*’ 0.05 ‘.’ 0.1 ‘ ’ 1
##
## Approximate significance of smooth terms:
##              edf Ref.df       F p-value
## s(age_z)   5.427  5.427 326.384  <2e-16 ***
## s(sleep_z) 1.000  1.000   0.397   0.529
## ---
## Signif. codes:  0 ‘***’ 0.001 ‘**’ 0.01 ‘*’ 0.05 ‘.’ 0.1 ‘ ’ 1
##
## R-sq.(adj) =   0.11
## lmer.REML = 3.9835e+05  Scale est. = 779.43    n = 33329
```

The plot below shows the sleep-volume curve for the original model and for the model with main effects of SES.

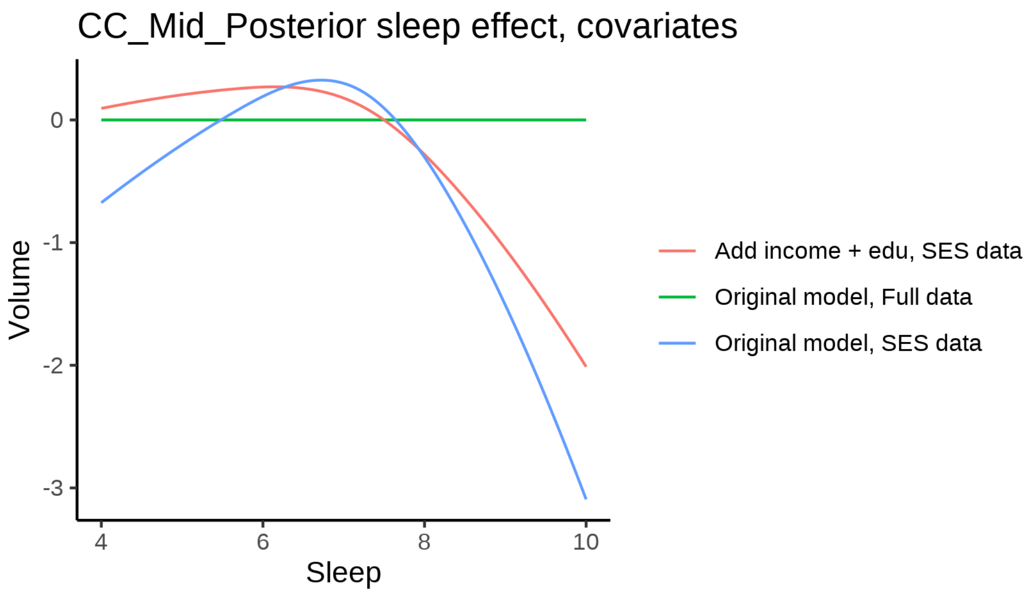

The plot below shows the sleep-volume curve for the original model and for the model with main effects of BMI.

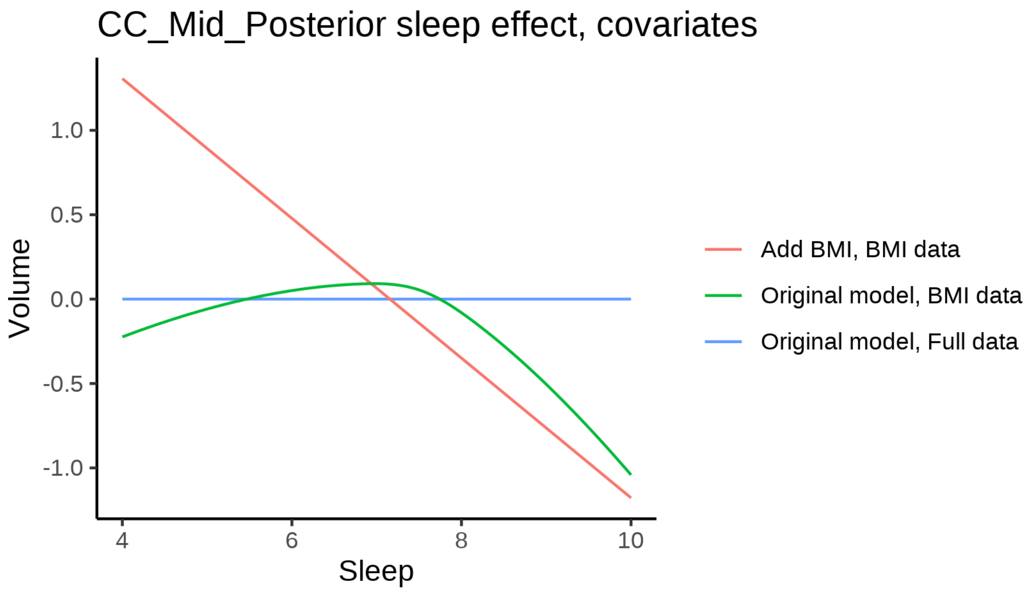

The plot below shows the sleep-volume curve for the original model and for the model with main effects of depression.

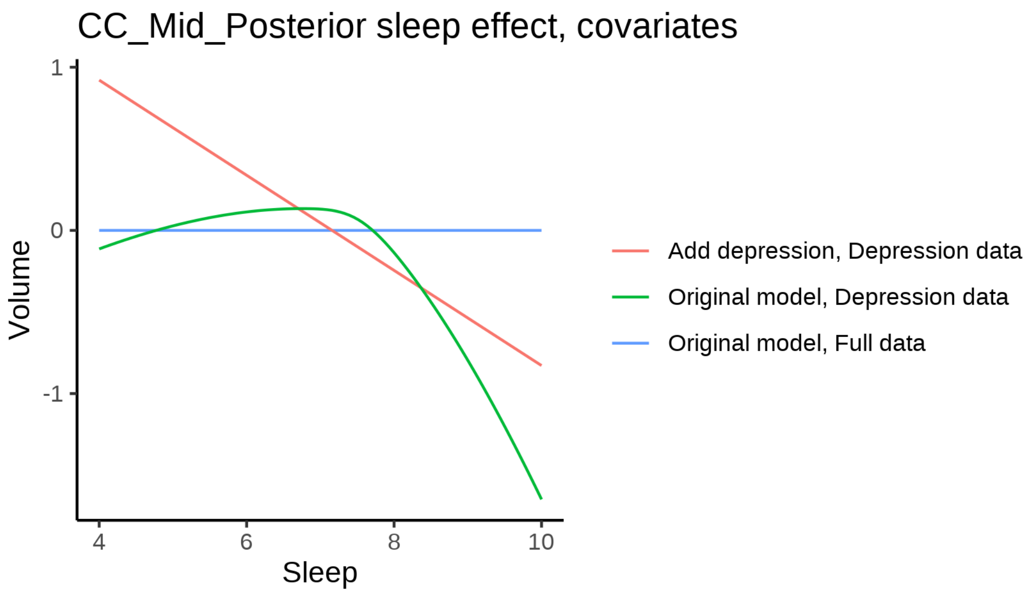

Models Not controling for ICV

Model outputs

Model without sleep term

```
##
## Family: gaussian
## Link function: identity
##
## Formula:
## value ~ sex + site + s(age_z, k = 10, bs = "cr")
## <environment: 0x55896058cc10>
##
## Parametric coefficients:
##                Estimate Std. Error t value Pr(>|t|)
## (Intercept)    510.8419     5.7428  88.953  < 2e-16 ***
## sexmale         12.5381     0.9883  12.687  < 2e-16 ***
## siteMPIB       -28.6913     7.3997  -3.877 0.000106 ***
## siteousAvanto -48.8229     5.9382  -8.222  < 2e-16 ***
## siteousPrisma -14.7692    10.1066  -1.461 0.143928
## siteousSkyra   -74.1954     5.6728 -13.079  < 2e-16 ***
## siteUB         -51.7313    17.6559  -2.930 0.003391 **
## siteUCAM       -27.1617     6.4503  -4.211 2.55e-05 ***
## siteUKB         25.0515     5.8962   4.249 2.15e-05 ***
## siteUmU        -24.4672     8.3543  -2.929 0.003405 **
## siteUOXF       -12.4063     7.0322  -1.764 0.077702 .
## ---
## Signif. codes:  0 '***' 0.001 '**' 0.01 '*' 0.05 '.' 0.1 ' ' 1
##
## Approximate significance of smooth terms:
##             edf Ref.df     F p-value
## s(age_z) 6.054  6.054 551.8  <2e-16 ***
```

```
## ---
## Signif. codes:  0 ‘***’ 0.001 ‘**’ 0.01 ‘*’ 0.05 ‘.’ 0.1 ‘ ’ 1
##
## R-sq.(adj) =  0.0866
## lmer.REML = 6.1483e+05  Scale est. = 751.3     n = 51248
```

Model with only main effects of age and sleep

```
##
## Family: gaussian
## Link function: identity
##
## Formula:
## value ~ sex + site + s(age_z, k = 10, bs = "cr") + s(sleep_z,
##     k = 5, bs = "cr")
## <environment: 0x55896058cc10>
##
## Parametric coefficients:
##               Estimate Std. Error t value Pr(>|t|)
## (Intercept)   511.3248     5.7480  88.957  < 2e-16 ***
## sexmale        12.4363     0.9887  12.578  < 2e-16 ***
## siteMPIB      -28.6308     7.4023  -3.868  0.00011 ***
## siteousAvanto -49.2183     5.9396  -8.286  < 2e-16 ***
## siteousPrisma -15.0449    10.1060  -1.489  0.13657
## siteousSkyra  -74.6195     5.6743 -13.151  < 2e-16 ***
## siteUB        -51.3830    17.6531  -2.911  0.00361 **
## siteUCAM      -27.1731     6.4502  -4.213 2.53e-05 ***
## siteUKB        24.5918     5.9013   4.167 3.09e-05 ***
## siteUmU       -24.4957     8.3718  -2.926  0.00344 **
## siteUOXF      -12.6253     7.0314  -1.796  0.07257 .
## ---
## Signif. codes:  0 ‘***’ 0.001 ‘**’ 0.01 ‘*’ 0.05 ‘.’ 0.1 ‘ ’ 1
##
## Approximate significance of smooth terms:
##              edf Ref.df       F p-value
## s(age_z)   6.040  6.040 546.542 < 2e-16 ***
## s(sleep_z) 2.884  2.884   3.997 0.00502 **
## ---
## Signif. codes:  0 ‘***’ 0.001 ‘**’ 0.01 ‘*’ 0.05 ‘.’ 0.1 ‘ ’ 1
##
## R-sq.(adj) =  0.0869
## lmer.REML = 6.1482e+05  Scale est. = 751.36    n = 51248
```

Model with full interaction between age and sleep

```
##
## Family: gaussian
## Link function: identity
##
## Formula:
## value ~ sex + site + t2(age_z, sleep_z, k = c(10, 4), bs = "cr")
## <environment: 0x55896058cc10>
##
## Parametric coefficients:
##               Estimate Std. Error t value Pr(>|t|)
## (Intercept)   511.7166     5.7615  88.816  < 2e-16 ***
```

```
## sexmale        12.5060     0.9906  12.624  < 2e-16 ***
## siteMPIB      -29.4742     7.4345  -3.965 7.36e-05 ***
## siteousAvanto -49.4583     5.9686  -8.286  < 2e-16 ***
## siteousPrisma -15.3773    10.1270  -1.518  0.12891
## siteousSkyra  -74.8122     5.7066 -13.110  < 2e-16 ***
## siteUB        -52.5624    17.6624  -2.976  0.00292 **
## siteUCAM      -27.8409     6.4798  -4.297 1.74e-05 ***
## siteUKB        24.1650     5.9138   4.086 4.39e-05 ***
## siteUmU       -25.2139     8.3918  -3.005  0.00266 **
## siteUOXF      -13.2783     7.0500  -1.883  0.05965 .
## ---
## Signif. codes:  0 '***' 0.001 '**' 0.01 '*' 0.05 '.' 0.1 ' ' 1
##
## Approximate significance of smooth terms:
##                      edf Ref.df     F p-value
## t2(age_z,sleep_z) 10.18  10.18 16.98  <2e-16 ***
## ---
## Signif. codes:  0 '***' 0.001 '**' 0.01 '*' 0.05 '.' 0.1 ' ' 1
##
## R-sq.(adj) =  0.0867
## lmer.REML = 6.1483e+05  Scale est. = 751.33    n = 51248
```

Model comparison

`mod_no_sleep` refers to model without sleep term, `mod_no_interaction` refers to model with only main effect of sleep, and `mod_full` refers to model with a full interaction between age and sleep. This is a nested model comparison, and the p-value at a given line refers to comparing the model at the line to the model on the line above. Hence, significance implies that the more complicated model is supported on statistical grounds.

To be even more specific, the p-value on the second row tests whether there is an association between sleep and volume. The p-value on the third row tests whether this association depends on age.

```
## Data: NULL
## Models:
## ml$mod_no_sleep$mer: NULL
## ml$mod_no_interaction$mer: NULL
## ml$mod_full$mer: NULL
##                           npar    AIC    BIC  logLik deviance  Chisq Df Pr(>Chisq)
## ml$mod_no_sleep$mer         15 614857 614990 -307414   614827
## ml$mod_no_interaction$mer   17 614853 615004 -307410   614819 8.0653  2    0.01773 *
## ml$mod_full$mer             19 614865 615033 -307413   614827 0.0000  2    1.00000
## ---
## Signif. codes:  0 '***' 0.001 '**' 0.01 '*' 0.05 '.' 0.1 ' ' 1
```

We chose the model based on the likelihood ratio test with 5 % significance level, which was `mod_no_interaction`.

Lifespan brain trajectory

The trajectory shown is from the chosen model `mod_no_interaction_no_icv`.

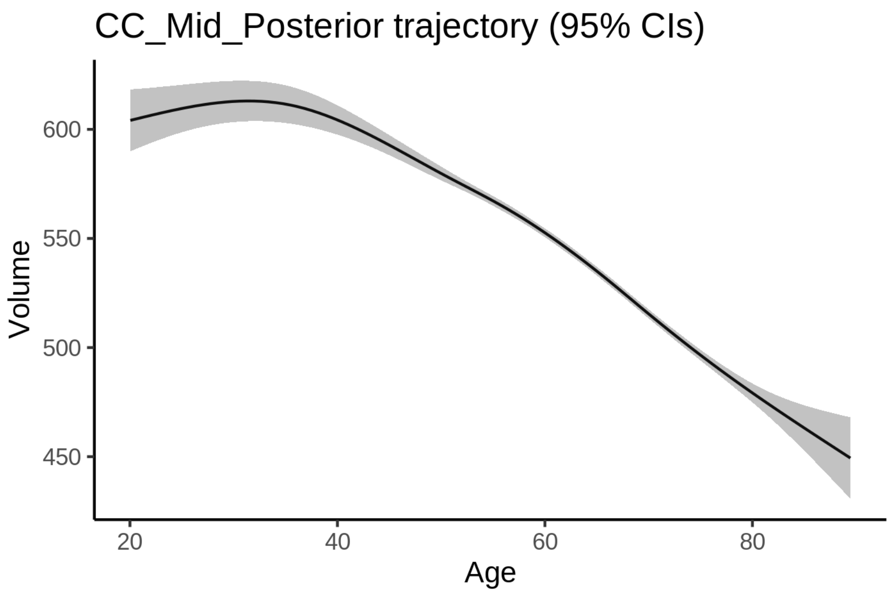

Effect of sleep

The chosen model only included the main effect of sleep, and hence the effect does not vary with age. The black dot shows the average sleep duration across all ages in the sample.

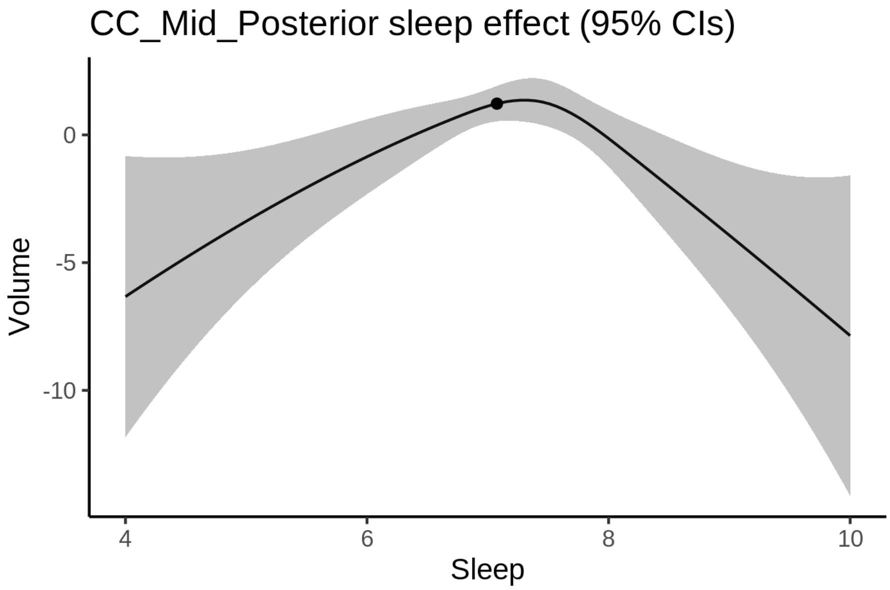

We also show the full interaction model for completeness, although it was not selected.

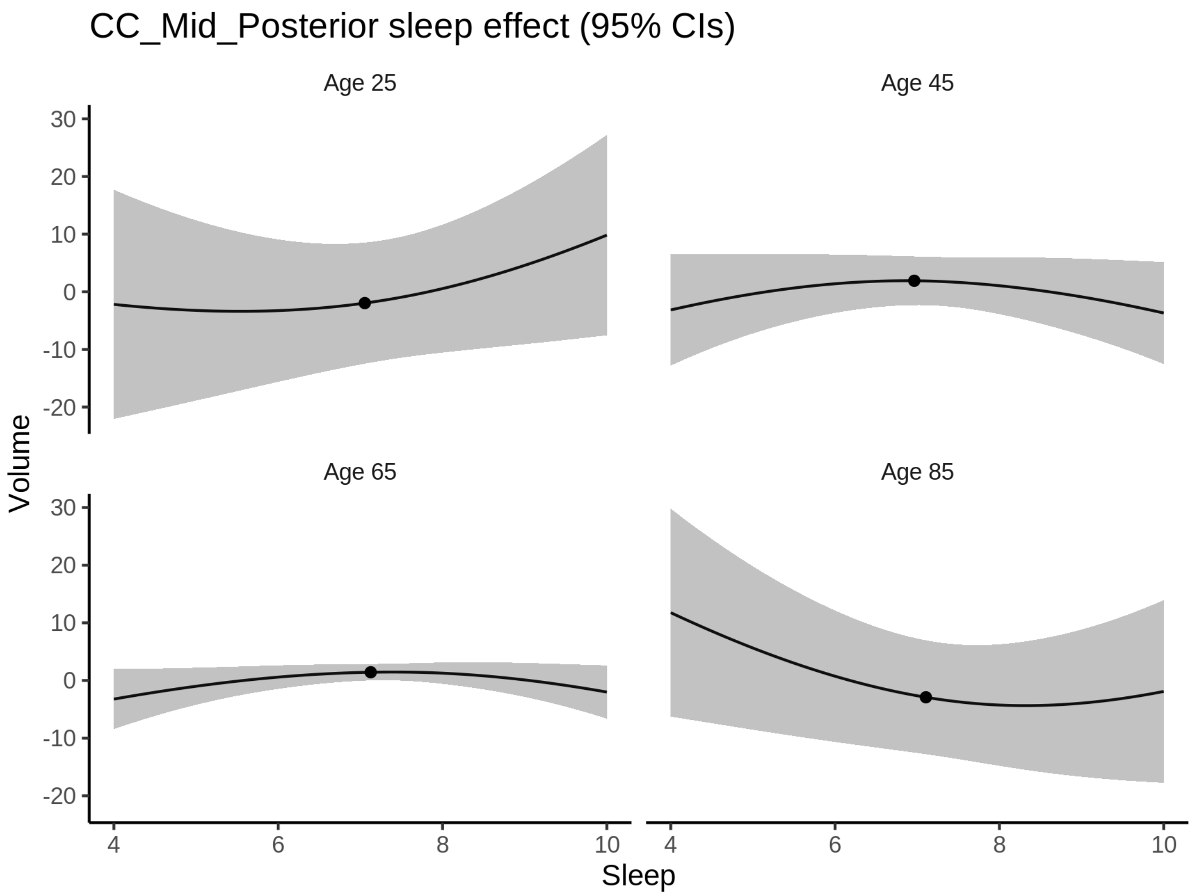

Deviation from sleep associated with maximal volume

Model with only main effect of sleep was chosen, so we show it for all ages at once. Maximum volume is attained at 7.3 hours of sleep. The percentage values in the plot are calculated as follows: The maximum at 100 % refers to a person at an arbitrary age with a sleep duration associated with maximum volume. For a female, this volume is 553 and for a male it is 565. The other percentage values show how large the expected volume is for someone with other sleep durations. For example, 99 % implies a 1 % reduction.

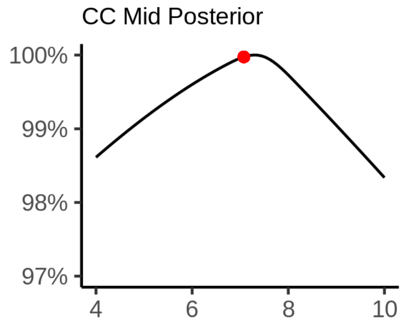

Comparison of mean sleep and sleep associated with maximum volume

A 95 % confidence interval for the sleep associated with maximum volume is [6.73, 7.58].

The plot below compares average sleep to the sleep associated with maximum volume.

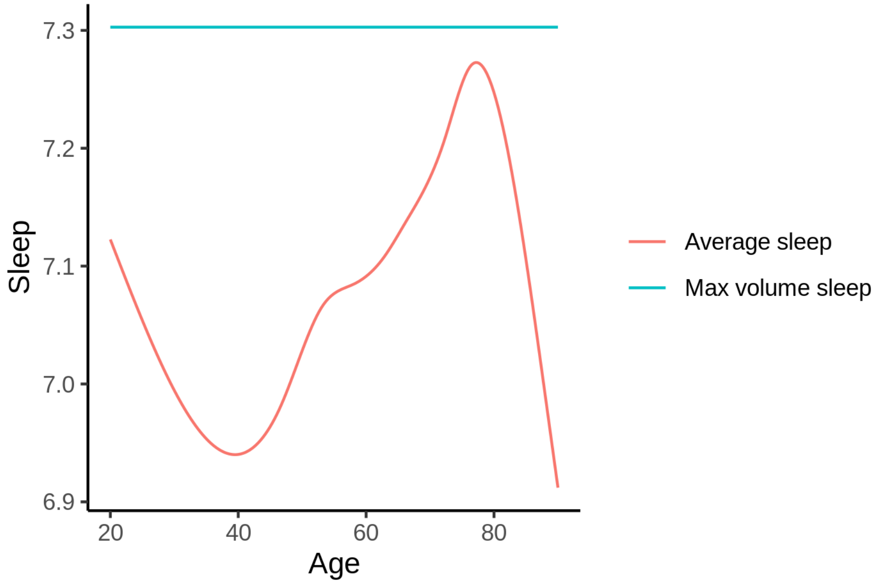

The next plot shows the difference between average sleep and sleep associated with maximum volume. The shaded region is a 95 % confidence interval.

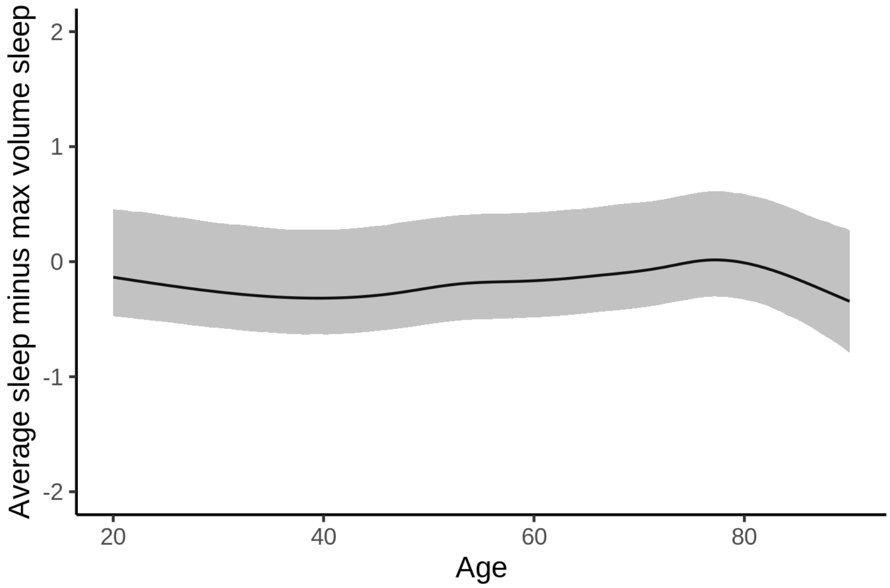

The next plot shows the probability that the sleep duration associated with maximum volume is longer than the average sleep duration, as a function of age. Probability below .05 can be interpreted as evidence that the sleep associated with maximum volume is shorter than the mean sleep, and probability above .95 can be interpreted the opposite way.

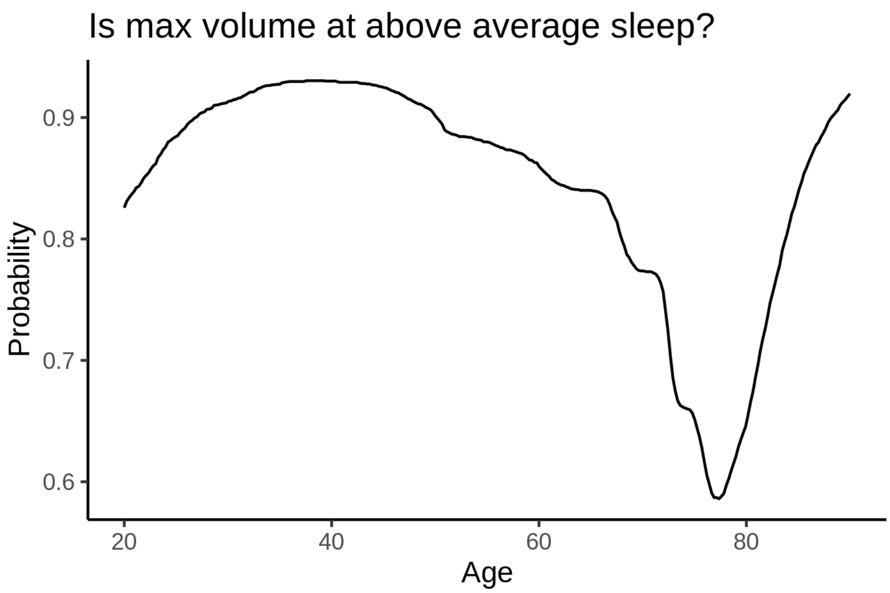

CC_Posterior

Descriptive statistics

Study	Observations	Unique IDs	Mean age	Age range
HCP	974	974	28.8	22 - 37
MPIB	675	390	63.1	24 - 83
UB	113	39	70.9	64 - 81
UCAM	878	630	54.9	20 - 88
UiO	1475	803	49.4	20 - 89
UKB	45941	43118	64.5	45 - 83
UmU	410	278	62.1	25 - 85
UOXF	769	769	69.8	60 - 85

Spaghetti plot

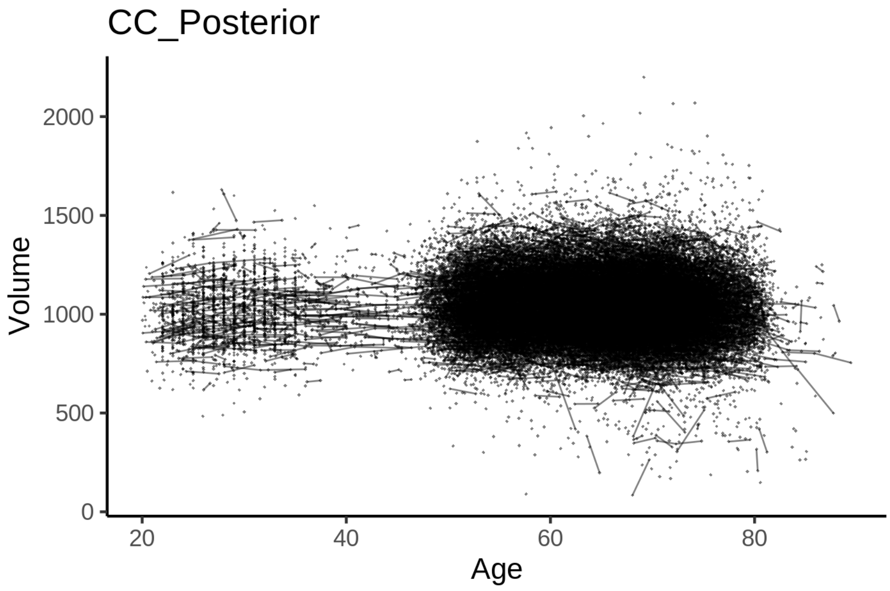

Models Controling for ICV

Model outputs

Model without sleep term

```
##
## Family: gaussian
## Link function: identity
##
## Formula:
## value ~ sex + site + icv + s(age_z, k = 10, bs = "cr")
## <environment: 0x558b286898f0>
##
## Parametric coefficients:
##                 Estimate Std. Error t value Pr(>|t|)
## (Intercept)   1048.3383     8.0603 130.062  < 2e-16 ***
## sexmale        -36.5920     1.6835 -21.736  < 2e-16 ***
## siteMPIB        30.6232    10.4580   2.928  0.00341 **
## siteousAvanto  -60.0045     8.2038  -7.314 2.63e-13 ***
## siteousPrisma  -15.6270    13.7892  -1.133  0.25710
## siteousSkyra   -45.6420     7.9401  -5.748 9.07e-09 ***
## siteUB         -74.7520    24.8480  -3.008  0.00263 **
## siteUCAM       -46.8578     9.0373  -5.185 2.17e-07 ***
## siteUKB         14.3572     8.2599   1.738  0.08218 .
## siteUmU         -3.5300    11.7219  -0.301  0.76330
## siteUOXF       -24.9164     9.8504  -2.529  0.01143 *
## icv             65.5903     0.8447  77.648  < 2e-16 ***
## ---
## Signif. codes:  0 ‘***’ 0.001 ‘**’ 0.01 ‘*’ 0.05 ‘.’ 0.1 ‘ ’ 1
##
```

```
## Approximate significance of smooth terms:
##             edf Ref.df     F p-value
## s(age_z) 7.801  7.801 15.83  <2e-16 ***
## ---
## Signif. codes:  0 '***' 0.001 '**' 0.01 '*' 0.05 '.' 0.1 ' ' 1
##
## R-sq.(adj) =  0.136
## lmer.REML = 6.4791e+05  Scale est. = 1086.4    n = 51235
```

Model with only main effects of age and sleep

```
##
## Family: gaussian
## Link function: identity
##
## Formula:
## value ~ sex + site + icv + s(age_z, k = 10, bs = "cr") + s(sleep_z,
##     k = 5, bs = "cr")
## <environment: 0x558b286898f0>
##
## Parametric coefficients:
##                 Estimate Std. Error t value Pr(>|t|)
## (Intercept)   1048.9325     8.0678 130.014  < 2e-16 ***
## sexmale        -36.5947     1.6834 -21.738  < 2e-16 ***
## siteMPIB        30.5104    10.4635   2.916  0.00355 **
## siteousAvanto  -60.4692     8.2059  -7.369 1.74e-13 ***
## siteousPrisma  -15.9644    13.7887  -1.158  0.24695
## siteousSkyra   -46.1880     7.9426  -5.815 6.09e-09 ***
## siteUB         -74.3108    24.8449  -2.991  0.00278 **
## siteUCAM       -46.8673     9.0378  -5.186 2.16e-07 ***
## siteUKB         13.7316     8.2683   1.661  0.09677 .
## siteUmU         -3.6474    11.7474  -0.310  0.75619
## siteUOXF       -25.2247     9.8507  -2.561  0.01045 *
## icv             65.4813     0.8455  77.444  < 2e-16 ***
## ---
## Signif. codes:  0 '***' 0.001 '**' 0.01 '*' 0.05 '.' 0.1 ' ' 1
##
## Approximate significance of smooth terms:
##               edf Ref.df      F p-value
## s(age_z)   7.795  7.795 15.522  <2e-16 ***
## s(sleep_z) 2.963  2.963  3.414   0.012 *
## ---
## Signif. codes:  0 '***' 0.001 '**' 0.01 '*' 0.05 '.' 0.1 ' ' 1
##
## R-sq.(adj) =  0.137
## lmer.REML = 6.4791e+05  Scale est. = 1086.5    n = 51235
```

Model with full interaction between age and sleep

```
##
## Family: gaussian
## Link function: identity
##
## Formula:
## value ~ sex + site + icv + t2(age_z, sleep_z, k = c(10, 4), bs = "cr")
## <environment: 0x558b286898f0>
```

```
##
## Parametric coefficients:
##                Estimate Std. Error t value Pr(>|t|)
## (Intercept)   1048.9628     8.0118 130.927  < 2e-16 ***
## sexmale        -36.5993     1.6870 -21.694  < 2e-16 ***
## siteMPIB        30.3028    10.4702   2.894  0.00380 **
## siteousAvanto  -60.5644     8.2250  -7.363 1.82e-13 ***
## siteousPrisma  -16.3177    13.8157  -1.181  0.23757
## siteousSkyra   -46.2002     7.9641  -5.801 6.63e-09 ***
## siteUB         -74.9726    24.8321  -3.019  0.00254 **
## siteUCAM       -47.2130     9.0399  -5.223 1.77e-07 ***
## siteUKB         13.7117     8.2060   1.671  0.09474 .
## siteUmU         -3.9105    11.7289  -0.333  0.73883
## siteUOXF       -25.4776     9.8144  -2.596  0.00944 **
## icv             65.5199     0.8452  77.519  < 2e-16 ***
## ---
## Signif. codes:  0 '***' 0.001 '**' 0.01 '*' 0.05 '.' 0.1 ' ' 1
##
## Approximate significance of smooth terms:
##                     edf Ref.df    F p-value
## t2(age_z,sleep_z) 12.85  12.85 9.24  <2e-16 ***
## ---
## Signif. codes:  0 '***' 0.001 '**' 0.01 '*' 0.05 '.' 0.1 ' ' 1
##
## R-sq.(adj) =  0.137
## lmer.REML = 6.4791e+05  Scale est. = 1086      n = 51235
```

Model comparison

`mod_no_sleep` refers to model without sleep term, `mod_no_interaction` refers to model with only main effect of sleep, and `mod_full` refers to model with a full interaction between age and sleep. This is a nested model comparison, and the p-value at a given line refers to comparing the model at the line to the model on the line above. Hence, significance implies that the more complicated model is supported on statistical grounds.

To be even more specific, the p-value on the second row tests whether there is an association between sleep and volume. The p-value on the third row tests whether this association depends on age.

```
## Data: NULL
## Models:
## ml$mod_no_sleep$mer: NULL
## ml$mod_no_interaction$mer: NULL
## ml$mod_full$mer: NULL
##                           npar    AIC    BIC  logLik deviance  Chisq Df Pr(>Chisq)
## ml$mod_no_sleep$mer         16 647946 648087 -323957   647914
## ml$mod_no_interaction$mer   18 647944 648103 -323954   647908 6.1148  2    0.04701 *
## ml$mod_full$mer             20 647953 648129 -323956   647913 0.0000  2    1.00000
## ---
## Signif. codes:  0 '***' 0.001 '**' 0.01 '*' 0.05 '.' 0.1 ' ' 1
```

We chose the model based on the likelihood ratio test with 5 % significance level, which was `mod_no_interaction`.

Lifespan brain trajectory

The trajectory shown is from the chosen model `mod_no_interaction`.

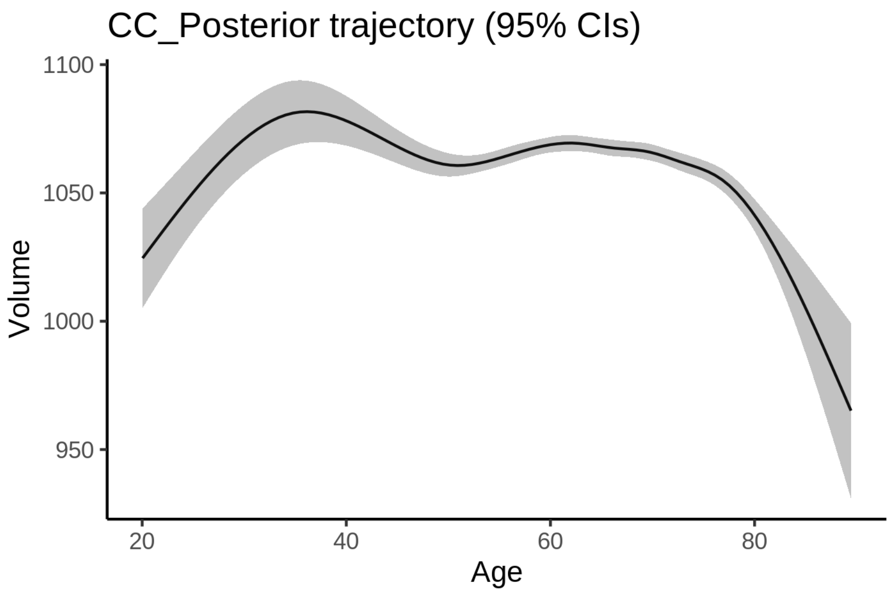

Effect of sleep

The chosen model only included the main effect of sleep, and hence the effect does not vary with age. The black dot shows the average sleep duration across all ages in the sample.

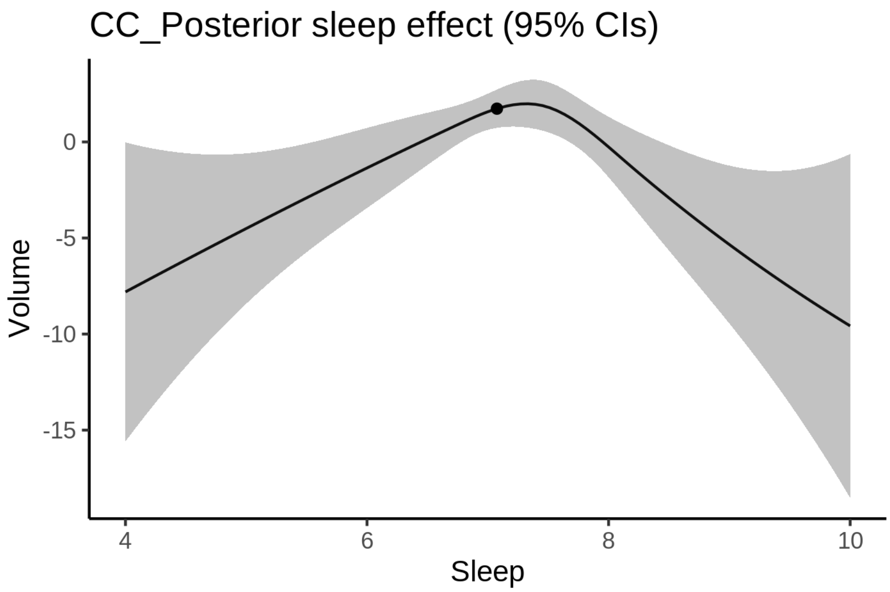

We also show the full interaction model for completeness, although it was not selected.

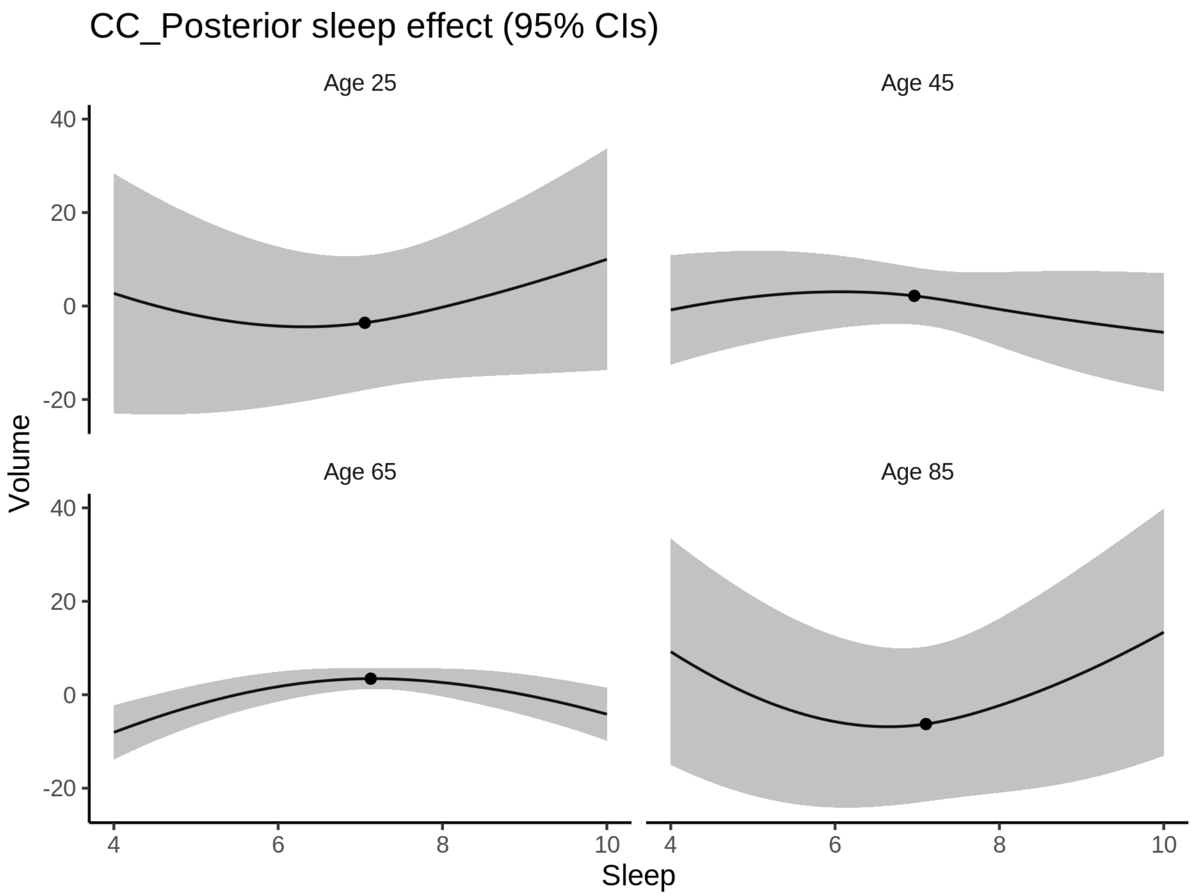

Deviation from sleep associated with maximal volume

Model with only main effect of sleep was chosen, so we show it for all ages at once. Maximum volume is attained at 7.3 hours of sleep. The percentage values in the plot are calculated as follows: The maximum at 100 % refers to a person at an arbitrary age with a sleep duration associated with maximum volume. For a female, this volume is 1055 and for a male it is 1018. The other percentage values show how large the expected volume is for someone with other sleep durations. For example, 99 % implies a 1 % reduction.

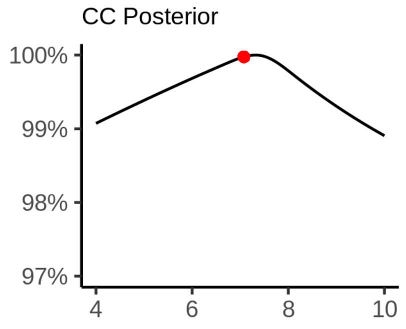

Comparison of mean sleep and sleep associated with maximum volume

A 95 % confidence interval for the sleep associated with maximum volume is [6.79, 7.58].

The plot below compares average sleep to the sleep associated with maximum volume.

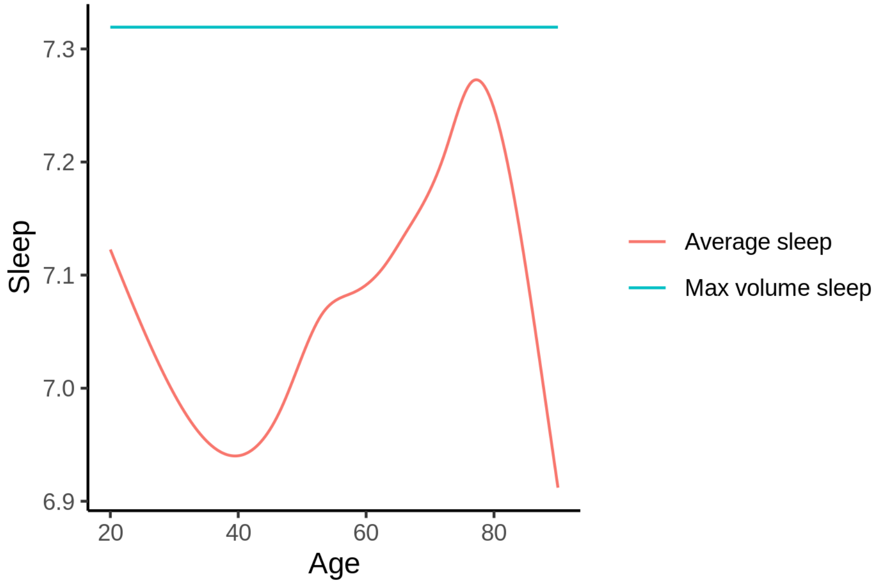

The next plot shows the difference between average sleep and sleep associated with maximum volume. The shaded region is a 95 % confidence interval.

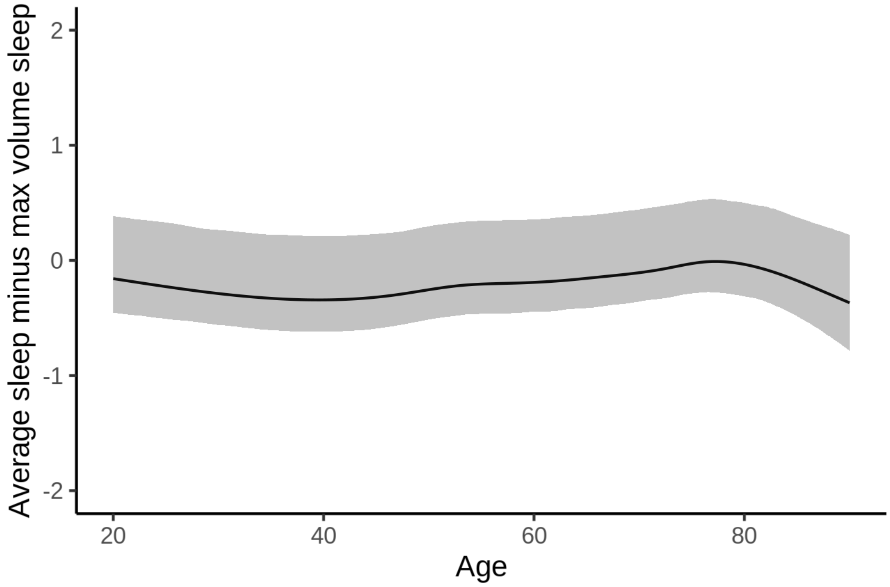

The next plot shows the probability that the sleep duration associated with maximum volume is longer than the average sleep duration, as a function of age. Probability below .05 can be interpreted as evidence that the sleep associated with maximum volume is shorter than the mean sleep, and probability above .95 can be interpreted the opposite way.

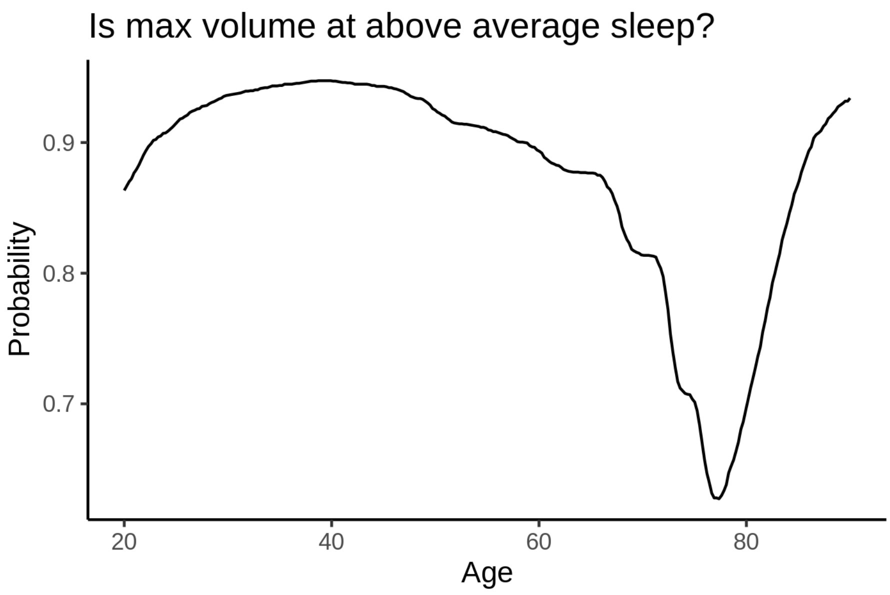

Controling for covariates

Below is the output for a model in which we only include data with income and education.

```
##
## Family: gaussian
## Link function: identity
##
## Formula:
## value ~ sex + site + s(age_z, k = 10, bs = "cr") + s(sleep_z,
##     k = 5, bs = "cr") + icv
## <environment: 0x558a479b9b88>
##
## Parametric coefficients:
##                Estimate Std. Error t value Pr(>|t|)
## (Intercept)    1114.752     19.322  57.693  < 2e-16 ***
## sexmale         -40.777      2.130 -19.148  < 2e-16 ***
## siteousAvanto -122.154     22.688  -5.384 7.33e-08 ***
## siteousPrisma  -57.543     23.653  -2.433  0.01499 *
## siteousSkyra  -102.897     20.993  -4.901 9.56e-07 ***
## siteUKB         -49.960     19.279  -2.591  0.00956 **
## siteUOXF        -71.296     21.715  -3.283  0.00103 **
## icv              69.072      1.092  63.253  < 2e-16 ***
## ---
## Signif. codes:  0 ‘***’ 0.001 ‘**’ 0.01 ‘*’ 0.05 ‘.’ 0.1 ‘ ’ 1
##
## Approximate significance of smooth terms:
##              edf Ref.df     F  p-value
## s(age_z)   4.507  4.507 4.923 0.000424 ***
## s(sleep_z) 2.472  2.472 2.181 0.210884
## ---
## Signif. codes:  0 ‘***’ 0.001 ‘**’ 0.01 ‘*’ 0.05 ‘.’ 0.1 ‘ ’ 1
##
```

```
## R-sq.(adj) =  0.137
## lmer.REML = 3.9407e+05  Scale est. = 1199.7    n = 31165
```

Below is the output for a model in which we control for the main effects of income and education.

```
##
## Family: gaussian
## Link function: identity
##
## Formula:
## value ~ sex + site + s(age_z, k = 10, bs = "cr") + s(sleep_z,
##     k = 5, bs = "cr") + icv + income_scaled + education_scaled
## <environment: 0x558a479b9b88>
##
## Parametric coefficients:
##                  Estimate Std. Error t value Pr(>|t|)
## (Intercept)      1109.680     19.443  57.072  < 2e-16 ***
## sexmale           -40.670      2.135 -19.045  < 2e-16 ***
## siteousAvanto    -122.396     22.694  -5.393 6.97e-08 ***
## siteousPrisma     -58.053     23.653  -2.454  0.01412 *
## siteousSkyra     -103.217     20.998  -4.916 8.90e-07 ***
## siteUKB           -50.363     19.289  -2.611  0.00903 **
## siteUOXF          -69.901     21.726  -3.217  0.00129 **
## icv                68.758      1.101  62.444  < 2e-16 ***
## income_scaled       2.012      2.536   0.793  0.42756
## education_scaled    5.734      3.001   1.910  0.05611 .
## ---
## Signif. codes:  0 ‘***’ 0.001 ‘**’ 0.01 ‘*’ 0.05 ‘.’ 0.1 ‘ ’ 1
##
## Approximate significance of smooth terms:
##              edf Ref.df     F  p-value
## s(age_z)   4.531  4.531 4.798 0.000527 ***
## s(sleep_z) 2.338  2.338 1.803 0.272692
## ---
## Signif. codes:  0 ‘***’ 0.001 ‘**’ 0.01 ‘*’ 0.05 ‘.’ 0.1 ‘ ’ 1
##
## R-sq.(adj) =  0.137
## lmer.REML = 3.9406e+05  Scale est. = 1200.2    n = 31165
```

We also included interaction effects between sleep duration and education and income, in another model. The output is shown below, and the interaction terms are `income_scaled:sleep_z` and `education_scaled:sleep_z`.

```
##
## Family: gaussian
## Link function: identity
##
## Formula:
## value ~ sex + site + s(age_z, k = 10, bs = "cr") + s(sleep_z,
##     k = 5, bs = "cr") + icv + income_scaled + education_scaled +
##     income_scaled:sleep_z + education_scaled:sleep_z
## <environment: 0x558a479b9b88>
##
## Parametric coefficients:
##                          Estimate Std. Error t value Pr(>|t|)
## (Intercept)              1109.517     19.445  57.061  < 2e-16 ***
```

```
## sexmale                    -40.602      2.137 -19.000  < 2e-16 ***
## siteousAvanto             -122.209     22.695  -5.385 7.30e-08 ***
## siteousPrisma              -57.703     23.656  -2.439  0.01472 *
## siteousSkyra              -103.036     20.999  -4.907 9.31e-07 ***
## siteUKB                    -50.177     19.291  -2.601  0.00930 **
## siteUOXF                   -69.637     21.732  -3.204  0.00135 **
## icv                         68.744      1.101  62.424  < 2e-16 ***
## income_scaled                1.951      2.537   0.769  0.44200
## education_scaled             5.789      3.002   1.928  0.05383 .
## income_scaled:sleep_z        1.514      2.524   0.600  0.54853
## education_scaled:sleep_z    -2.824      2.912  -0.970  0.33212
## ---
## Signif. codes:  0 '***' 0.001 '**' 0.01 '*' 0.05 '.' 0.1 ' ' 1
##
## Approximate significance of smooth terms:
##              edf Ref.df     F  p-value
## s(age_z)   4.525  4.525 4.805 0.000522 ***
## s(sleep_z) 2.291  2.291 1.540 0.336917
## ---
## Signif. codes:  0 '***' 0.001 '**' 0.01 '*' 0.05 '.' 0.1 ' ' 1
##
## R-sq.(adj) =  0.137
## lmer.REML = 3.9405e+05  Scale est. = 1200.2    n = 31165
```

We did the same controling for BMI. Below is the model with no covariates but only keeping data with BMI.

```
##
## Family: gaussian
## Link function: identity
##
## Formula:
## value ~ sex + site + s(age_z, k = 10, bs = "cr") + s(sleep_z,
##     k = 5, bs = "cr") + icv
## <environment: 0x558a47515c08>
##
## Parametric coefficients:
##              Estimate Std. Error t value Pr(>|t|)
## (Intercept)   1002.627     11.906  84.212  < 2e-16 ***
## sexmale        -40.157      2.071 -19.387  < 2e-16 ***
## siteousPrisma   39.256     15.946   2.462 0.013831 *
## siteousSkyra    12.311      8.494   1.449 0.147250
## siteUCAM         8.305     12.733   0.652 0.514224
## siteUKB         61.749     12.024   5.136 2.83e-07 ***
## siteUmU         50.288     14.757   3.408 0.000656 ***
## icv             68.994      1.066  64.752  < 2e-16 ***
## ---
## Signif. codes:  0 '***' 0.001 '**' 0.01 '*' 0.05 '.' 0.1 ' ' 1
##
## Approximate significance of smooth terms:
##              edf Ref.df     F  p-value
## s(age_z)   5.757  5.757 7.042 1.76e-06 ***
## s(sleep_z) 2.479  2.479 0.964    0.245
## ---
## Signif. codes:  0 '***' 0.001 '**' 0.01 '*' 0.05 '.' 0.1 ' ' 1
##
```

```
## R-sq.(adj) =  0.138
## lmer.REML = 4.2184e+05  Scale est. = 1119.3    n = 33403
```

Below is the model output with main effect.

```
##
## Family: gaussian
## Link function: identity
##
## Formula:
## value ~ sex + site + s(age_z, k = 10, bs = "cr") + s(sleep_z,
##     k = 5, bs = "cr") + icv + bmi
## <environment: 0x558a47515c08>
##
## Parametric coefficients:
##                Estimate Std. Error t value Pr(>|t|)
## (Intercept)   1044.4243    12.9385  80.722  < 2e-16 ***
## sexmale        -38.8309     2.0755 -18.709  < 2e-16 ***
## siteousPrisma   40.5813    15.9372   2.546 0.010891 *
## siteousSkyra    12.4462     8.4928   1.466 0.142792
## siteUCAM         9.1993    12.7237   0.723 0.469682
## siteUKB         63.1285    12.0158   5.254  1.5e-07 ***
## siteUmU         51.9688    14.7458   3.524 0.000425 ***
## icv             69.2610     1.0646  65.057  < 2e-16 ***
## bmi             -1.6607     0.2018  -8.229  < 2e-16 ***
## ---
## Signif. codes:  0 ‘***’ 0.001 ‘**’ 0.01 ‘*’ 0.05 ‘.’ 0.1 ‘ ’ 1
##
## Approximate significance of smooth terms:
##              edf Ref.df     F  p-value
## s(age_z)   5.737  5.737 7.605 1.29e-06 ***
## s(sleep_z) 1.915  1.915 0.908    0.462
## ---
## Signif. codes:  0 ‘***’ 0.001 ‘**’ 0.01 ‘*’ 0.05 ‘.’ 0.1 ‘ ’ 1
##
## R-sq.(adj) =   0.14
## lmer.REML = 4.2178e+05  Scale est. = 1119.1    n = 33403
```

Next is the model with BMI-sleep interaction.

```
##
## Family: gaussian
## Link function: identity
##
## Formula:
## value ~ sex + site + s(age_z, k = 10, bs = "cr") + s(sleep_z,
##     k = 5, bs = "cr") + icv + bmi + bmi:sleep_z
## <environment: 0x558a47515c08>
##
## Parametric coefficients:
##                Estimate Std. Error t value Pr(>|t|)
## (Intercept)   1044.6736    12.9457  80.696  < 2e-16 ***
## sexmale        -38.8353     2.0755 -18.711  < 2e-16 ***
## siteousPrisma   40.5812    15.9373   2.546 0.010892 *
## siteousSkyra    12.4293     8.4929   1.463 0.143340
## siteUCAM         9.1094    12.7248   0.716 0.474073
```

```
## siteUKB         63.0475    12.0171   5.246 1.56e-07 ***
## siteUmU         51.8878    14.7468   3.519 0.000434 ***
## icv             69.2568     1.0647  65.051  < 2e-16 ***
## bmi             -1.6650     0.2019  -8.246  < 2e-16 ***
## bmi:sleep_z     -0.1132     0.1912  -0.592 0.553960
## ---
## Signif. codes:  0 '***' 0.001 '**' 0.01 '*' 0.05 '.' 0.1 ' ' 1
##
## Approximate significance of smooth terms:
##               edf Ref.df     F  p-value
## s(age_z)   5.741  5.741 7.620 1.28e-06 ***
## s(sleep_z) 1.895  1.895 0.571    0.498
## ---
## Signif. codes:  0 '***' 0.001 '**' 0.01 '*' 0.05 '.' 0.1 ' ' 1
##
## R-sq.(adj) =   0.14
## lmer.REML = 4.2178e+05  Scale est. = 1119.1    n = 33403
```

We did the same controling for depression. Below is the model with no covariates but only keeping data with depression.

```
##
## Family: gaussian
## Link function: identity
##
## Formula:
## value ~ sex + site + s(age_z, k = 10, bs = "cr") + s(sleep_z,
##     k = 5, bs = "cr") + icv
## <environment: 0x5589612f93b0>
##
## Parametric coefficients:
##                Estimate Std. Error t value Pr(>|t|)
## (Intercept)   1058.208      9.954 106.307  < 2e-16 ***
## sexmale        -39.585      2.081 -19.018  < 2e-16 ***
## siteousAvanto  -59.567     19.233  -3.097  0.00196 **
## siteousPrisma   -7.392     18.560  -0.398  0.69044
## siteousSkyra   -35.425     14.706  -2.409  0.01601 *
## siteUCAM       -46.055     13.587  -3.390  0.00070 ***
## siteUKB          5.983      9.845   0.608  0.54342
## siteUmU         -7.158     13.386  -0.535  0.59283
## icv             68.573      1.069  64.143  < 2e-16 ***
## ---
## Signif. codes:  0 '***' 0.001 '**' 0.01 '*' 0.05 '.' 0.1 ' ' 1
##
## Approximate significance of smooth terms:
##               edf Ref.df     F  p-value
## s(age_z)   5.606  5.606 6.027 4.32e-05 ***
## s(sleep_z) 2.590  2.590 2.564    0.178
## ---
## Signif. codes:  0 '***' 0.001 '**' 0.01 '*' 0.05 '.' 0.1 ' ' 1
##
## R-sq.(adj) =  0.138
## lmer.REML = 4.2042e+05  Scale est. = 1085      n = 33313
```

Below is the model output with main effect.

```
##
## Family: gaussian
## Link function: identity
##
## Formula:
## value ~ sex + site + s(age_z, k = 10, bs = "cr") + s(sleep_z,
##     k = 5, bs = "cr") + icv + depression
## <environment: 0x5589612f93b0>
##
## Parametric coefficients:
##                Estimate Std. Error t value Pr(>|t|)
## (Intercept)    1058.897      9.997 105.923  < 2e-16 ***
## sexmale         -39.657      2.084 -19.033  < 2e-16 ***
## siteousAvanto   -59.194     19.237  -3.077 0.002092 **
## siteousPrisma    -7.056     18.564  -0.380 0.703870
## siteousSkyra    -35.010     14.714  -2.379 0.017347 *
## siteUCAM        -45.886     13.588  -3.377 0.000734 ***
## siteUKB           5.701      9.853   0.579 0.562834
## siteUmU          -5.055     13.667  -0.370 0.711477
## icv              68.584      1.069  64.150  < 2e-16 ***
## depression       -5.659      7.446  -0.760 0.447267
## ---
## Signif. codes:  0 ‘***’ 0.001 ‘**’ 0.01 ‘*’ 0.05 ‘.’ 0.1 ‘ ’ 1
##
## Approximate significance of smooth terms:
##               edf Ref.df     F  p-value
## s(age_z)    5.595  5.595 5.997 4.64e-05 ***
## s(sleep_z) 2.522  2.522 2.323    0.212
## ---
## Signif. codes:  0 ‘***’ 0.001 ‘**’ 0.01 ‘*’ 0.05 ‘.’ 0.1 ‘ ’ 1
##
## R-sq.(adj) =  0.138
## lmer.REML = 4.2041e+05  Scale est. = 1084.9    n = 33313
```

Next is the model with depression-sleep interaction.

```
##
## Family: gaussian
## Link function: identity
##
## Formula:
## value ~ sex + site + s(age_z, k = 10, bs = "cr") + s(sleep_z,
##     k = 5, bs = "cr") + icv + depression + depression:sleep_z
## <environment: 0x5589612f93b0>
##
## Parametric coefficients:
##                     Estimate Std. Error t value Pr(>|t|)
## (Intercept)         1058.892      9.997 105.922  < 2e-16 ***
## sexmale              -39.696      2.084 -19.049  < 2e-16 ***
## siteousAvanto        -59.419     19.237  -3.089 0.002012 **
## siteousPrisma         -7.285     18.564  -0.392 0.694748
## siteousSkyra         -35.225     14.714  -2.394 0.016674 *
## siteUCAM             -46.139     13.590  -3.395 0.000687 ***
## siteUKB                5.725      9.853   0.581 0.561220
## siteUmU               -3.261     13.784  -0.237 0.812990
```

```
## icv                  68.589      1.069  64.154  < 2e-16 ***
## depression           -6.168      7.458  -0.827 0.408219
## depression:sleep_z   -5.388      5.480  -0.983 0.325536
## ---
## Signif. codes:  0 '***' 0.001 '**' 0.01 '*' 0.05 '.' 0.1 ' ' 1
##
## Approximate significance of smooth terms:
##               edf Ref.df     F  p-value
## s(age_z)    5.577  5.577 5.975 4.86e-05 ***
## s(sleep_z) 2.442  2.442 2.105    0.235
## ---
## Signif. codes:  0 '***' 0.001 '**' 0.01 '*' 0.05 '.' 0.1 ' ' 1
##
## R-sq.(adj) =  0.138
## lmer.REML = 4.2041e+05  Scale est. = 1084.9    n = 33313
```

The plot below shows the sleep-volume curve for the original model and for the model with main effects of SES.

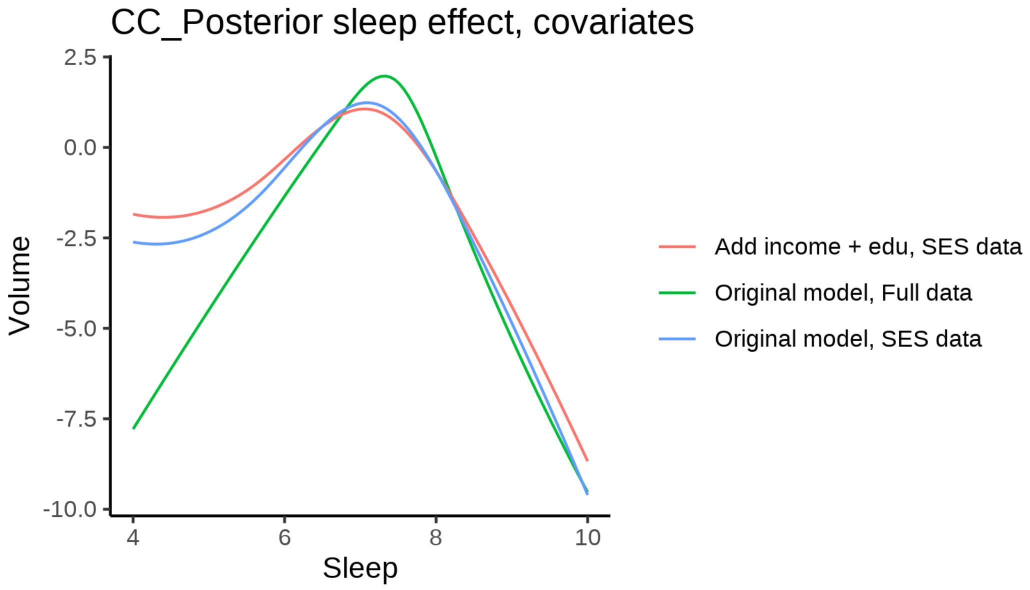

The plot below shows the sleep-volume curve for the original model and for the model with main effects of BMI.

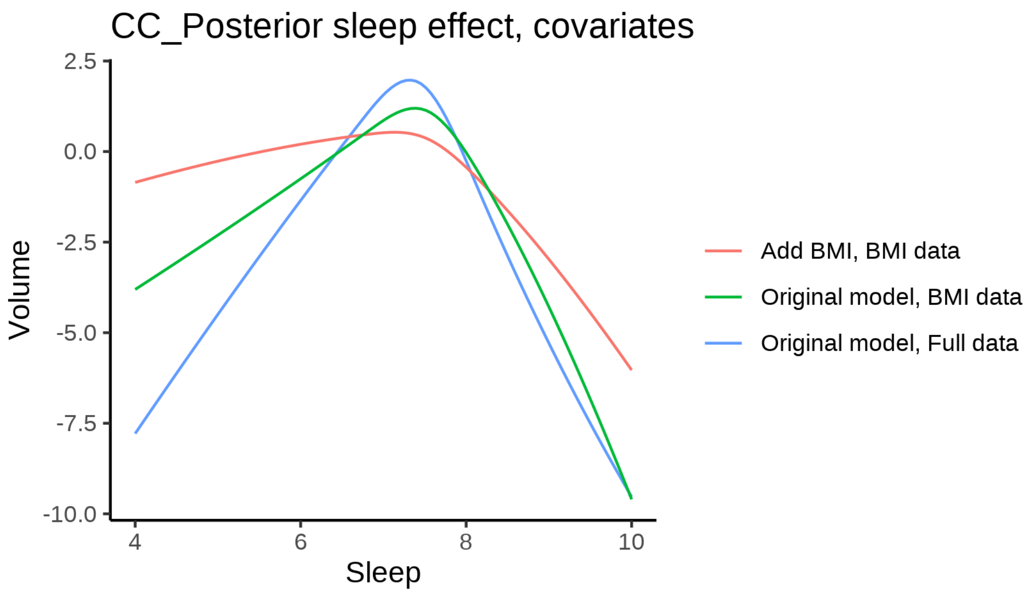

The plot below shows the sleep-volume curve for the original model and for the model with main effects of depression.

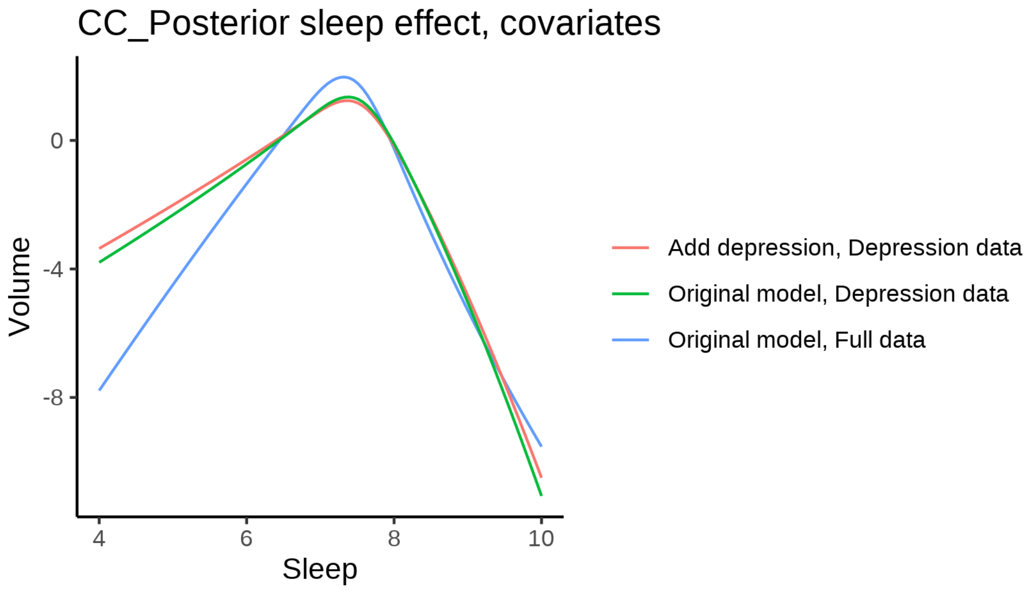

Models Not controling for ICV

Model outputs

Model without sleep term

```
##
## Family: gaussian
## Link function: identity
##
## Formula:
```

```
## value ~ sex + site + s(age_z, k = 10, bs = "cr")
## <environment: 0x558a4a807388>
##
## Parametric coefficients:
##                Estimate Std. Error t value Pr(>|t|)
## (Intercept)   1011.603      8.423 120.093  < 2e-16 ***
## sexmale         37.846      1.469  25.771  < 2e-16 ***
## siteMPIB       -74.095     10.956  -6.763 1.37e-11 ***
## siteousAvanto  -32.067      8.627  -3.717 0.000202 ***
## siteousPrisma    2.810     14.487   0.194 0.846216
## siteousSkyra   -41.430      8.384  -4.941 7.78e-07 ***
## siteUB         -93.371     26.359  -3.542 0.000397 ***
## siteUCAM       -43.281      9.533  -4.540 5.64e-06 ***
## siteUKB         17.051      8.645   1.972 0.048564 *
## siteUmU        -29.790     12.373  -2.408 0.016062 *
## siteUOXF       -39.489     10.353  -3.814 0.000137 ***
## ---
## Signif. codes:  0 '***' 0.001 '**' 0.01 '*' 0.05 '.' 0.1 ' ' 1
##
## Approximate significance of smooth terms:
##            edf Ref.df     F p-value
## s(age_z) 7.016  7.016 22.77  <2e-16 ***
## ---
## Signif. codes:  0 '***' 0.001 '**' 0.01 '*' 0.05 '.' 0.1 ' ' 1
##
## R-sq.(adj) =  0.0279
## lmer.REML = 6.536e+05  Scale est. = 1114.8    n = 51235
```

Model with only main effects of age and sleep

```
##
## Family: gaussian
## Link function: identity
##
## Formula:
## value ~ sex + site + s(age_z, k = 10, bs = "cr") + s(sleep_z,
##     k = 5, bs = "cr")
## <environment: 0x558a4a807388>
##
## Parametric coefficients:
##                Estimate Std. Error t value Pr(>|t|)
## (Intercept)   1013.732      8.427 120.291  < 2e-16 ***
## sexmale         37.556      1.469  25.570  < 2e-16 ***
## siteMPIB       -74.721     10.956  -6.820 9.19e-12 ***
## siteousAvanto  -33.520      8.626  -3.886 0.000102 ***
## siteousPrisma    1.735     14.482   0.120 0.904628
## siteousSkyra   -42.963      8.383  -5.125 2.99e-07 ***
## siteUB         -92.596     26.345  -3.515 0.000441 ***
## siteUCAM       -43.664      9.529  -4.582 4.62e-06 ***
## siteUKB         14.950      8.648   1.729 0.083878 .
## siteUmU        -31.605     12.394  -2.550 0.010776 *
## siteUOXF       -40.418     10.347  -3.906 9.39e-05 ***
## ---
## Signif. codes:  0 '***' 0.001 '**' 0.01 '*' 0.05 '.' 0.1 ' ' 1
##
```

```
## Approximate significance of smooth terms:
##              edf Ref.df     F p-value
## s(age_z)   6.978  6.978 21.96  <2e-16 ***
## s(sleep_z) 3.569  3.569 11.60  <2e-16 ***
## ---
## Signif. codes:  0 '***' 0.001 '**' 0.01 '*' 0.05 '.' 0.1 ' ' 1
##
## R-sq.(adj) =  0.0287
## lmer.REML = 6.5356e+05  Scale est. = 1114.9    n = 51235
```

Model with full interaction between age and sleep

```
##
## Family: gaussian
## Link function: identity
##
## Formula:
## value ~ sex + site + t2(age_z, sleep_z, k = c(10, 4), bs = "cr")
## <environment: 0x558a4a807388>
##
## Parametric coefficients:
##                Estimate Std. Error t value Pr(>|t|)
## (Intercept)   1013.717      8.409 120.555  < 2e-16 ***
## sexmale         37.737      1.472  25.636  < 2e-16 ***
## siteMPIB       -75.710     10.983  -6.893 5.51e-12 ***
## siteousAvanto  -33.725      8.659  -3.895 9.84e-05 ***
## siteousPrisma    1.262     14.515   0.087 0.930694
## siteousSkyra   -43.101      8.419  -5.120 3.07e-07 ***
## siteUB         -94.369     26.350  -3.581 0.000342 ***
## siteUCAM       -44.367      9.554  -4.644 3.43e-06 ***
## siteUKB         14.912      8.625   1.729 0.083843 .
## siteUmU        -32.167     12.401  -2.594 0.009493 **
## siteUOXF       -41.150     10.344  -3.978 6.95e-05 ***
## ---
## Signif. codes:  0 '***' 0.001 '**' 0.01 '*' 0.05 '.' 0.1 ' ' 1
##
## Approximate significance of smooth terms:
##                     edf Ref.df     F p-value
## t2(age_z,sleep_z) 13.13  13.13 2.777  0.0165 *
## ---
## Signif. codes:  0 '***' 0.001 '**' 0.01 '*' 0.05 '.' 0.1 ' ' 1
##
## R-sq.(adj) =  0.0285
## lmer.REML = 6.5358e+05  Scale est. = 1115      n = 51235
```

Model comparison

`mod_no_sleep` refers to model without sleep term, `mod_no_interaction` refers to model with only main effect of sleep, and `mod_full` refers to model with a full interaction between age and sleep. This is a nested model comparison, and the p-value at a given line refers to comparing the model at the line to the model on the line above. Hence, significance implies that the more complicated model is supported on statistical grounds.

To be even more specific, the p-value on the second row tests whether there is an association between sleep and volume. The p-value on the third row tests whether this association depends on age.

```
## Data: NULL
## Models:
## ml$mod_no_sleep$mer: NULL
## ml$mod_no_interaction$mer: NULL
## ml$mod_full$mer: NULL
##                            npar    AIC    BIC  logLik deviance  Chisq Df Pr(>Chisq)
## ml$mod_no_sleep$mer          15 653626 653759 -326798   653596
## ml$mod_no_interaction$mer    17 653594 653744 -326780   653560 36.195  2  1.382e-08 ***
## ml$mod_full$mer              19 653618 653786 -326790   653580  0.000  2          1
## ---
## Signif. codes:  0 '***' 0.001 '**' 0.01 '*' 0.05 '.' 0.1 ' ' 1
```

We chose the model based on the likelihood ratio test with 5 % significance level, which was `mod_no_interaction`.

Lifespan brain trajectory

The trajectory shown is from the chosen model `mod_no_interaction_no_icv`.

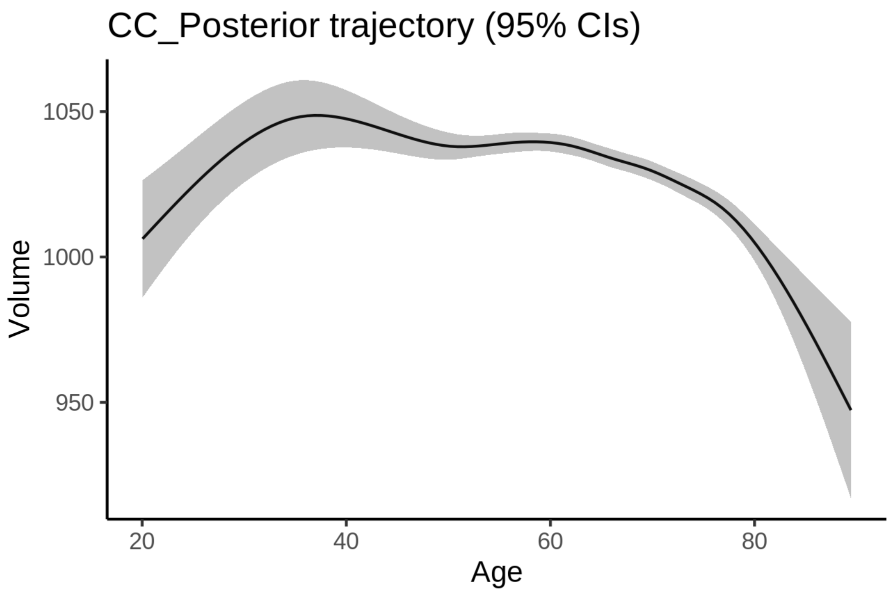

Effect of sleep

The chosen model only included the main effect of sleep, and hence the effect does not vary with age. The black dot shows the average sleep duration across all ages in the sample.

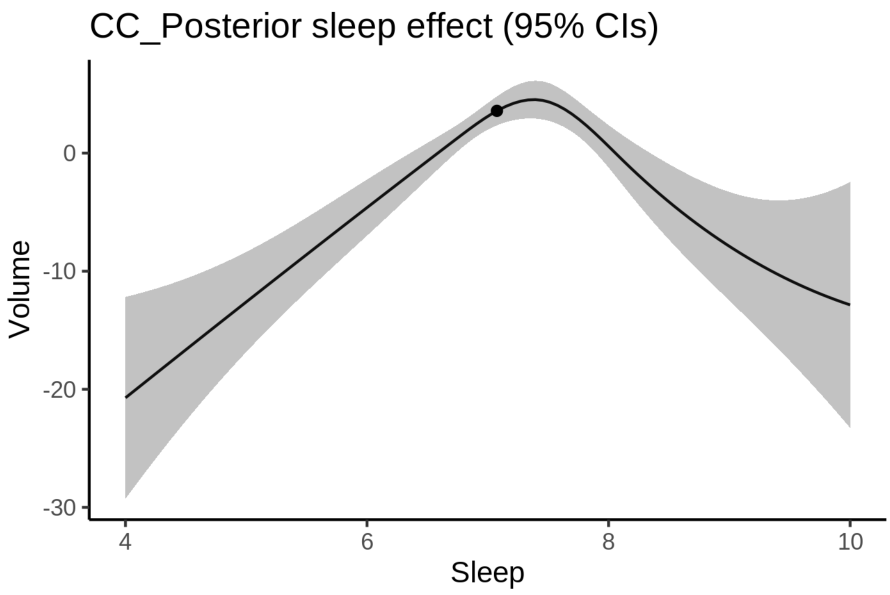

We also show the full interaction model for completeness, although it was not selected.

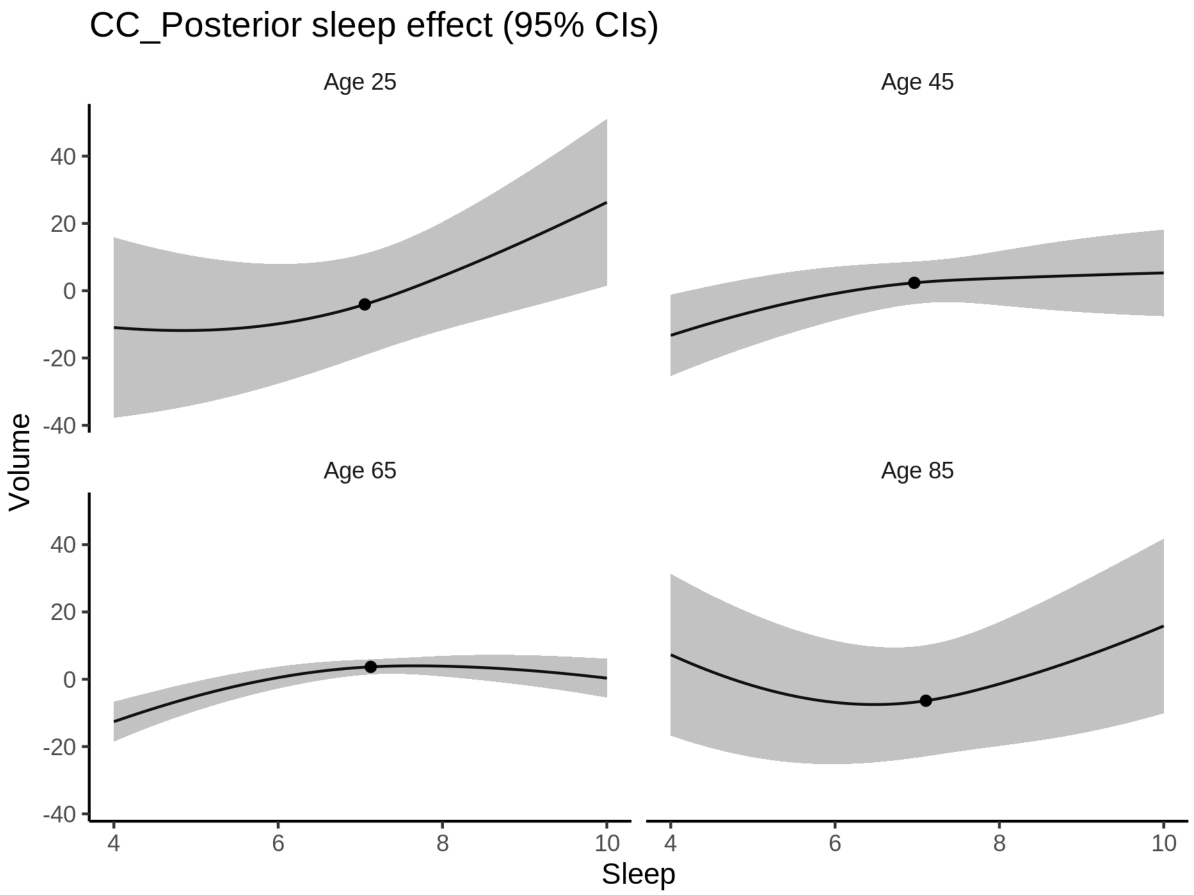

Deviation from sleep associated with maximal volume

Model with only main effect of sleep was chosen, so we show it for all ages at once. Maximum volume is attained at 7.4 hours of sleep. The percentage values in the plot are calculated as follows: The maximum at 100 % refers to a person at an arbitrary age with a sleep duration associated with maximum volume. For a female, this volume is 1025 and for a male it is 1063. The other percentage values show how large the expected volume is for someone with other sleep durations. For example, 99 % implies a 1 % reduction.

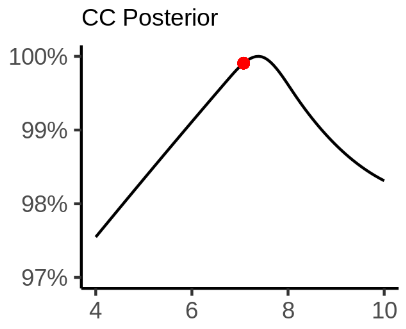

Comparison of mean sleep and sleep associated with maximum volume

A 95 % confidence interval for the sleep associated with maximum volume is [7.27, 7.45].

The plot below compares average sleep to the sleep associated with maximum volume.

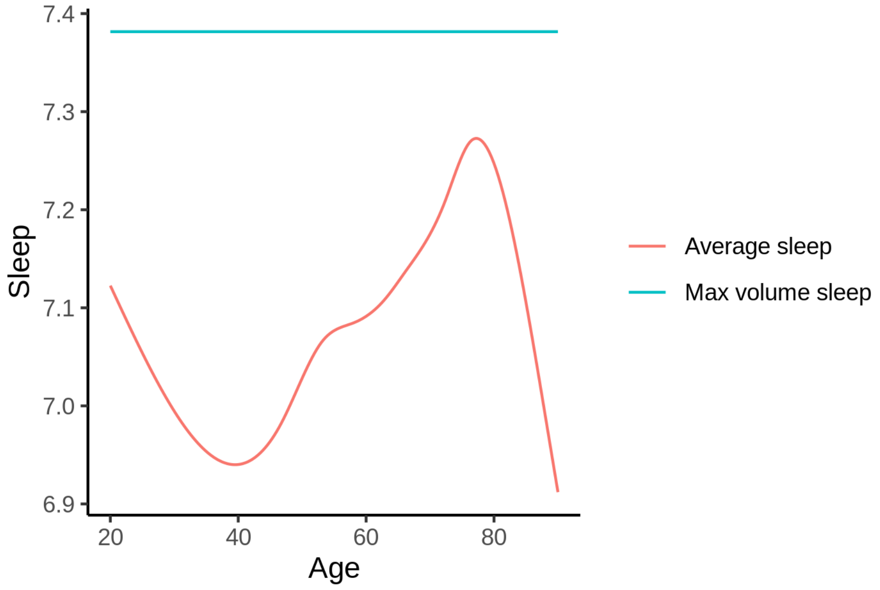

The next plot shows the difference between average sleep and sleep associated with maximum volume. The shaded region is a 95 % confidence interval.

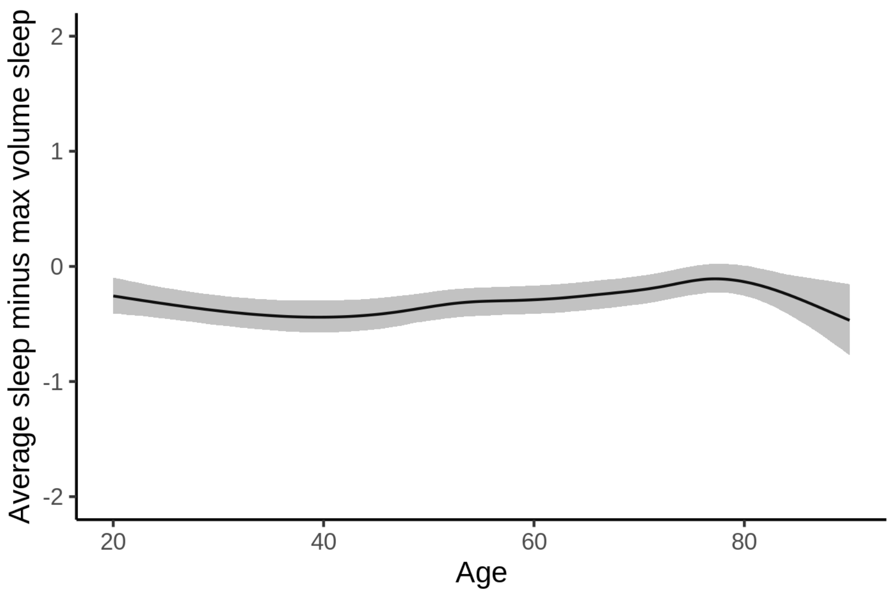

The next plot shows the probability that the sleep duration associated with maximum volume is longer than the average sleep duration, as a function of age. Probability below .05 can be interpreted as evidence that the sleep associated with maximum volume is shorter than the mean sleep, and probability above .95 can be interpreted the opposite way.

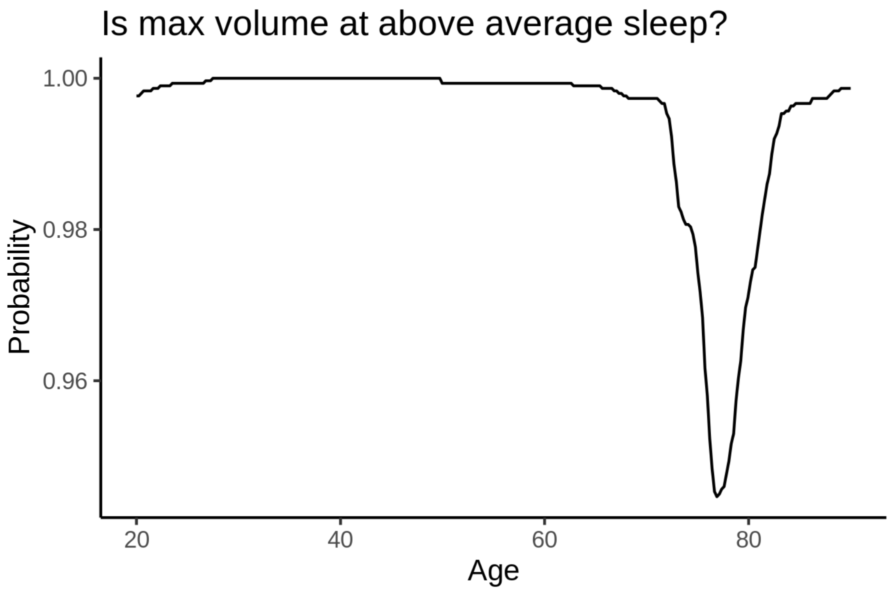

Cerebellum-Cortex

Descriptive statistics

Study	Observations	Unique IDs	Mean age	Age range
HCP	974	974	28.8	22 - 37
MPIB	677	391	63.1	24 - 83

Study	Observations	Unique IDs	Mean age	Age range
UB	113	39	70.9	64 - 81
UCAM	884	632	55.1	20 - 88
UiO	1474	803	49.3	20 - 89
UKB	45964	43128	64.5	45 - 83
UmU	405	276	62.1	25 - 85
UOXF	769	769	69.8	60 - 85

Spaghetti plot

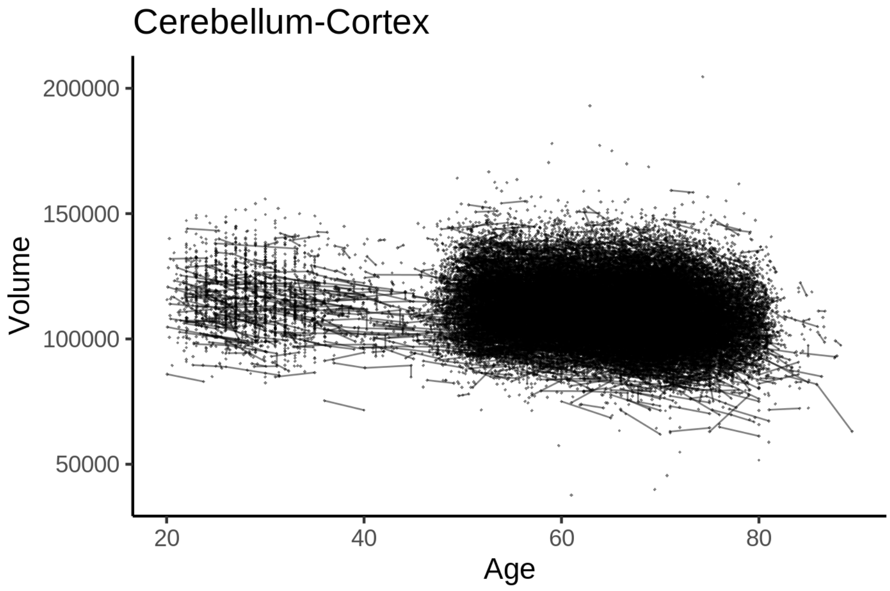

Models Controling for ICV

Model outputs

Model without sleep term

```
##
## Family: gaussian
## Link function: identity
##
## Formula:
## value ~ sex + site + icv + s(age_z, k = 10, bs = "cr")
## <environment: 0x558ad68c20f0>
##
## Parametric coefficients:
##                  Estimate Std. Error t value Pr(>|t|)
## (Intercept)     105008.45     492.70 213.129  < 2e-16 ***
## sexmale           5650.24     103.51  54.584  < 2e-16 ***
## siteMPIB          2227.29     641.50   3.472 0.000517 ***
## siteousAvanto    -4745.47     502.73  -9.439  < 2e-16 ***
## siteousPrisma     2585.10     844.41   3.061 0.002204 **
## siteousSkyra     -6909.69     487.22 -14.182  < 2e-16 ***
```

```
## siteUB          -5669.75    1527.92  -3.711 0.000207 ***
## siteUCAM         -589.69     554.11  -1.064 0.287233    
## siteUKB          4610.09     504.79   9.133  < 2e-16 ***
## siteUmU        -12066.02     720.63 -16.744  < 2e-16 ***
## siteUOXF        -4878.14     603.17  -8.088 6.22e-16 ***
## icv              5476.97      51.94 105.449  < 2e-16 ***
## ---
## Signif. codes:  0 '***' 0.001 '**' 0.01 '*' 0.05 '.' 0.1 ' ' 1
##
## Approximate significance of smooth terms:
##            edf Ref.df     F p-value    
## s(age_z) 7.404  7.404 345.2  <2e-16 ***
## ---
## Signif. codes:  0 '***' 0.001 '**' 0.01 '*' 0.05 '.' 0.1 ' ' 1
##
## R-sq.(adj) =  0.436   
## lmer.REML = 1.0703e+06  Scale est. = 3.944e+06  n = 51260
```

Model with only main effects of age and sleep

```
##
## Family: gaussian 
## Link function: identity 
##
## Formula:
## value ~ sex + site + icv + s(age_z, k = 10, bs = "cr") + s(sleep_z, 
##     k = 5, bs = "cr")
## <environment: 0x558ad68c20f0>
##
## Parametric coefficients:
##                 Estimate Std. Error t value Pr(>|t|)    
## (Intercept)    105023.57     493.12 212.978  < 2e-16 ***
## sexmale          5648.52     103.49  54.579  < 2e-16 ***
## siteMPIB         2261.49     641.73   3.524 0.000425 ***
## siteousAvanto   -4766.33     502.79  -9.480  < 2e-16 ***
## siteousPrisma    2568.95     844.26   3.043 0.002345 ** 
## siteousSkyra    -6938.02     487.31 -14.237  < 2e-16 ***
## siteUB          -5614.83    1527.45  -3.676 0.000237 ***
## siteUCAM         -567.72     554.06  -1.025 0.305529    
## siteUKB          4594.52     505.27   9.093  < 2e-16 ***
## siteUmU        -11980.80     722.08 -16.592  < 2e-16 ***
## siteUOXF        -4885.54     603.11  -8.101 5.59e-16 ***
## icv              5469.38      51.98 105.215  < 2e-16 ***
## ---
## Signif. codes:  0 '***' 0.001 '**' 0.01 '*' 0.05 '.' 0.1 ' ' 1
##
## Approximate significance of smooth terms:
##              edf Ref.df       F  p-value    
## s(age_z)   7.404  7.404 339.735  < 2e-16 ***
## s(sleep_z) 3.417  3.417   7.635 1.42e-05 ***
## ---
## Signif. codes:  0 '***' 0.001 '**' 0.01 '*' 0.05 '.' 0.1 ' ' 1
##
## R-sq.(adj) =  0.436   
## lmer.REML = 1.0703e+06  Scale est. = 3.9447e+06  n = 51260
```

Model with full interaction between age and sleep

```
##
## Family: gaussian
## Link function: identity
##
## Formula:
## value ~ sex + site + icv + t2(age_z, sleep_z, k = c(10, 4), bs = "cr")
## <environment: 0x558ad68c20f0>
##
## Parametric coefficients:
##                Estimate Std. Error t value Pr(>|t|)
## (Intercept)   105071.76     496.04 211.819  < 2e-16 ***
## sexmale         5643.56     103.72  54.411  < 2e-16 ***
## siteMPIB        2173.13     645.52   3.366 0.000762 ***
## siteousAvanto  -4810.61     506.06  -9.506  < 2e-16 ***
## siteousPrisma   2522.26     846.50   2.980 0.002887 **
## siteousSkyra   -6975.90     490.80 -14.213  < 2e-16 ***
## siteUB         -5723.83    1528.91  -3.744 0.000182 ***
## siteUCAM        -669.22     557.55  -1.200 0.230034
## siteUKB         4549.35     508.24   8.951  < 2e-16 ***
## siteUmU       -12071.90     724.84 -16.655  < 2e-16 ***
## siteUOXF       -4945.90     606.28  -8.158 3.49e-16 ***
## icv             5475.76      51.97 105.360  < 2e-16 ***
## ---
## Signif. codes:  0 ‘***’ 0.001 ‘**’ 0.01 ‘*’ 0.05 ‘.’ 0.1 ‘ ’ 1
##
## Approximate significance of smooth terms:
##                     edf Ref.df     F p-value
## t2(age_z,sleep_z) 13.72  13.72 11.95  <2e-16 ***
## ---
## Signif. codes:  0 ‘***’ 0.001 ‘**’ 0.01 ‘*’ 0.05 ‘.’ 0.1 ‘ ’ 1
##
## R-sq.(adj) =  0.436
## lmer.REML = 1.0703e+06  Scale est. = 3.9476e+06  n = 51260
```

Model comparison

`mod_no_sleep` refers to model without sleep term, `mod_no_interaction` refers to model with only main effect of sleep, and `mod_full` refers to model with a full interaction between age and sleep. This is a nested model comparison, and the p-value at a given line refers to comparing the model at the line to the model on the line above. Hence, significance implies that the more complicated model is supported on statistical grounds.

To be even more specific, the p-value on the second row tests whether there is an association between sleep and volume. The p-value on the third row tests whether this association depends on age.

```
## Data: NULL
## Models:
## ml$mod_no_sleep$mer: NULL
## ml$mod_no_interaction$mer: NULL
## ml$mod_full$mer: NULL
##                           npar     AIC     BIC  logLik deviance  Chisq Df Pr(>Chisq)
## ml$mod_no_sleep$mer         16 1070340 1070481 -535154  1070308
## ml$mod_no_interaction$mer   18 1070323 1070482 -535143  1070287 21.026  2  2.718e-05 ***
```

```
## ml$mod_full$mer             20 1070343 1070520 -535151  1070303  0.000  2          1
## ---
## Signif. codes:  0 ‘***’ 0.001 ‘**’ 0.01 ‘*’ 0.05 ‘.’ 0.1 ‘ ’ 1
```

We chose the model based on the likelihood ratio test with 5 % significance level, which was `mod_no_interaction`.

Lifespan brain trajectory

The trajectory shown is from the chosen model `mod_no_interaction`.

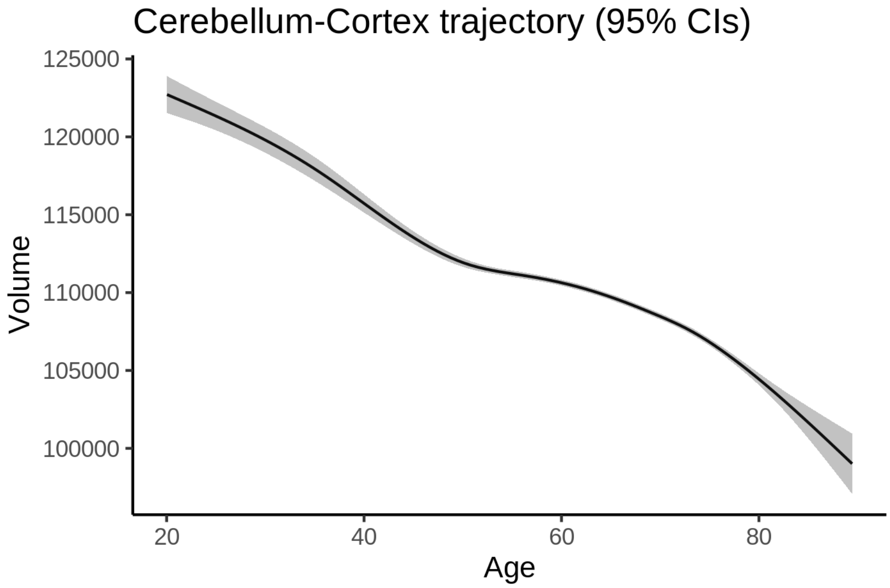

Effect of sleep

The chosen model only included the main effect of sleep, and hence the effect does not vary with age. The black dot shows the average sleep duration across all ages in the sample.

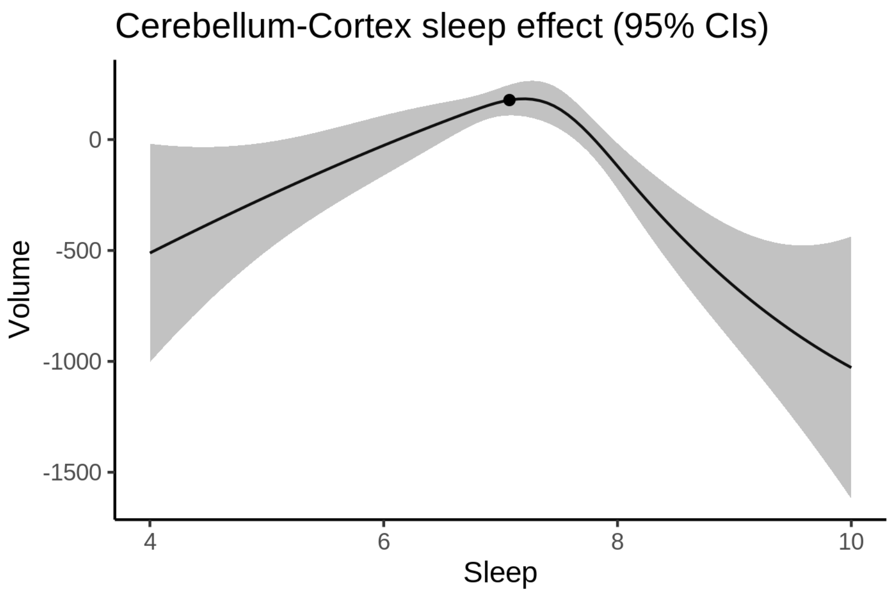

We also show the full interaction model for completeness, although it was not selected.

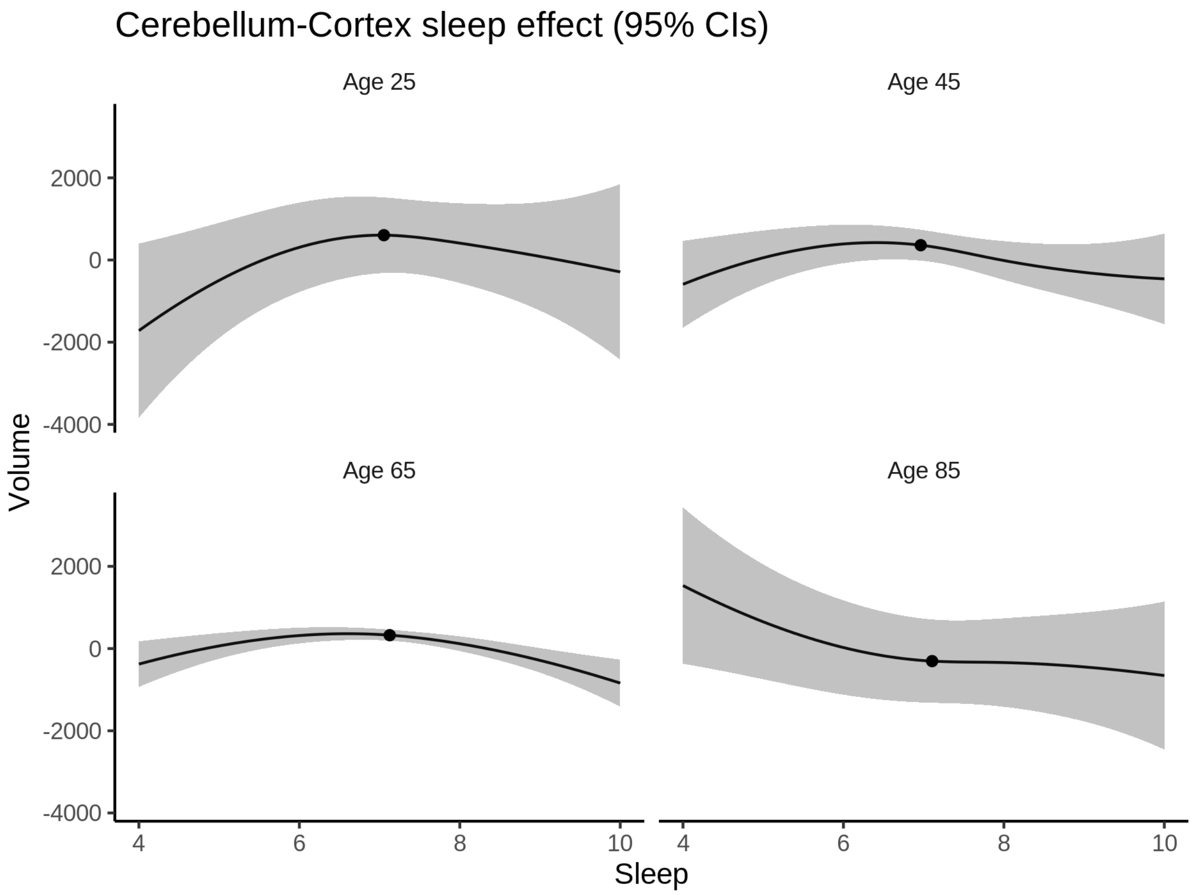

Deviation from sleep associated with maximal volume

Model with only main effect of sleep was chosen, so we show it for all ages at once. Maximum volume is attained at 7.2 hours of sleep. The percentage values in the plot are calculated as follows: The maximum at 100 % refers to a person at an arbitrary age with a sleep duration associated with maximum volume. For a female, this volume is 111685 and for a male it is 117333. The other percentage values show how large the expected volume is for someone with other sleep durations. For example, 99 % implies a 1 % reduction.

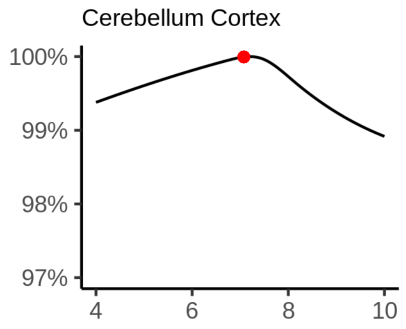

Comparison of mean sleep and sleep associated with maximum volume

A 95 % confidence interval for the sleep associated with maximum volume is [6.79, 7.33].

The plot below compares average sleep to the sleep associated with maximum volume.

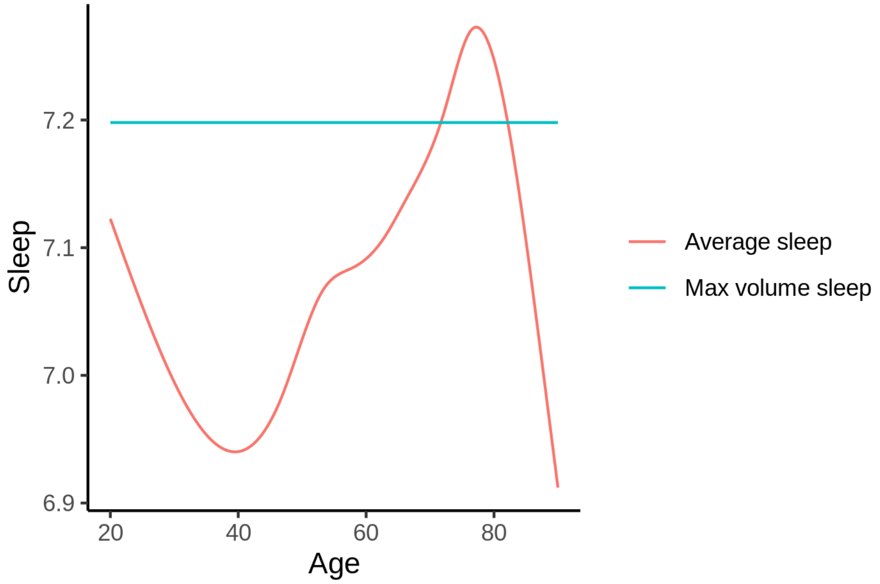

The next plot shows the difference between average sleep and sleep associated with maximum volume. The shaded region is a 95 % confidence interval.

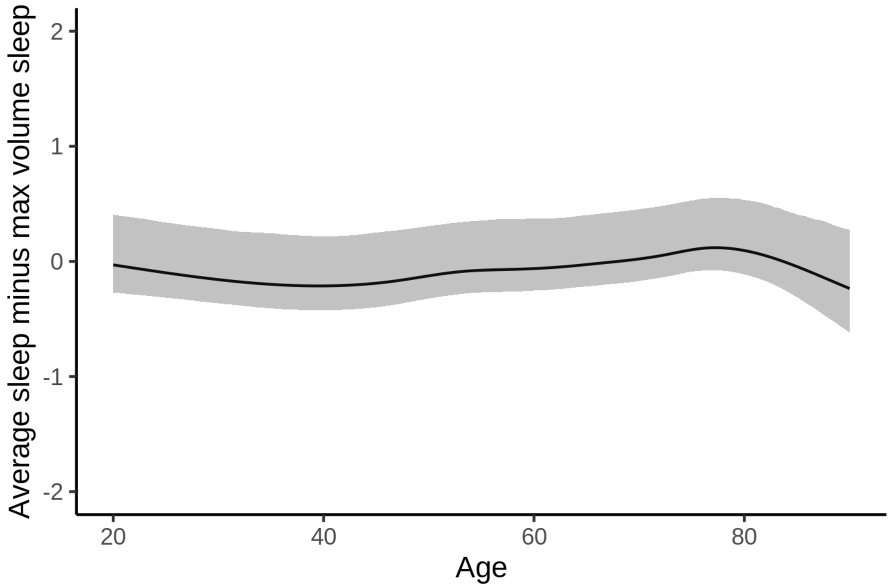

The next plot shows the probability that the sleep duration associated with maximum volume is longer than the average sleep duration, as a function of age. Probability below .05 can be interpreted as evidence that the sleep associated with maximum volume is shorter than the mean sleep, and probability above .95 can be interpreted the opposite way.

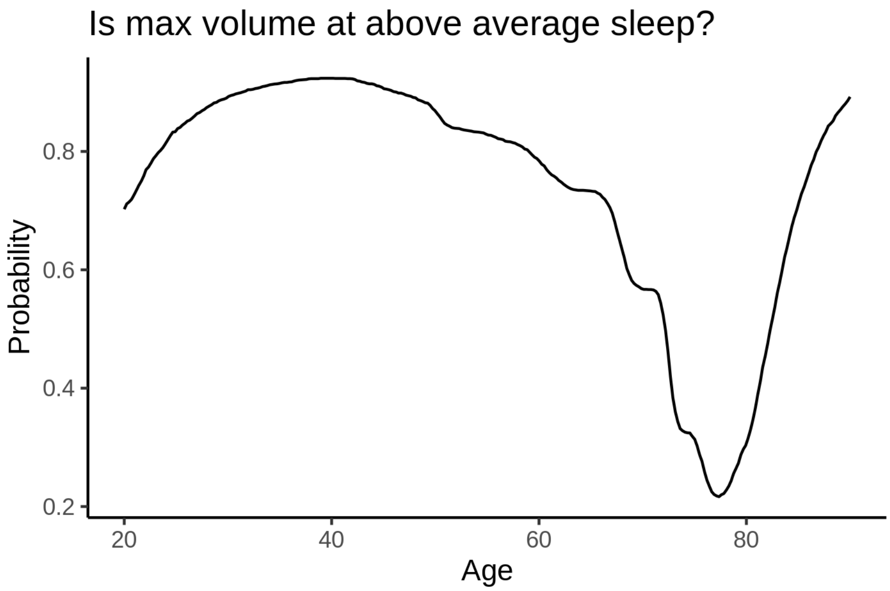

Controling for covariates

Below is the output for a model in which we only include data with income and education.

```
##
## Family: gaussian
## Link function: identity
##
## Formula:
```

```
## value ~ sex + site + s(age_z, k = 10, bs = "cr") + s(sleep_z,
##     k = 5, bs = "cr") + icv
## <environment: 0x558a4695c550>
##
## Parametric coefficients:
##                 Estimate Std. Error t value Pr(>|t|)
## (Intercept)    108960.76    1207.77  90.217  < 2e-16 ***
## sexmale          5582.76     132.41  42.163  < 2e-16 ***
## siteousAvanto -10148.35    1398.56  -7.256 4.07e-13 ***
## siteousPrisma    -14.98    1467.78  -0.010    0.992
## siteousSkyra  -11244.89    1306.60  -8.606  < 2e-16 ***
## siteUKB          534.83    1205.32   0.444    0.657
## siteUOXF       -7639.12    1356.11  -5.633 1.78e-08 ***
## icv             5663.68      67.88  83.433  < 2e-16 ***
## ---
## Signif. codes:  0 '***' 0.001 '**' 0.01 '*' 0.05 '.' 0.1 ' ' 1
##
## Approximate significance of smooth terms:
##               edf Ref.df       F p-value
## s(age_z)    6.036  6.036 168.237  <2e-16 ***
## s(sleep_z) 2.728  2.728   5.459  0.0019 **
## ---
## Signif. codes:  0 '***' 0.001 '**' 0.01 '*' 0.05 '.' 0.1 ' ' 1
##
## R-sq.(adj) =  0.408
## lmer.REML = 6.513e+05  Scale est. = 3.8427e+06  n = 31180
```

Below is the output for a model in which we control for the main effects of income and education.

```
##
## Family: gaussian
## Link function: identity
##
## Formula:
## value ~ sex + site + s(age_z, k = 10, bs = "cr") + s(sleep_z,
##     k = 5, bs = "cr") + icv + income_scaled + education_scaled
## <environment: 0x558a4695c550>
##
## Parametric coefficients:
##                    Estimate Std. Error t value Pr(>|t|)
## (Intercept)       107614.93    1213.67  88.669  < 2e-16 ***
## sexmale             5576.85     132.56  42.071  < 2e-16 ***
## siteousAvanto     -10113.57    1396.91  -7.240 4.59e-13 ***
## siteousPrisma       -121.76    1465.54  -0.083    0.934
## siteousSkyra      -11232.68    1304.71  -8.609  < 2e-16 ***
## siteUKB              567.24    1204.37   0.471    0.638
## siteUOXF           -7203.91    1354.87  -5.317 1.06e-07 ***
## icv                 5576.85      68.33  81.620  < 2e-16 ***
## income_scaled       1017.96     157.45   6.465 1.03e-10 ***
## education_scaled    1071.70     186.38   5.750 9.00e-09 ***
## ---
## Signif. codes:  0 '***' 0.001 '**' 0.01 '*' 0.05 '.' 0.1 ' ' 1
##
## Approximate significance of smooth terms:
##               edf Ref.df       F p-value
```

```
## s(age_z)   6.156  6.156 140.661  <2e-16 ***
## s(sleep_z) 2.284  2.284   4.245  0.0115 *
## ---
## Signif. codes:  0 ‘***’ 0.001 ‘**’ 0.01 ‘*’ 0.05 ‘.’ 0.1 ‘ ’ 1
##
## R-sq.(adj) =   0.41
## lmer.REML = 6.5117e+05  Scale est. = 3.8472e+06  n = 31180
```

We also included interaction effects between sleep duration and education and income, in another model. The output is shown below, and the interaction terms are `income_scaled:sleep_z` and `education_scaled:sleep_z`.

```
##
## Family: gaussian
## Link function: identity
##
## Formula:
## value ~ sex + site + s(age_z, k = 10, bs = "cr") + s(sleep_z,
##     k = 5, bs = "cr") + icv + income_scaled + education_scaled +
##     income_scaled:sleep_z + education_scaled:sleep_z
## <environment: 0x558a4695c550>
##
## Parametric coefficients:
##                           Estimate Std. Error t value Pr(>|t|)
## (Intercept)              107628.57    1213.72  88.677  < 2e-16 ***
## sexmale                    5565.78     132.64  41.960  < 2e-16 ***
## siteousAvanto            -10137.58    1396.88  -7.257 4.04e-13 ***
## siteousPrisma              -169.96    1465.61  -0.116   0.9077
## siteousSkyra             -11255.57    1304.68  -8.627  < 2e-16 ***
## siteUKB                     552.29    1204.45   0.459   0.6466
## siteUOXF                  -7219.16    1355.20  -5.327 1.01e-07 ***
## icv                        5578.45      68.33  81.639  < 2e-16 ***
## income_scaled              1028.34     157.53   6.528 6.77e-11 ***
## education_scaled           1063.98     186.40   5.708 1.15e-08 ***
## income_scaled:sleep_z      -291.52     156.73  -1.860   0.0629 .
## education_scaled:sleep_z    328.17     180.79   1.815   0.0695 .
## ---
## Signif. codes:  0 ‘***’ 0.001 ‘**’ 0.01 ‘*’ 0.05 ‘.’ 0.1 ‘ ’ 1
##
## Approximate significance of smooth terms:
##              edf Ref.df       F p-value
## s(age_z)   6.162  6.162 140.298  <2e-16 ***
## s(sleep_z) 2.332  2.332   3.349  0.0342 *
## ---
## Signif. codes:  0 ‘***’ 0.001 ‘**’ 0.01 ‘*’ 0.05 ‘.’ 0.1 ‘ ’ 1
##
## R-sq.(adj) =   0.41
## lmer.REML = 6.5114e+05  Scale est. = 3.8472e+06  n = 31180
```

We did the same controling for BMI. Below is the model with no covariates but only keeping data with BMI.

```
##
## Family: gaussian
## Link function: identity
##
## Formula:
```

```
## value ~ sex + site + s(age_z, k = 10, bs = "cr") + s(sleep_z,
##     k = 5, bs = "cr") + icv
## <environment: 0x55896137edd8>
##
## Parametric coefficients:
##               Estimate Std. Error t value Pr(>|t|)
## (Intercept)   98809.64     730.46 135.270  < 2e-16 ***
## sexmale        5572.67     128.36  43.414  < 2e-16 ***
## siteousPrisma  8219.31     974.87   8.431  < 2e-16 ***
## siteousSkyra   -966.00     511.87  -1.887   0.0591 .
## siteUCAM       5482.29     780.91   7.020 2.25e-12 ***
## siteUKB       10771.24     738.00  14.595  < 2e-16 ***
## siteUmU       -5757.06     905.16  -6.360 2.04e-10 ***
## icv            5643.96      66.04  85.466  < 2e-16 ***
## ---
## Signif. codes:  0 ‘***’ 0.001 ‘**’ 0.01 ‘*’ 0.05 ‘.’ 0.1 ‘ ’ 1
##
## Approximate significance of smooth terms:
##              edf Ref.df       F p-value
## s(age_z)   6.646  6.646 225.333 < 2e-16 ***
## s(sleep_z) 2.742  2.742   5.497 0.00116 **
## ---
## Signif. codes:  0 ‘***’ 0.001 ‘**’ 0.01 ‘*’ 0.05 ‘.’ 0.1 ‘ ’ 1
##
## R-sq.(adj) =  0.428
## lmer.REML = 6.9782e+05  Scale est. = 4.0433e+06  n = 33429
```

Below is the model output with main effect.

```
##
## Family: gaussian
## Link function: identity
##
## Formula:
## value ~ sex + site + s(age_z, k = 10, bs = "cr") + s(sleep_z,
##     k = 5, bs = "cr") + icv + bmi
## <environment: 0x55896137edd8>
##
## Parametric coefficients:
##                Estimate Std. Error t value Pr(>|t|)
## (Intercept)   102585.45     794.68 129.090  < 2e-16 ***
## sexmale         5691.92     128.46  44.311  < 2e-16 ***
## siteousPrisma   8342.91     973.66   8.569  < 2e-16 ***
## siteousSkyra    -955.31     511.75  -1.867   0.0619 .
## siteUCAM        5562.12     779.80   7.133 1.00e-12 ***
## siteUKB        10892.82     737.06  14.779  < 2e-16 ***
## siteUmU        -5600.19     903.74  -6.197 5.83e-10 ***
## icv             5667.54      65.91  85.989  < 2e-16 ***
## bmi             -149.90      12.50 -11.990  < 2e-16 ***
## ---
## Signif. codes:  0 ‘***’ 0.001 ‘**’ 0.01 ‘*’ 0.05 ‘.’ 0.1 ‘ ’ 1
##
## Approximate significance of smooth terms:
##              edf Ref.df       F p-value
## s(age_z)   6.634  6.634 225.845 < 2e-16 ***
```

```
## s(sleep_z) 2.382  2.382   5.905 0.00161 **
## ---
## Signif. codes:  0 ‘***’ 0.001 ‘**’ 0.01 ‘*’ 0.05 ‘.’ 0.1 ‘ ’ 1
##
## R-sq.(adj) =   0.43
## lmer.REML = 6.9767e+05  Scale est. = 4.0424e+06  n = 33429
```

Next is the model with BMI-sleep interaction.

```
##
## Family: gaussian
## Link function: identity
##
## Formula:
## value ~ sex + site + s(age_z, k = 10, bs = "cr") + s(sleep_z,
##     k = 5, bs = "cr") + icv + bmi + bmi:sleep_z
## <environment: 0x55896137edd8>
##
## Parametric coefficients:
##                 Estimate Std. Error t value Pr(>|t|)
## (Intercept)    1.026e+05  7.951e+02 129.019  < 2e-16 ***
## sexmale        5.692e+03  1.285e+02  44.309  < 2e-16 ***
## siteousPrisma  8.343e+03  9.737e+02   8.568  < 2e-16 ***
## siteousSkyra  -9.553e+02  5.118e+02  -1.867   0.0619 .
## siteUCAM       5.562e+03  7.799e+02   7.132 1.01e-12 ***
## siteUKB        1.089e+04  7.371e+02  14.777  < 2e-16 ***
## siteUmU       -5.600e+03  9.038e+02  -6.196 5.84e-10 ***
## icv            5.668e+03  6.591e+01  85.985  < 2e-16 ***
## bmi           -1.499e+02  1.251e+01 -11.983  < 2e-16 ***
## bmi:sleep_z   -1.392e-01  1.185e+01  -0.012   0.9906
## ---
## Signif. codes:  0 ‘***’ 0.001 ‘**’ 0.01 ‘*’ 0.05 ‘.’ 0.1 ‘ ’ 1
##
## Approximate significance of smooth terms:
##              edf Ref.df      F p-value
## s(age_z)   6.634  6.634 225.74  <2e-16 ***
## s(sleep_z) 2.381  2.381   2.22   0.095 .
## ---
## Signif. codes:  0 ‘***’ 0.001 ‘**’ 0.01 ‘*’ 0.05 ‘.’ 0.1 ‘ ’ 1
##
## R-sq.(adj) =   0.43
## lmer.REML = 6.9767e+05  Scale est. = 4.0424e+06  n = 33429
```

We did the same controling for depression. Below is the model with no covariates but only keeping data with depression.

```
##
## Family: gaussian
## Link function: identity
##
## Formula:
## value ~ sex + site + s(age_z, k = 10, bs = "cr") + s(sleep_z,
##     k = 5, bs = "cr") + icv
## <environment: 0x558962057e18>
##
## Parametric coefficients:
```

```
##                 Estimate Std. Error t value Pr(>|t|)
## (Intercept)   106215.39     615.75 172.498  < 2e-16 ***
## sexmale         5603.57     128.83  43.498  < 2e-16 ***
## siteousAvanto  -5079.60    1175.60  -4.321 1.56e-05 ***
## siteousPrisma   1630.21    1149.66   1.418  0.15620
## siteousSkyra   -7300.10     915.11  -7.977 1.54e-15 ***
## siteUCAM       -2195.05     842.51  -2.605  0.00918 **
## siteUKB         3279.97     609.01   5.386 7.26e-08 ***
## siteUmU       -13196.02     826.07 -15.974  < 2e-16 ***
## icv             5623.01      66.16  84.986  < 2e-16 ***
## ---
## Signif. codes:  0 ‘***’ 0.001 ‘**’ 0.01 ‘*’ 0.05 ‘.’ 0.1 ‘ ’ 1
##
## Approximate significance of smooth terms:
##               edf Ref.df      F  p-value
## s(age_z)   6.882  6.882 173.15  < 2e-16 ***
## s(sleep_z) 2.782  2.782   6.43 0.000825 ***
## ---
## Signif. codes:  0 ‘***’ 0.001 ‘**’ 0.01 ‘*’ 0.05 ‘.’ 0.1 ‘ ’ 1
##
## R-sq.(adj) =  0.425
## lmer.REML = 6.9552e+05  Scale est. = 3.795e+06  n = 33341
```

Below is the model output with main effect.

```
##
## Family: gaussian
## Link function: identity
##
## Formula:
## value ~ sex + site + s(age_z, k = 10, bs = "cr") + s(sleep_z,
##     k = 5, bs = "cr") + icv + depression
## <environment: 0x558962057e18>
##
## Parametric coefficients:
##                 Estimate Std. Error t value Pr(>|t|)
## (Intercept)   106436.61     618.22 172.167  < 2e-16 ***
## sexmale         5580.15     128.93  43.280  < 2e-16 ***
## siteousAvanto  -4961.26    1175.70  -4.220 2.45e-05 ***
## siteousPrisma   1738.82    1149.65   1.512    0.130
## siteousSkyra   -7167.82     915.40  -7.830 5.01e-15 ***
## siteUCAM       -2141.92     842.39  -2.543    0.011 *
## siteUKB         3189.83     609.32   5.235 1.66e-07 ***
## siteUmU       -12517.50     843.59 -14.838  < 2e-16 ***
## icv             5626.59      66.15  85.057  < 2e-16 ***
## depression     -1817.93     461.13  -3.942 8.09e-05 ***
## ---
## Signif. codes:  0 ‘***’ 0.001 ‘**’ 0.01 ‘*’ 0.05 ‘.’ 0.1 ‘ ’ 1
##
## Approximate significance of smooth terms:
##               edf Ref.df      F p-value
## s(age_z)   6.864  6.864 176.06 < 2e-16 ***
## s(sleep_z) 2.566  2.566   6.14 0.00132 **
## ---
## Signif. codes:  0 ‘***’ 0.001 ‘**’ 0.01 ‘*’ 0.05 ‘.’ 0.1 ‘ ’ 1
```

```
##
## R-sq.(adj) =  0.426
## lmer.REML = 6.9549e+05  Scale est. = 3.7947e+06  n = 33341
```

Next is the model with depression-sleep interaction.

```
##
## Family: gaussian
## Link function: identity
##
## Formula:
## value ~ sex + site + s(age_z, k = 10, bs = "cr") + s(sleep_z,
##     k = 5, bs = "cr") + icv + depression + depression:sleep_z
## <environment: 0x558962057e18>
##
## Parametric coefficients:
##                      Estimate Std. Error t value Pr(>|t|)
## (Intercept)         106436.96     618.21 172.169  < 2e-16 ***
## sexmale               5577.38     128.95  43.252  < 2e-16 ***
## siteousAvanto        -4980.18    1175.80  -4.236 2.29e-05 ***
## siteousPrisma         1719.59    1149.75   1.496   0.1348
## siteousSkyra         -7186.06     915.52  -7.849 4.31e-15 ***
## siteUCAM             -2161.59     842.55  -2.566   0.0103 *
## siteUKB               3190.41     609.32   5.236 1.65e-07 ***
## siteUmU             -12385.89     850.95 -14.555  < 2e-16 ***
## icv                   5626.74      66.15  85.058  < 2e-16 ***
## depression           -1849.86     462.10  -4.003 6.26e-05 ***
## depression:sleep_z    -403.02     339.93  -1.186   0.2358
## ---
## Signif. codes:  0 ‘***’ 0.001 ‘**’ 0.01 ‘*’ 0.05 ‘.’ 0.1 ‘ ’ 1
##
## Approximate significance of smooth terms:
##              edf Ref.df      F p-value
## s(age_z)   6.862  6.862 176.19  <2e-16 ***
## s(sleep_z) 2.597  2.597   4.05  0.0126 *
## ---
## Signif. codes:  0 ‘***’ 0.001 ‘**’ 0.01 ‘*’ 0.05 ‘.’ 0.1 ‘ ’ 1
##
## R-sq.(adj) =  0.426
## lmer.REML = 6.9547e+05  Scale est. = 3.7946e+06  n = 33341
```

The plot below shows the sleep-volume curve for the original model and for the model with main effects of SES.

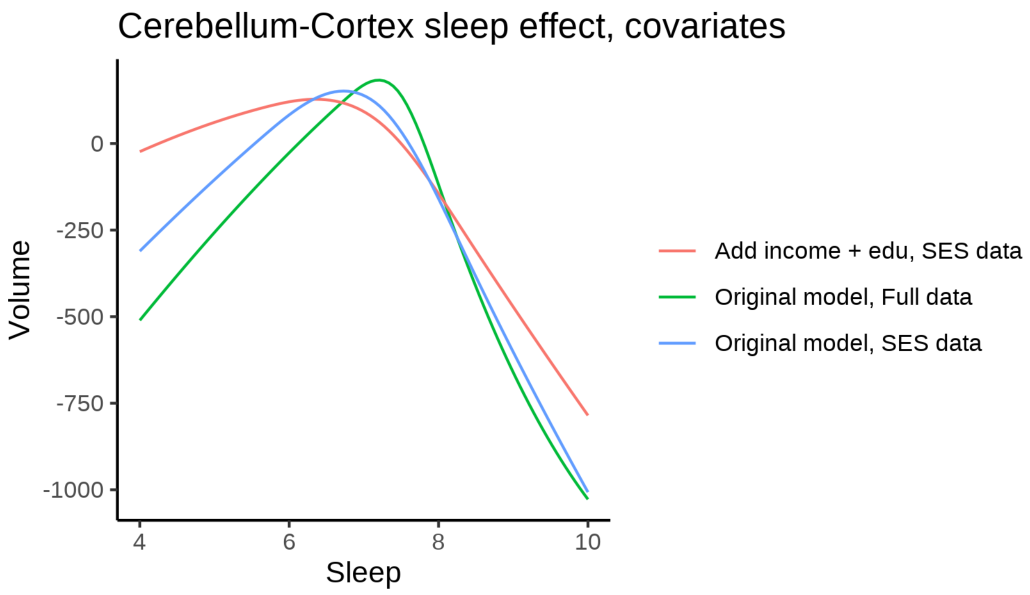

The plot below shows the sleep-volume curve for the original model and for the model with main effects of BMI.

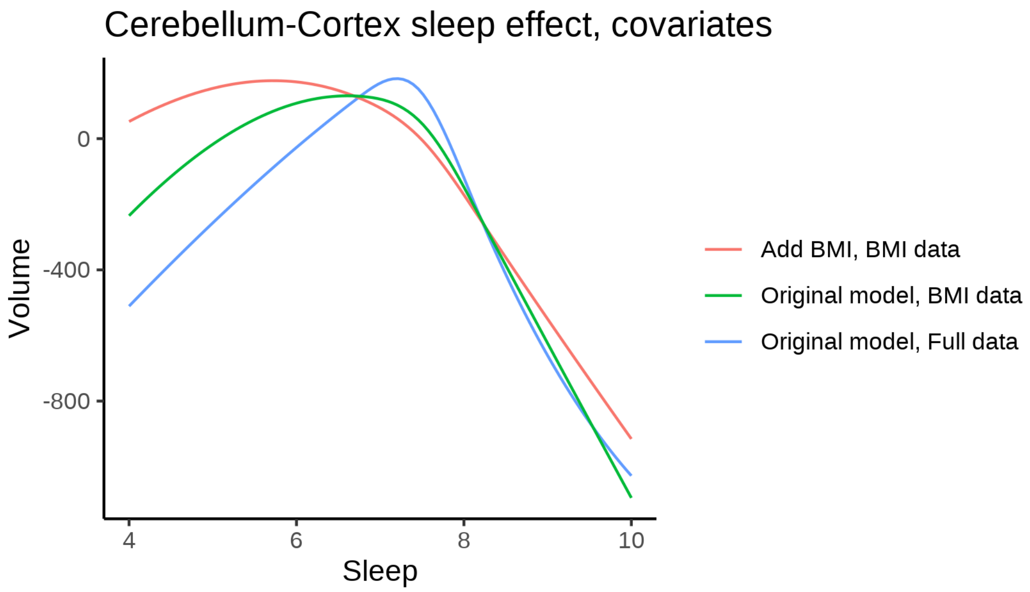

The plot below shows the sleep-volume curve for the original model and for the model with main effects of depression.

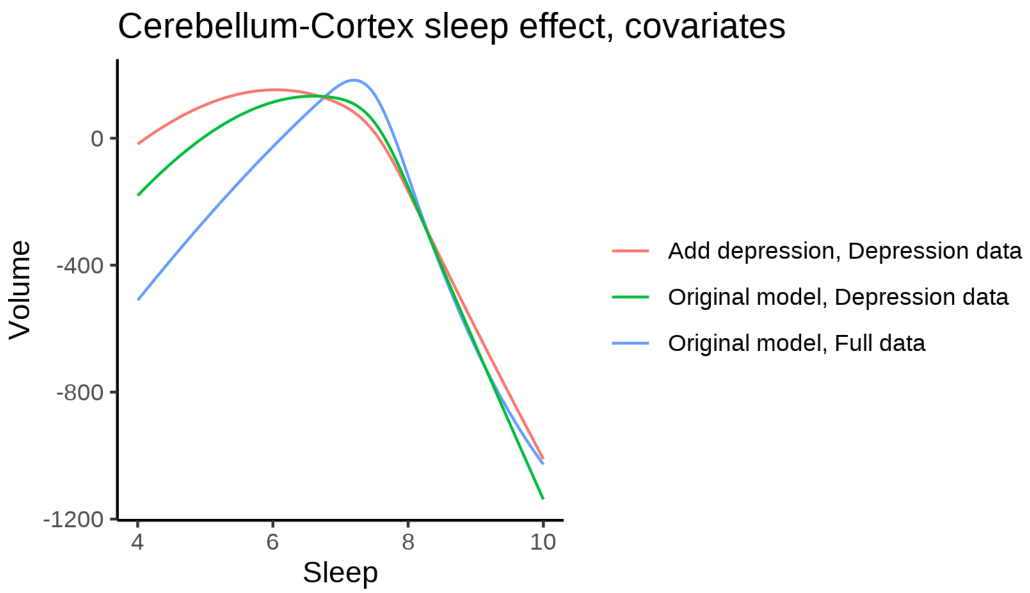

Models Not controling for ICV

Model outputs

Model without sleep term

```
##
## Family: gaussian
## Link function: identity
##
## Formula:
## value ~ sex + site + s(age_z, k = 10, bs = "cr")
## <environment: 0x558a46094a40>
##
## Parametric coefficients:
##                 Estimate Std. Error t value Pr(>|t|)
## (Intercept)   101973.96     534.27 190.865  < 2e-16 ***
## sexmale        11864.73      94.45 125.614  < 2e-16 ***
## siteMPIB       -6554.64     701.02  -9.350  < 2e-16 ***
## siteousAvanto  -2413.70     550.36  -4.386 1.16e-05 ***
## siteousPrisma   4065.09     920.89   4.414 1.02e-05 ***
## siteousSkyra   -6559.20     536.68 -12.222  < 2e-16 ***
## siteUB         -7265.62    1695.19  -4.286 1.82e-05 ***
## siteUCAM        -273.78     609.32  -0.449    0.653
## siteUKB         4801.39     548.08   8.760  < 2e-16 ***
## siteUmU       -14269.58     793.43 -17.985  < 2e-16 ***
## siteUOXF       -6128.31     659.43  -9.293  < 2e-16 ***
## ---
## Signif. codes:  0 '***' 0.001 '**' 0.01 '*' 0.05 '.' 0.1 ' ' 1
##
## Approximate significance of smooth terms:
##            edf Ref.df     F p-value
## s(age_z) 6.495  6.495 436.2  <2e-16 ***
```

```
## ---
## Signif. codes:  0 ‘***’ 0.001 ‘**’ 0.01 ‘*’ 0.05 ‘.’ 0.1 ‘ ’ 1
##
## R-sq.(adj) =  0.306
## lmer.REML = 1.0803e+06  Scale est. = 4.1449e+06  n = 51260
```

Model with only main effects of age and sleep

```
##
## Family: gaussian
## Link function: identity
##
## Formula:
## value ~ sex + site + s(age_z, k = 10, bs = "cr") + s(sleep_z,
##     k = 5, bs = "cr")
## <environment: 0x558a46094a40>
##
## Parametric coefficients:
##                Estimate Std. Error t value Pr(>|t|)
## (Intercept)   102110.04     534.53 191.029  < 2e-16 ***
## sexmale        11840.93      94.44 125.380  < 2e-16 ***
## siteMPIB       -6563.56     700.83  -9.365  < 2e-16 ***
## siteousAvanto  -2512.30     550.21  -4.566 4.98e-06 ***
## siteousPrisma   3990.25     920.40   4.335 1.46e-05 ***
## siteousSkyra   -6665.23     536.52 -12.423  < 2e-16 ***
## siteUB         -7183.60    1693.85  -4.241 2.23e-05 ***
## siteUCAM        -281.09     608.96  -0.462    0.644
## siteUKB         4669.32     548.33   8.516  < 2e-16 ***
## siteUmU       -14322.52     794.58 -18.025  < 2e-16 ***
## siteUOXF       -6183.70     659.02  -9.383  < 2e-16 ***
## ---
## Signif. codes:  0 ‘***’ 0.001 ‘**’ 0.01 ‘*’ 0.05 ‘.’ 0.1 ‘ ’ 1
##
## Approximate significance of smooth terms:
##              edf Ref.df      F p-value
## s(age_z)   6.485  6.485 429.04  <2e-16 ***
## s(sleep_z) 3.732  3.732  18.41  <2e-16 ***
## ---
## Signif. codes:  0 ‘***’ 0.001 ‘**’ 0.01 ‘*’ 0.05 ‘.’ 0.1 ‘ ’ 1
##
## R-sq.(adj) =  0.307
## lmer.REML = 1.0803e+06  Scale est. = 4.1455e+06  n = 51260
```

Model with full interaction between age and sleep

```
##
## Family: gaussian
## Link function: identity
##
## Formula:
## value ~ sex + site + t2(age_z, sleep_z, k = c(10, 4), bs = "cr")
## <environment: 0x558a46094a40>
##
## Parametric coefficients:
##                Estimate Std. Error t value Pr(>|t|)
## (Intercept)   102152.85     542.03 188.464  < 2e-16 ***
```

```
## sexmale        11859.92      94.67 125.280  < 2e-16 ***
## siteMPIB       -6741.70     706.83  -9.538  < 2e-16 ***
## siteousAvanto  -2552.52     555.22  -4.597 4.29e-06 ***
## siteousPrisma   3942.66     923.48   4.269 1.96e-05 ***
## siteousSkyra   -6701.24     541.85 -12.367  < 2e-16 ***
## siteUB         -7402.83    1697.32  -4.361 1.29e-05 ***
## siteUCAM        -424.54     614.93  -0.690     0.49
## siteUKB         4619.96     556.07   8.308  < 2e-16 ***
## siteUmU       -14454.04     799.98 -18.068  < 2e-16 ***
## siteUOXF       -6287.82     666.18  -9.439  < 2e-16 ***
## ---
## Signif. codes:  0 ‘***’ 0.001 ‘**’ 0.01 ‘*’ 0.05 ‘.’ 0.1 ‘ ’ 1
##
## Approximate significance of smooth terms:
##                     edf Ref.df     F p-value
## t2(age_z,sleep_z) 13.1   13.1 12.45  <2e-16 ***
## ---
## Signif. codes:  0 ‘***’ 0.001 ‘**’ 0.01 ‘*’ 0.05 ‘.’ 0.1 ‘ ’ 1
##
## R-sq.(adj) =  0.306
## lmer.REML = 1.0803e+06  Scale est. = 4.1484e+06  n = 51260
```

Model comparison

`mod_no_sleep` refers to model without sleep term, `mod_no_interaction` refers to model with only main effect of sleep, and `mod_full` refers to model with a full interaction between age and sleep. This is a nested model comparison, and the p-value at a given line refers to comparing the model at the line to the model on the line above. Hence, significance implies that the more complicated model is supported on statistical grounds.

To be even more specific, the p-value on the second row tests whether there is an association between sleep and volume. The p-value on the third row tests whether this association depends on age.

```
## Data: NULL
## Models:
## ml$mod_no_sleep$mer: NULL
## ml$mod_no_interaction$mer: NULL
## ml$mod_full$mer: NULL
##                           npar     AIC     BIC  logLik deviance  Chisq Df Pr(>Chisq)
## ml$mod_no_sleep$mer         15 1080353 1080485 -540161  1080323
## ml$mod_no_interaction$mer   17 1080294 1080445 -540130  1080260 62.498  2  2.684e-14 ***
## ml$mod_full$mer             19 1080342 1080510 -540152  1080304  0.000  2          1
## ---
## Signif. codes:  0 ‘***’ 0.001 ‘**’ 0.01 ‘*’ 0.05 ‘.’ 0.1 ‘ ’ 1
```

We chose the model based on the likelihood ratio test with 5 % significance level, which was `mod_no_interaction`.

Lifespan brain trajectory

The trajectory shown is from the chosen model `mod_no_interaction_no_icv`.

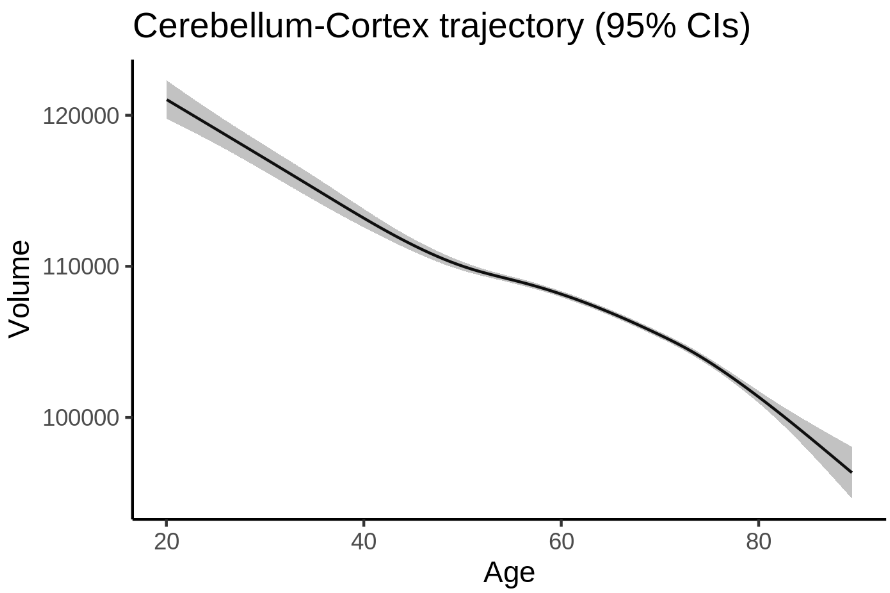

Effect of sleep

The chosen model only included the main effect of sleep, and hence the effect does not vary with age. The black dot shows the average sleep duration across all ages in the sample.

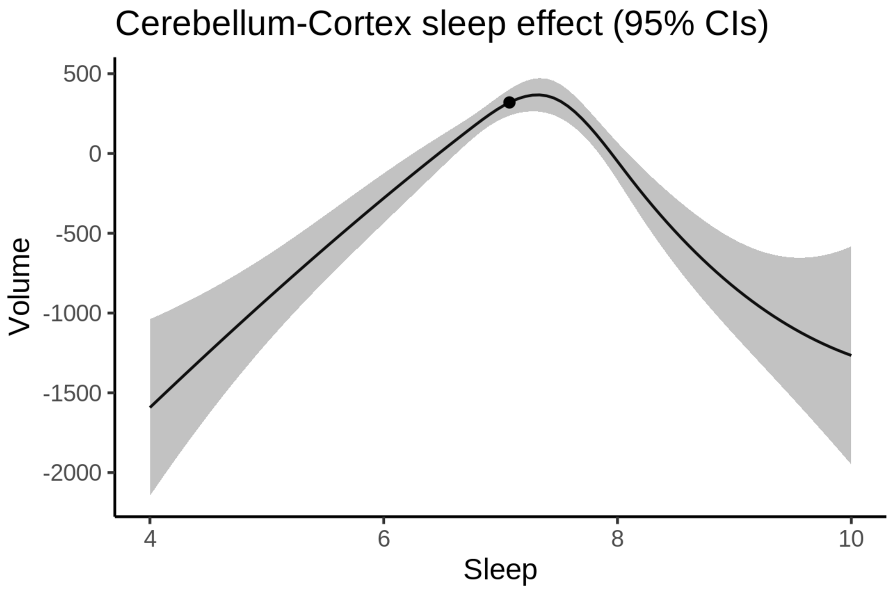

We also show the full interaction model for completeness, although it was not selected.

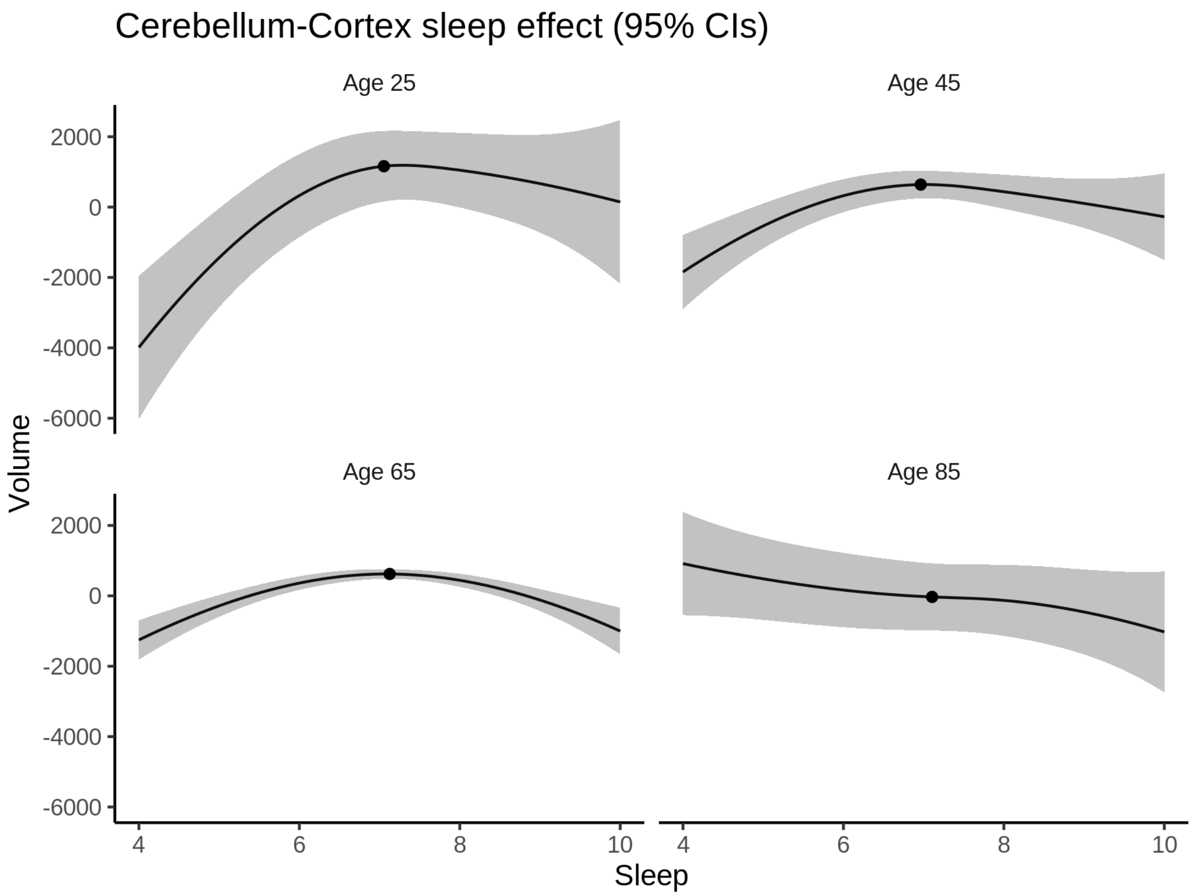

Deviation from sleep associated with maximal volume

Model with only main effect of sleep was chosen, so we show it for all ages at once. Maximum volume is attained at 7.3 hours of sleep. The percentage values in the plot are calculated as follows: The maximum at 100 % refers to a person at an arbitrary age with a sleep duration associated with maximum volume. For a female, this volume is 109118 and for a male it is 120959. The other percentage values show how large the expected volume is for someone with other sleep durations. For example, 99 % implies a 1 % reduction.

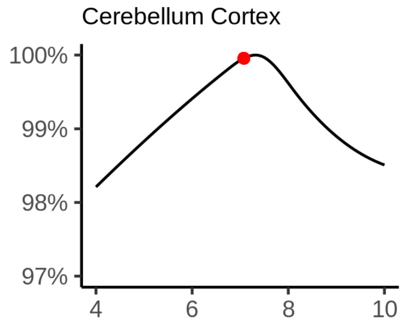

Comparison of mean sleep and sleep associated with maximum volume

A 95 % confidence interval for the sleep associated with maximum volume is [7.21, 7.39].

The plot below compares average sleep to the sleep associated with maximum volume.

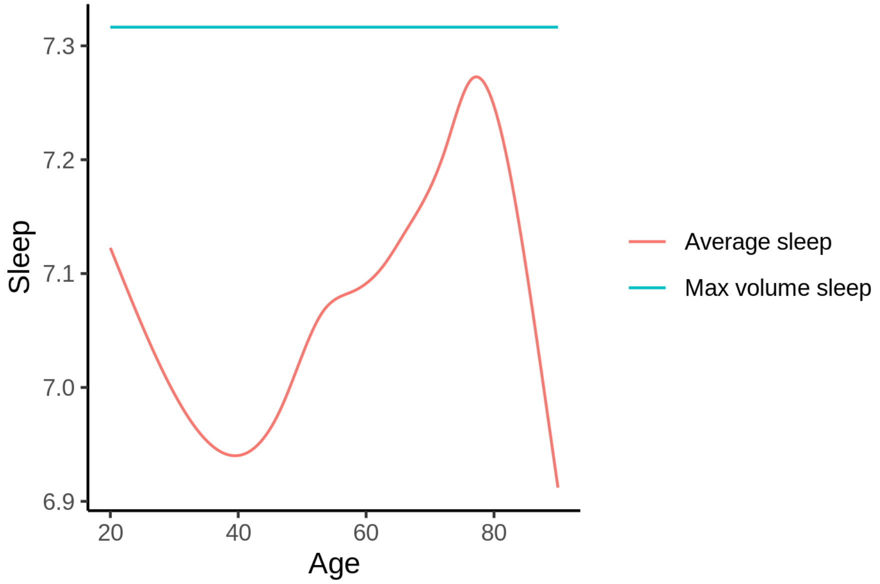

The next plot shows the difference between average sleep and sleep associated with maximum volume. The shaded region is a 95 % confidence interval.

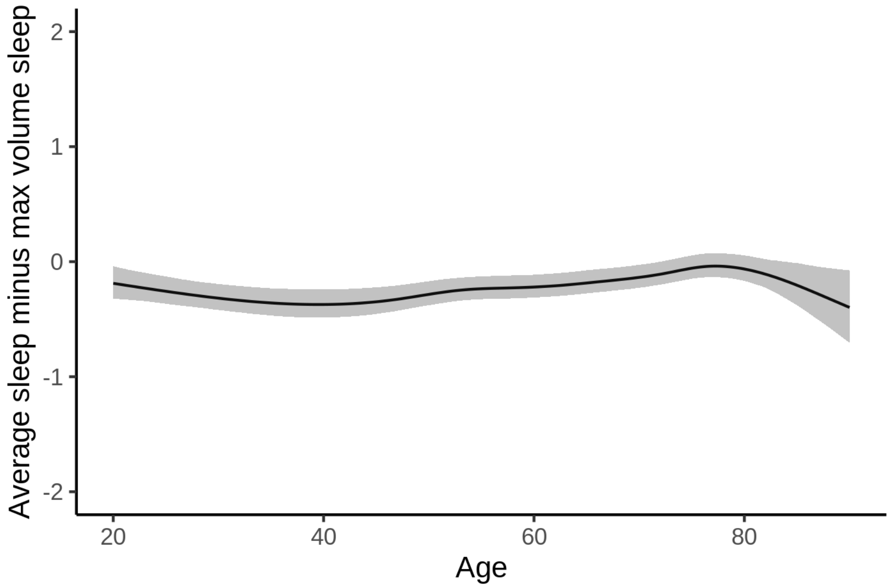

The next plot shows the probability that the sleep duration associated with maximum volume is longer than the average sleep duration, as a function of age. Probability below .05 can be interpreted as evidence that the sleep associated with maximum volume is shorter than the mean sleep, and probability above .95 can be interpreted the opposite way.

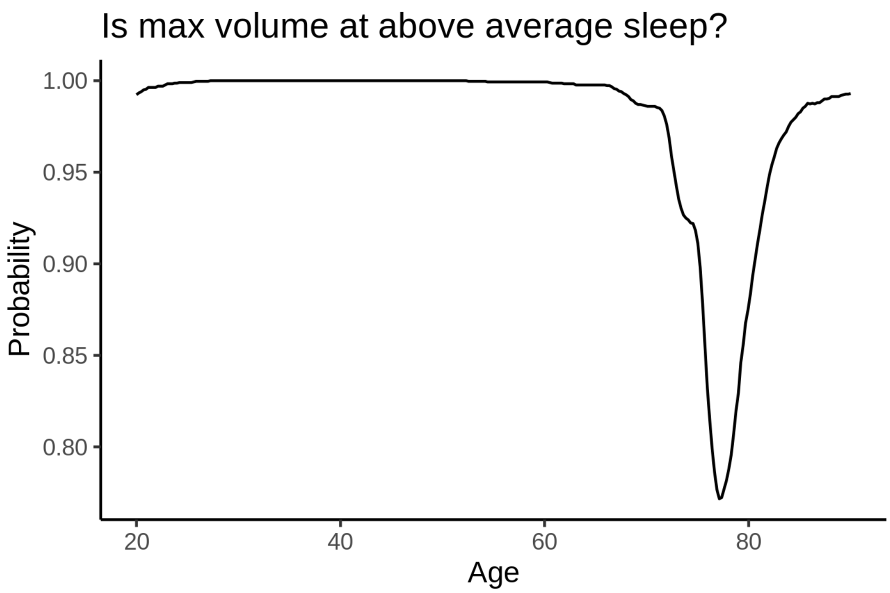

Cerebellum-White-Matter

Descriptive statistics

Study	Observations	Unique IDs	Mean age	Age range
HCP	974	974	28.8	22 - 37
MPIB	676	391	63.2	24 - 83
UB	113	39	70.9	64 - 81
UCAM	884	632	55.1	20 - 88
UiO	1475	803	49.4	20 - 89
UKB	45957	43127	64.5	45 - 83
UmU	407	278	62.2	25 - 85
UOXF	769	769	69.8	60 - 85

Spaghetti plot

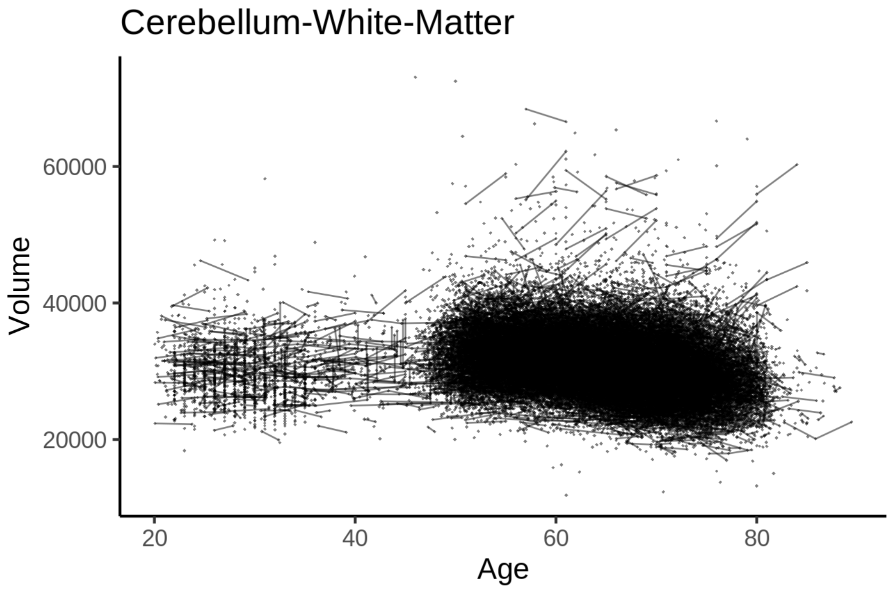

Models Controling for ICV

Model outputs

Model without sleep term

```
##
## Family: gaussian
## Link function: identity
##
## Formula:
## value ~ sex + site + icv + s(age_z, k = 10, bs = "cr")
## <environment: 0x558a49c89d70>
##
## Parametric coefficients:
##                Estimate Std. Error t value Pr(>|t|)
## (Intercept)    28007.63     200.60 139.619  < 2e-16 ***
## sexmale         -326.85      40.26  -8.118 4.83e-16 ***
## siteMPIB        2453.16     252.31   9.723  < 2e-16 ***
## siteousAvanto    768.40     210.75   3.646 0.000267 ***
## siteousPrisma   -905.74     352.70  -2.568 0.010232 *
## siteousSkyra    3589.32     192.91  18.606  < 2e-16 ***
## siteUB          -286.01     583.09  -0.491 0.623772
## siteUCAM         469.15     219.48   2.138 0.032560 *
## siteUKB         3267.10     205.84  15.872  < 2e-16 ***
## siteUmU        10774.80     283.42  38.017  < 2e-16 ***
## siteUOXF         786.44     242.39   3.245 0.001177 **
## icv             2067.58      20.27 102.006  < 2e-16 ***
## ---
## Signif. codes:  0 ‘***’ 0.001 ‘**’ 0.01 ‘*’ 0.05 ‘.’ 0.1 ‘ ’ 1
##
```

```
## Approximate significance of smooth terms:
##            edf Ref.df     F p-value
## s(age_z) 7.328  7.328 610.4  <2e-16 ***
## ---
## Signif. codes:  0 '***' 0.001 '**' 0.01 '*' 0.05 '.' 0.1 ' ' 1
##
## R-sq.(adj) =  0.324
## lmer.REML = 9.7747e+05  Scale est. = 1.6189e+06  n = 51255
```

Model with only main effects of age and sleep

```
##
## Family: gaussian
## Link function: identity
##
## Formula:
## value ~ sex + site + icv + s(age_z, k = 10, bs = "cr") + s(sleep_z,
##     k = 5, bs = "cr")
## <environment: 0x558a49c89d70>
##
## Parametric coefficients:
##                Estimate Std. Error t value Pr(>|t|)
## (Intercept)    27975.86     200.74 139.363  < 2e-16 ***
## sexmale         -328.56      40.25  -8.162 3.37e-16 ***
## siteMPIB        2492.98     252.43   9.876  < 2e-16 ***
## siteousAvanto    783.06     210.77   3.715 0.000203 ***
## siteousPrisma   -896.33     352.65  -2.542 0.011034 *
## siteousSkyra    3604.01     192.94  18.680  < 2e-16 ***
## siteUB          -269.59     582.96  -0.462 0.643761
## siteUCAM         489.63     219.47   2.231 0.025690 *
## siteUKB         3301.09     206.00  16.024  < 2e-16 ***
## siteUmU        10856.72     284.02  38.225  < 2e-16 ***
## siteUOXF         803.25     242.36   3.314 0.000919 ***
## icv             2068.89      20.28 102.000  < 2e-16 ***
## ---
## Signif. codes:  0 '***' 0.001 '**' 0.01 '*' 0.05 '.' 0.1 ' ' 1
##
## Approximate significance of smooth terms:
##              edf Ref.df       F  p-value
## s(age_z)   7.308  7.308 601.751  < 2e-16 ***
## s(sleep_z) 2.060  2.060   9.969 4.46e-05 ***
## ---
## Signif. codes:  0 '***' 0.001 '**' 0.01 '*' 0.05 '.' 0.1 ' ' 1
##
## R-sq.(adj) =  0.325
## lmer.REML = 9.7746e+05  Scale est. = 1.6186e+06  n = 51255
```

Model with full interaction between age and sleep

```
##
## Family: gaussian
## Link function: identity
##
## Formula:
## value ~ sex + site + icv + t2(age_z, sleep_z, k = c(10, 4), bs = "cr")
## <environment: 0x558a49c89d70>
```

```
##
## Parametric coefficients:
##               Estimate Std. Error t value Pr(>|t|)
## (Intercept)   27946.47     201.59 138.634  < 2e-16 ***
## sexmale        -330.97      40.34  -8.204 2.38e-16 ***
## siteMPIB       2522.62     253.85   9.937  < 2e-16 ***
## siteousAvanto   808.71     211.85   3.817 0.000135 ***
## siteousPrisma  -874.96     353.41  -2.476 0.013297 *
## siteousSkyra   3633.89     194.20  18.712  < 2e-16 ***
## siteUB         -245.32     583.39  -0.421 0.674121
## siteUCAM        522.66     220.77   2.367 0.017913 *
## siteUKB        3332.16     206.85  16.109  < 2e-16 ***
## siteUmU       10882.59     285.02  38.181  < 2e-16 ***
## siteUOXF        838.70     243.31   3.447 0.000567 ***
## icv            2069.54      20.28 102.041  < 2e-16 ***
## ---
## Signif. codes:  0 '***' 0.001 '**' 0.01 '*' 0.05 '.' 0.1 ' ' 1
##
## Approximate significance of smooth terms:
##                     edf Ref.df     F p-value
## t2(age_z,sleep_z) 12.85  12.85 7.989  <2e-16 ***
## ---
## Signif. codes:  0 '***' 0.001 '**' 0.01 '*' 0.05 '.' 0.1 ' ' 1
##
## R-sq.(adj) =  0.325
## lmer.REML = 9.7746e+05  Scale est. = 1.6184e+06  n = 51255
```

Model comparison

`mod_no_sleep` refers to model without sleep term, `mod_no_interaction` refers to model with only main effect of sleep, and `mod_full` refers to model with a full interaction between age and sleep. This is a nested model comparison, and the p-value at a given line refers to comparing the model at the line to the model on the line above. Hence, significance implies that the more complicated model is supported on statistical grounds.

To be even more specific, the p-value on the second row tests whether there is an association between sleep and volume. The p-value on the third row tests whether this association depends on age.

```
## Data: NULL
## Models:
## ml$mod_no_sleep$mer: NULL
## ml$mod_no_interaction$mer: NULL
## ml$mod_full$mer: NULL
##                           npar    AIC    BIC  logLik deviance  Chisq Df Pr(>Chisq)
## ml$mod_no_sleep$mer         16 977504 977646 -488736   977472
## ml$mod_no_interaction$mer   18 977491 977650 -488728   977455 17.453  2  0.0001622 ***
## ml$mod_full$mer             20 977499 977676 -488730   977459  0.000  2  1.0000000
## ---
## Signif. codes:  0 '***' 0.001 '**' 0.01 '*' 0.05 '.' 0.1 ' ' 1
```

We chose the model based on the likelihood ratio test with 5 % significance level, which was `mod_no_interaction`.

Lifespan brain trajectory

The trajectory shown is from the chosen model `mod_no_interaction`.

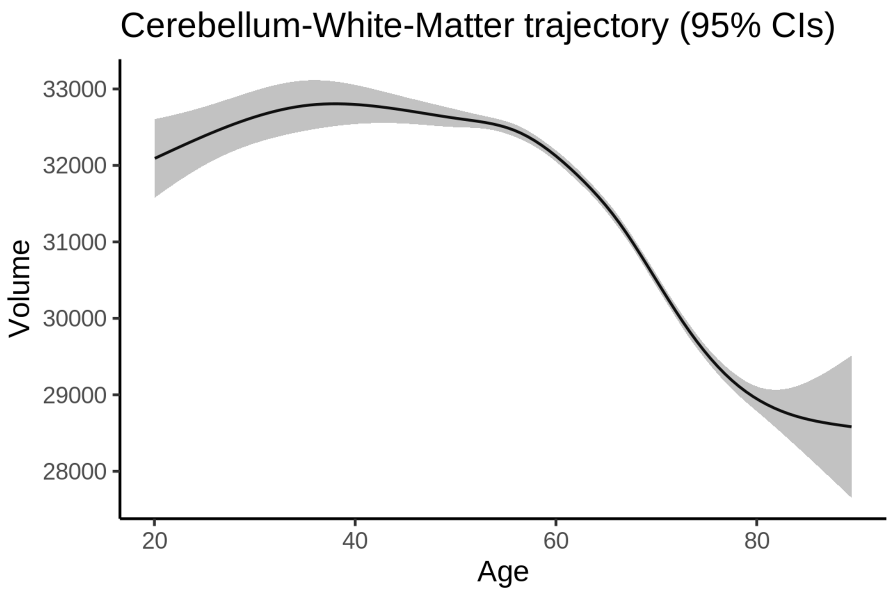

Effect of sleep

The chosen model only included the main effect of sleep, and hence the effect does not vary with age. The black dot shows the average sleep duration across all ages in the sample.

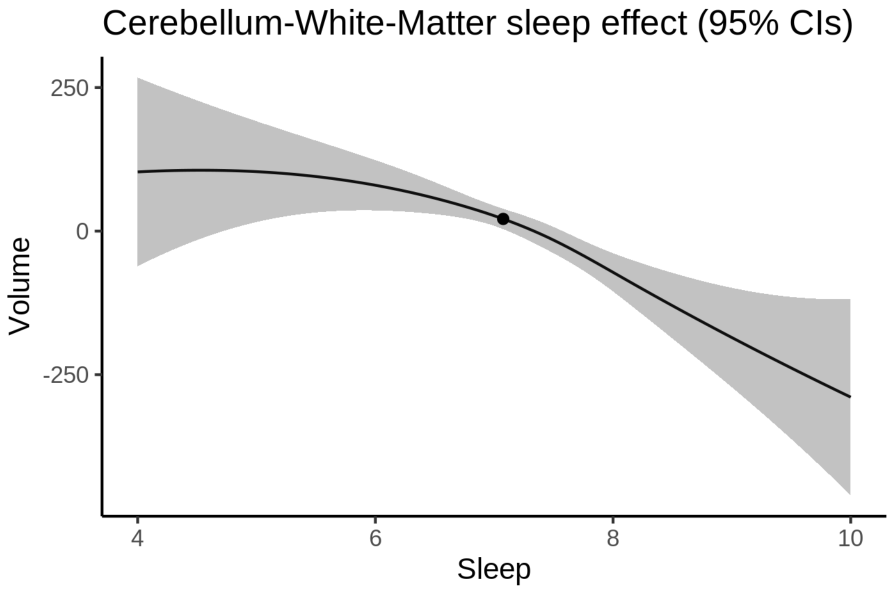

We also show the full interaction model for completeness, although it was not selected.

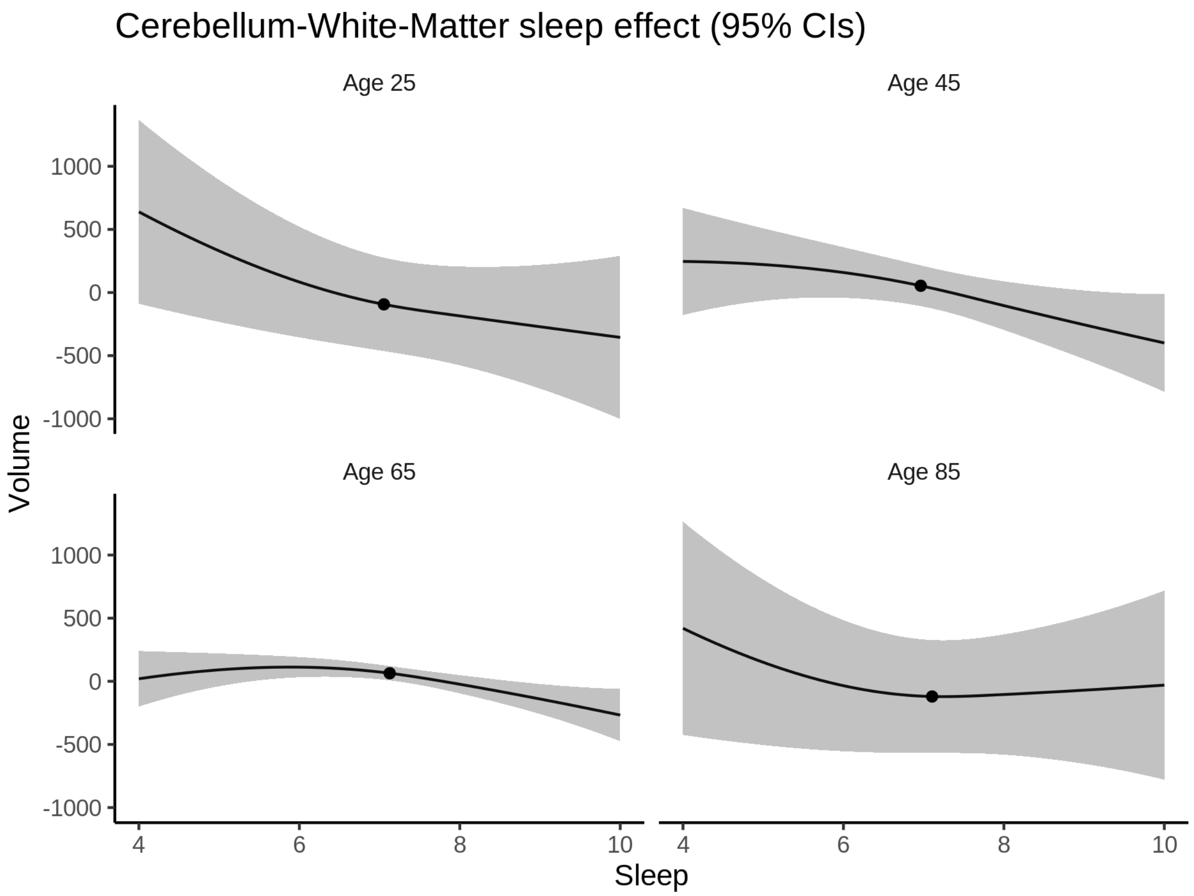

Deviation from sleep associated with maximal volume

Model with only main effect of sleep was chosen, so we show it for all ages at once. Maximum volume is attained at 4.5 hours of sleep. The percentage values in the plot are calculated as follows: The maximum at 100 % refers to a person at an arbitrary age with a sleep duration associated with maximum volume. For a female, this volume is 31423 and for a male it is 31095. The other percentage values show how large the expected volume is for someone with other sleep durations. For example, 99 % implies a 1 % reduction.

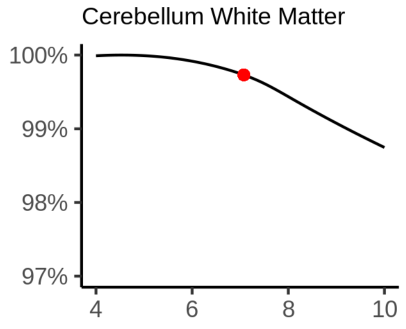

Comparison of mean sleep and sleep associated with maximum volume

A 95 % confidence interval for the sleep associated with maximum volume is [4, 6.06].

The plot below compares average sleep to the sleep associated with maximum volume.

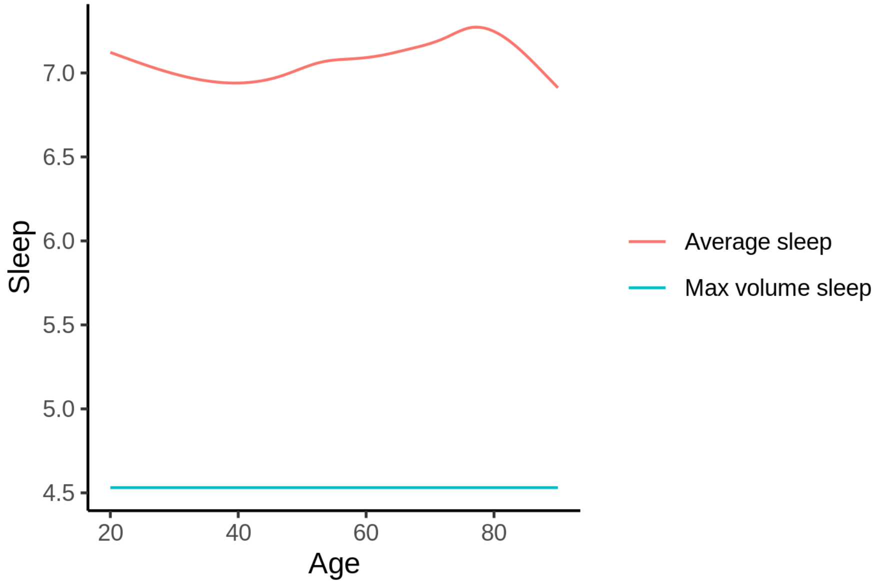

The next plot shows the difference between average sleep and sleep associated with maximum volume. The shaded region is a 95 % confidence interval.

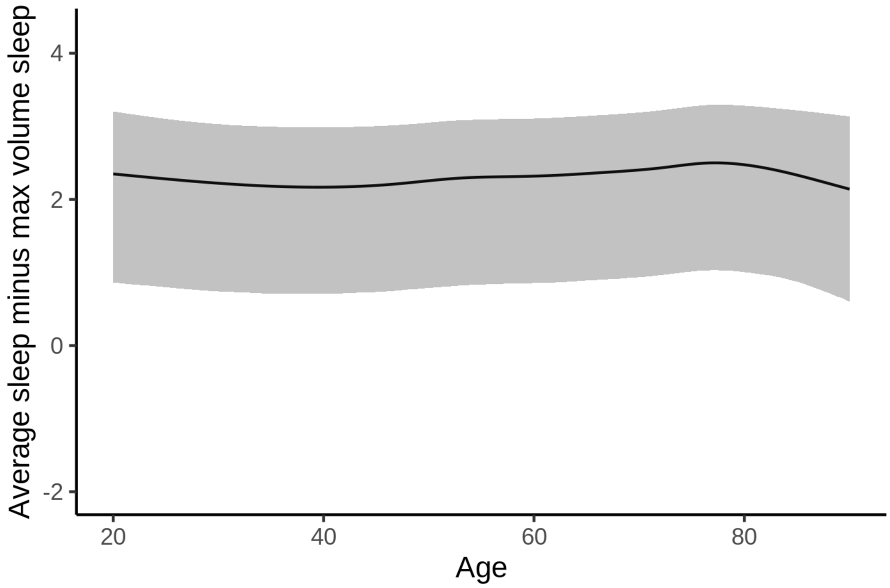

The next plot shows the probability that the sleep duration associated with maximum volume is longer than the average sleep duration, as a function of age. Probability below .05 can be interpreted as evidence that the sleep associated with maximum volume is shorter than the mean sleep, and probability above .95 can be interpreted the opposite way.

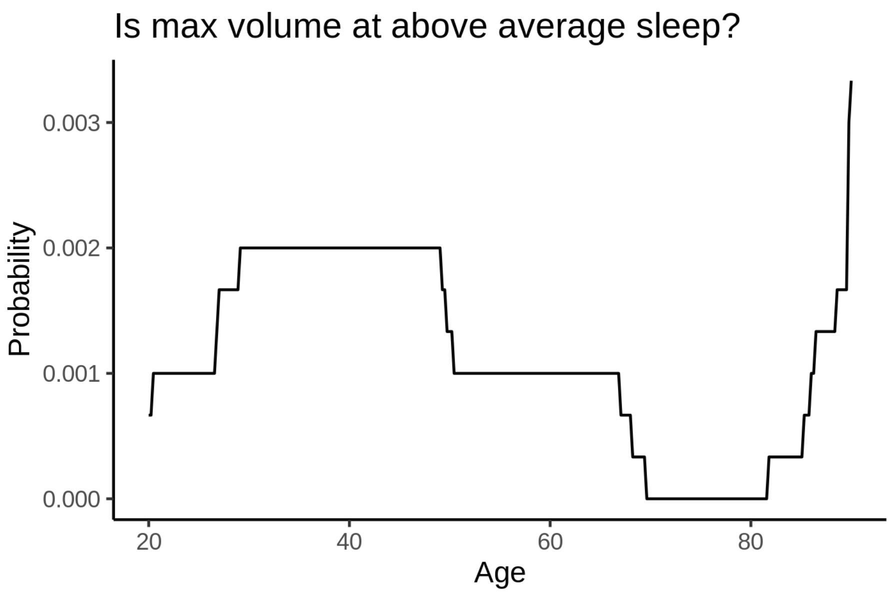

Controling for covariates

Below is the output for a model in which we only include data with income and education.

```
##
## Family: gaussian
## Link function: identity
##
## Formula:
## value ~ sex + site + s(age_z, k = 10, bs = "cr") + s(sleep_z,
##     k = 5, bs = "cr") + icv
## <environment: 0x558ad757b790>
##
## Parametric coefficients:
##                Estimate Std. Error t value Pr(>|t|)
## (Intercept)    30837.74     454.76  67.811  < 2e-16 ***
## sexmale         -409.89      50.22  -8.163 3.40e-16 ***
## siteousAvanto -3033.27     573.54  -5.289 1.24e-07 ***
## siteousPrisma -3764.78     562.38  -6.694 2.20e-11 ***
## siteousSkyra    547.36     491.22   1.114  0.26517
## siteUKB         462.96     454.05   1.020  0.30791
## siteUOXF      -1362.15     511.55  -2.663  0.00775 **
## icv            2150.91      25.83  83.270  < 2e-16 ***
## ---
## Signif. codes:  0 ‘***’ 0.001 ‘**’ 0.01 ‘*’ 0.05 ‘.’ 0.1 ‘ ’ 1
##
## Approximate significance of smooth terms:
##              edf Ref.df       F  p-value
## s(age_z)   6.400  6.400 423.807  < 2e-16 ***
## s(sleep_z) 2.694  2.694   6.072 0.000664 ***
## ---
## Signif. codes:  0 ‘***’ 0.001 ‘**’ 0.01 ‘*’ 0.05 ‘.’ 0.1 ‘ ’ 1
##
```

```
## R-sq.(adj) =  0.303
## lmer.REML = 5.9336e+05  Scale est. = 1.6984e+06  n = 31179
```

Below is the output for a model in which we control for the main effects of income and education.

```
##
## Family: gaussian
## Link function: identity
##
## Formula:
## value ~ sex + site + s(age_z, k = 10, bs = "cr") + s(sleep_z,
##     k = 5, bs = "cr") + icv + income_scaled + education_scaled
## <environment: 0x558ad757b790>
##
## Parametric coefficients:
##                  Estimate Std. Error t value Pr(>|t|)
## (Intercept)      30635.97     457.64  66.943  < 2e-16 ***
## sexmale           -407.59      50.35  -8.095 5.94e-16 ***
## siteousAvanto    -3044.18     573.56  -5.308 1.12e-07 ***
## siteousPrisma    -3790.45     562.29  -6.741 1.60e-11 ***
## siteousSkyra       535.21     491.23   1.090  0.27593
## siteUKB            457.44     454.17   1.007  0.31385
## siteUOXF         -1301.09     511.70  -2.543  0.01100 *
## icv               2138.26      26.04  82.101  < 2e-16 ***
## income_scaled      103.74      59.91   1.732  0.08333 .
## education_scaled   201.71      70.66   2.855  0.00431 **
## ---
## Signif. codes:  0 '***' 0.001 '**' 0.01 '*' 0.05 '.' 0.1 ' ' 1
##
## Approximate significance of smooth terms:
##              edf Ref.df       F  p-value
## s(age_z)   6.355  6.355 380.965  < 2e-16 ***
## s(sleep_z) 2.449  2.449   6.306 0.000783 ***
## ---
## Signif. codes:  0 '***' 0.001 '**' 0.01 '*' 0.05 '.' 0.1 ' ' 1
##
## R-sq.(adj) =  0.304
## lmer.REML = 5.9333e+05  Scale est. = 1.6988e+06  n = 31179
```

We also included interaction effects between sleep duration and education and income, in another model. The output is shown below, and the interaction terms are `income_scaled:sleep_z` and `education_scaled:sleep_z`.

```
##
## Family: gaussian
## Link function: identity
##
## Formula:
## value ~ sex + site + s(age_z, k = 10, bs = "cr") + s(sleep_z,
##     k = 5, bs = "cr") + icv + income_scaled + education_scaled +
##     income_scaled:sleep_z + education_scaled:sleep_z
## <environment: 0x558ad757b790>
##
## Parametric coefficients:
##                          Estimate Std. Error t value Pr(>|t|)
## (Intercept)              30644.43     457.66  66.959  < 2e-16 ***
```

```
## sexmale                   -410.16      50.39  -8.140 4.09e-16 ***
## siteousAvanto            -3052.49     573.55  -5.322 1.03e-07 ***
## siteousPrisma            -3805.89     562.33  -6.768 1.33e-11 ***
## siteousSkyra               527.13     491.23   1.073  0.28325
## siteUKB                    447.61     454.20   0.985  0.32440
## siteUOXF                 -1316.26     511.82  -2.572  0.01012 *
## icv                       2138.89      26.05  82.119  < 2e-16 ***
## income_scaled              105.96      59.94   1.768  0.07713 .
## education_scaled           199.38      70.67   2.821  0.00478 **
## income_scaled:sleep_z      -47.60      59.50  -0.800  0.42373
## education_scaled:sleep_z   131.74      68.57   1.921  0.05470 .
## ---
## Signif. codes:  0 '***' 0.001 '**' 0.01 '*' 0.05 '.' 0.1 ' ' 1
##
## Approximate significance of smooth terms:
##              edf Ref.df       F p-value
## s(age_z)   6.349  6.349 380.679 < 2e-16 ***
## s(sleep_z) 2.536  2.536   4.063 0.00947 **
## ---
## Signif. codes:  0 '***' 0.001 '**' 0.01 '*' 0.05 '.' 0.1 ' ' 1
##
## R-sq.(adj) =  0.304
## lmer.REML = 5.933e+05  Scale est. = 1.6988e+06  n = 31179
```

We did the same controling for BMI. Below is the model with no covariates but only keeping data with BMI.

```
##
## Family: gaussian
## Link function: identity
##
## Formula:
## value ~ sex + site + s(age_z, k = 10, bs = "cr") + s(sleep_z,
##     k = 5, bs = "cr") + icv
## <environment: 0x558ad923f0e8>
##
## Parametric coefficients:
##               Estimate Std. Error t value Pr(>|t|)
## (Intercept)   29123.37     376.69  77.313  < 2e-16 ***
## sexmale        -456.22      50.32  -9.066  < 2e-16 ***
## siteousPrisma -1686.88     480.76  -3.509 0.000451 ***
## siteousSkyra   2851.11     323.58   8.811  < 2e-16 ***
## siteUCAM       -429.40     391.11  -1.098 0.272260
## siteUKB        2190.09     379.10   5.777 7.67e-09 ***
## siteUmU        9969.42     429.41  23.216  < 2e-16 ***
## icv            2182.75      25.97  84.063  < 2e-16 ***
## ---
## Signif. codes:  0 '***' 0.001 '**' 0.01 '*' 0.05 '.' 0.1 ' ' 1
##
## Approximate significance of smooth terms:
##              edf Ref.df       F  p-value
## s(age_z)   7.237  7.237 367.765  < 2e-16 ***
## s(sleep_z) 2.037  2.037   6.878 0.000926 ***
## ---
## Signif. codes:  0 '***' 0.001 '**' 0.01 '*' 0.05 '.' 0.1 ' ' 1
##
```

```
## R-sq.(adj) =  0.309
## lmer.REML = 6.3794e+05  Scale est. = 1.7505e+06  n = 33428
```

Below is the model output with main effect.

```
##
## Family: gaussian
## Link function: identity
##
## Formula:
## value ~ sex + site + s(age_z, k = 10, bs = "cr") + s(sleep_z,
##     k = 5, bs = "cr") + icv + bmi
## <environment: 0x558ad923f0e8>
##
## Parametric coefficients:
##               Estimate Std. Error t value Pr(>|t|)
## (Intercept)   30342.89     396.07  76.610  < 2e-16 ***
## sexmale        -417.76      50.40  -8.290  < 2e-16 ***
## siteousPrisma -1629.25     480.36  -3.392 0.000695 ***
## siteousSkyra   2862.95     323.50   8.850  < 2e-16 ***
## siteUCAM       -398.20     390.82  -1.019 0.308265
## siteUKB        2231.40     378.85   5.890  3.9e-09 ***
## siteUmU       10025.16     429.06  23.365  < 2e-16 ***
## icv            2190.64      25.93  84.491  < 2e-16 ***
## bmi             -48.50       4.89  -9.918  < 2e-16 ***
## ---
## Signif. codes:  0 ‘***’ 0.001 ‘**’ 0.01 ‘*’ 0.05 ‘.’ 0.1 ‘ ’ 1
##
## Approximate significance of smooth terms:
##              edf Ref.df      F  p-value
## s(age_z)   7.242  7.242 371.46  < 2e-16 ***
## s(sleep_z) 1.402  1.402  12.25 0.000383 ***
## ---
## Signif. codes:  0 ‘***’ 0.001 ‘**’ 0.01 ‘*’ 0.05 ‘.’ 0.1 ‘ ’ 1
##
## R-sq.(adj) =  0.311
## lmer.REML = 6.3784e+05  Scale est. = 1.7502e+06  n = 33428
```

Next is the model with BMI-sleep interaction.

```
##
## Family: gaussian
## Link function: identity
##
## Formula:
## value ~ sex + site + s(age_z, k = 10, bs = "cr") + s(sleep_z,
##     k = 5, bs = "cr") + icv + bmi + bmi:sleep_z
## <environment: 0x558ad923f0e8>
##
## Parametric coefficients:
##                Estimate Std. Error t value Pr(>|t|)
## (Intercept)   30337.329    396.221  76.567  < 2e-16 ***
## sexmale        -417.583     50.397  -8.286  < 2e-16 ***
## siteousPrisma -1628.690    480.366  -3.391 0.000698 ***
## siteousSkyra   2864.400    323.509   8.854  < 2e-16 ***
## siteUCAM       -396.501    390.853  -1.014 0.310375
```

```
## siteUKB        2235.141    378.880   5.899 3.68e-09 ***
## siteUmU       10026.875    429.087  23.368  < 2e-16 ***
## icv            2191.119     25.921  84.529  < 2e-16 ***
## bmi             -48.488      4.890  -9.916  < 2e-16 ***
## bmi:sleep_z       2.963      4.628   0.640 0.522039
## ---
## Signif. codes:  0 '***' 0.001 '**' 0.01 '*' 0.05 '.' 0.1 ' ' 1
##
## Approximate significance of smooth terms:
##              edf Ref.df      F p-value
## s(age_z)   7.245  7.245 372.06  <2e-16 ***
## s(sleep_z) 1.000  1.000   1.65   0.199
## ---
## Signif. codes:  0 '***' 0.001 '**' 0.01 '*' 0.05 '.' 0.1 ' ' 1
##
## R-sq.(adj) =  0.311
## lmer.REML = 6.3783e+05  Scale est. = 1.7502e+06  n = 33428
```

We did the same controling for depression. Below is the model with no covariates but only keeping data with depression.

```
##
## Family: gaussian
## Link function: identity
##
## Formula:
## value ~ sex + site + s(age_z, k = 10, bs = "cr") + s(sleep_z,
##     k = 5, bs = "cr") + icv
## <environment: 0x558ad80e6430>
##
## Parametric coefficients:
##                Estimate Std. Error t value Pr(>|t|)
## (Intercept)    29743.85     236.40 125.818  < 2e-16 ***
## sexmale         -439.89      50.51  -8.710  < 2e-16 ***
## siteousAvanto   -269.42     585.92  -0.460 0.645650
## siteousPrisma -1610.45     467.68  -3.443 0.000575 ***
## siteousSkyra    2972.65     362.61   8.198 2.53e-16 ***
## siteUCAM       -1350.52     325.14  -4.154 3.28e-05 ***
## siteUKB         1540.93     233.71   6.593 4.37e-11 ***
## siteUmU         9346.59     318.37  29.358  < 2e-16 ***
## icv             2159.80      26.02  83.006  < 2e-16 ***
## ---
## Signif. codes:  0 '***' 0.001 '**' 0.01 '*' 0.05 '.' 0.1 ' ' 1
##
## Approximate significance of smooth terms:
##              edf Ref.df       F p-value
## s(age_z)   7.046  7.046 368.904 < 2e-16 ***
## s(sleep_z) 2.068  2.068   6.073 0.00193 **
## ---
## Signif. codes:  0 '***' 0.001 '**' 0.01 '*' 0.05 '.' 0.1 ' ' 1
##
## R-sq.(adj) =  0.319
## lmer.REML = 6.3601e+05  Scale est. = 1.702e+06  n = 33340
```

Below is the model output with main effect.

```
##
## Family: gaussian
## Link function: identity
##
## Formula:
## value ~ sex + site + s(age_z, k = 10, bs = "cr") + s(sleep_z,
##     k = 5, bs = "cr") + icv + depression
## <environment: 0x558ad80e6430>
##
## Parametric coefficients:
##               Estimate Std. Error t value Pr(>|t|)
## (Intercept)   29839.51     237.39 125.699  < 2e-16 ***
## sexmale        -449.62      50.54  -8.896  < 2e-16 ***
## siteousAvanto  -221.06     585.99  -0.377 0.705991
## siteousPrisma -1566.76     467.68  -3.350 0.000809 ***
## siteousSkyra   3028.34     362.76   8.348  < 2e-16 ***
## siteUCAM      -1329.01     325.10  -4.088 4.36e-05 ***
## siteUKB        1501.22     233.83   6.420 1.38e-10 ***
## siteUmU        9634.00     325.15  29.630  < 2e-16 ***
## icv            2161.30      26.01  83.097  < 2e-16 ***
## depression     -777.89     179.77  -4.327 1.51e-05 ***
## ---
## Signif. codes:  0 ‘***’ 0.001 ‘**’ 0.01 ‘*’ 0.05 ‘.’ 0.1 ‘ ’ 1
##
## Approximate significance of smooth terms:
##              edf Ref.df       F p-value
## s(age_z)   7.062  7.062 370.757 < 2e-16 ***
## s(sleep_z) 1.661  1.661   9.159 0.00191 **
## ---
## Signif. codes:  0 ‘***’ 0.001 ‘**’ 0.01 ‘*’ 0.05 ‘.’ 0.1 ‘ ’ 1
##
## R-sq.(adj) =  0.319
## lmer.REML = 6.3598e+05  Scale est. = 1.7023e+06  n = 33340
```

Next is the model with depression-sleep interaction.

```
##
## Family: gaussian
## Link function: identity
##
## Formula:
## value ~ sex + site + s(age_z, k = 10, bs = "cr") + s(sleep_z,
##     k = 5, bs = "cr") + icv + depression + depression:sleep_z
## <environment: 0x558ad80e6430>
##
## Parametric coefficients:
##                    Estimate Std. Error t value Pr(>|t|)
## (Intercept)        29839.14     237.38 125.700  < 2e-16 ***
## sexmale             -448.42      50.55  -8.871  < 2e-16 ***
## siteousAvanto       -211.74     586.03  -0.361 0.717866
## siteousPrisma      -1557.13     467.71  -3.329 0.000872 ***
## siteousSkyra        3037.66     362.80   8.373  < 2e-16 ***
## siteUCAM           -1320.15     325.15  -4.060 4.92e-05 ***
## siteUKB             1501.48     233.82   6.422 1.37e-10 ***
## siteUmU             9576.41     327.81  29.214  < 2e-16 ***
```

```
## icv                  2161.38      26.01  83.111  < 2e-16 ***
## depression           -767.19     179.79  -4.267 1.99e-05 ***
## depression:sleep_z    183.62     131.12   1.400 0.161400
## ---
## Signif. codes:  0 '***' 0.001 '**' 0.01 '*' 0.05 '.' 0.1 ' ' 1
##
## Approximate significance of smooth terms:
##               edf Ref.df      F  p-value
## s(age_z)    7.057  7.057 371.40  < 2e-16 ***
## s(sleep_z) 1.406  1.406  11.47 0.000745 ***
## ---
## Signif. codes:  0 '***' 0.001 '**' 0.01 '*' 0.05 '.' 0.1 ' ' 1
##
## R-sq.(adj) =  0.319
## lmer.REML = 6.3597e+05  Scale est. = 1.7024e+06  n = 33340
```

The plot below shows the sleep-volume curve for the original model and for the model with main effects of SES.

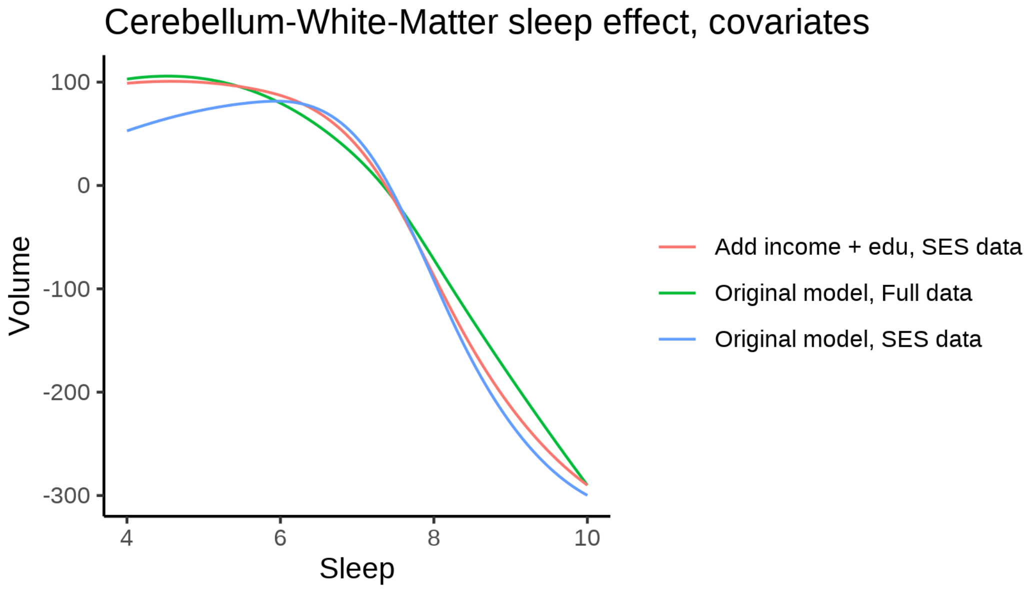

The plot below shows the sleep-volume curve for the original model and for the model with main effects of BMI.

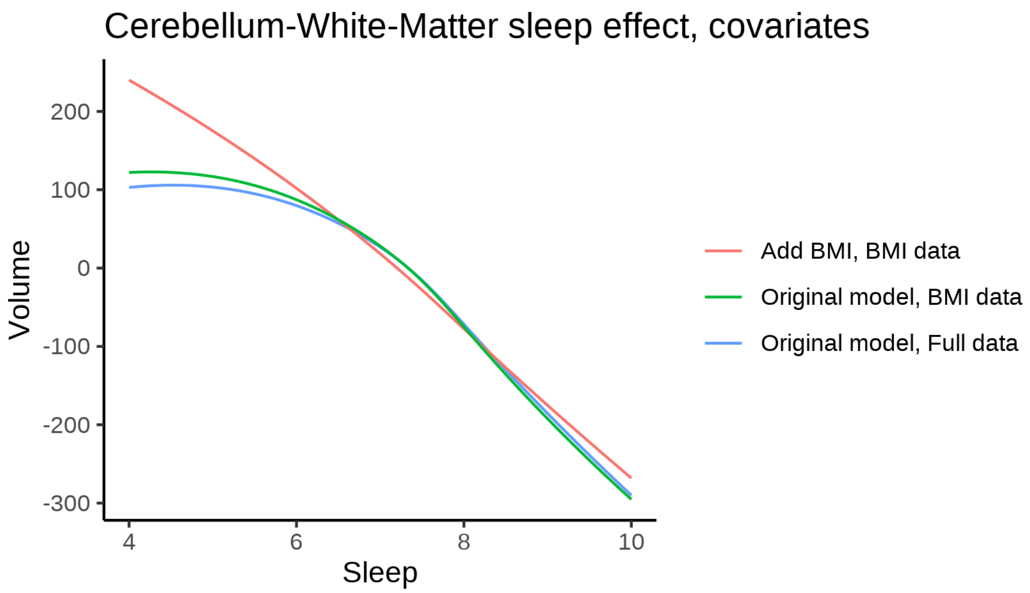

The plot below shows the sleep-volume curve for the original model and for the model with main effects of depression.

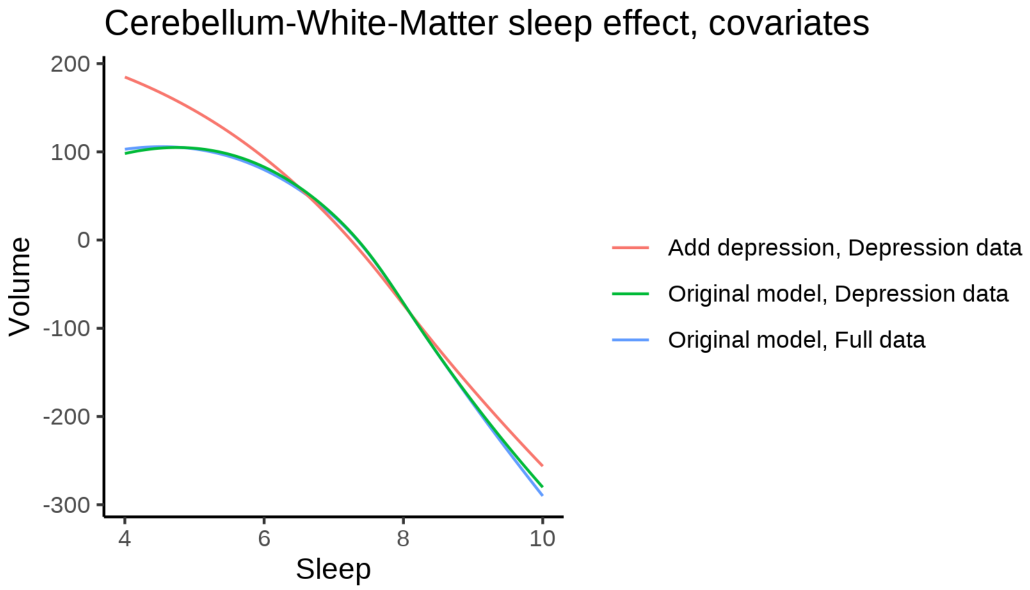

Models Not controling for ICV

Model outputs

Model without sleep term

```
##
## Family: gaussian
## Link function: identity
##
## Formula:
```

```
## value ~ sex + site + s(age_z, k = 10, bs = "cr")
## <environment: 0x5589589b2b80>
##
## Parametric coefficients:
##               Estimate Std. Error t value Pr(>|t|)
## (Intercept)    26860.8      220.1 122.053  < 2e-16 ***
## sexmale         2018.5       36.5  55.307  < 2e-16 ***
## siteMPIB        -850.3      276.3  -3.078  0.00209 **
## siteousAvanto   1703.3      228.8   7.444 9.96e-14 ***
## siteousPrisma   -583.6      385.8  -1.513  0.13036
## siteousSkyra    3754.9      212.6  17.665  < 2e-16 ***
## siteUB          -886.9      647.4  -1.370  0.17072
## siteUCAM         595.4      242.0   2.461  0.01387 *
## siteUKB         3341.1      226.2  14.773  < 2e-16 ***
## siteUmU         9931.9      312.6  31.771  < 2e-16 ***
## siteUOXF         314.8      266.7   1.181  0.23776
## ---
## Signif. codes:  0 '***' 0.001 '**' 0.01 '*' 0.05 '.' 0.1 ' ' 1
##
## Approximate significance of smooth terms:
##            edf Ref.df     F p-value
## s(age_z) 7.404  7.404 633.5  <2e-16 ***
## ---
## Signif. codes:  0 '***' 0.001 '**' 0.01 '*' 0.05 '.' 0.1 ' ' 1
##
## R-sq.(adj) =  0.177
## lmer.REML = 9.8688e+05  Scale est. = 1.6472e+06  n = 51255
```

Model with only main effects of age and sleep

```
##
## Family: gaussian
## Link function: identity
##
## Formula:
## value ~ sex + site + s(age_z, k = 10, bs = "cr") + s(sleep_z,
##     k = 5, bs = "cr")
## <environment: 0x5589589b2b80>
##
## Parametric coefficients:
##               Estimate Std. Error t value Pr(>|t|)
## (Intercept)   26874.23     220.27 122.006  < 2e-16 ***
## sexmale        2012.49      36.51  55.126  < 2e-16 ***
## siteMPIB       -833.11     276.33  -3.015  0.00257 **
## siteousAvanto  1688.64     228.86   7.379 1.62e-13 ***
## siteousPrisma  -594.44     385.68  -1.541  0.12325
## siteousSkyra   3738.34     212.59  17.585  < 2e-16 ***
## siteUB         -860.70     647.20  -1.330  0.18357
## siteUCAM        605.36     241.94   2.502  0.01235 *
## siteUKB        3329.61     226.36  14.709  < 2e-16 ***
## siteUmU        9961.54     313.23  31.802  < 2e-16 ***
## siteUOXF        311.34     266.62   1.168  0.24291
## ---
## Signif. codes:  0 '***' 0.001 '**' 0.01 '*' 0.05 '.' 0.1 ' ' 1
##
```

```
## Approximate significance of smooth terms:
##               edf Ref.df       F  p-value
## s(age_z)   7.398  7.398 621.325  < 2e-16 ***
## s(sleep_z) 3.453  3.453   9.445 1.86e-06 ***
## ---
## Signif. codes:  0 ‘***’ 0.001 ‘**’ 0.01 ‘*’ 0.05 ‘.’ 0.1 ‘ ’ 1
##
## R-sq.(adj) =  0.178
## lmer.REML = 9.8686e+05  Scale est. = 1.647e+06  n = 51255
```

Model with full interaction between age and sleep

```
##
## Family: gaussian
## Link function: identity
##
## Formula:
## value ~ sex + site + t2(age_z, sleep_z, k = c(10, 4), bs = "cr")
## <environment: 0x5589589b2b80>
##
## Parametric coefficients:
##                Estimate Std. Error t value Pr(>|t|)
## (Intercept)    26852.13     221.56 121.195  < 2e-16 ***
## sexmale         2016.99      36.59  55.120  < 2e-16 ***
## siteMPIB        -842.90     278.05  -3.031  0.00243 **
## siteousAvanto  1711.18     230.22   7.433 1.08e-13 ***
## siteousPrisma  -582.80     386.63  -1.507  0.13172
## siteousSkyra   3764.14     214.13  17.579  < 2e-16 ***
## siteUB         -884.97     647.91  -1.366  0.17198
## siteUCAM        604.70     243.55   2.483  0.01304 *
## siteUKB        3350.78     227.66  14.718  < 2e-16 ***
## siteUmU        9958.59     314.54  31.661  < 2e-16 ***
## siteUOXF        320.78     267.99   1.197  0.23132
## ---
## Signif. codes:  0 ‘***’ 0.001 ‘**’ 0.01 ‘*’ 0.05 ‘.’ 0.1 ‘ ’ 1
##
## Approximate significance of smooth terms:
##                     edf Ref.df     F p-value
## t2(age_z,sleep_z) 13.56  13.56 5.723  <2e-16 ***
## ---
## Signif. codes:  0 ‘***’ 0.001 ‘**’ 0.01 ‘*’ 0.05 ‘.’ 0.1 ‘ ’ 1
##
## R-sq.(adj) =  0.178
## lmer.REML = 9.8688e+05  Scale est. = 1.6473e+06  n = 51255
```

Model comparison

`mod_no_sleep` refers to model without sleep term, `mod_no_interaction` refers to model with only main effect of sleep, and `mod_full` refers to model with a full interaction between age and sleep. This is a nested model comparison, and the p-value at a given line refers to comparing the model at the line to the model on the line above. Hence, significance implies that the more complicated model is supported on statistical grounds.

To be even more specific, the p-value on the second row tests whether there is an association between sleep and volume. The p-value on the third row tests whether this association depends on age.

```
## Data: NULL
## Models:
## ml$mod_no_sleep$mer: NULL
## ml$mod_no_interaction$mer: NULL
## ml$mod_full$mer: NULL
##                            npar    AIC    BIC  logLik deviance  Chisq Df Pr(>Chisq)
## ml$mod_no_sleep$mer         15 986914 987047 -493442   986884
## ml$mod_no_interaction$mer   17 986892 987043 -493429   986858 25.985  2  2.277e-06 ***
## ml$mod_full$mer             19 986915 987083 -493439   986877  0.000  2          1
## ---
## Signif. codes:  0 ‘***’ 0.001 ‘**’ 0.01 ‘*’ 0.05 ‘.’ 0.1 ‘ ’ 1
```

We chose the model based on the likelihood ratio test with 5 % significance level, which was `mod_no_interaction`.

Lifespan brain trajectory

The trajectory shown is from the chosen model `mod_no_interaction_no_icv`.

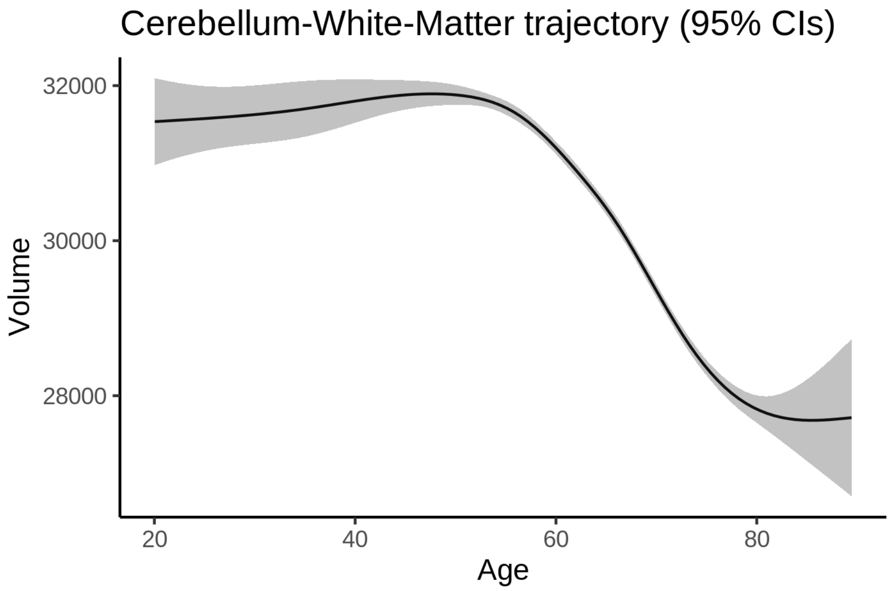

Effect of sleep

The chosen model only included the main effect of sleep, and hence the effect does not vary with age. The black dot shows the average sleep duration across all ages in the sample.

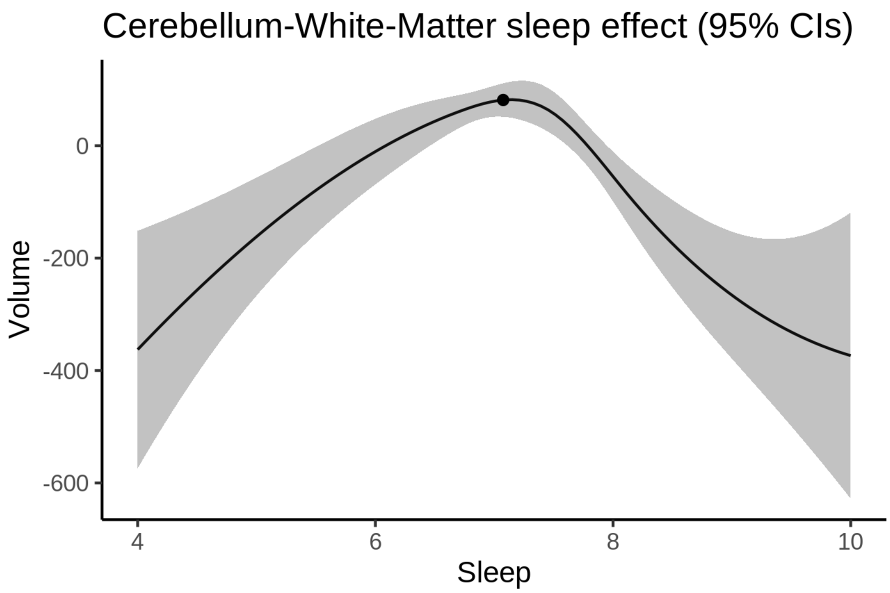

We also show the full interaction model for completeness, although it was not selected.

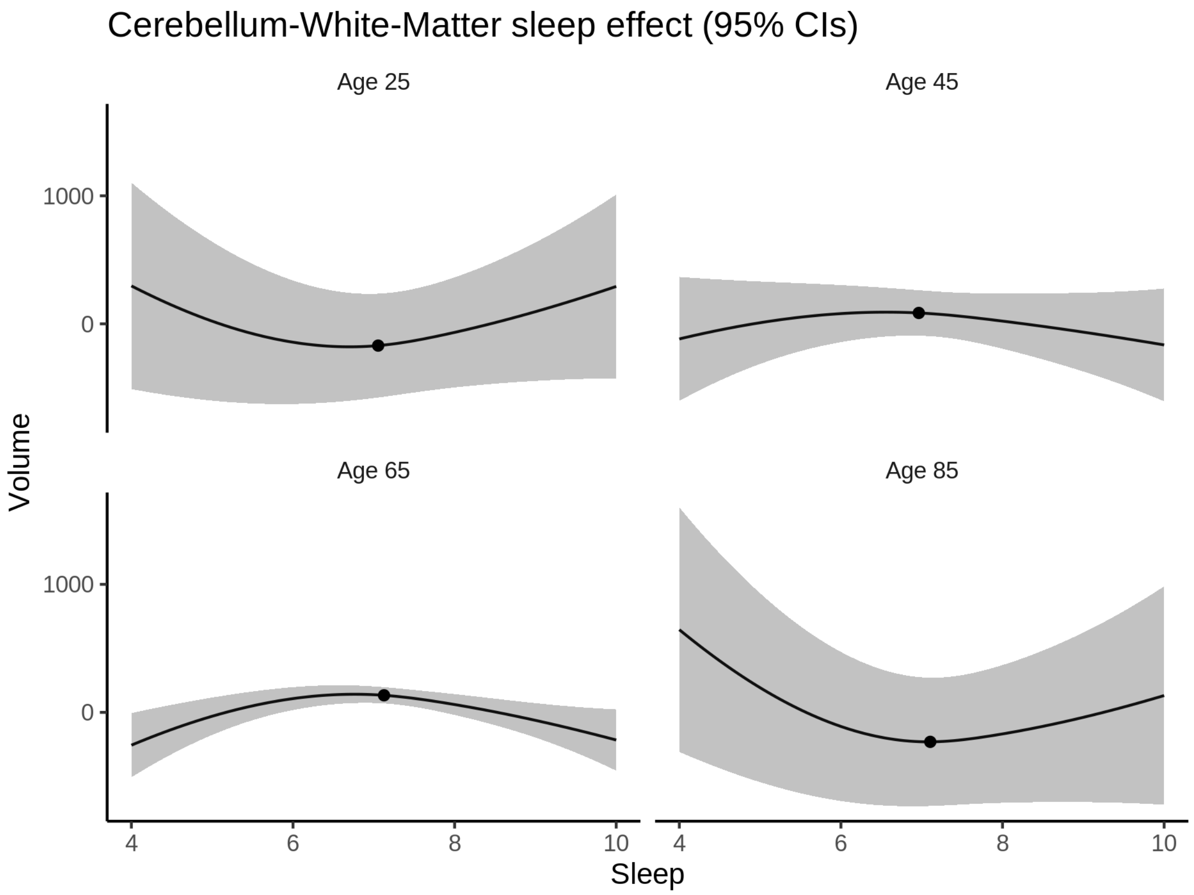

Deviation from sleep associated with maximal volume

Model with only main effect of sleep was chosen, so we show it for all ages at once. Maximum volume is attained at 7.1 hours of sleep. The percentage values in the plot are calculated as follows: The maximum at 100 % refers to a person at an arbitrary age with a sleep duration associated with maximum volume. For a female, this volume is 30469 and for a male it is 32482. The other percentage values show how large the expected volume is for someone with other sleep durations. For example, 99 % implies a 1 % reduction.

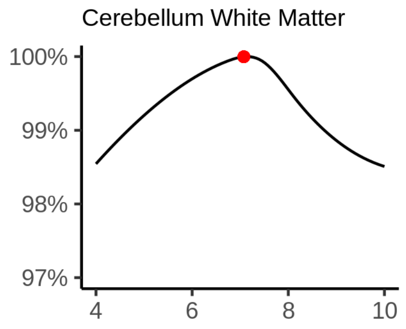

Comparison of mean sleep and sleep associated with maximum volume

A 95 % confidence interval for the sleep associated with maximum volume is [6.79, 7.33].

The plot below compares average sleep to the sleep associated with maximum volume.

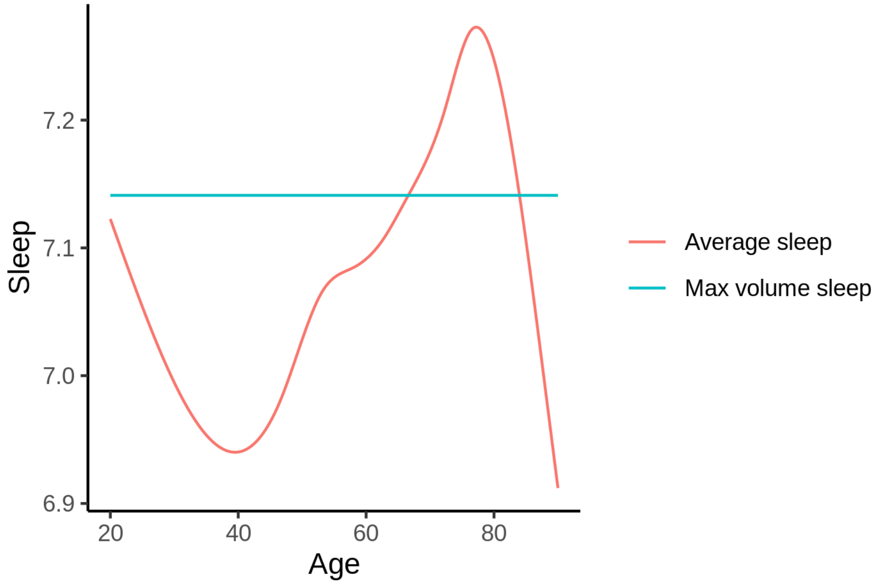

The next plot shows the difference between average sleep and sleep associated with maximum volume. The shaded region is a 95 % confidence interval.

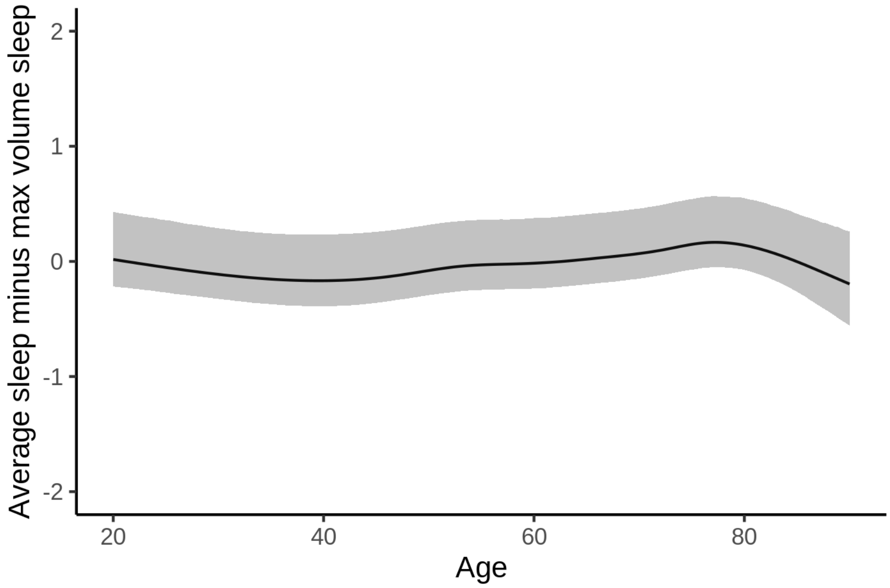

The next plot shows the probability that the sleep duration associated with maximum volume is longer than the average sleep duration, as a function of age. Probability below .05 can be interpreted as evidence that the sleep associated with maximum volume is shorter than the mean sleep, and probability above .95 can be interpreted the opposite way.

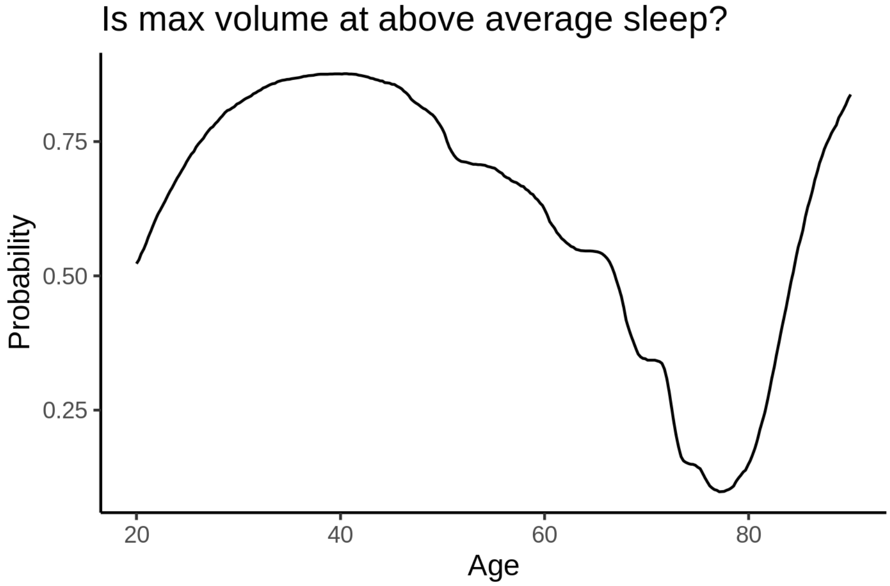

CerebralWhiteMatterVol

Descriptive statistics

Study	Observations	Unique IDs	Mean age	Age range
HCP	974	974	28.8	22 - 37
MPIB	677	391	63.1	24 - 83

Study	Observations	Unique IDs	Mean age	Age range
UB	113	39	70.9	64 - 81
UCAM	881	631	55.1	20 - 88
UiO	1475	803	49.4	20 - 89
UKB	45979	43135	64.5	45 - 83
UmU	412	279	62.2	25 - 85
UOXF	769	769	69.8	60 - 85

Spaghetti plot

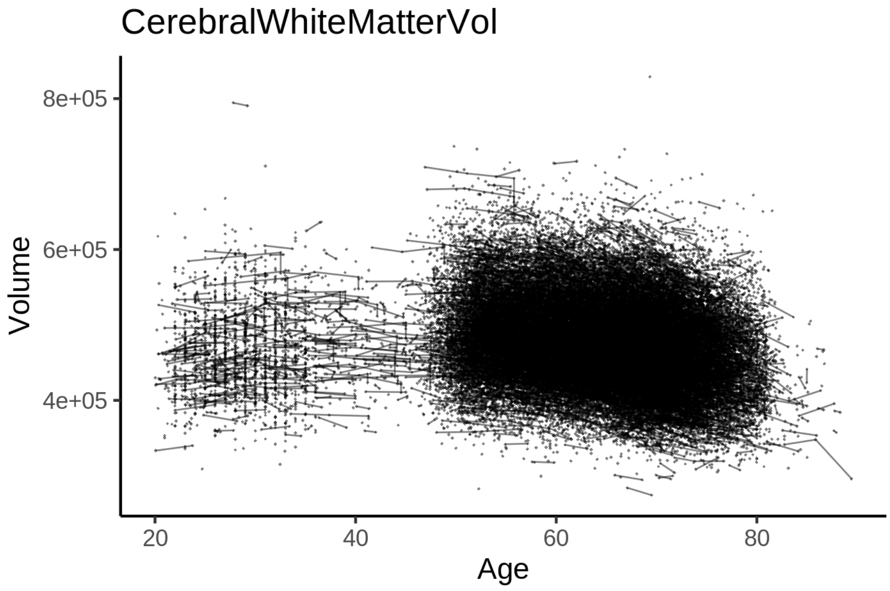

Models Controling for ICV

Model outputs

Model without sleep term

```
##
## Family: gaussian
## Link function: identity
##
## Formula:
## value ~ sex + site + icv + s(age_z, k = 10, bs = "cr")
## <environment: 0x558bed02fcd8>
##
## Parametric coefficients:
##                Estimate Std. Error t value Pr(>|t|)
## (Intercept)    449372.9     1636.1 274.668  < 2e-16 ***
## sexmale          8572.7      340.3  25.194  < 2e-16 ***
## siteMPIB        61369.1     2116.1  29.001  < 2e-16 ***
## siteousAvanto    2141.7     1661.4   1.289   0.1974
## siteousPrisma   -6242.1     2791.2  -2.236   0.0253 *
## siteousSkyra    -1612.7     1607.5  -1.003   0.3158
```

```
## siteUB        -11021.1     5024.8  -2.193   0.0283 *
## siteUCAM       -9216.5     1830.1  -5.036 4.77e-07 ***
## siteUKB        23312.2     1676.8  13.903  < 2e-16 ***
## siteUmU        13993.5     2371.0   5.902 3.62e-09 ***
## siteUOXF        1281.9     1997.0   0.642   0.5209
## icv            45620.5      170.7 267.228  < 2e-16 ***
## ---
## Signif. codes:  0 ‘***’ 0.001 ‘**’ 0.01 ‘*’ 0.05 ‘.’ 0.1 ‘ ’ 1
##
## Approximate significance of smooth terms:
##            edf Ref.df     F p-value
## s(age_z) 8.334  8.334 876.8  <2e-16 ***
## ---
## Signif. codes:  0 ‘***’ 0.001 ‘**’ 0.01 ‘*’ 0.05 ‘.’ 0.1 ‘ ’ 1
##
## R-sq.(adj) =  0.729
## lmer.REML = 1.193e+06  Scale est. = 4.489e+07  n = 51280
```

Model with only main effects of age and sleep

```
##
## Family: gaussian
## Link function: identity
##
## Formula:
## value ~ sex + site + icv + s(age_z, k = 10, bs = "cr") + s(sleep_z,
##     k = 5, bs = "cr")
## <environment: 0x558bed02fcd8>
##
## Parametric coefficients:
##               Estimate Std. Error t value Pr(>|t|)
## (Intercept)   449113.2     1637.4 274.291  < 2e-16 ***
## sexmale         8558.0      340.2  25.158  < 2e-16 ***
## siteMPIB       61723.5     2116.9  29.158  < 2e-16 ***
## siteousAvanto   2259.0     1661.6   1.360   0.1740
## siteousPrisma  -6152.9     2790.8  -2.205   0.0275 *
## siteousSkyra   -1500.4     1607.8  -0.933   0.3507
## siteUB        -10878.1     5023.2  -2.166   0.0303 *
## siteUCAM       -9056.0     1829.9  -4.949 7.49e-07 ***
## siteUKB        23590.6     1678.2  14.057  < 2e-16 ***
## siteUmU        14743.0     2375.8   6.205 5.50e-10 ***
## siteUOXF        1403.4     1996.8   0.703   0.4822
## icv            45631.6      170.8 267.117  < 2e-16 ***
## ---
## Signif. codes:  0 ‘***’ 0.001 ‘**’ 0.01 ‘*’ 0.05 ‘.’ 0.1 ‘ ’ 1
##
## Approximate significance of smooth terms:
##              edf Ref.df      F  p-value
## s(age_z)   8.333  8.333 863.69  < 2e-16 ***
## s(sleep_z) 2.292  2.292  11.25 4.49e-06 ***
## ---
## Signif. codes:  0 ‘***’ 0.001 ‘**’ 0.01 ‘*’ 0.05 ‘.’ 0.1 ‘ ’ 1
##
## R-sq.(adj) =  0.729
## lmer.REML = 1.193e+06  Scale est. = 4.4917e+07  n = 51280
```

Model with full interaction between age and sleep

```
##
## Family: gaussian
## Link function: identity
##
## Formula:
## value ~ sex + site + icv + t2(age_z, sleep_z, k = c(10, 4), bs = "cr")
## <environment: 0x558bed02fcd8>
##
## Parametric coefficients:
##                Estimate Std. Error t value Pr(>|t|)
## (Intercept)   448998.9     1648.5 272.365  < 2e-16 ***
## sexmale         8555.2      340.9  25.095  < 2e-16 ***
## siteMPIB       61844.3     2130.1  29.033  < 2e-16 ***
## siteousAvanto   2383.6     1673.1   1.425   0.1543
## siteousPrisma  -6065.5     2798.1  -2.168   0.0302 *
## siteousSkyra   -1354.1     1620.0  -0.836   0.4032
## siteUB        -10855.8     5028.2  -2.159   0.0309 *
## siteUCAM       -8972.6     1842.5  -4.870 1.12e-06 ***
## siteUKB        23708.2     1689.7  14.031  < 2e-16 ***
## siteUmU        14869.8     2386.1   6.232 4.64e-10 ***
## siteUOXF        1492.7     2008.4   0.743   0.4573
## icv            45636.1      170.8 267.169  < 2e-16 ***
## ---
## Signif. codes:  0 '***' 0.001 '**' 0.01 '*' 0.05 '.' 0.1 ' ' 1
##
## Approximate significance of smooth terms:
##                     edf Ref.df     F p-value
## t2(age_z,sleep_z) 15.53  15.53 16.69  <2e-16 ***
## ---
## Signif. codes:  0 '***' 0.001 '**' 0.01 '*' 0.05 '.' 0.1 ' ' 1
##
## R-sq.(adj) =  0.729
## lmer.REML = 1.193e+06  Scale est. = 4.4923e+07  n = 51280
```

Model comparison

`mod_no_sleep` refers to model without sleep term, `mod_no_interaction` refers to model with only main effect of sleep, and `mod_full` refers to model with a full interaction between age and sleep. This is a nested model comparison, and the p-value at a given line refers to comparing the model at the line to the model on the line above. Hence, significance implies that the more complicated model is supported on statistical grounds.

To be even more specific, the p-value on the second row tests whether there is an association between sleep and volume. The p-value on the third row tests whether this association depends on age.

```
## Data: NULL
## Models:
## ml$mod_no_sleep$mer: NULL
## ml$mod_no_interaction$mer: NULL
## ml$mod_full$mer: NULL
##                           npar     AIC     BIC  logLik deviance  Chisq Df Pr(>Chisq)
## ml$mod_no_sleep$mer         16 1193015 1193156 -596491  1192983
## ml$mod_no_interaction$mer   18 1192995 1193154 -596480  1192959 23.502  2  7.884e-06 ***
```

```
## ml$mod_full$mer              20 1193008 1193185 -596484  1192968  0.000  2          1
## ---
## Signif. codes:  0 ‘***’ 0.001 ‘**’ 0.01 ‘*’ 0.05 ‘.’ 0.1 ‘ ’ 1
```

We chose the model based on the likelihood ratio test with 5 % significance level, which was `mod_no_interaction`.

Lifespan brain trajectory

The trajectory shown is from the chosen model `mod_no_interaction`.

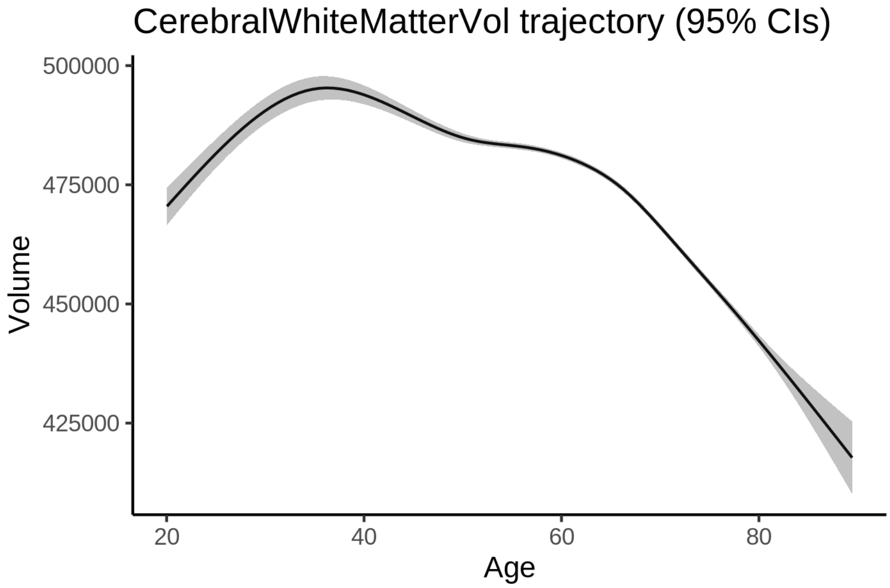

Effect of sleep

The chosen model only included the main effect of sleep, and hence the effect does not vary with age. The black dot shows the average sleep duration across all ages in the sample.

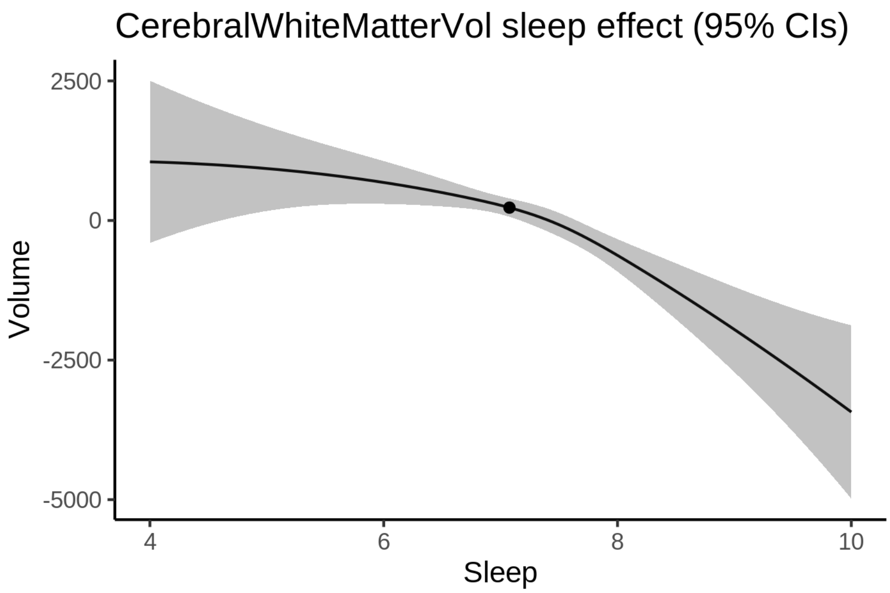

We also show the full interaction model for completeness, although it was not selected.

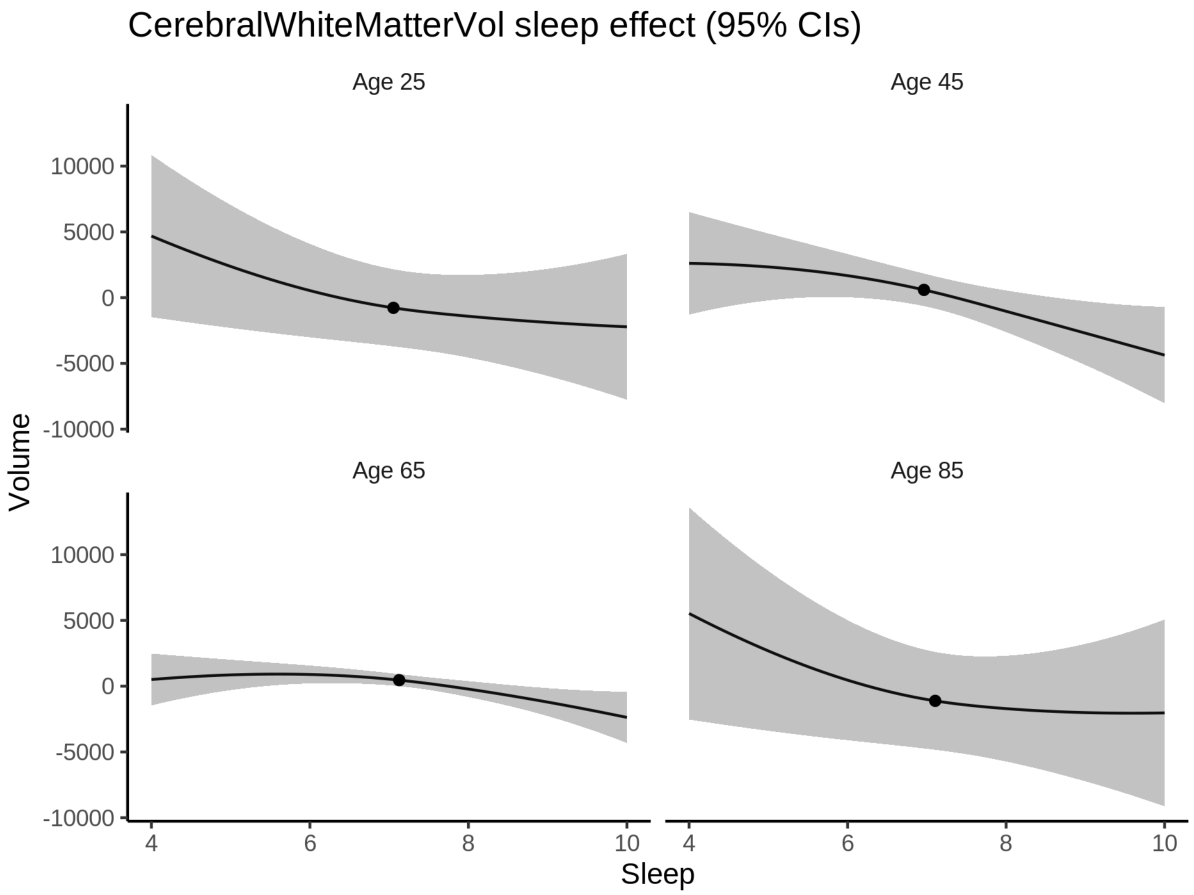

Deviation from sleep associated with maximal volume

Model with only main effect of sleep was chosen, so we show it for all ages at once. Maximum volume is attained at 4 hours of sleep. The percentage values in the plot are calculated as follows: The maximum at 100 % refers to a person at an arbitrary age with a sleep duration associated with maximum volume. For a female, this volume is 471997 and for a male it is 480555. The other percentage values show how large the expected volume is for someone with other sleep durations. For example, 99 % implies a 1 % reduction.

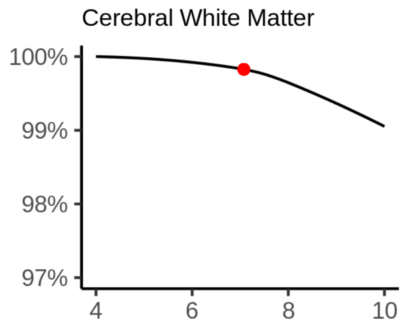

Comparison of mean sleep and sleep associated with maximum volume

A 95 % confidence interval for the sleep associated with maximum volume is [4, 6.06].

The plot below compares average sleep to the sleep associated with maximum volume.

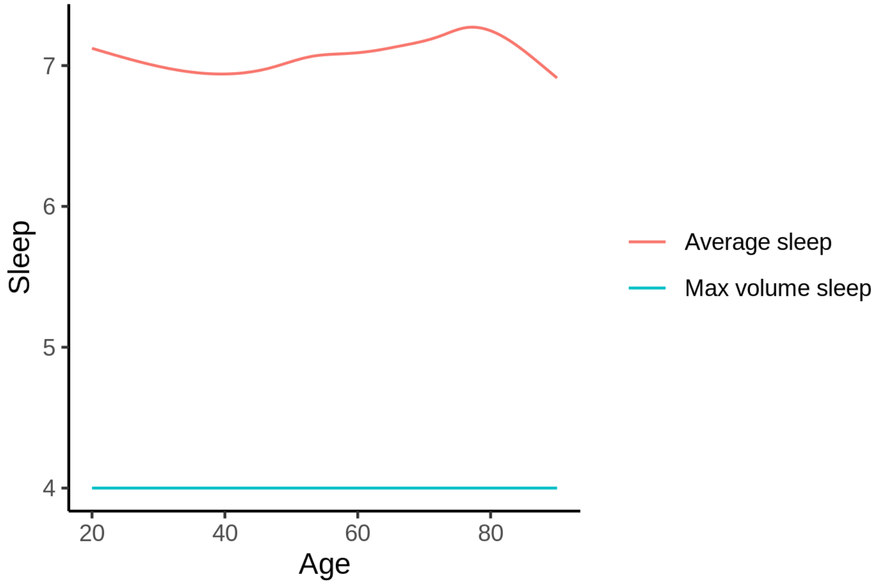

The next plot shows the difference between average sleep and sleep associated with maximum volume. The shaded region is a 95 % confidence interval.

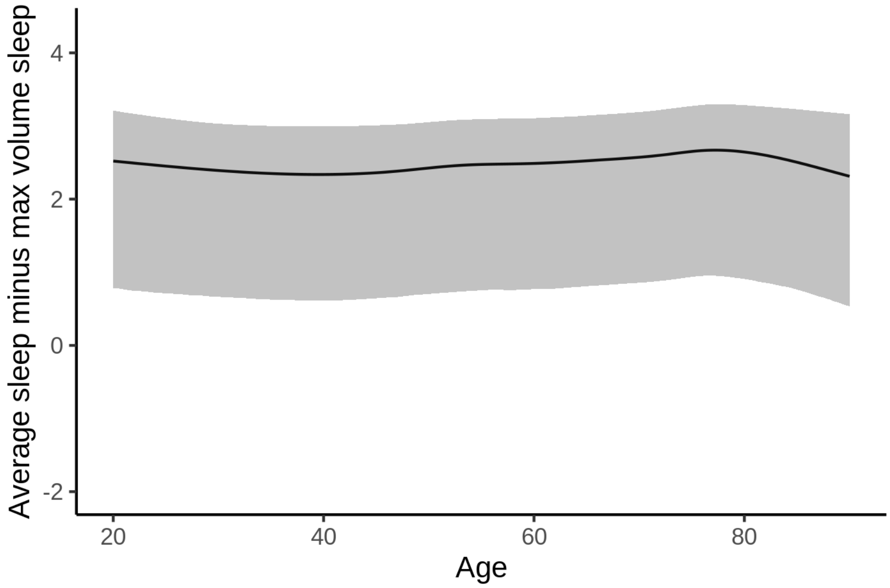

The next plot shows the probability that the sleep duration associated with maximum volume is longer than the average sleep duration, as a function of age. Probability below .05 can be interpreted as evidence that the sleep associated with maximum volume is shorter than the mean sleep, and probability above .95 can be interpreted the opposite way.

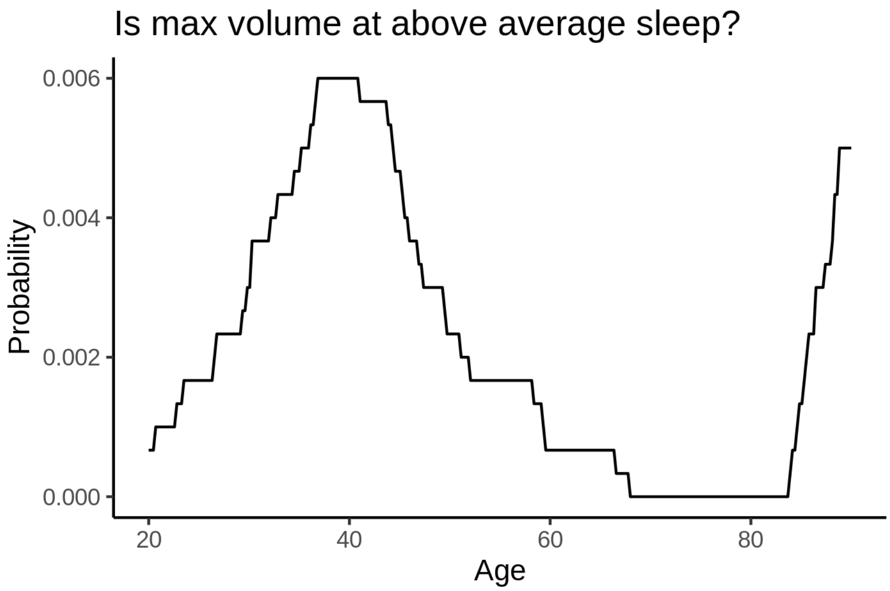

Controling for covariates

Below is the output for a model in which we only include data with income and education.

```
##
## Family: gaussian
## Link function: identity
##
## Formula:
```

```
## value ~ sex + site + s(age_z, k = 10, bs = "cr") + s(sleep_z,
##     k = 5, bs = "cr") + icv
## <environment: 0x558a47f00bb0>
##
## Parametric coefficients:
##              Estimate Std. Error t value Pr(>|t|)
## (Intercept)   521949.5     3900.2  133.83   <2e-16 ***
## sexmale         7792.6      425.4   18.32   <2e-16 ***
## siteousAvanto -77988.9     4526.8  -17.23   <2e-16 ***
## siteousPrisma -74182.5     4731.4  -15.68   <2e-16 ***
## siteousSkyra  -75120.2     4198.2  -17.89   <2e-16 ***
## siteUKB       -48770.3     3893.6  -12.53   <2e-16 ***
## siteUOXF      -59368.6     4376.1  -13.57   <2e-16 ***
## icv            47017.2      218.0  215.66   <2e-16 ***
## ---
## Signif. codes:  0 ‘***’ 0.001 ‘**’ 0.01 ‘*’ 0.05 ‘.’ 0.1 ‘ ’ 1
##
## Approximate significance of smooth terms:
##             edf Ref.df       F p-value
## s(age_z)   7.754  7.754 475.792  <2e-16 ***
## s(sleep_z) 1.478  1.478   2.587  0.0555 .
## ---
## Signif. codes:  0 ‘***’ 0.001 ‘**’ 0.01 ‘*’ 0.05 ‘.’ 0.1 ‘ ’ 1
##
## R-sq.(adj) =  0.735
## lmer.REML = 7.2471e+05  Scale est. = 4.5032e+07  n = 31196
```

Below is the output for a model in which we control for the main effects of income and education.

```
##
## Family: gaussian
## Link function: identity
##
## Formula:
## value ~ sex + site + s(age_z, k = 10, bs = "cr") + s(sleep_z,
##     k = 5, bs = "cr") + icv + income_scaled + education_scaled
## <environment: 0x558a47f00bb0>
##
## Parametric coefficients:
##                 Estimate Std. Error t value Pr(>|t|)
## (Intercept)      522573.0     3924.4 133.160   <2e-16 ***
## sexmale            7727.1      426.6  18.114   <2e-16 ***
## siteousAvanto    -77788.1     4527.8 -17.180   <2e-16 ***
## siteousPrisma    -74081.3     4731.3 -15.658   <2e-16 ***
## siteousSkyra     -74918.5     4199.1 -17.841   <2e-16 ***
## siteUKB          -48498.7     3895.8 -12.449   <2e-16 ***
## siteUOXF         -59441.5     4378.2 -13.577   <2e-16 ***
## icv               47052.2      219.9 214.002   <2e-16 ***
## income_scaled       464.4      506.5   0.917   0.3592
## education_scaled  -1386.4      599.5  -2.313   0.0207 *
## ---
## Signif. codes:  0 ‘***’ 0.001 ‘**’ 0.01 ‘*’ 0.05 ‘.’ 0.1 ‘ ’ 1
##
## Approximate significance of smooth terms:
##             edf Ref.df     F p-value
```

```
## s(age_z)   7.761  7.761 437.3  <2e-16 ***
## s(sleep_z) 1.563  1.563   2.3  0.0667 .
## ---
## Signif. codes:  0 '***' 0.001 '**' 0.01 '*' 0.05 '.' 0.1 ' ' 1
##
## R-sq.(adj) =  0.735
## lmer.REML = 7.2468e+05  Scale est. = 4.5048e+07  n = 31196
```

We also included interaction effects between sleep duration and education and income, in another model. The output is shown below, and the interaction terms are `income_scaled:sleep_z` and `education_scaled:sleep_z`.

```
##
## Family: gaussian
## Link function: identity
##
## Formula:
## value ~ sex + site + s(age_z, k = 10, bs = "cr") + s(sleep_z,
##     k = 5, bs = "cr") + icv + income_scaled + education_scaled +
##     income_scaled:sleep_z + education_scaled:sleep_z
## <environment: 0x558a47f00bb0>
##
## Parametric coefficients:
##                          Estimate Std. Error t value Pr(>|t|)
## (Intercept)              522642.1     3924.6 133.170   <2e-16 ***
## sexmale                    7743.5      426.9  18.140   <2e-16 ***
## siteousAvanto            -77799.4     4527.8 -17.182   <2e-16 ***
## siteousPrisma            -74054.8     4731.7 -15.651   <2e-16 ***
## siteousSkyra             -74929.1     4199.2 -17.844   <2e-16 ***
## siteUKB                  -48579.2     3896.1 -12.469   <2e-16 ***
## siteUOXF                 -59591.4     4379.4 -13.607   <2e-16 ***
## icv                       47053.1      219.9 213.983   <2e-16 ***
## income_scaled               443.8      506.9   0.876   0.3812
## education_scaled          -1382.7      599.6  -2.306   0.0211 *
## income_scaled:sleep_z       702.0      502.7   1.396   0.1626
## education_scaled:sleep_z    269.9      581.0   0.464   0.6423
## ---
## Signif. codes:  0 '***' 0.001 '**' 0.01 '*' 0.05 '.' 0.1 ' ' 1
##
## Approximate significance of smooth terms:
##              edf Ref.df       F p-value
## s(age_z)   7.757  7.757 437.496  <2e-16 ***
## s(sleep_z) 1.649  1.649   1.772   0.108
## ---
## Signif. codes:  0 '***' 0.001 '**' 0.01 '*' 0.05 '.' 0.1 ' ' 1
##
## R-sq.(adj) =  0.735
## lmer.REML = 7.2465e+05  Scale est. = 4.5049e+07  n = 31196
```

We did the same controling for BMI. Below is the model with no covariates but only keeping data with BMI.

```
##
## Family: gaussian
## Link function: identity
##
## Formula:
```

```
## value ~ sex + site + s(age_z, k = 10, bs = "cr") + s(sleep_z,
##     k = 5, bs = "cr") + icv
## <environment: 0x558a47a58568>
##
## Parametric coefficients:
##               Estimate Std. Error t value Pr(>|t|)
## (Intercept)   452757.0     2365.7 191.384  < 2e-16 ***
## sexmale         7085.3      408.9  17.329  < 2e-16 ***
## siteousPrisma  -6114.8     3145.7  -1.944   0.0519 .
## siteousSkyra    -470.0     1675.2  -0.281   0.7791
## siteUCAM      -10345.7     2519.3  -4.107 4.02e-05 ***
## siteUKB        20720.8     2390.0   8.670  < 2e-16 ***
## siteUmU        13205.8     2908.5   4.540 5.63e-06 ***
## icv            47511.1      210.1 226.096  < 2e-16 ***
## ---
## Signif. codes:  0 '***' 0.001 '**' 0.01 '*' 0.05 '.' 0.1 ' ' 1
##
## Approximate significance of smooth terms:
##              edf Ref.df       F p-value
## s(age_z)   7.697  7.697 555.050  <2e-16 ***
## s(sleep_z) 1.002  1.002   5.707  0.0169 *
## ---
## Signif. codes:  0 '***' 0.001 '**' 0.01 '*' 0.05 '.' 0.1 ' ' 1
##
## R-sq.(adj) =  0.742
## lmer.REML = 7.7586e+05  Scale est. = 4.3344e+07  n = 33449
```

Below is the model output with main effect.

```
##
## Family: gaussian
## Link function: identity
##
## Formula:
## value ~ sex + site + s(age_z, k = 10, bs = "cr") + s(sleep_z,
##     k = 5, bs = "cr") + icv + bmi
## <environment: 0x558a47a58568>
##
## Parametric coefficients:
##                Estimate Std. Error t value Pr(>|t|)
## (Intercept)   469696.67    2564.10 183.182  < 2e-16 ***
## sexmale         7615.37     408.21  18.655  < 2e-16 ***
## siteousPrisma  -5479.31    3138.56  -1.746   0.0809 .
## siteousSkyra    -426.20    1675.55  -0.254   0.7992
## siteUCAM       -9935.84    2512.80  -3.954 7.70e-05 ***
## siteUKB        21227.30    2384.67   8.902  < 2e-16 ***
## siteUmU        13945.37    2899.77   4.809 1.52e-06 ***
## icv            47609.94     209.25 227.528  < 2e-16 ***
## bmi             -671.05      39.65 -16.926  < 2e-16 ***
## ---
## Signif. codes:  0 '***' 0.001 '**' 0.01 '*' 0.05 '.' 0.1 ' ' 1
##
## Approximate significance of smooth terms:
##              edf Ref.df      F p-value
## s(age_z)   7.695  7.695 563.32 < 2e-16 ***
```

```
## s(sleep_z) 1.000  1.000  10.01 0.00156 **
## ---
## Signif. codes:  0 '***' 0.001 '**' 0.01 '*' 0.05 '.' 0.1 ' ' 1
##
## R-sq.(adj) =  0.745
## lmer.REML = 7.7557e+05  Scale est. = 4.3385e+07  n = 33449
```

Next is the model with BMI-sleep interaction.

```
##
## Family: gaussian
## Link function: identity
##
## Formula:
## value ~ sex + site + s(age_z, k = 10, bs = "cr") + s(sleep_z,
##     k = 5, bs = "cr") + icv + bmi + bmi:sleep_z
## <environment: 0x558a47a58568>
##
## Parametric coefficients:
##                Estimate Std. Error t value Pr(>|t|)
## (Intercept)   469730.54    2565.47 183.097  < 2e-16 ***
## sexmale         7614.71     408.22  18.653  < 2e-16 ***
## siteousPrisma  -5479.25    3138.58  -1.746   0.0809 .
## siteousSkyra    -428.75    1675.57  -0.256   0.7980
## siteUCAM       -9948.24    2513.01  -3.959 7.55e-05 ***
## siteUKB        21215.35    2384.88   8.896  < 2e-16 ***
## siteUmU        13934.63    2899.92   4.805 1.55e-06 ***
## icv            47609.37     209.26 227.517  < 2e-16 ***
## bmi             -671.59      39.67 -16.930  < 2e-16 ***
## bmi:sleep_z      -15.28      37.54  -0.407   0.6839
## ---
## Signif. codes:  0 '***' 0.001 '**' 0.01 '*' 0.05 '.' 0.1 ' ' 1
##
## Approximate significance of smooth terms:
##              edf Ref.df       F p-value
## s(age_z)   7.695  7.695 563.147  <2e-16 ***
## s(sleep_z) 1.000  1.000   0.016   0.899
## ---
## Signif. codes:  0 '***' 0.001 '**' 0.01 '*' 0.05 '.' 0.1 ' ' 1
##
## R-sq.(adj) =  0.745
## lmer.REML = 7.7556e+05  Scale est. = 4.3385e+07  n = 33449
```

We did the same controling for depression. Below is the model with no covariates but only keeping data with depression.

```
##
## Family: gaussian
## Link function: identity
##
## Formula:
## value ~ sex + site + s(age_z, k = 10, bs = "cr") + s(sleep_z,
##     k = 5, bs = "cr") + icv
## <environment: 0x558a473ca2d8>
##
## Parametric coefficients:
```

```
##                Estimate Std. Error t value Pr(>|t|)
## (Intercept)   502308.9     1973.3  254.55   <2e-16 ***
## sexmale         7244.3      413.3   17.53   <2e-16 ***
## siteousAvanto -46978.7     3847.4  -12.21   <2e-16 ***
## siteousPrisma -51554.6     3717.9  -13.87   <2e-16 ***
## siteousSkyra  -49103.8     2954.4  -16.62   <2e-16 ***
## siteUCAM      -61968.8     2709.1  -22.88   <2e-16 ***
## siteUKB       -28983.2     1951.7  -14.85   <2e-16 ***
## siteUmU       -36652.0     2644.1  -13.86   <2e-16 ***
## icv            47241.6      212.1  222.70   <2e-16 ***
## ---
## Signif. codes:  0 '***' 0.001 '**' 0.01 '*' 0.05 '.' 0.1 ' ' 1
##
## Approximate significance of smooth terms:
##              edf Ref.df     F p-value
## s(age_z)   7.703  7.703 545.5  <2e-16 ***
## s(sleep_z) 1.408  1.408   5.2  0.0301 *
## ---
## Signif. codes:  0 '***' 0.001 '**' 0.01 '*' 0.05 '.' 0.1 ' ' 1
##
## R-sq.(adj) =  0.736
## lmer.REML = 7.7393e+05  Scale est. = 4.2768e+07  n = 33361
```

Below is the model output with main effect.

```
##
## Family: gaussian
## Link function: identity
##
## Formula:
## value ~ sex + site + s(age_z, k = 10, bs = "cr") + s(sleep_z,
##     k = 5, bs = "cr") + icv + depression
## <environment: 0x558a473ca2d8>
##
## Parametric coefficients:
##                Estimate Std. Error t value Pr(>|t|)
## (Intercept)   503027.8     1981.5 253.865  < 2e-16 ***
## sexmale         7170.9      413.6  17.338  < 2e-16 ***
## siteousAvanto -46607.7     3847.5 -12.114  < 2e-16 ***
## siteousPrisma -51218.5     3718.0 -13.776  < 2e-16 ***
## siteousSkyra  -48688.6     2955.6 -16.473  < 2e-16 ***
## siteUCAM      -61794.6     2708.9 -22.812  < 2e-16 ***
## siteUKB       -29282.9     1952.8 -14.995  < 2e-16 ***
## siteUmU       -34490.8     2699.0 -12.779  < 2e-16 ***
## icv            47251.0      212.0 222.837  < 2e-16 ***
## depression     -5826.8     1467.3  -3.971 7.17e-05 ***
## ---
## Signif. codes:  0 '***' 0.001 '**' 0.01 '*' 0.05 '.' 0.1 ' ' 1
##
## Approximate significance of smooth terms:
##            edf Ref.df       F p-value
## s(age_z)   7.7    7.7 547.274 < 2e-16 ***
## s(sleep_z) 1.0    1.0   7.296 0.00692 **
## ---
## Signif. codes:  0 '***' 0.001 '**' 0.01 '*' 0.05 '.' 0.1 ' ' 1
```

```
##
## R-sq.(adj) =  0.736
## lmer.REML = 7.7389e+05  Scale est. = 4.2739e+07  n = 33361
```

Next is the model with depression-sleep interaction.

```
##
## Family: gaussian
## Link function: identity
##
## Formula:
## value ~ sex + site + s(age_z, k = 10, bs = "cr") + s(sleep_z,
##     k = 5, bs = "cr") + icv + depression + depression:sleep_z
## <environment: 0x558a473ca2d8>
##
## Parametric coefficients:
##                    Estimate Std. Error t value Pr(>|t|)
## (Intercept)        503027.6     1981.5 253.860  < 2e-16 ***
## sexmale              7169.3      413.7  17.331  < 2e-16 ***
## siteousAvanto      -46618.2     3847.8 -12.115  < 2e-16 ***
## siteousPrisma      -51228.9     3718.4 -13.777  < 2e-16 ***
## siteousSkyra       -48698.7     2956.0 -16.474  < 2e-16 ***
## siteUCAM           -61805.8     2709.4 -22.811  < 2e-16 ***
## siteUKB            -29282.2     1952.9 -14.994  < 2e-16 ***
## siteUmU            -34415.5     2721.0 -12.648  < 2e-16 ***
## icv                 47251.2      212.0 222.832  < 2e-16 ***
## depression          -5844.7     1469.6  -3.977 6.99e-05 ***
## depression:sleep_z   -233.8     1072.4  -0.218    0.827
## ---
## Signif. codes:  0 ‘***’ 0.001 ‘**’ 0.01 ‘*’ 0.05 ‘.’ 0.1 ‘ ’ 1
##
## Approximate significance of smooth terms:
##              edf Ref.df       F p-value
## s(age_z)   7.699  7.699 547.349  <2e-16 ***
## s(sleep_z) 1.000  1.000   4.682  0.0305 *
## ---
## Signif. codes:  0 ‘***’ 0.001 ‘**’ 0.01 ‘*’ 0.05 ‘.’ 0.1 ‘ ’ 1
##
## R-sq.(adj) =  0.736
## lmer.REML = 7.7388e+05  Scale est. = 4.2737e+07  n = 33361
```

The plot below shows the sleep-volume curve for the original model and for the model with main effects of SES.

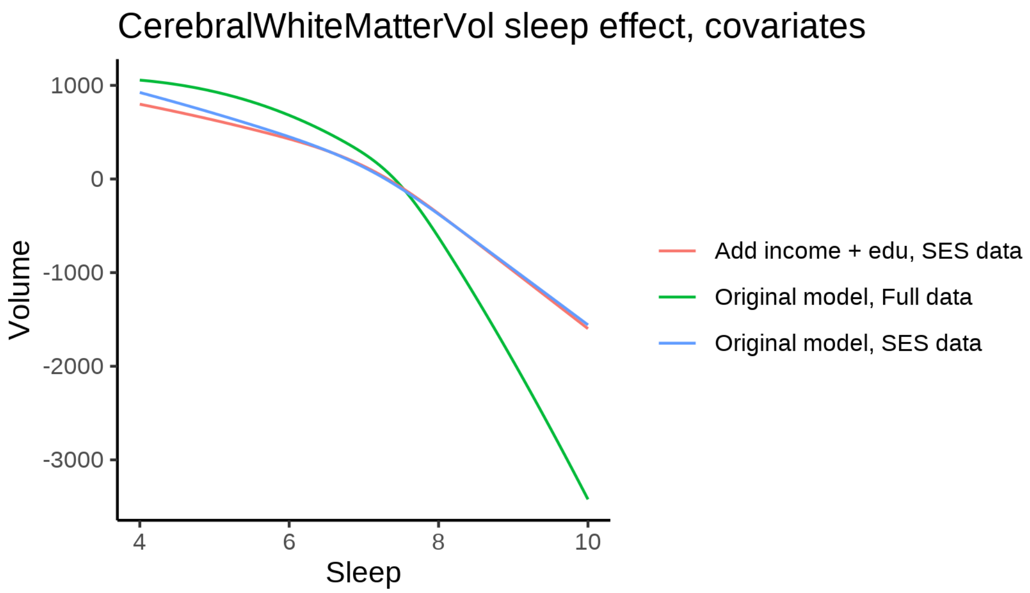

The plot below shows the sleep-volume curve for the original model and for the model with main effects of BMI.

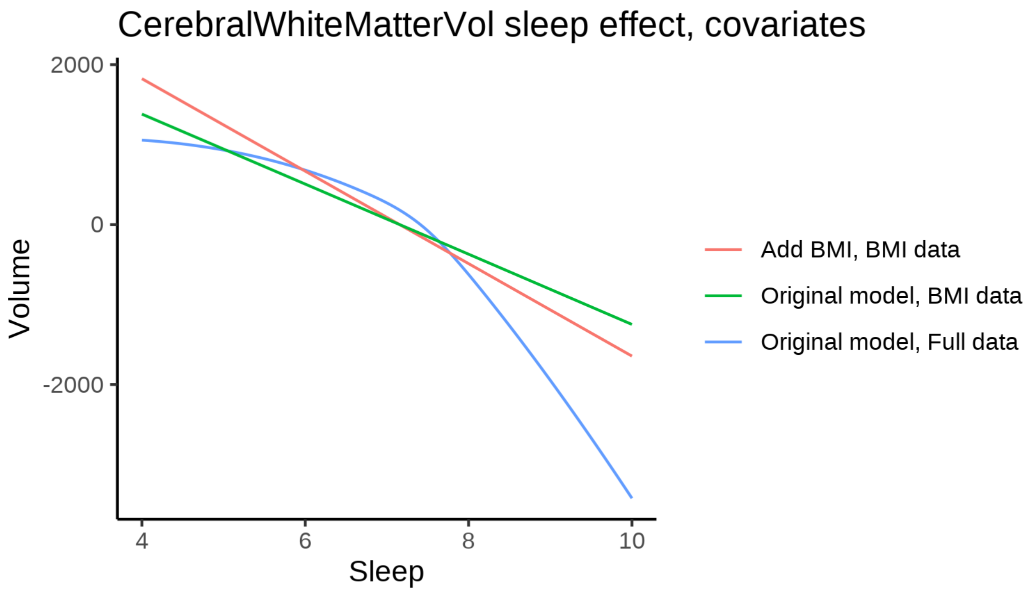

The plot below shows the sleep-volume curve for the original model and for the model with main effects of depression.

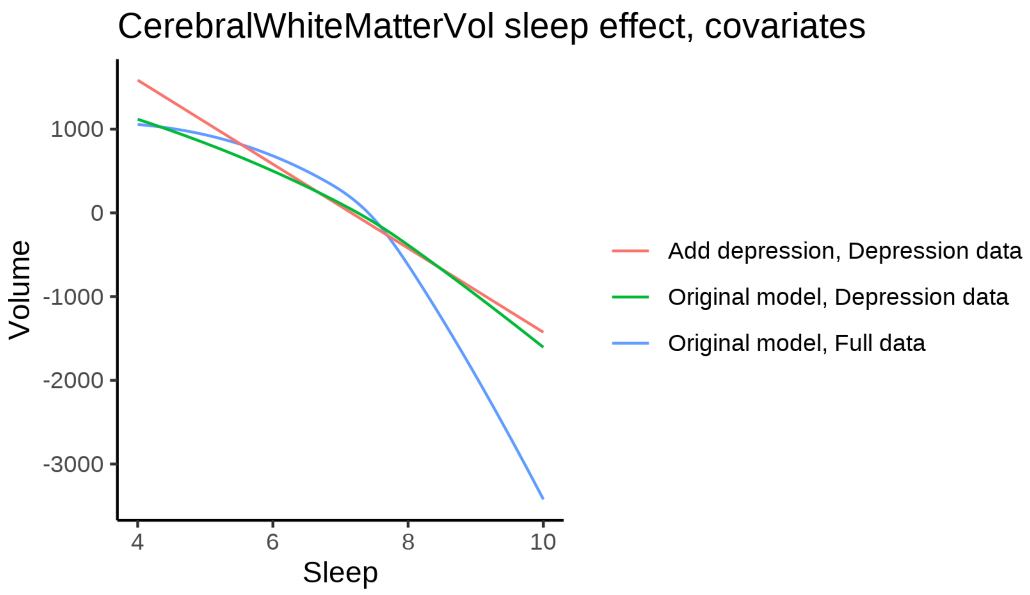

Models Not controling for ICV

Model outputs

Model without sleep term

```
##
## Family: gaussian
## Link function: identity
##
## Formula:
## value ~ sex + site + s(age_z, k = 10, bs = "cr")
## <environment: 0x558ad98ce1c8>
##
## Parametric coefficients:
##                Estimate Std. Error t value Pr(>|t|)
## (Intercept)    423160.1     2365.3 178.906  < 2e-16 ***
## sexmale         60429.5      443.9 136.125  < 2e-16 ***
## siteMPIB       -10704.8     3225.5  -3.319 0.000905 ***
## siteousAvanto   21812.8     2492.9   8.750  < 2e-16 ***
## siteousPrisma    6656.8     3920.4   1.698 0.089514 .
## siteousSkyra     1744.6     2472.6   0.706 0.480461
## siteUB         -23016.2     7969.8  -2.888 0.003880 **
## siteUCAM        -5725.5     2792.6  -2.050 0.040346 *
## siteUKB         25817.8     2421.5  10.662  < 2e-16 ***
## siteUmU         -3286.8     3640.1  -0.903 0.366563
## siteUOXF        -7869.6     2974.4  -2.646 0.008153 **
## ---
## Signif. codes:  0 ‘***’ 0.001 ‘**’ 0.01 ‘*’ 0.05 ‘.’ 0.1 ‘ ’ 1
##
## Approximate significance of smooth terms:
##             edf Ref.df     F p-value
## s(age_z) 7.606  7.606 730.5  <2e-16 ***
```

```
## ---
## Signif. codes:  0 ‘***’ 0.001 ‘**’ 0.01 ‘*’ 0.05 ‘.’ 0.1 ‘ ’ 1
##
## R-sq.(adj) =   0.32
## lmer.REML = 1.2364e+06  Scale est. = 4.3755e+07  n = 51280
```

Model with only main effects of age and sleep

```
##
## Family: gaussian
## Link function: identity
##
## Formula:
## value ~ sex + site + s(age_z, k = 10, bs = "cr") + s(sleep_z,
##     k = 5, bs = "cr")
## <environment: 0x558ad98ce1c8>
##
## Parametric coefficients:
##               Estimate Std. Error t value Pr(>|t|)
## (Intercept)   423866.3     2366.7 179.096  < 2e-16 ***
## sexmale        60315.2      443.9 135.887  < 2e-16 ***
## siteMPIB      -10839.0     3224.7  -3.361 0.000776 ***
## siteousAvanto  21311.0     2492.3   8.551  < 2e-16 ***
## siteousPrisma   6237.1     3919.0   1.591 0.111507
## siteousSkyra    1216.2     2471.9   0.492 0.622728
## siteUB        -22684.9     7963.9  -2.848 0.004395 **
## siteUCAM       -5819.5     2791.0  -2.085 0.037065 *
## siteUKB        25129.8     2422.9  10.372  < 2e-16 ***
## siteUmU        -3770.5     3645.4  -1.034 0.300988
## siteUOXF       -8172.4     2972.9  -2.749 0.005979 **
## ---
## Signif. codes:  0 ‘***’ 0.001 ‘**’ 0.01 ‘*’ 0.05 ‘.’ 0.1 ‘ ’ 1
##
## Approximate significance of smooth terms:
##              edf Ref.df      F p-value
## s(age_z)   7.598  7.598 721.10  <2e-16 ***
## s(sleep_z) 3.583  3.583  18.76  <2e-16 ***
## ---
## Signif. codes:  0 ‘***’ 0.001 ‘**’ 0.01 ‘*’ 0.05 ‘.’ 0.1 ‘ ’ 1
##
## R-sq.(adj) =  0.321
## lmer.REML = 1.2363e+06  Scale est. = 4.3776e+07  n = 51280
```

Model with full interaction between age and sleep

```
##
## Family: gaussian
## Link function: identity
##
## Formula:
## value ~ sex + site + t2(age_z, sleep_z, k = c(10, 4), bs = "cr")
## <environment: 0x558ad98ce1c8>
##
## Parametric coefficients:
##               Estimate Std. Error t value Pr(>|t|)
## (Intercept)   423694.0     2368.6 178.878  < 2e-16 ***
```

```
## sexmale        60429.3      444.7 135.879  < 2e-16 ***
## siteMPIB      -11289.5     3235.7  -3.489 0.000485 ***
## siteousAvanto  21481.8     2504.8   8.576  < 2e-16 ***
## siteousPrisma   6288.6     3930.5   1.600 0.109618
## siteousSkyra    1387.5     2484.8   0.558 0.576564
## siteUB        -23441.0     7968.0  -2.942 0.003264 **
## siteUCAM       -6116.2     2801.2  -2.183 0.029006 *
## siteUKB        25268.1     2424.2  10.423  < 2e-16 ***
## siteUmU        -3965.6     3651.1  -1.086 0.277429
## siteUOXF       -8372.2     2977.9  -2.811 0.004933 **
## ---
## Signif. codes:  0 ‘***’ 0.001 ‘**’ 0.01 ‘*’ 0.05 ‘.’ 0.1 ‘ ’ 1
##
## Approximate significance of smooth terms:
##                       edf Ref.df     F p-value
## t2(age_z,sleep_z) 12.65  12.65 13.19  <2e-16 ***
## ---
## Signif. codes:  0 ‘***’ 0.001 ‘**’ 0.01 ‘*’ 0.05 ‘.’ 0.1 ‘ ’ 1
##
## R-sq.(adj) =   0.32
## lmer.REML = 1.2363e+06  Scale est. = 4.3867e+07  n = 51280
```

Model comparison

`mod_no_sleep` refers to model without sleep term, `mod_no_interaction` refers to model with only main effect of sleep, and `mod_full` refers to model with a full interaction between age and sleep. This is a nested model comparison, and the p-value at a given line refers to comparing the model at the line to the model on the line above. Hence, significance implies that the more complicated model is supported on statistical grounds.

To be even more specific, the p-value on the second row tests whether there is an association between sleep and volume. The p-value on the third row tests whether this association depends on age.

```
## Data: NULL
## Models:
## ml$mod_no_sleep$mer: NULL
## ml$mod_no_interaction$mer: NULL
## ml$mod_full$mer: NULL
##                           npar     AIC     BIC  logLik deviance  Chisq Df Pr(>Chisq)
## ml$mod_no_sleep$mer         15 1236381 1236514 -618176  1236351
## ml$mod_no_interaction$mer   17 1236325 1236475 -618145  1236291 60.381  2  7.736e-14 ***
## ml$mod_full$mer             19 1236362 1236530 -618162  1236324  0.000  2          1
## ---
## Signif. codes:  0 ‘***’ 0.001 ‘**’ 0.01 ‘*’ 0.05 ‘.’ 0.1 ‘ ’ 1
```

We chose the model based on the likelihood ratio test with 5 % significance level, which was `mod_no_interaction`.

Lifespan brain trajectory

The trajectory shown is from the chosen model `mod_no_interaction_no_icv`.

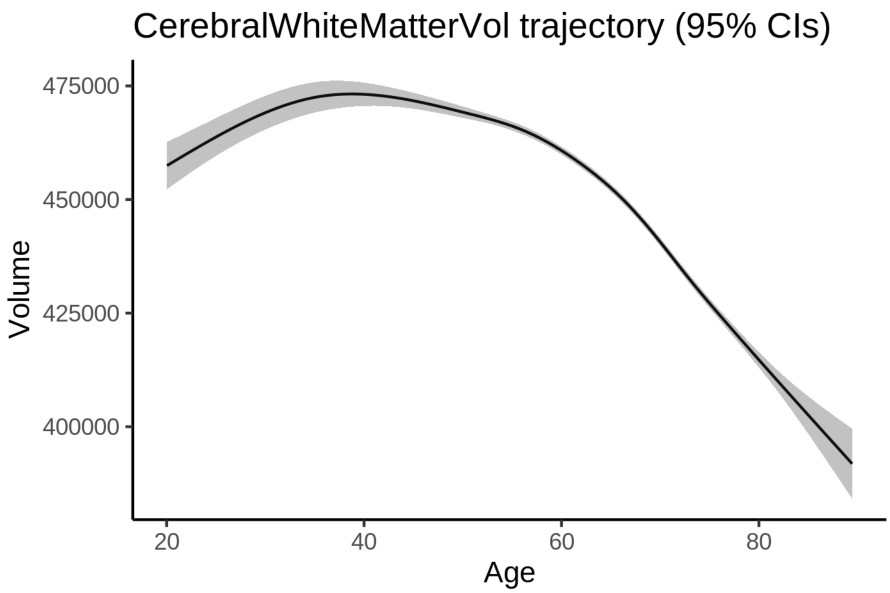

Effect of sleep

The chosen model only included the main effect of sleep, and hence the effect does not vary with age. The black dot shows the average sleep duration across all ages in the sample.

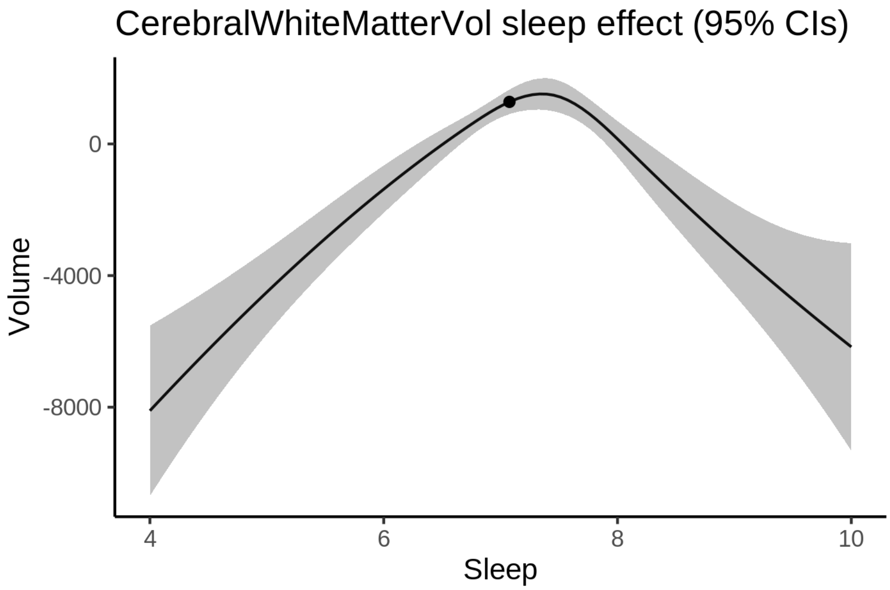

We also show the full interaction model for completeness, although it was not selected.

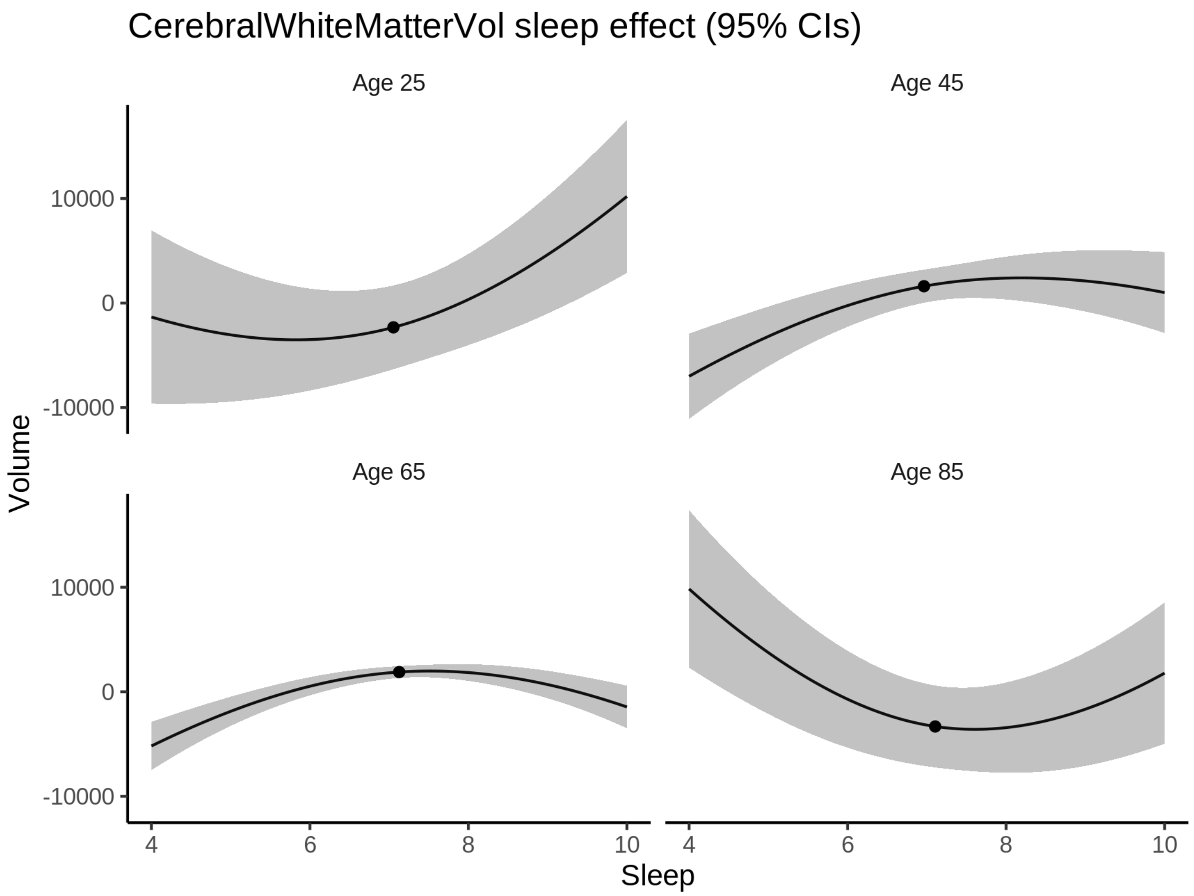

Deviation from sleep associated with maximal volume

Model with only main effect of sleep was chosen, so we show it for all ages at once. Maximum volume is attained at 7.4 hours of sleep. The percentage values in the plot are calculated as follows: The maximum at 100 % refers to a person at an arbitrary age with a sleep duration associated with maximum volume. For a female, this volume is 450342 and for a male it is 510657. The other percentage values show how large the expected volume is for someone with other sleep durations. For example, 99 % implies a 1 % reduction.

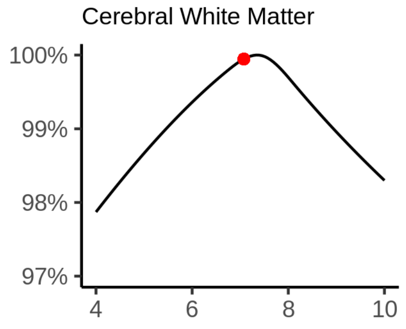

Comparison of mean sleep and sleep associated with maximum volume

A 95 % confidence interval for the sleep associated with maximum volume is [7.27, 7.45].

The plot below compares average sleep to the sleep associated with maximum volume.

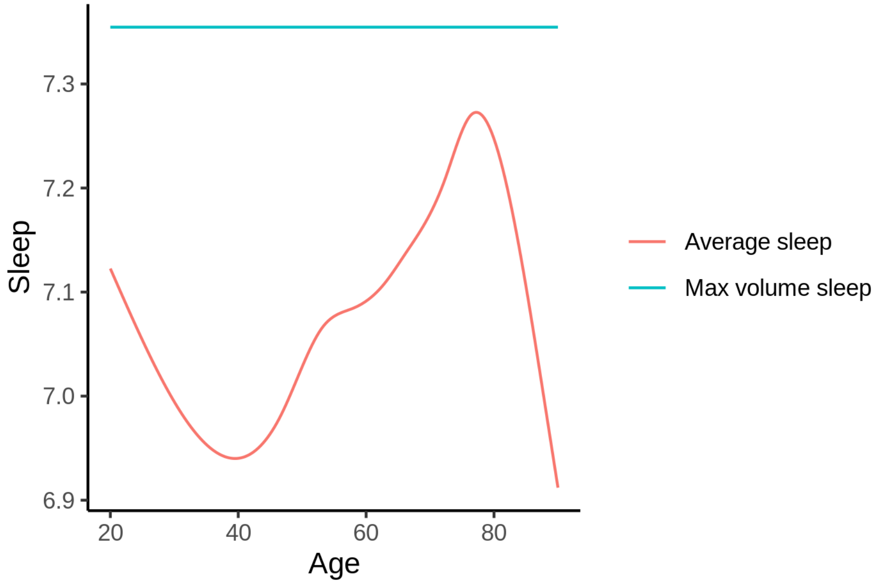

The next plot shows the difference between average sleep and sleep associated with maximum volume. The shaded region is a 95 % confidence interval.

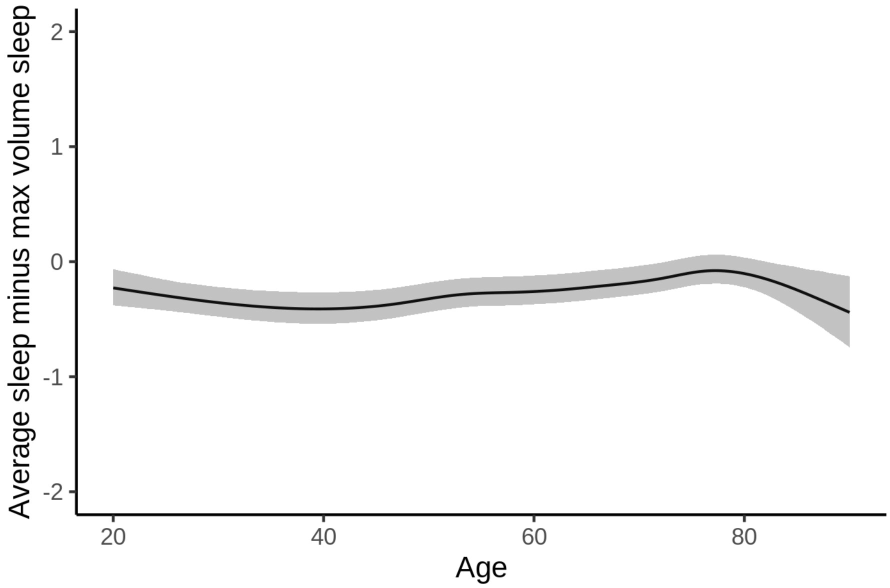

The next plot shows the probability that the sleep duration associated with maximum volume is longer than the average sleep duration, as a function of age. Probability below .05 can be interpreted as evidence that the sleep associated with maximum volume is shorter than the mean sleep, and probability above .95 can be interpreted the opposite way.

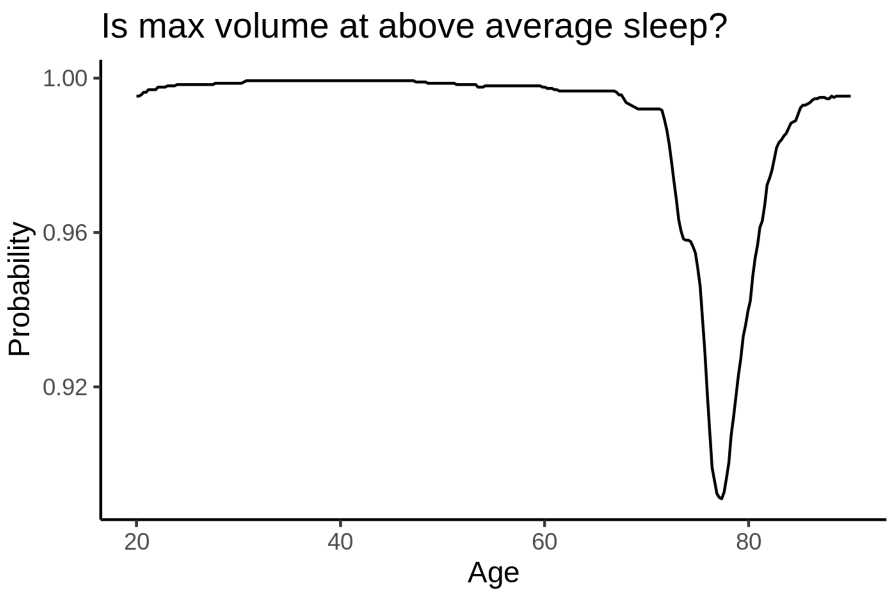

EstimatedTotalIntraCranialVol

Descriptive statistics

Study	Observations	Unique IDs	Mean age	Age range
HCP	974	974	28.8	22 - 37
MPIB	677	391	63.1	24 - 83
UB	113	39	70.9	64 - 81
UCAM	884	632	55.1	20 - 88
UiO	1475	803	49.4	20 - 89
UKB	45985	43138	64.5	45 - 83
UmU	412	279	62.2	25 - 85
UOXF	769	769	69.8	60 - 85

Spaghetti plot

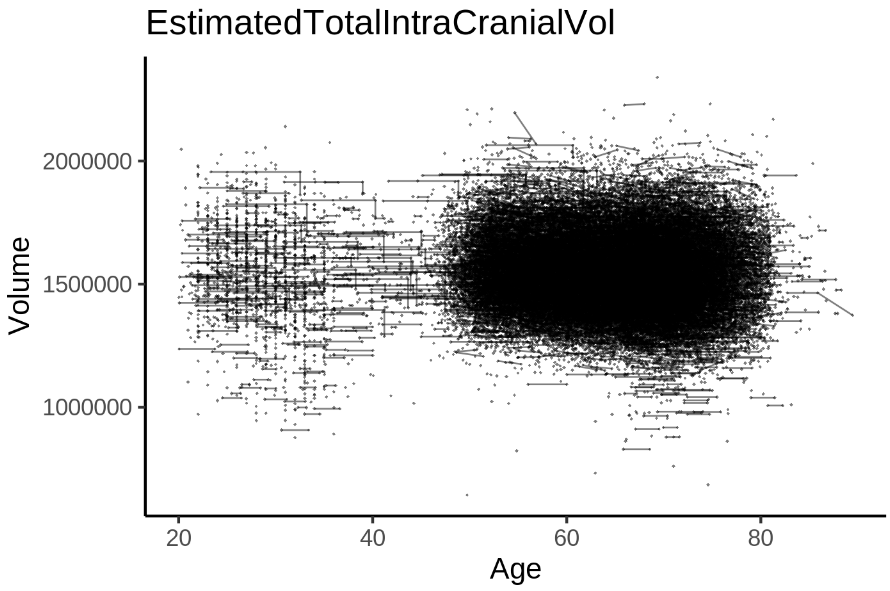

Models Controling for ICV

Model outputs

Model without sleep term

```
##
## Family: gaussian
## Link function: identity
##
## Formula:
## value ~ sex + site + s(age_z, k = 10, bs = "cr")
## <environment: 0x558ad6bc7f48>
##
## Parametric coefficients:
##                Estimate Std. Error t value Pr(>|t|)
## (Intercept)     1443795       5748 251.179  < 2e-16 ***
## sexmale          180533       1190 151.639  < 2e-16 ***
## siteMPIB        -245193       8372 -29.286  < 2e-16 ***
## siteousAvanto     66386       6467  10.265  < 2e-16 ***
## siteousPrisma     97945       9270  10.565  < 2e-16 ***
## siteousSkyra      10263       6458   1.589 0.112033
## siteUB           -33005      21265  -1.552 0.120654
## siteUCAM          19146       7206   2.657 0.007890 **
## siteUKB           19392       5863   3.307 0.000942 ***
## siteUmU          -51384       9450  -5.437 5.43e-08 ***
## siteUOXF         -22646       7479  -3.028 0.002462 **
## ---
## Signif. codes:  0 ‘***’ 0.001 ‘**’ 0.01 ‘*’ 0.05 ‘.’ 0.1 ‘ ’ 1
##
## Approximate significance of smooth terms:
```

```
##            edf Ref.df     F p-value
## s(age_z) 6.822  6.822 78.66  <2e-16 ***
## ---
## Signif. codes:  0 ‘***’ 0.001 ‘**’ 0.01 ‘*’ 0.05 ‘.’ 0.1 ‘ ’ 1
##
## R-sq.(adj) =   0.35
## lmer.REML = 1.3347e+06  Scale est. = 1.5253e+08  n = 51289
```

Model with only main effects of age and sleep

```
##
## Family: gaussian
## Link function: identity
##
## Formula:
## value ~ sex + site + s(age_z, k = 10, bs = "cr") + s(sleep_z,
##     k = 5, bs = "cr")
## <environment: 0x558ad6bc7f48>
##
## Parametric coefficients:
##                Estimate Std. Error t value Pr(>|t|)
## (Intercept)     1446700       5752 251.535  < 2e-16 ***
## sexmale          180150       1190 151.405  < 2e-16 ***
## siteMPIB        -246504       8367 -29.462  < 2e-16 ***
## siteousAvanto     64476       6463   9.976  < 2e-16 ***
## siteousPrisma     96173       9265  10.380  < 2e-16 ***
## siteousSkyra       8314       6454   1.288  0.19765
## siteUB           -32065      21241  -1.510  0.13116
## siteUCAM          18573       7200   2.580  0.00989 **
## siteUKB           16522       5866   2.816  0.00486 **
## siteUmU          -55183       9460  -5.833 5.46e-09 ***
## siteUOXF         -23711       7473  -3.173  0.00151 **
## ---
## Signif. codes:  0 ‘***’ 0.001 ‘**’ 0.01 ‘*’ 0.05 ‘.’ 0.1 ‘ ’ 1
##
## Approximate significance of smooth terms:
##              edf Ref.df     F p-value
## s(age_z)   6.850  6.850 77.75  <2e-16 ***
## s(sleep_z) 3.622  3.622 30.13  <2e-16 ***
## ---
## Signif. codes:  0 ‘***’ 0.001 ‘**’ 0.01 ‘*’ 0.05 ‘.’ 0.1 ‘ ’ 1
##
## R-sq.(adj) =  0.352
## lmer.REML = 1.3346e+06  Scale est. = 1.5253e+08  n = 51289
```

Model with full interaction between age and sleep

```
##
## Family: gaussian
## Link function: identity
##
## Formula:
## value ~ sex + site + t2(age_z, sleep_z, k = c(10, 4), bs = "cr")
## <environment: 0x558ad6bc7f48>
##
## Parametric coefficients:
```

```
##                Estimate Std. Error t value Pr(>|t|)
## (Intercept)    1447712       5782 250.401  < 2e-16 ***
## sexmale         180398       1192 151.370  < 2e-16 ***
## siteMPIB       -248528       8403 -29.577  < 2e-16 ***
## siteousAvanto    63187       6502   9.718  < 2e-16 ***
## siteousPrisma    94488       9293  10.167  < 2e-16 ***
## siteousSkyra      6842       6494   1.054 0.292060
## siteUB          -34496      21254  -1.623 0.104583
## siteUCAM         16386       7238   2.264 0.023578 *
## siteUKB          15423       5897   2.616 0.008909 **
## siteUmU         -56423       9489  -5.946 2.76e-09 ***
## siteUOXF        -25742       7505  -3.430 0.000604 ***
## ---
## Signif. codes:  0 ‘***’ 0.001 ‘**’ 0.01 ‘*’ 0.05 ‘.’ 0.1 ‘ ’ 1
##
## Approximate significance of smooth terms:
##                      edf Ref.df     F p-value
## t2(age_z,sleep_z) 13.63  13.63 5.027  <2e-16 ***
## ---
## Signif. codes:  0 ‘***’ 0.001 ‘**’ 0.01 ‘*’ 0.05 ‘.’ 0.1 ‘ ’ 1
##
## R-sq.(adj) =  0.352
## lmer.REML = 1.3346e+06  Scale est. = 1.5249e+08  n = 51289
```

Model comparison

`mod_no_sleep` refers to model without sleep term, `mod_no_interaction` refers to model with only main effect of sleep, and `mod_full` refers to model with a full interaction between age and sleep. This is a nested model comparison, and the p-value at a given line refers to comparing the model at the line to the model on the line above. Hence, significance implies that the more complicated model is supported on statistical grounds.

To be even more specific, the p-value on the second row tests whether there is an association between sleep and volume. The p-value on the third row tests whether this association depends on age.

```
## Data: NULL
## Models:
## ml$mod_no_sleep$mer: NULL
## ml$mod_no_interaction$mer: NULL
## ml$mod_full$mer: NULL
##                           npar     AIC     BIC  logLik deviance Chisq Df Pr(>Chisq)
## ml$mod_no_sleep$mer         15 1334752 1334885 -667361  1334722
## ml$mod_no_interaction$mer   17 1334654 1334804 -667310  1334620   102  2     <2e-16 ***
## ml$mod_full$mer             19 1334668 1334836 -667315  1334630     0  2          1
## ---
## Signif. codes:  0 ‘***’ 0.001 ‘**’ 0.01 ‘*’ 0.05 ‘.’ 0.1 ‘ ’ 1
```

We chose the model based on the likelihood ratio test with 5 % significance level, which was `mod_no_interaction`.

Lifespan brain trajectory

The trajectory shown is from the chosen model `mod_no_interaction`.

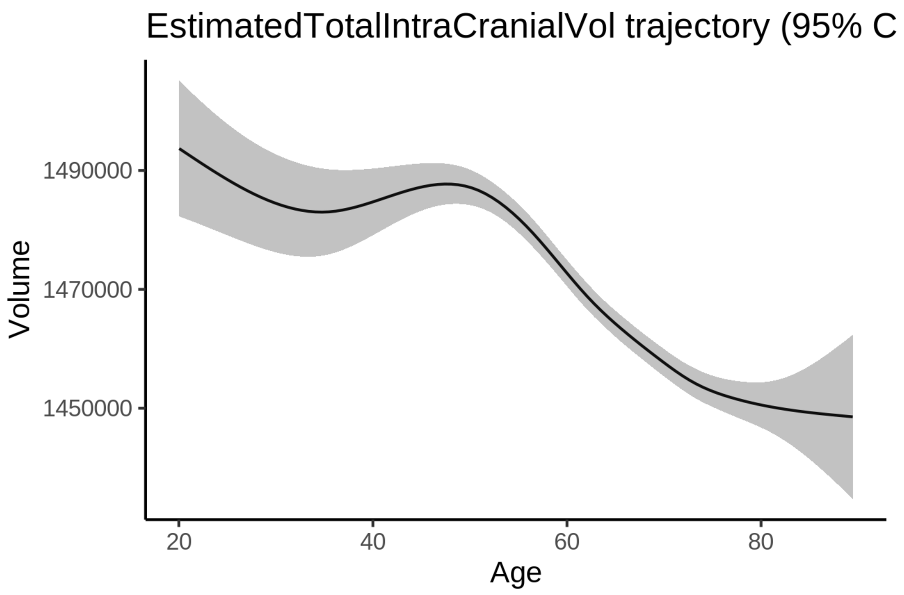

Effect of sleep

The chosen model only included the main effect of sleep, and hence the effect does not vary with age. The black dot shows the average sleep duration across all ages in the sample.

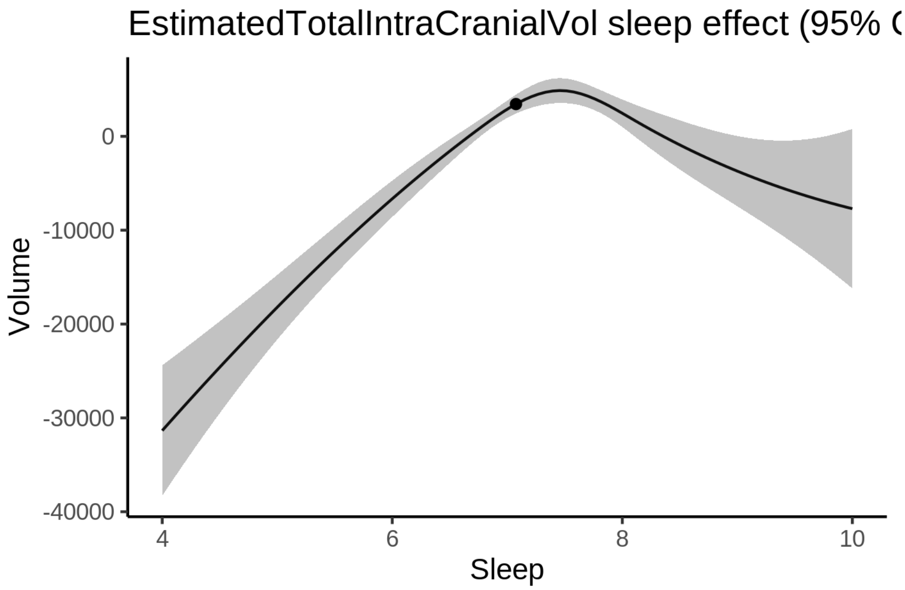

We also show the full interaction model for completeness, although it was not selected.

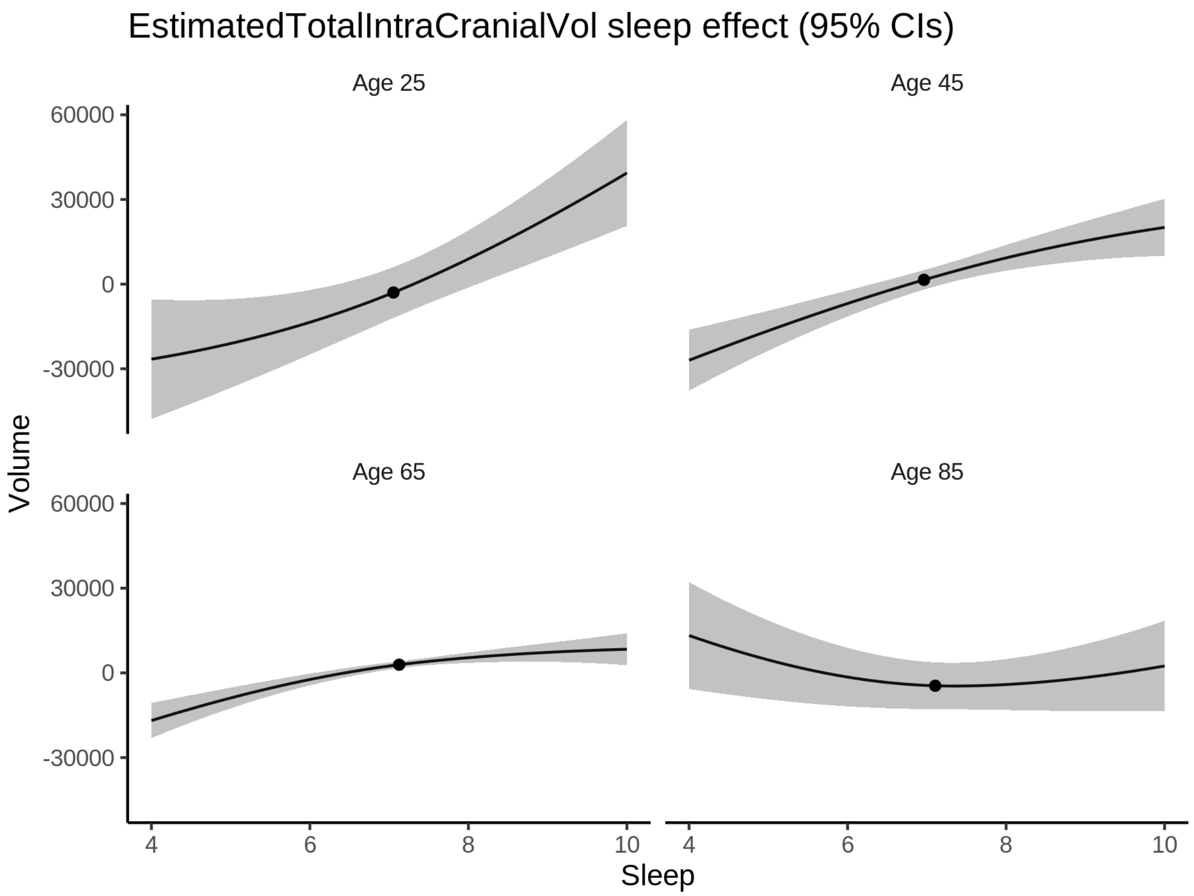

Deviation from sleep associated with maximal volume

Model with only main effect of sleep was chosen, so we show it for all ages at once. Maximum volume is attained at 7.5 hours of sleep. The percentage values in the plot are calculated as follows: The maximum at 100 % refers to a person at an arbitrary age with a sleep duration associated with maximum volume. For a female, this volume is 1472521 and for a male it is 1652671. The other percentage values show how large the expected volume is for someone with other sleep durations. For example, 99 % implies a 1 % reduction.

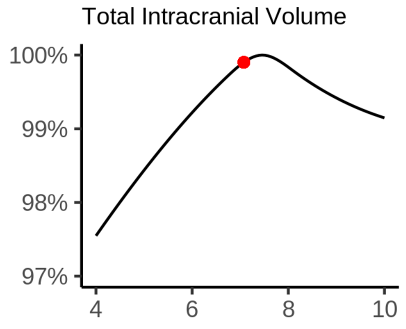

Comparison of mean sleep and sleep associated with maximum volume

A 95 % confidence interval for the sleep associated with maximum volume is [7.39, 7.58].

The plot below compares average sleep to the sleep associated with maximum volume.

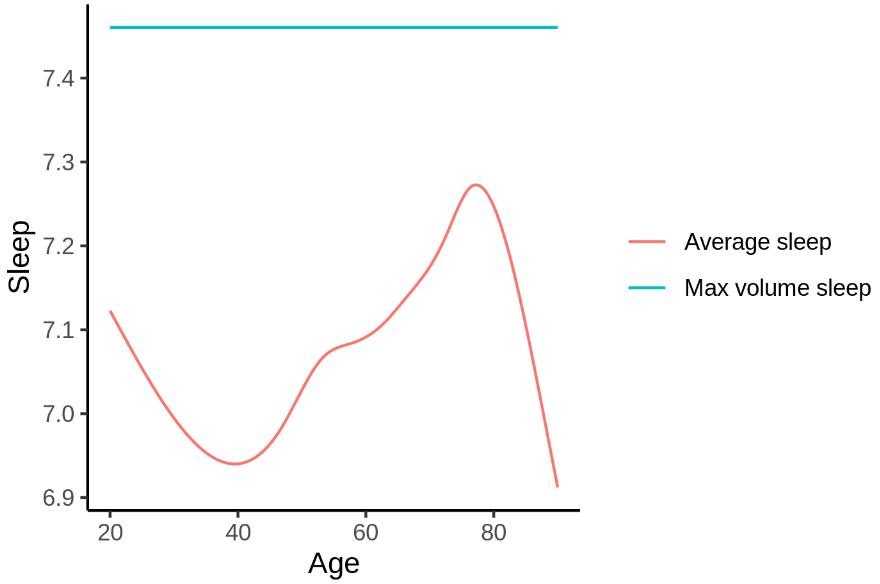

The next plot shows the difference between average sleep and sleep associated with maximum volume. The shaded region is a 95 % confidence interval.

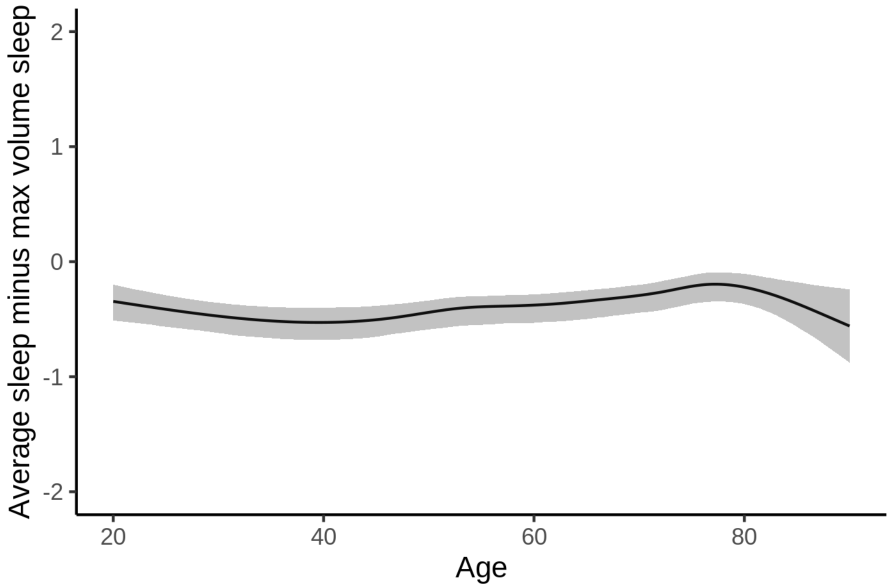

The next plot shows the probability that the sleep duration associated with maximum volume is longer than the average sleep duration, as a function of age. Probability below .05 can be interpreted as evidence that the sleep associated with maximum volume is shorter than the mean sleep, and probability above .95 can be interpreted the opposite way.

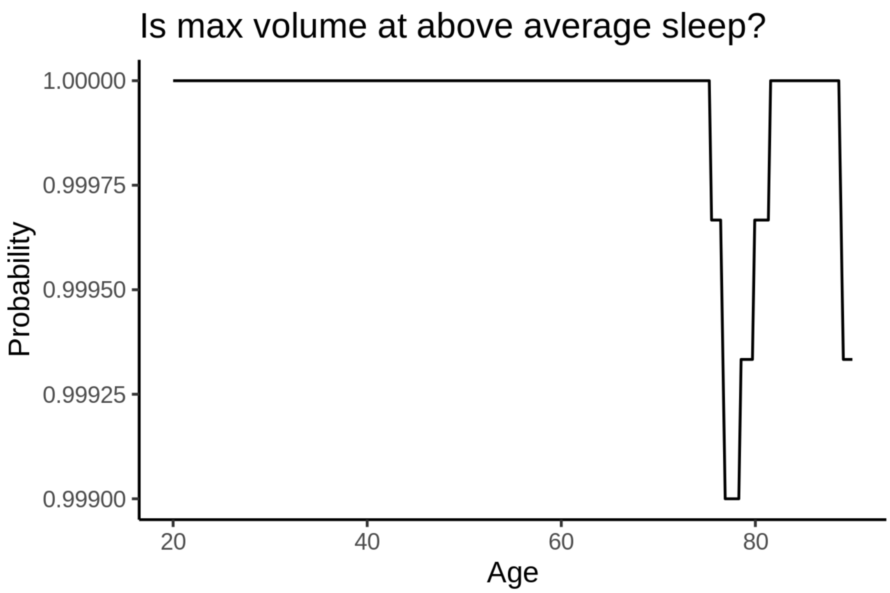

Controling for covariates

Below is the output for a model in which we only include data with income and education.

```
##
## Family: gaussian
## Link function: identity
##
## Formula:
## value ~ sex + site + s(age_z, k = 10, bs = "cr") + s(sleep_z,
##     k = 5, bs = "cr")
## <environment: 0x558ada633e00>
##
## Parametric coefficients:
##                Estimate Std. Error t value Pr(>|t|)
## (Intercept)     1170189      16243   72.04   <2e-16 ***
## sexmale          177837       1484  119.86   <2e-16 ***
## siteousAvanto    330643      18015   18.35   <2e-16 ***
## siteousPrisma    338578      19048   17.77   <2e-16 ***
## siteousSkyra     266856      17662   15.11   <2e-16 ***
## siteUKB          297548      16250   18.31   <2e-16 ***
## siteUOXF         217283      18377   11.82   <2e-16 ***
## ---
## Signif. codes:  0 '***' 0.001 '**' 0.01 '*' 0.05 '.' 0.1 ' ' 1
##
## Approximate significance of smooth terms:
##              edf Ref.df     F p-value
## s(age_z)   6.580  6.580 63.34  <2e-16 ***
## s(sleep_z) 3.619  3.619 19.82  <2e-16 ***
## ---
## Signif. codes:  0 '***' 0.001 '**' 0.01 '*' 0.05 '.' 0.1 ' ' 1
##
## R-sq.(adj) =  0.339
```

```
## lmer.REML = 8.1142e+05  Scale est. = 1.6633e+08  n = 31198
```

Below is the output for a model in which we control for the main effects of income and education.

```
##
## Family: gaussian
## Link function: identity
##
## Formula:
## value ~ sex + site + s(age_z, k = 10, bs = "cr") + s(sleep_z,
##     k = 5, bs = "cr") + income_scaled + education_scaled
## <environment: 0x558ada633e00>
##
## Parametric coefficients:
##                  Estimate Std. Error t value Pr(>|t|)
## (Intercept)       1137576      16159   70.40   <2e-16 ***
## sexmale            175337       1482  118.34   <2e-16 ***
## siteousAvanto      326634      17882   18.27   <2e-16 ***
## siteousPrisma      331656      18908   17.54   <2e-16 ***
## siteousSkyra       262689      17524   14.99   <2e-16 ***
## siteUKB            291247      16107   18.08   <2e-16 ***
## siteUOXF           225307      18212   12.37   <2e-16 ***
## income_scaled       24950       2114   11.80   <2e-16 ***
## education_scaled    36120       2525   14.31   <2e-16 ***
## ---
## Signif. codes:  0 ‘***’ 0.001 ‘**’ 0.01 ‘*’ 0.05 ‘.’ 0.1 ‘ ’ 1
##
## Approximate significance of smooth terms:
##              edf Ref.df     F p-value
## s(age_z)   5.906  5.906 42.96  <2e-16 ***
## s(sleep_z) 3.349  3.349 12.77  <2e-16 ***
## ---
## Signif. codes:  0 ‘***’ 0.001 ‘**’ 0.01 ‘*’ 0.05 ‘.’ 0.1 ‘ ’ 1
##
## R-sq.(adj) =   0.35
## lmer.REML = 8.1092e+05  Scale est. = 1.6785e+08  n = 31198
```

We also included interaction effects between sleep duration and education and income, in another model. The output is shown below, and the interaction terms are `income_scaled:sleep_z` and `education_scaled:sleep_z`.

```
##
## Family: gaussian
## Link function: identity
##
## Formula:
## value ~ sex + site + s(age_z, k = 10, bs = "cr") + s(sleep_z,
##     k = 5, bs = "cr") + income_scaled + education_scaled + income_scaled:sleep_z +
##     education_scaled:sleep_z
## <environment: 0x558ada633e00>
##
## Parametric coefficients:
##                          Estimate Std. Error t value Pr(>|t|)
## (Intercept)               1137216      16159  70.378   <2e-16 ***
## sexmale                    175397       1483 118.288   <2e-16 ***
## siteousAvanto              326930      17881  18.283   <2e-16 ***
```

```
## siteousPrisma              332110      18908  17.564   <2e-16 ***
## siteousSkyra               262985      17524  15.007   <2e-16 ***
## siteUKB                    291707      16107  18.110   <2e-16 ***
## siteUOXF                   226094      18216  12.412   <2e-16 ***
## income_scaled               24872       2115  11.761   <2e-16 ***
## education_scaled            36199       2525  14.336   <2e-16 ***
## income_scaled:sleep_z        1292       2142   0.603   0.5465
## education_scaled:sleep_z    -5869       2460  -2.386   0.0171 *
## ---
## Signif. codes:  0 ‘***’ 0.001 ‘**’ 0.01 ‘*’ 0.05 ‘.’ 0.1 ‘ ’ 1
##
## Approximate significance of smooth terms:
##              edf Ref.df     F  p-value
## s(age_z)   5.914  5.914 43.03  < 2e-16 ***
## s(sleep_z) 3.342  3.342 10.82 5.99e-08 ***
## ---
## Signif. codes:  0 ‘***’ 0.001 ‘**’ 0.01 ‘*’ 0.05 ‘.’ 0.1 ‘ ’ 1
##
## R-sq.(adj) =   0.35
## lmer.REML = 8.1088e+05  Scale est. = 1.6783e+08  n = 31198
```

We did the same controling for BMI. Below is the model with no covariates but only keeping data with BMI.

```
##
## Family: gaussian
## Link function: identity
##
## Formula:
## value ~ sex + site + s(age_z, k = 10, bs = "cr") + s(sleep_z,
##     k = 5, bs = "cr")
## <environment: 0x558ad7a13438>
##
## Parametric coefficients:
##               Estimate Std. Error t value Pr(>|t|)
## (Intercept)    1503984       7265 207.027  < 2e-16 ***
## sexmale         178117       1430 124.570  < 2e-16 ***
## siteousPrisma    28500       8342   3.416 0.000636 ***
## siteousSkyra    -61544       3176 -19.378  < 2e-16 ***
## siteUCAM        -39016       8475  -4.604 4.16e-06 ***
## siteUKB         -36357       7359  -4.941 7.83e-07 ***
## siteUmU        -112001      10365 -10.805  < 2e-16 ***
## ---
## Signif. codes:  0 ‘***’ 0.001 ‘**’ 0.01 ‘*’ 0.05 ‘.’ 0.1 ‘ ’ 1
##
## Approximate significance of smooth terms:
##              edf Ref.df     F p-value
## s(age_z)   6.208  6.208 73.14  <2e-16 ***
## s(sleep_z) 3.507  3.507 23.24  <2e-16 ***
## ---
## Signif. codes:  0 ‘***’ 0.001 ‘**’ 0.01 ‘*’ 0.05 ‘.’ 0.1 ‘ ’ 1
##
## R-sq.(adj) =  0.338
## lmer.REML = 8.6864e+05  Scale est. = 1.4743e+08  n = 33453
```

Below is the model output with main effect.

```
##
## Family: gaussian
## Link function: identity
##
## Formula:
## value ~ sex + site + s(age_z, k = 10, bs = "cr") + s(sleep_z,
##     k = 5, bs = "cr") + bmi
## <environment: 0x558ad7a13438>
##
## Parametric coefficients:
##                 Estimate Std. Error t value Pr(>|t|)
## (Intercept)   1482076.0     8408.4 176.261  < 2e-16 ***
## sexmale        177266.3     1438.7 123.214  < 2e-16 ***
## siteousPrisma   28222.8     8341.9   3.383 0.000717 ***
## siteousSkyra   -61511.1     3176.0 -19.367  < 2e-16 ***
## siteUCAM       -39578.2     8472.2  -4.672 3.00e-06 ***
## siteUKB        -37344.3     7358.1  -5.075 3.89e-07 ***
## siteUmU       -113078.3    10363.2 -10.912  < 2e-16 ***
## bmi               882.5      170.8   5.167 2.39e-07 ***
## ---
## Signif. codes:  0 ‘***’ 0.001 ‘**’ 0.01 ‘*’ 0.05 ‘.’ 0.1 ‘ ’ 1
##
## Approximate significance of smooth terms:
##               edf Ref.df     F p-value
## s(age_z)   6.187  6.187 73.18  <2e-16 ***
## s(sleep_z) 3.539  3.539 25.12  <2e-16 ***
## ---
## Signif. codes:  0 ‘***’ 0.001 ‘**’ 0.01 ‘*’ 0.05 ‘.’ 0.1 ‘ ’ 1
##
## R-sq.(adj) =  0.338
## lmer.REML = 8.686e+05  Scale est. = 1.4745e+08  n = 33453
```

Next is the model with BMI-sleep interaction.

```
##
## Family: gaussian
## Link function: identity
##
## Formula:
## value ~ sex + site + s(age_z, k = 10, bs = "cr") + s(sleep_z,
##     k = 5, bs = "cr") + bmi + bmi:sleep_z
## <environment: 0x558ad7a13438>
##
## Parametric coefficients:
##                 Estimate Std. Error t value Pr(>|t|)
## (Intercept)   1482526.6     8414.7 176.184  < 2e-16 ***
## sexmale        177247.2     1438.7 123.198  < 2e-16 ***
## siteousPrisma   28217.7     8342.0   3.383 0.000719 ***
## siteousSkyra   -61524.0     3176.0 -19.371  < 2e-16 ***
## siteUCAM       -39701.0     8472.5  -4.686 2.80e-06 ***
## siteUKB        -37461.8     7358.3  -5.091 3.58e-07 ***
## siteUmU       -113161.1    10363.1 -10.920  < 2e-16 ***
## bmi               874.3      170.9   5.116 3.15e-07 ***
## bmi:sleep_z      -223.3      162.6  -1.373 0.169745
## ---
```

```
## Signif. codes:  0 ‘***’ 0.001 ‘**’ 0.01 ‘*’ 0.05 ‘.’ 0.1 ‘ ’ 1
##
## Approximate significance of smooth terms:
##              edf Ref.df    F p-value
## s(age_z)   6.182  6.182 73.0  <2e-16 ***
## s(sleep_z) 3.549  3.549 17.5  <2e-16 ***
## ---
## Signif. codes:  0 ‘***’ 0.001 ‘**’ 0.01 ‘*’ 0.05 ‘.’ 0.1 ‘ ’ 1
##
## R-sq.(adj) =  0.338
## lmer.REML = 8.6858e+05  Scale est. = 1.4745e+08  n = 33453
```

We did the same controling for depression. Below is the model with no covariates but only keeping data with depression.

```
##
## Family: gaussian
## Link function: identity
##
## Formula:
## value ~ sex + site + s(age_z, k = 10, bs = "cr") + s(sleep_z,
##     k = 5, bs = "cr")
## <environment: 0x558a4613f810>
##
## Parametric coefficients:
##                Estimate Std. Error t value Pr(>|t|)
## (Intercept)     1226926       8272  148.33   <2e-16 ***
## sexmale          178444       1436  124.30   <2e-16 ***
## siteousAvanto    272632      12544   21.73   <2e-16 ***
## siteousPrisma    285879      13410   21.32   <2e-16 ***
## siteousSkyra     210583      11514   18.29   <2e-16 ***
## siteUCAM         247976      11372   21.81   <2e-16 ***
## siteUKB          240213       8262   29.08   <2e-16 ***
## siteUmU          167224      11266   14.84   <2e-16 ***
## ---
## Signif. codes:  0 ‘***’ 0.001 ‘**’ 0.01 ‘*’ 0.05 ‘.’ 0.1 ‘ ’ 1
##
## Approximate significance of smooth terms:
##              edf Ref.df     F p-value
## s(age_z)   6.397  6.397 71.05  <2e-16 ***
## s(sleep_z) 3.438  3.438 21.74  <2e-16 ***
## ---
## Signif. codes:  0 ‘***’ 0.001 ‘**’ 0.01 ‘*’ 0.05 ‘.’ 0.1 ‘ ’ 1
##
## R-sq.(adj) =  0.357
## lmer.REML = 8.658e+05  Scale est. = 1.4235e+08  n = 33365
```

Below is the model output with main effect.

```
##
## Family: gaussian
## Link function: identity
##
## Formula:
## value ~ sex + site + s(age_z, k = 10, bs = "cr") + s(sleep_z,
##     k = 5, bs = "cr") + depression
```

```
## <environment: 0x558a4613f810>
##
## Parametric coefficients:
##                Estimate Std. Error t value Pr(>|t|)
## (Intercept)     1225404       8303 147.579   <2e-16 ***
## sexmale          178594       1437 124.250   <2e-16 ***
## siteousAvanto    271638      12549  21.645   <2e-16 ***
## siteousPrisma    284868      13416  21.234   <2e-16 ***
## siteousSkyra     209525      11521  18.187   <2e-16 ***
## siteUCAM         247479      11373  21.761   <2e-16 ***
## siteUKB          240796       8266  29.132   <2e-16 ***
## siteUmU          162259      11511  14.096   <2e-16 ***
## depression        13159       6289   2.092   0.0364 *
## ---
## Signif. codes:  0 ‘***’ 0.001 ‘**’ 0.01 ‘*’ 0.05 ‘.’ 0.1 ‘ ’ 1
##
## Approximate significance of smooth terms:
##              edf Ref.df     F p-value
## s(age_z)   6.369  6.369 68.46  <2e-16 ***
## s(sleep_z) 3.462  3.462 22.55  <2e-16 ***
## ---
## Signif. codes:  0 ‘***’ 0.001 ‘**’ 0.01 ‘*’ 0.05 ‘.’ 0.1 ‘ ’ 1
##
## R-sq.(adj) =  0.357
## lmer.REML = 8.6578e+05  Scale est. = 1.4238e+08  n = 33365
```

Next is the model with depression-sleep interaction.

```
##
## Family: gaussian
## Link function: identity
##
## Formula:
## value ~ sex + site + s(age_z, k = 10, bs = "cr") + s(sleep_z,
##     k = 5, bs = "cr") + depression + depression:sleep_z
## <environment: 0x558a4613f810>
##
## Parametric coefficients:
##                     Estimate Std. Error t value Pr(>|t|)
## (Intercept)          1225403       8304 147.577   <2e-16 ***
## sexmale               178609       1438 124.232   <2e-16 ***
## siteousAvanto         271716      12550  21.650   <2e-16 ***
## siteousPrisma         284949      13417  21.238   <2e-16 ***
## siteousSkyra          209600      11522  18.191   <2e-16 ***
## siteUCAM              247585      11375  21.766   <2e-16 ***
## siteUKB               240790       8266  29.131   <2e-16 ***
## siteUmU               161488      11616  13.902   <2e-16 ***
## depression             13368       6303   2.121   0.0339 *
## depression:sleep_z      2324       4695   0.495   0.6206
## ---
## Signif. codes:  0 ‘***’ 0.001 ‘**’ 0.01 ‘*’ 0.05 ‘.’ 0.1 ‘ ’ 1
##
## Approximate significance of smooth terms:
##              edf Ref.df     F p-value
## s(age_z)   6.368  6.368 68.46  <2e-16 ***
```

```
## s(sleep_z) 3.447  3.447 19.25  <2e-16 ***
## ---
## Signif. codes:  0 '***' 0.001 '**' 0.01 '*' 0.05 '.' 0.1 ' ' 1
##
## R-sq.(adj) =  0.357
## lmer.REML = 8.6576e+05  Scale est. = 1.4238e+08  n = 33365
```

The plot below shows the sleep-volume curve for the original model and for the model with main effects of SES.

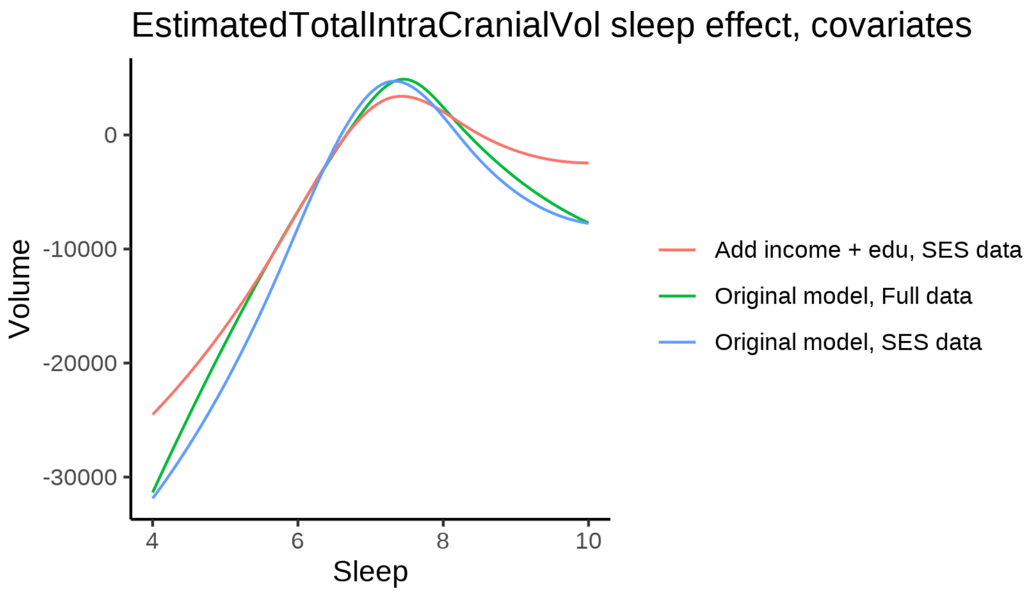

The plot below shows the sleep-volume curve for the original model and for the model with main effects of BMI.

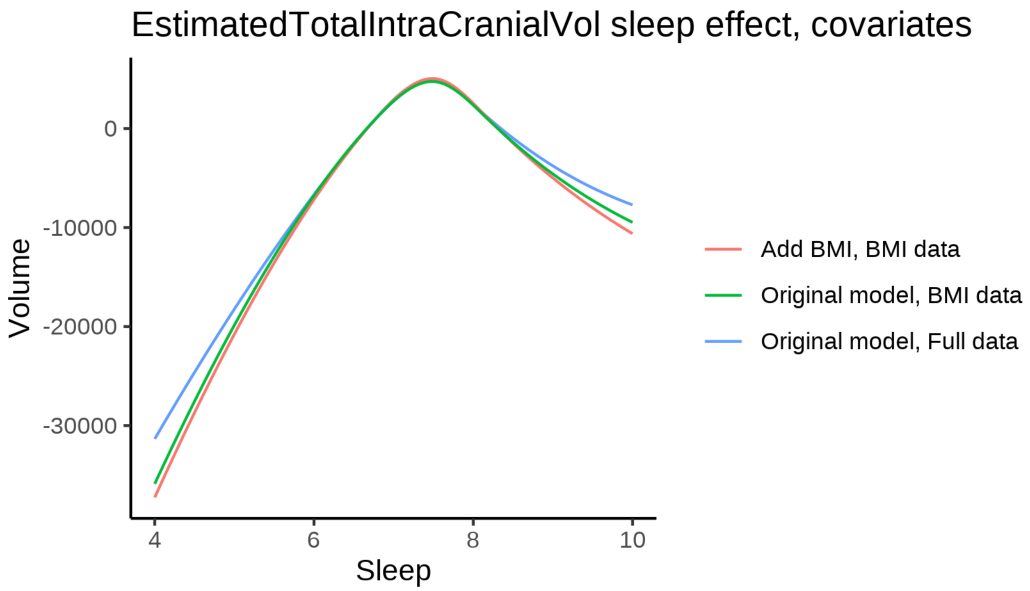

The plot below shows the sleep-volume curve for the original model and for the model with main effects of depression.

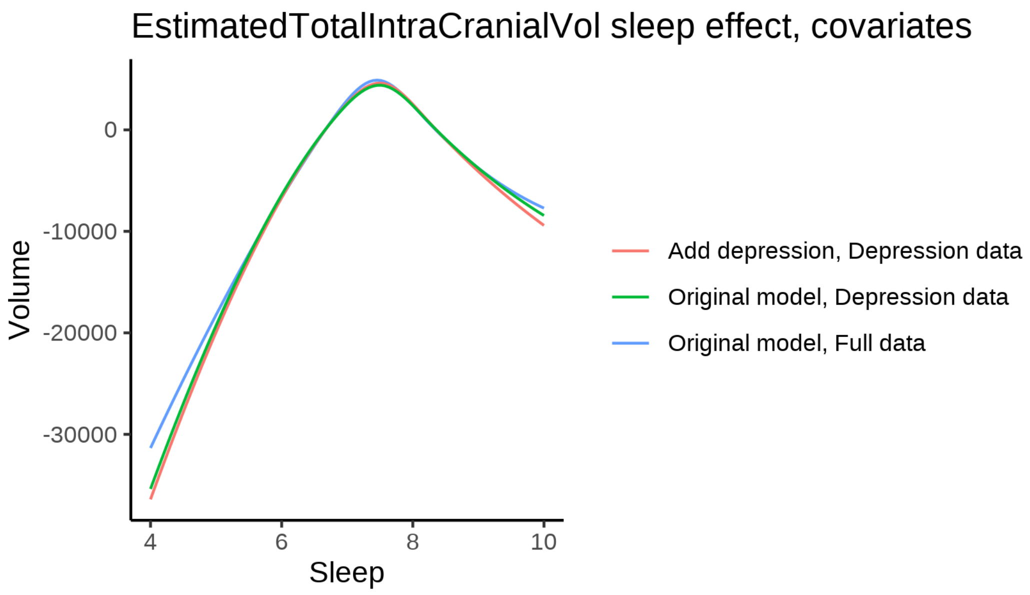

Models Not controling for ICV

Model outputs

Model without sleep term

```
##
## Family: gaussian
## Link function: identity
##
## Formula:
## value ~ sex + site + s(age_z, k = 10, bs = "cr")
## <environment: 0x558ad7ef87a8>
##
## Parametric coefficients:
##                Estimate Std. Error t value Pr(>|t|)
## (Intercept)     1443795       5748 251.179  < 2e-16 ***
## sexmale          180533       1190 151.639  < 2e-16 ***
## siteMPIB        -245193       8372 -29.286  < 2e-16 ***
## siteousAvanto     66386       6467  10.265  < 2e-16 ***
## siteousPrisma     97945       9270  10.565  < 2e-16 ***
## siteousSkyra      10263       6458   1.589 0.112033
## siteUB           -33005      21265  -1.552 0.120654
## siteUCAM          19146       7206   2.657 0.007890 **
## siteUKB           19392       5863   3.307 0.000942 ***
## siteUmU          -51384       9450  -5.437 5.43e-08 ***
## siteUOXF         -22646       7479  -3.028 0.002462 **
## ---
## Signif. codes:  0 ‘***’ 0.001 ‘**’ 0.01 ‘*’ 0.05 ‘.’ 0.1 ‘ ’ 1
##
## Approximate significance of smooth terms:
##             edf Ref.df     F p-value
## s(age_z) 6.822  6.822 78.66  <2e-16 ***
```

```
## ---
## Signif. codes:  0 '***' 0.001 '**' 0.01 '*' 0.05 '.' 0.1 ' ' 1
##
## R-sq.(adj) =   0.35
## lmer.REML = 1.3347e+06  Scale est. = 1.5253e+08  n = 51289
```

Model with only main effects of age and sleep

```
##
## Family: gaussian
## Link function: identity
##
## Formula:
## value ~ sex + site + s(age_z, k = 10, bs = "cr") + s(sleep_z,
##     k = 5, bs = "cr")
## <environment: 0x558ad7ef87a8>
##
## Parametric coefficients:
##                Estimate Std. Error t value Pr(>|t|)
## (Intercept)     1446700       5752 251.535  < 2e-16 ***
## sexmale          180150       1190 151.405  < 2e-16 ***
## siteMPIB        -246504       8367 -29.462  < 2e-16 ***
## siteousAvanto     64476       6463   9.976  < 2e-16 ***
## siteousPrisma     96173       9265  10.380  < 2e-16 ***
## siteousSkyra       8314       6454   1.288  0.19765
## siteUB           -32065      21241  -1.510  0.13116
## siteUCAM          18573       7200   2.580  0.00989 **
## siteUKB           16522       5866   2.816  0.00486 **
## siteUmU          -55183       9460  -5.833 5.46e-09 ***
## siteUOXF         -23711       7473  -3.173  0.00151 **
## ---
## Signif. codes:  0 '***' 0.001 '**' 0.01 '*' 0.05 '.' 0.1 ' ' 1
##
## Approximate significance of smooth terms:
##              edf Ref.df     F p-value
## s(age_z)   6.850  6.850 77.75  <2e-16 ***
## s(sleep_z) 3.622  3.622 30.13  <2e-16 ***
## ---
## Signif. codes:  0 '***' 0.001 '**' 0.01 '*' 0.05 '.' 0.1 ' ' 1
##
## R-sq.(adj) =  0.352
## lmer.REML = 1.3346e+06  Scale est. = 1.5253e+08  n = 51289
```

Model with full interaction between age and sleep

```
##
## Family: gaussian
## Link function: identity
##
## Formula:
## value ~ sex + site + t2(age_z, sleep_z, k = c(10, 4), bs = "cr")
## <environment: 0x558ad7ef87a8>
##
## Parametric coefficients:
##                Estimate Std. Error t value Pr(>|t|)
## (Intercept)     1447712       5782 250.401  < 2e-16 ***
```

```
## sexmale         180398       1192 151.370  < 2e-16 ***
## siteMPIB       -248528       8403 -29.577  < 2e-16 ***
## siteousAvanto    63187       6502   9.718  < 2e-16 ***
## siteousPrisma    94488       9293  10.167  < 2e-16 ***
## siteousSkyra      6842       6494   1.054 0.292060
## siteUB          -34496      21254  -1.623 0.104583
## siteUCAM         16386       7238   2.264 0.023578 *
## siteUKB          15423       5897   2.616 0.008909 **
## siteUmU         -56423       9489  -5.946 2.76e-09 ***
## siteUOXF        -25742       7505  -3.430 0.000604 ***
## ---
## Signif. codes:  0 '***' 0.001 '**' 0.01 '*' 0.05 '.' 0.1 ' ' 1
##
## Approximate significance of smooth terms:
##                      edf Ref.df     F p-value
## t2(age_z,sleep_z) 13.63  13.63 5.027  <2e-16 ***
## ---
## Signif. codes:  0 '***' 0.001 '**' 0.01 '*' 0.05 '.' 0.1 ' ' 1
##
## R-sq.(adj) =  0.352
## lmer.REML = 1.3346e+06  Scale est. = 1.5249e+08  n = 51289
```

Model comparison

`mod_no_sleep` refers to model without sleep term, `mod_no_interaction` refers to model with only main effect of sleep, and `mod_full` refers to model with a full interaction between age and sleep. This is a nested model comparison, and the p-value at a given line refers to comparing the model at the line to the model on the line above. Hence, significance implies that the more complicated model is supported on statistical grounds.

To be even more specific, the p-value on the second row tests whether there is an association between sleep and volume. The p-value on the third row tests whether this association depends on age.

```
## Data: NULL
## Models:
## ml$mod_no_sleep$mer: NULL
## ml$mod_no_interaction$mer: NULL
## ml$mod_full$mer: NULL
##                           npar     AIC     BIC  logLik deviance Chisq Df Pr(>Chisq)
## ml$mod_no_sleep$mer         15 1334752 1334885 -667361  1334722
## ml$mod_no_interaction$mer   17 1334654 1334804 -667310  1334620   102  2     <2e-16 ***
## ml$mod_full$mer             19 1334668 1334836 -667315  1334630     0  2          1
## ---
## Signif. codes:  0 '***' 0.001 '**' 0.01 '*' 0.05 '.' 0.1 ' ' 1
```

We chose the model based on the likelihood ratio test with 5 % significance level, which was `mod_no_interaction`.

Lifespan brain trajectory

The trajectory shown is from the chosen model `mod_no_interaction_no_icv`.

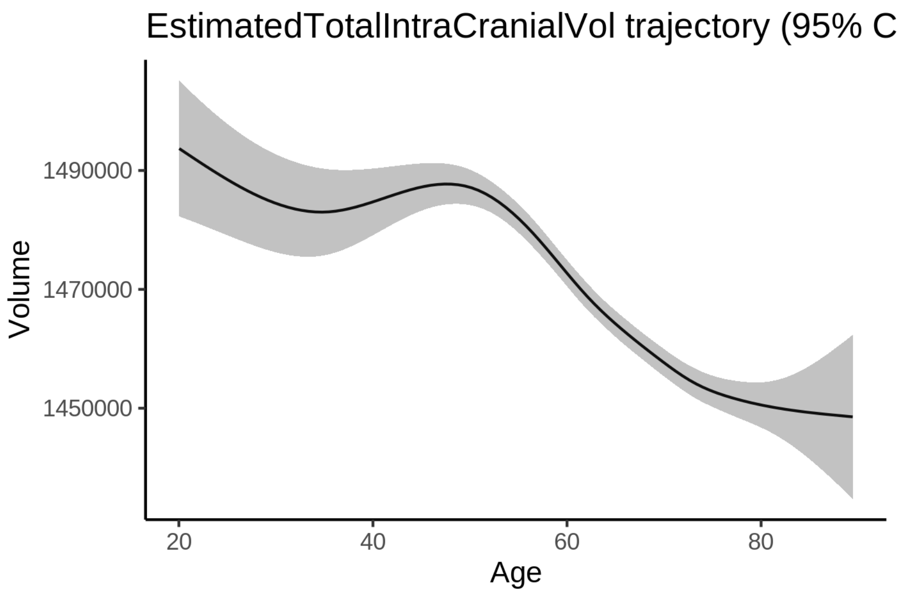

Effect of sleep

The chosen model only included the main effect of sleep, and hence the effect does not vary with age. The black dot shows the average sleep duration across all ages in the sample.

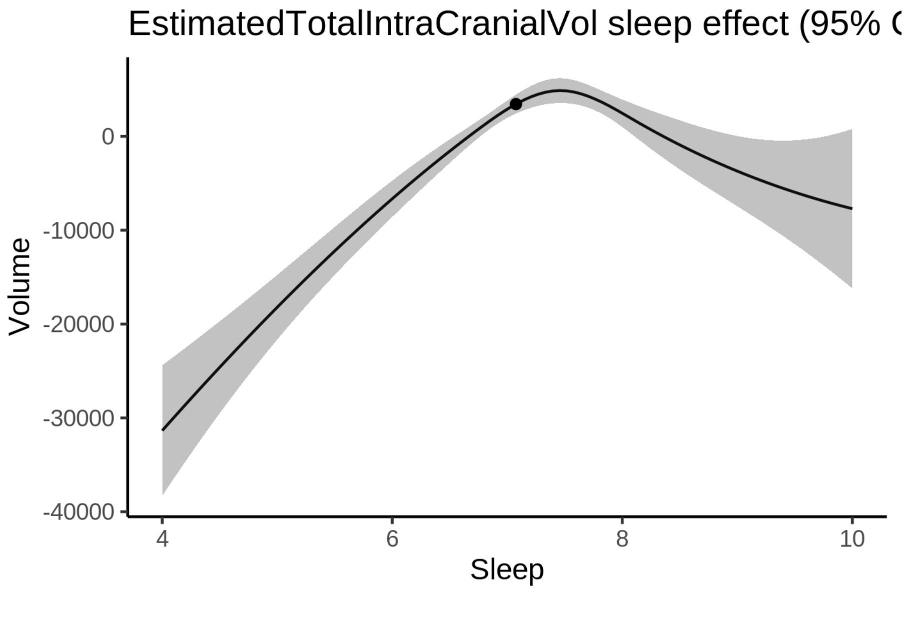

We also show the full interaction model for completeness, although it was not selected.

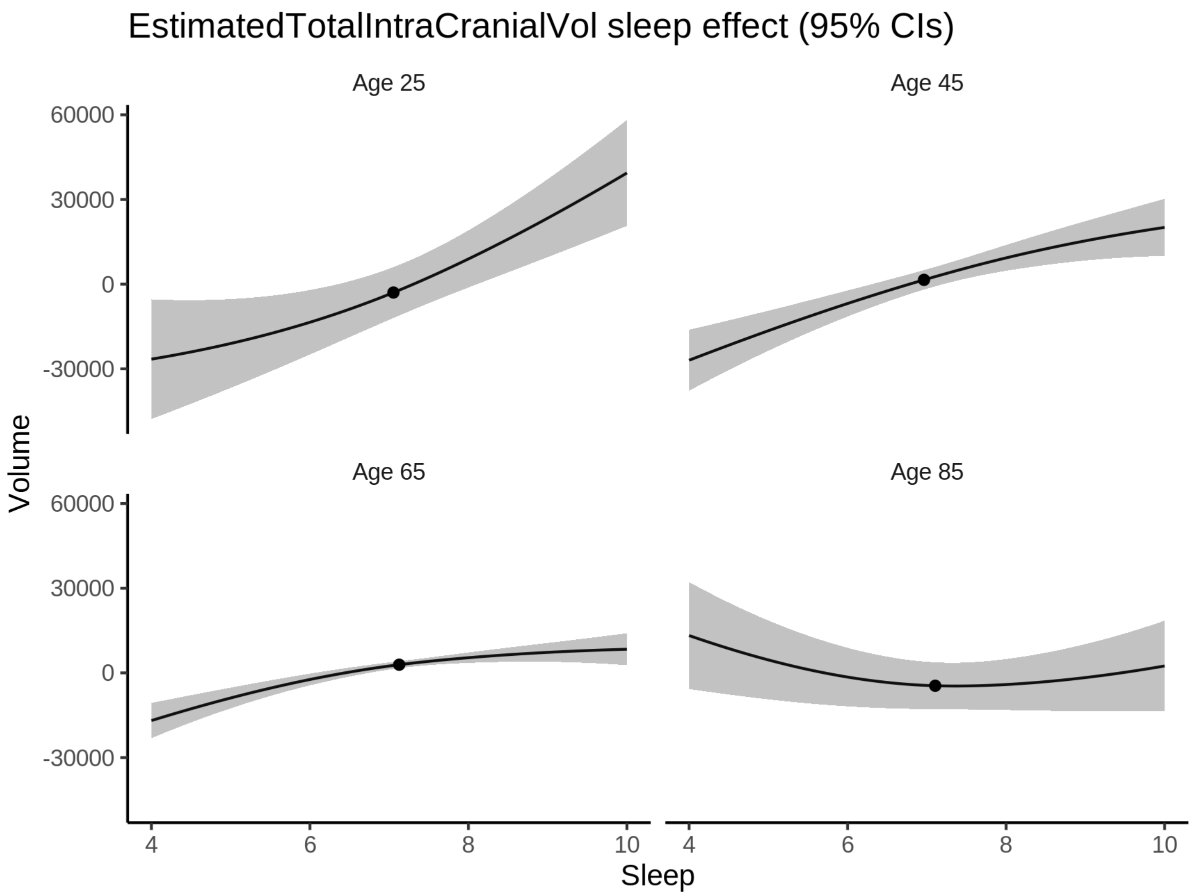

Deviation from sleep associated with maximal volume

Model with only main effect of sleep was chosen, so we show it for all ages at once. Maximum volume is attained at 7.5 hours of sleep. The percentage values in the plot are calculated as follows: The maximum at 100 % refers to a person at an arbitrary age with a sleep duration associated with maximum volume. For a female, this volume is 1472521 and for a male it is 1652671. The other percentage values show how large the expected volume is for someone with other sleep durations. For example, 99 % implies a 1 % reduction.

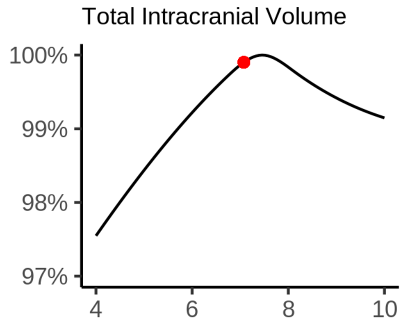

Comparison of mean sleep and sleep associated with maximum volume

A 95 % confidence interval for the sleep associated with maximum volume is [7.39, 7.58].

The plot below compares average sleep to the sleep associated with maximum volume.

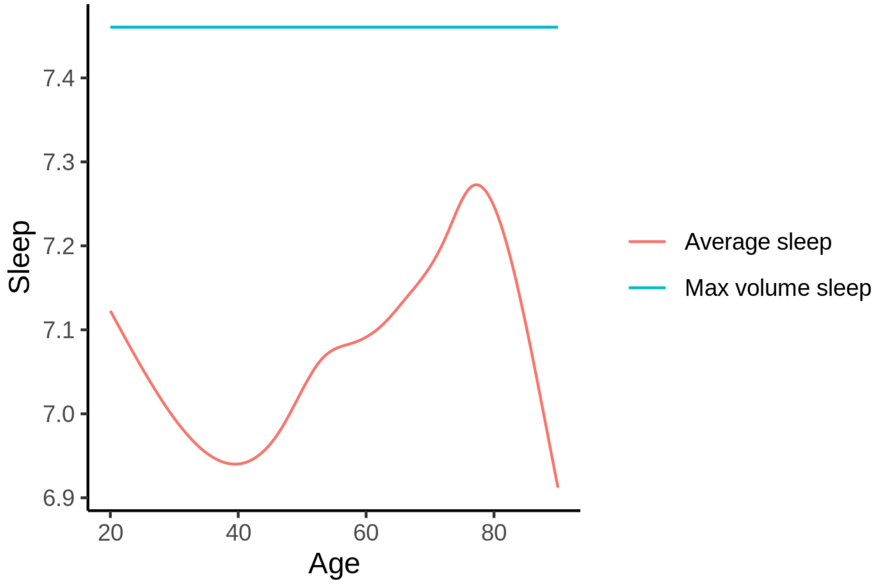

The next plot shows the difference between average sleep and sleep associated with maximum volume. The shaded region is a 95 % confidence interval.

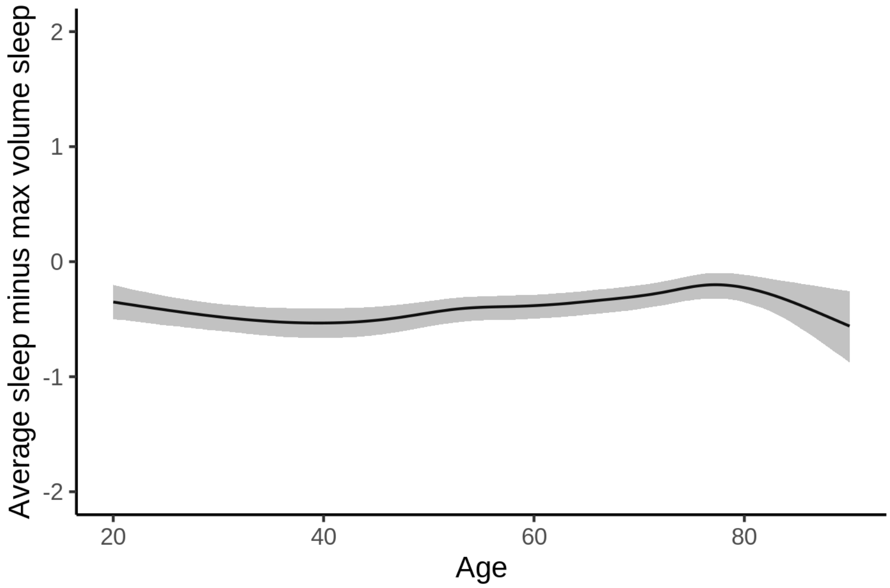

The next plot shows the probability that the sleep duration associated with maximum volume is longer than the average sleep duration, as a function of age. Probability below .05 can be interpreted as evidence that the sleep associated with maximum volume is shorter than the mean sleep, and probability above .95 can be interpreted the opposite way.

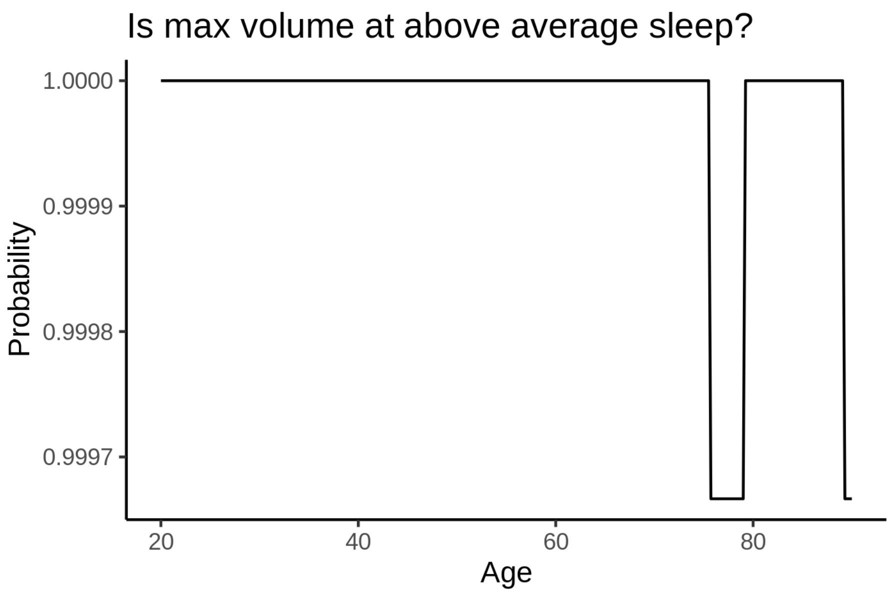

Hippocampus

Descriptive statistics

Study	Observations	Unique IDs	Mean age	Age range
HCP	974	974	28.8	22 - 37
MPIB	677	391	63.1	24 - 83
UB	113	39	70.9	64 - 81
UCAM	884	632	55.1	20 - 88
UiO	1475	803	49.4	20 - 89
UKB	45975	43133	64.5	45 - 83
UmU	412	279	62.2	25 - 85
UOXF	769	769	69.8	60 - 85

Spaghetti plot

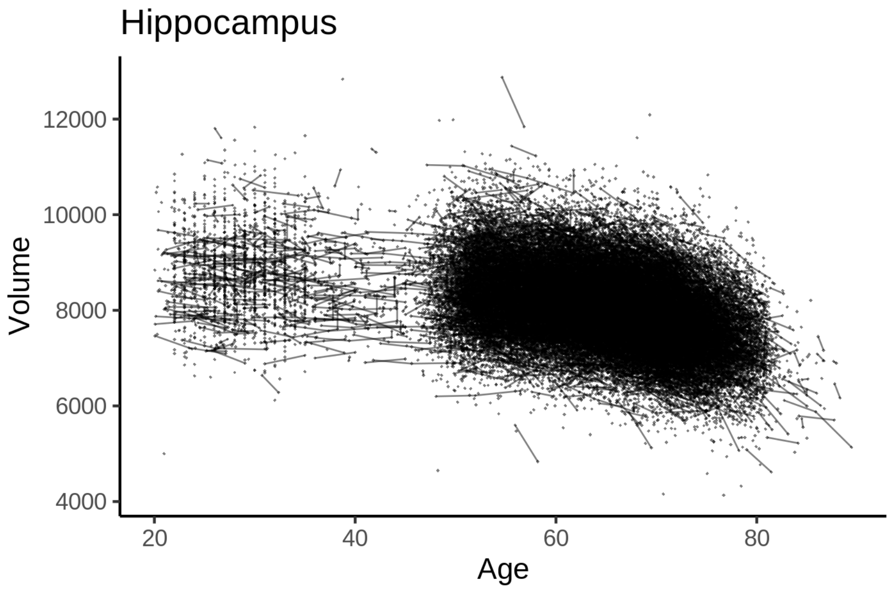

Models Controling for ICV

Model outputs

Model without sleep term

```
##
## Family: gaussian
## Link function: identity
##
## Formula:
## value ~ sex + site + icv + s(age_z, k = 10, bs = "cr")
## <environment: 0x5589769b2d88>
##
## Parametric coefficients:
##                Estimate Std. Error t value Pr(>|t|)
## (Intercept)    8113.326     34.449 235.518  < 2e-16 ***
## sexmale         122.795      7.124  17.236  < 2e-16 ***
## siteMPIB        274.609     44.378   6.188 6.14e-10 ***
## siteousAvanto -490.715     35.048 -14.001  < 2e-16 ***
## siteousPrisma  -92.809     59.127  -1.570  0.11650
## siteousSkyra  -193.298     33.728  -5.731 1.00e-08 ***
## siteUB        -139.796    105.037  -1.331  0.18322
## siteUCAM      -104.409     38.396  -2.719  0.00655 **
## siteUKB       -103.256     35.313  -2.924  0.00346 **
## siteUmU       -102.548     49.729  -2.062  0.03920 *
## siteUOXF      -304.011     41.979  -7.242 4.48e-13 ***
## icv            395.980      3.577 110.698  < 2e-16 ***
## ---
## Signif. codes:  0 ‘***’ 0.001 ‘**’ 0.01 ‘*’ 0.05 ‘.’ 0.1 ‘ ’ 1
##
```

```
## Approximate significance of smooth terms:
##             edf Ref.df    F p-value
## s(age_z) 7.951  7.951 1516  <2e-16 ***
## ---
## Signif. codes:  0 ‘***’ 0.001 ‘**’ 0.01 ‘*’ 0.05 ‘.’ 0.1 ‘ ’ 1
##
## R-sq.(adj) =  0.423
## lmer.REML = 7.9696e+05  Scale est. = 22402     n = 51279
```

Model with only main effects of age and sleep

```
##
## Family: gaussian
## Link function: identity
##
## Formula:
## value ~ sex + site + icv + s(age_z, k = 10, bs = "cr") + s(sleep_z,
##     k = 5, bs = "cr")
## <environment: 0x5589769b2d88>
##
## Parametric coefficients:
##                Estimate Std. Error t value Pr(>|t|)
## (Intercept)    8109.595     34.474 235.238  < 2e-16 ***
## sexmale         122.387      7.122  17.185  < 2e-16 ***
## siteMPIB        281.606     44.389   6.344 2.26e-10 ***
## siteousAvanto -489.706     35.049 -13.972  < 2e-16 ***
## siteousPrisma  -92.156     59.111  -1.559  0.11899
## siteousSkyra  -192.644     33.730  -5.711 1.13e-08 ***
## siteUB        -135.503    104.990  -1.291  0.19684
## siteUCAM      -100.882     38.388  -2.628  0.00859 **
## siteUKB        -99.174     35.342  -2.806  0.00502 **
## siteUmU        -87.373     49.825  -1.754  0.07950 .
## siteUOXF      -302.332     41.970  -7.203 5.95e-13 ***
## icv            395.828      3.579 110.584  < 2e-16 ***
## ---
## Signif. codes:  0 ‘***’ 0.001 ‘**’ 0.01 ‘*’ 0.05 ‘.’ 0.1 ‘ ’ 1
##
## Approximate significance of smooth terms:
##               edf Ref.df       F p-value
## s(age_z)   7.952  7.952 1494.16  <2e-16 ***
## s(sleep_z) 2.960  2.960   13.72  <2e-16 ***
## ---
## Signif. codes:  0 ‘***’ 0.001 ‘**’ 0.01 ‘*’ 0.05 ‘.’ 0.1 ‘ ’ 1
##
## R-sq.(adj) =  0.424
## lmer.REML = 7.9692e+05  Scale est. = 22414     n = 51279
```

Model with full interaction between age and sleep

```
##
## Family: gaussian
## Link function: identity
##
## Formula:
## value ~ sex + site + icv + t2(age_z, sleep_z, k = c(10, 4), bs = "cr")
## <environment: 0x5589769b2d88>
```

```
##
## Parametric coefficients:
##                Estimate Std. Error t value Pr(>|t|)
## (Intercept)   8109.374     34.554 234.690  < 2e-16 ***
## sexmale        122.108      7.137  17.109  < 2e-16 ***
## siteMPIB       281.105     44.594   6.304 2.93e-10 ***
## siteousAvanto -488.933     35.229 -13.879  < 2e-16 ***
## siteousPrisma  -93.748     59.244  -1.582  0.11356
## siteousSkyra  -191.597     33.925  -5.648 1.64e-08 ***
## siteUB        -136.380    105.038  -1.298  0.19416
## siteUCAM      -102.202     38.565  -2.650  0.00805 **
## siteUKB        -98.794     35.418  -2.789  0.00528 **
## siteUmU        -90.416     49.946  -1.810  0.07026 .
## siteUOXF      -301.345     42.082  -7.161 8.12e-13 ***
## icv            395.882      3.578 110.633  < 2e-16 ***
## ---
## Signif. codes:  0 '***' 0.001 '**' 0.01 '*' 0.05 '.' 0.1 ' ' 1
##
## Approximate significance of smooth terms:
##                      edf Ref.df     F p-value
## t2(age_z,sleep_z) 14.22  14.22 37.67  <2e-16 ***
## ---
## Signif. codes:  0 '***' 0.001 '**' 0.01 '*' 0.05 '.' 0.1 ' ' 1
##
## R-sq.(adj) =  0.424
## lmer.REML = 7.9692e+05  Scale est. = 22402     n = 51279
```

Model comparison

`mod_no_sleep` refers to model without sleep term, `mod_no_interaction` refers to model with only main effect of sleep, and `mod_full` refers to model with a full interaction between age and sleep. This is a nested model comparison, and the p-value at a given line refers to comparing the model at the line to the model on the line above. Hence, significance implies that the more complicated model is supported on statistical grounds.

To be even more specific, the p-value on the second row tests whether there is an association between sleep and volume. The p-value on the third row tests whether this association depends on age.

```
## Data: NULL
## Models:
## ml$mod_no_sleep$mer: NULL
## ml$mod_no_interaction$mer: NULL
## ml$mod_full$mer: NULL
##                           npar    AIC    BIC  logLik deviance   Chisq Df Pr(>Chisq)
## ml$mod_no_sleep$mer         16 796991 797132 -398479   796959
## ml$mod_no_interaction$mer   18 796960 797119 -398462   796924 34.6305  2  3.021e-08 ***
## ml$mod_full$mer             20 796963 797140 -398462   796923  1.0449  2     0.5931
## ---
## Signif. codes:  0 '***' 0.001 '**' 0.01 '*' 0.05 '.' 0.1 ' ' 1
```

We chose the model based on the likelihood ratio test with 5 % significance level, which was `mod_no_interaction`.

Lifespan brain trajectory

The trajectory shown is from the chosen model `mod_no_interaction`.

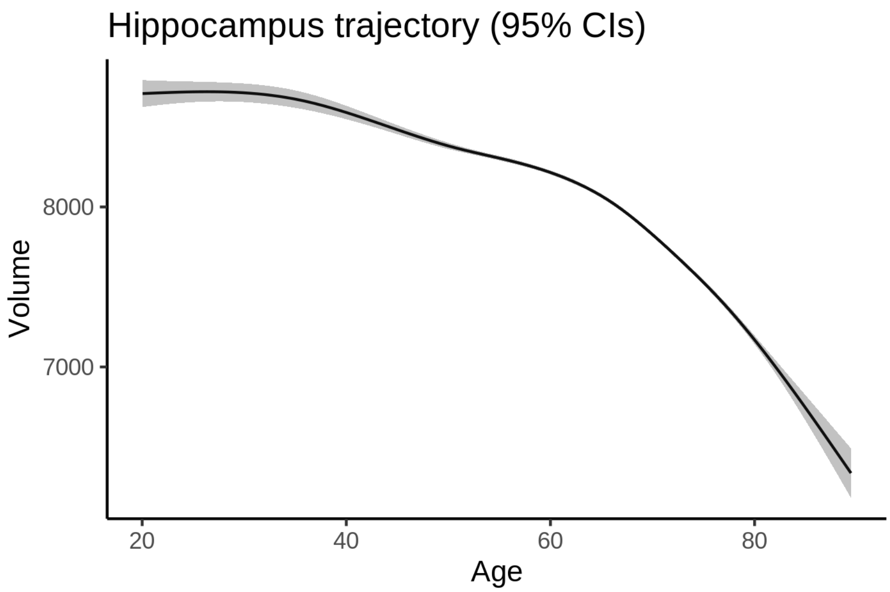

Effect of sleep

The chosen model only included the main effect of sleep, and hence the effect does not vary with age. The black dot shows the average sleep duration across all ages in the sample.

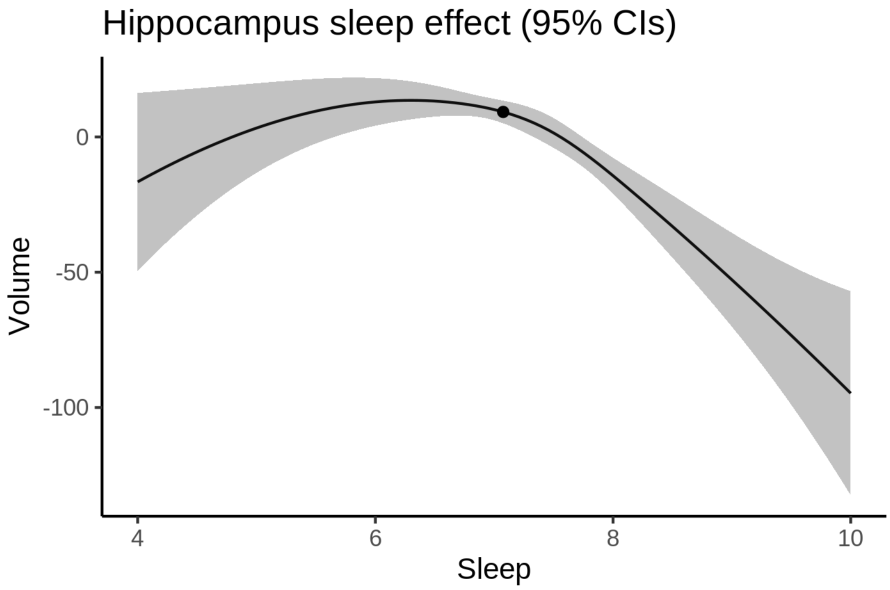

We also show the full interaction model for completeness, although it was not selected.

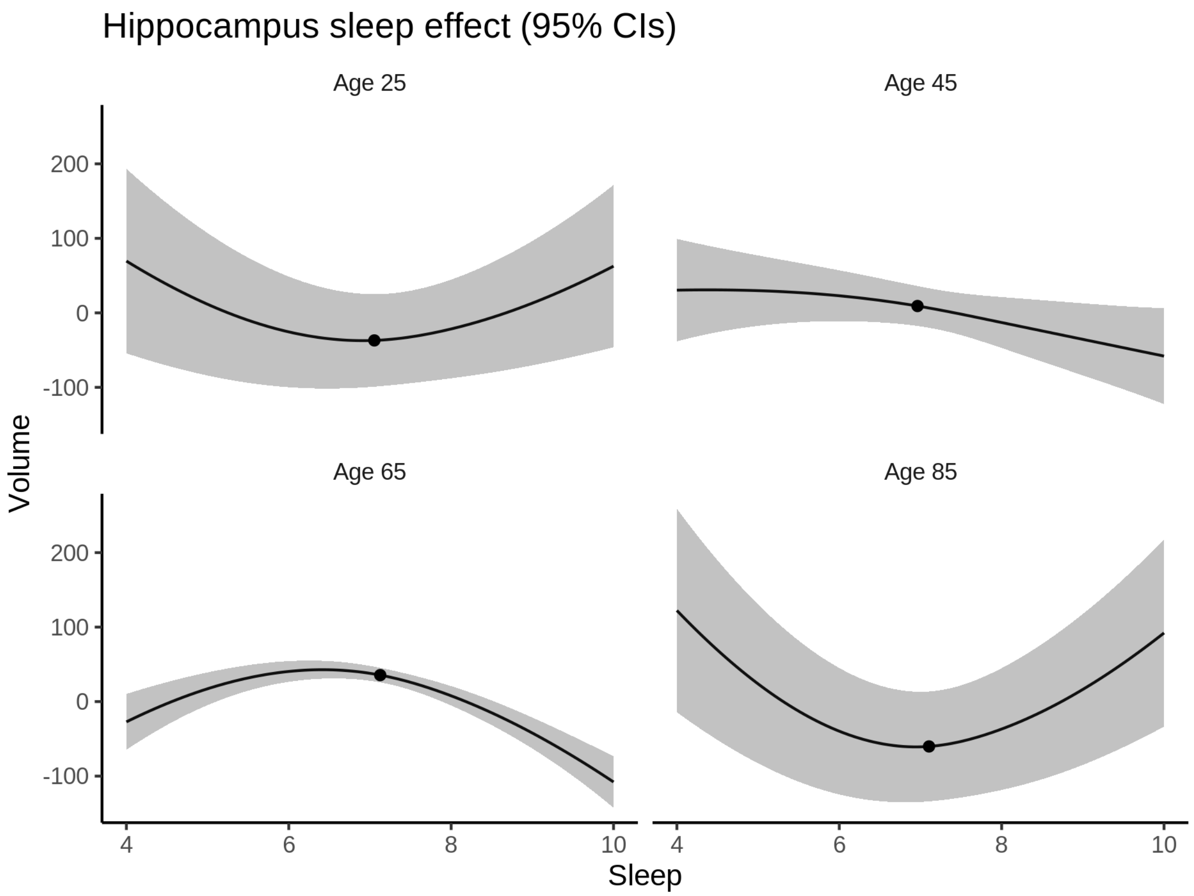

Deviation from sleep associated with maximal volume

Model with only main effect of sleep was chosen, so we show it for all ages at once. Maximum volume is attained at 6.3 hours of sleep. The percentage values in the plot are calculated as follows: The maximum at 100 % refers to a person at an arbitrary age with a sleep duration associated with maximum volume. For a female, this volume is 8060 and for a male it is 8182. The other percentage values show how large the expected volume is for someone with other sleep durations. For example, 99 % implies a 1 % reduction.

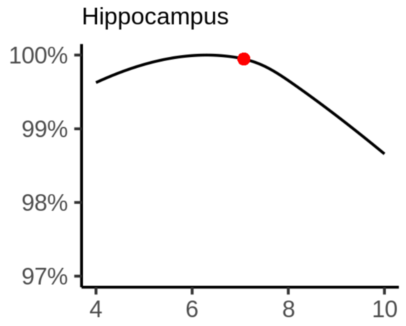

Comparison of mean sleep and sleep associated with maximum volume

A 95 % confidence interval for the sleep associated with maximum volume is [5.33, 7.15].

The plot below compares average sleep to the sleep associated with maximum volume.

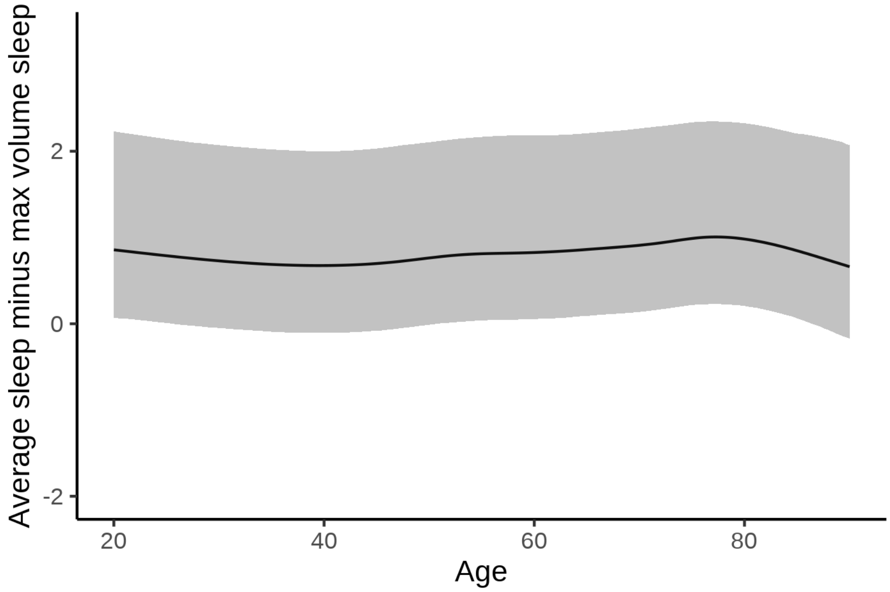

The next plot shows the difference between average sleep and sleep associated with maximum volume. The shaded region is a 95 % confidence interval.

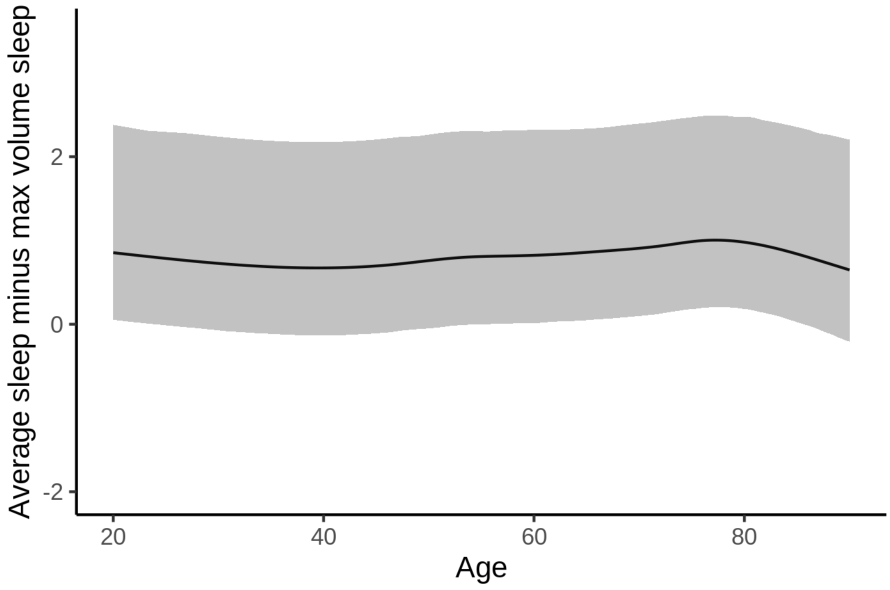

The next plot shows the probability that the sleep duration associated with maximum volume is longer than the average sleep duration, as a function of age. Probability below .05 can be interpreted as evidence that the sleep associated with maximum volume is shorter than the mean sleep, and probability above .95 can be interpreted the opposite way.

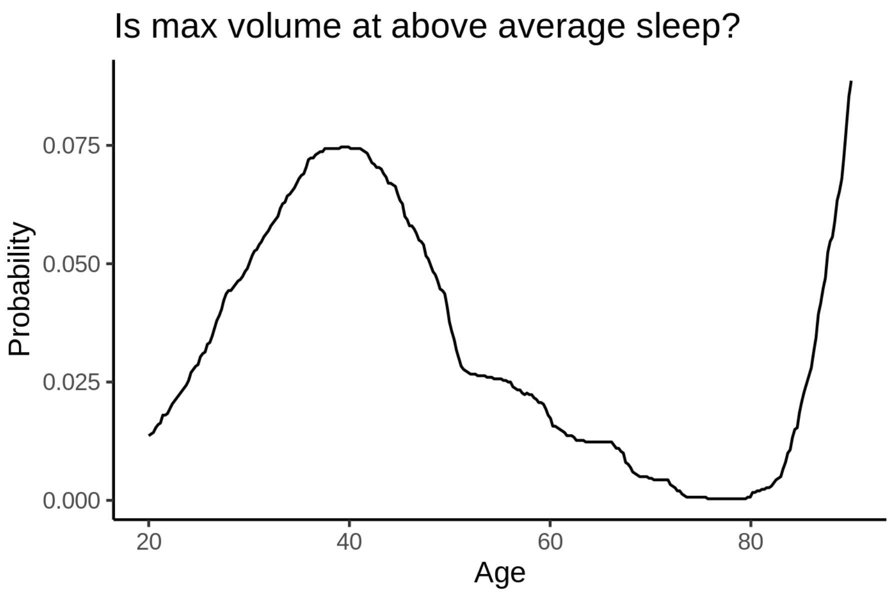

Controling for covariates

Below is the output for a model in which we only include data with income and education.

```
##
## Family: gaussian
## Link function: identity
##
## Formula:
## value ~ sex + site + s(age_z, k = 10, bs = "cr") + s(sleep_z,
##     k = 5, bs = "cr") + icv
## <environment: 0x558a48667170>
##
## Parametric coefficients:
##                Estimate Std. Error t value Pr(>|t|)
## (Intercept)    8469.311     82.446 102.725  < 2e-16 ***
## sexmale         102.521      9.018  11.368  < 2e-16 ***
## siteousAvanto -898.627     96.829  -9.281  < 2e-16 ***
## siteousPrisma -344.268    100.492  -3.426 0.000614 ***
## siteousSkyra  -556.398     88.938  -6.256 4.00e-10 ***
## siteUKB       -457.516     82.303  -5.559 2.74e-08 ***
## siteUOXF      -543.695     92.544  -5.875 4.27e-09 ***
## icv            412.033      4.628  89.035  < 2e-16 ***
## ---
## Signif. codes:  0 ‘***’ 0.001 ‘**’ 0.01 ‘*’ 0.05 ‘.’ 0.1 ‘ ’ 1
##
## Approximate significance of smooth terms:
##              edf Ref.df       F  p-value
## s(age_z)   6.898  6.898 906.250  < 2e-16 ***
## s(sleep_z) 2.701  2.701   7.544 0.000231 ***
## ---
## Signif. codes:  0 ‘***’ 0.001 ‘**’ 0.01 ‘*’ 0.05 ‘.’ 0.1 ‘ ’ 1
##
```

```
## R-sq.(adj) =  0.401
## lmer.REML = 4.846e+05  Scale est. = 23769     n = 31190
```

Below is the output for a model in which we control for the main effects of income and education.

```
##
## Family: gaussian
## Link function: identity
##
## Formula:
## value ~ sex + site + s(age_z, k = 10, bs = "cr") + s(sleep_z,
##     k = 5, bs = "cr") + icv + income_scaled + education_scaled
## <environment: 0x558a48667170>
##
## Parametric coefficients:
##                   Estimate Std. Error t value Pr(>|t|)
## (Intercept)       8438.563     82.959 101.720  < 2e-16 ***
## sexmale            102.719      9.043  11.359  < 2e-16 ***
## siteousAvanto     -898.983     96.844  -9.283  < 2e-16 ***
## siteousPrisma     -347.177    100.481  -3.455 0.000551 ***
## siteousSkyra      -557.210     88.947  -6.265 3.79e-10 ***
## siteUKB           -457.907     82.338  -5.561 2.70e-08 ***
## siteUOXF          -534.197     92.579  -5.770 7.99e-09 ***
## icv                410.078      4.666  87.893  < 2e-16 ***
## income_scaled       18.482     10.750   1.719 0.085569 .
## education_scaled    28.624     12.707   2.253 0.024284 *
## ---
## Signif. codes:  0 '***' 0.001 '**' 0.01 '*' 0.05 '.' 0.1 ' ' 1
##
## Approximate significance of smooth terms:
##              edf Ref.df       F  p-value
## s(age_z)   6.897  6.897 825.692  < 2e-16 ***
## s(sleep_z) 2.595  2.595   7.106 0.000452 ***
## ---
## Signif. codes:  0 '***' 0.001 '**' 0.01 '*' 0.05 '.' 0.1 ' ' 1
##
## R-sq.(adj) =  0.402
## lmer.REML = 4.8458e+05  Scale est. = 23791     n = 31190
```

We also included interaction effects between sleep duration and education and income, in another model. The output is shown below, and the interaction terms are `income_scaled:sleep_z` and `education_scaled:sleep_z`.

```
##
## Family: gaussian
## Link function: identity
##
## Formula:
## value ~ sex + site + s(age_z, k = 10, bs = "cr") + s(sleep_z,
##     k = 5, bs = "cr") + icv + income_scaled + education_scaled +
##     income_scaled:sleep_z + education_scaled:sleep_z
## <environment: 0x558a48667170>
##
## Parametric coefficients:
##                           Estimate Std. Error t value Pr(>|t|)
## (Intercept)               8438.866     82.963 101.718  < 2e-16 ***
```

```
## sexmale                   102.082      9.049  11.281  < 2e-16 ***
## siteousAvanto            -900.126     96.845  -9.295  < 2e-16 ***
## siteousPrisma            -349.758    100.489  -3.481 0.000501 ***
## siteousSkyra             -558.326     88.947  -6.277 3.50e-10 ***
## siteUKB                  -458.180     82.345  -5.564 2.66e-08 ***
## siteUOXF                 -534.034     92.603  -5.767 8.15e-09 ***
## icv                       410.146      4.666  87.901  < 2e-16 ***
## income_scaled              19.087     10.755   1.775 0.075968 .
## education_scaled           28.215     12.708   2.220 0.026413 *
## income_scaled:sleep_z     -18.477     10.703  -1.726 0.084315 .
## education_scaled:sleep_z   14.270     12.331   1.157 0.247173
## ---
## Signif. codes:  0 '***' 0.001 '**' 0.01 '*' 0.05 '.' 0.1 ' ' 1
##
## Approximate significance of smooth terms:
##              edf Ref.df       F p-value
## s(age_z)   6.897  6.897 824.909  <2e-16 ***
## s(sleep_z) 2.635  2.635   4.576  0.0111 *
## ---
## Signif. codes:  0 '***' 0.001 '**' 0.01 '*' 0.05 '.' 0.1 ' ' 1
##
## R-sq.(adj) =  0.402
## lmer.REML = 4.8456e+05  Scale est. = 23791     n = 31190
```

We did the same controling for BMI. Below is the model with no covariates but only keeping data with BMI.

```
##
## Family: gaussian
## Link function: identity
##
## Formula:
## value ~ sex + site + s(age_z, k = 10, bs = "cr") + s(sleep_z,
##     k = 5, bs = "cr") + icv
## <environment: 0x558a47e37130>
##
## Parametric coefficients:
##               Estimate Std. Error t value Pr(>|t|)
## (Intercept)   7606.759     52.116 145.959  < 2e-16 ***
## sexmale         96.281      8.758  10.994  < 2e-16 ***
## siteousPrisma  471.568     69.283   6.806 1.02e-11 ***
## siteousSkyra   387.221     38.042  10.179  < 2e-16 ***
## siteUCAM       430.543     55.307   7.785 7.20e-15 ***
## siteUKB        411.476     52.624   7.819 5.47e-15 ***
## siteUmU        435.593     63.458   6.864 6.80e-12 ***
## icv            417.266      4.507  92.578  < 2e-16 ***
## ---
## Signif. codes:  0 '***' 0.001 '**' 0.01 '*' 0.05 '.' 0.1 ' ' 1
##
## Approximate significance of smooth terms:
##              edf Ref.df      F  p-value
## s(age_z)   7.289  7.289 987.33  < 2e-16 ***
## s(sleep_z) 2.596  2.596   7.91 0.000148 ***
## ---
## Signif. codes:  0 '***' 0.001 '**' 0.01 '*' 0.05 '.' 0.1 ' ' 1
##
```

```
## R-sq.(adj) =  0.412
## lmer.REML = 5.1907e+05  Scale est. = 22530     n = 33445
```

Below is the model output with main effect.

```
##
## Family: gaussian
## Link function: identity
##
## Formula:
## value ~ sex + site + s(age_z, k = 10, bs = "cr") + s(sleep_z,
##     k = 5, bs = "cr") + icv + bmi
## <environment: 0x558a47e37130>
##
## Parametric coefficients:
##                Estimate Std. Error t value Pr(>|t|)
## (Intercept)   7589.5638    56.4029 134.560  < 2e-16 ***
## sexmale         95.7381     8.7840  10.899  < 2e-16 ***
## siteousPrisma  471.0061    69.2870   6.798 1.08e-11 ***
## siteousSkyra   387.1637    38.0431  10.177  < 2e-16 ***
## siteUCAM       430.1804    55.3100   7.778 7.60e-15 ***
## siteUKB        410.9495    52.6289   7.808 5.96e-15 ***
## siteUmU        434.8825    63.4645   6.852 7.39e-12 ***
## icv            417.1578     4.5092  92.514  < 2e-16 ***
## bmi              0.6817     0.8548   0.797    0.425
## ---
## Signif. codes:  0 ‘***’ 0.001 ‘**’ 0.01 ‘*’ 0.05 ‘.’ 0.1 ‘ ’ 1
##
## Approximate significance of smooth terms:
##              edf Ref.df      F  p-value
## s(age_z)   7.288  7.288 987.09  < 2e-16 ***
## s(sleep_z) 2.614  2.614   7.61 0.000144 ***
## ---
## Signif. codes:  0 ‘***’ 0.001 ‘**’ 0.01 ‘*’ 0.05 ‘.’ 0.1 ‘ ’ 1
##
## R-sq.(adj) =  0.412
## lmer.REML = 5.1907e+05  Scale est. = 22531     n = 33445
```

Next is the model with BMI-sleep interaction.

```
##
## Family: gaussian
## Link function: identity
##
## Formula:
## value ~ sex + site + s(age_z, k = 10, bs = "cr") + s(sleep_z,
##     k = 5, bs = "cr") + icv + bmi + bmi:sleep_z
## <environment: 0x558a47e37130>
##
## Parametric coefficients:
##                Estimate Std. Error t value Pr(>|t|)
## (Intercept)   7587.9785    56.4324 134.461  < 2e-16 ***
## sexmale         95.7691     8.7841  10.903  < 2e-16 ***
## siteousPrisma  471.0122    69.2870   6.798 1.08e-11 ***
## siteousSkyra   387.2929    38.0431  10.180  < 2e-16 ***
## siteUCAM       430.7383    55.3136   7.787 7.05e-15 ***
```

```
## siteUKB        411.5115    52.6327   7.819 5.50e-15 ***
## siteUmU        435.3608    63.4669   6.860 7.02e-12 ***
## icv            417.1870     4.5093  92.517  < 2e-16 ***
## bmi              0.7073     0.8553   0.827    0.408
## bmi:sleep_z      0.7032     0.8108   0.867    0.386
## ---
## Signif. codes:  0 '***' 0.001 '**' 0.01 '*' 0.05 '.' 0.1 ' ' 1
##
## Approximate significance of smooth terms:
##              edf Ref.df       F p-value
## s(age_z)   7.287  7.287 987.291 < 2e-16 ***
## s(sleep_z) 2.610  2.610   4.226 0.00772 **
## ---
## Signif. codes:  0 '***' 0.001 '**' 0.01 '*' 0.05 '.' 0.1 ' ' 1
##
## R-sq.(adj) =  0.412
## lmer.REML = 5.1907e+05  Scale est. = 22530     n = 33445
```

We did the same controling for depression. Below is the model with no covariates but only keeping data with depression.

```
##
## Family: gaussian
## Link function: identity
##
## Formula:
## value ~ sex + site + s(age_z, k = 10, bs = "cr") + s(sleep_z,
##     k = 5, bs = "cr") + icv
## <environment: 0x558a46be14b8>
##
## Parametric coefficients:
##                Estimate Std. Error t value Pr(>|t|)
## (Intercept)    8352.007     41.973 198.984  < 2e-16 ***
## sexmale          95.391      8.806  10.833  < 2e-16 ***
## siteousAvanto  -710.959     83.921  -8.472  < 2e-16 ***
## siteousPrisma  -194.873     79.554  -2.450   0.0143 *
## siteousSkyra   -296.464     62.943  -4.710 2.49e-06 ***
## siteUCAM       -351.896     57.537  -6.116 9.70e-10 ***
## siteUKB        -338.155     41.512  -8.146 3.89e-16 ***
## siteUmU        -305.071     56.245  -5.424 5.87e-08 ***
## icv             414.420      4.525  91.578  < 2e-16 ***
## ---
## Signif. codes:  0 '***' 0.001 '**' 0.01 '*' 0.05 '.' 0.1 ' ' 1
##
## Approximate significance of smooth terms:
##              edf Ref.df       F  p-value
## s(age_z)   7.219  7.219 954.439  < 2e-16 ***
## s(sleep_z) 2.670  2.670   9.018 4.83e-05 ***
## ---
## Signif. codes:  0 '***' 0.001 '**' 0.01 '*' 0.05 '.' 0.1 ' ' 1
##
## R-sq.(adj) =  0.406
## lmer.REML = 5.1749e+05  Scale est. = 22060     n = 33357
```

Below is the model output with main effect.

```
##
## Family: gaussian
## Link function: identity
##
## Formula:
## value ~ sex + site + s(age_z, k = 10, bs = "cr") + s(sleep_z,
##     k = 5, bs = "cr") + icv + depression
## <environment: 0x558a46be14b8>
##
## Parametric coefficients:
##               Estimate Std. Error t value Pr(>|t|)
## (Intercept)   8368.205     42.142 198.572  < 2e-16 ***
## sexmale         93.712      8.813  10.634  < 2e-16 ***
## siteousAvanto -702.588     83.923  -8.372  < 2e-16 ***
## siteousPrisma -187.324     79.553  -2.355   0.0185 *
## siteousSkyra  -287.038     62.965  -4.559 5.17e-06 ***
## siteUCAM      -348.098     57.529  -6.051 1.46e-09 ***
## siteUKB       -344.803     41.533  -8.302  < 2e-16 ***
## siteUmU       -255.878     57.431  -4.455 8.40e-06 ***
## icv            414.674      4.524  91.655  < 2e-16 ***
## depression    -132.626     31.489  -4.212 2.54e-05 ***
## ---
## Signif. codes:  0 '***' 0.001 '**' 0.01 '*' 0.05 '.' 0.1 ' ' 1
##
## Approximate significance of smooth terms:
##              edf Ref.df       F  p-value
## s(age_z)   7.210  7.210 953.699  < 2e-16 ***
## s(sleep_z) 2.447  2.447   9.146 5.27e-05 ***
## ---
## Signif. codes:  0 '***' 0.001 '**' 0.01 '*' 0.05 '.' 0.1 ' ' 1
##
## R-sq.(adj) =  0.406
## lmer.REML = 5.1747e+05  Scale est. = 22051     n = 33357
```

Next is the model with depression-sleep interaction.

```
##
## Family: gaussian
## Link function: identity
##
## Formula:
## value ~ sex + site + s(age_z, k = 10, bs = "cr") + s(sleep_z,
##     k = 5, bs = "cr") + icv + depression + depression:sleep_z
## <environment: 0x558a46be14b8>
##
## Parametric coefficients:
##                    Estimate Std. Error t value Pr(>|t|)
## (Intercept)        8368.208     42.142 198.572  < 2e-16 ***
## sexmale              93.554      8.814  10.614  < 2e-16 ***
## siteousAvanto      -703.682     83.930  -8.384  < 2e-16 ***
## siteousPrisma      -188.428     79.560  -2.368   0.0179 *
## siteousSkyra       -288.090     62.973  -4.575 4.78e-06 ***
## siteUCAM           -349.229     57.540  -6.069 1.30e-09 ***
## siteUKB            -344.746     41.533  -8.301  < 2e-16 ***
## siteUmU            -248.433     57.923  -4.289 1.80e-05 ***
```

```
## icv                  414.688      4.524  91.657  < 2e-16 ***
## depression          -134.501     31.548  -4.263 2.02e-05 ***
## depression:sleep_z  -22.906     23.170  -0.989   0.3229
## ---
## Signif. codes:  0 '***' 0.001 '**' 0.01 '*' 0.05 '.' 0.1 ' ' 1
##
## Approximate significance of smooth terms:
##              edf Ref.df       F p-value
## s(age_z)   7.208  7.208 954.084 < 2e-16 ***
## s(sleep_z) 2.437  2.437   5.718 0.00154 **
## ---
## Signif. codes:  0 '***' 0.001 '**' 0.01 '*' 0.05 '.' 0.1 ' ' 1
##
## R-sq.(adj) =  0.406
## lmer.REML = 5.1746e+05  Scale est. = 22050     n = 33357
```

The plot below shows the sleep-volume curve for the original model and for the model with main effects of SES.

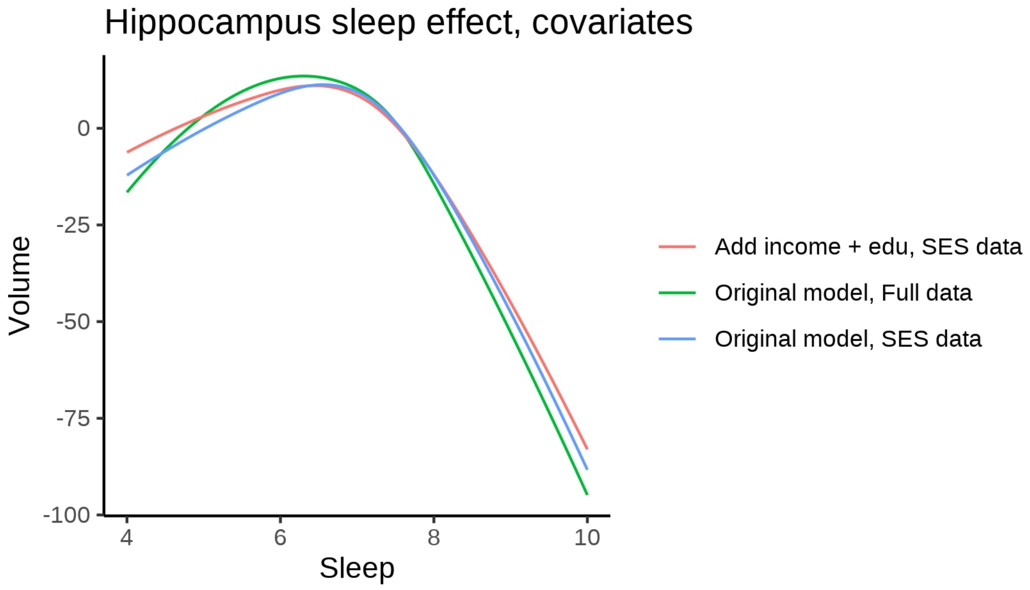

The plot below shows the sleep-volume curve for the original model and for the model with main effects of BMI.

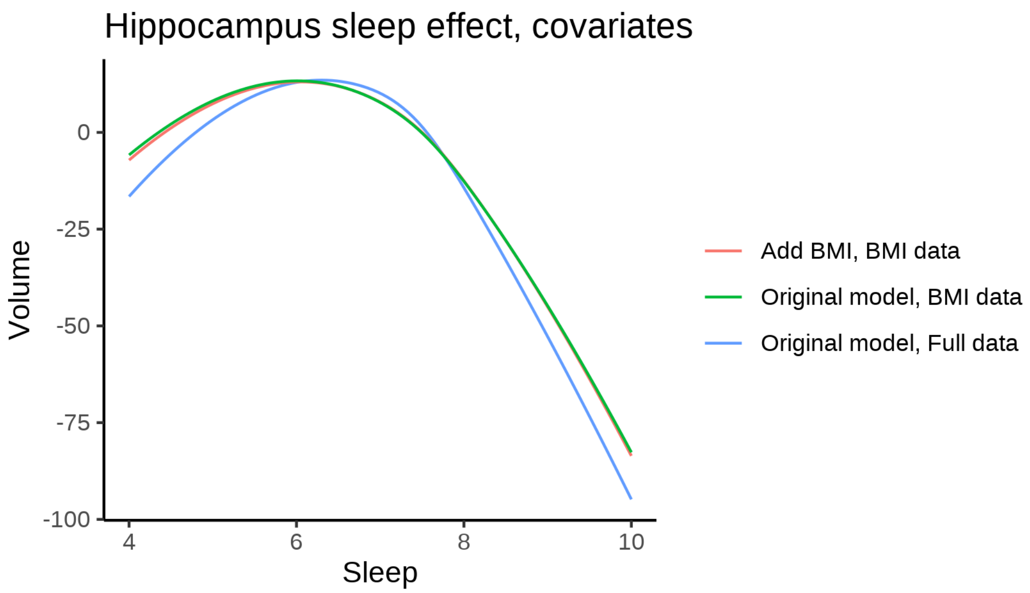

The plot below shows the sleep-volume curve for the original model and for the model with main effects of depression.

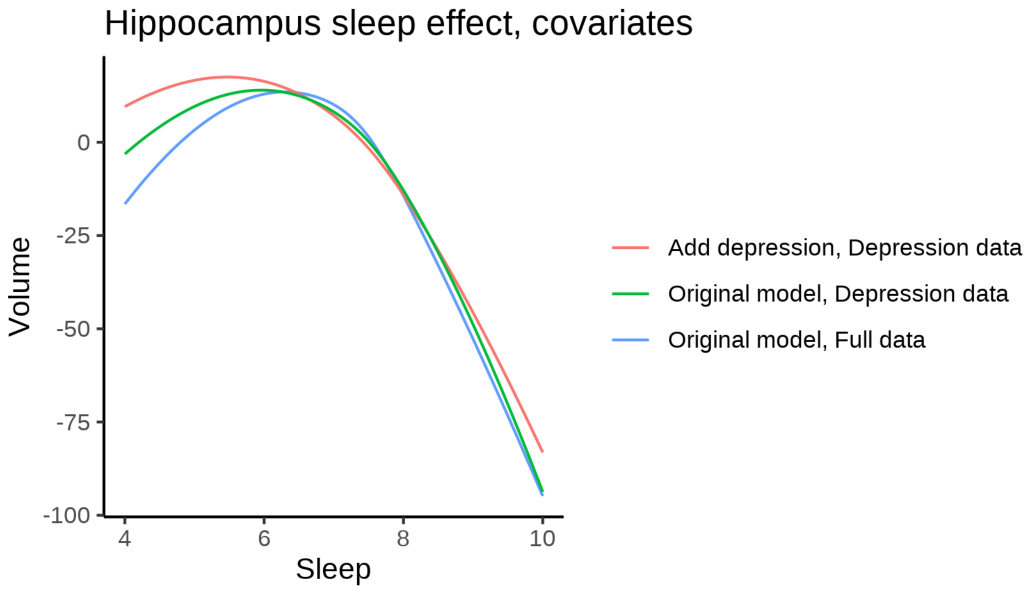

Models Not controling for ICV

Model outputs

Model without sleep term

```
##
## Family: gaussian
## Link function: identity
##
## Formula:
```

```
## value ~ sex + site + s(age_z, k = 10, bs = "cr")
## <environment: 0x558965378b70>
##
## Parametric coefficients:
##                Estimate Std. Error t value Pr(>|t|)
## (Intercept)    7890.184     37.974 207.780  < 2e-16 ***
## sexmale         572.297      6.562  87.219  < 2e-16 ***
## siteMPIB       -356.342     49.083  -7.260 3.93e-13 ***
## siteousAvanto -319.047     38.744  -8.235  < 2e-16 ***
## siteousPrisma     4.699     65.126   0.072   0.9425
## siteousSkyra  -164.791     37.586  -4.384 1.17e-05 ***
## siteUB        -250.702    117.823  -2.128   0.0334 *
## siteUCAM       -78.029     42.751  -1.825   0.0680 .
## siteUKB        -85.667     38.980  -2.198   0.0280 *
## siteUmU       -257.559     55.418  -4.648 3.37e-06 ***
## siteUOXF      -389.833     46.549  -8.375  < 2e-16 ***
## ---
## Signif. codes:  0 '***' 0.001 '**' 0.01 '*' 0.05 '.' 0.1 ' ' 1
##
## Approximate significance of smooth terms:
##            edf Ref.df    F p-value
## s(age_z) 7.553  7.553 1521  <2e-16 ***
## ---
## Signif. codes:  0 '***' 0.001 '**' 0.01 '*' 0.05 '.' 0.1 ' ' 1
##
## R-sq.(adj) =  0.276
## lmer.REML = 8.0789e+05  Scale est. = 23528     n = 51279
```

Model with only main effects of age and sleep

```
##
## Family: gaussian
## Link function: identity
##
## Formula:
## value ~ sex + site + s(age_z, k = 10, bs = "cr") + s(sleep_z,
##     k = 5, bs = "cr")
## <environment: 0x558965378b70>
##
## Parametric coefficients:
##                Estimate Std. Error t value Pr(>|t|)
## (Intercept)    7895.006     37.995 207.789  < 2e-16 ***
## sexmale         570.709      6.561  86.985  < 2e-16 ***
## siteMPIB       -353.222     49.075  -7.198 6.21e-13 ***
## siteousAvanto -323.584     38.737  -8.353  < 2e-16 ***
## siteousPrisma     1.231     65.096   0.019   0.9849
## siteousSkyra  -169.820     37.578  -4.519 6.22e-06 ***
## siteUB        -244.918    117.738  -2.080   0.0375 *
## siteUCAM       -76.844     42.731  -1.798   0.0721 .
## siteUKB        -89.993     39.001  -2.307   0.0210 *
## siteUmU       -252.812     55.506  -4.555 5.26e-06 ***
## siteUOXF      -391.929     46.525  -8.424  < 2e-16 ***
## ---
## Signif. codes:  0 '***' 0.001 '**' 0.01 '*' 0.05 '.' 0.1 ' ' 1
##
```

```
## Approximate significance of smooth terms:
##               edf Ref.df       F p-value
## s(age_z)   7.543  7.543 1499.75  <2e-16 ***
## s(sleep_z) 3.492  3.492   18.63  <2e-16 ***
## ---
## Signif. codes:  0 ‘***’ 0.001 ‘**’ 0.01 ‘*’ 0.05 ‘.’ 0.1 ‘ ’ 1
##
## R-sq.(adj) =  0.277
## lmer.REML = 8.0783e+05  Scale est. = 23540     n = 51279
```

Model with full interaction between age and sleep

```
##
## Family: gaussian
## Link function: identity
##
## Formula:
## value ~ sex + site + t2(age_z, sleep_z, k = c(10, 4), bs = "cr")
## <environment: 0x558965378b70>
##
## Parametric coefficients:
##                  Estimate Std. Error t value Pr(>|t|)
## (Intercept)   7893.37056   37.75700 209.057  < 2e-16 ***
## sexmale        571.54360    6.57358  86.945  < 2e-16 ***
## siteMPIB      -358.15036   49.09817  -7.295 3.04e-13 ***
## siteousAvanto -321.46608   38.83774  -8.277  < 2e-16 ***
## siteousPrisma    0.03865   65.22855   0.001   0.9995
## siteousSkyra  -167.53186   37.68760  -4.445 8.80e-06 ***
## siteUB        -251.84540  117.68892  -2.140   0.0324 *
## siteUCAM       -80.03449   42.75006  -1.872   0.0612 .
## siteUKB        -88.56632   38.72929  -2.287   0.0222 *
## siteUmU       -256.95138   55.42601  -4.636 3.56e-06 ***
## siteUOXF      -392.59384   46.37095  -8.466  < 2e-16 ***
## ---
## Signif. codes:  0 ‘***’ 0.001 ‘**’ 0.01 ‘*’ 0.05 ‘.’ 0.1 ‘ ’ 1
##
## Approximate significance of smooth terms:
##                     edf Ref.df     F p-value
## t2(age_z,sleep_z) 12.43  12.43 150.5  <2e-16 ***
## ---
## Signif. codes:  0 ‘***’ 0.001 ‘**’ 0.01 ‘*’ 0.05 ‘.’ 0.1 ‘ ’ 1
##
## R-sq.(adj) =  0.277
## lmer.REML = 8.0784e+05  Scale est. = 23536     n = 51279
```

Model comparison

`mod_no_sleep` refers to model without sleep term, `mod_no_interaction` refers to model with only main effect of sleep, and `mod_full` refers to model with a full interaction between age and sleep. This is a nested model comparison, and the p-value at a given line refers to comparing the model at the line to the model on the line above. Hence, significance implies that the more complicated model is supported on statistical grounds.

To be even more specific, the p-value on the second row tests whether there is an association between sleep and volume. The p-value on the third row tests whether this association depends on age.

```
## Data: NULL
## Models:
## ml$mod_no_sleep$mer: NULL
## ml$mod_no_interaction$mer: NULL
## ml$mod_full$mer: NULL
##                             npar    AIC    BIC  logLik deviance  Chisq Df Pr(>Chisq)
## ml$mod_no_sleep$mer           15 807917 808049 -403943   807887
## ml$mod_no_interaction$mer     17 807864 808014 -403915   807830 56.941  2  4.319e-13 ***
## ml$mod_full$mer               19 807876 808044 -403919   807838  0.000  2          1
## ---
## Signif. codes:  0 '***' 0.001 '**' 0.01 '*' 0.05 '.' 0.1 ' ' 1
```

We chose the model based on the likelihood ratio test with 5 % significance level, which was `mod_no_interaction`.

Lifespan brain trajectory

The trajectory shown is from the chosen model `mod_no_interaction_no_icv`.

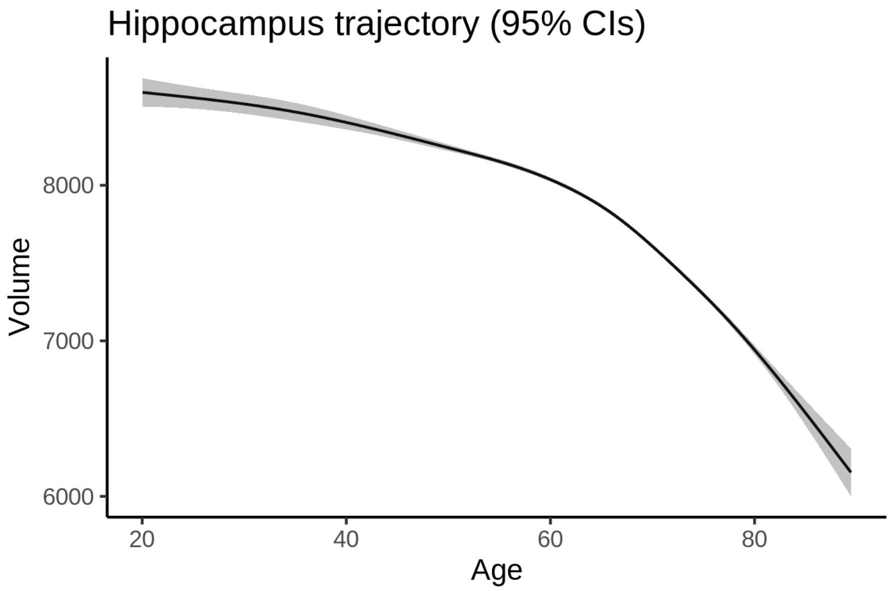

Effect of sleep

The chosen model only included the main effect of sleep, and hence the effect does not vary with age. The black dot shows the average sleep duration across all ages in the sample.

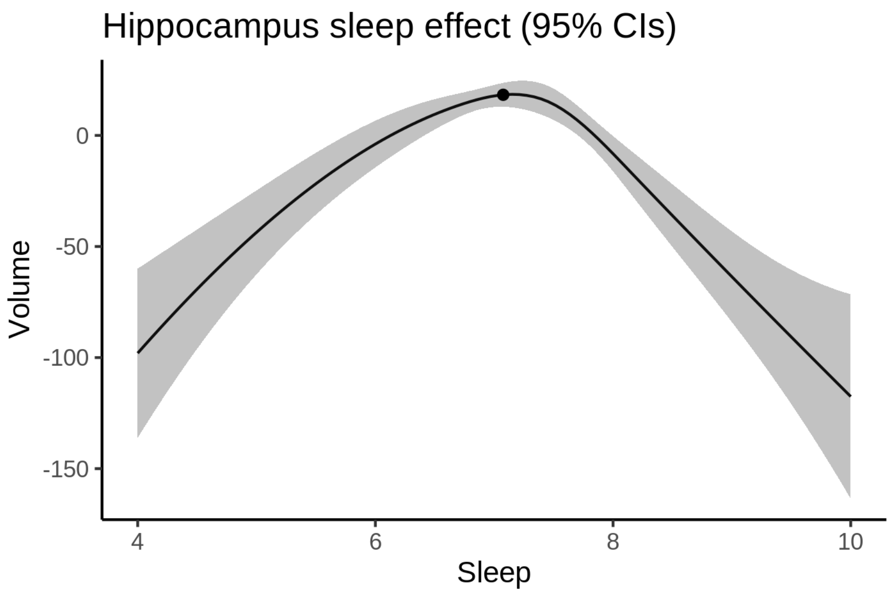

We also show the full interaction model for completeness, although it was not selected.

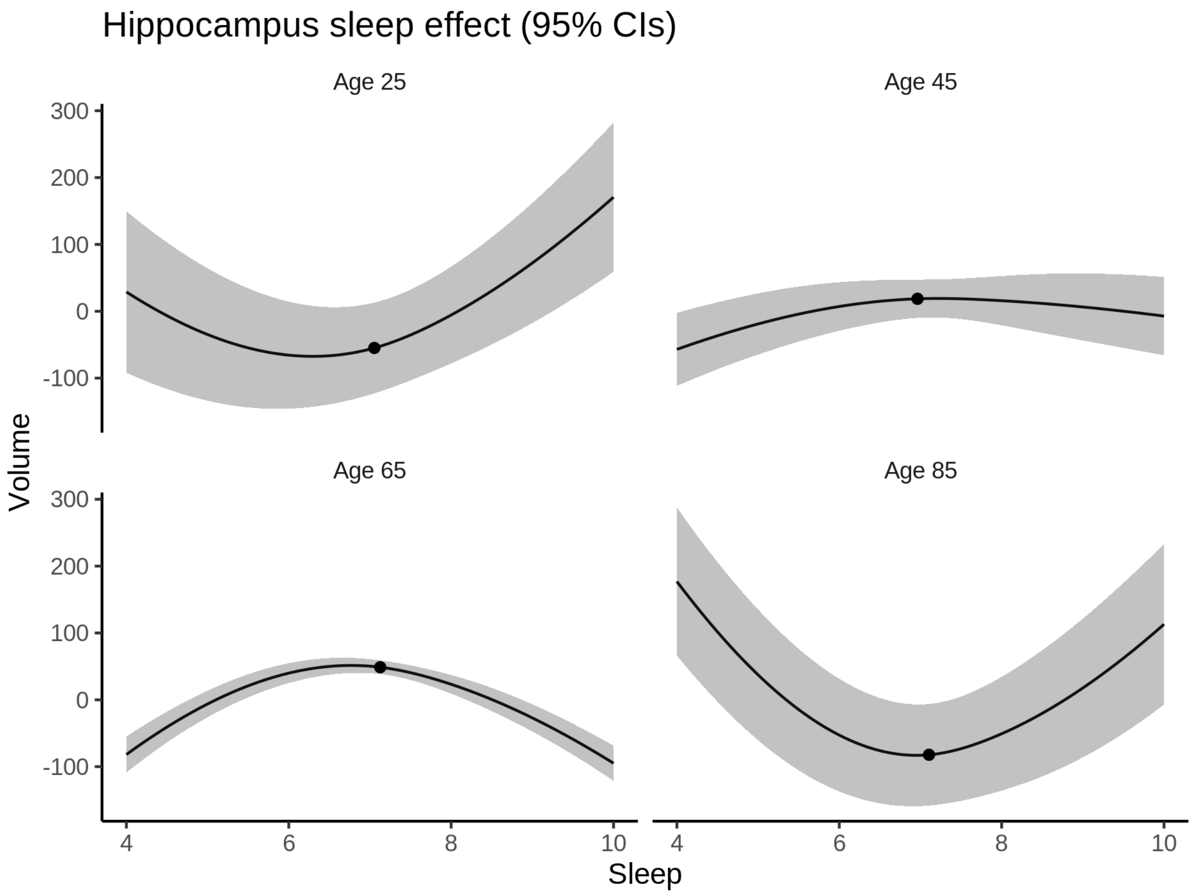

Deviation from sleep associated with maximal volume

Model with only main effect of sleep was chosen, so we show it for all ages at once. Maximum volume is attained at 7.2 hours of sleep. The percentage values in the plot are calculated as follows: The maximum at 100 % refers to a person at an arbitrary age with a sleep duration associated with maximum volume. For a female, this volume is 7875 and for a male it is 8445. The other percentage values show how large the expected volume is for someone with other sleep durations. For example, 99 % implies a 1 % reduction.

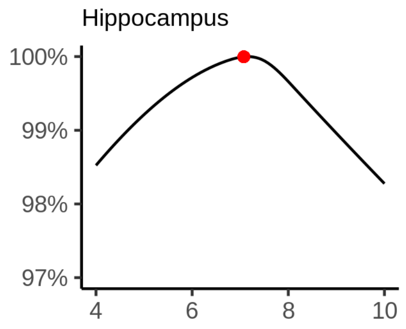

Comparison of mean sleep and sleep associated with maximum volume

A 95 % confidence interval for the sleep associated with maximum volume is [6.91, 7.33].

The plot below compares average sleep to the sleep associated with maximum volume.

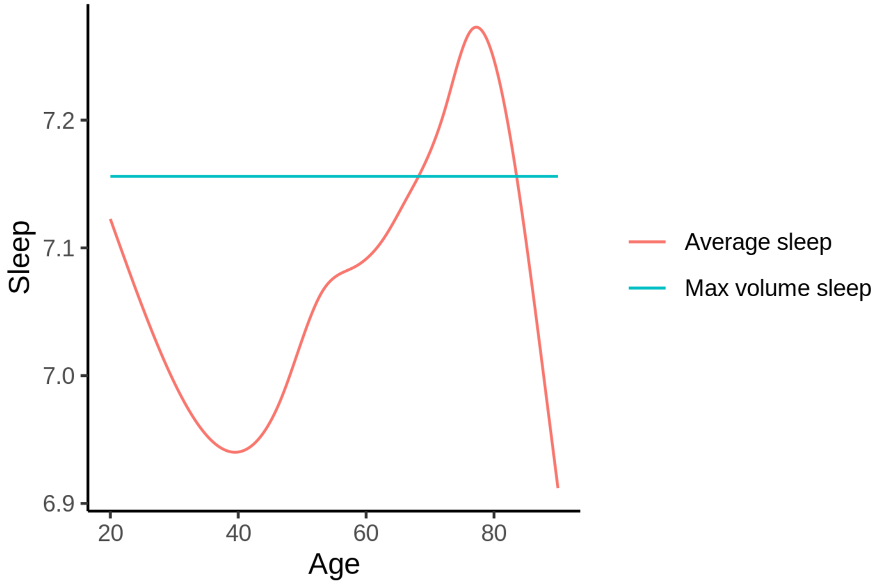

The next plot shows the difference between average sleep and sleep associated with maximum volume. The shaded region is a 95 % confidence interval.

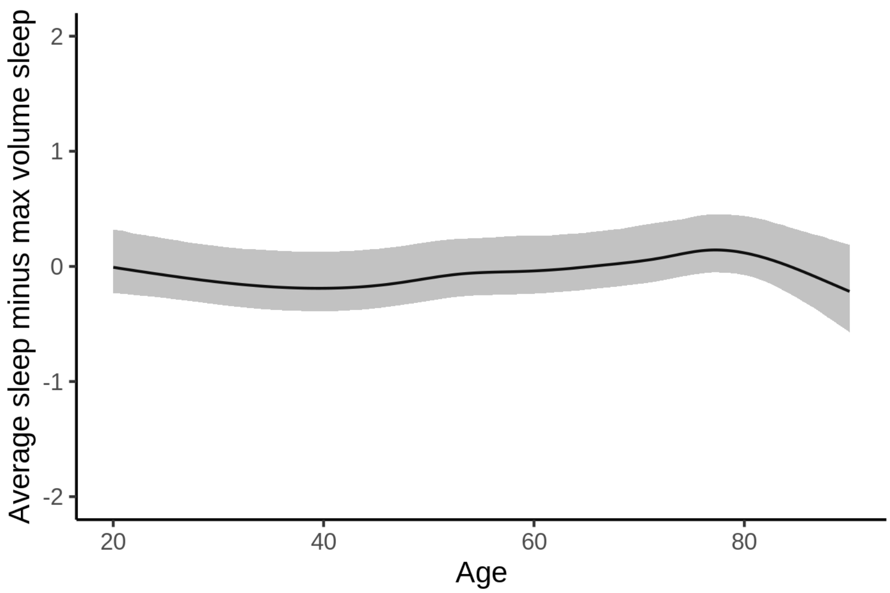

The next plot shows the probability that the sleep duration associated with maximum volume is longer than the average sleep duration, as a function of age. Probability below .05 can be interpreted as evidence that the sleep associated with maximum volume is shorter than the mean sleep, and probability above .95 can be interpreted the opposite way.

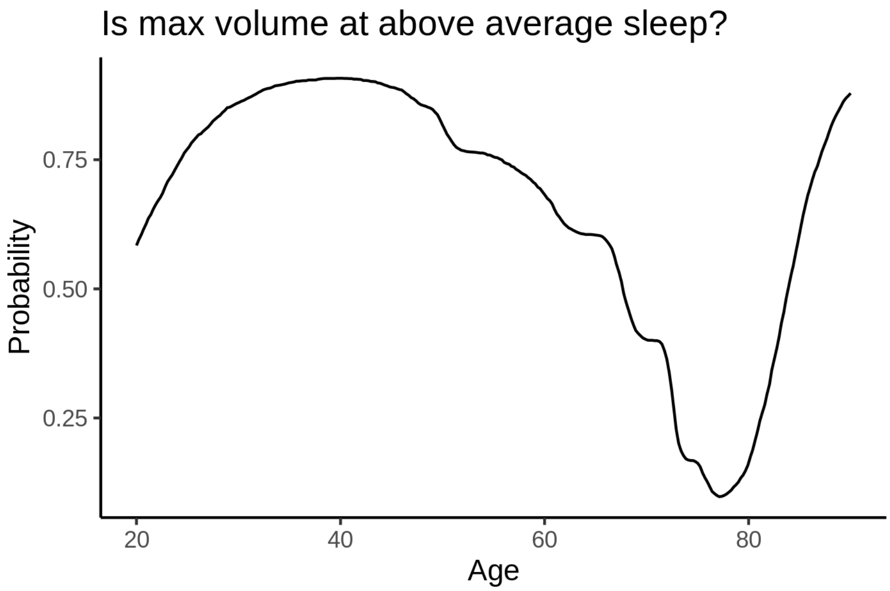

Pallidum

Descriptive statistics

Study	Observations	Unique IDs	Mean age	Age range
HCP	974	974	28.8	22 - 37
MPIB	677	391	63.1	24 - 83

Study	Observations	Unique IDs	Mean age	Age range
UB	113	39	70.9	64 - 81
UCAM	884	632	55.1	20 - 88
UiO	1474	803	49.3	20 - 89
UKB	45983	43137	64.5	45 - 83
UmU	412	279	62.2	25 - 85
UOXF	769	769	69.8	60 - 85

Spaghetti plot

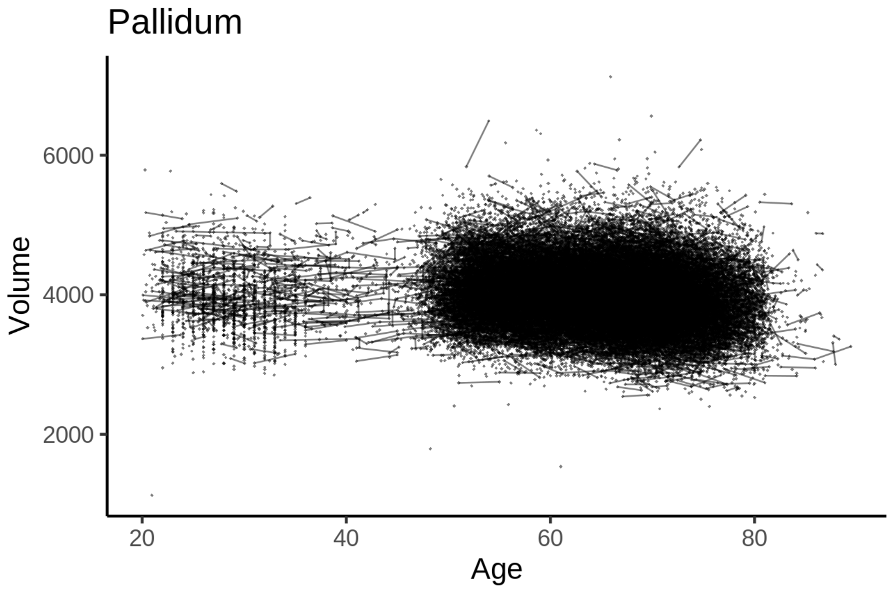

Models Controling for ICV

Model outputs

Model without sleep term

```
##
## Family: gaussian
## Link function: identity
##
## Formula:
## value ~ sex + site + icv + s(age_z, k = 10, bs = "cr")
## <environment: 0x5589571ece60>
##
## Parametric coefficients:
##                Estimate Std. Error t value Pr(>|t|)
## (Intercept)    3623.343     19.038 190.323  < 2e-16 ***
## sexmale          84.740      3.870  21.896  < 2e-16 ***
## siteMPIB        516.546     24.195  21.350  < 2e-16 ***
## siteousAvanto   113.472     19.767   5.740 9.50e-09 ***
## siteousPrisma   229.824     33.415   6.878 6.15e-12 ***
## siteousSkyra    231.801     18.449  12.565  < 2e-16 ***
```

```
## siteUB          79.105     56.467   1.401    0.161
## siteUCAM       260.690     20.996  12.416  < 2e-16 ***
## siteUKB        346.852     19.527  17.762  < 2e-16 ***
## siteUmU        145.754     27.131   5.372 7.81e-08 ***
## siteUOXF       104.884     23.085   4.543 5.55e-06 ***
## icv            260.238      1.947 133.663  < 2e-16 ***
## ---
## Signif. codes:  0 ‘***’ 0.001 ‘**’ 0.01 ‘*’ 0.05 ‘.’ 0.1 ‘ ’ 1
##
## Approximate significance of smooth terms:
##            edf Ref.df     F p-value
## s(age_z) 6.897  6.897 197.9  <2e-16 ***
## ---
## Signif. codes:  0 ‘***’ 0.001 ‘**’ 0.01 ‘*’ 0.05 ‘.’ 0.1 ‘ ’ 1
##
## R-sq.(adj) =  0.423
## lmer.REML = 7.3671e+05  Scale est. = 11441     n = 51286
```

Model with only main effects of age and sleep

```
##
## Family: gaussian
## Link function: identity
##
## Formula:
## value ~ sex + site + icv + s(age_z, k = 10, bs = "cr") + s(sleep_z,
##     k = 5, bs = "cr")
## <environment: 0x5589571ece60>
##
## Parametric coefficients:
##               Estimate Std. Error t value Pr(>|t|)
## (Intercept)   3624.196     19.056 190.183  < 2e-16 ***
## sexmale         84.770      3.870  21.903  < 2e-16 ***
## siteMPIB       515.617     24.212  21.296  < 2e-16 ***
## siteousAvanto  113.020     19.772   5.716 1.10e-08 ***
## siteousPrisma  229.521     33.416   6.869 6.56e-12 ***
## siteousSkyra   231.333     18.454  12.536  < 2e-16 ***
## siteUB          78.844     56.467   1.396    0.163
## siteUCAM       260.254     21.000  12.393  < 2e-16 ***
## siteUKB        345.945     19.548  17.697  < 2e-16 ***
## siteUmU        143.861     27.195   5.290 1.23e-07 ***
## siteUOXF       104.445     23.089   4.524 6.09e-06 ***
## icv            260.178      1.948 133.572  < 2e-16 ***
## ---
## Signif. codes:  0 ‘***’ 0.001 ‘**’ 0.01 ‘*’ 0.05 ‘.’ 0.1 ‘ ’ 1
##
## Approximate significance of smooth terms:
##              edf Ref.df      F p-value
## s(age_z)   6.897  6.897 197.91  <2e-16 ***
## s(sleep_z) 1.000  1.000   1.03    0.31
## ---
## Signif. codes:  0 ‘***’ 0.001 ‘**’ 0.01 ‘*’ 0.05 ‘.’ 0.1 ‘ ’ 1
##
## R-sq.(adj) =  0.423
## lmer.REML = 7.3671e+05  Scale est. = 11441     n = 51286
```

Model with full interaction between age and sleep

```
##
## Family: gaussian
## Link function: identity
##
## Formula:
## value ~ sex + site + icv + t2(age_z, sleep_z, k = c(10, 4), bs = "cr")
## <environment: 0x5589571ece60>
##
## Parametric coefficients:
##                Estimate Std. Error t value Pr(>|t|)
## (Intercept)   3627.809     18.887 192.084  < 2e-16 ***
## sexmale         85.273      3.878  21.987  < 2e-16 ***
## siteMPIB       512.051     24.205  21.155  < 2e-16 ***
## siteousAvanto  110.769     19.794   5.596 2.20e-08 ***
## siteousPrisma  227.337     33.471   6.792 1.12e-11 ***
## siteousSkyra   228.739     18.490  12.371  < 2e-16 ***
## siteUB          74.623     56.416   1.323    0.186
## siteUCAM       255.924     20.983  12.197  < 2e-16 ***
## siteUKB        341.998     19.362  17.664  < 2e-16 ***
## siteUmU        141.453     27.127   5.215 1.85e-07 ***
## siteUOXF        99.462     22.969   4.330 1.49e-05 ***
## icv            260.053      1.948 133.490  < 2e-16 ***
## ---
## Signif. codes:  0 ‘***’ 0.001 ‘**’ 0.01 ‘*’ 0.05 ‘.’ 0.1 ‘ ’ 1
##
## Approximate significance of smooth terms:
##                      edf Ref.df     F p-value
## t2(age_z,sleep_z) 11.08  11.08 26.33  <2e-16 ***
## ---
## Signif. codes:  0 ‘***’ 0.001 ‘**’ 0.01 ‘*’ 0.05 ‘.’ 0.1 ‘ ’ 1
##
## R-sq.(adj) =  0.424
## lmer.REML = 7.367e+05  Scale est. = 11432     n = 51286
```

Model comparison

`mod_no_sleep` refers to model without sleep term, `mod_no_interaction` refers to model with only main effect of sleep, and `mod_full` refers to model with a full interaction between age and sleep. This is a nested model comparison, and the p-value at a given line refers to comparing the model at the line to the model on the line above. Hence, significance implies that the more complicated model is supported on statistical grounds.

To be even more specific, the p-value on the second row tests whether there is an association between sleep and volume. The p-value on the third row tests whether this association depends on age.

```
## Data: NULL
## Models:
## ml$mod_no_sleep$mer: NULL
## ml$mod_no_interaction$mer: NULL
## ml$mod_full$mer: NULL
##                           npar    AIC    BIC  logLik deviance  Chisq Df Pr(>Chisq)
## ml$mod_no_sleep$mer         16 736738 736880 -368353   736706
## ml$mod_no_interaction$mer   18 736741 736900 -368353   736705 1.0303  2     0.5974
```

```
## ml$mod_full$mer            20 736742 736919 -368351   736702 3.4103  2     0.1817
```

We chose the model based on the likelihood ratio test with 5 % significance level, which was `mod_no_sleep`.

Lifespan brain trajectory

The trajectory shown is from the chosen model `mod_no_sleep`.

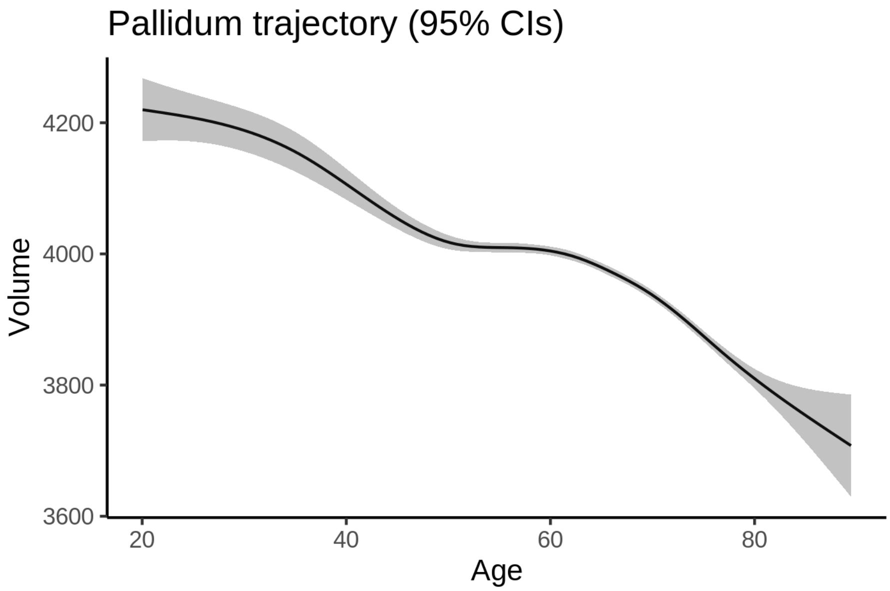

Effect of sleep

The chosen model did not include a sleep term, and hence we don't have any estimated effect of sleep.

We show the full interaction model for completeness, although it was not selected.

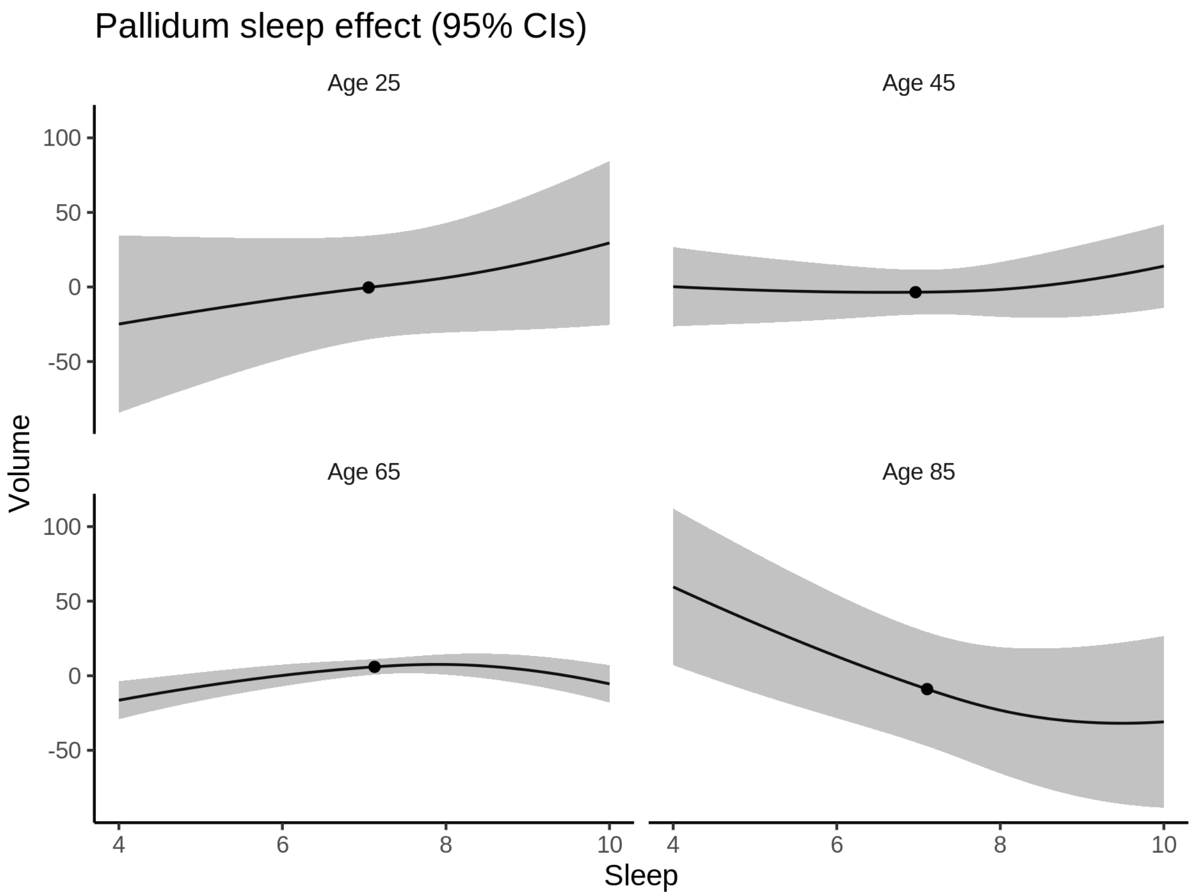

Deviation from sleep associated with maximal volume

Model with no sleep term was selected. No plots to show. (Although we can of course dig up the plots, which will be pretty flat).

Comparison of mean sleep and sleep associated with maximum volume

Nothing to show, as we did not find an association between sleep and volume.

Controling for covariates

Below is the output for a model in which we only include data with income and education.

```
##
## Family: gaussian
## Link function: identity
##
## Formula:
## value ~ sex + site + s(age_z, k = 10, bs = "cr") + s(sleep_z,
##     k = 5, bs = "cr") + icv
## <environment: 0x558ad64982c0>
##
## Parametric coefficients:
```

```
##                Estimate Std. Error t value Pr(>|t|)
## (Intercept)   4189.598     44.416  94.325  < 2e-16 ***
## sexmale         78.037      4.891  15.955  < 2e-16 ***
## siteousAvanto -441.819     54.577  -8.095 5.92e-16 ***
## siteousPrisma -318.789     54.758  -5.822 5.88e-09 ***
## siteousSkyra  -318.784     48.035  -6.637 3.26e-11 ***
## siteUKB       -219.792     44.340  -4.957 7.20e-07 ***
## siteUOXF      -408.274     49.929  -8.177 3.02e-16 ***
## icv            269.535      2.513 107.264  < 2e-16 ***
## ---
## Signif. codes:  0 ‘***’ 0.001 ‘**’ 0.01 ‘*’ 0.05 ‘.’ 0.1 ‘ ’ 1
##
## Approximate significance of smooth terms:
##               edf Ref.df       F p-value
## s(age_z)   5.989  5.989 115.067  <2e-16 ***
## s(sleep_z) 1.000  1.000   0.645   0.422
## ---
## Signif. codes:  0 ‘***’ 0.001 ‘**’ 0.01 ‘*’ 0.05 ‘.’ 0.1 ‘ ’ 1
##
## R-sq.(adj) =  0.423
## lmer.REML = 4.4782e+05  Scale est. = 12216     n = 31198
```

Below is the output for a model in which we control for the main effects of income and education.

```
##
## Family: gaussian
## Link function: identity
##
## Formula:
## value ~ sex + site + s(age_z, k = 10, bs = "cr") + s(sleep_z,
##     k = 5, bs = "cr") + icv + income_scaled + education_scaled
## <environment: 0x558ad64982c0>
##
## Parametric coefficients:
##                   Estimate Std. Error t value Pr(>|t|)
## (Intercept)      4167.611     44.691  93.254  < 2e-16 ***
## sexmale            78.433      4.904  15.994  < 2e-16 ***
## siteousAvanto    -443.357     54.578  -8.123 4.70e-16 ***
## siteousPrisma    -321.594     54.745  -5.874 4.29e-09 ***
## siteousSkyra     -320.521     48.033  -6.673 2.55e-11 ***
## siteUKB          -221.083     44.357  -4.984 6.26e-07 ***
## siteUOXF         -401.760     49.945  -8.044 9.00e-16 ***
## icv               268.109      2.534 105.793  < 2e-16 ***
## income_scaled       9.837      5.821   1.690 0.091034 .
## education_scaled   23.659      6.880   3.439 0.000585 ***
## ---
## Signif. codes:  0 ‘***’ 0.001 ‘**’ 0.01 ‘*’ 0.05 ‘.’ 0.1 ‘ ’ 1
##
## Approximate significance of smooth terms:
##               edf Ref.df       F p-value
## s(age_z)   6.008  6.008 102.528  <2e-16 ***
## s(sleep_z) 1.000  1.000   0.538   0.463
## ---
## Signif. codes:  0 ‘***’ 0.001 ‘**’ 0.01 ‘*’ 0.05 ‘.’ 0.1 ‘ ’ 1
##
```

```
## R-sq.(adj) =  0.424
## lmer.REML = 4.4779e+05  Scale est. = 12220     n = 31198
```

We also included interaction effects between sleep duration and education and income, in another model. The output is shown below, and the interaction terms are `income_scaled:sleep_z` and `education_scaled:sleep_z`.

```
##
## Family: gaussian
## Link function: identity
##
## Formula:
## value ~ sex + site + s(age_z, k = 10, bs = "cr") + s(sleep_z,
##     k = 5, bs = "cr") + icv + income_scaled + education_scaled +
##     income_scaled:sleep_z + education_scaled:sleep_z
## <environment: 0x558ad64982c0>
##
## Parametric coefficients:
##                          Estimate Std. Error t value Pr(>|t|)
## (Intercept)              4167.552     44.695  93.244  < 2e-16 ***
## sexmale                    78.145      4.907  15.924  < 2e-16 ***
## siteousAvanto            -443.762     54.580  -8.131 4.43e-16 ***
## siteousPrisma            -322.779     54.751  -5.895 3.78e-09 ***
## siteousSkyra             -320.929     48.034  -6.681 2.41e-11 ***
## siteUKB                  -221.039     44.363  -4.983 6.31e-07 ***
## siteUOXF                 -401.548     49.960  -8.037 9.50e-16 ***
## icv                       268.145      2.535 105.796  < 2e-16 ***
## income_scaled              10.147      5.824   1.742 0.081481 .
## education_scaled           23.497      6.880   3.415 0.000639 ***
## income_scaled:sleep_z      -8.279      5.761  -1.437 0.150701
## education_scaled:sleep_z    6.279      6.660   0.943 0.345794
## ---
## Signif. codes:  0 ‘***’ 0.001 ‘**’ 0.01 ‘*’ 0.05 ‘.’ 0.1 ‘ ’ 1
##
## Approximate significance of smooth terms:
##              edf Ref.df       F p-value
## s(age_z)   6.018  6.018 102.212  <2e-16 ***
## s(sleep_z) 1.000  1.000   0.002   0.966
## ---
## Signif. codes:  0 ‘***’ 0.001 ‘**’ 0.01 ‘*’ 0.05 ‘.’ 0.1 ‘ ’ 1
##
## R-sq.(adj) =  0.424
## lmer.REML = 4.4777e+05  Scale est. = 12220     n = 31198
```

We did the same controling for BMI. Below is the model with no covariates but only keeping data with BMI.

```
##
## Family: gaussian
## Link function: identity
##
## Formula:
## value ~ sex + site + s(age_z, k = 10, bs = "cr") + s(sleep_z,
##     k = 5, bs = "cr") + icv
## <environment: 0x558ad78498a8>
##
## Parametric coefficients:
```

```
##                Estimate Std. Error t value Pr(>|t|)
## (Intercept)   3720.417     32.793 113.452  < 2e-16 ***
## sexmale         75.663      4.760  15.897  < 2e-16 ***
## siteousPrisma  164.905     42.735   3.859 0.000114 ***
## siteousSkyra   177.662     26.977   6.586 4.60e-11 ***
## siteUCAM       173.722     34.320   5.062 4.18e-07 ***
## siteUKB        251.512     33.031   7.614 2.72e-14 ***
## siteUmU         56.722     38.202   1.485 0.137606
## icv            272.942      2.453 111.274  < 2e-16 ***
## ---
## Signif. codes:  0 ‘***’ 0.001 ‘**’ 0.01 ‘*’ 0.05 ‘.’ 0.1 ‘ ’ 1
##
## Approximate significance of smooth terms:
##              edf Ref.df       F p-value
## s(age_z)   6.085  6.085 132.435  <2e-16 ***
## s(sleep_z) 1.148  1.148   0.881    0.41
## ---
## Signif. codes:  0 ‘***’ 0.001 ‘**’ 0.01 ‘*’ 0.05 ‘.’ 0.1 ‘ ’ 1
##
## R-sq.(adj) =  0.428
## lmer.REML = 4.7992e+05  Scale est. = 11839     n = 33453
```

Below is the model output with main effect.

```
##
## Family: gaussian
## Link function: identity
##
## Formula:
## value ~ sex + site + s(age_z, k = 10, bs = "cr") + s(sleep_z,
##     k = 5, bs = "cr") + icv + bmi
## <environment: 0x558ad78498a8>
##
## Parametric coefficients:
##                 Estimate Std. Error t value Pr(>|t|)
## (Intercept)   3857.5496    34.7690 110.948  < 2e-16 ***
## sexmale         79.9958     4.7633  16.794  < 2e-16 ***
## siteousPrisma  170.7034    42.6810   4.000 6.36e-05 ***
## siteousSkyra   178.6425    26.9673   6.624 3.54e-11 ***
## siteUCAM       176.9618    34.2812   5.162 2.46e-07 ***
## siteUKB        256.0837    32.9959   7.761 8.66e-15 ***
## siteUmU         62.7090    38.1517   1.644      0.1
## icv            273.8375     2.4496 111.788  < 2e-16 ***
## bmi             -5.4508     0.4628 -11.777  < 2e-16 ***
## ---
## Signif. codes:  0 ‘***’ 0.001 ‘**’ 0.01 ‘*’ 0.05 ‘.’ 0.1 ‘ ’ 1
##
## Approximate significance of smooth terms:
##              edf Ref.df       F p-value
## s(age_z)   6.070  6.070 133.949  <2e-16 ***
## s(sleep_z) 2.069  2.069   1.191   0.274
## ---
## Signif. codes:  0 ‘***’ 0.001 ‘**’ 0.01 ‘*’ 0.05 ‘.’ 0.1 ‘ ’ 1
##
## R-sq.(adj) =  0.431
```

```
## lmer.REML = 4.7978e+05  Scale est. = 11834     n = 33453
```

Next is the model with BMI-sleep interaction.

```
##
## Family: gaussian
## Link function: identity
##
## Formula:
## value ~ sex + site + s(age_z, k = 10, bs = "cr") + s(sleep_z,
##     k = 5, bs = "cr") + icv + bmi + bmi:sleep_z
## <environment: 0x558ad78498a8>
##
## Parametric coefficients:
##                Estimate Std. Error t value Pr(>|t|)
## (Intercept)   3856.0984    34.7843 110.857  < 2e-16 ***
## sexmale         80.0223     4.7632  16.800  < 2e-16 ***
## siteousPrisma  170.7523    42.6813   4.001 6.33e-05 ***
## siteousSkyra   178.8093    26.9685   6.630 3.40e-11 ***
## siteUCAM       177.5262    34.2838   5.178 2.25e-07 ***
## siteUKB        256.6403    32.9985   7.777 7.62e-15 ***
## siteUmU         63.2037    38.1533   1.657   0.0976 .
## icv            273.8622     2.4496 111.797  < 2e-16 ***
## bmi             -5.4286     0.4631 -11.723  < 2e-16 ***
## bmi:sleep_z      0.6180     0.4385   1.409   0.1587
## ---
## Signif. codes:  0 ‘***’ 0.001 ‘**’ 0.01 ‘*’ 0.05 ‘.’ 0.1 ‘ ’ 1
##
## Approximate significance of smooth terms:
##              edf Ref.df       F p-value
## s(age_z)   6.067  6.067 134.171  <2e-16 ***
## s(sleep_z) 2.057  2.057   1.993   0.123
## ---
## Signif. codes:  0 ‘***’ 0.001 ‘**’ 0.01 ‘*’ 0.05 ‘.’ 0.1 ‘ ’ 1
##
## R-sq.(adj) =  0.431
## lmer.REML = 4.7978e+05  Scale est. = 11835     n = 33453
```

We did the same controling for depression. Below is the model with no covariates but only keeping data with depression.

```
##
## Family: gaussian
## Link function: identity
##
## Formula:
## value ~ sex + site + s(age_z, k = 10, bs = "cr") + s(sleep_z,
##     k = 5, bs = "cr") + icv
## <environment: 0x55895e81b3b8>
##
## Parametric coefficients:
##               Estimate Std. Error t value Pr(>|t|)
## (Intercept)   4087.141     22.608 180.786  < 2e-16 ***
## sexmale         76.372      4.792  15.938  < 2e-16 ***
## siteousAvanto -222.679     51.555  -4.319 1.57e-05 ***
## siteousPrisma -157.505     43.970  -3.582 0.000341 ***
```

```
## siteousSkyra  -161.431     34.270  -4.711 2.48e-06 ***
## siteUCAM      -216.399     30.993  -6.982 2.96e-12 ***
## siteUKB       -116.869     22.353  -5.228 1.72e-07 ***
## siteUmU       -312.669     30.354 -10.301  < 2e-16 ***
## icv            270.672      2.466 109.781  < 2e-16 ***
## ---
## Signif. codes:  0 ‘***’ 0.001 ‘**’ 0.01 ‘*’ 0.05 ‘.’ 0.1 ‘ ’ 1
##
## Approximate significance of smooth terms:
##              edf Ref.df       F p-value
## s(age_z)   6.219  6.219 118.626  <2e-16 ***
## s(sleep_z) 1.000  1.000   0.787   0.375
## ---
## Signif. codes:  0 ‘***’ 0.001 ‘**’ 0.01 ‘*’ 0.05 ‘.’ 0.1 ‘ ’ 1
##
## R-sq.(adj) =  0.423
## lmer.REML = 4.7855e+05  Scale est. = 11416     n = 33365
```

Below is the model output with main effect.

```
##
## Family: gaussian
## Link function: identity
##
## Formula:
## value ~ sex + site + s(age_z, k = 10, bs = "cr") + s(sleep_z,
##     k = 5, bs = "cr") + icv + depression
## <environment: 0x55895e81b3b8>
##
## Parametric coefficients:
##               Estimate Std. Error t value Pr(>|t|)
## (Intercept)   4090.507     22.709 180.125  < 2e-16 ***
## sexmale         76.036      4.796  15.852  < 2e-16 ***
## siteousAvanto -220.957     51.563  -4.285 1.83e-05 ***
## siteousPrisma -155.975     43.977  -3.547 0.000391 ***
## siteousSkyra  -159.489     34.288  -4.651 3.31e-06 ***
## siteUCAM      -215.598     30.995  -6.956 3.57e-12 ***
## siteUKB       -118.306     22.372  -5.288 1.24e-07 ***
## siteUmU       -302.711     30.999  -9.765  < 2e-16 ***
## icv            270.709      2.466 109.789  < 2e-16 ***
## depression     -26.873     17.007  -1.580 0.114091
## ---
## Signif. codes:  0 ‘***’ 0.001 ‘**’ 0.01 ‘*’ 0.05 ‘.’ 0.1 ‘ ’ 1
##
## Approximate significance of smooth terms:
##              edf Ref.df       F p-value
## s(age_z)   6.208  6.208 118.929  <2e-16 ***
## s(sleep_z) 1.093  1.093   0.646   0.469
## ---
## Signif. codes:  0 ‘***’ 0.001 ‘**’ 0.01 ‘*’ 0.05 ‘.’ 0.1 ‘ ’ 1
##
## R-sq.(adj) =  0.423
## lmer.REML = 4.7854e+05  Scale est. = 11415     n = 33365
```

Next is the model with depression-sleep interaction.

```
##
## Family: gaussian
## Link function: identity
##
## Formula:
## value ~ sex + site + s(age_z, k = 10, bs = "cr") + s(sleep_z,
##     k = 5, bs = "cr") + icv + depression + depression:sleep_z
## <environment: 0x55895e81b3b8>
##
## Parametric coefficients:
##                    Estimate Std. Error t value Pr(>|t|)
## (Intercept)        4090.507     22.710 180.123  < 2e-16 ***
## sexmale              76.084      4.797  15.860  < 2e-16 ***
## siteousAvanto      -220.601     51.567  -4.278 1.89e-05 ***
## siteousPrisma      -155.633     43.982  -3.539 0.000403 ***
## siteousSkyra       -159.148     34.294  -4.641 3.49e-06 ***
## siteUCAM           -215.251     31.002  -6.943 3.91e-12 ***
## siteUKB            -118.318     22.373  -5.289 1.24e-07 ***
## siteUmU            -304.959     31.253  -9.758  < 2e-16 ***
## icv                 270.706      2.466 109.784  < 2e-16 ***
## depression          -26.385     17.037  -1.549 0.121450
## depression:sleep_z    7.066     12.424   0.569 0.569562
## ---
## Signif. codes:  0 ‘***’ 0.001 ‘**’ 0.01 ‘*’ 0.05 ‘.’ 0.1 ‘ ’ 1
##
## Approximate significance of smooth terms:
##              edf Ref.df       F p-value
## s(age_z)   6.211  6.211 118.812  <2e-16 ***
## s(sleep_z) 1.118  1.118   0.172   0.801
## ---
## Signif. codes:  0 ‘***’ 0.001 ‘**’ 0.01 ‘*’ 0.05 ‘.’ 0.1 ‘ ’ 1
##
## R-sq.(adj) =  0.423
## lmer.REML = 4.7853e+05  Scale est. = 11415     n = 33365
```

The plot below shows the sleep-volume curve for the original model and for the model with main effects of SES.

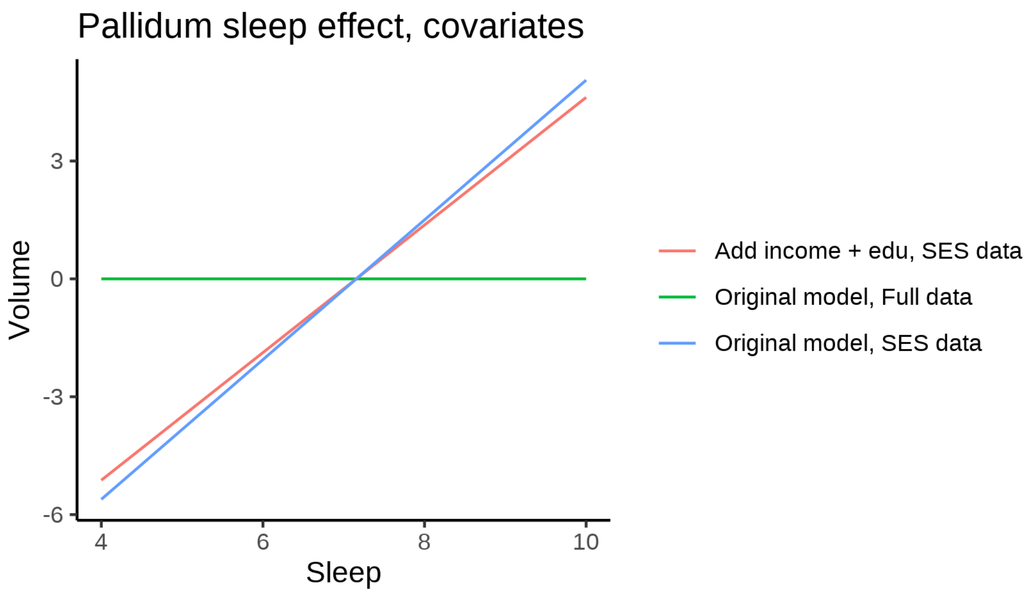

The plot below shows the sleep-volume curve for the original model and for the model with main effects of BMI.

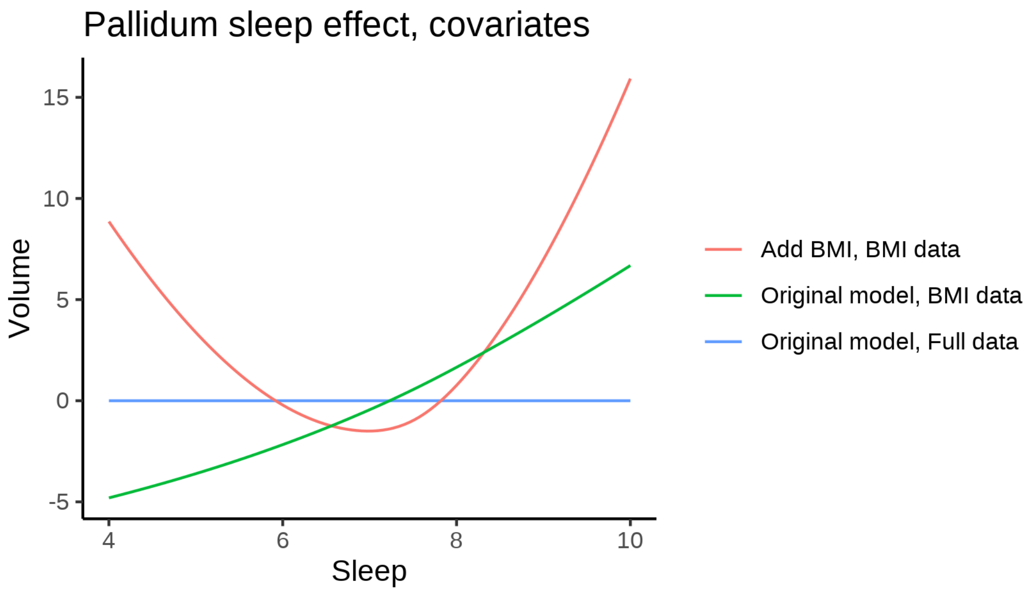

The plot below shows the sleep-volume curve for the original model and for the model with main effects of depression.

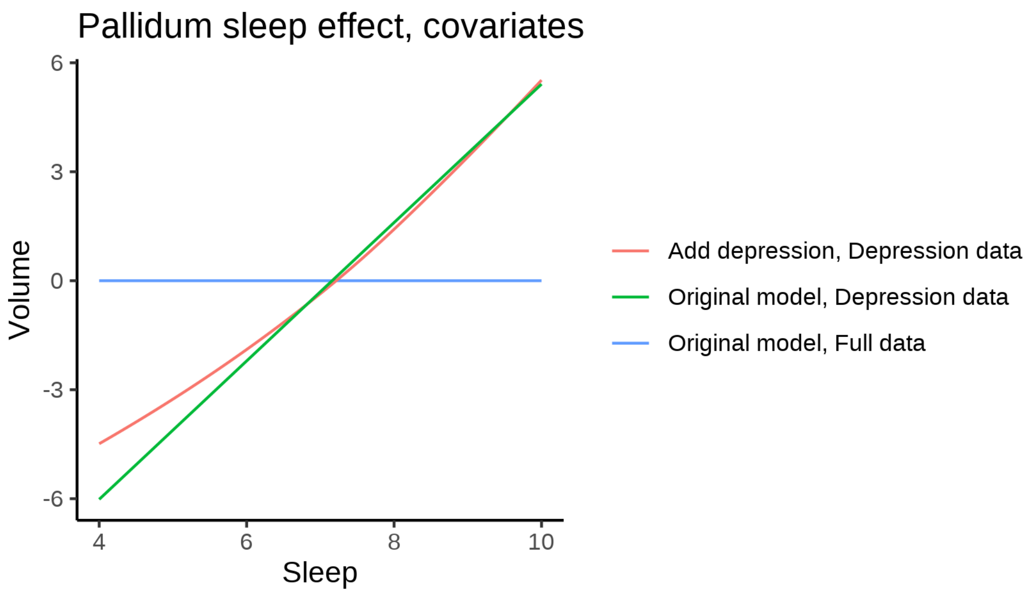

Models Not controling for ICV

Model outputs

Model without sleep term

```
##
## Family: gaussian
## Link function: identity
##
## Formula:
## value ~ sex + site + s(age_z, k = 10, bs = "cr")
## <environment: 0x558a4956ba70>
##
## Parametric coefficients:
##                Estimate Std. Error t value Pr(>|t|)
## (Intercept)    3485.197     21.582 161.484  < 2e-16 ***
## sexmale         379.961      3.729 101.893  < 2e-16 ***
## siteMPIB         94.820     27.878   3.401 0.000671 ***
## siteousAvanto   225.706     22.506  10.029  < 2e-16 ***
## siteousPrisma   277.428     38.410   7.223 5.16e-13 ***
## siteousSkyra    248.506     21.384  11.621  < 2e-16 ***
## siteUB           -2.812     66.477  -0.042 0.966260
## siteUCAM        271.905     24.299  11.190  < 2e-16 ***
## siteUKB         349.668     22.156  15.782  < 2e-16 ***
## siteUmU          35.660     31.472   1.133 0.257182
## siteUOXF         39.548     26.463   1.494 0.135066
## ---
## Signif. codes:  0 ‘***’ 0.001 ‘**’ 0.01 ‘*’ 0.05 ‘.’ 0.1 ‘ ’ 1
##
## Approximate significance of smooth terms:
##            edf Ref.df     F p-value
## s(age_z) 5.497  5.497 293.3  <2e-16 ***
```

```
## ---
## Signif. codes:  0 ‘***’ 0.001 ‘**’ 0.01 ‘*’ 0.05 ‘.’ 0.1 ‘ ’ 1
##
## R-sq.(adj) =  0.207
## lmer.REML = 7.5188e+05  Scale est. = 11768     n = 51286
```

Model with only main effects of age and sleep

```
##
## Family: gaussian
## Link function: identity
##
## Formula:
## value ~ sex + site + s(age_z, k = 10, bs = "cr") + s(sleep_z,
##     k = 5, bs = "cr")
## <environment: 0x558a4956ba70>
##
## Parametric coefficients:
##              Estimate Std. Error t value Pr(>|t|)
## (Intercept)    3491.43      21.60 161.633  < 2e-16 ***
## sexmale         379.35       3.73 101.707  < 2e-16 ***
## siteMPIB         91.26      27.88   3.273  0.00106 **
## siteousAvanto   221.80      22.51   9.855  < 2e-16 ***
## siteousPrisma   274.78      38.40   7.156 8.42e-13 ***
## siteousSkyra    244.50      21.39  11.433  < 2e-16 ***
## siteUB           -2.08      66.45  -0.031  0.97503
## siteUCAM        270.03      24.30  11.115  < 2e-16 ***
## siteUKB         343.42      22.18  15.487  < 2e-16 ***
## siteUmU          26.88      31.53   0.852  0.39400
## siteUOXF         36.83      26.46   1.392  0.16388
## ---
## Signif. codes:  0 ‘***’ 0.001 ‘**’ 0.01 ‘*’ 0.05 ‘.’ 0.1 ‘ ’ 1
##
## Approximate significance of smooth terms:
##             edf Ref.df      F  p-value
## s(age_z)   5.494  5.494 291.77  < 2e-16 ***
## s(sleep_z) 3.307  3.307  10.44 1.62e-07 ***
## ---
## Signif. codes:  0 ‘***’ 0.001 ‘**’ 0.01 ‘*’ 0.05 ‘.’ 0.1 ‘ ’ 1
##
## R-sq.(adj) =  0.207
## lmer.REML = 7.5185e+05  Scale est. = 11766     n = 51286
```

Model with full interaction between age and sleep

```
##
## Family: gaussian
## Link function: identity
##
## Formula:
## value ~ sex + site + t2(age_z, sleep_z, k = c(10, 4), bs = "cr")
## <environment: 0x558a4956ba70>
##
## Parametric coefficients:
##              Estimate Std. Error t value Pr(>|t|)
## (Intercept)   3500.705     21.471 163.047  < 2e-16 ***
```

```
## sexmale        380.534      3.736 101.854  < 2e-16 ***
## siteMPIB        79.114     27.898   2.836  0.00457 **
## siteousAvanto  216.581     22.551   9.604  < 2e-16 ***
## siteousPrisma  270.236     38.457   7.027 2.14e-12 ***
## siteousSkyra   238.796     21.443  11.137  < 2e-16 ***
## siteUB         -17.351     66.423  -0.261  0.79393
## siteUCAM       258.935     24.302  10.655  < 2e-16 ***
## siteUKB        333.359     22.028  15.133  < 2e-16 ***
## siteUmU         17.364     31.490   0.551  0.58136
## siteUOXF        23.926     26.377   0.907  0.36437
## ---
## Signif. codes:  0 '***' 0.001 '**' 0.01 '*' 0.05 '.' 0.1 ' ' 1
##
## Approximate significance of smooth terms:
##                     edf Ref.df     F p-value
## t2(age_z,sleep_z) 8.89   8.89 26.83  <2e-16 ***
## ---
## Signif. codes:  0 '***' 0.001 '**' 0.01 '*' 0.05 '.' 0.1 ' ' 1
##
## R-sq.(adj) =  0.207
## lmer.REML = 7.5184e+05  Scale est. = 11747     n = 51286
```

Model comparison

`mod_no_sleep` refers to model without sleep term, `mod_no_interaction` refers to model with only main effect of sleep, and `mod_full` refers to model with a full interaction between age and sleep. This is a nested model comparison, and the p-value at a given line refers to comparing the model at the line to the model on the line above. Hence, significance implies that the more complicated model is supported on statistical grounds.

To be even more specific, the p-value on the second row tests whether there is an association between sleep and volume. The p-value on the third row tests whether this association depends on age.

```
## Data: NULL
## Models:
## ml$mod_no_sleep$mer: NULL
## ml$mod_no_interaction$mer: NULL
## ml$mod_full$mer: NULL
##                           npar    AIC    BIC  logLik deviance  Chisq Df Pr(>Chisq)
## ml$mod_no_sleep$mer         15 751908 752040 -375939   751878
## ml$mod_no_interaction$mer   17 751882 752032 -375924   751848 30.014  2  3.038e-07 ***
## ml$mod_full$mer             19 751875 752043 -375918   751837 10.849  2   0.004408 **
## ---
## Signif. codes:  0 '***' 0.001 '**' 0.01 '*' 0.05 '.' 0.1 ' ' 1
```

We chose the model based on the likelihood ratio test with 5 % significance level, which was `mod_full`.

Lifespan brain trajectory

The trajectory shown is from the chosen model `mod_full_no_icv`.

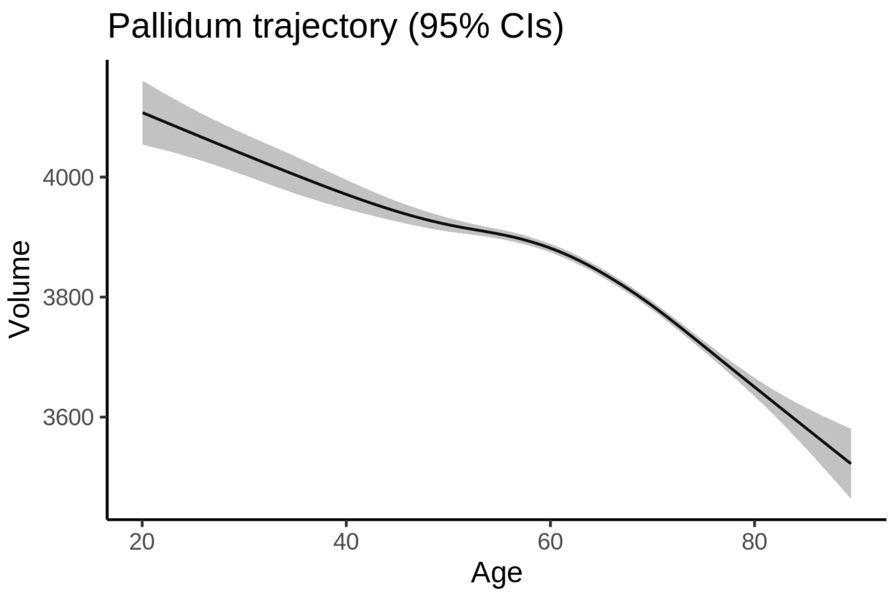

Effect of sleep

The chosen model included a full interaction between age and sleep, and the effect of sleep is hence plotted for a set of different ages. For comparability across ages, the sleep-volume curves at each age have been standardized so that they sum to zero.

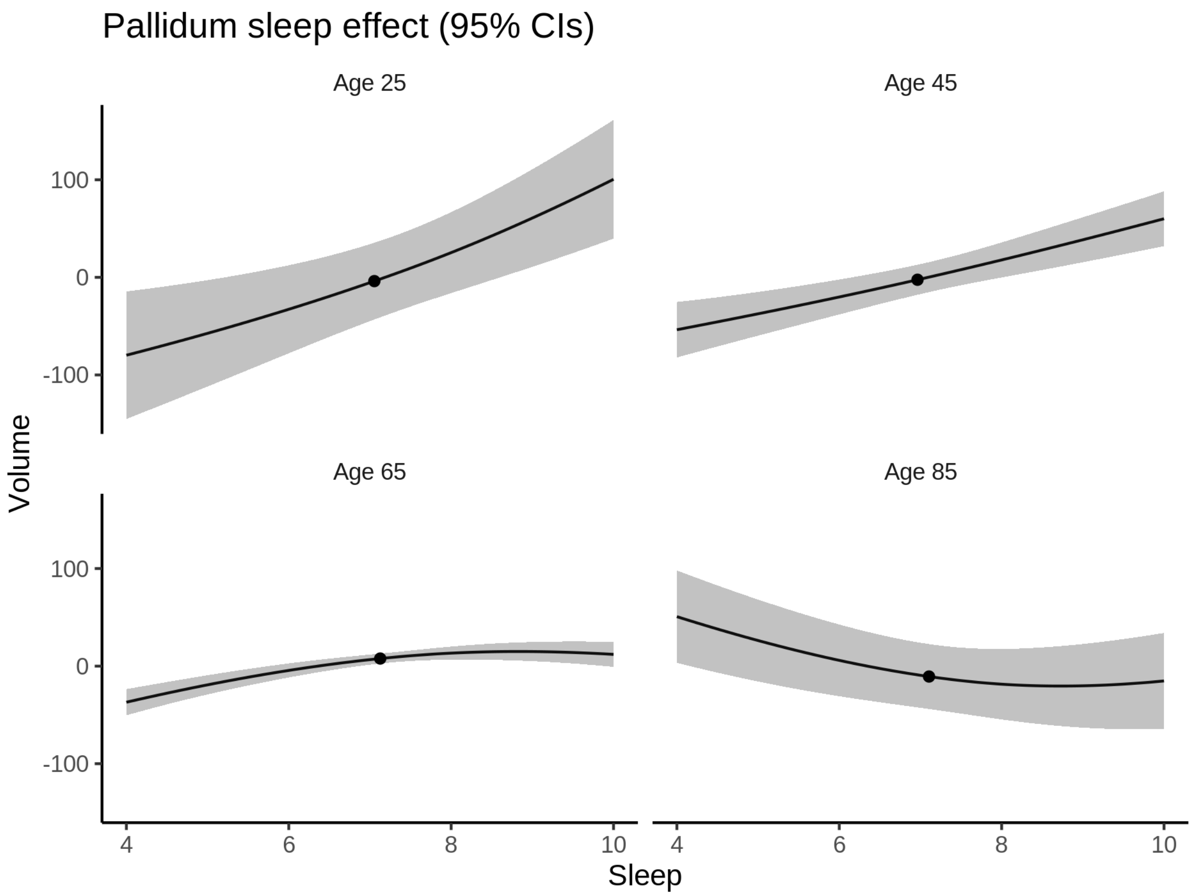

Deviation from sleep associated with maximal volume

The table show the sleep associated with maximum volume at chosen ages.

Age	Sleep at max vol
25	10.0
45	10.0
65	8.8
85	4.0

Model with sleep-age interaction was chosen, so we show it for four selected ages. The percentage values in the plot are calculated as follows: The maximum at 100 % refers to a person at the given age with a sleep duration associated with maximum volume. The other percentage values show how large the expected volume is for someone with other sleep durations. For example, 99 % implies a 1 % reduction.

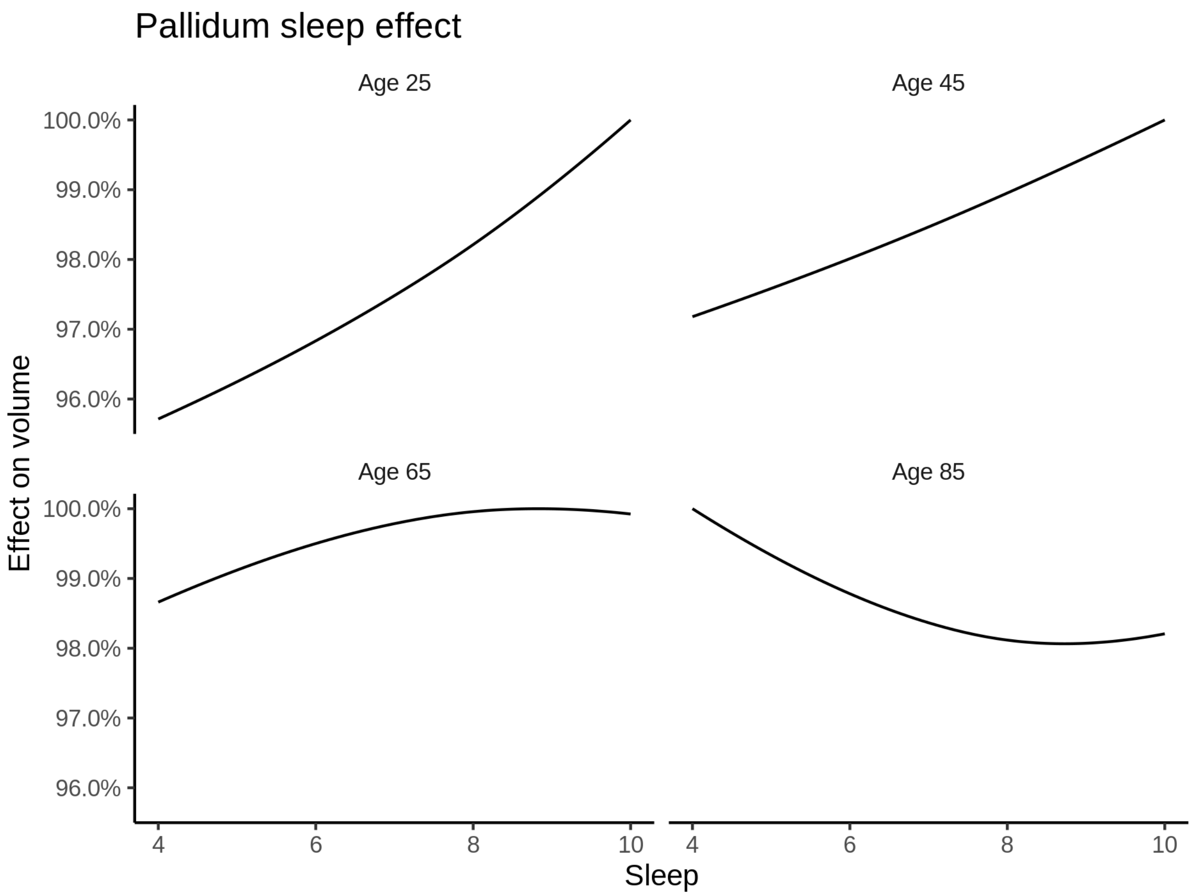

Comparison of mean sleep and sleep associated with maximum volume

The plot below compares average sleep to the sleep associated with maximum volume.

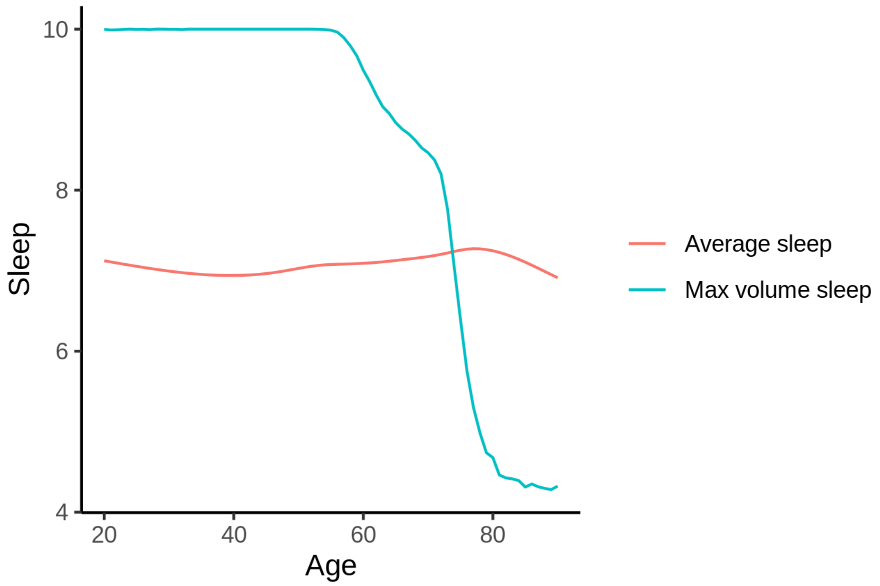

The next plot shows the difference between average sleep and sleep associated with maximum volume. The shaded region is a 95 % confidence interval.

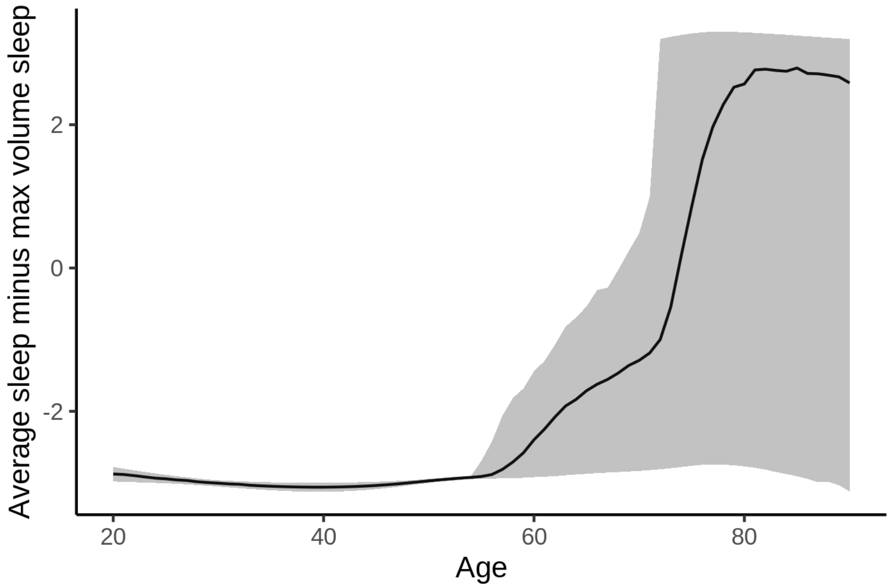

The next plot shows the probability that the sleep duration associated with maximum volume is longer than the average sleep duration, as a function of age. Probility below .05 can be interpreted as evidence that the sleep associated with maximum volume is shorter than the mean sleep, and probability above .95 can be interpreted the opposite way.

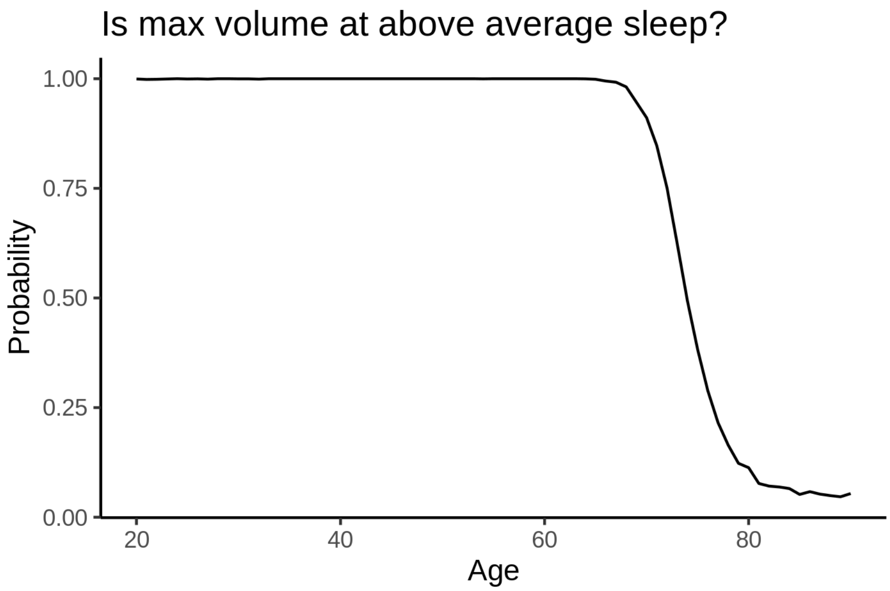

Putamen

Descriptive statistics

Study	Observations	Unique IDs	Mean age	Age range
HCP	974	974	28.8	22 - 37
MPIB	677	391	63.1	24 - 83
UB	113	39	70.9	64 - 81
UCAM	884	632	55.1	20 - 88
UiO	1469	802	49.3	20 - 88
UKB	45969	43130	64.5	45 - 83
UmU	412	279	62.2	25 - 85
UOXF	769	769	69.8	60 - 85

Spaghetti plot

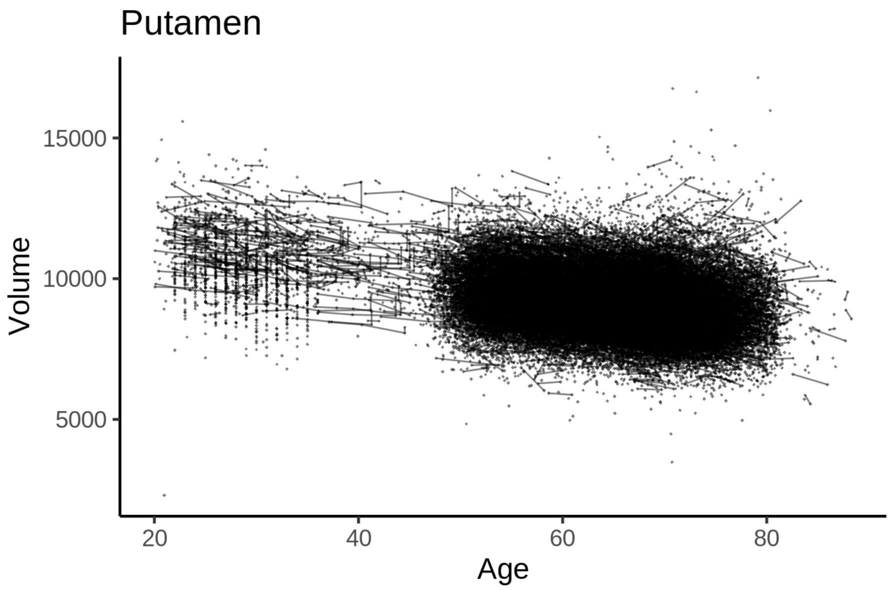

Models Controling for ICV

Model outputs

Model without sleep term

```
##
## Family: gaussian
## Link function: identity
##
## Formula:
## value ~ sex + site + icv + s(age_z, k = 10, bs = "cr")
## <environment: 0x5589874b5b48>
##
## Parametric coefficients:
##                Estimate Std. Error t value Pr(>|t|)
## (Intercept)   8660.652     45.857 188.864  < 2e-16 ***
## sexmale        356.351      9.805  36.344  < 2e-16 ***
## siteMPIB      1261.327     60.397  20.884  < 2e-16 ***
## siteousAvanto  601.474     47.403  12.689  < 2e-16 ***
```

```
## siteousPrisma  768.816     79.984   9.612  < 2e-16 ***
## siteousSkyra  1240.701     45.884  27.040  < 2e-16 ***
## siteUB         778.117    144.440   5.387 7.19e-08 ***
## siteUCAM       667.616     52.094  12.816  < 2e-16 ***
## siteUKB        350.620     46.950   7.468 8.28e-14 ***
## siteUmU        741.738     67.624  10.969  < 2e-16 ***
## siteUOXF       865.579     56.431  15.339  < 2e-16 ***
## icv            494.424      4.918 100.535  < 2e-16 ***
## ---
## Signif. codes:  0 ‘***’ 0.001 ‘**’ 0.01 ‘*’ 0.05 ‘.’ 0.1 ‘ ’ 1
##
## Approximate significance of smooth terms:
##            edf Ref.df     F p-value
## s(age_z) 5.841  5.841 882.5  <2e-16 ***
## ---
## Signif. codes:  0 ‘***’ 0.001 ‘**’ 0.01 ‘*’ 0.05 ‘.’ 0.1 ‘ ’ 1
##
## R-sq.(adj) =  0.408
## lmer.REML = 8.2883e+05  Scale est. = 35709     n = 51267
```

Model with only main effects of age and sleep

```
##
## Family: gaussian
## Link function: identity
##
## Formula:
## value ~ sex + site + icv + s(age_z, k = 10, bs = "cr") + s(sleep_z,
##     k = 5, bs = "cr")
## <environment: 0x5589874b5b48>
##
## Parametric coefficients:
##               Estimate Std. Error t value Pr(>|t|)
## (Intercept)   8657.776     45.905 188.601  < 2e-16 ***
## sexmale        356.256      9.805  36.335  < 2e-16 ***
## siteMPIB      1264.461     60.438  20.921  < 2e-16 ***
## siteousAvanto  603.022     47.416  12.718  < 2e-16 ***
## siteousPrisma  769.907     79.987   9.625  < 2e-16 ***
## siteousSkyra  1242.282     45.898  27.066  < 2e-16 ***
## siteUB         778.965    144.440   5.393 6.96e-08 ***
## siteUCAM       669.041     52.105  12.840  < 2e-16 ***
## siteUKB        353.674     47.004   7.524 5.38e-14 ***
## siteUmU        748.207     67.781  11.039  < 2e-16 ***
## siteUOXF       867.024     56.444  15.361  < 2e-16 ***
## icv            494.629      4.920 100.533  < 2e-16 ***
## ---
## Signif. codes:  0 ‘***’ 0.001 ‘**’ 0.01 ‘*’ 0.05 ‘.’ 0.1 ‘ ’ 1
##
## Approximate significance of smooth terms:
##              edf Ref.df       F p-value
## s(age_z)   5.852  5.852 877.902  <2e-16 ***
## s(sleep_z) 1.000  1.000   1.905   0.168
## ---
## Signif. codes:  0 ‘***’ 0.001 ‘**’ 0.01 ‘*’ 0.05 ‘.’ 0.1 ‘ ’ 1
##
```

```
## R-sq.(adj) =  0.408
## lmer.REML = 8.2883e+05  Scale est. = 35714     n = 51267
```

Model with full interaction between age and sleep

```
##
## Family: gaussian
## Link function: identity
##
## Formula:
## value ~ sex + site + icv + t2(age_z, sleep_z, k = c(10, 4), bs = "cr")
## <environment: 0x5589874b5b48>
##
## Parametric coefficients:
##               Estimate Std. Error t value Pr(>|t|)
## (Intercept)   8665.700     46.086 188.032  < 2e-16 ***
## sexmale        356.116      9.823  36.253  < 2e-16 ***
## siteMPIB      1257.823     60.737  20.709  < 2e-16 ***
## siteousAvanto  597.436     47.680  12.530  < 2e-16 ***
## siteousPrisma  765.089     80.154   9.545  < 2e-16 ***
## siteousSkyra  1236.626     46.191  26.772  < 2e-16 ***
## siteUB         771.723    144.527   5.340 9.35e-08 ***
## siteUCAM       661.904     52.387  12.635  < 2e-16 ***
## siteUKB        345.564     47.185   7.324 2.45e-13 ***
## siteUmU        740.230     67.979  10.889  < 2e-16 ***
## siteUOXF       859.264     56.654  15.167  < 2e-16 ***
## icv            494.579      4.921 100.508  < 2e-16 ***
## ---
## Signif. codes:  0 '***' 0.001 '**' 0.01 '*' 0.05 '.' 0.1 ' ' 1
##
## Approximate significance of smooth terms:
##                     edf Ref.df     F p-value
## t2(age_z,sleep_z) 8.985  8.985 50.32  <2e-16 ***
## ---
## Signif. codes:  0 '***' 0.001 '**' 0.01 '*' 0.05 '.' 0.1 ' ' 1
##
## R-sq.(adj) =  0.408
## lmer.REML = 8.2883e+05  Scale est. = 35710     n = 51267
```

Model comparison

`mod_no_sleep` refers to model without sleep term, `mod_no_interaction` refers to model with only main effect of sleep, and `mod_full` refers to model with a full interaction between age and sleep. This is a nested model comparison, and the p-value at a given line refers to comparing the model at the line to the model on the line above. Hence, significance implies that the more complicated model is supported on statistical grounds.

To be even more specific, the p-value on the second row tests whether there is an association between sleep and volume. The p-value on the third row tests whether this association depends on age.

```
## Data: NULL
## Models:
## ml$mod_no_sleep$mer: NULL
## ml$mod_no_interaction$mer: NULL
## ml$mod_full$mer: NULL
```

```
##                             npar    AIC    BIC  logLik deviance  Chisq Df Pr(>Chisq)
## ml$mod_no_sleep$mer         16 828865 829007 -414417   828833
## ml$mod_no_interaction$mer   18 828867 829026 -414416   828831 1.9049  2     0.3858
## ml$mod_full$mer             20 828875 829052 -414417   828835 0.0000  2     1.0000
```

We chose the model based on the likelihood ratio test with 5 % significance level, which was `mod_no_sleep`.

Lifespan brain trajectory

The trajectory shown is from the chosen model `mod_no_sleep`.

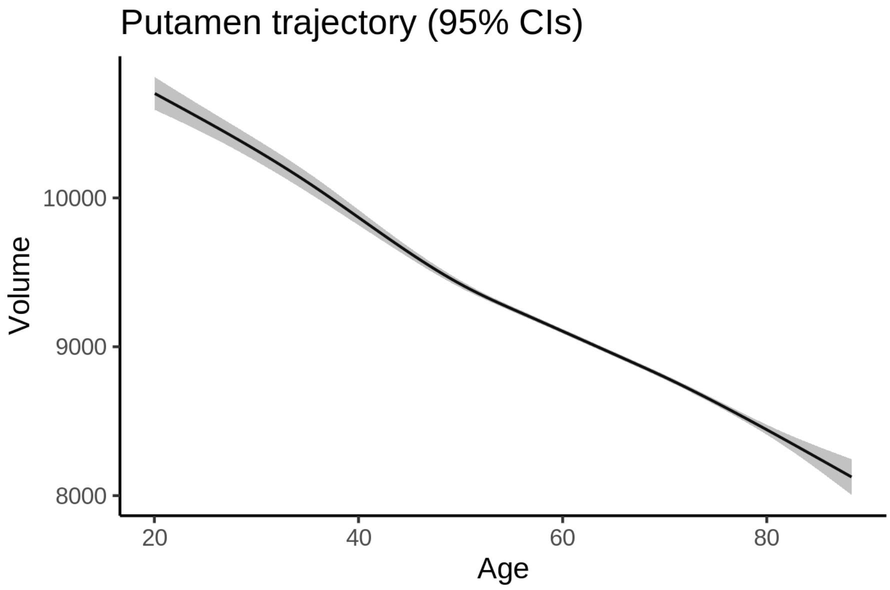

Effect of sleep

The chosen model did not include a sleep term, and hence we don't have any estimated effect of sleep.

We show the full interaction model for completeness, although it was not selected.

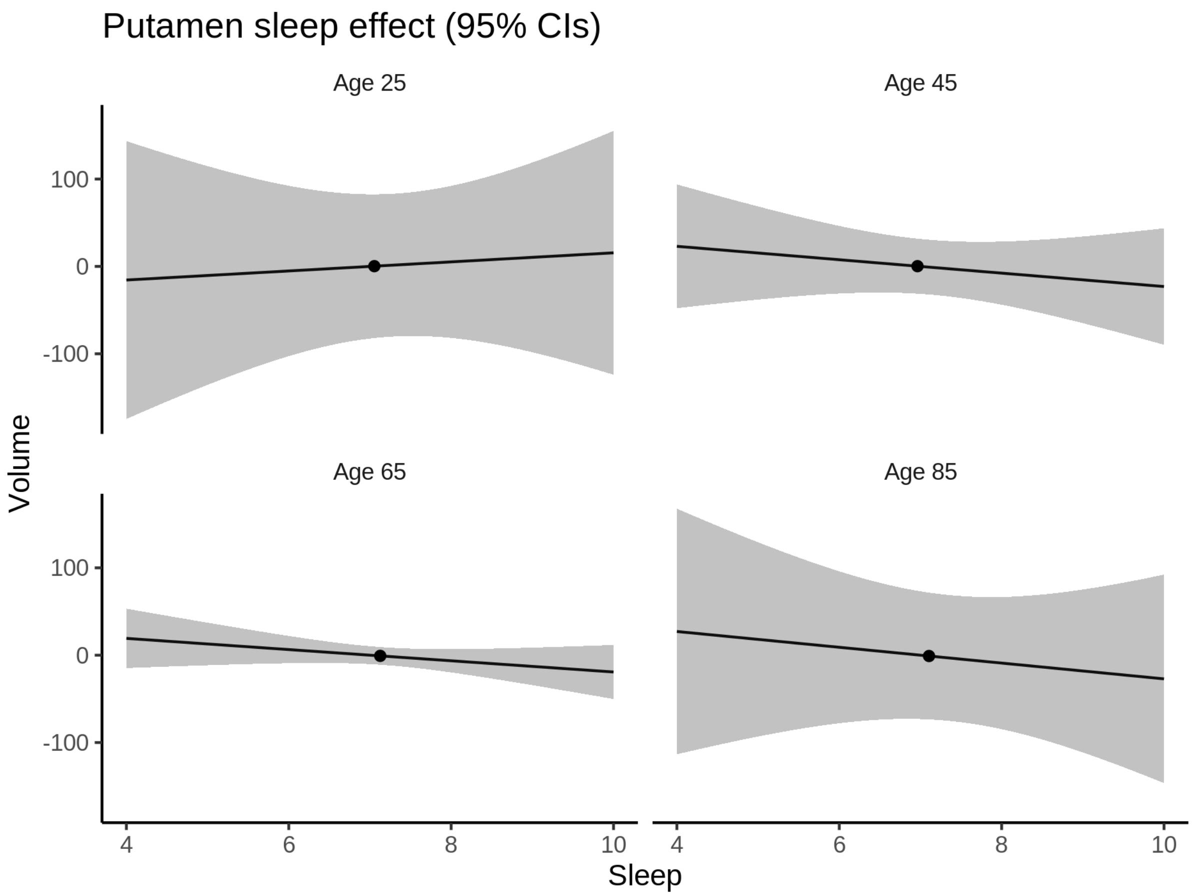

Deviation from sleep associated with maximal volume

Model with no sleep term was selected. No plots to show. (Although we can of course dig up the plots, which will be pretty flat).

Comparison of mean sleep and sleep associated with maximum volume

Nothing to show, as we did not find an association between sleep and volume.

Controling for covariates

Below is the output for a model in which we only include data with income and education.

```
##
## Family: gaussian
## Link function: identity
##
## Formula:
## value ~ sex + site + s(age_z, k = 10, bs = "cr") + s(sleep_z,
##     k = 5, bs = "cr") + icv
## <environment: 0x558a32330c40>
##
## Parametric coefficients:
```

```
##                Estimate Std. Error t value Pr(>|t|)
## (Intercept)   10049.328    112.262  89.517  < 2e-16 ***
## sexmale         354.242     12.399  28.571  < 2e-16 ***
## siteousAvanto  -872.332    130.804  -6.669 2.62e-11 ***
## siteousPrisma  -498.491    137.233  -3.632 0.000281 ***
## siteousSkyra    -79.852    122.246  -0.653 0.513627
## siteUKB       -1068.380    111.980  -9.541  < 2e-16 ***
## siteUOXF       -432.870    126.229  -3.429 0.000606 ***
## icv             507.249      6.353  79.850  < 2e-16 ***
## ---
## Signif. codes:  0 '***' 0.001 '**' 0.01 '*' 0.05 '.' 0.1 ' ' 1
##
## Approximate significance of smooth terms:
##               edf Ref.df       F p-value
## s(age_z)    3.752  3.752 651.974  <2e-16 ***
## s(sleep_z) 2.217  2.217   2.369    0.11
## ---
## Signif. codes:  0 '***' 0.001 '**' 0.01 '*' 0.05 '.' 0.1 ' ' 1
##
## R-sq.(adj) =  0.387
## lmer.REML = 5.0382e+05  Scale est. = 34651     n = 31186
```

Below is the output for a model in which we control for the main effects of income and education.

```
##
## Family: gaussian
## Link function: identity
##
## Formula:
## value ~ sex + site + s(age_z, k = 10, bs = "cr") + s(sleep_z,
##     k = 5, bs = "cr") + icv + income_scaled + education_scaled
## <environment: 0x558a32330c40>
##
## Parametric coefficients:
##                   Estimate Std. Error t value Pr(>|t|)
## (Intercept)      10061.955    112.932  89.098  < 2e-16 ***
## sexmale            354.119     12.434  28.481  < 2e-16 ***
## siteousAvanto     -871.914    130.835  -6.664 2.71e-11 ***
## siteousPrisma     -497.218    137.236  -3.623 0.000292 ***
## siteousSkyra       -79.371    122.283  -0.649 0.516294
## siteUKB          -1067.918    112.018  -9.533  < 2e-16 ***
## siteUOXF          -436.508    126.272  -3.457 0.000547 ***
## icv                508.028      6.406  79.302  < 2e-16 ***
## income_scaled       -6.705     14.749  -0.455 0.649383
## education_scaled   -12.645     17.483  -0.723 0.469495
## ---
## Signif. codes:  0 '***' 0.001 '**' 0.01 '*' 0.05 '.' 0.1 ' ' 1
##
## Approximate significance of smooth terms:
##               edf Ref.df       F p-value
## s(age_z)    3.719  3.719 617.592  <2e-16 ***
## s(sleep_z) 2.042  2.042   2.232   0.115
## ---
## Signif. codes:  0 '***' 0.001 '**' 0.01 '*' 0.05 '.' 0.1 ' ' 1
##
```

```
## R-sq.(adj) =  0.386
## lmer.REML = 5.0381e+05  Scale est. = 34643     n = 31186
```

We also included interaction effects between sleep duration and education and income, in another model. The output is shown below, and the interaction terms are `income_scaled:sleep_z` and `education_scaled:sleep_z`.

```
##
## Family: gaussian
## Link function: identity
##
## Formula:
## value ~ sex + site + s(age_z, k = 10, bs = "cr") + s(sleep_z,
##     k = 5, bs = "cr") + icv + income_scaled + education_scaled +
##     income_scaled:sleep_z + education_scaled:sleep_z
## <environment: 0x558a32330c40>
##
## Parametric coefficients:
##                            Estimate Std. Error t value Pr(>|t|)
## (Intercept)              10062.1118   112.9220  89.107  < 2e-16 ***
## sexmale                    353.4411    12.4417  28.408  < 2e-16 ***
## siteousAvanto             -873.3061   130.8372  -6.675 2.52e-11 ***
## siteousPrisma             -499.4219   137.2486  -3.639 0.000274 ***
## siteousSkyra               -80.7757   122.2793  -0.661 0.508884
## siteUKB                  -1067.0967   112.0261  -9.525  < 2e-16 ***
## siteUOXF                  -432.5819   126.3073  -3.425 0.000616 ***
## icv                        507.9083     6.4054  79.293  < 2e-16 ***
## income_scaled               -6.4504    14.7334  -0.438 0.661527
## education_scaled           -13.4372    17.4750  -0.769 0.441935
## income_scaled:sleep_z      -30.1961    14.6258  -2.065 0.038971 *
## education_scaled:sleep_z    -0.9861    16.9172  -0.058 0.953516
## ---
## Signif. codes:  0 ‘***’ 0.001 ‘**’ 0.01 ‘*’ 0.05 ‘.’ 0.1 ‘ ’ 1
##
## Approximate significance of smooth terms:
##              edf Ref.df       F p-value
## s(age_z)   3.737  3.737 613.922  <2e-16 ***
## s(sleep_z) 1.039  1.039   0.027   0.901
## ---
## Signif. codes:  0 ‘***’ 0.001 ‘**’ 0.01 ‘*’ 0.05 ‘.’ 0.1 ‘ ’ 1
##
## R-sq.(adj) =  0.386
## lmer.REML = 5.0379e+05  Scale est. = 34644     n = 31186
```

We did the same controling for BMI. Below is the model with no covariates but only keeping data with BMI.

```
##
## Family: gaussian
## Link function: identity
##
## Formula:
## value ~ sex + site + s(age_z, k = 10, bs = "cr") + s(sleep_z,
##     k = 5, bs = "cr") + icv
## <environment: 0x558a492785d8>
##
## Parametric coefficients:
```

```
##               Estimate Std. Error t value Pr(>|t|)
## (Intercept)   9191.331     67.326 136.519  < 2e-16 ***
## sexmale        348.376     12.049  28.912  < 2e-16 ***
## siteousPrisma  231.745     90.517   2.560  0.01046 *
## siteousSkyra   726.704     46.917  15.489  < 2e-16 ***
## siteUCAM       127.327     72.429   1.758  0.07877 .
## siteUKB       -192.377     68.006  -2.829  0.00467 **
## siteUmU        213.690     84.006   2.544  0.01097 *
## icv            514.064      6.193  83.011  < 2e-16 ***
## ---
## Signif. codes:  0 ‘***’ 0.001 ‘**’ 0.01 ‘*’ 0.05 ‘.’ 0.1 ‘ ’ 1
##
## Approximate significance of smooth terms:
##              edf Ref.df       F p-value
## s(age_z)   5.242  5.242 636.470  <2e-16 ***
## s(sleep_z) 1.946  1.946   2.821  0.0432 *
## ---
## Signif. codes:  0 ‘***’ 0.001 ‘**’ 0.01 ‘*’ 0.05 ‘.’ 0.1 ‘ ’ 1
##
## R-sq.(adj) =  0.412
## lmer.REML = 5.3976e+05  Scale est. = 33998     n = 33441
```

Below is the model output with main effect.

```
##
## Family: gaussian
## Link function: identity
##
## Formula:
## value ~ sex + site + s(age_z, k = 10, bs = "cr") + s(sleep_z,
##     k = 5, bs = "cr") + icv + bmi
## <environment: 0x558a492785d8>
##
## Parametric coefficients:
##               Estimate Std. Error t value Pr(>|t|)
## (Intercept)   8987.949     73.524 122.246  < 2e-16 ***
## sexmale        341.964     12.077  28.316  < 2e-16 ***
## siteousPrisma  226.004     90.490   2.498  0.01251 *
## siteousSkyra   726.161     46.921  15.476  < 2e-16 ***
## siteUCAM       123.561     72.408   1.706  0.08793 .
## siteUKB       -198.991     68.007  -2.926  0.00344 **
## siteUmU        205.474     83.985   2.447  0.01443 *
## icv            512.709      6.188  82.859  < 2e-16 ***
## bmi              8.076      1.173   6.882 5.99e-12 ***
## ---
## Signif. codes:  0 ‘***’ 0.001 ‘**’ 0.01 ‘*’ 0.05 ‘.’ 0.1 ‘ ’ 1
##
## Approximate significance of smooth terms:
##              edf Ref.df       F p-value
## s(age_z)   5.284  5.284 633.476  <2e-16 ***
## s(sleep_z) 1.121  1.121   3.087   0.063 .
## ---
## Signif. codes:  0 ‘***’ 0.001 ‘**’ 0.01 ‘*’ 0.05 ‘.’ 0.1 ‘ ’ 1
##
## R-sq.(adj) =  0.413
```

```
## lmer.REML = 5.3972e+05  Scale est. = 34005     n = 33441
```

Next is the model with BMI-sleep interaction.

```
##
## Family: gaussian
## Link function: identity
##
## Formula:
## value ~ sex + site + s(age_z, k = 10, bs = "cr") + s(sleep_z,
##     k = 5, bs = "cr") + icv + bmi + bmi:sleep_z
## <environment: 0x558a492785d8>
##
## Parametric coefficients:
##               Estimate Std. Error t value Pr(>|t|)
## (Intercept)   8984.548     73.563 122.134  < 2e-16 ***
## sexmale        342.034     12.077  28.322  < 2e-16 ***
## siteousPrisma  225.966     90.489   2.497  0.01252 *
## siteousSkyra   726.407     46.921  15.482  < 2e-16 ***
## siteUCAM       124.752     72.412   1.723  0.08493 .
## siteUKB       -197.790     68.010  -2.908  0.00364 **
## siteUmU        206.498     83.986   2.459  0.01395 *
## icv            512.778      6.188  82.867  < 2e-16 ***
## bmi              8.129      1.174   6.923 4.49e-12 ***
## bmi:sleep_z      1.573      1.110   1.417  0.15658
## ---
## Signif. codes:  0 ‘***’ 0.001 ‘**’ 0.01 ‘*’ 0.05 ‘.’ 0.1 ‘ ’ 1
##
## Approximate significance of smooth terms:
##              edf Ref.df      F p-value
## s(age_z)   5.282  5.282 634.13  <2e-16 ***
## s(sleep_z) 1.165  1.165   2.27   0.104
## ---
## Signif. codes:  0 ‘***’ 0.001 ‘**’ 0.01 ‘*’ 0.05 ‘.’ 0.1 ‘ ’ 1
##
## R-sq.(adj) =  0.413
## lmer.REML = 5.3971e+05  Scale est. = 34003     n = 33441
```

We did the same controling for depression. Below is the model with no covariates but only keeping data with depression.

```
##
## Family: gaussian
## Link function: identity
##
## Formula:
## value ~ sex + site + s(age_z, k = 10, bs = "cr") + s(sleep_z,
##     k = 5, bs = "cr") + icv
## <environment: 0x558a477b4d88>
##
## Parametric coefficients:
##               Estimate Std. Error t value Pr(>|t|)
## (Intercept)   9820.411     57.732 170.102  < 2e-16 ***
## sexmale        350.715     12.081  29.030  < 2e-16 ***
## siteousAvanto -650.538    110.289  -5.898 3.70e-09 ***
## siteousPrisma -333.675    107.387  -3.107  0.00189 **
```

```
## siteousSkyra   139.312     85.224   1.635  0.10213
## siteUCAM      -501.535     78.792  -6.365 1.97e-10 ***
## siteUKB       -830.330     57.098 -14.542  < 2e-16 ***
## siteUmU       -423.561     77.286  -5.480 4.27e-08 ***
## icv            510.361      6.201  82.300  < 2e-16 ***
## ---
## Signif. codes:  0 '***' 0.001 '**' 0.01 '*' 0.05 '.' 0.1 ' ' 1
##
## Approximate significance of smooth terms:
##              edf Ref.df       F p-value
## s(age_z)   5.705  5.705 503.751  <2e-16 ***
## s(sleep_z) 2.070  2.070   3.436  0.0366 *
## ---
## Signif. codes:  0 '***' 0.001 '**' 0.01 '*' 0.05 '.' 0.1 ' ' 1
##
## R-sq.(adj) =  0.402
## lmer.REML = 5.3801e+05  Scale est. = 34333     n = 33353
```

Below is the model output with main effect.

```
##
## Family: gaussian
## Link function: identity
##
## Formula:
## value ~ sex + site + s(age_z, k = 10, bs = "cr") + s(sleep_z,
##     k = 5, bs = "cr") + icv + depression
## <environment: 0x558a477b4d88>
##
## Parametric coefficients:
##                Estimate Std. Error t value Pr(>|t|)
## (Intercept)   9817.464     57.979 169.328  < 2e-16 ***
## sexmale        351.032     12.094  29.026  < 2e-16 ***
## siteousAvanto -652.237    110.329  -5.912 3.42e-09 ***
## siteousPrisma -335.222    107.421  -3.121  0.00181 **
## siteousSkyra   137.423     85.284   1.611  0.10711
## siteUCAM      -502.277     78.805  -6.374 1.87e-10 ***
## siteUKB       -829.151     57.142 -14.510  < 2e-16 ***
## siteUmU       -432.697     78.926  -5.482 4.23e-08 ***
## icv            510.307      6.201  82.288  < 2e-16 ***
## depression      24.531     43.146   0.569  0.56967
## ---
## Signif. codes:  0 '***' 0.001 '**' 0.01 '*' 0.05 '.' 0.1 ' ' 1
##
## Approximate significance of smooth terms:
##              edf Ref.df       F p-value
## s(age_z)   5.714  5.714 493.225  <2e-16 ***
## s(sleep_z) 1.971  1.971   2.882  0.0442 *
## ---
## Signif. codes:  0 '***' 0.001 '**' 0.01 '*' 0.05 '.' 0.1 ' ' 1
##
## R-sq.(adj) =  0.402
## lmer.REML = 5.38e+05  Scale est. = 34337     n = 33353
```

Next is the model with depression-sleep interaction.

```
##
## Family: gaussian
## Link function: identity
##
## Formula:
## value ~ sex + site + s(age_z, k = 10, bs = "cr") + s(sleep_z,
##     k = 5, bs = "cr") + icv + depression + depression:sleep_z
## <environment: 0x558a477b4d88>
##
## Parametric coefficients:
##                    Estimate Std. Error t value Pr(>|t|)
## (Intercept)        9817.443     57.980 169.325  < 2e-16 ***
## sexmale             350.946     12.096  29.014  < 2e-16 ***
## siteousAvanto      -652.692    110.338  -5.915 3.34e-09 ***
## siteousPrisma      -335.679    107.431  -3.125  0.00178 **
## siteousSkyra        136.999     85.295   1.606  0.10824
## siteUCAM           -502.832     78.821  -6.379 1.80e-10 ***
## siteUKB            -829.078     57.143 -14.509  < 2e-16 ***
## siteUmU            -428.918     79.588  -5.389 7.12e-08 ***
## icv                 510.325      6.202  82.288  < 2e-16 ***
## depression           23.341     43.231   0.540  0.58926
## depression:sleep_z  -11.297     31.614  -0.357  0.72084
## ---
## Signif. codes:  0 ‘***’ 0.001 ‘**’ 0.01 ‘*’ 0.05 ‘.’ 0.1 ‘ ’ 1
##
## Approximate significance of smooth terms:
##              edf Ref.df       F p-value
## s(age_z)   5.710  5.710 493.568  <2e-16 ***
## s(sleep_z) 2.016  2.016   2.093   0.134
## ---
## Signif. codes:  0 ‘***’ 0.001 ‘**’ 0.01 ‘*’ 0.05 ‘.’ 0.1 ‘ ’ 1
##
## R-sq.(adj) =  0.402
## lmer.REML = 5.3799e+05  Scale est. = 34336     n = 33353
```

The plot below shows the sleep-volume curve for the original model and for the model with main effects of SES.

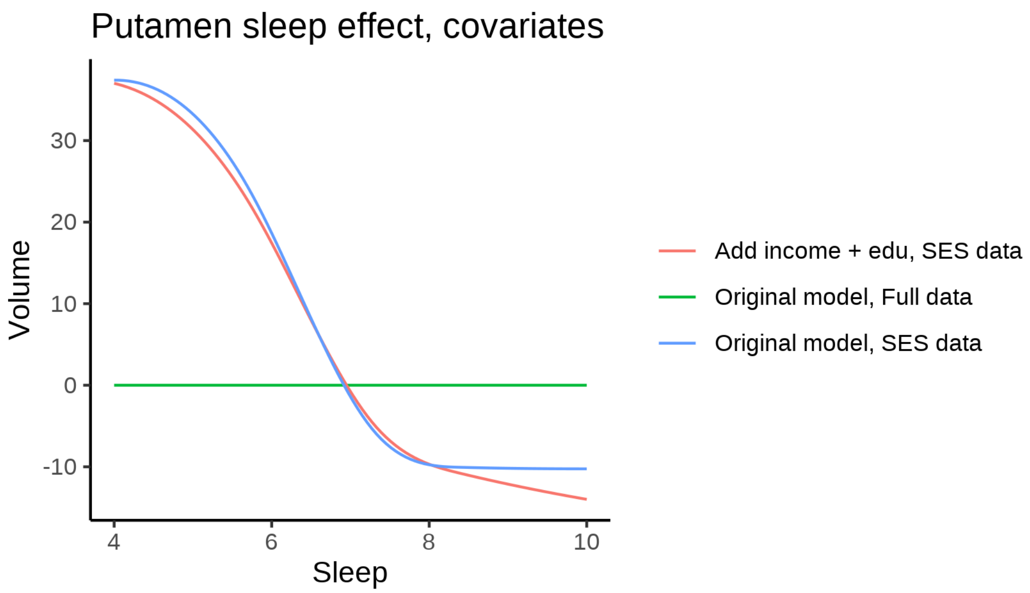

The plot below shows the sleep-volume curve for the original model and for the model with main effects of BMI.

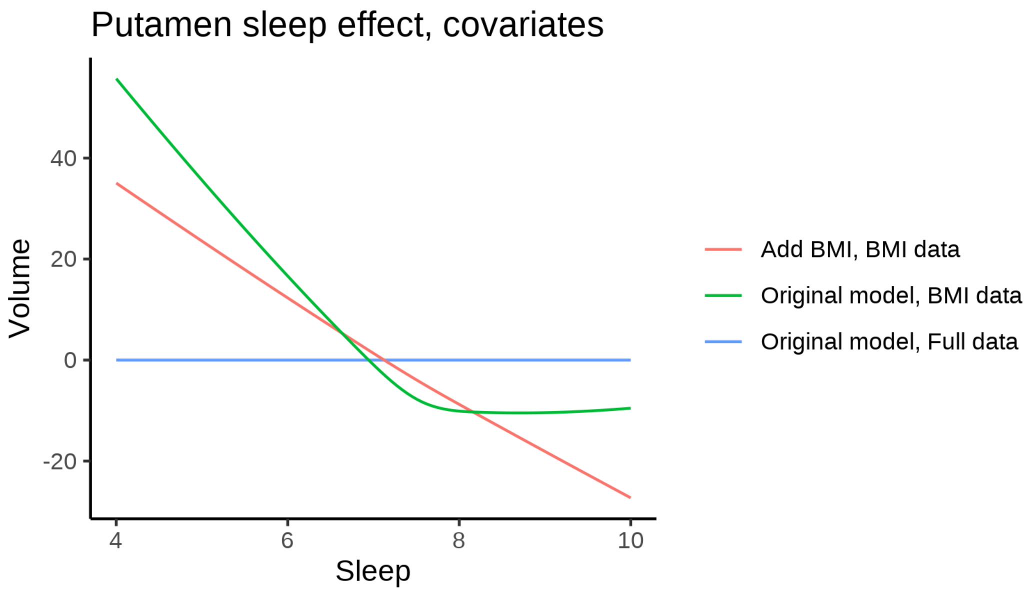

The plot below shows the sleep-volume curve for the original model and for the model with main effects of depression.

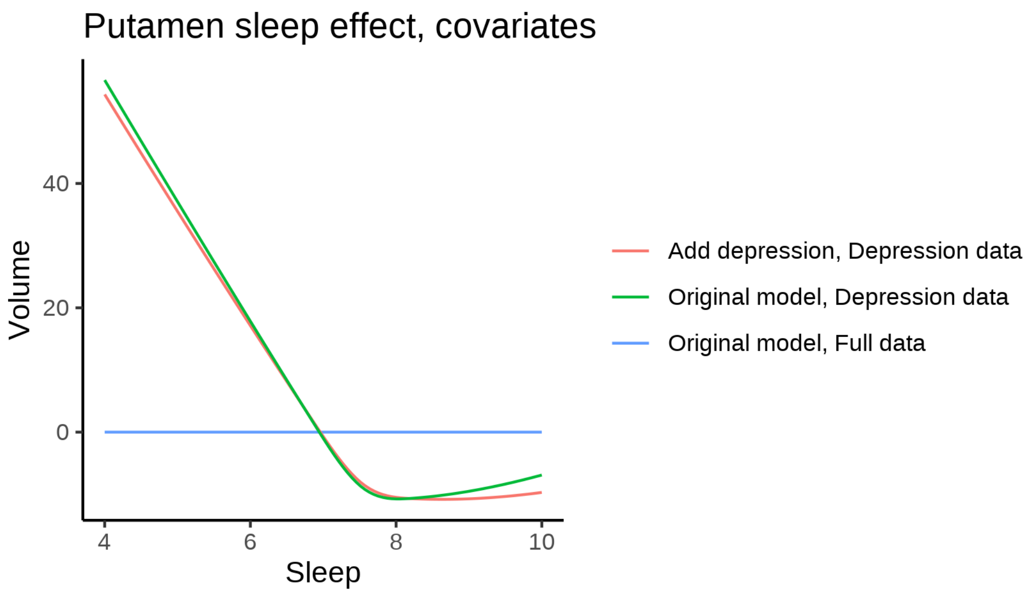

Models Not controling for ICV

Model outputs

Model without sleep term

```
##
## Family: gaussian
## Link function: identity
##
## Formula:
## value ~ sex + site + s(age_z, k = 10, bs = "cr")
## <environment: 0x558a5ec4d420>
##
## Parametric coefficients:
##                Estimate Std. Error t value Pr(>|t|)
## (Intercept)    8394.850     46.279 181.396  < 2e-16 ***
## sexmale         917.731      8.866 103.506  < 2e-16 ***
## siteMPIB        460.767     64.023   7.197 6.25e-13 ***
## siteousAvanto   807.199     50.782  15.895  < 2e-16 ***
## siteousPrisma   908.611     86.455  10.510  < 2e-16 ***
## siteousSkyra   1268.613     49.354  25.704  < 2e-16 ***
## siteUB          626.912    157.898   3.970 7.19e-05 ***
## siteUCAM        686.643     55.255  12.427  < 2e-16 ***
## siteUKB         359.302     47.344   7.589 3.27e-14 ***
## siteUmU         534.742     72.140   7.413 1.26e-13 ***
## siteUOXF        745.105     58.726  12.688  < 2e-16 ***
## ---
## Signif. codes:  0 ‘***’ 0.001 ‘**’ 0.01 ‘*’ 0.05 ‘.’ 0.1 ‘ ’ 1
##
## Approximate significance of smooth terms:
##             edf Ref.df    F p-value
## s(age_z) 3.122  3.122 1727  <2e-16 ***
```

```
## ---
## Signif. codes:  0 ‘***’ 0.001 ‘**’ 0.01 ‘*’ 0.05 ‘.’ 0.1 ‘ ’ 1
##
## R-sq.(adj) =  0.284
## lmer.REML = 8.3802e+05  Scale est. = 37539     n = 51267
```

Model with only main effects of age and sleep

```
##
## Family: gaussian
## Link function: identity
##
## Formula:
## value ~ sex + site + s(age_z, k = 10, bs = "cr") + s(sleep_z,
##     k = 5, bs = "cr")
## <environment: 0x558a5ec4d420>
##
## Parametric coefficients:
##               Estimate Std. Error t value Pr(>|t|)
## (Intercept)    8400.02      46.30 181.418  < 2e-16 ***
## sexmale         916.89       8.87 103.368  < 2e-16 ***
## siteMPIB        458.28      64.03   7.157 8.37e-13 ***
## siteousAvanto   803.74      50.79  15.824  < 2e-16 ***
## siteousPrisma   905.91      86.45  10.479  < 2e-16 ***
## siteousSkyra   1265.06      49.36  25.628  < 2e-16 ***
## siteUB          627.80     157.88   3.976 7.00e-05 ***
## siteUCAM        685.49      55.24  12.408  < 2e-16 ***
## siteUKB         354.29      47.36   7.480 7.54e-14 ***
## siteUmU         528.21      72.27   7.309 2.73e-13 ***
## siteUOXF        742.84      58.71  12.653  < 2e-16 ***
## ---
## Signif. codes:  0 ‘***’ 0.001 ‘**’ 0.01 ‘*’ 0.05 ‘.’ 0.1 ‘ ’ 1
##
## Approximate significance of smooth terms:
##              edf Ref.df        F p-value
## s(age_z)   3.111  3.111 1724.659  <2e-16 ***
## s(sleep_z) 2.299  2.299    4.241  0.0222 *
## ---
## Signif. codes:  0 ‘***’ 0.001 ‘**’ 0.01 ‘*’ 0.05 ‘.’ 0.1 ‘ ’ 1
##
## R-sq.(adj) =  0.284
## lmer.REML = 8.3801e+05  Scale est. = 37541     n = 51267
```

Model with full interaction between age and sleep

```
##
## Family: gaussian
## Link function: identity
##
## Formula:
## value ~ sex + site + t2(age_z, sleep_z, k = c(10, 4), bs = "cr")
## <environment: 0x558a5ec4d420>
##
## Parametric coefficients:
##               Estimate Std. Error t value Pr(>|t|)
## (Intercept)    8416.87      45.56 184.758  < 2e-16 ***
```

```
## sexmale         918.46       8.88 103.436  < 2e-16 ***
## siteMPIB        438.88      63.84   6.874 6.30e-12 ***
## siteousAvanto   793.82      50.81  15.623  < 2e-16 ***
## siteousPrisma   897.06      86.54  10.366  < 2e-16 ***
## siteousSkyra   1254.29      49.40  25.388  < 2e-16 ***
## siteUB          605.02     157.71   3.836 0.000125 ***
## siteUCAM        666.94      55.00  12.126  < 2e-16 ***
## siteUKB         336.23      46.57   7.221 5.25e-13 ***
## siteUmU         509.92      71.89   7.093 1.33e-12 ***
## siteUOXF        722.31      58.21  12.408  < 2e-16 ***
## ---
## Signif. codes:  0 '***' 0.001 '**' 0.01 '*' 0.05 '.' 0.1 ' ' 1
##
## Approximate significance of smooth terms:
##                     edf Ref.df     F p-value
## t2(age_z,sleep_z) 4.959  4.959 377.2  <2e-16 ***
## ---
## Signif. codes:  0 '***' 0.001 '**' 0.01 '*' 0.05 '.' 0.1 ' ' 1
##
## R-sq.(adj) =  0.284
## lmer.REML = 8.3801e+05  Scale est. = 37523     n = 51267
```

Model comparison

`mod_no_sleep` refers to model without sleep term, `mod_no_interaction` refers to model with only main effect of sleep, and `mod_full` refers to model with a full interaction between age and sleep. This is a nested model comparison, and the p-value at a given line refers to comparing the model at the line to the model on the line above. Hence, significance implies that the more complicated model is supported on statistical grounds.

To be even more specific, the p-value on the second row tests whether there is an association between sleep and volume. The p-value on the third row tests whether this association depends on age.

```
## Data: NULL
## Models:
## ml$mod_no_sleep$mer: NULL
## ml$mod_no_interaction$mer: NULL
## ml$mod_full$mer: NULL
##                           npar    AIC    BIC  logLik deviance  Chisq Df Pr(>Chisq)
## ml$mod_no_sleep$mer         15 838046 838179 -419008   838016
## ml$mod_no_interaction$mer   17 838045 838196 -419006   838011 4.9073  2    0.08598 .
## ml$mod_full$mer             19 838050 838218 -419006   838012 0.0000  2    1.00000
## ---
## Signif. codes:  0 '***' 0.001 '**' 0.01 '*' 0.05 '.' 0.1 ' ' 1
```

We chose the model based on the likelihood ratio test with 5 % significance level, which was `mod_no_sleep`.

Lifespan brain trajectory

The trajectory shown is from the chosen model `mod_no_sleep_no_icv`.

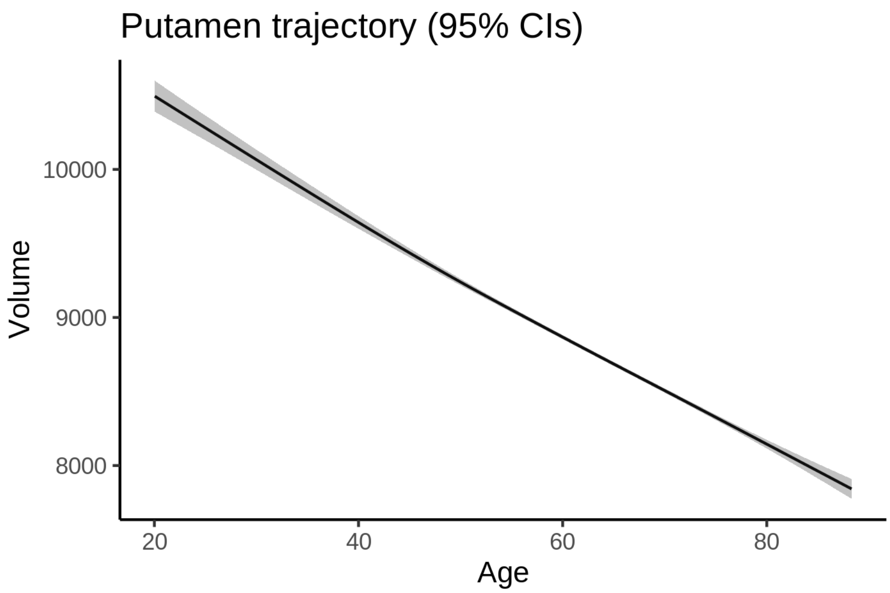

Effect of sleep

The chosen model did not include a sleep term, and hence we don't have any estimated effect of sleep. We show the full interaction model for completeness, although it was not selected.

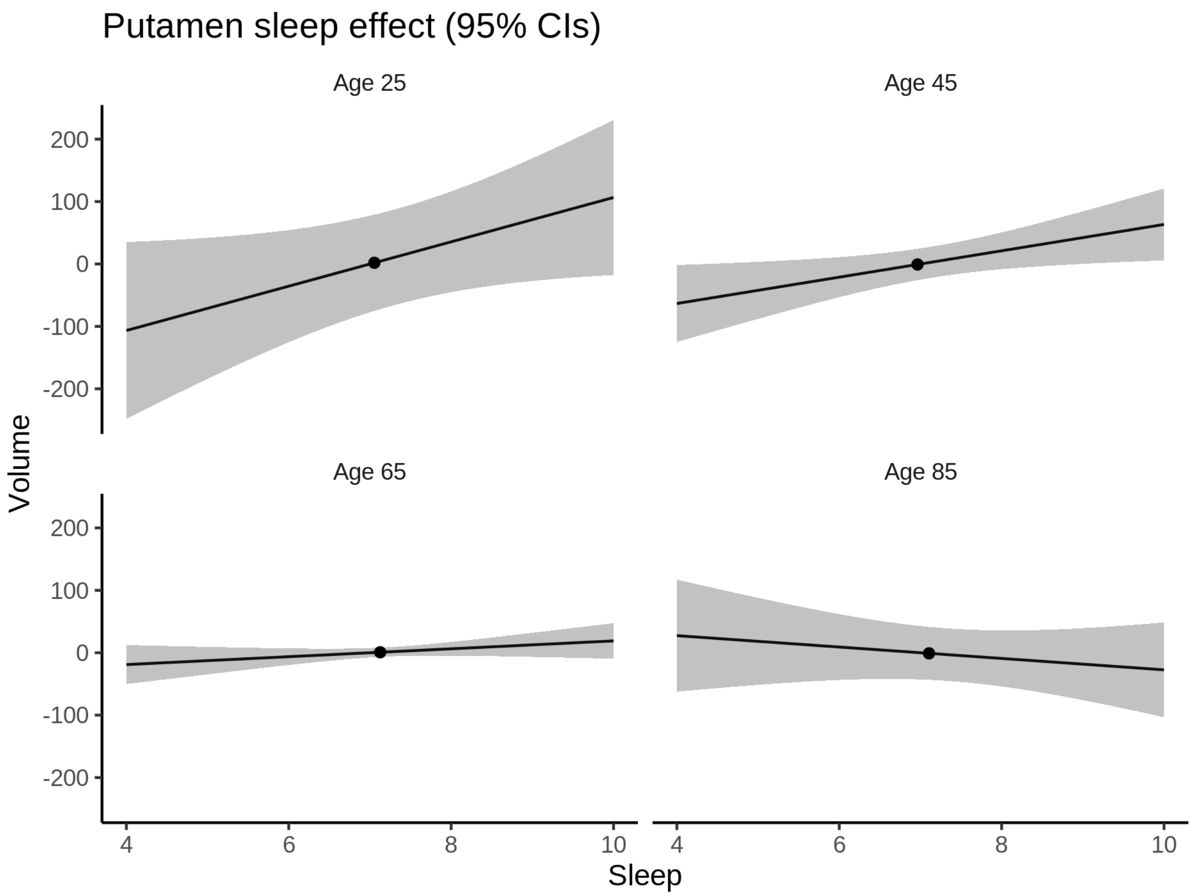

Deviation from sleep associated with maximal volume

Model with no sleep term was selected. No plots to show. (Although we can of course dig up the plots, which will be pretty flat).

Comparison of mean sleep and sleep associated with maximum volume

Nothing to show, as we did not find an association between sleep and volume.

Thalamus

Descriptive statistics

Study	Observations	Unique IDs	Mean age	Age range
HCP	974	974	28.8	22 - 37
MPIB	677	391	63.1	24 - 83
UB	113	39	70.9	64 - 81
UCAM	884	632	55.1	20 - 88
UiO	1475	803	49.4	20 - 89
UKB	45975	43133	64.5	45 - 83

Study	Observations	Unique IDs	Mean age	Age range
UmU	412	279	62.2	25 - 85
UOXF	769	769	69.8	60 - 85

Spaghetti plot

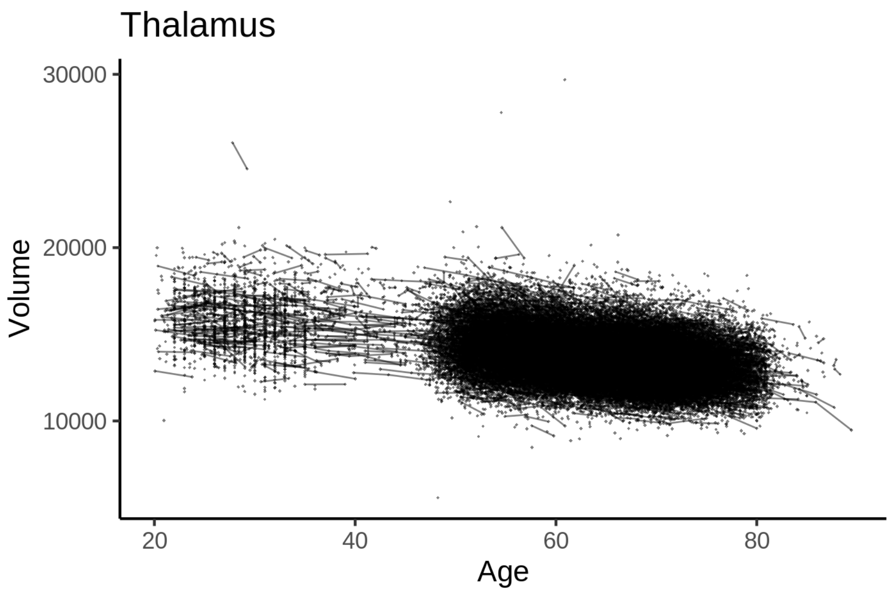

Models Controling for ICV

Model outputs

Model without sleep term

```
##
## Family: gaussian
## Link function: identity
##
## Formula:
## value ~ sex + site + icv + s(age_z, k = 10, bs = "cr")
## <environment: 0x558a46b9f400>
##
## Parametric coefficients:
##                  Estimate Std. Error t value Pr(>|t|)
## (Intercept)   13374.197     52.690 253.829  < 2e-16 ***
## sexmale         196.535     10.656  18.443  < 2e-16 ***
## siteMPIB       1615.000     66.774  24.186  < 2e-16 ***
## siteousAvanto   306.728     54.190   5.660 1.52e-08 ***
## siteousPrisma  1062.084     91.547  11.601  < 2e-16 ***
## siteousSkyra    409.248     50.894   8.041 9.08e-16 ***
## siteUB         1054.097    155.949   6.759 1.40e-11 ***
## siteUCAM       1053.651     57.958  18.180  < 2e-16 ***
## siteUKB         206.072     54.054   3.812 0.000138 ***
## siteUmU          -5.011     74.885  -0.067 0.946653
```

```
## siteUOXF        911.661     63.799  14.290  < 2e-16 ***
## icv             880.554      5.360 164.285  < 2e-16 ***
## ---
## Signif. codes:  0 ‘***’ 0.001 ‘**’ 0.01 ‘*’ 0.05 ‘.’ 0.1 ‘ ’ 1
##
## Approximate significance of smooth terms:
##            edf Ref.df    F p-value
## s(age_z) 7.872  7.872 1553  <2e-16 ***
## ---
## Signif. codes:  0 ‘***’ 0.001 ‘**’ 0.01 ‘*’ 0.05 ‘.’ 0.1 ‘ ’ 1
##
## R-sq.(adj) =  0.581
## lmer.REML = 8.4013e+05  Scale est. = 79442     n = 51279
```

Model with only main effects of age and sleep

```
##
## Family: gaussian
## Link function: identity
##
## Formula:
## value ~ sex + site + icv + s(age_z, k = 10, bs = "cr") + s(sleep_z,
##     k = 5, bs = "cr")
## <environment: 0x558a46b9f400>
##
## Parametric coefficients:
##                 Estimate Std. Error t value Pr(>|t|)
## (Intercept)   13368.382     52.736 253.498  < 2e-16 ***
## sexmale         196.118     10.654  18.408  < 2e-16 ***
## siteMPIB       1624.839     66.800  24.324  < 2e-16 ***
## siteousAvanto   308.695     54.198   5.696 1.24e-08 ***
## siteousPrisma  1063.405     91.530  11.618  < 2e-16 ***
## siteousSkyra    410.849     50.902   8.071 7.10e-16 ***
## siteUB         1060.364    155.893   6.802 1.04e-11 ***
## siteUCAM       1058.914     57.954  18.272  < 2e-16 ***
## siteUKB         212.312     54.106   3.924 8.72e-05 ***
## siteUmU          15.872     75.041   0.212    0.832
## siteUOXF        914.842     63.795  14.340  < 2e-16 ***
## icv             880.447      5.364 164.139  < 2e-16 ***
## ---
## Signif. codes:  0 ‘***’ 0.001 ‘**’ 0.01 ‘*’ 0.05 ‘.’ 0.1 ‘ ’ 1
##
## Approximate significance of smooth terms:
##              edf Ref.df       F  p-value
## s(age_z)   7.877  7.877 1535.12  < 2e-16 ***
## s(sleep_z) 3.022  3.022    9.21 4.36e-06 ***
## ---
## Signif. codes:  0 ‘***’ 0.001 ‘**’ 0.01 ‘*’ 0.05 ‘.’ 0.1 ‘ ’ 1
##
## R-sq.(adj) =  0.582
## lmer.REML = 8.401e+05  Scale est. = 79512     n = 51279
```

Model with full interaction between age and sleep

```
##
## Family: gaussian
```

```
## Link function: identity
##
## Formula:
## value ~ sex + site + icv + t2(age_z, sleep_z, k = c(10, 4), bs = "cr")
## <environment: 0x558a46b9f400>
##
## Parametric coefficients:
##                Estimate Std. Error t value Pr(>|t|)
## (Intercept)   13370.914     52.932 252.606  < 2e-16 ***
## sexmale         195.794     10.678  18.336  < 2e-16 ***
## siteMPIB       1622.533     67.162  24.159  < 2e-16 ***
## siteousAvanto   309.117     54.486   5.673 1.41e-08 ***
## siteousPrisma  1062.759     91.746  11.584  < 2e-16 ***
## siteousSkyra    412.162     51.227   8.046 8.75e-16 ***
## siteUB         1053.712    156.009   6.754 1.45e-11 ***
## siteUCAM       1056.239     58.271  18.126  < 2e-16 ***
## siteUKB         209.811     54.300   3.864 0.000112 ***
## siteUmU          11.574     75.281   0.154 0.877813
## siteUOXF        911.530     64.031  14.236  < 2e-16 ***
## icv             880.730      5.363 164.223  < 2e-16 ***
## ---
## Signif. codes:  0 ‘***’ 0.001 ‘**’ 0.01 ‘*’ 0.05 ‘.’ 0.1 ‘ ’ 1
##
## Approximate significance of smooth terms:
##                     edf Ref.df     F p-value
## t2(age_z,sleep_z) 14.55  14.55 33.02  <2e-16 ***
## ---
## Signif. codes:  0 ‘***’ 0.001 ‘**’ 0.01 ‘*’ 0.05 ‘.’ 0.1 ‘ ’ 1
##
## R-sq.(adj) =  0.582
## lmer.REML = 8.4011e+05  Scale est. = 79483     n = 51279
```

Model comparison

`mod_no_sleep` refers to model without sleep term, `mod_no_interaction` refers to model with only main effect of sleep, and `mod_full` refers to model with a full interaction between age and sleep. This is a nested model comparison, and the p-value at a given line refers to comparing the model at the line to the model on the line above. Hence, significance implies that the more complicated model is supported on statistical grounds.

To be even more specific, the p-value on the second row tests whether there is an association between sleep and volume. The p-value on the third row tests whether this association depends on age.

```
## Data: NULL
## Models:
## ml$mod_no_sleep$mer: NULL
## ml$mod_no_interaction$mer: NULL
## ml$mod_full$mer: NULL
##                           npar    AIC    BIC  logLik deviance  Chisq Df Pr(>Chisq)
## ml$mod_no_sleep$mer         16 840158 840299 -420063   840126
## ml$mod_no_interaction$mer   18 840139 840298 -420051   840103 22.987  2   1.02e-05 ***
## ml$mod_full$mer             20 840153 840329 -420056   840113  0.000  2          1
## ---
## Signif. codes:  0 ‘***’ 0.001 ‘**’ 0.01 ‘*’ 0.05 ‘.’ 0.1 ‘ ’ 1
```

We chose the model based on the likelihood ratio test with 5 % significance level, which was `mod_no_interaction`.

Lifespan brain trajectory

The trajectory shown is from the chosen model `mod_no_interaction`.

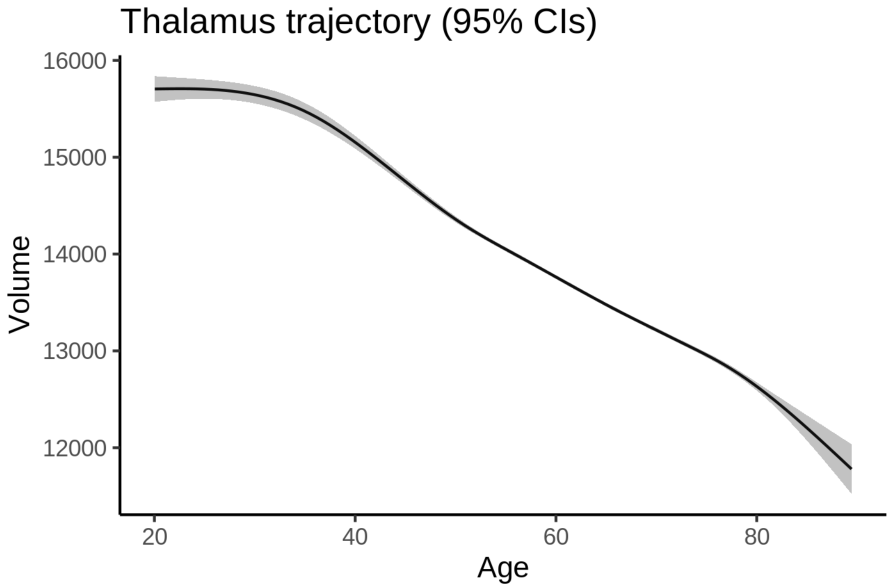

Effect of sleep

The chosen model only included the main effect of sleep, and hence the effect does not vary with age. The black dot shows the average sleep duration across all ages in the sample.

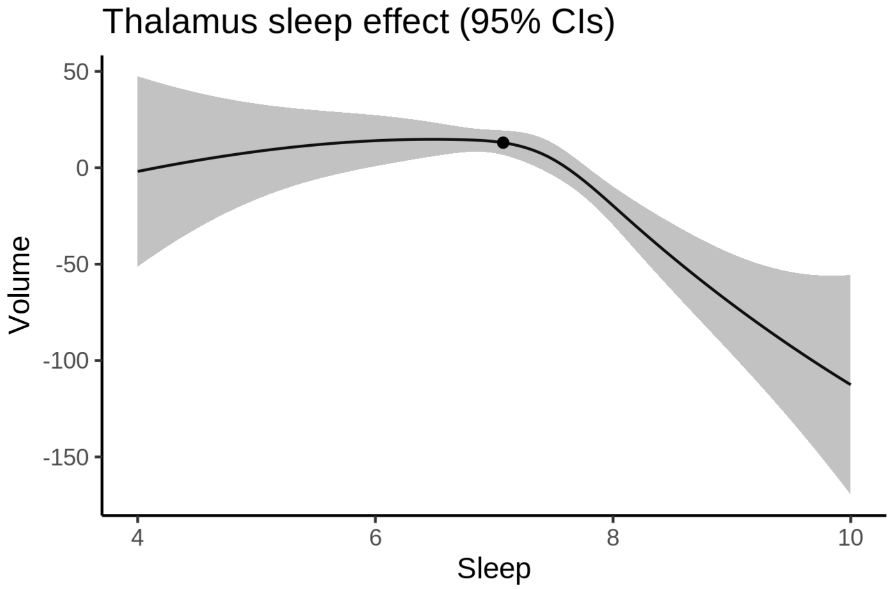

We also show the full interaction model for completeness, although it was not selected.

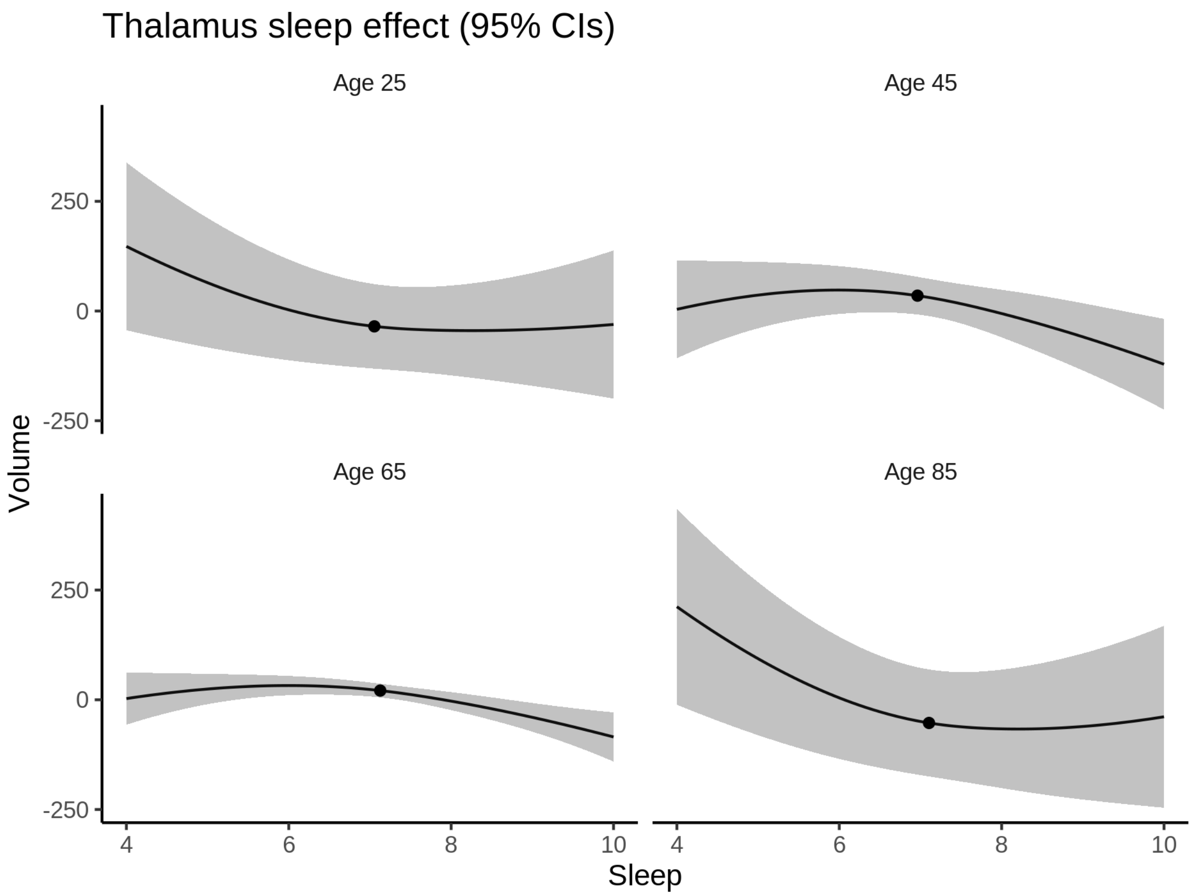

Deviation from sleep associated with maximal volume

Model with only main effect of sleep was chosen, so we show it for all ages at once. Maximum volume is attained at 6.5 hours of sleep. The percentage values in the plot are calculated as follows: The maximum at 100 % refers to a person at an arbitrary age with a sleep duration associated with maximum volume. For a female, this volume is 14077 and for a male it is 14273. The other percentage values show how large the expected volume is for someone with other sleep durations. For example, 99 % implies a 1 % reduction.

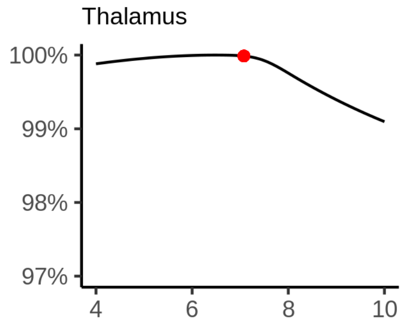

Comparison of mean sleep and sleep associated with maximum volume

A 95 % confidence interval for the sleep associated with maximum volume is [4, 7.15].

The plot below compares average sleep to the sleep associated with maximum volume.

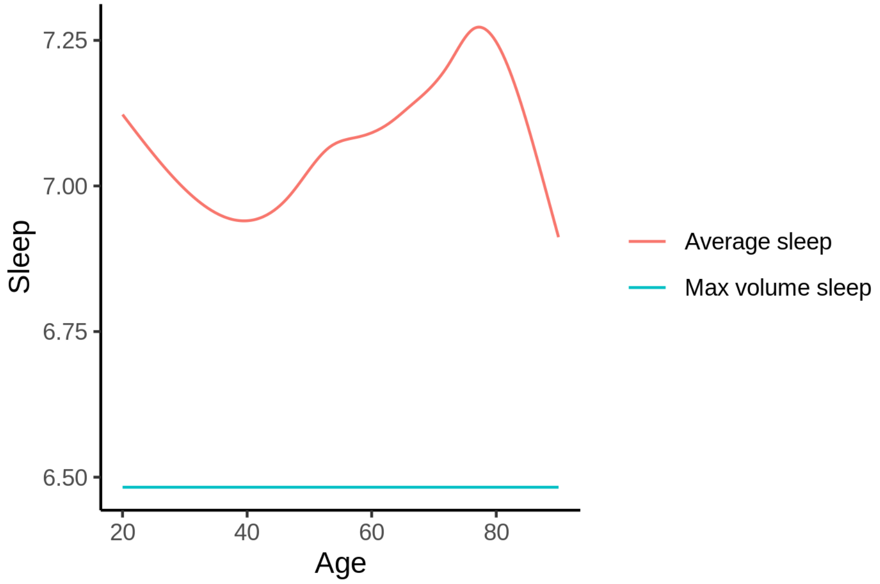

The next plot shows the difference between average sleep and sleep associated with maximum volume. The shaded region is a 95 % confidence interval.

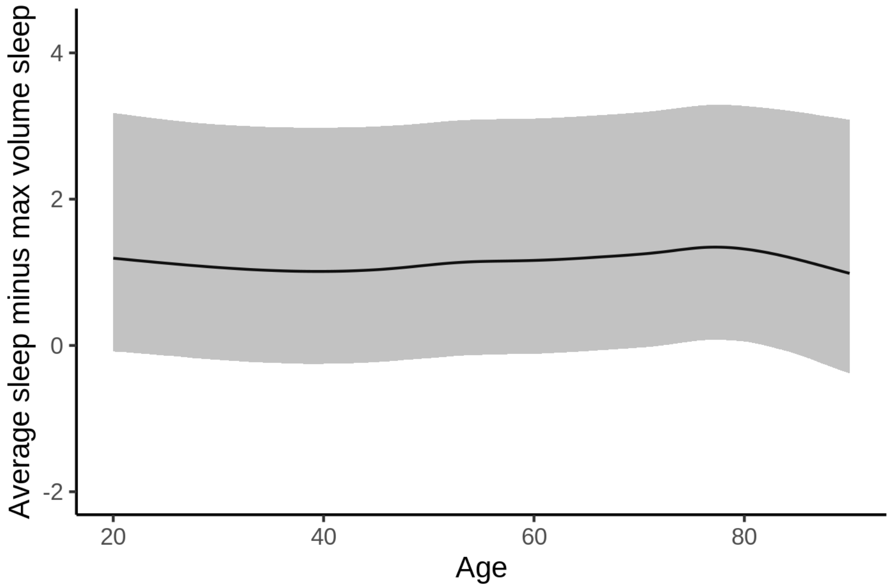

The next plot shows the probability that the sleep duration associated with maximum volume is longer than the average sleep duration, as a function of age. Probability below .05 can be interpreted as evidence that the sleep associated with maximum volume is shorter than the mean sleep, and probability above .95 can be interpreted the opposite way.

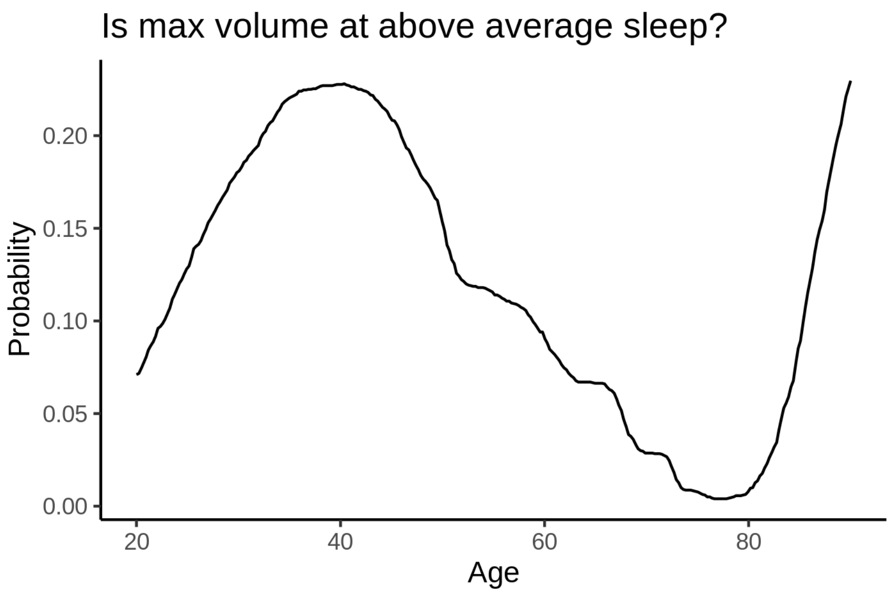

Controling for covariates

Below is the output for a model in which we only include data with income and education.

```
##
## Family: gaussian
## Link function: identity
##
## Formula:
## value ~ sex + site + s(age_z, k = 10, bs = "cr") + s(sleep_z,
##     k = 5, bs = "cr") + icv
## <environment: 0x55896133bb60>
##
## Parametric coefficients:
##                Estimate Std. Error t value Pr(>|t|)
## (Intercept)   15076.901    120.778 124.832  < 2e-16 ***
## sexmale         174.596     13.418  13.012  < 2e-16 ***
## siteousAvanto -1709.261    147.616 -11.579  < 2e-16 ***
## siteousPrisma  -406.556    149.583  -2.718  0.00657 **
## siteousSkyra  -1297.356    131.679  -9.852  < 2e-16 ***
## siteUKB       -1536.755    120.485 -12.755  < 2e-16 ***
## siteUOXF       -711.377    135.975  -5.232 1.69e-07 ***
## icv             898.979      6.896 130.364  < 2e-16 ***
## ---
## Signif. codes:  0 ‘***’ 0.001 ‘**’ 0.01 ‘*’ 0.05 ‘.’ 0.1 ‘ ’ 1
##
## Approximate significance of smooth terms:
##              edf Ref.df        F  p-value
## s(age_z)   3.331  3.331 1791.515  < 2e-16 ***
## s(sleep_z) 2.771  2.771    7.336 0.000221 ***
## ---
## Signif. codes:  0 ‘***’ 0.001 ‘**’ 0.01 ‘*’ 0.05 ‘.’ 0.1 ‘ ’ 1
##
```

```
## R-sq.(adj) =  0.555
## lmer.REML = 5.1044e+05  Scale est. = 81739     n = 31194
```

Below is the output for a model in which we control for the main effects of income and education.

```
##
## Family: gaussian
## Link function: identity
##
## Formula:
## value ~ sex + site + s(age_z, k = 10, bs = "cr") + s(sleep_z,
##     k = 5, bs = "cr") + icv + income_scaled + education_scaled
## <environment: 0x55896133bb60>
##
## Parametric coefficients:
##                   Estimate Std. Error t value Pr(>|t|)
## (Intercept)      15046.075    121.464 123.873  < 2e-16 ***
## sexmale            174.729     13.455  12.986  < 2e-16 ***
## siteousAvanto    -1709.996    147.605 -11.585  < 2e-16 ***
## siteousPrisma     -409.784    149.538  -2.740  0.00614 **
## siteousSkyra     -1298.148    131.688  -9.858  < 2e-16 ***
## siteUKB          -1537.457    120.467 -12.763  < 2e-16 ***
## siteUOXF          -702.322    135.969  -5.165 2.42e-07 ***
## icv                896.973      6.954 128.982  < 2e-16 ***
## income_scaled       19.899     15.974   1.246  0.21288
## education_scaled    28.297     18.897   1.497  0.13429
## ---
## Signif. codes:  0 '***' 0.001 '**' 0.01 '*' 0.05 '.' 0.1 ' ' 1
##
## Approximate significance of smooth terms:
##              edf Ref.df        F  p-value
## s(age_z)   3.241  3.241 1688.365  < 2e-16 ***
## s(sleep_z) 2.665  2.665    7.206 0.000326 ***
## ---
## Signif. codes:  0 '***' 0.001 '**' 0.01 '*' 0.05 '.' 0.1 ' ' 1
##
## R-sq.(adj) =  0.555
## lmer.REML = 5.1042e+05  Scale est. = 81800     n = 31194
```

We also included interaction effects between sleep duration and education and income, in another model. The output is shown below, and the interaction terms are `income_scaled:sleep_z` and `education_scaled:sleep_z`.

```
##
## Family: gaussian
## Link function: identity
##
## Formula:
## value ~ sex + site + s(age_z, k = 10, bs = "cr") + s(sleep_z,
##     k = 5, bs = "cr") + icv + income_scaled + education_scaled +
##     income_scaled:sleep_z + education_scaled:sleep_z
## <environment: 0x55896133bb60>
##
## Parametric coefficients:
##                           Estimate Std. Error t value Pr(>|t|)
## (Intercept)              15045.297    121.455 123.875  < 2e-16 ***
```

```
## sexmale                    174.148     13.464  12.934  < 2e-16 ***
## siteousAvanto            -1710.595    147.596 -11.590  < 2e-16 ***
## siteousPrisma             -411.496    149.542  -2.752  0.00593 **
## siteousSkyra             -1298.685    131.686  -9.862  < 2e-16 ***
## siteUKB                  -1536.414    120.462 -12.754  < 2e-16 ***
## siteUOXF                  -699.674    135.993  -5.145 2.69e-07 ***
## icv                        896.974      6.955 128.971  < 2e-16 ***
## income_scaled               20.515     15.981   1.284  0.19926
## education_scaled            28.010     18.900   1.482  0.13834
## income_scaled:sleep_z      -21.628     15.926  -1.358  0.17445
## education_scaled:sleep_z     1.352     18.344   0.074  0.94124
## ---
## Signif. codes:  0 '***' 0.001 '**' 0.01 '*' 0.05 '.' 0.1 ' ' 1
##
## Approximate significance of smooth terms:
##              edf Ref.df        F p-value
## s(age_z)   3.217  3.217 1699.202  <2e-16 ***
## s(sleep_z) 2.673  2.673    2.868   0.069 .
## ---
## Signif. codes:  0 '***' 0.001 '**' 0.01 '*' 0.05 '.' 0.1 ' ' 1
##
## R-sq.(adj) =  0.555
## lmer.REML = 5.104e+05  Scale est. = 81806     n = 31194
```

We did the same controling for BMI. Below is the model with no covariates but only keeping data with BMI.

```
##
## Family: gaussian
## Link function: identity
##
## Formula:
## value ~ sex + site + s(age_z, k = 10, bs = "cr") + s(sleep_z,
##     k = 5, bs = "cr") + icv
## <environment: 0x5589602edd20>
##
## Parametric coefficients:
##               Estimate Std. Error t value Pr(>|t|)
## (Intercept)   13635.869     85.714 159.085  < 2e-16 ***
## sexmale         163.769     13.030  12.569  < 2e-16 ***
## siteousPrisma   822.343    112.901   7.284 3.32e-13 ***
## siteousSkyra    232.174     68.558   3.387 0.000709 ***
## siteUCAM        814.823     90.175   9.036  < 2e-16 ***
## siteUKB         -67.346     86.384  -0.780 0.435624
## siteUmU        -233.709    101.216  -2.309 0.020949 *
## icv             909.969      6.716 135.495  < 2e-16 ***
## ---
## Signif. codes:  0 '***' 0.001 '**' 0.01 '*' 0.05 '.' 0.1 ' ' 1
##
## Approximate significance of smooth terms:
##              edf Ref.df        F  p-value
## s(age_z)   5.651  5.651 1366.485  < 2e-16 ***
## s(sleep_z) 2.589  2.589    6.966 0.000428 ***
## ---
## Signif. codes:  0 '***' 0.001 '**' 0.01 '*' 0.05 '.' 0.1 ' ' 1
##
```

```
## R-sq.(adj) =  0.582
## lmer.REML = 5.4679e+05  Scale est. = 75474     n = 33449
```

Below is the model output with main effect.

```
##
## Family: gaussian
## Link function: identity
##
## Formula:
## value ~ sex + site + s(age_z, k = 10, bs = "cr") + s(sleep_z,
##     k = 5, bs = "cr") + icv + bmi
## <environment: 0x5589602edd20>
##
## Parametric coefficients:
##                Estimate Std. Error t value Pr(>|t|)
## (Intercept)   13614.2523    91.4995 148.790  < 2e-16 ***
## sexmale         163.0917    13.0687  12.480  < 2e-16 ***
## siteousPrisma   821.5215   112.9095   7.276 3.52e-13 ***
## siteousSkyra    232.0673    68.5592   3.385 0.000713 ***
## siteUCAM        814.3570    90.1799   9.030  < 2e-16 ***
## siteUKB         -67.9988    86.3937  -0.787 0.431240
## siteUmU        -234.5920   101.2282  -2.317 0.020485 *
## icv             909.8307     6.7189 135.414  < 2e-16 ***
## bmi               0.8566     1.2711   0.674 0.500377
## ---
## Signif. codes:  0 ‘***’ 0.001 ‘**’ 0.01 ‘*’ 0.05 ‘.’ 0.1 ‘ ’ 1
##
## Approximate significance of smooth terms:
##             edf Ref.df        F  p-value
## s(age_z)   5.656  5.656 1365.113  < 2e-16 ***
## s(sleep_z) 2.617  2.617    6.669 0.000438 ***
## ---
## Signif. codes:  0 ‘***’ 0.001 ‘**’ 0.01 ‘*’ 0.05 ‘.’ 0.1 ‘ ’ 1
##
## R-sq.(adj) =  0.582
## lmer.REML = 5.4678e+05  Scale est. = 75476     n = 33449
```

Next is the model with BMI-sleep interaction.

```
##
## Family: gaussian
## Link function: identity
##
## Formula:
## value ~ sex + site + s(age_z, k = 10, bs = "cr") + s(sleep_z,
##     k = 5, bs = "cr") + icv + bmi + bmi:sleep_z
## <environment: 0x5589602edd20>
##
## Parametric coefficients:
##               Estimate Std. Error t value Pr(>|t|)
## (Intercept)   13610.616     91.537 148.690  < 2e-16 ***
## sexmale         163.160     13.069  12.485  < 2e-16 ***
## siteousPrisma   821.591    112.907   7.277  3.5e-13 ***
## siteousSkyra    232.435     68.558   3.390 0.000699 ***
## siteUCAM        815.697     90.183   9.045  < 2e-16 ***
```

```
## siteUKB         -66.647     86.394  -0.771 0.440462
## siteUmU        -233.461    101.229  -2.306 0.021101 *
## icv             909.910      6.719 135.425  < 2e-16 ***
## bmi               0.912      1.272   0.717 0.473281
## bmi:sleep_z       1.640      1.206   1.360 0.173772
## ---
## Signif. codes:  0 '***' 0.001 '**' 0.01 '*' 0.05 '.' 0.1 ' ' 1
##
## Approximate significance of smooth terms:
##              edf Ref.df        F p-value
## s(age_z)   5.646  5.646 1368.029  <2e-16 ***
## s(sleep_z) 2.565  2.565    3.456    0.02 *
## ---
## Signif. codes:  0 '***' 0.001 '**' 0.01 '*' 0.05 '.' 0.1 ' ' 1
##
## R-sq.(adj) =  0.582
## lmer.REML = 5.4678e+05  Scale est. = 75472     n = 33449
```

We did the same controling for depression. Below is the model with no covariates but only keeping data with depression.

```
##
## Family: gaussian
## Link function: identity
##
## Formula:
## value ~ sex + site + s(age_z, k = 10, bs = "cr") + s(sleep_z,
##     k = 5, bs = "cr") + icv
## <environment: 0x55895e4d75d0>
##
## Parametric coefficients:
##                 Estimate Std. Error t value Pr(>|t|)
## (Intercept)   14864.399     61.807 240.498  < 2e-16 ***
## sexmale         165.624     13.056  12.686  < 2e-16 ***
## siteousAvanto -1109.929    135.769  -8.175 3.06e-16 ***
## siteousPrisma  -324.664    119.706  -2.712  0.00669 **
## siteousSkyra   -755.799     93.726  -8.064 7.64e-16 ***
## siteUCAM       -448.938     84.864  -5.290 1.23e-07 ***
## siteUKB       -1311.541     61.119 -21.459  < 2e-16 ***
## siteUmU       -1469.083     82.941 -17.712  < 2e-16 ***
## icv             902.973      6.719 134.387  < 2e-16 ***
## ---
## Signif. codes:  0 '***' 0.001 '**' 0.01 '*' 0.05 '.' 0.1 ' ' 1
##
## Approximate significance of smooth terms:
##              edf Ref.df       F  p-value
## s(age_z)   7.099  7.099 951.524  < 2e-16 ***
## s(sleep_z) 2.594  2.594   7.873 0.000155 ***
## ---
## Signif. codes:  0 '***' 0.001 '**' 0.01 '*' 0.05 '.' 0.1 ' ' 1
##
## R-sq.(adj) =   0.57
## lmer.REML = 5.4493e+05  Scale est. = 72753     n = 33361
```

Below is the model output with main effect.

```
##
## Family: gaussian
## Link function: identity
##
## Formula:
## value ~ sex + site + s(age_z, k = 10, bs = "cr") + s(sleep_z,
##     k = 5, bs = "cr") + icv + depression
## <environment: 0x55895e4d75d0>
##
## Parametric coefficients:
##                Estimate Std. Error t value Pr(>|t|)
## (Intercept)   14886.219     62.064 239.853  < 2e-16 ***
## sexmale         163.360     13.067  12.502  < 2e-16 ***
## siteousAvanto -1098.740    135.767  -8.093 6.02e-16 ***
## siteousPrisma  -314.774    119.712  -2.629 0.008557 **
## siteousSkyra   -743.116     93.769  -7.925 2.35e-15 ***
## siteUCAM       -444.007     84.860  -5.232 1.68e-07 ***
## siteUKB       -1320.492     61.155 -21.593  < 2e-16 ***
## siteUmU       -1402.974     84.707 -16.563  < 2e-16 ***
## icv             903.327      6.718 134.464  < 2e-16 ***
## depression     -178.587     46.642  -3.829 0.000129 ***
## ---
## Signif. codes:  0 ‘***’ 0.001 ‘**’ 0.01 ‘*’ 0.05 ‘.’ 0.1 ‘ ’ 1
##
## Approximate significance of smooth terms:
##              edf Ref.df       F p-value
## s(age_z)   7.102  7.102 945.970 < 2e-16 ***
## s(sleep_z) 2.347  2.347   8.394 0.00012 ***
## ---
## Signif. codes:  0 ‘***’ 0.001 ‘**’ 0.01 ‘*’ 0.05 ‘.’ 0.1 ‘ ’ 1
##
## R-sq.(adj) =   0.57
## lmer.REML = 5.4491e+05  Scale est. = 72703     n = 33361
```

Next is the model with depression-sleep interaction.

```
##
## Family: gaussian
## Link function: identity
##
## Formula:
## value ~ sex + site + s(age_z, k = 10, bs = "cr") + s(sleep_z,
##     k = 5, bs = "cr") + icv + depression + depression:sleep_z
## <environment: 0x55895e4d75d0>
##
## Parametric coefficients:
##                     Estimate Std. Error t value Pr(>|t|)
## (Intercept)        14886.264     62.065 239.851  < 2e-16 ***
## sexmale              163.187     13.069  12.486  < 2e-16 ***
## siteousAvanto      -1099.863    135.774  -8.101 5.65e-16 ***
## siteousPrisma       -315.911    119.720  -2.639 0.008325 **
## siteousSkyra        -744.221     93.779  -7.936 2.15e-15 ***
## siteUCAM            -445.164     84.875  -5.245 1.57e-07 ***
## siteUKB            -1320.498     61.155 -21.592  < 2e-16 ***
## siteUmU            -1394.521     85.437 -16.322  < 2e-16 ***
```

```
## icv                    903.328      6.718 134.460  < 2e-16 ***
## depression            -180.478     46.739  -3.861 0.000113 ***
## depression:sleep_z     -26.174     34.302  -0.763 0.445446
## ---
## Signif. codes:  0 '***' 0.001 '**' 0.01 '*' 0.05 '.' 0.1 ' ' 1
##
## Approximate significance of smooth terms:
##               edf Ref.df       F p-value
## s(age_z)    7.091  7.091 947.297  <2e-16 ***
## s(sleep_z) 2.396  2.396   5.078  0.0031 **
## ---
## Signif. codes:  0 '***' 0.001 '**' 0.01 '*' 0.05 '.' 0.1 ' ' 1
##
## R-sq.(adj) =   0.57
## lmer.REML = 5.449e+05  Scale est. = 72702     n = 33361
```

The plot below shows the sleep-volume curve for the original model and for the model with main effects of SES.

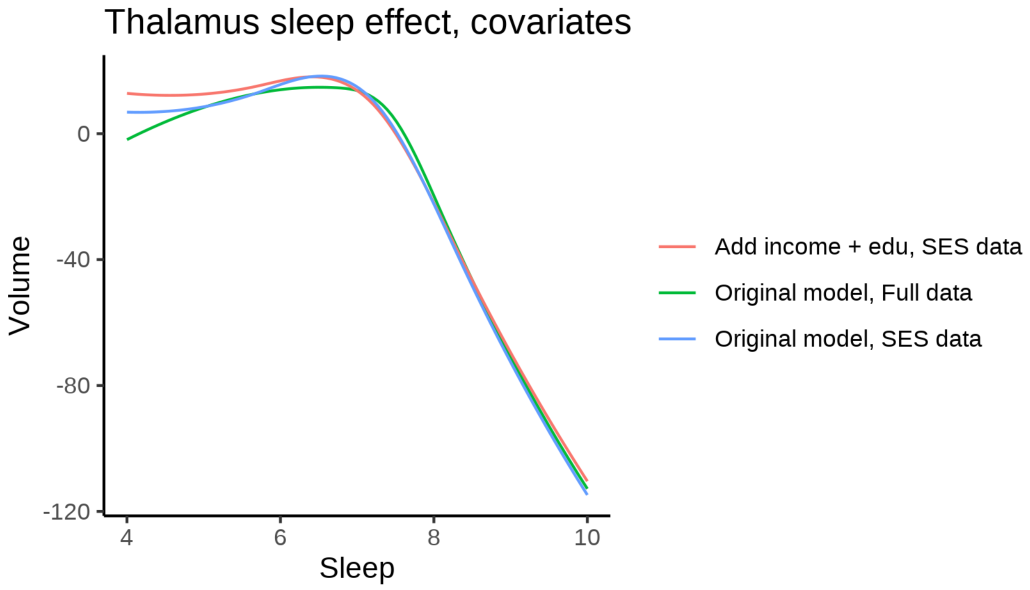

The plot below shows the sleep-volume curve for the original model and for the model with main effects of BMI.

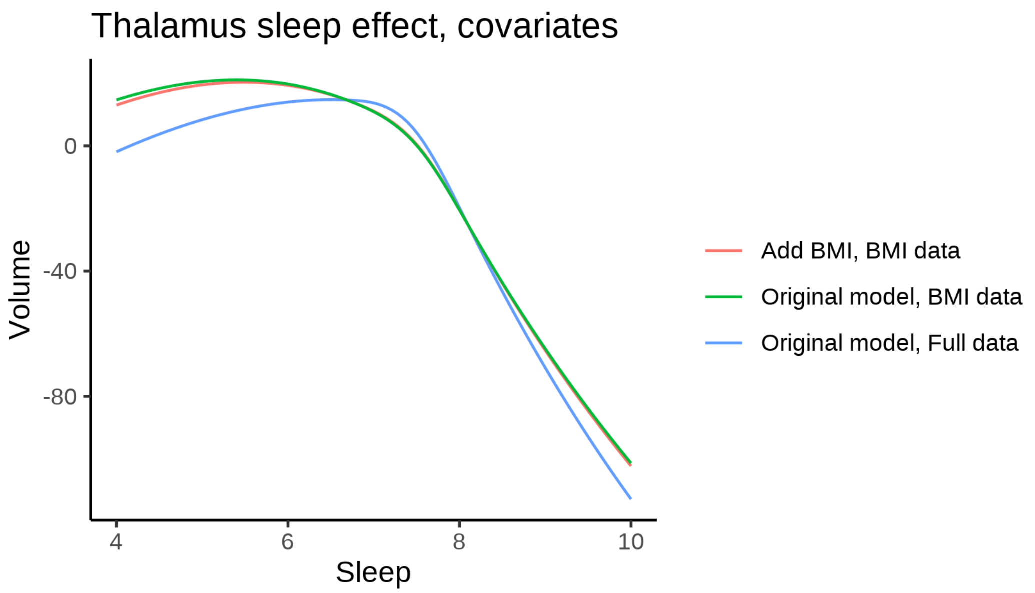

The plot below shows the sleep-volume curve for the original model and for the model with main effects of depression.

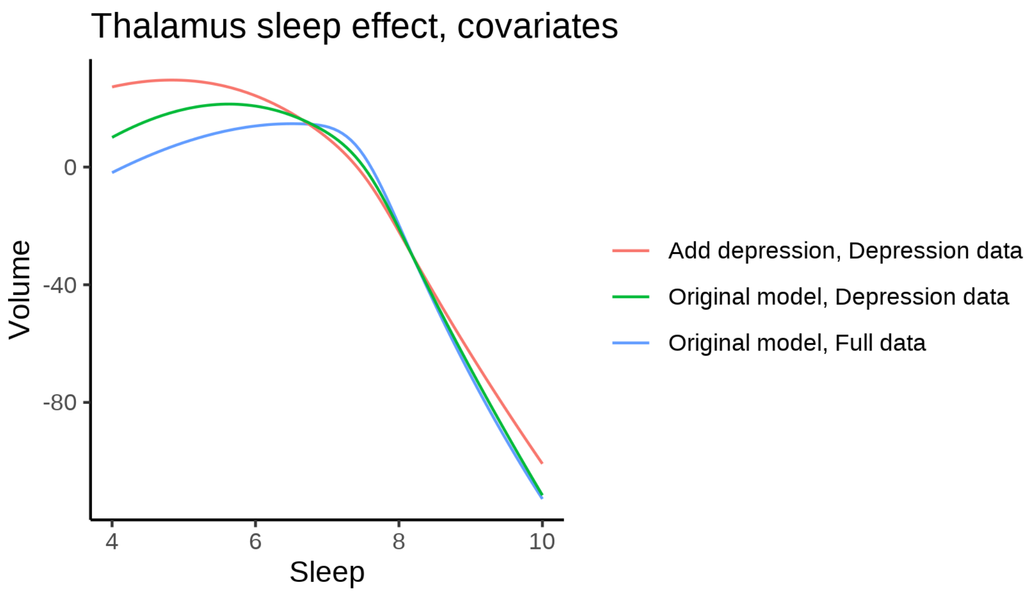

Models Not controling for ICV

Model outputs

Model without sleep term

```
##
## Family: gaussian
## Link function: identity
##
## Formula:
```

```
## value ~ sex + site + s(age_z, k = 10, bs = "cr")
## <environment: 0x558961ed7108>
##
## Parametric coefficients:
##                Estimate Std. Error t value Pr(>|t|)
## (Intercept)   12928.56      63.39 203.961  < 2e-16 ***
## sexmale        1196.94      10.97 109.157  < 2e-16 ***
## siteMPIB        176.45      81.98   2.152 0.031376 *
## siteousAvanto   671.85      65.40  10.273  < 2e-16 ***
## siteousPrisma  1238.89     111.09  11.152  < 2e-16 ***
## siteousSkyra    449.19      62.82   7.151 8.76e-13 ***
## siteUB          759.35     196.21   3.870 0.000109 ***
## siteUCAM       1074.91      71.41  15.052  < 2e-16 ***
## siteUKB         192.05      65.07   2.951 0.003164 **
## siteUmU        -395.22      92.54  -4.271 1.95e-05 ***
## siteUOXF        669.75      77.74   8.615  < 2e-16 ***
## ---
## Signif. codes:  0 '***' 0.001 '**' 0.01 '*' 0.05 '.' 0.1 ' ' 1
##
## Approximate significance of smooth terms:
##             edf Ref.df    F p-value
## s(age_z) 6.128  6.128 1681  <2e-16 ***
## ---
## Signif. codes:  0 '***' 0.001 '**' 0.01 '*' 0.05 '.' 0.1 ' ' 1
##
## R-sq.(adj) =  0.344
## lmer.REML = 8.6151e+05  Scale est. = 82533     n = 51279
```

Model with only main effects of age and sleep

```
##
## Family: gaussian
## Link function: identity
##
## Formula:
## value ~ sex + site + s(age_z, k = 10, bs = "cr") + s(sleep_z,
##     k = 5, bs = "cr")
## <environment: 0x558961ed7108>
##
## Parametric coefficients:
##                Estimate Std. Error t value Pr(>|t|)
## (Intercept)   12942.40      63.41 204.092  < 2e-16 ***
## sexmale        1194.24      10.96 108.924  < 2e-16 ***
## siteMPIB        176.78      81.96   2.157  0.03102 *
## siteousAvanto   661.36      65.38  10.115  < 2e-16 ***
## siteousPrisma  1231.13     111.04  11.088  < 2e-16 ***
## siteousSkyra    437.87      62.80   6.973 3.15e-12 ***
## siteUB          768.98     196.05   3.922 8.78e-05 ***
## siteUCAM       1074.88      71.37  15.060  < 2e-16 ***
## siteUKB         178.74      65.09   2.746  0.00604 **
## siteUmU        -398.28      92.69  -4.297 1.73e-05 ***
## siteUOXF        664.22      77.69   8.550  < 2e-16 ***
## ---
## Signif. codes:  0 '***' 0.001 '**' 0.01 '*' 0.05 '.' 0.1 ' ' 1
##
```

```
## Approximate significance of smooth terms:
##              edf Ref.df       F p-value
## s(age_z)   6.112  6.112 1664.17  <2e-16 ***
## s(sleep_z) 3.701  3.701   17.38  <2e-16 ***
## ---
## Signif. codes:  0 '***' 0.001 '**' 0.01 '*' 0.05 '.' 0.1 ' ' 1
##
## R-sq.(adj) =  0.345
## lmer.REML = 8.6145e+05  Scale est. = 82602     n = 51279
```

Model with full interaction between age and sleep

```
##
## Family: gaussian
## Link function: identity
##
## Formula:
## value ~ sex + site + t2(age_z, sleep_z, k = c(10, 4), bs = "cr")
## <environment: 0x558961ed7108>
##
## Parametric coefficients:
##                Estimate Std. Error t value Pr(>|t|)
## (Intercept)    12955.15      63.03 205.552  < 2e-16 ***
## sexmale         1195.58      10.99 108.786  < 2e-16 ***
## siteMPIB         160.13      82.09   1.951 0.051093 .
## siteousAvanto    653.12      65.55   9.964  < 2e-16 ***
## siteousPrisma   1222.37     111.25  10.988  < 2e-16 ***
## siteousSkyra     429.29      62.99   6.816 9.50e-12 ***
## siteUB           743.40     196.03   3.792 0.000149 ***
## siteUCAM        1059.31      71.47  14.822  < 2e-16 ***
## siteUKB          165.12      64.67   2.553 0.010668 *
## siteUmU         -413.16      92.65  -4.459 8.23e-06 ***
## siteUOXF         644.14      77.47   8.315  < 2e-16 ***
## ---
## Signif. codes:  0 '***' 0.001 '**' 0.01 '*' 0.05 '.' 0.1 ' ' 1
##
## Approximate significance of smooth terms:
##                      edf Ref.df     F p-value
## t2(age_z,sleep_z) 12.58  12.58 53.56  <2e-16 ***
## ---
## Signif. codes:  0 '***' 0.001 '**' 0.01 '*' 0.05 '.' 0.1 ' ' 1
##
## R-sq.(adj) =  0.345
## lmer.REML = 8.6148e+05  Scale est. = 82612     n = 51279
```

Model comparison

`mod_no_sleep` refers to model without sleep term, `mod_no_interaction` refers to model with only main effect of sleep, and `mod_full` refers to model with a full interaction between age and sleep. This is a nested model comparison, and the p-value at a given line refers to comparing the model at the line to the model on the line above. Hence, significance implies that the more complicated model is supported on statistical grounds.

To be even more specific, the p-value on the second row tests whether there is an association between sleep and volume. The p-value on the third row tests whether this association depends on age.

```
## Data: NULL
## Models:
## ml$mod_no_sleep$mer: NULL
## ml$mod_no_interaction$mer: NULL
## ml$mod_full$mer: NULL
##                            npar    AIC    BIC  logLik deviance  Chisq Df Pr(>Chisq)
## ml$mod_no_sleep$mer         15 861538 861671 -430754   861508
## ml$mod_no_interaction$mer   17 861484 861635 -430725   861450 57.921  2  2.647e-13 ***
## ml$mod_full$mer             19 861514 861682 -430738   861476  0.000  2          1
## ---
## Signif. codes:  0 '***' 0.001 '**' 0.01 '*' 0.05 '.' 0.1 ' ' 1
```

We chose the model based on the likelihood ratio test with 5 % significance level, which was `mod_no_interaction`.

Lifespan brain trajectory

The trajectory shown is from the chosen model `mod_no_interaction_no_icv`.

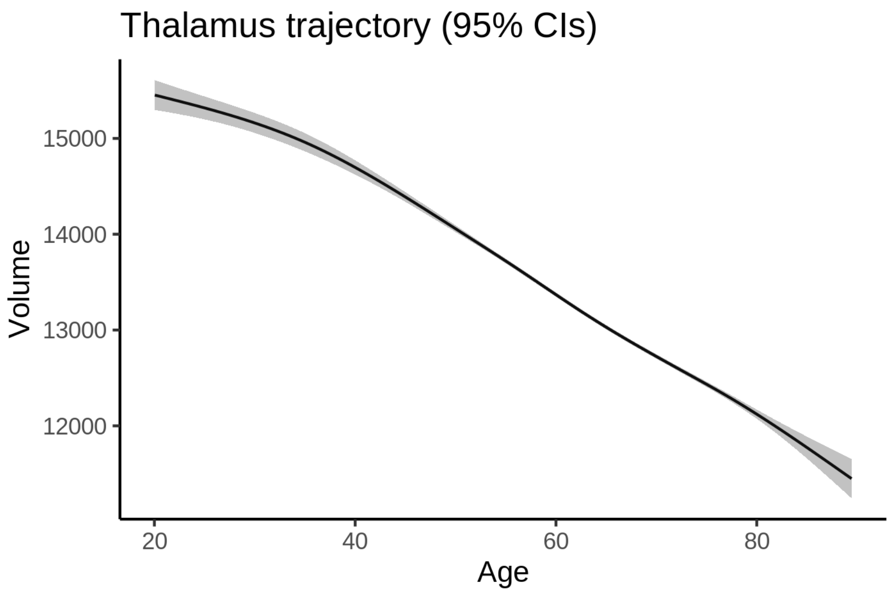

Effect of sleep

The chosen model only included the main effect of sleep, and hence the effect does not vary with age. The black dot shows the average sleep duration across all ages in the sample.

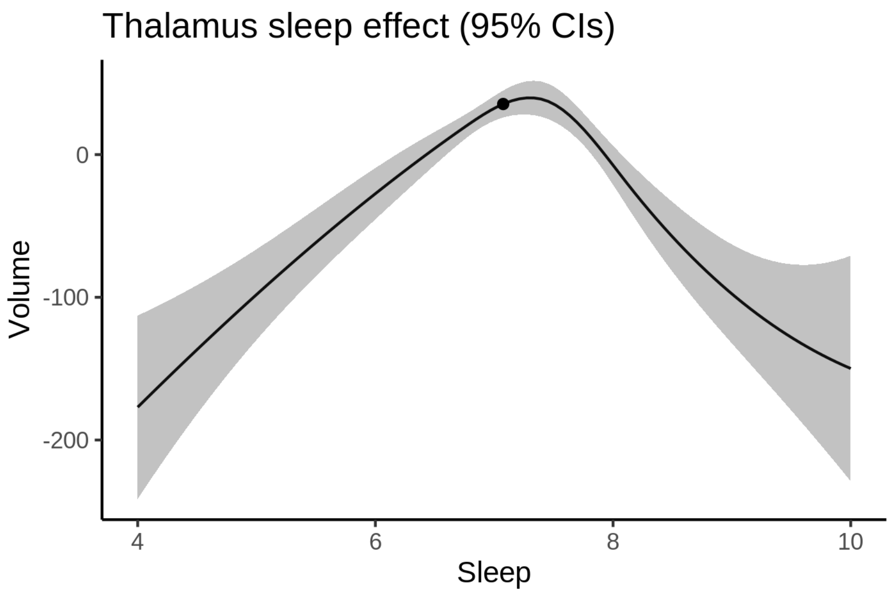

We also show the full interaction model for completeness, although it was not selected.

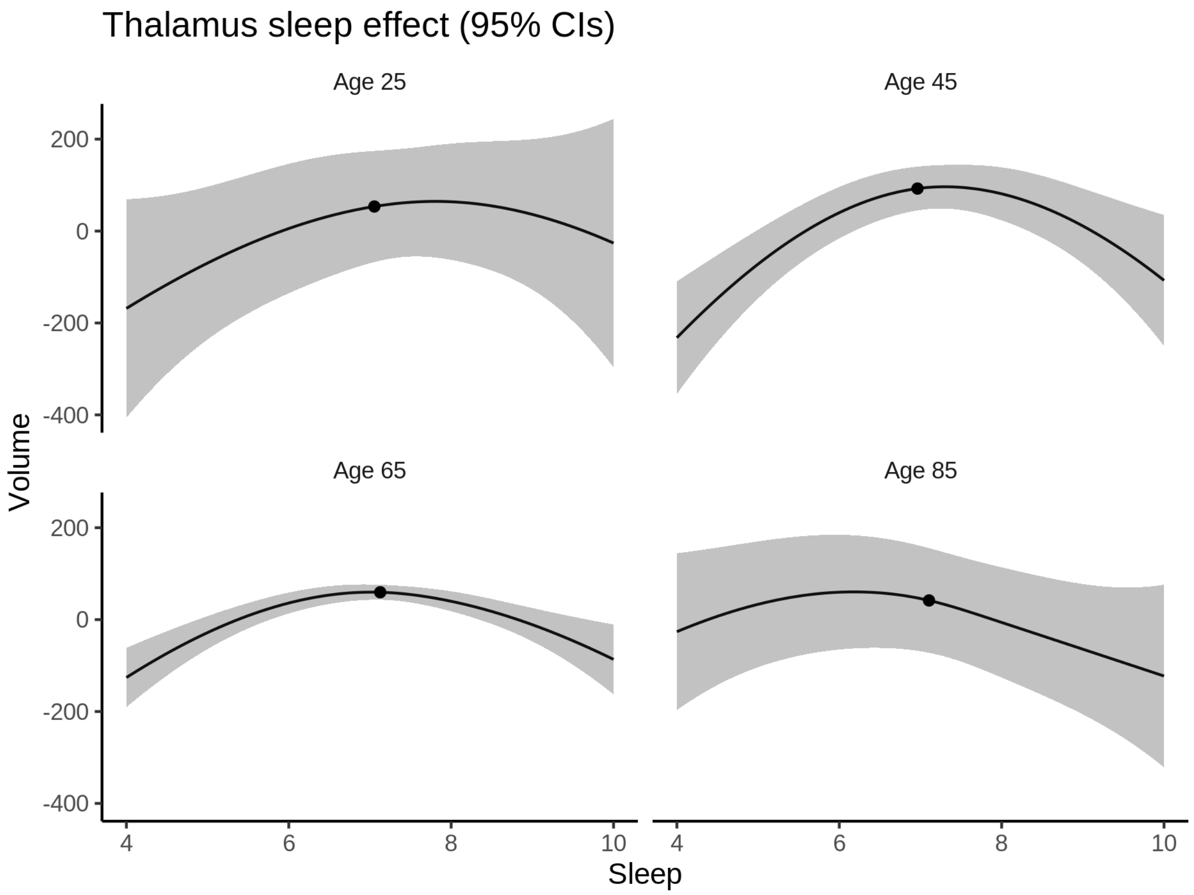

Deviation from sleep associated with maximal volume

Model with only main effect of sleep was chosen, so we show it for all ages at once. Maximum volume is attained at 7.3 hours of sleep. The percentage values in the plot are calculated as follows: The maximum at 100 % refers to a person at an arbitrary age with a sleep duration associated with maximum volume. For a female, this volume is 13655 and for a male it is 14849. The other percentage values show how large the expected volume is for someone with other sleep durations. For example, 99 % implies a 1 % reduction.

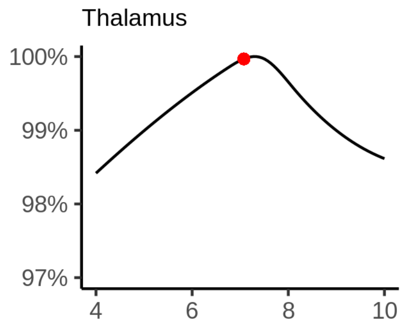

Comparison of mean sleep and sleep associated with maximum volume

A 95 % confidence interval for the sleep associated with maximum volume is [7.21, 7.39].

The plot below compares average sleep to the sleep associated with maximum volume.

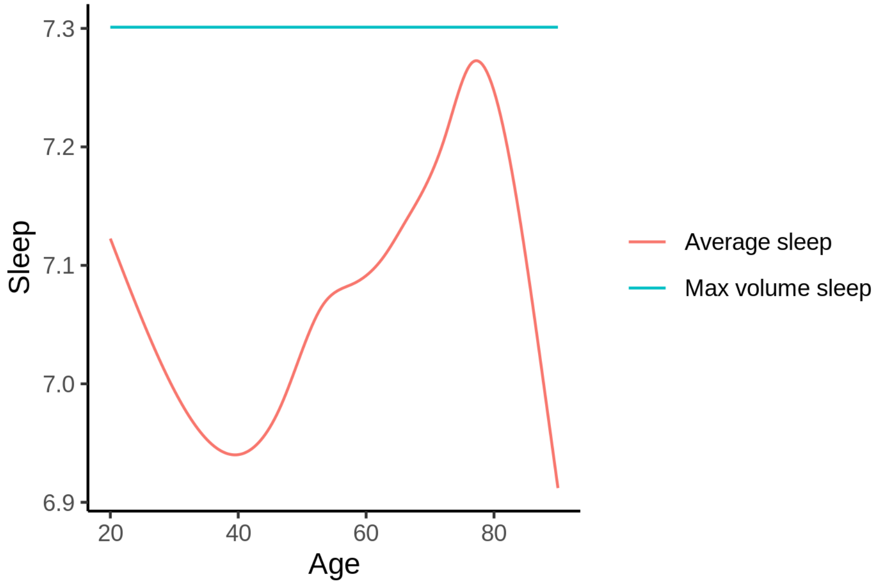

The next plot shows the difference between average sleep and sleep associated with maximum volume. The shaded region is a 95 % confidence interval.

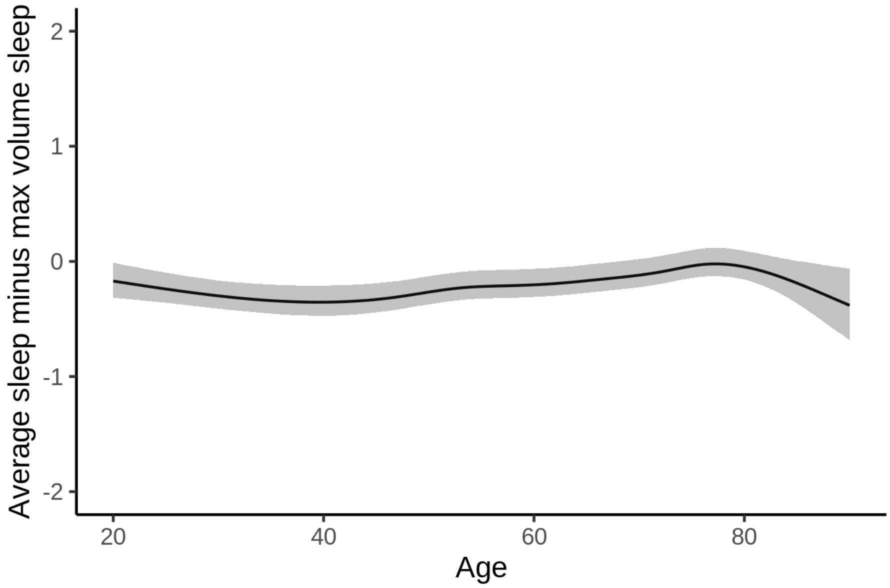

The next plot shows the probability that the sleep duration associated with maximum volume is longer than the average sleep duration, as a function of age. Probability below .05 can be interpreted as evidence that the sleep associated with maximum volume is shorter than the mean sleep, and probability above .95 can be interpreted the opposite way.

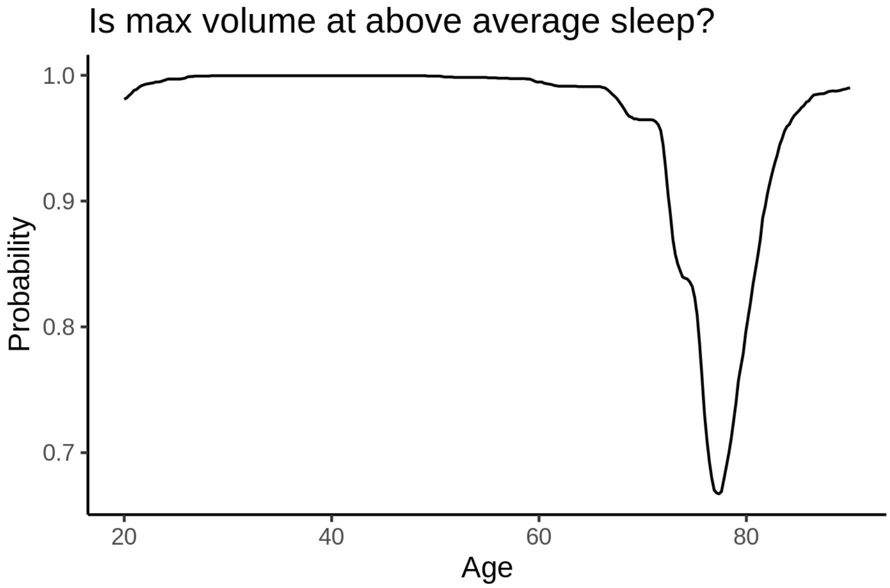

TotalGrayVol

Descriptive statistics

Study	Observations	Unique IDs	Mean age	Age range
HCP	974	974	28.8	22 - 37
MPIB	677	391	63.1	24 - 83

Study	Observations	Unique IDs	Mean age	Age range
UB	113	39	70.9	64 - 81
UCAM	884	632	55.1	20 - 88
UiO	1475	803	49.4	20 - 89
UKB	45978	43135	64.5	45 - 83
UmU	412	279	62.2	25 - 85
UOXF	769	769	69.8	60 - 85

Spaghetti plot

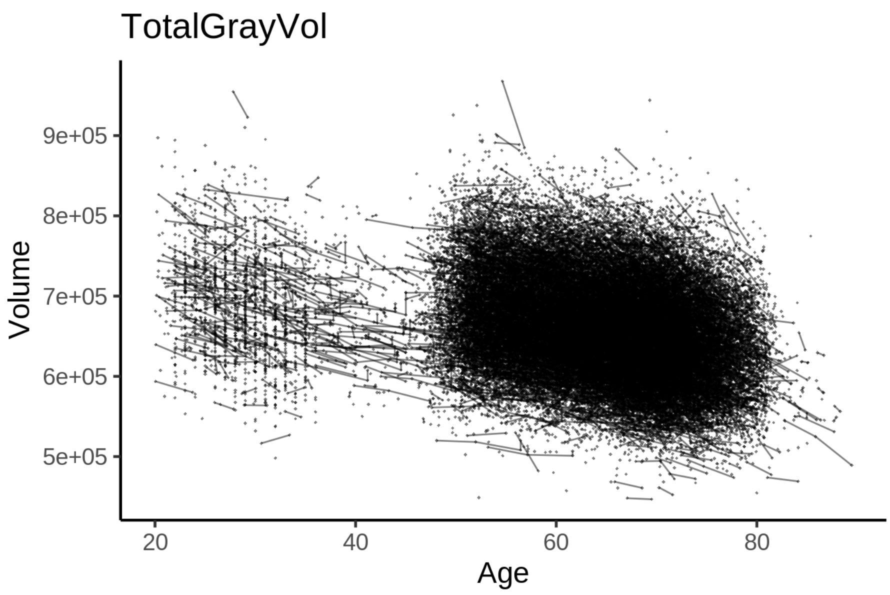

Models Controling for ICV

Model outputs

Model without sleep term

```
##
## Family: gaussian
## Link function: identity
##
## Formula:
## value ~ sex + site + icv + s(age_z, k = 10, bs = "cr")
## <environment: 0x5589627443f0>
##
## Parametric coefficients:
##                Estimate Std. Error t value Pr(>|t|)
## (Intercept)    606887.8     1672.2 362.919  < 2e-16 ***
## sexmale         15934.2      334.6  47.617  < 2e-16 ***
## siteMPIB        56130.9     2100.4  26.724  < 2e-16 ***
## siteousAvanto   -4998.6     1744.7  -2.865  0.00417 **
## siteousPrisma   31174.8     2923.3  10.664  < 2e-16 ***
## siteousSkyra    20133.2     1605.4  12.541  < 2e-16 ***
```

```
## siteUB         13390.5     4860.0   2.755  0.00587 **
## siteUCAM       14878.3     1827.3   8.142 3.97e-16 ***
## siteUKB        51681.0     1716.1  30.115  < 2e-16 ***
## siteUmU        26393.1     2357.3  11.196  < 2e-16 ***
## siteUOXF       10142.0     2019.0   5.023 5.10e-07 ***
## icv            45504.0      168.5 270.132  < 2e-16 ***
## ---
## Signif. codes:  0 ‘***’ 0.001 ‘**’ 0.01 ‘*’ 0.05 ‘.’ 0.1 ‘ ’ 1
##
## Approximate significance of smooth terms:
##            edf Ref.df    F p-value
## s(age_z) 7.941  7.941 1797  <2e-16 ***
## ---
## Signif. codes:  0 ‘***’ 0.001 ‘**’ 0.01 ‘*’ 0.05 ‘.’ 0.1 ‘ ’ 1
##
## R-sq.(adj) =  0.766
## lmer.REML = 1.1949e+06  Scale est. = 1.0496e+08  n = 51282
```

Model with only main effects of age and sleep

```
##
## Family: gaussian
## Link function: identity
##
## Formula:
## value ~ sex + site + icv + s(age_z, k = 10, bs = "cr") + s(sleep_z,
##     k = 5, bs = "cr")
## <environment: 0x5589627443f0>
##
## Parametric coefficients:
##               Estimate Std. Error t value Pr(>|t|)
## (Intercept)   607086.4     1672.6 362.958  < 2e-16 ***
## sexmale        15923.5      334.3  47.626  < 2e-16 ***
## siteMPIB       56214.6     2099.9  26.771  < 2e-16 ***
## siteousAvanto  -5206.9     1744.0  -2.986  0.00283 **
## siteousPrisma  31043.8     2920.9  10.628  < 2e-16 ***
## siteousSkyra   19884.2     1604.7  12.392  < 2e-16 ***
## siteUB         13687.7     4854.9   2.819  0.00481 **
## siteUCAM       14947.8     1826.0   8.186 2.76e-16 ***
## siteUKB        51475.6     1716.7  29.986  < 2e-16 ***
## siteUmU        26623.9     2360.7  11.278  < 2e-16 ***
## siteUOXF       10040.2     2017.5   4.977 6.50e-07 ***
## icv            45448.5      168.5 269.750  < 2e-16 ***
## ---
## Signif. codes:  0 ‘***’ 0.001 ‘**’ 0.01 ‘*’ 0.05 ‘.’ 0.1 ‘ ’ 1
##
## Approximate significance of smooth terms:
##              edf Ref.df       F p-value
## s(age_z)   7.937  7.937 1776.07  <2e-16 ***
## s(sleep_z) 3.674  3.674   24.31  <2e-16 ***
## ---
## Signif. codes:  0 ‘***’ 0.001 ‘**’ 0.01 ‘*’ 0.05 ‘.’ 0.1 ‘ ’ 1
##
## R-sq.(adj) =  0.767
## lmer.REML = 1.1948e+06  Scale est. = 1.0499e+08  n = 51282
```

Model with full interaction between age and sleep

```
##
## Family: gaussian
## Link function: identity
##
## Formula:
## value ~ sex + site + icv + t2(age_z, sleep_z, k = c(10, 4), bs = "cr")
## <environment: 0x5589627443f0>
##
## Parametric coefficients:
##                Estimate Std. Error t value Pr(>|t|)
## (Intercept)    607490.4     1680.2 361.557  < 2e-16 ***
## sexmale         15949.1      335.1  47.595  < 2e-16 ***
## siteMPIB        55703.9     2112.6  26.367  < 2e-16 ***
## siteousAvanto  -5575.6     1754.5  -3.178  0.00148 **
## siteousPrisma  30633.9     2928.1  10.462  < 2e-16 ***
## siteousSkyra   19532.4     1616.7  12.081  < 2e-16 ***
## siteUB         12972.0     4859.0   2.670  0.00759 **
## siteUCAM       14388.3     1838.1   7.828 5.06e-15 ***
## siteUKB        51055.8     1724.3  29.610  < 2e-16 ***
## siteUmU        26177.1     2370.2  11.044  < 2e-16 ***
## siteUOXF        9469.2     2026.3   4.673 2.97e-06 ***
## icv            45452.0      168.5 269.770  < 2e-16 ***
## ---
## Signif. codes:  0 ‘***’ 0.001 ‘**’ 0.01 ‘*’ 0.05 ‘.’ 0.1 ‘ ’ 1
##
## Approximate significance of smooth terms:
##                     edf Ref.df     F p-value
## t2(age_z,sleep_z) 17.59  17.59 28.11  <2e-16 ***
## ---
## Signif. codes:  0 ‘***’ 0.001 ‘**’ 0.01 ‘*’ 0.05 ‘.’ 0.1 ‘ ’ 1
##
## R-sq.(adj) =  0.767
## lmer.REML = 1.1948e+06  Scale est. = 1.05e+08  n = 51282
```

Model comparison

`mod_no_sleep` refers to model without sleep term, `mod_no_interaction` refers to model with only main effect of sleep, and `mod_full` refers to model with a full interaction between age and sleep. This is a nested model comparison, and the p-value at a given line refers to comparing the model at the line to the model on the line above. Hence, significance implies that the more complicated model is supported on statistical grounds.

To be even more specific, the p-value on the second row tests whether there is an association between sleep and volume. The p-value on the third row tests whether this association depends on age.

```
## Data: NULL
## Models:
## ml$mod_no_sleep$mer: NULL
## ml$mod_no_interaction$mer: NULL
## ml$mod_full$mer: NULL
##                           npar     AIC     BIC  logLik deviance  Chisq Df Pr(>Chisq)
## ml$mod_no_sleep$mer         16 1194944 1195085 -597456  1194912
## ml$mod_no_interaction$mer   18 1194866 1195025 -597415  1194830 81.882  2     <2e-16 ***
```

```
## ml$mod_full$mer             20 1194885 1195061 -597422  1194845  0.000  2          1
## ---
## Signif. codes:  0 ‘***’ 0.001 ‘**’ 0.01 ‘*’ 0.05 ‘.’ 0.1 ‘ ’ 1
```

We chose the model based on the likelihood ratio test with 5 % significance level, which was `mod_no_interaction`.

Lifespan brain trajectory

The trajectory shown is from the chosen model `mod_no_interaction`.

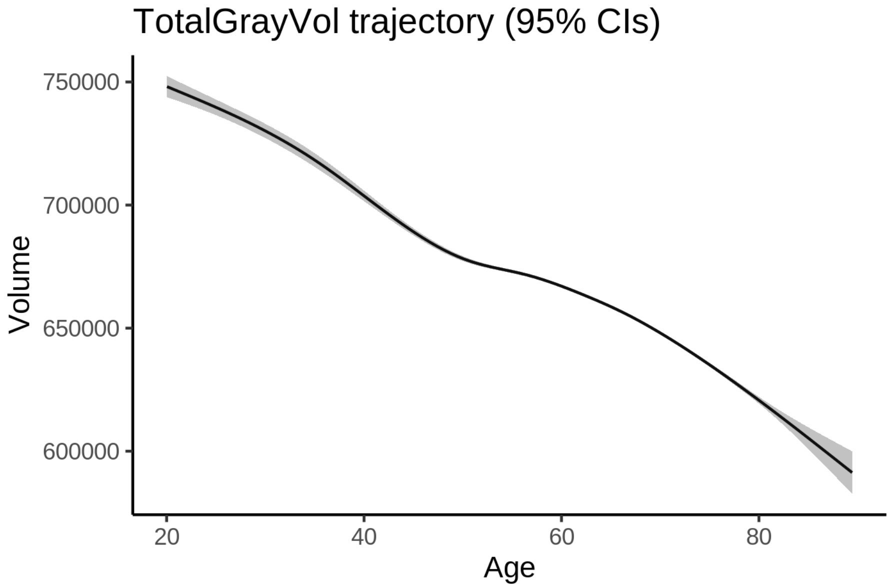

Effect of sleep

The chosen model only included the main effect of sleep, and hence the effect does not vary with age. The black dot shows the average sleep duration across all ages in the sample.

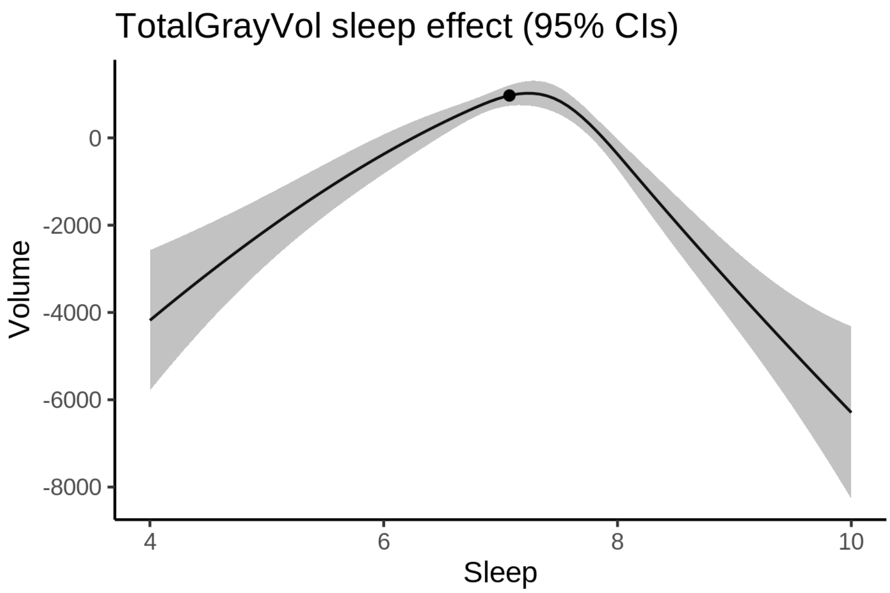

We also show the full interaction model for completeness, although it was not selected.

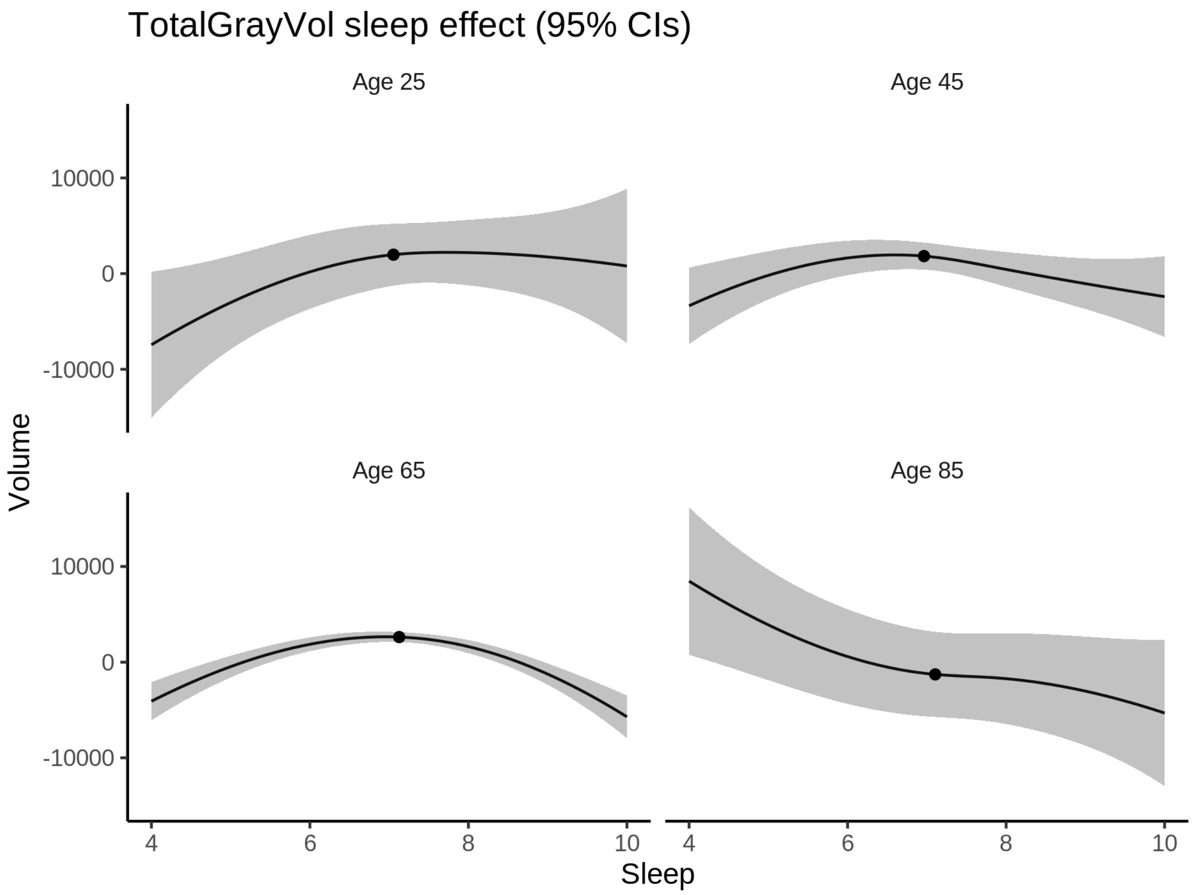

Deviation from sleep associated with maximal volume

Model with only main effect of sleep was chosen, so we show it for all ages at once. Maximum volume is attained at 7.2 hours of sleep. The percentage values in the plot are calculated as follows: The maximum at 100 % refers to a person at an arbitrary age with a sleep duration associated with maximum volume. For a female, this volume is 674017 and for a male it is 689940. The other percentage values show how large the expected volume is for someone with other sleep durations. For example, 99 % implies a 1 % reduction.

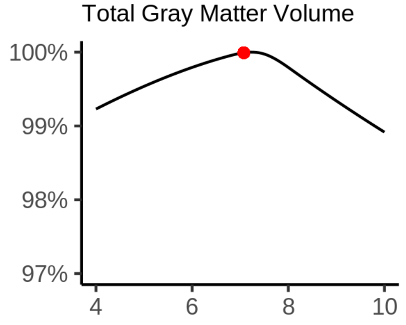

Comparison of mean sleep and sleep associated with maximum volume

A 95 % confidence interval for the sleep associated with maximum volume is [7.09, 7.33].

The plot below compares average sleep to the sleep associated with maximum volume.

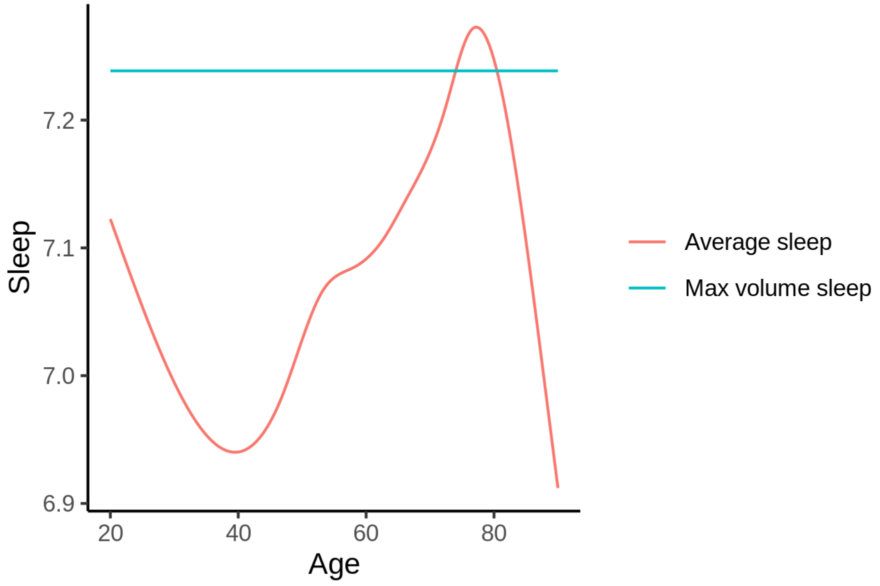

The next plot shows the difference between average sleep and sleep associated with maximum volume. The shaded region is a 95 % confidence interval.

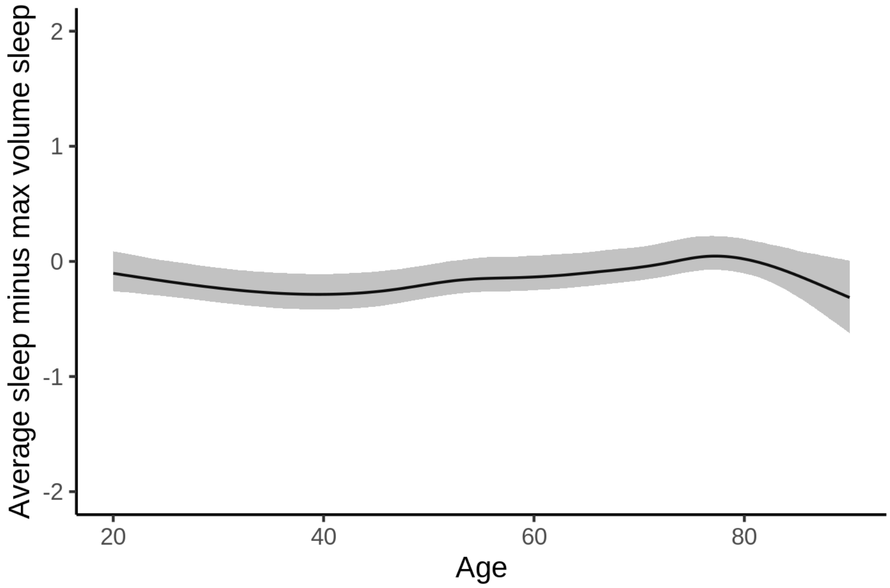

The next plot shows the probability that the sleep duration associated with maximum volume is longer than the average sleep duration, as a function of age. Probability below .05 can be interpreted as evidence that the sleep associated with maximum volume is shorter than the mean sleep, and probability above .95 can be interpreted the opposite way.

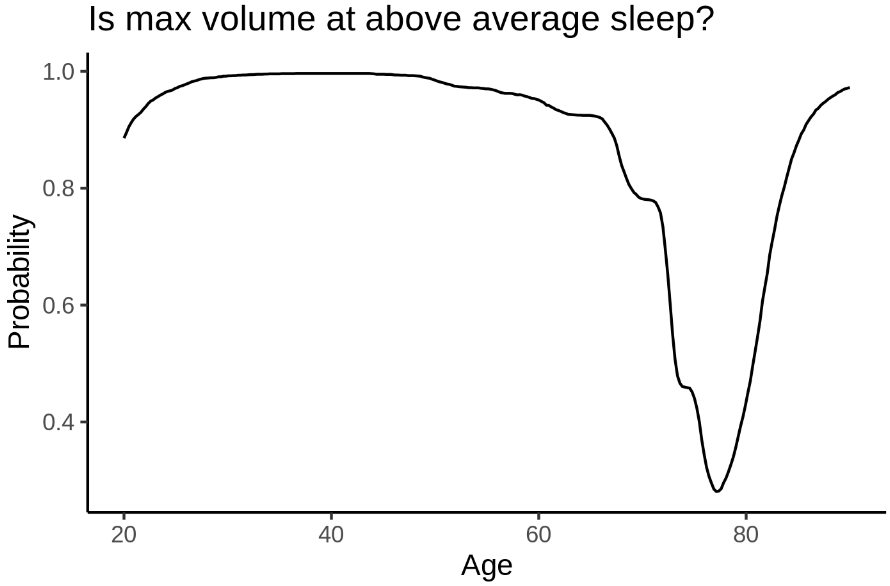

Controling for covariates

Below is the output for a model in which we only include data with income and education.

```
##
## Family: gaussian
## Link function: identity
##
## Formula:
```

```
## value ~ sex + site + s(age_z, k = 10, bs = "cr") + s(sleep_z,
##     k = 5, bs = "cr") + icv
## <environment: 0x558ad7a9d8f8>
##
## Parametric coefficients:
##               Estimate Std. Error t value Pr(>|t|)
## (Intercept)   675222.2     3798.7 177.751  < 2e-16 ***
## sexmale        15652.0      419.1  37.350  < 2e-16 ***
## siteousAvanto -80433.2     4797.6 -16.765  < 2e-16 ***
## siteousPrisma -32342.0     4693.8  -6.890 5.67e-12 ***
## siteousSkyra  -49323.3     4098.2 -12.035  < 2e-16 ***
## siteUKB       -17877.4     3793.0  -4.713 2.45e-06 ***
## siteUOXF      -50294.6     4272.6 -11.771  < 2e-16 ***
## icv            46477.7      215.6 215.572  < 2e-16 ***
## ---
## Signif. codes:  0 '***' 0.001 '**' 0.01 '*' 0.05 '.' 0.1 ' ' 1
##
## Approximate significance of smooth terms:
##              edf Ref.df       F p-value
## s(age_z)   6.812  6.812 1046.94  <2e-16 ***
## s(sleep_z) 3.511  3.511   15.32  <2e-16 ***
## ---
## Signif. codes:  0 '***' 0.001 '**' 0.01 '*' 0.05 '.' 0.1 ' ' 1
##
## R-sq.(adj) =  0.763
## lmer.REML = 7.2597e+05  Scale est. = 1.2113e+08  n = 31192
```

Below is the output for a model in which we control for the main effects of income and education.

```
##
## Family: gaussian
## Link function: identity
##
## Formula:
## value ~ sex + site + s(age_z, k = 10, bs = "cr") + s(sleep_z,
##     k = 5, bs = "cr") + icv + income_scaled + education_scaled
## <environment: 0x558ad7a9d8f8>
##
## Parametric coefficients:
##                   Estimate Std. Error t value Pr(>|t|)
## (Intercept)       672500.4     3821.9 175.958  < 2e-16 ***
## sexmale            15615.2      420.0  37.179  < 2e-16 ***
## siteousAvanto     -80362.9     4796.3 -16.755  < 2e-16 ***
## siteousPrisma     -32672.6     4690.9  -6.965 3.35e-12 ***
## siteousSkyra      -49284.0     4096.4 -12.031  < 2e-16 ***
## siteUKB           -17634.0     3793.2  -4.649 3.35e-06 ***
## siteUOXF          -49321.5     4272.6 -11.544  < 2e-16 ***
## icv                46302.5      217.3 213.104  < 2e-16 ***
## income_scaled       2316.7      499.9   4.634 3.60e-06 ***
## education_scaled    1803.3      589.4   3.059  0.00222 **
## ---
## Signif. codes:  0 '***' 0.001 '**' 0.01 '*' 0.05 '.' 0.1 ' ' 1
##
## Approximate significance of smooth terms:
##              edf Ref.df      F p-value
```

```
## s(age_z)   6.835  6.835 940.52  <2e-16 ***
## s(sleep_z) 3.440  3.440  13.04  <2e-16 ***
## ---
## Signif. codes:  0 ‘***’ 0.001 ‘**’ 0.01 ‘*’ 0.05 ‘.’ 0.1 ‘ ’ 1
##
## R-sq.(adj) =  0.763
## lmer.REML = 7.259e+05  Scale est. = 1.212e+08  n = 31192
```

We also included interaction effects between sleep duration and education and income, in another model. The output is shown below, and the interaction terms are `income_scaled:sleep_z` and `education_scaled:sleep_z`.

```
##
## Family: gaussian
## Link function: identity
##
## Formula:
## value ~ sex + site + s(age_z, k = 10, bs = "cr") + s(sleep_z,
##     k = 5, bs = "cr") + icv + income_scaled + education_scaled +
##     income_scaled:sleep_z + education_scaled:sleep_z
## <environment: 0x558ad7a9d8f8>
##
## Parametric coefficients:
##                          Estimate Std. Error t value Pr(>|t|)
## (Intercept)              672525.8     3822.0 175.963  < 2e-16 ***
## sexmale                   15577.5      420.3  37.065  < 2e-16 ***
## siteousAvanto            -80433.9     4796.2 -16.770  < 2e-16 ***
## siteousPrisma            -32842.8     4691.2  -7.001 2.59e-12 ***
## siteousSkyra             -49357.9     4096.2 -12.050  < 2e-16 ***
## siteUKB                  -17662.3     3793.4  -4.656 3.24e-06 ***
## siteUOXF                 -49341.6     4273.6 -11.546  < 2e-16 ***
## icv                       46307.6      217.3 213.123  < 2e-16 ***
## income_scaled              2353.3      500.2   4.705 2.55e-06 ***
## education_scaled           1778.9      589.5   3.018  0.00255 **
## income_scaled:sleep_z     -1027.0      498.8  -2.059  0.03949 *
## education_scaled:sleep_z   1006.5      572.4   1.758  0.07870 .
## ---
## Signif. codes:  0 ‘***’ 0.001 ‘**’ 0.01 ‘*’ 0.05 ‘.’ 0.1 ‘ ’ 1
##
## Approximate significance of smooth terms:
##              edf Ref.df      F p-value
## s(age_z)   6.837  6.837 939.60  <2e-16 ***
## s(sleep_z) 3.438  3.438  12.75  <2e-16 ***
## ---
## Signif. codes:  0 ‘***’ 0.001 ‘**’ 0.01 ‘*’ 0.05 ‘.’ 0.1 ‘ ’ 1
##
## R-sq.(adj) =  0.763
## lmer.REML = 7.2587e+05  Scale est. = 1.212e+08  n = 31192
```

We did the same controling for BMI. Below is the model with no covariates but only keeping data with BMI.

```
##
## Family: gaussian
## Link function: identity
##
## Formula:
```

```
## value ~ sex + site + s(age_z, k = 10, bs = "cr") + s(sleep_z,
##     k = 5, bs = "cr") + icv
## <environment: 0x558b60154c48>
##
## Parametric coefficients:
##               Estimate Std. Error t value Pr(>|t|)
## (Intercept)   599356.3     3023.1 198.258  < 2e-16 ***
## sexmale        15038.4      402.3  37.384  < 2e-16 ***
## siteousPrisma  40722.1     3853.5  10.568  < 2e-16 ***
## siteousSkyra   30061.1     2600.7  11.559  < 2e-16 ***
## siteUCAM       23364.4     3137.3   7.447 9.76e-14 ***
## siteUKB        58816.1     3042.4  19.332  < 2e-16 ***
## siteUmU        34980.3     3440.7  10.167  < 2e-16 ***
## icv            46962.7      207.6 226.210  < 2e-16 ***
## ---
## Signif. codes:  0 '***' 0.001 '**' 0.01 '*' 0.05 '.' 0.1 ' ' 1
##
## Approximate significance of smooth terms:
##              edf Ref.df       F p-value
## s(age_z)   7.496  7.496 1222.88  <2e-16 ***
## s(sleep_z) 3.452  3.452   13.17  <2e-16 ***
## ---
## Signif. codes:  0 '***' 0.001 '**' 0.01 '*' 0.05 '.' 0.1 ' ' 1
##
## R-sq.(adj) =  0.772
## lmer.REML = 7.7733e+05  Scale est. = 1.1321e+08  n = 33447
```

Below is the model output with main effect.

```
##
## Family: gaussian
## Link function: identity
##
## Formula:
## value ~ sex + site + s(age_z, k = 10, bs = "cr") + s(sleep_z,
##     k = 5, bs = "cr") + icv + bmi
## <environment: 0x558b60154c48>
##
## Parametric coefficients:
##                Estimate Std. Error t value Pr(>|t|)
## (Intercept)   611000.29    3176.46 192.353  < 2e-16 ***
## sexmale        15404.24     402.57  38.265  < 2e-16 ***
## siteousPrisma  41271.46    3848.60  10.724  < 2e-16 ***
## siteousSkyra   30177.10    2599.45  11.609  < 2e-16 ***
## siteUCAM       23671.95    3133.81   7.554 4.34e-14 ***
## siteUKB        59214.27    3039.21  19.483  < 2e-16 ***
## siteUmU        35513.62    3436.36  10.335  < 2e-16 ***
## icv            47036.96     207.24 226.971  < 2e-16 ***
## bmi             -463.24      39.15 -11.832  < 2e-16 ***
## ---
## Signif. codes:  0 '***' 0.001 '**' 0.01 '*' 0.05 '.' 0.1 ' ' 1
##
## Approximate significance of smooth terms:
##              edf Ref.df       F p-value
## s(age_z)   7.495  7.495 1227.91  <2e-16 ***
```

```
## s(sleep_z) 3.348  3.348   11.03  <2e-16 ***
## ---
## Signif. codes:  0 ‘***’ 0.001 ‘**’ 0.01 ‘*’ 0.05 ‘.’ 0.1 ‘ ’ 1
##
## R-sq.(adj) =  0.773
## lmer.REML = 7.7718e+05  Scale est. = 1.1315e+08  n = 33447
```

Next is the model with BMI-sleep interaction.

```
##
## Family: gaussian
## Link function: identity
##
## Formula:
## value ~ sex + site + s(age_z, k = 10, bs = "cr") + s(sleep_z,
##     k = 5, bs = "cr") + icv + bmi + bmi:sleep_z
## <environment: 0x558b60154c48>
##
## Parametric coefficients:
##                Estimate Std. Error t value Pr(>|t|)
## (Intercept)   610963.92    3177.79 192.261  < 2e-16 ***
## sexmale        15404.81     402.58  38.266  < 2e-16 ***
## siteousPrisma  41273.74    3848.64  10.724  < 2e-16 ***
## siteousSkyra   30182.38    2599.50  11.611  < 2e-16 ***
## siteUCAM       23686.17    3134.04   7.558 4.21e-14 ***
## siteUKB        59229.13    3039.45  19.487  < 2e-16 ***
## siteUmU        35526.21    3436.54  10.338  < 2e-16 ***
## icv            47037.65     207.25 226.965  < 2e-16 ***
## bmi             -462.70      39.18 -11.811  < 2e-16 ***
## bmi:sleep_z       14.89      37.22   0.400    0.689
## ---
## Signif. codes:  0 ‘***’ 0.001 ‘**’ 0.01 ‘*’ 0.05 ‘.’ 0.1 ‘ ’ 1
##
## Approximate significance of smooth terms:
##              edf Ref.df        F  p-value
## s(age_z)   7.495  7.495 1227.412  < 2e-16 ***
## s(sleep_z) 3.340  3.340    9.383 2.05e-06 ***
## ---
## Signif. codes:  0 ‘***’ 0.001 ‘**’ 0.01 ‘*’ 0.05 ‘.’ 0.1 ‘ ’ 1
##
## R-sq.(adj) =  0.773
## lmer.REML = 7.7718e+05  Scale est. = 1.1315e+08  n = 33447
```

We did the same controling for depression. Below is the model with no covariates but only keeping data with depression.

```
##
## Family: gaussian
## Link function: identity
##
## Formula:
## value ~ sex + site + s(age_z, k = 10, bs = "cr") + s(sleep_z,
##     k = 5, bs = "cr") + icv
## <environment: 0x55897646f050>
##
## Parametric coefficients:
```

```
##                Estimate Std. Error t value Pr(>|t|)
## (Intercept)   656906.3     1903.8 345.054  < 2e-16 ***
## sexmale        15204.8      406.5  37.408  < 2e-16 ***
## siteousAvanto -48905.1     4695.3 -10.416  < 2e-16 ***
## siteousPrisma -13980.9     3767.9  -3.711 0.000207 ***
## siteousSkyra  -23131.1     2923.7  -7.912 2.62e-15 ***
## siteUCAM      -34194.1     2621.2 -13.045  < 2e-16 ***
## siteUKB          748.2     1882.2   0.397 0.691005
## siteUmU       -22989.7     2560.8  -8.978  < 2e-16 ***
## icv            46674.0      209.4 222.865  < 2e-16 ***
## ---
## Signif. codes:  0 ‘***’ 0.001 ‘**’ 0.01 ‘*’ 0.05 ‘.’ 0.1 ‘ ’ 1
##
## Approximate significance of smooth terms:
##               edf Ref.df       F p-value
## s(age_z)   7.504  7.504 1057.92  <2e-16 ***
## s(sleep_z) 3.492  3.492   15.14  <2e-16 ***
## ---
## Signif. codes:  0 ‘***’ 0.001 ‘**’ 0.01 ‘*’ 0.05 ‘.’ 0.1 ‘ ’ 1
##
## R-sq.(adj) =  0.769
## lmer.REML = 7.754e+05  Scale est. = 1.0814e+08  n = 33359
```

Below is the model output with main effect.

```
##
## Family: gaussian
## Link function: identity
##
## Formula:
## value ~ sex + site + s(age_z, k = 10, bs = "cr") + s(sleep_z,
##     k = 5, bs = "cr") + icv + depression
## <environment: 0x55897646f050>
##
## Parametric coefficients:
##                Estimate Std. Error t value Pr(>|t|)
## (Intercept)   657842.6     1911.4 344.172  < 2e-16 ***
## sexmale        15108.4      406.7  37.149  < 2e-16 ***
## siteousAvanto -48423.6     4694.8 -10.314  < 2e-16 ***
## siteousPrisma -13559.7     3767.2  -3.599 0.000319 ***
## siteousSkyra  -22583.1     2924.3  -7.722 1.17e-14 ***
## siteUCAM      -33980.5     2620.3 -12.968  < 2e-16 ***
## siteUKB          361.0     1882.9   0.192 0.847943
## siteUmU       -20164.5     2615.8  -7.709 1.31e-14 ***
## icv            46688.5      209.4 223.009  < 2e-16 ***
## depression     -7629.3     1455.0  -5.244 1.58e-07 ***
## ---
## Signif. codes:  0 ‘***’ 0.001 ‘**’ 0.01 ‘*’ 0.05 ‘.’ 0.1 ‘ ’ 1
##
## Approximate significance of smooth terms:
##               edf Ref.df       F p-value
## s(age_z)   7.500  7.500 1057.83  <2e-16 ***
## s(sleep_z) 3.421  3.421   12.87  <2e-16 ***
## ---
## Signif. codes:  0 ‘***’ 0.001 ‘**’ 0.01 ‘*’ 0.05 ‘.’ 0.1 ‘ ’ 1
```

```
##
## R-sq.(adj) =  0.769
## lmer.REML = 7.7536e+05  Scale est. = 1.081e+08  n = 33359
```

Next is the model with depression-sleep interaction.

```
##
## Family: gaussian
## Link function: identity
##
## Formula:
## value ~ sex + site + s(age_z, k = 10, bs = "cr") + s(sleep_z,
##     k = 5, bs = "cr") + icv + depression + depression:sleep_z
## <environment: 0x55897646f050>
##
## Parametric coefficients:
##                     Estimate Std. Error t value Pr(>|t|)
## (Intercept)         657843.8     1911.4 344.170  < 2e-16 ***
## sexmale              15103.8      406.8  37.131  < 2e-16 ***
## siteousAvanto       -48456.0     4695.0 -10.321  < 2e-16 ***
## siteousPrisma       -13594.0     3767.6  -3.608 0.000309 ***
## siteousSkyra        -22614.9     2924.8  -7.732 1.09e-14 ***
## siteUCAM            -34012.0     2620.8 -12.978  < 2e-16 ***
## siteUKB                361.8     1882.9   0.192 0.847631
## siteUmU             -19938.0     2639.9  -7.553 4.38e-14 ***
## icv                  46688.8      209.4 223.007  < 2e-16 ***
## depression           -7690.4     1458.4  -5.273 1.35e-07 ***
## depression:sleep_z    -690.1     1082.0  -0.638 0.523591
## ---
## Signif. codes:  0 ‘***’ 0.001 ‘**’ 0.01 ‘*’ 0.05 ‘.’ 0.1 ‘ ’ 1
##
## Approximate significance of smooth terms:
##               edf Ref.df       F p-value
## s(age_z)    7.497  7.497 1058.13  <2e-16 ***
## s(sleep_z) 3.426  3.426   11.93  <2e-16 ***
## ---
## Signif. codes:  0 ‘***’ 0.001 ‘**’ 0.01 ‘*’ 0.05 ‘.’ 0.1 ‘ ’ 1
##
## R-sq.(adj) =  0.769
## lmer.REML = 7.7534e+05  Scale est. = 1.081e+08  n = 33359
```

The plot below shows the sleep-volume curve for the original model and for the model with main effects of SES.

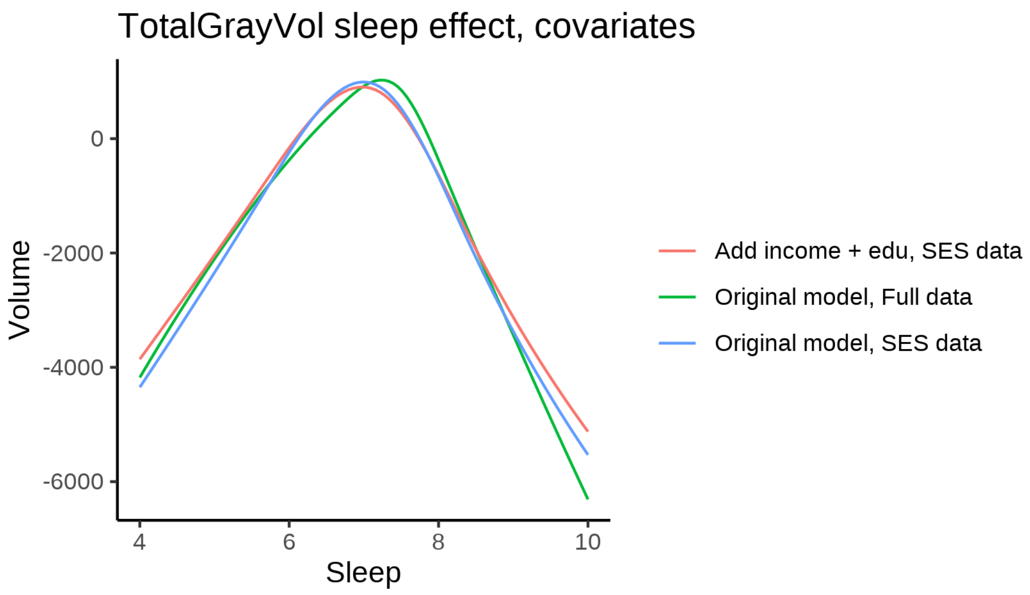

The plot below shows the sleep-volume curve for the original model and for the model with main effects of BMI.

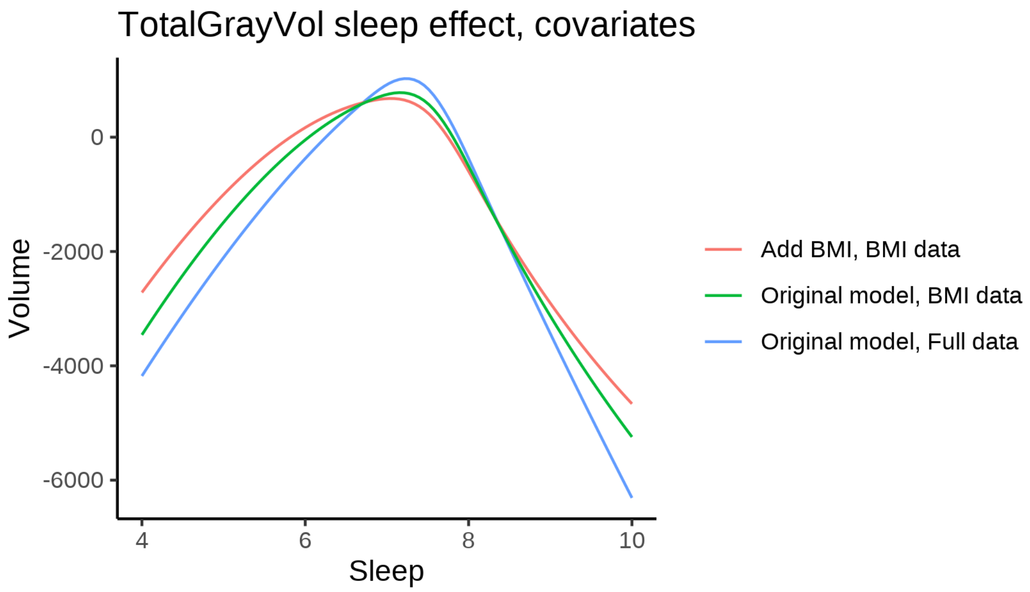

The plot below shows the sleep-volume curve for the original model and for the model with main effects of depression.

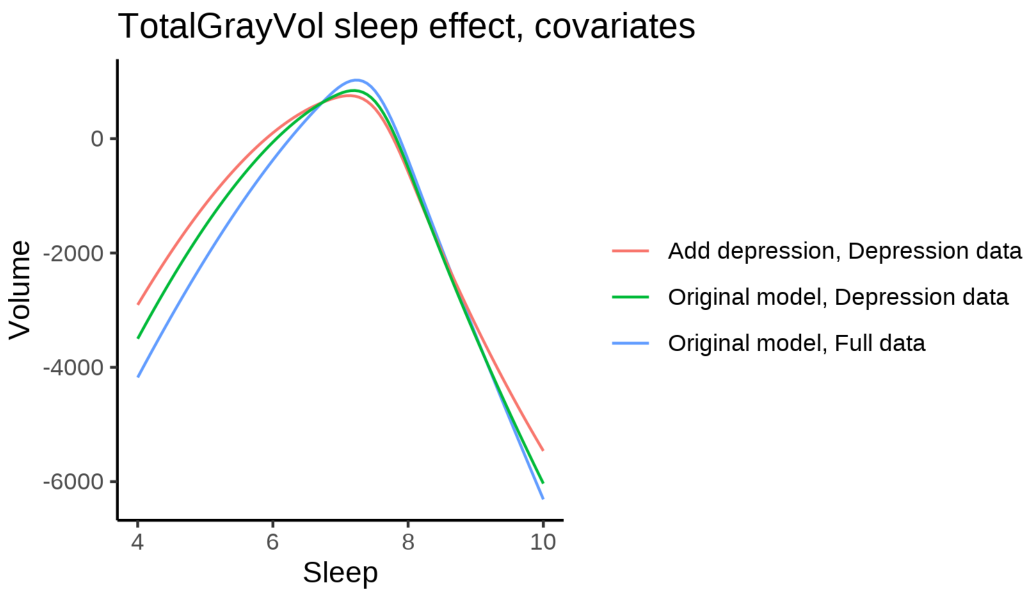

Models Not controling for ICV

Model outputs

Model without sleep term

```
##
## Family: gaussian
## Link function: identity
##
## Formula:
## value ~ sex + site + s(age_z, k = 10, bs = "cr")
## <environment: 0x55895c6bba00>
##
## Parametric coefficients:
##                Estimate Std. Error t value Pr(>|t|)
## (Intercept)    581423.9     2518.4 230.871  < 2e-16 ***
## sexmale         67598.0      438.3 154.231  < 2e-16 ***
## siteMPIB       -16256.1     3270.6  -4.970 6.71e-07 ***
## siteousAvanto   15039.2     2588.3   5.811 6.26e-09 ***
## siteousPrisma   39956.1     4371.5   9.140  < 2e-16 ***
## siteousSkyra    23528.2     2504.8   9.393  < 2e-16 ***
## siteUB            597.0     7858.4   0.076   0.9394
## siteUCAM        18001.0     2846.9   6.323 2.59e-10 ***
## siteUKB         53501.5     2584.7  20.699  < 2e-16 ***
## siteUmU          8532.8     3691.8   2.311   0.0208 *
## siteUOXF          194.5     3093.9   0.063   0.9499
## ---
## Signif. codes:  0 '***' 0.001 '**' 0.01 '*' 0.05 '.' 0.1 ' ' 1
##
## Approximate significance of smooth terms:
##             edf Ref.df    F p-value
## s(age_z) 6.487  6.487 1304  <2e-16 ***
```

```
## ---
## Signif. codes:  0 '***' 0.001 '**' 0.01 '*' 0.05 '.' 0.1 ' ' 1
##
## R-sq.(adj) =  0.406
## lmer.REML = 1.2391e+06  Scale est. = 1.1093e+08  n = 51282
```

Model with only main effects of age and sleep

```
##
## Family: gaussian
## Link function: identity
##
## Formula:
## value ~ sex + site + s(age_z, k = 10, bs = "cr") + s(sleep_z,
##     k = 5, bs = "cr")
## <environment: 0x55895c6bba00>
##
## Parametric coefficients:
##               Estimate Std. Error t value Pr(>|t|)
## (Intercept)   582618.1     2517.2 231.459  < 2e-16 ***
## sexmale        67414.4      437.7 154.003  < 2e-16 ***
## siteMPIB      -16563.5     3266.4  -5.071 3.97e-07 ***
## siteousAvanto  14200.0     2585.1   5.493 3.97e-08 ***
## siteousPrisma  39339.2     4365.3   9.012  < 2e-16 ***
## siteousSkyra   22644.2     2501.4   9.053  < 2e-16 ***
## siteUB          1079.3     7843.3   0.138   0.8906
## siteUCAM       17807.5     2842.3   6.265 3.75e-10 ***
## siteUKB        52333.9     2583.3  20.258  < 2e-16 ***
## siteUmU         7580.8     3693.5   2.052   0.0401 *
## siteUOXF        -321.3     3088.9  -0.104   0.9171
## ---
## Signif. codes:  0 '***' 0.001 '**' 0.01 '*' 0.05 '.' 0.1 ' ' 1
##
## Approximate significance of smooth terms:
##              edf Ref.df       F p-value
## s(age_z)   6.474  6.474 1290.78  <2e-16 ***
## s(sleep_z) 3.814  3.814   45.04  <2e-16 ***
## ---
## Signif. codes:  0 '***' 0.001 '**' 0.01 '*' 0.05 '.' 0.1 ' ' 1
##
## R-sq.(adj) =  0.408
## lmer.REML = 1.2389e+06  Scale est. = 1.1097e+08  n = 51282
```

Model with full interaction between age and sleep

```
##
## Family: gaussian
## Link function: identity
##
## Formula:
## value ~ sex + site + t2(age_z, sleep_z, k = c(10, 4), bs = "cr")
## <environment: 0x55895c6bba00>
##
## Parametric coefficients:
##               Estimate Std. Error t value Pr(>|t|)
## (Intercept)   583617.3     2503.6 233.111  < 2e-16 ***
```

```
## sexmale        67521.4      438.8 153.892  < 2e-16 ***
## siteMPIB      -17947.3     3272.4  -5.484 4.17e-08 ***
## siteousAvanto  13349.8     2592.8   5.149 2.63e-07 ***
## siteousPrisma  38530.1     4373.6   8.810  < 2e-16 ***
## siteousSkyra   21736.6     2509.8   8.661  < 2e-16 ***
## siteUB          -657.5     7842.4  -0.084   0.9332
## siteUCAM       16391.0     2847.5   5.756 8.65e-09 ***
## siteUKB        51276.8     2568.3  19.965  < 2e-16 ***
## siteUmU         6478.1     3693.3   1.754   0.0794 .
## siteUOXF       -1804.6     3081.5  -0.586   0.5581
## ---
## Signif. codes:  0 ‘***’ 0.001 ‘**’ 0.01 ‘*’ 0.05 ‘.’ 0.1 ‘ ’ 1
##
## Approximate significance of smooth terms:
##                     edf Ref.df    F p-value
## t2(age_z,sleep_z) 13.65  13.65 59.2  <2e-16 ***
## ---
## Signif. codes:  0 ‘***’ 0.001 ‘**’ 0.01 ‘*’ 0.05 ‘.’ 0.1 ‘ ’ 1
##
## R-sq.(adj) =  0.408
## lmer.REML = 1.239e+06  Scale est. = 1.1099e+08  n = 51282
```

Model comparison

`mod_no_sleep` refers to model without sleep term, `mod_no_interaction` refers to model with only main effect of sleep, and `mod_full` refers to model with a full interaction between age and sleep. This is a nested model comparison, and the p-value at a given line refers to comparing the model at the line to the model on the line above. Hence, significance implies that the more complicated model is supported on statistical grounds.

To be even more specific, the p-value on the second row tests whether there is an association between sleep and volume. The p-value on the third row tests whether this association depends on age.

```
## Data: NULL
## Models:
## ml$mod_no_sleep$mer: NULL
## ml$mod_no_interaction$mer: NULL
## ml$mod_full$mer: NULL
##                           npar     AIC     BIC  logLik deviance  Chisq Df Pr(>Chisq)
## ml$mod_no_sleep$mer         15 1239131 1239263 -619550  1239101
## ml$mod_no_interaction$mer   17 1238971 1239121 -619468  1238937 163.77  2     <2e-16 ***
## ml$mod_full$mer             19 1238992 1239160 -619477  1238954   0.00  2          1
## ---
## Signif. codes:  0 ‘***’ 0.001 ‘**’ 0.01 ‘*’ 0.05 ‘.’ 0.1 ‘ ’ 1
```

We chose the model based on the likelihood ratio test with 5 % significance level, which was `mod_no_interaction`.

Lifespan brain trajectory

The trajectory shown is from the chosen model `mod_no_interaction_no_icv`.

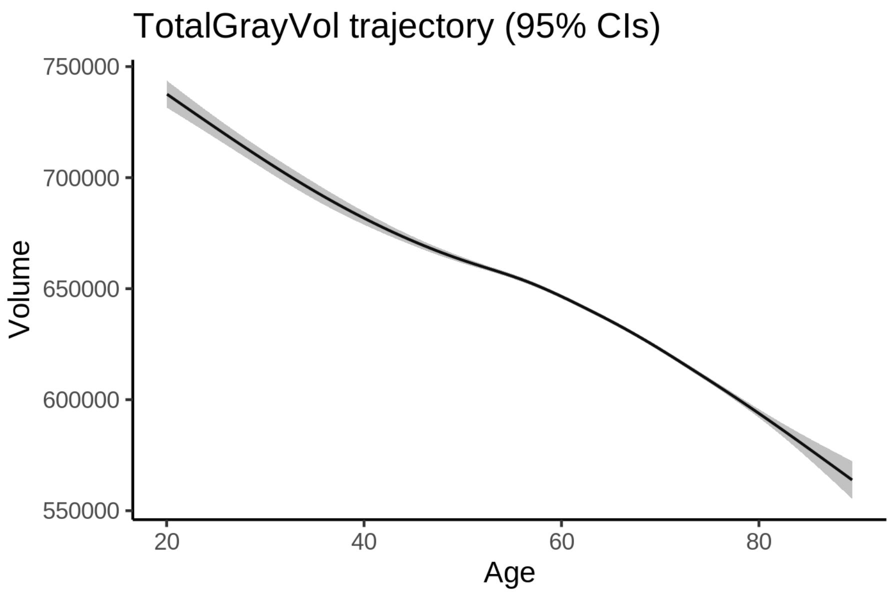

Effect of sleep

The chosen model only included the main effect of sleep, and hence the effect does not vary with age. The black dot shows the average sleep duration across all ages in the sample.

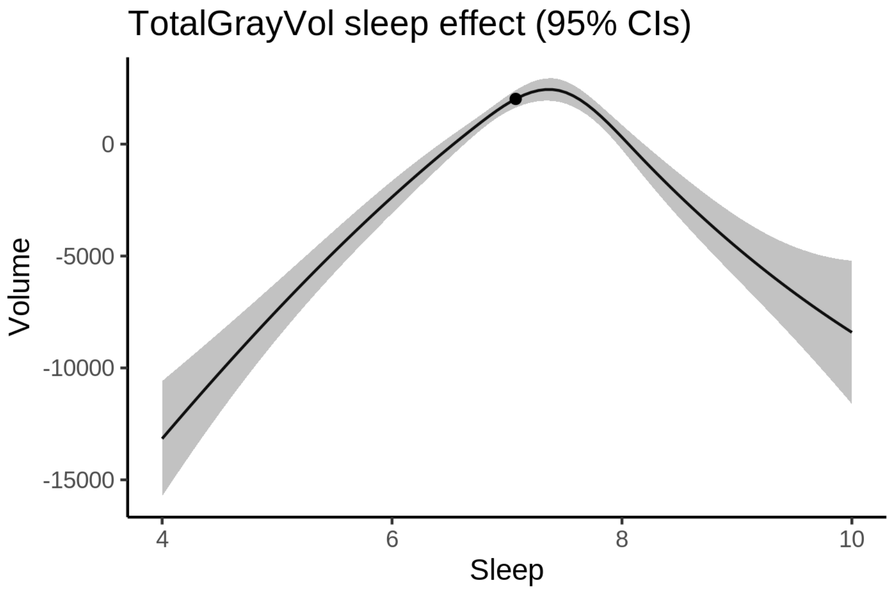

We also show the full interaction model for completeness, although it was not selected.

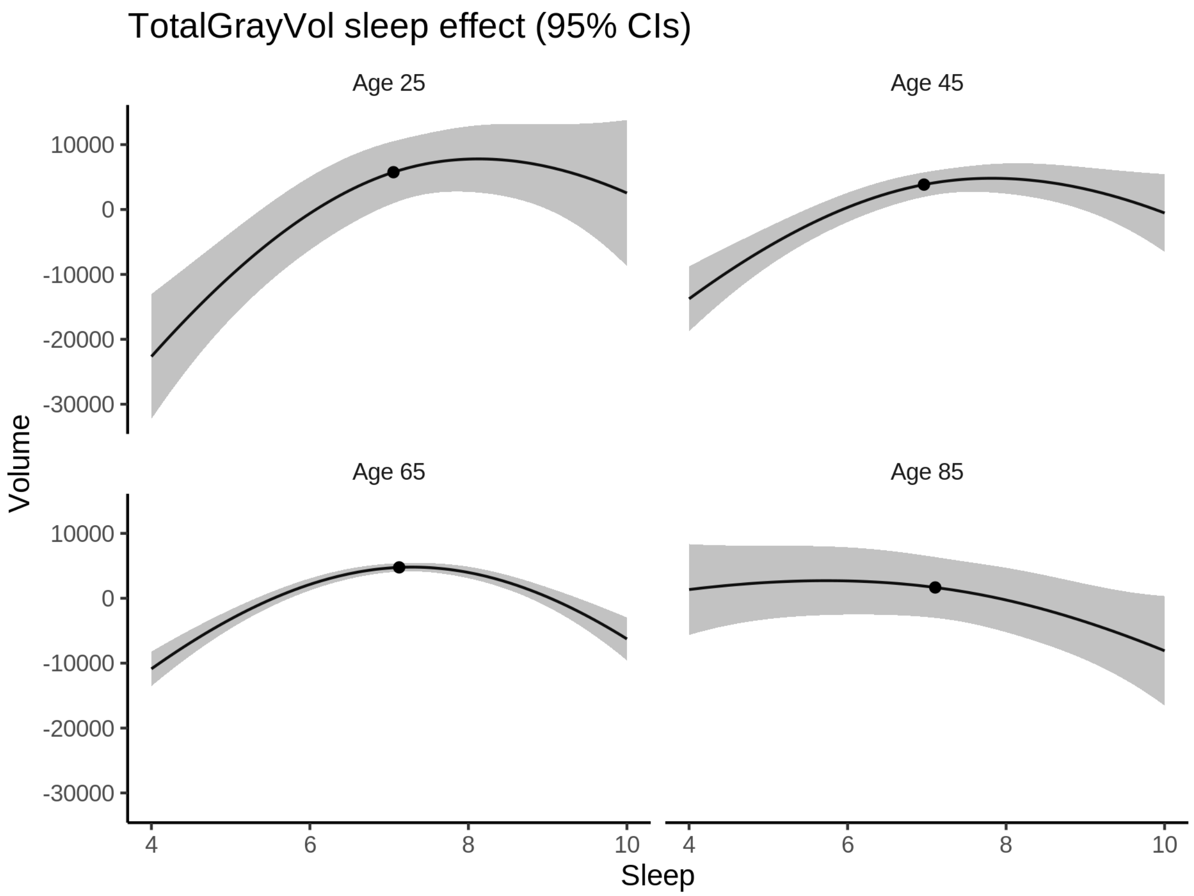

Deviation from sleep associated with maximal volume

Model with only main effect of sleep was chosen, so we show it for all ages at once. Maximum volume is attained at 7.4 hours of sleep. The percentage values in the plot are calculated as follows: The maximum at 100 % refers to a person at an arbitrary age with a sleep duration associated with maximum volume. For a female, this volume is 652185 and for a male it is 719599. The other percentage values show how large the expected volume is for someone with other sleep durations. For example, 99 % implies a 1 % reduction.

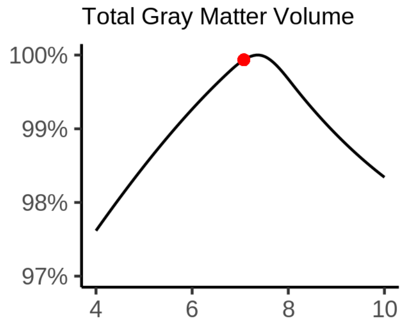

Comparison of mean sleep and sleep associated with maximum volume

A 95 % confidence interval for the sleep associated with maximum volume is [7.27, 7.39].

The plot below compares average sleep to the sleep associated with maximum volume.

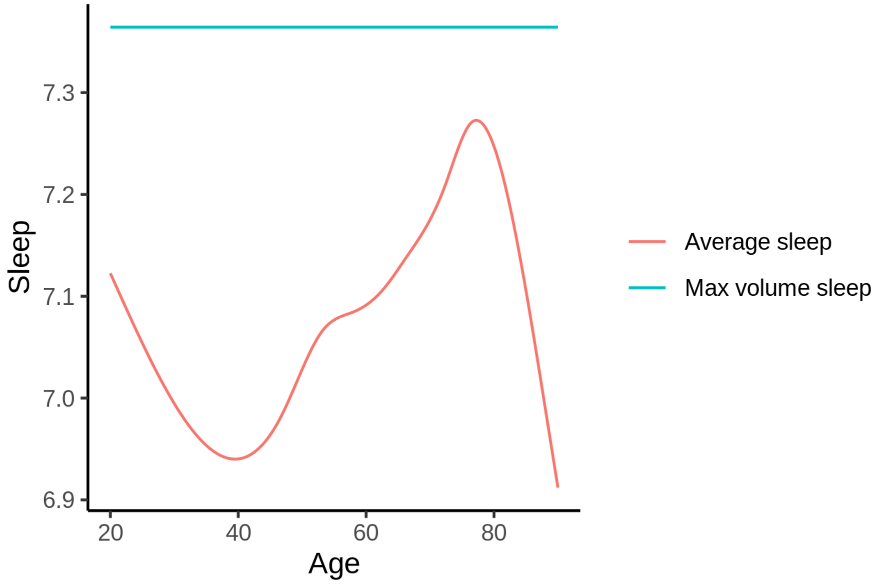

The next plot shows the difference between average sleep and sleep associated with maximum volume. The shaded region is a 95 % confidence interval.

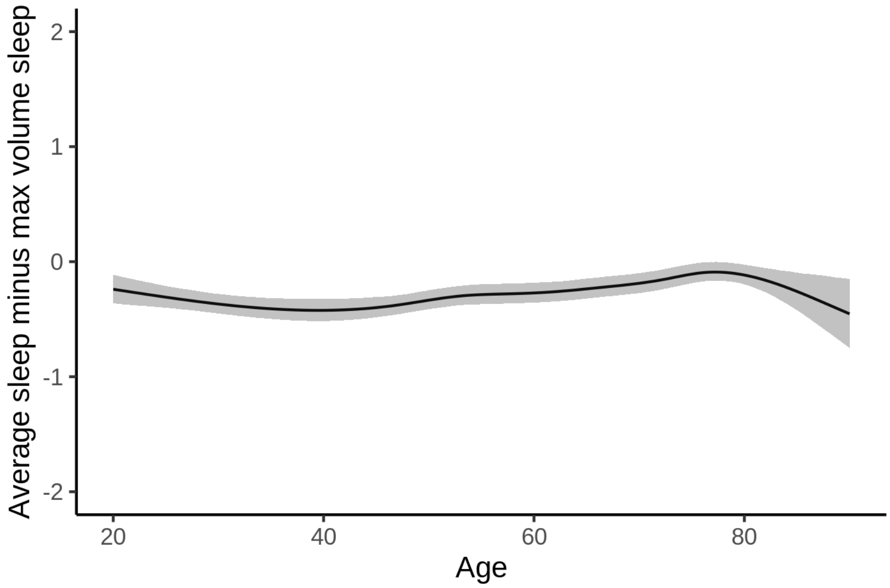

The next plot shows the probability that the sleep duration associated with maximum volume is longer than the average sleep duration, as a function of age. Probability below .05 can be interpreted as evidence that the sleep associated with maximum volume is shorter than the mean sleep, and probability above .95 can be interpreted the opposite way.

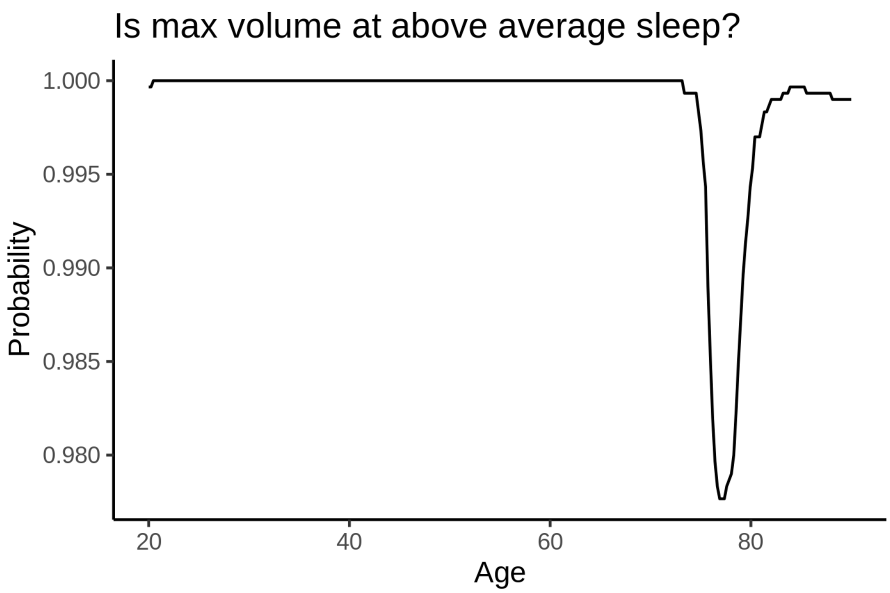

Ventricles

Descriptive statistics

Study	Observations	Unique IDs	Mean age	Age range
HCP	974	974	28.8	22 - 37
MPIB	677	391	63.1	24 - 83
UB	113	39	70.9	64 - 81
UCAM	884	632	55.1	20 - 88
UiO	1460	803	49.2	20 - 89
UKB	45979	43135	64.5	45 - 83
UmU	412	279	62.2	25 - 85
UOXF	769	769	69.8	60 - 85

Spaghetti plot

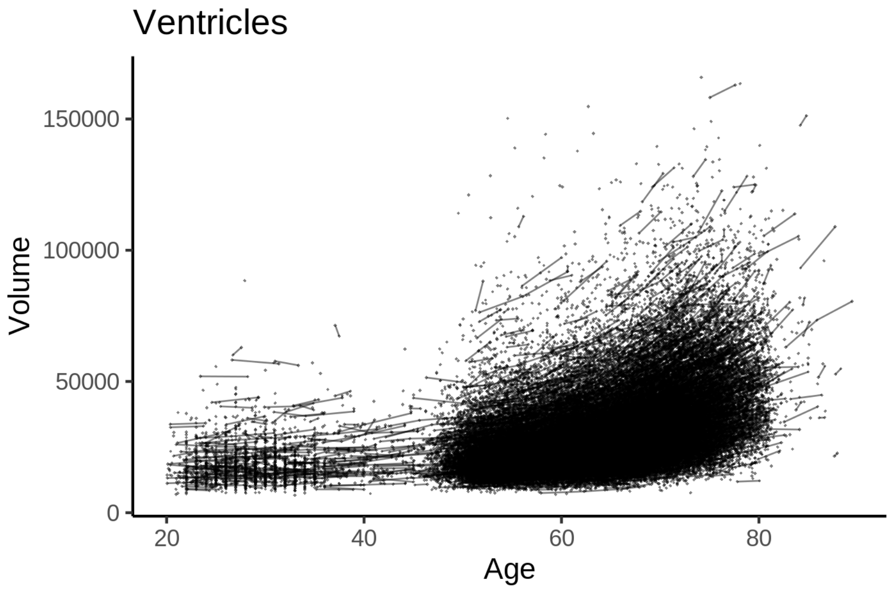

Models Controling for ICV

Model outputs

Model without sleep term

```
##
## Family: gaussian
## Link function: identity
##
## Formula:
## value ~ sex + site + icv + s(age_z, k = 10, bs = "cr")
## <environment: 0x558963bebd30>
##
## Parametric coefficients:
##               Estimate Std. Error t value Pr(>|t|)
## (Intercept)   31235.34     600.16  52.045  < 2e-16 ***
## sexmale        1887.28     141.49  13.339  < 2e-16 ***
## siteMPIB      11324.82     845.86  13.388  < 2e-16 ***
## siteousAvanto   834.77     647.56   1.289   0.1974
## siteousPrisma   360.15     970.56   0.371   0.7106
## siteousSkyra   4347.81     644.51   6.746 1.54e-11 ***
## siteUB        -1098.79    2098.99  -0.523   0.6006
## siteUCAM        128.31     724.35   0.177   0.8594
## siteUKB        -571.38     611.99  -0.934   0.3505
## siteUmU        5022.57     947.10   5.303 1.14e-07 ***
## siteUOXF       1804.74     763.36   2.364   0.0181 *
## icv            6211.59      69.92  88.835  < 2e-16 ***
## ---
## Signif. codes:  0 ‘***’ 0.001 ‘**’ 0.01 ‘*’ 0.05 ‘.’ 0.1 ‘ ’ 1
##
```

```
## Approximate significance of smooth terms:
##            edf Ref.df    F p-value
## s(age_z) 8.373  8.373 2510  <2e-16 ***
## ---
## Signif. codes:  0 ‘***’ 0.001 ‘**’ 0.01 ‘*’ 0.05 ‘.’ 0.1 ‘ ’ 1
##
## R-sq.(adj) =  0.391
## lmer.REML = 1.0979e+06  Scale est. = 2.0658e+06  n = 51268
```

Model with only main effects of age and sleep

```
##
## Family: gaussian
## Link function: identity
##
## Formula:
## value ~ sex + site + icv + s(age_z, k = 10, bs = "cr") + s(sleep_z,
##     k = 5, bs = "cr")
## <environment: 0x558963bebd30>
##
## Parametric coefficients:
##                Estimate Std. Error t value Pr(>|t|)
## (Intercept)   31264.34     600.68  52.048  < 2e-16 ***
## sexmale        1890.23     141.47  13.362  < 2e-16 ***
## siteMPIB      11249.10     846.15  13.294  < 2e-16 ***
## siteousAvanto   828.13     647.66   1.279   0.2010
## siteousPrisma   358.52     970.60   0.369   0.7118
## siteousSkyra   4345.16     644.64   6.740 1.60e-11 ***
## siteUB        -1132.56    2098.58  -0.540   0.5894
## siteUCAM        104.29     724.29   0.144   0.8855
## siteUKB        -602.99     612.57  -0.984   0.3249
## siteUmU        4844.60     948.95   5.105 3.32e-07 ***
## siteUOXF       1804.37     763.33   2.364   0.0181 *
## icv            6212.42      69.98  88.775  < 2e-16 ***
## ---
## Signif. codes:  0 ‘***’ 0.001 ‘**’ 0.01 ‘*’ 0.05 ‘.’ 0.1 ‘ ’ 1
##
## Approximate significance of smooth terms:
##              edf Ref.df        F  p-value
## s(age_z)   8.373  8.373 2489.484  < 2e-16 ***
## s(sleep_z) 2.624  2.624    5.613 0.000926 ***
## ---
## Signif. codes:  0 ‘***’ 0.001 ‘**’ 0.01 ‘*’ 0.05 ‘.’ 0.1 ‘ ’ 1
##
## R-sq.(adj) =  0.392
## lmer.REML = 1.0979e+06  Scale est. = 2.0668e+06  n = 51268
```

Model with full interaction between age and sleep

```
##
## Family: gaussian
## Link function: identity
##
## Formula:
## value ~ sex + site + icv + t2(age_z, sleep_z, k = c(10, 4), bs = "cr")
## <environment: 0x558963bebd30>
```

```
##
## Parametric coefficients:
##                Estimate Std. Error t value Pr(>|t|)
## (Intercept)   31361.08     603.38  51.976  < 2e-16 ***
## sexmale        1906.49     141.72  13.453  < 2e-16 ***
## siteMPIB      11172.38     850.01  13.144  < 2e-16 ***
## siteousAvanto   783.99     651.48   1.203   0.2288
## siteousPrisma   282.01     972.66   0.290   0.7719
## siteousSkyra   4285.38     648.63   6.607 3.96e-11 ***
## siteUB        -1253.56    2099.88  -0.597   0.5505
## siteUCAM         54.52     728.00   0.075   0.9403
## siteUKB        -712.01     615.31  -1.157   0.2472
## siteUmU        4772.10     951.53   5.015 5.32e-07 ***
## siteUOXF       1683.41     766.37   2.197   0.0281 *
## icv            6211.60      69.96  88.792  < 2e-16 ***
## ---
## Signif. codes:  0 '***' 0.001 '**' 0.01 '*' 0.05 '.' 0.1 ' ' 1
##
## Approximate significance of smooth terms:
##                       edf Ref.df     F p-value
## t2(age_z,sleep_z) 15.69  15.69 115.7  <2e-16 ***
## ---
## Signif. codes:  0 '***' 0.001 '**' 0.01 '*' 0.05 '.' 0.1 ' ' 1
##
## R-sq.(adj) =  0.392
## lmer.REML = 1.0978e+06  Scale est. = 2.0562e+06  n = 51268
```

Model comparison

`mod_no_sleep` refers to model without sleep term, `mod_no_interaction` refers to model with only main effect of sleep, and `mod_full` refers to model with a full interaction between age and sleep. This is a nested model comparison, and the p-value at a given line refers to comparing the model at the line to the model on the line above. Hence, significance implies that the more complicated model is supported on statistical grounds.

To be even more specific, the p-value on the second row tests whether there is an association between sleep and volume. The p-value on the third row tests whether this association depends on age.

```
## Data: NULL
## Models:
## ml$mod_no_sleep$mer: NULL
## ml$mod_no_interaction$mer: NULL
## ml$mod_full$mer: NULL
##                           npar     AIC     BIC  logLik deviance  Chisq Df Pr(>Chisq)
## ml$mod_no_sleep$mer         16 1097903 1098045 -548936  1097871
## ml$mod_no_interaction$mer   18 1097894 1098053 -548929  1097858 12.898  2  0.0015825 **
## ml$mod_full$mer             20 1097884 1098061 -548922  1097844 13.837  2  0.0009895 ***
## ---
## Signif. codes:  0 '***' 0.001 '**' 0.01 '*' 0.05 '.' 0.1 ' ' 1
```

We chose the model based on the likelihood ratio test with 5 % significance level, which was `mod_full`.

Lifespan brain trajectory

The trajectory shown is from the chosen model `mod_full`.

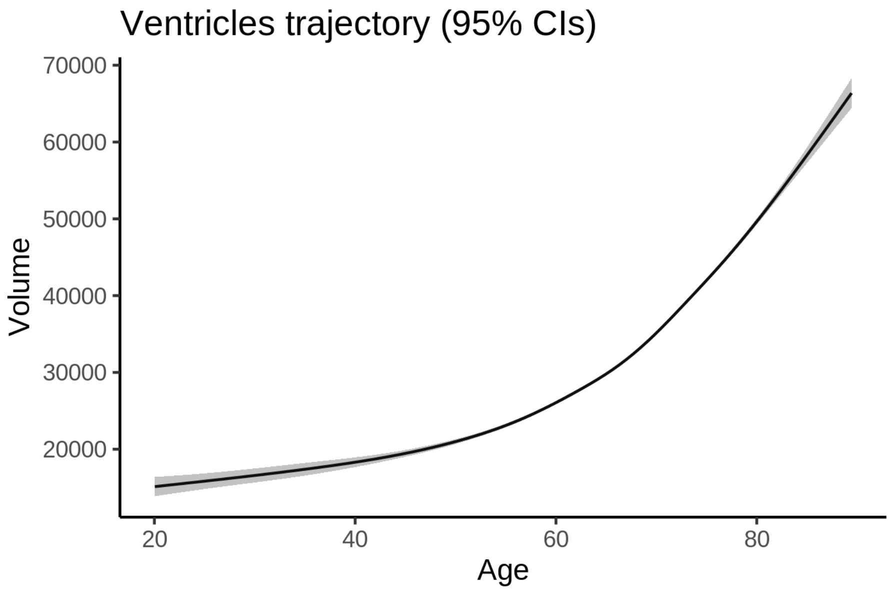

Effect of sleep

The chosen model included a full interaction between age and sleep, and the effect of sleep is hence plotted for a set of different ages. For comparability across ages, the sleep-volume curves at each age have been standardized so that they sum to zero.

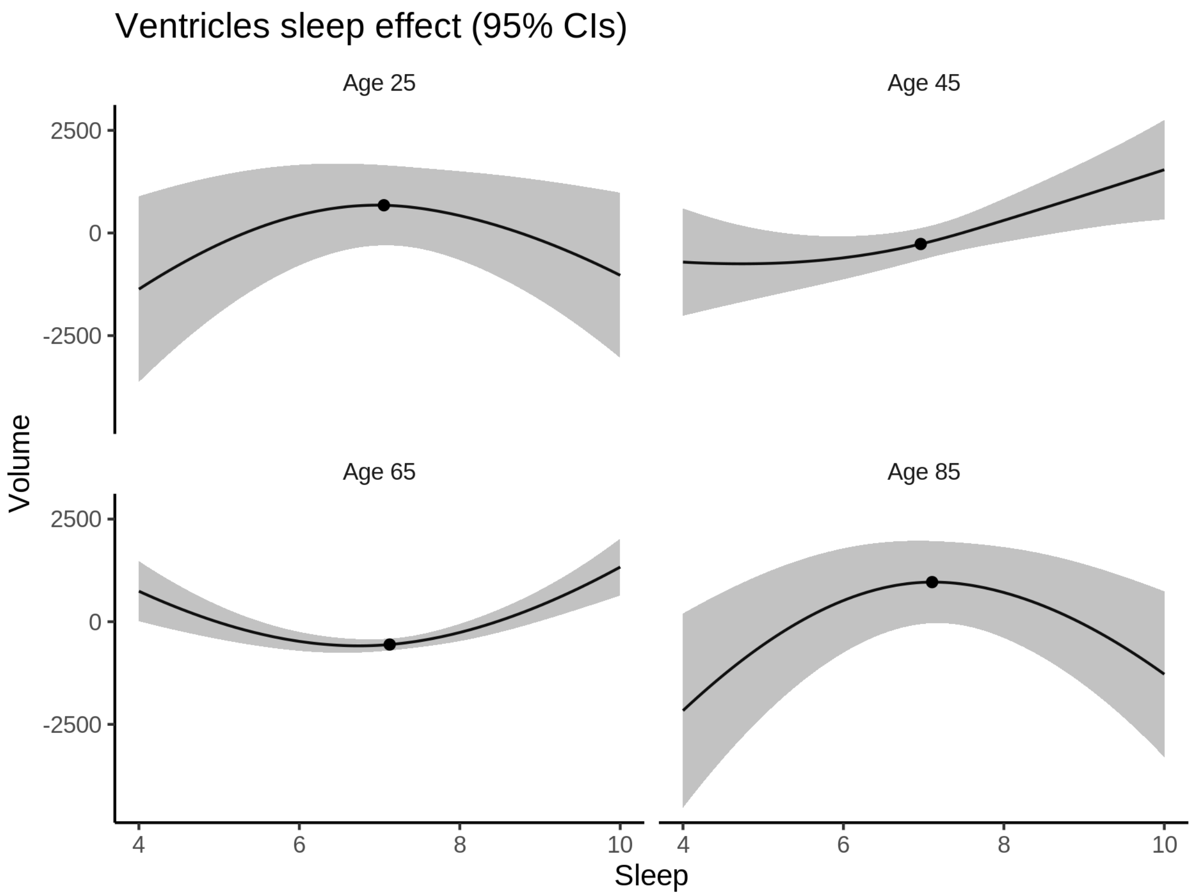

Deviation from sleep associated with maximal volume

The table show the sleep associated with maximum volume at chosen ages.

Age	Sleep at max vol
25	7.0
45	10.0
65	10.0
85	7.1

Model with sleep-age interaction was chosen, so we show it for four selected ages. The percentage values in the plot are calculated as follows: The maximum at 100 % refers to a person at the given age with a sleep duration associated with maximum volume. The other percentage values show how large the expected volume is for someone with other sleep durations. For example, 99 % implies a 1 % reduction.

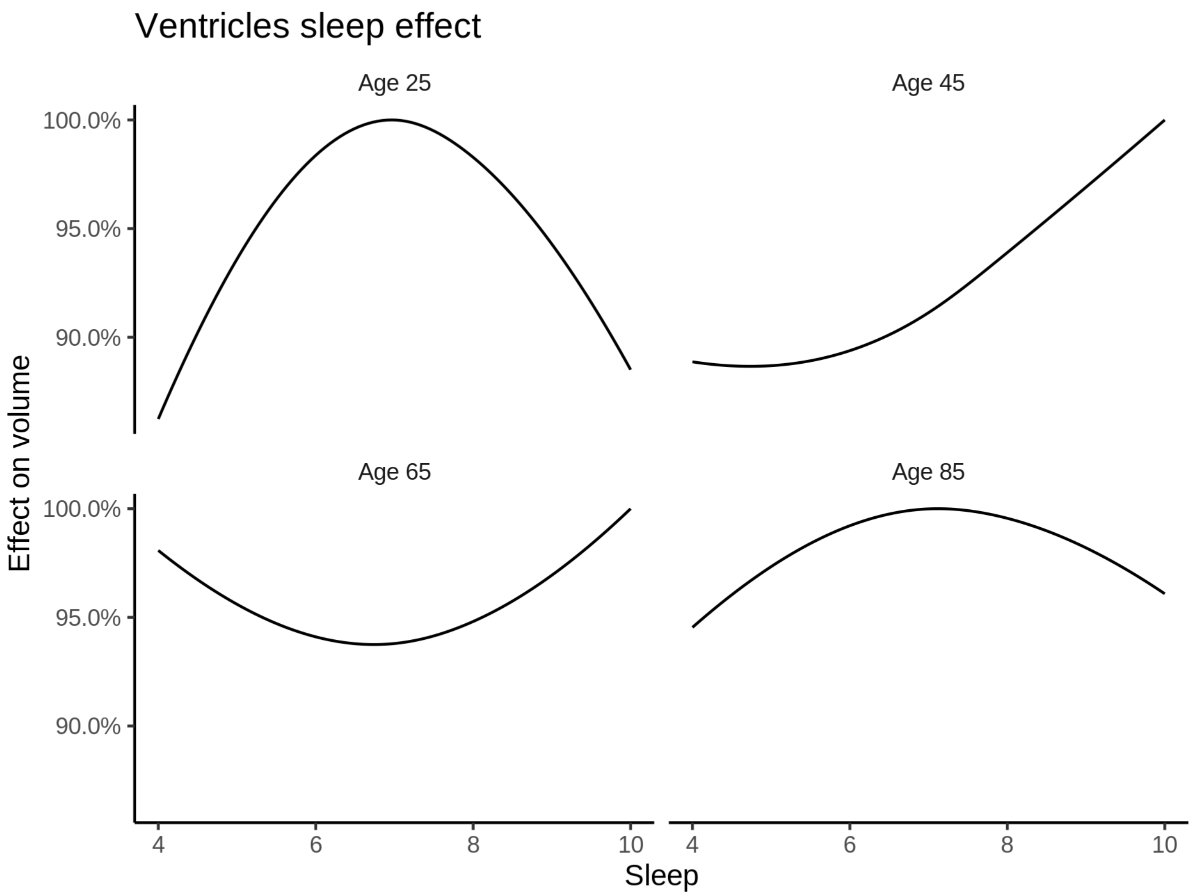

Comparison of mean sleep and sleep associated with maximum volume

The plot below compares average sleep to the sleep associated with maximum volume.

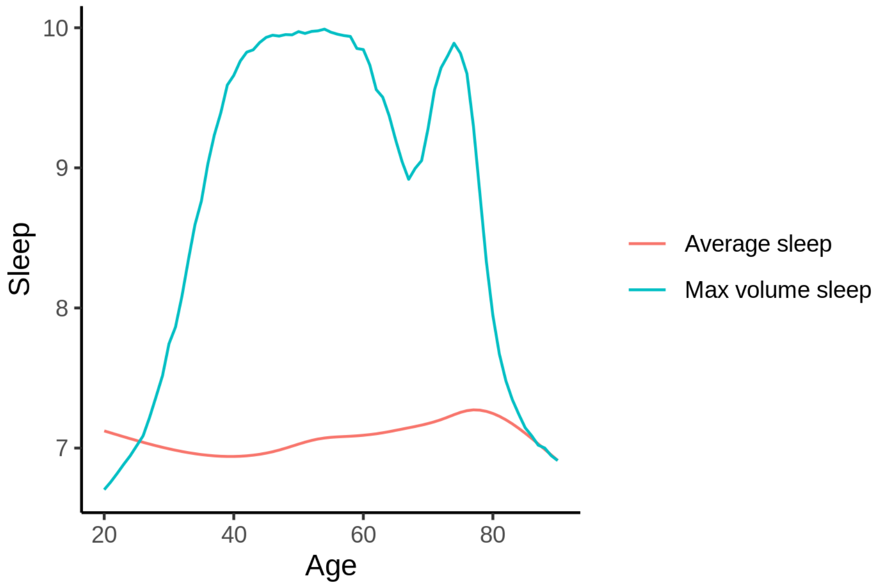

The next plot shows the difference between average sleep and sleep associated with maximum volume. The shaded region is a 95 % confidence interval.

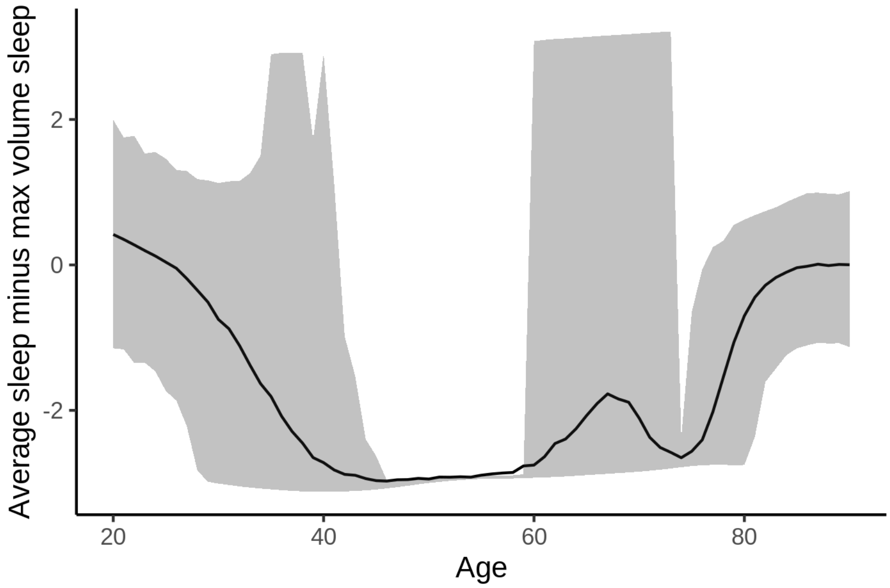

The next plot shows the probability that the sleep duration associated with maximum volume is longer than the average sleep duration, as a function of age. Probility below .05 can be interpreted as evidence that the sleep associated with maximum volume is shorter than the mean sleep, and probability above .95 can be interpreted the opposite way.

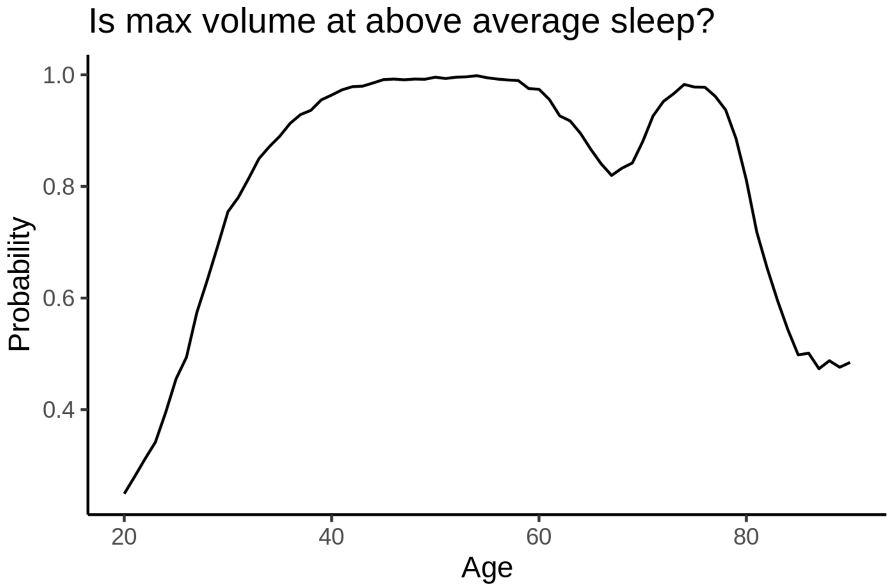

Controling for covariates

Below is the output for a model in which we only include data with income and education.

```
##
## Family: gaussian
## Link function: identity
```

```
##
## Formula:
## value ~ sex + site + s(age_z, k = 10, bs = "cr") + s(sleep_z,
##     k = 5, bs = "cr") + icv
## <environment: 0x5589f2747f60>
##
## Parametric coefficients:
##                Estimate Std. Error t value Pr(>|t|)
## (Intercept)    45162.03    1653.54  27.312  < 2e-16 ***
## sexmale         1849.62     180.62  10.240  < 2e-16 ***
## siteousAvanto -13829.90    1826.06  -7.574 3.73e-14 ***
## siteousPrisma -14574.87    1932.56  -7.542 4.76e-14 ***
## siteousSkyra   -9655.39    1785.28  -5.408 6.41e-08 ***
## siteUKB       -14334.03    1649.32  -8.691  < 2e-16 ***
## siteUOXF       -8985.34    1858.24  -4.835 1.34e-06 ***
## icv             6377.54      90.86  70.192  < 2e-16 ***
## ---
## Signif. codes:  0 ‘***’ 0.001 ‘**’ 0.01 ‘*’ 0.05 ‘.’ 0.1 ‘ ’ 1
##
## Approximate significance of smooth terms:
##              edf Ref.df      F p-value
## s(age_z)   7.878  7.878 1519.6  <2e-16 ***
## s(sleep_z) 2.225  2.225    2.2   0.141
## ---
## Signif. codes:  0 ‘***’ 0.001 ‘**’ 0.01 ‘*’ 0.05 ‘.’ 0.1 ‘ ’ 1
##
## R-sq.(adj) =  0.378
## lmer.REML = 6.6824e+05  Scale est. = 1.7799e+06  n = 31194
```

Below is the output for a model in which we control for the main effects of income and education.

```
##
## Family: gaussian
## Link function: identity
##
## Formula:
## value ~ sex + site + s(age_z, k = 10, bs = "cr") + s(sleep_z,
##     k = 5, bs = "cr") + icv + income_scaled + education_scaled
## <environment: 0x5589f2747f60>
##
## Parametric coefficients:
##                   Estimate Std. Error t value Pr(>|t|)
## (Intercept)       44674.19    1662.77  26.867  < 2e-16 ***
## sexmale            1889.36     181.07  10.434  < 2e-16 ***
## siteousAvanto    -13949.88    1826.27  -7.638 2.26e-14 ***
## siteousPrisma    -14655.55    1932.34  -7.584 3.43e-14 ***
## siteousSkyra      -9782.46    1785.46  -5.479 4.31e-08 ***
## siteUKB          -14503.28    1649.78  -8.791  < 2e-16 ***
## siteUOXF          -8908.30    1858.61  -4.793 1.65e-06 ***
## icv                6351.06      91.53  69.386  < 2e-16 ***
## income_scaled      -237.23     215.00  -1.103 0.269877
## education_scaled    960.39     256.99   3.737 0.000186 ***
## ---
## Signif. codes:  0 ‘***’ 0.001 ‘**’ 0.01 ‘*’ 0.05 ‘.’ 0.1 ‘ ’ 1
##
```

```
## Approximate significance of smooth terms:
##              edf Ref.df       F p-value
## s(age_z)   7.878  7.878 1425.37  <2e-16 ***
## s(sleep_z) 2.302  2.302    2.39   0.122
## ---
## Signif. codes:  0 ‘***’ 0.001 ‘**’ 0.01 ‘*’ 0.05 ‘.’ 0.1 ‘ ’ 1
##
## R-sq.(adj) =  0.379
## lmer.REML = 6.682e+05  Scale est. = 1.7793e+06  n = 31194
```

We also included interaction effects between sleep duration and education and income, in another model. The output is shown below, and the interaction terms are `income_scaled:sleep_z` and `education_scaled:sleep_z`.

```
##
## Family: gaussian
## Link function: identity
##
## Formula:
## value ~ sex + site + s(age_z, k = 10, bs = "cr") + s(sleep_z,
##     k = 5, bs = "cr") + icv + income_scaled + education_scaled +
##     income_scaled:sleep_z + education_scaled:sleep_z
## <environment: 0x5589f2747f60>
##
## Parametric coefficients:
##                           Estimate Std. Error t value Pr(>|t|)
## (Intercept)               44638.20    1662.85  26.844  < 2e-16 ***
## sexmale                    1898.12     181.20  10.476  < 2e-16 ***
## siteousAvanto            -13918.82    1826.28  -7.621 2.58e-14 ***
## siteousPrisma            -14610.13    1932.43  -7.561 4.13e-14 ***
## siteousSkyra              -9752.30    1785.46  -5.462 4.74e-08 ***
## siteUKB                  -14461.88    1649.90  -8.765  < 2e-16 ***
## siteUOXF                  -8845.63    1859.07  -4.758 1.96e-06 ***
## icv                        6348.79      91.54  69.356  < 2e-16 ***
## income_scaled              -244.14     215.11  -1.135 0.256405
## education_scaled            968.16     257.02   3.767 0.000166 ***
## income_scaled:sleep_z       156.86     216.18   0.726 0.468088
## education_scaled:sleep_z   -482.09     249.34  -1.933 0.053193 .
## ---
## Signif. codes:  0 ‘***’ 0.001 ‘**’ 0.01 ‘*’ 0.05 ‘.’ 0.1 ‘ ’ 1
##
## Approximate significance of smooth terms:
##              edf Ref.df        F p-value
## s(age_z)   7.877  7.877 1424.477  <2e-16 ***
## s(sleep_z) 2.361  2.361    3.619  0.0355 *
## ---
## Signif. codes:  0 ‘***’ 0.001 ‘**’ 0.01 ‘*’ 0.05 ‘.’ 0.1 ‘ ’ 1
##
## R-sq.(adj) =  0.379
## lmer.REML = 6.6817e+05  Scale est. = 1.7793e+06  n = 31194
```

We did the same controling for BMI. Below is the model with no covariates but only keeping data with BMI.

```
##
## Family: gaussian
## Link function: identity
```

```
##
## Formula:
## value ~ sex + site + s(age_z, k = 10, bs = "cr") + s(sleep_z,
##     k = 5, bs = "cr") + icv
## <environment: 0x558ad9d536b0>
##
## Parametric coefficients:
##               Estimate Std. Error t value Pr(>|t|)
## (Intercept)   31523.00     773.72  40.742  < 2e-16 ***
## sexmale        1807.60     175.33  10.309  < 2e-16 ***
## siteousPrisma  -103.29     909.13  -0.114    0.910
## siteousSkyra   4074.79     365.77  11.140  < 2e-16 ***
## siteUCAM       -601.01     881.36  -0.682    0.495
## siteUKB        -752.41     784.06  -0.960    0.337
## siteUmU        4712.91    1074.07   4.388 1.15e-05 ***
## icv            6429.37      88.61  72.561  < 2e-16 ***
## ---
## Signif. codes:  0 ‘***’ 0.001 ‘**’ 0.01 ‘*’ 0.05 ‘.’ 0.1 ‘ ’ 1
##
## Approximate significance of smooth terms:
##              edf Ref.df        F p-value
## s(age_z)   8.056  8.056 1670.639  <2e-16 ***
## s(sleep_z) 2.462  2.462    1.985  0.0776 .
## ---
## Signif. codes:  0 ‘***’ 0.001 ‘**’ 0.01 ‘*’ 0.05 ‘.’ 0.1 ‘ ’ 1
##
## R-sq.(adj) =  0.383
## lmer.REML = 7.1575e+05  Scale est. = 1.8476e+06  n = 33446
```

Below is the model output with main effect.

```
##
## Family: gaussian
## Link function: identity
##
## Formula:
## value ~ sex + site + s(age_z, k = 10, bs = "cr") + s(sleep_z,
##     k = 5, bs = "cr") + icv + bmi
## <environment: 0x558ad9d536b0>
##
## Parametric coefficients:
##               Estimate Std. Error t value Pr(>|t|)
## (Intercept)   30016.64     885.63  33.893  < 2e-16 ***
## sexmale        1759.31     175.85  10.005  < 2e-16 ***
## siteousPrisma  -121.22     908.98  -0.133 0.893908
## siteousSkyra   4074.48     365.70  11.142  < 2e-16 ***
## siteUCAM       -637.63     881.28  -0.724 0.469363
## siteUKB        -815.78     784.13  -1.040 0.298178
## siteUmU        4638.68    1074.12   4.319 1.57e-05 ***
## icv            6420.30      88.62  72.445  < 2e-16 ***
## bmi              60.35      17.26   3.496 0.000472 ***
## ---
## Signif. codes:  0 ‘***’ 0.001 ‘**’ 0.01 ‘*’ 0.05 ‘.’ 0.1 ‘ ’ 1
##
## Approximate significance of smooth terms:
```

```
##              edf Ref.df        F p-value
## s(age_z)   8.060  8.060 1671.561  <2e-16 ***
## s(sleep_z) 2.302  2.302    1.944  0.0932 .
## ---
## Signif. codes:  0 '***' 0.001 '**' 0.01 '*' 0.05 '.' 0.1 ' ' 1
##
## R-sq.(adj) =  0.383
## lmer.REML = 7.1573e+05  Scale est. = 1.8469e+06  n = 33446
```

Next is the model with BMI-sleep interaction.

```
##
## Family: gaussian
## Link function: identity
##
## Formula:
## value ~ sex + site + s(age_z, k = 10, bs = "cr") + s(sleep_z,
##     k = 5, bs = "cr") + icv + bmi + bmi:sleep_z
## <environment: 0x558ad9d536b0>
##
## Parametric coefficients:
##               Estimate Std. Error t value Pr(>|t|)
## (Intercept)   29921.50     886.18  33.764  < 2e-16 ***
## sexmale        1761.28     175.83  10.017  < 2e-16 ***
## siteousPrisma  -120.80     908.97  -0.133 0.894274
## siteousSkyra   4078.44     365.72  11.152  < 2e-16 ***
## siteUCAM       -607.91     881.27  -0.690 0.490315
## siteUKB        -789.31     784.14  -1.007 0.314137
## siteUmU        4661.36    1074.05   4.340 1.43e-05 ***
## icv            6422.04      88.61  72.472  < 2e-16 ***
## bmi              62.08      17.27   3.595 0.000325 ***
## bmi:sleep_z      46.45      16.36   2.839 0.004526 **
## ---
## Signif. codes:  0 '***' 0.001 '**' 0.01 '*' 0.05 '.' 0.1 ' ' 1
##
## Approximate significance of smooth terms:
##              edf Ref.df        F p-value
## s(age_z)   8.060  8.060 1670.285 < 2e-16 ***
## s(sleep_z) 2.253  2.253    4.127 0.00951 **
## ---
## Signif. codes:  0 '***' 0.001 '**' 0.01 '*' 0.05 '.' 0.1 ' ' 1
##
## R-sq.(adj) =  0.384
## lmer.REML = 7.1572e+05  Scale est. = 1.8471e+06  n = 33446
```

We did the same controling for depression. Below is the model with no covariates but only keeping data with depression.

```
##
## Family: gaussian
## Link function: identity
##
## Formula:
## value ~ sex + site + s(age_z, k = 10, bs = "cr") + s(sleep_z,
##     k = 5, bs = "cr") + icv
## <environment: 0x558ad797ced8>
```

```
##
## Parametric coefficients:
##                 Estimate Std. Error t value Pr(>|t|)
## (Intercept)    42470.07     855.21  49.660  < 2e-16 ***
## sexmale         1732.95     176.32   9.828  < 2e-16 ***
## siteousAvanto -10141.60    1332.35  -7.612 2.77e-14 ***
## siteousPrisma  -8964.20    1419.68  -6.314 2.75e-10 ***
## siteousSkyra   -6026.97    1200.89  -5.019 5.23e-07 ***
## siteUCAM      -10479.83    1162.79  -9.013  < 2e-16 ***
## siteUKB       -11575.50     846.22 -13.679  < 2e-16 ***
## siteUmU        -6117.45    1143.76  -5.349 8.92e-08 ***
## icv             6501.23      89.03  73.019  < 2e-16 ***
## ---
## Signif. codes:  0 ‘***’ 0.001 ‘**’ 0.01 ‘*’ 0.05 ‘.’ 0.1 ‘ ’ 1
##
## Approximate significance of smooth terms:
##               edf Ref.df        F p-value
## s(age_z)   7.967  7.967 1667.330  <2e-16 ***
## s(sleep_z) 2.648  2.648    4.306  0.0274 *
## ---
## Signif. codes:  0 ‘***’ 0.001 ‘**’ 0.01 ‘*’ 0.05 ‘.’ 0.1 ‘ ’ 1
##
## R-sq.(adj) =  0.384
## lmer.REML = 7.1363e+05  Scale est. = 1.9e+06   n = 33360
```

Below is the model output with main effect.

```
##
## Family: gaussian
## Link function: identity
##
## Formula:
## value ~ sex + site + s(age_z, k = 10, bs = "cr") + s(sleep_z,
##     k = 5, bs = "cr") + icv + depression
## <environment: 0x558ad797ced8>
##
## Parametric coefficients:
##                 Estimate Std. Error t value Pr(>|t|)
## (Intercept)    42156.19     858.47  49.106  < 2e-16 ***
## sexmale         1767.78     176.48  10.017  < 2e-16 ***
## siteousAvanto -10318.68    1332.64  -7.743 9.98e-15 ***
## siteousPrisma  -9140.39    1419.91  -6.437 1.23e-10 ***
## siteousSkyra   -6220.40    1201.48  -5.177 2.26e-07 ***
## siteUCAM      -10565.75    1162.74  -9.087  < 2e-16 ***
## siteUKB       -11452.13     846.59 -13.527  < 2e-16 ***
## siteUmU        -7104.05    1167.91  -6.083 1.19e-09 ***
## icv             6496.31      89.01  72.982  < 2e-16 ***
## depression      2635.56     634.78   4.152 3.31e-05 ***
## ---
## Signif. codes:  0 ‘***’ 0.001 ‘**’ 0.01 ‘*’ 0.05 ‘.’ 0.1 ‘ ’ 1
##
## Approximate significance of smooth terms:
##               edf Ref.df        F p-value
## s(age_z)   7.965  7.965 1661.722  <2e-16 ***
## s(sleep_z) 2.323  2.323    3.537  0.0444 *
```

```
## ---
## Signif. codes:  0 ‘***’ 0.001 ‘**’ 0.01 ‘*’ 0.05 ‘.’ 0.1 ‘ ’ 1
##
## R-sq.(adj) =  0.384
## lmer.REML = 7.1359e+05  Scale est. = 1.898e+06  n = 33360
```

Next is the model with depression-sleep interaction.

```
##
## Family: gaussian
## Link function: identity
##
## Formula:
## value ~ sex + site + s(age_z, k = 10, bs = "cr") + s(sleep_z,
##     k = 5, bs = "cr") + icv + depression + depression:sleep_z
## <environment: 0x558ad797ced8>
##
## Parametric coefficients:
##                    Estimate Std. Error t value Pr(>|t|)
## (Intercept)         42157.5      858.4  49.113  < 2e-16 ***
## sexmale              1777.0      176.5  10.068  < 2e-16 ***
## siteousAvanto      -10271.2     1332.6  -7.707 1.32e-14 ***
## siteousPrisma       -9093.1     1419.9  -6.404 1.53e-10 ***
## siteousSkyra        -6175.4     1201.5  -5.140 2.77e-07 ***
## siteUCAM           -10503.1     1162.8  -9.032  < 2e-16 ***
## siteUKB            -11457.4      846.5 -13.535  < 2e-16 ***
## siteUmU             -7529.6     1177.6  -6.394 1.64e-10 ***
## icv                  6495.2       89.0  72.979  < 2e-16 ***
## depression           2751.2      635.5   4.329 1.50e-05 ***
## depression:sleep_z   1293.8      466.6   2.773  0.00556 **
## ---
## Signif. codes:  0 ‘***’ 0.001 ‘**’ 0.01 ‘*’ 0.05 ‘.’ 0.1 ‘ ’ 1
##
## Approximate significance of smooth terms:
##              edf Ref.df       F p-value
## s(age_z)   7.964  7.964 1663.00  <2e-16 ***
## s(sleep_z) 2.183  2.183    1.82   0.195
## ---
## Signif. codes:  0 ‘***’ 0.001 ‘**’ 0.01 ‘*’ 0.05 ‘.’ 0.1 ‘ ’ 1
##
## R-sq.(adj) =  0.384
## lmer.REML = 7.1357e+05  Scale est. = 1.8979e+06  n = 33360
```

The plot below shows the sleep-volume curve for the original model and for the model with main effects of SES.

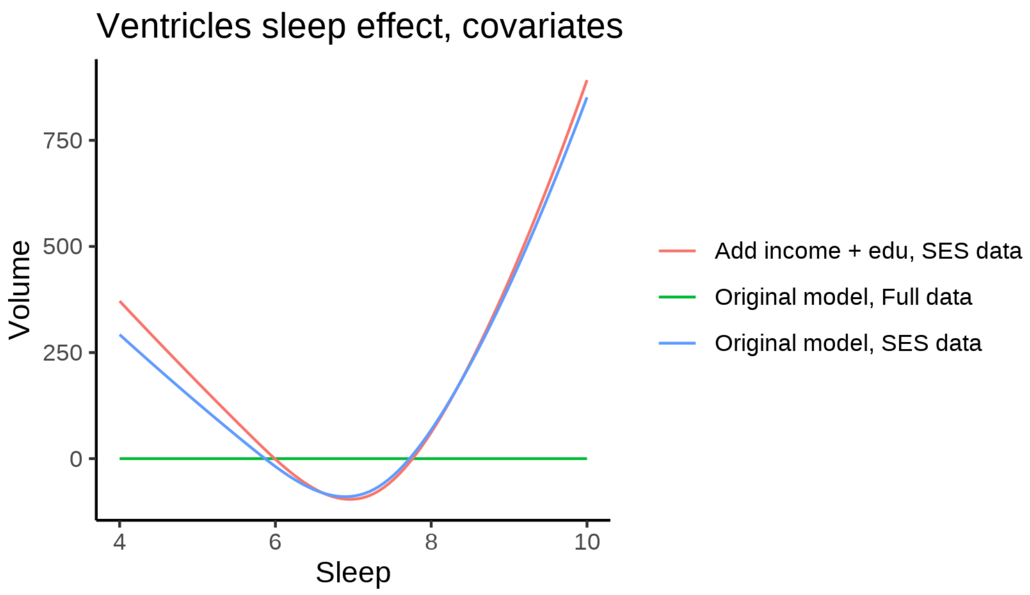

The plot below shows the sleep-volume curve for the original model and for the model with main effects of BMI.

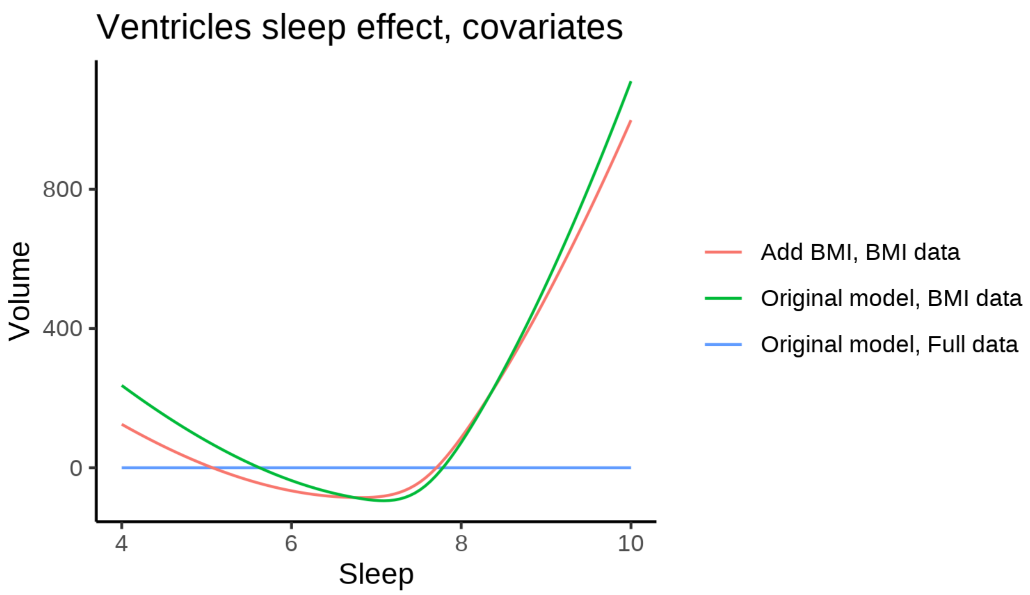

The plot below shows the sleep-volume curve for the original model and for the model with main effects of depression.

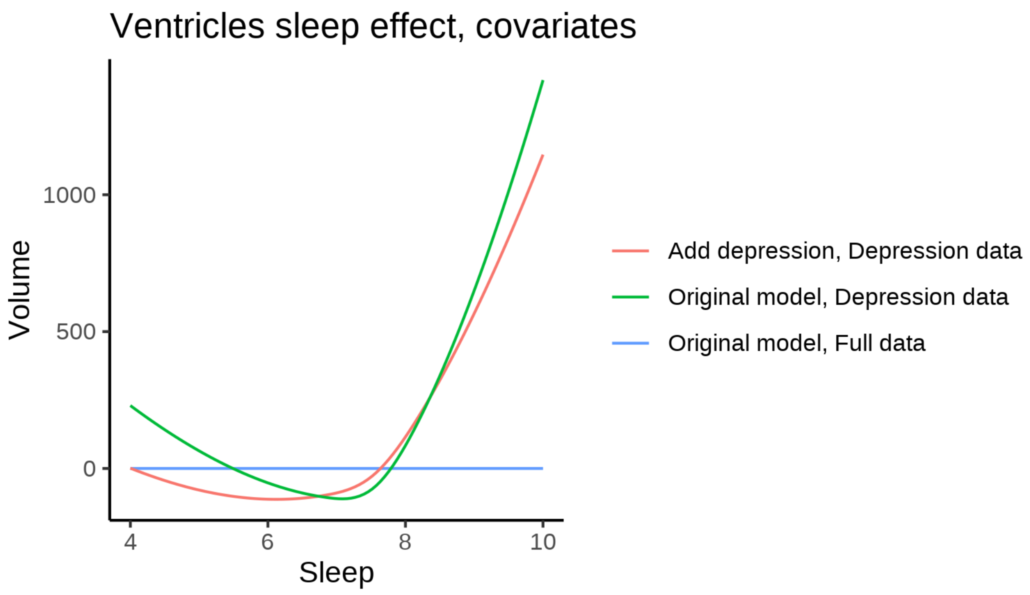

Models Not controling for ICV

Model outputs

Model without sleep term

```
##
## Family: gaussian
## Link function: identity
##
## Formula:
## value ~ sex + site + s(age_z, k = 10, bs = "cr")
## <environment: 0x55896133b770>
##
## Parametric coefficients:
##                Estimate Std. Error t value Pr(>|t|)
## (Intercept)    27453.18     632.21  43.424  < 2e-16 ***
## sexmale         8933.36     126.66  70.529  < 2e-16 ***
## siteMPIB        1594.29     899.98   1.771 0.076490 .
## siteousAvanto   3418.00     694.18   4.924 8.51e-07 ***
## siteousPrisma   3778.21    1015.66   3.720 0.000199 ***
## siteousSkyra    4700.19     692.62   6.786 1.16e-11 ***
## siteUB         -2587.40    2267.02  -1.141 0.253741
## siteUCAM         740.96     776.16   0.955 0.339757
## siteUKB            1.39     645.70   0.002 0.998282
## siteUmU         2844.61    1015.95   2.800 0.005113 **
## siteUOXF         721.38     812.72   0.888 0.374755
## ---
## Signif. codes:  0 '***' 0.001 '**' 0.01 '*' 0.05 '.' 0.1 ' ' 1
##
## Approximate significance of smooth terms:
##            edf Ref.df    F p-value
## s(age_z) 8.386  8.386 2067  <2e-16 ***
```

```
## ---
## Signif. codes:  0 ‘***’ 0.001 ‘**’ 0.01 ‘*’ 0.05 ‘.’ 0.1 ‘ ’ 1
##
## R-sq.(adj) =  0.286
## lmer.REML = 1.1052e+06  Scale est. = 2.0169e+06  n = 51268
```

Model with only main effects of age and sleep

```
##
## Family: gaussian
## Link function: identity
##
## Formula:
## value ~ sex + site + s(age_z, k = 10, bs = "cr") + s(sleep_z,
##     k = 5, bs = "cr")
## <environment: 0x55896133b770>
##
## Parametric coefficients:
##                Estimate Std. Error t value Pr(>|t|)
## (Intercept)     27582.5      632.6  43.601  < 2e-16 ***
## sexmale          8924.5      126.6  70.474  < 2e-16 ***
## siteMPIB         1466.0      900.1   1.629 0.103378
## siteousAvanto    3343.6      694.2   4.817 1.46e-06 ***
## siteousPrisma    3718.5     1015.6   3.661 0.000251 ***
## siteousSkyra     4628.9      692.6   6.684 2.36e-11 ***
## siteUB          -2590.8     2266.4  -1.143 0.252981
## siteUCAM          692.3      776.0   0.892 0.372313
## siteUKB          -129.8      646.1  -0.201 0.840833
## siteUmU          2513.2     1017.7   2.469 0.013537 *
## siteUOXF          684.3      812.6   0.842 0.399754
## ---
## Signif. codes:  0 ‘***’ 0.001 ‘**’ 0.01 ‘*’ 0.05 ‘.’ 0.1 ‘ ’ 1
##
## Approximate significance of smooth terms:
##              edf Ref.df       F  p-value
## s(age_z)   8.387  8.387 2054.44  < 2e-16 ***
## s(sleep_z) 1.000  1.000   26.33 2.75e-07 ***
## ---
## Signif. codes:  0 ‘***’ 0.001 ‘**’ 0.01 ‘*’ 0.05 ‘.’ 0.1 ‘ ’ 1
##
## R-sq.(adj) =  0.286
## lmer.REML = 1.1051e+06  Scale est. = 2.0179e+06  n = 51268
```

Model with full interaction between age and sleep

```
##
## Family: gaussian
## Link function: identity
##
## Formula:
## value ~ sex + site + t2(age_z, sleep_z, k = c(10, 4), bs = "cr")
## <environment: 0x55896133b770>
##
## Parametric coefficients:
##                Estimate Std. Error t value Pr(>|t|)
## (Intercept)     27664.5      636.6  43.458  < 2e-16 ***
```

```
## sexmale         8951.4      126.9  70.539  < 2e-16 ***
## siteMPIB        1337.8      904.4   1.479  0.13912
## siteousAvanto   3301.1      698.6   4.726  2.3e-06 ***
## siteousPrisma   3617.3     1017.9   3.554  0.00038 ***
## siteousSkyra    4565.0      697.2   6.548  5.9e-11 ***
## siteUB         -2759.1     2268.0  -1.217  0.22379
## siteUCAM         582.5      780.4   0.746  0.45545
## siteUKB         -225.9      650.2  -0.347  0.72826
## siteUmU         2430.8     1020.9   2.381  0.01727 *
## siteUOXF         540.4      816.6   0.662  0.50807
## ---
## Signif. codes:  0 ‘***’ 0.001 ‘**’ 0.01 ‘*’ 0.05 ‘.’ 0.1 ‘ ’ 1
##
## Approximate significance of smooth terms:
##                     edf Ref.df     F p-value
## t2(age_z,sleep_z) 15.86  15.86 87.92  <2e-16 ***
## ---
## Signif. codes:  0 ‘***’ 0.001 ‘**’ 0.01 ‘*’ 0.05 ‘.’ 0.1 ‘ ’ 1
##
## R-sq.(adj) =  0.286
## lmer.REML = 1.1051e+06  Scale est. = 2.0061e+06  n = 51268
```

Model comparison

`mod_no_sleep` refers to model without sleep term, `mod_no_interaction` refers to model with only main effect of sleep, and `mod_full` refers to model with a full interaction between age and sleep. This is a nested model comparison, and the p-value at a given line refers to comparing the model at the line to the model on the line above. Hence, significance implies that the more complicated model is supported on statistical grounds.

To be even more specific, the p-value on the second row tests whether there is an association between sleep and volume. The p-value on the third row tests whether this association depends on age.

```
## Data: NULL
## Models:
## ml$mod_no_sleep$mer: NULL
## ml$mod_no_interaction$mer: NULL
## ml$mod_full$mer: NULL
##                            npar     AIC     BIC  logLik deviance   Chisq Df Pr(>Chisq)
## ml$mod_no_sleep$mer          15 1105181 1105314 -552575  1105151
## ml$mod_no_interaction$mer    17 1105159 1105309 -552562  1105125 26.3185  2  1.928e-06 ***
## ml$mod_full$mer              19 1105154 1105322 -552558  1105116  8.9767  2    0.01124 *
## ---
## Signif. codes:  0 ‘***’ 0.001 ‘**’ 0.01 ‘*’ 0.05 ‘.’ 0.1 ‘ ’ 1
```

We chose the model based on the likelihood ratio test with 5 % significance level, which was `mod_full`.

Lifespan brain trajectory

The trajectory shown is from the chosen model `mod_full_no_icv`.

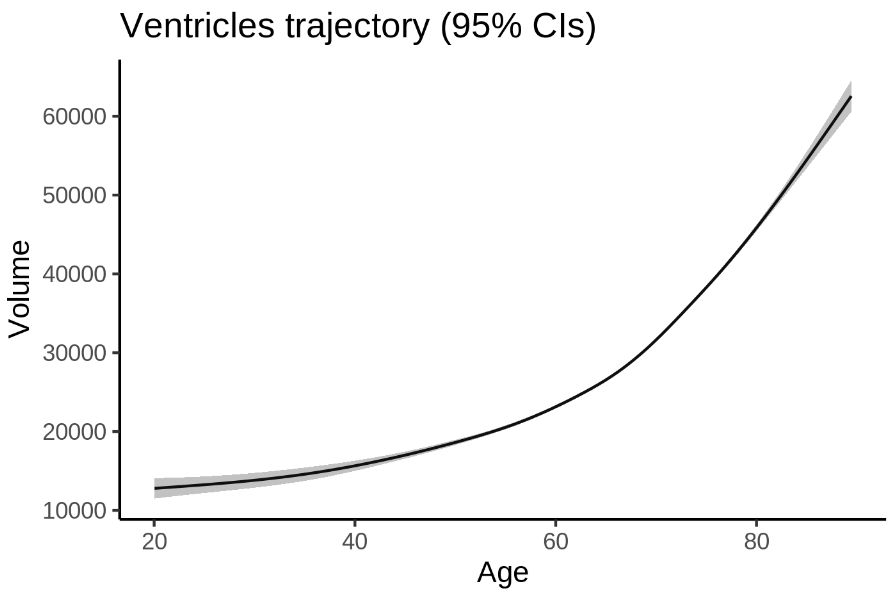

Effect of sleep

The chosen model included a full interaction between age and sleep, and the effect of sleep is hence plotted for a set of different ages. For comparability across ages, the sleep-volume curves at each age have been standardized so that they sum to zero.

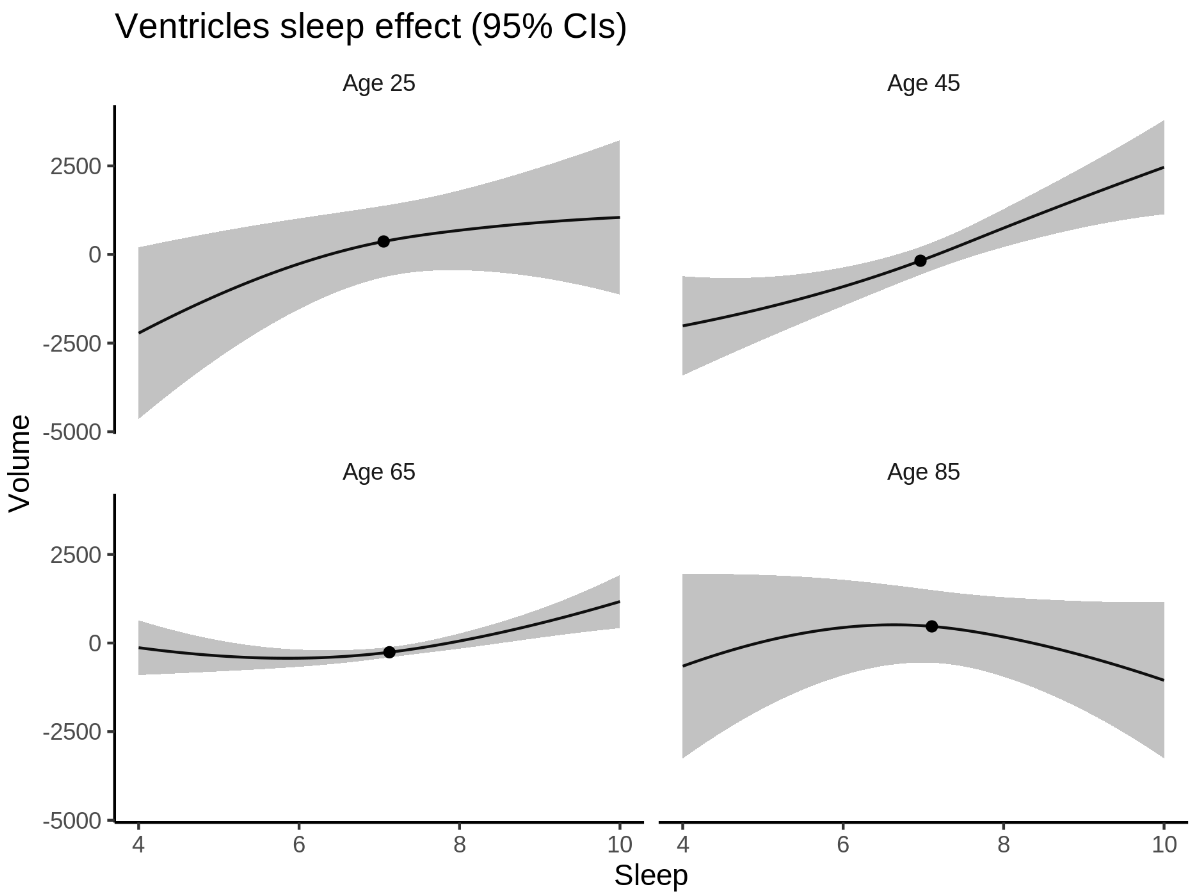

Deviation from sleep associated with maximal volume

The table show the sleep associated with maximum volume at chosen ages.

Age	Sleep at max vol
25	10.0
45	10.0
65	10.0
85	6.6

Model with sleep-age interaction was chosen, so we show it for four selected ages. The percentage values in the plot are calculated as follows: The maximum at 100 % refers to a person at the given age with a sleep duration associated with maximum volume. The other percentage values show how large the expected volume is for someone with other sleep durations. For example, 99 % implies a 1 % reduction.

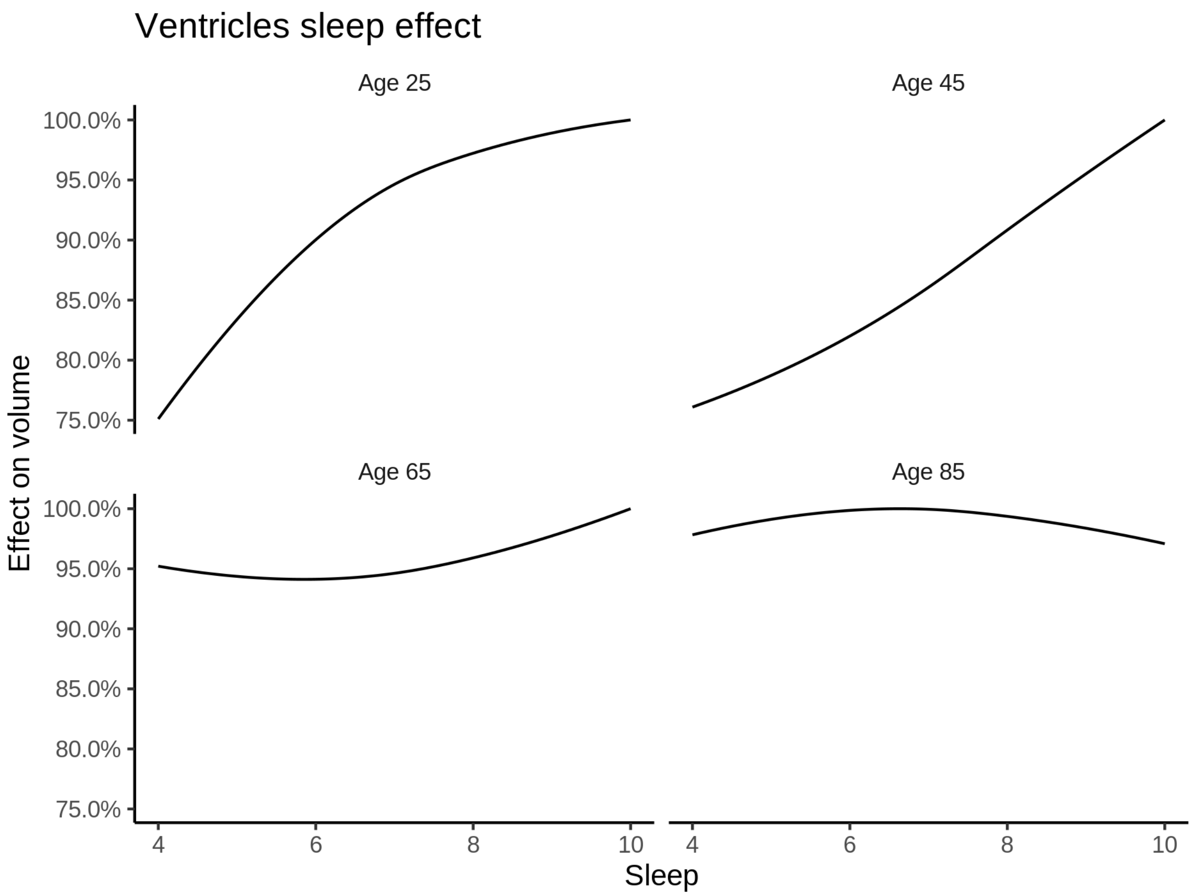

Comparison of mean sleep and sleep associated with maximum volume

The plot below compares average sleep to the sleep associated with maximum volume.

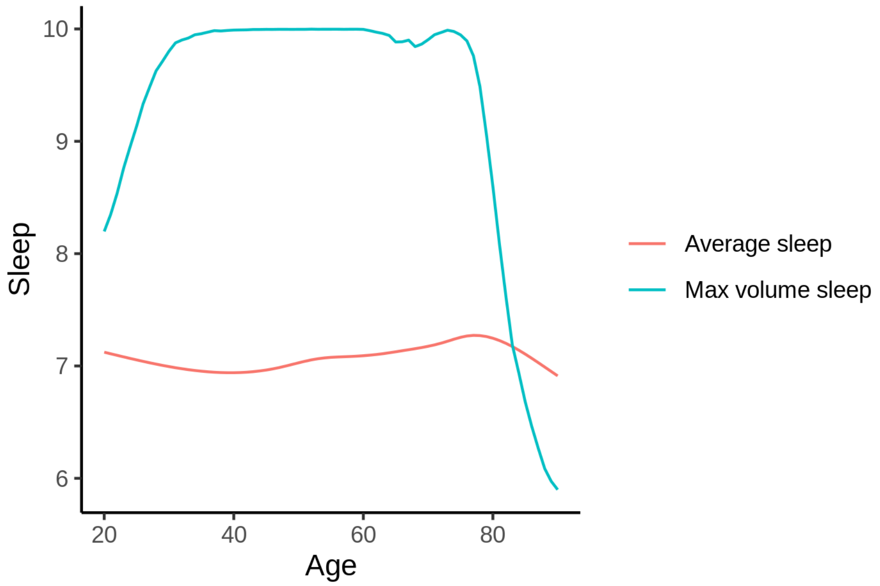

The next plot shows the difference between average sleep and sleep associated with maximum volume. The shaded region is a 95 % confidence interval.

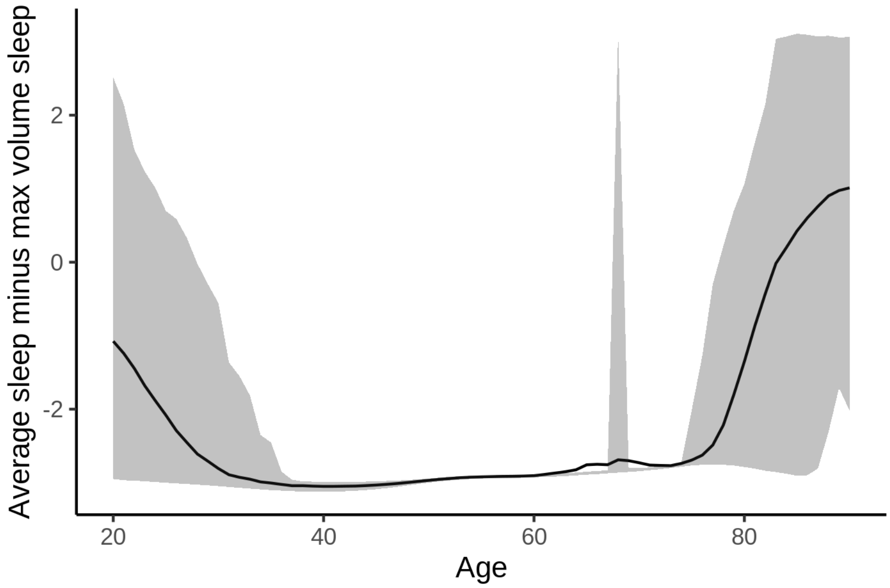

The next plot shows the probability that the sleep duration associated with maximum volume is longer than the average sleep duration, as a function of age. Probility below .05 can be interpreted as evidence that the sleep associated with maximum volume is shorter than the mean sleep, and probability above .95 can be interpreted the opposite way.

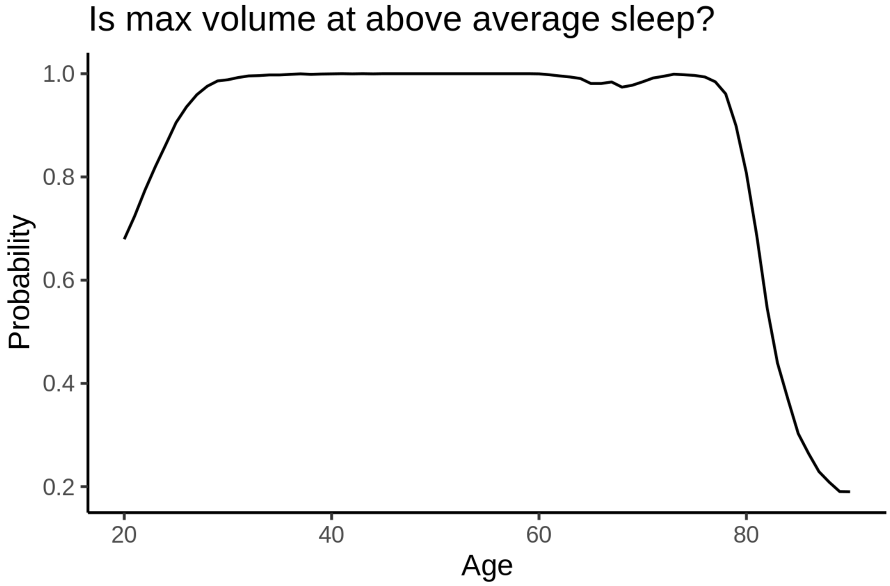

Summary

The plot below shows estimated sleep at maximum volume and 95 % confidence intervals for selected regions.

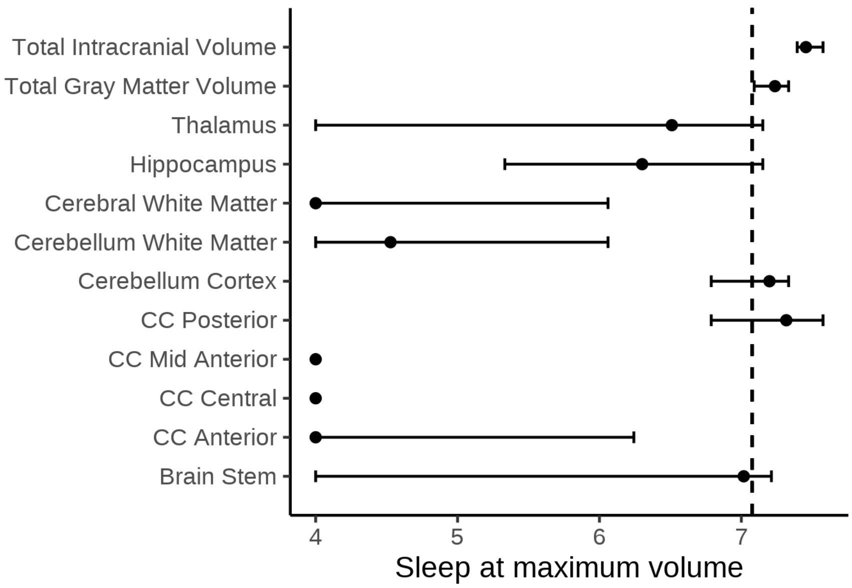

The next plot shows the effect of including sleep quality as covariates in the model.

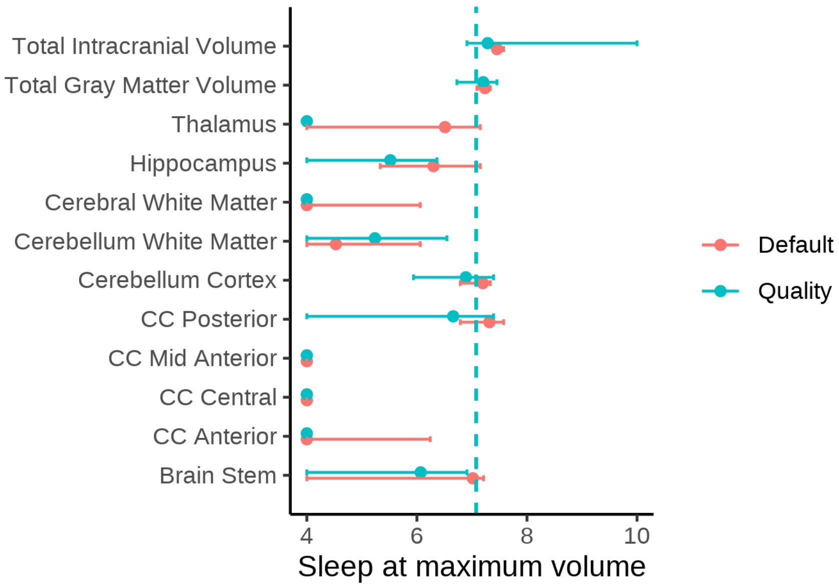

Multiple comparisons correction

Models controling for ICV

We performed correction for multiple comparisons for the main effect of sleep in each of the 19 subcortical regions considered above, using the Benjamini-Hochberg procedure.

The figure below shows a scatter plot of adjusted vs unadjusted p-values.

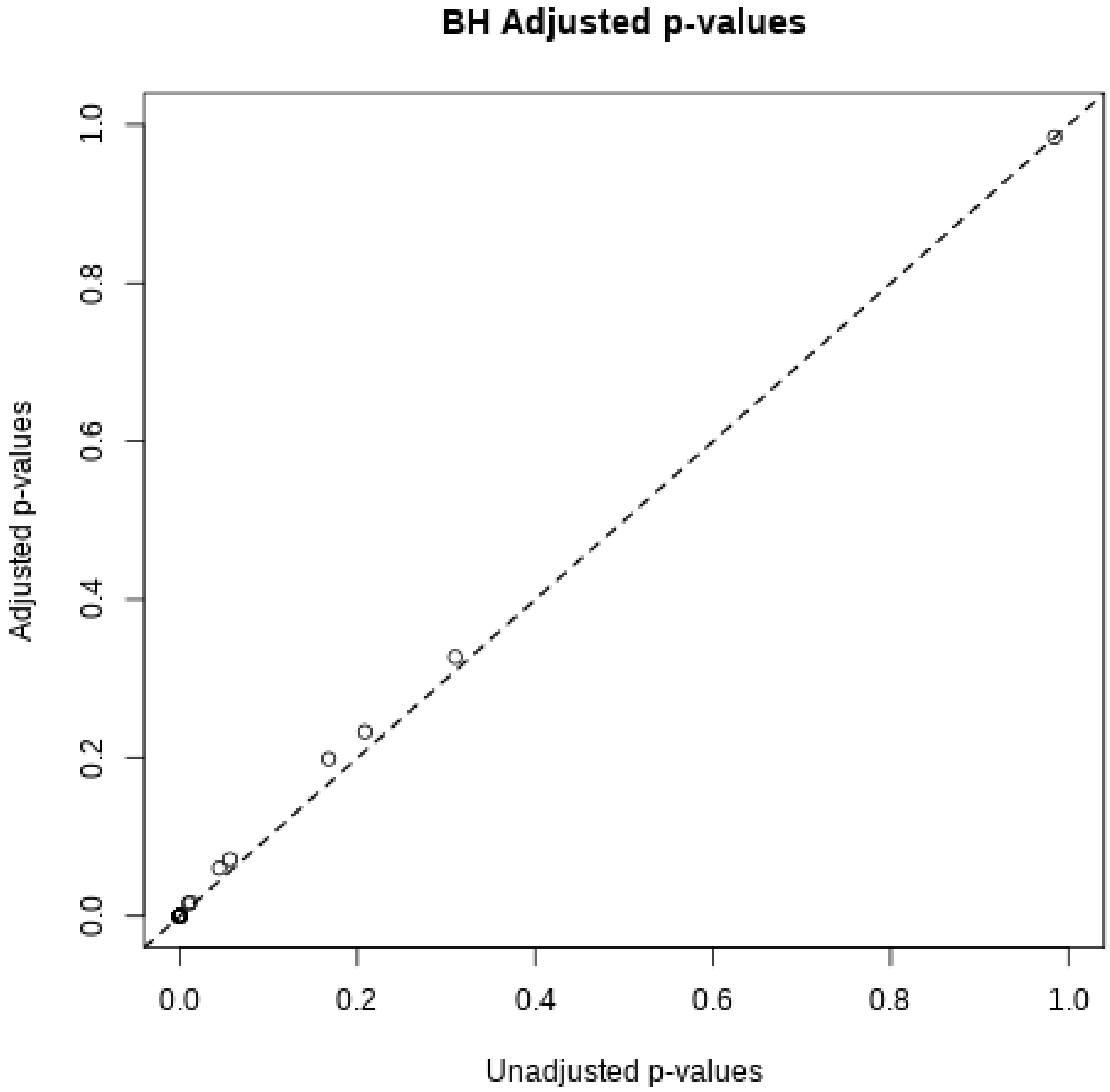

The table below shows the unadjusted and adjusted p-values for the main effect of sleep.

```
##
##
## |region                        | Unadjusted p-value| Adjusted p-value|
## |:-----------------------------|------------------:|----------------:|
## |Accumbens-area                |          0.9844851|        0.9844851|
## |Amygdala                      |          0.0566558|        0.0717640|
## |Brain-Stem                    |          0.0000002|        0.0000008|
## |Caudate                       |          0.0446225|        0.0605591|
## |CC_Anterior                   |          0.0100563|        0.0159226|
## |CC_Central                    |          0.0000362|        0.0000765|
## |CC_Mid_Anterior               |          0.0000024|        0.0000090|
## |CC_Mid_Posterior              |          0.2084835|        0.2330109|
## |CC_Posterior                  |          0.0119779|        0.0175062|
```

```
## |Cerebellum-Cortex              |          0.0000142|        0.0000336|
## |Cerebellum-White-Matter        |          0.0000446|        0.0000848|
## |CerebralWhiteMatterVol         |          0.0000045|        0.0000122|
## |EstimatedTotalIntraCranialVol  |          0.0000000|        0.0000000|
## |Hippocampus                    |          0.0000000|        0.0000000|
## |Pallidum                       |          0.3100803|        0.3273070|
## |Putamen                        |          0.1675098|        0.1989179|
## |Thalamus                       |          0.0000044|        0.0000122|
## |TotalGrayVol                   |          0.0000000|        0.0000000|
## |Ventricles                     |          0.0009262|        0.0015998|
```

Models not controling for ICV

We performed correction for multiple comparisons for the main effect of sleep in each of the 19 subcortical regions considered above, using the Benjamini-Hochberg procedure.

The figure below shows a scatter plot of adjusted vs unadjusted p-values.

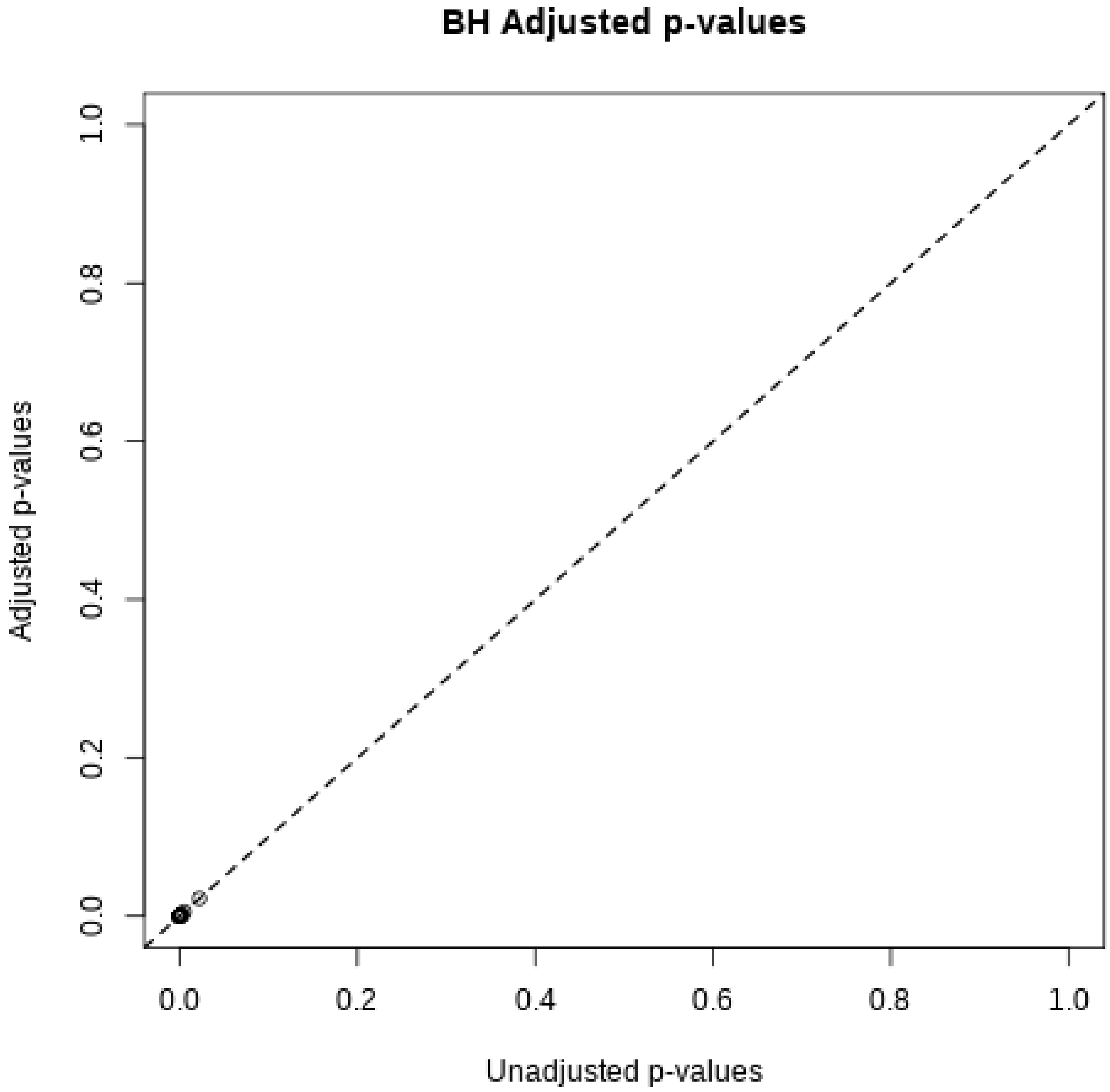

The table below shows the unadjusted and adjusted p-values for the main effect of sleep.

```
##
##
## |region                        | Unadjusted p-value| Adjusted p-value|
## |:-----------------------------|------------------:|----------------:|
## |Accumbens-area                |          0.0006969|        0.0009458|
## |Amygdala                      |          0.0007790|        0.0009868|
## |Brain-Stem                    |          0.0000000|        0.0000000|
## |Caudate                       |          0.0000010|        0.0000018|
## |CC_Anterior                   |          0.0000087|        0.0000128|
## |CC_Central                    |          0.0023553|        0.0026324|
## |CC_Mid_Anterior               |          0.0012699|        0.0015080|
## |CC_Mid_Posterior              |          0.0050213|        0.0053003|
## |CC_Posterior                  |          0.0000000|        0.0000000|
```

```
## |Cerebellum-Cortex            |          0.0000000|        0.0000000|
## |Cerebellum-White-Matter      |          0.0000019|        0.0000029|
## |CerebralWhiteMatterVol       |          0.0000000|        0.0000000|
## |EstimatedTotalIntraCranialVol |          0.0000000|        0.0000000|
## |Hippocampus                  |          0.0000000|        0.0000000|
## |Pallidum                     |          0.0000002|        0.0000003|
## |Putamen                      |          0.0222175|        0.0222175|
## |Thalamus                     |          0.0000000|        0.0000000|
## |TotalGrayVol                 |          0.0000000|        0.0000000|
## |Ventricles                   |          0.0000003|        0.0000005|
```

More details on covariates

Raw data

UKB

For the UKB, we used the income in US dollars. The distribution of this variable is shown in the histogram below.

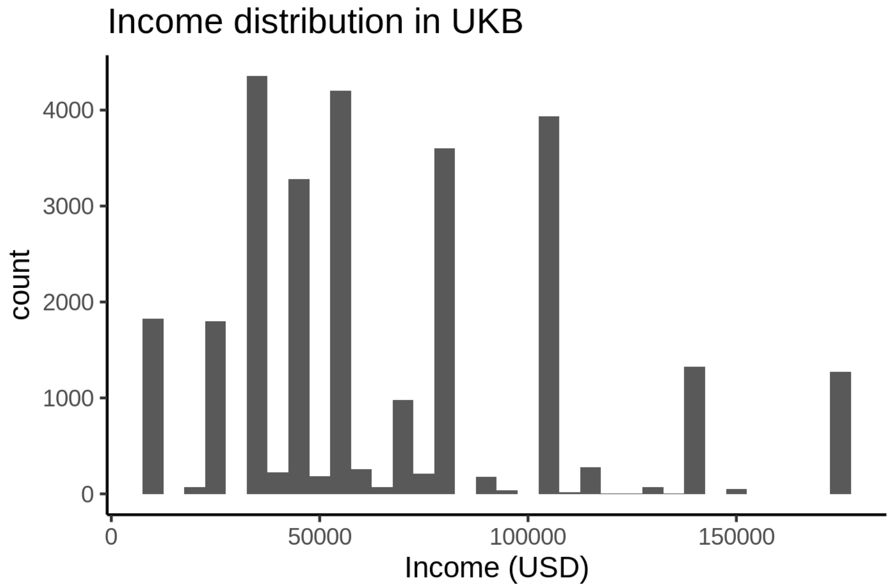

Education took four different categorical values in UKB, shown below.

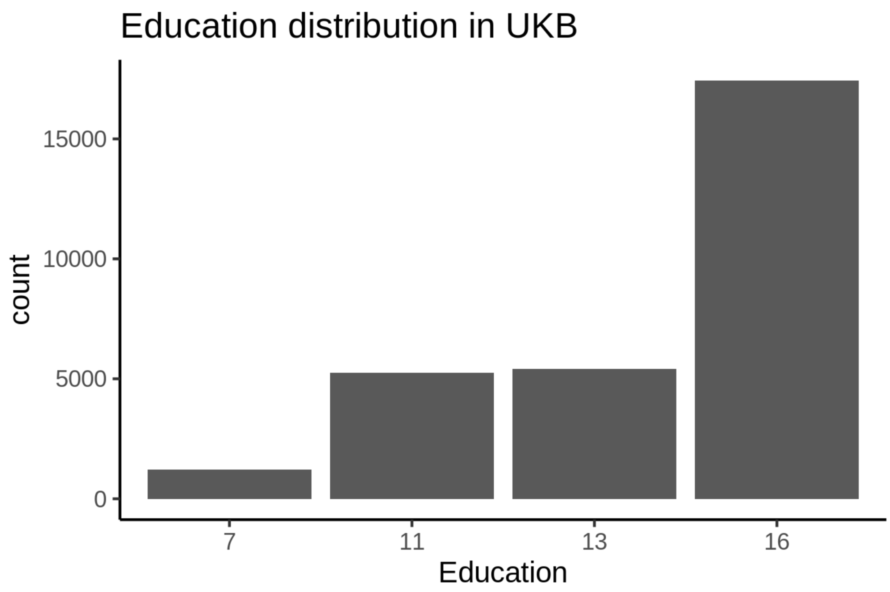

BMI distribution is shown below.

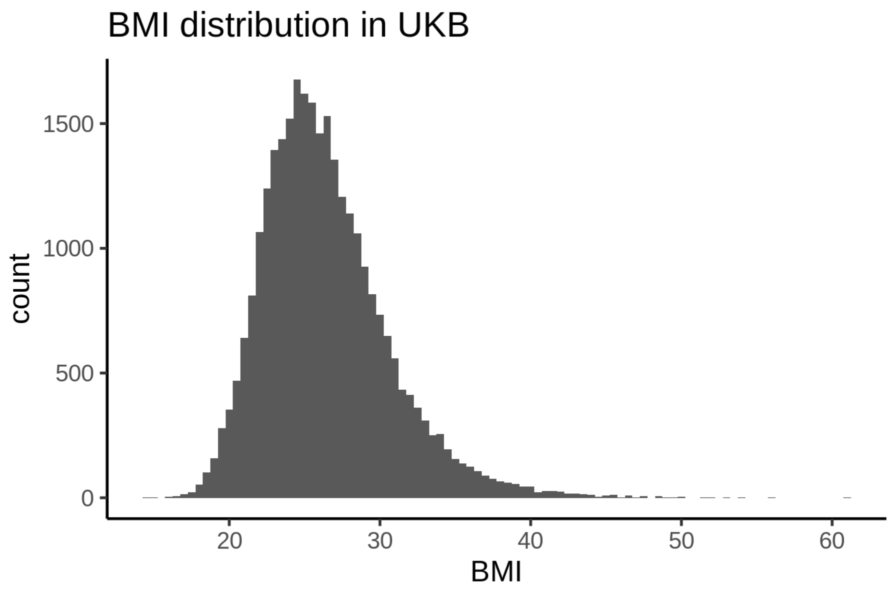

Depression distribution is shown below. This is based on the first principal component of depression covariates.

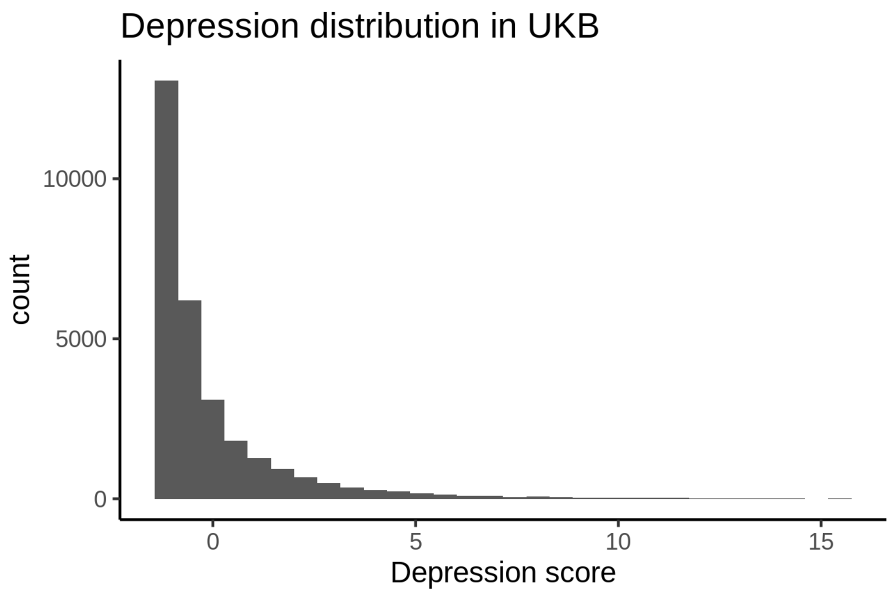

BASEII

Income is taken from the variable named “pnett,” which measures monthly income after tax.

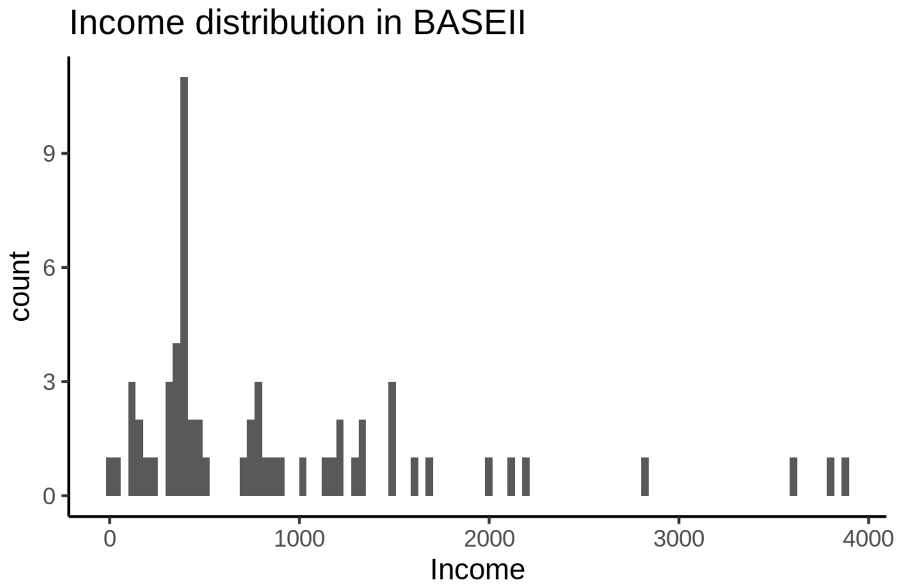

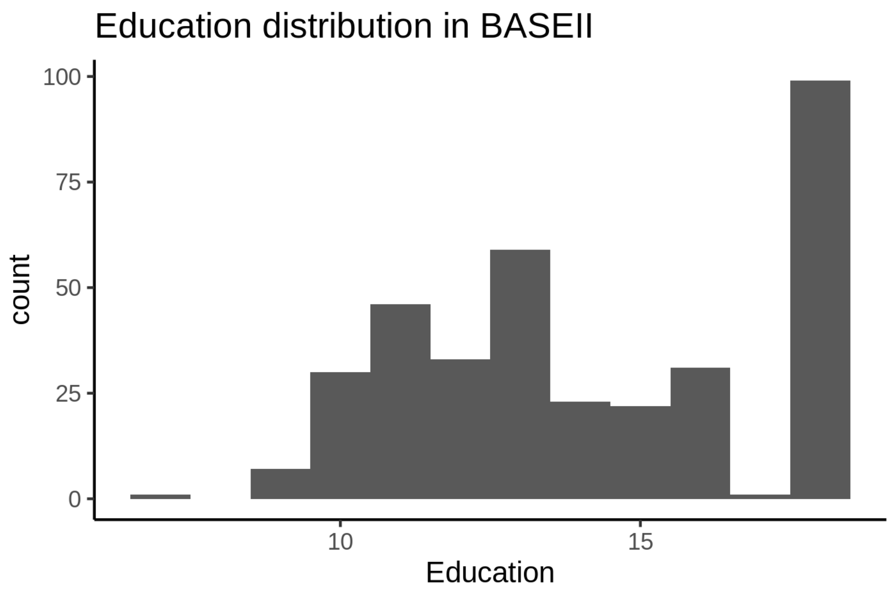

We did not have BMI information in BASEII. Depression scores are shown below.

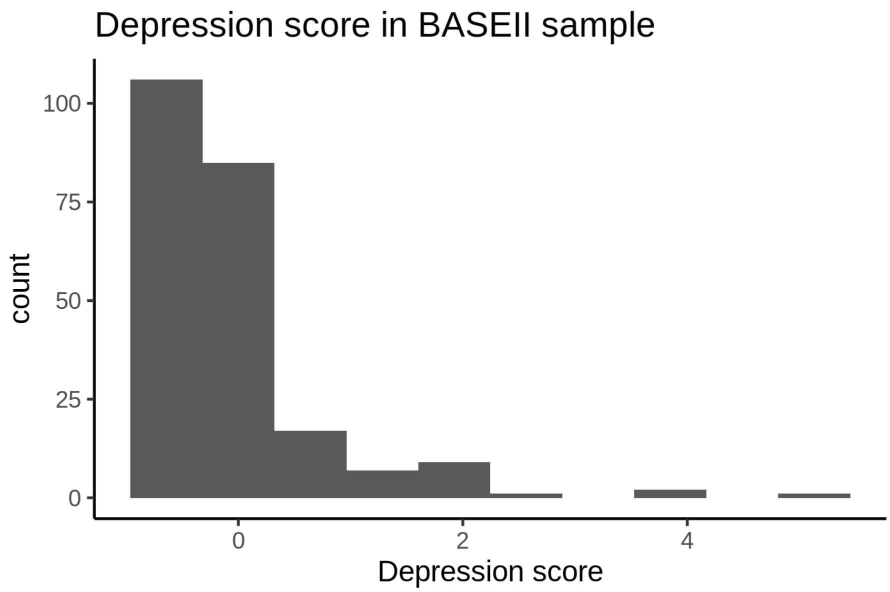

Betula

We did not have identifiable education and income variables for Betula, as they were anonymized. We did however have BMI and depression.

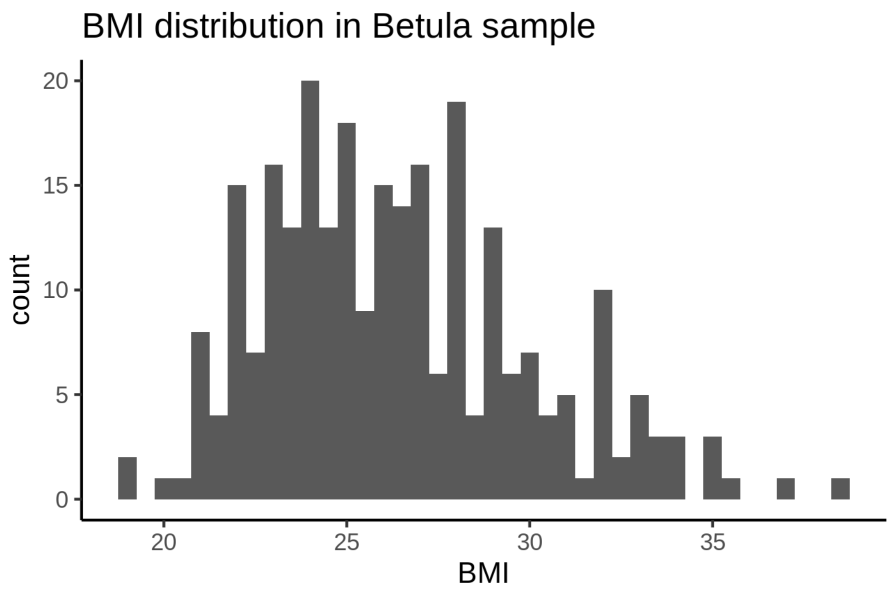

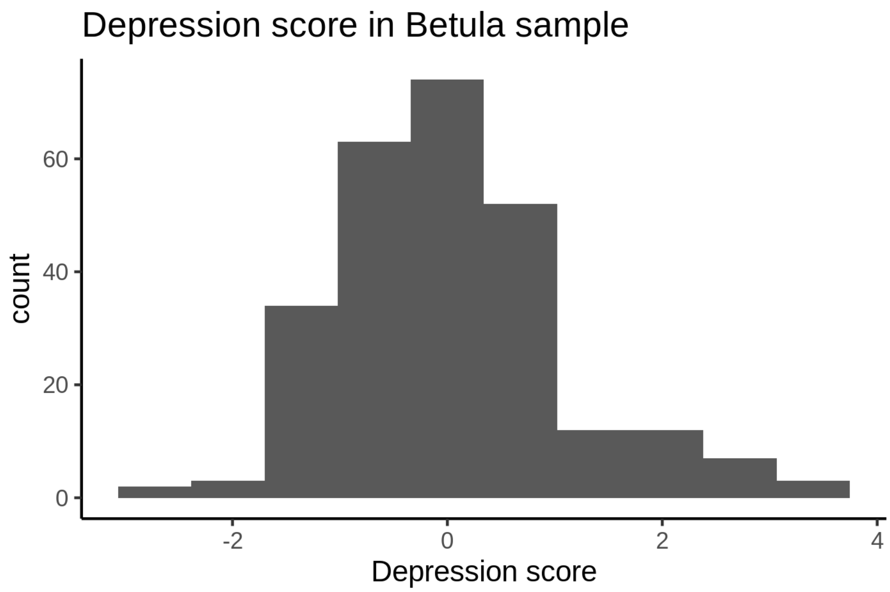

Cam-CAN

The income is household income.

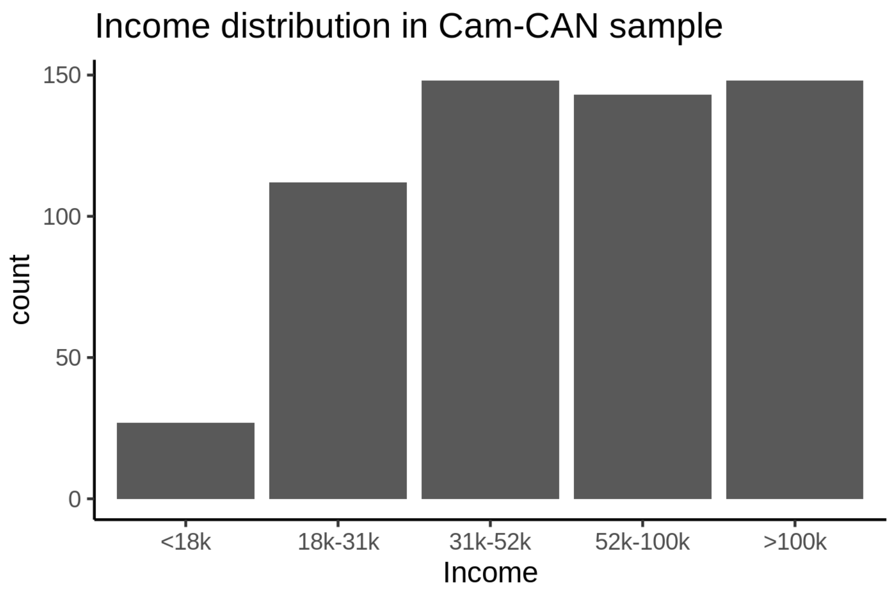

Age at completed education is shown below. The outlier with age above 50 should probably be removed.

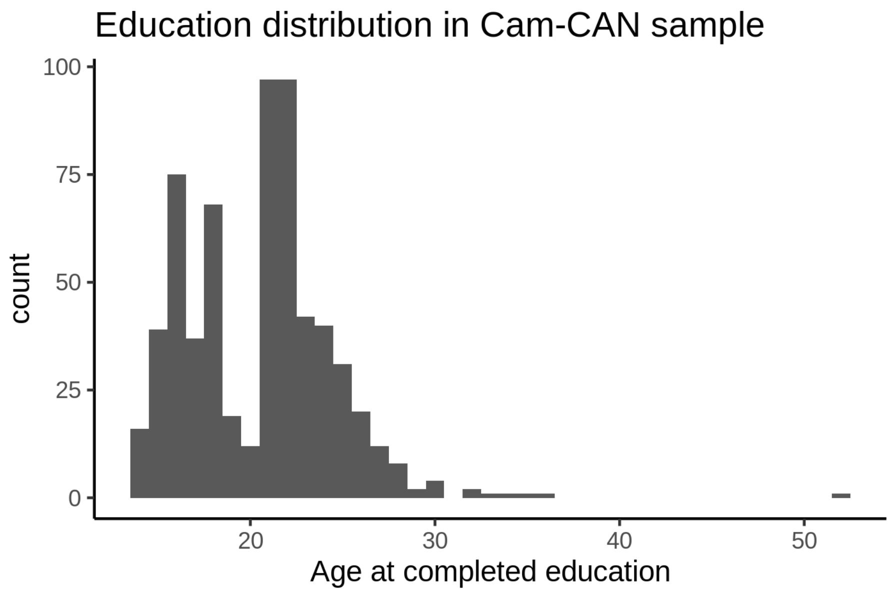

BMI distribution is shown below.

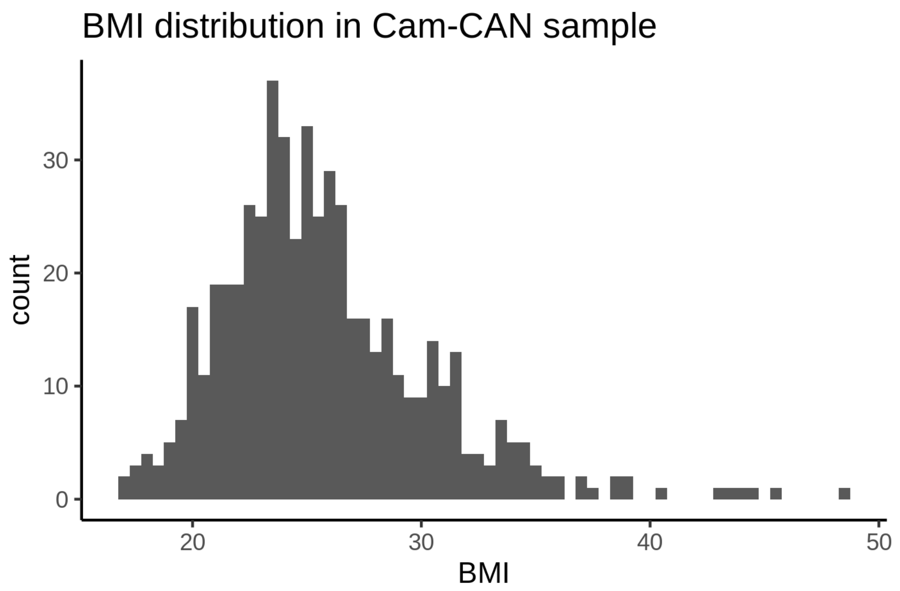
BMI distribution in Cam-CAN sample
count
30
20
10
0
20
30
40
50
BMI

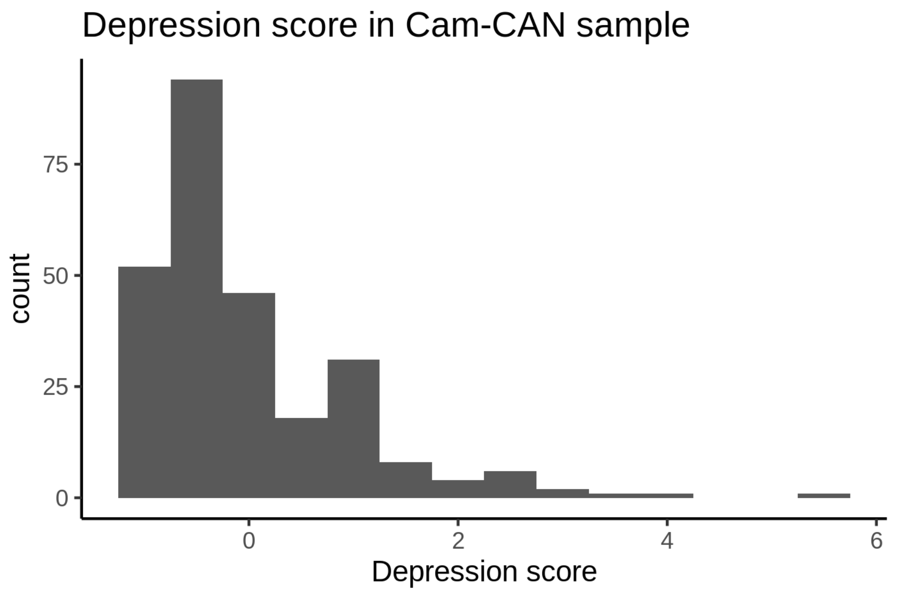
Depression score in Cam-CAN sample
count
75
50
25
0
0
2
4
6
Depression score

LCBC

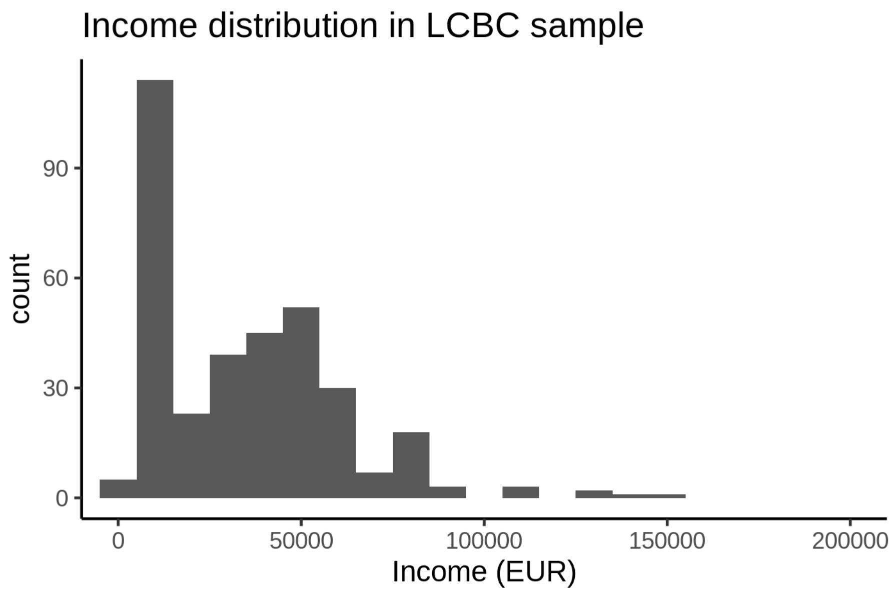
Income distribution in LCBC sample
count
90
60
30
0
0
50000
100000
150000
200000
Income (EUR)

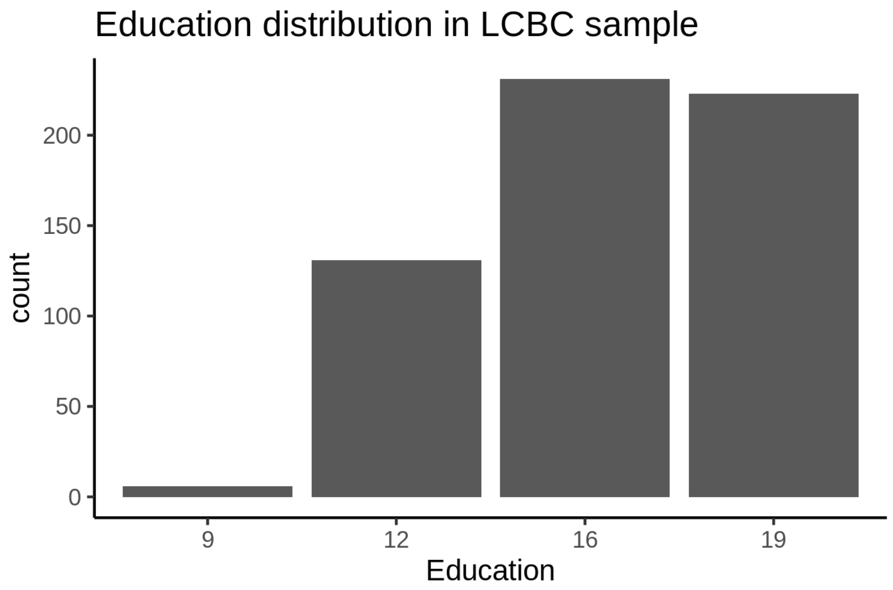
Education distribution in LCBC sample
count
200
150
100
50
0
9
12
16
19
Education

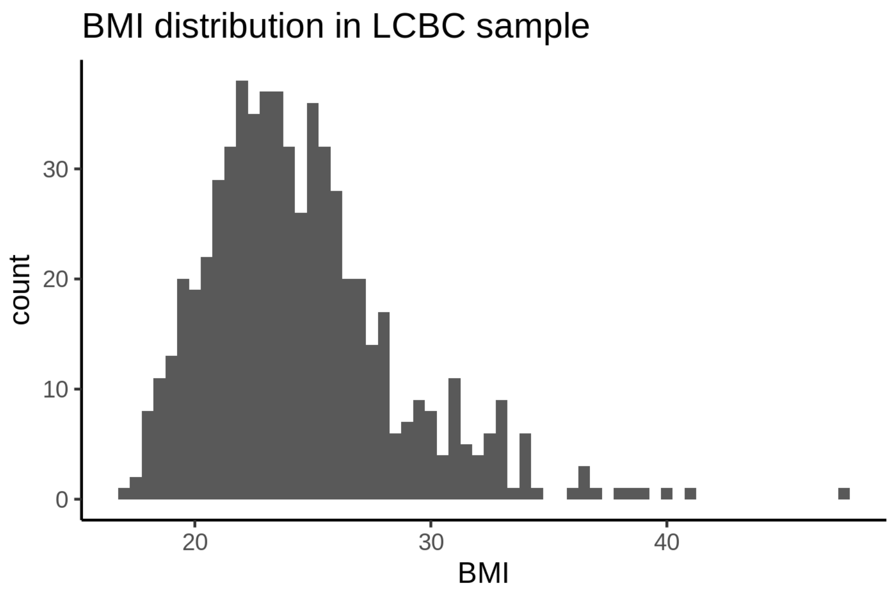

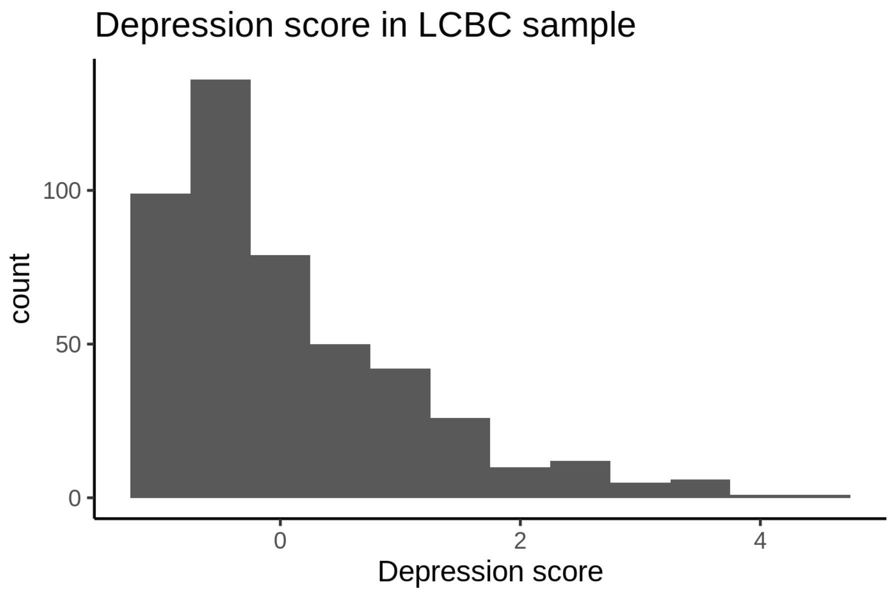

Oxford

Household income is on a ten-point categorical scale, cf. supplementary material to SES paper.

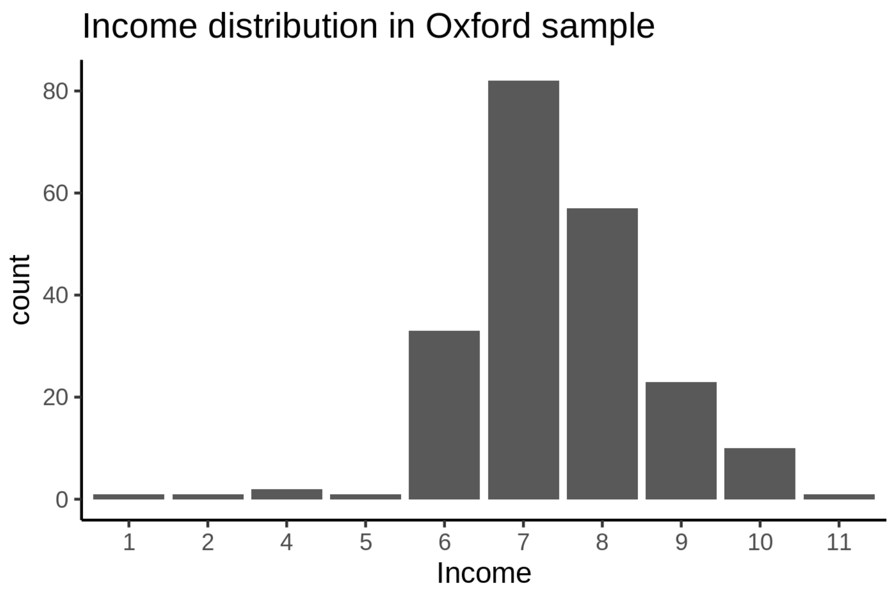

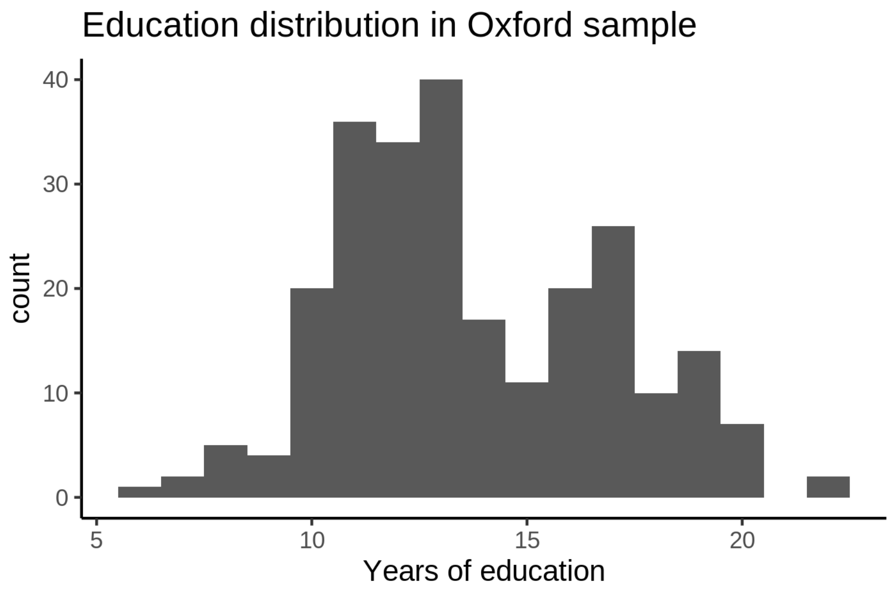

Data harmonization

Since the income and education variables at each site are not directly comparable, we had to convert them to something which can be compared across sites. This is described here.

Income

The plot below illustrates the data harmonization issue. The horizontal axis shows education levels mapped to the interval $[0, 1]$. While some sites have continuous income with very fat tail to the right (UiO, MPIB), others have predefined categories with very similar numbers in each. Since we are not able to split up the most coarse numbers into finer grained ones, we instead make the fine grained ones coarser.

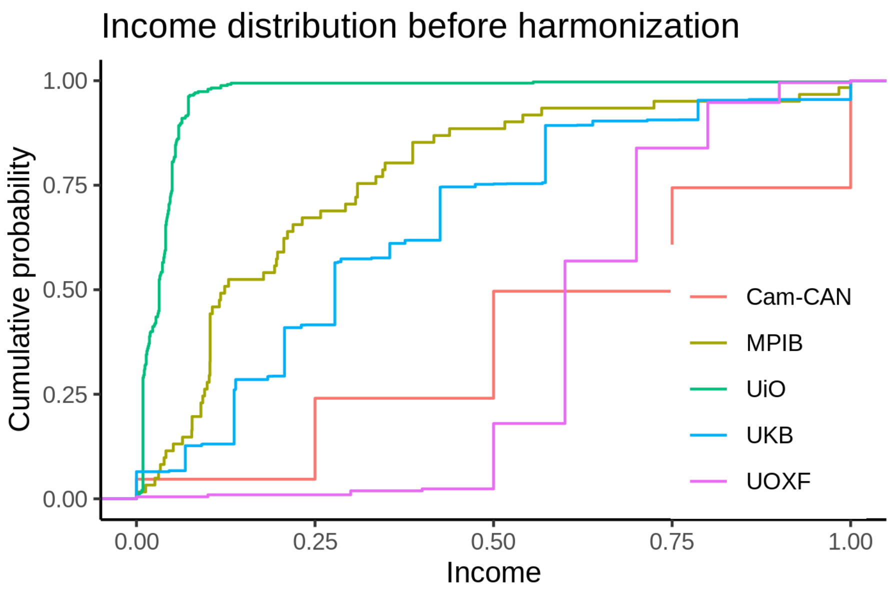

For each study separately, the income values were split into four equally sized bins, except for UOXF and Cam-CAN, for which we only had sufficient data to create three bins. The resulting distribution of values is shown in the plot below, and contains the income values used in subsequent analyses.

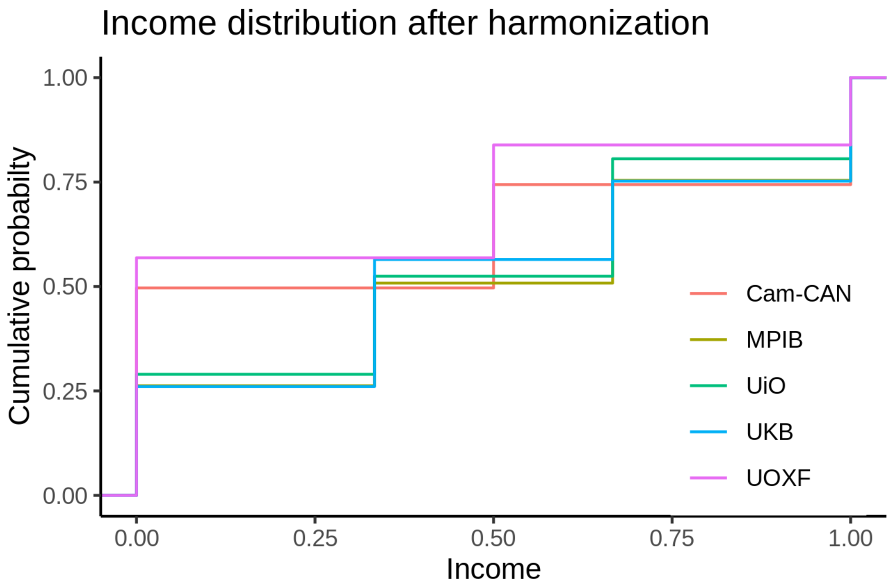

Education

A similar strategy was used to harmonize the education data. The plot below shows the original values.

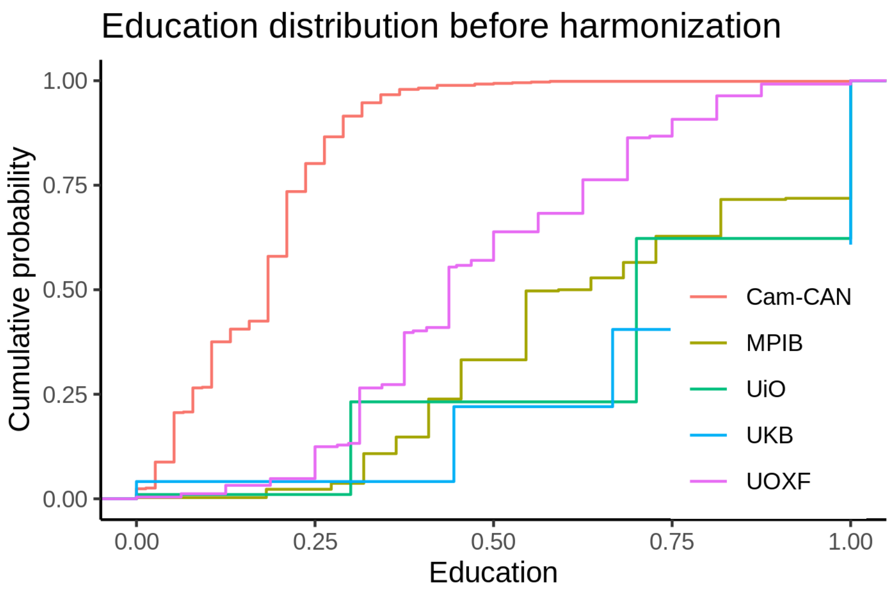

The next plot shows the distribution after harmonization. We are not able to make the distributions very similar, but at least it's an improvement over the raw values.

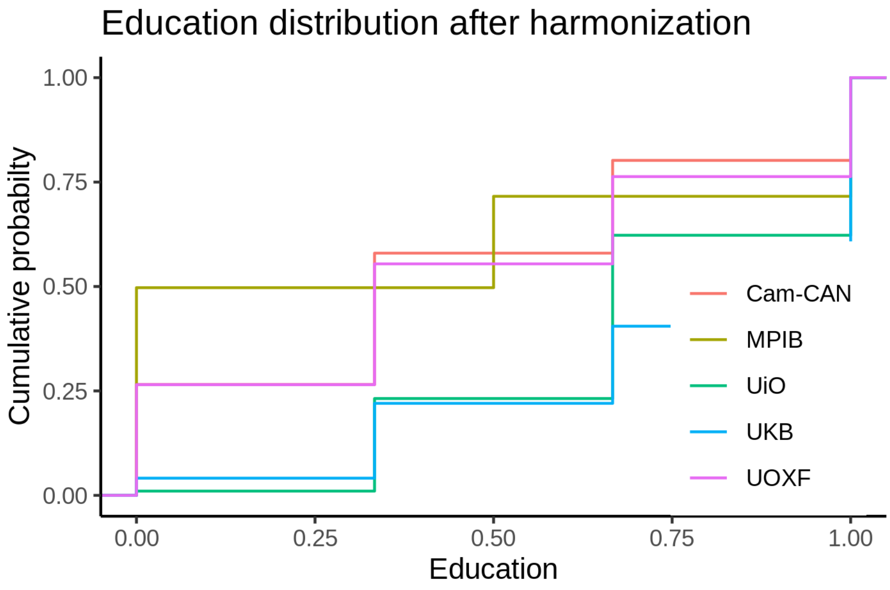

BMI

BMI is measured exactly the same way in each sample, so it represents the same thing. We hence use this variable as it is.

Depression

Distribution of depression covariates per site is shown below. The data are fairly harmonized.

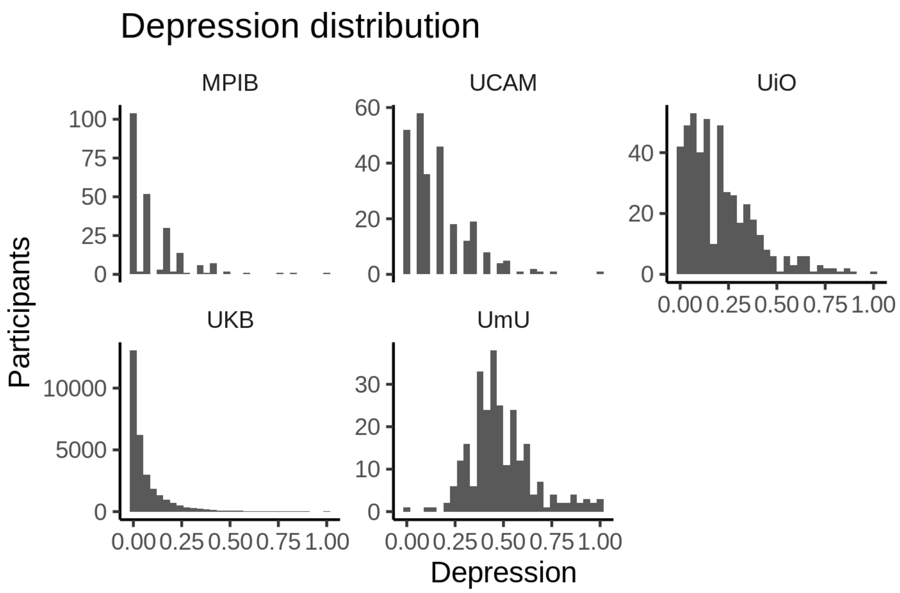

Relationship between covariates and sleep

Education and sleep

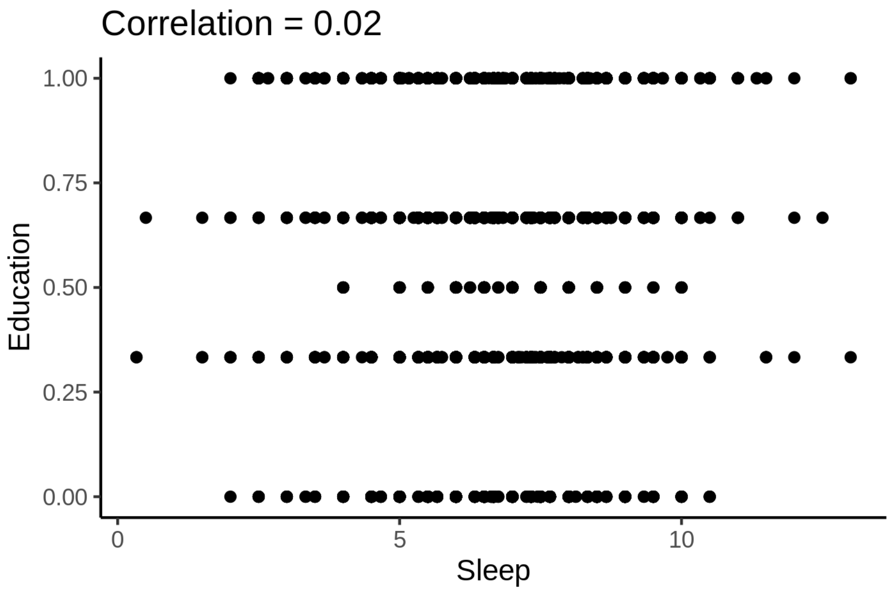

Income and sleep

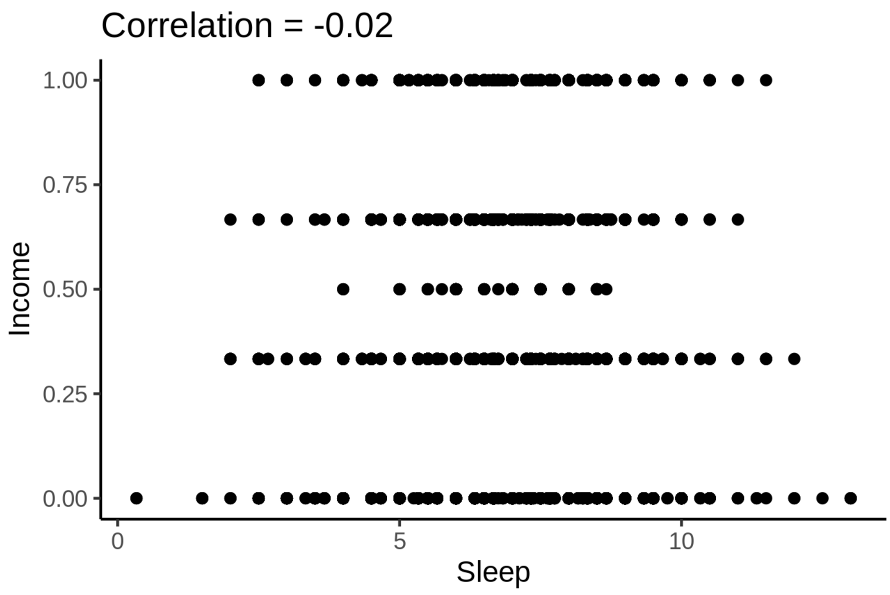

BMI and sleep

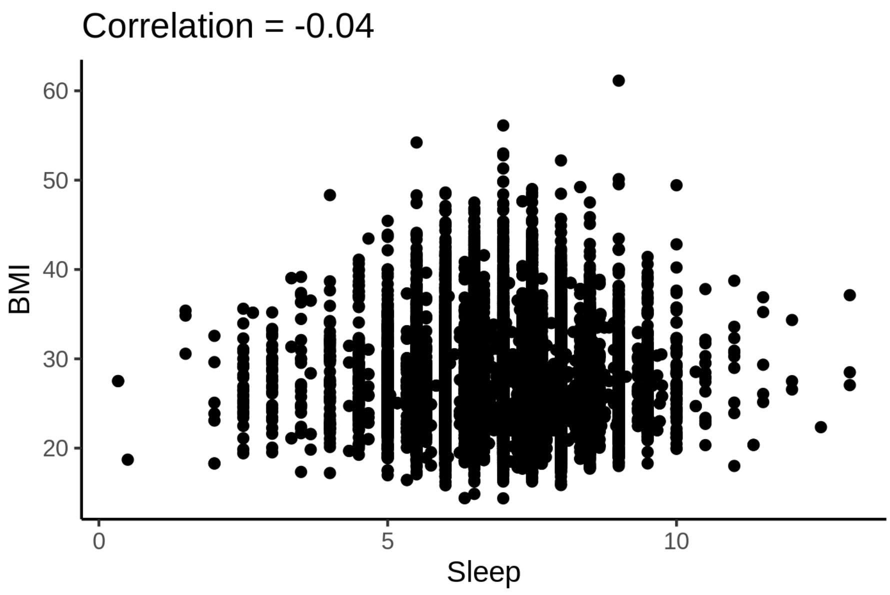

Depression and sleep

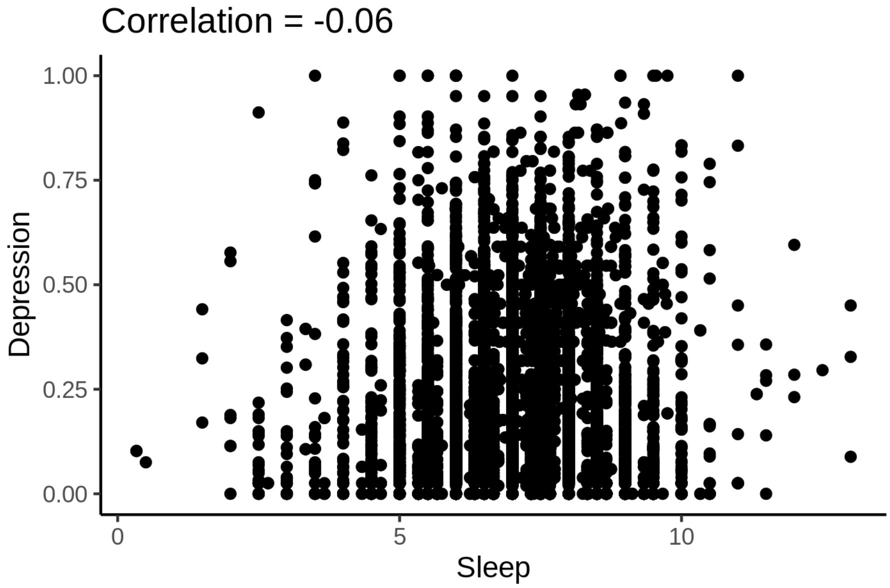

References

Benjamini, Yoav, and Yosef Hochberg. 1995. "Controlling the False Discovery Rate: A Practical and Powerful Approach to Multiple Testing." *Journal of the Royal Statistical Society: Series B (Methodological)* 57 (1): 289–300. https://doi.org/10.1111/j.2517-6161.1995.tb02031.x.

Henry, Lionel, and Hadley Wickham. 2020. *Purrr: Functional Programming Tools.* https://CRAN.R-project.org/package=purrr.

Hester, Jim. 2020. *Glue: Interpreted String Literals.* https://CRAN.R-project.org/package=glue.

R Core Team. 2020. *R: A Language and Environment for Statistical Computing.* Vienna, Austria: R Foundation for Statistical Computing. https://www.R-project.org/.

Ruppert, David, M. P. Wand, and R. J. Carroll. 2003. *Semiparametric Regression.* Cambridge University Press, Cambridge, U.K.

Sørensen, Øystein, Kristine B. Walhovd, and Anders M. Fjell. 2021. "A Recipe for Accurate Estimation of Lifespan Brain Trajectories, Distinguishing Longitudinal and Cohort Effects." *NeuroImage* 226 (February): 117596. https://doi.org/10.1016/j.neuroimage.2020.117596.

Wickham, Hadley. 2016. *Ggplot2: Elegant Graphics for Data Analysis.* Springer-Verlag New York. https://ggplot2.tidyverse.org.

———. 2019. *Stringr: Simple, Consistent Wrappers for Common String Operations.* https://CRAN.R-project.org/package=stringr.

———. 2021. *Tidyr: Tidy Messy Data.* https://CRAN.R-project.org/package=tidyr.

Wickham, Hadley, Mara Averick, Jennifer Bryan, Winston Chang, Lucy D'Agostino McGowan, Romain François, Garrett Grolemund, et al. 2019. "Welcome to the tidyverse." *Journal of Open Source Software* 4 (43): 1686. https://doi.org/10.21105/joss.01686.

Wickham, Hadley, Romain François, Lionel Henry, and Kirill Müller. 2021. *Dplyr: A Grammar of Data Manipulation.* https://CRAN.R-project.org/package=dplyr.

Wood, S. N. 2017. *Generalized Additive Models: An Introduction with R*. Second. Chapman and Hall/CRC.

Wood, Simon, and Fabian Scheipl. 2020. *Gamm4: Generalized Additive Mixed Models Using 'Mgcv' and 'Lme4'*. https://CRAN.R-project.org/package=gamm4.

Meta analysis

The analyses to be described were conducted in R version 4.1.0 (R Core Team 2020). We used the `metafor` package (Viechtbauer 2010) for the meta analysis and `tidyverse` (Wickham et al. 2019) for data manipulation and visualization.

```
library(tidyverse)
library(metafor)
```

Controling for ICV

We start the meta analysis using the models for subcortical volumes which controled for intracranial volume.

We performed a meta-analytic fit in order to find the average across both cortical and subcortical regions of the sleep associated with maximum cortical thickness and subcortical volume. Corpos callosum structures were excluded because with one exception their estimated maximum volume sleep were fixed at the lower boundary of four hours. Including them would hence lead to division by zero in the meta analysis. This can also be seen in the supplementary material for subcortical volumes. We also excluded total gray matter volume and intracranial volume, and weighted the three cortical clusters and the seven subcortical regions such that cortex and subcortex contributed equally. Random effects meta analysis was used, since we did not expect each region to have the same sleep duration associated with maximum thickness/volume.

To be able to compare estimates across all regions, we used the model with a smooth term for sleep and no age interaction for all subcortical regions. In the supplementary material for subcortical volumes, this model specification is titled `mod_no_interaction`. For the ventricles, we searched for the number of hours of sleep corresponding to minimal volume, while for all other regions we search for the sleep duration that maximized the volumes. The posterior means (estimates) and standard errors were computed by sampling 5000 Monte Carlo samples from the empirical Bayes posterior distribution of the model for each region, constraining the number of hours of sleep to be between 4 and 12.

The complete data are shown below, where Cluster 1, 2, and 3 refer to the cortical clusters.

Region	Posterior mean	Standard error	ci_lower	ci_upper	Weight
Accumbens-area	7.121	2.997	4.000	10.000	0.083
Amygdala	4.144	0.918	4.000	4.000	0.083
Brain-Stem	6.253	1.227	4.000	7.225	0.083
Caudate	9.856	0.918	10.000	10.000	0.083
Cerebellum-Cortex	7.153	0.254	6.750	7.350	0.083
Cerebellum-White-Matter	4.729	0.781	4.000	6.275	0.083
CerebralWhiteMatterVol	4.578	0.777	4.000	6.325	0.083
Hippocampus	6.260	0.533	4.699	7.050	0.083
Pallidum	9.042	2.197	4.000	10.000	0.083
Putamen	4.474	1.619	4.000	10.000	0.083
Thalamus	5.955	1.135	4.000	7.200	0.083
Ventricles	5.582	1.267	4.000	7.275	0.083
Cluster 1	6.440	0.355	5.720	7.140	0.333
Cluster 2	6.740	0.190	6.320	7.080	0.333
Cluster 3	7.020	0.205	6.400	7.220	0.333

The following call was used to fit the model.

```
meta_mod <- rma(yi = estimate, sei = standard_error, weights = weight,
                method = "REML", data = meta_dat)
```

And next is the model summary. The estimate refers to the average of the sleep associated with maximum subcortical volume and cortical thickness. The p-value is with respect to a null hypothesis that zero hours of sleep is optimal, and is hence not relevant. The confidence intervals, on the other hand, can be interpreted as usual.

```
summary(meta_mod)
```

```
##
## Random-Effects Model (k = 15; tau^2 estimator: REML)
##
##   logLik  deviance       AIC       BIC      AICc
## -26.3276   52.6552   56.6552   57.9333   57.7461
##
## tau^2 (estimated amount of total heterogeneity): 1.2872 (SE = 0.7348)
## tau (square root of estimated tau^2 value):      1.1346
## I^2 (total heterogeneity / total variability):   86.17%
## H^2 (total variability / sampling variability):  7.23
##
## Test for Heterogeneity:
## Q(df = 14) = 44.4388, p-val < .0001
##
## Model Results:
##
## estimate      se     zval    pval   ci.lb   ci.ub
##   6.4978  0.4261  15.2492  <.0001  5.6627  7.3330  ***
##
## ---
## Signif. codes:  0 '***' 0.001 '**' 0.01 '*' 0.05 '.' 0.1 ' ' 1
```

Additional sensitivity check

Since we use a random effects meta analysis it is also possible to include the corpus callosum structures, despite them having zero standard errors. We show this below, as a check of the robustness of the conclusions.

We weighted the corpus callosum structures such that they in total contributed as much as a single other subcortical region. The complete data are shown below.

```
meta_dat %>%
  rename(
    Region = region,
    Estimate = estimate,
    `Standard error` = standard_error,
    Weight = weight
  ) %>%
  select(-weight0) %>%
  knitr::kable(digits = 3)
```

Region	Estimate	Standard error	ci_lower	ci_upper	Weight
Accumbens-area	7.121	2.997	4.000	10.000	0.059
Amygdala	4.144	0.918	4.000	4.000	0.059

Region	Estimate	Standard error	ci_lower	ci_upper	Weight
Brain-Stem	6.253	1.227	4.000	7.225	0.059
Caudate	9.856	0.918	10.000	10.000	0.059
CC_Anterior	4.547	0.815	4.000	6.451	0.012
CC_Central	4.000	0.000	4.000	4.000	0.012
CC_Mid_Anterior	4.000	0.000	4.000	4.000	0.012
CC_Mid_Posterior	5.690	1.443	4.000	8.026	0.012
CC_Posterior	7.267	0.461	6.650	7.550	0.012
Cerebellum-Cortex	7.153	0.254	6.750	7.350	0.059
Cerebellum-White-Matter	4.729	0.781	4.000	6.275	0.059
CerebralWhiteMatterVol	4.578	0.777	4.000	6.325	0.059
Hippocampus	6.260	0.533	4.699	7.050	0.059
Pallidum	9.042	2.197	4.000	10.000	0.059
Putamen	4.474	1.619	4.000	10.000	0.059
Thalamus	5.955	1.135	4.000	7.200	0.059
Ventricles	5.582	1.267	4.000	7.275	0.059
Cluster 1	6.440	0.355	5.720	7.140	0.333
Cluster 2	6.740	0.190	6.320	7.080	0.333
Cluster 3	7.020	0.205	6.400	7.220	0.333

Next we fit the model. Note the warnings, which are expected.

```
meta_mod <- rma(yi = estimate, sei = standard_error, weights = weight,
                method = "REML", data = meta_dat)
```

```
## Warning: There are outcomes with non-positive sampling variances.

## Warning: Cannot compute Q-test, I^2, or H^2 when there are non-positive sampling
## variances in the data.
```

Here is the summary, which shows estimates close to what we got when excluding corpus callosum.

```
summary(meta_mod)
```

```
##
## Random-Effects Model (k = 20; tau^2 estimator: REML)
##
##   logLik  deviance       AIC       BIC      AICc
## -37.5276   75.0552   79.0552   80.9441   79.8052
##
## tau^2 (estimated amount of total heterogeneity): 1.8823 (SE = 0.8144)
## tau (square root of estimated tau^2 value):      1.3720
##
## Model Results:
##
## estimate      se     zval    pval   ci.lb   ci.ub
##   6.4905  0.5110  12.7004  <.0001  5.4889  7.4921  ***
##
## ---
## Signif. codes:  0 '***' 0.001 '**' 0.01 '*' 0.05 '.' 0.1 ' ' 1
```

Not Controling for ICV

We next use the subcortical models which did not contain ICV. In this case only the ventricles had zero standard error, but to make the results comparable to above, we performed one analysis excluding the corpos callosum and one analysis including corpos callosum.

The complete data are shown below.

Region	Estimate	Standard error	ci_lower	ci_upper	Weight
Accumbens-area	7.597	0.527	6.999	10.000	0.083
Amygdala	7.359	0.620	6.725	10.000	0.083
Brain-Stem	7.265	0.051	7.150	7.350	0.083
Caudate	9.467	0.783	7.650	10.000	0.083
Cerebellum-Cortex	7.313	0.049	7.200	7.400	0.083
Cerebellum-White-Matter	7.114	0.167	6.700	7.325	0.083
CerebralWhiteMatterVol	7.347	0.060	7.225	7.450	0.083
Hippocampus	7.133	0.125	6.825	7.300	0.083
Pallidum	7.831	0.833	7.375	10.000	0.083
Putamen	8.100	1.285	6.499	10.000	0.083
Thalamus	7.294	0.055	7.175	7.375	0.083
Ventricles	4.000	0.000	4.000	4.000	0.083
Cluster 1	6.440	0.355	5.720	7.140	0.333
Cluster 2	6.740	0.190	6.320	7.080	0.333
Cluster 3	7.020	0.205	6.400	7.220	0.333

The following call was used to fit the model.

```
meta_mod <- rma(yi = estimate, sei = standard_error, weights = weight,
                method = "REML", data = meta_dat)
```

```
## Warning: There are outcomes with non-positive sampling variances.
```

```
## Warning: Cannot compute Q-test, I^2, or H^2 when there are non-positive sampling
## variances in the data.
```

And next is the model summary.

```
summary(meta_mod)
```

```
##
## Random-Effects Model (k = 15; tau^2 estimator: REML)
##
##   logLik  deviance       AIC       BIC      AICc
## -21.5339   43.0678   47.0678   48.3459   48.1587
##
## tau^2 (estimated amount of total heterogeneity): 1.0814 (SE = 0.4649)
## tau (square root of estimated tau^2 value):      1.0399
##
## Model Results:
##
## estimate      se     zval    pval   ci.lb   ci.ub
##   7.0259  0.3531  19.8968  <.0001  6.3338  7.7180  ***
##
## ---
## Signif. codes:  0 '***' 0.001 '**' 0.01 '*' 0.05 '.' 0.1 ' ' 1
```

Next, we included the corpos callosum in the same way as in the sensitivity check above.

We weighted the corpus callosum structures such that they in total contributed as much as a single other subcortical region. The complete data are shown below.

```r
meta_dat %>%
  rename(
    Region = region,
    Estimate = estimate,
    `Standard error` = standard_error,
    Weight = weight
  ) %>%
  knitr::kable(digits = 3)
```

Region	Estimate	Standard error	ci_lower	ci_upper	weight0	Weight
Accumbens-area	7.597	0.527	6.999	10.000	1.0	0.059
Amygdala	7.359	0.620	6.725	10.000	1.0	0.059
Brain-Stem	7.265	0.051	7.150	7.350	1.0	0.059
Caudate	9.467	0.783	7.650	10.000	1.0	0.059
CC_Anterior	7.236	0.127	6.950	7.400	0.2	0.012
CC_Central	4.817	0.940	4.000	6.850	0.2	0.012
CC_Mid_Anterior	4.742	0.973	4.000	6.975	0.2	0.012
CC_Mid_Posterior	7.264	0.322	6.725	7.550	0.2	0.012
CC_Posterior	7.379	0.101	7.250	7.475	0.2	0.012
Cerebellum-Cortex	7.313	0.049	7.200	7.400	1.0	0.059
Cerebellum-White-Matter	7.114	0.167	6.700	7.325	1.0	0.059
CerebralWhiteMatterVol	7.347	0.060	7.225	7.450	1.0	0.059
Hippocampus	7.133	0.125	6.825	7.300	1.0	0.059
Pallidum	7.831	0.833	7.375	10.000	1.0	0.059
Putamen	8.100	1.285	6.499	10.000	1.0	0.059
Thalamus	7.294	0.055	7.175	7.375	1.0	0.059
Ventricles	4.000	0.000	4.000	4.000	1.0	0.059
Cluster 1	6.440	0.355	5.720	7.140	1.0	0.333
Cluster 2	6.740	0.190	6.320	7.080	1.0	0.333
Cluster 3	7.020	0.205	6.400	7.220	1.0	0.333

Next we fit the model.

```r
meta_mod <- rma(yi = estimate, sei = standard_error, weights = weight,
                method = "REML", data = meta_dat)
```

```
## Warning: There are outcomes with non-positive sampling variances.

## Warning: Cannot compute Q-test, I^2, or H^2 when there are non-positive sampling
## variances in the data.
```

Here is the summary, which shows estimates close to what we got when excluding corpus callosum.

```r
summary(meta_mod)
```

```
##
## Random-Effects Model (k = 20; tau^2 estimator: REML)
##
##   logLik  deviance       AIC       BIC      AICc
## -29.5571   59.1142   63.1142   65.0031   63.8642
##
```

```
## tau^2 (estimated amount of total heterogeneity): 0.9820 (SE = 0.3715)
## tau (square root of estimated tau^2 value):      0.9910
##
## Model Results:
##
## estimate      se     zval    pval   ci.lb   ci.ub
##   6.9525  0.3604  19.2918  <.0001  6.2462  7.6589  ***
##
## ---
## Signif. codes:  0 '***' 0.001 '**' 0.01 '*' 0.05 '.' 0.1 ' ' 1
```

References

R Core Team. 2020. *R: A Language and Environment for Statistical Computing.* Vienna, Austria: R Foundation for Statistical Computing. https://www.R-project.org/.

Viechtbauer, Wolfgang. 2010. "Conducting Meta-Analyses in R with the metafor Package." *Journal of Statistical Software* 36 (3): 1–48. https://doi.org/10.18637/jss.v036.i03.

Wickham, Hadley, Mara Averick, Jennifer Bryan, Winston Chang, Lucy D'Agostino McGowan, Romain François, Garrett Grolemund, et al. 2019. "Welcome to the tidyverse." *Journal of Open Source Software* 4 (43): 1686. https://doi.org/10.21105/joss.01686.

Vertex-wise cortical thickness results

We tested associations between sleep duration and cortical thickness by vertex-wise spatiotemporal linear mixed-effect models (LME)[1,2]. The hierarchical nature of the data - repeated measurements nested within participants - was accounted for using a random intercept term for subject ID. Surface results were corrected for multiple testing by 10,000 Z Monte Carlo simulations using $p < .01$ as cluster generation threshold and $p < .05$ as cluster threshold[3]. As we previously have seen that sleep-cortex relationships tend to be stronger with higher age, we ran separate analyses in young/ middle-aged (20-60 years) and older adults (>60 years), and for participants with below vs. above average reported sleep duration. Analyses were thus run in four groups: young/ middle-aged with below (n = 9464) vs. above (n = 6421) average sleep, and older adults with below (n = 17119) vs. above (n = 15053) average sleep. The spatially strongest effects were found in the older adults sleeping above average, where longer sleep duration was associated with thinner cortex across most of the brain surface. There were positive associations between sleep duration and thickness in the younger and older below average sleep groups, and no effects in the young group sleeping above average.

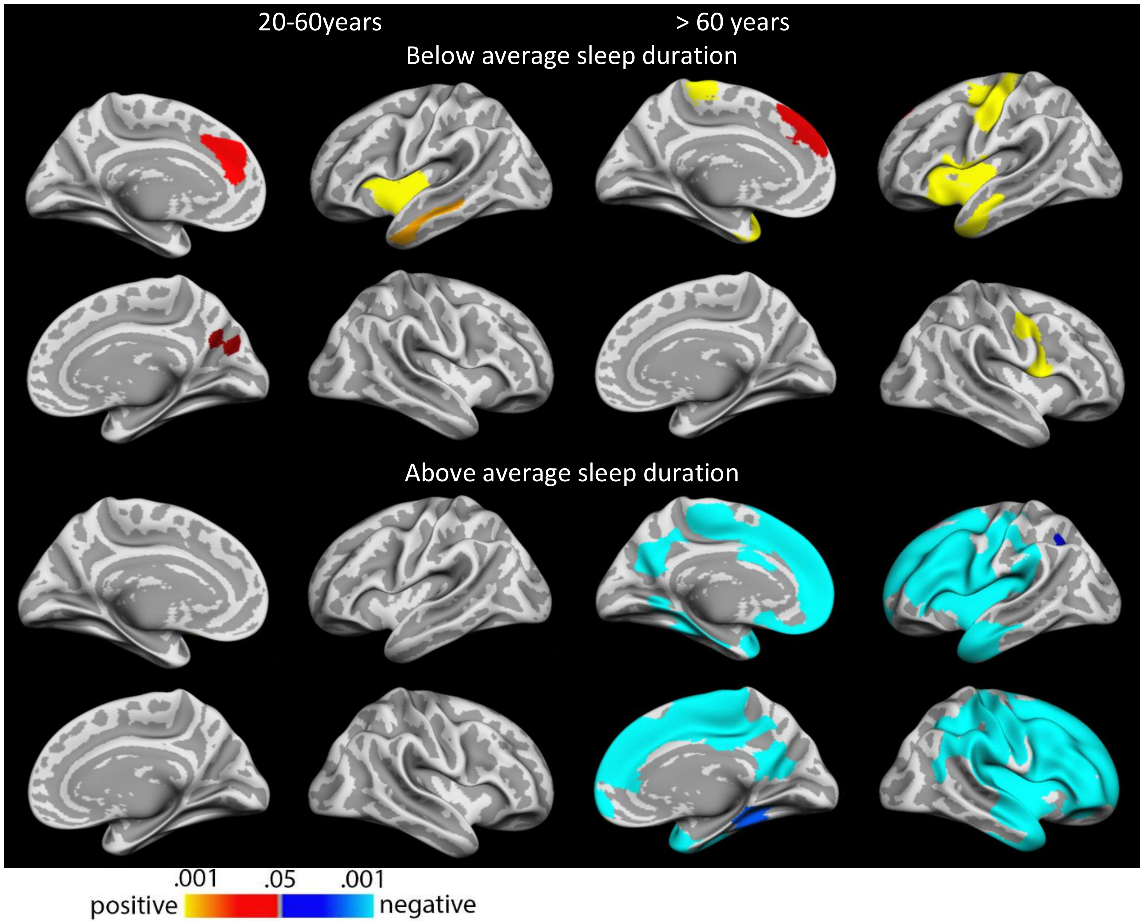

Associations between sleep duration and cortical thickness (p-value maps). Spatiotemporal LMEs were used to take advantage of both the longitudinal and cross-sectional data. Only clusters surviving corrections for multiple comparisons are shown. Across all panels, warm colors indicate that longer sleep is associated with thicker cortex and cold colors indicate that shorter sleep is associated with thicker cortex.

References

1 Bernal-Rusiel, J. L. *et al.* Statistical analysis of longitudinal neuroimage data with Linear Mixed Effects models. *Neuroimage* **66**, 249-260, doi:10.1016/j.neuroimage.2012.10.065 (2013).

2 Bernal-Rusiel, J. L. *et al.* Spatiotemporal linear mixed effects modeling for the mass-univariate analysis of longitudinal neuroimage data. *Neuroimage* **81**, 358-370, doi:10.1016/j.neuroimage.2013.05.049 (2013).

3 Greve, D. N. & Fischl, B. False positive rates in surface-based anatomical analysis. *Neuroimage* **171**, 6-14, doi:10.1016/j.neuroimage.2017.12.072 (2018).

Comparing mega and meta-analytic estimates for sleep – cortex relationships

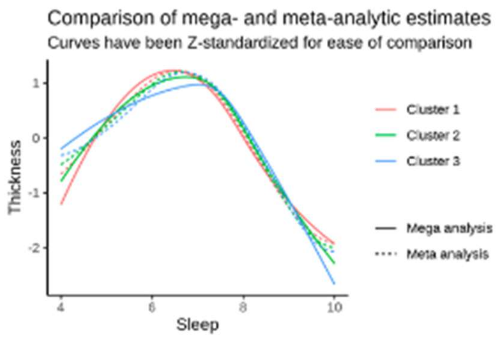

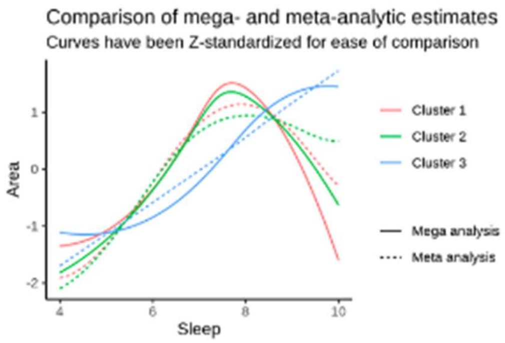

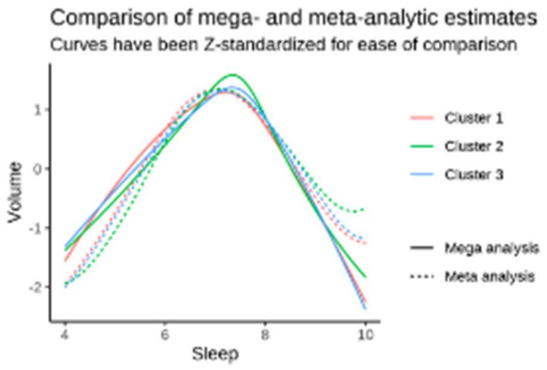

SI Genetic analyses

Genome-Wide Association studies (GWAS) Using UK Biobank data

We accessed the UK Biobank genetic data (https://www.ukbiobank.ac.uk/about-biobank-uk/ released in February 2020 through the application no. 32048. Ethical approval of UK Biobank project was obtained from the National Health Service National Research Ethics Service (Ref 11/NW/0382) and all participants provided written informed consent. Among the 502,507 participants, 40,682 had undergone MRI and 487,409 had DNA genotypes. The detailed information on genotyping, imputation and quality controls was published by[1]. Before performing GWAS, we excluded participants who are not self-reported white-British (n=92 900), have relatives in the biobank (n=148 689), had been labelled as outliers in missingness or heterozygosity(n=968) or had conflicting self-reported vs. genetic sex (n=378) by UK Biobank team. Among the remaining 339696 participants, 125 800 reported sleep duration longer than 7 hours, and 213 331 reported sleep duration less or equal to 7 hours. 29 155 of these participants had undergone MRI. Genotypes of the UK Biobank participants were filtered by removing variants that are non-SNP, had a minor allele frequency less than 0.01, failed the Hardy-Weinberg equilibrium test ($p<1e^{-6}$), or had large missingness (>0.05). In total, 9 153 887 autosomal SNPs were analyzed.

GWAS for sleep duration among participants sleeping <= 7hours

We performed GWAS for sleep durations for UKB participants who reported less than or equal to 7 hours of sleep and had not undergone MRI (n=197,137). The sleep duration was scaled by subtracting the mean and divided by the standard deviations in this subsample. A linear regression model was used to identify SNPs that were associated with scaled sleep durations. Age at baseline, sex and the top 10 genetic principal components (PC, obtained from UK Biobank) were included as covariates in the model. The PLINK2 command –glm was used for this analysis. A genomic inflation factor was estimated to be 1.03 based on the GWAS summary statistics, i.e., no discernable inflation of the associations was observed. The association quantile-quantile plot, and the Manhattan plot are shown below.

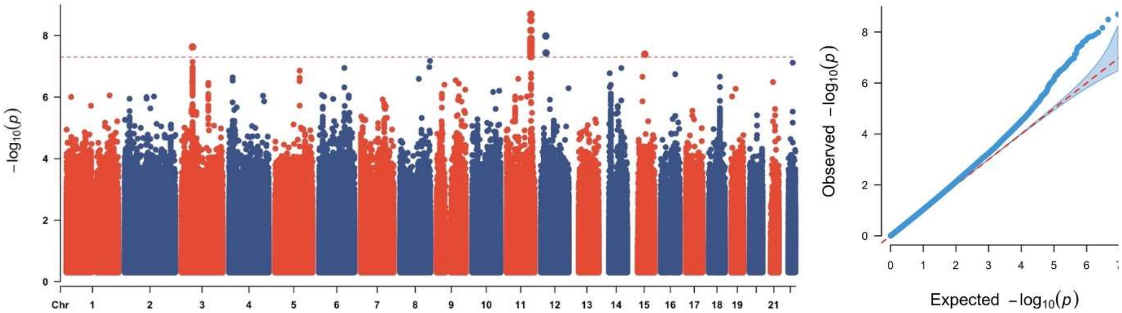

Left panel: Manhattan plot of GWAS for sleep duration in participants sleeping ≤ 7 hours, with the dotted red line showing the genome-wide significance level ($p < 5\times10^{-8}$). Right panel: QQ plot of GWAS for sleep duration.

GWAS for sleep duration among participants sleeping > 7 hours

We also performed GWAS for sleep durations for UKB participants who reported more than 7 hours of sleep and had not undergone MRI (n=112,839). The sleep duration was scaled by subtracting the

mean and divided by the standard deviations in this subsample. A linear regression model was used for identified SNPs that associated with scaled sleep durations. Age at baseline, sex and the top 10 genetic principal components (obtained from UK Biobank) were included as covariates in the model. The PLINK2[2] command –glm was used for this analysis. A genomic inflation factor was estimated to 1.02 based on the GWAS summary statistics, i.e., no discernable inflation of the associations was observed. The association quantile-quantile plot, and the Manhattan plot are shown below.

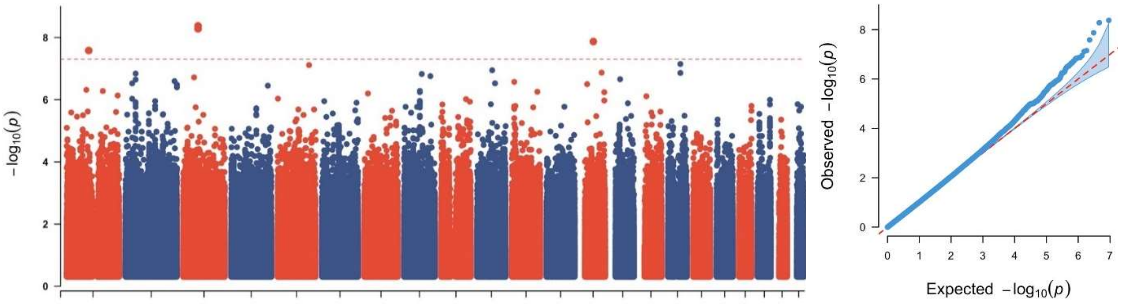

Left panel: Manhattan plot of GWAS for sleep duration in participants sleeping > 7 hours, with the dotted red line showing the genome-wide significance level (p < 5×10^{-8}). Right panel: QQ plot of GWAS for sleep duration.

GWAS for Hippocampal volume, total grey matter volume and intracranial volume

For the 29 155 participants, GWAS for total hippocampal volume (HippV), total grey matter volume (TGV) and estimated intracranial volume (ICV) were performed using the –glm function from PLINK2, separately for each MRI derived measure. In each GWAS, the phenotype was first scaled as for sleep duration. In addition to age at MRI, sex and the top 10 genetic PCs, ICV was included as a covariate for the GWAS of HippV and TGV. Genomic inflation factors were estimated to 1.05, 1.06 and 1.02 for the GWAS summary statistics for HippV, ICV and TGV, respectively. The association quantile-quantile plots, and the Manhattan plots are shown below.

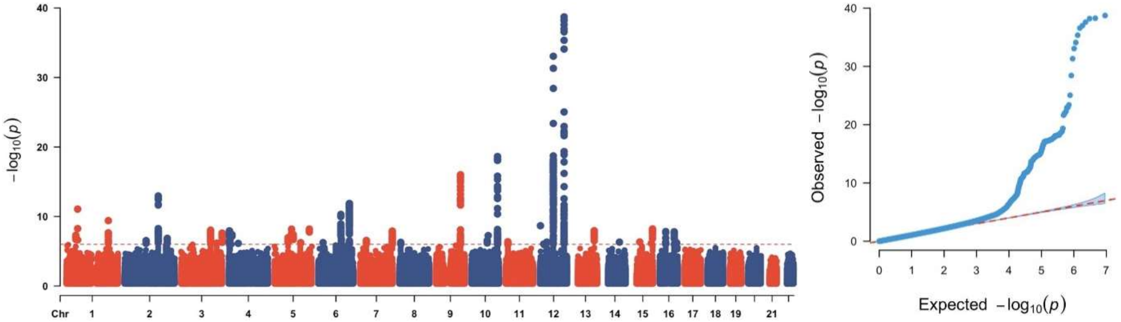

Left panel: Manhattan plot of GWAS for hippocampal volume, with the dotted red line showing the genome-wide significance level (p < 5×10^{-8}). Right panel: QQ plot of GWAS for hippocampal volume.

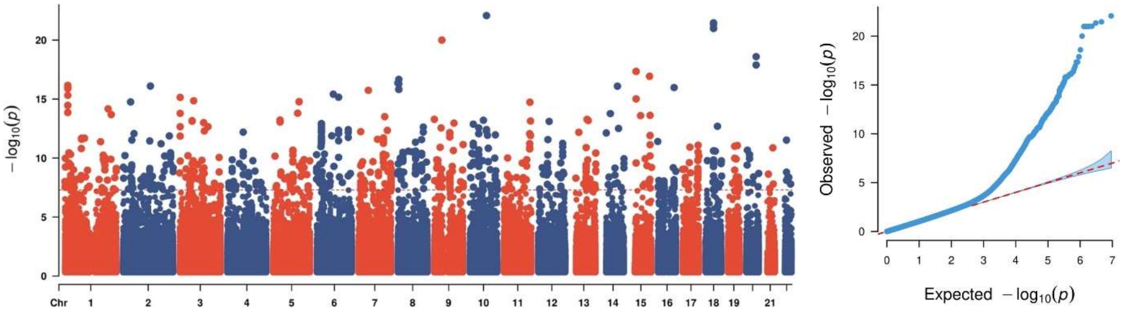

Left panel: Manhattan plot of GWAS for total gray matter volume, with the dotted red line showing the genome-wide significance level ($p < 5 \times 10^{-8}$). Right panel: QQ plot of GWAS for total gray matter volume.

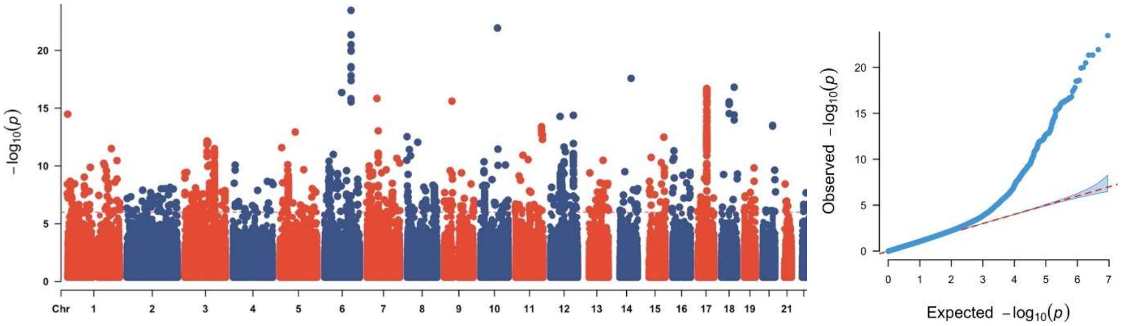

Left panel: Manhattan plot of GWAS for intracranial volume, with the dotted red line showing the genome-wide significance level ($p < 5 \times 10^{-8}$). Right panel: QQ plot of GWAS for intracranial volume.

SNP heritability and genetic correlations

The Linkage Disequilibrium score regression (LD)[3] model was used for estimating SNP heritability and between-traits genetic correlations using GWAS summary statistics. Association statistics were first aligned with high quality HapMap 3 SNPs, and then the LD scores for these set SNPs were used. The signed-sumstats were assigned to regression beta, 0. The SNP heritabilities for hippocampal volume, ICV and total gray matter volume were estimated to 0.29 (se=0.03), 0.35 (se=0.03) and 0.22 (se=0.02), respectively. The numbers for Sleep durations were 0.04 (se=0.003) and 0.02 (se=0.005) for participants sleeping less than or equal to versus longer than 7 hours, respectively. The genetic correlations between sleep duration (<=7 hours) and hippocampal volume, ICV and total gray matter volume were estimated to be 0.04 (se=0.05, p=0.47), 0.1 (se=0.05, p=0.05) and 0.09 (se=0.06, p=0.10), respectively. The genetic correlations between sleep duration (>7 hours) and hippocampal volume, ICV and total gray matter volume were estimated to be -0.08 (se=0.10, p=0.43), -0.07 (se=0.08, p=0.44) and 0.04 (se=0.10, p=0.70), respectively.

Polygenic scores

To accurately estimate the polygenic scores for a trait, we first computed the posterior effect size per SNP using the Bayesian mixture model implemented in PRS-CS[4]. The Polygenic risk score via continuous shrinkage priors (PRS-CS) model is a widely used method for computing polygenic score for highly polygenic traits1. PRS-CS shrinks effect sizes estimated from GWAS using linkage-

disequilibrium (LD) correlations in a Bayesian framework, assuming a two-component mixture prior distribution. The LD correlations provided by PRS-CS were based on the 1000 Genomes phase 3 European population. In total, about 1.3 million high quality SNPs were used. In addition, PRS-CS does not need information from the target sample where the estimated posterior effect will be used for computing polygenic score. Thus, the GWAS sample and the target sample were treated fully independently in the polygenic score computation. Furthermore, in light of ours and previously published GWAS results, we assumed a highly polygenic genetic architecture for both MRI derived traits and sleep duration, by setting the parameter phi to 0.01, instead of a grid-search strategy proposed by the model. We believe our choice, though conservative, further reduce the overfitting risk. For other parameters in PRS-CS, we used the default values.

The posterior effect sizes obtained by running PRS-CS on each GWAS were then used separately to compute polygenic scores. We did not use p-values or LD threshold to select SNPs. Rather, genome wide SNPs were used for computing the polygenic scores. After removing rare variants (minor allele frequency < 0.01) in UK biobank, variants not in the HapMap 3 data, and variants that are not on the Hardy-Weinberg equilibrium (p<10-6), we used the remaining 615297 SNPs for computing the polygenic scores for each trait. Recent methodology studies all point to the advantage of using shrinkage based methods over p-value based thresholding methods, for example LDpred2, PRS-CS, and the lasso-based models3, particularly for highly polygenic traits. The computed posterior effects were used as weights in the computation of PGSs for a trait by using the score function from PLINK2. To examine the associations between PGS for a trait with a second trait, linear regression models were used. The same covariates included in the GWAS analysis were included as covariates in addition to PGSs in these models.

PGS	Trait	Beta	Se	t-score	P	FDR
ICV	Sleep<7	1.91×10^{-2}	2.56×10^{-3}	8.47	$<2 \times 10^{-16}$	2.40×10^{-15}
	Sleep>7	-6.23×10^{-3}	2.98×10^{-3}	-2.09	0.04	0.32
HippV	Sleep<7	2.43×10^{-3}	2.26×10^{-3}	1.07	0.28	0.66
	Sleep>7	-4.16×10^{-3}	2.98×10^{-3}	-1.40	0.16	0.66
TGV	Sleep<7	1.05×10^{-2}	2.56×10^{-3}	4.65	3.28×10^{-6}	3.28×10^{-5}
	Sleep>7	-4.23×10^{-3}	2.97×10^{-3}	-1.42	0.16	0.66
Sleep<7	ICV	3.33×10^{-2}	4.77×10^{-3}	6.99	2.75×10^{-12}	3.03×10^{-11}
	HippV	3.25×10^{-3}	4.56×10^{-3}	0.713	0.48	0.66
	TGV	7.84×10^{-3}	2.91×10^{-3}	2.69	7.13×10^{-3}	6.42×10^{-2}
Sleep>7	ICV	-8.42×10^{-3}	4.78×10^{-3}	-1.76	7.81×10^{-2}	0.51
	HippV	-7.30×10^{-3}	4.56×10^{-3}	-1.60	0.11	0.66
	TGV	-4.50×10^{-3}	2.90×10^{-3}	-1.55	0.12	0.66

Polygenic score (PGS) associations between sleep duration and HippV, ICV and TGV. PGS were tested for associations with measured traits (Traits) in independent samples. Linear regression models were used to estimate the effect sizes of PGS to sleep duration for those sleeping less than or equal 7 hours per day (Sleep<7) and those sleeping more than 7 hours per day (Sleep>7). Generalized additive models were used for estimating effects of PGS to total Hippocampal volume (HippV), total grey matter volume (TGV) and estimated intracranial volume (ICV), taking age at scan as the smoothed variable. Age, sex and the top 10 genetic principal components were included as covariates in all models. For HippV and TGV, ICV was included as an additional covariate. Note, effect sizes (beta) indicate the amount of change in standard deviation of a trait by one standard deviate increase of a PGS.

Mendelian randomization

Instrumental variants selection

To determine the SNP-phenotype associations, the subset of SNPs associated with the phenotype having p <1x10^{-6} and also existing in the GWAS results were used. Using p<10-6 instead of the GWAS threshold (p<5x10-8) was done because of the relative low power of our GWAS for sleep duration, which used at most one third of the sample size used in previous sleep-duration studies. The comparatively low-powered GWAS combined with high polygenicity and low heritability estimates for sleep duration yields very few genome-wide significant loci/SNPs (4 for short-sleeper and 3 for long-sleeper). These small numbers of instruments explain only a minor portion of the variance. Hence, we had to use a trade-off between strength of the instrument and risk of horizontal pleiotropy, and therefore employed the suggestive p value thresholds for selecting instrument SNPs.

SNPs with minor allele frequency (MAF) < 0.05 in the GWAS sample, or having ambiguous allelic coding, i.e., A/T or C/G, were excluded from the instrument set. To remove correlated SNPs, the linkage disequilibrium (LD) clumping method implemented by PLINK was used. The following parameters in PLINK were set, clump-kb 10,000 kb, and --clump-r2 0.1. The LD structure from the corresponding GWAS sample were used for clumping. The full list of SNPs selected as instruments for each analysis are presented in SI Genetics supplementary tables. The strength of the instruments to an outcome was evaluated using the F statistic.

As we did not find significant causal effects of sleep duration on MRI-derived traits using p <10-6, we reasoned that the instrument strengths were still too low, and the exposure variance explained by instruments was 0.0014 for below-average and 0.0007 for above-average sleepers, estimated by the Steiger test, and reperformed MR using SNPs with an association p<10-5, where the explained variances increased to 0.003 for shorter than average sleepers and 0.005 for longer than average sleepers. We found no significant causal effects by this even more liberal threshold neither.

Exposure	outcome	Beta	Se	P
ICV	Sleep<7	5.99x10^{-2}	1.73x10^{-2}	5.36x10^{-4}
	Sleep>7	-3.67x10^{-3}	2.57x10^{-2}	0.87
HippV	Sleep<7	1.13x10^{-2}	1.45x10^{-2}	0.44
	Sleep>7	9.61x10^{-3}	2.18x10^{-2}	0.66
TGV	Sleep<7	5.18x10^{-2}	3.22x10^{-2}	0.11
	Sleep>7	-8.99x10^{-3}	6.59x10^{-2}	0.89
Sleep<7	ICV	4.38x10-2	0.14	0.75
	HippV	0.16	0.15	0.30
	TGV	2.66x10^{-2}	8.35x10^{-2}	0.75
Sleep>7	ICV	6.14x10^{-2}	0.38	0.87
	HippV	-0.16	0.22	0.48
	TGV	-0.35	0.14	1.22x10^{-2}

Inverse variance weighted Mendelian randomization results between sleep duration and HippV, ICV and TGV.

Finally, we performed two-sample Mendelian randomization analysis using several popular methods, the inverse variance weighted (IVW), the Egger regression, the weighted median and the robust adjusted profile score (RAPS) methods. The RAPs was only applied to weaker instruments, i.e.

P <10^{-5}. Full report for each pair of traits and each method were presented in SI Genetics supplementary notes.

Exposure	outcome	Beta	Se	P
Sleep<7	ICV	-7.90x10-3	8.47x10-2	0.93
	HippV	2.72x10-3	8.10x10-2	0.97
	TGV	6.96x10-3	5.15x10-2	0.89
Sleep>7	ICV	5.29×10^{-3}	7.29x10-2	0.94
	HippV	-6.95×10^{-2}	6.89×10^{-2}	0.31
	TGV	-2.10×10^{-2}	4.36×10^{-2}	0.63

Mendelian randomization by RAPS between sleep duration and HippV, ICV and TGV.

References

1. Sugden, K., *et al.* Establishing a generalized polyepigenetic biomarker for tobacco smoking. *Translational psychiatry* **9**, 92 (2019).
2. Chang, C.C., *et al.* Second-generation PLINK: rising to the challenge of larger and richer datasets. *Gigascience* **4**, 7 (2015).
3. Bulik-Sullivan, B.K., *et al.* LD Score regression distinguishes confounding from polygenicity in genome-wide association studies. *Nat Genet* **47**, 291-295 (2015).
4. Castellano, J.M., *et al.* Human apoE Isoforms Differentially Regulate Brain Amyloid-β Peptide Clearance. *Science translational medicine* **3**, 89ra57-89ra57 (2011).

Two sample MR report

HippV against SleepGt7

Date: **24 september, 2021** P<10-6

Results from two sample MR:

method	nsnp	b	se	pval
MR Egger	76	0.0675178	0.0370249	0.0722541
Weighted median	76	-0.0061928	0.0211743	0.7699293
Inverse variance weighted	76	-0.0057491	0.0149908	0.7013456
Simple mode	76	0.0223998	0.0475225	0.6387583
Weighted mode	76	0.0163095	0.0347411	0.6401034

Heterogeneity tests

method	Q	Q_df	Q_pval
MR Egger	89.81645	74	0.1017810
Inverse variance weighted	95.45002	75	0.0557322

Test for directional horizontal pleiotropy

egger_intercept	se	pval
-0.0038145	0.0017706	0.0344626

Test that the exposure is upstream of the outcome

snp_r2.exposure	snp_r2.outcome	correct_causal_direction	steiger_pval
0.0937249	0.0011498	TRUE	0

Note - R^2 values are approximate

Forest plot of single SNP MR

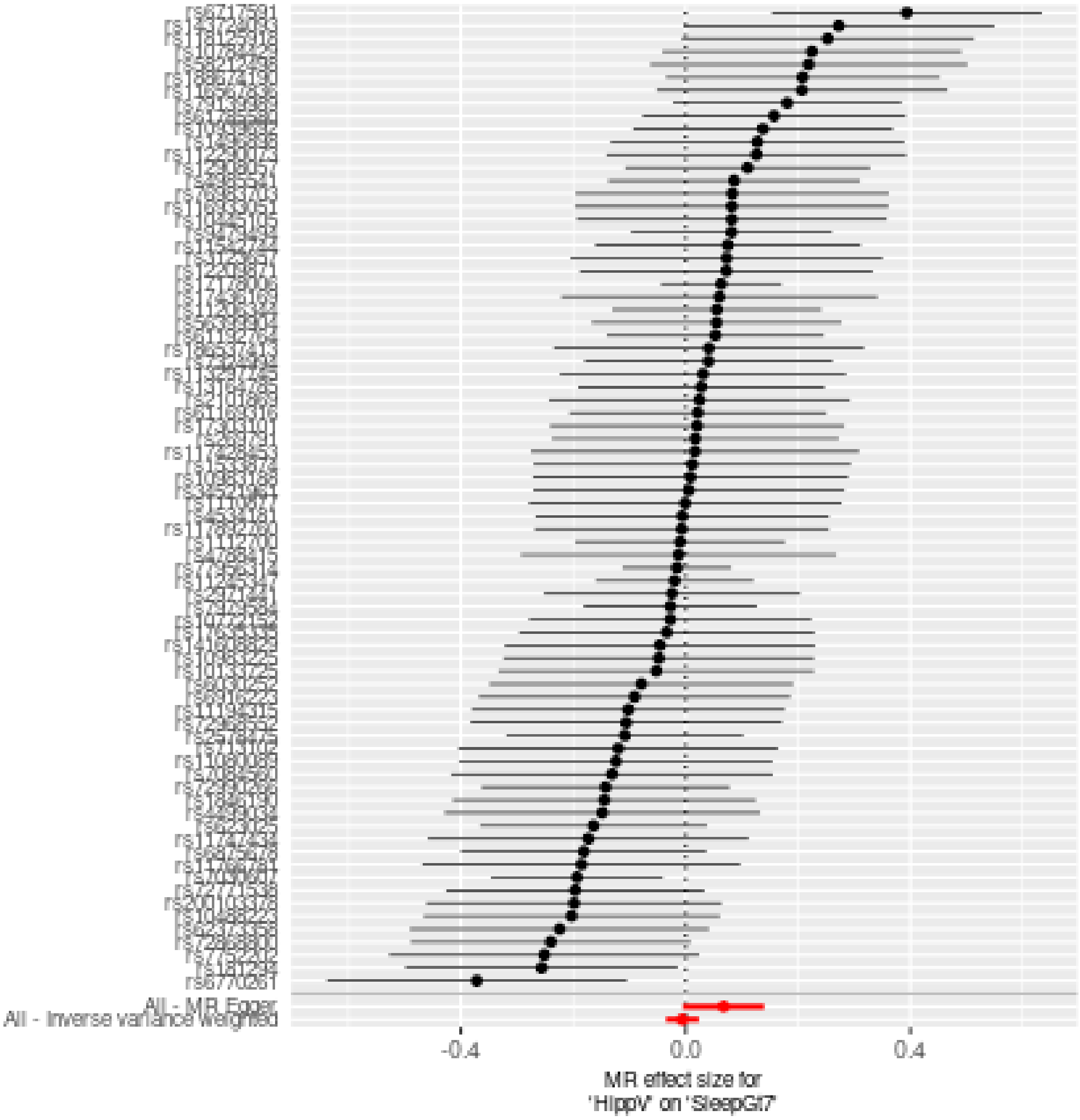

Comparison of results using different MR methods

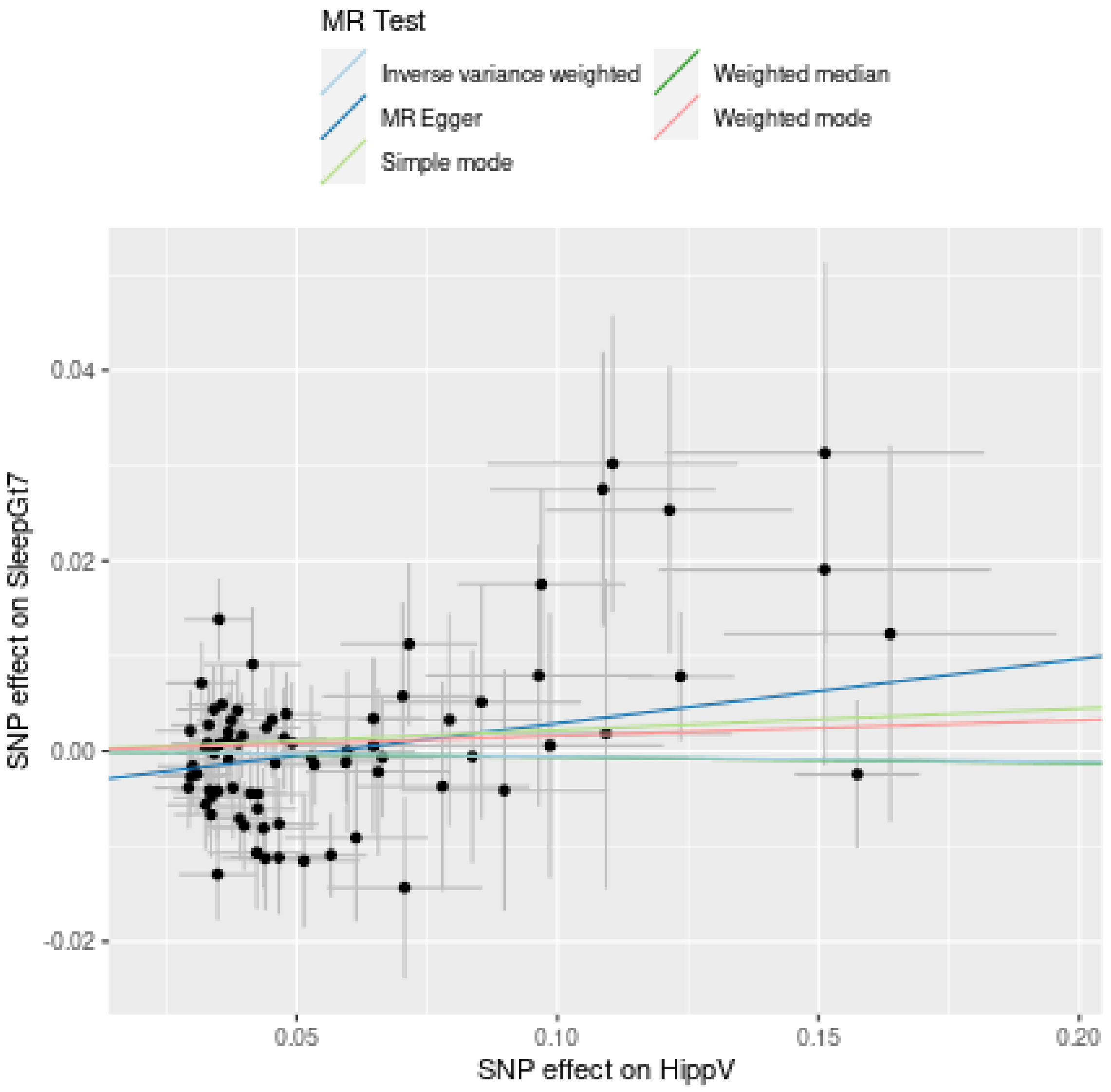

Funnel plot

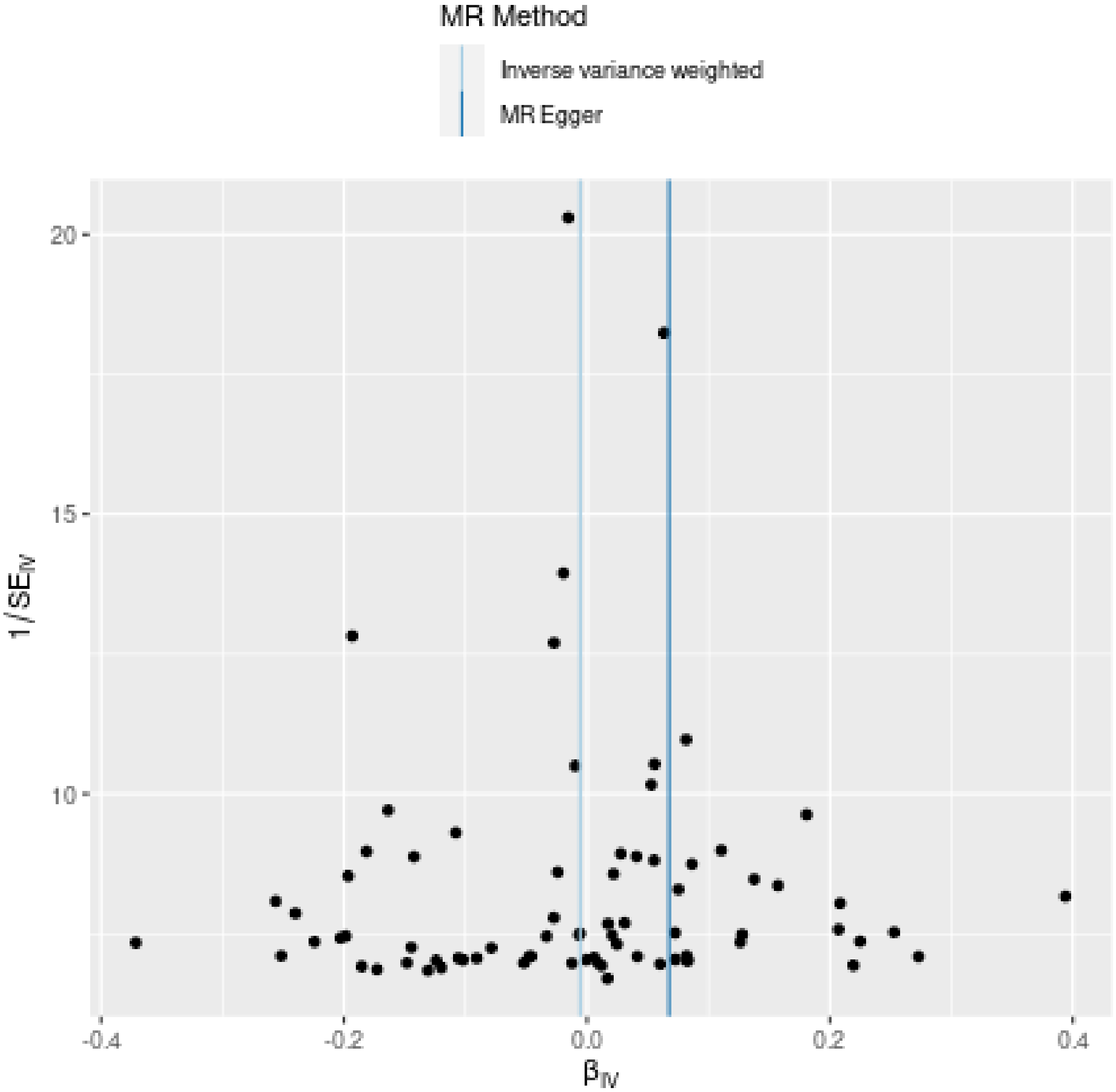

MR Method
Inverse variance weighted
MR Egger
20
15
10
1/SE$_{IV}$
-0.4
-0.2
0.0
0.2
0.4
β$_{IV}$

Leave-one-out sensitivity analysis

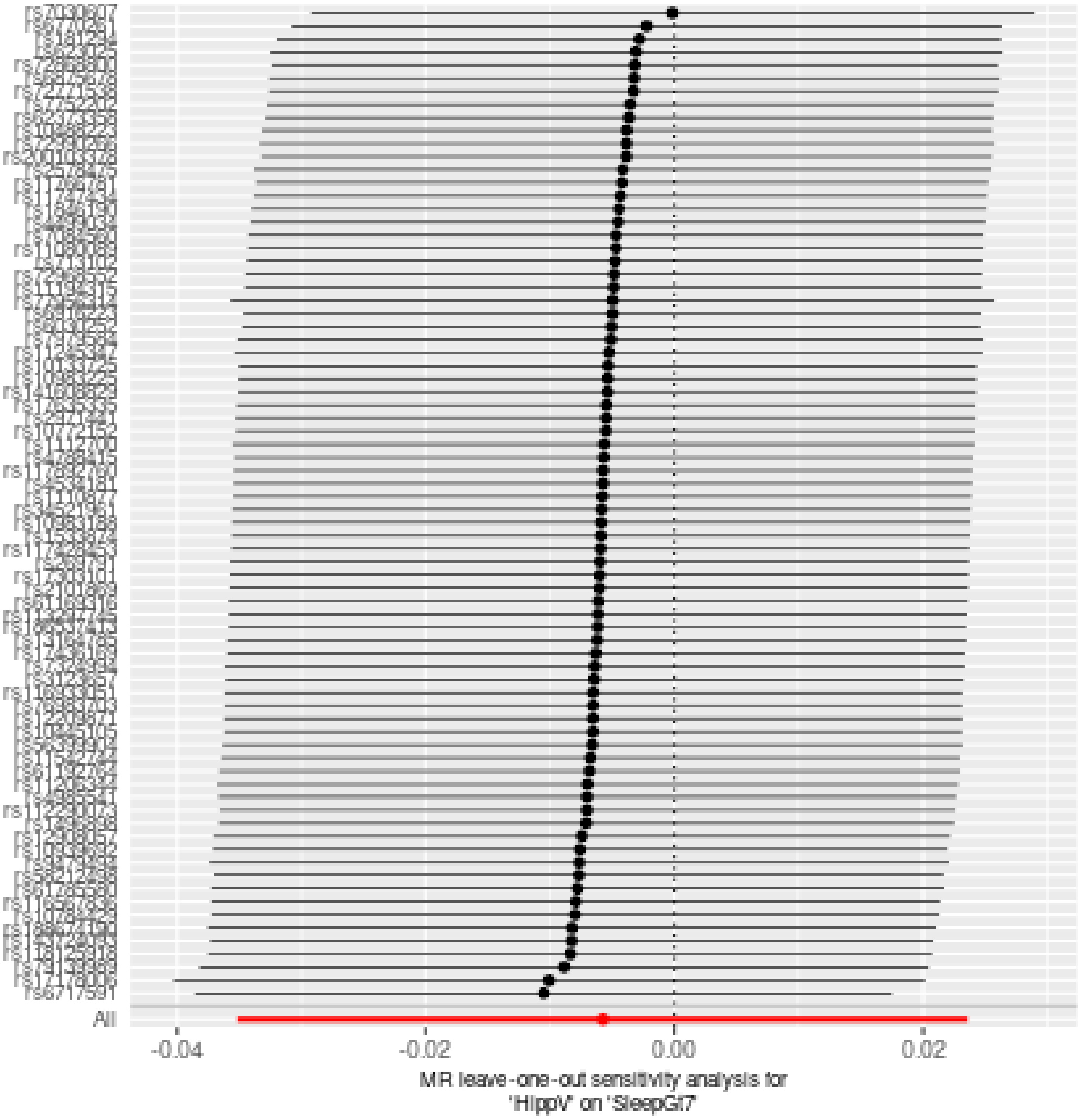

Two sample MR report

HippV against SleepLe7

P<10-6

Date: **24 september, 2021**

Results from two sample MR:

method	nsnp	b	se	pval
MR Egger	76	0.0169608	0.0265944	0.5255981
Weighted median	76	0.0014014	0.0156517	0.9286559
Inverse variance weighted	76	0.0105060	0.0104622	0.3152862
Simple mode	76	-0.0103722	0.0392096	0.7920952
Weighted mode	76	0.0097953	0.0251273	0.6977701

Heterogeneity tests

method	Q	Q_df	Q_pval
MR Egger	80.97263	74	0.2707679
Inverse variance weighted	81.04907	75	0.2962110

Test for directional horizontal pleiotropy

egger_intercept	se	pval
-0.0003362	0.0012719	0.79228

Test that the exposure is upstream of the outcome

snp_r2.exposure	snp_r2.outcome	correct_causal_direction	steiger_pval
0.0937249	0.0004956	TRUE	0

Note - R^2 values are approximate

Forest plot of single SNP MR

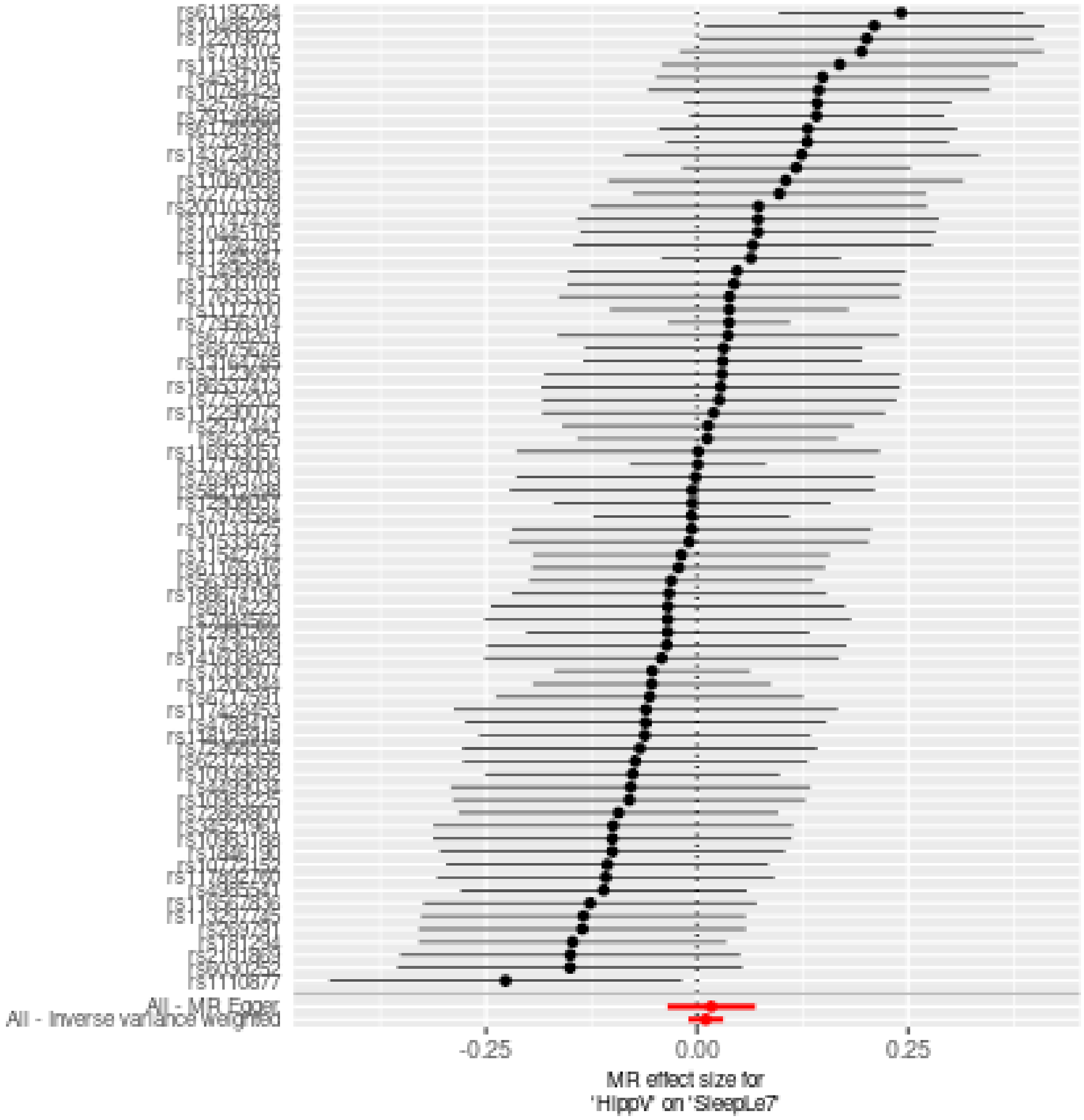

Comparison of results using different MR methods

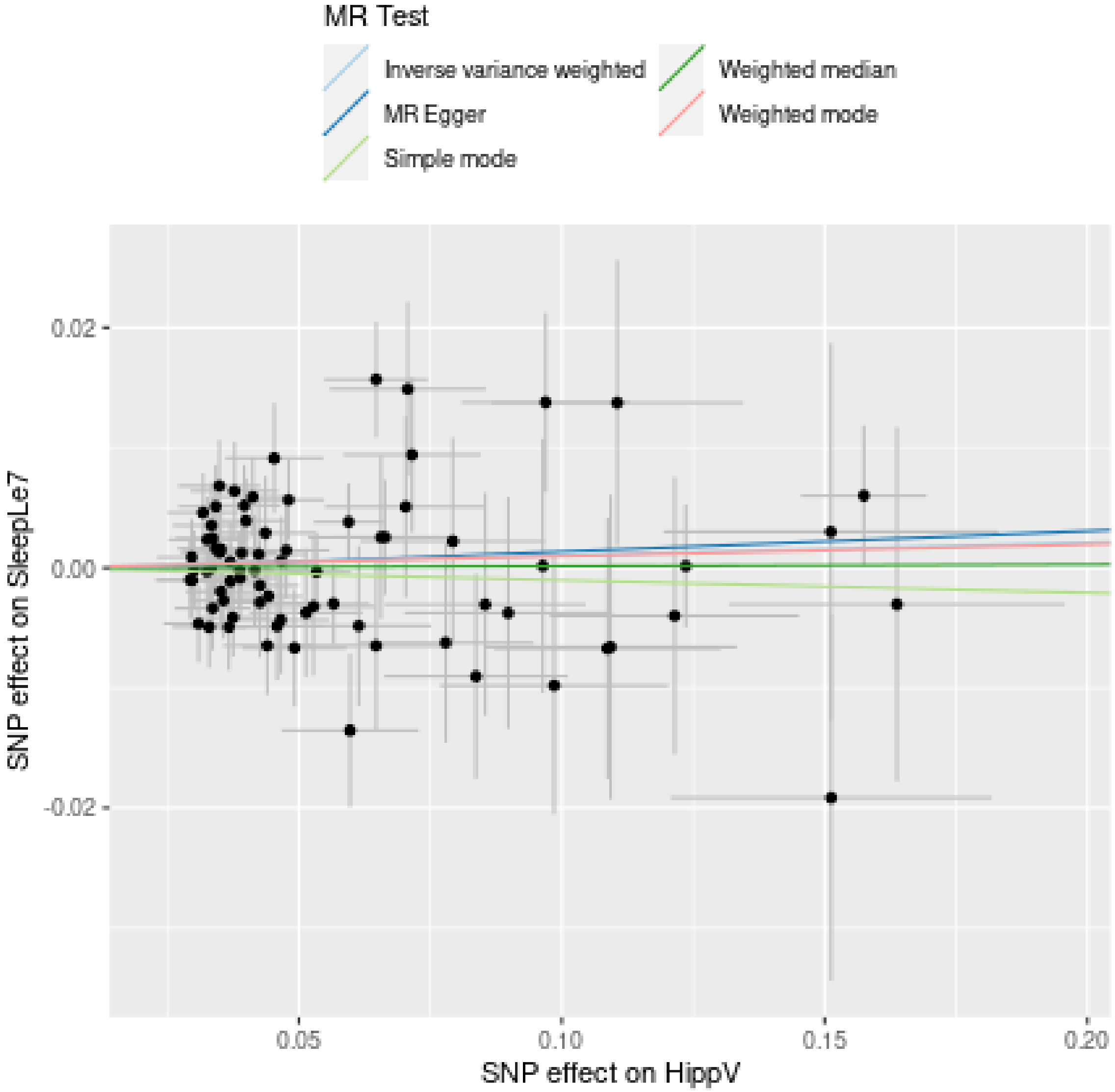

Funnel plot

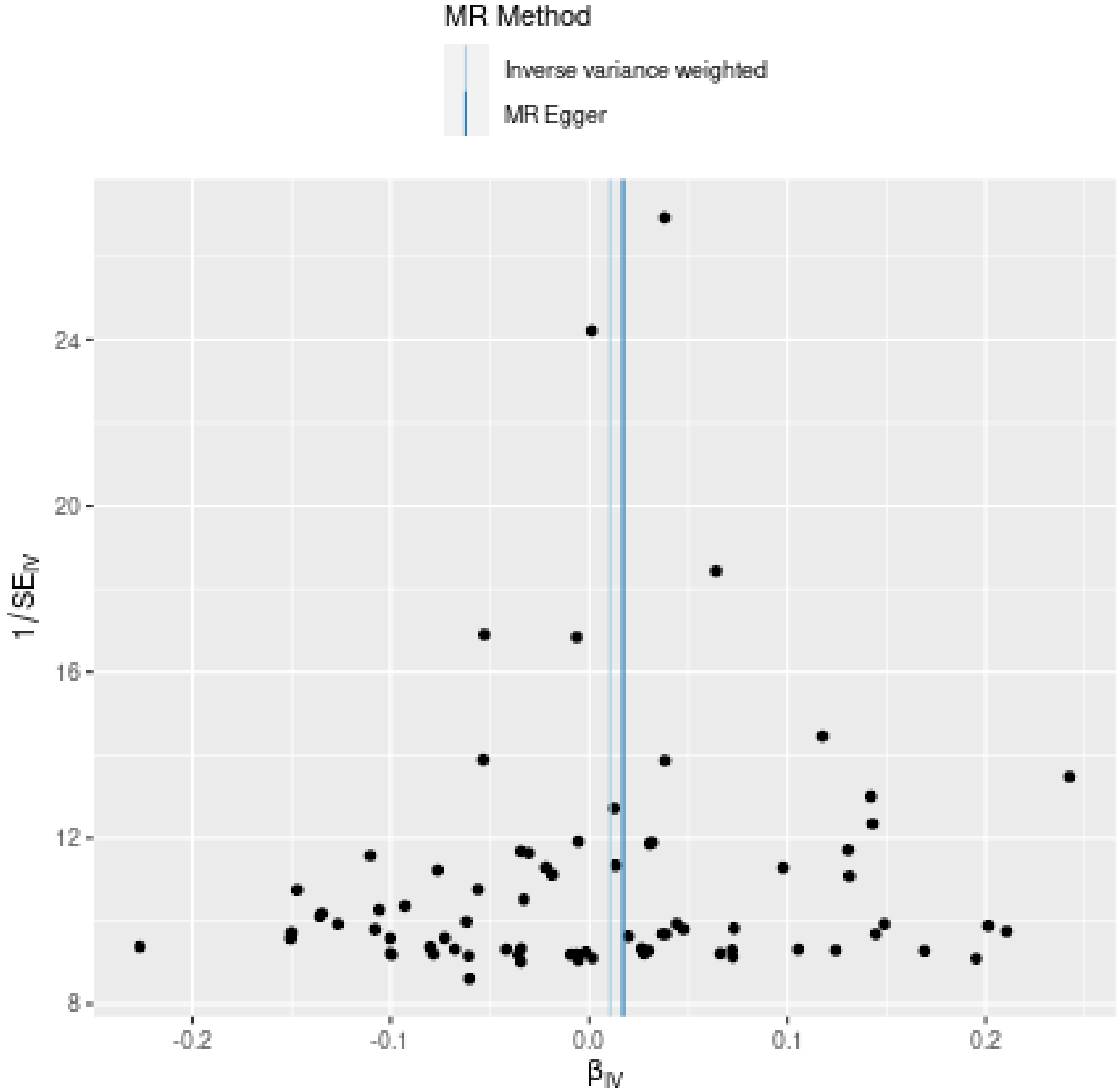

MR Method
Inverse variance weighted
MR Egger
24
20
16
12
8
1/SE_IV
-0.2
-0.1
0.0
0.1
0.2
β_IV

Leave-one-out sensitivity analysis

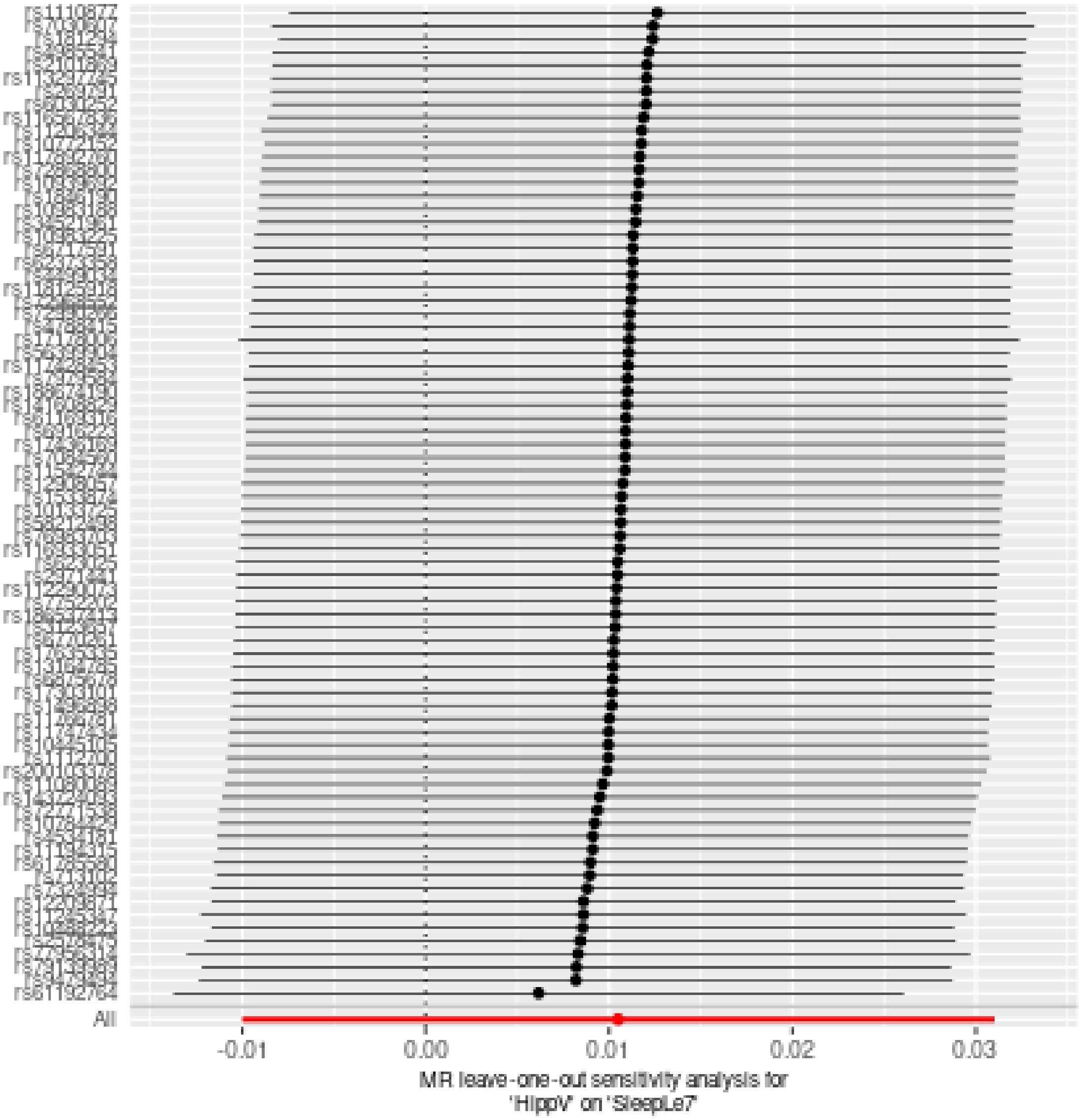

Two sample MR report

ICV against SleepGt7

P<10-6

Date: **24 september, 2021**

Results from two sample MR:

method	nsnp	b	se	pval
MR Egger	88	0.0339083	0.0396457	0.3947706
Weighted median	88	-0.0173681	0.0195531	0.3744033
Inverse variance weighted	88	-0.0070838	0.0147017	0.6299251
Simple mode	88	-0.0274712	0.0485554	0.5730056
Weighted mode	88	-0.0150815	0.0406290	0.7113908

Heterogeneity tests

method	Q	Q_df	Q_pval
MR Egger	113.5486	86	0.0249814
Inverse variance weighted	115.1845	87	0.0232427

Test for directional horizontal pleiotropy

egger_intercept	se	pval
-0.0021613	0.0019417	0.2687687

Test that the exposure is upstream of the outcome

snp_r2.exposure	snp_r2.outcome	correct_causal_direction	steiger_pval
0.097292	0.001197	TRUE	0

Note - R^2 values are approximate

Forest plot of single SNP MR

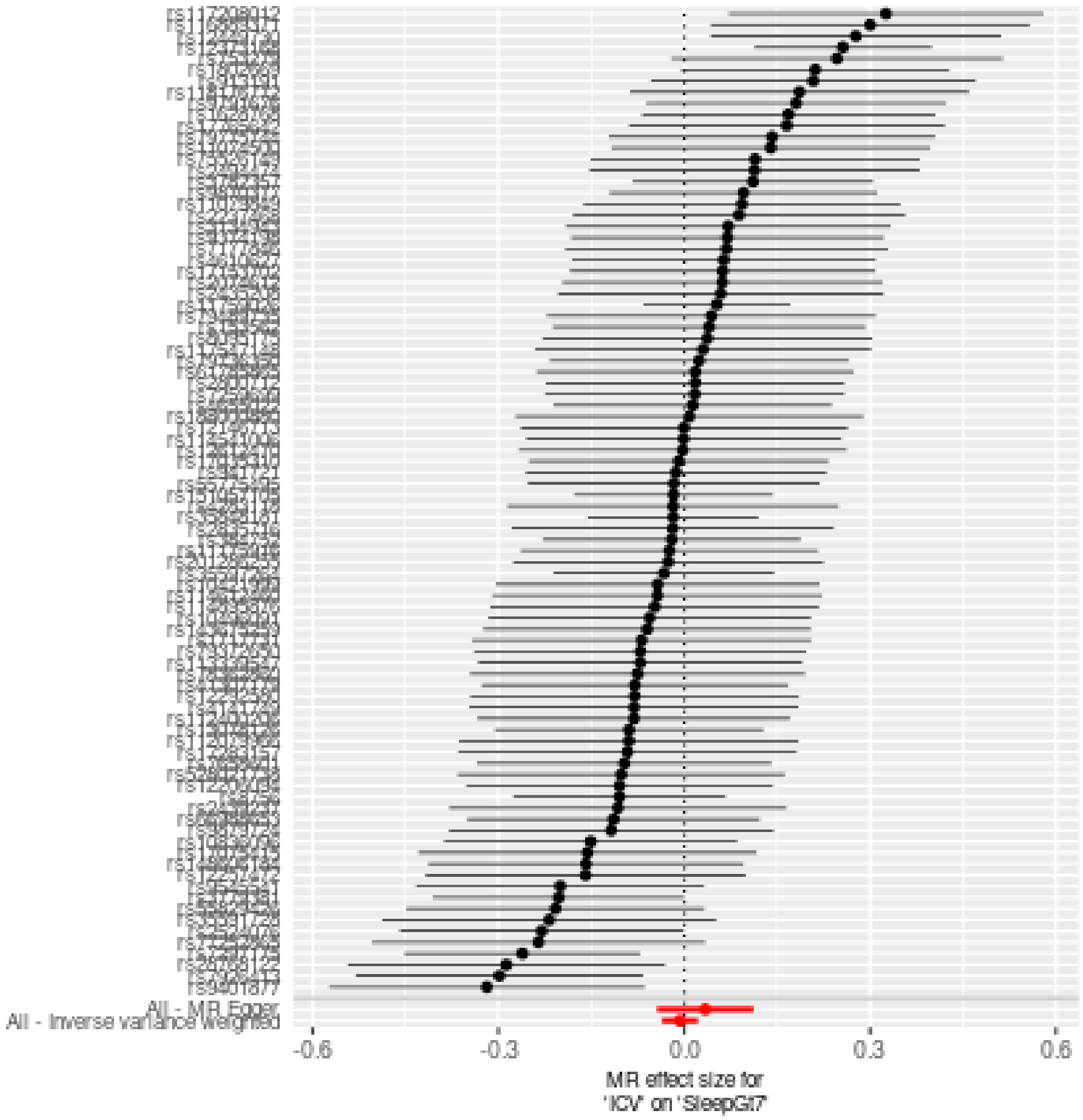

Comparison of results using different MR methods

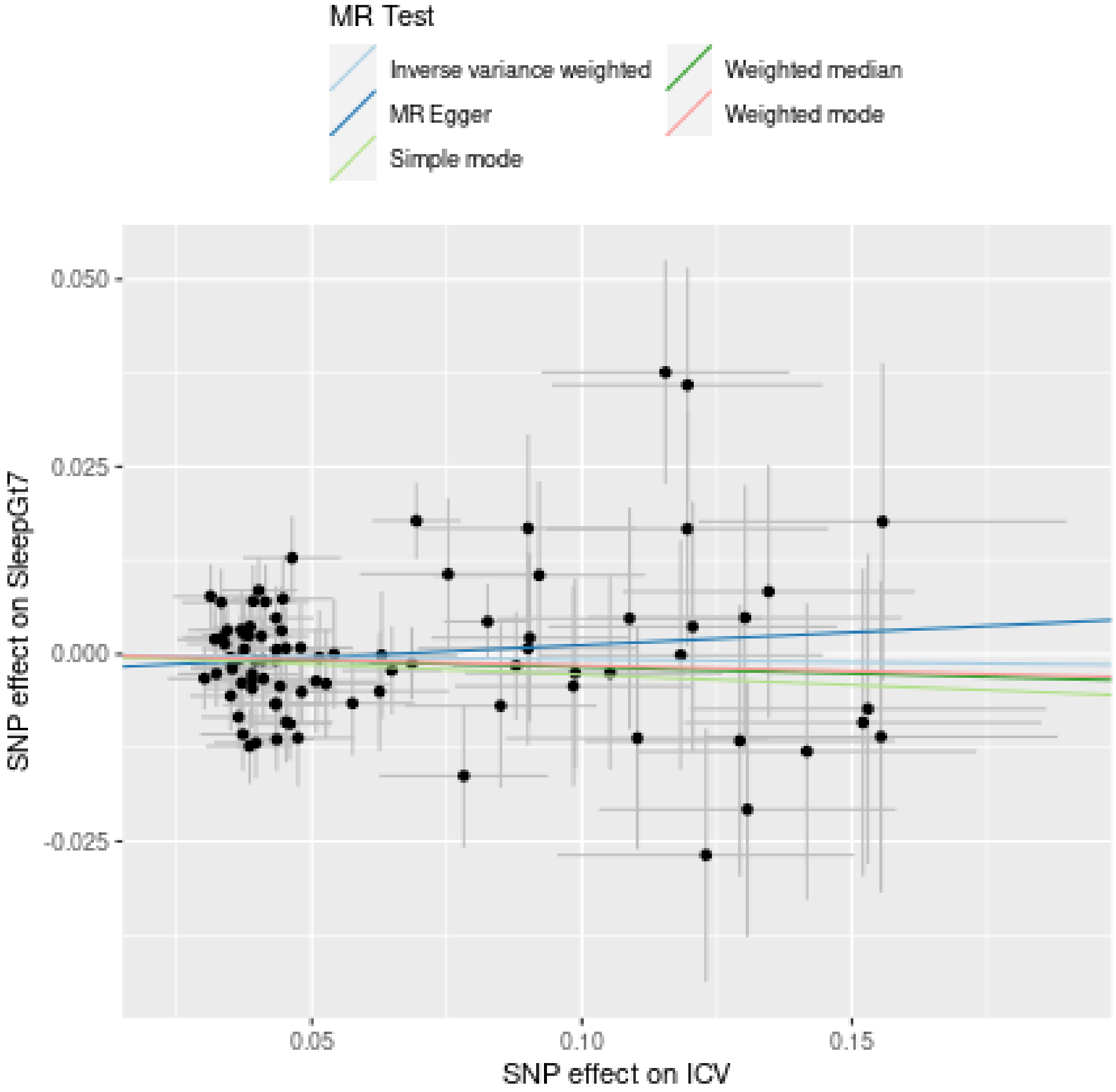

Funnel plot

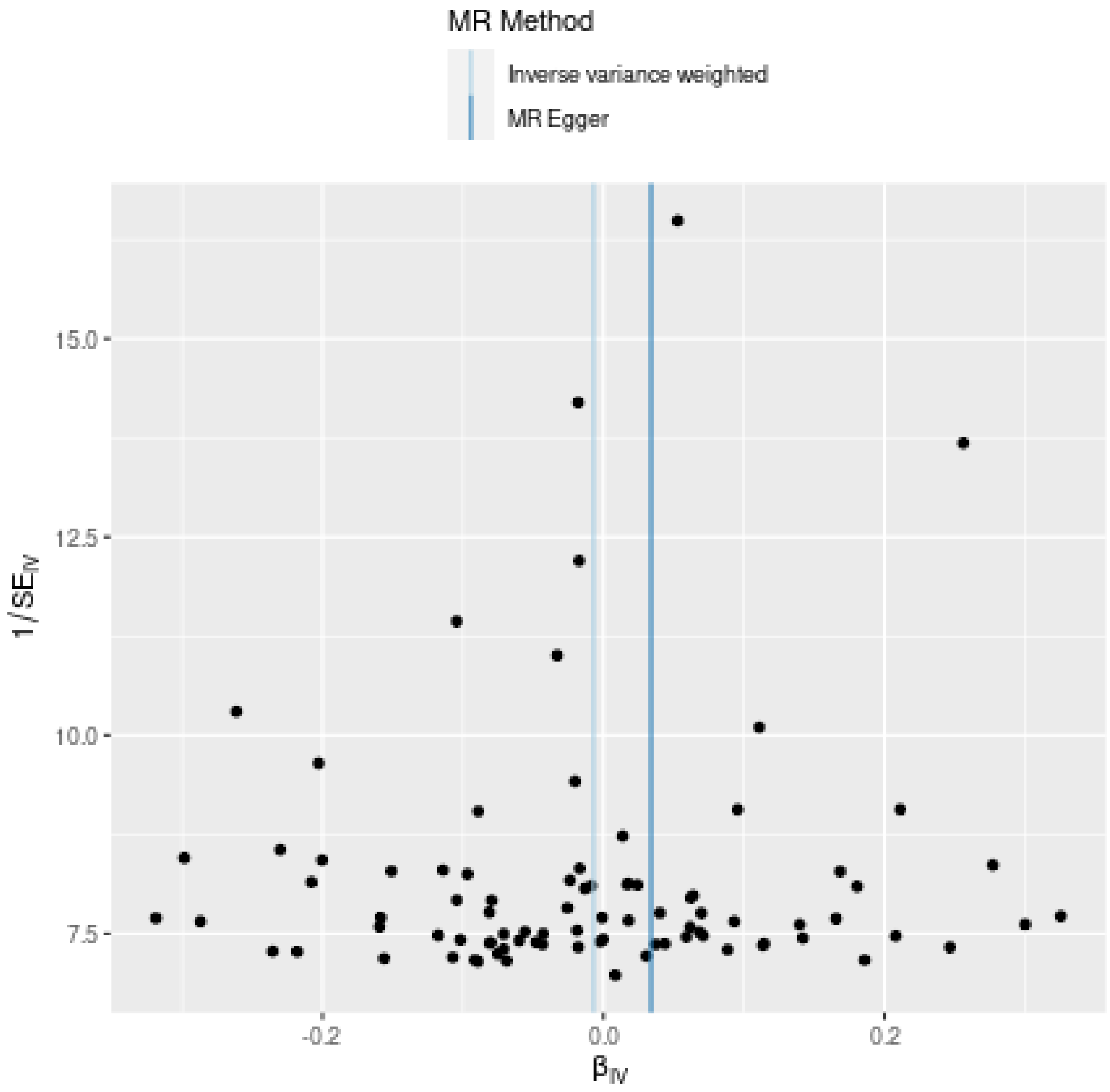
MR Method
Inverse variance weighted
MR Egger
15.0
12.5
10.0
7.5
1/SE_IV
-0.2
0.0
0.2
β_IV

Leave-one-out sensitivity analysis

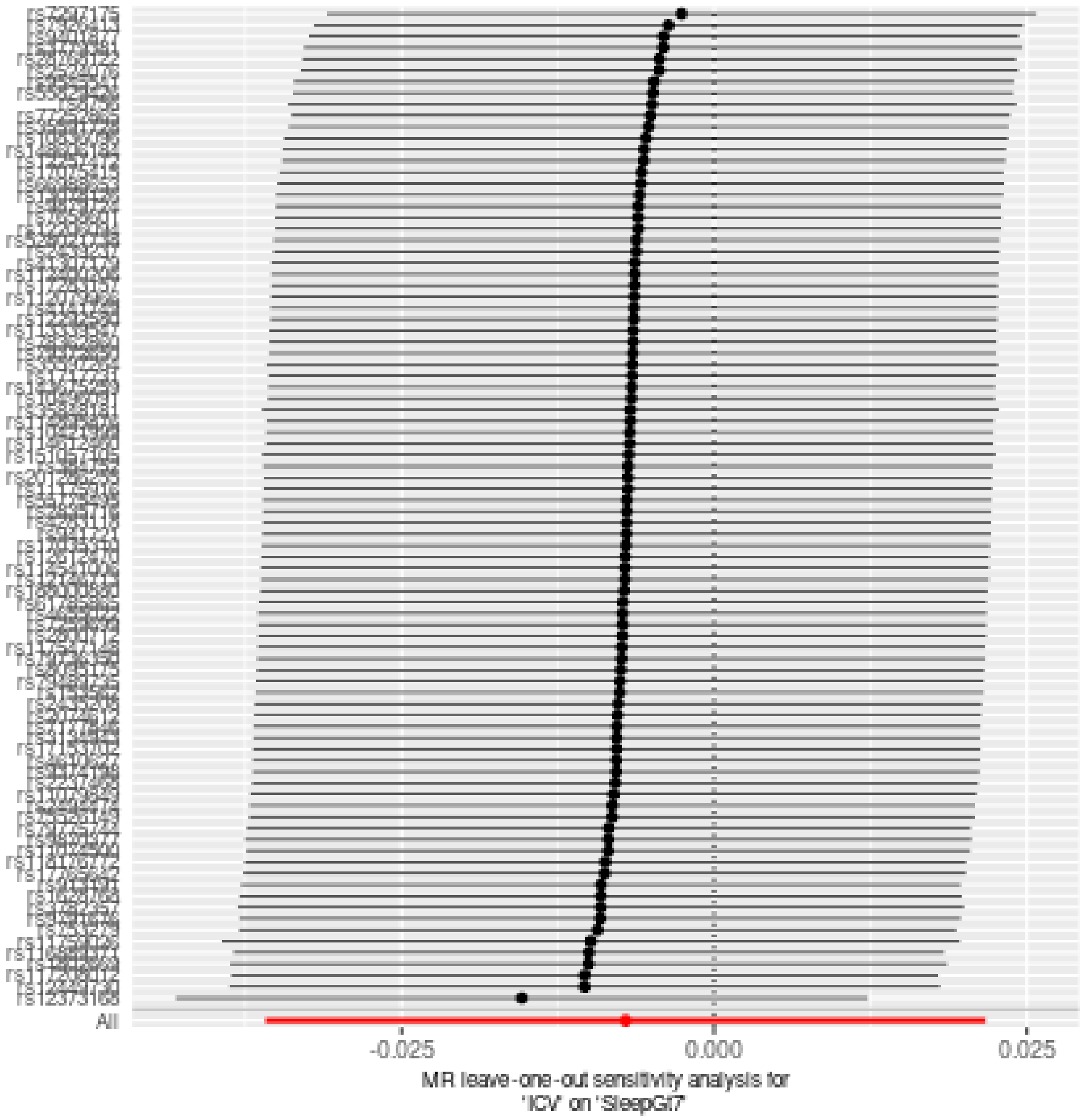

Two sample MR report

ICV against SleepLe7

Date: **24 september, 2021** P<10-6

Results from two sample MR:

method	nsnp	b	se	pval
MR Egger	88	-0.0023802	0.0318884	0.9406727
Weighted median	88	0.0427685	0.0150103	0.0043818
Inverse variance weighted	88	0.0408021	0.0118785	0.0005926
Simple mode	88	0.0482814	0.0384868	0.2130235
Weighted mode	88	0.0414786	0.0337732	0.2227018

Heterogeneity tests

method	Q	Q_df	Q_pval
MR Egger	127.3433	86	0.0025303
Inverse variance weighted	130.4896	87	0.0017729

Test for directional horizontal pleiotropy

egger_intercept	se	pval
0.0022751	0.0015608	0.1485691

Test that the exposure is upstream of the outcome

snp_r2.exposure	snp_r2.outcome	correct_causal_direction	steiger_pval
0.097292	0.0008324	TRUE	0

Note - R^2 values are approximate

Forest plot of single SNP MR

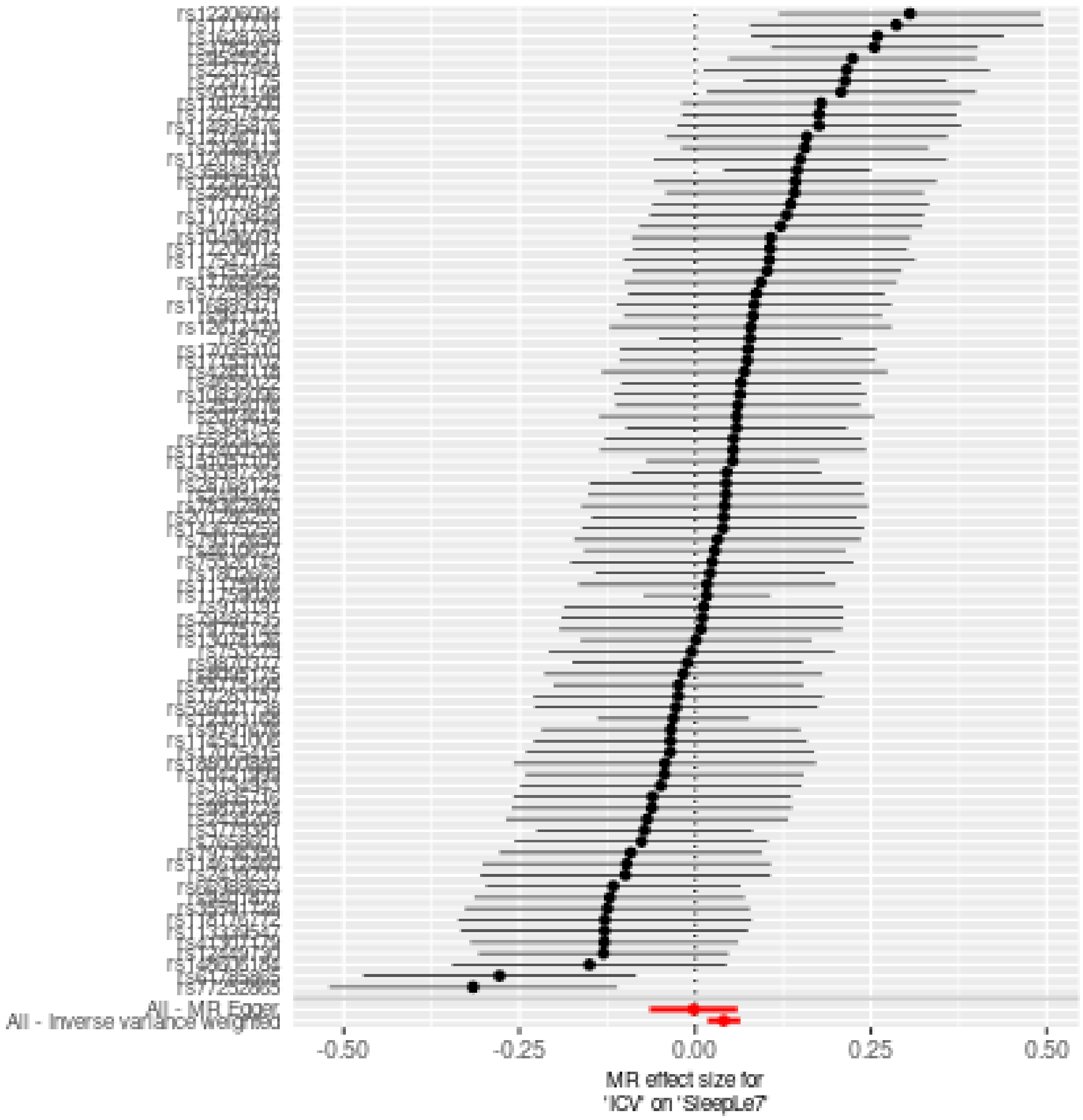

Comparison of results using different MR methods

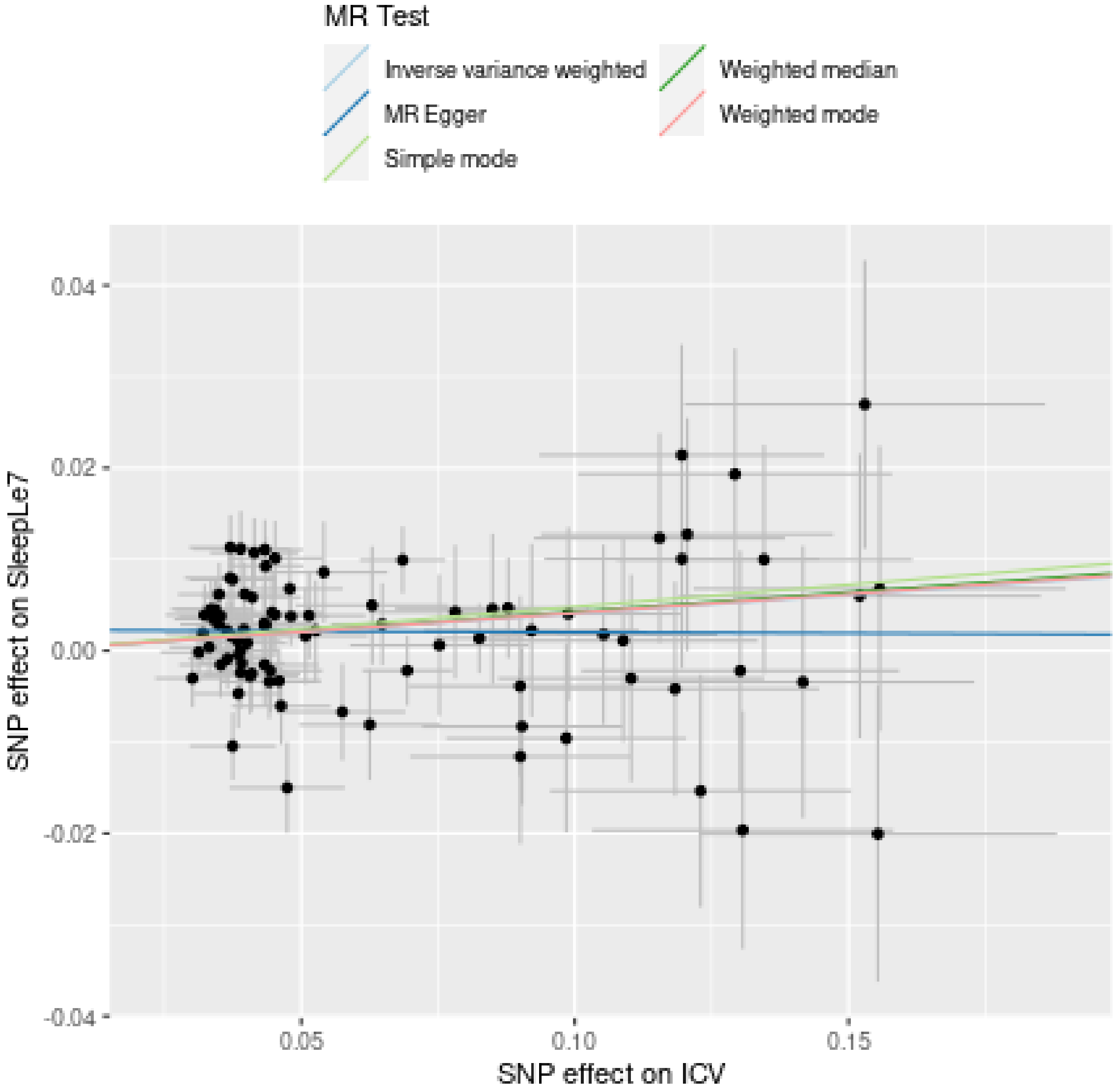

Funnel plot

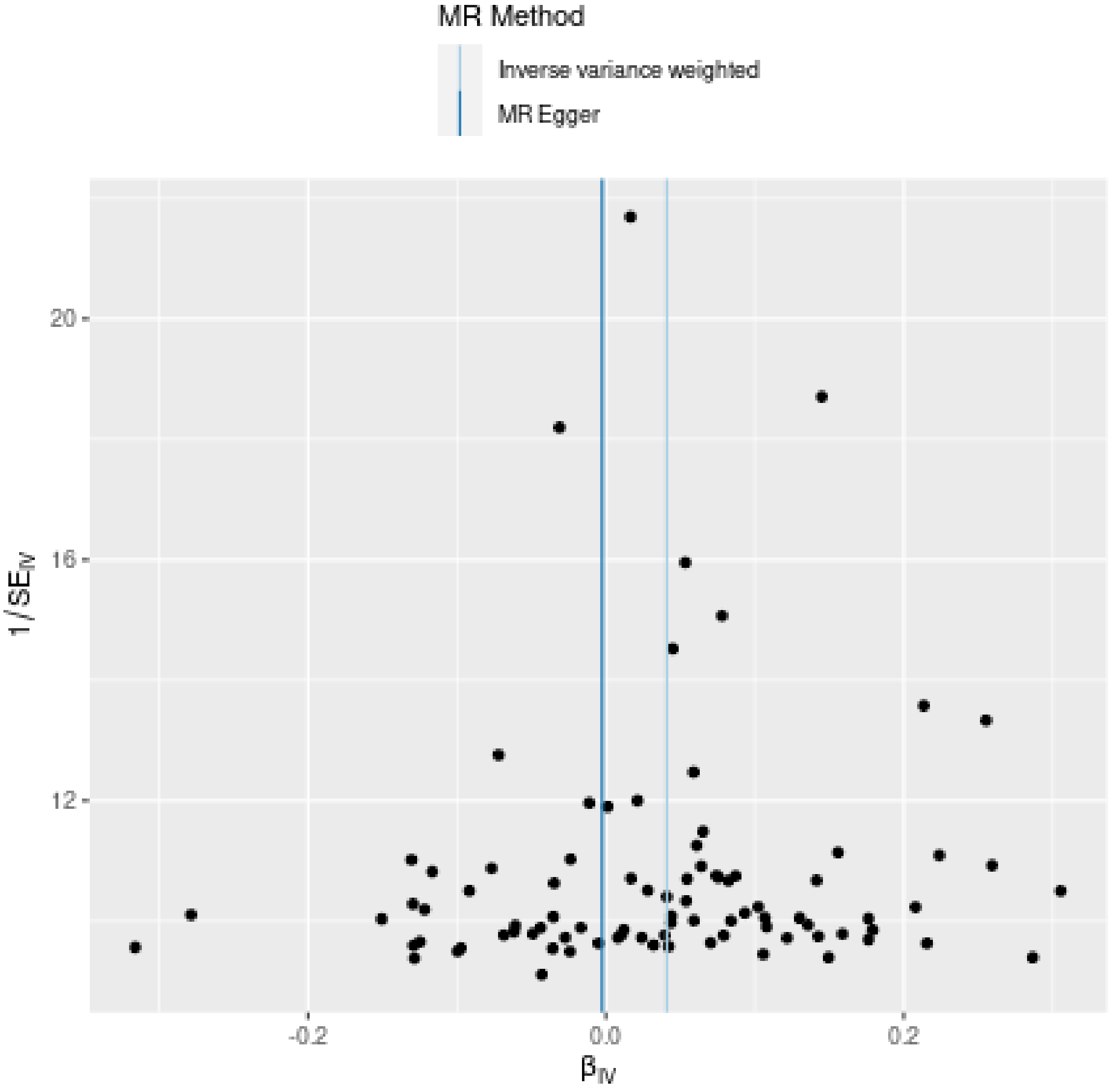
MR Method
Inverse variance weighted
MR Egger
20
16
12
$1/SE_{IV}$
-0.2
0.0
0.2
β_{IV}

Leave-one-out sensitivity analysis

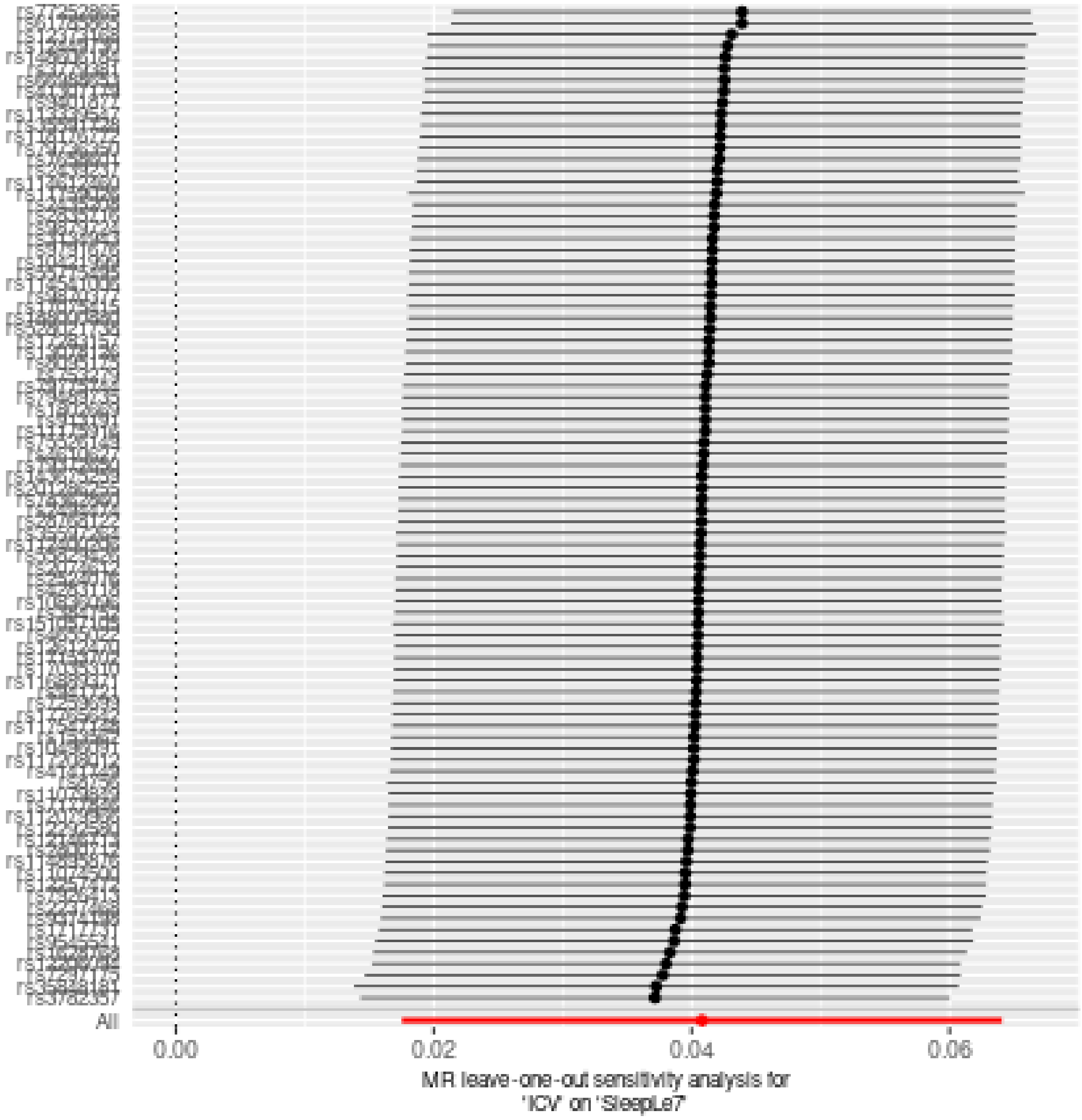

Two sample MR report

SleepGt7 against HippV P<10-6

Date: **24 september, 2021**

Results from two sample MR:

method	nsnp	b	se	pval
MR Egger	21	-0.0925011	0.1512298	0.5480129
Weighted median	21	-0.0458650	0.1029470	0.6559435
Inverse variance weighted	21	-0.0746943	0.0728970	0.3055257
Simple mode	21	-0.0566075	0.1817977	0.7587370
Weighted mode	21	-0.0606288	0.1772525	0.7358833

Heterogeneity tests

method	Q	Q_df	Q_pval
MR Egger	16.35114	19	0.6337366
Inverse variance weighted	16.36920	20	0.6934719

Test for directional horizontal pleiotropy

egger_intercept	se	pval
0.0006559	0.0048807	0.8945073

Test that the exposure is upstream of the outcome

snp_r2.exposure	snp_r2.outcome	correct_causal_direction	steiger_pval
0.0052712	0.0010812	TRUE	0

Note - R^2 values are approximate

Forest plot of single SNP MR

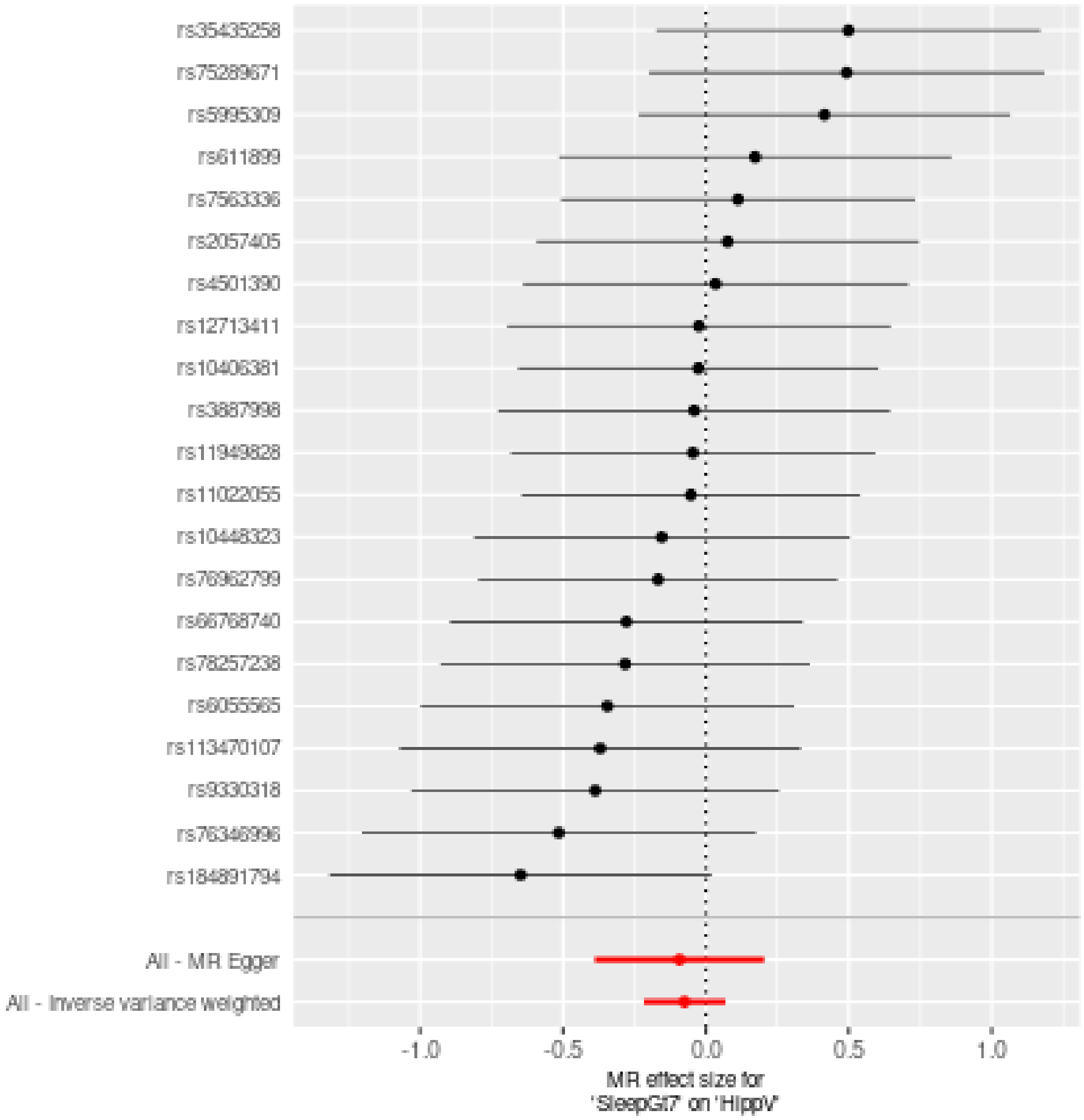

Comparison of results using different MR methods

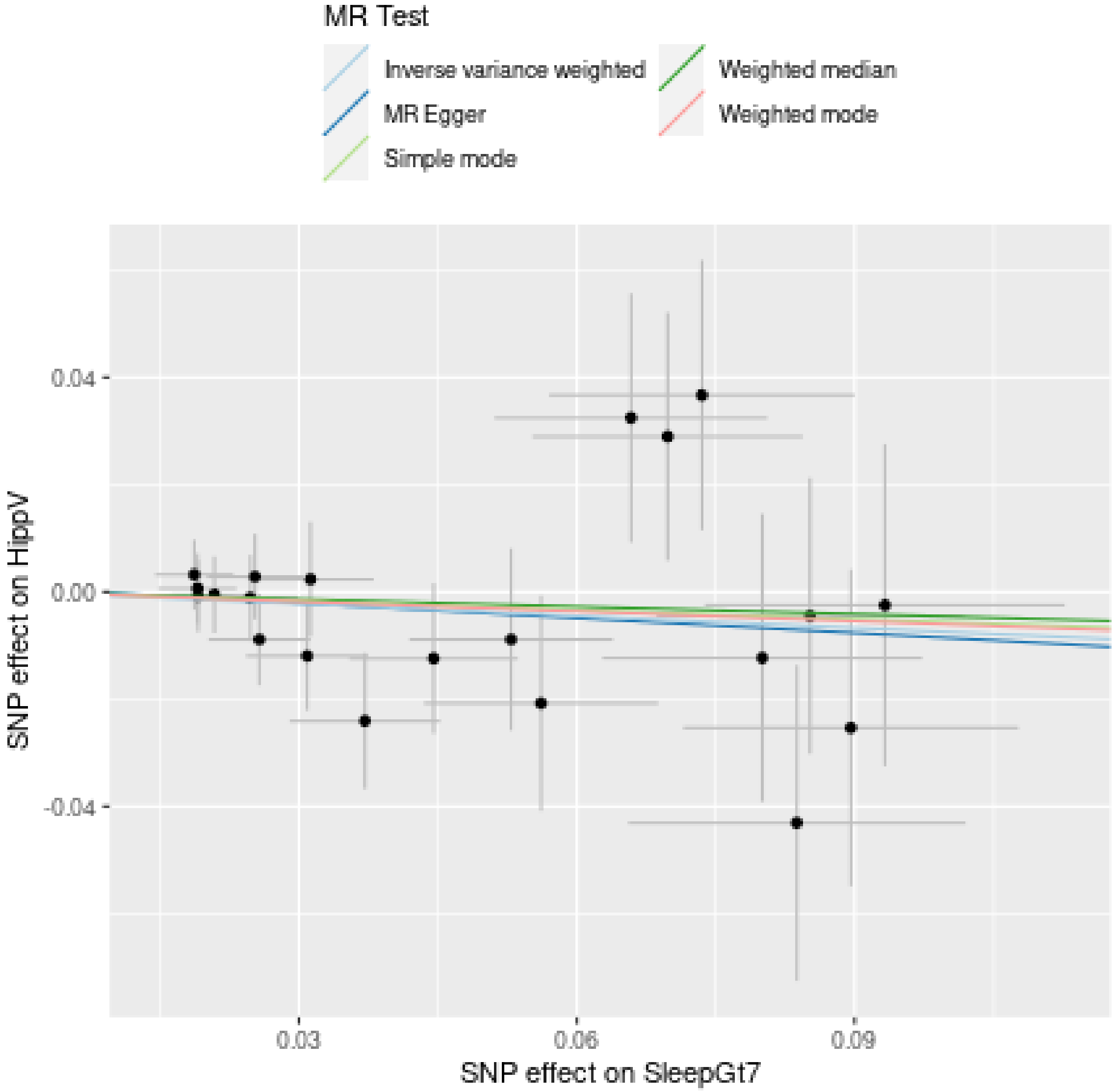

Funnel plot

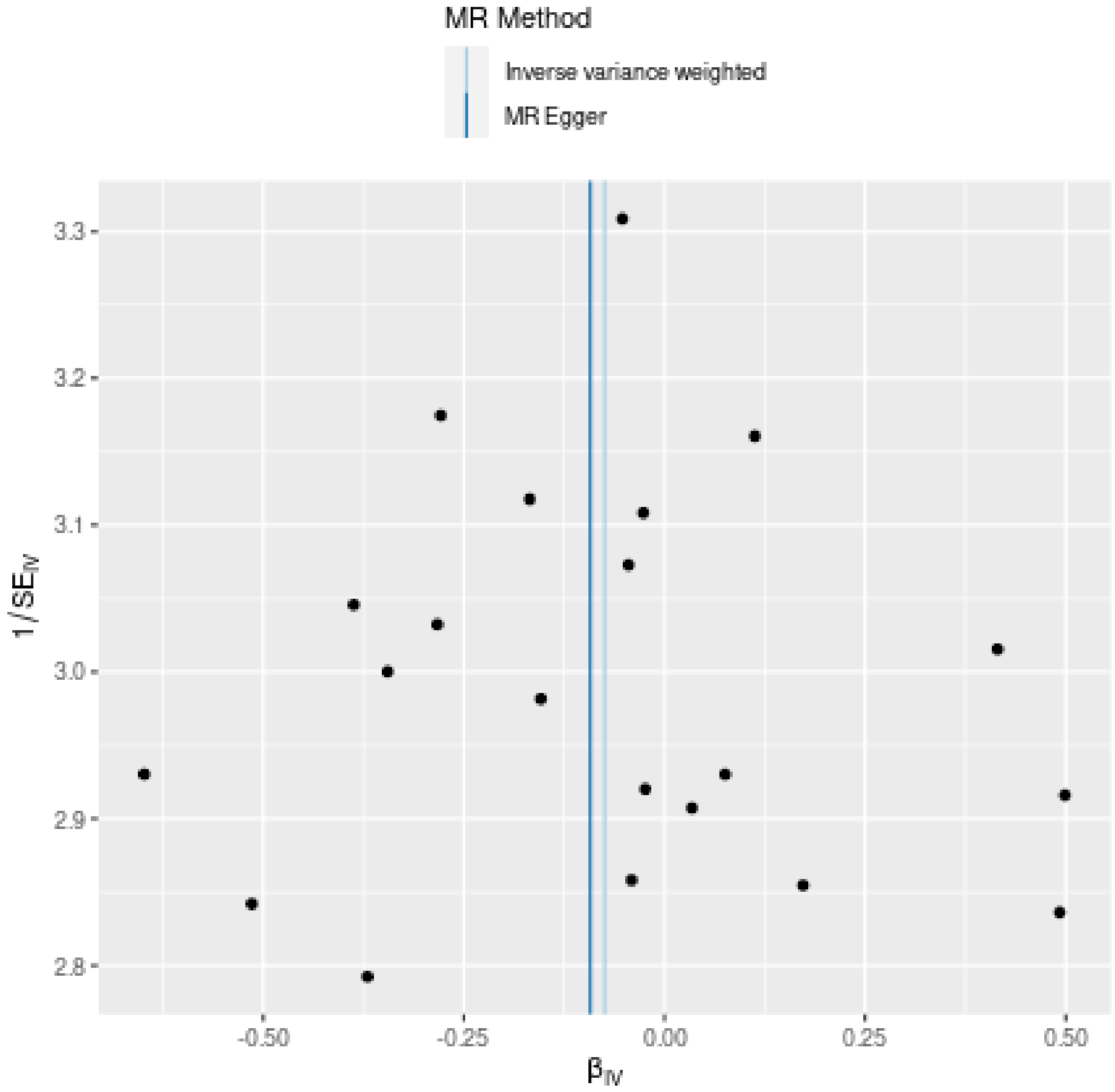
MR Method
Inverse variance weighted
MR Egger
3.3
3.2
3.1
3.0
2.9
2.8
1/SE$_{IV}$
-0.50
-0.25
0.00
0.25
0.50
β_{IV}

Leave-one-out sensitivity analysis

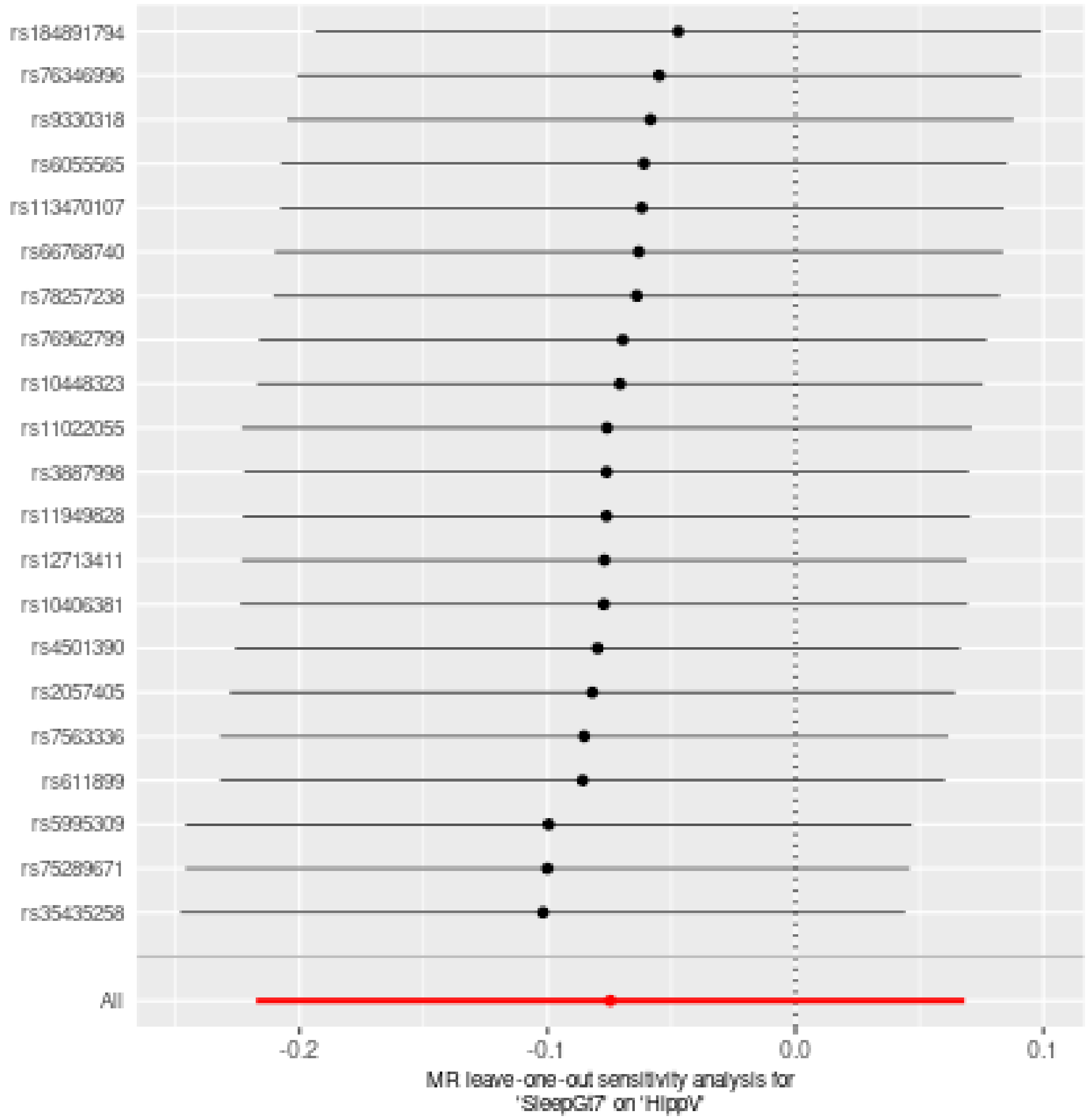

Two sample MR report

SleepGt7 against ICV

P<10-6

Date: **24 september, 2021**

Results from two sample MR:

method	nsnp	b	se	pval
MR Egger	21	-0.0290303	0.1581733	0.8563230
Weighted median	21	-0.0036594	0.1063354	0.9725472
Inverse variance weighted	21	0.0243909	0.0760816	0.7485224
Simple mode	21	0.0004752	0.1914223	0.9980439
Weighted mode	21	-0.0031448	0.1987966	0.9875353

Heterogeneity tests

method	Q	Q_df	Q_pval
MR Egger	12.19716	19	0.8770258
Inverse variance weighted	12.34556	20	0.9036687

Test for directional horizontal pleiotropy

egger_intercept	se	pval
0.0019644	0.0050993	0.7043465

Test that the exposure is upstream of the outcome

snp_r2.exposure	snp_r2.outcome	correct_causal_direction	steiger_pval
0.0052712	0.0005336	TRUE	0

Note - R^2 values are approximate

Forest plot of single SNP MR

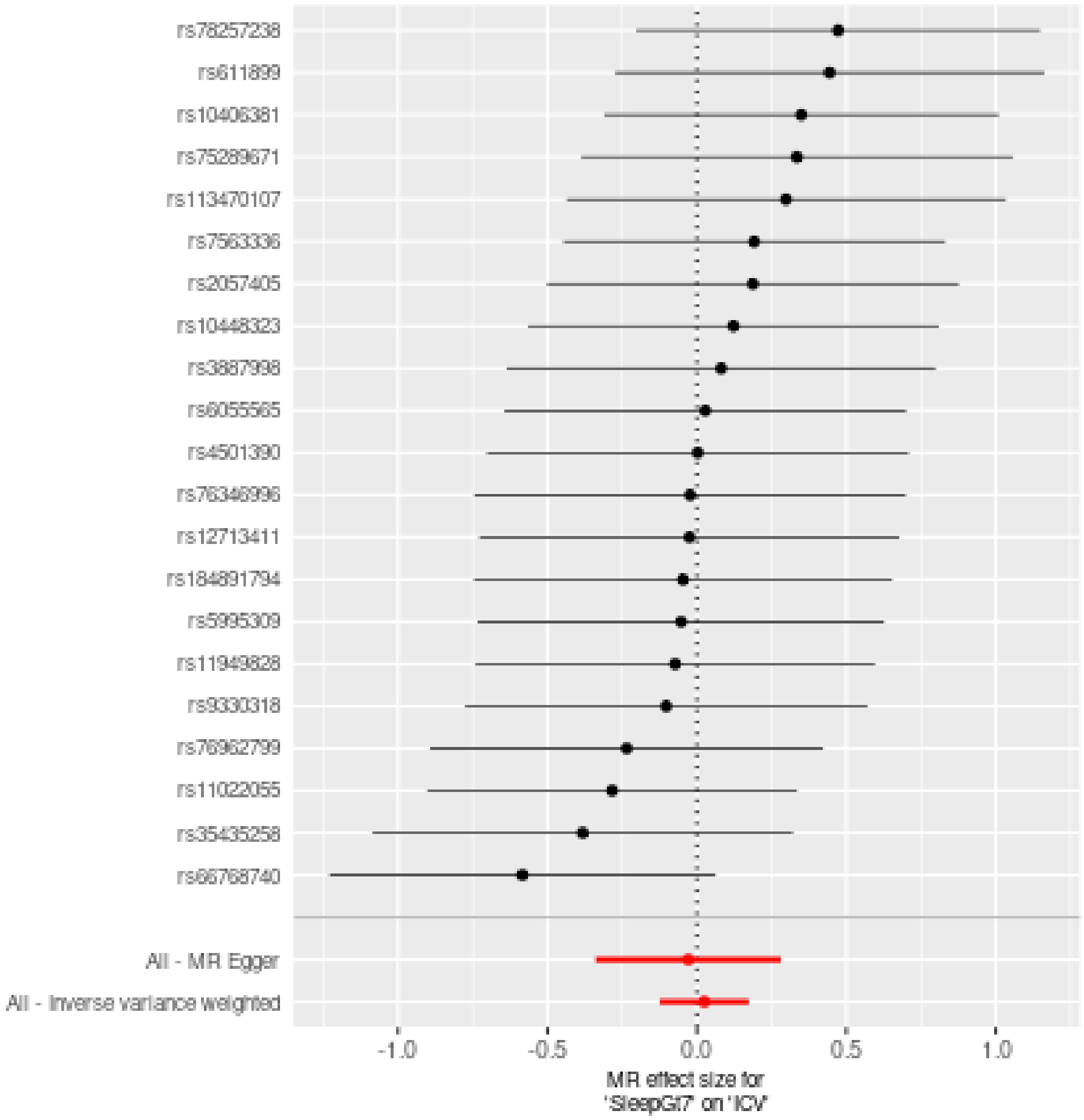

Comparison of results using different MR methods

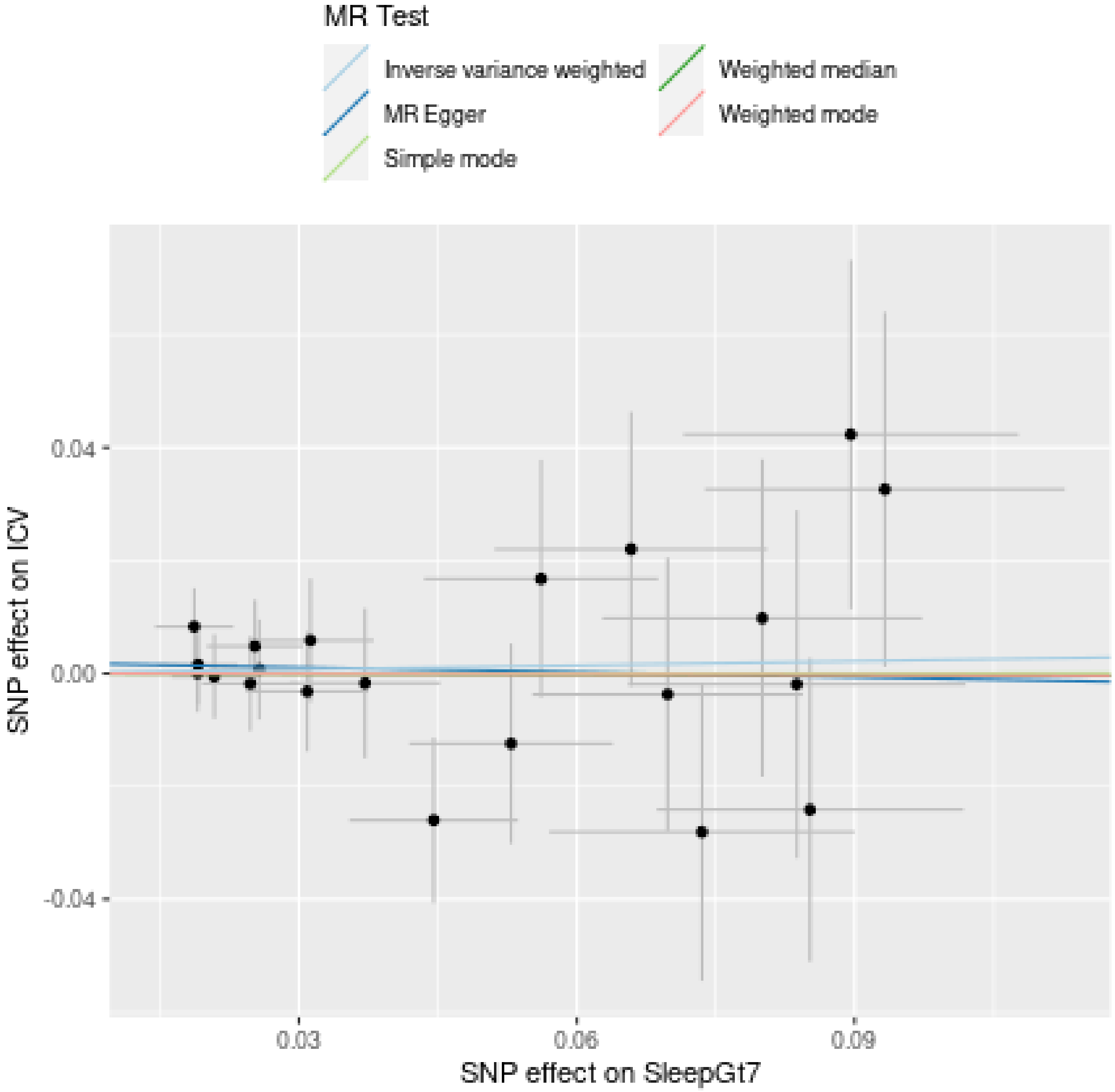

Funnel plot

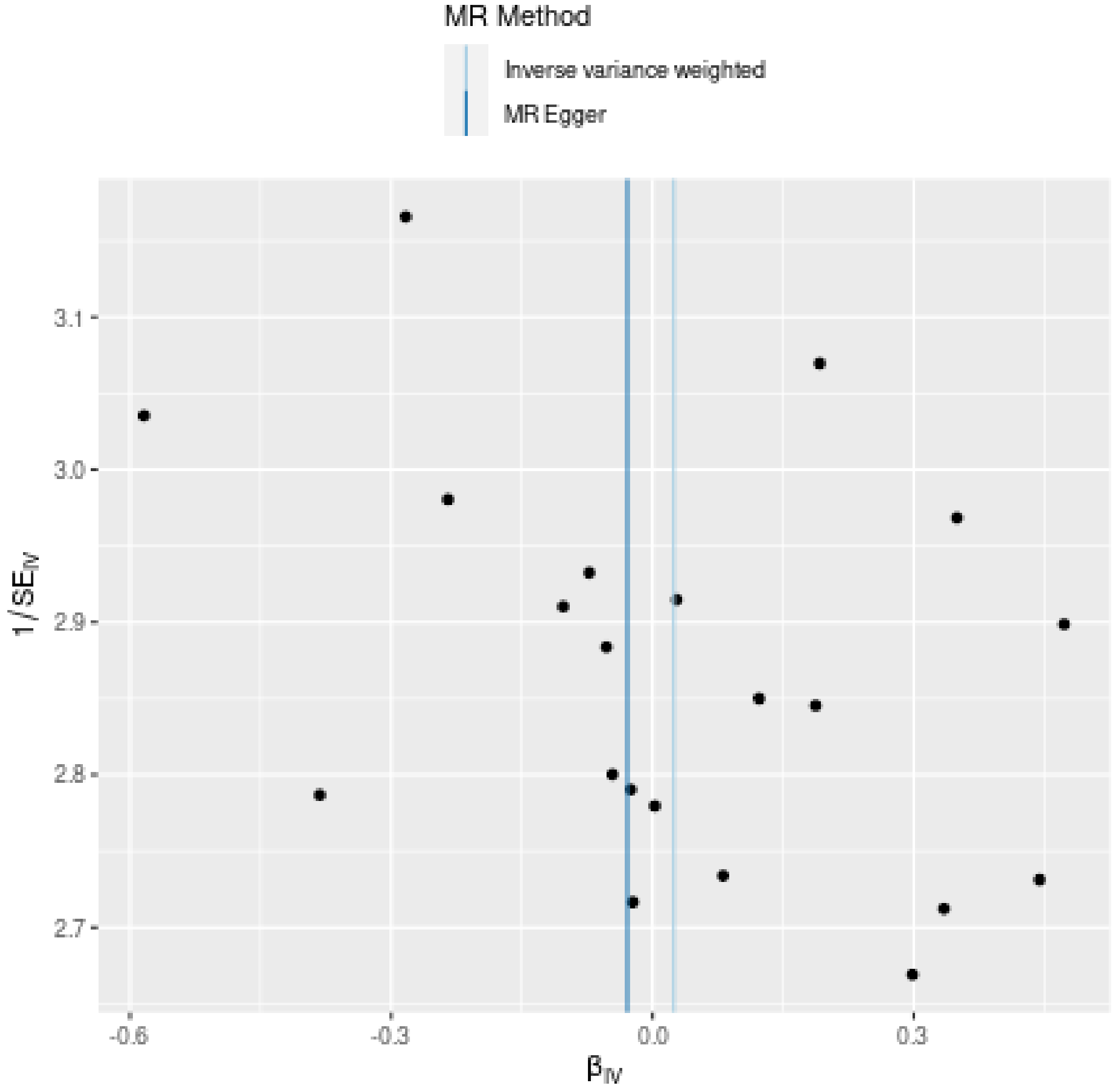
MR Method
Inverse variance weighted
MR Egger
3.1
3.0
2.9
2.8
2.7
1/SE_IV
-0.6
-0.3
0.0
0.3
β_IV

Leave-one-out sensitivity analysis

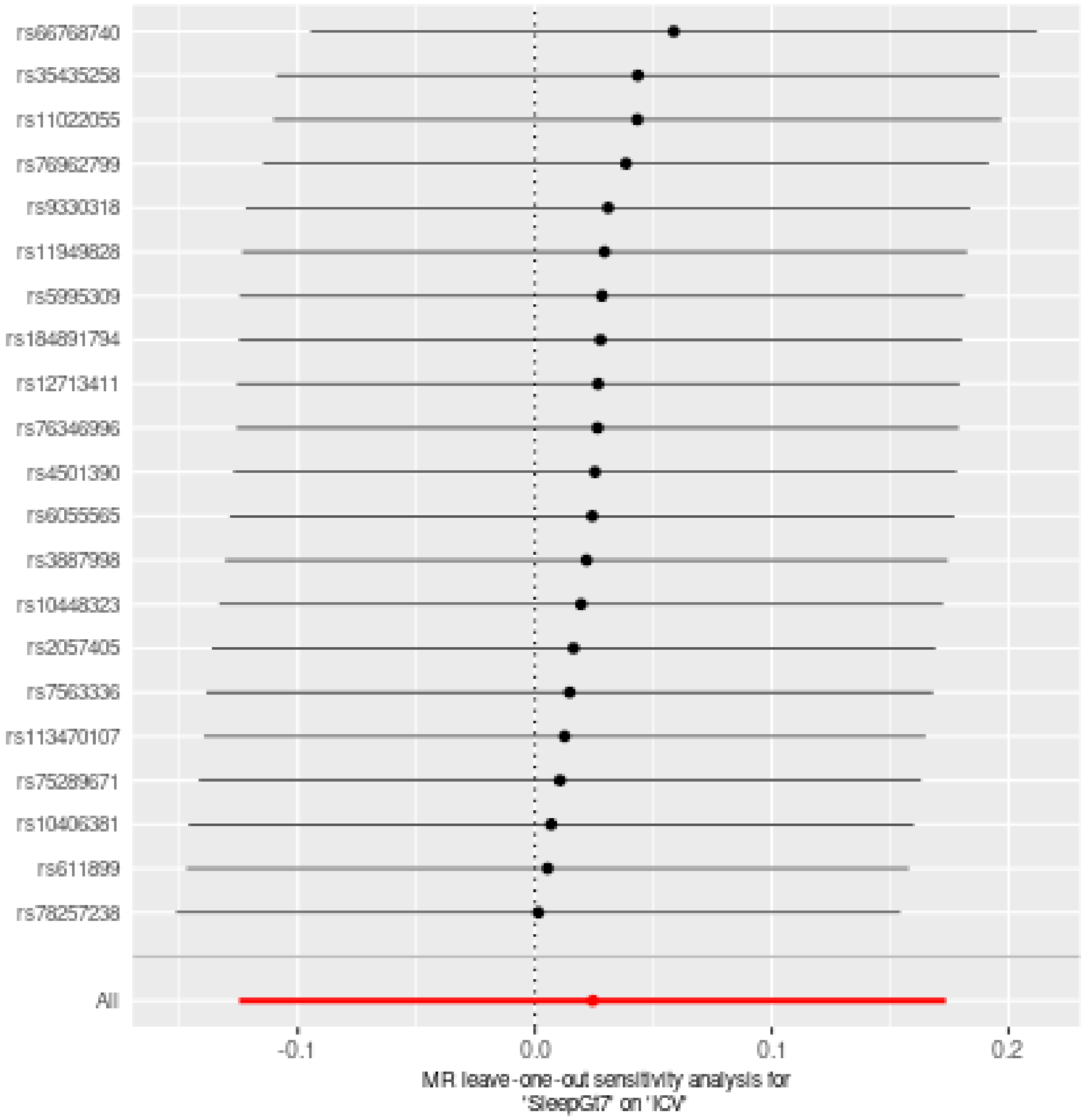

Two sample MR report

SleepGt7 against TGV

P<10-6

Date: **24 september, 2021**

Results from two sample MR:

method	nsnp	b	se	pval
MR Egger	21	-0.1411426	0.0960387	0.1580233
Weighted median	21	-0.0374634	0.0657610	0.5688877
Inverse variance weighted	21	-0.0540507	0.0461293	0.2413081
Simple mode	21	0.0416703	0.1285359	0.7491586
Weighted mode	21	0.0566952	0.1393968	0.6885346

Heterogeneity tests

method	Q	Q_df	Q_pval
MR Egger	16.74293	19	0.6072791
Inverse variance weighted	17.81191	20	0.5997972

Test for directional horizontal pleiotropy

egger_intercept	se	pval
0.0031989	0.0030939	0.3141566

Test that the exposure is upstream of the outcome

snp_r2.exposure	snp_r2.outcome	correct_causal_direction	steiger_pval
0.0052712	0.0010403	TRUE	0

Note - R^2 values are approximate

Forest plot of single SNP MR

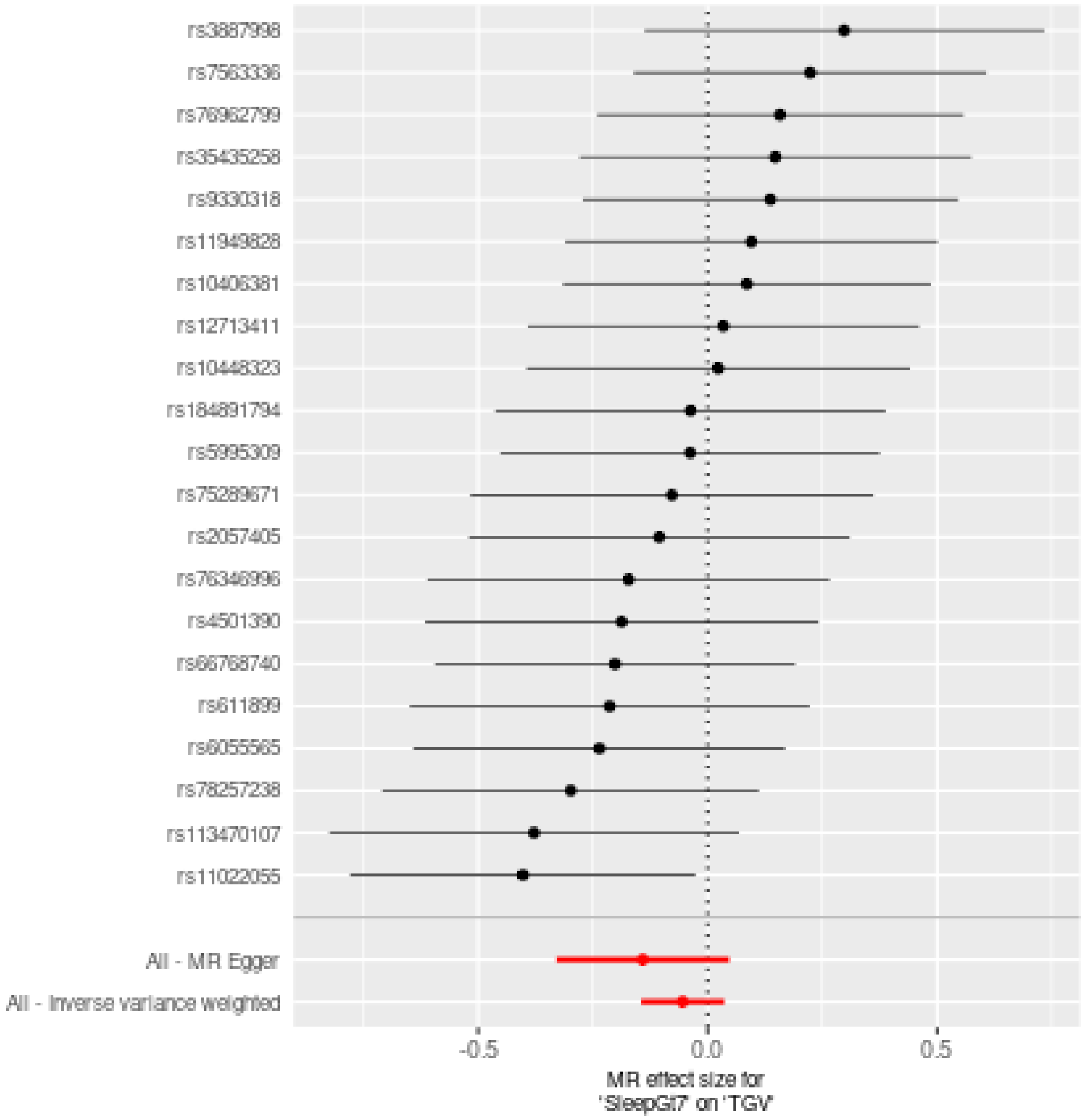

Comparison of results using different MR methods

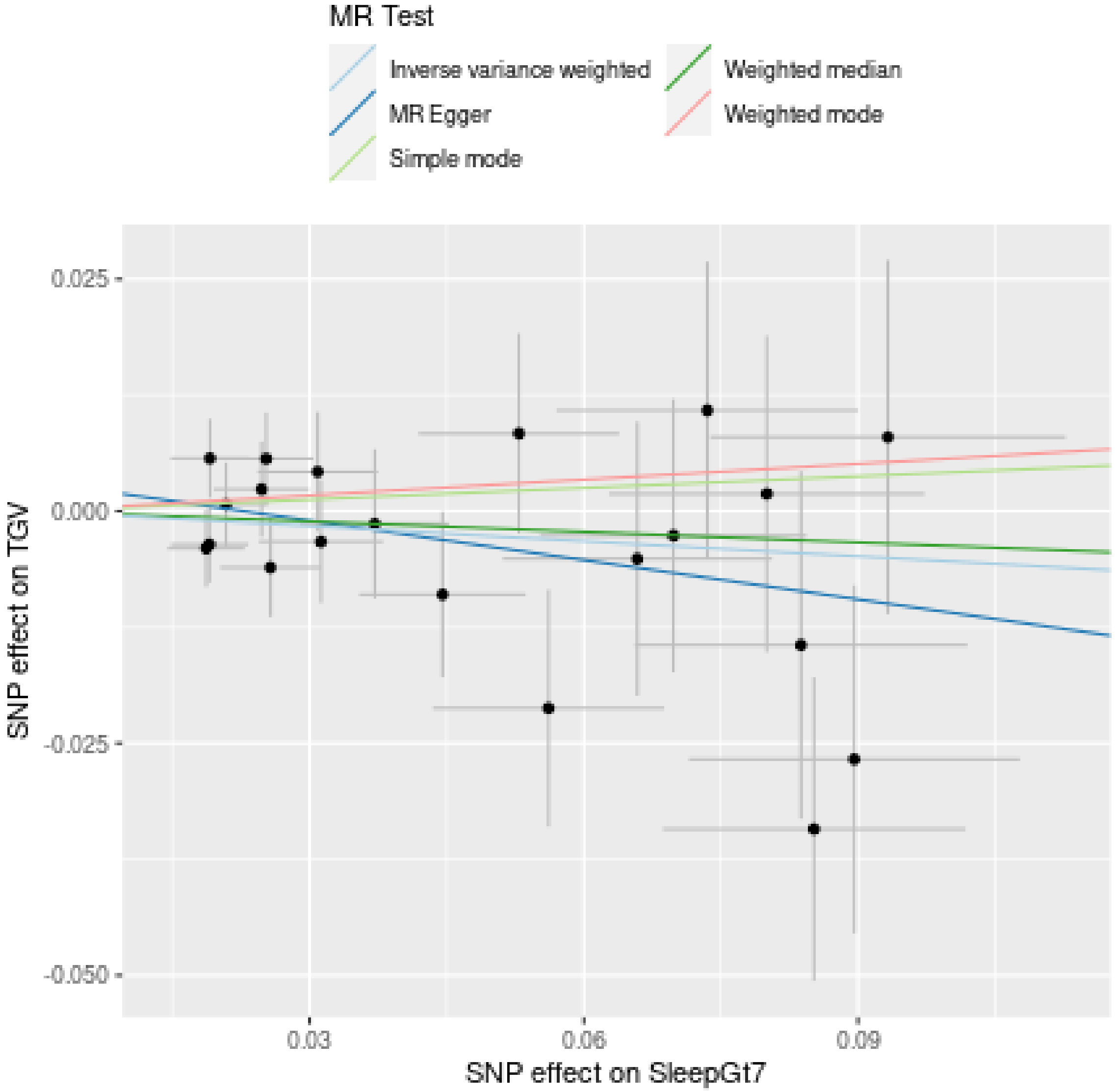

Funnel plot

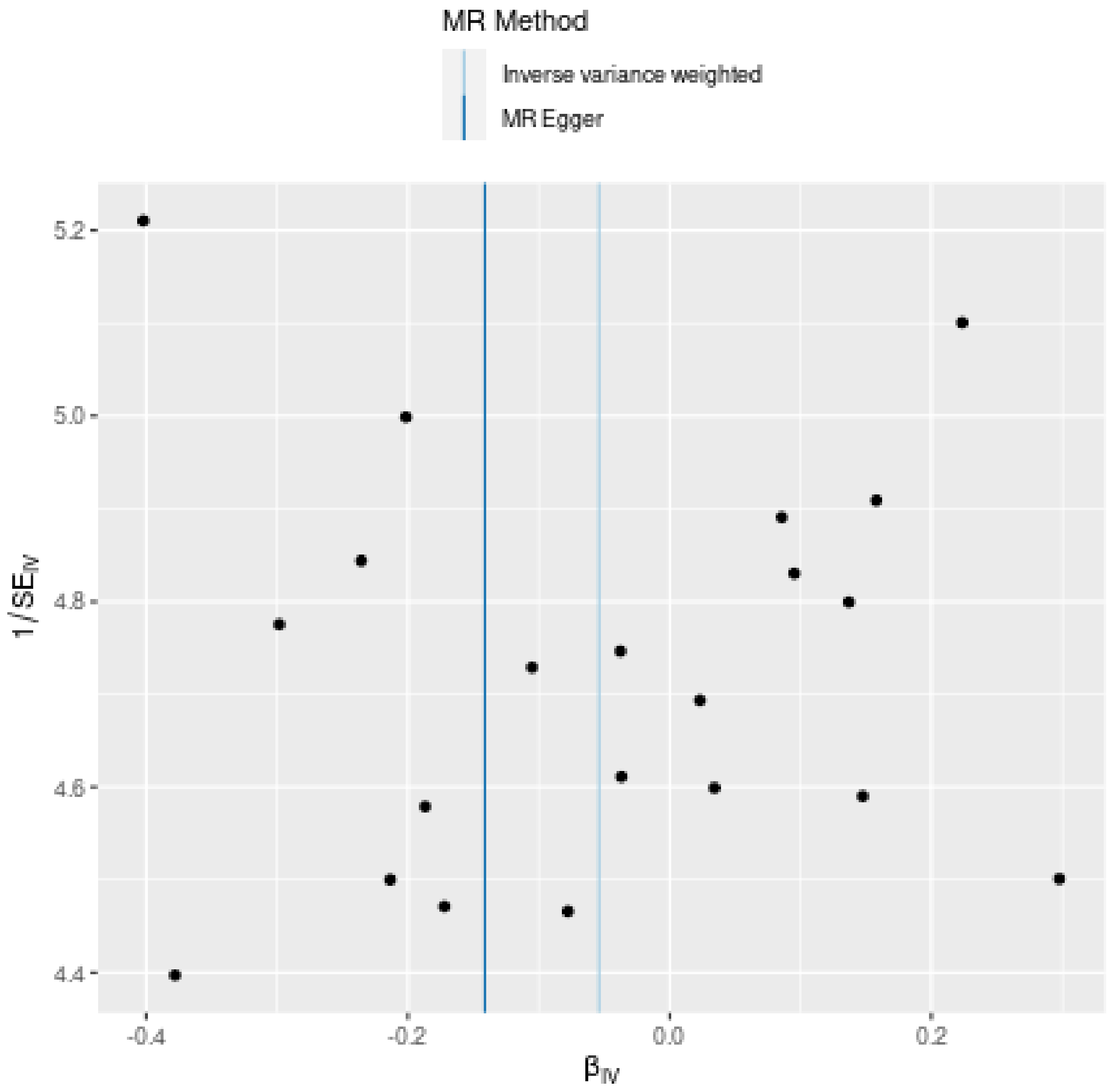

MR Method
Inverse variance weighted
MR Egger
5.2
5.0
4.8
4.6
4.4
1/SE$_{IV}$
-0.4
-0.2
0.0
0.2
β$_{IV}$

Leave-one-out sensitivity analysis

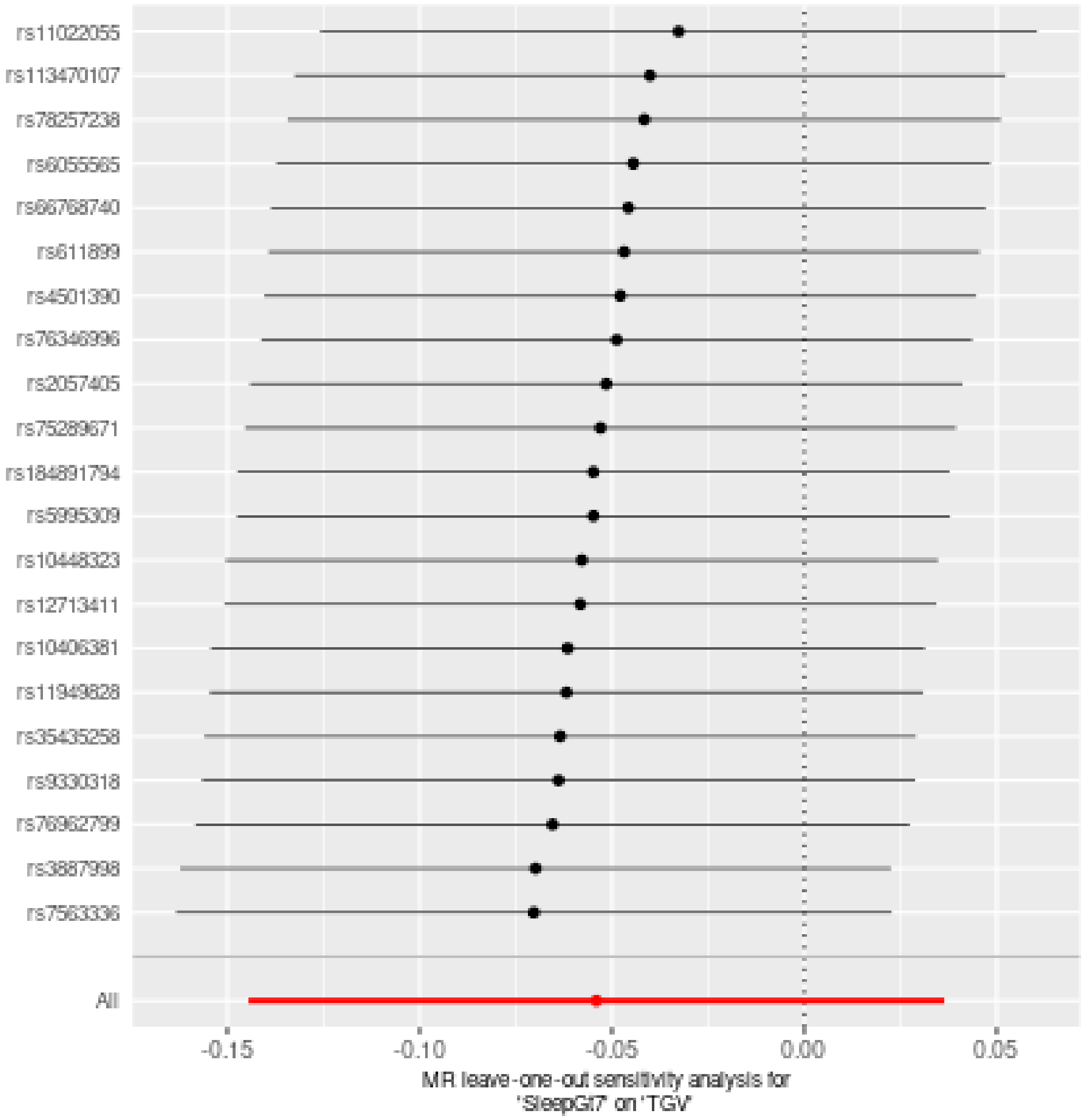

Two sample MR report

SleepLe7 against HippV

P<10-6

Date: **24 september, 2021**

Results from two sample MR:

method	nsnp	b	se	pval
MR Egger	30	-0.0678012	0.2229604	0.7633039
Weighted median	30	-0.0519938	0.1143050	0.6492035
Inverse variance weighted	30	0.0022258	0.0790990	0.9775514
Simple mode	30	-0.1871948	0.2508017	0.4614427
Weighted mode	30	-0.2103634	0.2740287	0.4488894

Heterogeneity tests

method	Q	Q_df	Q_pval
MR Egger	29.85086	28	0.3703822
Inverse variance weighted	29.97172	29	0.4154165

Test for directional horizontal pleiotropy

egger_intercept	se	pval
0.0015696	0.0046618	0.7388584

Test that the exposure is upstream of the outcome

snp_r2.exposure	snp_r2.outcome	correct_causal_direction	steiger_pval
0.0037888	0.0010377	TRUE	3.2e-06

Note - R^2 values are approximate

Forest plot of single SNP MR

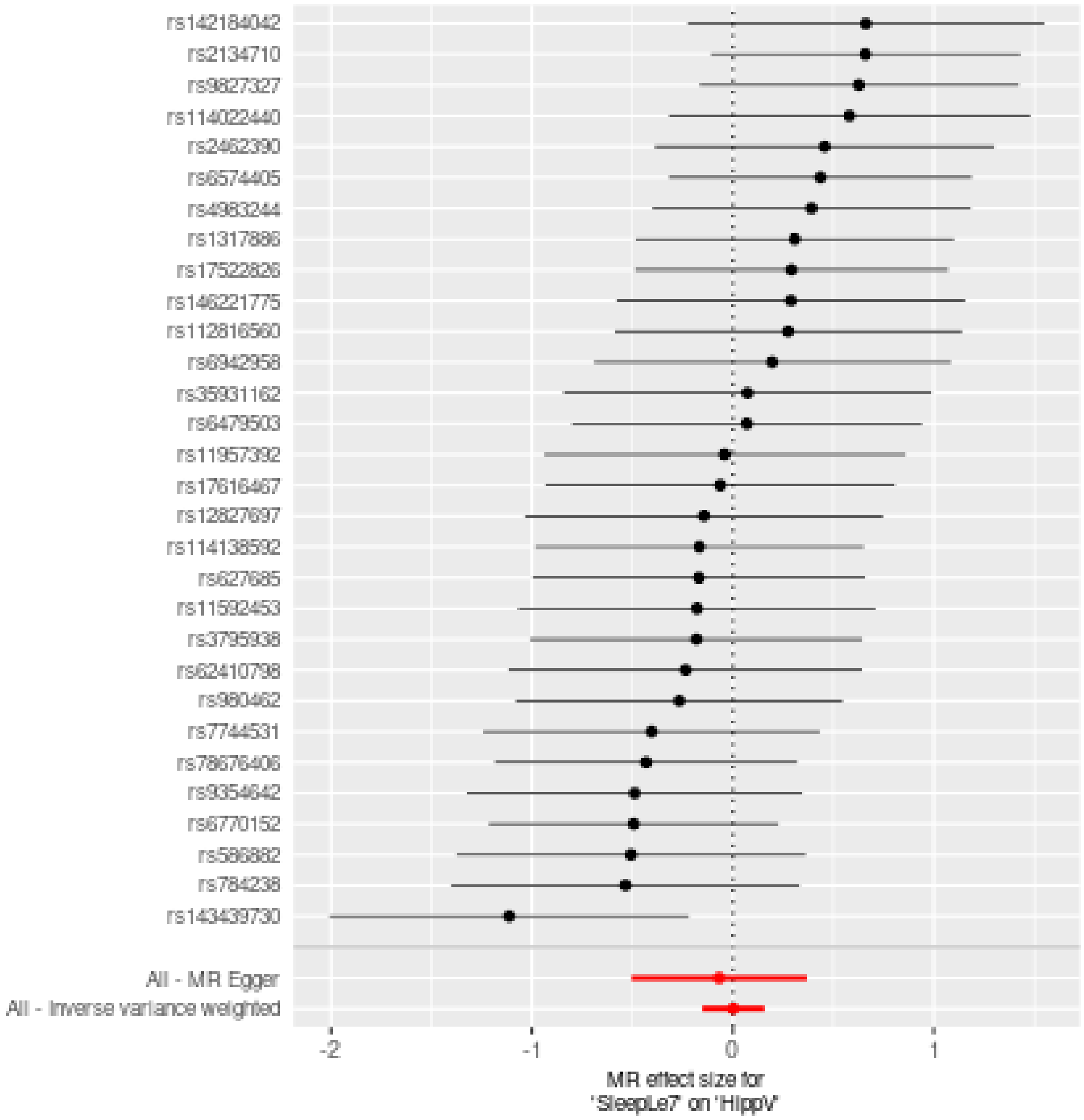

Comparison of results using different MR methods

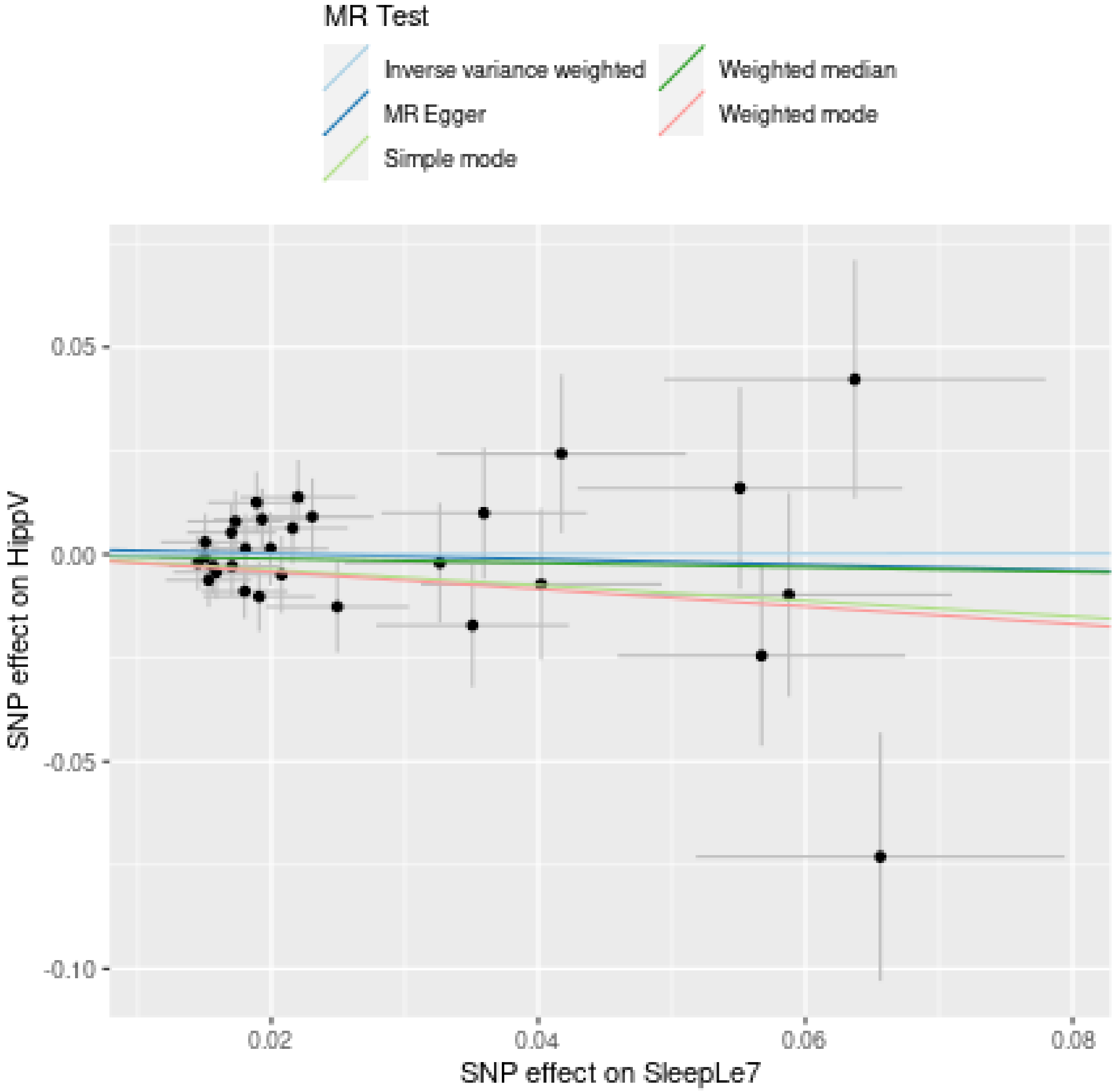

Funnel plot

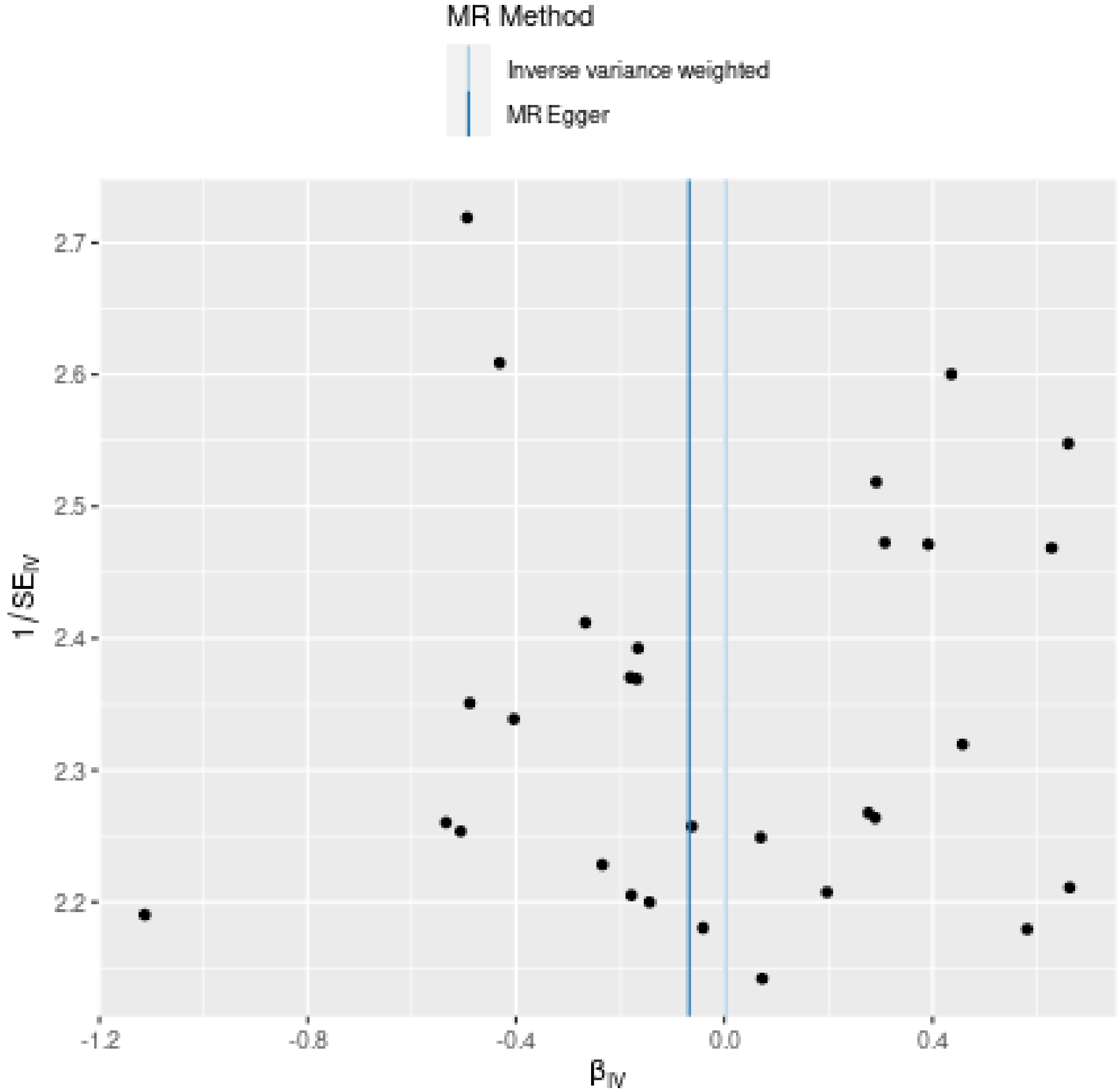

MR Method
Inverse variance weighted
MR Egger
2.7
2.6
2.5
2.4
2.3
2.2
1/SE$_{IV}$
-1.2
-0.8
-0.4
0.0
0.4
β$_{IV}$

Leave-one-out sensitivity analysis

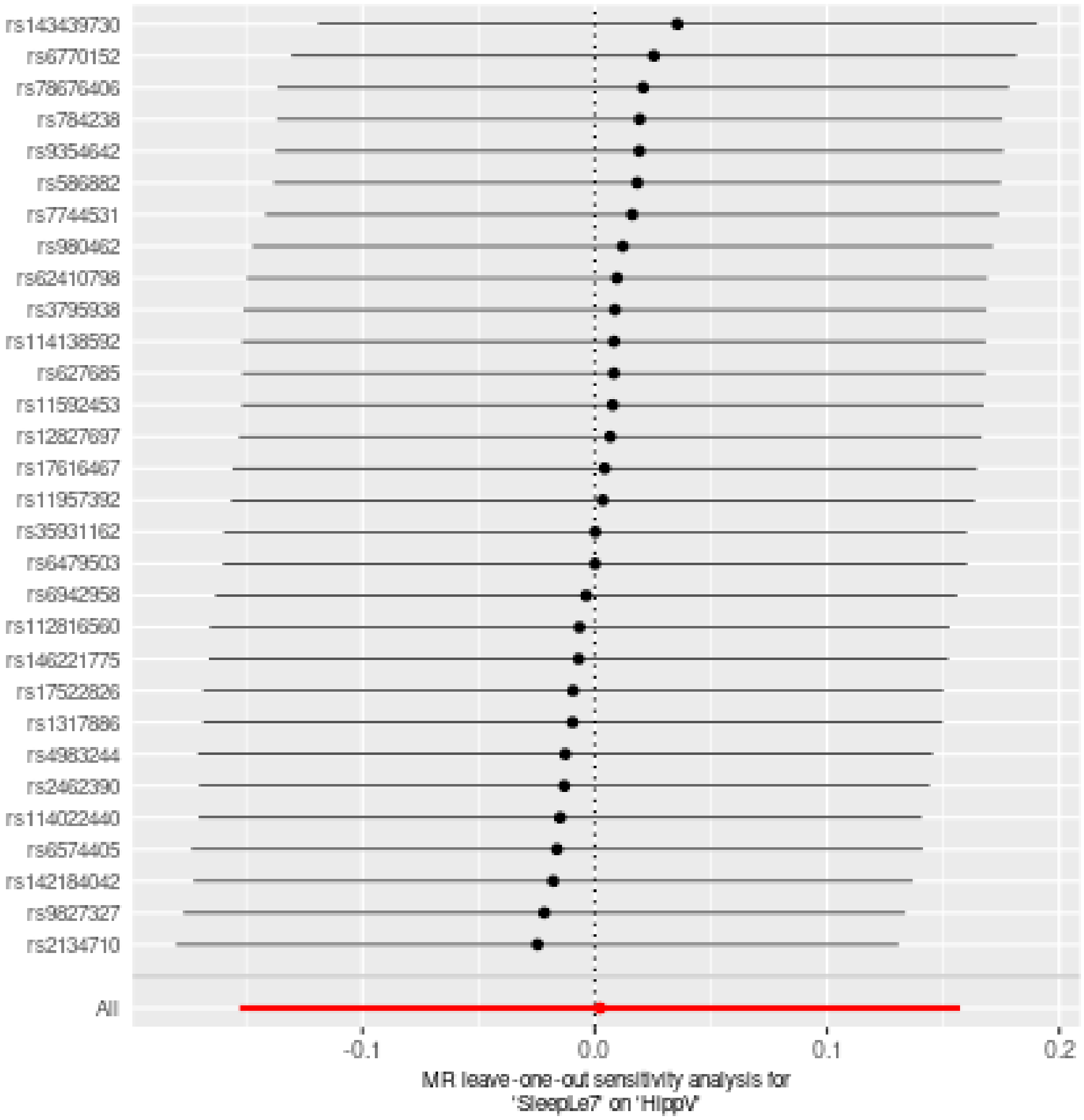

Two sample MR report

SleepLe7 against ICV

P<10-6

Date: **24 september, 2021**

Results from two sample MR:

method	nsnp	b	se	pval
MR Egger	30	0.0849180	0.2261827	0.7101625
Weighted median	30	-0.0461790	0.1143781	0.6864042
Inverse variance weighted	30	0.0318054	0.0813939	0.6959755
Simple mode	30	-0.0890807	0.2332230	0.7052794
Weighted mode	30	-0.0890807	0.2263548	0.6967960

Heterogeneity tests

method	Q	Q_df	Q_pval
MR Egger	28.07049	28	0.4607169
Inverse variance weighted	28.13401	29	0.5107562

Test for directional horizontal pleiotropy

egger_intercept	se	pval
-0.0011905	0.0047292	0.8030881

Test that the exposure is upstream of the outcome

snp_r2.exposure	snp_r2.outcome	correct_causal_direction	steiger_pval
0.0037888	0.0010717	TRUE	4.8e-06

Note - R^2 values are approximate

Forest plot of single SNP MR

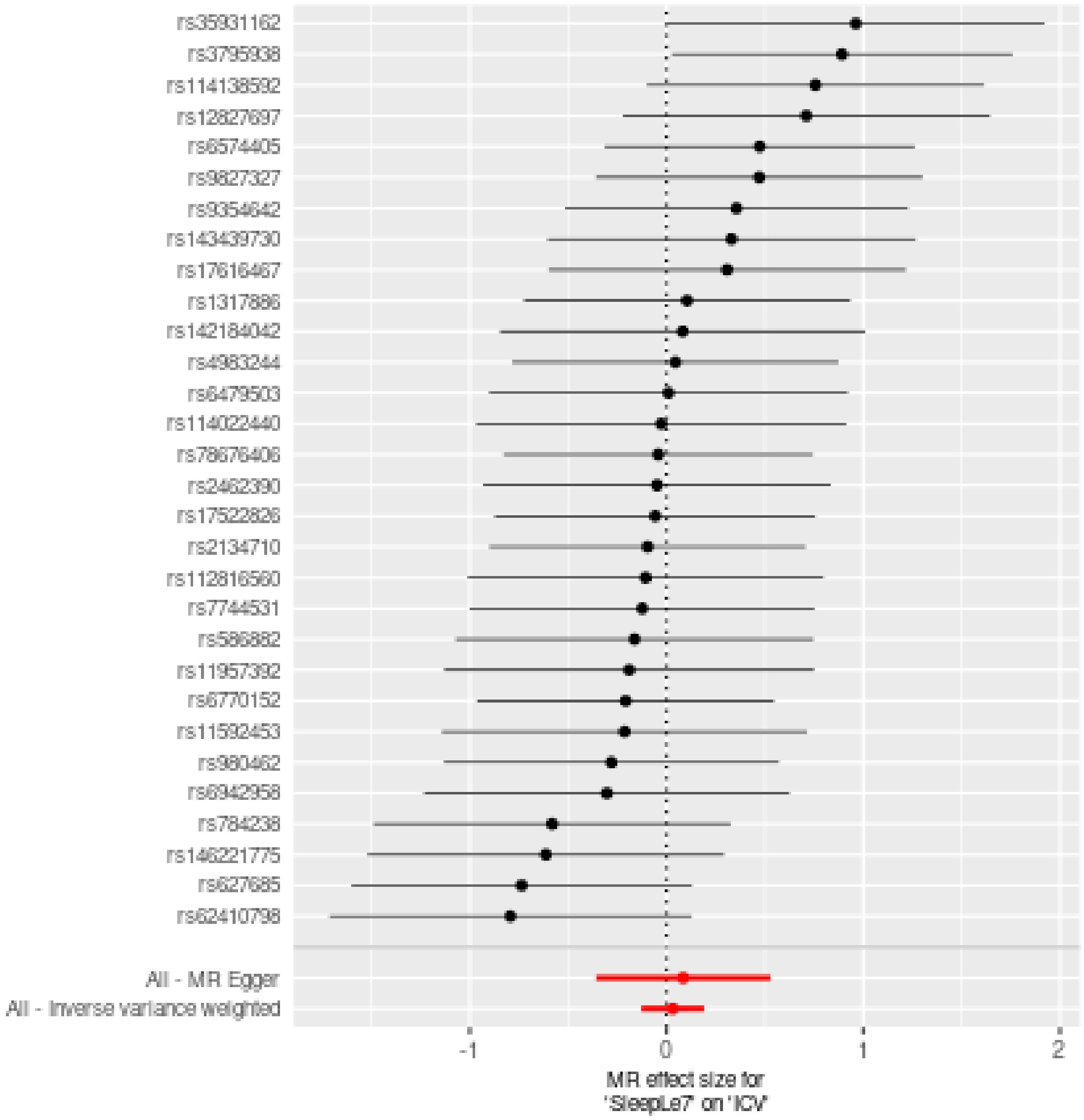

Comparison of results using different MR methods

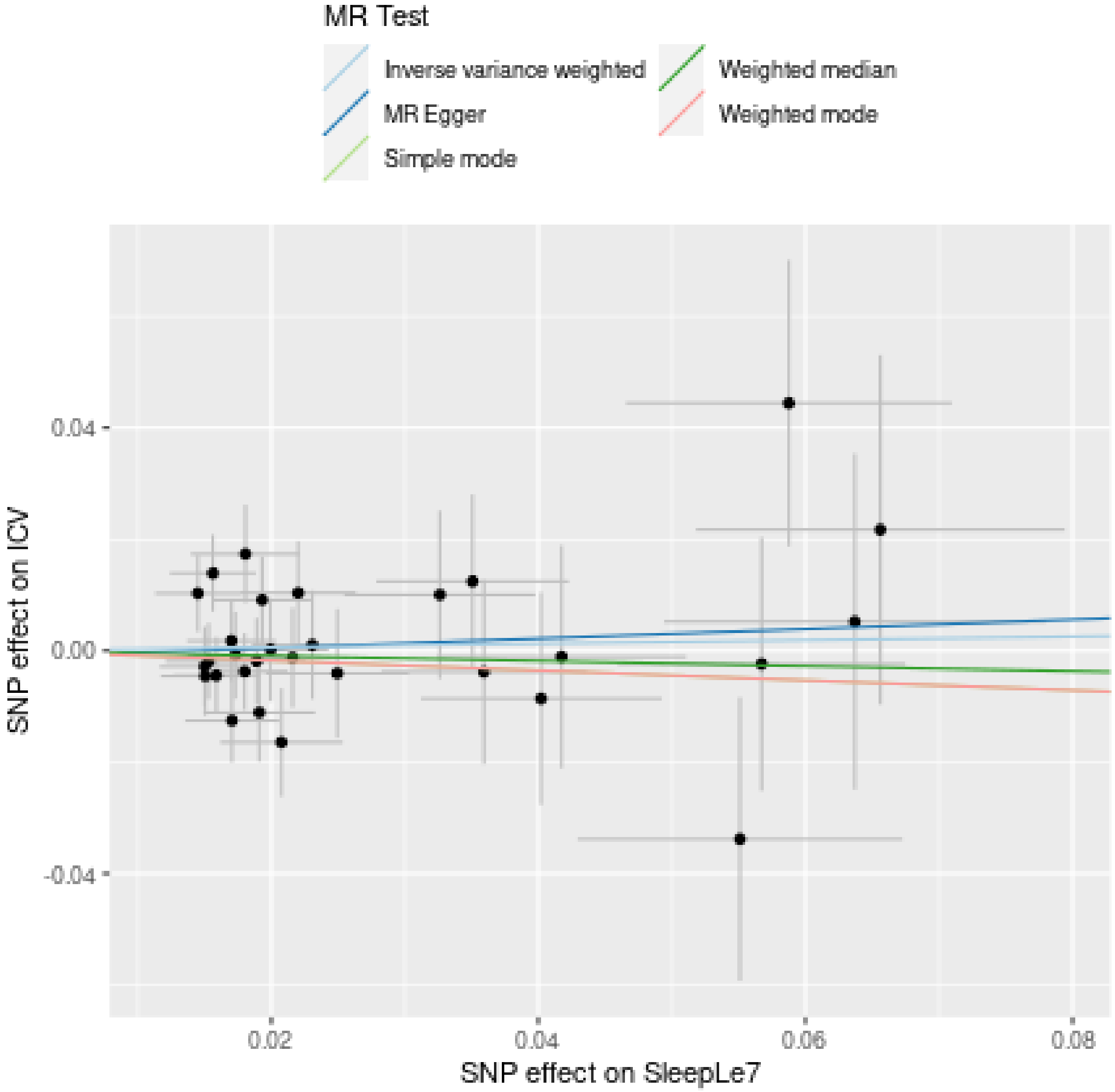

Funnel plot

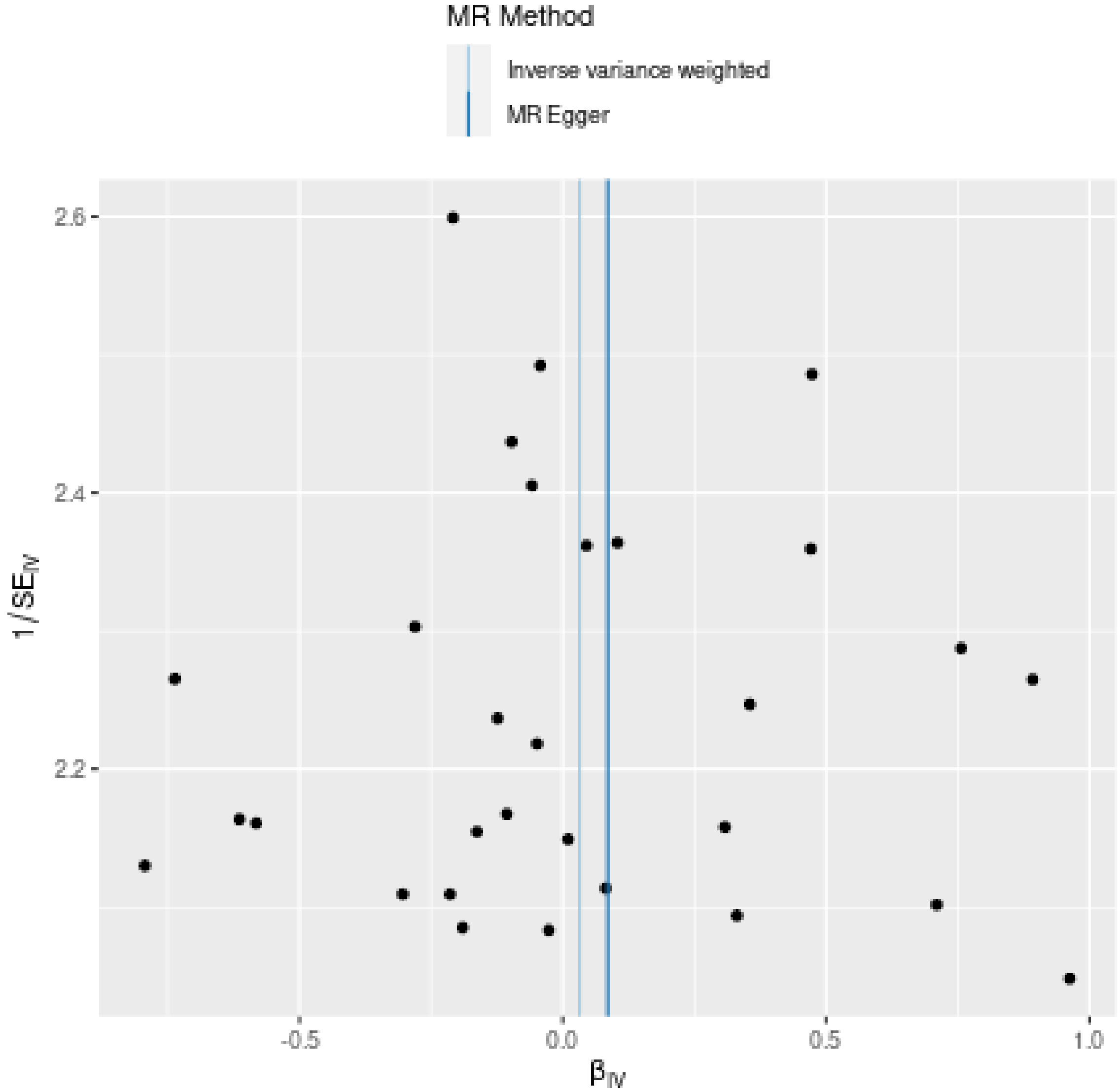

MR Method
Inverse variance weighted
MR Egger
2.6
2.4
2.2
-0.5
0.0
0.5
1.0
β_{IV}
$1/SE_{IV}$

Leave-one-out sensitivity analysis

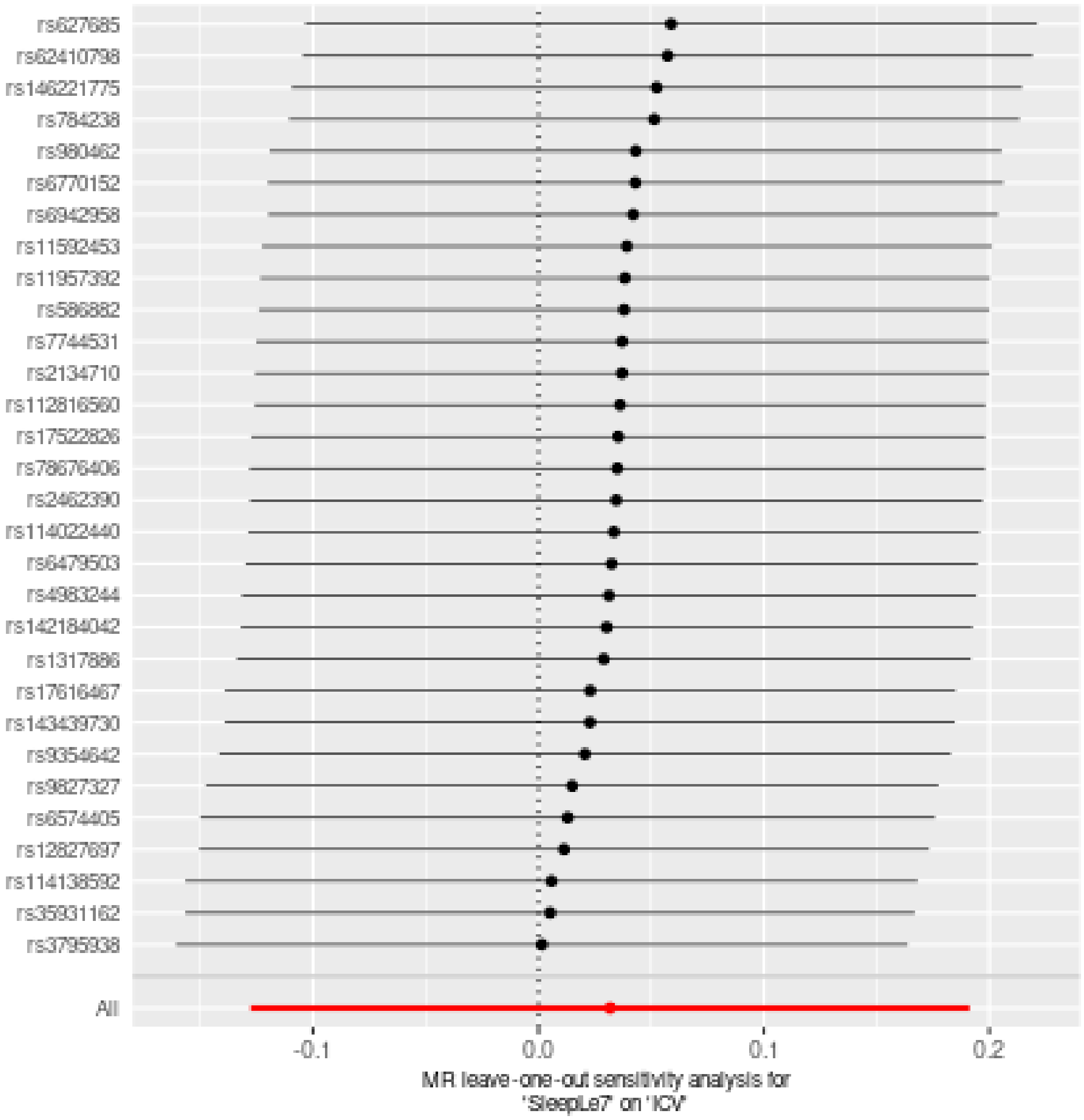

Two sample MR report

SleepLe7 against TGV

Date: **24 september, 2021**

Results from two sample MR:

method	nsnp	b	se	pval
MR Egger	30	-0.0961532	0.1379010	0.4913878
Weighted median	30	-0.0319099	0.0698793	0.6479281
Inverse variance weighted	30	-0.0103353	0.0494239	0.8343585
Simple mode	30	-0.0908140	0.1403078	0.5225623
Weighted mode	30	-0.0835624	0.1423746	0.5617996

Heterogeneity tests

method	Q	Q_df	Q_pval
MR Egger	28.29881	28	0.4487004
Inverse variance weighted	28.74861	29	0.4782141

Test for directional horizontal pleiotropy

egger_intercept	se	pval
0.0019235	0.0028832	0.5101537

Test that the exposure is upstream of the outcome

snp_r2.exposure	snp_r2.outcome	correct_causal_direction	steiger_pval
0.0037888	0.0010039	TRUE	2.1e-06

Note - R^2 values are approximate

Forest plot of single SNP MR

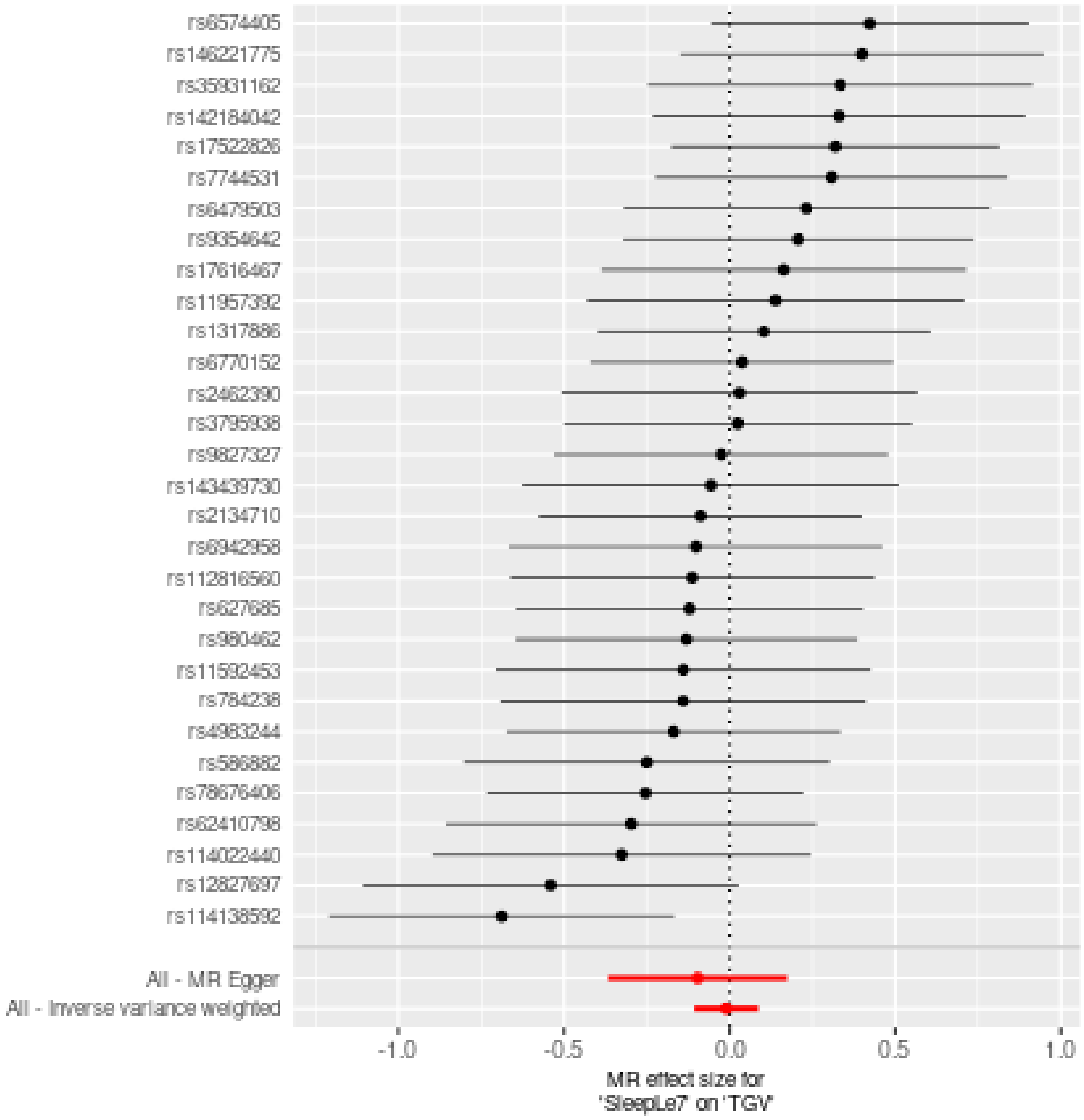

Comparison of results using different MR methods

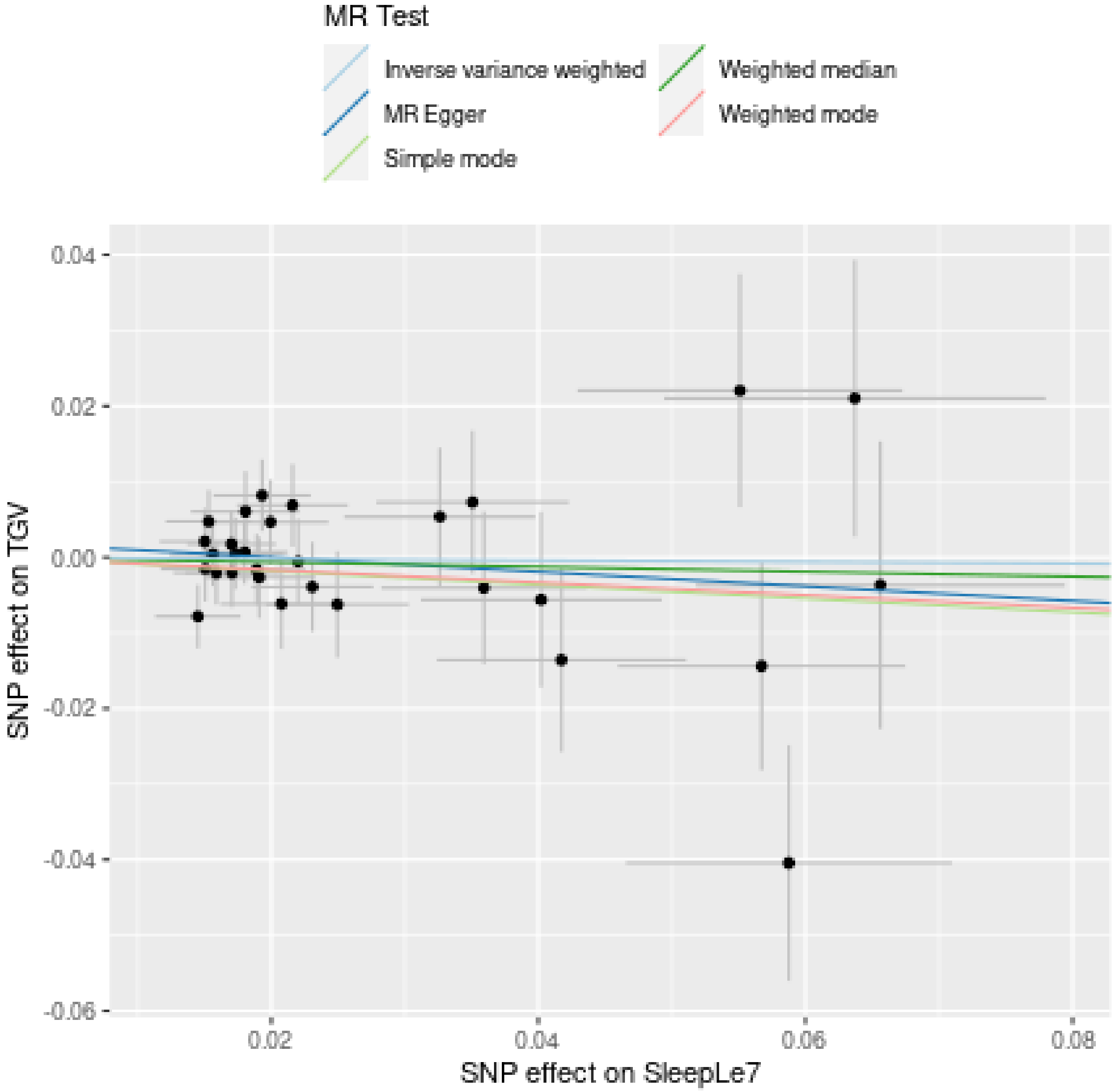

Funnel plot

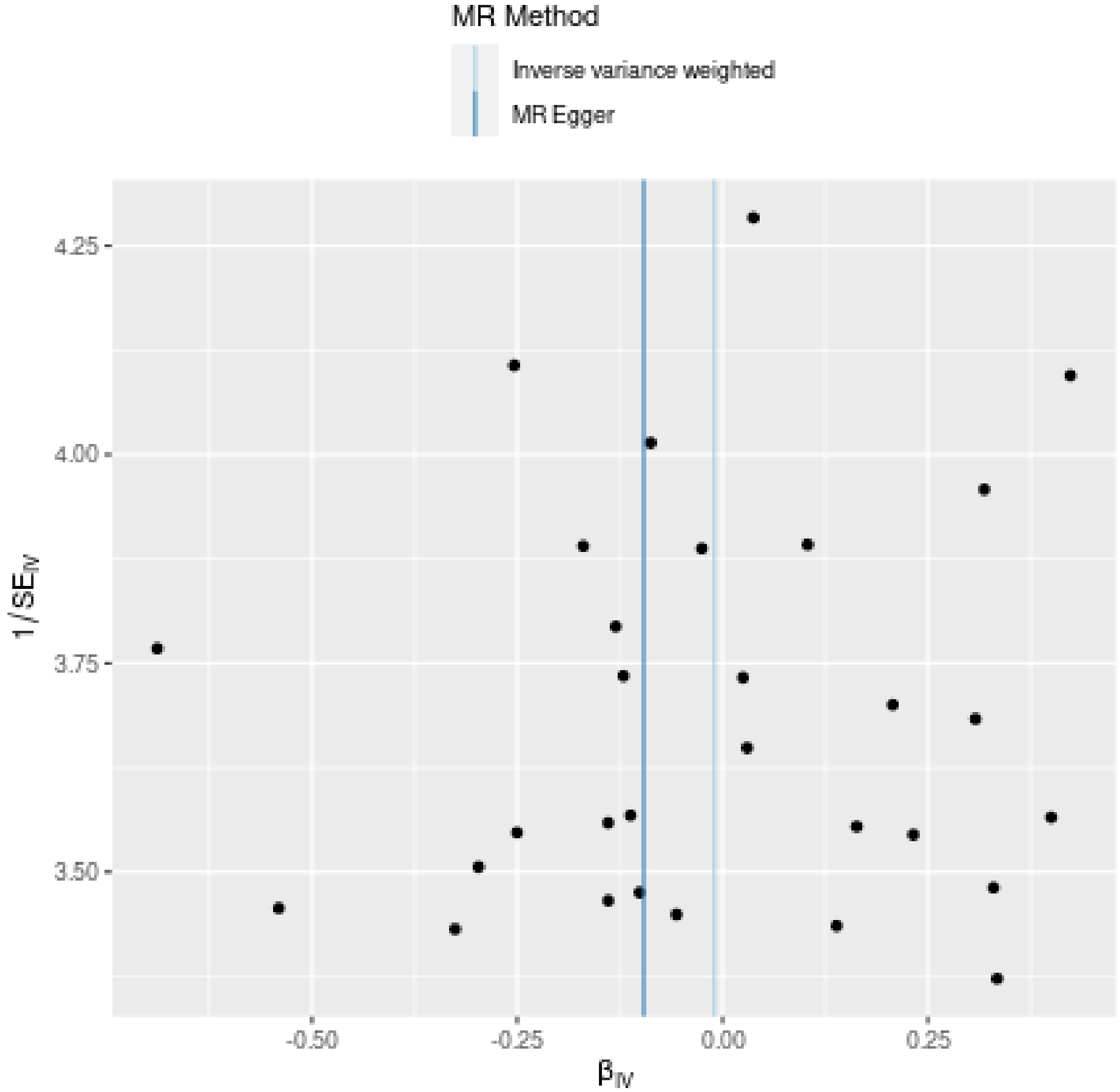
MR Method
Inverse variance weighted
MR Egger
4.25
4.00
3.75
3.50
1/SE$_{IV}$
-0.50
-0.25
0.00
0.25
β$_{IV}$

Leave-one-out sensitivity analysis

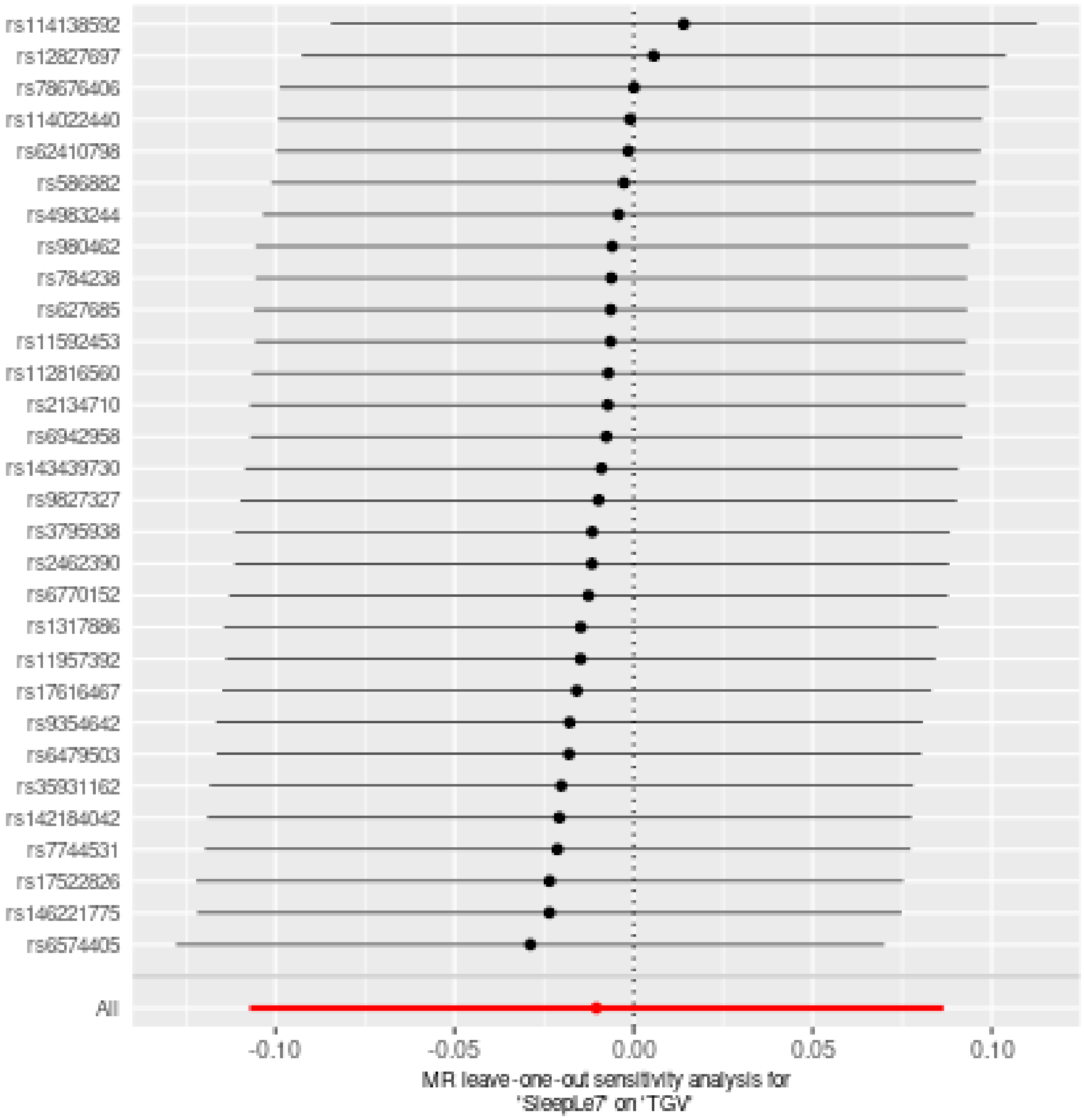

Two sample MR report

TGV against SleepGt7

P<10-6

Date: **24 september, 2021**

Results from two sample MR:

method	nsnp	b	se	pval
MR Egger	67	0.1054601	0.0837270	0.2123280
Weighted median	67	-0.0008322	0.0383481	0.9826867
Inverse variance weighted	67	-0.0001759	0.0331697	0.9957690
Simple mode	67	0.0111666	0.0984704	0.9100569
Weighted mode	67	0.0147731	0.0977878	0.8803788

Heterogeneity tests

method	Q	Q_df	Q_pval
MR Egger	109.1096	65	0.0005061
Inverse variance weighted	112.2713	66	0.0003332

Test for directional horizontal pleiotropy

egger_intercept	se	pval
-0.0032295	0.0023531	0.1746514

Test that the exposure is upstream of the outcome

snp_r2.exposure	snp_r2.outcome	correct_causal_direction	steiger_pval
0.0620032	0.0011137	TRUE	0

Note - R^2 values are approximate

Forest plot of single SNP MR

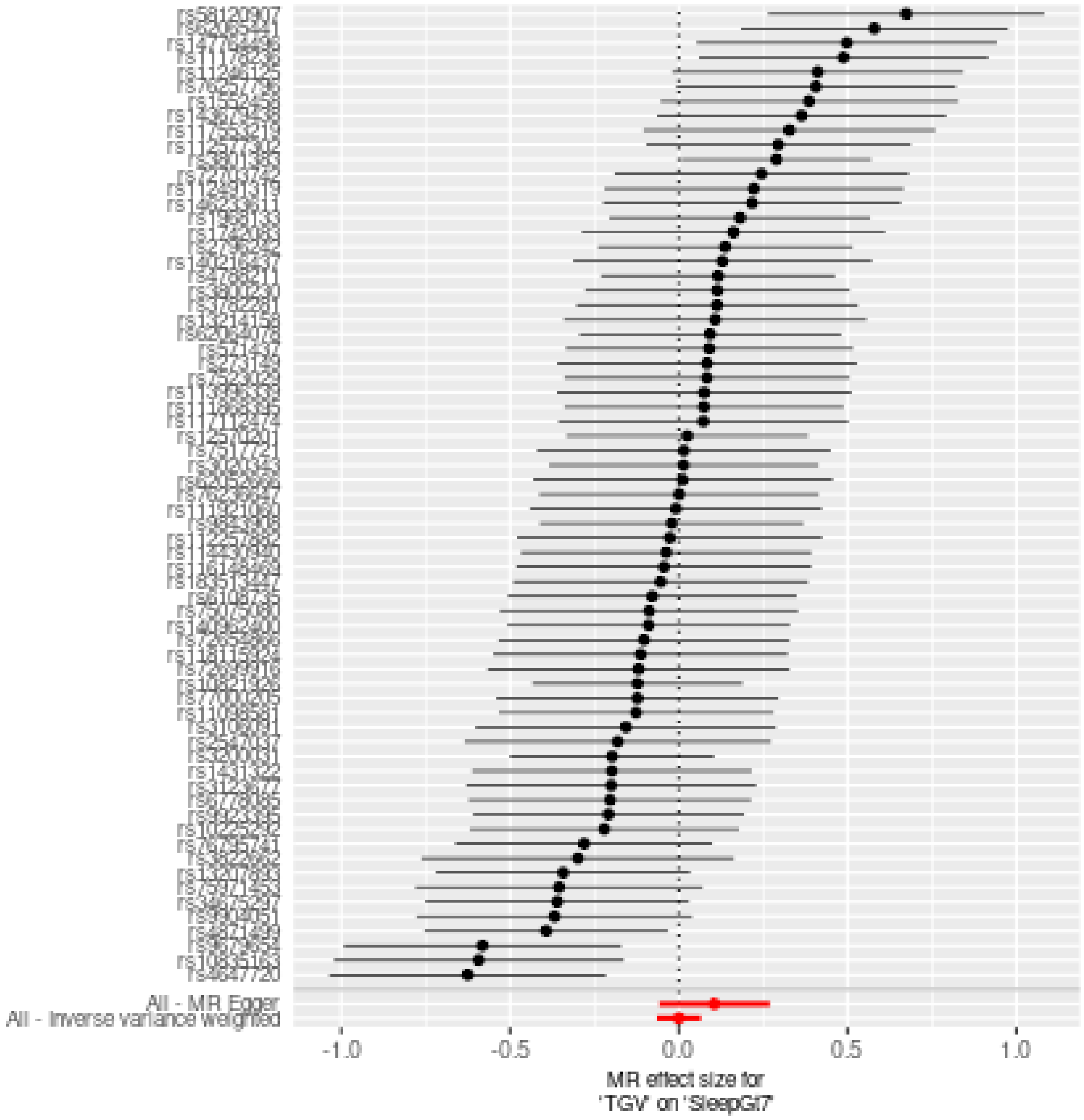

Comparison of results using different MR methods

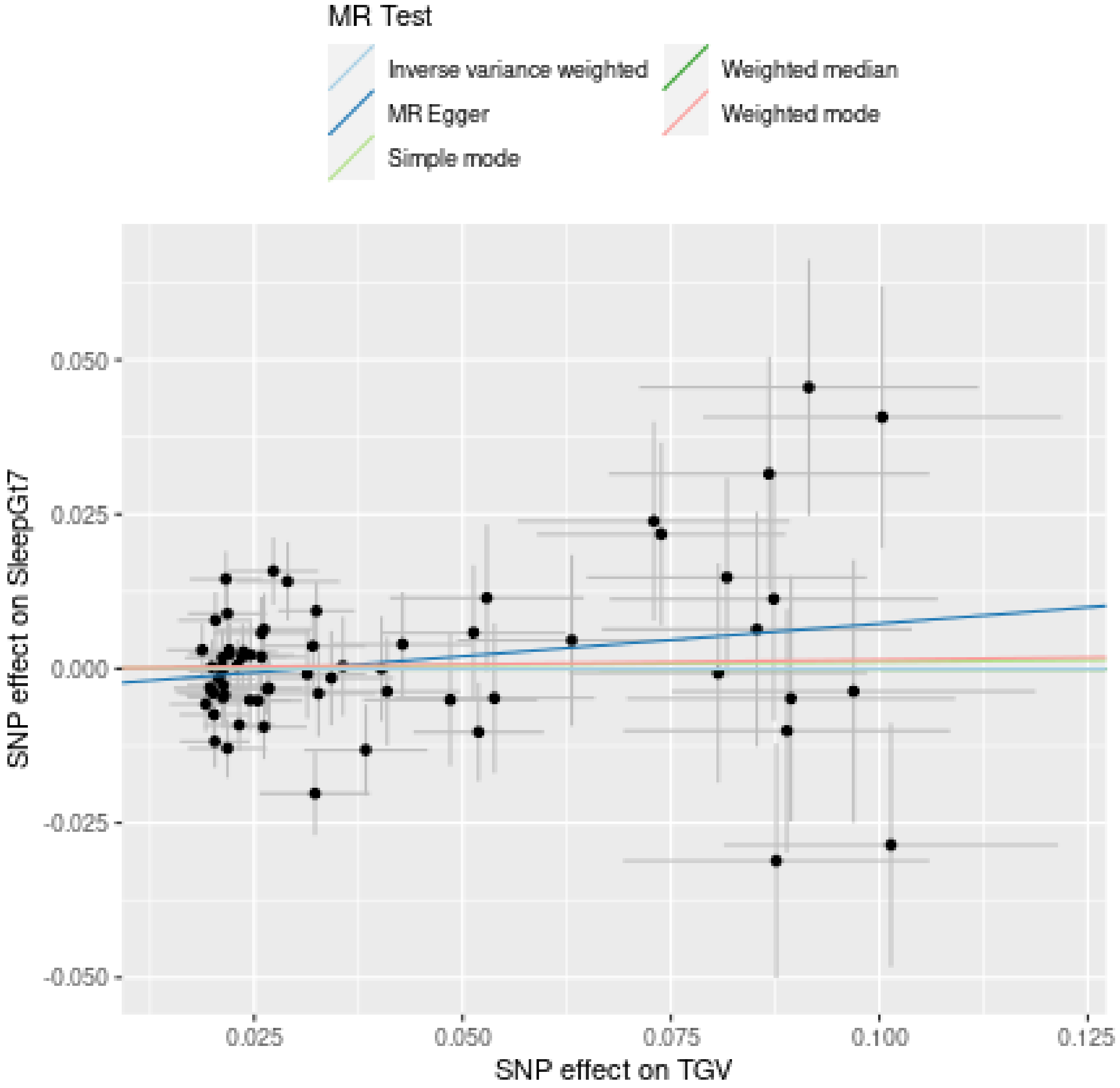

Funnel plot

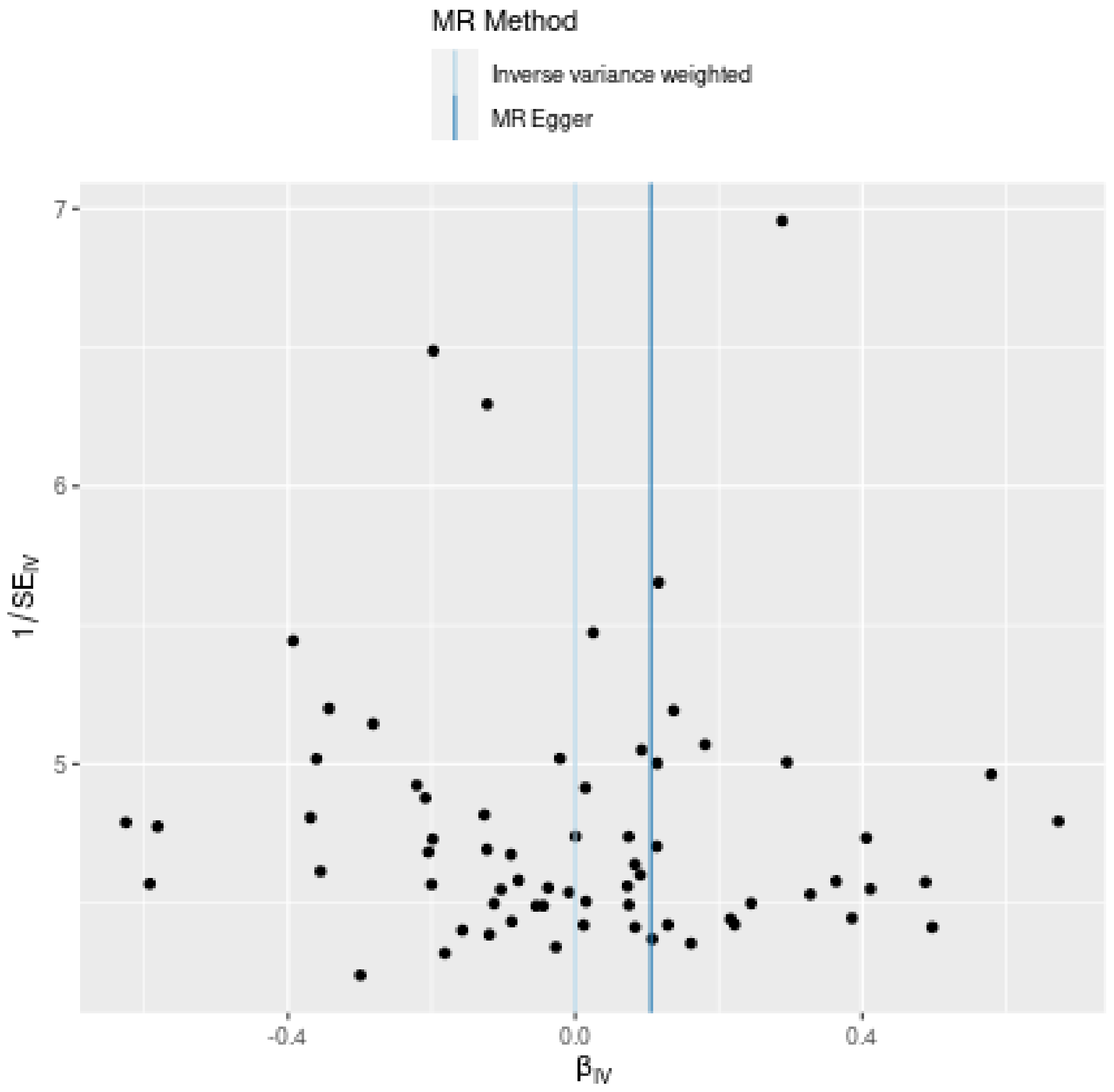
MR Method
Inverse variance weighted
MR Egger
7
6
5
1/SE$_{IV}$
-0.4
0.0
0.4
β$_{IV}$

Leave-one-out sensitivity analysis

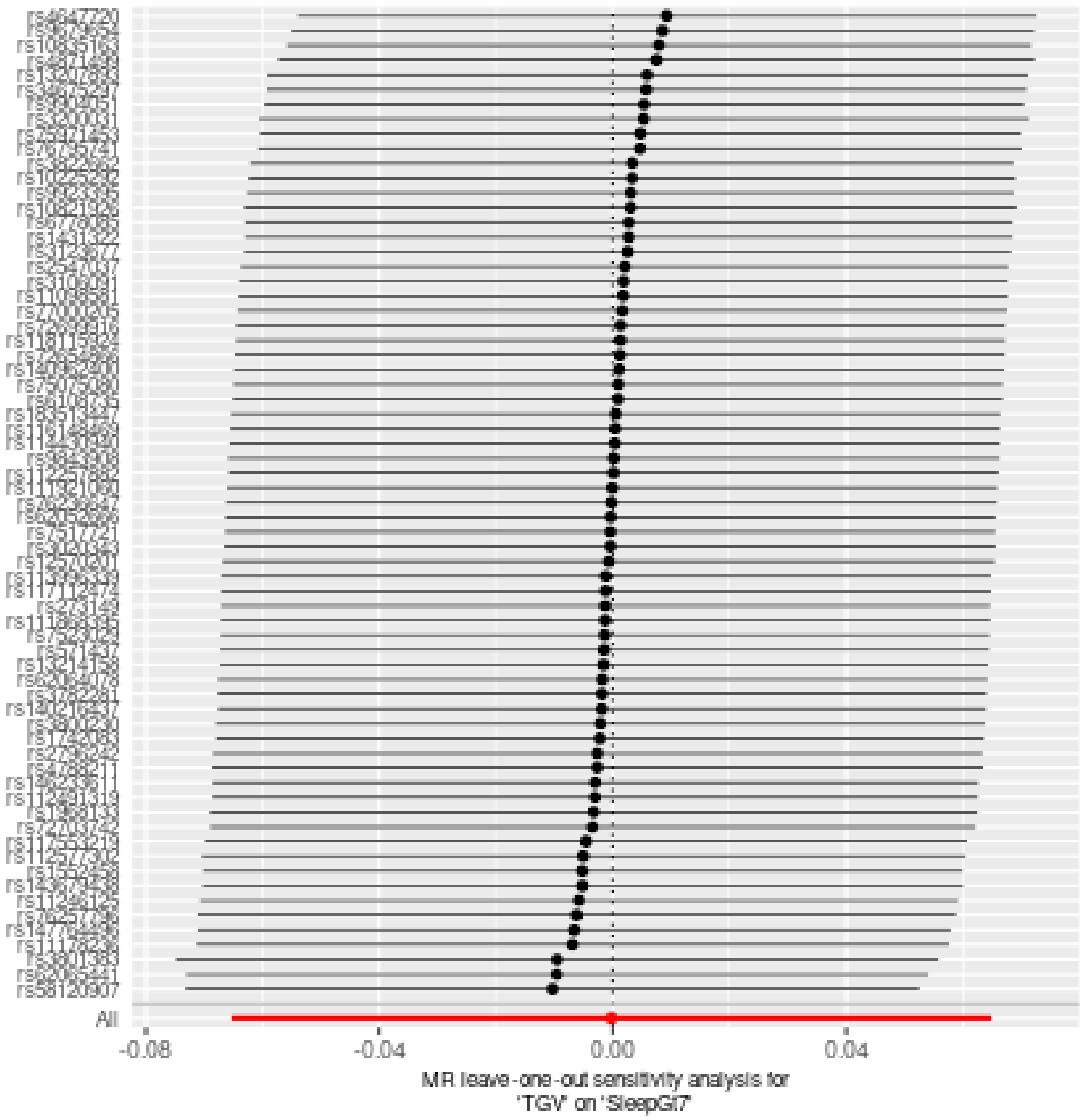

Two sample MR report

TGV against SleepLe7

P<10-6

Date: **24 september, 2021**

Results from two sample MR:

method	nsnp	b	se	pval
MR Egger	67	-0.0178969	0.0540929	0.7418183
Weighted median	67	0.0223517	0.0285948	0.4344087
Inverse variance weighted	67	0.0361167	0.0213874	0.0912793
Simple mode	67	-0.0029460	0.0643265	0.9636094
Weighted mode	67	0.0199181	0.0593531	0.7382476

Heterogeneity tests

method	Q	Q_df	Q_pval
MR Egger	80.03294	65	0.0991988
Inverse variance weighted	81.48735	66	0.0947592

Test for directional horizontal pleiotropy

egger_intercept	se	pval
0.0016544	0.0015222	0.2811208

Test that the exposure is upstream of the outcome

snp_r2.exposure	snp_r2.outcome	correct_causal_direction	steiger_pval
0.0620032	0.0004911	TRUE	0

Note - R^2 values are approximate

Forest plot of single SNP MR

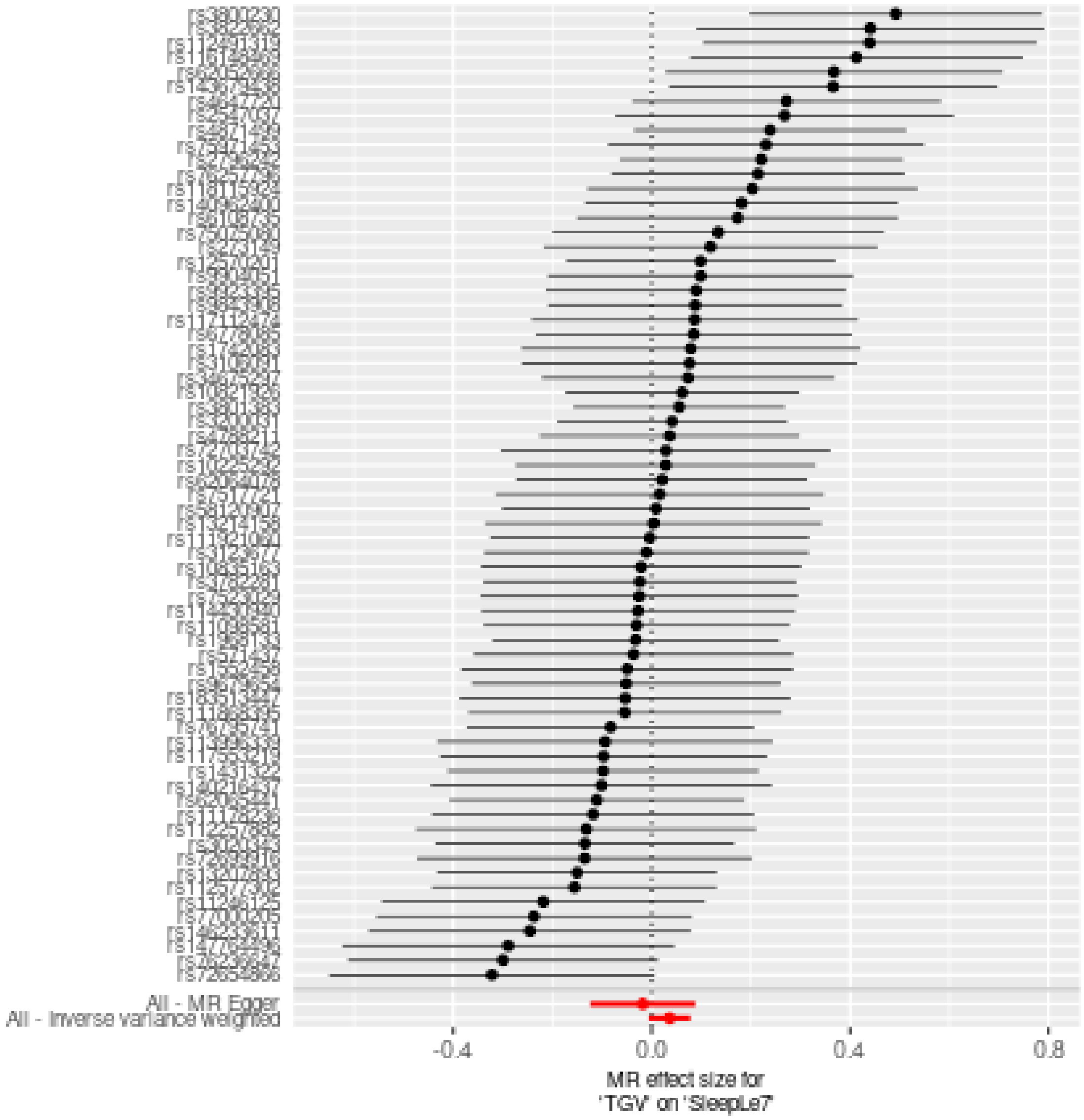

Comparison of results using different MR methods

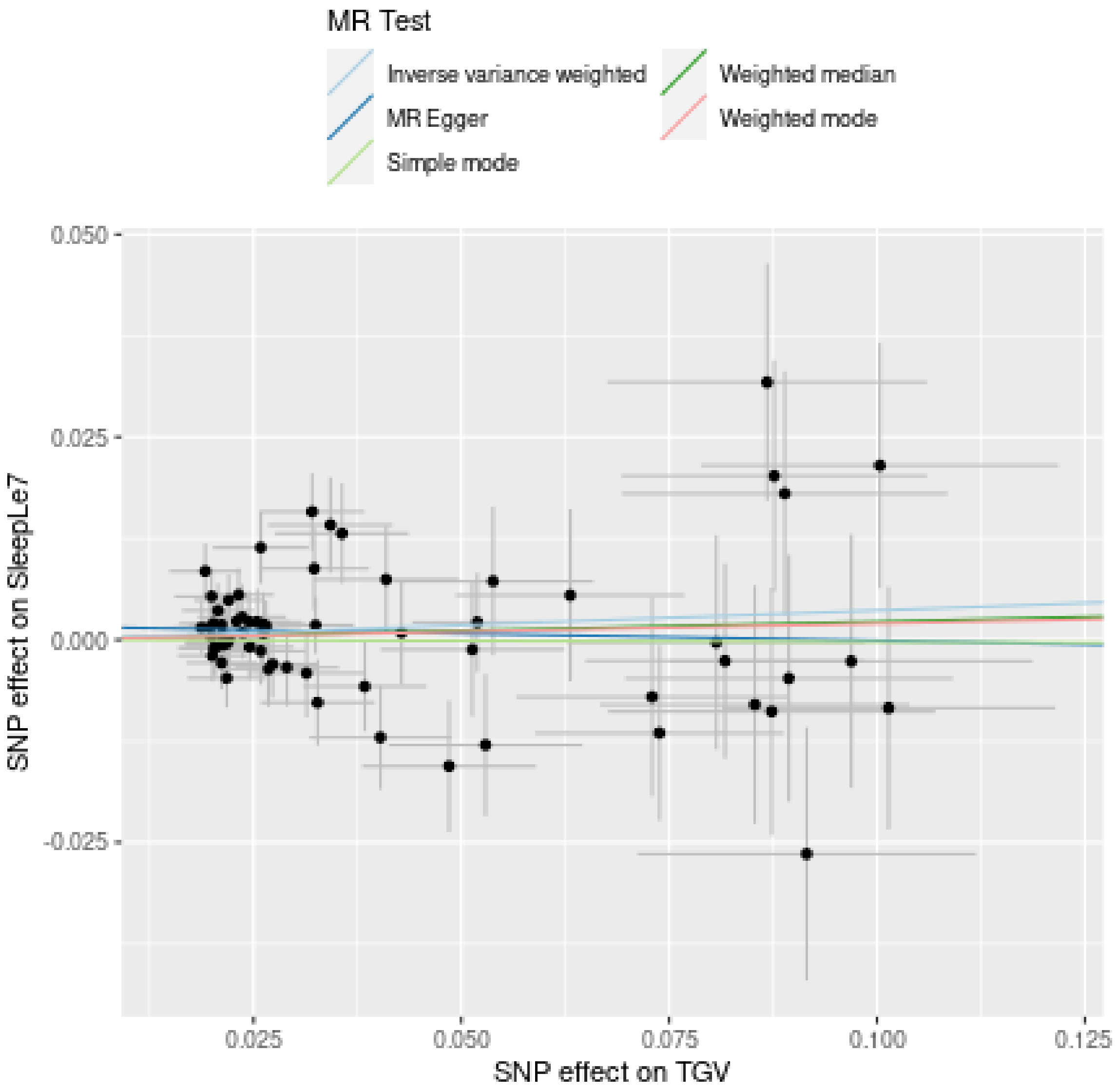

Funnel plot

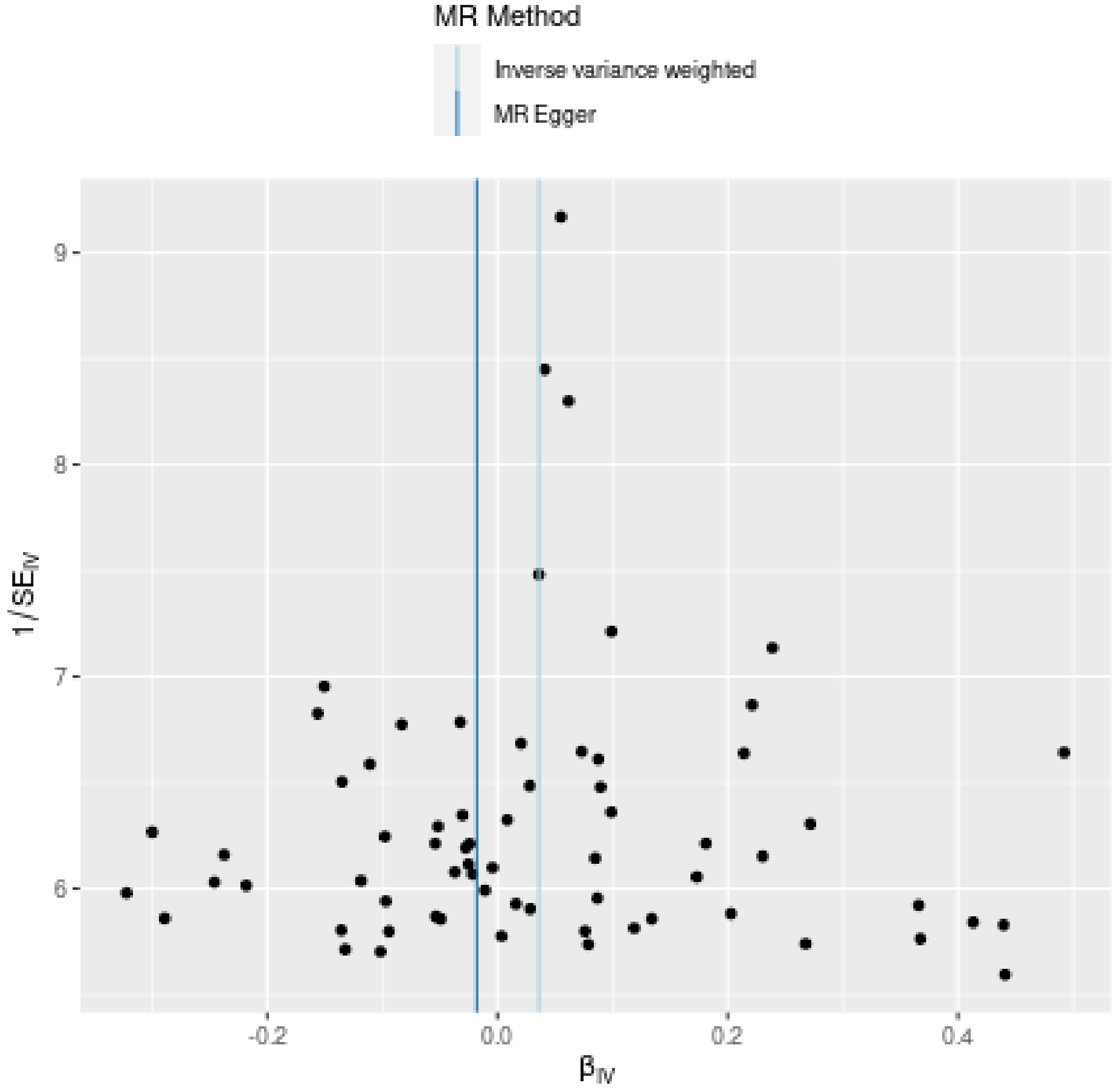

MR Method
Inverse variance weighted
MR Egger
9
8
7
6
1/SE$_{IV}$
-0.2
0.0
0.2
0.4
β$_{IV}$

Leave-one-out sensitivity analysis

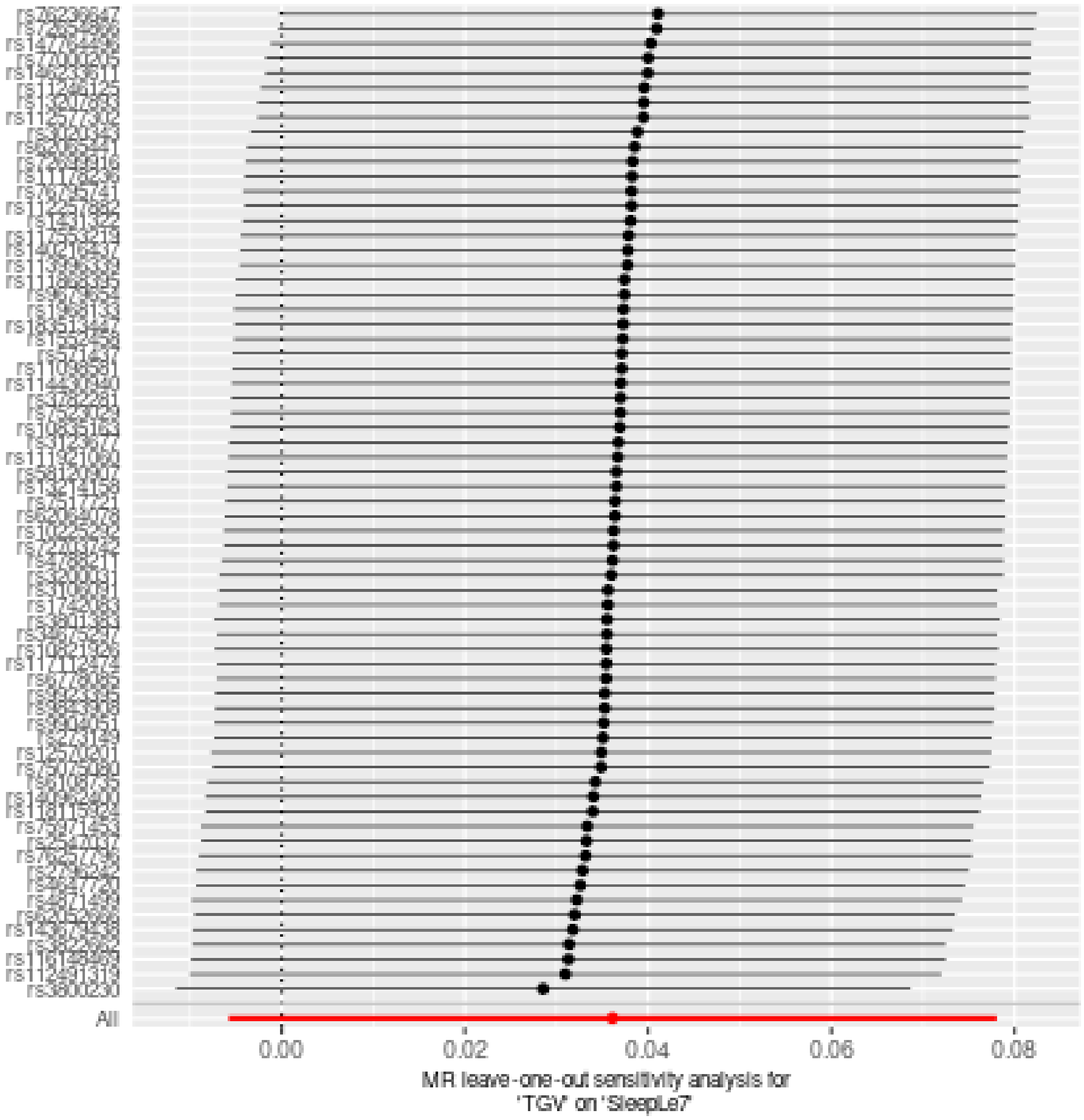

Two sample MR report

ICV against SleepLe7

Date: **17 juni, 2022**

Results from two sample MR:

method	nsnp	b	se	pval
MR Egger	12	-0.0305489	0.0907573	0.7433758
Weighted median	12	0.0202472	0.0280124	0.4698069
Inverse variance weighted	12	0.0468568	0.0255613	0.0667851
Simple mode	12	0.0289360	0.0414359	0.4994675
Weighted mode	12	0.0259266	0.0336285	0.4569656

Heterogeneity tests

method	Q	Q_df	Q_pval
MR Egger	17.33912	10	0.0671889
Inverse variance weighted	18.71135	11	0.0664759

Test for directional horizontal pleiotropy

egger_intercept	se	pval
0.0041922	0.0047125	0.3945729

Test that the exposure is upstream of the outcome

snp_r2.exposure	snp_r2.outcome	correct_causal_direction	steiger_pval
0.0222255	0.0001328	TRUE	0

Note - R^2 values are approximate

Forest plot of single SNP MR

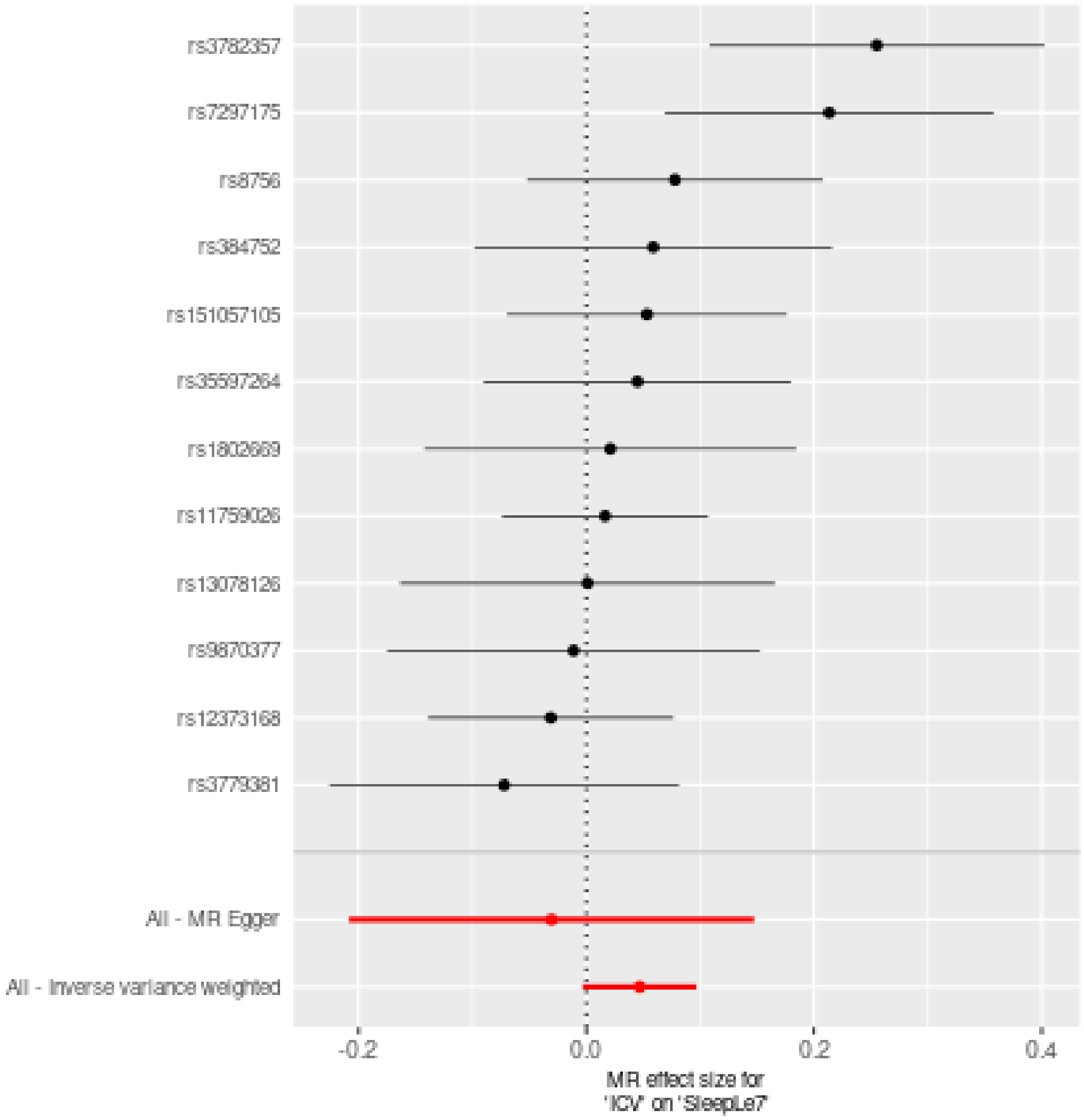

Comparison of results using different MR methods

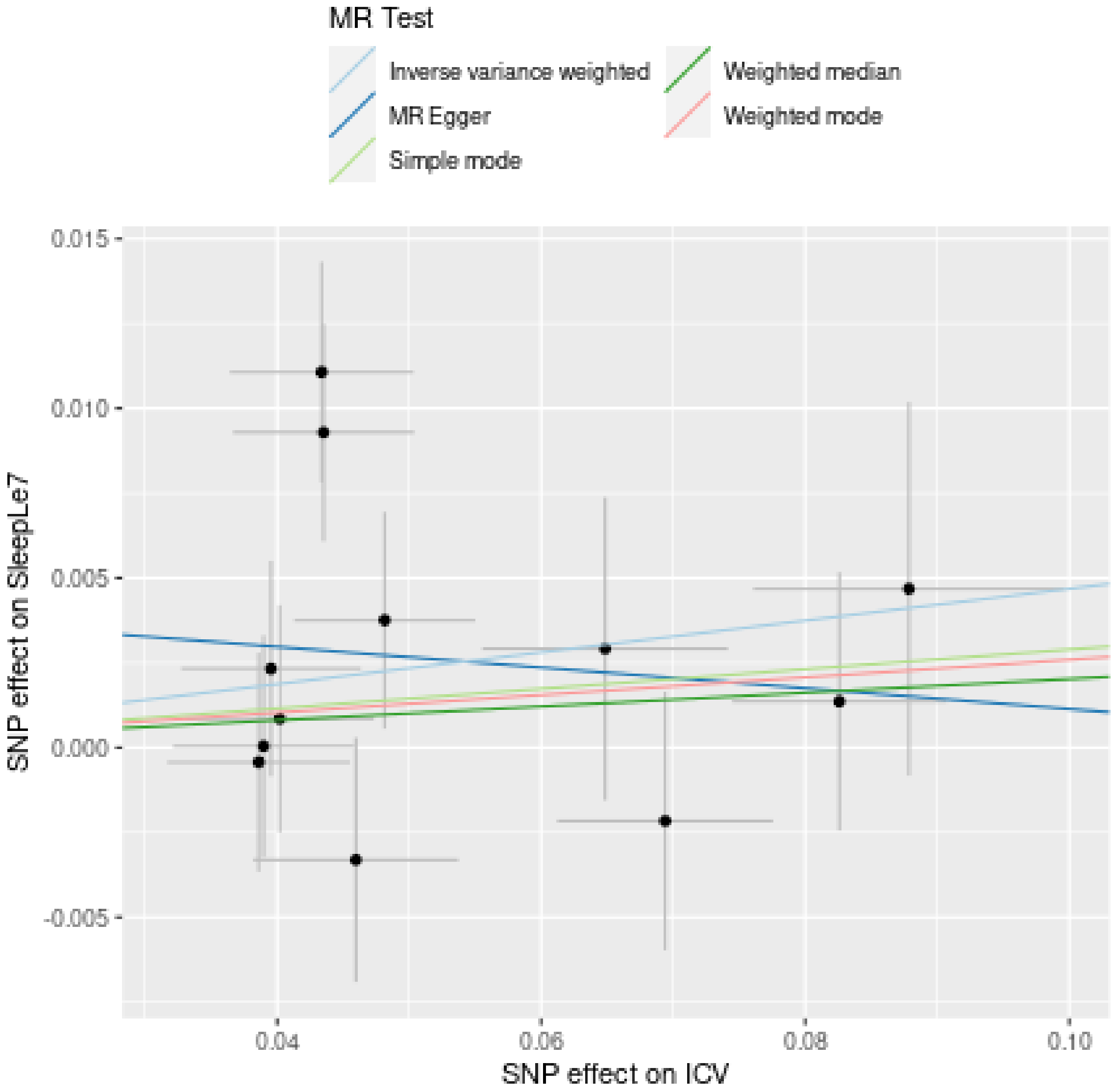

Funnel plot

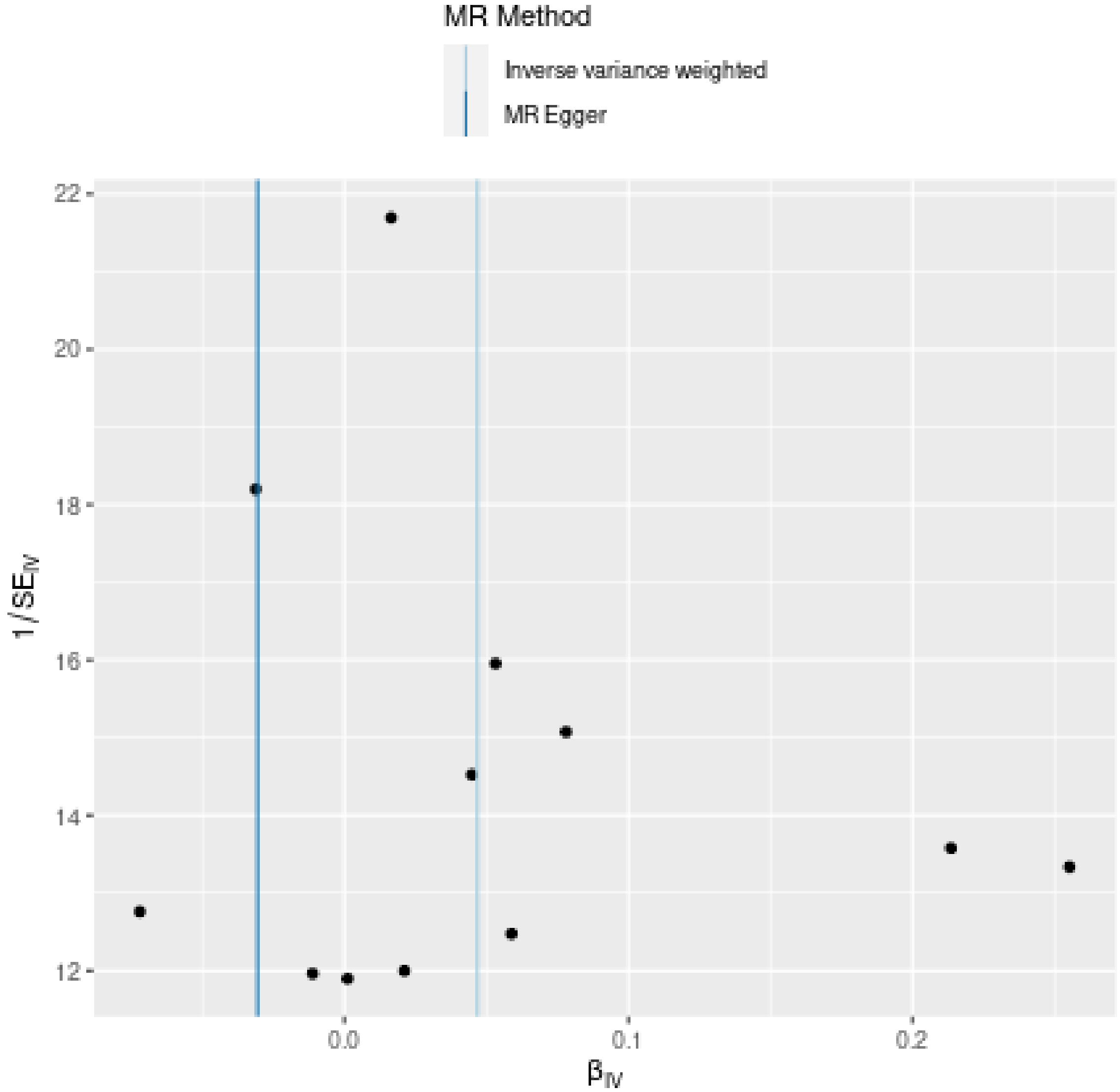
MR Method
Inverse variance weighted
MR Egger
22
20
18
16
14
12
$1/SE_{IV}$
0.0
0.1
0.2
β_{IV}

Leave-one-out sensitivity analysis

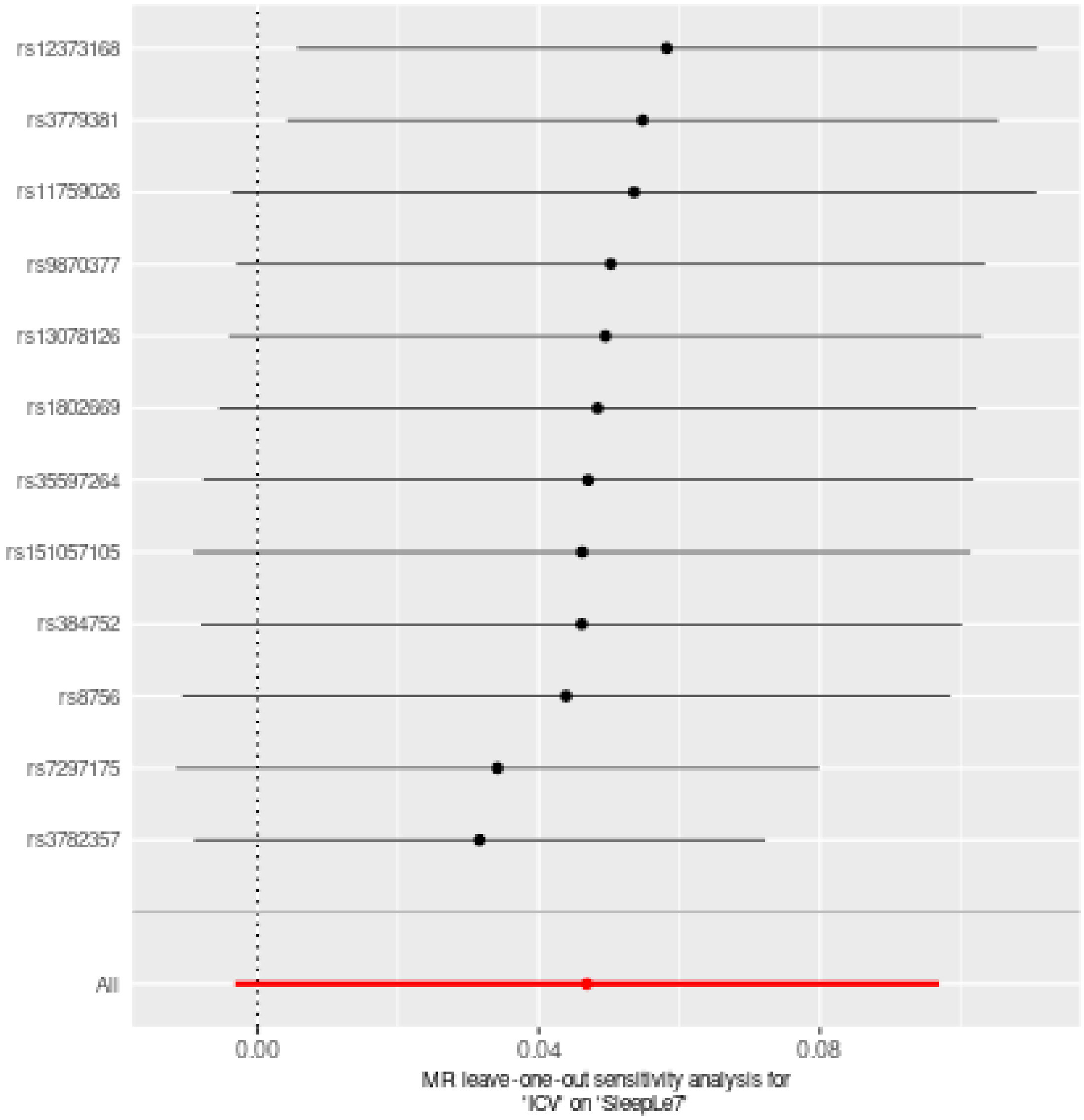

STROBE-MR checklist of recommended items to address in reports of Mendelian randomization studies[1 2]

Item No.	Section	Checklist item	Page No.	Relevant text from manuscript
1	**TITLE and ABSTRACT**	Indicate Mendelian randomization (MR) as the study's design in the title and/or the abstract if that is a main purpose of the study	3	Mendelian randomizaton did not yield evidence for causal impacts of sleep on brain structure.
	**INTRODUCTION**			
2	**Background**	Explain the scientific background and rationale for the reported study. What is the exposure? Is a potential causal relationship between exposure and outcome plausible? Justify why MR is a helpful method to address the study question	5	Cross-sectional relationships can be further investigated using genetic information to assess possible causality. Twin and genome wide association studies (GWAS) have demonstrated heritability and polygenic influences on sleep duration, although GWAS heritability is modest 50-56. Single nucleotide polymorphism heritability (SNP-h2) for sleep duration is typically below 10%57. To date, up to 78 independent genetic loci have been associated with sleep duration50, among which the thyroid-specific transcription factor gene (PAX8) and the Vaccinia related kinase 2 (VRK2) gene have been considered as the most robust findings. Besides gene discovery, genetic overlap between sleep duration and other conditions have been studied50,52,58, suggesting pleiotropy between sleep duration, somatic disorders, and neuropsychiatric health. However, no studies have investigated whether genes affect sleep duration uniformly for below vs. above average sleepers. Sleep duration tends to be positively related to health in below-average sleepers and negatively related to health in above average sleepers. If the same is true for brain characteristics, it is interesting to investigate genetic differences between these participants and to use Mendelian randomization analyses to examine the possible causal relationships between sleep duration and brain health as indexed by MRI.
3	**Objectives**	State specific objectives clearly, including pre-specified causal hypotheses (if any). State that MR is a method that, under specific assumptions, intends to estimate causal effects	6	Genetic analyses were undertaken to further investigate the sleep – brain relationships, and to make tentative inferences about causality. We took advantage of measured variation in genes for each trait of interest and used Mendelian

				randomization60 to test the direction of causality between sleep duration and brain structure.
	**METHODS**			
4	**Study design and data sources**	Present key elements of the study design early in the article. Consider including a table listing sources of data for all phases of the study. For each data source contributing to the analysis, describe the following:	5,6,13	To determine the plausible direction of causality between brain structure and sleep duration, we performed a series of genetic analyses using cross-sectional data from UK Biobank. Hippocampus, total gray matter volume and ICV were chosen as regions of interest as they showed the typical inverted U-shaped relationship to sleep duration. For details about selection of participant, quality control procedures and genetic analyses, see SI Genetic analyses, SI Genetics notes and SI Genetics tables.
	a)	Setting: Describe the study design and the underlying population, if possible. Describe the setting, locations, and relevant dates, including periods of recruitment, exposure, follow-up, and data collection, when available.	13	SI Genetic analyses
	b)	Participants: Give the eligibility criteria, and the sources and methods of selection of participants. Report the sample size, and whether any power or sample size calculations were carried out prior to the main analysis	25	SI Sample characteristics
	c)	Describe measurement, quality control and selection of genetic variants	29,30	The TwoSampleMR R package[118] was used to investigate the existence of causal relations between sleep duration and the brain variables. Independent instrumental SNPs were selected using the following parameters: association p value $<=10^{-6}$, MAF >=0.05, LD-r2 <=0.1, and LD-distance=10kb. The LD structure was derived from 10,000 independent European participants randomly selected from UKBB. The powerful inverse variance weighted (IVW) model from TwoSampleMR was used as the main model. Other models implemented in the software were also run as sensitivity analysis. To further support the results, the analysis was reperformed with p value $<=10^{-5}$, which would increase the strength of instrumenting for the less powerful sleep duration traits. For the only significant causal relation, i.e., ICV to sleep duration for shorter than average sleepers, a third analysis with p $<= 5x10^{-8}$ used for selecting instrumental SNP was performed. The standard output from TwoSampleMR is shown in SI Genetics notes.

		d) For each exposure, outcome, and other relevant variables, describe methods of assessment and diagnostic criteria for diseases	26,29,30	See above, and "For UKB, sleep was measured through multiple questions. … For UKB, we calculated a sum score of different sleep-related measures (sleeplessness [field 1200], problems getting up in the morning [field 1170], daytime dozing [field 1220], snoring [field 1210] and chronotype [field 1180])."
		e) Provide details of ethics committee approval and participant informed consent, if relevant	25	For UKB, ethics approval was obtained from the National Health Service National Research Ethics Service (Ref 11/NW/0382)
5	**Assumptions**	Explicitly state the three core IV assumptions for the main analysis (relevance, independence and exclusion restriction) as well assumptions for any additional or sensitivity analysis		
6	**Statistical methods: main analysis**	Describe statistical methods and statistics used	28-30	The TwoSampleMR R package128 was used to investigate the existence of causal relations between sleep duration and the brain variables. Independent instrumental SNPs were selected using the following parameters: association p value <=10-6, MAF >=0.05, LD-r2 <=0.1, and LD-distance=10kb. The LD structure was derived from 10,000 independent European participants randomly selected from UKBB. The powerful inverse variance weighted (IVW) model from TwoSampleMR was used as the main model. Other models implemented in the software were also run as sensitivity analysis. To further support the results, the analysis was reperformed with p value <=10-5, which would increase the strength of instrumenting for the less powerful sleep duration traits. For the only significant causal relation, i.e., ICV to sleep duration for shorter than average sleepers, a third analysis with p <= 5x10-8 used for selecting instrumental SNP was performed. The standard output from TwoSampleMR is shown in SI Genetics notes.
		a) Describe how quantitative variables were handled in the analyses (i.e., scale, units, model)		
		b) Describe how genetic variants were handled in the analyses and, if applicable, how their weights were selected		

		c)	Describe the MR estimator (e.g. two-stage least squares, Wald ratio) and related statistics. Detail the included covariates and, in case of two-sample MR, whether the same covariate set was used for adjustment in the two samples		
		d)	Explain how missing data were addressed		
		e)	If applicable, indicate how multiple testing was addressed		
7	**Assessment of assumptions**		Describe any methods or prior knowledge used to assess the assumptions or justify their validity		
8	**Sensitivity analyses and additional analyses**		Describe any sensitivity analyses or additional analyses performed (e.g. comparison of effect estimates from different approaches, independent replication, bias analytic techniques, validation of instruments, simulations)		
9	**Software and pre-registration**				
		a)	Name statistical software and package(s), including version and settings used	29	The TwoSampleMR R package128
		b)	State whether the study protocol and details were pre-registered (as well as when and where)		No
	**RESULTS**				
10	**Descriptive data**				
		a)	Report the numbers of individuals at each stage of included studies and reasons for exclusion. Consider use of a flow diagram		Supplemental Information
		b)	Report summary statistics for phenotypic exposure(s), outcome(s), and other relevant variables (e.g. means, SDs, proportions)		Supplemental Information
		c)	If the data sources include meta-analyses of previous studies, provide the assessments of heterogeneity across these studies		NaN
		d)	For two-sample MR:<br>i. Provide justification of the similarity of the genetic variant-exposure associations between the exposure and outcome samples<br>ii. Provide information on the number of individuals who overlap between the exposure and outcome studies		Supplemental Information
11	**Main results**				
		a)	Report the associations between genetic variant and exposure, and between genetic variant and outcome, preferably on an interpretable scale	15,16	Among the 12 pairs, ICV showed a significant causal effect (34 instrumental SNP, minimal F

					stats > 24; inverse-variance weighted beta = 0.060, se = 0.017, p = 5.36x10-4) on sleep duration for the shorter than average sleepers (Figure 6a and SI Instrumental variables), with no causal effects of sleep on ICV.
		b)	Report MR estimates of the relationship between exposure and outcome, and the measures of uncertainty from the MR analysis, on an interpretable scale, such as odds ratio or relative risk per SD difference		Supplemental Information
		c)	If relevant, consider translating estimates of relative risk into absolute risk for a meaningful time period		
		d)	Consider plots to visualize results (e.g. forest plot, scatterplot of associations between genetic variants and outcome versus between genetic variants and exposure)	15,16	Figure 5. Genetic relations between sleep duration and brain structure. a. Distribution of PGS-ICV in different sleep duration strata among shorter than average sleepers (< 7 hours). b. ICV for shorter than average sleepers with one standard deviation above (blue) and below (red) average PGS for sleep duration c. Distribution of PGS for total gray matter volume in different sleep duration strata among shorter than average sleepers. d. Total gray matter volume for shorter than average sleepers with one standard deviation above (blue) and below (red) average PGS for sleep duration.<br>Figure 6 SNP effects on MRI-derived measures in standard deviations. SNP effects on intracranial volume (ICV) (x-axis) and sleep duration (y-axis) for the shorter than average sleepers.
12	**Assessment of assumptions**				
		a)	Report the assessment of the validity of the assumptions		Supplemental Information for details
		b)	Report any additional statistics (e.g., assessments of heterogeneity across genetic variants, such as I^2, Q statistic or E-value)		Supplemental Information for details
13	**Sensitivity analyses and additional analyses**				
		a)	Report any sensitivity analyses to assess the robustness of the main results to violations of the assumptions		Supplemental Information for details
		b)	Report results from other sensitivity analyses or additional analyses		Supplemental Information for details

		c)	Report any assessment of direction of causal relationship (e.g., bidirectional MR)	15.16	We performed bidirectional Mendelian randomization analysis for each brain volumetric trait to sleep duration (see SI STROBE-MR-checklist for details, reporting according to best practice for MR studies).
		d)	When relevant, report and compare with estimates from non-MR analyses		NaN
		e)	Consider additional plots to visualize results (e.g., leave-one-out analyses)		Supplemental Information for details
	**DISCUSSION**				
14	**Key results**		Summarize key results with reference to study objectives	17,19-20	Aligning with the longitudinal results, the Mendelian randomization analyses did not reveal evidence for a causal impact of short sleep on brain structure. Taken together, the longitudinal, cross-sectional and genetic results suggest that short habitual sleep duration is not related to poorer brain health in healthy adults as indexed by structural brain measures, and that somewhat less than 7 hours of sleep is associated with the most favourable features, in line with converging evidence from research on mortality, health and cognition.<br>Finally, the Mendelian randomization analyses showed causal effects of ICV on sleep duration but no causal effects of sleep duration on any MRI-derived brain measure. Hence, in the current samples, people with larger heads on average report to sleep longer, and this relationship partly depends on genetics. The lack of an inverse influence of sleep duration on ICV was given, as ICV does not change in adults and hence cannot be affected by sleep. Still, the genetic results suggested that there may be a mechanistic relationship between ICV and sleep duration which could warrant further explorations. This effect was removed from the estimated sleep duration – brain volume relationships by covarying for ICV, which may contribute to explain that the nominally significant relationship between total gray matter volume and sleep duration in the Mendelian randomization analysis did not survive corrections. In sum, the genetic results were in coherence with a view of average and “optimal” sleep duration as relatively well aligned and did not provide evidence

					for a causal relationship of sleep duration on brain structural features.
15	**Limitations**		Discuss limitations of the study, taking into account the validity of the IV assumptions, other sources of potential bias, and imprecision. Discuss both direction and magnitude of any potential bias and any efforts to address them	21-22	Fifth, in order to study differential genetic influences on sleep duration in participants with different habitual sleep patterns, we stratified the sample by 7 hours, a strategy which made our GWAS underpowered. While the findings are promising, large-scale independent validation is needed. Furthermore, given the limited power of the GWAS, the causal relations suggested by the Mendelian randomization analysis will also need future replication.
16	**Interpretation**				
		a)	Meaning: Give a cautious overall interpretation of results in the context of their limitations and in comparison with other studies	19-20	
		b)	Mechanism: Discuss underlying biological mechanisms that could drive a potential causal relationship between the investigated exposure and the outcome, and whether the gene-environment equivalence assumption is reasonable. Use causal language carefully, clarifying that IV estimates may provide causal effects only under certain assumptions	19-20	
		c)	Clinical relevance: Discuss whether the results have clinical or public policy relevance, and to what extent they inform effect sizes of possible interventions		NaN
17	**Generalizability**		Discuss the generalizability of the study results (a) to other populations, (b) across other exposure periods/timings, and (c) across other levels of exposure	22	Finally, although some of the samples are population based, no MRI study is fully representative of the population from which it is sampled. Despite including studies from multiple European countries and the US, we can not exclude the possibility that other sleep-brain patterns exist in other populations from Europe or elsewhere.
	**OTHER INFORMATION**				
18	**Funding**		Describe sources of funding and the role of funders in the present study and, if applicable, sources of funding for the databases and original study or studies on which the present study is based	22-23	The Lifebrain consortium is funded by the EU Horizon 2020 Grant agreement number 732592 (Lifebrain). The different sub-studies are supported by different sources: LCBC: The European Research Council under grant agreements 283634, 725025 (to A.M.F.) and 313440 (to K.B.W.), as well as the Norwegian Research Council (to A.M.F., K.B.W.), The National

				Association for Public Health's dementia research program, Norway (to A.M.F). Betula: a scholar grant from the Knut and Alice Wallenberg (KAW) foundation to L.N. Barcelona: Partially supported by a Spanish Ministry of Economy and Competitiveness (MINECO) grant to D-BF [grant number PSI2015-64227-R (AEI/FEDER, UE)]; by the Walnuts and Healthy Aging study (http://www.clinicaltrials.gov; Grant NCT01634841) funded by the California Walnut Commission, Sacramento, California; and an ICREA Academia 2019 award. BASE-II has been supported by the German Federal Ministry of Education and Research under grant numbers 16SV5537/ 16SV5837/ 16SV5538/ 16SV5536K/ 01UW0808/ 01UW0706/ 01GL1716A/ 01GL1716B, the European Research Council under grant agreement 677804 (to S.K.). Work on the Whitehall II Imaging Substudy was mainly funded by Lifelong Health and Well-being Programme Grant G1001354 from the UK Medical Research Council ("Predicting MRI Abnormalities with Longitudinal Data of the Whitehall II Substudy") to K.E. The Wellcome Centre for Integrative Neuroimaging is supported by core funding from award 203139/Z/16/Z from the Wellcome Trust. Data were provided [in part] by the Human Connectome Project, WU-Minn Consortium (Principal Investigators: David Van Essen and Kamil Ugurbil; 1U54MH091657) funded by the 16 NIH Institutes and Centers that support the NIH Blueprint for Neuroscience Research; and by the McDonnell Center for Systems Neuroscience at Washington University. Part of the research was conducted using the UK Biobank resource under application number 32048.
19	**Data and data sharing**	Provide the data used to perform all analyses or report where and how the data can be accessed, and reference these sources in the article. Provide the statistical code needed to reproduce the results in the article, or report whether the code is publicly accessible and if so, where	30	Data supporting the results of the current study are available from the PI of each sub-study on request, given appropriate ethics and data protection approvals. Contact information can be obtained from the corresponding authors. UK Biobank data requests can be submitted to http://www.ukbiobank.ac.uk. Most of the r-code for the statistical analyses are provided in SI.

20	**Conflicts of Interest**	All authors should declare all potential conflicts of interest	30	Claire E Sexton reports consulting fees from Jazz Pharmaceuticals and is now a full-time employee of the U.S. Alzheimer’s Association. Christian A Drevon is a cofounder, stock-owner, board member and consultant in the contract laboratory Vitas AS, performing personalized analyses of blood biomarkers. The rest of the authors report no conflicts of interest.

This checklist is copyrighted by the Equator Network under the Creative Commons Attribution 3.0 Unported (CC BY 3.0) license.

1. Skrivankova VW, Richmond RC, Woolf BAR, Yarmolinsky J, Davies NM, Swanson SA, et al. Strengthening the Reporting of Observational Studies in Epidemiology using Mendelian Randomization (STROBE-MR) Statement. JAMA. 2021;under review.

2. Skrivankova VW, Richmond RC, Woolf BAR, Davies NM, Swanson SA, VanderWeele TJ, et al. Strengthening the Reporting of Observational Studies in Epidemiology using Mendelian Randomisation (STROBE-MR): Explanation and Elaboration. BMJ. 2021;375:n2233.
