# Supplementary material for: No phenotypic or genotypic evidence for a link between sleep duration and brain atrophy
Source: Nat Hum Behav. 2023 Oct 5;7(11):2008–22. doi: 10.1038/s41562-023-01707-5 (PMC10663160; doi:10.1038/s41562-023-01707-5)
Supplement: Supplementary file 2 — Reporting Summary [file 41562_2023_1707_MOESM2_ESM.pdf]

nature portfolio

Corresponding author(s): Anders M Fjell

Last updated by author(s): Jun 7, 2023

Reporting Summary

Nature Portfolio wishes to improve the reproducibility of the work that we publish. This form provides structure for consistency and transparency in reporting. For further information on Nature Portfolio policies, see our Editorial Policies and the Editorial Policy Checklist.

Statistics

For all statistical analyses, confirm that the following items are present in the figure legend, table legend, main text, or Methods section.

n/a	Confirmed	
☐	☒	The exact sample size (*n*) for each experimental group/condition, given as a discrete number and unit of measurement
☐	☒	A statement on whether measurements were taken from distinct samples or whether the same sample was measured repeatedly
☐	☒	The statistical test(s) used AND whether they are one- or two-sided<br>*Only common tests should be described solely by name; describe more complex techniques in the Methods section.*
☐	☒	A description of all covariates tested
☐	☒	A description of any assumptions or corrections, such as tests of normality and adjustment for multiple comparisons
☐	☒	A full description of the statistical parameters including central tendency (e.g. means) or other basic estimates (e.g. regression coefficient) AND variation (e.g. standard deviation) or associated estimates of uncertainty (e.g. confidence intervals)
☐	☒	For null hypothesis testing, the test statistic (e.g. *F*, *t*, *r*) with confidence intervals, effect sizes, degrees of freedom and *P* value noted<br>*Give P values as exact values whenever suitable.*
☒	☐	For Bayesian analysis, information on the choice of priors and Markov chain Monte Carlo settings
☐	☒	For hierarchical and complex designs, identification of the appropriate level for tests and full reporting of outcomes
☐	☒	Estimates of effect sizes (e.g. Cohen's *d*, Pearson's *r*), indicating how they were calculated

Our web collection on statistics for biologists contains articles on many of the points above.

Software and code

Policy information about availability of computer code

Data collection	No software was used for data collection for the current study.
Data analysis	FreeSurfer v6.0; R version 4.0.0 ("gamm4" version 0.2-26, "mgcv" version 1.8-28, "ggseg"), PLINK2

For manuscripts utilizing custom algorithms or software that are central to the research but not yet described in published literature, software must be made available to editors and reviewers. We strongly encourage code deposition in a community repository (e.g. GitHub). See the Nature Portfolio guidelines for submitting code & software for further information.

Data

Policy information about availability of data

All manuscripts must include a data availability statement. This statement should provide the following information, where applicable:

- Accession codes, unique identifiers, or web links for publicly available datasets
- A description of any restrictions on data availability
- For clinical datasets or third party data, please ensure that the statement adheres to our policy

Data supporting the results of the current study are available by requests to the PIs of each sub-study, given appropriate ethics and data protection approvals. Specific limitations on data access applies to some samples. Contact information can be obtained from the corresponding authors. UK Biobank data requests can be submitted to http://www.ukbiobank.ac.uk.

Research involving human participants, their data, or biological material

Policy information about studies with human participants or human data. See also policy information about sex, gender (identity/presentation), and sexual orientation and race, ethnicity and racism.

Reporting on sex and gender	Sex was included as covariate in the analyses. In addition, post hoc analyses were run split by sex to assess whether specific relationships were seen for males or females, and a formal sex-interaction analysis was further run directly to test effects of sex.
Reporting on race, ethnicity, or other socially relevant groupings	For the genetic analyses, participants who were not self-reported white-British were excluded. In addition, the top 10 genetic principal components were included as covariates to control for population structure. The main statistical analyses were repeated controlling for socioeconomic status, based on self-reported income and education. No other social, race or ethnicity variables were used.
Population characteristics	Community-dwelling participants from multiple countries in Europe and the US. Some were convenience samples, whereas others were contacted based on population registries. In total, data from 47,039 participants (20.0-89.4 years) with information about sleep duration and MRI of the brain were included. For 3,910, two or more MRI examinations were available, yielding a total of 51,320 MRIs (mean follow-up interval 2.5 years, range 0.005-11.2, 26,811 female/ 24,509 male observations).
Recruitment	Sample<br>Community-dwelling participants were recruited from multiple countries in Europe and the US. Some were convenience samples, whereas others were contacted based on population registries. No MRI sample is fully representative of the populations from which they are drawn. Which effects this may have on the results are unknown. For all reported results, relevant population characteristics such as age, sex, education, income, BMI and depression symptoms were covaried.
Ethics oversight	All procedures were approved by a relevant ethics review board. For Lifebrain, approval was given by the Regional Ethical Committee for South Norway, and all sub-studies were approved by the relevant national review boards. For UKB, ethics approval was obtained from the National Health Service National Research Ethics Service (Ref 11/NW/0382).

Note that full information on the approval of the study protocol must also be provided in the manuscript.

Field-specific reporting

Please select the one below that is the best fit for your research. If you are not sure, read the appropriate sections before making your selection.

☒ Life sciences ☐ Behavioural & social sciences ☐ Ecological, evolutionary & environmental sciences

For a reference copy of the document with all sections, see nature.com/documents/nr-reporting-summary-flat.pdf

Life sciences study design

All studies must disclose on these points even when the disclosure is negative.

Sample size	We used all available data points to maximize sample size, so this was not defined before the study. Sample size much larger than any existing study, yielding excellent statistical power.
Data exclusions	Exclusion criteria were predefined, and a detailed description is provided in the manuscript/ SI. Volumetric outliers were defined by having a residual more than four times the magnitude of the residuals standard error in an analysis of age effects and removed from the analyses. Each individual study feeding data into this work used different exclusion criteria before data were entered into the present study, detailed in the manuscript. For genetic analyses, we excluded participants who are not self-reported white-British (n=92.900), have relatives in the biobank (n=148.689), had been labeled as outliers in missingness or heterozygosity (n=968) or had conflicting self-reported vs genetic sex (n=378) by the UK Biobank team.
Replication	We did not have an independent replication sample. Running analyses on the full ample yielded maximal statistical power to detect miniute effects. As we did not find evidence for a relationship between sleep duration and brain change in our very big sample, replication was deemed unnecessary. Instead, permutation tests were used to assess stability. Results were evaluated based on effect sizes, p-values and confidence intervals. Proper statistical corrections for multiple comparisons were applied.
Randomization	This is an observational study, hence randomization is not relevant.
Blinding	This is an observational study, hence blinding is not relevant.

Reporting for specific materials, systems and methods

We require information from authors about some types of materials, experimental systems and methods used in many studies. Here, indicate whether each material, system or method listed is relevant to your study. If you are not sure if a list item applies to your research, read the appropriate section before selecting a response.

Materials & experimental systems

n/a	Involved in the study
☒	☐ Antibodies
☒	☐ Eukaryotic cell lines
☒	☐ Palaeontology and archaeology
☒	☐ Animals and other organisms
☒	☐ Clinical data
☒	☐ Dual use research of concern
☒	☐ Plants

Methods

n/a	Involved in the study
☒	☐ ChIP-seq
☒	☐ Flow cytometry
☐	☒ MRI-based neuroimaging

Magnetic resonance imaging

Experimental design

Design type: Structural (T1w)

Design specifications: Structural (T1w)

Behavioral performance measures: No task during scanning

Acquisition

Imaging type(s): Structural (T1w)

Field strength: 1.5T and 3.0T

Sequence & imaging parameters:
BASE-II Tim Trio Siemens 3.0 TR: 2500 ms, TE: 4.77 ms, TI: 1100 ms, flip angle: 7°, slice thickness: 1.0 mm, FoV 256×256 mm, 176 slices
Betula Discovery GE 3.0 TR: 8.19 ms, TE: 3.2 ms, TI: 450 ms, flip angle: 12°, slice thickness: 1 mm, FOV 250×250 mm, 180 slices
Cam-CAN Tim Trio
Siemens 3.0 TR: 2250 ms, TE: 2.98 ms, TI: 900 ms, flip angle: 9°, slice thickness 1 mm, FOV 256×240 mm, 192 slices
LCBC Avanto Siemens 1.5 TR: 2400 ms, TE: 3.61 ms, TI: 1000 ms, flip angle: 8°, slice thickness: 1.2 mm, FoV: 240×240 m, 160 slices, iPat = 2
Avanto Siemens 1.5 TR: 2400 ms, TE = 3.79 ms, TI = 1000 ms, flip angle = 8, slice thickness: 1.2 mm, FoV: 240 x 240 mm, 160 slices
Skyra Siemens 3.0 TR: 2300 ms, TE: 2.98 ms, TI: 850 ms, flip angle: 8°, slice thickness: 1 mm, FoV: 256×256 mm, 176 slices
Prisma Siemens 3.0 TR: 2400 ms, TE: 2.22 ms, TI: 1000 ms, flip angle: 8°, slice thickness: 0.8 mm, FoV: 240×256 mm, 208 slices, iPat = 2
UB Tim Trio Siemens 3.0 TR: 2300 ms, TE: 2.98, TI: 900 ms, slice thickness 1 mm, flip angle: 9°, FoV 256×256 mm, 240 slices
WH-II Verio Siemens 3.0 TR: 2530 ms, TE: 1.79/3.65/5.51/7.37 ms, TI: 1380 ms, flip angle: 7°, slice thickness: 1.0 mm, FOV: 256×256 mm
HCP Connectome
Skyra
Siemens* 3.0 TR: 2400 ms, TE: 2.14 ms, TI: 1000 ms, flip angle: 8°, slice thickness: 0.7 mm, FOV: 224 mm, 256 slices, GRAPPA = 2
UKB Skyra
Siemens 3.0 TR: 2000 ms, TI: 880 ms, slice thickness: 1 mm, FoV: 208×256 mm, 256 slices, iPAT=2

Area of acquisition: Whole brain coverage

Diffusion MRI: ☐ Used ☒ Not used

Preprocessing

Preprocessing software: FreeSurfer 6.0

Normalization: linear, T1

Normalization template: Talairach

Noise and artifact removal: To avoid introducing site-specific biases, quality control measures were imposed and no manual editing was done.

Volume censoring: Not used (astructural scans only)

Statistical modeling & inference

Model type and settings	Generalized additive mixed models; Spatio-temporal linear mixed models
Effect(s) tested	No tasks or stimulus conditions were used (structural only)

Specify type of analysis: ☐ Whole brain ☐ ROI-based ☒ Both

Anatomical location(s)	Vertex-wise, subcortical ROIs from FreeSurfer, Desikan-Killiany cortical parcellations
Statistic type for inference (See Eklund et al. 2016)	Vertex-wise spatio-temporal linear mixed-effects models.
Correction	Z Monte Carlo simulations with a cluster forming threshold of p < .01 and a cluster threshold of .05 for vertex-wise analyses; FDR for ROI analyses

Models & analysis

n/a	Involved in the study
☒	☐ Functional and/or effective connectivity
☒	☐ Graph analysis
☒	☐ Multivariate modeling or predictive analysis
